# Comparative proteomics of common allergenic tree pollens of birch, alder, and hazel

**DOI:** 10.1111/all.14694

**Published:** 2021-01-15

**Authors:** Barbara Darnhofer, Tamara Tomin, Laura Liesinger, Matthias Schittmayer, Peter Valentin Tomazic, Ruth Birner‐Gruenberger

**Affiliations:** ^1^ Diagnostic and Research Institute of Pathology Medical University of Graz Graz Austria; ^2^ Omics Center Graz BiotechMed‐Graz Graz Austria; ^3^ Faculty of Technical Chemistry Institute of Chemical Technologies and Analytics Technische Universität Wien (TU Wien) Vienna Austria; ^4^ Division of Phoniatrics Department of Otorhinolaryngology Medical University of Graz Graz Austria

**Keywords:** alder, birch, hazel, pollen, proteomics

## Abstract

**Background:**

In addition to known allergens, other proteins in pollen can aid the development of an immune response in allergic individuals. The contribution of the “unknown” protein allergens is apparent in phylogenetically related species where, despite of high homology of the lead allergens, the degree of allergenic potential can vary greatly. The aim of this study was to identify other potentially allergenic proteins in pollen of three common and highly related allergenic tree species: birch (*Betula pendula*), hazel (*Corylus avellana*) and alder (*Alnus glutinosa*).

**Methods:**

For that purpose, we carried out a comprehensive, comparative proteomic screening of the pollen from the three species. In order to maximize protein recovery and coverage, different protein extraction and isolation strategies during sample preparation were employed.

**Results:**

As a result, we report 2500–3000 identified proteins per each of the pollen species. Identified proteins were further used for a number of annotation steps, providing insight into differential distribution of peptidases, peptidase inhibitors and other potential allergenic proteins across the three species. Moreover, we carried out functional enrichment analyses that, interestingly, corroborated high species similarity in spite of their relatively distinct protein profiles.

**Conclusion:**

We provide to our knowledge first insight into proteomes of two very important allergenic pollen types, hazel and alder, where not even transcriptomics data are available, and compared them to birch. Datasets from this study can be readily used as protein databases and as such serve as basis for further functional studies.

## Graphical abstract

This study aims to identify and annotate novel potentially allergenic proteins in the three highly related pollen species: birch, hazel, and alder. Immunoblotting of pollen protein extracts with serum from allergenic patients corroborates differential allergenic potential despite of the high similarity of the three species. Comprehensive proteomics analysis provides the first available protein database of pollens of alder and hazel and enables thorough annotation of proteases and allergens as well as comparative functional analysis of expressed proteins across the three pollens.

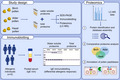

Abbreviations: LC‐MS, liquid chromatography‐mass spectrometry; SDS‐PAGE, sodium dodecyl sulfate polyacrylamide gel electrophoresis.

## 1 INTRODUCTION

Allergic respiratory diseases such as rhinitis and asthma represent a major healthcare burden and are the most common chronic diseases among young adults.^1^ Of the total number of allergic individuals, pollen allergies account for approximately 40% of all cases. The main body of allergenic pollen is produced by trees, of which the most dominant ones belong to the orders of *Fagales*, *Lamiales*, *Proteales* and *Pinales*.^2^ Out of the four orders, *Fagales* is leading in the number of allergenic species officially recognized by the World Health Organization and International Union of Immunological Societies (WHO/IUIS) (http://www.allergen.org/). Correspondingly, members of the *Fagales* order are the main cause of spring pollinosis in the northern hemisphere.^3^ Major *Fagales* pollen allergens (e.g., Bet v 1 in birch, Aln g 1 in alder and Cor a 1 in hazel) belong to the pathogenesis‐related protein class 10 (PR‐10), which also includes a large group of other food and aeroallergens.^2^

However, not all *Fagales* species cause the same degree and type of allergic reaction. This is, on the one hand, to some extent due to the physical properties of pollen^3^ as well as the potency of the lead allergen. For example, Bet v 1, the main allergen of birch pollen, is by the far most potent allergen compared to other allergenic proteins from related species.^3^, ^4^ On the other hand, different allergenic properties of pollen can also be due to different profiles of other non‐allergenic constituents of pollen, in particular enzymes that can aid the allergic response.^5^ For instance, different types of pollen proteases that are normally anchored to the pollen wall are known to be able to disrupt the tight junctions of respiratory epithelial cells, increasing the sensitization to allergens.^6^, ^7^ Next to proteases, different protease inhibitors that have been identified in pollen might also play a role in modulating the activity of pollen as well as of host proteases.^8^ In addition, intrinsic nicotinamide adenine dinucleotide phosphate (NADPH) oxidases of allergenic pollens have also emerged in recent years as potential inflammatory mediators.^9^ This implies that often synergistic activities of allergenic and non‐allergenic pollen proteins appear to be necessary to fully trigger the host's allergic response. Immunotherapy with isolated recombinant pollen allergens seems to be promising, as shown for both plant‐ and *E. coli*‐derived recombinant Bet v 1,^10^ but in some cases the isolated treatment fails to meet the efficiency of immunization with total pollen extracts.^11^ Such discrepancies might be in large part due to the host organism used for recombinant protein production. Naturally, plant‐derived recombinant proteins have a conformation more similar to the native ones, and thus should be the preferred option whenever possible.^12^

In this study, we focused on elucidation of potential allergenic and non‐allergenic protein contributors in pollen of the highly relevant and highly related allergenic *Fagales* species: birch (*Betula pendula*, BP), alder (*Alnus glutionsa*, AG) and hazel (*Corylus avellana*, CA). Of note, we considered several aspects: In addition to the above‐mentioned factors determining the allergenic potency of pollen, another important element is bioavailability of the allergenic components, for example, the efficiency of pollen grain rupture.^13^ The rupture can occur within several minutes in hypotonic solutions such rainwater or tears,^14^ or might take up to several hours, as shown for birch pollen in water.^13^ This means that different proteins are released over time by pollen particles depending on the environment the pollen is exposed to. To account for that, we employed two different protein extraction strategies. In the first approach we attempted to mimic the physiological environment of nasal mucus by incubating the pollen for prolonged time (4–10 h) at physiological pH in phosphate‐buffer saline (PBS), which has similar sodium, potassium and chloride ion content as nasal mucus.^15^ To aid protein solubilization and prevent formation of protein‐protein coagulates, PBS was supplied with a very low amount of a mild non‐ionic detergent (0.1% Triton X100) and the resulting protein extract was considered “water‐soluble protein fraction.” In the second extraction approach, in order to exclude any allergen‐release‐efficiency bias and recover as many proteins as possible, pollen grains were lysed using harsh bead‐treatment and solubilized in a potent anionic detergent (1% sodium‐dodecyl sulphate [SDS]). Proteins extracted in this way were referred to as the “total proteome.” Next to protein extraction, further protein preparation steps of the water‐soluble fraction were also carefully considered, as a much lower protein yield was expected there and it is known that pollen trapped in the nasal mucus not only releases protein allergens, but also a plethora of bioactive lipids^16^ which must be fully removed prior proteomics analysis. For that reason, we employed different protein preparation strategies of the soluble protein fractions to achieve optimal protein recovery and coverage.

Overall, we carried out a comprehensive proteomics screening which enabled us to annotate and compare the proteomics profiles of pollen from the three species (birch [*Betula pendula*, BP], alder [*Alnus glutinosa*, AG] and hazel [*Corylus avellana*, CA]). To our knowledge, this is the first study to tackle the yet not annotated alder and hazel pollen proteomes. In addition, we provide a comparative insight that despite their very high phylogenetic similarity, these three species have distinctive pollen proteome profiles, which may also to some extent contribute to their differential allergenic potentials.

## 2 MATERIALS AND METHODS

### 2.1 Pollen extract preparation with mild lysis conditions (water‐soluble proteome)

Pollen from birch (*Betula pendula*, BP), alder (*Alnus glutinosa*, AG) and hazel (*Corylus avellana*, CA) were collected in Styria, Austria during the pollen season. Water‐soluble protein pollen extracts were prepared by shaking for 4 h at room temperature in PBS containing 0.1% Triton X‐100, except for birch pollen, which had to be shaken for 10 h to extract comparable protein amounts. Pollen extracts were then left overnight at −20°C. The following day, extracts were centrifuged at 18,000 *g* for 20 min at 4°C, and the supernatants were collected for proteomics analysis (see Appendix S1).

### 2.2 Pollen extract preparation with harsh lysis conditions (total proteome extract)

Additionally, pollen from all 3 species (each in triplicates) were lysed with 500 µm Zirconium beads (Sigma) and MagNAlyser (Roche) for 3 min in 500 µL of 100 mM Tris‐HCl (containing 10 mM TCEP and 40 mM CAA). After removal of the beads, the extracts were adjusted to 1% SDS, left shaking for 10 min, followed by 10 min incubation at 95°C. Consequently, extracts were centrifuged at 18,000 *g* for 20 min at 4°C and the supernatants were collected for proteomics analysis.

## 3 RESULTS

### 3.1 Pollen extracts from three related Fagales species show distinctive protein profiles and allergenic responses

**FIGURE 1:**
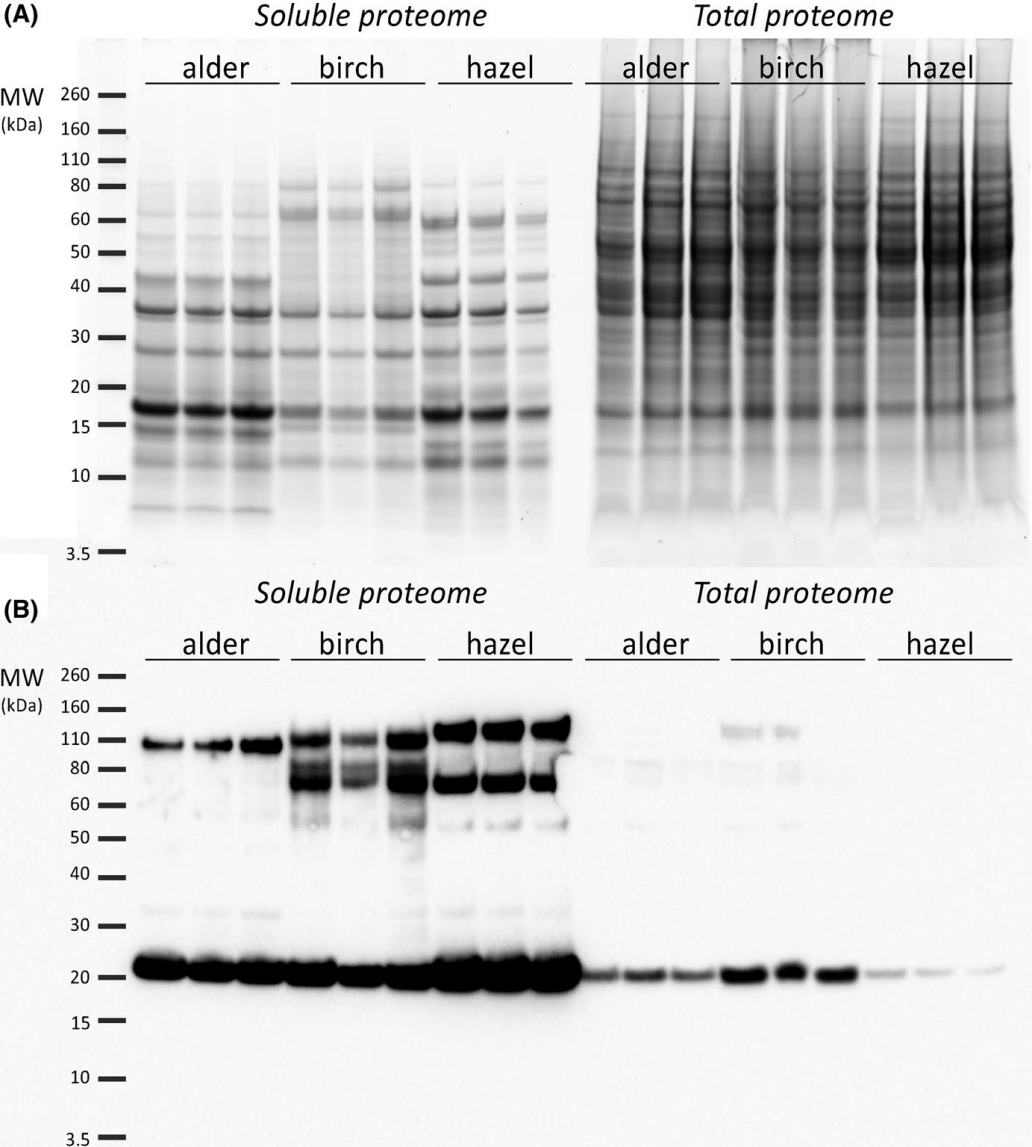
Pollen of three highly related *Fagales* species (birch [*Betula Pendula*], alder [*Alnus glutinosa*] and hazel [*Corylus avellana*]) display distinctive protein profiles and different allergenic potential. A, SDS‐PAGE of individual pollen protein extracts. Protein bands were visualized by fluorescent staining. Individual wells represent biological replicates of the same pollen species extracted either under mild (0.1% Triton‐X 100 in PBS; *Soluble Proteome*; n = 3 per pollen species) or harsh extraction conditions (Zr^2+^ beads, 1% SDS; *Total Proteome*; n = 3 per pollen species). B, Anti‐IgE Western blot of the soluble proteome (n = 3 per pollen species) and total proteome (n = 3 per pollen species) incubated with a pool of serum collected from five allergenic individuals

To address and visualize overall protein expression patterns of the water‐soluble and total proteome fractions of the three related *Fagales* pollen types, we first performed SDS‐PAGE followed by fluorescent staining of proteins. Due to technical challenges, birch pollen soluble extract had to be prepared differently than the other two (see materials and methods). Still, already a simple gel analysis pointed out prominent distinctions in the protein profiles of the soluble protein fraction of the three allergenic pollen species (Figure 1A). Besides the divergence in birch extract preparation (which needed to be shaken for a longer time [10 vs. regular 4 h] to yield comparable amounts of protein), this may be due to different protein release efficiencies of the pollen. It also is noteworthy to mention that the dominant allergenic PR‐10 protein members have a size of 17 kDa, which places them on the lower end of the observed mass range, most likely corresponding to the thick protein band just below 20 kDa (Figure 1A). On the other hand, harsh lysis conditions resulted in rather similar protein distributions across all three species, corroborating the usefulness of these extracts as total protein fractions (Figure 1A).

**FIGURE 2:**
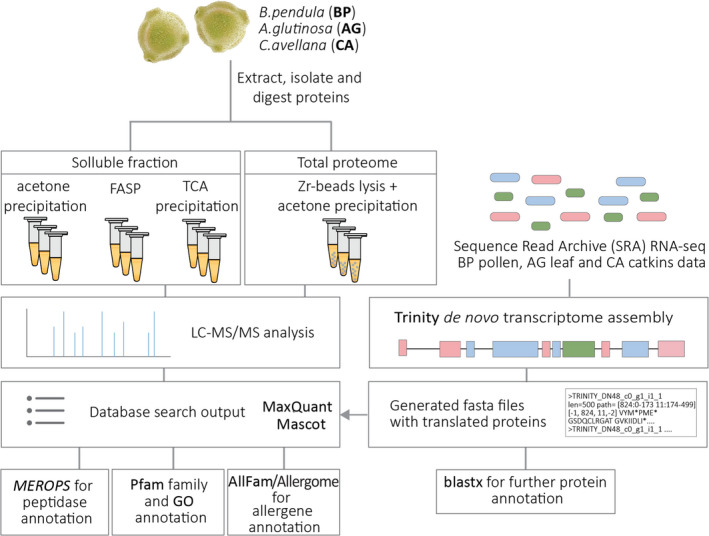
Proteomic workflow. Proteins were isolated from pollen of *B. pendula* (birch), *A. glutinosa* (alder) and *C. avellana* (hazel) to obtain soluble fractions (mild extraction in 0.1% Triton‐X in PBS) and the total proteomes (harsh mechanical lysis using Zirconium‐beads with acetone precipitation). Soluble extracts were processed using three different approaches, after which they were digested and subjected to LC‐MS/MS analysis. Protein databases were created from publicly available pollen (for birch), leaf (for alder) and catkins (for hazel) RNA‐seq data using Trinity de novo transcriptome assembly and Emboss alignment. Generated fasta files were used as databases for protein identification by Mascot and MaxQuant as well as annotation by blastx. Protein‐database matching (search output) resulting from data pooled from the different protein preparations (soluble fraction and total proteome) was then used for Pfam, MEROPS and Gene Ontology (GO) an AllFam annotations. FASP, Filter Aided Sample Preparation; TCA, trichloroacetic acid

In order to investigate the allergenic potential of the protein extracts from the three pollen species, we addressed their specific immunoglobulin (IgE) antibody responses. For that purpose, proteins from the soluble and total protein fractions were transferred to a nitrocellulose membrane and incubated with patients’ serum (pooled from five individuals allergic either only to birch or to all three species). Consequently, allergen bound IgE was detected and visualized using a reporter‐linked specific antibody against human IgE. As expected, in the soluble fraction most potent allergens were located in the area just below 20 kDa, corresponding to the size of PR‐10 protein family members (Figure 1B). However, despite high similarity between species, the soluble extracts of the three pollen types had surprisingly distinctive distributions of allergens, especially in the area between 50 and 110 kDa (Figure 1B). In the total proteome fraction, only the protein allergen group corresponding to PR‐10 proteins was able to bind IgE, albeit to a lesser extent. The reason for this most likely was the harsh lysis, which could have led to stronger denaturation and loss of the allergens’ protein conformation, rendering them less recognizable by IgE.

Considering the distinctive protein and allergen profile of the three species, we next performed a more detailed proteomic analysis employing LC‐MS/MS. In the lack of existing protein databases, we applied a multi‐step analysis workflow depicted in Figure 2 to identify and annotate the pollen proteomes of the three species. Given the different protein profiles obtained from the soluble and total proteome fractions, in order to achieve optimal proteome coverage, we used two different extraction approaches as well as three different protein sample preparation methods for consequent LC‐MS/MS analysis.

### 3.2 Complementarity of protein sample preparation methods

**FIGURE 3:**
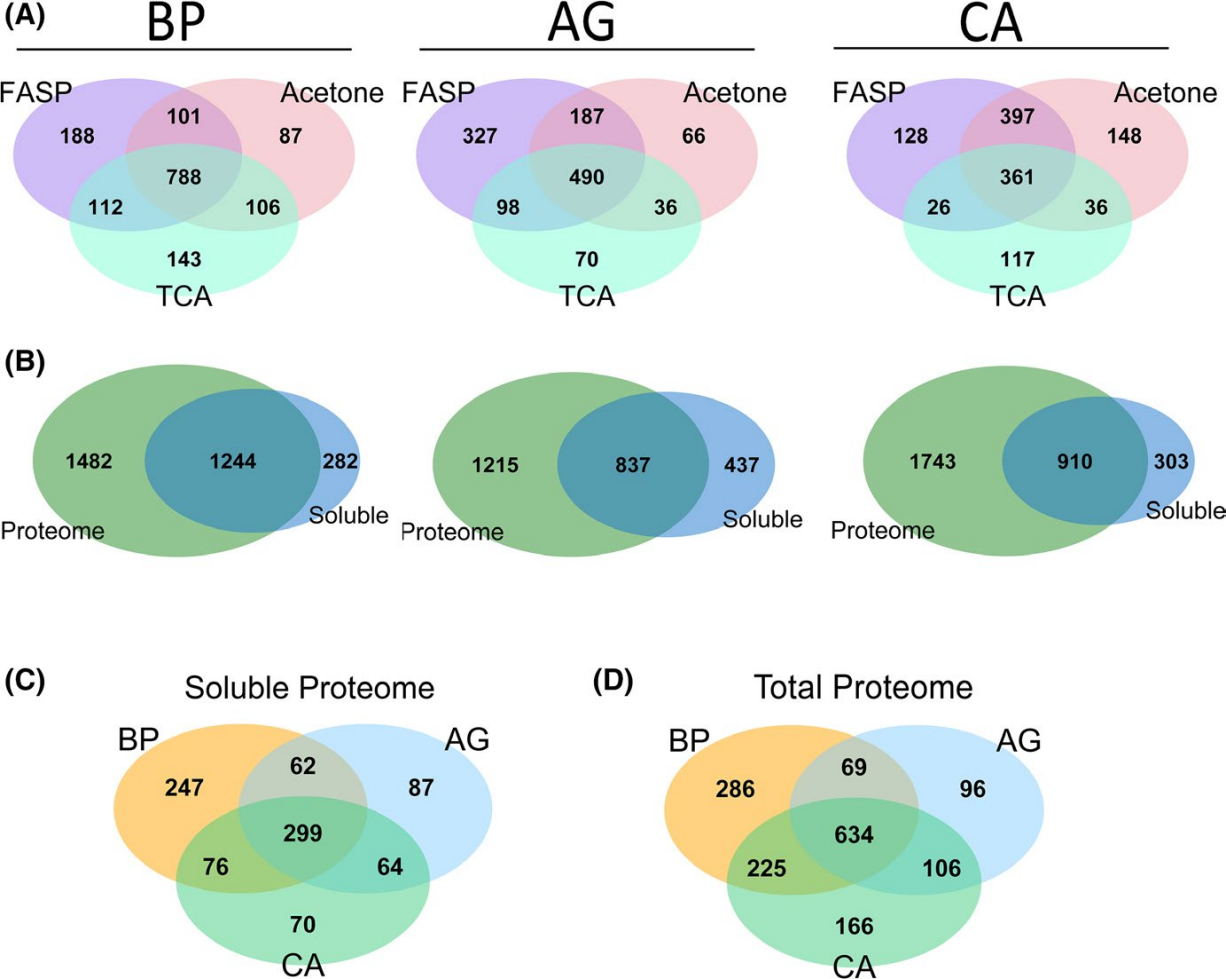
Complementarity of different protein sample preparation methods. A, Analysis of “soluble fraction” of the pollen proteomes (extracted by incubation in PBS containing 0.1% Triton X‐100) using different protein precipitation and re‐solubilization approaches. Venn diagrams show numbers of identified proteins by each method. B, Comparison of identified proteins in soluble (PBS, 0.1% Triton X‐100) vs. total protein fraction (zirconium beads, 1% SDS). C‐D: Pfam families of identified proteins of three *Fagales* pollen species. Venn diagram depicting overlap of annotated Pfam families in soluble proteome (C) and total proteome (D). Detailed list of identifications can be found in Tables [Link], [Link], [Link], [Link], [Link], [Link], [Link], [Link], [Link], [Link]. AG—*Alnus glutinosa*; alder, BP—*Betula pendula*; birch, CA—*Corylus avellana*; hazel, TCA—trichloroacetic acid, FASP—Filter Aided Sample Preparation

After water‐soluble pollen protein isolation, we tested three different yet commonly used sample preparation approaches.^17^ Proteins were either precipitated in triplicates (for hazel) or quadruplicates (birch and alder) from individual pollen extracts using acetone, trichloroacetic acid (TCA) or purified by applying the FASP protocol^18^ (Figure 2). The sample preparation method affected the overall number of identified proteins in the water‐soluble fraction of the three pollen species to a different extent. With 1189, 1082 and 1149 identified proteins in the pollen proteomes of birch by FASP, acetone and TCA precipitation respectively, the effect on overall obtained protein numbers appeared to be minor (Figure 3A). On the other hand, analysis of the soluble fraction of alder pollen extract resulted in 1102, 779 and 694 proteins identified by FASP, acetone and TCA precipitation respectively. For hazel, the FASP protocol resulted in 929 identified proteins, acetone precipitation in 942, while after TCA precipitation we could identify only 540 proteins. In addition, data analysis revealed that different preparations in all three species resulted in the extraction of distinct proteins (Figure 3A). In each of the three pollen species about 38%–72% of all identified proteins per preparation (TCA, acetone or FASP) were actually detected in all three preparation procedures. Correspondingly, the contribution of uniquely identified proteins per preparation method was rather high—spanning from 8%–30% across different pollen species and precipitation techniques (Figure 3A). Therefore, in order to ensure maximal information coverage, for all consequent analysis steps we used data pooled from all three sample preparation approaches for each pollen species.

Merged together, all three protein preparations resulted in 1525 identified proteins in the water‐soluble fraction of birch, 1274 in alder and 1213 in hazel. The large majority of proteins identified in the water‐soluble fraction (1244 (82%), 837 (66%) and 910 (75%) proteins in birch, alder and hazel, respectively) were also identified in the “total proteome” (solubilized proteome after harsh lysis) as shown in Figure 3B. Still, a significant number of proteins were identified exclusively in the water‐soluble protein fraction (282 [birch], 437 [alder] and 303 [hazel]; Figure 3B), which were potentially released only after prolonged exposure to water.

### 3.3 Database annotation and protein classification

For identification of proteins from the different pollen species, two search engines, Andromeda (MaxQuant) and Mascot, were employed. In all cases raw proteomics data was searched against protein databases (.fasta files in Appendix S1) created from Trinity de novo transcriptome assemblies of publicly available RNA‐seq data from the Sequence Read Archive (SRA) as described in the supplement (Figure 2). When creating the fasta protein database file, in order to provide a protein name for each Trinity identifier, de novo assembled Trinity databases were annotated using the blastx tool by matching the assembly to existing NCBI non‐redundant (nr) protein sequences of several phylogenetically closely related species. The top two hits per each Trinity identifier with an e‐value of <0.001 were listed as protein names. For more reliable peptidase annotations, de novo transcripts were also blasted via blastx over the manually curated MEROPS peptidase database with a maximum e‐value of 10^−4^ allowed for matching. In both cases (blastx search for related NCBI nr proteins and blastx‐MEROPS^19^ search for peptidase annotation) the obtained results were added as additional annotations to the list of Trinity identifiers.

Results from protein‐database matching were then subjected to further functional annotation employing Pfam,^20^ AllFam,^21^ Allergome,^22^ MEROPS,^19^ and GO enrichment.^23^

#### 3.3.1 Pfam family and Allergome annotation

The Pfam database recognizes sequence similarities that indicate homology and accordingly assigns similar proteins to a protein family.^20^ As a result of this approach, in the soluble protein fraction we could annotate 684 Pfam families in birch, 512 in alder and 509 in hazel (Figure 3C, Tables [Link], [Link], [Link], [Link], [Link], [Link], [Link], [Link], [Link], [Link]). On the other hand, annotation of the total proteome resulted in 1214 Pfam families from birch and 905 and 1131 from alder and hazel, respectively (Figure 3D, Tables [Link], [Link], [Link], [Link], [Link], [Link], [Link], [Link], [Link], [Link]). In both soluble and total proteomes, Pfam analysis of birch yielded in the highest number of annotations. This is not surprising, as in case of birch the transcript of pollen could be used for creation of the de novo assembled Trinity database, which were not available for alder and hazel. For alder, the transcriptome of leaves and for hazel, the transcriptome of catkins was used instead.

Combined datasets obtained from soluble and total protein fractions per each pollen species containing Pfam annotation were then searched for known allergens (mainly PR‐10 protein family members), whose allergenic nature was further corroborated using the Allergome platform, the database of allergenic molecules. Lists of Allergome annotated allergens for hazel, birch and alder are displayed in Tables [Link], [Link], [Link], [Link], respectively.

Among the annotated Pfam families, we identified 98 and 173 different protease families across the three pollen species in their soluble and total proteomes, respectively. Strikingly, out of the 98 protease families found in the soluble fraction, proteins from 34 different families could be identified exclusively in the soluble fractions (Tables [Link], [Link], [Link], [Link], [Link], [Link], [Link], [Link], [Link], [Link]).

#### 3.3.2 Peptidase and allergen annotation

**FIGURE 4:**
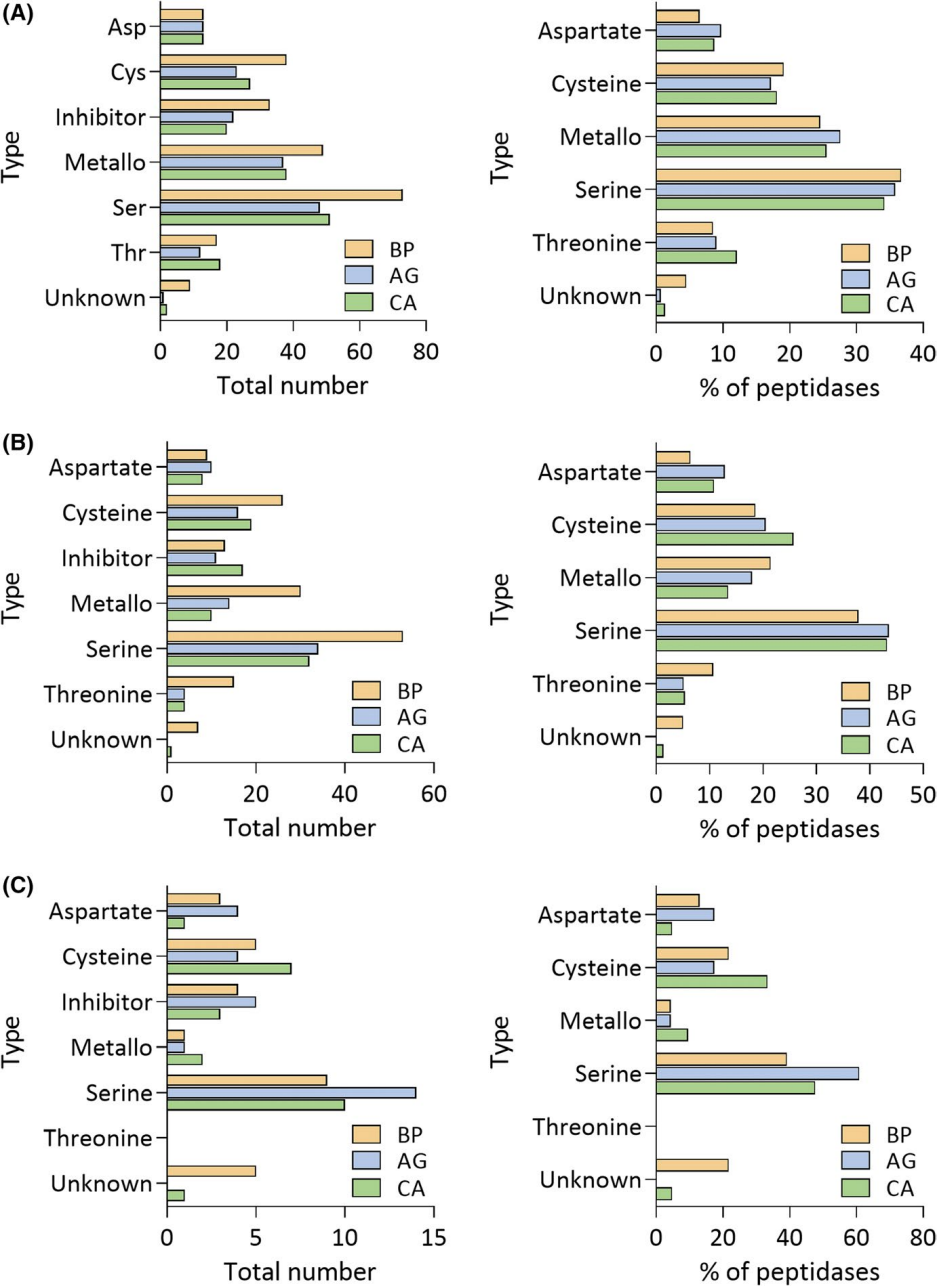
Distribution of MEROPS annotated identified peptidase subfamilies as well as peptidase inhibitors in three different pollen species. Panels represent total number of MEROPS annotated peptidases and peptidase inhibitors as well as the relative distribution (percentage) of different types of peptidases compared to the total number of MEROPS annotated peptidases per each species. A, Distribution of identified peptidases and peptidase inhibitors in combined total and soluble proteome fractions (peptidases and peptidase inhibitors from soluble fractions and bead lysis) (left: exact number of identified peptidases/peptidase inhibitors; right: distribution of peptidase types compared to total number of identified peptidases in this proteome fraction (in percent), (B) distribution of identified peptidases and peptidase inhibitors in soluble fraction (peptidases and peptidase inhibitors from soluble fractions) (left: exact number of identified peptidases/peptidase inhibitors; right: distribution of peptidase types compared to total number of identified peptidases in this proteome fraction (in percent), (C) distribution of identified peptidases and peptidase inhibitors peptidases and peptidase inhibitors identified exclusively in the soluble fraction (but not in the total proteome/bead lysis; left: exact number of identified peptidases/peptidase inhibitors; right: distribution of peptidase types compared to total number of identified peptidases in this proteome fraction [in percent]). Detailed list of identifications can be found in Tables [Link], [Link], [Link], [Link], [Link], [Link], [Link], [Link], [Link], [Link]. AG, *Alnus glutinosa*; BP, *Betula Pendula*; CA, *Corylus avellana*

In order to have an even closer look into peptidases, proteins that were identified as peptidases in MEROPS with high confidence were extracted from the result list of identified proteins with their corresponding blastx protein names, Pfam^20^ and MEROPS^19^ identifications. This extracted list of peptidases and peptidase inhibitors was then filtered to remove MEROPS entries labeled as “non‐peptidase homologues.” Based on the mechanism of their catalytic activity, peptidases were further allocated to six classes, namely aspartate‐, cysteine‐, metallo‐, serine‐, threonine‐peptidases and peptidases of unknown origin (Figure 4, Tables [Link], [Link], [Link], [Link], [Link], [Link], [Link], [Link], [Link], [Link]). Intriguingly, the distribution of proteases in the total proteome (Figure 4A) and the whole soluble fraction (Figure 4B), differed compared to the distribution of the proteases exclusively found in the soluble fraction (Figure 4C). This is of particular interest as the soluble fraction is more likely to represent the physiological state of how the nasal mucus and endothelium are exposed to pollen.

In the total proteome across the three pollen species the most abundant is the serine hydrolase family, accounting for 32%–36% of all annotated proteases (Figure 4A). Comparatively, the relatively as well as absolutely highest serine protease content was observed in the total proteome of birch. The same was true for the combined soluble fraction (Figure 4B). However, when proteases exclusive to the soluble fraction were separately observed, the trend was different: the relative abundance of serine hydrolases compared to other peptidases was much higher in alder (61%) than in birch (39%) (Figure 4C).

One of the most prominent serine hydrolase families detected in the soluble fraction was the Pfam family of subtilases (PF00082), which are serine hydrolases with conserved Asp/Ser/His catalytical triad.^24^ From this family alone, we detected 14 proteases in birch, 10 in hazel and two in alder, of which a total of five (two in birch, three in hazel) were identified only in the soluble fractions. According to the AllFam Database,^21^ 23 allergens are currently known for this Pfam family. Next to subtilases, another serine hydrolase family, namely carboxypeptidases (PF00450), stands out. In the soluble fraction of birch, we could detect 12 members of this family, in alder 11 and in hazel three, of which a total of six (three each in alder and birch) were found exclusively in the soluble fraction. AllFam currently only lists two allergens from this family (Api m 9 and Tri a CPDW‐II).

While in the total proteome the second most abundant hydrolase class are the metallopeptidases (Figure 4A), in the soluble fractions the next highest rank is taken by cysteine hydrolases (Figure 4B,C). Correspondingly, different members of cysteine protease family (PF00112) were identified in the soluble fraction (14, six and 12 in birch, alder and hazel, respectively) of which a total of seven were found in the soluble part only. A role for cysteine proteases in allergies has already been proposed, as they can disrupt tight junctions in epithelial cells.^6^, ^7^ AllFam lists 13 allergens deriving from this Pfam family, such as Der p 1 from house dust mites, which was shown to exhibit cysteine protease activity and also to be involved in disruption of the epithelial barrier.^25^

We moreover detected a number of different metallopeptidases (assigned to 16, four and eight different Pfam families in soluble fraction of birch, alder and hazel, respectively). Of these, arguably the most dominant family was the Pfam family of aminopeptidases (PF01433). Pollen aminopeptidases have also been reported to be able to disrupt the integrity of epithelium.^26^ However, AllFam does not list any allergens associated with this family.

Lastly, in addition to different proteases, we further report a comprehensive list of protease inhibitors belonging to 12 different Pfam families in the soluble fraction of birch, six in alder and 12 in hazel pollen (Tables [Link], [Link], [Link], [Link], [Link], [Link], [Link], [Link], [Link], [Link]). Pollen protease inhibitors can influence the activity of proteases, and therefore modulate the allergic response.^8^ For example, in alder and hazel we detected more inhibitors belonging the family of cystatins (cysteine protease inhibitors, PF16845) than in birch. The same was true for serine protease inhibitors (belonging to families of serpins [PF00079], I9 [PF05922] and potato inhibitor I [PF00280]),^27^ which were collectively more abundant in alder and hazel than in birch. This might suggest that cysteine and serine hydrolases are more active in birch than in the other two species, which could contribute to its higher allergenic potential.

#### 3.3.3 Functional profiling

Lastly, all identified proteins were subjected to functional profiling by GO^23^ to obtain a first indication on potential functional diversity. Up‐levelling of the GO terms to level 2 provides an overview of the distribution of proteins across GO classes. To estimate the similarities of the functional profiles (displayed in Figure [Link], [Link]) we calculated Spearman rank correlations. Interestingly, obtained correlations of the profiles were almost identical across all GO classes. This is also depicted by overall very similar GO profiles of birch, alder and hazel despite of their different proteomic compositions (Table 1 and Figure [Link], [Link]).

**TABLE 1:** Spearman rank correlation between functional profiles of soluble proteins

	GO total		GOBP		GOMF		GOCC	
	BP	AG	BP	AG	BP	AG	BP	AG
BP		0.98		0.98		0.98		0.98
CA	0.99	0.97	0.99	0.98	0.98	1.00	1.00	0.98

Abbreviations: AG, *Alnus glutinosa*; BP, *Betula pendula*; CA, *Corylus avellana*; GO total, Level 2 GO term classes; GOBP, GO Biological process; GOCC, Cellular compartment; GOMF, GO Molecular function.

## 4 DISCUSSION

Allergies are a great economic and health burden worldwide. Determination of the causes for differential allergenic responses provoked by different allergenic species was long based only on investigation of their lead protein allergens (such as members of the PR‐10 protein family in case of the *Fagales* order). However, with closer related species the structural homology and cross‐reactivity of their lead allergens is rather high, but still their allergenic potential can be completely different. For example, 79% to 83% of amino acid sequence identity is exhibited by the main allergens of alder (Aln g 1) and hazel (Cor a 1) compared to the sequence of Bet v 1,^4^ respectively. Nevertheless, birch pollen is still the most potent allergen in Europe,^4^ suggesting that potentially other, non‐allergenic pollen proteins may contribute to differential allergic responses. We as well demonstrated that the three pollen species display different allergenic responses (Figure 1B) and our next step will be an even more thorough analysis and annotation of these novel, potent allergens. This represents a challenge for itself, as sometimes identification of new, potentially potent allergens can be masked by other, more dominant ones. This was recently shown for the newly discovered olive allergen, Ole e 15 (cyclophilin) whose dimeric form has the same molecular mass as the glycosylated form of the most prevalent olive allergen, Ole e 1.^28^ The need for detailed analysis of both allergenic and non‐allergenic pollen proteins arises from the fact that success rates with allergen immunotherapy have not been satisfying, despite correlating it to the specific sensitization pattern. While in some cases treatment with recombinant allergenic proteins has been successful,^29^ another study showed that for children allergic to grass‐pollen, when matching the obtained sensitization profiles with a preparation of eight *Phleum pratense* molecules for specific immunotherapy, only 4% of the patients had a sensitization profile matching exactly the one proposed in the experimental allergen specific preparation. In the other 96% of the patients, a mismatch between the sensitization profile of each patient and the composition of the allergen specific preparation was observed, suggesting a wide heterogeneity of sensitization profiles.^30^ Thus, this could indicate that important allergens may be missed by the treatment with recombinant proteins, resulting in underpowered and overpowered immunization.^30^, ^31^

In recent years, different classes of proteases have gained attention with regard to allergies, and some allergens themselves have been described as proteases, for example Der p1 from house dust mite as a cysteine protease involved in disruption of the epithelial barrier^32^ and Der p3, 6 and 9^33^ as serine proteases. Additionally, proteases were found within allergenic sources, for example, white birch diffusate,^6^, ^34^ and hazel pollen diffusate.^6^ Here as well of particular importance are serine and cysteine proteases. As previously shown, serine proteases can degrade tight junctions in epithelial cells when exposed to diffusates from white birch,^34^ which could be blocked by addition of the serine protease inhibitor AEBSF (4‐(2‐aminoethyl)benzenesulfonyl fluoride). Similar results were obtained for cysteine proteases and the E‐64 cysteine protease inhibitor.^6^, ^7^ Since the loss of epithelial barrier function represents an opportunity for allergens for entry and development of allergic reactions,^35^ further investigation of identified cysteine and serine proteases are of high interest. In this study we give a detailed overview of protease distribution across the three highly relevant pollen species. For birch, in accordance with the published protein analysis of the water extract from commercially available birch pollen,^36^ we as well detect serine hydrolases as the most abundant protease family in birch pollen (Figure 4A). In addition, we report a comprehensive lists of distinctive Pfam protease families for the three pollen species (e. g. 75 in the soluble and 142 in the total protein fraction of birch), of which 13 matched those previously reported for the water extract of commercially available birch pollen.^36^ Similarly, in our data we also identify almost all identified proteins from the 1D/2D proteomics study of birch pollens from different origins reported by Erler et al 2011, both allergens and other metabolic enzymes.^5^ In addition to proteases, it is important to mention that different protease inhibitors (present also in pollen) can modulate the activity of proteases and influence the allergenic response.^8^, ^27^ In this regard, we as well report a detailed list of different protease inhibitors detected across the three species, which can serve as solid ground for future allergenic studies (Figure 4 and Tables [Link], [Link], [Link], [Link], [Link], [Link], [Link], [Link], [Link], [Link]). Moreover, it was recently shown that the epithelium itself actively regulates down‐stream allergic mechanisms via innate lymphoid cells type 2 by secreting IL‐33 and thymic stromal lymphopoietin among other cytokines.^37^ Thus, further knowledge about pollen protein content, their function and interaction with the nasal mucus and epithelial cell proteome harbors the potential of blocking stimuli exerted by the proteins on epithelial cells and the subsequent allergic cascade.

## 5 CONCLUSION

In this study, we employed different extraction and protein preparation approaches, which all together resulted in a comprehensive proteome annotation of pollen from three *Fagales* tree species, birch, alder and hazel, with 2500–3000 proteins identified per species (supplementary fasta files in Appendix S1). We give a detailed overview of protein families, with a special focus on proteases and protease inhibitors, which may contribute to their different allergenic potential. While for birch pollen an existing RNAseq dataset was available,^8^ this was not the case for the other two species used in this study, for which sequencing data of other plant parts had to be used for de novo database assembly (catkins and leaf for hazel and alder, respectively). When RNAseq datasets of hazel and alder pollen become available, the identified proteomes can be refined by using them as database for researching our LC‐MS/MS data. Therefore, we present here the first available alder and hazel pollen proteomes in comparison to birch as solid foundation for further research.

## CONFLICT OF INTEREST

None declared.

## AUTHOR CONTRIBUTIONS

R.B.‐G. designed the study. B.D., TT, and L.L. performed experiments. B.D., T.T., and M.S. analyzed data. B.D., T.T., P.V.T., and R.B.‐G. wrote the manuscript.

## Supporting information

Fig S1

Appendix S1

Table S1

Table S2

Table S3

Table S4

Table S5

Table S6

Table S7

Table S8

Table S9

Table S10

Table S11

Table S12

Table S13

Table S14

Table S15

Table S16

Table S17

Table S18

Table S19

Table S20

Table S21
