# Supplementary figures and images for: Comparative proteomics of common allergenic tree pollens of birch, alder, and hazel
Source: Allergy. 2021 Jan 15;76(6):1743–53. doi: 10.1111/all.14694 (PMC8248232; doi:10.1111/all.14694)

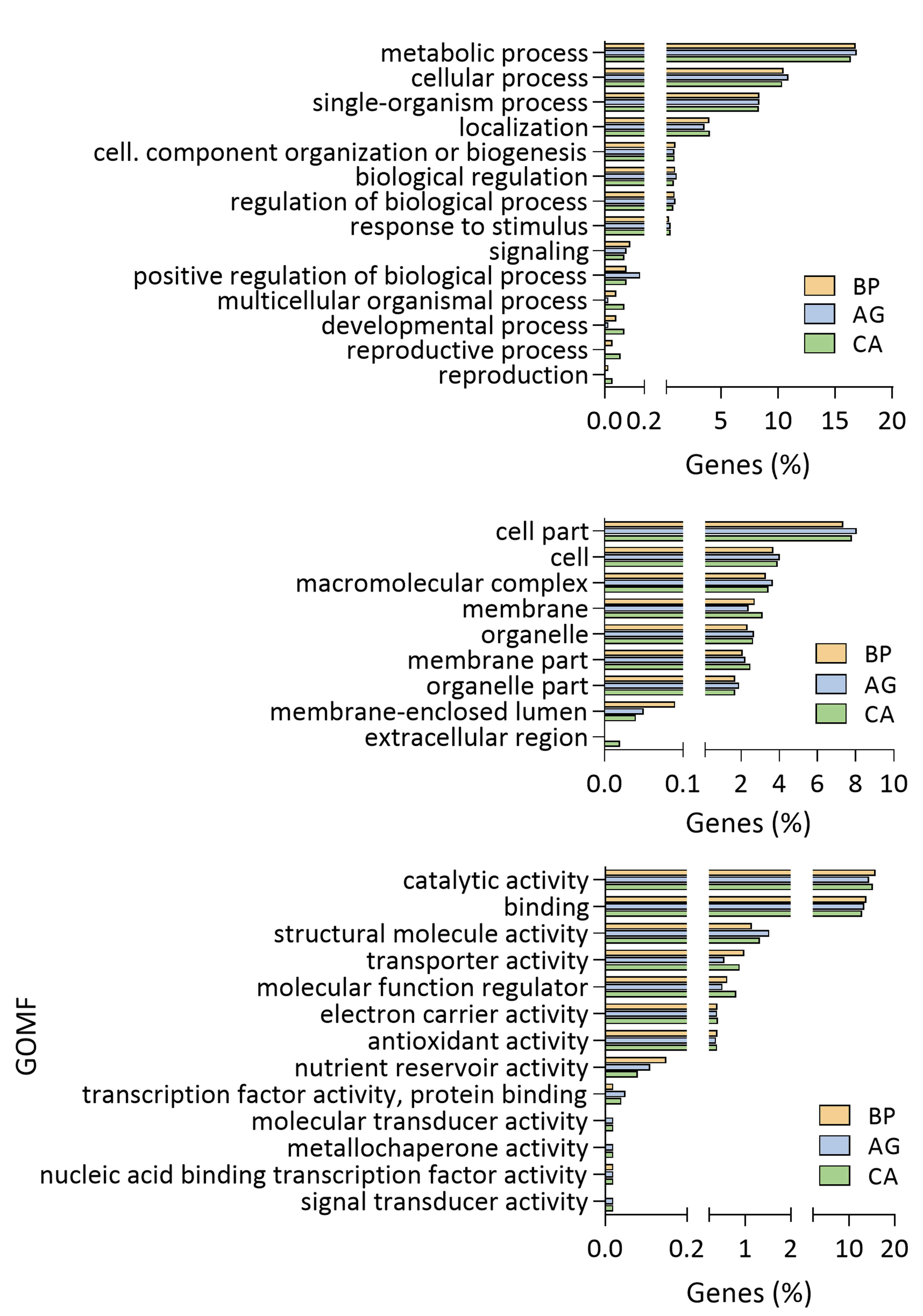

Supplement: Supplementary file 1 — Fig S1 [file ALL-76-1743-s001.tif]
