# Supplementary material for: Comparative proteomics of common allergenic tree pollens of birch, alder, and hazel
Source: Allergy. 2021 Jan 15;76(6):1743–53. doi: 10.1111/all.14694 (PMC8248232; doi:10.1111/all.14694)
Supplement: Supplementary file 2 — Appendix S1 [file ALL-76-1743-s022.docx]

**Comparative proteomics of common allergenic tree pollens of birch, alder and hazel**

Short title: Comparative proteomics of birch, alder and hazel pollens

Barbara Darnhofer^1,2^*, Tamara Tomin^1,2,3^*, Laura Liesinger^1,2^, Matthias Schittmayer^1,2,3^, Peter Valentin Tomazic^4#^, Ruth Birner-Gruenberger^1,2,3#^

^1^ Diagnostic and Research Institute of Pathology, Medical University of Graz, Stiftingtalstrasse 6, 8010 Graz, Austria

^2^ Omics Center Graz, BiotechMed-Graz, Stiftingtalstrasse 24, 8010 Graz, Austria

^3^ Institute of Chemical Technologies and Analytics, Faculty of Technical Chemistry, Technische Universität Wien, Getreidemarkt 9/164, 1060 Vienna, Austria

^4^ Division of Phoniatrics, Department of Otorhinolaryngology, Medical University of Graz, Auenbruggerplatz 26, 8036 Graz, Austria

* Contributed equally and thus should be considered co-first authors

^#^ Co-corresponding authors: Ruth Birner-Gruenberger, Institute of Chemical Technologies and Analytics, Faculty of Technical Chemistry, Vienna University of Technology - TU Wien, Getreidemarkt 9/164, 1060 Vienna, Austria, and Peter Valentin Tomazic, Division of Phoniatrics, Department of General Otorynolaryngology, Auenbruggerplatz 26, 8036 Graz, Austria; emails: [ruth.birner-gruenberger@tuwien.ac.at](mailto:ruth.birner-gruenberger@tuwien.ac.at) and [peter.tomazic@medunigraz.at](mailto:peter.tomazic@medunigraz.at)

# SUPPLEMENTARY METHODS

If not stated otherwise, all materials were obtained from Sigma Aldrich.

## Protein sample preparation from water-soluble fraction

Aliquots of 50 µg protein were subjected to either acetone or trichloroacetic acid (TCA) precipitation or modified Filter Aided Sample Preparation (FASP) ^1^ using Amicon Ultra 3kDa cut-off filters (Millipore, USA). For acetone precipitation, samples were diluted with four volumes of acetone, then incubated over-night at - 20 °C. For TCA precipitation, samples were diluted up to 100 µl with 50 % TCA prior overnight incubation at - 20 °C. The following day, protein pellets from acetone and TCA precipitations obtained after centrifugation were re-solubilized in TFE buffer (25 % trifluoroethanol (TFE), 100 mM Tris-HCl, 10 mM Tris(2-carboxyethyl) phosphine (TCEP) and 40 mM chloroacetamide (CAA)), then incubated for 10 min at 95 °C. Consequently, samples were diluted with 25 mM ammonium bicarbonate to reach 10 % TFE and digested overnight with trypsin. For FASP, in brief, the acetone precipitate was re-solubilized with FASP buffer (100 mM TrisHCl pH 8.5, 1 % SDS, 10 mM TCEP and 40 mM CAA). The FASP buffer (on cut-off filters) was exchanged three times with 8 M Urea, 100 mM Tris-HCl (pH 8.5), after which samples were predigested with rLysC (Promega, USA; enzyme/protein 1:100) for 4 hours at 37 °C. Samples were then diluted to 2 M Urea with 100 mM ammonium bicarbonate and digested with trypsin (Promega, USA; enzyme/ protein 1:50) overnight. Peptides were collected by centrifugation for 45 min at 14,000 *g* at room temperature (RT) and acidified with formic acid (FA; final concentration 0.1 %). For desalting, 3 µg of all digests were brought to a volume of 100 μl with 1 % trifluoroacetic acid in water and loaded onto self-packed 2-layer (18 gauge) 200 μl SDB-RPS Stage Tips (Empore SPE Disks, Sigma-Aldrich). The samples were passed through the stage tips by centrifugation (5 min, 1,500 *g*, RT), washed with 0.2 % trifluoroacetic acid, eluted with 5 % NH_4_OH / 80 % acetonitrile into a new tube and dried using a vacuum concentrator.

## Protein sample preparation from harsh lysis (beads)

Proteins were precipitated with four volumes of acetone over-night and obtained protein pellets were re-solubilized in TFE buffer (25 % TFE, 100 mM Tris HCl pH 8.5), then diluted with 50 mM ammonium bicarbonate (to reach 10 % TFE). Next, diluted samples were predigested with rLysC (Promega, USA; enzyme/protein 1:100) for 4 hours at 37 °C and, lastly, digested overnight with trypsin (Promega, USA; enzyme/ protein 1:50) overnight. Desalting was performed as described above.

## Gel electrophoresis and Western Blot analysis of pollens’ allergenic potential

## Soluble and total protein extracts from three different pollen species were prepared as described above. Consequently, 30 µg of protein extract per each replicate/species was separated on SDS polyacrylamide gels using MES SDS running buffer (Invitrogen-Thermo Fisher Scientific, USA). For the SDS-PAGE analysis of protein profiles, gel-separated proteins were stained with Krypton fluorescent protein stain (Thermo Fisher Scientific, USA) according to manufacturer’s instruction and visualized using Bio-Rad FX Imager (Bio-Rad, USA). For Western Blot analysis of allergenic potential of protein extracts, gel-separated proteins were transferred onto a nitrocellulose membrane (Amersham Protran, Cytiva, USA), which was then blocked for one hour at RT in 5 % milk in tris-buffer saline supplied with 1 % Tween 20 (TBST), and consequently incubated over night at 4 ° C with pooled human serum collected from five allergenic individuals (1 – 1.5 ml per individual). On the following day, after several rounds of washing with TBST, membrane was incubated for one hour at the RT with mouse monoclonal horseradish peroxidase (HRP) linked antibody against human immunoglobulin E (IgE; 1:1000 dilution in TBST; ab99806, Abcam, UK). Membrane was then again repeatedly washed with TBST, incubated with enhanced chemiluminescent (ECL) substrate kit (Pierce, Thermo Fisher Scientific, USA) and visualized using chemiluminescence channel on Bio-Rad FX Imager (Bio-Rad, USA).Liquid chromatography coupled mass spectrometry (LC-MS/MS) analyses

500 ng per sample (re-dissolved in 2 % acetonitrile / 0.1 % formic acid in water) was subjected to LC-MS/MS analysis. Protein digests were separated by nano-HPLC (Dionex Ultimate 3000, Thermo Fisher Scientific, USA) equipped with an Aurora (Ionoptics, Australia) nanocolumn (C18, 1.6 µm, 250 x 0.075 mm) at a flow rate of 300 nl/min at 50 °C using the following gradient, where solvent A is 0.1 % FA in water and solvent B is acetonitrile containing 0.1 % FA: 0-18 min: 2 % B; 18-100 min: 2-25 % B; 100-107 min: 25-35 % B; 107-108 min: 35-95 % B; 108-118 min: 95 % B; 118-118 min: 95-2 % B; 118-133 min: 2 % B. The maXis II ETD mass spectrometer (Bruker Daltonics, Germany) was operated with the captive source in positive mode employing following settings: mass range: 200 - 2000 m/z, 2 Hz, capillary 1600 V, dry gas flow 3 L/min at 150 °C, nanoBooster 0.2 bar, precursor acquisition control top 20 (collision induced dissociation (CID)). Mass spectrometry of FASP prepared samples was carried out on an Orbitrap Velos Pro (Thermo Fisher Scientific, USA) operated in positive mode with top 10 precursors fragmentation mode by alternating full scan MS (m/z 300 to 2000, 60000 resolution) in the ion cyclotron resonance (ICR) cell and MS/MS by CID of the 10 most intense peaks in the ion trap with dynamic exclusion enabled. The mass spectrometry proteomics data were deposited to the ProteomeXchange Consortium ^2^ via the partner repository with the dataset identifier PXD021133.(username: [reviewer_pxd021133@ebi.ac.uk](mailto:reviewer_pxd021133@ebi.ac.uk), password: wAcP2dY5)

## LC-MS/MS data analysis

As there were no comprehensive pollen protein databases available, pollen transcriptomes obtained from public RNA-seq runs from NCBI Sequence Read Archive (SRA) had to be taken as sources for database generation: SRR5485724 for birch ^3^, SRR513138 for hazel ^4^ and for alder SRR3486362, SRR3486361 (from project: White Alder - pooled seedling leaf RNAs from ozone treatments), SRR3486364 and SRR3486363 (from project: Red Alder - pooled seedling leaf RNAs from ozone treatments). Trinity ^5^ pipeline was employed for *de novo* transcriptome assembly. After six frame translation with Emboss, the generated protein databases were used for data analysis using MaxQuant 1.6.6.0 ^6^. Additionally, common contaminants were considered in the search. The following search settings were used: Cysteine carbamidomethylation was set as fixed modification and methionine oxidation was considered as variable. Digestion enzyme was trypsin with a maximum of two missed-cleavage sites allowed. Acceptance parameters for identification were set to 1 % false discovery rate (FDR) for peptide-spectrum matching (PSM), protein as well as site decoy fraction. In order to obtain further information regarding the role of “translated” proteins, the assembled transcripts were annotated by blastx using the NCBI non-redundant protein sequences of the following taxonomies (TXID: 3505 [*Betula pendula*], TXID: 3517: [*Alnus glutinosa*], TXID 3814 [*Papilionoideae*], TXID 13451 [*Corylus avellana*], TXID 16718 [*Juglans*], TXID 171637 [*Amygdaloideae*], TXID 235631 [[*Crotonoideae*](https://www.ncbi.nlm.nih.gov/Taxonomy/Browser/wwwtax.cgi?id=235631)] and TXID 238069 [*Saliceae*]) as database. The e-value cut off for blastx was set to 0.001. For the Pfam (version 30) based annotations we used the Pfam annotator (PfamScan). For the gene ontology (GO) classification the Pfam2go mapping was used (<http://geneontology.org/Pfam2go>). The GO terms were tracked back up to GO level 2 using CateGOrizer ^7^. For the MEROPS ^8^ based annotations a blastx was done against all peptides classified, with an e-value cut off of 0.001. Allergens were further annotated using the AllFam database ^9^. It is noteworthy to mention that for the different sample preparation methods of the soluble fraction as well as for the total proteome, the reported number of identified proteins represents the sum of all identified protein obtained from measuring triplicates or quadruplicates per each pollen species.

# Ethical aspects

The use of human biomaterials was approved by the Ethics committee of the Medical University of Graz (29-392 ex 16/17) and conformed with all pertaining regulations and the principles of the Declaration of Helsinki ^10^. Patients provided written informed consent to participate in the study.

SUPPLEMENTARY REFERENCES

1. Wiśniewski JR, Zougman A, Nagaraj N, Mann M. Universal sample preparation method for proteome analysis. Nat Methods 2009;6:359–362.

2. Vizcaíno JA, Deutsch EW, Wang R, Csordas A, Reisinger F, Ríos D, Dianes JA, Sun Z, Farrah T, Bandeira N, Binz P-A, Xenarios I, Eisenacher M, Mayer G, Gatto L, Campos A, Chalkley RJ, Kraus H-J, Albar JP, Martinez-Bartolomé S, Apweiler R, Omenn GS, Martens L, Jones AR, Hermjakob H. ProteomeXchange provides globally coordinated proteomics data submission and dissemination. Nat Biotechnol 2014;32:223–226.

3. Höllbacher B, Schmitt AO, Hofer H, Ferreira F, Lackner P. Identification of Proteases and Protease Inhibitors in Allergenic and Non-Allergenic Pollen. Int J Mol Sci 2017;18.

4. Rowley ER, Fox SE, Bryant DW, Sullivan CM, Priest HD, Givan SA, Mehlenbacher SA, Mockler TC. Assembly and Characterization of the European Hazelnut ‘Jefferson’ Transcriptome. Crop Science 2012;52:2679–2686.

5. Grabherr MG, Haas BJ, Yassour M, Levin JZ, Thompson DA, Amit I, Adiconis X, Fan L, Raychowdhury R, Zeng Q, Chen Z, Mauceli E, Hacohen N, Gnirke A, Rhind N, Di Palma F, Birren BW, Nusbaum C, Lindblad-Toh K, Friedman N, Regev A. Full-length transcriptome assembly from RNA-Seq data without a reference genome. Nat Biotechnol 2011;29:644–652.

6. Tyanova S, Temu T, Cox J. The MaxQuant computational platform for mass spectrometry-based shotgun proteomics. Nat Protoc 2016;11:2301–2319.

7. Na D, Son H, Gsponer J. Categorizer: a tool to categorize genes into user-defined biological groups based on semantic similarity. BMC Genomics 2014;15:1091.

8. Rawlings ND, Barrett AJ, Thomas PD, Huang X, Bateman A, Finn RD. The MEROPS database of proteolytic enzymes, their substrates and inhibitors in 2017 and a comparison with peptidases in the PANTHER database. Nucleic Acids Res 2018;46:D624-D632.

9. Radauer C, Bublin M, Wagner S, Mari A, Breiteneder H. Allergens are distributed into few protein families and possess a restricted number of biochemical functions. J Allergy Clin Immunol 2008;121:847-52.e7.

10. World Medical Association Declaration of Helsinki. JAMA 2013;310:2191.

# SUPPLEMENTARY FIGURES


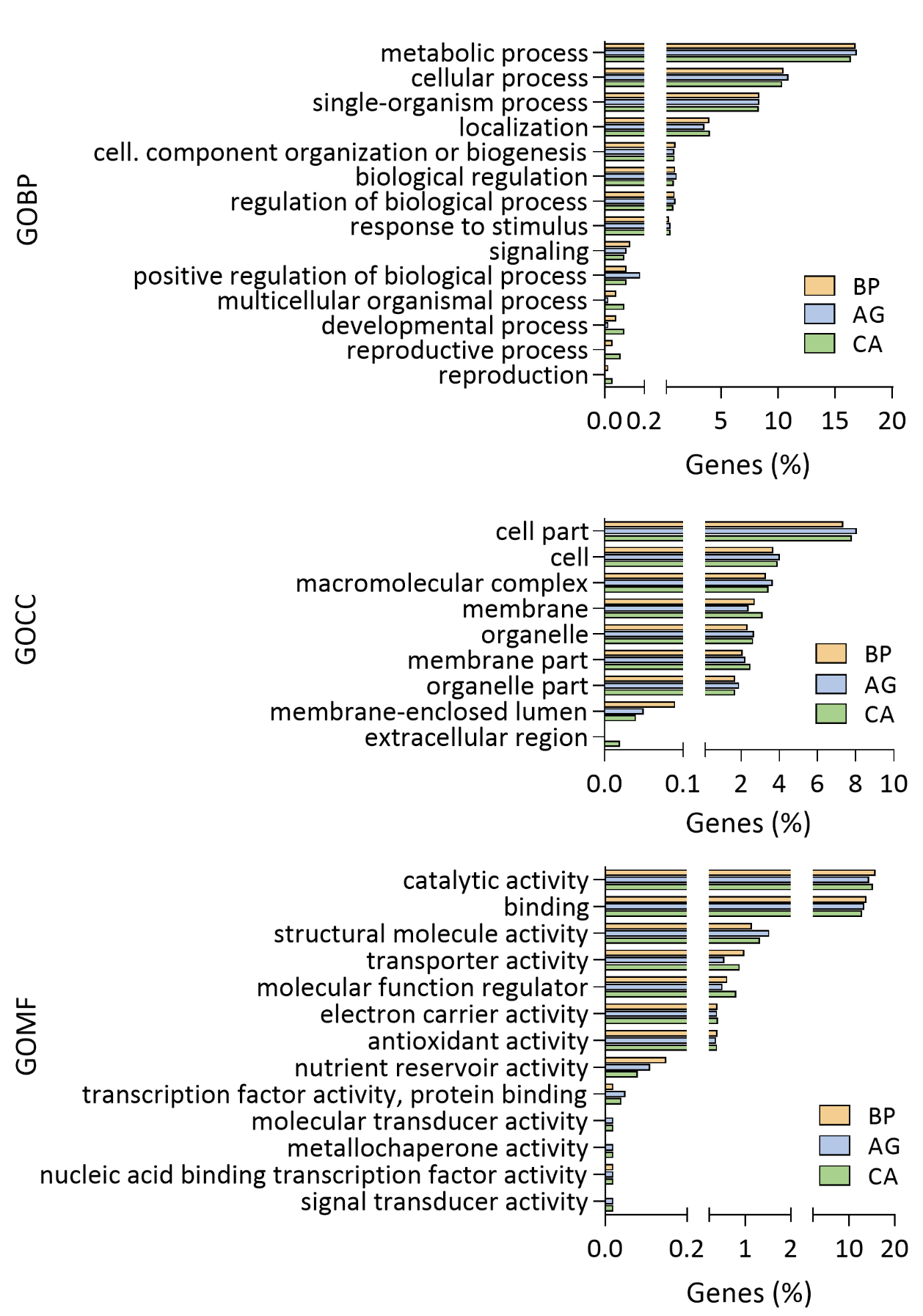


**Figure S1. Gene ontology annotation of identified proteins as input**. GOBP – Gene ontology annotation of biological process, GOMF – Gene ontology annotation of molecular function, GOCC – Gene ontology annotation of cellular compartment. AG - *Alnus glutinosa,* BP - *Betula pendula,* CA - *Corylus avellana*

# SUPPLEMENTARY FASTA files

The protein and RNA-sequences from all identified proteins from all 3 species can be downloaded here:

<http://omicscentergraz.at/resources.html>

# SUPPLEMENTARY TABLES

S1_Corylus_pfam.pdf

This table lists all proteins identified from *Corylus avellana* in the total proteome and the soluble proteome with Pfam annotation. Where MQ Score = max quant score, bitscore = For each Pfam family, a bit score gathering (GA) threshold I set by hand, such that all sequences scoring at or above this threshold appear in the full alignment;, e-value [Pfam] = The E-value is the number of hits that would be expected to have a score equal to or better than this value by chance alone. A good E-value is much less than 1.

S2_Corylus_Intensities.pdf

This table lists all proteins identified from *Corylus avellana* in the total proteome and the soluble proteome including the raw-intensities. Where fasp = Filter Aided Sample Preparation; tca = precipitation with tricloroacetic acid; zc = lysis with zirconium beads; Intensity = raw intensities from max quant search;

S3_Corylus_blastx.pdf

This table lists all proteins identified from *Corylus avellana* in the total proteome and the soluble proteome including blastx annonation. Hit1 = blastx top hit; hit2 = blastx top2 hit; e-value = The BLAST E-value is the number of expected hits of similar quality (score) that could be found just by chance; Bit score = The higher the bit-score, the better the sequence similarity. The bit-score is the requires size of a sequence database in which the current match could be found just by chance. The bit-score is a log2 scaled and normalized raw-score. Each increase by one doubles the required database size (2bit-score).

S4_Betula_pfam.pdf

This table lists all proteins identified from *Betula pendula* in the total proteome and the soluble proteome with Pfam annotation. Where MQ Score = max quant score, bitscore = For each Pfam family, a bit score gathering (GA) threshold I set by hand, such that all sequences scoring at or above this threshold appear in the full alignment, e-value [Pfam] = The E-value is the number of hits that would be expected to have a score equal to or better than this value by chance alone. A good E-value is much less than 1.

S5_Betula_Intensities.pdf

This table lists all proteins identified from *Betula pendula* in the total proteome and the soluble proteome including the raw-intensities. Where fasp = Filter Aided Sample Preparation; tca = precipitation with tricloroacetic acid; zc = lysis with zirconium beads; Intensity = raw intensities from max quant search;

S6_Betula_blastx.pdf

This table lists all proteins identified from *Betula pendula* in the total proteome and the soluble proteome including blastx annonation. hit1 = blastx top hit; hit2 = blastx top2 hit; e-value = The BLAST E-value is the number of expected hits of similar quality (score) that could be found just by chance; Bit score = The higher the bit-score, the better the sequence similarity. The bit-score is the requires size of a sequence database in which the current match could be found just by chance. The bit-score is a log2 scaled and normalized raw-score. Each increase by one doubles the required database size (2bit-score).

S7_Alnus_pfam.pdf

This table lists all proteins identified from *Alnus glutinosa* in the total proteome and the soluble proteome with Pfam annotation. Where MQ Score = max quant score, bitscore = For each Pfam family, a bit score gathering (GA) threshold I set by hand, such that all sequences scoring at or above this threshold appear in the full alignment, e-value [Pfam] = The E-value is the number of hits that would be expected to have a score equal to or better than this value by chance alone. A good E-value is much less than 1.

S8_Alnus_Intensities.pdf

This table lists all proteins identified from *Alnus glutinosa* in the total proteome and the soluble proteome including the raw-intensities. Where fasp = Filter Aided Sample Preparation; tca = precipitation with tricloroacetic acid; zc = lysis with zirconium beads; Intensity = raw intensities from max quant search;

S9_Alnus_blastx.pdf

This table lists all proteins identified from *Alnus glutinosa* in the total proteome and the soluble proteome including blastx annonation. Hit1 = blastx top hit; hit2 = blastx top2 hit; e-value = The BLAST E-value is the number of expected hits of similar quality (score) that could be found just by chance; Bit score = The higher the bit-score, the better the sequence similarity. The bit-score is the requires size of a sequence database in which the current match could be found just by chance. The bit-score is a log2 scaled and normalized raw-score. Each increase by one doubles the required database size (2bit-score).

S10_Corylusavellana_allergens

Known allergens detected in the proteome of *Corylus avellana* (total and soluble) additionally annotated using Allergome database.

S11_Betulapendula_allergens

Known allergens detected in the proteome of *Betula pendula* (total and soluble) additionally annotated using Allergome database.

S12_Alnusglutinosa_allergens

Known allergens detected in the proteome of *Alnus glutinosa* (total and soluble) additionally annotated using Allergome database.

S13_Betula_totalpeptidases.pdf

All proteins (total and soluble) from *Betula pendula* annotated as peptidases with the help of MEROPS can be found in this table.

S14_Alnus_totalpeptidases.pdf

All proteins (total and soluble) from *Alnus glutinosa* annotated as peptidases with the help of MEROPS can be found in this table.

S15_Corylus_totalpeptidases.pdf

All proteins (total and soluble) from *Corylus avellana* annotated as peptidases with the help of MEROPS can be found in this table.

S16_Betula_soluble_peptidases.pdf

All proteins from the soluble fraction from *Betula pendula* annotated as peptidases with the help of MEROPS can be found in this table.

S17_Betula_exclusively_soluble_peptidases.pdf

All proteins exclusively found in the soluble fraction from *Betula pendula* annotated as peptidases with the help of MEROPS can be found in this table.

S18_Alnus_soluble_peptidases.pdf

All proteins from the soluble fraction from *Alnus glutinosa* annotated as peptidases with the help of MEROPS can be found in this table.

S19_Alnus_exclusively_soluble_peptidases.pdf

All proteins exclusively found in the soluble fraction from *Alnus glutinosa* annotated as peptidases with the help of MEROPS can be found in this table.

S20_Corylus_soluble_peptidases.pdf

All proteins from the soluble fraction from *Corylus avellana* annotated as peptidases with the help of MEROPS can be found in this table.

S21_Corylus_exclusively_soluble_peptidases.pdf

All proteins exclusively found in the soluble fraction from *Corylus avellana* annotated as peptidases with the help of MEROPS can be found in this table.
