# Supplementary material for: Comparative proteomics of common allergenic tree pollens of birch, alder, and hazel
Source: Allergy. 2021 Jan 15;76(6):1743–53. doi: 10.1111/all.14694 (PMC8248232; doi:10.1111/all.14694)
Supplement: Supplementary file 3 — Table S1 [file ALL-76-1743-s018.pdf]

Supplementary Table S1: Pfam annotation of identified Corylus pollen proteins

Protein IDs	Peptide counts (all)	Sequence coverage [%]	Mol. weight [kDa]	MQ Score	Pfam accession	Pfam family name	bitscore	e-value [Pfam]	clan
TRINITY_DN8608_c2_g1_i2_5	11	47,9	23,435	323,31	PF00407.18	Bet_v_1	88,9	2,90E-25	CL0209
TRINITY_DN11207_c0_g1_i2_3	13	52,3	24,044	323,31	PF00407.18	Bet_v_1	88,1	4,80E-25	CL0209
TRINITY_DN9484_c0_g1_i1_5	27	56,4	55,056	323,31	PF00162.18	PGK	552,7	3,50E-166	No_clan
TRINITY_DN7872_c0_g1_i3_6	9	44,4	26,278	323,31	PF00076.21	RRM_1	75,9	1,40E-21	CL0221
TRINITY_DN10821_c0_g1_i5_2	15	41,2	41,714	323,31	PF05368.12	NmrA	289,3	1,90E-86	CL0063
TRINITY_DN11055_c0_g2_i3_5	27	60,2	53,494	323,31	PF07992.13	Pyr_redox_2	194	3,00E-57	CL0063
TRINITY_DN9053_c0_g1_i5_1	31	65,6	61,234	323,31	PF01704.17	UDPGP	612,1	3,50E-184	CL0110
TRINITY_DN10419_c0_g1_i1_1	29	49,1	75,178	323,31	PF01676.17	Metalloenzyme	345,7	1,20E-103	CL0088
TRINITY_DN11591_c0_g3_i5_3	9	36,8	27,18	323,31	PF00160.20	Pro_isomerase	160,7	3,10E-47	CL0475
TRINITY_DN11331_c0_g10_i2_1	10	53,5	16,89	323,31					
TRINITY_DN8476_c0_g1_i1_3	16	43,7	35,132	323,31	PF13417.5	GST_N_3	76,3	1,70E-21	CL0172
TRINITY_DN10442_c0_g1_i1_2	17	58,8	34,484	323,31	PF00248.20	Aldo_ket_red	157,5	3,60E-46	No_clan
TRINITY_DN9319_c0_g1_i1_2	19	48,7	51,488	323,31	PF00295.16	Glyco_hydro_28	335,5	2,80E-100	CL0268
TRINITY_DN9571_c0_g1_i2_3	9	29,4	40,827	323,31	PF00903.24	Glyoxalase	80	1,60E-22	CL0104
TRINITY_DN13451_c0_g2_i1_3	3	23,4	16,546	87,209					
TRINITY_DN8018_c0_g1_i2_4	6	22,3	24,579	114,01	PF00234.21	Tryp_alpha_amyl	28,4	1,60E-06	CL0482
TRINITY_DN10804_c2_g1_i1_3	12	36,9	42,601	323,31	PF00121.17	TIM	291	5,80E-87	CL0036
TRINITY_DN10890_c0_g3_i4_6	8	15,1	64,671	323,31	PF00295.16	Glyco_hydro_28	113,3	1,10E-32	CL0268
TRINITY_DN8608_c2_g1_i1_5	8	47,6	15,827	323,31	PF00407.18	Bet_v_1	58,3	7,70E-16	CL0209
TRINITY_DN6026_c0_g1_i1_2	8	34,7	24,033	323,31	PF00240.22	ubiquitin	115,1	7,90E-34	CL0072
TRINITY_DN11331_c0_g10_i3_1	10	51,7	15,737	323,31					
TRINITY_DN13307_c0_g1_i1_1	5	34	22,059	260,65	PF00235.18	Profilin	143,7	3,20E-42	CL0431
TRINITY_DN11501_c0_g1_i5_2	29	46,5	90,605	323,31	PF08267.11	Meth_synt_1	394,3	4,20E-118	CL0160
TRINITY_DN9487_c0_g3_i2_5	27	57,5	57,73	323,31	PF03952.15	Enolase_N	183	2,30E-54	CL0227
TRINITY_DN10587_c0_g1_i1_3	8	32,4	33,384	323,31	PF00254.27	FKBP_C	115,6	9,10E-34	CL0487
TRINITY_DN10479_c0_g2_i1_3	8	39,5	22,164	323,31	PF04043.14	PMEI	96,4	2,00E-27	No_clan
TRINITY_DN11616_c0_g1_i2_1	24	44,4	73,957	323,31	PF02878.15	PGM_PMM_I	114,4	2,90E-33	No_clan
TRINITY_DN4595_c0_g1_i1_3	2	23	11,12	95,701					
TRINITY_DN3727_c0_g2_i1_1	7	24,8	29,592	323,31	PF00838.16	TCTP	190	3,30E-56	CL0080
TRINITY_DN9731_c0_g1_i1_1	25	50,3	57,717	323,31	PF00262.17	Calreticulin	201,5	1,70E-59	CL0004

TRINITY_DN3709_c0_g1_i1_3	15	39,8	46,895	323,31	PF00056.22	Ldh_1_N	112,9	1,10E-32	CL0063
TRINITY_DN9065_c0_g1_i1_1	31	55,4	71,591	323,31	PF10509.8	GalKase_gal_bdg	76,2	9,40E-22	CL0329
TRINITY_DN11320_c0_g1_i11_3	22	42,7	46,219	323,31					
TRINITY_DN6201_c0_g1_i1_1	13	31,3	32,405	323,31	PF13499.5	EF-hand_7	54,5	1,10E-14	CL0220
TRINITY_DN6019_c0_g1_i1_3	21	44,3	56,627	323,31	PF00923.18	TAL_FSA	257,1	1,90E-76	CL0036
TRINITY_DN10785_c0_g1_i3_3	6	46,9	20,729	323,31	PF04043.14	PMEI	104,5	6,10E-30	No_clan
TRINITY_DN10341_c0_g2_i1_1	20	45	47,411	323,31	PF08240.11	ADH_N	37,7	1,30E-09	CL0296
TRINITY_DN10479_c0_g1_i2_1	12	42,7	29,353	323,31	PF04043.14	PMEI	115,4	2,70E-33	No_clan
TRINITY_DN10144_c0_g1_i2_2	11	43,6	22,646	323,31	PF01190.16	Pollen_Ole_e_I	61	1,00E-16	No_clan
TRINITY_DN5870_c0_g1_i2_4	7	47,5	21,339	323,31	PF00462.23	Glutaredoxin	73,8	8,60E-21	CL0172
TRINITY_DN10438_c0_g2_i1_2	12	42,3	42,265	323,31	PF01263.19	Aldose_epim	324,6	5,40E-97	CL0103
TRINITY_DN8953_c0_g1_i1_1	11	52,7	22,055	323,31	PF00903.24	Glyoxalase	30,9	2,40E-07	CL0104
TRINITY_DN9146_c0_g3_i2_3	31	41,4	87,956	323,31	PF00012.19	HSP70	871,8	1,80E-262	CL0108
TRINITY_DN10397_c0_g1_i2_5	8	35,2	23,909	227,92	PF00160.20	Pro_isomerase	156,3	7,00E-46	CL0475
TRINITY_DN10647_c0_g1_i1_4	11	25,2	43,928	204,38	PF02798.19	GST_N	58,1	7,80E-16	CL0172
TRINITY_DN11581_c0_g2_i3_2	8	39,6	24,444	323,31	PF00141.22	peroxidase	60,8	1,40E-16	CL0617
TRINITY_DN7958_c0_g4_i1_6	8	23,9	40,468	301,69	PF00071.21	Ras	167,3	1,90E-49	CL0023
TRINITY_DN10683_c0_g1_i3_5	8	41,4	30,004	228,93	PF00255.18	GSHPx	143,4	1,60E-42	CL0172
TRINITY_DN11604_c0_g1_i6_2	11	42,4	34,234	323,31	PF08241.11	Methyltransf_11	37,8	2,00E-09	CL0063
TRINITY_DN9279_c0_g1_i1_1	13	52,3	26,866	323,31	PF13405.5	EF-hand_6	28,2	9,70E-07	CL0220
TRINITY_DN11174_c0_g1_i5_1	10	30,1	50,288	323,31	PF00112.22	Peptidase_C1	280,3	1,30E-83	CL0125
TRINITY_DN11394_c0_g1_i1_2	22	46,2	60,912	323,31	PF00996.17	GDI	741,8	1,70E-223	CL0063
TRINITY_DN10920_c0_g1_i2_3	17	48,9	45,664	323,31	PF00294.23	PfkB	262,3	5,40E-78	CL0118
TRINITY_DN13244_c0_g1_i1_3	5	33,7	18,455	323,31	PF04043.14	PMEI	49,2	6,50E-13	No_clan
TRINITY_DN10804_c1_g1_i1_3	7	34,8	16,854	120,61	PF00121.17	TIM	127,6	4,70E-37	CL0036
TRINITY_DN10010_c0_g1_i2_4	10	27,3	50,872	323,31	PF00903.24	Glyoxalase	78,9	3,50E-22	CL0104
TRINITY_DN11304_c0_g1_i5_2	13	29,4	49,872	323,31	PF05221.16	AdoHcyase	226,5	3,70E-67	CL0325
TRINITY_DN14318_c0_g1_i1_5	1	7,8	14,25	6,4776					
TRINITY_DN11227_c0_g1_i5_5	9	33,8	35,227	306,54	PF01738.17	DLH	193,5	3,10E-57	CL0028
TRINITY_DN4231_c0_g1_i1_1	4	21,9	17,61	110,73	PF11976.7	Rad60-SLD	85	2,20E-24	CL0072
TRINITY_DN10618_c0_g1_i1_3	17	42,3	53,033	280,58	PF01965.23	DJ-1_PfpI	141,9	1,40E-41	CL0014
TRINITY_DN9827_c0_g1_i1_5	2	6,6	25,992	35,804	PF00550.24	PP-binding	42,8	4,80E-11	CL0314
TRINITY_DN7126_c0_g3_i1_3	9	29	25,513	323,31	PF02970.15	TBCA	93,4	7,30E-27	No_clan

TRINITY_DN11590_c1_g1_i1_3	11	30,2	44,936	309,6	PF01370.20	Epimerase	83,4	1,50E-23	CL0063
TRINITY_DN11652_c0_g1_i2_1	27	34,8	91,823	323,31	PF02518.25	HATPase_c	59,3	4,40E-16	CL0025
TRINITY_DN5250_c0_g1_i1_1	7	33	23,306	323,31	PF16845.4	SQAPI	80,7	7,10E-23	CL0121
TRINITY_DN11459_c1_g2_i2_1	27	44,3	79,297	323,31	PF00012.19	HSP70	873	7,90E-263	CL0108
TRINITY_DN10499_c0_g1_i1_2	16	40	52,119	323,31	PF08240.11	ADH_N	82,7	1,50E-23	CL0296
TRINITY_DN8634_c0_g1_i1_1	10	36,9	36,039	177,67	PF00257.18	Dehydrin	36,5	6,50E-09	No_clan
TRINITY_DN10893_c0_g1_i1_2	5	28,8	20,655	323,31	PF04043.14	PMEI	68,6	6,70E-19	No_clan
TRINITY_DN9811_c0_g1_i2_3	5	24,1	28,385	177,07	PF00080.19	Sod_Cu	165,5	7,20E-49	No_clan
TRINITY_DN8650_c0_g2_i3_2	20	52,1	46,868	318,17	PF03214.12	RGP	592,8	1,80E-178	CL0110
TRINITY_DN11653_c0_g1_i19_5	13	41,1	47,717	304,72	PF00022.18	Actin	490,7	2,40E-147	CL0108
TRINITY_DN8041_c0_g1_i2_3	23	57,4	49,238	323,31	PF00274.18	Glycolytic	573,6	1,00E-172	CL0035
TRINITY_DN14244_c0_g1_i1_2	4	36,7	15,05	191,09	PF03168.12	LEA_2	69,8	2,10E-19	CL0159
TRINITY_DN10590_c0_g2_i1_2	11	41,1	41,459	323,31	PF14543.5	TAXi_N	159,2	1,20E-46	CL0129
TRINITY_DN3503_c0_g1_i2_1	5	82,1	13,905	243,95	PF00036.31	EF-hand_1	31,6	6,10E-08	CL0220
TRINITY_DN10155_c0_g1_i1_2	12	44,5	37,157	217,34	PF13419.5	HAD_2	107,1	9,50E-31	CL0137
TRINITY_DN11019_c0_g1_i1_2	21	47,1	65,249	323,31	PF03721.13	UDPG_MGDP_dh_N	227,3	9,70E-68	CL0063
TRINITY_DN10859_c0_g1_i1_2	6	31,5	20,699	323,31	PF00254.27	FKBP_C	87,1	6,70E-25	CL0487
TRINITY_DN10804_c0_g1_i1_2	7	51	21,799	153,47	PF00121.17	TIM	157,4	3,60E-46	CL0036
TRINITY_DN7252_c0_g4_i3_3	29	37,4	101,56	323,31	PF02518.25	HATPase_c	47	3,10E-12	CL0025
TRINITY_DN11092_c0_g1_i2_3	11	51,2	36,118	323,31	PF13460.5	NAD_binding_10	116,2	1,50E-33	CL0063
TRINITY_DN11258_c0_g1_i2_3	14	44,2	38,645	323,31	PF00244.19	14-3-3	346,3	6,00E-104	No_clan
TRINITY_DN8200_c0_g1_i1_1	15	52	37,56	323,31	PF00484.18	Pro_CA	147,7	2,90E-43	No_clan
TRINITY_DN9523_c0_g1_i1_4	11	35,8	39,493	200,3					
TRINITY_DN10459_c0_g1_i1_3	9	37,8	30,545	323,31	PF08534.9	Redoxin	111,1	3,40E-32	CL0172
TRINITY_DN11248_c1_g1_i1_3	11	39	37,199	323,31	PF00736.18	EF1_GNE	110,1	4,10E-32	No_clan
TRINITY_DN10833_c1_g2_i1_1	4	34	17,367	232,78	PF02136.19	NTF2	128,8	1,40E-37	CL0051
TRINITY_DN9054_c0_g1_i2_1	6	30,4	23,21	275,72	PF03168.12	LEA_2	64,2	1,20E-17	CL0159
TRINITY_DN11641_c0_g1_i13_1	20	42,5	68,082	323,31	PF00085.19	Thioredoxin	103,6	4,30E-30	CL0172
TRINITY_DN9275_c0_g1_i1_1	14	45,7	33,455	323,31	PF08718.10	GLTP	152,5	8,90E-45	No_clan
TRINITY_DN11496_c0_g1_i1_2	15	38,2	52,775	323,31	PF00056.22	Ldh_1_N	162,9	4,40E-48	CL0063
TRINITY_DN10665_c0_g1_i1_2	18	48,5	56,021	323,31	PF00006.24	ATP-synt_ab	209,3	4,80E-62	CL0023
TRINITY_DN11667_c0_g1_i3_3	33	36,7	109,61	323,31	PF00009.26	GTP_EFTU	218,7	4,60E-65	CL0023

TRINITY_DN2899_c0_g1_i1_1	6	36,6	22,217	205,82	PF00235.18	Profilin	127,3	3,80E-37	CL0431
TRINITY_DN5468_c0_g2_i2_1	6	20,8	27,425	323,31					
TRINITY_DN11591_c0_g3_i6_3	4	54,5	11,81	28,199	PF00160.20	Pro_isomerase	67,5	1,50E-18	CL0475
TRINITY_DN11204_c0_g1_i1_3	24	31,8	86,703	323,31	PF16363.4	GDP_Man_Dehyd	232,4	8,40E-69	CL0063
TRINITY_DN10703_c1_g1_i1_1	5	41,3	22,465	244,74	PF00241.19	Cofilin_ADF	127,6	2,90E-37	CL0092
TRINITY_DN11207_c0_g1_i1_3	12	45,5	25,964	297,16	PF00407.18	Bet_v_1	87,5	7,60E-25	CL0209
TRINITY_DN10743_c0_g1_i1_2	10	28,9	38,425	255,09	PF00076.21	RRM_1	28,1	1,20E-06	CL0221
TRINITY_DN8812_c0_g1_i2_1	4	27,6	23,257	60,049	PF01187.17	MIF	78,8	3,40E-22	CL0082
TRINITY_DN11581_c0_g1_i4_3	7	47,3	20,281	68,262	PF00141.22	peroxidase	117,6	6,10E-34	CL0617
TRINITY_DN9487_c0_g2_i1_5	24	48,8	61,921	323,31	PF03952.15	Enolase_N	167,7	1,20E-49	CL0227
TRINITY_DN11173_c0_g3_i1_4	11	20,2	54,292	123,7	PF00153.26	Mito_carr	80,1	7,70E-23	No_clan
TRINITY_DN5663_c0_g2_i1_3	5	31	22,337	168,99	PF00462.23	Glutaredoxin	70	1,40E-19	CL0172
TRINITY_DN9210_c0_g1_i1_1	6	24,7	21,025	289,85	PF00173.27	Cyt-b5	34,5	1,50E-08	No_clan
TRINITY_DN9779_c0_g1_i1_1	19	36,9	68,486	323,31					
TRINITY_DN16006_c0_g1_i1_2	5	52	10,596	180,28	PF04043.14	PMEI	33	6,50E-08	No_clan
TRINITY_DN5448_c0_g2_i1_3	8	34,6	26,991	323,31	PF00179.25	UQ_con	50,1	1,90E-13	CL0208
TRINITY_DN10761_c0_g1_i2_2	21	39,2	55,951	323,31	PF02798.19	GST_N	61,1	9,10E-17	CL0172
TRINITY_DN11378_c0_g1_i1_4	14	31,7	61,451	323,31	PF00464.18	SHMT	621,8	3,40E-187	CL0061
TRINITY_DN10720_c0_g2_i2_6	15	39,2	49,716	323,31	PF00044.23	Gp_dh_N	116,2	6,70E-34	CL0063
TRINITY_DN11028_c0_g2_i2_3	9	33,9	34,994	282,63	PF00071.21	Ras	220,7	7,30E-66	CL0023
TRINITY_DN10686_c0_g1_i1_3	12	21,6	73,12	223,78	PF07732.14	Cu-oxidase_3	125	1,40E-36	CL0026
TRINITY_DN10496_c0_g1_i5_3	14	44,7	45,475	323,31	PF16363.4	GDP_Man_Dehyd	191,8	1,80E-56	CL0063
TRINITY_DN11432_c0_g1_i3_6	13	50,8	42,146	323,31	PF08240.11	ADH_N	92,5	1,30E-26	CL0296
TRINITY_DN4601_c0_g1_i1_1	5	25	23,015	123,79	PF00034.20	Cytochrom_C	46,5	6,00E-12	CL0318
TRINITY_DN10681_c0_g1_i1_2	22	25,8	122,04	323,31	PF00330.19	Aconitase	600,7	1,80E-180	No_clan
TRINITY_DN10442_c0_g2_i1_4	3	29,4	15,134	150,5					
TRINITY_DN9458_c0_g1_i2_2	15	36,4	49,944	234,55	PF00085.19	Thioredoxin	107,7	2,30E-31	CL0172
TRINITY_DN8250_c0_g1_i2_3	12	32,9	46,033	206,97	PF16884.4	ADH_N_2	91,9	1,90E-26	CL0296
TRINITY_DN10239_c0_g1_i1_2	10	41,7	32,872	323,31	PF00903.24	Glyoxalase	75,6	3,60E-21	CL0104
TRINITY_DN11469_c0_g2_i3_2	1	2,8	27,599	6,3296	PF03000.13	NPH3	103,6	1,30E-29	No_clan
TRINITY_DN7240_c0_g1_i2_1	3	13,4	15,774	263,37					
TRINITY_DN4014_c0_g1_i1_2	6	24,7	28,544	114,4	PF01597.18	GCV_H	164	1,10E-48	CL0105
TRINITY_DN10537_c0_g1_i2_4	14	50	41,037	323,31	PF00056.22	Ldh_1_N	114	5,30E-33	CL0063

TRINITY_DN5324_c1_g1_i2_1	3	30,2	16,441	185,17	PF00722.20	Glyco_hydro_16	33,2	3,10E-08	CL0004
TRINITY_DN5563_c0_g1_i1_6	1	3,9	20,035	94,929					
TRINITY_DN8045_c0_g1_i1_5	6	30,5	19,209	243,51	PF00085.19	Thioredoxin	113,8	3,00E-33	CL0172
TRINITY_DN8960_c0_g1_i1_2	8	43,9	20,672	323,31	PF01042.20	Ribonuc_L-PSP	121	2,50E-35	CL0534
TRINITY_DN4028_c1_g1_i1_2	8	30,7	26,973	180,26	PF00160.20	Pro_isomerase	162,6	8,20E-48	CL0475
TRINITY_DN11269_c2_g1_i10_3	17	34,4	60,934	216,39	PF00270.28	DEAD	135,5	1,50E-39	CL0023
TRINITY_DN11656_c0_g1_i6_2	11	26,4	63,551	205,93	PF00009.26	GTP_EFTU	180	3,40E-53	CL0023
TRINITY_DN10568_c0_g3_i2_1	18	30,6	71,617	224,4	PF00262.17	Calreticulin	485,3	9,20E-146	CL0004
TRINITY_DN9271_c0_g1_i1_1	5	25,1	31,595	323,31	PF06521.10	PAR1	241	3,70E-72	No_clan
TRINITY_DN10339_c0_g2_i1_4	5	26,6	27,02	323,31	PF00173.27	Cyt-b5	90,1	7,10E-26	No_clan
TRINITY_DN8726_c0_g2_i1_5	8	27,9	42,079	124,11	PF00160.20	Pro_isomerase	166,2	6,30E-49	CL0475
TRINITY_DN11504_c0_g1_i1_3	4	11,2	33,252	87,854					
TRINITY_DN10183_c0_g2_i2_2	14	41,3	43,052	323,31	PF00121.17	TIM	301	5,10E-90	CL0036
TRINITY_DN1820_c0_g1_i1_6	6	34,2	21,857	273,94	PF04043.14	PMEI	59,7	3,70E-16	No_clan
TRINITY_DN8787_c0_g2_i1_4	2	14,9	14,314	78,592					
TRINITY_DN10196_c0_g1_i1_1	12	38,3	42,496	323,31	PF07859.12	Abhydrolase_3	148,2	2,70E-43	CL0028
TRINITY_DN10535_c0_g1_i2_3	13	41,6	30,98	189,72	PF00406.21	ADK	161	2,00E-47	CL0023
TRINITY_DN11343_c0_g1_i7_2	16	22,2	72,823	173,59	PF00006.24	ATP-synt_ab	245	5,50E-73	CL0023
TRINITY_DN9170_c0_g1_i1_1	8	31,5	35,087	152,22	PF01738.17	DLH	100,2	1,10E-28	CL0028
TRINITY_DN9813_c0_g1_i1_2	36	42,8	125,04	323,31	PF00565.16	SNase	38,8	9,80E-10	CL0049
TRINITY_DN11430_c0_g2_i4_6	16	37,2	64,231	140	PF00456.20	Transketolase_N	184,5	2,20E-54	CL0254
TRINITY_DN9475_c0_g1_i1_3	15	38,1	51,557	323,31	PF16113.4	ECH_2	367,2	9,20E-110	CL0127
TRINITY_DN11040_c0_g1_i6_1	10	24,3	55,514	186,01	PF00190.21	Cupin_1	27,5	1,80E-06	CL0029
TRINITY_DN10148_c0_g1_i6_3	11	28,1	43,926	323,31	PF00481.20	PP2C	226,5	3,90E-67	CL0238
TRINITY_DN5484_c0_g2_i1_4	1	9,8	10,723	65,712					
TRINITY_DN7458_c0_g1_i3_2	3	27,7	16,014	176,57	PF05938.10	Self-incomp_S1	42,6	6,20E-11	No_clan
TRINITY_DN7727_c0_g2_i1_3	12	37,1	47,392	323,31	PF00112.22	Peptidase_C1	281,5	5,50E-84	CL0125
TRINITY_DN18091_c0_g1_i1_4	6	42,2	12,621	86,671	PF04043.14	PMEI	48,4	1,10E-12	No_clan
TRINITY_DN10656_c0_g1_i1_2	15	42,9	60,516	323,31	PF05694.10	SBP56	722,8	1,50E-217	CL0186
TRINITY_DN7279_c0_g1_i2_5	3	27,7	22,254	323,31	PF00076.21	RRM_1	82,9	9,40E-24	CL0221
TRINITY_DN11640_c0_g2_i6_2	20	23,7	106,91	251,48	PF01301.18	Glyco_hydro_35	382	2,60E-114	CL0058
TRINITY_DN1640_c0_g2_i1_1	6	83,2	10,337	259,86	PF02874.22	ATP-synt_ab_N	64	1,30E-17	CL0275
TRINITY_DN7420_c0_g1_i1_1	6	31,5	23,698	323,31	PF06110.10	DUF953	126,7	3,20E-37	CL0172

TRINITY_DN11604_c0_g1_i5_2	10	38,7	36,068	225,82	PF08241.11	Methyltransf_11	40,2	3,70E-10	CL0063
TRINITY_DN7220_c0_g1_i2_2	20	32,3	82,054	272,22	PF00012.19	HSP70	905,9	8,80E-273	CL0108
TRINITY_DN11320_c0_g1_i7_2	11	56,2	14,479	72,315					
TRINITY_DN17793_c0_g1_i1_3	1	10,7	12,646	6,7486					
TRINITY_DN128_c0_g1_i1_1	6	28,8	19,365	67,187	PF00240.22	ubiquitin	93,2	5,40E-27	CL0072
TRINITY_DN7906_c0_g1_i1_2	6	30	23,979	55,733	PF01230.22	HIT	98	3,80E-28	CL0265
TRINITY_DN9529_c0_g1_i4_3	3	29,2	13,486	209,04	PF03330.17	DPBB_1	35,3	1,00E-08	CL0199
TRINITY_DN10188_c0_g1_i1_1	13	30,6	50,595	184,51	PF06999.11	Suc_Fer-like	145,8	1,30E-42	No_clan
TRINITY_DN10809_c0_g1_i3_1	10	24,1	49,055	103,06	PF00141.22	peroxidase	163,1	7,40E-48	CL0617
TRINITY_DN10625_c0_g1_i2_2	12	33,1	48,274	231,18	PF00438.19	S-AdoMet_synt_N	133	4,70E-39	No_clan
TRINITY_DN11635_c2_g1_i4_2	13	32,5	44,04	235,41	PF00248.20	Aldo_ket_red	247,6	1,30E-73	No_clan
TRINITY_DN11639_c0_g1_i6_3	25	42,9	78,879	323,31	PF02874.22	ATP-synt_ab_N	51,1	1,30E-13	CL0275
TRINITY_DN10727_c0_g1_i1_2	11	39,4	35,493	108,07	PF00736.18	EF1_GNE	110,2	3,80E-32	No_clan
TRINITY_DN8828_c0_g1_i1_2	2	9,3	32,318	12,667					
TRINITY_DN9853_c0_g2_i1_2	9	29	37,524	273,17	PF01095.18	Pectinesterase	379,6	6,20E-114	CL0268
TRINITY_DN17353_c0_g1_i1_1	6	21,7	37,832	46,681	PF13640.5	2OG-FeII_Oxy_3	75,3	5,00E-21	CL0029
TRINITY_DN11024_c0_g2_i1_3	7	21,6	38,529	64,788	PF00106.24	adh_short	97,8	5,10E-28	CL0063
TRINITY_DN7121_c0_g2_i1_5	13	24,6	54,94	305,54	PF00274.18	Glycolytic	523,8	1,50E-157	CL0035
TRINITY_DN8295_c0_g1_i1_3	1	11	15,933	323,31					
TRINITY_DN7552_c0_g1_i2_3	15	32,3	52,167	136,56	PF16113.4	ECH_2	367,5	7,30E-110	CL0127
TRINITY_DN11568_c0_g1_i1_2	18	32,8	78,583	209,34	PF08323.10	Glyco_transf_5	242,1	6,40E-72	CL0113
TRINITY_DN11106_c0_g1_i3_1	20	34,9	68,067	191,27	PF00085.19	Thioredoxin	107,8	2,20E-31	CL0172
TRINITY_DN8501_c0_g1_i1_2	18	35	59,519	219,02	PF00155.20	Aminotran_1_2	315,5	4,60E-94	CL0061
TRINITY_DN9729_c0_g1_i1_5	15	40,9	52,001	323,31	PF08240.11	ADH_N	103,2	6,30E-30	CL0296
TRINITY_DN11231_c0_g1_i2_3	12	32,6	53,423	266,15	PF00573.21	Ribosomal_L4	134,4	3,40E-39	No_clan
TRINITY_DN11267_c0_g1_i1_2	9	27,1	39,398	101,43	PF12481.7	DUF3700	360,9	2,10E-108	CL0052
TRINITY_DN10955_c0_g1_i6_2	11	42,1	32,362	147,57	PF00071.21	Ras	193,5	1,60E-57	CL0023
TRINITY_DN10695_c0_g1_i3_1	13	21,4	83,824	323,31	PF00076.21	RRM_1	61,5	4,70E-17	CL0221
TRINITY_DN10388_c0_g2_i5_4	13	23,4	89,715	189,5	PF00082.21	Peptidase_S8	167,5	3,70E-49	No_clan
TRINITY_DN10780_c1_g2_i1_2	5	19,7	30,577	316,02	PF00334.18	NDK	181,5	6,30E-54	No_clan
TRINITY_DN4152_c0_g3_i1_2	7	37,4	22,701	82,938					
TRINITY_DN11590_c1_g2_i1_3	7	30,3	29,736	102,86	PF01370.20	Epimerase	71,9	4,80E-20	CL0063
TRINITY_DN11603_c0_g2_i4_3	13	30,2	61,751	236,47	PF00928.20	Adap_comp_sub	111	5,80E-32	CL0448

TRINITY_DN9993_c0_g1_i2_4	12	18,6	77,715	130,71	PF00082.21	Peptidase_S8	154,2	4,20E-45	No_clan
TRINITY_DN8821_c0_g1_i2_2	9	34,6	31,186	160,09	PF00071.21	Ras	207,6	7,60E-62	CL0023
TRINITY_DN11484_c0_g1_i1_1	16	18,9	104,63	133,67	PF02854.18	MIF4G	180,1	4,10E-53	CL0020
TRINITY_DN10975_c0_g1_i1_3	10	37,5	35,052	300,84	PF01287.19	eIF-5a	99,9	5,90E-29	CL0021
TRINITY_DN908_c0_g1_i1_5	5	52,2	17,832	215,08	PF00241.19	Cofilin_ADF	123,3	5,90E-36	CL0092
TRINITY_DN4171_c0_g2_i2_3	9	27,5	49,266	221,51	PF14226.5	DIOX_N	77,3	1,40E-21	CL0029
TRINITY_DN11158_c0_g1_i1_2	8	31	34,556	323,31	PF01557.17	FAA_hydrolase	201,1	1,60E-59	CL0377
TRINITY_DN11613_c0_g1_i1_1	12	32	47,669	178,15	PF00248.20	Aldo_ket_red	255,2	6,50E-76	No_clan
TRINITY_DN10470_c0_g1_i2_2	7	29,3	34,542	323,31	PF02431.14	Chalcone	108,5	2,60E-31	CL0560
TRINITY_DN12697_c0_g1_i1_3	10	43,6	33,052	82,426	PF13472.5	Lipase_GDSL_2	107,9	6,90E-31	CL0264
TRINITY_DN6523_c0_g1_i1_1	6	30,4	28,808	103,81	PF00179.25	UQ_con	163,1	2,70E-48	CL0208
TRINITY_DN7341_c0_g1_i1_3	7	21,8	41,461	323,31	PF03079.13	ARD	216,9	1,50E-64	CL0029
TRINITY_DN8404_c0_g1_i1_3	10	37,9	39,027	323,31	PF00293.27	NUDIX	76,5	1,70E-21	CL0261
TRINITY_DN11519_c0_g1_i1_1	23	30,5	110,35	289,76	PF01602.19	Adaptin_N	438,5	2,90E-131	CL0020
TRINITY_DN9737_c2_g2_i1_1	6	23,6	37,377	323,31	PF00025.20	Arf	266,2	8,70E-80	CL0023
TRINITY_DN11533_c0_g1_i2_3	19	27,3	107,86	266,31	PF00168.29	C2	51	1,30E-13	CL0154
TRINITY_DN8820_c0_g1_i1_1	16	32,4	68,021	303,67	PF03446.14	NAD_binding_2	161	2,30E-47	CL0063
TRINITY_DN5484_c0_g1_i1_2	5	44,7	11,512	45,368	PF00887.18	ACBP	77,5	6,30E-22	CL0632
TRINITY_DN10447_c0_g1_i1_3	2	20	10,225	114,49					
TRINITY_DN431_c0_g1_i1_6	10	27,4	38,872	323,31					
TRINITY_DN9847_c0_g1_i1_1	5	29,6	25,847	295,19	PF07876.11	Dabb	71,8	5,10E-20	CL0032
TRINITY_DN11173_c0_g2_i3_4	10	22	49,436	71,112	PF00153.26	Mito_carr	82,1	1,90E-23	No_clan
TRINITY_DN6616_c0_g1_i1_4	10	29	57,799	323,31	PF00626.21	Gelsolin	26,3	4,70E-06	CL0092
TRINITY_DN5630_c0_g1_i1_3	14	42,5	38,57	190,56	PF00248.20	Aldo_ket_red	167,7	2,90E-49	No_clan
TRINITY_DN4574_c0_g1_i2_2	7	55,4	24,926	323,31	PF08534.9	Redoxin	119,8	6,90E-35	CL0172
TRINITY_DN8931_c0_g1_i1_2	7	29,1	36,045	323,31	PF00719.18	Pyrophosphatase	159,6	5,10E-47	No_clan
TRINITY_DN11512_c1_g1_i2_2	17	39,8	50,974	323,31	PF00155.20	Aminotran_1_2	313,3	2,10E-93	CL0061
TRINITY_DN5614_c0_g2_i1_2	1	5,1	25,231	6,3598					
TRINITY_DN10149_c0_g1_i2_3	14	38,3	45,845	213,41	PF00244.19	14-3-3	353,1	5,00E-106	No_clan
TRINITY_DN9287_c0_g1_i1_3	4	17,4	45,014	100,39	PF02265.15	S1-P1_nuclease	266,1	3,30E-79	CL0368
TRINITY_DN13093_c0_g1_i1_3	13	41,5	46,681	216,66	PF01704.17	UDPGP	58,8	3,40E-16	CL0110
TRINITY_DN10610_c0_g2_i1_2	15	30,1	71,218	162,11	PF02874.22	ATP-synt_ab_N	51,1	1,30E-13	CL0275
TRINITY_DN4192_c0_g2_i1_1	5	23,6	26,639	201,83	PF02036.16	SCP2	69,7	2,30E-19	CL0311

TRINITY_DN11220_c1_g2_i2_2	7	25,8	38,348	158,54	PF13561.5	adh_short_C2	197	3,00E-58	CL0063
TRINITY_DN10317_c0_g1_i3_2	10	38,5	31,512	181,02	PF00459.24	Inositol_P	246,3	3,50E-73	CL0171
TRINITY_DN9083_c0_g1_i2_2	5	24,3	33,95	54,019	PF00173.27	Cyt-b5	52,9	2,80E-14	No_clan
TRINITY_DN6144_c0_g1_i2_2	7	50,3	21,154	223,2	PF04043.14	PMEI	68,2	9,30E-19	No_clan
TRINITY_DN13238_c0_g1_i1_4	2	28,2	8,4276	76,106	PF00182.18	Glyco_hydro_19	118,7	3,30E-34	CL0037
TRINITY_DN21237_c0_g1_i1_2	1	8,8	11,838	-2					
TRINITY_DN10003_c1_g1_i5_2	10	32,3	44,682	121,04	PF01842.24	ACT	35,3	6,40E-09	CL0070
TRINITY_DN11618_c0_g1_i1_2	24	35	99,705	323,31	PF02359.17	CDC48_N	80,8	6,00E-23	CL0332
TRINITY_DN4347_c0_g1_i1_6	13	24,2	81,078	272,61	PF01321.17	Creatinase_N	59,6	4,40E-16	CL0356
TRINITY_DN5723_c0_g1_i1_2	6	32,6	26,523	323,31	PF01625.20	PMSR	197,7	1,00E-58	No_clan
TRINITY_DN9234_c0_g1_i1_2	10	21,5	55,513	66,144	PF00297.21	Ribosomal_L3	651,4	3,40E-196	CL0575
TRINITY_DN13643_c0_g1_i1_2	9	58,1	23,888	323,31	PF00295.16	Glyco_hydro_28	173,2	6,70E-51	CL0268
TRINITY_DN6792_c0_g1_i2_5	9	35,7	34,973	323,31	PF02338.18	OTU	26,4	7,50E-06	CL0125
TRINITY_DN7574_c0_g1_i5_2	9	24	56,969	111,69	PF12708.6	Pectate_lyase_3	36,2	5,40E-09	CL0268
TRINITY_DN11191_c0_g1_i2_6	17	33	64,897	188,23	PF00883.20	Peptidase_M17	381,2	3,50E-114	CL0035
TRINITY_DN9754_c0_g1_i1_3	14	36,4	56,214	290,29	PF00108.22	Thiolase_N	267,2	1,30E-79	CL0046
TRINITY_DN5779_c0_g1_i1_2	7	22,7	34,883	122,37	PF00561.19	Abhydrolase_1	58,5	7,40E-16	CL0028
TRINITY_DN11323_c0_g1_i1_2	28	37,3	111,81	268,16	PF00012.19	HSP70	535,1	1,60E-160	CL0108
TRINITY_DN14709_c0_g1_i1_5	5	59,5	12,309	121	PF01657.16	Stress-antifung	81,8	4,10E-23	No_clan
TRINITY_DN9829_c0_g1_i3_2	5	38,4	26,582	196,23	PF00080.19	Sod_Cu	159,4	5,50E-47	No_clan
TRINITY_DN6396_c0_g1_i1_2	4	27,8	21,603	37,522	PF03671.13	Ufm1	139,4	2,70E-41	CL0072
TRINITY_DN8644_c0_g2_i1_1	4	29,7	15,566	323,31	PF16845.4	SQAPI	130	2,90E-38	CL0121
TRINITY_DN11241_c0_g1_i3_2	8	16,3	57,768	61,223	PF00153.26	Mito_carr	71,9	2,90E-20	No_clan
TRINITY_DN9392_c0_g1_i1_2	11	35	40,703	194,51	PF00638.17	Ran_BP1	138,6	1,10E-40	CL0266
TRINITY_DN10095_c0_g1_i1_3	12	28,9	66,041	137,6	PF00675.19	Peptidase_M16	126,4	8,00E-37	CL0094
TRINITY_DN9335_c0_g1_i1_4	12	39	48,064	148,37	PF00190.21	Cupin_1	99,7	1,10E-28	CL0029
TRINITY_DN10130_c0_g2_i6_2	13	32,7	64,745	158,65	PF00171.21	Aldedh	445,2	2,00E-133	CL0099
TRINITY_DN11642_c0_g1_i3_4	20	44,4	67,108	235,56	PF00118.23	Cpn60_TCP1	352,4	3,50E-105	No_clan
TRINITY_DN7240_c0_g1_i1_3	2	13,4	15,456	13,544					
TRINITY_DN11079_c0_g1_i1_5	11	32,1	38,269	77,98	PF15704.4	Mt_ATP_synt	264,5	4,90E-79	No_clan
TRINITY_DN10242_c0_g1_i3_2	5	26,1	24,163	42,178	PF13563.5	2_5_RNA_ligase2	41,7	9,60E-11	CL0247
TRINITY_DN3644_c0_g1_i2_3	4	43,7	14,476	58,877	PF04969.15	CS	39	1,10E-09	CL0190
TRINITY_DN11383_c0_g1_i4_1	6	13,2	60,238	222,59	PF00295.16	Glyco_hydro_28	130,2	7,90E-38	CL0268

TRINITY_DN9173_c0_g1_i1_4	6	43,2	28,031	177,91	PF00082.21	Peptidase_S8	47,3	1,50E-12	No_clan
TRINITY_DN4403_c0_g1_i1_1	13	39,3	48,653	323,31	PF01263.19	Aldose_epim	243,8	2,20E-72	CL0103
TRINITY_DN10720_c0_g1_i4_5	13	39,2	57,612	229,31	PF00044.23	Gp_dh_N	111	2,70E-32	CL0063
TRINITY_DN11647_c1_g1_i1_1	8	25,9	49,278	323,31	PF00150.17	Cellulase	75,3	4,80E-21	CL0058
TRINITY_DN12510_c0_g1_i1_1	7	24,1	36,081	228,58	PF13964.5	Kelch_6	26	7,40E-06	CL0186
TRINITY_DN1355_c0_g3_i1_5	1	10,6	13,37	6,8083					
TRINITY_DN11053_c0_g1_i4_6	13	42,6	52,4	323,31	PF00657.21	Lipase_GDSL	44,9	1,20E-11	CL0264
TRINITY_DN5919_c0_g1_i1_3	3	48,3	12,075	102,06					
TRINITY_DN11546_c0_g1_i6_5	16	22,4	97,974	160,41	PF00690.25	Cation_ATPase_N	25,2	9,10E-06	No_clan
TRINITY_DN11141_c0_g1_i6_1	20	39,1	76,775	231,55	PF00483.22	NTP_transferase	271,3	7,30E-81	CL0110
TRINITY_DN2708_c0_g2_i1_1	8	23,8	41,497	72,587	PF00450.21	Peptidase_S10	334,2	1,40E-99	CL0028
TRINITY_DN9530_c0_g3_i4_1	13	40,6	46,131	143,07	PF16363.4	GDP_Man_Dehyd	181,1	3,30E-53	CL0063
TRINITY_DN5278_c0_g1_i1_2	2	38,7	8,0774	16,679	PF00179.25	UQ_con	98,9	1,80E-28	CL0208
TRINITY_DN10989_c0_g2_i2_1	12	29,8	61,857	154,66	PF00370.20	FGGY_N	236,7	2,50E-70	CL0108
TRINITY_DN11653_c0_g2_i1_5	11	42,5	45,109	57,534	PF00022.18	Actin	411,2	3,40E-123	CL0108
TRINITY_DN7029_c0_g1_i1_3	11	52,4	25,321	52,604	PF00162.18	PGK	297,9	9,30E-89	No_clan
TRINITY_DN10674_c0_g1_i3_4	11	27,6	53,843	124,02	PF00481.20	PP2C	220,1	3,50E-65	CL0238
TRINITY_DN10049_c0_g1_i1_3	9	25	55,521	172,22	PF00266.18	Aminotran_5	177,1	4,70E-52	CL0061
TRINITY_DN7937_c0_g1_i2_5	5	26,9	23,549	35,364					
TRINITY_DN1722_c0_g2_i1_3	5	55,1	11,962	59,985	PF00085.19	Thioredoxin	94,9	2,30E-27	CL0172
TRINITY_DN11314_c0_g1_i3_2	13	33	58,805	139,74	PF03214.12	RGP	622,7	1,50E-187	CL0110
TRINITY_DN9588_c0_g1_i1_2	19	33,5	76,261	260,48	PF00365.19	PFK	100	1,30E-28	CL0240
TRINITY_DN10926_c1_g1_i3_3	1	2,8	27,502	23,305	PF01190.16	Pollen_Ole_e_I	65,2	5,10E-18	No_clan
TRINITY_DN5721_c0_g4_i1_3	3	29,5	15,718	160,02	PF10714.8	LEA_6	122,7	4,30E-36	No_clan
TRINITY_DN10576_c0_g1_i1_3	6	33,1	27,015	273,01	PF03358.14	FMN_red	46,6	2,70E-12	CL0042
TRINITY_DN11459_c1_g4_i8_2	26	43,2	78,864	98,115	PF00012.19	HSP70	842,6	1,20E-253	CL0108
TRINITY_DN10492_c1_g1_i2_6	9	28,6	44,634	251	PF00753.26	Lactamase_B	38	1,60E-09	CL0381
TRINITY_DN11529_c0_g1_i2_3	11	22,2	81,422	291,13	PF05817.13	Ribophorin_II	675,4	7,80E-203	No_clan
TRINITY_DN10583_c0_g2_i2_1	9	31,8	41,012	157,76	PF00012.19	HSP70	304,7	9,60E-91	CL0108
TRINITY_DN12655_c0_g1_i1_2	1	11,1	10,19	-2					
TRINITY_DN8865_c0_g2_i1_5	9	44,7	33,472	120,13	PF03721.13	UDPG_MGDP_dh_N	227,8	6,80E-68	CL0063
TRINITY_DN6870_c0_g1_i5_1	16	36,5	68,476	165,07	PF00224.20	PK	306	2,70E-91	CL0151

TRINITY_DN5791_c0_g1_i1_5	14	37,3	51,325	323,31	PF00370.20	FGGY_N	62,4	4,20E-17	CL0108
TRINITY_DN11572_c0_g1_i1_6	11	31,9	40,567	102,7	PF01015.17	Ribosomal_S3Ae	322,8	7,80E-97	No_clan
TRINITY_DN10346_c0_g2_i6_3	14	30,1	65,815	159,23	PF00483.22	NTP_transferase	226,5	3,50E-67	CL0110
TRINITY_DN11809_c0_g1_i1_1	5	32	16,573	42,903	PF01657.16	Stress-antifung	49,4	4,90E-13	No_clan
TRINITY_DN4290_c0_g1_i1_3	8	16,7	55,416	118,8					
TRINITY_DN10866_c0_g1_i1_1	12	34,2	58,183	162,16	PF00091.24	Tubulin	233,8	1,80E-69	CL0566
TRINITY_DN11465_c0_g1_i2_2	9	16,6	71,794	93,394	PF08245.11	Mur_ligase_M	29,7	5,50E-07	No_clan
TRINITY_DN10466_c0_g2_i3_1	17	29,2	70,924	209,17	PF00118.23	Cpn60_TCP1	328,9	4,60E-98	No_clan
TRINITY_DN3729_c0_g2_i1_1	6	43,9	21,943	266,2	PF00241.19	Cofilin_ADF	121,9	1,60E-35	CL0092
TRINITY_DN10917_c0_g1_i1_1	18	26	111,37	152,21	PF00676.19	E1_dh	219,1	5,90E-65	CL0254
TRINITY_DN4960_c0_g1_i2_6	12	36,3	53,41	143,03	PF00056.22	Ldh_1_N	160,3	2,70E-47	CL0063
TRINITY_DN10616_c0_g1_i2_2	4	32,2	24,956	323,31	PF13499.5	EF-hand_7	49,9	3,00E-13	CL0220
TRINITY_DN11240_c0_g2_i1_2	20	27,2	98,064	196,08	PF01433.19	Peptidase_M1	316,8	7,00E-95	CL0126
TRINITY_DN11190_c0_g1_i2_3	18	43,4	65,96	323,31	PF00224.20	PK	520,3	1,70E-156	CL0151
TRINITY_DN11571_c4_g2_i1_1	13	37,6	57,912	298,48	PF00091.24	Tubulin	211,4	1,30E-62	CL0566
TRINITY_DN11669_c393_g1_i1_1	1	6,5	16,644	23,643					
TRINITY_DN10467_c0_g1_i1_1	9	14,1	54,829	100,96	PF14543.5	TAXi_N	178	1,90E-52	CL0129
TRINITY_DN9500_c0_g2_i1_2	20	40,3	64,172	140,76	PF00118.23	Cpn60_TCP1	288,4	8,80E-86	No_clan
TRINITY_DN10347_c0_g1_i1_3	12	30,8	55,935	231,82	PF00180.19	Iso_dh	308,1	7,10E-92	CL0270
TRINITY_DN5968_c0_g1_i1_3	11	27,7	48,926	155,67	PF00153.26	Mito_carr	56,2	2,20E-15	No_clan
TRINITY_DN5544_c0_g1_i1_3	8	31	31,32	104,41	PF00347.22	Ribosomal_L6	45	1,20E-11	No_clan
TRINITY_DN153_c0_g1_i1_1	8	22	44,138	64,978	PF00240.22	ubiquitin	60,3	1,00E-16	CL0072
TRINITY_DN7265_c0_g2_i5_2	15	34,9	47,227	153,63	PF00656.21	Peptidase_C14	258,4	8,00E-77	CL0093
TRINITY_DN9812_c0_g1_i3_4	11	18,3	87,01	184,09	PF00076.21	RRM_1	69,7	1,30E-19	CL0221
TRINITY_DN8811_c0_g1_i3_5	16	38	46,447	148,96	PF00191.19	Annexin	71,1	5,50E-20	No_clan
TRINITY_DN10516_c0_g1_i1_2	9	34,5	42,476	172,58	PF00459.24	Inositol_P	192,6	8,40E-57	CL0171
TRINITY_DN4095_c0_g1_i1_4	7	30,7	32,788	77,469	PF13417.5	GST_N_3	63,7	1,40E-17	CL0172
TRINITY_DN14849_c0_g1_i1_6	1	19,7	8,0198	55,587	PF00141.22	peroxidase	58,6	6,60E-16	CL0617
TRINITY_DN11228_c0_g2_i1_3	4	16,4	19,564	323,31	PF06825.11	HSBP1	78,4	2,50E-22	No_clan
TRINITY_DN10795_c1_g1_i1_6	19	38,9	55,074	323,31	PF02798.19	GST_N	55,4	5,50E-15	CL0172
TRINITY_DN11220_c1_g2_i3_3	2	16,2	11,847	21,075	PF00106.24	adh_short	60,9	9,80E-17	CL0063
TRINITY_DN11391_c0_g2_i2_4	9	29,7	42,393	145,84	PF00231.18	ATP-synt	250,4	2,50E-74	No_clan
TRINITY_DN2394_c0_g1_i2_6	5	29	20,214	57,883	PF00034.20	Cytochrom_C	48,9	1,00E-12	CL0318

TRINITY_DN10841_c0_g2_i1_1	13	15,3	105,69	161,29	PF03141.15	Methyltransf_29	773,2	9,20E-233	CL0063
TRINITY_DN9488_c0_g2_i1_2	9	27,8	42,025	323,31	PF01182.19	Glucosamine_iso	242,5	4,50E-72	CL0246
TRINITY_DN959_c0_g1_i1_1	7	50,5	21,189	125,24	PF00295.16	Glyco_hydro_28	162,8	9,80E-48	CL0268
TRINITY_DN5607_c0_g1_i1_3	9	34,7	34,264	174,65	PF00071.21	Ras	186,5	2,40E-55	CL0023
TRINITY_DN11493_c0_g1_i5_3	8	29	35,113	173,92	PF00071.21	Ras	221,1	5,10E-66	CL0023
TRINITY_DN18554_c0_g1_i1_2	12	33,7	54,045	323,31	PF16499.4	Melibiase_2	261,8	5,80E-78	CL0058
TRINITY_DN11428_c0_g1_i2_3	13	31,5	60,583	121,57	PF07992.13	Pyr_redox_2	249,9	2,80E-74	CL0063
TRINITY_DN23504_c0_g1_i1_4	1	12,3	7,9071	6,6621	PF00141.22	peroxidase	40,2	2,70E-10	CL0617
TRINITY_DN11239_c1_g2_i1_6	6	24,2	26,136	69,504	PF00281.18	Ribosomal_L5	58,3	6,50E-16	CL0652
TRINITY_DN19480_c0_g1_i1_2	2	30,4	7,7355	27,538	PF00332.17	Glyco_hydro_17	57,1	1,70E-15	CL0058
TRINITY_DN12058_c0_g1_i1_3	8	38,6	19,257	52,316	PF07650.16	KH_2	49,7	2,20E-13	CL0007
TRINITY_DN4635_c0_g1_i1_6	5	18,4	40,416	68,759	PF00210.23	Ferritin	106,7	8,60E-31	CL0044
TRINITY_DN6720_c0_g1_i1_1	10	24	59,903	166,8	PF03405.13	FA_desaturase_2	533,8	1,10E-160	CL0044
TRINITY_DN15356_c0_g1_i1_3	5	35,3	15,544	94,73	PF02298.16	Cu_bind_like	74,7	4,10E-21	CL0026
TRINITY_DN10115_c0_g1_i5_2	6	38,2	21,477	51,794	PF01588.19	tRNA_bind	112,2	9,10E-33	CL0021
TRINITY_DN7162_c0_g1_i1_6	5	47,1	16,65	53,12	PF00462.23	Glutaredoxin	58,5	5,30E-16	CL0172
TRINITY_DN668_c0_g2_i1_6	6	21,4	37,5	59,738	PF00687.20	Ribosomal_L1	145,5	1,50E-42	No_clan
TRINITY_DN11756_c0_g2_i2_2	11	25,9	59,388	88,523	PF02878.15	PGM_PMM_I	77,2	9,00E-22	No_clan
TRINITY_DN3503_c0_g1_i1_2	4	68,8	10,615	68,429	PF13499.5	EF-hand_7	61,3	8,80E-17	CL0220
TRINITY_DN10567_c0_g1_i1_3	9	25,8	33,268	124,68	PF05873.11	Mt_ATP-synt_D	66,5	2,20E-18	No_clan
TRINITY_DN9859_c0_g2_i1_3	28	20,9	178	233,68	PF01326.18	PPDK_N	67,6	1,00E-18	CL0179
TRINITY_DN11119_c0_g1_i2_2	8	29,7	38,816	172,61	PF00318.19	Ribosomal_S2	49,5	2,80E-13	CL0067
TRINITY_DN6617_c0_g1_i1_4	5	52,3	21,677	136,76	PF03951.18	Gln-synt_N	40,9	1,20E-10	No_clan
TRINITY_DN8937_c0_g1_i1_1	15	25,7	72,406	142,25	PF00171.21	Aldedh	121,2	3,70E-35	CL0099
TRINITY_DN8063_c0_g1_i1_3	4	14,7	39,851	67,952	PF00719.18	Pyrophosphatase	183,4	2,40E-54	No_clan
TRINITY_DN3510_c0_g1_i2_1	4	10,7	46,958	103,36	PF13181.5	TPR_8	15	0,019	CL0020
TRINITY_DN3885_c0_g1_i1_3	12	57,9	27,567	81,053	PF04043.14	PMEI	66,9	2,30E-18	No_clan
TRINITY_DN11597_c1_g2_i2_1	9	13,7	90,8	74,918	PF03030.15	H_PPase	854,8	4,40E-257	No_clan
TRINITY_DN4297_c0_g1_i1_2	26	28,7	123,47	193,23	PF00311.16	PEPcase	1011,4	0	CL0151
TRINITY_DN11427_c0_g1_i3_2	10	26,2	44,444	153,05	PF05193.20	Peptidase_M16_C	121,3	4,40E-35	CL0094
TRINITY_DN8289_c0_g1_i2_3	17	20,4	105,12	276,93	PF00012.19	HSP70	315,9	3,90E-94	CL0108
TRINITY_DN9771_c0_g1_i4_2	15	31,1	68,279	147,04	PF03141.15	Methyltransf_29	763,4	9,00E-230	CL0063
TRINITY_DN11254_c0_g1_i1_3	12	24,5	72,104	111,89	PF00009.26	GTP_EFTU	146,2	7,70E-43	CL0023

TRINITY_DN11103_c0_g1_i2_3	11	25,9	68,809	167,53	PF00364.21	Biotin_lipoyl	68,1	4,00E-19	CL0105
TRINITY_DN11548_c1_g1_i1_3	7	42,8	27,905	204,12					
TRINITY_DN10949_c0_g1_i1_1	11	16,1	91,417	180,41	PF00890.23	FAD_binding_2	413,7	8,30E-124	CL0063
TRINITY_DN11059_c0_g1_i1_1	9	35,2	39,247	80,843	PF01459.21	Porin_3	214,6	1,60E-63	CL0193
TRINITY_DN3142_c1_g1_i1_1	6	36,4	24,561	84,763	PF12515.7	CaATP_NAI	68,3	2,90E-19	No_clan
TRINITY_DN10281_c0_g2_i1_2	4	18,2	24,443	54,752	PF00828.18	Ribosomal_L27A	77,4	1,40E-21	CL0588
TRINITY_DN10506_c0_g1_i1_2	10	27,5	44,209	323,31	PF00248.20	Aldo_ket_red	168,7	1,40E-49	No_clan
TRINITY_DN9505_c0_g2_i1_1	15	20,2	107,86	169,26	PF00862.18	Sucrose_synth	963,7	2,00E-290	CL0113
TRINITY_DN11355_c0_g1_i2_1	9	22,2	66,91	133,96	PF00400.31	WD40	13,8	0,079	CL0186
TRINITY_DN6984_c0_g1_i1_1	7	23,1	40,785	55,114	PF03152.13	UFD1	269,3	9,30E-81	CL0402
TRINITY_DN9211_c0_g2_i1_1	6	22,6	40,502	323,31	PF01625.20	PMSR	204,9	6,40E-61	No_clan
TRINITY_DN3640_c0_g1_i2_2	7	45,1	21,409	66,534	PF02115.16	Rho_GDI	184,1	2,20E-54	No_clan
TRINITY_DN11606_c0_g1_i1_4	10	32,7	41,932	108,15	PF01370.20	Epimerase	82,8	2,40E-23	CL0063
TRINITY_DN9177_c0_g2_i1_2	20	23,1	114,74	173,61	PF01602.19	Adaptin_N	300,9	1,50E-89	CL0020
TRINITY_DN11073_c0_g2_i1_2	8	26,6	43,764	89,508	PF00201.17	UDPGT	23,7	1,80E-05	CL0113
TRINITY_DN9688_c0_g1_i3_5	5	18,9	31,878	56,324	PF00177.20	Ribosomal_S7	125,5	1,20E-36	No_clan
TRINITY_DN5149_c0_g1_i1_1	2	39,7	8,5726	254,89	PF04526.12	DUF568	43,7	3,20E-11	No_clan
TRINITY_DN9163_c0_g1_i2_2	9	25,7	56,544	101,66	PF00085.19	Thioredoxin	99,6	7,60E-29	CL0172
TRINITY_DN9921_c0_g1_i1_2	17	37,2	67,382	141,59	PF00118.23	Cpn60_TCP1	507,3	3,60E-152	No_clan
TRINITY_DN8108_c0_g1_i1_2	19	37,3	64,968	279,62	PF03009.16	GDPD	87,2	1,30E-24	CL0384
TRINITY_DN11594_c0_g2_i5_1	7	31,2	35,27	62,689	PF00627.30	UBA	36,6	2,80E-09	CL0214
TRINITY_DN7706_c0_g2_i2_2	7	32	28,309	98,563	PF00900.19	Ribosomal_S4e	123,7	1,90E-36	No_clan
TRINITY_DN11322_c1_g1_i2_3	5	16,5	32,826	38,22	PF00252.17	Ribosomal_L16	143,1	4,40E-42	No_clan
TRINITY_DN6946_c0_g1_i1_2	11	30,1	55,86	134,44	PF03088.15	Str_synth	102,4	9,90E-30	CL0186
TRINITY_DN11349_c1_g2_i4_3	12	29,6	49,826	151,25	PF00012.19	HSP70	624,6	1,30E-187	CL0108
TRINITY_DN5652_c0_g3_i1_4	3	38,8	7,4504	40,866					
TRINITY_DN11033_c0_g1_i4_5	4	21,1	24,829	169,74	PF00334.18	NDK	176,9	1,70E-52	No_clan
TRINITY_DN3347_c0_g1_i1_2	2	9,2	32,461	33,997	PF00082.21	Peptidase_S8	64,2	1,10E-17	No_clan
TRINITY_DN6901_c0_g1_i1_3	2	16,7	24,456	87,886	PF00235.18	Profilin	144,6	1,60E-42	CL0431
TRINITY_DN5552_c0_g1_i1_1	6	23,3	33,487	121,77	PF05008.14	V-SNARE	88,9	2,00E-25	CL0147
TRINITY_DN10237_c0_g3_i1_1	17	22	115,6	217,27	PF00122.19	E1-E2_ATPase	167,8	1,50E-49	No_clan
TRINITY_DN11143_c0_g1_i5_1	8	26	55,933	275,31	PF00076.21	RRM_1	56,5	1,60E-15	CL0221
TRINITY_DN1882_c0_g1_i2_3	6	19,4	48,65	90,309	PF00108.22	Thiolase_N	308,8	2,60E-92	CL0046

TRINITY_DN10939_c0_g1_i1_1	11	26,6	59,961	102,71	PF00285.20	Citrate_synt	345,7	2,80E-103	No_clan
TRINITY_DN7127_c0_g1_i1_1	18	35,2	77,203	227,76	PF00069.24	Pkinase	229,1	5,60E-68	CL0016
TRINITY_DN11501_c0_g1_i3_2	19	45,8	57,618	203,36	PF08267.11	Meth_synt_1	393,1	9,80E-118	CL0160
TRINITY_DN9883_c0_g1_i1_1	10	24,9	45,602	89,214	PF17144.3	Ribosomal_L5e	274,9	2,00E-82	CL0267
TRINITY_DN10759_c0_g1_i2_5	7	22,2	46,081	68,894	PF02779.23	Transket_pyr	160,2	3,70E-47	CL0254
TRINITY_DN11019_c0_g1_i2_2	21	49,5	66,149	64,39	PF03721.13	UDPG_MGDP_dh_N	227,3	9,90E-68	CL0063
TRINITY_DN10818_c0_g1_i3_1	7	22,8	39,259	93,884	PF08079.11	Ribosomal_L30_N	88,6	2,10E-25	No_clan
TRINITY_DN20642_c0_g1_i1_4	4	19,6	23,028	46,526	PF00251.19	Glyco_hydro_32N	74,4	1,00E-20	CL0143
TRINITY_DN9556_c1_g1_i2_1	8	30,7	32,215	228,51	PF00179.25	UQ_con	144,5	1,50E-42	CL0208
TRINITY_DN10502_c0_g2_i8_3	19	27	123,21	161,93	PF02347.15	GDC-P	609	4,10E-183	CL0061
TRINITY_DN10701_c0_g1_i1_2	8	29,5	36,29	95,807	PF00333.19	Ribosomal_S5	108	1,60E-31	CL0196
TRINITY_DN11433_c0_g1_i2_1	14	18,2	117,43	205,25	PF00400.31	WD40	26,4	8,00E-06	CL0186
TRINITY_DN6503_c0_g2_i1_1	5	19,9	42,177	50,902	PF00466.19	Ribosomal_L10	63,9	1,10E-17	No_clan
TRINITY_DN6738_c0_g1_i1_3	3	9,4	26,342	167,99	PF02298.16	Cu_bind_like	65	4,30E-18	CL0026
TRINITY_DN9643_c0_g1_i1_2	8	22,5	54,836	75,78	PF00175.20	NAD_binding_1	93,5	1,10E-26	CL0091
TRINITY_DN2791_c0_g1_i1_3	8	41,7	30,407	173,2	PF00156.26	Pribosyltran	66,1	2,30E-18	CL0533
TRINITY_DN11325_c0_g1_i1_1	4	22,5	27,054	72,454	PF01248.25	Ribosomal_L7Ae	89,7	7,20E-26	CL0101
TRINITY_DN7495_c0_g1_i1_1	4	24,1	34,124	145,58	PF01849.17	NAC	80,2	7,30E-23	No_clan
TRINITY_DN2602_c0_g1_i2_3	12	32,1	58,605	106,25	PF00224.20	PK	392,8	1,10E-117	CL0151
TRINITY_DN11605_c1_g2_i1_4	3	22,1	27,973	93,285	PF02466.18	Tim17	44,8	1,10E-11	No_clan
TRINITY_DN6250_c0_g1_i2_3	12	25,5	72,201	95,462	PF01565.22	FAD_binding_4	56,5	2,30E-15	CL0077
TRINITY_DN6615_c0_g2_i1_4	8	58,7	20,941	47,605	PF00022.18	Actin	211,9	1,10E-62	CL0108
TRINITY_DN1128_c0_g2_i1_2	1	4,2	20,769	-2					
TRINITY_DN4481_c0_g1_i3_5	6	20,5	31,125	70,585	PF00827.16	Ribosomal_L15e	315,3	1,20E-94	CL0652
TRINITY_DN1332_c0_g1_i1_2	4	25	25,138	297,45	PF02265.15	S1-P1_nuclease	198,3	1,70E-58	CL0368
TRINITY_DN10179_c0_g1_i1_1	12	27,4	63,592	263,82	PF04043.14	PMEI	95,3	4,20E-27	No_clan
TRINITY_DN5568_c0_g1_i1_2	8	39,9	31,086	163,39	PF00076.21	RRM_1	56,2	2,10E-15	CL0221
TRINITY_DN11161_c0_g2_i1_1	6	50	13,738	89,054	PF05922.15	Inhibitor_I9	45,9	6,40E-12	CL0570
TRINITY_DN4141_c0_g1_i1_2	10	20,6	65,381	106,87	PF00289.21	Biotin_carb_N	135,5	9,50E-40	CL0483
TRINITY_DN15874_c0_g1_i1_6	3	21,9	17,069	81,459	PF04043.14	PMEI	51,8	9,90E-14	No_clan
TRINITY_DN10532_c0_g1_i1_4	4	21,3	31,234	205	PF00834.18	Ribul_P_3_epim	231,3	5,80E-69	CL0036
TRINITY_DN6875_c0_g1_i1_3	11	33,1	43,271	76,151	PF00244.19	14-3-3	351	2,10E-105	No_clan

TRINITY_DN7117_c0_g2_i2_1	5	23,7	25,74	45,284	PF03946.13	Ribosomal_L11_N	81,7	2,20E-23	No_clan
TRINITY_DN19584_c0_g1_i1_1	7	44,7	28,198	323,31	PF00887.18	ACBP	59,7	2,20E-16	CL0632
TRINITY_DN11441_c0_g1_i2_3	14	26,4	77,483	108,12	PF02219.16	MTHFR	403,6	4,10E-121	CL0086
TRINITY_DN11585_c0_g1_i2_3	16	23,3	88,044	118,12	PF00378.19	ECH_1	135,8	1,50E-39	CL0127
TRINITY_DN9778_c0_g1_i2_1	16	27,6	82,49	153,09	PF04053.13	Coatomer_WDAD	283,5	3,10E-84	CL0186
TRINITY_DN10084_c0_g1_i3_3	12	32,3	54,792	171,74	PF00141.22	peroxidase	149,7	9,20E-44	CL0617
TRINITY_DN8707_c0_g1_i1_4	11	30,7	39,647	108,12	PF01201.21	Ribosomal_S8e	192,2	3,80E-57	No_clan
TRINITY_DN8413_c0_g1_i1_3	4	19,1	25,409	32,716	PF01090.18	Ribosomal_S19e	191,3	5,10E-57	CL0123
TRINITY_DN11449_c0_g2_i5_3	19	28,5	95,965	153,48	PF00311.16	PEPcase	748,7	6,80E-225	CL0151
TRINITY_DN21888_c0_g1_i1_3	2	28,8	11,379	42,503					
TRINITY_DN10361_c1_g2_i1_1	6	17,9	35,258	41,941	PF03868.14	Ribosomal_L6e_N	55,5	4,20E-15	No_clan
TRINITY_DN7454_c0_g1_i1_5	4	27,8	19,666	49,324	PF00293.27	NUDIX	90,1	1,10E-25	CL0261
TRINITY_DN11044_c0_g1_i1_3	12	24,7	65,24	101,41	PF00996.17	GDI	766,9	4,10E-231	CL0063
TRINITY_DN10694_c0_g1_i1_2	7	26,8	40,563	310,86	PF00756.19	Esterase	188,2	1,80E-55	CL0028
TRINITY_DN7765_c0_g1_i1_3	5	21,9	28,816	62,114	PF00411.18	Ribosomal_S11	157,8	1,00E-46	CL0267
TRINITY_DN10259_c0_g1_i1_4	3	8	38,697	18,341	PF00213.17	OSCP	145,4	1,60E-42	CL0255
TRINITY_DN10666_c0_g1_i1_3	12	29,4	61,365	112,07	PF00330.19	Aconitase	404,3	7,30E-121	No_clan
TRINITY_DN8743_c0_g1_i2_2	1	4,4	25,585	7,8668	PF03760.14	LEA_1	73,8	1,20E-20	No_clan
TRINITY_DN7469_c0_g1_i1_3	8	30	35,8	139,53	PF13419.5	HAD_2	82	5,10E-23	CL0137
TRINITY_DN9596_c0_g1_i2_1	16	26,4	76,258	145,8					
TRINITY_DN9658_c0_g1_i1_4	8	21	54,767	57,899	PF03345.13	DDOST_48kD	434,3	4,00E-130	No_clan
TRINITY_DN7483_c0_g2_i2_4	4	17,1	26,16	25,796	PF02823.15	ATP-synt_DE_N	56,9	1,30E-15	No_clan
TRINITY_DN10418_c0_g1_i1_1	9	29,7	43,887	263,02	PF09032.10	Siah-Interact_N	28,3	1,50E-06	No_clan
TRINITY_DN6749_c0_g1_i3_2	8	15,8	68,178	77,305	PF00365.19	PFK	124,3	5,40E-36	CL0240
TRINITY_DN10064_c0_g1_i1_6	6	28,3	30,729	153,16	PF04969.15	CS	36,7	5,90E-09	CL0190
TRINITY_DN10803_c0_g1_i1_1	2	24,8	11,369	22,797					
TRINITY_DN10303_c0_g1_i1_3	5	12,3	52,013	70,155	PF00112.22	Peptidase_C1	281,5	5,60E-84	CL0125
TRINITY_DN2784_c0_g1_i1_3	9	36,4	29,789	63,645					
TRINITY_DN12921_c0_g1_i1_6	6	30,2	24,102	71,209	PF01088.20	Peptidase_C12	161,9	1,60E-47	CL0125
TRINITY_DN9516_c0_g1_i2_6	12	19	96,69	112,11	PF02990.15	EMP70	616,2	4,10E-185	No_clan
TRINITY_DN9036_c0_g1_i1_2	4	22	20,151	62,043	PF00085.19	Thioredoxin	108,4	1,40E-31	CL0172
TRINITY_DN20791_c0_g1_i1_4	1	10,9	9,5834	6,3403					
TRINITY_DN6266_c0_g1_i2_5	8	30,7	40,671	89,681	PF01266.23	DAO	29,3	5,70E-07	CL0063

TRINITY_DN11086_c0_g1_i1_1	6	24,4	38,394	107,71	PF10584.8	Proteasome_A_N	49,5	2,20E-13	CL0052
TRINITY_DN3111_c0_g2_i1_2	11	25,3	62,233	207,91	PF01432.19	Peptidase_M3	455	3,50E-136	CL0126
TRINITY_DN10597_c0_g2_i1_2	17	23,7	115,21	159,23	PF00899.20	ThiF	91	7,00E-26	CL0063
TRINITY_DN10098_c0_g1_i3_1	9	23,8	64,497	83,62	PF00240.22	ubiquitin	61,2	5,50E-17	CL0072
TRINITY_DN6445_c0_g1_i1_3	7	22,6	43,695	52,811	PF00657.21	Lipase_GDSL	66,8	2,30E-18	CL0264
TRINITY_DN10634_c0_g1_i2_4	4	16,7	38,311	28,215	PF13417.5	GST_N_3	67,8	7,70E-19	CL0172
TRINITY_DN5741_c0_g1_i2_3	5	22,1	25,395	42,58	PF03501.14	S10_plectin	147,2	1,10E-43	No_clan
TRINITY_DN7128_c0_g2_i1_1	6	20,7	41,187	106,97	PF01765.18	RRF	207,5	1,00E-61	No_clan
TRINITY_DN11601_c0_g2_i2_5	16	29,1	63,747	157,93	PF01433.19	Peptidase_M1	182,6	6,90E-54	CL0126
TRINITY_DN9962_c0_g2_i1_2	3	5,1	61,565	21,737	PF01565.22	FAD_binding_4	91,6	3,20E-26	CL0077
TRINITY_DN10845_c0_g1_i2_1	9	20,8	58,75	105,14	PF00180.19	Iso_dh	342,9	1,90E-102	CL0270
TRINITY_DN10542_c0_g1_i1_2	5	19,8	35,418	115,82	PF00076.21	RRM_1	80,7	4,70E-23	CL0221
TRINITY_DN7441_c0_g1_i1_6	7	57,6	15,705	139,39					
TRINITY_DN10521_c0_g1_i1_3	13	31,8	58,5	124,57	PF08442.9	ATP-grasp_2	199,1	5,10E-59	CL0179
TRINITY_DN10791_c2_g1_i3_2	6	30,7	31,486	69,663	PF00724.19	Oxidored_FMN	251,1	1,60E-74	CL0036
TRINITY_DN11097_c0_g1_i2_6	6	13,1	51,286	93,222	PF00079.19	Serpin	340,7	1,20E-101	No_clan
TRINITY_DN8226_c0_g1_i1_1	15	38,9	61,455	132,5	PF07992.13	Pyr_redox_2	189,1	9,60E-56	CL0063
TRINITY_DN8836_c0_g1_i1_2	8	26,4	48,688	70,282	PF01588.19	tRNA_bind	86,2	1,20E-24	CL0021
TRINITY_DN9745_c0_g1_i2_1	13	19,7	99,35	108,78	PF02847.16	MA3	84,3	5,00E-24	CL0020
TRINITY_DN6745_c0_g1_i6_1	7	32,6	39,722	165,59	PF00076.21	RRM_1	49,5	2,60E-13	CL0221
TRINITY_DN10651_c0_g1_i1_1	12	18,3	71,018	82,558	PF00254.27	FKBP_C	118	1,60E-34	CL0487
TRINITY_DN3758_c0_g1_i1_5	1	7,8	21,371	12,516	PF00428.18	Ribosomal_60s	90,6	6,70E-26	No_clan
TRINITY_DN9555_c0_g1_i2_4	1	8	12,028	6,7675					
TRINITY_DN8044_c0_g1_i1_2	11	24,1	70,527	98,925	PF00224.20	PK	530,3	1,70E-159	CL0151
TRINITY_DN5302_c0_g2_i1_4	3	17	16,129	23,533	PF00248.20	Aldo_ket_red	74,9	5,50E-21	No_clan
TRINITY_DN9331_c0_g1_i5_4	7	18,2	64,406	66,74	PF04597.13	Ribophorin_I	481	3,90E-144	No_clan
TRINITY_DN10842_c0_g1_i1_3	3	19,4	18,623	26,008	PF03760.14	LEA_1	45,8	6,70E-12	No_clan
TRINITY_DN10737_c0_g1_i2_1	7	19,9	45,941	73,745	PF00248.20	Aldo_ket_red	246,2	3,50E-73	No_clan
TRINITY_DN11246_c0_g1_i1_1	3	11,5	28,897	62,34	PF08069.11	Ribosomal_S13_N	110	3,70E-32	No_clan
TRINITY_DN10802_c0_g1_i2_2	6	14,9	50,299	83,248	PF13181.5	TPR_8	11,9	0,2	CL0020
TRINITY_DN10937_c0_g1_i1_3	5	19,6	33,343	46,271	PF00181.22	Ribosomal_L2	49,5	2,80E-13	CL0021
TRINITY_DN5821_c0_g1_i2_2	2	7,6	30,302	29,707	PF04043.14	PMEI	98,1	5,80E-28	No_clan
TRINITY_DN7491_c0_g1_i1_1	8	21,9	43,383	49,545	PF01248.25	Ribosomal_L7Ae	88,7	1,40E-25	CL0101

TRINITY_DN10883_c0_g1_i2_4	8	30,7	40,92	18,087	PF00160.20	Pro_isomerase	162,7	7,30E-48	CL0475
TRINITY_DN9369_c0_g1_i1_2	3	14,4	37,776	40,35	PF03896.15	TRAP_alpha	94,8	4,80E-27	No_clan
TRINITY_DN15371_c0_g1_i1_1	4	31,4	12,484	48,64	PF04043.14	PMEI	28,6	1,40E-06	No_clan
TRINITY_DN8535_c0_g1_i1_3	12	24,2	65,265	107,76	PF00676.19	E1_dh	271,8	5,30E-81	CL0254
TRINITY_DN11283_c0_g1_i3_1	6	21,8	41,497	73,348	PF10584.8	Proteasome_A_N	49,8	1,70E-13	CL0052
TRINITY_DN10469_c0_g2_i1_3	4	13,5	31,793	79,478	PF17135.3	Ribosomal_L18	314,5	1,60E-94	CL0588
TRINITY_DN6972_c0_g1_i1_5	6	18,7	38,269	302,04	PF00635.25	Motile_Sperm	107,7	2,50E-31	CL0556
TRINITY_DN9766_c0_g1_i2_1	9	24,9	51,647	108,12	PF00132.23	Hexapep	18,8	0,0009	CL0536
TRINITY_DN6771_c0_g1_i1_1	8	29	41,377	80,874	PF00224.20	PK	152,8	1,00E-44	CL0151
TRINITY_DN11054_c0_g1_i1_2	10	27,1	64,448	91,005	PF00330.19	Aconitase	251,1	2,00E-74	No_clan
TRINITY_DN11476_c0_g1_i2_1	11	27,3	54,915	103,58	PF02020.17	W2	69,3	2,20E-19	CL0020
TRINITY_DN9150_c0_g1_i3_1	5	36,4	22,766	65,373	PF00173.27	Cyt-b5	92,2	1,60E-26	No_clan
TRINITY_DN8849_c0_g2_i3_2	3	11,5	22,981	323,31	PF13499.5	EF-hand_7	36,4	5,00E-09	CL0220
TRINITY_DN7905_c0_g1_i1_5	11	41,9	32,238	94,995	PF14290.5	DUF4370	373	6,90E-112	No_clan
TRINITY_DN9056_c0_g1_i1_6	3	24,2	24,141	323,31	PF00111.26	Fer2	66,6	1,30E-18	CL0486
TRINITY_DN8426_c0_g1_i2_3	2	9,2	36,019	16,066	PF00141.22	peroxidase	271,7	4,70E-81	CL0617
TRINITY_DN4385_c0_g1_i1_2	12	29,5	58,511	91,182	PF11543.7	UN_NPL4	30,1	4,60E-07	CL0072
TRINITY_DN21126_c0_g1_i1_1	3	29,1	13,086	28,605					
TRINITY_DN11515_c0_g1_i3_1	10	27,2	57,529	108,6	PF00004.28	AAA	144,2	2,70E-42	CL0023
TRINITY_DN12722_c0_g1_i1_1	1	3,4	45,44	191	PF01095.18	Pectinesterase	238,9	4,50E-71	CL0268
TRINITY_DN11139_c0_g1_i1_3	8	11,6	79,64	58,291	PF03219.13	TLC	658,5	5,60E-198	CL0015
TRINITY_DN10080_c0_g1_i1_1	8	22,7	48,782	86,953	PF01937.18	DUF89	185,3	1,50E-54	No_clan
TRINITY_DN8178_c0_g1_i2_3	6	35,5	25,126	107,59	PF00294.23	PfkB	123,5	1,00E-35	CL0118
TRINITY_DN130_c0_g1_i1_2	11	31,3	46,513	151,47	PF00009.26	GTP_EFTU	147,8	2,50E-43	CL0023
TRINITY_DN11280_c0_g1_i4_3	18	37,4	70,198	129,48	PF00587.24	tRNA-synt_2b	65,1	7,20E-18	CL0040
TRINITY_DN11620_c0_g1_i1_2	8	23,7	38,271	52,371	PF10584.8	Proteasome_A_N	48,2	5,50E-13	CL0052
TRINITY_DN11411_c1_g1_i3_5	11	34,8	58,509	113,91	PF00438.19	S-AdoMet_synt_N	143	3,50E-42	No_clan
TRINITY_DN9704_c0_g1_i1_2	5	24	23,523	49,788	PF00410.18	Ribosomal_S8	75,9	2,30E-21	No_clan
TRINITY_DN2711_c0_g1_i1_3	9	22,7	52,336	249,16	PF00450.21	Peptidase_S10	249,3	8,90E-74	CL0028
TRINITY_DN10623_c0_g1_i2_5	2	10,3	26,188	59,192	PF01283.18	Ribosomal_S26e	179,6	1,50E-53	No_clan
TRINITY_DN9395_c0_g2_i1_3	6	14,7	53,224	93,465	PF09298.10	FAA_hydrolase_N	110,5	4,00E-32	No_clan
TRINITY_DN10777_c0_g1_i2_2	4	16,7	26,209	65,769	PF08694.10	UFC1	275,7	7,10E-83	CL0208
TRINITY_DN8939_c0_g1_i1_1	13	30,6	50,947	323,31	PF13489.5	Methyltransf_23	76,8	1,50E-21	CL0063

TRINITY_DN14055_c0_g1_i1_6	2	15,9	15,009	15,692					
TRINITY_DN5112_c0_g2_i3_3	11	38	50,065	137,09	PF00240.22	ubiquitin	53,4	1,50E-14	CL0072
TRINITY_DN3053_c0_g1_i1_6	4	31,5	13,75	323,31	PF00201.17	UDPGT	36,9	1,70E-09	CL0113
TRINITY_DN3542_c0_g2_i1_1	3	23,1	26,467	288,69	PF06201.12	PITH	133,8	5,30E-39	CL0202
TRINITY_DN8778_c0_g1_i2_2	6	21,9	37,611	51,421	PF12146.7	Hydrolase_4	41,5	8,20E-11	CL0028
TRINITY_DN8889_c0_g1_i1_1	6	25,3	43,588	72,183	PF14226.5	DIOX_N	86,3	2,20E-24	CL0029
TRINITY_DN6086_c0_g1_i1_1	9	35,6	30,928	64,893	PF04669.12	Polysacc_synt_4	240,1	1,20E-71	No_clan
TRINITY_DN9418_c0_g1_i2_5	9	30,9	47,087	101,31	PF00349.20	Hexokinase_1	79,5	3,00E-22	CL0108
TRINITY_DN9810_c0_g1_i1_3	9	21,9	56,126	64,039	PF00676.19	E1_dh	400	4,50E-120	CL0254
TRINITY_DN6193_c0_g1_i1_1	5	26,5	23,394	52,002	PF01778.16	Ribosomal_L28e	87,7	8,10E-25	No_clan
TRINITY_DN9474_c1_g1_i1_5	5	24,7	26,754	66,599	PF00179.25	UQ_con	162,4	4,30E-48	CL0208
TRINITY_DN10292_c0_g1_i2_6	4	17	29,298	323,31	PF00708.17	Acylphosphatase	79,4	1,80E-22	CL0622
TRINITY_DN11230_c0_g2_i2_6	6	35,8	26,073	257,38	PF03931.14	Skp1_POZ	106,5	5,10E-31	CL0033
TRINITY_DN10344_c0_g1_i1_2	7	28,5	50,98	67,413	PF12796.6	Ank_2	58,2	8,60E-16	CL0465
TRINITY_DN11434_c0_g1_i1_2	6	20,9	31,204	51,655	PF00416.21	Ribosomal_S13	190	1,70E-56	CL0303
TRINITY_DN11361_c0_g1_i1_2	10	20,3	59,659	79,996	PF01370.20	Epimerase	198,7	9,20E-59	CL0063
TRINITY_DN10628_c0_g1_i1_3	7	24,2	38,105	49,648	PF00571.27	CBS	30,1	4,50E-07	No_clan
TRINITY_DN5840_c0_g1_i1_1	6	42,1	15,617	56,952	PF01287.19	eIF-5a	62	4,00E-17	CL0021
TRINITY_DN10700_c0_g1_i1_3	4	33,3	16,474	96,744	PF01095.18	Pectinesterase	232,7	3,40E-69	CL0268
TRINITY_DN10484_c2_g1_i2_1	4	12,1	38,41	36,337	PF13774.5	Longin	77,9	4,00E-22	No_clan
TRINITY_DN11431_c0_g1_i3_5	1	6,2	22,595	50,111	PF01277.16	Oleosin	135,4	6,20E-40	No_clan
TRINITY_DN9680_c0_g1_i1_1	5	22	25,469	34,254	PF06108.11	DUF952	83,4	7,80E-24	CL0084
TRINITY_DN53_c0_g1_i1_5	1	8,1	13,662	7,2012	PF00082.21	Peptidase_S8	36,2	3,70E-09	No_clan
TRINITY_DN13553_c0_g1_i1_2	7	88,9	10,023	47,146	PF00626.21	Gelsolin	46,1	3,20E-12	CL0092
TRINITY_DN10106_c0_g1_i2_1	10	27	41,475	59,668	PF00244.19	14-3-3	345,5	1,00E-103	No_clan
TRINITY_DN10104_c0_g2_i1_2	7	21	42,801	54,695	PF00201.17	UDPGT	94	8,70E-27	CL0113
TRINITY_DN11228_c0_g1_i1_3	3	17,5	18,456	288,1	PF06825.11	HSBP1	81,7	2,40E-23	No_clan
TRINITY_DN9356_c0_g1_i2_6	7	15,3	60,735	64,155					
TRINITY_DN10740_c0_g1_i5_5	9	20,8	52,938	66,666	PF03223.14	V-ATPase_C	420,1	9,30E-126	No_clan
TRINITY_DN15242_c0_g1_i1_5	2	22,4	11,925	20,841	PF00578.20	AhpC-TSA	66,8	1,50E-18	CL0172
TRINITY_DN10478_c0_g2_i1_1	2	10,1	15,673	142,54					
TRINITY_DN10321_c0_g1_i2_2	3	13,9	27,848	23,248	PF16906.4	Ribosomal_L26	128,5	9,10E-38	No_clan
TRINITY_DN17896_c0_g1_i1_5	10	35,4	39,656	80,905	PF01145.24	Band_7	103,3	1,40E-29	CL0433

TRINITY_DN5702_c0_g1_i1_3	2	10,4	20,993	32,594					
TRINITY_DN6940_c0_g9_i5_6	3	14,1	28,008	32,239	PF01280.19	Ribosomal_L19e	221,4	4,10E-66	No_clan
TRINITY_DN10205_c0_g1_i1_2	7	15	74,079	323,31	PF00240.22	ubiquitin	67,6	5,30E-19	CL0072
TRINITY_DN10892_c0_g1_i2_4	12	18,8	99,767	107,89	PF01496.18	V_ATPase_I	975,6	1,50E-293	No_clan
TRINITY_DN11295_c0_g2_i3_1	9	23,1	71,591	139,32	PF07992.13	Pyr_redox_2	224,9	1,20E-66	CL0063
TRINITY_DN10858_c0_g1_i1_5	12	29	51,103	103,3	PF01145.24	Band_7	68,2	8,30E-19	CL0433
TRINITY_DN10871_c0_g1_i3_2	11	27,1	59,993	103,54	PF00171.21	Aldedh	576,7	2,60E-173	CL0099
TRINITY_DN6968_c0_g1_i1_1	5	13,9	37,368	33,139	PF00166.20	Cpn10	95,2	1,70E-27	CL0296
TRINITY_DN6689_c0_g1_i1_2	3	10,7	41,815	55,802	PF13561.5	adh_short_C2	227,3	1,70E-67	CL0063
TRINITY_DN10111_c0_g1_i3_2	10	24,5	62,456	111,93	PF00118.23	Cpn60_TCP1	494,1	3,80E-148	No_clan
TRINITY_DN9976_c0_g4_i1_4	3	16	24,915	45,852	PF00120.23	Gln-synt_C	55,9	3,20E-15	CL0286
TRINITY_DN11601_c0_g1_i1_1	15	43,6	48,437	169,22	PF11940.7	DUF3458	36,6	4,20E-09	No_clan
TRINITY_DN4015_c0_g1_i1_2	3	24,4	21,18	20,891	PF02298.16	Cu_bind_like	85,6	1,60E-24	CL0026
TRINITY_DN10140_c0_g1_i1_2	10	27,6	47,722	200,29	PF00248.20	Aldo_ket_red	191,6	1,50E-56	No_clan
TRINITY_DN2592_c0_g1_i1_1	12	32,5	55,448	134,73	PF03952.15	Enolase_N	188,2	5,40E-56	CL0227
TRINITY_DN10378_c0_g2_i2_1	6	17,9	47,116	120,19	PF08240.11	ADH_N	85,9	1,40E-24	CL0296
TRINITY_DN8575_c0_g2_i3_2	5	20,5	24,727	159,09	PF00255.18	GSHPx	146,2	2,10E-43	CL0172
TRINITY_DN11259_c0_g1_i2_5	15	27,1	87,305	131,72	PF02225.21	PA	43,2	3,00E-11	CL0364
TRINITY_DN11269_c2_g1_i2_3	16	27	64,296	104,62	PF00270.28	DEAD	135,3	1,70E-39	CL0023
TRINITY_DN9965_c1_g1_i1_3	3	32,5	15,92	112,76					
TRINITY_DN9622_c1_g1_i2_2	1	23,8	8,7298	152,99	PF05498.10	RALF	29	1,40E-06	No_clan
TRINITY_DN10079_c0_g1_i1_3	10	33,2	41,518	84,195	PF08240.11	ADH_N	35,7	5,70E-09	CL0296
TRINITY_DN3545_c0_g1_i2_1	5	31,8	12,648	31,385	PF01479.24	S4	47,5	1,00E-12	CL0492
TRINITY_DN5659_c0_g1_i1_1	18	29,9	100,81	159,96	PF00343.19	Phosphorylase	1009,6	6,90E-304	CL0113
TRINITY_DN5550_c0_g1_i2_4	5	54,9	16,231	172,75	PF00240.22	ubiquitin	71,6	3,00E-20	CL0072
TRINITY_DN11000_c0_g1_i1_2	7	23,8	42,201	61,196	PF08240.11	ADH_N	36,9	2,50E-09	CL0296
TRINITY_DN9087_c0_g1_i2_1	7	20,9	43,432	49,35	PF00635.25	Motile_Sperm	99,6	7,70E-29	CL0556
TRINITY_DN9399_c0_g1_i2_1	7	36	32,329	166,72	PF01048.19	PNP_UDP_1	71,4	5,90E-20	CL0408
TRINITY_DN11102_c0_g1_i4_6	4	12,9	40,677	86,032	PF00561.19	Abhydrolase_1	82,2	4,20E-23	CL0028
TRINITY_DN7857_c0_g1_i4_2	12	21,3	87,629	84,032	PF00378.19	ECH_1	145,6	1,40E-42	CL0127
TRINITY_DN5480_c0_g1_i2_3	6	21,1	42,176	43,84	PF00005.26	ABC_tran	54,6	1,50E-14	CL0023
TRINITY_DN3630_c0_g1_i1_5	8	25,8	50,294	60,847	PF00108.22	Thiolase_N	302	3,10E-90	CL0046
TRINITY_DN3986_c0_g2_i1_2	10	26,7	54,212	122,58	PF07992.13	Pyr_redox_2	159	1,40E-46	CL0063

TRINITY_DN10586_c0_g1_i1_1	5	32,2	25,915	102,6	PF00173.27	Cyt-b5	87,4	4,80E-25	No_clan
TRINITY_DN10813_c1_g1_i3_3	9	39,7	31,386	67,206	PF16363.4	GDP_Man_Dehyd	161,6	2,80E-47	CL0063
TRINITY_DN21280_c0_g1_i1_3	3	39,5	8,5615	22,309					
TRINITY_DN7020_c0_g1_i1_2	6	17,7	33,396	82,173	PF02297.16	COX6B	57,4	1,20E-15	CL0351
TRINITY_DN10799_c0_g2_i1_6	4	9,8	54,165	38,502	PF13181.5	TPR_8	17,4	0,0034	CL0020
TRINITY_DN8664_c0_g1_i3_3	5	18,2	41,47	41,515	PF01263.19	Aldose_epim	325,4	3,10E-97	CL0103
TRINITY_DN9559_c0_g1_i1_3	11	30,4	62,206	323,31	PF00790.18	VHS	88,4	3,40E-25	CL0009
TRINITY_DN11471_c0_g1_i3_3	5	16,1	31,191	88,324	PF01775.16	Ribosomal_L18A	182,2	2,90E-54	No_clan
TRINITY_DN7380_c0_g3_i1_1	3	19,2	16,445	73,416	PF07876.11	Dabb	100,7	4,90E-29	CL0032
TRINITY_DN9887_c0_g1_i1_2	4	23,5	25,568	15,255	PF01248.25	Ribosomal_L7Ae	88,4	1,80E-25	CL0101
TRINITY_DN10568_c0_g3_i5_1	9	22,6	60,785	73,872	PF00262.17	Calreticulin	440,2	4,50E-132	CL0004
TRINITY_DN10508_c0_g1_i1_5	1	4,5	25,051	9,4693	PF00248.20	Aldo_ket_red	108,9	2,30E-31	No_clan
TRINITY_DN9574_c0_g1_i3_1	10	32,9	46,093	78,604	PF00056.22	Ldh_1_N	157,7	1,70E-46	CL0063
TRINITY_DN6731_c0_g1_i1_1	16	27,1	91,936	187,2	PF03255.13	ACCA	181,3	8,00E-54	CL0127
TRINITY_DN10793_c0_g1_i1_1	4	15,2	40,211	64,255	PF01459.21	Porin_3	248,1	1,00E-73	CL0193
TRINITY_DN10605_c0_g1_i4_2	9	20,4	73,254	107,51	PF02985.21	HEAT	20,8	0,00028	CL0020
TRINITY_DN7923_c0_g1_i1_1	7	17,7	39,462	59,663	PF01092.18	Ribosomal_S6e	193,2	1,20E-57	No_clan
TRINITY_DN8263_c0_g1_i2_2	10	16,1	82,616	72,432	PF03141.15	Methyltransf_29	756	1,60E-227	CL0063
TRINITY_DN5337_c0_g1_i1_4	7	22,2	54,663	74,427	PF00561.19	Abhydrolase_1	53,6	2,20E-14	CL0028
TRINITY_DN11163_c0_g1_i8_3	9	29,9	38,368	74,539	PF13460.5	NAD_binding_10	29,3	6,80E-07	CL0063
TRINITY_DN10512_c1_g1_i1_2	4	32	24,49	33,293	PF02221.14	E1_DerP2_DerF2	71,5	8,20E-20	CL0532
TRINITY_DN5432_c0_g1_i1_5	4	12,4	40,788	44,224	PF01459.21	Porin_3	252,8	3,70E-75	CL0193
TRINITY_DN10108_c0_g1_i2_5	9	20,4	75,079	89,143	PF02383.17	Syja_N	293,1	2,50E-87	CL0031
TRINITY_DN10975_c0_g2_i1_3	7	23,4	36,121	66,606	PF01287.19	eIF-5a	98,1	2,10E-28	CL0021
TRINITY_DN10736_c0_g1_i2_1	9	19,7	69,8	82,67	PF00342.18	PGI	725,6	2,30E-218	CL0067
TRINITY_DN7422_c0_g2_i1_2	9	27,5	57,306	106,44	PF01293.19	PEPCK_ATP	30,5	1,40E-07	CL0374
TRINITY_DN10308_c0_g1_i1_1	5	19,9	31,007	35,083	PF00572.17	Ribosomal_L13	34,7	1,70E-08	No_clan
TRINITY_DN10384_c0_g3_i1_3	12	30,5	59,994	77,962	PF00091.24	Tubulin	233,6	2,10E-69	CL0566
TRINITY_DN3139_c0_g1_i1_2	6	24,3	32,628	50,211	PF00227.25	Proteasome	200,3	1,90E-59	CL0052
TRINITY_DN10364_c0_g1_i3_6	4	41,2	15,22	29,242	PF05938.10	Self-incomp_S1	77,6	8,30E-22	No_clan
TRINITY_DN20886_c0_g1_i1_5	3	12,8	27,411	63,305	PF00085.19	Thioredoxin	63	1,90E-17	CL0172
TRINITY_DN10883_c0_g1_i3_5	8	31,8	39,176	90,704	PF00160.20	Pro_isomerase	164,2	2,50E-48	CL0475
TRINITY_DN17539_c0_g2_i1_3	8	31	40,334	80,968					

TRINITY_DN10477_c0_g1_i1_1	10	21,3	69,139	87,954	PF00118.23	Cpn60_TCP1	556,3	5,10E-167	No_clan
TRINITY_DN6686_c0_g1_i1_2	9	30,9	50,176	80,968	PF04053.13	Coatomer_WDAD	345,7	4,20E-103	CL0186
TRINITY_DN11212_c0_g1_i1_6	5	24,9	28,513	68,485	PF13085.5	Fer2_3	94,4	3,60E-27	CL0486
TRINITY_DN11023_c0_g1_i2_1	9	14,8	111,89	74,539	PF02922.17	CBM_48	65,9	2,90E-18	CL0369
TRINITY_DN11458_c0_g1_i1_1	10	21,1	74,844	77,347	PF00118.23	Cpn60_TCP1	499,7	7,50E-150	No_clan
TRINITY_DN11615_c0_g1_i1_1	12	20,5	95,006	80,315	PF00076.21	RRM_1	28,2	1,10E-06	CL0221
TRINITY_DN4509_c0_g2_i1_3	6	39,8	20,208	50,426	PF05907.12	DUF866	166	5,10E-49	No_clan
TRINITY_DN11335_c0_g1_i1_1	12	27,4	58,764	82,116	PF00118.23	Cpn60_TCP1	402,9	1,70E-120	No_clan
TRINITY_DN5118_c0_g2_i1_6	9	48,2	22,283	69,752	PF00270.28	DEAD	122	2,10E-35	CL0023
TRINITY_DN9738_c2_g1_i2_3	7	32,8	30,253	52,876	PF16845.4	SQAPI	73,1	1,60E-20	CL0121
TRINITY_DN11571_c2_g3_i1_1	9	22	61,042	35,328	PF00091.24	Tubulin	227,4	1,60E-67	CL0566
TRINITY_DN17391_c0_g1_i1_5	3	27,5	13,205	84,243	PF14368.5	LTP_2	30	3,90E-07	CL0482
TRINITY_DN10823_c0_g1_i1_1	10	24,8	68,696	96,599	PF00118.23	Cpn60_TCP1	530,2	4,10E-159	No_clan
TRINITY_DN11383_c0_g1_i1_2	6	25,2	36,255	59,501	PF00295.16	Glyco_hydro_28	86,7	1,40E-24	CL0268
TRINITY_DN6993_c0_g1_i3_2	8	15,9	64,672	72,754	PF00285.20	Citrate_synt	446,9	4,70E-134	No_clan
TRINITY_DN11668_c1_g1_i1_2	4	6	76,287	49,491	PF02326.14	YMF19	53,5	3,00E-14	CL0255
TRINITY_DN3731_c0_g1_i2_3	9	38,5	30,732	151,94	PF00162.18	PGK	253,9	2,20E-75	No_clan
TRINITY_DN10138_c0_g1_i1_3	2	3,7	81,879	13,865	PF02516.13	STT3	213,9	4,00E-63	CL0111
TRINITY_DN8211_c0_g1_i2_3	6	19,5	42,765	164,2	PF00561.19	Abhydrolase_1	74,2	1,20E-20	CL0028
TRINITY_DN20888_c0_g1_i1_3	3	49,4	9,0523	38,299					
TRINITY_DN11494_c0_g1_i2_5	3	16,4	31,171	19,297	PF00719.18	Pyrophosphatase	178,4	8,00E-53	No_clan
TRINITY_DN7194_c1_g1_i2_2	5	25,1	28,113	63,618	PF02775.20	TPP_enzyme_C	54,4	1,10E-14	CL0254
TRINITY_DN8262_c0_g1_i1_1	5	25,1	37,684	47,023	PF00044.23	Gp_dh_N	129,9	3,60E-38	CL0063
TRINITY_DN14510_c0_g1_i1_2	1	13,8	10,226	54,879	PF00332.17	Glyco_hydro_17	95,2	4,60E-27	CL0058
TRINITY_DN9908_c0_g1_i2_1	4	12,8	34,684	25,243	PF00702.25	Hydrolase	60,1	3,20E-16	CL0137
TRINITY_DN5370_c0_g1_i1_1	1	11,2	15,686	49,993					
TRINITY_DN2867_c0_g1_i2_6	4	29,9	15,642	25,359	PF00481.20	PP2C	127,8	5,00E-37	CL0238
TRINITY_DN10919_c0_g1_i5_1	1	10,4	10,629	6,9387	PF00112.22	Peptidase_C1	56,5	3,70E-15	CL0125
TRINITY_DN8081_c0_g1_i3_3	10	19,6	59,505	82,77	PF01370.20	Epimerase	151,9	1,80E-44	CL0063
TRINITY_DN10953_c0_g1_i1_4	5	25,7	41,988	44,505	PF00109.25	ketoacyl-synt	146,7	8,40E-43	CL0046
TRINITY_DN3942_c0_g1_i3_3	7	22,9	45,049	71,583	PF10584.8	Proteasome_A_N	51,3	6,00E-14	CL0052
TRINITY_DN6284_c0_g1_i1_3	4	19,5	22,719	51,993					
TRINITY_DN6272_c0_g1_i1_2	7	20,6	50,479	69,913	PF00013.28	KH_1	50,1	1,70E-13	CL0007

TRINITY_DN10835_c0_g1_i2_3	6	23,7	32,46	37,151	PF04774.14	HABP4_PAI-RBP1	97,4	8,80E-28	No_clan
TRINITY_DN3173_c0_g3_i1_1	7	27,7	26,847	64,102					
TRINITY_DN8566_c0_g1_i2_1	10	19,7	70,228	79,24	PF00118.23	Cpn60_TCP1	507,7	2,70E-152	No_clan
TRINITY_DN18516_c0_g1_i1_1	3	31,8	14,217	79,396					
TRINITY_DN10724_c0_g1_i4_1	5	14,2	47,284	62,789	PF01263.19	Aldose_epim	317,6	7,20E-95	CL0103
TRINITY_DN10229_c0_g1_i2_1	3	15,1	23,775	19,203	PF01776.16	Ribosomal_L22e	156,1	3,30E-46	No_clan
TRINITY_DN2734_c0_g1_i1_1	2	12,9	28,605	14,129	PF00290.19	Trp_syntA	240,3	1,40E-71	CL0036
TRINITY_DN2645_c0_g2_i1_3	5	22,1	27,942	92,393	PF13417.5	GST_N_3	64,6	7,40E-18	CL0172
TRINITY_DN11051_c0_g2_i2_2	10	22,7	59,201	70,048	PF16363.4	GDP_Man_Dehyd	177,7	3,50E-52	CL0063
TRINITY_DN6332_c0_g2_i1_3	4	18,7	31,734	70,161	PF10584.8	Proteasome_A_N	34,2	1,40E-08	CL0052
TRINITY_DN10847_c0_g1_i5_1	3	16	32,718	70,879	PF01217.19	Clat_adaptor_s	76,4	2,00E-21	CL0212
TRINITY_DN9500_c0_g1_i4_1	12	35	44,077	42,939	PF00118.23	Cpn60_TCP1	177,1	4,90E-52	No_clan
TRINITY_DN9205_c0_g1_i1_1	4	22,1	28,905	26,309	PF01849.17	NAC	68,6	3,10E-19	No_clan
TRINITY_DN10268_c0_g1_i1_1	9	18,8	71,781	66,994	PF07991.11	IlvN	108,5	2,20E-31	CL0063
TRINITY_DN11412_c0_g1_i3_3	18	37,2	70,751	31,289	PF00004.28	AAA	162	8,40E-48	CL0023
TRINITY_DN4589_c0_g1_i2_4	3	20,8	19,395	230,76	PF14368.5	LTP_2	48,9	4,90E-13	CL0482
TRINITY_DN11511_c2_g1_i3_5	2	16,5	13,133	12,832	PF02798.19	GST_N	70,7	9,30E-20	CL0172
TRINITY_DN3847_c0_g1_i1_1	11	36,5	41,138	200,1	PF00150.17	Cellulase	52,6	4,00E-14	CL0058
TRINITY_DN11039_c0_g1_i5_1	9	16,6	88,113	97,714	PF00501.27	AMP-binding	342,5	2,60E-102	CL0378
TRINITY_DN10019_c0_g2_i1_2	11	21,1	64,645	82,718	PF09326.10	NADH_dhqG_C	47,2	2,50E-12	No_clan
TRINITY_DN10360_c0_g3_i2_3	10	16,5	102,83	79,55	PF02922.17	CBM_48	74,7	5,30E-21	CL0369
TRINITY_DN11478_c0_g2_i1_2	8	21,6	52,257	54,272	PF00199.18	Catalase	579,5	3,10E-174	No_clan
TRINITY_DN16366_c0_g1_i1_4	2	20,2	13,988	82,582	PF00933.20	Glyco_hydro_3	55,6	4,80E-15	CL0058
TRINITY_DN9354_c0_g2_i1_2	8	31,2	37,17	64,761	PF00346.18	Complex1_49kDa	397,1	2,80E-119	No_clan
TRINITY_DN6770_c0_g1_i2_3	8	25,6	43,673	61,501	PF12697.6	Abhydrolase_6	29,8	7,80E-07	CL0028
TRINITY_DN5978_c0_g1_i1_4	4	23,5	19,356	61,292	PF00403.25	HMA	62,3	4,10E-17	No_clan
TRINITY_DN10247_c0_g2_i1_3	4	23,1	27,899	76,013	PF01661.20	Macro	125,4	1,00E-36	CL0223
TRINITY_DN8913_c1_g2_i1_1	7	18,9	55,174	66,433	PF08545.9	ACP_syn_III	96,5	5,50E-28	CL0046
TRINITY_DN11338_c0_g1_i2_4	4	21,3	31,201	69,919	PF02271.15	UCR_14kD	94,7	2,40E-27	No_clan
TRINITY_DN11312_c0_g1_i1_1	3	12,7	52,825	60,629	PF02167.14	Cytochrom_C1	323,8	5,10E-97	CL0318
TRINITY_DN10708_c0_g1_i1_2	7	33	29,358	114,61	PF00400.31	WD40	33,1	6,20E-08	CL0186
TRINITY_DN10573_c0_g1_i2_2	7	12,1	82,134	47,339	PF00637.19	Clathrin	94,5	4,60E-27	CL0020
TRINITY_DN6429_c0_g1_i3_5	9	36,8	32,736	85,128	PF06957.10	COPI_C	464,2	3,10E-139	CL0020

TRINITY_DN8180_c0_g1_i1_1	4	11,4	46,056	71,404	PF02900.17	LigB	196,2	5,00E-58	CL0283
TRINITY_DN11655_c0_g1_i2_2	13	15,5	117,87	123,79	PF00690.25	Cation_ATPase_N	50,6	1,10E-13	No_clan
TRINITY_DN8076_c0_g1_i7_3	4	17,8	30,195	42,04	PF00248.20	Aldo_ket_red	90,1	1,30E-25	No_clan
TRINITY_DN9094_c0_g1_i3_6	7	16,2	62,434	249,74	PF00790.18	VHS	97	7,60E-28	CL0009
TRINITY_DN9800_c0_g1_i1_1	5	8,5	68,08	33,997	PF10559.8	Plug_translocon	65,2	3,10E-18	No_clan
TRINITY_DN21582_c0_g1_i1_3	3	38,5	10,729	167,93	PF08327.10	AHSA1	36,2	5,40E-09	CL0209
TRINITY_DN11557_c0_g2_i2_1	3	16,2	34,188	47,139	PF01722.17	BolA	45,7	5,40E-12	No_clan
TRINITY_DN9080_c0_g1_i2_2	13	27,7	65,448	110,07	PF00155.20	Aminotran_1_2	123,2	1,30E-35	CL0061
TRINITY_DN19006_c0_g1_i1_5	10	36,8	30,894	79,363	PF02933.16	CDC48_2	26,7	3,20E-06	CL0402
TRINITY_DN2622_c0_g1_i1_2	4	19,6	20,796	88,776					
TRINITY_DN1894_c0_g2_i1_5	2	38,5	8,7636	12,884	PF07944.11	Glyco_hydro_127	52,4	2,90E-14	CL0059
TRINITY_DN10750_c0_g1_i1_2	6	21,6	34,292	62,445	PF00227.25	Proteasome	157,9	1,80E-46	CL0052
TRINITY_DN336_c0_g1_i1_1	8	22	48,356	79,114	PF14306.5	PUA_2	118,3	2,20E-34	CL0178
TRINITY_DN10712_c0_g1_i2_6	5	20,9	26,555	31,927	PF00380.18	Ribosomal_S9	107,7	4,70E-31	CL0329
TRINITY_DN2749_c0_g1_i1_2	13	30,5	77,788	112,05	PF02770.18	Acyl-CoA_dh_M	55	6,70E-15	No_clan
TRINITY_DN6944_c1_g2_i2_2	2	3,3	58,657	11,641	PF07765.11	KIP1	44,8	9,80E-12	No_clan
TRINITY_DN10708_c1_g1_i1_1	7	41,7	28,555	105,5	PF00400.31	WD40	22,7	0,00012	CL0186
TRINITY_DN8860_c0_g2_i3_2	5	18,2	44,501	108,84	PF08597.9	eIF3_subunit	174	4,40E-51	No_clan
TRINITY_DN2935_c0_g1_i1_4	4	46,8	10,484	26,601					
TRINITY_DN5345_c0_g1_i1_1	9	29,7	48,104	89,199	PF00782.19	DSPc	82,4	2,20E-23	CL0031
TRINITY_DN2696_c0_g1_i1_2	3	15,6	19,234	21,477	PF01199.17	Ribosomal_L34e	123,4	3,10E-36	No_clan
TRINITY_DN6268_c0_g2_i1_1	5	23	41,408	50,706	PF00005.26	ABC_tran	51,3	1,50E-13	CL0023
TRINITY_DN20535_c0_g1_i1_2	4	46,4	14,744	39,895	PF00076.21	RRM_1	25,7	6,70E-06	CL0221
TRINITY_DN6734_c0_g1_i2_4	7	28,7	48,862	75,657	PF00759.18	Glyco_hydro_9	336,2	3,40E-100	CL0059
TRINITY_DN10790_c0_g1_i1_2	2	12,6	25,296	16,439	PF01282.18	Ribosomal_S24e	125,3	6,20E-37	No_clan
TRINITY_DN11422_c0_g1_i2_2	5	21,3	35,039	34,858	PF00227.25	Proteasome	113,4	8,30E-33	CL0052
TRINITY_DN4447_c0_g1_i1_4	2	27	10,428	66,045	PF14368.5	LTP_2	33,9	2,40E-08	CL0482
TRINITY_DN10526_c0_g1_i1_1	2	20,4	11,515	95,696					
TRINITY_DN11588_c0_g1_i3_2	3	9,2	40,278	20,013	PF00400.31	WD40	16,8	0,0086	CL0186
TRINITY_DN11110_c0_g3_i1_1	3	12,6	26,878	66,851	PF00085.19	Thioredoxin	82,6	1,50E-23	CL0172
TRINITY_DN3777_c0_g1_i2_4	9	53,5	26,604	66,675	PF02545.13	Maf	147,3	3,40E-43	CL0269
TRINITY_DN17601_c0_g1_i1_6	3	24,2	13,211	29,722	PF00109.25	ketoacyl-synt	28,9	7,60E-07	CL0046
TRINITY_DN11006_c0_g1_i1_2	6	19,4	48,344	44,136	PF03168.12	LEA_2	55,4	6,40E-15	CL0159

TRINITY_DN16803_c0_g1_i1_5	2	27,5	9,8782	22,841	PF00637.19	Clathrin	37,9	1,40E-09	CL0020	
TRINITY_DN22655_c0_g1_i1_2	4	53,2	8,5277	41,818	PF00182.18	Glyco_hydro_19	49,1	5,90E-13	CL0037	
TRINITY_DN9863_c2_g1_i1_1	6	27,9	31,191	92,182	PF00071.21	Ras	203,7	1,20E-60	CL0023	
TRINITY_DN7934_c0_g2_i1_1	5	25,3	29,399	61,17	PF01168.19	Ala_racemase_N	73,7	1,50E-20	CL0036	
TRINITY_DN11256_c0_g1_i2_2	9	19,1	60,541	63,23	PF05470.11	eIF-3c_N	315,9	4,20E-94	No_clan	
TRINITY_DN11403_c0_g1_i2_2	6	12,5	85,956	41,063	PF02990.15	EMP70	547,4	2,90E-164	No_clan	
TRINITY_DN11547_c0_g1_i4_3	2	12,2	18,689	64,089	PF02109.15	DAD	129,7	5,00E-38	No_clan	
TRINITY_DN8795_c0_g3_i1_4	3	19,5	13,487	30,487	PF01200.17	Ribosomal_S28e	83,3	8,30E-24	CL0021	
TRINITY_DN5882_c0_g3_i2_3	4	29,5	20,207	63,232	PF00238.18	Ribosomal_L14	121,4	2,00E-35	No_clan	
TRINITY_DN6879_c0_g1_i2_2	5	15,2	41,012	35,73	PF13176.5	TPR_7	14,4	0,027	CL0020	
TRINITY_DN8601_c0_g1_i1_2	2	8,2	20,035	12,123						
TRINITY_DN11298_c0_g1_i2_4	8	15,4	77,215	81,856	PF14555.5	UBA_4	47,3	1,20E-12	CL0214	
TRINITY_DN8357_c0_g1_i1_1	4	14,5	30,337	109,62	PF01652.17	IF4E	180	2,50E-53	CL0625	
TRINITY_DN9107_c0_g1_i2_2	1	4	23,936	8,6627	PF01277.16	Oleosin	165,2	3,40E-49	No_clan	
TRINITY_DN11330_c0_g1_i3_6	2	15,1	15,465	11,869	PF01667.16	Ribosomal_S27e	92,8	7,20E-27	CL0167	
TRINITY_DN10810_c0_g1_i4_1	3	12,3	27,347	20,82						
TRINITY_DN8957_c0_g1_i1_1	1	2,7	36,034	6,6924						
TRINITY_DN8356_c0_g2_i1_1	6	15,5	51,193	41,911	PF02136.19	NTF2	58,2	1,00E-15	CL0051	
TRINITY_DN10807_c0_g1_i5_1	9	24,8	51,428	57,62	PF00557.23	Peptidase_M24	74,5	8,60E-21	No_clan	
TRINITY_DN11558_c0_g1_i1_1	14	16,2	116,67	97,532	PF01399.26	PCI	77,9	6,60E-22	CL0123	
TRINITY_DN9852_c0_g1_i1_1	8	21,3	55,375	323,31	PF13519.5	VWA_2	96,1	1,70E-27	CL0128	
TRINITY_DN11418_c0_g2_i2_5	6	26,4	36,836	51,059	PF01946.16	Thi4	377,6	1,60E-113	CL0063	
TRINITY_DN5567_c0_g1_i3_3	2	8,7	18,659	7,5668	PF04568.11	IATP	25,3	1,40E-05	No_clan	
TRINITY_DN10055_c0_g1_i2_3	5	6,6	74,611	29,958	PF04597.13	Ribophorin_I	477,1	6,00E-143	No_clan	
TRINITY_DN8773_c0_g2_i2_4	7	17,6	56,394	86,969	PF05116.12	S6PP	339,9	7,70E-102	CL0137	
TRINITY_DN2809_c0_g1_i1_6	5	55	12,378	217,73	PF00293.27	NUDIX	46,5	3,30E-12	CL0261	
TRINITY_DN19230_c0_g1_i1_1	1	1,2	66,436	7,0086	PF01544.17	CorA	38,5	7,60E-10	No_clan	
TRINITY_DN11224_c0_g1_i1_1	6	21,1	36,003	72,912	PF01991.17	vATP-synt_E	236,7	1,40E-70	CL0255	
TRINITY_DN3638_c0_g1_i1_2	5	43,7	17,231	80,41	PF06094.11	GGACT	57,4	1,90E-15	CL0278	
TRINITY_DN11495_c0_g1_i1_2	2	9,3	24,455	15,965	PF01929.16	Ribosomal_L14e	88,1	3,70E-25	CL0107	
TRINITY_DN10903_c0_g1_i6_2	1	3,3	37,809	9,0443	PF00082.21	Peptidase_S8	72,4	3,50E-20	No_clan	
TRINITY_DN4170_c0_g3_i2_2	7	21,6	54,974	54,744	PF01179.19	Cu_amine_oxid	441,9	2,00E-132	No_clan	
TRINITY_DN10453_c0_g2_i2_3	3	11	32,505	18,908	PF00237.18	Ribosomal_L22	132	7,90E-39	No_clan	

TRINITY_DN10548_c1_g1_i1_3	5	17,2	39,374	35,119	PF01459.21	Porin_3	206,3	5,30E-61	CL0193
TRINITY_DN9786_c0_g1_i2_4	4	14	36,986	28,37	PF00481.20	PP2C	192,4	1,00E-56	CL0238
TRINITY_DN307_c0_g2_i1_6	3	20,7	18,354	19,871	PF07944.11	Glyco_hydro_127	118,1	3,60E-34	CL0059
TRINITY_DN3566_c0_g1_i1_1	6	10,6	64,856	38,264	PF00364.21	Biotin_lipoyl	63,4	1,20E-17	CL0105
TRINITY_DN6229_c0_g1_i1_2	3	21,7	29,066	80,142	PF06747.12	CHCH	35,7	6,20E-09	CL0351
TRINITY_DN10335_c1_g2_i2_2	9	19,7	67,719	85,152	PF01336.24	tRNA_anti-codon	46,5	2,50E-12	CL0021
TRINITY_DN10820_c1_g1_i1_3	7	17,5	64,751	79,917	PF01512.16	Complex1_51K	161,4	1,20E-47	CL0105
TRINITY_DN4435_c0_g1_i1_1	1	15,1	7,23	46,704					
TRINITY_DN9270_c0_g2_i2_2	3	9,6	27,683	23,346	PF00833.17	Ribosomal_S17e	190,1	9,50E-57	No_clan
TRINITY_DN9549_c0_g1_i1_2	6	34,5	26,38	40,158	PF06703.10	SPC25	134,1	3,60E-39	No_clan
TRINITY_DN11397_c0_g1_i5_2	7	32,8	31,62	94,443	PF13012.5	MitMem_reg	81,5	5,20E-23	No_clan
TRINITY_DN11446_c0_g1_i1_2	11	18,4	100,03	79,359	PF05193.20	Peptidase_M16_C	95	5,10E-27	CL0094
TRINITY_DN11125_c0_g9_i1_3	3	17,8	24,653	26,245	PF01251.17	Ribosomal_S7e	264	6,00E-79	CL0652
TRINITY_DN7263_c1_g1_i1_1	10	34,6	33,121	70,656					
TRINITY_DN11387_c0_g1_i4_2	15	17,7	115,14	95,344	PF01851.21	PC_rep	21,9	0,00016	CL0020
TRINITY_DN11199_c0_g5_i8_6	2	5,9	45,981	51,621	PF01912.17	eIF-6	268,1	3,10E-80	CL0197
TRINITY_DN8461_c0_g1_i1_1	10	26,9	58,588	68,722	PF00067.21	p450	24,3	1,10E-05	No_clan
TRINITY_DN3481_c0_g1_i1_6	4	21,2	30,232	26,492	PF00227.25	Proteasome	135,7	1,20E-39	CL0052
TRINITY_DN5908_c0_g1_i1_3	4	10,8	39,9	24,944	PF01145.24	Band_7	91,6	5,30E-26	CL0433
TRINITY_DN8185_c0_g1_i1_1	2	13,7	24,676	22,727	PF03939.12	Ribosomal_L23eN	68,6	3,60E-19	No_clan
TRINITY_DN4115_c0_g1_i1_2	5	39,3	20,683	103,67					
TRINITY_DN5077_c0_g1_i1_1	10	32	50,758	69,351	PF00291.24	PALP	217,4	2,50E-64	No_clan
TRINITY_DN7999_c0_g1_i1_3	7	19,8	40,327	32,8	PF08079.11	Ribosomal_L30_N	93	9,20E-27	No_clan
TRINITY_DN12921_c0_g1_i1_3	1	6,2	24,645	6,3324					
TRINITY_DN2387_c0_g2_i1_1	5	40	21,077	44,889	PF16363.4	GDP_Man_Dehyd	34,3	1,50E-08	CL0063
TRINITY_DN9350_c0_g2_i2_2	4	16,7	25,686	46,823	PF01777.17	Ribosomal_L27e	105,1	1,70E-30	No_clan
TRINITY_DN10819_c0_g1_i1_3	7	17,5	58,025	43,259	PF10250.8	O-FucT	107,7	9,40E-31	CL0113
TRINITY_DN9863_c1_g1_i2_5	6	31,4	22,738	37,232	PF00071.21	Ras	145,1	1,30E-42	CL0023
TRINITY_DN9866_c0_g1_i1_1	4	16,4	30,021	25,371	PF16205.4	Ribosomal_S17_N	108,1	1,90E-31	No_clan
TRINITY_DN11562_c0_g1_i1_2	10	21,7	57,62	71,76	PF01399.26	PCI	76,5	1,80E-21	CL0123
TRINITY_DN11571_c4_g3_i1_2	12	32,5	59,849	43,856	PF00091.24	Tubulin	226,4	3,30E-67	CL0566
TRINITY_DN9464_c0_g1_i4_3	11	22,7	77,18	87,982	PF00549.18	Ligase_CoA	45,9	4,70E-12	CL0506
TRINITY_DN8433_c1_g1_i2_6	7	20,5	37,781	49,115	PF05755.11	REF	320,5	4,40E-96	No_clan

TRINITY_DN10036_c0_g1_i3_6	3	22,7	24,012	21,705	PF00428.18	Ribosomal_60s	84	7,50E-24	No_clan
TRINITY_DN10785_c0_g1_i1_3	4	17	21,745	7,1153	PF04043.14	PMEI	99,3	2,40E-28	No_clan
TRINITY_DN4427_c0_g2_i1_1	10	21,1	74,394	73,591	PF03485.15	Arg_tRNA_synt_N	56,9	2,30E-15	No_clan
TRINITY_DN8833_c0_g1_i2_2	4	16,7	27,974	32,317	PF03737.14	RraA-like	143,2	6,20E-42	CL0364
TRINITY_DN10152_c0_g1_i1_1	3	20,9	19,248	35,079	PF03297.14	Ribosomal_S25	133,6	2,20E-39	CL0123
TRINITY_DN11018_c0_g1_i2_1	6	16,1	59,367	48,18	PF00155.20	Aminotran_1_2	123	1,40E-35	CL0061
TRINITY_DN8141_c0_g2_i1_3	4	11,5	54,557	28,052	PF02779.23	Transket_pyr	158,1	1,60E-46	CL0254
TRINITY_DN18656_c0_g1_i1_2	3	41,3	8,3087	21,371					
TRINITY_DN9214_c0_g1_i3_1	10	22,1	60,478	82,304	PF00240.22	ubiquitin	30,5	2,00E-07	CL0072
TRINITY_DN5754_c0_g1_i1_1	5	28,2	27,96	323,31	PF00314.16	Thaumatin	205,8	6,30E-61	CL0293
TRINITY_DN4395_c0_g1_i1_4	10	31,4	43,739	91,025	PF00004.28	AAA	40,2	3,70E-10	CL0023
TRINITY_DN10088_c0_g2_i1_3	8	28,9	43,319	52,87	PF14226.5	DIOX_N	114,7	3,50E-33	CL0029
TRINITY_DN18481_c0_g2_i1_2	8	52,1	21,072	60,22	PF00071.21	Ras	128,7	1,40E-37	CL0023
TRINITY_DN3240_c0_g1_i1_2	3	15,5	30,751	199	PF12796.6	Ank_2	45,3	9,10E-12	CL0465
TRINITY_DN9575_c0_g1_i1_1	9	24,1	51,726	68,456	PF00226.30	DnaJ	78,4	2,90E-22	CL0392
TRINITY_DN8266_c0_g1_i1_1	4	28,9	15,852	24,312	PF01991.17	vATP-synt_E	87,3	8,30E-25	CL0255
TRINITY_DN3684_c0_g3_i1_2	9	55,4	19,83	31,956	PF01201.21	Ribosomal_S8e	117,6	4,10E-34	No_clan
TRINITY_DN27_c0_g2_i1_5	3	22,7	16,344	22,6	PF00248.20	Aldo_ket_red	65,7	3,40E-18	No_clan
TRINITY_DN14743_c0_g1_i1_2	5	54	12,909	32,928	PF00311.16	PEPcase	120	8,00E-35	CL0151
TRINITY_DN11580_c0_g1_i4_3	11	16	87,576	80,308	PF03141.15	Methyltransf_29	764,6	3,70E-230	CL0063
TRINITY_DN10635_c0_g1_i3_5	3	10,8	28,463	19,873	PF01157.17	Ribosomal_L21e	146,4	1,70E-43	CL0107
TRINITY_DN11567_c1_g2_i1_2	5	12,9	56,506	70,489					
TRINITY_DN11631_c0_g1_i3_2	3	5,5	86,718	18,245	PF00116.19	COX2	178,6	3,20E-53	CL0026
TRINITY_DN1621_c0_g2_i2_2	3	17,5	22,07	22,951	PF03947.17	Ribosomal_L2_C	119,2	1,10E-34	CL0107
TRINITY_DN8095_c0_g1_i1_1	2	10,3	20,305	12,083					
TRINITY_DN5554_c0_g2_i1_3	5	27,6	24,561	34,583	PF00543.21	P-II	112,2	1,50E-32	CL0089
TRINITY_DN11457_c0_g1_i1_3	9	24,2	55,29	89,299	PF01399.26	PCI	49,7	3,90E-13	CL0123
TRINITY_DN6500_c0_g2_i1_1	1	4,1	34,535	67,594	PF01472.19	PUA	67,6	6,20E-19	CL0178
TRINITY_DN7157_c0_g1_i1_3	7	17,5	46,495	46,772	PF00764.18	Arginosuc_synth	517,7	2,10E-155	CL0039
TRINITY_DN9819_c1_g2_i1_2	5	16,2	41,161	47,214	PF01145.24	Band_7	84,9	6,00E-24	CL0433
TRINITY_DN11038_c0_g2_i1_2	9	36,9	41,999	58,699	PF01412.17	ArfGap	119,8	5,70E-35	No_clan
TRINITY_DN9121_c0_g1_i2_6	7	18,1	49,38	51,288	PF13561.5	adh_short_C2	170	5,50E-50	CL0063
TRINITY_DN11575_c0_g1_i5_2	3	9,2	43,381	91,463	PF04756.12	OST3_OST6	250,1	2,60E-74	CL0172

TRINITY_DN11451_c0_g1_i1_2	8	15,6	72,694	82,083	PF00005.26	ABC_tran	80,5	1,50E-22	CL0023
TRINITY_DN11459_c1_g2_i1_2	23	35,6	82,244	14,499	PF00012.19	HSP70	874,7	2,30E-263	CL0108
TRINITY_DN9611_c0_g1_i1_6	4	20,3	30,29	30,166	PF01294.17	Ribosomal_L13e	260,2	8,90E-78	No_clan
TRINITY_DN118_c0_g1_i1_3	2	16,9	15,392	14,843	PF08244.11	Glyco_hydro_32C	84,1	9,90E-24	CL0004
TRINITY_DN7932_c0_g1_i8_5	6	16,1	65,168	120,23	PF01749.19	IBB	31,6	1,50E-07	CL0020
TRINITY_DN9613_c0_g2_i2_4	2	9,2	26,809	30,825	PF00254.27	FKBP_C	98,1	2,60E-28	CL0487
TRINITY_DN7103_c0_g2_i2_2	7	22,5	47,398	68,029	PF00226.30	DnaJ	88,7	1,90E-25	CL0392
TRINITY_DN11303_c0_g1_i1_1	5	12,1	58,221	37,434	PF09598.9	Stm1_N	75,8	3,20E-21	No_clan
TRINITY_DN10641_c0_g1_i1_6	8	23,4	47,797	53,511	PF00316.19	FBPase	265,3	2,10E-79	CL0171
TRINITY_DN11410_c0_g1_i1_4	4	15,9	31,676	30,005	PF00582.25	Usp	100,2	1,30E-28	CL0039
TRINITY_DN6205_c0_g1_i1_2	3	21,6	22,657	75,458	PF03179.14	V-ATPase_G	113	8,10E-33	CL0255
TRINITY_DN11159_c1_g1_i8_5	7	19,9	49,019	46,187	PF00201.17	UDPGT	87	1,10E-24	CL0113
TRINITY_DN9714_c0_g1_i1_1	12	34,3	54,429	92,733	PF02127.14	Peptidase_M18	542,4	6,60E-163	CL0035
TRINITY_DN4382_c0_g1_i1_1	8	21,4	60,143	61,025	PF00004.28	AAA	134,2	3,40E-39	CL0023
TRINITY_DN11626_c0_g2_i1_5	8	10,6	90,925	69,681	PF02990.15	EMP70	740,1	1,20E-222	No_clan
TRINITY_DN9896_c0_g1_i5_1	4	15,1	26,594	30,354	PF04667.16	Endosulfine	72,6	2,00E-20	No_clan
TRINITY_DN10351_c0_g1_i3_1	7	16,9	60,853	58,128	PF03224.13	V-ATPase_H_N	271	1,30E-80	CL0020
TRINITY_DN7143_c0_g1_i1_1	2	5,8	37,137	14,511	PF00248.20	Aldo_ket_red	175,7	1,10E-51	No_clan
TRINITY_DN11432_c0_g1_i1_6	7	22,1	41,781	14,517	PF08240.11	ADH_N	96,8	5,80E-28	CL0296
TRINITY_DN9820_c0_g1_i3_3	4	8,3	66,567	35,68	PF00394.21	Cu-oxidase	137,8	3,00E-40	CL0026
TRINITY_DN10026_c0_g1_i1_5	6	36	32,037	67,539	PF02115.16	Rho_GDI	270,6	6,70E-81	No_clan
TRINITY_DN11576_c1_g1_i1_2	3	10,7	52,172	91,741	PF00112.22	Peptidase_C1	259,1	4,20E-77	CL0125
TRINITY_DN18710_c0_g1_i1_2	6	36	23,871	53,943	PF04752.11	ChaC	172,7	7,30E-51	CL0278
TRINITY_DN11480_c0_g1_i1_2	6	19,3	44,225	41,552	PF13640.5	2OG-FeII_Oxy_3	76,7	1,90E-21	CL0029
TRINITY_DN16427_c0_g1_i1_4	4	38,3	14,945	93,932	PF00106.24	adh_short	139,5	8,00E-41	CL0063
TRINITY_DN21781_c0_g1_i1_6	4	67,1	7,7839	114,65	PF00141.22	peroxidase	25,1	1,20E-05	CL0617
TRINITY_DN840_c0_g2_i1_1	7	27,5	36,554	50,547	PF03081.14	Exo70	349,5	2,10E-104	CL0295
TRINITY_DN9473_c0_g1_i1_3	4	23,3	26,458	46,072	PF05564.11	Auxin_repressed	181	9,50E-54	No_clan
TRINITY_DN11010_c0_g1_i2_2	3	11,8	23,578	7,0909	PF02823.15	ATP-synt_DE_N	60	1,50E-16	No_clan
TRINITY_DN11411_c1_g1_i1_5	10	38,4	47,991	67,174	PF00438.19	S-AdoMet_synt_N	143,5	2,40E-42	No_clan
TRINITY_DN13141_c0_g1_i1_2	2	35,7	9,6717	12,07	PF01263.19	Aldose_epim	57,3	1,40E-15	CL0103
TRINITY_DN10770_c0_g1_i1_1	8	18,7	74,059	67,397	PF01326.18	PPDK_N	111,7	4,00E-32	CL0179
TRINITY_DN1431_c0_g2_i1_2	8	28	45,938	66,824	PF00343.19	Phosphorylase	282,9	4,90E-84	CL0113

TRINITY_DN19678_c0_g1_i1_1	6	17,6	36,974	39,873	PF10151.8	TMEM214	65,1	4,10E-18	No_clan
TRINITY_DN249_c0_g2_i1_1	6	26,1	32,841	59,464					
TRINITY_DN17357_c0_g1_i1_3	4	13,5	46,014	56,197	PF00291.24	PALP	225,9	6,30E-67	No_clan
TRINITY_DN5130_c1_g1_i1_6	7	27,8	44,1	85,247	PF10509.8	GalKase_gal_bdg	28,6	7,20E-07	CL0329
TRINITY_DN10160_c0_g1_i3_6	8	17,3	69,174	63,459	PF00390.18	malic	252,9	1,50E-75	CL0603
TRINITY_DN5845_c0_g1_i3_2	10	29,9	52,089	106,41	PF02403.21	Seryl_tRNA_N	76,3	1,80E-21	CL0298
TRINITY_DN8910_c0_g1_i1_1	5	22,2	39,725	48,607	PF00464.18	SHMT	427,5	3,60E-128	CL0061
TRINITY_DN11500_c0_g1_i1_3	8	18,4	53,492	55,816	PF00400.31	WD40	32,3	1,10E-07	CL0186
TRINITY_DN11156_c0_g1_i1_3	5	12,9	59,273	36,319	PF00202.20	Aminotran_3	303,4	1,90E-90	CL0061
TRINITY_DN10981_c0_g2_i2_6	4	15,7	53,228	30,662	PF03951.18	Gln-synt_N	38,8	5,30E-10	No_clan
TRINITY_DN10644_c0_g1_i1_2	4	14	28,925	26,966	PF00025.20	Arf	222,3	2,70E-66	CL0023
TRINITY_DN21106_c0_g2_i1_1	3	32,2	16,253	150,93	PF00188.25	CAP	39,9	6,00E-10	CL0659
TRINITY_DN7447_c0_g1_i1_2	2	8	23,221	29,686	PF00505.18	HMG_box	80	1,20E-22	CL0114
TRINITY_DN10179_c0_g2_i1_1	1	7,6	14,565	9,5185	PF01095.18	Pectinesterase	43,7	1,30E-11	CL0268
TRINITY_DN10614_c0_g1_i2_2	7	17,7	68,974	47,526	PF07994.11	NAD_binding_5	472	9,40E-142	CL0063
TRINITY_DN14470_c0_g1_i1_4	6	47,7	12,26	42,275	PF00012.19	HSP70	157,4	3,80E-46	CL0108
TRINITY_DN5970_c0_g1_i1_3	7	25,7	34,732	47,675	PF00191.19	Annexin	72,3	2,30E-20	No_clan
TRINITY_DN10916_c0_g1_i1_6	5	18,2	44,913	40,063	PF00491.20	Arginase	225	1,20E-66	CL0302
TRINITY_DN9868_c0_g1_i1_3	7	14,9	71,88	50,662	PF10255.8	Paf67	500,1	4,30E-150	CL0020
TRINITY_DN11613_c0_g1_i2_2	7	20,9	45,319	16,929	PF00248.20	Aldo_ket_red	265,6	4,40E-79	No_clan
TRINITY_DN11620_c1_g1_i4_6	4	13,1	46,085	42,378	PF16158.4	N_BRCA1_IG	108,8	1,70E-31	No_clan
TRINITY_DN17153_c0_g1_i1_1	2	14,9	23,146	323,31					
TRINITY_DN8763_c0_g2_i1_1	7	12,3	85,375	104,26	PF00501.27	AMP-binding	337,3	9,90E-101	CL0378
TRINITY_DN11270_c0_g2_i1_1	6	19,2	48,654	45,591	PF01926.22	MMR_HSR1	77	1,10E-21	CL0023
TRINITY_DN3573_c0_g1_i1_2	6	17,9	52,546	50,62	PF01571.20	GCV_T	294	8,30E-88	CL0289
TRINITY_DN7327_c0_g1_i2_5	6	18,9	44,388	38,853	PF00762.18	Ferrochelatase	367,5	4,70E-110	CL0043
TRINITY_DN6062_c0_g2_i3_1	3	10,6	40,839	22,506	PF00076.21	RRM_1	76,6	9,00E-22	CL0221
TRINITY_DN5393_c0_g1_i1_2	2	7,8	30,974	12,98					
TRINITY_DN21568_c0_g1_i2_1	4	25,6	22,749	27,707	PF01213.18	CAP_N	182,5	1,30E-53	No_clan
TRINITY_DN11022_c0_g1_i1_1	8	17,7	84,957	78,26					
TRINITY_DN9756_c0_g1_i3_1	9	30,1	51,285	93,455	PF01546.27	Peptidase_M20	78,5	5,00E-22	CL0035
TRINITY_DN8806_c0_g1_i5_2	12	19,1	100,56	88,443	PF02518.25	HATPase_c	49,9	3,70E-13	CL0025
TRINITY_DN7109_c0_g1_i1_2	4	20,4	29,643	46,433	PF00080.19	Sod_Cu	164,9	1,10E-48	No_clan

TRINITY_DN3282_c0_g2_i1_4	5	22,8	37,106	38,309	PF07992.13	Pyr_redox_2	142	2,00E-41	CL0063
TRINITY_DN6144_c0_g1_i1_3	7	58,4	17,579	7,8712	PF04043.14	PMEI	52,4	6,60E-14	No_clan
TRINITY_DN10924_c0_g1_i1_3	4	19,8	27,566	30,005	PF00081.21	Sod_Fe_N	44,3	1,70E-11	No_clan
TRINITY_DN8033_c0_g1_i1_2	9	28,8	47,127	112,55	PF03141.15	Methyltransf_29	417	9,10E-125	CL0063
TRINITY_DN11083_c0_g1_i2_1	6	16,4	41,478	37,681	PF10151.8	TMEM214	53,9	1,00E-14	No_clan
TRINITY_DN9815_c0_g1_i1_2	7	23,9	53,017	100,83	PF00956.17	NAP	288,7	3,10E-86	No_clan
TRINITY_DN9347_c0_g1_i1_1	3	9,5	44,59	31,518	PF02921.13	UCR_TM	45,7	6,20E-12	CL0300
TRINITY_DN10872_c0_g1_i8_4	5	8,5	79,483	38,293	PF02990.15	EMP70	570,3	3,40E-171	No_clan
TRINITY_DN12354_c0_g1_i1_2	1	27,1	11,95	10,188	PF02115.16	Rho_GDI	118,6	2,60E-34	No_clan
TRINITY_DN6584_c0_g2_i1_1	5	30,1	19,335	15,058	PF00248.20	Aldo_ket_red	95,9	2,10E-27	No_clan
TRINITY_DN10462_c0_g1_i1_2	3	8,7	44,779	20,524	PF02629.18	CoA_binding	110,6	4,10E-32	CL0063
TRINITY_DN9076_c0_g2_i1_2	6	16,4	45,435	40,678	PF01546.27	Peptidase_M20	83,5	1,50E-23	CL0035
TRINITY_DN527_c0_g2_i1_3	2	23,7	14,545	20,742	PF00411.18	Ribosomal_S11	91,2	4,80E-26	CL0267
TRINITY_DN19375_c0_g1_i1_3	2	21,4	8,68	11,817	PF02826.18	2-Hacid_dh_C	70,6	9,80E-20	CL0063
TRINITY_DN9641_c0_g1_i1_2	8	31,2	43,47	113,82					
TRINITY_DN22097_c0_g1_i1_5	3	25,4	13,443	45,202					
TRINITY_DN12939_c0_g1_i1_5	7	44,9	25,321	52,713	PF00295.16	Glyco_hydro_28	229,6	4,60E-68	CL0268
TRINITY_DN12440_c0_g1_i1_2	5	43,7	18,274	34,294	PF00180.19	Iso_dh	113,2	1,30E-32	CL0270
TRINITY_DN21623_c0_g1_i1_1	2	50	8,3445	64,316	PF00141.22	peroxidase	74	1,30E-20	CL0617
TRINITY_DN10464_c0_g1_i1_6	7	18,3	64,768	50,985	PF00171.21	Aldedh	569	5,60E-171	CL0099
TRINITY_DN9302_c0_g1_i1_2	4	18,4	27,526	78,437	PF05938.10	Self-incomp_S1	57,1	1,90E-15	No_clan
TRINITY_DN1467_c0_g1_i1_4	6	26	31,591	61,906					
TRINITY_DN10869_c1_g3_i1_5	3	17,2	25,117	78,332	PF00314.16	Thaumatin	272,1	3,40E-81	CL0293
TRINITY_DN11008_c0_g1_i3_3	9	18,4	62,726	113,41	PF00464.18	SHMT	693,4	6,30E-209	CL0061
TRINITY_DN5035_c0_g1_i1_1	4	20,7	33,799	30,121	PF00300.21	His_Phos_1	73,9	1,30E-20	CL0071
TRINITY_DN11340_c0_g1_i2_2	8	13,8	84,812	72,399	PF14749.5	Acyl-CoA_ox_N	116,4	1,00E-33	CL0544
TRINITY_DN10734_c1_g1_i1_1	2	5,8	38,789	14,207					
TRINITY_DN10680_c4_g1_i3_4	7	63,5	10,772	8,1156	PF00240.22	ubiquitin	44,6	8,40E-12	CL0072
TRINITY_DN11420_c0_g1_i4_2	7	17,5	58,07	63,606	PF01532.19	Glyco_hydro_47	518,3	1,70E-155	CL0059
TRINITY_DN1806_c0_g1_i1_6	5	36,4	17,236	39,435	PF02990.15	EMP70	95,2	3,30E-27	No_clan
TRINITY_DN9463_c0_g1_i1_2	6	23,2	40,55	46,139	PF00118.23	Cpn60_TCP1	326,8	1,90E-97	No_clan
TRINITY_DN2883_c0_g1_i1_1	2	50	7,89	24,676					
TRINITY_DN11614_c0_g1_i5_1	7	22,1	53	54,368	PF01237.17	Oxysterol_BP	330,4	1,10E-98	No_clan

TRINITY_DN8774_c0_g2_i2_6	6	14,7	71,75	48,856	PF13905.5	Thioredoxin_8	93,1	9,50E-27	CL0172
TRINITY_DN9582_c0_g2_i1_5	8	30,2	39,4	71,984	PF00227.25	Proteasome	163,1	4,70E-48	CL0052
TRINITY_DN8902_c0_g1_i2_5	7	20,7	48,238	109,92	PF01363.20	FYVE	72,6	2,00E-20	CL0390
TRINITY_DN11545_c0_g1_i3_2	4	8,3	60,459	163,94	PF16499.4	Melibiase_2	284	1,00E-84	CL0058
TRINITY_DN11179_c0_g1_i4_3	6	18,1	50,213	46,26	PF01063.18	Aminotran_4	145,9	1,50E-42	No_clan
TRINITY_DN10263_c1_g1_i2_1	2	6,4	33,268	16,217	PF00179.25	UQ_con	127,5	2,60E-37	CL0208
TRINITY_DN9576_c0_g1_i2_5	3	19,9	19,699	18,972	PF02953.14	zf-Tim10_DDP	67,9	4,10E-19	No_clan
TRINITY_DN7960_c0_g1_i1_2	12	16	117,55	92,397	PF02861.19	Clp_N	62,3	3,10E-17	No_clan
TRINITY_DN5577_c0_g1_i1_5	1	3,7	26,326	16,53	PF00658.17	PABP	96,1	9,50E-28	No_clan
TRINITY_DN4472_c0_g1_i1_5	2	10,8	28,085	13,683	PF01248.25	Ribosomal_L7Ae	85,5	1,40E-24	CL0101
TRINITY_DN10370_c0_g1_i1_3	6	17	54,127	47,559	PF13646.5	HEAT_2	50,3	2,20E-13	CL0020
TRINITY_DN8106_c0_g1_i1_5	3	10,5	43,356	20,301	PF01182.19	Glucosamine_iso	244,2	1,40E-72	CL0246
TRINITY_DN11090_c0_g2_i6_4	2	13,8	19,922	11,64	PF00428.18	Ribosomal_60s	48,1	1,20E-12	No_clan
TRINITY_DN10461_c0_g1_i3_3	4	9,4	66,238	51,78	PF00450.21	Peptidase_S10	427,5	7,20E-128	CL0028
TRINITY_DN579_c0_g1_i1_3	1	3,7	29,47	6,7249					
TRINITY_DN14915_c0_g1_i1_3	2	26	7,9229	16,283	PF06094.11	GGACT	28,7	1,50E-06	CL0278
TRINITY_DN11178_c0_g1_i2_1	2	13,8	22,852	17,745	PF03227.15	GILT	33,8	3,00E-08	CL0172
TRINITY_DN8448_c0_g1_i1_1	6	25,5	33,677	46,842	PF06552.11	TOM20_plant	326,7	3,80E-98	CL0020
TRINITY_DN11626_c0_g1_i4_5	7	12,6	83,015	39,204	PF02990.15	EMP70	713,2	1,70E-214	No_clan
TRINITY_DN6292_c0_g2_i1_4	4	28,4	21,206	41,343	PF02800.19	Gp_dh_C	149,7	4,60E-44	CL0139
TRINITY_DN5457_c0_g1_i1_3	3	12,1	30,69	27,151	PF00857.19	Isochorismatase	113,8	9,00E-33	No_clan
TRINITY_DN6422_c0_g1_i1_2	5	14,8	44,466	48,891	PF00288.25	GHMP_kinases_N	42,6	4,80E-11	CL0329
TRINITY_DN7995_c0_g2_i1_1	4	22	28,52	21,463	PF00071.21	Ras	217,7	5,90E-65	CL0023
TRINITY_DN8330_c0_g1_i1_3	2	10,2	23,048	58,786	PF00550.24	PP-binding	44	1,90E-11	CL0314
TRINITY_DN10167_c0_g2_i1_3	3	12,4	28,128	20,804	PF01246.19	Ribosomal_L24e	99,8	6,60E-29	CL0175
TRINITY_DN9776_c0_g1_i4_2	4	9,9	47,908	26,214	PF01813.16	ATP-synt_D	231,6	6,40E-69	No_clan
TRINITY_DN11503_c0_g1_i1_1	3	15,1	32,378	42,547	PF07859.12	Abhydrolase_3	148,8	1,70E-43	CL0028
TRINITY_DN11383_c0_g1_i3_1	4	23,7	22,724	28,354	PF00295.16	Glyco_hydro_28	33,9	1,60E-08	CL0268
TRINITY_DN10659_c0_g1_i3_2	5	22,5	37,426	39,949	PF01583.19	APS_kinase	234,3	5,00E-70	CL0023
TRINITY_DN10371_c0_g1_i1_2	4	25	29,118	33,715	PF00582.25	Usp	98,4	4,50E-28	CL0039
TRINITY_DN6420_c0_g1_i1_4	3	12,7	25,242	17,979	PF04718.14	ATP-synt_G	79,3	2,80E-22	No_clan
TRINITY_DN10875_c0_g1_i2_2	6	18	58,688	58,842	PF00004.28	AAA	142,9	6,90E-42	CL0023
TRINITY_DN9826_c0_g1_i1_3	6	23,4	37,986	39,036	PF00004.28	AAA	138,3	1,90E-40	CL0023

TRINITY_DN8975_c0_g1_i1_3	6	18,4	44,404	56,196	PF00206.19	Lyase_1	206,7	5,20E-61	No_clan
TRINITY_DN8607_c0_g1_i2_1	1	5,1	26,83	8,6938	PF00190.21	Cupin_1	168,1	9,30E-50	CL0029
TRINITY_DN11635_c2_g1_i9_3	6	55,3	12,841	7,9892	PF00248.20	Aldo_ket_red	123,3	9,60E-36	No_clan
TRINITY_DN17821_c0_g1_i1_3	1	3,8	29,293	6,2728	PF00076.21	RRM_1	36,7	2,50E-09	CL0221
TRINITY_DN3258_c0_g1_i1_2	7	46,1	24,652	130,32	PF00400.31	WD40	17,9	0,0039	CL0186
TRINITY_DN10762_c0_g1_i1_1	4	21,3	25,202	85,637	PF00234.21	Tryp_alpha_amyl	30,8	2,70E-07	CL0482
TRINITY_DN10328_c0_g2_i1_2	7	20,1	54,03	51,434	PF02784.15	Orn_Arg_deC_N	164,4	2,70E-48	CL0036
TRINITY_DN11289_c0_g2_i2_2	6	13,2	53,359	45,869	PF10602.8	RPN7	195,1	7,00E-58	CL0020
TRINITY_DN4969_c0_g1_i1_3	6	23,9	45,437	43,545	PF01459.21	Porin_3	269,3	3,50E-80	CL0193
TRINITY_DN12289_c0_g1_i1_5	8	48,7	21,329	161,8					
TRINITY_DN11225_c0_g1_i1_3	6	16,8	49,395	44,621	PF05368.12	NmrA	266	2,50E-79	CL0063
TRINITY_DN20631_c0_g1_i1_5	2	31,9	7,7726	18,264					
TRINITY_DN15095_c0_g1_i1_4	6	36,6	18,42	110,17	PF00076.21	RRM_1	59,8	1,60E-16	CL0221
TRINITY_DN10798_c0_g1_i1_3	4	5,5	78,158	28,571	PF02516.13	STT3	323,1	3,10E-96	CL0111
TRINITY_DN17510_c0_g1_i1_2	6	24	29,245	43,961	PF01026.20	TatD_DNase	96,4	1,60E-27	CL0034
TRINITY_DN6021_c0_g1_i1_3	3	13,8	29,199	35,427	PF06417.11	DUF1077	124,1	2,60E-36	No_clan
TRINITY_DN9758_c0_g1_i1_3	1	4,1	26,394	7,0352					
TRINITY_DN2208_c0_g1_i1_1	4	19,9	32,216	71,573	PF02729.20	OTCace_N	137,2	3,80E-40	No_clan
TRINITY_DN11722_c0_g1_i1_5	3	33	12,665	31,376	PF00141.22	peroxidase	90,9	9,10E-26	CL0617
TRINITY_DN17467_c0_g1_i1_1	6	43,3	17,165	39,516	PF00152.19	tRNA-synt_2	50,1	1,90E-13	CL0040
TRINITY_DN3823_c0_g1_i1_6	2	6,4	48,072	15,344	PF03661.12	UPF0121	61,4	7,60E-17	No_clan
TRINITY_DN11535_c0_g1_i2_3	3	17,6	19,928	18,522	PF00338.21	Ribosomal_S10	94,9	2,50E-27	No_clan
TRINITY_DN8730_c1_g1_i1_2	6	20,7	51,583	320,16	PF00581.19	Rhodanese	52,5	5,30E-14	CL0031
TRINITY_DN2631_c0_g3_i1_2	1	13	21,988	37,238					
TRINITY_DN9280_c0_g2_i4_2	5	21,4	44,035	68,486	PF05091.11	eIF-3_zeta	398	7,50E-119	CL0236
TRINITY_DN5611_c0_g4_i1_3	6	25,6	35,899	39,825	PF13774.5	Longin	82,7	1,30E-23	No_clan
TRINITY_DN4378_c0_g1_i2_2	13	21	98,797	92,859	PF01411.18	tRNA-synt_2c	572,8	6,30E-172	CL0040
TRINITY_DN4596_c0_g1_i1_5	4	41,5	13,166	20,952	PF00724.19	Oxidored_FMN	43,2	2,70E-11	CL0036
TRINITY_DN11195_c0_g1_i1_2	2	4,2	60,588	13,338	PF00067.21	p450	346,9	1,60E-103	No_clan
TRINITY_DN4293_c0_g1_i1_4	6	19,5	52,316	52,685					
TRINITY_DN8155_c0_g1_i2_3	6	25,5	47,084	50,226	PF02771.15	Acyl-CoA_dh_N	120,1	6,70E-35	CL0544
TRINITY_DN9942_c0_g1_i1_4	11	22,2	68,016	95,993	PF12580.7	TPPII	213,3	1,80E-63	No_clan
TRINITY_DN11521_c0_g1_i3_1	6	17,1	49,238	55,276	PF00575.22	S1	49,1	4,80E-13	CL0021

TRINITY_DN10407_c0_g1_i1_3	6	16	50,563	46,738	PF00295.16	Glyco_hydro_28	321,6	4,60E-96	CL0268
TRINITY_DN11080_c0_g1_i1_1	3	18,7	22,269	27,013	PF01655.17	Ribosomal_L32e	170,1	1,40E-50	No_clan
TRINITY_DN12642_c0_g1_i1_3	5	25,6	34,092	38,156	PF09177.10	Syntaxin-6_N	85,1	3,60E-24	No_clan
TRINITY_DN10964_c0_g1_i1_1	2	12,3	19,779	12,488	PF00831.22	Ribosomal_L29	62,7	2,20E-17	CL0346
TRINITY_DN11121_c0_g1_i1_6	2	9,2	37,806	38,79	PF01596.16	Methyltransf_3	286,2	9,90E-86	CL0063
TRINITY_DN8201_c0_g2_i1_1	6	18,3	56,392	45,462	PF01370.20	Epimerase	79,9	1,80E-22	CL0063
TRINITY_DN2407_c0_g1_i1_1	5	18,8	36,94	31,284	PF13848.5	Thioredoxin_6	55,9	4,80E-15	CL0172
TRINITY_DN18360_c0_g1_i1_1	3	24,3	20,299	23,836	PF06747.12	CHCH	27,8	1,80E-06	CL0351
TRINITY_DN7023_c0_g1_i2_1	4	20,3	26,008	173,25					
TRINITY_DN10334_c0_g1_i2_3	4	21,7	36,811	36,955	PF01039.21	Carboxyl_trans	70	1,50E-19	CL0127
TRINITY_DN3765_c0_g1_i1_1	1	11,7	8,8628	7,5065					
TRINITY_DN6855_c0_g1_i1_1	5	17,1	46,468	41,253	PF05470.11	eIF-3c_N	447,2	7,10E-134	No_clan
TRINITY_DN9376_c1_g3_i1_4	1	1,4	71,101	6,4226					
TRINITY_DN13181_c0_g1_i1_4	5	56,1	13,43	29,968	PF00224.20	PK	119	1,90E-34	CL0151
TRINITY_DN6915_c0_g2_i2_2	4	23,5	12,462	27,464	PF02704.13	GASA	82,1	2,20E-23	No_clan
TRINITY_DN8184_c1_g1_i1_1	5	12,8	53,616	89,783	PF02878.15	PGM_PMM_I	86,6	1,10E-24	No_clan
TRINITY_DN19070_c0_g1_i1_2	1	9,6	12,052	12,778	PF08991.9	MTCP1	28,9	9,10E-07	CL0351
TRINITY_DN20708_c0_g1_i1_4	2	24,2	10,308	13,463	PF01918.20	Alba	73,2	1,00E-20	CL0441
TRINITY_DN11287_c0_g1_i2_3	5	23,5	38,291	49,76	PF03446.14	NAD_binding_2	141,6	2,10E-41	CL0063
TRINITY_DN9456_c0_g1_i4_3	3	9,8	46,86	32,455	PF12701.6	LSM14	108,5	1,20E-31	CL0527
TRINITY_DN8791_c0_g1_i1_3	3	15,9	29,472	17,745	PF00071.21	Ras	136,4	5,90E-40	CL0023
TRINITY_DN4375_c0_g3_i1_3	8	34,2	32,531	34,415	PF00071.21	Ras	198	6,60E-59	CL0023
TRINITY_DN10343_c0_g1_i4_1	5	14,7	35,238	29,883	PF01213.18	CAP_N	70,5	1,60E-19	No_clan
TRINITY_DN18958_c0_g1_i1_3	1	4,3	27,793	6,5558					
TRINITY_DN4412_c0_g1_i1_3	11	26,6	61,323	74,438	PF13943.5	WPP	99,9	7,80E-29	No_clan
TRINITY_DN3095_c0_g1_i2_2	5	22,4	25,521	40,397	PF11938.7	DUF3456	118,2	4,20E-34	No_clan
TRINITY_DN10358_c0_g1_i1_1	6	20,8	37,612	47,644	PF01873.16	eIF-5_eIF-2B	134,1	1,80E-39	No_clan
TRINITY_DN16061_c0_g1_i1_3	2	43,1	7,5406	124,95					
TRINITY_DN5302_c0_g1_i1_6	3	12	22,636	27,738	PF00248.20	Aldo_ket_red	71,6	5,70E-20	No_clan
TRINITY_DN11246_c1_g4_i2_1	3	19,6	17,066	11,584	PF08069.11	Ribosomal_S13_N	109	8,00E-32	No_clan
TRINITY_DN19132_c0_g1_i1_5	1	12	10,337	6,8018	PF13668.5	Ferritin_2	46,9	2,60E-12	CL0044
TRINITY_DN9764_c0_g1_i1_3	8	10,7	86,832	55,126	PF04068.14	RLI	50,9	9,40E-14	CL0344
TRINITY_DN7237_c0_g2_i1_3	3	13,5	30,626	161,9	PF03912.13	Psb28	144,9	8,50E-43	No_clan

TRINITY_DN11279_c0_g2_i3_5	4	22,7	35,497	29,431	PF00112.22	Peptidase_C1	249	4,80E-74	CL0125
TRINITY_DN10730_c0_g1_i1_1	9	14,5	84,751	72,017	PF06964.11	Alpha-L-AF_C	119,1	2,00E-34	CL0369
TRINITY_DN11396_c1_g1_i3_2	1	4,8	31,498	6,9537					
TRINITY_DN10977_c0_g1_i1_6	6	17,4	62,99	47,402	PF00270.28	DEAD	129,7	8,60E-38	CL0023
TRINITY_DN9743_c0_g2_i2_1	4	16,8	43,327	49,953	PF10075.8	CSN8_PSD8_EIF3K	88,6	3,40E-25	CL0123
TRINITY_DN10127_c0_g1_i2_5	1	10,2	19,717	38,513	PF01247.17	Ribosomal_L35Ae	151	7,50E-45	CL0575
TRINITY_DN10738_c0_g1_i2_5	7	11,7	79,049	55,255	PF13537.5	GATase_7	148,5	7,70E-44	CL0052
TRINITY_DN6947_c0_g1_i5_4	2	32,4	7,7025	14,263	PF00262.17	Calreticulin	56,8	1,60E-15	CL0004
TRINITY_DN4336_c0_g1_i1_3	6	24,3	37,228	45,915	PF03447.15	NAD_binding_3	49,5	5,40E-13	CL0063
TRINITY_DN11277_c0_g1_i2_2	8	16,6	64,005	78,611	PF00282.18	Pyridoxal_deC	346,4	1,40E-103	CL0061
TRINITY_DN3905_c0_g2_i2_3	4	17,9	41,293	27,663	PF08511.10	COQ9	98,5	1,30E-28	No_clan
TRINITY_DN5130_c0_g2_i1_3	3	41,4	9,1602	24,863					
TRINITY_DN21282_c0_g1_i1_3	3	36,5	11,633	20,765	PF00626.21	Gelsolin	24,3	2,00E-05	CL0092
TRINITY_DN7375_c0_g1_i1_1	4	34,9	22,16	29,015					
TRINITY_DN10410_c0_g1_i1_3	2	5,6	37,859	12,815	PF01257.18	2Fe-2S_thioredx	186,5	2,00E-55	CL0172
TRINITY_DN10668_c0_g1_i2_2	2	6,3	45,542	12,965					
TRINITY_DN1129_c0_g1_i1_2	7	31	38,699	55,824	PF13460.5	NAD_binding_10	73,7	1,60E-20	CL0063
TRINITY_DN9363_c0_g1_i2_2	6	20,5	35,938	49,899	PF00406.21	ADK	196,7	1,90E-58	CL0023
TRINITY_DN8678_c0_g3_i5_1	4	24,7	23,644	26,667	PF12353.7	eIF3g	33,2	5,20E-08	CL0511
TRINITY_DN9856_c0_g1_i2_6	5	13,8	49,396	36,353	PF16363.4	GDP_Man_Dehyd	230,5	3,00E-68	CL0063
TRINITY_DN16281_c0_g1_i1_4	3	31,7	10,597	26,524					
TRINITY_DN8090_c1_g1_i1_6	6	31,7	26,516	41,052	PF02575.15	YbaB_DNA_bd	61,4	6,50E-17	No_clan
TRINITY_DN18348_c0_g1_i1_1	3	32,1	12,961	22,994					
TRINITY_DN11641_c0_g1_i5_1	1	2,8	46,195	6,6712					
TRINITY_DN13961_c0_g1_i1_1	1	11	7,5687	-2					
TRINITY_DN1808_c0_g1_i1_6	6	30,2	22,211	12,58	PF00240.22	ubiquitin	63,1	1,40E-17	CL0072
TRINITY_DN10282_c0_g1_i6_1	5	31,9	22,472	30,693	PF00462.23	Glutaredoxin	62,3	3,30E-17	CL0172
TRINITY_DN11194_c0_g1_i1_2	4	21	26,145	29,049					
TRINITY_DN4777_c0_g2_i2_3	2	5,6	32,021	16,391	PF02453.16	Reticulon	193,3	2,10E-57	No_clan
TRINITY_DN11197_c0_g1_i1_5	3	15,3	30,287	49,742	PF02466.18	Tim17	48,3	9,30E-13	No_clan
TRINITY_DN1802_c0_g1_i1_4	4	25,1	26,7	21,117	PF01991.17	vATP-synt_E	142,3	1,10E-41	CL0255
TRINITY_DN10578_c0_g2_i1_1	2	7,5	39,94	13,438	PF01086.16	Clathrin_lg_ch	45,3	1,00E-11	No_clan
TRINITY_DN17430_c0_g1_i1_5	3	20,7	17,758	21,677	PF01161.19	PBP	95,1	3,80E-27	No_clan

TRINITY_DN9138_c0_g1_i1_4	2	8,8	29,024	26,551	PF00160.20	Pro_isomerase	175,6	7,90E-52	CL0475
TRINITY_DN10772_c1_g1_i1_2	5	15,2	55,568	41,645	PF00149.27	Metallophos	123,4	1,60E-35	CL0163
TRINITY_DN11439_c0_g1_i2_4	3	14,2	37,029	103,37	PF00227.25	Proteasome	101,4	4,00E-29	CL0052
TRINITY_DN7730_c0_g1_i3_4	6	16,5	44,733	38,541	PF00291.24	PALP	224,2	2,10E-66	No_clan
TRINITY_DN10562_c0_g1_i5_3	3	6,9	60,237	22,855	PF00557.23	Peptidase_M24	185,9	6,80E-55	No_clan
TRINITY_DN8163_c0_g1_i1_1	6	16,7	54,078	43,415	PF01546.27	Peptidase_M20	126	1,50E-36	CL0035
TRINITY_DN10420_c0_g1_i3_2	5	27,4	37,776	45,362	PF00970.23	FAD_binding_6	109,1	1,00E-31	CL0076
TRINITY_DN10122_c0_g1_i1_3	3	22,5	20,63	54,491	PF00462.23	Glutaredoxin	79,5	1,50E-22	CL0172
TRINITY_DN7204_c0_g1_i1_1	1	6,4	23,537	31,421					
TRINITY_DN11311_c0_g1_i2_2	6	16,4	48,071	110,17	PF00128.23	Alpha-amylase	54,3	1,40E-14	CL0058
TRINITY_DN11251_c0_g1_i2_1	10	22,3	70,987	73,784	PF07724.13	AAA_2	185,8	6,30E-55	CL0023
TRINITY_DN2678_c0_g1_i1_1	2	28	8,0644	13,764					
TRINITY_DN9042_c0_g1_i1_3	7	36,5	36,703	70,641	PF03807.16	F420_oxidored	68,8	4,30E-19	CL0063
TRINITY_DN134_c0_g1_i3_5	6	22,5	47,374	323,31	PF14686.5	fn3_3	89,3	9,50E-26	CL0287
TRINITY_DN7354_c1_g1_i1_2	3	24,5	27,65	20,031	PF06172.10	Cupin_5	147,5	2,10E-43	CL0029
TRINITY_DN7673_c0_g1_i1_3	4	22	25,578	24,454	PF00690.25	Cation_ATPase_N	40	2,10E-10	No_clan
TRINITY_DN5029_c0_g1_i1_6	9	12,7	92,295	79,14					
TRINITY_DN4009_c0_g1_i2_3	3	29,4	18,461	20,823	PF00343.19	Phosphorylase	148,7	1,80E-43	CL0113
TRINITY_DN4424_c0_g1_i3_3	7	24,8	48,673	68,952	PF08240.11	ADH_N	97,9	2,70E-28	CL0296
TRINITY_DN17664_c0_g2_i1_3	5	55,2	14,667	44,249					
TRINITY_DN7316_c0_g1_i1_3	6	29,9	40,494	147,9	PF00206.19	Lyase_1	272,6	4,30E-81	No_clan
TRINITY_DN9014_c0_g1_i1_3	1	7,2	35	57,486	PF13833.5	EF-hand_8	18,4	0,0013	CL0220
TRINITY_DN17081_c0_g1_i1_1	3	40,2	11,562	36,56	PF00311.16	PEPcase	30,2	1,10E-07	CL0151
TRINITY_DN7215_c0_g1_i1_5	2	24	15,088	14,395	PF03911.15	Sec61_beta	64,6	5,40E-18	No_clan
TRINITY_DN11034_c0_g1_i3_3	1	4,1	26,444	6,5762	PF02221.14	E1_DerP2_DerF2	76,4	2,50E-21	CL0532
TRINITY_DN21713_c0_g1_i1_2	4	43,7	13,943	36,108					
TRINITY_DN7461_c0_g1_i1_6	5	11,2	43,611	31,831	PF01025.18	GrpE	153,3	4,20E-45	No_clan
TRINITY_DN10238_c0_g2_i1_1	6	12,4	80,39	47,859	PF03141.15	Methyltransf_29	727,7	6,00E-219	CL0063
TRINITY_DN2462_c0_g2_i1_2	5	13,1	50,306	35,957	PF00627.30	UBA	31,3	1,20E-07	CL0214
TRINITY_DN16740_c0_g1_i1_5	2	28,2	7,4792	13,741					
TRINITY_DN7713_c0_g1_i1_6	4	31	20,826	34,862	PF01722.17	BolA	70,8	7,80E-20	No_clan
TRINITY_DN7723_c0_g1_i2_2	7	26,9	35,865	48,047	PF00294.23	PfkB	239	6,50E-71	CL0118
TRINITY_DN10456_c0_g1_i4_1	3	13,9	30,049	24,019	PF04979.13	IPP-2	83,2	2,30E-23	No_clan

TRINITY_DN10811_c0_g1_i1_2	2	11,5	31,372	13,539	PF00412.21	LIM	45,8	4,90E-12	CL0167
TRINITY_DN10241_c0_g2_i1_1	2	9,5	39,871	18,054	PF00026.22	Asp	266,2	4,00E-79	CL0129
TRINITY_DN10843_c0_g2_i2_2	3	24,6	28,784	39,375					
TRINITY_DN888_c0_g1_i1_1	3	20,9	31,228	33,648	PF07986.11	TBCC	36,6	2,90E-09	CL0391
TRINITY_DN9357_c0_g1_i1_4	1	0,9	91,018	-2					
TRINITY_DN10551_c0_g1_i1_3	5	10,8	65,937	35,949	PF00479.21	G6PD_N	201,1	1,90E-59	CL0063
TRINITY_DN9412_c0_g1_i1_2	5	16,6	64,377	35,415	PF01301.18	Glyco_hydro_35	90,9	1,00E-25	CL0058
TRINITY_DN14016_c0_g2_i1_2	5	11	48,2	32,764	PF00174.18	Oxidored_molyb	193	2,70E-57	CL0072
TRINITY_DN5499_c0_g1_i1_3	2	12,1	24,71	33,331					
TRINITY_DN10701_c0_g1_i2_1	5	19,3	28,441	25,364	PF00333.19	Ribosomal_S5	52,1	4,40E-14	CL0196
TRINITY_DN5059_c0_g1_i1_5	6	25,2	23,456	32,761	PF00464.18	SHMT	277,2	1,60E-82	CL0061
TRINITY_DN6933_c0_g1_i1_2	12	35,3	60,056	111,2					
TRINITY_DN10774_c0_g1_i1_1	6	21,9	45,818	45,873	PF01398.20	JAB	50,4	1,80E-13	CL0366
TRINITY_DN10849_c0_g1_i2_2	1	3,8	22,85	6,6206					
TRINITY_DN10042_c0_g1_i2_2	4	12	48,25	27,652	PF00923.18	TAL_FSA	165	2,10E-48	CL0036
TRINITY_DN8175_c0_g1_i1_2	3	19,5	26,629	40,378					
TRINITY_DN11149_c0_g1_i1_3	9	28,5	49,126	71,339	PF01172.17	SBDS	102,3	1,10E-29	No_clan
TRINITY_DN14909_c0_g1_i1_3	9	28,5	47,187	66,458	PF10143.8	PhosphMutase	170,7	2,20E-50	No_clan
TRINITY_DN6906_c0_g1_i2_2	5	18,3	44,332	73,165	PF00793.19	DAHP_synth_1	203,9	2,00E-60	CL0036
TRINITY_DN4515_c0_g2_i1_2	3	58,9	7,7354	17,906	PF02136.19	NTF2	47,6	2,00E-12	CL0051
TRINITY_DN11230_c0_g2_i7_6	4	23,5	26,088	-2	PF03931.14	Skp1_POZ	106,5	5,20E-31	CL0033
TRINITY_DN19409_c0_g1_i1_3	4	34	15,738	29,348	PF00106.24	adh_short	107,3	6,20E-31	CL0063
TRINITY_DN10897_c0_g1_i1_1	6	24,1	38,981	44,916	PF00627.30	UBA	54,8	5,50E-15	CL0214
TRINITY_DN7208_c0_g1_i1_2	3	7,5	39,373	18,212	PF00462.23	Glutaredoxin	62,8	2,30E-17	CL0172
TRINITY_DN7184_c0_g1_i2_3	2	15	14,214	11,931	PF10890.7	Cyt_b-c1_8	124,9	1,20E-36	CL0429
TRINITY_DN6124_c0_g1_i2_1	5	14,5	51,774	34,079	PF07244.14	POTRA	22,3	0,00013	CL0191
TRINITY_DN17685_c0_g1_i1_2	6	35,5	17,529	9,6538	PF07650.16	KH_2	50	1,80E-13	CL0007
TRINITY_DN10323_c0_g3_i1_1	6	13,5	91,223	48,391	PF08323.10	Glyco_transf_5	221,3	1,40E-65	CL0113
TRINITY_DN9450_c0_g1_i1_2	4	14,4	35,71	28,395	PF04733.13	Coatomer_E	453,6	2,70E-136	CL0020
TRINITY_DN11814_c0_g1_i1_6	3	7,2	54,95	20,609	PF00291.24	PALP	160,9	4,20E-47	No_clan
TRINITY_DN22082_c0_g1_i1_3	2	28	10,235	22,31	PF00450.21	Peptidase_S10	103,3	1,90E-29	CL0028
TRINITY_DN7979_c0_g1_i2_2	5	21,6	39,885	31,437	PF01263.19	Aldose_epim	147,4	5,10E-43	CL0103
TRINITY_DN1283_c0_g1_i1_3	1	17	17,989	31,62	PF00188.25	CAP	74,6	1,20E-20	CL0659

TRINITY_DN9248_c0_g1_i2_3	7	23,3	42,893	48,555	PF08712.10	Nfu_N	107,3	2,80E-31	No_clan
TRINITY_DN10710_c2_g1_i2_2	6	25,6	44,109	42,554	PF00076.21	RRM_1	67,7	5,20E-19	CL0221
TRINITY_DN5831_c1_g1_i1_5	4	20,4	24,7	25,331	PF00501.27	AMP-binding	153,6	5,40E-45	CL0378
TRINITY_DN8425_c0_g1_i1_1	3	26,4	19,418	44,632	PF03179.14	V-ATPase_G	103,7	6,60E-30	CL0255
TRINITY_DN1080_c0_g1_i1_2	1	8,3	14,245	7,1787	PF00164.24	Ribosom_S12_S23	53,7	1,30E-14	CL0021
TRINITY_DN10249_c0_g1_i1_1	3	15,6	24,737	57,919	PF01370.20	Epimerase	28,9	6,70E-07	CL0063
TRINITY_DN2941_c0_g1_i1_2	2	43,7	7,0041	15,546	PF00920.20	ILVD_EDD	101,2	5,10E-29	No_clan
TRINITY_DN17262_c0_g1_i1_3	8	26,6	50,053	58,662	PF00675.19	Peptidase_M16	122,8	1,10E-35	CL0094
TRINITY_DN6331_c0_g1_i2_6	5	12,1	51,161	47,071	PF00169.28	PH	44,1	2,20E-11	CL0266
TRINITY_DN9436_c0_g1_i2_2	2	17,2	17,812	28,142	PF02320.15	UCR_hinge	80	1,00E-22	No_clan
TRINITY_DN11363_c0_g2_i3_3	6	10,7	69,176	46,879	PF00171.21	Aldedh	475,8	1,00E-142	CL0099
TRINITY_DN16910_c0_g1_i1_4	1	14,1	9,6335	11,844	PF02265.15	S1-P1_nuclease	28,2	1,40E-06	CL0368
TRINITY_DN791_c0_g1_i1_6	6	28,2	27,33	38,23	PF03949.14	Malic_M	225,1	8,00E-67	CL0063
TRINITY_DN12144_c0_g1_i1_4	4	22	31,565	39,253	PF03141.15	Methyltransf_29	378,4	4,60E-113	CL0063
TRINITY_DN3683_c0_g2_i2_1	4	10,5	55,997	32,378	PF04107.12	GCS2	306,8	1,40E-91	CL0286
TRINITY_DN10116_c1_g2_i1_2	3	10,9	46,923	22,058	PF02812.17	ELFV_dehydrog_N	130,9	2,40E-38	CL0603
TRINITY_DN4525_c0_g1_i1_2	5	24,7	24,768	25,478	PF00210.23	Ferritin	79,9	1,60E-22	CL0044
TRINITY_DN5136_c0_g1_i1_3	8	38,4	33,051	57,871	PF08240.11	ADH_N	114,6	1,80E-33	CL0296
TRINITY_DN7885_c0_g1_i8_1	8	21,1	57,623	69,048	PF00004.28	AAA	145,9	8,40E-43	CL0023
TRINITY_DN6908_c0_g1_i1_6	5	22,1	32,743	59,761	PF13774.5	Longin	41,9	7,00E-11	No_clan
TRINITY_DN6313_c0_g1_i1_1	6	31,1	24,38	16,492	PF00071.21	Ras	177,5	1,30E-52	CL0023
TRINITY_DN8703_c0_g1_i1_3	5	13,4	51,846	40,578	PF00571.27	CBS	30,7	3,00E-07	No_clan
TRINITY_DN8590_c0_g1_i1_2	2	10,9	25,907	19,84	PF04398.11	DUF538	114,3	3,50E-33	No_clan
TRINITY_DN8759_c0_g1_i1_1	6	12,6	59,097	41,093	PF00226.30	DnaJ	80,4	7,30E-23	CL0392
TRINITY_DN6356_c0_g1_i1_3	3	21	32,979	44,981	PF00400.31	WD40	23,8	5,40E-05	CL0186
TRINITY_DN9997_c0_g1_i1_5	5	10,7	61,42	30,629	PF00343.19	Phosphorylase	557,9	3,10E-167	CL0113
TRINITY_DN10922_c0_g1_i1_2	6	16,5	43,452	12,412	PF00071.21	Ras	220,2	1,00E-65	CL0023
TRINITY_DN18757_c0_g1_i1_1	2	54,4	7,4812	15,989					
TRINITY_DN11571_c4_g3_i5_2	11	31	57,619	66,049	PF00091.24	Tubulin	226,5	3,00E-67	CL0566
TRINITY_DN7455_c0_g1_i3_2	5	17	52,903	39,456	PF00587.24	tRNA-synt_2b	133,9	5,40E-39	CL0040
TRINITY_DN7736_c0_g1_i2_1	9	19,9	63,159	59,865	PF00988.21	CPSase_sm_chain	160,9	1,10E-47	CL0364
TRINITY_DN6430_c0_g1_i2_2	6	23,8	34,174	38,831	PF03405.13	FA_desaturase_2	381,8	2,00E-114	CL0044
TRINITY_DN11913_c0_g1_i1_2	4	17,2	27,829	26,603	PF03959.12	FSH1	164,4	2,70E-48	CL0028

TRINITY_DN11663_c2_g1_i1_3	6	19,7	56,422	64,374					
TRINITY_DN5983_c0_g1_i1_2	2	11,2	23,795	18,422					
TRINITY_DN8699_c1_g1_i2_2	1	8,5	19,058	10,41					
TRINITY_DN6592_c0_g1_i1_1	4	20,5	35,511	44,564	PF06552.11	TOM20_plant	320,4	3,20E-96	CL0020
TRINITY_DN10388_c0_g2_i6_4	11	21,3	80,684	21,927	PF00082.21	Peptidase_S8	162,2	1,50E-47	No_clan
TRINITY_DN11021_c0_g1_i1_2	3	11,9	40,987	31,886	PF13452.5	MaoC_dehydrat_N	24,3	2,50E-05	CL0050
TRINITY_DN3815_c0_g1_i1_1	7	17,8	58,542	58,248	PF00009.26	GTP_EFTU	87,6	7,00E-25	CL0023
TRINITY_DN10218_c0_g1_i2_2	7	14,5	70,207	77,178	PF05450.14	Nicastrin	29,2	5,50E-07	CL0035
TRINITY_DN6921_c0_g1_i1_1	3	14,5	43,617	100,06	PF08033.11	Sec23_BS	59,4	4,20E-16	No_clan
TRINITY_DN8720_c0_g1_i1_2	2	12,7	23,735	21,118	PF01918.20	Alba	46,8	1,80E-12	CL0441
TRINITY_DN8463_c0_g2_i1_5	10	18	81,28	99,551	PF00133.21	tRNA-synt_1	58,5	3,60E-16	CL0039
TRINITY_DN9325_c0_g2_i1_5	7	24,1	47,072	69,934	PF00501.27	AMP-binding	233,3	3,60E-69	CL0378
TRINITY_DN10162_c0_g1_i1_5	4	10,7	48,202	25,327	PF00466.19	Ribosomal_L10	66,8	1,30E-18	No_clan
TRINITY_DN9738_c1_g1_i1_3	1	11,5	12,541	91,401	PF00031.20	Cystatin	42,2	6,70E-11	CL0121
TRINITY_DN10335_c0_g1_i1_1	2	28,1	10,768	17,178					
TRINITY_DN4334_c0_g1_i1_3	4	18,9	26,975	48,291	PF01253.21	SUI1	72,2	3,50E-20	No_clan
TRINITY_DN11667_c0_g1_i5_1	10	38,6	40,185	7,5964	PF00009.26	GTP_EFTU	218,3	6,20E-65	CL0023
TRINITY_DN21463_c0_g1_i1_5	3	47	9,2121	23,295	PF00626.21	Gelsolin	45,8	4,00E-12	CL0092
TRINITY_DN9495_c0_g1_i1_2	3	5,9	79,633	20,641	PF01293.19	PEPCK_ATP	710,3	9,30E-214	CL0374
TRINITY_DN8648_c0_g1_i4_3	4	11,3	52,462	34,145	PF01070.17	FMN_dh	453,9	3,10E-136	CL0036
TRINITY_DN20196_c0_g1_i1_2	2	54,7	10,121	17,994					
TRINITY_DN369_c0_g2_i1_2	3	46,6	11,016	38,867					
TRINITY_DN4975_c0_g2_i1_1	3	20,1	24,498	22,138	PF02466.18	Tim17	44	2,10E-11	No_clan
TRINITY_DN10898_c0_g1_i1_3	2	10,8	29,076	15,085	PF05753.13	TRAP_beta	92,1	2,40E-26	CL0159
TRINITY_DN19644_c0_g1_i1_6	2	17,9	16,691	15,352	PF00251.19	Glyco_hydro_32N	68,3	7,60E-19	CL0143
TRINITY_DN10905_c1_g1_i1_1	4	16,2	37,894	37,123	PF00270.28	DEAD	53	3,30E-14	CL0023
TRINITY_DN11102_c1_g1_i1_1	2	23,1	9,9412	13,297					
TRINITY_DN8311_c0_g1_i1_6	5	22,3	30,519	36,105	PF07859.12	Abhydrolase_3	139	1,80E-40	CL0028
TRINITY_DN8932_c0_g2_i2_2	8	18,2	69,952	86,138	PF08492.11	SRP72	59,9	2,20E-16	No_clan
TRINITY_DN7024_c0_g1_i1_2	3	5,4	73,834	58,468	PF01602.19	Adaptin_N	383,7	1,20E-114	CL0020
TRINITY_DN5823_c0_g1_i1_2	3	13,8	34,292	20,258					
TRINITY_DN7700_c0_g1_i1_1	2	19,1	12,253	14,182					

TRINITY_DN5993_c0_g2_i1_6	3	35,1	12,194	94,618	PF00085.19	Thioredoxin	80,8	5,30E-23	CL0172
TRINITY_DN9585_c0_g1_i1_2	8	23,6	49,72	58,297	PF00561.19	Abhydrolase_1	100,2	1,30E-28	CL0028
TRINITY_DN10250_c0_g1_i1_4	2	11,3	25,193	87,929	PF01984.19	dsDNA_bind	104,6	3,20E-30	No_clan
TRINITY_DN11946_c0_g1_i1_6	4	19,3	32,902	33,593	PF02734.16	Dak2	146,4	6,90E-43	No_clan
TRINITY_DN6080_c0_g1_i1_2	3	17,7	39,334	22,515	PF00180.19	Iso_dh	418,2	2,40E-125	CL0270
TRINITY_DN7401_c0_g1_i1_3	6	12,7	75,921	47,403	PF12899.6	Glyco_hydro_100	721,5	2,70E-217	CL0059
TRINITY_DN2864_c0_g1_i1_4	3	37,6	10,376	20,246	PF00400.31	WD40	26,6	7,10E-06	CL0186
TRINITY_DN9953_c0_g1_i1_3	3	18,1	27,897	19,29	PF01217.19	Clat_adaptor_s	77,7	7,70E-22	CL0212
TRINITY_DN4450_c0_g2_i1_2	1	6,6	16,24	6,6363	PF00658.17	PABP	75,2	3,20E-21	No_clan
TRINITY_DN18876_c0_g1_i1_5	2	19,4	13,92	12,482	PF00483.22	NTP_transferase	112,5	2,20E-32	CL0110
TRINITY_DN10281_c0_g1_i1_1	3	13	25,361	20,639	PF00828.18	Ribosomal_L27A	75,5	4,90E-21	CL0588
TRINITY_DN7840_c0_g1_i1_3	3	25,9	13,347	18,591	PF00152.19	tRNA-synt_2	78,6	3,90E-22	CL0040
TRINITY_DN8316_c0_g1_i1_3	4	16,3	34,48	14,323	PF00687.20	Ribosomal_L1	148,9	1,40E-43	No_clan
TRINITY_DN15493_c0_g1_i1_5	2	42,7	9,7981	42,958	PF00637.19	Clathrin	36,3	4,20E-09	CL0020
TRINITY_DN8497_c0_g1_i1_2	6	18	55,379	38,461	PF01546.27	Peptidase_M20	113,5	1,00E-32	CL0035
TRINITY_DN9872_c0_g1_i2_5	2	10,2	22,146	13,835	PF01990.16	ATP-synt_F	99	1,40E-28	No_clan
TRINITY_DN5492_c0_g2_i1_3	5	11,2	99,204	43,413	PF02148.18	zf-UBP	56,8	1,90E-15	CL0229
TRINITY_DN10815_c0_g2_i1_3	4	17	23,941	26,248	PF00160.20	Pro_isomerase	151,4	2,20E-44	CL0475
TRINITY_DN9629_c0_g1_i1_1	2	7,6	50,62	268,21	PF05721.12	PhyH	196,4	6,20E-58	CL0029
TRINITY_DN9337_c0_g1_i3_1	5	19,1	40,463	34,986	PF00106.24	adh_short	93,8	8,40E-27	CL0063
TRINITY_DN2350_c0_g1_i1_3	2	25	7,4624	16,325					
TRINITY_DN4704_c0_g1_i1_2	5	45,2	20,249	23,162	PF00091.24	Tubulin	201,3	1,70E-59	CL0566
TRINITY_DN10796_c0_g1_i2_5	7	23,4	43,492	56,735	PF14938.5	SNAP	376,9	6,00E-113	CL0020
TRINITY_DN9670_c0_g1_i1_4	2	8,2	28,185	16,856	PF00085.19	Thioredoxin	80,9	5,10E-23	CL0172
TRINITY_DN2761_c0_g2_i1_2	4	24,8	23,473	29,435	PF00171.21	Aldedh	268,1	1,00E-79	CL0099
TRINITY_DN7182_c0_g1_i2_2	3	29,1	16,532	18,024	PF08267.11	Meth_synt_1	138,4	3,30E-40	CL0160
TRINITY_DN11525_c0_g1_i1_2	7	19,8	41,251	18,901	PF00153.26	Mito_carr	81,3	3,20E-23	No_clan
TRINITY_DN4779_c0_g2_i1_1	2	20,5	14,021	22,062	PF04674.11	Phi_1	125,4	2,40E-36	No_clan
TRINITY_DN11487_c0_g2_i1_1	3	10,4	34,032	18,751	PF08241.11	Methyltransf_11	72,5	3,20E-20	CL0063
TRINITY_DN11353_c0_g1_i2_3	5	19,6	34,881	33,057	PF01398.20	JAB	48	1,00E-12	CL0366
TRINITY_DN4833_c0_g1_i1_1	5	10,4	48,868	24,796	PF00282.18	Pyridoxal_deC	321,1	6,90E-96	CL0061
TRINITY_DN7188_c0_g2_i3_2	2	10	27,417	12,501	PF03647.12	Tmemb_14	62,8	3,70E-17	No_clan
TRINITY_DN10558_c0_g1_i2_4	3	14,1	41,813	19,999	PF00227.25	Proteasome	158,1	1,60E-46	CL0052

TRINITY_DN10992_c0_g1_i1_2	6	8,2	81,296	43,186	PF00749.20	tRNA-synt_1c	339,1	1,70E-101	CL0039
TRINITY_DN23494_c0_g1_i1_6	2	15,3	16,663	13,881	PF00332.17	Glyco_hydro_17	30,2	2,70E-07	CL0058
TRINITY_DN6209_c0_g1_i3_2	6	19,5	39,378	29,024	PF00244.19	14-3-3	322,2	1,40E-96	No_clan
TRINITY_DN1928_c0_g1_i2_3	3	20,5	15,081	16,524	PF04969.15	CS	50,1	4,00E-13	CL0190
TRINITY_DN22312_c0_g1_i1_4	3	39,8	11,493	46,576	PF05822.11	UMPH-1	86,8	1,50E-24	CL0137
TRINITY_DN137_c0_g1_i1_1	1	13,3	8,4372	6,9985					
TRINITY_DN7231_c0_g1_i1_1	3	16,5	25,179	24,564					
TRINITY_DN8793_c0_g1_i1_4	4	17,7	26,728	11,496	PF03737.14	RraA-like	141,6	2,00E-41	CL0364
TRINITY_DN16084_c0_g1_i1_4	2	16,9	9,2024	14,519	PF02518.25	HATPase_c	35,8	8,90E-09	CL0025
TRINITY_DN10831_c0_g1_i1_3	1	5,1	21,656	7,9585	PF16845.4	SQAPI	59,8	2,40E-16	CL0121
TRINITY_DN15011_c0_g1_i1_2	5	24,1	36,704	36,96	PF00133.21	tRNA-synt_1	40,1	1,30E-10	CL0039
TRINITY_DN11284_c0_g2_i1_3	5	25,1	31,053	41,302	PF03332.12	PMM	362,2	9,50E-109	CL0137
TRINITY_DN18948_c1_g1_i1_2	1	22,4	8,1612	40,602					
TRINITY_DN11157_c0_g2_i3_3	8	19,7	67,271	54,431	PF01474.15	DAHP_synth_2	650,7	7,90E-196	CL0036
TRINITY_DN2503_c0_g2_i1_1	4	16,5	40,061	43,505	PF00076.21	RRM_1	43,1	2,50E-11	CL0221
TRINITY_DN22430_c0_g1_i1_3	3	46,7	8,0531	19,609					
TRINITY_DN9526_c1_g1_i1_2	3	16,9	26,726	28,307	PF04716.13	ETC_C1_NDUFA5	96,7	4,80E-28	No_clan
TRINITY_DN11455_c0_g1_i6_2	4	15,7	43,069	27,4	PF01992.15	vATP-synt_AC39	370,3	9,80E-111	No_clan
TRINITY_DN10158_c0_g1_i1_1	3	11,8	22,634	22,244	PF01124.17	MAPEG	69	3,20E-19	No_clan
TRINITY_DN3458_c0_g1_i1_4	2	40,4	9,9202	17,705	PF01182.19	Glucosamine_iso	82,8	2,90E-23	CL0246
TRINITY_DN11286_c0_g1_i3_2	5	13,6	53,429	30,582	PF00390.18	malic	200,2	2,20E-59	CL0603
TRINITY_DN5958_c0_g2_i1_1	1	18,3	13,281	13,026					
TRINITY_DN9297_c0_g3_i2_1	3	5,6	53,021	16,886					
TRINITY_DN5161_c0_g2_i1_2	5	17,5	45,105	48,526	PF02861.19	Clp_N	57,1	1,30E-15	No_clan
TRINITY_DN11263_c0_g1_i1_3	5	7,7	75,288	30,004	PF03463.14	eRF1_1	70	1,70E-19	No_clan
TRINITY_DN2170_c0_g1_i1_5	4	42,1	12,892	38,311	PF00637.19	Clathrin	44,4	1,40E-11	CL0020
TRINITY_DN8027_c0_g1_i1_1	3	14,7	49,503	23,369	PF00790.18	VHS	117,2	4,40E-34	CL0009
TRINITY_DN10305_c0_g1_i5_3	4	13,7	50,487	43,366	PF03647.12	Tmemb_14	41,7	1,30E-10	No_clan
TRINITY_DN7619_c0_g2_i1_3	7	26,4	40,599	48,9	PF01014.17	Uricase	64	1,60E-17	CL0334
TRINITY_DN10995_c0_g2_i4_3	5	15,1	35,702	32,939	PF16491.4	Peptidase_M48_N	27,9	1,70E-06	No_clan
TRINITY_DN10905_c0_g1_i1_4	6	31,5	29,675	37,688	PF00270.28	DEAD	56,1	3,60E-15	CL0023
TRINITY_DN10393_c0_g1_i1_2	5	14,4	38,118	192,75	PF13462.5	Thioredoxin_4	23,5	4,60E-05	CL0172
TRINITY_DN13635_c0_g1_i3_1	4	9,6	43,487	25,186	PF00152.19	tRNA-synt_2	207,2	2,90E-61	CL0040

TRINITY_DN9622_c1_g3_i1_2	1	18,5	11,778	16,766	PF05498.10	RALF	32,1	1,40E-07	No_clan
TRINITY_DN10585_c0_g2_i1_2	3	16,3	14,142	204,93					
TRINITY_DN7164_c0_g1_i2_1	6	19,4	49,582	45,378	PF00069.24	Pkinase	237,2	1,80E-70	CL0016
TRINITY_DN9538_c0_g1_i1_2	5	7,9	72,807	34,968	PF05577.11	Peptidase_S28	280,4	2,10E-83	CL0028
TRINITY_DN9261_c0_g1_i2_1	10	16,2	91,019	58,651	PF02225.21	PA	42,6	4,60E-11	CL0364
TRINITY_DN3879_c0_g1_i1_5	5	65,1	9,4597	15,046	PF01370.20	Epimerase	56,5	2,50E-15	CL0063
TRINITY_DN74_c0_g1_i1_5	2	10,1	32,792	15,575	PF00025.20	Arf	217,3	9,00E-65	CL0023
TRINITY_DN5513_c0_g1_i1_2	3	9,2	49,73	31,86	PF17284.1	Spermine_synt_N	84,2	4,00E-24	No_clan
TRINITY_DN7914_c0_g2_i3_5	2	16,9	16,334	12,753					
TRINITY_DN1457_c0_g1_i1_1	3	24	15,319	52,76	PF00076.21	RRM_1	51,7	5,20E-14	CL0221
TRINITY_DN14031_c0_g1_i1_2	7	47,8	20,095	55,786					
TRINITY_DN11004_c0_g1_i4_1	3	9,2	43,847	47,22	PF00083.23	Sugar_tr	267	2,80E-79	CL0015
TRINITY_DN239_c0_g1_i1_4	3	10,5	44,052	19,707	PF00970.23	FAD_binding_6	85,8	1,90E-24	CL0076
TRINITY_DN6479_c0_g2_i1_3	3	14,9	21,96	18,5					
TRINITY_DN9536_c0_g1_i1_3	4	7,3	67,855	24,43	PF01399.26	PCI	71,5	6,50E-20	CL0123
TRINITY_DN8870_c0_g1_i1_2	1	19,1	14,934	11,731					
TRINITY_DN13262_c0_g1_i1_3	4	19,4	39,91	32,728	PF01979.19	Amidohydro_1	147	7,90E-43	CL0034
TRINITY_DN11425_c1_g1_i2_1	4	14,9	35,908	38,021	PF03665.12	UPF0172	191,6	1,30E-56	CL0366
TRINITY_DN11659_c1_g1_i1_5	4	10	67,222	31,444	PF00171.21	Aldedh	601,1	1,00E-180	CL0099
TRINITY_DN15964_c0_g1_i1_3	1	13,7	10,199	17,019	PF00240.22	ubiquitin	72	2,30E-20	CL0072
TRINITY_DN5268_c0_g1_i1_2	1	4,6	41,102	7,2539	PF03151.15	TPT	379,7	8,70E-114	CL0184
TRINITY_DN20307_c0_g1_i1_4	2	29,5	8,5207	14,018	PF13847.5	Methyltransf_31	34,2	1,90E-08	CL0063
TRINITY_DN2136_c0_g2_i1_2	4	11,6	40,397	32,1	PF01975.16	SurE	178,6	1,00E-52	No_clan
TRINITY_DN10948_c0_g1_i6_4	7	15,7	64,679	41,979	PF01399.26	PCI	70,8	1,10E-19	CL0123
TRINITY_DN7010_c0_g2_i1_5	5	16,6	35,436	19,082	PF00297.21	Ribosomal_L3	429,2	1,20E-128	CL0575
TRINITY_DN18488_c0_g1_i1_2	2	23,8	13,976	190,19	PF06201.12	PITH	45,6	7,60E-12	CL0202
TRINITY_DN10697_c0_g1_i5_1	5	24,4	34,964	66,423	PF13561.5	adh_short_C2	195,8	7,30E-58	CL0063
TRINITY_DN8284_c0_g1_i1_2	5	17,2	41,616	184,67	PF02668.15	TauD	121,8	4,00E-35	CL0029
TRINITY_DN6841_c0_g1_i2_2	7	22	49,101	53,592	PF00218.20	IGPS	267,1	1,10E-79	CL0036
TRINITY_DN8688_c0_g2_i1_5	3	19,7	23,744	29,413					
TRINITY_DN10771_c0_g1_i1_5	5	10,8	48,193	37,725	PF13432.5	TPR_16	22,6	0,00012	CL0020
TRINITY_DN8280_c0_g2_i1_2	3	10,1	34,383	20,578	PF02230.15	Abhydrolase_2	163,5	5,50E-48	CL0028
TRINITY_DN2036_c0_g2_i1_1	2	11,3	28,397	20,232	PF07977.12	FabA	118,7	1,30E-34	CL0050

TRINITY_DN8545_c0_g2_i1_1	6	12,6	65,795	40,426	PF02881.18	SRP54_N	56,7	2,00E-15	No_clan
TRINITY_DN9929_c0_g2_i1_1	4	11,7	40,256	26,976	PF14560.5	Ubiquitin_2	96,3	9,90E-28	CL0072
TRINITY_DN16390_c0_g1_i1_1	2	36,2	8,9881	15,835	PF00400.31	WD40	20,4	0,00062	CL0186
TRINITY_DN9968_c0_g1_i3_3	10	21,4	69,584	62,655	PF13905.5	Thioredoxin_8	98,5	2,00E-28	CL0172
TRINITY_DN6363_c0_g1_i2_3	3	14,2	26,108	22,943	PF00215.23	OMPdecase	206	5,40E-61	CL0036
TRINITY_DN10584_c0_g1_i2_3	2	11,4	23,923	195,26	PF14368.5	LTP_2	54,4	1,00E-14	CL0482
TRINITY_DN10580_c0_g1_i1_3	3	17	46,112	38,85	PF02446.16	Glyco_hydro_77	137,7	4,90E-40	CL0058
TRINITY_DN320_c0_g1_i1_6	6	41,9	22,332	35,031	PF13246.5	Cation_ATPase	66,7	1,30E-18	CL0137
TRINITY_DN4506_c0_g1_i1_6	4	14,5	45,065	68,512	PF01070.17	FMN_dh	461,8	1,20E-138	CL0036
TRINITY_DN11518_c0_g3_i1_3	5	35,6	24,744	44,775					
TRINITY_DN8574_c0_g2_i2_3	4	33,3	28,495	75,242	PF07992.13	Pyr_redox_2	97,4	8,10E-28	CL0063
TRINITY_DN7917_c0_g1_i3_2	2	6,9	35,934	16,152	PF00076.21	RRM_1	59,9	1,40E-16	CL0221
TRINITY_DN11331_c0_g10_i5_1	7	44,9	20,014	20,077					
TRINITY_DN305_c1_g1_i1_2	3	22,6	19,289	24,969	PF03081.14	Exo70	160,9	4,20E-47	CL0295
TRINITY_DN10976_c0_g1_i2_2	4	9,2	59,14	26,695	PF00571.27	CBS	16,7	0,0071	No_clan
TRINITY_DN4215_c0_g2_i1_1	1	10,7	8,5989	-2					
TRINITY_DN11518_c0_g1_i3_1	5	14,6	53,531	35,687	PF00587.24	tRNA-synt_2b	36,7	3,70E-09	CL0040
TRINITY_DN8989_c0_g2_i2_3	1	5,1	20,026	6,4894					
TRINITY_DN18517_c0_g1_i1_6	2	26,9	11,46	21,792	PF00076.21	RRM_1	69,1	1,90E-19	CL0221
TRINITY_DN3918_c0_g1_i1_3	6	37,9	28,451	45,607	PF01118.23	Semialdhyde_dh	106,5	1,10E-30	CL0063
TRINITY_DN8387_c0_g1_i1_3	6	17,5	52,645	42,869	PF00251.19	Glyco_hydro_32N	128,4	3,90E-37	CL0143
TRINITY_DN8291_c1_g1_i2_1	2	9	17,923	13,448	PF03208.18	PRA1	147,7	1,50E-43	No_clan
TRINITY_DN4038_c0_g2_i1_1	6	14,6	65,25	47,808	PF01487.14	DHquinase_I	237,7	1,80E-70	CL0036
TRINITY_DN8117_c0_g1_i1_1	5	19,3	36,054	29,112	PF01412.17	ArfGap	141,4	1,10E-41	No_clan
TRINITY_DN11962_c0_g1_i1_4	2	5,7	52,434	14,486	PF00781.23	DAGK_cat	27,2	2,20E-06	CL0240
TRINITY_DN10349_c0_g1_i2_2	5	13,4	62,425	38,141	PF09440.9	eIF3_N	167,5	1,70E-49	No_clan
TRINITY_DN4294_c0_g2_i1_3	3	12,9	27,444	26,983	PF01915.21	Glyco_hydro_3_C	136,9	7,80E-40	No_clan
TRINITY_DN10817_c0_g2_i5_3	7	9,1	136,73	44,354	PF00862.18	Sucrose_synth	41,8	4,20E-11	CL0113
TRINITY_DN15272_c0_g1_i1_6	3	20,8	15,977	18,015					
TRINITY_DN2207_c0_g1_i1_5	3	17,6	18,526	18,944	PF15511.5	CENP-T_C	32,8	5,50E-08	CL0012
TRINITY_DN10776_c0_g2_i2_1	3	8,3	55,387	19,405	PF00201.17	UDPGT	108,1	4,60E-31	CL0113
TRINITY_DN9409_c0_g1_i1_2	5	25	24,459	41,181	PF14852.5	Fis1_TPR_N	43,5	1,60E-11	CL0020
TRINITY_DN4376_c0_g1_i3_3	4	8,3	63,792	34,409	PF01077.21	NIR_SIR	175,2	6,40E-52	No_clan

TRINITY_DN13346_c0_g1_i1_4	5	32,6	24,409	36,282	PF08534.9	Redoxin	85,3	3,00E-24	CL0172
TRINITY_DN19634_c0_g1_i1_3	1	8,6	13,842	6,6679	PF00179.25	UQ_con	162,8	3,30E-48	CL0208
TRINITY_DN8342_c0_g2_i2_2	2	6,3	38,118	14,936	PF05648.13	PEX11	167,7	2,40E-49	No_clan
TRINITY_DN8274_c0_g2_i2_2	4	20,5	28,854	71,179	PF00795.21	CN_hydrolase	152,7	1,10E-44	No_clan
TRINITY_DN11230_c0_g2_i11_6	5	27,7	29,99	17,531	PF03931.14	Skp1_POZ	106,4	5,50E-31	CL0033
TRINITY_DN6850_c0_g1_i1_2	4	12,4	38,139	24,187					
TRINITY_DN4513_c0_g1_i1_3	4	24	29,126	25,47	PF02359.17	CDC48_N	81,9	2,60E-23	CL0332
TRINITY_DN11374_c0_g1_i7_3	3	13,6	41,12	19,782	PF04199.12	Cyclase	76,8	1,60E-21	CL0364
TRINITY_DN11011_c0_g1_i3_3	3	17,3	30,102	24,54	PF01323.19	DSBA	118,6	2,70E-34	CL0172
TRINITY_DN8107_c0_g1_i1_3	4	30,2	20,825	31,768	PF00082.21	Peptidase_S8	34,4	1,30E-08	No_clan
TRINITY_DN9485_c0_g1_i2_4	1	2,5	44,059	6,4388					
TRINITY_DN7556_c0_g1_i1_1	6	26	31,646	48,329	PF00071.21	Ras	153,5	3,20E-45	CL0023
TRINITY_DN14011_c0_g1_i1_1	2	31,6	11,03	23,993					
TRINITY_DN8957_c0_g1_i1_2	3	9,5	37,307	17,736	PF01873.16	eIF-5_eIF-2B	125,3	9,60E-37	No_clan
TRINITY_DN11390_c1_g1_i2_2	1	15,4	12,158	6,994	PF00137.20	ATP-synt_C	39,9	3,60E-10	No_clan
TRINITY_DN17748_c0_g1_i1_5	1	14,5	7,9647	6,7341	PF13855.5	LRR_8	35	8,00E-09	CL0022
TRINITY_DN6342_c0_g1_i2_3	6	20,6	49,677	54,998	PF00155.20	Aminotran_1_2	147,2	6,30E-43	CL0061
TRINITY_DN11664_c0_g1_i2_3	6	8,1	102,77	48,367	PF04734.12	Ceramidase_alk	783,8	6,10E-236	No_clan
TRINITY_DN9943_c0_g1_i2_6	7	13,3	87,193	46,649	PF06480.14	FtsH_ext	51	1,40E-13	No_clan
TRINITY_DN13988_c0_g1_i1_6	5	17,3	40,902	34,18					
TRINITY_DN19538_c0_g1_i1_4	2	10,1	27,482	96,441					
TRINITY_DN10766_c0_g1_i8_2	3	7,9	48,452	38,272	PF02990.15	EMP70	385,3	4,00E-115	No_clan
TRINITY_DN11385_c0_g1_i2_1	4	26,8	24,209	26,359					
TRINITY_DN10112_c0_g1_i1_1	2	10,7	30,048	15,739	PF04573.11	SPC22	171,4	1,00E-50	No_clan
TRINITY_DN7707_c0_g1_i1_4	5	31,8	25,149	46,749	PF01717.17	Meth_synt_2	361,1	5,10E-108	CL0160
TRINITY_DN15157_c0_g1_i1_5	1	27,5	8,8108	7,8433					
TRINITY_DN1898_c0_g1_i1_4	2	32,1	12,463	15,876	PF00282.18	Pyridoxal_deC	61,4	5,50E-17	CL0061
TRINITY_DN12752_c0_g1_i1_1	2	25,5	10,374	12,462	PF05879.11	RHD3	94,6	4,00E-27	CL0023
TRINITY_DN10808_c0_g1_i2_1	9	23	63,624	59,588	PF00365.19	PFK	205,5	9,80E-61	CL0240
TRINITY_DN10486_c0_g1_i1_1	6	15,3	65,24	39,758	PF01417.19	ENTH	143,4	3,30E-42	CL0009
TRINITY_DN1527_c0_g1_i1_2	2	13,9	25,396	34,92	PF16363.4	GDP_Man_Dehyd	116,6	1,40E-33	CL0063
TRINITY_DN19189_c0_g1_i1_3	4	42	15,835	15,95	PF00198.22	2-oxoacid_dh	39	5,50E-10	CL0149
TRINITY_DN8363_c0_g1_i1_2	2	7,6	36,896	12,564	PF03949.14	Malic_M	335,2	2,00E-100	CL0063

TRINITY_DN11604_c0_g1_i7_2	6	22,3	36,098	323,31	PF08241.11	Methyltransf_11	40,2	3,70E-10	CL0063
TRINITY_DN4772_c0_g1_i1_2	5	15,5	44,9	33,104	PF02817.16	E3_binding	30,5	3,10E-07	No_clan
TRINITY_DN8086_c0_g1_i1_2	4	29,4	23,707	29,132	PF13669.5	Glyoxalase_4	34,9	1,40E-08	CL0104
TRINITY_DN3945_c0_g1_i1_3	3	13,6	26,508	21,035	PF00639.20	Rotamase	80,6	1,20E-22	CL0487
TRINITY_DN9766_c0_g2_i1_3	7	22,8	42,574	25,327	PF00132.23	Hexapep	20,3	0,0003	CL0536
TRINITY_DN8049_c0_g1_i1_2	4	12,6	57,73	26,665	PF02889.15	Sec63	90,8	7,10E-26	No_clan
TRINITY_DN11285_c0_g1_i5_6	2	11,2	15,546	12,887	PF02798.19	GST_N	69,3	2,50E-19	CL0172
TRINITY_DN9993_c0_g1_i4_4	2	22,7	14,627	9,9569					
TRINITY_DN18979_c0_g1_i1_4	2	35,6	9,3973	15,396					
TRINITY_DN9241_c0_g1_i1_2	3	6,4	66,317	27,802	PF04499.14	SAPS	28,4	6,60E-07	No_clan
TRINITY_DN17053_c0_g1_i1_3	2	25,8	9,9203	14,439					
TRINITY_DN8570_c0_g1_i2_1	3	11,9	33,182	21,408	PF02230.15	Abhydrolase_2	137,1	6,90E-40	CL0028
TRINITY_DN11109_c0_g2_i3_1	7	11,2	95,888	30,362	PF02990.15	EMP70	596,9	3,00E-179	No_clan
TRINITY_DN23174_c0_g1_i1_1	1	13,5	9,5238	6,496					
TRINITY_DN11127_c0_g1_i1_3	2	5,2	56,177	15,785	PF00085.19	Thioredoxin	89,4	1,10E-25	CL0172
TRINITY_DN3682_c0_g1_i1_3	1	4,2	28,275	7,1967	PF00885.18	DMRL_synthase	167,5	1,40E-49	No_clan
TRINITY_DN11049_c0_g1_i2_6	4	6,2	65,936	23,734	PF01979.19	Amidohydro_1	98,7	3,80E-28	CL0034
TRINITY_DN4610_c0_g1_i1_3	4	26,4	23,102	24,03					
TRINITY_DN3669_c0_g2_i1_1	3	27	13,142	6,3399	PF00153.26	Mito_carr	52,8	2,60E-14	No_clan
TRINITY_DN374_c0_g2_i1_2	4	19,8	21,183	27,397	PF00224.20	PK	133	1,10E-38	CL0151
TRINITY_DN1993_c0_g1_i1_1	3	35,2	10,036	22,231					
TRINITY_DN11635_c2_g1_i3_2	8	24	41,57	12,807	PF00248.20	Aldo_ket_red	241,6	9,10E-72	No_clan
TRINITY_DN5082_c0_g1_i1_6	5	33,5	18,915	62,155	PF00085.19	Thioredoxin	30,1	3,30E-07	CL0172
TRINITY_DN8123_c0_g1_i1_1	3	16,2	28,701	60,245	PF04398.11	DUF538	92,5	2,00E-26	No_clan
TRINITY_DN10102_c0_g1_i2_4	4	12,3	50,255	31,306	PF01363.20	FYVE	51	1,10E-13	CL0390
TRINITY_DN15522_c0_g1_i1_1	2	30,9	8,8301	16,285	PF00076.21	RRM_1	50,5	1,20E-13	CL0221
TRINITY_DN5853_c0_g1_i2_1	4	13,4	60,838	49,359	PF06552.11	TOM20_plant	25,9	6,70E-06	CL0020
TRINITY_DN4080_c1_g1_i1_3	5	26,3	33,169	39,15	PF05633.10	BPS1	399	2,00E-119	CL0133
TRINITY_DN2512_c0_g1_i1_1	3	16,5	22,125	18,507	PF00206.19	Lyase_1	111,2	5,70E-32	No_clan
TRINITY_DN8982_c0_g1_i3_1	3	11	39,391	27,611	PF00156.26	Pribosyltran	36	4,20E-09	CL0533
TRINITY_DN5937_c0_g1_i1_6	4	8,9	82,85	40,288	PF13620.5	CarboxypepD_reg	35,5	8,40E-09	CL0287
TRINITY_DN10930_c0_g2_i2_1	3	12,8	30,108	22,123	PF05042.12	Caleosin	266,1	1,20E-79	CL0220
TRINITY_DN13030_c0_g1_i1_6	2	11,3	24,678	13,631					

TRINITY_DN5505_c0_g1_i1_3	6	10,9	70,215	47,059	PF01344.24	Kelch_1	40,8	1,10E-10	CL0186
TRINITY_DN11234_c0_g1_i2_1	2	10,4	22,256	7,7385	PF01929.16	Ribosomal_L14e	89,8	1,10E-25	CL0107
TRINITY_DN3811_c0_g2_i1_6	3	19,2	21,527	106,71	PF01491.15	Frataxin_Cyay	121,2	1,70E-35	No_clan
TRINITY_DN12438_c0_g1_i2_2	1	3,9	26,272	6,5225	PF04573.11	SPC22	180,6	1,60E-53	No_clan
TRINITY_DN21231_c0_g1_i1_4	2	16,8	15,027	17,742					
TRINITY_DN22880_c0_g1_i1_1	1	26,3	10,784	11,759	PF01643.16	Acyl-ACP_TE	48,1	8,30E-13	CL0050
TRINITY_DN209_c0_g1_i1_6	1	5,3	34,272	-2	PF10184.8	DUF2358	129,4	6,30E-38	CL0051
TRINITY_DN11070_c0_g1_i6_2	4	8,3	71,849	33,196	PF05602.11	CLPTM1	507,4	3,20E-152	No_clan
TRINITY_DN6751_c0_g1_i1_5	1	12,7	10,779	12,741					
TRINITY_DN9669_c0_g1_i1_5	2	21,5	16,795	14,393					
TRINITY_DN6123_c0_g1_i1_4	5	9,4	74,385	30,464	PF00069.24	Pkinase	244	1,50E-72	CL0016
TRINITY_DN9646_c0_g1_i1_2	6	10,7	82,229	52,492	PF07774.12	DUF1620	224,6	9,90E-67	No_clan
TRINITY_DN3063_c0_g2_i1_5	3	8,2	51,022	20,89	PF08241.11	Methyltransf_11	70,4	1,40E-19	CL0063
TRINITY_DN8427_c0_g1_i1_3	1	7	23,186	8,0316	PF00230.19	MIP	183,8	3,60E-54	No_clan
TRINITY_DN10246_c1_g1_i1_3	2	24,4	28,849	80,208	PF00111.26	Fer2	64,3	7,10E-18	CL0486
TRINITY_DN9816_c0_g1_i2_1	3	15,1	30,721	24,07	PF00227.25	Proteasome	145,7	1,10E-42	CL0052
TRINITY_DN6842_c0_g1_i1_5	3	52,2	9,9092	20,387	PF00248.20	Aldo_ket_red	45,4	5,20E-12	No_clan
TRINITY_DN4755_c0_g2_i1_6	4	26,9	14,823	24,147	PF06212.11	GRIM-19	73,7	1,20E-20	No_clan
TRINITY_DN8066_c0_g1_i1_3	7	35,2	36,486	18,097	PF00091.24	Tubulin	235,6	5,10E-70	CL0566
TRINITY_DN7303_c0_g1_i1_3	2	8,6	20,844	11,644					
TRINITY_DN9503_c0_g1_i1_2	5	19,2	46,367	35,274	PF07714.16	Pkinase_Tyr	150,9	3,50E-44	CL0016
TRINITY_DN4985_c0_g1_i1_3	2	14,7	24,478	15,35	PF01641.17	SelR	128,3	1,40E-37	CL0080
TRINITY_DN11329_c0_g1_i2_1	2	10,6	35,194	14,314	PF02771.15	Acyl-CoA_dh_N	85,4	4,00E-24	CL0544
TRINITY_DN4045_c0_g1_i1_1	4	17,5	35,773	15,103	PF01294.17	Ribosomal_L13e	257,9	4,60E-77	No_clan
TRINITY_DN11005_c0_g1_i2_2	6	16,7	60,868	43,177	PF02854.18	MIF4G	104	7,90E-30	CL0020
TRINITY_DN6033_c0_g1_i1_3	4	20,5	33,846	29,922	PF08543.11	Phos_pyr_kin	53,3	2,20E-14	CL0118
TRINITY_DN10177_c0_g1_i1_1	3	16,1	24,583	20,957					
TRINITY_DN2451_c0_g1_i1_2	7	22,8	50,397	45,591	PF00326.20	Peptidase_S9	234,8	6,60E-70	CL0028
TRINITY_DN2230_c0_g2_i1_1	6	16,5	51,691	42,529	PF01634.17	HisG	124	4,90E-36	CL0177
TRINITY_DN6871_c0_g1_i1_3	3	13,7	22,789	11,352	PF04718.14	ATP-synt_G	79,5	2,50E-22	No_clan
TRINITY_DN12604_c0_g1_i1_3	8	15,7	73,874	56,039	PF00686.18	CBM_20	78	3,40E-22	CL0369
TRINITY_DN11543_c0_g1_i1_3	1	1	82,212	-2					
TRINITY_DN8511_c0_g2_i1_6	4	14,1	40,919	30,473	PF01268.18	FTHFS	467,7	3,50E-140	CL0023

TRINITY_DN7492_c0_g1_i1_6	1	5,6	22,062	6,6914	PF01249.17	Ribosomal_S21e	121,2	1,30E-35	No_clan
TRINITY_DN10983_c0_g1_i3_5	3	10,6	50,183	18,401					
TRINITY_DN20990_c0_g1_i1_1	1	12	11,571	6,6114					
TRINITY_DN7067_c0_g1_i1_3	4	22	43,155	36,887	PF00106.24	adh_short	93,7	8,80E-27	CL0063
TRINITY_DN10272_c0_g1_i2_4	1	8,9	14,523	8,0681					
TRINITY_DN7028_c0_g1_i1_3	4	6,3	78,284	25,536	PF00933.20	Glyco_hydro_3	234,5	1,80E-69	CL0058
TRINITY_DN9822_c0_g1_i2_3	6	24	25,853	35,594	PF01145.24	Band_7	93	2,00E-26	CL0433
TRINITY_DN10973_c0_g1_i2_3	4	15,3	41,856	27,972	PF08355.11	EF_assoc_1	63,2	1,20E-17	No_clan
TRINITY_DN8002_c0_g1_i1_2	3	8,3	39,129	27,107	PF02338.18	OTU	116,6	9,30E-34	CL0125
TRINITY_DN10262_c0_g2_i1_1	1	3,6	42,066	9,7138	PF00574.22	CLP_protease	266,1	1,40E-79	CL0127
TRINITY_DN5679_c0_g1_i1_2	4	9,3	61,322	40,314					
TRINITY_DN728_c0_g1_i2_3	3	23,3	17,462	27,782					
TRINITY_DN11853_c0_g1_i1_1	5	20,9	28,269	28,743					
TRINITY_DN11868_c0_g1_i1_5	4	16,6	35,753	27,817	PF02786.16	CPSase_L_D2	119	1,80E-34	CL0179
TRINITY_DN10119_c0_g1_i1_1	5	7,5	90,673	52,999					
TRINITY_DN2584_c0_g2_i1_3	1	8,6	16,893	11,936	PF02672.14	CP12	89,6	1,50E-25	No_clan
TRINITY_DN9619_c0_g2_i2_2	4	10,6	51,883	26,857	PF01233.18	NMT	261,2	2,80E-78	CL0257
TRINITY_DN10243_c0_g1_i1_3	4	12,8	52,65	30,242	PF01150.16	GDA1_CD39	348,9	3,10E-104	CL0108
TRINITY_DN17816_c0_g1_i1_6	1	6,1	16,23	-2					
TRINITY_DN23502_c0_g1_i1_3	1	20	8,8499	8,0362					
TRINITY_DN4242_c0_g1_i3_4	3	46,1	12,215	242,6					
TRINITY_DN11128_c0_g1_i1_3	6	25,5	44,673	43,099	PF08240.11	ADH_N	33,6	2,50E-08	CL0296
TRINITY_DN6438_c0_g3_i1_3	5	22,3	31,573	46,963	PF13561.5	adh_short_C2	209,7	4,20E-62	CL0063
TRINITY_DN8591_c0_g1_i2_5	5	11,2	55,269	34,198	PF01633.19	Choline_kinase	238,9	4,10E-71	CL0016
TRINITY_DN13303_c0_g1_i1_6	3	21,6	14,142	12,847	PF00118.23	Cpn60_TCP1	71,4	5,40E-20	No_clan
TRINITY_DN11162_c0_g1_i3_4	5	21,7	32,722	37,309	PF13774.5	Longin	86,6	8,00E-25	No_clan
TRINITY_DN17711_c0_g1_i1_5	4	33,5	17,773	24,332					
TRINITY_DN7181_c0_g1_i1_6	6	22,7	28,807	13,15	PF00189.19	Ribosomal_S3_C	86,9	9,40E-25	No_clan
TRINITY_DN8712_c0_g1_i1_2	5	18,7	43,043	36,175	PF01678.18	DAP_epimerase	124,7	2,10E-36	CL0288
TRINITY_DN11271_c1_g2_i1_1	7	69,1	16,588	179,98	PF00240.22	ubiquitin	95,2	1,30E-27	CL0072
TRINITY_DN10739_c0_g1_i1_3	9	15,2	78,177	24,576	PF00012.19	HSP70	886	9,30E-267	CL0108
TRINITY_DN11930_c0_g1_i1_6	2	35,4	9,045	16,399					
TRINITY_DN19914_c0_g1_i1_2	5	17,1	37,313	36,76	PF00709.20	Adenylsucc_synt	351	8,30E-105	CL0023

TRINITY_DN9037_c0_g1_i2_3	5	12,4	72,571	44,321	PF13850.5	ERGIC_N	83,1	1,20E-23	No_clan
TRINITY_DN9789_c0_g1_i1_2	2	2,8	66,749	12,512	PF05631.13	MFS_5	634,4	3,80E-191	CL0015
TRINITY_DN11534_c1_g1_i2_2	3	6,5	63,617	21,463	PF10508.8	Proteasom_PSMB	55,1	4,20E-15	CL0020
TRINITY_DN5090_c0_g1_i1_1	3	44,5	16,959	24,329	PF07983.12	X8	86,6	1,20E-24	No_clan
TRINITY_DN11564_c1_g1_i1_3	4	8,3	58,971	27,231	PF03690.12	UPF0160	444,1	3,40E-133	No_clan
TRINITY_DN13530_c0_g1_i1_2	3	40,6	11,462	37,718	PF00122.19	E1-E2_ATPase	76,8	1,40E-21	No_clan
TRINITY_DN11513_c0_g1_i18_2	3	12	25,298	46,527	PF00043.24	GST_C	30,4	3,10E-07	CL0497
TRINITY_DN9031_c0_g2_i2_1	2	6,1	39,862	16,177	PF03127.13	GAT	54,5	9,90E-15	No_clan
TRINITY_DN9718_c0_g1_i1_1	4	17,9	43,149	100,07	PF02893.19	GRAM	97,9	2,80E-28	CL0266
TRINITY_DN7063_c0_g1_i1_2	9	25	61,96	62,843	PF02696.13	UPF0061	438,9	2,00E-131	No_clan
TRINITY_DN22484_c0_g1_i1_1	3	44,9	8,4181	20,009					
TRINITY_DN10475_c1_g1_i1_4	5	19,7	31,813	23,631	PF00071.21	Ras	204,6	6,10E-61	CL0023
TRINITY_DN11134_c0_g1_i2_1	5	10,7	61,388	44,24	PF02786.16	CPSase_L_D2	128,4	2,30E-37	CL0179
TRINITY_DN7509_c0_g1_i1_1	1	4,4	30,317	7,1393	PF06814.12	Lung_7-TM_R	84,6	6,60E-24	CL0192
TRINITY_DN15062_c0_g1_i1_1	4	31,6	17,621	16,413	PF00733.20	Asn_synthase	38,4	1,10E-09	CL0039
TRINITY_DN22190_c0_g1_i1_5	2	24,1	9,0371	47,233					
TRINITY_DN8143_c0_g1_i3_2	4	6,7	102,17	27,129	PF01636.22	APH	155,6	1,80E-45	CL0016
TRINITY_DN8488_c0_g1_i1_3	5	10,9	65,374	38,239	PF02878.15	PGM_PMM_I	25,6	7,70E-06	No_clan
TRINITY_DN7081_c0_g2_i1_1	3	25,2	17,41	18,439	PF02775.20	TPP_enzyme_C	38,7	7,70E-10	CL0254
TRINITY_DN5728_c0_g1_i1_3	4	10,7	57,653	26,111					
TRINITY_DN10017_c0_g1_i1_5	5	13,2	43,471	35,88	PF00300.21	His_Phos_1	25,8	7,00E-06	CL0071
TRINITY_DN10253_c0_g1_i2_6	5	15,4	58,015	30,65	PF00155.20	Aminotran_1_2	221,9	1,30E-65	CL0061
TRINITY_DN17872_c0_g1_i1_2	2	20,8	12,261	13,116	PF00171.21	Aldedh	178,3	1,90E-52	CL0099
TRINITY_DN20473_c0_g1_i1_1	2	25	10,345	12,612	PF04718.14	ATP-synt_G	33,8	4,20E-08	No_clan
TRINITY_DN5188_c0_g1_i2_1	4	10,2	68,203	26,43	PF08623.9	TIP120	193,2	2,40E-57	CL0020
TRINITY_DN3281_c0_g1_i1_6	2	13,3	16,214	12,921					
TRINITY_DN12704_c0_g1_i1_1	4	15,1	47,139	30,778	PF02358.15	Trehalose_PPase	236,9	1,50E-70	CL0137
TRINITY_DN11185_c0_g1_i1_1	4	10,5	64,81	29,25	PF09335.10	SNARE_assoc	33,2	5,50E-08	No_clan
TRINITY_DN10615_c1_g1_i1_1	1	3,3	40,053	11,372	PF02815.18	MIR	49,5	4,10E-13	CL0066
TRINITY_DN63_c0_g1_i1_3	5	31,6	23,874	34,573	PF01918.20	Alba	67,4	6,90E-19	CL0441
TRINITY_DN6258_c0_g1_i2_6	1	7	24,51	16,208					
TRINITY_DN6195_c0_g2_i1_2	2	5,3	26,511	11,426	PF01918.20	Alba	50	1,80E-13	CL0441
TRINITY_DN6481_c0_g1_i1_1	1	5,9	25,617	7,7879	PF00160.20	Pro_isomerase	172,4	7,70E-51	CL0475

TRINITY_DN14793_c0_g1_i1_1	6	26,1	42,877	48,454	PF09334.10	tRNA-synt_1g	437,1	4,60E-131	CL0039
TRINITY_DN4384_c0_g1_i1_6	1	9,9	12,515	145,93	PF02298.16	Cu_bind_like	65,3	3,50E-18	CL0026
TRINITY_DN9364_c0_g1_i1_1	4	17,8	48,082	40,427	PF00226.30	DnaJ	99,1	1,10E-28	CL0392
TRINITY_DN9162_c0_g3_i1_4	2	11,7	24,418	61,573	PF01641.17	SelR	160,9	1,20E-47	CL0080
TRINITY_DN7289_c0_g1_i1_3	7	21,1	64,337	45,038	PF14829.5	GPAT_N	120,1	2,70E-35	No_clan
TRINITY_DN3061_c0_g2_i1_3	2	6,2	39,611	12,075	PF00657.21	Lipase_GDSL	50	3,30E-13	CL0264
TRINITY_DN10426_c0_g1_i1_5	6	11,8	86,76	48,142	PF02922.17	CBM_48	48,5	7,80E-13	CL0369
TRINITY_DN10788_c0_g1_i1_1	3	6,6	41,009	23,82	PF08059.12	SEP	92,3	2,00E-26	No_clan
TRINITY_DN11653_c0_g1_i11_5	12	39,2	47,827	9,7006	PF00022.18	Actin	491,2	1,70E-147	CL0108
TRINITY_DN8256_c0_g1_i1_1	2	9,5	33,173	15,421	PF03381.14	CDC50	293,9	1,20E-87	No_clan
TRINITY_DN19311_c0_g1_i1_5	5	29,7	36,928	37,276	PF02212.17	GED	95,4	1,50E-27	No_clan
TRINITY_DN11315_c0_g1_i1_2	2	5,9	40,67	11,123	PF10075.8	CSN8_PSD8_EIF3K	65,9	3,50E-18	CL0123
TRINITY_DN9466_c0_g1_i1_2	1	10,1	21,077	11,765	PF06232.10	ATS3	180,9	7,10E-54	CL0321
TRINITY_DN10194_c0_g1_i1_3	2	6,7	50,29	15,742	PF00026.22	Asp	385,3	2,20E-115	CL0129
TRINITY_DN6989_c0_g1_i1_2	4	14,7	32,607	24,196	PF02824.20	TGS	55	5,70E-15	CL0072
TRINITY_DN14960_c0_g1_i1_3	5	15,6	40,102	32	PF00180.19	Iso_dh	157,5	4,60E-46	CL0270
TRINITY_DN6781_c0_g2_i1_1	4	6,8	78,519	26,482	PF00083.23	Sugar_tr	160,6	5,40E-47	CL0015
TRINITY_DN4623_c0_g2_i2_1	4	10,7	58,244	42,233	PF00332.17	Glyco_hydro_17	276,4	3,10E-82	CL0058
TRINITY_DN10600_c0_g1_i1_5	6	11,2	88,824	44,103	PF04424.12	MINDY_DUB	124,5	2,00E-36	No_clan
TRINITY_DN1407_c0_g1_i2_4	1	7,2	19,199	11,413	PF00637.19	Clathrin	37,6	1,60E-09	CL0020
TRINITY_DN2265_c0_g1_i1_5	3	15,8	23,902	19,134					
TRINITY_DN8192_c0_g2_i2_5	4	20,3	28,632	32,004	PF00149.27	Metallophos	48,6	1,30E-12	CL0163
TRINITY_DN6164_c0_g2_i1_1	5	35,6	20,856	41,23	PF12999.6	PRKCSH-like	135,6	1,40E-39	No_clan
TRINITY_DN715_c0_g1_i1_6	2	18,4	11,287	49,852	PF10203.8	Pet191_N	67,5	8,60E-19	No_clan
TRINITY_DN18948_c0_g1_i1_2	2	39,4	11,914	19,042	PF00005.26	ABC_tran	52,6	6,10E-14	CL0023
TRINITY_DN20147_c0_g1_i1_4	4	33,9	14,009	25,582	PF00781.23	DAGK_cat	50,8	1,10E-13	CL0240
TRINITY_DN8171_c0_g1_i1_2	4	14	38,419	25,984	PF00501.27	AMP-binding	155,1	2,00E-45	CL0378
TRINITY_DN6538_c0_g1_i1_4	5	32,8	19,2	11,341	PF01717.17	Meth_synt_2	142,7	1,40E-41	CL0160
TRINITY_DN6949_c0_g1_i2_3	5	10,1	58,254	26,609	PF00294.23	PfkB	264,5	1,10E-78	CL0118
TRINITY_DN15219_c0_g1_i1_6	6	40,6	19,434	36,318	PF00183.17	HSP90	183,5	6,60E-54	No_clan
TRINITY_DN11665_c0_g1_i3_2	9	11,3	100,26	18,107	PF00122.19	E1-E2_ATPase	164	2,40E-48	No_clan
TRINITY_DN17687_c0_g1_i1_2	2	32,9	9,1632	15,101					
TRINITY_DN9097_c0_g1_i1_2	5	21,7	38,069	34,737	PF00153.26	Mito_carr	62,8	1,90E-17	No_clan

TRINITY_DN17840_c0_g1_i1_2	3	24,6	14,718	17,173	PF01031.19	Dynamin_M	50	2,20E-13	No_clan
TRINITY_DN2942_c0_g1_i1_5	1	7,7	16,064	6,5426	PF16363.4	GDP_Man_Dehyd	123,2	1,40E-35	CL0063
TRINITY_DN4861_c0_g3_i1_4	2	9,7	25,881	6,5762	PF03939.12	Ribosomal_L23eN	71,2	5,80E-20	No_clan
TRINITY_DN1788_c0_g2_i1_3	2	5,7	38,972	17,227	PF02921.13	UCR_TM	45,7	6,20E-12	CL0300
TRINITY_DN6718_c0_g1_i1_5	2	13,7	11,446	12,657					
TRINITY_DN2144_c0_g2_i1_3	4	26,3	38,463	36,223	PF02893.19	GRAM	95,4	1,70E-27	CL0266
TRINITY_DN4927_c0_g1_i1_6	1	9,5	12,774	7,2919	PF13802.5	Gal_mutarotas_2	71,2	6,40E-20	CL0103
TRINITY_DN11271_c0_g1_i1_6	7	37,9	15,708	15,851	PF00240.22	ubiquitin	103,6	3,10E-30	CL0072
TRINITY_DN8924_c0_g1_i1_1	4	13,6	50,23	29,643	PF01429.18	MBD	48,4	5,40E-13	CL0081
TRINITY_DN779_c0_g1_i1_1	4	17,6	28,833	47,557	PF01026.20	TatD_DNase	194,3	2,20E-57	CL0034
TRINITY_DN938_c0_g1_i1_5	3	20,8	22,757	117,94	PF08597.9	eIF3_subunit	135,8	2,10E-39	No_clan
TRINITY_DN9792_c1_g2_i1_6	3	14,6	28,976	20,742	PF00171.21	Aldedh	193,3	5,10E-57	CL0099
TRINITY_DN9457_c0_g1_i2_1	3	8,8	57,829	23,09	PF00995.22	Sec1	202,6	1,50E-59	No_clan
TRINITY_DN4143_c0_g1_i1_1	8	37,9	36,746	7,2876	PF00091.24	Tubulin	236,3	3,00E-70	CL0566
TRINITY_DN13762_c0_g1_i1_3	2	17,4	12,063	11,705	PF03719.14	Ribosomal_S5_C	27,9	1,10E-06	CL0329
TRINITY_DN8478_c1_g1_i1_2	3	18,5	16,162	25,179					
TRINITY_DN7608_c0_g1_i1_4	5	19	37,73	40,948	PF00789.19	UBX	72	3,20E-20	CL0072
TRINITY_DN4512_c0_g1_i1_4	1	7,4	14,921	6,7198					
TRINITY_DN9675_c0_g1_i1_1	6	17,4	41,321	25,219	PF00294.23	PfkB	275,9	3,70E-82	CL0118
TRINITY_DN7474_c0_g2_i1_2	4	19,3	32,636	44,415	PF00635.25	Motile_Sperm	94,1	4,00E-27	CL0556
TRINITY_DN10982_c0_g1_i1_1	1	2,4	40,202	6,6564	PF01070.17	FMN_dh	413,6	5,40E-124	CL0036
TRINITY_DN1335_c0_g1_i1_2	3	12,3	40,651	20,464	PF05212.11	DUF707	419,4	7,20E-126	No_clan
TRINITY_DN10530_c0_g1_i4_3	2	4,6	63,729	16,682	PF01409.19	tRNA-synt_2d	311	5,00E-93	CL0040
TRINITY_DN8417_c0_g1_i2_2	2	15,3	23,476	13,47	PF12796.6	Ank_2	40,5	2,90E-10	CL0465
TRINITY_DN11265_c0_g1_i1_3	3	8,8	37,122	17,464	PF01501.19	Glyco_transf_8	292	3,80E-87	CL0110
TRINITY_DN2984_c0_g2_i1_3	4	14,5	43,833	32,525	PF00571.27	CBS	20,5	0,00045	No_clan
TRINITY_DN1687_c0_g1_i1_2	4	12,6	39,897	14,449	PF01602.19	Adaptin_N	196,6	5,80E-58	CL0020
TRINITY_DN8065_c0_g1_i1_1	7	30	43,004	46,929	PF00928.20	Adap_comp_sub	239,7	3,30E-71	CL0448
TRINITY_DN11452_c0_g2_i2_2	7	23,6	49,764	48,479	PF14226.5	DIOX_N	62,6	5,10E-17	CL0029
TRINITY_DN6644_c0_g1_i1_2	2	15	20,059	14,125					
TRINITY_DN3807_c1_g1_i1_3	2	39,3	9,9731	21,833	PF12799.6	LRR_4	30,5	3,10E-07	CL0022
TRINITY_DN7004_c0_g2_i1_2	2	13	20,197	13,199	PF04281.12	Tom22	32,3	7,00E-08	No_clan
TRINITY_DN10332_c0_g2_i1_4	4	12,5	57,333	39,078	PF00202.20	Aminotran_3	312,3	3,70E-93	CL0061

TRINITY_DN8478_c0_g1_i1_1	3	22,1	14,867	19,478					
TRINITY_DN11043_c0_g1_i1_6	1	2,6	56,452	12,033	PF01222.16	ERG4_ERG24	288,4	7,90E-86	CL0115
TRINITY_DN6414_c0_g2_i2_1	3	17,1	32,576	20,532	PF01725.15	Ham1p_like	179,4	5,10E-53	CL0269
TRINITY_DN12528_c0_g1_i1_1	3	20,7	32,203	20,307					
TRINITY_DN2126_c0_g1_i1_4	4	30,2	17,102	13,153	PF03953.16	Tubulin_C	107,6	4,50E-31	CL0442
TRINITY_DN8608_c1_g1_i1_4	3	13,6	25,778	19,89	PF00407.18	Bet_v_1	85,8	2,50E-24	CL0209
TRINITY_DN10518_c0_g1_i3_1	2	6,9	45,077	23,861	PF03492.14	Methyltransf_7	318,7	4,00E-95	CL0063
TRINITY_DN10154_c0_g1_i1_3	4	10,9	52,027	28,95	PF01180.20	DHO_dh	110	1,20E-31	CL0036
TRINITY_DN4990_c0_g2_i1_5	2	18,4	23,564	13,005	PF00232.17	Glyco_hydro_1	210,1	3,70E-62	CL0058
TRINITY_DN8848_c0_g1_i1_2	6	18	59,234	28,507	PF00514.22	Arm	44,2	1,20E-11	CL0020
TRINITY_DN2750_c0_g2_i1_2	2	6,2	29,994	12,62	PF13419.5	HAD_2	80,7	1,20E-22	CL0137
TRINITY_DN6078_c0_g1_i2_4	3	13	40,518	21,44					
TRINITY_DN8790_c0_g2_i2_2	1	4,4	24,229	6,4446	PF03650.12	MPC	123,4	4,00E-36	CL0141
TRINITY_DN6367_c0_g1_i1_3	2	12,3	30,76	15,023	PF01652.17	IF4E	184,1	1,40E-54	CL0625
TRINITY_DN11511_c1_g1_i1_6	1	7,1	14,377	6,3072					
TRINITY_DN6200_c0_g1_i1_6	2	21,4	19,307	17,892	PF00291.24	PALP	78,1	7,10E-22	No_clan
TRINITY_DN8259_c0_g1_i1_2	4	14,3	36,689	54,828	PF04414.11	tRNA_deacylase	229,5	2,70E-68	CL0408
TRINITY_DN6098_c0_g1_i1_2	3	7,4	50,58	17,869	PF00348.16	polyprenyl_synt	292,6	1,70E-87	CL0613
TRINITY_DN17635_c0_g1_i1_1	5	17,6	35,058	11,355	PF00330.19	Aconitase	202,1	1,50E-59	No_clan
TRINITY_DN21752_c0_g1_i1_3	2	55,7	8,8966	20,439					
TRINITY_DN11371_c0_g2_i1_1	3	7,6	55,643	20,732	PF00202.20	Aminotran_3	296,4	2,40E-88	CL0061
TRINITY_DN4480_c0_g1_i2_2	3	11,7	38,481	21,773	PF00153.26	Mito_carr	67,3	7,80E-19	No_clan
TRINITY_DN6825_c0_g1_i1_1	3	8,9	44,988	21,037	PF13561.5	adh_short_C2	161,5	2,10E-47	CL0063
TRINITY_DN15184_c0_g1_i1_6	2	14,3	16,376	12,678					
TRINITY_DN2842_c0_g1_i1_6	3	19,7	25,807	29,779	PF14226.5	DIOX_N	94,5	6,90E-27	CL0029
TRINITY_DN7658_c0_g1_i1_3	6	19,5	32,178	13,322	PF00071.21	Ras	204	9,90E-61	CL0023
TRINITY_DN6555_c0_g1_i2_5	1	5,1	29,795	8,3485	PF03151.15	TPT	273	2,80E-81	CL0184
TRINITY_DN7691_c0_g1_i2_3	3	11,7	36,879	19,012	PF02705.15	K_trans	115,2	2,80E-33	CL0062
TRINITY_DN11075_c0_g1_i1_1	5	7	73,32	6,2723	PF00076.21	RRM_1	55,5	3,50E-15	CL0221
TRINITY_DN8971_c0_g1_i1_4	1	24	10,895	27,206	PF16114.4	Citrate_bind	69	3,30E-19	CL0506
TRINITY_DN11579_c0_g1_i2_1	2	17,3	24,33	19,877	PF01592.15	NifU_N	183,5	1,40E-54	CL0233
TRINITY_DN3015_c0_g1_i1_1	4	15,3	40,462	30,78	PF01031.19	Dynamin_M	202,4	8,10E-60	No_clan
TRINITY_DN1893_c0_g1_i1_2	1	25,7	8,2732	20,251					

TRINITY_DN51_c0_g1_i1_2	3	19,8	20,1	22,69	PF00180.19	Iso_dh	153,4	8,10E-45	CL0270
TRINITY_DN9907_c0_g2_i2_3	2	4,7	44,196	11,691	PF13640.5	2OG-FeII_Oxy_3	73	2,70E-20	CL0029
TRINITY_DN12604_c0_g1_i1_2	3	5,3	73,357	18,231	PF02446.16	Glyco_hydro_77	238,5	1,30E-70	CL0058
TRINITY_DN16223_c0_g1_i1_1	2	42,7	8,1734	11,531	PF13246.5	Cation_ATPase	41,6	9,30E-11	CL0137
TRINITY_DN1060_c0_g1_i1_1	3	11,5	23,784	19,979	PF00226.30	DnaJ	37,5	1,80E-09	CL0392
TRINITY_DN10488_c0_g1_i1_2	5	14,7	45,762	37,396	PF04845.12	PurA	72,2	3,50E-20	CL0609
TRINITY_DN9024_c1_g1_i2_5	4	14,9	32,892	24,12	PF03937.15	Sdh5	74	6,50E-21	No_clan
TRINITY_DN10216_c0_g1_i1_3	2	13,5	23,737	15,187	PF00378.19	ECH_1	79,2	2,80E-22	CL0127
TRINITY_DN4751_c0_g1_i1_3	3	14,4	37,806	35,205	PF13266.5	DUF4057	515,7	4,60E-155	No_clan
TRINITY_DN3627_c0_g2_i1_4	4	12,7	53,928	26,616	PF04137.14	ERO1	418,3	2,30E-125	No_clan
TRINITY_DN5091_c0_g1_i1_4	5	16,7	43,759	31,733	PF05701.10	WEMBL	399,4	2,40E-119	No_clan
TRINITY_DN10763_c0_g1_i4_1	1	14,9	9,4027	6,5876					
TRINITY_DN11635_c2_g1_i6_2	8	20,9	45,031	20,759	PF00248.20	Aldo_ket_red	240	2,70E-71	No_clan
TRINITY_DN19987_c0_g1_i1_3	2	19,5	9,9091	7,0101	PF00244.19	14-3-3	85,6	3,00E-24	No_clan
TRINITY_DN10849_c0_g1_i2_5	1	8,1	21,818	6,613					
TRINITY_DN9489_c0_g2_i1_2	2	9,9	39,259	19,26	PF00112.22	Peptidase_C1	236,7	3,00E-70	CL0125
TRINITY_DN4080_c0_g1_i1_3	3	20,6	18,864	21,561	PF05633.10	BPS1	129,3	1,70E-37	CL0133
TRINITY_DN6613_c0_g1_i1_4	3	15,2	35,69	33,685	PF00293.27	NUDIX	81	6,90E-23	CL0261
TRINITY_DN8186_c0_g1_i3_1	7	23,8	41,598	27,996	PF00224.20	PK	241,3	1,30E-71	CL0151
TRINITY_DN11291_c0_g1_i2_3	3	13,8	33,549	23,587	PF02737.17	3HCDH_N	117,4	6,10E-34	CL0063
TRINITY_DN11520_c0_g1_i1_2	3	7,5	66,385	19,016	PF00328.21	His_Phos_2	169,9	9,60E-50	CL0071
TRINITY_DN8996_c0_g1_i1_4	1	5,8	31,704	10,048	PF00179.25	UQ_con	175	5,80E-52	CL0208
TRINITY_DN10798_c1_g1_i1_2	2	10,6	18,402	11,337	PF02516.13	STT3	74,7	6,30E-21	CL0111
TRINITY_DN4765_c0_g3_i1_1	5	20,2	39,632	32,664	PF00069.24	Pkinase	92,9	2,00E-26	CL0016
TRINITY_DN8594_c0_g1_i1_1	3	47,5	10,898	25,199					
TRINITY_DN10973_c0_g3_i2_1	4	13,3	30,982	31,179	PF00071.21	Ras	31,3	1,20E-07	CL0023
TRINITY_DN17195_c0_g1_i1_1	2	13,7	18,368	13,043	PF00168.29	C2	63,1	2,20E-17	CL0154
TRINITY_DN7057_c1_g1_i1_2	3	7,7	52,58	19,34	PF07859.12	Abhydrolase_3	58,4	8,20E-16	CL0028
TRINITY_DN23079_c0_g1_i1_4	2	37,7	8,2407	10,978	PF00702.25	Hydrolase	33,1	6,20E-08	CL0137
TRINITY_DN1992_c0_g1_i1_1	2	18,8	15,213	7,6057					
TRINITY_DN13881_c0_g1_i1_1	4	20,6	35,299	28,962	PF04321.16	RmlD_sub_bind	112,2	2,20E-32	CL0063
TRINITY_DN14656_c0_g1_i1_2	3	52,7	8,2872	20,016					
TRINITY_DN3861_c0_g1_i2_3	4	21,4	27,902	25,96	PF07944.11	Glyco_hydro_127	29,4	2,70E-07	CL0059

TRINITY_DN8241_c0_g1_i1_2	2	17,1	31,795	13,082	PF00274.18	Glycolytic	446	6,50E-134	CL0035
TRINITY_DN17884_c0_g1_i1_4	2	40	7,6419	38,583	PF00133.21	tRNA-synt_1	81,6	3,60E-23	CL0039
TRINITY_DN7822_c0_g1_i3_2	1	4,7	47,56	27,227	PF04144.12	SCAMP	182,8	6,30E-54	No_clan
TRINITY_DN11037_c0_g1_i1_3	1	4,3	43,876	9,3249	PF00364.21	Biotin_lipoyl	30,7	1,90E-07	CL0105
TRINITY_DN5712_c0_g2_i2_1	3	14,2	43,452	25,754	PF00501.27	AMP-binding	166	9,30E-49	CL0378
TRINITY_DN22171_c0_g1_i1_5	3	38,9	13,833	19,694	PF08502.9	LeuA_dimer	45	8,70E-12	No_clan
TRINITY_DN12955_c0_g1_i1_1	1	21,4	9,4475	7,8602					
TRINITY_DN10631_c0_g1_i1_4	2	6,3	51,659	13,982	PF07851.12	TMPIT	335,3	3,50E-100	No_clan
TRINITY_DN6543_c0_g1_i1_4	3	17,6	22,099	19,564					
TRINITY_DN10109_c0_g1_i3_2	1	5,2	25,188	7,1508					
TRINITY_DN11659_c0_g1_i1_1	1	7,4	17,869	7,0109	PF00171.21	Aldedh	53,6	1,20E-14	CL0099
TRINITY_DN21796_c0_g1_i1_1	2	24	15,875	16,104	PF00271.30	Helicase_C	57,7	1,20E-15	CL0023
TRINITY_DN10608_c0_g1_i4_3	2	8,8	31,289	15,417	PF02826.18	2-Hacid_dh_C	178,7	6,20E-53	CL0063
TRINITY_DN8882_c0_g1_i3_2	3	10	53,397	28,457	PF00917.25	MATH	33,9	2,60E-08	CL0389
TRINITY_DN8417_c1_g1_i1_6	2	34,3	7,4932	13,372					
TRINITY_DN285_c0_g1_i1_6	2	9	34,606	13,939	PF16752.4	TBCC_N	92,9	1,70E-26	No_clan
TRINITY_DN8647_c0_g1_i1_4	1	5,9	19,852	6,6033					
TRINITY_DN1686_c0_g1_i1_2	1	8,6	13,003	6,5766	PF02297.16	COX6B	61	8,90E-17	CL0351
TRINITY_DN16666_c0_g1_i1_3	1	22,3	10,158	10,533					
TRINITY_DN8134_c0_g1_i1_2	1	5,6	21,533	6,5533	PF08768.10	DUF1794	114,8	3,10E-33	CL0116
TRINITY_DN1558_c0_g1_i1_3	4	33,1	17,65	43,975	PF01504.17	PIP5K	75,2	4,40E-21	CL0016
TRINITY_DN7263_c0_g1_i1_2	2	10,7	21,835	12,691	PF13418.5	Kelch_4	33,6	2,50E-08	CL0186
TRINITY_DN9028_c0_g1_i1_3	2	4,7	71,209	17,59	PF13639.5	zf-RING_2	42	8,20E-11	CL0229
TRINITY_DN10191_c0_g1_i1_3	1	11,3	15,324	16,775					
TRINITY_DN9202_c0_g1_i1_5	1	5,7	19,278	6,3482	PF08122.11	NDUF_B12	28,5	1,10E-06	No_clan
TRINITY_DN3322_c0_g2_i1_2	3	14,6	45,159	29,507	PF01960.17	ArgJ	465,5	9,70E-140	CL0635
TRINITY_DN661_c0_g1_i1_1	2	13,4	30,647	15,293	PF00977.20	His_biosynth	120,1	8,70E-35	CL0036
TRINITY_DN284_c0_g2_i1_5	2	27,2	15,668	23,09	PF01300.17	Sua5_yciO_yrdC	82,2	2,90E-23	No_clan
TRINITY_DN804_c0_g1_i1_1	2	18,8	16,898	23,44					
TRINITY_DN14006_c0_g1_i1_3	2	26,1	12,023	11,805	PF00109.25	ketoacyl-synt	73	2,60E-20	CL0046
TRINITY_DN21069_c0_g1_i1_4	3	21	23,615	24,043	PF07944.11	Glyco_hydro_127	135,3	2,20E-39	CL0059
TRINITY_DN9015_c0_g1_i1_4	4	25,8	19,994	38,35	PF09066.9	B2-adapt-app_C	110,7	3,60E-32	CL0545
TRINITY_DN10966_c0_g1_i1_3	4	25,4	26,349	32,587	PF03483.16	B3_4	36	4,80E-09	CL0383

TRINITY_DN3062_c0_g1_i1_3	4	13,5	34,617	27,824	PF03071.14	GNT-I	375,9	2,20E-112	CL0110	
TRINITY_DN15393_c0_g1_i1_5	1	18,1	13,277	9,556	PF06045.10	Rhamnogal_lyase	92,5	2,30E-26	CL0103	
TRINITY_DN7397_c0_g1_i2_1	2	13,4	40,363	13,394	PF09439.9	SRPRB	121	3,40E-35	CL0023	
TRINITY_DN843_c0_g1_i1_5	5	13,4	48,826	36,227	PF13432.5	TPR_16	15,5	0,018	CL0020	
TRINITY_DN13608_c0_g1_i1_6	2	27,8	11,803	24,218						
TRINITY_DN3889_c0_g3_i1_3	2	10,5	15,523	11,781	PF06201.12	PITH	88,2	5,70E-25	CL0202	
TRINITY_DN6029_c0_g1_i1_1	4	26,9	23,055	32,503	PF00026.22	Asp	195,7	1,20E-57	CL0129	
TRINITY_DN5896_c0_g1_i2_3	2	16,5	27,114	14,813						
TRINITY_DN12487_c0_g1_i1_1	9	45,9	24,278	40,464	PF13246.5	Cation_ATPase	27,2	2,90E-06	CL0137	
TRINITY_DN21101_c0_g1_i1_4	2	26,9	11,59	27,297	PF04321.16	RmlD_sub_bind	37,9	9,50E-10	CL0063	
TRINITY_DN20395_c0_g2_i1_2	2	29,4	17,522	26,757						
TRINITY_DN11574_c0_g1_i5_3	4	7,4	64,601	27,689	PF01565.22	FAD_binding_4	91,5	3,50E-26	CL0077	
TRINITY_DN16156_c0_g1_i1_2	1	20	8,4896	46,803						
TRINITY_DN10717_c1_g1_i3_1	1	1,8	58,615	6,7271	PF02136.19	NTF2	109	2,00E-31	CL0051	
TRINITY_DN11531_c0_g1_i3_3	4	7,1	65,942	25,545	PF00632.24	HECT	304,5	8,50E-91	CL0552	
TRINITY_DN7911_c0_g1_i3_1	3	12,6	27,513	130,34	PF00085.19	Thioredoxin	103,6	4,20E-30	CL0172	
TRINITY_DN5389_c0_g1_i1_5	1	4,6	29,851	9,5259	PF14533.5	USP7_C2	127,3	6,00E-37	CL0072	
TRINITY_DN7964_c0_g4_i1_6	3	21,6	21,069	21,942	PF07714.16	Pkinase_Tyr	94	8,50E-27	CL0016	
TRINITY_DN12169_c0_g1_i1_2	2	9,7	18,392	15,779	PF00076.21	RRM_1	46,1	3,00E-12	CL0221	
TRINITY_DN23339_c0_g1_i1_1	1	16,1	10,432	7,5318	PF01274.21	Malate_synthase	34,5	6,30E-09	CL0151	
TRINITY_DN9250_c0_g1_i1_3	2	2,8	49,771	13,008	PF00226.30	DnaJ	94,6	2,60E-27	CL0392	
TRINITY_DN18399_c0_g1_i1_1	2	18,9	13,866	12,597	PF13246.5	Cation_ATPase	29,3	6,10E-07	CL0137	
TRINITY_DN9486_c0_g1_i1_2	3	9	55,221	68,912	PF00076.21	RRM_1	49,9	1,90E-13	CL0221	
TRINITY_DN5674_c0_g1_i1_1	4	12,3	60,184	30,2	PF01595.19	DUF21	129,1	1,30E-37	No_clan	
TRINITY_DN6255_c0_g3_i1_1	3	22,1	27,131	28,533	PF00560.32	LRR_1	11,6	0,3	CL0022	
TRINITY_DN10766_c0_g1_i3_3	4	7,9	74,746	25,787	PF02990.15	EMP70	564,7	1,60E-169	No_clan	
TRINITY_DN10543_c0_g1_i1_6	2	8,6	42,28	18,451	PF00300.21	His_Phos_1	26,7	3,50E-06	CL0071	
TRINITY_DN2263_c0_g2_i1_6	1	6,3	23,174	7,7685	PF08033.11	Sec23_BS	75	5,70E-21	No_clan	
TRINITY_DN11003_c0_g2_i5_2	5	15,5	58,372	30,525	PF00282.18	Pyridoxal_deC	72,2	2,90E-20	CL0061	
TRINITY_DN9687_c1_g1_i2_2	2	4,6	36,575	11,459	PF06273.10	eIF-4B	348,6	6,90E-104	No_clan	
TRINITY_DN9737_c1_g1_i1_4	6	27,9	32,025	6,9917	PF00025.20	Arf	265,6	1,30E-79	CL0023	
TRINITY_DN11475_c0_g1_i1_2	1	3,3	56,965	-2						
TRINITY_DN6747_c0_g1_i1_2	2	6,1	38,039	11,159						

TRINITY_DN2919_c0_g1_i1_5	3	28	20,26	6,5685	PF02773.15	S-AdoMet_synt_C	210,7	6,60E-63	No_clan
TRINITY_DN9215_c0_g1_i1_2	2	6	42,04	12,969	PF03088.15	Str_synth	117,2	2,30E-34	CL0186
TRINITY_DN5123_c0_g1_i2_4	1	13,1	19,339	56,849	PF01920.19	Prefoldin_2	77,2	7,40E-22	CL0200
TRINITY_DN2276_c0_g2_i1_6	6	23,8	55,406	36,82	PF00686.18	CBM_20	64,4	6,10E-18	CL0369
TRINITY_DN8639_c0_g1_i1_6	2	8,9	30,499	17,276	PF08540.9	HMG_CoA_synt_C	263,5	2,30E-78	CL0046
TRINITY_DN6651_c0_g1_i1_3	2	4,5	62,61	14,315	PF00854.20	PTR2	236,8	3,40E-70	CL0015
TRINITY_DN8585_c0_g1_i1_4	6	22,6	61,812	44,353	PF00202.20	Aminotran_3	369,9	1,10E-110	CL0061
TRINITY_DN21541_c0_g1_i1_4	1	14,3	14,664	7,5763	PF00450.21	Peptidase_S10	187,4	5,30E-55	CL0028
TRINITY_DN8030_c0_g1_i1_2	1	5,7	21,477	7,3427					
TRINITY_DN7519_c0_g2_i1_2	6	15	52,195	21,195	PF01399.26	PCI	81,5	5,20E-23	CL0123
TRINITY_DN11415_c0_g1_i1_1	2	8,8	25,514	13,143	PF08523.9	MBF1	85,2	2,90E-24	No_clan
TRINITY_DN9353_c0_g1_i1_3	2	6,6	43,586	20,795	PF02453.16	Reticulon	200,8	1,10E-59	No_clan
TRINITY_DN5812_c0_g1_i1_1	4	15,5	34,06	25,477	PF00227.25	Proteasome	132,9	8,40E-39	CL0052
TRINITY_DN11107_c1_g1_i4_3	7	15,1	78,463	42,453					
TRINITY_DN3448_c0_g1_i1_2	1	4,2	38,104	8,6161	PF03083.15	MtN3_slv	83,2	9,10E-24	CL0141
TRINITY_DN9452_c0_g1_i1_1	2	6,7	40,567	12,629					
TRINITY_DN8582_c0_g2_i1_3	6	18,2	46,847	37,575	PF00365.19	PFK	187,1	3,80E-55	CL0240
TRINITY_DN15392_c0_g1_i1_5	2	22,5	12,468	15,167	PF01602.19	Adaptin_N	132,3	1,80E-38	CL0020
TRINITY_DN2089_c0_g1_i1_5	2	23,9	15,1	16,116					
TRINITY_DN8917_c0_g2_i1_3	7	18,2	60,036	50,779	PF00085.19	Thioredoxin	54,9	6,10E-15	CL0172
TRINITY_DN16117_c0_g1_i1_6	1	16,2	7,7507	7,3117	PF01301.18	Glyco_hydro_35	71,5	8,40E-20	CL0058
TRINITY_DN8546_c0_g1_i1_2	5	12,1	75,378	30,281	PF00501.27	AMP-binding	345,5	3,20E-103	CL0378
TRINITY_DN3962_c0_g1_i2_3	2	6,5	74,441	23,891	PF01602.19	Adaptin_N	168,9	1,50E-49	CL0020
TRINITY_DN2992_c0_g1_i1_3	4	18,6	41,239	33,399	PF03952.15	Enolase_N	120,8	3,90E-35	CL0227
TRINITY_DN11605_c1_g1_i1_5	2	15,3	21,004	11,199	PF02466.18	Tim17	49,6	3,70E-13	No_clan
TRINITY_DN401_c0_g1_i1_3	4	16,5	31,961	7,9454	PF00071.21	Ras	203,5	1,40E-60	CL0023
TRINITY_DN18414_c0_g1_i1_2	3	11,6	44,6	19,183	PF01408.21	GFO_IDH_MocA	41,4	2,00E-10	CL0063
TRINITY_DN9346_c0_g1_i2_1	4	8,3	62,924	24,012	PF01842.24	ACT	45	6,00E-12	CL0070
TRINITY_DN8618_c0_g2_i2_4	3	4,8	73,737	27,412	PF16499.4	Melibiase_2	24,2	1,50E-05	CL0058
TRINITY_DN5167_c0_g1_i1_5	2	5,2	55,923	18,781					
TRINITY_DN15469_c0_g1_i1_6	2	28	11,7	13,611	PF00350.22	Dynamin_N	76,8	1,80E-21	CL0023
TRINITY_DN19298_c0_g1_i1_1	1	14,5	13,369	15,299	PF01979.19	Amidohydro_1	62,1	5,10E-17	CL0034
TRINITY_DN11193_c0_g2_i3_2	3	13,4	39,365	20,382	PF01087.21	GalP_UDP_transf	71,5	1,10E-19	CL0265

TRINITY_DN11599_c1_g1_i4_1	3	34	11,764	19,499					
TRINITY_DN6675_c0_g1_i4_2	4	10,8	81,162	44,815	PF00270.28	DEAD	162,2	9,60E-48	CL0023
TRINITY_DN9665_c0_g1_i1_2	1	6,9	17,491	6,5689					
TRINITY_DN10222_c0_g1_i5_2	4	8	85,124	39,147	PF14438.5	SM-ATX	89,7	9,10E-26	CL0527
TRINITY_DN8244_c0_g1_i1_2	3	16,8	28,933	18,998	PF00085.19	Thioredoxin	79,3	1,60E-22	CL0172
TRINITY_DN6988_c0_g2_i1_1	3	13,3	46,133	23,121	PF00956.17	NAP	291,4	4,90E-87	No_clan
TRINITY_DN21693_c0_g1_i1_3	2	46,2	8,8806	15,708					
TRINITY_DN6721_c0_g1_i1_2	5	16,2	47,572	31,089	PF06068.12	TIP49	487,1	3,60E-146	CL0023
TRINITY_DN10523_c1_g1_i1_2	3	18	22,837	13,413	PF01776.16	Ribosomal_L22e	156	3,40E-46	No_clan
TRINITY_DN8754_c0_g1_i1_1	3	13,1	27,796	6,9356	PF00237.18	Ribosomal_L22	133,3	3,10E-39	No_clan
TRINITY_DN21226_c0_g1_i1_1	2	26	8,3075	12,179					
TRINITY_DN18056_c0_g1_i1_1	4	22,2	31,685	88,204	PF02469.21	Fasciclin	51,2	1,30E-13	No_clan
TRINITY_DN11502_c0_g1_i1_2	5	11,9	57,526	31,339	PF06911.11	Senescence	156,4	8,20E-46	No_clan
TRINITY_DN8308_c0_g2_i1_2	3	9,2	64,719	21,402	PF00112.22	Peptidase_C1	282,1	3,60E-84	CL0125
TRINITY_DN5126_c0_g1_i2_2	3	10,5	40,99	20,15	PF01875.16	Memo	278,1	5,50E-83	CL0283
TRINITY_DN7908_c0_g1_i1_3	3	13,6	42,162	21,206	PF08241.11	Methyltransf_11	22	0,00018	CL0063
TRINITY_DN10955_c0_g2_i3_1	10	40,5	32,078	26,663	PF00071.21	Ras	191,1	8,90E-57	CL0023
TRINITY_DN8800_c0_g1_i1_3	3	13,4	33,48	19,473	PF01467.25	CTP_transf_like	59,4	4,00E-16	CL0039
TRINITY_DN4365_c0_g1_i1_1	2	22,1	12,279	16,297	PF04842.11	DUF639	42	6,20E-11	No_clan
TRINITY_DN14633_c0_g1_i1_2	2	9,3	32,456	16,559	PF01380.21	SIS	114,9	2,00E-33	CL0067
TRINITY_DN3011_c0_g2_i1_3	3	27,3	20,773	21,088	PF05811.12	DUF842	122,2	1,10E-35	No_clan
TRINITY_DN2343_c0_g1_i1_1	3	21	22,689	20,315					
TRINITY_DN9033_c1_g1_i1_6	2	13,1	18,114	14,367	PF01105.23	EMP24_GP25L	72,2	4,80E-20	CL0521
TRINITY_DN2502_c0_g2_i1_2	2	12,7	25,256	12,723	PF02990.15	EMP70	178,4	2,10E-52	No_clan
TRINITY_DN7907_c0_g1_i4_4	1	7,4	23,097	12,015					
TRINITY_DN642_c0_g1_i1_2	4	13,8	46,24	27,443	PF01379.19	Porphobil_deam	244,2	8,20E-73	CL0177
TRINITY_DN701_c0_g2_i1_6	4	8,3	56,051	27,613	PF00403.25	HMA	42,6	5,70E-11	No_clan
TRINITY_DN6896_c0_g1_i1_1	1	6,7	18,616	7,2288	PF17250.1	NDUFB11	176,6	1,00E-52	No_clan
TRINITY_DN12989_c1_g1_i1_1	1	11,4	11,561	7,8755					
TRINITY_DN11038_c0_g1_i1_3	2	29,8	9,7543	7,9169					
TRINITY_DN6818_c0_g1_i1_3	2	8,8	28,723	25,614	PF03517.12	Voldacs	86,1	2,20E-24	CL0266
TRINITY_DN18372_c0_g1_i1_2	3	66,2	7,4115	27,517	PF02826.18	2-Hacid_dh_C	90,2	8,80E-26	CL0063
TRINITY_DN19362_c0_g1_i1_3	3	21	17,538	19,36					

TRINITY_DN13345_c0_g1_i1_4	1	13,6	9,727	6,8652	PF00458.19	WHEP-TRS	33,2	4,00E-08	CL0600
TRINITY_DN8412_c0_g1_i2_1	1	7,5	21,344	37,307	PF00179.25	UQ_con	130,8	2,50E-38	CL0208
TRINITY_DN15351_c0_g1_i1_3	3	22,1	27,416	19,745	PF03909.16	BSD	61,2	6,50E-17	No_clan
TRINITY_DN19403_c0_g1_i1_1	3	14,2	36,38	19,611	PF14510.5	ABC_trans_N	46,7	3,20E-12	No_clan
TRINITY_DN4912_c0_g1_i1_3	2	6,5	66,525	29,576	PF01039.21	Carboxyl_trans	522,7	8,30E-157	CL0127
TRINITY_DN9231_c0_g1_i3_3	2	11,1	18,143	12,077	PF00550.24	PP-binding	36,7	3,80E-09	CL0314
TRINITY_DN10613_c0_g1_i1_2	3	11,7	44,633	27,145	PF08241.11	Methyltransf_11	63,7	1,70E-17	CL0063
TRINITY_DN3524_c0_g1_i1_2	2	9,1	34,136	16,366	PF00226.30	DnaJ	92,1	1,60E-26	CL0392
TRINITY_DN3963_c0_g1_i1_1	3	5,1	75,993	23,137	PF00326.20	Peptidase_S9	77,3	1,00E-21	CL0028
TRINITY_DN1013_c0_g2_i1_6	3	9,6	44,362	17,102	PF01238.20	PMI_typeI	333,8	1,40E-99	CL0029
TRINITY_DN6811_c0_g2_i3_2	3	7,7	50,643	27,326	PF00579.24	tRNA-synt_1b	54	1,40E-14	CL0039
TRINITY_DN12201_c0_g2_i1_2	4	9,6	51,093	10,358	PF00076.21	RRM_1	57,5	8,30E-16	CL0221
TRINITY_DN8972_c0_g2_i1_1	3	5,9	87,582	28,66	PF03169.14	OPT	559,7	8,10E-168	No_clan
TRINITY_DN4764_c0_g2_i1_1	4	15	41,453	26,283	PF00226.30	DnaJ	83,7	6,60E-24	CL0392
TRINITY_DN2457_c0_g1_i1_1	1	18,7	13,303	13,246					
TRINITY_DN11036_c0_g1_i5_1	2	5,4	45,493	40,251	PF00083.23	Sugar_tr	291,9	7,90E-87	CL0015
TRINITY_DN10961_c0_g1_i3_3	3	16,9	28,507	23,444					
TRINITY_DN4707_c0_g3_i1_1	3	10,1	45,746	17,893	PF00704.27	Glyco_hydro_18	52,5	5,50E-14	CL0058
TRINITY_DN10673_c0_g1_i2_3	3	4,4	88,794	19,055	PF00888.21	Cullin	575	2,00E-172	No_clan
TRINITY_DN9408_c0_g1_i1_6	4	9,2	60,548	28,06	PF00749.20	tRNA-synt_1c	296,1	2,10E-88	CL0039
TRINITY_DN1822_c0_g2_i1_4	3	24,9	19,874	14,727	PF00183.17	HSP90	247,8	2,20E-73	No_clan
TRINITY_DN18094_c0_g1_i1_4	2	6,6	42,804	12,497	PF08033.11	Sec23_BS	55,6	6,10E-15	No_clan
TRINITY_DN3816_c0_g1_i1_4	3	13,4	39,708	21,28	PF08100.10	Dimerisation	49,5	3,00E-13	CL0123
TRINITY_DN10296_c0_g1_i1_1	1	4	35,122	7,3125	PF01849.17	NAC	77,3	6,20E-22	No_clan
TRINITY_DN5361_c0_g2_i1_1	4	10,2	58,324	26,133	PF07748.12	Glyco_hydro_38C	177,6	4,10E-52	CL0103
TRINITY_DN512_c0_g1_i1_4	4	12,8	42,777	25,564	PF00076.21	RRM_1	37,5	1,50E-09	CL0221
TRINITY_DN6953_c0_g1_i1_5	5	15,8	52,617	36,014	PF00155.20	Aminotran_1_2	240,8	2,30E-71	CL0061
TRINITY_DN22332_c0_g1_i1_1	2	29,5	9,8066	31,235					
TRINITY_DN8109_c0_g1_i2_3	3	12,3	31,575	20,033	PF09229.10	Aha1_N	87,4	9,00E-25	CL0648
TRINITY_DN9320_c0_g1_i1_1	3	25,8	13,586	18,279	PF14705.5	Costars	84,4	4,00E-24	No_clan
TRINITY_DN3842_c0_g1_i1_3	1	5,8	22,068	7,9578	PF06592.12	DUF1138	145,2	4,20E-43	No_clan
TRINITY_DN19595_c0_g1_i1_5	3	37,1	13,205	15,654	PF01095.18	Pectinesterase	148,6	1,40E-43	CL0268
TRINITY_DN12651_c0_g2_i1_3	2	7,1	52,083	13,276	PF00155.20	Aminotran_1_2	163,5	7,10E-48	CL0061

TRINITY_DN5781_c0_g1_i1_5	5	18,9	41,255	34,612	PF16940.4	Tic110	33,4	1,70E-08	No_clan
TRINITY_DN14210_c0_g1_i1_2	4	27,7	24,835	41,572					
TRINITY_DN5618_c0_g1_i1_1	2	17,5	18,806	20,752	PF00106.24	adh_short	121,1	3,70E-35	CL0063
TRINITY_DN8203_c0_g2_i1_6	3	32,8	13,562	27,442	PF02953.14	zf-Tim10_DDP	69,3	1,50E-19	No_clan
TRINITY_DN4496_c1_g1_i1_2	2	22,8	17,132	13,306	PF00342.18	PGI	43,6	1,40E-11	CL0067
TRINITY_DN13442_c0_g1_i1_1	2	21,4	12,944	13,231					
TRINITY_DN7081_c1_g1_i1_1	2	26,2	8,4717	13,094					
TRINITY_DN2528_c0_g1_i2_1	5	14,1	52,032	30,224	PF00270.28	DEAD	151,8	1,50E-44	CL0023
TRINITY_DN6839_c0_g2_i1_4	2	6,7	57,416	16,786	PF02705.15	K_trans	532	1,40E-159	CL0062
TRINITY_DN13687_c0_g1_i1_2	1	11	19,451	7,6866	PF02298.16	Cu_bind_like	76,8	9,00E-22	CL0026
TRINITY_DN7339_c0_g1_i1_1	2	10,9	20,638	12,998	PF01918.20	Alba	47,1	1,50E-12	CL0441
TRINITY_DN5933_c0_g1_i3_1	3	26,5	20,141	17,06	PF02597.19	ThiS	51,8	8,90E-14	CL0072
TRINITY_DN10134_c0_g1_i3_5	3	9,4	44,211	20,46	PF08241.11	Methyltransf_11	69,5	2,70E-19	CL0063
TRINITY_DN5831_c0_g1_i1_3	2	20,3	12,704	14,874					
TRINITY_DN8464_c0_g1_i3_6	2	13,9	26,729	11,228	PF04667.16	Endosulfine	84,2	4,70E-24	No_clan
TRINITY_DN11887_c0_g2_i1_4	1	11,3	14,04	26,023	PF00899.20	ThiF	32,9	3,70E-08	CL0063
TRINITY_DN3833_c0_g1_i1_2	3	14,1	28,277	32,17	PF04832.11	SOUL	177,2	2,80E-52	CL0319
TRINITY_DN10145_c0_g1_i1_3	3	15,2	25,919	19,556	PF11969.7	DcpS_C	95,6	2,30E-27	CL0265
TRINITY_DN7100_c0_g1_i2_3	3	17	25,324	14,953	PF00635.25	Motile_Sperm	98,2	2,10E-28	CL0556
TRINITY_DN23044_c0_g1_i1_1	1	14,5	9,1023	7,5232					
TRINITY_DN13518_c0_g1_i1_4	3	30,2	16,776	18,743	PF06427.10	UDP-g_GGTase	229,3	9,40E-68	No_clan
TRINITY_DN19196_c0_g1_i1_5	1	17,7	8,7178	6,9199	PF00141.22	peroxidase	37,4	2,00E-09	CL0617
TRINITY_DN3817_c0_g1_i1_5	1	10,5	12,999	7,1412	PF13943.5	WPP	121,4	1,50E-35	No_clan
TRINITY_DN4458_c0_g1_i1_2	3	28,4	16,267	21,367	PF00266.18	Aminotran_5	107,8	5,30E-31	CL0061
TRINITY_DN7255_c0_g1_i1_3	2	7,3	48,305	13,979					
TRINITY_DN6045_c0_g1_i3_1	2	8,4	33,202	14,041	PF16653.4	Sacchrp_dh_C	140,8	7,30E-41	No_clan
TRINITY_DN11230_c0_g1_i2_6	1	2,1	46,328	11,469	PF00847.19	AP2	44,3	1,40E-11	CL0081
TRINITY_DN3420_c0_g1_i2_3	3	18,3	26,567	19,73	PF04073.14	tRNA_edit	28	1,70E-06	No_clan
TRINITY_DN11146_c0_g1_i2_3	4	10,4	62,135	24,715	PF07714.16	Pkinase_Tyr	159,9	6,60E-47	CL0016
TRINITY_DN9423_c0_g3_i2_1	1	6,4	23,215	7,1204					
TRINITY_DN9788_c0_g1_i1_3	3	9,6	56,775	47,015	PF00004.28	AAA	150,1	4,20E-44	CL0023
TRINITY_DN2628_c0_g2_i1_1	1	15,2	9,9799	31,01					
TRINITY_DN9299_c0_g2_i1_3	3	9,3	47,291	20,487	PF09296.10	NUDIX-like	35,7	9,50E-09	CL0261

TRINITY_DN11235_c0_g2_i1_1	5	10,6	68,249	29,194					
TRINITY_DN5744_c1_g1_i2_2	2	22	19,133	18,69					
TRINITY_DN5626_c0_g2_i1_2	3	9,5	36,995	18,861	PF01025.18	GrpE	156,8	3,40E-46	No_clan
TRINITY_DN11440_c0_g1_i3_4	2	5,5	47,831	11,441	PF01399.26	PCI	41,9	1,10E-10	CL0123
TRINITY_DN11350_c0_g1_i3_2	3	12,3	45,25	27,637	PF01761.19	DHQ_synthase	352,2	1,30E-105	CL0224
TRINITY_DN11359_c0_g1_i1_2	3	9,3	46,672	20,873	PF00782.19	DSPc	40,8	1,60E-10	CL0031
TRINITY_DN9373_c0_g1_i1_6	1	16	8,9543	9,112					
TRINITY_DN12458_c0_g3_i1_3	6	17,6	52,03	33,79	PF00270.28	DEAD	181,1	1,50E-53	CL0023
TRINITY_DN5495_c0_g1_i2_2	2	4,1	49,265	11,198	PF02374.14	ArsA_ATPase	348,7	2,50E-104	CL0023
TRINITY_DN12738_c0_g1_i1_1	2	12,8	19,913	12,355	PF05222.14	AlaDh_PNT_N	92,2	3,10E-26	CL0325
TRINITY_DN7345_c0_g1_i1_4	4	11,1	48,762	28,02	PF10602.8	RPN7	190	2,50E-56	CL0020
TRINITY_DN6917_c0_g1_i1_3	4	13,2	54,151	23,1	PF00202.20	Aminotran_3	228	1,40E-67	CL0061
TRINITY_DN9153_c0_g1_i1_3	2	3	63,173	11,848	PF01055.25	Glyco_hydro_31	347,5	1,30E-103	CL0058
TRINITY_DN19500_c0_g1_i1_1	2	14,3	16,204	15,687	PF00575.22	S1	49	5,50E-13	CL0021
TRINITY_DN12333_c0_g1_i1_2	3	14,2	33,465	20,041					
TRINITY_DN10789_c0_g1_i1_1	5	17,8	47,873	31,241	PF01429.18	MBD	36,1	3,50E-09	CL0081
TRINITY_DN2912_c0_g2_i1_4	4	18,1	26,836	21,896	PF04278.11	Tic22	275,9	2,40E-82	No_clan
TRINITY_DN14787_c0_g1_i1_6	6	41	23,013	52,079	PF03109.15	ABC1	114,4	3,10E-33	CL0016
TRINITY_DN11096_c0_g1_i1_1	2	10,6	22,269	13,295	PF01920.19	Prefoldin_2	68,6	3,70E-19	CL0200
TRINITY_DN5451_c0_g1_i1_6	4	28,2	18,205	12,591	PF00411.18	Ribosomal_S11	110,3	5,70E-32	CL0267
TRINITY_DN11157_c0_g2_i5_4	1	4,1	26,021	-2					
TRINITY_DN8090_c0_g3_i1_2	5	25	26,893	19,67	PF02575.15	YbaB_DNA_bd	58,3	6,50E-16	No_clan
TRINITY_DN5532_c0_g1_i1_3	3	7,4	55,613	19,975	PF08442.9	ATP-grasp_2	60,5	1,40E-16	CL0179
TRINITY_DN8331_c0_g2_i1_3	2	12,7	30,168	16,287	PF03763.12	Remorin_C	108,8	1,10E-31	No_clan
TRINITY_DN7840_c1_g1_i1_1	2	7,6	27,574	16,497	PF00152.19	tRNA-synt_2	107,7	5,50E-31	CL0040
TRINITY_DN1523_c0_g1_i1_1	4	9,7	56,361	28,572	PF01053.19	Cys_Met_Meta_PP	483,1	3,80E-145	CL0061
TRINITY_DN14177_c0_g1_i1_3	1	15,7	9,6699	6,8332	PF01266.23	DAO	34,7	1,30E-08	CL0063
TRINITY_DN10869_c1_g1_i2_5	3	18,4	26,034	6,8154	PF00314.16	Thaumatin	267,6	8,00E-80	CL0293
TRINITY_DN5473_c0_g1_i1_5	3	12,2	34,237	18,596					
TRINITY_DN21224_c0_g1_i1_2	3	20	17,809	17,901					
TRINITY_DN6919_c0_g2_i1_3	2	13	21,045	13,448	PF01095.18	Pectinesterase	65,2	3,70E-18	CL0268
TRINITY_DN6224_c0_g1_i1_1	5	11,2	62,839	32,354	PF00400.31	WD40	12,9	0,15	CL0186

TRINITY_DN1121_c0_g1_i1_3	1	8,9	21,141	-2					
TRINITY_DN1480_c0_g1_i1_2	5	48,4	17,284	33,358					
TRINITY_DN12167_c0_g1_i1_5	1	9	17,791	8,5331	PF13520.5	AA_permease_2	40	2,00E-10	CL0062
TRINITY_DN458_c0_g1_i1_5	1	11	11,333	7,1125					
TRINITY_DN2404_c0_g1_i1_2	3	8,9	60,279	18,795	PF09743.8	E3_UFM1_ligase	310,3	1,20E-92	No_clan
TRINITY_DN9978_c0_g1_i1_2	1	4,8	22,844	17,04	PF06747.12	CHCH	38	1,20E-09	CL0351
TRINITY_DN8843_c0_g1_i3_1	3	8,1	42,872	22,822	PF00102.26	Y_phosphatase	237,1	1,80E-70	CL0031
TRINITY_DN6703_c0_g1_i1_2	3	15,8	32,6	43,541	PF01259.17	SAICAR_synt	241,7	8,70E-72	No_clan
TRINITY_DN11300_c0_g1_i1_3	5	14	70,826	35,73	PF00501.27	AMP-binding	285	7,10E-85	CL0378
TRINITY_DN3617_c0_g3_i1_5	1	6,5	22,326	6,7172					
TRINITY_DN10446_c0_g2_i1_1	2	2,9	80,087	11,732	PF00350.22	Dynamin_N	187,3	2,10E-55	CL0023
TRINITY_DN14501_c0_g1_i1_3	2	15,2	15,723	15,943	PF00295.16	Glyco_hydro_28	72,2	3,60E-20	CL0268
TRINITY_DN13753_c0_g1_i1_5	2	12,2	32,475	16,263	PF01412.17	ArfGap	67,5	8,80E-19	No_clan
TRINITY_DN18831_c0_g1_i1_1	2	35,1	12,052	15,723	PF07802.10	GCK	42	8,70E-11	No_clan
TRINITY_DN7892_c0_g1_i3_1	1	9,8	13,513	28,64					
TRINITY_DN6230_c0_g1_i1_1	2	8,1	32,591	14,392	PF05832.11	DUF846	162,6	4,90E-48	No_clan
TRINITY_DN18774_c0_g1_i1_4	3	23,6	18,388	18,741	PF05701.10	WEMBL	211,9	1,50E-62	No_clan
TRINITY_DN1997_c0_g1_i1_3	3	12,7	18,496	19,177	PF00067.21	p450	30,2	1,70E-07	No_clan
TRINITY_DN7250_c0_g1_i1_5	5	26,3	28,381	54,014	PF01583.19	APS_kinase	240,1	8,80E-72	CL0023
TRINITY_DN15321_c0_g1_i1_5	2	24,2	9,6377	13,35	PF08240.11	ADH_N	44,3	1,20E-11	CL0296
TRINITY_DN19751_c0_g1_i1_1	2	15,4	17,954	11,627	PF00122.19	E1-E2_ATPase	95,2	3,00E-27	No_clan
TRINITY_DN3119_c0_g1_i1_5	3	15,3	33,051	20,588	PF08662.10	eIF2A	43,1	3,90E-11	CL0186
TRINITY_DN2586_c0_g2_i1_1	1	12,4	10,379	6,8062	PF08240.11	ADH_N	36,7	2,90E-09	CL0296
TRINITY_DN18819_c0_g1_i1_4	3	24,4	21,321	17,475	PF01253.21	SUI1	87,7	5,30E-25	No_clan
TRINITY_DN13993_c0_g1_i1_4	1	18,1	14,289	6,5702					
TRINITY_DN20785_c0_g1_i1_2	2	30,4	12,087	13,665	PF00790.18	VHS	33,5	3,10E-08	CL0009
TRINITY_DN11337_c0_g1_i4_2	1	5,2	28,733	11,77					
TRINITY_DN8955_c0_g1_i4_2	1	5,1	26,946	7,2166	PF00190.21	Cupin_1	149,5	5,00E-44	CL0029
TRINITY_DN12162_c0_g1_i1_3	3	13	39,217	38,972					
TRINITY_DN9518_c0_g2_i2_1	1	8,3	14,971	7,2517	PF00574.22	CLP_protease	66,7	2,10E-18	CL0127
TRINITY_DN23667_c0_g1_i1_6	1	24,2	11,021	6,5176	PF00155.20	Aminotran_1_2	29,5	3,90E-07	CL0061
TRINITY_DN15391_c0_g1_i1_3	2	12,3	27,236	14,005					
TRINITY_DN8805_c0_g1_i2_1	3	5,1	81,716	21,191	PF03398.13	Ist1	196,1	3,30E-58	No_clan

TRINITY_DN7176_c0_g1_i3_3	2	17,6	25,088	14,091	PF00928.20	Adap_comp_sub	136,9	7,20E-40	CL0448
TRINITY_DN5898_c0_g1_i1_1	1	7,6	20,169	10,098	PF02953.14	zf-Tim10_DDP	69,7	1,20E-19	No_clan
TRINITY_DN8672_c0_g2_i1_3	4	26,6	25,123	31,474	PF02996.16	Prefoldin	98,9	1,60E-28	CL0200
TRINITY_DN1176_c0_g1_i1_6	2	27,6	9,3193	14,13					
TRINITY_DN4088_c0_g1_i1_6	4	10,3	63,833	24,936	PF00924.17	MS_channel	128,8	1,80E-37	No_clan
TRINITY_DN7361_c0_g1_i1_3	3	42,3	17,134	51,063	PF00173.27	Cyt-b5	87,2	5,60E-25	No_clan
TRINITY_DN6371_c0_g1_i2_5	3	9,1	47,046	20,44					
TRINITY_DN152_c0_g1_i1_1	2	16,1	26,408	31,094	PF05116.12	S6PP	49,8	2,90E-13	CL0137
TRINITY_DN211_c0_g1_i1_2	3	9,6	32,813	18,581	PF00179.25	UQ_con	133,8	2,90E-39	CL0208
TRINITY_DN10187_c0_g2_i3_3	3	9,7	43,065	18,402	PF04190.12	DUF410	229,8	4,30E-68	No_clan
TRINITY_DN10966_c1_g2_i1_2	3	20,7	20,026	22,204	PF03483.16	B3_4	52,2	5,40E-14	CL0383
TRINITY_DN17047_c0_g1_i1_4	4	28,1	24,255	27,074					
TRINITY_DN2953_c0_g1_i1_6	3	9,9	36,093	28,079					
TRINITY_DN10123_c0_g1_i1_2	5	13	54,236	30,424	PF00579.24	tRNA-synt_1b	218,6	1,00E-64	CL0039
TRINITY_DN6691_c0_g1_i1_3	2	4,9	52,896	14,022	PF13949.5	ALIX_LYPXL_bnd	234,1	1,90E-69	No_clan
TRINITY_DN9992_c0_g1_i2_2	2	10,3	27,076	13,12	PF00583.24	Acetyltransf_1	57,1	1,70E-15	CL0257
TRINITY_DN10481_c0_g2_i1_2	1	7,5	22,939	10,343	PF00834.18	Ribul_P_3_epim	198,1	8,70E-59	CL0036
TRINITY_DN23047_c0_g1_i1_3	2	32,6	9,0722	82,076	PF01230.22	HIT	22,3	0,00016	CL0265
TRINITY_DN12530_c0_g1_i1_3	1	15,6	10,343	9,7844	PF16656.4	Pur_ac_phosph_N	34,4	2,30E-08	CL0159
TRINITY_DN5479_c0_g1_i1_1	2	8	53,384	17,231	PF00202.20	Aminotran_3	394,4	4,20E-118	CL0061
TRINITY_DN14670_c0_g1_i1_5	2	17,6	18,34	15,439	PF03071.14	GNT-I	113,3	1,10E-32	CL0110
TRINITY_DN7295_c0_g1_i1_1	2	5,6	42,754	11,475	PF00155.20	Aminotran_1_2	175,3	1,90E-51	CL0061
TRINITY_DN9388_c0_g1_i2_3	4	11,1	53,513	27,031	PF00787.23	PX	84,5	4,70E-24	No_clan
TRINITY_DN9217_c0_g1_i2_3	4	11,5	49,532	29,717	PF03839.15	Sec62	48,3	8,30E-13	No_clan
TRINITY_DN11220_c1_g2_i10_1	1	8	20,453	8,8872	PF00106.24	adh_short	115,9	1,40E-33	CL0063
TRINITY_DN7324_c0_g1_i1_3	3	6,1	52,937	16,687	PF00004.28	AAA	133,8	4,50E-39	CL0023
TRINITY_DN8489_c0_g2_i1_1	2	11,7	23,246	12,865	PF00696.27	AA_kinase	69,7	2,60E-19	No_clan
TRINITY_DN9496_c0_g2_i1_6	2	13,5	24,142	17,538					
TRINITY_DN8940_c0_g2_i1_4	2	12,9	22,894	51,084	PF04398.11	DUF538	112,6	1,10E-32	No_clan
TRINITY_DN2829_c0_g1_i1_4	3	9,4	33,352	17,751	PF13419.5	HAD_2	67,1	1,90E-18	CL0137
TRINITY_DN8775_c0_g1_i1_6	2	4,2	52,28	12,031					
TRINITY_DN4667_c0_g1_i2_3	1	11	11,387	6,496	PF01300.17	Sua5_yciO_yrdC	69,8	1,90E-19	No_clan
TRINITY_DN22872_c0_g1_i1_1	2	21,8	11,566	12,496	PF00076.21	RRM_1	35,8	4,80E-09	CL0221

TRINITY_DN19824_c0_g1_i1_3	3	27,8	20,903	41,14	PF00982.20	Glyco_transf_20	118,7	2,50E-34	CL0113
TRINITY_DN9699_c0_g1_i1_4	3	9,7	56,927	21,418	PF13793.5	Pribosyltran_N	25,8	7,60E-06	CL0533
TRINITY_DN3755_c0_g2_i1_3	4	11,8	63,737	27,354	PF00106.24	adh_short	108,7	2,20E-31	CL0063
TRINITY_DN11202_c0_g1_i2_2	2	11,7	23,753	20,635	PF05047.15	L51_S25_CI-B8	50,5	1,40E-13	CL0172
TRINITY_DN6447_c0_g1_i1_4	2	3,6	56,169	12,048	PF01546.27	Peptidase_M20	76,7	1,80E-21	CL0035
TRINITY_DN5808_c0_g1_i1_2	3	9,8	55,731	23,845					
TRINITY_DN10647_c0_g2_i1_5	7	18,9	34,141	23,45	PF02798.19	GST_N	64,1	1,00E-17	CL0172
TRINITY_DN14457_c0_g1_i1_4	1	6,4	20,818	7,7385					
TRINITY_DN7706_c0_g1_i1_3	4	12,3	40,546	-2	PF08071.11	RS4NT	68,9	2,70E-19	No_clan
TRINITY_DN2695_c0_g1_i1_6	2	11,2	30,121	14,255	PF03009.16	GDPD	105,9	2,70E-30	CL0384
TRINITY_DN3350_c0_g1_i1_2	3	32,6	19,255	22,681	PF04043.14	PMEI	84,1	1,20E-23	No_clan
TRINITY_DN11297_c1_g1_i1_2	1	4,5	35,914	7,0138	PF13883.5	Pyrid_oxidase_2	191,6	9,10E-57	CL0336
TRINITY_DN11354_c1_g1_i3_1	3	10,5	48,902	19,866					
TRINITY_DN12695_c0_g2_i1_3	3	11,2	58,284	25,208					
TRINITY_DN12034_c0_g1_i1_1	2	12,1	22,229	16,167	PF13432.5	TPR_16	25,4	1,50E-05	CL0020
TRINITY_DN4302_c0_g1_i1_3	3	6,7	68,111	17,879	PF00759.18	Glyco_hydro_9	449,9	1,10E-134	CL0059
TRINITY_DN18986_c0_g1_i1_2	1	11	10,321	6,3984	PF01842.24	ACT	31,8	8,40E-08	CL0070
TRINITY_DN16321_c0_g1_i1_6	2	29	11,267	12,787					
TRINITY_DN10277_c0_g1_i1_3	5	13,9	48,169	33,132					
TRINITY_DN16791_c0_g1_i1_4	1	17,6	9,3887	7,9169	PF11837.7	DUF3357	42,6	6,20E-11	No_clan
TRINITY_DN7314_c0_g1_i1_1	2	14,3	30,258	15,608	PF05564.11	Auxin_repressed	34,7	2,20E-08	No_clan
TRINITY_DN11345_c0_g1_i1_5	1	7,1	19,859	6,5161	PF06592.12	DUF1138	150,1	1,20E-44	No_clan
TRINITY_DN23429_c0_g1_i1_1	2	35,6	10,091	22,768	PF04811.14	Sec23_trunk	72,7	3,00E-20	CL0128
TRINITY_DN4338_c0_g2_i1_1	3	10,4	36,637	19,006	PF05879.11	RHD3	176,1	9,30E-52	CL0023
TRINITY_DN13879_c0_g1_i1_4	2	15,6	18,411	13,365	PF04227.11	Indigoidine_A	132,2	2,10E-38	No_clan
TRINITY_DN1790_c0_g1_i1_2	2	28,7	11,333	12,648					
TRINITY_DN5067_c0_g1_i1_3	3	10,7	39,603	20,762	PF00400.31	WD40	32,3	1,10E-07	CL0186
TRINITY_DN9550_c0_g1_i1_3	4	10,7	47,072	23,799	PF00013.28	KH_1	48,4	5,80E-13	CL0007
TRINITY_DN5828_c0_g1_i2_4	2	8,3	40,644	15,074	PF03357.20	Snf7	140	5,40E-41	CL0235
TRINITY_DN20109_c0_g1_i1_4	1	9,1	12,023	6,7699	PF00699.19	Urease_beta	114,5	1,60E-33	No_clan
TRINITY_DN11375_c0_g1_i1_1	2	14	21,172	16,414	PF00166.20	Cpn10	56,3	2,30E-15	CL0296
TRINITY_DN4236_c0_g1_i1_1	3	30,9	14,825	17,796					
TRINITY_DN4156_c0_g1_i1_1	2	18,7	12,157	47,293	PF00076.21	RRM_1	32,9	3,80E-08	CL0221

TRINITY_DN11786_c0_g1_i1_3	3	17	19,882	17,706	PF00012.19	HSP70	32,3	2,90E-08	CL0108
TRINITY_DN15397_c0_g1_i1_3	2	20,8	8,4155	11,689	PF00696.27	AA_kinase	58,6	6,20E-16	No_clan
TRINITY_DN5555_c0_g1_i2_1	3	7,9	64,694	23,876	PF00501.27	AMP-binding	346,3	1,80E-103	CL0378
TRINITY_DN6669_c0_g2_i2_2	3	18,5	28,704	19,841					
TRINITY_DN5818_c0_g1_i1_3	2	12,9	29,165	12,962	PF00291.24	PALP	136,6	1,10E-39	No_clan
TRINITY_DN10622_c0_g1_i3_1	4	7,7	84,546	29,319	PF00013.28	KH_1	39,9	2,60E-10	CL0007
TRINITY_DN6444_c0_g1_i1_1	1	4,5	28,681	6,4668					
TRINITY_DN8167_c0_g1_i2_2	1	2,7	60,45	19,528	PF00076.21	RRM_1	49,6	2,40E-13	CL0221
TRINITY_DN10005_c0_g1_i2_3	2	17,1	13,76	12,148	PF00076.21	RRM_1	59,7	1,70E-16	CL0221
TRINITY_DN18506_c0_g1_i1_1	3	46,7	13,843	29,264	PF02330.15	MAM33	101,2	6,80E-29	No_clan
TRINITY_DN9802_c0_g1_i3_2	4	16,3	27,054	23,572	PF01095.18	Pectinesterase	90,5	7,30E-26	CL0268
TRINITY_DN11201_c1_g1_i1_2	2	6,4	50,963	18,303	PF02544.15	Steroid_dh	94,7	4,70E-27	CL0115
TRINITY_DN2574_c0_g1_i1_2	1	4,8	22,191	6,9985	PF02485.20	Branch	39,2	5,00E-10	CL0110
TRINITY_DN2926_c0_g2_i1_1	4	39,7	12,961	22,438	PF01450.18	IlvC	82,1	4,20E-23	CL0106
TRINITY_DN1172_c0_g1_i1_3	2	28,7	12,135	21,882	PF00069.24	Pkinase	105,1	3,50E-30	CL0016
TRINITY_DN15441_c0_g1_i1_4	1	12,8	21,719	8,964					
TRINITY_DN2072_c0_g1_i1_5	2	27,3	21,817	19,58	PF08282.11	Hydrolase_3	22,6	7,10E-05	CL0137
TRINITY_DN11308_c0_g1_i1_4	2	6,1	26,52	14,128	PF02966.15	DIM1	222,3	1,30E-66	CL0172
TRINITY_DN11604_c0_g1_i4_3	3	14,2	35,529	14,429	PF08241.11	Methyltransf_11	38,2	1,50E-09	CL0063
TRINITY_DN6973_c0_g1_i2_3	3	9	56,176	25,721	PF01553.20	Acyltransferase	59,1	3,20E-16	CL0228
TRINITY_DN19024_c0_g1_i1_1	1	6	25,944	9,6666					
TRINITY_DN8174_c0_g1_i1_3	1	6,8	39,752	27,747					
TRINITY_DN6495_c0_g1_i1_1	4	21,8	38,454	30,692	PF00156.26	Pribosyltran	35,7	5,30E-09	CL0533
TRINITY_DN14955_c0_g1_i1_1	2	28,4	8,5637	11,028					
TRINITY_DN5794_c0_g1_i3_3	3	8,2	47,649	20,884	PF00483.22	NTP_transferase	95	5,00E-27	CL0110
TRINITY_DN5657_c0_g1_i1_3	4	11,5	37,722	32,136	PF01997.15	Translin	166,4	7,60E-49	No_clan
TRINITY_DN20134_c0_g1_i1_2	1	10,1	10,546	6,7699					
TRINITY_DN5823_c1_g1_i1_3	2	23,9	7,7539	11,924					
TRINITY_DN10611_c0_g2_i1_3	4	11,7	55,757	24,529	PF05577.11	Peptidase_S28	257,3	2,20E-76	CL0028
TRINITY_DN8028_c1_g2_i2_1	2	11,9	26,242	15,138					
TRINITY_DN20414_c0_g1_i1_1	1	11,5	17,328	25,907					
TRINITY_DN18416_c0_g1_i1_1	3	9,9	36,017	20,477	PF14523.5	Syntaxin_2	77,5	7,30E-22	CL0445
TRINITY_DN5396_c0_g2_i1_1	1	12,4	20,602	40,854	PF00515.27	TPR_1	28,1	1,10E-06	CL0020

TRINITY_DN4301_c0_g1_i1_1	4	15,4	32,795	24,676	PF12799.6	LRR_4	36,9	2,90E-09	CL0022
TRINITY_DN6535_c0_g1_i1_1	2	10,6	23,744	11,837	PF00271.30	Helicase_C	107,5	4,30E-31	CL0023
TRINITY_DN5580_c0_g1_i1_4	4	14,6	52,027	29,685	PF14604.5	SH3_9	41,2	9,80E-11	CL0010
TRINITY_DN10847_c0_g1_i3_3	2	20,7	19,711	11,087	PF01217.19	Clat_adaptor_s	66	3,10E-18	CL0212
TRINITY_DN14525_c0_g1_i1_6	2	14,3	22,332	12,755	PF01370.20	Epimerase	24,9	1,20E-05	CL0063
TRINITY_DN10966_c1_g1_i1_2	1	4,2	36,059	7,7462	PF03484.14	B5	35,4	8,90E-09	No_clan
TRINITY_DN12386_c0_g1_i1_4	4	11,7	45,491	24,836	PF06414.11	Zeta_toxin	58	6,90E-16	CL0023
TRINITY_DN3579_c0_g1_i1_4	1	10,5	13,145	8,48	PF03061.21	4HBT	43,1	3,70E-11	CL0050
TRINITY_DN19791_c0_g1_i1_5	2	17,2	12,802	12,51	PF04053.13	Coatomer_WDAD	87	1,30E-24	CL0186
TRINITY_DN10085_c0_g1_i1_3	3	8,4	60,82	29,175	PF12796.6	Ank_2	33,5	4,40E-08	CL0465
TRINITY_DN5782_c0_g1_i1_1	1	5,8	21,024	7,4081	PF02943.14	FeThRed_B	138,2	8,50E-41	No_clan
TRINITY_DN13590_c0_g1_i1_3	3	13,7	26,582	16,456					
TRINITY_DN1136_c0_g2_i1_1	1	7,1	28,076	6,6922	PF04871.12	Uso1_p115_C	107,6	4,90E-31	No_clan
TRINITY_DN9839_c0_g1_i1_1	2	3,2	94,196	13,118	PF00005.26	ABC_tran	68,4	8,30E-19	CL0023
TRINITY_DN3828_c0_g1_i2_1	1	7,7	26,256	9,4052	PF01012.20	ETF	46,2	4,20E-12	CL0039
TRINITY_DN800_c0_g1_i1_1	2	21,4	12,286	14,955	PF00342.18	PGI	31,5	6,50E-08	CL0067
TRINITY_DN10682_c0_g1_i2_5	2	6,2	53,634	13,593	PF07063.12	DUF1338	267,7	1,20E-79	No_clan
TRINITY_DN11406_c0_g2_i1_5	2	4,3	56,104	15,016	PF00481.20	PP2C	42,4	5,70E-11	CL0238
TRINITY_DN9265_c0_g1_i1_4	2	10,9	26,545	14,44	PF00076.21	RRM_1	50,7	1,10E-13	CL0221
TRINITY_DN3622_c0_g1_i1_2	4	11,1	59,04	22,539	PF01179.19	Cu_amine_oxid	516,2	5,50E-155	No_clan
TRINITY_DN4087_c0_g2_i1_1	3	20,6	27,221	19,842	PF01593.23	Amino_oxidase	83,2	2,20E-23	CL0063
TRINITY_DN10280_c0_g2_i1_3	1	6,6	21,088	7,5686	PF09446.9	VMA21	72,5	2,00E-20	No_clan
TRINITY_DN17659_c0_g1_i1_1	2	15,1	10,326	16,519	PF00920.20	ILVD_EDD	82	3,40E-23	No_clan
TRINITY_DN391_c0_g2_i1_3	2	13,5	22,992	29,521	PF08324.10	PUL	82,2	3,20E-23	No_clan
TRINITY_DN18625_c0_g1_i1_3	1	5,8	18,462	-2					
TRINITY_DN15558_c0_g1_i1_3	2	16,2	19,414	11,246	PF05879.11	RHD3	224,9	1,60E-66	CL0023
TRINITY_DN8990_c0_g1_i6_4	2	9	34,623	11,811	PF01256.16	Carb_kinase	86,8	1,40E-24	CL0118
TRINITY_DN9713_c0_g1_i1_2	3	8,1	63,33	18,462	PF14559.5	TPR_19	26,3	7,60E-06	CL0020
TRINITY_DN4_c0_g2_i1_2	2	32,4	8,0491	12,175	PF00637.19	Clathrin	42,3	5,90E-11	CL0020
TRINITY_DN3956_c0_g2_i1_1	2	13,4	19,36	12,924					
TRINITY_DN2951_c0_g1_i1_3	2	13,8	17,345	12,618	PF10780.8	MRP_L53	53,2	2,30E-14	No_clan
TRINITY_DN8715_c0_g1_i2_3	3	13,1	27,613	16,932	PF04051.15	TRAPP	124,5	2,50E-36	CL0210
TRINITY_DN11521_c0_g3_i1_1	1	11,5	11,542	7,5759	PF07541.11	EIF_2_alpha	102,7	1,20E-29	No_clan

TRINITY_DN7431_c0_g1_i1_3	2	10	31,795	11,657	PF00160.20	Pro_isomerase	114,2	6,10E-33	CL0475
TRINITY_DN20738_c0_g1_i1_3	2	47,2	9,1544	13,935	PF00501.27	AMP-binding	58,5	4,00E-16	CL0378
TRINITY_DN11007_c0_g1_i2_1	3	7,1	62,711	45,505	PF00275.19	EPSP_synthase	482,6	7,90E-145	CL0290
TRINITY_DN18675_c0_g1_i1_5	3	20,1	18,548	24,369	PF03031.17	NIF	55,3	5,80E-15	CL0137
TRINITY_DN4569_c0_g1_i2_2	2	10,9	28,553	12,981	PF05383.16	La	78,6	2,50E-22	CL0123
TRINITY_DN4303_c0_g1_i1_5	3	16,9	25,803	17,544	PF00118.23	Cpn60_TCP1	129,4	1,40E-37	No_clan
TRINITY_DN11099_c0_g1_i3_2	2	7,3	42,645	15,193	PF10058.8	zinc_ribbon_10	68,4	3,00E-19	CL0167
TRINITY_DN5386_c0_g1_i1_2	2	11,4	41,315	16,171	PF02883.19	Alpha_adaptinC2	106	1,20E-30	CL0159
TRINITY_DN3521_c0_g1_i1_6	5	21	43,119	30,8	PF04012.11	PspA_IM30	181,7	1,30E-53	CL0235
TRINITY_DN13705_c0_g1_i1_5	1	5,9	35,36	31,726					
TRINITY_DN3201_c0_g1_i1_6	2	3,9	62,843	17,433	PF13850.5	ERGIC_N	104,1	3,50E-30	No_clan
TRINITY_DN9124_c0_g4_i1_6	2	6,1	47,527	14,044	PF04548.15	AIG1	274,8	3,30E-82	CL0023
TRINITY_DN10878_c0_g1_i1_2	4	9,7	85,718	25,695	PF03141.15	Methyltransf_29	764,5	4,00E-230	CL0063
TRINITY_DN8588_c0_g1_i3_2	3	13,8	34,267	12,992	PF00632.24	HECT	278,9	5,20E-83	CL0552
TRINITY_DN8864_c0_g1_i1_1	2	9,8	29,455	14,716	PF00561.19	Abhydrolase_1	30,2	3,10E-07	CL0028
TRINITY_DN18025_c0_g1_i1_5	1	9,3	16,774	14,731	PF02953.14	zf-Tim10_DDP	88,4	1,70E-25	No_clan
TRINITY_DN15533_c0_g1_i1_2	4	22	23,205	24,742	PF00350.22	Dynamin_N	53,4	2,80E-14	CL0023
TRINITY_DN17372_c0_g1_i1_4	2	9,4	44,226	18,093	PF00294.23	PfkB	199,4	7,20E-59	CL0118
TRINITY_DN10448_c0_g1_i2_3	3	8,4	72,536	28,995	PF04258.12	Peptidase_A22B	273,1	2,70E-81	CL0130
TRINITY_DN2556_c0_g1_i2_1	3	10,3	55,696	17,943	PF00226.30	DnaJ	94,2	3,40E-27	CL0392
TRINITY_DN11115_c0_g1_i1_2	1	3	52,68	15,034	PF05637.11	Glyco_transf_34	245,5	5,60E-73	CL0110
TRINITY_DN6126_c0_g1_i2_6	2	9,3	41,986	12,967	PF01398.20	JAB	71,9	3,90E-20	CL0366
TRINITY_DN18060_c0_g1_i1_4	2	14,4	19,503	6,7521	PF00248.20	Aldo_ket_red	174,1	3,20E-51	No_clan
TRINITY_DN8332_c0_g1_i1_1	2	11,6	23,793	11,518	PF13015.5	PRKCSH_1	100,3	7,60E-29	CL0226
TRINITY_DN7043_c0_g3_i1_3	4	13,9	41,699	26,092	PF00596.20	Aldolase_II	133,7	6,50E-39	No_clan
TRINITY_DN15047_c0_g1_i1_2	1	8,6	17,152	7,7072	PF02630.13	SCO1-SenC	108,6	2,70E-31	CL0172
TRINITY_DN4679_c0_g1_i1_4	2	14,8	30,318	24,276					
TRINITY_DN5533_c1_g1_i1_2	2	11	25,292	13,091	PF08172.11	CASP_C	190,7	2,50E-56	No_clan
TRINITY_DN3362_c0_g1_i1_3	2	19	15,236	13,163	PF02774.17	Semialdhyde_dhC	26,4	5,80E-06	CL0139
TRINITY_DN11453_c1_g2_i1_1	3	4,3	90,528	20,563	PF00005.26	ABC_tran	86,8	1,60E-24	CL0023
TRINITY_DN21448_c0_g1_i1_2	2	35,6	9,0685	11,822	PF03759.12	PRONE	78,2	4,60E-22	No_clan
TRINITY_DN4482_c0_g1_i2_6	2	14,1	18,729	14,074	PF04099.11	Sybindin	79,7	1,90E-22	CL0212
TRINITY_DN3062_c1_g1_i1_5	2	37,1	14,109	30,6	PF03071.14	GNT-I	187,4	3,50E-55	CL0110

TRINITY_DN21074_c0_g1_i1_5	2	46,1	8,4204	44,082					
TRINITY_DN6202_c0_g1_i1_4	3	37,3	16,731	20,266	PF00076.21	RRM_1	70,2	9,20E-20	CL0221
TRINITY_DN9665_c1_g1_i2_1	2	11,5	27,722	12,445	PF01842.24	ACT	34,1	1,60E-08	CL0070
TRINITY_DN23089_c0_g1_i1_2	1	13	7,5043	6,7736					
TRINITY_DN4752_c0_g1_i1_6	3	7,7	47,022	17,798	PF00085.19	Thioredoxin	87,4	4,90E-25	CL0172
TRINITY_DN6323_c0_g1_i1_4	1	2,2	40,605	-2	PF01650.17	Peptidase_C13	150,4	6,10E-44	CL0093
TRINITY_DN7547_c0_g1_i1_2	5	30,8	23,001	-2	PF03953.16	Tubulin_C	104,4	4,50E-30	CL0442
TRINITY_DN11503_c0_g1_i5_1	2	8	32,335	6,6263	PF07859.12	Abhydrolase_3	154,6	2,90E-45	CL0028
TRINITY_DN6410_c0_g2_i1_3	3	13,6	25,911	6,496	PF01246.19	Ribosomal_L24e	101,4	2,20E-29	CL0175
TRINITY_DN17585_c0_g1_i1_4	2	7,9	39,286	13,138	PF02114.15	Phosducin	58,5	4,30E-16	CL0172
TRINITY_DN7272_c0_g1_i2_2	3	20,9	22,929	21,03	PF08662.10	eIF2A	76,2	2,70E-21	CL0186
TRINITY_DN8313_c0_g1_i1_1	1	23,9	12,368	15,713					
TRINITY_DN10899_c1_g1_i9_2	3	16,5	24,497	12,224	PF05529.11	Bap31	45,4	6,20E-12	No_clan
TRINITY_DN10964_c0_g2_i1_1	1	8,7	10,475	6,3861	PF00831.22	Ribosomal_L29	52,1	4,30E-14	CL0346
TRINITY_DN5766_c0_g1_i1_2	3	10,2	37,137	20,531	PF05739.18	SNARE	43,1	3,00E-11	No_clan
TRINITY_DN16709_c0_g1_i1_5	2	25,9	8,6728	14,671	PF00731.19	AIRC	66,9	1,30E-18	No_clan
TRINITY_DN20234_c0_g1_i1_2	2	43,3	10,427	12,569					
TRINITY_DN13627_c0_g1_i1_4	3	18,7	23,856	19,357	PF04136.14	Sec34	161,3	1,30E-47	No_clan
TRINITY_DN22360_c0_g1_i1_5	1	11	11,677	7,2364					
TRINITY_DN5465_c0_g1_i1_6	1	7,4	15,032	6,4652	PF05768.13	DUF836	61,6	6,70E-17	CL0172
TRINITY_DN14137_c1_g1_i1_2	1	13	13,26	7,0607	PF01593.23	Amino_oxidase	55	7,90E-15	CL0063
TRINITY_DN4218_c0_g1_i1_3	2	11,4	28,6	6,4331	PF00291.24	PALP	81,3	7,50E-23	No_clan
TRINITY_DN405_c0_g2_i1_3	2	10,1	35,714	15,129					
TRINITY_DN2745_c0_g1_i1_1	2	13	21,776	10,884	PF05368.12	NmrA	114,9	3,80E-33	CL0063
TRINITY_DN15009_c0_g1_i1_3	2	10,2	26,208	11,447	PF02548.14	Pantoate_transf	240,5	1,80E-71	CL0151
TRINITY_DN20316_c0_g1_i1_4	1	10,1	13,575	7,1898					
TRINITY_DN11294_c0_g1_i2_1	2	8,2	50,723	30,927	PF04177.11	TAP42	279,7	2,70E-83	No_clan
TRINITY_DN19054_c0_g1_i1_3	3	17,1	25,656	18,916	PF13414.5	TPR_11	41,5	7,40E-11	CL0020
TRINITY_DN4034_c0_g2_i1_6	2	15,8	19,118	13,073	PF02151.18	UVR	22,1	8,30E-05	No_clan
TRINITY_DN12290_c0_g1_i1_4	1	5,7	26,797	9,6831	PF00696.27	AA_kinase	101,3	5,90E-29	No_clan
TRINITY_DN21049_c0_g1_i1_5	1	14,9	10,551	9,808	PF05938.10	Self-incomp_S1	61,1	1,10E-16	No_clan
TRINITY_DN10192_c0_g2_i2_4	1	3,1	47,251	6,4022	PF13409.5	GST_N_2	69,3	2,30E-19	CL0172
TRINITY_DN5313_c0_g1_i1_6	3	10,5	38,502	20,552					

TRINITY_DN1722_c0_g1_i1_6	1	9,3	11,636	81,108					
TRINITY_DN594_c0_g1_i1_2	3	12,7	33,707	18,549	PF02781.15	G6PD_C	336,4	1,20E-100	No_clan
TRINITY_DN14750_c0_g1_i1_3	2	24,4	10,636	12,46	PF02897.14	Peptidase_S9_N	95,8	2,30E-27	CL0186
TRINITY_DN10152_c0_g1_i1_3	1	5,7	18,36	6,4522					
TRINITY_DN7940_c0_g1_i2_6	2	9,4	22,036	10,872	PF00168.29	C2	50,9	1,40E-13	CL0154
TRINITY_DN3239_c0_g2_i1_3	1	10,9	16,464	10,04	PF00690.25	Cation_ATPase_N	67,8	4,60E-19	No_clan
TRINITY_DN4761_c0_g1_i1_3	2	7,2	37,86	34,962	PF05903.13	Peptidase_C97	152,6	6,20E-45	CL0125
TRINITY_DN8530_c0_g1_i3_3	3	23,8	27,842	19,537	PF08553.9	VID27	113	1,50E-32	No_clan
TRINITY_DN16028_c0_g1_i1_6	3	20,5	18,871	20,27					
TRINITY_DN515_c0_g1_i1_1	1	18,6	10,253	12,884	PF00692.18	dUTPase	62,8	2,30E-17	CL0153
TRINITY_DN9271_c0_g1_i2_1	3	30,1	15,288	-2	PF06521.10	PAR1	219,7	1,30E-65	No_clan
TRINITY_DN9769_c0_g1_i2_3	5	14,8	62,137	30,053					
TRINITY_DN17789_c0_g1_i1_2	3	6,2	48,258	12,206	PF00160.20	Pro_isomerase	141,9	1,90E-41	CL0475
TRINITY_DN17147_c0_g3_i1_3	1	4,4	26,904	7,6365	PF00383.22	dCMP_cyt_deam_1	83,7	6,10E-24	CL0109
TRINITY_DN18723_c0_g1_i1_3	2	28,2	16,765	13,253	PF01459.21	Porin_3	119,6	1,50E-34	CL0193
TRINITY_DN13968_c0_g1_i1_1	4	10,5	60,191	27,653					
TRINITY_DN1050_c0_g1_i1_3	4	18,3	36,668	27,026	PF00004.28	AAA	148,5	1,30E-43	CL0023
TRINITY_DN630_c0_g1_i2_2	2	8,8	29,15	11,243					
TRINITY_DN10963_c0_g1_i1_3	3	14,6	38,088	24,871	PF12838.6	Fer4_7	48,6	8,70E-13	CL0344
TRINITY_DN10629_c0_g1_i1_1	2	11,2	30,047	25,343	PF00255.18	GSHPx	136,9	1,60E-40	CL0172
TRINITY_DN2614_c0_g1_i1_5	4	13,4	46,152	35,195	PF08264.12	Anticodon_1	124,2	4,00E-36	CL0258
TRINITY_DN11634_c0_g1_i3_1	3	7,7	68,521	17,498	PF08156.12	NOP5NT	69,3	2,50E-19	No_clan
TRINITY_DN10531_c0_g2_i1_3	2	7,4	39,839	12,651	PF02492.18	cobW	109,2	1,50E-31	CL0023
TRINITY_DN2332_c0_g1_i1_2	3	5,9	71,914	18,517	PF00291.24	PALP	274,3	1,20E-81	No_clan
TRINITY_DN17272_c0_g1_i1_3	3	9,3	59,741	18,775	PF00852.18	Glyco_transf_10	113,7	7,10E-33	CL0113
TRINITY_DN6275_c0_g2_i2_1	3	19,9	33,819	19,012	PF14523.5	Syntaxin_2	92,5	1,60E-26	CL0445
TRINITY_DN7918_c1_g1_i1_2	2	10,6	35,145	7,8331	PF00255.18	GSHPx	144,5	7,10E-43	CL0172
TRINITY_DN10401_c0_g1_i2_1	5	20,7	35,386	12,604	PF00071.21	Ras	184,4	1,10E-54	CL0023
TRINITY_DN6828_c0_g1_i1_3	2	9	37,112	34,189	PF02020.17	W2	79,7	1,20E-22	CL0020
TRINITY_DN3006_c0_g1_i2_2	4	8,1	68,053	24,498	PF00888.21	Cullin	534	5,00E-160	No_clan
TRINITY_DN11284_c0_g1_i1_2	1	23,4	7,8732	9,8089					
TRINITY_DN7192_c0_g1_i1_3	3	12,4	41,111	20,135	PF13883.5	Pyrid_oxidase_2	64,3	1,20E-17	CL0336

TRINITY_DN8534_c0_g1_i2_3	3	11,2	42,638	19,198	PF01915.21	Glyco_hydro_3_C	141,6	3,00E-41	No_clan
TRINITY_DN7045_c0_g1_i2_1	3	10,1	33,854	20,686					
TRINITY_DN7890_c0_g1_i3_3	2	8,4	32,206	11,714	PF13561.5	adh_short_C2	128,6	2,50E-37	CL0063
TRINITY_DN11617_c0_g1_i1_3	3	7,7	64,285	17,29	PF00155.20	Aminotran_1_2	115,8	2,30E-33	CL0061
TRINITY_DN15269_c0_g1_i1_3	2	22,2	15,532	16,285	PF00675.19	Peptidase_M16	35,1	1,10E-08	CL0094
TRINITY_DN10161_c0_g1_i1_2	1	2,7	41,412	6,4948	PF01063.18	Aminotran_4	126,7	1,10E-36	No_clan
TRINITY_DN819_c0_g1_i1_5	2	14,2	19,575	10,675	PF00155.20	Aminotran_1_2	134,5	4,80E-39	CL0061
TRINITY_DN13462_c0_g1_i1_3	3	25,2	15,398	18,383	PF04176.12	TIP41	63,3	1,80E-17	No_clan
TRINITY_DN4339_c0_g1_i1_1	2	8,7	19,079	11,536					
TRINITY_DN19938_c0_g1_i1_2	1	21,5	8,9191	6,6903					
TRINITY_DN10594_c0_g1_i4_3	2	9,5	26,587	11,127	PF00717.22	Peptidase_S24	42,4	4,50E-11	CL0299
TRINITY_DN7751_c0_g1_i1_3	1	6,9	26,697	17,026	PF00168.29	C2	74	8,90E-21	CL0154
TRINITY_DN9327_c0_g1_i3_2	3	15,2	29,707	22,339	PF12352.7	V-SNARE_C	56,9	1,80E-15	CL0147
TRINITY_DN8047_c0_g1_i1_3	4	12,3	42,389	28,312	PF03357.20	Snf7	154,7	1,60E-45	CL0235
TRINITY_DN10285_c0_g1_i1_1	1	3	36,861	6,7797	PF08212.11	Lipocalin_2	178,1	8,80E-53	CL0116
TRINITY_DN8681_c0_g1_i1_3	2	3,7	64,455	13,739	PF07223.10	DUF1421	82,9	9,00E-24	CL0214
TRINITY_DN5586_c0_g1_i1_1	3	6,8	61,434	20,451	PF13812.5	PPR_3	27,3	2,60E-06	CL0020
TRINITY_DN2976_c0_g1_i1_3	1	4,4	37,965	7,3616	PF07714.16	Pkinase_Tyr	83,8	1,00E-23	CL0016
TRINITY_DN7053_c1_g1_i1_1	2	10,5	24,709	12,359	PF07946.13	DUF1682	157,3	5,00E-46	No_clan
TRINITY_DN9308_c0_g2_i1_6	3	5,1	63,002	11,508	PF01412.17	ArfGap	138,4	9,50E-41	No_clan
TRINITY_DN7166_c0_g1_i1_1	2	13	19,368	13,312	PF02148.18	zf-UBP	59,1	3,70E-16	CL0229
TRINITY_DN17861_c0_g1_i1_6	2	6,6	40,098	14,204	PF00153.26	Mito_carr	43,8	1,70E-11	No_clan
TRINITY_DN23265_c0_g1_i1_1	1	13,8	11,795	10,949	PF04755.11	PAP_fibrillin	56,8	2,70E-15	No_clan
TRINITY_DN11443_c1_g1_i3_3	2	12,6	39,386	12,956	PF00389.29	2-Hacid_dh	53,5	1,80E-14	CL0325
TRINITY_DN9627_c0_g2_i1_1	2	18,3	15,666	13,392					
TRINITY_DN20236_c0_g1_i1_5	1	8,1	13,709	9,0989	PF00583.24	Acetyltransf_1	28,6	1,30E-06	CL0257
TRINITY_DN12747_c0_g1_i1_3	1	11,8	9,3967	6,8816					
TRINITY_DN4648_c0_g1_i1_3	1	12,7	13,579	9,7006	PF00378.19	ECH_1	63,7	1,50E-17	CL0127
TRINITY_DN396_c0_g1_i1_1	1	20,7	12,144	6,6813	PF01645.16	Glu_synthase	178,1	2,70E-52	CL0036
TRINITY_DN3770_c0_g1_i1_3	1	8,2	25,364	11,439	PF12068.7	DUF3548	82,4	3,00E-23	No_clan
TRINITY_DN7965_c1_g1_i1_2	3	14,4	37,5	21,083	PF01704.17	UDPGP	137,2	5,40E-40	CL0110
TRINITY_DN5625_c0_g2_i1_1	2	6,3	41,581	13,49	PF13847.5	Methyltransf_31	67,4	1,10E-18	CL0063
TRINITY_DN9598_c0_g1_i1_1	2	6,7	32,436	11,248	PF00378.19	ECH_1	152,9	8,80E-45	CL0127

TRINITY_DN6936_c0_g1_i1_3	1	2,2	61,487	7,5759	PF01490.17	Aa_trans	349,5	2,00E-104	CL0062
TRINITY_DN22611_c0_g1_i1_5	1	22,1	7,6036	13,312	PF01749.19	IBB	25	1,70E-05	CL0020
TRINITY_DN4497_c1_g1_i1_1	4	46,9	11,065	6,9707	PF00240.22	ubiquitin	28,1	1,20E-06	CL0072
TRINITY_DN16255_c0_g2_i1_1	1	3,5	32,901	6,9846	PF03981.11	Ubiq_cyt_C_chap	119,9	7,90E-35	No_clan
TRINITY_DN3029_c0_g2_i1_3	2	14,5	19,621	12,291	PF03767.13	Acid_phosphat_B	56,2	3,30E-15	CL0137
TRINITY_DN7010_c0_g2_i2_5	5	22	26,563	6,4948	PF00297.21	Ribosomal_L3	287,1	1,70E-85	CL0575
TRINITY_DN8504_c0_g1_i1_2	2	21,7	16,603	13,716					
TRINITY_DN3130_c0_g2_i2_1	3	6,7	55,371	20,229	PF10509.8	GalKase_gal_bdg	29,3	4,20E-07	CL0329
TRINITY_DN13283_c0_g1_i1_5	2	26,9	10,277	13,405	PF01074.21	Glyco_hydro_38	49,1	4,40E-13	CL0158
TRINITY_DN3329_c0_g1_i1_6	1	7,1	22,388	7,8307	PF00013.28	KH_1	67,3	7,10E-19	CL0007
TRINITY_DN10602_c1_g1_i2_3	1	7,3	17,983	6,4615	PF05365.11	UCR_UQCRX_QCR9	69,7	1,40E-19	No_clan
TRINITY_DN5356_c0_g1_i1_3	1	3	68,601	6,696	PF00069.24	Pkinase	222,2	7,00E-66	CL0016
TRINITY_DN8024_c0_g1_i1_1	1	6,3	27,343	7,3437	PF05071.15	NDUFA12	87,1	1,00E-24	No_clan
TRINITY_DN9300_c0_g1_i1_5	3	6,3	65,14	18,844	PF08911.10	NUP50	61,1	1,10E-16	No_clan
TRINITY_DN9177_c0_g3_i1_3	7	22,4	43,203	13,739	PF07718.11	Coatamer_beta_C	204,7	4,60E-61	CL0159
TRINITY_DN14061_c0_g1_i1_4	3	25	14,943	18,697	PF14226.5	DIOX_N	86,7	1,80E-24	CL0029
TRINITY_DN8441_c0_g2_i2_3	3	5,7	62,698	19,315	PF00789.19	UBX	61,3	7,00E-17	CL0072
TRINITY_DN7456_c0_g2_i1_3	2	7,5	51,716	15,422	PF00483.22	NTP_transferase	177,3	3,50E-52	CL0110
TRINITY_DN4946_c0_g2_i1_3	2	5,9	53,132	12,151	PF04715.12	Anth_synt_I_N	60,9	1,40E-16	No_clan
TRINITY_DN18912_c0_g1_i1_2	2	9,5	39,012	12,102	PF00069.24	Pkinase	214,5	1,50E-63	CL0016
TRINITY_DN12766_c0_g1_i1_5	3	33,1	14,491	21,619					
TRINITY_DN4468_c0_g1_i1_6	3	10,8	54,5	22,846	PF00899.20	ThiF	86,3	1,90E-24	CL0063
TRINITY_DN12470_c0_g1_i1_3	2	7,3	35,61	12,001	PF00686.18	CBM_20	58,6	4,00E-16	CL0369
TRINITY_DN10609_c0_g1_i2_2	2	4,4	65,803	11,736	PF00067.21	p450	211,4	1,90E-62	No_clan
TRINITY_DN11550_c0_g1_i3_3	2	6,5	44,603	13,661	PF00795.21	CN_hydrolase	184,6	1,90E-54	No_clan
TRINITY_DN18034_c0_g1_i1_3	1	12,8	8,8452	6,4446					
TRINITY_DN10769_c0_g1_i1_1	1	4,2	40,783	12,288	PF03878.14	YIF1	197,6	2,10E-58	CL0112
TRINITY_DN11604_c0_g1_i8_1	5	47,6	19,162	6,6489	PF08241.11	Methyltransf_11	23,5	6,00E-05	CL0063
TRINITY_DN11302_c0_g1_i2_1	2	6	68,366	15,473	PF16656.4	Pur_ac_phosph_N	84,1	7,20E-24	CL0159
TRINITY_DN12632_c0_g1_i1_3	2	21,3	18,156	22,949	PF13489.5	Methyltransf_23	68,2	6,60E-19	CL0063
TRINITY_DN9378_c0_g1_i4_2	3	5,6	74,884	17,8	PF17047.4	SMP_LBD	62,4	3,40E-17	CL0648
TRINITY_DN9423_c0_g2_i1_4	3	18,2	20,933	17,91					

TRINITY_DN7649_c0_g1_i2_4	1	5,7	22,559	7,0181	PF04061.13	ORMDL	156,5	2,90E-46	No_clan
TRINITY_DN18448_c0_g1_i1_3	1	10,9	11,507	7,3536	PF01553.20	Acyltransferase	23,2	4,00E-05	CL0228
TRINITY_DN11658_c0_g1_i3_3	3	3,5	129,45	21,816	PF12515.7	CaATP_NAI	62,8	1,50E-17	No_clan
TRINITY_DN18880_c0_g1_i1_1	2	26,8	13,915	11,729	PF05879.11	RHD3	98,7	2,30E-28	CL0023
TRINITY_DN10400_c0_g1_i3_1	3	8	72,498	25,842	PF08156.12	NOP5NT	66,7	1,60E-18	No_clan
TRINITY_DN1374_c0_g1_i1_1	2	10,8	18,751	6,3861					
TRINITY_DN3233_c0_g1_i1_1	4	20,6	24,634	21,704	PF00160.20	Pro_isomerase	138,4	2,20E-40	CL0475
TRINITY_DN16037_c0_g1_i1_6	1	13,5	10,783	6,9537					
TRINITY_DN4496_c0_g1_i1_6	3	20,3	17,991	18,025	PF00342.18	PGI	69,2	2,40E-19	CL0067
TRINITY_DN3498_c0_g1_i1_3	7	20,2	39,675	13,31	PF00557.23	Peptidase_M24	73,7	1,50E-20	No_clan
TRINITY_DN8015_c0_g2_i1_1	2	5,5	51,589	12,236	PF00155.20	Aminotran_1_2	170,1	7,00E-50	CL0061
TRINITY_DN8457_c0_g1_i2_3	1	6,1	30,093	6,7628					
TRINITY_DN8936_c0_g2_i1_3	1	14	12,54	6,834					
TRINITY_DN4836_c0_g2_i2_1	2	7,7	54,621	16,028	PF02929.16	Bgal_small_N	258,4	6,10E-77	CL0103
TRINITY_DN4305_c0_g1_i1_2	2	11,3	28,232	19,631	PF01521.19	Fe-S_biosyn	48,9	5,70E-13	No_clan
TRINITY_DN912_c0_g1_i1_3	2	10,1	25,155	104,69					
TRINITY_DN2769_c0_g1_i1_2	1	2,6	56,369	7,0983	PF00995.22	Sec1	359,5	4,70E-107	No_clan
TRINITY_DN7152_c0_g1_i2_3	2	9,5	35,787	15,509					
TRINITY_DN7966_c1_g1_i1_1	2	17,6	17,516	14,342					
TRINITY_DN4959_c0_g1_i1_6	1	10,6	9,7869	7,3545					
TRINITY_DN11436_c0_g1_i3_2	4	13,4	51,869	18,422	PF02874.22	ATP-synt_ab_N	42,9	5,00E-11	CL0275
TRINITY_DN6999_c0_g1_i1_5	2	12	22,837	13,052					
TRINITY_DN19644_c0_g1_i1_5	1	10,5	16,713	-2					
TRINITY_DN12673_c0_g1_i1_2	1	14,4	12,688	8,1351	PF00481.20	PP2C	89,6	2,40E-25	CL0238
TRINITY_DN10039_c0_g1_i2_2	1	6,6	24,893	7,273					
TRINITY_DN6311_c0_g1_i1_1	3	11,6	27,421	12,725	PF00224.20	PK	147,1	5,50E-43	CL0151
TRINITY_DN19028_c0_g1_i1_2	1	13,6	8,9972	7,3459	PF00349.20	Hexokinase_1	96,2	2,30E-27	CL0108
TRINITY_DN10956_c0_g1_i2_3	3	5,4	64,64	18,912	PF01154.16	HMG_CoA_synt_N	268,3	2,60E-80	CL0046
TRINITY_DN10484_c2_g1_i1_1	3	8,1	37,089	38,985	PF13774.5	Longin	74,3	5,40E-21	No_clan
TRINITY_DN17185_c0_g1_i1_2	1	5	27,113	7,1508	PF00254.27	FKBP_C	79,5	1,60E-22	CL0487
TRINITY_DN11454_c0_g1_i2_6	2	9,4	39,032	12,68	PF01105.23	EMP24_GP25L	106,6	1,30E-30	CL0521
TRINITY_DN588_c0_g1_i1_4	1	24,3	11,616	6,6783	PF01417.19	ENTH	64,6	7,90E-18	CL0009
TRINITY_DN12701_c0_g1_i1_3	3	16,2	20,918	17,979	PF00534.19	Glycos_transf_1	108	3,60E-31	CL0113

TRINITY_DN7373_c0_g2_i1_3	4	10,5	50,864	24,31					
TRINITY_DN11305_c0_g1_i2_2	2	8	47,482	17,796	PF00179.25	UQ_con	72,1	3,20E-20	CL0208
TRINITY_DN5684_c0_g1_i1_3	3	12,3	40,45	18,196	PF14681.5	UPRTase	231	8,50E-69	CL0533
TRINITY_DN864_c0_g1_i1_1	2	8,7	52,833	14,588	PF04280.14	Tim44	117,7	4,10E-34	CL0051
TRINITY_DN4602_c0_g1_i1_1	1	3	49,765	7,7685	PF01399.26	PCI	66	3,40E-18	CL0123
TRINITY_DN10884_c0_g1_i1_3	2	7,5	39,515	14,198	PF00106.24	adh_short	84,5	5,90E-24	CL0063
TRINITY_DN14631_c0_g1_i1_2	1	12	10,247	6,3439	PF01789.15	PsbP	62,3	5,30E-17	CL0619
TRINITY_DN10399_c0_g1_i1_1	1	3,6	40,847	7,0735	PF01956.15	DUF106	174,3	1,60E-51	CL0376
TRINITY_DN11619_c0_g2_i6_1	1	3,6	44,629	15,391	PF03169.14	OPT	256,5	5,20E-76	No_clan
TRINITY_DN5247_c0_g2_i1_5	2	3,1	108,38	13,811					
TRINITY_DN2861_c0_g1_i1_2	2	13,4	22,875	12,867	PF00933.20	Glyco_hydro_3	170,8	4,40E-50	CL0058
TRINITY_DN6453_c0_g1_i1_2	3	12,9	43,669	20,819	PF00191.19	Annexin	52,8	2,80E-14	No_clan
TRINITY_DN13293_c0_g1_i1_6	1	9,8	15,233	7,2625					
TRINITY_DN5843_c0_g1_i1_3	3	23,6	29,053	40,616	PF02801.21	Ketoacyl-synt_C	100,4	5,80E-29	CL0046
TRINITY_DN3985_c0_g1_i1_5	2	10,3	27,896	14,17	PF12352.7	V-SNARE_C	45,1	8,70E-12	CL0147
TRINITY_DN5098_c0_g2_i1_2	2	5,4	53,986	13,937	PF01764.24	Lipase_3	118,4	2,10E-34	CL0028
TRINITY_DN6975_c0_g2_i1_1	2	7,3	52,657	12,217	PF01926.22	MMR_HSR1	73,4	1,30E-20	CL0023
TRINITY_DN3955_c0_g1_i1_6	1	4,8	36,117	9,4865					
TRINITY_DN21162_c0_g1_i1_1	1	20,7	9,874	25,853					
TRINITY_DN13980_c0_g1_i1_4	1	6,2	25,604	6,5786	PF00995.22	Sec1	157,7	6,00E-46	No_clan
TRINITY_DN6436_c0_g2_i1_3	7	26,2	34,698	20,662	PF00071.21	Ras	206,3	1,90E-61	CL0023
TRINITY_DN7382_c0_g1_i1_5	2	5,9	55,046	16,538	PF00153.26	Mito_carr	44,4	1,10E-11	No_clan
TRINITY_DN8895_c0_g1_i3_1	3	6,3	47,296	16,222	PF01212.20	Beta_elim_lyase	319,9	1,40E-95	CL0061
TRINITY_DN11523_c0_g1_i1_2	3	9,1	68,328	17,377	PF03055.14	RPE65	409,4	1,80E-122	No_clan
TRINITY_DN6472_c0_g1_i1_3	1	13,9	17,955	12,973					
TRINITY_DN8927_c0_g1_i1_3	4	10	53,707	23,022	PF01399.26	PCI	65,3	5,60E-18	CL0123
TRINITY_DN22235_c0_g1_i1_2	2	22,7	14,609	11,953					
TRINITY_DN22096_c0_g1_i1_6	1	12,3	14,525	9,4491					
TRINITY_DN10384_c0_g1_i1_3	10	26,9	60,604	7,7269	PF00091.24	Tubulin	233,1	2,90E-69	CL0566
TRINITY_DN12671_c0_g1_i1_1	2	4,9	56,61	14,51	PF14555.5	UBA_4	27,1	2,50E-06	CL0214
TRINITY_DN15659_c0_g1_i1_5	2	28,1	9,9162	7,1508	PF00637.19	Clathrin	63,1	2,40E-17	CL0020
TRINITY_DN3596_c0_g1_i1_4	1	14,1	17,24	6,7476	PF00241.19	Cofilin_ADF	103,5	8,10E-30	CL0092
TRINITY_DN8121_c0_g1_i1_3	1	6,5	26,232	7,8509	PF00335.19	Tetraspannin	32	8,90E-08	CL0347

TRINITY_DN20264_c0_g1_i1_2	1	19,7	7,7277	8,0941					
TRINITY_DN18042_c0_g1_i1_3	1	11,7	17,448	6,9313					
TRINITY_DN18961_c0_g1_i1_1	2	24,7	9,8148	11,857	PF09070.10	PFU	104,4	3,60E-30	No_clan
TRINITY_DN3104_c0_g2_i1_5	2	14,2	14,487	12,322	PF00307.30	CH	23,6	4,10E-05	CL0188
TRINITY_DN17865_c0_g1_i1_1	2	10,8	37,5	15,628	PF14492.5	EFG_II	108,9	9,00E-32	CL0437
TRINITY_DN11260_c0_g1_i2_3	3	8,7	52,639	21,769	PF00291.24	PALP	114,8	4,60E-33	No_clan
TRINITY_DN6677_c0_g2_i1_6	2	38,3	8,4584	14,084	PF00280.17	potato_inhibit	86	1,90E-24	CL0367
TRINITY_DN14239_c0_g1_i1_6	1	9,3	16,371	7,122					
TRINITY_DN12081_c0_g1_i1_6	3	6,5	58,724	21,163	PF13540.5	RCC1_2	29,6	4,00E-07	CL0186
TRINITY_DN18664_c0_g1_i1_1	3	23,6	24,302	18,75	PF01012.20	ETF	90,6	9,80E-26	CL0039
TRINITY_DN3353_c0_g1_i1_5	2	21,3	15,532	20,343	PF09127.10	Leuk-A4-hydro_C	99,3	1,20E-28	CL0020
TRINITY_DN13649_c0_g1_i1_6	3	13,8	23,502	21,949	PF00012.19	HSP70	76,9	8,70E-22	CL0108
TRINITY_DN21861_c0_g1_i1_3	1	14,7	12,624	9,6282					
TRINITY_DN22107_c0_g1_i1_1	1	19,8	11,21	8,6931					
TRINITY_DN19998_c0_g1_i1_4	2	35,7	10,931	13,872	PF00682.18	HMGL-like	93,4	1,60E-26	CL0036
TRINITY_DN4490_c0_g2_i2_2	1	4,6	51,561	8,2974	PF01490.17	Aa_trans	160	6,50E-47	CL0062
TRINITY_DN22424_c0_g2_i1_6	3	26,5	19,365	14,615	PF01474.15	DAHP_synth_2	208,3	1,40E-61	CL0036
TRINITY_DN1144_c0_g1_i1_3	2	5,3	48,474	12,444	PF02885.16	Glycos_trans_3N	41,3	9,40E-11	No_clan
TRINITY_DN4661_c0_g1_i3_3	2	11	34,408	12,342	PF00400.31	WD40	16,1	0,015	CL0186
TRINITY_DN3820_c0_g2_i1_3	3	5,6	73,378	17,544	PF01399.26	PCI	64,5	1,00E-17	CL0123
TRINITY_DN10264_c0_g1_i1_2	3	7,3	54,44	12,682	PF00149.27	Metallophos	124,5	7,20E-36	CL0163
TRINITY_DN8158_c0_g1_i1_1	1	5,2	40,731	7,8404	PF01398.20	JAB	93,3	9,20E-27	CL0366
TRINITY_DN9206_c0_g2_i1_1	4	14,7	37,941	26,271	PF01112.17	Asparaginase_2	387,7	3,00E-116	CL0052
TRINITY_DN9226_c0_g1_i2_3	2	5,4	53,442	13,463	PF07712.11	SURNod19	642,4	1,80E-193	No_clan
TRINITY_DN585_c0_g1_i1_6	1	13,8	9,2842	6,4921	PF16363.4	GDP_Man_Dehyd	55	7,70E-15	CL0063
TRINITY_DN21456_c0_g1_i1_2	1	10,7	11,334	7,2517					
TRINITY_DN5447_c0_g2_i1_2	1	3,7	59,135	11,758	PF00076.21	RRM_1	27	2,70E-06	CL0221
TRINITY_DN23101_c0_g1_i1_6	1	11,6	13,207	7,0191					
TRINITY_DN1220_c0_g1_i1_6	4	9,3	49,428	22,645	PF01425.20	Amidase	93,3	1,50E-26	No_clan
TRINITY_DN13227_c0_g1_i1_1	1	16,5	12,326	6,8939	PF11987.7	IF-2	69,9	1,60E-19	No_clan
TRINITY_DN5512_c0_g3_i1_3	1	7	34,645	8,1448					
TRINITY_DN4033_c0_g2_i1_3	1	4,7	33,834	9,741	PF12812.6	PDZ_1	38,8	6,60E-10	CL0466

TRINITY_DN4640_c0_g1_i1_2	2	8,6	29,461	14,313	PF00383.22	dCMP_cyt_deam_1	83,8	5,80E-24	CL0109
TRINITY_DN5850_c0_g1_i1_1	1	10,3	14,988	9,8235					
TRINITY_DN11339_c0_g1_i3_1	1	3	35,998	6,5762	PF01965.23	DJ-1_PfpI	165	1,10E-48	CL0014
TRINITY_DN2739_c0_g1_i1_1	1	6,3	26,244	7,4474	PF04628.12	Sedlin_N	147	3,00E-43	CL0212
TRINITY_DN6721_c1_g1_i1_1	4	43,7	12,698	25,312	PF06068.12	TIP49	123,5	9,10E-36	CL0023
TRINITY_DN7299_c0_g1_i1_1	1	5,2	18,941	6,4894	PF05051.12	COX17	70,1	1,40E-19	CL0351
TRINITY_DN2661_c0_g2_i1_1	2	9,1	49,239	15,326	PF13839.5	PC-Esterase	260,8	1,80E-77	CL0264
TRINITY_DN7083_c0_g2_i2_1	2	6	62,118	14,198	PF01063.18	Aminotran_4	148,6	2,20E-43	No_clan
TRINITY_DN18425_c0_g1_i1_2	1	9,1	12,531	6,3674	PF01031.19	Dynamin_M	82,2	3,40E-23	No_clan
TRINITY_DN11416_c0_g1_i1_3	2	5,7	57,76	18,282					
TRINITY_DN9924_c0_g1_i1_2	2	20,1	14,522	12,589					
TRINITY_DN8093_c0_g1_i1_2	2	6,3	37,947	11,179	PF01467.25	CTP_transf_like	114,7	3,60E-33	CL0039
TRINITY_DN766_c0_g1_i1_2	3	23	16,463	12,97	PF03358.14	FMN_red	46,9	2,20E-12	CL0042
TRINITY_DN10380_c0_g1_i2_1	1	7,6	17,648	7,0453	PF07719.16	TPR_2	30,4	2,20E-07	CL0020
TRINITY_DN2440_c0_g1_i2_2	3	4,7	70,781	18,245	PF01406.18	tRNA-synt_1e	411,4	2,10E-123	CL0039
TRINITY_DN18420_c0_g1_i1_3	1	14,7	15,818	7,3996	PF10408.8	Ufd2P_core	88,9	2,70E-25	No_clan
TRINITY_DN7383_c0_g1_i1_3	1	4,7	34,047	7,0302	PF03357.20	Snf7	123,8	5,00E-36	CL0235
TRINITY_DN14144_c0_g1_i1_6	2	12,5	22,082	13,744					
TRINITY_DN16953_c0_g1_i1_2	1	20,8	8,4516	12,183					
TRINITY_DN10509_c0_g1_i1_3	2	5,9	33,117	11,731	PF01738.17	DLH	102,2	2,60E-29	CL0028
TRINITY_DN7183_c0_g1_i2_1	2	7	41,267	12,849	PF01926.22	MMR_HSR1	55,7	4,20E-15	CL0023
TRINITY_DN17895_c0_g1_i1_1	2	17	18,688	36,964	PF00982.20	Glyco_transf_20	25,6	4,30E-06	CL0113
TRINITY_DN9983_c0_g1_i1_1	2	6,6	57,02	14,38	PF04209.12	HgmA	700,7	5,40E-211	CL0029
TRINITY_DN1054_c0_g1_i1_1	4	13,6	27,246	17,326	PF13417.5	GST_N_3	30,8	2,60E-07	CL0172
TRINITY_DN1267_c0_g1_i1_6	1	13,6	10,985	7,7946	PF00132.23	Hexapep	18,6	0,001	CL0536
TRINITY_DN10960_c0_g1_i1_3	2	6,4	48,381	12,412	PF00574.22	CLP_protease	147,7	3,10E-43	CL0127
TRINITY_DN20312_c0_g1_i1_4	2	21,3	16,732	18,358	PF00800.17	PDT	30,5	2,70E-07	CL0177
TRINITY_DN18128_c0_g1_i1_2	2	32,1	9,2557	13,074					
TRINITY_DN8563_c0_g1_i1_2	2	7,4	42,569	12,886	PF04893.16	Yip1	52,8	3,50E-14	CL0112
TRINITY_DN6232_c0_g1_i3_2	2	5,7	70,789	13,489					
TRINITY_DN5437_c0_g1_i1_2	2	5,9	60,632	15,224	PF03141.15	Methyltransf_29	404,4	5,80E-121	CL0063
TRINITY_DN3837_c0_g1_i1_3	2	12	24,287	28,138					

TRINITY_DN4426_c0_g1_i1_2	2	7,4	48,53	12,426	PF14416.5	PMR5N	71,7	4,10E-20	No_clan
TRINITY_DN13216_c0_g1_i1_3	1	34,5	8,7808	6,6533					
TRINITY_DN8517_c0_g1_i3_3	3	7,4	72,212	18,702	PF12819.6	Malectin_like	210,3	3,70E-62	CL0468
TRINITY_DN17094_c0_g1_i1_4	3	18,5	21,397	19,249	PF12763.6	EF-hand_4	43,9	1,60E-11	CL0220
TRINITY_DN20326_c0_g1_i1_2	1	7,1	17,71	6,6156	PF00443.28	UCH	68,4	6,00E-19	CL0125
TRINITY_DN7535_c0_g2_i1_1	1	5,4	42,729	6,6489	PF01694.21	Rhomboid	137,8	2,50E-40	CL0207
TRINITY_DN7228_c0_g1_i2_1	2	6,9	43,41	12,361	PF10408.8	Ufd2P_core	279,4	5,90E-83	No_clan
TRINITY_DN16259_c0_g1_i1_3	2	33	9,5437	12,847	PF00133.21	tRNA-synt_1	75,5	2,50E-21	CL0039
TRINITY_DN21689_c0_g1_i1_3	1	4,7	22,273	6,5225	PF00076.21	RRM_1	61,6	4,40E-17	CL0221
TRINITY_DN8651_c0_g2_i1_2	2	7,4	48,095	17,308	PF03643.14	Vps26	412,2	7,00E-124	CL0135
TRINITY_DN11262_c0_g1_i1_3	1	4	37,958	6,3132	PF00071.21	Ras	199,4	2,40E-59	CL0023
TRINITY_DN2292_c0_g3_i1_5	2	19,1	10,655	23,834	PF00428.18	Ribosomal_60s	39,5	6,10E-10	No_clan
TRINITY_DN12820_c0_g1_i1_2	2	20,8	16,199	13,029	PF00307.30	CH	30	4,40E-07	CL0188
TRINITY_DN21154_c0_g1_i1_4	1	8,4	16,993	7,0983					
TRINITY_DN17026_c0_g1_i1_1	1	4,6	27,745	55,155	PF02140.17	Gal_Lectin	74	9,30E-21	No_clan
TRINITY_DN14578_c0_g1_i1_4	2	13	17,677	12,873	PF00560.32	LRR_1	11,8	0,27	CL0022
TRINITY_DN5368_c0_g1_i2_5	6	22,4	35,968	12,251	PF00244.19	14-3-3	346,4	5,50E-104	No_clan
TRINITY_DN17464_c0_g1_i1_1	2	16	19,677	11,26	PF01380.21	SIS	55,7	4,10E-15	CL0067
TRINITY_DN8742_c0_g1_i1_3	1	4,2	37,583	7,8326	PF00025.20	Arf	167,9	1,30E-49	CL0023
TRINITY_DN14137_c0_g1_i1_2	2	28,8	8,3896	13,812	PF01593.23	Amino_oxidase	30,4	2,20E-07	CL0063
TRINITY_DN2434_c0_g1_i1_5	2	5,8	45,318	11,59					
TRINITY_DN10474_c1_g1_i1_2	3	6,3	74,006	16,607	PF00069.24	Pkinase	268,3	5,90E-80	CL0016
TRINITY_DN12774_c0_g1_i1_1	1	10,8	17,607	6,801	PF04909.13	Amidohydro_2	35,8	7,00E-09	CL0034
TRINITY_DN3904_c0_g1_i1_3	2	12,2	16,074	12,29					
TRINITY_DN19463_c0_g1_i1_1	1	9,8	13,65	8,0601					
TRINITY_DN8904_c0_g2_i1_3	3	8,3	48,994	19,105	PF14604.5	SH3_9	40	2,40E-10	CL0010
TRINITY_DN8767_c0_g1_i1_3	2	7,8	56,318	15,522					
TRINITY_DN3578_c0_g1_i1_2	5	30,3	17,215	6,5762	PF00347.22	Ribosomal_L6	42,6	6,70E-11	No_clan
TRINITY_DN15270_c0_g1_i1_2	2	17,1	21,144	13,814					
TRINITY_DN1008_c0_g2_i1_2	1	6,1	17,815	6,7699					
TRINITY_DN13945_c0_g1_i1_1	2	10,2	25,342	12,271	PF00069.24	Pkinase	80	1,60E-22	CL0016
TRINITY_DN7325_c0_g1_i1_1	2	9,3	21,479	11,733	PF00240.22	ubiquitin	25,2	9,20E-06	CL0072
TRINITY_DN11924_c0_g1_i1_2	2	9,4	41,24	13,931					

TRINITY_DN10508_c0_g1_i3_5	3	9,8	38,718	16,222	PF00248.20	Aldo_ket_red	168,3	1,90E-49	No_clan
TRINITY_DN7011_c0_g1_i1_2	2	5,1	38,014	12,753	PF00307.30	CH	38,6	9,00E-10	CL0188
TRINITY_DN3619_c0_g1_i1_2	2	15,6	16,006	12,474	PF07748.12	Glyco_hydro_38C	30	2,50E-07	CL0103
TRINITY_DN14686_c0_g1_i1_1	1	19,8	9,789	6,7863	PF00759.18	Glyco_hydro_9	91,3	8,00E-26	CL0059
TRINITY_DN9581_c0_g1_i2_2	2	12,1	24,196	36,854	PF03061.21	4HBT	29,7	5,50E-07	CL0050
TRINITY_DN9707_c0_g1_i1_1	2	6,7	42,528	16,512	PF00701.21	DHDPS	306	1,60E-91	CL0036
TRINITY_DN8938_c0_g1_i1_2	2	6	53,967	13,983					
TRINITY_DN10825_c0_g1_i4_1	1	9,6	18,078	8,1567					
TRINITY_DN7033_c0_g2_i1_2	3	5,2	69,38	17,057					
TRINITY_DN10999_c0_g1_i5_2	3	6,1	96,338	14,147	PF03030.15	H_PPase	879,1	2,10E-264	No_clan
TRINITY_DN3792_c0_g1_i1_2	4	17,3	36,384	24,471					
TRINITY_DN15541_c0_g1_i1_1	1	10,3	16,289	7,3253					
TRINITY_DN2393_c0_g1_i1_3	2	6,4	48,815	15,429					
TRINITY_DN10184_c0_g1_i1_3	2	3,6	51,805	16,374	PF01073.18	3Beta_HSD	160,7	3,40E-47	CL0063
TRINITY_DN7705_c0_g1_i2_6	3	7,2	66,351	19,799	PF00076.21	RRM_1	44,1	1,30E-11	CL0221
TRINITY_DN7315_c0_g1_i1_4	2	8,9	42,346	24,011	PF01612.19	DNA_pol_A_exo1	80,8	9,00E-23	CL0219
TRINITY_DN8439_c0_g1_i1_6	3	9,6	40,739	11,204	PF00141.22	peroxidase	158,5	1,90E-46	CL0617
TRINITY_DN3610_c0_g2_i1_2	1	7,9	15,56	6,8876	PF07798.10	DUF1640	140,7	4,60E-41	No_clan
TRINITY_DN3307_c0_g1_i1_1	2	16,4	23,329	12,744	PF01535.19	PPR	24,7	1,60E-05	CL0020
TRINITY_DN18256_c0_g1_i1_4	3	10,6	49,182	17,324					
TRINITY_DN11948_c0_g1_i1_6	1	3,3	34,325	6,916	PF13622.5	4HBT_3	201,9	1,80E-59	CL0050
TRINITY_DN5672_c0_g1_i1_6	1	1,7	66,159	-2					
TRINITY_DN138_c0_g1_i1_3	2	26,6	15,232	11,85					
TRINITY_DN10435_c0_g1_i1_1	2	5,8	77,751	12,497	PF03630.13	Fumble	306,3	2,40E-91	CL0108
TRINITY_DN10493_c0_g1_i1_1	6	20,7	33,317	12,885	PF00071.21	Ras	222,1	2,60E-66	CL0023
TRINITY_DN10290_c0_g1_i1_1	2	5,6	51,766	16,35	PF08569.10	Mo25	413,2	7,90E-124	CL0020
TRINITY_DN12636_c0_g1_i1_3	2	18,2	15,173	13,9	PF00107.25	ADH_zinc_N	66,9	1,40E-18	CL0063
TRINITY_DN14818_c0_g1_i1_6	2	21,6	17,459	14,096	PF00300.21	His_Phos_1	52,5	4,60E-14	CL0071
TRINITY_DN360_c0_g1_i1_4	2	12,5	33,64	17,621	PF05686.11	Glyco_transf_90	362,6	2,10E-108	CL0113
TRINITY_DN7953_c0_g2_i1_2	2	4,1	55,659	11,879	PF01494.18	FAD_binding_3	36,1	4,00E-09	CL0063
TRINITY_DN8751_c1_g1_i1_3	2	7,9	53,605	15,612	PF00400.31	WD40	13	0,14	CL0186
TRINITY_DN19806_c0_g1_i1_2	2	25	13,142	12,08	PF02733.16	Dak1	160,2	4,80E-47	CL0245
TRINITY_DN18164_c0_g1_i1_4	1	12,5	14,854	7,3518	PF05193.20	Peptidase_M16_C	30,4	3,20E-07	CL0094

TRINITY_DN7968_c0_g1_i1_1	1	4,8	22,972	7,1275	PF01894.16	UPF0047	130,3	3,20E-38	No_clan
TRINITY_DN7925_c0_g2_i1_4	2	10,1	32,777	12,646	PF00956.17	NAP	27,1	2,10E-06	No_clan
TRINITY_DN8225_c0_g1_i1_3	2	6,5	63,684	13,669	PF01650.17	Peptidase_C13	379,2	8,80E-114	CL0093
TRINITY_DN7814_c0_g1_i1_2	2	5,4	54,822	12,933	PF06886.10	TPX2	70,7	8,20E-20	No_clan
TRINITY_DN21393_c0_g1_i1_4	2	20	18,12	20,215	PF03759.12	PRONE	91,3	4,90E-26	No_clan
TRINITY_DN20157_c0_g1_i1_3	2	31,1	8,4173	11,595	PF13639.5	zf-RING_2	47,9	1,10E-12	CL0229
TRINITY_DN4739_c0_g2_i1_6	2	26,6	20,133	16,15	PF13365.5	Trypsin_2	89,3	4,20E-25	CL0124
TRINITY_DN6504_c0_g1_i1_3	2	3,5	45,315	11,281	PF00612.26	IQ	23	3,70E-05	CL0220
TRINITY_DN9930_c0_g1_i3_4	2	15,3	24,077	18,129	PF01588.19	tRNA_bind	86,8	7,50E-25	CL0021
TRINITY_DN11687_c0_g1_i1_1	2	35,4	8,7137	10,88					
TRINITY_DN968_c0_g1_i1_2	1	5,7	25,709	8,5462	PF00067.21	p450	156,7	7,60E-46	No_clan
TRINITY_DN8156_c0_g1_i1_6	2	5,9	41,568	12,055	PF05686.11	Glyco_transf_90	552,9	3,50E-166	CL0113
TRINITY_DN9403_c0_g1_i2_1	3	10,5	45,882	13,212	PF08240.11	ADH_N	108,6	1,30E-31	CL0296
TRINITY_DN10308_c0_g2_i1_1	2	10,7	23,839	6,4287					
TRINITY_DN5427_c0_g1_i1_3	1	12,1	18,759	20,547					
TRINITY_DN10971_c0_g1_i2_2	3	9,1	53,469	19,589	PF07676.11	PD40	26	5,80E-06	CL0186
TRINITY_DN21130_c0_g1_i1_6	2	11,1	22,382	10,738					
TRINITY_DN7507_c0_g1_i1_6	1	12,3	18,306	9,5183					
TRINITY_DN8691_c0_g1_i1_4	2	13,1	17,355	11,866					
TRINITY_DN13949_c0_g1_i2_2	2	11,1	35,504	13,721	PF00106.24	adh_short	165,8	7,00E-49	CL0063
TRINITY_DN17228_c0_g1_i1_3	1	19,3	9,4239	11,972					
TRINITY_DN15289_c0_g1_i1_4	1	9,8	13,01	7,1926	PF08207.11	EFP_N	63,2	1,60E-17	CL0107
TRINITY_DN3649_c0_g1_i1_2	1	5,9	23,468	7,842	PF00300.21	His_Phos_1	22,9	5,30E-05	CL0071
TRINITY_DN3740_c0_g2_i1_2	1	9,3	23,753	7,1331	PF01121.19	CoaE	123,8	5,60E-36	CL0023
TRINITY_DN18927_c0_g1_i1_2	2	10,9	34,54	12,77	PF01467.25	CTP_transf_like	28,7	1,20E-06	CL0039
TRINITY_DN8933_c1_g1_i1_2	1	20,2	9,2906	6,7734	PF00004.28	AAA	81	9,40E-23	CL0023
TRINITY_DN16700_c0_g1_i1_4	1	17,9	7,4043	8,9071					
TRINITY_DN709_c0_g2_i1_2	2	6,8	32,639	11,244	PF00400.31	WD40	14,8	0,036	CL0186
TRINITY_DN813_c0_g1_i1_3	1	11	20,248	6,3526					
TRINITY_DN20089_c0_g1_i1_1	1	11,7	8,3746	6,4757	PF13202.5	EF-hand_5	26,6	2,70E-06	CL0220
TRINITY_DN19940_c0_g1_i1_4	1	7,7	18,588	7,679	PF01031.19	Dynamin_M	54,9	6,90E-15	No_clan
TRINITY_DN16604_c0_g1_i1_3	2	17	16,314	17,699	PF07719.16	TPR_2	28,1	1,20E-06	CL0020
TRINITY_DN9343_c0_g1_i1_2	2	6,8	74,733	19,179	PF07714.16	Pkinase_Tyr	107,3	7,10E-31	CL0016

TRINITY_DN8561_c0_g1_i4_1	4	27,5	29,506	24,773					
TRINITY_DN21803_c0_g1_i1_1	1	19,4	12,662	7,562					
TRINITY_DN4286_c0_g1_i1_4	2	12,7	33,306	15,24	PF00069.24	Pkinase	123,2	1,10E-35	CL0016
TRINITY_DN5644_c1_g3_i1_6	1	22,8	15,48	6,5392					
TRINITY_DN11599_c0_g1_i3_2	2	16,3	31,609	11,849					
TRINITY_DN17497_c0_g1_i1_2	2	9,8	50,66	12,069	PF02786.16	CPSase_L_D2	98,6	3,10E-28	CL0179
TRINITY_DN3550_c0_g2_i1_2	2	4	48,67	11,8	PF10191.8	COG7	431,6	5,50E-129	CL0294
TRINITY_DN4310_c0_g1_i1_1	1	5,9	18,702	6,4157	PF00724.19	Oxidored_FMN	42	6,20E-11	CL0036
TRINITY_DN21144_c0_g1_i1_4	3	14	31,477	21,253	PF00795.21	CN_hydrolase	150,3	5,60E-44	No_clan
TRINITY_DN23569_c0_g1_i1_2	1	23,7	7,9162	6,7389					
TRINITY_DN2786_c0_g1_i1_5	1	13,3	21,737	9,1134	PF00657.21	Lipase_GDSL	31,9	1,10E-07	CL0264
TRINITY_DN5005_c0_g1_i1_1	2	7,4	43,484	21,114	PF00581.19	Rhodanese	31,1	2,40E-07	CL0031
TRINITY_DN5974_c0_g1_i1_3	2	5,5	39,906	10,745	PF02485.20	Branch	199,7	4,90E-59	CL0110
TRINITY_DN6400_c0_g1_i4_1	4	17,8	26,673	11,567	PF00071.21	Ras	130	5,60E-38	CL0023
TRINITY_DN11632_c0_g1_i2_5	2	3,8	81,885	7,8946	PF01873.16	eIF-5_eIF-2B	125,7	7,60E-37	No_clan
TRINITY_DN17813_c0_g1_i1_1	1	13,2	19,963	13,232	PF00069.24	Pkinase	71,3	7,60E-20	CL0016
TRINITY_DN17235_c0_g1_i1_3	1	7,9	23,856	8,1107					
TRINITY_DN6926_c0_g1_i1_6	2	11,7	17,183	16,596	PF10961.7	SelK_SelG	72,4	3,30E-20	No_clan
TRINITY_DN17803_c0_g2_i1_3	2	7,2	50,786	11,721	PF13320.5	DUF4091	63,6	1,30E-17	No_clan
TRINITY_DN2011_c0_g1_i1_4	2	30,3	8,5633	12,894					
TRINITY_DN8075_c0_g1_i4_2	2	7,2	45,937	13,235					
TRINITY_DN2579_c0_g1_i1_2	2	34,1	9,545	12,883	PF12436.7	USP7_ICP0_bdg	74,5	7,80E-21	CL0072
TRINITY_DN19468_c0_g1_i1_2	1	5,3	23,27	7,2127	PF00995.22	Sec1	164,7	4,60E-48	No_clan
TRINITY_DN3475_c0_g2_i1_2	2	9,9	28,132	11,257					
TRINITY_DN4140_c0_g1_i1_5	1	4,4	30,727	6,9305	PF00445.17	Ribonuclease_T2	209,8	3,50E-62	No_clan
TRINITY_DN545_c0_g1_i1_2	2	26,4	17,203	18,077	PF07766.12	LETM1	139,7	9,70E-41	No_clan
TRINITY_DN6995_c0_g1_i1_6	1	4,7	23,094	6,9846	PF01423.21	LSM	65,1	3,20E-18	CL0527
TRINITY_DN9117_c0_g1_i2_2	2	6,7	36,43	13,072	PF00168.29	C2	80	1,30E-22	CL0154
TRINITY_DN14629_c0_g1_i1_3	1	14,9	11,124	7,3181					
TRINITY_DN8532_c0_g1_i1_2	2	16,8	16,75	10,824	PF00574.22	CLP_protease	36,1	5,20E-09	CL0127
TRINITY_DN9825_c0_g1_i1_3	2	5,6	78,282	12,037	PF01434.17	Peptidase_M41	252,4	3,00E-75	CL0126
TRINITY_DN2131_c0_g1_i1_6	1	4,4	40,4	11,754	PF02540.16	NAD_synthase	69,4	2,20E-19	CL0039
TRINITY_DN2699_c0_g1_i1_6	2	4,9	60,618	9,3007	PF00479.21	G6PD_N	168,5	2,00E-49	CL0063

TRINITY_DN10766_c0_g1_i8_3	1	2,5	46,906	-2					
TRINITY_DN6812_c0_g1_i7_1	2	6,8	48,707	10,888	PF00069.24	Pkinase	103,5	1,10E-29	CL0016
TRINITY_DN19649_c0_g1_i1_5	1	19,7	13,67	7,3023					
TRINITY_DN10388_c0_g2_i1_5	3	5,9	78,894	12,664	PF00082.21	Peptidase_S8	164,4	3,30E-48	No_clan
TRINITY_DN8750_c0_g2_i1_3	3	8,5	52,799	11,566	PF14306.5	PUA_2	149,7	4,80E-44	CL0178
TRINITY_DN17662_c0_g2_i1_1	1	8,8	27,262	32,257	PF00574.22	CLP_protease	166,1	7,10E-49	CL0127
TRINITY_DN5306_c0_g1_i1_1	2	9,5	32,605	12,449	PF12899.6	Glyco_hydro_100	490,4	3,80E-147	CL0059
TRINITY_DN4670_c0_g1_i1_2	4	13,1	54,279	15,025	PF07766.12	LETM1	178,4	1,60E-52	No_clan
TRINITY_DN9078_c0_g1_i2_3	2	6,7	42,511	12,878					
TRINITY_DN8091_c0_g1_i1_6	2	11,9	29,503	11,871	PF03357.20	Snf7	147,1	3,50E-43	CL0235
TRINITY_DN12406_c0_g1_i1_2	2	18,2	16,758	7,6126	PF12701.6	LSM14	106	7,40E-31	CL0527
TRINITY_DN17368_c0_g1_i1_2	1	15	18,885	9,6769	PF00534.19	Glycos_transf_1	32,9	4,20E-08	CL0113
TRINITY_DN9727_c0_g1_i1_3	4	12,7	37,019	43,495	PF00657.21	Lipase_GDSL	59,1	5,10E-16	CL0264
TRINITY_DN17339_c0_g1_i1_3	1	26	8,8874	35,531	PF00036.31	EF-hand_1	33,8	1,20E-08	CL0220
TRINITY_DN18284_c0_g1_i1_2	2	10,9	26,515	11,71	PF16113.4	ECH_2	272,9	4,30E-81	CL0127
TRINITY_DN10226_c0_g1_i1_1	1	3,8	39,146	6,418	PF16561.4	AMPK1_CBM	57,6	1,00E-15	CL0369
TRINITY_DN8973_c0_g1_i1_1	2	7,3	38,806	13,803	PF05773.21	RWD	82,8	2,00E-23	CL0208
TRINITY_DN16264_c0_g1_i1_2	2	32,9	8,4927	12,958	PF00733.20	Asn_synthase	71,7	7,90E-20	CL0039
TRINITY_DN12122_c0_g1_i1_3	3	9,9	48,045	22,394	PF14802.5	TMEM192	54,8	6,20E-15	No_clan
TRINITY_DN4741_c0_g1_i1_2	2	4,9	54,722	12,441	PF16891.4	STPPase_N	70,9	8,20E-20	No_clan
TRINITY_DN20857_c0_g1_i1_2	1	5,8	19,265	6,7121	PF13419.5	HAD_2	53,3	3,20E-14	CL0137
TRINITY_DN13782_c0_g1_i1_1	2	26,7	8,843	11,928					
TRINITY_DN4532_c0_g1_i1_2	1	2,7	54,783	6,5299	PF01148.19	CTP_transf_1	287,9	8,70E-86	CL0234
TRINITY_DN39_c0_g2_i1_6	2	11,6	22,691	12,388					
TRINITY_DN20415_c0_g1_i1_2	1	14,1	8,9973	6,5642					
TRINITY_DN9710_c1_g1_i1_6	2	5,3	64,909	10,87	PF01535.19	PPR	23,6	3,60E-05	CL0020
TRINITY_DN371_c0_g1_i1_2	1	3,4	49,167	7,0317	PF05918.10	API5	359,2	3,70E-107	CL0020
TRINITY_DN20208_c0_g1_i1_1	1	17,1	8,0021	7,1508	PF01843.18	DIL	54	1,60E-14	No_clan
TRINITY_DN11629_c0_g1_i3_3	3	3,9	102,32	108,49	PF00933.20	Glyco_hydro_3	133,9	7,40E-39	CL0058
TRINITY_DN12689_c0_g1_i1_2	2	4,7	54,663	14,685	PF13764.5	E3_UbLigase_R4	478,3	4,30E-143	No_clan
TRINITY_DN8223_c0_g1_i4_6	1	4,5	36,495	-2					
TRINITY_DN20465_c0_g1_i1_6	1	19,2	7,4231	8,1522	PF00370.20	FGGY_N	32,6	5,40E-08	CL0108
TRINITY_DN7564_c1_g2_i2_4	2	14,1	19,683	13,214					

TRINITY_DN10977_c0_g2_i3_5	5	13,5	60,258	13,998	PF00270.28	DEAD	132,5	1,20E-38	CL0023
TRINITY_DN23633_c0_g1_i1_2	1	22,7	7,9653	6,3807	PF13840.5	ACT_7	39,4	3,60E-10	CL0070
TRINITY_DN6603_c0_g1_i1_3	3	8,5	57,472	19,02	PF03635.16	Vps35	386,1	3,40E-115	No_clan
TRINITY_DN9814_c0_g1_i2_2	3	7,3	49,726	18,148	PF00112.22	Peptidase_C1	216,5	4,30E-64	CL0125
TRINITY_DN7007_c0_g1_i1_6	2	8,4	45,473	11,475	PF10609.8	ParA	275	4,90E-82	CL0023
TRINITY_DN14463_c0_g1_i1_1	1	10,2	20,315	6,7651	PF00149.27	Metallophos	41,1	2,50E-10	CL0163
TRINITY_DN6833_c0_g1_i1_2	1	4,8	29,9	7,1747	PF00160.20	Pro_isomerase	116,8	9,80E-34	CL0475
TRINITY_DN839_c0_g1_i2_1	1	14,2	12,198	7,0925	PF01780.18	Ribosomal_L37ae	78,4	2,90E-22	CL0167
TRINITY_DN20539_c0_g1_i1_2	2	18,2	18,134	25,846	PF00012.19	HSP70	163,8	4,30E-48	CL0108
TRINITY_DN3431_c0_g1_i1_2	2	15	14,641	12,189					
TRINITY_DN11964_c0_g1_i1_3	1	4	28,232	6,7638	PF01301.18	Glyco_hydro_35	296,4	3,00E-88	CL0058
TRINITY_DN4866_c0_g2_i1_1	4	6,8	75,23	26,533	PF16969.4	SRP68	546,4	9,50E-164	No_clan
TRINITY_DN16866_c0_g1_i1_6	1	17,5	8,823	6,8661					
TRINITY_DN13956_c0_g1_i1_2	1	13,8	10,927	8,5748	PF01417.19	ENTH	48,7	6,70E-13	CL0009
TRINITY_DN4399_c0_g1_i1_6	2	30,2	11,419	6,472	PF14523.5	Syntaxin_2	26,3	6,70E-06	CL0445
TRINITY_DN10671_c0_g1_i5_2	2	7	46,486	16,379	PF04981.12	NMD3	143,3	8,00E-42	No_clan
TRINITY_DN3450_c0_g1_i1_1	2	8,6	39,678	11,413	PF13507.5	GATase_5	351	2,70E-105	CL0014
TRINITY_DN19533_c0_g1_i1_2	2	18,2	12,181	14,443					
TRINITY_DN14184_c0_g1_i1_2	2	34,9	11,762	11,819					
TRINITY_DN10011_c0_g2_i1_5	2	6,4	36,51	11,98	PF00098.22	zf-CCHC	31,2	1,40E-07	CL0511
TRINITY_DN10051_c0_g1_i3_2	2	4,8	58,151	15,143	PF00226.30	DnaJ	81,1	4,30E-23	CL0392
TRINITY_DN9084_c1_g1_i1_6	1	2,7	37,203	6,2973	PF01871.16	AMMECR1	160,3	2,80E-47	No_clan
TRINITY_DN13392_c0_g1_i1_2	2	11,9	27,301	11,541	PF00515.27	TPR_1	31,5	9,00E-08	CL0020
TRINITY_DN5114_c0_g1_i1_5	1	4,3	32,117	7,1701	PF07724.13	AAA_2	105,5	3,00E-30	CL0023
TRINITY_DN18316_c0_g1_i1_1	1	6,4	24,542	7,6517	PF03070.15	TENA_THI-4	33,3	4,00E-08	CL0230
TRINITY_DN764_c0_g1_i1_4	3	15,2	34,615	16,745	PF00786.27	PBD	37,4	2,30E-09	No_clan
TRINITY_DN10979_c0_g1_i3_3	10	21,1	49,919	9,7138	PF03214.12	RGP	584,9	4,70E-176	CL0110
TRINITY_DN10800_c0_g1_i2_2	2	8,3	55,205	12,179	PF00288.25	GHMP_kinases_N	33,5	3,50E-08	CL0329
TRINITY_DN14978_c0_g1_i1_4	1	15,5	7,8048	7,3011	PF04815.14	Sec23_helical	34,9	9,70E-09	No_clan
TRINITY_DN6214_c0_g1_i1_1	1	4,4	35,56	7,5174	PF12352.7	V-SNARE_C	66,9	1,40E-18	CL0147
TRINITY_DN14988_c0_g2_i1_4	1	12	16,139	6,3362	PF08502.9	LeuA_dimer	75,1	4,20E-21	No_clan
TRINITY_DN6278_c0_g1_i1_2	2	6,6	39,058	12,581	PF05770.10	Ins134_P3_kin	317,1	1,00E-94	CL0179
TRINITY_DN6964_c0_g1_i1_1	2	10,7	27,01	12,531	PF00091.24	Tubulin	24,9	1,80E-05	CL0566

TRINITY_DN3722_c0_g1_i1_4	2	10,3	29,69	11,374	PF00753.26	Lactamase_B	38,6	1,00E-09	CL0381
TRINITY_DN22328_c0_g1_i1_3	1	29,8	8,9288	10,93					
TRINITY_DN18052_c0_g2_i1_1	1	5,3	29,027	6,7821					
TRINITY_DN11467_c0_g1_i1_1	1	5,6	21,895	6,5129					
TRINITY_DN15189_c0_g1_i1_3	1	10,6	21,345	7,339					
TRINITY_DN14289_c0_g2_i1_2	1	9,8	14,027	7,1747	PF02769.21	AIRS_C	56	4,60E-15	No_clan
TRINITY_DN1005_c0_g1_i2_3	1	10,6	15,857	8,1262	PF02150.15	RNA_POL_M_15KD	44	1,40E-11	CL0167
TRINITY_DN3819_c0_g1_i2_3	2	7,8	53,719	13,535	PF03853.14	YjeF_N	116,6	9,90E-34	CL0063
TRINITY_DN7943_c0_g1_i2_2	2	4,6	72,343	6,777	PF00122.19	E1-E2_ATPase	24,3	1,70E-05	No_clan
TRINITY_DN2112_c0_g2_i1_6	1	11	13,637	8,4741					
TRINITY_DN10350_c1_g1_i4_2	1	3,2	37,807	7,1787	PF03398.13	Ist1	99,6	1,50E-28	No_clan
TRINITY_DN6884_c1_g3_i1_2	1	3,4	38,329	6,522	PF00106.24	adh_short	165	1,20E-48	CL0063
TRINITY_DN12265_c0_g1_i1_2	2	20,3	14,635	11,771	PF05822.11	UMPH-1	176	8,80E-52	CL0137
TRINITY_DN21124_c0_g1_i3_3	1	11,4	9,7812	6,5762					
TRINITY_DN9012_c0_g1_i1_4	2	8,3	46,943	12,779	PF00389.29	2-Hacid_dh	55,9	3,10E-15	CL0325
TRINITY_DN9946_c0_g2_i4_3	1	1,4	99,481	6,2907	PF00862.18	Sucrose_synth	951,3	1,10E-286	CL0113
TRINITY_DN5727_c0_g2_i1_6	1	9,4	14,536	-2					
TRINITY_DN12393_c0_g1_i1_4	2	8,3	33,842	14,006	PF07992.13	Pyr_redox_2	101,4	5,00E-29	CL0063
TRINITY_DN4111_c0_g2_i1_2	1	5,7	27,998	8,0508	PF00107.25	ADH_zinc_N	45,3	7,10E-12	CL0063
TRINITY_DN11057_c0_g8_i1_3	1	6,6	20,125	7,1508	PF00016.19	RuBisCO_large	277,7	9,50E-83	No_clan
TRINITY_DN2598_c0_g2_i1_3	1	1,4	86,872	6,6096	PF01301.18	Glyco_hydro_35	183,9	5,10E-54	CL0058
TRINITY_DN9900_c0_g1_i2_2	1	5,8	35,342	9,0926	PF02845.15	CUE	21,3	0,00014	CL0214
TRINITY_DN3402_c0_g1_i1_5	1	23,1	9,9723	6,8275					
TRINITY_DN9871_c0_g1_i2_2	1	5,5	27,555	6,8332	PF08695.9	Coa1	34	1,90E-08	CL0455
TRINITY_DN9274_c0_g1_i1_2	2	10,2	36,37	14,792	PF00445.17	Ribonuclease_T2	171,8	1,60E-50	No_clan
TRINITY_DN20612_c0_g1_i1_1	1	10,9	11,217	7,2635	PF00076.21	RRM_1	48,9	3,90E-13	CL0221
TRINITY_DN21955_c0_g1_i1_1	1	15,2	12,291	8,089	PF13393.5	tRNA-synt_His	34,2	1,50E-08	CL0040
TRINITY_DN7460_c0_g1_i1_6	1	4,4	28,376	7,2876	PF02492.18	cobW	26,5	3,80E-06	CL0023
TRINITY_DN6570_c0_g1_i1_6	1	7,6	21,854	6,5316	PF05347.14	Complex1_LYR	30,3	3,00E-07	CL0491
TRINITY_DN12116_c0_g1_i1_5	1	9,5	18,705	8,1712					
TRINITY_DN18165_c0_g1_i1_3	1	8,1	22,768	6,6551	PF07086.11	Jagunal	30,3	3,90E-07	No_clan
TRINITY_DN19285_c0_g1_i1_2	2	12,9	33,258	13,106	PF00459.24	Inositol_P	181,4	2,20E-53	CL0171

TRINITY_DN4092_c0_g1_i1_3	1	5,7	20,682	7,0055					
TRINITY_DN4871_c0_g1_i1_3	1	9,6	15,386	6,2893	PF03171.19	2OG-FeII_Oxy	60,9	1,20E-16	CL0029
TRINITY_DN23190_c0_g1_i1_4	1	20,7	10,596	6,5826	PF00067.21	p450	88,9	2,70E-25	No_clan
TRINITY_DN2267_c0_g1_i1_5	1	1,3	100,01	7,1569	PF00400.31	WD40	12,3	0,23	CL0186
TRINITY_DN12570_c0_g1_i1_4	2	34,8	12,48	11,973					
TRINITY_DN19686_c0_g1_i1_5	2	29	12,052	13,355	PF00675.19	Peptidase_M16	110	9,30E-32	CL0094
TRINITY_DN15214_c0_g1_i1_3	1	12,9	10,581	7,1412	PF08100.10	Dimerisation	75,8	1,80E-21	CL0123
TRINITY_DN18839_c0_g1_i1_6	1	16	11,298	9,6314	PF00156.26	Pribosyltran	52,5	3,60E-14	CL0533
TRINITY_DN12937_c0_g1_i1_3	1	12,4	17,405	7,1278	PF02020.17	W2	41,2	1,20E-10	CL0020
TRINITY_DN3038_c0_g1_i1_4	1	6,3	27,125	6,418	PF02485.20	Branch	156,3	9,00E-46	CL0110
TRINITY_DN3285_c0_g1_i1_1	2	8,9	24,878	9,048	PF00632.24	HECT	41,5	1,00E-10	CL0552
TRINITY_DN4341_c0_g1_i1_2	1	18,2	10,734	7,2971	PF01008.16	IF-2B	82,9	2,10E-23	CL0246
TRINITY_DN5664_c0_g1_i1_3	2	6,4	38,036	14,627					
TRINITY_DN3970_c0_g1_i1_1	1	7,1	18,001	7,1569	PF00892.19	EamA	29,3	7,40E-07	CL0184
TRINITY_DN12410_c0_g1_i1_6	3	8,3	42,015	18,813	PF01370.20	Epimerase	97,8	6,00E-28	CL0063
TRINITY_DN4354_c0_g1_i1_4	1	11,9	10,721	6,5199	PF15801.4	zf-C6H2	57,9	8,40E-16	CL0175
TRINITY_DN9720_c0_g1_i6_2	1	19,4	17,113	7,312	PF00076.21	RRM_1	54,7	6,10E-15	CL0221
TRINITY_DN22980_c0_g1_i1_3	1	13,3	10,988	9,9					
TRINITY_DN9256_c0_g1_i4_1	2	6,7	40,639	16,658					
TRINITY_DN8333_c0_g1_i1_3	1	7,8	17,827	20,017					
TRINITY_DN294_c0_g1_i1_2	1	4,8	37,295	8,2264	PF00443.28	UCH	134	5,80E-39	CL0125
TRINITY_DN8005_c0_g1_i2_2	3	14,3	33,549	6,5734	PF00071.21	Ras	187,9	8,60E-56	CL0023
TRINITY_DN17859_c0_g1_i1_3	1	8,2	18,451	10,395					
TRINITY_DN1542_c0_g1_i1_6	2	21,1	15,345	16,334	PF14008.5	Metallophos_C	33,6	4,80E-08	No_clan
TRINITY_DN7035_c1_g2_i1_3	2	5,9	53,811	12,482	PF01388.20	ARID	59,5	3,40E-16	CL0123
TRINITY_DN19252_c0_g1_i1_4	2	18,8	16,477	12,54					
TRINITY_DN1690_c0_g1_i2_5	2	5,6	46,543	11,429	PF01008.16	IF-2B	28,2	9,60E-07	CL0246
TRINITY_DN18166_c0_g1_i1_4	1	11,6	9,7533	6,7653					
TRINITY_DN9981_c1_g1_i1_3	2	8	39,025	12,63	PF03997.11	VPS28	230	1,60E-68	CL0596
TRINITY_DN8032_c0_g1_i1_2	1	3,4	52,986	11,676	PF01545.20	Cation_efflux	92,9	2,00E-26	No_clan
TRINITY_DN10046_c0_g2_i1_4	3	13,3	33,533	21,074	PF00576.20	Transthyretin	107,1	6,70E-31	CL0287
TRINITY_DN21517_c0_g1_i1_4	2	8	22,241	11,934	PF00254.27	FKBP_C	121,4	1,40E-35	CL0487
TRINITY_DN316_c0_g1_i1_1	1	12,4	12,146	7,7879					

TRINITY_DN8057_c0_g1_i2_5	1	3,9	36,302	6,6029	PF14938.5	SNAP	90,6	1,00E-25	CL0020
TRINITY_DN22718_c0_g1_i1_2	1	8	12,031	9,4545	PF01412.17	ArfGap	47,5	1,40E-12	No_clan
TRINITY_DN5217_c0_g1_i1_4	1	6,2	32,75	7,1846	PF04551.13	GcpE	166,4	8,80E-49	No_clan
TRINITY_DN8159_c0_g2_i1_1	1	13,1	19,446	27,08	PF00441.23	Acyl-CoA_dh_1	50,4	2,50E-13	CL0087
TRINITY_DN2797_c0_g2_i1_4	1	3,5	45,901	7,345	PF04815.14	Sec23_helical	73,7	7,80E-21	No_clan
TRINITY_DN3030_c0_g1_i1_6	1	2,3	52,003	7,296	PF02636.16	Methyltransf_28	243,7	2,20E-72	CL0063
TRINITY_DN16052_c0_g1_i1_2	2	20,3	16,954	12,114	PF00694.18	Aconitase_C	108,1	3,80E-31	CL0364
TRINITY_DN8771_c0_g1_i1_2	2	8	40,65	12,913	PF09756.8	DDRGK	174,1	2,20E-51	CL0123
TRINITY_DN5711_c0_g2_i1_5	1	8,5	23,155	9,5812	PF04442.13	CtaG_Cox11	179,3	4,40E-53	No_clan
TRINITY_DN11595_c1_g1_i1_3	2	4,4	73,9	12,388	PF00854.20	PTR2	458,6	1,40E-137	CL0015
TRINITY_DN18730_c0_g1_i1_1	1	11,1	16,481	9,8655	PF05187.12	ETF_QO	71,5	5,00E-20	CL0344
TRINITY_DN5685_c0_g1_i4_5	2	10,1	31,172	13,998	PF00149.27	Metallophos	25,3	1,70E-05	CL0163
TRINITY_DN11028_c0_g3_i1_3	7	33,9	31,913	13,769	PF00071.21	Ras	223	1,30E-66	CL0023
TRINITY_DN5255_c1_g1_i1_4	1	18,4	10,8	7,6671	PF00464.18	SHMT	169,3	8,80E-50	CL0061
TRINITY_DN10013_c0_g1_i4_2	2	9,5	27,754	12,937	PF00106.24	adh_short	133,1	7,30E-39	CL0063
TRINITY_DN6316_c0_g1_i4_3	2	7,2	53,484	14,87	PF00022.18	Actin	506,5	3,90E-152	CL0108
TRINITY_DN7363_c0_g1_i1_3	2	6,4	57,056	15,379					
TRINITY_DN18596_c0_g1_i1_5	1	12	17,729	7,1779	PF00069.24	Pkinase	156,5	7,70E-46	CL0016
TRINITY_DN17114_c0_g1_i1_1	1	13,2	12,095	6,9822					
TRINITY_DN19946_c0_g1_i1_4	2	8,1	33,575	12,621	PF08240.11	ADH_N	34,7	1,20E-08	CL0296
TRINITY_DN8995_c0_g1_i1_3	3	7,3	57,759	16,95	PF11919.7	DUF3437	100,9	2,60E-29	No_clan
TRINITY_DN9650_c0_g1_i1_1	1	4	66,946	7,8046	PF06886.10	TPX2	75,3	3,00E-21	No_clan
TRINITY_DN7338_c0_g3_i1_2	2	10,6	21,985	11,486					
TRINITY_DN4072_c0_g2_i1_2	2	5,4	56,242	14,245					
TRINITY_DN2062_c0_g1_i1_6	4	23,4	21,045	6,6464	PF03030.15	H_PPase	316,3	4,30E-94	No_clan
TRINITY_DN5791_c1_g1_i1_2	1	12,1	15,103	6,703					
TRINITY_DN6320_c0_g2_i1_1	2	10,6	36,376	6,5263	PF14686.5	fn3_3	91,1	2,50E-26	CL0287
TRINITY_DN6069_c0_g2_i1_6	1	7,4	25,13	6,4428	PF11712.7	Vma12	35,1	1,10E-08	No_clan
TRINITY_DN18724_c0_g1_i1_4	1	15,9	15,491	9,6436	PF00069.24	Pkinase	49,8	2,70E-13	CL0016
TRINITY_DN4227_c0_g1_i1_3	1	4,5	55,723	6,6565	PF01435.17	Peptidase_M48	109,2	2,00E-31	CL0126
TRINITY_DN18944_c0_g1_i1_1	2	19,7	16,67	13,11	PF01535.19	PPR	10,8	0,46	CL0020
TRINITY_DN9604_c0_g1_i3_3	2	7,1	42,704	12,529					
TRINITY_DN19600_c0_g1_i1_6	1	6,6	26,304	6,9213					

TRINITY_DN2329_c0_g1_i2_6	1	6,1	18,888	7,3018	PF00994.23	MoCF_biosynth	85,6	2,30E-24	No_clan
TRINITY_DN827_c0_g1_i1_1	1	3	49,554	6,2753					
TRINITY_DN8908_c0_g1_i2_1	2	5,3	61,655	13,218	PF03407.15	Nucleotid_trans	184,9	1,70E-54	CL0110
TRINITY_DN18109_c0_g1_i1_5	1	8,2	19,016	10,081	PF01425.20	Amidase	66,8	1,60E-18	No_clan
TRINITY_DN11206_c0_g1_i2_4	1	10,4	17,119	9,5111					
TRINITY_DN18626_c1_g2_i1_2	2	11,5	39,548	14,994	PF04683.12	Proteasom_Rpn13	82,7	1,90E-23	CL0266
TRINITY_DN21756_c0_g1_i1_1	2	39,4	11,674	11,474					
TRINITY_DN2355_c0_g1_i1_2	1	9,3	16,797	9,741	PF07714.16	Pkinase_Tyr	110,6	7,10E-32	CL0016
TRINITY_DN2509_c0_g1_i1_1	2	4,9	47,583	11,336	PF04030.13	ALO	39,6	4,80E-10	CL0277
TRINITY_DN19715_c0_g1_i1_5	2	27,3	8,148	19,097					
TRINITY_DN4748_c0_g1_i1_2	1	2,7	49,321	6,3853	PF00149.27	Metallophos	143,3	1,30E-41	CL0163
TRINITY_DN21521_c1_g1_i1_3	1	18,6	11,006	7,323	PF11145.7	DUF2921	28,4	3,40E-07	No_clan
TRINITY_DN5242_c0_g3_i1_6	1	2,4	45,636	6,7699	PF03764.17	EFG_IV	85,6	2,00E-24	CL0329
TRINITY_DN19260_c0_g1_i1_1	1	23	10,876	10,03					
TRINITY_DN3078_c0_g1_i1_3	2	6,4	41,394	11,394	PF00557.23	Peptidase_M24	190,4	2,90E-56	No_clan
TRINITY_DN10865_c0_g2_i2_2	1	4,4	29,41	6,4238	PF09585.9	Lin0512_fam	134,3	1,60E-39	No_clan
TRINITY_DN17892_c1_g1_i1_5	1	8,5	25,007	7,9779	PF00450.21	Peptidase_S10	118,5	4,50E-34	CL0028
TRINITY_DN2656_c0_g1_i1_2	1	12,7	12,575	10,197					
TRINITY_DN22169_c0_g1_i1_5	1	12,2	9,7761	7,4081					
TRINITY_DN10693_c1_g1_i1_3	1	4,4	68,107	10,185	PF00400.31	WD40	29	1,20E-06	CL0186
TRINITY_DN7588_c0_g1_i2_5	1	6,5	27,45	9,7084	PF01105.23	EMP24_GP25L	167,9	1,90E-49	CL0521
TRINITY_DN1809_c0_g1_i1_3	2	6,3	40,903	11,411	PF03571.14	Peptidase_M49	32,9	2,10E-08	CL0126
TRINITY_DN10089_c0_g3_i1_3	1	2,7	59,881	7,0616	PF00076.21	RRM_1	47	1,50E-12	CL0221
TRINITY_DN2082_c1_g1_i1_3	1	12,8	13,1	8,0247	PF07714.16	Pkinase_Tyr	64,7	7,20E-18	CL0016
TRINITY_DN238_c0_g1_i1_3	2	27,1	7,5017	6,6494	PF08240.11	ADH_N	31,6	1,10E-07	CL0296
TRINITY_DN5724_c0_g2_i1_1	2	2,6	104,66	11,615	PF04727.12	ELMO_CED12	159,6	7,90E-47	No_clan
TRINITY_DN21573_c0_g1_i1_1	1	18,4	11,39	12,14					
TRINITY_DN11569_c0_g1_i2_3	1	5,8	33,132	15,892	PF10252.8	PP28	94,3	4,20E-27	No_clan
TRINITY_DN5094_c0_g1_i1_4	4	36,1	17,348	-2	PF00022.18	Actin	144,8	2,50E-42	CL0108
TRINITY_DN19637_c0_g1_i1_4	2	12,7	20,544	12,756	PF00365.19	PFK	134,6	3,90E-39	CL0240
TRINITY_DN4599_c0_g1_i1_1	1	2	61,444	6,4862	PF13091.5	PLDc_2	41	1,50E-10	CL0479
TRINITY_DN11898_c0_g1_i1_4	2	6,6	37,791	12,292	PF00117.27	GATase	191,5	1,10E-56	CL0014
TRINITY_DN13648_c0_g1_i1_3	1	9	16,596	6,7019					

TRINITY_DN22384_c0_g1_i1_6	2	28,9	13,439	14,115	PF00930.20	DPPIV_N	92,3	2,40E-26	CL0186
TRINITY_DN8593_c0_g1_i1_3	2	5,6	39,412	10,845					
TRINITY_DN6624_c0_g2_i2_2	2	6,9	48,256	12,504	PF11416.7	Syntaxin-5_N	21,7	7,70E-05	No_clan
TRINITY_DN8264_c0_g1_i2_1	2	5,3	58,59	13,381	PF16294.4	RSB_motif	52,6	4,50E-14	No_clan
TRINITY_DN7248_c0_g1_i1_5	2	16,2	12,855	10,752	PF00067.21	p450	37,4	1,20E-09	No_clan
TRINITY_DN19832_c0_g1_i1_3	2	24	13,029	11,348					
TRINITY_DN23704_c0_g1_i1_6	1	32,4	7,4204	6,5559	PF00205.21	TPP_enzyme_M	41,2	1,20E-10	CL0085
TRINITY_DN15338_c0_g1_i1_3	1	22,1	8,2351	7,8001					
TRINITY_DN9948_c0_g2_i1_3	1	10,8	20,491	20,335					
TRINITY_DN13388_c0_g1_i1_5	1	13,3	14,398	6,7395	PF03016.14	Exostosin	140,7	5,10E-41	No_clan
TRINITY_DN5559_c0_g1_i1_4	2	5,5	55,155	14,319	PF03367.12	zf-ZPR1	179,2	4,60E-53	CL0167
TRINITY_DN17697_c0_g1_i1_3	1	14,3	10,783	7,0844	PF03446.14	NAD_binding_2	54,9	1,00E-14	CL0063
TRINITY_DN5154_c0_g1_i1_5	1	4,2	47,317	7,9032	PF00076.21	RRM_1	49,3	3,00E-13	CL0221
TRINITY_DN20546_c0_g1_i1_1	1	9,8	19,169	7,7743	PF01645.16	Glu_synthase	168,6	2,10E-49	CL0036
TRINITY_DN6573_c0_g1_i1_4	2	7,2	48,971	11,719	PF01694.21	Rhomboid	83,9	1,00E-23	CL0207
TRINITY_DN3293_c0_g1_i1_2	2	7,8	34,861	11,367	PF09787.8	Golgin_A5	34,2	1,70E-08	No_clan
TRINITY_DN9459_c0_g2_i1_1	1	1,6	82,917	7,5232	PF00232.17	Glyco_hydro_1	112,1	2,00E-32	CL0058
TRINITY_DN4865_c0_g1_i1_1	1	9,5	32,216	10,293	PF07651.15	ANTH	31,7	6,90E-08	CL0009
TRINITY_DN3689_c0_g2_i1_5	2	5,7	40,504	12,008	PF04278.11	Tic22	367,8	2,10E-110	No_clan
TRINITY_DN2686_c0_g1_i1_5	1	2,9	36,38	6,5762	PF09835.8	DUF2062	56,9	1,90E-15	No_clan
TRINITY_DN4162_c0_g1_i1_5	2	7,3	39,645	10,864	PF04097.13	Nic96	98,5	3,10E-28	No_clan
TRINITY_DN14842_c0_g1_i1_3	2	26,4	9,3687	7,1569					
TRINITY_DN1392_c0_g2_i1_1	1	3,3	39,655	6,6941	PF10408.8	Ufd2P_core	147,7	4,50E-43	No_clan
TRINITY_DN3303_c0_g1_i1_3	1	7,3	23,662	15,463	PF09763.8	Sec3_C	72,9	1,80E-20	CL0295
TRINITY_DN5303_c0_g1_i1_3	1	5,7	30,141	7,0453	PF16897.4	MMR_HSR1_Xtn	143,5	2,00E-42	No_clan
TRINITY_DN3626_c0_g3_i1_6	1	8,8	17,018	7,6675	PF01981.15	PTH2	78,6	3,90E-22	CL0305
TRINITY_DN12852_c0_g1_i1_2	1	5,4	30,063	7,0351					
TRINITY_DN16500_c0_g1_i1_3	1	15,1	8,3025	7,1103					
TRINITY_DN13231_c0_g1_i1_1	2	12,7	27,708	20,186					
TRINITY_DN9164_c0_g1_i1_1	1	5,3	29,687	15,106	PF02941.14	FeThRed_A	95,8	1,20E-27	CL0610
TRINITY_DN6110_c0_g1_i2_1	2	7	33,805	11,242	PF13646.5	HEAT_2	40,8	2,00E-10	CL0020
TRINITY_DN9679_c0_g2_i6_4	2	4,9	56,322	11,714	PF00494.18	SQS_PSY	157,6	3,70E-46	CL0613
TRINITY_DN185_c0_g1_i1_1	1	2,3	37,766	6,3396	PF00999.20	Na_H_Exchanger	154,5	3,10E-45	CL0064

TRINITY_DN16939_c0_g1_i1_3	1	28,8	7,7616	9,0769	PF00501.27	AMP-binding	62,2	3,00E-17	CL0378
TRINITY_DN10156_c0_g1_i1_1	1	5,1	34,267	8,3548	PF01964.17	ThiC_Rad_SAM	47,4	1,10E-12	CL0036
TRINITY_DN9970_c0_g1_i3_1	1	6,7	20,355	7,1125					
TRINITY_DN11182_c0_g1_i1_5	2	12,4	26,331	12,438					
TRINITY_DN6813_c0_g2_i1_6	2	3,9	70,882	13,513	PF00171.21	Aldedh	413,1	1,10E-123	CL0099
TRINITY_DN18653_c0_g1_i1_5	1	6,4	25,449	7,7614					
TRINITY_DN17522_c0_g1_i1_4	1	7,6	12,69	6,7736	PF00153.26	Mito_carr	86,3	9,50E-25	No_clan
TRINITY_DN14413_c0_g1_i1_4	1	16,3	10,971	8,3608	PF06068.12	TIP49	168,2	2,50E-49	CL0023
TRINITY_DN5635_c0_g1_i1_5	2	7,9	48,782	13,361	PF00069.24	Pkinase	204,6	1,60E-60	CL0016
TRINITY_DN10294_c0_g1_i2_1	2	6,1	44,812	11,152					
TRINITY_DN19410_c0_g2_i1_2	2	15,7	17,517	11,643	PF10604.8	Polyketide_cyc2	85,8	2,90E-24	CL0209
TRINITY_DN3519_c0_g1_i1_4	1	4,3	30,599	6,4791					
TRINITY_DN10650_c0_g1_i3_2	1	7,1	16,816	6,6096	PF02325.16	YGGT	38,6	9,80E-10	No_clan
TRINITY_DN3798_c0_g1_i2_1	2	12,6	30,449	11,436	PF00928.20	Adap_comp_sub	295,5	3,00E-88	CL0448
TRINITY_DN2524_c0_g1_i1_2	1	13,2	17,857	6,9379	PF01841.18	Transglut_core	47,8	1,50E-12	CL0125
TRINITY_DN14304_c0_g1_i1_3	1	16,4	7,7214	7,1508					
TRINITY_DN10946_c0_g1_i1_3	2	4,2	61,613	11,811	PF00815.19	Histidinol_dh	535,2	1,00E-160	CL0099
TRINITY_DN4824_c0_g1_i1_6	1	7,4	13,322	6,5675	PF06487.11	SAP18	96	1,90E-27	CL0072
TRINITY_DN1455_c0_g1_i1_5	2	6,9	31,275	11,745	PF13561.5	adh_short_C2	141,6	2,70E-41	CL0063
TRINITY_DN19943_c0_g1_i1_1	1	18,1	11,214	6,8897					
TRINITY_DN7659_c0_g1_i2_1	1	10,1	16,082	64,119	PF00076.21	RRM_1	85,5	1,50E-24	CL0221
TRINITY_DN18303_c0_g1_i1_2	1	9,9	15,634	10,081					
TRINITY_DN7752_c0_g1_i1_1	2	10,1	32,433	12,27	PF09335.10	SNARE_assoc	71,3	8,80E-20	No_clan
TRINITY_DN3113_c0_g1_i1_2	1	9,8	20,031	7,3014	PF00443.28	UCH	49,4	3,80E-13	CL0125
TRINITY_DN13954_c0_g1_i1_5	1	6,3	17,522	6,4446	PF00248.20	Aldo_ket_red	97,2	8,60E-28	No_clan
TRINITY_DN9113_c0_g1_i1_6	1	2,3	73,14	7,9169	PF06472.14	ABC_membrane_2	221,5	1,30E-65	CL0241
TRINITY_DN2642_c0_g1_i1_1	1	3,3	43,047	7,2108	PF02574.15	S-methyl_trans	262,8	3,90E-78	No_clan
TRINITY_DN9696_c0_g1_i1_5	2	12,3	27,758	12,394	PF08661.10	Rep_fac-A_3	66,1	2,90E-18	CL0021
TRINITY_DN2508_c0_g1_i1_3	1	5,1	34,853	9,8405	PF00291.24	PALP	251,6	9,40E-75	No_clan
TRINITY_DN8157_c0_g1_i1_3	1	4,8	31,23	8,144	PF00571.27	CBS	48,5	8,00E-13	No_clan
TRINITY_DN20773_c0_g1_i1_5	1	12,1	14,124	7,421	PF02897.14	Peptidase_S9_N	90	1,20E-25	CL0186
TRINITY_DN2427_c0_g1_i1_5	2	8,8	33,871	10,836					

TRINITY_DN566_c0_g2_i1_1	1	3,3	41,01	6,6046	PF00535.25	Glycos_transf_2	80,3	1,30E-22	CL0110
TRINITY_DN1856_c0_g2_i1_3	1	11,8	15,541	6,801					
TRINITY_DN3611_c0_g1_i2_3	1	2,8	55,875	6,5212	PF01535.19	PPR	26,3	5,00E-06	CL0020
TRINITY_DN8137_c0_g1_i1_6	1	12,2	16,592	7,401					
TRINITY_DN4839_c0_g2_i1_2	1	19,1	14,958	7,8127					
TRINITY_DN10092_c0_g1_i1_3	2	3,2	58,53	10,61	PF00646.32	F-box	24,7	1,40E-05	CL0271
TRINITY_DN8102_c0_g1_i1_6	2	5,1	68,223	13,899	PF03152.13	UFD1	160,1	3,20E-47	CL0402
TRINITY_DN2436_c0_g1_i1_2	1	4,9	32,959	7,4152	PF13602.5	ADH_zinc_N_2	78,1	1,10E-21	CL0063
TRINITY_DN9750_c0_g2_i3_3	1	5,3	26,797	6,5161	PF00076.21	RRM_1	23,8	2,60E-05	CL0221
TRINITY_DN13772_c0_g1_i1_5	1	15,1	7,9869	7,7693					
TRINITY_DN20973_c0_g1_i1_1	1	30,7	8,5286	25,411	PF00069.24	Pkinase	66,8	1,70E-18	CL0016
TRINITY_DN13500_c0_g1_i1_1	1	3	39,647	6,5426					
TRINITY_DN19696_c0_g1_i1_1	1	11,2	13,668	9,8302					
TRINITY_DN18663_c0_g1_i1_3	4	20,4	31,08	6,4619	PF00311.16	PEPcase	102,8	1,30E-29	CL0151
TRINITY_DN10941_c0_g1_i2_2	1	6	24,808	6,8825	PF00252.17	Ribosomal_L16	127	4,10E-37	No_clan
TRINITY_DN205_c0_g2_i1_3	2	6,4	34,194	13,232	PF00697.21	PRAI	139,2	1,30E-40	CL0036
TRINITY_DN10620_c0_g1_i1_3	2	6,1	43,003	12,852	PF00153.26	Mito_carr	64	8,40E-18	No_clan
TRINITY_DN12943_c0_g1_i1_3	1	18,9	13,192	6,3313	PF13589.5	HATPase_c_3	32,7	5,30E-08	CL0025
TRINITY_DN2735_c0_g1_i1_2	1	4,9	24,676	6,4331					
TRINITY_DN15045_c0_g1_i1_3	1	13,3	10,233	6,5415	PF03097.17	BRO1	69,9	1,60E-19	No_clan
TRINITY_DN17024_c0_g1_i1_1	1	11,2	11,383	7,1508	PF00202.20	Aminotran_3	92,7	1,70E-26	CL0061
TRINITY_DN4004_c0_g1_i1_6	1	5,6	29,667	-2	PF08711.10	Med26	46,5	2,50E-12	No_clan
TRINITY_DN12269_c0_g2_i1_2	1	6,6	20,551	6,6145					
TRINITY_DN9455_c0_g1_i1_6	1	3,7	29,262	6,3538	PF02678.15	Pirin	47,3	1,60E-12	CL0029
TRINITY_DN7173_c0_g2_i1_4	1	5	30,917	6,3209					
TRINITY_DN5388_c0_g2_i1_3	1	5,7	20,547	6,3799	PF09360.9	zf-CDGSH	38,1	1,20E-09	No_clan
TRINITY_DN10533_c0_g1_i4_1	2	5,8	65,751	13,052	PF00013.28	KH_1	27,3	2,10E-06	CL0007
TRINITY_DN15880_c0_g1_i1_6	1	17,1	8,0058	6,418					
TRINITY_DN21652_c0_g1_i1_3	1	21,9	8,122	9,8655	PF01116.19	F_bP_aldolase	49,8	3,00E-13	CL0036
TRINITY_DN22321_c0_g1_i1_6	1	10,5	14,842	6,7012					
TRINITY_DN12098_c0_g2_i1_2	1	13	14,723	7,7636					
TRINITY_DN6146_c0_g1_i2_4	1	5,7	27,398	7,0616	PF04430.13	DUF498	107	4,50E-31	No_clan
TRINITY_DN8428_c0_g1_i1_3	2	9	38,808	12,45	PF00933.20	Glyco_hydro_3	119,7	1,50E-34	CL0058

TRINITY_DN17847_c0_g2_i1_2	1	4,6	38,866	7,0887					
TRINITY_DN4796_c0_g1_i1_2	2	7,7	31,716	13,055					
TRINITY_DN5072_c0_g1_i1_1	1	5,7	27,322	7,3411	PF00297.21	Ribosomal_L3	68,6	4,30E-19	CL0575
TRINITY_DN6088_c0_g1_i4_1	2	10,6	33,893	13,549	PF05742.11	TANGO2	242,4	6,80E-72	No_clan
TRINITY_DN15797_c0_g1_i1_2	1	8,8	12,414	6,3549	PF01963.16	TraB	28,8	1,00E-06	CL0572
TRINITY_DN18297_c0_g2_i1_1	2	7,5	45,065	11,753	PF00288.25	GHMP_kinases_N	73,2	1,40E-20	CL0329
TRINITY_DN17041_c0_g1_i1_1	1	4	36,605	6,418	PF12146.7	Hydrolase_4	181,9	1,10E-53	CL0028
TRINITY_DN7113_c0_g1_i3_4	1	6,2	23,284	7,3751	PF03656.12	Pam16	78,5	4,00E-22	CL0392
TRINITY_DN12379_c0_g1_i1_3	1	16,5	9,2894	6,6689	PF05761.13	5_nucleotid	88,5	4,10E-25	CL0137
TRINITY_DN15487_c0_g1_i1_3	1	16,3	10,679	12,055					
TRINITY_DN3915_c0_g1_i1_3	1	8	19,855	6,9426	PF04178.11	Got1	103,7	6,80E-30	No_clan
TRINITY_DN11583_c1_g3_i2_2	2	5,1	56,012	6,5161					
TRINITY_DN11439_c0_g1_i3_4	3	16,5	35,446	7,5342	PF00227.25	Proteasome	107,1	7,10E-31	CL0052
TRINITY_DN7466_c0_g3_i1_3	2	2,4	85,033	11,639	PF01434.17	Peptidase_M41	139,3	1,20E-40	CL0126
TRINITY_DN11372_c0_g1_i1_1	2	5,9	48,906	12,539	PF02176.17	zf-TRAF	41,5	1,50E-10	CL0389
TRINITY_DN2241_c0_g1_i1_2	1	21,1	15,469	7,3234	PF00698.20	Acyl_transf_1	47,5	1,60E-12	CL0323
TRINITY_DN2564_c0_g1_i1_3	2	3,2	90,747	13,43					
TRINITY_DN4503_c0_g1_i1_2	1	5,9	22,296	6,567	PF01370.20	Epimerase	47,1	1,90E-12	CL0063
TRINITY_DN8115_c0_g1_i1_2	2	2,8	113,6	11,564	PF04065.14	Not3	280,3	9,50E-84	No_clan
TRINITY_DN271_c0_g1_i1_6	1	16,5	9,2734	6,3527	PF00933.20	Glyco_hydro_3	39,6	3,70E-10	CL0058
TRINITY_DN12627_c0_g1_i1_5	1	6,4	16,743	6,3777	PF02458.14	Transferase	58,1	5,70E-16	CL0149
TRINITY_DN6168_c0_g2_i1_3	1	4,9	19,799	6,6902	PF05173.13	DapB_C	72,5	2,80E-20	CL0063
TRINITY_DN15083_c0_g1_i1_4	1	17,3	9,0149	6,9744					
TRINITY_DN22088_c0_g1_i1_2	2	37,5	7,8627	12,724					
TRINITY_DN10295_c0_g1_i4_2	1	11,3	21,86	6,3785					
TRINITY_DN13908_c0_g1_i1_1	2	19,1	15,248	12,244	PF05761.13	5_nucleotid	123,1	1,30E-35	CL0137
TRINITY_DN250_c0_g2_i1_3	1	11,7	13,999	10,25	PF02263.18	GBP	25,4	7,20E-06	CL0023
TRINITY_DN9708_c0_g1_i1_2	2	3,5	60,575	12,802	PF00450.21	Peptidase_S10	475,7	1,60E-142	CL0028
TRINITY_DN14410_c0_g1_i1_1	1	12,8	13,922	6,6498					
TRINITY_DN2548_c0_g2_i1_4	1	5,1	45,387	9,9891	PF00999.20	Na_H_Exchanger	99,5	1,60E-28	CL0064
TRINITY_DN6286_c0_g1_i1_1	1	17,5	10,594	7,982					
TRINITY_DN17369_c0_g1_i1_5	1	4	37,115	8,1387					
TRINITY_DN8472_c0_g1_i1_2	3	16,9	19,025	11,433	PF00076.21	RRM_1	32,6	5,00E-08	CL0221

TRINITY_DN4972_c0_g1_i4_1	2	5,3	61,78	13,268	PF00149.27	Metallophos	116,9	1,60E-33	CL0163
TRINITY_DN9379_c0_g1_i3_2	1	8,9	18,673	64,313	PF04614.11	Pex19	77,9	9,00E-22	No_clan
TRINITY_DN23442_c0_g1_i1_5	1	16	8,4648	6,6239	PF09285.10	Elong-fact-P_C	52,6	2,60E-14	CL0021
TRINITY_DN20268_c0_g1_i1_5	1	22,5	8,986	6,7177					
TRINITY_DN2087_c1_g1_i1_3	1	5,1	33,131	6,4984	PF02713.13	DUF220	114,2	2,00E-33	No_clan
TRINITY_DN9218_c0_g1_i7_3	1	2,6	70,743	6,5211	PF08606.10	Prp19	107,1	3,00E-31	No_clan
TRINITY_DN12527_c0_g1_i1_2	1	6,1	25,393	7,3556	PF00534.19	Glycos_transf_1	32,1	7,30E-08	CL0113
TRINITY_DN22389_c0_g1_i1_6	1	13,5	11,481	9,53					
TRINITY_DN9732_c0_g1_i3_2	1	9,3	28,429	7,6393	PF04051.15	TRAPP	147,7	1,80E-43	CL0210
TRINITY_DN9091_c0_g1_i1_3	1	13,8	16,44	7,1576					
TRINITY_DN1560_c0_g1_i1_1	1	4,5	21,762	6,6564					
TRINITY_DN14904_c0_g1_i1_6	1	19	10,518	9,7179	PF07992.13	Pyr_redox_2	30	2,80E-07	CL0063
TRINITY_DN12383_c0_g1_i1_2	1	14,5	9,2853	6,5642	PF00249.30	Myb_DNA-binding	29,5	5,80E-07	CL0123
TRINITY_DN5000_c0_g1_i1_6	2	5	66,89	13,964					
TRINITY_DN2849_c0_g1_i1_4	2	5,1	40,325	10,676	PF00899.20	ThiF	203,5	3,00E-60	CL0063
TRINITY_DN11583_c1_g1_i2_1	2	4,3	64,793	11,692	PF00069.24	Pkinase	93,4	1,40E-26	CL0016
TRINITY_DN11555_c0_g1_i1_4	1	0,8	143,46	-2					
TRINITY_DN11211_c0_g2_i4_2	1	4,9	24,48	6,4706	PF05755.11	REF	167,6	3,00E-49	No_clan
TRINITY_DN10288_c0_g2_i1_3	1	2,8	54,485	7,6772					
TRINITY_DN16723_c0_g1_i1_1	2	28,6	8,5176	14,146					
TRINITY_DN5420_c0_g1_i1_2	1	3,4	68,77	6,6052	PF01501.19	Glyco_transf_8	26,5	3,80E-06	CL0110
TRINITY_DN15186_c0_g1_i1_2	1	14,2	12,791	7,3367	PF06644.10	ATP11	66,9	2,20E-18	No_clan
TRINITY_DN6233_c0_g1_i4_1	1	8,9	28,849	6,6219					
TRINITY_DN4088_c0_g1_i1_1	1	2,7	61,92	6,354					
TRINITY_DN1341_c0_g1_i2_3	1	15,2	11,532	6,8661	PF12874.6	zf-met	21,3	0,00023	CL0361
TRINITY_DN5675_c0_g1_i3_1	1	4,1	46,39	7,7783					
TRINITY_DN5431_c0_g2_i1_2	1	2,5	47,894	7,2977	PF13920.5	zf-C3HC4_3	57,3	9,70E-16	CL0229
TRINITY_DN9005_c1_g1_i3_1	1	3,9	44,046	9,3077	PF01758.15	SBF	170,9	2,30E-50	CL0064
TRINITY_DN22172_c0_g1_i1_3	1	18,1	10,281	7,7743					
TRINITY_DN5643_c0_g2_i1_3	2	6,1	51,799	12,583	PF00091.24	Tubulin	132,7	1,70E-38	CL0566
TRINITY_DN18093_c0_g1_i1_1	1	6,1	28,615	6,5541					
TRINITY_DN8505_c0_g1_i1_3	1	5,9	26,87	7,7964	PF01230.22	HIT	87,1	9,80E-25	CL0265

TRINITY_DN3938_c0_g1_i1_2	1	3,6	54,813	8,5182	PF00620.26	RhoGAP	103,6	7,70E-30	CL0409
TRINITY_DN11081_c0_g1_i2_3	1	4,3	58,635	6,6538					
TRINITY_DN8657_c0_g1_i1_3	2	11,6	18,149	12,035	PF13637.5	Ank_4	62,2	4,00E-17	CL0465
TRINITY_DN18007_c0_g1_i1_2	1	5,7	19,708	6,385					
TRINITY_DN11493_c0_g1_i3_3	1	7,6	18,353	6,3527					
TRINITY_DN11659_c1_g1_i3_5	4	10,1	66,479	6,8939	PF00171.21	Aldedh	602,1	5,20E-181	CL0099
TRINITY_DN11367_c0_g1_i1_2	1	3,8	37,353	6,4022	PF02567.15	PhzC-PhzF	293,7	1,30E-87	CL0288
TRINITY_DN14035_c0_g1_i1_1	1	16,8	10,114	-2					
TRINITY_DN7672_c0_g2_i3_6	1	5,5	36,395	6,4576	PF01399.26	PCI	91,1	5,30E-26	CL0123
TRINITY_DN4363_c0_g1_i1_2	1	3,9	61,999	7,8636	PF00501.27	AMP-binding	305	6,20E-91	CL0378
TRINITY_DN9570_c0_g1_i2_6	1	2,4	61,769	7,7462					
TRINITY_DN12047_c0_g1_i1_5	1	10,8	15,951	6,401					
TRINITY_DN8161_c0_g1_i1_2	1	4,2	31,702	6,496	PF00572.17	Ribosomal_L13	153,1	3,80E-45	No_clan
TRINITY_DN9497_c1_g1_i1_5	1	16,5	12,353	8,865	PF05347.14	Complex1_LYR	43,3	2,50E-11	CL0491
TRINITY_DN18064_c0_g1_i1_5	1	9,8	14,455	6,2753	PF08302.10	tRNA_lig_CPD	22,3	6,70E-05	No_clan
TRINITY_DN3901_c0_g1_i1_6	1	8,6	17,623	7,9065	PF06747.12	CHCH	32,8	4,90E-08	CL0351
TRINITY_DN9544_c0_g1_i2_3	1	3,3	30,976	6,6564	PF00583.24	Acetyltransf_1	54,6	1,10E-14	CL0257
TRINITY_DN5823_c2_g1_i1_1	1	8,6	15,229	7,0109					
TRINITY_DN10520_c0_g1_i1_2	1	3	84,024	13,523	PF06419.10	COG6	748	6,10E-225	CL0295
TRINITY_DN11739_c0_g2_i1_5	1	7,4	25,671	6,9085	PF13516.5	LRR_6	15,9	0,009	CL0022
TRINITY_DN950_c0_g2_i1_5	2	15,3	20,255	11,35	PF00098.22	zf-CCHC	27,1	2,80E-06	CL0511
TRINITY_DN1342_c0_g2_i1_1	2	6,7	37,846	-2	PF00270.28	DEAD	74,9	6,00E-21	CL0023
TRINITY_DN10787_c0_g2_i1_6	1	3,6	59,26	6,5997	PF00201.17	UDPGT	73,1	1,80E-20	CL0113
TRINITY_DN15996_c0_g1_i1_5	1	6,9	18,17	6,4862	PF08699.9	ArgoL1	66,8	8,50E-19	No_clan
TRINITY_DN13943_c0_g1_i1_2	1	14,4	13,11	7,0602					
TRINITY_DN4307_c0_g2_i2_3	1	1,7	77,962	6,5199	PF01661.20	Macro	91,9	2,60E-26	CL0223
TRINITY_DN13850_c0_g1_i1_2	2	26,5	9,4353	7,382	PF00076.21	RRM_1	39,1	4,50E-10	CL0221
TRINITY_DN22319_c0_g1_i1_6	1	15,2	12,561	9,5812					
TRINITY_DN19381_c0_g1_i1_1	1	3,8	43,003	6,9889					
TRINITY_DN17549_c0_g1_i1_3	1	5,9	18,897	6,6841	PF01103.22	Bac_surface_Ag	101,4	6,10E-29	CL0193
TRINITY_DN15080_c0_g2_i1_2	1	4,6	33,305	6,2823	PF05071.15	NDUFA12	44,1	2,50E-11	No_clan
TRINITY_DN18817_c0_g1_i1_6	1	11,5	9,595	6,4446	PF05368.12	NmrA	54,1	1,40E-14	CL0063
TRINITY_DN11009_c0_g1_i1_1	1	10,8	16,243	7,0607	PF14541.5	TAXi_C	79,4	2,40E-22	CL0129

TRINITY_DN8994_c0_g2_i2_3	1	5	26,002	6,3787	PF00582.25	Usp	99,8	1,70E-28	CL0039
TRINITY_DN17962_c0_g1_i1_6	1	8,9	11,903	6,4166					
TRINITY_DN7296_c0_g1_i1_3	2	3,6	69,775	12,087	PF00076.21	RRM_1	58,8	3,10E-16	CL0221
TRINITY_DN17826_c0_g2_i1_1	2	8,4	61,171	11,583	PF00069.24	Pkinase	216	5,40E-64	CL0016
TRINITY_DN12326_c0_g1_i1_4	1	5,7	25,014	6,3399					
TRINITY_DN2620_c0_g2_i2_2	1	8,9	26,774	6,6935	PF01423.21	LSM	56	2,10E-15	CL0527
TRINITY_DN2361_c0_g1_i1_2	1	15,3	13,004	6,6536	PF05653.13	Mg_trans_NIPA	47	1,70E-12	CL0184
TRINITY_DN1411_c0_g1_i1_5	1	3,6	33,05	6,6096	PF01268.18	FTHFS	329,1	3,40E-98	CL0023
TRINITY_DN11963_c0_g1_i1_5	1	20,8	7,3995	7,1228					
TRINITY_DN9986_c0_g1_i2_1	1	2,1	64,68	6,6039	PF01545.20	Cation_efflux	118,8	2,20E-34	No_clan
TRINITY_DN3850_c0_g1_i1_2	1	3,2	46,827	6,418	PF02446.16	Glyco_hydro_77	418,2	3,80E-125	CL0058
TRINITY_DN13162_c0_g1_i1_5	2	17,1	23,032	-2	PF16190.4	E1_FCCH	97,9	2,60E-28	No_clan
TRINITY_DN6999_c1_g1_i1_1	1	7,1	24,777	8,3918	PF01975.16	SurE	148,5	1,80E-43	No_clan
TRINITY_DN6417_c0_g2_i1_1	2	5,6	45,937	11,927	PF01633.19	Choline_kinase	213,3	2,80E-63	CL0016
TRINITY_DN11852_c0_g1_i1_2	1	3,7	35,315	7,1393	PF00763.22	THF_DHG_CYH	96,8	8,60E-28	CL0603
TRINITY_DN3994_c0_g1_i1_6	1	8,7	17,887	7,1141					
TRINITY_DN10757_c0_g1_i6_3	2	9,1	28,317	11,055	PF00255.18	GSHPx	152,2	2,90E-45	CL0172
TRINITY_DN858_c0_g2_i1_3	2	8,4	40,784	7,7935	PF00483.22	NTP_transferase	50	2,60E-13	CL0110
TRINITY_DN23297_c0_g1_i1_4	1	12,6	9,617	7,2012					
TRINITY_DN19876_c0_g1_i1_6	1	7,9	13,503	6,2896					
TRINITY_DN11014_c0_g1_i1_4	1	8,4	23,683	6,5656	PF00582.25	Usp	82,3	4,20E-23	CL0039
TRINITY_DN13986_c1_g1_i1_2	1	12,2	10,375	6,9417					
TRINITY_DN4916_c0_g2_i1_4	1	10,9	16,879	9,5812					
TRINITY_DN13205_c0_g1_i1_2	1	6,1	25,254	6,8586					
TRINITY_DN2844_c0_g1_i1_3	1	8,6	18,776	7,4689					
TRINITY_DN19026_c0_g1_i1_2	1	9,4	13,8	6,5283	PF08569.10	Mo25	106,6	1,50E-30	CL0020
TRINITY_DN10596_c0_g1_i1_3	1	1,7	78,527	6,2669	PF13855.5	LRR_8	36,4	3,00E-09	CL0022
TRINITY_DN11089_c0_g1_i1_6	1	3	44,518	6,5642	PF00076.21	RRM_1	63,7	9,30E-18	CL0221
TRINITY_DN1625_c0_g1_i1_1	1	23,7	10,208	8,2909	PF07983.12	X8	69	3,90E-19	No_clan
TRINITY_DN4295_c0_g1_i2_2	1	14,6	16,968	7,821					
TRINITY_DN21571_c0_g1_i1_3	1	14,5	8,3188	6,7271					
TRINITY_DN9775_c0_g1_i1_1	2	7,8	33,121	13,183	PF00407.18	Bet_v_1	44,9	1,00E-11	CL0209
TRINITY_DN17721_c0_g1_i1_3	1	5,7	38,354	6,7956	PF00364.21	Biotin_lipoyl	84,4	3,40E-24	CL0105

TRINITY_DN11860_c0_g1_i1_4	1	9	23,564	6,5581	PF09742.8	Dymeclin	80,1	1,20E-22	CL0456
TRINITY_DN19531_c0_g1_i1_1	1	8	21,912	9,4491					
TRINITY_DN194_c0_g2_i1_1	1	5,2	30,547	6,9104	PF13812.5	PPR_3	25,6	8,60E-06	CL0020
TRINITY_DN426_c0_g1_i1_2	1	4,5	26,568	6,8372					
TRINITY_DN23380_c0_g1_i1_2	1	10,5	11,275	6,5426	PF01116.19	F_bP_aldolase	81,7	5,50E-23	CL0036
TRINITY_DN7432_c0_g1_i1_5	1	3,6	35,795	7,2438	PF00266.18	Aminotran_5	349	2,60E-104	CL0061
TRINITY_DN9824_c0_g2_i1_2	1	5,4	20,609	10,358	PF00462.23	Glutaredoxin	72,9	1,70E-20	CL0172
TRINITY_DN14943_c0_g1_i1_3	1	14	13,885	7,7191	PF02878.15	PGM_PMM_I	37,6	1,50E-09	No_clan
TRINITY_DN4390_c0_g1_i2_1	5	12,9	45,275	8,2121	PF00364.21	Biotin_lipoyl	56,8	1,30E-15	CL0105
TRINITY_DN3895_c0_g2_i1_2	1	4,8	32,684	7,2119					
TRINITY_DN2101_c0_g1_i1_2	1	41	8,9646	18,462					
TRINITY_DN22364_c0_g1_i1_6	1	14,9	7,8548	7,2689	PF09763.8	Sec3_C	33,8	1,10E-08	CL0295
TRINITY_DN3021_c0_g2_i1_6	1	3,4	50,262	7,2445	PF00294.23	PfkB	94,5	6,70E-27	CL0118
TRINITY_DN1919_c0_g1_i1_1	1	3,8	45,693	6,7027	PF01593.23	Amino_oxidase	138,5	3,70E-40	CL0063
TRINITY_DN11220_c1_g3_i1_3	1	4,7	24,908	6,4331	PF13561.5	adh_short_C2	174,1	3,10E-51	CL0063
TRINITY_DN8579_c0_g2_i1_1	1	3,3	50,322	10,004	PF00562.27	RNA_pol_Rpb2_6	281,8	8,40E-84	CL0410
TRINITY_DN7867_c0_g1_i4_3	1	5,5	25,502	6,786	PF10607.8	CLTH	128,6	1,60E-37	No_clan
TRINITY_DN11046_c0_g1_i2_2	1	2	80,982	7,8712	PF00651.30	BTB	30,3	3,70E-07	CL0033
TRINITY_DN8640_c0_g1_i1_1	1	5,8	26,874	6,9022	PF00168.29	C2	82,5	2,10E-23	CL0154
TRINITY_DN2390_c0_g2_i1_1	1	6,6	21,208	7,1548	PF16123.4	HAGH_C	88,2	3,40E-25	CL0381
TRINITY_DN12575_c0_g1_i1_3	1	4,6	28,663	6,496	PF00999.20	Na_H_Exchanger	54,6	6,90E-15	CL0064
TRINITY_DN10417_c0_g2_i1_1	1	3	46,055	7,1393	PF00566.17	RabGAP-TBC	173,5	4,40E-51	No_clan
TRINITY_DN17426_c0_g1_i1_6	1	5,6	21,752	7,3011					
TRINITY_DN9926_c0_g1_i2_5	1	7,4	14,86	6,5762					
TRINITY_DN2534_c0_g1_i1_4	1	12,2	17,295	6,7115	PF13890.5	Rab3-GTPase_cat	173,7	2,20E-51	No_clan
TRINITY_DN2915_c0_g1_i1_3	1	18,4	8,2212	7,0616	PF01039.21	Carboxyl_trans	42,6	2,90E-11	CL0127
TRINITY_DN11633_c0_g1_i3_5	1	3,4	64,55	7,312					
TRINITY_DN15398_c0_g1_i1_5	1	14,6	15,056	6,5997	PF16940.4	Tic110	226	7,60E-67	No_clan
TRINITY_DN5905_c0_g1_i1_3	1	4,3	38,982	6,5757					
TRINITY_DN9808_c0_g1_i1_1	1	6,6	42,76	6,9133	PF01027.19	Bax1-I	160,2	5,30E-47	CL0453
TRINITY_DN10830_c0_g1_i1_1	1	2,8	39,459	6,3161					
TRINITY_DN5571_c0_g1_i2_2	1	5,9	28,807	7,0453					
TRINITY_DN14099_c0_g1_i1_1	1	8	21,546	7,0453					

TRINITY_DN1513_c0_g1_i1_1	1	7,1	23,814	7,7943	PF13041.5	PPR_2	50,6	1,50E-13	CL0020
TRINITY_DN9703_c0_g1_i1_1	1	5,8	34,852	16,322	PF08507.9	COPI_assoc	67,3	1,20E-18	No_clan
TRINITY_DN30_c0_g1_i1_2	1	3,7	41,921	6,9103	PF00069.24	Pkinase	104,4	5,80E-30	CL0016
TRINITY_DN4271_c0_g2_i1_6	1	7,4	24,208	8,0317	PF00248.20	Aldo_ket_red	110,9	5,80E-32	No_clan
TRINITY_DN9637_c0_g1_i2_1	1	2,7	50,299	6,4921	PF03463.14	eRF1_1	174,3	9,70E-52	No_clan
TRINITY_DN14624_c0_g1_i1_5	1	11,7	11,86	6,3399	PF00664.22	ABC_membrane	39,6	4,40E-10	CL0241
TRINITY_DN7843_c0_g1_i2_1	1	11,7	12,529	7,1412	PF03179.14	V-ATPase_G	91,9	2,90E-26	CL0255
TRINITY_DN14064_c0_g1_i1_1	1	9	13,638	7,1926					
TRINITY_DN19958_c0_g1_i1_2	1	33,3	7,7041	6,3022					
TRINITY_DN12317_c0_g2_i1_2	1	10,6	16,424	7,557	PF00248.20	Aldo_ket_red	46	3,50E-12	No_clan
TRINITY_DN106_c0_g1_i1_6	1	4,4	30,431	6,4238	PF00485.17	PRK	30,3	3,00E-07	CL0023
TRINITY_DN10121_c0_g1_i2_2	1	2,9	45,307	7,1941					
TRINITY_DN16265_c0_g1_i1_6	1	9,7	15,335	7,5944					
TRINITY_DN22994_c0_g1_i1_2	1	24,4	9,4857	6,6368					
TRINITY_DN16148_c0_g1_i1_2	1	15,2	10,501	6,9889	PF01565.22	FAD_binding_4	52,5	3,80E-14	CL0077
TRINITY_DN10093_c0_g1_i1_4	1	2,4	50,687	6,5426					
TRINITY_DN13095_c0_g1_i1_2	1	3,4	42,2	6,9537	PF00781.23	DAGK_cat	73,4	1,10E-20	CL0240
TRINITY_DN6032_c0_g1_i1_1	1	4,3	31,479	6,3399	PF04695.12	Pex14_N	91,8	6,50E-26	No_clan
TRINITY_DN4104_c0_g1_i2_2	1	15,5	19,856	7,215	PF07059.11	DUF1336	55,9	5,60E-15	No_clan
TRINITY_DN11490_c0_g1_i4_6	1	9,9	15,441	6,8825					
TRINITY_DN11358_c0_g1_i2_2	1	2,7	54,866	7,3751	PF06273.10	eIF-4B	28,7	6,30E-07	No_clan
TRINITY_DN5926_c0_g1_i1_3	2	10	21,662	-2					
TRINITY_DN10755_c0_g1_i3_1	1	6,7	20,844	6,3282					
TRINITY_DN13112_c0_g1_i1_5	1	14,4	9,6462	6,3118					
TRINITY_DN1601_c0_g1_i1_6	1	6,3	16,997	6,5762	PF11282.7	DUF3082	101	3,00E-29	No_clan
TRINITY_DN20005_c0_g1_i1_6	2	13,7	21,229	11,717	PF02883.19	Alpha_adaptinC2	51,8	7,60E-14	CL0159
TRINITY_DN9298_c0_g1_i2_6	1	4,8	27,736	6,7013	PF03141.15	Methyltransf_29	205	1,60E-60	CL0063
TRINITY_DN10791_c2_g1_i1_2	5	25,9	33,379	11,154	PF00724.19	Oxidored_FMN	250,7	2,10E-74	CL0036
TRINITY_DN12338_c0_g1_i1_2	1	8,4	16,687	7,1747	PF08323.10	Glyco_transf_5	35,4	8,30E-09	CL0113
TRINITY_DN2894_c0_g1_i1_6	1	11,4	11,73	6,654	PF00443.28	UCH	28,8	7,70E-07	CL0125
TRINITY_DN9321_c0_g1_i1_1	1	3,7	38,202	6,3399					
TRINITY_DN8786_c1_g1_i1_3	1	12,1	23,921	7,8979	PF03016.14	Exostosin	30,8	1,60E-07	No_clan
TRINITY_DN8717_c0_g1_i1_1	1	2,4	79,982	6,6409	PF00899.20	ThiF	245,9	3,50E-73	CL0063

TRINITY_DN266_c0_g2_i2_2	1	3,2	60,824	6,7237	PF09412.9	XendoU	331,6	2,80E-99	No_clan
TRINITY_DN10315_c0_g1_i3_3	1	3,9	45,85	6,3285	PF04144.12	SCAMP	182,7	6,80E-54	No_clan
TRINITY_DN19933_c0_g1_i1_3	1	11,3	16,291	9,3323					
TRINITY_DN9610_c0_g1_i5_6	1	2,8	43,73	7,3117	PF00076.21	RRM_1	41,3	9,30E-11	CL0221
TRINITY_DN3076_c0_g1_i1_4	1	2,7	50,709	6,9658	PF13519.5	VWA_2	98,2	3,60E-28	CL0128
TRINITY_DN10575_c0_g1_i1_1	1	3,2	41,149	7,1941	PF06364.11	DUF1068	276,3	7,80E-83	No_clan
TRINITY_DN14305_c0_g1_i1_5	1	8,6	16,074	6,6046	PF02121.17	IP_trans	163,7	5,00E-48	CL0209
TRINITY_DN11352_c0_g1_i1_1	1	3,6	72,115	6,6876	PF12220.7	U1snRNP70_N	71,5	6,40E-20	CL0221
TRINITY_DN6147_c0_g1_i2_6	1	9,4	19,468	6,3287					
TRINITY_DN10057_c1_g1_i2_3	1	13,1	16,685	7,5406	PF00203.20	Ribosomal_S19	66,9	1,00E-18	No_clan
TRINITY_DN13010_c0_g1_i1_1	1	19,5	8,7248	6,2965					
TRINITY_DN2392_c0_g1_i1_2	1	3,1	47,647	6,9631					
TRINITY_DN9690_c0_g1_i2_3	1	2,9	42,62	6,8372	PF05739.18	SNARE	43,7	1,90E-11	No_clan
TRINITY_DN11443_c0_g1_i1_3	1	2,7	41,451	6,6112	PF01370.20	Epimerase	88,8	3,50E-25	CL0063
TRINITY_DN14199_c0_g1_i1_6	1	7,9	16,844	6,2996	PF00924.17	MS_channel	24,4	1,70E-05	No_clan
TRINITY_DN7984_c0_g1_i2_3	1	7,9	19,459	7,7193					
TRINITY_DN15785_c0_g1_i1_6	1	19,8	9,8775	6,6763	PF01031.19	Dynamin_M	38,6	6,40E-10	No_clan
TRINITY_DN21396_c0_g1_i1_3	1	9,7	15,011	6,6239	PF01762.20	Galactosyl_T	36,1	5,20E-09	CL0110
TRINITY_DN7330_c0_g1_i1_3	1	3,1	50,858	6,3209	PF00270.28	DEAD	145,8	1,00E-42	CL0023
TRINITY_DN9918_c0_g2_i5_3	3	5,7	57,711	11,83	PF02874.22	ATP-synt_ab_N	58,9	5,00E-16	CL0275
TRINITY_DN10206_c0_g8_i1_3	1	3,4	50,523	6,4751	PF00069.24	Pkinase	247,2	1,60E-73	CL0016
TRINITY_DN21905_c0_g1_i1_6	1	16,2	8,5256	6,3677					
TRINITY_DN20071_c0_g1_i1_6	1	15	10,555	6,3931					
TRINITY_DN8834_c0_g1_i4_2	1	7,4	27,023	6,6479	PF01521.19	Fe-S_biosyn	70,4	1,20E-19	No_clan
TRINITY_DN10839_c0_g1_i3_6	1	14,7	23,853	6,8404	PF04800.11	ETC_C1_NDUFA4	109,5	6,80E-32	No_clan
TRINITY_DN2321_c0_g1_i1_6	1	4	30,805	6,5097	PF04263.15	TPK_catalytic	133,4	3,80E-39	No_clan
TRINITY_DN12272_c0_g1_i1_2	1	16,5	10,56	6,3105					
TRINITY_DN8223_c1_g1_i1_3	1	24,7	9,8358	7,5002					
TRINITY_DN11343_c0_g1_i7_1	1	2,2	72,139	6,3858					
TRINITY_DN20424_c0_g1_i1_1	1	10,1	14,146	6,7753					
TRINITY_DN3551_c0_g1_i1_3	1	4,1	33,045	7,1941					
TRINITY_DN9616_c0_g1_i1_1	5	21,5	29,632	8,1891	PF00416.21	Ribosomal_S13	188	6,90E-56	CL0303
TRINITY_DN13664_c0_g1_i1_1	1	22,1	9,4684	-2					

TRINITY_DN9032_c0_g1_i5_4	1	4,1	34,044	-2					
TRINITY_DN10274_c0_g1_i3_2	1	5,4	23,07	6,3909	PF13616.5	Rotamase_3	69,2	3,90E-19	CL0487
TRINITY_DN10289_c0_g1_i1_3	1	4,8	29,885	6,7388	PF00535.25	Glycos_transf_2	127,7	3,50E-37	CL0110
TRINITY_DN1145_c0_g2_i1_6	1	8,9	18,591	-2					
TRINITY_DN11535_c0_g1_i3_2	2	12,3	22,244	8,0373	PF00338.21	Ribosomal_S10	95,6	1,50E-27	No_clan
TRINITY_DN14223_c0_g1_i1_5	1	8,8	12,764	6,3717	PF00076.21	RRM_1	77,4	4,90E-22	CL0221
TRINITY_DN18123_c0_g2_i1_2	1	4,1	36,457	7,2492					
TRINITY_DN18755_c0_g1_i1_2	1	10,7	14,9	6,6074					
TRINITY_DN20629_c0_g1_i1_1	1	8	18,57	6,2981	PF03407.15	Nucleotid_trans	72,9	3,30E-20	CL0110
TRINITY_DN20794_c0_g1_i1_5	1	16,1	9,198	6,645					
TRINITY_DN21254_c0_g1_i1_2	1	5,5	24,82	6,8105	PF13499.5	EF-hand_7	43,7	2,70E-11	CL0220
TRINITY_DN22136_c0_g1_i1_1	1	17,1	8,0454	6,2922					
TRINITY_DN22623_c0_g1_i1_1	1	25,7	7,7902	12,562					
TRINITY_DN2924_c0_g1_i1_3	1	4,5	29,877	6,8105	PF01997.15	Translin	193,7	3,20E-57	No_clan
TRINITY_DN493_c0_g1_i1_1	1	2,6	45,316	6,9574	PF00013.28	KH_1	56,9	1,20E-15	CL0007
TRINITY_DN6077_c0_g2_i1_2	1	3,7	34,286	14,394	PF01008.16	IF-2B	245,2	6,80E-73	CL0246
TRINITY_DN6267_c0_g1_i1_2	1	17,1	7,7717	47,523					
TRINITY_DN6751_c0_g1_i1_3	1	11,9	10,521	-2					
TRINITY_DN9010_c0_g1_i2_3	1	3,8	36,721	8,8627	PF01470.16	Peptidase_C15	64,2	1,50E-17	CL0379
