# Supplementary material for: Comparative proteomics of common allergenic tree pollens of birch, alder, and hazel
Source: Allergy. 2021 Jan 15;76(6):1743–53. doi: 10.1111/all.14694 (PMC8248232; doi:10.1111/all.14694)
Supplement: Supplementary file 4 — Table S2 [file ALL-76-1743-s017.pdf]

Supplementary Table S2: Intensities of identified Corylus pollen proteins in different preparations

Protein IDs	Intensity fasp1	Intensity fasp2	Intensity fasp3	Intensity acetone1	Intensity acetone 2	Intensity acetone 3	Intensity tca1	Intensity tca2	Intensity tca3	Intensity zc1	Intensity zc2	Intensity zc3
TRINITY_DN8608_c2_g1_i2_5	461560000	1504900000	1069800000	110450000	473800000	671090000	0	0	0	19351000	14571000	16655000
TRINITY_DN11207_c0_g1_i2_3	823390000	546540000	506860000	132820000	221410000	345100000	0	0	0	50187000	41037000	45594000
TRINITY_DN9484_c0_g1_i1_5	377560000	353740000	387990000	169660000	287210000	410480000	83866000	47337000	186800000	71395000	74399000	92316000
TRINITY_DN7872_c0_g1_i3_6	559500000	612990000	586610000	44216000	111150000	148740000	36082000	26832000	46256000	16007000	44459000	17136000
TRINITY_DN10821_c0_g1_i5_2	373300000	704830000	600390000	28468000	91393000	132660000	0	3651400	50413000	24822000	26394000	29224000
TRINITY_DN11055_c0_g2_i3_5	375630000	329640000	434460000	77628000	83664000	288380000	38353000	54448000	51906000	56495000	51018000	33570000
TRINITY_DN9053_c0_g1_i5_1	247470000	194830000	269310000	150640000	176230000	173330000	77793000	89836000	178170000	102740000	68357000	58304000
TRINITY_DN10419_c0_g1_i1_1	316730000	271150000	288660000	107860000	133220000	105940000	39362000	27973000	86312000	56904000	43037000	46298000
TRINITY_DN11591_c0_g3_i5_3	237140000	204590000	294600000	42843000	143220000	150470000	93563000	90041000	96497000	48314000	31731000	21595000
TRINITY_DN11331_c0_g10_i2_1	61834000	393000000	513400000	965650	44430000	35672000	0	0	0	118280000	157810000	86044000
TRINITY_DN8476_c0_g1_i1_3	270190000	163840000	347320000	36017000	144100000	205540000	18468000	15506000	0	17350000	41627000	32485000
TRINITY_DN10442_c0_g1_i1_2	183700000	173120000	161130000	95934000	101000000	106340000	57335000	67184000	175730000	28990000	23976000	19744000
TRINITY_DN9319_c0_g1_i1_2	143550000	163620000	205970000	48656000	107950000	131510000	0	38844000	0	96962000	114770000	130160000
TRINITY_DN9571_c0_g1_i2_3	131800000	157040000	163440000	32733000	163750000	182870000	20010000	28721000	166800000	27340000	22821000	14637000
TRINITY_DN13451_c0_g2_i1_3	233010000	475370000	250470000	20584000	55137000	0	0	0	0	0	0	10684000
TRINITY_DN8018_c0_g1_i2_4	126840000	257310000	182780000	5557500	181670000	260180000	0	0	0	8005900	7581100	7204700
TRINITY_DN10804_c2_g1_i1_3	168860000	142820000	144290000	74779000	83194000	86800000	26532000	9028700	75123000	49982000	36442000	39298000
TRINITY_DN10890_c0_g3_i4_6	133390000	229080000	275090000	30860000	61938000	121450000	0	0	0	19501000	21145000	23119000
TRINITY_DN8608_c2_g1_i1_5	265690000	216840000	202290000	1985200	102200000	39735000	0	0	0	24804000	10195000	10332000
TRINITY_DN6026_c0_g1_i1_2	199710000	171320000	183910000	13257000	68230000	45047000	48415000	57759000	28535000	4912000	19658000	1885500
TRINITY_DN11331_c0_g10_i3_1	111990000	118990000	120890000	7895500	64361000	64431000	0	0	0	151570000	150830000	51235000
TRINITY_DN13307_c0_g1_i1_1	109550000	70222000	120300000	62834000	142480000	148280000	5830700	56441000	90762000	7714800	4084000	5940800
TRINITY_DN11501_c0_g1_i5_2	37888000	14393000	12763000	41024000	39032000	26071000	25481000	20479000	43432000	188110000	141810000	144890000
TRINITY_DN9487_c0_g3_i2_5	49688000	101040000	96516000	41518000	53523000	67725000	17497000	10131000	14372000	101110000	89582000	79839000
TRINITY_DN10587_c0_g1_i1_3	108200000	92856000	98325000	33177000	125710000	119670000	4495000	33849000	43999000	26519000	18715000	16012000
TRINITY_DN10479_c0_g2_i1_3	119170000	96860000	111170000	44066000	181910000	101260000	0	0	0	18699000	15111000	14556000
TRINITY_DN11616_c0_g1_i2_1	57454000	47658000	52489000	59783000	57806000	37350000	66071000	43078000	89131000	59552000	56183000	47422000
TRINITY_DN4595_c0_g1_i1_3	37990000	160400000	143820000	17375000	139070000	109470000	0	0	0	41874000	219400	23388000
TRINITY_DN3727_c0_g2_i1_1	168490000	140360000	125130000	29198000	58945000	28649000	35663000	19081000	31222000	8551300	6476700	6402800
TRINITY_DN9731_c0_g1_i1_1	50562000	39148000	39769000	36148000	58492000	44930000	12734000	9683500	14352000	132040000	105550000	96670000

TRINITY_DN3709_c0_g1_i1_3	60205000	54165000	70355000	30187000	19226000	21585000	33670000	43885000	75925000	96289000	59547000	53406000
TRINITY_DN9065_c0_g1_i1_1	75458000	28521000	27858000	60605000	67858000	53040000	39854000	59600000	71291000	43783000	38052000	33313000
TRINITY_DN11320_c0_g1_i11_3	65469000	48841000	47186000	33148000	35284000	32033000	0	0	0	117990000	101880000	114740000
TRINITY_DN6201_c0_g1_i1_1	67018000	101940000	119580000	14721000	76353000	52527000	54110000	17123000	70384000	3981200	7783500	2612300
TRINITY_DN6019_c0_g1_i1_3	70444000	57258000	62082000	43039000	44841000	26386000	61876000	85811000	57410000	26872000	25877000	25113000
TRINITY_DN10785_c0_g1_i3_3	78100000	131290000	137030000	63575000	79513000	72280000	0	0	0	14912000	0	7345900
TRINITY_DN10341_c0_g2_i1_1	97518000	116130000	120030000	73823000	65283000	68967000	4025600	0	1350700	11317000	9977200	12381000
TRINITY_DN10479_c0_g1_i2_1	30850000	31352000	43830000	52487000	75956000	105890000	41386000	67251000	61211000	39795000	13416000	15800000
TRINITY_DN10144_c0_g1_i2_2	65169000	64279000	82459000	47168000	136110000	84146000	10299000	0	0	30084000	23537000	24350000
TRINITY_DN5870_c0_g1_i2_4	40093000	44362000	61760000	41628000	61480000	51176000	62129000	72579000	91063000	12749000	8941000	10037000
TRINITY_DN10438_c0_g2_i1_2	42144000	27206000	39391000	38921000	45926000	34340000	3749200	9677000	208700000	15400000	12326000	10901000
TRINITY_DN8953_c0_g1_i1_1	31200000	24081000	92245000	52660000	133730000	109930000	0	0	0	11695000	9931800	9416000
TRINITY_DN9146_c0_g3_i2_3	1058000	1418500	728240	3121900	3164600	952440	1621200	2291300	0	156190000	163490000	104080000
TRINITY_DN10397_c0_g1_i2_5	75867000	66791000	39325000	42446000	37911000	25139000	34564000	28423000	43610000	19256000	8323800	10527000
TRINITY_DN10647_c0_g1_i1_4	91807000	33435000	46790000	79484000	29857000	49375000	0	0	0	35225000	13521000	39206000
TRINITY_DN11581_c0_g2_i3_2	63807000	62084000	89245000	42210000	39082000	45263000	4059000	2327800	4233000	24028000	18480000	20430000
TRINITY_DN7958_c0_g4_i1_6	33113000	31300000	28390000	25026000	25208000	18774000	46335000	66145000	70027000	25633000	22114000	20167000
TRINITY_DN10683_c0_g1_i3_5	63460000	48078000	70596000	13330000	21015000	30319000	36712000	38379000	49606000	4770700	11797000	15739000
TRINITY_DN11604_c0_g1_i6_2	57261000	54719000	54562000	50063000	72149000	52180000	0	0	0	22955000	18878000	15070000
TRINITY_DN9279_c0_g1_i1_1	52428000	40474000	42578000	36719000	42053000	26898000	38287000	48682000	61473000	3756700	892990	2628600
TRINITY_DN11174_c0_g1_i5_1	68969000	67461000	62587000	61538000	51999000	50458000	0	0	0	11371000	9463900	11056000
TRINITY_DN11394_c0_g1_i1_2	43233000	36005000	31858000	20077000	22812000	18505000	14733000	3265900	14451000	90530000	53909000	41884000
TRINITY_DN10920_c0_g1_i2_3	37705000	29421000	54081000	37725000	57576000	31424000	14927000	9118500	24882000	33615000	28835000	28522000
TRINITY_DN13244_c0_g1_i1_3	80928000	83185000	78971000	43045000	44600000	36650000	0	0	0	6042500	5682700	5341700
TRINITY_DN10804_c1_g1_i1_3	61471000	81415000	80246000	20677000	13742000	19985000	11950000	16960000	32126000	13024000	15918000	10025000
TRINITY_DN10010_c0_g1_i2_4	25432000	37485000	35430000	31768000	36564000	26473000	38063000	20599000	78522000	10901000	9747300	8166500
TRINITY_DN11304_c0_g1_i5_2	2221500	2478700	2040200	1300700	1311100	514110	3920400	0	2524300	114150000	112470000	115730000
TRINITY_DN14318_c0_g1_i1_5	0	0	0	0	0	0	111470000	111110000	118700000	0	0	0
TRINITY_DN11227_c0_g1_i5_5	15887000	21199000	22903000	49823000	53384000	44279000	6319200	55415000	30321000	11283000	14225000	11100000
TRINITY_DN4231_c0_g1_i1_1	47994000	47356000	35240000	25319000	26589000	20915000	33154000	39309000	43691000	3435500	2800100	2660700
TRINITY_DN10618_c0_g1_i1_3	70771000	55437000	56330000	24573000	39111000	23706000	10202000	9412100	14573000	6917200	3377600	3756200
TRINITY_DN9827_c0_g1_i1_5	51756000	61059000	61003000	4424900	49491000	40382000	0	0	0	15982000	12076000	11889000
TRINITY_DN7126_c0_g3_i1_3	51999000	34127000	50904000	46443000	27354000	33292000	21993000	16334000	14045000	2755800	1862100	2758300

TRINITY_DN11590_c1_g1_i1_3	29664000	26494000	29143000	23555000	25460000	21837000	15902000	16194000	92490000	8200300	5698900	5319500
TRINITY_DN11652_c0_g1_i2_1	13902000	13815000	14785000	10750000	17827000	13208000	2424800	3713400	745540	79003000	56349000	72271000
TRINITY_DN5250_c0_g1_i1_1	68293000	87219000	79936000	15739000	9357300	12895000	0	0	2500200	4205300	5632600	4041300
TRINITY_DN11459_c1_g2_i2_1	4204800	4107900	4023900	3493800	1903400	3151400	4361800	0	3514700	99250000	85106000	76401000
TRINITY_DN10499_c0_g1_i1_2	22361000	28783000	21648000	9417400	8088100	17617000	15236000	33430000	32508000	35659000	33010000	27409000
TRINITY_DN8634_c0_g1_i1_1	44226000	52422000	22261000	1930000	58041000	61291000	0	0	0	27992000	8811600	4054400
TRINITY_DN10893_c0_g1_i1_2	24479000	34685000	44130000	25871000	52565000	31563000	0	0	0	24844000	24657000	18037000
TRINITY_DN9811_c0_g1_i2_3	32083000	41821000	51322000	3691800	53881000	66400000	0	1958000	2439000	3667500	2418300	2588000
TRINITY_DN8650_c0_g2_i3_2	692660	880000	671500	701090	878980	133280	803860	1946100	0	119020000	70702000	65389000
TRINITY_DN11653_c0_g1_i19_5	12701000	10459000	9670400	1656800	2246400	2443200	6500700	6357500	9295400	67736000	58312000	72416000
TRINITY_DN8041_c0_g1_i2_3	1226400	1173400	840390	695560	558840	462060	2718300	0	5161700	95303000	58993000	91942000
TRINITY_DN14244_c0_g1_i1_2	41933000	50963000	48981000	33812000	41005000	32025000	0	0	0	0	5016000	1245700
TRINITY_DN10590_c0_g2_i1_2	26833000	22725000	24295000	42160000	27072000	41225000	3352300	6494600	6921900	17835000	15976000	15074000
TRINITY_DN3503_c0_g1_i2_1	10778000	10863000	16673000	8726900	101180000	96838000	0	0	0	1953000	1214400	1358500
TRINITY_DN10155_c0_g1_i1_2	30537000	31031000	31648000	27398000	50992000	33501000	706090	7381200	25236000	4135000	1271800	1317900
TRINITY_DN11019_c0_g1_i1_2	180120	0	0	0	116860	70139	0	746340	0	100320000	66308000	77155000
TRINITY_DN10859_c0_g1_i1_2	13011000	71267000	15573000	32854000	30966000	13761000	7743900	40880000	9186700	3579300	1607100	3799500
TRINITY_DN10804_c0_g1_i1_2	46941000	34599000	33303000	8595600	23815000	25149000	12672000	15910000	13987000	9908300	9207700	9248700
TRINITY_DN7252_c0_g4_i3_3	5413400	6554200	6026800	538800	4495500	4440500	6223400	6693200	7134100	79145000	55077000	61274000
TRINITY_DN11092_c0_g1_i2_3	16450000	11945000	17421000	15646000	15519000	18456000	37142000	35783000	66789000	1688200	725380	1052800
TRINITY_DN11258_c0_g1_i2_3	1906000	1034400	1817300	763550	1892100	1924100	2903800	4247300	1726000	74277000	63575000	82304000
TRINITY_DN8200_c0_g1_i1_1	21541000	17590000	18406000	11168000	21203000	13633000	0	0	0	54712000	51430000	25296000
TRINITY_DN9523_c0_g1_i1_4	23886000	26073000	24354000	19634000	22784000	18811000	27926000	24080000	34916000	5506900	4153400	1906700
TRINITY_DN10459_c0_g1_i1_3	10507000	58655000	18650000	11018000	24767000	19345000	14522000	10274000	9376000	22495000	12277000	17837000
TRINITY_DN11248_c1_g1_i1_3	14173000	10398000	18681000	16981000	23056000	16349000	5620700	13428000	21086000	29058000	27667000	29601000
TRINITY_DN10833_c1_g2_i1_1	46768000	33226000	17691000	14931000	15688000	12466000	16193000	10693000	49504000	1369000	4841500	2604300
TRINITY_DN9054_c0_g1_i2_1	33754000	34790000	27950000	26051000	29983000	48877000	0	0	0	3497600	9789900	8518800
TRINITY_DN11641_c0_g1_i13_1	11006000	10188000	9260200	9135800	11844000	10407000	1264900	1948200	695610	57849000	53901000	42532000
TRINITY_DN9275_c0_g1_i1_1	20352000	24435000	32491000	23689000	29293000	24570000	12155000	15032000	12906000	9469300	5783300	7724600
TRINITY_DN11496_c0_g1_i1_2	1953900	3015500	3219900	4254800	6766500	2223600	5875000	8944400	3928800	64367000	62343000	49506000
TRINITY_DN10665_c0_g1_i1_2	13316	264760	518950	0	410280	0	288880	0	487070	78889000	59105000	72989000
TRINITY_DN11667_c0_g1_i3_3	228350	272240	0	362180	756450	743880	1165600	625630	812820	74605000	62589000	68398000
TRINITY_DN2899_c0_g1_i1_1	20187000	28833000	28794000	20903000	22853000	17010000	7763700	25237000	28723000	3283300	1318800	1286300

TRINITY_DN5468_c0_g2_i2_1	21141000	12366000	13243000	6381900	17452000	14404000	0	0	0	43984000	37855000	38679000
TRINITY_DN11591_c0_g3_i6_3	1983400	4282700	4262500	37589000	60881000	39126000	11816000	13013000	16894000	4995800	5283000	4602800
TRINITY_DN11204_c0_g1_i1_3	0	0	0	241110	331800	187850	0	0	0	66047000	69463000	64029000
TRINITY_DN10703_c1_g1_i1_1	831770	768950	2798100	25860000	27694000	25963000	33257000	18728000	40055000	5495100	7999700	6053000
TRINITY_DN11207_c0_g1_i1_3	27516000	28063000	33451000	30637000	36679000	28246000	0	0	0	4020200	3434900	2952800
TRINITY_DN10743_c0_g1_i1_2	39157000	37700000	31054000	12688000	21147000	9612700	3433500	3564900	3901000	12041000	7619000	10086000
TRINITY_DN8812_c0_g1_i2_1	10822000	14434000	12683000	42929000	47112000	31150000	16344000	3644300	0	4496700	4498700	917950
TRINITY_DN11581_c0_g1_i4_3	28534000	14581000	26606000	2680500	0	7462600	37858000	31677000	36408000	482120	1282700	0
TRINITY_DN9487_c0_g2_i1_5	15476000	15468000	13986000	12222000	9870200	6880700	0	3043500	6196700	34213000	36688000	31770000
TRINITY_DN11173_c0_g3_i1_4	40515	136570	61267	0	0	82880	0	0	0	74997000	69675000	39884000
TRINITY_DN5663_c0_g2_i1_3	21958000	27966000	27561000	32277000	26720000	19633000	5299200	6389400	7339300	4248800	2209500	2037700
TRINITY_DN9210_c0_g1_i1_1	20286000	16360000	16657000	37648000	41944000	35337000	0	0	6188200	3243900	3081700	2223500
TRINITY_DN9779_c0_g1_i1_1	139360	37318	0	0	134110	0	413980	413600	0	62782000	56248000	58507000
TRINITY_DN16006_c0_g1_i1_2	20724000	31114000	15473000	30910000	42572000	28375000	0	0	0	1117500	775920	2655600
TRINITY_DN5448_c0_g2_i1_3	8187700	12698000	9748400	20475000	19210000	14826000	17421000	38936000	23869000	2562300	2280300	2263200
TRINITY_DN10761_c0_g1_i2_2	12147000	10191000	10208000	6974900	8104700	2598600	9301300	23851000	23928000	23224000	22426000	17075000
TRINITY_DN11378_c0_g1_i1_4	0	0	0	219490	498070	0	0	0	0	67359000	58147000	42971000
TRINITY_DN10720_c0_g2_i2_6	563350	1142500	893380	218700	823970	180300	535670	7197500	4252100	46234000	56008000	50651000
TRINITY_DN11028_c0_g2_i2_3	7020100	14367000	14909000	14862000	14970000	12306000	10437000	25460000	10363000	12994000	16050000	14771000
TRINITY_DN10686_c0_g1_i1_3	0	411920	0	0	128540	0	0	0	0	64698000	49795000	50626000
TRINITY_DN10496_c0_g1_i5_3	1158300	196940	752950	3392200	2385300	953140	2899800	1315200	1406200	56564000	42763000	50644000
TRINITY_DN11432_c0_g1_i3_6	11197000	8385800	7272600	21797000	19507000	16060000	0	2523700	0	31168000	27157000	18904000
TRINITY_DN4601_c0_g1_i1_1	24621000	25047000	15617000	14867000	25842000	5921900	3222000	27697000	11146000	4092000	0	4139300
TRINITY_DN10681_c0_g1_i1_2	1357600	1307100	970900	269380	1235800	771340	0	1194100	0	59312000	43530000	48560000
TRINITY_DN10442_c0_g2_i1_4	37197000	35309000	34555000	2467100	19618000	10763000	0	0	0	4216200	6489000	5674100
TRINITY_DN9458_c0_g1_i2_2	6314600	7907900	6164200	3764400	8815400	3531300	1128000	8125500	2388100	37858000	36012000	33374000
TRINITY_DN8250_c0_g1_i2_3	6428000	16068000	13304000	6527100	6097200	9293600	20940000	18190000	17573000	14331000	14145000	12285000
TRINITY_DN10239_c0_g1_i1_2	19308000	17437000	6310900	28830000	23679000	24704000	12148000	318980	15469000	2573500	1551000	2718600
TRINITY_DN11469_c0_g2_i3_2	0	0	0	56429000	55273000	42259000	0	0	0	0	0	0
TRINITY_DN7240_c0_g1_i2_1	4245000	5316000	5121900	4869900	4824300	4612000	0	0	0	42618000	42294000	39866000
TRINITY_DN4014_c0_g1_i1_2	43154000	1834200	14666000	12799000	14505000	13454000	13294000	0	16703000	8623000	7862100	6064500
TRINITY_DN10537_c0_g1_i2_4	9516200	10896000	11199000	7307100	9606900	7531700	9576300	1998400	8735600	28952000	24890000	22624000
TRINITY_DN5324_c1_g1_i2_1	12002000	12052000	12908000	35817000	40385000	31910000	0	0	0	2989000	2251300	2189800

TRINITY_DN5563_c0_g1_i1_6	0	47720000	36728000	16078000	20109000	18626000	0	0	0	3323200	3067800	2733100
TRINITY_DN8045_c0_g1_i1_5	63144000	36465000	10286000	20696000	2397300	3646700	0	5081300	5651100	0	0	0
TRINITY_DN8960_c0_g1_i1_2	14625000	11632000	9138400	4803000	11508000	7629200	27785000	15573000	25177000	7644900	5766100	5449100
TRINITY_DN4028_c1_g1_i1_2	27974000	19775000	17153000	24509000	23958000	9792800	0	0	17452000	1119200	2110200	1037200
TRINITY_DN11269_c2_g1_i10_3	1705800	1273300	1896900	1127400	589410	971140	6118500	6417000	6798700	48610000	38397000	30926000
TRINITY_DN11656_c0_g1_i6_2	4703000	5083000	4181900	214340	1922500	1289400	4414400	6984000	11800000	45608000	24838000	33017000
TRINITY_DN10568_c0_g3_i2_1	0	0	0	0	0	0	0	0	528770	55072000	47321000	40865000
TRINITY_DN9271_c0_g1_i1_1	25520000	19185000	20132000	18744000	23789000	18790000	0	0	0	4405400	5925700	4702500
TRINITY_DN10339_c0_g2_i1_4	7325500	12478000	12278000	22591000	24221000	20340000	0	13732000	19315000	3815200	3021000	1822500
TRINITY_DN8726_c0_g2_i1_5	7717600	4517100	4531800	10721000	7410200	7361100	28412000	27841000	33956000	4573000	2217800	1488200
TRINITY_DN11504_c0_g1_i1_3	43252000	9226000	25186000	12520000	8846400	8187700	2464100	1717600	16653000	2893800	2959600	3387400
TRINITY_DN10183_c0_g2_i2_2	4286000	2135100	3927500	4255300	3212000	14130000	15034000	9190000	54084000	3980200	12770000	9144900
TRINITY_DN1820_c0_g1_i1_6	35608000	22641000	21257000	7468000	28365000	13570000	0	0	0	2151100	1427500	3300900
TRINITY_DN8787_c0_g2_i1_4	6874900	80517000	39730000	0	4978800	562840	0	0	0	0	0	0
TRINITY_DN10196_c0_g1_i1_1	23601000	17441000	26077000	12188000	19263000	13850000	0	0	0	7115400	8178300	3043600
TRINITY_DN10535_c0_g1_i2_3	5374300	4663400	8451700	12105000	19793000	12811000	9366400	25427000	9534700	8844500	7115100	5948700
TRINITY_DN11343_c0_g1_i7_2	194150	393270	0	0	192660	84863	0	0	0	48535000	44416000	34298000
TRINITY_DN9170_c0_g1_i1_1	34099000	33153000	26030000	13652000	6958900	4248700	0	0	0	2902700	2678000	4203700
TRINITY_DN9813_c0_g1_i1_2	2334300	1925000	2053800	1374800	2491100	861700	0	0	817590	40421000	39202000	32869000
TRINITY_DN11430_c0_g2_i4_6	0	0	0	0	0	0	0	0	0	46155000	41492000	36290000
TRINITY_DN9475_c0_g1_i1_3	23585000	16278000	13407000	13916000	19554000	12876000	4145600	4752700	5091900	3443200	3066600	3057600
TRINITY_DN11040_c0_g1_i6_1	180330	204300	0	0	227730	0	1345200	1399800	1407800	43962000	37743000	36128000
TRINITY_DN10148_c0_g1_i6_3	8902700	8333600	9840600	7602500	5937400	5040300	17615000	8697800	33034000	7089200	5384600	5000000
TRINITY_DN5484_c0_g2_i1_4	18494000	23849000	22048000	19076000	16544000	16110000	0	0	0	0	2746100	2786500
TRINITY_DN7458_c0_g1_i3_2	4816800	5413500	7583200	31989000	22693000	35933000	0	0	0	5734700	3310100	4025800
TRINITY_DN7727_c0_g2_i1_3	15816000	13122000	20324000	13383000	39124000	12561000	0	0	0	1709600	1690500	3376100
TRINITY_DN18091_c0_g1_i1_4	24873000	19029000	17676000	20855000	22600000	9321300	0	0	0	1212300	3103400	0
TRINITY_DN10656_c0_g1_i1_2	4873800	5663800	7078300	11816000	15544000	10033000	4062000	8840100	12560000	15722000	12707000	9024400
TRINITY_DN7279_c0_g1_i2_5	4121800	7169000	7010900	11325000	20575000	13976000	16148000	16632000	18377000	1055000	607320	385320
TRINITY_DN11640_c0_g2_i6_2	0	0	0	0	0	0	0	0	0	45998000	27897000	43401000
TRINITY_DN1640_c0_g2_i1_1	0	0	0	0	0	0	0	0	0	41310000	39558000	35270000
TRINITY_DN7420_c0_g1_i1_1	1291800	382360	1483700	2842100	3993600	4061100	11894000	54058000	30922000	1307200	1183800	1483300
TRINITY_DN11604_c0_g1_i5_2	12333000	13121000	13427000	18779000	29355000	20375000	0	0	0	2833400	1893000	2677500

TRINITY_DN7220_c0_g1_i2_2	171590	188070	130710	0	77249	0	0	0	0	42073000	42232000	29879000
TRINITY_DN11320_c0_g1_i7_2	30949000	15122000	17270000	19109000	12409000	18401000	0	0	0	0	1232500	0
TRINITY_DN17793_c0_g1_i1_3	0	0	1619700	671410	56302000	48473000	0	0	0	0	3289600	3479500
TRINITY_DN128_c0_g1_i1_1	17362000	10411000	11801000	22374000	24192000	25914000	0	0	1598000	0	0	0
TRINITY_DN7906_c0_g1_i1_2	8454500	817500	5751600	19919000	31539000	11397000	0	10969000	11518000	4941100	4169600	4175200
TRINITY_DN9529_c0_g1_i4_3	22436000	21413000	18809000	8305300	20748000	15871000	0	0	0	2292100	1790400	1448100
TRINITY_DN10188_c0_g1_i1_1	13024000	10588000	11509000	7121200	9561500	5053900	9415000	6405000	12139000	8935300	9235800	9847700
TRINITY_DN10809_c0_g1_i3_1	7568600	2841300	2498500	15612000	16967000	12309000	2492000	2373400	2812500	20780000	13249000	9844300
TRINITY_DN10625_c0_g1_i2_2	5810000	2811800	3078600	8370500	11211000	9115200	450570	866780	2913400	30480000	15841000	16954000
TRINITY_DN11635_c2_g1_i4_2	11113000	13446000	13901000	6528200	7940500	5124700	12329000	11808000	19525000	2926600	556260	2692400
TRINITY_DN11639_c0_g1_i6_3	0	0	0	0	0	0	0	0	0	39539000	33119000	34629000
TRINITY_DN10727_c0_g1_i1_2	4174600	3715600	4522800	4027400	9071400	10914000	0	0	0	24989000	26780000	18997000
TRINITY_DN8828_c0_g1_i1_2	0	0	0	0	106120000	0	0	0	0	99905	88655	0
TRINITY_DN9853_c0_g2_i1_2	16757000	7037900	9614700	3536800	5156100	5737400	2298900	2296600	2452100	24371000	7100200	19666000
TRINITY_DN17353_c0_g1_i1_1	0	0	0	63895000	0	0	0	0	0	22175000	169180	18404000
TRINITY_DN11024_c0_g2_i1_3	2419800	648320	3066400	5867100	3684200	3798100	26277000	28473000	30101000	0	0	0
TRINITY_DN7121_c0_g2_i1_5	111610	204850	127430	0	0	0	0	533650	0	45581000	26405000	31121000
TRINITY_DN8295_c0_g1_i1_3	22192000	17998000	22762000	9403300	6176700	14266000	0	0	0	3598300	2999700	4136200
TRINITY_DN7552_c0_g1_i2_3	26660000	17233000	23425000	9566700	14080000	7878000	0	0	0	2086600	1475900	1106400
TRINITY_DN11568_c0_g1_i1_2	0	0	0	0	0	0	0	0	0	36454000	34378000	31101000
TRINITY_DN11106_c0_g1_i3_1	124800	0	128330	94454	316460	0	0	0	0	41842000	29497000	28313000
TRINITY_DN8501_c0_g1_i1_2	31920	43462	27734	0	0	0	0	0	0	35343000	31367000	33151000
TRINITY_DN9729_c0_g1_i1_5	9159900	4905000	3803600	11768000	11572000	7287700	0	0	0	23394000	11420000	15855000
TRINITY_DN11231_c0_g1_i2_3	0	0	0	0	0	0	0	0	0	38747000	32811000	27105000
TRINITY_DN11267_c0_g1_i1_2	4544200	3257800	2350700	3506700	2389000	1810500	21717000	18588000	10574000	10614000	7285800	10145000
TRINITY_DN10955_c0_g1_i6_2	13738000	13743000	13235000	4218700	3878700	2704900	13122000	1257600	13749000	8918700	4391200	3550000
TRINITY_DN10695_c0_g1_i3_1	14412000	12178000	13349000	5285600	12545000	10900000	8408800	728580	12173000	2619800	2252600	1341100
TRINITY_DN10388_c0_g2_i5_4	984050	978910	1529700	10820000	10396000	11335000	3727000	4200200	6351000	17084000	13510000	14804000
TRINITY_DN10780_c1_g2_i1_2	4625300	4566400	4941800	4317800	2034500	1942400	6315600	0	5221700	22813000	20096000	18253000
TRINITY_DN4152_c0_g3_i1_2	7268000	3755500	6625900	3880900	5723600	3369400	18691000	22966000	18103000	2206400	1951600	410040
TRINITY_DN11590_c1_g2_i1_3	5424100	8909100	9240700	18915000	17798000	14784000	0	0	0	7523300	4935100	7166900
TRINITY_DN11603_c0_g2_i4_3	7793300	9243900	8042200	4779300	7851300	3211200	2643100	9782000	9809900	12416000	10360000	8723200
TRINITY_DN9993_c0_g1_i2_4	169830	202270	164130	661860	276710	125120	0	0	0	34892000	30155000	27642000

TRINITY_DN8821_c0_g1_i2_2	6355300	5114100	5812100	1312400	2045900	1684700	7453200	12159000	9467900	14755000	13253000	13634000
TRINITY_DN11484_c0_g1_i1_1	20725000	21241000	10449000	7406800	16162000	7218600	0	0	0	3665800	2916100	3003400
TRINITY_DN10975_c0_g1_i1_3	6670100	17026000	16303000	9068200	17724000	10820000	378340	0	0	5705800	3950800	5001700
TRINITY_DN908_c0_g1_i1_5	7966500	5109100	6305800	17885000	17585000	12819000	10598000	905100	9162500	1178800	897080	2235500
TRINITY_DN4171_c0_g2_i2_3	609090	247400	539370	2122700	2416900	1141200	21500000	18877000	42031000	1415400	817810	478240
TRINITY_DN11158_c0_g1_i1_2	2930700	3473500	10136000	15854000	16119000	9970200	1947500	16255000	6469900	3149600	4101100	1090400
TRINITY_DN11613_c0_g1_i1_1	6527000	5149900	4000900	4418500	3920900	5139700	14805000	16611000	18624000	4414400	4235500	3638000
TRINITY_DN10470_c0_g1_i2_2	20982000	21659000	21190000	5031100	11008000	7644500	0	0	0	1559900	419930	1727200
TRINITY_DN12697_c0_g1_i1_3	14559000	16482000	8068000	15005000	17176000	8071200	0	0	0	5857200	2435500	3420600
TRINITY_DN6523_c0_g1_i1_1	366550	691810	621370	5465100	6351300	7493600	15010000	11290000	25642000	7219300	5614100	4741300
TRINITY_DN7341_c0_g1_i1_3	6978100	7333200	8267500	12524000	19609000	12626000	6305900	6478000	6365200	1664200	1009900	813940
TRINITY_DN8404_c0_g1_i1_3	18447000	15954000	18113000	13288000	9359300	8277900	0	0	0	2230500	1826500	1496200
TRINITY_DN11519_c0_g1_i1_1	1792100	1082200	1070200	1984300	3295300	3103100	2566500	2881900	3137200	27031000	20308000	20539000
TRINITY_DN9737_c2_g2_i1_1	2467700	2158200	2693900	2549000	2778100	2820000	7579300	10453000	15631000	19289000	8605800	10175000
TRINITY_DN11533_c0_g1_i2_3	269030	168350	138320	0	258160	0	0	0	0	32924000	22677000	30501000
TRINITY_DN8820_c0_g1_i1_1	0	0	0	164480	354260	0	0	0	0	33970000	24669000	27174000
TRINITY_DN5484_c0_g1_i1_2	11347000	4308400	16748000	16723000	21806000	14766000	0	0	0	0	0	0
TRINITY_DN10447_c0_g1_i1_3	11408000	14379000	3911800	1193400	4840800	1204300	0	0	0	0	0	48322000
TRINITY_DN431_c0_g1_i1_6	18886000	13485000	9183600	13074000	15741000	12131000	0	0	0	1004100	1579700	0
TRINITY_DN9847_c0_g1_i1_1	30532000	15235000	15728000	6499000	7207200	5807600	0	0	0	1246800	1272100	1127300
TRINITY_DN11173_c0_g2_i3_4	85631	133310	0	159380	210060	0	0	0	0	32644000	24754000	26458000
TRINITY_DN6616_c0_g1_i1_4	6644900	5355200	6844500	13896000	11425000	10281000	0	0	833940	10732000	7785200	9526800
TRINITY_DN5630_c0_g1_i1_3	4895800	2688500	4473800	1198900	1519000	1055200	0	1177500	0	24059000	18727000	23492000
TRINITY_DN4574_c0_g1_i2_2	3319000	4051800	4353600	8123500	9358100	8637900	0	8511100	14868000	7334500	8194500	5732300
TRINITY_DN8931_c0_g1_i1_2	6057700	5474800	5863800	8599700	12399000	10426000	8291400	0	10229000	5524500	4164800	5271800
TRINITY_DN11512_c1_g1_i2_2	567250	575880	507620	88543	0	0	0	0	0	31743000	28507000	19816000
TRINITY_DN5614_c0_g2_i1_2	0	0	0	0	0	0	0	81772000	0	0	0	0
TRINITY_DN10149_c0_g1_i2_3	89500	0	0	297040	328080	197220	913760	935780	0	27794000	21261000	29605000
TRINITY_DN9287_c0_g1_i1_3	0	0	0	0	381960	118660	17008000	29066000	33884000	292240	242380	123090
TRINITY_DN13093_c0_g1_i1_3	5026000	3145400	2553700	6967000	7869800	5007000	3945700	2856500	6950900	17239000	9154700	10181000
TRINITY_DN10610_c0_g2_i1_2	0	0	0	0	0	0	0	0	0	28750000	30363000	21173000
TRINITY_DN4192_c0_g2_i1_1	1395600	538130	1110000	4207600	4374100	5636100	14073000	25877000	16378000	3986200	1601300	966040
TRINITY_DN11220_c1_g2_i2_2	519780	389870	0	0	533640	0	0	0	0	25224000	36181000	16998000

TRINITY_DN10317_c0_g1_i3_2	14521000	9819200	9921300	12531000	7455300	8225000	5907300	0	0	3716300	2161800	5551000
TRINITY_DN9083_c0_g1_i2_2	137390	163240	625890	10544000	19484000	15453000	0	0	12470000	8182100	6043600	6656200
TRINITY_DN6144_c0_g1_i2_2	1795300	3371100	1270300	7640100	11859000	9286200	0	0	0	16488000	14530000	13377000
TRINITY_DN13238_c0_g1_i1_4	167330	226520	14153000	4859400	4673100	3819100	15430000	17861000	18352000	0	0	0
TRINITY_DN21237_c0_g1_i1_2	0	0	0	0	0	0	0	77964000	0	0	0	0
TRINITY_DN10003_c1_g1_i5_2	0	160990	210260	0	0	0	0	0	0	28086000	23253000	26120000
TRINITY_DN11618_c0_g1_i1_2	0	0	0	0	0	0	0	0	0	30235000	26421000	20554000
TRINITY_DN4347_c0_g1_i1_6	0	0	0	0	0	0	0	0	0	31449000	21158000	24323000
TRINITY_DN5723_c0_g1_i1_2	13758000	10620000	10811000	6108800	5926400	824140	6790700	6983000	14511000	0	0	0
TRINITY_DN9234_c0_g1_i1_2	0	0	0	0	0	0	0	0	0	28739000	24360000	23111000
TRINITY_DN13643_c0_g1_i1_2	689860	6933400	5959700	0	3131900	3204900	0	0	0	18413000	21069000	16787000
TRINITY_DN6792_c0_g1_i2_5	12688000	11878000	12436000	7490000	9022000	7782300	5818100	0	6627700	390270	299470	1187700
TRINITY_DN7574_c0_g1_i5_2	0	50296	44268	0	168800	70528	25551000	9352000	25874000	7230600	4063000	3165500
TRINITY_DN11191_c0_g1_i2_6	0	0	0	0	0	0	0	339870	0	28947000	23981000	22121000
TRINITY_DN9754_c0_g1_i1_3	0	0	0	0	0	244460	336140	256170	1617800	27016000	24444000	21385000
TRINITY_DN5779_c0_g1_i1_2	5865200	7602800	10653000	12950000	15026000	12379000	0	0	3474900	2408500	2599400	2273000
TRINITY_DN11323_c0_g1_i1_2	0	0	0	0	450780	373420	0	0	0	30725000	22226000	21370000
TRINITY_DN14709_c0_g1_i1_5	7807400	7301900	5704800	6489200	5736600	4484600	5579600	11574000	8009900	4644900	3584500	4200200
TRINITY_DN9829_c0_g1_i3_2	11396000	9125000	12500000	3068200	8477300	3084700	8234600	0	10443000	1281300	3918000	3371800
TRINITY_DN6396_c0_g1_i1_2	0	1682900	0	4685800	5346800	4139600	13609000	2274800	37402000	3095700	0	2324100
TRINITY_DN8644_c0_g2_i1_1	27859000	15608000	8854300	2619100	4366100	11241000	0	0	186530	2443500	682330	573600
TRINITY_DN11241_c0_g1_i3_2	0	0	0	0	0	0	0	0	0	22656000	23656000	28106000
TRINITY_DN9392_c0_g1_i1_2	5224700	3681000	1744900	9452600	7364700	3230400	12415000	0	13199000	6355800	4901700	6814300
TRINITY_DN10095_c0_g1_i1_3	0	0	0	0	0	0	0	0	0	29239000	19933000	25118000
TRINITY_DN9335_c0_g1_i1_4	1119700	583190	617830	0	565880	456310	5338200	7755900	6024300	16460000	20536000	14027000
TRINITY_DN10130_c0_g2_i6_2	0	0	0	149050	157770	0	287580	0	0	23755000	23430000	25577000
TRINITY_DN11642_c0_g1_i3_4	0	0	0	0	0	0	0	0	0	27420000	21687000	22414000
TRINITY_DN7240_c0_g1_i1_3	2015600	191610	1674000	3718900	3031700	3082500	0	0	0	29986000	4328500	23171000
TRINITY_DN11079_c0_g1_i1_5	0	0	0	0	0	0	0	0	0	23132000	25185000	22789000
TRINITY_DN10242_c0_g1_i3_2	7990200	7549700	6434800	7199400	4752400	5982500	8545100	11039000	10699000	91846	0	584930
TRINITY_DN3644_c0_g1_i2_3	8789200	11538000	11076000	15975000	10207000	8794500	0	0	0	0	2854900	1284300
TRINITY_DN11383_c0_g1_i4_1	13627000	14584000	1303000	8507200	9821700	2119200	0	0	0	6027600	9375100	5027200
TRINITY_DN9173_c0_g1_i1_4	4599800	4058700	7714500	7886200	8387700	5938400	0	0	0	11384000	10393000	9785500

TRINITY_DN4403_c0_g1_i1_1	5383700	3312600	3444400	8358900	6693900	7816700	2828600	10338000	17071000	1432800	2140000	1208900
TRINITY_DN10720_c0_g1_i4_5	324000	25585	37846	0	794170	611490	0	0	0	20330000	20726000	26926000
TRINITY_DN11647_c1_g1_i1_1	9398400	7463400	4158200	9567700	11877000	14470000	3423500	1062000	4210200	2073500	1292700	771350
TRINITY_DN12510_c0_g1_i1_1	714530	369870	454720	600030	322440	611250	28706000	19022000	18540000	151220	262140	0
TRINITY_DN1355_c0_g3_i1_5	0	0	0	0	0	0	0	69331000	0	0	0	0
TRINITY_DN11053_c0_g1_i4_6	538150	1299100	1357400	1821000	941750	925000	0	0	0	20346000	20377000	21606000
TRINITY_DN5919_c0_g1_i1_3	988580	938560	1689700	3344400	3160200	3636100	12212000	16736000	16371000	3859500	3267300	3002500
TRINITY_DN11546_c0_g1_i6_5	0	0	108540	212420	0	100080	0	0	0	26954000	21038000	20710000
TRINITY_DN11141_c0_g1_i6_1	0	0	0	0	0	0	0	0	0	25455000	18786000	24804000
TRINITY_DN2708_c0_g2_i1_1	763320	1189900	889210	0	0	81428	0	7205700	18020000	11806000	13266000	15819000
TRINITY_DN9530_c0_g3_i4_1	0	0	0	0	0	0	0	0	0	25216000	18230000	25472000
TRINITY_DN5278_c0_g1_i1_2	0	0	0	0	15322000	13096000	0	3275100	36690000	344230	0	0
TRINITY_DN10989_c0_g2_i2_1	1176600	907920	1394100	1276200	1308200	1160700	0	470020	0	21912000	20589000	18225000
TRINITY_DN11653_c0_g2_i1_5	357780	172010	0	0	6273700	0	0	0	2329600	20586000	20036000	18216000
TRINITY_DN7029_c0_g1_i1_3	8469000	14447000	23232000	0	681130	587500	1747400	0	0	2273400	8344300	7017500
TRINITY_DN10674_c0_g1_i3_4	4568300	4013100	3259600	3570600	3072100	2433300	13784000	13353000	14321000	652400	1560700	1576600
TRINITY_DN10049_c0_g1_i1_3	0	0	0	0	127650	0	3199000	4113900	4495600	24376000	12474000	17012000
TRINITY_DN7937_c0_g1_i2_5	108380	0	0	991360	763060	1067500	0	0	0	20103000	26952000	15555000
TRINITY_DN1722_c0_g2_i1_3	1768400	1412300	1094900	18133000	17152000	15242000	0	3677700	3572500	1856900	0	1280900
TRINITY_DN11314_c0_g1_i3_2	0	13428	0	0	0	0	0	0	0	20454000	23651000	20601000
TRINITY_DN9588_c0_g1_i1_2	0	0	0	0	0	0	0	0	0	24604000	21952000	17982000
TRINITY_DN10926_c1_g1_i3_3	0	0	0	0	0	0	0	0	64393000	0	0	0
TRINITY_DN5721_c0_g4_i1_3	2105300	2046700	3837400	6880500	9918700	9078900	0	0	0	10316000	10179000	9338500
TRINITY_DN10576_c0_g1_i1_3	5848700	4535100	4219500	463860	1639300	1632200	2116700	2479800	3755700	15803000	12601000	7574800
TRINITY_DN11459_c1_g4_i8_2	252090	306110	209900	0	0	0	0	1479700	1553400	18841000	20497000	19435000
TRINITY_DN10492_c1_g1_i2_6	14990000	7055100	5312000	12548000	11731000	10222000	0	0	0	172650	0	403710
TRINITY_DN11529_c0_g1_i2_3	0	0	0	0	0	0	0	0	0	19778000	21635000	20981000
TRINITY_DN10583_c0_g2_i2_1	1328500	1283700	1269200	812700	803650	0	0	1115700	6642400	21047000	8246300	19295000
TRINITY_DN12655_c0_g1_i1_2	0	0	0	0	0	0	61744000	0	0	0	0	0
TRINITY_DN8865_c0_g2_i1_5	0	0	0	0	0	0	0	0	0	27609000	19524000	13610000
TRINITY_DN6870_c0_g1_i5_1	0	0	0	0	0	0	0	0	0	21930000	21353000	16464000
TRINITY_DN5791_c0_g1_i1_5	2101400	1527500	2513400	1687500	2165900	1143000	1067500	1092000	3412100	17395000	13085000	12035000
TRINITY_DN11572_c0_g1_i1_6	0	0	0	0	0	0	0	0	0	21074000	13693000	24112000

TRINITY_DN10346_c0_g2_i6_3	0	0	0	0	99900	0	0	0	0	21160000	18662000	18634000
TRINITY_DN11809_c0_g1_i1_1	1139200	1614900	1780800	3129200	5316700	4782700	5642100	15758000	7529800	5606100	2992500	2934200
TRINITY_DN4290_c0_g1_i1_3	8989800	8981500	8144700	3600400	7499900	5128100	0	0	0	6031900	2450500	6792600
TRINITY_DN10866_c0_g1_i1_1	0	0	0	0	0	0	0	0	377130	22093000	20469000	14382000
TRINITY_DN11465_c0_g1_i2_2	22309000	11598000	12239000	0	164350	0	0	0	0	4500400	3316500	3133500
TRINITY_DN10466_c0_g2_i3_1	0	0	0	0	0	0	0	0	0	19730000	20243000	17191000
TRINITY_DN3729_c0_g2_i1_1	7725000	3648300	4271100	4296600	5945300	5544000	5853600	6292700	6593400	2786800	1777300	2353300
TRINITY_DN10917_c0_g1_i1_1	0	0	0	0	0	0	0	0	0	22728000	15110000	18420000
TRINITY_DN4960_c0_g1_i2_6	791440	531100	505380	1033000	355180	0	2214300	1619700	4437500	17512000	14817000	11524000
TRINITY_DN10616_c0_g1_i2_2	8053900	1848900	6684000	13603000	7146400	9096300	0	0	0	4222400	836780	3198800
TRINITY_DN11240_c0_g2_i1_2	2738700	2650400	2597000	1556700	1405500	950580	0	324060	327830	15465000	12283000	13814000
TRINITY_DN11190_c0_g1_i2_3	0	0	0	0	0	0	0	0	0	19116000	19675000	14962000
TRINITY_DN11571_c4_g2_i1_1	0	0	0	0	0	0	0	0	0	20519000	16775000	16240000
TRINITY_DN11669_c393_g1_i1_1	26743000	19033000	3989600	1523900	867710	826080	0	0	0	0	0	475950
TRINITY_DN10467_c0_g1_i1_1	5885200	5800000	5502500	93190	2073800	0	0	0	0	14407000	10651000	8834400
TRINITY_DN9500_c0_g2_i1_2	0	0	0	0	0	0	0	0	0	24603000	14406000	13905000
TRINITY_DN10347_c0_g1_i1_3	415120	300100	529540	204660	262840	914820	0	0	0	20559000	15983000	13562000
TRINITY_DN5968_c0_g1_i1_3	0	0	0	0	0	0	0	0	0	20322000	14955000	17161000
TRINITY_DN5544_c0_g1_i1_3	0	0	0	0	0	0	0	0	0	20796000	15347000	16271000
TRINITY_DN153_c0_g1_i1_1	635920	275420	979180	2062700	3345000	6523800	16729000	7962000	10507000	1617600	1176900	548190
TRINITY_DN7265_c0_g2_i5_2	464210	304080	209950	769230	0	0	0	0	0	19475000	13082000	17265000
TRINITY_DN9812_c0_g1_i3_4	3370000	4798300	4017800	2265500	7117600	7021000	5277100	3013400	7934700	2771100	1637300	2322300
TRINITY_DN8811_c0_g1_i3_5	1366600	962110	909850	879300	433100	865730	0	0	0	17558000	19016000	9177800
TRINITY_DN10516_c0_g1_i1_2	1236800	732160	789330	1228100	672390	2774900	9109000	14555000	18458000	379800	569350	408770
TRINITY_DN4095_c0_g1_i1_4	4602900	4327200	5679700	6549700	4779200	2338700	0	0	0	6693200	10507000	5339900
TRINITY_DN14849_c0_g1_i1_6	0	0	0	0	0	0	18558000	449690	31789000	0	0	0
TRINITY_DN11228_c0_g2_i1_3	6116000	7476100	6058500	5161400	4465100	2242300	0	0	6162400	4519400	3492200	4055100
TRINITY_DN10795_c1_g1_i1_6	5233200	4136400	3919200	2815400	1620000	2430700	2374300	2039000	9500000	8015300	6042700	1316500
TRINITY_DN11220_c1_g2_i3_3	76780	50563	0	425270	589790	437430	0	0	0	17817000	15497000	14421000
TRINITY_DN11391_c0_g2_i2_4	0	0	0	0	0	0	0	0	0	19235000	13667000	16140000
TRINITY_DN2394_c0_g1_i2_6	4767900	6816800	5373100	4523400	5778900	2091200	7382100	0	9055600	856870	761630	711190
TRINITY_DN10841_c0_g2_i1_1	0	0	0	511970	505230	0	0	0	0	18689000	17071000	11104000
TRINITY_DN9488_c0_g2_i1_2	1990800	2059500	2297900	7786500	7089200	8185400	2824300	9301800	3396400	985210	846840	1117200

TRINITY_DN959_c0_g1_i1_1	163490	219520	0	0	103470	168980	0	0	0	17832000	16499000	12834000
TRINITY_DN5607_c0_g1_i1_3	8845800	6970900	2937600	3877400	739420	4827000	10027000	0	0	5097600	441030	4005200
TRINITY_DN11493_c0_g1_i5_3	2752800	2913700	3020600	1380100	1572700	0	6419600	0	13775000	6329600	3856000	5397400
TRINITY_DN18554_c0_g1_i1_2	1429800	959830	948480	2614200	981840	644470	8475100	4322900	15453000	5795100	2127300	3344100
TRINITY_DN11428_c0_g1_i2_3	0	0	0	0	0	0	0	0	0	16040000	17485000	13512000
TRINITY_DN23504_c0_g1_i1_4	0	0	0	0	0	0	19868000	0	26855000	0	0	0
TRINITY_DN11239_c1_g2_i1_6	0	0	0	0	0	0	0	0	0	15921000	15927000	14664000
TRINITY_DN19480_c0_g1_i1_2	11901000	9309100	9244800	2343600	6126900	5043400	737890	0	0	842870	0	653830
TRINITY_DN12058_c0_g1_i1_3	0	0	0	0	0	0	0	0	0	17028000	15170000	13688000
TRINITY_DN4635_c0_g1_i1_6	0	0	0	0	0	0	0	0	0	19958000	15072000	10663000
TRINITY_DN6720_c0_g1_i1_1	0	0	0	0	0	0	0	0	0	19293000	12603000	13597000
TRINITY_DN15356_c0_g1_i1_3	10542000	11577000	12622000	1077600	1404200	3522700	0	0	2969900	629470	320370	581940
TRINITY_DN10115_c0_g1_i5_2	6927800	5234900	4227600	3521300	3498800	4306300	3090500	3332500	9406000	0	652000	768200
TRINITY_DN7162_c0_g1_i1_6	3541200	8038800	7985600	2119900	6027500	1584100	2975400	6430100	3298500	1185000	0	1764400
TRINITY_DN668_c0_g2_i1_6	0	0	0	0	0	0	0	0	0	17117000	14378000	12756000
TRINITY_DN11756_c0_g2_i2_2	167440	100720	73976	110180	0	0	10974000	2435600	7434200	8872400	6932700	6666000
TRINITY_DN3503_c0_g1_i1_2	150100	44230	403990	15768000	16672000	10090000	0	0	0	0	0	474110
TRINITY_DN10567_c0_g1_i1_3	0	0	0	0	0	0	0	0	0	17034000	14264000	12262000
TRINITY_DN9859_c0_g2_i1_3	255260	106160	260120	0	0	306680	0	0	0	16483000	15401000	10685000
TRINITY_DN11119_c0_g1_i2_2	0	0	0	0	0	0	0	0	0	15477000	13930000	13733000
TRINITY_DN6617_c0_g1_i1_4	0	0	0	0	0	0	0	0	0	22154000	14067000	6796200
TRINITY_DN8937_c0_g1_i1_1	0	0	0	0	0	0	0	0	0	19511000	10469000	12970000
TRINITY_DN8063_c0_g1_i1_3	0	0	0	157760	0	0	0	0	0	19029000	9793300	13956000
TRINITY_DN3510_c0_g1_i2_1	2140600	1755600	2169500	1520100	1051300	2028100	12817000	11096000	4636600	1466300	941830	1231100
TRINITY_DN3885_c0_g1_i1_3	383760	351170	411520	0	0	0	557260	0	0	17740000	12855000	10408000
TRINITY_DN11597_c1_g2_i2_1	0	0	0	0	0	0	0	0	0	19160000	9734300	13591000
TRINITY_DN4297_c0_g1_i1_2	0	0	0	0	0	0	0	0	0	19566000	9879600	12952000
TRINITY_DN11427_c0_g1_i3_2	0	0	0	0	0	0	0	0	0	14830000	16583000	10863000
TRINITY_DN8289_c0_g1_i2_3	0	0	0	0	0	0	0	0	0	18660000	11378000	12201000
TRINITY_DN9771_c0_g1_i4_2	0	0	0	0	0	0	0	0	0	20709000	10312000	10558000
TRINITY_DN11254_c0_g1_i1_3	1378200	829780	1061200	6730900	3492400	5890700	2982500	0	2694300	7746300	3371600	5297800
TRINITY_DN11103_c0_g1_i2_3	263430	0	151700	0	0	0	0	0	261970	18415000	10561000	11760000
TRINITY_DN11548_c1_g1_i1_3	2178300	1528400	1243000	1391100	3009400	2288100	10619000	0	17443000	825670	568280	293630

TRINITY_DN10949_c0_g1_i1_1	0	31678	0	0	0	0	0	0	0	16983000	11542000	12657000
TRINITY_DN11059_c0_g1_i1_1	0	0	0	0	0	0	0	0	0	15434000	11418000	14167000
TRINITY_DN3142_c1_g1_i1_1	0	0	0	0	0	0	0	0	0	15106000	14696000	10955000
TRINITY_DN10281_c0_g2_i1_2	0	0	0	0	0	0	0	0	0	13814000	15339000	11566000
TRINITY_DN10506_c0_g1_i1_2	6330700	3611000	3158300	1741400	4341200	4767000	0	0	2103700	4614100	5994500	3675400
TRINITY_DN9505_c0_g2_i1_1	0	0	0	0	0	0	0	0	0	15026000	11902000	13390000
TRINITY_DN11355_c0_g1_i2_1	0	0	0	0	0	0	0	0	145550	11942000	9212300	18990000
TRINITY_DN6984_c0_g1_i1_1	3689500	4177500	6539200	2023600	2550800	3928800	3152200	6973500	5950000	0	1274300	0
TRINITY_DN9211_c0_g2_i1_1	3938200	5615000	4494600	435230	2718800	3515600	2809000	4874500	9940400	1042600	0	816520
TRINITY_DN3640_c0_g1_i2_2	7685000	1722000	1751900	2792300	3010800	4877800	5532600	1756200	1999300	4712700	3106400	1230500
TRINITY_DN11606_c0_g1_i1_4	3855800	3798800	8760800	2719300	2515800	2431700	5121300	771230	8635000	392620	522110	323850
TRINITY_DN9177_c0_g2_i1_2	0	0	0	0	0	0	0	0	0	17398000	11206000	11145000
TRINITY_DN11073_c0_g2_i1_2	7612200	6910500	5855700	3245000	4606100	3789300	0	0	0	3092600	2080200	2474800
TRINITY_DN9688_c0_g1_i3_5	0	0	0	0	0	0	0	0	0	12984000	10012000	16574000
TRINITY_DN5149_c0_g1_i1_1	1438100	1304400	2510400	9534000	10056000	7154600	0	0	0	3075200	1919200	1992400
TRINITY_DN9163_c0_g1_i2_2	0	0	0	0	0	0	0	0	0	12914000	13897000	12088000
TRINITY_DN9921_c0_g1_i1_2	0	0	0	0	0	0	0	0	0	14952000	11775000	12120000
TRINITY_DN8108_c0_g1_i1_2	2585500	2452300	2565600	1434500	381840	1023000	0	0	0	9471700	9206400	9708600
TRINITY_DN11594_c0_g2_i5_1	845900	648680	1065000	1966700	3015100	1776000	13112000	9482700	3037500	1759900	639730	890750
TRINITY_DN7706_c0_g2_i2_2	0	0	0	0	0	0	0	0	0	15136000	12448000	10477000
TRINITY_DN11322_c1_g1_i2_3	0	0	0	0	0	0	0	0	301510	15409000	10218000	12005000
TRINITY_DN6946_c0_g1_i1_2	0	0	0	0	0	0	0	0	0	15355000	11579000	10777000
TRINITY_DN11349_c1_g2_i4_3	635910	210930	185780	0	0	0	1434000	1236800	1389900	13280000	9593300	9674900
TRINITY_DN5652_c0_g3_i1_4	1335500	1449500	4189900	5799500	6076900	4723500	0	4266700	4545700	2043300	1620400	1561900
TRINITY_DN11033_c0_g1_i4_5	0	0	0	0	0	0	0	0	0	13551000	11132000	12695000
TRINITY_DN3347_c0_g1_i1_2	0	0	0	0	0	0	11595000	12404000	12891000	0	0	0
TRINITY_DN6901_c0_g1_i1_3	3721600	4044300	4013500	4446600	9618200	7247100	1155900	0	0	1416100	756530	352830
TRINITY_DN5552_c0_g1_i1_1	4398900	5829500	5713800	1621400	3104600	1291100	2860500	7105100	3190800	688030	427640	381490
TRINITY_DN10237_c0_g3_i1_1	0	0	0	0	0	0	0	0	0	15090000	8640600	12748000
TRINITY_DN11143_c0_g1_i5_1	325330	933300	1588800	6574600	8210900	7845500	3348900	0	6403900	493230	197110	340080
TRINITY_DN1882_c0_g1_i2_3	0	0	0	0	0	0	0	0	0	13486000	11208000	11341000
TRINITY_DN10939_c0_g1_i1_1	0	0	0	0	0	0	0	0	0	13993000	12040000	9950100
TRINITY_DN7127_c0_g1_i1_1	161750	0	155210	1814700	1249400	1646100	0	0	0	14828000	9515400	6432900

TRINITY_DN11501_c0_g1_i3_2	828180	1020800	903560	340830	896700	412060	0	0	0	12409000	9523700	9349000
TRINITY_DN9883_c0_g1_i1_1	0	0	0	0	0	0	0	0	0	11529000	9194200	14842000
TRINITY_DN10759_c0_g1_i2_5	0	0	0	0	0	0	0	0	0	13349000	11299000	10902000
TRINITY_DN11019_c0_g1_i2_2	0	0	0	0	116860	0	0	0	0	15638000	9769100	9919800
TRINITY_DN10818_c0_g1_i3_1	0	0	0	0	0	0	0	0	0	13027000	12251000	10076000
TRINITY_DN20642_c0_g1_i1_4	2648700	1312200	1076900	466450	428770	327910	11505000	16580000	0	555280	303960	103110
TRINITY_DN9556_c1_g1_i2_1	3582100	5073200	4351000	3303900	3415300	2197000	3335000	4323900	3855000	583080	562210	467610
TRINITY_DN10502_c0_g2_i8_3	0	0	0	0	0	0	0	0	0	13829000	10628000	10591000
TRINITY_DN10701_c0_g1_i1_2	0	0	0	0	0	0	0	0	0	11948000	11388000	11566000
TRINITY_DN11433_c0_g1_i2_1	0	0	0	0	0	0	0	0	0	11755000	14102000	9019500
TRINITY_DN6503_c0_g2_i1_1	0	582520	0	92933	0	0	0	0	0	12652000	11882000	9390900
TRINITY_DN6738_c0_g1_i1_3	167950	368430	238580	897160	924360	836380	8529900	0	22540000	0	0	0
TRINITY_DN9643_c0_g1_i1_2	844840	1528400	1441700	838340	1743400	1075700	4110400	7178700	10398000	1808400	871230	2360800
TRINITY_DN2791_c0_g1_i1_3	3334000	3639200	3688500	3535000	3611700	2948400	895870	5362500	5566500	873040	277450	402460
TRINITY_DN11325_c0_g1_i1_1	0	0	0	0	0	0	1454800	0	2705400	12356000	9253100	8266100
TRINITY_DN7495_c0_g1_i1_1	983830	1142900	1430900	1395400	1860900	1598600	0	4908100	1020900	5965400	6621400	7062500
TRINITY_DN2602_c0_g1_i2_3	0	0	0	0	0	0	0	0	0	15213000	9275400	9453000
TRINITY_DN11605_c1_g2_i1_4	0	0	0	0	0	0	0	0	0	12471000	11317000	10067000
TRINITY_DN6250_c0_g1_i2_3	0	0	0	0	0	0	0	0	0	15481000	8536700	9809700
TRINITY_DN6615_c0_g2_i1_4	0	0	0	0	0	0	0	0	0	12953000	10380000	10457000
TRINITY_DN1128_c0_g2_i1_2	18160000	0	15486000	0	0	0	0	0	0	0	0	0
TRINITY_DN4481_c0_g1_i3_5	0	0	0	0	0	0	0	0	0	6536100	16333000	10733000
TRINITY_DN1332_c0_g1_i1_2	2925800	947700	2079900	5702600	11543000	9097600	799440	0	0	267520	0	0
TRINITY_DN10179_c0_g1_i1_1	901390	834590	875620	0	123840	115020	0	0	0	9137100	11265000	10055000
TRINITY_DN5568_c0_g1_i1_2	3861600	1627400	2140000	5302200	1684500	3134900	5813600	4028100	4285400	271040	856900	186000
TRINITY_DN11161_c0_g2_i1_1	867240	978840	1241000	974390	2157100	915200	0	2721800	3325100	9738800	5055700	5028200
TRINITY_DN4141_c0_g1_i1_2	0	0	0	0	0	0	0	0	0	11573000	11681000	9526200
TRINITY_DN15874_c0_g1_i1_6	2795300	714960	1953000	310830	355830	270240	7367700	0	7322600	1029400	3142900	7506900
TRINITY_DN10532_c0_g1_i1_4	614900	627710	1101700	1688900	7408200	2072100	8608400	3480200	3292600	1437400	1164500	1114900
TRINITY_DN6875_c0_g1_i1_3	0	0	0	0	0	0	0	0	0	14827000	3334100	14438000
TRINITY_DN7117_c0_g2_i2_1	75548	111560	0	0	0	0	0	0	0	10399000	12447000	9501800
TRINITY_DN19584_c0_g1_i1_1	2087900	1880300	2698200	3998600	5341400	3619200	1018400	1020700	1287900	3652500	2312100	3497700
TRINITY_DN11441_c0_g1_i2_3	883790	528530	536050	1164500	502890	522450	0	0	0	10815000	9529700	7821600

TRINITY_DN11585_c0_g1_i2_3	0	0	0	0	0	0	0	0	0	12604000	9935500	9764700
TRINITY_DN9778_c0_g1_i2_1	0	0	0	0	0	0	0	0	0	10457000	12122000	9561200
TRINITY_DN10084_c0_g1_i3_3	4407600	5782700	6242200	1922200	2711600	4362400	2117800	0	0	534580	2077100	1976100
TRINITY_DN8707_c0_g1_i1_4	0	0	0	0	0	0	0	0	0	15148000	6722100	10153000
TRINITY_DN8413_c0_g1_i1_3	0	0	0	0	0	0	0	0	0	11955000	12999000	7051400
TRINITY_DN11449_c0_g2_i5_3	0	0	0	0	0	0	0	0	0	13144000	8739000	10070000
TRINITY_DN21888_c0_g1_i1_3	5097300	5157800	5616300	4271700	4925600	4294800	0	0	0	994000	1573500	0
TRINITY_DN10361_c1_g2_i1_1	0	0	0	0	0	0	0	0	0	13109000	8270300	9666300
TRINITY_DN7454_c0_g1_i1_5	1134400	764090	1282000	2160100	3274200	3595000	5693600	3078300	9264600	557560	206330	0
TRINITY_DN11044_c0_g1_i1_3	0	0	0	0	128560	0	0	0	0	12427000	9067000	9320200
TRINITY_DN10694_c0_g1_i1_2	1585400	2502000	2889800	2268800	2449400	2541100	2056500	2263500	3083800	4277900	2056100	2964200
TRINITY_DN7765_c0_g1_i1_3	0	0	0	0	0	0	496330	0	0	12388000	9298800	8571700
TRINITY_DN10259_c0_g1_i1_4	0	0	0	0	0	0	0	0	0	10789000	11479000	8451700
TRINITY_DN10666_c0_g1_i1_3	630910	0	0	227770	229920	160780	0	0	0	10028000	10443000	8944000
TRINITY_DN8743_c0_g1_i2_2	0	0	0	0	0	0	0	0	0	11060000	9928300	9662100
TRINITY_DN7469_c0_g1_i1_3	2292400	3340900	3751900	4223600	3232200	1328100	2352100	3215300	3491100	1381500	1171300	847350
TRINITY_DN9596_c0_g1_i2_1	1004400	1104100	914790	1122200	1071100	727130	0	0	0	9731900	5431900	8903500
TRINITY_DN9658_c0_g1_i1_4	0	0	0	0	0	0	0	0	0	11166000	8849500	9985400
TRINITY_DN7483_c0_g2_i2_4	0	0	0	0	0	0	0	0	0	11514000	7885200	10462000
TRINITY_DN10418_c0_g1_i1_1	4099800	6929700	4797500	2356100	4633200	2789100	0	0	0	1531200	1758800	884120
TRINITY_DN6749_c0_g1_i3_2	0	0	0	0	0	0	0	0	0	12467000	8932500	8317100
TRINITY_DN10064_c0_g1_i1_6	1747900	1975400	1892500	7745800	9079100	6627200	0	0	0	0	454890	182640
TRINITY_DN10803_c0_g1_i1_1	711910	778370	750490	2708800	10145000	2364800	0	0	0	3800300	2879300	5302800
TRINITY_DN10303_c0_g1_i1_3	1090900	1670300	1751500	0	2657000	1285500	300740	0	0	8888000	6481000	4988700
TRINITY_DN2784_c0_g1_i1_3	2478500	1585900	2008200	1116600	1702200	390500	4989700	0	3134400	4319600	3908900	3407100
TRINITY_DN12921_c0_g1_i1_6	269970	1583700	1591100	1102300	4537700	3039700	1712400	0	2413100	4919200	3045500	4716600
TRINITY_DN9516_c0_g1_i2_6	0	0	0	0	0	0	0	0	0	11517000	9435600	7770300
TRINITY_DN9036_c0_g1_i1_2	3684400	1975300	3459100	1856300	3362500	1633200	5037300	5817600	0	919320	442950	453490
TRINITY_DN20791_c0_g1_i1_4	14294000	14259000	0	0	0	0	0	0	0	0	0	0
TRINITY_DN6266_c0_g1_i2_5	0	0	0	0	0	0	0	0	0	11150000	10728000	6502200
TRINITY_DN11086_c0_g1_i1_1	0	0	0	0	0	0	0	0	0	10349000	8156000	9864200
TRINITY_DN3111_c0_g2_i1_2	1337000	1117400	1370200	1260000	160730	202040	0	0	340270	8907500	6753900	6838900
TRINITY_DN10597_c0_g2_i1_2	0	0	0	0	0	0	0	0	0	9629400	9969300	8576200

TRINITY_DN10098_c0_g1_i3_1	1073800	2218600	3612100	0	6616800	4897700	0	0	3043600	2951600	1807200	1932200
TRINITY_DN6445_c0_g1_i1_3	0	0	0	0	0	0	1489800	1312500	1618300	7062600	9309000	7233000
TRINITY_DN10634_c0_g1_i2_4	4285400	5297100	6318700	2473100	2815200	1741600	0	0	0	1984100	1447300	1635900
TRINITY_DN5741_c0_g1_i2_3	0	0	0	0	0	0	0	0	0	10612000	8917300	8238200
TRINITY_DN7128_c0_g2_i1_1	1370400	1247800	958260	943770	808670	1824400	1808400	9402900	6382400	1381700	542890	998850
TRINITY_DN11601_c0_g2_i2_5	485430	300110	473730	417000	109730	108990	181160	0	0	11070000	6328900	8111400
TRINITY_DN9962_c0_g2_i1_2	5532500	8155700	0	2213500	3607600	2693300	0	3456000	0	0	0	1928800
TRINITY_DN10845_c0_g1_i2_1	0	0	0	0	0	0	0	320380	285270	11891000	6849200	8068200
TRINITY_DN10542_c0_g1_i1_2	1195400	623590	841450	797380	1948300	2087200	0	0	0	7745800	6463900	5658600
TRINITY_DN7441_c0_g1_i1_6	615450	3816900	1819800	560870	5508400	4429900	0	0	0	3859000	3583100	3134000
TRINITY_DN10521_c0_g1_i1_3	0	0	0	0	0	0	0	0	0	12402000	7491400	7408200
TRINITY_DN10791_c2_g1_i3_2	1128200	659430	779110	1139600	1044000	565510	6517700	7394600	7578600	0	130360	251560
TRINITY_DN11097_c0_g1_i2_6	2997300	4297100	3814200	0	3067800	881810	0	4707300	4753600	1192700	315350	972860
TRINITY_DN8226_c0_g1_i1_1	1094900	535010	1107900	2544300	1288700	787400	1792200	0	607700	5755100	6453800	4927700
TRINITY_DN8836_c0_g1_i1_2	375290	2929900	773090	2286000	4465400	5059500	470400	3560500	3071900	1404400	1438900	982110
TRINITY_DN9745_c0_g1_i2_1	0	0	0	0	61116	164580	0	0	0	12267000	6654000	7596500
TRINITY_DN6745_c0_g1_i6_1	1358700	1414500	280670	0	1367500	1295400	0	7091000	3609200	3964600	3112100	3227300
TRINITY_DN10651_c0_g1_i1_1	2272700	1550000	1627200	600770	759450	504450	941790	0	0	7505400	7241400	3584200
TRINITY_DN3758_c0_g1_i1_5	0	0	0	0	0	0	0	0	0	10458000	8921100	7080600
TRINITY_DN9555_c0_g1_i2_4	0	0	0	0	0	0	0	0	0	15210000	11032000	0
TRINITY_DN8044_c0_g1_i1_2	0	0	0	0	0	0	0	0	0	10930000	9455400	5722900
TRINITY_DN5302_c0_g2_i1_4	0	0	0	484560	0	0	0	0	24759000	451640	0	401890
TRINITY_DN9331_c0_g1_i5_4	0	0	0	0	0	0	0	0	0	11016000	8194900	6703200
TRINITY_DN10842_c0_g1_i1_3	0	0	0	2290600	2681900	0	0	0	0	8840400	3240900	8809400
TRINITY_DN10737_c0_g1_i2_1	999500	683600	482310	163460	0	233440	1200400	11201000	10895000	0	0	0
TRINITY_DN11246_c0_g1_i1_1	0	0	0	0	0	0	0	0	0	7871300	9336400	8630100
TRINITY_DN10802_c0_g1_i2_2	3843200	4323100	3657800	2275900	3387800	1816900	4350900	0	0	682400	709630	635020
TRINITY_DN10937_c0_g1_i1_3	0	0	0	0	0	0	0	0	0	11539000	7491200	6643000
TRINITY_DN5821_c0_g1_i2_2	6551100	4447200	5371600	1950800	0	1614700	0	0	0	2196800	1779500	1750900
TRINITY_DN7491_c0_g1_i1_1	0	0	0	0	0	0	0	0	0	9857000	6554500	9112200
TRINITY_DN10883_c0_g1_i2_4	16311	0	0	1073900	751000	562200	7176000	8161700	7612300	0	0	0
TRINITY_DN9369_c0_g1_i1_2	0	0	0	0	0	0	0	0	0	9509200	8670100	7070600
TRINITY_DN15371_c0_g1_i1_1	0	0	0	0	0	0	0	0	0	8597300	8463900	8003800

TRINITY_DN8535_c0_g1_i1_3	0	0	0	0	0	0	0	0	0	11320000	4408000	9325400
TRINITY_DN11283_c0_g1_i3_1	0	0	0	0	0	0	0	0	0	10012000	6507500	8473900
TRINITY_DN10469_c0_g2_i1_3	0	0	0	0	0	0	0	0	0	11625000	7919600	5447100
TRINITY_DN6972_c0_g1_i1_5	4471700	2724000	2822500	555940	90417	1805500	1247500	3582900	6722000	0	587030	259310
TRINITY_DN9766_c0_g1_i2_1	0	0	0	0	0	0	0	0	0	10215000	6794200	7788700
TRINITY_DN6771_c0_g1_i1_1	0	0	0	0	0	0	0	0	0	9820000	7741000	7139000
TRINITY_DN11054_c0_g1_i1_2	0	0	0	0	0	0	0	0	0	6486800	11279000	6929900
TRINITY_DN11476_c0_g1_i2_1	1801200	1598500	1935400	354440	1848200	658680	0	0	0	7042600	4811700	4618100
TRINITY_DN9150_c0_g1_i3_1	66192	67986	39858	0	769840	0	7503300	0	13185000	1446900	1117000	468530
TRINITY_DN8849_c0_g2_i3_2	3318700	4051800	4305300	2485300	3009200	2312500	0	0	5092500	0	0	0
TRINITY_DN7905_c0_g1_i1_5	0	0	0	0	0	0	0	0	0	10394000	7309600	6789100
TRINITY_DN9056_c0_g1_i1_6	589230	549270	758000	1417500	1536400	1344100	3067000	4176300	9772100	406550	182360	687620
TRINITY_DN8426_c0_g1_i2_3	0	0	0	0	64977	0	5088600	9088200	10139000	0	0	0
TRINITY_DN4385_c0_g1_i1_2	5266300	3328500	2602000	1085500	3611300	1591800	0	0	5459300	559580	645580	0
TRINITY_DN21126_c0_g1_i1_1	242880	245470	435150	5927700	5162800	5738800	0	0	0	2435900	1960700	1934800
TRINITY_DN11515_c0_g1_i3_1	0	0	0	0	0	0	0	0	0	9193700	7833500	6891200
TRINITY_DN12722_c0_g1_i1_1	2666400	2881400	3086400	5079400	4343400	5182300	0	0	0	625960	0	0
TRINITY_DN11139_c0_g1_i1_3	0	0	0	0	0	0	0	0	0	9072300	9247400	5491500
TRINITY_DN10080_c0_g1_i1_1	619870	2156100	557450	1352300	0	0	3918200	5914700	7348200	375120	857360	622640
TRINITY_DN8178_c0_g1_i2_3	1553600	1541400	1821200	803000	1274600	813000	1070900	0	1612500	7300100	2981500	2949600
TRINITY_DN130_c0_g1_i1_2	0	0	0	0	0	0	0	0	0	9667500	6078500	7959900
TRINITY_DN11280_c0_g1_i4_3	0	0	0	0	0	0	0	0	0	9802100	9284500	4527900
TRINITY_DN11620_c0_g1_i1_2	0	0	0	0	0	0	0	0	0	9605900	5848400	7977300
TRINITY_DN11411_c1_g1_i3_5	157790	0	48943	166770	176710	0	233270	0	0	3116900	9906500	9591100
TRINITY_DN9704_c0_g1_i1_2	0	0	0	0	0	0	0	0	0	3415900	6445900	13506000
TRINITY_DN2711_c0_g1_i1_3	2680400	1967700	2265200	2284500	3541600	1186100	0	0	0	3967400	2425700	2987400
TRINITY_DN10623_c0_g1_i2_5	0	0	0	0	0	0	0	0	0	8107300	8298200	6856400
TRINITY_DN9395_c0_g2_i1_3	959810	1810800	2039500	1337400	1309900	1017500	0	0	0	6252400	4491500	3995300
TRINITY_DN10777_c0_g1_i2_2	4212500	3229400	2763300	2543200	2309000	0	1895400	2051900	3237900	375680	0	531630
TRINITY_DN8939_c0_g1_i1_1	2182900	2754200	2400100	2287800	3369500	1533000	1362500	1389400	1515800	1506300	1634500	1164600
TRINITY_DN14055_c0_g1_i1_6	0	0	0	0	0	0	0	0	0	22870000	73453	0
TRINITY_DN5112_c0_g2_i3_3	1463900	1898700	2039600	1498600	2436000	3357600	0	0	0	4246800	3129400	2826400
TRINITY_DN3053_c0_g1_i1_6	1963600	3590200	2955200	5506600	4475200	2417300	0	0	0	561480	520310	904300

TRINITY_DN3542_c0_g2_i1_1	4193800	4903800	5663000	2416500	2495300	1984000	0	0	715400	342620	162570	0
TRINITY_DN8778_c0_g1_i2_2	439580	0	651810	2458100	3229500	2090400	3392900	6180300	4320300	0	0	0
TRINITY_DN8889_c0_g1_i1_1	0	0	0	0	0	0	0	0	0	9590700	6135400	6775400
TRINITY_DN6086_c0_g1_i1_1	0	0	0	0	0	0	0	0	0	8933300	6189300	7374200
TRINITY_DN9418_c0_g1_i2_5	0	0	0	0	0	0	0	0	0	9293800	6369500	6818100
TRINITY_DN9810_c0_g1_i1_3	0	0	0	0	0	0	0	0	0	8591300	8303500	5504200
TRINITY_DN6193_c0_g1_i1_1	0	0	0	0	0	0	0	0	0	8435800	7049700	6901000
TRINITY_DN9474_c1_g1_i1_5	305860	0	212360	491640	0	0	5180600	7252000	7751300	283510	699600	202260
TRINITY_DN10292_c0_g1_i2_6	3055500	2817900	3185000	3807100	4069300	4474500	0	813270	0	0	0	0
TRINITY_DN11230_c0_g2_i2_6	3489900	280600	4419500	1105700	1149000	2023900	649350	1413200	2449400	1059600	2483200	1639000
TRINITY_DN10344_c0_g1_i1_2	660890	114930	1144300	2765600	2528100	2241900	0	0	8347800	701500	1080400	2565600
TRINITY_DN11434_c0_g1_i1_2	0	0	0	0	0	0	0	0	0	7362800	7970100	6740600
TRINITY_DN11361_c0_g1_i1_2	166110	0	125760	1023400	791260	648700	0	0	0	7296500	3972000	8040500
TRINITY_DN10628_c0_g1_i1_3	0	0	0	0	0	0	0	1637400	3599900	8163900	4169600	4481400
TRINITY_DN5840_c0_g1_i1_1	1295600	2391500	1240700	595150	1312100	1473100	2673700	4354800	3596100	1335400	836160	891250
TRINITY_DN10700_c0_g1_i1_3	0	0	0	0	0	0	0	0	0	8937200	7012000	5884400
TRINITY_DN10484_c2_g1_i2_1	5521100	3333300	3680500	2609500	1254500	1844600	1941700	0	0	0	0	1604800
TRINITY_DN11431_c0_g1_i3_5	0	0	0	0	0	0	0	0	0	5522000	8395000	7853600
TRINITY_DN9680_c0_g1_i1_1	1907700	1952600	2163800	1802200	627210	1224800	6984300	5089200	0	0	0	0
TRINITY_DN53_c0_g1_i1_5	6302500	6448100	8999300	0	0	0	0	0	0	0	0	0
TRINITY_DN13553_c0_g1_i1_2	2333300	3472100	3077400	1264000	1892100	167190	0	0	0	2406900	1994600	4964900
TRINITY_DN10106_c0_g1_i2_1	0	172800	0	0	0	0	0	0	0	6113700	4935500	10316000
TRINITY_DN10104_c0_g2_i1_2	0	0	0	0	0	0	0	0	0	9514500	6342300	5664300
TRINITY_DN11228_c0_g1_i1_3	1342200	1369500	841600	1225600	1315600	1153200	4083500	5939900	3739200	221530	0	234170
TRINITY_DN9356_c0_g1_i2_6	0	0	0	0	0	0	0	0	0	10538000	6459000	4362100
TRINITY_DN10740_c0_g1_i5_5	0	0	20728	0	141250	0	0	0	0	7031900	7161100	6993700
TRINITY_DN15242_c0_g1_i1_5	0	130080	166070	0	0	0	6254800	6464800	7699900	273780	213370	0
TRINITY_DN10478_c0_g2_i1_1	1340100	1106500	1150700	0	2780000	4784200	762690	4064900	0	2338800	667300	2079300
TRINITY_DN10321_c0_g1_i2_2	0	0	0	0	0	0	0	0	0	8129800	6705800	6130000
TRINITY_DN17896_c0_g1_i1_5	0	0	0	0	0	0	0	0	0	7280500	6755200	6906800
TRINITY_DN5702_c0_g1_i1_3	0	0	0	92770	0	0	0	0	0	9906300	5508300	5429400
TRINITY_DN6940_c0_g9_i5_6	0	0	0	0	0	0	0	0	0	7736400	6281200	6773300
TRINITY_DN10205_c0_g1_i1_2	2816800	3235900	2934500	1299700	4493700	3648400	0	0	0	831150	778720	696660

TRINITY_DN10892_c0_g1_i2_4	0	0	0	0	0	0	0	0	0	7675700	6691700	6319200
TRINITY_DN11295_c0_g2_i3_1	0	0	0	0	0	0	0	0	0	6533900	9737100	4380000
TRINITY_DN10858_c0_g1_i1_5	0	0	0	0	0	0	0	0	0	7091700	7533100	6017600
TRINITY_DN10871_c0_g1_i3_2	0	0	0	0	0	0	0	0	0	6830800	6254000	7551600
TRINITY_DN6968_c0_g1_i1_1	0	0	0	0	0	0	0	0	0	8518700	4989000	7110000
TRINITY_DN6689_c0_g1_i1_2	0	0	0	0	0	0	0	0	0	7356100	7253200	6000200
TRINITY_DN10111_c0_g1_i3_2	0	0	0	0	0	0	0	0	0	9323500	6842200	4378500
TRINITY_DN9976_c0_g4_i1_4	0	0	0	0	0	0	0	0	0	11178000	0	9360900
TRINITY_DN11601_c0_g1_i1_1	230820	57751	195840	235080	0	0	0	0	0	11279000	4685900	3703900
TRINITY_DN4015_c0_g1_i1_2	3382700	3093200	5228100	3297800	2970600	2089600	0	0	0	0	0	236660
TRINITY_DN10140_c0_g1_i1_2	1613900	1603100	1928700	4507000	4132400	2292600	757080	959480	1438400	361850	0	596920
TRINITY_DN2592_c0_g1_i1_1	0	0	0	0	0	0	0	0	0	7332300	5437700	7349500
TRINITY_DN10378_c0_g2_i2_1	1904600	2871200	2920900	1149800	1114900	0	0	0	1700900	2282300	3723100	2256700
TRINITY_DN8575_c0_g2_i3_2	3968900	4889200	3932600	0	3361200	1924800	0	0	0	663350	588050	567130
TRINITY_DN11259_c0_g1_i2_5	219290	122450	74721	635990	0	618120	0	0	0	6287700	6701200	5204800
TRINITY_DN11269_c2_g1_i2_3	126860	143770	103030	0	0	0	0	0	821030	7416400	7165400	3904400
TRINITY_DN9965_c1_g1_i1_3	1322500	554390	391710	0	0	0	0	0	0	6265200	7186700	3910900
TRINITY_DN9622_c1_g1_i2_2	0	0	0	0	0	0	0	0	0	6592700	7556700	5379200
TRINITY_DN10079_c0_g1_i1_3	1156700	842430	1434500	1679800	1835100	500100	572370	0	0	5714700	2528700	3229800
TRINITY_DN3545_c0_g1_i2_1	0	0	0	0	0	0	0	0	0	5776300	3566400	10109000
TRINITY_DN5659_c0_g1_i1_1	0	0	0	0	0	0	0	0	0	8757700	6729200	3959500
TRINITY_DN5550_c0_g1_i2_4	2409900	1980800	2834400	2549900	4219200	689430	1002200	1017500	1120100	862680	257150	387690
TRINITY_DN11000_c0_g1_i1_2	30357	73434	64736	0	103010	0	0	0	0	7757100	6628300	4630500
TRINITY_DN9087_c0_g1_i2_1	4277800	5583900	3766400	2003900	0	1867400	0	0	0	587350	451490	736140
TRINITY_DN9399_c0_g1_i2_1	1876900	2396000	818820	4099600	2850700	1357100	3173100	0	0	1167500	154840	1356300
TRINITY_DN11102_c0_g1_i4_6	1632300	2191000	2584200	3657800	3600400	3952500	0	0	0	0	691730	919520
TRINITY_DN7857_c0_g1_i4_2	0	0	0	0	0	0	0	0	0	7790700	5336100	6101300
TRINITY_DN5480_c0_g1_i2_3	0	0	0	0	0	0	0	0	0	7757800	6440000	4975300
TRINITY_DN3630_c0_g1_i1_5	0	0	0	0	0	0	0	0	0	7619700	7202700	4311800
TRINITY_DN3986_c0_g2_i1_2	0	0	0	0	0	0	0	0	0	8989100	5214800	4914500
TRINITY_DN10586_c0_g1_i1_1	854430	239910	746810	2818800	2608400	2713400	0	0	0	3127800	2688900	3250600
TRINITY_DN10813_c1_g1_i3_3	0	0	0	0	0	0	0	0	0	7753900	4915500	6284200
TRINITY_DN21280_c0_g1_i1_3	1569500	4470800	1538900	3115300	2962300	3637300	0	0	0	455250	418050	743180

TRINITY_DN7020_c0_g1_i1_2	2013800	2397100	2273300	0	548350	0	0	0	0	4558900	4640400	2440100
TRINITY_DN10799_c0_g2_i1_6	110430	0	1080300	2639000	2572900	3721100	741290	0	0	4223200	1354600	2362200
TRINITY_DN8664_c0_g1_i3_3	42312	182700	51254	0	0	0	0	0	0	6149200	5804200	6396500
TRINITY_DN9559_c0_g1_i1_3	2074000	2445100	2029800	2951600	3247600	2618600	0	0	0	769540	863550	1452900
TRINITY_DN11471_c0_g1_i3_3	0	0	0	0	0	0	0	0	0	6346000	4802500	7291400
TRINITY_DN7380_c0_g3_i1_1	871340	1141500	1001000	4233500	6645800	4473200	0	0	0	0	0	0
TRINITY_DN9887_c0_g1_i1_2	0	0	0	0	0	0	1495600	1779300	3999900	2535600	4487500	4047900
TRINITY_DN10568_c0_g3_i5_1	0	0	0	217560	0	0	0	0	0	8459900	5263800	4387000
TRINITY_DN10508_c0_g1_i1_5	0	0	0	0	0	0	5467100	5836600	6962000	0	0	0
TRINITY_DN9574_c0_g1_i3_1	0	0	0	0	0	0	0	0	0	7099100	4517700	6556800
TRINITY_DN6731_c0_g1_i1_1	0	0	0	0	0	0	0	0	0	7275800	3706100	7183600
TRINITY_DN10793_c0_g1_i1_1	0	0	0	0	0	0	0	0	0	7256100	5438000	5461300
TRINITY_DN10605_c0_g1_i4_2	0	0	0	0	0	0	0	0	0	7922000	6423400	3627000
TRINITY_DN7923_c0_g1_i1_1	0	0	0	0	0	0	0	0	0	7237500	5954800	4713700
TRINITY_DN8263_c0_g1_i2_2	0	0	0	0	0	0	0	0	0	5593100	8281200	4013100
TRINITY_DN5337_c0_g1_i1_4	0	0	0	0	0	0	0	0	0	7055700	6570000	4238100
TRINITY_DN11163_c0_g1_i8_3	0	0	0	0	0	0	0	0	0	7072700	7179500	3599300
TRINITY_DN10512_c1_g1_i1_2	1532900	1792600	1683300	5392600	3287900	2406000	0	0	0	0	859980	872310
TRINITY_DN5432_c0_g1_i1_5	0	0	0	0	0	0	0	0	0	5850400	6236100	5723000
TRINITY_DN10108_c0_g1_i2_5	0	0	0	0	0	0	0	0	0	7170400	4720800	5898800
TRINITY_DN10975_c0_g2_i1_3	1292100	2145600	2371900	1025200	2778600	2578300	2069500	298200	2151800	0	520590	554920
TRINITY_DN10736_c0_g1_i2_1	0	0	0	0	0	0	0	0	0	6054400	6054600	5659700
TRINITY_DN7422_c0_g2_i1_2	0	0	0	0	0	0	0	0	0	5770400	7010800	4982800
TRINITY_DN10308_c0_g1_i1_1	0	0	0	0	0	0	0	0	0	6877900	5278000	5594400
TRINITY_DN10384_c0_g3_i1_3	0	0	0	0	0	0	147370	0	0	7254400	5776800	4553100
TRINITY_DN3139_c0_g1_i1_2	0	0	0	0	0	0	0	0	0	5777000	5808200	6050300
TRINITY_DN10364_c0_g1_i3_6	280290	533640	660010	0	0	690510	0	0	0	2558800	5344500	7369500
TRINITY_DN20886_c0_g1_i1_5	1937700	1658900	1251300	1068600	2032600	941450	1583100	2142700	2028900	666530	1494700	606620
TRINITY_DN10883_c0_g1_i3_5	58044	22194	48488	1685400	2166600	1391600	2489200	0	9526200	0	0	0
TRINITY_DN17539_c0_g2_i1_3	0	0	0	0	88342	0	0	0	0	6136000	4569900	6542200
TRINITY_DN10477_c0_g1_i1_1	0	0	0	0	0	0	0	0	0	6314600	6875300	4125400
TRINITY_DN6686_c0_g1_i1_2	0	0	0	0	0	0	0	0	0	6780900	5832600	4617400
TRINITY_DN11212_c0_g1_i1_6	0	0	0	0	0	0	0	0	0	6684300	4923500	5574800

TRINITY_DN11023_c0_g1_i2_1	0	0	0	244130	312240	252560	0	0	0	7137000	4480400	4740900
TRINITY_DN11458_c0_g1_i1_1	0	0	0	0	0	0	0	0	0	8245100	4587800	4288800
TRINITY_DN11615_c0_g1_i1_1	734740	2764900	1566400	0	0	0	0	0	0	6639700	4242400	1063100
TRINITY_DN4509_c0_g2_i1_3	2740000	2569300	1926600	2457900	1448400	625470	0	1712200	2898200	237480	201290	173500
TRINITY_DN11335_c0_g1_i1_1	0	0	0	0	0	0	0	0	0	8022700	4441600	4507400
TRINITY_DN5118_c0_g2_i1_6	0	0	0	0	0	0	0	0	0	7998700	6135400	2811600
TRINITY_DN9738_c2_g1_i2_3	514340	104000	259130	0	0	0	0	1792000	0	4995500	3524800	5732200
TRINITY_DN11571_c2_g3_i1_1	0	0	0	0	0	0	0	0	0	6232700	7521400	3069300
TRINITY_DN17391_c0_g1_i1_5	4719000	1686600	2709100	772160	2110900	4749800	0	0	0	0	0	0
TRINITY_DN10823_c0_g1_i1_1	0	0	0	0	0	0	0	0	0	5659800	5632500	5402500
TRINITY_DN11383_c0_g1_i1_2	3729300	2601200	2727300	1483400	2336500	0	0	0	0	1970600	1399500	413490
TRINITY_DN6993_c0_g1_i3_2	0	0	0	0	0	0	0	0	0	5719300	4835300	6081800
TRINITY_DN11668_c1_g1_i1_2	0	0	0	0	0	0	0	0	0	5930900	5987900	4674800
TRINITY_DN3731_c0_g1_i2_3	0	0	0	175510	0	139850	0	0	0	5856700	5137300	5160800
TRINITY_DN10138_c0_g1_i1_3	0	0	0	0	0	0	0	0	0	5969600	5570400	4894100
TRINITY_DN8211_c0_g1_i2_3	2174700	1853700	2230600	1700600	2374200	1615300	1752000	0	2263700	0	280790	91169
TRINITY_DN20888_c0_g1_i1_3	562970	182540	317090	1379400	1531200	0	4379200	0	5871800	992880	670950	420720
TRINITY_DN11494_c0_g1_i2_5	0	0	0	0	0	0	0	1509100	1419500	5287700	4004500	4057600
TRINITY_DN7194_c1_g1_i2_2	0	0	0	0	0	0	364240	0	0	5796100	5269700	4819700
TRINITY_DN8262_c0_g1_i1_1	0	0	0	0	0	0	0	0	0	6581600	5009100	4560600
TRINITY_DN14510_c0_g1_i1_2	7014000	3580700	3102800	0	1066000	875120	0	0	0	180300	157640	146770
TRINITY_DN9908_c0_g1_i2_1	0	0	177000	0	902300	0	4903000	0	10064000	0	0	0
TRINITY_DN5370_c0_g1_i1_1	1347600	1160200	2137400	0	776650	1484500	0	0	0	3167000	2426800	3515000
TRINITY_DN2867_c0_g1_i2_6	374670	269370	229500	437760	0	357720	4749100	3004100	5694400	310630	249570	251920
TRINITY_DN10919_c0_g1_i5_1	0	0	0	0	0	0	5019400	5104700	5804100	0	0	0
TRINITY_DN8081_c0_g1_i3_3	207630	0	0	226740	126630	0	0	0	0	6098400	3910600	5306100
TRINITY_DN10953_c0_g1_i1_4	0	0	0	0	0	0	0	0	0	5995400	4967000	4913000
TRINITY_DN3942_c0_g1_i3_3	0	0	0	0	0	0	0	0	0	7213400	4575700	3962300
TRINITY_DN6284_c0_g1_i1_3	120490	59464	212290	636630	670480	801140	0	0	0	4346400	4308100	4495300
TRINITY_DN6272_c0_g1_i1_2	226540	652660	239960	0	111510	0	1840200	0	3240100	2708500	3166900	3424600
TRINITY_DN10835_c0_g1_i2_3	1775700	955770	811210	4975700	3591500	174960	0	0	0	1141300	1292300	774200
TRINITY_DN3173_c0_g3_i1_1	670830	814610	429600	0	562050	0	0	0	1138500	4997500	3093600	3780300
TRINITY_DN8566_c0_g1_i2_1	0	0	0	0	0	0	0	0	0	6956800	4230700	4273000

TRINITY_DN18516_c0_g1_i1_1	1270700	3551600	1977500	990390	1505500	2560700	0	0	0	788810	1540600	1187800
TRINITY_DN10724_c0_g1_i4_1	392600	506100	437860	0	0	0	0	0	0	4707800	5557000	3763600
TRINITY_DN10229_c0_g1_i2_1	0	0	0	0	0	0	0	0	0	6871900	3246600	5245800
TRINITY_DN2734_c0_g1_i1_1	0	13830	67661	0	0	0	6552900	4655300	0	1381300	1427200	1178800
TRINITY_DN2645_c0_g2_i1_3	2852700	1560800	1516700	1308400	1844700	1177800	2054400	0	2609800	338750	0	0
TRINITY_DN11051_c0_g2_i2_2	0	0	0	0	0	0	0	0	0	6412900	4832500	3974500
TRINITY_DN6332_c0_g2_i1_3	0	0	0	0	0	0	0	0	0	5979300	4714900	4417000
TRINITY_DN10847_c0_g1_i5_1	234400	463760	384430	0	0	796870	1696300	1624300	0	2390700	3984400	3513300
TRINITY_DN9500_c0_g1_i4_1	0	0	0	0	0	0	0	0	0	7054800	4146900	3873400
TRINITY_DN9205_c0_g1_i1_1	2817000	1486400	1808300	880250	0	851940	0	0	0	4021200	581440	2620400
TRINITY_DN10268_c0_g1_i1_1	0	0	0	0	0	0	0	0	0	5482000	4283200	5301300
TRINITY_DN11412_c0_g1_i3_3	0	0	0	0	0	0	0	0	0	5753100	4993900	4270600
TRINITY_DN4589_c0_g1_i2_4	2151500	1543700	1593000	4765300	2926600	1425800	0	0	0	213970	267930	121170
TRINITY_DN11511_c2_g1_i3_5	4112300	3452700	3719800	1196900	1083100	1036500	0	0	0	216110	0	154110
TRINITY_DN3847_c0_g1_i1_1	1752000	1398300	1417900	1979900	2471500	2665900	0	0	0	1106800	1168500	917920
TRINITY_DN11039_c0_g1_i5_1	0	0	0	0	0	0	0	0	0	5793900	4868500	4191100
TRINITY_DN10019_c0_g2_i1_2	0	0	0	0	0	0	0	0	0	4470500	6159500	4211300
TRINITY_DN10360_c0_g3_i2_3	0	0	0	0	0	0	0	0	0	7350100	3227300	4243300
TRINITY_DN11478_c0_g2_i1_2	0	0	0	0	0	0	0	0	0	6671000	4848500	3247000
TRINITY_DN16366_c0_g1_i1_4	1156500	2429800	1891500	2556100	3142500	2106000	0	0	0	575240	475210	431810
TRINITY_DN9354_c0_g2_i1_2	0	0	0	0	0	0	0	0	0	5890500	4529000	4307300
TRINITY_DN6770_c0_g1_i2_3	1132500	1005500	965620	1557000	1663700	1375500	391940	1250800	4752200	313390	189580	111400
TRINITY_DN5978_c0_g1_i1_4	477650	108300	218560	1504100	1028100	499440	325530	0	0	3718400	3623200	3136000
TRINITY_DN10247_c0_g2_i1_3	801480	542340	633140	676070	1310300	1072500	1021800	4584000	2733500	402250	408730	335150
TRINITY_DN8913_c1_g2_i1_1	0	0	0	0	0	0	0	0	0	4477700	5634700	4384900
TRINITY_DN11338_c0_g1_i2_4	0	0	0	0	0	0	0	0	0	6316300	4659600	3506200
TRINITY_DN11312_c0_g1_i1_1	0	0	0	0	0	0	0	0	0	5502900	4744000	4207000
TRINITY_DN10708_c0_g1_i1_2	245630	330930	351670	598990	129240	155410	1253300	1183800	2138200	2638100	2204200	3223500
TRINITY_DN10573_c0_g1_i2_2	0	0	0	0	0	0	0	0	0	5982500	3221600	5244800
TRINITY_DN6429_c0_g1_i3_5	0	0	0	0	0	0	0	0	0	5354200	5967600	3125700
TRINITY_DN8180_c0_g1_i1_1	616660	74528	415620	1373000	1266900	0	4287100	0	5696800	692230	0	0
TRINITY_DN11655_c0_g1_i2_2	0	0	0	0	0	0	0	0	0	5405100	4122000	4876500
TRINITY_DN8076_c0_g1_i7_3	0	0	0	0	0	0	0	0	0	4836500	6747400	2814800

TRINITY_DN9094_c0_g1_i3_6	2162500	2825200	2971100	1566900	2198800	835620	0	0	0	649400	369050	718340
TRINITY_DN9800_c0_g1_i1_1	0	0	0	0	0	0	0	0	0	5987500	4242400	4052800
TRINITY_DN21582_c0_g1_i1_3	1145500	602720	1190300	375310	356600	450320	2080700	4437400	2513900	601610	520590	0
TRINITY_DN11557_c0_g2_i2_1	2741900	954510	1298300	2431300	3934000	0	0	0	0	628650	1781300	472670
TRINITY_DN9080_c0_g1_i2_2	0	0	0	0	0	0	0	0	0	4320700	3739600	6154800
TRINITY_DN19006_c0_g1_i1_5	153020	0	0	0	403330	273940	1302600	1366900	1812700	4666800	1828800	2374600
TRINITY_DN2622_c0_g1_i1_2	0	0	0	0	0	0	0	0	0	5025300	3463900	5652100
TRINITY_DN1894_c0_g2_i1_5	0	0	0	0	0	0	6083900	0	7890900	0	132680	0
TRINITY_DN10750_c0_g1_i1_2	0	0	0	0	0	0	0	0	0	5341700	4721200	4036300
TRINITY_DN336_c0_g1_i1_1	0	0	0	0	0	0	0	0	0	3541600	3661300	6892600
TRINITY_DN10712_c0_g1_i2_6	0	0	932320	0	0	0	0	0	0	5328000	4191600	3554400
TRINITY_DN2749_c0_g1_i1_2	0	0	0	0	0	0	0	0	0	5180400	5447500	3303500
TRINITY_DN6944_c1_g2_i2_2	0	0	0	0	0	0	13827000	0	0	0	88442	0
TRINITY_DN10708_c1_g1_i1_1	679930	586700	325070	411950	725320	211840	1178500	0	1327700	3051400	2094900	3288100
TRINITY_DN8860_c0_g2_i3_2	1649100	2196600	2884000	1138500	1286200	1262900	0	0	0	1247000	671170	1529500
TRINITY_DN2935_c0_g1_i1_4	0	0	0	0	0	0	0	0	0	4303500	6161700	3395700
TRINITY_DN5345_c0_g1_i1_1	0	149150	0	3172600	3299700	2001400	491000	984680	0	944600	1297700	1481700
TRINITY_DN2696_c0_g1_i1_2	0	0	0	0	0	0	0	0	0	5187300	2463400	6157100
TRINITY_DN6268_c0_g2_i1_1	0	0	0	0	0	0	0	0	0	5273500	2936300	5589600
TRINITY_DN20535_c0_g1_i1_2	0	0	0	3697100	4247300	3530900	0	0	0	1033700	725590	537920
TRINITY_DN6734_c0_g1_i2_4	0	0	0	0	0	0	0	0	0	4588100	4257500	4850200
TRINITY_DN10790_c0_g1_i1_2	0	0	0	0	0	0	0	0	0	6445600	5359200	1860400
TRINITY_DN11422_c0_g1_i2_2	0	0	0	0	0	0	0	0	0	5130300	4571600	3924900
TRINITY_DN4447_c0_g1_i1_4	0	0	0	1532100	4018100	7693600	0	0	0	141860	172320	0
TRINITY_DN10526_c0_g1_i1_1	642110	609240	675260	2338200	1712200	2095000	0	0	0	1882000	2189700	1365500
TRINITY_DN11588_c0_g1_i3_2	0	0	0	0	0	0	1503500	1462100	0	5460600	1544800	3522900
TRINITY_DN11110_c0_g3_i1_1	1275400	2392900	2321400	1534300	2137200	2392900	0	0	0	707980	581070	0
TRINITY_DN3777_c0_g1_i2_4	2195800	1410700	1131000	0	0	1010400	2050500	3623500	1684600	0	0	166090
TRINITY_DN17601_c0_g1_i1_6	0	0	0	0	0	0	0	0	0	5072500	3814000	4382500
TRINITY_DN11006_c0_g1_i1_2	0	0	0	162260	0	0	351560	0	0	4380500	3541400	4766200
TRINITY_DN16803_c0_g1_i1_5	0	0	0	0	0	0	0	0	0	5388200	3946100	3867400
TRINITY_DN22655_c0_g1_i1_2	146040	5491700	1457500	0	3469700	2621200	0	0	0	0	0	0
TRINITY_DN9863_c2_g1_i1_1	142740	617790	719060	695050	847500	770170	769170	737760	742790	2700400	2655200	1771800

TRINITY_DN7934_c0_g2_i1_1	1175200	1156100	1526100	1121400	589700	1586900	1369500	1565500	1774200	511320	347680	432610
TRINITY_DN11256_c0_g1_i2_2	0	0	0	0	0	0	0	0	0	4963800	4124900	4009300
TRINITY_DN11403_c0_g1_i2_2	0	0	0	0	0	0	0	0	0	4226900	3765300	5104300
TRINITY_DN11547_c0_g1_i4_3	0	0	0	0	0	0	0	0	0	5136600	4256100	3678500
TRINITY_DN8795_c0_g3_i1_4	0	0	0	0	0	0	0	1465200	4054800	3035800	1796600	2700200
TRINITY_DN5882_c0_g3_i2_3	0	0	0	0	0	0	0	0	3057000	4700200	3983600	1308900
TRINITY_DN6879_c0_g1_i2_2	388040	475290	489050	2279700	2409200	2432400	0	0	0	2138600	1022100	1413300
TRINITY_DN8601_c0_g1_i1_2	0	0	0	0	0	0	0	0	0	6973600	6041700	0
TRINITY_DN11298_c0_g1_i2_4	133890	161340	519410	3154100	3108500	2267600	0	0	1635900	538530	1187600	288960
TRINITY_DN8357_c0_g1_i1_1	1189500	1328000	766850	725000	364940	515160	1224600	0	1822600	2222100	1649200	1051100
TRINITY_DN9107_c0_g1_i2_2	0	0	0	0	0	0	0	0	0	0	6475900	6355100
TRINITY_DN11330_c0_g1_i3_6	0	0	0	0	0	0	0	0	0	1039500	6096800	5673300
TRINITY_DN10810_c0_g1_i4_1	2867500	2052100	3281200	1317000	1537200	1226100	0	0	0	237850	229490	0
TRINITY_DN8957_c0_g1_i1_1	0	0	0	0	0	0	0	0	12749000	0	0	0
TRINITY_DN8356_c0_g2_i1_1	723740	0	0	1087700	2852300	1253100	4066600	0	0	1078700	889560	789120
TRINITY_DN10807_c0_g1_i5_1	0	0	94753	0	0	0	0	0	0	5155600	4291700	3069200
TRINITY_DN11558_c0_g1_i1_1	0	0	0	0	0	0	0	0	0	5913400	3073600	3550300
TRINITY_DN9852_c0_g1_i1_1	302740	319320	244140	0	365770	0	0	0	0	3306300	5157100	2821600
TRINITY_DN11418_c0_g2_i2_5	0	0	0	0	0	0	0	0	0	5696500	3165500	3546000
TRINITY_DN5567_c0_g1_i3_3	0	0	0	0	4201100	1682300	0	0	0	3220200	0	3297700
TRINITY_DN10055_c0_g1_i2_3	0	0	0	0	0	0	0	0	0	6139700	3773700	2399900
TRINITY_DN8773_c0_g2_i2_4	0	0	0	136700	0	0	0	0	0	4633700	3618400	3915300
TRINITY_DN2809_c0_g1_i1_6	1491600	1178500	1379700	0	1560600	1688400	2573100	2424900	0	0	0	0
TRINITY_DN19230_c0_g1_i1_1	0	0	0	0	0	0	0	0	0	4471500	4037600	3732700
TRINITY_DN11224_c0_g1_i1_1	0	0	0	0	0	0	0	0	0	4114800	5534400	2555700
TRINITY_DN3638_c0_g1_i1_2	2516400	1980700	2373300	1128300	1950400	658740	0	0	0	898780	624960	0
TRINITY_DN11495_c0_g1_i1_2	0	0	0	0	0	0	0	0	0	5620200	3970900	2535000
TRINITY_DN10903_c0_g1_i6_2	0	0	0	0	0	0	0	6229600	5880100	0	0	0
TRINITY_DN4170_c0_g3_i2_2	0	0	0	0	0	0	1542000	0	0	4079500	3603000	2804200
TRINITY_DN10453_c0_g2_i2_3	0	0	0	0	0	0	0	0	0	5360300	3430700	3228000
TRINITY_DN10548_c1_g1_i1_3	0	0	0	0	0	0	0	0	0	4154100	4471500	3367800
TRINITY_DN9786_c0_g1_i2_4	0	0	0	0	0	0	1376700	3144800	1816000	2243800	2025400	1361100
TRINITY_DN307_c0_g2_i1_6	0	0	0	0	0	0	3361700	3610600	4290000	230080	318600	134650

TRINITY_DN3566_c0_g1_i1_1	0	0	0	0	0	0	0	0	0	3520000	4784900	3562400
TRINITY_DN6229_c0_g1_i1_2	248400	402940	293780	0	1455400	2457700	2718200	0	1869900	434840	1035700	895460
TRINITY_DN10335_c1_g2_i2_2	0	0	0	0	0	0	0	0	0	6267000	1929500	3614800
TRINITY_DN10820_c1_g1_i1_3	0	0	0	0	0	0	0	0	0	5085200	2459700	4244000
TRINITY_DN4435_c0_g1_i1_1	0	0	0	0	0	0	0	0	0	4929300	3935600	2852300
TRINITY_DN9270_c0_g2_i2_2	0	0	0	0	0	0	0	0	0	6081400	2116400	3516200
TRINITY_DN9549_c0_g1_i1_2	0	0	0	0	0	0	0	0	0	2930600	4402200	4338900
TRINITY_DN11397_c0_g1_i5_2	0	0	0	0	0	0	0	0	0	5933700	3347200	2379600
TRINITY_DN11446_c0_g1_i1_2	0	0	0	0	0	0	0	0	0	4491600	4436700	2725900
TRINITY_DN11125_c0_g9_i1_3	0	0	0	0	0	0	0	0	0	4471100	3468300	3678200
TRINITY_DN7263_c1_g1_i1_1	703360	523020	565110	558350	259110	0	0	0	0	3956600	1947500	3071400
TRINITY_DN11387_c0_g1_i4_2	0	0	0	0	0	0	0	0	0	2353600	4133700	5050800
TRINITY_DN11199_c0_g5_i8_6	1974400	2744800	3082800	0	0	587840	0	0	0	1135100	1023500	973880
TRINITY_DN8461_c0_g1_i1_1	0	0	0	0	0	0	0	0	0	5090200	3128400	3261600
TRINITY_DN3481_c0_g1_i1_6	0	0	0	0	0	0	0	0	0	4759300	2537400	4121100
TRINITY_DN5908_c0_g1_i1_3	28054	20172	42057	0	0	0	0	0	0	5769300	4972600	579350
TRINITY_DN8185_c0_g1_i1_1	0	0	0	0	0	0	0	0	0	4394900	3609300	3287300
TRINITY_DN4115_c0_g1_i1_2	923940	1130900	750030	1426800	1204900	611790	414950	1115800	529880	1422000	1095600	631420
TRINITY_DN5077_c0_g1_i1_1	0	0	0	0	0	0	0	454220	0	5204000	3306600	2274300
TRINITY_DN7999_c0_g1_i1_3	0	0	0	0	0	0	0	0	0	2725200	6401900	2105100
TRINITY_DN12921_c0_g1_i1_3	0	0	0	0	0	0	11115000	0	0	0	0	0
TRINITY_DN2387_c0_g2_i1_1	0	0	0	0	0	0	0	0	0	5452300	3827200	1826700
TRINITY_DN9350_c0_g2_i2_2	0	0	0	0	0	0	0	0	0	5827300	2695400	2578500
TRINITY_DN10819_c0_g1_i1_3	0	4062700	0	0	0	0	0	0	0	1583500	3990100	1416600
TRINITY_DN9863_c1_g1_i2_5	0	0	0	361270	0	0	1762800	1924900	2178300	1762800	1612900	1418800
TRINITY_DN9866_c0_g1_i1_1	0	0	0	0	0	0	0	0	0	5055300	1329100	4633800
TRINITY_DN11562_c0_g1_i1_2	0	0	0	0	0	0	0	0	0	4196100	2634600	4175900
TRINITY_DN11571_c4_g3_i1_2	81283	0	25544	0	0	0	0	0	0	4419300	3688300	2784400
TRINITY_DN9464_c0_g1_i4_3	0	0	0	0	0	0	0	0	0	3055700	3225400	4715900
TRINITY_DN8433_c1_g1_i2_6	0	0	0	0	175210	185340	0	0	0	3164700	2282300	5184000
TRINITY_DN10036_c0_g1_i3_6	0	0	0	0	0	0	493380	0	0	3897900	3274500	3317000
TRINITY_DN10785_c0_g1_i1_3	1632600	645720	1363800	1867500	1566400	2032600	0	0	0	846200	0	941070
TRINITY_DN4427_c0_g2_i1_1	0	0	0	0	0	0	0	0	0	3309500	3705000	3854400

TRINITY_DN8833_c0_g1_i2_2	316070	429960	413090	410630	166790	0	0	0	0	2896500	3040800	3169200
TRINITY_DN10152_c0_g1_i1_1	0	0	0	0	0	0	0	0	0	4774000	3585900	2450200
TRINITY_DN11018_c0_g1_i2_1	0	0	0	0	0	0	0	0	0	3102900	5043100	2629300
TRINITY_DN8141_c0_g2_i1_3	0	0	0	0	0	0	0	0	0	5310700	3978900	1482700
TRINITY_DN18656_c0_g1_i1_2	1400300	216190	40762	606410	665470	1346500	0	0	0	266140	3228500	2887000
TRINITY_DN9214_c0_g1_i3_1	102920	1472900	1059900	2066900	617200	658830	0	0	0	1869600	969450	1831100
TRINITY_DN5754_c0_g1_i1_1	1501700	1392300	1604600	1524600	1698700	1815500	129460	0	972390	0	0	0
TRINITY_DN4395_c0_g1_i1_4	0	0	0	0	0	0	0	0	0	4963800	2859600	2808800
TRINITY_DN10088_c0_g2_i1_3	1233100	711090	784230	816820	1227300	956140	0	0	0	1928700	1402200	1549600
TRINITY_DN18481_c0_g2_i1_2	30601	0	81622	104890	0	0	0	1700800	1807600	3384100	2865700	622050
TRINITY_DN3240_c0_g1_i1_2	944000	878260	1108700	1077300	1790500	2025500	0	0	0	831810	1276100	632440
TRINITY_DN9575_c0_g1_i1_1	120700	135470	154810	327850	0	296240	569220	0	0	3907200	1989900	2998200
TRINITY_DN8266_c0_g1_i1_1	0	0	0	0	0	0	0	0	0	4721400	2371400	3392200
TRINITY_DN3684_c0_g3_i1_2	0	0	0	0	0	0	0	0	0	4113400	3599700	2763800
TRINITY_DN27_c0_g2_i1_5	0	0	0	0	0	0	0	0	0	4663600	2124500	3540900
TRINITY_DN14743_c0_g1_i1_2	0	0	0	0	0	0	0	0	0	3518700	3029600	3754800
TRINITY_DN11580_c0_g1_i4_3	0	0	0	0	0	0	0	0	0	5163600	2010500	3120700
TRINITY_DN10635_c0_g1_i3_5	0	0	0	0	0	0	0	0	0	5187900	2050500	3024000
TRINITY_DN11567_c1_g2_i1_2	1950100	392380	553840	3141100	1841300	1506000	0	0	286490	254340	0	305440
TRINITY_DN11631_c0_g1_i3_2	0	0	0	0	0	0	0	0	0	4220500	2380000	3599200
TRINITY_DN1621_c0_g2_i2_2	0	0	0	0	0	0	0	0	0	3261200	3218700	3615000
TRINITY_DN8095_c0_g1_i1_1	0	0	0	0	0	0	0	0	0	5123800	1026000	3931100
TRINITY_DN5554_c0_g2_i1_3	0	0	0	0	0	0	0	1170000	1555700	3801200	1476400	2019700
TRINITY_DN11457_c0_g1_i1_3	0	0	0	0	0	0	0	0	0	3598500	3309200	3105400
TRINITY_DN6500_c0_g2_i1_1	768570	507920	599860	405660	409770	0	2448200	2474900	2393400	0	0	0
TRINITY_DN7157_c0_g1_i1_3	0	0	0	0	0	0	0	0	0	7569300	1138400	1288800
TRINITY_DN9819_c1_g2_i1_2	0	0	0	0	0	0	0	0	0	3585900	4071100	2308200
TRINITY_DN11038_c0_g2_i1_2	956860	0	0	0	953650	738560	0	0	0	2309600	2399200	2598000
TRINITY_DN9121_c0_g1_i2_6	0	0	0	0	0	0	0	0	0	3294500	3027200	3597900
TRINITY_DN11575_c0_g1_i5_2	0	0	0	0	0	0	0	0	0	2310300	3835800	3768100
TRINITY_DN11451_c0_g1_i1_2	0	0	0	0	0	0	0	0	0	4037900	3808500	2049500
TRINITY_DN11459_c1_g2_i1_2	0	0	0	0	0	0	0	0	0	5567100	4325400	0
TRINITY_DN9611_c0_g1_i1_6	0	0	0	0	0	0	0	0	0	1271300	5685000	2908300

TRINITY_DN118_c0_g1_i1_3	0	0	0	0	0	0	1417900	3334600	5107200	0	0	0
TRINITY_DN7932_c0_g1_i8_5	0	0	0	0	0	0	0	0	0	3923800	2668300	3252200
TRINITY_DN9613_c0_g2_i2_4	523700	936780	1104200	1711000	2239100	0	1632800	1680000	0	0	0	0
TRINITY_DN7103_c0_g2_i2_2	0	0	0	0	0	0	0	0	0	3482400	4419700	1918500
TRINITY_DN11303_c0_g1_i1_1	751490	397040	0	938520	2617700	3603400	0	0	878570	0	540100	92267
TRINITY_DN10641_c0_g1_i1_6	0	0	0	0	0	0	0	0	0	4182200	2269300	3354400
TRINITY_DN11410_c0_g1_i1_4	294910	0	304210	549380	556380	0	0	0	0	3096400	2432900	2562000
TRINITY_DN6205_c0_g1_i1_2	0	0	0	0	0	0	0	0	0	2958200	1953800	4845900
TRINITY_DN11159_c1_g1_i8_5	0	0	0	0	0	0	0	0	0	4188000	1484000	4054800
TRINITY_DN9714_c0_g1_i1_1	0	0	0	0	0	0	0	0	0	3403700	3098400	3218100
TRINITY_DN4382_c0_g1_i1_1	0	0	0	0	0	0	0	0	0	4516300	2560200	2620200
TRINITY_DN11626_c0_g2_i1_5	246370	203300	137450	0	0	79947	0	0	0	2201200	3294300	3511700
TRINITY_DN9896_c0_g1_i5_1	0	0	430740	3394800	1990100	3539200	0	0	0	177830	140000	0
TRINITY_DN10351_c0_g1_i3_1	0	0	0	0	0	0	0	0	0	2743700	3646000	3274900
TRINITY_DN7143_c0_g1_i1_1	0	0	0	0	0	0	3921700	768730	4970600	0	0	0
TRINITY_DN11432_c0_g1_i1_6	495420	665060	285570	869350	899740	640980	0	0	0	3344700	2449100	0
TRINITY_DN9820_c0_g1_i3_3	75744	144470	125490	0	0	0	1926600	1654800	1957100	1627000	846570	1242200
TRINITY_DN10026_c0_g1_i1_5	74652	613180	843810	301810	0	480220	1021100	1250300	1713300	1308000	912850	1065900
TRINITY_DN11576_c1_g1_i1_2	0	2582500	2697200	1335100	1292100	0	0	0	1339700	0	149640	183570
TRINITY_DN18710_c0_g1_i1_2	1418800	2901500	2021300	1412100	1286500	536920	0	0	0	0	0	0
TRINITY_DN11480_c0_g1_i1_2	0	0	0	0	429820	270900	0	0	0	3417500	2132500	3324700
TRINITY_DN16427_c0_g1_i1_4	0	0	0	0	0	0	0	0	0	2181900	5100200	2273900
TRINITY_DN21781_c0_g1_i1_6	247420	254460	0	548510	370780	515770	0	0	0	3152700	2314300	2131800
TRINITY_DN840_c0_g2_i1_1	0	0	0	0	0	0	0	0	0	3256100	3234700	3008900
TRINITY_DN9473_c0_g1_i1_3	0	0	0	0	0	0	0	0	0	3030100	3288200	3179400
TRINITY_DN11010_c0_g1_i2_2	0	0	0	0	0	0	0	0	0	3521800	3157000	2811300
TRINITY_DN11411_c1_g1_i1_5	200400	125040	314720	1600900	1705300	1924000	0	0	0	1268500	1129800	1174100
TRINITY_DN13141_c0_g1_i1_2	0	0	0	0	837330	654890	0	2937700	3907700	447840	349600	306050
TRINITY_DN10770_c0_g1_i1_1	0	0	0	0	0	0	0	0	0	2725100	4197700	2485300
TRINITY_DN1431_c0_g2_i1_2	0	0	0	0	0	0	0	0	0	2396000	3813300	3149500
TRINITY_DN19678_c0_g1_i1_1	0	0	0	0	0	0	0	0	0	2129500	5058100	2135500
TRINITY_DN249_c0_g2_i1_1	1073800	156130	826330	3202900	1594400	1917700	0	0	0	0	0	534790
TRINITY_DN17357_c0_g1_i1_3	0	0	0	0	0	0	0	0	0	4342100	2189600	2764900

TRINITY_DN5130_c1_g1_i1_6	0	161970	0	0	0	0	0	0	0	4520900	3481300	1123200
TRINITY_DN10160_c0_g1_i3_6	0	0	0	0	0	0	0	0	0	3012700	3268200	2954900
TRINITY_DN5845_c0_g1_i3_2	0	0	0	0	0	0	0	0	0	2890300	3022200	3294400
TRINITY_DN8910_c0_g1_i1_1	0	0	0	0	0	0	0	0	0	3084300	3237200	2862800
TRINITY_DN11500_c0_g1_i1_3	0	0	0	410380	0	0	0	0	0	4147300	2111300	2492700
TRINITY_DN11156_c0_g1_i1_3	0	0	0	0	0	0	0	0	0	4717400	2259300	2152200
TRINITY_DN10981_c0_g2_i2_6	0	0	0	0	0	0	0	0	0	1950800	595510	6575800
TRINITY_DN10644_c0_g1_i1_2	39454	0	51986	0	0	0	0	0	0	4094500	2801200	2120200
TRINITY_DN21106_c0_g2_i1_1	4127000	783630	3287700	0	0	790450	0	0	0	0	0	118500
TRINITY_DN7447_c0_g1_i1_2	0	0	194640	1638100	464840	1425800	1316400	1739500	2295000	0	0	0
TRINITY_DN10179_c0_g2_i1_1	985210	1111800	911530	0	422010	0	0	0	0	2049600	1822000	1742500
TRINITY_DN10614_c0_g1_i2_2	0	0	0	0	0	0	0	0	0	4869700	2311200	1855500
TRINITY_DN14470_c0_g1_i1_4	0	0	0	0	0	0	0	0	0	2304900	3041900	3653000
TRINITY_DN5970_c0_g1_i1_3	0	0	0	0	0	0	0	0	0	3507900	3192600	2294900
TRINITY_DN10916_c0_g1_i1_6	0	0	0	0	0	0	0	0	0	4132200	2387500	2474700
TRINITY_DN9868_c0_g1_i1_3	0	0	0	0	0	0	0	0	0	3923200	1607900	3456400
TRINITY_DN11613_c0_g1_i2_2	0	214720	270390	553620	728530	784880	0	0	5899200	523910	0	0
TRINITY_DN11620_c1_g1_i4_6	863030	1320900	1157300	1709300	1770000	908340	0	0	0	436460	370150	436270
TRINITY_DN17153_c0_g1_i1_1	1178000	1174700	1007400	299450	1119000	420860	1018900	1303700	1434100	0	0	0
TRINITY_DN8763_c0_g2_i1_1	0	436960	358710	291170	297580	252010	0	0	0	3248800	1878600	2156000
TRINITY_DN11270_c0_g2_i1_1	0	0	0	0	0	0	0	0	0	2592100	3536200	2783400
TRINITY_DN3573_c0_g1_i1_2	0	45341	91126	147610	0	108200	0	0	0	3279800	2677300	2532900
TRINITY_DN7327_c0_g1_i2_5	0	0	0	0	0	0	0	0	0	3569900	1943900	3332000
TRINITY_DN6062_c0_g2_i3_1	525540	865140	1067000	1547000	2680700	1872400	0	0	0	144540	126890	0
TRINITY_DN5393_c0_g1_i1_2	0	0	0	0	0	0	0	0	0	3430000	2640100	2751200
TRINITY_DN21568_c0_g1_i2_1	915300	1505200	1152500	0	467380	505270	0	0	0	1409600	1836800	998540
TRINITY_DN11022_c0_g1_i1_1	0	156350	156400	305790	0	985600	0	0	0	2287100	2283000	2581800
TRINITY_DN9756_c0_g1_i3_1	0	0	0	0	0	0	0	0	0	3417200	2888300	2440500
TRINITY_DN8806_c0_g1_i5_2	0	0	0	0	0	0	0	0	0	4216600	2114100	2401600
TRINITY_DN7109_c0_g1_i1_2	398010	375780	530510	1295300	1408200	1370700	990610	1044600	1139800	0	0	174050
TRINITY_DN3282_c0_g2_i1_4	0	0	0	0	0	0	0	0	0	3885700	2606700	2233200
TRINITY_DN6144_c0_g1_i1_3	0	0	0	1192300	1868800	1149800	0	0	0	2570700	0	1943000
TRINITY_DN10924_c0_g1_i1_3	0	0	0	0	0	0	0	0	0	3274600	1678500	3770500

TRINITY_DN8033_c0_g1_i1_2	0	0	0	0	0	0	0	0	0	3595300	2922800	2199800
TRINITY_DN11083_c0_g1_i2_1	0	0	0	0	0	0	0	0	0	3553900	2653400	2488000
TRINITY_DN9815_c0_g1_i1_2	0	0	0	0	0	0	0	0	0	1781200	2304400	4581300
TRINITY_DN9347_c0_g1_i1_1	0	0	0	0	0	0	0	0	601880	2726900	1582300	3749100
TRINITY_DN10872_c0_g1_i8_4	0	0	0	0	0	0	0	0	0	3516200	1759300	3364400
TRINITY_DN12354_c0_g1_i1_2	0	0	0	3225700	3173100	2205300	0	0	0	0	0	0
TRINITY_DN6584_c0_g2_i1_1	1998000	1749900	2193800	696090	822300	1142900	0	0	0	0	0	0
TRINITY_DN10462_c0_g1_i1_2	0	0	0	0	0	0	0	0	0	3957000	1429700	3206700
TRINITY_DN9076_c0_g2_i1_2	224110	254970	211530	0	227470	0	0	0	0	4952800	2716900	0
TRINITY_DN527_c0_g2_i1_3	0	0	0	0	0	0	0	0	0	3063000	2712100	2808700
TRINITY_DN19375_c0_g1_i1_3	0	0	0	0	0	0	0	0	0	4817200	0	3752000
TRINITY_DN9641_c0_g1_i1_2	0	0	0	0	0	0	0	0	0	3545900	3036900	1980300
TRINITY_DN22097_c0_g1_i1_5	276770	293360	391090	1449400	1340400	1558700	0	0	0	1400800	905570	946960
TRINITY_DN12939_c0_g1_i1_5	0	0	0	0	0	0	0	0	0	3042800	2501100	3009500
TRINITY_DN12440_c0_g1_i1_2	0	0	0	0	0	0	0	0	0	4049200	522130	3969900
TRINITY_DN21623_c0_g1_i1_1	0	0	36739	2634200	2779700	2101000	0	0	0	496310	476730	0
TRINITY_DN10464_c0_g1_i1_6	0	0	0	0	0	0	0	0	0	2971200	3091000	2457700
TRINITY_DN9302_c0_g1_i1_2	188510	225780	348040	0	0	249290	0	0	0	3409600	1553400	2539100
TRINITY_DN1467_c0_g1_i1_4	0	0	0	0	0	0	0	0	0	3139100	2050500	3319800
TRINITY_DN10869_c1_g3_i1_5	956750	916260	713720	1693700	2394500	1721800	0	0	0	0	106450	0
TRINITY_DN11008_c0_g1_i3_3	0	0	0	0	0	0	0	0	0	3396000	1880000	3202900
TRINITY_DN5035_c0_g1_i1_1	739470	1152300	754530	1974200	2127100	927880	445420	0	0	145980	190190	0
TRINITY_DN11340_c0_g1_i2_2	0	0	0	0	0	0	0	0	0	2871900	2923800	2602700
TRINITY_DN10734_c1_g1_i1_1	0	0	0	0	0	0	0	0	0	1962800	2664400	3754100
TRINITY_DN10680_c4_g1_i3_4	1276000	1563900	1193800	0	0	4319800	0	0	0	0	0	0
TRINITY_DN11420_c0_g1_i4_2	0	0	0	0	0	0	0	0	0	3822100	2531000	2000300
TRINITY_DN1806_c0_g1_i1_6	0	0	0	0	0	0	0	0	0	2735200	2374600	3241000
TRINITY_DN9463_c0_g1_i1_2	0	0	0	0	0	0	0	0	0	3868600	1435500	2998900
TRINITY_DN2883_c0_g1_i1_1	0	0	0	0	0	0	0	0	0	5030200	2232800	1030000
TRINITY_DN11614_c0_g1_i5_1	0	0	0	0	0	0	0	0	0	3284100	2329400	2652000
TRINITY_DN8774_c0_g2_i2_6	0	246870	273030	340490	0	0	0	0	0	1760800	3634800	1985300
TRINITY_DN9582_c0_g2_i1_5	0	0	0	0	0	0	0	0	0	4898500	1304700	2020500
TRINITY_DN8902_c0_g1_i2_5	560890	862990	1343000	324730	1489600	607180	0	526550	2209200	112940	81218	98675

TRINITY_DN11545_c0_g1_i3_2	1642700	2041900	2143600	663200	651650	186300	0	0	0	401660	258530	226040
TRINITY_DN11179_c0_g1_i4_3	0	0	0	0	0	0	0	0	0	2807400	3442500	1962900
TRINITY_DN10263_c1_g1_i2_1	1106900	1647000	1096000	0	1136100	0	3195000	0	0	0	0	0
TRINITY_DN9576_c0_g1_i2_5	0	0	0	0	0	0	0	0	0	3499000	2037500	2620100
TRINITY_DN7960_c0_g1_i1_2	0	0	0	0	0	0	0	0	0	3397500	2314500	2435100
TRINITY_DN5577_c0_g1_i1_5	1094500	1305100	1426500	1375400	1530800	1412000	0	0	0	0	0	0
TRINITY_DN4472_c0_g1_i1_5	0	0	0	0	0	0	0	0	0	5112900	3028700	0
TRINITY_DN10370_c0_g1_i1_3	0	0	0	0	0	0	0	0	0	3397500	2867900	1846000
TRINITY_DN8106_c0_g1_i1_5	0	0	0	0	141140	0	323540	3685600	3954000	0	0	0
TRINITY_DN11090_c0_g2_i6_4	0	0	0	0	0	0	0	0	0	3307800	0	4771400
TRINITY_DN10461_c0_g1_i3_3	0	0	0	0	0	0	0	0	0	2990600	3048600	2031200
TRINITY_DN579_c0_g1_i1_3	0	0	0	0	0	0	0	0	0	0	0	8063900
TRINITY_DN14915_c0_g1_i1_3	0	0	0	0	0	0	0	5024700	3037900	0	0	0
TRINITY_DN11178_c0_g1_i2_1	0	86700	0	635010	620730	514070	1256200	1346300	1471800	839370	631990	650350
TRINITY_DN8448_c0_g1_i1_1	132690	179330	142040	0	0	0	562850	0	568120	1995900	2227000	2220200
TRINITY_DN11626_c0_g1_i4_5	0	0	0	0	0	0	0	0	0	3259800	2582000	2169900
TRINITY_DN6292_c0_g2_i1_4	0	0	0	0	0	0	0	0	0	2483200	3722900	1748500
TRINITY_DN5457_c0_g1_i1_3	192290	162760	122130	484360	387450	662310	0	2850900	3092100	0	0	0
TRINITY_DN6422_c0_g1_i1_2	747000	392600	517540	171790	146190	304070	0	0	0	3373600	1327400	948880
TRINITY_DN7995_c0_g2_i1_1	458430	521870	822840	129080	0	119970	352180	2452800	1492000	609970	515890	452830
TRINITY_DN8330_c0_g1_i1_3	120730	127020	169590	0	836370	647980	0	0	0	0	6020500	0
TRINITY_DN10167_c0_g2_i1_3	0	0	0	0	0	0	0	0	0	3570000	2438800	1908000
TRINITY_DN9776_c0_g1_i4_2	0	0	0	0	0	0	0	0	0	3499700	2174700	2183000
TRINITY_DN11503_c0_g1_i1_1	1255000	1002200	1999300	552300	596800	1101600	0	0	0	963220	363320	0
TRINITY_DN11383_c0_g1_i3_1	74278	31195	270550	0	0	1429800	0	0	0	2306000	2077400	1639000
TRINITY_DN10659_c0_g1_i3_2	274840	381700	0	201450	332490	0	0	0	0	2983200	1924500	1718000
TRINITY_DN10371_c0_g1_i1_2	0	0	0	83502	76586	0	0	0	0	3241400	2652600	1724600
TRINITY_DN6420_c0_g1_i1_4	0	0	0	0	0	0	0	0	0	2470800	1720500	3586300
TRINITY_DN10875_c0_g1_i2_2	0	0	0	0	0	0	0	0	0	3472400	2771500	1510000
TRINITY_DN9826_c0_g1_i1_3	0	0	0	0	0	0	0	0	0	2791300	2201100	2708000
TRINITY_DN8975_c0_g1_i1_3	0	0	0	0	0	0	0	0	0	3477500	1557800	2650400
TRINITY_DN8607_c0_g1_i2_1	0	0	0	0	0	0	3714500	3962600	0	0	0	0
TRINITY_DN11635_c2_g1_i9_3	0	0	0	0	230110	0	2510100	2434700	2488400	0	0	0

TRINITY_DN17821_c0_g1_i1_3	0	0	0	0	0	0	0	0	7661400	0	0	0
TRINITY_DN3258_c0_g1_i1_2	2024700	798390	902890	341610	440980	299660	0	0	0	634300	988860	1204500
TRINITY_DN10762_c0_g1_i1_1	2760100	655800	540660	0	1594100	222350	0	0	0	623100	492630	725110
TRINITY_DN10328_c0_g2_i1_2	0	0	0	0	0	0	0	0	0	1723600	1746700	4136000
TRINITY_DN11289_c0_g2_i2_2	0	0	0	0	0	0	0	0	0	2544800	2079500	2975100
TRINITY_DN4969_c0_g1_i1_3	0	0	0	0	0	0	0	0	0	2303900	3061700	2225700
TRINITY_DN12289_c0_g1_i1_5	1021000	894870	677450	438190	478100	180640	0	0	0	2237400	959300	703050
TRINITY_DN11225_c0_g1_i1_3	2209800	1115200	1643000	0	1020700	861510	0	0	0	274780	227280	231610
TRINITY_DN20631_c0_g1_i1_5	1513600	48366	84827	2166400	903660	918420	857070	0	1087900	0	0	0
TRINITY_DN15095_c0_g1_i1_4	305890	430000	707520	1122000	1362800	1247600	0	0	0	913450	1229100	254860
TRINITY_DN10798_c0_g1_i1_3	0	0	0	0	0	0	0	0	0	3597400	1694200	2260400
TRINITY_DN17510_c0_g1_i1_2	1048900	1670700	1596400	502290	1029700	785270	0	0	541120	0	200370	143240
TRINITY_DN6021_c0_g1_i1_3	0	0	0	0	0	0	0	0	0	2681900	2307400	2456300
TRINITY_DN9758_c0_g1_i1_3	0	0	0	0	0	0	3330400	0	4073000	0	0	0
TRINITY_DN2208_c0_g1_i1_1	0	0	0	0	0	0	0	0	0	2211000	3319600	1857500
TRINITY_DN11722_c0_g1_i1_5	475580	302550	426890	395550	1600500	1232200	0	0	0	1093400	957850	898280
TRINITY_DN17467_c0_g1_i1_1	0	0	0	0	0	0	0	0	0	3265500	1513500	2600100
TRINITY_DN3823_c0_g1_i1_6	401930	482430	819590	790390	877510	749990	1324300	1075100	0	269720	204210	372580
TRINITY_DN11535_c0_g1_i2_3	0	0	0	0	0	0	325240	0	0	2072000	3788600	1179100
TRINITY_DN8730_c1_g1_i1_2	505790	374620	681970	798700	290040	504310	0	0	0	1172700	1707700	1326900
TRINITY_DN2631_c0_g3_i1_2	0	0	0	0	0	0	0	0	0	3951800	551220	2849100
TRINITY_DN9280_c0_g2_i4_2	0	0	0	0	0	0	0	0	0	3741800	1628800	1969500
TRINITY_DN5611_c0_g4_i1_3	0	0	0	0	0	0	0	0	0	3195900	2616800	1523500
TRINITY_DN4378_c0_g1_i2_2	131240	0	75430	0	0	0	0	0	0	3357600	2647500	1040400
TRINITY_DN4596_c0_g1_i1_5	632800	683730	610690	0	673670	0	0	4480800	0	0	143560	0
TRINITY_DN11195_c0_g1_i1_2	0	0	0	0	0	0	0	0	0	3076700	1951500	2188800
TRINITY_DN4293_c0_g1_i1_4	1412900	834010	1126400	475670	1798000	267790	0	0	0	645220	78068	561390
TRINITY_DN8155_c0_g1_i2_3	0	0	0	0	0	0	0	0	0	2780300	2229900	2187300
TRINITY_DN9942_c0_g1_i1_4	0	0	0	0	0	0	0	0	0	2684500	2560600	1946700
TRINITY_DN11521_c0_g1_i3_1	0	0	0	0	0	0	0	0	0	3162000	1756000	2257500
TRINITY_DN10407_c0_g1_i1_3	0	0	0	0	0	0	0	0	0	3007500	951710	3214600
TRINITY_DN11080_c0_g1_i1_1	0	0	0	0	0	0	0	0	0	1885200	1840800	3434400
TRINITY_DN12642_c0_g1_i1_3	268920	625250	847710	1549600	1701900	1579900	0	0	0	470650	113040	0

TRINITY_DN10964_c0_g1_i1_1	0	0	0	0	0	0	0	0	0	2908900	1886500	2338800
TRINITY_DN11121_c0_g1_i1_6	159160	0	203740	1184400	0	0	0	0	0	2962700	1309400	1314000
TRINITY_DN8201_c0_g2_i1_1	0	0	0	0	0	0	0	0	0	2486600	3006500	1629200
TRINITY_DN2407_c0_g1_i1_1	0	0	0	0	0	0	0	0	0	3310900	3420900	383590
TRINITY_DN18360_c0_g1_i1_1	422080	0	469520	2227400	1657500	1923200	0	0	0	410840	0	0
TRINITY_DN7023_c0_g1_i2_1	554730	792080	1320300	909400	2022500	813530	0	0	0	253360	196660	233390
TRINITY_DN10334_c0_g1_i2_3	0	0	0	0	0	0	0	0	0	2775800	2561900	1751400
TRINITY_DN3765_c0_g1_i1_1	1641200	1566500	1354400	0	962700	659530	0	0	0	0	462550	429710
TRINITY_DN6855_c0_g1_i1_1	0	0	0	0	0	0	0	0	0	2884900	2175500	1976900
TRINITY_DN9376_c1_g3_i1_4	0	0	0	0	0	0	0	7035600	0	0	0	0
TRINITY_DN13181_c0_g1_i1_4	0	0	0	0	0	0	0	0	0	3093300	2387100	1525300
TRINITY_DN6915_c0_g2_i2_2	524560	0	155080	3021400	2793200	142820	355420	0	0	0	0	0
TRINITY_DN8184_c1_g1_i1_1	0	183410	107420	216840	377480	181180	878810	1579800	1036600	1439300	653280	335670
TRINITY_DN19070_c0_g1_i1_2	131010	152170	132250	1200700	1420800	978950	1125900	910180	936400	0	0	0
TRINITY_DN20708_c0_g1_i1_4	0	0	0	0	0	0	0	0	0	2370700	2713500	1834900
TRINITY_DN11287_c0_g1_i2_3	0	0	0	0	0	0	0	0	0	2601800	2332100	1983000
TRINITY_DN9456_c0_g1_i4_3	243840	91431	69757	2062600	1411200	1348400	0	815420	872290	0	0	0
TRINITY_DN8791_c0_g1_i1_3	182290	217860	323070	0	0	0	0	0	874060	2489800	1731700	1080400
TRINITY_DN4375_c0_g3_i1_3	0	0	0	0	0	0	0	0	0	2956100	999370	2941300
TRINITY_DN10343_c0_g1_i4_1	0	0	0	0	0	0	0	0	0	2434700	1929000	2525100
TRINITY_DN18958_c0_g1_i1_3	0	0	0	0	0	0	0	0	6870500	0	0	0
TRINITY_DN4412_c0_g1_i1_3	326580	682720	591750	0	177120	0	0	0	0	1947500	1753100	1387300
TRINITY_DN3095_c0_g1_i2_2	1179800	127150	163580	452210	679710	450080	1090700	1370700	0	588880	752730	0
TRINITY_DN10358_c0_g1_i1_1	0	0	0	538250	348260	292050	0	0	0	1947600	2326000	1382000
TRINITY_DN16061_c0_g1_i1_3	200000	178980	433260	92303	908110	684920	0	0	0	1601900	1471800	1232100
TRINITY_DN5302_c0_g1_i1_6	3100400	1631000	424680	468610	358200	680480	0	0	0	125560	0	0
TRINITY_DN11246_c1_g4_i2_1	0	0	0	0	0	0	0	0	0	2464200	2334400	1989600
TRINITY_DN19132_c0_g1_i1_5	0	0	0	0	0	0	0	0	6781800	0	0	0
TRINITY_DN9764_c0_g1_i1_3	0	0	0	0	0	0	507250	0	0	2177800	2315800	1758000
TRINITY_DN7237_c0_g2_i1_3	1530000	1545800	1541600	898980	0	895900	0	0	0	175090	171250	0
TRINITY_DN11279_c0_g2_i3_5	0	86671	0	585070	623140	70265	0	0	302840	2134800	1952100	990250
TRINITY_DN10730_c0_g1_i1_1	0	0	0	0	0	0	0	0	0	3057900	1011600	2671400
TRINITY_DN11396_c1_g1_i3_2	0	0	0	0	0	0	0	0	0	0	0	6735900

TRINITY_DN10977_c0_g1_i1_6	0	0	0	0	0	0	1415500	0	1922900	873170	1471200	1031700
TRINITY_DN9743_c0_g2_i2_1	0	0	0	0	0	0	0	0	0	2977300	1726900	1995100
TRINITY_DN10127_c0_g1_i2_5	0	0	0	0	0	0	0	0	0	2815700	2191000	1677300
TRINITY_DN10738_c0_g1_i2_5	0	0	0	0	0	0	0	0	0	2545100	1988400	2140700
TRINITY_DN6947_c0_g1_i5_4	0	0	0	0	0	0	0	0	0	2569500	2268200	1808400
TRINITY_DN4336_c0_g1_i1_3	0	0	0	0	0	0	0	0	0	2746500	2070600	1804300
TRINITY_DN11277_c0_g1_i2_2	0	116860	0	0	0	0	0	0	0	3497900	1268600	1733200
TRINITY_DN3905_c0_g2_i2_3	0	0	0	0	60973	0	0	0	0	2642200	1979000	1929800
TRINITY_DN5130_c0_g2_i1_3	0	0	0	0	0	0	0	0	0	2640600	872640	3098100
TRINITY_DN21282_c0_g1_i1_3	466770	34346	225340	1544700	261240	539510	0	0	0	2629200	0	878770
TRINITY_DN7375_c0_g1_i1_1	0	0	0	0	0	0	0	0	0	1844200	1814900	2887100
TRINITY_DN10410_c0_g1_i1_3	0	0	0	0	0	0	0	0	0	2768200	1656800	2116600
TRINITY_DN10668_c0_g1_i2_2	192810	214420	305620	1407800	1463300	1124300	0	0	0	1116100	0	699400
TRINITY_DN1129_c0_g1_i1_2	0	0	0	0	0	0	0	0	0	2090400	3274200	1157000
TRINITY_DN9363_c0_g1_i2_2	0	0	0	0	76146	0	0	0	0	3339700	1236400	1865800
TRINITY_DN8678_c0_g3_i5_1	1006200	0	0	0	1441900	0	254300	2401000	0	0	693590	706420
TRINITY_DN9856_c0_g1_i2_6	0	0	43852	0	0	0	0	0	0	2141800	2010300	2304900
TRINITY_DN16281_c0_g1_i1_4	0	0	0	0	0	0	0	0	0	2800500	1604000	2090100
TRINITY_DN8090_c1_g1_i1_6	0	0	0	0	109490	64578	450500	1484900	1043900	1404600	823350	1098600
TRINITY_DN18348_c0_g1_i1_1	0	0	0	0	0	0	0	0	0	2873700	1418400	2182500
TRINITY_DN11641_c0_g1_i5_1	0	0	0	0	0	0	0	0	0	6454800	0	0
TRINITY_DN13961_c0_g1_i1_1	1378500	741510	4292400	0	0	0	0	0	0	0	0	0
TRINITY_DN1808_c0_g1_i1_6	0	0	0	0	0	0	0	0	0	2686500	1823800	1901600
TRINITY_DN10282_c0_g1_i6_1	0	0	0	394820	407090	306700	0	0	1203700	1312900	1465700	1307100
TRINITY_DN11194_c0_g1_i1_2	0	0	0	0	0	0	0	0	0	1569100	2071600	2753900
TRINITY_DN4777_c0_g2_i2_3	0	0	0	0	0	0	0	0	0	3517300	1142700	1734500
TRINITY_DN11197_c0_g1_i1_5	0	0	0	0	0	0	0	0	0	4287100	1043400	1046100
TRINITY_DN1802_c0_g1_i1_4	0	0	0	0	0	0	0	0	0	1992600	1625700	2757300
TRINITY_DN10578_c0_g2_i1_1	0	0	0	0	0	0	0	0	0	2641200	1387000	2329900
TRINITY_DN17430_c0_g1_i1_5	632980	639190	859840	2127700	1353700	0	0	0	0	0	735620	0
TRINITY_DN9138_c0_g1_i1_4	1128600	1055800	821170	0	0	1476900	230790	1240900	0	216980	0	172360
TRINITY_DN10772_c1_g1_i1_2	0	0	0	0	0	0	0	0	0	2645200	1685600	1969900
TRINITY_DN11439_c0_g1_i2_4	0	0	0	0	0	0	0	0	0	2884200	1735200	1676100

TRINITY_DN7730_c0_g1_i3_4	0	0	0	0	0	0	0	0	0	2732200	1703900	1858000
TRINITY_DN10562_c0_g1_i5_3	0	0	0	0	0	0	0	0	0	3149600	1336000	1807900
TRINITY_DN8163_c0_g1_i1_1	0	0	0	0	0	0	0	0	0	1893900	1399900	2989200
TRINITY_DN10420_c0_g1_i3_2	0	0	0	0	0	0	0	0	0	2337500	1512900	2418100
TRINITY_DN10122_c0_g1_i1_3	2230000	0	135320	1343300	918190	478200	0	0	0	699940	202580	236100
TRINITY_DN7204_c0_g1_i1_1	297820	343090	476730	1561600	1867800	1690200	0	0	0	0	0	0
TRINITY_DN11311_c0_g1_i2_2	946700	1139600	1188200	508740	1158200	709180	0	0	0	327390	0	251870
TRINITY_DN11251_c0_g1_i2_1	0	0	0	0	0	0	0	0	0	3046100	1421100	1754600
TRINITY_DN2678_c0_g1_i1_1	0	0	0	0	0	0	0	0	0	2849900	2263600	1104400
TRINITY_DN9042_c0_g1_i1_3	0	0	0	0	0	0	0	0	0	3259900	2016400	934430
TRINITY_DN134_c0_g1_i3_5	763160	747050	779160	1256100	951790	1608300	0	0	0	0	0	101940
TRINITY_DN7354_c1_g1_i1_2	0	1518700	1547000	821260	1641500	672750	0	0	0	0	0	0
TRINITY_DN7673_c0_g1_i1_3	0	0	0	0	0	0	0	0	0	1084100	1698900	3396500
TRINITY_DN5029_c0_g1_i1_6	0	0	0	0	0	0	0	0	0	2153100	1993100	2016500
TRINITY_DN4009_c0_g1_i2_3	0	0	0	0	0	0	0	0	0	2889100	1685600	1584300
TRINITY_DN4424_c0_g1_i3_3	0	0	0	0	0	0	0	0	0	3271300	1006700	1880100
TRINITY_DN17664_c0_g2_i1_3	832170	401280	805700	1422400	846910	847330	0	0	0	320350	238940	426430
TRINITY_DN7316_c0_g1_i1_3	0	0	0	0	0	0	0	0	0	2764200	1452000	1916600
TRINITY_DN9014_c0_g1_i1_3	0	0	0	249350	392870	175130	0	0	0	1906900	1766200	1640100
TRINITY_DN17081_c0_g1_i1_1	0	0	0	0	0	0	0	0	0	2210700	2006300	1904500
TRINITY_DN7215_c0_g1_i1_5	0	0	0	0	0	0	0	0	0	3092400	141720	2881400
TRINITY_DN11034_c0_g1_i3_3	0	0	1794500	0	2143800	0	0	0	0	819870	702840	639980
TRINITY_DN21713_c0_g1_i1_2	463650	234160	314070	1153300	1338100	1148500	0	0	0	517710	538610	390910
TRINITY_DN7461_c0_g1_i1_6	0	0	0	0	0	0	0	0	0	2154000	2104100	1836400
TRINITY_DN10238_c0_g2_i1_1	0	0	0	0	0	0	0	0	0	3586800	919120	1569700
TRINITY_DN2462_c0_g2_i1_2	838570	47105	585610	0	0	708800	1774800	1657400	0	0	0	424380
TRINITY_DN16740_c0_g1_i1_5	0	0	0	0	0	0	0	0	0	2500100	2269800	1261000
TRINITY_DN7713_c0_g1_i1_6	119550	0	190890	1885700	1938800	1834100	0	0	0	0	0	0
TRINITY_DN7723_c0_g1_i2_2	151810	172980	120750	0	183010	0	2017000	1112800	0	827740	514030	867010
TRINITY_DN10456_c0_g1_i4_1	831240	458050	894370	454640	0	392570	0	951040	1212100	620140	0	140910
TRINITY_DN10811_c0_g1_i1_2	138220	179650	380870	0	1402700	0	0	0	0	1808700	2027300	0
TRINITY_DN10241_c0_g2_i1_1	0	0	0	0	0	0	0	0	0	1902700	2642900	1388700
TRINITY_DN10843_c0_g2_i2_2	0	0	0	0	0	0	0	0	0	3498500	782450	1613300

TRINITY_DN888_c0_g1_i1_1	0	0	0	0	0	0	0	0	0	2729500	1475500	1687600
TRINITY_DN9357_c0_g1_i1_4	0	0	5881100	0	0	0	0	0	0	0	0	0
TRINITY_DN10551_c0_g1_i1_3	0	0	0	0	0	0	0	0	0	3535600	1270800	1058900
TRINITY_DN9412_c0_g1_i1_2	0	313200	273910	286050	158940	0	0	0	0	1742200	2411500	676020
TRINITY_DN14016_c0_g2_i1_2	0	0	0	0	0	0	0	0	0	2359900	2077400	1418900
TRINITY_DN5499_c0_g1_i1_3	24337	0	0	0	249000	308030	0	0	0	2378900	1280700	1570300
TRINITY_DN10701_c0_g1_i2_1	0	0	0	0	0	0	0	0	0	2260700	1695800	1835900
TRINITY_DN5059_c0_g1_i1_5	0	0	0	0	0	0	0	0	0	1717100	1408900	2664200
TRINITY_DN6933_c0_g1_i1_2	120140	186630	169460	0	0	125580	231920	0	0	1767100	1588100	1589600
TRINITY_DN10774_c0_g1_i1_1	0	0	0	0	0	0	0	0	0	3259800	1449500	1069200
TRINITY_DN10849_c0_g1_i2_2	0	0	0	0	0	0	0	0	0	0	5762000	0
TRINITY_DN10042_c0_g1_i2_2	0	0	0	0	0	0	0	0	0	3366200	1520100	868450
TRINITY_DN8175_c0_g1_i1_2	0	0	0	0	0	0	0	0	0	1963500	2049700	1714300
TRINITY_DN11149_c0_g1_i1_3	472720	721340	746930	254740	295250	532430	408060	0	0	1095000	653430	543580
TRINITY_DN14909_c0_g1_i1_3	0	0	0	0	0	0	0	0	0	3007800	1752400	961480
TRINITY_DN6906_c0_g1_i2_2	0	0	0	0	0	0	0	0	0	2106100	2262500	1314800
TRINITY_DN4515_c0_g2_i1_2	485420	556410	945410	980610	834760	1550000	0	0	0	0	0	306710
TRINITY_DN11230_c0_g2_i7_6	0	0	0	0	0	0	0	0	0	3176700	0	2481300
TRINITY_DN19409_c0_g1_i1_3	0	0	0	0	0	0	0	0	0	1341700	3042700	1247700
TRINITY_DN10897_c0_g1_i1_1	932860	602370	667040	384870	827050	977590	0	0	0	421070	433700	376190
TRINITY_DN7208_c0_g1_i1_2	0	0	0	0	0	0	1052300	1302300	3260300	0	0	0
TRINITY_DN7184_c0_g1_i2_3	0	0	0	0	0	0	0	0	0	3070500	2530900	0
TRINITY_DN6124_c0_g1_i2_1	0	0	0	0	0	0	0	0	0	2056700	2082200	1455200
TRINITY_DN17685_c0_g1_i1_2	0	0	0	0	0	0	0	0	0	2094500	1817400	1664600
TRINITY_DN10323_c0_g3_i1_1	0	0	0	0	0	0	0	0	0	2095500	2370800	1087800
TRINITY_DN9450_c0_g1_i1_2	0	0	0	0	0	0	0	0	0	2692700	808590	2052400
TRINITY_DN11814_c0_g1_i1_6	0	0	0	0	0	0	0	0	0	2910000	1389100	1251000
TRINITY_DN22082_c0_g1_i1_3	51394	72232	78425	0	0	0	0	0	2217200	1680400	1387200	0
TRINITY_DN7979_c0_g1_i2_2	3381000	0	120280	169980	0	1784700	0	0	0	0	0	0
TRINITY_DN1283_c0_g1_i1_3	0	0	0	1818200	1984200	1643100	0	0	0	0	0	0
TRINITY_DN9248_c0_g1_i2_3	0	340000	312140	0	521680	0	0	0	0	2507300	887230	870720
TRINITY_DN10710_c2_g1_i2_2	45450	93857	69022	210830	881340	482460	0	0	392410	1690600	950340	617410
TRINITY_DN5831_c1_g1_i1_5	0	0	0	0	0	0	0	0	0	1253700	2284400	1895300

TRINITY_DN8425_c0_g1_i1_1	0	0	0	0	0	0	0	0	0	2163200	1631600	1610600
TRINITY_DN1080_c0_g1_i1_2	0	0	0	0	0	0	0	0	0	2167800	1317000	1889000
TRINITY_DN10249_c0_g1_i1_1	445880	389910	617430	372240	369020	315470	0	1182100	1335400	191270	140790	0
TRINITY_DN2941_c0_g1_i1_2	0	0	0	0	0	0	0	0	0	1720000	2535300	1101200
TRINITY_DN17262_c0_g1_i1_3	0	0	0	0	0	0	0	0	0	2721400	1870400	761880
TRINITY_DN6331_c0_g1_i2_6	0	0	0	0	131960	90690	0	0	0	2138700	1249900	1738700
TRINITY_DN9436_c0_g1_i2_2	0	0	0	0	324390	0	0	324150	0	1909600	1542900	1246600
TRINITY_DN11363_c0_g2_i3_3	0	0	0	0	0	0	0	0	0	2524800	1701300	1117700
TRINITY_DN16910_c0_g1_i1_4	315310	343370	471710	1507400	1392700	1307400	0	0	0	0	0	0
TRINITY_DN791_c0_g1_i1_6	0	0	0	0	0	0	0	0	0	3092800	903970	1339600
TRINITY_DN12144_c0_g1_i1_4	0	0	0	0	0	0	0	0	0	2787500	968570	1571400
TRINITY_DN3683_c0_g2_i2_1	0	0	35562	0	0	0	0	0	0	1387800	1999200	1901600
TRINITY_DN10116_c1_g2_i1_2	0	0	0	0	0	0	0	0	0	2651200	1815400	857400
TRINITY_DN4525_c0_g1_i1_2	0	0	0	0	0	0	0	0	0	1283500	2542000	1467300
TRINITY_DN5136_c0_g1_i1_3	0	0	0	0	0	0	0	0	0	1620000	2430200	1221500
TRINITY_DN7885_c0_g1_i8_1	0	0	0	0	0	0	0	0	0	2399100	1305500	1549900
TRINITY_DN6908_c0_g1_i1_6	63119	0	58708	228930	339170	239480	0	1161700	0	1035700	626000	1497500
TRINITY_DN6313_c0_g1_i1_1	0	0	0	288060	219340	607960	1379600	0	927190	1021800	0	798450
TRINITY_DN8703_c0_g1_i1_3	0	0	0	0	0	0	0	0	0	1804900	1717500	1715900
TRINITY_DN8590_c0_g1_i1_2	0	0	0	0	0	0	1254900	2691100	1290500	0	0	0
TRINITY_DN8759_c0_g1_i1_1	0	0	0	208160	0	0	0	0	0	1568900	1778600	1678200
TRINITY_DN6356_c0_g1_i1_3	75439	110120	0	391410	369820	0	0	0	0	1612700	1474300	1184600
TRINITY_DN9997_c0_g1_i1_5	0	0	0	0	0	0	0	0	0	2433100	2019200	757190
TRINITY_DN10922_c0_g1_i1_2	0	0	0	0	0	0	0	0	0	2247300	1640500	1290100
TRINITY_DN18757_c0_g1_i1_1	0	0	0	0	0	0	0	0	0	1363000	2286600	1522400
TRINITY_DN11571_c4_g3_i5_2	147250	0	0	0	0	0	0	0	0	2113500	1714200	1161200
TRINITY_DN7455_c0_g1_i3_2	0	0	0	0	0	0	0	0	0	2261800	1407800	1459900
TRINITY_DN7736_c0_g1_i2_1	0	0	0	0	0	0	0	0	0	1820400	1643900	1660100
TRINITY_DN6430_c0_g1_i2_2	0	0	0	0	0	0	0	0	0	3384800	728560	1004400
TRINITY_DN11913_c0_g1_i1_2	609780	532300	1446100	773750	870030	676310	0	0	0	202770	0	0
TRINITY_DN11663_c2_g1_i1_3	0	0	0	0	0	0	0	0	0	1891400	1121000	2085300
TRINITY_DN5983_c0_g1_i1_2	0	0	0	0	0	0	0	0	0	1707300	1315400	2073000
TRINITY_DN8699_c1_g1_i2_2	0	0	0	0	0	0	0	0	0	5078500	0	0

TRINITY_DN6592_c0_g1_i1_1	0	0	0	0	0	0	0	0	0	2157000	1156600	1760200
TRINITY_DN10388_c0_g2_i6_4	0	0	0	0	0	0	2463400	0	0	1056000	942510	611260
TRINITY_DN11021_c0_g1_i1_2	0	0	0	0	0	0	0	0	0	1913300	1768500	1383200
TRINITY_DN3815_c0_g1_i1_1	0	0	0	0	0	0	0	0	0	2204700	1924300	922670
TRINITY_DN10218_c0_g1_i2_2	0	0	0	0	0	0	0	0	0	943330	2828500	1277900
TRINITY_DN6921_c0_g1_i1_1	0	0	0	0	0	0	0	0	0	2209300	1899800	919030
TRINITY_DN8720_c0_g1_i1_2	0	0	28226	0	421490	0	0	0	0	2141400	876930	1559700
TRINITY_DN8463_c0_g2_i1_5	0	0	0	0	0	0	0	0	0	1374600	1917500	1728300
TRINITY_DN9325_c0_g2_i1_5	0	0	0	0	0	0	0	0	0	2150200	1596600	1264400
TRINITY_DN10162_c0_g1_i1_5	0	0	0	0	0	0	0	0	0	1514100	1258400	2233000
TRINITY_DN9738_c1_g1_i1_3	552720	0	0	611540	603130	0	0	0	0	1206400	1075000	955430
TRINITY_DN10335_c0_g1_i1_1	0	0	0	0	218500	174770	0	0	0	2182800	2426300	0
TRINITY_DN4334_c0_g1_i1_3	1670400	289430	699370	0	731550	0	0	0	0	716990	258500	631740
TRINITY_DN11667_c0_g1_i5_1	0	0	0	0	0	0	0	0	0	1825600	1657700	1514200
TRINITY_DN21463_c0_g1_i1_5	0	0	0	0	0	0	0	0	0	1212700	2332300	1445800
TRINITY_DN9495_c0_g1_i1_2	0	0	0	0	0	0	0	0	0	1993500	1518500	1468300
TRINITY_DN8648_c0_g1_i4_3	0	0	0	0	0	0	0	0	0	991130	1502400	2467100
TRINITY_DN20196_c0_g1_i1_2	0	0	0	0	0	0	0	0	0	1998900	1280400	1677100
TRINITY_DN369_c0_g2_i1_2	474110	434430	536140	792230	858970	387650	0	0	0	200720	527890	740020
TRINITY_DN4975_c0_g2_i1_1	0	0	0	0	0	0	0	0	0	1380900	1878600	1692400
TRINITY_DN10898_c0_g1_i1_3	0	0	0	0	0	0	0	0	0	3564300	0	1379200
TRINITY_DN19644_c0_g1_i1_6	0	0	0	0	0	0	1205900	1860900	1876000	0	0	0
TRINITY_DN10905_c1_g1_i1_1	53887	63738	0	0	228180	0	0	0	0	2074000	2002000	510290
TRINITY_DN11102_c1_g1_i1_1	0	0	0	0	0	0	0	2652500	2274600	0	0	0
TRINITY_DN8311_c0_g1_i1_6	939620	792790	1264200	484870	417790	0	0	0	0	344430	293850	386800
TRINITY_DN8932_c0_g2_i2_2	0	0	0	0	0	0	0	0	0	2184800	1917600	818590
TRINITY_DN7024_c0_g1_i1_2	372300	580700	490420	181830	0	475800	0	0	0	537810	781750	1495300
TRINITY_DN5823_c0_g1_i1_2	0	0	0	0	0	0	0	0	0	1402300	2370500	1142200
TRINITY_DN7700_c0_g1_i1_1	0	0	0	0	0	148990	2156700	2600300	0	0	0	0
TRINITY_DN5993_c0_g2_i1_6	0	560990	164390	640820	643110	534840	944430	863240	0	216890	190860	144800
TRINITY_DN9585_c0_g1_i1_2	50778	0	0	670970	254030	64426	330430	0	0	1087000	1470600	975720
TRINITY_DN10250_c0_g1_i1_4	1252200	1391100	1593500	279470	374810	0	0	0	0	0	0	0
TRINITY_DN11946_c0_g1_i1_6	0	0	0	0	0	0	0	0	0	1895100	2521300	433890

TRINITY_DN6080_c0_g1_i1_2	0	0	0	0	0	0	0	0	0	2153200	1206600	1437700
TRINITY_DN7401_c0_g1_i1_3	0	0	0	0	0	0	0	0	0	2238000	1334200	1219800
TRINITY_DN2864_c0_g1_i1_4	0	0	0	0	0	0	0	0	0	2182500	1062000	1534200
TRINITY_DN9953_c0_g1_i1_3	159740	343750	286780	0	0	0	0	0	0	1986800	1040400	950550
TRINITY_DN4450_c0_g2_i1_2	1165300	1526900	1339800	0	0	0	0	0	0	0	721430	0
TRINITY_DN18876_c0_g1_i1_5	0	0	0	0	0	0	0	0	0	1613900	1594600	1540400
TRINITY_DN10281_c0_g1_i1_1	0	0	0	0	0	0	0	0	0	1673500	1647400	1419300
TRINITY_DN7840_c0_g1_i1_3	0	0	0	0	0	0	0	0	0	1586000	1271400	1871500
TRINITY_DN8316_c0_g1_i1_3	0	0	0	0	0	0	0	0	0	2000400	1218500	1504500
TRINITY_DN15493_c0_g1_i1_5	0	0	0	0	0	0	0	0	0	2253400	1722900	743840
TRINITY_DN8497_c0_g1_i1_2	0	0	0	0	0	0	0	0	0	1987900	1397200	1333400
TRINITY_DN9872_c0_g1_i2_5	0	0	0	0	0	0	0	0	0	2570800	0	2113700
TRINITY_DN5492_c0_g2_i1_3	0	0	0	0	0	0	0	0	0	1984800	1655100	1036400
TRINITY_DN10815_c0_g2_i1_3	576780	614040	912950	1002700	647820	511830	0	0	0	185940	218270	0
TRINITY_DN9629_c0_g1_i1_1	468720	461010	885150	487850	1039400	886730	0	0	0	237770	197810	0
TRINITY_DN9337_c0_g1_i3_1	0	0	0	0	0	0	0	0	0	1473200	2024900	1154300
TRINITY_DN2350_c0_g1_i1_3	0	0	109610	0	2455700	1320500	0	0	746070	0	0	0
TRINITY_DN4704_c0_g1_i1_2	0	0	0	0	0	0	0	0	0	1666900	1619600	1334600
TRINITY_DN10796_c0_g1_i2_5	0	0	0	0	0	0	0	0	0	1789500	1212200	1610800
TRINITY_DN9670_c0_g1_i1_4	0	0	0	0	0	0	1293100	0	3307700	0	0	0
TRINITY_DN2761_c0_g2_i1_2	0	0	0	0	0	0	0	0	0	1654500	1487400	1444400
TRINITY_DN7182_c0_g1_i2_2	0	0	0	0	0	0	0	0	0	913650	496020	3175800
TRINITY_DN11525_c0_g1_i1_2	0	0	0	0	0	0	0	0	0	2716000	922810	940880
TRINITY_DN4779_c0_g2_i1_1	0	0	0	0	0	0	0	0	0	1672700	1712800	1184100
TRINITY_DN11487_c0_g2_i1_1	0	0	0	0	0	0	0	0	0	1683800	1534800	1343200
TRINITY_DN11353_c0_g1_i2_3	0	0	0	0	0	0	0	0	0	1926300	955170	1671200
TRINITY_DN4833_c0_g1_i1_1	0	0	0	0	0	0	0	0	0	1892600	909450	1748900
TRINITY_DN7188_c0_g2_i3_2	0	0	0	0	0	0	0	0	0	1581500	1497800	1462300
TRINITY_DN10558_c0_g1_i2_4	0	0	0	0	0	0	0	0	0	2597600	803210	1123100
TRINITY_DN10992_c0_g1_i1_2	0	0	0	0	0	0	0	0	0	1431900	1797100	1279500
TRINITY_DN23494_c0_g1_i1_6	441160	507820	0	542660	682540	452270	0	0	0	913100	824330	142830
TRINITY_DN6209_c0_g1_i3_2	0	0	0	0	0	0	0	0	0	1236300	1036200	2231000
TRINITY_DN1928_c0_g1_i2_3	2054100	0	1953700	163620	156090	158160	0	0	0	0	0	0

TRINITY_DN22312_c0_g1_i1_4	0	0	21293	950180	584620	1328700	0	0	806630	423500	361890	0
TRINITY_DN137_c0_g1_i1_1	0	0	0	0	0	0	0	0	0	1558800	1501900	1409700
TRINITY_DN7231_c0_g1_i1_1	869330	747340	823860	673300	707810	642820	0	0	0	0	0	0
TRINITY_DN8793_c0_g1_i1_4	0	0	0	0	0	0	0	0	0	1686400	1379900	1396200
TRINITY_DN16084_c0_g1_i1_4	0	0	0	0	0	0	0	0	0	0	1801900	2652400
TRINITY_DN10831_c0_g1_i1_3	533340	417290	398220	1003300	791060	752110	0	0	0	558340	0	0
TRINITY_DN15011_c0_g1_i1_2	0	0	0	0	0	0	0	0	0	2283900	1386100	782300
TRINITY_DN11284_c0_g2_i1_3	0	127450	377330	827040	930110	408350	0	0	0	1113500	666400	0
TRINITY_DN18948_c1_g1_i1_2	0	0	0	0	0	0	0	0	0	1731600	1355300	1351000
TRINITY_DN11157_c0_g2_i3_3	0	0	0	0	0	0	0	0	0	1065500	1616300	1749800
TRINITY_DN2503_c0_g2_i1_1	0	99218	151690	606710	690360	578840	0	0	0	696560	1058600	543740
TRINITY_DN22430_c0_g1_i1_3	738460	0	0	0	0	722570	845250	981120	930090	202190	0	0
TRINITY_DN9526_c1_g1_i1_2	0	0	0	0	0	0	0	0	0	1332800	2065200	1020200
TRINITY_DN11455_c0_g1_i6_2	0	0	0	0	0	0	0	0	0	1618600	1406000	1391000
TRINITY_DN10158_c0_g1_i1_1	0	0	0	0	0	0	0	0	0	240960	2533600	1634400
TRINITY_DN3458_c0_g1_i1_4	0	0	0	1865600	1343700	1197400	0	0	0	0	0	0
TRINITY_DN11286_c0_g1_i3_2	0	0	0	0	0	0	0	0	0	1504000	1516800	1382500
TRINITY_DN5958_c0_g2_i1_1	0	0	0	543670	622120	0	0	0	0	1286500	1115700	829890
TRINITY_DN9297_c0_g3_i2_1	0	0	0	0	192560	0	0	0	0	908440	1796100	1499400
TRINITY_DN5161_c0_g2_i1_2	0	0	0	0	0	0	0	0	0	2032400	1127400	1234700
TRINITY_DN11263_c0_g1_i1_3	0	0	0	0	188060	0	415060	0	0	2425300	751400	603870
TRINITY_DN2170_c0_g1_i1_5	0	0	0	0	0	0	0	0	0	1612300	1452000	1319000
TRINITY_DN8027_c0_g1_i1_1	0	0	0	885320	216910	929680	772620	254540	377270	379540	167700	377820
TRINITY_DN10305_c0_g1_i5_3	0	0	0	0	0	0	0	0	0	1654100	1373100	1320800
TRINITY_DN7619_c0_g2_i1_3	0	0	0	0	0	0	0	0	0	1537400	1913000	893970
TRINITY_DN10995_c0_g2_i4_3	0	0	0	0	0	0	0	0	0	1861400	1128700	1350800
TRINITY_DN10905_c0_g1_i1_4	0	0	0	357920	303850	329890	0	0	0	1073500	1236800	1037400
TRINITY_DN10393_c0_g1_i1_2	1314100	956970	874680	0	0	0	0	0	0	0	0	1191100
TRINITY_DN13635_c0_g1_i3_1	0	0	0	0	0	0	0	0	0	1094300	2454200	782080
TRINITY_DN9622_c1_g3_i1_2	386490	0	0	0	0	0	0	0	0	3941200	0	0
TRINITY_DN10585_c0_g2_i1_2	276040	537120	346690	0	1126000	1390000	0	0	0	221990	152980	266980
TRINITY_DN7164_c0_g1_i2_1	0	0	0	0	0	0	0	0	0	2352000	1133900	830340
TRINITY_DN9538_c0_g1_i1_2	0	0	0	0	0	0	0	0	0	2131800	741040	1428000

TRINITY_DN9261_c0_g1_i2_1	0	0	0	0	0	0	0	0	0	1271300	2017400	1009600
TRINITY_DN3879_c0_g1_i1_5	0	0	0	0	0	0	0	0	0	2397800	1752100	135660
TRINITY_DN74_c0_g1_i1_5	0	0	0	0	0	0	0	0	0	1637100	1390400	1257500
TRINITY_DN5513_c0_g1_i1_2	508290	796180	870330	0	0	659690	0	0	0	343580	708450	377570
TRINITY_DN7914_c0_g2_i3_5	0	0	0	0	0	0	0	0	0	2027200	1196300	1018500
TRINITY_DN1457_c0_g1_i1_1	182430	492120	674980	962470	1027400	894920	0	0	0	0	0	0
TRINITY_DN14031_c0_g1_i1_2	0	0	0	0	0	0	0	0	0	1287500	1350200	1570400
TRINITY_DN11004_c0_g1_i4_1	0	0	0	0	0	0	0	0	0	1618000	1378400	1209100
TRINITY_DN239_c0_g1_i1_4	0	0	0	0	0	0	0	0	0	2330300	0	1864000
TRINITY_DN6479_c0_g2_i1_3	0	0	0	0	0	0	0	0	0	1355300	1055500	1781200
TRINITY_DN9536_c0_g1_i1_3	0	0	0	0	0	0	0	0	0	1344400	2410700	436340
TRINITY_DN8870_c0_g1_i1_2	0	0	0	0	0	0	0	0	0	1688800	1398300	1093400
TRINITY_DN13262_c0_g1_i1_3	0	0	0	0	0	0	0	0	0	1260000	1578900	1340400
TRINITY_DN11425_c1_g1_i2_1	56608	0	0	0	0	0	0	0	0	1859700	1173100	1083500
TRINITY_DN11659_c1_g1_i1_5	0	0	0	0	0	0	0	0	0	801060	1922100	1438700
TRINITY_DN15964_c0_g1_i1_3	967270	851450	915250	0	156460	132950	0	0	1137100	0	0	0
TRINITY_DN5268_c0_g1_i1_2	0	0	0	0	0	0	0	0	0	1642800	1208700	1302800
TRINITY_DN20307_c0_g1_i1_4	0	0	0	0	93711	0	474410	0	602990	1525000	759210	698300
TRINITY_DN2136_c0_g2_i1_2	118830	132840	143110	0	449830	0	0	0	0	1116200	883500	1306900
TRINITY_DN10948_c0_g1_i6_4	0	0	0	0	0	0	0	0	0	1469100	675790	1997100
TRINITY_DN7010_c0_g2_i1_5	0	0	0	0	0	0	0	0	0	2180600	916230	1037500
TRINITY_DN18488_c0_g1_i1_2	382440	538070	982310	424210	815040	0	0	0	0	681050	157720	150940
TRINITY_DN10697_c0_g1_i5_1	0	0	0	0	0	0	0	0	0	2603700	1249900	263390
TRINITY_DN8284_c0_g1_i1_2	241010	143210	414170	0	587570	526280	0	658440	716990	170140	291180	361190
TRINITY_DN6841_c0_g1_i2_2	0	0	0	0	156890	0	545080	0	0	1619800	491070	1285200
TRINITY_DN8688_c0_g2_i1_5	0	0	0	0	0	0	0	0	0	2581300	204620	1301100
TRINITY_DN10771_c0_g1_i1_5	0	0	0	0	0	0	0	0	0	655030	1582300	1841100
TRINITY_DN8280_c0_g2_i1_2	646970	558070	270150	0	1886500	295710	419870	0	0	0	0	0
TRINITY_DN2036_c0_g2_i1_1	0	0	0	0	0	0	0	0	0	2127000	244900	1705000
TRINITY_DN8545_c0_g2_i1_1	0	0	0	0	0	0	0	0	0	1813500	936310	1309400
TRINITY_DN9929_c0_g2_i1_1	176150	199130	0	0	0	0	0	0	3301800	0	210220	164160
TRINITY_DN16390_c0_g1_i1_1	0	0	0	0	0	0	0	0	0	1575400	1299200	1176700
TRINITY_DN9968_c0_g1_i3_3	40177	0	79220	120810	0	0	0	0	0	1275100	1171800	1359900

TRINITY_DN6363_c0_g1_i2_3	0	0	190130	1187800	0	356760	0	0	0	923300	718770	665720
TRINITY_DN10584_c0_g1_i2_3	456020	600520	609490	263570	1206500	905360	0	0	0	0	0	0
TRINITY_DN10580_c0_g1_i1_3	0	0	0	0	0	0	0	0	0	1717900	1036400	1277100
TRINITY_DN320_c0_g1_i1_6	0	0	0	0	0	0	0	0	0	1941100	1116900	951930
TRINITY_DN4506_c0_g1_i1_6	0	0	0	0	0	0	0	0	0	1454100	1429000	1126700
TRINITY_DN11518_c0_g3_i1_3	0	0	0	0	0	0	0	0	0	435820	2106800	1461100
TRINITY_DN8574_c0_g2_i2_3	0	0	0	0	0	0	0	0	0	2739200	1051700	201240
TRINITY_DN7917_c0_g1_i3_2	0	0	0	293690	0	248390	1837200	1171700	0	187240	109250	144030
TRINITY_DN11331_c0_g10_i5_1	0	0	0	0	0	0	0	0	0	1292000	1034400	1664100
TRINITY_DN305_c1_g1_i1_2	0	0	0	0	0	0	0	0	0	1704200	1172100	1112500
TRINITY_DN10976_c0_g1_i2_2	0	0	0	0	0	0	0	0	0	1797900	473280	1707600
TRINITY_DN4215_c0_g2_i1_1	0	0	3977400	0	0	0	0	0	0	0	0	0
TRINITY_DN11518_c0_g1_i3_1	0	0	0	0	0	0	0	358530	0	1333800	909320	1367000
TRINITY_DN8989_c0_g2_i2_3	0	0	0	0	0	0	0	0	0	1527700	1248300	1187800
TRINITY_DN18517_c0_g1_i1_6	0	171180	0	303490	482070	275510	0	0	0	1967200	225420	533610
TRINITY_DN3918_c0_g1_i1_3	0	0	0	0	0	0	0	0	0	1733800	1271000	943020
TRINITY_DN8387_c0_g1_i1_3	0	0	0	0	0	0	0	0	0	2161500	759250	1024500
TRINITY_DN8291_c1_g1_i2_1	0	0	0	0	0	0	0	0	0	2065000	1672200	204540
TRINITY_DN4038_c0_g2_i1_1	0	0	0	0	0	0	0	0	0	1522300	1380700	1009400
TRINITY_DN8117_c0_g1_i1_1	0	168170	320740	515810	856980	0	0	0	0	905570	472220	672040
TRINITY_DN11962_c0_g1_i1_4	0	0	0	0	0	0	0	0	0	1849500	968830	1083600
TRINITY_DN10349_c0_g1_i2_2	0	0	0	0	0	0	0	0	0	1086700	1612400	1202700
TRINITY_DN4294_c0_g2_i1_3	108800	136930	252060	1198000	899980	1298800	0	0	0	0	0	0
TRINITY_DN10817_c0_g2_i5_3	0	0	0	0	0	0	0	0	0	1608000	669900	1612600
TRINITY_DN15272_c0_g1_i1_6	0	0	0	0	0	0	0	0	0	1233700	1025900	1625900
TRINITY_DN2207_c0_g1_i1_5	85072	0	0	0	0	74856	0	0	0	1106100	814920	1798700
TRINITY_DN10776_c0_g2_i2_1	0	0	0	148580	170090	160290	0	0	0	1926200	551420	918530
TRINITY_DN9409_c0_g1_i1_2	0	0	0	213870	760840	383570	0	0	0	818400	504620	1178500
TRINITY_DN4376_c0_g1_i3_3	0	0	0	0	0	0	0	0	0	1493700	881710	1470700
TRINITY_DN13346_c0_g1_i1_4	117400	125910	131610	266260	173570	231570	0	0	0	1300500	642090	828170
TRINITY_DN19634_c0_g1_i1_3	0	0	2146900	0	1669900	0	0	0	0	0	0	0
TRINITY_DN8342_c0_g2_i2_2	0	0	0	0	0	0	0	0	0	1706900	350860	1757700
TRINITY_DN8274_c0_g2_i2_2	514780	501200	534690	636580	457120	466430	0	0	0	697610	0	0

TRINITY_DN11230_c0_g2_i11_6	0	0	21432	596250	0	655540	770880	0	717970	581660	0	458610
TRINITY_DN6850_c0_g1_i1_2	243290	178200	434400	806920	1576000	277450	0	0	0	282680	0	0
TRINITY_DN4513_c0_g1_i1_3	0	0	0	0	0	0	0	0	0	1527100	1340900	928360
TRINITY_DN11374_c0_g1_i7_3	0	0	0	0	0	0	0	0	0	1909900	960510	922010
TRINITY_DN11011_c0_g1_i3_3	0	0	0	0	0	0	690710	1108700	1979400	0	0	0
TRINITY_DN8107_c0_g1_i1_3	0	0	0	0	0	0	0	0	0	1721600	687710	1350500
TRINITY_DN9485_c0_g1_i2_4	0	0	0	0	0	0	0	3757300	0	0	0	0
TRINITY_DN7556_c0_g1_i1_1	207710	423630	407160	276030	267580	267640	0	0	0	1182600	342950	374790
TRINITY_DN14011_c0_g1_i1_1	0	0	0	0	0	0	0	0	0	1452800	1282900	1012600
TRINITY_DN8957_c0_g1_i1_2	0	51455	0	0	0	0	0	1162900	1189200	0	510560	827620
TRINITY_DN11390_c1_g1_i2_2	0	0	0	0	0	0	0	0	0	2106700	0	1634900
TRINITY_DN17748_c0_g1_i1_5	0	0	0	0	0	0	0	0	0	2004800	1718700	0
TRINITY_DN6342_c0_g1_i2_3	0	0	0	0	0	0	0	0	0	1665500	1428800	621560
TRINITY_DN11664_c0_g1_i2_3	166530	129600	0	321670	129810	104990	471880	629960	0	382950	727390	650660
TRINITY_DN9943_c0_g1_i2_6	0	0	0	0	0	0	0	0	0	1648400	1014700	1048700
TRINITY_DN13988_c0_g1_i1_6	0	0	0	0	0	0	0	0	0	1732600	841110	1122900
TRINITY_DN19538_c0_g1_i1_4	627360	927580	1002900	313640	0	0	0	384270	438980	0	0	0
TRINITY_DN10766_c0_g1_i8_2	0	0	0	0	0	0	0	0	0	1538100	1012200	1140300
TRINITY_DN11385_c0_g1_i2_1	721020	0	0	0	1430400	665100	0	0	0	600820	272460	0
TRINITY_DN10112_c0_g1_i1_1	0	0	0	0	0	0	0	0	0	1686400	683790	1308500
TRINITY_DN7707_c0_g1_i1_4	132270	248780	237410	70618	0	0	0	0	0	1402100	348700	1234000
TRINITY_DN15157_c0_g1_i1_5	0	0	0	332260	324460	0	318920	0	0	920850	1100700	676380
TRINITY_DN1898_c0_g1_i1_4	0	0	0	0	0	0	0	0	0	1535100	1046200	1082000
TRINITY_DN12752_c0_g1_i1_1	0	0	0	0	0	0	0	0	0	1213700	1066800	1382200
TRINITY_DN10808_c0_g1_i2_1	0	0	0	0	0	0	0	0	0	1394500	662400	1603500
TRINITY_DN10486_c0_g1_i1_1	22768	208180	258590	0	295160	0	0	0	0	2025900	411600	437710
TRINITY_DN1527_c0_g1_i1_2	0	0	0	0	0	0	0	0	0	1683700	1299700	671690
TRINITY_DN19189_c0_g1_i1_3	0	0	0	0	0	0	0	0	0	1138000	992960	1514600
TRINITY_DN8363_c0_g1_i1_2	0	0	0	0	0	0	0	0	0	1163300	1278600	1199100
TRINITY_DN11604_c0_g1_i7_2	380800	351010	455240	731890	983410	736090	0	0	0	0	0	0
TRINITY_DN4772_c0_g1_i1_2	0	0	0	0	0	0	0	0	0	1923500	626560	1087200
TRINITY_DN8086_c0_g1_i1_2	0	0	141530	0	0	3042500	0	0	0	448550	0	0
TRINITY_DN3945_c0_g1_i1_3	0	0	0	0	0	0	0	0	0	1024800	0	2606500

TRINITY_DN9766_c0_g2_i1_3	0	0	0	0	0	0	0	0	0	2186500	771270	668650
TRINITY_DN8049_c0_g1_i1_2	0	0	0	0	0	0	0	0	0	1993800	1090600	537100
TRINITY_DN11285_c0_g1_i5_6	959680	659800	774560	634070	0	0	0	0	0	564890	0	0
TRINITY_DN9993_c0_g1_i4_4	0	0	0	0	0	0	0	0	0	1345900	1254000	989720
TRINITY_DN18979_c0_g1_i1_4	0	0	0	0	288770	0	225030	209080	0	734560	930090	1192500
TRINITY_DN9241_c0_g1_i1_2	0	101240	0	350250	1184600	0	0	0	0	920430	310560	701560
TRINITY_DN17053_c0_g1_i1_3	0	0	0	431970	230440	239580	2393200	0	0	265660	0	0
TRINITY_DN8570_c0_g1_i2_1	0	0	0	0	0	0	1198600	0	1444600	306950	163320	429300
TRINITY_DN11109_c0_g2_i3_1	0	0	0	0	0	0	0	0	0	1746500	1170000	620980
TRINITY_DN23174_c0_g1_i1_1	0	0	0	0	0	0	0	0	0	1347800	1144000	1045100
TRINITY_DN11127_c0_g1_i1_3	0	0	0	0	0	0	0	0	0	1514400	1054400	967520
TRINITY_DN3682_c0_g1_i1_3	0	0	0	0	0	0	0	0	0	1368100	1102600	1060900
TRINITY_DN11049_c0_g1_i2_6	0	0	0	0	0	0	0	0	0	847600	1325000	1357700
TRINITY_DN4610_c0_g1_i1_3	0	0	0	0	0	0	0	0	0	1792700	1203600	520330
TRINITY_DN3669_c0_g2_i1_1	0	0	0	0	0	0	0	0	0	1357100	1036500	1121800
TRINITY_DN374_c0_g2_i1_2	0	0	0	0	0	0	0	0	0	1852100	700230	958370
TRINITY_DN1993_c0_g1_i1_1	0	0	0	0	147830	0	0	0	0	1044200	1622000	695960
TRINITY_DN11635_c2_g1_i3_2	0	0	0	0	0	0	0	3055400	0	230340	207450	0
TRINITY_DN5082_c0_g1_i1_6	625950	707000	796890	0	636450	618990	0	0	0	107120	0	0
TRINITY_DN8123_c0_g1_i1_1	0	0	309180	523920	445250	420680	0	0	0	941980	565230	275380
TRINITY_DN10102_c0_g1_i2_4	67311	0	0	630290	0	1049200	828270	489740	0	0	265270	140770
TRINITY_DN15522_c0_g1_i1_1	265930	399290	148150	643110	717350	659120	311780	0	302420	0	0	0
TRINITY_DN5853_c0_g1_i2_1	0	0	0	0	0	0	0	0	0	1560000	1075500	808610
TRINITY_DN4080_c1_g1_i1_3	0	0	0	0	0	0	0	0	0	1102600	1319200	1018300
TRINITY_DN2512_c0_g1_i1_1	0	0	0	0	0	0	0	0	0	127420	1708200	1604400
TRINITY_DN8982_c0_g1_i3_1	0	0	0	0	0	0	0	0	0	1365200	1154900	918420
TRINITY_DN5937_c0_g1_i1_6	0	0	0	0	0	100640	0	0	0	813340	1047100	1460800
TRINITY_DN10930_c0_g2_i2_1	0	0	0	0	0	0	0	0	0	319090	1649200	1450700
TRINITY_DN13030_c0_g1_i1_6	0	0	0	0	0	0	0	0	0	1147700	1304400	951580
TRINITY_DN5505_c0_g1_i1_3	311690	432550	497100	0	184530	133880	0	0	0	612970	551050	675880
TRINITY_DN11234_c0_g1_i2_1	0	0	0	0	0	0	0	0	0	1361100	1078200	956300
TRINITY_DN3811_c0_g2_i1_6	648160	889140	784850	0	0	274710	0	605340	0	0	180430	0
TRINITY_DN12438_c0_g1_i2_2	0	0	0	0	0	0	0	0	0	1316100	1070700	993640

TRINITY_DN21231_c0_g1_i1_4	0	0	0	906400	913470	971640	0	0	0	250790	0	329350
TRINITY_DN22880_c0_g1_i1_1	0	0	0	0	0	0	0	0	0	1265900	1053700	1043000
TRINITY_DN209_c0_g1_i1_6	0	0	0	0	0	0	0	3353700	0	0	0	0
TRINITY_DN11070_c0_g1_i6_2	0	0	0	0	0	0	0	0	0	1054200	469500	1825300
TRINITY_DN6751_c0_g1_i1_5	101690	0	0	797260	853840	906270	0	0	0	128090	321120	238970
TRINITY_DN9669_c0_g1_i1_5	0	0	0	1727800	250940	1146400	0	0	0	108760	107830	0
TRINITY_DN6123_c0_g1_i1_4	0	0	65345	0	181640	0	0	0	0	1705200	0	1369900
TRINITY_DN9646_c0_g1_i1_2	0	0	0	0	0	0	0	0	0	1242100	1097300	982680
TRINITY_DN3063_c0_g2_i1_5	0	0	0	0	0	0	0	0	0	1269600	1092000	954520
TRINITY_DN8427_c0_g1_i1_3	0	0	0	0	0	0	0	0	0	3313500	0	0
TRINITY_DN10246_c1_g1_i1_3	82634	194270	436690	293040	394560	1526600	0	0	0	166480	0	215460
TRINITY_DN9816_c0_g1_i2_1	0	0	0	0	0	0	0	0	0	1927100	0	1381200
TRINITY_DN6842_c0_g1_i1_5	151400	137780	236580	931220	917500	0	0	0	0	385810	338140	203130
TRINITY_DN4755_c0_g2_i1_6	0	0	0	0	0	0	0	0	0	1450600	462810	1378700
TRINITY_DN8066_c0_g1_i1_3	0	0	0	0	0	0	0	0	0	1480600	882170	914580
TRINITY_DN7303_c0_g1_i1_3	0	0	0	0	0	0	0	0	0	941100	1497300	828980
TRINITY_DN9503_c0_g1_i1_2	0	0	0	0	0	0	0	0	0	2198500	634300	431970
TRINITY_DN4985_c0_g1_i1_3	0	0	0	469130	824160	494540	0	0	0	594630	459930	420350
TRINITY_DN11329_c0_g1_i2_1	0	0	0	0	0	0	0	0	0	1980200	474980	806100
TRINITY_DN4045_c0_g1_i1_1	0	0	0	0	0	0	0	0	0	2326700	473600	458920
TRINITY_DN11005_c0_g1_i2_2	0	0	0	0	0	0	0	0	0	1403700	1298300	556270
TRINITY_DN6033_c0_g1_i1_3	0	0	0	253570	0	694950	0	0	0	1074400	691070	542860
TRINITY_DN10177_c0_g1_i1_1	0	0	0	0	0	0	0	0	0	1756100	650570	844120
TRINITY_DN2451_c0_g1_i1_2	59441	87270	47799	0	0	0	0	0	0	971600	1145500	929980
TRINITY_DN2230_c0_g2_i1_1	0	0	0	0	0	0	0	0	0	1137500	1473000	630770
TRINITY_DN6871_c0_g1_i1_3	0	0	0	0	0	0	0	0	0	895280	1231400	1106300
TRINITY_DN12604_c0_g1_i1_3	0	0	0	0	0	0	0	0	0	1385200	1417300	427270
TRINITY_DN11543_c0_g1_i1_3	0	1449100	1779300	0	0	0	0	0	0	0	0	0
TRINITY_DN8511_c0_g2_i1_6	0	0	0	0	0	0	0	0	0	890430	1255200	1082800
TRINITY_DN7492_c0_g1_i1_6	0	0	0	0	0	0	0	0	0	3225600	0	0
TRINITY_DN10983_c0_g1_i3_5	0	0	0	0	0	0	0	0	0	1492900	1575700	149160
TRINITY_DN20990_c0_g1_i1_1	569780	351560	471880	0	1255100	563430	0	0	0	0	0	0
TRINITY_DN7067_c0_g1_i1_3	0	0	0	0	0	0	0	0	0	794970	1024400	1387600

TRINITY_DN10272_c0_g1_i2_4	0	0	0	0	0	0	0	0	0	1240600	859300	1102000
TRINITY_DN7028_c0_g1_i1_3	0	0	0	0	0	69825	0	1491200	1240900	0	377870	0
TRINITY_DN9822_c0_g1_i2_3	0	0	0	0	0	0	0	0	0	1365200	0	1791400
TRINITY_DN10973_c0_g1_i2_3	0	0	0	0	0	0	0	0	0	1346400	752960	1047800
TRINITY_DN8002_c0_g1_i1_2	122910	0	0	0	496020	76999	0	1485500	0	356430	298840	306230
TRINITY_DN10262_c0_g2_i1_1	0	0	0	0	0	0	0	0	0	1365000	979120	790620
TRINITY_DN5679_c0_g1_i1_2	0	0	0	0	0	0	0	0	0	727480	1869500	530660
TRINITY_DN728_c0_g1_i2_3	0	0	0	0	0	0	0	0	0	1217600	1387600	515860
TRINITY_DN11853_c0_g1_i1_1	0	0	0	0	0	0	0	0	0	1954000	1023600	137320
TRINITY_DN11868_c0_g1_i1_5	0	0	0	0	0	0	0	0	0	1176000	1067000	865260
TRINITY_DN10119_c0_g1_i1_1	54789	0	335770	733790	1209000	0	0	0	0	0	335770	436940
TRINITY_DN2584_c0_g2_i1_3	0	0	0	777570	565280	733490	385460	418720	0	0	95126	129640
TRINITY_DN9619_c0_g2_i2_2	170020	0	237600	0	0	0	0	0	0	527580	917870	1216400
TRINITY_DN10243_c0_g1_i1_3	0	0	0	0	0	0	0	0	0	1283200	1140700	643580
TRINITY_DN17816_c0_g1_i1_6	0	1713600	1352700	0	0	0	0	0	0	0	0	0
TRINITY_DN23502_c0_g1_i1_3	0	0	0	0	0	0	0	0	0	1094500	1054600	912670
TRINITY_DN4242_c0_g1_i3_4	500620	852760	742220	0	957220	0	0	0	0	0	0	0
TRINITY_DN11128_c0_g1_i1_3	0	0	0	0	0	0	0	0	0	1517600	354850	1178100
TRINITY_DN6438_c0_g3_i1_3	0	0	0	0	0	0	0	0	0	604110	1144500	1295500
TRINITY_DN8591_c0_g1_i2_5	1081700	311840	1013300	493680	0	0	0	0	0	0	136710	0
TRINITY_DN13303_c0_g1_i1_6	0	0	0	0	0	0	0	0	0	3033200	0	0
TRINITY_DN11162_c0_g1_i3_4	0	0	0	261120	303230	245390	0	0	0	799810	550230	870780
TRINITY_DN17711_c0_g1_i1_5	0	0	0	0	0	0	0	0	0	1330100	0	1693200
TRINITY_DN7181_c0_g1_i1_6	0	0	0	0	0	0	0	0	0	2341000	670800	0
TRINITY_DN8712_c0_g1_i1_2	0	53057	0	0	130180	0	0	0	0	1632000	467670	723460
TRINITY_DN11271_c1_g2_i1_1	281700	463670	525090	460300	647740	618890	0	0	0	0	0	0
TRINITY_DN10739_c0_g1_i1_3	0	0	0	0	0	0	794570	849060	0	727710	294310	326570
TRINITY_DN11930_c0_g1_i1_6	0	0	0	0	0	0	0	0	0	1499100	381110	1104700
TRINITY_DN19914_c0_g1_i1_2	0	0	0	0	0	0	0	0	0	1110000	1379900	494150
TRINITY_DN9037_c0_g1_i2_3	0	0	0	0	0	0	0	0	0	503050	1731800	743370
TRINITY_DN9789_c0_g1_i1_2	0	0	0	0	0	0	0	0	0	1550100	1418600	0
TRINITY_DN11534_c1_g1_i2_2	0	0	0	0	0	0	0	0	0	1247600	1106700	611570
TRINITY_DN5090_c0_g1_i1_1	80593	0	68527	1123600	95836	1120400	0	0	0	229000	246320	0

TRINITY_DN11564_c1_g1_i1_3	0	57045	57144	0	0	0	0	0	0	1634600	116390	1097400
TRINITY_DN13530_c0_g1_i1_2	0	0	0	0	0	0	0	0	0	1403800	1057600	498600
TRINITY_DN11513_c0_g1_i18_2	846690	977860	0	317530	323360	494360	0	0	0	0	0	0
TRINITY_DN9031_c0_g2_i2_1	581890	137150	0	931310	709910	594220	0	0	0	0	0	0
TRINITY_DN9718_c0_g1_i1_1	364100	334210	137680	0	0	0	0	0	0	977020	459410	674730
TRINITY_DN7063_c0_g1_i1_2	0	0	0	0	0	0	0	0	0	894360	1473500	579190
TRINITY_DN22484_c0_g1_i1_1	0	0	0	0	0	0	0	0	0	917470	1050800	971600
TRINITY_DN10475_c1_g1_i1_4	150180	191010	210090	0	0	0	0	0	1402500	177060	134710	647320
TRINITY_DN11134_c0_g1_i2_1	0	0	0	0	0	0	0	0	0	964470	1306200	633690
TRINITY_DN7509_c0_g1_i1_1	0	0	0	0	0	0	0	0	0	1092000	846660	959620
TRINITY_DN15062_c0_g1_i1_1	0	0	0	0	0	0	0	0	0	1330000	1339300	218830
TRINITY_DN22190_c0_g1_i1_5	996900	361030	440670	820950	0	0	0	0	0	0	267560	0
TRINITY_DN8143_c0_g1_i3_2	0	0	0	0	0	0	0	0	0	1360000	978670	542760
TRINITY_DN8488_c0_g1_i1_3	0	0	0	0	0	0	117820	0	0	1479500	815380	465420
TRINITY_DN7081_c0_g2_i1_1	0	0	0	0	0	0	0	0	0	1080100	995970	801190
TRINITY_DN5728_c0_g1_i1_3	0	0	0	0	0	0	0	0	0	1453300	545340	878460
TRINITY_DN10017_c0_g1_i1_5	0	0	0	388200	261650	312080	0	0	0	883770	105610	923750
TRINITY_DN10253_c0_g1_i2_6	0	0	0	0	0	0	0	0	0	2039400	566650	266190
TRINITY_DN17872_c0_g1_i1_2	0	0	0	0	0	0	0	0	0	472380	1033100	1360400
TRINITY_DN20473_c0_g1_i1_1	0	0	0	0	0	0	0	0	0	984750	1225700	649210
TRINITY_DN5188_c0_g1_i2_1	0	0	0	0	0	0	0	0	0	1406500	964820	485430
TRINITY_DN3281_c0_g1_i1_6	0	0	0	0	0	0	0	1055200	1801500	0	0	0
TRINITY_DN12704_c0_g1_i1_1	0	0	0	0	0	0	0	0	0	899890	1144200	809760
TRINITY_DN11185_c0_g1_i1_1	0	0	0	0	0	0	0	0	0	1089900	636500	1123600
TRINITY_DN10615_c1_g1_i1_1	0	0	0	0	0	0	0	0	0	1025200	990370	832780
TRINITY_DN63_c0_g1_i1_3	0	0	0	0	0	0	0	0	0	672160	899400	1276000
TRINITY_DN6258_c0_g1_i2_6	0	0	0	0	0	0	0	0	0	1761100	861990	217360
TRINITY_DN6195_c0_g2_i1_2	0	0	0	0	0	0	0	0	0	2834500	0	0
TRINITY_DN6481_c0_g1_i1_1	0	0	0	0	0	0	1209900	0	1476500	0	0	147920
TRINITY_DN14793_c0_g1_i1_1	0	0	0	0	0	0	0	0	0	1079600	711020	1038900
TRINITY_DN4384_c0_g1_i1_6	669160	706700	535710	751610	0	166230	0	0	0	0	0	0
TRINITY_DN9364_c0_g1_i1_1	0	0	0	0	0	0	0	0	0	923010	1427600	477130
TRINITY_DN9162_c0_g3_i1_4	73103	102110	221450	0	704880	560200	0	0	0	556700	454240	151690

TRINITY_DN7289_c0_g1_i1_3	167160	0	0	93815	0	92990	0	0	0	525990	810750	1126100
TRINITY_DN3061_c0_g2_i1_3	992770	559800	609650	0	653760	0	0	0	0	0	0	0
TRINITY_DN10426_c0_g1_i1_5	0	0	0	0	0	0	0	0	0	1041400	1496100	276860
TRINITY_DN10788_c0_g1_i1_1	59174	0	0	546500	345380	458910	0	513600	879400	0	0	0
TRINITY_DN11653_c0_g1_i11_5	0	0	0	0	0	0	0	0	0	901040	917120	979530
TRINITY_DN8256_c0_g1_i1_1	0	0	0	0	0	0	0	0	0	1564800	609120	622410
TRINITY_DN19311_c0_g1_i1_5	0	0	0	0	0	0	0	0	0	1539400	407880	847260
TRINITY_DN11315_c0_g1_i1_2	0	0	0	0	0	0	0	0	0	1700400	542970	545890
TRINITY_DN9466_c0_g1_i1_2	0	0	0	0	0	0	0	0	0	1022800	855750	905670
TRINITY_DN10194_c0_g1_i1_3	425710	449490	547200	0	0	0	0	0	0	575890	330210	453980
TRINITY_DN6989_c0_g1_i1_2	0	0	0	0	0	0	0	0	0	1150700	1078900	549590
TRINITY_DN14960_c0_g1_i1_3	0	0	0	0	0	0	0	0	0	762120	1263900	751480
TRINITY_DN6781_c0_g2_i1_1	0	0	0	154470	0	0	0	0	0	1322000	1286000	0
TRINITY_DN4623_c0_g2_i2_1	239160	677680	799730	230900	711750	0	0	0	0	0	52029	43654
TRINITY_DN10600_c0_g1_i1_5	327180	215210	298980	0	399120	0	0	0	0	494760	100190	904450
TRINITY_DN1407_c0_g1_i2_4	0	0	0	0	0	0	0	0	0	1154000	655640	926690
TRINITY_DN2265_c0_g1_i1_5	112250	270180	331490	1179900	473700	368740	0	0	0	0	0	0
TRINITY_DN8192_c0_g2_i2_5	0	0	0	0	0	0	0	0	0	1511700	500580	717230
TRINITY_DN6164_c0_g2_i1_1	0	0	0	0	0	0	0	0	0	831110	600560	1297000
TRINITY_DN715_c0_g1_i1_6	244350	391560	320330	879800	891870	0	0	0	0	0	0	0
TRINITY_DN18948_c0_g1_i1_2	0	0	0	0	0	0	0	0	0	767260	839880	1118700
TRINITY_DN20147_c0_g1_i1_4	0	0	0	0	0	0	0	0	0	840780	671820	1207700
TRINITY_DN8171_c0_g1_i1_2	299520	0	0	0	0	601780	1292400	0	0	206230	0	316200
TRINITY_DN6538_c0_g1_i1_4	466320	0	0	0	0	0	0	0	0	0	2239600	0
TRINITY_DN6949_c0_g1_i2_3	0	0	0	0	0	0	0	0	0	1709800	121930	860860
TRINITY_DN15219_c0_g1_i1_6	0	0	0	0	0	0	0	0	0	665420	1497700	527730
TRINITY_DN11665_c0_g1_i3_2	0	0	0	0	0	0	0	0	0	1769600	150840	763120
TRINITY_DN17687_c0_g1_i1_2	199920	395310	267270	317630	643080	448910	0	0	0	207390	197230	0
TRINITY_DN9097_c0_g1_i1_2	0	0	0	0	0	0	0	0	0	646240	1703200	326200
TRINITY_DN17840_c0_g1_i1_2	0	0	0	0	0	0	0	0	0	1109500	1166500	385460
TRINITY_DN2942_c0_g1_i1_5	0	0	0	0	0	0	0	0	0	1013600	941160	706510
TRINITY_DN4861_c0_g3_i1_4	0	0	0	0	0	0	0	0	0	0	1495400	1158800
TRINITY_DN1788_c0_g2_i1_3	0	0	0	0	0	0	0	0	0	1477200	903620	272300

TRINITY_DN6718_c0_g1_i1_5	0	0	0	0	0	0	0	0	0	1303700	689850	659420
TRINITY_DN2144_c0_g2_i1_3	0	0	0	259620	0	0	0	0	0	800640	756430	834460
TRINITY_DN4927_c0_g1_i1_6	0	0	0	0	0	0	0	0	0	1026100	814900	804410
TRINITY_DN11271_c0_g1_i1_6	0	131480	362420	589970	1289700	265400	0	0	0	0	0	0
TRINITY_DN8924_c0_g1_i1_1	0	0	0	801410	949440	885620	0	0	0	0	0	0
TRINITY_DN779_c0_g1_i1_1	496950	240570	327060	782640	282030	490410	0	0	0	0	0	0
TRINITY_DN938_c0_g1_i1_5	182110	168120	175190	828230	96801	729160	0	0	0	164130	138900	130670
TRINITY_DN9792_c1_g2_i1_6	0	0	0	0	0	0	0	0	0	964580	842950	803420
TRINITY_DN9457_c0_g1_i2_1	0	0	0	0	0	0	0	0	0	934930	795550	875430
TRINITY_DN4143_c0_g1_i1_1	0	0	0	0	0	0	0	0	0	919560	892790	789090
TRINITY_DN13762_c0_g1_i1_3	0	0	0	0	0	0	0	0	0	0	1805100	782850
TRINITY_DN8478_c1_g1_i1_2	0	0	0	0	0	0	0	0	0	843960	1645700	96239
TRINITY_DN7608_c0_g1_i1_4	287440	290230	333450	199120	0	0	0	0	0	0	407630	1063200
TRINITY_DN4512_c0_g1_i1_4	1184800	742590	653260	0	0	0	0	0	0	0	0	0
TRINITY_DN9675_c0_g1_i1_1	482370	0	203890	0	0	0	0	0	0	660240	505910	723580
TRINITY_DN7474_c0_g2_i1_2	0	50144	68475	0	0	0	0	0	0	393050	1242600	816760
TRINITY_DN10982_c0_g1_i1_1	0	0	0	0	0	0	0	0	0	1394700	0	1171900
TRINITY_DN1335_c0_g1_i1_2	0	0	0	0	0	0	0	0	0	1185000	782650	596380
TRINITY_DN10530_c0_g1_i4_3	0	0	0	0	0	0	0	0	0	912430	843750	804720
TRINITY_DN8417_c0_g1_i2_2	0	0	0	0	0	0	0	0	0	449750	837850	1271100
TRINITY_DN11265_c0_g1_i1_3	0	0	0	0	0	0	0	0	0	926830	695910	928620
TRINITY_DN2984_c0_g2_i1_3	0	0	0	0	0	0	0	0	0	817890	658060	1074900
TRINITY_DN1687_c0_g1_i1_2	0	0	0	0	0	0	0	0	0	917090	817490	812930
TRINITY_DN8065_c0_g1_i1_1	0	0	0	0	0	0	0	0	0	1446100	396890	701010
TRINITY_DN11452_c0_g2_i2_2	0	0	0	0	0	126610	0	0	0	893800	677410	843400
TRINITY_DN6644_c0_g1_i1_2	0	0	136050	404460	840410	394730	0	0	760640	0	0	0
TRINITY_DN3807_c1_g1_i1_3	0	0	0	0	0	0	0	0	0	1583700	0	946430
TRINITY_DN7004_c0_g2_i1_2	0	0	0	0	0	0	0	0	0	1241300	167790	1118700
TRINITY_DN10332_c0_g2_i1_4	0	0	0	0	0	0	0	0	0	624240	892190	1010600
TRINITY_DN8478_c0_g1_i1_1	226820	154530	134300	0	0	0	127190	0	0	595800	350990	929260
TRINITY_DN11043_c0_g1_i1_6	0	0	0	0	0	0	0	0	0	0	1144600	1369000
TRINITY_DN6414_c0_g2_i2_1	0	0	0	0	223020	358730	0	791560	1140100	0	0	0
TRINITY_DN12528_c0_g1_i1_1	0	0	0	1215600	0	538360	0	0	0	562210	0	193290

TRINITY_DN2126_c0_g1_i1_4	0	0	0	0	0	0	0	0	0	1408500	763020	333410
TRINITY_DN8608_c1_g1_i1_4	1092100	770290	624910	0	0	0	0	0	0	0	0	0
TRINITY_DN10518_c0_g1_i3_1	0	0	0	0	0	0	0	0	0	987020	727540	765290
TRINITY_DN10154_c0_g1_i1_3	0	0	0	0	0	0	0	0	0	1428700	834460	208870
TRINITY_DN4990_c0_g2_i1_5	244990	381240	254570	867540	0	720500	0	0	0	0	0	0
TRINITY_DN8848_c0_g1_i1_2	0	0	0	0	0	0	0	0	0	1291600	931280	245500
TRINITY_DN2750_c0_g2_i1_2	180470	255330	222140	0	0	1803600	0	0	0	0	0	0
TRINITY_DN6078_c0_g1_i2_4	0	0	0	0	0	0	0	0	0	1022900	854830	582310
TRINITY_DN8790_c0_g2_i2_2	0	0	0	0	0	0	0	0	0	1274400	1162800	0
TRINITY_DN6367_c0_g1_i1_3	375850	314700	143070	0	0	0	0	0	0	474140	850900	272330
TRINITY_DN11511_c1_g1_i1_6	508490	0	407130	797570	715770	0	0	0	0	0	0	0
TRINITY_DN6200_c0_g1_i1_6	0	0	0	0	0	0	0	0	0	955390	752050	719980
TRINITY_DN8259_c0_g1_i1_2	632480	346440	510730	0	826090	110260	0	0	0	0	0	0
TRINITY_DN6098_c0_g1_i1_2	0	0	0	0	0	0	0	0	0	482280	1154100	786290
TRINITY_DN17635_c0_g1_i1_1	128890	0	0	0	0	0	0	0	0	716200	1576400	0
TRINITY_DN21752_c0_g1_i1_3	0	0	0	0	0	0	0	0	0	1315200	700680	405440
TRINITY_DN11371_c0_g2_i1_1	0	0	0	0	0	0	0	0	0	1180900	409910	826170
TRINITY_DN4480_c0_g1_i2_2	0	0	0	0	0	0	0	0	0	823180	712590	877170
TRINITY_DN6825_c0_g1_i1_1	0	0	0	0	0	0	0	0	0	667990	490640	1231600
TRINITY_DN15184_c0_g1_i1_6	0	0	0	0	0	0	0	0	0	1470800	485740	431720
TRINITY_DN2842_c0_g1_i1_6	98353	86739	108140	612710	443220	407770	0	0	0	346980	108460	173630
TRINITY_DN7658_c0_g1_i1_3	0	0	228690	0	0	0	0	0	0	877860	632600	643090
TRINITY_DN6555_c0_g1_i2_5	0	0	0	0	0	0	0	0	0	1009900	753070	617970
TRINITY_DN7691_c0_g1_i2_3	0	0	0	0	0	0	0	0	0	966540	579700	829060
TRINITY_DN11075_c0_g1_i1_1	0	0	0	0	0	0	0	1172200	1199900	0	0	0
TRINITY_DN8971_c0_g1_i1_4	0	0	0	0	0	0	0	0	0	1171700	486120	713710
TRINITY_DN11579_c0_g1_i2_1	0	0	0	72816	0	0	0	0	0	926830	938700	425190
TRINITY_DN3015_c0_g1_i1_1	0	0	0	0	0	0	0	0	0	1492800	869390	0
TRINITY_DN1893_c0_g1_i1_2	0	0	0	0	0	0	0	0	0	897080	786530	678370
TRINITY_DN51_c0_g1_i1_2	0	0	0	0	0	0	0	0	0	1201100	0	1154700
TRINITY_DN9907_c0_g2_i2_3	0	0	0	0	0	0	0	0	0	0	1087600	1265700
TRINITY_DN12604_c0_g1_i1_2	0	0	0	0	0	0	0	0	0	1144200	609210	596840
TRINITY_DN16223_c0_g1_i1_1	0	0	0	0	0	0	0	0	0	1589000	0	759240

TRINITY_DN1060_c0_g1_i1_1	0	0	0	0	0	0	0	0	0	1395800	605350	343850
TRINITY_DN10488_c0_g1_i1_2	0	0	0	0	0	0	0	0	0	483520	918280	932860
TRINITY_DN9024_c1_g1_i2_5	0	0	0	0	0	0	0	0	0	694810	902690	734810
TRINITY_DN10216_c0_g1_i1_3	0	0	0	0	0	0	0	0	0	1305600	573800	450930
TRINITY_DN4751_c0_g1_i1_3	0	0	0	0	0	0	0	0	0	798500	706160	823400
TRINITY_DN3627_c0_g2_i1_4	0	0	0	0	0	0	0	0	0	546640	579290	1200900
TRINITY_DN5091_c0_g1_i1_4	0	0	0	0	0	0	0	0	0	1044900	880160	386580
TRINITY_DN10763_c0_g1_i4_1	0	0	0	0	0	0	0	0	0	2303000	0	0
TRINITY_DN11635_c2_g1_i6_2	35914	0	0	0	509300	418600	0	0	0	504180	284940	549310
TRINITY_DN19987_c0_g1_i1_3	0	0	0	0	0	0	0	0	0	861670	612620	827100
TRINITY_DN10849_c0_g1_i2_5	0	0	0	0	0	0	0	0	0	0	1229100	1064400
TRINITY_DN9489_c0_g2_i1_2	0	0	0	0	0	0	1619100	655290	0	0	0	0
TRINITY_DN4080_c0_g1_i1_3	0	0	0	0	0	0	0	0	0	1750600	270070	248310
TRINITY_DN6613_c0_g1_i1_4	0	0	0	0	0	0	0	0	0	822770	760010	674160
TRINITY_DN8186_c0_g1_i3_1	0	0	0	0	0	0	0	0	0	527100	1019000	706660
TRINITY_DN11291_c0_g1_i2_3	0	0	0	0	0	0	0	0	0	626000	854220	772090
TRINITY_DN11520_c0_g1_i1_2	0	0	0	0	0	0	0	0	0	1440200	251500	547540
TRINITY_DN8996_c0_g1_i1_4	0	0	0	926440	0	865960	0	0	0	214810	229340	0
TRINITY_DN10798_c1_g1_i1_2	0	0	0	0	0	0	0	0	0	1291400	0	935490
TRINITY_DN4765_c0_g3_i1_1	0	0	0	0	0	0	0	0	0	820140	201720	1201500
TRINITY_DN8594_c0_g1_i1_1	0	0	0	0	1136800	0	0	0	0	154770	337460	592730
TRINITY_DN10973_c0_g3_i2_1	0	0	0	0	0	0	0	0	0	1201600	627250	373660
TRINITY_DN17195_c0_g1_i1_1	0	0	0	0	0	0	0	0	0	974230	743950	479980
TRINITY_DN7057_c1_g1_i1_2	0	0	0	0	0	0	0	0	0	1314000	263030	620290
TRINITY_DN23079_c0_g1_i1_4	0	0	0	0	0	0	0	0	0	863290	723280	607640
TRINITY_DN1992_c0_g1_i1_1	0	0	0	0	0	0	0	0	0	828960	697740	666920
TRINITY_DN13881_c0_g1_i1_1	409080	0	0	0	0	0	641380	797790	0	176490	0	167570
TRINITY_DN14656_c0_g1_i1_2	0	0	0	0	0	0	0	0	0	1190100	218790	777190
TRINITY_DN3861_c0_g1_i2_3	0	0	0	0	0	0	0	0	0	978160	381290	824320
TRINITY_DN8241_c0_g1_i1_2	0	0	0	0	0	0	0	0	0	78118	0	2102900
TRINITY_DN17884_c0_g1_i1_4	0	0	0	0	0	0	0	0	0	1018800	925490	233480
TRINITY_DN7822_c0_g1_i3_2	0	0	0	0	271440	354850	0	0	0	652590	428180	469660
TRINITY_DN11037_c0_g1_i1_3	0	0	0	0	0	0	0	0	0	1086300	0	1071600

TRINITY_DN5712_c0_g2_i2_1	0	0	0	0	0	0	0	0	0	552680	954900	646500
TRINITY_DN22171_c0_g1_i1_5	0	0	0	0	0	0	0	0	0	1047200	585130	519590
TRINITY_DN12955_c0_g1_i1_1	0	0	0	0	0	0	0	0	0	818630	637090	681720
TRINITY_DN10631_c0_g1_i1_4	0	0	0	0	0	0	0	0	0	1160200	324790	650150
TRINITY_DN6543_c0_g1_i1_4	0	0	0	0	0	0	0	0	0	1062300	691860	379730
TRINITY_DN10109_c0_g1_i3_2	0	0	0	0	0	0	0	0	0	849810	669930	611880
TRINITY_DN11659_c0_g1_i1_1	0	0	0	0	0	0	0	0	0	801550	717340	611730
TRINITY_DN21796_c0_g1_i1_1	0	0	0	0	0	0	0	0	0	1074500	170460	882890
TRINITY_DN10608_c0_g1_i4_3	0	0	0	0	0	0	0	0	0	675740	963170	483540
TRINITY_DN8882_c0_g1_i3_2	0	0	0	0	0	0	0	0	0	1228400	423100	469790
TRINITY_DN8417_c1_g1_i1_6	0	0	0	0	0	180590	0	0	0	899760	335340	693730
TRINITY_DN285_c0_g1_i1_6	50704	0	0	268050	274360	278760	748080	0	488980	0	0	0
TRINITY_DN8647_c0_g1_i1_4	0	0	0	0	0	0	0	0	2108300	0	0	0
TRINITY_DN1686_c0_g1_i1_2	0	0	0	0	0	0	0	0	0	1091900	1014900	0
TRINITY_DN16666_c0_g1_i1_3	0	0	0	0	0	0	0	0	0	1221800	883050	0
TRINITY_DN8134_c0_g1_i1_2	722220	455030	521520	0	0	404410	0	0	0	0	0	0
TRINITY_DN1558_c0_g1_i1_3	0	0	0	0	0	0	0	0	0	1162000	566150	373160
TRINITY_DN7263_c0_g1_i1_2	0	0	0	173460	0	0	0	0	0	661130	635880	629600
TRINITY_DN9028_c0_g1_i1_3	0	0	0	0	0	0	0	0	0	865560	570260	659310
TRINITY_DN10191_c0_g1_i1_3	72507	62936	0	269810	0	243250	0	0	0	519220	489460	435200
TRINITY_DN9202_c0_g1_i1_5	0	0	0	0	0	0	0	0	0	1192700	0	894430
TRINITY_DN3322_c0_g2_i1_2	0	0	0	0	0	0	0	0	0	668870	621860	794490
TRINITY_DN661_c0_g1_i1_1	0	0	0	126530	0	0	0	0	0	938370	413590	604630
TRINITY_DN284_c0_g2_i1_5	0	40153	103760	357940	833940	582040	0	0	0	0	165000	0
TRINITY_DN804_c0_g1_i1_1	0	0	0	248200	0	310920	0	0	0	577730	462250	472390
TRINITY_DN14006_c0_g1_i1_3	0	0	0	0	0	0	0	0	0	815800	718020	535690
TRINITY_DN21069_c0_g1_i1_4	0	0	0	0	0	0	494740	553950	0	329350	221220	469060
TRINITY_DN9015_c0_g1_i1_4	0	353250	254350	1342500	0	109460	0	0	0	0	0	0
TRINITY_DN10966_c0_g1_i1_3	0	0	0	0	0	0	0	0	0	764320	714370	579900
TRINITY_DN3062_c0_g1_i1_3	0	0	0	0	0	0	0	0	0	1052000	654020	345140
TRINITY_DN15393_c0_g1_i1_5	0	0	0	0	1013200	1032900	0	0	0	0	0	0
TRINITY_DN7397_c0_g1_i2_1	0	0	0	0	0	0	0	0	0	841530	324890	879480
TRINITY_DN843_c0_g1_i1_5	0	119270	76284	0	0	208180	731280	0	0	619130	291290	0

TRINITY_DN13608_c0_g1_i1_6	0	0	0	0	0	0	0	0	0	767510	655240	617540
TRINITY_DN3889_c0_g3_i1_3	209650	1093800	736800	0	0	0	0	0	0	0	0	0
TRINITY_DN6029_c0_g1_i1_1	0	0	0	0	0	0	0	0	0	723680	816410	499640
TRINITY_DN5896_c0_g1_i2_3	0	0	0	0	0	0	0	0	0	408190	843190	783610
TRINITY_DN12487_c0_g1_i1_1	0	0	0	0	0	0	0	0	0	0	752960	1282000
TRINITY_DN21101_c0_g1_i1_4	0	0	0	0	0	0	0	0	0	1103100	488840	441990
TRINITY_DN20395_c0_g2_i1_2	0	0	0	610790	552810	102980	0	0	0	445830	311820	0
TRINITY_DN11574_c0_g1_i5_3	626970	763570	576400	0	0	0	0	0	0	57057	0	0
TRINITY_DN16156_c0_g1_i1_2	360440	192640	208840	0	341550	0	0	0	0	341890	285840	286310
TRINITY_DN10717_c1_g1_i3_1	0	18128	0	0	811450	547350	0	0	0	0	352560	286770
TRINITY_DN11531_c0_g1_i3_3	0	0	0	0	0	0	0	0	0	1358800	270340	385610
TRINITY_DN7911_c0_g1_i3_1	279720	261800	113910	0	0	0	537790	0	635950	184280	0	0
TRINITY_DN5389_c0_g1_i1_5	0	0	0	0	0	0	0	0	0	782910	653070	577260
TRINITY_DN7964_c0_g4_i1_6	0	0	0	0	0	0	0	490640	0	763520	593700	162770
TRINITY_DN12169_c0_g1_i1_2	0	36336	73730	0	328180	0	1438200	0	0	0	128640	0
TRINITY_DN23339_c0_g1_i1_1	0	0	0	0	0	0	0	0	0	704100	686590	609780
TRINITY_DN9250_c0_g1_i1_3	0	0	0	243850	316130	479250	959520	0	0	0	0	0
TRINITY_DN18399_c0_g1_i1_1	0	0	0	0	0	0	0	0	0	523260	740960	721300
TRINITY_DN9486_c0_g1_i1_2	123890	285610	464650	583600	526730	0	0	0	0	0	0	0
TRINITY_DN5674_c0_g1_i1_1	0	0	0	0	0	0	0	0	0	579600	610540	793490
TRINITY_DN6255_c0_g3_i1_1	63878	80554	0	594710	368030	138360	0	0	0	413430	0	319390
TRINITY_DN10766_c0_g1_i3_3	0	0	0	0	0	0	0	0	0	1295500	207870	474440
TRINITY_DN10543_c0_g1_i1_6	0	0	0	0	0	0	0	0	0	832710	647740	482880
TRINITY_DN2263_c0_g2_i1_6	0	0	0	0	0	0	0	0	0	765370	591600	600560
TRINITY_DN11003_c0_g2_i5_2	0	0	0	0	854420	228940	0	0	0	0	872150	0
TRINITY_DN9687_c1_g1_i2_2	0	0	1357200	0	0	596570	0	0	0	0	0	0
TRINITY_DN9737_c1_g1_i1_4	456360	570070	679170	0	0	247960	0	0	0	0	0	0
TRINITY_DN11475_c0_g1_i1_2	894600	1056200	0	0	0	0	0	0	0	0	0	0
TRINITY_DN6747_c0_g1_i1_2	0	0	1187300	0	0	0	0	0	0	760630	0	0
TRINITY_DN2919_c0_g1_i1_5	0	0	0	0	0	0	0	0	0	1317200	0	630200
TRINITY_DN9215_c0_g1_i1_2	0	0	0	0	0	0	0	0	0	598460	927130	419980
TRINITY_DN5123_c0_g1_i2_4	0	0	0	0	0	0	0	0	0	814560	509180	616720
TRINITY_DN2276_c0_g2_i1_6	0	0	0	0	0	0	0	0	0	632840	624910	679200

TRINITY_DN8639_c0_g1_i1_6	0	0	0	0	0	0	0	0	0	579610	707350	647410
TRINITY_DN6651_c0_g1_i1_3	0	0	0	0	0	0	0	0	0	870010	738040	325820
TRINITY_DN8585_c0_g1_i1_4	0	0	0	0	0	0	0	0	0	483290	645200	803240
TRINITY_DN21541_c0_g1_i1_4	0	0	0	0	0	0	0	0	0	785410	624020	512520
TRINITY_DN8030_c0_g1_i1_2	0	0	0	0	0	0	0	0	0	1141000	780490	0
TRINITY_DN7519_c0_g2_i1_2	0	0	0	0	0	0	0	0	0	405410	599300	911230
TRINITY_DN11415_c0_g1_i1_1	0	0	0	0	0	0	1134900	0	0	318840	0	461840
TRINITY_DN9353_c0_g1_i1_3	314770	0	0	1155000	0	0	0	0	0	0	238500	206670
TRINITY_DN5812_c0_g1_i1_1	0	0	0	0	0	0	0	0	0	416390	1494600	0
TRINITY_DN11107_c1_g1_i4_3	0	0	0	0	0	0	0	0	0	1459700	100310	347110
TRINITY_DN3448_c0_g1_i1_2	0	0	0	0	0	0	0	0	0	546650	660250	699450
TRINITY_DN9452_c0_g1_i1_1	0	0	0	0	0	0	0	0	0	539250	844400	520000
TRINITY_DN8582_c0_g2_i1_3	0	0	0	0	0	0	0	0	0	1051400	657650	189600
TRINITY_DN15392_c0_g1_i1_5	0	0	0	0	0	0	0	0	0	1068800	410390	414360
TRINITY_DN2089_c0_g1_i1_5	0	0	0	0	0	0	0	0	0	617820	749290	518300
TRINITY_DN8917_c0_g2_i1_3	17013	34377	38416	143990	0	0	0	0	0	1165100	128120	357240
TRINITY_DN16117_c0_g1_i1_6	0	0	0	0	0	0	0	0	0	1018000	865500	0
TRINITY_DN8546_c0_g1_i1_2	0	0	0	0	0	0	0	0	0	152380	1080100	649660
TRINITY_DN3962_c0_g1_i2_3	0	0	0	0	0	0	0	0	0	652440	649940	573910
TRINITY_DN2992_c0_g1_i1_3	0	0	0	0	0	0	0	0	0	753810	621510	499620
TRINITY_DN11605_c1_g1_i1_5	0	0	0	0	0	0	0	0	0	1034500	0	839380
TRINITY_DN401_c0_g1_i1_3	0	0	0	0	0	0	0	0	0	697610	607600	554230
TRINITY_DN18414_c0_g1_i1_2	0	0	0	0	0	0	0	0	0	661120	0	1195300
TRINITY_DN9346_c0_g1_i2_1	0	0	0	0	0	0	0	0	0	1082600	626060	139650
TRINITY_DN8618_c0_g2_i2_4	438310	488260	413860	249100	255770	0	0	0	0	0	0	0
TRINITY_DN5167_c0_g1_i1_5	0	0	0	0	0	0	434280	0	1408900	0	0	0
TRINITY_DN15469_c0_g1_i1_6	0	0	0	0	0	0	0	0	0	1065500	0	775860
TRINITY_DN19298_c0_g1_i1_1	0	0	0	0	0	0	0	0	0	0	1008000	826340
TRINITY_DN11193_c0_g2_i3_2	0	0	0	0	0	0	0	0	0	1329700	499690	0
TRINITY_DN11599_c1_g1_i4_1	260580	407810	663510	121420	154710	0	0	0	0	221390	0	0
TRINITY_DN6675_c0_g1_i4_2	0	0	0	0	0	0	0	0	0	739270	600730	488060
TRINITY_DN9665_c0_g1_i1_2	0	0	0	0	0	0	0	0	0	714630	632490	480030
TRINITY_DN10222_c0_g1_i5_2	0	0	0	0	0	0	0	0	0	755360	327440	730370

TRINITY_DN8244_c0_g1_i1_2	81393	0	197820	0	0	0	0	0	0	0	944480	587020
TRINITY_DN6988_c0_g2_i1_1	0	0	0	0	0	0	0	0	0	430430	611540	765650
TRINITY_DN21693_c0_g1_i1_3	0	0	0	0	0	0	0	0	0	774320	401180	631170
TRINITY_DN6721_c0_g1_i1_2	0	0	0	0	0	0	0	0	0	781290	647210	378030
TRINITY_DN10523_c1_g1_i1_2	0	0	0	0	0	0	0	0	0	0	843630	962460
TRINITY_DN8754_c0_g1_i1_1	0	0	0	0	0	0	0	0	0	1099200	705720	0
TRINITY_DN21226_c0_g1_i1_1	0	0	0	0	0	0	0	0	0	351620	776870	672170
TRINITY_DN18056_c0_g1_i1_1	235420	136490	264600	0	828360	119710	0	0	0	120690	0	95014
TRINITY_DN11502_c0_g1_i1_2	379280	0	431640	158550	0	0	335800	0	0	217020	0	276760
TRINITY_DN8308_c0_g2_i1_2	150350	137970	0	711380	794880	0	0	0	0	0	0	0
TRINITY_DN5126_c0_g1_i2_2	0	0	0	334840	277350	151310	0	0	0	497540	0	532850
TRINITY_DN7908_c0_g1_i1_3	0	0	0	0	425940	220240	0	0	0	351100	271710	524890
TRINITY_DN10955_c0_g2_i3_1	236300	331700	418390	294020	243240	270080	0	0	0	0	0	0
TRINITY_DN8800_c0_g1_i1_3	0	0	0	0	0	0	0	0	0	806650	415990	570030
TRINITY_DN4365_c0_g1_i1_1	0	0	0	0	0	0	0	0	0	641840	694610	454480
TRINITY_DN14633_c0_g1_i1_2	0	0	0	0	0	0	0	0	0	1353600	356190	80093
TRINITY_DN3011_c0_g2_i1_3	0	0	0	0	0	0	0	0	0	709760	560090	519900
TRINITY_DN2343_c0_g1_i1_1	0	169050	146810	0	140250	0	267290	0	403250	181930	338270	142460
TRINITY_DN9033_c1_g1_i1_6	0	0	0	0	0	0	0	0	0	910750	865650	0
TRINITY_DN2502_c0_g2_i1_2	0	0	0	0	0	0	0	0	0	256820	781000	735710
TRINITY_DN7907_c0_g1_i4_4	0	0	0	0	0	0	0	0	0	475660	376320	920950
TRINITY_DN642_c0_g1_i1_2	0	0	0	0	0	0	457010	984640	329990	0	0	0
TRINITY_DN701_c0_g2_i1_6	456280	211850	229400	203140	0	129080	292330	0	0	131970	116730	0
TRINITY_DN6896_c0_g1_i1_1	0	0	0	0	0	0	0	0	0	717340	562210	489680
TRINITY_DN12989_c1_g1_i1_1	0	0	0	0	0	0	0	0	0	0	918520	848900
TRINITY_DN11038_c0_g1_i1_3	0	0	0	0	0	0	0	0	0	873300	260600	632410
TRINITY_DN6818_c0_g1_i1_3	498170	390600	366210	196990	156620	0	0	0	0	0	157170	0
TRINITY_DN18372_c0_g1_i1_2	0	0	0	0	0	0	0	0	0	831820	300330	631560
TRINITY_DN19362_c0_g1_i1_3	0	0	0	0	0	0	0	0	0	917200	298210	542530
TRINITY_DN13345_c0_g1_i1_4	0	0	0	0	0	0	0	0	0	626410	601020	527100
TRINITY_DN8412_c0_g1_i2_1	129990	207330	194290	0	0	0	0	0	1221600	0	0	0
TRINITY_DN15351_c0_g1_i1_3	0	0	0	564730	357790	443560	381220	0	0	0	0	0
TRINITY_DN19403_c0_g1_i1_1	0	0	0	0	0	0	0	0	0	1039300	234370	472880

TRINITY_DN4912_c0_g1_i1_3	0	0	0	0	0	0	0	0	0	764190	628560	353760
TRINITY_DN9231_c0_g1_i3_3	0	0	0	838430	907190	0	0	0	0	0	0	0
TRINITY_DN10613_c0_g1_i1_2	0	0	0	0	0	0	0	0	0	717430	584680	438040
TRINITY_DN3524_c0_g1_i1_2	0	0	0	0	0	0	0	0	0	1078900	323170	333880
TRINITY_DN3963_c0_g1_i1_1	0	0	0	0	0	0	0	0	0	772280	707060	252150
TRINITY_DN1013_c0_g2_i1_6	0	0	0	170900	165340	0	0	0	601870	378420	165970	247120
TRINITY_DN6811_c0_g2_i3_2	0	0	0	0	0	0	0	0	0	438350	740690	549970
TRINITY_DN12201_c0_g2_i1_2	104540	101640	178870	0	1049500	0	0	0	0	0	0	291940
TRINITY_DN8972_c0_g2_i1_1	0	0	0	0	0	0	0	0	0	301900	191900	1232600
TRINITY_DN4764_c0_g2_i1_1	0	0	0	0	0	0	0	0	0	463400	780790	477300
TRINITY_DN2457_c0_g1_i1_1	0	0	0	0	0	0	0	0	0	692140	496220	529220
TRINITY_DN11036_c0_g1_i5_1	0	0	0	0	0	0	0	0	0	1208200	182760	318480
TRINITY_DN10961_c0_g1_i3_3	0	0	0	0	0	0	0	0	0	779950	682400	233310
TRINITY_DN4707_c0_g3_i1_1	0	0	0	1304300	0	0	0	0	0	0	389560	0
TRINITY_DN10673_c0_g1_i2_3	0	0	0	0	0	0	0	0	0	787330	184530	719390
TRINITY_DN9408_c0_g1_i1_6	0	0	0	0	0	0	0	0	0	813090	374050	497790
TRINITY_DN1822_c0_g2_i1_4	0	0	0	0	0	0	0	0	0	675110	615420	393890
TRINITY_DN18094_c0_g1_i1_4	0	0	0	0	0	0	0	0	0	745820	584760	352700
TRINITY_DN3816_c0_g1_i1_4	0	0	0	167700	0	0	0	0	0	317570	613570	584480
TRINITY_DN10296_c0_g1_i1_1	0	0	0	0	0	0	0	0	0	0	983050	698530
TRINITY_DN5361_c0_g2_i1_1	0	0	0	0	0	0	0	0	0	1246100	223410	211230
TRINITY_DN512_c0_g1_i1_4	0	436410	448170	0	260390	207060	0	0	0	208170	59737	60113
TRINITY_DN6953_c0_g1_i1_5	0	0	0	0	0	0	0	0	0	805970	529890	343660
TRINITY_DN22332_c0_g1_i1_1	153580	336070	100460	0	0	620850	0	0	0	0	467130	0
TRINITY_DN8109_c0_g1_i2_3	392190	408670	392040	259380	0	225090	0	0	0	0	0	0
TRINITY_DN9320_c0_g1_i1_1	298530	285510	269400	726080	93108	0	0	0	0	0	0	0
TRINITY_DN3842_c0_g1_i1_3	0	0	0	0	0	0	0	0	0	957960	710530	0
TRINITY_DN19595_c0_g1_i1_5	703060	0	265120	0	0	0	0	0	0	0	696580	0
TRINITY_DN12651_c0_g2_i1_3	0	0	0	0	0	0	0	0	0	1098800	210150	349780
TRINITY_DN5781_c0_g1_i1_5	0	0	0	0	0	0	0	0	0	475830	708080	470010
TRINITY_DN14210_c0_g1_i1_2	0	0	0	0	0	0	0	0	0	393900	723590	533240
TRINITY_DN5618_c0_g1_i1_1	0	0	0	0	0	0	0	0	0	1321000	326870	0
TRINITY_DN8203_c0_g2_i1_6	0	0	0	0	0	0	0	0	0	381370	302500	963590

TRINITY_DN4496_c1_g1_i1_2	0	0	0	0	0	0	0	0	0	827000	0	816030
TRINITY_DN13442_c0_g1_i1_1	0	0	0	0	0	0	0	0	0	255380	595840	791570
TRINITY_DN7081_c1_g1_i1_1	0	0	0	0	0	0	0	0	0	471280	398870	772330
TRINITY_DN2528_c0_g1_i2_1	0	0	0	0	0	0	0	0	0	1097300	290100	251460
TRINITY_DN6839_c0_g2_i1_4	0	0	0	0	0	0	0	0	0	911690	721590	0
TRINITY_DN13687_c0_g1_i1_2	0	0	0	396460	0	432950	0	0	0	360840	199630	238330
TRINITY_DN7339_c0_g1_i1_1	0	0	0	0	0	0	0	0	0	1627900	0	0
TRINITY_DN5933_c0_g1_i3_1	0	207800	0	609060	808130	0	0	0	0	0	0	0
TRINITY_DN10134_c0_g1_i3_5	0	0	0	0	0	0	0	0	0	705220	597780	319340
TRINITY_DN5831_c0_g1_i1_3	0	0	0	0	0	0	0	0	0	276450	708350	637350
TRINITY_DN8464_c0_g1_i3_6	0	0	0	0	406380	0	0	0	0	441640	387520	383150
TRINITY_DN11887_c0_g2_i1_4	0	0	0	0	0	0	0	0	0	639280	492210	486080
TRINITY_DN3833_c0_g1_i1_2	0	0	0	0	0	0	0	0	0	0	998480	617700
TRINITY_DN10145_c0_g1_i1_3	0	0	0	0	0	0	0	0	0	0	536440	1079300
TRINITY_DN7100_c0_g1_i2_3	142820	158890	141940	239790	310080	210040	0	0	0	180390	131130	100240
TRINITY_DN23044_c0_g1_i1_1	0	0	0	0	0	0	0	0	0	612630	483270	503130
TRINITY_DN13518_c0_g1_i1_4	0	0	0	0	0	0	0	0	0	704210	316230	576290
TRINITY_DN19196_c0_g1_i1_5	408560	520670	666300	0	0	0	0	0	0	0	0	0
TRINITY_DN3817_c0_g1_i1_5	0	0	0	0	0	0	0	0	0	0	837590	754360
TRINITY_DN4458_c0_g1_i1_2	0	0	0	0	0	0	0	0	0	760340	574540	253300
TRINITY_DN7255_c0_g1_i1_3	0	0	0	0	0	0	0	0	0	516350	765620	306000
TRINITY_DN6045_c0_g1_i3_1	0	0	0	0	0	0	0	0	0	1200900	384750	0
TRINITY_DN11230_c0_g1_i2_6	888880	691990	0	0	0	0	0	0	0	0	0	0
TRINITY_DN3420_c0_g1_i2_3	0	0	0	0	224880	0	0	0	0	612620	0	733430
TRINITY_DN11146_c0_g1_i2_3	0	0	0	0	0	0	0	0	0	580620	473470	513940
TRINITY_DN9423_c0_g3_i2_1	0	0	0	0	1567800	0	0	0	0	0	0	0
TRINITY_DN9788_c0_g1_i1_3	0	0	0	0	0	0	0	0	0	1235300	331360	0
TRINITY_DN2628_c0_g2_i1_1	265420	117370	166150	374790	474490	0	0	0	0	167910	0	0
TRINITY_DN9299_c0_g2_i1_3	0	0	0	0	0	0	0	0	0	458700	338720	766250
TRINITY_DN11235_c0_g2_i1_1	0	0	0	0	0	0	0	364020	0	289050	680470	230030
TRINITY_DN5744_c1_g1_i2_2	0	0	0	0	0	0	0	0	0	774580	374800	412290
TRINITY_DN5626_c0_g2_i1_2	0	0	0	0	0	0	0	0	0	960510	94074	506720
TRINITY_DN11440_c0_g1_i3_4	0	0	0	0	0	0	0	0	0	0	399170	1159400

TRINITY_DN11350_c0_g1_i3_2	0	0	0	0	0	0	0	0	0	48648	778030	729560
TRINITY_DN11359_c0_g1_i1_2	0	0	0	0	0	0	0	0	0	733780	338390	480890
TRINITY_DN9373_c0_g1_i1_6	0	0	0	0	0	0	0	0	0	889780	0	661890
TRINITY_DN12458_c0_g3_i1_3	0	0	0	0	0	0	0	0	0	777820	194420	575580
TRINITY_DN5495_c0_g1_i2_2	0	0	0	0	0	0	0	0	0	563900	981880	0
TRINITY_DN12738_c0_g1_i1_1	0	0	0	0	0	0	0	0	0	650080	358070	535940
TRINITY_DN7345_c0_g1_i1_4	0	0	0	0	0	0	0	0	0	854770	688050	0
TRINITY_DN6917_c0_g1_i1_3	0	0	0	0	0	0	0	0	0	985500	412210	140090
TRINITY_DN9153_c0_g1_i1_3	0	0	0	0	0	0	0	0	0	896290	303290	338070
TRINITY_DN19500_c0_g1_i1_1	0	0	0	0	0	0	0	0	0	627980	478760	428570
TRINITY_DN12333_c0_g1_i1_2	0	0	0	0	0	0	0	0	0	572400	577700	379820
TRINITY_DN10789_c0_g1_i1_1	0	0	0	453380	535150	538090	0	0	0	0	0	0
TRINITY_DN2912_c0_g2_i1_4	0	0	0	0	0	0	0	0	0	1011700	322450	191830
TRINITY_DN14787_c0_g1_i1_6	0	0	0	0	0	0	0	0	0	627330	563840	334310
TRINITY_DN11096_c0_g1_i1_1	0	0	0	0	0	0	0	0	0	297770	595980	628070
TRINITY_DN5451_c0_g1_i1_6	0	0	0	0	0	0	0	0	0	458420	380110	668970
TRINITY_DN11157_c0_g2_i5_4	685920	819570	0	0	0	0	0	0	0	0	0	0
TRINITY_DN8090_c0_g3_i1_2	0	0	114280	0	109450	0	0	0	0	656220	429080	191810
TRINITY_DN5532_c0_g1_i1_3	0	0	0	0	0	0	0	0	0	251990	668740	575720
TRINITY_DN8331_c0_g2_i1_3	0	0	0	271070	0	470890	0	0	0	567960	0	179750
TRINITY_DN7840_c1_g1_i1_1	0	0	0	0	0	0	0	0	0	1027700	190120	271570
TRINITY_DN1523_c0_g1_i1_1	0	0	0	0	0	0	0	0	0	833630	241160	414320
TRINITY_DN14177_c0_g1_i1_3	0	0	0	0	0	0	0	0	0	576650	448640	462920
TRINITY_DN10869_c1_g1_i2_5	0	0	0	0	0	0	1485700	0	0	0	0	0
TRINITY_DN5473_c0_g1_i1_5	0	0	0	0	0	0	0	0	0	552510	450760	481850
TRINITY_DN21224_c0_g1_i1_2	0	0	0	0	0	0	0	0	0	953580	271580	259850
TRINITY_DN6919_c0_g2_i1_3	0	0	0	0	0	0	0	0	0	681100	390380	411830
TRINITY_DN6224_c0_g1_i1_1	0	0	0	0	0	0	0	0	0	496550	450450	535410
TRINITY_DN1121_c0_g1_i1_3	1480300	0	0	0	0	0	0	0	0	0	0	0
TRINITY_DN1480_c0_g1_i1_2	0	0	0	0	0	0	0	0	0	534060	686550	259610
TRINITY_DN12167_c0_g1_i1_5	0	0	0	0	0	0	0	0	0	624850	429110	416610
TRINITY_DN458_c0_g1_i1_5	0	0	0	0	0	0	0	0	0	510650	516090	443790
TRINITY_DN2404_c0_g1_i1_2	0	0	0	0	0	0	0	0	0	559330	516110	392460

TRINITY_DN9978_c0_g1_i1_2	447650	513480	505600	0	0	0	0	0	0	0	0	0
TRINITY_DN8843_c0_g1_i3_1	526730	0	569260	0	0	366030	0	0	0	0	0	0
TRINITY_DN6703_c0_g1_i1_2	0	0	0	0	0	0	0	0	663550	545860	0	250240
TRINITY_DN11300_c0_g1_i1_3	0	0	0	0	0	0	0	0	0	498290	373140	587120
TRINITY_DN3617_c0_g3_i1_5	0	0	0	0	0	0	0	0	0	506580	489610	459020
TRINITY_DN10446_c0_g2_i1_1	0	0	0	0	0	0	0	0	0	684810	211100	558550
TRINITY_DN14501_c0_g1_i1_3	0	0	0	0	0	0	0	0	0	0	257700	1193700
TRINITY_DN13753_c0_g1_i1_5	0	0	0	0	0	0	0	0	0	562150	395900	491370
TRINITY_DN18831_c0_g1_i1_1	0	0	0	0	291440	0	0	0	0	326490	233480	593800
TRINITY_DN7892_c0_g1_i3_1	287890	306500	245710	255640	0	344310	0	0	0	0	0	0
TRINITY_DN6230_c0_g1_i1_1	0	0	0	0	0	0	0	0	0	0	993290	440230
TRINITY_DN18774_c0_g1_i1_4	0	0	0	0	0	0	0	0	0	357070	248720	825520
TRINITY_DN1997_c0_g1_i1_3	0	0	0	0	0	0	0	0	0	920600	0	508830
TRINITY_DN7250_c0_g1_i1_5	0	0	0	0	0	0	119250	0	0	574830	350810	370470
TRINITY_DN15321_c0_g1_i1_5	193630	238750	305860	245350	238470	192560	0	0	0	0	0	0
TRINITY_DN19751_c0_g1_i1_1	0	0	0	0	0	0	0	0	0	1218400	0	195350
TRINITY_DN3119_c0_g1_i1_5	95031	27217	42047	204470	242210	0	0	0	0	276980	420240	104320
TRINITY_DN2586_c0_g2_i1_1	0	0	0	0	0	0	0	0	0	807570	604810	0
TRINITY_DN18819_c0_g1_i1_4	0	0	0	0	1079900	0	0	0	0	328220	0	0
TRINITY_DN13993_c0_g1_i1_4	0	0	0	0	0	0	0	0	0	853860	0	553860
TRINITY_DN20785_c0_g1_i1_2	117260	84948	89925	420120	541460	0	0	0	0	0	82461	71095
TRINITY_DN11337_c0_g1_i4_2	0	0	0	0	0	0	0	0	0	565600	423160	417340
TRINITY_DN8955_c0_g1_i4_2	0	0	0	0	0	0	0	0	0	576370	391710	437810
TRINITY_DN12162_c0_g1_i1_3	0	0	0	0	0	0	0	0	0	528630	477290	394440
TRINITY_DN9518_c0_g2_i2_1	0	0	0	0	0	0	0	0	0	534350	437830	425380
TRINITY_DN23667_c0_g1_i1_6	0	0	0	0	0	0	0	0	0	0	782630	611370
TRINITY_DN15391_c0_g1_i1_3	0	0	0	231290	221870	446940	0	0	0	315170	84488	91571
TRINITY_DN8805_c0_g1_i2_1	0	0	0	0	199160	206570	0	0	0	317460	284730	382560
TRINITY_DN7176_c0_g1_i3_3	0	0	0	0	0	0	0	0	0	336300	708550	343060
TRINITY_DN5898_c0_g1_i1_1	0	0	0	0	0	0	0	0	0	538640	392380	440150
TRINITY_DN8672_c0_g2_i1_3	0	0	0	0	0	0	0	0	0	753530	209550	402930
TRINITY_DN1176_c0_g1_i1_6	0	0	0	0	0	0	0	0	0	540110	410960	414370
TRINITY_DN4088_c0_g1_i1_6	0	0	0	0	0	0	0	0	0	568020	566000	230210

TRINITY_DN7361_c0_g1_i1_3	0	0	0	0	0	0	0	0	0	0	304900	1055500
TRINITY_DN6371_c0_g1_i2_5	0	0	0	0	0	0	0	0	0	768740	382770	207140
TRINITY_DN152_c0_g1_i1_1	0	0	0	0	0	0	0	0	0	789820	226880	338700
TRINITY_DN211_c0_g1_i1_2	670450	346140	331090	0	0	0	0	0	0	0	0	0
TRINITY_DN10187_c0_g2_i3_3	0	0	0	0	0	0	0	0	0	715300	408290	219560
TRINITY_DN10966_c1_g2_i1_2	0	0	0	0	0	0	0	0	0	635980	238170	455830
TRINITY_DN17047_c0_g1_i1_4	0	0	0	0	0	0	0	0	0	676870	242130	409590
TRINITY_DN2953_c0_g1_i1_6	0	0	0	0	104860	0	0	0	0	565310	464710	189180
TRINITY_DN10123_c0_g1_i1_2	0	0	0	0	0	0	0	0	0	847490	407300	64891
TRINITY_DN6691_c0_g1_i1_3	0	0	0	0	0	0	0	0	0	655400	199530	464380
TRINITY_DN9992_c0_g1_i2_2	0	0	0	0	0	0	0	0	0	579940	401490	336460
TRINITY_DN10481_c0_g2_i1_2	0	0	0	0	0	0	0	0	0	530170	465740	320610
TRINITY_DN23047_c0_g1_i1_3	0	308170	0	0	0	232460	334940	436150	0	0	0	0
TRINITY_DN12530_c0_g1_i1_3	75158	162010	101850	519280	0	453050	0	0	0	0	0	0
TRINITY_DN5479_c0_g1_i1_1	0	0	0	0	0	0	0	0	0	468990	573220	265930
TRINITY_DN14670_c0_g1_i1_5	0	0	0	0	0	0	0	0	0	677200	305500	321190
TRINITY_DN7295_c0_g1_i1_1	0	0	0	0	0	0	0	0	0	210640	620160	472310
TRINITY_DN9388_c0_g1_i2_3	0	0	0	0	0	0	0	0	0	181560	633290	488040
TRINITY_DN9217_c0_g1_i2_3	0	0	0	0	0	0	0	0	0	153710	994770	149930
TRINITY_DN11220_c1_g2_i10_1	0	0	0	0	0	0	0	929110	0	137630	126950	103350
TRINITY_DN7324_c0_g1_i1_3	0	0	0	0	0	0	0	0	0	0	791300	497350
TRINITY_DN8489_c0_g2_i1_1	0	0	0	0	0	0	0	0	0	692510	358750	233670
TRINITY_DN9496_c0_g2_i1_6	0	0	0	0	0	0	0	0	0	574330	532910	177070
TRINITY_DN8940_c0_g2_i1_4	303520	436030	404280	0	0	139650	0	0	0	0	0	0
TRINITY_DN2829_c0_g1_i1_4	983830	265890	33556	0	0	0	0	0	0	0	0	0
TRINITY_DN8775_c0_g1_i1_6	0	0	0	0	0	0	0	0	0	376320	465720	439340
TRINITY_DN4667_c0_g1_i2_3	0	0	0	702880	0	577460	0	0	0	0	0	0
TRINITY_DN22872_c0_g1_i1_1	0	525280	127200	311800	0	314570	0	0	0	0	0	0
TRINITY_DN19824_c0_g1_i1_3	0	0	0	0	0	0	0	0	0	443960	397050	434130
TRINITY_DN9699_c0_g1_i1_4	0	0	0	0	0	0	0	0	0	609640	0	665510
TRINITY_DN3755_c0_g2_i1_3	0	0	0	0	0	0	0	0	0	509140	0	765570
TRINITY_DN11202_c0_g1_i2_2	0	0	0	0	0	0	0	0	0	274110	740900	258920
TRINITY_DN6447_c0_g1_i1_4	0	0	0	0	0	0	0	0	0	293880	476670	502610

TRINITY_DN5808_c0_g1_i1_2	0	0	0	0	0	0	0	0	0	715520	244540	312050
TRINITY_DN10647_c0_g2_i1_5	303280	347510	267720	0	180400	172930	0	0	0	0	0	0
TRINITY_DN14457_c0_g1_i1_4	0	0	0	0	0	0	0	0	0	0	658400	613140
TRINITY_DN7706_c0_g1_i1_3	0	0	0	0	0	0	0	0	0	772050	499450	0
TRINITY_DN2695_c0_g1_i1_6	0	0	26311	0	0	0	0	0	0	180940	531290	532270
TRINITY_DN3350_c0_g1_i1_2	58733	41657	101620	352520	194240	321360	0	0	0	198240	0	0
TRINITY_DN11297_c1_g1_i1_2	0	0	0	0	0	0	343330	450660	474030	0	0	0
TRINITY_DN11354_c1_g1_i3_1	0	0	0	0	84315	64726	0	0	0	418200	524430	174630
TRINITY_DN12695_c0_g2_i1_3	0	0	0	0	0	0	0	0	0	150180	967870	148140
TRINITY_DN12034_c0_g1_i1_1	0	0	0	0	0	0	0	0	0	356490	576660	332750
TRINITY_DN4302_c0_g1_i1_3	0	0	0	0	0	0	0	0	0	348850	620960	292320
TRINITY_DN18986_c0_g1_i1_2	0	0	0	0	0	0	0	0	0	704080	554370	0
TRINITY_DN16321_c0_g1_i1_6	0	0	0	0	0	0	0	0	0	567790	473290	211590
TRINITY_DN10277_c0_g1_i1_3	0	0	0	0	0	0	0	0	0	554020	211390	486040
TRINITY_DN16791_c0_g1_i1_4	0	0	0	0	0	0	0	0	0	473200	416130	361210
TRINITY_DN7314_c0_g1_i1_1	0	0	0	0	0	0	0	0	0	648590	600280	0
TRINITY_DN11345_c0_g1_i1_5	0	0	0	0	0	0	0	0	0	0	0	1248000
TRINITY_DN23429_c0_g1_i1_1	0	0	0	0	0	0	0	0	0	570690	440440	236710
TRINITY_DN4338_c0_g2_i1_1	0	0	0	0	0	0	0	0	0	638200	527040	78057
TRINITY_DN13879_c0_g1_i1_4	0	0	0	0	0	0	0	0	0	480060	438320	324060
TRINITY_DN1790_c0_g1_i1_2	0	0	0	0	0	0	0	0	0	450370	456990	334850
TRINITY_DN5067_c0_g1_i1_3	138580	366510	139380	264150	0	175560	0	0	0	0	78234	79202
TRINITY_DN9550_c0_g1_i1_3	0	0	0	125830	138660	0	0	0	0	413030	230620	330490
TRINITY_DN5828_c0_g1_i2_4	0	0	0	0	471380	279020	0	0	0	180880	305390	0
TRINITY_DN20109_c0_g1_i1_4	0	0	0	0	0	0	0	0	0	670940	563090	0
TRINITY_DN11375_c0_g1_i1_1	0	0	0	0	0	0	0	0	0	335490	0	898210
TRINITY_DN4236_c0_g1_i1_1	0	0	0	0	0	0	0	0	0	1132600	0	100160
TRINITY_DN4156_c0_g1_i1_1	285750	113990	158260	0	0	0	0	0	0	267420	225660	180890
TRINITY_DN11786_c0_g1_i1_3	14242	21081	37809	0	0	0	0	0	0	587200	388880	182400
TRINITY_DN15397_c0_g1_i1_3	0	0	0	0	0	0	0	0	0	758080	0	466060
TRINITY_DN5555_c0_g1_i2_1	0	0	0	0	0	0	0	0	0	265380	560770	396690
TRINITY_DN6669_c0_g2_i2_2	46392	271390	264820	121800	518320	0	0	0	0	0	0	0
TRINITY_DN5818_c0_g1_i1_3	0	0	0	0	0	0	0	0	0	845350	0	376270

TRINITY_DN10622_c0_g1_i3_1	0	0	0	0	0	0	168100	220470	0	424210	244870	162100
TRINITY_DN6444_c0_g1_i1_1	0	0	0	0	0	0	0	0	0	483470	375470	359940
TRINITY_DN8167_c0_g1_i2_2	0	0	117790	227000	0	0	0	0	871970	0	0	0
TRINITY_DN10005_c0_g1_i2_3	0	0	0	421640	0	0	0	604960	0	0	0	188160
TRINITY_DN18506_c0_g1_i1_1	0	0	0	0	0	0	0	0	0	658970	0	555490
TRINITY_DN9802_c0_g1_i3_2	0	0	0	0	0	0	0	0	0	838330	373340	0
TRINITY_DN11201_c1_g1_i1_2	0	0	0	0	0	0	0	0	0	670190	263640	274180
TRINITY_DN2574_c0_g1_i1_2	0	0	0	0	0	0	0	0	0	449440	408280	348040
TRINITY_DN2926_c0_g2_i1_1	0	0	0	0	0	0	0	0	0	602340	172390	430710
TRINITY_DN1172_c0_g1_i1_3	0	0	0	0	0	0	0	0	0	654580	0	550060
TRINITY_DN15441_c0_g1_i1_4	0	0	0	0	0	0	0	0	0	484040	358400	360730
TRINITY_DN2072_c0_g1_i1_5	0	0	0	0	0	0	0	0	0	546550	286010	369350
TRINITY_DN11308_c0_g1_i1_4	35526	0	0	392730	378710	394650	0	0	0	0	0	0
TRINITY_DN11604_c0_g1_i4_3	109820	40201	71921	527440	0	450740	0	0	0	0	0	0
TRINITY_DN6973_c0_g1_i2_3	0	0	0	0	0	0	0	0	0	204600	832790	159750
TRINITY_DN19024_c0_g1_i1_1	0	0	0	0	0	0	0	0	0	608030	138260	447340
TRINITY_DN8174_c0_g1_i1_3	0	0	0	0	0	0	0	0	0	564510	403460	224080
TRINITY_DN6495_c0_g1_i1_1	0	0	0	0	0	0	0	0	0	368560	520300	300950
TRINITY_DN14955_c0_g1_i1_1	0	0	0	0	0	0	0	0	0	0	404650	782540
TRINITY_DN5794_c0_g1_i3_3	0	0	0	0	0	0	0	0	0	416230	405830	356910
TRINITY_DN5657_c0_g1_i1_3	0	0	0	0	0	0	0	0	0	388100	242340	540970
TRINITY_DN20134_c0_g1_i1_2	0	0	0	0	0	0	0	0	0	428640	380950	361430
TRINITY_DN5823_c1_g1_i1_3	0	0	0	0	0	0	0	0	0	828370	338720	0
TRINITY_DN10611_c0_g2_i1_3	0	0	0	0	0	0	0	0	0	646730	206320	312230
TRINITY_DN8028_c1_g2_i2_1	0	0	84417	323490	383520	330870	0	0	0	0	0	40865
TRINITY_DN20414_c0_g1_i1_1	0	0	0	0	0	0	0	0	0	480400	412900	268150
TRINITY_DN18416_c0_g1_i1_1	79362	74910	93943	0	146770	0	0	0	0	316840	449030	0
TRINITY_DN5396_c0_g2_i1_1	0	0	0	0	0	0	0	0	0	464420	374830	318500
TRINITY_DN4301_c0_g1_i1_1	210990	49036	0	0	0	0	0	0	0	671210	226180	0
TRINITY_DN6535_c0_g1_i1_1	0	0	0	0	0	0	0	0	0	411850	424010	319570
TRINITY_DN5580_c0_g1_i1_4	0	0	0	0	0	0	0	0	0	744620	203140	199000
TRINITY_DN10847_c0_g1_i3_3	0	0	0	0	0	0	0	0	0	446130	350850	347460
TRINITY_DN14525_c0_g1_i1_6	0	0	0	0	0	0	0	394440	0	420710	0	328180

TRINITY_DN10966_c1_g1_i1_2	0	0	0	0	0	0	0	0	0	485660	333680	320990
TRINITY_DN12386_c0_g1_i1_4	0	0	0	0	0	0	0	0	0	237730	702400	199810
TRINITY_DN3579_c0_g1_i1_4	0	0	0	0	0	0	0	0	0	421920	362740	351250
TRINITY_DN19791_c0_g1_i1_5	0	0	0	0	0	0	0	0	0	405420	410700	319510
TRINITY_DN10085_c0_g1_i1_3	0	0	0	0	0	0	0	0	0	776200	204730	151150
TRINITY_DN5782_c0_g1_i1_1	0	0	0	0	0	0	0	0	1130100	0	0	0
TRINITY_DN13590_c0_g1_i1_3	0	0	0	0	0	0	0	0	0	1129600	0	0
TRINITY_DN1136_c0_g2_i1_1	0	0	0	0	0	0	0	0	0	442910	365910	313530
TRINITY_DN9839_c0_g1_i1_1	95282	129530	121780	0	0	0	0	0	0	205840	362710	206060
TRINITY_DN3828_c0_g1_i2_1	0	0	0	0	0	0	0	0	0	630730	0	489540
TRINITY_DN800_c0_g1_i1_1	0	0	0	0	0	0	0	0	0	538900	393030	187980
TRINITY_DN10682_c0_g1_i2_5	0	0	0	0	0	0	0	0	0	475320	103750	539240
TRINITY_DN11406_c0_g2_i1_5	0	0	0	0	0	0	0	0	0	445140	355460	317620
TRINITY_DN9265_c0_g1_i1_4	56648	91757	77395	147040	0	0	0	0	743810	0	0	0
TRINITY_DN3622_c0_g1_i1_2	0	0	0	0	0	0	0	0	0	0	691830	420660
TRINITY_DN4087_c0_g2_i1_1	0	0	0	0	0	0	0	0	0	656850	154340	298610
TRINITY_DN10280_c0_g2_i1_3	0	0	0	0	0	0	0	0	0	449040	304320	351750
TRINITY_DN17659_c0_g1_i1_1	0	0	0	0	0	0	0	0	0	446610	370870	286320
TRINITY_DN391_c0_g2_i1_3	61004	53289	149310	164960	86929	87060	0	0	0	262720	236050	0
TRINITY_DN18625_c0_g1_i1_3	0	609650	491440	0	0	0	0	0	0	0	0	0
TRINITY_DN15558_c0_g1_i1_3	0	0	0	0	0	0	0	0	0	936130	0	164320
TRINITY_DN8990_c0_g1_i6_4	0	0	0	0	0	0	0	0	0	0	588290	510090
TRINITY_DN9713_c0_g1_i1_2	0	0	0	0	0	0	0	0	0	315600	481650	298410
TRINITY_DN4_c0_g2_i1_2	0	0	0	0	0	0	0	0	0	227990	660270	205720
TRINITY_DN3956_c0_g2_i1_1	0	0	0	0	0	0	0	0	0	595440	281870	211910
TRINITY_DN2951_c0_g1_i1_3	0	0	0	0	0	0	0	0	0	490540	468000	127780
TRINITY_DN8715_c0_g1_i2_3	0	0	0	0	0	0	0	0	0	0	705330	380990
TRINITY_DN11521_c0_g3_i1_1	0	0	0	0	0	0	0	0	0	460550	309540	316050
TRINITY_DN7431_c0_g1_i1_3	0	0	0	0	0	0	0	0	0	1082600	0	0
TRINITY_DN20738_c0_g1_i1_3	0	0	0	0	0	0	0	0	0	687170	0	395100
TRINITY_DN11007_c0_g1_i2_1	127570	0	0	0	0	0	271110	0	0	187670	309510	179670
TRINITY_DN18675_c0_g1_i1_5	0	0	0	0	0	0	0	0	0	362340	333620	370010
TRINITY_DN4569_c0_g1_i2_2	0	0	0	0	0	0	0	0	0	535610	315890	210610

TRINITY_DN4303_c0_g1_i1_5	0	0	0	0	0	0	0	0	0	162800	495050	399850
TRINITY_DN11099_c0_g1_i3_2	0	0	0	0	0	0	0	0	0	567040	487950	0
TRINITY_DN5386_c0_g1_i1_2	0	0	0	361520	392560	0	0	0	0	199940	0	100840
TRINITY_DN3521_c0_g1_i1_6	0	0	0	0	0	0	0	0	0	629400	424290	0
TRINITY_DN13705_c0_g1_i1_5	0	0	0	0	0	0	0	0	0	566910	179760	304000
TRINITY_DN3201_c0_g1_i1_6	0	0	0	0	0	0	0	0	0	470330	95978	484070
TRINITY_DN9124_c0_g4_i1_6	0	0	0	0	0	0	0	0	0	376730	252890	419930
TRINITY_DN10878_c0_g1_i1_2	0	0	0	0	0	0	0	0	0	382330	255740	410920
TRINITY_DN8588_c0_g1_i3_2	0	0	0	0	0	0	0	0	0	539500	0	508900
TRINITY_DN8864_c0_g1_i1_1	0	0	0	0	0	0	0	0	0	160910	451490	435380
TRINITY_DN18025_c0_g1_i1_5	0	0	0	0	0	0	0	0	0	518940	527400	0
TRINITY_DN15533_c0_g1_i1_2	0	0	0	0	0	0	0	0	0	949820	0	95774
TRINITY_DN17372_c0_g1_i1_4	0	0	0	0	0	0	0	0	0	391030	309250	342530
TRINITY_DN10448_c0_g1_i2_3	0	0	0	0	0	0	0	0	0	289780	512970	238940
TRINITY_DN2556_c0_g1_i2_1	0	0	0	0	162730	0	0	0	0	0	555680	320920
TRINITY_DN11115_c0_g1_i1_2	0	0	0	0	0	0	0	0	0	403830	331680	303640
TRINITY_DN6126_c0_g1_i2_6	0	0	0	0	0	0	0	0	0	326230	457410	255180
TRINITY_DN18060_c0_g1_i1_4	0	0	0	0	0	0	209300	0	0	0	828460	0
TRINITY_DN8332_c0_g1_i1_1	0	0	0	0	0	0	0	0	0	312390	0	724610
TRINITY_DN7043_c0_g3_i1_3	0	0	0	158930	0	0	0	0	0	189000	0	686350
TRINITY_DN15047_c0_g1_i1_2	0	0	0	0	0	0	0	0	0	406530	357430	270060
TRINITY_DN4679_c0_g1_i1_4	0	0	0	0	0	0	0	0	0	380780	329370	322200
TRINITY_DN5533_c1_g1_i1_2	0	0	0	0	803470	0	0	0	0	0	226900	0
TRINITY_DN3362_c0_g1_i1_3	0	0	0	0	0	0	0	0	0	752760	135120	142390
TRINITY_DN11453_c1_g2_i1_1	0	43324	0	255680	0	0	0	0	0	123780	301050	304870
TRINITY_DN21448_c0_g1_i1_2	0	0	0	0	0	0	0	0	0	747160	278430	0
TRINITY_DN4482_c0_g1_i2_6	0	0	0	0	0	0	0	0	0	205720	670900	143090
TRINITY_DN3062_c1_g1_i1_5	0	0	0	0	0	0	0	0	0	336150	190600	491370
TRINITY_DN21074_c0_g1_i1_5	0	0	0	0	0	0	0	0	0	483950	209660	324100
TRINITY_DN6202_c0_g1_i1_4	0	0	0	0	0	172620	350870	0	0	241330	252210	0
TRINITY_DN9665_c1_g1_i2_1	0	0	0	0	0	0	0	0	0	325010	482800	209160
TRINITY_DN23089_c0_g1_i1_2	0	0	0	0	0	0	0	0	0	0	490410	525110
TRINITY_DN4752_c0_g1_i1_6	0	0	0	0	0	0	0	0	0	573590	222070	217030

TRINITY_DN6323_c0_g1_i1_4	0	1011100	0	0	0	0	0	0	0	0	0	0
TRINITY_DN7547_c0_g1_i1_2	0	0	0	0	0	0	0	0	0	0	1011000	0
TRINITY_DN11503_c0_g1_i5_1	318340	382730	308820	0	0	0	0	0	0	0	0	0
TRINITY_DN6410_c0_g2_i1_3	0	0	0	0	0	0	0	0	0	0	509940	498450
TRINITY_DN17585_c0_g1_i1_4	98498	0	0	0	0	0	0	0	0	437790	346780	124770
TRINITY_DN7272_c0_g1_i2_2	66191	0	0	0	123640	85660	274750	0	0	266940	106500	82982
TRINITY_DN8313_c0_g1_i1_1	0	0	0	0	0	0	0	0	0	571710	433960	0
TRINITY_DN10899_c1_g1_i9_2	0	0	0	0	0	0	0	0	0	486320	272050	246290
TRINITY_DN10964_c0_g2_i1_1	0	0	0	0	0	0	0	0	0	0	0	997800
TRINITY_DN5766_c0_g1_i1_2	0	0	0	0	0	0	282770	0	0	220840	493790	0
TRINITY_DN16709_c0_g1_i1_5	0	0	0	0	0	0	0	0	0	453600	197700	345200
TRINITY_DN20234_c0_g1_i1_2	0	0	0	0	0	201200	0	0	0	196580	311480	286590
TRINITY_DN13627_c0_g1_i1_4	0	0	0	0	0	0	0	0	0	166300	683110	144360
TRINITY_DN22360_c0_g1_i1_5	0	0	0	0	0	0	0	0	0	608800	384970	0
TRINITY_DN5465_c0_g1_i1_6	0	231450	226750	0	0	399860	0	0	0	134950	0	0
TRINITY_DN14137_c1_g1_i1_2	0	0	0	0	0	0	0	0	0	346550	344480	300420
TRINITY_DN4218_c0_g1_i1_3	0	0	0	0	0	0	0	0	0	586170	0	404470
TRINITY_DN405_c0_g2_i1_3	0	0	0	0	0	0	0	0	0	453480	403030	124950
TRINITY_DN2745_c0_g1_i1_1	0	0	0	0	0	0	0	0	0	0	178120	800970
TRINITY_DN15009_c0_g1_i1_3	0	0	0	0	0	0	0	0	0	522290	231360	224360
TRINITY_DN20316_c0_g1_i1_4	0	0	0	0	0	0	0	0	0	447550	278850	249800
TRINITY_DN11294_c0_g1_i2_1	0	0	0	0	0	0	0	0	0	508470	256560	209300
TRINITY_DN19054_c0_g1_i1_3	0	0	411700	0	0	0	0	0	0	134060	105850	321370
TRINITY_DN4034_c0_g2_i1_6	0	0	0	0	0	0	0	0	0	396820	302100	272650
TRINITY_DN12290_c0_g1_i1_4	0	0	0	0	0	0	0	0	0	358490	314700	294350
TRINITY_DN21049_c0_g1_i1_5	0	0	0	0	0	0	0	0	0	478940	487900	0
TRINITY_DN10192_c0_g2_i2_4	0	0	0	0	138510	0	0	0	0	330430	224550	271900
TRINITY_DN5313_c0_g1_i1_6	0	0	0	0	0	0	0	0	0	391510	277690	293900
TRINITY_DN1722_c0_g1_i1_6	0	364720	598320	0	0	0	0	0	0	0	0	0
TRINITY_DN594_c0_g1_i1_2	0	0	0	0	0	0	0	0	0	141780	701280	119970
TRINITY_DN14750_c0_g1_i1_3	0	0	0	0	0	0	0	0	0	287530	221880	452960
TRINITY_DN10152_c0_g1_i1_3	0	961940	0	0	0	0	0	0	0	0	0	0
TRINITY_DN7940_c0_g1_i2_6	0	0	0	0	0	0	0	0	0	387010	0	574510

TRINITY_DN3239_c0_g2_i1_3	0	0	0	0	0	0	0	0	0	532920	0	428110
TRINITY_DN4761_c0_g1_i1_3	271030	181440	280540	0	0	227390	0	0	0	0	0	0
TRINITY_DN8530_c0_g1_i3_3	0	0	0	0	0	0	0	0	0	357640	302800	298260
TRINITY_DN16028_c0_g1_i1_6	0	0	0	0	0	0	0	0	0	584860	373810	0
TRINITY_DN515_c0_g1_i1_1	0	0	0	187030	154150	156210	0	0	0	253210	207280	0
TRINITY_DN9271_c0_g1_i2_1	0	0	0	0	953960	0	0	0	0	0	0	0
TRINITY_DN9769_c0_g1_i2_3	0	0	0	0	0	161840	0	0	0	177410	233590	381090
TRINITY_DN17789_c0_g1_i1_2	0	126650	234740	0	0	0	0	0	0	207100	189900	193150
TRINITY_DN17147_c0_g3_i1_3	194660	0	0	260540	0	206930	0	0	0	0	287250	0
TRINITY_DN18723_c0_g1_i1_3	0	0	0	0	0	0	0	0	0	309250	0	639140
TRINITY_DN13968_c0_g1_i1_1	0	0	0	0	0	0	0	0	0	414510	441940	90814
TRINITY_DN1050_c0_g1_i1_3	0	0	0	0	0	0	0	0	0	0	509690	437500
TRINITY_DN630_c0_g1_i2_2	0	0	0	0	0	0	0	0	0	945860	0	0
TRINITY_DN10963_c0_g1_i1_3	0	0	0	0	0	0	0	0	0	487320	158300	299530
TRINITY_DN10629_c0_g1_i1_1	0	0	0	0	0	0	0	0	0	319150	540820	84553
TRINITY_DN2614_c0_g1_i1_5	0	0	0	0	0	0	0	0	0	310350	0	631910
TRINITY_DN11634_c0_g1_i3_1	0	0	0	0	0	0	0	0	0	502530	159320	278330
TRINITY_DN10531_c0_g2_i1_3	0	0	0	0	0	0	0	0	0	641490	298680	0
TRINITY_DN2332_c0_g1_i1_2	0	0	0	0	0	0	0	0	0	235120	469460	235350
TRINITY_DN17272_c0_g1_i1_3	0	0	0	0	0	0	0	0	0	406940	329760	201070
TRINITY_DN6275_c0_g2_i2_1	0	0	0	0	0	0	0	0	0	0	356550	572600
TRINITY_DN7918_c1_g1_i1_2	0	0	0	0	0	0	0	0	0	412890	289290	226930
TRINITY_DN10401_c0_g1_i2_1	0	0	0	0	0	0	0	0	0	397070	256070	274600
TRINITY_DN6828_c0_g1_i1_3	119220	132950	94016	304570	0	0	0	0	0	276290	0	0
TRINITY_DN3006_c0_g1_i2_2	0	0	0	0	0	0	0	0	0	346410	348800	229800
TRINITY_DN11284_c0_g1_i1_2	0	0	0	255330	277890	192080	0	0	0	0	198690	0
TRINITY_DN7192_c0_g1_i1_3	0	0	0	0	0	0	0	0	0	395380	314600	211220
TRINITY_DN8534_c0_g1_i2_3	0	0	0	0	0	0	0	0	0	397890	358270	158630
TRINITY_DN7045_c0_g1_i2_1	0	0	0	0	0	0	0	0	0	615880	0	298840
TRINITY_DN7890_c0_g1_i3_3	0	0	0	0	0	0	0	0	0	0	0	913650
TRINITY_DN11617_c0_g1_i1_3	0	0	0	0	0	0	0	0	0	600560	211140	101850
TRINITY_DN15269_c0_g1_i1_3	0	0	0	0	0	0	0	0	0	372700	317320	222130
TRINITY_DN10161_c0_g1_i1_2	0	0	0	0	0	0	0	0	0	478090	0	433710

TRINITY_DN819_c0_g1_i1_5	0	0	0	0	0	0	0	0	0	626020	285150	0
TRINITY_DN13462_c0_g1_i1_3	287740	0	52862	0	0	373810	0	0	0	196110	0	0
TRINITY_DN4339_c0_g1_i1_1	0	0	0	0	0	0	0	0	0	638510	0	271960
TRINITY_DN19938_c0_g1_i1_2	0	0	0	0	0	0	0	0	0	0	905870	0
TRINITY_DN10594_c0_g1_i4_3	0	0	0	0	0	0	0	0	0	644820	257840	0
TRINITY_DN7751_c0_g1_i1_3	0	0	0	0	0	0	0	0	0	365120	252410	283620
TRINITY_DN9327_c0_g1_i3_2	0	0	0	0	0	0	0	0	0	605070	109540	185600
TRINITY_DN8047_c0_g1_i1_3	0	0	0	0	0	0	0	0	0	395170	389900	112150
TRINITY_DN10285_c0_g1_i1_1	0	0	0	0	0	0	0	393720	503060	0	0	0
TRINITY_DN8681_c0_g1_i1_3	0	0	0	0	0	0	0	0	0	252120	641010	0
TRINITY_DN5586_c0_g1_i1_1	0	0	0	0	0	0	0	0	0	750920	142020	0
TRINITY_DN2976_c0_g1_i1_3	0	0	0	0	0	0	0	0	0	441300	0	449540
TRINITY_DN7053_c1_g1_i1_1	0	0	0	0	0	0	0	0	0	283120	390220	216340
TRINITY_DN9308_c0_g2_i1_6	0	160550	171890	0	215440	0	0	336560	0	0	0	0
TRINITY_DN7166_c0_g1_i1_1	126700	116060	88894	302450	249270	0	0	0	0	0	0	0
TRINITY_DN17861_c0_g1_i1_6	0	0	0	0	0	0	0	0	0	343480	279560	257960
TRINITY_DN23265_c0_g1_i1_1	0	0	0	0	0	0	0	0	0	335750	292450	249960
TRINITY_DN11443_c1_g1_i3_3	0	0	0	0	0	0	0	0	0	589590	150140	136410
TRINITY_DN9627_c0_g2_i1_1	0	135350	180260	0	0	0	0	0	0	373620	185050	0
TRINITY_DN20236_c0_g1_i1_5	162580	379000	331020	0	0	0	0	0	0	0	0	0
TRINITY_DN12747_c0_g1_i1_3	0	0	0	0	0	0	0	0	0	773120	98040	0
TRINITY_DN4648_c0_g1_i1_3	0	0	0	0	0	0	0	0	0	502580	0	368320
TRINITY_DN396_c0_g1_i1_1	0	0	0	0	0	0	0	0	0	462090	0	408050
TRINITY_DN3770_c0_g1_i1_3	0	0	0	0	0	0	0	0	0	495470	202200	169840
TRINITY_DN7965_c1_g1_i1_2	0	0	0	0	131820	0	0	0	0	416760	0	318900
TRINITY_DN5625_c0_g2_i1_1	0	0	0	0	379630	0	0	0	0	57847	221660	208280
TRINITY_DN9598_c0_g1_i1_1	0	0	0	0	0	0	0	0	0	431210	433040	0
TRINITY_DN6936_c0_g1_i1_3	0	0	0	0	0	0	0	0	0	484520	0	379450
TRINITY_DN22611_c0_g1_i1_5	0	0	0	0	0	0	0	0	0	470750	205380	187370
TRINITY_DN4497_c1_g1_i1_1	0	0	0	0	0	0	0	0	0	0	463990	396050
TRINITY_DN16255_c0_g2_i1_1	0	0	0	0	0	0	0	0	0	340610	290930	226820
TRINITY_DN3029_c0_g2_i1_3	193160	0	0	338380	0	0	0	0	0	326690	0	0
TRINITY_DN7010_c0_g2_i2_5	0	0	0	0	0	0	0	0	0	0	0	853480

TRINITY_DN8504_c0_g1_i1_2	0	0	0	0	0	0	0	0	0	508830	121720	221760
TRINITY_DN3130_c0_g2_i2_1	0	0	0	0	198780	192310	0	0	0	248390	0	212540
TRINITY_DN13283_c0_g1_i1_5	0	0	0	0	0	0	0	0	0	308220	0	542320
TRINITY_DN3329_c0_g1_i1_6	0	0	0	0	0	0	0	0	0	435640	413480	0
TRINITY_DN10602_c1_g1_i2_3	0	0	0	0	0	0	0	0	0	382140	183600	282300
TRINITY_DN5356_c0_g1_i1_3	0	0	0	0	846490	0	0	0	0	0	0	0
TRINITY_DN8024_c0_g1_i1_1	0	0	0	0	0	0	0	0	0	0	0	845750
TRINITY_DN9300_c0_g1_i1_5	0	0	0	142060	208650	0	313160	0	0	102270	0	79055
TRINITY_DN9177_c0_g3_i1_3	0	0	0	0	0	0	0	0	0	394320	239530	210110
TRINITY_DN14061_c0_g1_i1_4	0	0	0	0	0	0	0	802990	0	0	39710	0
TRINITY_DN8441_c0_g2_i2_3	108240	173090	288650	0	0	0	0	0	0	159290	0	105220
TRINITY_DN7456_c0_g2_i1_3	0	0	0	0	0	0	0	0	0	420300	296770	116500
TRINITY_DN4946_c0_g2_i1_3	0	0	0	0	0	0	0	0	0	249570	411330	168790
TRINITY_DN18912_c0_g1_i1_2	0	0	0	0	0	0	0	0	0	476940	351300	0
TRINITY_DN12766_c0_g1_i1_5	0	0	0	0	0	0	0	0	0	485530	115410	227280
TRINITY_DN4468_c0_g1_i1_6	0	0	0	0	0	0	0	0	0	496200	169600	156760
TRINITY_DN12470_c0_g1_i1_3	0	0	0	0	0	0	0	0	0	0	634410	187380
TRINITY_DN10609_c0_g1_i2_2	0	0	0	0	0	0	0	0	0	211660	437100	170470
TRINITY_DN11550_c0_g1_i3_3	0	0	0	0	0	0	0	0	0	137870	356350	324260
TRINITY_DN18034_c0_g1_i1_3	0	0	0	0	0	0	0	0	0	472770	0	342590
TRINITY_DN10769_c0_g1_i1_1	0	0	0	0	0	0	0	0	0	287590	268550	258960
TRINITY_DN11604_c0_g1_i8_1	0	0	0	0	0	693680	0	0	0	0	0	120800
TRINITY_DN11302_c0_g1_i2_1	0	22468	0	0	0	0	0	0	0	589280	113250	88900
TRINITY_DN12632_c0_g1_i1_3	0	0	0	0	0	0	0	0	0	544830	268800	0
TRINITY_DN9378_c0_g1_i4_2	0	0	0	0	0	0	0	0	0	421940	388520	0
TRINITY_DN9423_c0_g2_i1_4	0	0	0	0	0	0	0	0	0	400480	408140	0
TRINITY_DN7649_c0_g1_i2_4	0	0	0	0	0	0	0	0	0	302500	262830	240200
TRINITY_DN18448_c0_g1_i1_3	0	0	0	0	0	0	0	0	0	310380	244490	250200
TRINITY_DN11658_c0_g1_i3_3	0	0	0	0	0	0	0	0	0	478090	114100	211780
TRINITY_DN18880_c0_g1_i1_1	0	0	0	0	0	0	0	0	0	116870	0	687000
TRINITY_DN10400_c0_g1_i3_1	0	0	0	0	0	0	0	0	0	110130	253350	439910
TRINITY_DN1374_c0_g1_i1_1	0	0	0	0	700380	0	0	0	0	0	0	102820
TRINITY_DN3233_c0_g1_i1_1	0	0	0	0	0	0	0	0	0	301720	104140	397190

TRINITY_DN16037_c0_g1_i1_6	0	0	0	0	0	0	0	0	0	468540	331530	0
TRINITY_DN4496_c0_g1_i1_6	0	0	0	0	0	0	0	0	0	155860	642090	0
TRINITY_DN3498_c0_g1_i1_3	0	0	0	0	0	0	0	0	0	621490	0	175440
TRINITY_DN8015_c0_g2_i1_1	136380	99125	64045	0	132180	0	0	0	0	365050	0	0
TRINITY_DN8457_c0_g1_i2_3	0	0	0	0	0	0	0	0	0	327270	262120	205330
TRINITY_DN8936_c0_g2_i1_3	0	0	0	0	0	0	0	0	0	415330	0	376870
TRINITY_DN4836_c0_g2_i2_1	0	0	0	0	0	0	0	0	0	317960	264660	209090
TRINITY_DN4305_c0_g1_i1_2	0	0	0	0	0	0	0	0	0	448870	341380	0
TRINITY_DN912_c0_g1_i1_3	24884	357960	407200	0	0	0	0	0	0	0	0	0
TRINITY_DN2769_c0_g1_i1_2	0	0	0	0	0	0	0	0	0	315260	234600	238030
TRINITY_DN7152_c0_g1_i2_3	0	0	0	0	0	0	0	0	0	220200	163890	403760
TRINITY_DN7966_c1_g1_i1_1	0	0	0	0	0	0	0	0	0	517770	0	269220
TRINITY_DN4959_c0_g1_i1_6	785250	0	0	0	0	0	0	0	0	0	0	0
TRINITY_DN11436_c0_g1_i3_2	0	0	0	0	0	0	0	0	0	364680	418510	0
TRINITY_DN6999_c0_g1_i1_5	0	0	0	0	0	0	0	0	0	187730	409530	185040
TRINITY_DN19644_c0_g1_i1_5	389480	0	392250	0	0	0	0	0	0	0	0	0
TRINITY_DN12673_c0_g1_i1_2	41591	0	0	374170	0	365610	0	0	0	0	0	0
TRINITY_DN10039_c0_g1_i2_2	0	0	0	0	0	63952	0	0	0	464260	248760	0
TRINITY_DN6311_c0_g1_i1_1	0	0	0	0	0	0	0	0	0	0	117240	658190
TRINITY_DN19028_c0_g1_i1_2	0	0	0	0	0	0	0	0	0	775260	0	0
TRINITY_DN10956_c0_g1_i2_3	0	0	0	0	0	0	0	0	0	274010	0	499480
TRINITY_DN10484_c2_g1_i1_1	0	379750	0	0	0	0	0	0	0	0	393380	0
TRINITY_DN17185_c0_g1_i1_2	0	0	0	0	0	0	0	0	0	314120	232180	226820
TRINITY_DN11454_c0_g1_i2_6	0	0	0	0	0	0	0	0	0	241220	530870	0
TRINITY_DN588_c0_g1_i1_4	0	0	0	0	0	0	0	0	0	435510	0	336250
TRINITY_DN12701_c0_g1_i1_3	0	0	0	0	0	0	0	0	0	452940	0	316360
TRINITY_DN7373_c0_g2_i1_3	0	0	0	0	0	0	0	0	0	333560	0	435090
TRINITY_DN11305_c0_g1_i2_2	0	0	0	0	0	0	0	0	0	282510	297750	187870
TRINITY_DN5684_c0_g1_i1_3	0	0	0	0	0	0	0	0	0	188280	417880	159590
TRINITY_DN864_c0_g1_i1_1	0	0	0	0	0	0	0	0	0	270670	129010	362960
TRINITY_DN4602_c0_g1_i1_1	0	0	0	0	0	0	0	0	0	264700	228970	268320
TRINITY_DN10884_c0_g1_i1_3	0	0	0	0	0	0	0	0	0	429970	329260	0
TRINITY_DN14631_c0_g1_i1_2	0	0	0	0	0	0	758620	0	0	0	0	0

TRINITY_DN10399_c0_g1_i1_1	0	0	0	0	0	0	0	0	0	185870	166980	402740
TRINITY_DN11619_c0_g2_i6_1	0	0	0	0	0	0	0	0	0	461930	0	292960
TRINITY_DN5247_c0_g2_i1_5	0	0	0	0	0	0	0	0	0	367870	224560	160920
TRINITY_DN2861_c0_g1_i1_2	0	0	0	0	0	0	0	514770	0	125860	112410	0
TRINITY_DN6453_c0_g1_i1_2	0	0	0	0	0	0	0	0	0	0	409640	343210
TRINITY_DN13293_c0_g1_i1_6	0	0	0	0	0	0	294560	0	0	0	255810	197430
TRINITY_DN5843_c0_g1_i1_3	0	0	0	0	0	0	0	0	0	414830	0	332830
TRINITY_DN3985_c0_g1_i1_5	0	0	0	218960	0	0	0	0	0	212600	315540	0
TRINITY_DN5098_c0_g2_i1_2	0	0	0	0	0	0	0	0	0	483800	124770	135660
TRINITY_DN6975_c0_g2_i1_1	0	0	0	0	0	0	0	0	0	236720	318580	185850
TRINITY_DN3955_c0_g1_i1_6	0	0	0	0	0	0	0	0	0	330400	215570	187680
TRINITY_DN21162_c0_g1_i1_1	0	0	0	0	0	0	0	0	0	313610	215650	201630
TRINITY_DN13980_c0_g1_i1_4	0	0	0	0	0	0	0	0	0	280650	228090	218050
TRINITY_DN6436_c0_g2_i1_3	0	0	0	191220	183200	139170	0	0	0	211300	0	0
TRINITY_DN7382_c0_g1_i1_5	0	0	0	0	0	0	0	0	0	218610	261270	244930
TRINITY_DN8895_c0_g1_i3_1	0	0	0	0	0	0	0	0	0	0	539100	185710
TRINITY_DN11523_c0_g1_i1_2	0	0	0	0	0	0	0	0	0	0	427120	296820
TRINITY_DN6472_c0_g1_i1_3	0	0	0	0	0	0	0	0	0	365040	0	357360
TRINITY_DN8927_c0_g1_i1_3	0	0	0	0	0	0	0	0	0	381190	207100	133100
TRINITY_DN22235_c0_g1_i1_2	110130	179650	0	0	0	428560	0	0	0	0	0	0
TRINITY_DN22096_c0_g1_i1_6	0	0	0	0	0	0	0	0	0	265190	228800	223850
TRINITY_DN10384_c0_g1_i1_3	0	0	0	0	0	0	0	0	0	0	403860	312700
TRINITY_DN12671_c0_g1_i1_1	0	0	0	0	0	72786	0	0	0	236130	216410	190180
TRINITY_DN15659_c0_g1_i1_5	0	0	0	0	0	0	0	0	0	421190	291540	0
TRINITY_DN3596_c0_g1_i1_4	0	0	0	0	0	91277	0	0	620020	0	0	0
TRINITY_DN8121_c0_g1_i1_3	0	0	0	0	0	0	0	0	0	262480	227520	220020
TRINITY_DN20264_c0_g1_i1_2	0	0	0	0	0	0	0	0	0	266600	227400	215010
TRINITY_DN18042_c0_g1_i1_3	0	0	0	0	0	0	0	0	0	439300	0	269010
TRINITY_DN18961_c0_g1_i1_1	0	0	0	0	197000	506690	0	0	0	0	0	0
TRINITY_DN3104_c0_g2_i1_5	0	0	0	0	0	0	0	0	0	225870	328400	149400
TRINITY_DN17865_c0_g1_i1_1	0	0	0	0	0	0	0	0	0	416080	287590	0
TRINITY_DN11260_c0_g1_i2_3	0	0	0	0	0	0	0	0	0	284400	277040	141300
TRINITY_DN6677_c0_g2_i1_6	88118	109300	0	0	165410	339780	0	0	0	0	0	0

TRINITY_DN14239_c0_g1_i1_6	0	0	0	0	0	0	0	0	0	265600	198030	235790
TRINITY_DN12081_c0_g1_i1_6	0	0	0	0	0	0	0	0	0	464780	153480	81123
TRINITY_DN18664_c0_g1_i1_1	0	0	0	0	0	0	0	0	0	458170	240050	0
TRINITY_DN3353_c0_g1_i1_5	0	0	0	0	0	0	0	0	0	253860	269030	175080
TRINITY_DN13649_c0_g1_i1_6	0	0	0	0	0	0	0	0	0	253880	200400	243510
TRINITY_DN21861_c0_g1_i1_3	0	0	0	0	0	0	0	0	0	262040	238520	196620
TRINITY_DN22107_c0_g1_i1_1	0	0	0	0	0	0	0	0	0	696350	0	0
TRINITY_DN19998_c0_g1_i1_4	0	0	0	0	0	0	0	0	0	274170	0	418790
TRINITY_DN4490_c0_g2_i2_2	0	0	0	0	0	0	0	0	0	277680	220270	194050
TRINITY_DN22424_c0_g2_i1_6	0	0	0	0	0	0	0	0	0	331360	212440	147360
TRINITY_DN1144_c0_g1_i1_3	0	0	0	0	0	0	0	0	0	365640	318270	0
TRINITY_DN4661_c0_g1_i3_3	0	0	0	0	0	0	0	0	0	312630	112630	256850
TRINITY_DN3820_c0_g2_i1_3	0	0	0	0	0	0	0	0	0	463220	161280	55711
TRINITY_DN10264_c0_g1_i1_2	0	0	0	0	0	0	0	0	0	0	402090	274300
TRINITY_DN8158_c0_g1_i1_1	0	0	0	0	0	0	0	0	0	263820	199340	213180
TRINITY_DN9206_c0_g2_i1_1	222550	104950	52400	0	0	0	0	0	0	0	176740	118790
TRINITY_DN9226_c0_g1_i2_3	0	0	0	119000	134800	0	0	0	0	144900	148740	126130
TRINITY_DN585_c0_g1_i1_6	0	0	0	0	0	0	0	0	0	381120	0	292160
TRINITY_DN21456_c0_g1_i1_2	0	0	0	0	0	0	0	0	0	272580	191380	206420
TRINITY_DN5447_c0_g2_i1_2	0	0	0	0	0	0	0	0	0	334420	184760	148730
TRINITY_DN23101_c0_g1_i1_6	0	0	0	0	0	0	0	0	0	355690	311010	0
TRINITY_DN1220_c0_g1_i1_6	0	0	0	0	0	0	0	0	0	66618	430500	169040
TRINITY_DN13227_c0_g1_i1_1	0	0	0	0	0	0	0	0	0	391080	0	274450
TRINITY_DN5512_c0_g3_i1_3	0	0	0	339700	324620	0	0	0	0	0	0	0
TRINITY_DN4033_c0_g2_i1_3	0	0	0	0	0	0	0	0	0	399070	0	265020
TRINITY_DN4640_c0_g1_i1_2	86956	84524	101120	0	0	0	0	0	0	390080	0	0
TRINITY_DN5850_c0_g1_i1_1	0	0	0	0	0	0	0	0	0	277160	198310	186810
TRINITY_DN11339_c0_g1_i3_1	0	0	0	0	0	0	0	0	0	273300	201940	186380
TRINITY_DN2739_c0_g1_i1_1	0	0	0	0	0	0	0	0	0	368430	0	292490
TRINITY_DN6721_c1_g1_i1_1	0	0	0	0	0	0	0	0	0	660830	0	0
TRINITY_DN7299_c0_g1_i1_1	0	0	128130	529920	0	0	0	0	0	0	0	0
TRINITY_DN2661_c0_g2_i1_1	0	0	0	0	0	0	0	0	0	296560	227640	133150
TRINITY_DN7083_c0_g2_i2_1	0	0	0	0	0	0	0	0	0	309030	0	346630

TRINITY_DN18425_c0_g1_i1_2	0	0	0	0	0	0	0	0	0	376060	0	278370
TRINITY_DN11416_c0_g1_i1_3	0	0	0	0	0	0	0	0	0	266490	162620	223820
TRINITY_DN9924_c0_g1_i1_2	37526	0	0	0	0	0	0	0	527470	85956	0	0
TRINITY_DN8093_c0_g1_i1_2	0	0	0	0	0	0	0	0	0	0	409650	240970
TRINITY_DN766_c0_g1_i1_2	64404	83323	107380	223550	171140	0	0	0	0	0	0	0
TRINITY_DN10380_c0_g1_i2_1	0	0	0	0	0	0	0	526170	0	0	121020	0
TRINITY_DN2440_c0_g1_i2_2	0	0	0	0	0	0	0	0	0	208820	0	438030
TRINITY_DN18420_c0_g1_i1_3	0	0	0	0	0	0	0	0	0	331670	147980	167010
TRINITY_DN7383_c0_g1_i1_3	0	0	0	0	0	0	0	0	0	0	328120	318220
TRINITY_DN14144_c0_g1_i1_6	0	0	0	0	0	0	0	0	0	208800	190310	246800
TRINITY_DN16953_c0_g1_i1_2	0	0	0	0	0	0	0	0	0	271620	187740	185500
TRINITY_DN10509_c0_g1_i1_3	136100	207920	210190	0	0	0	0	0	0	89219	0	0
TRINITY_DN7183_c0_g1_i2_1	0	0	0	0	0	0	0	0	0	362540	280790	0
TRINITY_DN17895_c0_g1_i1_1	0	0	0	0	0	0	0	0	0	469890	173090	0
TRINITY_DN9983_c0_g1_i1_1	0	0	0	0	0	0	0	0	0	68948	239360	334600
TRINITY_DN1054_c0_g1_i1_1	0	101680	108220	193490	144820	92722	0	0	0	0	0	0
TRINITY_DN1267_c0_g1_i1_6	0	0	0	0	0	0	0	0	0	249460	217410	173920
TRINITY_DN10960_c0_g1_i1_3	0	0	0	0	0	0	0	0	0	228560	411610	0
TRINITY_DN20312_c0_g1_i1_4	0	0	0	0	0	0	0	0	0	0	0	639520
TRINITY_DN18128_c0_g1_i1_2	0	0	0	0	0	0	0	0	0	636540	0	0
TRINITY_DN8563_c0_g1_i1_2	0	0	0	0	0	0	0	0	0	0	0	636000
TRINITY_DN6232_c0_g1_i3_2	0	0	0	0	0	0	0	0	0	236600	101630	297750
TRINITY_DN5437_c0_g1_i1_2	0	0	0	0	0	0	0	0	0	153490	102260	378660
TRINITY_DN3837_c0_g1_i1_3	0	0	0	0	0	0	0	0	0	153440	246720	231100
TRINITY_DN4426_c0_g1_i1_2	0	0	0	0	0	0	0	0	0	286840	192510	151600
TRINITY_DN13216_c0_g1_i1_3	0	0	0	0	0	0	0	629830	0	0	0	0
TRINITY_DN8517_c0_g1_i3_3	0	0	0	0	0	0	0	0	0	208190	322470	94162
TRINITY_DN17094_c0_g1_i1_4	0	0	46284	0	366820	210780	0	0	0	0	0	0
TRINITY_DN20326_c0_g1_i1_2	0	0	0	0	0	0	0	0	0	267750	0	355620
TRINITY_DN7535_c0_g2_i1_1	0	0	0	0	0	0	0	0	0	328220	294680	0
TRINITY_DN7228_c0_g1_i2_1	0	0	0	0	0	0	0	0	0	282540	149650	190650
TRINITY_DN16259_c0_g1_i1_3	0	0	0	0	0	0	0	0	0	123170	499550	0
TRINITY_DN21689_c0_g1_i1_3	103090	106730	132390	0	278990	0	0	0	0	0	0	0

TRINITY_DN8651_c0_g2_i1_2	0	0	0	0	0	0	0	0	0	0	0	620450
TRINITY_DN11262_c0_g1_i1_3	0	0	0	0	0	0	0	0	0	222770	218830	177830
TRINITY_DN2292_c0_g3_i1_5	99270	123490	395900	0	0	0	0	0	0	0	0	0
TRINITY_DN12820_c0_g1_i1_2	0	0	0	0	0	131930	0	0	0	355710	130460	0
TRINITY_DN21154_c0_g1_i1_4	0	0	0	0	0	0	0	0	0	200910	223680	192450
TRINITY_DN17026_c0_g1_i1_1	215150	0	0	0	0	0	401760	0	0	0	0	0
TRINITY_DN14578_c0_g1_i1_4	0	531780	0	0	84459	0	0	0	0	0	0	0
TRINITY_DN5368_c0_g1_i2_5	0	0	0	0	0	0	0	0	0	614960	0	0
TRINITY_DN17464_c0_g1_i1_1	0	0	0	0	0	0	0	0	0	257150	356730	0
TRINITY_DN8742_c0_g1_i1_3	0	0	0	0	0	0	0	0	0	225420	201480	186940
TRINITY_DN14137_c0_g1_i1_2	0	0	0	0	0	0	0	0	0	191790	152060	268910
TRINITY_DN2434_c0_g1_i1_5	0	0	0	0	0	0	0	0	0	322890	289120	0
TRINITY_DN10474_c1_g1_i1_2	0	0	0	0	0	0	0	0	0	461540	149860	0
TRINITY_DN12774_c0_g1_i1_1	0	0	0	0	0	0	0	0	611310	0	0	0
TRINITY_DN3904_c0_g1_i1_3	0	0	0	0	0	0	0	0	0	0	67857	543430
TRINITY_DN19463_c0_g1_i1_1	0	0	0	0	0	0	0	0	0	220520	199010	190010
TRINITY_DN8904_c0_g2_i1_3	0	0	0	0	0	0	0	0	0	385400	0	223330
TRINITY_DN8767_c0_g1_i1_3	0	0	0	0	0	0	0	0	0	248530	239600	120580
TRINITY_DN3578_c0_g1_i1_2	0	0	0	0	0	0	0	0	0	0	0	598590
TRINITY_DN15270_c0_g1_i1_2	0	0	0	0	0	0	0	0	0	309610	287980	0
TRINITY_DN1008_c0_g2_i1_2	0	0	0	0	0	0	0	0	0	0	594040	0
TRINITY_DN13945_c0_g1_i1_1	0	0	0	0	0	0	0	0	0	593070	0	0
TRINITY_DN7325_c0_g1_i1_1	0	0	0	0	0	138110	0	0	0	0	0	454910
TRINITY_DN11924_c0_g1_i1_2	0	0	0	0	0	0	0	0	0	290830	106190	195050
TRINITY_DN10508_c0_g1_i3_5	0	62903	0	0	0	0	0	0	0	315150	213420	0
TRINITY_DN7011_c0_g1_i1_2	0	0	0	0	0	0	0	0	0	204220	386960	0
TRINITY_DN3619_c0_g1_i1_2	0	0	0	0	0	0	0	0	0	0	394820	194870
TRINITY_DN14686_c0_g1_i1_1	0	0	0	0	0	0	0	0	0	0	0	585170
TRINITY_DN9581_c0_g1_i2_2	0	0	0	0	0	0	0	0	0	421700	0	163190
TRINITY_DN9707_c0_g1_i1_1	0	0	0	0	0	0	0	0	0	584680	0	0
TRINITY_DN8938_c0_g1_i1_2	0	0	0	0	0	0	0	0	0	432270	150710	0
TRINITY_DN10825_c0_g1_i4_1	79766	0	0	0	0	0	0	0	0	187650	168220	146400
TRINITY_DN7033_c0_g2_i1_2	0	0	0	0	0	0	0	0	0	192480	388670	0

TRINITY_DN10999_c0_g1_i5_2	0	0	0	0	0	0	0	0	0	179570	399550	0
TRINITY_DN3792_c0_g1_i1_2	0	0	0	0	0	0	0	0	0	392060	0	186170
TRINITY_DN15541_c0_g1_i1_1	0	0	0	0	0	0	0	0	0	578030	0	0
TRINITY_DN2393_c0_g1_i1_3	0	0	0	0	0	0	0	0	0	434390	141040	0
TRINITY_DN10184_c0_g1_i1_3	0	0	0	0	0	0	0	0	0	224280	351050	0
TRINITY_DN7705_c0_g1_i2_6	0	0	0	0	0	0	0	0	0	344620	93537	137140
TRINITY_DN7315_c0_g1_i1_4	0	0	0	0	0	0	0	0	0	375430	0	198200
TRINITY_DN8439_c0_g1_i1_6	0	0	0	292250	0	0	0	0	0	135690	142990	0
TRINITY_DN3610_c0_g2_i1_2	0	0	0	0	0	0	0	0	0	324630	0	245830
TRINITY_DN3307_c0_g1_i1_1	0	0	0	0	0	0	0	0	0	568930	0	0
TRINITY_DN18256_c0_g1_i1_4	0	0	0	0	79385	181060	0	0	0	308110	0	0
TRINITY_DN11948_c0_g1_i1_6	0	0	0	0	0	0	0	0	0	214230	187040	167010
TRINITY_DN5672_c0_g1_i1_6	0	266290	298280	0	0	0	0	0	0	0	0	0
TRINITY_DN138_c0_g1_i1_3	0	0	0	0	0	0	0	0	0	0	465640	98276
TRINITY_DN10435_c0_g1_i1_1	0	0	0	0	0	0	0	0	0	352220	211150	0
TRINITY_DN10493_c0_g1_i1_1	0	0	89624	0	0	0	0	0	51461	201680	0	219250
TRINITY_DN10290_c0_g1_i1_1	0	0	0	0	0	0	0	0	0	226900	334150	0
TRINITY_DN12636_c0_g1_i1_3	0	0	0	0	0	90935	0	469520	0	0	0	0
TRINITY_DN14818_c0_g1_i1_6	0	0	0	0	0	0	0	0	0	386330	174000	0
TRINITY_DN360_c0_g1_i1_4	0	0	0	0	0	0	0	0	0	328970	127390	103720
TRINITY_DN7953_c0_g2_i1_2	0	0	0	0	0	0	0	0	0	217350	340760	0
TRINITY_DN8751_c1_g1_i1_3	0	0	184570	0	0	0	372210	0	0	0	0	0
TRINITY_DN19806_c0_g1_i1_2	0	0	0	0	0	0	0	0	0	555960	0	0
TRINITY_DN18164_c0_g1_i1_4	0	0	0	0	0	0	0	0	0	308680	247270	0
TRINITY_DN7968_c0_g1_i1_1	0	0	0	0	0	0	0	0	0	0	281090	272730
TRINITY_DN7925_c0_g2_i1_4	0	0	0	0	0	0	0	0	0	291770	0	261660
TRINITY_DN8225_c0_g1_i1_3	0	0	0	0	0	0	0	0	0	0	0	553400
TRINITY_DN7814_c0_g1_i1_2	0	0	0	0	0	0	337000	0	0	214450	0	0
TRINITY_DN21393_c0_g1_i1_4	0	0	0	0	0	0	0	0	0	166940	148080	236360
TRINITY_DN20157_c0_g1_i1_3	0	0	0	383330	0	167640	0	0	0	0	0	0
TRINITY_DN4739_c0_g2_i1_6	0	0	0	0	0	0	0	0	0	550270	0	0
TRINITY_DN6504_c0_g1_i1_3	0	351010	0	198320	0	0	0	0	0	0	0	0
TRINITY_DN9930_c0_g1_i3_4	258100	0	0	0	290900	0	0	0	0	0	0	0

TRINITY_DN11687_c0_g1_i1_1	0	0	0	0	0	0	0	0	0	0	162300	385880
TRINITY_DN968_c0_g1_i1_2	0	0	0	0	0	0	0	0	0	0	277110	270310
TRINITY_DN8156_c0_g1_i1_6	0	0	0	0	0	0	0	0	0	539770	0	0
TRINITY_DN9403_c0_g1_i2_1	0	0	0	0	0	0	0	0	0	0	241550	297900
TRINITY_DN10308_c0_g2_i1_1	0	0	0	0	0	0	0	0	0	537420	0	0
TRINITY_DN5427_c0_g1_i1_3	0	0	0	0	0	0	0	0	0	536700	0	0
TRINITY_DN10971_c0_g1_i2_2	0	0	0	0	0	0	194640	0	0	98001	128240	115320
TRINITY_DN21130_c0_g1_i1_6	0	0	0	0	0	0	0	0	0	460290	0	75216
TRINITY_DN7507_c0_g1_i1_6	0	0	0	0	0	0	0	0	0	202520	165250	167110
TRINITY_DN8691_c0_g1_i1_4	0	0	0	67762	0	0	0	0	0	279250	0	187780
TRINITY_DN13949_c0_g1_i2_2	0	0	0	0	0	0	0	0	0	0	336200	198330
TRINITY_DN17228_c0_g1_i1_3	0	0	0	0	0	0	0	0	0	294900	0	239300
TRINITY_DN15289_c0_g1_i1_4	69817	47632	0	0	99574	0	314870	0	0	0	0	0
TRINITY_DN3649_c0_g1_i1_2	0	0	0	0	0	0	0	0	0	293880	0	237410
TRINITY_DN3740_c0_g2_i1_2	0	0	0	0	0	0	0	0	0	294490	0	236430
TRINITY_DN18927_c0_g1_i1_2	0	0	0	0	0	0	0	0	0	231270	299530	0
TRINITY_DN8933_c1_g1_i1_2	0	0	0	0	0	0	0	0	0	259700	0	269850
TRINITY_DN16700_c0_g1_i1_4	0	0	0	0	0	0	0	0	0	0	0	529390
TRINITY_DN709_c0_g2_i1_2	0	0	0	0	0	0	0	0	0	106180	422500	0
TRINITY_DN813_c0_g1_i1_3	0	0	0	0	526990	0	0	0	0	0	0	0
TRINITY_DN20089_c0_g1_i1_1	234120	0	147420	144980	0	0	0	0	0	0	0	0
TRINITY_DN19940_c0_g1_i1_4	0	0	0	0	0	0	0	0	0	199780	132690	193020
TRINITY_DN16604_c0_g1_i1_3	0	0	0	0	0	0	0	0	0	525080	0	0
TRINITY_DN9343_c0_g1_i1_2	0	0	0	0	0	0	0	0	0	267990	131290	124370
TRINITY_DN8561_c0_g1_i4_1	0	0	0	0	0	0	0	0	0	161240	84491	276470
TRINITY_DN21803_c0_g1_i1_1	0	0	0	0	0	0	0	0	0	257260	118070	145560
TRINITY_DN4286_c0_g1_i1_4	0	0	0	0	0	0	0	0	0	148930	255950	115890
TRINITY_DN5644_c1_g3_i1_6	0	0	0	0	518930	0	0	0	0	0	0	0
TRINITY_DN11599_c0_g1_i3_2	0	54170	68821	0	394940	0	0	0	0	0	0	0
TRINITY_DN17497_c0_g1_i1_2	0	0	0	0	0	0	0	0	0	400700	0	116790
TRINITY_DN3550_c0_g2_i1_2	0	0	0	0	0	0	0	0	0	418620	0	98199
TRINITY_DN4310_c0_g1_i1_1	167420	183970	163420	0	0	0	0	0	0	0	0	0
TRINITY_DN21144_c0_g1_i1_4	0	51041	0	0	0	0	0	0	0	463140	0	0

TRINITY_DN23569_c0_g1_i1_2	0	0	0	0	0	0	0	0	0	203870	144630	165510
TRINITY_DN2786_c0_g1_i1_5	0	0	0	0	0	0	0	0	0	287410	225150	0
TRINITY_DN5005_c0_g1_i1_1	0	0	0	0	0	0	0	0	0	153810	241080	117620
TRINITY_DN5974_c0_g1_i1_3	0	0	0	0	0	0	0	0	0	0	368740	143580
TRINITY_DN6400_c0_g1_i4_1	0	0	0	0	0	0	0	0	0	305480	204880	0
TRINITY_DN11632_c0_g1_i2_5	0	0	0	0	154330	113640	0	0	0	0	124650	116900
TRINITY_DN17813_c0_g1_i1_1	0	0	0	0	0	0	0	0	0	304620	0	204250
TRINITY_DN17235_c0_g1_i1_3	0	0	0	0	0	0	0	0	0	186350	166890	155470
TRINITY_DN6926_c0_g1_i1_6	0	0	0	0	0	0	0	0	0	150210	259600	98889
TRINITY_DN17803_c0_g2_i1_3	0	0	0	0	0	0	0	0	0	408000	98568	0
TRINITY_DN2011_c0_g1_i1_4	0	0	0	0	0	0	0	0	0	339410	0	166680
TRINITY_DN8075_c0_g1_i4_2	0	0	0	0	0	0	0	0	0	197010	0	306840
TRINITY_DN2579_c0_g1_i1_2	0	0	0	0	0	0	0	0	0	288870	0	213360
TRINITY_DN19468_c0_g1_i1_2	0	0	0	0	0	0	0	0	0	283370	0	218190
TRINITY_DN3475_c0_g2_i1_2	0	0	0	0	0	0	0	0	0	202430	0	296780
TRINITY_DN4140_c0_g1_i1_5	0	0	0	0	0	0	499110	0	0	0	0	0
TRINITY_DN545_c0_g1_i1_2	0	0	0	0	0	0	0	0	0	0	0	498630
TRINITY_DN6995_c0_g1_i1_6	0	0	0	0	0	0	0	0	0	207680	146070	144780
TRINITY_DN9117_c0_g1_i2_2	0	0	0	0	0	0	0	0	0	349290	0	148090
TRINITY_DN14629_c0_g1_i1_3	0	0	0	0	0	0	0	0	0	267600	229100	0
TRINITY_DN8532_c0_g1_i1_2	0	0	0	0	0	0	0	0	0	0	496370	0
TRINITY_DN9825_c0_g1_i1_3	0	0	0	0	0	0	0	0	0	136400	264780	91062
TRINITY_DN2131_c0_g1_i1_6	0	0	0	0	0	0	0	0	0	273260	0	218870
TRINITY_DN2699_c0_g1_i1_6	0	0	0	0	0	0	0	0	0	206990	163150	120250
TRINITY_DN10766_c0_g1_i8_3	0	489370	0	0	0	0	0	0	0	0	0	0
TRINITY_DN6812_c0_g1_i7_1	0	0	0	0	0	0	0	0	0	149220	0	339750
TRINITY_DN19649_c0_g1_i1_5	0	0	0	0	0	0	0	0	0	0	134870	353710
TRINITY_DN10388_c0_g2_i1_5	0	0	0	0	0	0	0	0	0	122010	194340	170750
TRINITY_DN8750_c0_g2_i1_3	0	0	0	0	0	0	0	0	0	484480	0	0
TRINITY_DN17662_c0_g2_i1_1	0	0	0	0	0	0	0	0	0	0	263330	221100
TRINITY_DN5306_c0_g1_i1_1	0	0	0	0	0	0	0	0	0	151030	161000	171860
TRINITY_DN4670_c0_g1_i1_2	0	0	0	0	0	0	0	0	0	302990	176780	0
TRINITY_DN9078_c0_g1_i2_3	0	0	0	0	142800	336370	0	0	0	0	0	0

TRINITY_DN8091_c0_g1_i1_6	0	0	0	0	0	0	0	0	0	239750	79744	158450
TRINITY_DN12406_c0_g1_i1_2	0	0	0	245160	0	232630	0	0	0	0	0	0
TRINITY_DN17368_c0_g1_i1_2	0	0	0	0	0	0	0	0	0	275150	0	202590
TRINITY_DN9727_c0_g1_i1_3	229760	0	91926	0	0	0	0	0	0	0	153590	0
TRINITY_DN17339_c0_g1_i1_3	38093	0	436710	0	0	0	0	0	0	0	0	0
TRINITY_DN18284_c0_g1_i1_2	0	0	0	0	0	0	0	0	0	230980	243560	0
TRINITY_DN10226_c0_g1_i1_1	0	0	0	0	0	0	0	0	0	0	474080	0
TRINITY_DN8973_c0_g1_i1_1	243340	114610	115910	0	0	0	0	0	0	0	0	0
TRINITY_DN16264_c0_g1_i1_2	0	0	0	0	0	0	0	0	0	0	473780	0
TRINITY_DN12122_c0_g1_i1_3	0	0	0	0	0	0	0	0	0	145830	119690	207960
TRINITY_DN4741_c0_g1_i1_2	0	0	0	0	0	0	0	0	0	0	367440	104760
TRINITY_DN20857_c0_g1_i1_2	0	0	0	0	0	0	0	470100	0	0	0	0
TRINITY_DN13782_c0_g1_i1_1	186970	0	0	0	0	157380	0	0	0	0	0	125040
TRINITY_DN4532_c0_g1_i1_2	0	0	0	0	0	0	0	0	0	203850	152310	112940
TRINITY_DN39_c0_g2_i1_6	106490	124710	121250	0	0	116140	0	0	0	0	0	0
TRINITY_DN20415_c0_g1_i1_2	0	0	0	0	0	0	0	0	0	252630	214340	0
TRINITY_DN9710_c1_g1_i1_6	0	0	0	0	0	0	0	0	0	320670	0	143680
TRINITY_DN371_c0_g1_i1_2	0	0	0	0	0	0	0	0	0	257640	206200	0
TRINITY_DN20208_c0_g1_i1_1	0	0	0	0	0	0	0	0	0	174450	147860	139880
TRINITY_DN11629_c0_g1_i3_3	116630	0	153670	0	190030	0	0	0	0	0	0	0
TRINITY_DN12689_c0_g1_i1_2	0	0	0	0	0	0	0	0	0	248150	112020	99706
TRINITY_DN8223_c0_g1_i4_6	0	0	0	0	0	0	0	0	0	0	0	459680
TRINITY_DN20465_c0_g1_i1_6	0	0	0	0	0	0	0	0	0	0	261010	197920
TRINITY_DN7564_c1_g2_i2_4	0	0	0	0	0	0	0	0	0	0	209460	249270
TRINITY_DN10977_c0_g2_i3_5	0	0	0	0	0	0	0	0	0	0	293510	163890
TRINITY_DN23633_c0_g1_i1_2	0	0	0	0	0	0	0	0	0	456270	0	0
TRINITY_DN6603_c0_g1_i1_3	0	0	0	0	0	0	0	0	0	291280	164260	0
TRINITY_DN9814_c0_g1_i2_2	0	0	0	0	0	0	0	0	335950	0	117060	0
TRINITY_DN7007_c0_g1_i1_6	0	0	0	0	0	0	0	0	0	166860	148600	137320
TRINITY_DN14463_c0_g1_i1_1	0	0	0	0	0	0	0	0	451970	0	0	0
TRINITY_DN6833_c0_g1_i1_2	0	0	0	0	0	0	0	0	0	0	234430	217490
TRINITY_DN839_c0_g1_i2_1	0	0	0	0	0	0	0	0	0	161650	109860	180020
TRINITY_DN20539_c0_g1_i1_2	0	0	0	0	0	0	0	0	0	272540	0	178990

TRINITY_DN3431_c0_g1_i1_2	0	0	0	0	0	0	0	0	0	451190	0	0
TRINITY_DN11964_c0_g1_i1_3	0	0	0	0	0	0	0	0	0	450420	0	0
TRINITY_DN4866_c0_g2_i1_1	0	0	0	0	0	0	0	0	0	150350	91639	207410
TRINITY_DN16866_c0_g1_i1_6	67939	67335	110560	0	203100	0	0	0	0	0	0	0
TRINITY_DN13956_c0_g1_i1_2	0	0	0	0	0	0	0	0	0	0	214370	233860
TRINITY_DN4399_c0_g1_i1_6	0	0	0	0	0	0	0	0	0	0	234340	213420
TRINITY_DN10671_c0_g1_i5_2	0	0	0	0	0	0	0	0	0	180150	129750	137440
TRINITY_DN3450_c0_g1_i1_1	0	0	0	0	0	0	0	0	0	136530	0	306080
TRINITY_DN19533_c0_g1_i1_2	0	0	0	0	0	0	0	0	0	146180	91247	205170
TRINITY_DN14184_c0_g1_i1_2	0	0	0	0	0	0	0	0	0	198500	71891	171030
TRINITY_DN10011_c0_g2_i1_5	0	0	253600	0	0	0	0	186000	0	0	0	0
TRINITY_DN10051_c0_g1_i3_2	0	0	0	0	0	0	0	0	0	284090	154970	0
TRINITY_DN9084_c1_g1_i1_6	0	246220	192470	0	0	0	0	0	0	0	0	0
TRINITY_DN13392_c0_g1_i1_2	0	0	0	0	0	139950	0	0	0	298320	0	0
TRINITY_DN5114_c0_g1_i1_5	0	0	0	0	0	0	0	0	0	245180	0	192780
TRINITY_DN18316_c0_g1_i1_1	0	0	0	0	0	0	0	0	0	235220	201730	0
TRINITY_DN764_c0_g1_i1_4	0	0	0	0	0	0	0	0	0	316100	0	119890
TRINITY_DN10979_c0_g1_i3_3	0	0	0	0	0	0	0	0	0	0	221550	212500
TRINITY_DN10800_c0_g1_i2_2	0	0	0	0	0	0	0	0	0	145280	0	287780
TRINITY_DN14978_c0_g1_i1_4	0	0	0	0	0	0	0	0	0	0	432850	0
TRINITY_DN6214_c0_g1_i1_1	0	0	0	0	0	0	0	0	0	189980	95579	146820
TRINITY_DN14988_c0_g2_i1_4	0	0	0	0	0	0	0	0	0	236320	195960	0
TRINITY_DN6278_c0_g1_i1_2	0	96853	0	114440	126970	0	0	0	0	0	92910	0
TRINITY_DN6964_c0_g1_i1_1	0	0	0	0	0	0	192110	0	0	130640	108290	0
TRINITY_DN3722_c0_g1_i1_4	0	0	0	0	0	0	0	0	0	140570	0	290420
TRINITY_DN22328_c0_g1_i1_3	0	0	0	0	0	0	0	0	0	185060	122960	119830
TRINITY_DN18052_c0_g2_i1_1	0	218370	208820	0	0	0	0	0	0	0	0	0
TRINITY_DN11467_c0_g1_i1_1	0	425580	0	0	0	0	0	0	0	0	0	0
TRINITY_DN15189_c0_g1_i1_3	0	0	0	0	0	0	0	0	0	241600	0	183820
TRINITY_DN14289_c0_g2_i1_2	0	0	0	0	0	0	0	0	0	223830	201190	0
TRINITY_DN1005_c0_g1_i2_3	0	0	0	0	0	0	0	0	0	159220	138980	125260
TRINITY_DN3819_c0_g1_i2_3	0	0	0	0	0	0	0	0	0	104200	318620	0
TRINITY_DN7943_c0_g1_i2_2	0	0	0	0	0	0	0	0	0	202460	99083	119590

TRINITY_DN2112_c0_g2_i1_6	0	0	0	0	0	0	0	0	0	227680	0	192890
TRINITY_DN10350_c1_g1_i4_2	0	0	0	0	0	0	0	0	0	241940	0	178140
TRINITY_DN6884_c1_g3_i1_2	0	0	0	0	0	0	0	0	0	230680	189040	0
TRINITY_DN12265_c0_g1_i1_2	0	0	61553	0	0	0	0	0	0	0	198800	159050
TRINITY_DN21124_c0_g1_i3_3	0	0	0	0	0	0	0	0	0	418810	0	0
TRINITY_DN9012_c0_g1_i1_4	0	0	0	0	0	0	0	0	0	157720	0	260580
TRINITY_DN9946_c0_g2_i4_3	0	0	0	0	0	0	0	0	0	176350	111840	129580
TRINITY_DN5727_c0_g2_i1_6	0	0	0	0	0	0	0	0	0	0	229610	185670
TRINITY_DN12393_c0_g1_i1_4	0	0	0	0	0	0	0	0	0	167800	118340	127620
TRINITY_DN4111_c0_g2_i1_2	0	0	0	0	0	0	0	0	0	169930	129210	112130
TRINITY_DN11057_c0_g8_i1_3	0	0	0	0	0	0	0	0	0	168950	105830	136040
TRINITY_DN2598_c0_g2_i1_3	0	0	0	0	0	0	410130	0	0	0	0	0
TRINITY_DN9900_c0_g1_i2_2	0	0	0	0	0	0	0	0	0	136400	141260	132160
TRINITY_DN3402_c0_g1_i1_5	0	0	0	0	0	0	0	0	0	0	409670	0
TRINITY_DN9871_c0_g1_i2_2	0	0	0	0	0	0	0	0	0	147950	142210	119390
TRINITY_DN9274_c0_g1_i1_2	0	0	0	0	0	0	0	0	0	0	162710	246120
TRINITY_DN20612_c0_g1_i1_1	47752	55424	53672	0	0	251930	0	0	0	0	0	0
TRINITY_DN21955_c0_g1_i1_1	0	0	0	0	0	0	0	0	0	168810	239700	0
TRINITY_DN7460_c0_g1_i1_6	0	0	0	0	0	0	0	0	0	261260	147040	0
TRINITY_DN6570_c0_g1_i1_6	0	0	0	0	0	0	0	0	0	408140	0	0
TRINITY_DN12116_c0_g1_i1_5	0	0	0	0	0	0	0	0	0	266750	0	140510
TRINITY_DN18165_c0_g1_i1_3	0	0	0	0	0	0	0	0	0	0	406370	0
TRINITY_DN19285_c0_g1_i1_2	0	0	0	0	0	0	0	0	0	0	283030	122050
TRINITY_DN4092_c0_g1_i1_3	0	0	0	0	0	0	0	0	0	403610	0	0
TRINITY_DN4871_c0_g1_i1_3	0	26502	0	0	0	0	0	0	0	376800	0	0
TRINITY_DN23190_c0_g1_i1_4	0	0	0	0	0	0	0	0	0	242910	0	159690
TRINITY_DN2267_c0_g1_i1_5	0	0	0	0	0	0	0	0	0	0	243100	158500
TRINITY_DN12570_c0_g1_i1_4	0	0	0	400720	0	0	0	0	0	0	0	0
TRINITY_DN19686_c0_g1_i1_5	0	0	0	0	0	0	0	0	0	262730	136420	0
TRINITY_DN15214_c0_g1_i1_3	0	0	0	0	0	0	0	0	0	0	395520	0
TRINITY_DN18839_c0_g1_i1_6	0	0	0	243780	0	148680	0	0	0	0	0	0
TRINITY_DN12937_c0_g1_i1_3	0	0	0	0	0	0	0	0	0	213510	0	178920
TRINITY_DN3038_c0_g1_i1_4	0	0	0	0	0	0	0	0	0	222890	168160	0

TRINITY_DN3285_c0_g1_i1_1	0	0	0	0	0	0	0	0	0	146300	116950	126480
TRINITY_DN4341_c0_g1_i1_2	0	0	0	0	0	0	0	0	0	221680	167940	0
TRINITY_DN5664_c0_g1_i1_3	0	0	0	0	0	0	0	0	0	268780	120710	0
TRINITY_DN3970_c0_g1_i1_1	0	0	0	0	0	0	0	0	0	0	0	389210
TRINITY_DN12410_c0_g1_i1_6	0	0	45271	0	138050	0	0	0	0	0	0	205580
TRINITY_DN4354_c0_g1_i1_4	0	197010	0	101090	90119	0	0	0	0	0	0	0
TRINITY_DN9720_c0_g1_i6_2	0	0	0	0	0	0	0	386240	0	0	0	0
TRINITY_DN22980_c0_g1_i1_3	0	0	0	0	0	0	0	0	0	215950	167890	0
TRINITY_DN9256_c0_g1_i4_1	0	0	0	0	0	0	0	0	0	187800	192590	0
TRINITY_DN8333_c0_g1_i1_3	0	95706	77065	0	207140	0	0	0	0	0	0	0
TRINITY_DN294_c0_g1_i1_2	0	0	0	0	0	0	0	0	0	148880	110740	118830
TRINITY_DN8005_c0_g1_i2_2	0	0	0	0	0	0	0	0	0	0	0	377860
TRINITY_DN17859_c0_g1_i1_3	0	0	0	0	0	0	0	0	0	166090	91841	115350
TRINITY_DN1542_c0_g1_i1_6	63560	74776	102020	0	0	131460	0	0	0	0	0	0
TRINITY_DN7035_c1_g2_i1_3	156560	0	0	0	214310	0	0	0	0	0	0	0
TRINITY_DN19252_c0_g1_i1_4	0	0	0	0	0	0	0	0	0	102820	167910	99617
TRINITY_DN1690_c0_g1_i2_5	0	0	0	0	0	0	0	0	0	0	0	370100
TRINITY_DN18166_c0_g1_i1_4	0	0	0	0	0	0	0	0	369840	0	0	0
TRINITY_DN9981_c1_g1_i1_3	0	0	0	0	0	92796	0	0	0	0	276550	0
TRINITY_DN8032_c0_g1_i1_2	0	0	0	0	0	0	0	0	0	133120	90774	144820
TRINITY_DN10046_c0_g2_i1_4	0	0	0	0	0	0	0	0	0	259620	0	108540
TRINITY_DN21517_c0_g1_i1_4	201180	0	0	0	165660	0	0	0	0	0	0	0
TRINITY_DN316_c0_g1_i1_1	0	0	0	0	0	0	0	0	0	187070	177930	0
TRINITY_DN8057_c0_g1_i2_5	0	0	0	0	0	0	0	0	0	220690	143000	0
TRINITY_DN22718_c0_g1_i1_2	0	165470	197160	0	0	0	0	0	0	0	0	0
TRINITY_DN5217_c0_g1_i1_4	0	0	0	0	0	0	0	0	0	145570	131620	85177
TRINITY_DN8159_c0_g2_i1_1	0	0	0	0	0	0	0	0	0	237130	121280	0
TRINITY_DN2797_c0_g2_i1_4	0	0	0	0	0	0	0	0	0	0	175860	182200
TRINITY_DN3030_c0_g1_i1_6	0	0	0	0	0	0	0	0	0	144100	102680	107380
TRINITY_DN16052_c0_g1_i1_2	0	0	0	0	0	0	0	0	0	98812	65319	188930
TRINITY_DN8771_c0_g1_i1_2	0	0	0	0	0	0	0	0	0	352490	0	0
TRINITY_DN5711_c0_g2_i1_5	0	0	0	0	0	0	0	0	0	196460	0	155990
TRINITY_DN11595_c1_g1_i1_3	0	0	0	0	0	0	0	0	0	160340	135820	56271

TRINITY_DN18730_c0_g1_i1_1	0	0	0	0	0	0	0	0	0	133720	111680	106430
TRINITY_DN5685_c0_g1_i4_5	67474	35270	45522	0	0	0	0	0	0	203520	0	0
TRINITY_DN11028_c0_g3_i1_3	349950	0	0	0	0	0	0	0	0	0	0	0
TRINITY_DN5255_c1_g1_i1_4	0	0	0	0	0	0	0	0	0	189590	159740	0
TRINITY_DN10013_c0_g1_i4_2	0	0	0	0	0	0	0	0	0	209590	0	138500
TRINITY_DN6316_c0_g1_i4_3	0	0	0	0	0	0	0	0	0	145300	80208	120850
TRINITY_DN7363_c0_g1_i1_3	0	0	0	0	0	0	0	0	0	91553	174220	80240
TRINITY_DN18596_c0_g1_i1_5	0	0	0	0	0	0	0	0	0	185410	0	159800
TRINITY_DN17114_c0_g1_i1_1	0	0	0	0	0	0	0	0	0	0	177750	166260
TRINITY_DN19946_c0_g1_i1_4	0	0	0	0	0	0	0	0	0	0	152260	191290
TRINITY_DN8995_c0_g1_i1_3	0	0	0	0	0	0	0	0	0	106700	151770	84822
TRINITY_DN9650_c0_g1_i1_1	0	0	0	0	0	343080	0	0	0	0	0	0
TRINITY_DN7338_c0_g3_i1_2	115320	114810	112340	0	0	0	0	0	0	0	0	0
TRINITY_DN4072_c0_g2_i1_2	0	0	0	0	0	0	0	0	0	171360	86722	82940
TRINITY_DN2062_c0_g1_i1_6	0	0	0	0	0	0	0	0	0	0	339970	0
TRINITY_DN5791_c1_g1_i1_2	0	0	0	0	0	0	0	0	0	339810	0	0
TRINITY_DN6320_c0_g2_i1_1	0	0	0	0	0	0	0	0	0	208790	0	129650
TRINITY_DN6069_c0_g2_i1_6	0	0	0	0	0	0	0	0	0	184810	0	153370
TRINITY_DN18724_c0_g1_i1_4	0	0	0	0	0	0	0	0	0	214930	0	122180
TRINITY_DN4227_c0_g1_i1_3	0	0	0	0	0	0	0	0	0	142250	94900	99168
TRINITY_DN18944_c0_g1_i1_1	0	0	0	0	0	0	0	0	0	0	265520	70302
TRINITY_DN9604_c0_g1_i3_3	0	0	0	0	0	0	0	0	0	112940	123580	97979
TRINITY_DN19600_c0_g1_i1_6	0	0	0	0	0	0	0	0	0	0	155160	176060
TRINITY_DN2329_c0_g1_i2_6	0	0	0	0	0	0	0	0	0	194520	0	136180
TRINITY_DN827_c0_g1_i1_1	0	0	0	0	0	0	0	0	0	0	169040	161640
TRINITY_DN8908_c0_g1_i2_1	0	0	0	0	0	0	0	0	0	133980	0	196180
TRINITY_DN18109_c0_g1_i1_5	0	0	0	0	0	0	0	0	0	174960	155040	0
TRINITY_DN11206_c0_g1_i2_4	0	0	0	0	0	0	0	0	0	170620	158350	0
TRINITY_DN18626_c1_g2_i1_2	0	0	0	0	0	0	0	0	0	0	0	327420
TRINITY_DN21756_c0_g1_i1_1	0	0	0	0	0	0	0	0	0	327420	0	0
TRINITY_DN2355_c0_g1_i1_2	0	0	0	0	0	0	0	0	0	115570	101630	110070
TRINITY_DN2509_c0_g1_i1_1	158680	0	0	0	0	168110	0	0	0	0	0	0
TRINITY_DN19715_c0_g1_i1_5	0	0	0	0	0	0	0	0	0	236270	0	90244

TRINITY_DN4748_c0_g1_i1_2	0	0	0	0	0	0	0	0	0	0	176630	148070
TRINITY_DN21521_c1_g1_i1_3	0	0	0	0	0	0	0	0	0	127090	104410	92175
TRINITY_DN5242_c0_g3_i1_6	0	0	0	0	0	0	0	0	0	172740	150880	0
TRINITY_DN19260_c0_g1_i1_1	0	0	0	0	0	0	0	0	0	101220	115420	106870
TRINITY_DN3078_c0_g1_i1_3	0	0	0	0	0	0	0	0	0	117230	97865	107480
TRINITY_DN10865_c0_g2_i2_2	0	0	0	182530	0	140020	0	0	0	0	0	0
TRINITY_DN17892_c1_g1_i1_5	0	0	0	0	0	0	0	0	0	196080	125750	0
TRINITY_DN2656_c0_g1_i1_2	0	0	0	0	0	0	0	0	0	135420	108590	77746
TRINITY_DN22169_c0_g1_i1_5	0	0	0	0	0	0	0	0	0	190600	0	131060
TRINITY_DN10693_c1_g1_i1_3	0	0	0	0	0	0	0	0	0	123040	102870	93309
TRINITY_DN7588_c0_g1_i2_5	0	0	0	0	0	0	0	0	0	0	318320	0
TRINITY_DN1809_c0_g1_i1_3	0	0	0	0	0	0	0	0	0	150560	0	166400
TRINITY_DN10089_c0_g3_i1_3	0	0	0	0	0	0	0	0	0	109760	94940	111390
TRINITY_DN2082_c1_g1_i1_3	0	0	0	0	0	0	0	0	0	170890	0	145140
TRINITY_DN238_c0_g1_i1_3	86300	123950	104600	0	0	0	0	0	0	0	0	0
TRINITY_DN5724_c0_g2_i1_1	0	0	0	0	0	0	0	0	0	0	206190	108400
TRINITY_DN21573_c0_g1_i1_1	0	0	0	0	0	0	0	0	0	196190	0	118230
TRINITY_DN11569_c0_g1_i2_3	0	0	0	0	0	0	0	0	0	0	0	313530
TRINITY_DN5094_c0_g1_i1_4	0	0	0	0	0	313140	0	0	0	0	0	0
TRINITY_DN19637_c0_g1_i1_4	0	0	0	0	0	0	0	0	0	0	209060	102620
TRINITY_DN4599_c0_g1_i1_1	0	0	0	0	0	0	0	0	0	173450	138190	0
TRINITY_DN11898_c0_g1_i1_4	0	0	0	0	0	0	0	0	0	311560	0	0
TRINITY_DN13648_c0_g1_i1_3	0	0	0	0	0	0	0	0	0	179860	0	131660
TRINITY_DN22384_c0_g1_i1_6	0	0	0	0	0	0	0	0	0	141050	74975	95274
TRINITY_DN8593_c0_g1_i1_3	0	0	0	0	0	0	0	0	0	120230	188440	0
TRINITY_DN6624_c0_g2_i2_2	0	0	0	0	0	0	0	0	0	124980	182450	0
TRINITY_DN8264_c0_g1_i2_1	0	0	0	0	73207	0	0	0	0	0	234090	0
TRINITY_DN7248_c0_g1_i1_5	0	0	0	0	0	0	0	0	0	0	159700	146280
TRINITY_DN19832_c0_g1_i1_3	0	0	0	0	0	0	0	0	0	122700	182680	0
TRINITY_DN23704_c0_g1_i1_6	0	0	0	0	0	0	0	0	0	0	0	304990
TRINITY_DN15338_c0_g1_i1_3	0	0	0	0	0	0	0	0	0	175660	0	126930
TRINITY_DN9948_c0_g2_i1_3	0	0	0	0	0	0	0	0	0	170790	130870	0
TRINITY_DN13388_c0_g1_i1_5	0	0	0	0	0	0	0	0	0	163570	137020	0

TRINITY_DN5559_c0_g1_i1_4	0	0	0	0	0	0	0	0	0	108190	98744	92953
TRINITY_DN17697_c0_g1_i1_3	0	0	0	0	0	0	0	0	0	159410	0	140160
TRINITY_DN5154_c0_g1_i1_5	0	0	0	0	0	0	0	0	0	113270	103010	82676
TRINITY_DN20546_c0_g1_i1_1	0	0	0	0	0	0	0	0	0	0	0	298880
TRINITY_DN6573_c0_g1_i1_4	0	0	0	0	0	75715	0	0	0	154840	0	68248
TRINITY_DN3293_c0_g1_i1_2	0	0	0	0	0	0	0	0	0	129460	67513	100810
TRINITY_DN9459_c0_g2_i1_1	0	0	0	0	0	0	0	0	0	161910	0	135710
TRINITY_DN4865_c0_g1_i1_1	0	0	0	0	0	0	0	0	0	203850	0	93012
TRINITY_DN3689_c0_g2_i1_5	0	0	0	0	0	0	0	0	0	294050	0	0
TRINITY_DN2686_c0_g1_i1_5	0	0	0	0	0	0	0	0	0	184750	108830	0
TRINITY_DN4162_c0_g1_i1_5	0	0	0	0	0	0	0	0	0	98720	0	194640
TRINITY_DN14842_c0_g1_i1_3	0	0	0	0	0	0	0	0	0	293000	0	0
TRINITY_DN1392_c0_g2_i1_1	0	0	0	0	0	0	0	0	0	102750	99190	90388
TRINITY_DN3303_c0_g1_i1_3	0	0	0	0	0	0	0	0	0	102440	92177	95231
TRINITY_DN5303_c0_g1_i1_3	0	0	0	0	0	0	0	0	0	0	151570	137950
TRINITY_DN3626_c0_g3_i1_6	38658	42672	55257	0	0	151940	0	0	0	0	0	0
TRINITY_DN12852_c0_g1_i1_2	0	0	0	0	0	0	0	0	0	0	287790	0
TRINITY_DN16500_c0_g1_i1_3	0	0	0	0	0	0	0	0	0	286620	0	0
TRINITY_DN13231_c0_g1_i1_1	0	0	0	0	0	0	0	0	0	138230	148140	0
TRINITY_DN9164_c0_g1_i1_1	0	0	0	0	0	0	0	0	0	165560	119930	0
TRINITY_DN6110_c0_g1_i2_1	0	0	0	0	0	0	0	0	0	139950	0	143820
TRINITY_DN9679_c0_g2_i6_4	0	0	0	0	0	0	0	0	0	0	283160	0
TRINITY_DN185_c0_g1_i1_1	0	0	0	0	0	0	0	0	0	0	142200	140800
TRINITY_DN16939_c0_g1_i1_3	0	0	0	0	0	0	0	0	0	105890	108890	66633
TRINITY_DN10156_c0_g1_i1_1	0	0	0	0	0	0	0	0	0	141440	139510	0
TRINITY_DN9970_c0_g1_i3_1	0	0	0	0	0	0	0	0	0	0	280720	0
TRINITY_DN11182_c0_g1_i1_5	0	0	0	0	0	0	0	0	0	0	53681	226320
TRINITY_DN6813_c0_g2_i1_6	0	0	0	0	0	0	0	0	0	55268	224650	0
TRINITY_DN18653_c0_g1_i1_5	0	0	0	0	0	0	0	0	0	279390	0	0
TRINITY_DN17522_c0_g1_i1_4	0	0	0	0	0	0	0	0	0	155300	123370	0
TRINITY_DN14413_c0_g1_i1_4	0	0	0	0	0	0	0	0	0	165870	0	112600
TRINITY_DN5635_c0_g1_i1_5	0	0	0	0	0	0	0	0	0	202590	75765	0
TRINITY_DN10294_c0_g1_i2_1	0	0	0	0	0	0	0	0	0	0	102690	174790

TRINITY_DN19410_c0_g2_i1_2	0	157380	0	0	0	119430	0	0	0	0	0	0
TRINITY_DN3519_c0_g1_i1_4	0	0	0	0	0	0	0	0	0	0	0	276780
TRINITY_DN10650_c0_g1_i3_2	0	0	0	0	0	0	0	0	0	0	0	275680
TRINITY_DN3798_c0_g1_i2_1	0	0	0	0	0	0	0	0	0	126910	148460	0
TRINITY_DN2524_c0_g1_i1_2	0	0	0	0	0	0	0	0	0	274900	0	0
TRINITY_DN14304_c0_g1_i1_3	0	0	0	0	0	0	0	0	0	0	0	274830
TRINITY_DN10946_c0_g1_i1_3	0	0	0	0	0	0	0	0	0	172280	102080	0
TRINITY_DN4824_c0_g1_i1_6	0	0	0	0	0	0	0	0	0	274170	0	0
TRINITY_DN1455_c0_g1_i1_5	0	0	0	0	0	0	0	0	0	274030	0	0
TRINITY_DN19943_c0_g1_i1_1	0	0	0	0	0	0	0	0	0	137180	0	135820
TRINITY_DN7659_c0_g1_i2_1	96649	82657	93173	0	0	0	0	0	0	0	0	0
TRINITY_DN18303_c0_g1_i1_2	0	0	0	0	0	0	0	0	0	143150	129150	0
TRINITY_DN7752_c0_g1_i1_1	0	0	0	0	0	0	0	0	0	166560	0	103570
TRINITY_DN3113_c0_g1_i1_2	0	0	0	0	0	0	0	0	0	145280	0	123780
TRINITY_DN13954_c0_g1_i1_5	0	0	0	0	0	0	0	0	0	268730	0	0
TRINITY_DN9113_c0_g1_i1_6	0	0	0	0	0	0	0	0	0	150050	0	118240
TRINITY_DN2642_c0_g1_i1_1	0	0	0	0	0	0	267580	0	0	0	0	0
TRINITY_DN9696_c0_g1_i1_5	0	0	82323	0	0	0	0	0	0	0	0	182950
TRINITY_DN2508_c0_g1_i1_3	0	0	0	0	0	0	0	0	0	98750	82316	83833
TRINITY_DN8157_c0_g1_i1_3	0	0	0	0	0	0	0	0	0	154920	0	109590
TRINITY_DN20773_c0_g1_i1_5	0	0	0	0	0	0	0	0	0	263880	0	0
TRINITY_DN2427_c0_g1_i1_5	0	0	0	0	0	0	0	0	0	0	261590	0
TRINITY_DN566_c0_g2_i1_1	0	0	0	0	0	0	0	0	0	260650	0	0
TRINITY_DN1856_c0_g2_i1_3	0	0	0	0	0	0	0	0	0	0	127930	132000
TRINITY_DN3611_c0_g1_i2_3	0	0	0	0	0	0	0	0	0	0	259080	0
TRINITY_DN8137_c0_g1_i1_6	0	0	0	0	0	0	0	0	0	259060	0	0
TRINITY_DN4839_c0_g2_i1_2	0	0	0	0	0	0	0	0	0	0	257840	0
TRINITY_DN10092_c0_g1_i1_3	0	0	0	0	0	0	0	0	0	155060	0	102680
TRINITY_DN8102_c0_g1_i1_6	0	0	0	0	0	0	0	0	0	87702	92262	77584
TRINITY_DN2436_c0_g1_i1_2	0	0	0	0	0	0	0	0	0	0	0	257530
TRINITY_DN9750_c0_g2_i3_3	0	0	0	0	0	0	0	0	0	257390	0	0
TRINITY_DN13772_c0_g1_i1_5	0	0	0	0	0	0	0	0	0	93345	85320	77062
TRINITY_DN20973_c0_g1_i1_1	0	0	0	0	0	0	0	0	0	145190	0	110400

TRINITY_DN13500_c0_g1_i1_1	0	0	0	0	255270	0	0	0	0	0	0	0
TRINITY_DN19696_c0_g1_i1_1	0	0	0	0	0	0	0	0	0	140710	114190	0
TRINITY_DN18663_c0_g1_i1_3	0	0	0	0	0	0	0	0	0	0	253490	0
TRINITY_DN10941_c0_g1_i2_2	0	0	0	0	0	0	0	0	0	253020	0	0
TRINITY_DN205_c0_g2_i1_3	0	0	0	0	0	90272	0	0	0	162400	0	0
TRINITY_DN10620_c0_g1_i1_3	0	0	0	0	0	0	0	0	0	0	98329	153660
TRINITY_DN12943_c0_g1_i1_3	0	0	0	0	0	0	0	0	0	127830	0	122630
TRINITY_DN2735_c0_g1_i1_2	0	0	0	0	0	0	0	0	0	0	249660	0
TRINITY_DN15045_c0_g1_i1_3	0	0	0	0	0	0	0	0	0	249480	0	0
TRINITY_DN17024_c0_g1_i1_1	0	0	0	0	0	0	0	0	0	0	0	247650
TRINITY_DN4004_c0_g1_i1_6	0	0	0	0	0	0	0	0	247420	0	0	0
TRINITY_DN12269_c0_g2_i1_2	0	32148	0	0	0	214780	0	0	0	0	0	0
TRINITY_DN9455_c0_g1_i1_6	246100	0	0	0	0	0	0	0	0	0	0	0
TRINITY_DN7173_c0_g2_i1_4	0	0	0	0	0	0	0	0	0	0	245460	0
TRINITY_DN5388_c0_g2_i1_3	0	0	0	0	0	244770	0	0	0	0	0	0
TRINITY_DN10533_c0_g1_i4_1	0	0	0	0	0	0	0	0	0	0	140180	104210
TRINITY_DN15880_c0_g1_i1_6	0	0	0	0	0	0	0	0	0	243590	0	0
TRINITY_DN21652_c0_g1_i1_3	0	0	0	0	0	0	0	0	0	242970	0	0
TRINITY_DN22321_c0_g1_i1_6	0	0	0	0	0	0	0	0	0	125680	0	117250
TRINITY_DN12098_c0_g2_i1_2	0	0	0	0	0	0	0	0	0	0	0	242080
TRINITY_DN6146_c0_g1_i2_4	0	0	0	0	0	0	0	0	0	239520	0	0
TRINITY_DN8428_c0_g1_i1_3	0	0	0	0	0	0	0	0	0	155130	0	84146
TRINITY_DN17847_c0_g2_i1_2	0	0	0	0	0	0	0	0	0	130400	107820	0
TRINITY_DN4796_c0_g1_i1_2	0	0	0	0	0	0	0	0	0	236650	0	0
TRINITY_DN5072_c0_g1_i1_1	0	0	0	0	0	0	0	0	0	138270	97647	0
TRINITY_DN6088_c0_g1_i4_1	0	0	0	114210	0	121360	0	0	0	0	0	0
TRINITY_DN15797_c0_g1_i1_2	0	0	0	0	0	0	0	0	0	234840	0	0
TRINITY_DN18297_c0_g2_i1_1	0	0	0	0	0	0	0	0	0	234630	0	0
TRINITY_DN17041_c0_g1_i1_1	0	0	0	0	0	0	0	0	0	0	233710	0
TRINITY_DN7113_c0_g1_i3_4	0	0	0	0	0	0	0	0	0	92285	39929	99714
TRINITY_DN12379_c0_g1_i1_3	0	0	0	0	0	0	0	0	0	122890	107090	0
TRINITY_DN15487_c0_g1_i1_3	0	0	0	0	0	0	0	0	0	121660	106720	0
TRINITY_DN3915_c0_g1_i1_3	0	0	0	0	0	0	0	0	0	0	226400	0

TRINITY_DN11583_c1_g3_i2_2	0	0	0	0	0	0	0	0	0	125170	0	100150
TRINITY_DN11439_c0_g1_i3_4	0	0	0	0	0	0	0	0	0	0	123360	101870
TRINITY_DN7466_c0_g3_i1_3	0	0	0	0	0	0	0	0	0	123890	0	101040
TRINITY_DN11372_c0_g1_i1_1	0	0	0	0	0	0	0	0	0	0	0	224010
TRINITY_DN2241_c0_g1_i1_2	0	0	0	0	0	0	0	0	0	223950	0	0
TRINITY_DN2564_c0_g1_i1_3	0	0	0	0	0	0	0	0	0	75112	145850	0
TRINITY_DN4503_c0_g1_i1_2	0	0	0	0	0	0	0	0	0	219520	0	0
TRINITY_DN8115_c0_g1_i1_2	0	0	0	0	0	89883	0	0	0	128310	0	0
TRINITY_DN271_c0_g1_i1_6	0	0	0	85902	131920	0	0	0	0	0	0	0
TRINITY_DN12627_c0_g1_i1_5	131820	0	0	0	0	0	0	0	0	0	0	85218
TRINITY_DN6168_c0_g2_i1_3	0	0	0	0	0	0	0	0	0	216490	0	0
TRINITY_DN15083_c0_g1_i1_4	0	0	0	0	0	0	0	0	0	122290	94124	0
TRINITY_DN22088_c0_g1_i1_2	0	0	0	0	0	0	0	0	0	0	113330	102580
TRINITY_DN10295_c0_g1_i4_2	0	0	0	0	0	0	0	0	0	0	212570	0
TRINITY_DN13908_c0_g1_i1_1	0	0	0	0	0	0	0	0	0	212190	0	0
TRINITY_DN250_c0_g2_i1_3	0	0	0	0	0	0	0	0	0	111370	100770	0
TRINITY_DN9708_c0_g1_i1_2	0	0	0	0	0	0	0	0	0	0	0	212130
TRINITY_DN14410_c0_g1_i1_1	0	0	0	0	0	0	0	0	0	0	210320	0
TRINITY_DN2548_c0_g2_i1_4	0	0	0	0	0	0	0	0	0	123000	0	86845
TRINITY_DN6286_c0_g1_i1_1	0	0	0	0	0	0	0	0	0	0	0	209400
TRINITY_DN17369_c0_g1_i1_5	0	0	0	0	0	0	0	0	0	105860	102690	0
TRINITY_DN8472_c0_g1_i1_2	0	208410	0	0	0	0	0	0	0	0	0	0
TRINITY_DN4972_c0_g1_i4_1	0	0	0	0	0	0	0	0	0	93704	0	114290
TRINITY_DN9379_c0_g1_i3_2	0	92038	115730	0	0	0	0	0	0	0	0	0
TRINITY_DN23442_c0_g1_i1_5	0	0	0	0	0	0	0	0	0	0	0	204830
TRINITY_DN20268_c0_g1_i1_5	0	0	0	0	0	0	0	0	0	0	0	204140
TRINITY_DN2087_c1_g1_i1_3	0	0	0	0	0	0	0	0	0	96880	106840	0
TRINITY_DN9218_c0_g1_i7_3	0	0	0	0	0	0	0	0	0	117300	0	86420
TRINITY_DN12527_c0_g1_i1_2	0	0	0	0	0	0	0	0	0	76169	65437	58467
TRINITY_DN22389_c0_g1_i1_6	0	0	0	0	0	0	0	0	0	109890	0	90160
TRINITY_DN9732_c0_g1_i3_2	0	0	0	0	0	0	0	0	0	118430	81101	0
TRINITY_DN9091_c0_g1_i1_3	0	0	0	0	0	196180	0	0	0	0	0	0
TRINITY_DN1560_c0_g1_i1_1	0	0	0	0	0	196040	0	0	0	0	0	0

TRINITY_DN14904_c0_g1_i1_6	0	0	0	0	0	0	0	0	0	0	195980	0
TRINITY_DN12383_c0_g1_i1_2	0	0	0	193910	0	0	0	0	0	0	0	0
TRINITY_DN5000_c0_g1_i1_6	0	0	0	0	0	0	0	0	0	88912	104700	0
TRINITY_DN2849_c0_g1_i1_4	0	0	0	0	0	0	0	0	0	193360	0	0
TRINITY_DN11583_c1_g1_i2_1	0	0	0	0	0	0	0	0	0	117140	75834	0
TRINITY_DN11555_c0_g1_i1_4	0	0	0	0	0	0	0	0	192860	0	0	0
TRINITY_DN11211_c0_g2_i4_2	0	0	0	0	0	0	0	0	0	192700	0	0
TRINITY_DN10288_c0_g2_i1_3	0	0	0	0	0	0	0	0	0	191390	0	0
TRINITY_DN16723_c0_g1_i1_1	0	0	0	0	0	0	0	0	0	0	0	190350
TRINITY_DN5420_c0_g1_i1_2	0	0	0	0	0	0	0	0	0	189070	0	0
TRINITY_DN15186_c0_g1_i1_2	0	0	0	0	0	0	0	0	0	99098	89949	0
TRINITY_DN6233_c0_g1_i4_1	0	0	0	0	0	0	0	0	0	188570	0	0
TRINITY_DN4088_c0_g1_i1_1	0	0	0	0	0	0	0	0	186850	0	0	0
TRINITY_DN1341_c0_g1_i2_3	0	0	0	186120	0	0	0	0	0	0	0	0
TRINITY_DN5675_c0_g1_i3_1	0	0	0	0	0	0	185220	0	0	0	0	0
TRINITY_DN5431_c0_g2_i1_2	0	0	0	0	0	0	184980	0	0	0	0	0
TRINITY_DN9005_c1_g1_i3_1	0	0	0	0	0	0	0	0	0	0	184170	0
TRINITY_DN22172_c0_g1_i1_3	0	0	0	0	0	0	0	0	0	183980	0	0
TRINITY_DN5643_c0_g2_i1_3	0	0	0	0	0	0	0	0	0	0	183550	0
TRINITY_DN18093_c0_g1_i1_1	0	0	0	0	0	0	0	0	0	0	183450	0
TRINITY_DN8505_c0_g1_i1_3	0	0	0	0	0	0	0	0	0	0	0	183060
TRINITY_DN3938_c0_g1_i1_2	0	0	0	0	0	0	0	0	0	182610	0	0
TRINITY_DN11081_c0_g1_i2_3	0	0	0	0	0	0	0	182270	0	0	0	0
TRINITY_DN8657_c0_g1_i1_3	181090	0	0	0	0	0	0	0	0	0	0	0
TRINITY_DN18007_c0_g1_i1_2	97862	82970	0	0	0	0	0	0	0	0	0	0
TRINITY_DN11493_c0_g1_i3_3	0	0	0	179630	0	0	0	0	0	0	0	0
TRINITY_DN11659_c1_g1_i3_5	0	0	0	0	0	0	0	0	0	0	0	179170
TRINITY_DN11367_c0_g1_i1_2	0	0	0	0	0	0	0	0	0	0	0	178270
TRINITY_DN14035_c0_g1_i1_1	0	0	0	0	0	0	177600	0	0	0	0	0
TRINITY_DN7672_c0_g2_i3_6	0	0	0	0	0	0	0	0	0	177370	0	0
TRINITY_DN4363_c0_g1_i1_2	0	0	0	0	0	0	0	0	0	176940	0	0
TRINITY_DN9570_c0_g1_i2_6	0	0	0	0	0	0	0	0	0	0	176800	0
TRINITY_DN12047_c0_g1_i1_5	0	0	0	0	0	0	0	0	0	108820	0	67655

TRINITY_DN8161_c0_g1_i1_2	0	0	0	0	0	0	0	0	0	174830	0	0
TRINITY_DN9497_c1_g1_i1_5	0	0	0	0	0	0	0	0	0	0	173360	0
TRINITY_DN18064_c0_g1_i1_5	0	0	0	0	0	0	0	0	0	0	173290	0
TRINITY_DN3901_c0_g1_i1_6	0	0	0	0	172890	0	0	0	0	0	0	0
TRINITY_DN9544_c0_g1_i2_3	0	0	0	0	0	0	0	0	0	172740	0	0
TRINITY_DN5823_c2_g1_i1_1	0	0	0	0	0	0	0	0	0	0	172620	0
TRINITY_DN10520_c0_g1_i1_2	0	0	0	0	0	0	0	0	0	0	172390	0
TRINITY_DN11739_c0_g2_i1_5	0	0	0	0	0	0	0	0	0	171870	0	0
TRINITY_DN950_c0_g2_i1_5	0	0	0	0	97294	0	0	0	0	74308	0	0
TRINITY_DN1342_c0_g2_i1_1	0	0	0	0	0	0	0	0	0	171510	0	0
TRINITY_DN10787_c0_g2_i1_6	0	0	0	0	0	0	0	0	0	169960	0	0
TRINITY_DN15996_c0_g1_i1_5	0	0	0	0	0	0	0	0	0	169410	0	0
TRINITY_DN13943_c0_g1_i1_2	0	0	0	0	0	0	0	0	0	91199	0	77826
TRINITY_DN4307_c0_g2_i2_3	0	0	0	0	0	0	165930	0	0	0	0	0
TRINITY_DN13850_c0_g1_i1_2	0	0	0	0	0	0	0	0	0	0	0	165510
TRINITY_DN22319_c0_g1_i1_6	0	0	0	0	0	0	0	0	0	0	165470	0
TRINITY_DN19381_c0_g1_i1_1	0	0	0	0	0	0	0	0	0	88218	0	75118
TRINITY_DN17549_c0_g1_i1_3	0	0	0	0	0	0	0	0	0	0	163150	0
TRINITY_DN15080_c0_g2_i1_2	0	0	0	0	0	0	0	0	0	0	161700	0
TRINITY_DN18817_c0_g1_i1_6	0	0	0	0	0	0	0	0	0	161380	0	0
TRINITY_DN11009_c0_g1_i1_1	0	0	0	0	0	0	0	0	0	161150	0	0
TRINITY_DN8994_c0_g2_i2_3	0	0	0	0	0	0	0	0	0	100380	59687	0
TRINITY_DN17962_c0_g1_i1_6	49172	57343	53499	0	0	0	0	0	0	0	0	0
TRINITY_DN7296_c0_g1_i1_3	0	0	0	0	0	0	0	0	0	159880	0	0
TRINITY_DN17826_c0_g2_i1_1	0	0	0	0	0	0	0	0	0	74583	0	83182
TRINITY_DN12326_c0_g1_i1_4	0	0	0	0	0	0	0	0	0	90756	0	64507
TRINITY_DN2620_c0_g2_i2_2	0	0	0	0	0	0	0	0	0	154360	0	0
TRINITY_DN2361_c0_g1_i1_2	0	0	0	0	0	0	0	0	153820	0	0	0
TRINITY_DN1411_c0_g1_i1_5	0	0	0	0	0	0	0	0	0	0	153470	0
TRINITY_DN11963_c0_g1_i1_5	0	0	0	0	0	0	0	0	0	0	152790	0
TRINITY_DN9986_c0_g1_i2_1	0	0	0	0	0	0	0	0	0	0	0	152760
TRINITY_DN3850_c0_g1_i1_2	0	0	0	0	0	0	0	0	0	0	0	152710
TRINITY_DN13162_c0_g1_i1_5	0	0	0	0	0	0	0	0	0	152520	0	0

TRINITY_DN6999_c1_g1_i1_1	0	0	0	0	0	0	0	0	0	152260	0	0
TRINITY_DN6417_c0_g2_i1_1	152160	0	0	0	0	0	0	0	0	0	0	0
TRINITY_DN11852_c0_g1_i1_2	0	0	0	0	0	0	0	0	0	0	150530	0
TRINITY_DN3994_c0_g1_i1_6	0	0	0	0	0	0	0	0	150380	0	0	0
TRINITY_DN10757_c0_g1_i6_3	99839	0	0	0	0	49906	0	0	0	0	0	0
TRINITY_DN858_c0_g2_i1_3	0	0	0	0	0	0	0	0	0	0	149230	0
TRINITY_DN23297_c0_g1_i1_4	0	0	0	0	0	0	0	0	0	0	0	149170
TRINITY_DN19876_c0_g1_i1_6	0	0	0	0	0	0	0	0	0	149110	0	0
TRINITY_DN11014_c0_g1_i1_4	0	0	0	0	0	0	0	0	0	0	0	148070
TRINITY_DN13986_c1_g1_i1_2	147640	0	0	0	0	0	0	0	0	0	0	0
TRINITY_DN4916_c0_g2_i1_4	0	0	0	0	0	0	0	0	0	147320	0	0
TRINITY_DN13205_c0_g1_i1_2	0	0	0	0	0	0	0	0	0	0	0	146740
TRINITY_DN2844_c0_g1_i1_3	0	0	0	0	0	0	0	0	0	145910	0	0
TRINITY_DN19026_c0_g1_i1_2	0	0	0	0	0	0	0	0	0	0	87474	58024
TRINITY_DN10596_c0_g1_i1_3	0	0	0	0	0	0	0	0	0	83171	61978	0
TRINITY_DN11089_c0_g1_i1_6	0	0	0	0	0	0	0	0	0	0	0	143970
TRINITY_DN1625_c0_g1_i1_1	0	0	0	0	0	142510	0	0	0	0	0	0
TRINITY_DN4295_c0_g1_i2_2	0	0	0	0	0	0	0	0	0	0	0	142230
TRINITY_DN21571_c0_g1_i1_3	0	0	0	0	0	0	0	0	0	0	142090	0
TRINITY_DN9775_c0_g1_i1_1	0	70066	0	0	0	71745	0	0	0	0	0	0
TRINITY_DN17721_c0_g1_i1_3	0	0	0	0	0	0	0	0	0	141720	0	0
TRINITY_DN11860_c0_g1_i1_4	0	0	0	0	0	0	0	0	0	141350	0	0
TRINITY_DN19531_c0_g1_i1_1	0	0	0	0	0	0	0	0	0	139700	0	0
TRINITY_DN194_c0_g2_i1_1	38795	35207	63900	0	0	0	0	0	0	0	0	0
TRINITY_DN426_c0_g1_i1_2	0	0	0	0	0	0	0	0	0	0	137640	0
TRINITY_DN23380_c0_g1_i1_2	0	0	0	0	0	0	0	0	0	0	0	137500
TRINITY_DN7432_c0_g1_i1_5	0	0	0	0	0	0	0	0	0	0	0	137130
TRINITY_DN9824_c0_g2_i1_2	0	0	135680	0	0	0	0	0	0	0	0	0
TRINITY_DN14943_c0_g1_i1_3	0	0	0	0	0	0	0	0	0	74766	57572	0
TRINITY_DN4390_c0_g1_i2_1	0	0	0	0	0	0	0	0	0	0	129810	0
TRINITY_DN3895_c0_g2_i1_2	0	0	0	0	0	0	0	0	0	129770	0	0
TRINITY_DN2101_c0_g1_i1_2	0	0	0	0	0	0	0	0	0	128650	0	0
TRINITY_DN22364_c0_g1_i1_6	0	0	0	0	0	0	0	0	0	0	127870	0

TRINITY_DN3021_c0_g2_i1_6	0	0	0	0	0	0	0	0	0	0	73191	53014
TRINITY_DN1919_c0_g1_i1_1	0	0	0	0	0	0	0	0	0	124730	0	0
TRINITY_DN11220_c1_g3_i1_3	0	0	0	0	0	0	0	0	0	0	124210	0
TRINITY_DN8579_c0_g2_i1_1	0	0	0	0	0	0	0	0	0	62647	61399	0
TRINITY_DN7867_c0_g1_i4_3	0	0	0	0	0	0	0	0	0	0	0	123480
TRINITY_DN11046_c0_g1_i2_2	0	0	0	0	0	0	0	0	0	0	120050	0
TRINITY_DN8640_c0_g1_i1_1	0	0	0	0	0	0	0	0	0	0	118570	0
TRINITY_DN2390_c0_g2_i1_1	0	0	0	0	0	0	0	0	0	0	118510	0
TRINITY_DN12575_c0_g1_i1_3	0	0	0	0	0	0	0	0	0	118210	0	0
TRINITY_DN10417_c0_g2_i1_1	0	0	0	0	0	0	0	0	0	0	0	116100
TRINITY_DN17426_c0_g1_i1_6	0	0	0	0	0	0	0	0	0	0	0	113910
TRINITY_DN9926_c0_g1_i2_5	0	0	0	0	0	0	0	0	0	113230	0	0
TRINITY_DN2534_c0_g1_i1_4	0	0	0	0	0	0	0	0	0	0	0	111930
TRINITY_DN2915_c0_g1_i1_3	0	0	0	0	0	0	0	0	0	111200	0	0
TRINITY_DN11633_c0_g1_i3_5	0	0	0	110020	0	0	0	0	0	0	0	0
TRINITY_DN15398_c0_g1_i1_5	0	0	0	0	0	0	0	0	0	109750	0	0
TRINITY_DN5905_c0_g1_i1_3	0	0	0	0	108260	0	0	0	0	0	0	0
TRINITY_DN9808_c0_g1_i1_1	0	0	0	0	0	0	0	0	0	0	0	107160
TRINITY_DN10830_c0_g1_i1_1	0	0	0	0	0	0	0	0	0	0	107130	0
TRINITY_DN5571_c0_g1_i2_2	0	0	0	0	0	0	0	0	0	106630	0	0
TRINITY_DN14099_c0_g1_i1_1	0	0	0	0	0	0	0	0	0	105980	0	0
TRINITY_DN1513_c0_g1_i1_1	0	0	0	0	0	0	0	0	0	0	0	105860
TRINITY_DN9703_c0_g1_i1_1	0	0	0	0	0	105710	0	0	0	0	0	0
TRINITY_DN30_c0_g1_i1_2	0	0	0	0	0	0	0	0	0	105560	0	0
TRINITY_DN4271_c0_g2_i1_6	0	0	0	0	0	0	0	0	0	103610	0	0
TRINITY_DN9637_c0_g1_i2_1	0	0	0	0	0	0	0	0	0	103220	0	0
TRINITY_DN14624_c0_g1_i1_5	0	0	0	0	0	0	0	0	0	102900	0	0
TRINITY_DN7843_c0_g1_i2_1	0	0	0	0	0	0	0	0	0	0	101750	0
TRINITY_DN14064_c0_g1_i1_1	0	0	0	0	0	0	0	0	0	101300	0	0
TRINITY_DN19958_c0_g1_i1_2	0	0	0	0	0	0	0	0	0	0	0	101160
TRINITY_DN12317_c0_g2_i1_2	0	0	0	0	0	0	0	0	0	0	100510	0
TRINITY_DN106_c0_g1_i1_6	0	0	0	0	0	0	0	0	0	98996	0	0
TRINITY_DN10121_c0_g1_i2_2	0	0	0	0	0	0	0	0	0	0	98545	0

TRINITY_DN16265_c0_g1_i1_6	0	0	0	0	0	0	0	0	0	0	97162	0
TRINITY_DN22994_c0_g1_i1_2	0	0	0	0	0	0	0	0	0	0	97047	0
TRINITY_DN16148_c0_g1_i1_2	0	0	0	0	0	0	0	0	0	0	95069	0
TRINITY_DN10093_c0_g1_i1_4	0	0	0	0	0	0	0	0	0	0	94593	0
TRINITY_DN13095_c0_g1_i1_2	0	0	0	0	0	0	0	0	0	0	94350	0
TRINITY_DN6032_c0_g1_i1_1	0	0	0	0	0	0	0	0	0	0	94324	0
TRINITY_DN4104_c0_g1_i2_2	0	0	0	0	0	0	0	0	0	0	94066	0
TRINITY_DN11490_c0_g1_i4_6	0	0	0	0	0	0	0	0	0	93106	0	0
TRINITY_DN11358_c0_g1_i2_2	0	0	0	0	0	0	0	0	0	0	92928	0
TRINITY_DN5926_c0_g1_i1_3	41642	50749	0	0	0	0	0	0	0	0	0	0
TRINITY_DN10755_c0_g1_i3_1	0	0	0	0	0	0	0	0	0	92364	0	0
TRINITY_DN13112_c0_g1_i1_5	0	0	0	0	0	0	0	0	0	0	0	92015
TRINITY_DN1601_c0_g1_i1_6	0	0	0	0	0	0	0	0	0	0	91919	0
TRINITY_DN20005_c0_g1_i1_6	10079	0	79666	0	0	0	0	0	0	0	0	0
TRINITY_DN9298_c0_g1_i2_6	0	0	0	0	0	0	0	0	0	0	88927	0
TRINITY_DN10791_c2_g1_i1_2	0	70097	18313	0	0	0	0	0	0	0	0	0
TRINITY_DN12338_c0_g1_i1_2	0	0	0	0	0	0	0	0	0	88380	0	0
TRINITY_DN2894_c0_g1_i1_6	0	0	0	0	0	0	0	0	0	88104	0	0
TRINITY_DN9321_c0_g1_i1_1	0	0	0	0	0	0	0	0	0	0	0	85743
TRINITY_DN8786_c1_g1_i1_3	0	0	0	0	0	0	0	0	0	85145	0	0
TRINITY_DN8717_c0_g1_i1_1	0	0	0	0	0	0	0	0	0	84764	0	0
TRINITY_DN266_c0_g2_i2_2	0	0	0	0	0	0	0	0	0	82427	0	0
TRINITY_DN10315_c0_g1_i3_3	0	0	0	0	0	0	0	0	0	0	81732	0
TRINITY_DN19933_c0_g1_i1_3	0	0	0	0	0	0	0	0	0	0	80795	0
TRINITY_DN9610_c0_g1_i5_6	0	0	0	0	0	0	0	0	0	0	79631	0
TRINITY_DN3076_c0_g1_i1_4	0	0	0	0	0	0	0	0	0	0	79159	0
TRINITY_DN10575_c0_g1_i1_1	0	0	0	0	0	0	0	0	0	0	0	76848
TRINITY_DN14305_c0_g1_i1_5	0	0	0	0	0	0	0	0	0	73359	0	0
TRINITY_DN11352_c0_g1_i1_1	0	0	0	0	0	0	0	0	0	0	0	70555
TRINITY_DN6147_c0_g1_i2_6	0	0	0	0	0	0	0	0	0	38003	27879	0
TRINITY_DN10057_c1_g1_i2_3	0	0	0	0	0	0	0	0	0	0	0	65118
TRINITY_DN13010_c0_g1_i1_1	0	0	0	0	0	0	0	0	63248	0	0	0
TRINITY_DN2392_c0_g1_i1_2	0	0	0	0	60335	0	0	0	0	0	0	0

TRINITY_DN9690_c0_g1_i2_3	0	0	0	0	0	0	0	0	0	0	59821	0
TRINITY_DN11443_c0_g1_i1_3	25463	31951	0	0	0	0	0	0	0	0	0	0
TRINITY_DN14199_c0_g1_i1_6	0	0	0	0	0	0	0	0	0	0	57006	0
TRINITY_DN7984_c0_g1_i2_3	0	0	0	0	0	0	0	0	0	55800	0	0
TRINITY_DN15785_c0_g1_i1_6	0	0	0	0	0	0	0	0	0	0	0	54977
TRINITY_DN21396_c0_g1_i1_3	0	0	0	0	0	0	0	0	0	53921	0	0
TRINITY_DN7330_c0_g1_i1_3	0	0	0	0	0	0	0	0	0	51134	0	0
TRINITY_DN9918_c0_g2_i5_3	0	0	0	0	0	0	0	0	0	50035	0	0
TRINITY_DN10206_c0_g8_i1_3	0	0	49321	0	0	0	0	0	0	0	0	0
TRINITY_DN21905_c0_g1_i1_6	0	0	49187	0	0	0	0	0	0	0	0	0
TRINITY_DN20071_c0_g1_i1_6	0	0	0	0	0	0	0	0	0	0	48749	0
TRINITY_DN8834_c0_g1_i4_2	0	0	0	0	0	0	0	0	0	0	45098	0
TRINITY_DN10839_c0_g1_i3_6	0	0	0	0	0	0	0	0	0	44060	0	0
TRINITY_DN2321_c0_g1_i1_6	42992	0	0	0	0	0	0	0	0	0	0	0
TRINITY_DN12272_c0_g1_i1_2	0	0	0	0	0	0	0	0	0	0	41753	0
TRINITY_DN8223_c1_g1_i1_3	0	0	0	0	0	0	0	0	0	40544	0	0
TRINITY_DN11343_c0_g1_i7_1	0	0	33951	0	0	0	0	0	0	0	0	0
TRINITY_DN20424_c0_g1_i1_1	0	0	32906	0	0	0	0	0	0	0	0	0
TRINITY_DN3551_c0_g1_i1_3	0	0	0	0	0	0	0	0	0	0	0	32562
TRINITY_DN9616_c0_g1_i1_1	0	0	0	0	0	0	0	0	0	0	26570	0
TRINITY_DN13664_c0_g1_i1_1	0	26261	0	0	0	0	0	0	0	0	0	0
TRINITY_DN9032_c0_g1_i5_4	0	0	0	0	0	0	0	0	0	0	12085	0
TRINITY_DN10274_c0_g1_i3_2	0	0	0	0	0	0	0	0	0	0	0	0
TRINITY_DN10289_c0_g1_i1_3	0	0	0	0	0	0	0	0	0	0	0	0
TRINITY_DN1145_c0_g2_i1_6	0	0	0	0	0	0	0	0	0	0	0	0
TRINITY_DN11535_c0_g1_i3_2	0	0	0	0	0	0	0	0	0	0	0	0
TRINITY_DN14223_c0_g1_i1_5	0	0	0	0	0	0	0	0	0	0	0	0
TRINITY_DN18123_c0_g2_i1_2	0	0	0	0	0	0	0	0	0	0	0	0
TRINITY_DN18755_c0_g1_i1_2	0	0	0	0	0	0	0	0	0	0	0	0
TRINITY_DN20629_c0_g1_i1_1	0	0	0	0	0	0	0	0	0	0	0	0
TRINITY_DN20794_c0_g1_i1_5	0	0	0	0	0	0	0	0	0	0	0	0
TRINITY_DN21254_c0_g1_i1_2	0	0	0	0	0	0	0	0	0	0	0	0
TRINITY_DN22136_c0_g1_i1_1	0	0	0	0	0	0	0	0	0	0	0	0

TRINITY_DN22623_c0_g1_i1_1	0	0	0	0	0	0	0	0	0	0	0	0
TRINITY_DN2924_c0_g1_i1_3	0	0	0	0	0	0	0	0	0	0	0	0
TRINITY_DN493_c0_g1_i1_1	0	0	0	0	0	0	0	0	0	0	0	0
TRINITY_DN6077_c0_g2_i1_2	0	0	0	0	0	0	0	0	0	0	0	0
TRINITY_DN6267_c0_g1_i1_2	0	0	0	0	0	0	0	0	0	0	0	0
TRINITY_DN6751_c0_g1_i1_3	0	0	0	0	0	0	0	0	0	0	0	0
TRINITY_DN9010_c0_g1_i2_3	0	0	0	0	0	0	0	0	0	0	0	0
