# Supplementary material for: Comparative proteomics of common allergenic tree pollens of birch, alder, and hazel
Source: Allergy. 2021 Jan 15;76(6):1743–53. doi: 10.1111/all.14694 (PMC8248232; doi:10.1111/all.14694)
Supplement: Supplementary file 5 — Table S3 [file ALL-76-1743-s005.pdf]

Supplementary Table S3: Top BLAST hits of identified Corylus pollen proteins

Protein IDs	e-value [blastx hit 1]	bitscore [blastx hit 1]	accession [blastx hit 1]	name [blastx hit 1]	organism [blastx hit 1]	accession [blastx hit 2]
TRINITY_DN8608_c2_g1_i2_5	5,39E-101	293	emb|CAA50325.1	major allergen	Corylus avellana	CAA50327.1
TRINITY_DN11207_c0_g1_i2_3	1,62E-98	287	emb|CAA96549.1	major allergen Cor a 1	Corylus avellana	XP_018825721.1
TRINITY_DN9484_c0_g1_i1_5	0	726	XP_008383158.1	phosphoglycerate kinase, cytosolic	Malus domestica	XP_008226131.1
TRINITY_DN7872_c0_g1_i3_6	1,31E-49	163	XP_016650474.1	glycine-rich RNA-binding protein GRP1A	Prunus mume	XP_018847818.1
TRINITY_DN10821_c0_g1_i5_2	0	620	AGU09563.1	isoflavone reductase	Corylus avellana	AAG22740.1
TRINITY_DN11055_c0_g2_i3_5	0	720	XP_008241272.1	monodehydroascorbate reductase	Prunus mume	XP_009374749.1
TRINITY_DN9053_c0_g1_i5_1	0	822	XP_018811449.1	UTP--glucose-1-phosphate uridylyltransferase	Juglans regia	XP_018816885.1
TRINITY_DN10419_c0_g1_i1_1	0	1015	XP_018819373.1	2,3-bisphosphoglycerate-independent phosphoglycerate mutase	Juglans regia	XP_018819374.1
TRINITY_DN11591_c0_g3_i5_3	3,83E-108	313	21886603|emb|CAC84116.1	peptidylprolyl isomerase (cyclophilin)	Betula pendula	|219736587|emb|CAW66894.1
TRINITY_DN11331_c0_g10_i2_1	3,49E-45	147	emb|CAC39160.1	putative LEA III protein isoform 1	Corylus avellana	CAC39110.1
TRINITY_DN8476_c0_g1_i1_3	1,60E-155	438	AHF71027.1	glutathione S-transferase	Betula pendula	XP_018812288.1
TRINITY_DN10442_c0_g1_i1_2	1,16E-179	502	XP_018839113.1	aldo-keto reductase family 4 member C9-like	Juglans regia	XP_018839114.1
TRINITY_DN9319_c0_g1_i1_2	3,81E-157	456	XP_018849923.1	exopolygalacturonase-like	Juglans regia	XP_018849924.1
TRINITY_DN9571_c0_g1_i2_3	0	551	XP_018841935.1	putative lactoylglutathione lyase	Juglans regia	KHN33024.1
TRINITY_DN13451_c0_g2_i1_3	6,80E-13	64,3	KOM34373.1	hypothetical protein LR48_Vigan02g052300	Vigna angularis	ONI23844.1
TRINITY_DN8018_c0_g1_i2_4	1,10E-31	117	XP_018813928.1	non-specific lipid-transfer protein A-like	Juglans regia	XP_019438918.1
TRINITY_DN10804_c2_g1_i1_3	2,84E-151	431	XP_018814537.1	triosephosphate isomerase, cytosolic	Juglans regia	OAY46102.1
TRINITY_DN10890_c0_g3_i4_6	0	634	XP_008239046.1	probable polygalacturonase	Prunus mume	ONI07400.1
TRINITY_DN8608_c2_g1_i1_5	4,69E-65	199	emb|CAA50328.1	major allergen	Corylus avellana	CAA50326.1
TRINITY_DN6026_c0_g1_i1_2	4,39E-92	270	XP_008241145.1	ubiquitin-NEDD8-like protein RUB2	Prunus mume	ONH95703.1
TRINITY_DN11331_c0_g10_i3_1	2,86E-41	136	emb|CAC39110.1	putative LEA III protein isoform 2	Corylus avellana	CAC39160.1
TRINITY_DN13307_c0_g1_i1_1	4,01E-81	241	ABG81302.1	pollen profilin variant 7	Corylus avellana	ABG81303.1
TRINITY_DN11501_c0_g1_i5_2	0	1476	ONH94297.1	hypothetical protein PRUPE_7G009200	Prunus persica	ONH94298.1
TRINITY_DN9487_c0_g3_i2_5	0	838	XP_008241309.1	enolase	Prunus mume	ONH96293.1
TRINITY_DN10587_c0_g1_i1_3	1,07E-90	271	XP_018844175.1	peptidyl-prolyl cis-trans isomerase FKBP15-1	Juglans regia	KDP45276.1
TRINITY_DN10479_c0_g2_i1_3	7,90E-35	125	XP_018841685.1	putative invertase inhibitor	Juglans regia	XP_018853991.1
TRINITY_DN11616_c0_g1_i2_1	0	1087	XP_018837851.1	phosphoglucomutase, cytoplasmic	Juglans regia	XP_018826631.1
TRINITY_DN4595_c0_g1_i1_3	4,18E-17	73,6	XP_018814700.1	major pollen allergen Ole e 6-like	Juglans regia	KDP44441.1
TRINITY_DN3727_c0_g2_i1_1	4,52E-97	285	XP_015939773.1	translationally-controlled tumor protein homolog	Arachis duranensis	ABI84255.1

TRINITY_DN9731_c0_g1_i1_1	0	605	XP_018816251.1	calreticulin	Juglans regia	XP_018856844.1
TRINITY_DN3709_c0_g1_i1_3	0	560	XP_018807661.1	malate dehydrogenase	Juglans regia	ALI57374.1
TRINITY_DN9065_c0_g1_i1_1	0	890	XP_018825758.1	galactokinase-like isoform X1	Juglans regia	XP_018825759.1
TRINITY_DN11320_c0_g1_i11_3	7,20E-74	238	XP_018815580.1	uncharacterized protein LOC108987164	Juglans regia	XP_015964166.1
TRINITY_DN6201_c0_g1_i1_1	1,84E-103	304	ONI14208.1	hypothetical protein PRUPE_4G269100	Prunus persica	ADR80688.1
TRINITY_DN6019_c0_g1_i1_3	0	760	XP_018841632.1	uncharacterized protein LOC109006717	Juglans regia	XP_018829078.1
TRINITY_DN10785_c0_g1_i3_3	2,74E-39	136	XP_018841685.1	putative invertase inhibitor	Juglans regia	XP_018853991.1
TRINITY_DN10341_c0_g2_i1_1	1,40E-158	454	XP_018823047.1	2-methylene-furan-3-one reductase-like	Juglans regia	XP_004509819.1
TRINITY_DN10479_c0_g1_i2_1	8,97E-43	148	XP_018806754.1	putative invertase inhibitor	Juglans regia	XP_018815938.1
TRINITY_DN10144_c0_g1_i2_2	2,49E-82	246	CAA74365.1	putative Ole e 1 protein	Betula pendula	O49813.1
TRINITY_DN5870_c0_g1_i2_4	2,00E-63	195	XP_018842397.1	glutaredoxin-like	Juglans regia	XP_009334103.1
TRINITY_DN10438_c0_g2_i1_2	0	622	XP_018806786.1	aldose 1-epimerase	Juglans regia	XP_008374865.1
TRINITY_DN8953_c0_g1_i1_1	2,75E-79	237	XP_018823225.1	uncharacterized protein LOC108992954	Juglans regia	XP_009353814.1
TRINITY_DN9146_c0_g3_i2_3	0	1253	emb|CAC14168.1	putative luminal binding protein	Corylus avellana	CAW63975.1
TRINITY_DN10397_c0_g1_i2_5	5,38E-111	321	KDP46494.1	hypothetical protein JCGZ_08466	Jatropha curcas	XP_012067558.1
TRINITY_DN10647_c0_g1_i1_4	2,50E-115	340	XP_008221708.1	glutathione S-transferase U17-like	Prunus mume	ONI30654.1
TRINITY_DN11581_c0_g2_i3_2	2,88E-56	183	ACV50426.1	cytosolic ascorbate peroxidase-1	Jatropha curcas	KDP44134.1
TRINITY_DN7958_c0_g4_i1_6	4,28E-164	461	KOM41958.1	hypothetical protein LR48_Vigan04g215600	Vigna angularis	KOM46459.1
TRINITY_DN10683_c0_g1_i3_5	7,88E-123	354	XP_018852474.1	probable phospholipid hydroperoxide glutathione peroxidase	Juglans regia	XP_008238855.1
TRINITY_DN11604_c0_g1_i6_2	1,43E-146	416	XP_018846260.1	putative methyltransferase DDB_G0268948	Juglans regia	XP_018838664.1
TRINITY_DN9279_c0_g1_i1_1	3,11E-101	294	XP_018847774.1	probable calcium-binding protein CML13	Juglans regia	XP_018831699.1
TRINITY_DN11174_c0_g1_i5_1	0	516	XP_018814951.1	senescence-specific cysteine protease SAG39-like	Juglans regia	XP_018830491.1
TRINITY_DN11394_c0_g1_i1_2	0	831	XP_018818327.1	guanosine nucleotide diphosphate dissociation inhibitor At5g09550	Juglans regia	ONH90692.1
TRINITY_DN10920_c0_g1_i2_3	0	651	ONI03043.1	hypothetical protein PRUPE_6G235100	Prunus persica	XP_008243957.1
TRINITY_DN13244_c0_g1_i1_3	5,71E-16	77	XP_018847235.1	cell wall / vacuolar inhibitor of fructosidase 1	Juglans regia	ONI14778.1
TRINITY_DN10804_c1_g1_i1_3	6,43E-56	180	XP_018823301.1	triosephosphate isomerase, cytosolic	Juglans regia	ONI00223.1
TRINITY_DN10010_c0_g1_i2_4	0	612	XP_018814928.1	probable lactoylglutathione lyase, chloroplastic	Juglans regia	OAY27259.1
TRINITY_DN11304_c0_g1_i5_2	0	619	OAY28308.1	hypothetical protein MANES_15G057000	Manihot esculenta	KDP39327.1
TRINITY_DN14318_c0_g1_i1_5	7,51E-70	218	ONH92634.1	hypothetical protein PRUPE_8G185500	Prunus persica	ONH92633.1
TRINITY_DN11227_c0_g1_i5_5	4,07E-163	459	XP_018807198.1	uncharacterized protein LOC108980651	Juglans regia	OAY33079.1
TRINITY_DN4231_c0_g1_i1_1	1,02E-55	174	XP_018847733.1	small ubiquitin-related modifier 1-like	Juglans regia	XP_018818051.1
TRINITY_DN10618_c0_g1_i1_3	0	785	XP_018849508.1	protein DJ-1 homolog B-like	Juglans regia	KDP32522.1
TRINITY_DN9827_c0_g1_i1_5	3,19E-57	182	XP_018826050.1	acyl carrier protein 1, chloroplastic-like	Juglans regia	XP_018840595.1
TRINITY_DN7126_c0_g3_i1_3	3,84E-67	206	XP_018831298.1	tubulin-folding cofactor A-like	Juglans regia	XP_018831299.1
TRINITY_DN11590_c1_g1_i1_3	0	561	XP_018839732.1	cinnamoyl-CoA reductase 1-like	Juglans regia	BAE48658.1

TRINITY_DN11652_c0_g1_i2_1	0	1158	XP_018825745.1	heat shock cognate protein 80-like	Juglans regia	XP_018825752.1
TRINITY_DN5250_c0_g1_i1_1	1,54E-46	153	XP_018833110.1	cysteine proteinase inhibitor A-like	Juglans regia	XP_018833111.1
TRINITY_DN11459_c1_g2_i2_1	0	1185	OAY46075.1	hypothetical protein MANES_07G114800	Manihot esculenta	KDP44981.1
TRINITY_DN10499_c0_g1_i1_2	0	694	CAJ21172.1 alcohol dehydrogenase	Alnus glutinosa		XP_018839886.1
TRINITY_DN8634_c0_g1_i1_1						
TRINITY_DN10893_c0_g1_i1_2	2,38E-21	90,5	XP_018806754.1	putative invertase inhibitor	Juglans regia	XP_018815938.1
TRINITY_DN9811_c0_g1_i2_3	3,90E-94	278	KDP23592.1	hypothetical protein JCGZ_23425	Jatropha curcas	XP_012089157.1
TRINITY_DN8650_c0_g2_i3_2	0	711	XP_018818659.1	alpha-1,4-glucan-protein synthase [UDP-forming] 2	Juglans regia	XP_018817015.1
TRINITY_DN11653_c0_g1_i19_5	0	781	XP_008241048.1	actin	Prunus mume	XP_008385127.1
TRINITY_DN8041_c0_g1_i2_3	0	650	ONH96252.1	hypothetical protein PRUPE_7G116600	Prunus persica	XP_007202259.1
TRINITY_DN14244_c0_g1_i1_2	2,16E-66	202	XP_018830082.1	desiccation protectant protein Lea14 homolog	Juglans regia	XP_018847006.1
TRINITY_DN10590_c0_g2_i1_2	1,22E-147	428	ONI04889.1	hypothetical protein PRUPE_6G346300	Prunus persica	XP_007208154.2
TRINITY_DN3503_c0_g1_i2_1	4,56E-39	130	CAA73147.1	Bet v 4	Betula pendula	CAA73147.1
TRINITY_DN10155_c0_g1_i1_2	2,31E-141	404	XP_018860753.1	haloacid dehalogenase-like hydrolase domain-containing protein Sgpp	Juglans regia	KDP45952.1
TRINITY_DN11019_c0_g1_i1_2	0	954	XP_018831898.1	UDP-glucose 6-dehydrogenase 3	Juglans regia	XP_018818494.1
TRINITY_DN10859_c0_g1_i1_2	1,93E-72	218	XP_018846792.1	peptidyl-prolyl cis-trans isomerase FKBP12	Juglans regia	AFK34981.1
TRINITY_DN10804_c0_g1_i1_2	5,02E-82	248	XP_008223629.1	triosephosphate isomerase, cytosolic	Prunus mume	XP_009353661.1
TRINITY_DN7252_c0_g4_i3_3	0	1279	KYP38780.1	Endoplasmin isogeny	Cajanus cajan	XP_020203653.1
TRINITY_DN11092_c0_g1_i2_3	1,88E-166	470	XP_012074957.1	uncharacterized protein At5g02240	Jatropha curcas	KDP35646.1
TRINITY_DN11258_c0_g1_i2_3	1,41E-167	471	XP_018808590.1	14-3-3-like protein	Juglans regia	XP_018806495.1
TRINITY_DN8200_c0_g1_i1_1	3,74E-154	437	KHN12106.1	Carbonic anhydrase, chloroplastic	Glycine soja	KRH74596.1
TRINITY_DN9523_c0_g1_i1_4	1,06E-169	476	XP_018838373.1	protein CDI-like	Juglans regia	XP_015964723.1
TRINITY_DN10459_c0_g1_i1_3	8,00E-93	275	XP_018848789.1	peroxiredoxin-2B	Juglans regia	XP_018857900.1
TRINITY_DN11248_c1_g1_i1_3	9,06E-111	326	XP_018825058.1	elongation factor 1-delta-like	Juglans regia	XP_018825059.1
TRINITY_DN10833_c1_g2_i1_1	5,57E-85	249	XP_018840197.1	nuclear transport factor 2-like	Juglans regia	ONI29953.1
TRINITY_DN9054_c0_g1_i2_1	3,04E-84	250	XP_018830082.1	desiccation protectant protein Lea14 homolog	Juglans regia	XP_018847006.1
TRINITY_DN11641_c0_g1_i13_1	0	823	XP_018858114.1	protein disulfide-isomerase-like	Juglans regia	XP_018858121.1
TRINITY_DN9275_c0_g1_i1_1	1,39E-130	373	XP_018807996.1	glycolipid transfer protein 1-like	Juglans regia	XP_018808001.1
TRINITY_DN11496_c0_g1_i1_2	0	612	XP_018849818.1	malate dehydrogenase, mitochondrial	Juglans regia	XP_018822629.1
TRINITY_DN10665_c0_g1_i1_2	0	808	XP_008243316.1	ATP synthase subunit beta, mitochondrial	Prunus mume	XP_018849756.1
TRINITY_DN11667_c0_g1_i3_3	0	1652	XP_018815585.1	elongation factor 2-like isoform X1	Juglans regia	XP_018815586.1
TRINITY_DN2899_c0_g1_i1_1	9,07E-84	248	ABG81298.1	pollen profilin variant 3	Corylus avellana	A4KA41.1
TRINITY_DN5468_c0_g2_i2_1	5,14E-56	181	XP_018829795.1	uncharacterized protein At4g13230	Juglans regia	XP_020234770.1
TRINITY_DN11591_c0_g3_i6_3	9,55E-53	167	21886603|emb|CAC84116.1	peptidylprolyl isomerase (cyclophilin)	Betula pendula	|219736587|emb|CAW66894.1
TRINITY_DN11204_c0_g1_i1_3	0	1310	XP_018848851.1	trifunctional UDP-glucose 4,6-dehydratase/UDP-4-keto-6-deoxy-D-glucose 3,5-epimerase/UDP-4-keto-L-rhamnose-reductase RHM1	Juglans regia	KHN36936.1

TRINITY_DN10703_c1_g1_i1_1	4,40E-94	274	ONI23028.1	hypothetical protein PRUPE_2G165300	Prunus persica	XP_007218574.1
TRINITY_DN11207_c0_g1_i1_3	1,97E-101	295	emb|CAA96549.1	major allergen Cor a 1	Corylus avellana	XP_018825721.1
TRINITY_DN10743_c0_g1_i1_2	1,34E-139	402	XP_018829015.1	binding partner of ACD11 1-like	Juglans regia	KRH74377.1
TRINITY_DN8812_c0_g1_i2_1	1,77E-72	219	XP_012089770.1	macrophage migration inhibitory factor homolog isoform X2	Jatropha curcas	XP_009373089.1
TRINITY_DN11581_c0_g1_i4_3	1,34E-94	279	XP_018818881.1	L-ascorbate peroxidase, cytosolic	Juglans regia	XP_018818882.1
TRINITY_DN9487_c0_g2_i1_5	0	854	XP_008376333.1	enolase	Malus domestica	ONI25116.1
TRINITY_DN11173_c0_g3_i1_4	0	610	XP_008229069.1	ADP,ATP carrier protein 3, mitochondrial	Prunus mume	OAY34034.1
TRINITY_DN5663_c0_g2_i1_3	7,47E-61	190	XP_018845447.1	glutaredoxin-C4	Juglans regia	KDP46838.1
TRINITY_DN9210_c0_g1_i1_1	3,11E-44	146	XP_018839522.1	probable steroid-binding protein 3	Juglans regia	XP_018839569.1
TRINITY_DN9779_c0_g1_i1_1	0	878	XP_018820845.1	xylose isomerase	Juglans regia	XP_012065287.1
TRINITY_DN16006_c0_g1_i1_2	6,21E-25	96,7	XP_018837516.1	uncharacterized protein LOC109003708	Juglans regia	ONI14778.1
TRINITY_DN5448_c0_g2_i1_3	1,42E-100	293	ADU56174.1	ubiquitin-conjugating family protein	Jatropha curcas	ADV04060.1
TRINITY_DN10761_c0_g1_i2_2	0	698	XP_018811005.1	elongation factor 1-gamma-like isoform X1	Juglans regia	XP_018817874.1
TRINITY_DN11378_c0_g1_i1_4	0	936	XP_018844065.1	serine hydroxymethyltransferase 4	Juglans regia	OAY28306.1
TRINITY_DN10720_c0_g2_i2_6	0	621	XP_014494377.1	glyceraldehyde-3-phosphate dehydrogenase GAPC1, cytosolic	Vigna radiata var. radiata	ACU19758.1
TRINITY_DN11028_c0_g2_i2_3	2,16E-142	404	XP_018825159.1	GTP-binding protein YPTM2	Juglans regia	XP_008238395.1
TRINITY_DN10686_c0_g1_i1_3	0	974	XP_018816425.1	L-ascorbate oxidase homolog	Juglans regia	XP_018819385.1
TRINITY_DN10496_c0_g1_i5_3	0	618	XP_018857524.1	UDP-glucose 4-epimerase GEPI48-like	Juglans regia	XP_018824378.1
TRINITY_DN11432_c0_g1_i3_6	0	562	XP_008236513.1	probable mannitol dehydrogenase	Prunus mume	ONI02572.1
TRINITY_DN4601_c0_g1_i1_1	8,42E-76	228	ONI35591.1	hypothetical protein PRUPE_1G544400	Prunus persica	XP_007225902.1
TRINITY_DN10681_c0_g1_i1_2	0	1818	XP_018841811.1	aconitate hydratase, cytoplasmic isoform X1	Juglans regia	KDP22711.1
TRINITY_DN10442_c0_g2_i1_4	5,01E-27	106	XP_018839113.1	aldo-keto reductase family 4 member C9-like	Juglans regia	XP_018839114.1
TRINITY_DN9458_c0_g1_i2_2	0	624	XP_018807246.1	probable protein disulfide-isomerase A6	Juglans regia	KDP28233.1
TRINITY_DN8250_c0_g1_i2_3	0	599	XP_018851336.1	2-alkenal reductase (NADP(+)-dependent)-like isoform X2	Juglans regia	XP_018851335.1
TRINITY_DN10239_c0_g1_i1_2	1,95E-132	379	XP_018858784.1	lactoylglutathione lyase isoform X1	Juglans regia	ONI00873.1
TRINITY_DN11469_c0_g2_i3_2	2,21E-120	359	XP_018814905.1	root phototropism protein 2	Juglans regia	KRH30675.1
TRINITY_DN7240_c0_g1_i2_1	8,91E-16	71,6	XP_008341835.1	stress-induced protein KIN2-like	Malus domestica	XP_009373724.1
TRINITY_DN4014_c0_g1_i1_2	2,97E-86	257	XP_018837329.1	glycine cleavage system H protein 2, mitochondrial-like	Juglans regia	XP_018859042.1
TRINITY_DN10537_c0_g1_i2_4	0	617	XP_020538312.1	malate dehydrogenase, cytoplasmic isoform X1	Jatropha curcas	KDP28817.1
TRINITY_DN5324_c1_g1_i2_1	1,75E-60	192	OAY32796.1	hypothetical protein MANES_13G046500	Manihot esculenta	KDP26372.1
TRINITY_DN5563_c0_g1_i1_6						
TRINITY_DN8045_c0_g1_i1_5	1,59E-75	225	XP_018821975.1	thioredoxin H-type	Juglans regia	XP_018821976.1
TRINITY_DN8960_c0_g1_i1_2	7,25E-96	280	XP_018824314.1	reactive Intermediate Deaminase A, chloroplastic-like	Juglans regia	XP_018811490.1
TRINITY_DN4028_c1_g1_i1_2	5,07E-103	300	emb|CAW66836.1	unnamed protein product	Glycine max	CAW52404.1
TRINITY_DN11269_c2_g1_i10_3	0	830	XP_018835118.1	eukaryotic initiation factor 4A-15	Juglans regia	XP_018835119.1
TRINITY_DN11656_c0_g1_i6_2	0	888	XP_018856355.1	elongation factor 1-alpha	Juglans regia	XP_018805761.1

TRINITY_DN10568_c0_g3_i2_1	0	778	OAY30593.1	hypothetical protein MANES_14G043300	Manihot esculenta	XP_014505844.1
TRINITY_DN9271_c0_g1_i1_1	4,77E-74	229	AFK35102.1	unknown	Lotus japonicus	KHN12948.1
TRINITY_DN10339_c0_g2_i1_4	3,29E-81	244	XP_018842805.1	cytochrome b5-like	Juglans regia	XP_018842806.1
TRINITY_DN8726_c0_g2_i1_5	4,73E-136	393	XP_018813322.1	photosynthetic NDH subunit of lumenal location 5, chloroplastic-like	Juglans regia	XP_018811825.1
TRINITY_DN11504_c0_g1_i1_3	1,93E-82	249	XP_018852846.1	uncharacterized protein At4g28440-like	Juglans regia	KDP26583.1
TRINITY_DN10183_c0_g2_i2_2	0	535	XP_018823791.1	triosephosphate isomerase, chloroplastic	Juglans regia	XP_020425754.1
TRINITY_DN1820_c0_g1_i1_6	3,20E-18	82,4	XP_018806754.1	putative invertase inhibitor	Juglans regia	XP_018815938.1
TRINITY_DN8787_c0_g2_i1_4	1,41E-24	93,6	XP_008341834.1	stress-induced protein KIN2-like	Malus domestica	XP_008341836.1
TRINITY_DN10196_c0_g1_i1_1	1,88E-149	429	XP_018830611.1	carboxylesterase 1-like	Juglans regia	XP_016649976.1
TRINITY_DN10535_c0_g1_i2_3	1,67E-128	367	XP_018843515.1	UMP-CMP kinase 3-like	Juglans regia	XP_018818963.1
TRINITY_DN11343_c0_g1_i7_2	0	872	dbj|BAO50884.1	ATP synthase F1 subunit 1 (mitochondrion)	Hevea brasiliensis	BAO50925.1
TRINITY_DN9170_c0_g1_i1_1	1,45E-144	411	XP_018826002.1	endo-1,3;1,4-beta-D-glucanase isoform X2	Juglans regia	XP_018826001.1
TRINITY_DN9813_c0_g1_i1_2	0	1726	XP_018832881.1	staphylococcal nuclease domain-containing protein 1-like	Juglans regia	XP_018832889.1
TRINITY_DN11430_c0_g2_i4_6	0	894	OAY49326.1	hypothetical protein MANES_05G047000	Manihot esculenta	XP_018856789.1
TRINITY_DN9475_c0_g1_i1_3	0	720	XP_018838095.1	3-hydroxyisobutyryl-CoA hydrolase-like protein 3, mitochondrial isoform X1	Juglans regia	XP_018838098.1
TRINITY_DN11040_c0_g1_i6_1	0	654	XP_018845321.1	vicilin-like seed storage protein At2g28490 isoform X3	Juglans regia	XP_018845319.1
TRINITY_DN10148_c0_g1_i6_3	0	567	XP_018839537.1	probable protein phosphatase 2C 59	Juglans regia	XP_018839538.1
TRINITY_DN5484_c0_g2_i1_4	3,40E-09	53,9	KOM45422.1	hypothetical protein LR48_Vigan06g072800	Vigna angularis	XP_017426562.1
TRINITY_DN7458_c0_g1_i3_2	1,02E-13	68,2	KDP44437.1	hypothetical protein JCGZ_16270	Jatropha curcas	KOM25386.1
TRINITY_DN7727_c0_g2_i1_3	1,01E-176	501	XP_018824181.1	ervatamin-B-like	Juglans regia	XP_008229136.1
TRINITY_DN18091_c0_g1_i1_4	6,79E-15	71,2	XP_018806754.1	putative invertase inhibitor	Juglans regia	XP_018815938.1
TRINITY_DN10656_c0_g1_i1_2	0	934	XP_018844057.1	selenium-binding protein 2-like	Juglans regia	XP_008380460.1
TRINITY_DN7279_c0_g1_i2_5	2,92E-60	189	XP_018844068.1	glycine-rich RNA-binding protein 2, mitochondrial-like	Juglans regia	XP_018844069.1
TRINITY_DN11640_c0_g2_i6_2	0	1446	XP_018841471.1	beta-galactosidase 13-like	Juglans regia	XP_009374578.2
TRINITY_DN1640_c0_g2_i1_1	3,69E-52	174	dbj|BAO50884.1	ATP synthase F1 subunit 1 (mitochondrion)	Hevea brasiliensis	BAO50925.1
TRINITY_DN7420_c0_g1_i1_1	1,34E-84	250	XP_008231331.1	thioredoxin-like protein Clot	Prunus mume	ONI20288.1
TRINITY_DN11604_c0_g1_i5_2	5,21E-146	416	XP_018846260.1	putative methyltransferase DDB_G0268948	Juglans regia	XP_018807016.1
TRINITY_DN7220_c0_g1_i2_2	0	1222	XP_018826868.1	heat shock 70 kDa protein, mitochondrial	Juglans regia	XP_018846283.1
TRINITY_DN11320_c0_g1_i7_2	6,40E-19	86,3	XP_008344290.1	uncharacterized protein LOC103407118	Malus domestica	XP_008344290.1
TRINITY_DN17793_c0_g1_i1_3						
TRINITY_DN128_c0_g1_i1_1	8,48E-79	234	XP_019433617.1	ubiquitin-60S ribosomal protein L40 isoform X1	Lupinus angustifolius	ACJ84010.1
TRINITY_DN7906_c0_g1_i1_2	1,91E-97	284	XP_018822739.1	14 kDa zinc-binding protein	Juglans regia	XP_007202700.2
TRINITY_DN9529_c0_g1_i4_3	3,04E-40	135	ONI34270.1	hypothetical protein PRUPE_1G472300	Prunus persica	ONI34271.1
TRINITY_DN10188_c0_g1_i1_1	0	525	XP_018859879.1	altered inheritance of mitochondria protein 32-like	Juglans regia	ONI19698.1
TRINITY_DN10809_c0_g1_i3_1	0	522	XP_018841469.1	L-ascorbate peroxidase 3, peroxisomal-like	Juglans regia	XP_018823838.1
TRINITY_DN10625_c0_g1_i2_2	0	729	OAY42731.1	hypothetical protein MANES_08G011500	Manihot esculenta	XP_018856900.1
TRINITY_DN11635_c2_g1_i4_2	0	644	XP_018824429.1	probable aldo-keto reductase 2	Juglans regia	XP_018858774.1

TRINITY_DN11639_c0_g1_i6_3	0	1235	XP_018838281.1	V-type proton ATPase catalytic subunit A	Juglans regia	OAY48691.1
TRINITY_DN10727_c0_g1_i1_2	4,47E-100	298	XP_018833734.1	elongation factor 1-beta 2	Juglans regia	XP_007146187.1
TRINITY_DN8828_c0_g1_i1_2	3,07E-171	497	XP_018831158.1	conserved oligomeric Golgi complex subunit 3	Juglans regia	ONH97445.1
TRINITY_DN9853_c0_g2_i1_2	0	537	XP_018859093.1	probable pectinesterase/pectinesterase inhibitor 51	Juglans regia	XP_018831968.1
TRINITY_DN17353_c0_g1_i1_1	3,01E-176	495	XP_018823126.1	probable prolyl 4-hydroxylase 6	Juglans regia	XP_018844780.1
TRINITY_DN11024_c0_g2_i1_3	1,40E-150	429	XP_018858487.1	(+)-neomenthol dehydrogenase-like isoform X1	Juglans regia	XP_020218534.1
TRINITY_DN7121_c0_g2_i1_5	0	752	XP_018843179.1	fructose-bisphosphate aldolase 3, chloroplastic	Juglans regia	ONI31556.1
TRINITY_DN8295_c0_g1_i1_3						
TRINITY_DN7552_c0_g1_i2_3	0	699	XP_018837780.1	3-hydroxyisobutyryl-CoA hydrolase-like protein 5 isoform X1	Juglans regia	XP_018837782.1
TRINITY_DN11568_c0_g1_i1_2	0	1068	XP_018824334.1	granule-bound starch synthase 1, chloroplastic/amyloplastic-like isoform X1	Juglans regia	XP_018824335.1
TRINITY_DN11106_c0_g1_i3_1	0	792	XP_018819528.1	protein disulfide isomerase-like 1-4	Juglans regia	OAY38003.1
TRINITY_DN8501_c0_g1_i1_2	0	849	XP_018822246.1	aspartate aminotransferase P2, mitochondrial-like	Juglans regia	XP_018822247.1
TRINITY_DN9729_c0_g1_i1_5	0	597	XP_018824948.1	probable cinnamyl alcohol dehydrogenase 9	Juglans regia	ONI01833.1
TRINITY_DN11231_c0_g1_i2_3	0	634	XP_018822731.1	60S ribosomal protein L4	Juglans regia	XP_018850207.1
TRINITY_DN11267_c0_g1_i1_2	3,01E-162	458	XP_018839423.1	stem-specific protein TSJT1-like	Juglans regia	XP_008224229.1
TRINITY_DN10955_c0_g1_i6_2	8,16E-145	409	XP_012083760.1	ras-related protein RABH1b	Jatropha curcas	ONI22221.1
TRINITY_DN10695_c0_g1_i3_1	0	952	XP_020533781.1	polyadenylate-binding protein 2	Jatropha curcas	KYP48173.1
TRINITY_DN10388_c0_g2_i5_4	0	1026	XP_008218932.1	subtilisin-like protease SBT5.4	Prunus mume	XP_008384613.1
TRINITY_DN10780_c1_g2_i1_2	1,02E-97	287	XP_018819438.1	nucleoside diphosphate kinase B	Juglans regia	ADB85102.1
TRINITY_DN4152_c0_g3_i1_2	2,90E-103	308	XP_018828611.1	uncharacterized protein LOC108996994	Juglans regia	XP_018819156.1
TRINITY_DN11590_c1_g2_i1_3	1,70E-161	455	XP_018839732.1	cinnamoyl-CoA reductase 1-like	Juglans regia	BAE48658.1
TRINITY_DN11603_c0_g2_i4_3	0	944	XP_018859052.1	coatomer subunit delta	Juglans regia	XP_018859053.1
TRINITY_DN9993_c0_g1_i2_4	0	1145	XP_018839760.1	subtilisin-like protease SBT4.15	Juglans regia	OAY42882.1
TRINITY_DN8821_c0_g1_i2_2	1,04E-155	436	XP_018838488.1	ras-related protein RABA1f	Juglans regia	XP_018860734.1
TRINITY_DN11484_c0_g1_i1_1	0	1230	XP_018823485.1	eukaryotic translation initiation factor-like isoform X2	Juglans regia	XP_018823484.1
TRINITY_DN10975_c0_g1_i1_3	3,11E-110	321	KYP55475.1	Eukaryotic translation initiation factor 5A-2	Cajanus cajan	XP_020228782.1
TRINITY_DN908_c0_g1_i1_5	1,30E-91	266	XP_018830768.1	actin-depolymerizing factor 7	Juglans regia	XP_018828430.1
TRINITY_DN4171_c0_g2_i2_3	0	654	XP_018829624.1	naringenin,2-oxoglutarate 3-dioxygenase	Juglans regia	ARV78456.1
TRINITY_DN11158_c0_g1_i1_2	1,22E-145	413	XP_018815728.1	acylpyruvase FAHD1, mitochondrial-like	Juglans regia	XP_018806160.1
TRINITY_DN11613_c0_g1_i1_1	0	610	XP_018811967.1	probable aldo-keto reductase 1	Juglans regia	XP_018826312.1
TRINITY_DN10470_c0_g1_i2_2	1,90E-116	338	KDP21650.1	hypothetical protein JCGZ_03321	Jatropha curcas	XP_012091217.1
TRINITY_DN12697_c0_g1_i1_3	3,27E-139	397	XP_008225202.1	GDSL esterase/lipase At5g45920	Prunus mume	XP_018849879.1
TRINITY_DN6523_c0_g1_i1_1	2,47E-108	313	ADV04063.1	protein binding/ubiquitin-protein ligase 2	Hevea brasiliensis	AGQ57016.1
TRINITY_DN7341_c0_g1_i1_3	1,20E-133	384	XP_018818615.1	1,2-dihydroxy-3-keto-5-methylthiopentene dioxygenase 2	Juglans regia	XP_018839409.1

TRINITY_DN8404_c0_g1_i1_3	0	534	ABW06959.1	isopentenyl pyrophosphate isomerase	Corylus avellana	XP_018815655.1
TRINITY_DN11519_c0_g1_i1_1	0	1680	XP_018847842.1	coatomer subunit gamma-2	Juglans regia	OAY25507.1
TRINITY_DN9737_c2_g2_i1_1	9,81E-131	375	OIV93727.1	hypothetical protein TanjilG_16578	Lupinus angustifolius	XP_007157443.1
TRINITY_DN11533_c0_g1_i2_3	0	1559	XP_018851707.1	phospholipase D alpha 1	Juglans regia	XP_018850650.1
TRINITY_DN8820_c0_g1_i1_1	0	947	OAY56999.1	hypothetical protein MANES_02G062200	Manihot esculenta	XP_018816810.1
TRINITY_DN5484_c0_g1_i1_2	2,48E-42	137	KDP39763.1	hypothetical protein JCGZ_02783	Jatropha curcas	AJE63430.1
TRINITY_DN10447_c0_g1_i1_3	3,75E-16	70,9	XP_018834136.1	late embryogenesis abundant protein 2-like	Juglans regia	ONI18849.1
TRINITY_DN431_c0_g1_i1_6	1,95E-165	467	XP_018850456.1	uncharacterized protein LOC109013009	Juglans regia	AFK36402.1
TRINITY_DN9847_c0_g1_i1_1	2,23E-111	323	XP_018832824.1	stress-response A/B barrel domain-containing protein UP3-like	Juglans regia	XP_018844473.1
TRINITY_DN11173_c0_g2_i3_4	0	681	XP_018816668.1	ADP,ATP carrier protein 1, mitochondrial	Juglans regia	CAA05979.1
TRINITY_DN6616_c0_g1_i1_4	0	543	XP_018857281.1	villin-3-like isoform X1	Juglans regia	XP_018857282.1
TRINITY_DN5630_c0_g1_i1_3	0	542	XP_018820284.1	aldo-keto reductase family 4 member C9-like	Juglans regia	XP_018820285.1
TRINITY_DN4574_c0_g1_i2_2	1,49E-111	323	XP_018812389.1	peroxiredoxin-2E-2, chloroplastic-like	Juglans regia	XP_018854230.1
TRINITY_DN8931_c0_g1_i1_2	5,78E-170	478	XP_018826881.1	soluble inorganic pyrophosphatase 6, chloroplastic-like	Juglans regia	XP_018844455.1
TRINITY_DN11512_c1_g1_i2_2	0	842	XP_018811769.1	aspartate aminotransferase, cytoplasmic	Juglans regia	AGF95095.1
TRINITY_DN5614_c0_g2_i1_2	1,82E-98	299	XP_018823830.1	DDB1- and CUL4-associated factor 8-like	Juglans regia	XP_018823831.1
TRINITY_DN10149_c0_g1_i2_3	0	514	XP_018818507.1	14-3-3-like protein GF14 iota isoform X1	Juglans regia	XP_018852339.1
TRINITY_DN9287_c0_g1_i1_3	0	561	XP_018807335.1	endonuclease 4-like	Juglans regia	ONI10288.1
TRINITY_DN13093_c0_g1_i1_3	0	717	XP_018849179.1	UDP-sugar pyrophosphorylase-like isoform X2	Juglans regia	XP_018849178.1
TRINITY_DN10610_c0_g2_i1_2	0	975	XP_004492897.1	V-type proton ATPase subunit B 2-like	Cicer arietinum	AES80476.1
TRINITY_DN4192_c0_g2_i1_1	2,60E-75	228	KYP50897.1	Peroxisomal multifunctional enzyme type 2	Cajanus cajan	XP_020231842.1
TRINITY_DN11220_c1_g2_i2_2	1,56E-132	383	XP_018841297.1	tropinone reductase homolog At5g06060-like	Juglans regia	XP_018850680.1
TRINITY_DN10317_c0_g1_i3_2	0	525	XP_018838677.1	inositol-phosphate phosphatase-like	Juglans regia	XP_015958999.1
TRINITY_DN9083_c0_g1_i2_2	1,79E-116	338	XP_018842921.1	membrane steroid-binding protein 1-like	Juglans regia	XP_018830725.1
TRINITY_DN6144_c0_g1_i2_2	4,73E-14	71,2	XP_018853991.1	pectinesterase inhibitor-like	Juglans regia	XP_018806753.1
TRINITY_DN13238_c0_g1_i1_4	9,10E-48	157	XP_018858281.1	chitinase 10-like	Juglans regia	OAY48827.1
TRINITY_DN21237_c0_g1_i1_2	6,86E-63	202	XP_018837443.1	UDP-glycosyltransferase 74E1-like	Juglans regia	XP_018810592.1
TRINITY_DN10003_c1_g1_i5_2	0	651	XP_018827473.1	D-3-phosphoglycerate dehydrogenase 1, chloroplastic-like	Juglans regia	XP_008353434.1
TRINITY_DN11618_c0_g1_i1_2	0	1480	XP_004493989.1	cell division control protein 48 homolog D	Cicer arietinum	XP_018836272.1
TRINITY_DN4347_c0_g1_i1_6	0	1174	XP_018826381.1	probable Xaa-Pro aminopeptidase P	Juglans regia	XP_018829411.1
TRINITY_DN5723_c0_g1_i1_2	6,39E-121	345	KDP30996.1	hypothetical protein JCGZ_11372	Jatropha curcas	XP_012079937.1
TRINITY_DN9234_c0_g1_i1_2	0	737	ADR71240.1	60S ribosomal protein L3B	Hevea brasiliensis	XP_018817903.1
TRINITY_DN13643_c0_g1_i1_2	2,23E-65	211	XP_015945795.1	polygalacturonase-like	Arachis duranensis	XP_008379967.1
TRINITY_DN6792_c0_g1_i2_5	1,50E-142	404	XP_018811458.1	ubiquitin thioesterase OTU1-like	Juglans regia	XP_018811464.1
TRINITY_DN7574_c0_g1_i5_2	0	692	XP_018841184.1	polygalacturonase QRT3-like	Juglans regia	XP_009363254.1
TRINITY_DN11191_c0_g1_i2_6	0	973	XP_018823509.1	leucine aminopeptidase 1-like	Juglans regia	XP_018810862.1
TRINITY_DN9754_c0_g1_i1_3	0	787	XP_018817260.1	3-ketoacyl-CoA thiolase 2, peroxisomal	Juglans regia	XP_018809682.1
TRINITY_DN5779_c0_g1_i1_2	1,47E-160	452	XP_018822871.1	strigolactone esterase D14	Juglans regia	XP_018838099.1

TRINITY_DN11323_c0_g1_i1_2	0	1412	XP_018840813.1	heat shock 70 kDa protein 15-like	Juglans regia	OAY36779.1
TRINITY_DN14709_c0_g1_i1_5	5,38E-59	185	XP_018843858.1	cysteine-rich repeat secretory protein 38-like isoform X1	Juglans regia	XP_018843859.1
TRINITY_DN9829_c0_g1_i3_2	4,55E-100	295	XP_018818935.1	superoxide dismutase [Cu-Zn], chloroplastic	Juglans regia	XP_009372495.1
TRINITY_DN6396_c0_g1_i1_2	2,48E-54	172	XP_018851363.1	ubiquitin-fold modifier 1	Juglans regia	XP_004505718.1
TRINITY_DN8644_c0_g2_i1_1	1,90E-42	140	XP_018838679.1	cysteine proteinase inhibitor 5	Juglans regia	KDP21895.1
TRINITY_DN11241_c0_g1_i3_2	0	581	XP_018834071.1	mitochondrial phosphate carrier protein 3, mitochondrial-like	Juglans regia	XP_018834879.1
TRINITY_DN9392_c0_g1_i1_2	1,05E-115	339	XP_018819182.1	ran-binding protein 1 homolog a-like	Juglans regia	XP_018835624.1
TRINITY_DN10095_c0_g1_i1_3	0	886	XP_018811089.1	mitochondrial-processing peptidase subunit alpha-like isoform X1	Juglans regia	XP_018843285.1
TRINITY_DN9335_c0_g1_i1_4	0	611	XP_018828122.1	glutelin type-B 5-like	Juglans regia	XP_018828123.1
TRINITY_DN10130_c0_g2_i6_2	0	919	XP_018844904.1	aldehyde dehydrogenase family 7 member A1 isoform X1	Juglans regia	XP_018844905.1
TRINITY_DN11642_c0_g1_i3_4	0	1089	ONH98963.1	hypothetical protein PRUPE_6G002500	Prunus persica	ONH98964.1
TRINITY_DN7240_c0_g1_i1_3	7,97E-19	79,3	ONI18849.1	hypothetical protein PRUPE_3G243400	Prunus persica	XP_007216872.2
TRINITY_DN11079_c0_g1_i1_5	1,24E-122	356	XP_018807368.1	probable ATP synthase 24 kDa subunit, mitochondrial isoform X2	Juglans regia	XP_018807367.1
TRINITY_DN10242_c0_g1_i3_2	1,96E-93	277	XP_018830075.1	cyclic phosphodiesterase-like	Juglans regia	XP_009360792.1
TRINITY_DN3644_c0_g1_i2_3	1,34E-59	187	XP_018813581.1	uncharacterized protein Os08g0359500	Juglans regia	KDP28144.1
TRINITY_DN11383_c0_g1_i4_1	0	780	XP_018847358.1	probable polygalacturonase	Juglans regia	XP_018847365.1
TRINITY_DN9173_c0_g1_i1_4	7,88E-98	306	ONH99465.1	hypothetical protein PRUPE_6G031400	Prunus persica	XP_007207992.2
TRINITY_DN4403_c0_g1_i1_1	0	579	XP_018805947.1	putative glucose-6-phosphate 1-epimerase isoform X1	Juglans regia	XP_018805948.1
TRINITY_DN10720_c0_g1_i4_5	0	624	XP_019434027.1	glyceraldehyde-3-phosphate dehydrogenase, cytosolic-like	Lupinus angustifolius	OIV89251.1
TRINITY_DN11647_c1_g1_i1_1	0	700	KDP26669.1	hypothetical protein JCGZ_17827	Jatropha curcas	XP_020538969.1
TRINITY_DN12510_c0_g1_i1_1	0	520	XP_018847494.1	nitrile-specifier protein 5 isoform X2	Juglans regia	XP_018847493.1
TRINITY_DN1355_c0_g3_i1_5	1,75E-32	122	XP_018820031.1	transcription initiation factor IIA large subunit-like	Juglans regia	XP_018855526.1
TRINITY_DN11053_c0_g1_i4_6	5,32E-159	457	XP_018818750.1	GDSL esterase/lipase At3g48460-like	Juglans regia	KDP42125.1
TRINITY_DN5919_c0_g1_i1_3	8,44E-47	155	XP_018829689.1	3-isopropylmalate dehydratase small subunit 3-like	Juglans regia	XP_016175139.1
TRINITY_DN11546_c0_g1_i6_5	0	1615	XP_018845356.1	calcium-transporting ATPase 2, plasma membrane-type-like isoform X1	Juglans regia	XP_018845357.1
TRINITY_DN11141_c0_g1_i6_1	0	951	XP_018848687.1	glucose-1-phosphate adenylyltransferase large subunit 1-like isoform X1	Juglans regia	XP_018821124.1
TRINITY_DN2708_c0_g2_i1_1	0	566	XP_018844468.1	serine carboxypeptidase-like 40	Juglans regia	XP_020413400.1
TRINITY_DN9530_c0_g3_i4_1	0	670	XP_018814476.1	UDP-glucuronic acid decarboxylase 6	Juglans regia	XP_018814477.1
TRINITY_DN5278_c0_g1_i1_2	3,69E-41	135	KRH00483.1	hypothetical protein GLYMA_18G216000	Glycine max	AFK41154.1
TRINITY_DN10989_c0_g2_i2_1	0	942	XP_018806799.1	glycerol kinase	Juglans regia	XP_007215813.2
TRINITY_DN11653_c0_g2_i1_5	0	634	XP_018848909.1	actin-like	Juglans regia	XP_008240928.1
TRINITY_DN7029_c0_g1_i1_3	1,45E-145	419	OIW11687.1	hypothetical protein TanjilG_12206	Lupinus angustifolius	XP_019443705.1
TRINITY_DN10674_c0_g1_i3_4	0	529	XP_018809730.1	probable protein phosphatase 2C 76	Juglans regia	XP_018809734.1

TRINITY_DN10049_c0_g1_i1_3	0	742	XP_018855304.1	phosphoserine aminotransferase 1, chloroplastic-like	Juglans regia	XP_018852010.1
TRINITY_DN7937_c0_g1_i2_5	3,30E-47	156	XP_018839196.1	uncharacterized protein LOC109004950	Juglans regia	ONI26983.1
TRINITY_DN1722_c0_g2_i1_3	1,65E-55	172	AEC03317.1	thioredoxin H-type 3	Hevea brasiliensis	AAZ32865.1
TRINITY_DN11314_c0_g1_i3_2	0	615	XP_018829808.1	probable UDP-arabinopyranose mutase 5 isoform X1	Juglans regia	XP_018829809.1
TRINITY_DN9588_c0_g1_i1_2	0	1152	XP_018843066.1	pyrophosphate--fructose 6-phosphate 1-phosphotransferase subunit alpha	Juglans regia	XP_018832985.1
TRINITY_DN10926_c1_g1_i3_3	2,36E-81	245	XP_018828917.1	pollen-specific protein C13-like	Juglans regia	XP_018849651.1
TRINITY_DN5721_c0_g4_i1_3	2,47E-25	96,7	XP_018851682.1	uncharacterized protein LOC109013892	Juglans regia	GAU12555.1
TRINITY_DN10576_c0_g1_i1_3	1,76E-119	343	OAY54996.1	hypothetical protein MANES_03G119600	Manihot esculenta	XP_018823733.1
TRINITY_DN11459_c1_g4_i8_2	0	1163	KDP35613.1	hypothetical protein JCGZ_09051	Jatropha curcas	XP_012074911.1
TRINITY_DN10492_c1_g1_i2_6	4,19E-165	467	XP_018833512.1	hydroxyacylglutathione hydrolase cytoplasmic	Juglans regia	XP_019430954.1
TRINITY_DN11529_c0_g1_i2_3	0	1022	XP_018816364.1	dolichyl-diphosphooligosaccharide--protein glycosyltransferase subunit 2-like	Juglans regia	XP_018827273.1
TRINITY_DN10583_c0_g2_i2_1	2,61E-170	494	XP_018814481.1	heat shock cognate 70 kDa protein 2-like	Juglans regia	KDP44981.1
TRINITY_DN12655_c0_g1_i1_2						
TRINITY_DN8865_c0_g2_i1_5	0	576	XP_018816673.1	UDP-glucose 6-dehydrogenase 1	Juglans regia	XP_018816674.1
TRINITY_DN6870_c0_g1_i5_1	0	1028	XP_018805935.1	pyruvate kinase 1, cytosolic isoform X2	Juglans regia	KDP29239.1
TRINITY_DN5791_c0_g1_i1_5	0	842	XP_018822123.1	xylulose kinase	Juglans regia	XP_018822124.1
TRINITY_DN11572_c0_g1_i1_6	6,25E-171	480	XP_018818651.1	40S ribosomal protein S3a-like	Juglans regia	XP_018826766.1
TRINITY_DN10346_c0_g2_i6_3	0	937	XP_018833268.1	glucose-1-phosphate adenylyltransferase small subunit, chloroplastic/amyloplastic	Juglans regia	AES71731.1
TRINITY_DN11809_c0_g1_i1_1	5,76E-81	243	XP_018843858.1	cysteine-rich repeat secretory protein 38-like isoform X1	Juglans regia	XP_018843859.1
TRINITY_DN4290_c0_g1_i1_3	0	652	XP_018809774.1	protein transport protein SEC31 homolog B-like isoform X1	Juglans regia	XP_018809776.1
TRINITY_DN10866_c0_g1_i1_1	0	861	XP_008221256.1	tubulin beta chain-like	Prunus mume	KDP36918.1
TRINITY_DN11465_c0_g1_i2_2	0	860	XP_018848980.1	folylpolyglutamate synthase	Juglans regia	OAY38548.1
TRINITY_DN10466_c0_g2_i3_1	0	1026	XP_018815415.1	ruBisCO large subunit-binding protein subunit alpha	Juglans regia	XP_009376066.1
TRINITY_DN3729_c0_g2_i1_1	1,78E-78	234	XP_008392560.1	actin-depolymerizing factor 7-like	Malus domestica	XP_003532683.1
TRINITY_DN10917_c0_g1_i1_1	0	1779	XP_018810025.1	2-oxoglutarate dehydrogenase, mitochondrial-like	Juglans regia	XP_018810026.1
TRINITY_DN4960_c0_g1_i2_6	0	756	XP_018826582.1	malate dehydrogenase, chloroplastic	Juglans regia	XP_018826583.1
TRINITY_DN10616_c0_g1_i2_2	1,52E-82	247	XP_018855792.1	probable calcium-binding protein CML27	Juglans regia	XP_018810240.1
TRINITY_DN11240_c0_g2_i1_2	0	1545	XP_018836580.1	aminopeptidase M1-like	Juglans regia	XP_018836578.1
TRINITY_DN11190_c0_g1_i2_3	0	956	XP_018845845.1	pyruvate kinase, cytosolic isozyme	Juglans regia	ONH98946.1
TRINITY_DN11571_c4_g2_i1_1	0	830	XP_008379344.1	tubulin alpha-2 chain	Malus domestica	XP_008230754.1
TRINITY_DN11669_c393_g1_i1_1	5,37E-12	63,5	XP_018831494.1	protein PXR1	Juglans regia	XP_018831495.1
TRINITY_DN10467_c0_g1_i1_1	1,10E-154	452	XP_018817112.1	aspartic proteinase nepenthesin-1-like	Juglans regia	XP_004503002.1
TRINITY_DN9500_c0_g2_i1_2	0	1034	XP_018839307.1	chaperonin CPN60-2, mitochondrial	Juglans regia	XP_018850271.1
TRINITY_DN10347_c0_g1_i1_3	0	809	KDP35739.1	hypothetical protein JCGZ_10511	Jatropha curcas	XP_012074735.1

TRINITY_DN5968_c0_g1_i1_3	0	550	OAY54741.1	hypothetical protein MANES_03G097900	Manihot esculenta	XP_018834543.1
TRINITY_DN5544_c0_g1_i1_3	3,69E-129	368	XP_008234622.1	60S ribosomal protein L9-1	Prunus mume	ONI25805.1
TRINITY_DN153_c0_g1_i1_1	0	620	XP_018813794.1	ubiquitin receptor RAD23b-like	Juglans regia	ONI16551.1
TRINITY_DN7265_c0_g2_i5_2	0	584	ADM52185.1	type II metacaspase	Hevea brasiliensis	ANB41198.1
TRINITY_DN9812_c0_g1_i3_4	0	990	XP_018813723.1	polyadenylate-binding protein 2-like	Juglans regia	XP_018813723.1
TRINITY_DN8811_c0_g1_i3_5	0	551	XP_008219675.1	annexin D1 isoform X3	Prunus mume	ONI35090.1
TRINITY_DN10516_c0_g1_i1_2	0	585	XP_018827577.1	SAL1 phosphatase-like isoform X2	Juglans regia	XP_018827576.1
TRINITY_DN4095_c0_g1_i1_4	8,41E-122	352	XP_018860721.1	glutathione S-transferase DHAR2-like	Juglans regia	XP_018860724.1
TRINITY_DN14849_c0_g1_i1_6	1,95E-40	138	XP_008374580.1	peroxidase P7-like	Malus domestica	XP_008354170.1
TRINITY_DN11228_c0_g2_i1_3	7,76E-45	147	XP_019419272.1	heat shock factor-binding protein 1-like	Lupinus angustifolius	XP_018857823.1
TRINITY_DN10795_c1_g1_i1_6	0	733	XP_018850665.1	elongation factor 1-gamma-like	Juglans regia	ONI35669.1
TRINITY_DN11220_c1_g2_i3_3	3,91E-47	155	XP_018841297.1	tropinone reductase homolog At5g06060-like	Juglans regia	XP_018837198.1
TRINITY_DN11391_c0_g2_i2_4	0	612	XP_018824861.1	ATP synthase subunit gamma, mitochondrial	Juglans regia	XP_018824860.1
TRINITY_DN2394_c0_g1_i2_6	3,27E-73	220	XP_018834238.1	cytochrome c	Juglans regia	XP_018831209.1
TRINITY_DN10841_c0_g2_i1_1	0	1134	XP_018844383.1	probable methyltransferase PMT27	Juglans regia	XP_018844383.1
TRINITY_DN9488_c0_g2_i1_2	4,09E-175	494	XP_018818800.1	probable 6-phosphogluconolactonase 4, chloroplastic	Juglans regia	KDP37759.1
TRINITY_DN959_c0_g1_i1_1	9,69E-78	242	XP_018850081.1	exopolygalacturonase-like	Juglans regia	XP_018823962.1
TRINITY_DN5607_c0_g1_i1_3	6,28E-128	367	XP_018827839.1	ras-related protein RABC1 isoform X1	Juglans regia	XP_018827840.1
TRINITY_DN11493_c0_g1_i5_3	1,40E-125	362	XP_018841699.1	ras-related protein RABE1c-like	Juglans regia	XP_018841700.1
TRINITY_DN18554_c0_g1_i1_2	0	696	XP_018857901.1	alpha-galactosidase 1-like	Juglans regia	XP_008221110.1
TRINITY_DN11428_c0_g1_i2_3	0	922	XP_018814251.1	dihydrolipoyl dehydrogenase 1, mitochondrial	Juglans regia	XP_018814252.1
TRINITY_DN23504_c0_g1_i1_4	3,32E-35	125	XP_018821921.1	peroxidase P7	Juglans regia	XP_016181866.1
TRINITY_DN11239_c1_g2_i1_6	6,27E-101	295	XP_008366039.1	60S ribosomal protein L11-1	Malus domestica	XP_009352738.1
TRINITY_DN19480_c0_g1_i1_2	2,09E-36	130	XP_018854675.1	glucan endo-1,3-beta-glucosidase 13-like, partial	Juglans regia	XP_018812075.1
TRINITY_DN12058_c0_g1_i1_3	4,15E-93	274	XP_018827015.1	40S ribosomal protein S3-2-like	Juglans regia	XP_018844535.1
TRINITY_DN4635_c0_g1_i1_6	1,31E-131	381	XP_018821858.1	ferritin-3, chloroplastic	Juglans regia	OAY46317.1
TRINITY_DN6720_c0_g1_i1_1	0	763	XP_018839373.1	stearoyl-[acyl-carrier-protein 9-desaturase, chloroplastic-like	Juglans regia	XP_018812159.1
TRINITY_DN15356_c0_g1_i1_3	3,82E-67	205	XP_018844610.1	mavicyanin-like	Juglans regia	OAY29562.1
TRINITY_DN10115_c0_g1_i5_2	3,68E-98	306	XP_018822921.1	methionine--tRNA ligase, cytoplasmic	Juglans regia	OAY28281.1
TRINITY_DN7162_c0_g1_i1_6	1,97E-62	193	XP_008235553.1	monothiol glutaredoxin-S10	Prunus mume	ONH93069.1
TRINITY_DN668_c0_g2_i1_6	1,87E-135	387	XP_018859632.1	60S ribosomal protein L10a	Juglans regia	XP_018813838.1
TRINITY_DN11756_c0_g2_i2_2	0	930	XP_019427299.1	phosphoglucomutase, chloroplastic	Lupinus angustifolius	XP_019427300.1
TRINITY_DN3503_c0_g1_i1_2	4,74E-36	121	CAA73147.1	Bet v 4	Betula pendula	CAA76831.1
TRINITY_DN10567_c0_g1_i1_3	5,78E-101	297	XP_018858751.1	ATP synthase subunit d, mitochondrial	Juglans regia	XP_018836470.1
TRINITY_DN9859_c0_g2_i1_3	0	2552	XP_018805213.1	alpha-glucan water dikinase, chloroplastic isoform X1	Juglans regia	XP_018805214.1
TRINITY_DN11119_c0_g1_i2_2	3,17E-166	469	KDP29482.1	hypothetical protein JCGZ_19441	Jatropha curcas	XP_012081951.1
TRINITY_DN6617_c0_g1_i1_4	7,27E-114	332	XP_018823640.1	glutamine synthetase nodule isozyme-like	Juglans regia	XP_014495844.1

TRINITY_DN8937_c0_g1_i1_1	0	1080	XP_018831656.1	probable aldehyde dehydrogenase isoform X2	Juglans regia	XP_018831655.1
TRINITY_DN8063_c0_g1_i1_3	2,73E-152	432	XP_018807637.1	soluble inorganic pyrophosphatase 4 isoform X1	Juglans regia	XP_018807638.1
TRINITY_DN3510_c0_g1_i2_1	4,21E-134	392	XP_018841836.1	protein SLOW GREEN 1, chloroplastic	Juglans regia	ONI31974.1
TRINITY_DN3885_c0_g1_i1_3	6,30E-126	373	XP_018825590.1	probable pectinesterase/pectinesterase inhibitor 21	Juglans regia	ONI28224.1
TRINITY_DN11597_c1_g2_i2_1	0	1415	ALO24186.1	H+-pyrophosphatase	Ammopiptanthus nanus	GAU11922.1
TRINITY_DN4297_c0_g1_i1_2	0	1884	XP_018849376.1	phosphoenolpyruvate carboxylase, housekeeping isozyme	Juglans regia	XP_018822521.1
TRINITY_DN11427_c0_g1_i3_2	0	572	OAY60240.1	hypothetical protein MANES_01G097600	Manihot esculenta	XP_018816849.1
TRINITY_DN8289_c0_g1_i2_3	0	1476	XP_018835062.1	heat shock 70 kDa protein 17	Juglans regia	ONI17954.1
TRINITY_DN9771_c0_g1_i4_2	0	1078	XP_018809366.1	probable methyltransferase PMT18	Juglans regia	XP_018844923.1
TRINITY_DN11254_c0_g1_i1_3	0	827	XP_018829690.1	eukaryotic peptide chain release factor GTP-binding subunit ERF3A isoform X1	Juglans regia	XP_018829691.1
TRINITY_DN11103_c0_g1_i2_3	0	930	XP_018848571.1	dihydrolipoyllysine-residue acetyltransferase component 2 of pyruvate dehydrogenase complex, mitochondrial-like	Juglans regia	XP_018835126.1
TRINITY_DN11548_c1_g1_i1_3	2,72E-62	197	XP_018837465.1	uncharacterized protein At5g48480-like	Juglans regia	XP_018858259.1
TRINITY_DN10949_c0_g1_i1_1	0	1170	XP_018826449.1	succinate dehydrogenase [ubiquinone] flavoprotein subunit 1, mitochondrial	Juglans regia	OAY57384.1
TRINITY_DN11059_c0_g1_i1_1	3,85E-171	481	XP_018845359.1	mitochondrial outer membrane protein porin 2-like	Juglans regia	XP_018849544.1
TRINITY_DN3142_c1_g1_i1_1	3,17E-82	265	OAY35576.1	hypothetical protein MANES_12G113500	Manihot esculenta	OAY35575.1
TRINITY_DN10281_c0_g2_i1_2	4,15E-94	275	XP_018823169.1	60S ribosomal protein L27a-3-like	Juglans regia	KDP31039.1
TRINITY_DN10506_c0_g1_i1_2	0	540	XP_018815367.1	NADP-dependent D-sorbitol-6-phosphate dehydrogenase-like	Juglans regia	OAY52428.1
TRINITY_DN9505_c0_g2_i1_1	0	1518	XP_018824768.1	sucrose synthase 2	Juglans regia	AGQ57013.1
TRINITY_DN11355_c0_g1_i2_1	0	611	XP_018829694.1	guanine nucleotide-binding protein subunit beta-like protein	Juglans regia	XP_018860423.1
TRINITY_DN6984_c0_g1_i1_1	5,04E-176	496	XP_018815358.1	ubiquitin fusion degradation protein 1 homolog	Juglans regia	XP_015957218.1
TRINITY_DN9211_c0_g2_i1_1	2,57E-151	431	XP_018810444.1	peptide methionine sulfoxide reductase A1-like	Juglans regia	XP_020962619.1
TRINITY_DN3640_c0_g1_i2_2	9,00E-102	297	XP_018849930.1	rho GDP-dissociation inhibitor 1-like	Juglans regia	KOM46347.1
TRINITY_DN11606_c0_g1_i1_4	0	578	XP_018819193.1	cinnamoyl-CoA reductase 1	Juglans regia	ONH99805.1
TRINITY_DN9177_c0_g2_i1_2	0	1811	OAY27565.1	hypothetical protein MANES_16G135200	Manihot esculenta	OAY27566.1
TRINITY_DN11073_c0_g2_i1_2	0	587	XP_018838486.1	UDP-glycosyltransferase 79B30 isoform X2	Juglans regia	XP_018838487.1
TRINITY_DN9688_c0_g1_i3_5	7,64E-143	404	XP_018851128.1	40S ribosomal protein S5	Juglans regia	XP_008380691.1
TRINITY_DN5149_c0_g1_i1_1	3,25E-26	100	XP_018844150.1	auxin-induced in root cultures protein 12-like	Juglans regia	AES78980.1
TRINITY_DN9163_c0_g1_i2_2	0	744	XP_018840769.1	protein disulfide isomerase-like 2-3	Juglans regia	XP_018827031.1

TRINITY_DN9921_c0_g1_i1_2	0	1028	XP_018810822.1	T-complex protein 1 subunit theta	Juglans regia	KHN00674.1
TRINITY_DN8108_c0_g1_i1_2	0	970	XP_018833427.1	glycerophosphodiester phosphodiesterase GDPDL7	Juglans regia	XP_018850343.1
TRINITY_DN11594_c0_g2_i5_1	1,32E-120	353	OAY31304.1	hypothetical protein MANES_14G101300	Manihot esculenta	KYP42200.1
TRINITY_DN7706_c0_g2_i2_2	1,92E-118	340	KHN10552.1	40S ribosomal protein S4	Glycine soja	XP_006576177.1
TRINITY_DN11322_c1_g1_i2_3	4,41E-158	443	AFK38655.1	unknown	Lotus japonicus	XP_004502806.1
TRINITY_DN6946_c0_g1_i1_2	0	672	XP_018851843.1	protein STRICTOSIDINE SYNTHASE-LIKE 3-like	Juglans regia	XP_018844479.1
TRINITY_DN11349_c1_g2_i4_3	0	785	KYP63219.1	hypothetical protein KK1_017786	Cajanus cajan	XP_020220874.1
TRINITY_DN5652_c0_g3_i1_4	6,53E-27	99,4	XP_018830082.1	desiccation protectant protein Lea14 homolog	Juglans regia	XP_018845104.1
TRINITY_DN11033_c0_g1_i4_5	7,54E-110	319	OAY59659.1	hypothetical protein MANES_01G048900	Manihot esculenta	OAY56354.1
TRINITY_DN3347_c0_g1_i1_2	1,37E-156	454	XP_018807664.1	subtilisin-like protease SBT1.7, partial	Juglans regia	XP_018851647.1
TRINITY_DN6901_c0_g1_i1_3	1,25E-79	238	AAK01235.1|AF327622_1 minor allergen hazelnut profilin	Corylus avellana		AAK01236.1|AF327623_1 minor allergen hazelnut profilin
TRINITY_DN5552_c0_g1_i1_1	2,09E-136	389	XP_018813989.1	vesicle transport v-SNARE 13-like	Juglans regia	XP_018813990.1
TRINITY_DN10237_c0_g3_i1_1	0	1726	OAY37891.1	hypothetical protein MANES_11G137000	Manihot esculenta	KDP44163.1
TRINITY_DN11143_c0_g1_i5_1	3,25E-160	467	XP_018847175.1	heterogeneous nuclear ribonucleoprotein 1-like	Juglans regia	XP_018827257.1
TRINITY_DN1882_c0_g1_i2_3	0	713	XP_018848751.1	acetyl-CoA acetyltransferase, cytosolic 1 isoform X2	Juglans regia	XP_018848747.1
TRINITY_DN10939_c0_g1_i1_1	0	824	XP_018858191.1	citrate synthase, mitochondrial	Juglans regia	XP_018858196.1
TRINITY_DN7127_c0_g1_i1_1	0	868	ONI09316.1	hypothetical protein PRUPE_5G231400	Prunus persica	XP_007210883.1
TRINITY_DN11501_c0_g1_i3_2	0	854	XP_018851852.1	5-methyltetrahydropteroyltriglutamate--homocysteine methyltransferase-like	Juglans regia	ONH94297.1
TRINITY_DN9883_c0_g1_i1_1	4,70E-163	463	XP_018852330.1	60S ribosomal protein L5	Juglans regia	XP_018840429.1
TRINITY_DN10759_c0_g1_i2_5	0	684	XP_012088757.1	pyruvate dehydrogenase E1 component subunit beta-1, mitochondrial isoform X2	Jatropha curcas	XP_012088758.1
TRINITY_DN11019_c0_g1_i2_2	0	955	XP_018818494.1	UDP-glucose 6-dehydrogenase 4	Juglans regia	XP_018831898.1
TRINITY_DN10818_c0_g1_i3_1	1,22E-155	440	XP_018840931.1	60S ribosomal protein L7-2-like	Juglans regia	XP_018840932.1
TRINITY_DN20642_c0_g1_i1_4	5,40E-117	339	XP_018856378.1	beta-fructofuranosidase, insoluble isoenzyme 1-like, partial	Juglans regia	XP_018837352.1
TRINITY_DN9556_c1_g1_i2_1	4,86E-134	382	KDP22561.1	hypothetical protein JCGZ_26392	Jatropha curcas	XP_012090621.1
TRINITY_DN10502_c0_g2_i8_3	0	1958	XP_018845797.1	glycine dehydrogenase (decarboxylating), mitochondrial	Juglans regia	XP_009361869.1
TRINITY_DN10701_c0_g1_i1_2	3,65E-158	447	XP_018834440.1	40S ribosomal protein S2-4-like	Juglans regia	XP_018837209.1
TRINITY_DN11433_c0_g1_i2_1	0	1726	XP_018845914.1	coatomer subunit beta'-2-like isoform X1	Juglans regia	XP_018845915.1
TRINITY_DN6503_c0_g2_i1_1	1,92E-172	488	XP_018846015.1	60S acidic ribosomal protein P0-like	Juglans regia	OIW02717.1
TRINITY_DN6738_c0_g1_i1_3	1,74E-81	246	XP_018836953.1	early nodulin-like protein 1	Juglans regia	XP_018832258.1
TRINITY_DN9643_c0_g1_i1_2	0	672	XP_014518753.1	ferredoxin--NADP reductase, root isozyme, chloroplastic	Vigna radiata var. radiata	XP_020221626.1
TRINITY_DN2791_c0_g1_i1_3	3,25E-117	341	XP_018808748.1	adenine phosphoribosyltransferase 1-like	Juglans regia	XP_004501529.1
TRINITY_DN11325_c0_g1_i1_1	2,09E-84	253	XP_018833404.1	40S ribosomal protein S12-like	Juglans regia	XP_018835263.1

TRINITY_DN7495_c0_g1_i1_1	2,28E-93	279	XP_018849369.1	nascent polypeptide-associated complex subunit alpha-like protein 1	Juglans regia	XP_008231597.1
TRINITY_DN2602_c0_g1_i2_3	0	906	XP_018842622.1	pyruvate kinase isozyme G, chloroplastic isoform X2	Juglans regia	XP_018842621.1
TRINITY_DN11605_c1_g2_i1_4	2,22E-50	166	XP_009364401.1	outer envelope pore protein 16, chloroplastic-like	Pyrus x bretschneideri	XP_009364415.1
TRINITY_DN6250_c0_g1_i2_3	0	1090	XP_018805414.1	delta(24)-sterol reductase	Juglans regia	XP_018805415.1
TRINITY_DN6615_c0_g2_i1_4	1,08E-137	388	AFP43693.1	actin 1, partial	Eriobotrya japonica	ABU68265.1
TRINITY_DN1128_c0_g2_i1_2	2,44E-47	155	XP_018838929.1	uncharacterized protein LOC109004735 isoform X5	Juglans regia	XP_018838927.1
TRINITY_DN4481_c0_g1_i3_5	1,98E-125	359	XP_018825022.1	60S ribosomal protein L15-1 isoform X1	Juglans regia	XP_018825023.1
TRINITY_DN1332_c0_g1_i1_2	2,25E-136	389	XP_018858051.1	endonuclease 2 isoform X2	Juglans regia	XP_008339659.1
TRINITY_DN10179_c0_g1_i1_1	0	748	XP_018860760.1	probable pectinesterase/pectinesterase inhibitor 21	Juglans regia	KDP23449.1
TRINITY_DN5568_c0_g1_i1_2	4,01E-136	397	XP_018807889.1	heterogeneous nuclear ribonucleoprotein 1-like	Juglans regia	XP_018810068.1
TRINITY_DN11161_c0_g2_i1_1	6,33E-43	141	OAY23711.1	hypothetical protein MANES_18G101100	Manihot esculenta	XP_018821061.1
TRINITY_DN4141_c0_g1_i1_2	0	1016	XP_018842240.1	biotin carboxylase 1, chloroplastic isoform X1	Juglans regia	XP_018842244.1
TRINITY_DN15874_c0_g1_i1_6	1,06E-80	253	XP_018859093.1	probable pectinesterase/pectinesterase inhibitor 51	Juglans regia	XP_018842710.1
TRINITY_DN10532_c0_g1_i1_4	6,91E-157	440	XP_018849247.1	ribulose-phosphate 3-epimerase, cytoplasmic isoform	Juglans regia	XP_008379127.1
TRINITY_DN6875_c0_g1_i1_3	4,53E-171	481	KDP41387.1	hypothetical protein JCGZ_15794	Jatropha curcas	XP_012067879.1
TRINITY_DN7117_c0_g2_i2_1	2,43E-94	277	OAY42674.1	hypothetical protein MANES_08G007000	Manihot esculenta	OAY38010.1
TRINITY_DN19584_c0_g1_i1_1	1,12E-150	439	XP_018850062.1	acyl-CoA-binding domain-containing protein 4	Juglans regia	ONI04459.1
TRINITY_DN11441_c0_g1_i2_3	0	1121	XP_018839329.1	methylenetetrahydrofolate reductase 2-like	Juglans regia	XP_018858229.1
TRINITY_DN11585_c0_g1_i2_3	0	1316	XP_018815731.1	glyoxysomal fatty acid beta-oxidation multifunctional protein MFP-a-like	Juglans regia	XP_018825769.1
TRINITY_DN9778_c0_g1_i2_1	0	1135	ONI11196.1	hypothetical protein PRUPE_4G091800	Prunus persica	ONI11197.1
TRINITY_DN10084_c0_g1_i3_3	0	609	XP_018855479.1	probable L-ascorbate peroxidase 6, chloroplastic isoform X2	Juglans regia	XP_018855478.1
TRINITY_DN8707_c0_g1_i1_4	2,80E-114	335	OAY32872.1	hypothetical protein MANES_13G052100	Manihot esculenta	XP_009371092.1
TRINITY_DN8413_c0_g1_i1_3	6,32E-94	275	XP_018816977.1	40S ribosomal protein S19-3	Juglans regia	KHN12325.1
TRINITY_DN11449_c0_g2_i5_3	0	1406	XP_018809529.1	phosphoenolpyruvate carboxylase 4-like isoform X1	Juglans regia	XP_018809531.1
TRINITY_DN21888_c0_g1_i1_3	2,50E-19	85,9	XP_018824867.1	ras GTPase-activating protein-binding protein 2 isoform X1	Juglans regia	XP_018824868.1
TRINITY_DN10361_c1_g2_i1_1	3,42E-122	354	XP_018834379.1	60S ribosomal protein L6	Juglans regia	XP_009354690.1
TRINITY_DN7454_c0_g1_i1_5	2,02E-88	262	XP_009351382.2	nudix hydrolase 26, chloroplastic-like	Pyrus x bretschneideri	XP_009351383.2
TRINITY_DN11044_c0_g1_i1_3	0	865	OAY58939.1	hypothetical protein MANES_02G218200	Manihot esculenta	XP_008243091.1

TRINITY_DN10694_c0_g1_i1_2	0	530	XP_018839634.1	S-formylglutathione hydrolase	Juglans regia	OAY43221.1
TRINITY_DN7765_c0_g1_i1_3	2,50E-82	248	XP_018819687.1	40S ribosomal protein S14-3-like	Juglans regia	XP_018859543.1
TRINITY_DN10259_c0_g1_i1_4	1,27E-123	359	XP_018834430.1	ATP synthase subunit O, mitochondrial-like	Juglans regia	XP_008231281.1
TRINITY_DN10666_c0_g1_i1_3	0	1115	XP_018807321.1	aconitate hydratase 1	Juglans regia	KRH28848.1
TRINITY_DN8743_c0_g1_i2_2	3,57E-27	107	ONH95541.1	hypothetical protein PRUPE_7G076400	Prunus persica	ONH95542.1
TRINITY_DN7469_c0_g1_i1_3	1,37E-161	454	XP_018820822.1	(DL)-glycerol-3-phosphatase 2	Juglans regia	XP_008223698.1
TRINITY_DN9596_c0_g1_i2_1	0	758	XP_018823042.1	proton pump-interactor 1-like	Juglans regia	XP_018823042.1
TRINITY_DN9658_c0_g1_i1_4	0	775	XP_018849591.1	dolichyl-diphosphooligosaccharide--protein glycosyltransferase 48 kDa subunit-like isoform X2	Juglans regia	XP_004507412.1
TRINITY_DN7483_c0_g2_i2_4	1,31E-112	325	OAY49459.1	hypothetical protein MANES_05G057900	Manihot esculenta	XP_018819121.1
TRINITY_DN10418_c0_g1_i1_1	3,64E-127	369	XP_018809757.1	calcyclin-binding protein-like	Juglans regia	XP_018819738.1
TRINITY_DN6749_c0_g1_i3_2	0	979	XP_018836262.1	pyrophosphate--fructose 6-phosphate 1-phosphotransferase subunit beta-like	Juglans regia	XP_018829538.1
TRINITY_DN10064_c0_g1_i1_6	3,74E-74	229	XP_018832799.1	uncharacterized protein At3g03773-like	Juglans regia	XP_008237430.1
TRINITY_DN10803_c0_g1_i1_1	1,64E-09	56,2	XP_018811921.1	elicitor-responsive protein 3	Juglans regia	XP_009340615.1
TRINITY_DN10303_c0_g1_i1_3	0	732	XP_018837049.1	cysteine proteinase RD21A-like	Juglans regia	XP_018837050.1
TRINITY_DN2784_c0_g1_i1_3	1,47E-146	428	XP_018839074.1	UDP-sugar pyrophosphorylase-like	Juglans regia	XP_018849179.1
TRINITY_DN12921_c0_g1_i1_6	1,28E-120	346	XP_018825134.1	ubiquitin carboxyl-terminal hydrolase 3-like	Juglans regia	XP_018825135.1
TRINITY_DN9516_c0_g1_i2_6	0	1238	XP_018840650.1	transmembrane 9 superfamily member 11 isoform X1	Juglans regia	XP_018840651.1
TRINITY_DN9036_c0_g1_i1_2	2,76E-71	217	XP_018822894.1	uncharacterized protein LOC108992718	Juglans regia	XP_018850381.1
TRINITY_DN20791_c0_g1_i1_4	3,84E-43	147	XP_018849298.1	E3 ubiquitin-protein ligase RING1-like	Juglans regia	XP_020224288.1
TRINITY_DN6266_c0_g1_i2_5	0	553	XP_018851614.1	glycerol-3-phosphate dehydrogenase SDP6, mitochondrial	Juglans regia	XP_018822930.1
TRINITY_DN11086_c0_g1_i1_1	7,63E-173	484	XP_018837325.1	proteasome subunit alpha type-5	Juglans regia	XP_018837326.1
TRINITY_DN3111_c0_g2_i1_2	0	974	XP_018813437.1	probable cytosolic oligopeptidase A	Juglans regia	XP_018850785.1
TRINITY_DN10597_c0_g2_i1_2	0	1867	XP_018819183.1	ubiquitin-activating enzyme E1 1-like	Juglans regia	XP_018828478.1
TRINITY_DN10098_c0_g1_i3_1	0	671	KDP43753.1	hypothetical protein JCGZ_22380	Jatropha curcas	OAY60978.1
TRINITY_DN6445_c0_g1_i1_3	1,20E-153	442	ONI10836.1	hypothetical protein PRUPE_4G070900	Prunus persica	XP_007213451.2
TRINITY_DN10634_c0_g1_i2_4	4,81E-126	364	OAY46654.1	hypothetical protein MANES_06G016600	Manihot esculenta	XP_008218606.1
TRINITY_DN5741_c0_g1_i2_3	6,10E-60	191	KDP22223.1	hypothetical protein JCGZ_26054	Jatropha curcas	XP_012090181.1
TRINITY_DN7128_c0_g2_i1_1	1,68E-152	435	XP_018856017.1	ribosome-recycling factor, chloroplastic isoform X1	Juglans regia	XP_018856023.1
TRINITY_DN11601_c0_g2_i2_5	0	970	XP_018852509.1	puromycin-sensitive aminopeptidase isoform X2	Juglans regia	XP_018852512.1
TRINITY_DN9962_c0_g2_i1_2	0	850	XP_018814064.1	reticuline oxidase-like protein	Juglans regia	XP_018808169.1
TRINITY_DN10845_c0_g1_i2_1	0	707	XP_018851483.1	isocitrate dehydrogenase [NAD] catalytic subunit 5, mitochondrial isoform X1	Juglans regia	ONI03044.1
TRINITY_DN10542_c0_g1_i1_2	1,58E-63	208	XP_018824896.1	glycine-rich RNA-binding protein 3, mitochondrial-like	Juglans regia	XP_018824896.1
TRINITY_DN7441_c0_g1_i1_6	2,94E-11	63,2	AGC51777.1	dehydrin protein	Manihot esculenta	OAY50493.1
TRINITY_DN10521_c0_g1_i1_3	0	775	XP_018827302.1	succinate--CoA ligase [ADP-forming] subunit beta, mitochondrial	Juglans regia	XP_018847166.1

TRINITY_DN10791_c2_g1_i3_2	2,93E-174	490	XP_018841148.1	12-oxophytodienoate reductase 2-like	Juglans regia	OAY23951.1
TRINITY_DN11097_c0_g1_i2_6	0	603	OAY50881.1	hypothetical protein MANES_05G169500	Manihot esculenta	XP_009364054.1
TRINITY_DN8226_c0_g1_i1_1	0	836	XP_018829248.1	monodehydroascorbate reductase 5, mitochondrial	Juglans regia	XP_012086827.1
TRINITY_DN8836_c0_g1_i1_2	0	617	XP_018846192.1	aminoacyl tRNA synthase complex-interacting multifunctional protein 1 isoform X1	Juglans regia	XP_018846193.1
TRINITY_DN9745_c0_g1_i2_1	0	1227	XP_018849604.1	programmed cell death protein 4-like	Juglans regia	XP_018849604.1
TRINITY_DN6745_c0_g1_i6_1	5,09E-170	483	XP_018811421.1	polyadenylate-binding protein RBP45C-like	Juglans regia	XP_018811421.1
TRINITY_DN10651_c0_g1_i1_1	0	892	XP_018844173.1	peptidyl-prolyl cis-trans isomerase FKBP62-like isoform X1	Juglans regia	KDP45275.1
TRINITY_DN3758_c0_g1_i1_5	5,69E-33	118	XP_009366604.1	60S acidic ribosomal protein P1-like	Pyrus x bretschneideri	XP_008376223.1
TRINITY_DN9555_c0_g1_i2_4	4,38E-42	141	KRH17781.1	hypothetical protein GLYMA_13G015400	Glycine max	BAU03535.1
TRINITY_DN8044_c0_g1_i1_2	0	1007	XP_018858088.1	pyruvate kinase, cytosolic isozyme-like	Juglans regia	XP_018858157.1
TRINITY_DN5302_c0_g2_i1_4	2,23E-84	254	XP_015950188.1	NADPH-dependent aldo-keto reductase, chloroplastic	Arachis duranensis	XP_016183769.1
TRINITY_DN9331_c0_g1_i5_4	0	817	XP_018828790.1	dolichyl-diphosphooligosaccharide--protein glycosyltransferase subunit 1B	Juglans regia	KDP44377.1
TRINITY_DN10842_c0_g1_i1_3						
TRINITY_DN10737_c0_g1_i2_1	0	613	XP_018839416.1	perakine reductase-like	Juglans regia	XP_018818666.1
TRINITY_DN11246_c0_g1_i1_1	2,45E-104	303	dbj|BAT97743.1	hypothetical protein VIGAN_09127500	Vigna angularis var. angularis	XP_017441415.1
TRINITY_DN10802_c0_g1_i2_2	0	546	XP_018838600.1	protein SGT1 homolog	Juglans regia	KDP22207.1
TRINITY_DN10937_c0_g1_i1_3	1,27E-158	447	XP_018860017.1	60S ribosomal protein L8-1-like	Juglans regia	XP_018822032.1
TRINITY_DN5821_c0_g1_i2_2	2,16E-79	243	XP_018815170.1	21 kDa protein	Juglans regia	KOM39461.1
TRINITY_DN7491_c0_g1_i1_1	3,41E-158	449	XP_018812091.1	60S ribosomal protein L7a-1	Juglans regia	XP_018815479.1
TRINITY_DN10883_c0_g1_i2_4	4,47E-159	451	XP_018832945.1	peptidyl-prolyl cis-trans isomerase A1 isoform X1	Juglans regia	XP_018832946.1
TRINITY_DN9369_c0_g1_i1_2	3,54E-146	417	XP_018823879.1	translocon-associated protein subunit alpha-like	Juglans regia	XP_018841445.1
TRINITY_DN15371_c0_g1_i1_1	4,33E-32	119	XP_018832135.1	probable pectinesterase/pectinesterase inhibitor 21, partial	Juglans regia	XP_018825590.1
TRINITY_DN8535_c0_g1_i1_3	0	760	XP_018822783.1	pyruvate dehydrogenase E1 component subunit alpha-3, chloroplastic	Juglans regia	OAY48935.1
TRINITY_DN11283_c0_g1_i3_1	2,94E-171	481	XP_018824212.1	proteasome subunit alpha type-4	Juglans regia	XP_008371922.1
TRINITY_DN10469_c0_g2_i1_3	3,42E-106	310	XP_018841792.1	60S ribosomal protein L18-2-like	Juglans regia	XP_018815940.1
TRINITY_DN6972_c0_g1_i1_5	1,75E-112	331	XP_018820307.1	vesicle-associated protein 1-2-like isoform X2	Juglans regia	XP_018853856.1
TRINITY_DN9766_c0_g1_i2_1	0	541	XP_018821649.1	gamma carbonic anhydrase 1, mitochondrial-like	Juglans regia	XP_018843856.1
TRINITY_DN6771_c0_g1_i1_1	1,83E-175	505	XP_018839434.1	pyruvate kinase isozyme A, chloroplastic-like	Juglans regia	XP_008353310.1

TRINITY_DN11054_c0_g1_i1_2	0	1008	XP_018807686.1	3-isopropylmalate dehydratase large subunit, chloroplastic-like	Juglans regia	XP_018820100.1
TRINITY_DN11476_c0_g1_i2_1	0	755	OAY34237.1	hypothetical protein MANES_12G005200	Manihot esculenta	XP_018815635.1
TRINITY_DN9150_c0_g1_i3_1	4,53E-81	241	OAY55416.1	hypothetical protein MANES_03G152300	Manihot esculenta	AAT84459.1
TRINITY_DN8849_c0_g2_i3_2	2,21E-71	218	XP_008354935.1	uncharacterized protein LOC103418606	Malus domestica	XP_018504852.1
TRINITY_DN7905_c0_g1_i1_5	1,84E-114	333	XP_018816103.1	succinate dehydrogenase subunit 5, mitochondrial-like	Juglans regia	XP_009367369.1
TRINITY_DN9056_c0_g1_i1_6	8,79E-98	285	XP_018813840.1	ferredoxin, root R-B1-like	Juglans regia	ONI01252.1
TRINITY_DN8426_c0_g1_i2_3	0	547	XP_018828606.1	peroxidase 4-like	Juglans regia	XP_018806705.1
TRINITY_DN4385_c0_g1_i1_2	0	697	XP_018843013.1	NPL4-like protein 1	Juglans regia	XP_008234969.1
TRINITY_DN21126_c0_g1_i1_1						
TRINITY_DN11515_c0_g1_i3_1	0	818	OAY44035.1	hypothetical protein MANES_08G117200	Manihot esculenta	XP_018841804.1
TRINITY_DN12722_c0_g1_i1_1	7,15E-164	469	XP_009343360.1	putative pectinesterase 63	Pyrus x bretschneideri	XP_008391732.1
TRINITY_DN11139_c0_g1_i1_3	0	986	XP_018823517.1	plastidic ATP/ADP-transporter-like	Juglans regia	XP_018827696.1
TRINITY_DN10080_c0_g1_i1_1	0	667	XP_018841575.1	uncharacterized protein At2g17340-like	Juglans regia	XP_018841576.1
TRINITY_DN8178_c0_g1_i2_3	1,66E-116	339	XP_018822554.1	probable fructokinase-4	Juglans regia	KHN06200.1
TRINITY_DN130_c0_g1_i1_2	0	663	XP_018845963.1	elongation factor Tu, mitochondrial-like	Juglans regia	XP_018847444.1
TRINITY_DN11280_c0_g1_i4_3	0	960	XP_018843166.1	proline--tRNA ligase, cytoplasmic-like isoform X1	Juglans regia	XP_018843167.1
TRINITY_DN11620_c0_g1_i1_2	7,41E-172	482	XP_018835046.1	proteasome subunit alpha type-6 isoform X1	Juglans regia	XP_018835047.1
TRINITY_DN11411_c1_g1_i3_5	0	771	XP_018832890.1	S-adenosylmethionine synthase 1	Juglans regia	XP_009363082.1
TRINITY_DN9704_c0_g1_i1_2	2,53E-92	272	XP_019463034.1	40S ribosomal protein S15a-1 isoform X3	Lupinus angustifolius	XP_012081549.1
TRINITY_DN2711_c0_g1_i1_3	0	665	XP_018833157.1	serine carboxypeptidase-like 51	Juglans regia	OAY51501.1
TRINITY_DN10623_c0_g1_i2_5	4,65E-47	156	XP_018821920.1	40S ribosomal protein S26-1-like	Juglans regia	XP_018810475.1
TRINITY_DN9395_c0_g2_i1_3	0	783	XP_018827585.1	fumarylacetoacetase	Juglans regia	KDP37782.1
TRINITY_DN10777_c0_g1_i2_2	1,51E-126	359	XP_018808681.1	ubiquitin-fold modifier-conjugating enzyme 1	Juglans regia	KDP41535.1
TRINITY_DN8939_c0_g1_i1_1	0	718	XP_018814264.1	phosphomethylethanolamine N-methyltransferase-like	Juglans regia	XP_018857980.1
TRINITY_DN14055_c0_g1_i1_6	5,27E-41	149	XP_018819183.1	ubiquitin-activating enzyme E1 1-like	Juglans regia	XP_018828478.1
TRINITY_DN5112_c0_g2_i3_3	0	710	XP_009339277.1	DNA damage-inducible protein 1	Pyrus x bretschneideri	XP_008384617.1
TRINITY_DN3053_c0_g1_i1_6	1,29E-54	176	XP_018854383.1	UDP-glycosyltransferase 79B30-like	Juglans regia	XP_018838486.1
TRINITY_DN3542_c0_g2_i1_1	2,68E-137	389	XP_018844636.1	PITH domain-containing protein 1	Juglans regia	AFK35651.1
TRINITY_DN8778_c0_g1_i2_2	5,01E-141	405	XP_008242359.1	uncharacterized protein LOC103340695 isoform X4	Prunus mume	ONH97760.1
TRINITY_DN8889_c0_g1_i1_1	0	527	XP_018820940.1	flavonol synthase/flavanone 3-hydroxylase-like	Juglans regia	XP_009367022.1
TRINITY_DN6086_c0_g1_i1_1	3,78E-156	440	XP_018860145.1	probable methyltransferase At1g27930	Juglans regia	KDP46273.1
TRINITY_DN9418_c0_g1_i2_5	0	588	XP_018859966.1	hexokinase-1-like isoform X2	Juglans regia	KYP67483.1
TRINITY_DN9810_c0_g1_i1_3	0	743	XP_018844405.1	pyruvate dehydrogenase E1 component subunit alpha-1, mitochondrial-like	Juglans regia	KDP21276.1

TRINITY_DN6193_c0_g1_i1_1	9,03E-74	224	XP_008242440.1	60S ribosomal protein L28-1	Prunus mume	ONH97860.1
TRINITY_DN9474_c1_g1_i1_5	2,77E-105	305	AFK36220.1	unknown	Lotus japonicus	XP_004489035.1
TRINITY_DN10292_c0_g1_i2_6	1,42E-63	202	XP_018820585.1	uncharacterized protein LOC108990917	Juglans regia	XP_018814019.1
TRINITY_DN11230_c0_g2_i2_6	3,77E-96	281	emb|CAW56310.1	unnamed protein product	Glycine max	CBC31240.1
TRINITY_DN10344_c0_g1_i1_2	0	627	XP_018829145.1	ankyrin repeat domain-containing protein 2B-like	Juglans regia	XP_018841596.1
TRINITY_DN11434_c0_g1_i1_2	1,38E-95	281	XP_008224314.1	40S ribosomal protein S18	Prunus mume	XP_008390888.1
TRINITY_DN11361_c0_g1_i1_2	0	764	XP_018833171.1	UDP-D-apiose/UDP-D-xylose synthase 2	Juglans regia	XP_008385469.1
TRINITY_DN10628_c0_g1_i1_3	6,93E-138	394	XP_018815110.1	CBS domain-containing protein CBSX3, mitochondrial-like	Juglans regia	OAY58828.1
TRINITY_DN5840_c0_g1_i1_1	9,44E-89	259	AAQ08198.1	eukaryotic translation initiation factor 5A isoform VIII, partial	Hevea brasiliensis	XP_018823424.1
TRINITY_DN10700_c0_g1_i1_3	6,34E-82	251	XP_018832602.1	probable pectinesterase/pectinesterase inhibitor 21	Juglans regia	XP_008351186.1
TRINITY_DN10484_c2_g1_i2_1	4,66E-141	402	OAY32771.1	hypothetical protein MANES_13G044600	Manihot esculenta	XP_018840734.1
TRINITY_DN11431_c0_g1_i3_5	3,35E-44	149	KDP20078.1	hypothetical protein JCGZ_05847	Jatropha curcas	XP_012092953.1
TRINITY_DN9680_c0_g1_i1_1	5,28E-75	227	XP_018838135.1	uncharacterized protein LOC109004143 isoform X1	Juglans regia	XP_018838136.1
TRINITY_DN53_c0_g1_i1_5	6,29E-57	191	XP_017180254.1	subtilisin-like protease SBT5.4	Malus domestica	XP_008353281.1
TRINITY_DN13553_c0_g1_i1_2	2,78E-48	167	XP_018816352.1	villin-3-like isoform X3	Juglans regia	XP_018816351.1
TRINITY_DN10106_c0_g1_i2_1	0	521	XP_018816665.1	14-3-3-like protein A	Juglans regia	XP_018816666.1
TRINITY_DN10104_c0_g2_i1_2	2,23E-134	397	OAY38507.1	hypothetical protein MANES_10G020400	Manihot esculenta	XP_009346996.2
TRINITY_DN11228_c0_g1_i1_3	4,65E-34	119	XP_008223181.1	heat shock factor-binding protein 1	Prunus mume	ONI28303.1
TRINITY_DN9356_c0_g1_i2_6	3,23E-177	511	XP_018856959.1	coiled-coil domain-containing protein 18-like	Juglans regia	ONI33943.1
TRINITY_DN10740_c0_g1_i5_5	0	672	ONI20047.1	hypothetical protein PRUPE_3G312200	Prunus persica	XP_007215551.1
TRINITY_DN15242_c0_g1_i1_5	1,42E-52	168	XP_018833870.1	1-Cys peroxiredoxin	Juglans regia	XP_016190426.1
TRINITY_DN10478_c0_g2_i1_1						
TRINITY_DN10321_c0_g1_i2_2	2,72E-74	226	OAY33633.1	hypothetical protein MANES_13G112100	Manihot esculenta	KDP32073.1
TRINITY_DN17896_c0_g1_i1_5	0	642	XP_018842745.1	stomatin-like protein 2, mitochondrial	Juglans regia	XP_018828153.1
TRINITY_DN5702_c0_g1_i1_3						
TRINITY_DN6940_c0_g9_i5_6	6,46E-113	326	KDP28201.1	hypothetical protein JCGZ_13972	Jatropha curcas	XP_012082823.1
TRINITY_DN10205_c0_g1_i1_2	0	684	XP_018841544.1	ubiquitin domain-containing protein DSK2a-like isoform X3	Juglans regia	XP_018841547.1
TRINITY_DN10892_c0_g1_i2_4	0	1444	XP_018840323.1	V-type proton ATPase subunit a3-like	Juglans regia	ONH91014.1
TRINITY_DN11295_c0_g2_i3_1	0	902	XP_018857292.1	glutathione reductase, cytosolic-like	Juglans regia	XP_018857293.1
TRINITY_DN10858_c0_g1_i1_5	0	644	XP_018818486.1	erlin-2-B	Juglans regia	XP_018818487.1
TRINITY_DN10871_c0_g1_i3_2	0	933	AHJ79156.1	betaine-aldehyde dehydrogenase	Juglans regia	XP_018831659.1
TRINITY_DN6968_c0_g1_i1_1	1,26E-158	449	XP_018844949.1	20 kDa chaperonin, chloroplastic-like	Juglans regia	XP_018844947.1
TRINITY_DN6689_c0_g1_i1_2	1,58E-164	468	XP_018814780.1	3-oxoacyl-[acyl-carrier-protein reductase 4 isoform X2	Juglans regia	XP_018814779.1
TRINITY_DN10111_c0_g1_i3_2	0	941	KDP26716.1	hypothetical protein JCGZ_17874	Jatropha curcas	XP_012085554.1
TRINITY_DN9976_c0_g4_i1_4	3,55E-119	347	AAB61597.1	glutamine synthetase	Hevea brasiliensis	CBN69820.1

TRINITY_DN11601_c0_g1_i1_1	0	676	XP_018852509.1	puromycin-sensitive aminopeptidase isoform X2	Juglans regia	XP_018852512.1
TRINITY_DN4015_c0_g1_i1_2	2,44E-70	213	XP_018842089.1	basic blue protein-like	Juglans regia	AET04469.1
TRINITY_DN10140_c0_g1_i1_2	0	600	XP_018826879.1	L-galactose dehydrogenase	Juglans regia	XP_008236174.1
TRINITY_DN2592_c0_g1_i1_1	0	902	XP_018806299.1	enolase 1, chloroplastic	Juglans regia	XP_008244075.1
TRINITY_DN10378_c0_g2_i2_1	0	678	ONH93092.1	hypothetical protein PRUPE_8G212700	Prunus persica	XP_020425931.1
TRINITY_DN8575_c0_g2_i3_2	1,67E-114	329	XP_018806533.1	probable glutathione peroxidase 2	Juglans regia	BAT72709.1
TRINITY_DN11259_c0_g1_i2_5	0	1168	XP_018806016.1	vacuolar-sorting receptor 3-like	Juglans regia	XP_018846135.1
TRINITY_DN11269_c2_g1_i2_3	0	839	XP_018845033.1	eukaryotic initiation factor 4A-15-like	Juglans regia	XP_018844689.1
TRINITY_DN9965_c1_g1_i1_3	1,21E-56	177	XP_018814660.1	uncharacterized protein At2g27730, mitochondrial	Juglans regia	XP_018814661.1
TRINITY_DN9622_c1_g1_i2_2	1,22E-07	48,9	KDP45878.1	hypothetical protein JCGZ_15322	Jatropha curcas	OAY34547.1
TRINITY_DN10079_c0_g1_i1_3	0	564	XP_018856841.1	quinone oxidoreductase PIG3-like	Juglans regia	XP_016179633.1
TRINITY_DN3545_c0_g1_i2_1	9,81E-71	213	XP_018817673.1	40S ribosomal protein S9-2-like	Juglans regia	XP_018845180.1
TRINITY_DN5659_c0_g1_i1_1	0	1528	XP_018837598.1	alpha-glucan phosphorylase, H isozyme-like isoform X1	Juglans regia	XP_018837601.1
TRINITY_DN5550_c0_g1_i2_4	3,75E-41	145	XP_018816861.1	ubiquitin receptor RAD23c-like	Juglans regia	XP_018815467.1
TRINITY_DN11000_c0_g1_i1_2	2,04E-180	508	XP_018844199.1	putative quinone-oxidoreductase homolog, chloroplastic	Juglans regia	XP_018850172.1
TRINITY_DN9087_c0_g1_i2_1	8,77E-104	310	XP_018813170.1	vesicle-associated protein 1-2-like	Juglans regia	KHN04359.1
TRINITY_DN9399_c0_g1_i2_1	7,10E-138	394	XP_018841431.1	5'-methylthioadenosine/S-adenosylhomocysteine nucleosidase 2-like isoform X1	Juglans regia	XP_018815344.1
TRINITY_DN11102_c0_g1_i4_6	1,02E-172	487	XP_008225042.1	AB hydrolase superfamily protein YfhM-like	Prunus mume	ONI10358.1
TRINITY_DN7857_c0_g1_i4_2	0	1188	XP_018815161.1	peroxisomal fatty acid beta-oxidation multifunctional protein AIM1	Juglans regia	ONH98171.1
TRINITY_DN5480_c0_g1_i2_3	0	535	XP_018816315.1	ABC transporter I family member 20-like isoform X1	Juglans regia	AIU41667.1
TRINITY_DN3630_c0_g1_i1_5	0	703	XP_018837387.1	acetyl-CoA acetyltransferase, cytosolic 1	Juglans regia	XP_008224638.1
TRINITY_DN3986_c0_g2_i1_2	0	863	XP_018820151.1	external alternative NAD(P)H-ubiquinone oxidoreductase B2, mitochondrial-like	Juglans regia	XP_015943513.1
TRINITY_DN10586_c0_g1_i1_1	9,11E-79	237	XP_018840715.1	cytochrome b5, seed isoform	Juglans regia	XP_018821473.1
TRINITY_DN10813_c1_g1_i3_3	0	518	XP_018825586.1	UDP-glucuronic acid decarboxylase 4-like	Juglans regia	XP_018857609.1
TRINITY_DN21280_c0_g1_i1_3	1,82E-37	136	KDP30704.1	hypothetical protein JCGZ_16402	Jatropha curcas	XP_012080785.1
TRINITY_DN7020_c0_g1_i1_2	1,58E-71	223	XP_018833902.1	cytochrome c oxidase subunit 6b-1-like isoform X2	Juglans regia	XP_018833901.1
TRINITY_DN10799_c0_g2_i1_6	4,40E-115	349	XP_018831678.1	FAM10 family protein At4g22670-like	Juglans regia	XP_018831678.1
TRINITY_DN8664_c0_g1_i3_3	0	540	XP_018826547.1	aldose 1-epimerase	Juglans regia	ONI08609.1
TRINITY_DN9559_c0_g1_i1_3	0	709	XP_018817113.1	TOM1-like protein 2	Juglans regia	XP_018817114.1
TRINITY_DN11471_c0_g1_i3_3	2,84E-126	360	XP_018850586.1	60S ribosomal protein L18a	Juglans regia	OAY29135.1
TRINITY_DN7380_c0_g3_i1_1	4,01E-62	190	OAY21768.1	hypothetical protein MANES_S058900	Manihot esculenta	KDP37139.1
TRINITY_DN9887_c0_g1_i1_2	2,92E-86	256	XP_018853126.1	40S ribosomal protein S12-like	Juglans regia	XP_018808087.1
TRINITY_DN10568_c0_g3_i5_1	0	643	XP_018829196.1	calnexin homolog	Juglans regia	XP_018829125.1

TRINITY_DN10508_c0_g1_i1_5	7,40E-121	350	XP_018823258.1	non-functional NADPH-dependent codeinone reductase 2-like	Juglans regia	KDP38187.1
TRINITY_DN9574_c0_g1_i3_1	0	683	XP_018829628.1	malate dehydrogenase, glyoxysomal	Juglans regia	XP_018819958.1
TRINITY_DN6731_c0_g1_i1_1	0	1090	XP_018833363.1	acetyl-coenzyme A carboxylase carboxyl transferase subunit alpha, chloroplastic-like	Juglans regia	XP_018833364.1
TRINITY_DN10793_c0_g1_i1_1	0	508	XP_018823011.1	mitochondrial outer membrane protein porin of 34 kDa	Juglans regia	XP_018835940.1
TRINITY_DN10605_c0_g1_i4_2	0	1176	XP_018851318.1	serine/threonine-protein phosphatase 2A 65 kDa regulatory subunit A beta isoform	Juglans regia	XP_018829949.1
TRINITY_DN7923_c0_g1_i1_1	1,32E-141	405	XP_018817983.1	40S ribosomal protein S6-like	Juglans regia	XP_018808592.1
TRINITY_DN8263_c0_g1_i2_2	0	1072	XP_018839045.1	probable methyltransferase PMT26	Juglans regia	XP_018839046.1
TRINITY_DN5337_c0_g1_i1_4	0	850	XP_018841219.1	uncharacterized protein LOC109006407	Juglans regia	ONI23255.1
TRINITY_DN11163_c0_g1_i8_3	0	602	ONH93127.1	hypothetical protein PRUPE_8G214700	Prunus persica	ONH93128.1
TRINITY_DN10512_c1_g1_i1_2	2,26E-60	191	XP_008230471.1	putative phosphatidylglycerol/phosphatidylinositol transfer protein DDB_G0282179	Prunus mume	ONI01573.1
TRINITY_DN5432_c0_g1_i1_5	0	524	XP_018812299.1	mitochondrial outer membrane protein porin of 36 kDa	Juglans regia	XP_008339931.1
TRINITY_DN10108_c0_g1_i2_5	0	1100	XP_018842661.1	phosphoinositide phosphatase SAC6-like	Juglans regia	XP_018842662.1
TRINITY_DN10975_c0_g2_i1_3	2,34E-110	322	XP_018847195.1	eukaryotic translation initiation factor 5A-2	Juglans regia	XP_018829201.1
TRINITY_DN10736_c0_g1_i2_1	0	1055	XP_018826687.1	glucose-6-phosphate isomerase, cytosolic	Juglans regia	KRH61180.1
TRINITY_DN7422_c0_g2_i1_2	0	689	XP_018849575.1	uncharacterized protein LOC109012411 isoform X1	Juglans regia	XP_018849576.1
TRINITY_DN10308_c0_g1_i1_1	2,05E-133	379	XP_018860302.1	60S ribosomal protein L13a-4	Juglans regia	XP_018860308.1
TRINITY_DN10384_c0_g3_i1_3	0	861	XP_016204269.1	tubulin beta-1 chain	Arachis ipaensis	XP_015967408.1
TRINITY_DN3139_c0_g1_i1_2	3,19E-137	392	XP_008337994.1	proteasome subunit alpha type-7	Malus domestica	XP_009363477.1
TRINITY_DN10364_c0_g1_i3_6	3,99E-16	73,9	XP_004515192.1	uncharacterized protein LOC101514444	Cicer arietinum	ONI26968.1
TRINITY_DN20886_c0_g1_i1_5	1,29E-79	242	XP_018835853.1	thioredoxin O2, mitochondrial-like isoform X1	Juglans regia	XP_018835854.1
TRINITY_DN10883_c0_g1_i3_5	5,89E-161	455	XP_018832945.1	peptidyl-prolyl cis-trans isomerase A1 isoform X1	Juglans regia	XP_018832946.1
TRINITY_DN17539_c0_g2_i1_3	4,53E-89	289	XP_018827628.1	probable methyltransferase PMT26	Juglans regia	XP_018827629.1
TRINITY_DN10477_c0_g1_i1_1	0	1060	XP_008230393.1	T-complex protein 1 subunit zeta 1	Prunus mume	ONI18783.1
TRINITY_DN6686_c0_g1_i1_2	0	793	XP_018815316.1	coatomer subunit alpha-1	Juglans regia	XP_018840319.1
TRINITY_DN11212_c0_g1_i1_6	5,67E-148	418	XP_018814321.1	succinate dehydrogenase [ubiquinone] iron-sulfur subunit 2, mitochondrial-like	Juglans regia	XP_004488937.1
TRINITY_DN11023_c0_g1_i2_1	0	1410	ONH96238.1	hypothetical protein PRUPE_7G115400	Prunus persica	XP_007204282.1
TRINITY_DN11458_c0_g1_i1_1	0	996	XP_018812048.1	T-complex protein 1 subunit beta	Juglans regia	OAY50499.1
TRINITY_DN11615_c0_g1_i1_1	0	1195	ONI05147.1	hypothetical protein PRUPE_6G358600	Prunus persica	XP_007208057.1
TRINITY_DN4509_c0_g2_i1_3	2,93E-105	303	XP_018852574.1	UPF0587 protein C1orf123 homolog	Juglans regia	XP_014503583.1
TRINITY_DN11335_c0_g1_i1_1	0	882	XP_018837266.1	T-complex protein 1 subunit gamma isoform X1	Juglans regia	XP_018837267.1

TRINITY_DN5118_c0_g2_i1_6	4,76E-123	364	XP_018848856.1	DEAD-box ATP-dependent RNA helicase 37-like	Juglans regia	XP_018848857.1
TRINITY_DN9738_c2_g1_i2_3	1,41E-104	306	XP_018818478.1	cysteine proteinase inhibitor 6-like	Juglans regia	XP_012068733.1
TRINITY_DN11571_c2_g3_i1_1	0	821	OAY54743.1	hypothetical protein MANES_03G098100	Manihot esculenta	XP_018834544.1
TRINITY_DN17391_c0_g1_i1_5	5,26E-32	113	OAY49409.1	hypothetical protein MANES_05G054100	Manihot esculenta	KDP34869.1
TRINITY_DN10823_c0_g1_i1_1	0	986	XP_018837477.1	T-complex protein 1 subunit eta isoform X1	Juglans regia	OAY37541.1
TRINITY_DN11383_c0_g1_i1_2	9,46E-157	451	XP_018847358.1	probable polygalacturonase	Juglans regia	XP_018847365.1
TRINITY_DN6993_c0_g1_i3_2	0	986	XP_018814240.1	citrate synthase, glyoxysomal	Juglans regia	XP_018828184.1
TRINITY_DN11668_c1_g1_i1_2	1,49E-68	226	emb|CBX33398.1	atp8 (mitochondrion)	Malus domestica	YP_006666136.1
TRINITY_DN3731_c0_g1_i2_3	1,17E-144	419	XP_018847910.1	phosphoglycerate kinase, chloroplastic	Juglans regia	ONI11909.1
TRINITY_DN10138_c0_g1_i1_3	0	974	ONH97993.1	hypothetical protein PRUPE_7G222300	Prunus persica	ONH97994.1
TRINITY_DN8211_c0_g1_i2_3	0	558	XP_018850910.1	bifunctional epoxide hydrolase 2-like	Juglans regia	ONI17394.1
TRINITY_DN20888_c0_g1_i1_3	9,50E-14	69,3	XP_018843078.1	calponin homology domain-containing protein DDB_G0272472	Juglans regia	XP_018843079.1
TRINITY_DN11494_c0_g1_i2_5	7,20E-141	399	XP_018814564.1	soluble inorganic pyrophosphatase 1	Juglans regia	XP_018834262.1
TRINITY_DN7194_c1_g1_i2_2	4,64E-128	373	emb|CAA91445.1	pyruvate decarboxylase, partial	Pisum sativum	|P51851.1|PDC2_PEA RecName: Full=Pyruvate decarboxylase 2; Short=PDC
TRINITY_DN8262_c0_g1_i1_1	3,09E-163	464	XP_018840354.1	glyceraldehyde-3-phosphate dehydrogenase GAPCP2, chloroplastic-like isoform X1	Juglans regia	XP_018840355.1
TRINITY_DN14510_c0_g1_i1_2	5,89E-54	176	XP_018824621.1	glucan endo-1,3-beta-glucosidase 14-like	Juglans regia	XP_018824593.1
TRINITY_DN9908_c0_g1_i2_1	0	521	XP_018824887.1	phosphoserine phosphatase, chloroplastic isoform X1	Juglans regia	XP_018824888.1
TRINITY_DN5370_c0_g1_i1_1	1,75E-49	158	ONH96817.1	hypothetical protein PRUPE_7G153900	Prunus persica	XP_007204141.1
TRINITY_DN2867_c0_g1_i2_6	2,53E-85	254	OAY58377.1	hypothetical protein MANES_02G172800	Manihot esculenta	OAY58378.1
TRINITY_DN10919_c0_g1_i5_1	2,37E-34	124	XP_018806957.1	vignain-like	Juglans regia	AFP20583.1
TRINITY_DN8081_c0_g1_i3_3	0	771	XP_018830218.1	GDP-mannose 3,5-epimerase 2	Juglans regia	XP_018826982.1
TRINITY_DN10953_c0_g1_i1_4	0	637	XP_018819716.1	3-oxoacyl-[acyl-carrier-protein synthase I, chloroplastic	Juglans regia	XP_018819717.1
TRINITY_DN3942_c0_g1_i3_3	3,09E-173	489	XP_018821628.1	proteasome subunit alpha type-1-B-like	Juglans regia	XP_018845049.1
TRINITY_DN6284_c0_g1_i1_3	3,68E-06	50,4	AES73502.1	hypothetical protein MTR_3g105560	Medicago truncatula	XP_003603251.1
TRINITY_DN6272_c0_g1_i1_2	1,54E-171	488	XP_008232049.1	protein BTR1 isoform X2	Prunus mume	ONI21855.1
TRINITY_DN10835_c0_g1_i2_3	4,50E-65	212	XP_014516606.1	plasminogen activator inhibitor 1 RNA-binding protein-like	Vigna radiata var. radiata	KOM58621.1
TRINITY_DN3173_c0_g3_i1_1	1,05E-110	339	XP_018813437.1	probable cytosolic oligopeptidase A	Juglans regia	OAY33523.1
TRINITY_DN8566_c0_g1_i2_1	0	995	XP_018854045.1	T-complex protein 1 subunit alpha	Juglans regia	XP_018854052.1
TRINITY_DN18516_c0_g1_i1_1	4,06E-74	227	XP_018815980.1	uncharacterized protein LOC108987512	Juglans regia	XP_009365361.1
TRINITY_DN10724_c0_g1_i4_1	0	610	XP_018813802.1	aldose 1-epimerase-like	Juglans regia	XP_009347058.1
TRINITY_DN10229_c0_g1_i2_1	6,99E-55	175	XP_008232650.1	60S ribosomal protein L22-3	Prunus mume	ONI22680.1
TRINITY_DN2734_c0_g1_i1_1	1,30E-135	387	XP_018827347.1	tryptophan synthase alpha chain, chloroplastic-like isoform X2	Juglans regia	XP_018827345.1

TRINITY_DN2645_c0_g2_i1_3	2,78E-140	399	XP_018843199.1	glutathione S-transferase DHAR3, chloroplastic-like	Juglans regia	XP_018816866.1
TRINITY_DN11051_c0_g2_i2_2	0	749	XP_008225692.1	UDP-arabinose 4-epimerase 1	Prunus mume	AGH25534.1
TRINITY_DN6332_c0_g2_i1_3	2,71E-171	477	XP_018837327.1	proteasome subunit alpha type-2-B	Juglans regia	ACU21161.1
TRINITY_DN10847_c0_g1_i5_1	2,71E-104	305	XP_018835110.1	coatomer subunit zeta-2-like isoform X2	Juglans regia	XP_009372405.1
TRINITY_DN9500_c0_g1_i4_1	0	716	XP_018840764.1	chaperonin CPN60-2, mitochondrial-like	Juglans regia	ONH93030.1
TRINITY_DN9205_c0_g1_i1_1	1,28E-80	244	XP_016651213.1	basic transcription factor 3 isoform X1	Prunus mume	XP_008240335.1
TRINITY_DN10268_c0_g1_i1_1	0	1047	XP_018809878.1	ketol-acid reductoisomerase, chloroplastic	Juglans regia	XP_018840362.1
TRINITY_DN11412_c0_g1_i3_3	0	1088	KDP29157.1	hypothetical protein JCGZ_16546	Jatropha curcas	KDP29157.1
TRINITY_DN4589_c0_g1_i2_4	1,17E-35	125	OAY30128.1	hypothetical protein MANES_14G006000	Manihot esculenta	XP_020540656.1
TRINITY_DN11511_c2_g1_i3_5	6,30E-54	172	XP_018807308.1	probable glutathione S-transferase	Juglans regia	XP_009344342.1
TRINITY_DN3847_c0_g1_i1_1	1,63E-179	508	XP_018821126.1	mannan endo-1,4-beta-mannosidase 7-like	Juglans regia	ONI32614.1
TRINITY_DN11039_c0_g1_i5_1	0	1211	XP_018848760.1	long chain acyl-CoA synthetase 8	Juglans regia	XP_018848761.1
TRINITY_DN10019_c0_g2_i1_2	0	938	XP_018816636.1	NADH dehydrogenase [ubiquinone] iron-sulfur protein 1, mitochondrial	Juglans regia	XP_008381363.1
TRINITY_DN10360_c0_g3_i2_3	0	1399	XP_018836405.1	1,4-alpha-glucan-branching enzyme 1, chloroplastic/amyloplastic-like	Juglans regia	OAY50415.1
TRINITY_DN11478_c0_g2_i1_2	0	886	XP_018836411.1	catalase isozyme 1	Juglans regia	CAD42908.1
TRINITY_DN16366_c0_g1_i1_4	1,12E-79	253	XP_008339210.1	beta-xylosidase/alpha-L-arabinofuranosidase 1-like	Malus domestica	XP_009354080.1
TRINITY_DN9354_c0_g2_i1_2	0	675	AET62933.1	NADH dehydrogenase subunit 7 (mitochondrion)	Lotus japonicus	YP_005090473.1
TRINITY_DN6770_c0_g1_i2_3	0	613	XP_018845549.1	uncharacterized protein LOC109009507 isoform X2	Juglans regia	XP_012077420.1
TRINITY_DN5978_c0_g1_i1_4	1,67E-39	133	ADD69807.1	copper transport protein ATOX1	Hevea brasiliensis	OAY45760.1
TRINITY_DN10247_c0_g2_i1_3	1,09E-115	336	XP_018813211.1	uncharacterized protein LOC108985386 isoform X1	Juglans regia	XP_004498498.1
TRINITY_DN8913_c1_g2_i1_1	0	750	XP_018848180.1	3-oxoacyl-[acyl-carrier-protein synthase 3 A, chloroplastic-like	Juglans regia	XP_018844419.1
TRINITY_DN11338_c0_g1_i2_4	7,72E-58	184	XP_018834799.1	cytochrome b-c1 complex subunit 7-2-like	Juglans regia	XP_008219895.1
TRINITY_DN11312_c0_g1_i1_1	0	560	XP_008231164.1	cytochrome c1-2, heme protein, mitochondrial	Prunus mume	ONI19859.1
TRINITY_DN10708_c0_g1_i1_2	1,16E-99	299	XP_018847873.1	serine-threonine kinase receptor-associated protein-like	Juglans regia	XP_016179643.1
TRINITY_DN10573_c0_g1_i2_2	0	1055	XP_018846325.1	clathrin heavy chain 1	Juglans regia	XP_018846323.1
TRINITY_DN6429_c0_g1_i3_5	0	586	XP_018815316.1	coatomer subunit alpha-1	Juglans regia	XP_018840319.1
TRINITY_DN8180_c0_g1_i1_1	3,60E-168	475	XP_018809229.1	extradiol ring-cleavage dioxygenase-like	Juglans regia	XP_018844729.1
TRINITY_DN11655_c0_g1_i2_2	0	1672	XP_018857326.1	ATPase 8, plasma membrane-type-like	Juglans regia	XP_018833676.1
TRINITY_DN8076_c0_g1_i7_3	1,32E-116	342	XP_018840255.1	probable voltage-gated potassium channel subunit beta	Juglans regia	XP_016183426.1
TRINITY_DN9094_c0_g1_i3_6	0	667	XP_018847401.1	TOM1-like protein 2	Juglans regia	XP_018847402.1
TRINITY_DN9800_c0_g1_i1_1	0	961	XP_018840407.1	protein transport protein Sec61 subunit alpha-like	Juglans regia	XP_018840408.1

TRINITY_DN21582_c0_g1_i1_3	5,59E-48	159	XP_018809717.1	activator of 90 kDa heat shock protein ATPase homolog 1-like	Juglans regia	OAY40634.1
TRINITY_DN11557_c0_g2_i2_1	1,34E-69	218	XP_018842727.1	protein BOLA4, chloroplastic/mitochondrial-like	Juglans regia	XP_018828128.1
TRINITY_DN9080_c0_g1_i2_2	0	943	XP_018850554.1	alanine aminotransferase 2-like	Juglans regia	ONI15222.1
TRINITY_DN19006_c0_g1_i1_5	0	539	KDP20974.1	hypothetical protein JCGZ_21445	Jatropha curcas	XP_012091606.1
TRINITY_DN2622_c0_g1_i1_2	1,28E-54	174	KDP39545.1	hypothetical protein JCGZ_02565	Jatropha curcas	XP_012070250.1
TRINITY_DN1894_c0_g2_i1_5	4,58E-37	135	XP_018807473.1	uncharacterized protein LOC108980890	Juglans regia	ONH99280.1
TRINITY_DN10750_c0_g1_i1_2	2,26E-160	451	XP_018845601.1	proteasome subunit beta type-6	Juglans regia	OAY53494.1
TRINITY_DN336_c0_g1_i1_1	0	709	XP_018812595.1	ATP sulfurylase 2-like	Juglans regia	ONI32439.1
TRINITY_DN10712_c0_g1_i2_6	3,75E-100	291	XP_008222957.1	40S ribosomal protein S16	Prunus mume	ONI28680.1
TRINITY_DN2749_c0_g1_i1_2	0	1225	XP_018824245.1	acyl-coenzyme A oxidase 3, peroxisomal-like	Juglans regia	XP_018824246.1
TRINITY_DN6944_c1_g2_i2_2	0	715	XP_018828540.1	protein NETWORKED 4A-like isoform X4	Juglans regia	XP_018828537.1
TRINITY_DN10708_c1_g1_i1_1	3,24E-147	419	XP_018847873.1	serine-threonine kinase receptor-associated protein-like	Juglans regia	XP_007154947.1
TRINITY_DN8860_c0_g2_i3_2	1,07E-92	282	XP_018843123.1	eukaryotic translation initiation factor 3 subunit J-like	Juglans regia	OAY46647.1
TRINITY_DN2935_c0_g1_i1_4	1,39E-48	169	XP_018809529.1	phosphoenolpyruvate carboxylase 4-like isoform X1	Juglans regia	KDP29342.1
TRINITY_DN5345_c0_g1_i1_1	0	607	XP_018850953.1	dual specificity protein phosphatase 12-like	Juglans regia	XP_018850954.1
TRINITY_DN2696_c0_g1_i1_2	3,67E-61	189	XP_008239951.1	60S ribosomal protein L34-like	Prunus mume	ONI08672.1
TRINITY_DN6268_c0_g2_i1_1	1,42E-180	507	XP_018827184.1	ABC transporter I family member 19-like	Juglans regia	AIU41666.1
TRINITY_DN20535_c0_g1_i1_2	9,85E-50	169	XP_018824867.1	ras GTPase-activating protein-binding protein 2 isoform X1	Juglans regia	XP_018824868.1
TRINITY_DN6734_c0_g1_i2_4	0	572	XP_008220580.1	endoglucanase 16	Prunus mume	ONI33063.1
TRINITY_DN10790_c0_g1_i1_2	3,09E-82	245	XP_008243052.1	40S ribosomal protein S24-1-like	Prunus mume	ONH98838.1
TRINITY_DN11422_c0_g1_i2_2	7,43E-153	431	XP_018840891.1	proteasome subunit beta type-1	Juglans regia	XP_018809677.1
TRINITY_DN4447_c0_g1_i1_4	2,53E-27	100	AES80599.1	Lipid transfer protein	Medicago truncatula	XP_003624381.1
TRINITY_DN10526_c0_g1_i1_1	2,14E-13	64,3	XP_018844967.1	uncharacterized protein LOC109009078	Juglans regia	XP_017192435.1
TRINITY_DN11588_c0_g1_i3_2	0	588	XP_018847856.1	protein transport protein SEC13 homolog B	Juglans regia	XP_018839977.1
TRINITY_DN11110_c0_g3_i1_1	1,87E-75	230	XP_018817862.1	thioredoxin M3, chloroplastic-like	Juglans regia	AEC03325.1
TRINITY_DN3777_c0_g1_i2_4	6,90E-132	374	XP_018821677.1	maf-like protein DDB_G0281937 isoform X6	Juglans regia	XP_018821674.1
TRINITY_DN17601_c0_g1_i1_6	7,44E-47	160	KDP37953.1	hypothetical protein JCGZ_04596	Jatropha curcas	XP_020534892.1
TRINITY_DN11006_c0_g1_i1_2	7,02E-172	488	OAY51355.1	hypothetical protein MANES_05G208200	Manihot esculenta	OAY51356.1
TRINITY_DN16803_c0_g1_i1_5	1,42E-44	156	XP_018846323.1	clathrin heavy chain 1-like isoform X1	Juglans regia	XP_018846324.1
TRINITY_DN22655_c0_g1_i1_2	3,38E-24	95,9	OIW07050.1	hypothetical protein TanjilG_02684	Lupinus angustifolius	XP_019451454.1
TRINITY_DN9863_c2_g1_i1_1	3,11E-154	432	OAY25631.1	hypothetical protein MANES_17G110100	Manihot esculenta	XP_018845779.1
TRINITY_DN7934_c0_g2_i1_1	8,73E-144	407	XP_018857393.1	proline synthase co-transcribed bacterial homolog protein-like	Juglans regia	XP_008224704.1
TRINITY_DN11256_c0_g1_i2_2	0	744	XP_018842400.1	eukaryotic translation initiation factor 3 subunit C isoform X1	Juglans regia	XP_018842401.1
TRINITY_DN11403_c0_g1_i2_2	0	904	XP_008218557.1	transmembrane 9 superfamily member 1	Prunus mume	ONI36176.1

TRINITY_DN11547_c0_g1_i4_3	3,04E-75	224	6723463|emb|CAB66329.1	defender against apoptotic cell death	Betula pendula	20138029
TRINITY_DN8795_c0_g3_i1_4	3,42E-14	66,6	XP_008342573.1	40S ribosomal protein S28-like	Malus domestica	ONI35609.1
TRINITY_DN5882_c0_g3_i2_3	2,05E-94	275	XP_008218349.1	60S ribosomal protein L23	Prunus mume	XP_008224171.1
TRINITY_DN6879_c0_g1_i2_2	0	606	XP_018812241.1	hsp70-Hsp90 organizing protein 3-like isoform X1	Juglans regia	XP_018812241.1
TRINITY_DN8601_c0_g1_i1_2	8,55E-56	175	XP_018859049.1	uncharacterized protein LOC109020967	Juglans regia	XP_018859050.1
TRINITY_DN11298_c0_g1_i2_4	0	760	XP_018811933.1	plant UBX domain-containing protein 8	Juglans regia	XP_018828814.1
TRINITY_DN8357_c0_g1_i1_1	2,24E-115	333	XP_018810064.1	eukaryotic translation initiation factor	Juglans regia	XP_009334209.1
TRINITY_DN9107_c0_g1_i2_2	7,77E-61	191	XP_018845470.1	oleosin 1	Juglans regia	XP_020212629.1
TRINITY_DN11330_c0_g1_i3_6	9,89E-47	150	OAY42355.1	hypothetical protein MANES_09G173400	Manihot esculenta	KDP23246.1
TRINITY_DN10810_c0_g1_i4_1	1,06E-75	236	XP_018807734.1	probable proteasome inhibitor isoform X1	Juglans regia	KDP24535.1
TRINITY_DN8957_c0_g1_i1_1	2,01E-95	295	OIW17270.1	hypothetical protein TanjilG_22382	Lupinus angustifolius	XP_018835420.1
TRINITY_DN8356_c0_g2_i1_1	1,08E-166	481	XP_018807873.1	putative G3BP-like protein isoform X4	Juglans regia	XP_018807872.1
TRINITY_DN10807_c0_g1_i5_1	0	595	XP_008243362.1	ERBB-3 BINDING PROTEIN 1	Prunus mume	ONI03759.1
TRINITY_DN11558_c0_g1_i1_1	0	1365	XP_018839647.1	eukaryotic translation initiation factor 3 subunit A-like	Juglans regia	XP_018839648.1
TRINITY_DN9852_c0_g1_i1_1	0	551	XP_020227721.1	26S proteasome non-ATPase regulatory subunit 4 homolog isoform X1	Cajanus cajan	XP_018829606.1
TRINITY_DN11418_c0_g2_i2_5	0	568	CAA66064.1 thaizole biosynthetic enzmye	Alnus glutinosa		Q38709.1
TRINITY_DN5567_c0_g1_i3_3	1,44E-14	69,7	XP_018807685.1	ATPase inhibitor, mitochondrial-like	Juglans regia	XP_018813700.1
TRINITY_DN10055_c0_g1_i2_3	0	1023	XP_018860642.1	dolichyl-diphosphooligosaccharide--protein glycosyltransferase subunit 1A-like isoform X1	Juglans regia	XP_008237940.1
TRINITY_DN8773_c0_g2_i2_4	0	728	XP_018851148.1	probable sucrose-phosphatase 2 isoform X1	Juglans regia	XP_009362647.1
TRINITY_DN2809_c0_g1_i1_6	4,18E-69	208	XP_018849412.1	nudix hydrolase 16, mitochondrial isoform X3	Juglans regia	XP_018849410.1
TRINITY_DN19230_c0_g1_i1_1	0	753	XP_008229423.1	uncharacterized protein LOC103328788	Prunus mume	XP_008229422.2
TRINITY_DN11224_c0_g1_i1_1	3,16E-148	419	AHA83526.1	V-type proton ATPase subunit E	Hevea brasiliensis	XP_018831865.1
TRINITY_DN3638_c0_g1_i1_2	3,55E-76	228	XP_018845602.1	AIG2-like protein	Juglans regia	OAY53495.1
TRINITY_DN11495_c0_g1_i1_2	2,88E-76	229	XP_018820939.1	60S ribosomal protein L14-1	Juglans regia	XP_018808011.1
TRINITY_DN10903_c0_g1_i6_2	3,56E-143	428	XP_018851647.1	subtilisin-like protease SBT1.7 isoform X1	Juglans regia	XP_018851649.1
TRINITY_DN4170_c0_g3_i2_2	0	779	XP_018840459.1	primary amine oxidase	Juglans regia	XP_008224551.1
TRINITY_DN10453_c0_g2_i2_3	1,13E-112	327	KHN12396.1	60S ribosomal protein L17-2	Glycine soja	KRH42660.1
TRINITY_DN10548_c1_g1_i1_3	2,66E-165	467	XP_018820853.1	mitochondrial outer membrane protein porin 4	Juglans regia	XP_018831181.1
TRINITY_DN9786_c0_g1_i2_4	8,96E-174	487	XP_018823175.1	probable protein phosphatase 2C 39	Juglans regia	XP_018850794.1
TRINITY_DN307_c0_g2_i1_6	5,21E-90	280	ONH99280.1	hypothetical protein PRUPE_6G022600	Prunus persica	XP_018807473.1
TRINITY_DN3566_c0_g1_i1_1	0	775	XP_018852266.1	dihydrolipoyllysine-residue succinyltransferase component of 2-oxoglutarate dehydrogenase complex 2, mitochondrial-like	Juglans regia	XP_018852267.1

TRINITY_DN6229_c0_g1_i1_2	3,28E-49	164	XP_018826924.1	uncharacterized protein C6C3.02c-like	Juglans regia	XP_009343812.1
TRINITY_DN10335_c1_g2_i2_2	0	1027	XP_018806897.1	lysine--tRNA ligase isoform X1	Juglans regia	XP_018806898.1
TRINITY_DN10820_c1_g1_i1_3	0	924	XP_018857649.1	NADH dehydrogenase [ubiquinone] flavoprotein 1, mitochondrial-like	Juglans regia	XP_018836688.1
TRINITY_DN4435_c0_g1_i1_1						
TRINITY_DN9270_c0_g2_i2_2	1,17E-84	253	XP_018835344.1	40S ribosomal protein S17-like	Juglans regia	XP_018855694.1
TRINITY_DN9549_c0_g1_i1_2	1,42E-114	329	XP_016166939.1	probable signal peptidase complex subunit 2	Arachis ipaensis	XP_015931364.1
TRINITY_DN11397_c0_g1_i5_2	1,30E-105	313	ONI24561.1	hypothetical protein PRUPE_2G247200	Prunus persica	XP_007218776.1
TRINITY_DN11446_c0_g1_i1_2	0	1514	XP_018835573.1	presequence protease 1, chloroplastic/mitochondrial-like	Juglans regia	ONH91536.1
TRINITY_DN11125_c0_g9_i1_3	6,45E-131	370	XP_007160383.1	hypothetical protein PHAVU_002G317200g	Phaseolus vulgaris	XP_007160384.1
TRINITY_DN7263_c1_g1_i1_1	6,69E-135	401	XP_018850062.1	acyl-CoA-binding domain-containing protein 4	Juglans regia	ONI04458.1
TRINITY_DN11387_c0_g1_i4_2	0	1551	XP_018843471.1	26S proteasome non-ATPase regulatory subunit 2 homolog A	Juglans regia	XP_018834279.1
TRINITY_DN11199_c0_g5_i8_6	8,36E-176	494	OIV90699.1	hypothetical protein TanjilG_15085	Lupinus angustifolius	OIW07225.1
TRINITY_DN8461_c0_g1_i1_1	0	807	XP_018821861.1	cytochrome P450 90A1 isoform X1	Juglans regia	XP_018810085.1
TRINITY_DN3481_c0_g1_i1_6	1,44E-119	346	XP_018831769.1	proteasome subunit alpha type-3	Juglans regia	XP_018822732.1
TRINITY_DN5908_c0_g1_i1_3	0	568	OAY48170.1	hypothetical protein MANES_06G137400	Manihot esculenta	KDP22391.1
TRINITY_DN8185_c0_g1_i1_1	1,00E-76	232	XP_014496997.1	60S ribosomal protein L23A	Vigna radiata var. radiata	XP_020224350.1
TRINITY_DN4115_c0_g1_i1_2	8,55E-78	238	XP_018846161.1	uncharacterized protein LOC109009947	Juglans regia	ONH97840.1
TRINITY_DN5077_c0_g1_i1_1	0	694	XP_018840433.1	cysteine synthase, chloroplastic/chromoplastic isoform X1	Juglans regia	XP_018840434.1
TRINITY_DN7999_c0_g1_i1_3	1,29E-131	380	XP_008227948.1	60S ribosomal protein L7-2	Prunus mume	ONI14974.1
TRINITY_DN12921_c0_g1_i1_3	1,28E-120	346	XP_018825134.1	ubiquitin carboxyl-terminal hydrolase 3-like	Juglans regia	XP_018825135.1
TRINITY_DN2387_c0_g2_i1_1	3,64E-67	209	ONI00303.1	hypothetical protein PRUPE_6G081100	Prunus persica	XP_008223244.1
TRINITY_DN9350_c0_g2_i2_2	1,26E-76	231	XP_018813383.1	60S ribosomal protein L27	Juglans regia	XP_018827822.1
TRINITY_DN10819_c0_g1_i1_3	0	732	XP_018859227.1	uncharacterized protein At1g04910	Juglans regia	XP_008353648.1
TRINITY_DN9863_c1_g1_i2_5	4,52E-93	273	OIV96039.1	hypothetical protein TanjilG_27143	Lupinus angustifolius	KOM53238.1
TRINITY_DN9866_c0_g1_i1_1	2,88E-98	288	XP_018808968.1	40S ribosomal protein S11	Juglans regia	XP_018822277.1
TRINITY_DN11562_c0_g1_i1_2	0	728	ONI30885.1	hypothetical protein PRUPE_1G279500	Prunus persica	XP_007222547.1
TRINITY_DN11571_c4_g3_i1_2	0	863	6723478|emb|CAB66336.1	alpha-tubulin	Betula pendula	KRH62139.1
TRINITY_DN9464_c0_g1_i4_3	0	1195	XP_018858095.1	ATP-citrate synthase beta chain protein 2	Juglans regia	XP_018858096.1
TRINITY_DN8433_c1_g1_i2_6	2,07E-140	402	XP_018850051.1	REF/SRPP-like protein At3g05500	Juglans regia	XP_018850053.1
TRINITY_DN10036_c0_g1_i3_6	1,69E-31	115	ABH03379.1	60S acidic ribosomal protein	Prunus dulcis	AAL91663.1
TRINITY_DN10785_c0_g1_i1_3	1,56E-36	129	XP_018841685.1	putative invertase inhibitor	Juglans regia	XP_018853991.1
TRINITY_DN4427_c0_g2_i1_1	0	1101	XP_018836294.1	arginine--tRNA ligase, cytoplasmic-like isoform X1	Juglans regia	XP_018836295.1
TRINITY_DN8833_c0_g1_i2_2	1,87E-104	305	ONH99570.1	hypothetical protein PRUPE_6G036500	Prunus persica	KDP29516.1
TRINITY_DN10152_c0_g1_i1_1	5,45E-44	145	OAY45736.1	hypothetical protein MANES_07G087100	Manihot esculenta	OAY51398.1

TRINITY_DN11018_c0_g1_i2_1	0	834	XP_018847079.1	LL-diaminopimelate aminotransferase, chloroplastic-like	Juglans regia	XP_009378578.1
TRINITY_DN8141_c0_g2_i1_3	0	729	XP_018810396.1	pyruvate dehydrogenase E1 component subunit beta-3, chloroplastic	Juglans regia	XP_018810397.1
TRINITY_DN18656_c0_g1_i1_2	5,71E-26	96,7	XP_018839525.1	chorismate mutase 2-like	Juglans regia	XP_018833669.1
TRINITY_DN9214_c0_g1_i3_1	0	861	XP_018843339.1	ubiquitin carboxyl-terminal hydrolase 6-like isoform X1	Juglans regia	XP_018843340.1
TRINITY_DN5754_c0_g1_i1_1	5,28E-132	377	XP_018828872.1	protein P21-like	Juglans regia	KDP46638.1
TRINITY_DN4395_c0_g1_i1_4	0	671	XP_018860049.1	vesicle-fusing ATPase	Juglans regia	XP_018860050.1
TRINITY_DN10088_c0_g2_i1_3	0	613	XP_018846556.1	protein DMR6-LIKE OXYGENASE 2-like	Juglans regia	XP_007201373.1
TRINITY_DN18481_c0_g2_i1_2	3,78E-112	322	XP_008237447.1	ras-related protein Rab7	Prunus mume	ONH90275.1
TRINITY_DN3240_c0_g1_i1_2	3,28E-101	298	XP_018820087.1	tankyrase	Juglans regia	KOM34811.1
TRINITY_DN9575_c0_g1_i1_1	0	619	ONI21561.1	hypothetical protein PRUPE_2G072900	Prunus persica	XP_018852654.1
TRINITY_DN8266_c0_g1_i1_1	3,09E-55	176	XP_018819326.1	V-type proton ATPase subunit E2 isoform X1	Juglans regia	XP_018819328.1
TRINITY_DN3684_c0_g3_i1_2	9,37E-96	281	XP_018812582.1	40S ribosomal protein S8-like	Juglans regia	XP_018812589.1
TRINITY_DN27_c0_g2_i1_5	3,86E-73	226	XP_018840255.1	probable voltage-gated potassium channel subunit beta	Juglans regia	XP_018818414.1
TRINITY_DN14743_c0_g1_i1_2	1,80E-69	225	XP_020987312.1	phosphoenolpyruvate carboxylase 4 isoform X2	Arachis duranensis	XP_018809529.1
TRINITY_DN11580_c0_g1_i4_3	0	1075	XP_018842287.1	probable methyltransferase PMT3	Juglans regia	XP_018808117.1
TRINITY_DN10635_c0_g1_i3_5	1,56E-110	319	XP_018815794.1	60S ribosomal protein L21-1-like	Juglans regia	OAY43087.1
TRINITY_DN11567_c1_g2_i1_2	1,56E-118	357	XP_018833217.1	uncharacterized protein LOC109000707	Juglans regia	XP_018833218.1
TRINITY_DN11631_c0_g1_i3_2	0	934	AET62894.1	cytochrome c oxidase subunit 1 (mitochondrion)	Millettia pinnata	YP_005090434.1
TRINITY_DN1621_c0_g2_i2_2	7,15E-84	250	XP_008358599.1	60S ribosomal protein L8-3, partial	Malus domestica	ONI29567.1
TRINITY_DN8095_c0_g1_i1_1	2,87E-30	108	XP_018836020.1	uncharacterized protein LOC109002634	Juglans regia	XP_018823193.1
TRINITY_DN5554_c0_g2_i1_3	1,79E-100	294	XP_018838134.1	nitrogen regulatory protein P-II homolog	Juglans regia	XP_018824177.1
TRINITY_DN11457_c0_g1_i1_3	0	776	XP_018816946.1	eukaryotic translation initiation factor 3 subunit M-like	Juglans regia	KDP27264.1
TRINITY_DN6500_c0_g2_i1_1	2,76E-115	334	XP_018815941.1	malignant T-cell-amplified sequence 1 homolog	Juglans regia	OAY44020.1
TRINITY_DN7157_c0_g1_i1_3	0	718	XP_018825130.1	argininosuccinate synthase, chloroplastic-like	Juglans regia	XP_016198696.1
TRINITY_DN9819_c1_g2_i1_2	0	534	XP_018849554.1	prohibitin-1, mitochondrial isoform X1	Juglans regia	XP_018849555.1
TRINITY_DN11038_c0_g2_i1_2	0	607	XP_018817568.1	probable ADP-ribosylation factor GTPase-activating protein AGD9 isoform X1	Juglans regia	XP_018817570.1
TRINITY_DN9121_c0_g1_i2_6	0	690	XP_018825271.1	enoyl-[acyl-carrier-protein reductase [NADH], chloroplastic-like	Juglans regia	ONH99164.1
TRINITY_DN11575_c0_g1_i5_2	1,13E-176	499	XP_018860327.1	probable dolichyl-diphosphooligosaccharide--protein glycosyltransferase subunit 3B	Juglans regia	OAY25973.1
TRINITY_DN11451_c0_g1_i1_2	0	951	KDP43990.1	hypothetical protein JCGZ_05457	Jatropha curcas	XP_012064729.1
TRINITY_DN11459_c1_g2_i1_2	0	1198	XP_018823263.1	probable mediator of RNA polymerase II transcription subunit 37c	Juglans regia	CAW63949.1

TRINITY_DN9611_c0_g1_i1_6	5,22E-116	335	OAY39836.1	hypothetical protein MANES_10G126300	Manihot esculenta	XP_018845301.1
TRINITY_DN118_c0_g1_i1_3	3,37E-62	206	XP_018815884.1	acid beta-fructofuranosidase-like	Juglans regia	OAY48150.1
TRINITY_DN7932_c0_g1_i8_5	0	903	XP_018816499.1	importin subunit alpha-4	Juglans regia	OAY42413.1
TRINITY_DN9613_c0_g2_i2_4	1,76E-93	276	XP_018814519.1	peptidyl-prolyl cis-trans isomerase FKBP20-1	Juglans regia	KDP44932.1
TRINITY_DN7103_c0_g2_i2_2	0	565	XP_008236939.1	chaperone protein dnaJ 10	Prunus mume	ONH91068.1
TRINITY_DN11303_c0_g1_i1_1	3,60E-85	272	XP_018825127.1	plasminogen activator inhibitor 1 RNA-binding protein	Juglans regia	OAY60106.1
TRINITY_DN10641_c0_g1_i1_6	0	660	KDP42999.1	hypothetical protein JCGZ_25185	Jatropha curcas	XP_012066022.1
TRINITY_DN11410_c0_g1_i1_4	2,62E-77	236	XP_018812979.1	universal stress protein PHOS32-like	Juglans regia	XP_018858703.1
TRINITY_DN6205_c0_g1_i1_2	3,35E-43	144	XP_018850188.1	V-type proton ATPase subunit G-like	Juglans regia	XP_018850189.1
TRINITY_DN11159_c1_g1_i8_5	0	637	XP_018851345.1	UDP-glycosyltransferase 74E2-like isoform X1	Juglans regia	XP_018851346.1
TRINITY_DN9714_c0_g1_i1_1	0	855	XP_018811308.1	probable aspartyl aminopeptidase	Juglans regia	XP_018814786.1
TRINITY_DN4382_c0_g1_i1_1	0	871	XP_018839280.1	26S protease regulatory subunit 7A	Juglans regia	OAY36310.1
TRINITY_DN11626_c0_g2_i1_5	0	1123	XP_018823588.1	transmembrane 9 superfamily member 8-like	Juglans regia	XP_018810860.1
TRINITY_DN9896_c0_g1_i5_1	3,18E-36	128	XP_018836815.1	uncharacterized protein LOC109003223	Juglans regia	XP_018836816.1
TRINITY_DN10351_c0_g1_i3_1	0	838	XP_012070239.1	V-type proton ATPase subunit H	Jatropha curcas	ONI25948.1
TRINITY_DN7143_c0_g1_i1_1	0	513	XP_018844177.1	non-functional NADPH-dependent codeinone reductase 2-like isoform X1	Juglans regia	ONI30338.1
TRINITY_DN11432_c0_g1_i1_6	0	605	ONI02574.1	hypothetical protein PRUPE_6G207500	Prunus persica	XP_007205414.1
TRINITY_DN9820_c0_g1_i3_3	0	993	XP_018812045.1	L-ascorbate oxidase homolog	Juglans regia	XP_018838329.1
TRINITY_DN10026_c0_g1_i1_5	3,84E-157	442	XP_018809557.1	rho GDP-dissociation inhibitor 1-like isoform X1	Juglans regia	XP_018809558.1
TRINITY_DN11576_c1_g1_i1_2	0	639	XP_018844184.1	thiol protease aleurain-like	Juglans regia	KDP33917.1
TRINITY_DN18710_c0_g1_i1_2	9,08E-109	314	XP_018822169.1	gamma-glutamylcyclotransferase 2-3-like	Juglans regia	XP_008219660.1
TRINITY_DN11480_c0_g1_i1_2	0	534	XP_018840568.1	probable prolyl 4-hydroxylase 4	Juglans regia	OAY27838.1
TRINITY_DN16427_c0_g1_i1_4	2,48E-83	251	AGT95889.1	beta-ketoacyl-ACP reductase	Vernicia fordii	OIW14657.1
TRINITY_DN21781_c0_g1_i1_6	2,33E-27	104	XP_018805205.1	peroxidase 5-like	Juglans regia	XP_008221883.1
TRINITY_DN840_c0_g2_i1_1	0	562	XP_018834105.1	exocyst complex component EXO70B1-like	Juglans regia	XP_008341257.1
TRINITY_DN9473_c0_g1_i1_3	1,95E-44	149	XP_018858854.1	auxin-repressed 12.5 kDa protein-like	Juglans regia	XP_018811601.1
TRINITY_DN11010_c0_g1_i2_2	1,09E-72	223	XP_018848209.1	ATP synthase subunit delta', mitochondrial-like	Juglans regia	XP_018848210.1
TRINITY_DN11411_c1_g1_i1_5	0	763	XP_018832890.1	S-adenosylmethionine synthase 1	Juglans regia	XP_012084185.1
TRINITY_DN13141_c0_g1_i1_2	8,54E-51	164	OAY60149.1	hypothetical protein MANES_01G089200	Manihot esculenta	OAY60147.1
TRINITY_DN10770_c0_g1_i1_1	0	1143	XP_018815758.1	phosphoglucan, water dikinase, chloroplastic isoform X2	Juglans regia	XP_018815757.1
TRINITY_DN1431_c0_g2_i1_2	0	671	XP_018816788.1	alpha-1,4 glucan phosphorylase L isozyme, chloroplastic/amyloplastic	Juglans regia	XP_018503860.1
TRINITY_DN19678_c0_g1_i1_1	7,31E-160	462	XP_018807104.1	uncharacterized protein LOC108980590 isoform X2	Juglans regia	XP_018807103.1
TRINITY_DN249_c0_g2_i1_1	3,80E-123	358	XP_018832807.1	velvet complex subunit B	Juglans regia	KDP20282.1
TRINITY_DN17357_c0_g1_i1_3	0	555	XP_018842448.1	cysteine synthase	Juglans regia	XP_018842449.1

TRINITY_DN5130_c1_g1_i1_6	0	670	XP_018845600.1	L-arabinokinase-like	Juglans regia	XP_018845086.1
TRINITY_DN10160_c0_g1_i3_6	0	971	XP_018831786.1	NAD-dependent malic enzyme 59 kDa isoform, mitochondrial	Juglans regia	KDP28665.1
TRINITY_DN5845_c0_g1_i3_2	0	813	XP_018811146.1	serine--tRNA ligase-like	Juglans regia	XP_018841783.1
TRINITY_DN8910_c0_g1_i1_1	0	580	XP_018807956.1	serine hydroxymethyltransferase, mitochondrial-like	Juglans regia	KDP41520.1
TRINITY_DN11500_c0_g1_i1_3	0	637	XP_018828827.1	eukaryotic translation initiation factor 3 subunit I-like	Juglans regia	XP_018818014.1
TRINITY_DN11156_c0_g1_i1_3	0	920	KDP30431.1	hypothetical protein JCGZ_16670	Jatropha curcas	XP_012081034.1
TRINITY_DN10981_c0_g2_i2_6	0	638	XP_018816762.1	glutamine synthetase cytosolic isozyme	Juglans regia	ONI17549.1
TRINITY_DN10644_c0_g1_i1_2	1,79E-110	320	XP_008340621.1	GTP-binding protein SAR1A	Malus domestica	XP_008353168.1
TRINITY_DN21106_c0_g2_i1_1	9,72E-34	118	KDP25905.1	hypothetical protein JCGZ_22976	Jatropha curcas	KDP25906.1
TRINITY_DN7447_c0_g1_i1_2	2,04E-70	216	XP_018855746.1	HMG1/2-like protein	Juglans regia	XP_018809064.1
TRINITY_DN10179_c0_g2_i1_1	1,93E-23	99	XP_018860760.1	probable pectinesterase/pectinesterase inhibitor 21	Juglans regia	KDP23449.1
TRINITY_DN10614_c0_g1_i2_2	0	1023	XP_018850821.1	inositol-3-phosphate synthase	Juglans regia	XP_018850822.1
TRINITY_DN14470_c0_g1_i1_4	5,46E-74	224	KRH26302.1	hypothetical protein GLYMA_12G1662002, partial	Glycine max	KHN39675.1
TRINITY_DN5970_c0_g1_i1_3	0	537	XP_018850787.1	annexin D2-like	Juglans regia	XP_018834475.1
TRINITY_DN10916_c0_g1_i1_6	0	619	OAY49327.1	hypothetical protein MANES_05G047100	Manihot esculenta	KDP28773.1
TRINITY_DN9868_c0_g1_i1_3	0	1011	XP_018845552.1	eukaryotic translation initiation factor 3 subunit L-like	Juglans regia	KDP34205.1
TRINITY_DN11613_c0_g1_i2_2	0	592	XP_018811967.1	probable aldo-keto reductase 1	Juglans regia	XP_018826312.1
TRINITY_DN11620_c1_g1_i4_6	3,13E-146	436	ONI08225.1	hypothetical protein PRUPE_5G165800	Prunus persica	XP_020419755.1
TRINITY_DN17153_c0_g1_i1_1	4,34E-109	316	XP_018810863.1	26S proteasome non-ATPase regulatory subunit 9	Juglans regia	XP_019432553.1
TRINITY_DN8763_c0_g2_i1_1	0	1199	XP_018815548.1	long chain acyl-CoA synthetase 4-like	Juglans regia	XP_018824382.1
TRINITY_DN11270_c0_g2_i1_1	0	752	XP_018850292.1	obg-like ATPase 1	Juglans regia	ONI11169.1
TRINITY_DN3573_c0_g1_i1_2	0	788	XP_018849865.1	aminomethyltransferase, mitochondrial	Juglans regia	XP_018849809.1
TRINITY_DN7327_c0_g1_i2_5	0	726	XP_018812439.1	ferrochelatase-2, chloroplastic-like	Juglans regia	OAY54085.1
TRINITY_DN6062_c0_g2_i3_1	2,23E-61	200	XP_018851019.1	glycine-rich RNA-binding protein RZ1A-like	Juglans regia	KDP37561.1
TRINITY_DN5393_c0_g1_i1_2	4,37E-85	255	XP_018829153.1	uncharacterized protein LOC108997378	Juglans regia	AES93703.1
TRINITY_DN21568_c0_g1_i2_1	1,40E-119	351	XP_018807064.1	cyclase-associated protein 1-like	Juglans regia	XP_018847838.1
TRINITY_DN11022_c0_g1_i1_1	0	654	XP_018843078.1	calponin homology domain-containing protein DDB_G0272472	Juglans regia	XP_018843078.1
TRINITY_DN9756_c0_g1_i3_1	0	736	XP_018813395.1	ureidoglycolate hydrolase	Juglans regia	ONI26562.1
TRINITY_DN8806_c0_g1_i5_2	0	1313	XP_018818540.1	heat shock protein 90-5, chloroplastic	Juglans regia	ONI31143.1
TRINITY_DN7109_c0_g1_i1_2	1,15E-87	262	XP_018820088.1	superoxide dismutase [Cu-Zn] 2	Juglans regia	ADR70869.1
TRINITY_DN3282_c0_g2_i1_4	0	682	XP_018805409.1	dihydrolipoyl dehydrogenase 2, chloroplastic-like	Juglans regia	XP_008223314.1
TRINITY_DN6144_c0_g1_i1_3	2,40E-11	63,2	XP_018806754.1	putative invertase inhibitor	Juglans regia	XP_018815938.1
TRINITY_DN10924_c0_g1_i1_3	2,38E-101	298	XP_007137617.1	hypothetical protein PHAVU_009G141600g	Phaseolus vulgaris	ESW09611.1
TRINITY_DN8033_c0_g1_i1_2	0	601	XP_018826576.1	probable methyltransferase PMT2	Juglans regia	ONI08656.1
TRINITY_DN11083_c0_g1_i2_1	1,43E-151	444	XP_018816929.1	uncharacterized protein LOC108988207	Juglans regia	XP_018843198.1

TRINITY_DN9815_c0_g1_i1_2	1,76E-173	495	XP_018845516.1	nucleosome assembly protein 1;2-like	Juglans regia	XP_018845505.1
TRINITY_DN9347_c0_g1_i1_1	2,25E-167	474	XP_018834050.1	cytochrome b-c1 complex subunit Rieske-4, mitochondrial-like	Juglans regia	XP_018834872.1
TRINITY_DN10872_c0_g1_i8_4	0	1034	XP_018849128.1	transmembrane 9 superfamily member 3	Juglans regia	XP_018823528.1
TRINITY_DN12354_c0_g1_i1_2	2,96E-45	150	KDP30853.1	hypothetical protein JCGZ_13796	Jatropha curcas	XP_012080696.1
TRINITY_DN6584_c0_g2_i1_1	5,01E-83	251	AFK48248.1	unknown	Lotus japonicus	XP_018839113.1
TRINITY_DN10462_c0_g1_i1_2	0	587	XP_018849296.1	succinate--CoA ligase [ADP-forming] subunit alpha-1, mitochondrial	Juglans regia	XP_009355925.1
TRINITY_DN9076_c0_g2_i1_2	0	799	XP_018824705.1	acetylornithine deacetylase	Juglans regia	XP_008238268.1
TRINITY_DN527_c0_g2_i1_3	4,07E-65	199	XP_018814658.1	40S ribosomal protein S14-3-like	Juglans regia	XP_018814659.1
TRINITY_DN19375_c0_g1_i1_3	5,43E-47	159	ACJ85543.1	unknown, partial	Medicago truncatula	XP_018810128.1
TRINITY_DN9641_c0_g1_i1_2	1,65E-100	306	XP_018818310.1	uncharacterized protein LOC108989227 isoform X2	Juglans regia	OAY57847.1
TRINITY_DN22097_c0_g1_i1_5	6,89E-05	45,4	XP_018845634.1	suppressor protein SRP40-like	Juglans regia	
TRINITY_DN12939_c0_g1_i1_5	1,95E-109	325	XP_018825627.1	exopolygalacturonase-like	Juglans regia	XP_018809534.1
TRINITY_DN12440_c0_g1_i1_2	2,60E-98	295	KDP45313.1	hypothetical protein JCGZ_09562	Jatropha curcas	OAY25717.1
TRINITY_DN21623_c0_g1_i1_1	9,10E-43	144	XP_018831704.1	peroxidase 65-like isoform X2	Juglans regia	XP_018831703.1
TRINITY_DN10464_c0_g1_i1_6	0	904	XP_008226002.1	succinate-semialdehyde dehydrogenase, mitochondrial	Prunus mume	ONI11823.1
TRINITY_DN9302_c0_g1_i1_2	9,23E-57	181	XP_018856804.1	uncharacterized protein LOC109019033	Juglans regia	ONI26978.1
TRINITY_DN1467_c0_g1_i1_4	7,41E-142	420	XP_018845708.1	beta-galactosidase-like	Juglans regia	XP_018845720.1
TRINITY_DN10869_c1_g3_i1_5	1,56E-137	390	XP_018850618.1	thaumatin-like protein 1	Juglans regia	XP_018811059.1
TRINITY_DN11008_c0_g1_i3_3	0	1002	OAY33522.1	hypothetical protein MANES_13G103800	Manihot esculenta	OAY35689.1
TRINITY_DN5035_c0_g1_i1_1	1,59E-133	382	XP_018841531.1	metal-independent phosphoserine phosphatase-like	Juglans regia	XP_018841533.1
TRINITY_DN11340_c0_g1_i2_2	0	1269	XP_018825075.1	peroxisomal acyl-coenzyme A oxidase 1	Juglans regia	XP_018819219.1
TRINITY_DN10734_c1_g1_i1_1	2,41E-122	356	ONI25352.1	hypothetical protein PRUPE_2G297800	Prunus persica	XP_007218846.1
TRINITY_DN10680_c4_g1_i3_4	1,87E-64	194	ABD61727.1	polyubiquitin, partial	Lupinus albus	ABD61727.1
TRINITY_DN11420_c0_g1_i4_2	0	943	XP_018820403.1	mannosyl-oligosaccharide 1,2-alpha-mannosidase MNS1	Juglans regia	XP_012573113.1
TRINITY_DN1806_c0_g1_i1_6	3,53E-74	237	XP_018817514.1	transmembrane 9 superfamily member 7-like	Juglans regia	XP_018843226.1
TRINITY_DN9463_c0_g1_i1_2	0	653	XP_018854693.1	T-complex protein 1 subunit delta	Juglans regia	KYP61474.1
TRINITY_DN2883_c0_g1_i1_1	4,21E-38	136	XP_018820151.1	external alternative NAD(P)H-ubiquinone oxidoreductase B2, mitochondrial-like	Juglans regia	XP_009375317.1
TRINITY_DN11614_c0_g1_i5_1	0	695	XP_018841744.1	oxysterol-binding protein-related protein 3A	Juglans regia	XP_018807417.1
TRINITY_DN8774_c0_g2_i2_6	0	835	XP_018846385.1	probable nucleoredoxin 1	Juglans regia	OAY34529.1
TRINITY_DN9582_c0_g2_i1_5	0	528	OAY32440.1	hypothetical protein MANES_13G017700	Manihot esculenta	KDP26783.1
TRINITY_DN8902_c0_g1_i2_5	0	652	XP_018837262.1	protein FREE1	Juglans regia	KDP36892.1
TRINITY_DN11545_c0_g1_i3_2	0	769	XP_018821818.1	alpha-galactosidase 3 isoform X1	Juglans regia	OAY46379.1
TRINITY_DN11179_c0_g1_i4_3	0	702	XP_018842503.1	branched-chain-amino-acid aminotransferase 3, chloroplastic-like	Juglans regia	XP_009342188.1
TRINITY_DN10263_c1_g1_i2_1	1,34E-129	370	XP_018836944.1	NEDD8-conjugating enzyme Ubc12-like	Juglans regia	XP_018836945.1

TRINITY_DN9576_c0_g1_i2_5	8,85E-61	187	XP_018836049.1	mitochondrial import inner membrane translocase subunit Tim9	Juglans regia	XP_008357211.1
TRINITY_DN7960_c0_g1_i1_2	0	1595	XP_018836595.1	chaperone protein ClpC, chloroplastic	Juglans regia	XP_018832706.1
TRINITY_DN5577_c0_g1_i1_5	6,41E-60	202	XP_018844109.1	polyadenylate-binding protein 7 isoform X2	Juglans regia	XP_018844108.1
TRINITY_DN4472_c0_g1_i1_5	1,06E-71	219	KOM56056.1	hypothetical protein LR48_Vigan10g194800	Vigna angularis	XP_014506213.1
TRINITY_DN10370_c0_g1_i1_3	0	738	OAY28247.1	hypothetical protein MANES_15G052800	Manihot esculenta	XP_008219009.1
TRINITY_DN8106_c0_g1_i1_5	1,74E-170	482	XP_018818519.1	probable 6-phosphogluconolactonase 1 isoform X1	Juglans regia	XP_018818520.1
TRINITY_DN11090_c0_g2_i6_4	1,91E-20	86,7	XP_018852998.1	60S acidic ribosomal protein P3-like	Juglans regia	XP_018860534.1
TRINITY_DN10461_c0_g1_i3_3	0	920	XP_018836913.1	serine carboxypeptidase-like	Juglans regia	ONI23789.1
TRINITY_DN579_c0_g1_i1_3	1,07E-126	385	KDP33292.1	hypothetical protein JCGZ_13079	Jatropha curcas	XP_012077722.1
TRINITY_DN14915_c0_g1_i1_3	8,92E-34	117	XP_009346482.1	putative gamma-glutamylcyclotransferase At3g02910	Pyrus x bretschneideri	XP_009347907.1
TRINITY_DN11178_c0_g1_i2_1	3,51E-95	282	XP_018828615.1	gamma-interferon-inducible lysosomal thiol reductase-like	Juglans regia	XP_008234981.2
TRINITY_DN8448_c0_g1_i1_1	3,94E-114	332	XP_018815442.1	mitochondrial import receptor subunit TOM20-like	Juglans regia	XP_018860144.1
TRINITY_DN11626_c0_g1_i4_5	0	1061	XP_018839574.1	transmembrane 9 superfamily member 8-like	Juglans regia	OAY27492.1
TRINITY_DN6292_c0_g2_i1_4	1,14E-88	269	XP_018838773.1	glyceraldehyde-3-phosphate dehydrogenase GAPCP2, chloroplastic-like	Juglans regia	XP_018840356.1
TRINITY_DN5457_c0_g1_i1_3	2,43E-139	396	XP_018841293.1	nicotinamidase 1-like	Juglans regia	XP_018841294.1
TRINITY_DN6422_c0_g1_i1_2	0	655	XP_018809442.1	glucuronokinase 1-like	Juglans regia	XP_018846472.1
TRINITY_DN7995_c0_g2_i1_1	4,38E-146	410	OAY45046.1	hypothetical protein MANES_07G026200	Manihot esculenta	OAY39696.1
TRINITY_DN8330_c0_g1_i1_3	5,75E-58	183	XP_018833952.1	acyl carrier protein 1, chloroplastic-like	Juglans regia	XP_018840027.1
TRINITY_DN10167_c0_g2_i1_3	3,95E-78	237	XP_018836192.1	60S ribosomal protein L24	Juglans regia	XP_007145271.1
TRINITY_DN9776_c0_g1_i4_2	1,17E-169	480	XP_018814237.1	V-type proton ATPase subunit D-like	Juglans regia	XP_018814238.1
TRINITY_DN11503_c0_g1_i1_1	4,28E-141	404	XP_018835591.1	2-hydroxyisoflavanone dehydratase-like	Juglans regia	XP_018835592.1
TRINITY_DN11383_c0_g1_i3_1	1,19E-111	331	XP_018847358.1	probable polygalacturonase	Juglans regia	XP_018809647.1
TRINITY_DN10659_c0_g1_i3_2	1,31E-144	410	XP_018810806.1	adenylyl-sulfate kinase 3	Juglans regia	XP_018810807.1
TRINITY_DN10371_c0_g1_i1_2	1,32E-95	281	XP_018825729.1	universal stress protein PHOS32-like isoform X2	Juglans regia	KDP27925.1
TRINITY_DN6420_c0_g1_i1_4	2,16E-77	232	XP_018812440.1	uncharacterized protein LOC108984828	Juglans regia	XP_018812441.1
TRINITY_DN10875_c0_g1_i2_2	0	779	XP_018815323.1	26S protease regulatory subunit S10B homolog B-like	Juglans regia	KEH36195.1
TRINITY_DN9826_c0_g1_i1_3	0	546	KHN05911.1	26S proteasome regulatory subunit 4 like A	Glycine soja	KHN48977.1
TRINITY_DN8975_c0_g1_i1_3	0	723	XP_018837796.1	argininosuccinate lyase, chloroplastic	Juglans regia	XP_009350549.1
TRINITY_DN8607_c0_g1_i2_1	6,74E-125	357	XP_018837970.1	germin-like protein subfamily 2 member 1	Juglans regia	XP_018806639.1
TRINITY_DN11635_c2_g1_i9_3	1,55E-74	228	XP_016194603.1	probable aldo-keto reductase 2	Arachis ipaensis	XP_018858776.1
TRINITY_DN17821_c0_g1_i1_3	7,16E-105	307	XP_018806260.1	uncharacterized protein LOC108979922 isoform X1	Juglans regia	XP_018806284.1
TRINITY_DN3258_c0_g1_i1_2	1,10E-130	390	XP_018858135.1	LOW QUALITY PROTEIN: phospholipase A-2-activating protein	Juglans regia	XP_018858135.1
TRINITY_DN10762_c0_g1_i1_1	3,62E-41	142	KEH29195.1	Lipid transfer protein, partial	Medicago truncatula	XP_013455191.1

TRINITY_DN10328_c0_g2_i1_2	0	854	XP_008236927.1	diaminopimelate decarboxylase 1, chloroplastic-like	Prunus mume	ONH91092.1
TRINITY_DN11289_c0_g2_i2_2	0	691	KDP30971.1	hypothetical protein JCGZ_11347	Jatropha curcas	XP_012079902.1
TRINITY_DN4969_c0_g1_i1_3	0	584	XP_018815797.1	mitochondrial import receptor subunit TOM40-1-like	Juglans regia	XP_018824435.1
TRINITY_DN12289_c0_g1_i1_5	5,59E-117	352	XP_018846638.1	uncharacterized protein LOC109010309	Juglans regia	OIV93417.1
TRINITY_DN11225_c0_g1_i1_3	4,58E-173	491	XP_018820980.1	isoflavone reductase-like protein	Juglans regia	XP_018820986.1
TRINITY_DN20631_c0_g1_i1_5	1,39E-35	125	XP_018856033.1	fatty-acid-binding protein 1	Juglans regia	OAY49712.1
TRINITY_DN15095_c0_g1_i1_4	1,76E-108	321	XP_018812652.1	heterogeneous nuclear ribonucleoprotein 1-like	Juglans regia	XP_018812652.1
TRINITY_DN10798_c0_g1_i1_3	0	1155	XP_008240342.1	dolichyl-diphosphooligosaccharide--protein glycosyltransferase subunit STT3B	Prunus mume	ONI09533.1
TRINITY_DN17510_c0_g1_i1_2	1,35E-112	331	XP_018851182.1	putative deoxyribonuclease TATDN1	Juglans regia	OAY43006.1
TRINITY_DN6021_c0_g1_i1_3	1,34E-119	343	XP_008220646.1	ER membrane protein complex subunit 4	Prunus mume	OAY53579.1
TRINITY_DN9758_c0_g1_i1_3	6,36E-87	260	XP_018846860.1	uncharacterized GPI-anchored protein At5g19250-like	Juglans regia	XP_018840499.1
TRINITY_DN2208_c0_g1_i1_1	0	563	XP_008220830.1	ornithine carbamoyltransferase, chloroplastic	Prunus mume	ONI32617.1
TRINITY_DN11722_c0_g1_i1_5	2,99E-58	186	XP_018805662.1	peroxidase 65-like	Juglans regia	XP_018831704.1
TRINITY_DN17467_c0_g1_i1_1	8,91E-96	291	XP_018845710.1	aspartate--tRNA ligase 2, cytoplasmic	Juglans regia	AES59117.1
TRINITY_DN3823_c0_g1_i1_6	0	532	XP_018834911.1	transmembrane protein 33 homolog	Juglans regia	XP_018834117.1
TRINITY_DN11535_c0_g1_i2_3	2,12E-82	243	XP_018815860.1	40S ribosomal protein S20-2	Juglans regia	XP_018815861.1
TRINITY_DN8730_c1_g1_i1_2	0	682	XP_018832781.1	thiosulfate/3-mercaptopyruvate sulfurtransferase 1, mitochondrial-like isoform X1	Juglans regia	KDP25080.1
TRINITY_DN2631_c0_g3_i1_2						
TRINITY_DN9280_c0_g2_i4_2	0	544	XP_018852431.1	eukaryotic translation initiation factor 3 subunit D-like	Juglans regia	XP_018852432.1
TRINITY_DN5611_c0_g4_i1_3	1,50E-154	436	ONI15307.1	hypothetical protein PRUPE_3G036800	Prunus persica	XP_018839178.1
TRINITY_DN4378_c0_g1_i2_2	0	1489	XP_018840722.1	alanine--tRNA ligase-like	Juglans regia	XP_018840731.1
TRINITY_DN4596_c0_g1_i1_5	2,66E-73	226	OAY23950.1	hypothetical protein MANES_18G120300	Manihot esculenta	OAY23951.1
TRINITY_DN11195_c0_g1_i1_2	0	665	XP_018810634.1	cytochrome P450 71D11-like	Juglans regia	XP_018810643.1
TRINITY_DN4293_c0_g1_i1_4	0	643	XP_018806029.1	zinc finger protein-like 1 homolog isoform X2	Juglans regia	XP_018806028.1
TRINITY_DN8155_c0_g1_i2_3	0	776	XP_018845323.1	isovaleryl-CoA dehydrogenase, mitochondrial	Juglans regia	ONI00604.1
TRINITY_DN9942_c0_g1_i1_4	0	921	XP_018830702.1	LOW QUALITY PROTEIN: tripeptidyl-peptidase 2-like	Juglans regia	XP_018828368.1
TRINITY_DN11521_c0_g1_i3_1	0	565	XP_018811219.1	eukaryotic translation initiation factor 2 subunit alpha homolog	Juglans regia	OAY54482.1
TRINITY_DN10407_c0_g1_i1_3	4,95E-175	500	XP_018823962.1	polygalacturonase-like	Juglans regia	XP_018810971.1
TRINITY_DN11080_c0_g1_i1_1	3,87E-82	244	XP_018833220.1	60S ribosomal protein L32-1-like	Juglans regia	XP_018833221.1
TRINITY_DN12642_c0_g1_i1_3	3,21E-137	392	XP_018840400.1	syntaxin-61-like	Juglans regia	XP_018840401.1
TRINITY_DN10964_c0_g1_i1_1	2,05E-72	218	XP_008222785.1	60S ribosomal protein L35-like	Prunus mume	ONI00395.1

TRINITY_DN11121_c0_g1_i1_6	1,05E-146	417	XP_018819049.1	probable caffeoyl-CoA O-methyltransferase At4g26220 isoform X1	Juglans regia	XP_018819050.1
TRINITY_DN8201_c0_g2_i1_1	0	827	XP_018858503.1	UDP-sulfoquinovose synthase, chloroplastic	Juglans regia	XP_018858504.1
TRINITY_DN2407_c0_g1_i1_1	0	545	XP_018837500.1	protein disulfide isomerase-like 1-6	Juglans regia	KDP37240.1
TRINITY_DN18360_c0_g1_i1_1	2,24E-48	158	XP_012068558.1	mitochondrial intermembrane space import and assembly protein 40 homolog	Jatropha curcas	KDP40464.1
TRINITY_DN7023_c0_g1_i2_1	1,94E-60	191	XP_018807798.1	uncharacterized protein LOC108981167	Juglans regia	XP_018807799.1
TRINITY_DN10334_c0_g1_i2_3	0	605	AXB70991.1	acetyl-CoA carboxylase carboxyltransferase beta subunit (chloroplast)	Betula pendula var. carelica	YP_009700057.1
TRINITY_DN3765_c0_g1_i1_1	1,75E-36	125	XP_018807308.1	probable glutathione S-transferase	Juglans regia	XP_012084509.1
TRINITY_DN6855_c0_g1_i1_1	1,79E-179	528	XP_018842400.1	eukaryotic translation initiation factor 3 subunit C isoform X1	Juglans regia	XP_018842401.1
TRINITY_DN9376_c1_g3_i1_4	0	878	XP_018810413.1	calcium-dependent protein kinase 26	Juglans regia	XP_018810414.1
TRINITY_DN13181_c0_g1_i1_4	3,54E-82	244	ACJ85830.1	unknown	Medicago truncatula	KRH38304.1
TRINITY_DN6915_c0_g2_i2_2	6,53E-47	150	OAY23002.1	hypothetical protein MANES_18G043700	Manihot esculenta	XP_018857441.1
TRINITY_DN8184_c1_g1_i1_1	0	825	XP_018828806.1	uncharacterized protein LOC108997121 isoform X3	Juglans regia	XP_018828805.1
TRINITY_DN19070_c0_g1_i1_2	2,23E-16	72	XP_018835558.1	cx9C motif-containing protein 4	Juglans regia	XP_019434942.1
TRINITY_DN20708_c0_g1_i1_4	4,72E-45	149	XP_012571951.1	ribonuclease P protein subunit p25-like protein isoform X2	Cicer arietinum	XP_004502885.1
TRINITY_DN11287_c0_g1_i2_3	2,13E-171	483	XP_018820882.1	glyoxylate/succinic semialdehyde reductase 1 isoform X1	Juglans regia	XP_018820891.1
TRINITY_DN9456_c0_g1_i4_3	7,63E-172	499	XP_018836156.1	protein decapping 5 isoform X1	Juglans regia	XP_018836164.1
TRINITY_DN8791_c0_g1_i1_3	6,12E-108	314	XP_018829585.1	rac-like GTP-binding protein ARAC1	Juglans regia	XP_018829587.1
TRINITY_DN4375_c0_g3_i1_3	9,49E-144	407	XP_018848977.1	ras-related protein Rab2BV-like	Juglans regia	XP_018848979.1
TRINITY_DN10343_c0_g1_i4_1	1,06E-147	428	XP_018847838.1	cyclase-associated protein 1-like	Juglans regia	XP_018807064.1
TRINITY_DN18958_c0_g1_i1_3	3,14E-103	309	XP_018842913.1	beta-glucuronosyltransferase GlcAT14A-like, partial	Juglans regia	XP_008243337.1
TRINITY_DN4412_c0_g1_i1_3	0	823	XP_018846853.1	RAN GTPase-activating protein 2	Juglans regia	XP_018846854.1
TRINITY_DN3095_c0_g1_i2_2	9,57E-90	266	XP_018860616.1	protein canopy-1	Juglans regia	XP_008237962.1
TRINITY_DN10358_c0_g1_i1_1	2,31E-174	488	XP_018828541.1	eukaryotic translation initiation factor 2 subunit beta like	Juglans regia	XP_018828542.1
TRINITY_DN16061_c0_g1_i1_3	4,32E-33	123	XP_018857281.1	villin-3-like isoform X1	Juglans regia	XP_018857282.1
TRINITY_DN5302_c0_g1_i1_6	6,72E-102	297	XP_018817591.1	aldo-keto reductase family 4 member C9-like	Juglans regia	OAY43567.1
TRINITY_DN11246_c1_g4_i2_1	1,68E-89	261	XP_018848065.1	40S ribosomal protein S13	Juglans regia	XP_018815859.1
TRINITY_DN19132_c0_g1_i1_5	2,61E-29	110	XP_018847246.1	desiccation-related protein PCC13-62-like	Juglans regia	XP_018855451.1
TRINITY_DN9764_c0_g1_i1_3	0	1223	AIU41641.1	ABC transporter family protein	Hevea brasiliensis	OAY52361.1
TRINITY_DN7237_c0_g2_i1_3	7,02E-103	301	XP_018827351.1	photosystem II reaction center PSB28 protein, chloroplastic	Juglans regia	XP_008235587.1
TRINITY_DN11279_c0_g2_i3_5	2,46E-180	507	XP_018840325.1	cysteine protease RD19A-like	Juglans regia	XP_018855046.1
TRINITY_DN10730_c0_g1_i1_1	0	1137	XP_018822704.1	alpha-L-arabinofuranosidase 1	Juglans regia	XP_018822706.1
TRINITY_DN11396_c1_g1_i3_2	4,28E-90	271	XP_018831695.1	two-component response regulator ARR5	Juglans regia	BAT76136.1

TRINITY_DN10977_c0_g1_i1_6	0	836	XP_018850655.1	DEAD-box ATP-dependent RNA helicase 56-like	Juglans regia	XP_018851705.1
TRINITY_DN9743_c0_g2_i2_1	1,23E-170	480	XP_019464492.1	26S proteasome non-ATPase regulatory subunit 8 homolog A-like	Lupinus angustifolius	XP_019464493.1
TRINITY_DN10127_c0_g1_i2_5	4,62E-73	219	XP_018857781.1	60S ribosomal protein L35a-1-like	Juglans regia	XP_018821837.1
TRINITY_DN10738_c0_g1_i2_5	0	1072	XP_018834473.1	asparagine synthetase [glutamine-hydrolyzing] 3	Juglans regia	XP_018847046.1
TRINITY_DN6947_c0_g1_i5_4	1,80E-29	112	XP_018829126.1	calnexin homolog	Juglans regia	XP_018829196.1
TRINITY_DN4336_c0_g1_i1_3	1,40E-180	505	XP_008358263.1	bifunctional aspartokinase/homoserine dehydrogenase 2, chloroplastic-like	Malus domestica	XP_018834214.1
TRINITY_DN11277_c0_g1_i2_2	0	845	XP_018849108.1	glutamate decarboxylase-like	Juglans regia	XP_018823508.1
TRINITY_DN3905_c0_g2_i2_3	1,85E-163	464	XP_018814733.1	ubiquinone biosynthesis protein COQ9, mitochondrial-like	Juglans regia	ONI21607.1
TRINITY_DN5130_c0_g2_i1_3	7,19E-26	103	XP_018845086.1	L-arabinokinase-like	Juglans regia	ACU14214.1
TRINITY_DN21282_c0_g1_i1_3	8,64E-64	210	XP_018816352.1	villin-3-like isoform X3	Juglans regia	XP_018816352.1
TRINITY_DN7375_c0_g1_i1_1	7,33E-70	214	KYP70253.1	hypothetical protein KK1_009464	Cajanus cajan	XP_020211581.1
TRINITY_DN10410_c0_g1_i1_3	1,71E-179	501	XP_018817323.1	NADH dehydrogenase [ubiquinone] flavoprotein 2, mitochondrial-like	Juglans regia	XP_018830173.1
TRINITY_DN10668_c0_g1_i2_2	6,78E-133	389	XP_018826990.1	uncharacterized protein LOC108995816	Juglans regia	OAY42107.1
TRINITY_DN1129_c0_g1_i1_2	1,52E-159	452	OAY27542.1	hypothetical protein MANES_16G133500	Manihot esculenta	XP_008379609.1
TRINITY_DN9363_c0_g1_i2_2	1,61E-169	475	XP_018811310.1	adenylate kinase 4	Juglans regia	XP_008239440.1
TRINITY_DN8678_c0_g3_i5_1	1,74E-94	281	OIV93915.1	hypothetical protein TanjilG_05618	Lupinus angustifolius	XP_019421602.1
TRINITY_DN9856_c0_g1_i2_6	0	678	XP_018828640.1	bifunctional UDP-glucose 4-epimerase and UDP-xylose 4-epimerase 1	Juglans regia	XP_018811940.1
TRINITY_DN16281_c0_g1_i1_4	1,23E-30	118	XP_018820283.1	delta-1-pyrroline-5-carboxylate synthase-like	Juglans regia	XP_018839115.1
TRINITY_DN8090_c1_g1_i1_6	2,23E-106	309	XP_018828370.1	nucleoid-associated protein At4g30620, chloroplastic-like	Juglans regia	XP_015933002.1
TRINITY_DN18348_c0_g1_i1_1	3,96E-62	208	XP_018815316.1	coatomer subunit alpha-1	Juglans regia	KHN47074.1
TRINITY_DN11641_c0_g1_i5_1	0	567	XP_018858114.1	protein disulfide-isomerase-like	Juglans regia	XP_018858114.1
TRINITY_DN13961_c0_g1_i1_1	1,89E-27	107	XP_008354813.1	4-coumarate--CoA ligase-like 5	Malus domestica	XP_008232324.1
TRINITY_DN1808_c0_g1_i1_6	3,73E-67	207	XP_008227802.1	ubiquitin-40S ribosomal protein S27a	Prunus mume	XP_008389677.1
TRINITY_DN10282_c0_g1_i6_1	6,25E-98	285	XP_018827597.1	monothiol glutaredoxin-S15, mitochondrial	Juglans regia	XP_018827598.1
TRINITY_DN11194_c0_g1_i1_2	4,22E-76	231	OAY37464.1	hypothetical protein MANES_11G103800	Manihot esculenta	XP_018851840.1
TRINITY_DN4777_c0_g2_i2_3	4,36E-116	338	emb|CAX05442.1	unnamed protein product	Glycine max	CAX05469.1
TRINITY_DN11197_c0_g1_i1_5	9,42E-78	237	XP_018808548.1	outer envelope pore protein 16-3, chloroplastic/mitochondrial	Juglans regia	XP_008367365.1
TRINITY_DN1802_c0_g1_i1_4	3,91E-83	251	XP_018819328.1	V-type proton ATPase subunit E2 isoform X2	Juglans regia	XP_018832413.1
TRINITY_DN10578_c0_g2_i1_1	4,26E-85	264	XP_018838441.1	clathrin light chain 1-like	Juglans regia	ONI17384.1
TRINITY_DN17430_c0_g1_i1_5	5,14E-79	235	KDP22858.1	hypothetical protein JCGZ_00445	Jatropha curcas	AID51396.1
TRINITY_DN9138_c0_g1_i1_4	3,67E-107	311	XP_018835316.1	peptidyl-prolyl cis-trans isomerase CYP18-2	Juglans regia	ONH96008.1
TRINITY_DN10772_c1_g1_i1_2	0	645	XP_018807984.1	serine/threonine-protein phosphatase PP2A catalytic subunit-like	Juglans regia	XP_008351677.1
TRINITY_DN11439_c0_g1_i2_4	5,20E-169	474	XP_018824239.1	proteasome subunit beta type-4-like	Juglans regia	XP_018844541.1

TRINITY_DN7730_c0_g1_i3_4	0	657	259432124|emb|CBF70796.1	unnamed protein product	Betula pendula	|259455288|emb|CBF86074.1
TRINITY_DN10562_c0_g1_i5_3	0	891	XP_018819043.1	xaa-Pro dipeptidase	Juglans regia	XP_008242428.1
TRINITY_DN8163_c0_g1_i1_1	0	761	XP_018829218.1	aminoacylase-1	Juglans regia	XP_009340792.1
TRINITY_DN10420_c0_g1_i3_2	4,88E-161	455	ONH94455.1	hypothetical protein PRUPE_7G017700	Prunus persica	XP_007204707.1
TRINITY_DN10122_c0_g1_i1_3	6,67E-59	184	XP_018821252.1	glutaredoxin-like	Juglans regia	XP_018837755.1
TRINITY_DN7204_c0_g1_i1_1	5,92E-18	84,7	XP_018852304.1	putative HVA22-like protein g	Juglans regia	AES73999.1
TRINITY_DN11311_c0_g1_i2_2	0	689	KHN12782.1	Alpha-amylase	Glycine soja	KRH05687.1
TRINITY_DN11251_c0_g1_i2_1	0	1127	XP_018821368.1	chaperone protein ClpB3, chloroplastic-like	Juglans regia	XP_018816799.1
TRINITY_DN2678_c0_g1_i1_1	8,49E-36	131	XP_018839115.1	delta-1-pyrroline-5-carboxylate synthase-like	Juglans regia	XP_018820283.1
TRINITY_DN9042_c0_g1_i1_3	6,11E-154	437	ONH95000.1	hypothetical protein PRUPE_7G045500	Prunus persica	XP_007202429.1
TRINITY_DN134_c0_g1_i3_5	0	666	XP_018836276.1	probable rhamnogalacturonate lyase B, partial	Juglans regia	KDP41508.1
TRINITY_DN7354_c1_g1_i1_2	2,41E-112	324	XP_018836279.1	uncharacterized protein LOC109002826	Juglans regia	XP_018836280.1
TRINITY_DN7673_c0_g1_i1_3	3,27E-122	353	XP_018809846.1	ATPase 2, plasma membrane-type-like, partial	Juglans regia	XP_018844325.1
TRINITY_DN5029_c0_g1_i1_6	0	1365	XP_018836327.1	uncharacterized protein LOC109002866	Juglans regia	OAY56146.1
TRINITY_DN4009_c0_g1_i2_3	2,70E-78	239	OAY56694.1	hypothetical protein MANES_02G037700	Manihot esculenta	XP_008230854.1
TRINITY_DN4424_c0_g1_i3_3	0	738	XP_018850112.1	uncharacterized protein LOC109012766	Juglans regia	XP_019432415.1
TRINITY_DN17664_c0_g2_i1_3	7,91E-07	51,6	XP_018856751.1	eukaryotic translation initiation factor 5B-like, partial	Juglans regia	XP_018856751.1
TRINITY_DN7316_c0_g1_i1_3	0	635	ONH93998.1	hypothetical protein PRUPE_8G265300	Prunus persica	XP_007200983.1
TRINITY_DN9014_c0_g1_i1_3	2,54E-137	392	XP_018813944.1	probable calcium-binding protein CML21	Juglans regia	XP_018813954.1
TRINITY_DN17081_c0_g1_i1_1	1,67E-60	187	AFK44147.1	unknown	Medicago truncatula	XP_018809529.1
TRINITY_DN7215_c0_g1_i1_5	2,67E-24	94	XP_004504298.1	protein transport protein Sec61 subunit beta-like	Cicer arietinum	XP_014490350.1
TRINITY_DN11034_c0_g1_i3_3	8,69E-76	230	XP_018851174.1	putative phosphatidylglycerol/phosphatidylinositol transfer protein DDB_G0282179	Juglans regia	XP_018836592.1
TRINITY_DN21713_c0_g1_i1_2	3,23E-20	90,1	XP_018843078.1	calponin homology domain-containing protein DDB_G0272472	Juglans regia	XP_018843079.1
TRINITY_DN7461_c0_g1_i1_6	1,35E-137	400	XP_018824670.1	uncharacterized protein LOC108994051 isoform X1	Juglans regia	XP_018824678.1
TRINITY_DN10238_c0_g2_i1_1	0	1147	XP_018852174.1	probable methyltransferase PMT21	Juglans regia	XP_018852176.1
TRINITY_DN2462_c0_g2_i1_2	1,91E-117	353	XP_018837102.1	chromatin assembly factor 1 subunit A isoform X1	Juglans regia	XP_018837102.1
TRINITY_DN16740_c0_g1_i1_5						
TRINITY_DN7713_c0_g1_i1_6	1,47E-46	152	XP_018860422.1	protein BOLA2	Juglans regia	ACU15761.1
TRINITY_DN7723_c0_g1_i2_2	2,99E-162	461	XP_008232832.1	probable fructokinase-7	Prunus mume	XP_009375843.1
TRINITY_DN10456_c0_g1_i4_1	8,44E-88	263	XP_018830835.1	protein phosphatase inhibitor 2 isoform X2	Juglans regia	XP_018830836.1
TRINITY_DN10811_c0_g1_i1_2	6,13E-111	323	XP_019460659.1	LIM domain-containing protein PLIM2c-like	Lupinus angustifolius	ONI23012.1
TRINITY_DN10241_c0_g2_i1_1	0	606	XP_018836309.1	aspartic proteinase-like	Juglans regia	XP_018836310.1

TRINITY_DN10843_c0_g2_i2_2	6,04E-117	348	AXB70991.1	acetyl-CoA carboxylase carboxyltransferase beta subunit (chloroplast)	Betula pendula var. carelica	YP_009700057.1
TRINITY_DN888_c0_g1_i1_1	1,11E-108	330	XP_018808540.1	TBCC domain-containing protein 1-like	Juglans regia	XP_018843632.1
TRINITY_DN9357_c0_g1_i1_4	0	1189	XP_018806855.1	NF-X1-type zinc finger protein NFXL1	Juglans regia	XP_018806855.1
TRINITY_DN10551_c0_g1_i1_3	0	981	XP_018846380.1	glucose-6-phosphate 1-dehydrogenase, cytoplasmic isoform-like isoform X2	Juglans regia	XP_018846377.1
TRINITY_DN9412_c0_g1_i1_2	0	711	dbj|GAU15760.1	hypothetical protein TSUD_235760	Trifolium subterraneum	KYP78448.1
TRINITY_DN14016_c0_g2_i1_2	0	734	XP_008231125.1	sulfite oxidase	Prunus mume	ONI19931.1
TRINITY_DN5499_c0_g1_i1_3	4,32E-129	372	XP_018835130.1	uncharacterized oxidoreductase At4g09670-like	Juglans regia	KYP59370.1
TRINITY_DN10701_c0_g1_i2_1	3,73E-108	317	XP_018834440.1	40S ribosomal protein S2-4-like	Juglans regia	XP_018837209.1
TRINITY_DN5059_c0_g1_i1_5	8,79E-144	414	XP_018807956.1	serine hydroxymethyltransferase, mitochondrial-like	Juglans regia	OAY35689.1
TRINITY_DN6933_c0_g1_i1_2	1,46E-164	496	XP_018812044.1	large proline-rich protein BAG6-like isoform X2	Juglans regia	XP_018812043.1
TRINITY_DN10774_c0_g1_i1_1	0	606	XP_018815793.1	eukaryotic translation initiation factor 3 subunit H	Juglans regia	ONI29040.1
TRINITY_DN10849_c0_g1_i2_2	3,17E-20	86,3	XP_018852386.1	protein SPIRAL1-like 1	Juglans regia	XP_018852387.1
TRINITY_DN10042_c0_g1_i2_2	0	671	XP_018848132.1	uncharacterized protein LOC109011398	Juglans regia	XP_008238614.1
TRINITY_DN8175_c0_g1_i1_2	4,01E-140	417	XP_018844607.1	cation/H(+) antiporter 15-like	Juglans regia	KDP39397.1
TRINITY_DN11149_c0_g1_i1_3	0	587	XP_018807006.1	ribosome maturation protein SBDS	Juglans regia	KDP43068.1
TRINITY_DN14909_c0_g1_i1_3	0	811	XP_018834925.1	uncharacterized protein LOC109001888	Juglans regia	XP_018834926.1
TRINITY_DN6906_c0_g1_i2_2	0	555	XP_018819297.1	2-dehydro-3-deoxyphosphooctonate aldolase	Juglans regia	XP_019459201.1
TRINITY_DN4515_c0_g2_i1_2	1,08E-27	100	XP_018807039.1	nuclear transport factor 2 isoform X4	Juglans regia	XP_018807041.1
TRINITY_DN11230_c0_g2_i7_6	3,58E-54	176	XP_012088486.1	SKP1-like protein 1B	Jatropha curcas	KDP23982.1
TRINITY_DN19409_c0_g1_i1_3	9,01E-88	261	XP_018821879.1	tropinone reductase homolog At5g06060-like	Juglans regia	OAY38586.1
TRINITY_DN10897_c0_g1_i1_1	4,08E-150	432	XP_018816861.1	ubiquitin receptor RAD23c-like	Juglans regia	XP_018815486.1
TRINITY_DN7208_c0_g1_i1_2	3,26E-176	495	XP_018825934.1	bifunctional monothiol glutaredoxin-S16, chloroplastic	Juglans regia	XP_008243461.1
TRINITY_DN7184_c0_g1_i2_3	2,58E-33	115	XP_018815086.1	cytochrome b-c1 complex subunit 8	Juglans regia	OAY24007.1
TRINITY_DN6124_c0_g1_i2_1	0	812	XP_018812297.1	sorting and assembly machinery component 50 homolog B-like	Juglans regia	XP_018821909.1
TRINITY_DN17685_c0_g1_i1_2	1,94E-76	231	XP_018827015.1	40S ribosomal protein S3-2-like	Juglans regia	XP_018844535.1
TRINITY_DN10323_c0_g3_i1_1	0	1060	XP_018842853.1	soluble starch synthase 1, chloroplastic/amyloplastic	Juglans regia	KDP42052.1
TRINITY_DN9450_c0_g1_i1_2	0	555	XP_018860411.1	coatomer subunit epsilon-1	Juglans regia	XP_018828599.1
TRINITY_DN11814_c0_g1_i1_6	0	798	XP_018811056.1	tryptophan synthase beta chain 1-like	Juglans regia	XP_018824063.1
TRINITY_DN22082_c0_g1_i1_3	3,71E-35	129	XP_016188001.1	serine carboxypeptidase-like 40	Arachis ipaensis	XP_015952981.1
TRINITY_DN7979_c0_g1_i2_2	0	563	XP_018829441.1	putative glucose-6-phosphate 1-epimerase	Juglans regia	OAY57420.1
TRINITY_DN1283_c0_g1_i1_3	8,09E-54	171	XP_007148307.1	hypothetical protein PHAVU_006G197500g	Phaseolus vulgaris	ESW20301.1
TRINITY_DN9248_c0_g1_i2_3	7,83E-138	399	KDP30075.1	hypothetical protein JCGZ_18623	Jatropha curcas	XP_012081293.1

TRINITY_DN10710_c2_g1_i2_2	1,41E-153	441	XP_018846484.1	28 kDa ribonucleoprotein, chloroplastic-like	Juglans regia	XP_018845781.1
TRINITY_DN5831_c1_g1_i1_5	1,05E-139	410	XP_018840476.1	long chain acyl-CoA synthetase 6, peroxisomal-like	Juglans regia	XP_018846842.1
TRINITY_DN8425_c0_g1_i1_1	1,23E-46	152	XP_018852827.1	V-type proton ATPase subunit G-like	Juglans regia	XP_018852828.1
TRINITY_DN1080_c0_g1_i1_2	1,61E-22	89,4	XP_019444359.1	40S ribosomal protein S23-2-like	Lupinus angustifolius	XP_004493946.1
TRINITY_DN10249_c0_g1_i1_1	4,25E-107	315	XP_018859182.1	cinnamoyl-CoA reductase 2-like isoform X1	Juglans regia	KDP36740.1
TRINITY_DN2941_c0_g1_i1_2	4,82E-27	106	XP_020210577.1	dihydroxy-acid dehydratase, chloroplastic	Cajanus cajan	XP_008380606.1
TRINITY_DN17262_c0_g1_i1_3	0	679	XP_015954179.1	mitochondrial-processing peptidase subunit alpha	Arachis duranensis	XP_004503508.1
TRINITY_DN6331_c0_g1_i2_6	0	559	XP_018834193.1	dynamin-2A-like	Juglans regia	XP_018831147.1
TRINITY_DN9436_c0_g1_i2_2	1,21E-41	137	XP_012066031.1	cytochrome b-c1 complex subunit 6	Jatropha curcas	XP_008218833.1
TRINITY_DN11363_c0_g2_i3_3	0	1020	XP_018826910.1	methylmalonate-semialdehyde dehydrogenase acylating, [mitochondrial]	Juglans regia	XP_012074593.1
TRINITY_DN16910_c0_g1_i1_4	1,83E-30	112	XP_018858056.1	endonuclease 2 isoform X3	Juglans regia	XP_018858046.1
TRINITY_DN791_c0_g1_i1_6	1,54E-148	423	OAY62541.1	hypothetical protein MANES_01G275200, partial	Manihot esculenta	XP_018843513.1
TRINITY_DN12144_c0_g1_i1_4	0	564	XP_018826576.1	probable methyltransferase PMT2	Juglans regia	XP_008356626.1
TRINITY_DN3683_c0_g2_i2_1	0	879	XP_018829254.1	glutamate--cysteine ligase, chloroplastic	Juglans regia	XP_018829255.1
TRINITY_DN10116_c1_g2_i1_2	0	672	XP_018831026.1	glutamate dehydrogenase A	Juglans regia	XP_018808101.1
TRINITY_DN4525_c0_g1_i1_2	9,24E-66	208	XP_018825826.1	ferritin-3, chloroplastic-like	Juglans regia	OAY43763.1
TRINITY_DN5136_c0_g1_i1_3	0	587	XP_018830669.1	L-idonate 5-dehydrogenase-like	Juglans regia	OAY48229.1
TRINITY_DN7885_c0_g1_i8_1	0	786	XP_018841063.1	26S protease regulatory subunit 8 homolog A	Juglans regia	XP_018847874.1
TRINITY_DN6908_c0_g1_i1_6	3,85E-138	392	XP_018833476.1	VAMP-like protein YKT61	Juglans regia	XP_018850344.1
TRINITY_DN6313_c0_g1_i1_1	1,17E-125	358	emb|CAA98179.1	RAB11C	Lotus japonicus	|Q40193.1|RB11C_LOTJA RecName: Full=Ras-related protein Rab11C
TRINITY_DN8703_c0_g1_i1_3	0	550	XP_018812700.1	SNF1-related protein kinase regulatory subunit gamma-1-like	Juglans regia	XP_018812702.1
TRINITY_DN8590_c0_g1_i1_2	5,23E-72	222	XP_008246159.1	uncharacterized protein LOC103344327	Prunus mume	ONI04228.1
TRINITY_DN8759_c0_g1_i1_1	0	674	XP_018852657.1	dnaJ protein homolog	Juglans regia	XP_018805733.1
TRINITY_DN6356_c0_g1_i1_3	5,36E-151	440	XP_018813002.1	actin-interacting protein 1-2-like	Juglans regia	KDP41126.1
TRINITY_DN9997_c0_g1_i1_5	0	782	XP_018816788.1	alpha-1,4 glucan phosphorylase L isozyme, chloroplastic/amyloplastic	Juglans regia	KRH18405.1
TRINITY_DN10922_c0_g1_i1_2	3,53E-123	358	KDP29852.1	hypothetical protein JCGZ_18427	Jatropha curcas	XP_012081550.1
TRINITY_DN18757_c0_g1_i1_1	1,02E-34	128	XP_017184179.1	LOW QUALITY PROTEIN: coatomer subunit alpha-1-like	Malus domestica	ONI11196.1
TRINITY_DN11571_c4_g3_i5_2	0	820	KRH62139.1	hypothetical protein GLYMA_04G088500	Glycine max	XP_003522731.1
TRINITY_DN7455_c0_g1_i3_2	0	765	XP_018848499.1	threonine--tRNA ligase, mitochondrial 1-like	Juglans regia	XP_012092067.1
TRINITY_DN7736_c0_g1_i2_1	0	775	XP_018827777.1	carbamoyl-phosphate synthase small chain, chloroplastic	Juglans regia	XP_009364736.1
TRINITY_DN6430_c0_g1_i2_2	0	529	ONI05269.1	hypothetical protein PRUPE_6G365100	Prunus persica	XP_007205316.1

TRINITY_DN11913_c0_g1_i1_2	7,18E-140	396	OAY58075.1	hypothetical protein MANES_02G147600	Manihot esculenta	XP_018808841.1
TRINITY_DN11663_c2_g1_i1_3	0	669	XP_018833905.1	3-oxo-Delta(4,5)-steroid 5-beta-reductase-like	Juglans regia	XP_018836149.1
TRINITY_DN5983_c0_g1_i1_2	2,10E-69	211	XP_018823645.1	uncharacterized protein LOC108993249	Juglans regia	XP_018857024.1
TRINITY_DN8699_c1_g1_i2_2	2,17E-30	110	ONH98720.1	hypothetical protein PRUPE_7G263500	Prunus persica	AES92510.1
TRINITY_DN6592_c0_g1_i1_1	5,06E-110	322	XP_018836742.1	mitochondrial import receptor subunit TOM20-like	Juglans regia	XP_018852624.1
TRINITY_DN10388_c0_g2_i6_4	0	942	XP_008218932.1	subtilisin-like protease SBT5.4	Prunus mume	XP_008353281.1
TRINITY_DN11021_c0_g1_i1_2	0	553	XP_018856631.1	enoyl-CoA hydratase 2, peroxisomal	Juglans regia	OAY22551.1
TRINITY_DN3815_c0_g1_i1_1	0	934	XP_018825735.1	eukaryotic translation initiation factor 2 subunit gamma-like	Juglans regia	XP_018825736.1
TRINITY_DN10218_c0_g1_i2_2	0	994	XP_018839513.1	nicalin-1 isoform X1	Juglans regia	XP_018839514.1
TRINITY_DN6921_c0_g1_i1_1	0	648	XP_018848547.1	protein transport protein SEC23-like	Juglans regia	XP_018848548.1
TRINITY_DN8720_c0_g1_i1_2	1,56E-75	228	XP_018841009.1	uncharacterized protein At2g34160-like	Juglans regia	XP_018858938.1
TRINITY_DN8463_c0_g2_i1_5	0	1172	XP_018817805.1	leucine--tRNA ligase, cytoplasmic isoform X2	Juglans regia	XP_018817804.1
TRINITY_DN9325_c0_g2_i1_5	0	679	XP_018833047.1	malonate--CoA ligase-like isoform X4	Juglans regia	XP_018843242.1
TRINITY_DN10162_c0_g1_i1_5	0	514	XP_018806389.1	60S acidic ribosomal protein P0	Juglans regia	OAY46226.1
TRINITY_DN9738_c1_g1_i1_3	1,85E-29	107	XP_018856764.1	cysteine proteinase inhibitor	Juglans regia	XP_018843658.1
TRINITY_DN10335_c0_g1_i1_1						
TRINITY_DN4334_c0_g1_i1_3	2,62E-114	329	XP_018845596.1	translation machinery-associated protein 22	Juglans regia	OAY53925.1
TRINITY_DN11667_c0_g1_i5_1	0	601	XP_004488810.1	elongation factor 2	Cicer arietinum	XP_004488812.1
TRINITY_DN21463_c0_g1_i1_5	1,09E-48	168	XP_004513861.1	villin-3-like	Cicer arietinum	XP_016183542.1
TRINITY_DN9495_c0_g1_i1_2	0	1172	XP_018813183.1	phosphoenolpyruvate carboxykinase [ATP-like]	Juglans regia	XP_018813767.1
TRINITY_DN8648_c0_g1_i4_3	0	696	XP_018810395.1	peroxisomal (S)-2-hydroxy-acid oxidase GLO1	Juglans regia	XP_018819691.1
TRINITY_DN20196_c0_g1_i1_2	5,64E-46	161	XP_018809774.1	protein transport protein SEC31 homolog B-like isoform X1	Juglans regia	XP_018809776.1
TRINITY_DN369_c0_g2_i1_2	3,26E-38	132	XP_018833669.1	chorismate mutase 2	Juglans regia	XP_018848607.1
TRINITY_DN4975_c0_g2_i1_1	2,15E-102	301	OAY51228.1	hypothetical protein MANES_05G197500	Manihot esculenta	ONI25827.1
TRINITY_DN10898_c0_g1_i1_3	2,97E-46	157	XP_018834886.1	translocon-associated protein subunit beta-like isoform X1	Juglans regia	XP_018834081.1
TRINITY_DN19644_c0_g1_i1_6	1,01E-76	244	XP_018815884.1	acid beta-fructofuranosidase-like	Juglans regia	AFU56882.1
TRINITY_DN10905_c1_g1_i1_1	1,44E-179	510	XP_018844115.1	DEAD-box ATP-dependent RNA helicase 38	Juglans regia	OAY42787.1
TRINITY_DN11102_c1_g1_i1_1	1,79E-40	139	XP_008225042.1	AB hydrolase superfamily protein YfhM-like	Prunus mume	ONI10359.1
TRINITY_DN8311_c0_g1_i1_6	6,24E-165	463	XP_018835580.1	2-hydroxyisoflavanone dehydratase-like	Juglans regia	XP_018835581.1
TRINITY_DN8932_c0_g2_i2_2	0	877	XP_018806310.1	signal recognition particle subunit SRP72-like	Juglans regia	ONI30173.1
TRINITY_DN7024_c0_g1_i1_2	0	1138	XP_020230079.1	beta-adaptin-like protein C	Cajanus cajan	KYP52643.1
TRINITY_DN5823_c0_g1_i1_2	5,04E-130	402	XP_018806543.1	isoleucine--tRNA ligase, cytoplasmic	Juglans regia	XP_009337446.1
TRINITY_DN7700_c0_g1_i1_1	2,08E-35	127	XP_018853171.1	probable protein phosphatase 2C 11	Juglans regia	XP_018857120.1
TRINITY_DN5993_c0_g2_i1_6	2,33E-47	153	KRH26085.1	hypothetical protein GLYMA_12G151400	Glycine max	KRH26086.1

TRINITY_DN9585_c0_g1_i1_2	0	699	XP_018826923.1	proline iminopeptidase	Juglans regia	XP_008236170.1
TRINITY_DN10250_c0_g1_i1_4	2,09E-63	197	XP_018841906.1	DNA-binding protein DDB_G0278111-like isoform X2	Juglans regia	XP_018841907.1
TRINITY_DN11946_c0_g1_i1_6	3,35E-122	367	XP_018859162.1	putative 3,4-dihydroxy-2-butanone kinase, partial	Juglans regia	XP_018823489.1
TRINITY_DN6080_c0_g1_i1_2	0	677	XP_018844342.1	3-isopropylmalate dehydrogenase 2, chloroplastic-like	Juglans regia	OAY36953.1
TRINITY_DN7401_c0_g1_i1_3	0	1100	XP_018815365.1	probable alkaline/neutral invertase B	Juglans regia	XP_018815366.1
TRINITY_DN2864_c0_g1_i1_4	4,69E-56	190	OAY52464.1	hypothetical protein MANES_04G085400	Manihot esculenta	XP_018815316.1
TRINITY_DN9953_c0_g1_i1_3	7,10E-109	315	XP_018841912.1	coatomer subunit zeta-1-like	Juglans regia	XP_008225364.1
TRINITY_DN4450_c0_g2_i1_2	1,21E-34	131	XP_018807796.1	polyadenylate-binding protein 3-like	Juglans regia	XP_018848084.1
TRINITY_DN18876_c0_g1_i1_5	6,58E-82	251	XP_018500437.1	glucose-1-phosphate adenylyltransferase large subunit 1, chloroplastic	Pyrus x bretschneideri	KRH51054.1
TRINITY_DN10281_c0_g1_i1_1	3,28E-98	286	KDP26654.1	hypothetical protein JCGZ_17812	Jatropha curcas	XP_012085480.1
TRINITY_DN7840_c0_g1_i1_3	5,31E-65	209	XP_018845710.1	aspartate--tRNA ligase 2, cytoplasmic	Juglans regia	KRH15638.1
TRINITY_DN8316_c0_g1_i1_3	5,42E-134	382	XP_018813683.1	60S ribosomal protein L10a-like	Juglans regia	CAX02806.1
TRINITY_DN15493_c0_g1_i1_5	9,82E-47	163	dbj|BAT88327.1	hypothetical protein VIGAN_05178900	Vigna angularis var. angularis	XP_007146784.1
TRINITY_DN8497_c0_g1_i1_2	0	790	XP_018809942.1	IAA-amino acid hydrolase ILR1-like 4	Juglans regia	XP_008242448.1
TRINITY_DN9872_c0_g1_i2_5	4,10E-84	249	XP_008237928.1	V-type proton ATPase subunit F	Prunus mume	XP_018830250.1
TRINITY_DN5492_c0_g2_i1_3	0	1443	XP_018851882.1	ubiquitin carboxyl-terminal hydrolase 14 isoform X1	Juglans regia	OAY54976.1
TRINITY_DN10815_c0_g2_i1_3	4,90E-114	328	OAY53361.1	hypothetical protein MANES_04G157000	Manihot esculenta	OIW08118.1
TRINITY_DN9629_c0_g1_i1_1	0	525	XP_018824738.1	phytanoyl-CoA dioxygenase	Juglans regia	XP_008243375.1
TRINITY_DN9337_c0_g1_i3_1	0	592	XP_018848102.1	dehydrogenase/reductase SDR family member 12 isoform X2	Juglans regia	XP_018848101.1
TRINITY_DN2350_c0_g1_i1_3						
TRINITY_DN4704_c0_g1_i1_2	6,83E-117	343	XP_008240156.1	tubulin beta chain-like	Prunus mume	ONI09332.1
TRINITY_DN10796_c0_g1_i2_5	1,02E-175	494	XP_018821859.1	alpha-soluble NSF attachment protein 2-like	Juglans regia	KYP44939.1
TRINITY_DN9670_c0_g1_i1_4	5,16E-76	232	XP_018810316.1	thioredoxin F-type, chloroplastic-like	Juglans regia	XP_009340388.1
TRINITY_DN2761_c0_g2_i1_2	5,65E-132	385	KHN12100.1	Aldehyde dehydrogenase family 2 member B7, mitochondrial	Glycine soja	KRH74615.1
TRINITY_DN7182_c0_g1_i2_2	5,55E-71	232	XP_018851852.1	5-methyltetrahydropteroyltriglutamate--homocysteine methyltransferase-like	Juglans regia	BAT89114.1
TRINITY_DN11525_c0_g1_i1_2	0	567	XP_018830936.1	ADP,ATP carrier protein, mitochondrial	Juglans regia	XP_018830937.1
TRINITY_DN4779_c0_g2_i1_1	5,79E-47	155	OAY41761.1	hypothetical protein MANES_09G127600	Manihot esculenta	ONI25929.1
TRINITY_DN11487_c0_g2_i1_1	2,49E-157	447	XP_008221201.1	24-methylenesterol C-methyltransferase 2	Prunus mume	ONI32296.1
TRINITY_DN11353_c0_g1_i2_3	1,32E-141	406	XP_018838151.1	26S proteasome non-ATPase regulatory subunit 14 homolog	Juglans regia	XP_018857189.1
TRINITY_DN4833_c0_g1_i1_1	0	797	XP_018816807.1	glutamate decarboxylase 1	Juglans regia	BAF80895.1
TRINITY_DN7188_c0_g2_i3_2	3,14E-71	221	XP_018847055.1	protein FATTY ACID EXPORT 1, chloroplastic-like	Juglans regia	XP_018844008.1
TRINITY_DN10558_c0_g1_i2_4	0	526	XP_018846483.1	proteasome subunit beta type-7-B	Juglans regia	KDP46386.1

TRINITY_DN10992_c0_g1_i1_2	0	1290	XP_018806637.1	glutamate--tRNA ligase, cytoplasmic	Juglans regia	XP_018806638.1
TRINITY_DN23494_c0_g1_i1_6	1,83E-79	246	XP_018839811.1	glucan endo-1,3-beta-glucosidase 8	Juglans regia	AFK43211.1
TRINITY_DN6209_c0_g1_i3_2	4,85E-172	482	OAY23218.1	hypothetical protein MANES_18G060900	Manihot esculenta	XP_018812968.1
TRINITY_DN1928_c0_g1_i2_3	6,08E-81	244	XP_018850753.1	protein BOBBER 1	Juglans regia	XP_018809881.1
TRINITY_DN22312_c0_g1_i1_4	2,41E-54	176	XP_018857864.1	7-methylguanosine phosphate-specific 5'-nucleotidase A-like	Juglans regia	XP_018857865.1
TRINITY_DN137_c0_g1_i1_1	2,60E-24	99	XP_018817293.1	protein transport protein SEC31 homolog B-like isoform X1	Juglans regia	XP_018817298.1
TRINITY_DN7231_c0_g1_i1_1	1,41E-82	248	XP_018820994.1	phosphopantetheine adenylyltransferase	Juglans regia	XP_009371619.1
TRINITY_DN8793_c0_g1_i1_4	4,08E-110	318	OIW17591.1	hypothetical protein TanjilG_00219	Lupinus angustifolius	XP_019429396.1
TRINITY_DN16084_c0_g1_i1_4	1,85E-44	156	XP_018849887.1	heat shock protein 90-6, mitochondrial isoform X2	Juglans regia	XP_018849886.1
TRINITY_DN10831_c0_g1_i1_3	5,00E-46	152	XP_018848549.1	cysteine proteinase inhibitor B-like	Juglans regia	XP_018848556.1
TRINITY_DN15011_c0_g1_i1_2	0	567	XP_018817805.1	leucine--tRNA ligase, cytoplasmic isoform X2	Juglans regia	XP_018817804.1
TRINITY_DN11284_c0_g2_i1_3	8,74E-158	442	XP_018849141.1	phosphomannomutase-like	Juglans regia	XP_019426420.1
TRINITY_DN18948_c1_g1_i1_2	6,64E-34	117	AES64100.2	multidrug resistance-associated protein, putative	Medicago truncatula	XP_003593849.2
TRINITY_DN11157_c0_g2_i3_3	0	912	XP_008386115.1	phospho-2-dehydro-3-deoxyheptonate aldolase 1, chloroplastic-like	Malus domestica	ALE18236.1
TRINITY_DN2503_c0_g2_i1_1	6,72E-100	307	XP_018818477.1	putative G3BP-like protein	Juglans regia	XP_008243840.1
TRINITY_DN22430_c0_g1_i1_3	6,96E-45	148	ONI20307.1	hypothetical protein PRUPE_2G008200	Prunus persica	ONI20304.1
TRINITY_DN9526_c1_g1_i1_2	1,22E-103	301	XP_018847314.1	probable NADH dehydrogenase [ubiquinone] 1 alpha subcomplex subunit 5, mitochondrial	Juglans regia	XP_018847315.1
TRINITY_DN11455_c0_g1_i6_2	0	678	XP_008230735.1	V-type proton ATPase subunit d2	Prunus mume	ONI19309.1
TRINITY_DN10158_c0_g1_i1_1	1,58E-65	202	OAY57255.1	hypothetical protein MANES_02G082400	Manihot esculenta	XP_012074113.1
TRINITY_DN3458_c0_g1_i1_4	1,97E-45	152	XP_018818800.1	probable 6-phosphogluconolactonase 4, chloroplastic	Juglans regia	OIV98040.1
TRINITY_DN11286_c0_g1_i3_2	0	745	KDP33810.1	hypothetical protein JCGZ_07381	Jatropha curcas	XP_012076890.1
TRINITY_DN5958_c0_g2_i1_1						
TRINITY_DN9297_c0_g3_i2_1	7,15E-138	408	XP_018849569.1	multiple organellar RNA editing factor 8, chloroplastic/mitochondrial	Juglans regia	XP_008226518.1
TRINITY_DN5161_c0_g2_i1_2	0	703	XP_018821368.1	chaperone protein ClpB3, chloroplastic-like	Juglans regia	XP_008230845.1
TRINITY_DN11263_c0_g1_i1_3	0	820	XP_018834951.1	eukaryotic peptide chain release factor subunit 1-3-like	Juglans regia	XP_018834952.1
TRINITY_DN2170_c0_g1_i1_5	1,25E-67	224	KHN04772.1	Clathrin heavy chain 1	Glycine soja	XP_015931279.1
TRINITY_DN8027_c0_g1_i1_1	0	613	XP_018811770.1	target of Myb protein 1-like	Juglans regia	XP_018845550.1
TRINITY_DN10305_c0_g1_i5_3	3,54E-133	391	XP_018814379.1	protein FATTY ACID EXPORT 3, chloroplastic	Juglans regia	XP_008244826.1
TRINITY_DN7619_c0_g2_i1_3	0	554	XP_018835354.1	uricase-2 isozyme 2	Juglans regia	CAB77205.1
TRINITY_DN10995_c0_g2_i4_3	4,71E-162	462	XP_018837241.1	CAAX prenyl protease 1 homolog	Juglans regia	OAY49116.1
TRINITY_DN10905_c0_g1_i1_4	4,35E-111	333	XP_018844115.1	DEAD-box ATP-dependent RNA helicase 38	Juglans regia	KDP25247.1
TRINITY_DN10393_c0_g1_i1_2	2,73E-126	365	XP_018808145.1	uncharacterized protein LOC108981433 isoform X2	Juglans regia	XP_018808144.1

TRINITY_DN13635_c0_g1_i3_1	0	600	XP_018846328.1	asparagine--tRNA ligase, cytoplasmic 1 isoform X1	Juglans regia	XP_016189315.1
TRINITY_DN9622_c1_g3_i1_2	1,01E-08	52,4	KDP45878.1	hypothetical protein JCGZ_15322	Jatropha curcas	OAY34547.1
TRINITY_DN10585_c0_g2_i1_2	1,03E-27	102	XP_018821552.1	uncharacterized protein LOC108991675 isoform X1	Juglans regia	XP_018821553.1
TRINITY_DN7164_c0_g1_i2_1	0	607	XP_018842034.1	serine/threonine-protein kinase SRK2A	Juglans regia	KHN11243.1
TRINITY_DN9538_c0_g1_i1_2	0	822	OAY33567.1	hypothetical protein MANES_13G107500	Manihot esculenta	KDP32128.1
TRINITY_DN9261_c0_g1_i2_1	0	1126	XP_018814376.1	vacuolar-sorting receptor 1-like	Juglans regia	XP_018814377.1
TRINITY_DN3879_c0_g1_i1_5	4,69E-46	153	XP_008223244.1	UDP-glucuronic acid decarboxylase 5 isoform X2	Prunus mume	ONI00302.1
TRINITY_DN74_c0_g1_i1_5	4,68E-121	348	XP_018805410.1	GTP-binding protein SAR1A-like	Juglans regia	XP_018805411.1
TRINITY_DN5513_c0_g1_i1_2	0	607	XP_018815515.1	spermidine synthase 1-like	Juglans regia	OAY34570.1
TRINITY_DN7914_c0_g2_i3_5	6,96E-39	139	XP_018814398.1	protein RETICULATA-RELATED 3, chloroplastic-like	Juglans regia	XP_018819342.1
TRINITY_DN1457_c0_g1_i1_1	3,45E-66	205	XP_018836017.1	THO complex subunit 4A-like	Juglans regia	XP_018823252.1
TRINITY_DN14031_c0_g1_i1_2	2,35E-89	287	XP_018815758.1	phosphoglucan, water dikinase, chloroplastic isoform X2	Juglans regia	XP_018815757.1
TRINITY_DN11004_c0_g1_i4_1	0	610	XP_018814877.1	sugar transport protein 14-like	Juglans regia	XP_018823615.1
TRINITY_DN239_c0_g1_i1_4	0	557	XP_018841018.1	NADH-cytochrome b5 reductase-like protein	Juglans regia	XP_009368820.1
TRINITY_DN6479_c0_g2_i1_3	5,68E-51	174	XP_018815210.1	UDP-glucuronic acid decarboxylase 2-like	Juglans regia	XP_009348817.1
TRINITY_DN9536_c0_g1_i1_3	0	845	XP_018846215.1	26S proteasome non-ATPase regulatory subunit 12 homolog A	Juglans regia	OAY33256.1
TRINITY_DN8870_c0_g1_i1_2						
TRINITY_DN13262_c0_g1_i1_3	0	596	XP_018857605.1	urease	Juglans regia	XP_018857607.1
TRINITY_DN11425_c1_g1_i2_1	1,32E-116	339	XP_018813434.1	ER membrane protein complex subunit 8/9 homolog	Juglans regia	XP_018825614.1
TRINITY_DN11659_c1_g1_i1_5	0	977	XP_018831593.1	aldehyde dehydrogenase family 2 member B4, mitochondrial isoform X1	Juglans regia	XP_018831594.1
TRINITY_DN15964_c0_g1_i1_3	8,00E-46	160	XP_018812043.1	large proline-rich protein BAG6-like isoform X1	Juglans regia	XP_018812044.1
TRINITY_DN5268_c0_g1_i1_2	0	595	XP_018844048.1	xylulose 5-phosphate/phosphate translocator, chloroplastic-like	Juglans regia	XP_009348960.2
TRINITY_DN20307_c0_g1_i1_4	4,13E-42	146	XP_018857981.1	phosphomethylethanolamine N-methyltransferase isoform X2	Juglans regia	XP_018857980.1
TRINITY_DN2136_c0_g2_i1_2	1,75E-171	484	XP_018856419.1	uncharacterized protein LOC109018719	Juglans regia	XP_018856421.1
TRINITY_DN10948_c0_g1_i6_4	0	798	KYP46302.1	putative 26S proteasome non-ATPase regulatory subunit 3	Cajanus cajan	XP_020236013.1
TRINITY_DN7010_c0_g2_i1_5	0	511	XP_018806331.1	60S ribosomal protein L3-2	Juglans regia	XP_018806332.1
TRINITY_DN18488_c0_g1_i1_2	2,64E-50	162	KDP23652.1	hypothetical protein JCGZ_23485	Jatropha curcas	XP_012089246.1
TRINITY_DN10697_c0_g1_i5_1	2,49E-135	388	XP_018827848.1	tropinone reductase-like 3	Juglans regia	KDP20683.1
TRINITY_DN8284_c0_g1_i1_2	0	596	XP_018843274.1	clavaminate synthase-like protein At3g21360	Juglans regia	XP_009353541.1

TRINITY_DN6841_c0_g1_i2_2	0	693	XP_018848755.1	indole-3-glycerol phosphate synthase, chloroplastic-like	Juglans regia	XP_012081510.1
TRINITY_DN8688_c0_g2_i1_5	1,73E-83	248	XP_018849401.1	NADH dehydrogenase [ubiquinone] 1 alpha subcomplex subunit 6	Juglans regia	OAY36255.1
TRINITY_DN10771_c0_g1_i1_5	0	560	XP_018840009.1	ER membrane protein complex subunit 2	Juglans regia	ONI04815.1
TRINITY_DN8280_c0_g2_i1_2	6,35E-154	435	XP_018827791.1	probable carboxylesterase SOBER1-like	Juglans regia	OAY25864.1
TRINITY_DN2036_c0_g2_i1_1	1,08E-122	352	XP_018850818.1	uncharacterized protein LOC109013246	Juglans regia	ONI02161.1
TRINITY_DN8545_c0_g2_i1_1	0	948	OAY36897.1	hypothetical protein MANES_11G058200	Manihot esculenta	KDP37062.1
TRINITY_DN9929_c0_g2_i1_1	2,23E-154	438	XP_018835787.1	tubulin-folding cofactor B-like isoform X1	Juglans regia	OAY40244.1
TRINITY_DN16390_c0_g1_i1_1	1,03E-49	168	dbj|GAU21041.1	hypothetical protein TSUD_132530, partial	Trifolium subterraneum	GAU21041.1
TRINITY_DN9968_c0_g1_i3_3	0	900	XP_008221828.1	probable nucleoredoxin 1 isoform X1	Prunus mume	ONI28946.1
TRINITY_DN6363_c0_g1_i2_3	1,69E-140	407	XP_009371794.1	uridine 5'-monophosphate synthase-like	Pyrus x bretschneideri	XP_004514472.1
TRINITY_DN10584_c0_g1_i2_3	3,20E-45	152	XP_018825824.1	lipid transfer-like protein VAS isoform X2	Juglans regia	XP_018825823.1
TRINITY_DN10580_c0_g1_i1_3	0	567	XP_018843664.1	4-alpha-glucanotransferase DPE2 isoform X2	Juglans regia	XP_018843661.1
TRINITY_DN320_c0_g1_i1_6	6,77E-109	339	XP_018817247.1	calcium-transporting ATPase 1, endoplasmic reticulum-type-like	Juglans regia	XP_018818320.1
TRINITY_DN4506_c0_g1_i1_6	0	601	XP_018835024.1	peroxisomal (S)-2-hydroxy-acid oxidase GLO4-like isoform X3	Juglans regia	XP_009378364.1
TRINITY_DN11518_c0_g3_i1_3	1,32E-154	450	XP_018829229.1	glycine--tRNA ligase, mitochondrial 1-like	Juglans regia	XP_018841636.1
TRINITY_DN8574_c0_g2_i2_3	1,74E-134	389	XP_018834985.1	thioredoxin reductase 1-like	Juglans regia	KDP24919.1
TRINITY_DN7917_c0_g1_i3_2	4,40E-128	368	XP_018831192.1	polyadenylate-binding protein 1 isoform X1	Juglans regia	XP_018808102.1
TRINITY_DN11331_c0_g10_i5_1	2,95E-34	121	emb|CAC39160.1	putative LEA III protein isoform 1	Corylus avellana	CAC39110.1
TRINITY_DN305_c1_g1_i1_2	4,12E-89	277	XP_018811830.1	exocyst complex component EXO70B1	Juglans regia	KDP41809.1
TRINITY_DN10976_c0_g1_i2_2	0	684	XP_018825366.1	SNF1-related protein kinase regulatory subunit gamma-1	Juglans regia	XP_008239461.1
TRINITY_DN4215_c0_g2_i1_1						
TRINITY_DN11518_c0_g1_i3_1	0	820	XP_018829229.1	glycine--tRNA ligase, mitochondrial 1-like	Juglans regia	XP_018841636.1
TRINITY_DN8989_c0_g2_i2_3	4,13E-53	179	XP_018859947.1	molybdate-anion transporter-like	Juglans regia	XP_008346660.1
TRINITY_DN18517_c0_g1_i1_6	8,00E-54	181	OIW12575.1	hypothetical protein TanjilG_04739	Lupinus angustifolius	OIW12575.1
TRINITY_DN3918_c0_g1_i1_3	2,81E-174	489	XP_018846568.1	probable N-acetyl-gamma-glutamyl-phosphate reductase, chloroplastic	Juglans regia	XP_009344175.1
TRINITY_DN8387_c0_g1_i1_3	0	635	XP_018844424.1	acid beta-fructofuranosidase-like	Juglans regia	XP_018848119.1
TRINITY_DN8291_c1_g1_i2_1	4,96E-64	200	XP_018826247.1	PRA1 family protein B4-like	Juglans regia	XP_008370199.1
TRINITY_DN4038_c0_g2_i1_1	0	926	XP_018840585.1	bifunctional 3-dehydroquinate dehydratase/shikimate dehydrogenase, chloroplastic-like isoform X1	Juglans regia	XP_018840586.1
TRINITY_DN8117_c0_g1_i1_1	5,91E-116	339	XP_018503189.1	probable ADP-ribosylation factor GTPase-activating protein AGD15	Pyrus x bretschneideri	XP_008346894.1
TRINITY_DN11962_c0_g1_i1_4	0	617	KHN04292.1	Diacylglycerol kinase iota	Glycine soja	KOM43927.1
TRINITY_DN10349_c0_g1_i2_2	0	856	XP_018839623.1	eukaryotic translation initiation factor 3 subunit E	Juglans regia	XP_018808879.1

TRINITY_DN4294_c0_g2_i1_3	2,19E-158	462	XP_018842163.1	beta-xylosidase/alpha-L-arabinofuranosidase 2-like	Juglans regia	KDP30704.1
TRINITY_DN10817_c0_g2_i5_3	0	1864	XP_018826787.1	probable sucrose-phosphate synthase 1	Juglans regia	XP_018814107.1
TRINITY_DN15272_c0_g1_i1_6	5,07E-24	99	XP_018823987.1	peroxisomal and mitochondrial division factor 2-like	Juglans regia	XP_018823988.1
TRINITY_DN2207_c0_g1_i1_5	3,07E-51	162	AHV84766.1	histone 4, partial	Codiaeum variegatum	KRG95810.1
TRINITY_DN10776_c0_g2_i2_1	5,74E-165	479	XP_018811231.1	anthocyanidin 3-O-glucosyltransferase 7-like	Juglans regia	XP_020220316.1
TRINITY_DN9409_c0_g1_i1_2	5,92E-77	233	XP_018831781.1	mitochondrial fission 1 protein A-like	Juglans regia	XP_018831782.1
TRINITY_DN4376_c0_g1_i3_3	0	966	XP_018830969.1	sulfite reductase [ferredoxin], chloroplastic-like	Juglans regia	XP_018848022.1
TRINITY_DN13346_c0_g1_i1_4	6,13E-121	346	XP_018840507.1	peroxiredoxin-2F, mitochondrial-like	Juglans regia	XP_018846871.1
TRINITY_DN19634_c0_g1_i1_3	8,21E-81	238	OAY23706.1	hypothetical protein MANES_18G100700	Manihot esculenta	OAY23707.1
TRINITY_DN8342_c0_g2_i2_2	5,88E-144	410	XP_018830330.1	peroxisomal membrane protein 11C	Juglans regia	XP_018809573.1
TRINITY_DN8274_c0_g2_i2_2	1,04E-138	399	XP_018844545.1	omega-amidase, chloroplastic	Juglans regia	XP_004501439.1
TRINITY_DN11230_c0_g2_i11_6	1,97E-89	266	XP_018847894.1	SKP1-like protein 1B	Juglans regia	OAY50541.1
TRINITY_DN6850_c0_g1_i1_2	8,96E-109	323	XP_018842766.1	uncharacterized protein LOC109007529	Juglans regia	ONI25762.1
TRINITY_DN4513_c0_g1_i1_3	9,30E-149	439	XP_018819793.1	cell division cycle protein 48 homolog	Juglans regia	XP_018819791.1
TRINITY_DN11374_c0_g1_i7_3	5,13E-139	400	XP_018827477.1	uncharacterized protein LOC108996170 isoform X2	Juglans regia	XP_018827476.1
TRINITY_DN11011_c0_g1_i3_3	9,72E-157	440	XP_018816905.1	uncharacterized protein LOC108988199 isoform X1	Juglans regia	XP_018816906.1
TRINITY_DN8107_c0_g1_i1_3	8,53E-102	324	XP_018830702.1	LOW QUALITY PROTEIN: tripeptidyl-peptidase 2-like	Juglans regia	OAY48306.1
TRINITY_DN9485_c0_g1_i2_4	8,76E-146	421	XP_018845020.1	probable WRKY transcription factor 7	Juglans regia	ALU11214.1
TRINITY_DN7556_c0_g1_i1_1	1,03E-117	340	OIV93234.1	hypothetical protein TanjilG_27413	Lupinus angustifolius	XP_019423713.1
TRINITY_DN14011_c0_g1_i1_1	1,55E-37	137	OAY39576.1	hypothetical protein MANES_10G105800	Manihot esculenta	KDP40898.1
TRINITY_DN8957_c0_g1_i1_2	2,01E-95	295	OIW17270.1	hypothetical protein TanjilG_22382	Lupinus angustifolius	XP_018835420.1
TRINITY_DN11390_c1_g1_i2_2	7,37E-25	96,7	KDP27632.1	hypothetical protein JCGZ_19637	Jatropha curcas	KDP32809.1
TRINITY_DN17748_c0_g1_i1_5	4,86E-17	78,2	XP_018808617.1	receptor-like protein kinase HAIKU2	Juglans regia	XP_018808617.1
TRINITY_DN6342_c0_g1_i2_3	0	718	XP_018838516.1	long chain base biosynthesis protein 1	Juglans regia	XP_018838517.1
TRINITY_DN11664_c0_g1_i2_3	0	1387	XP_018842610.1	neutral ceramidase-like	Juglans regia	XP_018837606.1
TRINITY_DN9943_c0_g1_i2_6	0	1291	XP_018850454.1	ATP-dependent zinc metalloprotease FTSH 10, mitochondrial-like isoform X2	Juglans regia	XP_018850452.1
TRINITY_DN13988_c0_g1_i1_6	0	592	XP_018859288.1	E3 ubiquitin-protein ligase UPL2-like	Juglans regia	XP_018848705.1
TRINITY_DN19538_c0_g1_i1_4	4,25E-117	337	XP_018829348.1	uncharacterized protein At4g14100-like isoform X1	Juglans regia	XP_018841627.1
TRINITY_DN10766_c0_g1_i8_2	0	598	XP_018816803.1	transmembrane 9 superfamily member 3-like	Juglans regia	OAY29373.1
TRINITY_DN11385_c0_g1_i2_1	3,95E-06	50,1	XP_018828115.1	uncharacterized protein LOC108996593	Juglans regia	XP_018842687.1
TRINITY_DN10112_c0_g1_i1_1	7,45E-101	295	XP_016205498.1	signal peptidase complex subunit 3B	Arachis ipaensis	XP_015968566.1
TRINITY_DN7707_c0_g1_i1_4	7,58E-156	456	XP_018851852.1	5-methyltetrahydropteroyltriglutamate--homocysteine methyltransferase-like	Juglans regia	XP_007146040.1

TRINITY_DN15157_c0_g1_i1_5	3,11E-18	81,6	XP_018846638.1	uncharacterized protein LOC109010309	Juglans regia	XP_016183136.1
TRINITY_DN1898_c0_g1_i1_4	1,77E-68	206	ACU23663.1	unknown, partial	Glycine max	BAT88127.1
TRINITY_DN12752_c0_g1_i1_1	1,95E-42	145	XP_018858382.1	protein ROOT HAIR DEFECTIVE 3-like	Juglans regia	XP_018843379.1
TRINITY_DN10808_c0_g1_i2_1	0	862	XP_018845472.1	ATP-dependent 6-phosphofructokinase 3-like isoform X1	Juglans regia	XP_018837558.1
TRINITY_DN10486_c0_g1_i1_1	0	759	XP_018834513.1	clathrin interactor EPSIN 1	Juglans regia	XP_018834519.1
TRINITY_DN1527_c0_g1_i1_2	1,93E-128	374	XP_018841198.1	UDP-glucuronic acid decarboxylase 1	Juglans regia	ONI03264.1
TRINITY_DN19189_c0_g1_i1_3	1,10E-62	203	XP_018811788.1	dihydrolipoyllysine-residue succinyltransferase component of 2-oxoglutarate dehydrogenase complex 2, mitochondrial-like	Juglans regia	XP_008226865.1
TRINITY_DN8363_c0_g1_i1_2	0	553	XP_018839142.1	NADP-dependent malic enzyme-like	Juglans regia	XP_018843270.1
TRINITY_DN11604_c0_g1_i7_2	3,45E-147	418	XP_018846260.1	putative methyltransferase DDB_G0268948	Juglans regia	XP_018807016.1
TRINITY_DN4772_c0_g1_i1_2	0	609	XP_018853594.1	dihydrolipoyllysine-residue acetyltransferase component 1 of pyruvate dehydrogenase complex, mitochondrial	Juglans regia	XP_008233240.1
TRINITY_DN8086_c0_g1_i1_2	4,79E-85	252	XP_018842451.1	lactoylglutathione lyase-like	Juglans regia	XP_008390907.1
TRINITY_DN3945_c0_g1_i1_3	3,22E-73	223	XP_018822042.1	peptidyl-prolyl cis-trans isomerase Pin1	Juglans regia	KDP46719.1
TRINITY_DN9766_c0_g2_i1_3	0	531	XP_018829443.1	gamma carbonic anhydrase 1, mitochondrial-like isoform X1	Juglans regia	XP_018829444.1
TRINITY_DN8049_c0_g1_i1_2	0	709	XP_018848330.1	dnaJ protein ERDJ2A	Juglans regia	XP_018848331.1
TRINITY_DN11285_c0_g1_i5_6	7,27E-77	231	XP_018849016.1	glutathione S-transferase U8-like	Juglans regia	XP_018849018.1
TRINITY_DN9993_c0_g1_i4_4	2,44E-49	171	XP_018839760.1	subtilisin-like protease SBT4.15	Juglans regia	GAU16058.1
TRINITY_DN18979_c0_g1_i1_4	8,79E-32	120	ONH97426.1	hypothetical protein PRUPE_7G189500	Prunus persica	ONH97424.1
TRINITY_DN9241_c0_g1_i1_2	0	764	XP_018847866.1	serine/threonine-protein phosphatase 6 regulatory subunit 3-like isoform X1	Juglans regia	XP_018828049.1
TRINITY_DN17053_c0_g1_i1_3	4,57E-37	123	ONI23348.1	hypothetical protein PRUPE_2G183600	Prunus persica	OAY49054.1
TRINITY_DN8570_c0_g1_i2_1	3,72E-157	443	XP_018841038.1	acyl-protein thioesterase 2-like isoform X1	Juglans regia	XP_020207225.1
TRINITY_DN11109_c0_g2_i3_1	0	1198	OAY32959.1	hypothetical protein MANES_13G058800	Manihot esculenta	OAY34927.1
TRINITY_DN23174_c0_g1_i1_1	3,04E-24	99,4	XP_016183778.1	delta-1-pyrroline-5-carboxylate synthase isoform X2	Arachis ipaensis	XP_015950169.1
TRINITY_DN11127_c0_g1_i1_3	0	702	XP_018848695.1	protein disulfide-isomerase 5-2-like	Juglans regia	XP_018821144.1
TRINITY_DN3682_c0_g1_i1_3	1,10E-99	294	XP_017442803.1	6,7-dimethyl-8-ribityllumazine synthase, chloroplastic isoform X1	Vigna angularis	XP_017442804.1
TRINITY_DN11049_c0_g1_i2_6	0	959	XP_018833063.1	dihydropyrimidinase-like	Juglans regia	XP_018842508.1
TRINITY_DN4610_c0_g1_i1_3	3,57E-78	236	XP_018818244.1	uncharacterized protein LOC108989175	Juglans regia	KDP45236.1
TRINITY_DN3669_c0_g2_i1_1	3,14E-47	152	AFK48077.1	unknown	Lotus japonicus	XP_018830936.1
TRINITY_DN374_c0_g2_i1_2	2,69E-98	300	XP_018847349.1	plastidial pyruvate kinase 2	Juglans regia	OAY48391.1
TRINITY_DN1993_c0_g1_i1_1	3,59E-19	84,7	ONI04862.1	hypothetical protein PRUPE_6G344400	Prunus persica	XP_020421508.1
TRINITY_DN11635_c2_g1_i3_2	0	585	XP_018824429.1	probable aldo-keto reductase 2	Juglans regia	XP_018858774.1
TRINITY_DN5082_c0_g1_i1_6	1,72E-82	246	XP_018824069.1	thioredoxin domain-containing protein PLP3B-like	Juglans regia	XP_018824070.1
TRINITY_DN8123_c0_g1_i1_1	1,26E-111	322	XP_018826216.1	uncharacterized protein At5g01610-like	Juglans regia	XP_018809179.1

TRINITY_DN10102_c0_g1_i2_4	2,29E-159	456	XP_018824108.1	hepatocyte growth factor-regulated tyrosine kinase substrate isoform X3	Juglans regia	XP_018824107.1
TRINITY_DN15522_c0_g1_i1_1	9,29E-27	105	ONI13583.1	hypothetical protein PRUPE_4G231900	Prunus persica	ONI13583.1
TRINITY_DN5853_c0_g1_i2_1	0	791	XP_018824342.1	uncharacterized protein LOC108993777	Juglans regia	XP_018824343.1
TRINITY_DN4080_c1_g1_i1_3	9,37E-172	484	XP_018838198.1	uncharacterized protein LOC109004190	Juglans regia	XP_018829345.1
TRINITY_DN2512_c0_g1_i1_1	2,55E-117	346	XP_018824803.1	fumarate hydratase 1, mitochondrial	Juglans regia	ONH93997.1
TRINITY_DN8982_c0_g1_i3_1	0	631	XP_018815404.1	ribose-phosphate pyrophosphokinase 4-like isoform X2	Juglans regia	XP_018815403.1
TRINITY_DN5937_c0_g1_i1_6	0	1190	XP_018842317.1	nodal modulator 1	Juglans regia	XP_008227880.1
TRINITY_DN10930_c0_g2_i2_1	7,26E-145	409	XP_009379738.1	peroxygenase-like	Pyrus x bretschneideri	XP_008218348.1
TRINITY_DN13030_c0_g1_i1_6	6,02E-58	197	XP_018837506.1	glucosidase 2 subunit beta	Juglans regia	XP_008221020.1
TRINITY_DN5505_c0_g1_i1_3	0	863	XP_018848045.1	acyl-CoA-binding domain-containing protein 4	Juglans regia	XP_018848046.1
TRINITY_DN11234_c0_g1_i2_1	6,77E-76	228	KDP40355.1	hypothetical protein JCGZ_02353	Jatropha curcas	XP_012069863.1
TRINITY_DN3811_c0_g2_i1_6	4,03E-98	286	XP_018821966.1	frataxin, mitochondrial-like isoform X2	Juglans regia	XP_018821965.1
TRINITY_DN12438_c0_g1_i2_2	2,36E-119	340	KYP71247.1	Signal peptidase complex subunit 3B	Cajanus cajan	XP_020212447.1
TRINITY_DN21231_c0_g1_i1_4	2,31E-32	124	XP_018843078.1	calponin homology domain-containing protein DDB_G0272472	Juglans regia	XP_018843079.1
TRINITY_DN22880_c0_g1_i1_1	2,51E-40	140	XP_018851581.1	oleoyl-acyl carrier protein thioesterase 1, chloroplastic-like	Juglans regia	XP_018830356.1
TRINITY_DN209_c0_g1_i1_6	7,68E-145	411	XP_018836108.1	uncharacterized protein LOC109002706	Juglans regia	XP_008232957.1
TRINITY_DN11070_c0_g1_i6_2	0	1030	XP_018809133.1	cleft lip and palate transmembrane protein 1 homolog	Juglans regia	XP_009379523.1
TRINITY_DN6751_c0_g1_i1_5	9,02E-06	46,2	XP_018819823.1	MFP1 attachment factor 1-like	Juglans regia	XP_018824955.1
TRINITY_DN9669_c0_g1_i1_5						
TRINITY_DN6123_c0_g1_i1_4	0	1059	XP_018829137.1	calcium-dependent protein kinase 26	Juglans regia	XP_018829138.1
TRINITY_DN9646_c0_g1_i1_2	0	1058	XP_018842577.1	ER membrane protein complex subunit 1-like	Juglans regia	XP_018845533.1
TRINITY_DN3063_c0_g2_i1_5	0	668	XP_018857224.1	cycloartenol-C-24-methyltransferase	Juglans regia	XP_008231464.1
TRINITY_DN8427_c0_g1_i1_3	1,30E-114	332	XP_008233574.1	aquaporin TIP1-3	Prunus mume	XP_008343557.1
TRINITY_DN10246_c1_g1_i1_3	5,01E-92	273	XP_018842585.1	ferredoxin, root R-B2-like	Juglans regia	XP_007142856.1
TRINITY_DN9816_c0_g1_i2_1	1,21E-143	405	XP_018844705.1	proteasome subunit beta type-3-A	Juglans regia	KDP32836.1
TRINITY_DN6842_c0_g1_i1_5	8,24E-30	111	XP_018839113.1	aldo-keto reductase family 4 member C9-like	Juglans regia	XP_018839114.1
TRINITY_DN4755_c0_g2_i1_6	1,75E-38	131	XP_018839306.1	NADH dehydrogenase [ubiquinone] 1 alpha subcomplex subunit 13-A	Juglans regia	OAY55450.1
TRINITY_DN8066_c0_g1_i1_3	0	606	XP_018836442.1	tubulin beta chain-like	Juglans regia	XP_018854022.1
TRINITY_DN7303_c0_g1_i1_3	2,16E-18	81,6	XP_007139705.1	hypothetical protein PHAVU_008G052400g	Phaseolus vulgaris	ESW11699.1
TRINITY_DN9503_c0_g1_i1_2	0	585	ONH96801.1	hypothetical protein PRUPE_7G153100	Prunus persica	XP_007204613.1
TRINITY_DN4985_c0_g1_i1_3	2,52E-118	340	XP_018844456.1	peptide methionine sulfoxide reductase B5-like	Juglans regia	XP_018855277.1
TRINITY_DN11329_c0_g1_i2_1	8,95E-180	508	KDP24432.1	hypothetical protein JCGZ_24996	Jatropha curcas	XP_012087835.1
TRINITY_DN4045_c0_g1_i1_1	6,70E-130	372	OAY39836.1	hypothetical protein MANES_10G126300	Manihot esculenta	XP_018845301.1

TRINITY_DN11005_c0_g1_i2_2	0	850	XP_018819162.1	eukaryotic translation initiation factor 4G-like isoform X1	Juglans regia	XP_018819170.1
TRINITY_DN6033_c0_g1_i1_3	0	556	XP_018816770.1	pyridoxal kinase-like isoform X1	Juglans regia	OAY45362.1
TRINITY_DN10177_c0_g1_i1_1	1,84E-61	192	XP_018824894.1	uncharacterized protein LOC108994217	Juglans regia	OAY47489.1
TRINITY_DN2451_c0_g1_i1_2	0	734	XP_018858250.1	uncharacterized protein LOC109020252	Juglans regia	XP_020421355.1
TRINITY_DN2230_c0_g2_i1_1	0	682	XP_018835559.1	ATP phosphoribosyltransferase 2, chloroplastic isoform X1	Juglans regia	OAY44990.1
TRINITY_DN6871_c0_g1_i1_3	2,54E-81	241	XP_018846174.1	uncharacterized protein LOC109009954	Juglans regia	XP_018846183.1
TRINITY_DN12604_c0_g1_i1_3	0	749	XP_018843664.1	4-alpha-glucanotransferase DPE2 isoform X2	Juglans regia	XP_018843664.1
TRINITY_DN11543_c0_g1_i1_3	0	807	XP_018809975.1	protein LEO1 homolog isoform X1	Juglans regia	XP_018809984.1
TRINITY_DN8511_c0_g2_i1_6	0	615	XP_008227769.1	formate--tetrahydrofolate ligase	Prunus mume	ONH99130.1
TRINITY_DN7492_c0_g1_i1_6	1,21E-53	169	XP_018825951.1	40S ribosomal protein S21-2-like	Juglans regia	XP_018825952.1
TRINITY_DN10983_c0_g1_i3_5	0	548	XP_018837992.1	uncharacterized protein LOC109004054	Juglans regia	XP_018837993.1
TRINITY_DN20990_c0_g1_i1_1						
TRINITY_DN7067_c0_g1_i1_3	0	543	XP_018838640.1	short-chain dehydrogenase TIC 32, chloroplastic-like	Juglans regia	ONI25356.1
TRINITY_DN10272_c0_g1_i2_4	2,47E-27	100	XP_018824900.1	mitochondrial import receptor subunit TOM5 homolog	Juglans regia	OAY47495.1
TRINITY_DN7028_c0_g1_i1_3	0	1189	XP_018852049.1	uncharacterized protein LOC109014152 isoform X2	Juglans regia	XP_018852048.1
TRINITY_DN9822_c0_g1_i2_3	1,70E-135	386	XP_018835931.1	hypersensitive-induced response protein 2-like	Juglans regia	XP_018835932.1
TRINITY_DN10973_c0_g1_i2_3	3,36E-176	509	XP_018843210.1	mitochondrial Rho GTPase 1-like	Juglans regia	XP_018841787.1
TRINITY_DN8002_c0_g1_i1_2	2,85E-154	440	XP_018843798.1	OTU domain-containing protein 6B	Juglans regia	XP_018843799.1
TRINITY_DN10262_c0_g2_i1_1	0	548	XP_008235142.1	ATP-dependent Clp protease proteolytic subunit 5, chloroplastic	Prunus mume	ONH94009.1
TRINITY_DN5679_c0_g1_i1_2	0	888	XP_018833940.1	probable manganese-transporting ATPase PDR2	Juglans regia	XP_018833946.1
TRINITY_DN728_c0_g1_i2_3	3,57E-56	194	XP_018820348.1	myosin-11 isoform X2	Juglans regia	XP_018820347.1
TRINITY_DN11853_c0_g1_i1_1	9,41E-117	340	XP_018820273.1	bifunctional dTDP-4-dehydrorhamnose 3,5-epimerase/dTDP-4-dehydrorhamnose reductase-like	Juglans regia	XP_018854480.1
TRINITY_DN11868_c0_g1_i1_5	0	635	XP_008224708.1	carbamoyl-phosphate synthase large chain, chloroplastic	Prunus mume	XP_008224708.1
TRINITY_DN10119_c0_g1_i1_1	0	834	XP_018858974.1	clathrin interactor EPSIN 2 isoform X2	Juglans regia	XP_018858973.1
TRINITY_DN2584_c0_g2_i1_3	1,59E-61	189	XP_018843076.1	calvin cycle protein CP12-3, chloroplastic	Juglans regia	OAY50363.1
TRINITY_DN9619_c0_g2_i2_2	0	808	XP_009349374.1	glycylpeptide N-tetradecanoyltransferase 1 isoform X1	Pyrus x bretschneideri	XP_008337728.1
TRINITY_DN10243_c0_g1_i1_3	0	646	XP_018846976.1	LOW QUALITY PROTEIN: apyrase 1-like	Juglans regia	XP_008240451.1
TRINITY_DN17816_c0_g1_i1_6	3,41E-75	236	XP_018829704.1	pentatricopeptide repeat-containing protein At1g05750, chloroplastic	Juglans regia	KHN17971.1
TRINITY_DN23502_c0_g1_i1_3	1,35E-21	90,1	XP_008391767.1	GDSL esterase/lipase At5g03610-like	Malus domestica	OAY42884.1
TRINITY_DN4242_c0_g1_i3_4	2,61E-54	175	XP_018836111.1	hevamine-A-like isoform X1	Juglans regia	XP_018836112.1

TRINITY_DN11128_c0_g1_i1_3	0	558	XP_018844572.1	uncharacterized protein LOC109008795	Juglans regia	XP_012090472.1
TRINITY_DN6438_c0_g3_i1_3	1,23E-143	409	KDP39714.1	hypothetical protein JCGZ_02734	Jatropha curcas	XP_012070471.1
TRINITY_DN8591_c0_g1_i2_5	0	619	XP_018818410.1	probable choline kinase 2	Juglans regia	ONI21005.1
TRINITY_DN13303_c0_g1_i1_6	8,18E-77	242	XP_018840764.1	chaperonin CPN60-2, mitochondrial-like	Juglans regia	KDP33373.1
TRINITY_DN11162_c0_g1_i3_4	2,43E-144	408	XP_008219517.1	vesicle-associated membrane protein 711	Prunus mume	ONI34446.1
TRINITY_DN17711_c0_g1_i1_5	2,37E-86	275	XP_018810540.1	probable glucan 1,3-alpha-glucosidase	Juglans regia	XP_018810541.1
TRINITY_DN7181_c0_g1_i1_6	2,66E-92	276	XP_018808381.1	40S ribosomal protein S3-2	Juglans regia	XP_018827015.1
TRINITY_DN8712_c0_g1_i1_2	0	536	XP_008364783.1	LOW QUALITY PROTEIN: diaminopimelate epimerase, chloroplastic-like	Malus domestica	XP_008375702.1
TRINITY_DN11271_c1_g2_i1_1	3,19E-103	297	AAZ32851.1	pentameric polyubiquitin, partial	Medicago sativa	AAZ32851.1
TRINITY_DN10739_c0_g1_i1_3	0	1118	KDP23486.1	hypothetical protein JCGZ_23319	Jatropha curcas	XP_012089020.1
TRINITY_DN11930_c0_g1_i1_6	2,41E-25	102	XP_018842577.1	ER membrane protein complex subunit 1-like	Juglans regia	XP_018845532.1
TRINITY_DN19914_c0_g1_i1_2	0	553	XP_018842344.1	adenylosuccinate synthetase 2, chloroplastic	Juglans regia	OAY40523.1
TRINITY_DN9037_c0_g1_i2_3	0	872	XP_018810986.1	protein disulfide-isomerase 5-3-like	Juglans regia	XP_009378812.1
TRINITY_DN9789_c0_g1_i1_2	0	827	XP_018842813.1	molybdate-anion transporter	Juglans regia	XP_018818792.1
TRINITY_DN11534_c1_g1_i2_2	0	803	XP_018850329.1	uncharacterized protein LOC109012887 isoform X3	Juglans regia	XP_018850309.1
TRINITY_DN5090_c0_g1_i1_1	1,49E-63	206	XP_018824593.1	glucan endo-1,3-beta-glucosidase 12-like	Juglans regia	XP_018812075.1
TRINITY_DN11564_c1_g1_i1_3	0	633	XP_018824341.1	UPF0160 protein	Juglans regia	XP_018859956.1
TRINITY_DN13530_c0_g1_i1_2	4,13E-58	196	dbj|GAU35356.1	hypothetical protein TSUD_337480	Trifolium subterraneum	XP_018817247.1
TRINITY_DN11513_c0_g1_i18_2	4,47E-65	204	XP_018807308.1	probable glutathione S-transferase	Juglans regia	XP_018805444.1
TRINITY_DN9031_c0_g2_i2_1	1,16E-158	454	XP_018827261.1	TOM1-like protein 2	Juglans regia	XP_008235674.1
TRINITY_DN9718_c0_g1_i1_1	1,15E-151	434	XP_018808845.1	GEM-like protein 1	Juglans regia	KOM41786.1
TRINITY_DN7063_c0_g1_i1_2	0	993	XP_018859772.1	uncharacterized protein LOC109021570	Juglans regia	ONH99202.1
TRINITY_DN22484_c0_g1_i1_1	4,68E-15	72,4	OAY62514.1	hypothetical protein MANES_01G273000	Manihot esculenta	XP_018819170.1
TRINITY_DN10475_c1_g1_i1_4	1,58E-125	360	XP_018834389.1	ras-related protein RABA4c	Juglans regia	ONI20318.1
TRINITY_DN11134_c0_g1_i2_1	0	1008	XP_018818858.1	carbamoyl-phosphate synthase large chain, chloroplastic	Juglans regia	XP_018818858.1
TRINITY_DN7509_c0_g1_i1_1	6,46E-134	389	XP_008350414.1	protein GPR107-like	Malus domestica	XP_008366712.1
TRINITY_DN15062_c0_g1_i1_1	4,45E-92	282	XP_018835576.1	asparagine synthetase [glutamine-hydrolyzing]	Juglans regia	XP_018819154.1
TRINITY_DN22190_c0_g1_i1_5	7,65E-30	109	XP_018819148.1	glutathione S-transferase U17-like	Juglans regia	XP_018819149.1
TRINITY_DN8143_c0_g1_i3_2	0	1477	XP_018815732.1	probable acyl-CoA dehydrogenase IBR3	Juglans regia	OAY56055.1
TRINITY_DN8488_c0_g1_i1_3	0	947	XP_018849269.1	phosphoacetylglucosamine mutase-like	Juglans regia	XP_018838256.1
TRINITY_DN7081_c0_g2_i1_1	9,77E-63	206	XP_018816821.1	2-hydroxyacyl-CoA lyase	Juglans regia	XP_008358155.1
TRINITY_DN5728_c0_g1_i1_3	0	822	XP_018833569.1	brefeldin A-inhibited guanine nucleotide-exchange protein 5 isoform X1	Juglans regia	XP_018833570.1
TRINITY_DN10017_c0_g1_i1_5	0	514	ONI35507.1	hypothetical protein PRUPE_1G540200	Prunus persica	XP_007222567.1
TRINITY_DN10253_c0_g1_i2_6	0	830	XP_018817131.1	uncharacterized protein LOC108988346 isoform X2	Juglans regia	XP_018817130.1
TRINITY_DN17872_c0_g1_i1_2	2,41E-69	216	KRH74616.1	hypothetical protein GLYMA_01G031500	Glycine max	XP_009363532.1

TRINITY_DN20473_c0_g1_i1_1	4,17E-49	155	XP_018809915.1	uncharacterized protein LOC108982896	Juglans regia	XP_018809916.1
TRINITY_DN5188_c0_g1_i2_1	0	867	OAY45096.1	hypothetical protein MANES_07G030800	Manihot esculenta	OAY45097.1
TRINITY_DN3281_c0_g1_i1_6	2,48E-71	216	XP_018822137.1	uncharacterized protein LOC108992124	Juglans regia	XP_007199293.2
TRINITY_DN12704_c0_g1_i1_1	5,68E-146	420	XP_008345924.1	probable alpha,alpha-trehalose-phosphate synthase [UDP-forming] 7	Malus domestica	XP_018812523.1
TRINITY_DN11185_c0_g1_i1_1	0	686	XP_018840464.1	vacuole membrane protein KMS1-like	Juglans regia	XP_018846833.1
TRINITY_DN10615_c1_g1_i1_1	4,93E-138	398	KOM42046.1	hypothetical protein LR48_Vigan04g224400, partial	Vigna angularis	KRH61685.1
TRINITY_DN63_c0_g1_i1_3	3,45E-90	269	XP_018851775.1	ribonuclease P protein subunit p25-like protein	Juglans regia	XP_014504552.1
TRINITY_DN6258_c0_g1_i2_6	4,62E-80	241	XP_018848279.1	uncharacterized protein LOC109011491	Juglans regia	XP_018860648.1
TRINITY_DN6195_c0_g2_i1_2	8,21E-67	207	XP_018809743.1	uncharacterized protein At2g34160-like	Juglans regia	KOM29975.1
TRINITY_DN6481_c0_g1_i1_1	7,05E-104	301	XP_018846675.1	peptidyl-prolyl cis-trans isomerase CYP18-1-like	Juglans regia	XP_018806960.1
TRINITY_DN14793_c0_g1_i1_1	0	688	XP_018822921.1	methionine--tRNA ligase, cytoplasmic	Juglans regia	KDP39347.1
TRINITY_DN4384_c0_g1_i1_6	5,79E-64	196	XP_018836953.1	early nodulin-like protein 1	Juglans regia	XP_018832258.1
TRINITY_DN9364_c0_g1_i1_1	0	592	XP_018821210.1	dnaJ protein ERDJ3B	Juglans regia	XP_018843883.1
TRINITY_DN9162_c0_g3_i1_4	2,20E-95	280	XP_018826083.1	peptide methionine sulfoxide reductase B5-like isoform X1	Juglans regia	XP_018826084.1
TRINITY_DN7289_c0_g1_i1_3	0	671	XP_018830095.1	glycerol-3-phosphate acyltransferase, chloroplastic isoform X2	Juglans regia	XP_018830093.1
TRINITY_DN3061_c0_g2_i1_3	0	548	XP_009379345.1	GDSL esterase/lipase EXL3-like	Pyrus x bretschneideri	XP_008242093.1
TRINITY_DN10426_c0_g1_i1_5	0	1179	XP_008342522.1	isoamylase 3, chloroplastic-like	Malus domestica	ONI35223.1
TRINITY_DN10788_c0_g1_i1_1	3,40E-172	486	OAY25879.1	hypothetical protein MANES_16G002900	Manihot esculenta	KYP62724.1
TRINITY_DN11653_c0_g1_i11_5	0	778	XP_008385127.1	actin	Malus domestica	XP_017191420.1
TRINITY_DN8256_c0_g1_i1_1	1,65E-172	485	XP_018816558.1	ALA-interacting subunit 1-like	Juglans regia	XP_018846350.1
TRINITY_DN19311_c0_g1_i1_5	4,26E-166	487	XP_018811234.1	dynamin-related protein 3A	Juglans regia	XP_018826873.1
TRINITY_DN11315_c0_g1_i1_2	9,74E-153	432	XP_018811802.1	eukaryotic translation initiation factor 3 subunit K-like	Juglans regia	KDP33976.1
TRINITY_DN9466_c0_g1_i1_2	3,85E-84	250	XP_008234339.1	uncharacterized protein LOC103333300	Prunus mume	ONI25146.1
TRINITY_DN10194_c0_g1_i1_3	0	704	XP_018811868.1	aspartic proteinase-like isoform X2	Juglans regia	XP_018811875.1
TRINITY_DN6989_c0_g1_i1_2	4,96E-176	507	XP_018848499.1	threonine--tRNA ligase, mitochondrial 1-like	Juglans regia	OAY49849.1
TRINITY_DN14960_c0_g1_i1_3	0	577	XP_018842395.1	isocitrate dehydrogenase [NADP]	Juglans regia	ONI19628.1
TRINITY_DN6781_c0_g2_i1_1	0	1099	XP_018841565.1	monosaccharide-sensing protein 2-like	Juglans regia	XP_018841567.1
TRINITY_DN4623_c0_g2_i2_1	0	704	XP_018836920.1	glucan endo-1,3-beta-glucosidase 7	Juglans regia	XP_018807062.1
TRINITY_DN10600_c0_g1_i1_5	0	952	XP_018820267.1	ubiquitin carboxyl-terminal hydrolase MINDY-2-like	Juglans regia	XP_018820490.1
TRINITY_DN1407_c0_g1_i2_4	2,95E-68	229	XP_018835615.1	clathrin heavy chain 2	Juglans regia	XP_018846323.1
TRINITY_DN2265_c0_g1_i1_5	9,39E-81	258	XP_018831429.1	HIPL1 protein-like	Juglans regia	XP_018831975.1
TRINITY_DN8192_c0_g2_i2_5	3,63E-121	350	AES60048.1	serine/threonine protein phosphatase 2A	Medicago truncatula	XP_003589797.1
TRINITY_DN6164_c0_g2_i1_1	1,19E-89	278	XP_018837506.1	glucosidase 2 subunit beta	Juglans regia	ONH98947.1
TRINITY_DN715_c0_g1_i1_6	1,60E-36	122	XP_018838938.1	cytochrome c oxidase assembly factor 5	Juglans regia	XP_018838939.1

TRINITY_DN18948_c0_g1_i1_2	7,62E-44	156	XP_018830041.1	ABC transporter B family member 9 isoform X1	Juglans regia	XP_018830041.1
TRINITY_DN20147_c0_g1_i1_4	3,98E-51	172	XP_009350530.1	diacylglycerol kinase 5-like	Pyrus x bretschneideri	XP_018501644.1
TRINITY_DN8171_c0_g1_i1_2	0	553	XP_018852464.1	oxalate--CoA ligase-like	Juglans regia	OAY59825.1
TRINITY_DN6538_c0_g1_i1_4	2,78E-69	228	XP_018829853.1	5-methyltetrahydropteroyltriglutamate--homocysteine methyltransferase	Juglans regia	XP_018837618.1
TRINITY_DN6949_c0_g1_i2_3	0	605	XP_018860003.1	probable fructokinase-6, chloroplastic	Juglans regia	XP_008229214.1
TRINITY_DN15219_c0_g1_i1_6	1,37E-102	315	XP_020962497.1	heat shock protein 90-6, mitochondrial isoform X3	Arachis ipaensis	XP_020962496.1
TRINITY_DN11665_c0_g1_i3_2	0	1537	KDP33445.1	hypothetical protein JCGZ_07016	Jatropha curcas	XP_012076337.1
TRINITY_DN17687_c0_g1_i1_2	2,58E-42	142	XP_018846809.1	haloacid dehalogenase-like hydrolase domain-containing protein At2g33255	Juglans regia	OAY28174.1
TRINITY_DN9097_c0_g1_i1_2	0	527	XP_018811469.1	mitochondrial carnitine/acylcarnitine carrier-like protein	Juglans regia	XP_018811470.1
TRINITY_DN17840_c0_g1_i1_2	2,09E-80	251	XP_018818346.1	dynamin-related protein 5A	Juglans regia	XP_018824324.1
TRINITY_DN2942_c0_g1_i1_5	2,59E-86	254	XP_018857291.1	GDP-mannose 4,6 dehydratase 1, partial	Juglans regia	XP_018805499.1
TRINITY_DN4861_c0_g3_i1_4	1,39E-67	209	XP_014496997.1	60S ribosomal protein L23A	Vigna radiata var. radiata	XP_020224350.1
TRINITY_DN1788_c0_g2_i1_3	8,86E-160	452	ONI29783.1	hypothetical protein PRUPE_1G214500	Prunus persica	XP_007222511.1
TRINITY_DN6718_c0_g1_i1_5	1,84E-46	156	KRH51652.1	hypothetical protein GLYMA_06G020700	Glycine max	ACS93764.1
TRINITY_DN2144_c0_g2_i1_3	8,46E-134	387	XP_018820983.1	GLABRA2 expression modulator-like	Juglans regia	XP_018830258.1
TRINITY_DN4927_c0_g1_i1_6	2,10E-62	206	XP_018827664.1	probable glucan 1,3-alpha-glucosidase isoform X2	Juglans regia	GAU19561.1
TRINITY_DN11271_c0_g1_i1_6	3,08E-42	139	KDP42861.1	hypothetical protein JCGZ_23803	Jatropha curcas	CAA31627.1
TRINITY_DN8924_c0_g1_i1_1	8,16E-44	159	XP_008337280.1	methyl-CpG-binding domain-containing protein 11-like	Malus domestica	XP_008337277.1
TRINITY_DN779_c0_g1_i1_1	1,48E-162	457	XP_018828138.1	uncharacterized protein LOC108996608	Juglans regia	OAY44531.1
TRINITY_DN938_c0_g1_i1_5	3,41E-65	204	XP_018843123.1	eukaryotic translation initiation factor 3 subunit J-like	Juglans regia	XP_008238161.1
TRINITY_DN9792_c1_g2_i1_6	3,88E-162	462	XP_018825543.1	aldehyde dehydrogenase family 3 member H1	Juglans regia	XP_008219617.2
TRINITY_DN9457_c0_g1_i2_1	0	694	XP_018809732.1	SEC1 family transport protein SLY1-like	Juglans regia	XP_018841532.1
TRINITY_DN4143_c0_g1_i1_1	0	633	KOM53141.1	hypothetical protein LR48_Vigan09g180000	Vigna angularis	BAT87699.1
TRINITY_DN13762_c0_g1_i1_3	7,78E-41	139	XP_018834440.1	40S ribosomal protein S2-4-like	Juglans regia	XP_018837209.1
TRINITY_DN8478_c1_g1_i1_2	7,80E-71	216	XP_018836308.1	uncharacterized protein LOC109002851	Juglans regia	XP_008224696.1
TRINITY_DN7608_c0_g1_i1_4	1,66E-121	356	KHN04999.1	FAS-associated factor 2-B	Glycine soja	XP_018844497.1
TRINITY_DN4512_c0_g1_i1_4	4,99E-57	187	XP_018821126.1	mannan endo-1,4-beta-mannosidase 7-like	Juglans regia	XP_008220834.1
TRINITY_DN9675_c0_g1_i1_1	0	558	XP_018830828.1	probable fructokinase-7	Juglans regia	XP_008234319.1
TRINITY_DN7474_c0_g2_i1_2	1,44E-114	333	XP_018825107.1	vesicle-associated protein 2-1-like isoform X3	Juglans regia	XP_018825108.1
TRINITY_DN10982_c0_g1_i1_1	0	613	XP_009354832.1	peroxisomal (S)-2-hydroxy-acid oxidase-like isoform X1	Pyrus x bretschneideri	ONI12161.1

TRINITY_DN1335_c0_g1_i1_2	0	614	XP_018819539.1	uncharacterized protein LOC108990124 isoform X2	Juglans regia	XP_018819538.1
TRINITY_DN10530_c0_g1_i4_3	0	928	XP_018847824.1	phenylalanine--tRNA ligase alpha subunit, cytoplasmic	Juglans regia	XP_008236892.1
TRINITY_DN8417_c0_g1_i2_2	1,97E-126	361	XP_018836212.1	26S proteasome non-ATPase regulatory subunit 10	Juglans regia	KDP41272.1
TRINITY_DN11265_c0_g1_i1_3	0	550	XP_018850162.1	galacturonosyltransferase 8-like	Juglans regia	OAY32840.1
TRINITY_DN2984_c0_g2_i1_3	0	538	XP_018824151.1	CBS domain-containing protein CBSX6	Juglans regia	KYP47638.1
TRINITY_DN1687_c0_g1_i1_2	0	622	KRH47017.1	hypothetical protein GLYMA_07G004100	Glycine max	XP_003529523.1
TRINITY_DN8065_c0_g1_i1_1	0	587	XP_018821177.1	AP-4 complex subunit mu-like	Juglans regia	XP_018851429.1
TRINITY_DN11452_c0_g2_i2_2	0	620	XP_018837874.1	1-aminocyclopropane-1-carboxylate oxidase homolog 4-like	Juglans regia	XP_018807675.1
TRINITY_DN6644_c0_g1_i1_2	2,70E-39	139	XP_018822954.1	uncharacterized protein LOC108992770	Juglans regia	OAY21302.1
TRINITY_DN3807_c1_g1_i1_3	4,21E-45	155	dbj|BAT79464.1	hypothetical protein VIGAN_02235800	Vigna angularis var. angularis	BAT79464.1
TRINITY_DN7004_c0_g2_i1_2	8,42E-34	120	XP_018820092.1	mitochondrial import receptor subunit TOM9-2-like	Juglans regia	OAY55497.1
TRINITY_DN10332_c0_g2_i1_4	0	889	XP_018828572.1	alanine--glyoxylate aminotransferase 2 homolog 2, mitochondrial-like	Juglans regia	OAY41602.1
TRINITY_DN8478_c0_g1_i1_1	7,04E-17	77,8	XP_018836308.1	uncharacterized protein LOC109002851	Juglans regia	OAY37734.1
TRINITY_DN11043_c0_g1_i1_6	0	808	XP_018838284.1	7-dehydrocholesterol reductase	Juglans regia	KDP25052.1
TRINITY_DN6414_c0_g2_i2_1	3,02E-131	375	XP_018840252.1	inosine triphosphate pyrophosphatase isoform X2	Juglans regia	KHN24364.1
TRINITY_DN12528_c0_g1_i1_1	9,79E-56	189	XP_018836693.1	uncharacterized protein LOC109003142	Juglans regia	XP_004504464.1
TRINITY_DN2126_c0_g1_i1_4	1,34E-105	303	XP_008393423.1	tubulin beta-1 chain-like	Malus domestica	AFK39823.1
TRINITY_DN8608_c1_g1_i1_4	6,93E-100	291	AAG40330.1|AF323974_1 major allergen variant Cor a 1.0403	Corylus avellana		AAG40329.1|AF323973_1 major allergen variant Cor a 1.0402
TRINITY_DN10518_c0_g1_i3_1	6,33E-140	408	XP_018843448.1	probable S-adenosylmethionine-dependent methyltransferase At5g38780	Juglans regia	XP_009347740.1
TRINITY_DN10154_c0_g1_i1_3	0	775	XP_018810134.1	dihydropyrimidine dehydrogenase (NADP(+)), chloroplastic-like	Juglans regia	XP_018823611.1
TRINITY_DN4990_c0_g2_i1_5	6,90E-132	384	ONI30961.1	hypothetical protein PRUPE_1G284600	Prunus persica	XP_007224102.1
TRINITY_DN8848_c0_g1_i1_2	0	712	XP_018847993.1	importin subunit alpha-1-like	Juglans regia	XP_018847994.1
TRINITY_DN2750_c0_g2_i1_2	6,60E-172	481	XP_018842902.1	haloacid dehalogenase-like hydrolase domain-containing protein At4g39970 isoform X1	Juglans regia	XP_018860336.1
TRINITY_DN6078_c0_g1_i2_4	8,29E-87	274	XP_018831343.1	probable ADP-ribosylation factor GTPase-activating protein AGD6	Juglans regia	OAY41270.1
TRINITY_DN8790_c0_g2_i2_2	6,79E-66	202	XP_008374266.1	mitochondrial pyruvate carrier 1-like	Malus domestica	XP_008374267.1
TRINITY_DN6367_c0_g1_i1_3	5,18E-111	324	KRH11114.1	hypothetical protein GLYMA_15G089800	Glycine max	XP_003546060.1
TRINITY_DN11511_c1_g1_i1_6	5,60E-20	85,5	XP_008226264.1	probable glutathione S-transferase	Prunus mume	XP_020417337.1
TRINITY_DN6200_c0_g1_i1_6	5,30E-114	337	OAY27086.1	hypothetical protein MANES_16G098600	Manihot esculenta	KDP33877.1
TRINITY_DN8259_c0_g1_i1_2	0	532	XP_018852406.1	D-aminoacyl-tRNA deacylase	Juglans regia	XP_018852407.1

TRINITY_DN6098_c0_g1_i1_2	0	644	XP_018813512.1	farnesyl pyrophosphate synthase 1-like	Juglans regia	XP_018813518.1
TRINITY_DN17635_c0_g1_i1_1	3,93E-153	456	XP_018807321.1	aconitate hydratase 1	Juglans regia	OAY57605.1
TRINITY_DN21752_c0_g1_i1_3	7,00E-16	75,5	XP_018819170.1	eukaryotic translation initiation factor 4G-like isoform X2	Juglans regia	XP_018819162.1
TRINITY_DN11371_c0_g2_i1_1	0	803	XP_018809613.1	alanine--glyoxylate aminotransferase 2 homolog 1, mitochondrial	Juglans regia	XP_019437590.1
TRINITY_DN4480_c0_g1_i2_2	0	534	XP_008239736.1	mitochondrial uncoupling protein 1	Prunus mume	KDP24500.1
TRINITY_DN6825_c0_g1_i1_1	0	531	XP_018847917.1	peroxisomal 2,4-dienoyl-CoA reductase	Juglans regia	XP_008383145.1
TRINITY_DN15184_c0_g1_i1_6	9,48E-24	96,7	XP_018855089.1	protein disulfide isomerase-like 1-6, partial	Juglans regia	XP_018837500.1
TRINITY_DN2842_c0_g1_i1_6	5,33E-97	289	ONI06500.1	hypothetical protein PRUPE_5G064700	Prunus persica	XP_007209384.1
TRINITY_DN7658_c0_g1_i1_3	1,02E-151	427	XP_018832029.1	ras-related protein RABA1b-like	Juglans regia	XP_018849174.1
TRINITY_DN6555_c0_g1_i2_5	4,60E-118	348	XP_018838239.1	triose phosphate/phosphate translocator, non-green plastid, chloroplastic-like	Juglans regia	XP_018821285.1
TRINITY_DN7691_c0_g1_i2_3	1,59E-122	368	OAY49551.1	hypothetical protein MANES_05G065200	Manihot esculenta	AES82113.1
TRINITY_DN11075_c0_g1_i1_1	0	651	XP_018828379.1	heterogeneous nuclear ribonucleoprotein 1-like	Juglans regia	XP_018823852.1
TRINITY_DN8971_c0_g1_i1_4	6,73E-36	130	XP_018816525.1	ATP-citrate synthase alpha chain protein 3	Juglans regia	XP_008360239.2
TRINITY_DN11579_c0_g1_i2_1	1,20E-86	258	KDP31091.1	hypothetical protein JCGZ_11467	Jatropha curcas	XP_012080054.1
TRINITY_DN3015_c0_g1_i1_1	0	710	XP_018837882.1	dynamin-related protein 1C	Juglans regia	XP_018814632.1
TRINITY_DN1893_c0_g1_i1_2	6,94E-30	114	XP_018840883.1	protein transport Sec1a-like	Juglans regia	XP_008232655.1
TRINITY_DN51_c0_g1_i1_2	2,54E-111	326	ONH92468.1	hypothetical protein PRUPE_8G177400	Prunus persica	XP_007199907.1
TRINITY_DN9907_c0_g2_i2_3	2,68E-177	499	XP_018840900.1	probable prolyl 4-hydroxylase 10	Juglans regia	XP_018807322.1
TRINITY_DN12604_c0_g1_i1_2	0	749	XP_018843664.1	4-alpha-glucanotransferase DPE2 isoform X2	Juglans regia	XP_018843664.1
TRINITY_DN16223_c0_g1_i1_1	1,11E-23	97,1	XP_018846984.1	calcium-transporting ATPase 4, plasma membrane-type-like isoform X2	Juglans regia	XP_018846983.1
TRINITY_DN1060_c0_g1_i1_1	5,64E-98	300	XP_018829351.1	uncharacterized protein LOC108997478 isoform X2	Juglans regia	XP_018829342.1
TRINITY_DN10488_c0_g1_i1_2	0	533	ONH98930.1	hypothetical protein PRUPE_6G000600	Prunus persica	XP_007205625.1
TRINITY_DN9024_c1_g1_i2_5	6,03E-93	277	XP_018845871.1	succinate dehydrogenase assembly factor 2, mitochondrial isoform X1	Juglans regia	KDP22524.1
TRINITY_DN10216_c0_g1_i1_3	1,08E-111	323	XP_018825523.1	enoyl-CoA delta isomerase 1, peroxisomal-like	Juglans regia	XP_018825524.1
TRINITY_DN4751_c0_g1_i1_3	0	508	XP_018834471.1	uncharacterized protein LOC109001574 isoform X1	Juglans regia	XP_018834472.1
TRINITY_DN3627_c0_g2_i1_4	0	728	XP_018819725.1	endoplasmic reticulum oxidoreductin-2-like	Juglans regia	XP_018819726.1
TRINITY_DN5091_c0_g1_i1_4	5,08E-138	419	XP_018805796.1	protein WEAK CHLOROPLAST MOVEMENT UNDER BLUE LIGHT 1-like	Juglans regia	XP_018805797.1
TRINITY_DN10763_c0_g1_i4_1						
TRINITY_DN11635_c2_g1_i6_2	0	595	XP_018824429.1	probable aldo-keto reductase 2	Juglans regia	XP_018858774.1
TRINITY_DN19987_c0_g1_i1_3	1,62E-46	152	ONI35294.1	hypothetical protein PRUPE_1G528100	Prunus persica	XP_007223705.1
TRINITY_DN10849_c0_g1_i2_5	3,17E-20	86,3	XP_018852386.1	protein SPIRAL1-like 1	Juglans regia	XP_018852387.1
TRINITY_DN9489_c0_g2_i1_2	0	528	XP_018813719.1	cysteine proteinase COT44-like	Juglans regia	XP_018845023.1
TRINITY_DN4080_c0_g1_i1_3	7,57E-42	148	XP_018838198.1	uncharacterized protein LOC109004190	Juglans regia	XP_008238951.1

TRINITY_DN6613_c0_g1_i1_4	7,49E-152	433	XP_018847491.1	nudix hydrolase 2-like isoform X1	Juglans regia	XP_018833252.1
TRINITY_DN8186_c0_g1_i3_1	0	630	XP_018845363.1	pyruvate kinase 1, cytosolic-like	Juglans regia	XP_009361441.1
TRINITY_DN11291_c0_g1_i2_3	1,49E-141	405	XP_018850746.1	uncharacterized protein LOC109013191 isoform X2	Juglans regia	OAY54849.1
TRINITY_DN11520_c0_g1_i1_2	0	808	XP_018811216.1	multiple inositol polyphosphate phosphatase 1	Juglans regia	XP_008233804.1
TRINITY_DN8996_c0_g1_i1_4	2,85E-110	320	KEH19791.1	ubiquitin-conjugating enzyme	Medicago truncatula	XP_013445765.1
TRINITY_DN10798_c1_g1_i1_2	3,30E-58	197	XP_018844510.1	dolichyl-diphosphooligosaccharide--protein glycosyltransferase subunit STT3B	Juglans regia	XP_018808402.1
TRINITY_DN4765_c0_g3_i1_1	0	565	XP_018811976.1	CBL-interacting serine/threonine-protein kinase 9	Juglans regia	XP_008233067.1
TRINITY_DN8594_c0_g1_i1_1	5,16E-11	58,2	KDP41684.1	hypothetical protein JCGZ_16091	Jatropha curcas	KDP41684.1
TRINITY_DN10973_c0_g3_i2_1	2,01E-163	472	XP_018843210.1	mitochondrial Rho GTPase 1-like	Juglans regia	XP_018841787.1
TRINITY_DN17195_c0_g1_i1_1	9,91E-87	254	XP_018808351.1	elicitor-responsive protein 1-like	Juglans regia	XP_018808352.1
TRINITY_DN7057_c1_g1_i1_2	0	683	XP_018834116.1	probable isoprenylcysteine alpha-carbonyl methylesterase ICMEL2	Juglans regia	XP_009357013.1
TRINITY_DN23079_c0_g1_i1_4	1,88E-42	150	XP_018846983.1	calcium-transporting ATPase 4, plasma membrane-type-like isoform X1	Juglans regia	XP_018846984.1
TRINITY_DN1992_c0_g1_i1_1	5,98E-76	246	XP_015938020.1	coatomer subunit beta-1	Arachis duranensis	XP_016174940.1
TRINITY_DN13881_c0_g1_i1_1	2,20E-174	489	XP_018859762.1	LOW QUALITY PROTEIN: methionine adenosyltransferase 2 subunit beta	Juglans regia	XP_008232770.1
TRINITY_DN14656_c0_g1_i1_2	5,19E-43	145	ONH95538.1	hypothetical protein PRUPE_7G076200	Prunus persica	ONH95537.1
TRINITY_DN3861_c0_g1_i2_3	1,24E-107	334	XP_018807473.1	uncharacterized protein LOC108980890	Juglans regia	KDP22063.1
TRINITY_DN8241_c0_g1_i1_2	2,44E-178	500	XP_018825788.1	fructose-bisphosphate aldolase, cytoplasmic isozyme 1	Juglans regia	KDP32624.1
TRINITY_DN17884_c0_g1_i1_4	7,93E-41	145	XP_018806543.1	isoleucine--tRNA ligase, cytoplasmic	Juglans regia	ONI29194.1
TRINITY_DN7822_c0_g1_i3_2	0	521	XP_018860709.1	secretory carrier-associated membrane protein 1 isoform X1	Juglans regia	XP_018860711.1
TRINITY_DN11037_c0_g1_i1_3	4,98E-130	380	XP_018838963.1	uncharacterized protein LOC109004752	Juglans regia	XP_018821762.1
TRINITY_DN5712_c0_g2_i2_1	0	681	XP_018827690.1	acetyl-coenzyme A synthetase, chloroplastic/glyoxysomal isoform X1	Juglans regia	XP_018827691.1
TRINITY_DN22171_c0_g1_i1_5	9,03E-78	245	ONI02007.1	hypothetical protein PRUPE_6G172500	Prunus persica	ONI02008.1
TRINITY_DN12955_c0_g1_i1_1	2,55E-36	132	XP_018829351.1	uncharacterized protein LOC108997478 isoform X2	Juglans regia	XP_018829342.1
TRINITY_DN10631_c0_g1_i1_4	0	591	XP_018844917.1	transmembrane protein 120 homolog	Juglans regia	XP_018809367.1
TRINITY_DN6543_c0_g1_i1_4	2,28E-42	156	XP_018835518.1	UDP-glucose:glycoprotein glucosyltransferase	Juglans regia	ONI36146.1
TRINITY_DN10109_c0_g1_i3_2	3,61E-70	217	XP_018842596.1	tobamovirus multiplication protein 2A-like, partial	Juglans regia	XP_018838258.1
TRINITY_DN11659_c0_g1_i1_1	4,06E-34	129	XP_008221788.1	aldehyde dehydrogenase family 2 member B7, mitochondrial	Prunus mume	KDP26565.1
TRINITY_DN21796_c0_g1_i1_1	1,73E-45	150	XP_008344672.1	DEAD-box ATP-dependent RNA helicase 52C-like, partial	Malus domestica	KDP30456.1
TRINITY_DN10608_c0_g1_i4_3	1,06E-126	367	XP_018837520.1	hydroxyphenylpyruvate reductase	Juglans regia	XP_008342684.1

TRINITY_DN8882_c0_g1_i3_2	0	861	XP_018857854.1	ubiquitin carboxyl-terminal hydrolase 13-like	Juglans regia	XP_018852065.1
TRINITY_DN8417_c1_g1_i1_6	5,25E-23	91,7	XP_018836212.1	26S proteasome non-ATPase regulatory subunit 10	Juglans regia	XP_007157422.1
TRINITY_DN285_c0_g1_i1_6	4,11E-163	462	XP_018847038.1	tubulin-folding cofactor C	Juglans regia	XP_017188064.1
TRINITY_DN8647_c0_g1_i1_4	8,93E-57	186	KHN11010.1	F-box protein	Glycine soja	XP_018833073.1
TRINITY_DN1686_c0_g1_i1_2	1,20E-38	128	XP_014507355.1	cytochrome c oxidase subunit 6b-2	Vigna radiata var. radiata	ACU17340.1
TRINITY_DN16666_c0_g1_i1_3	3,58E-24	99	XP_018847012.1	ATP sulfurylase 2-like	Juglans regia	XP_018812595.1
TRINITY_DN8134_c0_g1_i1_2	2,68E-79	238	XP_018830449.1	UPF0678 fatty acid-binding protein-like protein At1g79260 isoform X1	Juglans regia	XP_018830450.1
TRINITY_DN1558_c0_g1_i1_3	1,62E-87	275	XP_009336194.1	phosphatidylinositol 4-phosphate 5-kinase 5-like	Pyrus x bretschneideri	XP_009348280.1
TRINITY_DN7263_c0_g1_i1_2	1,33E-127	378	XP_018850062.1	acyl-CoA-binding domain-containing protein 4	Juglans regia	XP_018850062.1
TRINITY_DN9028_c0_g1_i1_3	0	893	XP_018849693.1	ERAD-associated E3 ubiquitin-protein ligase HRD1B-like	Juglans regia	XP_018849695.1
TRINITY_DN10191_c0_g1_i1_3	1,32E-36	126	OAY35302.1	hypothetical protein MANES_12G089400	Manihot esculenta	XP_018850228.1
TRINITY_DN9202_c0_g1_i1_5	3,78E-32	114	XP_018855976.1	NADH dehydrogenase [ubiquinone] 1 beta subcomplex subunit 3-A-like	Juglans regia	XP_008218629.1
TRINITY_DN3322_c0_g2_i1_2	0	644	XP_018807641.1	arginine biosynthesis bifunctional protein ArgJ, chloroplastic	Juglans regia	XP_008369856.1
TRINITY_DN661_c0_g1_i1_1	1,18E-148	429	XP_018811850.1	imidazole glycerol phosphate synthase hisHF, chloroplastic isoform X3	Juglans regia	XP_018811844.1
TRINITY_DN284_c0_g2_i1_5	1,74E-67	210	XP_018819519.1	yrdC domain-containing protein, mitochondrial isoform X2	Juglans regia	XP_018819515.1
TRINITY_DN804_c0_g1_i1_1	1,39E-20	87,8	XP_018825836.1	uncharacterized protein LOC108994893	Juglans regia	XP_018825834.1
TRINITY_DN14006_c0_g1_i1_3	1,53E-73	221	AFK45051.1	unknown	Lotus japonicus	XP_018820205.1
TRINITY_DN21069_c0_g1_i1_4	4,13E-118	359	XP_018807473.1	uncharacterized protein LOC108980890	Juglans regia	ONH99278.1
TRINITY_DN9015_c0_g1_i1_4	4,17E-78	253	OAY57931.1	hypothetical protein MANES_02G136100	Manihot esculenta	XP_018829157.1
TRINITY_DN10966_c0_g1_i1_3	2,73E-127	373	XP_018819428.1	phenylalanine--tRNA ligase beta subunit, cytoplasmic-like	Juglans regia	XP_018814927.1
TRINITY_DN3062_c0_g1_i1_3	3,17E-143	414	XP_018841559.1	alpha-1,3-mannosyl-glycoprotein 2-beta-N-acetylglucosaminyltransferase-like isoform X1	Juglans regia	XP_018841560.1
TRINITY_DN15393_c0_g1_i1_5	6,51E-74	223	XP_018835474.1	uncharacterized protein LOC109002264	Juglans regia	XP_018808497.1
TRINITY_DN7397_c0_g1_i2_1	1,23E-155	442	XP_018805220.1	signal recognition particle receptor subunit beta-like	Juglans regia	XP_018805221.1
TRINITY_DN843_c0_g1_i1_5	9,85E-122	362	XP_018821230.1	protein SLOW GREEN 1, chloroplastic-like	Juglans regia	XP_016647139.1
TRINITY_DN13608_c0_g1_i1_6	2,61E-56	190	OAY33315.1	hypothetical protein MANES_13G085700	Manihot esculenta	XP_018844607.1
TRINITY_DN3889_c0_g3_i1_3	3,01E-70	213	XP_015940120.1	PITH domain-containing protein At3g04780	Arachis duranensis	XP_015940121.1
TRINITY_DN6029_c0_g1_i1_1	4,02E-121	357	XP_018836309.1	aspartic proteinase-like	Juglans regia	XP_018836310.1
TRINITY_DN5896_c0_g1_i2_3	2,30E-91	226	XP_018829342.1	uncharacterized protein LOC108997478 isoform X1	Juglans regia	XP_018829342.1

TRINITY_DN12487_c0_g1_i1_1	5,26E-138	414	XP_018844325.1	ATPase 9, plasma membrane-type	Juglans regia	XP_019448064.1
TRINITY_DN21101_c0_g1_i1_4	6,69E-67	207	XP_018854480.1	bifunctional dTDP-4-dehydrorhamnose 3,5-epimerase/dTDP-4-dehydrorhamnose reductase	Juglans regia	XP_018820521.1
TRINITY_DN20395_c0_g2_i1_2	2,60E-26	108	XP_018835918.1	myb-like protein X	Juglans regia	XP_018835919.1
TRINITY_DN11574_c0_g1_i5_3	0	837	XP_018808164.1	flavin-dependent oxidoreductase FOX2-like	Juglans regia	XP_008225656.1
TRINITY_DN16156_c0_g1_i1_2	9,29E-38	127	KRH41969.1	hypothetical protein GLYMA_08G061200	Glycine max	KRH41968.1
TRINITY_DN10717_c1_g1_i3_1	0	545	XP_018807382.1	putative G3BP-like protein	Juglans regia	XP_018850163.1
TRINITY_DN11531_c0_g1_i3_3	0	952	OAY28337.1	hypothetical protein MANES_15G059000, partial	Manihot esculenta	OAY28338.1
TRINITY_DN7911_c0_g1_i3_1	7,63E-93	274	XP_018848154.1	thioredoxin Y1, chloroplastic-like	Juglans regia	ONH90075.1
TRINITY_DN5389_c0_g1_i1_5	1,60E-108	322	XP_017184434.1	ubiquitin carboxyl-terminal hydrolase 13-like, partial	Malus domestica	OAY40681.1
TRINITY_DN7964_c0_g4_i1_6	1,76E-118	342	XP_018848922.1	probable protein kinase At2g41970 isoform X2	Juglans regia	XP_018848920.1
TRINITY_DN12169_c0_g1_i1_2	9,36E-76	238	XP_018814628.1	putative G3BP-like protein	Juglans regia	XP_018816596.1
TRINITY_DN23339_c0_g1_i1_1	3,59E-45	156	XP_018821986.1	malate synthase, glyoxysomal	Juglans regia	XP_020216328.1
TRINITY_DN9250_c0_g1_i1_3	1,64E-171	489	XP_018828279.1	dnaJ homolog subfamily B member 4-like	Juglans regia	XP_020223904.1
TRINITY_DN18399_c0_g1_i1_1	4,81E-75	245	XP_018809070.1	probable manganese-transporting ATPase PDR2	Juglans regia	XP_018809074.1
TRINITY_DN9486_c0_g1_i1_2	0	637	XP_018835583.1	polyadenylate-binding protein RBP47-like isoform X1	Juglans regia	XP_018841963.1
TRINITY_DN5674_c0_g1_i1_1	0	712	XP_018840528.1	DUF21 domain-containing protein At4g14240-like	Juglans regia	XP_018826137.1
TRINITY_DN6255_c0_g3_i1_1	9,31E-102	308	XP_018859113.1	uncharacterized protein At4g06744-like	Juglans regia	XP_018859113.1
TRINITY_DN10766_c0_g1_i3_3	0	985	KHN28892.1	Putative phagocytic receptor 1b	Glycine soja	KHN25962.1
TRINITY_DN10543_c0_g1_i1_6	0	523	XP_018859854.1	phosphoglycerate mutase-like protein 1 isoform X6	Juglans regia	XP_018859855.1
TRINITY_DN2263_c0_g2_i1_6	8,86E-112	332	XP_008342628.1	protein transport protein Sec24-like At4g32640	Malus domestica	XP_017185211.1
TRINITY_DN11003_c0_g2_i5_2	0	833	XP_004485538.1	serine decarboxylase 1	Cicer arietinum	XP_004485539.1
TRINITY_DN9687_c1_g1_i2_2	6,70E-86	272	XP_018821713.1	eukaryotic translation initiation factor 4B2-like	Juglans regia	XP_018821321.1
TRINITY_DN9737_c1_g1_i1_4	2,47E-132	376	AEA92306.1	Arf2	Hevea brasiliensis	OAY48870.1
TRINITY_DN11475_c0_g1_i1_2	0	659	XP_018836507.1	ankyrin repeat-containing protein ITN1-like	Juglans regia	XP_016175448.1
TRINITY_DN6747_c0_g1_i1_2	5,67E-167	486	XP_018826882.1	protein EXECUTER 1, chloroplastic-like	Juglans regia	XP_008373217.1
TRINITY_DN2919_c0_g1_i1_5	3,72E-118	344	XP_018816480.1	S-adenosylmethionine synthase 5-like	Juglans regia	OAY43921.1
TRINITY_DN9215_c0_g1_i1_2	0	605	XP_018848409.1	protein STRICTOSIDINE SYNTHASE-LIKE 10-like	Juglans regia	XP_008244837.1
TRINITY_DN5123_c0_g1_i2_4	2,73E-78	233	OAY42085.1	hypothetical protein MANES_09G151800	Manihot esculenta	OAY42086.1
TRINITY_DN2276_c0_g2_i1_6	7,84E-179	513	XP_018825905.1	uncharacterized protein LOC108994927 isoform X3	Juglans regia	XP_018825903.1
TRINITY_DN8639_c0_g1_i1_6	5,85E-131	383	KDP25525.1	hypothetical protein JCGZ_20681	Jatropha curcas	XP_012087024.1
TRINITY_DN6651_c0_g1_i1_3	0	627	XP_018811480.1	protein NRT1/ PTR FAMILY 8.1-like	Juglans regia	XP_018811480.1

TRINITY_DN8585_c0_g1_i1_4	0	744	XP_008226105.1	acetylornithine aminotransferase, mitochondrial-like	Prunus mume	ONI11967.1
TRINITY_DN21541_c0_g1_i1_4	3,04E-83	256	XP_009358971.1	serine carboxypeptidase-like 20 isoform X1	Pyrus x bretschneideri	XP_009358972.1
TRINITY_DN8030_c0_g1_i1_2	7,58E-47	152	XP_018833990.1	uncharacterized protein LOC109001246	Juglans regia	XP_018824533.1
TRINITY_DN7519_c0_g2_i1_2	0	712	XP_018823857.1	probable 26S proteasome non-ATPase regulatory subunit 3	Juglans regia	XP_018841498.1
TRINITY_DN11415_c0_g1_i1_1	8,38E-78	235	KYP55926.1	Multiprotein-bridging factor 1a	Cajanus cajan	KYP67761.1
TRINITY_DN9353_c0_g1_i1_3	4,39E-113	335	emb|CAX05443.1	unnamed protein product	Glycine max	CAX05471.1
TRINITY_DN5812_c0_g1_i1_1	5,08E-142	402	XP_018839374.1	proteasome subunit beta type-2-A	Juglans regia	XP_018812168.1
TRINITY_DN11107_c1_g1_i4_3	0	1040	XP_018831636.1	phosphatidylinositol 3,4,5-trisphosphate 3-phosphatase and protein-tyrosine-phosphatase PTEN2A-like	Juglans regia	XP_018831637.1
TRINITY_DN3448_c0_g1_i1_2	4,62E-139	398	XP_018828828.1	bidirectional sugar transporter SWEET4 isoform X1	Juglans regia	XP_008230949.1
TRINITY_DN9452_c0_g1_i1_1	1,02E-129	374	OAY41624.1	hypothetical protein MANES_09G116800	Manihot esculenta	XP_018812032.1
TRINITY_DN8582_c0_g2_i1_3	0	695	XP_018837412.1	ATP-dependent 6-phosphofructokinase 2	Juglans regia	OAY41242.1
TRINITY_DN15392_c0_g1_i1_5	2,69E-69	226	XP_016187872.1	beta-adaptin-like protein B isoform X2	Arachis ipaensis	ONI07170.1
TRINITY_DN2089_c0_g1_i1_5	2,80E-79	251	XP_018809084.1	delta-1-pyrroline-5-carboxylate synthase isoform X2	Juglans regia	XP_018809083.1
TRINITY_DN8917_c0_g2_i1_3	0	858	XP_018820175.1	monothiol glutaredoxin-S17	Juglans regia	XP_018820175.1
TRINITY_DN16117_c0_g1_i1_6	3,51E-31	117	XP_018845708.1	beta-galactosidase-like	Juglans regia	XP_018845720.1
TRINITY_DN8546_c0_g1_i1_2	0	1264	XP_018817103.1	long chain acyl-CoA synthetase 9, chloroplastic	Juglans regia	XP_018812536.1
TRINITY_DN3962_c0_g1_i2_3	0	1045	XP_018845571.1	beta-adaptin-like protein A	Juglans regia	XP_009367922.1
TRINITY_DN2992_c0_g1_i1_3	0	714	XP_018842611.1	cytosolic enolase 3	Juglans regia	ONI00028.1
TRINITY_DN11605_c1_g1_i1_5	4,09E-77	231	KDP34497.1	hypothetical protein JCGZ_11047	Jatropha curcas	XP_012075960.1
TRINITY_DN401_c0_g1_i1_3	3,63E-135	385	XP_015949837.1	ras-related protein RABA4d	Arachis duranensis	XP_012073057.1
TRINITY_DN18414_c0_g1_i1_2	0	597	XP_018835247.1	uncharacterized protein YMR315W	Juglans regia	KDP20201.1
TRINITY_DN9346_c0_g1_i2_1	0	731	XP_018851337.1	acetolactate synthase small subunit 2, chloroplastic-like isoform X1	Juglans regia	ONH98842.1
TRINITY_DN8618_c0_g2_i2_4	0	1080	XP_018821744.1	probable alpha-galactosidase B	Juglans regia	ONI30366.1
TRINITY_DN5167_c0_g1_i1_5	0	877	XP_018810974.1	uncharacterized protein LOC108983704	Juglans regia	XP_018855417.1
TRINITY_DN15469_c0_g1_i1_6	4,33E-66	211	AET01305.2	dynamin 1E-like protein	Medicago truncatula	XP_003626829.2
TRINITY_DN19298_c0_g1_i1_1	3,04E-59	197	KRH31453.1	hypothetical protein GLYMA_11G248700	Glycine max	ONI30124.1
TRINITY_DN11193_c0_g2_i3_2	0	510	XP_018846970.1	ADP-glucose phosphorylase-like isoform X1	Juglans regia	OAY41942.1
TRINITY_DN11599_c1_g1_i4_1	1,84E-37	135	XP_018830823.1	anthocyanidin 3-O-glucosyltransferase 2-like isoform X2	Juglans regia	XP_018815994.1
TRINITY_DN6675_c0_g1_i4_2	0	802	XP_018843891.1	DEAD-box ATP-dependent RNA helicase 53-like	Juglans regia	ONI18008.1
TRINITY_DN9665_c0_g1_i1_2	1,20E-04	46,2	OIW04983.1	hypothetical protein TanjilG_24455	Lupinus angustifolius	XP_019455534.1
TRINITY_DN10222_c0_g1_i5_2	0	822	XP_018835087.1	polyadenylate-binding protein-interacting protein 3-like isoform X1	Juglans regia	XP_018835088.1

TRINITY_DN8244_c0_g1_i1_2	3,38E-59	189	XP_009358246.1	protein disulfide isomerase-like 5-1	Pyrus x bretschneideri	XP_009338286.1
TRINITY_DN6988_c0_g2_i1_1	1,10E-156	451	XP_018821556.1	nucleosome assembly protein 1;4-like	Juglans regia	ONI15531.1
TRINITY_DN21693_c0_g1_i1_3	1,28E-40	145	XP_018829558.1	alpha-mannosidase	Juglans regia	XP_007156173.1
TRINITY_DN6721_c0_g1_i1_2	0	722	XP_018844889.1	ruvB-like protein 1 isoform X1	Juglans regia	XP_018844890.1
TRINITY_DN10523_c1_g1_i1_2	7,96E-55	174	XP_018815191.1	60S ribosomal protein L22-2-like	Juglans regia	XP_018858309.1
TRINITY_DN8754_c0_g1_i1_1	2,10E-117	337	OIV98465.1	hypothetical protein TanjilG_16792	Lupinus angustifolius	XP_019414713.1
TRINITY_DN21226_c0_g1_i1_1	1,83E-42	150	XP_018817814.1	cullin-associated NEDD8-dissociated protein 1	Juglans regia	XP_020539505.1
TRINITY_DN18056_c0_g1_i1_1	2,02E-96	294	XP_018813115.1	fasciclin-like arabinogalactan protein 1	Juglans regia	KDP39270.1
TRINITY_DN11502_c0_g1_i1_2	0	615	XP_018823768.1	protein EARLY-RESPONSIVE TO DEHYDRATION 7, chloroplastic-like	Juglans regia	XP_018823769.1
TRINITY_DN8308_c0_g2_i1_2	0	777	XP_018852257.1	cysteine proteinase RD21A-like	Juglans regia	XP_018819893.1
TRINITY_DN5126_c0_g1_i2_2	0	509	XP_009355788.1	protein MEMO1	Pyrus x bretschneideri	XP_012076496.1
TRINITY_DN7908_c0_g1_i1_3	2,28E-138	399	XP_018821130.1	anamorsin homolog isoform X1	Juglans regia	XP_018821131.1
TRINITY_DN10955_c0_g2_i3_1	2,04E-150	423	XP_018843526.1	ras-related protein RABH1b-like isoform X1	Juglans regia	XP_018843527.1
TRINITY_DN8800_c0_g1_i1_3	6,01E-142	410	XP_018819392.1	ethanolamine-phosphate cytidylyltransferase	Juglans regia	XP_018819392.1
TRINITY_DN4365_c0_g1_i1_1	1,56E-38	140	XP_018812893.1	uncharacterized protein LOC108985160 isoform X2	Juglans regia	XP_018812892.1
TRINITY_DN14633_c0_g1_i1_2	0	589	XP_018834060.1	glutamine--fructose-6-phosphate aminotransferase [isomerizing 2]	Juglans regia	KDP26882.1
TRINITY_DN3011_c0_g2_i1_3	1,23E-86	255	XP_018832728.1	protein FAM136A-like	Juglans regia	XP_018840436.1
TRINITY_DN2343_c0_g1_i1_1	2,51E-112	339	KOM29550.1	hypothetical protein LR48_Vigan727s000900	Vigna angularis	KOM45043.1
TRINITY_DN9033_c1_g1_i1_6	1,24E-57	183	XP_018839419.1	transmembrane emp24 domain-containing protein p24beta3-like isoform X1	Juglans regia	XP_014511241.1
TRINITY_DN2502_c0_g2_i1_2	2,32E-131	385	XP_018816803.1	transmembrane 9 superfamily member 3-like	Juglans regia	KRH38977.1
TRINITY_DN7907_c0_g1_i4_4	2,89E-79	239	XP_018834938.1	uncharacterized protein LOC109001895 isoform X1	Juglans regia	XP_018834946.1
TRINITY_DN642_c0_g1_i1_2	0	654	XP_018808362.1	porphobilinogen deaminase, chloroplastic isoform X1	Juglans regia	XP_012072847.1
TRINITY_DN701_c0_g2_i1_6	4,18E-177	505	XP_018847248.1	copper chaperone for superoxide dismutase, chloroplastic/cytosolic isoform X1	Juglans regia	XP_018847253.1
TRINITY_DN6896_c0_g1_i1_1	3,61E-61	188	XP_018836754.1	uncharacterized protein LOC109003181	Juglans regia	XP_018836756.1
TRINITY_DN12989_c1_g1_i1_1	1,22E-50	162	ONI28085.1	hypothetical protein PRUPE_1G122200	Prunus persica	XP_016647418.1
TRINITY_DN11038_c0_g1_i1_3	2,10E-17	79,7	XP_018817568.1	probable ADP-ribosylation factor GTPase-activating protein AGD9 isoform X1	Juglans regia	XP_018817570.1
TRINITY_DN6818_c0_g1_i1_3	5,44E-108	315	XP_018815857.1	chloride conductance regulatory protein ICln isoform X1	Juglans regia	KDP22395.1
TRINITY_DN18372_c0_g1_i1_2	5,64E-36	131	ONI17578.1	hypothetical protein PRUPE_3G168100	Prunus persica	XP_007217629.1

TRINITY_DN19362_c0_g1_i1_3	1,87E-92	284	XP_018832925.1	protein ABC transporter 1, mitochondrial	Juglans regia	KDP28361.1
TRINITY_DN13345_c0_g1_i1_4	1,64E-30	117	XP_018829229.1	glycine--tRNA ligase, mitochondrial 1-like	Juglans regia	XP_018841637.1
TRINITY_DN8412_c0_g1_i2_1	9,54E-104	299	XP_018822047.1	probable ubiquitin-conjugating enzyme E2 16	Juglans regia	OAY60809.1
TRINITY_DN15351_c0_g1_i1_3	7,06E-79	248	OAY49651.1	hypothetical protein MANES_05G072200	Manihot esculenta	ONH93689.1
TRINITY_DN19403_c0_g1_i1_1	0	545	XP_018827627.1	pleiotropic drug resistance protein 1-like	Juglans regia	XP_018827627.1
TRINITY_DN4912_c0_g1_i1_3	0	1057	XP_018817860.1	methylcrotonoyl-CoA carboxylase beta chain, mitochondrial isoform X2	Juglans regia	XP_018817859.1
TRINITY_DN9231_c0_g1_i3_3	3,04E-63	194	XP_018857951.1	acyl carrier protein 3, mitochondrial-like isoform X2	Juglans regia	XP_018857952.1
TRINITY_DN10613_c0_g1_i1_2	0	616	XP_018829679.1	2-methyl-6-phytyl-1,4-hydroquinone methyltransferase, chloroplastic-like isoform X1	Juglans regia	XP_018829680.1
TRINITY_DN3524_c0_g1_i1_2	5,81E-135	395	XP_018847795.1	dnaJ protein P58IPK homolog	Juglans regia	XP_008369755.1
TRINITY_DN3963_c0_g1_i1_1	0	1107	XP_018842470.1	probable glutamyl endopeptidase, chloroplastic isoform X2	Juglans regia	XP_018842472.1
TRINITY_DN1013_c0_g2_i1_6	0	691	XP_018816814.1	mannose-6-phosphate isomerase 1	Juglans regia	KDP32959.1
TRINITY_DN6811_c0_g2_i3_2	0	731	XP_018836625.1	tryptophan--tRNA ligase, cytoplasmic	Juglans regia	XP_019462109.1
TRINITY_DN12201_c0_g2_i1_2	0	542	XP_018849912.1	heterogeneous nuclear ribonucleoprotein 1 isoform X3	Juglans regia	XP_018849912.1
TRINITY_DN8972_c0_g2_i1_1	0	1244	XP_018813427.1	oligopeptide transporter 1-like	Juglans regia	XP_018813417.1
TRINITY_DN4764_c0_g2_i1_1	0	512	XP_009357399.1	chaperone protein dnaJ 50-like	Pyrus x bretschneideri	XP_009340937.1
TRINITY_DN2457_c0_g1_i1_1	5,35E-52	178	XP_018848022.1	sulfite reductase [ferredoxin], chloroplastic	Juglans regia	OAY30100.1
TRINITY_DN11036_c0_g1_i5_1	2,63E-175	503	XP_018821141.1	sugar transport protein 10-like	Juglans regia	XP_018805622.1
TRINITY_DN10961_c0_g1_i3_3	1,01E-88	283	XP_018835052.1	subtilisin-like protease SBT1.4	Juglans regia	XP_008228179.1
TRINITY_DN4707_c0_g3_i1_1	0	636	XP_018827904.1	chitinase domain-containing protein 1	Juglans regia	ONI23379.1
TRINITY_DN10673_c0_g1_i2_3	0	1274	XP_018840640.1	cullin-1-like	Juglans regia	XP_018826086.1
TRINITY_DN9408_c0_g1_i1_6	0	910	XP_018849698.1	glutamine--tRNA ligase-like	Juglans regia	XP_018849699.1
TRINITY_DN1822_c0_g2_i1_4	4,51E-106	324	KYP48152.1	Heat shock protein 90	Cajanus cajan	XP_020234319.1
TRINITY_DN18094_c0_g1_i1_4	0	600	XP_018816462.1	protein transport protein Sec24-like At3g07100	Juglans regia	XP_018816463.1
TRINITY_DN3816_c0_g1_i1_4	0	527	XP_018818080.1	(R,S)-reticuline 7-O-methyltransferase-like	Juglans regia	XP_018832989.1
TRINITY_DN10296_c0_g1_i1_1	1,82E-59	194	XP_018833432.1	nascent polypeptide-associated complex subunit alpha-like protein 2	Juglans regia	XP_018842755.1
TRINITY_DN5361_c0_g2_i1_1	0	715	XP_018823060.1	alpha-mannosidase 2	Juglans regia	XP_008230682.1
TRINITY_DN512_c0_g1_i1_4	8,82E-165	474	XP_018807711.1	putative G3BP-like protein	Juglans regia	XP_018820107.1
TRINITY_DN6953_c0_g1_i1_5	0	787	XP_018850824.1	bifunctional aspartate aminotransferase and glutamate/aspartate-prephenate aminotransferase-like	Juglans regia	XP_018816075.1
TRINITY_DN22332_c0_g1_i1_1	2,96E-35	130	XP_018819162.1	eukaryotic translation initiation factor 4G-like isoform X1	Juglans regia	XP_018819170.1
TRINITY_DN8109_c0_g1_i2_3	2,59E-121	349	XP_018833575.1	uncharacterized protein LOC109000956 isoform X1	Juglans regia	XP_008380623.1

TRINITY_DN9320_c0_g1_i1_1	2,10E-41	136	AFK48356.1	unknown	Medicago truncatula	OIV93135.1
TRINITY_DN3842_c0_g1_i1_3	2,28E-46	152	XP_018830962.1	uncharacterized protein LOC108998731 isoform X1	Juglans regia	XP_018830963.1
TRINITY_DN19595_c0_g1_i1_5	5,34E-68	208	ACJ85775.1	unknown	Medicago truncatula	KHN34065.1
TRINITY_DN12651_c0_g2_i1_3	0	769	XP_018815003.1	histidinol-phosphate aminotransferase, chloroplastic like	Juglans regia	XP_018815004.1
TRINITY_DN5781_c0_g1_i1_5	0	553	XP_018828503.1	protein TIC110, chloroplastic isoform X1	Juglans regia	XP_018828504.1
TRINITY_DN14210_c0_g1_i1_2	9,02E-68	226	ONH98837.1	hypothetical protein PRUPE_7G268000	Prunus persica	XP_020423648.1
TRINITY_DN5618_c0_g1_i1_1	4,13E-94	277	XP_018817906.1	NADPH-dependent pterin aldehyde reductase	Juglans regia	XP_012066739.1
TRINITY_DN8203_c0_g2_i1_6	9,49E-29	104	OAY56244.1	hypothetical protein MANES_02G000300	Manihot esculenta	XP_018839983.1
TRINITY_DN4496_c1_g1_i1_2	1,98E-94	277	XP_017421783.1	glucose-6-phosphate isomerase 1, chloroplastic	Vigna angularis	KHN26125.1
TRINITY_DN13442_c0_g1_i1_1						
TRINITY_DN7081_c1_g1_i1_1	8,67E-40	141	XP_018816821.1	2-hydroxyacyl-CoA lyase	Juglans regia	XP_008353818.1
TRINITY_DN2528_c0_g1_i2_1	0	874	XP_008232788.1	DEAD-box ATP-dependent RNA helicase 8	Prunus mume	ONI23005.1
TRINITY_DN6839_c0_g2_i1_4	0	727	AJA36505.1	KUP15	Prunus persica	AJA36506.1
TRINITY_DN13687_c0_g1_i1_2	3,25E-62	194	XP_018839161.1	early nodulin-like protein 1	Juglans regia	OAY53057.1
TRINITY_DN7339_c0_g1_i1_1	3,14E-81	241	XP_018812852.1	uncharacterized protein At2g34160-like	Juglans regia	OAY27368.1
TRINITY_DN5933_c0_g1_i3_1	1,55E-55	175	XP_018834211.1	molybdopterin synthase sulfur carrier subunit	Juglans regia	XP_018834212.1
TRINITY_DN10134_c0_g1_i3_5	0	532	XP_018837640.1	probable tocopherol O-methyltransferase, chloroplastic isoform X4	Juglans regia	XP_018837639.1
TRINITY_DN5831_c0_g1_i1_3	1,74E-47	165	XP_018840476.1	long chain acyl-CoA synthetase 6, peroxisomal-like	Juglans regia	OAY42281.1
TRINITY_DN8464_c0_g1_i3_6	8,16E-65	201	XP_018805629.1	uncharacterized protein LOC108979405, partial	Juglans regia	XP_008339881.1
TRINITY_DN11887_c0_g2_i1_4	7,81E-54	178	XP_018825160.1	ubiquitin-like modifier-activating enzyme 5	Juglans regia	XP_009368530.1
TRINITY_DN3833_c0_g1_i1_2	1,07E-106	311	XP_018838204.1	heme-binding protein 1	Juglans regia	ONI03605.1
TRINITY_DN10145_c0_g1_i1_3	1,35E-81	244	XP_018813174.1	bifunctional adenosine 5'-phosphosulfate phosphorylase/adenylylsulfatase HINT4	Juglans regia	XP_009334507.1
TRINITY_DN7100_c0_g1_i2_3	6,00E-95	281	XP_018853745.1	vesicle-associated protein 1-3-like	Juglans regia	XP_018820386.1
TRINITY_DN23044_c0_g1_i1_1	5,28E-42	149	XP_018830702.1	LOW QUALITY PROTEIN: tripeptidyl-peptidase 2-like	Juglans regia	XP_018828368.1
TRINITY_DN13518_c0_g1_i1_4	5,33E-91	292	XP_016179809.2	UDP-glucose:glycoprotein glucosyltransferase isoform X1	Arachis ipaensis	XP_016179810.1
TRINITY_DN19196_c0_g1_i1_5	3,25E-27	104	XP_008242033.1	peroxidase 64	Prunus mume	XP_018829560.1
TRINITY_DN3817_c0_g1_i1_5	5,91E-44	145	XP_018819823.1	MFP1 attachment factor 1-like	Juglans regia	KOM51468.1
TRINITY_DN4458_c0_g1_i1_2	5,24E-68	208	ACU15091.1	unknown, partial	Glycine max	XP_012076009.1
TRINITY_DN7255_c0_g1_i1_3	0	525	XP_018833509.1	uncharacterized protein LOC109000915 isoform X1	Juglans regia	ONI24868.1
TRINITY_DN6045_c0_g1_i3_1	3,49E-133	392	XP_018824101.1	alpha-aminoadipic semialdehyde synthase isoform X3	Juglans regia	XP_018824100.1

TRINITY_DN11230_c0_g1_i2_6	2,30E-105	318	XP_018824793.1	ethylene-responsive transcription factor 1-like	Juglans regia	XP_018817988.1
TRINITY_DN3420_c0_g1_i2_3	6,78E-144	408	XP_018828952.1	uncharacterized protein LOC108997227 isoform X3	Juglans regia	XP_018828950.1
TRINITY_DN11146_c0_g1_i2_3	0	680	XP_008222614.2	PTI1-like tyrosine-protein kinase 1 isoform X1	Prunus mume	ONI29237.1
TRINITY_DN9423_c0_g3_i2_1	7,00E-99	288	XP_018851087.1	uncharacterized protein At3g49720-like	Juglans regia	XP_018816519.1
TRINITY_DN9788_c0_g1_i1_3	0	810	XP_018835689.1	26S protease regulatory subunit 6B homolog	Juglans regia	XP_018828576.1
TRINITY_DN2628_c0_g2_i1_1	7,96E-33	124	XP_018809515.1	pantothenate kinase 2 isoform X2	Juglans regia	XP_018809511.1
TRINITY_DN9299_c0_g2_i1_3	0	618	XP_018841021.1	nudix hydrolase 19, chloroplastic isoform X2	Juglans regia	XP_018841020.1
TRINITY_DN11235_c0_g2_i1_1	0	699	XP_018843251.1	VHS domain-containing protein At3g16270	Juglans regia	XP_018843252.1
TRINITY_DN5744_c1_g1_i2_2	8,78E-97	284	XP_018825081.1	gamma carbonic anhydrase-like 2, mitochondrial	Juglans regia	XP_008239494.1
TRINITY_DN5626_c0_g2_i1_2	1,98E-138	400	XP_018822742.1	uncharacterized protein LOC108992601 isoform X1	Juglans regia	XP_008240630.1
TRINITY_DN11440_c0_g1_i3_4	0	681	XP_018852211.1	26S proteasome non-ATPase regulatory subunit 13 homolog A	Juglans regia	XP_018852212.1
TRINITY_DN11350_c0_g1_i3_2	0	678	XP_018834582.1	3-dehydroquinate synthase, chloroplastic-like	Juglans regia	XP_018832026.1
TRINITY_DN11359_c0_g1_i1_2	0	612	XP_018848058.1	phosphoglucan phosphatase DSP4, amyloplastic-like isoform X2	Juglans regia	XP_018807428.1
TRINITY_DN9373_c0_g1_i1_6	1,69E-07	48,9	XP_018823891.1	NADH-ubiquinone oxidoreductase 20.9 kDa subunit-like	Juglans regia	XP_016196445.1
TRINITY_DN12458_c0_g3_i1_3	0	827	XP_018835696.1	DEAD-box ATP-dependent RNA helicase 52A-like isoform X1	Juglans regia	ONH99629.1
TRINITY_DN5495_c0_g1_i2_2	0	578	ONI22818.1	hypothetical protein PRUPE_2G153000	Prunus persica	XP_007218197.1
TRINITY_DN12738_c0_g1_i1_1	2,69E-106	331	XP_018824098.1	alpha-aminoadipic semialdehyde synthase isoform X1	Juglans regia	XP_008363056.1
TRINITY_DN7345_c0_g1_i1_4	0	665	XP_018810471.1	COP9 signalosome complex subunit 1-like	Juglans regia	OAY49230.1
TRINITY_DN6917_c0_g1_i1_3	0	816	XP_018830113.1	glutamate-1-semialdehyde 2,1-aminomutase 2, chloroplastic	Juglans regia	OAY47995.1
TRINITY_DN9153_c0_g1_i1_3	0	880	XP_018810540.1	probable glucan 1,3-alpha-glucosidase	Juglans regia	XP_018810541.1
TRINITY_DN19500_c0_g1_i1_1	1,42E-82	250	KDP42130.1	hypothetical protein JCGZ_01918	Jatropha curcas	XP_012067122.1
TRINITY_DN12333_c0_g1_i1_2	2,50E-140	403	XP_018836373.1	uncharacterized protein LOC109002903 isoform X1	Juglans regia	XP_008340413.1
TRINITY_DN10789_c0_g1_i1_1	8,07E-113	338	XP_018851719.1	methyl-CpG-binding domain-containing protein 11-like isoform X1	Juglans regia	XP_018851720.1
TRINITY_DN2912_c0_g2_i1_4	9,21E-119	345	XP_008222776.1	protein TIC 22-like, chloroplastic	Prunus mume	ONI28935.1
TRINITY_DN14787_c0_g1_i1_6	1,51E-131	387	OIW13021.1	hypothetical protein TanjilG_15470	Lupinus angustifolius	XP_019441253.1
TRINITY_DN11096_c0_g1_i1_1	1,99E-71	216	XP_018823473.1	prefoldin subunit 1	Juglans regia	XP_008351369.1
TRINITY_DN5451_c0_g1_i1_6	1,27E-41	140	CAZ77321.1	unnamed protein product	Lupinus luteus	CBD28206.1

TRINITY_DN11157_c0_g2_i5_4	2,72E-121	349	XP_018848970.1	phospho-2-dehydro-3-deoxyheptonate aldolase 1, chloroplastic-like	Juglans regia	XP_018858624.1
TRINITY_DN8090_c0_g3_i1_2	1,14E-94	280	ONI32925.1	hypothetical protein PRUPE_1G394300	Prunus persica	XP_007223519.1
TRINITY_DN5532_c0_g1_i1_3	0	796	XP_018857848.1	ATP-citrate synthase alpha chain protein 2	Juglans regia	XP_018805227.1
TRINITY_DN8331_c0_g2_i1_3	9,82E-58	187	XP_015933877.1	remorin	Arachis duranensis	XP_016178691.1
TRINITY_DN7840_c1_g1_i1_1	5,63E-103	313	XP_008219827.1	aspartate--tRNA ligase 2, cytoplasmic	Prunus mume	KYP72364.1
TRINITY_DN1523_c0_g1_i1_1	0	772	ONH94961.1	hypothetical protein PRUPE_7G042400	Prunus persica	XP_007202012.1
TRINITY_DN14177_c0_g1_i1_3	4,66E-39	140	XP_018822930.1	glycerol-3-phosphate dehydrogenase SDP6, mitochondrial-like	Juglans regia	XP_018851614.1
TRINITY_DN10869_c1_g1_i2_5	1,77E-139	395	XP_018850618.1	thaumatin-like protein 1	Juglans regia	XP_018851425.1
TRINITY_DN5473_c0_g1_i1_5	0	514	XP_018842494.1	uncharacterized protein LOC109007319	Juglans regia	ONH94022.1
TRINITY_DN21224_c0_g1_i1_2	1,15E-54	185	XP_018814176.1	mitochondrial Rho GTPase 1-like isoform X2	Juglans regia	XP_018814175.1
TRINITY_DN6919_c0_g2_i1_3	3,17E-56	185	XP_018825658.1	probable pectinesterase 67	Juglans regia	KYP40464.1
TRINITY_DN6224_c0_g1_i1_1	0	771	XP_004486807.1	serine/threonine protein phosphatase 2A 55 kDa regulatory subunit B beta isoform-like isoform X2	Cicer arietinum	XP_008228325.1
TRINITY_DN1121_c0_g1_i1_3	6,36E-67	207	XP_018845676.1	putative RNA methyltransferase At5g10620 isoform X2	Juglans regia	XP_019447505.1
TRINITY_DN1480_c0_g1_i1_2	2,47E-88	278	ONI00157.1	hypothetical protein PRUPE_6G071100	Prunus persica	XP_020420760.1
TRINITY_DN12167_c0_g1_i1_5	2,43E-70	223	OAY52283.1	hypothetical protein MANES_04G070800	Manihot esculenta	OAY52284.1
TRINITY_DN458_c0_g1_i1_5	6,18E-39	142	KEH28616.1	coatomer subunit beta	Medicago truncatula	XP_013454585.1
TRINITY_DN2404_c0_g1_i1_2	0	883	XP_018824574.1	E3 UFM1-protein ligase 1 homolog isoform X1	Juglans regia	XP_018824575.1
TRINITY_DN9978_c0_g1_i1_2	2,94E-68	208	XP_018826018.1	NADH dehydrogenase [ubiquinone 1] alpha subcomplex subunit 8-B-like	Juglans regia	XP_018840578.1
TRINITY_DN8843_c0_g1_i3_1	1,21E-170	483	XP_018815020.1	protein-tyrosine-phosphatase PTP1 isoform X1	Juglans regia	XP_018815022.1
TRINITY_DN6703_c0_g1_i1_2	0	536	XP_008219371.1	phosphoribosylaminoimidazole-succinocarboxamide synthase, chloroplastic	Prunus mume	XP_008338902.1
TRINITY_DN11300_c0_g1_i1_3	0	1034	XP_018809451.1	acetate/butyrate--CoA ligase AAE7, peroxisomal	Juglans regia	XP_016197951.1
TRINITY_DN3617_c0_g3_i1_5	4,38E-71	215	XP_018827356.1	bet1-like SNARE 1-1	Juglans regia	XP_020213032.1
TRINITY_DN10446_c0_g2_i1_1	0	1178	XP_018824324.1	dynamin-related protein 5A	Juglans regia	XP_004503616.1
TRINITY_DN14501_c0_g1_i1_3	1,13E-73	230	XP_018825627.1	exopolygalacturonase-like	Juglans regia	XP_018809534.1
TRINITY_DN13753_c0_g1_i1_5	1,07E-168	474	XP_009344408.1	ADP-ribosylation factor GTPase-activating protein AGD12-like	Pyrus x bretschneideri	XP_009344410.1
TRINITY_DN18831_c0_g1_i1_1	4,67E-14	69,3	XP_018805269.1	uncharacterized protein LOC108979131	Juglans regia	XP_018836814.1
TRINITY_DN7892_c0_g1_i3_1	3,57E-27	107	XP_018844545.1	omega-amidase, chloroplastic	Juglans regia	XP_008377810.1
TRINITY_DN6230_c0_g1_i1_1	4,06E-111	322	XP_018832001.1	Golgi apparatus membrane protein-like protein ECHIDNA isoform X1	Juglans regia	XP_018816227.1
TRINITY_DN18774_c0_g1_i1_4	7,02E-75	244	XP_009359468.1	protein WEAK CHLOROPLAST MOVEMENT UNDER BLUE LIGHT 1-like	Pyrus x bretschneideri	XP_008220219.1
TRINITY_DN1997_c0_g1_i1_3	1,07E-79	238	XP_018855392.1	cytochrome P450 CYP82H23-like, partial	Juglans regia	XP_018857298.1

TRINITY_DN7250_c0_g1_i1_5	1,14E-143	408	XP_018806025.1	adenylyl-sulfate kinase 3-like isoform X2	Juglans regia	XP_018806026.1
TRINITY_DN15321_c0_g1_i1_5	2,80E-42	145	KDP25182.1	hypothetical protein JCGZ_20338	Jatropha curcas	XP_012086572.1
TRINITY_DN19751_c0_g1_i1_1	2,34E-72	238	XP_018846984.1	calcium-transporting ATPase 4, plasma membrane-type-like isoform X2	Juglans regia	XP_018846983.1
TRINITY_DN3119_c0_g1_i1_5	5,76E-176	501	XP_018809797.1	eukaryotic translation initiation factor 2A	Juglans regia	XP_018809798.1
TRINITY_DN2586_c0_g2_i1_1	2,12E-43	147	OAY34831.1	hypothetical protein MANES_12G050000	Manihot esculenta	OAY34825.1
TRINITY_DN18819_c0_g1_i1_4	1,06E-77	231	XP_018821680.1	protein translation factor SUI1 homolog 1	Juglans regia	XP_018853744.1
TRINITY_DN13993_c0_g1_i1_4	2,35E-66	221	XP_018817804.1	leucine--tRNA ligase, cytoplasmic isoform X1	Juglans regia	XP_018817805.1
TRINITY_DN20785_c0_g1_i1_2	4,42E-45	154	ONH93280.1	hypothetical protein PRUPE_8G222800	Prunus persica	XP_007199901.1
TRINITY_DN11337_c0_g1_i4_2	1,75E-95	283	XP_015951555.1	uncharacterized protein LOC107476272	Arachis duranensis	KHN35544.1
TRINITY_DN8955_c0_g1_i4_2	1,77E-104	306	XP_018831621.1	germin-like protein subfamily T member 2	Juglans regia	XP_018832655.1
TRINITY_DN12162_c0_g1_i1_3	0	612	XP_018809437.1	golgin candidate 6 isoform X2	Juglans regia	XP_018809435.1
TRINITY_DN9518_c0_g2_i2_1	1,48E-43	147	XP_018817358.1	ATP-dependent Clp protease proteolytic subunit 2, mitochondrial	Juglans regia	KYP68403.1
TRINITY_DN23667_c0_g1_i1_6	2,74E-33	121	ONI16679.1	hypothetical protein PRUPE_3G115000	Prunus persica	ONI16680.1
TRINITY_DN15391_c0_g1_i1_3	2,31E-52	187	XP_018854869.1	eukaryotic translation initiation factor 4G-like isoform X1	Juglans regia	XP_018854875.1
TRINITY_DN8805_c0_g1_i2_1	0	632	XP_018821343.1	IST1 homolog	Juglans regia	XP_018809491.1
TRINITY_DN7176_c0_g1_i3_3	3,57E-88	261	AFK36986.1	unknown	Lotus japonicus	KDP31052.1
TRINITY_DN5898_c0_g1_i1_1	1,78E-54	171	XP_018830593.1	mitochondrial import inner membrane translocase subunit TIM10-like	Juglans regia	XP_018830594.1
TRINITY_DN8672_c0_g2_i1_3	7,76E-97	283	XP_018828972.1	probable prefoldin subunit 5	Juglans regia	KDP37534.1
TRINITY_DN1176_c0_g1_i1_6	1,71E-40	145	XP_008221172.1	uncharacterized protein LOC103321167 isoform X3	Prunus mume	XP_020420490.1
TRINITY_DN4088_c0_g1_i1_6	0	843	XP_018808493.1	mechanosensitive ion channel protein 1, mitochondrial	Juglans regia	XP_018808494.1
TRINITY_DN7361_c0_g1_i1_3	6,08E-69	208	XP_018852394.1	cytochrome b5	Juglans regia	OAY59358.1
TRINITY_DN6371_c0_g1_i2_5	0	619	XP_018835872.1	SEC12-like protein 2	Juglans regia	XP_018835880.1
TRINITY_DN152_c0_g1_i1_1	6,67E-132	401	XP_018837083.1	probable sucrose-phosphate synthase 2	Juglans regia	ONI28760.1
TRINITY_DN211_c0_g1_i1_2	2,00E-110	322	XP_008393051.1	ubiquitin-conjugating enzyme E2-23 kDa-like	Malus domestica	XP_008393052.1
TRINITY_DN10187_c0_g2_i3_3	0	580	XP_018840945.1	Golgi to ER traffic protein 4 homolog	Juglans regia	ONI24006.1
TRINITY_DN10966_c1_g2_i1_2	4,33E-105	314	XP_018819428.1	phenylalanine--tRNA ligase beta subunit, cytoplasmic-like	Juglans regia	XP_018806301.1
TRINITY_DN17047_c0_g1_i1_4	8,33E-88	274	XP_018851837.1	outer envelope protein 61	Juglans regia	XP_008230607.1
TRINITY_DN2953_c0_g1_i1_6	3,38E-143	447	XP_018850497.1	uncharacterized protein LOC109013033	Juglans regia	XP_018850498.1
TRINITY_DN10123_c0_g1_i1_2	0	708	XP_018829285.1	tyrosine--tRNA ligase 1, cytoplasmic	Juglans regia	KOM45120.1
TRINITY_DN6691_c0_g1_i1_3	0	654	XP_018849759.1	ALG-2 interacting protein X-like	Juglans regia	XP_018849760.1
TRINITY_DN9992_c0_g1_i2_2	1,12E-111	323	XP_018844469.1	N-alpha-acetyltransferase daf-31	Juglans regia	XP_016171058.1
TRINITY_DN10481_c0_g2_i1_2	3,64E-112	326	XP_018850886.1	ribulose-phosphate 3-epimerase, chloroplastic	Juglans regia	AAM19354.1
TRINITY_DN23047_c0_g1_i1_3	1,05E-25	97,8	XP_018837336.1	14 kDa zinc-binding protein	Juglans regia	ONI11561.1

TRINITY_DN12530_c0_g1_i1_3	3,04E-52	175	XP_018820769.1	probable inactive purple acid phosphatase 27 isoform X3	Juglans regia	XP_018820768.1
TRINITY_DN5479_c0_g1_i1_1	0	837	XP_018847636.1	ornithine aminotransferase, mitochondrial-like	Juglans regia	XP_018819712.1
TRINITY_DN14670_c0_g1_i1_5	7,24E-65	206	ACU18645.1	unknown	Glycine max	XP_003523980.1
TRINITY_DN7295_c0_g1_i1_1	0	721	OAY30633.1	hypothetical protein MANES_14G046600	Manihot esculenta	ONI07879.1
TRINITY_DN9388_c0_g1_i2_3	0	729	XP_018849256.1	sorting nexin 1-like isoform X1	Juglans regia	XP_018849257.1
TRINITY_DN9217_c0_g1_i2_3	0	637	XP_018830975.1	uncharacterized protein LOC108998739	Juglans regia	ONI13861.1
TRINITY_DN11220_c1_g2_i10_1	2,18E-72	223	XP_018841297.1	tropinone reductase homolog At5g06060-like	Juglans regia	XP_008223763.1
TRINITY_DN7324_c0_g1_i1_3	0	738	XP_018809755.1	katanin p60 ATPase-containing subunit A-like 2 isoform X2	Juglans regia	XP_018809754.1
TRINITY_DN8489_c0_g2_i1_1	1,07E-85	260	KDP26135.1	hypothetical protein JCGZ_22236	Jatropha curcas	XP_012086081.1
TRINITY_DN9496_c0_g2_i1_6	4,17E-106	308	XP_018829252.1	uncharacterized protein LOC108997431	Juglans regia	XP_008390244.1
TRINITY_DN8940_c0_g2_i1_4	1,44E-88	262	XP_018817485.1	uncharacterized protein LOC108988625	Juglans regia	ONI10985.1
TRINITY_DN2829_c0_g1_i1_4	1,88E-180	504	XP_018812633.1	haloacid dehalogenase-like hydrolase domain-containing protein At3g48420	Juglans regia	XP_018812634.1
TRINITY_DN8775_c0_g1_i1_6	0	717	ONI03611.1	hypothetical protein PRUPE_6G269000	Prunus persica	XP_007205244.1
TRINITY_DN4667_c0_g1_i2_3	3,98E-64	198	XP_018819521.1	yrdC domain-containing protein, mitochondrial isoform X4	Juglans regia	XP_018819522.1
TRINITY_DN22872_c0_g1_i1_1	1,26E-53	179	XP_007153579.1	hypothetical protein PHAVU_003G0475001g, partial	Phaseolus vulgaris	XP_007153579.1
TRINITY_DN19824_c0_g1_i1_3	1,38E-108	317	KDP24985.1	hypothetical protein JCGZ_24314	Jatropha curcas	XP_012087520.2
TRINITY_DN9699_c0_g1_i1_4	0	676	XP_018813478.1	ribose-phosphate pyrophosphokinase 4-like isoform X1	Juglans regia	ONI30368.1
TRINITY_DN3755_c0_g2_i1_3	0	1023	XP_018808112.1	zinc-binding alcohol dehydrogenase domain-containing protein 2 isoform X1	Juglans regia	XP_018808113.1
TRINITY_DN11202_c0_g1_i2_2	4,66E-61	190	XP_018829244.1	NADH dehydrogenase [ubiquinone 1] alpha subcomplex subunit 2-like	Juglans regia	XP_018841602.1
TRINITY_DN6447_c0_g1_i1_4	0	800	XP_018841179.1	allantoate deiminase	Juglans regia	XP_012088571.1
TRINITY_DN5808_c0_g1_i1_2	0	919	XP_018844988.1	nicotinate phosphoribosyltransferase 1	Juglans regia	OIW09714.1
TRINITY_DN10647_c0_g2_i1_5	2,37E-109	321	XP_008221708.1	glutathione S-transferase U17-like	Prunus mume	XP_018819149.1
TRINITY_DN14457_c0_g1_i1_4	7,15E-116	353	XP_018824311.1	importin subunit beta-1-like	Juglans regia	XP_018835204.1
TRINITY_DN7706_c0_g1_i1_3	4,45E-171	481	XP_018819194.1	40S ribosomal protein S4-3	Juglans regia	AES86765.1
TRINITY_DN2695_c0_g1_i1_6	2,35E-136	394	XP_018824898.1	glycerophosphodiester phosphodiesterase GDPD6	Juglans regia	XP_008374638.1
TRINITY_DN3350_c0_g1_i1_2	5,68E-51	166	XP_018847236.1	cell wall / vacuolar inhibitor of fructosidase 1-like	Juglans regia	KHN03269.1
TRINITY_DN11297_c1_g1_i1_2	1,98E-106	313	XP_018828854.1	protein CREG1	Juglans regia	ONI19412.1
TRINITY_DN11354_c1_g1_i3_1	0	546	XP_018806278.1	hsp70-binding protein 1	Juglans regia	KDP35664.1
TRINITY_DN12695_c0_g2_i1_3	8,51E-179	513	XP_018835532.1	multiple organellar RNA editing factor 1, mitochondrial	Juglans regia	XP_009355259.1
TRINITY_DN12034_c0_g1_i1_1	1,29E-101	308	XP_018808625.1	serine/threonine-protein phosphatase 5 isoform X1	Juglans regia	XP_018808633.1

TRINITY_DN4302_c0_g1_i1_3	0	817	XP_018852306.1	endoglucanase 10	Juglans regia	OAY50597.1
TRINITY_DN18986_c0_g1_i1_2	7,02E-54	179	KOM31605.1	hypothetical protein LR48_Vigan01g116000	Vigna angularis	XP_018834214.1
TRINITY_DN16321_c0_g1_i1_6	2,62E-62	209	XP_018846305.1	BEACH domain-containing protein C2 isoform X2	Juglans regia	XP_018846304.1
TRINITY_DN10277_c0_g1_i1_3	0	597	XP_016181742.1	uncharacterized protein LOC107623862	Arachis ipaensis	XP_015943541.1
TRINITY_DN16791_c0_g1_i1_4	2,28E-32	122	XP_018848119.1	acid beta-fructofuranosidase-like	Juglans regia	XP_018844424.1
TRINITY_DN7314_c0_g1_i1_1	6,74E-56	180	XP_018839925.1	dormancy-associated protein homolog 4 isoform X1	Juglans regia	XP_018839926.1
TRINITY_DN11345_c0_g1_i1_5	4,08E-46	150	XP_018830962.1	uncharacterized protein LOC108998731 isoform X1	Juglans regia	XP_017192091.1
TRINITY_DN23429_c0_g1_i1_1	1,00E-42	151	XP_018816462.1	protein transport protein Sec24-like At3g07100	Juglans regia	XP_018816463.1
TRINITY_DN4338_c0_g2_i1_1	4,64E-123	377	XP_018843379.1	protein ROOT HAIR DEFECTIVE 3-like	Juglans regia	XP_018859440.1
TRINITY_DN13879_c0_g1_i1_4	1,13E-74	231	XP_018825514.1	uncharacterized protein LOC108994665	Juglans regia	KDP43637.1
TRINITY_DN1790_c0_g1_i1_2	8,02E-51	169	OAY58248.1	hypothetical protein MANES_02G161800	Manihot esculenta	KDP32782.1
TRINITY_DN5067_c0_g1_i1_3	2,12E-168	478	XP_018813685.1	protein CIA1-like	Juglans regia	XP_018813685.1
TRINITY_DN9550_c0_g1_i1_3	0	640	XP_018859242.1	flowering locus K homology domain-like	Juglans regia	XP_008218287.1
TRINITY_DN5828_c0_g1_i2_4	1,49E-99	298	XP_018856589.1	vacuolar protein sorting-associated protein 20 homolog 2-like	Juglans regia	XP_018827788.1
TRINITY_DN20109_c0_g1_i1_4	1,25E-61	201	XP_018852796.1	urease-like, partial	Juglans regia	XP_018857605.1
TRINITY_DN11375_c0_g1_i1_1	1,29E-65	202	XP_018854926.1	uncharacterized protein LOC109017055	Juglans regia	XP_018819075.1
TRINITY_DN4236_c0_g1_i1_1	8,46E-25	98,2	XP_018810319.1	uncharacterized protein LOC108983212	Juglans regia	XP_009340616.1
TRINITY_DN4156_c0_g1_i1_1	1,47E-48	156	KDP41325.1	hypothetical protein JCGZ_15732	Jatropha curcas	XP_012067811.1
TRINITY_DN11786_c0_g1_i1_3	2,17E-91	286	XP_018811352.1	heat shock 70 kDa protein 16-like	Juglans regia	XP_018811359.1
TRINITY_DN15397_c0_g1_i1_3	1,04E-42	143	XP_008372179.1	bifunctional aspartokinase/homoserine dehydrogenase 1, chloroplastic-like	Malus domestica	KOM31605.1
TRINITY_DN5555_c0_g1_i2_1	0	996	AIR95612.1	4-coumarate:CoA ligase-like protein	Betula pendula	XP_018858801.1
TRINITY_DN6669_c0_g2_i2_2	6,22E-148	418	XP_018859721.1	uncharacterized protein LOC109021532	Juglans regia	XP_020218942.1
TRINITY_DN5818_c0_g1_i1_3	2,21E-123	355	XP_018805705.1	cysteine synthase	Juglans regia	OAY44160.1
TRINITY_DN10622_c0_g1_i3_1	0	902	XP_018819971.1	KH domain-containing protein HEN4-like isoform X1	Juglans regia	XP_018819972.1
TRINITY_DN6444_c0_g1_i1_1	7,62E-69	219	XP_018852566.1	uncharacterized protein LOC109014492 isoform X1	Juglans regia	XP_018836633.1
TRINITY_DN8167_c0_g1_i2_2	0	692	KDP33409.1	hypothetical protein JCGZ_06980	Jatropha curcas	XP_018845609.1
TRINITY_DN10005_c0_g1_i2_3	3,60E-37	127	XP_018852254.1	glycine-rich RNA-binding protein 4, mitochondrial	Juglans regia	XP_009355558.1
TRINITY_DN18506_c0_g1_i1_1	5,32E-40	137	XP_018821736.1	uncharacterized protein At2g39795, mitochondrial-like	Juglans regia	XP_015931449.1
TRINITY_DN9802_c0_g1_i3_2	6,47E-102	303	XP_018841149.1	pectinesterase 31 isoform X1	Juglans regia	XP_008230805.1
TRINITY_DN11201_c1_g1_i1_2	0	585	XP_008382818.1	very-long-chain enoyl-CoA reductase	Malus domestica	XP_018814522.1
TRINITY_DN2574_c0_g1_i1_2	1,28E-78	245	XP_018844777.1	beta-glucuronosyltransferase GlcAT14B	Juglans regia	KDP45456.1
TRINITY_DN2926_c0_g2_i1_1	9,90E-66	207	XP_008353737.1	ketol-acid reductoisomerase, chloroplastic	Malus domestica	XP_018838530.1
TRINITY_DN1172_c0_g1_i1_3	5,20E-57	182	XP_018831728.1	CBL-interacting serine/threonine-protein kinase 9-like isoform X5	Juglans regia	XP_018831726.1

TRINITY_DN15441_c0_g1_i1_4	1,06E-69	234	XP_008232710.1	BEACH domain-containing protein C2	Prunus mume	XP_016650092.1
TRINITY_DN2072_c0_g1_i1_5	5,36E-125	379	XP_018846984.1	calcium-transporting ATPase 4, plasma membrane-type-like isoform X2	Juglans regia	XP_018846983.1
TRINITY_DN11308_c0_g1_i1_4	9,38E-104	301	XP_007147409.1	hypothetical protein PHAVU_006G122000g	Phaseolus vulgaris	ESW19403.1
TRINITY_DN11604_c0_g1_i4_3	1,64E-157	444	XP_018838664.1	putative methyltransferase DDB_G0268948	Juglans regia	XP_018807016.1
TRINITY_DN6973_c0_g1_i2_3	0	680	XP_018811852.1	1-acyl-sn-glycerol-3-phosphate acyltransferase 2	Juglans regia	BAE48660.1
TRINITY_DN19024_c0_g1_i1_1	1,04E-115	356	KDP45840.1	hypothetical protein JCGZ_17447	Jatropha curcas	XP_012079840.1
TRINITY_DN8174_c0_g1_i1_3	1,44E-141	407	XP_018828995.1	ER membrane protein complex subunit 10	Juglans regia	KDP26890.1
TRINITY_DN6495_c0_g1_i1_1	0	585	XP_018815300.1	amidophosphoribosyltransferase, chloroplastic	Juglans regia	XP_018841407.1
TRINITY_DN14955_c0_g1_i1_1	8,01E-35	128	XP_008361531.1	bifunctional aspartokinase/homoserine dehydrogenase 1, chloroplastic-like isoform X2	Malus domestica	XP_008361530.1
TRINITY_DN5794_c0_g1_i3_3	0	781	XP_008239702.1	mannose-1-phosphate guanyltransferase alpha	Prunus mume	ONI08471.1
TRINITY_DN5657_c0_g1_i1_3	7,75E-156	441	XP_018829841.1	translin-like	Juglans regia	XP_008243962.1
TRINITY_DN20134_c0_g1_i1_2	9,06E-25	100	XP_018825160.1	ubiquitin-like modifier-activating enzyme 5	Juglans regia	XP_009368530.1
TRINITY_DN5823_c1_g1_i1_3	8,40E-39	139	XP_018806543.1	isoleucine--tRNA ligase, cytoplasmic	Juglans regia	KHN05507.1
TRINITY_DN10611_c0_g2_i1_3	0	876	XP_018822003.1	probable serine protease EDA2	Juglans regia	OAY60835.1
TRINITY_DN8028_c1_g2_i2_1	7,45E-41	143	ONI09830.1	hypothetical protein PRUPE_4G012100	Prunus persica	XP_007213842.1
TRINITY_DN20414_c0_g1_i1_1	3,64E-89	268	XP_018843094.1	uncharacterized protein LOC109007740 isoform X2	Juglans regia	XP_018843093.1
TRINITY_DN18416_c0_g1_i1_1	1,63E-125	364	XP_018834855.1	syntaxin-22-like	Juglans regia	KYP63599.1
TRINITY_DN5396_c0_g2_i1_1	3,77E-38	134	XP_018843535.1	uncharacterized protein LOC109008046	Juglans regia	XP_018832195.1
TRINITY_DN4301_c0_g1_i1_1	2,10E-120	352	XP_018814804.1	protein phosphatase 1 regulatory subunit pprA	Juglans regia	XP_004503473.1
TRINITY_DN6535_c0_g1_i1_1	1,21E-100	308	XP_018848856.1	DEAD-box ATP-dependent RNA helicase 37-like	Juglans regia	XP_018848857.1
TRINITY_DN5580_c0_g1_i1_4	0	674	XP_018815364.1	SH3 domain-containing protein 2-like	Juglans regia	XP_018841364.1
TRINITY_DN10847_c0_g1_i3_3	7,95E-92	269	XP_018835110.1	coatomer subunit zeta-2-like isoform X2	Juglans regia	XP_018835109.1
TRINITY_DN14525_c0_g1_i1_6	4,39E-116	337	ANG56496.1	cinnamoyl-CoA reductase	Hevea brasiliensis subsp. brasiliensis	OAY46635.1
TRINITY_DN10966_c1_g1_i1_2	2,40E-167	483	XP_018814927.1	phenylalanine--tRNA ligase beta subunit, cytoplasmic-like	Juglans regia	XP_018806301.1
TRINITY_DN12386_c0_g1_i1_4	0	620	XP_018805889.1	uncharacterized protein LOC108979636	Juglans regia	XP_016650601.1
TRINITY_DN3579_c0_g1_i1_4	4,07E-47	153	XP_008343872.1	acyl-coenzyme A thioesterase 13-like	Malus domestica	XP_008383304.1
TRINITY_DN19791_c0_g1_i1_5	1,14E-58	199	XP_018840319.1	coatomer subunit alpha-1-like	Juglans regia	XP_018840320.1
TRINITY_DN10085_c0_g1_i1_3	0	791	XP_018823182.1	serine/threonine-protein kinase STY8-like isoform X1	Juglans regia	XP_018836819.1
TRINITY_DN5782_c0_g1_i1_1	1,51E-91	268	XP_018823137.1	ferredoxin-thioredoxin reductase catalytic chain, chloroplastic	Juglans regia	XP_008230669.1
TRINITY_DN13590_c0_g1_i1_3	4,04E-150	434	XP_018832601.1	ABC transporter G family member 40-like, partial	Juglans regia	XP_018823393.1

TRINITY_DN1136_c0_g2_i1_1	5,48E-106	327	XP_018809439.1	golgin candidate 6 isoform X4	Juglans regia	XP_018809438.1
TRINITY_DN9839_c0_g1_i1_1	0	1237	XP_018835197.1	ABC transporter F family member 3	Juglans regia	OAY56307.1
TRINITY_DN3828_c0_g1_i2_1	7,93E-133	383	XP_018810997.1	electron transfer flavoprotein subunit alpha, mitochondrial	Juglans regia	ONI13147.1
TRINITY_DN800_c0_g1_i1_1	1,47E-61	202	OAY23211.1	hypothetical protein MANES_18G060600	Manihot esculenta	OAY23212.1
TRINITY_DN10682_c0_g1_i2_5	0	659	XP_018812300.1	uncharacterized protein LOC108984712	Juglans regia	ONI30704.1
TRINITY_DN11406_c0_g2_i1_5	0	644	XP_018843822.1	probable protein phosphatase 2C 60 isoform X1	Juglans regia	XP_018843823.1
TRINITY_DN9265_c0_g1_i1_4	1,48E-123	358	XP_018825622.1	heterogeneous nuclear ribonucleoprotein 1	Juglans regia	KDP39313.1
TRINITY_DN3622_c0_g1_i1_2	0	931	XP_018819003.1	uncharacterized protein LOC108989738 isoform X1	Juglans regia	XP_018819004.1
TRINITY_DN4087_c0_g2_i1_1	2,42E-101	305	OIV91038.1	hypothetical protein TanjilG_16998	Lupinus angustifolius	GAU40565.1
TRINITY_DN10280_c0_g2_i1_3	2,81E-59	187	XP_018850000.1	uncharacterized protein LOC109012689	Juglans regia	XP_008371919.1
TRINITY_DN17659_c0_g1_i1_1	1,13E-43	153	XP_018827094.1	dihydroxy-acid dehydratase, chloroplastic-like	Juglans regia	AFK41004.1
TRINITY_DN391_c0_g2_i1_3	5,30E-45	163	OAY25488.1	hypothetical protein MANES_17G099000	Manihot esculenta	OAY25489.1
TRINITY_DN18625_c0_g1_i1_3	6,48E-53	182	XP_018835049.1	uncharacterized protein LOC109001971 isoform X2	Juglans regia	XP_018835048.1
TRINITY_DN15558_c0_g1_i1_3	1,64E-98	292	XP_018817511.1	protein ROOT HAIR DEFECTIVE 3-like	Juglans regia	XP_020224988.1
TRINITY_DN8990_c0_g1_i6_4	8,61E-125	366	XP_018840513.1	ATP-dependent (S)-NAD(P)H-hydrate dehydratase isoform X2	Juglans regia	XP_018840512.1
TRINITY_DN9713_c0_g1_i1_2	2,51E-180	518	XP_018820105.1	uncharacterized protein LOC108990561	Juglans regia	ONI26834.1
TRINITY_DN4_c0_g2_i1_2	1,01E-38	139	XP_016179779.2	clathrin heavy chain 1	Arachis ipaensis	OAY38388.1
TRINITY_DN3956_c0_g2_i1_1	1,87E-94	300	OIW18588.1	hypothetical protein TanjilG_13340	Lupinus angustifolius	XP_019452623.1
TRINITY_DN2951_c0_g1_i1_3	7,74E-79	233	XP_018807777.1	uncharacterized protein LOC108981148	Juglans regia	XP_012073843.1
TRINITY_DN8715_c0_g1_i2_3	9,08E-132	374	XP_008244363.1	trafficking protein particle complex subunit 3	Prunus mume	XP_008345058.1
TRINITY_DN11521_c0_g3_i1_1	1,46E-64	202	KDP42130.1	hypothetical protein JCGZ_01918	Jatropha curcas	XP_012067122.1
TRINITY_DN7431_c0_g1_i1_3	1,13E-103	306	XP_018842322.1	peptidyl-prolyl cis-trans isomerase CYP21-4-like isoform X1	Juglans regia	XP_018822168.1
TRINITY_DN20738_c0_g1_i1_3	5,19E-44	154	XP_018846842.1	long chain acyl-CoA synthetase 6, peroxisomal-like	Juglans regia	XP_018840476.1
TRINITY_DN11007_c0_g1_i2_1	0	888	XP_018807730.1	3-phosphoshikimate 1-carboxyvinyltransferase 2	Juglans regia	ONI22671.1
TRINITY_DN18675_c0_g1_i1_5	5,20E-81	248	XP_018812975.1	mitochondrial import inner membrane translocase subunit TIM50	Juglans regia	XP_018825124.1
TRINITY_DN4569_c0_g1_i2_2	6,37E-93	288	XP_018829629.1	la-related protein 1C-like	Juglans regia	KDP23970.1
TRINITY_DN4303_c0_g1_i1_5	3,13E-140	407	XP_018821634.1	chaperonin CPN60-like 2, mitochondrial isoform X3	Juglans regia	XP_018821632.1
TRINITY_DN11099_c0_g1_i3_2	0	572	XP_018845677.1	uncharacterized protein At2g24330	Juglans regia	XP_018813351.1
TRINITY_DN5386_c0_g1_i1_2	4,17E-136	414	XP_018808480.1	AP-1 complex subunit gamma-2-like isoform X1	Juglans regia	XP_018808481.1
TRINITY_DN3521_c0_g1_i1_6	0	561	XP_018807462.1	membrane-associated 30 kDa protein, chloroplastic-like	Juglans regia	XP_018806924.1

TRINITY_DN13705_c0_g1_i1_5	2,09E-180	504	ONI06351.1	hypothetical protein PRUPE_5G055300	Prunus persica	XP_007209414.1
TRINITY_DN3201_c0_g1_i1_6	0	757	KHN21828.1	Endoplasmic reticulum-Golgi intermediate compartment protein 3	Glycine soja	KRH16700.1
TRINITY_DN9124_c0_g4_i1_6	0	565	XP_018841131.1	protein AIG1-like	Juglans regia	XP_012073509.1
TRINITY_DN10878_c0_g1_i1_2	0	1154	XP_018817683.1	probable methyltransferase PMT2	Juglans regia	XP_018845206.1
TRINITY_DN8588_c0_g1_i3_2	3,33E-170	501	XP_007141284.1	hypothetical protein PHAVU_008G183200g	Phaseolus vulgaris	ESW13278.1
TRINITY_DN8864_c0_g1_i1_1	6,90E-118	350	XP_018859548.1	protein AUXIN RESPONSE 4	Juglans regia	XP_018859549.1
TRINITY_DN18025_c0_g1_i1_5	4,50E-39	131	XP_018830814.1	mitochondrial import inner membrane translocase subunit TIM8	Juglans regia	XP_008239525.1
TRINITY_DN15533_c0_g1_i1_2	1,38E-125	381	XP_018831147.1	dynamin-2A-like	Juglans regia	XP_018834193.1
TRINITY_DN17372_c0_g1_i1_4	0	570	XP_018848560.1	uncharacterized protein LOC109011716	Juglans regia	ONI12261.1
TRINITY_DN10448_c0_g1_i2_3	0	731	XP_018815772.1	signal peptide peptidase-like 3	Juglans regia	XP_018824430.1
TRINITY_DN2556_c0_g1_i2_1	7,85E-166	476	XP_018834738.1	dnaJ homolog subfamily B member 1	Juglans regia	ONI34180.1
TRINITY_DN11115_c0_g1_i1_2	0	706	XP_018813560.1	putative glycosyltransferase 7	Juglans regia	KRH28334.1
TRINITY_DN6126_c0_g1_i2_6	8,67E-179	503	XP_018814173.1	COP9 signalosome complex subunit 6a	Juglans regia	KDP29518.1
TRINITY_DN18060_c0_g1_i1_4	1,45E-112	327	KYP71807.1	Auxin-induced protein PCNT115 family, partial	Cajanus cajan	XP_020212112.1
TRINITY_DN8332_c0_g1_i1_1	4,05E-113	340	XP_018837506.1	glucosidase 2 subunit beta	Juglans regia	XP_017191383.1
TRINITY_DN7043_c0_g3_i1_3	0	632	XP_018823086.1	probable bifunctional methylthioribulose-1-phosphate dehydratase/enolase-phosphatase E1 isoform X1	Juglans regia	XP_018823094.1
TRINITY_DN15047_c0_g1_i1_2	7,09E-70	217	KRH00278.1	hypothetical protein GLYMA_18G203300	Glycine max	KHN20313.1
TRINITY_DN4679_c0_g1_i1_4	2,16E-109	322	XP_018812502.1	putative SNAP25 homologous protein SNAP30	Juglans regia	XP_018859370.1
TRINITY_DN5533_c1_g1_i1_2	2,22E-128	381	XP_018847198.1	protein CASP	Juglans regia	XP_007152793.1
TRINITY_DN3362_c0_g1_i1_3	9,66E-78	239	XP_018846568.1	probable N-acetyl-gamma-glutamyl-phosphate reductase, chloroplastic	Juglans regia	OAY29429.1
TRINITY_DN11453_c1_g2_i1_1	0	1081	KDP44499.1	hypothetical protein JCGZ_16332	Jatropha curcas	XP_012093127.1
TRINITY_DN21448_c0_g1_i1_2	1,06E-30	117	XP_018856633.1	rop guanine nucleotide exchange factor 1-like isoform X2	Juglans regia	XP_018856632.1
TRINITY_DN4482_c0_g1_i2_6	1,09E-36	129	ACJ84157.1	unknown	Medicago truncatula	AFK34625.1
TRINITY_DN3062_c1_g1_i1_5	1,15E-69	219	AFK33869.1	unknown	Lotus japonicus	KRH51322.1
TRINITY_DN21074_c0_g1_i1_5	9,34E-41	145	XP_018842420.1	uncharacterized protein LOC109007267	Juglans regia	XP_018842421.1
TRINITY_DN6202_c0_g1_i1_4	1,93E-95	285	XP_018857622.1	polyadenylate-binding protein RBP45-like	Juglans regia	XP_018857622.1
TRINITY_DN9665_c1_g1_i2_1	1,71E-128	382	XP_018810128.1	D-3-phosphoglycerate dehydrogenase 2, chloroplastic-like	Juglans regia	OIW16971.1
TRINITY_DN23089_c0_g1_i1_2	8,64E-28	108	KDP44879.1	hypothetical protein JCGZ_01379	Jatropha curcas	ADK37758.1
TRINITY_DN4752_c0_g1_i1_6	0	559	XP_018823596.1	TPR repeat-containing thioredoxin TDX	Juglans regia	ONI09381.1
TRINITY_DN6323_c0_g1_i1_4	0	571	XP_018832755.1	putative GPI-anchor transamidase	Juglans regia	XP_019417178.1
TRINITY_DN7547_c0_g1_i1_2	1,74E-106	308	AFK39823.1	unknown	Lotus japonicus	BAN42598.1
TRINITY_DN11503_c0_g1_i5_1	2,54E-144	412	XP_018835591.1	2-hydroxyisoflavanone dehydratase-like	Juglans regia	XP_018835592.1
TRINITY_DN6410_c0_g2_i1_3	3,39E-69	214	XP_018828731.1	60S ribosomal protein L24-like	Juglans regia	XP_018811954.1
TRINITY_DN17585_c0_g1_i1_4	2,79E-127	369	XP_018845112.1	phosducin-like protein 3	Juglans regia	KDP46320.1
TRINITY_DN7272_c0_g1_i2_2	3,34E-85	265	XP_018809797.1	eukaryotic translation initiation factor 2A	Juglans regia	XP_018809798.1

TRINITY_DN8313_c0_g1_i1_1	5,05E-24	95,9	XP_018841597.1	uncharacterized protein LOC109006689	Juglans regia	XP_018829186.1
TRINITY_DN10899_c1_g1_i9_2	3,42E-63	200	XP_018848462.1	B-cell receptor-associated protein 31	Juglans regia	AFK41440.1
TRINITY_DN10964_c0_g2_i1_1	1,55E-30	108	XP_008233544.1	60S ribosomal protein L35	Prunus mume	ONI24141.1
TRINITY_DN5766_c0_g1_i1_2	2,22E-142	407	XP_018849008.1	syntaxin-52-like isoform X1	Juglans regia	XP_018849009.1
TRINITY_DN16709_c0_g1_i1_5	2,06E-30	114	XP_020422794.1	phosphoribosylaminoimidazole carboxylase, chloroplastic isoform X4	Prunus persica	XP_008218293.1
TRINITY_DN20234_c0_g1_i1_2	1,56E-46	162	XP_018858135.1	LOW QUALITY PROTEIN: phospholipase A-2-activating protein	Juglans regia	KDP46216.1
TRINITY_DN13627_c0_g1_i1_4	2,42E-118	358	XP_018831158.1	conserved oligomeric Golgi complex subunit 3	Juglans regia	XP_016651945.1
TRINITY_DN22360_c0_g1_i1_5	1,59E-34	129	XP_018830674.1	uncharacterized protein LOC108998567 isoform X2	Juglans regia	XP_018830673.1
TRINITY_DN5465_c0_g1_i1_6	2,89E-50	160	XP_018822177.1	uncharacterized protein LOC108992164	Juglans regia	OAY23399.1
TRINITY_DN14137_c1_g1_i1_2	2,93E-70	221	OAY48825.1	hypothetical protein MANES_05G008200	Manihot esculenta	OAY48824.1
TRINITY_DN4218_c0_g1_i1_3	1,01E-154	445	OAY36457.1	hypothetical protein MANES_11G022700	Manihot esculenta	XP_018835103.1
TRINITY_DN405_c0_g2_i1_3	1,14E-161	456	XP_018837929.1	uncharacterized protein LOC109004012	Juglans regia	XP_018837016.1
TRINITY_DN2745_c0_g1_i1_1	2,02E-117	340	XP_018852251.1	bifunctional pinoresinol-lariciresinol reductase-like	Juglans regia	AFK36279.1
TRINITY_DN15009_c0_g1_i1_3	6,30E-137	394	KDP36668.1	hypothetical protein JCGZ_07886	Jatropha curcas	XP_012073897.1
TRINITY_DN20316_c0_g1_i1_4	1,77E-70	225	XP_018829351.1	uncharacterized protein LOC108997478 isoform X2	Juglans regia	XP_018829342.1
TRINITY_DN11294_c0_g1_i2_1	0	526	XP_018842849.1	PP2A regulatory subunit TAP46-like isoform X1	Juglans regia	XP_007136517.1
TRINITY_DN19054_c0_g1_i1_3	1,50E-133	383	XP_018818642.1	RNA polymerase II-associated protein 3	Juglans regia	OAY27988.1
TRINITY_DN4034_c0_g2_i1_6	4,16E-70	229	XP_018843108.1	protein lsd90	Juglans regia	XP_007157244.1
TRINITY_DN12290_c0_g1_i1_4	5,32E-140	413	XP_018809084.1	delta-1-pyrroline-5-carboxylate synthase isoform X2	Juglans regia	XP_018809083.1
TRINITY_DN21049_c0_g1_i1_5	8,44E-16	72	XP_014522582.1	uncharacterized protein LOC106779065	Vigna radiata var. radiata	ONH89732.1
TRINITY_DN10192_c0_g2_i2_4	0	626	XP_012081268.1	uncharacterized protein LOC105641356	Jatropha curcas	KDP30151.1
TRINITY_DN5313_c0_g1_i1_6	0	557	XP_018845293.1	outer envelope pore protein 37, chloroplastic-like	Juglans regia	XP_018839352.1
TRINITY_DN1722_c0_g1_i1_6	8,33E-08	50,8	AEC03320.1	thioredoxin H-type 5	Hevea brasiliensis	ACU14710.1
TRINITY_DN594_c0_g1_i1_2	0	543	XP_008222207.1	glucose-6-phosphate 1-dehydrogenase, chloroplastic	Prunus mume	ONI29917.1
TRINITY_DN14750_c0_g1_i1_3	4,10E-48	166	XP_007142735.1	hypothetical protein PHAVU_007G012500g	Phaseolus vulgaris	ESW14729.1
TRINITY_DN10152_c0_g1_i1_3	5,45E-44	145	OAY45736.1	hypothetical protein MANES_07G087100	Manihot esculenta	OAY51398.1
TRINITY_DN7940_c0_g1_i2_6	6,75E-110	320	KDP29643.1	hypothetical protein JCGZ_18805	Jatropha curcas	OAY52335.1
TRINITY_DN3239_c0_g2_i1_3	2,49E-36	136	XP_018817247.1	calcium-transporting ATPase 1, endoplasmic reticulum-type-like	Juglans regia	OAY37891.1
TRINITY_DN4761_c0_g1_i1_3	5,35E-148	422	XP_018821662.1	desumoylating isopeptidase 1-like	Juglans regia	XP_018821664.1
TRINITY_DN8530_c0_g1_i3_3	1,50E-152	443	KDP42293.1	hypothetical protein JCGZ_01617	Jatropha curcas	XP_012066878.1
TRINITY_DN16028_c0_g1_i1_6	7,33E-94	293	XP_018846155.1	vacuolar protein sorting-associated protein 51 homolog	Juglans regia	ONI05445.1

TRINITY_DN515_c0_g1_i1_1	2,74E-48	157	OAY22813.1	hypothetical protein MANES_18G027900	Manihot esculenta	XP_007225947.2
TRINITY_DN9271_c0_g1_i2_1	1,24E-65	201	AFK35102.1	unknown	Lotus japonicus	AFK33904.1
TRINITY_DN9769_c0_g1_i2_3	0	864	XP_018851305.1	switch-associated protein 70	Juglans regia	XP_008229573.1
TRINITY_DN17789_c0_g1_i1_2	0	576	XP_018806102.1	peptidyl-prolyl cis-trans isomerase CYP40-like	Juglans regia	XP_009350556.1
TRINITY_DN17147_c0_g3_i1_3	1,14E-122	350	KDP23628.1	hypothetical protein JCGZ_23461	Jatropha curcas	XP_012089213.1
TRINITY_DN18723_c0_g1_i1_3	6,70E-81	243	XP_018849537.1	mitochondrial outer membrane protein porin 2-like isoform X2	Juglans regia	XP_018849534.1
TRINITY_DN13968_c0_g1_i1_1	0	755	XP_018825846.1	enhancer of mRNA-decapping protein 4-like	Juglans regia	KDP32637.1
TRINITY_DN1050_c0_g1_i1_3	0	580	KDP37591.1	hypothetical protein JCGZ_07937	Jatropha curcas	XP_012072805.1
TRINITY_DN630_c0_g1_i2_2	2,56E-118	341	XP_018813065.1	uncharacterized protein LOC108985285	Juglans regia	XP_009340998.1
TRINITY_DN10963_c0_g1_i1_3	8,72E-144	409	OAY29673.1	hypothetical protein MANES_15G163400	Manihot esculenta	XP_018847825.1
TRINITY_DN10629_c0_g1_i1_1	1,03E-104	305	XP_009347705.1	probable glutathione peroxidase 5 isoform X2	Pyrus x bretschneideri	XP_018501031.1
TRINITY_DN2614_c0_g1_i1_5	0	602	XP_018808786.1	valine--tRNA ligase, mitochondrial 1	Juglans regia	XP_018808787.1
TRINITY_DN11634_c0_g1_i3_1	0	835	XP_018845274.1	probable nucleolar protein 5-2	Juglans regia	XP_018845272.1
TRINITY_DN10531_c0_g2_i1_3	2,16E-174	490	XP_018850584.1	urease accessory protein G	Juglans regia	XP_008225465.1
TRINITY_DN2332_c0_g1_i1_2	0	938	XP_018844104.1	threonine dehydratase biosynthetic, chloroplastic	Juglans regia	XP_008241338.1
TRINITY_DN17272_c0_g1_i1_3	0	804	XP_018817811.1	putative fucosyltransferase-like protein	Juglans regia	KOM52073.1
TRINITY_DN6275_c0_g2_i2_1	5,91E-131	377	XP_018809603.1	syntaxin-22-like	Juglans regia	XP_018809113.1
TRINITY_DN7918_c1_g1_i1_2	3,29E-141	402	XP_018833655.1	phospholipid hydroperoxide glutathione peroxidase 1, chloroplastic-like	Juglans regia	XP_018839583.1
TRINITY_DN10401_c0_g1_i2_1	9,51E-152	427	XP_018849330.1	ras-related protein Rab7	Juglans regia	CAA98171.1
TRINITY_DN6828_c0_g1_i1_3	6,11E-91	282	XP_009371822.1	eukaryotic translation initiation factor 5-like	Pyrus x bretschneideri	XP_009371823.1
TRINITY_DN3006_c0_g1_i2_2	0	1020	KDP37982.1	hypothetical protein JCGZ_04625	Jatropha curcas	XP_012072113.1
TRINITY_DN11284_c0_g1_i1_2	2,71E-14	69,3	OAY49206.1	hypothetical protein MANES_05G037500	Manihot esculenta	OAY49207.1
TRINITY_DN7192_c0_g1_i1_3	0	611	XP_018810921.1	uncharacterized protein LOC108983664	Juglans regia	ONI15001.1
TRINITY_DN8534_c0_g1_i2_3	0	588	XP_018813445.1	probable beta-D-xylosidase 6	Juglans regia	OAY33527.1
TRINITY_DN7045_c0_g1_i2_1	1,08E-137	414	XP_018824574.1	E3 UFM1-protein ligase 1 homolog isoform X1	Juglans regia	XP_015952319.1
TRINITY_DN7890_c0_g1_i3_3	6,00E-172	480	XP_018815495.1	uncharacterized protein LOC108987089	Juglans regia	XP_009344930.1
TRINITY_DN11617_c0_g1_i1_3	0	937	XP_018813477.1	glutamate--glyoxylate aminotransferase 2-like	Juglans regia	XP_018819005.1
TRINITY_DN15269_c0_g1_i1_3	2,21E-79	247	XP_007146874.1	hypothetical protein PHAVU_006G0775000g, partial	Phaseolus vulgaris	ESW18868.1
TRINITY_DN10161_c0_g1_i1_2	0	566	XP_018850255.1	D-amino-acid transaminase, chloroplastic-like	Juglans regia	XP_008226423.1
TRINITY_DN819_c0_g1_i1_5	3,37E-116	340	XP_018828199.1	aspartate aminotransferase, mitochondrial-like isoform X1	Juglans regia	XP_018828201.1
TRINITY_DN13462_c0_g1_i1_3	2,22E-69	214	XP_018809406.1	TIP41-like protein isoform X3	Juglans regia	XP_018809404.1

TRINITY_DN4339_c0_g1_i1_1	1,06E-63	208	XP_016649401.1	serine/threonine protein phosphatase 2A 55 kDa regulatory subunit B beta isoform isoform X2	Prunus mume	XP_008228323.1
TRINITY_DN19938_c0_g1_i1_2	2,28E-22	93,6	XP_008228505.1	MAR-binding filament-like protein 1-1	Prunus mume	XP_018845059.1
TRINITY_DN10594_c0_g1_i4_3	1,33E-124	355	XP_014499883.1	signal peptidase complex catalytic subunit SEC11A-like	Vigna radiata var. radiata	BAT91522.1
TRINITY_DN7751_c0_g1_i1_3	4,10E-86	257	XP_020215321.1	protein C2-DOMAIN ABA-RELATED 9-like	Cajanus cajan	ONI31760.1
TRINITY_DN9327_c0_g1_i3_2	4,88E-165	460	XP_018853705.1	Golgi SNAP receptor complex member 1-2	Juglans regia	OAY62437.1
TRINITY_DN8047_c0_g1_i1_3	4,51E-95	285	XP_018843992.1	vacuolar protein sorting-associated protein 32 homolog 2	Juglans regia	XP_018846598.1
TRINITY_DN10285_c0_g1_i1_1	4,02E-110	322	XP_018850349.1	temperature-induced lipocalin-1	Juglans regia	XP_018850350.1
TRINITY_DN8681_c0_g1_i1_3	0	691	XP_018832099.1	actin cytoskeleton-regulatory complex protein PAN1-like	Juglans regia	XP_018838276.1
TRINITY_DN5586_c0_g1_i1_1	0	855	XP_018850957.1	pentatricopeptide repeat-containing protein At1g80270, mitochondrial-like	Juglans regia	OAY40209.1
TRINITY_DN2976_c0_g1_i1_3	0	585	XP_018840746.1	putative kinase-like protein TMKL1	Juglans regia	KDP26419.1
TRINITY_DN7053_c1_g1_i1_1	1,99E-120	354	XP_018817873.1	uncharacterized protein At5g49945-like	Juglans regia	XP_018809158.1
TRINITY_DN9308_c0_g2_i1_6	0	728	XP_018851632.1	probable ADP-ribosylation factor GTPase-activating protein AGD5 isoform X1	Juglans regia	XP_018851640.1
TRINITY_DN7166_c0_g1_i1_1	1,13E-80	239	XP_008373046.1	histone deacetylase 6	Malus domestica	XP_008356371.1
TRINITY_DN17861_c0_g1_i1_6	1,75E-176	498	XP_018839810.1	S-adenosylmethionine carrier 1, chloroplastic/mitochondrial isoform X2	Juglans regia	XP_018839807.1
TRINITY_DN23265_c0_g1_i1_1	9,60E-36	128	XP_018852253.1	plastoglobulin-1, chloroplastic-like	Juglans regia	KDP30383.1
TRINITY_DN11443_c1_g1_i3_3	0	586	XP_018823000.1	formate dehydrogenase, mitochondrial	Juglans regia	XP_018828647.1
TRINITY_DN9627_c0_g2_i1_1	3,58E-53	167	dbj|GAU18587.1	hypothetical protein TSUD_124170	Trifolium subterraneum	GAU18586.1
TRINITY_DN20236_c0_g1_i1_5	1,77E-33	118	XP_018813647.1	glucosamine 6-phosphate N-acetyltransferase-like	Juglans regia	XP_018813648.1
TRINITY_DN12747_c0_g1_i1_3	6,02E-18	75,9	dbj|GAU16285.1	hypothetical protein TSUD_299170	Trifolium subterraneum	KHN23719.1
TRINITY_DN4648_c0_g1_i1_3	1,75E-51	167	XP_018859347.1	probable enoyl-CoA hydratase 1, peroxisomal	Juglans regia	XP_008369667.1
TRINITY_DN396_c0_g1_i1_1	7,17E-64	213	OAY40227.1	hypothetical protein MANES_09G005600	Manihot esculenta	XP_018826303.1
TRINITY_DN3770_c0_g1_i1_3	1,01E-115	345	XP_018826783.1	TBC1 domain family member 17-like isoform X3	Juglans regia	XP_018836649.1
TRINITY_DN7965_c1_g1_i1_2	0	602	XP_018827562.1	UDP-N-acetylglucosamine diphosphorylase 2 isoform X1	Juglans regia	XP_018827564.1
TRINITY_DN5625_c0_g2_i1_1	0	548	XP_018835418.1	protein-lysine N-methyltransferase Mettl10-like	Juglans regia	XP_018817005.1
TRINITY_DN9598_c0_g1_i1_1	3,71E-156	441	XP_018838997.1	delta(3,5)-Delta(2,4)-dienoyl-CoA isomerase, peroxisomal	Juglans regia	XP_008222222.1
TRINITY_DN6936_c0_g1_i1_3	0	795	XP_018845130.1	lysine histidine transporter 1-like	Juglans regia	XP_018845129.1
TRINITY_DN22611_c0_g1_i1_5	1,90E-37	134	XP_018812785.1	importin subunit alpha-2-like	Juglans regia	XP_018825763.1
TRINITY_DN4497_c1_g1_i1_1	1,58E-45	147	XP_018854928.1	ubiquitin-40S ribosomal protein S27a-like	Juglans regia	XP_018805239.1

TRINITY_DN16255_c0_g2_i1_1	6,44E-171	478	XP_018843402.1	ubiquinol-cytochrome-c reductase complex assembly factor 1	Juglans regia	XP_018843404.1
TRINITY_DN3029_c0_g2_i1_3	2,41E-74	169	XP_018846418.1	acid phosphatase 1-like	Juglans regia	XP_018846418.1
TRINITY_DN7010_c0_g2_i2_5	2,58E-130	377	XP_008218725.1	60S ribosomal protein L3-2	Prunus mume	ONI36287.1
TRINITY_DN8504_c0_g1_i1_2	1,22E-25	104	XP_018848912.1	delta(12)-fatty-acid desaturase FAD2 isoform X1	Juglans regia	XP_018848914.1
TRINITY_DN3130_c0_g2_i2_1	0	702	XP_018822117.1	galacturonokinase	Juglans regia	XP_012079700.1
TRINITY_DN13283_c0_g1_i1_5	1,14E-50	174	KDP45423.1	hypothetical protein JCGZ_09672	Jatropha curcas	XP_012082412.1
TRINITY_DN3329_c0_g1_i1_6	1,29E-92	289	XP_018844641.1	far upstream element-binding protein 1 isoform X2	Juglans regia	XP_018844641.1
TRINITY_DN10602_c1_g1_i2_3	9,89E-34	118	XP_008233937.1	cytochrome b-c1 complex subunit 9-like	Prunus mume	ONI34935.1
TRINITY_DN5356_c0_g1_i1_3	0	1027	XP_018808146.1	serine/threonine-protein kinase tricorner-like isoform X1	Juglans regia	XP_018808154.1
TRINITY_DN8024_c0_g1_i1_1	9,93E-99	288	XP_018849696.1	probable NADH dehydrogenase [ubiquinone] 1 alpha subcomplex subunit 12	Juglans regia	XP_008234575.1
TRINITY_DN9300_c0_g1_i1_5	2,11E-175	507	XP_018845068.1	nuclear pore complex protein NUP50A-like	Juglans regia	XP_018845069.1
TRINITY_DN9177_c0_g3_i1_3	0	585	XP_007142133.1	hypothetical protein PHAVU_008G255400g	Phaseolus vulgaris	ESW14127.1
TRINITY_DN14061_c0_g1_i1_4	6,28E-59	188	XP_018845017.1	probable 2-oxoglutarate-dependent dioxygenase At3g49630 isoform X2	Juglans regia	XP_018845016.1
TRINITY_DN8441_c0_g2_i2_3	0	720	XP_018816062.1	plant UBX domain-containing protein 11	Juglans regia	XP_018816063.1
TRINITY_DN7456_c0_g2_i1_3	0	708	XP_018857484.1	mannose-1-phosphate guanylyltransferase 1	Juglans regia	XP_018857485.1
TRINITY_DN4946_c0_g2_i1_3	0	761	XP_018844867.1	anthranilate synthase alpha subunit 2, chloroplastic-like	Juglans regia	XP_004497238.1
TRINITY_DN18912_c0_g1_i1_2	0	659	XP_018848690.1	calcium-dependent protein kinase 11-like	Juglans regia	XP_018821057.1
TRINITY_DN12766_c0_g1_i1_5	1,78E-63	213	XP_018825846.1	enhancer of mRNA-decapping protein 4-like	Juglans regia	XP_016650129.1
TRINITY_DN4468_c0_g1_i1_6	0	944	XP_018858508.1	NEDD8-activating enzyme E1 regulatory subunit AXR1-like	Juglans regia	XP_018858509.1
TRINITY_DN12470_c0_g1_i1_3	0	564	XP_018815757.1	phosphoglucan, water dikinase, chloroplastic isoform X1	Juglans regia	XP_018815758.1
TRINITY_DN10609_c0_g1_i2_2	0	922	XP_018829077.1	sterol 14-demethylase	Juglans regia	XP_018841630.1
TRINITY_DN11550_c0_g1_i3_3	0	726	XP_018826883.1	beta-ureidopropionase-like	Juglans regia	XP_009368303.1
TRINITY_DN18034_c0_g1_i1_3	2,47E-47	164	XP_009359673.1	brefeldin A-inhibited guanine nucleotide-exchange protein 5-like	Pyrus x bretschneideri	XP_018503710.1
TRINITY_DN10769_c0_g1_i1_1	1,96E-172	484	XP_018839985.1	protein YIF1B-like	Juglans regia	XP_018839986.1
TRINITY_DN11604_c0_g1_i8_1	9,09E-82	246	XP_018846260.1	putative methyltransferase DDB_G0268948	Juglans regia	XP_018838664.1
TRINITY_DN11302_c0_g1_i2_1	0	886	XP_018826789.1	bifunctional purple acid phosphatase 26-like	Juglans regia	XP_008242837.1
TRINITY_DN12632_c0_g1_i1_3	4,60E-103	302	XP_018812444.1	ubiquinone biosynthesis O-methyltransferase, mitochondrial	Juglans regia	AFK39361.1
TRINITY_DN9378_c0_g1_i4_2	0	892	XP_018808947.1	synaptotagmin-2	Juglans regia	XP_018808948.1
TRINITY_DN9423_c0_g2_i1_4	1,36E-81	243	XP_018851087.1	uncharacterized protein At3g49720-like	Juglans regia	XP_018816519.1
TRINITY_DN7649_c0_g1_i2_4	2,05E-95	278	OAY48980.1	hypothetical protein MANES_05G020500	Manihot esculenta	XP_009334366.1

TRINITY_DN18448_c0_g1_i1_3	1,12E-55	178	KRG95307.1	hypothetical protein GLYMA_19G142500	Glycine max	XP_004513649.1
TRINITY_DN11658_c0_g1_i3_3	0	1732	XP_018847260.1	calcium-transporting ATPase 10, plasma membrane-type-like	Juglans regia	XP_018847261.1
TRINITY_DN18880_c0_g1_i1_1	2,44E-69	216	XP_018858382.1	protein ROOT HAIR DEFECTIVE 3-like	Juglans regia	XP_018848604.1
TRINITY_DN10400_c0_g1_i3_1	0	905	XP_018811309.1	nucleolar protein 56-like	Juglans regia	XP_018823191.1
TRINITY_DN1374_c0_g1_i1_1	5,14E-61	187	XP_016182321.1	upstream activation factor subunit UAF30	Arachis ipaensis	XP_020969280.1
TRINITY_DN3233_c0_g1_i1_1	2,58E-132	394	XP_018834801.1	peptidyl-prolyl cis-trans isomerase CYP95 isoform X2	Juglans regia	XP_018834802.1
TRINITY_DN16037_c0_g1_i1_6	1,11E-47	166	XP_018833351.1	protein DEFECTIVE IN EXINE FORMATION 1	Juglans regia	XP_009370211.1
TRINITY_DN4496_c0_g1_i1_6	4,66E-103	306	KHN26125.1	Glucose-6-phosphate isomerase, partial	Glycine soja	OAY23212.1
TRINITY_DN3498_c0_g1_i1_3	0	524	XP_018825910.1	ERBB-3 BINDING PROTEIN 1 isoform X1	Juglans regia	XP_018825911.1
TRINITY_DN8015_c0_g2_i1_1	0	757	XP_018827773.1	uncharacterized protein LOC108996364	Juglans regia	XP_018827774.1
TRINITY_DN8457_c0_g1_i2_3	1,12E-64	203	XP_018844015.1	protein EARLY RESPONSIVE TO DEHYDRATION 15-like	Juglans regia	XP_018852944.1
TRINITY_DN8936_c0_g2_i1_3	2,05E-24	97,8	XP_008232561.1	uncharacterized protein LOC103331692	Prunus mume	ONI22544.1
TRINITY_DN4836_c0_g2_i2_1	0	652	XP_018849426.1	uncharacterized protein LOC109012316	Juglans regia	XP_008363290.1
TRINITY_DN4305_c0_g1_i1_2	3,94E-70	217	XP_018813688.1	iron-sulfur assembly protein IscA-like 2, mitochondrial isoform X1	Juglans regia	KDP42748.1
TRINITY_DN912_c0_g1_i1_3	1,27E-124	355	XP_018809955.1	eukaryotic initiation factor 4A	Juglans regia	KDP31923.1
TRINITY_DN2769_c0_g1_i1_2	0	863	XP_018859383.1	vacuolar protein sorting-associated protein 45 homolog isoform X2	Juglans regia	XP_018839879.1
TRINITY_DN7152_c0_g1_i2_3	7,40E-135	385	XP_018827308.1	uncharacterized protein LOC108996034 isoform X1	Juglans regia	XP_018827309.1
TRINITY_DN7966_c1_g1_i1_1	1,25E-47	154	XP_018826615.1	uncharacterized protein LOC108995493	Juglans regia	XP_018845960.1
TRINITY_DN4959_c0_g1_i1_6	2,58E-10	59,7	OIW21969.1	hypothetical protein TanjilG_18289	Lupinus angustifolius	OIW21969.1
TRINITY_DN11436_c0_g1_i3_2	0	865	ARI45576.1	ATP synthase CF1 beta subunit (chloroplast)	Corylus avellana	AUG61323.1
TRINITY_DN6999_c0_g1_i1_5	1,35E-93	283	XP_018821643.1	uncharacterized protein LOC108991725	Juglans regia	XP_018848582.1
TRINITY_DN19644_c0_g1_i1_5	1,01E-76	244	XP_018815884.1	acid beta-fructofuranosidase-like	Juglans regia	AFU56882.1
TRINITY_DN12673_c0_g1_i1_2	2,23E-58	186	XP_018851375.1	probable protein phosphatase 2C 11	Juglans regia	XP_018857896.1
TRINITY_DN10039_c0_g1_i2_2	1,69E-36	132	XP_012569846.1	multiple organellar RNA editing factor 2, chloroplastic-like	Cicer arietinum	XP_008224177.2
TRINITY_DN6311_c0_g1_i1_1	3,83E-162	465	OAY48391.1	hypothetical protein MANES_06G155200	Manihot esculenta	XP_018847349.1
TRINITY_DN19028_c0_g1_i1_2	1,13E-45	154	XP_017191518.1	hexokinase-2, chloroplastic isoform X2	Malus domestica	OAY22639.1
TRINITY_DN10956_c0_g1_i2_3	0	877	XP_018820512.1	hydroxymethylglutaryl-CoA synthase-like	Juglans regia	OAY59897.1
TRINITY_DN10484_c2_g1_i1_1	9,48E-137	391	KDP26403.1	hypothetical protein JCGZ_17561	Jatropha curcas	XP_012085134.1
TRINITY_DN17185_c0_g1_i1_2	8,31E-121	359	XP_018828206.1	peptidyl-prolyl cis-trans isomerase FKBP62-like	Juglans regia	XP_018828206.1
TRINITY_DN11454_c0_g1_i2_6	3,17E-125	362	ONH99322.1	hypothetical protein PRUPE_6G024700	Prunus persica	ONH99323.1
TRINITY_DN588_c0_g1_i1_4	3,67E-51	176	XP_016647703.1	LOW QUALITY PROTEIN: clathrin interactor EPSIN 2-like	Prunus mume	XP_020409996.1
TRINITY_DN12701_c0_g1_i1_3	5,53E-98	293	XP_018849203.1	alpha-1,3/1,6-mannosyltransferase ALG2 isoform X2	Juglans regia	XP_018849195.1
TRINITY_DN7373_c0_g2_i1_3	0	752	XP_018833292.1	uncharacterized protein LOC109000757	Juglans regia	OAY32974.1
TRINITY_DN11305_c0_g1_i2_2	1,81E-168	479	XP_018825692.1	ubiquitin-conjugating enzyme E2 32-like	Juglans regia	OAY27662.1

TRINITY_DN5684_c0_g1_i1_3	9,13E-152	433	XP_018858478.1	uracil phosphoribosyltransferase isoform X1	Juglans regia	XP_018858479.1
TRINITY_DN864_c0_g1_i1_1	0	737	XP_018852537.1	mitochondrial import inner membrane translocase subunit TIM44-2-like	Juglans regia	KDP26532.1
TRINITY_DN4602_c0_g1_i1_1	0	773	XP_018821666.1	COP9 signalosome complex subunit 4 isoform X2	Juglans regia	KDP38481.1
TRINITY_DN10884_c0_g1_i1_3	0	538	XP_018852504.1	short-chain dehydrogenase TIC 32, chloroplastic-like	Juglans regia	ONI07132.1
TRINITY_DN14631_c0_g1_i1_2	2,41E-32	116	KDP37390.1	hypothetical protein JCGZ_08401	Jatropha curcas	XP_012072933.1
TRINITY_DN10399_c0_g1_i1_1	7,19E-173	484	XP_018809559.1	ER membrane protein complex subunit 3-like	Juglans regia	XP_018842205.1
TRINITY_DN11619_c0_g2_i6_1	0	570	XP_018834293.1	probable metal-nicotianamine transporter YSL7	Juglans regia	XP_018834942.1
TRINITY_DN5247_c0_g2_i1_5	0	1271	XP_018822020.1	uncharacterized protein LOC108992033	Juglans regia	OAY57474.1
TRINITY_DN2861_c0_g1_i1_2	1,26E-128	376	XP_018842938.1	uncharacterized protein LOC109007633	Juglans regia	XP_018812490.1
TRINITY_DN6453_c0_g1_i1_2	0	540	XP_018837250.1	annexin D5	Juglans regia	XP_004503249.1
TRINITY_DN13293_c0_g1_i1_6	4,32E-50	175	XP_018851380.1	uncharacterized protein LOC109013676	Juglans regia	XP_018856765.1
TRINITY_DN5843_c0_g1_i1_3	3,14E-67	211	AAW31716.1	plastid beta-ketoacyl ACP synthase, partial	Betula pendula	KYP57812.1
TRINITY_DN3985_c0_g1_i1_5	2,55E-117	338	XP_018834170.1	membrin-11-like	Juglans regia	XP_018839683.1
TRINITY_DN5098_c0_g2_i1_2	0	629	XP_018860675.1	uncharacterized protein LOC109022268	Juglans regia	XP_018856468.1
TRINITY_DN6975_c0_g2_i1_1	0	717	OAY53197.1	hypothetical protein MANES_04G143600	Manihot esculenta	OAY53198.1
TRINITY_DN3955_c0_g1_i1_6	1,61E-176	503	XP_018807977.1	uncharacterized protein LOC108981316	Juglans regia	XP_004498174.1
TRINITY_DN21162_c0_g1_i1_1	2,63E-41	147	XP_018859288.1	E3 ubiquitin-protein ligase UPL2-like	Juglans regia	XP_018848707.1
TRINITY_DN13980_c0_g1_i1_4	4,82E-149	430	XP_018808656.1	SNARE-interacting protein KEULE-like	Juglans regia	XP_018847688.1
TRINITY_DN6436_c0_g2_i1_3	6,76E-153	431	OAY39697.1	hypothetical protein MANES_10G115900	Manihot esculenta	KDP42462.1
TRINITY_DN7382_c0_g1_i1_5	0	795	XP_018836467.1	mitochondrial substrate carrier family protein C isoform X1	Juglans regia	XP_018836468.1
TRINITY_DN8895_c0_g1_i3_1	0	633	XP_018838226.1	probable low-specificity L-threonine aldolase 1	Juglans regia	XP_018838227.1
TRINITY_DN11523_c0_g1_i1_2	0	1031	XP_018856581.1	carotenoid 9,10(9',10')-cleavage dioxygenase 1	Juglans regia	ONI20422.1
TRINITY_DN6472_c0_g1_i1_3	3,22E-35	122	XP_008241390.1	uncharacterized protein LOC103339816	Prunus mume	XP_008387535.1
TRINITY_DN8927_c0_g1_i1_3	0	765	XP_018845117.1	COP9 signalosome complex subunit 3 isoform X1	Juglans regia	XP_018845118.1
TRINITY_DN22235_c0_g1_i1_2	9,20E-70	216	XP_008223254.1	uncharacterized protein LOC103323072	Prunus mume	ONI28186.1
TRINITY_DN22096_c0_g1_i1_6	6,27E-38	140	XP_018841295.1	kinase-interacting protein 1-like	Juglans regia	XP_020216020.1
TRINITY_DN10384_c0_g1_i1_3	0	874	OAY51257.1	hypothetical protein MANES_05G200200	Manihot esculenta	KDP36971.1
TRINITY_DN12671_c0_g1_i1_1	0	761	XP_018841273.1	plant UBX domain-containing protein 7	Juglans regia	OAY25414.1
TRINITY_DN15659_c0_g1_i1_5	8,08E-53	181	KHN48575.1	Clathrin heavy chain 2	Glycine soja	XP_004491025.1
TRINITY_DN3596_c0_g1_i1_4	8,25E-65	198	XP_018831984.1	actin-depolymerizing factor 2-like	Juglans regia	XP_018814539.1
TRINITY_DN8121_c0_g1_i1_3	4,31E-78	241	OAY32068.1	hypothetical protein MANES_14G163800	Manihot esculenta	KDP38755.1
TRINITY_DN20264_c0_g1_i1_2	3,05E-38	138	XP_018855488.1	protein TOC75-3, chloroplastic-like	Juglans regia	OIW03716.1
TRINITY_DN18042_c0_g1_i1_3	6,91E-97	299	KDP35792.1	hypothetical protein JCGZ_10428	Jatropha curcas	XP_012074678.1
TRINITY_DN18961_c0_g1_i1_1	3,91E-51	174	KDP46216.1	hypothetical protein JCGZ_10056	Jatropha curcas	XP_012069525.1

TRINITY_DN3104_c0_g2_i1_5	1,32E-83	262	XP_018838880.1	fimbrin-5-like	Juglans regia	KEH28484.1
TRINITY_DN17865_c0_g1_i1_1	0	617	XP_018854140.1	elongation factor G-2, mitochondrial	Juglans regia	XP_008232545.1
TRINITY_DN11260_c0_g1_i2_3	0	709	XP_018823451.1	bifunctional D-cysteine desulfhydrase/1-aminocyclopropane-1-carboxylate deaminase, mitochondrial	Juglans regia	ONI09471.1
TRINITY_DN6677_c0_g2_i1_6	3,57E-33	113	XP_018813390.1	glu S.griseus protease inhibitor-like	Juglans regia	ONI26581.1
TRINITY_DN14239_c0_g1_i1_6	1,32E-62	207	XP_018846458.1	apoptotic chromatin condensation inducer in the nucleus-like isoform X1	Juglans regia	XP_018846459.1
TRINITY_DN12081_c0_g1_i1_6	0	800	XP_018821827.1	ultraviolet-B receptor UVR8-like isoform X2	Juglans regia	XP_018821826.1
TRINITY_DN18664_c0_g1_i1_1	1,64E-105	308	XP_018814230.1	electron transfer flavoprotein subunit beta, mitochondrial	Juglans regia	ONI26653.1
TRINITY_DN3353_c0_g1_i1_5	2,78E-74	236	KDP46145.1	hypothetical protein JCGZ_06656	Jatropha curcas	XP_012073647.1
TRINITY_DN13649_c0_g1_i1_6	2,17E-56	184	KRH26303.1	hypothetical protein GLYMA_12G1662001, partial	Glycine max	XP_018809001.1
TRINITY_DN21861_c0_g1_i1_3	1,53E-49	170	XP_018832925.1	protein ABC transporter 1, mitochondrial	Juglans regia	XP_009344790.1
TRINITY_DN22107_c0_g1_i1_1	2,85E-29	113	XP_018851050.1	uncharacterized protein LOC109013419	Juglans regia	XP_018842090.1
TRINITY_DN19998_c0_g1_i1_4	1,49E-57	191	XP_018816061.1	2-isopropylmalate synthase 1, chloroplastic-like	Juglans regia	XP_008392601.1
TRINITY_DN4490_c0_g2_i2_2	0	691	XP_018814136.1	vacuolar amino acid transporter 1-like isoform X1	Juglans regia	XP_018814137.1
TRINITY_DN22424_c0_g2_i1_6	8,92E-112	332	XP_018810164.1	phospho-2-dehydro-3-deoxyheptonate aldolase 2, chloroplastic-like isoform X1	Juglans regia	OAY60153.1
TRINITY_DN1144_c0_g1_i1_3	0	634	XP_018837754.1	anthranilate phosphoribosyltransferase, chloroplastic-like	Juglans regia	OAY47794.1
TRINITY_DN4661_c0_g1_i3_3	1,23E-180	509	XP_018821344.1	angio-associated migratory cell protein	Juglans regia	XP_018821344.1
TRINITY_DN3820_c0_g2_i1_3	0	544	XP_018841709.1	eukaryotic translation initiation factor 3 subunit A-like	Juglans regia	XP_018809947.1
TRINITY_DN10264_c0_g1_i1_2	0	640	KDP21266.1	hypothetical protein JCGZ_21737	Jatropha curcas	XP_012091995.1
TRINITY_DN8158_c0_g1_i1_1	0	550	XP_018845644.1	COP9 signalosome complex subunit 5b-like	Juglans regia	XP_007137114.1
TRINITY_DN9206_c0_g2_i1_1	2,59E-175	494	XP_018824534.1	isoaspartyl peptidase/L-asparaginase-like	Juglans regia	XP_018824524.1
TRINITY_DN9226_c0_g1_i2_3	0	702	XP_018826456.1	uncharacterized protein LOC108995353	Juglans regia	KYP49632.1
TRINITY_DN585_c0_g1_i1_6	6,23E-45	154	XP_018844067.1	UDP-glucuronate 4-epimerase 6	Juglans regia	XP_016187710.1
TRINITY_DN21456_c0_g1_i1_2	2,45E-43	153	XP_018811830.1	exocyst complex component EXO70B1	Juglans regia	XP_009336452.1
TRINITY_DN5447_c0_g2_i1_2	0	869	XP_018811673.1	polypyrimidine tract-binding protein homolog 3	Juglans regia	XP_018811674.1
TRINITY_DN23101_c0_g1_i1_6	1,06E-31	120	XP_018837412.1	ATP-dependent 6-phosphofructokinase 2	Juglans regia	XP_018854632.1
TRINITY_DN1220_c0_g1_i1_6	0	691	ONI09345.1	hypothetical protein PRUPE_5G233600	Prunus persica	XP_007211280.1
TRINITY_DN13227_c0_g1_i1_1	1,88E-54	186	OAY58446.1	hypothetical protein MANES_02G178500	Manihot esculenta	XP_008361615.2
TRINITY_DN5512_c0_g3_i1_3	4,40E-114	335	XP_018817110.1	uncharacterized protein LOC108988328	Juglans regia	XP_009346349.1
TRINITY_DN4033_c0_g2_i1_3	3,73E-176	507	ONI17441.1	hypothetical protein PRUPE_3G159400	Prunus persica	ONI17442.1
TRINITY_DN4640_c0_g1_i1_2	5,20E-110	319	AFK37233.1	unknown	Lotus japonicus	XP_004508073.1
TRINITY_DN5850_c0_g1_i1_1	7,47E-49	167	XP_018850159.1	protein ZINC INDUCED FACILITATOR-LIKE 1-like	Juglans regia	KDP27074.1
TRINITY_DN11339_c0_g1_i3_1	0	578	XP_018816749.1	protein DJ-1 homolog D-like	Juglans regia	XP_018816749.1

TRINITY_DN2739_c0_g1_i1_1	2,34E-94	276	XP_018827376.1	trafficking protein particle complex subunit 2-like	Juglans regia	XP_008218711.1
TRINITY_DN6721_c1_g1_i1_1	1,23E-61	199	XP_018844889.1	ruvB-like protein 1 isoform X1	Juglans regia	XP_018844890.1
TRINITY_DN7299_c0_g1_i1_1	2,80E-43	142	XP_018822933.1	cytochrome c oxidase copper chaperone 1-like	Juglans regia	XP_018850576.1
TRINITY_DN2661_c0_g2_i1_1	0	590	XP_018836286.1	protein trichome birefringence-like 16	Juglans regia	XP_018836287.1
TRINITY_DN7083_c0_g2_i2_1	0	905	XP_018817752.1	branched-chain-amino-acid aminotransferase-like protein 1	Juglans regia	XP_020212375.1
TRINITY_DN18425_c0_g1_i1_2	3,31E-63	206	XP_018818346.1	dynamin-related protein 5A	Juglans regia	OAY49013.1
TRINITY_DN11416_c0_g1_i1_3	0	665	XP_018835846.1	polygalacturonase QRT3	Juglans regia	XP_018835847.1
TRINITY_DN9924_c0_g1_i1_2	2,03E-28	103	XP_015962951.1	uncharacterized protein LOC107486893	Arachis duranensis	XP_014497407.1
TRINITY_DN8093_c0_g1_i1_2	9,86E-171	481	XP_018830672.1	choline-phosphate cytidylyltransferase 2-like	Juglans regia	XP_018828350.1
TRINITY_DN766_c0_g1_i1_2	2,32E-66	204	OAY54996.1	hypothetical protein MANES_03G119600	Manihot esculenta	OAY28606.1
TRINITY_DN10380_c0_g1_i2_1	3,13E-66	207	OAY31883.1	hypothetical protein MANES_14G148900	Manihot esculenta	KYP53704.1
TRINITY_DN2440_c0_g1_i2_2	0	917	XP_018859103.1	cysteine--tRNA ligase 2, cytoplasmic-like isoform X1	Juglans regia	XP_018849702.1
TRINITY_DN18420_c0_g1_i1_3	3,00E-62	210	XP_018815848.1	probable ubiquitin conjugation factor E4	Juglans regia	KRG93599.1
TRINITY_DN7383_c0_g1_i1_3	8,63E-121	349	XP_018827251.1	vacuolar protein sorting-associated protein 2 homolog 3	Juglans regia	XP_008235640.1
TRINITY_DN14144_c0_g1_i1_6	7,89E-83	248	XP_018832787.1	uncharacterized protein LOC109000390	Juglans regia	ONH90281.1
TRINITY_DN16953_c0_g1_i1_2	4,61E-09	55,5	XP_018852024.1	uncharacterized protein LOC109014136	Juglans regia	XP_008241938.1
TRINITY_DN10509_c0_g1_i1_3	2,52E-138	394	XP_018826140.1	endo-1,3;1,4-beta-D-glucanase-like isoform X1	Juglans regia	XP_008223020.1
TRINITY_DN7183_c0_g1_i2_1	1,66E-178	513	XP_018817029.1	GTPase LSG1-2	Juglans regia	XP_018817030.1
TRINITY_DN17895_c0_g1_i1_1	9,22E-75	243	XP_018812523.1	probable alpha,alpha-trehalose-phosphate synthase [UDP-forming 7]	Juglans regia	XP_018841187.1
TRINITY_DN9983_c0_g1_i1_1	0	848	XP_018822700.1	homogentisate 1,2-dioxygenase-like	Juglans regia	XP_018849997.1
TRINITY_DN1054_c0_g1_i1_1	2,33E-99	295	XP_017188304.1	protein IN2-1 homolog B-like	Malus domestica	ONI24059.1
TRINITY_DN1267_c0_g1_i1_6	2,76E-57	191	XP_018825638.1	translation initiation factor eIF-2B subunit epsilon isoform X5	Juglans regia	XP_018825637.1
TRINITY_DN10960_c0_g1_i1_3	0	560	XP_018841808.1	ATP-dependent Clp protease proteolytic subunit-related protein 3, chloroplastic	Juglans regia	XP_007147022.1
TRINITY_DN20312_c0_g1_i1_4	1,07E-59	192	XP_018818889.1	arogenate dehydratase/prephenate dehydratase 2, chloroplastic isoform X3	Juglans regia	XP_018818888.1
TRINITY_DN18128_c0_g1_i1_2	2,63E-30	116	KDP34198.1	hypothetical protein JCGZ_07769	Jatropha curcas	XP_012077428.1
TRINITY_DN8563_c0_g1_i1_2	5,06E-168	475	XP_004494622.1	protein YIPF1 homolog	Cicer arietinum	OAY42270.1
TRINITY_DN6232_c0_g1_i3_2	0	855	ONI24548.1	hypothetical protein PRUPE_2G246000	Prunus persica	XP_007220435.1
TRINITY_DN5437_c0_g1_i1_2	0	935	XP_018852337.1	probable methyltransferase PMT5	Juglans regia	XP_018852338.1
TRINITY_DN3837_c0_g1_i1_3	8,07E-67	207	XP_018843373.1	uncharacterized protein LOC109007919	Juglans regia	XP_008228306.1
TRINITY_DN4426_c0_g1_i1_2	0	716	XP_018830807.1	protein trichome birefringence-like 18	Juglans regia	XP_018830808.1
TRINITY_DN13216_c0_g1_i1_3	8,03E-36	124	XP_008348817.1	copper-transporting ATPase PAA1, chloroplastic-like isoform X1	Malus domestica	XP_008348818.1

TRINITY_DN8517_c0_g1_i3_3	0	766	XP_018840482.1	probable LRR receptor-like serine/threonine-protein kinase At1g67720	Juglans regia	XP_018846848.1
TRINITY_DN17094_c0_g1_i1_4	3,93E-86	277	XP_018848266.1	epidermal growth factor receptor substrate 15-like 1 isoform X2	Juglans regia	XP_018848266.1
TRINITY_DN20326_c0_g1_i1_2	5,80E-98	283	XP_014492833.1	ubiquitin carboxyl-terminal hydrolase 12-like	Vigna radiata var. radiata	KYP36624.1
TRINITY_DN7535_c0_g2_i1_1	1,31E-153	440	XP_018828729.1	RHOMBOID-like protein 2	Juglans regia	KDP25494.1
TRINITY_DN7228_c0_g1_i2_1	0	663	ONI19115.1	hypothetical protein PRUPE_3G259400	Prunus persica	XP_007214914.1
TRINITY_DN16259_c0_g1_i1_3	8,43E-51	174	XP_018808786.1	valine--tRNA ligase, mitochondrial 1	Juglans regia	XP_018808787.1
TRINITY_DN21689_c0_g1_i1_3	3,34E-70	222	XP_018859875.1	glycine-rich RNA-binding protein 3, mitochondrial	Juglans regia	XP_018859875.1
TRINITY_DN8651_c0_g2_i1_2	0	596	XP_018842811.1	vacuolar protein sorting-associated protein 26B-like	Juglans regia	XP_018842812.1
TRINITY_DN11262_c0_g1_i1_3	7,04E-132	379	XP_018858783.1	ras-related protein RABF2a	Juglans regia	AGL52580.1
TRINITY_DN2292_c0_g3_i1_5	5,90E-33	115	OAY51431.1	hypothetical protein MANES_04G006200	Manihot esculenta	XP_018848651.1
TRINITY_DN12820_c0_g1_i1_2	1,97E-47	167	XP_018838880.1	fimbrin-5-like	Juglans regia	XP_008342163.1
TRINITY_DN21154_c0_g1_i1_4	4,76E-74	238	XP_018824493.1	uncharacterized protein LOC108993891	Juglans regia	ONI13349.1
TRINITY_DN17026_c0_g1_i1_1	5,08E-130	373	XP_018819460.1	beta-galactosidase 6-like	Juglans regia	XP_018819501.1
TRINITY_DN14578_c0_g1_i1_4	3,92E-73	231	XP_018834206.1	leucine-rich repeat receptor-like protein kinase PXC2	Juglans regia	XP_018834206.1
TRINITY_DN5368_c0_g1_i2_5	1,86E-168	472	XP_018830497.1	14-3-3-like protein C isoform X1	Juglans regia	XP_008225282.1
TRINITY_DN17464_c0_g1_i1_1	1,17E-83	248	dbj|GAU32431.1	hypothetical protein TSUD_158290	Trifolium subterraneum	XP_008345781.1
TRINITY_DN8742_c0_g1_i1_3	2,98E-139	397	XP_008234264.1	ADP-ribosylation factor-related protein 1	Prunus mume	XP_018842923.1
TRINITY_DN14137_c0_g1_i1_2	5,46E-23	95,1	XP_008227771.1	protoporphyrinogen oxidase 1, chloroplastic	Prunus mume	ONI14680.1
TRINITY_DN2434_c0_g1_i1_5	0	593	XP_018835586.1	uncharacterized protein LOC109002344 isoform X2	Juglans regia	XP_018835588.1
TRINITY_DN10474_c1_g1_i1_2	0	986	XP_018813982.1	SNF1-related protein kinase catalytic subunit alpha KIN10-like	Juglans regia	XP_018813984.1
TRINITY_DN12774_c0_g1_i1_1	1,06E-99	294	XP_018811826.1	uncharacterized protein LOC108984353	Juglans regia	ONI29911.1
TRINITY_DN3904_c0_g1_i1_3	3,50E-57	188	dbj|BAT79239.1	hypothetical protein VIGAN_02208400	Vigna angularis var. angularis	XP_018827765.1
TRINITY_DN19463_c0_g1_i1_1	2,13E-48	167	XP_017191425.1	rab3 GTPase-activating protein catalytic subunit-like isoform X2	Malus domestica	XP_008385868.1
TRINITY_DN8904_c0_g2_i1_3	0	548	XP_018830043.1	SH3 domain-containing protein 3 isoform X1	Juglans regia	OAY25056.1
TRINITY_DN8767_c0_g1_i1_3	0	606	XP_018834921.1	protein FLX-like 2	Juglans regia	OAY60372.1
TRINITY_DN3578_c0_g1_i1_2	8,00E-75	225	XP_018809376.1	60S ribosomal protein L9-like	Juglans regia	XP_018809377.1
TRINITY_DN15270_c0_g1_i1_2	1,84E-85	276	XP_018841295.1	kinase-interacting protein 1-like	Juglans regia	XP_009364270.1
TRINITY_DN1008_c0_g2_i1_2	3,87E-13	70,9	XP_008231927.1	importin-5	Prunus mume	XP_009375844.1
TRINITY_DN13945_c0_g1_i1_1	5,29E-137	397	XP_018815129.1	cyclin-dependent kinase F-1	Juglans regia	ONH98065.1
TRINITY_DN7325_c0_g1_i1_1	6,05E-45	147	ONI00719.1	hypothetical protein PRUPE_6G102700	Prunus persica	ONI00720.1
TRINITY_DN11924_c0_g1_i1_2	4,43E-155	456	XP_018848367.1	SPX domain-containing membrane protein At4g22990-like isoform X3	Juglans regia	XP_018848364.1

TRINITY_DN10508_c0_g1_i3_5	0	525	XP_018823258.1	non-functional NADPH-dependent codeinone reductase 2-like	Juglans regia	XP_018836052.1
TRINITY_DN7011_c0_g1_i1_2	4,76E-160	453	XP_018826575.1	microtubule-associated protein RP/EB family member 1A isoform X2	Juglans regia	XP_008239808.1
TRINITY_DN3619_c0_g1_i1_2	6,25E-46	164	XP_018834055.1	probable alpha-mannosidase At5g13980 isoform X3	Juglans regia	XP_018834056.1
TRINITY_DN14686_c0_g1_i1_1	1,13E-43	151	XP_018845260.1	endoglucanase 16	Juglans regia	XP_012077550.1
TRINITY_DN9581_c0_g1_i2_2	1,94E-92	272	XP_018807341.1	acyl-coenzyme A thioesterase 13	Juglans regia	KDP46555.1
TRINITY_DN9707_c0_g1_i1_1	0	608	XP_018846669.1	4-hydroxy-tetrahydrodipicolinate synthase, chloroplastic-like	Juglans regia	XP_018806961.1
TRINITY_DN8938_c0_g1_i1_2	1,44E-168	484	XP_018829162.1	uncharacterized protein LOC108997384 isoform X2	Juglans regia	XP_018829161.1
TRINITY_DN10825_c0_g1_i4_1	1,16E-39	138	XP_009368696.1	uncharacterized protein LOC103958177	Pyrus x bretschneideri	XP_009352068.1
TRINITY_DN7033_c0_g2_i1_2	0	971	XP_018852332.1	probable phosphoinositide phosphatase SAC9	Juglans regia	XP_008243038.1
TRINITY_DN10999_c0_g1_i5_2	0	1280	XP_018850254.1	pyrophosphate-energized membrane proton pump 3 isoform X1	Juglans regia	XP_018850256.1
TRINITY_DN3792_c0_g1_i1_2	0	665	XP_018845113.1	protein TPLATE isoform X1	Juglans regia	XP_018845114.1
TRINITY_DN15541_c0_g1_i1_1	3,43E-73	239	XP_018841510.1	transportin-1-like isoform X1	Juglans regia	XP_018841511.1
TRINITY_DN2393_c0_g1_i1_3	4,13E-113	358	XP_018810529.1	auxilin-related protein 2-like	Juglans regia	XP_018827617.1
TRINITY_DN10184_c0_g1_i1_3	0	654	ONI33038.1	hypothetical protein PRUPE_1G401700	Prunus persica	XP_007222217.2
TRINITY_DN7705_c0_g1_i2_6	0	588	XP_018854773.1	heterogeneous nuclear ribonucleoprotein R-like isoform X2	Juglans regia	XP_018854759.1
TRINITY_DN7315_c0_g1_i1_4	0	600	XP_018834505.1	uncharacterized protein LOC109001605	Juglans regia	XP_008220433.1
TRINITY_DN8439_c0_g1_i1_6	2,08E-158	450	XP_018807846.1	L-ascorbate peroxidase 2, cytosolic-like	Juglans regia	XP_009342360.1
TRINITY_DN3610_c0_g2_i1_2	3,84E-81	243	XP_018821997.1	protein FMP32, mitochondrial-like	Juglans regia	XP_018821998.1
TRINITY_DN3307_c0_g1_i1_1	9,99E-112	329	XP_018846091.1	pentatricopeptide repeat-containing protein At1g61870, mitochondrial-like	Juglans regia	XP_018810112.1
TRINITY_DN18256_c0_g1_i1_4	0	638	XP_018836506.1	uncharacterized protein LOC109003012	Juglans regia	XP_008233533.1
TRINITY_DN11948_c0_g1_i1_6	1,00E-176	499	XP_018837315.1	uncharacterized protein LOC109003573	Juglans regia	KDP28635.1
TRINITY_DN5672_c0_g1_i1_6	0	955	XP_018851667.1	FRIGIDA-like protein 3	Juglans regia	OAY33680.1
TRINITY_DN138_c0_g1_i1_3	2,38E-50	172	XP_018807197.1	uncharacterized protein LOC108980650	Juglans regia	XP_008230044.1
TRINITY_DN10435_c0_g1_i1_1	0	1229	XP_018809511.1	pantothenate kinase 2 isoform X1	Juglans regia	XP_018809513.1
TRINITY_DN10493_c0_g1_i1_1	4,40E-114	332	KEH42355.1	RAB GTPase-like protein A5B	Medicago truncatula	XP_013468318.1
TRINITY_DN10290_c0_g1_i1_1	0	647	XP_008355915.1	putative MO25-like protein At5g47540	Malus domestica	XP_008227465.1
TRINITY_DN12636_c0_g1_i1_3	4,62E-72	218	AFK39482.1	unknown	Lotus japonicus	XP_018822564.1
TRINITY_DN14818_c0_g1_i1_6	8,80E-104	304	XP_018828900.1	uncharacterized protein LOC108997197	Juglans regia	XP_018828901.1
TRINITY_DN360_c0_g1_i1_4	6,76E-167	476	XP_018835381.1	protein O-glucosyltransferase 1-like	Juglans regia	XP_018810046.1
TRINITY_DN7953_c0_g2_i1_2	0	838	XP_018827095.1	ubiquinone biosynthesis monooxygenase COQ6, mitochondrial isoform X1	Juglans regia	OAY55385.1
TRINITY_DN8751_c1_g1_i1_3	0	655	XP_018836391.1	WD repeat-containing protein DWA2-like	Juglans regia	ONI32229.1
TRINITY_DN19806_c0_g1_i1_2	8,44E-64	207	XP_018859162.1	putative 3,4-dihydroxy-2-butanone kinase, partial	Juglans regia	XP_020215528.1

TRINITY_DN18164_c0_g1_i1_4	5,03E-72	236	XP_018828527.1	nardilysin-like	Juglans regia	XP_016646759.1
TRINITY_DN7968_c0_g1_i1_1	1,48E-110	319	XP_018832963.1	uncharacterized protein LOC109000515	Juglans regia	XP_018843096.1
TRINITY_DN7925_c0_g2_i1_4	2,03E-149	423	XP_018826593.1	NAP1-related protein 2	Juglans regia	XP_018833970.1
TRINITY_DN8225_c0_g1_i1_3	0	858	XP_018844632.1	vacuolar-processing enzyme-like	Juglans regia	XP_018844633.1
TRINITY_DN7814_c0_g1_i1_2	6,91E-159	461	XP_018843188.1	protein WVD2-like 2	Juglans regia	XP_018826282.1
TRINITY_DN21393_c0_g1_i1_4	3,46E-67	217	XP_018858362.1	rop guanine nucleotide exchange factor 12-like	Juglans regia	XP_008228557.1
TRINITY_DN20157_c0_g1_i1_3	1,96E-43	147	XP_018849298.1	E3 ubiquitin-protein ligase RING1-like	Juglans regia	XP_007152550.1
TRINITY_DN4739_c0_g2_i1_6	1,08E-112	332	OAY44057.1	hypothetical protein MANES_08G119000	Manihot esculenta	KDP23488.1
TRINITY_DN6504_c0_g1_i1_3	6,71E-120	357	XP_018839613.1	uncharacterized protein LOC109005243 isoform X1	Juglans regia	XP_018839614.1
TRINITY_DN9930_c0_g1_i3_4	7,86E-104	305	XP_018839330.1	aminoacyl tRNA synthase complex-interacting multifunctional protein 1	Juglans regia	XP_009348571.1
TRINITY_DN11687_c0_g1_i1_1	2,59E-27	107	XP_018810128.1	D-3-phosphoglycerate dehydrogenase 2, chloroplastic-like	Juglans regia	XP_007142145.1
TRINITY_DN968_c0_g1_i1_2	9,77E-111	329	XP_018847080.1	cytochrome P450 81E8-like, partial	Juglans regia	XP_018842054.1
TRINITY_DN8156_c0_g1_i1_6	0	614	XP_018830767.1	O-glucosyltransferase rumi homolog	Juglans regia	OAY48296.1
TRINITY_DN9403_c0_g1_i2_1	0	667	XP_018830668.1	sorbitol dehydrogenase	Juglans regia	KYP38685.1
TRINITY_DN10308_c0_g2_i1_1	1,87E-92	273	KDP26388.1	hypothetical protein JCGZ_17546	Jatropha curcas	XP_012085110.1
TRINITY_DN5427_c0_g1_i1_3	9,41E-34	119	XP_018853827.1	uncharacterized protein LOC109015827	Juglans regia	XP_018853937.1
TRINITY_DN10971_c0_g1_i2_2	0	806	XP_018836549.1	uncharacterized protein LOC109003048	Juglans regia	KDP32822.1
TRINITY_DN21130_c0_g1_i1_6	4,67E-120	368	XP_018815773.1	importin beta-like SAD2	Juglans regia	KRH01530.1
TRINITY_DN7507_c0_g1_i1_6	6,41E-39	131	XP_018850773.1	uncharacterized protein LOC109013215 isoform X2	Juglans regia	XP_018850772.1
TRINITY_DN8691_c0_g1_i1_4	7,64E-55	172	XP_008229999.1	uncharacterized protein LOC103329321	Prunus mume	XP_009368392.1
TRINITY_DN13949_c0_g1_i2_2	2,95E-171	480	XP_018850845.1	NADPH-dependent 1-acyldihydroxyacetone phosphate reductase-like	Juglans regia	XP_018846462.1
TRINITY_DN17228_c0_g1_i1_3	8,14E-24	97,8	XP_018828527.1	nardilysin-like	Juglans regia	XP_008359044.2
TRINITY_DN15289_c0_g1_i1_4	3,00E-71	216	XP_018814411.1	uncharacterized protein LOC108986289	Juglans regia	KDP21869.1
TRINITY_DN3649_c0_g1_i1_2	9,58E-83	251	XP_018854291.1	uncharacterized protein LOC109016313	Juglans regia	OAY62495.1
TRINITY_DN3740_c0_g2_i1_2	9,72E-109	315	XP_008235034.1	dephospho-CoA kinase	Prunus mume	XP_008368962.1
TRINITY_DN18927_c0_g1_i1_2	0	525	XP_018858396.1	uncharacterized protein LOC109020402 isoform X1	Juglans regia	XP_018806239.1
TRINITY_DN8933_c1_g1_i1_2	1,69E-46	154	dbj|BAU02187.1	hypothetical protein VIGAN_11165700	Vigna angularis var. angularis	XP_014624238.1
TRINITY_DN16700_c0_g1_i1_4						
TRINITY_DN709_c0_g2_i1_2	1,79E-177	499	XP_018853661.1	SEC12-like protein 1	Juglans regia	XP_009373824.1
TRINITY_DN813_c0_g1_i1_3	4,57E-101	303	XP_018853528.1	GABA transporter 1-like	Juglans regia	XP_009350466.1
TRINITY_DN20089_c0_g1_i1_1	4,75E-16	75,5	XP_018809065.1	uncharacterized TPR repeat-containing protein At1g05150-like	Juglans regia	OAY50637.1
TRINITY_DN19940_c0_g1_i1_4	7,02E-110	338	XP_018834193.1	dynamin-2A-like	Juglans regia	XP_018831147.1
TRINITY_DN16604_c0_g1_i1_3	3,59E-69	216	XP_018812823.1	dnaJ protein P58IPK homolog, partial	Juglans regia	XP_018847795.1
TRINITY_DN9343_c0_g1_i1_2	0	994	XP_018851735.1	E3 ubiquitin-protein ligase KEG-like	Juglans regia	XP_018851736.1
TRINITY_DN8561_c0_g1_i4_1	4,34E-83	251	XP_018825585.1	uncharacterized protein LOC108994714	Juglans regia	OAY27757.1

TRINITY_DN21803_c0_g1_i1_1	5,58E-65	207	XP_018855937.1	serine/threonine protein phosphatase 2A regulatory subunit B''beta-like	Juglans regia	XP_018818295.1
TRINITY_DN4286_c0_g1_i1_4	0	553	XP_018845608.1	U-box domain-containing protein 34-like isoform X2	Juglans regia	XP_018845606.1
TRINITY_DN5644_c1_g3_i1_6	1,41E-06	50,8	XP_018834731.1	uncharacterized protein LOC109001780	Juglans regia	KHN10293.1
TRINITY_DN11599_c0_g1_i3_2	1,34E-136	398	XP_018830827.1	anthocyanidin 3-O-glucosyltransferase 2-like isoform X6	Juglans regia	XP_018830826.1
TRINITY_DN17497_c0_g1_i1_2	0	801	XP_018856249.1	methylcrotonoyl-CoA carboxylase subunit alpha, mitochondrial isoform X2	Juglans regia	XP_018856242.1
TRINITY_DN3550_c0_g2_i1_2	0	704	XP_018815864.1	conserved oligomeric Golgi complex subunit 7	Juglans regia	XP_008234406.1
TRINITY_DN4310_c0_g1_i1_1	3,94E-61	197	OAY23950.1	hypothetical protein MANES_18G120300	Manihot esculenta	XP_018841146.1
TRINITY_DN21144_c0_g1_i1_4	2,24E-140	402	XP_018850569.1	nitrilase-like protein 2	Juglans regia	XP_015942307.1
TRINITY_DN23569_c0_g1_i1_2	2,01E-17	79	XP_008234398.1	trehalose-phosphate phosphatase A	Prunus mume	XP_016650210.1
TRINITY_DN2786_c0_g1_i1_5	5,41E-93	279	XP_018807742.1	GDSL esterase/lipase At5g03610-like isoform X1	Juglans regia	OAY42883.1
TRINITY_DN5005_c0_g1_i1_1	0	514	XP_018833335.1	rhodanese-like domain-containing protein 11, chloroplastic	Juglans regia	XP_008374369.1
TRINITY_DN5974_c0_g1_i1_3	9,80E-171	484	XP_018808798.1	uncharacterized protein LOC108981991	Juglans regia	XP_018843303.1
TRINITY_DN6400_c0_g1_i4_1	2,87E-101	297	XP_018860314.1	ras-related protein RABA1f-like	Juglans regia	OIV93234.1
TRINITY_DN11632_c0_g1_i2_5	0	570	XP_018860284.1	probable eukaryotic translation initiation factor 5-1	Juglans regia	AHA83527.1
TRINITY_DN17813_c0_g1_i1_1	4,61E-115	341	XP_008372356.1	serine/threonine-protein kinase tricorner	Malus domestica	ONH93245.1
TRINITY_DN17235_c0_g1_i1_3	2,01E-81	260	XP_018850837.1	germinal center kinase 1-like isoform X2	Juglans regia	XP_018850820.1
TRINITY_DN6926_c0_g1_i1_6	3,25E-41	137	OAY58238.1	hypothetical protein MANES_02G160800	Manihot esculenta	XP_008385565.1
TRINITY_DN17803_c0_g2_i1_3	0	820	XP_018821646.1	uncharacterized protein LOC108991727 isoform X2	Juglans regia	XP_018821645.1
TRINITY_DN2011_c0_g1_i1_4	2,14E-28	109	XP_018806881.1	GTPase-activating protein GYP1-like	Juglans regia	KRH24570.1
TRINITY_DN8075_c0_g1_i4_2	0	610	XP_018818640.1	uncharacterized protein LOC108989478	Juglans regia	ONI26425.1
TRINITY_DN2579_c0_g1_i1_2	9,11E-40	143	OAY45686.1	hypothetical protein MANES_07G083000	Manihot esculenta	KDP27531.1
TRINITY_DN19468_c0_g1_i1_2	6,16E-124	358	OAY57019.1	hypothetical protein MANES_02G064000	Manihot esculenta	OAY57009.1
TRINITY_DN3475_c0_g2_i1_2	1,00E-89	275	XP_018848680.1	eukaryotic translation initiation factor 5B-like	Juglans regia	XP_018848181.1
TRINITY_DN4140_c0_g1_i1_5	3,65E-135	384	XP_008221612.1	ribonuclease 3-like	Prunus mume	ONI30823.1
TRINITY_DN545_c0_g1_i1_2	2,09E-70	229	XP_018851343.1	LETM1 and EF-hand domain-containing protein 1, mitochondrial	Juglans regia	XP_018851344.1
TRINITY_DN6995_c0_g1_i1_6	3,87E-62	192	XP_014503527.1	small nuclear ribonucleoprotein Sm D2-like	Vigna radiata var. radiata	XP_014503537.1
TRINITY_DN9117_c0_g1_i2_2	1,12E-105	323	XP_018808629.1	synaptotagmin-5-like	Juglans regia	OAY49277.1
TRINITY_DN14629_c0_g1_i1_3	4,61E-58	186	XP_018843094.1	uncharacterized protein LOC109007740 isoform X2	Juglans regia	XP_018843093.1
TRINITY_DN8532_c0_g1_i1_2	4,65E-33	122	XP_018821438.1	ATP-dependent Clp protease proteolytic subunit-related protein 4, chloroplastic-like	Juglans regia	KDP39950.1
TRINITY_DN9825_c0_g1_i1_3	0	1075	OAY54110.1	hypothetical protein MANES_03G049000	Manihot esculenta	XP_012077273.1

TRINITY_DN2131_c0_g1_i1_6	0	625	XP_018835199.1	glutamine-dependent NAD(+) synthetase	Juglans regia	XP_018835200.1
TRINITY_DN2699_c0_g1_i1_6	0	912	XP_018808908.1	glucose-6-phosphate 1-dehydrogenase, chloroplastic isoform X1	Juglans regia	XP_018808909.1
TRINITY_DN10766_c0_g1_i8_3	0	598	XP_018816803.1	transmembrane 9 superfamily member 3-like	Juglans regia	OAY29373.1
TRINITY_DN6812_c0_g1_i7_1	0	647	XP_018805195.1	probable serine/threonine-protein kinase DDB_G0291350	Juglans regia	ONI09009.1
TRINITY_DN19649_c0_g1_i1_5	3,58E-55	189	XP_009366253.1	type I inositol polyphosphate 5-phosphatase 12-like isoform X2	Pyrus x bretschneideri	XP_009366252.1
TRINITY_DN10388_c0_g2_i1_5	0	902	XP_018810465.1	subtilisin-like protease SBT5.4	Juglans regia	XP_008218932.1
TRINITY_DN8750_c0_g2_i1_3	0	805	XP_018818643.1	ATP sulfurylase 1, chloroplastic-like	Juglans regia	XP_018839429.1
TRINITY_DN17662_c0_g2_i1_1	1,70E-107	317	XP_018837945.1	ATP-dependent Clp protease proteolytic subunit 4, chloroplastic	Juglans regia	KYP66493.1
TRINITY_DN5306_c0_g1_i1_1	0	580	OAY58628.1	hypothetical protein MANES_02G194100	Manihot esculenta	OAY58629.1
TRINITY_DN4670_c0_g1_i1_2	0	599	XP_018832866.1	LETM1 and EF-hand domain-containing protein 1, mitochondrial-like	Juglans regia	XP_018832867.1
TRINITY_DN9078_c0_g1_i2_3	1,15E-09	65,9	XP_018848181.1	eukaryotic translation initiation factor 5E	Juglans regia	XP_018848182.1
TRINITY_DN8091_c0_g1_i1_6	5,68E-132	376	XP_018827022.1	vacuolar protein sorting-associated protein 2 homolog 1	Juglans regia	XP_015931320.1
TRINITY_DN12406_c0_g1_i1_2	2,44E-82	257	XP_018815451.1	protein decapping 5-like	Juglans regia	OAY31246.1
TRINITY_DN17368_c0_g1_i1_2	4,52E-89	280	XP_018813997.1	granule-bound starch synthase 2, chloroplastic/amyloplastic-like	Juglans regia	XP_018815969.1
TRINITY_DN9727_c0_g1_i1_3	0	530	XP_018847896.1	GDSL esterase/lipase EXL3-like	Juglans regia	XP_018846820.1
TRINITY_DN17339_c0_g1_i1_3	5,09E-16	70,1	2051993|emb|CAA73147.1	Bet v 4	Betula pendula	CAA76831.1
TRINITY_DN18284_c0_g1_i1_2	2,00E-131	380	XP_018818624.1	3-hydroxyisobutyryl-CoA hydrolase 1-like	Juglans regia	OAY38989.1
TRINITY_DN10226_c0_g1_i1_1	7,66E-167	471	XP_018816923.1	protein PTST, chloroplastic isoform X1	Juglans regia	XP_018816924.1
TRINITY_DN8973_c0_g1_i1_1	8,67E-121	352	XP_018809935.1	RWD domain-containing protein 1	Juglans regia	XP_012069022.1
TRINITY_DN16264_c0_g1_i1_2	2,10E-44	154	OAY58864.1	hypothetical protein MANES_02G212800	Manihot esculenta	KDP45791.1
TRINITY_DN12122_c0_g1_i1_3	0	548	XP_018844413.1	uncharacterized protein LOC109008682	Juglans regia	ONH97583.1
TRINITY_DN4741_c0_g1_i1_2	0	597	XP_018841794.1	serine/threonine-protein phosphatase PP1	Juglans regia	XP_008388516.1
TRINITY_DN20857_c0_g1_i1_2	4,28E-112	323	XP_018812734.1	haloacid dehalogenase-like hydrolase domain-containing protein 3	Juglans regia	XP_018812735.1
TRINITY_DN13782_c0_g1_i1_1	8,38E-41	146	XP_015952377.1	LOW QUALITY PROTEIN: beta-galactosidase 8-like	Arachis duranensis	ONI00641.1
TRINITY_DN4532_c0_g1_i1_2	0	769	KDP20674.1	hypothetical protein JCGZ_21145	Jatropha curcas	XP_012091244.1
TRINITY_DN39_c0_g2_i1_6	1,37E-56	188	XP_018816046.1	uncharacterized protein LOC108987537	Juglans regia	XP_008388391.1
TRINITY_DN20415_c0_g1_i1_2	3,24E-44	155	XP_018822938.1	exocyst complex component SEC8-like	Juglans regia	XP_018822939.1
TRINITY_DN9710_c1_g1_i1_6	0	838	XP_018805234.1	pentatricopeptide repeat-containing protein At1g60770 isoform X1	Juglans regia	XP_008368256.1
TRINITY_DN371_c0_g1_i1_2	0	530	XP_018838495.1	apoptosis inhibitor 5-like protein API5	Juglans regia	XP_018833552.1
TRINITY_DN20208_c0_g1_i1_1	3,49E-43	145	XP_008344029.1	myosin-17-like	Malus domestica	XP_018846185.1
TRINITY_DN11629_c0_g1_i3_3	0	1211	OAY27675.1	hypothetical protein MANES_15G006300	Manihot esculenta	KDP27887.1
TRINITY_DN12689_c0_g1_i1_2	0	689	XP_018816643.1	auxin transport protein BIG	Juglans regia	ONI18935.1

TRINITY_DN8223_c0_g1_i4_6	0	591	XP_018833203.1	uncharacterized protein LOC109000690 isoform X1	Juglans regia	KYP54237.1
TRINITY_DN20465_c0_g1_i1_6	3,33E-30	114	XP_008223661.1	FGGY carbohydrate kinase domain-containing protein isoform X2	Prunus mume	ONI27478.1
TRINITY_DN7564_c1_g2_i2_4	5,48E-49	161	XP_018851171.1	uncharacterized protein LOC109013510	Juglans regia	ONH95810.1
TRINITY_DN10977_c0_g2_i3_5	0	796	XP_018822222.1	DEAD-box ATP-dependent RNA helicase 56	Juglans regia	XP_018812870.1
TRINITY_DN23633_c0_g1_i1_2	1,74E-42	149	KRH58836.1	hypothetical protein GLYMA_05G151100	Glycine max	AAC05981.1
TRINITY_DN6603_c0_g1_i1_3	0	762	XP_018829609.1	vacuolar protein sorting-associated protein 35A-like	Juglans regia	XP_020540155.1
TRINITY_DN9814_c0_g1_i2_2	0	615	XP_018843117.1	cathepsin B-like	Juglans regia	ONI14643.1
TRINITY_DN7007_c0_g1_i1_6	0	685	XP_018857638.1	cytosolic Fe-S cluster assembly factor NBP35	Juglans regia	XP_008219249.1
TRINITY_DN14463_c0_g1_i1_1	1,16E-100	301	ONH96206.1	hypothetical protein PRUPE_7G113300	Prunus persica	XP_007202051.1
TRINITY_DN6833_c0_g1_i1_2	1,61E-144	408	XP_018857020.1	peptidyl-prolyl cis-trans isomerase CYP23	Juglans regia	KDP41170.1
TRINITY_DN839_c0_g1_i2_1	7,35E-36	122	XP_018854860.1	60S ribosomal protein L37a-1	Juglans regia	XP_012075387.1
TRINITY_DN20539_c0_g1_i1_2	6,11E-97	300	XP_018811352.1	heat shock 70 kDa protein 16-like	Juglans regia	XP_018811359.1
TRINITY_DN3431_c0_g1_i1_2	1,34E-65	218	OAY59027.1	hypothetical protein MANES_02G225500	Manihot esculenta	KDP45840.1
TRINITY_DN11964_c0_g1_i1_3	3,28E-138	412	KYP52193.1	Beta-galactosidase 7	Cajanus cajan	XP_020230592.1
TRINITY_DN4866_c0_g2_i1_1	0	1061	XP_018824522.1	signal recognition particle subunit SRP68	Juglans regia	XP_018824523.1
TRINITY_DN16866_c0_g1_i1_6	3,58E-36	125	XP_018848532.1	fatty-acid-binding protein 3, chloroplastic isoform X3	Juglans regia	XP_018848525.1
TRINITY_DN13956_c0_g1_i1_2	6,74E-46	154	XP_018820907.1	clathrin interactor 1	Juglans regia	ONI26712.1
TRINITY_DN4399_c0_g1_i1_6	1,96E-30	113	XP_018809603.1	syntaxin-22-like	Juglans regia	XP_018809113.1
TRINITY_DN10671_c0_g1_i5_2	0	708	XP_018825891.1	60S ribosomal export protein NMD3-like	Juglans regia	XP_018825892.1
TRINITY_DN3450_c0_g1_i1_1	0	596	XP_018814445.1	probable phosphoribosylformylglycinamidine synthase, chloroplastic/mitochondrial	Juglans regia	XP_018814446.1
TRINITY_DN19533_c0_g1_i1_2	1,31E-64	213	XP_018830097.1	exocyst complex component SEC10-like	Juglans regia	OAY49100.1
TRINITY_DN14184_c0_g1_i1_2	5,26E-65	216	OAY37958.1	hypothetical protein MANES_11G141000	Manihot esculenta	KRG89037.1
TRINITY_DN10011_c0_g2_i1_5	8,23E-42	147	XP_018817613.1	serine/arginine-rich splicing factor RSZ22-like	Juglans regia	KRH16159.1
TRINITY_DN10051_c0_g1_i3_2	0	529	XP_018858935.1	chaperone protein dnaJ 49-like	Juglans regia	XP_018858936.1
TRINITY_DN9084_c1_g1_i1_6	5,98E-147	416	XP_018813699.1	uncharacterized protein At2g38710-like	Juglans regia	XP_018835340.1
TRINITY_DN13392_c0_g1_i1_2	2,54E-110	333	XP_018851837.1	outer envelope protein 61	Juglans regia	KDP42955.1
TRINITY_DN5114_c0_g1_i1_5	0	565	XP_018834958.1	CLP protease regulatory subunit CLPX1, mitochondrial-like isoform X1	Juglans regia	XP_018834169.1
TRINITY_DN18316_c0_g1_i1_1	3,01E-124	369	XP_018832758.1	probable aminopyrimidine aminohydrolase, mitochondrial isoform X1	Juglans regia	XP_018832759.1
TRINITY_DN764_c0_g1_i1_4	2,02E-77	241	XP_018847165.1	CRIB domain-containing protein RIC7	Juglans regia	XP_012569758.1
TRINITY_DN10979_c0_g1_i3_3	0	672	XP_014490708.1	alpha-1,4-glucan-protein synthase [UDP-forming]	Vigna radiata var. radiata	KYP69807.1
TRINITY_DN10800_c0_g1_i2_2	0	787	XP_018813340.1	diphosphomevalonate decarboxylase MVD2-like isoform X2	Juglans regia	XP_018813339.1

TRINITY_DN14978_c0_g1_i1_4	5,38E-33	117	XP_018857909.1	protein transport protein Sec23A-like, partial	Juglans regia	XP_017185486.1
TRINITY_DN6214_c0_g1_i1_1	1,30E-148	421	XP_018812149.1	Golgi SNAP receptor complex member 1-1-like	Juglans regia	XP_008228765.1
TRINITY_DN14988_c0_g2_i1_4	4,15E-57	181	XP_008393190.1	2-isopropylmalate synthase 1, chloroplastic-like	Malus domestica	XP_008232677.1
TRINITY_DN6278_c0_g1_i1_2	8,01E-162	461	XP_018838848.1	inositol-tetrakisphosphate 1-kinase 1-like	Juglans regia	XP_018838851.1
TRINITY_DN6964_c0_g1_i1_1	2,12E-155	442	KDP39208.1	hypothetical protein JCGZ_00965	Jatropha curcas	XP_012070932.1
TRINITY_DN3722_c0_g1_i1_4	2,10E-167	469	XP_018857023.1	persulfide dioxygenase ETHE1 homolog, mitochondrial	Juglans regia	XP_018857027.1
TRINITY_DN22328_c0_g1_i1_3						
TRINITY_DN18052_c0_g2_i1_1	8,78E-157	442	XP_008228852.1	uncharacterized protein LOC103328234 isoform X1	Prunus mume	ONI16530.1
TRINITY_DN11467_c0_g1_i1_1	8,09E-45	162	OAY31944.1	hypothetical protein MANES_14G154000	Manihot esculenta	XP_018820842.1
TRINITY_DN15189_c0_g1_i1_3	4,27E-72	238	XP_018830674.1	uncharacterized protein LOC108998567 isoform X2	Juglans regia	XP_018830675.1
TRINITY_DN14289_c0_g2_i1_2	2,00E-63	213	XP_018814445.1	probable phosphoribosylformylglycinamidine synthase, chloroplastic/mitochondrial	Juglans regia	XP_018814446.1
TRINITY_DN1005_c0_g1_i2_3	3,12E-77	228	XP_007138997.1	hypothetical protein PHAVU_009G256100g	Phaseolus vulgaris	ESW10991.1
TRINITY_DN3819_c0_g1_i2_3	0	824	XP_018808839.1	pyridoxine/pyridoxamine 5'-phosphate oxidase 1, chloroplastic isoform X1	Juglans regia	XP_018808840.1
TRINITY_DN7943_c0_g1_i2_2	0	932	XP_018859360.1	calcium-transporting ATPase 9, plasma membrane-type-like isoform X2	Juglans regia	OIW09320.1
TRINITY_DN2112_c0_g2_i1_6	1,00E-40	148	XP_018831617.1	transportin MOS14-like isoform X2	Juglans regia	XP_018831616.1
TRINITY_DN10350_c1_g1_i4_2	4,11E-110	328	XP_018814268.1	IST1 homolog isoform X2	Juglans regia	XP_018814267.1
TRINITY_DN6884_c1_g3_i1_2	0	555	XP_018833006.1	dehydrogenase/reductase SDR family member 7 isoform X1	Juglans regia	XP_018833007.1
TRINITY_DN12265_c0_g1_i1_2	5,88E-81	245	XP_018857864.1	7-methylguanosine phosphate-specific 5'-nucleotidase A-like	Juglans regia	XP_018857865.1
TRINITY_DN21124_c0_g1_i3_3	2,60E-07	51,2	AES98826.1	sorting nexin 2B	Medicago truncatula	XP_003615868.1
TRINITY_DN9012_c0_g1_i1_4	0	580	XP_018839527.1	uncharacterized protein LOC109005179 isoform X1	Juglans regia	XP_018839528.1
TRINITY_DN9946_c0_g2_i4_3	0	1552	XP_018824886.1	sucrose synthase	Juglans regia	AIJ28960.1
TRINITY_DN5727_c0_g2_i1_6	2,11E-45	153	XP_018846161.1	uncharacterized protein LOC109009947	Juglans regia	KDP33741.1
TRINITY_DN12393_c0_g1_i1_4	4,43E-171	483	XP_018829738.1	apoptosis-inducing factor homolog B-like	Juglans regia	XP_008229851.1
TRINITY_DN4111_c0_g2_i1_2	5,60E-74	228	AFK39482.1	unknown	Lotus japonicus	XP_009361550.1
TRINITY_DN11057_c0_g8_i1_3	5,55E-133	375	AJT37390.1	ribulose-1,5-bisphosphate carboxylase/oxygenase large subunit, partial (chloroplast)	Prunus davidiana	AJT37338.1
TRINITY_DN2598_c0_g2_i1_3	0	1206	XP_020411192.1	beta-galactosidase 9 isoform X2	Prunus persica	ONI28773.1
TRINITY_DN9900_c0_g1_i2_2	2,02E-86	262	XP_018834736.1	polyadenylate-binding protein-interacting protein 5-like	Juglans regia	XP_018834737.1

TRINITY_DN3402_c0_g1_i1_5	4,62E-34	124	XP_016202338.1	long chain acyl-CoA synthetase 6, peroxisomal	Arachis ipaensis	XP_015964764.1
TRINITY_DN9871_c0_g1_i2_2	3,10E-100	294	XP_018807876.1	uncharacterized protein LOC108981223	Juglans regia	XP_018827114.1
TRINITY_DN9274_c0_g1_i1_2	8,19E-155	439	XP_018849297.1	ribonuclease 2	Juglans regia	OAY34914.1
TRINITY_DN20612_c0_g1_i1_1	6,70E-46	155	XP_018859875.1	glycine-rich RNA-binding protein 3, mitochondrial	Juglans regia	XP_018859875.1
TRINITY_DN21955_c0_g1_i1_1	2,27E-60	202	XP_018834202.1	histidine--tRNA ligase, cytoplasmic	Juglans regia	KDP45604.1
TRINITY_DN7460_c0_g1_i1_6	7,21E-108	323	XP_008228667.1	COBW domain-containing protein 1	Prunus mume	KDP37034.1
TRINITY_DN6570_c0_g1_i1_6	2,82E-68	208	ACU13557.1	unknown	Glycine max	KRH60759.1
TRINITY_DN12116_c0_g1_i1_5	1,21E-60	206	XP_018848266.1	epidermal growth factor receptor substrate 15-like 1 isoform X2	Juglans regia	XP_018848264.1
TRINITY_DN18165_c0_g1_i1_3	2,00E-74	226	XP_018851955.1	uncharacterized protein LOC109014089	Juglans regia	XP_018806985.1
TRINITY_DN19285_c0_g1_i1_2	3,79E-176	494	XP_018850920.1	bifunctional phosphatase IMPL2, chloroplastic-like	Juglans regia	XP_018815280.1
TRINITY_DN4092_c0_g1_i1_3	9,65E-18	82,4	XP_018847499.1	ATP-dependent Clp protease ATP-binding subunit CLPT2, chloroplastic	Juglans regia	KHN42727.1
TRINITY_DN4871_c0_g1_i1_3	1,20E-75	232	XP_018815686.1	2-oxoglutarate-Fe(II) type oxidoreductase-like	Juglans regia	XP_018821188.1
TRINITY_DN23190_c0_g1_i1_4	3,29E-60	187	1818937063|gb|QIH97370.1	CYP01, partial	Betula pendula	XP_020215963.1
TRINITY_DN2267_c0_g1_i1_5	0	1376	KDP23611.1	hypothetical protein JCGZ_23444	Jatropha curcas	XP_012089192.1
TRINITY_DN12570_c0_g1_i1_4	4,78E-67	211	XP_018844982.1	PI-PLC X domain-containing protein At5g67130	Juglans regia	KDP31946.1
TRINITY_DN19686_c0_g1_i1_5	3,20E-65	216	XP_018838814.1	insulin-degrading enzyme-like 1, peroxisomal	Juglans regia	GAU30047.1
TRINITY_DN15214_c0_g1_i1_3	3,65E-41	136	XP_018822768.1	caffeic acid 3-O-methyltransferase-like	Juglans regia	XP_018829884.1
TRINITY_DN18839_c0_g1_i1_6	2,40E-66	201	KRH28044.1	hypothetical protein GLYMA_11G031200	Glycine max	CAY07947.1
TRINITY_DN12937_c0_g1_i1_3	6,71E-58	196	XP_018825635.1	translation initiation factor eIF-2B subunit epsilon isoform X2	Juglans regia	XP_018825636.1
TRINITY_DN3038_c0_g1_i1_4	7,63E-153	435	XP_018850543.1	beta-glucuronosyltransferase GlcAT14B-like	Juglans regia	XP_018850551.1
TRINITY_DN3285_c0_g1_i1_1	9,78E-129	404	XP_018859288.1	E3 ubiquitin-protein ligase UPL2-like	Juglans regia	XP_007141284.1
TRINITY_DN4341_c0_g1_i1_2	1,82E-62	198	XP_018839261.1	translation initiation factor eIF-2B subunit alpha	Juglans regia	OAY36332.1
TRINITY_DN5664_c0_g1_i1_3	5,36E-117	359	XP_018843108.1	protein lsd90	Juglans regia	XP_008224596.1
TRINITY_DN3970_c0_g1_i1_1	4,33E-79	243	XP_018835712.1	uncharacterized membrane protein At1g06890-like	Juglans regia	XP_018835713.1
TRINITY_DN12410_c0_g1_i1_6	0	579	XP_018846318.1	cinnamoyl-CoA reductase 1-like	Juglans regia	KDP41979.1
TRINITY_DN4354_c0_g1_i1_4	8,27E-49	163	KDP28778.1	hypothetical protein JCGZ_14549	Jatropha curcas	XP_012083605.1
TRINITY_DN9720_c0_g1_i6_2	1,95E-50	165	XP_018815216.1	U1 small nuclear ribonucleoprotein A-like	Juglans regia	XP_018843610.1
TRINITY_DN22980_c0_g1_i1_3	1,25E-30	118	XP_018830674.1	uncharacterized protein LOC108998567 isoform X2	Juglans regia	XP_018830673.1
TRINITY_DN9256_c0_g1_i4_1	0	615	XP_018826937.1	uncharacterized protein LOC108995769	Juglans regia	XP_008229750.1
TRINITY_DN8333_c0_g1_i1_3	1,72E-70	222	XP_018826609.1	activating signal cointegrator 1	Juglans regia	ACU17315.1
TRINITY_DN294_c0_g1_i1_2	0	595	XP_018824318.1	ubiquitin carboxyl-terminal hydrolase 9-like	Juglans regia	XP_018817088.1

TRINITY_DN8005_c0_g1_i2_2	1,53E-151	426	XP_018847846.1	ras-related protein Rab7 isoform X1	Juglans regia	XP_018847847.1
TRINITY_DN17859_c0_g1_i1_3	1,75E-12	63,2	XP_018841427.1	uncharacterized protein LOC109006562	Juglans regia	GAU32297.1
TRINITY_DN1542_c0_g1_i1_6	1,06E-62	203	XP_008231569.2	probable inactive purple acid phosphatase 27	Prunus mume	XP_018820767.1
TRINITY_DN7035_c1_g2_i1_3	0	746	XP_018843045.1	AT-rich interactive domain-containing protein 6 isoform X1	Juglans regia	XP_018843046.1
TRINITY_DN19252_c0_g1_i1_4	4,16E-58	196	XP_018830059.1	alpha,alpha-trehalose-phosphate synthase [UDP-forming] 1-like isoform X4	Juglans regia	XP_018830058.1
TRINITY_DN1690_c0_g1_i2_5	0	703	XP_018820372.1	translation initiation factor eIF-2B subunit beta-like isoform X1	Juglans regia	XP_018820373.1
TRINITY_DN18166_c0_g1_i1_4	6,06E-17	77,8	AES99208.1	zinc ion-binding protein	Medicago truncatula	XP_003616250.1
TRINITY_DN9981_c1_g1_i1_3	6,51E-127	366	XP_018815961.1	vacuolar protein sorting-associated protein 28 homolog 2	Juglans regia	XP_018815962.1
TRINITY_DN8032_c0_g1_i1_2	0	611	XP_018818273.1	metal tolerance protein 4-like	Juglans regia	XP_018834606.1
TRINITY_DN10046_c0_g2_i1_4	2,51E-162	459	XP_018845168.1	uric acid degradation bifunctional protein TTL isoform X2	Juglans regia	XP_018845167.1
TRINITY_DN21517_c0_g1_i1_4	8,40E-99	301	XP_018828206.1	peptidyl-prolyl cis-trans isomerase FKBP62-like	Juglans regia	XP_018828206.1
TRINITY_DN316_c0_g1_i1_1	2,91E-64	201	XP_018812502.1	putative SNAP25 homologous protein SNAP30	Juglans regia	KHN48724.1
TRINITY_DN8057_c0_g1_i2_5	0	533	XP_018806434.1	gamma-soluble NSF attachment protein-like	Juglans regia	XP_008225700.1
TRINITY_DN22718_c0_g1_i1_2	1,80E-24	99	XP_018844938.1	ADP-ribosylation factor GTPase-activating protein AGD12-like	Juglans regia	XP_018844939.1
TRINITY_DN5217_c0_g1_i1_4	0	575	XP_018819506.1	4-hydroxy-3-methylbut-2-en-1-yl diphosphate synthase (ferredoxin), chloroplastic	Juglans regia	XP_018819507.1
TRINITY_DN8159_c0_g2_i1_1	1,51E-75	227	AFK38803.1	unknown	Lotus japonicus	KRH00272.1
TRINITY_DN2797_c0_g2_i1_4	1,10E-163	463	XP_008360906.1	uncharacterized protein LOC103424589	Malus domestica	XP_018858950.1
TRINITY_DN3030_c0_g1_i1_6	0	819	XP_018823023.1	NADH dehydrogenase [ubiquinone] complex I, assembly factor 7	Juglans regia	KDP45470.1
TRINITY_DN16052_c0_g1_i1_2	1,70E-86	253	XP_007132547.1	hypothetical protein PHAVU_011G1037000g, partial	Phaseolus vulgaris	ESW04541.1
TRINITY_DN8771_c0_g1_i1_2	2,83E-93	285	KDP39067.1	hypothetical protein JCGZ_00824	Jatropha curcas	XP_008365610.1
TRINITY_DN5711_c0_g2_i1_5	2,26E-84	255	XP_016166363.1	cytochrome c oxidase assembly protein COX11, mitochondrial	Arachis ipaensis	XP_016166364.1
TRINITY_DN11595_c1_g1_i1_3	0	1071	XP_018828763.1	protein NRT1/ PTR FAMILY 8.3-like	Juglans regia	ONI31929.1
TRINITY_DN18730_c0_g1_i1_1	9,14E-88	272	XP_018822599.1	electron transfer flavoprotein-ubiquinone oxidoreductase, mitochondrial isoform X2	Juglans regia	XP_018822598.1
TRINITY_DN5685_c0_g1_i4_5	6,81E-100	295	ACU18395.1	unknown	Glycine max	XP_012084998.1
TRINITY_DN11028_c0_g3_i1_3	4,46E-141	399	OAY62531.1	hypothetical protein MANES_01G274300	Manihot esculenta	AFK48486.1
TRINITY_DN5255_c1_g1_i1_4	2,26E-59	184	XP_008246172.1	serine hydroxymethyltransferase 3, chloroplastic-like, partial	Prunus mume	KYP52446.1
TRINITY_DN10013_c0_g1_i4_2	2,01E-165	464	XP_018820232.1	very-long-chain 3-oxoacyl-CoA reductase 1	Juglans regia	OAY32455.1

TRINITY_DN6316_c0_g1_i4_3	0	838	XP_018840171.1	actin-related protein 4-like	Juglans regia	XP_018825389.1
TRINITY_DN7363_c0_g1_i1_3	0	804	XP_018816542.1	uncharacterized protein LOC108987901 isoform X1	Juglans regia	KDP21813.1
TRINITY_DN18596_c0_g1_i1_5	1,05E-108	326	OIV90433.1	hypothetical protein TanjilG_01911	Lupinus angustifolius	XP_019426881.1
TRINITY_DN17114_c0_g1_i1_1	3,16E-38	134	XP_018828255.1	LOW QUALITY PROTEIN: protein TIC 22-like, chloroplastic	Juglans regia	XP_009373582.1
TRINITY_DN19946_c0_g1_i1_4	0	536	XP_018818905.1	probable trans-2-enoyl-CoA reductase, mitochondrial isoform X1	Juglans regia	OAY37907.1
TRINITY_DN8995_c0_g1_i1_3	0	841	XP_018813899.1	proteasome activator subunit 4-like	Juglans regia	XP_008359029.2
TRINITY_DN9650_c0_g1_i1_1	0	542	XP_018822229.1	protein WVD2-like 6 isoform X2	Juglans regia	XP_018822227.1
TRINITY_DN7338_c0_g3_i1_2	3,58E-78	257	XP_008237688.1	putative transcription elongation factor SPT5 homolog 1	Prunus mume	ONH89815.1
TRINITY_DN4072_c0_g2_i1_2	3,94E-172	530	XP_018837736.1	protein MODIFIER OF SNC1 1-like isoform X2	Juglans regia	XP_018837729.1
TRINITY_DN2062_c0_g1_i1_6	2,87E-136	403	OAY52772.1	hypothetical protein MANES_04G109900	Manihot esculenta	XP_009334631.1
TRINITY_DN5791_c1_g1_i1_2	2,45E-71	227	XP_018822123.1	xylulose kinase	Juglans regia	XP_018822124.1
TRINITY_DN6320_c0_g2_i1_1	0	541	XP_018839054.1	probable rhamnogalacturonate lyase B	Juglans regia	XP_018842495.1
TRINITY_DN6069_c0_g2_i1_6	4,02E-93	275	XP_018808825.1	uncharacterized protein LOC108982016	Juglans regia	XP_008238218.1
TRINITY_DN18724_c0_g1_i1_4	1,21E-86	259	OAY40689.1	hypothetical protein MANES_09G041800	Manihot esculenta	ONH95766.1
TRINITY_DN4227_c0_g1_i1_3	0	736	XP_018830819.1	uncharacterized protein LOC108998648	Juglans regia	ONI25150.1
TRINITY_DN18944_c0_g1_i1_1	2,90E-92	287	XP_018837769.1	pentatricopeptide repeat-containing protein At2g37230	Juglans regia	XP_018837770.1
TRINITY_DN9604_c0_g1_i3_3	4,04E-151	431	XP_018851110.1	uncharacterized protein At3g49720-like	Juglans regia	XP_018851111.1
TRINITY_DN19600_c0_g1_i1_6	4,85E-68	228	XP_018815969.1	granule-bound starch synthase 2, chloroplastic/amyloplastic-like	Juglans regia	XP_018813997.1
TRINITY_DN2329_c0_g1_i2_6	6,20E-82	259	XP_018851853.1	molybdopterin biosynthesis protein CNX1	Juglans regia	XP_018851854.1
TRINITY_DN827_c0_g1_i1_1	0	731	XP_018828741.1	regulator of nonsense transcripts UPF2	Juglans regia	XP_008233383.1
TRINITY_DN8908_c0_g1_i2_1	0	766	XP_018821636.1	arabinosyltransferase RRA3-like	Juglans regia	ONI32725.1
TRINITY_DN18109_c0_g1_i1_5	3,65E-62	206	KDP23937.1	hypothetical protein JCGZ_27097	Jatropha curcas	XP_012088555.1
TRINITY_DN11206_c0_g1_i2_4	2,70E-58	182	XP_018834275.1	uncharacterized protein At4g28440	Juglans regia	XP_018805198.1
TRINITY_DN18626_c1_g2_i1_2	1,03E-168	477	XP_018818706.1	26S proteasome regulatory subunit RPN13 isoform X2	Juglans regia	XP_018818704.1
TRINITY_DN21756_c0_g1_i1_1	9,83E-18	80,9	XP_018822873.1	nudix hydrolase 20, chloroplastic-like isoform X1	Juglans regia	XP_018822874.1
TRINITY_DN2355_c0_g1_i1_2	2,95E-92	276	XP_018831876.1	serine/threonine-protein kinase STY46-like isoform X2	Juglans regia	XP_018831875.1
TRINITY_DN2509_c0_g1_i1_1	0	702	XP_018805322.1	L-gulonolactone oxidase 2-like	Juglans regia	KRH06390.1
TRINITY_DN19715_c0_g1_i1_5	5,95E-25	98,2	XP_018845106.1	uncharacterized protein LOC109009176	Juglans regia	XP_008231154.1
TRINITY_DN4748_c0_g1_i1_2	0	719	XP_018851863.1	serine/threonine-protein phosphatase 7	Juglans regia	XP_012087578.1
TRINITY_DN21521_c1_g1_i1_3	1,56E-22	94,7	XP_018820196.1	uncharacterized protein LOC108990636	Juglans regia	KYP62920.1
TRINITY_DN5242_c0_g3_i1_6	0	719	XP_018833828.1	110 kDa U5 small nuclear ribonucleoprotein component CLO	Juglans regia	KYP57369.1
TRINITY_DN19260_c0_g1_i1_1	1,29E-52	181	XP_018807838.1	uncharacterized protein LOC108981175	Juglans regia	ONH92663.1
TRINITY_DN3078_c0_g1_i1_3	0	641	XP_018853672.1	methionine aminopeptidase 1A	Juglans regia	OAY62431.1
TRINITY_DN10865_c0_g2_i2_2	1,11E-87	263	XP_018838514.1	uncharacterized protein LOC109004430	Juglans regia	XP_019422537.1

TRINITY_DN17892_c1_g1_i1_5	8,88E-134	389	XP_018818227.1	serine carboxypeptidase-like 20	Juglans regia	XP_019449310.1
TRINITY_DN2656_c0_g1_i1_2	2,62E-50	175	XP_018851186.1	trafficking protein particle complex subunit 8 isoform X3	Juglans regia	XP_018851184.1
TRINITY_DN22169_c0_g1_i1_5	9,01E-29	109	OAY41043.1	hypothetical protein MANES_09G069500, partial	Manihot esculenta	XP_018812823.1
TRINITY_DN10693_c1_g1_i1_3	0	705	XP_018840893.1	transducin beta-like protein 2	Juglans regia	XP_018809678.1
TRINITY_DN7588_c0_g1_i2_5	9,29E-115	331	XP_018835502.1	transmembrane emp24 domain-containing protein p24delta3-like	Juglans regia	XP_008339346.1
TRINITY_DN1809_c0_g1_i1_3	0	679	XP_018847511.1	nudix hydrolase 3 isoform X1	Juglans regia	XP_018847513.1
TRINITY_DN10089_c0_g3_i1_3	0	751	XP_008228163.1	oligouridylate-binding protein 1B	Prunus mume	ONI15331.1
TRINITY_DN2082_c1_g1_i1_3	1,10E-53	184	XP_018826267.1	receptor-like protein kinase ANXUR2	Juglans regia	OAY27654.1
TRINITY_DN238_c0_g1_i1_3	6,04E-24	96,3	XP_018815084.1	2-methylene-furan-3-one reductase-like	Juglans regia	ACU18144.1
TRINITY_DN5724_c0_g2_i1_1	0	777	XP_018845876.1	probable beta-1,4-xylosyltransferase IRX10L isoform X1	Juglans regia	KHN17455.1
TRINITY_DN21573_c0_g1_i1_1	3,37E-15	73,9	XP_018808385.1	probable alkaline/neutral invertase D	Juglans regia	XP_008378785.1
TRINITY_DN11569_c0_g1_i2_3	4,31E-62	197	KDP21030.1	hypothetical protein JCGZ_21501	Jatropha curcas	XP_012091694.1
TRINITY_DN5094_c0_g1_i1_4	1,23E-63	194	XP_017434911.1	actin	Vigna angularis	AFP43695.1
TRINITY_DN19637_c0_g1_i1_4	5,06E-118	348	XP_018830337.1	ATP-dependent 6-phosphofructokinase 5, chloroplastic-like	Juglans regia	XP_018820958.1
TRINITY_DN4599_c0_g1_i1_1	0	835	XP_018839577.1	phospholipase D Z-like	Juglans regia	ONI32918.1
TRINITY_DN11898_c0_g1_i1_4	0	547	XP_018824800.1	CTP synthase-like	Juglans regia	XP_018824801.1
TRINITY_DN13648_c0_g1_i1_3	1,56E-52	176	XP_018815870.1	BAG family molecular chaperone regulator 7-like	Juglans regia	XP_018851941.1
TRINITY_DN22384_c0_g1_i1_6	1,39E-72	234	XP_018824673.1	uncharacterized protein LOC108994054 isoform X2	Juglans regia	XP_018824674.1
TRINITY_DN8593_c0_g1_i1_3	0	543	XP_018840607.1	uncharacterized protein LOC109005947	Juglans regia	XP_018826121.1
TRINITY_DN6624_c0_g2_i2_2	1,26E-151	437	XP_018834729.1	syntaxin-32	Juglans regia	XP_018834730.1
TRINITY_DN8264_c0_g1_i2_1	8,29E-127	392	XP_018850843.1	apoptotic chromatin condensation inducer in the nucleus-like isoform X3	Juglans regia	XP_018850843.1
TRINITY_DN7248_c0_g1_i1_5	2,44E-50	171	XP_018816030.1	cytochrome P450 CYP82D47-like	Juglans regia	XP_018846611.1
TRINITY_DN19832_c0_g1_i1_3	2,23E-67	223	KDP22648.1	hypothetical protein JCGZ_02490	Jatropha curcas	XP_012089904.1
TRINITY_DN23704_c0_g1_i1_6	1,05E-24	99,4	XP_018816821.1	2-hydroxyacyl-CoA lyase	Juglans regia	XP_008229283.1
TRINITY_DN15338_c0_g1_i1_3	1,38E-42	142	XP_018854121.1	pentatricopeptide repeat-containing protein At1g26460, mitochondrial-like, partial	Juglans regia	XP_018859839.1
TRINITY_DN9948_c0_g2_i1_3	1,30E-57	187	XP_018841100.1	chorismate mutase 1, chloroplastic-like	Juglans regia	XP_018840102.1
TRINITY_DN13388_c0_g1_i1_5	9,98E-71	223	XP_018809497.1	probable arabinosyltransferase ARAD1	Juglans regia	OAY58565.1
TRINITY_DN5559_c0_g1_i1_4	0	890	XP_018827750.1	zinc finger protein ZPR1-like	Juglans regia	XP_020202613.1
TRINITY_DN17697_c0_g1_i1_3	6,61E-64	201	XP_008228276.1	glyoxylate/succinic semialdehyde reductase 2, chloroplastic	Prunus mume	XP_018821557.1
TRINITY_DN5154_c0_g1_i1_5	7,00E-74	241	XP_018848228.1	serine/arginine-rich splicing factor SR45 isoform X2	Juglans regia	XP_018848227.1
TRINITY_DN20546_c0_g1_i1_1	1,07E-104	331	XP_018826303.1	ferredoxin-dependent glutamate synthase, chloroplastic-like isoform X2	Juglans regia	XP_018826298.1

TRINITY_DN6573_c0_g1_i1_4	0	662	XP_018848829.1	rhomboid-like protein 15	Juglans regia	XP_018851134.1
TRINITY_DN3293_c0_g1_i1_2	1,81E-166	483	XP_018841822.1	golgin candidate 2-like	Juglans regia	KDP41144.1
TRINITY_DN9459_c0_g2_i1_1	0	1064	XP_018831622.1	beta-glucosidase-like SFR2, chloroplastic isoform X1	Juglans regia	XP_018831623.1
TRINITY_DN4865_c0_g1_i1_1	2,77E-101	312	XP_018842148.1	putative clathrin assembly protein At4g02650	Juglans regia	XP_018851018.1
TRINITY_DN3689_c0_g2_i1_5	3,03E-152	434	XP_018813758.1	protein TIC 22, chloroplastic	Juglans regia	OAY22914.1
TRINITY_DN2686_c0_g1_i1_5	5,53E-96	286	XP_018834185.1	uncharacterized protein LOC109001389 isoform X1	Juglans regia	OAY58732.1
TRINITY_DN4162_c0_g1_i1_5	0	675	XP_018852272.1	nuclear pore complex protein NUP93A-like	Juglans regia	OAY36979.1
TRINITY_DN14842_c0_g1_i1_3	1,40E-37	137	XP_008233492.1	delta-1-pyrroline-5-carboxylate synthase	Prunus mume	ALT55651.1
TRINITY_DN1392_c0_g2_i1_1	0	598	XP_018815848.1	probable ubiquitin conjugation factor E4	Juglans regia	ONI19115.1
TRINITY_DN3303_c0_g1_i1_3	8,21E-118	353	KRH38150.1	hypothetical protein GLYMA_09G114500	Glycine max	KDP38447.1
TRINITY_DN5303_c0_g1_i1_3	5,01E-134	387	XP_018850136.1	developmentally-regulated G-protein 3	Juglans regia	XP_016162010.1
TRINITY_DN3626_c0_g3_i1_6	2,74E-74	224	XP_018845630.1	putative peptidyl-tRNA hydrolase PTRHD1	Juglans regia	XP_018817605.1
TRINITY_DN12852_c0_g1_i1_2	2,36E-137	404	XP_018808302.1	probable 26S protease regulatory subunit 10B isoform X2	Juglans regia	XP_018808301.1
TRINITY_DN16500_c0_g1_i1_3	2,23E-39	141	XP_018835573.1	presequence protease 1, chloroplastic/mitochondrial-like	Juglans regia	XP_004511282.1
TRINITY_DN13231_c0_g1_i1_1	3,24E-71	228	XP_018843888.1	transmembrane protein 184B	Juglans regia	XP_018843889.1
TRINITY_DN9164_c0_g1_i1_1	1,47E-47	161	XP_018836471.1	ferredoxin-thioredoxin reductase, variable chain, chloroplastic	Juglans regia	XP_018836479.1
TRINITY_DN6110_c0_g1_i2_1	2,55E-133	385	XP_018827350.1	deoxyhypusine hydroxylase	Juglans regia	XP_018827350.1
TRINITY_DN9679_c0_g2_i6_4	0	803	XP_018841362.1	squalene synthase-like isoform X1	Juglans regia	XP_018815354.1
TRINITY_DN185_c0_g1_i1_1	0	539	XP_018827553.1	cation/H(+) antiporter 15-like	Juglans regia	ONI14265.1
TRINITY_DN16939_c0_g1_i1_3	2,27E-38	137	AES72328.1	4-coumarate:CoA ligase-like protein	Medicago truncatula	XP_003602077.1
TRINITY_DN10156_c0_g1_i1_1	6,77E-71	235	XP_018805424.1	phosphomethylpyrimidine synthase, chloroplastic	Juglans regia	XP_018805432.1
TRINITY_DN9970_c0_g1_i3_1	2,07E-04	45,4	XP_018836017.1	THO complex subunit 4A-like	Juglans regia	XP_018823251.1
TRINITY_DN11182_c0_g1_i1_5	2,33E-67	208	XP_018825198.1	uncharacterized protein LOC108994434	Juglans regia	XP_018825199.1
TRINITY_DN6813_c0_g2_i1_6	0	1066	XP_018825750.1	aldehyde dehydrogenase 22A1	Juglans regia	XP_008223354.1
TRINITY_DN18653_c0_g1_i1_5	6,41E-23	101	XP_018834253.1	AP-4 complex subunit epsilon-like	Juglans regia	XP_008375399.1
TRINITY_DN17522_c0_g1_i1_4	1,66E-53	179	OIW18230.1	hypothetical protein TanjilG_06314	Lupinus angustifolius	XP_019458044.1
TRINITY_DN14413_c0_g1_i1_4	5,05E-49	158	XP_017183078.1	ruvB-like 2	Malus domestica	XP_017180148.1
TRINITY_DN5635_c0_g1_i1_5	0	763	OAY61248.1	hypothetical protein MANES_01G175100	Manihot esculenta	OAY61249.1
TRINITY_DN10294_c0_g1_i2_1	7,96E-154	439	XP_018811873.1	peroxisome biogenesis protein 22-like isoform X1	Juglans regia	KDP25866.1
TRINITY_DN19410_c0_g2_i1_2	1,70E-64	199	XP_018845496.1	lachrymatory-factor synthase-like	Juglans regia	XP_004501141.1
TRINITY_DN3519_c0_g1_i1_4	2,14E-144	414	XP_008243353.1	uncharacterized protein LOC103341587	Prunus mume	ONI31661.1
TRINITY_DN10650_c0_g1_i3_2	1,95E-28	108	XP_018827787.1	ylmG homolog protein 1-2, chloroplastic-like	Juglans regia	XP_008227970.1
TRINITY_DN3798_c0_g1_i2_1	0	531	XP_018813476.1	AP-1 complex subunit mu-2-like	Juglans regia	XP_016190265.1

TRINITY_DN2524_c0_g1_i1_2	1,46E-98	302	XP_018826310.1	peptide-N(4)-(N-acetyl-beta-glucosaminyl)asparagine amidase isoform X1	Juglans regia	XP_018826311.1
TRINITY_DN14304_c0_g1_i1_3	9,17E-06	45,8	XP_018830807.1	protein trichome birefringence-like 18	Juglans regia	XP_018830808.1
TRINITY_DN10946_c0_g1_i1_3	0	886	XP_018846000.1	histidinol dehydrogenase, chloroplastic isoform X1	Juglans regia	XP_018846001.1
TRINITY_DN4824_c0_g1_i1_6	5,59E-61	187	XP_018806648.1	histone deacetylase complex subunit SAP18-like, partial	Juglans regia	XP_014522945.1
TRINITY_DN1455_c0_g1_i1_5	8,59E-144	409	XP_018812413.1	uncharacterized protein LOC108984806	Juglans regia	OAY21731.1
TRINITY_DN19943_c0_g1_i1_1	1,73E-19	84,7	XP_018823987.1	peroxisomal and mitochondrial division factor 2-like	Juglans regia	XP_018823988.1
TRINITY_DN7659_c0_g1_i2_1	3,03E-65	199	XP_018835303.1	glycine-rich RNA-binding protein 4, mitochondrial-like	Juglans regia	XP_018835298.1
TRINITY_DN18303_c0_g1_i1_2	3,72E-16	79	XP_018805226.1	protein trichome birefringence-like 2	Juglans regia	ONI30408.1
TRINITY_DN7752_c0_g1_i1_1	6,08E-140	399	XP_018829221.1	uncharacterized protein LOC108997414	Juglans regia	OAY42346.1
TRINITY_DN3113_c0_g1_i1_2	8,77E-114	338	KDP40491.1	hypothetical protein JCGZ_24490	Jatropha curcas	XP_012068599.1
TRINITY_DN13954_c0_g1_i1_5	8,95E-105	310	XP_018842294.1	uncharacterized protein LOC109007179	Juglans regia	OIW20924.1
TRINITY_DN9113_c0_g1_i1_6	0	1004	XP_018850790.1	ABC transporter D family member 1-like isoform X3	Juglans regia	XP_018850790.1
TRINITY_DN2642_c0_g1_i1_1	0	566	OAY49731.1	hypothetical protein MANES_05G078500	Manihot esculenta	OAY61640.1
TRINITY_DN9696_c0_g1_i1_5	1,82E-64	200	XP_018856972.1	replication protein A 14 kDa subunit B-like	Juglans regia	XP_018856973.1
TRINITY_DN2508_c0_g1_i1_3	0	528	XP_018852462.1	serine racemase	Juglans regia	XP_008238865.1
TRINITY_DN8157_c0_g1_i1_3	3,94E-105	310	XP_018817798.1	CBS domain-containing protein CBSX1, chloroplastic-like	Juglans regia	XP_018817799.1
TRINITY_DN20773_c0_g1_i1_5	1,54E-69	226	XP_018858250.1	uncharacterized protein LOC109020252	Juglans regia	KHN23130.1
TRINITY_DN2427_c0_g1_i1_5	1,21E-93	282	XP_018807354.1	uncharacterized protein LOC108980800	Juglans regia	XP_018807362.1
TRINITY_DN566_c0_g2_i1_1	0	613	XP_018805839.1	dolichyl-phosphate beta-glucosyltransferase-like	Juglans regia	XP_018814490.1
TRINITY_DN1856_c0_g2_i1_3	9,73E-65	212	XP_018811690.1	neutral/alkaline invertase 3, chloroplastic-like	Juglans regia	XP_018811691.1
TRINITY_DN3611_c0_g1_i2_3	0	773	XP_018815648.1	pentatricopeptide repeat-containing protein At2g15690-like	Juglans regia	XP_018819012.1
TRINITY_DN8137_c0_g1_i1_6	3,99E-91	282	OAY49267.1	hypothetical protein MANES_05G042200	Manihot esculenta	OAY49266.1
TRINITY_DN4839_c0_g2_i1_2	1,30E-32	114	XP_018819611.1	uncharacterized protein LOC108990185	Juglans regia	OAY25146.1
TRINITY_DN10092_c0_g1_i1_3	0	687	XP_018812786.1	F-box protein SKIP16-like isoform X1	Juglans regia	XP_018822588.1
TRINITY_DN8102_c0_g1_i1_6	0	951	XP_018810077.1	ubiquitin fusion degradation protein 1	Juglans regia	XP_008229992.1
TRINITY_DN2436_c0_g1_i1_2	1,59E-154	440	XP_018850153.1	reticulon-4-interacting protein 1, mitochondrial-like isoform X1	Juglans regia	XP_018822796.1
TRINITY_DN9750_c0_g2_i3_3	4,42E-110	326	XP_009344066.1	polyadenylate-binding protein-interacting protein 12-like	Pyrus x bretschneideri	XP_009344066.1
TRINITY_DN13772_c0_g1_i1_5	3,24E-39	140	KHN38006.1	Topless-related protein 1	Glycine soja	XP_018840348.1
TRINITY_DN20973_c0_g1_i1_1	5,95E-37	130	XP_018810129.1	mitogen-activated protein kinase kinase 5-like	Juglans regia	XP_008229371.1
TRINITY_DN13500_c0_g1_i1_1	4,95E-88	272	XP_018838255.1	dnaJ homolog subfamily C member 2	Juglans regia	XP_008229529.1

TRINITY_DN19696_c0_g1_i1_1	1,17E-49	174	XP_018812876.1	ABC transporter C family member 3-like	Juglans regia	XP_018812887.1
TRINITY_DN18663_c0_g1_i1_3	1,64E-167	493	XP_018808616.1	phosphoenolpyruvate carboxylase 2	Juglans regia	AJP17168.1
TRINITY_DN10941_c0_g1_i2_2	6,04E-61	192	XP_018841839.1	uncharacterized protein LOC109006880	Juglans regia	XP_018841840.1
TRINITY_DN205_c0_g2_i1_3	1,73E-148	422	XP_018842270.1	N-(5'-phosphoribosyl)anthranilate isomerase 1, chloroplastic-like isoform X2	Juglans regia	XP_018842269.1
TRINITY_DN10620_c0_g1_i1_3	0	511	XP_018843207.1	peroxisomal adenine nucleotide carrier 1-like	Juglans regia	XP_018843209.1
TRINITY_DN12943_c0_g1_i1_3	8,56E-65	214	KDP29908.1	hypothetical protein JCGZ_18477	Jatropha curcas	XP_012081434.1
TRINITY_DN2735_c0_g1_i1_2	4,83E-115	348	XP_018826310.1	peptide-N(4)-(N-acetyl-beta-glucosaminyl)asparagine amidase isoform X1	Juglans regia	XP_018826311.1
TRINITY_DN15045_c0_g1_i1_3	5,31E-38	138	XP_018849759.1	ALG-2 interacting protein X-like	Juglans regia	XP_018849760.1
TRINITY_DN17024_c0_g1_i1_1	1,24E-66	211	XP_018819405.1	alanine--glyoxylate aminotransferase 2 homolog 2, mitochondrial-like	Juglans regia	XP_018813062.1
TRINITY_DN4004_c0_g1_i1_6	7,38E-116	340	XP_018824946.1	probable mediator of RNA polymerase II transcription subunit 26c	Juglans regia	XP_018824947.1
TRINITY_DN12269_c0_g2_i1_2	2,68E-123	357	XP_018841143.1	uncharacterized protein LOC109006342 isoform X1	Juglans regia	XP_018841144.1
TRINITY_DN9455_c0_g1_i1_6	9,01E-124	359	KDP42878.1	hypothetical protein JCGZ_23820	Jatropha curcas	XP_012066253.1
TRINITY_DN7173_c0_g2_i1_4	1,17E-177	516	XP_018824311.1	importin subunit beta-1-like	Juglans regia	XP_018835204.1
TRINITY_DN5388_c0_g2_i1_3	8,11E-45	149	XP_018830706.1	CDGSH iron-sulfur domain-containing protein NEET	Juglans regia	XP_020221377.1
TRINITY_DN10533_c0_g1_i4_1	0	755	XP_018834221.1	far upstream element-binding protein 2-like isoform X2	Juglans regia	XP_018834221.1
TRINITY_DN15880_c0_g1_i1_6	7,95E-37	134	XP_018816812.1	transportin MOS14 isoform X1	Juglans regia	XP_018816813.1
TRINITY_DN21652_c0_g1_i1_3	1,82E-32	122	XP_018825842.1	uncharacterized protein LOC108994897 isoform X4	Juglans regia	XP_018825838.1
TRINITY_DN22321_c0_g1_i1_6	1,04E-12	68,9	XP_018809403.1	homeobox protein HAT3.1	Juglans regia	XP_018809403.1
TRINITY_DN12098_c0_g2_i1_2	3,50E-63	205	XP_018807977.1	uncharacterized protein LOC108981316	Juglans regia	XP_008236697.1
TRINITY_DN6146_c0_g1_i2_4	3,61E-90	267	XP_009363132.1	NADH dehydrogenase [ubiquinone] 1 alpha subcomplex assembly factor 3-like isoform X1	Pyrus x bretschneideri	XP_009363133.1
TRINITY_DN8428_c0_g1_i1_3	0	648	XP_018813445.1	probable beta-D-xylosidase 6	Juglans regia	XP_008236660.1
TRINITY_DN17847_c0_g2_i1_2	3,28E-170	508	XP_018841626.1	uncharacterized protein LOC109006713	Juglans regia	KDP23248.1
TRINITY_DN4796_c0_g1_i1_2	2,84E-77	236	XP_018847950.1	uncharacterized protein LOC109011278	Juglans regia	XP_018831509.1
TRINITY_DN5072_c0_g1_i1_1	4,42E-110	324	XP_018808361.1	50S ribosomal protein L3-2, chloroplastic-like	Juglans regia	XP_008223504.1
TRINITY_DN6088_c0_g1_i4_1	5,47E-150	424	XP_018829327.1	transport and Golgi organization 2 homolog	Juglans regia	XP_008372850.1
TRINITY_DN15797_c0_g1_i1_2	1,03E-59	190	XP_018858693.1	traB domain-containing protein-like	Juglans regia	XP_018820413.1
TRINITY_DN18297_c0_g2_i1_1	0	577	XP_018811142.1	mevalonate kinase	Juglans regia	XP_008246488.1
TRINITY_DN17041_c0_g1_i1_1	2,43E-150	429	XP_018836516.1	caffeoylshikimate esterase-like	Juglans regia	XP_018828026.1
TRINITY_DN7113_c0_g1_i3_4	2,00E-72	219	XP_018858971.1	mitochondrial import inner membrane translocase subunit Tim16	Juglans regia	KDP42870.1

TRINITY_DN12379_c0_g1_i1_3	5,87E-47	159	XP_018833231.1	5'-nucleotidase domain-containing protein DDB_G0275467 isoform X3	Juglans regia	XP_018833232.1
TRINITY_DN15487_c0_g1_i1_3	2,60E-31	120	XP_018831313.1	WD repeat-containing protein 44-like	Juglans regia	AES79256.1
TRINITY_DN3915_c0_g1_i1_3	3,06E-111	320	XP_018813585.1	protein transport protein SFT2-like	Juglans regia	XP_018825801.1
TRINITY_DN11583_c1_g3_i2_2	0	556	XP_018817582.1	casein kinase 1-like protein 1	Juglans regia	XP_018845405.1
TRINITY_DN11439_c0_g1_i3_4	2,37E-169	474	XP_018824239.1	proteasome subunit beta type-4-like	Juglans regia	XP_018844541.1
TRINITY_DN7466_c0_g3_i1_3	0	1048	XP_018842944.1	ATP-dependent zinc metalloprotease FTSH 9, chloroplastic-like	Juglans regia	ONH99982.1
TRINITY_DN11372_c0_g1_i1_1	0	602	XP_018848836.1	uncharacterized protein LOC109011909 isoform X1	Juglans regia	XP_018848837.1
TRINITY_DN2241_c0_g1_i1_2	1,05E-79	245	XP_018828611.1	uncharacterized protein LOC108996994	Juglans regia	XP_018819156.1
TRINITY_DN2564_c0_g1_i1_3	0	936	XP_018816881.1	WPP domain-interacting tail-anchored protein 1-like	Juglans regia	XP_018816882.1
TRINITY_DN4503_c0_g1_i1_2	1,58E-72	226	ONI27712.1	hypothetical protein PRUPE_1G100900	Prunus persica	XP_007222683.1
TRINITY_DN8115_c0_g1_i1_2	0	1428	XP_018832818.1	general negative regulator of transcription subunit 3 isoform X2	Juglans regia	XP_018832816.1
TRINITY_DN271_c0_g1_i1_6	2,20E-34	127	XP_018842163.1	beta-xylosidase/alpha-L-arabinofuranosidase 2-like	Juglans regia	OAY52100.1
TRINITY_DN12627_c0_g1_i1_5	5,08E-70	222	XP_018837869.1	coumaroyl-CoA:anthocyanidin 3-O-glucoside-6''-O-coumaroyltransferase 1-like	Juglans regia	KRH22466.1
TRINITY_DN6168_c0_g2_i1_3	6,65E-110	320	XP_018845283.1	4-hydroxy-tetrahydrodipicolinate reductase 2, chloroplastic-like	Juglans regia	KDP26187.1
TRINITY_DN15083_c0_g1_i1_4	1,26E-30	116	XP_018807197.1	uncharacterized protein LOC108980650	Juglans regia	KHN15602.1
TRINITY_DN22088_c0_g1_i1_2	7,30E-26	103	XP_018841295.1	kinase-interacting protein 1-like	Juglans regia	XP_020216020.1
TRINITY_DN10295_c0_g1_i4_2	6,79E-38	132	XP_018845364.1	uncharacterized protein LOC109009365	Juglans regia	XP_008232032.1
TRINITY_DN13908_c0_g1_i1_1	1,03E-80	248	XP_009375745.2	5'-nucleotidase domain-containing protein DDB_G0275467 isoform X2	Pyrus x bretschneideri	XP_017187537.1
TRINITY_DN250_c0_g2_i1_3	1,07E-35	133	ONH93446.1	hypothetical protein PRUPE_8G233000	Prunus persica	XP_020426420.1
TRINITY_DN9708_c0_g1_i1_2	0	875	XP_018818699.1	serine carboxypeptidase II-2	Juglans regia	XP_008243035.1
TRINITY_DN14410_c0_g1_i1_1	1,85E-34	129	OIW08904.1	hypothetical protein TanjilG_05880	Lupinus angustifolius	XP_019448393.1
TRINITY_DN2548_c0_g2_i1_4	0	673	KDP28658.1	hypothetical protein JCGZ_14429	Jatropha curcas	XP_012083434.1
TRINITY_DN6286_c0_g1_i1_1	2,13E-39	137	XP_018812884.1	putative UDP-sugar transporter DDB_G0278631	Juglans regia	XP_018822183.1
TRINITY_DN17369_c0_g1_i1_5	3,98E-72	237	XP_018822483.1	uncharacterized protein DDB_G0283697-like	Juglans regia	XP_018826899.1
TRINITY_DN8472_c0_g1_i1_2	9,61E-86	258	XP_018809112.1	binding partner of ACD11 1-like	Juglans regia	KDP28936.1
TRINITY_DN4972_c0_g1_i4_1	0	785	XP_009368358.1	serine/threonine-protein phosphatase BSL3	Pyrus x bretschneideri	XP_008385576.1
TRINITY_DN9379_c0_g1_i3_2	2,04E-57	184	XP_018859928.1	peroxisome biogenesis protein 19-2-like	Juglans regia	XP_015933903.1
TRINITY_DN23442_c0_g1_i1_5	5,23E-28	103	XP_018837143.1	uncharacterized protein LOC109003443 isoform X5	Juglans regia	XP_018837137.1
TRINITY_DN20268_c0_g1_i1_5	8,58E-45	157	XP_018842469.1	probable glutamyl endopeptidase, chloroplastic isoform X1	Juglans regia	XP_018842470.1
TRINITY_DN2087_c1_g1_i1_3	4,02E-158	448	XP_018851393.1	uncharacterized protein LOC109013683	Juglans regia	XP_018851394.1

TRINITY_DN9218_c0_g1_i7_3	0	1042	XP_018857360.1	pre-mRNA-processing factor 19-like isoform X1	Juglans regia	XP_018807788.1
TRINITY_DN12527_c0_g1_i1_2	5,29E-109	328	XP_018833558.1	uncharacterized protein LOC109000940	Juglans regia	XP_008352247.1
TRINITY_DN22389_c0_g1_i1_6	1,44E-06	49,7	XP_018849371.1	protein ELC-like	Juglans regia	
TRINITY_DN9732_c0_g1_i3_2	4,88E-116	333	XP_018859435.1	trafficking protein particle complex subunit 6B	Juglans regia	XP_020208519.1
TRINITY_DN9091_c0_g1_i1_3	9,51E-11	60,1	XP_018856978.1	uncharacterized protein LOC109019191 isoform X2	Juglans regia	XP_018856977.1
TRINITY_DN1560_c0_g1_i1_1	6,48E-101	300	XP_018816582.1	uncharacterized protein LOC108987951	Juglans regia	XP_009365025.1
TRINITY_DN14904_c0_g1_i1_6	4,91E-50	166	XP_018834985.1	thioredoxin reductase 1-like	Juglans regia	KRH28954.1
TRINITY_DN12383_c0_g1_i1_2	4,09E-37	135	XP_018835537.1	dnaJ homolog subfamily C member 2-like	Juglans regia	XP_018842014.1
TRINITY_DN5000_c0_g1_i1_6	0	1019	XP_018814165.1	peroxisome biogenesis protein 5	Juglans regia	KDP29519.1
TRINITY_DN2849_c0_g1_i1_4	0	637	XP_018824599.1	adenylyltransferase and sulfurtransferase MOCS3	Juglans regia	XP_015973067.1
TRINITY_DN11583_c1_g1_i2_1	0	793	XP_018835116.1	casein kinase 1-like protein 2	Juglans regia	ONI15496.1
TRINITY_DN11555_c0_g1_i1_4	0	2027	XP_008231249.1	cellulose synthase A catalytic subunit 3 [UDP-forming]	Prunus mume	XP_008231250.1
TRINITY_DN11211_c0_g2_i4_2	5,48E-81	246	XP_018845966.1	REF/SRPP-like protein At1g67360 isoform X1	Juglans regia	XP_018845967.1
TRINITY_DN10288_c0_g2_i1_3	4,76E-107	326	XP_018819413.1	uncharacterized protein LOC108990025 isoform X2	Juglans regia	XP_018819412.1
TRINITY_DN16723_c0_g1_i1_1	3,07E-31	118	XP_018841898.1	probable starch synthase 4, chloroplastic/amyloplastic	Juglans regia	XP_014490146.1
TRINITY_DN5420_c0_g1_i1_2	0	815	XP_018832761.1	inositol phosphorylceramide glucuronosyltransferase 1	Juglans regia	ONI05028.1
TRINITY_DN15186_c0_g1_i1_2	4,57E-70	213	XP_009352822.1	uncharacterized protein LOC103944136	Pyrus x bretschneideri	XP_018838494.1
TRINITY_DN6233_c0_g1_i4_1	2,13E-128	376	XP_018815872.1	probable mitochondrial saccharopine dehydrogenase-like oxidoreductase At5g39410	Juglans regia	ONI19128.1
TRINITY_DN4088_c0_g1_i1_1	0	843	XP_018808493.1	mechanosensitive ion channel protein 1, mitochondrial	Juglans regia	XP_018808494.1
TRINITY_DN1341_c0_g1_i2_3	3,09E-19	79	KYP46136.1	hypothetical protein KK1_032303	Cajanus cajan	KDP39739.1
TRINITY_DN5675_c0_g1_i3_1	1,40E-176	498	XP_018855654.1	survival of motor neuron-related-splicing factor 30 isoform X1	Juglans regia	XP_008218638.1
TRINITY_DN5431_c0_g2_i1_2	4,06E-138	406	XP_018818099.1	probable E3 ubiquitin-protein ligase LOG2	Juglans regia	OAY42666.1
TRINITY_DN9005_c1_g1_i3_1	0	644	XP_018822849.1	sodium/pyruvate cotransporter BASS2, chloroplastic isoform X1	Juglans regia	XP_018822850.1
TRINITY_DN22172_c0_g1_i1_3	9,05E-19	84	XP_018856996.1	golgin candidate 5-like	Juglans regia	XP_018837529.1
TRINITY_DN5643_c0_g2_i1_3	0	708	XP_018813681.1	cell division protein FtsZ homolog 2-2, chloroplastic	Juglans regia	XP_018813682.1
TRINITY_DN18093_c0_g1_i1_1	2,24E-127	365	XP_017182893.1	exocyst complex component SEC8-like	Malus domestica	OAY59728.1
TRINITY_DN8505_c0_g1_i1_3	8,18E-92	272	XP_018818253.1	bifunctional bis(5'-adenosyl)-triphosphatase/adenylylsulfatase FHIT	Juglans regia	XP_019439570.1

TRINITY_DN3938_c0_g1_i1_2	0	690	XP_018831176.1	uncharacterized Rho GTPase-activating protein At5g61530-like isoform X2	Juglans regia	XP_018831175.1
TRINITY_DN11081_c0_g1_i2_3	0	624	XP_018823256.1	protein kinase APK1A, chloroplastic-like isoform X3	Juglans regia	XP_018823257.1
TRINITY_DN8657_c0_g1_i1_3	4,23E-71	215	XP_018825467.1	ankyrin repeat-containing protein P16F5.05c isoform X2	Juglans regia	XP_020418677.1
TRINITY_DN18007_c0_g1_i1_2	1,43E-87	261	XP_018848880.1	uncharacterized protein LOC109011934	Juglans regia	XP_018848881.1
TRINITY_DN11493_c0_g1_i3_3	8,48E-86	255	XP_008241493.1	ras-related protein RABE1a	Prunus mume	XP_008387728.1
TRINITY_DN11659_c1_g1_i3_5	0	979	XP_018831593.1	aldehyde dehydrogenase family 2 member B4, mitochondrial isoform X1	Juglans regia	XP_018831594.1
TRINITY_DN11367_c0_g1_i1_2	1,04E-167	473	XP_018805726.1	uncharacterized protein LOC108979494	Juglans regia	OAY61686.1
TRINITY_DN14035_c0_g1_i1_1	1,55E-19	86,3	XP_009350736.1	protein STRUBBELIG-RECEPTOR FAMILY 3-like isoform X1	Pyrus x bretschneideri	XP_009350742.1
TRINITY_DN7672_c0_g2_i3_6	0	548	XP_018831761.1	COP9 signalosome complex subunit 2	Juglans regia	XP_004493278.1
TRINITY_DN4363_c0_g1_i1_2	0	1005	XP_018836397.1	probable acyl-activating enzyme 1, peroxisomal isoform X1	Juglans regia	ONH90064.1
TRINITY_DN9570_c0_g1_i2_6	0	873	XP_018810880.1	GBF-interacting protein 1-like isoform X2	Juglans regia	XP_018810879.1
TRINITY_DN12047_c0_g1_i1_5	9,56E-68	216	XP_018835604.1	armadillo repeat-containing protein 6	Juglans regia	XP_004498168.1
TRINITY_DN8161_c0_g1_i1_2	1,13E-104	307	XP_018845119.1	uncharacterized protein LOC109009183 isoform X1	Juglans regia	XP_018845108.1
TRINITY_DN9497_c1_g1_i1_5	5,60E-38	127	XP_018815228.1	mitochondrial zinc maintenance protein 1, mitochondrial	Juglans regia	XP_008235091.1
TRINITY_DN18064_c0_g1_i1_5	3,44E-64	215	XP_018818865.1	uncharacterized protein LOC108989641	Juglans regia	XP_007132178.1
TRINITY_DN3901_c0_g1_i1_6	3,05E-50	163	XP_018859305.1	uncharacterized protein C6C3.02c-like isoform X1	Juglans regia	XP_018859307.1
TRINITY_DN9544_c0_g1_i2_3	2,91E-109	317	OAY27343.1	hypothetical protein MANES_16G118300	Manihot esculenta	KDP33636.1
TRINITY_DN5823_c2_g1_i1_1	1,82E-49	173	XP_018806543.1	isoleucine--tRNA ligase, cytoplasmic	Juglans regia	XP_008222579.1
TRINITY_DN10520_c0_g1_i1_2	0	1111	XP_018814583.1	conserved oligomeric Golgi complex subunit 6	Juglans regia	KDP37812.1
TRINITY_DN11739_c0_g2_i1_5	1,07E-60	203	XP_018837001.1	RAN GTPase-activating protein 1	Juglans regia	XP_018837002.1
TRINITY_DN950_c0_g2_i1_5	8,17E-88	281	XP_018825599.1	nuclear export mediator factor NEMF-like	Juglans regia	XP_018856628.1
TRINITY_DN1342_c0_g2_i1_1	1,92E-162	472	OAY47836.1	hypothetical protein MANES_06G109500	Manihot esculenta	KHN34349.1
TRINITY_DN10787_c0_g2_i1_6	0	652	XP_018844870.1	UDP-glycosyltransferase 71K1-like	Juglans regia	KYP72899.1
TRINITY_DN15996_c0_g1_i1_5	4,04E-98	307	ONI09503.1	hypothetical protein PRUPE_5G241600	Prunus persica	ONI09502.1
TRINITY_DN13943_c0_g1_i1_2	4,19E-69	214	XP_016203508.1	prostaglandin E synthase 2	Arachis ipaensis	XP_020958475.1
TRINITY_DN4307_c0_g2_i2_3	0	990	KDP41268.1	hypothetical protein JCGZ_15675	Jatropha curcas	XP_012067735.1
TRINITY_DN13850_c0_g1_i1_2	3,13E-44	154	XP_018817793.1	polyadenylate-binding protein 8-like	Juglans regia	XP_018817793.1
TRINITY_DN22319_c0_g1_i1_6	1,68E-51	178	XP_018849426.1	uncharacterized protein LOC109012316	Juglans regia	OIV96914.1
TRINITY_DN19381_c0_g1_i1_1	0	640	XP_018829523.1	nuclear pore complex protein NUP155	Juglans regia	XP_008221462.1
TRINITY_DN17549_c0_g1_i1_3	9,36E-81	257	XP_009379451.1	outer envelope protein 80, chloroplastic-like	Pyrus x bretschneideri	XP_009376166.1
TRINITY_DN15080_c0_g2_i1_2	1,45E-87	264	XP_018811585.1	uncharacterized protein LOC108984165 isoform X2	Juglans regia	XP_018811577.1
TRINITY_DN18817_c0_g1_i1_6	5,87E-33	119	XP_018833631.1	eugenol synthase 1-like	Juglans regia	OAY25173.1

TRINITY_DN11009_c0_g1_i1_1	1,20E-50	172	XP_018805570.1	aspartyl protease family protein 2	Juglans regia	XP_008371957.1
TRINITY_DN8994_c0_g2_i2_3	6,32E-74	226	XP_018806489.1	universal stress protein A-like protein	Juglans regia	XP_009356253.1
TRINITY_DN17962_c0_g1_i1_6	1,46E-56	182	XP_018837748.1	diphthine methyltransferase homolog	Juglans regia	XP_018837749.1
TRINITY_DN7296_c0_g1_i1_3	3,61E-102	330	XP_018830148.1	nucleolin 2-like isoform X2	Juglans regia	XP_018830147.1
TRINITY_DN17826_c0_g2_i1_1	0	807	XP_018820470.1	3-phosphoinositide-dependent protein kinase 1-like	Juglans regia	XP_018853620.1
TRINITY_DN12326_c0_g1_i1_4	1,83E-82	254	XP_018815852.1	GDP-mannose transporter GONST3 isoform X2	Juglans regia	XP_018815851.1
TRINITY_DN2620_c0_g2_i2_2	3,11E-62	195	XP_008377079.1	small nuclear ribonucleoprotein SmD3b	Malus domestica	XP_008387870.1
TRINITY_DN2361_c0_g1_i1_2	2,81E-39	138	KDP47108.1	hypothetical protein JCGZ_03916	Jatropha curcas	XP_012069973.1
TRINITY_DN1411_c0_g1_i1_5	3,05E-172	495	XP_018834612.1	formate--tetrahydrofolate ligase	Juglans regia	KHN42246.1
TRINITY_DN11963_c0_g1_i1_5	7,68E-36	130	XP_018821632.1	chaperonin CPN60-like 2, mitochondrial isoform X1	Juglans regia	XP_008228292.1
TRINITY_DN9986_c0_g1_i2_1	0	683	XP_018807809.1	metal tolerance protein 10-like	Juglans regia	XP_018857914.1
TRINITY_DN3850_c0_g1_i1_2	0	753	XP_018850129.1	4-alpha-glucanotransferase, chloroplastic/amyloplastic	Juglans regia	XP_008232738.1
TRINITY_DN13162_c0_g1_i1_5	6,08E-126	384	XP_018825830.1	ubiquitin-activating enzyme E1 1-like isoform X2	Juglans regia	XP_018825828.1
TRINITY_DN6999_c1_g1_i1_1	5,63E-123	358	XP_018821643.1	uncharacterized protein LOC108991725	Juglans regia	XP_018848582.1
TRINITY_DN6417_c0_g2_i1_1	0	600	XP_018841012.1	probable ethanolamine kinase	Juglans regia	ONH98290.1
TRINITY_DN11852_c0_g1_i1_2	5,53E-171	483	XP_018845391.1	bifunctional protein FolD 4, chloroplastic	Juglans regia	OAY53286.1
TRINITY_DN3994_c0_g1_i1_6	1,85E-55	186	XP_004491215.1	stomatal closure-related actin-binding protein 1	Cicer arietinum	XP_006595782.1
TRINITY_DN10757_c0_g1_i6_3	5,90E-109	315	XP_018852475.1	probable glutathione peroxidase 8	Juglans regia	AFK48933.1
TRINITY_DN858_c0_g2_i1_3	0	682	XP_018823141.1	mannose-1-phosphate guanyltransferase alpha-like	Juglans regia	XP_018823142.1
TRINITY_DN23297_c0_g1_i1_4	1,74E-52	179	KDP42514.1	hypothetical protein JCGZ_00311	Jatropha curcas	XP_012066762.1
TRINITY_DN19876_c0_g1_i1_6	1,62E-66	214	XP_018847448.1	uncharacterized protein LOC109010934	Juglans regia	XP_018825310.1
TRINITY_DN11014_c0_g1_i1_4	5,08E-83	248	XP_018849005.1	universal stress protein PHOS32-like	Juglans regia	XP_018849006.1
TRINITY_DN13986_c1_g1_i1_2	7,92E-52	177	XP_018827526.1	beta-galactosidase 10	Juglans regia	OAY36002.1
TRINITY_DN4916_c0_g2_i1_4	6,56E-61	188	XP_018816863.1	uncharacterized protein LOC108988171	Juglans regia	XP_018816864.1
TRINITY_DN13205_c0_g1_i1_2	2,58E-72	243	XP_018837736.1	protein MODIFIER OF SNC1 1-like isoform X2	Juglans regia	XP_018837729.1
TRINITY_DN2844_c0_g1_i1_3	1,43E-101	300	dbj|BAT85501.1	hypothetical protein VIGAN_04305800	Vigna angularis var. angularis	XP_014627214.1
TRINITY_DN19026_c0_g1_i1_2	5,05E-28	109	XP_009339937.1	calcium-binding protein 39-like	Pyrus x bretschneideri	XP_008390799.1
TRINITY_DN10596_c0_g1_i1_3	0	948	XP_018821947.1	uncharacterized protein LOC108991975	Juglans regia	XP_008230188.1
TRINITY_DN11089_c0_g1_i1_6	3,24E-108	323	XP_018815200.1	serine/arginine-rich-splicing factor SR34-like isoform X3	Juglans regia	XP_018815196.1
TRINITY_DN1625_c0_g1_i1_1	3,35E-43	149	XP_018806507.1	glucan endo-1,3-beta-glucosidase-like	Juglans regia	XP_018814434.1
TRINITY_DN4295_c0_g1_i2_2	2,63E-34	120	XP_018858723.1	tetraspanin-19 isoform X5	Juglans regia	XP_018858724.1
TRINITY_DN21571_c0_g1_i1_3	9,11E-42	148	ONI35915.1	hypothetical protein PRUPE_1G560300	Prunus persica	XP_020534686.1
TRINITY_DN9775_c0_g1_i1_1	5,30E-56	182	OAY41762.1	hypothetical protein MANES_09G127700	Manihot esculenta	KDP39526.1

TRINITY_DN17721_c0_g1_i1_3	4,19E-83	258	XP_018815636.1	biotin carboxyl carrier protein of acetyl-CoA carboxylase, chloroplastic isoform X1	Juglans regia	ADN52613.1
TRINITY_DN11860_c0_g1_i1_4	1,03E-118	353	XP_018839411.1	dymeclin-like isoform X2	Juglans regia	XP_018818649.1
TRINITY_DN19531_c0_g1_i1_1	9,15E-122	373	XP_018822938.1	exocyst complex component SEC8-like	Juglans regia	XP_018822939.1
TRINITY_DN194_c0_g2_i1_1	2,76E-163	467	XP_018816985.1	pentatricopeptide repeat-containing protein At1g77360, mitochondrial-like isoform X5	Juglans regia	XP_018816984.1
TRINITY_DN426_c0_g1_i1_2	1,56E-121	365	XP_018809084.1	delta-1-pyrroline-5-carboxylate synthase isoform X2	Juglans regia	XP_018809083.1
TRINITY_DN23380_c0_g1_i1_2	2,71E-27	108	XP_020987369.1	LOW QUALITY PROTEIN: uncharacterized protein LOC107464626	Arachis duranensis	XP_009360071.1
TRINITY_DN7432_c0_g1_i1_5	0	530	XP_018833108.1	cysteine desulfurase 1, chloroplastic	Juglans regia	KOM53194.1
TRINITY_DN9824_c0_g2_i1_2	6,19E-77	231	XP_018829803.1	uncharacterized protein LOC108997870	Juglans regia	XP_008218429.1
TRINITY_DN14943_c0_g1_i1_3	5,82E-41	147	XP_015941468.1	phosphoglucomutase, chloroplastic	Arachis duranensis	AES62609.1
TRINITY_DN4390_c0_g1_i2_1	0	546	XP_018849861.1	dihydrolipoyllysine-residue succinyltransferase component of 2-oxoglutarate dehydrogenase complex 1, mitochondrial-like isoform X1	Juglans regia	XP_018849862.1
TRINITY_DN3895_c0_g2_i1_2	7,87E-114	350	XP_018806735.1	mechanosensitive ion channel protein 2, chloroplastic	Juglans regia	XP_018806736.1
TRINITY_DN2101_c0_g1_i1_2	6,54E-22	92,4	XP_018809774.1	protein transport protein SEC31 homolog B-like isoform X1	Juglans regia	XP_018809776.1
TRINITY_DN22364_c0_g1_i1_6	5,18E-39	140	XP_009365627.1	exocyst complex component SEC3A-like	Pyrus x bretschneideri	ONI32790.1
TRINITY_DN3021_c0_g2_i1_6	0	629	XP_018849969.1	ketohexokinase-like isoform X1	Juglans regia	ONH93084.1
TRINITY_DN1919_c0_g1_i1_1	0	513	XP_018849245.1	protoporphyrinogen oxidase, mitochondrial isoform X1	Juglans regia	XP_018849245.1
TRINITY_DN11220_c1_g3_i1_3	3,74E-132	377	XP_018841297.1	tropinone reductase homolog At5g06060-like	Juglans regia	XP_018850680.1
TRINITY_DN8579_c0_g2_i1_1	0	665	XP_012091599.1	DNA-directed RNA polymerase II subunit RPB2 isoform X1	Jatropha curcas	XP_020541368.1
TRINITY_DN7867_c0_g1_i4_3	3,24E-110	320	XP_018809788.1	glucose-induced degradation protein 8 homolog	Juglans regia	XP_018809789.1
TRINITY_DN11046_c0_g1_i2_2	0	1017	XP_018828658.1	BTB/POZ domain-containing protein At5g03250-like	Juglans regia	XP_018844832.1
TRINITY_DN8640_c0_g1_i1_1	4,14E-103	300	XP_008374850.1	protein C2-DOMAIN ABA-RELATED 4-like	Malus domestica	XP_018857996.1
TRINITY_DN2390_c0_g2_i1_1	7,25E-78	239	XP_018822547.1	probable hydroxyacylglutathione hydrolase 2, chloroplastic isoform X2	Juglans regia	XP_018812777.1
TRINITY_DN12575_c0_g1_i1_3	1,38E-124	370	XP_020973003.1	K(+) efflux antiporter 4, partial	Arachis ipaensis	XP_016651595.1
TRINITY_DN10417_c0_g2_i1_1	0	731	XP_018826781.1	TBC1 domain family member 17-like isoform X1	Juglans regia	XP_018826782.1
TRINITY_DN17426_c0_g1_i1_6	4,67E-48	166	XP_018816046.1	uncharacterized protein LOC108987537	Juglans regia	OAY44637.1
TRINITY_DN9926_c0_g1_i2_5	2,52E-23	94	XP_018836523.1	beta-hexosaminidase 1-like	Juglans regia	KDP37399.1
TRINITY_DN2534_c0_g1_i1_4	2,64E-92	286	XP_018824493.1	uncharacterized protein LOC108993891	Juglans regia	KRG98729.1

TRINITY_DN2915_c0_g1_i1_3	2,62E-43	152	XP_018849647.1	acetyl-CoA carboxylase 1-like	Juglans regia	XP_018849648.1
TRINITY_DN11633_c0_g1_i3_5	0	930	XP_018844883.1	non-specific phospholipase C1-like	Juglans regia	XP_018846016.1
TRINITY_DN15398_c0_g1_i1_5	1,83E-81	262	XP_018828504.1	protein TIC110, chloroplastic isoform X2	Juglans regia	XP_018828503.1
TRINITY_DN5905_c0_g1_i1_3	0	573	ONI09011.1	hypothetical protein PRUPE_5G211900	Prunus persica	XP_008240052.1
TRINITY_DN9808_c0_g1_i1_1	2,11E-117	345	XP_018826112.1	BI1-like protein	Juglans regia	XP_018840552.1
TRINITY_DN10830_c0_g1_i1_1	0	516	XP_018851799.1	ACT domain-containing protein ACR12	Juglans regia	XP_018846054.1
TRINITY_DN5571_c0_g1_i2_2	6,04E-96	298	XP_018854351.1	cingulin-like	Juglans regia	XP_018806130.1
TRINITY_DN14099_c0_g1_i1_1	4,69E-105	326	XP_018828510.1	rab3 GTPase-activating protein catalytic subunit isoform X1	Juglans regia	XP_018828511.1
TRINITY_DN1513_c0_g1_i1_1	2,27E-134	388	XP_018845774.1	pentatricopeptide repeat-containing protein At4g35850, mitochondrial-like isoform X1	Juglans regia	XP_018845777.1
TRINITY_DN9703_c0_g1_i1_1	4,37E-89	268	XP_018826884.1	uncharacterized protein LOC108995726	Juglans regia	XP_018818332.1
TRINITY_DN30_c0_g1_i1_2	0	595	XP_018847661.1	CBL-interacting serine/threonine-protein kinase 23 isoform X3	Juglans regia	XP_018819681.1
TRINITY_DN4271_c0_g2_i1_6	4,38E-117	343	XP_018842294.1	uncharacterized protein LOC109007179	Juglans regia	KDP40340.1
TRINITY_DN9637_c0_g1_i2_1	0	704	XP_018809628.1	protein PELOTA 1	Juglans regia	XP_009337981.1
TRINITY_DN14624_c0_g1_i1_5	1,48E-41	149	XP_012090136.1	ABC transporter C family member 10	Jatropha curcas	KDP22187.1
TRINITY_DN7843_c0_g1_i2_1	5,58E-33	115	OAY45897.1	hypothetical protein MANES_07G101100	Manihot esculenta	ADB85087.1
TRINITY_DN14064_c0_g1_i1_1	1,87E-42	150	XP_018829333.1	uncharacterized protein LOC108997475	Juglans regia	XP_018829334.1
TRINITY_DN19958_c0_g1_i1_2	1,11E-20	84,7	XP_018807289.1	CRIB domain-containing protein RIC5-like	Juglans regia	KDP26504.1
TRINITY_DN12317_c0_g2_i1_2	5,77E-77	237	OAY38183.1	hypothetical protein MANES_11G159800	Manihot esculenta	OAY38182.1
TRINITY_DN106_c0_g1_i1_6	5,53E-148	420	XP_018827300.1	putative uridine kinase C227.14	Juglans regia	ONH93435.1
TRINITY_DN10121_c0_g1_i2_2	0	527	XP_018848619.1	protein FLX-like 1	Juglans regia	XP_018813162.1
TRINITY_DN16265_c0_g1_i1_6	2,47E-35	133	XP_018820691.1	protein S-acyltransferase 24	Juglans regia	ONH98508.1
TRINITY_DN22994_c0_g1_i1_2	2,28E-46	159	XP_018807599.1	EH domain-containing protein 1-like	Juglans regia	OAY28437.1
TRINITY_DN16148_c0_g1_i1_2	5,42E-41	145	KHN15894.1	L-gulonolactone oxidase	Glycine soja	KRH06390.1
TRINITY_DN10093_c0_g1_i1_4	2,21E-117	374	XP_018819031.1	uncharacterized protein LOC108989762 isoform X2	Juglans regia	XP_018819030.1
TRINITY_DN13095_c0_g1_i1_2	0	639	ONI08824.1	hypothetical protein PRUPE_5G203000	Prunus persica	XP_007210336.1
TRINITY_DN6032_c0_g1_i1_1	3,94E-95	295	XP_018821273.1	peroxisomal membrane protein PEX14	Juglans regia	XP_018821274.1
TRINITY_DN4104_c0_g1_i2_2	3,25E-59	191	XP_018839964.1	protein ENHANCED DISEASE RESISTANCE 2-like isoform X3	Juglans regia	XP_018839960.1
TRINITY_DN11490_c0_g1_i4_6	1,85E-58	184	ABW22414.1	S1 ribosomal protein (mitochondrion)	Glycine max	CBX33386.1
TRINITY_DN11358_c0_g1_i2_2	1,86E-149	444	XP_018838909.1	uncharacterized protein LOC109004724 isoform X2	Juglans regia	XP_018838910.1
TRINITY_DN5926_c0_g1_i1_3	4,44E-78	234	XP_018811596.1	uncharacterized protein At4g28440-like	Juglans regia	KDP21003.1
TRINITY_DN10755_c0_g1_i3_1	4,41E-54	172	XP_018846043.1	protein KRTCAP2 homolog	Juglans regia	OAY23478.1
TRINITY_DN13112_c0_g1_i1_5	1,23E-23	97,8	OAY35140.1	hypothetical protein MANES_12G075600	Manihot esculenta	OAY35139.1
TRINITY_DN1601_c0_g1_i1_6	1,51E-64	202	XP_018822833.1	uncharacterized protein LOC108992673	Juglans regia	KHN28085.1
TRINITY_DN20005_c0_g1_i1_6	9,24E-118	361	XP_018813272.1	AP-2 complex subunit alpha-1-like	Juglans regia	XP_018833174.1
TRINITY_DN9298_c0_g1_i2_6	1,51E-82	258	XP_018855556.1	probable methyltransferase PMT13	Juglans regia	XP_018855557.1
TRINITY_DN10791_c2_g1_i1_2	0	521	XP_018841148.1	12-oxophytodienoate reductase 2-like	Juglans regia	OAY23951.1
TRINITY_DN12338_c0_g1_i1_2	1,27E-51	176	ONI01534.1	hypothetical protein PRUPE_6G144700	Prunus persica	XP_008230284.1

TRINITY_DN2894_c0_g1_i1_6	2,68E-22	94,7	XP_018817532.1	ubiquitin carboxyl-terminal hydrolase 24 isoform X2	Juglans regia	XP_018817528.1
TRINITY_DN9321_c0_g1_i1_1	1,47E-125	385	XP_018859491.1	uncharacterized protein LOC109021330 isoform X4	Juglans regia	XP_018859489.1
TRINITY_DN8786_c1_g1_i1_3	4,61E-59	195	XP_018806595.1	probable glycosyltransferase At5g03795	Juglans regia	XP_009364236.1
TRINITY_DN8717_c0_g1_i1_1	0	1162	XP_018815330.1	SUMO-activating enzyme subunit 2-like isoform X1	Juglans regia	XP_018807405.1
TRINITY_DN266_c0_g2_i2_2	0	642	XP_018821455.1	poly(U)-specific endoribonuclease-B-like isoform X1	Juglans regia	XP_018821456.1
TRINITY_DN10315_c0_g1_i3_3	0	548	XP_018822721.1	secretory carrier-associated membrane protein 3-like	Juglans regia	XP_018839942.1
TRINITY_DN19933_c0_g1_i1_3	7,89E-46	162	OIV90433.1	hypothetical protein TanjilG_01911	Lupinus angustifolius	XP_019426881.1
TRINITY_DN9610_c0_g1_i5_6	2,47E-140	403	XP_018842815.1	RNA-binding protein 1-like	Juglans regia	XP_018842816.1
TRINITY_DN3076_c0_g1_i1_4	3,46E-162	468	XP_008372906.1	26S proteasome non-ATPase regulatory subunit 4 homolog isoform X1	Malus domestica	ONH92223.1
TRINITY_DN10575_c0_g1_i1_1	1,01E-77	241	XP_018809258.1	uncharacterized protein LOC108982363	Juglans regia	ONI18405.1
TRINITY_DN14305_c0_g1_i1_5	9,34E-90	265	XP_018817440.1	phosphatidylinositol transfer protein 2-like isoform X1	Juglans regia	XP_018817441.1
TRINITY_DN11352_c0_g1_i1_1	2,92E-132	400	XP_018826436.1	U1 small nuclear ribonucleoprotein 70 kDa-like	Juglans regia	XP_018826436.1
TRINITY_DN6147_c0_g1_i2_6	3,64E-54	172	ONH97119.1	hypothetical protein PRUPE_7G170900	Prunus persica	XP_007202770.1
TRINITY_DN10057_c1_g1_i2_3	6,45E-66	201	XP_008224427.1	40S ribosomal protein S15-4	Prunus mume	XP_008391094.1
TRINITY_DN13010_c0_g1_i1_1	4,52E-25	95,9	KYP69972.1	hypothetical protein KK1_009179	Cajanus cajan	XP_020212031.1
TRINITY_DN2392_c0_g1_i1_2	0	632	OAY28989.1	hypothetical protein MANES_15G109300	Manihot esculenta	KDP30591.1
TRINITY_DN9690_c0_g1_i2_3	2,46E-140	404	XP_020240158.1	syntaxin-71-like	Cajanus cajan	OAY42657.1
TRINITY_DN11443_c0_g1_i1_3	1,42E-177	500	KDP28756.1	hypothetical protein JCGZ_14527	Jatropha curcas	XP_012083577.1
TRINITY_DN14199_c0_g1_i1_6	2,65E-69	226	XP_018806735.1	mechanosensitive ion channel protein 2, chloroplastic	Juglans regia	XP_018806736.1
TRINITY_DN7984_c0_g1_i2_3	7,26E-49	171	XP_018831324.1	H/ACA ribonucleoprotein complex subunit 4	Juglans regia	XP_014518907.1
TRINITY_DN15785_c0_g1_i1_6	9,38E-51	162	XP_020206789.1	dynamin-2B-like	Cajanus cajan	GAU44658.1
TRINITY_DN21396_c0_g1_i1_3	2,28E-76	236	XP_020212887.1	beta-1,3-galactosyltransferase 7-like isoform X1	Cajanus cajan	KYP70021.1
TRINITY_DN7330_c0_g1_i1_3	0	880	XP_018827923.1	DEAD-box ATP-dependent RNA helicase 31-like isoform X1	Juglans regia	XP_018827924.1
TRINITY_DN9918_c0_g2_i5_3	0	932	YP_009318148.1	ATP synthase CF1 alpha subunit (chloroplast)	Corylus avellana	AOZ20350.1
TRINITY_DN10206_c0_g8_i1_3	0	543	XP_018828519.1	CBL-interacting serine/threonine-protein kinase 11	Juglans regia	XP_018835724.1
TRINITY_DN21905_c0_g1_i1_6	4,27E-38	138	XP_018813845.1	uncharacterized protein LOC108985854 isoform X2	Juglans regia	XP_018813843.1
TRINITY_DN20071_c0_g1_i1_6	2,13E-33	125	XP_018845025.1	phosphatidylinositol 3,4,5-trisphosphate 3-phosphatase and protein-tyrosine-phosphatase PTEN2A-like	Juglans regia	KHN36257.1

TRINITY_DN8834_c0_g1_i4_2	1,17E-75	230	XP_004494031.1	iron-sulfur assembly protein IscA-like 1, mitochondrial	Cicer arietinum	KRH67908.1
TRINITY_DN10839_c0_g1_i3_6	7,03E-95	278	XP_008237795.1	NADH dehydrogenase [ubiquinone] iron-sulfur protein 4, mitochondrial	Prunus mume	ONI02745.1
TRINITY_DN2321_c0_g1_i1_6	4,96E-152	429	XP_018815159.1	thiamine pyrophosphokinase 1 isoform X1	Juglans regia	ONH93890.1
TRINITY_DN12272_c0_g1_i1_2	5,69E-28	110	XP_012087612.1	arginyl-tRNA--protein transferase 2 isoform X2	Jatropha curcas	KDP44783.1
TRINITY_DN8223_c1_g1_i1_3	1,51E-13	68,9	XP_018833203.1	uncharacterized protein LOC109000690 isoform X1	Juglans regia	XP_018843246.1
TRINITY_DN11343_c0_g1_i7_1	0	872	dbj|BAO50884.1	ATP synthase F1 subunit 1 (mitochondrion)	Hevea brasiliensis	BAO50925.1
TRINITY_DN20424_c0_g1_i1_1	6,05E-45	159	XP_018840549.1	protein FAR1-RELATED SEQUENCE 7-like	Juglans regia	XP_008353242.1
TRINITY_DN3551_c0_g1_i1_3	1,89E-113	346	XP_018851703.1	polygalacturonate 4-alpha-galacturonosyltransferase-like isoform X2	Juglans regia	XP_018851702.1
TRINITY_DN9616_c0_g1_i1_1	2,00E-99	293	ONI26263.1	hypothetical protein PRUPE_1G013500	Prunus persica	KDP36554.1
TRINITY_DN13664_c0_g1_i1_1	3,71E-42	150	XP_018849684.1	protein TPR2-like	Juglans regia	XP_018828941.1
TRINITY_DN9032_c0_g1_i5_4	1,61E-134	384	emb|CAX86785.1	unnamed protein product	Glycine max	CAY07604.1
TRINITY_DN10274_c0_g1_i3_2	6,40E-74	224	XP_004500009.1	peptidyl-prolyl cis-trans isomerase NIMA-interacting 4	Cicer arietinum	ACJ86035.1
TRINITY_DN10289_c0_g1_i1_3	3,23E-168	468	XP_018836403.1	dolichol-phosphate mannosyltransferase subunit 1	Juglans regia	KDP36797.1
TRINITY_DN1145_c0_g2_i1_6	1,38E-85	269	XP_018834399.1	uncharacterized protein LOC109001525	Juglans regia	XP_018816504.1
TRINITY_DN11535_c0_g1_i3_2	1,23E-82	244	XP_018815860.1	40S ribosomal protein S20-2	Juglans regia	XP_018815861.1
TRINITY_DN14223_c0_g1_i1_5	1,26E-48	157	XP_018810638.1	glycine-rich RNA-binding protein RZ1C-like, partial	Juglans regia	XP_018811403.1
TRINITY_DN18123_c0_g2_i1_2	1,68E-163	479	ONH97567.1	hypothetical protein PRUPE_7G197100	Prunus persica	XP_007204263.1
TRINITY_DN18755_c0_g1_i1_2	9,21E-21	87	OAY52867.1	hypothetical protein MANES_04G117400	Manihot esculenta	OAY52866.1
TRINITY_DN20629_c0_g1_i1_1	6,92E-95	282	XP_018806320.1	UDP-D-xylose:L-fucose alpha-1,3-D-xylosyltransferase MGP4-like, partial	Juglans regia	XP_018836102.1
TRINITY_DN20794_c0_g1_i1_5	9,85E-20	86,3	XP_008219968.1	U-box domain-containing protein 52	Prunus mume	ONI33983.1
TRINITY_DN21254_c0_g1_i1_2	6,78E-91	267	XP_018857098.1	calmodulin-like protein 3	Juglans regia	XP_020538862.1
TRINITY_DN22136_c0_g1_i1_1	4,18E-18	75,9	dbj|GAU23459.1	hypothetical protein TSUD_331570	Trifolium subterraneum	KHN35208.1
TRINITY_DN22623_c0_g1_i1_1	4,99E-30	110	OAY57022.1	hypothetical protein MANES_02G064300	Manihot esculenta	GAU26654.1
TRINITY_DN2924_c0_g1_i1_3	2,99E-146	414	ONI31113.1	hypothetical protein PRUPE_1G293300	Prunus persica	XP_020411372.1
TRINITY_DN493_c0_g1_i1_1	0	551	XP_018827207.1	flowering locus K homology domain-like	Juglans regia	XP_018827208.1
TRINITY_DN6077_c0_g2_i1_2	0	514	XP_018836291.1	methylthioribose-1-phosphate isomerase	Juglans regia	KDP33930.1
TRINITY_DN6267_c0_g1_i1_2	6,82E-25	97,4	XP_018850061.1	protein SRC2 homolog	Juglans regia	XP_018845907.1
TRINITY_DN6751_c0_g1_i1_3	9,02E-06	46,2	XP_018819823.1	MFP1 attachment factor 1-like	Juglans regia	XP_018824955.1
TRINITY_DN9010_c0_g1_i2_3	2,01E-129	373	XP_018816307.1	uncharacterized protein LOC108987761	Juglans regia	XP_018816308.1
