# Supplementary material for: Comparative proteomics of common allergenic tree pollens of birch, alder, and hazel
Source: Allergy. 2021 Jan 15;76(6):1743–53. doi: 10.1111/all.14694 (PMC8248232; doi:10.1111/all.14694)
Supplement: Supplementary file 6 — Table S4 [file ALL-76-1743-s003.pdf]

Supplementary Table S4: Pfam annotation of identified Betula pollen proteins

Protein IDs	Peptide counts (all)	Sequence coverage [%]	Mol. weight [kDa]	MQ Score	Pfam accession	Pfam family name	bitscore	e-value [Pfam]	clan
TRINITY_DN10036_c0_g1::TRINITY_DN10036_c0_g1_i2::g.4202::m.4202	1	7,8	18,092	2,7266	PF01026.20	TatD_DNase	90,6	9,60E-26	CL0034
TRINITY_DN12431_c0_g1::TRINITY_DN12431_c0_g1_i5::g.12785::m.12785	3	19,6	17,333	5,8852	PF03939.12	Ribosomal_L23eN	70,3	1,10E-19	No_clan
TRINITY_DN1011_c0_g1::TRINITY_DN1011_c0_g1_i1::g.255::m.255	1	4,3	27,47	2,1061	PF07748.12	Glyco_hydro_38C	104,3	7,40E-30	CL0103
TRINITY_DN10140_c0_g1::TRINITY_DN10140_c0_g1_i2::g.4317::m.4317	8	38,7	38,161	158,1	PF00112.22	Peptidase_C1	282,3	3,20E-84	CL0125
TRINITY_DN10146_c0_g1::TRINITY_DN10146_c0_g1_i1::g.4308::m.4308	1	11,4	13,716	5,7992	PF02953.14	zf-Tim10_DDP	88,5	1,60E-25	No_clan
TRINITY_DN10166_c0_g1::TRINITY_DN10166_c0_g1_i3::g.4379::m.4379	6	29,6	27,02	23,624	PF00166.20	Cpn10	97,2	4,20E-28	CL0296
TRINITY_DN10182_c0_g1::TRINITY_DN10182_c0_g1_i1::g.4394::m.4394	2	12,9	19,243	2,0797	PF00168.29	C2	76	2,20E-21	CL0154
TRINITY_DN10225_c0_g1::TRINITY_DN10225_c0_g1_i1::g.4474::m.4474	5	16,5	29,779	7,7568	PF00578.20	AhpC-TSA	134,3	2,00E-39	CL0172
TRINITY_DN10290_c0_g3::TRINITY_DN10290_c0_g3_i1::g.4573::m.4573	2	17,3	33,509	4,7599	PF00290.19	Trp_syntA	327,9	2,70E-98	CL0036
TRINITY_DN10291_c0_g2::TRINITY_DN10291_c0_g2_i1::g.4575::m.4575	1	6,6	17,351	2,1289	PF02777.17	Sod_Fe_C	106,4	6,60E-31	No_clan
TRINITY_DN10293_c0_g1::TRINITY_DN10293_c0_g1_i1::g.4492::m.4492	1	5,6	29,189	2,2317	PF01765.18	RRF	178,5	8,60E-53	No_clan
TRINITY_DN10295_c0_g1::TRINITY_DN10295_c0_g1_i2::g.4577::m.4577	3	16,9	21,393	5,7478	PF04752.11	ChaC	182,2	8,70E-54	CL0278
TRINITY_DN10320_c0_g1::TRINITY_DN10320_c0_g1_i1::g.4626::m.4626	2	26,2	13,422	4,4358	PF05564.11	Auxin_repressed	195,5	2,90E-58	No_clan
TRINITY_DN10326_c0_g1::TRINITY_DN10326_c0_g1_i1::g.4634::m.4634	4	14,9	37,74	-2	PF00224.20	PK	319,1	2,70E-95	CL0151
TRINITY_DN10328_c0_g1::TRINITY_DN10328_c0_g1_i1::g.4642::m.4642	1	9,6	16,672	8,2999	PF02221.14	E1_DerP2_DerF2	72,4	4,40E-20	CL0532
TRINITY_DN10333_c0_g1::TRINITY_DN10333_c0_g1_i1::g.4649::m.4649	3	8,8	49,341	4,7748	PF00009.26	GTP_EFTU	190,9	1,60E-56	CL0023
TRINITY_DN10355_c0_g1::TRINITY_DN10355_c0_g1_i1::g.4689::m.4689	3	35,3	11,426	4,662					
TRINITY_DN10355_c0_g1::TRINITY_DN10355_c0_g1_i2::g.4690::m.4690	3	33	12,112	9,3135					
TRINITY_DN10373_c0_g1::TRINITY_DN10373_c0_g1_i1::g.4691::m.4691	4	21,9	21,959	29,956					
TRINITY_DN10374_c0_g1::TRINITY_DN10374_c0_g1_i1::g.4693::m.4693	2	4,4	57,312	3,4647	PF10508.8	Proteasom_PSMB	43	2,00E-11	CL0020

TRINITY_DN10431_c0_g1::TRINITY_DN10431_c0_g1_i2::g.4811::m.4811	4	17,3	30,821	32,796	PF06552.11	TOM20_plant	332,2	7,40E-100	CL0020
TRINITY_DN10432_c0_g1::TRINITY_DN10432_c0_g1_i2::g.4807::m.4807	3	20,9	15,546	3,6181	PF01929.16	Ribosomal_L14e	91,9	2,50E-26	CL0107
TRINITY_DN10468_c0_g2::TRINITY_DN10468_c0_g2_i1::g.4854::m.4854	2	4	56,705	2,7044	PF01487.14	DHquinase_I	175	2,60E-51	CL0036
TRINITY_DN10469_c0_g1::TRINITY_DN10469_c0_g1_i1::g.4851::m.4851	2	21,6	17,001	16,863	PF05811.12	DUF842	127	3,50E-37	No_clan
TRINITY_DN10567_c0_g1::TRINITY_DN10567_c0_g1_i2::g.5026::m.5026	2	29,9	11,846	3,8758					
TRINITY_DN10578_c0_g1::TRINITY_DN10578_c0_g1_i5::g.5050::m.5050	7	17,6	59,491	11,731	PF00270.28	DEAD	182,6	5,20E-54	CL0023
TRINITY_DN10610_c0_g1::TRINITY_DN10610_c0_g1_i2::g.5110::m.5110	1	5,3	26,916	2,2859	PF02894.16	GFO_IDH_MocA_C	57,1	1,50E-15	CL0218
TRINITY_DN10617_c0_g1::TRINITY_DN10617_c0_g1_i2::g.5120::m.5120	1	7,6	20,716	2,2564	PF05916.10	Sld5	36,7	4,30E-09	No_clan
TRINITY_DN10621_c0_g1::TRINITY_DN10621_c0_g1_i2::g.5142::m.5142	1	5,5	22,52	2,1432	PF06127.10	DUF962	105,5	1,00E-30	No_clan
TRINITY_DN10627_c0_g1::TRINITY_DN10627_c0_g1_i1::g.5151::m.5151	7	19,3	65,231	14,927	PF02776.17	TPP_enzyme_N	120,4	5,50E-35	CL0254
TRINITY_DN10654_c0_g1::TRINITY_DN10654_c0_g1_i1::g.5213::m.5213	2	9,3	26,202	3,3883	PF02330.15	MAM33	70,9	1,30E-19	No_clan
TRINITY_DN10660_c0_g1::TRINITY_DN10660_c0_g1_i1::g.5237::m.5237	4	19	35,245	48,388	PF05773.21	RWD	81,7	4,20E-23	CL0208
TRINITY_DN10668_c0_g1::TRINITY_DN10668_c0_g1_i3::g.5273::m.5273	2	4,4	69,622	3,5303	PF00400.31	WD40	15,3	0,025	CL0186
TRINITY_DN10700_c0_g1::TRINITY_DN10700_c0_g1_i1::g.5317::m.5317	4	17,6	27,789	4,6665	PF00889.18	EF_TS	173,1	5,40E-51	No_clan
TRINITY_DN10710_c0_g1::TRINITY_DN10710_c0_g1_i1::g.5355::m.5355	3	13,6	26,639	5,8536					
TRINITY_DN10714_c0_g1::TRINITY_DN10714_c0_g1_i7::g.5396::m.5396	3	8,1	63,023	4,8388	PF13537.5	GATase_7	83,1	1,30E-23	CL0052
TRINITY_DN10714_c0_g1::TRINITY_DN10714_c0_g1_i4::g.5392::m.5392	2	21,2	12,651	3,9349					
TRINITY_DN10737_c0_g1::TRINITY_DN10737_c0_g1_i1::g.5423::m.5423	3	19,8	18,373	6,6117	PF00583.24	Acetyltransf_1	56,4	3,00E-15	CL0257
TRINITY_DN10747_c0_g1::TRINITY_DN10747_c0_g1_i1::g.5399::m.5399	1	4,8	32,546	1,8418	PF01612.19	DNA_pol_A_exo1	63	2,60E-17	CL0219
TRINITY_DN10767_c0_g1::TRINITY_DN10767_c0_g1_i1::g.5469::m.5469	2	10,6	38,341	2,4357	PF01212.20	Beta_elim_lyase	316,7	1,30E-94	CL0061
TRINITY_DN10774_c0_g1::TRINITY_DN10774_c0_g1_i7::g.5503::m.5503	1	6,2	21,903	2,2779	PF13012.5	MitMem_reg	91,6	3,80E-26	No_clan

TRINITY_DN10779_c0_g1::TRINITY_DN10779_c0_g1_i3::g.5511::m.5511	9	38	33,521	152,42	PF00293.27	NUDIX	75,9	2,70E-21	CL0261
TRINITY_DN10818_c0_g1::TRINITY_DN10818_c0_g1_i2::g.5587::m.5587	3	30,1	11,952	45,304	PF14368.5	LTP_2	50,6	1,50E-13	CL0482
TRINITY_DN10819_c0_g1::TRINITY_DN10819_c0_g1_i3::g.5615::m.5615	1	15,7	11,636	2,3806					
TRINITY_DN10821_c0_g1::TRINITY_DN10821_c0_g1_i1::g.5589::m.5589	2	14,8	22,468	8,7493	PF06552.11	TOM20_plant	323,5	3,40E-97	CL0020
TRINITY_DN10830_c0_g1::TRINITY_DN10830_c0_g1_i1::g.5629::m.5629	5	28,5	26,606	16,124	PF13417.5	GST_N_3	62,5	3,40E-17	CL0172
TRINITY_DN10845_c0_g1::TRINITY_DN10845_c0_g1_i1::g.5649::m.5649	1	9	23,338	2,2759					
TRINITY_DN10846_c0_g1::TRINITY_DN10846_c0_g1_i1::g.5657::m.5657	1	5,6	20,569	1,9409					
TRINITY_DN10853_c0_g1::TRINITY_DN10853_c0_g1_i1::g.5658::m.5658	2	12,6	18,966	2,8655	PF04398.11	DUF538	116	1,10E-33	No_clan
TRINITY_DN10855_c0_g2::TRINITY_DN10855_c0_g2_i3::g.5689::m.5689	2	12,6	19,959	2,8302	PF09360.9	zf-CDGSH	38	1,30E-09	No_clan
TRINITY_DN10864_c0_g1::TRINITY_DN10864_c0_g1_i1::g.5678::m.5678	1	4	44,693	2,3659	PF17177.3	PPR_long	29	5,80E-07	CL0020
TRINITY_DN10875_c0_g1::TRINITY_DN10875_c0_g1_i3::g.5717::m.5717	1	3	45,581	2,2987	PF00850.18	Hist_deacetyl	271,1	1,30E-80	CL0302
TRINITY_DN10893_c0_g1::TRINITY_DN10893_c0_g1_i1::g.5751::m.5751	2	11	37,324	59,821	PF00332.17	Glyco_hydro_17	377,7	4,30E-113	CL0058
TRINITY_DN10896_c0_g1::TRINITY_DN10896_c0_g1_i3::g.5759::m.5759	1	10,9	12,896	2,0405	PF00484.18	Pro_CA	37,6	2,30E-09	No_clan
TRINITY_DN11870_c0_g1::TRINITY_DN11870_c0_g1_i2::g.9275::m.9275	2	12,5	13,765	12,492	PF01199.17	Ribosomal_L34e	131,2	1,20E-38	No_clan
TRINITY_DN10911_c0_g2::TRINITY_DN10911_c0_g2_i2::g.5805::m.5805	7	31,2	29,421	22,505	PF00227.25	Proteasome	164,3	2,10E-48	CL0052
TRINITY_DN10935_c0_g1::TRINITY_DN10935_c0_g1_i5::g.5847::m.5847	1	14,1	11,286	2,61	PF00240.22	ubiquitin	48	7,00E-13	CL0072
TRINITY_DN10955_c0_g1::TRINITY_DN10955_c0_g1_i1::g.5913::m.5913	1	11,7	12,041	1,8653	PF05486.11	SRP9-21	46,2	4,10E-12	CL0623
TRINITY_DN10960_c0_g1::TRINITY_DN10960_c0_g1_i2::g.5906::m.5906	2	5,9	39,075	1,888	PF00012.19	HSP70	401,1	6,20E-120	CL0108
TRINITY_DN10969_c0_g1::TRINITY_DN10969_c0_g1_i1::g.5933::m.5933	6	19,7	49,112	47,008	PF07732.14	Cu-oxidase_3	41,6	9,90E-11	CL0026
TRINITY_DN10989_c0_g1::TRINITY_DN10989_c0_g1_i3::g.6012::m.6012	5	25,2	29,165	12,846					
TRINITY_DN10990_c0_g1::TRINITY_DN10990_c0_g1_i1::g.6030::m.6030	2	7,6	47,62	4,3438	PF03151.15	TPT	352,2	2,00E-105	CL0184

TRINITY_DN11003_c0_g3::TRINITY_DN11003_c0_g3_i1::g.6096::m.6096	3	25,5	16,066	4,6887	PF00166.20	Cpn10	87,7	3,60E-25	CL0296
TRINITY_DN11011_c0_g1::TRINITY_DN11011_c0_g1_i3::g.6100::m.6100	2	2,2	109,46	6,1775	PF03810.18	IBN_N	48,4	5,90E-13	CL0020
TRINITY_DN11014_c0_g1::TRINITY_DN11014_c0_g1_i2::g.6114::m.6114	3	27,8	21,697	5,7773					
TRINITY_DN11023_c0_g2::TRINITY_DN11023_c0_g2_i1::g.6151::m.6151	1	2,1	76,032	1,9089					
TRINITY_DN11029_c0_g1::TRINITY_DN11029_c0_g1_i1::g.6154::m.6154	4	9,3	71,297	8,3996	PF00501.27	AMP-binding	285,2	6,40E-85	CL0378
TRINITY_DN11049_c0_g1::TRINITY_DN11049_c0_g1_i1::g.6211::m.6211	3	27,5	15,905	5,7668	PF01161.19	PBP	82,5	2,90E-23	No_clan
TRINITY_DN11053_c0_g1::TRINITY_DN11053_c0_g1_i3::g.6092::m.6092	3	23,6	16,83	14,07	PF01247.17	Ribosomal_L35Ae	150,8	8,40E-45	CL0575
TRINITY_DN11064_c0_g2::TRINITY_DN11064_c0_g2_i1::g.6238::m.6238	1	5,3	32,446	2,7706	PF01680.16	SOR_SNZ	285,6	2,10E-85	CL0036
TRINITY_DN11068_c0_g1::TRINITY_DN11068_c0_g1_i1::g.6241::m.6241	2	22,1	12,109	5,11	PF00428.18	Ribosomal_60s	51,4	1,20E-13	No_clan
TRINITY_DN11073_c0_g1::TRINITY_DN11073_c0_g1_i1::g.6247::m.6247	4	19,1	22,762	22,589	PF00177.20	Ribosomal_S7	126,8	4,70E-37	No_clan
TRINITY_DN11076_c0_g1::TRINITY_DN11076_c0_g1_i2::g.6263::m.6263	1	1,9	71,607	1,9343	PF13812.5	PPR_3	17,9	0,0022	CL0020
TRINITY_DN11079_c0_g1::TRINITY_DN11079_c0_g1_i1::g.6259::m.6259	3	9,8	35,619	3,7796	PF01344.24	Kelch_1	25,8	5,50E-06	CL0186
TRINITY_DN11090_c0_g1::TRINITY_DN11090_c0_g1_i1::g.6289::m.6289	3	20,2	24,293	7,1393	PF01280.19	Ribosomal_L19e	222	2,70E-66	No_clan
TRINITY_DN11092_c0_g1::TRINITY_DN11092_c0_g1_i5::g.6307::m.6307	2	12,8	20,106	3,1979	PF03009.16	GDPD	44,7	1,30E-11	CL0384
TRINITY_DN11097_c0_g1::TRINITY_DN11097_c0_g1_i1::g.6303::m.6303	9	30,7	34,933	34,099	PF13668.5	Ferritin_2	118,8	1,60E-34	CL0044
TRINITY_DN11104_c0_g1::TRINITY_DN11104_c0_g1_i1::g.6331::m.6331	1	2,8	65,455	2,207	PF12899.6	Glyco_hydro_100	721,6	2,60E-217	CL0059
TRINITY_DN11123_c0_g1::TRINITY_DN11123_c0_g1_i4::g.6381::m.6381	13	44,8	42,446	52,391	PF01588.19	tRNA_bind	89,2	1,30E-25	CL0021
TRINITY_DN11125_c0_g1::TRINITY_DN11125_c0_g1_i2::g.6392::m.6392	1	1,7	95,873	2,145					
TRINITY_DN11142_c1_g1::TRINITY_DN11142_c1_g1_i2::g.6468::m.6468	3	16,4	22,377	4,6298	PF10075.8	CSN8_PSD8_EIF3K	66,9	1,70E-18	CL0123
TRINITY_DN11160_c0_g1::TRINITY_DN11160_c0_g1_i1::g.6484::m.6484	4	26,4	30,058	65,956	PF00722.20	Glyco_hydro_16	177,7	1,30E-52	CL0004
TRINITY_DN11182_c0_g1::TRINITY_DN11182_c0_g1_i3::g.6546::m.6546	4	23,5	29,045	22,229	PF00459.24	Inositol_P	103,7	1,10E-29	CL0171

TRINITY_DN11191_c0_g1::TRINITY_DN11191_c0_g1_i2::g.6575::m.6575	4	30,1	15,925	110,63	PF13499.5	EF-hand_7	36,8	3,90E-09	CL0220
TRINITY_DN11195_c0_g1::TRINITY_DN11195_c0_g1_i1::g.6571::m.6571	2	2,5	125,72	3,8204	PF05664.10	DUF810	810,4	1,40E-243	No_clan
TRINITY_DN11202_c0_g1::TRINITY_DN11202_c0_g1_i1::g.6604::m.6604	2	6,3	35,398	3,0112	PF00191.19	Annexin	55	6,00E-15	No_clan
TRINITY_DN11209_c0_g1::TRINITY_DN11209_c0_g1_i1::g.6616::m.6616	3	12	29,936	4,4748	PF00725.21	3HCDH	72,8	2,50E-20	CL0106
TRINITY_DN11213_c0_g1::TRINITY_DN11213_c0_g1_i2::g.6642::m.6642	5	21,9	26,221	16,946	PF03868.14	Ribosomal_L6e_N	61,8	4,80E-17	No_clan
TRINITY_DN11216_c0_g1::TRINITY_DN11216_c0_g1_i3::g.6674::m.6674	2	5,2	72,042	6,6668	PF00232.17	Glyco_hydro_1	113,5	7,80E-33	CL0058
TRINITY_DN11228_c0_g1::TRINITY_DN11228_c0_g1_i4::g.6693::m.6693	1	10,4	13,298	87,187	PF01984.19	dsDNA_bind	82,3	2,70E-23	No_clan
TRINITY_DN11231_c0_g1::TRINITY_DN11231_c0_g1_i2::g.6700::m.6700	10	20,7	65,38	38,096	PF00920.20	ILVD_EDD	718	5,80E-216	No_clan
TRINITY_DN11247_c0_g1::TRINITY_DN11247_c0_g1_i5::g.6785::m.6785	6	22,7	27,587	323,31	PF05368.12	NmrA	208,4	1,00E-61	CL0063
TRINITY_DN11253_c0_g1::TRINITY_DN11253_c0_g1_i2::g.6760::m.6760	3	17,9	20,476	24,133	PF01491.15	Frataxin_Cyay	121,3	1,70E-35	No_clan
TRINITY_DN11269_c0_g1::TRINITY_DN11269_c0_g1_i4::g.6758::m.6758	1	11,9	11,865	3,1917	PF05676.12	NDUF_B7	101,6	1,30E-29	CL0351
TRINITY_DN11271_c0_g1::TRINITY_DN11271_c0_g1_i1::g.6832::m.6832	2	15,7	21,563	5,6995	PF12796.6	Ank_2	48,8	7,50E-13	CL0465
TRINITY_DN11273_c0_g1::TRINITY_DN11273_c0_g1_i1::g.6839::m.6839	1	4,8	24,727	2,399	PF01728.18	FtsJ	150,4	5,00E-44	CL0063
TRINITY_DN11277_c0_g1::TRINITY_DN11277_c0_g1_i2::g.6842::m.6842	1	10,1	16,72	3,1959					
TRINITY_DN11278_c0_g2::TRINITY_DN11278_c0_g2_i1::g.6843::m.6843	2	7,4	23,431	97,767	PF00550.24	PP-binding	44,5	1,40E-11	CL0314
TRINITY_DN11289_c0_g1::TRINITY_DN11289_c0_g1_i1::g.6862::m.6862	1	4	33,397	2,9618	PF00795.21	CN_hydrolase	179	1,00E-52	No_clan
TRINITY_DN11290_c1_g1::TRINITY_DN11290_c1_g1_i4::g.6884::m.6884	1	11,5	14,52	5,8688	PF01918.20	Alba	47,8	8,50E-13	CL0441
TRINITY_DN11301_c0_g1::TRINITY_DN11301_c0_g1_i2::g.6892::m.6892	2	12	23,119	2,8915	PF08154.11	NLE	41,8	1,10E-10	CL0072
TRINITY_DN11319_c0_g2::TRINITY_DN11319_c0_g2_i2::g.7052::m.7052	11	76,9	16,678	265,03	PF13410.5	GST_C_2	38,1	1,10E-09	CL0497
TRINITY_DN11323_c0_g1::TRINITY_DN11323_c0_g1_i5::g.7006::m.7006	2	13,6	24,69	5,3667	PF03357.20	Snf7	147	3,70E-43	CL0235
TRINITY_DN11329_c0_g1::TRINITY_DN11329_c0_g1_i1::g.6996::m.6996	1	4,7	34,386	-2	PF12776.6	Myb_DNA-bind_3	86,9	1,30E-24	CL0123

TRINITY_DN11331_c0_g1::TRINITY_DN11331_c0_g1_i1::g.7013::m.7013	2	9,7	32,667	4,0201					
TRINITY_DN11338_c0_g1::TRINITY_DN11338_c0_g1_i3::g.7037::m.7037	6	14,5	46,784	11,198	PF00988.21	CPSase_sm_chain	159,9	2,30E-47	CL0364
TRINITY_DN11345_c0_g1::TRINITY_DN11345_c0_g1_i1::g.7064::m.7064	5	23,4	32,604	30,786	PF01875.16	Memo	280,5	1,10E-83	CL0283
TRINITY_DN11347_c0_g1::TRINITY_DN11347_c0_g1_i2::g.7021::m.7021	1	11,6	21,289	14,587					
TRINITY_DN11351_c0_g4::TRINITY_DN11351_c0_g4_i3::g.7095::m.7095	2	10,1	25,526	6,2503	PF03517.12	Voldacs	88,4	4,00E-25	CL0266
TRINITY_DN11353_c0_g1::TRINITY_DN11353_c0_g1_i1::g.7089::m.7089	4	20,9	32,503	6,9778	PF14580.5	LRR_9	261,3	2,80E-78	CL0022
TRINITY_DN11356_c0_g1::TRINITY_DN11356_c0_g1_i1::g.7113::m.7113	3	18,8	17,596	29,514	PF03760.14	LEA_1	67,5	1,10E-18	No_clan
TRINITY_DN11357_c0_g2::TRINITY_DN11357_c0_g2_i1::g.7096::m.7096	4	58	14,013	46,198	PF00235.18	Profilin	148,4	1,10E-43	CL0431
TRINITY_DN11371_c0_g2::TRINITY_DN11371_c0_g2_i2::g.7148::m.7148	4	14,3	37,804	7,2376	PF00400.31	WD40	22,9	0,0001	CL0186
TRINITY_DN11377_c0_g1::TRINITY_DN11377_c0_g1_i3::g.7158::m.7158	1	6,7	18,534	2,4426	PF01327.20	Pep_deformylase	148,5	1,10E-43	No_clan
TRINITY_DN11382_c0_g1::TRINITY_DN11382_c0_g1_i1::g.7166::m.7166	3	10,4	44,237	4,9874	PF02779.23	Transket_pyr	158,2	1,50E-46	CL0254
TRINITY_DN11389_c0_g1::TRINITY_DN11389_c0_g1_i1::g.7193::m.7193	4	19,3	38,382	8,358	PF00400.31	WD40	20,5	0,00057	CL0186
TRINITY_DN11391_c0_g1::TRINITY_DN11391_c0_g1_i3::g.7245::m.7245	1	6,6	39,25	3,3661	PF10609.8	ParA	275,5	3,30E-82	CL0023
TRINITY_DN11399_c0_g1::TRINITY_DN11399_c0_g1_i3::g.6893::m.6893	9	45,6	29,827	68,483	PF01015.17	Ribosomal_S3Ae	325,2	1,40E-97	No_clan
TRINITY_DN11403_c0_g1::TRINITY_DN11403_c0_g1_i1::g.7256::m.7256	2	5,8	43,673	3,1091	PF01535.19	PPR	24,8	1,50E-05	CL0020
TRINITY_DN11410_c0_g1::TRINITY_DN11410_c0_g1_i1::g.7283::m.7283	2	7,6	34,592	3,9161	PF00400.31	WD40	16,5	0,011	CL0186
TRINITY_DN11411_c0_g1::TRINITY_DN11411_c0_g1_i2::g.7300::m.7300	5	19,6	27,783	51,821	PF00827.16	Ribosomal_L15e	312,9	6,80E-94	CL0652
TRINITY_DN11412_c0_g1::TRINITY_DN11412_c0_g1_i1::g.7292::m.7292	3	12,1	38,832	6,3583	PF03446.14	NAD_binding_2	132,2	1,60E-38	CL0063
TRINITY_DN11423_c0_g1::TRINITY_DN11423_c0_g1_i1::g.7308::m.7308	3	16,1	23,727	2,4768	PF00687.20	Ribosomal_L1	133,8	6,10E-39	No_clan
TRINITY_DN12374_c0_g1::TRINITY_DN12374_c0_g1_i5::g.12261::m.12261	4	26,3	19,712	42,289	PF00281.18	Ribosomal_L5	65,6	3,30E-18	CL0652
TRINITY_DN11445_c0_g1::TRINITY_DN11445_c0_g1_i3::g.7277::m.7277	1	6,5	20,775	2,3666	PF00153.26	Mito_carr	52,9	2,50E-14	No_clan
TRINITY_DN11447_c0_g1::TRINITY_DN11447_c0_g1_i1::g.7417::m.7417	1	3,4	40,836	1,8827	PF01633.19	Choline_kinase	213,2	3,00E-63	CL0016

TRINITY_DN11448_c0_g1::TRINITY_DN11448_c0_g1_i1::g.7372::m.7372	2	15,9	17,968	2,7591	PF13943.5	WPP	128,4	1,00E-37	No_clan
TRINITY_DN11457_c0_g1::TRINITY_DN11457_c0_g1_i1::g.7427::m.7427	1	4,9	36,037	4,8088	PF01798.17	Nop	220,7	1,60E-65	No_clan
TRINITY_DN11464_c0_g1::TRINITY_DN11464_c0_g1_i3::g.7465::m.7465	8	11,9	97,803	14,991	PF03097.17	BRO1	359,1	2,30E-107	No_clan
TRINITY_DN11465_c0_g1::TRINITY_DN11465_c0_g1_i2::g.7454::m.7454	19	48,7	55,061	156,49	PF07992.13	Pyr_redox_2	187	4,10E-55	CL0063
TRINITY_DN11467_c0_g1::TRINITY_DN11467_c0_g1_i3::g.7480::m.7480	7	16,8	65,512	12,649					
TRINITY_DN11479_c0_g1::TRINITY_DN11479_c0_g1_i6::g.7503::m.7503	2	15,9	19,709	3,5889	PF09835.8	DUF2062	58	8,60E-16	No_clan
TRINITY_DN11480_c0_g1::TRINITY_DN11480_c0_g1_i7::g.7531::m.7531	2	13,4	25,312	3,1374					
TRINITY_DN11484_c0_g1::TRINITY_DN11484_c0_g1_i2::g.7552::m.7552	3	27,8	12,307	3,896	PF01776.16	Ribosomal_L22e	81,5	4,90E-23	No_clan
TRINITY_DN11500_c0_g1::TRINITY_DN11500_c0_g1_i2::g.7570::m.7570	1	4,6	32,15	-2	PF01373.16	Glyco_hydro_14	239,8	4,40E-71	CL0058
TRINITY_DN11501_c0_g1::TRINITY_DN11501_c0_g1_i5::g.7580::m.7580	4	33,1	16,066	20,282	PF13669.5	Glyoxalase_4	35,4	9,70E-09	CL0104
TRINITY_DN11519_c0_g1::TRINITY_DN11519_c0_g1_i2::g.7693::m.7693	3	6,4	69,811	6,2331	PF12037.7	DUF3523	336,9	7,40E-101	No_clan
TRINITY_DN11520_c0_g2::TRINITY_DN11520_c0_g2_i2::g.7719::m.7719	3	21,7	25,598	4,3178	PF02374.14	ArsA_ATPase	155,2	2,20E-45	CL0023
TRINITY_DN11523_c0_g1::TRINITY_DN11523_c0_g1_i1::g.7740::m.7740	6	23,4	29,949	15,912	PF08071.11	RS4NT	69,5	1,80E-19	No_clan
TRINITY_DN11523_c0_g3::TRINITY_DN11523_c0_g3_i2::g.7748::m.7748	5	19,3	29,918	3,8675	PF08071.11	RS4NT	69,5	1,80E-19	No_clan
TRINITY_DN11534_c0_g1::TRINITY_DN11534_c0_g1_i5::g.7773::m.7773	2	10,8	29,975	2,5817	PF00106.24	adh_short	167,8	1,80E-49	CL0063
TRINITY_DN11539_c0_g1::TRINITY_DN11539_c0_g1_i5::g.7794::m.7794	6	26,6	39,497	17,533	PF09598.9	Stm1_N	76,6	1,90E-21	No_clan
TRINITY_DN11548_c0_g1::TRINITY_DN11548_c0_g1_i1::g.7714::m.7714	5	17,5	68,635	17,126					
TRINITY_DN11552_c0_g1::TRINITY_DN11552_c0_g1_i1::g.7817::m.7817	3	6,3	75,327	4,7657	PF06964.11	Alpha-L-AF_C	124,7	3,70E-36	CL0369
TRINITY_DN11558_c0_g1::TRINITY_DN11558_c0_g1_i1::g.7858::m.7858	6	33,6	23,402	323,31	PF00197.17	Kunitz_legume	207,3	1,50E-61	CL0066
TRINITY_DN11560_c0_g1::TRINITY_DN11560_c0_g1_i1::g.7865::m.7865	4	13,9	39,344	7,5284	PF08240.11	ADH_N	109,7	5,80E-32	CL0296
TRINITY_DN11562_c0_g1::TRINITY_DN11562_c0_g1_i2::g.7869::m.7869	2	13,9	19,018	22,379	PF00085.19	Thioredoxin	102,4	1,10E-29	CL0172

TRINITY_DN11568_c0_g1::TRINITY_DN11568_c0_g1_i1::g.7589::m.7589	2	18,2	21,769	7,4696	PF02466.18	Tim17	62,2	4,40E-17	No_clan
TRINITY_DN11577_c0_g1::TRINITY_DN11577_c0_g1_i3::g.7931::m.7931	2	19,8	13,526	2,4893	PF00338.21	Ribosomal_S10	100,7	3,80E-29	No_clan
TRINITY_DN11580_c0_g1::TRINITY_DN11580_c0_g1_i1::g.7933::m.7933	8	24,4	40,366	82,74	PF03690.12	UPF0160	451,3	2,10E-135	No_clan
TRINITY_DN11631_c0_g1::TRINITY_DN11631_c0_g1_i1::g.8169::m.8169	2	8,9	32,672	47,999	PF01975.16	SurE	180,3	3,30E-53	No_clan
TRINITY_DN11631_c1_g1::TRINITY_DN11631_c1_g1_i4::g.8173::m.8173	2	11,6	27,458	2,9852	PF08513.10	LisH	35,4	6,30E-09	CL0561
TRINITY_DN11635_c0_g1::TRINITY_DN11635_c0_g1_i7::g.8217::m.8217	11	37,8	42,385	58,517	PF00274.18	Glycolytic	524,1	1,20E-157	CL0035
TRINITY_DN11637_c0_g1::TRINITY_DN11637_c0_g1_i3::g.8078::m.8078	10	26,5	52,194	125,7					
TRINITY_DN11638_c0_g1::TRINITY_DN11638_c0_g1_i1::g.8051::m.8051	2	5,6	42,63	1,9233					
TRINITY_DN11643_c0_g1::TRINITY_DN11643_c0_g1_i1::g.8230::m.8230	5	26	27,021	8,592	PF01398.20	JAB	43,9	1,80E-11	CL0366
TRINITY_DN11646_c0_g1::TRINITY_DN11646_c0_g1_i1::g.8053::m.8053	2	14,6	18,269	3,181					
TRINITY_DN11648_c0_g3::TRINITY_DN11648_c0_g3_i1::g.8240::m.8240	4	38,9	13,063	6,9739	PF01920.19	Prefoldin_2	67	1,10E-18	CL0200
TRINITY_DN11662_c0_g1::TRINITY_DN11662_c0_g1_i1::g.8281::m.8281	4	20,7	20,552	9,4443	PF00160.20	Pro_isomerase	152,2	1,30E-44	CL0475
TRINITY_DN11669_c0_g1::TRINITY_DN11669_c0_g1_i1::g.8337::m.8337	4	18,5	23,255	7,0596	PF01282.18	Ribosomal_S24e	125,7	4,90E-37	No_clan
TRINITY_DN11674_c0_g1::TRINITY_DN11674_c0_g1_i3::g.8359::m.8359	1	5,8	22,957	1,905	PF00071.21	Ras	182	5,70E-54	CL0023
TRINITY_DN11684_c0_g1::TRINITY_DN11684_c0_g1_i1::g.8363::m.8363	2	11,7	30,569	10,84	PF13460.5	NAD_binding_10	68	8,80E-19	CL0063
TRINITY_DN11687_c0_g1::TRINITY_DN11687_c0_g1_i2::g.8407::m.8407	6	17	46,165	13,734	PF00627.30	UBA	29,7	3,90E-07	CL0214
TRINITY_DN11691_c0_g1::TRINITY_DN11691_c0_g1_i1::g.8068::m.8068	6	16,9	52,272	19,52					
TRINITY_DN11695_c0_g2::TRINITY_DN11695_c0_g2_i5::g.8192::m.8192	2	12,6	27,333	4,8362	PF02544.15	Steroid_dh	23,9	3,00E-05	CL0115
TRINITY_DN11696_c0_g1::TRINITY_DN11696_c0_g1_i4::g.8428::m.8428	10	43,1	37,281	117,45	PF00248.20	Aldo_ket_red	166,2	8,50E-49	No_clan
TRINITY_DN13507_c0_g2::TRINITY_DN13507_c0_g2_i4::g.24688::m.24688	2	23,1	12,769	3,2539	PF01655.17	Ribosomal_L32e	152,9	3,20E-45	No_clan
TRINITY_DN11725_c0_g2::TRINITY_DN11725_c0_g2_i1::g.8540::m.8540	4	25,6	17,245	12,806					

TRINITY_DN11729_c1_g1::TRINITY_DN11729_c1_g1_i5::g.8590::m.8590	1	10,7	16,327	1,9177	PF06984.12	MRP-L47	122,2	6,40E-36	CL0346
TRINITY_DN11737_c0_g1::TRINITY_DN11737_c0_g1_i1::g.8601::m.8601	5	32,4	16,482	254,02	PF00334.18	NDK	186,2	2,30E-55	No_clan
TRINITY_DN11739_c0_g1::TRINITY_DN11739_c0_g1_i1::g.8613::m.8613	2	9,9	38,847	5,1441	PF00827.16	Ribosomal_L15e	33,7	2,60E-08	CL0652
TRINITY_DN11760_c0_g1::TRINITY_DN11760_c0_g1_i1::g.8722::m.8722	6	27,1	34,244	203,06	PF00466.19	Ribosomal_L10	67	1,20E-18	No_clan
TRINITY_DN11771_c0_g1::TRINITY_DN11771_c0_g1_i2::g.8609::m.8609	4	12,3	39,884	6,6477	PF02885.16	Glycos_trans_3N	46,8	1,80E-12	No_clan
TRINITY_DN11779_c0_g1::TRINITY_DN11779_c0_g1_i1::g.8787::m.8787	6	24,4	45,19	44,712	PF00180.19	Iso_dh	460,2	4,00E-138	CL0270
TRINITY_DN11784_c0_g1::TRINITY_DN11784_c0_g1_i1::g.8804::m.8804	3	30	20,961	6,2831					
TRINITY_DN11789_c0_g1::TRINITY_DN11789_c0_g1_i4::g.8830::m.8830	12	43,3	32,494	323,31	PF00903.24	Glyoxalase	80,3	1,20E-22	CL0104
TRINITY_DN11799_c0_g1::TRINITY_DN11799_c0_g1_i1::g.8868::m.8868	2	7,3	53,615	3,088	PF01409.19	tRNA-synt_2d	165,2	1,60E-48	CL0040
TRINITY_DN11806_c0_g1::TRINITY_DN11806_c0_g1_i1::g.8989::m.8989	2	5,2	63,286	3,3968					
TRINITY_DN11842_c0_g1::TRINITY_DN11842_c0_g1_i5::g.9125::m.9125	6	17,5	54,786	16,881	PF13432.5	TPR_16	24,4	3,30E-05	CL0020
TRINITY_DN11846_c0_g3::TRINITY_DN11846_c0_g3_i2::g.9155::m.9155	2	30,8	13,192	19,474					
TRINITY_DN11853_c0_g1::TRINITY_DN11853_c0_g1_i3::g.9201::m.9201	3	12,1	38,385	6,9393	PF08241.11	Methyltransf_11	62,7	3,60E-17	CL0063
TRINITY_DN11854_c0_g1::TRINITY_DN11854_c0_g1_i1::g.9190::m.9190	2	17	15,972	323,31	PF00085.19	Thioredoxin	108,2	1,70E-31	CL0172
TRINITY_DN11860_c0_g1::TRINITY_DN11860_c0_g1_i2::g.9213::m.9213	4	46,5	10,936	2,19	PF00022.18	Actin	148,7	1,70E-43	CL0108
TRINITY_DN11865_c0_g1::TRINITY_DN11865_c0_g1_i7::g.9262::m.9262	3	4	116,34	6,9156	PF05222.14	AlaDh_PNT_N	89,3	2,40E-25	CL0325
TRINITY_DN11876_c0_g1::TRINITY_DN11876_c0_g1_i1::g.9282::m.9282	1	10,3	15,927	2,3413					
TRINITY_DN11883_c0_g2::TRINITY_DN11883_c0_g2_i5::g.9377::m.9377	3	6,8	69,409	6,4991	PF00149.27	Metallophos	72,4	6,60E-20	CL0163
TRINITY_DN11884_c0_g1::TRINITY_DN11884_c0_g1_i2::g.9315::m.9315	1	6,9	34,271	7,017	PF00574.22	CLP_protease	234,4	7,70E-70	CL0127
TRINITY_DN11895_c0_g1::TRINITY_DN11895_c0_g1_i1::g.9352::m.9352	2	9,5	50,838	48,74	PF00332.17	Glyco_hydro_17	347	9,60E-104	CL0058
TRINITY_DN11898_c0_g1::TRINITY_DN11898_c0_g1_i2::g.9380::m.9380	10	23,7	76,319	235,79	PF00326.20	Peptidase_S9	150,2	4,90E-44	CL0028

TRINITY_DN11905_c0_g1::TRINITY_DN11905_c0_g1_i3::g.9416::m.9416	6	30,4	34,798	13,954	PF01459.21	Porin_3	271	1,00E-80	CL0193
TRINITY_DN11909_c0_g1::TRINITY_DN11909_c0_g1_i2::g.9470::m.9470	5	13,6	56,719	38,393	PF00561.19	Abhydrolase_1	54,7	1,10E-14	CL0028
TRINITY_DN11919_c0_g1::TRINITY_DN11919_c0_g1_i1::g.9495::m.9495	4	25,7	25,746	10,697	PF00334.18	NDK	177,8	9,30E-53	No_clan
TRINITY_DN11922_c0_g1::TRINITY_DN11922_c0_g1_i1::g.9511::m.9511	1	5,1	35,818	1,924	PF07859.12	Abhydrolase_3	138,2	3,10E-40	CL0028
TRINITY_DN11924_c0_g1::TRINITY_DN11924_c0_g1_i1::g.9513::m.9513	9	31,9	43,558	162,83	PF08240.11	ADH_N	87,1	6,00E-25	CL0296
TRINITY_DN11935_c0_g1::TRINITY_DN11935_c0_g1_i2::g.9568::m.9568	3	10,8	48,344	6,1653	PF01180.20	DHO_dh	327,2	7,90E-98	CL0036
TRINITY_DN11950_c0_g1::TRINITY_DN11950_c0_g1_i3::g.9633::m.9633	2	10,2	26,585	2,2982	PF00443.28	UCH	102,6	2,30E-29	CL0125
TRINITY_DN11951_c0_g1::TRINITY_DN11951_c0_g1_i4::g.9610::m.9610	4	9	54,682	6,1629	PF08245.11	Mur_ligase_M	28,2	1,50E-06	No_clan
TRINITY_DN11955_c0_g1::TRINITY_DN11955_c0_g1_i1::g.9671::m.9671	6	21	44,879	26,012	PF13561.5	adh_short_C2	225,6	5,90E-67	CL0063
TRINITY_DN11955_c0_g2::TRINITY_DN11955_c0_g2_i1::g.9673::m.9673	4	12,6	41,519	3,1934	PF13561.5	adh_short_C2	225,2	7,80E-67	CL0063
TRINITY_DN11961_c0_g1::TRINITY_DN11961_c0_g1_i6::g.9678::m.9678	3	18,2	25,384	7,3457					
TRINITY_DN11973_c1_g1::TRINITY_DN11973_c1_g1_i7::g.9759::m.9759	6	16,8	55,91	17,617	PF16656.4	Pur_ac_phosph_N	86	1,80E-24	CL0159
TRINITY_DN11975_c0_g1::TRINITY_DN11975_c0_g1_i3::g.9738::m.9738	2	6,5	44,469	2,8207	PF01926.22	MMR_HSR1	73,9	9,70E-21	CL0023
TRINITY_DN11978_c1_g4::TRINITY_DN11978_c1_g4_i1::g.9533::m.9533	2	3,3	60,245	2,7186	PF00939.18	Na_sulph_symp	467,6	5,00E-140	CL0182
TRINITY_DN11984_c0_g1::TRINITY_DN11984_c0_g1_i17::g.9822::m.9822	4	12,4	40,832	4,7572	PF01256.16	Carb_kinase	172,3	1,10E-50	CL0118
TRINITY_DN11989_c0_g1::TRINITY_DN11989_c0_g1_i1::g.9805::m.9805	5	15,3	51,415	8,5474	PF00155.20	Aminotran_1_2	242,4	7,50E-72	CL0061
TRINITY_DN11991_c0_g1::TRINITY_DN11991_c0_g1_i1::g.9823::m.9823	5	13,6	50,567	9,3675	PF01399.26	PCI	72,4	3,40E-20	CL0123
TRINITY_DN12003_c0_g1::TRINITY_DN12003_c0_g1_i1::g.9933::m.9933	6	32,3	29,761	15,234	PF01459.21	Porin_3	207	3,30E-61	CL0193
TRINITY_DN12011_c0_g1::TRINITY_DN12011_c0_g1_i3::g.9976::m.9976	6	18,1	43,433	323,31	PF13181.5	TPR_8	15,7	0,012	CL0020
TRINITY_DN12016_c0_g1::TRINITY_DN12016_c0_g1_i3::g.10082::m.10082	5	26,4	21,386	15,457	PF01775.16	Ribosomal_L18A	181,4	5,10E-54	No_clan
TRINITY_DN12020_c1_g1::TRINITY_DN12020_c1_g1_i2::g.10043::m.10043	4	20,9	32,574	10,917	PF08059.12	SEP	94,4	4,40E-27	No_clan

TRINITY_DN12028_c1_g1::TRINITY_DN12028_c1_g1_i1::g.9901::m.9901	5	26,9	22,596	19,136	PF01565.22	FAD_binding_4	99,3	1,40E-28	CL0077
TRINITY_DN12030_c0_g1::TRINITY_DN12030_c0_g1_i2::g.10080::m.10080	8	20,4	46,907	28,952	PF01546.27	Peptidase_M20	80,4	1,30E-22	CL0035
TRINITY_DN12041_c0_g1::TRINITY_DN12041_c0_g1_i1::g.10132::m.10132	7	27,8	34,559	229,07	PF00719.18	Pyrophosphatase	158,4	1,20E-46	No_clan
TRINITY_DN12047_c0_g1::TRINITY_DN12047_c0_g1_i4::g.10037::m.10037	1	2,8	45,71	1,8697					
TRINITY_DN12060_c0_g1::TRINITY_DN12060_c0_g1_i2::g.10212::m.10212	2	7	40,195	3,8518	PF13516.5	LRR_6	30,9	1,40E-07	CL0022
TRINITY_DN12062_c0_g1::TRINITY_DN12062_c0_g1_i4::g.10228::m.10228	17	43,9	56,545	217,9	PF00483.22	NTP_transferase	228,5	8,70E-68	CL0110
TRINITY_DN12067_c0_g1::TRINITY_DN12067_c0_g1_i1::g.10231::m.10231	7	51,5	15,097	170,91	PF06110.10	DUF953	131,6	9,80E-39	CL0172
TRINITY_DN12068_c0_g1::TRINITY_DN12068_c0_g1_i4::g.10141::m.10141	1	2,7	48,742	1,8506	PF07478.12	Dala_Dala_lig_C	57,8	1,00E-15	CL0179
TRINITY_DN1207_c0_g1::TRINITY_DN1207_c0_g1_i1::g.307::m.307	1	1,5	84,873	2,1464	PF03644.12	Glyco_hydro_85	318,6	4,00E-95	CL0058
TRINITY_DN12086_c0_g1::TRINITY_DN12086_c0_g1_i1::g.10415::m.10415	1	14,2	14,176	2,8747	PF00550.24	PP-binding	48,1	1,00E-12	CL0314
TRINITY_DN12087_c0_g1::TRINITY_DN12087_c0_g1_i3::g.10442::m.10442	2	13,1	21,568	2,0883	PF01105.23	EMP24_GP25L	96,4	1,70E-27	CL0521
TRINITY_DN12093_c0_g1::TRINITY_DN12093_c0_g1_i8::g.10458::m.10458	7	19,1	61,128	10,239	PF07992.13	Pyr_redox_2	215,2	1,00E-63	CL0063
TRINITY_DN12098_c3_g1::TRINITY_DN12098_c3_g1_i8::g.10503::m.10503	2	12,8	25,248	96,319	PF00190.21	Cupin_1	151,4	1,20E-44	CL0029
TRINITY_DN12101_c0_g1::TRINITY_DN12101_c0_g1_i1::g.9937::m.9937	1	3,8	34,154	1,8997	PF04927.11	SMP	69,4	2,50E-19	No_clan
TRINITY_DN12116_c0_g1::TRINITY_DN12116_c0_g1_i3::g.10615::m.10615	4	36,8	13,106	8,9526	PF10417.8	1-cysPrx_C	49,3	3,00E-13	No_clan
TRINITY_DN12116_c0_g2::TRINITY_DN12116_c0_g2_i1::g.10617::m.10617	2	15,2	15,252	30,401	PF00578.20	AhpC-TSA	89,2	1,70E-25	CL0172
TRINITY_DN12118_c0_g1::TRINITY_DN12118_c0_g1_i1::g.10631::m.10631	3	8,1	52,951	5,3631	PF02784.15	Orn_Arg_deC_N	164,9	1,80E-48	CL0036
TRINITY_DN12130_c0_g1::TRINITY_DN12130_c0_g1_i1::g.10682::m.10682	2	18,5	15,277	3,2496					
TRINITY_DN12134_c9_g1::TRINITY_DN12134_c9_g1_i4::g.10752::m.10752	1	2,8	64,83	2,1171	PF01501.19	Glyco_transf_8	306,8	1,10E-91	CL0110
TRINITY_DN12135_c0_g1::TRINITY_DN12135_c0_g1_i2::g.10684::m.10684	2	10	27,299	23,074	PF00657.21	Lipase_GDSL	51	1,60E-13	CL0264
TRINITY_DN12136_c0_g1::TRINITY_DN12136_c0_g1_i2::g.10688::m.10688	4	11	52,808	7,3857	PF00266.18	Aminotran_5	469,8	5,40E-141	CL0061

TRINITY_DN12154_c0_g1::TRINITY_DN12154_c0_g1_i6::g.10864::m.10864	2	8,2	28,088	3,3652					
TRINITY_DN12161_c0_g1::TRINITY_DN12161_c0_g1_i4::g.10883::m.10883	3	35,2	13,853	7,1093					
TRINITY_DN12162_c0_g1::TRINITY_DN12162_c0_g1_i2::g.10879::m.10879	4	37,9	14,479	214,96	PF06094.11	GGACT	54,7	1,40E-14	CL0278
TRINITY_DN12167_c0_g1::TRINITY_DN12167_c0_g1_i7::g.10902::m.10902	2	12,2	28,001	4,5475	PF01956.15	DUF106	176	4,70E-52	CL0376
TRINITY_DN12181_c0_g1::TRINITY_DN12181_c0_g1_i6::g.10945::m.10945	1	7,2	16,763	1,8864	PF12146.7	Hydrolase_4	36,9	2,10E-09	CL0028
TRINITY_DN12184_c0_g1::TRINITY_DN12184_c0_g1_i1::g.10535::m.10535	3	9,1	37,514	3,8527	PF06694.10	Plant_NMP1	631,1	3,00E-190	No_clan
TRINITY_DN12195_c0_g1::TRINITY_DN12195_c0_g1_i1::g.10552::m.10552	4	21,1	25,51	36,622	PF13561.5	adh_short_C2	197	3,10E-58	CL0063
TRINITY_DN12196_c0_g1::TRINITY_DN12196_c0_g1_i3::g.11012::m.11012	2	8,2	27,262	3,6886	PF00230.19	MIP	261,6	5,80E-78	No_clan
TRINITY_DN12201_c0_g1::TRINITY_DN12201_c0_g1_i3::g.10701::m.10701	8	29,8	37,537	30,839	PF00056.22	Ldh_1_N	155,9	6,30E-46	CL0063
TRINITY_DN12213_c0_g1::TRINITY_DN12213_c0_g1_i3::g.11157::m.11157	2	11,1	28,002	7,487	PF13417.5	GST_N_3	51	1,30E-13	CL0172
TRINITY_DN12221_c0_g1::TRINITY_DN12221_c0_g1_i1::g.11196::m.11196	4	25,2	15,257	323,31	PF02298.16	Cu_bind_like	66,2	1,90E-18	CL0026
TRINITY_DN12226_c1_g1::TRINITY_DN12226_c1_g1_i9::g.11323::m.11323	3	30,5	13,063	7,592	PF04667.16	Endosulfine	71,9	3,30E-20	No_clan
TRINITY_DN12247_c0_g5::TRINITY_DN12247_c0_g5_i2::g.11476::m.11476	2	6	49,189	4,9323					
TRINITY_DN12248_c0_g2::TRINITY_DN12248_c0_g2_i1::g.11152::m.11152	5	17,3	48,406	13,347	PF00226.30	DnaJ	84,3	4,40E-24	CL0392
TRINITY_DN12250_c0_g1::TRINITY_DN12250_c0_g1_i2::g.11365::m.11365	8	19	48,948	50,08	PF00009.26	GTP_EFTU	195,8	4,90E-58	CL0023
TRINITY_DN12251_c0_g1::TRINITY_DN12251_c0_g1_i7::g.11382::m.11382	1	13,7	11,392	1,8893	PF10276.8	zf-CHCC	52,6	3,10E-14	CL0045
TRINITY_DN16521_c2_g4::TRINITY_DN16521_c2_g4_i1::g.68855::m.68855	1	9,8	12,384	2,5023	PF01248.25	Ribosomal_L7Ae	79	1,50E-22	CL0101
TRINITY_DN12257_c1_g1::TRINITY_DN12257_c1_g1_i2::g.11446::m.11446	2	4,3	46,865	2,4926	PF00928.20	Adap_comp_sub	263,6	1,60E-78	CL0448
TRINITY_DN12264_c2_g2::TRINITY_DN12264_c2_g2_i2::g.11522::m.11522	7	17,4	65,836	37,478	PF14555.5	UBA_4	51,2	7,20E-14	CL0214
TRINITY_DN12268_c0_g1::TRINITY_DN12268_c0_g1_i1::g.11491::m.11491	5	14,6	52,522	8,0811	PF04597.13	Ribophorin_I	481,1	3,80E-144	No_clan
TRINITY_DN12273_c2_g3::TRINITY_DN12273_c2_g3_i1::g.11683::m.11683	10	63,1	12,143	82,022	PF00244.19	14.03.2003	117,7	4,60E-34	No_clan

TRINITY_DN12275_c0_g1::TRINITY_DN12275_c0_g1_i2::g.11548::m.11548	4	22,9	30,028	4,718	PF01015.17	Ribosomal_S3Ae	323,9	3,50E-97	No_clan
TRINITY_DN15102_c1_g3::TRINITY_DN15102_c1_g3_i1::g.46949::m.46949	2	7,3	37,928	4,7295	PF01946.16	Thi4	381	1,60E-114	CL0063
TRINITY_DN12285_c1_g1::TRINITY_DN12285_c1_g1_i3::g.11619::m.11619	2	7	40,672	7,3401	PF03088.15	Str_synth	117,3	2,30E-34	CL0186
TRINITY_DN12307_c0_g1::TRINITY_DN12307_c0_g1_i3::g.11724::m.11724	5	8,9	70,951	6,3547	PF13812.5	PPR_3	30,8	2,20E-07	CL0020
TRINITY_DN12310_c0_g1::TRINITY_DN12310_c0_g1_i3::g.11787::m.11787	5	16,1	47,905	288,78	PF16499.4	Melibiase_2	280,9	8,80E-84	CL0058
TRINITY_DN12314_c0_g1::TRINITY_DN12314_c0_g1_i2::g.11797::m.11797	6	21,2	42,557	49,02	PF00141.22	peroxidase	151,6	2,50E-44	CL0617
TRINITY_DN12315_c0_g1::TRINITY_DN12315_c0_g1_i2::g.11830::m.11830	3	8,6	60,073	6,7972	PF12108.7	SF3a60_bindingd	47,1	1,50E-12	No_clan
TRINITY_DN12323_c0_g1::TRINITY_DN12323_c0_g1_i4::g.11964::m.11964	10	23,5	55,285	13,809	PF00224.20	PK	526	3,20E-158	CL0151
TRINITY_DN12323_c0_g2::TRINITY_DN12323_c0_g2_i9::g.11969::m.11969	6	13,9	58,782	9,0912	PF13943.5	WPP	122,1	9,20E-36	No_clan
TRINITY_DN12323_c0_g2::TRINITY_DN12323_c0_g2_i3::g.11963::m.11963	3	21,4	16,47	4,8041	PF00380.18	Ribosomal_S9	111,3	3,60E-32	CL0329
TRINITY_DN12324_c0_g2::TRINITY_DN12324_c0_g2_i2::g.11908::m.11908	5	22,6	26,176	22,633	PF01991.17	vATP-synt_E	230,6	1,00E-68	CL0255
TRINITY_DN12329_c1_g1::TRINITY_DN12329_c1_g1_i1::g.11925::m.11925	10	29,3	41,185	151,68	PF01937.18	DUF89	183,1	7,00E-54	No_clan
TRINITY_DN12332_c0_g1::TRINITY_DN12332_c0_g1_i2::g.11982::m.11982	5	22,8	31,412	148,32	PF04969.15	CS	76,3	2,70E-21	CL0190
TRINITY_DN12342_c1_g2::TRINITY_DN12342_c1_g2_i2::g.12106::m.12106	1	6,2	20,882	3,0972	PF02298.16	Cu_bind_like	82,7	1,30E-23	CL0026
TRINITY_DN12342_c1_g3::TRINITY_DN12342_c1_g3_i1::g.12105::m.12105	4	29,5	18,879	164,55	PF02298.16	Cu_bind_like	71,6	3,80E-20	CL0026
TRINITY_DN12343_c0_g1::TRINITY_DN12343_c0_g1_i5::g.11815::m.11815	3	20,4	22,179	8,4301	PF01251.17	Ribosomal_S7e	269,7	1,00E-80	CL0652
TRINITY_DN12343_c1_g1::TRINITY_DN12343_c1_g1_i5::g.11822::m.11822	3	14,7	21,938	28,575	PF01251.17	Ribosomal_S7e	266,1	1,30E-79	CL0652
TRINITY_DN12348_c0_g1::TRINITY_DN12348_c0_g1_i1::g.11710::m.11710	1	14	15,716	4,7837					
TRINITY_DN12349_c8_g2::TRINITY_DN12349_c8_g2_i1::g.12068::m.12068	4	30,9	16,085	66,679	PF00076.21	RRM_1	34,9	9,50E-09	CL0221
TRINITY_DN12349_c8_g2::TRINITY_DN12349_c8_g2_i3::g.12071::m.12071	4	29,9	17,934	7,0584					
TRINITY_DN12352_c0_g2::TRINITY_DN12352_c0_g2_i4::g.12096::m.12096	1	4,2	31,251	2,3194	PF01650.17	Peptidase_C13	333,4	8,50E-100	CL0093

TRINITY_DN12353_c0_g1::TRINITY_DN12353_c0_g1_i1::g.12504::m.12504	2	10	45,75	2,9809	PF00201.17	UDPGT	47	1,50E-12	CL0113
TRINITY_DN12358_c0_g1::TRINITY_DN12358_c0_g1_i4::g.12129::m.12129	5	14,4	51,233	12,976	PF14555.5	UBA_4	39,7	2,80E-10	CL0214
TRINITY_DN12363_c0_g1::TRINITY_DN12363_c0_g1_i2::g.12162::m.12162	3	7	72,923	6,1467	PF02446.16	Glyco_hydro_77	497,1	4,60E-149	CL0058
TRINITY_DN12367_c0_g1::TRINITY_DN12367_c0_g1_i3::g.12189::m.12189	6	23,6	47,179	35,992	PF08240.11	ADH_N	97,2	4,40E-28	CL0296
TRINITY_DN12375_c3_g1::TRINITY_DN12375_c3_g1_i9::g.12353::m.12353	4	11,1	57,868	5,3067	PF03141.15	Methyltransf_29	611,5	1,00E-183	CL0063
TRINITY_DN12375_c3_g5::TRINITY_DN12375_c3_g5_i1::g.12365::m.12365	7	16,6	69,166	19,274	PF03141.15	Methyltransf_29	763,5	8,60E-230	CL0063
TRINITY_DN12384_c0_g1::TRINITY_DN12384_c0_g1_i2::g.12333::m.12333	2	11,1	29,097	4,6285	PF01729.18	QRPTase_C	196,3	2,80E-58	CL0036
TRINITY_DN12386_c0_g1::TRINITY_DN12386_c0_g1_i3::g.12387::m.12387	3	7,1	64,819	6,2601	PF00995.22	Sec1	422,5	4,00E-126	No_clan
TRINITY_DN12393_c0_g1::TRINITY_DN12393_c0_g1_i2::g.12483::m.12483	4	12,5	39,579	41,781	PF01408.21	GFO_IDH_MocA	64,3	1,60E-17	CL0063
TRINITY_DN12397_c0_g1::TRINITY_DN12397_c0_g1_i3::g.12463::m.12463	23	38,5	95,342	323,31	PF01301.18	Glyco_hydro_35	394,7	3,70E-118	CL0058
TRINITY_DN12399_c0_g1::TRINITY_DN12399_c0_g1_i1::g.12011::m.12011	1	3	51,483	2,0848	PF00450.21	Peptidase_S10	264,3	2,50E-78	CL0028
TRINITY_DN12400_c3_g4::TRINITY_DN12400_c3_g4_i4::g.12578::m.12578	1	1,7	92,227	2,6861	PF06972.10	DUF1296	107,2	3,00E-31	CL0214
TRINITY_DN12400_c4_g2::TRINITY_DN12400_c4_g2_i2::g.12585::m.12585	1	6,3	14,119	2,2679	PF00173.27	Cyt-b5	63,6	1,30E-17	No_clan
TRINITY_DN12403_c0_g1::TRINITY_DN12403_c0_g1_i5::g.12601::m.12601	1	9,1	13,767	2,3962	PF00011.20	HSP20	67,4	8,90E-19	CL0190
TRINITY_DN12405_c0_g1::TRINITY_DN12405_c0_g1_i1::g.12602::m.12602	3	5,6	62,218	3,0815	PF00333.19	Ribosomal_S5	62,7	2,10E-17	CL0196
TRINITY_DN12422_c0_g1::TRINITY_DN12422_c0_g1_i2::g.12726::m.12726	6	24,8	31,221	13,067	PF13419.5	HAD_2	59,3	4,70E-16	CL0137
TRINITY_DN12426_c1_g1::TRINITY_DN12426_c1_g1_i1::g.12851::m.12851	8	41,6	30,351	168,05	PF06999.11	Suc_Fer-like	132,2	1,90E-38	No_clan
TRINITY_DN12436_c4_g2::TRINITY_DN12436_c4_g2_i4::g.12996::m.12996	7	15,9	59,145	14,754	PF00118.23	Cpn60_TCP1	520,5	3,70E-156	No_clan
TRINITY_DN12438_c0_g1::TRINITY_DN12438_c0_g1_i11::g.12896::m.12896	5	22,1	39,342	11,297	PF09412.9	XendoU	205,9	6,00E-61	No_clan
TRINITY_DN12438_c0_g2::TRINITY_DN12438_c0_g2_i1::g.12882::m.12882	2	11,3	23,404	2,3618					

TRINITY_DN12440_c1_g1::TRINITY_DN12440_c1_g1_i2::g.13042::m.13042	5	11,3	58,372	5,6909	PF00171.21	Aldedh	603,9	1,50E-181	CL0099
TRINITY_DN12440_c1_g2::TRINITY_DN12440_c1_g2_i1::g.13039::m.13039	13	35,1	58,592	45,751	PF00171.21	Aldedh	598,9	4,90E-180	CL0099
TRINITY_DN12441_c0_g1::TRINITY_DN12441_c0_g1_i1::g.12899::m.12899	9	19,2	62,901	39,664	PF02018.16	CBM_4_9	41,6	1,30E-10	CL0202
TRINITY_DN12445_c0_g2::TRINITY_DN12445_c0_g2_i13::g.12955::m.12955	5	60,1	15,633	14,757	PF02127.14	Peptidase_M18	159,4	1,20E-46	CL0035
TRINITY_DN12452_c0_g1::TRINITY_DN12452_c0_g1_i1::g.12978::m.12978	8	25,7	33,243	41,983	PF00389.29	2-Hacid_dh	52,4	3,90E-14	CL0325
TRINITY_DN12454_c0_g1::TRINITY_DN12454_c0_g1_i1::g.12984::m.12984	6	16,4	55,469	21,85	PF00240.22	ubiquitin	31,6	9,50E-08	CL0072
TRINITY_DN12460_c3_g3::TRINITY_DN12460_c3_g3_i1::g.13367::m.13367	2	16,6	16,368	7,3412					
TRINITY_DN12462_c4_g1::TRINITY_DN12462_c4_g1_i1::g.13140::m.13140	1	7,4	14,864	1,9505	PF00350.22	Dynamin_N	99,3	2,30E-28	CL0023
TRINITY_DN12468_c0_g1::TRINITY_DN12468_c0_g1_i1::g.13082::m.13082	7	19,7	48,87	22,617	PF03345.13	DDOST_48kD	432,9	1,10E-129	No_clan
TRINITY_DN12471_c0_g1::TRINITY_DN12471_c0_g1_i6::g.13129::m.13129	2	11,3	40,827	5,5462	PF04280.14	Tim44	148,5	1,30E-43	CL0051
TRINITY_DN12477_c0_g1::TRINITY_DN12477_c0_g1_i2::g.13209::m.13209	11	29,7	60,945	18,48	PF02776.17	TPP_enzyme_N	139,3	8,60E-41	CL0254
TRINITY_DN12478_c4_g4::TRINITY_DN12478_c4_g4_i5::g.13332::m.13332	3	15,5	28,002	7,1971					
TRINITY_DN12480_c0_g2::TRINITY_DN12480_c0_g2_i1::g.13216::m.13216	3	23	14,065	5,7112	PF04718.14	ATP-synt_G	80,2	1,50E-22	No_clan
TRINITY_DN12481_c0_g1::TRINITY_DN12481_c0_g1_i2::g.13223::m.13223	2	11,7	16,309	3,8665	PF01124.17	MAPEG	84	7,50E-24	No_clan
TRINITY_DN12499_c2_g2::TRINITY_DN12499_c2_g2_i3::g.13408::m.13408	4	36,3	14,053	8,199	PF01776.16	Ribosomal_L22e	154,1	1,30E-45	No_clan
TRINITY_DN12503_c2_g1::TRINITY_DN12503_c2_g1_i3::g.13440::m.13440	2	24,6	14,417	4,1064	PF00255.18	GSHPx	92,2	1,30E-26	CL0172
TRINITY_DN12509_c0_g1::TRINITY_DN12509_c0_g1_i1::g.13435::m.13435	4	23,4	32,321	6,7868	PF00300.21	His_Phos_1	26,2	5,30E-06	CL0071
TRINITY_DN12510_c1_g1::TRINITY_DN12510_c1_g1_i1::g.13448::m.13448	1	2	78,439	2,1367	PF07676.11	PD40	28,5	9,30E-07	CL0186
TRINITY_DN12512_c0_g1::TRINITY_DN12512_c0_g1_i7::g.13451::m.13451	1	8,2	13,898	2,0302	PF04718.14	ATP-synt_G	82,7	2,50E-23	No_clan
TRINITY_DN12515_c0_g1::TRINITY_DN12515_c0_g1_i3::g.13461::m.13461	5	16,7	44,31	8,9716	PF01571.20	GCV_T	293,6	1,00E-87	CL0289

TRINITY_DN12516_c0_g1::TRINITY_DN12516_c0_g1_i2::g.13472::m.13472	8	11,8	92,627	25,133	PF00862.18	Sucrose_synth	971,7	7,60E-293	CL0113
TRINITY_DN12519_c1_g2::TRINITY_DN12519_c1_g2_i2::g.13489::m.13489	2	17,8	15,587	3,2822	PF14008.5	Metallophos_C	40,4	3,70E-10	No_clan
TRINITY_DN12524_c0_g1::TRINITY_DN12524_c0_g1_i3::g.13484::m.13484	1	4,6	35,564	2,149	PF04755.11	PAP_fibrillin	263,7	1,00E-78	No_clan
TRINITY_DN12528_c1_g2::TRINITY_DN12528_c1_g2_i1::g.13492::m.13492	1	6	35,278	3,0289	PF01025.18	GrpE	142,1	1,10E-41	No_clan
TRINITY_DN12529_c0_g1::TRINITY_DN12529_c0_g1_i3::g.13512::m.13512	7	22	47,218	16,376	PF00571.27	CBS	28,6	1,30E-06	No_clan
TRINITY_DN12530_c0_g1::TRINITY_DN12530_c0_g1_i1::g.13568::m.13568	3	19,3	23,95	8,1316	PF01849.17	NAC	78,2	3,20E-22	No_clan
TRINITY_DN12537_c1_g2::TRINITY_DN12537_c1_g2_i4::g.13603::m.13603	7	36,9	16,961	164,9					
TRINITY_DN12537_c1_g2::TRINITY_DN12537_c1_g2_i7::g.13611::m.13611	5	33,1	14,369	11,647					
TRINITY_DN12537_c5_g1::TRINITY_DN12537_c5_g1_i1::g.13612::m.13612	1	12,2	13,244	26,079					
TRINITY_DN12538_c0_g3::TRINITY_DN12538_c0_g3_i3::g.13566::m.13566	12	48,9	37,853	323,31	PF16363.4	GDP_Man_Dehyd	216,3	6,60E-64	CL0063
TRINITY_DN12541_c0_g1::TRINITY_DN12541_c0_g1_i2::g.13592::m.13592	1	8,3	20,806	5,143	PF05753.13	TRAP_beta	190,1	2,00E-56	CL0159
TRINITY_DN12552_c0_g1::TRINITY_DN12552_c0_g1_i2::g.13425::m.13425	2	15,2	18,488	3,9279	PF12689.6	Acid_PPase	77,4	1,00E-21	CL0137
TRINITY_DN12560_c1_g1::TRINITY_DN12560_c1_g1_i3::g.13781::m.13781	2	12,9	22,972	15,696	PF00076.21	RRM_1	52,5	2,90E-14	CL0221
TRINITY_DN12561_c1_g1::TRINITY_DN12561_c1_g1_i2::g.13897::m.13897	5	40,4	11,098	269,47	PF01095.18	Pectinesterase	65,9	2,30E-18	CL0268
TRINITY_DN12563_c3_g1::TRINITY_DN12563_c3_g1_i1::g.13893::m.13893	2	18,7	16,386	3,9891					
TRINITY_DN12563_c5_g1::TRINITY_DN12563_c5_g1_i1::g.13894::m.13894	19	23,1	111,5	125,49	PF00686.18	CBM_20	74,6	4,20E-21	CL0369
TRINITY_DN12583_c1_g3::TRINITY_DN12583_c1_g3_i1::g.14008::m.14008	1	5,4	31,111	36,132	PF00069.24	Pkinase	103,1	1,50E-29	CL0016
TRINITY_DN12587_c0_g1::TRINITY_DN12587_c0_g1_i1::g.13954::m.13954	6	31	24,898	37,383	PF00106.24	adh_short	136,4	7,60E-40	CL0063
TRINITY_DN12588_c4_g1::TRINITY_DN12588_c4_g1_i6::g.14071::m.14071	10	48	31,388	37,743	PF03446.14	NAD_binding_2	137	5,70E-40	CL0063
TRINITY_DN12590_c1_g3::TRINITY_DN12590_c1_g3_i2::g.13976::m.13976	3	10,5	29,975	4,0223					
TRINITY_DN12590_c2_g2::TRINITY_DN12590_c2_g2_i2::g.13982::m.13982	4	22,1	20,544	248,88	PF11938.7	DUF3456	105,2	4,30E-30	No_clan

TRINITY_DN12592_c2_g1::TRINITY_DN12592_c2_g1_i5::g.14064::m.14064	2	32,4	10,996	41,937	PF08752.9	COP-gamma_platf	109,5	1,40E-31	CL0159
TRINITY_DN12593_c2_g1::TRINITY_DN12593_c2_g1_i1::g.14128::m.14128	2	10,6	16,617	2,6195					
TRINITY_DN12593_c3_g1::TRINITY_DN12593_c3_g1_i6::g.14142::m.14142	6	16	52,022	8,634	PF00365.19	PFK	210,5	2,80E-62	CL0240
TRINITY_DN12599_c0_g1::TRINITY_DN12599_c0_g1_i6::g.14058::m.14058	15	39,1	52,742	62,706	PF00118.23	Cpn60_TCP1	244,3	2,10E-72	No_clan
TRINITY_DN12608_c0_g1::TRINITY_DN12608_c0_g1_i2::g.14177::m.14177	1	2,8	28,293	1,9142	PF03876.16	SHS2_Rpb7-N	29,7	5,80E-07	CL0319
TRINITY_DN12611_c0_g2::TRINITY_DN12611_c0_g2_i7::g.14237::m.14237	2	9,4	26,17	3,2677	PF01135.18	PCMT	287,4	6,20E-86	CL0063
TRINITY_DN12614_c0_g1::TRINITY_DN12614_c0_g1_i2::g.14256::m.14256	11	26,8	61,412	47,683	PF00365.19	PFK	125,1	3,00E-36	CL0240
TRINITY_DN12615_c0_g2::TRINITY_DN12615_c0_g2_i14::g.14340::m.14340	2	52,3	11,314	29,029	PF00428.18	Ribosomal_60s	91,3	4,00E-26	No_clan
TRINITY_DN12618_c1_g1::TRINITY_DN12618_c1_g1_i9::g.14449::m.14449	1	4,3	40,164	1,8695	PF00076.21	RRM_1	50,1	1,70E-13	CL0221
TRINITY_DN12620_c0_g1::TRINITY_DN12620_c0_g1_i4::g.14272::m.14272	2	6,3	64,804	4,965					
TRINITY_DN12621_c1_g1::TRINITY_DN12621_c1_g1_i2::g.14309::m.14309	8	40,2	28,964	16,327	PF03767.13	Acid_phosphat_B	237	1,90E-70	CL0137
TRINITY_DN12624_c0_g1::TRINITY_DN12624_c0_g1_i1::g.14480::m.14480	6	17,6	52,469	9,1472	PF00702.25	Hydrolase	49,5	5,80E-13	CL0137
TRINITY_DN12625_c0_g2::TRINITY_DN12625_c0_g2_i2::g.14331::m.14331	4	14,6	62,19	13,321	PF03000.13	NPH3	308	5,50E-92	No_clan
TRINITY_DN12628_c0_g1::TRINITY_DN12628_c0_g1_i15::g.14380::m.14380	2	13,1	18,989	3,7978					
TRINITY_DN12632_c0_g1::TRINITY_DN12632_c0_g1_i1::g.14398::m.14398	2	9,8	34,293	4,9547	PF12263.7	DUF3611	186,5	3,60E-55	No_clan
TRINITY_DN12634_c0_g1::TRINITY_DN12634_c0_g1_i2::g.14412::m.14412	1	3,3	54,766	2,1789	PF00009.26	GTP_EFTU	105,9	1,80E-30	CL0023
TRINITY_DN12636_c0_g1::TRINITY_DN12636_c0_g1_i3::g.14427::m.14427	4	38,7	20,401	57,945					
TRINITY_DN12639_c0_g1::TRINITY_DN12639_c0_g1_i2::g.14158::m.14158	2	7,3	29,108	2,0607	PF02453.16	Reticulon	193,4	2,00E-57	No_clan
TRINITY_DN12640_c0_g1::TRINITY_DN12640_c0_g1_i4::g.14587::m.14587	9	32,5	44,576	28,192	PF00790.18	VHS	66,9	1,50E-18	CL0009
TRINITY_DN12647_c0_g1::TRINITY_DN12647_c0_g1_i2::g.14733::m.14733	6	41,4	20,705	55,129	PF08752.9	COP-gamma_platf	66,8	2,00E-18	CL0159
TRINITY_DN12648_c0_g1::TRINITY_DN12648_c0_g1_i4::g.15036::m.15036	9	54,5	22,693	18,347	PF00071.21	Ras	189,9	2,20E-56	CL0023
TRINITY_DN1265_c0_g1::TRINITY_DN1265_c0_g1_i1::g.324::m.324	5	29,2	34,112	11,577					

TRINITY_DN12654_c0_g3::TRINITY_DN12654_c0_g3_i5::g.14722::m.14722	7	14,1	73,54	16,866	PF01433.19	Peptidase_M1	150,8	3,70E-44	CL0126
TRINITY_DN12655_c1_g2::TRINITY_DN12655_c1_g2_i3::g.14763::m.14763	3	9,1	47,376	5,354	PF00400.31	WD40	13,4	0,1	CL0186
TRINITY_DN12658_c0_g1::TRINITY_DN12658_c0_g1_i2::g.14732::m.14732	4	39,3	13,644	9,4422	PF05347.14	Complex1_LYR	28,2	1,30E-06	CL0491
TRINITY_DN12675_c1_g1::TRINITY_DN12675_c1_g1_i4::g.14912::m.14912	5	21,7	33,27	5,0412	PF00575.22	S1	50,1	2,50E-13	CL0021
TRINITY_DN12675_c1_g1::TRINITY_DN12675_c1_g1_i2::g.14909::m.14909	7	27,6	38,736	12,913	PF00575.22	S1	48,8	6,30E-13	CL0021
TRINITY_DN12678_c0_g1::TRINITY_DN12678_c0_g1_i7::g.14864::m.14864	3	10	49,888	12,202	PF16969.4	SRP68	370,2	2,20E-110	No_clan
TRINITY_DN12690_c0_g1::TRINITY_DN12690_c0_g1_i3::g.14937::m.14937	29	61,4	61,127	323,31	PF01676.17	Metalloenzyme	347,5	3,20E-104	CL0088
TRINITY_DN12692_c0_g1::TRINITY_DN12692_c0_g1_i7::g.14951::m.14951	14	38,9	46,637	323,31	PF16113.4	ECH_2	358,2	5,00E-107	CL0127
TRINITY_DN12693_c0_g1::TRINITY_DN12693_c0_g1_i1::g.14939::m.14939	4	36,3	12,84	5,2838					
TRINITY_DN12693_c0_g1::TRINITY_DN12693_c0_g1_i3::g.14940::m.14940	3	30,5	14,524	134,24	PF01370.20	Epimerase	22,4	6,70E-05	CL0063
TRINITY_DN12696_c1_g1::TRINITY_DN12696_c1_g1_i6::g.15028::m.15028	1	7,7	20,448	2,4632	PF02190.15	LON_substr_bdg	64,6	1,00E-17	CL0178
TRINITY_DN12699_c0_g1::TRINITY_DN12699_c0_g1_i1::g.14170::m.14170	9	43,8	16,024	264,98	PF01190.16	Pollen_Ole_e_I	66,4	2,10E-18	No_clan
TRINITY_DN12699_c0_g1::TRINITY_DN12699_c0_g1_i7::g.14174::m.14174	8	45,5	13,891	323,31	PF01190.16	Pollen_Ole_e_I	38,5	1,00E-09	No_clan
TRINITY_DN12704_c0_g1::TRINITY_DN12704_c0_g1_i4::g.15091::m.15091	4	20,9	30,37	8,3129	PF00153.26	Mito_carr	62,5	2,40E-17	No_clan
TRINITY_DN12707_c0_g1::TRINITY_DN12707_c0_g1_i3::g.15086::m.15086	1	3,2	34,116	10,626	PF01156.18	IU_nuc_hydro	227,4	2,80E-67	No_clan
TRINITY_DN12711_c1_g1::TRINITY_DN12711_c1_g1_i2::g.15227::m.15227	9	36	41,447	114,98	PF00240.22	ubiquitin	68,2	3,50E-19	CL0072
TRINITY_DN12711_c1_g1::TRINITY_DN12711_c1_g1_i4::g.15231::m.15231	4	16,4	29,048	2,8238	PF00627.30	UBA	52,8	2,40E-14	CL0214
TRINITY_DN12713_c0_g2::TRINITY_DN12713_c0_g2_i2::g.15182::m.15182	6	18,3	40,952	13,3	PF03214.12	RGP	579,9	1,50E-174	CL0110
TRINITY_DN12714_c1_g2::TRINITY_DN12714_c1_g2_i1::g.15270::m.15270	1	3,4	50,215	2,6565	PF06814.12	Lung_7-TM_R	167,3	4,10E-49	CL0192
TRINITY_DN12721_c0_g3::TRINITY_DN12721_c0_g3_i6::g.15362::m.15362	4	36,7	12,345	63,436	PF07876.11	Dabb	107,1	5,10E-31	CL0032

TRINITY_DN12723_c0_g1::TRINITY_DN12723_c0_g1_i2::g.15224::m.15224	3	7,6	51,344	7,2983	PF10602.8	RPN7	194,3	1,20E-57	CL0020
TRINITY_DN12731_c3_g2::TRINITY_DN12731_c3_g2_i1::g.15534::m.15534	1	12,4	11,132	3,8158	PF00295.16	Glyco_hydro_28	120,9	5,20E-35	CL0268
TRINITY_DN12731_c3_g4::TRINITY_DN12731_c3_g4_i1::g.15536::m.15536	1	5,4	19,39	1,9945	PF00295.16	Glyco_hydro_28	43,5	1,90E-11	CL0268
TRINITY_DN12733_c0_g1::TRINITY_DN12733_c0_g1_i1::g.15369::m.15369	4	25,3	20,083	2,4186	PF00071.21	Ras	164,2	1,60E-48	CL0023
TRINITY_DN12745_c0_g1::TRINITY_DN12745_c0_g1_i6::g.15122::m.15122	1	9,6	15,105	2,2191	PF04367.12	DUF502	81,2	4,20E-23	No_clan
TRINITY_DN12750_c0_g2::TRINITY_DN12750_c0_g2_i1::g.15503::m.15503	2	9,7	36,666	3,9919	PF03283.12	PAE	463,3	4,80E-139	CL0028
TRINITY_DN12755_c3_g1::TRINITY_DN12755_c3_g1_i1::g.16006::m.16006	2	13,8	19,352	3,9022	PF13855.5	LRR_8	28	1,30E-06	CL0022
TRINITY_DN12761_c4_g1::TRINITY_DN12761_c4_g1_i4::g.15698::m.15698	6	23,9	26,681	102,8	PF00635.25	Motile_Sperm	104,8	2,00E-30	CL0556
TRINITY_DN12761_c4_g2::TRINITY_DN12761_c4_g2_i1::g.15691::m.15691	1	8,6	13,004	1,9804					
TRINITY_DN12765_c0_g2::TRINITY_DN12765_c0_g2_i9::g.15778::m.15778	2	9,7	25,693	3,2909	PF00160.20	Pro_isomerase	97,6	7,70E-28	CL0475
TRINITY_DN12769_c0_g3::TRINITY_DN12769_c0_g3_i1::g.15717::m.15717	5	56,2	14,972	323,31	PF00076.21	RRM_1	84,1	3,90E-24	CL0221
TRINITY_DN12776_c1_g2::TRINITY_DN12776_c1_g2_i6::g.15805::m.15805	2	22,4	11,802	323,31					
TRINITY_DN12779_c0_g1::TRINITY_DN12779_c0_g1_i3::g.15740::m.15740	5	11,2	62,843	69,402	PF01293.19	PEPCK_ATP	712,4	2,10E-214	CL0374
TRINITY_DN12789_c1_g2::TRINITY_DN12789_c1_g2_i26::g.15965::m.15965	2	13,1	20,932	40,035	PF08263.11	LRRNT_2	38,4	1,00E-09	No_clan
TRINITY_DN12789_c1_g2::TRINITY_DN12789_c1_g2_i12::g.15958::m.15958	1	8,9	14,737	16,581	PF00106.24	adh_short	76,8	1,30E-21	CL0063
TRINITY_DN12789_c1_g2::TRINITY_DN12789_c1_g2_i25::g.15964::m.15964	1	8,2	11,949	4,9304	PF00106.24	adh_short	72,7	2,40E-20	CL0063
TRINITY_DN12813_c0_g1::TRINITY_DN12813_c0_g1_i4::g.16165::m.16165	16	52,4	35,589	323,31	PF00056.22	Ldh_1_N	114	5,40E-33	CL0063
TRINITY_DN12824_c1_g1::TRINITY_DN12824_c1_g1_i1::g.16233::m.16233	2	11,3	24,145	4,8637					
TRINITY_DN12824_c2_g2::TRINITY_DN12824_c2_g2_i6::g.16244::m.16244	9	40,7	21,994	35,015	PF00160.20	Pro_isomerase	158	2,10E-46	CL0475
TRINITY_DN12826_c0_g1::TRINITY_DN12826_c0_g1_i1::g.16222::m.16222	4	23,6	31,202	9,737	PF02265.15	S1-P1_nuclease	238,3	1,00E-70	CL0368
TRINITY_DN12828_c0_g1::TRINITY_DN12828_c0_g1_i2::g.16430::m.16430	1	7,2	16,473	2,1061	PF00071.21	Ras	120,8	3,80E-35	CL0023

TRINITY_DN12829_c0_g1::TRINITY_DN12829_c0_g1_i5::g.16229::m.16229	2	20	17,221	5,1261	PF08240.11	ADH_N	32,3	6,50E-08	CL0296
TRINITY_DN12832_c2_g6::TRINITY_DN12832_c2_g6_i1::g.16307::m.16307	7	33,4	31,584	32,324	PF01145.24	Band_7	85,5	4,10E-24	CL0433
TRINITY_DN12832_c2_g7::TRINITY_DN12832_c2_g7_i2::g.16314::m.16314	5	21,4	31,776	8,1476	PF01145.24	Band_7	77,4	1,30E-21	CL0433
TRINITY_DN12837_c2_g1::TRINITY_DN12837_c2_g1_i5::g.16578::m.16578	3	22	12,709	8,1656					
TRINITY_DN12837_c2_g2::TRINITY_DN12837_c2_g2_i1::g.16572::m.16572	5	33,5	18,775	4,9411	PF00295.16	Glyco_hydro_28	38,2	7,80E-10	CL0268
TRINITY_DN12837_c2_g2::TRINITY_DN12837_c2_g2_i2::g.16573::m.16573	4	28,7	21,557	-2	PF00295.16	Glyco_hydro_28	36	3,80E-09	CL0268
TRINITY_DN12837_c2_g2::TRINITY_DN12837_c2_g2_i7::g.16580::m.16580	6	48,2	18,547	323,31	PF00295.16	Glyco_hydro_28	36,1	3,40E-09	CL0268
TRINITY_DN12837_c2_g6::TRINITY_DN12837_c2_g6_i1::g.16583::m.16583	1	14,1	11,024	67,241					
TRINITY_DN12843_c0_g1::TRINITY_DN12843_c0_g1_i3::g.16447::m.16447	3	3,8	115,52	5,125	PF08323.10	Glyco_transf_5	224,2	1,80E-66	CL0113
TRINITY_DN12846_c1_g3::TRINITY_DN12846_c1_g3_i2::g.16059::m.16059	2	21,6	18,372	5,9011	PF02560.13	Cyanate_lyase	102,9	5,60E-30	No_clan
TRINITY_DN12853_c0_g1::TRINITY_DN12853_c0_g1_i3::g.16604::m.16604	10	32,9	42,351	41,194	PF01145.24	Band_7	70,2	2,00E-19	CL0433
TRINITY_DN12855_c4_g1::TRINITY_DN12855_c4_g1_i7::g.16693::m.16693	4	10,6	53,898	2,4634	PF00485.17	PRK	153,5	5,30E-45	CL0023
TRINITY_DN12856_c0_g1::TRINITY_DN12856_c0_g1_i4::g.16613::m.16613	3	11,9	52,438	8,4217	PF04774.14	HABP4_PAI-RBP1	31,8	2,00E-07	No_clan
TRINITY_DN12857_c0_g1::TRINITY_DN12857_c0_g1_i2::g.16763::m.16763	4	16,4	39,068	6,7275	PF04755.11	PAP_fibrillin	229,6	3,00E-68	No_clan
TRINITY_DN12861_c0_g1::TRINITY_DN12861_c0_g1_i2::g.16621::m.16621	3	17,6	20,321	3,4408	PF14622.5	Ribonucleas_3_3	39,6	4,80E-10	CL0539
TRINITY_DN12877_c0_g1::TRINITY_DN12877_c0_g1_i2::g.16745::m.16745	3	16,6	23,799	323,31	PF07876.11	Dabb	66	3,40E-18	CL0032
TRINITY_DN12879_c0_g1::TRINITY_DN12879_c0_g1_i1::g.16746::m.16746	6	37	27,288	137,9	PF00756.19	Esterase	181,2	2,50E-53	CL0028
TRINITY_DN12880_c1_g2::TRINITY_DN12880_c1_g2_i3::g.16816::m.16816	9	45,8	26,677	41,77	PF14290.5	DUF4370	392,9	5,90E-118	No_clan
TRINITY_DN12883_c0_g1::TRINITY_DN12883_c0_g1_i3::g.16877::m.16877	7	39	25,909	27,726	PF00903.24	Glyoxalase	75	5,50E-21	CL0104
TRINITY_DN12883_c0_g1::TRINITY_DN12883_c0_g1_i6::g.16879::m.16879	7	39	25,885	156,85	PF00903.24	Glyoxalase	75	5,50E-21	CL0104
TRINITY_DN12888_c1_g1::TRINITY_DN12888_c1_g1_i1::g.16988::m.16988	4	30,7	22,811	16,805	PF04832.11	SOUL	68,8	5,70E-19	CL0319

TRINITY_DN12890_c2_g4::TRINITY_DN12890_c2_g4_i2::g.17047::m.17047	1	10,8	13,324	168,26					
TRINITY_DN12893_c0_g1::TRINITY_DN12893_c0_g1_i1::g.17069::m.17069	5	59,6	11,029	24,208	PF01722.17	BolA	73,3	1,30E-20	No_clan
TRINITY_DN12896_c0_g1::TRINITY_DN12896_c0_g1_i6::g.17068::m.17068	3	11,7	29,532	3,6372	PF02374.14	ArsA_ATPase	234,4	1,60E-69	CL0023
TRINITY_DN12897_c1_g1::TRINITY_DN12897_c1_g1_i8::g.17082::m.17082	3	14,8	32,849	4,7236	PF04176.12	TIP41	205,3	4,20E-61	No_clan
TRINITY_DN12914_c1_g1::TRINITY_DN12914_c1_g1_i4::g.17252::m.17252	2	24,5	11,09	5,5438	PF00403.25	HMA	62,2	4,20E-17	No_clan
TRINITY_DN12917_c0_g1::TRINITY_DN12917_c0_g1_i8::g.17363::m.17363	2	22,2	13,076	3,2462	PF00226.30	DnaJ	93,3	6,70E-27	CL0392
TRINITY_DN12927_c2_g2::TRINITY_DN12927_c2_g2_i9::g.17583::m.17583	9	17,8	97,572	19,704	PF02922.17	CBM_48	65,6	3,50E-18	CL0369
TRINITY_DN12931_c0_g1::TRINITY_DN12931_c0_g1_i4::g.17462::m.17462	4	25,6	21,704	9,6096	PF03665.12	UPF0172	177,1	3,70E-52	CL0366
TRINITY_DN12935_c1_g1::TRINITY_DN12935_c1_g1_i3::g.17528::m.17528	6	23	21,668	2,0062	PF00107.25	ADH_zinc_N	89,3	1,80E-25	CL0063
TRINITY_DN12952_c0_g1::TRINITY_DN12952_c0_g1_i2::g.17756::m.17756	5	18,1	38,692	52,202	PF01063.18	Aminotran_4	129	2,20E-37	No_clan
TRINITY_DN12955_c2_g1::TRINITY_DN12955_c2_g1_i5::g.17882::m.17882	3	6,8	98,004	10,644	PF04065.14	Not3	278,9	2,50E-83	No_clan
TRINITY_DN12966_c1_g1::TRINITY_DN12966_c1_g1_i2::g.17932::m.17932	15	43,4	47,808	323,31	PF00330.19	Aconitase	131,7	3,40E-38	No_clan
TRINITY_DN12966_c3_g1::TRINITY_DN12966_c3_g1_i1::g.17946::m.17946	21	44,8	74,473	140,93	PF00330.19	Aconitase	431,8	3,20E-129	No_clan
TRINITY_DN12971_c4_g2::TRINITY_DN12971_c4_g2_i4::g.17979::m.17979	5	24,9	27,167	20,772	PF10584.8	Proteasome_A_N	50,9	7,70E-14	CL0052
TRINITY_DN12974_c0_g1::TRINITY_DN12974_c0_g1_i2::g.18074::m.18074	2	4,2	80,408	2,7909					
TRINITY_DN12983_c5_g1::TRINITY_DN12983_c5_g1_i4::g.18189::m.18189	3	7	44,862	2,8508	PF00004.28	AAA	150,7	2,60E-44	CL0023
TRINITY_DN12986_c2_g1::TRINITY_DN12986_c2_g1_i7::g.17676::m.17676	3	4,9	84,775	3,2727	PF05691.11	Raffinose_syn	1125,4	0	CL0058
TRINITY_DN12991_c0_g2::TRINITY_DN12991_c0_g2_i1::g.18234::m.18234	5	12,3	54,391	19,846	PF13432.5	TPR_16	18,6	0,0021	CL0020
TRINITY_DN12993_c1_g1::TRINITY_DN12993_c1_g1_i9::g.18389::m.18389	2	15,1	16,285	19,81	PF16845.4	SQAPI	76,8	1,20E-21	CL0121
TRINITY_DN12993_c1_g5::TRINITY_DN12993_c1_g5_i1::g.18384::m.18384	3	8,8	52,077	4,9583	PF14306.5	PUA_2	147	3,40E-43	CL0178
TRINITY_DN13004_c2_g2::TRINITY_DN13004_c2_g2_i3::g.18474::m.18474	2	9,1	31,416	19,453	PF16035.4	Chalcone_2	38,2	1,20E-09	CL0560

TRINITY_DN13006_c0_g1::TRINITY_DN13006_c0_g1_i3::g.18571::m.18571	1	12	15,06	2,4972	PF01248.25	Ribosomal_L7Ae	90,1	5,10E-26	CL0101
TRINITY_DN13008_c2_g1::TRINITY_DN13008_c2_g1_i1::g.18585::m.18585	2	12	32,715	2,1871					
TRINITY_DN13010_c2_g2::TRINITY_DN13010_c2_g2_i9::g.18708::m.18708	1	9,6	13,309	2,478	PF01105.23	EMP24_GP25L	99,1	2,60E-28	CL0521
TRINITY_DN13014_c6_g2::TRINITY_DN13014_c6_g2_i4::g.18867::m.18867	12	38,8	49,553	106,66	PF00091.24	Tubulin	226	4,30E-67	CL0566
TRINITY_DN13021_c1_g1::TRINITY_DN13021_c1_g1_i1::g.18775::m.18775	1	6,6	32,729	2,8942	PF13668.5	Ferritin_2	114,6	3,50E-33	CL0044
TRINITY_DN13021_c1_g2::TRINITY_DN13021_c1_g2_i3::g.18779::m.18779	6	29,2	32,729	73,577	PF13668.5	Ferritin_2	116	1,20E-33	CL0044
TRINITY_DN13024_c1_g2::TRINITY_DN13024_c1_g2_i1::g.18973::m.18973	3	29	14,829	323,31	PF01095.18	Pectinesterase	177,5	2,20E-52	CL0268
TRINITY_DN13025_c0_g1::TRINITY_DN13025_c0_g1_i1::g.18736::m.18736	2	4,9	51,087	3,0017	PF00899.20	ThiF	207	2,70E-61	CL0063
TRINITY_DN13032_c1_g1::TRINITY_DN13032_c1_g1_i5::g.18813::m.18813	1	4,6	31,631	2,7377					
TRINITY_DN13033_c2_g1::TRINITY_DN13033_c2_g1_i6::g.18937::m.18937	5	52,3	14,207	224,98	PF00235.18	Profilin	98,1	4,00E-28	CL0431
TRINITY_DN16368_c1_g2::TRINITY_DN16368_c1_g2_i3::g.66564::m.66564	2	25,7	12,592	4,8424	PF00179.25	UQ_con	149,1	5,40E-44	CL0208
TRINITY_DN13047_c0_g2::TRINITY_DN13047_c0_g2_i4::g.19117::m.19117	3	8,3	46,068	4,2219	PF02729.20	OTCace_N	156,1	5,90E-46	No_clan
TRINITY_DN13051_c9_g1::TRINITY_DN13051_c9_g1_i1::g.19838::m.19838	1	19,6	12,296	32,807					
TRINITY_DN13052_c1_g1::TRINITY_DN13052_c1_g1_i8::g.19267::m.19267	3	12,8	25,817	4,2988	PF00076.21	RRM_1	50,2	1,60E-13	CL0221
TRINITY_DN13066_c0_g1::TRINITY_DN13066_c0_g1_i8::g.19396::m.19396	6	36,1	23,084	13,505	PF00071.21	Ras	188,8	4,70E-56	CL0023
TRINITY_DN13067_c1_g1::TRINITY_DN13067_c1_g1_i2::g.19355::m.19355	6	44,1	16,332	323,31	PF00254.27	FKBP_C	117,9	1,70E-34	CL0487
TRINITY_DN13073_c0_g1::TRINITY_DN13073_c0_g1_i2::g.19364::m.19364	4	8,6	56,279	6,1217	PF07994.11	NAD_binding_5	470,3	3,10E-141	CL0063
TRINITY_DN13076_c2_g1::TRINITY_DN13076_c2_g1_i6::g.19526::m.19526	1	6,7	31,011	2,8232	PF01053.19	Cys_Met_Meta_PP	153,1	6,90E-45	CL0061
TRINITY_DN13077_c0_g1::TRINITY_DN13077_c0_g1_i9::g.19580::m.19580	2	12,2	26,452	4,0496					
TRINITY_DN13082_c0_g1::TRINITY_DN13082_c0_g1_i2::g.19429::m.19429	4	26,6	30,217	34,133	PF02893.19	GRAM	101,6	2,10E-29	CL0266
TRINITY_DN13083_c4_g1::TRINITY_DN13083_c4_g1_i1::g.19609::m.19609	2	12,8	25,459	3,9845	PF04073.14	tRNA_edit	33,8	2,70E-08	No_clan

TRINITY_DN13091_c0_g1::TRINITY_DN13091_c0_g1_i4::g.19648::m.19648	8	47,2	20,246	9,4964	PF00724.19	Oxidored_FMN	119,8	1,30E-34	CL0036
TRINITY_DN13091_c0_g1::TRINITY_DN13091_c0_g1_i8::g.19651::m.19651	8	49,4	20,319	2,1814	PF00724.19	Oxidored_FMN	116,3	1,50E-33	CL0036
TRINITY_DN13091_c0_g2::TRINITY_DN13091_c0_g2_i2::g.19654::m.19654	3	19,6	16,744	2,6788	PF00724.19	Oxidored_FMN	153,2	9,70E-45	CL0036
TRINITY_DN13098_c2_g1::TRINITY_DN13098_c2_g1_i5::g.19700::m.19700	3	11,4	50,657	5,7658					
TRINITY_DN13102_c0_g1::TRINITY_DN13102_c0_g1_i5::g.19880::m.19880	2	5,2	58,61	5,1916	PF13855.5	LRR_8	29,4	4,50E-07	CL0022
TRINITY_DN13107_c5_g1::TRINITY_DN13107_c5_g1_i1::g.19946::m.19946	3	13	26,858	4,9161	PF01277.16	Oleosin	146,3	2,60E-43	No_clan
TRINITY_DN13110_c0_g1::TRINITY_DN13110_c0_g1_i3::g.20012::m.20012	1	12,7	17,65	3,3023	PF08784.10	RPA_C	70,8	1,20E-19	CL0123
TRINITY_DN13113_c1_g1::TRINITY_DN13113_c1_g1_i4::g.20046::m.20046	10	22,1	64,014	21,959	PF00224.20	PK	309,1	2,90E-92	CL0151
TRINITY_DN13121_c2_g1::TRINITY_DN13121_c2_g1_i1::g.20130::m.20130	3	10	39,501	7,0109	PF08606.10	Prp19	109,7	4,80E-32	No_clan
TRINITY_DN13122_c2_g4::TRINITY_DN13122_c2_g4_i4::g.20179::m.20179	4	13,5	29,776	8,9129	PF02790.14	COX2_TM	115,9	6,30E-34	No_clan
TRINITY_DN13122_c2_g7::TRINITY_DN13122_c2_g7_i5::g.20188::m.20188	2	17,6	13,946	4,0759					
TRINITY_DN13123_c3_g2::TRINITY_DN13123_c3_g2_i7::g.20202::m.20202	6	33,6	26,498	12,225	PF13419.5	HAD_2	81,5	7,10E-23	CL0137
TRINITY_DN13126_c1_g2::TRINITY_DN13126_c1_g2_i1::g.20156::m.20156	7	57,3	14,14	3,6846	PF00071.21	Ras	144,4	2,00E-42	CL0023
TRINITY_DN13126_c1_g4::TRINITY_DN13126_c1_g4_i1::g.20159::m.20159	7	61,7	13,686	146,19	PF00071.21	Ras	141,5	1,70E-41	CL0023
TRINITY_DN13127_c0_g1::TRINITY_DN13127_c0_g1_i1::g.20146::m.20146	6	22	41,405	14,755	PF16363.4	GDP_Man_Dehyd	456,5	5,40E-137	CL0063
TRINITY_DN13128_c0_g1::TRINITY_DN13128_c0_g1_i1::g.20153::m.20153	7	26,6	54,903	14,576	PF00270.28	DEAD	119,1	1,60E-34	CL0023
TRINITY_DN13129_c8_g5::TRINITY_DN13129_c8_g5_i1::g.20297::m.20297	5	12,3	65,993	10,993	PF07244.14	POTRA	22,8	9,80E-05	CL0191
TRINITY_DN13133_c2_g1::TRINITY_DN13133_c2_g1_i2::g.20342::m.20342	8	26,8	44,546	77,227	PF08669.10	GCV_T_C	32,6	6,60E-08	No_clan
TRINITY_DN13140_c0_g1::TRINITY_DN13140_c0_g1_i10::g.20388::m.20388	7	39,1	29,639	20,285	PF00132.23	Hexapep	22,9	4,60E-05	CL0536
TRINITY_DN13140_c0_g3::TRINITY_DN13140_c0_g3_i1::g.20389::m.20389	6	35,5	29,687	23,894	PF00132.23	Hexapep	18,5	0,0011	CL0536
TRINITY_DN13144_c0_g1::TRINITY_DN13144_c0_g1_i6::g.19931::m.19931	2	17,4	16,771	4,5645	PF09585.9	Lin0512_fam	136,5	3,50E-40	No_clan

TRINITY_DN13145_c0_g1::TRINITY_DN13145_c0_g1_i9::g.20440::m.20440	10	31,4	47,443	70,846	PF00004.28	AAA	144,7	1,90E-42	CL0023
TRINITY_DN13146_c0_g1::TRINITY_DN13146_c0_g1_i3::g.20394::m.20394	1	17,5	17,146	8,3544					
TRINITY_DN13149_c0_g1::TRINITY_DN13149_c0_g1_i7::g.20402::m.20402	2	6,4	44,687	4,0189	PF13793.5	Pribosyltran_N	25,7	8,10E-06	CL0533
TRINITY_DN13150_c1_g1::TRINITY_DN13150_c1_g1_i6::g.20550::m.20550	2	11	22,802	3,8794	PF00076.21	RRM_1	78,1	3,10E-22	CL0221
TRINITY_DN13153_c0_g3::TRINITY_DN13153_c0_g3_i1::g.20486::m.20486	2	21,8	14,168	4,1781	PF01373.16	Glyco_hydro_14	138,3	3,00E-40	CL0058
TRINITY_DN13159_c0_g1::TRINITY_DN13159_c0_g1_i1::g.20565::m.20565	5	20,9	19,76	5,2098	PF00160.20	Pro_isomerase	159,8	5,80E-47	CL0475
TRINITY_DN13161_c0_g1::TRINITY_DN13161_c0_g1_i3::g.20601::m.20601	1	13,7	21,663	4,5329	PF03330.17	DPBB_1	84,3	5,00E-24	CL0199
TRINITY_DN13161_c0_g2::TRINITY_DN13161_c0_g2_i6::g.20596::m.20596	3	23,4	18,896	3,8723	PF04573.11	SPC22	177	1,90E-52	No_clan
TRINITY_DN13164_c0_g2::TRINITY_DN13164_c0_g2_i1::g.20739::m.20739	14	32,1	55,764	94,297	PF00180.19	Iso_dh	286,8	2,20E-85	CL0270
TRINITY_DN13164_c0_g4::TRINITY_DN13164_c0_g4_i1::g.20741::m.20741	12	26,9	47,002	46,951	PF00180.19	Iso_dh	309,1	3,50E-92	CL0270
TRINITY_DN13171_c0_g1::TRINITY_DN13171_c0_g1_i3::g.20617::m.20617	4	32,2	15,904	23,834	PF01090.18	Ribosomal_S19e	193,1	1,40E-57	CL0123
TRINITY_DN13179_c1_g1::TRINITY_DN13179_c1_g1_i6::g.20799::m.20799	2	16,1	19,973	10,862	PF13499.5	EF-hand_7	58,9	4,70E-16	CL0220
TRINITY_DN13179_c1_g3::TRINITY_DN13179_c1_g3_i2::g.20801::m.20801	4	42,4	13,198	13,797					
TRINITY_DN13180_c0_g1::TRINITY_DN13180_c0_g1_i3::g.20757::m.20757	2	7,1	43,864	10,462	PF01676.17	Metalloenzyme	88,9	2,90E-25	CL0088
TRINITY_DN13193_c0_g2::TRINITY_DN13193_c0_g2_i7::g.20882::m.20882	2	16,9	28,534	4,406	PF00684.18	DnaJ_CXXCXGXG	44,4	1,50E-11	No_clan
TRINITY_DN13194_c3_g1::TRINITY_DN13194_c3_g1_i1::g.20967::m.20967	6	36,9	17,49	98,096					
TRINITY_DN13194_c4_g1::TRINITY_DN13194_c4_g1_i4::g.20971::m.20971	2	23,9	15,402	5,1649	PF00886.18	Ribosomal_S16	89,2	1,20E-25	No_clan
TRINITY_DN13194_c5_g1::TRINITY_DN13194_c5_g1_i1::g.20972::m.20972	3	11,4	34,32	77,767	PF13855.5	LRR_8	33,7	2,10E-08	CL0022
TRINITY_DN13198_c0_g2::TRINITY_DN13198_c0_g2_i2::g.20890::m.20890	3	21,5	21,962	5,0934	PF02365.14	NAM	91,3	7,60E-26	No_clan
TRINITY_DN13200_c1_g3::TRINITY_DN13200_c1_g3_i1::g.20941::m.20941	2	14	18,25	37,488	PF00160.20	Pro_isomerase	178,6	9,50E-53	CL0475
TRINITY_DN13201_c1_g1::TRINITY_DN13201_c1_g1_i6::g.20932::m.20932	3	7,9	38,594	37,523	PF00226.30	DnaJ	90,8	4,00E-26	CL0392
TRINITY_DN13203_c0_g1::TRINITY_DN13203_c0_g1_i1::g.21059::m.21059	3	26,8	12,328	132,34	PF00085.19	Thioredoxin	110,7	2,70E-32	CL0172

TRINITY_DN13204_c1_g1::TRINITY_DN13204_c1_g1_i4::g.21099::m.21099	5	43	12,852	104,03	PF05922.15	Inhibitor_I9	48,6	9,20E-13	CL0570
TRINITY_DN13205_c0_g1::TRINITY_DN13205_c0_g1_i3::g.21053::m.21053	1	9,6	13,293	2,7919	PF07859.12	Abhydrolase_3	53,6	2,50E-14	CL0028
TRINITY_DN13211_c0_g1::TRINITY_DN13211_c0_g1_i3::g.21098::m.21098	9	23,1	44,561	3,2584	PF00297.21	Ribosomal_L3	646,3	1,20E-194	CL0575
TRINITY_DN13211_c0_g2::TRINITY_DN13211_c0_g2_i3::g.21096::m.21096	10	27,5	44,521	18,883	PF00297.21	Ribosomal_L3	650,2	7,80E-196	CL0575
TRINITY_DN13215_c0_g6::TRINITY_DN13215_c0_g6_i3::g.21426::m.21426	3	27,3	14,852	17,535	PF08597.9	eIF3_subunit	105,9	2,80E-30	No_clan
TRINITY_DN13215_c0_g4::TRINITY_DN13215_c0_g4_i6::g.21439::m.21439	2	10,9	25,1	15,412	PF08597.9	eIF3_subunit	176,3	8,90E-52	No_clan
TRINITY_DN13217_c3_g3::TRINITY_DN13217_c3_g3_i1::g.21250::m.21250	1	7,1	30,592	5,5814					
TRINITY_DN13221_c2_g1::TRINITY_DN13221_c2_g1_i1::g.21206::m.21206	10	50,7	29,335	49,182	PF01459.21	Porin_3	218,5	1,00E-64	CL0193
TRINITY_DN13221_c3_g1::TRINITY_DN13221_c3_g1_i2::g.21212::m.21212	3	16	26,64	4,0321	PF00248.20	Aldo_ket_red	176,7	5,10E-52	No_clan
TRINITY_DN13227_c0_g1::TRINITY_DN13227_c0_g1_i8::g.21288::m.21288	19	40,6	68,364	121,7	PF01704.17	UDPGP	55,6	3,10E-15	CL0110
TRINITY_DN13227_c0_g6::TRINITY_DN13227_c0_g6_i1::g.21293::m.21293	1	10,4	12,126	27,306	PF01187.17	MIF	91,3	4,40E-26	CL0082
TRINITY_DN13228_c1_g1::TRINITY_DN13228_c1_g1_i1::g.21382::m.21382	4	27,3	20,405	16,007	PF00085.19	Thioredoxin	86,6	8,40E-25	CL0172
TRINITY_DN13233_c0_g4::TRINITY_DN13233_c0_g4_i3::g.21366::m.21366	2	6,4	67,108	2,1051	PF03765.14	CRAL_TRIO_N	37,9	1,40E-09	No_clan
TRINITY_DN13234_c0_g1::TRINITY_DN13234_c0_g1_i7::g.21346::m.21346	7	26,4	36,657	26,938	PF14559.5	TPR_19	27,3	3,60E-06	CL0020
TRINITY_DN13241_c2_g1::TRINITY_DN13241_c2_g1_i4::g.21412::m.21412	1	17,2	16,71	2,3037	PF03870.14	RNA_pol_Rpb8	161,5	1,20E-47	CL0021
TRINITY_DN17556_c3_g1::TRINITY_DN17556_c3_g1_i1::g.85912::m.85912	1	12,9	11,698	2,3756	PF04116.12	FA_hydroxylase	52,5	5,70E-14	No_clan
TRINITY_DN13244_c2_g1::TRINITY_DN13244_c2_g1_i6::g.21528::m.21528	6	52,3	17,192	52,56	PF00179.25	UQ_con	165,4	5,20E-49	CL0208
TRINITY_DN13244_c3_g1::TRINITY_DN13244_c3_g1_i1::g.21540::m.21540	12	40,8	36,34	287,19	PF00056.22	Ldh_1_N	161,4	1,20E-47	CL0063
TRINITY_DN13246_c1_g2::TRINITY_DN13246_c1_g2_i1::g.21483::m.21483	1	2,9	45,227	1,8518					
TRINITY_DN13249_c0_g1::TRINITY_DN13249_c0_g1_i1::g.21073::m.21073	1	6,2	29,042	2,5634	PF13561.5	adh_short_C2	218,5	8,50E-65	CL0063
TRINITY_DN13259_c0_g1::TRINITY_DN13259_c0_g1_i1::g.21585::m.21585	10	28,7	42,803	19,767	PF03223.14	V-ATPase_C	422,7	1,40E-126	No_clan

TRINITY_DN13260_c0_g1::TRINITY_DN13260_c0_g1_i2::g.21630::m.21630	2	16,3	14,255	3,3179					
TRINITY_DN13260_c0_g2::TRINITY_DN13260_c0_g2_i1::g.21634::m.21634	2	16,3	21,546	4,998					
TRINITY_DN13264_c3_g1::TRINITY_DN13264_c3_g1_i4::g.21666::m.21666	7	24,6	48,994	25,806	PF01546.27	Peptidase_M20	120,1	9,10E-35	CL0035
TRINITY_DN13265_c1_g1::TRINITY_DN13265_c1_g1_i6::g.21679::m.21679	10	47,4	28,914	119,88	PF00244.19	14.03.2003	352,7	6,40E-106	No_clan
TRINITY_DN13265_c1_g2::TRINITY_DN13265_c1_g2_i3::g.21681::m.21681	3	14,3	29,094	2,2584	PF00244.19	14.03.2003	343,8	3,40E-103	No_clan
TRINITY_DN13268_c1_g1::TRINITY_DN13268_c1_g1_i7::g.21886::m.21886	14	32,1	55,03	38,531	PF00764.18	Arginosuc_synth	523,2	4,60E-157	CL0039
TRINITY_DN13275_c3_g1::TRINITY_DN13275_c3_g1_i7::g.21820::m.21820	1	5,3	25,985	1,9508	PF00314.16	Thaumatin	260,2	1,40E-77	CL0293
TRINITY_DN13280_c0_g2::TRINITY_DN13280_c0_g2_i2::g.21976::m.21976	8	27,5	45,011	17,027	PF00462.23	Glutaredoxin	60,7	1,10E-16	CL0172
TRINITY_DN13283_c0_g1::TRINITY_DN13283_c0_g1_i3::g.21813::m.21813	11	35	44,879	66,435	PF00573.21	Ribosomal_L4	134,3	3,70E-39	No_clan
TRINITY_DN13284_c0_g1::TRINITY_DN13284_c0_g1_i3::g.21957::m.21957	3	8,4	48,398	68,295	PF00076.21	RRM_1	44,9	6,90E-12	CL0221
TRINITY_DN13284_c1_g1::TRINITY_DN13284_c1_g1_i7::g.21970::m.21970	8	43,5	21,168	102,04	PF12481.7	DUF3700	309,1	1,40E-92	CL0052
TRINITY_DN13288_c0_g1::TRINITY_DN13288_c0_g1_i6::g.22068::m.22068	3	10,6	36,997	4,0555	PF00332.17	Glyco_hydro_17	203,4	5,00E-60	CL0058
TRINITY_DN13288_c0_g3::TRINITY_DN13288_c0_g3_i3::g.22072::m.22072	6	13,2	55,792	48,756	PF00332.17	Glyco_hydro_17	329,1	2,80E-98	CL0058
TRINITY_DN13289_c1_g1::TRINITY_DN13289_c1_g1_i2::g.22086::m.22086	3	8,4	50,979	4,5878	PF07738.12	Sad1_UNC	136	7,00E-40	CL0202
TRINITY_DN13289_c1_g2::TRINITY_DN13289_c1_g2_i4::g.22093::m.22093	1	15,1	12,499	2,471					
TRINITY_DN13291_c0_g1::TRINITY_DN13291_c0_g1_i4::g.22017::m.22017	6	39	16,75	163,33	PF01597.18	GCV_H	164,4	8,70E-49	CL0105
TRINITY_DN13292_c1_g6::TRINITY_DN13292_c1_g6_i2::g.22041::m.22041	3	30,2	14,542	4,6839					
TRINITY_DN13300_c0_g1::TRINITY_DN13300_c0_g1_i5::g.22083::m.22083	2	5,5	42,336	4,6058	PF03917.16	GSH_synth_ATP	196,6	5,00E-58	CL0483
TRINITY_DN13302_c0_g2::TRINITY_DN13302_c0_g2_i1::g.22438::m.22438	6	26,1	37,113	41,225	PF08511.10	COQ9	101,9	1,10E-29	No_clan
TRINITY_DN13316_c0_g1::TRINITY_DN13316_c0_g1_i4::g.22236::m.22236	6	9,7	87,595	10,186	PF02922.17	CBM_48	43,7	2,40E-11	CL0369
TRINITY_DN13325_c1_g1::TRINITY_DN13325_c1_g1_i1::g.22310::m.22310	2	7,2	36,324	4,7898					

TRINITY_DN13334_c3_g1::TRINITY_DN13334_c3_g1_i2::g.22908::m.22908	2	15,7	14,482	2,9978	PF00082.21	Peptidase_S8	47	1,80E-12	No_clan
TRINITY_DN13334_c3_g4::TRINITY_DN13334_c3_g4_i1::g.22910::m.22910	3	18,5	20,541	72,996	PF00082.21	Peptidase_S8	50,3	1,80E-13	No_clan
TRINITY_DN13342_c2_g1::TRINITY_DN13342_c2_g1_i2::g.22558::m.22558	4	17,5	29,56	6,0231	PF00153.26	Mito_carr	66	2,00E-18	No_clan
TRINITY_DN13342_c3_g1::TRINITY_DN13342_c3_g1_i3::g.22570::m.22570	2	15	15,696	5,0696					
TRINITY_DN13342_c3_g2::TRINITY_DN13342_c3_g2_i4::g.22568::m.22568	10	19,4	69,641	19,582	PF01031.19	Dynamin_M	80,9	8,30E-23	No_clan
TRINITY_DN13348_c2_g1::TRINITY_DN13348_c2_g1_i3::g.22605::m.22605	4	30,7	16,228	5,2791	PF00411.18	Ribosomal_S11	158,7	5,20E-47	CL0267
TRINITY_DN13348_c4_g1::TRINITY_DN13348_c4_g1_i6::g.22618::m.22618	3	12,5	29,736	4,6566	PF05739.18	SNARE	44,4	1,10E-11	No_clan
TRINITY_DN13349_c1_g1::TRINITY_DN13349_c1_g1_i7::g.22584::m.22584	4	23,4	22,786	12,563	PF00571.27	CBS	31,3	1,90E-07	No_clan
TRINITY_DN13350_c3_g1::TRINITY_DN13350_c3_g1_i5::g.22631::m.22631	5	19,7	34,699	39,309	PF00450.21	Peptidase_S10	283,8	2,80E-84	CL0028
TRINITY_DN13374_c2_g1::TRINITY_DN13374_c2_g1_i4::g.22819::m.22819	3	7,2	59,764	3,8481	PF00109.25	ketoacyl-synt	199,8	5,00E-59	CL0046
TRINITY_DN13376_c2_g1::TRINITY_DN13376_c2_g1_i2::g.22983::m.22983	6	48,5	18,821	323,31	PF01625.20	PMSR	199,2	3,60E-59	No_clan
TRINITY_DN13376_c2_g2::TRINITY_DN13376_c2_g2_i3::g.22986::m.22986	8	49,4	34,409	323,31	PF00112.22	Peptidase_C1	239,5	3,90E-71	CL0125
TRINITY_DN13377_c3_g1::TRINITY_DN13377_c3_g1_i6::g.23084::m.23084	1	7	17,885	2,3989	PF06113.11	BRE	53,2	1,80E-14	No_clan
TRINITY_DN13377_c3_g2::TRINITY_DN13377_c3_g2_i9::g.23085::m.23085	8	32,2	25,51	323,31	PF02798.19	GST_N	61,7	6,00E-17	CL0172
TRINITY_DN13379_c2_g1::TRINITY_DN13379_c2_g1_i3::g.22962::m.22962	1	14,1	11,11	2,1576	PF00153.26	Mito_carr	43,8	1,70E-11	No_clan
TRINITY_DN13381_c2_g1::TRINITY_DN13381_c2_g1_i4::g.23007::m.23007	5	30,8	23,326	6,2532	PF00071.21	Ras	204,4	7,30E-61	CL0023
TRINITY_DN13381_c2_g2::TRINITY_DN13381_c2_g2_i20::g.23020::m.23020	6	40	17,999	37,813	PF00071.21	Ras	156,5	4,10E-46	CL0023
TRINITY_DN13383_c0_g1::TRINITY_DN13383_c0_g1_i9::g.23043::m.23043	1	15,7	28,298	3,1182	PF14523.5	Syntaxin_2	80,4	9,60E-23	CL0445
TRINITY_DN13383_c1_g4::TRINITY_DN13383_c1_g4_i2::g.23048::m.23048	6	17,6	47,07	3,9071	PF00464.18	SHMT	648,8	2,20E-195	CL0061
TRINITY_DN13389_c0_g2::TRINITY_DN13389_c0_g2_i2::g.23122::m.23122	7	33,2	31,598	112,94	PF00291.24	PALP	225,4	9,60E-67	No_clan
TRINITY_DN13397_c1_g1::TRINITY_DN13397_c1_g1_i6::g.22118::m.22118	1	9,7	16,012	2,1453	PF01208.16	URO-D	35,6	5,30E-09	CL0160

TRINITY_DN13398_c7_g1::TRINITY_DN13398_c7_g1_i2::g.23298::m.23298	2	6,7	45,299	4,6466	PF14580.5	LRR_9	51	1,10E-13	CL0022
TRINITY_DN13404_c2_g1::TRINITY_DN13404_c2_g1_i2::g.23444::m.23444	1	13,8	12,282	2,2507					
TRINITY_DN13408_c0_g1::TRINITY_DN13408_c0_g1_i4::g.23389::m.23389	2	13,9	28,495	3,4217	PF00702.25	Hydrolase	52,8	5,90E-14	CL0137
TRINITY_DN13409_c1_g1::TRINITY_DN13409_c1_g1_i8::g.23419::m.23419	18	58,4	38,181	57,604	PF00044.23	Gp_dh_N	125	1,20E-36	CL0063
TRINITY_DN13415_c1_g2::TRINITY_DN13415_c1_g2_i6::g.23608::m.23608	3	11,3	40,384	6,6744	PF05057.13	DUF676	216,3	3,20E-64	CL0028
TRINITY_DN13415_c1_g3::TRINITY_DN13415_c1_g3_i1::g.23602::m.23602	14	26,4	74,54	2,9614	PF00311.16	PEPcase	190,8	3,30E-56	CL0151
TRINITY_DN13415_c1_g3::TRINITY_DN13415_c1_g3_i4::g.23607::m.23607	19	20	124,36	38,976	PF00311.16	PEPcase	189,5	8,10E-56	CL0151
TRINITY_DN13419_c2_g1::TRINITY_DN13419_c2_g1_i6::g.23702::m.23702	9	23,5	53,073	26,325	PF00365.19	PFK	207,6	2,10E-61	CL0240
TRINITY_DN13420_c0_g1::TRINITY_DN13420_c0_g1_i1::g.23499::m.23499	1	6,4	22,652	2,6908	PF03357.20	Snf7	44,1	1,50E-11	CL0235
TRINITY_DN13421_c0_g4::TRINITY_DN13421_c0_g4_i1::g.23529::m.23529	2	2,3	112,23	2,5747	PF05664.10	DUF810	960,2	6,60E-289	No_clan
TRINITY_DN13422_c4_g1::TRINITY_DN13422_c4_g1_i9::g.23675::m.23675	6	31,3	27,313	85,392	PF10584.8	Proteasome_A_N	49,8	1,80E-13	CL0052
TRINITY_DN13423_c2_g1::TRINITY_DN13423_c2_g1_i2::g.23680::m.23680	1	11,8	15,303	2,9374					
TRINITY_DN13425_c1_g1::TRINITY_DN13425_c1_g1_i4::g.23719::m.23719	3	20,9	31,04	8,4137	PF00970.23	FAD_binding_6	112,3	1,10E-32	CL0076
TRINITY_DN13425_c1_g3::TRINITY_DN13425_c1_g3_i2::g.23712::m.23712	3	13,7	31,166	8,6422	PF00970.23	FAD_binding_6	107,3	3,80E-31	CL0076
TRINITY_DN13427_c0_g2::TRINITY_DN13427_c0_g2_i5::g.23653::m.23653	3	25,8	15,077	6,2863	PF01283.18	Ribosomal_S26e	181,9	3,10E-54	No_clan
TRINITY_DN13427_c0_g3::TRINITY_DN13427_c0_g3_i1::g.23641::m.23641	3	27,5	14,894	3,9223	PF01283.18	Ribosomal_S26e	182,1	2,70E-54	No_clan
TRINITY_DN13431_c11_g1::TRINITY_DN13431_c11_g1_i1::g.23860::m.23860	9	28,6	46,701	25,166	PF01293.19	PEPCK_ATP	31,5	6,90E-08	CL0374
TRINITY_DN13432_c1_g1::TRINITY_DN13432_c1_g1_i19::g.23847::m.23847	7	30,1	35,151	16,092	PF00481.20	PP2C	208,9	9,00E-62	CL0238
TRINITY_DN13433_c0_g1::TRINITY_DN13433_c0_g1_i4::g.23725::m.23725	7	15,6	62,581	26,099	PF05450.14	Nicastrin	33	3,90E-08	CL0035
TRINITY_DN13436_c3_g1::TRINITY_DN13436_c3_g1_i7::g.23925::m.23925	7	31,6	41,543	177,98	PF00108.22	Thiolase_N	307,3	7,10E-92	CL0046
TRINITY_DN13436_c3_g2::TRINITY_DN13436_c3_g2_i1::g.23914::m.23914	6	26	42,028	12,458	PF00108.22	Thiolase_N	300,9	6,30E-90	CL0046

TRINITY_DN13437_c2_g2::TRINITY_DN13437_c2_g2_i2::g.23355::m.23355	2	7,3	60,561	13,833	PF03398.13	Ist1	44,1	1,80E-11	No_clan
TRINITY_DN13437_c3_g1::TRINITY_DN13437_c3_g1_i7::g.23377::m.23377	2	5,3	60,297	6,0019	PF02990.15	EMP70	532,3	1,10E-159	No_clan
TRINITY_DN13437_c3_g3::TRINITY_DN13437_c3_g3_i4::g.23371::m.23371	3	7,7	64,106	12,668	PF02990.15	EMP70	563,4	4,10E-169	No_clan
TRINITY_DN13446_c0_g3::TRINITY_DN13446_c0_g3_i1::g.23949::m.23949	1	5,3	39,033	3,1786	PF16884.4	ADH_N_2	65,4	3,50E-18	CL0296
TRINITY_DN13456_c0_g1::TRINITY_DN13456_c0_g1_i3::g.24091::m.24091	2	4,3	67,001	3,2031					
TRINITY_DN13460_c3_g2::TRINITY_DN13460_c3_g2_i3::g.24327::m.24327	2	11,4	26,759	7,4875	PF13537.5	GATase_7	137,1	2,40E-40	CL0052
TRINITY_DN13463_c1_g1::TRINITY_DN13463_c1_g1_i3::g.24177::m.24177	5	29,6	27,096	12,761	PF02900.17	LigB	158,6	1,50E-46	CL0283
TRINITY_DN13465_c1_g2::TRINITY_DN13465_c1_g2_i1::g.24163::m.24163	9	40	28,506	37,105	PF00330.19	Aconitase	221,4	2,10E-65	No_clan
TRINITY_DN13465_c2_g1::TRINITY_DN13465_c2_g1_i6::g.24168::m.24168	3	22,3	19,46	6,099	PF02881.18	SRP54_N	61,4	6,60E-17	No_clan
TRINITY_DN13466_c1_g1::TRINITY_DN13466_c1_g1_i2::g.24484::m.24484	4	17,5	23,457	8,8485	PF00572.17	Ribosomal_L13	34,7	1,70E-08	No_clan
TRINITY_DN13471_c1_g1::TRINITY_DN13471_c1_g1_i4::g.24277::m.24277	1	10,6	14,179	2,1678					
TRINITY_DN13477_c2_g3::TRINITY_DN13477_c2_g3_i1::g.24414::m.24414	4	22,3	28,404	9,6646	PF00705.17	PCNA_N	181,1	5,90E-54	CL0060
TRINITY_DN13480_c0_g1::TRINITY_DN13480_c0_g1_i3::g.24330::m.24330	1	3,3	73,354	2,1063	PF02142.21	MGS	73,4	1,20E-20	No_clan
TRINITY_DN13485_c2_g2::TRINITY_DN13485_c2_g2_i2::g.24426::m.24426	5	24,4	27,095	24,813	PF01088.20	Peptidase_C12	202,2	7,60E-60	CL0125
TRINITY_DN13485_c2_g3::TRINITY_DN13485_c2_g3_i2::g.24428::m.24428	6	50,8	22,158	54,873	PF00390.18	malic	108,4	3,30E-31	CL0603
TRINITY_DN13487_c0_g1::TRINITY_DN13487_c0_g1_i1::g.24417::m.24417	2	3,7	61,414	2,244	PF03062.18	MBOAT	91,8	5,10E-26	CL0517
TRINITY_DN13494_c0_g2::TRINITY_DN13494_c0_g2_i2::g.24524::m.24524	4	19,3	23,08	1,9971	PF00163.18	Ribosomal_S4	32,2	1,40E-07	CL0492
TRINITY_DN13494_c0_g3::TRINITY_DN13494_c0_g3_i1::g.24527::m.24527	4	19,3	23,12	8,7631	PF00163.18	Ribosomal_S4	30,1	6,30E-07	CL0492
TRINITY_DN13503_c0_g1::TRINITY_DN13503_c0_g1_i7::g.24647::m.24647	3	14	24,583	6,7438	PF13450.5	NAD_binding_8	60	1,80E-16	CL0063
TRINITY_DN13512_c0_g1::TRINITY_DN13512_c0_g1_i2::g.24707::m.24707	1	10,6	11,632	5,0664	PF04450.11	BSP	63,1	2,80E-17	CL0126
TRINITY_DN13512_c0_g2::TRINITY_DN13512_c0_g2_i1::g.24706::m.24706	2	18,2	12,58	6,3502	PF04450.11	BSP	167,6	2,70E-49	CL0126

TRINITY_DN13521_c0_g1::TRINITY_DN13521_c0_g1_i1::g.24833::m.24833	1	23,5	10,903	4,7193	PF00198.22	2-oxoacid_dh	112,9	1,40E-32	CL0149
TRINITY_DN13521_c0_g4::TRINITY_DN13521_c0_g4_i1::g.24842::m.24842	7	25,3	40,429	27,184	PF00364.21	Biotin_lipoyl	60,1	1,30E-16	CL0105
TRINITY_DN13523_c0_g1::TRINITY_DN13523_c0_g1_i7::g.24832::m.24832	1	11,5	19,75	3,4226	PF03358.14	FMN_red	123,9	4,30E-36	CL0042
TRINITY_DN13524_c0_g1::TRINITY_DN13524_c0_g1_i8::g.24870::m.24870	1	20	14,524	2,2113	PF00917.25	MATH	62,8	2,70E-17	CL0389
TRINITY_DN13527_c0_g1::TRINITY_DN13527_c0_g1_i14::g.25011::m.25011	4	8,7	62,672	6,3453	PF00888.21	Cullin	569,6	8,30E-171	No_clan
TRINITY_DN13531_c1_g1::TRINITY_DN13531_c1_g1_i7::g.25242::m.25242	17	21,8	112,66	37,44	PF07765.11	KIP1	112,3	8,30E-33	No_clan
TRINITY_DN13535_c5_g1::TRINITY_DN13535_c5_g1_i1::g.25172::m.25172	4	17	24,957	27,216	PF02987.15	LEA_4	30,2	3,20E-07	No_clan
TRINITY_DN13537_c0_g1::TRINITY_DN13537_c0_g1_i2::g.25155::m.25155	2	15,6	18,834	4,3577					
TRINITY_DN13542_c0_g1::TRINITY_DN13542_c0_g1_i7::g.24740::m.24740	2	8,4	35,498	3,8882	PF01088.20	Peptidase_C12	180	4,70E-53	CL0125
TRINITY_DN13546_c0_g1::TRINITY_DN13546_c0_g1_i3::g.25193::m.25193	7	13,4	71,044	12,123	PF03141.15	Methyltransf_29	766,6	9,90E-231	CL0063
TRINITY_DN13561_c3_g1::TRINITY_DN13561_c3_g1_i11::g.25972::m.25972	3	10,1	54,185	5,1195	PF00282.18	Pyridoxal_deC	411,1	3,20E-123	CL0061
TRINITY_DN13563_c1_g1::TRINITY_DN13563_c1_g1_i8::g.25419::m.25419	25	27,4	136,62	90,177	PF00400.31	WD40	23,6	6,10E-05	CL0186
TRINITY_DN13564_c1_g1::TRINITY_DN13564_c1_g1_i6::g.26053::m.26053	3	31,4	15,063	104,06	PF00173.27	Cyt-b5	90,5	5,10E-26	No_clan
TRINITY_DN13566_c3_g2::TRINITY_DN13566_c3_g2_i6::g.25511::m.25511	3	14,2	38,718	11,653					
TRINITY_DN13569_c0_g2::TRINITY_DN13569_c0_g2_i1::g.25677::m.25677	2	8,4	33,371	7,0275	PF00005.26	ABC_tran	48,8	8,80E-13	CL0023
TRINITY_DN13571_c3_g1::TRINITY_DN13571_c3_g1_i9::g.25607::m.25607	4	14,8	39,055	12,933	PF00270.28	DEAD	120,5	6,10E-35	CL0023
TRINITY_DN13573_c3_g3::TRINITY_DN13573_c3_g3_i2::g.25665::m.25665	2	13,6	27,657	3,059	PF10058.8	zinc_ribbon_10	73,3	8,90E-21	CL0167
TRINITY_DN13585_c0_g1::TRINITY_DN13585_c0_g1_i3::g.25706::m.25706	1	6,7	27,626	2,2006					
TRINITY_DN13587_c1_g3::TRINITY_DN13587_c1_g3_i8::g.25768::m.25768	3	12,5	33,437	3,2646	PF00106.24	adh_short	97	9,00E-28	CL0063
TRINITY_DN13588_c3_g5::TRINITY_DN13588_c3_g5_i3::g.25866::m.25866	16	71,2	28,476	295,53	PF03214.12	RGP	427,8	2,80E-128	CL0110

TRINITY_DN13592_c0_g2::TRINITY_DN13592_c0_g2_i5::g.25790::m.25790	6	15,2	43,886	143,59	PF00790.18	VHS	90,1	1,00E-25	CL0009
TRINITY_DN13594_c1_g2::TRINITY_DN13594_c1_g2_i6::g.26004::m.26004	5	44,8	13,453	14,363	PF00462.23	Glutaredoxin	78,8	2,40E-22	CL0172
TRINITY_DN13599_c0_g2::TRINITY_DN13599_c0_g2_i3::g.24684::m.24684	3	15,7	24,584	65,834	PF00687.20	Ribosomal_L1	145,5	1,50E-42	No_clan
TRINITY_DN13600_c1_g7::TRINITY_DN13600_c1_g7_i6::g.25108::m.25108	6	25,2	26,982	16,913	PF03647.12	Tmemb_14	62,2	5,70E-17	No_clan
TRINITY_DN13605_c0_g1::TRINITY_DN13605_c0_g1_i3::g.26153::m.26153	1	4,9	27,236	2,7929	PF03151.15	TPT	72,9	2,50E-20	CL0184
TRINITY_DN13606_c2_g2::TRINITY_DN13606_c2_g2_i1::g.26262::m.26262	18	51,9	52,92	272,72	PF03721.13	UDPG_MGDP_dh_N	225,5	3,60E-67	CL0063
TRINITY_DN13606_c2_g2::TRINITY_DN13606_c2_g2_i2::g.26272::m.26272	17	52,2	52,85	79,583	PF03721.13	UDPG_MGDP_dh_N	228	6,30E-68	CL0063
TRINITY_DN13606_c2_g3::TRINITY_DN13606_c2_g3_i2::g.26267::m.26267	4	5,4	89,564	6,1995	PF04091.11	Sec15	257,5	1,80E-76	CL0295
TRINITY_DN13608_c0_g1::TRINITY_DN13608_c0_g1_i3::g.26161::m.26161	2	6,9	41,429	3,9273					
TRINITY_DN13610_c2_g1::TRINITY_DN13610_c2_g1_i1::g.26215::m.26215	1	3,8	48,884	2,5118	PF03151.15	TPT	379	1,40E-113	CL0184
TRINITY_DN13614_c0_g1::TRINITY_DN13614_c0_g1_i2::g.26309::m.26309	3	16,5	32,455	5,0715	PF03981.11	Ubiq_cyt_C_chap	116	1,30E-33	No_clan
TRINITY_DN13615_c2_g2::TRINITY_DN13615_c2_g2_i1::g.26300::m.26300	1	11,8	14,396	-2	PF03079.13	ARD	155,8	9,20E-46	CL0029
TRINITY_DN13616_c0_g1::TRINITY_DN13616_c0_g1_i3::g.26276::m.26276	2	11,6	17,938	10,293	PF08768.10	DUF1794	143,7	3,70E-42	CL0116
TRINITY_DN13617_c1_g1::TRINITY_DN13617_c1_g1_i9::g.26343::m.26343	3	7,4	64,781	4,8977	PF12701.6	LSM14	108,4	1,30E-31	CL0527
TRINITY_DN13619_c0_g1::TRINITY_DN13619_c0_g1_i2::g.26348::m.26348	3	29,3	18,189	34,085	PF00582.25	Usp	101,2	6,50E-29	CL0039
TRINITY_DN13623_c1_g1::TRINITY_DN13623_c1_g1_i6::g.26584::m.26584	1	2,6	47,496	1,8535	PF02705.15	K_trans	319,5	3,30E-95	CL0062
TRINITY_DN13624_c1_g6::TRINITY_DN13624_c1_g6_i6::g.26482::m.26482	23	40	72,931	323,31	PF02518.25	HATPase_c	43,4	3,80E-11	CL0025
TRINITY_DN13630_c0_g1::TRINITY_DN13630_c0_g1_i5::g.26390::m.26390	1	1,3	162,22	3,114					
TRINITY_DN13636_c0_g1::TRINITY_DN13636_c0_g1_i5::g.26555::m.26555	2	22,9	18,726	35,424	PF00106.24	adh_short	115,9	1,40E-33	CL0063
TRINITY_DN13637_c0_g1::TRINITY_DN13637_c0_g1_i7::g.26541::m.26541	1	14,2	14,154	3,0575					
TRINITY_DN13640_c0_g3::TRINITY_DN13640_c0_g3_i9::g.26621::m.26621	2	6,8	44,027	2,7487	PF13249.5	SQHop_cyclase_N	38,6	6,00E-10	CL0059

TRINITY_DN13642_c0_g1::TRINITY_DN13642_c0_g1_i1::g.26569::m.26569	3	5,2	71,346	3,4648	PF00071.21	Ras	50,3	1,80E-13	CL0023
TRINITY_DN13645_c2_g2::TRINITY_DN13645_c2_g2_i6::g.26141::m.26141	2	16	21,99	3,0632	PF01918.20	Alba	67,6	5,60E-19	CL0441
TRINITY_DN13650_c8_g1::TRINITY_DN13650_c8_g1_i8::g.26099::m.26099	3	44,1	14,009	10,456	PF06201.12	PITH	88	6,70E-25	CL0202
TRINITY_DN13650_c9_g1::TRINITY_DN13650_c9_g1_i8::g.26115::m.26115	2	4,7	68,692	2,9732	PF05193.20	Peptidase_M16_C	121,4	3,90E-35	CL0094
TRINITY_DN13652_c1_g1::TRINITY_DN13652_c1_g1_i1::g.26699::m.26699	1	14,4	12,965	2,1096					
TRINITY_DN13653_c3_g1::TRINITY_DN13653_c3_g1_i1::g.26703::m.26703	10	19,5	76,405	24,986	PF00501.27	AMP-binding	339,5	2,00E-101	CL0378
TRINITY_DN13661_c3_g4::TRINITY_DN13661_c3_g4_i1::g.26847::m.26847	4	21,2	17,498	8,0914	PF01287.19	eIF-5a	100,9	2,90E-29	CL0021
TRINITY_DN13666_c1_g1::TRINITY_DN13666_c1_g1_i1::g.26956::m.26956	2	2,4	100,7	31,349	PF08263.11	LRRNT_2	22,1	0,00013	No_clan
TRINITY_DN13666_c1_g1::TRINITY_DN13666_c1_g1_i6::g.26985::m.26985	8	27,7	38,203	48,397	PF08263.11	LRRNT_2	24,4	2,50E-05	No_clan
TRINITY_DN13674_c0_g1::TRINITY_DN13674_c0_g1_i2::g.26925::m.26925	12	19,4	110,61	33,48	PF00343.19	Phosphorylase	436,8	1,30E-130	CL0113
TRINITY_DN13676_c3_g1::TRINITY_DN13676_c3_g1_i3::g.27135::m.27135	1	9,6	15,078	21,491					
TRINITY_DN13683_c1_g1::TRINITY_DN13683_c1_g1_i4::g.27154::m.27154	4	9,3	79,698	16,262	PF02657.14	SufE	93,8	6,20E-27	CL0233
TRINITY_DN13688_c0_g1::TRINITY_DN13688_c0_g1_i3::g.27185::m.27185	2	7,7	32,232	3,4996					
TRINITY_DN13691_c2_g1::TRINITY_DN13691_c2_g1_i3::g.27222::m.27222	3	17	25,215	7,6786	PF07977.12	FabA	119,4	7,70E-35	CL0050
TRINITY_DN13692_c0_g1::TRINITY_DN13692_c0_g1_i6::g.26626::m.26626	7	18,4	42,822	4,2337	PF00153.26	Mito_carr	79,4	1,30E-22	No_clan
TRINITY_DN13692_c1_g1::TRINITY_DN13692_c1_g1_i1::g.26628::m.26628	8	23,6	37,283	36,584	PF00153.26	Mito_carr	82,1	1,90E-23	No_clan
TRINITY_DN13702_c0_g1::TRINITY_DN13702_c0_g1_i4::g.27394::m.27394	1	11,1	11,187	11,402	PF02148.18	zf-UBP	46,2	4,00E-12	CL0229
TRINITY_DN13705_c0_g1::TRINITY_DN13705_c0_g1_i7::g.27449::m.27449	8	27,6	45,975	26,319	PF00091.24	Tubulin	180,4	4,20E-53	CL0566
TRINITY_DN13705_c0_g2::TRINITY_DN13705_c0_g2_i4::g.27444::m.27444	7	33,7	32,327	13,743	PF04845.12	PurA	73,1	1,90E-20	CL0609
TRINITY_DN13706_c2_g1::TRINITY_DN13706_c2_g1_i3::g.27413::m.27413	1	5,1	44,217	2,0339	PF03095.14	PTPA	406,7	5,90E-122	No_clan

TRINITY_DN13712_c0_g1::TRINITY_DN13712_c0_g1_i1::g.27487::m.27487	7	30,1	32,821	21,383	PF14938.5	SNAP	376,6	7,30E-113	CL0020
TRINITY_DN13720_c0_g6::TRINITY_DN13720_c0_g6_i1::g.27649::m.27649	2	10	26,813	2,7944					
TRINITY_DN13721_c0_g1::TRINITY_DN13721_c0_g1_i7::g.27621::m.27621	6	14,3	59,439	31,762	PF02990.15	EMP70	529	1,10E-158	No_clan
TRINITY_DN13724_c5_g4::TRINITY_DN13724_c5_g4_i1::g.27589::m.27589	1	2,6	48,538	1,843	PF00917.25	MATH	67,7	8,30E-19	CL0389
TRINITY_DN13726_c0_g3::TRINITY_DN13726_c0_g3_i1::g.27596::m.27596	12	29,5	49,29	28,215	PF00009.26	GTP_EFTU	180,2	2,90E-53	CL0023
TRINITY_DN13727_c0_g1::TRINITY_DN13727_c0_g1_i1::g.27676::m.27676	6	23	38,173	16,224	PF00155.20	Aminotran_1_2	217	4,00E-64	CL0061
TRINITY_DN13727_c0_g2::TRINITY_DN13727_c0_g2_i11::g.27681::m.27681	2	22,6	13,744	4,5473					
TRINITY_DN13729_c0_g1::TRINITY_DN13729_c0_g1_i8::g.27674::m.27674	10	38,8	35,365	42,406	PF07859.12	Abhydrolase_3	159,8	7,40E-47	CL0028
TRINITY_DN13734_c2_g1::TRINITY_DN13734_c2_g1_i1::g.27715::m.27715	1	5,1	27,844	2,0284	PF12481.7	DUF3700	347	3,80E-104	CL0052
TRINITY_DN13736_c4_g2::TRINITY_DN13736_c4_g2_i5::g.27391::m.27391	6	28,2	29,321	17,571	PF00248.20	Aldo_ket_red	130,5	6,20E-38	No_clan
TRINITY_DN13739_c0_g1::TRINITY_DN13739_c0_g1_i1::g.27726::m.27726	1	9,7	15,533	2,2148	PF12796.6	Ank_2	53	3,70E-14	CL0465
TRINITY_DN13740_c0_g1::TRINITY_DN13740_c0_g1_i2::g.27757::m.27757	4	10,3	49,148	8,7481	PF03463.14	eRF1_1	71	8,30E-20	No_clan
TRINITY_DN13743_c6_g3::TRINITY_DN13743_c6_g3_i1::g.27816::m.27816	2	7,4	44,697	2,6451	PF02493.19	MORN	19,2	0,00073	CL0251
TRINITY_DN13752_c3_g3::TRINITY_DN13752_c3_g3_i1::g.27967::m.27967	2	3,9	64,838	3,2191	PF00854.20	PTR2	271,8	8,10E-81	CL0015
TRINITY_DN13753_c0_g1::TRINITY_DN13753_c0_g1_i3::g.27858::m.27858	2	6,2	37,001	57,772	PF01636.22	APH	44,8	1,30E-11	CL0016
TRINITY_DN13757_c0_g1::TRINITY_DN13757_c0_g1_i4::g.28204::m.28204	3	15,7	27,504	62,333	PF00333.19	Ribosomal_S5	108,2	1,30E-31	CL0196
TRINITY_DN13767_c0_g1::TRINITY_DN13767_c0_g1_i2::g.28089::m.28089	2	15,5	16,53	6,2844	PF00004.28	AAA	22,2	0,00013	CL0023
TRINITY_DN13776_c0_g1::TRINITY_DN13776_c0_g1_i2::g.28095::m.28095	10	77,6	16,236	323,01	PF00294.23	PfkB	121,4	4,20E-35	CL0118
TRINITY_DN13776_c0_g2::TRINITY_DN13776_c0_g2_i2::g.28097::m.28097	8	47,1	24,425	99,195	PF00294.23	PfkB	170,9	3,60E-50	CL0118
TRINITY_DN13778_c1_g3::TRINITY_DN13778_c1_g3_i1::g.28124::m.28124	2	4,7	51,143	3,4687	PF06068.12	TIP49	604,7	6,70E-182	CL0023

TRINITY_DN13782_c0_g3::TRINITY_DN13782_c0_g3_i2::g.28223::m.28223	1	9,3	18,763	2,2781	PF10252.8	PP28	97,2	5,40E-28	No_clan
TRINITY_DN15075_c1_g2::TRINITY_DN15075_c1_g2_i1::g.46516::m.46516	2	13,2	11,977	2,7263	PF00125.23	Histone	123,4	6,80E-36	CL0012
TRINITY_DN13787_c3_g4::TRINITY_DN13787_c3_g4_i2::g.28260::m.28260	5	24,6	29,387	21,403	PF01459.21	Porin_3	248,5	7,30E-74	CL0193
TRINITY_DN13787_c3_g4::TRINITY_DN13787_c3_g4_i3::g.28262::m.28262	6	28,9	32,536	29,071	PF01459.21	Porin_3	251,3	1,10E-74	CL0193
TRINITY_DN13790_c1_g1::TRINITY_DN13790_c1_g1_i1::g.28245::m.28245	5	16	41,621	8,3948	PF01370.20	Epimerase	155,8	1,20E-45	CL0063
TRINITY_DN13791_c2_g2::TRINITY_DN13791_c2_g2_i3::g.28238::m.28238	2	5,9	34,608	2,5061	PF13041.5	PPR_2	50,4	1,70E-13	CL0020
TRINITY_DN13792_c0_g1::TRINITY_DN13792_c0_g1_i4::g.28291::m.28291	1	13	12,889	2,1072					
TRINITY_DN13798_c2_g3::TRINITY_DN13798_c2_g3_i1::g.28387::m.28387	1	5,6	19,763	6,1786	PF00462.23	Glutaredoxin	73,8	8,70E-21	CL0172
TRINITY_DN13809_c0_g1::TRINITY_DN13809_c0_g1_i2::g.28550::m.28550	4	14,1	44,32	9,5852	PF00579.24	tRNA-synt_1b	219,7	4,80E-65	CL0039
TRINITY_DN13812_c2_g1::TRINITY_DN13812_c2_g1_i1::g.28623::m.28623	1	17,1	15,52	110,24	PF09598.9	Stm1_N	64	1,60E-17	No_clan
TRINITY_DN13815_c1_g1::TRINITY_DN13815_c1_g1_i21::g.28891::m.28891	11	34,4	39,111	5,0874	PF16363.4	GDP_Man_Dehyd	181,4	2,60E-53	CL0063
TRINITY_DN13815_c1_g2::TRINITY_DN13815_c1_g2_i8::g.28885::m.28885	2	10,9	12,464	11,305					
TRINITY_DN13815_c1_g2::TRINITY_DN13815_c1_g2_i6::g.28882::m.28882	11	42,5	38,728	78,151	PF16363.4	GDP_Man_Dehyd	181,6	2,30E-53	CL0063
TRINITY_DN13818_c0_g2::TRINITY_DN13818_c0_g2_i1::g.28967::m.28967	3	14,4	46,763	24,196	PF08442.9	ATP-grasp_2	60,8	1,20E-16	CL0179
TRINITY_DN13818_c0_g2::TRINITY_DN13818_c0_g2_i15::g.28994::m.28994	3	12,7	40,166	2,9172	PF08442.9	ATP-grasp_2	61,4	8,10E-17	CL0179
TRINITY_DN1382_c0_g1::TRINITY_DN1382_c0_g1_i1::g.348::m.348	2	12,9	23,495	2,5653	PF00022.18	Actin	253,5	2,50E-75	CL0108
TRINITY_DN13820_c2_g1::TRINITY_DN13820_c2_g1_i2::g.28694::m.28694	9	25,7	46,35	53,941	PF00226.30	DnaJ	81,4	3,50E-23	CL0392
TRINITY_DN13823_c0_g2::TRINITY_DN13823_c0_g2_i2::g.29073::m.29073	12	48,5	40,703	49,069	PF02136.19	NTF2	101,2	4,80E-29	CL0051
TRINITY_DN13825_c0_g1::TRINITY_DN13825_c0_g1_i1::g.28844::m.28844	1	5	17,771	3,3537	PF00248.20	Aldo_ket_red	49,8	2,30E-13	No_clan
TRINITY_DN13825_c0_g1::TRINITY_DN13825_c0_g1_i2::g.28846::m.28846	4	32,1	17,604	8,0659	PF00248.20	Aldo_ket_red	62	4,60E-17	No_clan
TRINITY_DN13825_c0_g1::TRINITY_DN13825_c0_g1_i3::g.28848::m.28848	2	10,8	17,661	2,3515	PF00248.20	Aldo_ket_red	52,5	3,60E-14	No_clan
TRINITY_DN13825_c0_g2::TRINITY_DN13825_c0_g2_i1::g.28850::m.28850	8	56,4	18,015	33,636	PF00248.20	Aldo_ket_red	102,3	2,40E-29	No_clan

TRINITY_DN13825_c0_g2::TRINITY_DN13825_c0_g2_i4::g.28851::m.28851	6	37,7	17,996	4,6231	PF00248.20	Aldo_ket_red	102,3	2,40E-29	No_clan
TRINITY_DN13825_c1_g1::TRINITY_DN13825_c1_g1_i2::g.28853::m.28853	16	63,6	31,09	181,21	PF00248.20	Aldo_ket_red	158,7	1,60E-46	No_clan
TRINITY_DN13826_c0_g2::TRINITY_DN13826_c0_g2_i4::g.28934::m.28934	2	21,8	18,801	10,021	PF12220.7	U1snRNP70_N	73,8	1,20E-20	CL0221
TRINITY_DN13827_c1_g1::TRINITY_DN13827_c1_g1_i4::g.28842::m.28842	3	13,7	31,96	7,5577					
TRINITY_DN13833_c0_g1::TRINITY_DN13833_c0_g1_i8::g.29930::m.29930	3	12,8	35,741	5,5195	PF00149.27	Metallophos	122,8	2,40E-35	CL0163
TRINITY_DN13833_c0_g2::TRINITY_DN13833_c0_g2_i5::g.29925::m.29925	2	29,6	13,18	3,5544					
TRINITY_DN13841_c4_g1::TRINITY_DN13841_c4_g1_i2::g.28420::m.28420	4	15,1	40,205	12,672	PF13432.5	TPR_16	16,6	0,0089	CL0020
TRINITY_DN13841_c4_g1::TRINITY_DN13841_c4_g1_i7::g.28424::m.28424	4	13,3	44,173	29,527	PF01965.23	DJ-1_PfpI	163,8	2,70E-48	CL0014
TRINITY_DN13846_c2_g2::TRINITY_DN13846_c2_g2_i2::g.28548::m.28548	3	25	22,919	11,095	PF00227.25	Proteasome	145,3	1,40E-42	CL0052
TRINITY_DN13849_c3_g3::TRINITY_DN13849_c3_g3_i8::g.28501::m.28501	1	5,6	33,465	3,5482	PF16543.4	DFRP_C	43,4	3,40E-11	No_clan
TRINITY_DN13851_c1_g1::TRINITY_DN13851_c1_g1_i8::g.29304::m.29304	3	10,1	55,972	12,137	PF00026.22	Asp	465,4	9,20E-140	CL0129
TRINITY_DN13851_c1_g2::TRINITY_DN13851_c1_g2_i2::g.29292::m.29292	2	6	31,408	2,4007	PF00026.22	Asp	287,8	1,00E-85	CL0129
TRINITY_DN13852_c0_g1::TRINITY_DN13852_c0_g1_i7::g.28442::m.28442	3	11,6	38,722	6,1001	PF01507.18	PAPS_reduct	46,6	3,60E-12	CL0039
TRINITY_DN13858_c5_g2::TRINITY_DN13858_c5_g2_i2::g.29432::m.29432	1	4,6	26,899	2,1585	PF04051.15	TRAPP	145,7	7,40E-43	CL0210
TRINITY_DN13858_c6_g1::TRINITY_DN13858_c6_g1_i3::g.29437::m.29437	2	11,8	20,035	3,7074	PF00702.25	Hydrolase	59,3	5,80E-16	CL0137
TRINITY_DN13860_c0_g1::TRINITY_DN13860_c0_g1_i1::g.29366::m.29366	10	22,9	60,453	29,09	PF00085.19	Thioredoxin	54,4	8,70E-15	CL0172
TRINITY_DN13861_c0_g1::TRINITY_DN13861_c0_g1_i7::g.29399::m.29399	2	9,4	25,791	4,0262	PF05648.13	PEX11	163,8	3,80E-48	No_clan
TRINITY_DN13865_c0_g1::TRINITY_DN13865_c0_g1_i3::g.29082::m.29082	13	31,8	57,946	66,825	PF00483.22	NTP_transferase	273,6	1,40E-81	CL0110
TRINITY_DN13869_c6_g1::TRINITY_DN13869_c6_g1_i2::g.29653::m.29653	6	23,7	34,938	13,631	PF01014.17	Uricase	64,6	1,10E-17	CL0334
TRINITY_DN13871_c3_g1::TRINITY_DN13871_c3_g1_i4::g.29817::m.29817	2	2,8	123,93	2,3535	PF08626.10	TRAPPC9-Trs120	66,5	7,70E-19	No_clan
TRINITY_DN13872_c1_g1::TRINITY_DN13872_c1_g1_i2::g.29678::m.29678	5	37,8	16,221	15,806	PF01230.22	HIT	95,9	1,70E-27	CL0265

TRINITY_DN13872_c3_g1::TRINITY_DN13872_c3_g1_i6::g.29683::m.29683	1	14,4	15,413	3,4548	PF02466.18	Tim17	47,8	1,40E-12	No_clan
TRINITY_DN13876_c0_g1::TRINITY_DN13876_c0_g1_i2::g.29614::m.29614	6	24,3	30,177	44,159	PF13417.5	GST_N_3	68,1	6,00E-19	CL0172
TRINITY_DN13878_c2_g2::TRINITY_DN13878_c2_g2_i3::g.29757::m.29757	2	16,7	18,224	32,718	PF01230.22	HIT	45,2	1,10E-11	CL0265
TRINITY_DN13884_c2_g1::TRINITY_DN13884_c2_g1_i6::g.29871::m.29871	3	15,2	41,37	8,857	PF07145.14	PAM2	23,3	2,90E-05	No_clan
TRINITY_DN13888_c0_g2::TRINITY_DN13888_c0_g2_i1::g.29877::m.29877	1	18,4	12,293	3,4498					
TRINITY_DN13889_c0_g1::TRINITY_DN13889_c0_g1_i2::g.29853::m.29853	2	3,9	86,874	3,8842	PF13812.5	PPR_3	36,1	4,60E-09	CL0020
TRINITY_DN13893_c0_g2::TRINITY_DN13893_c0_g2_i1::g.30000::m.30000	2	10	29,626	6,2635	PF09325.9	Vps5	89	3,20E-25	CL0145
TRINITY_DN13894_c1_g1::TRINITY_DN13894_c1_g1_i8::g.29999::m.29999	1	8	20,664	2,5064	PF00171.21	Aldedh	55,9	2,40E-15	CL0099
TRINITY_DN13896_c0_g4::TRINITY_DN13896_c0_g4_i4::g.30042::m.30042	2	9,3	28,989	2,1079	PF07651.15	ANTH	223,1	3,20E-66	CL0009
TRINITY_DN13898_c1_g1::TRINITY_DN13898_c1_g1_i2::g.30051::m.30051	5	20,1	30,555	7,1287	PF04548.15	AIG1	124,2	4,20E-36	CL0023
TRINITY_DN13901_c0_g1::TRINITY_DN13901_c0_g1_i11::g.28513::m.28513	6	30,4	20,408	151,29	PF00085.19	Thioredoxin	62,6	2,60E-17	CL0172
TRINITY_DN13903_c0_g3::TRINITY_DN13903_c0_g3_i6::g.30258::m.30258	10	40,7	37,126	24,119	PF00141.22	peroxidase	222,8	4,30E-66	CL0617
TRINITY_DN13912_c2_g1::TRINITY_DN13912_c2_g1_i3::g.30407::m.30407	1	2,5	52,421	2,314	PF00332.17	Glyco_hydro_17	242,6	5,80E-72	CL0058
TRINITY_DN13915_c0_g2::TRINITY_DN13915_c0_g2_i2::g.30373::m.30373	1	19,6	11,197	3,0483	PF00408.19	PGM_PMM_IV	52,1	5,30E-14	No_clan
TRINITY_DN13916_c2_g1::TRINITY_DN13916_c2_g1_i2::g.30380::m.30380	33	40,3	108,41	323,31	PF00565.16	SNase	39,4	6,40E-10	CL0049
TRINITY_DN13916_c3_g1::TRINITY_DN13916_c3_g1_i2::g.30393::m.30393	15	35,5	53,367	120,22	PF00262.17	Calreticulin	490,9	1,80E-147	CL0004
TRINITY_DN13916_c3_g3::TRINITY_DN13916_c3_g3_i3::g.30403::m.30403	7	15,5	61,58	30,495	PF00262.17	Calreticulin	480,9	1,90E-144	CL0004
TRINITY_DN13918_c1_g1::TRINITY_DN13918_c1_g1_i8::g.30345::m.30345	8	12,8	109,25	24,784	PF01851.21	PC_rep	27,4	2,70E-06	CL0020
TRINITY_DN13920_c2_g1::TRINITY_DN13920_c2_g1_i2::g.30365::m.30365	3	30,3	21,439	12,088	PF02734.16	Dak2	150,1	5,10E-44	No_clan
TRINITY_DN13921_c0_g1::TRINITY_DN13921_c0_g1_i2::g.30314::m.30314	2	11,6	30,885	5,2153	PF04144.12	SCAMP	157,1	5,10E-46	No_clan
TRINITY_DN13928_c0_g1::TRINITY_DN13928_c0_g1_i1::g.30448::m.30448	5	6	113,57	8,9071					

TRINITY_DN13930_c0_g1::TRINITY_DN13930_c0_g1_i1::g.30435::m.30435	3	15,1	33,021	5,1549	PF02729.20	OTCace_N	152,3	8,50E-45	No_clan
TRINITY_DN13940_c4_g1::TRINITY_DN13940_c4_g1_i3::g.30598::m.30598	1	10,4	12,31	14,403					
TRINITY_DN13943_c0_g2::TRINITY_DN13943_c0_g2_i1::g.30525::m.30525	3	33	12,518	6,6628	PF03650.12	MPC	139,6	3,70E-41	CL0141
TRINITY_DN13945_c0_g1::TRINITY_DN13945_c0_g1_i2::g.30107::m.30107	7	28	33,104	10,187	PF13640.5	2OG-FeII_Oxy_3	76,8	1,70E-21	CL0029
TRINITY_DN13947_c3_g5::TRINITY_DN13947_c3_g5_i8::g.30694::m.30694	10	47,3	28,991	196,74	PF00753.26	Lactamase_B	44,1	2,10E-11	CL0381
TRINITY_DN13959_c0_g2::TRINITY_DN13959_c0_g2_i7::g.30593::m.30593	2	7,5	52,446	2,0199	PF03810.18	IBN_N	63,5	1,20E-17	CL0020
TRINITY_DN13961_c0_g1::TRINITY_DN13961_c0_g1_i2::g.30621::m.30621	3	11,6	42,623	4,8794	PF01634.17	HisG	127,5	4,00E-37	CL0177
TRINITY_DN13975_c1_g4::TRINITY_DN13975_c1_g4_i3::g.30944::m.30944	2	8,5	12,63	33,011	PF01263.19	Aldose_epim	87,1	1,20E-24	CL0103
TRINITY_DN13980_c0_g1::TRINITY_DN13980_c0_g1_i1::g.30892::m.30892	3	13,9	38,71	8,2443	PF13489.5	Methyltransf_23	74,4	8,50E-21	CL0063
TRINITY_DN13981_c1_g3::TRINITY_DN13981_c1_g3_i1::g.30911::m.30911	2	10,4	37,493	6,857	PF10250.8	O-FucT	73,8	1,90E-20	CL0113
TRINITY_DN13992_c5_g1::TRINITY_DN13992_c5_g1_i1::g.31138::m.31138	2	9,6	12,399	8,2008	PF00407.18	Bet_v_1	41	1,60E-10	CL0209
TRINITY_DN14005_c0_g1::TRINITY_DN14005_c0_g1_i3::g.31329::m.31329	1	15,5	13,741	12,652					
TRINITY_DN14005_c1_g1::TRINITY_DN14005_c1_g1_i1::g.31333::m.31333	2	4,8	50,544	3,1601	PF00266.18	Aminotran_5	53,9	1,30E-14	CL0061
TRINITY_DN14010_c5_g1::TRINITY_DN14010_c5_g1_i2::g.31420::m.31420	3	21,1	12,353	6,6803	PF01158.17	Ribosomal_L36e	144,1	1,10E-42	No_clan
TRINITY_DN14011_c0_g1::TRINITY_DN14011_c0_g1_i1::g.31283::m.31283	6	24,9	30,023	8,0919	PF01025.18	GrpE	155,6	8,40E-46	No_clan
TRINITY_DN14014_c3_g1::TRINITY_DN14014_c3_g1_i1::g.31362::m.31362	2	23,4	14,383	7,4304	PF03080.14	Neprosin	71,3	7,10E-20	No_clan
TRINITY_DN14016_c0_g1::TRINITY_DN14016_c0_g1_i9::g.31308::m.31308	3	21,1	17,09	4,1537	PF12799.6	LRR_4	34	2,40E-08	CL0022
TRINITY_DN14021_c1_g2::TRINITY_DN14021_c1_g2_i5::g.31431::m.31431	10	29,7	42,589	218,02	PF00079.19	Serpin	344,9	6,10E-103	No_clan
TRINITY_DN14025_c2_g1::TRINITY_DN14025_c2_g1_i8::g.31522::m.31522	9	20	66,546	122,61	PF13898.5	DUF4205	203,1	5,30E-60	No_clan
TRINITY_DN14028_c2_g1::TRINITY_DN14028_c2_g1_i4::g.31476::m.31476	2	3,6	102,87	6,9495	PF08414.9	NADPH_Ox	123,4	3,50E-36	No_clan
TRINITY_DN14030_c0_g1::TRINITY_DN14030_c0_g1_i1::g.31442::m.31442	19	71,5	35,379	323,31	PF00294.23	PfkB	262,4	4,90E-78	CL0118

TRINITY_DN14036_c4_g1::TRINITY_DN14036_c4_g1_i2::g.31377::m.31377	1	11,5	19,695	4,3307	PF01722.17	BolA	50,8	1,40E-13	No_clan
TRINITY_DN14038_c1_g1::TRINITY_DN14038_c1_g1_i3::g.31643::m.31643	9	28,5	37,737	5,206	PF00270.28	DEAD	136,4	7,60E-40	CL0023
TRINITY_DN14038_c1_g2::TRINITY_DN14038_c1_g2_i5::g.31646::m.31646	13	38,2	41,892	255,49	PF00270.28	DEAD	138,7	1,50E-40	CL0023
TRINITY_DN14041_c2_g1::TRINITY_DN14041_c2_g1_i4::g.31794::m.31794	19	40,9	68,474	323,31	PF02878.15	PGM_PMM_I	125,7	9,60E-37	No_clan
TRINITY_DN14041_c3_g1::TRINITY_DN14041_c3_g1_i2::g.31810::m.31810	2	6,3	40,869	3,8184	PF00170.20	bZIP_1	35,2	8,80E-09	CL0018
TRINITY_DN14042_c0_g1::TRINITY_DN14042_c0_g1_i1::g.31618::m.31618	3	4,8	70,928	2,605	PF12037.7	DUF3523	364,8	2,30E-109	No_clan
TRINITY_DN14047_c1_g1::TRINITY_DN14047_c1_g1_i4::g.31668::m.31668	2	11,2	26,828	4,4458	PF00248.20	Aldo_ket_red	198,7	1,10E-58	No_clan
TRINITY_DN14048_c1_g1::TRINITY_DN14048_c1_g1_i1::g.31820::m.31820	1	8,2	22,849	2,3472	PF09430.9	DUF2012	80,5	1,00E-22	CL0287
TRINITY_DN14049_c0_g1::TRINITY_DN14049_c0_g1_i2::g.31233::m.31233	3	14,3	29,43	3,5394					
TRINITY_DN14063_c0_g1::TRINITY_DN14063_c0_g1_i1::g.31854::m.31854	1	9,4	28,244	2,2285	PF03029.16	ATP_bind_1	235,6	6,20E-70	CL0023
TRINITY_DN14069_c0_g3::TRINITY_DN14069_c0_g3_i3::g.32072::m.32072	1	2,5	49,782	2,3947					
TRINITY_DN14069_c0_g5::TRINITY_DN14069_c0_g5_i5::g.32070::m.32070	1	10	14,447	2,6406	PF00326.20	Peptidase_S9	24,1	2,00E-05	CL0028
TRINITY_DN14078_c2_g2::TRINITY_DN14078_c2_g2_i1::g.32075::m.32075	1	11,8	13,056	1,8944	PF00240.22	ubiquitin	33,1	3,10E-08	CL0072
TRINITY_DN14079_c0_g2::TRINITY_DN14079_c0_g2_i4::g.32111::m.32111	3	37,7	12,714	2,7404	PF02127.14	Peptidase_M18	131,2	4,10E-38	CL0035
TRINITY_DN14080_c2_g1::TRINITY_DN14080_c2_g1_i10::g.32162::m.32162	12	59,4	24,436	17,529	PF00012.19	HSP70	308,1	8,90E-92	CL0108
TRINITY_DN14080_c2_g2::TRINITY_DN14080_c2_g2_i1::g.32148::m.32148	7	41,3	19,977	3,9585	PF00012.19	HSP70	306,7	2,30E-91	CL0108
TRINITY_DN14082_c1_g1::TRINITY_DN14082_c1_g1_i3::g.32100::m.32100	4	19,2	34,919	10,407					
TRINITY_DN14089_c0_g1::TRINITY_DN14089_c0_g1_i1::g.32223::m.32223	6	20,4	41,408	2,4215	PF13561.5	adh_short_C2	173,5	4,80E-51	CL0063
TRINITY_DN14089_c0_g1::TRINITY_DN14089_c0_g1_i6::g.32232::m.32232	7	25,1	41,695	20,235	PF13561.5	adh_short_C2	168,7	1,40E-49	CL0063
TRINITY_DN14090_c0_g1::TRINITY_DN14090_c0_g1_i4::g.32240::m.32240	9	29,2	44,525	63,686	PF01926.22	MMR_HSR1	77,3	8,50E-22	CL0023
TRINITY_DN14091_c1_g2::TRINITY_DN14091_c1_g2_i1::g.32197::m.32197	16	28	78,485	30,998	PF00378.19	ECH_1	145,2	2,00E-42	CL0127

TRINITY_DN14096_c0_g2::TRINITY_DN14096_c0_g2_i2::g.32310::m.32310	2	6,5	48,502	3,2801	PF16113.4	ECH_2	387,9	4,50E-116	CL0127
TRINITY_DN14096_c0_g4::TRINITY_DN14096_c0_g4_i2::g.32317::m.32317	3	31,9	12,357	2,2709	PF01849.17	NAC	27,3	2,50E-06	No_clan
TRINITY_DN14097_c2_g2::TRINITY_DN14097_c2_g2_i1::g.31570::m.31570	7	27,8	44,38	38,786	PF03081.14	Exo70	150,8	5,00E-44	CL0295
TRINITY_DN14106_c1_g2::TRINITY_DN14106_c1_g2_i1::g.32414::m.32414	8	20,1	48,781	14,035	PF01761.19	DHQ_synthase	352	1,50E-105	CL0224
TRINITY_DN14107_c0_g1::TRINITY_DN14107_c0_g1_i1::g.32583::m.32583	3	8,2	47,874	6,3328	PF00013.28	KH_1	47,2	1,30E-12	CL0007
TRINITY_DN14108_c0_g1::TRINITY_DN14108_c0_g1_i6::g.32445::m.32445	9	27,1	58,483	88,484	PF00240.22	ubiquitin	72,9	1,20E-20	CL0072
TRINITY_DN14114_c1_g1::TRINITY_DN14114_c1_g1_i9::g.32755::m.32755	8	22	55,61	24,64	PF01399.26	PCI	80,6	9,70E-23	CL0123
TRINITY_DN14115_c3_g1::TRINITY_DN14115_c3_g1_i10::g.32650::m.32650	7	22,3	45,551	54,75	PF01532.19	Glyco_hydro_47	328,1	8,40E-98	CL0059
TRINITY_DN14119_c2_g1::TRINITY_DN14119_c2_g1_i7::g.32525::m.32525	1	9	16,382	3,0949	PF00579.24	tRNA-synt_1b	61,5	7,20E-17	CL0039
TRINITY_DN14120_c0_g1::TRINITY_DN14120_c0_g1_i4::g.32545::m.32545	10	44,9	23,24	323,31	PF00121.17	TIM	247,6	1,00E-73	CL0036
TRINITY_DN14120_c0_g3::TRINITY_DN14120_c0_g3_i2::g.32536::m.32536	13	51,7	31,151	323,31	PF00121.17	TIM	293,3	1,20E-87	CL0036
TRINITY_DN14137_c0_g1::TRINITY_DN14137_c0_g1_i2::g.33039::m.33039	4	13	53,496	5,9951	PF00291.24	PALP	273,9	1,60E-81	No_clan
TRINITY_DN14139_c1_g3::TRINITY_DN14139_c1_g3_i2::g.32774::m.32774	5	13	50,787	9,0998	PF00152.19	tRNA-synt_2	44,7	8,30E-12	CL0040
TRINITY_DN14141_c0_g1::TRINITY_DN14141_c0_g1_i5::g.32827::m.32827	4	35,7	13,446	9,0072	PF05873.11	Mt_ATP-synt_D	52,8	3,70E-14	No_clan
TRINITY_DN14143_c3_g2::TRINITY_DN14143_c3_g2_i2::g.32997::m.32997	19	31,6	67,933	38,184	PF00342.18	PGI	167	5,30E-49	CL0067
TRINITY_DN14145_c0_g4::TRINITY_DN14145_c0_g4_i3::g.33080::m.33080	2	13,3	21,674	7,6665	PF09320.10	DUF1977	38,5	1,00E-09	No_clan
TRINITY_DN14157_c1_g1::TRINITY_DN14157_c1_g1_i4::g.33107::m.33107	6	22,1	39,006	11,787	PF00076.21	RRM_1	54	9,90E-15	CL0221
TRINITY_DN14157_c2_g1::TRINITY_DN14157_c2_g1_i3::g.33123::m.33123	5	56,9	11,492	142,42	PF00428.18	Ribosomal_60s	84,3	6,40E-24	No_clan
TRINITY_DN14162_c4_g1::TRINITY_DN14162_c4_g1_i2::g.33191::m.33191	10	53,8	22,013	323,31	PF13419.5	HAD_2	74	1,40E-20	CL0137
TRINITY_DN14175_c1_g1::TRINITY_DN14175_c1_g1_i3::g.33680::m.33680	3	7,8	51,349	6,5393	PF09405.9	Btz	66,2	3,10E-18	No_clan
TRINITY_DN19343_c7_g5::TRINITY_DN19343_c7_g5_i1::g.116417::m.116417	2	19,9	15,602	3,2747	PF01373.16	Glyco_hydro_14	65,3	4,50E-18	CL0058

TRINITY_DN14180_c0_g1::TRINITY_DN14180_c0_g1_i2::g.33272::m.33272	1	3,4	45,511	3,3487	PF00128.23	Alpha-amylase	48,1	1,10E-12	CL0058
TRINITY_DN14181_c0_g3::TRINITY_DN14181_c0_g3_i1::g.33572::m.33572	5	9,4	57,944	8,0334	PF00171.21	Aldedh	475,9	9,70E-143	CL0099
TRINITY_DN14185_c5_g3::TRINITY_DN14185_c5_g3_i3::g.33391::m.33391	15	62	35,679	99,433	PF00056.22	Ldh_1_N	115,3	2,10E-33	CL0063
TRINITY_DN14186_c0_g1::TRINITY_DN14186_c0_g1_i2::g.33325::m.33325	2	14,6	24,077	4,4239	PF13793.5	Pribosyltran_N	150,3	1,80E-44	CL0533
TRINITY_DN14188_c2_g2::TRINITY_DN14188_c2_g2_i3::g.32373::m.32373	9	42,3	36,022	88,067	PF00400.31	WD40	14,9	0,034	CL0186
TRINITY_DN14193_c0_g1::TRINITY_DN14193_c0_g1_i4::g.33482::m.33482	1	11,6	13,901	2,1844	PF04836.11	IFRD_C	63,4	2,00E-17	No_clan
TRINITY_DN14194_c0_g2::TRINITY_DN14194_c0_g2_i9::g.33546::m.33546	5	15,5	50,699	8,896	PF00202.20	Aminotran_3	370	1,10E-110	CL0061
TRINITY_DN14194_c1_g1::TRINITY_DN14194_c1_g1_i9::g.33557::m.33557	1	12,4	22,292	6,0515					
TRINITY_DN14197_c0_g1::TRINITY_DN14197_c0_g1_i2::g.33493::m.33493	7	23,7	47,711	16,501	PF00004.28	AAA	134,8	2,20E-39	CL0023
TRINITY_DN14198_c0_g1::TRINITY_DN14198_c0_g1_i9::g.33644::m.33644	3	27,2	18,515	6,9668	PF00534.19	Glycos_transf_1	34,9	9,60E-09	CL0113
TRINITY_DN14198_c0_g1::TRINITY_DN14198_c0_g1_i5::g.33635::m.33635	6	26,9	39,065	15,899	PF08323.10	Glyco_transf_5	67,9	9,90E-19	CL0113
TRINITY_DN14198_c0_g1::TRINITY_DN14198_c0_g1_i4::g.33633::m.33633	2	13,8	23,894	2,711	PF08323.10	Glyco_transf_5	147,1	6,30E-43	CL0113
TRINITY_DN14200_c0_g1::TRINITY_DN14200_c0_g1_i2::g.33523::m.33523	6	11,9	68,386	10,586	PF00933.20	Glyco_hydro_3	242	8,90E-72	CL0058
TRINITY_DN14202_c2_g1::TRINITY_DN14202_c2_g1_i3::g.33869::m.33869	2	15,7	17,303	3,7293	PF05008.14	V-SNARE	88,4	2,70E-25	CL0147
TRINITY_DN14203_c2_g1::TRINITY_DN14203_c2_g1_i1::g.33817::m.33817	15	19,1	115,48	40,944	PF02861.19	Clp_N	56,5	2,00E-15	No_clan
TRINITY_DN14203_c2_g2::TRINITY_DN14203_c2_g2_i3::g.33825::m.33825	6	8,8	103,39	9,63	PF02861.19	Clp_N	47,8	1,10E-12	No_clan
TRINITY_DN14204_c0_g1::TRINITY_DN14204_c0_g1_i1::g.33924::m.33924	3	22,1	12,356	226,47	PF01095.18	Pectinesterase	73,3	1,20E-20	CL0268
TRINITY_DN14204_c0_g1::TRINITY_DN14204_c0_g1_i2::g.33925::m.33925	4	44,4	10,771	61,432	PF01095.18	Pectinesterase	55,9	2,50E-15	CL0268
TRINITY_DN14204_c1_g1::TRINITY_DN14204_c1_g1_i2::g.33927::m.33927	2	11,9	29,85	5,0875					
TRINITY_DN14209_c0_g1::TRINITY_DN14209_c0_g1_i2::g.34018::m.34018	2	6,7	38,161	5,1938					
TRINITY_DN14210_c0_g1::TRINITY_DN14210_c0_g1_i5::g.33918::m.33918	2	11,7	21,311	22,025	PF01912.17	eIF-6	229,2	2,60E-68	CL0197

TRINITY_DN14213_c1_g1::TRINITY_DN14213_c1_g1_i6::g.33993::m.33993	14	35,8	49,462	39,184	PF14306.5	PUA_2	148,1	1,50E-43	CL0178
TRINITY_DN14216_c0_g3::TRINITY_DN14216_c0_g3_i3::g.34216::m.34216	4	30,7	16,752	2,3726	PF00248.20	Aldo_ket_red	142,6	1,30E-41	No_clan
TRINITY_DN14219_c0_g1::TRINITY_DN14219_c0_g1_i18::g.33900::m.33900	4	14,7	26,595	6,5561	PF00535.25	Glycos_transf_2	127,1	5,60E-37	CL0110
TRINITY_DN14219_c0_g1::TRINITY_DN14219_c0_g1_i15::g.33893::m.33893	5	22,1	38,888	10,243	PF09229.10	Aha1_N	103,4	1,00E-29	CL0648
TRINITY_DN14233_c0_g1::TRINITY_DN14233_c0_g1_i2::g.34181::m.34181	7	18,6	55,906	17,1	PF00275.19	EPSP_synthase	480,7	3,00E-144	CL0290
TRINITY_DN14238_c0_g1::TRINITY_DN14238_c0_g1_i3::g.34351::m.34351	7	42,8	20,355	77,537	PF00160.20	Pro_isomerase	166,9	3,80E-49	CL0475
TRINITY_DN14239_c2_g2::TRINITY_DN14239_c2_g2_i2::g.34369::m.34369	2	6,1	45,161	8,0036	PF07393.10	Sec10	306,3	4,50E-91	CL0294
TRINITY_DN14242_c1_g2::TRINITY_DN14242_c1_g2_i9::g.34423::m.34423	4	20,9	32,289	182,49	PF00753.26	Lactamase_B	24,7	1,80E-05	CL0381
TRINITY_DN14245_c1_g1::TRINITY_DN14245_c1_g1_i8::g.34453::m.34453	2	22,3	15,665	5,553	PF02815.18	MIR	38,5	9,70E-10	CL0066
TRINITY_DN14246_c2_g1::TRINITY_DN14246_c2_g1_i1::g.34260::m.34260	4	11,9	38,505	8,7786	PF01087.21	GalP_UDP_transf	71	1,60E-19	CL0265
TRINITY_DN14251_c1_g2::TRINITY_DN14251_c1_g2_i7::g.34536::m.34536	5	26,2	29,6	8,3057	PF13023.5	HD_3	160	4,40E-47	CL0237
TRINITY_DN14258_c0_g1::TRINITY_DN14258_c0_g1_i5::g.34520::m.34520	5	10,8	86,279	11,146	PF07766.12	LETM1	338,2	2,80E-101	No_clan
TRINITY_DN14258_c0_g5::TRINITY_DN14258_c0_g5_i3::g.34521::m.34521	4	8,3	85,136	5,8443	PF07766.12	LETM1	358,8	1,50E-107	No_clan
TRINITY_DN14259_c3_g1::TRINITY_DN14259_c3_g1_i2::g.34652::m.34652	2	9,6	41,782	3,856	PF04371.14	PAD_porph	427,2	3,60E-128	CL0197
TRINITY_DN14259_c5_g1::TRINITY_DN14259_c5_g1_i8::g.34656::m.34656	1	10,3	16,649	3,0141					
TRINITY_DN14260_c1_g1::TRINITY_DN14260_c1_g1_i4::g.34485::m.34485	8	39,3	31,513	21,303	PF00793.19	DAHP_synth_1	206,1	4,20E-61	CL0036
TRINITY_DN14266_c0_g1::TRINITY_DN14266_c0_g1_i8::g.34769::m.34769	24	39,3	89,603	131,48	PF02359.17	CDC48_N	80,7	6,30E-23	CL0332
TRINITY_DN14266_c0_g5::TRINITY_DN14266_c0_g5_i1::g.34758::m.34758	9	48,7	25,782	6,0722	PF02933.16	CDC48_2	49,3	2,90E-13	CL0402
TRINITY_DN14268_c0_g1::TRINITY_DN14268_c0_g1_i4::g.34610::m.34610	6	23,8	44,552	21,618	PF02771.15	Acyl-CoA_dh_N	116,4	9,40E-34	CL0544
TRINITY_DN14274_c2_g1::TRINITY_DN14274_c2_g1_i1::g.34705::m.34705	1	8,4	24,611	2,8307	PF00282.18	Pyridoxal_deC	29,1	3,60E-07	CL0061

TRINITY_DN14279_c0_g2::TRINITY_DN14279_c0_g2_i1::g.34822::m.34822	7	18,4	68,029	34,759	PF03219.13	TLC	660,1	1,90E-198	CL0015
TRINITY_DN14281_c0_g1::TRINITY_DN14281_c0_g1_i1::g.34852::m.34852	3	9	47,999	4,891	PF01494.18	FAD_binding_3	29,6	3,70E-07	CL0063
TRINITY_DN14286_c1_g1::TRINITY_DN14286_c1_g1_i4::g.34874::m.34874	5	31,9	21,724	13,269	PF06703.10	SPC25	140,1	4,90E-41	No_clan
TRINITY_DN14291_c3_g7::TRINITY_DN14291_c3_g7_i2::g.35143::m.35143	3	15,7	26,082	23,767	PF00583.24	Acetyltransf_1	50,2	2,40E-13	CL0257
TRINITY_DN14304_c1_g1::TRINITY_DN14304_c1_g1_i6::g.35219::m.35219	1	8,5	15,707	1,9027					
TRINITY_DN14308_c0_g1::TRINITY_DN14308_c0_g1_i1::g.35383::m.35383	7	23,8	45,031	20,675	PF02136.19	NTF2	97,4	7,50E-28	CL0051
TRINITY_DN14308_c1_g1::TRINITY_DN14308_c1_g1_i5::g.35396::m.35396	3	13,5	37,704	6,2509	PF00076.21	RRM_1	46,8	1,80E-12	CL0221
TRINITY_DN14310_c2_g3::TRINITY_DN14310_c2_g3_i5::g.35345::m.35345	1	9	17,97	2,7688	PF01150.16	GDA1_CD39	92,5	2,10E-26	CL0108
TRINITY_DN14316_c0_g1::TRINITY_DN14316_c0_g1_i5::g.35352::m.35352	1	13,2	14,607	2,6785					
TRINITY_DN14318_c1_g3::TRINITY_DN14318_c1_g3_i3::g.35377::m.35377	1	7,6	12,981	2,0073	PF04043.14	PMEI	60,1	2,80E-16	No_clan
TRINITY_DN14320_c0_g6::TRINITY_DN14320_c0_g6_i4::g.35483::m.35483	2	22,7	19,643	4,0885	PF03127.13	GAT	67	1,30E-18	No_clan
TRINITY_DN14322_c1_g1::TRINITY_DN14322_c1_g1_i1::g.35379::m.35379	4	12,9	52,159	8,126	PF00202.20	Aminotran_3	292,5	3,70E-87	CL0061
TRINITY_DN14323_c0_g1::TRINITY_DN14323_c0_g1_i2::g.35566::m.35566	15	22,4	94,891	76,531	PF02518.25	HATPase_c	50	3,40E-13	CL0025
TRINITY_DN14323_c2_g3::TRINITY_DN14323_c2_g3_i2::g.35593::m.35593	4	17,1	43,357	8,7012	PF00004.28	AAA	123,4	7,30E-36	CL0023
TRINITY_DN14323_c3_g1::TRINITY_DN14323_c3_g1_i1::g.35598::m.35598	2	16	15,872	1,9425	PF06068.12	TIP49	188,4	1,80E-55	CL0023
TRINITY_DN14324_c0_g1::TRINITY_DN14324_c0_g1_i3::g.35430::m.35430	2	7,1	47,853	3,6457	PF01398.20	JAB	90,1	8,50E-26	CL0366
TRINITY_DN14325_c0_g1::TRINITY_DN14325_c0_g1_i1::g.35421::m.35421	13	63,4	32,214	323,31	PF00141.22	peroxidase	276,9	1,20E-82	CL0617
TRINITY_DN14326_c0_g1::TRINITY_DN14326_c0_g1_i2::g.35500::m.35500	2	16,9	17,841	5,8591					
TRINITY_DN14328_c3_g1::TRINITY_DN14328_c3_g1_i9::g.35530::m.35530	27	53,5	55,564	323,31	PF00085.19	Thioredoxin	104,8	1,80E-30	CL0172
TRINITY_DN14334_c2_g6::TRINITY_DN14334_c2_g6_i1::g.35159::m.35159	2	27,2	11,105	2,2427	PF05056.11	DUF674	39,9	2,10E-10	No_clan
TRINITY_DN14335_c0_g1::TRINITY_DN14335_c0_g1_i7::g.35543::m.35543	3	13,2	35,11	6,6855	PF03643.14	Vps26	413,4	2,90E-124	CL0135

TRINITY_DN14338_c3_g2::TRINITY_DN14338_c3_g2_i1::g.35555::m.35555	7	23,3	46,407	12,162	PF00232.17	Glyco_hydro_1	463,7	4,40E-139	CL0058
TRINITY_DN14341_c0_g1::TRINITY_DN14341_c0_g1_i1::g.35559::m.35559	19	46	53,383	139,43					
TRINITY_DN14353_c0_g2::TRINITY_DN14353_c0_g2_i2::g.35716::m.35716	3	23,8	20,574	7,5609	PF01086.16	Clathrin_lg_ch	24,5	2,20E-05	No_clan
TRINITY_DN14356_c4_g1::TRINITY_DN14356_c4_g1_i9::g.35853::m.35853	2	13,2	30,294	5,4151					
TRINITY_DN14356_c5_g1::TRINITY_DN14356_c5_g1_i2::g.35855::m.35855	2	4,6	42,02	2,6791	PF00035.25	dsrm	57,1	2,00E-15	CL0196
TRINITY_DN14357_c5_g2::TRINITY_DN14357_c5_g2_i13::g.35817::m.35817	2	16,3	13,958	2,398	PF01918.20	Alba	67,4	6,50E-19	CL0441
TRINITY_DN14360_c0_g2::TRINITY_DN14360_c0_g2_i2::g.35887::m.35887	11	31,9	50,292	24,639	PF00091.24	Tubulin	236,1	3,50E-70	CL0566
TRINITY_DN14361_c0_g1::TRINITY_DN14361_c0_g1_i5::g.35830::m.35830	7	13,2	91,705	13,118					
TRINITY_DN14373_c0_g1::TRINITY_DN14373_c0_g1_i5::g.36002::m.36002	1	10,1	16,169	1,8933	PF12643.6	MazG-like	29,7	5,30E-07	CL0231
TRINITY_DN14374_c0_g1::TRINITY_DN14374_c0_g1_i4::g.36023::m.36023	2	13,3	23,046	7,1444	PF04525.11	LOR	200,7	1,40E-59	CL0395
TRINITY_DN14376_c2_g1::TRINITY_DN14376_c2_g1_i4::g.36173::m.36173	2	20,5	12,578	2,5988					
TRINITY_DN14376_c2_g2::TRINITY_DN14376_c2_g2_i8::g.36166::m.36166	2	8,5	31,339	1,8694	PF01145.24	Band_7	89,6	2,20E-25	CL0433
TRINITY_DN14376_c2_g4::TRINITY_DN14376_c2_g4_i7::g.36164::m.36164	2	12	22,132	4,3122	PF01145.24	Band_7	25,6	9,50E-06	CL0433
TRINITY_DN14378_c0_g2::TRINITY_DN14378_c0_g2_i6::g.36119::m.36119	6	31,4	24,923	17,785	PF00252.17	Ribosomal_L16	143,2	4,30E-42	No_clan
TRINITY_DN14391_c2_g1::TRINITY_DN14391_c2_g1_i3::g.36277::m.36277	1	21	10,747	3,4831					
TRINITY_DN14391_c2_g2::TRINITY_DN14391_c2_g2_i2::g.36282::m.36282	4	39,6	12,818	30,996					
TRINITY_DN14391_c2_g3::TRINITY_DN14391_c2_g3_i1::g.36280::m.36280	1	15,6	14,198	3,4493					
TRINITY_DN14396_c0_g2::TRINITY_DN14396_c0_g2_i1::g.35371::m.35371	4	31,9	18,024	21,016	PF05899.11	Cupin_3	101,2	1,70E-29	CL0029
TRINITY_DN14398_c0_g1::TRINITY_DN14398_c0_g1_i7::g.36405::m.36405	13	51,2	46,081	323,31	PF11543.7	UN_NPL4	29,1	9,50E-07	CL0072
TRINITY_DN14403_c4_g2::TRINITY_DN14403_c4_g2_i1::g.36526::m.36526	15	16,7	122,39	31,836	PF00686.18	CBM_20	56,1	2,50E-15	CL0369
TRINITY_DN14404_c3_g2::TRINITY_DN14404_c3_g2_i3::g.36551::m.36551	4	12,7	63,205	8,154	PF00009.26	GTP_EFTU	207,1	1,70E-61	CL0023

TRINITY_DN14404_c3_g3::TRINITY_DN14404_c3_g3_i3::g.36554::m.36554	1	7,4	21,786	1,9569	PF03764.17	EFG_IV	109,7	6,70E-32	CL0329
TRINITY_DN14405_c4_g1::TRINITY_DN14405_c4_g1_i11::g.36585::m.36585	14	25,8	74,261	61,392	PF14749.5	Acyl-CoA_ox_N	112,9	1,30E-32	CL0544
TRINITY_DN14406_c2_g1::TRINITY_DN14406_c2_g1_i7::g.36607::m.36607	12	23,9	75,965	65,65	PF02770.18	Acyl-CoA_dh_M	53,8	1,60E-14	No_clan
TRINITY_DN14408_c0_g1::TRINITY_DN14408_c0_g1_i2::g.36628::m.36628	1	16	14,447	3,3	PF00995.22	Sec1	74,8	8,00E-21	No_clan
TRINITY_DN14408_c0_g2::TRINITY_DN14408_c0_g2_i5::g.36629::m.36629	2	4,1	58,441	3,0275	PF00995.22	Sec1	300,4	3,60E-89	No_clan
TRINITY_DN14410_c0_g6::TRINITY_DN14410_c0_g6_i1::g.36618::m.36618	2	18,3	12,575	28,61	PF02109.15	DAD	134	2,20E-39	No_clan
TRINITY_DN14413_c0_g1::TRINITY_DN14413_c0_g1_i13::g.36824::m.36824	9	31,8	38,538	42,214	PF02779.23	Transket_pyr	156,4	5,40E-46	CL0254
TRINITY_DN14413_c0_g2::TRINITY_DN14413_c0_g2_i2::g.36805::m.36805	2	4,7	48,834	3,1254	PF01053.19	Cys_Met_Meta_PP	278	7,90E-83	CL0061
TRINITY_DN14419_c1_g1::TRINITY_DN14419_c1_g1_i6::g.36874::m.36874	3	5,8	73,36	3,6757	PF16656.4	Pur_ac_phosph_N	67	1,50E-18	CL0159
TRINITY_DN14420_c1_g1::TRINITY_DN14420_c1_g1_i4::g.36850::m.36850	2	18,3	16,47	5,0345					
TRINITY_DN14421_c2_g1::TRINITY_DN14421_c2_g1_i4::g.36938::m.36938	5	37,9	17,694	231,58	PF00025.20	Arf	232,8	1,50E-69	CL0023
TRINITY_DN14427_c0_g1::TRINITY_DN14427_c0_g1_i8::g.36957::m.36957	1	4,6	44,006	3,048	PF01553.20	Acyltransferase	61,2	6,90E-17	CL0228
TRINITY_DN14430_c0_g1::TRINITY_DN14430_c0_g1_i7::g.37424::m.37424	5	12,8	59,087	13,972	PF00479.21	G6PD_N	197,5	2,60E-58	CL0063
TRINITY_DN14435_c3_g2::TRINITY_DN14435_c3_g2_i6::g.36467::m.36467	18	48,5	59,728	181,36	PF11421.7	Synthase_beta	59,1	4,90E-16	No_clan
TRINITY_DN14435_c4_g2::TRINITY_DN14435_c4_g2_i1::g.36484::m.36484	11	42,9	37,865	247,22	PF00141.22	peroxidase	229,3	4,40E-68	CL0617
TRINITY_DN14453_c2_g1::TRINITY_DN14453_c2_g1_i6::g.37399::m.37399	1	5,6	18,235	6,2716	PF05726.12	Pirin_C	121,5	1,60E-35	CL0029
TRINITY_DN14453_c2_g3::TRINITY_DN14453_c2_g3_i2::g.37393::m.37393	2	19	15,101	3,5513	PF02678.15	Pirin	94,6	3,10E-27	CL0029
TRINITY_DN14455_c0_g1::TRINITY_DN14455_c0_g1_i2::g.37451::m.37451	11	36,4	42,459	24,562	PF00162.18	PGK	431,3	2,80E-129	No_clan
TRINITY_DN14455_c0_g9::TRINITY_DN14455_c0_g9_i1::g.37463::m.37463	22	74,6	36,121	323,31	PF00162.18	PGK	480,2	3,80E-144	No_clan
TRINITY_DN14459_c0_g1::TRINITY_DN14459_c0_g1_i2::g.37381::m.37381	2	8	40,733	4,2158	PF00288.25	GHMP_kinases_N	73,6	1,10E-20	CL0329
TRINITY_DN14460_c2_g2::TRINITY_DN14460_c2_g2_i7::g.37472::m.37472	13	36,9	53,632	29,071	PF07992.13	Pyr_redox_2	232,2	6,70E-69	CL0063

TRINITY_DN14461_c1_g2::TRINITY_DN14461_c1_g2_i3::g.37522::m.37522	3	16,9	26,45	5,9643	PF02140.17	Gal_Lectin	31,9	1,20E-07	No_clan
TRINITY_DN14461_c1_g4::TRINITY_DN14461_c1_g4_i3::g.37527::m.37527	2	24,7	16,408	1,8883	PF01301.18	Glyco_hydro_35	55,7	5,30E-15	CL0058
TRINITY_DN14467_c3_g1::TRINITY_DN14467_c3_g1_i7::g.37643::m.37643	2	4,3	46,506	2,8372	PF01154.16	HMG_CoA_synt_N	205,4	5,10E-61	CL0046
TRINITY_DN14469_c0_g1::TRINITY_DN14469_c0_g1_i2::g.37789::m.37789	5	16,1	40,307	9,369	PF08241.11	Methyltransf_11	78,3	4,80E-22	CL0063
TRINITY_DN14469_c0_g2::TRINITY_DN14469_c0_g2_i3::g.37807::m.37807	4	10,4	50,873	14,71	PF14555.5	UBA_4	40	2,40E-10	CL0214
TRINITY_DN14475_c2_g1::TRINITY_DN14475_c2_g1_i7::g.36756::m.36756	2	14,8	20,979	3,9208	PF00494.18	SQS_PSY	85,9	2,80E-24	CL0613
TRINITY_DN14479_c0_g2::TRINITY_DN14479_c0_g2_i3::g.37670::m.37670	2	24,7	16,919	2,3947	PF00313.21	CSD	80,2	6,60E-23	CL0021
TRINITY_DN14480_c1_g1::TRINITY_DN14480_c1_g1_i3::g.37689::m.37689	1	10	11,73	1,958	PF07200.12	Mod_r	76,2	2,60E-21	CL0596
TRINITY_DN14482_c0_g1::TRINITY_DN14482_c0_g1_i1::g.37716::m.37716	1	3,5	61,69	3,1632					
TRINITY_DN14486_c0_g4::TRINITY_DN14486_c0_g4_i1::g.37922::m.37922	1	5,8	28,412	1,8714					
TRINITY_DN14491_c0_g1::TRINITY_DN14491_c0_g1_i2::g.37893::m.37893	1	7,4	26,046	2,0449	PF05633.10	BPS1	328,5	5,10E-98	CL0133
TRINITY_DN14491_c1_g3::TRINITY_DN14491_c1_g3_i2::g.37906::m.37906	2	7,7	32,252	2,659	PF02897.14	Peptidase_S9_N	210,6	3,10E-62	CL0186
TRINITY_DN14491_c1_g5::TRINITY_DN14491_c1_g5_i7::g.37912::m.37912	6	17,7	53,923	21,604	PF00326.20	Peptidase_S9	235,3	4,70E-70	CL0028
TRINITY_DN14491_c1_g5::TRINITY_DN14491_c1_g5_i8::g.37914::m.37914	3	25	16,748	7,2428	PF06747.12	CHCH	28,2	1,30E-06	CL0351
TRINITY_DN14491_c1_g6::TRINITY_DN14491_c1_g6_i1::g.37898::m.37898	2	12,5	21,551	3,5163	PF06888.11	Put_Phosphatase	243,3	2,00E-72	CL0137
TRINITY_DN14504_c3_g1::TRINITY_DN14504_c3_g1_i2::g.38234::m.38234	4	9,8	40,002	2,9293	PF00364.21	Biotin_lipoyl	63,1	1,50E-17	CL0105
TRINITY_DN14505_c2_g1::TRINITY_DN14505_c2_g1_i2::g.38114::m.38114	1	14,5	11,855	10,652	PF03357.20	Snf7	57,1	1,50E-15	CL0235
TRINITY_DN14508_c8_g1::TRINITY_DN14508_c8_g1_i4::g.38373::m.38373	5	47	16,351	75,928	PF03168.12	LEA_2	68,9	4,20E-19	CL0159
TRINITY_DN14508_c8_g1::TRINITY_DN14508_c8_g1_i7::g.38377::m.38377	5	49,6	14,87	284,11	PF03168.12	LEA_2	70,4	1,40E-19	CL0159
TRINITY_DN14508_c8_g1::TRINITY_DN14508_c8_g1_i8::g.38378::m.38378	5	43	16,325	45,788	PF03168.12	LEA_2	67,8	9,20E-19	CL0159
TRINITY_DN14511_c0_g1::TRINITY_DN14511_c0_g1_i3::g.38108::m.38108	7	76,1	12,064	323,31	PF00076.21	RRM_1	79,7	9,40E-23	CL0221
TRINITY_DN14513_c0_g3::TRINITY_DN14513_c0_g3_i9::g.38189::m.38189	4	34,4	17,711	89,285	PF00407.18	Bet_v_1	79,6	2,00E-22	CL0209

TRINITY_DN14516_c0_g1::TRINITY_DN14516_c0_g1_i7::g.38023::m.38023	1	5,1	21,608	5,247	PF02861.19	Clp_N	38,4	9,20E-10	No_clan
TRINITY_DN14517_c1_g1::TRINITY_DN14517_c1_g1_i15::g.38263::m.38263	7	19,8	44,944	124,96	PF00240.22	ubiquitin	60,1	1,20E-16	CL0072
TRINITY_DN14518_c1_g2::TRINITY_DN14518_c1_g2_i4::g.38282::m.38282	1	2,4	51,886	2,2509	PF01210.22	NAD_Gly3P_dh_N	39,6	4,70E-10	CL0063
TRINITY_DN14520_c0_g2::TRINITY_DN14520_c0_g2_i5::g.38465::m.38465	3	4,8	85,65	4,9337	PF00888.21	Cullin	738,5	6,30E-222	No_clan
TRINITY_DN14525_c1_g1::TRINITY_DN14525_c1_g1_i2::g.38384::m.38384	8	12,9	77,608	10,105	PF02770.18	Acyl-CoA_dh_M	52,1	5,30E-14	No_clan
TRINITY_DN14538_c2_g2::TRINITY_DN14538_c2_g2_i3::g.38504::m.38504	2	7,8	35,565	3,6488	PF00106.24	adh_short	125	2,40E-36	CL0063
TRINITY_DN14538_c2_g2::TRINITY_DN14538_c2_g2_i9::g.38508::m.38508	2	14,5	25,914	3,3881	PF12352.7	V-SNARE_C	45,7	5,40E-12	CL0147
TRINITY_DN14539_c1_g2::TRINITY_DN14539_c1_g2_i5::g.38538::m.38538	11	38,3	45,745	30,409	PF00091.24	Tubulin	182,6	9,10E-54	CL0566
TRINITY_DN14545_c0_g1::TRINITY_DN14545_c0_g1_i2::g.38714::m.38714	5	24,2	31,115	12,324	PF01168.19	Ala_racemase_N	80,7	1,10E-22	CL0036
TRINITY_DN14545_c0_g2::TRINITY_DN14545_c0_g2_i2::g.38716::m.38716	13	36,8	50,227	98,49	PF00091.24	Tubulin	234,2	1,40E-69	CL0566
TRINITY_DN14545_c0_g2::TRINITY_DN14545_c0_g2_i6::g.38726::m.38726	10	28,3	50,236	6,0217	PF00091.24	Tubulin	233,9	1,70E-69	CL0566
TRINITY_DN14547_c0_g1::TRINITY_DN14547_c0_g1_i3::g.38055::m.38055	5	29,5	31,282	25,614	PF01398.20	JAB	71,3	5,80E-20	CL0366
TRINITY_DN14553_c3_g1::TRINITY_DN14553_c3_g1_i5::g.38708::m.38708	3	6,9	63,745	5,4719	PF12819.6	Malectin_like	47,6	1,20E-12	CL0468
TRINITY_DN14555_c5_g1::TRINITY_DN14555_c5_g1_i2::g.38904::m.38904	1	8,3	16,533	2,8091	PF00515.27	TPR_1	36,8	1,90E-09	CL0020
TRINITY_DN14555_c5_g4::TRINITY_DN14555_c5_g4_i1::g.38907::m.38907	4	39,4	10,915	323,31					
TRINITY_DN14557_c1_g1::TRINITY_DN14557_c1_g1_i7::g.38742::m.38742	1	4,6	38,974	11,026	PF00300.21	His_Phos_1	64,6	8,50E-18	CL0071
TRINITY_DN14558_c2_g3::TRINITY_DN14558_c2_g3_i1::g.38817::m.38817	7	42,9	17,153	323,31	PF00240.22	ubiquitin	116,2	3,60E-34	CL0072
TRINITY_DN14563_c5_g1::TRINITY_DN14563_c5_g1_i5::g.38974::m.38974	2	9	23,141	3,7908	PF03151.15	TPT	197,5	2,80E-58	CL0184
TRINITY_DN14572_c4_g1::TRINITY_DN14572_c4_g1_i1::g.39016::m.39016	2	32,4	12,285	6,6911	PF01643.16	Acyl-ACP_TE	94,9	4,30E-27	CL0050
TRINITY_DN14576_c1_g5::TRINITY_DN14576_c1_g5_i7::g.39012::m.39012	6	63,8	15,115	191,86	PF00241.19	Cofilin_ADF	125,6	1,10E-36	CL0092
TRINITY_DN14578_c1_g1::TRINITY_DN14578_c1_g1_i1::g.39115::m.39115	8	27,3	42,886	42,285	PF00438.19	S-AdoMet_synt_N	142,4	5,40E-42	No_clan

TRINITY_DN14578_c1_g1::TRINITY_DN14578_c1_g1_i8::g.39125::m.39125	7	29,3	43,099	14,653	PF00438.19	S-AdoMet_synt_N	143,5	2,50E-42	No_clan
TRINITY_DN14578_c1_g3::TRINITY_DN14578_c1_g3_i1::g.39119::m.39119	4	15,6	44,159	-2	PF00438.19	S-AdoMet_synt_N	143,1	3,20E-42	No_clan
TRINITY_DN14585_c2_g1::TRINITY_DN14585_c2_g1_i7::g.39202::m.39202	3	23,5	21,013	8,0878	PF00657.21	Lipase_GDSL	82,8	3,00E-23	CL0264
TRINITY_DN14591_c4_g1::TRINITY_DN14591_c4_g1_i9::g.39216::m.39216	7	19,8	50,917	14,491	PF00464.18	SHMT	691	3,50E-208	CL0061
TRINITY_DN14591_c5_g1::TRINITY_DN14591_c5_g1_i3::g.39221::m.39221	1	7,1	27,558	2,1789	PF01553.20	Acyltransferase	61,4	6,30E-17	CL0228
TRINITY_DN14598_c1_g3::TRINITY_DN14598_c1_g3_i4::g.39295::m.39295	5	16,6	48,252	68,213	PF14829.5	GPAT_N	80,1	8,20E-23	No_clan
TRINITY_DN14600_c0_g1::TRINITY_DN14600_c0_g1_i7::g.38565::m.38565	13	31,1	60,69	23,052	PF00118.23	Cpn60_TCP1	540,4	3,40E-162	No_clan
TRINITY_DN14601_c2_g1::TRINITY_DN14601_c2_g1_i8::g.39287::m.39287	3	15,4	33,992	5,5508	PF03367.12	zf-ZPR1	181,3	1,10E-53	CL0167
TRINITY_DN14602_c3_g2::TRINITY_DN14602_c3_g2_i9::g.39596::m.39596	5	10	60,091	20,151	PF00501.27	AMP-binding	324	1,10E-96	CL0378
TRINITY_DN14603_c3_g1::TRINITY_DN14603_c3_g1_i1::g.39490::m.39490	6	7,6	113,27	8,5339	PF00004.28	AAA	123,3	7,60E-36	CL0023
TRINITY_DN14612_c0_g1::TRINITY_DN14612_c0_g1_i2::g.39550::m.39550	6	18	48,152	11,663	PF16656.4	Pur_ac_phosph_N	65,9	3,40E-18	CL0159
TRINITY_DN14617_c1_g1::TRINITY_DN14617_c1_g1_i9::g.39676::m.39676	2	5,9	52,008	3,2295	PF12899.6	Glyco_hydro_100	588,3	7,20E-177	CL0059
TRINITY_DN14624_c0_g1::TRINITY_DN14624_c0_g1_i4::g.39690::m.39690	1	4,8	24,532	4,7784	PF00885.18	DMRL_synthase	168,5	6,80E-50	No_clan
TRINITY_DN14626_c0_g2::TRINITY_DN14626_c0_g2_i6::g.39817::m.39817	1	4,9	26,909	2,0049	PF12796.6	Ank_2	68,2	6,60E-19	CL0465
TRINITY_DN14629_c0_g3::TRINITY_DN14629_c0_g3_i2::g.40344::m.40344	1	8,5	17,656	2,1641					
TRINITY_DN14630_c1_g1::TRINITY_DN14630_c1_g1_i1::g.39822::m.39822	4	21,3	27,797	19,838	PF00025.20	Arf	218,4	4,00E-65	CL0023
TRINITY_DN14630_c2_g8::TRINITY_DN14630_c2_g8_i1::g.39829::m.39829	4	28,6	19,829	1,9775	PF00025.20	Arf	210,7	9,70E-63	CL0023
TRINITY_DN14635_c2_g1::TRINITY_DN14635_c2_g1_i1::g.39353::m.39353	8	19,5	61,738	74,099	PF10255.8	Paf67	496,4	5,80E-149	CL0020
TRINITY_DN14645_c3_g1::TRINITY_DN14645_c3_g1_i7::g.39449::m.39449	5	8,3	84,977	13,77	PF00153.26	Mito_carr	42,1	5,60E-11	No_clan
TRINITY_DN14648_c0_g1::TRINITY_DN14648_c0_g1_i1::g.39924::m.39924	25	42,8	83,44	323,31	PF00933.20	Glyco_hydro_3	127,1	8,30E-37	CL0058

TRINITY_DN14649_c1_g6::TRINITY_DN14649_c1_g6_i3::g.39965::m.39965	3	17,9	24,519	11,917	PF00406.21	ADK	198,6	5,20E-59	CL0023
TRINITY_DN14654_c1_g1::TRINITY_DN14654_c1_g1_i1::g.40157::m.40157	4	7,2	72,461	6,1064	PF02696.13	UPF0061	441,9	2,50E-132	No_clan
TRINITY_DN14656_c1_g3::TRINITY_DN14656_c1_g3_i2::g.40036::m.40036	4	5,1	117,65	8,0468	PF00999.20	Na_H_Exchanger	200,2	4,20E-59	CL0064
TRINITY_DN14658_c2_g1::TRINITY_DN14658_c2_g1_i2::g.40152::m.40152	4	8,2	54,728	6,2604	PF05577.11	Peptidase_S28	259,1	6,50E-77	CL0028
TRINITY_DN14659_c0_g2::TRINITY_DN14659_c0_g2_i6::g.40081::m.40081	5	30,4	27,982	11,363	PF00227.25	Proteasome	102,9	1,40E-29	CL0052
TRINITY_DN14661_c1_g1::TRINITY_DN14661_c1_g1_i2::g.40114::m.40114	1	11,2	17,736	3,0017	PF02441.18	Flavoprotein	63,5	1,80E-17	No_clan
TRINITY_DN14669_c0_g2::TRINITY_DN14669_c0_g2_i2::g.40246::m.40246	1	1,8	88,919	1,9044	PF13087.5	AAA_12	195,5	6,80E-58	CL0023
TRINITY_DN14670_c0_g1::TRINITY_DN14670_c0_g1_i1::g.40393::m.40393	2	19,1	13,104	-2	PF00724.19	Oxidored_FMN	32,4	5,10E-08	CL0036
TRINITY_DN14670_c2_g3::TRINITY_DN14670_c2_g3_i4::g.40396::m.40396	1	7,5	13,636	15,246	PF04969.15	CS	40	5,60E-10	CL0190
TRINITY_DN14674_c1_g1::TRINITY_DN14674_c1_g1_i2::g.40399::m.40399	3	10,9	33,063	4,3301	PF01025.18	GrpE	88,4	3,70E-25	No_clan
TRINITY_DN14684_c2_g1::TRINITY_DN14684_c2_g1_i9::g.40676::m.40676	3	22,6	23,026	7,8974	PF01583.19	APS_kinase	238,5	2,60E-71	CL0023
TRINITY_DN14685_c0_g1::TRINITY_DN14685_c0_g1_i6::g.40758::m.40758	4	1,9	346,83	6,4064	PF06101.10	Vps62	41,8	4,10E-11	No_clan
TRINITY_DN14688_c0_g2::TRINITY_DN14688_c0_g2_i3::g.40630::m.40630	3	14,9	34,121	6,7979	PF00282.18	Pyridoxal_deC	33,7	1,50E-08	CL0061
TRINITY_DN14696_c0_g1::TRINITY_DN14696_c0_g1_i1::g.40665::m.40665	3	16,7	21,098	289,4	PF02298.16	Cu_bind_like	93,7	4,80E-27	CL0026
TRINITY_DN14696_c0_g1::TRINITY_DN14696_c0_g1_i2::g.40666::m.40666	3	18,3	21,583	12,384	PF02298.16	Cu_bind_like	90,7	4,00E-26	CL0026
TRINITY_DN14697_c1_g1::TRINITY_DN14697_c1_g1_i3::g.40725::m.40725	4	14,8	40,111	7,6594	PF01467.25	CTP_transf_like	29,1	8,90E-07	CL0039
TRINITY_DN14703_c2_g1::TRINITY_DN14703_c2_g1_i4::g.41069::m.41069	3	15,9	29,847	4,5801	PF01588.19	tRNA_bind	98,7	1,40E-28	CL0021
TRINITY_DN14704_c0_g1::TRINITY_DN14704_c0_g1_i5::g.40889::m.40889	5	14,6	41,663	28,814	PF03662.13	Glyco_hydro_79n	519,3	2,80E-156	CL0058
TRINITY_DN14704_c1_g3::TRINITY_DN14704_c1_g3_i1::g.40903::m.40903	3	8	60,348	7,3467	PF03662.13	Glyco_hydro_79n	516,9	1,40E-155	CL0058
TRINITY_DN14707_c0_g2::TRINITY_DN14707_c0_g2_i9::g.40985::m.40985	7	19,8	59,128	15,527	PF13943.5	WPP	123,6	3,20E-36	No_clan
TRINITY_DN14710_c1_g2::TRINITY_DN14710_c1_g2_i5::g.41059::m.41059	2	21	20,222	3,1768					

TRINITY_DN14710_c1_g4::TRINITY_DN14710_c1_g4_i3::g.41054::m.41054	9	15,6	89,781	20,761	PF05879.11	RHD3	992	1,50E-298	CL0023
TRINITY_DN14711_c0_g2::TRINITY_DN14711_c0_g2_i2::g.40961::m.40961	10	28,5	46,696	28,991	PF01145.24	Band_7	102,8	1,90E-29	CL0433
TRINITY_DN14711_c0_g4::TRINITY_DN14711_c0_g4_i1::g.40962::m.40962	2	6,6	41,425	-2	PF01145.24	Band_7	102,2	3,10E-29	CL0433
TRINITY_DN14717_c1_g1::TRINITY_DN14717_c1_g1_i9::g.42525::m.42525	6	30,1	23,987	9,0932	PF00071.21	Ras	222,7	1,70E-66	CL0023
TRINITY_DN14718_c0_g1::TRINITY_DN14718_c0_g1_i3::g.41289::m.41289	2	10,9	28,137	1,8575	PF00390.18	malic	221,2	8,20E-66	CL0603
TRINITY_DN14718_c0_g2::TRINITY_DN14718_c0_g2_i3::g.41293::m.41293	4	43,1	15,745	91,798	PF03949.14	Malic_M	153,1	7,50E-45	CL0063
TRINITY_DN14718_c0_g3::TRINITY_DN14718_c0_g3_i4::g.41295::m.41295	2	7,9	26,704	2,6047	PF03949.14	Malic_M	269,7	2,00E-80	CL0063
TRINITY_DN14723_c2_g4::TRINITY_DN14723_c2_g4_i2::g.41268::m.41268	6	19,3	43,55	10,855	PF05633.10	BPS1	532,4	6,10E-160	CL0133
TRINITY_DN14724_c2_g1::TRINITY_DN14724_c2_g1_i1::g.41239::m.41239	12	18,5	98,216	29,935	PF00696.27	AA_kinase	149,1	1,50E-43	No_clan
TRINITY_DN14724_c2_g3::TRINITY_DN14724_c2_g3_i6::g.41251::m.41251	1	3,5	46,449	2,8496	PF00571.27	CBS	21,9	0,00016	No_clan
TRINITY_DN14725_c0_g1::TRINITY_DN14725_c0_g1_i4::g.41213::m.41213	1	6,3	14,113	2,721	PF11595.7	DUF3245	47	3,30E-12	No_clan
TRINITY_DN14730_c2_g2::TRINITY_DN14730_c2_g2_i1::g.41382::m.41382	14	89,4	11,724	323,31					
TRINITY_DN14731_c0_g4::TRINITY_DN14731_c0_g4_i2::g.41282::m.41282	6	54,9	15,776	42,214	PF10584.8	Proteasome_A_N	53,2	1,50E-14	CL0052
TRINITY_DN14738_c2_g1::TRINITY_DN14738_c2_g1_i2::g.41499::m.41499	6	13,3	71,159	12,532	PF02776.17	TPP_enzyme_N	181,5	9,60E-54	CL0254
TRINITY_DN14741_c0_g1::TRINITY_DN14741_c0_g1_i2::g.41477::m.41477	4	7,9	65,328	28,418	PF12222.7	PNGaseA	384,6	5,40E-115	No_clan
TRINITY_DN14744_c0_g1::TRINITY_DN14744_c0_g1_i1::g.41504::m.41504	4	13,6	48,683	12,048	PF00085.19	Thioredoxin	90,2	6,40E-26	CL0172
TRINITY_DN14751_c0_g1::TRINITY_DN14751_c0_g1_i8::g.40814::m.40814	3	4,5	99,44	4,7964	PF04389.16	Peptidase_M28	122,1	2,10E-35	CL0035
TRINITY_DN14755_c2_g1::TRINITY_DN14755_c2_g1_i4::g.41671::m.41671	5	43,5	13,379	275,59	PF00107.25	ADH_zinc_N	37,6	1,70E-09	CL0063
TRINITY_DN14755_c2_g2::TRINITY_DN14755_c2_g2_i1::g.41670::m.41670	10	49,1	31,365	323,31	PF08240.11	ADH_N	101,9	1,60E-29	CL0296
TRINITY_DN14756_c11_g3::TRINITY_DN14756_c11_g3_i1::g.41922::m.41922	4	12,8	40,814	6,267	PF04043.14	PMEI	57,6	1,60E-15	No_clan
TRINITY_DN14757_c1_g1::TRINITY_DN14757_c1_g1_i7::g.41725::m.41725	2	10,7	45,684	3,7901					

TRINITY_DN14761_c1_g4::TRINITY_DN14761_c1_g4_i4::g.41839::m.41839	3	3,8	132,53	5,1554	PF00069.24	Pkinase	171,6	1,90E-50	CL0016
TRINITY_DN14763_c1_g1::TRINITY_DN14763_c1_g1_i5::g.41937::m.41937	2	4,6	48,024	2,4307	PF02671.20	PAH	62,3	3,20E-17	No_clan
TRINITY_DN14766_c7_g2::TRINITY_DN14766_c7_g2_i3::g.41862::m.41862	20	42	67,544	255,81	PF08323.10	Glyco_transf_5	250,5	1,60E-74	CL0113
TRINITY_DN14774_c0_g5::TRINITY_DN14774_c0_g5_i3::g.42116::m.42116	1	1,4	134,46	4,5161	PF10433.8	MMS1_N	568,6	9,50E-171	No_clan
TRINITY_DN14775_c0_g2::TRINITY_DN14775_c0_g2_i2::g.42036::m.42036	5	13,1	54,386	9,1211	PF00328.21	His_Phos_2	170,1	8,40E-50	CL0071
TRINITY_DN14776_c1_g1::TRINITY_DN14776_c1_g1_i2::g.42049::m.42049	31	71,5	54,52	323,31	PF10509.8	GalKase_gal_bdg	78,2	2,30E-22	CL0329
TRINITY_DN14777_c1_g1::TRINITY_DN14777_c1_g1_i9::g.42089::m.42089	5	15,6	53,423	10,484	PF00155.20	Aminotran_1_2	128,5	3,20E-37	CL0061
TRINITY_DN14777_c4_g4::TRINITY_DN14777_c4_g4_i3::g.42104::m.42104	1	1,3	83,685	18,375	PF00078.26	RVT_1	58,7	5,20E-16	CL0027
TRINITY_DN14781_c0_g1::TRINITY_DN14781_c0_g1_i21::g.42142::m.42142	5	11,3	56,537	16,668	PF00307.30	CH	60,8	1,10E-16	CL0188
TRINITY_DN14785_c0_g1::TRINITY_DN14785_c0_g1_i8::g.42289::m.42289	2	13,5	36,921	10,523	PF00085.19	Thioredoxin	55,9	3,00E-15	CL0172
TRINITY_DN14785_c0_g1::TRINITY_DN14785_c0_g1_i6::g.42288::m.42288	1	21,3	12,325	5,2731					
TRINITY_DN14786_c1_g2::TRINITY_DN14786_c1_g2_i1::g.42235::m.42235	1	0,8	284,34	2,1038	PF14237.5	DUF4339	44,7	7,60E-12	No_clan
TRINITY_DN14788_c0_g1::TRINITY_DN14788_c0_g1_i1::g.42248::m.42248	2	33,3	11,471	3,9517					
TRINITY_DN14788_c0_g2::TRINITY_DN14788_c0_g2_i4::g.42257::m.42257	4	20,7	22,249	6,5303	PF13472.5	Lipase_GDSL_2	66	5,20E-18	CL0264
TRINITY_DN14788_c0_g4::TRINITY_DN14788_c0_g4_i2::g.42252::m.42252	10	20,2	76,666	23,517	PF02824.20	TGS	53,8	1,40E-14	CL0072
TRINITY_DN14799_c0_g2::TRINITY_DN14799_c0_g2_i3::g.42387::m.42387	6	27,5	33,343	11,074	PF04278.11	Tic22	238,5	6,40E-71	No_clan
TRINITY_DN14801_c0_g1::TRINITY_DN14801_c0_g1_i4::g.42400::m.42400	3	17,2	36,962	3,5333	PF08700.10	Vps51	88,5	2,20E-25	CL0295
TRINITY_DN14802_c2_g1::TRINITY_DN14802_c2_g1_i3::g.42628::m.42628	1	4,9	30,953	2,4339	PF00297.21	Ribosomal_L3	58,1	6,90E-16	CL0575
TRINITY_DN14803_c0_g1::TRINITY_DN14803_c0_g1_i4::g.42638::m.42638	4	12,8	41,431	6,1313	PF00581.19	Rhodanese	53,3	3,10E-14	CL0031
TRINITY_DN14810_c0_g2::TRINITY_DN14810_c0_g2_i2::g.42832::m.42832	4	31,3	17,739	25,549	PF03946.13	Ribosomal_L11_N	82,6	1,10E-23	No_clan
TRINITY_DN14814_c1_g3::TRINITY_DN14814_c1_g3_i3::g.42810::m.42810	7	26,1	40,983	50,259	PF01118.23	Semialdhyde_dh	110,1	7,90E-32	CL0063

TRINITY_DN14828_c0_g3::TRINITY_DN14828_c0_g3_i7::g.43009::m.43009	2	9,7	37,505	4,5868	PF16656.4	Pur_ac_phosph_N	50	3,20E-13	CL0159
TRINITY_DN14830_c5_g1::TRINITY_DN14830_c5_g1_i9::g.42943::m.42943	3	9	51,966	2,9323	PF00856.27	SET	35,9	8,80E-09	No_clan
TRINITY_DN14831_c2_g1::TRINITY_DN14831_c2_g1_i4::g.42949::m.42949	8	44,3	21,791	323,31	PF00635.25	Motile_Sperm	98,9	1,30E-28	CL0556
TRINITY_DN14833_c0_g2::TRINITY_DN14833_c0_g2_i2::g.42977::m.42977	2	6,8	44,235	3,3265	PF00206.19	Lyase_1	175,9	1,20E-51	No_clan
TRINITY_DN14840_c3_g1::TRINITY_DN14840_c3_g1_i19::g.43227::m.43227	1	10,2	13,586	2,0405	PF01869.19	BcrAD_BadFG	45,2	7,60E-12	CL0108
TRINITY_DN14845_c0_g2::TRINITY_DN14845_c0_g2_i5::g.43176::m.43176	17	44,6	54,136	78,819	PF02874.22	ATP-synt_ab_N	51,7	8,80E-14	CL0275
TRINITY_DN14858_c0_g1::TRINITY_DN14858_c0_g1_i1::g.43399::m.43399	21	24,8	128,27	109,9	PF00899.20	ThiF	112,6	1,80E-32	CL0063
TRINITY_DN14858_c0_g5::TRINITY_DN14858_c0_g5_i1::g.43408::m.43408	9	11,1	119,86	-2	PF00899.20	ThiF	102,1	2,70E-29	CL0063
TRINITY_DN14860_c0_g1::TRINITY_DN14860_c0_g1_i1::g.43455::m.43455	1	2,9	75,775	2,9411	PF00324.20	AA_permease	132,2	1,90E-38	CL0062
TRINITY_DN14860_c0_g2::TRINITY_DN14860_c0_g2_i3::g.43462::m.43462	9	9,3	130,74	16,444	PF14833.5	NAD_binding_11	75,8	3,10E-21	CL0106
TRINITY_DN14862_c0_g2::TRINITY_DN14862_c0_g2_i3::g.43396::m.43396	3	26,1	22,181	6,077	PF04674.11	Phi_1	252,8	3,40E-75	No_clan
TRINITY_DN14869_c3_g5::TRINITY_DN14869_c3_g5_i3::g.43830::m.43830	8	53,4	15,05	29,156	PF00012.19	HSP70	62,8	1,60E-17	CL0108
TRINITY_DN14870_c0_g1::TRINITY_DN14870_c0_g1_i2::g.43479::m.43479	7	44,8	24,542	16,584	PF00227.25	Proteasome	114,7	3,10E-33	CL0052
TRINITY_DN14872_c2_g1::TRINITY_DN14872_c2_g1_i7::g.43673::m.43673	5	12,8	57,907	52,103	PF01487.14	DHquinase_I	235	1,10E-69	CL0036
TRINITY_DN14872_c2_g2::TRINITY_DN14872_c2_g2_i2::g.43670::m.43670	29	23,1	164,82	90,883	PF01326.18	PPDK_N	64,4	9,40E-18	CL0179
TRINITY_DN14875_c0_g2::TRINITY_DN14875_c0_g2_i7::g.43614::m.43614	9	26,1	50,976	16,141	PF00009.26	GTP_EFTU	88,2	4,60E-25	CL0023
TRINITY_DN14877_c0_g1::TRINITY_DN14877_c0_g1_i9::g.43584::m.43584	8	36	28,687	204,78	PF00076.21	RRM_1	37,1	2,00E-09	CL0221
TRINITY_DN14879_c3_g1::TRINITY_DN14879_c3_g1_i2::g.43728::m.43728	8	19,5	61,986	6,3214	PF01749.19	IBB	79,2	2,00E-22	CL0020
TRINITY_DN14879_c3_g3::TRINITY_DN14879_c3_g3_i2::g.43725::m.43725	10	24,9	58,611	37,374	PF01749.19	IBB	83,4	1,00E-23	CL0020
TRINITY_DN14879_c3_g4::TRINITY_DN14879_c3_g4_i1::g.43729::m.43729	7	45,4	23,887	90,609	PF00071.21	Ras	209,9	1,50E-62	CL0023
TRINITY_DN14880_c2_g1::TRINITY_DN14880_c2_g1_i5::g.43756::m.43756	2	14,8	20,03	3,8507					
TRINITY_DN14880_c3_g1::TRINITY_DN14880_c3_g1_i7::g.43762::m.43762	2	5,8	40,636	2,621	PF01172.17	SBDS	95,1	1,80E-27	No_clan

TRINITY_DN14881_c0_g1::TRINITY_DN14881_c0_g1_i4::g.43780::m.43780	2	5,5	50,659	3,681	PF00850.18	Hist_deacetyl	268,8	6,70E-80	CL0302
TRINITY_DN14887_c2_g3::TRINITY_DN14887_c2_g3_i1::g.43865::m.43865	2	6,5	47,723	2,1374	PF00485.17	PRK	88	6,10E-25	CL0023
TRINITY_DN14887_c2_g4::TRINITY_DN14887_c2_g4_i1::g.43866::m.43866	3	11,1	39,369	10,749					
TRINITY_DN14897_c3_g5::TRINITY_DN14897_c3_g5_i1::g.43938::m.43938	3	22,5	15,346	4,7947	PF00125.23	Histone	80,3	1,50E-22	CL0012
TRINITY_DN14898_c0_g1::TRINITY_DN14898_c0_g1_i2::g.43888::m.43888	2	7,4	38,348	3,5789	PF00106.24	adh_short	144,5	2,50E-42	CL0063
TRINITY_DN14898_c0_g2::TRINITY_DN14898_c0_g2_i1::g.43887::m.43887	1	7,3	22,32	5,9473	PF00107.25	ADH_zinc_N	101,3	3,40E-29	CL0063
TRINITY_DN14909_c1_g1::TRINITY_DN14909_c1_g1_i3::g.44168::m.44168	10	11,4	117,4	17,126	PF08700.10	Vps51	59,2	2,90E-16	CL0295
TRINITY_DN14909_c1_g2::TRINITY_DN14909_c1_g2_i4::g.44177::m.44177	24	34,7	94,434	264,7	PF02518.25	HATPase_c	47,8	1,70E-12	CL0025
TRINITY_DN14909_c1_g5::TRINITY_DN14909_c1_g5_i1::g.44180::m.44180	8	10,1	112,01	13,447	PF16940.4	Tic110	966,7	3,40E-291	No_clan
TRINITY_DN14912_c1_g1::TRINITY_DN14912_c1_g1_i4::g.44268::m.44268	5	23	28,17	28,92	PF13344.5	Hydrolase_6	75,6	2,50E-21	CL0137
TRINITY_DN14916_c3_g2::TRINITY_DN14916_c3_g2_i1::g.44503::m.44503	2	9,4	35,007	3,3038	PF01398.20	JAB	102,5	1,20E-29	CL0366
TRINITY_DN14920_c0_g1::TRINITY_DN14920_c0_g1_i8::g.44465::m.44465	7	20,1	37,474	43,503	PF00316.19	FBPase	261	4,70E-78	CL0171
TRINITY_DN14922_c2_g1::TRINITY_DN14922_c2_g1_i5::g.44553::m.44553	3	1,7	293,07	8,0502	PF10347.8	Fmp27_GFWDK	24,9	1,70E-05	No_clan
TRINITY_DN14927_c1_g1::TRINITY_DN14927_c1_g1_i11::g.44438::m.44438	3	23,6	19,438	7,8867					
TRINITY_DN14931_c0_g1::TRINITY_DN14931_c0_g1_i6::g.44458::m.44458	2	3,6	93,325	2,9535					
TRINITY_DN14932_c2_g1::TRINITY_DN14932_c2_g1_i5::g.44480::m.44480	1	7,5	11,477	1,945	PF00724.19	Oxidored_FMN	79,5	2,50E-22	CL0036
TRINITY_DN14933_c0_g2::TRINITY_DN14933_c0_g2_i3::g.44460::m.44460	6	46,7	18,357	12,937	PF06172.10	Cupin_5	147,5	2,20E-43	CL0029
TRINITY_DN14937_c1_g1::TRINITY_DN14937_c1_g1_i10::g.44580::m.44580	1	11,8	20,065	7,1945	PF00182.18	Glyco_hydro_19	333,9	5,60E-100	CL0037
TRINITY_DN14938_c0_g1::TRINITY_DN14938_c0_g1_i5::g.44776::m.44776	4	15,2	30,189	11,374	PF01873.16	eIF-5_eIF-2B	134,8	1,10E-39	No_clan
TRINITY_DN14939_c1_g2::TRINITY_DN14939_c1_g2_i3::g.44685::m.44685	5	36	22,054	108,52	PF00657.21	Lipase_GDSL	32,4	8,10E-08	CL0264
TRINITY_DN14939_c1_g3::TRINITY_DN14939_c1_g3_i3::g.44686::m.44686	3	13,7	34,305	4,9429	PF05368.12	NmrA	249,5	2,80E-74	CL0063

TRINITY_DN14942_c0_g1::TRINITY_DN14942_c0_g1_i6::g.44624::m.44624	1	1,5	91,043	2,0791	PF10536.8	PMD	336,7	1,50E-100	No_clan
TRINITY_DN14942_c2_g1::TRINITY_DN14942_c2_g1_i4::g.44640::m.44640	4	40,8	14,098	9,2964	PF02575.15	YbaB_DNA_bd	62,7	2,70E-17	No_clan
TRINITY_DN14948_c0_g1::TRINITY_DN14948_c0_g1_i2::g.43967::m.43967	2	5,7	60,728	4,693	PF14416.5	PMR5N	53,9	1,50E-14	No_clan
TRINITY_DN14955_c4_g2::TRINITY_DN14955_c4_g2_i6::g.44900::m.44900	2	11,6	26,939	4,9451	PF13202.5	EF-hand_5	18,9	0,00069	CL0220
TRINITY_DN14957_c1_g1::TRINITY_DN14957_c1_g1_i3::g.44723::m.44723	3	18,3	26,03	7,4304	PF08777.10	RRM_3	71	6,40E-20	CL0221
TRINITY_DN14965_c0_g1::TRINITY_DN14965_c0_g1_i3::g.45244::m.45244	9	19,4	66,842	48,779	PF02878.15	PGM_PMM_I	90,9	5,10E-26	No_clan
TRINITY_DN14967_c2_g1::TRINITY_DN14967_c2_g1_i8::g.44945::m.44945	3	15	28,265	7,1247	PF02453.16	Reticulon	198,3	6,20E-59	No_clan
TRINITY_DN14970_c1_g1::TRINITY_DN14970_c1_g1_i3::g.45037::m.45037	21	43,3	61,816	99,55	PF00118.23	Cpn60_TCP1	291,7	8,70E-87	No_clan
TRINITY_DN14971_c0_g1::TRINITY_DN14971_c0_g1_i2::g.44966::m.44966	3	11,9	36,087	17,442	PF00596.20	Aldolase_II	30,4	3,10E-07	No_clan
TRINITY_DN14972_c1_g2::TRINITY_DN14972_c1_g2_i4::g.44982::m.44982	8	51,4	23,579	249,52	PF02338.18	OTU	30,1	5,20E-07	CL0125
TRINITY_DN14973_c1_g2::TRINITY_DN14973_c1_g2_i1::g.45015::m.45015	5	30,7	18,674	11,448	PF04716.13	ETC_C1_NDUFA5	89,8	6,70E-26	No_clan
TRINITY_DN14977_c0_g1::TRINITY_DN14977_c0_g1_i14::g.45080::m.45080	1	6,6	21,073	2,8757	PF00149.27	Metallophos	36,4	7,00E-09	CL0163
TRINITY_DN14979_c0_g1::TRINITY_DN14979_c0_g1_i9::g.45197::m.45197	14	27	71,561	1,9596	PF00004.28	AAA	161,5	1,30E-47	CL0023
TRINITY_DN14982_c0_g1::TRINITY_DN14982_c0_g1_i9::g.45222::m.45222	2	8,5	38,544	5,2425	PF09177.10	Syntaxin-6_N	37,5	2,40E-09	No_clan
TRINITY_DN14985_c3_g2::TRINITY_DN14985_c3_g2_i1::g.45232::m.45232	3	6,3	86,575	8,6835	PF02516.13	STT3	348,6	6,00E-104	CL0111
TRINITY_DN14986_c2_g1::TRINITY_DN14986_c2_g1_i1::g.45365::m.45365	3	21,9	19,835	164,78	PF06201.12	PITH	139,7	7,90E-41	CL0202
TRINITY_DN14986_c4_g1::TRINITY_DN14986_c4_g1_i9::g.45372::m.45372	16	33,8	53,885	323,31	PF00370.20	FGGY_N	61,8	6,40E-17	CL0108
TRINITY_DN14993_c0_g1::TRINITY_DN14993_c0_g1_i2::g.45400::m.45400	1	4,5	40,73	2,5628	PF14802.5	TMEM192	53,3	1,90E-14	No_clan
TRINITY_DN15001_c1_g2::TRINITY_DN15001_c1_g2_i1::g.45482::m.45482	2	19,2	14,239	117,7	PF00076.21	RRM_1	27,2	2,30E-06	CL0221
TRINITY_DN15004_c1_g1::TRINITY_DN15004_c1_g1_i9::g.45691::m.45691	18	38	67,714	103,48	PF00365.19	PFK	95,5	3,30E-27	CL0240
TRINITY_DN15008_c0_g1::TRINITY_DN15008_c0_g1_i3::g.45737::m.45737	18	21,5	116,46	94,713	PF00690.25	Cation_ATPase_N	61,4	4,50E-17	No_clan

TRINITY_DN15008_c0_g2::TRINITY_DN15008_c0_g2_i1::g.45732::m.45732	10	12,2	117,02	5,3976	PF00690.25	Cation_ATPase_N	49,8	1,90E-13	No_clan
TRINITY_DN15008_c0_g3::TRINITY_DN15008_c0_g3_i2::g.45738::m.45738	11	22,2	77,271	13,218	PF00690.25	Cation_ATPase_N	52,1	3,60E-14	No_clan
TRINITY_DN15010_c0_g3::TRINITY_DN15010_c0_g3_i5::g.45655::m.45655	21	61,5	52,922	323,31	PF05694.10	SBP56	725,8	1,70E-218	CL0186
TRINITY_DN15011_c4_g2::TRINITY_DN15011_c4_g2_i4::g.45809::m.45809	2	11,5	24,517	3,6938	PF07714.16	Pkinase_Tyr	44,2	1,30E-11	CL0016
TRINITY_DN15012_c0_g1::TRINITY_DN15012_c0_g1_i2::g.45660::m.45660	2	10,1	30,087	3,2663	PF02894.16	GFO_IDH_MocA_C	23,8	3,20E-05	CL0218
TRINITY_DN15019_c3_g2::TRINITY_DN15019_c3_g2_i1::g.45833::m.45833	1	4,6	35,266	-2	PF12776.6	Myb_DNA-bind_3	82	4,30E-23	CL0123
TRINITY_DN15025_c0_g1::TRINITY_DN15025_c0_g1_i1::g.45918::m.45918	25	67,5	45,237	323,31	PF03952.15	Enolase_N	142	1,10E-41	CL0227
TRINITY_DN15025_c0_g1::TRINITY_DN15025_c0_g1_i8::g.45925::m.45925	23	64,9	48,346	323,31	PF03952.15	Enolase_N	177,9	8,70E-53	CL0227
TRINITY_DN15027_c0_g1::TRINITY_DN15027_c0_g1_i5::g.45870::m.45870	2	5,6	51,928	3,2367	PF01239.21	PPTA	24,1	2,00E-05	CL0020
TRINITY_DN15029_c2_g1::TRINITY_DN15029_c2_g1_i2::g.45973::m.45973	4	32,4	11,694	42,111	PF11976.7	Rad60-SLD	86,4	7,90E-25	CL0072
TRINITY_DN15029_c4_g1::TRINITY_DN15029_c4_g1_i3::g.45979::m.45979	2	15,9	17,662	10,669	PF05071.15	NDUFA12	89,8	1,50E-25	No_clan
TRINITY_DN15029_c5_g6::TRINITY_DN15029_c5_g6_i4::g.46001::m.46001	2	23,2	24,33	3,9525	PF03647.12	Tmemb_14	74,2	9,90E-21	No_clan
TRINITY_DN15030_c2_g10::TRINITY_DN15030_c2_g10_i10::g.46047::m.46047	2	3,3	95,085	2,9755	PF04841.12	Vps16_N	256,5	3,40E-76	No_clan
TRINITY_DN15036_c1_g1::TRINITY_DN15036_c1_g1_i9::g.46235::m.46235	4	24,1	16,157	271,82	PF00188.25	CAP	76,1	3,80E-21	CL0659
TRINITY_DN15045_c2_g1::TRINITY_DN15045_c2_g1_i7::g.46215::m.46215	6	18,2	51,578	20,701	PF03224.13	V-ATPase_H_N	264,9	8,90E-79	CL0020
TRINITY_DN15046_c3_g2::TRINITY_DN15046_c3_g2_i6::g.45963::m.45963	2	17,3	12,803	3,0278					
TRINITY_DN15060_c1_g1::TRINITY_DN15060_c1_g1_i6::g.46413::m.46413	2	3,6	68,662	3,3102	PF07714.16	Pkinase_Tyr	105,6	2,40E-30	CL0016
TRINITY_DN15066_c0_g1::TRINITY_DN15066_c0_g1_i4::g.46431::m.46431	4	9,5	54,131	2,7419	PF00485.17	PRK	167,3	3,10E-49	CL0023
TRINITY_DN15069_c0_g1::TRINITY_DN15069_c0_g1_i1::g.46535::m.46535	8	64,8	21,844	29,874	PF02115.16	Rho_GDI	267,2	7,20E-80	No_clan
TRINITY_DN15069_c1_g1::TRINITY_DN15069_c1_g1_i6::g.46552::m.46552	2	31	12,798	11,368	PF02115.16	Rho_GDI	53,7	2,00E-14	No_clan
TRINITY_DN15072_c0_g1::TRINITY_DN15072_c0_g1_i2::g.46447::m.46447	5	27,6	32,737	13,888	PF00400.31	WD40	16,9	0,0081	CL0186

TRINITY_DN15082_c1_g1::TRINITY_DN15082_c1_g1_i1::g.46708::m.46708	4	7,6	65,225	5,1006	PF07732.14	Cu-oxidase_3	124,8	1,60E-36	CL0026
TRINITY_DN15082_c2_g5::TRINITY_DN15082_c2_g5_i1::g.46709::m.46709	5	21,5	26,869	301,9	PF14543.5	TAXi_N	99,6	2,20E-28	CL0129
TRINITY_DN15085_c0_g2::TRINITY_DN15085_c0_g2_i3::g.46604::m.46604	5	33,6	14,867	68,524					
TRINITY_DN15089_c0_g1::TRINITY_DN15089_c0_g1_i2::g.46688::m.46688	12	32,3	59,234	29,507	PF00118.23	Cpn60_TCP1	559,3	6,20E-168	No_clan
TRINITY_DN15090_c0_g1::TRINITY_DN15090_c0_g1_i3::g.46611::m.46611	5	5,1	124,46	8,4181	PF13365.5	Trypsin_2	65,1	1,20E-17	CL0124
TRINITY_DN15100_c0_g1::TRINITY_DN15100_c0_g1_i1::g.45506::m.45506	2	9,3	42,632	4,3604					
TRINITY_DN15100_c3_g1::TRINITY_DN15100_c3_g1_i6::g.45535::m.45535	1	6,7	16,952	1,9843	PF01643.16	Acyl-ACP_TE	126,3	1,20E-36	CL0050
TRINITY_DN15101_c4_g1::TRINITY_DN15101_c4_g1_i4::g.45560::m.45560	4	10,3	55,603	2,7212	PF01399.26	PCI	69,4	3,00E-19	CL0123
TRINITY_DN15108_c2_g1::TRINITY_DN15108_c2_g1_i5::g.47064::m.47064	17	27,2	85,611	62,362	PF03255.13	ACCA	181,9	5,40E-54	CL0127
TRINITY_DN15110_c1_g1::TRINITY_DN15110_c1_g1_i4::g.47001::m.47001	1	3,7	38,372	2,6908	PF08240.11	ADH_N	91,4	2,80E-26	CL0296
TRINITY_DN15113_c1_g1::TRINITY_DN15113_c1_g1_i9::g.47525::m.47525	2	14,3	16,371	2,1775	PF02943.14	FeThRed_B	140,4	1,80E-41	No_clan
TRINITY_DN15115_c0_g4::TRINITY_DN15115_c0_g4_i3::g.47102::m.47102	2	8,5	35,663	2,7007	PF00561.19	Abhydrolase_1	91,9	4,50E-26	CL0028
TRINITY_DN15119_c0_g1::TRINITY_DN15119_c0_g1_i1::g.47105::m.47105	2	5,1	48,975	2,5652	PF01264.20	Chorismate_synt	465,7	5,00E-140	No_clan
TRINITY_DN15120_c1_g1::TRINITY_DN15120_c1_g1_i1::g.47172::m.47172	9	35,2	34,563	148,52	PF13460.5	NAD_binding_10	117,8	4,60E-34	CL0063
TRINITY_DN15121_c0_g1::TRINITY_DN15121_c0_g1_i1::g.47196::m.47196	2	2,5	81,473	3,1361	PF12755.6	Vac14_Fab1_bd	139,9	2,70E-41	CL0020
TRINITY_DN15127_c0_g2::TRINITY_DN15127_c0_g2_i1::g.47217::m.47217	6	7,9	103,79	10,025	PF01074.21	Glyco_hydro_38	189,6	7,00E-56	CL0158
TRINITY_DN15130_c6_g1::TRINITY_DN15130_c6_g1_i3::g.47299::m.47299	1	3,9	33,095	3,0688	PF03909.16	BSD	32,5	6,00E-08	No_clan
TRINITY_DN15130_c6_g3::TRINITY_DN15130_c6_g3_i3::g.47307::m.47307	8	28,7	38,559	17,946	PF00481.20	PP2C	222,3	7,50E-66	CL0238
TRINITY_DN15131_c1_g3::TRINITY_DN15131_c1_g3_i1::g.47487::m.47487	2	20,1	16,931	14,299					
TRINITY_DN15133_c0_g1::TRINITY_DN15133_c0_g1_i1::g.47252::m.47252	9	35,9	44,729	60,639	PF00561.19	Abhydrolase_1	98,1	6,00E-28	CL0028
TRINITY_DN15139_c1_g2::TRINITY_DN15139_c1_g2_i1::g.47376::m.47376	2	2,7	76,19	1,9533	PF00139.18	Lectin_legB	227,3	1,90E-67	CL0004

TRINITY_DN15141_c1_g1::TRINITY_DN15141_c1_g1_i2::g.47502::m.47502	3	9,5	29,615	3,1723	PF00227.25	Proteasome	159,2	7,60E-47	CL0052
TRINITY_DN15141_c2_g2::TRINITY_DN15141_c2_g2_i7::g.47512::m.47512	1	14,2	11,982	4,6447					
TRINITY_DN15142_c0_g1::TRINITY_DN15142_c0_g1_i16::g.47469::m.47469	2	20,1	22,898	9,0724	PF00933.20	Glyco_hydro_3	155,6	1,80E-45	CL0058
TRINITY_DN15145_c0_g1::TRINITY_DN15145_c0_g1_i10::g.46819::m.46819	1	14	13,412	2,956					
TRINITY_DN15145_c0_g2::TRINITY_DN15145_c0_g2_i2::g.46817::m.46817	3	9	53,217	6,8044	PF00561.19	Abhydrolase_1	38,7	7,90E-10	CL0028
TRINITY_DN15149_c3_g1::TRINITY_DN15149_c3_g1_i3::g.47576::m.47576	7	19,5	53,884	23,324	PF00005.26	ABC_tran	43,8	3,10E-11	CL0023
TRINITY_DN15165_c0_g1::TRINITY_DN15165_c0_g1_i4::g.47616::m.47616	7	50,2	20,522	27,826	PF04927.11	SMP	80,7	7,50E-23	No_clan
TRINITY_DN15166_c0_g1::TRINITY_DN15166_c0_g1_i7::g.47733::m.47733	4	18,9	28,694	6,7217	PF05648.13	PEX11	144,4	3,20E-42	No_clan
TRINITY_DN15169_c1_g1::TRINITY_DN15169_c1_g1_i14::g.47860::m.47860	2	16	25,613	4,262	PF01593.23	Amino_oxidase	119,6	2,00E-34	CL0063
TRINITY_DN15177_c0_g1::TRINITY_DN15177_c0_g1_i2::g.47833::m.47833	8	23,4	42,188	16,067	PF00202.20	Aminotran_3	336,8	1,30E-100	CL0061
TRINITY_DN15178_c0_g1::TRINITY_DN15178_c0_g1_i16::g.47873::m.47873	3	9	36,619	4,6681	PF16486.4	ArgoN	111,1	5,90E-32	No_clan
TRINITY_DN15180_c0_g2::TRINITY_DN15180_c0_g2_i2::g.47796::m.47796	5	22,1	40,325	19,605					
TRINITY_DN15183_c1_g3::TRINITY_DN15183_c1_g3_i4::g.48285::m.48285	2	7,3	32,295	24,801	PF04601.12	DUF569	244,6	2,00E-73	CL0066
TRINITY_DN15187_c1_g1::TRINITY_DN15187_c1_g1_i9::g.47932::m.47932	2	24,2	13,247	21,22	PF00076.21	RRM_1	64,4	5,90E-18	CL0221
TRINITY_DN15194_c1_g2::TRINITY_DN15194_c1_g2_i1::g.48344::m.48344	8	60,3	12,899	99,903	PF00085.19	Thioredoxin	103,4	4,90E-30	CL0172
TRINITY_DN15198_c1_g2::TRINITY_DN15198_c1_g2_i7::g.48337::m.48337	6	22,3	38,873	32,61	PF11891.7	RETICULATA-like	218,1	6,50E-65	No_clan
TRINITY_DN15198_c1_g3::TRINITY_DN15198_c1_g3_i1::g.48326::m.48326	1	4,6	52,625	4,8531	PF12265.7	CAF1C_H4-bd	65,7	3,00E-18	No_clan
TRINITY_DN15203_c1_g1::TRINITY_DN15203_c1_g1_i19::g.48562::m.48562	1	8,2	16,891	2,0854	PF03398.13	Ist1	103,6	9,10E-30	No_clan
TRINITY_DN15203_c1_g1::TRINITY_DN15203_c1_g1_i8::g.48552::m.48552	6	16,6	48,988	14,657	PF16363.4	GDP_Man_Dehyd	204,5	2,50E-60	CL0063
TRINITY_DN15203_c1_g4::TRINITY_DN15203_c1_g4_i2::g.48560::m.48560	3	9,3	48,3	5,1111	PF16363.4	GDP_Man_Dehyd	210,4	4,10E-62	CL0063
TRINITY_DN15205_c1_g1::TRINITY_DN15205_c1_g1_i3::g.48499::m.48499	2	17,6	19,691	8,8414	PF00112.22	Peptidase_C1	182,8	8,90E-54	CL0125

TRINITY_DN15208_c2_g1::TRINITY_DN15208_c2_g1_i1::g.48542::m.48542	6	14,8	59,094	10,872	PF00224.20	PK	392,3	1,50E-117	CL0151
TRINITY_DN15211_c1_g1::TRINITY_DN15211_c1_g1_i7::g.48526::m.48526	6	35,5	27,53	128,37	PF01048.19	PNP_UDP_1	83,7	1,00E-23	CL0408
TRINITY_DN15217_c1_g2::TRINITY_DN15217_c1_g2_i9::g.48873::m.48873	2	7,5	33,912	2,8159	PF03141.15	Methyltransf_29	187,1	4,40E-55	CL0063
TRINITY_DN15221_c1_g3::TRINITY_DN15221_c1_g3_i1::g.48708::m.48708	5	11,3	66,771	7,6993	PF16499.4	Melibiase_2	46,1	3,30E-12	CL0058
TRINITY_DN15222_c4_g2::TRINITY_DN15222_c4_g2_i2::g.48684::m.48684	4	16,2	38,01	88,656	PF04756.12	OST3_OST6	263,4	2,30E-78	CL0172
TRINITY_DN15232_c2_g1::TRINITY_DN15232_c2_g1_i5::g.49050::m.49050	4	29,3	16,06	10,674	PF08069.11	Ribosomal_S13_N	108,3	1,30E-31	No_clan
TRINITY_DN15236_c1_g2::TRINITY_DN15236_c1_g2_i4::g.49063::m.49063	7	27,9	45,956	24,121	PF00400.31	WD40	17,7	0,0046	CL0186
TRINITY_DN15250_c0_g1::TRINITY_DN15250_c0_g1_i1::g.49205::m.49205	4	22,3	34,027	10,01	PF00795.21	CN_hydrolase	134,8	3,00E-39	No_clan
TRINITY_DN15257_c0_g2::TRINITY_DN15257_c0_g2_i2::g.49306::m.49306	1	4,4	30,694	1,9017	PF00583.24	Acetyltransf_1	56,7	2,30E-15	CL0257
TRINITY_DN15261_c0_g1::TRINITY_DN15261_c0_g1_i6::g.49258::m.49258	12	34,5	47,943	130,96	PF00085.19	Thioredoxin	101	2,80E-29	CL0172
TRINITY_DN15263_c1_g2::TRINITY_DN15263_c1_g2_i4::g.49263::m.49263	3	8,1	50,046	4,2685	PF00201.17	UDPGT	95,7	2,60E-27	CL0113
TRINITY_DN15264_c2_g1::TRINITY_DN15264_c2_g1_i9::g.49352::m.49352	6	37,1	14,93	36,583					
TRINITY_DN15264_c3_g1::TRINITY_DN15264_c3_g1_i8::g.49356::m.49356	3	33,6	15,82	5,288	PF13649.5	Methyltransf_25	55,8	5,40E-15	CL0063
TRINITY_DN15264_c3_g2::TRINITY_DN15264_c3_g2_i3::g.49355::m.49355	3	22,5	17,184	3,7738	PF06803.11	DUF1232	34,1	1,60E-08	No_clan
TRINITY_DN15274_c1_g2::TRINITY_DN15274_c1_g2_i1::g.49397::m.49397	3	44,6	12,563	5,7416					
TRINITY_DN15276_c1_g1::TRINITY_DN15276_c1_g1_i6::g.49461::m.49461	8	37,8	33,457	84,911	PF00400.31	WD40	21,5	0,00029	CL0186
TRINITY_DN15290_c1_g1::TRINITY_DN15290_c1_g1_i13::g.49834::m.49834	27	62	61,462	323,31	PF02878.15	PGM_PMM_I	107,1	5,10E-31	No_clan
TRINITY_DN15292_c0_g3::TRINITY_DN15292_c0_g3_i4::g.49797::m.49797	1	3,8	34,233	2,8778					
TRINITY_DN15292_c1_g3::TRINITY_DN15292_c1_g3_i1::g.49799::m.49799	2	12,1	27,491	4,3158	PF00168.29	C2	47,7	1,40E-12	CL0154
TRINITY_DN15294_c5_g1::TRINITY_DN15294_c5_g1_i4::g.49817::m.49817	7	17,6	56,651	15,504	PF00282.18	Pyridoxal_deC	364,2	5,40E-109	CL0061
TRINITY_DN15294_c5_g3::TRINITY_DN15294_c5_g3_i1::g.49818::m.49818	6	16,3	56,702	31,399	PF00282.18	Pyridoxal_deC	350,5	8,30E-105	CL0061

TRINITY_DN15297_c0_g1::TRINITY_DN15297_c0_g1_i10::g.49743::m.49743	6	18,3	36,34	4,437	PF00316.19	FBPase	257	7,60E-77	CL0171
TRINITY_DN15298_c0_g1::TRINITY_DN15298_c0_g1_i1::g.49745::m.49745	3	25,3	16,623	6,1867	PF05529.11	Bap31	45,8	4,90E-12	No_clan
TRINITY_DN15301_c2_g1::TRINITY_DN15301_c2_g1_i12::g.49765::m.49765	1	8,2	19,25	2,0804	PF00025.20	Arf	151,6	1,30E-44	CL0023
TRINITY_DN15302_c0_g1::TRINITY_DN15302_c0_g1_i3::g.49970::m.49970	2	9,4	32,779	3,0159					
TRINITY_DN15303_c0_g1::TRINITY_DN15303_c0_g1_i2::g.49908::m.49908	6	25,6	37,001	29,821	PF02502.17	LacAB_rpiB	100,1	9,50E-29	No_clan
TRINITY_DN15312_c1_g1::TRINITY_DN15312_c1_g1_i7::g.50104::m.50104	1	8,6	20,136	2,9709					
TRINITY_DN15315_c0_g1::TRINITY_DN15315_c0_g1_i1::g.50016::m.50016	14	38,2	47,593	24,458	PF00118.23	Cpn60_TCP1	196,5	6,30E-58	No_clan
TRINITY_DN15315_c0_g3::TRINITY_DN15315_c0_g3_i4::g.50023::m.50023	14	30	57,512	57,985	PF00155.20	Aminotran_1_2	132,5	1,90E-38	CL0061
TRINITY_DN15316_c4_g2::TRINITY_DN15316_c4_g2_i4::g.50087::m.50087	1	7,2	17,06	2,0874	PF00717.22	Peptidase_S24	43,4	2,30E-11	CL0299
TRINITY_DN15317_c1_g1::TRINITY_DN15317_c1_g1_i5::g.50064::m.50064	6	37,7	17,362	78,949	PF01287.19	eIF-5a	101,4	2,10E-29	CL0021
TRINITY_DN15323_c1_g4::TRINITY_DN15323_c1_g4_i7::g.50170::m.50170	2	16,5	20,792	5,5529	PF06232.10	ATS3	180,1	1,30E-53	CL0321
TRINITY_DN15328_c3_g2::TRINITY_DN15328_c3_g2_i4::g.50205::m.50205	4	12,4	46,654	6,5576	PF00687.20	Ribosomal_L1	119,1	1,90E-34	No_clan
TRINITY_DN15338_c2_g1::TRINITY_DN15338_c2_g1_i4::g.50559::m.50559	5	39,5	17,119	4,3482	PF00179.25	UQ_con	165,8	3,90E-49	CL0208
TRINITY_DN15338_c3_g1::TRINITY_DN15338_c3_g1_i1::g.50560::m.50560	4	15,4	25,487	5,5644	PF00334.18	NDK	180,8	1,10E-53	No_clan
TRINITY_DN15339_c4_g4::TRINITY_DN15339_c4_g4_i1::g.49925::m.49925	1	11,6	13,087	2,8744	PF03029.16	ATP_bind_1	58,6	7,30E-16	CL0023
TRINITY_DN15339_c5_g2::TRINITY_DN15339_c5_g2_i1::g.49931::m.49931	5	10	83,719	12,808	PF00564.23	PB1	66,2	1,70E-18	CL0072
TRINITY_DN15345_c2_g1::TRINITY_DN15345_c2_g1_i10::g.49844::m.49844	2	9,5	56,555	2,751	PF05691.11	Raffinose_syn	714,7	9,40E-215	CL0058
TRINITY_DN15347_c2_g1::TRINITY_DN15347_c2_g1_i4::g.50637::m.50637	3	16,1	23,755	10,592					
TRINITY_DN15348_c2_g2::TRINITY_DN15348_c2_g2_i4::g.50676::m.50676	13	46,7	38,576	323,31	PF03214.12	RGP	626,7	9,10E-189	CL0110
TRINITY_DN15352_c3_g1::TRINITY_DN15352_c3_g1_i9::g.50776::m.50776	11	23,5	71,8	181,75	PF00076.21	RRM_1	61,8	3,80E-17	CL0221
TRINITY_DN15353_c0_g3::TRINITY_DN15353_c0_g3_i2::g.50015::m.50015	13	20,4	86,367	85,069	PF02854.18	MIF4G	185,9	6,70E-55	CL0020

TRINITY_DN15354_c0_g1::TRINITY_DN15354_c0_g1_i1::g.50622::m.50622	5	7,8	89,622	10,84	PF00547.17	Urease_gamma	139,2	4,00E-41	No_clan
TRINITY_DN15356_c1_g1::TRINITY_DN15356_c1_g1_i4::g.50914::m.50914	2	16,9	23,433	2,4596	PF12678.6	zf-rbx1	35,9	6,50E-09	CL0229
TRINITY_DN15357_c0_g1::TRINITY_DN15357_c0_g1_i2::g.50651::m.50651	3	18,9	19,152	6,1818	PF02823.15	ATP-synt_DE_N	58,6	4,10E-16	No_clan
TRINITY_DN15365_c0_g2::TRINITY_DN15365_c0_g2_i4::g.50831::m.50831	2	15,6	23,282	8,0996	PF00483.22	NTP_transferase	41	1,50E-10	CL0110
TRINITY_DN15366_c1_g1::TRINITY_DN15366_c1_g1_i4::g.50810::m.50810	1	3,2	61,97	2,5638	PF06814.12	Lung_7-TM_R	333,8	7,80E-100	CL0192
TRINITY_DN15367_c2_g1::TRINITY_DN15367_c2_g1_i7::g.50786::m.50786	1	8,9	13,37	2,4589	PF02221.14	E1_DerP2_DerF2	52	8,90E-14	CL0532
TRINITY_DN15371_c0_g2::TRINITY_DN15371_c0_g2_i1::g.50837::m.50837	2	8,2	38,872	3,9965					
TRINITY_DN15372_c1_g1::TRINITY_DN15372_c1_g1_i7::g.50862::m.50862	1	12,4	17,167	2,1362					
TRINITY_DN16209_c0_g5::TRINITY_DN16209_c0_g5_i1::g.64031::m.64031	4	24,6	14,79	6,6802	PF00410.18	Ribosomal_S8	78	5,30E-22	No_clan
TRINITY_DN15384_c0_g1::TRINITY_DN15384_c0_g1_i3::g.51070::m.51070	5	16,6	41,231	9,961	PF01926.22	MMR_HSR1	74,7	5,60E-21	CL0023
TRINITY_DN15390_c3_g1::TRINITY_DN15390_c3_g1_i2::g.51061::m.51061	1	7	21,856	2,5444	PF00230.19	MIP	30,8	2,10E-07	No_clan
TRINITY_DN15394_c0_g1::TRINITY_DN15394_c0_g1_i2::g.49883::m.49883	30	46,7	95,917	310,58	PF00343.19	Phosphorylase	1006,4	6,20E-303	CL0113
TRINITY_DN15394_c0_g2::TRINITY_DN15394_c0_g2_i6::g.49878::m.49878	13	15	100,34	85,17	PF00012.19	HSP70	330,4	1,50E-98	CL0108
TRINITY_DN15397_c1_g1::TRINITY_DN15397_c1_g1_i5::g.51177::m.51177	6	10,9	82,514	20,598	PF02990.15	EMP70	748	4,80E-225	No_clan
TRINITY_DN15400_c1_g2::TRINITY_DN15400_c1_g2_i8::g.50620::m.50620	3	26,5	18,992	3,5419	PF04398.11	DUF538	95	3,30E-27	No_clan
TRINITY_DN15404_c3_g2::TRINITY_DN15404_c3_g2_i3::g.51327::m.51327	2	9,9	35,861	1,9017	PF09296.10	NUDIX-like	37,1	3,50E-09	CL0261
TRINITY_DN15405_c3_g1::TRINITY_DN15405_c3_g1_i6::g.51555::m.51555	1	9,1	16,576	2,2977	PF02212.17	GED	84,5	4,00E-24	No_clan
TRINITY_DN15405_c3_g1::TRINITY_DN15405_c3_g1_i4::g.51552::m.51552	1	16,4	13,811	3,1509	PF01031.19	Dynamin_M	42,8	3,30E-11	No_clan
TRINITY_DN15411_c1_g1::TRINITY_DN15411_c1_g1_i1::g.51593::m.51593	2	5,3	41,572	2,9112	PF00400.31	WD40	23,3	7,90E-05	CL0186
TRINITY_DN15411_c1_g2::TRINITY_DN15411_c1_g2_i10::g.51619::m.51619	2	21,3	16,521	2,8944	PF00076.21	RRM_1	41,4	8,80E-11	CL0221
TRINITY_DN15416_c2_g2::TRINITY_DN15416_c2_g2_i3::g.51536::m.51536	6	35,8	22,555	10,78	PF00227.25	Proteasome	135,5	1,40E-39	CL0052

TRINITY_DN15422_c1_g1::TRINITY_DN15422_c1_g1_i1::g.51639::m.51639	1	4,1	32,444	2,5078	PF05147.12	LANC_like	274,1	1,00E-81	CL0059
TRINITY_DN15424_c2_g3::TRINITY_DN15424_c2_g3_i3::g.51667::m.51667	6	15,6	57,212	29,72	PF00190.21	Cupin_1	132	1,20E-38	CL0029
TRINITY_DN15425_c0_g1::TRINITY_DN15425_c0_g1_i1::g.51653::m.51653	5	17,9	41,147	66,265	PF00724.19	Oxidored_FMN	302,1	4,70E-90	CL0036
TRINITY_DN15426_c0_g2::TRINITY_DN15426_c0_g2_i4::g.51819::m.51819	11	43,8	20,021	282,82	PF02970.15	TBCA	95,4	1,80E-27	No_clan
TRINITY_DN15427_c0_g1::TRINITY_DN15427_c0_g1_i2::g.51743::m.51743	5	16,2	57,356	7,923					
TRINITY_DN15428_c6_g1::TRINITY_DN15428_c6_g1_i5::g.51800::m.51800	2	18,9	14,041	3,542					
TRINITY_DN15433_c2_g1::TRINITY_DN15433_c2_g1_i2::g.51849::m.51849	2	11,2	30,843	3,5124					
TRINITY_DN15434_c1_g2::TRINITY_DN15434_c1_g2_i2::g.51814::m.51814	6	30,2	23,903	7,9124	PF00071.21	Ras	206,5	1,70E-61	CL0023
TRINITY_DN15434_c1_g3::TRINITY_DN15434_c1_g3_i3::g.51817::m.51817	5	27,2	24,997	4,8206	PF00071.21	Ras	205,6	3,10E-61	CL0023
TRINITY_DN15436_c5_g1::TRINITY_DN15436_c5_g1_i4::g.51870::m.51870	5	10,8	57,864	8,4097					
TRINITY_DN15438_c0_g1::TRINITY_DN15438_c0_g1_i13::g.52894::m.52894	11	30,8	53,813	22,552	PF03446.14	NAD_binding_2	158,5	1,40E-46	CL0063
TRINITY_DN15439_c1_g1::TRINITY_DN15439_c1_g1_i4::g.51919::m.51919	9	25,7	52,099	38,591	PF01394.19	Clathrin_propel	27,9	1,70E-06	CL0020
TRINITY_DN15441_c0_g1::TRINITY_DN15441_c0_g1_i3::g.51487::m.51487	5	47,3	12,285	5,0592	PF00034.20	Cytochrom_C	48,1	1,80E-12	CL0318
TRINITY_DN15441_c0_g2::TRINITY_DN15441_c0_g2_i3::g.51488::m.51488	5	43,1	13,614	22,399	PF00034.20	Cytochrom_C	48,3	1,60E-12	CL0318
TRINITY_DN15442_c0_g2::TRINITY_DN15442_c0_g2_i1::g.51251::m.51251	2	20	13,879	2,7616	PF02036.16	SCP2	71,6	5,70E-20	CL0311
TRINITY_DN15444_c2_g1::TRINITY_DN15444_c2_g1_i7::g.52052::m.52052	4	23,4	22,988	8,854					
TRINITY_DN15444_c2_g1::TRINITY_DN15444_c2_g1_i4::g.52046::m.52046	1	10,2	14,811	2,1437					
TRINITY_DN15444_c2_g1::TRINITY_DN15444_c2_g1_i2::g.52034::m.52034	5	25,8	26,603	11,249					
TRINITY_DN15444_c2_g2::TRINITY_DN15444_c2_g2_i4::g.52039::m.52039	4	12,6	36,386	6,0997	PF00076.21	RRM_1	49,8	2,10E-13	CL0221
TRINITY_DN15448_c0_g1::TRINITY_DN15448_c0_g1_i6::g.51979::m.51979	2	18,3	32,091	12,311	PF01960.17	ArgJ	377,9	3,80E-113	CL0635
TRINITY_DN15451_c0_g1::TRINITY_DN15451_c0_g1_i8::g.52025::m.52025	2	6,8	47,129	27,699	PF00899.20	ThiF	170,6	3,30E-50	CL0063

TRINITY_DN15452_c6_g1::TRINITY_DN15452_c6_g1_i5::g.52129::m.52129	17	43,2	47,57	323,31	PF00923.18	TAL_FSA	257,5	1,40E-76	CL0036
TRINITY_DN15454_c2_g1::TRINITY_DN15454_c2_g1_i4::g.52157::m.52157	1	2,7	49,094	1,8582	PF00459.24	Inositol_P	184,1	3,30E-54	CL0171
TRINITY_DN15456_c0_g1::TRINITY_DN15456_c0_g1_i1::g.52131::m.52131	1	3,7	32,737	9,0834	PF02770.18	Acyl-CoA_dh_M	81,1	4,60E-23	No_clan
TRINITY_DN15457_c3_g1::TRINITY_DN15457_c3_g1_i1::g.52194::m.52194	2	33,3	10,826	5,0198					
TRINITY_DN15457_c4_g1::TRINITY_DN15457_c4_g1_i5::g.52200::m.52200	5	31,8	19,066	7,1556	PF00006.24	ATP-synt_ab	36,7	3,00E-09	CL0023
TRINITY_DN15458_c1_g2::TRINITY_DN15458_c1_g2_i4::g.52100::m.52100	2	17,8	15,012	3,9115					
TRINITY_DN15458_c1_g3::TRINITY_DN15458_c1_g3_i6::g.52102::m.52102	1	9,3	16,167	3,1003	PF00574.22	CLP_protease	215,9	3,70E-64	CL0127
TRINITY_DN15460_c0_g1::TRINITY_DN15460_c0_g1_i9::g.52319::m.52319	2	12,9	31,617	8,0209	PF11566.7	PI31_Prot_N	70,6	1,20E-19	No_clan
TRINITY_DN15462_c1_g3::TRINITY_DN15462_c1_g3_i2::g.52234::m.52234	1	7	20,654	2,4918					
TRINITY_DN15464_c0_g1::TRINITY_DN15464_c0_g1_i3::g.52265::m.52265	13	52,9	30,484	7,9066	PF00012.19	HSP70	444,7	4,00E-133	CL0108
TRINITY_DN15464_c0_g2::TRINITY_DN15464_c0_g2_i1::g.52259::m.52259	13	43,4	39,227	264,69	PF00012.19	HSP70	400,9	6,80E-120	CL0108
TRINITY_DN15464_c0_g4::TRINITY_DN15464_c0_g4_i1::g.52272::m.52272	8	37,9	26,595	5,7052	PF00012.19	HSP70	223	5,10E-66	CL0108
TRINITY_DN15464_c0_g5::TRINITY_DN15464_c0_g5_i1::g.52274::m.52274	7	46,8	19,538	3,1946	PF00012.19	HSP70	234,4	1,80E-69	CL0108
TRINITY_DN15467_c6_g1::TRINITY_DN15467_c6_g1_i1::g.52486::m.52486	17	57,8	36,022	323,31	PF16884.4	ADH_N_2	81,4	3,70E-23	CL0296
TRINITY_DN15473_c0_g1::TRINITY_DN15473_c0_g1_i4::g.52564::m.52564	11	33,4	45,644	36,502	PF00903.24	Glyoxalase	77,7	8,20E-22	CL0104
TRINITY_DN15473_c1_g1::TRINITY_DN15473_c1_g1_i2::g.52569::m.52569	4	11,1	47,948	16,724	PF01979.19	Amidohydro_1	184,3	3,50E-54	CL0034
TRINITY_DN15475_c0_g1::TRINITY_DN15475_c0_g1_i2::g.52448::m.52448	5	17,1	44,864	11,15	PF02485.20	Branch	277,4	9,40E-83	CL0110
TRINITY_DN15478_c0_g1::TRINITY_DN15478_c0_g1_i13::g.52472::m.52472	1	9,1	19,664	2,1752	PF13920.5	zf-C3HC4_3	50,1	1,70E-13	CL0229
TRINITY_DN15478_c1_g1::TRINITY_DN15478_c1_g1_i1::g.52474::m.52474	2	5,9	43,642	3,9852	PF16113.4	ECH_2	370,6	8,40E-111	CL0127
TRINITY_DN15482_c4_g1::TRINITY_DN15482_c4_g1_i5::g.52502::m.52502	3	22	24,409	15,749	PF03896.15	TRAP_alpha	93	1,70E-26	No_clan
TRINITY_DN15485_c1_g1::TRINITY_DN15485_c1_g1_i8::g.52679::m.52679	2	9,8	24,22	2,8208	PF06273.10	eIF-4B	111,7	4,50E-32	No_clan

TRINITY_DN15486_c0_g3::TRINITY_DN15486_c0_g3_i1::g.52746::m.52746	1	9,2	18,946	2,6047	PF00201.17	UDPGT	24,8	8,00E-06	CL0113
TRINITY_DN15488_c1_g5::TRINITY_DN15488_c1_g5_i3::g.52738::m.52738	6	25,6	40,579	20,887	PF14226.5	DIOX_N	78,2	7,70E-22	CL0029
TRINITY_DN15498_c4_g2::TRINITY_DN15498_c4_g2_i2::g.51311::m.51311	1	10	29,147	13,592	PF04893.16	Yip1	52,3	4,90E-14	CL0112
TRINITY_DN15505_c1_g1::TRINITY_DN15505_c1_g1_i3::g.53026::m.53026	1	10,4	11,418	2,1432					
TRINITY_DN15507_c1_g1::TRINITY_DN15507_c1_g1_i1::g.53015::m.53015	3	17,9	25,931	29,486	PF16845.4	SQAPI	74	8,50E-21	CL0121
TRINITY_DN15510_c3_g3::TRINITY_DN15510_c3_g3_i2::g.53102::m.53102	2	5,1	53,952	4,1779	PF01496.18	V_ATPase_I	451,8	3,60E-135	No_clan
TRINITY_DN15513_c2_g1::TRINITY_DN15513_c2_g1_i6::g.53086::m.53086	1	11,3	14,416	2,8231					
TRINITY_DN15513_c2_g2::TRINITY_DN15513_c2_g2_i3::g.53087::m.53087	2	5,6	42,553	3,5278	PF12697.6	Abhydrolase_6	49,5	7,50E-13	CL0028
TRINITY_DN15514_c4_g2::TRINITY_DN15514_c4_g2_i2::g.53063::m.53063	5	9,6	66,49	7,9857					
TRINITY_DN15517_c0_g1::TRINITY_DN15517_c0_g1_i10::g.53139::m.53139	5	8	101,02	9,3543	PF04869.13	Uso1_p115_head	94,6	5,80E-27	No_clan
TRINITY_DN15518_c0_g1::TRINITY_DN15518_c0_g1_i2::g.53176::m.53176	2	1,8	165,21	3,6749	PF12783.6	Sec7_N	120,1	7,20E-35	No_clan
TRINITY_DN15518_c0_g2::TRINITY_DN15518_c0_g2_i2::g.53174::m.53174	2	1,6	164,25	3,1213	PF12783.6	Sec7_N	118,1	3,00E-34	No_clan
TRINITY_DN15521_c0_g1::TRINITY_DN15521_c0_g1_i5::g.53143::m.53143	12	28,5	57,391	106,51	PF00224.20	PK	308,6	4,30E-92	CL0151
TRINITY_DN15525_c0_g1::TRINITY_DN15525_c0_g1_i8::g.53299::m.53299	14	24,5	79,498	171,78	PF00082.21	Peptidase_S8	139,7	1,10E-40	No_clan
TRINITY_DN15527_c0_g1::TRINITY_DN15527_c0_g1_i4::g.53287::m.53287	1	4,4	39,857	2,2107	PF00240.22	ubiquitin	55,2	4,00E-15	CL0072
TRINITY_DN15531_c1_g1::TRINITY_DN15531_c1_g1_i14::g.53405::m.53405	6	55,2	14,406	294,14	PF00293.27	NUDIX	47,5	1,60E-12	CL0261
TRINITY_DN15531_c1_g1::TRINITY_DN15531_c1_g1_i8::g.53401::m.53401	4	37,8	13,11	4,726	PF00293.27	NUDIX	36	5,60E-09	CL0261
TRINITY_DN15542_c3_g2::TRINITY_DN15542_c3_g2_i4::g.53532::m.53532	1	17,8	14,647	5,7025	PF00892.19	EamA	28,9	9,70E-07	CL0184
TRINITY_DN15546_c0_g1::TRINITY_DN15546_c0_g1_i6::g.53570::m.53570	1	3,3	40,295	2,7846	PF02171.16	Piwi	343,7	8,20E-103	CL0219
TRINITY_DN15549_c2_g2::TRINITY_DN15549_c2_g2_i1::g.53640::m.53640	8	27,8	43,584	23,375	PF00076.21	RRM_1	61,4	5,10E-17	CL0221
TRINITY_DN15551_c3_g3::TRINITY_DN15551_c3_g3_i8::g.53664::m.53664	2	14,2	25,375	5,2727					

TRINITY_DN15556_c2_g3::TRINITY_DN15556_c2_g3_i7::g.53767::m.53767	11	35,3	46,628	74,99	PF01464.19	SLT	40,7	1,40E-10	CL0037
TRINITY_DN15558_c1_g3::TRINITY_DN15558_c1_g3_i8::g.53729::m.53729	6	15,5	41,078	8,5313	PF00153.26	Mito_carr	83,6	6,20E-24	No_clan
TRINITY_DN15558_c1_g3::TRINITY_DN15558_c1_g3_i7::g.53727::m.53727	6	15,4	41,087	-2	PF00153.26	Mito_carr	83,6	6,20E-24	No_clan
TRINITY_DN15571_c4_g3::TRINITY_DN15571_c4_g3_i4::g.54049::m.54049	3	9,5	45,464	5,8993	PF00483.22	NTP_transferase	94,2	8,50E-27	CL0110
TRINITY_DN15578_c0_g1::TRINITY_DN15578_c0_g1_i2::g.54153::m.54153	6	39,2	17,652	140,38	PF03737.14	RraA-like	147,3	3,30E-43	CL0364
TRINITY_DN15579_c1_g3::TRINITY_DN15579_c1_g3_i1::g.54029::m.54029	2	5,7	50,283	2,8059	PF00270.28	DEAD	150,5	3,80E-44	CL0023
TRINITY_DN15579_c2_g1::TRINITY_DN15579_c2_g1_i2::g.54035::m.54035	2	20,7	13,509	2,5878	PF00295.16	Glyco_hydro_28	70,7	1,00E-19	CL0268
TRINITY_DN15581_c1_g3::TRINITY_DN15581_c1_g3_i1::g.54122::m.54122	14	48,9	41,989	229,66	PF00266.18	Aminotran_5	176,3	7,90E-52	CL0061
TRINITY_DN15584_c3_g1::TRINITY_DN15584_c3_g1_i1::g.54058::m.54058	4	33,6	15,278	63,254					
TRINITY_DN15585_c1_g1::TRINITY_DN15585_c1_g1_i8::g.54308::m.54308	1	8,7	13,741	4,6622	PF13472.5	Lipase_GDSL_2	44,6	1,90E-11	CL0264
TRINITY_DN15585_c1_g1::TRINITY_DN15585_c1_g1_i4::g.54306::m.54306	6	35,2	21,512	244,86	PF00657.21	Lipase_GDSL	64,5	1,20E-17	CL0264
TRINITY_DN15589_c0_g1::TRINITY_DN15589_c0_g1_i4::g.54099::m.54099	25	56,1	59,543	323,31	PF00883.20	Peptidase_M17	382,7	1,20E-114	CL0035
TRINITY_DN15591_c0_g2::TRINITY_DN15591_c0_g2_i2::g.54135::m.54135	6	23,1	44,932	33,499					
TRINITY_DN15594_c4_g1::TRINITY_DN15594_c4_g1_i1::g.54180::m.54180	2	7,8	46,52	5,0376	PF00953.20	Glycos_transf_4	125,8	1,40E-36	No_clan
TRINITY_DN15598_c1_g1::TRINITY_DN15598_c1_g1_i2::g.54269::m.54269	2	4,8	62,789	3,227	PF13424.5	TPR_12	34,5	1,80E-08	CL0020
TRINITY_DN15598_c2_g1::TRINITY_DN15598_c2_g1_i7::g.54284::m.54284	6	18,8	41,838	40,161	PF13519.5	VWA_2	96,7	1,10E-27	CL0128
TRINITY_DN15599_c0_g1::TRINITY_DN15599_c0_g1_i1::g.54231::m.54231	2	24,2	12,161	5,0343	PF00234.21	Tryp_alpha_amyl	32,9	6,30E-08	CL0482
TRINITY_DN15601_c2_g1::TRINITY_DN15601_c2_g1_i1::g.52933::m.52933	3	22,6	12,437	5,4699					
TRINITY_DN15603_c1_g3::TRINITY_DN15603_c1_g3_i1::g.54361::m.54361	2	18,5	14,818	3,4378	PF01920.19	Prefoldin_2	79,7	1,30E-22	CL0200
TRINITY_DN15610_c2_g1::TRINITY_DN15610_c2_g1_i5::g.54395::m.54395	6	32,6	30,381	52,395	PF13460.5	NAD_binding_10	77,8	8,50E-22	CL0063
TRINITY_DN15614_c2_g1::TRINITY_DN15614_c2_g1_i7::g.54755::m.54755	8	66,9	16,755	21,816	PF00106.24	adh_short	28	1,20E-06	CL0063

TRINITY_DN15614_c2_g1::TRINITY_DN15614_c2_g1_i2::g.54750::m.54750	6	30,6	20,878	-2	PF00106.24	adh_short	97	8,50E-28	CL0063
TRINITY_DN15617_c2_g1::TRINITY_DN15617_c2_g1_i3::g.54449::m.54449	1	14,9	13,238	3,0861	PF00639.20	Rotamase	84,3	8,20E-24	CL0487
TRINITY_DN15619_c2_g2::TRINITY_DN15619_c2_g2_i4::g.54476::m.54476	3	16	20,646	4,6161	PF07851.12	TMPIT	72,3	3,70E-20	No_clan
TRINITY_DN15621_c1_g1::TRINITY_DN15621_c1_g1_i6::g.54532::m.54532	8	22	64,451	31,52	PF12999.6	PRKCSH-like	126,4	9,60E-37	No_clan
TRINITY_DN15624_c0_g1::TRINITY_DN15624_c0_g1_i1::g.54548::m.54548	4	6,8	93,01	9,3887	PF01636.22	APH	156,9	7,60E-46	CL0016
TRINITY_DN15625_c0_g3::TRINITY_DN15625_c0_g3_i1::g.54608::m.54608	4	23,3	24,983	8,3345	PF13774.5	Longin	85,8	1,40E-24	No_clan
TRINITY_DN15627_c0_g1::TRINITY_DN15627_c0_g1_i7::g.54594::m.54594	1	4,7	30,22	3,6928	PF01370.20	Epimerase	95,5	3,10E-27	CL0063
TRINITY_DN15627_c0_g2::TRINITY_DN15627_c0_g2_i1::g.54590::m.54590	2	6,2	49,048	4,7568	PF03407.15	Nucleotid_trans	188,6	1,20E-55	CL0110
TRINITY_DN15634_c1_g2::TRINITY_DN15634_c1_g2_i1::g.54735::m.54735	2	3,4	97,127	2,8951					
TRINITY_DN15635_c1_g1::TRINITY_DN15635_c1_g1_i2::g.54767::m.54767	3	5,8	57,375	6,7488	PF00083.23	Sugar_tr	379,4	2,20E-113	CL0015
TRINITY_DN15638_c0_g2::TRINITY_DN15638_c0_g2_i2::g.54677::m.54677	1	12,2	27,521	4,6723	PF00076.21	RRM_1	41,3	9,20E-11	CL0221
TRINITY_DN15641_c2_g1::TRINITY_DN15641_c2_g1_i3::g.54993::m.54993	6	22,8	34,093	15,518	PF04073.14	tRNA_edit	82,5	2,30E-23	No_clan
TRINITY_DN15642_c0_g3::TRINITY_DN15642_c0_g3_i1::g.55086::m.55086	1	9,8	15,521	2,9448	PF09180.10	ProRS-C_1	73,2	1,40E-20	No_clan
TRINITY_DN15656_c0_g5::TRINITY_DN15656_c0_g5_i2::g.55149::m.55149	3	11,1	43,966	6,6774	PF00091.24	Tubulin	140,1	9,00E-41	CL0566
TRINITY_DN15658_c0_g1::TRINITY_DN15658_c0_g1_i3::g.55055::m.55055	3	3,5	128,41	6,7628	PF05833.10	FbpA	60,8	9,40E-17	CL0303
TRINITY_DN15660_c2_g1::TRINITY_DN15660_c2_g1_i4::g.55101::m.55101	1	5,1	32,387	4,3478	PF01694.21	Rhomboid	25,2	1,20E-05	CL0207
TRINITY_DN15671_c0_g2::TRINITY_DN15671_c0_g2_i5::g.55277::m.55277	16	37,5	62,665	238,01	PF00342.18	PGI	725,3	2,90E-218	CL0067
TRINITY_DN15673_c2_g2::TRINITY_DN15673_c2_g2_i2::g.55381::m.55381	4	29,7	15,059	9,1581	PF01929.16	Ribosomal_L14e	92	2,30E-26	CL0107
TRINITY_DN15675_c4_g2::TRINITY_DN15675_c4_g2_i2::g.55509::m.55509	2	8,7	28,033	4,3565	PF00156.26	Pribosyltran	85,1	3,20E-24	CL0533
TRINITY_DN15675_c5_g2::TRINITY_DN15675_c5_g2_i2::g.55536::m.55536	10	33,9	48,482	44,852	PF00349.20	Hexokinase_1	201,3	1,40E-59	CL0108
TRINITY_DN15677_c1_g6::TRINITY_DN15677_c1_g6_i3::g.55549::m.55549	2	26	21,296	3,1247	PF00686.18	CBM_20	60,2	1,30E-16	CL0369

TRINITY_DN15683_c2_g1::TRINITY_DN15683_c2_g1_i10::g.55639::m.55639	1	6,9	22,47	13,554	PF08030.11	NAD_binding_6	136,2	9,20E-40	CL0091
TRINITY_DN15685_c2_g1::TRINITY_DN15685_c2_g1_i7::g.55504::m.55504	1	3,9	52,764	3,5137	PF01501.19	Glyco_transf_8	316,8	9,80E-95	CL0110
TRINITY_DN15687_c3_g1::TRINITY_DN15687_c3_g1_i1::g.55726::m.55726	5	52,9	15,166	20,35	PF01323.19	DSBA	68,5	6,10E-19	CL0172
TRINITY_DN15690_c0_g1::TRINITY_DN15690_c0_g1_i7::g.55685::m.55685	3	17,7	28,656	4,965	PF01156.18	IU_nuc_hydro	188,2	2,40E-55	No_clan
TRINITY_DN15691_c0_g1::TRINITY_DN15691_c0_g1_i9::g.55585::m.55585	1	2,9	66,279	3,9157	PF01902.16	Diphthami_syn_2	37,1	2,10E-09	CL0039
TRINITY_DN15693_c4_g1::TRINITY_DN15693_c4_g1_i3::g.55852::m.55852	2	9,2	35,778	4,9247	PF00005.26	ABC_tran	67,8	1,20E-18	CL0023
TRINITY_DN15694_c2_g2::TRINITY_DN15694_c2_g2_i2::g.54517::m.54517	5	46,7	22,239	16,341	PF02167.14	Cytochrom_C1	258	6,70E-77	CL0318
TRINITY_DN15696_c0_g5::TRINITY_DN15696_c0_g5_i1::g.55738::m.55738	2	9,5	27,295	93,008	PF00400.31	WD40	21,9	0,00021	CL0186
TRINITY_DN15699_c1_g3::TRINITY_DN15699_c1_g3_i5::g.55823::m.55823	5	9,7	87,538	9,5622	PF04810.14	zf-Sec23_Sec24	62,2	3,20E-17	No_clan
TRINITY_DN15700_c1_g1::TRINITY_DN15700_c1_g1_i3::g.54890::m.54890	10	14,9	110,36	22,936	PF13360.5	PQQ_2	46,6	2,90E-12	CL0186
TRINITY_DN15701_c0_g1::TRINITY_DN15701_c0_g1_i7::g.55836::m.55836	2	4,8	78,842	2,0079					
TRINITY_DN15702_c0_g3::TRINITY_DN15702_c0_g3_i1::g.55933::m.55933	2	6,9	28,24	2,3997	PF00181.22	Ribosomal_L2	63,6	1,20E-17	CL0021
TRINITY_DN15702_c0_g4::TRINITY_DN15702_c0_g4_i1::g.55937::m.55937	5	20,1	32,981	10,418	PF00181.22	Ribosomal_L2	60,2	1,30E-16	CL0021
TRINITY_DN15705_c0_g3::TRINITY_DN15705_c0_g3_i5::g.56096::m.56096	1	14,3	11,821	2,2972	PF04190.12	DUF410	42,9	4,60E-11	No_clan
TRINITY_DN15729_c0_g1::TRINITY_DN15729_c0_g1_i9::g.56378::m.56378	2	12,2	24,879	2,0736	PF00571.27	CBS	38,1	1,40E-09	No_clan
TRINITY_DN15732_c1_g2::TRINITY_DN15732_c1_g2_i3::g.56333::m.56333	13	36,8	54,309	26,109	PF00118.23	Cpn60_TCP1	501,5	2,10E-150	No_clan
TRINITY_DN15733_c1_g1::TRINITY_DN15733_c1_g1_i2::g.56414::m.56414	2	19	18,308	16,183	PF02996.16	Prefoldin	102,1	1,70E-29	CL0200
TRINITY_DN15733_c2_g3::TRINITY_DN15733_c2_g3_i4::g.56425::m.56425	1	1,4	121,69	2,0376	PF00168.29	C2	82,4	2,20E-23	CL0154
TRINITY_DN15741_c2_g1::TRINITY_DN15741_c2_g1_i4::g.55925::m.55925	1	13,4	16,999	11,376	PF00168.29	C2	38	1,50E-09	CL0154
TRINITY_DN15744_c2_g4::TRINITY_DN15744_c2_g4_i2::g.56499::m.56499	1	7,7	22,022	2,2355	PF04012.11	PspA_IM30	103,1	1,50E-29	CL0235
TRINITY_DN15754_c0_g4::TRINITY_DN15754_c0_g4_i1::g.56661::m.56661	1	9,9	15,918	2,0848	PF03168.12	LEA_2	28,2	2,00E-06	CL0159

TRINITY_DN15761_c1_g2::TRINITY_DN15761_c1_g2_i2::g.56714::m.56714	11	28,1	54,826	5,1521	PF00091.24	Tubulin	231,3	1,00E-68	CL0566
TRINITY_DN15762_c0_g1::TRINITY_DN15762_c0_g1_i1::g.56697::m.56697	3	3,1	154,58	4,3238	PF02769.21	AIRS_C	82,1	4,30E-23	No_clan
TRINITY_DN15763_c0_g1::TRINITY_DN15763_c0_g1_i1::g.56716::m.56716	6	11,3	75,163	7,7593	PF02990.15	EMP70	595	1,10E-178	No_clan
TRINITY_DN15771_c0_g1::TRINITY_DN15771_c0_g1_i5::g.56845::m.56845	6	25,4	31,505	16,548	PF00400.31	WD40	12,4	0,22	CL0186
TRINITY_DN15771_c0_g2::TRINITY_DN15771_c0_g2_i3::g.56846::m.56846	14	19,7	89,308	55,169	PF00400.31	WD40	26,9	5,70E-06	CL0186
TRINITY_DN15774_c1_g1::TRINITY_DN15774_c1_g1_i3::g.56941::m.56941	7	52,5	17,567	323,31	PF00903.24	Glyoxalase	39,3	5,90E-10	CL0104
TRINITY_DN15774_c2_g1::TRINITY_DN15774_c2_g1_i10::g.56953::m.56953	11	14,6	119,31	23,526	PF00328.21	His_Phos_2	451,9	2,00E-135	CL0071
TRINITY_DN15779_c0_g2::TRINITY_DN15779_c0_g2_i1::g.57109::m.57109	2	8,4	25,806	3,1252					
TRINITY_DN15783_c0_g1::TRINITY_DN15783_c0_g1_i4::g.56969::m.56969	3	15,7	22,255	5,447	PF01928.20	CYTH	85,8	3,00E-24	CL0273
TRINITY_DN15784_c0_g1::TRINITY_DN15784_c0_g1_i2::g.57047::m.57047	4	5,9	85,78	7,4561	PF04734.12	Ceramidase_alk	790,5	5,90E-238	No_clan
TRINITY_DN15793_c0_g1::TRINITY_DN15793_c0_g1_i1::g.57091::m.57091	3	46,5	11,293	8,9842	PF00928.20	Adap_comp_sub	88,6	3,90E-25	CL0448
TRINITY_DN15794_c0_g2::TRINITY_DN15794_c0_g2_i7::g.57165::m.57165	4	13,7	58,418	53,286	PF02383.17	Syja_N	290	2,30E-86	CL0031
TRINITY_DN15799_c0_g1::TRINITY_DN15799_c0_g1_i1::g.55952::m.55952	3	19,6	29,555	16,228	PF00182.18	Glyco_hydro_19	419,1	5,20E-126	CL0037
TRINITY_DN15799_c0_g1::TRINITY_DN15799_c0_g1_i13::g.55967::m.55967	3	24,1	23,864	10,588	PF00182.18	Glyco_hydro_19	330,8	4,90E-99	CL0037
TRINITY_DN15799_c0_g1::TRINITY_DN15799_c0_g1_i6::g.55958::m.55958	2	12,9	26,902	2,6109	PF00182.18	Glyco_hydro_19	405,3	8,70E-122	CL0037
TRINITY_DN15799_c0_g1::TRINITY_DN15799_c0_g1_i8::g.55961::m.55961	2	14,9	21,74	68,421	PF00182.18	Glyco_hydro_19	342,5	1,30E-102	CL0037
TRINITY_DN15805_c1_g2::TRINITY_DN15805_c1_g2_i2::g.57262::m.57262	6	7	167,7	13,024	PF16507.4	BLM10_mid	114,3	5,60E-33	No_clan
TRINITY_DN15810_c0_g1::TRINITY_DN15810_c0_g1_i2::g.57313::m.57313	1	6,9	18,039	1,9755	PF03908.12	Sec20	27,5	2,00E-06	CL0147
TRINITY_DN15811_c0_g2::TRINITY_DN15811_c0_g2_i2::g.57329::m.57329	6	42,7	17,398	23,105	PF10584.8	Proteasome_A_N	57,8	5,50E-16	CL0052
TRINITY_DN15811_c0_g5::TRINITY_DN15811_c0_g5_i2::g.57331::m.57331	5	33,8	18,148	5,8205	PF00227.25	Proteasome	129	1,30E-37	CL0052

TRINITY_DN15818_c1_g1::TRINITY_DN15818_c1_g1_i7::g.57419::m.57419	3	24,3	25,237	4,8292	PF10185.8	Mesd	25,3	1,10E-05	No_clan
TRINITY_DN15823_c4_g1::TRINITY_DN15823_c4_g1_i3::g.57508::m.57508	2	4,1	69,651	3,4745	PF04146.14	YTH	140,7	2,30E-41	CL0178
TRINITY_DN15824_c1_g1::TRINITY_DN15824_c1_g1_i3::g.57478::m.57478	2	9,2	39,703	2,2118					
TRINITY_DN15828_c0_g1::TRINITY_DN15828_c0_g1_i4::g.57452::m.57452	2	17,4	16,134	3,9175	PF04828.13	GFA	27,9	2,00E-06	CL0080
TRINITY_DN15831_c4_g2::TRINITY_DN15831_c4_g2_i2::g.57659::m.57659	1	2,3	58,497	2,2078	PF04185.13	Phosphoesterase	358,1	4,80E-107	CL0088
TRINITY_DN15832_c2_g1::TRINITY_DN15832_c2_g1_i7::g.57636::m.57636	2	6,2	43,283	3,3659	PF00106.24	adh_short	115,3	2,10E-33	CL0063
TRINITY_DN15833_c5_g1::TRINITY_DN15833_c5_g1_i8::g.57893::m.57893	2	18,6	17,991	2,0724					
TRINITY_DN15833_c5_g1::TRINITY_DN15833_c5_g1_i12::g.57896::m.57896	3	24,6	21,665	4,1901	PF01412.17	ArfGap	142,1	6,60E-42	No_clan
TRINITY_DN15836_c2_g1::TRINITY_DN15836_c2_g1_i8::g.57209::m.57209	11	24,1	63,826	26,672	PF13537.5	GATase_7	148,5	7,20E-44	CL0052
TRINITY_DN15837_c1_g2::TRINITY_DN15837_c1_g2_i3::g.57761::m.57761	4	16,1	43,557	8,3681	PF00202.20	Aminotran_3	229,7	4,30E-68	CL0061
TRINITY_DN15860_c0_g1::TRINITY_DN15860_c0_g1_i3::g.57901::m.57901	1	3,6	43,386	2,828	PF00854.20	PTR2	232,1	9,00E-69	CL0015
TRINITY_DN15863_c0_g1::TRINITY_DN15863_c0_g1_i12::g.58136::m.58136	5	35,3	23,161	11,414	PF01263.19	Aldose_epim	140,9	4,80E-41	CL0103
TRINITY_DN15863_c0_g2::TRINITY_DN15863_c0_g2_i4::g.58135::m.58135	14	55,6	36,09	316,55	PF01263.19	Aldose_epim	243,1	3,60E-72	CL0103
TRINITY_DN15865_c2_g1::TRINITY_DN15865_c2_g1_i9::g.58071::m.58071	1	12,8	15,611	2,1107	PF05348.10	UMP1	126,1	7,40E-37	No_clan
TRINITY_DN15873_c1_g1::TRINITY_DN15873_c1_g1_i7::g.58225::m.58225	1	16	11,606	2,0376					
TRINITY_DN15876_c0_g2::TRINITY_DN15876_c0_g2_i4::g.58231::m.58231	1	10,2	17,569	2,901	PF00076.21	RRM_1	48,4	5,80E-13	CL0221
TRINITY_DN15886_c0_g2::TRINITY_DN15886_c0_g2_i6::g.58296::m.58296	8	18,4	82,456	22,614	PF08323.10	Glyco_transf_5	219,5	5,10E-65	CL0113
TRINITY_DN15893_c1_g2::TRINITY_DN15893_c1_g2_i6::g.57605::m.57605	9	31,5	45,487	58,879	PF00180.19	Iso_dh	350,8	7,60E-105	CL0270
TRINITY_DN15894_c0_g1::TRINITY_DN15894_c0_g1_i1::g.58474::m.58474	1	8,8	17,783	2,0485	PF01575.18	MaoC_dehydratas	89,9	8,20E-26	CL0050
TRINITY_DN15895_c3_g1::TRINITY_DN15895_c3_g1_i1::g.57722::m.57722	3	14,6	38,708	5,2311	PF00076.21	RRM_1	69,4	1,60E-19	CL0221
TRINITY_DN15895_c3_g2::TRINITY_DN15895_c3_g2_i1::g.57724::m.57724	4	16,9	39,182	7,4874	PF00076.21	RRM_1	71,7	2,90E-20	CL0221

TRINITY_DN15895_c4_g2::TRINITY_DN15895_c4_g2_i3::g.57730::m.57730	1	8,5	22,422	2,0646	PF01936.17	NYN	57,6	1,80E-15	CL0280
TRINITY_DN15896_c1_g3::TRINITY_DN15896_c1_g3_i4::g.58593::m.58593	9	37,2	26,844	202,55	PF00141.22	peroxidase	211,5	1,20E-62	CL0617
TRINITY_DN15899_c1_g1::TRINITY_DN15899_c1_g1_i1::g.58501::m.58501	3	24,8	22,066	2,0219					
TRINITY_DN15900_c2_g1::TRINITY_DN15900_c2_g1_i1::g.57240::m.57240	4	7,8	89,574	9,0109	PF00326.20	Peptidase_S9	124,8	3,00E-36	CL0028
TRINITY_DN15903_c3_g2::TRINITY_DN15903_c3_g2_i1::g.58748::m.58748	5	36,2	13,923	6,8311					
TRINITY_DN15923_c1_g1::TRINITY_DN15923_c1_g1_i8::g.59029::m.59029	1	3	69,983	1,9876	PF08797.10	HIRAN	68,1	4,40E-19	No_clan
TRINITY_DN15924_c1_g1::TRINITY_DN15924_c1_g1_i2::g.59050::m.59050	5	15	50,099	167,36	PF00112.22	Peptidase_C1	282	4,10E-84	CL0125
TRINITY_DN15927_c0_g1::TRINITY_DN15927_c0_g1_i2::g.59092::m.59092	9	26,4	53,353	18,814	PF00349.20	Hexokinase_1	209,5	4,30E-62	CL0108
TRINITY_DN15929_c0_g1::TRINITY_DN15929_c0_g1_i6::g.59159::m.59159	2	2,4	120,25	3,3608	PF00628.28	PHD	38,7	6,10E-10	CL0390
TRINITY_DN1593_c0_g1::TRINITY_DN1593_c0_g1_i1::g.417::m.417	2	14,6	17,115	19,43	PF06108.11	DUF952	87,1	5,30E-25	CL0084
TRINITY_DN15930_c2_g2::TRINITY_DN15930_c2_g2_i5::g.59203::m.59203	7	30,7	39,522	15,494	PF03405.13	FA_desaturase_2	533,5	1,40E-160	CL0044
TRINITY_DN15930_c2_g3::TRINITY_DN15930_c2_g3_i6::g.59212::m.59212	8	28,6	37,957	14,865	PF03405.13	FA_desaturase_2	530,9	8,20E-160	CL0044
TRINITY_DN15931_c0_g1::TRINITY_DN15931_c0_g1_i5::g.59103::m.59103	2	6,3	36,834	5,8046	PF00450.21	Peptidase_S10	289,8	4,30E-86	CL0028
TRINITY_DN15932_c0_g3::TRINITY_DN15932_c0_g3_i5::g.59231::m.59231	16	44,5	41,158	208,62	PF08240.11	ADH_N	83	1,10E-23	CL0296
TRINITY_DN15932_c0_g5::TRINITY_DN15932_c0_g5_i5::g.59228::m.59228	4	11,5	59,539	7,9723	PF00009.26	GTP_EFTU	103,6	8,80E-30	CL0023
TRINITY_DN15935_c0_g1::TRINITY_DN15935_c0_g1_i1::g.59258::m.59258	6	15	44,97	43,076	PF00289.21	Biotin_carb_N	136,4	5,00E-40	CL0483
TRINITY_DN15944_c0_g3::TRINITY_DN15944_c0_g3_i1::g.58792::m.58792	1	6,8	18,481	2,3954	PF01249.17	Ribosomal_S21e	120,3	2,50E-35	No_clan
TRINITY_DN15946_c1_g1::TRINITY_DN15946_c1_g1_i2::g.59352::m.59352	5	15,2	49,841	9,6163	PF01233.18	NMT	258,5	1,80E-77	CL0257
TRINITY_DN15946_c1_g2::TRINITY_DN15946_c1_g2_i8::g.59360::m.59360	4	14,7	49,722	9,1807	PF01233.18	NMT	259,8	7,30E-78	CL0257
TRINITY_DN15949_c0_g1::TRINITY_DN15949_c0_g1_i6::g.59510::m.59510	13	19,5	99,946	35,898	PF00240.22	ubiquitin	74,3	4,20E-21	CL0072
TRINITY_DN15949_c1_g2::TRINITY_DN15949_c1_g2_i8::g.59514::m.59514	7	20,4	40,303	59,325	PF13181.5	TPR_8	12,3	0,15	CL0020

TRINITY_DN15951_c5_g2::TRINITY_DN15951_c5_g2_i3::g.59457::m.59457	6	23,6	39,174	11,484	PF01263.19	Aldose_epim	318,3	4,50E-95	CL0103
TRINITY_DN15954_c0_g1::TRINITY_DN15954_c0_g1_i1::g.59385::m.59385	15	48,7	35,959	323,31	PF00191.19	Annexin	71,4	4,40E-20	No_clan
TRINITY_DN15955_c0_g1::TRINITY_DN15955_c0_g1_i1::g.59530::m.59530	2	9,2	28,6	4,3909	PF00762.18	Ferrochelatase	166,1	9,10E-49	CL0043
TRINITY_DN15963_c1_g1::TRINITY_DN15963_c1_g1_i1::g.59605::m.59605	2	5,2	47,17	2,049	PF00479.21	G6PD_N	197,2	3,20E-58	CL0063
TRINITY_DN15963_c2_g1::TRINITY_DN15963_c2_g1_i4::g.59609::m.59609	6	24,5	39,46	323,31	PF00112.22	Peptidase_C1	257	1,80E-76	CL0125
TRINITY_DN15977_c4_g2::TRINITY_DN15977_c4_g2_i4::g.59843::m.59843	3	20,6	19,987	5,4886	PF04430.13	DUF498	107,5	3,20E-31	No_clan
TRINITY_DN15978_c1_g2::TRINITY_DN15978_c1_g2_i1::g.59851::m.59851	3	8,6	63,995	7,1471	PF01434.17	Peptidase_M41	249,9	1,70E-74	CL0126
TRINITY_DN15979_c0_g1::TRINITY_DN15979_c0_g1_i3::g.59847::m.59847	8	20,6	48,038	15,205	PF00676.19	E1_dh	275,2	4,70E-82	CL0254
TRINITY_DN15981_c2_g1::TRINITY_DN15981_c2_g1_i5::g.59872::m.59872	2	28,2	12,534	13,78	PF06825.11	HSBP1	45	6,80E-12	No_clan
TRINITY_DN15983_c2_g1::TRINITY_DN15983_c2_g1_i5::g.59899::m.59899	8	28,1	48,989	49,218	PF00675.19	Peptidase_M16	116,3	1,10E-33	CL0094
TRINITY_DN15984_c0_g3::TRINITY_DN15984_c0_g3_i1::g.59910::m.59910	2	15,1	22,853	2,6939	PF03088.15	Str_synth	32,9	4,70E-08	CL0186
TRINITY_DN15988_c0_g1::TRINITY_DN15988_c0_g1_i7::g.59999::m.59999	8	24,3	55,608	18,497	PF03109.15	ABC1	109,5	1,00E-31	CL0016
TRINITY_DN16000_c0_g1::TRINITY_DN16000_c0_g1_i10::g.60178::m.60178	8	29,3	59,997	66,878	PF01039.21	Carboxyl_trans	71	7,40E-20	CL0127
TRINITY_DN16005_c0_g3::TRINITY_DN16005_c0_g3_i6::g.60403::m.60403	5	16,8	44,768	13,763	PF02812.17	ELFV_dehydrog_N	170,5	1,40E-50	CL0603
TRINITY_DN16008_c3_g2::TRINITY_DN16008_c3_g2_i3::g.60431::m.60431	2	21,8	12,401	3,4224	PF03179.14	V-ATPase_G	111,7	2,10E-32	CL0255
TRINITY_DN16015_c1_g1::TRINITY_DN16015_c1_g1_i9::g.60551::m.60551	3	17,6	26,318	12,202	PF14523.5	Syntaxin_2	104,6	2,70E-30	CL0445
TRINITY_DN16016_c2_g1::TRINITY_DN16016_c2_g1_i9::g.60539::m.60539	2	15,5	14,408	2,734	PF01918.20	Alba	47,4	1,20E-12	CL0441
TRINITY_DN16017_c1_g2::TRINITY_DN16017_c1_g2_i2::g.60617::m.60617	8	20,7	60,916	30,436	PF00076.21	RRM_1	71,6	3,30E-20	CL0221
TRINITY_DN16019_c1_g2::TRINITY_DN16019_c1_g2_i3::g.60717::m.60717	1	5,6	20,608	1,8929					
TRINITY_DN16024_c0_g2::TRINITY_DN16024_c0_g2_i5::g.60694::m.60694	7	22,2	43,891	23,536	PF00676.19	E1_dh	400	4,60E-120	CL0254
TRINITY_DN16026_c0_g1::TRINITY_DN16026_c0_g1_i2::g.60976::m.60976	8	4,6	286,16	15,484					

TRINITY_DN16028_c0_g1::TRINITY_DN16028_c0_g1_i7::g.60828::m.60828	3	9,4	48,896	8,1989	PF06159.12	DUF974	230	3,40E-68	No_clan
TRINITY_DN16029_c2_g3::TRINITY_DN16029_c2_g3_i1::g.61032::m.61032	5	34,3	22,2	27,488	PF08534.9	Redoxin	82,1	2,90E-23	CL0172
TRINITY_DN16031_c0_g3::TRINITY_DN16031_c0_g3_i1::g.60905::m.60905	13	27	70,781	105,44	PF00400.31	WD40	16,6	0,0099	CL0186
TRINITY_DN16032_c0_g1::TRINITY_DN16032_c0_g1_i1::g.60895::m.60895	14	55,8	33,429	2,0863	PF00190.21	Cupin_1	72,6	2,40E-20	CL0029
TRINITY_DN16032_c0_g1::TRINITY_DN16032_c0_g1_i2::g.60897::m.60897	16	52	38,52	21,27	PF00190.21	Cupin_1	100,6	5,40E-29	CL0029
TRINITY_DN16032_c0_g1::TRINITY_DN16032_c0_g1_i3::g.60899::m.60899	16	52,8	38,532	323,31	PF00190.21	Cupin_1	100,6	5,40E-29	CL0029
TRINITY_DN16032_c0_g1::TRINITY_DN16032_c0_g1_i4::g.60901::m.60901	15	54,5	38,345	64,057	PF00190.21	Cupin_1	100,7	5,30E-29	CL0029
TRINITY_DN16035_c3_g1::TRINITY_DN16035_c3_g1_i2::g.61026::m.61026	3	11	53,598	8,717	PF03909.16	BSD	58,3	5,00E-16	No_clan
TRINITY_DN16038_c1_g1::TRINITY_DN16038_c1_g1_i5::g.61002::m.61002	9	26,6	57,399	30,115	PF00224.20	PK	387	6,10E-116	CL0151
TRINITY_DN16040_c0_g1::TRINITY_DN16040_c0_g1_i12::g.61056::m.61056	9	13,9	114,53	18,787	PF12515.7	CaATP_NAI	67,2	6,20E-19	No_clan
TRINITY_DN16041_c1_g2::TRINITY_DN16041_c1_g2_i6::g.61105::m.61105	2	12,4	28,066	8,1951	PF00291.24	PALP	85,1	5,10E-24	No_clan
TRINITY_DN16049_c0_g2::TRINITY_DN16049_c0_g2_i3::g.60387::m.60387	1	9,1	25,488	2,0518	PF00106.24	adh_short	170,2	3,30E-50	CL0063
TRINITY_DN16055_c0_g1::TRINITY_DN16055_c0_g1_i6::g.61225::m.61225	3	7,2	73,09	4,576	PF02841.13	GBP_C	32	7,70E-08	CL0023
TRINITY_DN16055_c0_g3::TRINITY_DN16055_c0_g3_i1::g.61219::m.61219	12	32,7	53,318	38,151	PF00171.21	Aldedh	577,9	1,10E-173	CL0099
TRINITY_DN16056_c1_g1::TRINITY_DN16056_c1_g1_i8::g.61254::m.61254	2	9	32,79	2,0054	PF04144.12	SCAMP	190,2	3,50E-56	No_clan
TRINITY_DN16058_c1_g1::TRINITY_DN16058_c1_g1_i7::g.61245::m.61245	5	17,5	43,417	9,0708	PF00787.23	PX	48,1	8,80E-13	No_clan
TRINITY_DN16061_c2_g1::TRINITY_DN16061_c2_g1_i1::g.61310::m.61310	9	22,1	49,948	97,11	PF00156.26	Pribosyltran	35,8	4,70E-09	CL0533
TRINITY_DN16061_c2_g2::TRINITY_DN16061_c2_g2_i3::g.61317::m.61317	10	49,6	29,643	77,857	PF00231.18	ATP-synt	241,2	1,60E-71	No_clan
TRINITY_DN16066_c2_g3::TRINITY_DN16066_c2_g3_i1::g.61892::m.61892	2	19,1	21,902	7,9392					
TRINITY_DN16074_c1_g1::TRINITY_DN16074_c1_g1_i7::g.61513::m.61513	4	5,6	100,73	34,332	PF13418.5	Kelch_4	26,7	3,80E-06	CL0186
TRINITY_DN16077_c0_g5::TRINITY_DN16077_c0_g5_i1::g.61752::m.61752	5	30,5	24,529	-2	PF00091.24	Tubulin	209,8	4,10E-62	CL0566

TRINITY_DN16082_c0_g1::TRINITY_DN16082_c0_g1_i2::g.61608::m.61608	4	9,1	58,458	3,102	PF00483.22	NTP_transferase	257,9	8,70E-77	CL0110
TRINITY_DN16086_c0_g2::TRINITY_DN16086_c0_g2_i6::g.61825::m.61825	1	3,8	51,807	2,3013					
TRINITY_DN16090_c0_g1::TRINITY_DN16090_c0_g1_i3::g.61630::m.61630	2	5,6	51,757	3,1287					
TRINITY_DN19086_c1_g1::TRINITY_DN19086_c1_g1_i6::g.111724::m.111724	1	10	12,053	2,211	PF00350.22	Dynamin_N	33,9	2,90E-08	CL0023
TRINITY_DN16101_c1_g1::TRINITY_DN16101_c1_g1_i2::g.61978::m.61978	13	18	105,07	34,224	PF00690.25	Cation_ATPase_N	50,1	1,50E-13	No_clan
TRINITY_DN16102_c2_g2::TRINITY_DN16102_c2_g2_i1::g.62195::m.62195	3	22,6	16,562	2,6125					
TRINITY_DN16102_c2_g6::TRINITY_DN16102_c2_g6_i1::g.62197::m.62197	5	26,5	21,723	11,579	PF00557.23	Peptidase_M24	85,8	3,00E-24	No_clan
TRINITY_DN16105_c7_g3::TRINITY_DN16105_c7_g3_i3::g.62193::m.62193	6	34,1	25,382	21,711	PF00635.25	Motile_Sperm	97,2	4,40E-28	CL0556
TRINITY_DN16110_c4_g1::TRINITY_DN16110_c4_g1_i10::g.62321::m.62321	2	9,5	30,272	2,7695	PF07250.10	Glyoxal_oxid_N	290,3	8,50E-87	No_clan
TRINITY_DN16110_c4_g1::TRINITY_DN16110_c4_g1_i11::g.62324::m.62324	1	11	17,176	2,9619	PF07250.10	Glyoxal_oxid_N	56,7	1,80E-15	No_clan
TRINITY_DN16112_c0_g1::TRINITY_DN16112_c0_g1_i8::g.62808::m.62808	25	63,7	52,163	323,31	PF01704.17	UDPGP	613,7	1,20E-184	CL0110
TRINITY_DN16112_c0_g1::TRINITY_DN16112_c0_g1_i3::g.62802::m.62802	8	47,5	19,887	87,836	PF01704.17	UDPGP	68,2	4,80E-19	CL0110
TRINITY_DN16113_c1_g1::TRINITY_DN16113_c1_g1_i2::g.62243::m.62243	9	21,1	58,348	20,407	PF00118.23	Cpn60_TCP1	286	4,50E-85	No_clan
TRINITY_DN16118_c1_g1::TRINITY_DN16118_c1_g1_i2::g.62411::m.62411	2	13,1	26,46	5,278	PF00153.26	Mito_carr	69,6	1,50E-19	No_clan
TRINITY_DN16119_c4_g1::TRINITY_DN16119_c4_g1_i1::g.62575::m.62575	2	6,3	61,563	3,6299	PF04810.14	zf-Sec23_Sec24	26,7	4,00E-06	No_clan
TRINITY_DN16119_c4_g3::TRINITY_DN16119_c4_g3_i2::g.62577::m.62577	1	9,8	21,853	2,7789	PF04815.14	Sec23_helical	40,1	2,30E-10	No_clan
TRINITY_DN16121_c0_g1::TRINITY_DN16121_c0_g1_i2::g.62465::m.62465	1	21	13,2	43,368	PF01217.19	Clat_adaptor_s	56,3	3,00E-15	CL0212
TRINITY_DN16121_c0_g1::TRINITY_DN16121_c0_g1_i8::g.62467::m.62467	2	20,8	11,942	48,904	PF01217.19	Clat_adaptor_s	46,3	3,70E-12	CL0212
TRINITY_DN16123_c0_g2::TRINITY_DN16123_c0_g2_i3::g.62404::m.62404	5	14,4	46,501	6,3046	PF00270.28	DEAD	143,8	4,20E-42	CL0023
TRINITY_DN16125_c0_g1::TRINITY_DN16125_c0_g1_i5::g.62472::m.62472	2	21,9	16,369	3,728	PF13023.5	HD_3	124,8	2,90E-36	CL0237
TRINITY_DN16126_c1_g1::TRINITY_DN16126_c1_g1_i17::g.62053::m.62053	2	11,1	24,922	3,2577	PF00202.20	Aminotran_3	108,1	3,60E-31	CL0061

TRINITY_DN16126_c1_g1::TRINITY_DN16126_c1_g1_i14::g.62052::m.62052	1	4,4	52,476	3,4716	PF00202.20	Aminotran_3	318,1	6,30E-95	CL0061
TRINITY_DN16128_c0_g1::TRINITY_DN16128_c0_g1_i5::g.62674::m.62674	2	5,2	65,871	2,3635	PF00388.18	PI-PLC-X	167,9	9,40E-50	CL0384
TRINITY_DN16131_c0_g1::TRINITY_DN16131_c0_g1_i1::g.62590::m.62590	2	6,5	68,891	2,67	PF10291.8	muHD	38,7	7,10E-10	CL0448
TRINITY_DN16132_c1_g1::TRINITY_DN16132_c1_g1_i4::g.62630::m.62630	8	30,4	18,085	259,47	PF13833.5	EF-hand_8	51,1	7,80E-14	CL0220
TRINITY_DN16132_c1_g2::TRINITY_DN16132_c1_g2_i1::g.62633::m.62633	9	41,6	16,967	323,31	PF13499.5	EF-hand_7	55,5	5,30E-15	CL0220
TRINITY_DN16133_c0_g4::TRINITY_DN16133_c0_g4_i5::g.62653::m.62653	3	11,9	25,636	4,915	PF00364.21	Biotin_lipoyl	50,1	1,70E-13	CL0105
TRINITY_DN16134_c1_g1::TRINITY_DN16134_c1_g1_i3::g.62771::m.62771	4	19,1	36,733	10,354	PF00005.26	ABC_tran	58,1	1,20E-15	CL0023
TRINITY_DN16136_c0_g1::TRINITY_DN16136_c0_g1_i4::g.62642::m.62642	9	43,1	32,955	42,182	PF02817.16	E3_binding	53,7	1,70E-14	No_clan
TRINITY_DN16138_c0_g1::TRINITY_DN16138_c0_g1_i2::g.62708::m.62708	3	13	40,9	9,7617	PF06068.12	TIP49	494,4	2,10E-148	CL0023
TRINITY_DN16140_c1_g1::TRINITY_DN16140_c1_g1_i18::g.63031::m.63031	1	19,2	13,51	7,8829					
TRINITY_DN16144_c0_g3::TRINITY_DN16144_c0_g3_i9::g.62076::m.62076	6	19,7	49,103	27,786					
TRINITY_DN16146_c1_g1::TRINITY_DN16146_c1_g1_i2::g.62212::m.62212	3	6,6	73,624	3,2977					
TRINITY_DN16157_c0_g1::TRINITY_DN16157_c0_g1_i7::g.62500::m.62500	2	5,1	55,187	4,2533	PF00067.21	p450	207,2	3,70E-61	No_clan
TRINITY_DN16163_c4_g1::TRINITY_DN16163_c4_g1_i1::g.63306::m.63306	5	29,1	28,698	61,285	PF00295.16	Glyco_hydro_28	229	7,00E-68	CL0268
TRINITY_DN16163_c6_g1::TRINITY_DN16163_c6_g1_i3::g.63330::m.63330	5	33,7	26,72	323,31	PF00295.16	Glyco_hydro_28	61,5	6,30E-17	CL0268
TRINITY_DN16164_c3_g2::TRINITY_DN16164_c3_g2_i1::g.62975::m.62975	2	13,6	24,637	3,6071	PF05755.11	REF	268,4	4,00E-80	No_clan
TRINITY_DN16168_c0_g1::TRINITY_DN16168_c0_g1_i6::g.63149::m.63149	15	22,1	97,214	32,893	PF01851.21	PC_rep	21,3	0,00024	CL0020
TRINITY_DN16170_c0_g1::TRINITY_DN16170_c0_g1_i3::g.63133::m.63133	4	9,7	76,767	5,6992	PF00501.27	AMP-binding	345,9	2,50E-103	CL0378
TRINITY_DN16171_c0_g1::TRINITY_DN16171_c0_g1_i2::g.63190::m.63190	1	2,5	51,298	2,1432	PF00173.27	Cyt-b5	73,9	8,00E-21	No_clan
TRINITY_DN16184_c0_g3::TRINITY_DN16184_c0_g3_i5::g.63369::m.63369	6	22	42,154	17,505	PF03088.15	Str_synth	105,6	1,00E-30	CL0186
TRINITY_DN16191_c2_g1::TRINITY_DN16191_c2_g1_i7::g.63681::m.63681	12	24,5	61,464	98,244	PF00307.30	CH	58,4	6,60E-16	CL0188

TRINITY_DN16192_c0_g2::TRINITY_DN16192_c0_g2_i1::g.63470::m.63470	5	8,5	70,878	11,321	PF02516.13	STT3	403,8	1,10E-120	CL0111
TRINITY_DN16195_c0_g1::TRINITY_DN16195_c0_g1_i3::g.63589::m.63589	1	17,3	12,426	4,8091	PF03080.14	Neprosin	82,7	2,30E-23	No_clan
TRINITY_DN16200_c0_g2::TRINITY_DN16200_c0_g2_i2::g.62796::m.62796	1	7,4	29,132	2,7994	PF13646.5	HEAT_2	58,5	5,90E-16	CL0020
TRINITY_DN16201_c0_g1::TRINITY_DN16201_c0_g1_i1::g.62689::m.62689	10	52,2	23,963	22,059	PF00347.22	Ribosomal_L6	47,7	1,80E-12	No_clan
TRINITY_DN16201_c3_g1::TRINITY_DN16201_c3_g1_i2::g.62696::m.62696	1	14,5	12,461	2,7782	PF01780.18	Ribosomal_L37ae	132,1	5,30E-39	CL0167
TRINITY_DN16210_c2_g2::TRINITY_DN16210_c2_g2_i2::g.64102::m.64102	7	44,8	22,24	68,689	PF00071.21	Ras	169,9	3,00E-50	CL0023
TRINITY_DN16211_c0_g2::TRINITY_DN16211_c0_g2_i6::g.63944::m.63944	2	2,7	57,452	1,9376	PF16876.4	Lipin_mid	51,2	9,70E-14	No_clan
TRINITY_DN16212_c0_g1::TRINITY_DN16212_c0_g1_i2::g.63955::m.63955	6	25,4	37,06	37,292	PF17284.1	Spermine_synt_N	86,5	7,60E-25	No_clan
TRINITY_DN16212_c0_g2::TRINITY_DN16212_c0_g2_i5::g.63967::m.63967	1	6,5	23,547	2,0684	PF05903.13	Peptidase_C97	155,6	7,50E-46	CL0125
TRINITY_DN16215_c5_g2::TRINITY_DN16215_c5_g2_i7::g.63985::m.63985	10	23,4	65,792	22,162	PF00549.18	Ligase_CoA	46,2	3,80E-12	CL0506
TRINITY_DN16216_c0_g1::TRINITY_DN16216_c0_g1_i6::g.64074::m.64074	6	7,3	109,32	10,658	PF01602.19	Adaptin_N	367,5	9,40E-110	CL0020
TRINITY_DN16217_c1_g2::TRINITY_DN16217_c1_g2_i6::g.64087::m.64087	2	6,1	43,543	3,3718	PF00400.31	WD40	13,3	0,11	CL0186
TRINITY_DN16217_c1_g2::TRINITY_DN16217_c1_g2_i5::g.64084::m.64084	1	5	50,666	3,3924	PF01435.17	Peptidase_M48	107,4	7,10E-31	CL0126
TRINITY_DN16218_c1_g2::TRINITY_DN16218_c1_g2_i6::g.64375::m.64375	4	20,9	25,635	8,5665	PF00571.27	CBS	50,6	1,80E-13	No_clan
TRINITY_DN16222_c0_g1::TRINITY_DN16222_c0_g1_i6::g.64118::m.64118	2	17,8	19,188	4,2575	PF11416.7	Syntaxin-5_N	28,5	5,70E-07	No_clan
TRINITY_DN16222_c1_g2::TRINITY_DN16222_c1_g2_i6::g.64132::m.64132	3	6,2	66,341	5,6569	PF00069.24	Pkinase	247,4	1,40E-73	CL0016
TRINITY_DN16223_c1_g2::TRINITY_DN16223_c1_g2_i2::g.64137::m.64137	4	35,8	17,321	10,597	PF08534.9	Redoxin	114,5	3,00E-33	CL0172
TRINITY_DN16227_c0_g1::TRINITY_DN16227_c0_g1_i8::g.64252::m.64252	6	43,5	19,874	106,03	PF01765.18	RRF	211,8	4,90E-63	No_clan
TRINITY_DN16231_c1_g1::TRINITY_DN16231_c1_g1_i4::g.64294::m.64294	4	11,5	47,591	9,2172	PF01238.20	PMI_typeI	362,7	2,20E-108	CL0029
TRINITY_DN16233_c2_g1::TRINITY_DN16233_c2_g1_i1::g.64327::m.64327	4	15,1	32,047	8,2136	PF00153.26	Mito_carr	56,1	2,40E-15	No_clan

TRINITY_DN16235_c0_g1::TRINITY_DN16235_c0_g1_i5::g.64344::m.64344	6	11,2	76,703	4,0267	PF00501.27	AMP-binding	349,8	1,50E-104	CL0378
TRINITY_DN16236_c1_g2::TRINITY_DN16236_c1_g2_i1::g.64353::m.64353	8	27,5	27,518	19,507					
TRINITY_DN16238_c3_g1::TRINITY_DN16238_c3_g1_i4::g.64390::m.64390	7	17,5	59,757	208,89	PF00150.17	Cellulase	82,9	2,40E-23	CL0058
TRINITY_DN16240_c0_g1::TRINITY_DN16240_c0_g1_i3::g.64405::m.64405	2	3,9	83,456	2,9502	PF02922.17	CBM_48	74,8	4,90E-21	CL0369
TRINITY_DN16241_c0_g1::TRINITY_DN16241_c0_g1_i2::g.63739::m.63739	1	5,9	26,295	2,0836	PF01370.20	Epimerase	48,5	7,20E-13	CL0063
TRINITY_DN16241_c1_g4::TRINITY_DN16241_c1_g4_i5::g.63753::m.63753	3	9,9	63,895	5,5944	PF03630.13	Fumble	307,9	7,60E-92	CL0108
TRINITY_DN16243_c0_g1::TRINITY_DN16243_c0_g1_i2::g.64445::m.64445	7	18,9	57,609	14,68	PF01496.18	V_ATPase_I	539	1,50E-161	No_clan
TRINITY_DN16244_c0_g2::TRINITY_DN16244_c0_g2_i4::g.64523::m.64523	1	14,3	18,466	3,8201					
TRINITY_DN16246_c0_g1::TRINITY_DN16246_c0_g1_i1::g.63895::m.63895	8	30	34,797	310,27	PF00248.20	Aldo_ket_red	194	2,90E-57	No_clan
TRINITY_DN16246_c1_g1::TRINITY_DN16246_c1_g1_i7::g.63898::m.63898	4	40,2	11,986	74,618	PF00254.27	FKBP_C	89,6	1,20E-25	CL0487
TRINITY_DN16248_c0_g1::TRINITY_DN16248_c0_g1_i2::g.64527::m.64527	7	22,1	45,803	14,635	PF09298.10	FAA_hydrolase_N	115,2	1,40E-33	No_clan
TRINITY_DN16248_c1_g2::TRINITY_DN16248_c1_g2_i8::g.64559::m.64559	9	60,1	15,079	28,74	PF00459.24	Inositol_P	98,1	5,80E-28	CL0171
TRINITY_DN16248_c1_g2::TRINITY_DN16248_c1_g2_i1::g.64537::m.64537	1	8,7	14,334	62,511	PF00459.24	Inositol_P	37	2,30E-09	CL0171
TRINITY_DN16257_c0_g1::TRINITY_DN16257_c0_g1_i2::g.64601::m.64601	2	4	75,994	3,9196	PF13532.5	2OG-FeII_Oxy_2	29,4	8,00E-07	CL0029
TRINITY_DN16262_c1_g2::TRINITY_DN16262_c1_g2_i1::g.64873::m.64873	3	24,3	11,865	7,5717	PF00759.18	Glyco_hydro_9	76,4	2,60E-21	CL0059
TRINITY_DN16262_c1_g3::TRINITY_DN16262_c1_g3_i2::g.64875::m.64875	1	14,8	13,155	3,1489	PF00759.18	Glyco_hydro_9	116,7	1,60E-33	CL0059
TRINITY_DN16267_c0_g1::TRINITY_DN16267_c0_g1_i3::g.64801::m.64801	3	6,9	59,046	6,1914	PF03398.13	Ist1	198,2	7,70E-59	No_clan
TRINITY_DN16268_c4_g1::TRINITY_DN16268_c4_g1_i16::g.64855::m.64855	5	11,4	72,342	9,6818	PF13320.5	DUF4091	62,8	2,40E-17	No_clan
TRINITY_DN16270_c0_g2::TRINITY_DN16270_c0_g2_i15::g.64899::m.64899	2	7,5	36,468	2,4277					
TRINITY_DN16271_c1_g1::TRINITY_DN16271_c1_g1_i4::g.64790::m.64790	1	11	12,508	2,0014					
TRINITY_DN16272_c1_g2::TRINITY_DN16272_c1_g2_i3::g.64808::m.64808	10	30,5	49,154	27,028	PF01546.27	Peptidase_M20	69,7	2,50E-19	CL0035

TRINITY_DN16277_c1_g3::TRINITY_DN16277_c1_g3_i4::g.64929::m.64929	3	33,1	19,544	11,861					
TRINITY_DN16278_c2_g2::TRINITY_DN16278_c2_g2_i8::g.65116::m.65116	1	7,1	28,564	2,6064	PF01556.17	DnaJ_C	152,4	8,30E-45	No_clan
TRINITY_DN16280_c0_g1::TRINITY_DN16280_c0_g1_i6::g.65080::m.65080	2	8,1	29,617	4,0495	PF13417.5	GST_N_3	52,6	4,00E-14	CL0172
TRINITY_DN16282_c1_g3::TRINITY_DN16282_c1_g3_i1::g.64973::m.64973	6	14,8	60,215	9,099	PF01474.15	DAHP_synth_2	651,3	5,10E-196	CL0036
TRINITY_DN16286_c1_g1::TRINITY_DN16286_c1_g1_i5::g.65098::m.65098	6	29,1	43,296	20,487	PF01412.17	ArfGap	117,8	2,40E-34	No_clan
TRINITY_DN16290_c1_g2::TRINITY_DN16290_c1_g2_i6::g.65201::m.65201	6	14,6	66,785	10,854	PF00390.18	malic	254,6	4,40E-76	CL0603
TRINITY_DN16295_c1_g2::TRINITY_DN16295_c1_g2_i1::g.65228::m.65228	3	15,8	32,022	6,8114	PF00069.24	Pkinase	174,6	2,20E-51	CL0016
TRINITY_DN16295_c1_g3::TRINITY_DN16295_c1_g3_i8::g.65237::m.65237	1	3,1	41,395	3,0264	PF01975.16	SurE	169,2	8,30E-50	No_clan
TRINITY_DN16296_c2_g1::TRINITY_DN16296_c2_g1_i8::g.65364::m.65364	2	14,6	34,444	8,1883	PF00106.24	adh_short	87	9,90E-25	CL0063
TRINITY_DN16298_c0_g1::TRINITY_DN16298_c0_g1_i6::g.65227::m.65227	4	29,8	28,824	6,8016	PF03807.16	F420_oxidored	69,9	1,90E-19	CL0063
TRINITY_DN16303_c1_g1::TRINITY_DN16303_c1_g1_i9::g.65704::m.65704	3	11,4	35,295	6,3274					
TRINITY_DN16303_c1_g2::TRINITY_DN16303_c1_g2_i1::g.65686::m.65686	4	24,2	20,71	6,7562					
TRINITY_DN16305_c0_g1::TRINITY_DN16305_c0_g1_i3::g.65508::m.65508	5	18,9	24,255	12,814	PF04969.15	CS	40	5,70E-10	CL0190
TRINITY_DN16305_c1_g1::TRINITY_DN16305_c1_g1_i3::g.65515::m.65515	1	3,6	47,869	3,2472	PF00226.30	DnaJ	92,1	1,60E-26	CL0392
TRINITY_DN16306_c1_g1::TRINITY_DN16306_c1_g1_i3::g.65480::m.65480	2	28,9	13,22	4,169					
TRINITY_DN16306_c1_g2::TRINITY_DN16306_c1_g2_i2::g.65481::m.65481	1	7,3	13,516	1,9272	PF00561.19	Abhydrolase_1	49	5,80E-13	CL0028
TRINITY_DN16307_c2_g1::TRINITY_DN16307_c2_g1_i9::g.65550::m.65550	9	19,4	63,293	18,243	PF02985.21	HEAT	17,7	0,0027	CL0020
TRINITY_DN16307_c2_g3::TRINITY_DN16307_c2_g3_i3::g.65541::m.65541	3	6,9	59,946	-2	PF02985.21	HEAT	21,1	0,00022	CL0020
TRINITY_DN16308_c0_g1::TRINITY_DN16308_c0_g1_i5::g.65986::m.65986	2	10,7	25,606	3,8267	PF00153.26	Mito_carr	57,2	1,10E-15	No_clan
TRINITY_DN16309_c0_g1::TRINITY_DN16309_c0_g1_i5::g.65915::m.65915	2	17,1	16,423	4,2341	PF04979.13	IPP-2	47,9	1,90E-12	No_clan
TRINITY_DN16310_c4_g1::TRINITY_DN16310_c4_g1_i21::g.65586::m.65586	2	14,8	22,057	10,889	PF00574.22	CLP_protease	225,3	4,70E-67	CL0127

TRINITY_DN16311_c1_g1::TRINITY_DN16311_c1_g1_i8::g.65782::m.65782	3	5,9	56,583	6,3388	PF00083.23	Sugar_tr	350,6	1,20E-104	CL0015
TRINITY_DN16315_c0_g1::TRINITY_DN16315_c0_g1_i3::g.65602::m.65602	2	5	61,304	4,5072	PF01073.18	3Beta_HSD	251,7	6,20E-75	CL0063
TRINITY_DN16317_c2_g1::TRINITY_DN16317_c2_g1_i4::g.65631::m.65631	8	25,7	42,821	64,051	PF01370.20	Epimerase	201,4	1,40E-59	CL0063
TRINITY_DN16319_c0_g3::TRINITY_DN16319_c0_g3_i4::g.65613::m.65613	1	11,8	16,798	2,6088					
TRINITY_DN16319_c0_g6::TRINITY_DN16319_c0_g6_i2::g.65617::m.65617	2	7,1	52,97	8,0847	PF02845.15	CUE	34	1,50E-08	CL0214
TRINITY_DN16321_c0_g3::TRINITY_DN16321_c0_g3_i5::g.65822::m.65822	3	10,9	40,526	6,8697	PF01070.17	FMN_dh	459	8,60E-138	CL0036
TRINITY_DN16324_c2_g1::TRINITY_DN16324_c2_g1_i11::g.65876::m.65876	8	15,4	81,351	53,612	PF00686.18	CBM_20	24,2	2,20E-05	CL0369
TRINITY_DN16328_c4_g1::TRINITY_DN16328_c4_g1_i3::g.65859::m.65859	4	36,3	13,286	17,229					
TRINITY_DN16328_c4_g1::TRINITY_DN16328_c4_g1_i2::g.65854::m.65854	3	38	10,989	4,9739					
TRINITY_DN16328_c4_g2::TRINITY_DN16328_c4_g2_i8::g.65867::m.65867	2	7,5	29,318	3,0313	PF13561.5	adh_short_C2	129,5	1,30E-37	CL0063
TRINITY_DN16330_c0_g1::TRINITY_DN16330_c0_g1_i8::g.66108::m.66108	4	18,5	34,758	6,7756	PF03081.14	Exo70	340,9	8,40E-102	CL0295
TRINITY_DN16337_c1_g1::TRINITY_DN16337_c1_g1_i12::g.66068::m.66068	3	12,5	34,054	26,333					
TRINITY_DN16337_c1_g3::TRINITY_DN16337_c1_g3_i2::g.66071::m.66071	2	6,2	43,944	20,578	PF00380.18	Ribosomal_S9	158,8	7,20E-47	CL0329
TRINITY_DN16339_c2_g1::TRINITY_DN16339_c2_g1_i6::g.65997::m.65997	6	25,9	33,045	16,317	PF03152.13	UFD1	270,4	4,20E-81	CL0402
TRINITY_DN16339_c2_g2::TRINITY_DN16339_c2_g2_i2::g.65998::m.65998	7	8,7	107,65	9,9773	PF02190.15	LON_substr_bdg	118,5	3,20E-34	CL0178
TRINITY_DN16342_c0_g1::TRINITY_DN16342_c0_g1_i1::g.66087::m.66087	9	30,5	54,465	65,275	PF00171.21	Aldedh	437,8	3,60E-131	CL0099
TRINITY_DN16344_c0_g1::TRINITY_DN16344_c0_g1_i6::g.66181::m.66181	3	13,9	39,459	4,4775	PF05822.11	UMPH-1	312,4	1,90E-93	CL0137
TRINITY_DN16345_c0_g1::TRINITY_DN16345_c0_g1_i2::g.66198::m.66198	27	36,6	92,147	323,31	PF00168.29	C2	56	3,60E-15	CL0154
TRINITY_DN16345_c2_g1::TRINITY_DN16345_c2_g1_i1::g.66206::m.66206	2	21	12,12	5,2468	PF08991.9	MTCP1	52,4	4,10E-14	CL0351
TRINITY_DN16356_c0_g1::TRINITY_DN16356_c0_g1_i13::g.66037::m.66037	2	9,1	25,558	2,9887	PF04791.15	LMBR1	98	5,50E-28	No_clan

TRINITY_DN16358_c2_g1::TRINITY_DN16358_c2_g1_i5::g.66392::m.66392	2	14,5	26,234	2,22	PF00490.20	ALAD	347,2	7,40E-104	CL0036
TRINITY_DN16361_c1_g1::TRINITY_DN16361_c1_g1_i5::g.66288::m.66288	12	27,5	60,754	64,314	PF00675.19	Peptidase_M16	127,7	3,10E-37	CL0094
TRINITY_DN16364_c0_g1::TRINITY_DN16364_c0_g1_i6::g.66368::m.66368	25	48,6	68,722	132,95	PF02874.22	ATP-synt_ab_N	51,4	1,10E-13	CL0275
TRINITY_DN16367_c2_g1::TRINITY_DN16367_c2_g1_i2::g.66527::m.66527	3	13,4	39,93	21,91	PF01678.18	DAP_epimerase	121,4	2,20E-35	CL0288
TRINITY_DN16369_c2_g1::TRINITY_DN16369_c2_g1_i6::g.66520::m.66520	3	7,2	59,397	27,953					
TRINITY_DN16369_c2_g2::TRINITY_DN16369_c2_g2_i2::g.66506::m.66506	5	50,9	12,259	36,553	PF00210.23	Ferritin	49,6	3,60E-13	CL0044
TRINITY_DN16369_c2_g3::TRINITY_DN16369_c2_g3_i1::g.66518::m.66518	3	18,1	18,213	8,7135	PF00210.23	Ferritin	52,6	4,00E-14	CL0044
TRINITY_DN16370_c1_g1::TRINITY_DN16370_c1_g1_i6::g.66493::m.66493	3	6,7	51,74	3,9848	PF03071.14	GNT-I	695,4	2,40E-209	CL0110
TRINITY_DN16376_c0_g2::TRINITY_DN16376_c0_g2_i2::g.66583::m.66583	24	33,4	97,919	323,31	PF01433.19	Peptidase_M1	312,2	1,80E-93	CL0126
TRINITY_DN16378_c0_g2::TRINITY_DN16378_c0_g2_i3::g.66649::m.66649	11	40,8	31,369	56,798	PF00481.20	PP2C	229	6,50E-68	CL0238
TRINITY_DN16381_c2_g1::TRINITY_DN16381_c2_g1_i3::g.66693::m.66693	17	22,6	120,34	44,119	PF05193.20	Peptidase_M16_C	94	1,00E-26	CL0094
TRINITY_DN16382_c2_g3::TRINITY_DN16382_c2_g3_i2::g.66735::m.66735	2	9	31,712	2,908	PF01266.23	DAO	126,6	1,50E-36	CL0063
TRINITY_DN16384_c1_g4::TRINITY_DN16384_c1_g4_i4::g.66906::m.66906	2	4,5	55,795	5,1647	PF17047.4	SMP_LBD	62,7	2,80E-17	CL0648
TRINITY_DN16387_c0_g7::TRINITY_DN16387_c0_g7_i3::g.66774::m.66774	1	8,1	14,919	2,3998	PF03908.12	Sec20	30,5	2,30E-07	CL0147
TRINITY_DN16390_c0_g1::TRINITY_DN16390_c0_g1_i2::g.66783::m.66783	5	14,2	50,675	9,0513	PF00013.28	KH_1	41,6	7,30E-11	CL0007
TRINITY_DN16399_c1_g2::TRINITY_DN16399_c1_g2_i5::g.67010::m.67010	7	20,4	52,388	15,688	PF00285.20	Citrate_synt	347	1,10E-103	No_clan
TRINITY_DN16402_c0_g3::TRINITY_DN16402_c0_g3_i1::g.67045::m.67045	2	15,1	20,039	5,9818	PF01494.18	FAD_binding_3	60,4	1,70E-16	CL0063
TRINITY_DN16403_c4_g2::TRINITY_DN16403_c4_g2_i2::g.67059::m.67059	1	12,7	13,755	2,5893	PF00507.18	Oxidored_q4	126,7	2,90E-37	No_clan
TRINITY_DN16406_c2_g3::TRINITY_DN16406_c2_g3_i1::g.67214::m.67214	4	24,6	23,913	16,679	PF01294.17	Ribosomal_L13e	265	3,00E-79	No_clan
TRINITY_DN16408_c1_g1::TRINITY_DN16408_c1_g1_i1::g.67131::m.67131	5	13,1	56,175	32,588					
TRINITY_DN16410_c3_g3::TRINITY_DN16410_c3_g3_i5::g.67165::m.67165	8	27,7	49,967	46,374	PF00201.17	UDPGT	46	3,00E-12	CL0113

TRINITY_DN16413_c0_g1::TRINITY_DN16413_c0_g1_i2::g.67167::m.67167	1	14,3	18,904	2,5269	PF00107.25	ADH_zinc_N	91,9	2,70E-26	CL0063
TRINITY_DN16420_c0_g1::TRINITY_DN16420_c0_g1_i2::g.67378::m.67378	15	41,3	47,915	52,879	PF02798.19	GST_N	56,8	2,00E-15	CL0172
TRINITY_DN16420_c0_g2::TRINITY_DN16420_c0_g2_i1::g.67374::m.67374	22	50,1	51,027	323,31	PF02798.19	GST_N	57,4	1,20E-15	CL0172
TRINITY_DN16420_c0_g2::TRINITY_DN16420_c0_g2_i4::g.67379::m.67379	10	39,2	30,276	39,47	PF02798.19	GST_N	58,4	6,40E-16	CL0172
TRINITY_DN16421_c0_g1::TRINITY_DN16421_c0_g1_i1::g.67240::m.67240	11	25	51,796	112,11	PF00251.19	Glyco_hydro_32N	347,4	7,70E-104	CL0143
TRINITY_DN16422_c1_g1::TRINITY_DN16422_c1_g1_i4::g.67346::m.67346	26	45,9	72,965	323,31	PF00012.19	HSP70	905,9	8,50E-273	CL0108
TRINITY_DN16423_c1_g1::TRINITY_DN16423_c1_g1_i12::g.67294::m.67294	3	4,2	125,22	7,2925	PF02854.18	MIF4G	105,2	3,50E-30	CL0020
TRINITY_DN16423_c1_g3::TRINITY_DN16423_c1_g3_i3::g.67278::m.67278	15	37,5	59,027	45,593	PF00587.24	tRNA-synt_2b	64,8	9,10E-18	CL0040
TRINITY_DN16429_c0_g2::TRINITY_DN16429_c0_g2_i3::g.67310::m.67310	2	12,5	24,199	4,2416	PF00887.18	ACBP	96,2	9,20E-28	CL0632
TRINITY_DN16437_c1_g2::TRINITY_DN16437_c1_g2_i4::g.67526::m.67526	14	39,7	51,791	70,172	PF00464.18	SHMT	626,8	1,00E-188	CL0061
TRINITY_DN16438_c0_g4::TRINITY_DN16438_c0_g4_i1::g.67637::m.67637	3	7,6	61,215	5,9057	PF05691.11	Raffinose_syn	858	4,00E-258	CL0058
TRINITY_DN16440_c0_g1::TRINITY_DN16440_c0_g1_i4::g.67503::m.67503	6	11	96,491	19,23	PF00982.20	Glyco_transf_20	606,1	3,90E-182	CL0113
TRINITY_DN16442_c0_g2::TRINITY_DN16442_c0_g2_i5::g.67615::m.67615	3	12,6	29,236	3,8237	PF00294.23	PfkB	202,2	1,00E-59	CL0118
TRINITY_DN16447_c0_g1::TRINITY_DN16447_c0_g1_i1::g.67047::m.67047	5	30,6	21,163	2,131	PF00327.19	Ribosomal_L30	76,3	1,10E-21	No_clan
TRINITY_DN16447_c0_g1::TRINITY_DN16447_c0_g1_i6::g.67050::m.67050	6	27,2	28,444	11,397	PF08079.11	Ribosomal_L30_N	89,3	1,30E-25	No_clan
TRINITY_DN16453_c1_g1::TRINITY_DN16453_c1_g1_i1::g.67791::m.67791	1	10,5	19,539	2,1966	PF00076.21	RRM_1	66	1,80E-18	CL0221
TRINITY_DN16453_c2_g1::TRINITY_DN16453_c2_g1_i14::g.67807::m.67807	3	7,4	55,725	7,5585	PF00557.23	Peptidase_M24	186,1	5,80E-55	No_clan
TRINITY_DN16454_c1_g1::TRINITY_DN16454_c1_g1_i2::g.67727::m.67727	8	25,3	45,853	14,219	PF02518.25	HATPase_c	48,1	1,30E-12	CL0025
TRINITY_DN16454_c2_g1::TRINITY_DN16454_c2_g1_i4::g.67739::m.67739	2	4	60,476	2,8065					
TRINITY_DN16456_c0_g2::TRINITY_DN16456_c0_g2_i7::g.67919::m.67919	2	13	38,356	7,328	PF06552.11	TOM20_plant	26,5	4,30E-06	CL0020

TRINITY_DN16461_c2_g1::TRINITY_DN16461_c2_g1_i2::g.67850::m.67850	1	12,1	19,325	1,9845	PF00501.27	AMP-binding	92,8	1,60E-26	CL0378
TRINITY_DN16462_c1_g1::TRINITY_DN16462_c1_g1_i7::g.68028::m.68028	4	17,1	27,134	40,782	PF02230.15	Abhydrolase_2	140,8	5,10E-41	CL0028
TRINITY_DN16464_c0_g1::TRINITY_DN16464_c0_g1_i9::g.67881::m.67881	1	5,6	48,132	7,0684					
TRINITY_DN16466_c0_g1::TRINITY_DN16466_c0_g1_i6::g.67994::m.67994	2	9,2	49,407	3,0526	PF06911.11	Senescence	161	3,20E-47	No_clan
TRINITY_DN16466_c0_g3::TRINITY_DN16466_c0_g3_i1::g.67992::m.67992	9	25,4	49,2	26,454	PF06911.11	Senescence	148,6	2,00E-43	No_clan
TRINITY_DN16469_c0_g1::TRINITY_DN16469_c0_g1_i7::g.67034::m.67034	2	1,9	127,94	32,909	PF00564.23	PB1	68,6	2,90E-19	CL0072
TRINITY_DN16471_c14_g1::TRINITY_DN16471_c14_g1_i3::g.68132::m.68132	3	9,8	45,418	3,0427					
TRINITY_DN16476_c3_g1::TRINITY_DN16476_c3_g1_i9::g.68231::m.68231	3	9	58,589	10,728	PF03016.14	Exostosin	207,2	2,90E-61	No_clan
TRINITY_DN16479_c1_g2::TRINITY_DN16479_c1_g2_i4::g.68347::m.68347	4	3,8	89,395	6,6239	PF13432.5	TPR_16	19	0,0015	CL0020
TRINITY_DN16483_c1_g1::TRINITY_DN16483_c1_g1_i8::g.68282::m.68282	9	8,3	194,86	21,153	PF16213.4	DCB	94,4	5,80E-27	No_clan
TRINITY_DN16485_c1_g1::TRINITY_DN16485_c1_g1_i5::g.68172::m.68172	4	13,7	39,373	2,5926	PF00365.19	PFK	184,3	2,80E-54	CL0240
TRINITY_DN16487_c1_g1::TRINITY_DN16487_c1_g1_i9::g.68393::m.68393	6	26,2	25,313	18,787					
TRINITY_DN16487_c1_g2::TRINITY_DN16487_c1_g2_i5::g.68395::m.68395	3	23,1	17,263	14,258	PF01849.17	NAC	69,9	1,20E-19	No_clan
TRINITY_DN16487_c1_g6::TRINITY_DN16487_c1_g6_i1::g.68386::m.68386	3	19	20,482	5,1505					
TRINITY_DN16490_c2_g1::TRINITY_DN16490_c2_g1_i1::g.68401::m.68401	5	16,6	45,693	27,051	PF07992.13	Pyr_redox_2	152,6	1,30E-44	CL0063
TRINITY_DN16497_c0_g2::TRINITY_DN16497_c0_g2_i8::g.67601::m.67601	8	14,6	66,157	17,372	PF04068.14	RLI	51,3	6,80E-14	CL0344
TRINITY_DN16497_c0_g5::TRINITY_DN16497_c0_g5_i1::g.67600::m.67600	2	4,6	49,154	2,522	PF01207.16	Dus	248,2	9,00E-74	CL0036
TRINITY_DN16501_c0_g1::TRINITY_DN16501_c0_g1_i7::g.68513::m.68513	4	13,2	48,562	8,4202	PF01370.20	Epimerase	169,8	6,40E-50	CL0063
TRINITY_DN16504_c2_g1::TRINITY_DN16504_c2_g1_i5::g.68713::m.68713	1	6,6	17,35	4,7305	PF00179.25	UQ_con	177,8	8,00E-53	CL0208
TRINITY_DN16506_c3_g1::TRINITY_DN16506_c3_g1_i1::g.68717::m.68717	1	19,2	13,825	14,819	PF01198.18	Ribosomal_L31e	146,5	1,90E-43	No_clan
TRINITY_DN16509_c1_g1::TRINITY_DN16509_c1_g1_i1::g.68768::m.68768	14	33,8	66,917	102,03	PF02219.16	MTHFR	404	3,20E-121	CL0086

TRINITY_DN16513_c0_g2::TRINITY_DN16513_c0_g2_i7::g.68926::m.68926	2	8	42,471	4,2256	PF13419.5	HAD_2	93,3	1,60E-26	CL0137
TRINITY_DN16513_c0_g3::TRINITY_DN16513_c0_g3_i2::g.68923::m.68923	3	15,4	34,72	7,7441	PF00804.24	Syntaxin	233,2	1,90E-69	CL0445
TRINITY_DN16523_c1_g1::TRINITY_DN16523_c1_g1_i4::g.69126::m.69126	4	21,6	33,499	7,6102					
TRINITY_DN16523_c1_g3::TRINITY_DN16523_c1_g3_i4::g.69129::m.69129	9	12,2	117,33	15,57					
TRINITY_DN16526_c1_g2::TRINITY_DN16526_c1_g2_i8::g.69034::m.69034	4	20,8	21,558	72,302	PF03079.13	ARD	208,7	4,70E-62	CL0029
TRINITY_DN16528_c1_g1::TRINITY_DN16528_c1_g1_i4::g.69093::m.69093	2	11,3	24,816	3,6126					
TRINITY_DN16528_c2_g1::TRINITY_DN16528_c2_g1_i3::g.69117::m.69117	3	15,1	26,438	5,0285	PF01301.18	Glyco_hydro_35	261,2	1,60E-77	CL0058
TRINITY_DN16533_c3_g1::TRINITY_DN16533_c3_g1_i1::g.69070::m.69070	3	13,2	40	5,1956					
TRINITY_DN16535_c0_g2::TRINITY_DN16535_c0_g2_i8::g.69172::m.69172	1	2,9	52,048	6,2311	PF00909.20	Ammonium_transp	461,8	1,40E-138	No_clan
TRINITY_DN16542_c2_g2::TRINITY_DN16542_c2_g2_i16::g.69553::m.69553	6	10,2	117,02	4,7442					
TRINITY_DN16542_c2_g2::TRINITY_DN16542_c2_g2_i8::g.69479::m.69479	6	9,3	114,27	11,24					
TRINITY_DN16544_c0_g2::TRINITY_DN16544_c0_g2_i7::g.68626::m.68626	3	21	22,398	14,336	PF02466.18	Tim17	47,5	1,70E-12	No_clan
TRINITY_DN16549_c1_g2::TRINITY_DN16549_c1_g2_i1::g.69658::m.69658	3	22,3	23,536	8,9558					
TRINITY_DN16550_c0_g1::TRINITY_DN16550_c0_g1_i1::g.69273::m.69273	2	6,4	67,483	2,7205	PF02130.16	UPF0054	111,9	1,60E-32	CL0126
TRINITY_DN16551_c0_g1::TRINITY_DN16551_c0_g1_i1::g.69240::m.69240	2	7,6	32,828	2,6907	PF00175.20	NAD_binding_1	61,2	1,20E-16	CL0091
TRINITY_DN16552_c0_g2::TRINITY_DN16552_c0_g2_i1::g.69236::m.69236	2	22,5	11,369	7,5845	PF05042.12	Caleosin	67,7	1,10E-18	CL0220
TRINITY_DN16552_c0_g2::TRINITY_DN16552_c0_g2_i2::g.69237::m.69237	2	16	16	2,7279	PF05042.12	Caleosin	141	3,10E-41	CL0220
TRINITY_DN16555_c1_g6::TRINITY_DN16555_c1_g6_i1::g.69342::m.69342	3	25,5	15,911	19,746	PF01095.18	Pectinesterase	91,8	3,00E-26	CL0268
TRINITY_DN16557_c0_g1::TRINITY_DN16557_c0_g1_i4::g.69370::m.69370	11	26,2	56,808	26,257	PF00013.28	KH_1	41,2	1,00E-10	CL0007
TRINITY_DN16558_c0_g1::TRINITY_DN16558_c0_g1_i2::g.69315::m.69315	16	19,9	140,04	37,058	PF02854.18	MIF4G	27,3	2,40E-06	CL0020
TRINITY_DN16571_c0_g1::TRINITY_DN16571_c0_g1_i1::g.69820::m.69820	2	14,1	26,261	4,2905	PF05172.12	Nup35_RRM	45,5	5,20E-12	CL0221

TRINITY_DN16571_c1_g1::TRINITY_DN16571_c1_g1_i8::g.69835::m.69835	11	61,4	22,404	323,31	PF08718.10	GLTP	153,2	5,60E-45	No_clan
TRINITY_DN16577_c0_g1::TRINITY_DN16577_c0_g1_i6::g.69863::m.69863	3	17,5	29,999	7,2744	PF12146.7	Hydrolase_4	42,6	3,70E-11	CL0028
TRINITY_DN16578_c2_g1::TRINITY_DN16578_c2_g1_i1::g.69884::m.69884	3	3,9	98,117	3,6574	PF02854.18	MIF4G	96,7	1,30E-27	CL0020
TRINITY_DN16580_c0_g3::TRINITY_DN16580_c0_g3_i6::g.69917::m.69917	2	3,9	66,54	3,4102	PF00013.28	KH_1	42,1	5,30E-11	CL0007
TRINITY_DN16581_c1_g3::TRINITY_DN16581_c1_g3_i2::g.69928::m.69928	13	20,1	83,298	99,549	PF00082.21	Peptidase_S8	161	3,60E-47	No_clan
TRINITY_DN17504_c0_g1::TRINITY_DN17504_c0_g1_i2::g.84827::m.84827	2	14,4	14,051	3,8767	PF00125.23	Histone	64,8	8,70E-18	CL0012
TRINITY_DN16589_c2_g3::TRINITY_DN16589_c2_g3_i3::g.69229::m.69229	6	50	15,243	266,59	PF00080.19	Sod_Cu	167,5	1,80E-49	No_clan
TRINITY_DN16594_c0_g2::TRINITY_DN16594_c0_g2_i1::g.69960::m.69960	2	7,1	34,068	4,347	PF00403.25	HMA	43,6	2,70E-11	No_clan
TRINITY_DN16600_c2_g1::TRINITY_DN16600_c2_g1_i15::g.68704::m.68704	1	12,5	13,008	8,6732					
TRINITY_DN16609_c0_g4::TRINITY_DN16609_c0_g4_i3::g.70404::m.70404	2	1,3	179,31	2,363	PF13639.5	zf-RING_2	29,1	8,20E-07	CL0229
TRINITY_DN16612_c3_g2::TRINITY_DN16612_c3_g2_i3::g.70349::m.70349	5	28,9	24,463	26,216	PF00690.25	Cation_ATPase_N	50,6	1,00E-13	No_clan
TRINITY_DN16612_c3_g7::TRINITY_DN16612_c3_g7_i2::g.70355::m.70355	5	23,9	33,241	5,4752	PF00122.19	E1-E2_ATPase	48,1	8,40E-13	No_clan
TRINITY_DN16613_c0_g1::TRINITY_DN16613_c0_g1_i5::g.70442::m.70442	3	6,7	57,235	4,252	PF07899.10	Frigida	313	1,70E-93	No_clan
TRINITY_DN16615_c1_g3::TRINITY_DN16615_c1_g3_i6::g.70426::m.70426	2	5,6	50,335	3,3967	PF00067.21	p450	288,8	6,40E-86	No_clan
TRINITY_DN16616_c1_g1::TRINITY_DN16616_c1_g1_i2::g.70640::m.70640	5	18,2	34,267	36,168					
TRINITY_DN16618_c2_g1::TRINITY_DN16618_c2_g1_i4::g.70494::m.70494	4	8,7	67,972	6,4149	PF04424.12	MINDY_DUB	124,5	1,90E-36	No_clan
TRINITY_DN16619_c0_g1::TRINITY_DN16619_c0_g1_i7::g.70514::m.70514	5	17,2	57,685	12,943	PF00291.24	PALP	163,6	6,20E-48	No_clan
TRINITY_DN16625_c2_g1::TRINITY_DN16625_c2_g1_i8::g.70673::m.70673	7	52	16,925	61,512	PF00071.21	Ras	116,6	7,30E-34	CL0023
TRINITY_DN16625_c3_g1::TRINITY_DN16625_c3_g1_i9::g.70678::m.70678	9	52,2	23,202	20,566	PF00071.21	Ras	186,1	3,00E-55	CL0023
TRINITY_DN16629_c1_g1::TRINITY_DN16629_c1_g1_i1::g.70763::m.70763	4	29,9	14,504	139,55					
TRINITY_DN16629_c2_g3::TRINITY_DN16629_c2_g3_i2::g.70768::m.70768	2	18,8	16,875	3,306					
TRINITY_DN16630_c2_g1::TRINITY_DN16630_c2_g1_i6::g.70777::m.70777	9	60,2	21,504	323,31	PF00244.19	14.03.2003	256,6	1,50E-76	No_clan

TRINITY_DN16630_c2_g2::TRINITY_DN16630_c2_g2_i3::g.70772::m.70772	3	10,5	47,116	7,7305	PF00291.24	PALP	123,3	1,20E-35	No_clan
TRINITY_DN16640_c0_g1::TRINITY_DN16640_c0_g1_i4::g.70902::m.70902	1	8,3	25,981	3,298	PF00642.23	zf-CCCH	30,5	2,30E-07	CL0537
TRINITY_DN16641_c0_g1::TRINITY_DN16641_c0_g1_i7::g.70870::m.70870	1	5,1	30,68	2,0684	PF07690.15	MFS_1	146	1,20E-42	CL0015
TRINITY_DN16642_c3_g1::TRINITY_DN16642_c3_g1_i8::g.71010::m.71010	2	23,2	13,429	9,1777	PF01593.23	Amino_oxidase	62,9	3,20E-17	CL0063
TRINITY_DN16643_c2_g1::TRINITY_DN16643_c2_g1_i2::g.70325::m.70325	1	11,2	18,314	2,7735	PF00569.16	ZZ	33,1	3,20E-08	CL0006
TRINITY_DN16644_c3_g1::TRINITY_DN16644_c3_g1_i2::g.70216::m.70216	4	56,8	12,414	122,19	PF00190.21	Cupin_1	38,7	6,40E-10	CL0029
TRINITY_DN16644_c3_g1::TRINITY_DN16644_c3_g1_i4::g.70221::m.70221	9	23,8	57,36	93,231	PF00190.21	Cupin_1	28,8	7,50E-07	CL0029
TRINITY_DN16649_c0_g2::TRINITY_DN16649_c0_g2_i3::g.71022::m.71022	3	3,5	129,48	5,4767					
TRINITY_DN16650_c2_g2::TRINITY_DN16650_c2_g2_i4::g.71040::m.71040	2	16,8	21,662	6,9982	PF01661.20	Macro	134,7	1,30E-39	CL0223
TRINITY_DN16659_c4_g1::TRINITY_DN16659_c4_g1_i7::g.71135::m.71135	7	14,9	76,035	11,594	PF01417.19	ENTH	29,9	4,50E-07	CL0009
TRINITY_DN16661_c1_g2::TRINITY_DN16661_c1_g2_i5::g.71230::m.71230	2	3,3	74,333	4,0843	PF12899.6	Glyco_hydro_100	722,1	1,90E-217	CL0059
TRINITY_DN16664_c4_g1::TRINITY_DN16664_c4_g1_i4::g.71662::m.71662	2	15,9	18,882	5,9676					
TRINITY_DN16667_c0_g2::TRINITY_DN16667_c0_g2_i1::g.71232::m.71232	13	20,4	81,145	40,017	PF00456.20	Transketolase_N	501,6	7,90E-151	CL0254
TRINITY_DN16668_c3_g1::TRINITY_DN16668_c3_g1_i1::g.71264::m.71264	13	16	113,9	42,815	PF02347.15	GDC-P	610	2,00E-183	CL0061
TRINITY_DN16674_c0_g1::TRINITY_DN16674_c0_g1_i2::g.71326::m.71326	5	7,7	76,688	6,9293					
TRINITY_DN16675_c0_g2::TRINITY_DN16675_c0_g2_i3::g.71545::m.71545	2	5,8	50,72	3,1102	PF04715.12	Anth_synt_I_N	65,5	5,50E-18	No_clan
TRINITY_DN16682_c4_g1::TRINITY_DN16682_c4_g1_i2::g.71560::m.71560	2	15,7	19,661	3,8545	PF13905.5	Thioredoxin_8	54	1,50E-14	CL0172
TRINITY_DN16683_c2_g2::TRINITY_DN16683_c2_g2_i1::g.71583::m.71583	1	13	12,639	2,5988	PF00627.30	UBA	39,5	3,50E-10	CL0214
TRINITY_DN16684_c1_g1::TRINITY_DN16684_c1_g1_i1::g.71521::m.71521	1	9	17,261	2,0715	PF00071.21	Ras	56,7	1,90E-15	CL0023
TRINITY_DN16687_c0_g1::TRINITY_DN16687_c0_g1_i2::g.71593::m.71593	1	2,8	58,421	2,6112					
TRINITY_DN16690_c0_g1::TRINITY_DN16690_c0_g1_i1::g.71666::m.71666	6	13,7	73,769	13,562	PF17004.4	SRP_TPR_like	56,3	2,70E-15	CL0020

TRINITY_DN16691_c0_g1::TRINITY_DN16691_c0_g1_i7::g.71695::m.71695	2	6,3	34,41	4,7763	PF02629.18	CoA_binding	112	1,60E-32	CL0063
TRINITY_DN16697_c1_g2::TRINITY_DN16697_c1_g2_i3::g.70483::m.70483	1	13,7	18,937	2,2911	PF02359.17	CDC48_N	85,1	2,60E-24	CL0332
TRINITY_DN16699_c3_g1::TRINITY_DN16699_c3_g1_i15::g.70256::m.70256	4	11,6	54,318	7,3729	PF01602.19	Adaptin_N	244,7	1,60E-72	CL0020
TRINITY_DN16700_c3_g3::TRINITY_DN16700_c3_g3_i4::g.71827::m.71827	4	27,5	17,647	4,9262	PF01201.21	Ribosomal_S8e	142,7	7,10E-42	No_clan
TRINITY_DN16700_c3_g5::TRINITY_DN16700_c3_g5_i2::g.71826::m.71826	2	18,2	18,795	4,6251	PF14226.5	DIOX_N	77,7	1,10E-21	CL0029
TRINITY_DN16705_c7_g1::TRINITY_DN16705_c7_g1_i5::g.72000::m.72000	1	2	96,031	2,9549	PF00133.21	tRNA-synt_1	621,9	9,20E-187	CL0039
TRINITY_DN16706_c0_g2::TRINITY_DN16706_c0_g2_i5::g.72057::m.72057	2	8,1	36,813	5,2553	PF00076.21	RRM_1	53,9	1,10E-14	CL0221
TRINITY_DN16711_c0_g1::TRINITY_DN16711_c0_g1_i4::g.72030::m.72030	5	16,5	72,642	23,361	PF12068.7	DUF3548	77,5	9,60E-22	No_clan
TRINITY_DN16713_c1_g2::TRINITY_DN16713_c1_g2_i6::g.72096::m.72096	3	10,4	53,827	6,5587	PF01363.20	FYVE	65,2	4,10E-18	CL0390
TRINITY_DN16713_c1_g3::TRINITY_DN16713_c1_g3_i3::g.72094::m.72094	4	32,8	20,615	6,0463	PF09423.9	PhoD	27,6	1,30E-06	CL0163
TRINITY_DN16718_c6_g1::TRINITY_DN16718_c6_g1_i4::g.72149::m.72149	5	28,1	23,695	29,534	PF00759.18	Glyco_hydro_9	143,5	1,10E-41	CL0059
TRINITY_DN16719_c0_g1::TRINITY_DN16719_c0_g1_i6::g.72195::m.72195	2	9,1	29,968	1,876	PF14389.5	Lzipper-MIP1	82,6	1,70E-23	No_clan
TRINITY_DN16719_c1_g1::TRINITY_DN16719_c1_g1_i3::g.72216::m.72216	8	17,9	59,457	25,86	PF02990.15	EMP70	475,6	1,70E-142	No_clan
TRINITY_DN16736_c3_g2::TRINITY_DN16736_c3_g2_i1::g.72521::m.72521	13	47,9	42,536	133,34	PF00056.22	Ldh_1_N	158,7	8,50E-47	CL0063
TRINITY_DN16740_c1_g1::TRINITY_DN16740_c1_g1_i2::g.72692::m.72692	3	8,3	44,688	2,9876	PF00400.31	WD40	13,9	0,07	CL0186
TRINITY_DN16741_c1_g1::TRINITY_DN16741_c1_g1_i4::g.72580::m.72580	2	3	93,98	2,4157	PF02847.16	MA3	105,9	1,00E-30	CL0020
TRINITY_DN16744_c0_g2::TRINITY_DN16744_c0_g2_i2::g.72603::m.72603	3	9,7	35,579	3,5188	PF10496.8	Syntaxin-18_N	57,7	9,60E-16	No_clan
TRINITY_DN16745_c0_g1::TRINITY_DN16745_c0_g1_i9::g.72756::m.72756	7	11,1	93,145	21,935					
TRINITY_DN16751_c0_g2::TRINITY_DN16751_c0_g2_i11::g.72773::m.72773	12	24	61,845	134,4	PF00171.21	Aldedh	121,4	3,30E-35	CL0099
TRINITY_DN16752_c9_g1::TRINITY_DN16752_c9_g1_i5::g.72819::m.72819	8	20,6	58,481	16,219	PF01749.19	IBB	87,5	5,40E-25	CL0020
TRINITY_DN16756_c2_g7::TRINITY_DN16756_c2_g7_i3::g.72811::m.72811	13	33,6	53,851	41,531	PF00224.20	PK	517,5	1,30E-155	CL0151

TRINITY_DN16763_c1_g3::TRINITY_DN16763_c1_g3_i3::g.72969::m.72969	8	23	47,116	16,972	PF01399.26	PCI	77	1,30E-21	CL0123
TRINITY_DN16766_c2_g2::TRINITY_DN16766_c2_g2_i3::g.73070::m.73070	1	9	24,081	3,304					
TRINITY_DN16768_c1_g1::TRINITY_DN16768_c1_g1_i8::g.72998::m.72998	5	8,8	84,986	7,4161	PF03030.15	H_PPase	880	1,10E-264	No_clan
TRINITY_DN16768_c1_g4::TRINITY_DN16768_c1_g4_i5::g.73029::m.73029	2	3,5	78,807	3,6011	PF00083.23	Sugar_tr	179,6	9,30E-53	CL0015
TRINITY_DN16770_c0_g2::TRINITY_DN16770_c0_g2_i2::g.73031::m.73031	2	18	20,607	4,5801	PF02798.19	GST_N	33,3	4,20E-08	CL0172
TRINITY_DN16772_c0_g1::TRINITY_DN16772_c0_g1_i3::g.72983::m.72983	2	3,5	77,18	2,7103	PF05691.11	Raffinose_syn	851,3	4,50E-256	CL0058
TRINITY_DN16773_c1_g1::TRINITY_DN16773_c1_g1_i5::g.73048::m.73048	2	8,2	36,501	6,0878	PF01412.17	ArfGap	127,5	2,30E-37	No_clan
TRINITY_DN16773_c2_g1::TRINITY_DN16773_c2_g1_i3::g.73056::m.73056	5	8,5	69,052	7,0988	PF04597.13	Ribophorin_I	478	3,20E-143	No_clan
TRINITY_DN16774_c0_g1::TRINITY_DN16774_c0_g1_i9::g.73138::m.73138	8	31,4	41,01	25,588	PF00782.19	DSPc	84,2	6,20E-24	CL0031
TRINITY_DN16782_c2_g1::TRINITY_DN16782_c2_g1_i3::g.73171::m.73171	1	9,8	15,234	17,132	PF03080.14	Neprosin	153,8	4,00E-45	No_clan
TRINITY_DN16782_c2_g2::TRINITY_DN16782_c2_g2_i4::g.73167::m.73167	2	24,6	20,81	2,7767	PF03080.14	Neprosin	207,6	1,40E-61	No_clan
TRINITY_DN16787_c1_g2::TRINITY_DN16787_c1_g2_i3::g.73271::m.73271	2	6	41,353	2,5965	PF00294.23	PfkB	268,6	6,20E-80	CL0118
TRINITY_DN16788_c0_g2::TRINITY_DN16788_c0_g2_i3::g.73187::m.73187	3	5,6	68,475	20,825	PF01301.18	Glyco_hydro_35	379,7	1,30E-113	CL0058
TRINITY_DN16789_c2_g2::TRINITY_DN16789_c2_g2_i2::g.73230::m.73230	4	12,8	42,173	9,7824	PF03031.17	NIF	121,5	2,50E-35	CL0137
TRINITY_DN16789_c2_g4::TRINITY_DN16789_c2_g4_i5::g.73232::m.73232	4	23,6	22,33	6,158	PF00438.19	S-AdoMet_synt_N	144,1	1,60E-42	No_clan
TRINITY_DN16789_c2_g5::TRINITY_DN16789_c2_g5_i1::g.73225::m.73225	2	7,4	49,721	5,3005	PF00117.27	GATase	93,4	1,40E-26	CL0014
TRINITY_DN16793_c3_g3::TRINITY_DN16793_c3_g3_i3::g.73369::m.73369	1	4,1	33,66	3,9704	PF02737.17	3HCDH_N	209,4	3,40E-62	CL0063
TRINITY_DN16802_c5_g3::TRINITY_DN16802_c5_g3_i3::g.73661::m.73661	1	5,3	29,113	2,7941	PF04146.14	YTH	57,2	1,40E-15	CL0178
TRINITY_DN16806_c1_g1::TRINITY_DN16806_c1_g1_i6::g.73621::m.73621	2	6,4	47,671	3,1818	PF00155.20	Aminotran_1_2	160,6	5,40E-47	CL0061
TRINITY_DN16808_c0_g1::TRINITY_DN16808_c0_g1_i2::g.73724::m.73724	5	24,6	25,883	99,961	PF01738.17	DLH	128,2	2,90E-37	CL0028

TRINITY_DN16814_c2_g1::TRINITY_DN16814_c2_g1_i8::g.73807::m.73807	10	54,9	22,836	77,521	PF15704.4	Mt_ATP_synt	253,1	1,50E-75	No_clan
TRINITY_DN16815_c5_g2::TRINITY_DN16815_c5_g2_i1::g.73817::m.73817	15	49,3	41,043	79,524	PF02874.22	ATP-synt_ab_N	69,5	2,40E-19	CL0275
TRINITY_DN16817_c0_g1::TRINITY_DN16817_c0_g1_i3::g.73774::m.73774	1	2,8	71,998	2,1966	PF11837.7	DUF3357	65,7	4,10E-18	No_clan
TRINITY_DN16818_c0_g1::TRINITY_DN16818_c0_g1_i9::g.73983::m.73983	7	27,5	41,608	33,423	PF00956.17	NAP	288	5,30E-86	No_clan
TRINITY_DN16820_c1_g1::TRINITY_DN16820_c1_g1_i7::g.73896::m.73896	2	17,8	15,091	41,924	PF00076.21	RRM_1	78,9	1,70E-22	CL0221
TRINITY_DN16821_c1_g1::TRINITY_DN16821_c1_g1_i1::g.73860::m.73860	3	30,5	23,149	28,986	PF07802.10	GCK	124,5	1,50E-36	No_clan
TRINITY_DN16821_c2_g1::TRINITY_DN16821_c2_g1_i2::g.73867::m.73867	7	7,9	119,65	12,198	PF12764.6	Gly-rich_Ago1	58,5	8,90E-16	No_clan
TRINITY_DN16821_c3_g1::TRINITY_DN16821_c3_g1_i2::g.73875::m.73875	6	10,8	68,628	8,9989	PF07765.11	KIP1	85,5	1,90E-24	No_clan
TRINITY_DN16826_c3_g1::TRINITY_DN16826_c3_g1_i6::g.74102::m.74102	3	5,7	94,431	5,2699					
TRINITY_DN16827_c1_g1::TRINITY_DN16827_c1_g1_i8::g.73997::m.73997	20	49,4	45,318	212,85	PF08442.9	ATP-grasp_2	197,8	1,30E-58	CL0179
TRINITY_DN16828_c0_g2::TRINITY_DN16828_c0_g2_i1::g.74105::m.74105	3	9,1	37,29	19,682					
TRINITY_DN16828_c3_g2::TRINITY_DN16828_c3_g2_i1::g.74126::m.74126	2	21,5	17,062	5,4299	PF01467.25	CTP_transf_like	57,4	1,60E-15	CL0039
TRINITY_DN16834_c1_g1::TRINITY_DN16834_c1_g1_i1::g.74148::m.74148	4	10,3	56,417	5,9561	PF00069.24	Pkinase	251,4	8,60E-75	CL0016
TRINITY_DN16836_c1_g1::TRINITY_DN16836_c1_g1_i8::g.74234::m.74234	6	5,3	134,29	8,9056	PF08623.9	TIP120	191,7	7,00E-57	CL0020
TRINITY_DN16836_c1_g3::TRINITY_DN16836_c1_g3_i3::g.74224::m.74224	14	31,1	58,026	227,94	PF01213.18	CAP_N	288,4	7,70E-86	No_clan
TRINITY_DN16837_c0_g2::TRINITY_DN16837_c0_g2_i4::g.74272::m.74272	4	13,1	59,946	23,593	PF05602.11	CLPTM1	486,6	6,50E-146	No_clan
TRINITY_DN16837_c1_g1::TRINITY_DN16837_c1_g1_i3::g.74278::m.74278	1	3,7	53,712	2,5256	PF03435.17	Sacchrp_dh_NADP	64,2	1,30E-17	CL0063
TRINITY_DN16840_c2_g1::TRINITY_DN16840_c2_g1_i1::g.74291::m.74291	14	34,3	41,603	323,31	PF00248.20	Aldo_ket_red	164,8	2,30E-48	No_clan
TRINITY_DN16840_c2_g3::TRINITY_DN16840_c2_g3_i5::g.74315::m.74315	7	22	52,481	47,856	PF03952.15	Enolase_N	188,6	4,20E-56	CL0227
TRINITY_DN16842_c1_g4::TRINITY_DN16842_c1_g4_i5::g.74459::m.74459	3	19,5	16,537	207,73	PF00111.26	Fer2	67,8	5,40E-19	CL0486
TRINITY_DN16842_c1_g6::TRINITY_DN16842_c1_g6_i1::g.74458::m.74458	3	33,2	21,494	158,53	PF00111.26	Fer2	65,5	2,80E-18	CL0486

TRINITY_DN16845_c1_g1::TRINITY_DN16845_c1_g1_i1::g.74565::m.74565	6	34,3	23,071	4,3078	PF00071.21	Ras	192,8	2,70E-57	CL0023
TRINITY_DN17999_c1_g1::TRINITY_DN17999_c1_g1_i8::g.91779::m.91779	1	5,7	20,933	2,3963	PF14541.5	TAXi_C	55,2	6,70E-15	CL0129
TRINITY_DN16852_c0_g1::TRINITY_DN16852_c0_g1_i5::g.74328::m.74328	2	22,6	14,609	40,414	PF00834.18	Ribul_P_3_epim	144,3	2,80E-42	CL0036
TRINITY_DN16859_c0_g3::TRINITY_DN16859_c0_g3_i1::g.74469::m.74469	3	24,6	13,821	83,801	PF13180.5	PDZ_2	30,9	2,30E-07	CL0466
TRINITY_DN16861_c5_g5::TRINITY_DN16861_c5_g5_i1::g.74534::m.74534	2	6,9	27,838	2,3268	PF04614.11	Pex19	135,6	2,20E-39	No_clan
TRINITY_DN16865_c3_g3::TRINITY_DN16865_c3_g3_i2::g.74745::m.74745	14	29,8	67,219	323,31	PF00887.18	ACBP	45,2	7,50E-12	CL0632
TRINITY_DN16867_c0_g1::TRINITY_DN16867_c0_g1_i8::g.74659::m.74659	2	6,5	38,296	1,9531	PF00415.17	RCC1	49,7	4,00E-13	CL0186
TRINITY_DN16872_c0_g1::TRINITY_DN16872_c0_g1_i9::g.74807::m.74807	5	18	44,901	13,716	PF01063.18	Aminotran_4	146	1,40E-42	No_clan
TRINITY_DN16875_c1_g2::TRINITY_DN16875_c1_g2_i1::g.74175::m.74175	15	33,2	70,892	91,845	PF00076.21	RRM_1	70,1	9,60E-20	CL0221
TRINITY_DN16879_c1_g4::TRINITY_DN16879_c1_g4_i3::g.74866::m.74866	8	10,9	101,07	15,573	PF02861.19	Clp_N	37,1	2,30E-09	No_clan
TRINITY_DN16896_c1_g1::TRINITY_DN16896_c1_g1_i19::g.75135::m.75135	2	19,6	14,935	3,6567	PF01641.17	SelR	160,2	1,90E-47	CL0080
TRINITY_DN16898_c0_g1::TRINITY_DN16898_c0_g1_i2::g.75056::m.75056	1	6,9	21,517	1,9975	PF08207.11	EFP_N	35,6	6,40E-09	CL0107
TRINITY_DN16901_c2_g1::TRINITY_DN16901_c2_g1_i2::g.75153::m.75153	1	1,8	98,148	2,4465	PF08263.11	LRRNT_2	22,9	7,30E-05	No_clan
TRINITY_DN16914_c0_g2::TRINITY_DN16914_c0_g2_i3::g.75483::m.75483	2	17,6	11,418	27,95	PF00254.27	FKBP_C	76,9	1,00E-21	CL0487
TRINITY_DN16924_c1_g1::TRINITY_DN16924_c1_g1_i1::g.75666::m.75666	2	8,8	31,39	4,6481	PF04893.16	Yip1	53,9	1,60E-14	CL0112
TRINITY_DN16925_c0_g1::TRINITY_DN16925_c0_g1_i1::g.75769::m.75769	2	5,2	50,6	2,7149	PF00443.28	UCH	126,2	1,50E-36	CL0125
TRINITY_DN16927_c0_g1::TRINITY_DN16927_c0_g1_i2::g.75647::m.75647	2	12,6	28,301	5,1081	PF04511.14	DER1	195,6	7,20E-58	CL0207
TRINITY_DN16927_c1_g1::TRINITY_DN16927_c1_g1_i7::g.75660::m.75660	2	8	36,23	4,1479	PF03328.13	HpcH_HpaI	177,8	1,50E-52	CL0151
TRINITY_DN16928_c0_g2::TRINITY_DN16928_c0_g2_i1::g.75662::m.75662	1	8,1	23,402	2,9585	PF03171.19	2OG-FeII_Oxy	65,7	4,00E-18	CL0029
TRINITY_DN16932_c1_g1::TRINITY_DN16932_c1_g1_i8::g.75833::m.75833	1	9,9	20,739	5,6925					
TRINITY_DN16932_c1_g2::TRINITY_DN16932_c1_g2_i8::g.75832::m.75832	4	27,6	15,409	6,6426					
TRINITY_DN16933_c4_g2::TRINITY_DN16933_c4_g2_i2::g.75851::m.75851	1	6,9	16,762	1,8883	PF01259.17	SAICAR_synt	89,5	2,40E-25	No_clan

TRINITY_DN16933_c5_g1::TRINITY_DN16933_c5_g1_i5::g.75858::m.75858	10	33,7	47,427	43,075	PF00155.20	Aminotran_1_2	298,6	6,00E-89	CL0061
TRINITY_DN16939_c0_g1::TRINITY_DN16939_c0_g1_i3::g.75902::m.75902	3	15,8	21,997	4,7111	PF00462.23	Glutaredoxin	57,8	8,70E-16	CL0172
TRINITY_DN16939_c1_g2::TRINITY_DN16939_c1_g2_i8::g.75928::m.75928	6	37,6	20,994	187,04	PF05008.14	V-SNARE	88,7	2,20E-25	CL0147
TRINITY_DN16940_c1_g1::TRINITY_DN16940_c1_g1_i2::g.75863::m.75863	3	35,1	12,336	5,251	PF01738.17	DLH	49,4	3,80E-13	CL0028
TRINITY_DN16940_c1_g2::TRINITY_DN16940_c1_g2_i2::g.75865::m.75865	6	34,3	25,64	47,67	PF01738.17	DLH	98,6	3,40E-28	CL0028
TRINITY_DN8216_c0_g1::TRINITY_DN8216_c0_g1_i1::g.2536::m.2536	1	6,2	25,344	2,0196	PF00078.26	RVT_1	70,5	1,30E-19	CL0027
TRINITY_DN16954_c2_g1::TRINITY_DN16954_c2_g1_i2::g.76223::m.76223	1	2,3	39,274	3,0707	PF00462.23	Glutaredoxin	48,6	6,60E-13	CL0172
TRINITY_DN16958_c1_g1::TRINITY_DN16958_c1_g1_i8::g.76184::m.76184	1	10,9	17,082	14,294	PF00505.18	HMG_box	81	5,90E-23	CL0114
TRINITY_DN16963_c2_g3::TRINITY_DN16963_c2_g3_i1::g.76239::m.76239	8	32,7	34,406	13,376	PF00004.28	AAA	144,5	2,30E-42	CL0023
TRINITY_DN16964_c2_g5::TRINITY_DN16964_c2_g5_i4::g.76427::m.76427	3	34,3	11,968	11,721	PF03297.14	Ribosomal_S25	136,8	2,40E-40	CL0123
TRINITY_DN16965_c3_g1::TRINITY_DN16965_c3_g1_i6::g.76283::m.76283	6	13,1	70,757	11,649	PF03321.12	GH3	666,3	3,30E-200	CL0378
TRINITY_DN16967_c3_g1::TRINITY_DN16967_c3_g1_i3::g.76257::m.76257	11	43,3	33,325	323,31	PF00121.17	TIM	300,5	7,20E-90	CL0036
TRINITY_DN16971_c2_g1::TRINITY_DN16971_c2_g1_i8::g.76469::m.76469	1	8,7	15,398	2,1858	PF08538.9	DUF1749	70,8	9,30E-20	CL0028
TRINITY_DN16973_c0_g1::TRINITY_DN16973_c0_g1_i3::g.76496::m.76496	2	4,9	61,817	5,0263	PF01501.19	Glyco_transf_8	265	6,40E-79	CL0110
TRINITY_DN16978_c1_g3::TRINITY_DN16978_c1_g3_i2::g.76542::m.76542	2	5,4	52,634	2,1546	PF13181.5	TPR_8	24,1	2,40E-05	CL0020
TRINITY_DN16980_c0_g3::TRINITY_DN16980_c0_g3_i2::g.76506::m.76506	2	3,2	82,366	2,3004	PF17177.3	PPR_long	36,2	3,60E-09	CL0020
TRINITY_DN16987_c2_g1::TRINITY_DN16987_c2_g1_i9::g.76589::m.76589	2	7	39,408	3,5357	PF00561.19	Abhydrolase_1	77,5	1,10E-21	CL0028
TRINITY_DN16988_c0_g1::TRINITY_DN16988_c0_g1_i6::g.76602::m.76602	17	52,1	38,042	130,2	PF00191.19	Annexin	75,4	2,60E-21	No_clan
TRINITY_DN16993_c0_g1::TRINITY_DN16993_c0_g1_i5::g.76723::m.76723	2	24,5	11,637	4,1302	PF01370.20	Epimerase	55,2	6,10E-15	CL0063
TRINITY_DN16993_c0_g2::TRINITY_DN16993_c0_g2_i2::g.76741::m.76741	10	18,5	83,01	259,84	PF00082.21	Peptidase_S8	165,6	1,40E-48	No_clan
TRINITY_DN16993_c0_g3::TRINITY_DN16993_c0_g3_i3::g.76730::m.76730	1	2,6	49,9	2,2509	PF01501.19	Glyco_transf_8	219,1	6,80E-65	CL0110

TRINITY_DN16995_c1_g3::TRINITY_DN16995_c1_g3_i3::g.76630::m.76630	5	33,9	19,091	41,9	PF00838.16	TCTP	189,7	4,10E-56	CL0080
TRINITY_DN16996_c2_g1::TRINITY_DN16996_c2_g1_i5::g.76760::m.76760	7	13,2	79,429	10,748	PF02897.14	Peptidase_S9_N	457,6	3,30E-137	CL0186
TRINITY_DN17004_c0_g1::TRINITY_DN17004_c0_g1_i2::g.76878::m.76878	1	8,1	24,291	2,1441					
TRINITY_DN17004_c0_g6::TRINITY_DN17004_c0_g6_i1::g.76895::m.76895	6	7,7	110,79	6,9666	PF16004.4	EFTUD2	121,9	2,10E-35	No_clan
TRINITY_DN17006_c0_g4::TRINITY_DN17006_c0_g4_i2::g.76859::m.76859	3	24,6	15,171	6,4307	PF01248.25	Ribosomal_L7Ae	94,1	3,00E-27	CL0101
TRINITY_DN17008_c0_g1::TRINITY_DN17008_c0_g1_i2::g.77040::m.77040	3	15,4	22,534	3,8712	PF00174.18	Oxidored_molyb	60,6	1,30E-16	CL0072
TRINITY_DN17008_c0_g1::TRINITY_DN17008_c0_g1_i7::g.77048::m.77048	3	19,6	20,551	7,6702	PF00174.18	Oxidored_molyb	105,5	2,10E-30	CL0072
TRINITY_DN17009_c5_g2::TRINITY_DN17009_c5_g2_i5::g.77303::m.77303	1	5,3	32,7	-2	PF13520.5	AA_permease_2	45,9	3,30E-12	CL0062
TRINITY_DN17010_c0_g1::TRINITY_DN17010_c0_g1_i4::g.76944::m.76944	2	1,9	139,74	2,938	PF04563.14	RNA_pol_Rpb2_1	251,2	5,50E-75	No_clan
TRINITY_DN17014_c1_g3::TRINITY_DN17014_c1_g3_i2::g.77015::m.77015	6	24,3	38,382	17,782	PF01398.20	JAB	51,8	6,60E-14	CL0366
TRINITY_DN17016_c4_g1::TRINITY_DN17016_c4_g1_i2::g.77096::m.77096	1	7,6	14,27	2,0968	PF00083.23	Sugar_tr	75,5	3,40E-21	CL0015
TRINITY_DN17017_c2_g1::TRINITY_DN17017_c2_g1_i7::g.77030::m.77030	5	24,1	26,994	70,97	PF00857.19	Isochorismatase	103,2	1,60E-29	No_clan
TRINITY_DN17019_c1_g3::TRINITY_DN17019_c1_g3_i1::g.77181::m.77181	6	28,2	24,603	19,088	PF00638.17	Ran_BP1	138,2	1,40E-40	CL0266
TRINITY_DN17020_c0_g1::TRINITY_DN17020_c0_g1_i5::g.77212::m.77212	5	20,2	34,683	14,373					
TRINITY_DN17024_c0_g5::TRINITY_DN17024_c0_g5_i1::g.77080::m.77080	4	15,4	28,938	8,2108					
TRINITY_DN17027_c2_g2::TRINITY_DN17027_c2_g2_i1::g.77239::m.77239	5	48,9	15,657	10,528	PF02785.18	Biotin_carb_C	134,6	1,20E-39	CL0105
TRINITY_DN17028_c2_g2::TRINITY_DN17028_c2_g2_i3::g.77278::m.77278	2	2,4	123,33	4,5861					
TRINITY_DN17028_c2_g2::TRINITY_DN17028_c2_g2_i3::g.77279::m.77279	2	9,6	25,32	4,1202					
TRINITY_DN17031_c2_g2::TRINITY_DN17031_c2_g2_i1::g.77290::m.77290	2	15,3	25,468	5,2875	PF13774.5	Longin	43,6	2,10E-11	No_clan
TRINITY_DN17036_c2_g3::TRINITY_DN17036_c2_g3_i3::g.77409::m.77409	1	17,1	13,612	2,3783	PF01121.19	CoaE	80,3	1,20E-22	CL0023
TRINITY_DN17040_c1_g7::TRINITY_DN17040_c1_g7_i1::g.77416::m.77416	3	33,3	14,262	78,054	PF01849.17	NAC	69,8	1,30E-19	No_clan

TRINITY_DN17041_c0_g1::TRINITY_DN17041_c0_g1_i4::g.77480::m.77480	8	8,5	129	15,783	PF02837.17	Glyco_hydro_2_N	158	1,80E-46	CL0202
TRINITY_DN17042_c1_g2::TRINITY_DN17042_c1_g2_i2::g.77496::m.77496	18	19,4	150,63	63,654	PF16529.4	Ge1_WD40	76,3	1,90E-21	CL0186
TRINITY_DN17051_c1_g1::TRINITY_DN17051_c1_g1_i6::g.77580::m.77580	2	4,1	73,902	3,6628	PF11443.7	DUF2828	733,5	1,40E-220	No_clan
TRINITY_DN17055_c3_g2::TRINITY_DN17055_c3_g2_i4::g.77693::m.77693	8	6,6	176,3	15,769					
TRINITY_DN17060_c1_g1::TRINITY_DN17060_c1_g1_i6::g.77747::m.77747	4	17	37,509	10,994	PF13847.5	Methyltransf_31	65	5,90E-18	CL0063
TRINITY_DN17060_c1_g1::TRINITY_DN17060_c1_g1_i4::g.77740::m.77740	16	24,4	93,33	83,224	PF03141.15	Methyltransf_29	762,1	2,10E-229	CL0063
TRINITY_DN17061_c4_g1::TRINITY_DN17061_c4_g1_i15::g.77796::m.77796	2	8,3	21,876	4,0318	PF09229.10	Aha1_N	92,3	2,70E-26	CL0648
TRINITY_DN17067_c1_g1::TRINITY_DN17067_c1_g1_i1::g.77883::m.77883	23	25	136,95	46,031	PF00400.31	WD40	23,6	6,10E-05	CL0186
TRINITY_DN17067_c1_g2::TRINITY_DN17067_c1_g2_i1::g.77892::m.77892	19	37,9	80,098	260,06	PF00082.21	Peptidase_S8	169,3	1,00E-49	No_clan
TRINITY_DN17067_c2_g2::TRINITY_DN17067_c2_g2_i1::g.77907::m.77907	5	13,2	48,042	5,4049	PF00364.21	Biotin_lipoyl	58,4	4,20E-16	CL0105
TRINITY_DN17070_c2_g4::TRINITY_DN17070_c2_g4_i5::g.77858::m.77858	11	58,6	25,967	107,09	PF10584.8	Proteasome_A_N	50,2	1,30E-13	CL0052
TRINITY_DN17074_c4_g2::TRINITY_DN17074_c4_g2_i4::g.77976::m.77976	10	31,6	53,272	29,469	PF00206.19	Lyase_1	386,9	6,90E-116	No_clan
TRINITY_DN17082_c3_g1::TRINITY_DN17082_c3_g1_i1::g.78154::m.78154	6	15	58,479	13,278	PF01053.19	Cys_Met_Meta_PP	473,9	2,40E-142	CL0061
TRINITY_DN17082_c4_g2::TRINITY_DN17082_c4_g2_i2::g.78169::m.78169	5	11,6	71,122	11,633	PF08172.11	CASP_C	136,8	7,20E-40	No_clan
TRINITY_DN17086_c3_g1::TRINITY_DN17086_c3_g1_i14::g.77558::m.77558	5	17,1	44,51	10,307	PF07714.16	Pkinase_Tyr	161,1	2,80E-47	CL0016
TRINITY_DN17089_c1_g2::TRINITY_DN17089_c1_g2_i5::g.78211::m.78211	4	11,3	63,495	7,3785	PF00515.27	TPR_1	29,9	2,90E-07	CL0020
TRINITY_DN17091_c0_g2::TRINITY_DN17091_c0_g2_i7::g.78224::m.78224	6	13	78,942	32,776	PF00226.30	DnaJ	33,6	3,00E-08	CL0392
TRINITY_DN17093_c0_g1::TRINITY_DN17093_c0_g1_i9::g.78387::m.78387	1	8,1	26,11	2,534					
TRINITY_DN17096_c1_g1::TRINITY_DN17096_c1_g1_i4::g.78249::m.78249	2	21,6	12,265	4,228					
TRINITY_DN17096_c2_g1::TRINITY_DN17096_c2_g1_i4::g.78253::m.78253	2	5,4	47,922	3,025	PF01399.26	PCI	67,2	1,50E-18	CL0123

TRINITY_DN17097_c0_g1::TRINITY_DN17097_c0_g1_i1::g.78356::m.78356	2	8,7	41,583	2,2674					
TRINITY_DN17099_c3_g2::TRINITY_DN17099_c3_g2_i2::g.76778::m.76778	7	16,6	60,538	12,302	PF16294.4	RSB_motif	53,3	2,70E-14	No_clan
TRINITY_DN17100_c3_g1::TRINITY_DN17100_c3_g1_i1::g.78525::m.78525	2	7,7	20,694	2,8523	PF00237.18	Ribosomal_L22	137,2	2,00E-40	No_clan
TRINITY_DN17109_c1_g4::TRINITY_DN17109_c1_g4_i1::g.78802::m.78802	6	38,8	17,593	16,185	PF00416.21	Ribosomal_S13	192,4	3,00E-57	CL0303
TRINITY_DN17113_c0_g1::TRINITY_DN17113_c0_g1_i1::g.78814::m.78814	2	2,1	119,54	2,7325	PF03810.18	IBN_N	24,6	1,60E-05	CL0020
TRINITY_DN17125_c1_g1::TRINITY_DN17125_c1_g1_i9::g.78918::m.78918	4	11,4	48,456	6,2312	PF01417.19	ENTH	30,8	2,30E-07	CL0009
TRINITY_DN17130_c2_g2::TRINITY_DN17130_c2_g2_i6::g.79154::m.79154	9	19,2	72,084	25,559					
TRINITY_DN17135_c0_g1::TRINITY_DN17135_c0_g1_i6::g.79192::m.79192	2	10,2	36,059	4,4183	PF07910.12	Peptidase_C78	174,4	1,90E-51	CL0125
TRINITY_DN17136_c2_g3::TRINITY_DN17136_c2_g3_i6::g.79230::m.79230	4	33,1	16,8	7,8313	PF00071.21	Ras	95,1	3,00E-27	CL0023
TRINITY_DN17136_c2_g4::TRINITY_DN17136_c2_g4_i1::g.79232::m.79232	2	12,1	36,45	13,472					
TRINITY_DN17142_c0_g1::TRINITY_DN17142_c0_g1_i3::g.79276::m.79276	9	29,9	37,249	44,235	PF01026.20	TatD_DNase	168,3	1,90E-49	CL0034
TRINITY_DN17142_c1_g1::TRINITY_DN17142_c1_g1_i7::g.79281::m.79281	1	8,2	19,074	2,3354	PF05212.11	DUF707	109,4	1,90E-31	No_clan
TRINITY_DN17145_c0_g1::TRINITY_DN17145_c0_g1_i4::g.79323::m.79323	14	18,6	91,699	6,5289	PF00637.19	Clathrin	82,1	3,20E-23	CL0020
TRINITY_DN17145_c0_g3::TRINITY_DN17145_c0_g3_i2::g.79319::m.79319	16	20,8	104,19	125,86	PF00637.19	Clathrin	40,7	1,90E-10	CL0020
TRINITY_DN17145_c0_g4::TRINITY_DN17145_c0_g4_i1::g.79322::m.79322	12	19,5	84,112	65,177	PF01394.19	Clathrin_propel	26,2	6,20E-06	CL0020
TRINITY_DN17147_c0_g1::TRINITY_DN17147_c0_g1_i4::g.79344::m.79344	6	43	18,575	143,27	PF01073.18	3Beta_HSD	43,3	2,00E-11	CL0063
TRINITY_DN17150_c2_g1::TRINITY_DN17150_c2_g1_i1::g.79329::m.79329	7	36,8	27,379	68,548	PF10584.8	Proteasome_A_N	50,5	1,10E-13	CL0052
TRINITY_DN17150_c2_g1::TRINITY_DN17150_c2_g1_i7::g.79332::m.79332	6	32,4	27,465	2,8155	PF10584.8	Proteasome_A_N	50,5	1,10E-13	CL0052
TRINITY_DN17157_c0_g1::TRINITY_DN17157_c0_g1_i2::g.79386::m.79386	2	12,9	18,734	2,9809	PF06364.11	DUF1068	226,5	1,60E-67	No_clan
TRINITY_DN17158_c8_g2::TRINITY_DN17158_c8_g2_i1::g.79404::m.79404	4	16,1	28,399	14,479	PF01092.18	Ribosomal_S6e	197,2	6,80E-59	No_clan
TRINITY_DN17159_c3_g1::TRINITY_DN17159_c3_g1_i2::g.79474::m.79474	1	16	12,32	2,1789	PF04442.13	CtaG_Cox11	161,6	1,20E-47	No_clan
TRINITY_DN17160_c1_g3::TRINITY_DN17160_c1_g3_i2::g.79397::m.79397	2	5,2	66,693	9,6932	PF07738.12	Sad1_UNC	111,2	3,20E-32	CL0202

TRINITY_DN17163_c2_g2::TRINITY_DN17163_c2_g2_i2::g.79515::m.79515	1	13,3	14,128	2,0264	PF00348.16	polyprenyl_synt	59,6	2,20E-16	CL0613
TRINITY_DN17167_c2_g1::TRINITY_DN17167_c2_g1_i1::g.79577::m.79577	4	45,8	14,024	16,879	PF00295.16	Glyco_hydro_28	115,4	2,50E-33	CL0268
TRINITY_DN17167_c4_g1::TRINITY_DN17167_c4_g1_i2::g.79586::m.79586	2	17,2	18,262	4,1142	PF13561.5	adh_short_C2	118,1	4,10E-34	CL0063
TRINITY_DN17169_c0_g2::TRINITY_DN17169_c0_g2_i1::g.79657::m.79657	2	19,9	14,769	6,8585	PF05093.12	CIAPIN1	59,8	2,80E-16	No_clan
TRINITY_DN17174_c0_g2::TRINITY_DN17174_c0_g2_i1::g.79619::m.79619	2	22,2	12,391	3,5601	PF01781.17	Ribosomal_L38e	117,4	1,90E-34	No_clan
TRINITY_DN17177_c3_g1::TRINITY_DN17177_c3_g1_i5::g.79706::m.79706	11	53,5	24,467	66,789	PF00736.18	EF1_GNE	112,5	7,30E-33	No_clan
TRINITY_DN17183_c0_g2::TRINITY_DN17183_c0_g2_i5::g.79774::m.79774	5	29,5	22,846	10,948	PF02545.13	Maf	93,2	1,40E-26	CL0269
TRINITY_DN17189_c3_g1::TRINITY_DN17189_c3_g1_i6::g.79844::m.79844	3	9,9	34,496	4,1065	PF00244.19	14.03.2003	243,2	1,90E-72	No_clan
TRINITY_DN17192_c0_g2::TRINITY_DN17192_c0_g2_i8::g.79905::m.79905	2	11,3	25,296	10,004	PF00076.21	RRM_1	53,1	1,90E-14	CL0221
TRINITY_DN17195_c1_g1::TRINITY_DN17195_c1_g1_i2::g.79932::m.79932	6	10,5	87,284	12,536	PF02493.19	MORN	29,1	5,20E-07	CL0251
TRINITY_DN17198_c0_g1::TRINITY_DN17198_c0_g1_i4::g.79878::m.79878	2	5	46,885	4,084	PF14543.5	TAXi_N	156,5	7,40E-46	CL0129
TRINITY_DN17200_c0_g1::TRINITY_DN17200_c0_g1_i7::g.79915::m.79915	1	10,8	17,48	2,1037					
TRINITY_DN17201_c0_g1::TRINITY_DN17201_c0_g1_i1::g.79917::m.79917	7	15,2	64,777	13,703	PF02776.17	TPP_enzyme_N	122	1,70E-35	CL0254
TRINITY_DN17202_c2_g1::TRINITY_DN17202_c2_g1_i4::g.80033::m.80033	10	17,5	66,232	18,915	PF10151.8	TMEM214	86,9	1,00E-24	No_clan
TRINITY_DN17204_c0_g1::TRINITY_DN17204_c0_g1_i8::g.80281::m.80281	10	34,4	38,899	35,217	PF00675.19	Peptidase_M16	144,5	2,10E-42	CL0094
TRINITY_DN17207_c1_g1::TRINITY_DN17207_c1_g1_i5::g.80226::m.80226	6	12,6	71,209	10,771	PF14845.5	Glycohydro_20b2	67,6	1,60E-18	CL0546
TRINITY_DN17213_c1_g1::TRINITY_DN17213_c1_g1_i3::g.80196::m.80196	2	12,7	25,236	2,5808	PF04968.11	CHORD	86,5	1,30E-24	No_clan
TRINITY_DN17213_c1_g3::TRINITY_DN17213_c1_g3_i3::g.80204::m.80204	2	5,9	63,185	5,9024	PF03901.16	Glyco_transf_22	345,1	6,40E-103	CL0111
TRINITY_DN17214_c1_g1::TRINITY_DN17214_c1_g1_i9::g.80183::m.80183	5	17,5	37,49	12,286	PF00579.24	tRNA-synt_1b	21,3	0,00013	CL0039
TRINITY_DN17217_c1_g1::TRINITY_DN17217_c1_g1_i9::g.80247::m.80247	1	9,5	18,688	2,6044					
TRINITY_DN17219_c0_g1::TRINITY_DN17219_c0_g1_i4::g.80391::m.80391	2	28,7	15,66	6,8877	PF02466.18	Tim17	42,6	5,60E-11	No_clan

TRINITY_DN17221_c0_g2::TRINITY_DN17221_c0_g2_i11::g.80299::m.80299	9	19,5	67,288	59,408	PF00009.26	GTP_EFTU	145,5	1,30E-42	CL0023
TRINITY_DN17227_c2_g4::TRINITY_DN17227_c2_g4_i1::g.80549::m.80549	19	39	59,616	142,23	PF01565.22	FAD_binding_4	85,4	2,70E-24	CL0077
TRINITY_DN17228_c0_g1::TRINITY_DN17228_c0_g1_i3::g.80433::m.80433	2	12,2	18,526	2,1947	PF01246.19	Ribosomal_L24e	99,6	7,60E-29	CL0175
TRINITY_DN17228_c0_g2::TRINITY_DN17228_c0_g2_i3::g.80435::m.80435	3	26,9	13,837	4,5574	PF01246.19	Ribosomal_L24e	99,6	7,70E-29	CL0175
TRINITY_DN17229_c1_g1::TRINITY_DN17229_c1_g1_i16::g.80496::m.80496	2	9,8	25,323	2,2086	PF02798.19	GST_N	70,5	1,00E-19	CL0172
TRINITY_DN17234_c0_g1::TRINITY_DN17234_c0_g1_i11::g.80741::m.80741	2	19,4	17,315	5,955					
TRINITY_DN17237_c2_g3::TRINITY_DN17237_c2_g3_i7::g.80360::m.80360	13	17,2	106,21	12,563	PF01602.19	Adaptin_N	305,8	4,70E-91	CL0020
TRINITY_DN17241_c0_g1::TRINITY_DN17241_c0_g1_i1::g.80827::m.80827	2	3,3	87,666	2,9425	PF00082.21	Peptidase_S8	181	2,90E-53	No_clan
TRINITY_DN17242_c0_g2::TRINITY_DN17242_c0_g2_i1::g.80718::m.80718	5	14,8	45,752	24,236	PF00201.17	UDPGT	34,9	7,00E-09	CL0113
TRINITY_DN17244_c0_g1::TRINITY_DN17244_c0_g1_i3::g.79946::m.79946	9	25,5	49,271	76,661	PF00332.17	Glyco_hydro_17	225,9	7,10E-67	CL0058
TRINITY_DN17471_c1_g1::TRINITY_DN17471_c1_g1_i8::g.84225::m.84225	1	2,9	69,684	2,6761	PF16209.4	PhoLip_ATPase_N	76,8	7,20E-22	No_clan
TRINITY_DN17250_c2_g1::TRINITY_DN17250_c2_g1_i27::g.80938::m.80938	3	26,6	19,463	16,216	PF01217.19	Clat_adaptor_s	82,9	1,90E-23	CL0212
TRINITY_DN17254_c0_g1::TRINITY_DN17254_c0_g1_i5::g.81103::m.81103	8	33,6	34,339	31,442	PF00013.28	KH_1	62,4	2,50E-17	CL0007
TRINITY_DN17259_c0_g1::TRINITY_DN17259_c0_g1_i9::g.80974::m.80974	1	5,2	25,939	2,2078	PF05739.18	SNARE	44,2	1,30E-11	No_clan
TRINITY_DN17268_c0_g1::TRINITY_DN17268_c0_g1_i4::g.80463::m.80463	2	6	48,167	3,3463	PF00566.17	RabGAP-TBC	181,8	1,30E-53	No_clan
TRINITY_DN17271_c3_g3::TRINITY_DN17271_c3_g3_i8::g.81059::m.81059	3	24,9	21,047	10,828	PF03357.20	Snf7	151,5	1,50E-44	CL0235
TRINITY_DN17275_c2_g1::TRINITY_DN17275_c2_g1_i6::g.81136::m.81136	6	22,3	34,626	79,51	PF17144.3	Ribosomal_L5e	277	4,50E-83	CL0267
TRINITY_DN17277_c1_g3::TRINITY_DN17277_c1_g3_i4::g.81251::m.81251	19	54,6	39,53	323,31	PF00085.19	Thioredoxin	106	7,80E-31	CL0172
TRINITY_DN17278_c1_g1::TRINITY_DN17278_c1_g1_i1::g.81211::m.81211	2	18,9	14,409	3,8448	PF06592.12	DUF1138	146,9	1,30E-43	No_clan
TRINITY_DN17279_c2_g2::TRINITY_DN17279_c2_g2_i30::g.80027::m.80027	3	6,2	99,608	13,575	PF15277.5	Sec3-PIP2_bind	61,5	6,00E-17	CL0266
TRINITY_DN17281_c2_g3::TRINITY_DN17281_c2_g3_i2::g.81288::m.81288	4	26,3	17,335	7,9825	PF01253.21	SUI1	45,9	5,90E-12	No_clan
TRINITY_DN17286_c0_g1::TRINITY_DN17286_c0_g1_i7::g.81470::m.81470	3	3,9	115,56	3,2128	PF01074.21	Glyco_hydro_38	321,8	3,60E-96	CL0158

TRINITY_DN17286_c1_g1::TRINITY_DN17286_c1_g1_i1::g.81477::m.81477	3	3,8	113,49	6,2211	PF01074.21	Glyco_hydro_38	315,2	3,80E-94	CL0158
TRINITY_DN17290_c1_g1::TRINITY_DN17290_c1_g1_i4::g.81361::m.81361	5	48,3	20,675	251,93	PF00080.19	Sod_Cu	159,8	4,20E-47	No_clan
TRINITY_DN17302_c1_g3::TRINITY_DN17302_c1_g3_i1::g.81624::m.81624	2	2,7	106,84	2,5005	PF00403.25	HMA	46,4	3,70E-12	No_clan
TRINITY_DN17303_c1_g1::TRINITY_DN17303_c1_g1_i7::g.81652::m.81652	2	9,2	42,877	2,4992	PF00892.19	EamA	46,1	4,80E-12	CL0184
TRINITY_DN17307_c3_g1::TRINITY_DN17307_c3_g1_i2::g.81691::m.81691	2	5,8	41,601	3,0217	PF01979.19	Amidohydro_1	88,7	4,10E-25	CL0034
TRINITY_DN17310_c0_g1::TRINITY_DN17310_c0_g1_i2::g.81834::m.81834	2	9,8	35,186	5,5257	PF00076.21	RRM_1	63,4	1,20E-17	CL0221
TRINITY_DN17310_c0_g2::TRINITY_DN17310_c0_g2_i2::g.81838::m.81838	5	16,9	34,903	16,02	PF00638.17	Ran_BP1	65,6	4,50E-18	CL0266
TRINITY_DN17312_c0_g3::TRINITY_DN17312_c0_g3_i1::g.81763::m.81763	14	27,5	62,965	54,428	PF00389.29	2-Hacid_dh	119	1,00E-34	CL0325
TRINITY_DN17313_c1_g1::TRINITY_DN17313_c1_g1_i12::g.81814::m.81814	5	19,7	32,977	66,001	PF00102.26	Y_phosphatase	218,8	7,00E-65	CL0031
TRINITY_DN17314_c1_g1::TRINITY_DN17314_c1_g1_i1::g.81914::m.81914	1	11,3	15,132	2,0138	PF00781.23	DAGK_cat	74,2	6,30E-21	CL0240
TRINITY_DN17314_c1_g2::TRINITY_DN17314_c1_g2_i7::g.81924::m.81924	3	19,5	23,887	5,5341	PF00609.18	DAGK_acc	83	2,70E-23	No_clan
TRINITY_DN17321_c2_g1::TRINITY_DN17321_c2_g1_i8::g.82045::m.82045	2	15,6	22,724	3,3477					
TRINITY_DN17321_c2_g2::TRINITY_DN17321_c2_g2_i3::g.82053::m.82053	3	11,3	33,609	4,7196	PF00210.23	Ferritin	113,5	6,80E-33	CL0044
TRINITY_DN17322_c1_g1::TRINITY_DN17322_c1_g1_i1::g.82026::m.82026	2	5,3	57,941	5,8956	PF12739.6	TRAPPC-Trs85	238,1	1,50E-70	No_clan
TRINITY_DN17327_c0_g4::TRINITY_DN17327_c0_g4_i4::g.82004::m.82004	2	15,2	17,992	4,0109					
TRINITY_DN17332_c3_g1::TRINITY_DN17332_c3_g1_i4::g.82072::m.82072	2	10,7	38,074	5,0997	PF05153.14	MIOX	409,7	4,20E-123	CL0237
TRINITY_DN17333_c5_g3::TRINITY_DN17333_c5_g3_i2::g.82453::m.82453	3	17,5	30,866	7,4055	PF00956.17	NAP	27,2	1,80E-06	No_clan
TRINITY_DN17336_c2_g1::TRINITY_DN17336_c2_g1_i3::g.82080::m.82080	3	5,9	67,464	4,6713	PF08323.10	Glyco_transf_5	181,9	1,60E-53	CL0113
TRINITY_DN17336_c6_g1::TRINITY_DN17336_c6_g1_i1::g.82089::m.82089	3	9,4	54,676	5,4287	PF01039.21	Carboxyl_trans	494,9	2,20E-148	CL0127
TRINITY_DN17340_c1_g1::TRINITY_DN17340_c1_g1_i17::g.82513::m.82513	1	14,9	17,571	2,235					
TRINITY_DN17343_c0_g2::TRINITY_DN17343_c0_g2_i1::g.82237::m.82237	4	6,8	97,362	8,5934	PF00962.21	A_deaminase	424,6	2,70E-127	CL0034

TRINITY_DN17345_c4_g3::TRINITY_DN17345_c4_g3_i2::g.81576::m.81576	2	24,6	12,801	4,4026					
TRINITY_DN17351_c1_g1::TRINITY_DN17351_c1_g1_i15::g.82398::m.82398	3	19,3	21,714	1,8645	PF13344.5	Hydrolase_6	121,4	1,40E-35	CL0137
TRINITY_DN17353_c2_g2::TRINITY_DN17353_c2_g2_i2::g.82341::m.82341	2	13,6	28,481	2,0458					
TRINITY_DN17355_c4_g1::TRINITY_DN17355_c4_g1_i4::g.82584::m.82584	23	34,6	84,78	323,31	PF08267.11	Meth_synt_1	395,9	1,40E-118	CL0160
TRINITY_DN17355_c4_g1::TRINITY_DN17355_c4_g1_i11::g.82598::m.82598	28	53,8	71,977	323,31	PF08267.11	Meth_synt_1	391,5	3,10E-117	CL0160
TRINITY_DN17356_c4_g2::TRINITY_DN17356_c4_g2_i4::g.82383::m.82383	1	2,4	52,406	2,0028	PF03062.18	MBOAT	211,2	2,30E-62	CL0517
TRINITY_DN17360_c0_g1::TRINITY_DN17360_c0_g1_i6::g.82556::m.82556	2	8,4	39,02	4,071					
TRINITY_DN17369_c1_g1::TRINITY_DN17369_c1_g1_i7::g.82610::m.82610	7	33,5	25,349	299,69	PF00227.25	Proteasome	163,2	4,50E-48	CL0052
TRINITY_DN17373_c0_g1::TRINITY_DN17373_c0_g1_i1::g.82647::m.82647	6	15,9	54,294	22,873	PF00350.22	Dynamin_N	74,7	8,10E-21	CL0023
TRINITY_DN17374_c2_g1::TRINITY_DN17374_c2_g1_i5::g.82712::m.82712	2	5,7	48,694	2,3974	PF00083.23	Sugar_tr	351,2	7,80E-105	CL0015
TRINITY_DN17375_c4_g1::TRINITY_DN17375_c4_g1_i2::g.82845::m.82845	4	9,6	69,23	6,2822	PF03453.16	MoeA_N	134,3	2,70E-39	No_clan
TRINITY_DN17380_c2_g1::TRINITY_DN17380_c2_g1_i4::g.82866::m.82866	3	4,4	96,987	4,7508	PF03810.18	IBN_N	42,8	3,40E-11	CL0020
TRINITY_DN17383_c0_g1::TRINITY_DN17383_c0_g1_i3::g.82754::m.82754	4	18,4	36,43	9,8646	PF00557.23	Peptidase_M24	149,6	8,60E-44	No_clan
TRINITY_DN17384_c3_g1::TRINITY_DN17384_c3_g1_i3::g.82818::m.82818	10	37,1	41,958	20,226	PF00291.24	PALP	223,2	4,40E-66	No_clan
TRINITY_DN17386_c2_g1::TRINITY_DN17386_c2_g1_i2::g.82885::m.82885	2	3,1	97,775	6,5689	PF00999.20	Na_H_Exchanger	154,7	2,60E-45	CL0064
TRINITY_DN17387_c1_g1::TRINITY_DN17387_c1_g1_i3::g.82928::m.82928	11	10,4	154,08	30,638	PF15044.5	CLU_N	72,2	3,20E-20	No_clan
TRINITY_DN17388_c0_g2::TRINITY_DN17388_c0_g2_i7::g.82945::m.82945	2	11	29,938	5,3748	PF00834.18	Ribul_P_3_epim	294,7	2,20E-88	CL0036
TRINITY_DN17394_c0_g1::TRINITY_DN17394_c0_g1_i5::g.83048::m.83048	1	3,9	33,92	2,777					
TRINITY_DN17396_c1_g1::TRINITY_DN17396_c1_g1_i2::g.82990::m.82990	13	12,6	184,24	30,425	PF06427.10	UDP-g_GGTase	1109,4	0	No_clan
TRINITY_DN17401_c1_g1::TRINITY_DN17401_c1_g1_i4::g.83099::m.83099	2	9,4	32,104	3,5004	PF00557.23	Peptidase_M24	117,2	7,20E-34	No_clan
TRINITY_DN17401_c2_g3::TRINITY_DN17401_c2_g3_i2::g.83107::m.83107	1	3,2	51,384	2,149	PF01490.17	Aa_trans	208,5	1,30E-61	CL0062

TRINITY_DN17405_c1_g3::TRINITY_DN17405_c1_g3_i1::g.83287::m.83287	9	34,3	35,924	38,243	PF00970.23	FAD_binding_6	86	1,60E-24	CL0076
TRINITY_DN17406_c4_g1::TRINITY_DN17406_c4_g1_i2::g.83243::m.83243	2	7,1	30,315	2,1456	PF00348.16	polyprenyl_synt	121,6	2,60E-35	CL0613
TRINITY_DN17406_c4_g4::TRINITY_DN17406_c4_g4_i2::g.83246::m.83246	11	21,2	77,917	31,655	PF02847.16	MA3	87,3	6,00E-25	CL0020
TRINITY_DN17420_c0_g1::TRINITY_DN17420_c0_g1_i9::g.83584::m.83584	14	58,7	37,063	7,9528	PF00044.23	Gp_dh_N	115,7	9,50E-34	CL0063
TRINITY_DN17420_c0_g1::TRINITY_DN17420_c0_g1_i7::g.83574::m.83574	15	65,5	35,66	142,52	PF00044.23	Gp_dh_N	97,4	4,60E-28	CL0063
TRINITY_DN17420_c0_g2::TRINITY_DN17420_c0_g2_i1::g.83570::m.83570	19	68,9	36,848	95,685	PF00044.23	Gp_dh_N	113,5	4,40E-33	CL0063
TRINITY_DN17420_c0_g2::TRINITY_DN17420_c0_g2_i5::g.83586::m.83586	14	68,7	26,574	-2	PF02800.19	Gp_dh_C	240,8	4,10E-72	CL0139
TRINITY_DN17420_c0_g3::TRINITY_DN17420_c0_g3_i2::g.83576::m.83576	19	72,1	36,682	284,85	PF00044.23	Gp_dh_N	114,2	2,70E-33	CL0063
TRINITY_DN17427_c0_g1::TRINITY_DN17427_c0_g1_i5::g.83626::m.83626	9	34,9	36,722	-2	PF00933.20	Glyco_hydro_3	175,4	1,70E-51	CL0058
TRINITY_DN17427_c0_g1::TRINITY_DN17427_c0_g1_i16::g.83642::m.83642	12	22,9	73,509	48,211	PF00933.20	Glyco_hydro_3	233,2	4,40E-69	CL0058
TRINITY_DN17427_c0_g1::TRINITY_DN17427_c0_g1_i3::g.83624::m.83624	6	17,1	42,401	45,489	PF00933.20	Glyco_hydro_3	57,5	1,30E-15	CL0058
TRINITY_DN17431_c5_g1::TRINITY_DN17431_c5_g1_i7::g.83828::m.83828	2	3	93,814	2,8622	PF00076.21	RRM_1	44,3	1,10E-11	CL0221
TRINITY_DN17433_c1_g2::TRINITY_DN17433_c1_g2_i1::g.83589::m.83589	2	16,7	14,563	3,6753	PF06747.12	CHCH	37,1	2,20E-09	CL0351
TRINITY_DN17435_c0_g1::TRINITY_DN17435_c0_g1_i2::g.83592::m.83592	3	20,4	25,438	6,7662	PF00160.20	Pro_isomerase	113,8	8,20E-33	CL0475
TRINITY_DN17436_c0_g1::TRINITY_DN17436_c0_g1_i7::g.83790::m.83790	2	3,4	119,32	4,0223					
TRINITY_DN17440_c0_g1::TRINITY_DN17440_c0_g1_i7::g.84097::m.84097	5	10,5	76,306	24,349	PF00226.30	DnaJ	67,8	6,20E-19	CL0392
TRINITY_DN17440_c0_g3::TRINITY_DN17440_c0_g3_i6::g.84091::m.84091	13	58,1	29,962	323,31	PF00996.17	GDI	466,7	5,20E-140	CL0063
TRINITY_DN17440_c0_g5::TRINITY_DN17440_c0_g5_i3::g.84089::m.84089	6	37,1	18,855	138,61	PF00996.17	GDI	218	1,50E-64	CL0063
TRINITY_DN17441_c2_g4::TRINITY_DN17441_c2_g4_i3::g.83805::m.83805	1	5,2	39,764	2,1896	PF13850.5	ERGIC_N	95,3	1,90E-27	No_clan
TRINITY_DN17443_c0_g6::TRINITY_DN17443_c0_g6_i1::g.83849::m.83849	3	6,2	63,955	5,6084	PF01565.22	FAD_binding_4	61,1	8,30E-17	CL0077
TRINITY_DN17448_c2_g2::TRINITY_DN17448_c2_g2_i8::g.83900::m.83900	10	50,6	34,828	77,013	PF02209.18	VHP	55,7	3,50E-15	No_clan

TRINITY_DN17448_c2_g3::TRINITY_DN17448_c2_g3_i9::g.83903::m.83903	21	40,6	70,429	323,31	PF00626.21	Gelsolin	61,6	4,40E-17	CL0092
TRINITY_DN17451_c1_g1::TRINITY_DN17451_c1_g1_i3::g.84062::m.84062	1	6,3	34,095	3,2977					
TRINITY_DN17453_c0_g6::TRINITY_DN17453_c0_g6_i1::g.83881::m.83881	1	13,7	10,883	1,8691					
TRINITY_DN17454_c3_g1::TRINITY_DN17454_c3_g1_i1::g.84103::m.84103	4	12,8	36,913	4,7346	PF07859.12	Abhydrolase_3	177,9	2,20E-52	CL0028
TRINITY_DN17456_c0_g1::TRINITY_DN17456_c0_g1_i8::g.83943::m.83943	4	23,7	21,594	9,9987	PF00719.18	Pyrophosphatase	160,8	2,10E-47	No_clan
TRINITY_DN17457_c4_g1::TRINITY_DN17457_c4_g1_i4::g.83960::m.83960	3	14,6	23,47	205,19	PF01652.17	IF4E	182	6,10E-54	CL0625
TRINITY_DN17461_c1_g1::TRINITY_DN17461_c1_g1_i12::g.84192::m.84192	5	10,9	46,099	16,166	PF00160.20	Pro_isomerase	133,8	5,70E-39	CL0475
TRINITY_DN17463_c2_g1::TRINITY_DN17463_c2_g1_i7::g.84144::m.84144	3	10,8	40,116	5,3882	PF00128.23	Alpha-amylase	44	1,90E-11	CL0058
TRINITY_DN17467_c0_g2::TRINITY_DN17467_c0_g2_i8::g.84133::m.84133	1	5,5	26,018	2,0014	PF13877.5	RPAP3_C	84,1	7,00E-24	No_clan
TRINITY_DN17469_c0_g1::TRINITY_DN17469_c0_g1_i8::g.84329::m.84329	6	40,1	21,931	24,193	PF00071.21	Ras	222,1	2,60E-66	CL0023
TRINITY_DN17475_c1_g1::TRINITY_DN17475_c1_g1_i10::g.84374::m.84374	4	9,1	77,928	7,7034	PF10408.8	Ufd2P_core	597,3	3,50E-179	No_clan
TRINITY_DN17483_c0_g1::TRINITY_DN17483_c0_g1_i3::g.84425::m.84425	3	3,3	142,17	8,6973	PF13001.6	Ecm29	293,1	3,50E-87	No_clan
TRINITY_DN17484_c0_g3::TRINITY_DN17484_c0_g3_i1::g.84435::m.84435	6	35,6	21,921	14,427	PF08712.10	Nfu_N	109,8	4,70E-32	No_clan
TRINITY_DN17490_c0_g3::TRINITY_DN17490_c0_g3_i5::g.84477::m.84477	1	5,4	25,108	1,8779	PF00069.24	Pkinase	62,3	4,20E-17	CL0016
TRINITY_DN17493_c2_g1::TRINITY_DN17493_c2_g1_i7::g.84644::m.84644	8	19,1	57,001	15,021	PF00171.21	Aldedh	573,6	2,30E-172	CL0099
TRINITY_DN17498_c2_g1::TRINITY_DN17498_c2_g1_i6::g.83121::m.83121	5	8,5	83,225	9,3271	PF00483.22	NTP_transferase	40,6	2,00E-10	CL0110
TRINITY_DN17501_c0_g2::TRINITY_DN17501_c0_g2_i6::g.84659::m.84659	10	28,3	69,454	26,996	PF02225.21	PA	45,1	7,40E-12	CL0364
TRINITY_DN17503_c0_g1::TRINITY_DN17503_c0_g1_i2::g.84892::m.84892	3	25,2	13,712	6,7338					
TRINITY_DN17503_c1_g2::TRINITY_DN17503_c1_g2_i5::g.84903::m.84903	5	9,2	65,743	8,627	PF05701.10	WEMBL	108,6	3,00E-31	No_clan
TRINITY_DN17506_c1_g1::TRINITY_DN17506_c1_g1_i6::g.84994::m.84994	21	28,6	91,906	49,502	PF03141.15	Methyltransf_29	777,9	3,60E-234	CL0063
TRINITY_DN17507_c1_g2::TRINITY_DN17507_c1_g2_i9::g.85053::m.85053	2	4,3	69,193	3,5754	PF03969.15	AFG1_ATPase	237,7	1,60E-70	CL0023

TRINITY_DN17508_c4_g1::TRINITY_DN17508_c4_g1_i3::g.85406::m.85406	1	7	25,471	2,5218					
TRINITY_DN17508_c5_g2::TRINITY_DN17508_c5_g2_i5::g.85417::m.85417	2	16,8	13,743	7,7982	PF00295.16	Glyco_hydro_28	149,5	1,10E-43	CL0268
TRINITY_DN17508_c5_g4::TRINITY_DN17508_c5_g4_i2::g.85425::m.85425	2	41	10,482	3,7623	PF12708.6	Pectate_lyase_3	29,9	4,70E-07	CL0268
TRINITY_DN17513_c0_g1::TRINITY_DN17513_c0_g1_i8::g.85061::m.85061	3	3,8	140,89	8,9376	PF12612.7	TFCD_C	181,8	1,10E-53	No_clan
TRINITY_DN17515_c0_g1::TRINITY_DN17515_c0_g1_i3::g.85102::m.85102	8	16,4	67,94	19,394	PF01268.18	FTHFS	844,1	3,30E-254	CL0023
TRINITY_DN17518_c0_g1::TRINITY_DN17518_c0_g1_i5::g.85741::m.85741	3	21,5	22,259	9,1185	PF03931.14	Skp1_POZ	107,2	3,20E-31	CL0033
TRINITY_DN17518_c0_g2::TRINITY_DN17518_c0_g2_i4::g.85745::m.85745	2	17,2	19,307	3,4562	PF03931.14	Skp1_POZ	105,4	1,20E-30	CL0033
TRINITY_DN17522_c3_g1::TRINITY_DN17522_c3_g1_i3::g.85131::m.85131	1	9,4	12,605	2,0874	PF00238.18	Ribosomal_L14	159,8	2,50E-47	No_clan
TRINITY_DN17524_c0_g2::TRINITY_DN17524_c0_g2_i3::g.85311::m.85311	2	9,1	34,251	2,8728	PF08543.11	Phos_pyr_kin	53,4	2,20E-14	CL0118
TRINITY_DN17527_c0_g3::TRINITY_DN17527_c0_g3_i1::g.85247::m.85247	3	5,7	50,55	4,751	PF05631.13	MFS_5	628,4	2,50E-189	CL0015
TRINITY_DN17528_c1_g1::TRINITY_DN17528_c1_g1_i8::g.85347::m.85347	2	17,2	19,447	5,0124	PF00350.22	Dynamin_N	106,1	1,80E-30	CL0023
TRINITY_DN17528_c1_g2::TRINITY_DN17528_c1_g2_i6::g.85342::m.85342	6	12,5	68,444	8,874	PF00350.22	Dynamin_N	188,2	1,00E-55	CL0023
TRINITY_DN17530_c3_g12::TRINITY_DN17530_c3_g12_i1::g.85464::m.85464	1	11	12,49	13,922	PF02326.14	YMF19	84,1	8,00E-24	CL0255
TRINITY_DN17531_c3_g1::TRINITY_DN17531_c3_g1_i2::g.85473::m.85473	2	15,5	17,009	3,3485					
TRINITY_DN17539_c0_g2::TRINITY_DN17539_c0_g2_i3::g.85610::m.85610	5	11,1	76,905	16,76	PF01103.22	Bac_surface_Ag	59,3	4,00E-16	CL0193
TRINITY_DN17541_c1_g1::TRINITY_DN17541_c1_g1_i11::g.86034::m.86034	1	2,2	99,52	2,8989					
TRINITY_DN17545_c0_g1::TRINITY_DN17545_c0_g1_i1::g.84935::m.84935	11	20,1	81,759	6,064	PF00458.19	WHEP-TRS	33,5	3,30E-08	CL0600
TRINITY_DN17545_c0_g1::TRINITY_DN17545_c0_g1_i11::g.84946::m.84946	11	20,9	77,353	2,0152	PF00458.19	WHEP-TRS	35,2	9,70E-09	CL0600
TRINITY_DN17545_c0_g1::TRINITY_DN17545_c0_g1_i5::g.84940::m.84940	12	23,4	77,263	29,386	PF00458.19	WHEP-TRS	35,2	9,70E-09	CL0600
TRINITY_DN17548_c3_g2::TRINITY_DN17548_c3_g2_i2::g.85798::m.85798	6	23,5	33,783	14,22	PF04321.16	RmlD_sub_bind	50,6	1,30E-13	CL0063
TRINITY_DN17549_c7_g7::TRINITY_DN17549_c7_g7_i1::g.85830::m.85830	2	28,2	11,347	25,806	PF11721.7	Malectin	29,1	8,70E-07	CL0468

TRINITY_DN17553_c1_g1::TRINITY_DN17553_c1_g1_i2::g.85833::m.85833	2	10,1	22,435	31,183	PF00255.18	GSHPx	142,6	2,80E-42	CL0172
TRINITY_DN17557_c0_g1::TRINITY_DN17557_c0_g1_i2::g.85865::m.85865	2	6,7	31,878	2,45	PF13519.5	VWA_2	97,8	4,80E-28	CL0128
TRINITY_DN17558_c0_g1::TRINITY_DN17558_c0_g1_i9::g.85984::m.85984	10	23,1	66,483	33,16	PF00389.29	2-Hacid_dh	118,2	1,70E-34	CL0325
TRINITY_DN17561_c0_g1::TRINITY_DN17561_c0_g1_i2::g.86132::m.86132	6	4,1	274,67	7,7336	PF08082.10	PRO8NT	283,2	4,20E-85	No_clan
TRINITY_DN17564_c2_g2::TRINITY_DN17564_c2_g2_i9::g.86086::m.86086	9	19	70,829	9,7716	PF00076.21	RRM_1	70,4	7,50E-20	CL0221
TRINITY_DN17564_c2_g2::TRINITY_DN17564_c2_g2_i5::g.86079::m.86079	8	18,6	53,371	15,75	PF01842.24	ACT	45,3	4,80E-12	CL0070
TRINITY_DN17566_c1_g1::TRINITY_DN17566_c1_g1_i8::g.86112::m.86112	8	24,2	59,201	18,32	PF00682.18	HMGL-like	302,8	2,10E-90	CL0036
TRINITY_DN17573_c2_g1::TRINITY_DN17573_c2_g1_i14::g.86248::m.86248	2	13,3	26,666	3,6355					
TRINITY_DN17576_c2_g1::TRINITY_DN17576_c2_g1_i10::g.86267::m.86267	2	24,6	12,96	4,6442					
TRINITY_DN17578_c2_g1::TRINITY_DN17578_c2_g1_i2::g.85652::m.85652	1	5,1	41,293	3,1935	PF01398.20	JAB	118,1	1,90E-34	CL0366
TRINITY_DN17578_c3_g1::TRINITY_DN17578_c3_g1_i5::g.85666::m.85666	13	17,5	119,74	37,309	PF00862.18	Sucrose_synth	40,4	1,10E-10	CL0113
TRINITY_DN17586_c3_g1::TRINITY_DN17586_c3_g1_i5::g.86439::m.86439	3	5,4	87,068	6,2634	PF04499.14	SAPS	107,8	5,70E-31	No_clan
TRINITY_DN17586_c3_g4::TRINITY_DN17586_c3_g4_i6::g.86457::m.86457	2	5,2	58,755	2,4843	PF04499.14	SAPS	143,7	7,10E-42	No_clan
TRINITY_DN17588_c1_g2::TRINITY_DN17588_c1_g2_i1::g.86528::m.86528	2	12,9	21,982	4,2696	PF01641.17	SelR	160,9	1,10E-47	CL0080
TRINITY_DN17602_c0_g2::TRINITY_DN17602_c0_g2_i1::g.86960::m.86960	2	1,1	229,56	2,9981	PF13764.5	E3_UbLigase_R4	1090,2	0	No_clan
TRINITY_DN17602_c0_g3::TRINITY_DN17602_c0_g3_i7::g.86972::m.86972	4	10,9	40,891	5,9301	PF01380.21	SIS	114,1	3,60E-33	CL0067
TRINITY_DN17602_c0_g7::TRINITY_DN17602_c0_g7_i2::g.86978::m.86978	3	2,6	184,26	6,1009	PF00569.16	ZZ	36,5	2,90E-09	CL0006
TRINITY_DN17610_c1_g1::TRINITY_DN17610_c1_g1_i7::g.87051::m.87051	1	12	18,418	2,5041					
TRINITY_DN17612_c1_g2::TRINITY_DN17612_c1_g2_i3::g.87024::m.87024	3	5,2	96,13	5,4543	PF03810.18	IBN_N	50,6	1,20E-13	CL0020
TRINITY_DN17612_c1_g4::TRINITY_DN17612_c1_g4_i2::g.87038::m.87038	2	6,1	59,426	4,5283	PF13513.5	HEAT_EZ	34,4	2,00E-08	CL0020
TRINITY_DN17613_c0_g1::TRINITY_DN17613_c0_g1_i11::g.87113::m.87113	4	8	87,617	9,9925	PF01434.17	Peptidase_M41	142,6	1,20E-41	CL0126
TRINITY_DN17614_c0_g2::TRINITY_DN17614_c0_g2_i5::g.87093::m.87093	20	42,9	61,676	230,15	PF00085.19	Thioredoxin	106,1	7,50E-31	CL0172

TRINITY_DN17618_c2_g2::TRINITY_DN17618_c2_g2_i9::g.87148::m.87148	2	27,1	13,265	7,0175	PF00067.21	p450	78,2	4,80E-22	No_clan
TRINITY_DN17619_c0_g2::TRINITY_DN17619_c0_g2_i5::g.87168::m.87168	2	4,4	74,895	3,0346	PF08553.9	VID27	166,7	8,80E-49	No_clan
TRINITY_DN17621_c1_g3::TRINITY_DN17621_c1_g3_i2::g.87259::m.87259	8	21,9	47,142	17,338	PF02020.17	W2	70,2	1,10E-19	CL0020
TRINITY_DN17623_c2_g1::TRINITY_DN17623_c2_g1_i4::g.87423::m.87423	3	33,6	14,225	91,099	PF06521.10	PAR1	142,9	5,50E-42	No_clan
TRINITY_DN17623_c4_g3::TRINITY_DN17623_c4_g3_i4::g.87434::m.87434	2	23,8	14,074	6,6821					
TRINITY_DN17625_c1_g2::TRINITY_DN17625_c1_g2_i5::g.87298::m.87298	1	2,9	53,233	2,0992					
TRINITY_DN17627_c2_g1::TRINITY_DN17627_c2_g1_i6::g.87313::m.87313	3	14,3	24,448	6,2436	PF14681.5	UPRTase	231,9	4,60E-69	CL0533
TRINITY_DN17630_c0_g1::TRINITY_DN17630_c0_g1_i8::g.87337::m.87337	7	50	14,722	323,31	PF00462.23	Glutaredoxin	68,1	5,40E-19	CL0172
TRINITY_DN17631_c3_g6::TRINITY_DN17631_c3_g6_i1::g.87349::m.87349	3	20,5	21,149	13,781	PF04969.15	CS	34	4,20E-08	CL0190
TRINITY_DN17638_c0_g5::TRINITY_DN17638_c0_g5_i1::g.87396::m.87396	6	45,3	16,868	14,047	PF00295.16	Glyco_hydro_28	113,9	7,30E-33	CL0268
TRINITY_DN17644_c1_g2::TRINITY_DN17644_c1_g2_i9::g.87544::m.87544	6	15,9	53,332	14,761	PF00199.18	Catalase	582,3	4,20E-175	No_clan
TRINITY_DN17658_c5_g3::TRINITY_DN17658_c5_g3_i2::g.87770::m.87770	2	11,3	20,862	2,462	PF00227.25	Proteasome	108,3	3,10E-31	CL0052
TRINITY_DN17662_c4_g1::TRINITY_DN17662_c4_g1_i8::g.87880::m.87880	10	38,3	40,044	141,26	PF00288.25	GHMP_kinases_N	40,7	1,90E-10	CL0329
TRINITY_DN17665_c0_g2::TRINITY_DN17665_c0_g2_i3::g.87796::m.87796	1	7,8	13,101	4,4317	PF13417.5	GST_N_3	72,4	2,70E-20	CL0172
TRINITY_DN17670_c0_g2::TRINITY_DN17670_c0_g2_i7::g.87973::m.87973	5	10,8	57,444	9,2305	PF10250.8	O-FucT	224,9	2,00E-66	CL0113
TRINITY_DN17673_c0_g1::TRINITY_DN17673_c0_g1_i5::g.88024::m.88024	1	2,2	60,42	2,2779	PF03552.13	Cellulose_synt	607,1	4,90E-182	CL0110
TRINITY_DN17673_c0_g1::TRINITY_DN17673_c0_g1_i8::g.88029::m.88029	1	24,1	11,805	1,8422					
TRINITY_DN17674_c1_g2::TRINITY_DN17674_c1_g2_i8::g.88061::m.88061	3	8,2	58,114	5,4841	PF08241.11	Methyltransf_11	29,1	1,10E-06	CL0063
TRINITY_DN17675_c2_g3::TRINITY_DN17675_c2_g3_i6::g.88141::m.88141	4	18,5	24,176	59,901	PF00213.17	OSCP	126,7	9,50E-37	CL0255
TRINITY_DN17680_c4_g1::TRINITY_DN17680_c4_g1_i22::g.88254::m.88254	4	8,7	65,306	6,7258	PF07651.15	ANTH	323,8	6,20E-97	CL0009
TRINITY_DN17687_c2_g1::TRINITY_DN17687_c2_g1_i6::g.88326::m.88326	6	24	34,738	14,405	PF08240.11	ADH_N	35,9	5,20E-09	CL0296

TRINITY_DN17688_c2_g2::TRINITY_DN17688_c2_g2_i2::g.88270::m.88270	1	8,7	14,025	2,2359	PF00759.18	Glyco_hydro_9	23,5	3,20E-05	CL0059
TRINITY_DN17688_c2_g3::TRINITY_DN17688_c2_g3_i4::g.88274::m.88274	1	9,1	10,755	10,174	PF00759.18	Glyco_hydro_9	88,6	5,20E-25	CL0059
TRINITY_DN17688_c2_g6::TRINITY_DN17688_c2_g6_i1::g.88272::m.88272	2	23,5	12,696	135,97	PF00759.18	Glyco_hydro_9	107,3	1,10E-30	CL0059
TRINITY_DN17690_c0_g3::TRINITY_DN17690_c0_g3_i4::g.88277::m.88277	8	68,8	13,378	294,48	PF01042.20	Ribonuc_L-PSP	139,3	5,30E-41	CL0534
TRINITY_DN17695_c2_g1::TRINITY_DN17695_c2_g1_i3::g.88380::m.88380	1	0,6	224,76	1,8997	PF03104.18	DNA_pol_B_exo1	278,1	8,70E-83	CL0219
TRINITY_DN17697_c2_g1::TRINITY_DN17697_c2_g1_i5::g.88410::m.88410	8	16,6	77,439	16,48	PF03460.16	NIR_SIR_ferr	58,8	3,20E-16	No_clan
TRINITY_DN17699_c0_g2::TRINITY_DN17699_c0_g2_i2::g.88416::m.88416	12	10,4	177,31	23,953	PF00310.20	GATase_2	587,6	1,10E-176	CL0052
TRINITY_DN17700_c1_g1::TRINITY_DN17700_c1_g1_i9::g.86845::m.86845	3	15,1	29,123	5,0753	PF00378.19	ECH_1	177,1	3,50E-52	CL0127
TRINITY_DN17700_c2_g2::TRINITY_DN17700_c2_g2_i11::g.86871::m.86871	1	11,9	13,451	4,1379					
TRINITY_DN17704_c0_g1::TRINITY_DN17704_c0_g1_i3::g.88593::m.88593	4	6,8	89,339	7,3981	PF04548.15	AIG1	102,9	1,40E-29	CL0023
TRINITY_DN17708_c1_g1::TRINITY_DN17708_c1_g1_i3::g.88548::m.88548	2	8,8	49,104	25,477	PF00022.18	Actin	510,3	2,80E-153	CL0108
TRINITY_DN17715_c1_g2::TRINITY_DN17715_c1_g2_i7::g.88682::m.88682	4	21,6	31,142	19,318	PF00923.18	TAL_FSA	163,5	6,30E-48	CL0036
TRINITY_DN17716_c3_g1::TRINITY_DN17716_c3_g1_i5::g.88746::m.88746	6	21,3	45,039	12,305	PF02403.21	Seryl_tRNA_N	30,6	2,80E-07	CL0298
TRINITY_DN17717_c2_g1::TRINITY_DN17717_c2_g1_i3::g.88728::m.88728	1	8,9	12,464	1,958					
TRINITY_DN17718_c6_g2::TRINITY_DN17718_c6_g2_i4::g.88774::m.88774	2	2,2	91,863	3,3245	PF14381.5	EDR1	189,1	6,70E-56	CL0125
TRINITY_DN17727_c1_g3::TRINITY_DN17727_c1_g3_i1::g.88887::m.88887	1	3,6	34,891	1,8657	PF00450.21	Peptidase_S10	325,9	4,90E-97	CL0028
TRINITY_DN17731_c6_g1::TRINITY_DN17731_c6_g1_i4::g.89011::m.89011	2	16,3	22,177	2,042					
TRINITY_DN17735_c1_g1::TRINITY_DN17735_c1_g1_i1::g.89033::m.89033	2	12,1	17,799	30,531	PF13602.5	ADH_zinc_N_2	75,7	6,50E-21	CL0063
TRINITY_DN17735_c1_g2::TRINITY_DN17735_c1_g2_i2::g.89035::m.89035	2	12,1	18,556	3,4276	PF08240.11	ADH_N	36,6	3,10E-09	CL0296
TRINITY_DN17739_c0_g2::TRINITY_DN17739_c0_g2_i3::g.89048::m.89048	1	7,8	19,052	20,191	PF05512.10	AWPM-19	180,1	2,30E-53	No_clan
TRINITY_DN17740_c0_g1::TRINITY_DN17740_c0_g1_i2::g.89044::m.89044	5	36,8	18,593	35,451	PF00155.20	Aminotran_1_2	53,7	1,70E-14	CL0061

TRINITY_DN17740_c0_g2::TRINITY_DN17740_c0_g2_i7::g.89045::m.89045	6	35,3	30,36	21,767	PF00155.20	Aminotran_1_2	62,6	3,40E-17	CL0061
TRINITY_DN17744_c7_g1::TRINITY_DN17744_c7_g1_i4::g.89103::m.89103	2	10,5	24,364	3,9818	PF14938.5	SNAP	77,4	1,10E-21	CL0020
TRINITY_DN17745_c1_g5::TRINITY_DN17745_c1_g5_i2::g.88501::m.88501	1	14,5	11,157	2,0359	PF00428.18	Ribosomal_60s	86,6	1,20E-24	No_clan
TRINITY_DN17751_c2_g3::TRINITY_DN17751_c2_g3_i5::g.89327::m.89327	22	36,7	72,353	167,71	PF16363.4	GDP_Man_Dehyd	206,7	5,20E-61	CL0063
TRINITY_DN17755_c1_g2::TRINITY_DN17755_c1_g2_i5::g.89168::m.89168	5	14	40,499	8,1959	PF00364.21	Biotin_lipoyl	60	1,40E-16	CL0105
TRINITY_DN17756_c1_g1::TRINITY_DN17756_c1_g1_i7::g.89229::m.89229	4	4,4	156,9	6,5878	PF14443.5	DBC1	144	2,10E-42	No_clan
TRINITY_DN17758_c1_g2::TRINITY_DN17758_c1_g2_i7::g.89243::m.89243	3	6,3	41,919	4,2894	PF03839.15	Sec62	49,2	4,10E-13	No_clan
TRINITY_DN17765_c0_g1::TRINITY_DN17765_c0_g1_i1::g.89341::m.89341	4	16,6	38,439	8,3268	PF02574.15	S-methyl_trans	262,3	5,80E-78	No_clan
TRINITY_DN17769_c1_g1::TRINITY_DN17769_c1_g1_i1::g.89345::m.89345	1	8,4	14,522	99,462					
TRINITY_DN17769_c1_g1::TRINITY_DN17769_c1_g1_i4::g.89348::m.89348	1	18,5	15,423	42,045					
TRINITY_DN17776_c0_g1::TRINITY_DN17776_c0_g1_i2::g.89550::m.89550	1	4,2	33,674	2,0768	PF07859.12	Abhydrolase_3	149,3	1,30E-43	CL0028
TRINITY_DN17776_c1_g1::TRINITY_DN17776_c1_g1_i2::g.89554::m.89554	3	32,6	14,219	11,943	PF07859.12	Abhydrolase_3	39	7,30E-10	CL0028
TRINITY_DN17784_c0_g1::TRINITY_DN17784_c0_g1_i3::g.89656::m.89656	2	7,9	37,086	4,2346					
TRINITY_DN17792_c0_g2::TRINITY_DN17792_c0_g2_i8::g.89735::m.89735	20	70,1	38,258	323,31	PF00274.18	Glycolytic	568,7	3,30E-171	CL0035
TRINITY_DN17792_c0_g2::TRINITY_DN17792_c0_g2_i6::g.89728::m.89728	7	72,5	13,736	2,0194	PF00274.18	Glycolytic	180,5	3,50E-53	CL0035
TRINITY_DN17792_c0_g5::TRINITY_DN17792_c0_g5_i3::g.89751::m.89751	1	19,4	17,556	2,7121					
TRINITY_DN17792_c0_g7::TRINITY_DN17792_c0_g7_i6::g.89759::m.89759	1	26	11,039	6,2723					
TRINITY_DN17794_c1_g1::TRINITY_DN17794_c1_g1_i3::g.89692::m.89692	2	12,9	20,919	3,4936	PF01138.20	RNase_PH	102,7	1,90E-29	CL0329
TRINITY_DN17797_c0_g1::TRINITY_DN17797_c0_g1_i20::g.88479::m.88479	1	3,3	23,802	2,2157	PF01544.17	CorA	35,9	4,70E-09	No_clan
TRINITY_DN17805_c0_g1::TRINITY_DN17805_c0_g1_i5::g.89980::m.89980	1	11,9	18,065	6,876	PF01592.15	NifU_N	185	5,20E-55	CL0233
TRINITY_DN17806_c2_g2::TRINITY_DN17806_c2_g2_i4::g.90030::m.90030	4	8,8	80,294	7,2263	PF12932.6	Sec16	84,2	9,30E-24	No_clan

TRINITY_DN17808_c3_g2::TRINITY_DN17808_c3_g2_i3::g.89986::m.89986	1	11,8	11,357	134,07					
TRINITY_DN17814_c1_g1::TRINITY_DN17814_c1_g1_i9::g.90451::m.90451	1	4,8	30,208	2,0981					
TRINITY_DN17814_c1_g2::TRINITY_DN17814_c1_g2_i3::g.90444::m.90444	6	46,3	16,871	113,66	PF00085.19	Thioredoxin	81,5	3,20E-23	CL0172
TRINITY_DN17821_c0_g1::TRINITY_DN17821_c0_g1_i3::g.91084::m.91084	1	12,1	11,443	1,899	PF07724.13	AAA_2	101,6	4,60E-29	CL0023
TRINITY_DN17826_c0_g3::TRINITY_DN17826_c0_g3_i6::g.90229::m.90229	3	16,8	19,582	30,686	PF03018.13	Dirigent	169,6	3,40E-50	CL0650
TRINITY_DN17827_c3_g3::TRINITY_DN17827_c3_g3_i1::g.90327::m.90327	1	3,2	48,104	5,3182	PF08263.11	LRRNT_2	30,7	2,60E-07	No_clan
TRINITY_DN17835_c3_g1::TRINITY_DN17835_c3_g1_i1::g.90619::m.90619	1	3,3	52,23	1,9165	PF00063.20	Myosin_head	448,6	3,20E-134	CL0023
TRINITY_DN17836_c0_g3::TRINITY_DN17836_c0_g3_i4::g.90474::m.90474	2	17,6	14,944	4,1281	PF01521.19	Fe-S_biosyn	42,5	5,50E-11	No_clan
TRINITY_DN17837_c2_g1::TRINITY_DN17837_c2_g1_i9::g.90611::m.90611	21	50,3	50,685	323,31	PF00155.20	Aminotran_1_2	316,4	2,30E-94	CL0061
TRINITY_DN17845_c1_g3::TRINITY_DN17845_c1_g3_i3::g.90638::m.90638	3	11,2	41,322	5,7796	PF00332.17	Glyco_hydro_17	294,1	1,20E-87	CL0058
TRINITY_DN17846_c4_g1::TRINITY_DN17846_c4_g1_i2::g.90658::m.90658	2	10,5	28,461	4,7875	PF00069.24	Pkinase	97,3	8,40E-28	CL0016
TRINITY_DN17846_c4_g3::TRINITY_DN17846_c4_g3_i1::g.90654::m.90654	3	9,5	35,421	4,4957	PF00326.20	Peptidase_S9	50,7	1,40E-13	CL0028
TRINITY_DN17847_c0_g1::TRINITY_DN17847_c0_g1_i7::g.90759::m.90759	13	38,5	42,685	22,701	PF00022.18	Actin	449	1,10E-134	CL0108
TRINITY_DN17851_c1_g3::TRINITY_DN17851_c1_g3_i2::g.90156::m.90156	4	20,7	36,796	8,5098	PF00491.20	Arginase	224,4	1,90E-66	CL0302
TRINITY_DN17853_c1_g4::TRINITY_DN17853_c1_g4_i19::g.90697::m.90697	6	33,6	23,967	319,48	PF01557.17	FAA_hydrolase	201,4	1,30E-59	CL0377
TRINITY_DN17857_c0_g1::TRINITY_DN17857_c0_g1_i1::g.90819::m.90819	11	25,3	74,31	66,073	PF05817.13	Ribophorin_II	615,5	1,10E-184	No_clan
TRINITY_DN17861_c1_g1::TRINITY_DN17861_c1_g1_i1::g.90803::m.90803	5	51,4	11,966	3,4094	PF00141.22	peroxidase	69,8	2,50E-19	CL0617
TRINITY_DN17861_c1_g1::TRINITY_DN17861_c1_g1_i8::g.90805::m.90805	6	50,6	16,841	148,3	PF00141.22	peroxidase	108,9	2,70E-31	CL0617
TRINITY_DN17863_c0_g3::TRINITY_DN17863_c0_g3_i3::g.91035::m.91035	14	22,4	71,932	30,274	PF00071.21	Ras	49,3	3,70E-13	CL0023
TRINITY_DN17874_c2_g1::TRINITY_DN17874_c2_g1_i9::g.91168::m.91168	7	12,7	73,773	20,095	PF02990.15	EMP70	740,2	1,10E-222	No_clan

TRINITY_DN17874_c2_g4::TRINITY_DN17874_c2_g4_i3::g.91179::m.91179	6	12,4	72,941	56,071	PF02990.15	EMP70	721,6	4,70E-217	No_clan
TRINITY_DN17875_c1_g1::TRINITY_DN17875_c1_g1_i11::g.91219::m.91219	1	12,4	13,521	2,6731	PF02263.18	GBP	37,3	1,70E-09	CL0023
TRINITY_DN17879_c1_g3::TRINITY_DN17879_c1_g3_i5::g.91260::m.91260	3	6,4	66,425	6,2205	PF00005.26	ABC_tran	68,2	9,00E-19	CL0023
TRINITY_DN17881_c0_g1::TRINITY_DN17881_c0_g1_i3::g.91227::m.91227	2	7,4	48,01	5,8525	PF12796.6	Ank_2	58,3	8,10E-16	CL0465
TRINITY_DN17882_c2_g1::TRINITY_DN17882_c2_g1_i14::g.91309::m.91309	1	5,3	29,24	2,0378	PF00501.27	AMP-binding	130	8,30E-38	CL0378
TRINITY_DN17884_c0_g1::TRINITY_DN17884_c0_g1_i1::g.91267::m.91267	2	22,8	15,342	8,1237					
TRINITY_DN17889_c3_g3::TRINITY_DN17889_c3_g3_i2::g.91373::m.91373	3	17,4	17,508	3,728	PF12146.7	Hydrolase_4	76,8	1,40E-21	CL0028
TRINITY_DN17891_c2_g1::TRINITY_DN17891_c2_g1_i4::g.91423::m.91423	4	29,4	16,137	15,588	PF01778.16	Ribosomal_L28e	116,7	8,30E-34	No_clan
TRINITY_DN17896_c3_g1::TRINITY_DN17896_c3_g1_i3::g.91440::m.91440	1	8,3	22,192	2,6178	PF03248.12	Rer1	246,5	1,10E-73	No_clan
TRINITY_DN17897_c2_g2::TRINITY_DN17897_c2_g2_i2::g.91535::m.91535	10	21,5	75,298	16,638	PF03081.14	Exo70	408,3	2,70E-122	CL0295
TRINITY_DN17898_c0_g1::TRINITY_DN17898_c0_g1_i6::g.91458::m.91458	7	4,4	233,48	12,929	PF07926.11	TPR_MLP1_2	93,8	7,80E-27	No_clan
TRINITY_DN17899_c1_g1::TRINITY_DN17899_c1_g1_i20::g.90008::m.90008	12	36	57,05	305,66	PF00202.20	Aminotran_3	306,1	2,70E-91	CL0061
TRINITY_DN17906_c0_g1::TRINITY_DN17906_c0_g1_i4::g.91749::m.91749	6	18	42,536	66,587	PF02883.19	Alpha_adaptinC2	30,9	2,40E-07	CL0159
TRINITY_DN17906_c0_g2::TRINITY_DN17906_c0_g2_i5::g.91748::m.91748	3	7,4	53,676	6,3102	PF01602.19	Adaptin_N	486,9	6,30E-146	CL0020
TRINITY_DN17907_c0_g1::TRINITY_DN17907_c0_g1_i1::g.91817::m.91817	1	2,4	67,759	2,0881	PF03164.13	Mon1	480,1	5,20E-144	No_clan
TRINITY_DN17908_c2_g1::TRINITY_DN17908_c2_g1_i5::g.91796::m.91796	4	13,8	42,243	7,9203	PF03283.12	PAE	524,1	1,60E-157	CL0028
TRINITY_DN17913_c4_g1::TRINITY_DN17913_c4_g1_i1::g.91856::m.91856	2	5,6	45,277	19,233	PF03009.16	GDPD	228	1,50E-67	CL0384
TRINITY_DN17918_c1_g1::TRINITY_DN17918_c1_g1_i4::g.91866::m.91866	1	12,1	15,567	2,2497	PF00106.24	adh_short	73,1	1,90E-20	CL0063
TRINITY_DN17919_c1_g1::TRINITY_DN17919_c1_g1_i2::g.91916::m.91916	3	3,8	123,31	4,9417	PF13646.5	HEAT_2	33,5	3,90E-08	CL0020
TRINITY_DN17919_c1_g4::TRINITY_DN17919_c1_g4_i5::g.91938::m.91938	4	5,4	122,56	9,3238	PF13646.5	HEAT_2	31,2	1,90E-07	CL0020

TRINITY_DN17921_c2_g1::TRINITY_DN17921_c2_g1_i7::g.92112::m.92112	21	25,8	111,54	145,03	PF01411.18	tRNA-synt_2c	739,5	2,00E-222	CL0040
TRINITY_DN17921_c3_g2::TRINITY_DN17921_c3_g2_i1::g.92117::m.92117	21	8	406,31	53,331	PF06012.11	DUF908	113,6	1,20E-32	No_clan
TRINITY_DN17922_c0_g1::TRINITY_DN17922_c0_g1_i13::g.92083::m.92083	2	1,5	135,79	2,4754	PF11262.7	Tho2	330,6	7,70E-99	No_clan
TRINITY_DN17923_c2_g2::TRINITY_DN17923_c2_g2_i2::g.91995::m.91995	3	4,4	91,851	5,2062	PF04100.11	Vps53_N	502,2	8,00E-151	CL0295
TRINITY_DN17924_c2_g1::TRINITY_DN17924_c2_g1_i1::g.91979::m.91979	2	12,5	29,8	4,9743	PF01336.24	tRNA_anti-codon	27,2	2,60E-06	CL0021
TRINITY_DN17925_c2_g1::TRINITY_DN17925_c2_g1_i5::g.91985::m.91985	3	29,3	15,664	5,8239					
TRINITY_DN17927_c1_g1::TRINITY_DN17927_c1_g1_i3::g.92125::m.92125	2	10,8	37,123	8,1322	PF01012.20	ETF	112	2,80E-32	CL0039
TRINITY_DN17928_c2_g1::TRINITY_DN17928_c2_g1_i2::g.92278::m.92278	4	19,3	29,8	7,8547	PF02230.15	Abhydrolase_2	164,3	3,30E-48	CL0028
TRINITY_DN17932_c0_g2::TRINITY_DN17932_c0_g2_i2::g.92648::m.92648	1	3,2	38,726	2,1317					
TRINITY_DN17933_c0_g2::TRINITY_DN17933_c0_g2_i1::g.92168::m.92168	3	35,3	14,818	11,512	PF00173.27	Cyt-b5	90,6	4,90E-26	No_clan
TRINITY_DN17936_c4_g1::TRINITY_DN17936_c4_g1_i9::g.92365::m.92365	1	15,3	13,605	1,9505	PF04142.14	Nuc_sug_transp	37,2	1,70E-09	CL0184
TRINITY_DN17937_c1_g5::TRINITY_DN17937_c1_g5_i2::g.92219::m.92219	2	21,2	12,383	3,5108	PF00742.18	Homoserine_dh	60,3	2,00E-16	No_clan
TRINITY_DN17937_c1_g6::TRINITY_DN17937_c1_g6_i1::g.92221::m.92221	1	8,8	12,155	1,9134	PF00742.18	Homoserine_dh	55,8	5,00E-15	No_clan
TRINITY_DN17938_c1_g2::TRINITY_DN17938_c1_g2_i1::g.92333::m.92333	1	3,3	36,575	5,5123	PF04107.12	GCS2	215,2	1,10E-63	CL0286
TRINITY_DN17938_c1_g3::TRINITY_DN17938_c1_g3_i1::g.92335::m.92335	4	22	23,812	7,6439	PF04107.12	GCS2	85,6	3,40E-24	CL0286
TRINITY_DN17942_c1_g1::TRINITY_DN17942_c1_g1_i6::g.91683::m.91683	5	9,1	57,333	9,8567	PF02990.15	EMP70	450,9	5,20E-135	No_clan
TRINITY_DN17944_c2_g2::TRINITY_DN17944_c2_g2_i3::g.92569::m.92569	5	12,5	69,584	12,209	PF07738.12	Sad1_UNC	108,3	2,60E-31	CL0202
TRINITY_DN17952_c0_g1::TRINITY_DN17952_c0_g1_i8::g.92586::m.92586	3	4,7	86,093	3,6845	PF00888.21	Cullin	606,6	5,30E-182	No_clan
TRINITY_DN17953_c1_g8::TRINITY_DN17953_c1_g8_i1::g.91726::m.91726	2	10	30,05	3,1424	PF02666.14	PS_Dcarbxylase	198,1	1,00E-58	No_clan
TRINITY_DN17957_c0_g1::TRINITY_DN17957_c0_g1_i11::g.92631::m.92631	8	3,2	405,77	11,355	PF06012.11	DUF908	51,9	7,10E-14	No_clan

TRINITY_DN17965_c0_g2::TRINITY_DN17965_c0_g2_i2::g.92668::m.92668	1	11	14,018	2,1022	PF00815.19	Histidinol_dh	163,9	5,20E-48	CL0099
TRINITY_DN17968_c0_g2::TRINITY_DN17968_c0_g2_i1::g.92847::m.92847	2	2,1	157,5	3,786	PF12783.6	Sec7_N	96,2	1,70E-27	No_clan
TRINITY_DN17970_c3_g1::TRINITY_DN17970_c3_g1_i6::g.92954::m.92954	5	12,1	59,26	9,9672	PF05577.11	Peptidase_S28	282,3	5,70E-84	CL0028
TRINITY_DN17973_c0_g1::TRINITY_DN17973_c0_g1_i1::g.92481::m.92481	8	19	50,516	4,5328	PF02518.25	HATPase_c	58,9	6,10E-16	CL0025
TRINITY_DN17973_c0_g2::TRINITY_DN17973_c0_g2_i4::g.92495::m.92495	4	26,8	20,164	12,079	PF03501.14	S10_plectin	146	2,50E-43	No_clan
TRINITY_DN17973_c0_g3::TRINITY_DN17973_c0_g3_i3::g.92497::m.92497	14	20,8	80,632	63,459	PF02518.25	HATPase_c	55,6	6,20E-15	CL0025
TRINITY_DN17973_c0_g5::TRINITY_DN17973_c0_g5_i1::g.92489::m.92489	4	14,5	29,153	3,4154	PF00183.17	HSP90	359,7	2,60E-107	No_clan
TRINITY_DN17978_c0_g1::TRINITY_DN17978_c0_g1_i12::g.93003::m.93003	3	10	51,541	23,007	PF01409.19	tRNA-synt_2d	156,2	9,40E-46	CL0040
TRINITY_DN17979_c4_g2::TRINITY_DN17979_c4_g2_i7::g.93178::m.93178	5	11,8	67,384	9,7933	PF00258.24	Flavodoxin_1	109,6	1,30E-31	CL0042
TRINITY_DN17980_c0_g1::TRINITY_DN17980_c0_g1_i2::g.92937::m.92937	1	11,1	25,886	25,697	PF09066.9	B2-adapt-app_C	98,5	2,10E-28	CL0545
TRINITY_DN17985_c0_g4::TRINITY_DN17985_c0_g4_i1::g.93309::m.93309	5	19,7	36,414	14,1	PF00346.18	Complex1_49kDa	406,6	3,70E-122	No_clan
TRINITY_DN17986_c0_g1::TRINITY_DN17986_c0_g1_i2::g.93139::m.93139	4	17,3	29,983	5,5002	PF12931.6	Sec16_C	28,2	1,30E-06	No_clan
TRINITY_DN17990_c1_g1::TRINITY_DN17990_c1_g1_i2::g.93320::m.93320	2	10,2	30,203	5,4422	PF00240.22	ubiquitin	38,1	8,40E-10	CL0072
TRINITY_DN17993_c0_g1::TRINITY_DN17993_c0_g1_i2::g.93217::m.93217	3	12,9	36,664	7,2579	PF00348.16	polyprenyl_synt	121,3	3,30E-35	CL0613
TRINITY_DN17996_c1_g1::TRINITY_DN17996_c1_g1_i2::g.93353::m.93353	2	7,4	26,624	2,4113					
TRINITY_DN18000_c3_g4::TRINITY_DN18000_c3_g4_i1::g.93345::m.93345	1	8,6	17,972	2,9448					
TRINITY_DN18002_c1_g3::TRINITY_DN18002_c1_g3_i1::g.93438::m.93438	4	8,4	57,144	6,6475	PF00759.18	Glyco_hydro_9	445,2	2,80E-133	CL0059
TRINITY_DN18003_c1_g3::TRINITY_DN18003_c1_g3_i3::g.93450::m.93450	8	32,1	35,838	34,876	PF03168.12	LEA_2	54,9	9,50E-15	CL0159
TRINITY_DN18004_c1_g11::TRINITY_DN18004_c1_g11_i1::g.93466::m.93466	3	24,2	17,554	9,3273	PF02798.19	GST_N	21,9	0,00016	CL0172
TRINITY_DN18004_c1_g6::TRINITY_DN18004_c1_g6_i1::g.93462::m.93462	6	26,4	16,954	97,054	PF00043.24	GST_C	67,9	6,10E-19	CL0497
TRINITY_DN18011_c2_g1::TRINITY_DN18011_c2_g1_i8::g.93702::m.93702	11	45,3	35,388	56,126	PF00294.23	PfkB	263,5	2,30E-78	CL0118

TRINITY_DN18015_c3_g4::TRINITY_DN18015_c3_g4_i1::g.93638::m.93638	9	22,5	57,039	64,859	PF08662.10	eIF2A	255,8	2,50E-76	CL0186
TRINITY_DN18017_c0_g1::TRINITY_DN18017_c0_g1_i4::g.93738::m.93738	2	6,2	44,357	3,1545	PF06741.12	LsmAD	86,2	1,60E-24	No_clan
TRINITY_DN18020_c2_g2::TRINITY_DN18020_c2_g2_i4::g.93764::m.93764	1	6,4	30,18	2,3544	PF13334.5	DUF4094	71,2	6,50E-20	No_clan
TRINITY_DN18021_c3_g2::TRINITY_DN18021_c3_g2_i4::g.93827::m.93827	1	20,7	12,259	2,4598					
TRINITY_DN18024_c1_g1::TRINITY_DN18024_c1_g1_i2::g.93774::m.93774	2	8,1	24,86	12,755	PF13561.5	adh_short_C2	176,5	5,50E-52	CL0063
TRINITY_DN18028_c1_g1::TRINITY_DN18028_c1_g1_i5::g.93929::m.93929	14	26,7	60,227	319,46	PF00254.27	FKBP_C	117,3	2,70E-34	CL0487
TRINITY_DN18029_c5_g1::TRINITY_DN18029_c5_g1_i6::g.93985::m.93985	21	57,9	49,096	153,79	PF00155.20	Aminotran_1_2	315,5	4,60E-94	CL0061
TRINITY_DN18029_c5_g2::TRINITY_DN18029_c5_g2_i4::g.93978::m.93978	10	26,2	63,73	32,1	PF02450.14	LCAT	215,6	1,10E-63	CL0028
TRINITY_DN18037_c1_g1::TRINITY_DN18037_c1_g1_i9::g.94092::m.94092	2	4,3	59,715	3,0092	PF07534.15	TLD	78,8	4,00E-22	No_clan
TRINITY_DN18038_c0_g1::TRINITY_DN18038_c0_g1_i7::g.94082::m.94082	5	36,9	13,705	10,663	PF00338.21	Ribosomal_S10	97,6	3,50E-28	No_clan
TRINITY_DN18040_c0_g1::TRINITY_DN18040_c0_g1_i2::g.94127::m.94127	2	13,4	25,807	3,1169	PF01008.16	IF-2B	194,6	1,80E-57	CL0246
TRINITY_DN18047_c0_g1::TRINITY_DN18047_c0_g1_i1::g.93485::m.93485	6	11,1	73,013	15,3	PF09731.8	Mitofilin	235,9	1,10E-69	No_clan
TRINITY_DN18048_c4_g4::TRINITY_DN18048_c4_g4_i5::g.94249::m.94249	10	25,1	49,039	16,796	PF01412.17	ArfGap	114	3,60E-33	No_clan
TRINITY_DN18049_c1_g1::TRINITY_DN18049_c1_g1_i7::g.94346::m.94346	6	18	49,539	2,101	PF00004.28	AAA	137,7	2,80E-40	CL0023
TRINITY_DN18049_c1_g3::TRINITY_DN18049_c1_g3_i3::g.94352::m.94352	6	20,9	49,618	23,092	PF00004.28	AAA	137,5	3,20E-40	CL0023
TRINITY_DN18053_c4_g1::TRINITY_DN18053_c4_g1_i4::g.94304::m.94304	8	19,2	51,864	14,259	PF10151.8	TMEM214	47,2	1,10E-12	No_clan
TRINITY_DN18055_c0_g1::TRINITY_DN18055_c0_g1_i4::g.94445::m.94445	8	5	242,17	14,352	PF00270.28	DEAD	100,7	7,30E-29	CL0023
TRINITY_DN18055_c0_g3::TRINITY_DN18055_c0_g3_i2::g.94441::m.94441	2	8,7	23,63	3,0216	PF01248.25	Ribosomal_L7Ae	78,3	2,60E-22	CL0101
TRINITY_DN18057_c0_g3::TRINITY_DN18057_c0_g3_i1::g.94357::m.94357	14	37,8	58,834	61,526	PF00118.23	Cpn60_TCP1	508,7	1,40E-152	No_clan
TRINITY_DN18060_c0_g2::TRINITY_DN18060_c0_g2_i3::g.94415::m.94415	2	10,5	24,632	3,3411	PF08718.10	GLTP	110,2	9,60E-32	No_clan
TRINITY_DN18061_c2_g1::TRINITY_DN18061_c2_g1_i8::g.94553::m.94553	6	16,3	43,138	11,33	PF01370.20	Epimerase	23,7	2,70E-05	CL0063

TRINITY_DN18061_c2_g3::TRINITY_DN18061_c2_g3_i1::g.94540::m.94540	4	16,7	16,345	6,043	PF00833.17	Ribosomal_S17e	195,6	1,90E-58	No_clan
TRINITY_DN18061_c2_g5::TRINITY_DN18061_c2_g5_i2::g.94542::m.94542	3	10,3	55,792	13,13	PF12819.6	Malectin_like	212,6	7,70E-63	CL0468
TRINITY_DN18066_c0_g3::TRINITY_DN18066_c0_g3_i5::g.94429::m.94429	2	7,7	20,099	323,31	PF01472.19	PUA	69,2	2,00E-19	CL0178
TRINITY_DN18074_c1_g1::TRINITY_DN18074_c1_g1_i2::g.94688::m.94688	2	2,8	70,467	1,9857	PF07651.15	ANTH	279,3	2,30E-83	CL0009
TRINITY_DN18083_c3_g1::TRINITY_DN18083_c3_g1_i4::g.94853::m.94853	3	12,3	32,914	5,0306	PF00574.22	CLP_protease	173,4	3,90E-51	CL0127
TRINITY_DN18083_c4_g1::TRINITY_DN18083_c4_g1_i1::g.94854::m.94854	7	43	27,905	16,081	PF04774.14	HABP4_PAI-RBP1	104,7	5,00E-30	No_clan
TRINITY_DN18086_c1_g2::TRINITY_DN18086_c1_g2_i3::g.94830::m.94830	3	33,9	12,743	198,65	PF14368.5	LTP_2	62,2	3,50E-17	CL0482
TRINITY_DN18091_c1_g1::TRINITY_DN18091_c1_g1_i6::g.94898::m.94898	2	9,3	24,127	2,5912	PF00795.21	CN_hydrolase	158,4	2,00E-46	No_clan
TRINITY_DN18096_c1_g1::TRINITY_DN18096_c1_g1_i2::g.95119::m.95119	4	6,8	69,087	5,7063	PF08238.11	Sel1	7,5	6,6	CL0020
TRINITY_DN18097_c14_g3::TRINITY_DN18097_c14_g3_i5::g.95141::m.95141	3	11,9	42,714	10,725	PF08545.9	ACP_syn_III	95,3	1,30E-27	CL0046
TRINITY_DN18113_c1_g2::TRINITY_DN18113_c1_g2_i2::g.95380::m.95380	5	16,7	34,469	17,333	PF05116.12	S6PP	324	5,70E-97	CL0137
TRINITY_DN18115_c1_g1::TRINITY_DN18115_c1_g1_i4::g.95413::m.95413	2	17,8	17,203	5,3936	PF01992.15	vATP-synt_AC39	150,1	9,80E-44	No_clan
TRINITY_DN18120_c2_g1::TRINITY_DN18120_c2_g1_i8::g.95450::m.95450	5	57	11,563	24,356	PF00248.20	Aldo_ket_red	45,3	5,80E-12	No_clan
TRINITY_DN18124_c0_g4::TRINITY_DN18124_c0_g4_i1::g.95606::m.95606	7	37,5	31,078	32,644	PF10075.8	CSN8_PSD8_EIF3K	92,5	2,10E-26	CL0123
TRINITY_DN18127_c0_g1::TRINITY_DN18127_c0_g1_i1::g.95817::m.95817	3	4,3	123,55	4,4933	PF02213.15	GYF	39,9	2,40E-10	No_clan
TRINITY_DN18132_c2_g1::TRINITY_DN18132_c2_g1_i3::g.95646::m.95646	3	28,7	13,079	4,8905	PF00085.19	Thioredoxin	70	1,30E-19	CL0172
TRINITY_DN18139_c1_g1::TRINITY_DN18139_c1_g1_i7::g.95745::m.95745	2	12,9	25,456	3,8159	PF16752.4	TBCC_N	101,4	3,70E-29	No_clan
TRINITY_DN18145_c2_g1::TRINITY_DN18145_c2_g1_i9::g.95178::m.95178	13	57,1	28,577	323,31	PF00484.18	Pro_CA	154,9	1,80E-45	No_clan
TRINITY_DN18146_c1_g5::TRINITY_DN18146_c1_g5_i1::g.95204::m.95204	2	21,9	17,176	21,096	PF02466.18	Tim17	50,3	2,30E-13	No_clan
TRINITY_DN18148_c1_g6::TRINITY_DN18148_c1_g6_i2::g.95979::m.95979	1	5,6	20,524	5,4115	PF00149.27	Metallophos	51,4	1,70E-13	CL0163
TRINITY_DN18149_c0_g1::TRINITY_DN18149_c0_g1_i3::g.95908::m.95908	4	18,7	32,205	8,7541	PF00112.22	Peptidase_C1	163,1	9,50E-48	CL0125
TRINITY_DN18151_c0_g1::TRINITY_DN18151_c0_g1_i1::g.95922::m.95922	6	17,7	36,547	14,041	PF00318.19	Ribosomal_S2	49,7	2,50E-13	CL0067

TRINITY_DN18151_c1_g8::TRINITY_DN18151_c1_g8_i1::g.95937::m.95937	2	12,7	18,015	2,2666	PF04969.15	CS	65,5	6,10E-18	CL0190
TRINITY_DN18156_c0_g1::TRINITY_DN18156_c0_g1_i18::g.96138::m.96138	3	9,6	58,296	9,3715	PF09743.8	E3_UFM1_ligase	228,3	1,20E-67	No_clan
TRINITY_DN18157_c0_g1::TRINITY_DN18157_c0_g1_i6::g.96079::m.96079	6	30,7	24,827	105,81	PF00112.22	Peptidase_C1	126,4	1,50E-36	CL0125
TRINITY_DN18164_c1_g1::TRINITY_DN18164_c1_g1_i4::g.96244::m.96244	20	29,6	75,727	248,93	PF00012.19	HSP70	875,5	1,40E-263	CL0108
TRINITY_DN18164_c1_g2::TRINITY_DN18164_c1_g2_i2::g.96243::m.96243	13	63,1	26,532	323,31	PF00012.19	HSP70	389,7	1,70E-116	CL0108
TRINITY_DN18168_c0_g1::TRINITY_DN18168_c0_g1_i2::g.96274::m.96274	1	20,7	12,335	4,3947					
TRINITY_DN18171_c1_g1::TRINITY_DN18171_c1_g1_i5::g.96291::m.96291	6	17,6	55,074	19,575	PF00069.24	Pkinase	253,9	1,50E-75	CL0016
TRINITY_DN18173_c2_g1::TRINITY_DN18173_c2_g1_i8::g.96624::m.96624	16	23,1	108,88	44,233	PF13528.5	Glyco_trans_1_3	28,6	7,70E-07	CL0113
TRINITY_DN18173_c2_g1::TRINITY_DN18173_c2_g1_i9::g.96627::m.96627	15	25,9	95,248	4,2542	PF13528.5	Glyco_trans_1_3	28,5	7,80E-07	CL0113
TRINITY_DN18179_c2_g1::TRINITY_DN18179_c2_g1_i5::g.96397::m.96397	11	30,5	52,704	72,264	PF02136.19	NTF2	109,2	1,70E-31	CL0051
TRINITY_DN18180_c0_g1::TRINITY_DN18180_c0_g1_i6::g.96457::m.96457	2	1,4	141,82	2,7181	PF13639.5	zf-RING_2	29,4	6,70E-07	CL0229
TRINITY_DN18181_c1_g1::TRINITY_DN18181_c1_g1_i2::g.96439::m.96439	9	36,6	29,889	100,94	PF12697.6	Abhydrolase_6	29,9	7,80E-07	CL0028
TRINITY_DN18181_c1_g1::TRINITY_DN18181_c1_g1_i3::g.96440::m.96440	7	24,5	31,061	16,729	PF12697.6	Abhydrolase_6	32,7	1,10E-07	CL0028
TRINITY_DN18182_c1_g1::TRINITY_DN18182_c1_g1_i5::g.96464::m.96464	10	41,9	26,23	41,373	PF07650.16	KH_2	48,7	4,50E-13	CL0007
TRINITY_DN18184_c1_g1::TRINITY_DN18184_c1_g1_i15::g.96527::m.96527	3	28	11,606	2,9269	PF00719.18	Pyrophosphatase	77	1,20E-21	No_clan
TRINITY_DN18185_c0_g2::TRINITY_DN18185_c0_g2_i8::g.96676::m.96676	4	7,9	87,295	6,1521	PF01839.22	FG-GAP	23,2	5,10E-05	CL0186
TRINITY_DN18186_c3_g1::TRINITY_DN18186_c3_g1_i8::g.96539::m.96539	2	2,5	110,82	3,7875	PF08389.11	Xpo1	72,2	4,20E-20	CL0020
TRINITY_DN18188_c2_g1::TRINITY_DN18188_c2_g1_i6::g.95147::m.95147	1	2,1	75,374	1,8834	PF09787.8	Golgin_A5	59,6	3,10E-16	No_clan
TRINITY_DN18192_c1_g2::TRINITY_DN18192_c1_g2_i5::g.96646::m.96646	6	30,5	40,427	186,07	PF00076.21	RRM_1	45,8	3,70E-12	CL0221
TRINITY_DN18196_c4_g4::TRINITY_DN18196_c4_g4_i1::g.96720::m.96720	1	10,5	12,01	1,9281	PF00412.21	LIM	45,2	7,30E-12	CL0167
TRINITY_DN18205_c0_g1::TRINITY_DN18205_c0_g1_i3::g.96958::m.96958	16	51,1	29,742	323,31	PF08241.11	Methyltransf_11	44,4	1,80E-11	CL0063

TRINITY_DN18208_c0_g1::TRINITY_DN18208_c0_g1_i5::g.96946::m.96946	2	8	37,467	3,855	PF08241.11	Methyltransf_11	72,2	3,80E-20	CL0063
TRINITY_DN18211_c0_g1::TRINITY_DN18211_c0_g1_i1::g.96953::m.96953	3	22,4	21,138	5,0204	PF13905.5	Thioredoxin_8	28,5	1,30E-06	CL0172
TRINITY_DN18223_c0_g1::TRINITY_DN18223_c0_g1_i1::g.97248::m.97248	5	7,1	103,78	9,3672	PF03810.18	IBN_N	66,3	1,50E-18	CL0020
TRINITY_DN18226_c4_g1::TRINITY_DN18226_c4_g1_i9::g.97134::m.97134	2	9,2	47,912	7,3139					
TRINITY_DN18227_c0_g1::TRINITY_DN18227_c0_g1_i4::g.97154::m.97154	1	8,2	26,74	3,979	PF02037.26	SAP	39,5	2,90E-10	CL0306
TRINITY_DN18228_c1_g3::TRINITY_DN18228_c1_g3_i10::g.97346::m.97346	3	6,9	63,193	2,8221	PF00270.28	DEAD	92,7	2,00E-26	CL0023
TRINITY_DN18229_c0_g2::TRINITY_DN18229_c0_g2_i1::g.97234::m.97234	3	26,7	15,354	10,568	PF01596.16	Methyltransf_3	202,2	4,90E-60	CL0063
TRINITY_DN18230_c1_g3::TRINITY_DN18230_c1_g3_i4::g.97286::m.97286	1	6	26,634	2,5881	PF03168.12	LEA_2	26,4	7,00E-06	CL0159
TRINITY_DN18231_c0_g2::TRINITY_DN18231_c0_g2_i7::g.97309::m.97309	4	3	213,59	7,4552	PF16418.4	CNOT1_HEAT	113,1	1,10E-32	No_clan
TRINITY_DN18232_c0_g1::TRINITY_DN18232_c0_g1_i6::g.97390::m.97390	12	12,5	136,2	19,503	PF00133.21	tRNA-synt_1	309,2	4,20E-92	CL0039
TRINITY_DN18239_c4_g4::TRINITY_DN18239_c4_g4_i1::g.97381::m.97381	7	28,7	28,492	14,747	PF08079.11	Ribosomal_L30_N	95,2	1,80E-27	No_clan
TRINITY_DN18240_c0_g1::TRINITY_DN18240_c0_g1_i3::g.97435::m.97435	4	15,2	41,028	6,7586	PF02883.19	Alpha_adaptinC2	108,5	2,00E-31	CL0159
TRINITY_DN18242_c1_g1::TRINITY_DN18242_c1_g1_i1::g.97405::m.97405	6	22	45,092	21,596	PF00076.21	RRM_1	54	1,00E-14	CL0221
TRINITY_DN18242_c3_g1::TRINITY_DN18242_c3_g1_i7::g.97423::m.97423	1	11,2	27,017	7,5696					
TRINITY_DN18242_c3_g2::TRINITY_DN18242_c3_g2_i3::g.97426::m.97426	4	29,3	21,446	7,6829	PF00076.21	RRM_1	56,2	2,00E-15	CL0221
TRINITY_DN18246_c0_g1::TRINITY_DN18246_c0_g1_i3::g.96857::m.96857	7	25,6	48,08	17,992	PF00709.20	Adenylsucc_synt	567,5	1,60E-170	CL0023
TRINITY_DN18252_c2_g3::TRINITY_DN18252_c2_g3_i2::g.97577::m.97577	3	8,1	53,632	10,687	PF01370.20	Epimerase	76,8	1,60E-21	CL0063
TRINITY_DN18255_c0_g1::TRINITY_DN18255_c0_g1_i22::g.97730::m.97730	4	16	36,214	9,1683	PF04212.17	MIT	76,6	1,10E-21	No_clan
TRINITY_DN18255_c1_g1::TRINITY_DN18255_c1_g1_i6::g.97743::m.97743	14	11,3	173,76	32,906	PF02736.18	Myosin_N	29,6	4,00E-07	No_clan
TRINITY_DN18258_c0_g1::TRINITY_DN18258_c0_g1_i1::g.97653::m.97653	2	17,4	12,087	2,3992	PF00107.25	ADH_zinc_N	61,9	5,30E-17	CL0063
TRINITY_DN18258_c0_g1::TRINITY_DN18258_c0_g1_i10::g.97660::m.97660	7	28,5	36,928	149,66	PF08240.11	ADH_N	96,9	5,60E-28	CL0296

TRINITY_DN18258_c0_g1::TRINITY_DN18258_c0_g1_i6::g.97657::m.97657	2	32,4	11,988	5,2519	PF08240.11	ADH_N	77,8	4,90E-22	CL0296
TRINITY_DN18266_c2_g1::TRINITY_DN18266_c2_g1_i1::g.97903::m.97903	3	28,4	12,627	5,6789	PF05755.11	REF	136,1	1,20E-39	No_clan
TRINITY_DN18268_c0_g1::TRINITY_DN18268_c0_g1_i8::g.98068::m.98068	5	21,9	41,087	11,064	PF03485.15	Arg_tRNA_synt_N	58,9	5,20E-16	No_clan
TRINITY_DN18272_c2_g1::TRINITY_DN18272_c2_g1_i1::g.97904::m.97904	15	20,9	102,74	42,476	PF02861.19	Clp_N	62,6	2,60E-17	No_clan
TRINITY_DN18272_c3_g3::TRINITY_DN18272_c3_g3_i3::g.97910::m.97910	7	14,2	62,635	19,321	PF01565.22	FAD_binding_4	64,5	7,50E-18	CL0077
TRINITY_DN18275_c0_g4::TRINITY_DN18275_c0_g4_i1::g.98310::m.98310	3	2,3	180,58	4,8617	PF02383.17	Syja_N	141,9	2,60E-41	CL0031
TRINITY_DN18281_c0_g1::TRINITY_DN18281_c0_g1_i4::g.98025::m.98025	2	1,4	134,3	1,9416	PF04563.14	RNA_pol_Rpb2_1	84,8	5,00E-24	No_clan
TRINITY_DN18283_c0_g1::TRINITY_DN18283_c0_g1_i12::g.98055::m.98055	1	5,2	21,908	2,0601	PF01963.16	TraB	73,2	2,80E-20	CL0572
TRINITY_DN18287_c2_g1::TRINITY_DN18287_c2_g1_i6::g.98094::m.98094	2	8,7	44,613	4,2216	PF03951.18	Gln-synt_N	39,5	3,30E-10	No_clan
TRINITY_DN18287_c3_g1::TRINITY_DN18287_c3_g1_i2::g.98102::m.98102	5	20,7	32,827	36,601	PF00383.22	dCMP_cyt_deam_1	35,4	7,10E-09	CL0109
TRINITY_DN18293_c3_g1::TRINITY_DN18293_c3_g1_i3::g.98277::m.98277	2	25,2	11,867	23,801					
TRINITY_DN18294_c2_g1::TRINITY_DN18294_c2_g1_i3::g.98438::m.98438	3	4,5	108,43	3,4107	PF13202.5	EF-hand_5	16,9	0,003	CL0220
TRINITY_DN18296_c0_g1::TRINITY_DN18296_c0_g1_i5::g.98309::m.98309	4	17,3	27,565	323,31					
TRINITY_DN18300_c1_g1::TRINITY_DN18300_c1_g1_i6::g.96808::m.96808	4	31,3	18,071	7,4312	PF14852.5	Fis1_TPR_N	44,6	7,60E-12	CL0020
TRINITY_DN18301_c0_g2::TRINITY_DN18301_c0_g2_i7::g.97543::m.97543	2	7,7	38,024	5,0208	PF04548.15	AIG1	274,3	4,80E-82	CL0023
TRINITY_DN18302_c1_g2::TRINITY_DN18302_c1_g2_i2::g.98507::m.98507	19	62,7	30,231	242,06	PF08240.11	ADH_N	36,1	4,50E-09	CL0296
TRINITY_DN18302_c1_g2::TRINITY_DN18302_c1_g2_i4::g.98509::m.98509	19	62,7	30,217	323,31	PF08240.11	ADH_N	36,1	4,50E-09	CL0296
TRINITY_DN18306_c2_g1::TRINITY_DN18306_c2_g1_i5::g.98735::m.98735	14	55,6	33,958	323,31	PF00180.19	Iso_dh	222,6	7,10E-66	CL0270
TRINITY_DN18306_c2_g1::TRINITY_DN18306_c2_g1_i6::g.98737::m.98737	6	52,5	11,634	323,31	PF00180.19	Iso_dh	66,1	2,80E-18	CL0270
TRINITY_DN18307_c0_g1::TRINITY_DN18307_c0_g1_i4::g.98617::m.98617	2	7,8	44,589	3,7615	PF04810.14	zf-Sec23_Sec24	64,4	6,70E-18	No_clan
TRINITY_DN18311_c1_g1::TRINITY_DN18311_c1_g1_i2::g.98629::m.98629	1	8,5	17,838	2,1022	PF00406.21	ADK	166,9	2,90E-49	CL0023
TRINITY_DN18313_c3_g2::TRINITY_DN18313_c3_g2_i2::g.98848::m.98848	8	24,9	50,345	19,379	PF01546.27	Peptidase_M20	127,6	4,80E-37	CL0035

TRINITY_DN18316_c4_g8::TRINITY_DN18316_c4_g8_i2::g.98671::m.98671	7	31,9	25,118	10,396	PF00071.21	Ras	207,3	9,00E-62	CL0023
TRINITY_DN18320_c3_g1::TRINITY_DN18320_c3_g1_i1::g.98804::m.98804	2	8	42,828	6,5665	PF00112.22	Peptidase_C1	216,9	3,20E-64	CL0125
TRINITY_DN18320_c3_g3::TRINITY_DN18320_c3_g3_i3::g.98813::m.98813	3	14	26,131	5,8038	PF00076.21	RRM_1	53,2	1,80E-14	CL0221
TRINITY_DN18321_c2_g1::TRINITY_DN18321_c2_g1_i1::g.98839::m.98839	3	23,6	17,863	46,517	PF00635.25	Motile_Sperm	103,8	4,00E-30	CL0556
TRINITY_DN18324_c2_g1::TRINITY_DN18324_c2_g1_i6::g.99024::m.99024	6	18,6	51,477	9,8728	PF01237.17	Oxysterol_BP	337,5	7,90E-101	No_clan
TRINITY_DN18328_c0_g1::TRINITY_DN18328_c0_g1_i9::g.98876::m.98876	12	68,2	24,292	79,325	PF00484.18	Pro_CA	124,9	3,00E-36	No_clan
TRINITY_DN18331_c2_g5::TRINITY_DN18331_c2_g5_i1::g.98900::m.98900	8	23,8	51,194	18,197	PF09440.9	eIF3_N	165,7	6,50E-49	No_clan
TRINITY_DN18333_c2_g6::TRINITY_DN18333_c2_g6_i1::g.98947::m.98947	5	45	11,487	192,28	PF04043.14	PMEI	54,1	2,00E-14	No_clan
TRINITY_DN18341_c1_g1::TRINITY_DN18341_c1_g1_i3::g.99097::m.99097	3	40,4	11,61	323,31	PF13833.5	EF-hand_8	55,4	3,50E-15	CL0220
TRINITY_DN18349_c3_g1::TRINITY_DN18349_c3_g1_i4::g.99210::m.99210	3	11	39,176	3,4076	PF00270.28	DEAD	162,7	6,50E-48	CL0023
TRINITY_DN18349_c4_g1::TRINITY_DN18349_c4_g1_i3::g.99227::m.99227	2	6,1	53,033	2,5593	PF04576.14	Zein-binding	115,5	9,40E-34	No_clan
TRINITY_DN18350_c0_g2::TRINITY_DN18350_c0_g2_i2::g.99155::m.99155	3	28,6	16,946	7,4017					
TRINITY_DN18350_c0_g4::TRINITY_DN18350_c0_g4_i3::g.99164::m.99164	3	11,3	44,133	11,236	PF02469.21	Fasciclin	23	6,80E-05	No_clan
TRINITY_DN18355_c0_g1::TRINITY_DN18355_c0_g1_i6::g.99167::m.99167	3	23,4	19,274	4,3397	PF00536.29	SAM_1	60,2	1,90E-16	CL0003
TRINITY_DN18356_c1_g1::TRINITY_DN18356_c1_g1_i9::g.99317::m.99317	4	10,3	51,633	4,5992					
TRINITY_DN18360_c0_g2::TRINITY_DN18360_c0_g2_i3::g.99190::m.99190	3	11,9	40,864	15,045	PF00012.19	HSP70	535,3	1,40E-160	CL0108
TRINITY_DN18360_c0_g4::TRINITY_DN18360_c0_g4_i2::g.99196::m.99196	10	42,9	32,081	229,33	PF00012.19	HSP70	305,6	5,10E-91	CL0108
TRINITY_DN18360_c0_g5::TRINITY_DN18360_c0_g5_i1::g.99194::m.99194	7	37,7	23,944	19,913	PF00012.19	HSP70	300,5	1,70E-89	CL0108
TRINITY_DN18360_c0_g7::TRINITY_DN18360_c0_g7_i2::g.99197::m.99197	18	58,9	39,367	323,31	PF00012.19	HSP70	540	5,20E-162	CL0108
TRINITY_DN18365_c0_g2::TRINITY_DN18365_c0_g2_i2::g.99254::m.99254	2	6,1	46,182	2,8639	PF00076.21	RRM_1	64,1	7,30E-18	CL0221
TRINITY_DN18366_c0_g2::TRINITY_DN18366_c0_g2_i3::g.99417::m.99417	2	12,6	17,784	3,9395	PF16205.4	Ribosomal_S17_N	110,2	4,30E-32	No_clan

TRINITY_DN18367_c3_g7::TRINITY_DN18367_c3_g7_i1::g.99480::m.99480	1	8,5	20,86	2,5309					
TRINITY_DN18371_c0_g1::TRINITY_DN18371_c0_g1_i2::g.99819::m.99819	2	8,5	23,446	2,2703	PF06244.11	Ccdc124	47,9	1,80E-12	CL0114
TRINITY_DN18372_c2_g2::TRINITY_DN18372_c2_g2_i3::g.99514::m.99514	2	7,2	42,281	4,3357	PF00782.19	DSPc	24,3	2,10E-05	CL0031
TRINITY_DN18372_c2_g6::TRINITY_DN18372_c2_g6_i1::g.99517::m.99517	2	18,5	24,994	9,2177					
TRINITY_DN18372_c2_g8::TRINITY_DN18372_c2_g8_i1::g.99519::m.99519	2	31,5	17,034	7,3359					
TRINITY_DN18378_c3_g4::TRINITY_DN18378_c3_g4_i1::g.99575::m.99575	3	23,8	22,46	21,148	PF00173.27	Cyt-b5	52	5,50E-14	No_clan
TRINITY_DN18383_c2_g3::TRINITY_DN18383_c2_g3_i1::g.99654::m.99654	11	29,3	56,907	109,49	PF13432.5	TPR_16	15,5	0,019	CL0020
TRINITY_DN18388_c1_g1::TRINITY_DN18388_c1_g1_i7::g.99758::m.99758	5	61,2	14,023	15,001					
TRINITY_DN18395_c1_g2::TRINITY_DN18395_c1_g2_i5::g.99070::m.99070	5	16,9	50,034	34,581	PF00240.22	ubiquitin	69,4	1,50E-19	CL0072
TRINITY_DN18395_c1_g2::TRINITY_DN18395_c1_g2_i6::g.99075::m.99075	4	21,6	24,931	3,5226	PF00627.30	UBA	40,8	1,40E-10	CL0214
TRINITY_DN18396_c2_g1::TRINITY_DN18396_c2_g1_i1::g.99882::m.99882	1	18,1	11,644	6,6731	PF00083.23	Sugar_tr	47,6	1,00E-12	CL0015
TRINITY_DN18397_c1_g1::TRINITY_DN18397_c1_g1_i2::g.99905::m.99905	4	4,7	110,56	6,1614	PF09324.9	DUF1981	109,8	4,20E-32	No_clan
TRINITY_DN18404_c1_g1::TRINITY_DN18404_c1_g1_i7::g.100040::m.100040	5	11,8	52,666	7,766	PF00479.21	G6PD_N	146	1,60E-42	CL0063
TRINITY_DN18406_c4_g1::TRINITY_DN18406_c4_g1_i5::g.99964::m.99964	8	14,4	92,747	23,299	PF00350.22	Dynamin_N	185,5	7,20E-55	CL0023
TRINITY_DN18412_c2_g2::TRINITY_DN18412_c2_g2_i2::g.100143::m.100143	5	11,6	52,188	9,4095	PF10559.8	Plug_translocon	65,7	2,20E-18	No_clan
TRINITY_DN18418_c0_g3::TRINITY_DN18418_c0_g3_i2::g.100138::m.100138	3	12,1	34,173	6,0781	PF07063.12	DUF1338	269,8	2,80E-80	No_clan
TRINITY_DN18419_c1_g1::TRINITY_DN18419_c1_g1_i5::g.100150::m.100150	13	15	130,43	20,234	PF02786.16	CPSase_L_D2	240,9	8,70E-72	CL0179
TRINITY_DN18422_c0_g3::TRINITY_DN18422_c0_g3_i1::g.100190::m.100190	16	45,2	53,314	155,36	PF03446.14	NAD_binding_2	162,2	1,00E-47	CL0063
TRINITY_DN18427_c0_g1::TRINITY_DN18427_c0_g1_i8::g.100422::m.100422	5	50	15,036	191,61	PF00173.27	Cyt-b5	94,3	3,30E-27	No_clan
TRINITY_DN18427_c1_g2::TRINITY_DN18427_c1_g2_i5::g.100438::m.100438	7	45,1	25,626	52,74	PF10584.8	Proteasome_A_N	34,6	1,00E-08	CL0052
TRINITY_DN18432_c0_g1::TRINITY_DN18432_c0_g1_i9::g.99987::m.99987	8	2,2	477,91	10,391	PF12624.6	Chorein_N	112,3	1,20E-32	No_clan
TRINITY_DN18433_c1_g3::TRINITY_DN18433_c1_g3_i2::g.100387::m.100387	10	34,1	33,442	15,406	PF12353.7	eIF3g	120,2	6,40E-35	CL0511

TRINITY_DN18433_c1_g4::TRINITY_DN18433_c1_g4_i1::g.100393::m.100393	7	30,1	31,971	3,1992	PF12353.7	eIF3g	117,6	4,10E-34	CL0511
TRINITY_DN18434_c0_g1::TRINITY_DN18434_c0_g1_i17::g.100395::m.100395	2	17,4	18,485	44,551	PF08324.10	PUL	112,6	1,70E-32	No_clan
TRINITY_DN18436_c0_g2::TRINITY_DN18436_c0_g2_i4::g.100110::m.100110	3	7,6	53,639	4,9705	PF10191.8	COG7	504,9	3,60E-151	CL0294
TRINITY_DN18436_c0_g2::TRINITY_DN18436_c0_g2_i2::g.100108::m.100108	2	6,4	38,29	3,316	PF10191.8	COG7	400,6	1,30E-119	CL0294
TRINITY_DN18437_c1_g1::TRINITY_DN18437_c1_g1_i4::g.100574::m.100574	1	2,8	63,687	3,1577	PF08799.10	PRP4	43,7	1,30E-11	No_clan
TRINITY_DN18437_c2_g2::TRINITY_DN18437_c2_g2_i5::g.100589::m.100589	3	1,9	172,72	4,9863	PF16213.4	DCB	93,6	9,80E-27	No_clan
TRINITY_DN18444_c3_g2::TRINITY_DN18444_c3_g2_i4::g.100535::m.100535	8	17,4	73,867	13,335	PF12569.7	NARP1	599,2	7,10E-180	CL0020
TRINITY_DN18446_c0_g1::TRINITY_DN18446_c0_g1_i3::g.100648::m.100648	2	12,1	22,343	8,4839					
TRINITY_DN18450_c3_g3::TRINITY_DN18450_c3_g3_i7::g.100614::m.100614	3	6	97,454	6,8252					
TRINITY_DN18452_c0_g2::TRINITY_DN18452_c0_g2_i5::g.100623::m.100623	2	18,2	13,771	9,3588	PF02733.16	Dak1	170,8	2,90E-50	CL0245
TRINITY_DN18452_c0_g3::TRINITY_DN18452_c0_g3_i8::g.100622::m.100622	1	3,5	24,231	2,684	PF02733.16	Dak1	123,7	6,30E-36	CL0245
TRINITY_DN18459_c3_g1::TRINITY_DN18459_c3_g1_i2::g.100697::m.100697	2	2,5	112,37	4,3567	PF00521.19	DNA_topoisoIV	499,3	8,50E-150	No_clan
TRINITY_DN18466_c3_g1::TRINITY_DN18466_c3_g1_i12::g.101047::m.101047	1	10,9	13,398	10,733					
TRINITY_DN18466_c3_g1::TRINITY_DN18466_c3_g1_i19::g.101050::m.101050	2	31,4	10,42	38,215					
TRINITY_DN18472_c4_g2::TRINITY_DN18472_c4_g2_i2::g.100983::m.100983	3	14	34,299	7,3322	PF00069.24	Pkinase	89,7	1,90E-25	CL0016
TRINITY_DN18476_c0_g1::TRINITY_DN18476_c0_g1_i4::g.100953::m.100953	2	25,4	12,95	223,24	PF04043.14	PMEI	28	2,20E-06	No_clan
TRINITY_DN18478_c2_g2::TRINITY_DN18478_c2_g2_i3::g.101133::m.101133	3	5	66,952	5,1787	PF00501.27	AMP-binding	336,2	2,00E-100	CL0378
TRINITY_DN18479_c0_g3::TRINITY_DN18479_c0_g3_i1::g.100965::m.100965	6	32	21,922	2,1944	PF00071.21	Ras	192,6	3,10E-57	CL0023
TRINITY_DN18479_c0_g5::TRINITY_DN18479_c0_g5_i1::g.100968::m.100968	5	30,9	19,672	2,6236	PF00071.21	Ras	165	9,50E-49	CL0023
TRINITY_DN18481_c3_g4::TRINITY_DN18481_c3_g4_i2::g.101020::m.101020	6	13,6	73,238	13,343	PF03639.12	Glyco_hydro_81	567,9	3,50E-170	No_clan

TRINITY_DN18482_c1_g1::TRINITY_DN18482_c1_g1_i7::g.100962::m.100962	1	11,9	21,491	2,4956	PF00542.18	Ribosomal_L12	81	5,40E-23	No_clan
TRINITY_DN18489_c2_g1::TRINITY_DN18489_c2_g1_i22::g.101262::m.101262	6	11,4	76,875	94,187	PF00258.24	Flavodoxin_1	120,7	4,90E-35	CL0042
TRINITY_DN18489_c2_g2::TRINITY_DN18489_c2_g2_i5::g.101255::m.101255	10	66,4	16,579	204,3	PF00179.25	UQ_con	52,1	4,90E-14	CL0208
TRINITY_DN18490_c1_g1::TRINITY_DN18490_c1_g1_i2::g.101177::m.101177	3	6,9	47,509	6,4887	PF00349.20	Hexokinase_1	199	7,40E-59	CL0108
TRINITY_DN18492_c0_g1::TRINITY_DN18492_c0_g1_i14::g.101403::m.101403	1	5,9	22,772	2,0285	PF13499.5	EF-hand_7	42,2	7,70E-11	CL0220
TRINITY_DN18494_c1_g1::TRINITY_DN18494_c1_g1_i7::g.101464::m.101464	2	12,7	28,058	6,8491	PF13561.5	adh_short_C2	204,8	1,30E-60	CL0063
TRINITY_DN18496_c3_g1::TRINITY_DN18496_c3_g1_i9::g.99949::m.99949	5	7,8	93,742	9,7382	PF05701.10	WEMBL	849,1	1,50E-255	No_clan
TRINITY_DN18497_c0_g1::TRINITY_DN18497_c0_g1_i7::g.101435::m.101435	2	17,9	20,013	3,5887	PF01174.18	SNO	24,4	2,10E-05	CL0014
TRINITY_DN18498_c3_g4::TRINITY_DN18498_c3_g4_i4::g.101572::m.101572	4	25,2	16,128	6,8228	PF06212.11	GRIM-19	142,9	5,00E-42	No_clan
TRINITY_DN18503_c1_g1::TRINITY_DN18503_c1_g1_i3::g.101658::m.101658	4	21,6	30,415	6,512	PF06026.13	Rib_5-P_isom_A	208,2	5,80E-62	CL0246
TRINITY_DN18504_c0_g1::TRINITY_DN18504_c0_g1_i8::g.101692::m.101692	3	5,7	73,53	3,7281	PF01841.18	Transglut_core	42,1	8,90E-11	CL0125
TRINITY_DN18518_c1_g1::TRINITY_DN18518_c1_g1_i12::g.103139::m.103139	2	4,4	71,913	3,2593	PF16923.4	Glyco_hydro_63N	142,2	2,40E-41	No_clan
TRINITY_DN18521_c6_g4::TRINITY_DN18521_c6_g4_i1::g.101901::m.101901	2	16,5	17,863	6,1295	PF02672.14	CP12	88	4,80E-25	No_clan
TRINITY_DN18521_c6_g6::TRINITY_DN18521_c6_g6_i1::g.101903::m.101903	4	32,5	16,687	32,056	PF00141.22	peroxidase	147,9	3,30E-43	CL0617
TRINITY_DN18521_c6_g6::TRINITY_DN18521_c6_g6_i4::g.101904::m.101904	4	28,5	15,146	-2	PF00141.22	peroxidase	120,4	8,60E-35	CL0617
TRINITY_DN18526_c1_g2::TRINITY_DN18526_c1_g2_i7::g.101942::m.101942	15	34,7	67,476	136,69	PF01321.17	Creatinase_N	57,4	2,20E-15	CL0356
TRINITY_DN18527_c2_g1::TRINITY_DN18527_c2_g1_i1::g.101980::m.101980	1	15,8	12,771	3,0869	PF00201.17	UDPGT	28,9	4,50E-07	CL0113
TRINITY_DN18531_c0_g1::TRINITY_DN18531_c0_g1_i4::g.102214::m.102214	35	48,9	93,216	323,31	PF01433.19	Peptidase_M1	181,7	1,40E-53	CL0126
TRINITY_DN18531_c0_g1::TRINITY_DN18531_c0_g1_i9::g.102220::m.102220	5	56	12,849	1,9151	PF17432.1	DUF3458_C	178,1	2,60E-52	CL0020
TRINITY_DN18532_c1_g1::TRINITY_DN18532_c1_g1_i2::g.102269::m.102269	6	9,6	101,8	18,624	PF12329.7	TMF_DNA_bd	52,9	2,60E-14	No_clan
TRINITY_DN18543_c1_g3::TRINITY_DN18543_c1_g3_i1::g.101857::m.101857	9	16,4	96,972	25,601	PF02148.18	zf-UBP	54,4	1,10E-14	CL0229

TRINITY_DN18544_c2_g2::TRINITY_DN18544_c2_g2_i1::g.102351::m.102351	1	3	76,121	2,3627	PF01434.17	Peptidase_M41	270,8	7,10E-81	CL0126
TRINITY_DN18545_c0_g2::TRINITY_DN18545_c0_g2_i6::g.102386::m.102386	11	26,1	71,143	63,379	PF00890.23	FAD_binding_2	413,5	9,90E-124	CL0063
TRINITY_DN18548_c0_g1::TRINITY_DN18548_c0_g1_i3::g.102416::m.102416	9	40,7	25,271	142,3	PF00736.18	EF1_GNE	111,4	1,60E-32	No_clan
TRINITY_DN18548_c0_g1::TRINITY_DN18548_c0_g1_i8::g.102421::m.102421	9	57,8	22,975	323,31	PF00736.18	EF1_GNE	97,5	3,50E-28	No_clan
TRINITY_DN18548_c0_g1::TRINITY_DN18548_c0_g1_i9::g.102424::m.102424	7	36,4	24,082	-2	PF00736.18	EF1_GNE	40	3,20E-10	No_clan
TRINITY_DN18551_c0_g2::TRINITY_DN18551_c0_g2_i5::g.102562::m.102562	6	7,7	75,896	19,907	PF03030.15	H_PPase	803	2,20E-241	No_clan
TRINITY_DN18561_c0_g1::TRINITY_DN18561_c0_g1_i3::g.102624::m.102624	4	3	184,66	6,1494	PF02213.15	GYF	36,4	3,00E-09	No_clan
TRINITY_DN18566_c1_g3::TRINITY_DN18566_c1_g3_i2::g.102668::m.102668	4	20	20,263	11,782	PF04733.13	Coatomer_E	298,9	3,70E-89	CL0020
TRINITY_DN18568_c1_g1::TRINITY_DN18568_c1_g1_i19::g.102809::m.102809	8	7,6	127,13	14,36	PF00133.21	tRNA-synt_1	666,7	2,40E-200	CL0039
TRINITY_DN18576_c1_g1::TRINITY_DN18576_c1_g1_i10::g.101619::m.101619	13	30	65,538	49,935	PF07992.13	Pyr_redox_2	196,5	5,10E-58	CL0063
TRINITY_DN18579_c2_g2::TRINITY_DN18579_c2_g2_i4::g.102900::m.102900	3	17,3	30,205	6,545	PF00620.26	RhoGAP	80,8	8,40E-23	CL0409
TRINITY_DN18581_c2_g1::TRINITY_DN18581_c2_g1_i1::g.102861::m.102861	5	33,7	22,682	27,285	PF00450.21	Peptidase_S10	150,6	8,10E-44	CL0028
TRINITY_DN18581_c2_g2::TRINITY_DN18581_c2_g2_i8::g.102871::m.102871	4	36,8	13,927	10,744	PF00450.21	Peptidase_S10	128,2	5,00E-37	CL0028
TRINITY_DN18581_c2_g2::TRINITY_DN18581_c2_g2_i16::g.102885::m.102885	8	49,3	16,532	203,68	PF00450.21	Peptidase_S10	67,6	1,20E-18	CL0028
TRINITY_DN18584_c1_g5::TRINITY_DN18584_c1_g5_i6::g.103010::m.103010	6	26,3	27,016	145,71	PF01657.16	Stress-antifung	75,1	4,90E-21	No_clan
TRINITY_DN18595_c0_g1::TRINITY_DN18595_c0_g1_i7::g.103121::m.103121	1	1,2	127,16	2,1844	PF01602.19	Adaptin_N	358,8	4,20E-107	CL0020
TRINITY_DN18601_c0_g1::TRINITY_DN18601_c0_g1_i1::g.101595::m.101595	3	16,1	18,781	2,722	PF00795.21	CN_hydrolase	35,2	7,40E-09	No_clan
TRINITY_DN18601_c0_g1::TRINITY_DN18601_c0_g1_i6::g.101598::m.101598	5	28,4	24,393	46,395	PF00795.21	CN_hydrolase	156,6	7,00E-46	No_clan
TRINITY_DN18605_c15_g2::TRINITY_DN18605_c15_g2_i1::g.103707::m.103707	3	18,9	16,488	5,8607	PF07731.13	Cu-oxidase_2	84,1	6,60E-24	CL0026
TRINITY_DN18610_c1_g2::TRINITY_DN18610_c1_g2_i3::g.103387::m.103387	10	12,7	105,55	38,085	PF13802.5	Gal_mutarotas_2	67	1,30E-18	CL0103

TRINITY_DN18611_c0_g1::TRINITY_DN18611_c0_g1_i4::g.103412::m.103412	5	10,5	66,624	6,8668	PF00005.26	ABC_tran	86,1	2,80E-24	CL0023
TRINITY_DN18612_c0_g3::TRINITY_DN18612_c0_g3_i1::g.103400::m.103400	3	16,3	25,35	26,06	PF00076.21	RRM_1	60,9	7,30E-17	CL0221
TRINITY_DN18615_c1_g3::TRINITY_DN18615_c1_g3_i2::g.103395::m.103395	2	17,1	11,419	5,9541	PF01738.17	DLH	56,9	2,00E-15	CL0028
TRINITY_DN18618_c2_g1::TRINITY_DN18618_c2_g1_i14::g.103865::m.103865	16	20,3	121	40,819	PF00082.21	Peptidase_S8	270,6	1,40E-80	No_clan
TRINITY_DN18618_c2_g3::TRINITY_DN18618_c2_g3_i2::g.103853::m.103853	4	25,2	24,174	5,6989					
TRINITY_DN18619_c0_g2::TRINITY_DN18619_c0_g2_i2::g.103437::m.103437	4	23	20,856	11,856	PF17135.3	Ribosomal_L18	314,8	1,30E-94	CL0588
TRINITY_DN18628_c2_g1::TRINITY_DN18628_c2_g1_i28::g.103627::m.103627	1	3,7	47,882	1,9738	PF00662.19	Proton_antipo_N	47,8	1,00E-12	No_clan
TRINITY_DN18631_c1_g1::TRINITY_DN18631_c1_g1_i3::g.103586::m.103586	5	20,4	46,053	16,959	PF08263.11	LRRNT_2	24,3	2,70E-05	No_clan
TRINITY_DN18631_c1_g1::TRINITY_DN18631_c1_g1_i1::g.103584::m.103584	1	5,8	29,319	3,0442	PF03878.14	YIF1	197,5	2,20E-58	CL0112
TRINITY_DN18634_c2_g1::TRINITY_DN18634_c2_g1_i4::g.104214::m.104214	8	13,4	84,519	43,355	PF00082.21	Peptidase_S8	164,8	2,40E-48	No_clan
TRINITY_DN18637_c1_g1::TRINITY_DN18637_c1_g1_i7::g.103721::m.103721	2	4,5	54,295	3,6895	PF13499.5	EF-hand_7	72,9	2,00E-20	CL0220
TRINITY_DN18639_c0_g2::TRINITY_DN18639_c0_g2_i1::g.103472::m.103472	2	18,1	17,068	6,0939					
TRINITY_DN18640_c0_g2::TRINITY_DN18640_c0_g2_i8::g.103688::m.103688	5	39,5	17,993	51,912	PF00293.27	NUDIX	95,3	2,60E-27	CL0261
TRINITY_DN18641_c0_g1::TRINITY_DN18641_c0_g1_i1::g.103822::m.103822	2	19,8	11,302	7,487	PF17181.3	EPF	53,6	1,50E-14	No_clan
TRINITY_DN18647_c1_g1::TRINITY_DN18647_c1_g1_i1::g.103820::m.103820	2	7,9	33,495	2,8473	PF00574.22	CLP_protease	222,2	4,10E-66	CL0127
TRINITY_DN18652_c1_g2::TRINITY_DN18652_c1_g2_i5::g.104071::m.104071	1	5,9	20,376	2,3924	PF08059.12	SEP	35,2	1,30E-08	No_clan
TRINITY_DN18652_c2_g1::TRINITY_DN18652_c2_g1_i2::g.104075::m.104075	2	5,5	76,436	5,5614	PF01532.19	Glyco_hydro_47	523,4	4,90E-157	CL0059
TRINITY_DN18657_c1_g1::TRINITY_DN18657_c1_g1_i4::g.104087::m.104087	2	9,8	25,431	3,1927					
TRINITY_DN18657_c3_g2::TRINITY_DN18657_c3_g2_i5::g.104104::m.104104	1	11,3	15,527	2,3071	PF02686.14	Glu-tRNAGln	43,9	2,10E-11	No_clan
TRINITY_DN18658_c0_g1::TRINITY_DN18658_c0_g1_i8::g.104114::m.104114	4	16,4	36,28	5,3232	PF00149.27	Metallophos	48,8	1,10E-12	CL0163
TRINITY_DN18658_c1_g1::TRINITY_DN18658_c1_g1_i9::g.104122::m.104122	23	69,5	42,999	323,31	PF07992.13	Pyr_redox_2	169,4	9,50E-50	CL0063

TRINITY_DN18663_c1_g1::TRINITY_DN18663_c1_g1_i1::g.104197::m.104197	4	27	19,232	18,601	PF04043.14	PMEI	80,6	1,40E-22	No_clan
TRINITY_DN18666_c0_g1::TRINITY_DN18666_c0_g1_i7::g.104181::m.104181	4	25,9	18,925	5,2303	PF16363.4	GDP_Man_Dehyd	76	3,20E-21	CL0063
TRINITY_DN18667_c1_g2::TRINITY_DN18667_c1_g2_i6::g.104311::m.104311	5	18,2	33,726	13,362	PF13432.5	TPR_16	24,3	3,40E-05	CL0020
TRINITY_DN18669_c1_g1::TRINITY_DN18669_c1_g1_i3::g.104346::m.104346	4	26,8	31,571	9,1895	PF09439.9	SRPRB	121,1	3,10E-35	CL0023
TRINITY_DN18673_c1_g1::TRINITY_DN18673_c1_g1_i5::g.104369::m.104369	2	14,9	19,741	10,418					
TRINITY_DN18674_c2_g2::TRINITY_DN18674_c2_g2_i1::g.104393::m.104393	2	9,3	38,071	6,2892					
TRINITY_DN18681_c1_g1::TRINITY_DN18681_c1_g1_i1::g.104540::m.104540	4	28,7	27,245	27,983	PF03358.14	FMN_red	42,4	5,30E-11	CL0042
TRINITY_DN18686_c0_g1::TRINITY_DN18686_c0_g1_i9::g.104732::m.104732	16	33,3	73,878	323,31	PF02727.15	Cu_amine_oxidN2	69,3	2,40E-19	CL0047
TRINITY_DN18687_c0_g1::TRINITY_DN18687_c0_g1_i17::g.103218::m.103218	2	5,3	55,254	2,6307	PF04097.13	Nic96	160,7	4,60E-47	No_clan
TRINITY_DN18690_c2_g2::TRINITY_DN18690_c2_g2_i1::g.104786::m.104786	2	4,2	52,938	2,6008	PF05637.11	Glyco_transf_34	247,8	1,10E-73	CL0110
TRINITY_DN18694_c1_g1::TRINITY_DN18694_c1_g1_i5::g.104667::m.104667	1	7	20,47	2,0854	PF13561.5	adh_short_C2	27,1	2,50E-06	CL0063
TRINITY_DN18697_c0_g2::TRINITY_DN18697_c0_g2_i2::g.103311::m.103311	6	15,4	62,636	8,7045	PF08156.12	NOP5NT	71,4	5,40E-20	No_clan
TRINITY_DN18701_c4_g10::TRINITY_DN18701_c4_g10_i2::g.103928::m.103928	5	20,9	41,132	7,589	PF14369.5	zinc_ribbon_9	50,8	1,40E-13	CL0167
TRINITY_DN18704_c0_g1::TRINITY_DN18704_c0_g1_i1::g.104889::m.104889	5	12,5	68,376	19,222	PF00270.28	DEAD	162,6	6,80E-48	CL0023
TRINITY_DN18707_c1_g1::TRINITY_DN18707_c1_g1_i8::g.104943::m.104943	1	5	24,517	2,0609	PF05529.11	Bap31	40,7	1,80E-10	No_clan
TRINITY_DN18707_c2_g1::TRINITY_DN18707_c2_g1_i4::g.104948::m.104948	14	15,2	132,91	37,357	PF13620.5	CarboxypepD_reg	36,2	5,20E-09	CL0287
TRINITY_DN18713_c3_g1::TRINITY_DN18713_c3_g1_i2::g.105054::m.105054	6	40	18,937	5,464	PF01965.23	DJ-1_PfpI	127,8	3,20E-37	CL0014
TRINITY_DN18713_c3_g1::TRINITY_DN18713_c3_g1_i8::g.105061::m.105061	14	40,7	45,921	306,29	PF01965.23	DJ-1_PfpI	143,5	4,60E-42	CL0014
TRINITY_DN18714_c0_g3::TRINITY_DN18714_c0_g3_i1::g.105033::m.105033	2	18,5	16,647	3,1392					
TRINITY_DN18718_c3_g5::TRINITY_DN18718_c3_g5_i3::g.105071::m.105071	2	14,8	15,496	2,8513	PF08523.9	MBF1	86,1	1,50E-24	No_clan
TRINITY_DN18726_c1_g1::TRINITY_DN18726_c1_g1_i9::g.105485::m.105485	8	9,6	176,46	20,074					
TRINITY_DN18727_c2_g1::TRINITY_DN18727_c2_g1_i8::g.105290::m.105290	2	12,1	34,422	4,4723	PF07059.11	DUF1336	218	1,50E-64	No_clan

TRINITY_DN18731_c0_g5::TRINITY_DN18731_c0_g5_i1::g.105388::m.105388	2	14,9	27,184	5,1038	PF01652.17	IF4E	179,4	4,00E-53	CL0625
TRINITY_DN18733_c1_g1::TRINITY_DN18733_c1_g1_i16::g.105945::m.105945	4	32,1	23,347	10,642					
TRINITY_DN18733_c1_g5::TRINITY_DN18733_c1_g5_i2::g.105940::m.105940	1	2	69,572	2,1201	PF05277.11	DUF726	333,4	1,20E-99	CL0028
TRINITY_DN18736_c0_g1::TRINITY_DN18736_c0_g1_i7::g.105711::m.105711	3	3,6	118,94	4,2082	PF04048.13	Sec8_exocyst	125,3	1,50E-36	CL0295
TRINITY_DN18739_c0_g2::TRINITY_DN18739_c0_g2_i10::g.105638::m.105638	7	9,4	102,15	12,185	PF01399.26	PCI	72,5	3,10E-20	CL0123
TRINITY_DN18739_c0_g3::TRINITY_DN18739_c0_g3_i2::g.105640::m.105640	4	9,8	60,161	5,9484	PF01399.26	PCI	80,1	1,40E-22	CL0123
TRINITY_DN18741_c1_g1::TRINITY_DN18741_c1_g1_i9::g.105752::m.105752	3	13,5	34,794	18,216	PF02114.15	Phosducin	58,6	4,20E-16	CL0172
TRINITY_DN18743_c1_g1::TRINITY_DN18743_c1_g1_i1::g.105300::m.105300	3	40,4	13,318	323,31	PF00313.21	CSD	91,8	1,70E-26	CL0021
TRINITY_DN18745_c0_g3::TRINITY_DN18745_c0_g3_i8::g.105419::m.105419	6	15	43,653	24,847	PF00076.21	RRM_1	62,1	3,00E-17	CL0221
TRINITY_DN18745_c0_g4::TRINITY_DN18745_c0_g4_i1::g.105401::m.105401	1	2,8	54,839	2,1405	PF00288.25	GHMP_kinases_N	60,7	1,10E-16	CL0329
TRINITY_DN18749_c0_g2::TRINITY_DN18749_c0_g2_i4::g.105830::m.105830	8	31,6	31,046	33,892	PF00175.20	NAD_binding_1	93,7	9,80E-27	CL0091
TRINITY_DN18753_c1_g3::TRINITY_DN18753_c1_g3_i14::g.106335::m.106335	2	4,9	61,983	4,4816	PF03055.14	RPE65	404,5	5,50E-121	No_clan
TRINITY_DN18754_c0_g1::TRINITY_DN18754_c0_g1_i2::g.106002::m.106002	1	2,5	58,143	1,8897	PF07223.10	DUF1421	84,9	2,20E-24	CL0214
TRINITY_DN18755_c3_g1::TRINITY_DN18755_c3_g1_i1::g.105920::m.105920	3	12,9	34,644	7,699	PF07714.16	Pkinase_Tyr	144,3	3,60E-42	CL0016
TRINITY_DN18760_c9_g1::TRINITY_DN18760_c9_g1_i2::g.106072::m.106072	9	35,2	34,328	26,07	PF01301.18	Glyco_hydro_35	107	1,30E-30	CL0058
TRINITY_DN18760_c9_g3::TRINITY_DN18760_c9_g3_i2::g.106074::m.106074	3	21,1	18,328	5,59	PF13364.5	BetaGal_dom4_5	26,3	7,80E-06	CL0202
TRINITY_DN18764_c1_g1::TRINITY_DN18764_c1_g1_i6::g.106059::m.106059	6	21,7	35,138	149,94	PF01370.20	Epimerase	87,1	1,10E-24	CL0063
TRINITY_DN18768_c2_g1::TRINITY_DN18768_c2_g1_i1::g.106105::m.106105	5	14,4	45,922	7,8052	PF06273.10	eIF-4B	599,6	5,50E-180	No_clan
TRINITY_DN18771_c1_g1::TRINITY_DN18771_c1_g1_i3::g.106137::m.106137	2	9	43,153	3,1631	PF00069.24	Pkinase	220,8	1,90E-65	CL0016
TRINITY_DN18772_c0_g1::TRINITY_DN18772_c0_g1_i5::g.106264::m.106264	2	5	67,655	4,8494	PF03483.16	B3_4	92	3,00E-26	CL0383
TRINITY_DN18781_c0_g5::TRINITY_DN18781_c0_g5_i3::g.106209::m.106209	6	39,8	22,868	14,873	PF00400.31	WD40	13	0,14	CL0186

TRINITY_DN18783_c5_g1::TRINITY_DN18783_c5_g1_i3::g.106356::m.106356	4	10,1	55,293	9,3019	PF00450.21	Peptidase_S10	490,3	6,00E-147	CL0028
TRINITY_DN18788_c0_g2::TRINITY_DN18788_c0_g2_i3::g.106445::m.106445	1	2,6	73,366	5,6722					
TRINITY_DN18792_c0_g2::TRINITY_DN18792_c0_g2_i6::g.106589::m.106589	3	10	56,535	4,8151	PF00069.24	Pkinase	217,1	2,50E-64	CL0016
TRINITY_DN18793_c0_g1::TRINITY_DN18793_c0_g1_i11::g.106461::m.106461	4	17,8	30,584	8,7743	PF00448.21	SRP54	85,6	3,20E-24	CL0023
TRINITY_DN18797_c1_g2::TRINITY_DN18797_c1_g2_i1::g.106436::m.106436	2	15,7	13,625	3,413	PF04199.12	Cyclase	23,9	3,60E-05	CL0364
TRINITY_DN18799_c1_g2::TRINITY_DN18799_c1_g2_i3::g.106472::m.106472	8	14,8	79,737	27,89	PF00501.27	AMP-binding	348	5,30E-104	CL0378
TRINITY_DN18805_c4_g1::TRINITY_DN18805_c4_g1_i2::g.106689::m.106689	2	8,8	20,357	2,5041	PF00406.21	ADK	146	8,10E-43	CL0023
TRINITY_DN18806_c2_g1::TRINITY_DN18806_c2_g1_i13::g.106758::m.106758	3	14,6	28,86	5,4785	PF00307.30	CH	39,4	5,10E-10	CL0188
TRINITY_DN18817_c0_g1::TRINITY_DN18817_c0_g1_i10::g.107314::m.107314	9	17,7	68,241	52,217					
TRINITY_DN18818_c1_g1::TRINITY_DN18818_c1_g1_i7::g.106901::m.106901	6	18,2	45,263	10,086	PF01218.17	Coprogen_oxidas	452,4	4,20E-136	No_clan
TRINITY_DN18820_c2_g1::TRINITY_DN18820_c2_g1_i7::g.106999::m.106999	3	12,2	36,083	6,3835	PF01344.24	Kelch_1	42,1	4,40E-11	CL0186
TRINITY_DN18821_c0_g1::TRINITY_DN18821_c0_g1_i8::g.106977::m.106977	2	6,9	47,555	4,415	PF02786.16	CPSase_L_D2	253,3	1,50E-75	CL0179
TRINITY_DN19648_c4_g4::TRINITY_DN19648_c4_g4_i4::g.120655::m.120655	2	23,7	17,251	6,1229	PF00203.20	Ribosomal_S19	116,3	3,70E-34	No_clan
TRINITY_DN18832_c0_g1::TRINITY_DN18832_c0_g1_i1::g.107098::m.107098	5	32,3	21,677	12,036	PF08212.11	Lipocalin_2	180,9	1,10E-53	CL0116
TRINITY_DN18833_c4_g2::TRINITY_DN18833_c4_g2_i4::g.107207::m.107207	3	13	27,685	5,5178	PF03629.17	SASA	310,6	5,50E-93	CL0264
TRINITY_DN18840_c3_g1::TRINITY_DN18840_c3_g1_i1::g.107249::m.107249	8	57,1	17,897	66,163					
TRINITY_DN18845_c2_g1::TRINITY_DN18845_c2_g1_i6::g.106645::m.106645	3	4,8	89,474	7,1687	PF03810.18	IBN_N	50,4	1,40E-13	CL0020
TRINITY_DN18848_c2_g2::TRINITY_DN18848_c2_g2_i6::g.107389::m.107389	2	24,5	16,064	16,104	PF00240.22	ubiquitin	66,3	1,40E-18	CL0072
TRINITY_DN18850_c2_g1::TRINITY_DN18850_c2_g1_i14::g.107185::m.107185	3	12,1	47,695	6,1025	PF00501.27	AMP-binding	250,4	2,40E-74	CL0378
TRINITY_DN18850_c2_g2::TRINITY_DN18850_c2_g2_i2::g.107178::m.107178	1	2,3	55,32	1,8534					
TRINITY_DN18852_c0_g2::TRINITY_DN18852_c0_g2_i1::g.107431::m.107431	2	6,3	41,796	2,097	PF02136.19	NTF2	105,3	2,70E-30	CL0051

TRINITY_DN18855_c1_g1::TRINITY_DN18855_c1_g1_i19::g.107600::m.107600	9	17	77,643	17,567	PF00862.18	Sucrose_synth	38,3	4,80E-10	CL0113
TRINITY_DN18855_c1_g1::TRINITY_DN18855_c1_g1_i51::g.107677::m.107677	7	11,6	96,263	5,5361	PF00862.18	Sucrose_synth	37,7	7,00E-10	CL0113
TRINITY_DN18857_c0_g1::TRINITY_DN18857_c0_g1_i9::g.107487::m.107487	2	12	25,428	2,789	PF00400.31	WD40	12,6	0,19	CL0186
TRINITY_DN18857_c0_g5::TRINITY_DN18857_c0_g5_i2::g.107485::m.107485	3	36,7	16,171	23,11					
TRINITY_DN18860_c2_g4::TRINITY_DN18860_c2_g4_i2::g.107536::m.107536	2	9	48,129	9,9882	PF13664.5	DUF4149	77,7	6,30E-22	CL0430
TRINITY_DN18862_c1_g2::TRINITY_DN18862_c1_g2_i1::g.107502::m.107502	3	8,1	46,789	5,6138	PF00206.19	Lyase_1	235,2	1,00E-69	No_clan
TRINITY_DN18865_c1_g1::TRINITY_DN18865_c1_g1_i8::g.107763::m.107763	3	23,6	30,145	64,194	PF00300.21	His_Phos_1	26,9	3,10E-06	CL0071
TRINITY_DN18870_c1_g1::TRINITY_DN18870_c1_g1_i1::g.107725::m.107725	8	35,6	34,788	57,486	PF01182.19	Glucosamine_iso	244,2	1,40E-72	CL0246
TRINITY_DN18870_c1_g2::TRINITY_DN18870_c1_g2_i1::g.107727::m.107727	4	15,7	28,008	9,0238	PF01182.19	Glucosamine_iso	242,6	4,20E-72	CL0246
TRINITY_DN18873_c0_g1::TRINITY_DN18873_c0_g1_i7::g.107966::m.107966	1	1,9	68,833	2,2987	PF02222.21	ATP-grasp	189,4	3,40E-56	CL0179
TRINITY_DN18875_c2_g2::TRINITY_DN18875_c2_g2_i2::g.107929::m.107929	15	61,6	36,757	222,82	PF00022.18	Actin	416,2	1,00E-124	CL0108
TRINITY_DN18875_c2_g2::TRINITY_DN18875_c2_g2_i3::g.107932::m.107932	15	63,9	35,399	10,84	PF00022.18	Actin	390,2	7,90E-117	CL0108
TRINITY_DN18875_c2_g5::TRINITY_DN18875_c2_g5_i2::g.107944::m.107944	7	65,8	17,809	2,5955	PF00022.18	Actin	216,7	3,70E-64	CL0108
TRINITY_DN18878_c2_g2::TRINITY_DN18878_c2_g2_i9::g.108021::m.108021	2	6,4	46,229	2,1125	PF00067.21	p450	138,3	2,80E-40	No_clan
TRINITY_DN18883_c1_g1::TRINITY_DN18883_c1_g1_i14::g.108273::m.108273	22	34	94,516	61,99	PF00012.19	HSP70	533,8	4,00E-160	CL0108
TRINITY_DN18883_c1_g1::TRINITY_DN18883_c1_g1_i17::g.108283::m.108283	20	39,4	72,952	12,794	PF00012.19	HSP70	516,6	6,60E-155	CL0108
TRINITY_DN18884_c1_g1::TRINITY_DN18884_c1_g1_i5::g.108202::m.108202	2	3,3	78,268	4,8021	PF08700.10	Vps51	45,9	4,20E-12	CL0295
TRINITY_DN18886_c3_g1::TRINITY_DN18886_c3_g1_i2::g.108068::m.108068	3	17,8	30,709	5,2556	PF08100.10	Dimerisation	25,9	7,00E-06	CL0123
TRINITY_DN18889_c2_g1::TRINITY_DN18889_c2_g1_i17::g.108140::m.108140	7	37	25,627	57,054	PF00702.25	Hydrolase	56,9	3,30E-15	CL0137
TRINITY_DN18894_c1_g1::TRINITY_DN18894_c1_g1_i9::g.108231::m.108231	7	50,9	12,357	76,254	PF00244.19	14.03.2003	118,3	3,10E-34	No_clan

TRINITY_DN18894_c1_g1::TRINITY_DN18894_c1_g1_i5::g.108225::m.108225	7	55,8	18,324	38,607	PF00244.19	14.03.2003	221,4	9,20E-66	No_clan
TRINITY_DN18894_c1_g3::TRINITY_DN18894_c1_g3_i1::g.108226::m.108226	8	32,1	33,982	20,224	PF00013.28	KH_1	49,2	3,20E-13	CL0007
TRINITY_DN18895_c1_g2::TRINITY_DN18895_c1_g2_i8::g.108488::m.108488	5	18,3	29,047	8,2485	PF00160.20	Pro_isomerase	164	2,90E-48	CL0475
TRINITY_DN18900_c0_g1::TRINITY_DN18900_c0_g1_i6::g.107340::m.107340	3	11,8	38,207	5,9245	PF05542.10	DUF760	72,4	2,70E-20	No_clan
TRINITY_DN18902_c0_g1::TRINITY_DN18902_c0_g1_i9::g.108609::m.108609	8	25,5	49,306	85,003	PF00331.19	Glyco_hydro_10	167,4	4,00E-49	CL0058
TRINITY_DN18903_c0_g1::TRINITY_DN18903_c0_g1_i9::g.108678::m.108678	2	4,9	46,675	2,7767	PF13393.5	tRNA-synt_His	55,2	6,00E-15	CL0040
TRINITY_DN18905_c1_g2::TRINITY_DN18905_c1_g2_i9::g.108635::m.108635	1	10,2	16,696	3,6522					
TRINITY_DN18909_c0_g1::TRINITY_DN18909_c0_g1_i4::g.108774::m.108774	7	16,5	66,473	18,234	PF08662.10	eIF2A	220,1	2,20E-65	CL0186
TRINITY_DN18913_c2_g1::TRINITY_DN18913_c2_g1_i5::g.108928::m.108928	4	6,1	81,894	7,1589	PF03635.16	Vps35	853,9	1,10E-256	No_clan
TRINITY_DN18919_c2_g1::TRINITY_DN18919_c2_g1_i5::g.108804::m.108804	2	3,6	75,671	2,1788					
TRINITY_DN18921_c0_g1::TRINITY_DN18921_c0_g1_i2::g.108916::m.108916	4	11,2	66,555	31,789	PF00995.22	Sec1	370,8	1,80E-110	No_clan
TRINITY_DN18925_c2_g2::TRINITY_DN18925_c2_g2_i2::g.109044::m.109044	1	16	14,347	5,0631	PF04431.12	Pec_lyase_N	78,2	3,80E-22	No_clan
TRINITY_DN18926_c2_g1::TRINITY_DN18926_c2_g1_i1::g.109132::m.109132	5	7,6	84,296	7,0869	PF08318.11	COG4	249,6	3,90E-74	CL0294
TRINITY_DN18930_c0_g5::TRINITY_DN18930_c0_g5_i4::g.109035::m.109035	2	11,9	32,539	4,4189	PF00067.21	p450	242,4	7,80E-72	No_clan
TRINITY_DN18932_c2_g2::TRINITY_DN18932_c2_g2_i2::g.109083::m.109083	2	9	37,535	5,4721	PF05770.10	Ins134_P3_kin	381,1	3,60E-114	CL0179
TRINITY_DN18939_c0_g2::TRINITY_DN18939_c0_g2_i7::g.109183::m.109183	1	3,6	37,037	4,6686	PF16863.4	NtCtMGAM_N	81,4	5,10E-23	No_clan
TRINITY_DN18940_c3_g1::TRINITY_DN18940_c3_g1_i4::g.109190::m.109190	1	15,1	13,41	6,3473	PF06480.14	FtsH_ext	49	5,80E-13	No_clan
TRINITY_DN18942_c3_g3::TRINITY_DN18942_c3_g3_i8::g.109006::m.109006	6	12,7	88,721	12,298	PF00076.21	RRM_1	68,6	2,80E-19	CL0221
TRINITY_DN18947_c6_g2::TRINITY_DN18947_c6_g2_i2::g.109306::m.109306	3	25,6	13,171	73,458	PF00112.22	Peptidase_C1	119,8	1,70E-34	CL0125
TRINITY_DN18947_c6_g5::TRINITY_DN18947_c6_g5_i2::g.109307::m.109307	2	11,8	16,196	5,1078	PF00112.22	Peptidase_C1	94,8	7,10E-27	CL0125
TRINITY_DN18952_c2_g1::TRINITY_DN18952_c2_g1_i9::g.109479::m.109479	2	5,1	48,498	2,9818	PF00067.21	p450	260,6	2,30E-77	No_clan

TRINITY_DN18954_c0_g3::TRINITY_DN18954_c0_g3_i4::g.109379::m.109379	13	41,4	50,269	50,45	PF00330.19	Aconitase	175,6	1,70E-51	No_clan
TRINITY_DN18955_c2_g1::TRINITY_DN18955_c2_g1_i2::g.109309::m.109309	1	17,5	14,624	6,3334	PF01920.19	Prefoldin_2	77,7	5,40E-22	CL0200
TRINITY_DN18955_c3_g1::TRINITY_DN18955_c3_g1_i8::g.109317::m.109317	3	9,3	58,415	5,69	PF00009.26	GTP_EFTU	61,4	7,30E-17	CL0023
TRINITY_DN18961_c1_g1::TRINITY_DN18961_c1_g1_i1::g.109399::m.109399	10	39,7	30,144	204,65					
TRINITY_DN18962_c1_g1::TRINITY_DN18962_c1_g1_i3::g.109730::m.109730	3	6,6	87,999	5,719	PF16486.4	ArgoN	48,8	1,00E-12	No_clan
TRINITY_DN18965_c3_g1::TRINITY_DN18965_c3_g1_i3::g.109455::m.109455	1	25,4	9,8984	43,297					
TRINITY_DN18966_c3_g3::TRINITY_DN18966_c3_g3_i5::g.109514::m.109514	1	4,6	42,594	1,8351	PF03153.12	TFIIA	48,4	1,20E-12	No_clan
TRINITY_DN18967_c1_g1::TRINITY_DN18967_c1_g1_i1::g.109547::m.109547	4	8,6	49,3	7,1453	PF01979.19	Amidohydro_1	100,7	9,40E-29	CL0034
TRINITY_DN18968_c1_g1::TRINITY_DN18968_c1_g1_i9::g.109574::m.109574	2	6,6	53,73	4,3028	PF00122.19	E1-E2_ATPase	80,5	1,00E-22	No_clan
TRINITY_DN18969_c3_g1::TRINITY_DN18969_c3_g1_i1::g.109636::m.109636	20	48,5	53,329	323,31	PF05221.16	AdoHcyase	477,2	1,60E-143	CL0325
TRINITY_DN18971_c1_g2::TRINITY_DN18971_c1_g2_i2::g.109643::m.109643	1	13,8	12,581	1,9377	PF07168.10	Ureide_permease	153	8,40E-45	CL0184
TRINITY_DN18972_c2_g2::TRINITY_DN18972_c2_g2_i2::g.109605::m.109605	3	25,8	40,792	15,379	PF12796.6	Ank_2	58,8	5,70E-16	CL0465
TRINITY_DN18973_c0_g1::TRINITY_DN18973_c0_g1_i1::g.109653::m.109653	7	32,7	24,884	51,241	PF01201.21	Ribosomal_S8e	197,2	1,00E-58	No_clan
TRINITY_DN18981_c5_g1::TRINITY_DN18981_c5_g1_i2::g.109832::m.109832	13	31,1	60,505	39,872	PF00118.23	Cpn60_TCP1	534,3	2,50E-160	No_clan
TRINITY_DN18982_c0_g1::TRINITY_DN18982_c0_g1_i8::g.109877::m.109877	9	30,4	43,833	95,548	PF00557.23	Peptidase_M24	96,2	1,90E-27	No_clan
TRINITY_DN18983_c0_g1::TRINITY_DN18983_c0_g1_i9::g.110074::m.110074	2	4,2	74,027	2,7482	PF00415.17	RCC1	38,3	1,40E-09	CL0186
TRINITY_DN18985_c1_g6::TRINITY_DN18985_c1_g6_i3::g.109896::m.109896	2	4,6	61,269	2,7493	PF17047.4	SMP_LBD	66,7	1,70E-18	CL0648
TRINITY_DN18987_c2_g1::TRINITY_DN18987_c2_g1_i1::g.110056::m.110056	4	7	76,14	4,4961	PF00933.20	Glyco_hydro_3	240,1	3,40E-71	CL0058
TRINITY_DN18987_c4_g3::TRINITY_DN18987_c4_g3_i1::g.110065::m.110065	7	40,2	23,795	65,986	PF00933.20	Glyco_hydro_3	135,9	1,90E-39	CL0058
TRINITY_DN18987_c4_g5::TRINITY_DN18987_c4_g5_i1::g.110067::m.110067	1	12	10,96	2,4605					
TRINITY_DN18988_c0_g2::TRINITY_DN18988_c0_g2_i2::g.109900::m.109900	5	50	12,125	290,89	PF00162.18	PGK	127,7	4,90E-37	No_clan

TRINITY_DN18990_c0_g1::TRINITY_DN18990_c0_g1_i1::g.110013::m.110013	1	7,9	25,647	2,8782					
TRINITY_DN18991_c0_g1::TRINITY_DN18991_c0_g1_i4::g.110139::m.110139	3	13,1	38,518	5,3375	PF00013.28	KH_1	56,1	2,20E-15	CL0007
TRINITY_DN18997_c1_g1::TRINITY_DN18997_c1_g1_i3::g.110273::m.110273	3	5,5	101,37	8,5304	PF14510.5	ABC_trans_N	38	1,70E-09	No_clan
TRINITY_DN18998_c2_g1::TRINITY_DN18998_c2_g1_i7::g.108559::m.108559	5	7,1	126,94	13,013	PF00018.27	SH3_1	23,6	2,60E-05	CL0010
TRINITY_DN18999_c4_g1::TRINITY_DN18999_c4_g1_i2::g.110202::m.110202	2	14,8	34,428	19,275	PF02893.19	GRAM	92,3	1,50E-26	CL0266
TRINITY_DN19001_c1_g1::TRINITY_DN19001_c1_g1_i22::g.110256::m.110256	8	50	19,031	7,4007	PF01263.19	Aldose_epim	125,9	1,80E-36	CL0103
TRINITY_DN19006_c2_g1::TRINITY_DN19006_c2_g1_i11::g.110719::m.110719	1	7,7	24,886	3,2561	PF00069.24	Pkinase	103,2	1,40E-29	CL0016
TRINITY_DN19009_c0_g1::TRINITY_DN19009_c0_g1_i8::g.110506::m.110506	2	10,9	20,836	2,0857	PF00179.25	UQ_con	134,6	1,70E-39	CL0208
TRINITY_DN19010_c0_g2::TRINITY_DN19010_c0_g2_i1::g.110437::m.110437	1	11,4	14,174	54,531	PF00076.21	RRM_1	84,7	2,60E-24	CL0221
TRINITY_DN19013_c0_g2::TRINITY_DN19013_c0_g2_i3::g.110484::m.110484	4	30,7	16,21	28,072	PF00411.18	Ribosomal_S11	160,5	1,50E-47	CL0267
TRINITY_DN19014_c2_g1::TRINITY_DN19014_c2_g1_i4::g.110590::m.110590	5	48,6	12,587	44,358	PF00311.16	PEPcase	74,8	3,90E-21	CL0151
TRINITY_DN19014_c2_g2::TRINITY_DN19014_c2_g2_i8::g.110573::m.110573	14	18	110,18	6,148	PF00311.16	PEPcase	1016,4	0	CL0151
TRINITY_DN19014_c2_g2::TRINITY_DN19014_c2_g2_i15::g.110588::m.110588	18	25,8	98,921	-2	PF00311.16	PEPcase	930,7	6,00E-280	CL0151
TRINITY_DN19014_c2_g2::TRINITY_DN19014_c2_g2_i4::g.110567::m.110567	19	26,7	98,89	42,485	PF00311.16	PEPcase	929,9	1,10E-279	CL0151
TRINITY_DN19016_c1_g5::TRINITY_DN19016_c1_g5_i1::g.110613::m.110613	1	12,7	12,624	2,1405	PF00348.16	polyprenyl_synt	64,3	8,30E-18	CL0613
TRINITY_DN19022_c0_g1::TRINITY_DN19022_c0_g1_i10::g.110657::m.110657	1	4,8	34,553	2,5868	PF00180.19	Iso_dh	203,9	3,40E-60	CL0270
TRINITY_DN19026_c0_g3::TRINITY_DN19026_c0_g3_i5::g.110636::m.110636	6	11,6	80,226	10,705	PF14570.5	zf-RING_4	52,5	2,80E-14	CL0229
TRINITY_DN19032_c2_g1::TRINITY_DN19032_c2_g1_i2::g.110806::m.110806	1	4,7	38,018	2,5674	PF00069.24	Pkinase	101,7	4,00E-29	CL0016
TRINITY_DN19032_c3_g1::TRINITY_DN19032_c3_g1_i17::g.110826::m.110826	1	2,3	64,283	2,3532	PF00069.24	Pkinase	145,8	1,40E-42	CL0016
TRINITY_DN19036_c0_g1::TRINITY_DN19036_c0_g1_i4::g.110800::m.110800	2	9,9	32,018	2,9273	PF00168.29	C2	53,8	1,80E-14	CL0154
TRINITY_DN19040_c3_g4::TRINITY_DN19040_c3_g4_i1::g.110853::m.110853	10	37,4	41,386	250,65	PF00698.20	Acyl_transf_1	89,8	2,20E-25	CL0323

TRINITY_DN19041_c0_g1::TRINITY_DN19041_c0_g1_i8::g.110918::m.110918	9	51,7	23,346	275,41	PF00071.21	Ras	188,4	6,00E-56	CL0023
TRINITY_DN19045_c3_g1::TRINITY_DN19045_c3_g1_i11::g.111100::m.111100	1	4,1	50,785	3,1164	PF04576.14	Zein-binding	66,6	1,60E-18	No_clan
TRINITY_DN19047_c1_g5::TRINITY_DN19047_c1_g5_i2::g.111027::m.111027	3	13,7	36,636	8,4719	PF14226.5	DIOX_N	120,3	6,90E-35	CL0029
TRINITY_DN19049_c0_g1::TRINITY_DN19049_c0_g1_i9::g.110547::m.110547	3	14,5	39,235	7,4995					
TRINITY_DN19058_c2_g1::TRINITY_DN19058_c2_g1_i7::g.111114::m.111114	4	12,5	45,321	7,1332	PF14543.5	TAXi_N	171,9	1,40E-50	CL0129
TRINITY_DN19061_c2_g1::TRINITY_DN19061_c2_g1_i2::g.111195::m.111195	5	18,1	28,385	8,2148	PF01813.16	ATP-synt_D	238,9	3,80E-71	No_clan
TRINITY_DN19061_c2_g6::TRINITY_DN19061_c2_g6_i3::g.111201::m.111201	4	16,1	29,132	3,3472	PF01813.16	ATP-synt_D	233,5	1,70E-69	No_clan
TRINITY_DN19063_c1_g2::TRINITY_DN19063_c1_g2_i13::g.111218::m.111218	3	14,6	36,352	6,2669	PF01467.25	CTP_transf_like	38,8	9,20E-10	CL0039
TRINITY_DN19064_c0_g1::TRINITY_DN19064_c0_g1_i3::g.111228::m.111228	1	3,3	54,132	1,924	PF00005.26	ABC_tran	85,7	3,70E-24	CL0023
TRINITY_DN19066_c1_g3::TRINITY_DN19066_c1_g3_i1::g.111236::m.111236	2	8,6	27,75	2,6423	PF13460.5	NAD_binding_10	156,6	5,90E-46	CL0063
TRINITY_DN19069_c0_g1::TRINITY_DN19069_c0_g1_i4::g.111231::m.111231	1	8,3	34,196	3,3337					
TRINITY_DN19070_c1_g2::TRINITY_DN19070_c1_g2_i7::g.110348::m.110348	7	31,7	24,709	296,99	PF09032.10	Siah-Interact_N	29,8	5,20E-07	No_clan
TRINITY_DN19075_c0_g2::TRINITY_DN19075_c0_g2_i2::g.111296::m.111296	4	8,1	66,179	7,9946	PF01565.22	FAD_binding_4	56,7	2,00E-15	CL0077
TRINITY_DN19075_c1_g2::TRINITY_DN19075_c1_g2_i3::g.111314::m.111314	1	22,6	12,011	2,8193					
TRINITY_DN19077_c0_g1::TRINITY_DN19077_c0_g1_i1::g.111277::m.111277	8	29,9	31,854	47,197	PF00141.22	peroxidase	164	3,90E-48	CL0617
TRINITY_DN19078_c0_g3::TRINITY_DN19078_c0_g3_i6::g.111344::m.111344	1	9,9	15,701	5,937	PF05184.14	SapB_1	58,3	4,80E-16	No_clan
TRINITY_DN19080_c0_g1::TRINITY_DN19080_c0_g1_i5::g.111810::m.111810	7	23,6	45,039	42,943	PF01180.20	DHO_dh	106,9	1,00E-30	CL0036
TRINITY_DN19080_c0_g2::TRINITY_DN19080_c0_g2_i7::g.111803::m.111803	5	7,7	105,73	9,1564	PF00326.20	Peptidase_S9	75,8	3,00E-21	CL0028
TRINITY_DN19082_c3_g1::TRINITY_DN19082_c3_g1_i2::g.111610::m.111610	1	7,6	17,654	3,1926	PF13664.5	DUF4149	92	2,20E-26	CL0430
TRINITY_DN19082_c3_g2::TRINITY_DN19082_c3_g2_i2::g.111602::m.111602	1	3,1	34,932	1,8827	PF00854.20	PTR2	118,9	2,10E-34	CL0015
TRINITY_DN19085_c0_g1::TRINITY_DN19085_c0_g1_i22::g.111744::m.111744	1	3,6	43,209	2,0848					
TRINITY_DN19087_c0_g3::TRINITY_DN19087_c0_g3_i1::g.111646::m.111646	1	17,1	11,451	3,5162	PF09446.9	VMA21	70,1	1,10E-19	No_clan

TRINITY_DN19089_c3_g2::TRINITY_DN19089_c3_g2_i5::g.111660::m.111660	2	5,7	44,434	2,5765	PF01399.26	PCI	40,3	3,30E-10	CL0123
TRINITY_DN19093_c2_g2::TRINITY_DN19093_c2_g2_i1::g.111778::m.111778	8	17	69,628	14,691	PF00390.18	malic	257,3	6,80E-77	CL0603
TRINITY_DN19095_c0_g1::TRINITY_DN19095_c0_g1_i1::g.111856::m.111856	5	25,6	17,482	33,208	PF01287.19	eIF-5a	99,5	7,90E-29	CL0021
TRINITY_DN19101_c2_g2::TRINITY_DN19101_c2_g2_i4::g.111950::m.111950	2	4,7	52,523	3,7678	PF03853.14	YjeF_N	115,6	1,90E-33	CL0063
TRINITY_DN19108_c2_g3::TRINITY_DN19108_c2_g3_i2::g.112222::m.112222	3	41	14,54	22,426	PF11721.7	Malectin	29,4	7,00E-07	CL0468
TRINITY_DN19110_c2_g1::TRINITY_DN19110_c2_g1_i2::g.112351::m.112351	2	29,7	24,873	2,7986					
TRINITY_DN19115_c1_g2::TRINITY_DN19115_c1_g2_i10::g.112400::m.112400	4	10,7	53,131	9,5089	PF00485.17	PRK	161,9	1,40E-47	CL0023
TRINITY_DN19119_c0_g1::TRINITY_DN19119_c0_g1_i6::g.112459::m.112459	4	24,4	32,238	10,614	PF00995.22	Sec1	109,7	2,10E-31	No_clan
TRINITY_DN19123_c0_g2::TRINITY_DN19123_c0_g2_i1::g.112423::m.112423	2	5,4	63,864	3,1822	PF03152.13	UFD1	155,8	6,70E-46	CL0402
TRINITY_DN19124_c2_g3::TRINITY_DN19124_c2_g3_i2::g.112438::m.112438	3	16,3	34,428	5,5022	PF01595.19	DUF21	133,1	7,50E-39	No_clan
TRINITY_DN19126_c0_g1::TRINITY_DN19126_c0_g1_i4::g.112486::m.112486	7	14,4	68,629	16,819	PF03141.15	Methyltransf_29	724,4	6,10E-218	CL0063
TRINITY_DN19133_c0_g1::TRINITY_DN19133_c0_g1_i8::g.112559::m.112559	2	25,8	18,255	6,3016					
TRINITY_DN19136_c0_g1::TRINITY_DN19136_c0_g1_i3::g.112661::m.112661	1	12,4	14,163	2,7655	PF00179.25	UQ_con	72,4	2,60E-20	CL0208
TRINITY_DN19141_c0_g1::TRINITY_DN19141_c0_g1_i8::g.112746::m.112746	6	20,5	47,994	11,43	PF00364.21	Biotin_lipoyl	71,4	3,70E-20	CL0105
TRINITY_DN19143_c2_g1::TRINITY_DN19143_c2_g1_i5::g.112885::m.112885	2	2,7	110,09	2,4614					
TRINITY_DN19145_c0_g2::TRINITY_DN19145_c0_g2_i2::g.112927::m.112927	10	59	18,999	323,31	PF04043.14	PMEI	46,5	4,40E-12	No_clan
TRINITY_DN19149_c0_g1::TRINITY_DN19149_c0_g1_i16::g.113757::m.113757	2	7,1	49,177	2,7117					
TRINITY_DN19149_c0_g1::TRINITY_DN19149_c0_g1_i14::g.113756::m.113756	1	17,2	13,142	2,8645	PF00957.20	Synaptobrevin	30,5	2,20E-07	CL0445
TRINITY_DN19153_c1_g1::TRINITY_DN19153_c1_g1_i1::g.112868::m.112868	2	14,7	24,309	5,6536	PF03765.14	CRAL_TRIO_N	32,3	7,90E-08	No_clan
TRINITY_DN19153_c1_g3::TRINITY_DN19153_c1_g3_i2::g.112871::m.112871	1	18,1	11,555	2,9405					
TRINITY_DN19154_c1_g1::TRINITY_DN19154_c1_g1_i1::g.113024::m.113024	5	25,8	25,092	62,201	PF00081.21	Sod_Fe_N	115,8	8,30E-34	No_clan

TRINITY_DN19156_c1_g1::TRINITY_DN19156_c1_g1_i7::g.113150::m.113150	3	20,4	22,099	5,1388	PF00445.17	Ribonuclease_T2	137,1	7,10E-40	No_clan
TRINITY_DN19158_c0_g2::TRINITY_DN19158_c0_g2_i3::g.112794::m.112794	6	16,6	61,596	23,097					
TRINITY_DN19159_c0_g2::TRINITY_DN19159_c0_g2_i3::g.113047::m.113047	3	27,4	16,197	15,047	PF00182.18	Glyco_hydro_19	209,9	4,40E-62	CL0037
TRINITY_DN19169_c1_g1::TRINITY_DN19169_c1_g1_i1::g.113168::m.113168	3	21	12,604	5,4527	PF01370.20	Epimerase	42,4	5,00E-11	CL0063
TRINITY_DN19169_c1_g3::TRINITY_DN19169_c1_g3_i6::g.113174::m.113174	4	33,1	16,896	198,96					
TRINITY_DN19174_c1_g3::TRINITY_DN19174_c1_g3_i1::g.113399::m.113399	5	21,3	31,453	15,849	PF13249.5	SQHop_cyclase_N	119,3	1,60E-34	CL0059
TRINITY_DN19176_c1_g1::TRINITY_DN19176_c1_g1_i6::g.113373::m.113373	1	11,7	11,186	1,9818					
TRINITY_DN19177_c0_g1::TRINITY_DN19177_c0_g1_i1::g.113288::m.113288	12	20,2	68,624	18,666	PF00749.20	tRNA-synt_1c	335,1	2,90E-100	CL0039
TRINITY_DN19181_c1_g1::TRINITY_DN19181_c1_g1_i4::g.113426::m.113426	3	10,9	38,286	11,824	PF00903.24	Glyoxalase	28,6	1,20E-06	CL0104
TRINITY_DN19182_c1_g1::TRINITY_DN19182_c1_g1_i6::g.113457::m.113457	12	32,5	49,41	154,95	PF00152.19	tRNA-synt_2	263,5	2,10E-78	CL0040
TRINITY_DN19186_c1_g1::TRINITY_DN19186_c1_g1_i2::g.113474::m.113474	4	43,5	13,841	29,839	PF01717.17	Meth_synt_2	176,7	6,40E-52	CL0160
TRINITY_DN19187_c0_g2::TRINITY_DN19187_c0_g2_i3::g.113547::m.113547	6	53,9	13,598	27,085	PF00627.30	UBA	36,1	4,10E-09	CL0214
TRINITY_DN19187_c2_g2::TRINITY_DN19187_c2_g2_i1::g.113564::m.113564	6	32,8	21,423	93,583	PF00543.21	P-II	114,3	3,10E-33	CL0089
TRINITY_DN19191_c0_g2::TRINITY_DN19191_c0_g2_i4::g.113786::m.113786	1	16,2	18,368	2,5804	PF00149.27	Metallophos	44	3,30E-11	CL0163
TRINITY_DN19193_c1_g1::TRINITY_DN19193_c1_g1_i1::g.113618::m.113618	3	33	12,902	31,454	PF16113.4	ECH_2	87,1	1,40E-24	CL0127
TRINITY_DN19193_c1_g3::TRINITY_DN19193_c1_g3_i5::g.113622::m.113622	1	7,8	20,978	2,7964	PF16113.4	ECH_2	250,5	2,80E-74	CL0127
TRINITY_DN19195_c4_g1::TRINITY_DN19195_c4_g1_i3::g.113639::m.113639	4	18,8	39,195	8,5879	PF01301.18	Glyco_hydro_35	403,8	5,90E-121	CL0058
TRINITY_DN19198_c2_g2::TRINITY_DN19198_c2_g2_i7::g.112135::m.112135	3	16,8	21,468	5,5657	PF13774.5	Longin	84,8	3,00E-24	No_clan
TRINITY_DN19199_c1_g1::TRINITY_DN19199_c1_g1_i2::g.113819::m.113819	2	0,7	274,02	2,6092					
TRINITY_DN19207_c4_g2::TRINITY_DN19207_c4_g2_i3::g.114212::m.114212	1	1,9	70,111	2,0394	PF00564.23	PB1	69,7	1,40E-19	CL0072
TRINITY_DN19209_c0_g1::TRINITY_DN19209_c0_g1_i7::g.114040::m.114040	3	15,9	32,024	4,8785	PF11987.7	IF-2	77,5	6,50E-22	No_clan

TRINITY_DN19210_c1_g1::TRINITY_DN19210_c1_g1_i13::g.114068::m.114068	4	7	96,563	9,9203	PF15469.5	Sec5	168,5	1,30E-49	CL0295
TRINITY_DN19212_c0_g1::TRINITY_DN19212_c0_g1_i8::g.114108::m.114108	1	3,8	42,017	2,4369	PF07714.16	Pkinase_Tyr	249,8	2,50E-74	CL0016
TRINITY_DN19212_c1_g1::TRINITY_DN19212_c1_g1_i8::g.114114::m.114114	3	10,6	30,317	3,9989	PF00012.19	HSP70	32,2	3,10E-08	CL0108
TRINITY_DN19219_c1_g1::TRINITY_DN19219_c1_g1_i14::g.114200::m.114200	6	16	63,726	10,019	PF08263.11	LRRNT_2	31,1	1,90E-07	No_clan
TRINITY_DN19222_c3_g1::TRINITY_DN19222_c3_g1_i5::g.114236::m.114236	4	12,4	49,584	8,4423	PF00201.17	UDPGT	114	7,60E-33	CL0113
TRINITY_DN19228_c2_g1::TRINITY_DN19228_c2_g1_i5::g.114244::m.114244	2	21,4	15,815	2,6772					
TRINITY_DN19231_c4_g1::TRINITY_DN19231_c4_g1_i8::g.114359::m.114359	4	11,1	56,78	7,3473	PF00501.27	AMP-binding	345,8	2,50E-103	CL0378
TRINITY_DN19235_c2_g1::TRINITY_DN19235_c2_g1_i2::g.114336::m.114336	2	22,7	21,884	6,2295	PF12819.6	Malectin_like	56,9	1,80E-15	CL0468
TRINITY_DN19235_c2_g2::TRINITY_DN19235_c2_g2_i3::g.114333::m.114333	4	21,9	27,012	42,123	PF14560.5	Ubiquitin_2	101,8	2,00E-29	CL0072
TRINITY_DN19239_c3_g1::TRINITY_DN19239_c3_g1_i4::g.113825::m.113825	1	2,8	49,994	1,8413	PF06814.12	Lung_7-TM_R	164,1	3,90E-48	CL0192
TRINITY_DN19240_c1_g1::TRINITY_DN19240_c1_g1_i5::g.114383::m.114383	2	26,1	15,351	6,3586	PF01704.17	UDPGP	26	3,10E-06	CL0110
TRINITY_DN19240_c1_g2::TRINITY_DN19240_c1_g2_i4::g.114388::m.114388	1	4,7	52,501	2,3833					
TRINITY_DN19241_c0_g1::TRINITY_DN19241_c0_g1_i6::g.115140::m.115140	1	13	12,246	4,9423					
TRINITY_DN19244_c2_g1::TRINITY_DN19244_c2_g1_i18::g.114657::m.114657	4	16,9	28,997	8,354	PF01918.20	Alba	65,5	2,70E-18	CL0441
TRINITY_DN19247_c0_g1::TRINITY_DN19247_c0_g1_i9::g.114529::m.114529	12	25,4	58,138	265,83	PF00928.20	Adap_comp_sub	108,2	4,20E-31	CL0448
TRINITY_DN19247_c0_g1::TRINITY_DN19247_c0_g1_i8::g.114528::m.114528	7	34,3	26,473	2,1676	PF00928.20	Adap_comp_sub	108,8	2,70E-31	CL0448
TRINITY_DN19251_c1_g2::TRINITY_DN19251_c1_g2_i24::g.114984::m.114984	12	31,4	60,22	63,99	PF00069.24	Pkinase	226,6	3,10E-67	CL0016
TRINITY_DN19251_c1_g2::TRINITY_DN19251_c1_g2_i17::g.114967::m.114967	7	45,8	16,898	9,1928					
TRINITY_DN19251_c1_g2::TRINITY_DN19251_c1_g2_i27::g.114997::m.114997	9	39,4	29,239	13,031					
TRINITY_DN19252_c1_g4::TRINITY_DN19252_c1_g4_i7::g.114562::m.114562	4	20,4	33,078	10,318	PF13616.5	Rotamase_3	58,1	1,10E-15	CL0487
TRINITY_DN19255_c4_g1::TRINITY_DN19255_c4_g1_i5::g.114861::m.114861	2	12,9	20,203	3,5737					

TRINITY_DN19257_c3_g1::TRINITY_DN19257_c3_g1_i1::g.114723::m.114723	1	5,9	42,324	1,9831	PF04526.12	DUF568	80,2	1,30E-22	No_clan
TRINITY_DN19260_c1_g1::TRINITY_DN19260_c1_g1_i11::g.114784::m.114784	12	38,7	41,331	67,786	PF00996.17	GDI	626,1	2,10E-188	CL0063
TRINITY_DN19261_c3_g1::TRINITY_DN19261_c3_g1_i6::g.113934::m.113934	7	28,3	36,426	18,405	PF02364.14	Glucan_synthase	455,3	3,70E-136	CL0111
TRINITY_DN19263_c0_g1::TRINITY_DN19263_c0_g1_i2::g.114802::m.114802	5	26,6	24,624	10,439	PF01432.19	Peptidase_M3	185,4	2,10E-54	CL0126
TRINITY_DN19263_c0_g1::TRINITY_DN19263_c0_g1_i6::g.114813::m.114813	4	15,1	31,693	2,0194	PF01432.19	Peptidase_M3	273	5,80E-81	CL0126
TRINITY_DN19263_c0_g3::TRINITY_DN19263_c0_g3_i3::g.114810::m.114810	13	45,3	41,434	2,6017	PF01432.19	Peptidase_M3	162	2,70E-47	CL0126
TRINITY_DN19263_c0_g3::TRINITY_DN19263_c0_g3_i4::g.114811::m.114811	16	46,6	48,427	237,79	PF01432.19	Peptidase_M3	167,3	6,80E-49	CL0126
TRINITY_DN19268_c1_g1::TRINITY_DN19268_c1_g1_i2::g.114841::m.114841	10	27	46,903	35,361	PF00118.23	Cpn60_TCP1	417,9	4,80E-125	No_clan
TRINITY_DN19270_c0_g2::TRINITY_DN19270_c0_g2_i4::g.115031::m.115031	5	24,2	30,584	7,8696	PF00656.21	Peptidase_C14	93,2	2,20E-26	CL0093
TRINITY_DN19277_c4_g1::TRINITY_DN19277_c4_g1_i8::g.115170::m.115170	2	9	43,03	1,9402	PF01734.21	Patatin	77,2	1,70E-21	CL0323
TRINITY_DN19280_c0_g1::TRINITY_DN19280_c0_g1_i3::g.115040::m.115040	1	12	12,402	1,8435					
TRINITY_DN19280_c0_g1::TRINITY_DN19280_c0_g1_i5::g.115042::m.115042	2	7,6	47,713	4,0833					
TRINITY_DN19281_c0_g3::TRINITY_DN19281_c0_g3_i5::g.115036::m.115036	16	55,4	31,125	310,4	PF00262.17	Calreticulin	199,9	5,00E-59	CL0004
TRINITY_DN19281_c0_g5::TRINITY_DN19281_c0_g5_i1::g.115035::m.115035	9	60,8	13,782	212,26	PF00262.17	Calreticulin	54,1	1,00E-14	CL0004
TRINITY_DN19286_c0_g1::TRINITY_DN19286_c0_g1_i3::g.115330::m.115330	11	15,6	88,556	64,427	PF00293.27	NUDIX	60	2,10E-16	CL0261
TRINITY_DN19289_c1_g2::TRINITY_DN19289_c1_g2_i6::g.115366::m.115366	2	3,8	62,652	3,3073					
TRINITY_DN19290_c6_g1::TRINITY_DN19290_c6_g1_i1::g.115224::m.115224	5	15,8	48,026	10,733	PF00266.18	Aminotran_5	289,8	2,50E-86	CL0061
TRINITY_DN19292_c1_g3::TRINITY_DN19292_c1_g3_i1::g.115388::m.115388	17	47,6	38,427	170,33	PF14492.5	EFG_II	35,4	8,00E-09	CL0437
TRINITY_DN19292_c1_g4::TRINITY_DN19292_c1_g4_i1::g.115390::m.115390	22	54,4	56,452	323,31	PF00009.26	GTP_EFTU	220,9	1,00E-65	CL0023
TRINITY_DN19301_c0_g1::TRINITY_DN19301_c0_g1_i6::g.115549::m.115549	3	20,3	27,58	6,9578	PF00786.27	PBD	37,3	2,40E-09	No_clan
TRINITY_DN19302_c0_g2::TRINITY_DN19302_c0_g2_i5::g.115749::m.115749	2	11,5	22,945	2,6124	PF00063.20	Myosin_head	244,4	2,00E-72	CL0023

TRINITY_DN19305_c2_g3::TRINITY_DN19305_c2_g3_i4::g.115690::m.115690	1	7,8	18,212	8,0791	PF01490.17	Aa_trans	120,9	5,10E-35	CL0062
TRINITY_DN19318_c3_g1::TRINITY_DN19318_c3_g1_i7::g.115859::m.115859	1	6,9	18,564	1,8779	PF01300.17	Sua5_yciO_yrdC	137,5	3,10E-40	No_clan
TRINITY_DN19319_c0_g3::TRINITY_DN19319_c0_g3_i9::g.115912::m.115912	2	11,3	20,853	3,535	PF00076.21	RRM_1	60,1	1,30E-16	CL0221
TRINITY_DN19326_c2_g1::TRINITY_DN19326_c2_g1_i5::g.116051::m.116051	5	6,3	119,13	10,639	PF13802.5	Gal_mutarotas_2	53	3,00E-14	CL0103
TRINITY_DN19330_c0_g1::TRINITY_DN19330_c0_g1_i8::g.116161::m.116161	2	29	10,98	5,8936	PF03143.16	GTP_EFTU_D3	97,9	3,90E-28	No_clan
TRINITY_DN19330_c0_g3::TRINITY_DN19330_c0_g3_i6::g.116162::m.116162	11	31	39,21	29,445	PF00009.26	GTP_EFTU	179,8	3,90E-53	CL0023
TRINITY_DN19332_c1_g1::TRINITY_DN19332_c1_g1_i1::g.116126::m.116126	5	15	53,508	12,344	PF00478.24	IMPDH	404,1	4,20E-121	CL0036
TRINITY_DN19332_c2_g1::TRINITY_DN19332_c2_g1_i2::g.116131::m.116131	8	18,7	51,717	22,449	PF04043.14	PMEI	89,6	2,30E-25	No_clan
TRINITY_DN19332_c2_g5::TRINITY_DN19332_c2_g5_i3::g.116136::m.116136	6	26,1	26,714	44,498	PF00857.19	Isochorismatase	115	4,00E-33	No_clan
TRINITY_DN19333_c1_g1::TRINITY_DN19333_c1_g1_i1::g.116168::m.116168	2	3,6	78,707	3,6009	PF00501.27	AMP-binding	133,5	7,20E-39	CL0378
TRINITY_DN19335_c0_g3::TRINITY_DN19335_c0_g3_i4::g.116290::m.116290	1	5,7	20,158	1,9028	PF01095.18	Pectinesterase	127,4	4,00E-37	CL0268
TRINITY_DN19343_c7_g1::TRINITY_DN19343_c7_g1_i3::g.116415::m.116415	1	4,8	23,449	2,8807	PF01373.16	Glyco_hydro_14	216,9	4,10E-64	CL0058
TRINITY_DN19343_c7_g2::TRINITY_DN19343_c7_g2_i2::g.116418::m.116418	1	11,7	11,589	2,5843	PF01373.16	Glyco_hydro_14	106	1,90E-30	CL0058
TRINITY_DN19344_c5_g1::TRINITY_DN19344_c5_g1_i3::g.115614::m.115614	5	26,5	28,688	11,209	PF07839.10	CaM_binding	67,4	1,60E-18	No_clan
TRINITY_DN19344_c5_g1::TRINITY_DN19344_c5_g1_i8::g.115623::m.115623	1	13,8	12,02	2,1965					
TRINITY_DN19344_c5_g2::TRINITY_DN19344_c5_g2_i3::g.115627::m.115627	2	9,3	21,371	3,475					
TRINITY_DN19345_c3_g1::TRINITY_DN19345_c3_g1_i6::g.117087::m.117087	1	19,3	12,509	3,7877					
TRINITY_DN19354_c2_g2::TRINITY_DN19354_c2_g2_i9::g.116448::m.116448	8	51,8	22,46	164,37	PF02798.19	GST_N	63,3	1,80E-17	CL0172
TRINITY_DN19354_c2_g2::TRINITY_DN19354_c2_g2_i8::g.116447::m.116447	10	63,4	19,189	115,03	PF02798.19	GST_N	32,7	6,60E-08	CL0172
TRINITY_DN19354_c2_g2::TRINITY_DN19354_c2_g2_i2::g.116436::m.116436	8	71,6	14,972	5,7339	PF00043.24	GST_C	54	1,30E-14	CL0497
TRINITY_DN19356_c0_g4::TRINITY_DN19356_c0_g4_i8::g.116588::m.116588	1	4,2	30,031	2,3998	PF16076.4	Acyltransf_C	69,4	2,00E-19	No_clan

TRINITY_DN19358_c6_g3::TRINITY_DN19358_c6_g3_i5::g.116477::m.116477	2	21,6	18,538	7,4551	PF05721.12	PhyH	139,7	1,50E-40	CL0029
TRINITY_DN19361_c0_g1::TRINITY_DN19361_c0_g1_i10::g.117059::m.117059	2	1,3	149,18	1,9343	PF04931.12	DNA_pol_phi	646	7,20E-194	No_clan
TRINITY_DN19366_c1_g3::TRINITY_DN19366_c1_g3_i1::g.116912::m.116912	2	13,3	13,917	4,4711	PF01248.25	Ribosomal_L7Ae	91,9	1,50E-26	CL0101
TRINITY_DN19372_c1_g1::TRINITY_DN19372_c1_g1_i2::g.116684::m.116684	7	17,5	59,117	17,447	PF00501.27	AMP-binding	312,6	3,00E-93	CL0378
TRINITY_DN19377_c2_g2::TRINITY_DN19377_c2_g2_i2::g.116852::m.116852	2	11,6	26,067	7,5869					
TRINITY_DN19379_c0_g1::TRINITY_DN19379_c0_g1_i4::g.116795::m.116795	11	17,7	72,743	1,9944	PF00012.19	HSP70	878,9	1,30E-264	CL0108
TRINITY_DN19385_c2_g1::TRINITY_DN19385_c2_g1_i11::g.117072::m.117072	2	9,9	31,842	4,5851	PF04842.11	DUF639	157,4	3,40E-46	No_clan
TRINITY_DN19385_c2_g1::TRINITY_DN19385_c2_g1_i5::g.117068::m.117068	1	7,5	19,458	2,4517					
TRINITY_DN19385_c2_g1::TRINITY_DN19385_c2_g1_i13::g.117074::m.117074	1	8,3	17,307	1,9971					
TRINITY_DN19385_c2_g2::TRINITY_DN19385_c2_g2_i1::g.117065::m.117065	2	15,1	13,369	5,6207					
TRINITY_DN19388_c2_g2::TRINITY_DN19388_c2_g2_i2::g.117003::m.117003	1	6,2	22,668	4,8923	PF07859.12	Abhydrolase_3	121	5,60E-35	CL0028
TRINITY_DN19390_c1_g1::TRINITY_DN19390_c1_g1_i8::g.117167::m.117167	1	1,2	138,67	2,5924	PF00664.22	ABC_membrane	80	2,00E-22	CL0241
TRINITY_DN19395_c0_g1::TRINITY_DN19395_c0_g1_i9::g.117248::m.117248	5	58,5	14,106	150,62	PF00407.18	Bet_v_1	69,4	2,90E-19	CL0209
TRINITY_DN19395_c1_g2::TRINITY_DN19395_c1_g2_i1::g.117249::m.117249	2	25,4	12,833	6,7098	PF14008.5	Metallophos_C	66,1	3,40E-18	No_clan
TRINITY_DN19395_c1_g4::TRINITY_DN19395_c1_g4_i2::g.117259::m.117259	1	3,7	53,309	3,3303	PF16656.4	Pur_ac_phosph_N	71,8	5,00E-20	CL0159
TRINITY_DN19396_c1_g1::TRINITY_DN19396_c1_g1_i8::g.116313::m.116313	22	32,1	119,55	109,94	PF00664.22	ABC_membrane	87,8	8,40E-25	CL0241
TRINITY_DN19396_c1_g2::TRINITY_DN19396_c1_g2_i11::g.116356::m.116356	20	29,6	111,28	4,8261	PF00664.22	ABC_membrane	181,5	2,40E-53	CL0241
TRINITY_DN19401_c0_g3::TRINITY_DN19401_c0_g3_i5::g.116386::m.116386	1	6	27,787	1,8857	PF00076.21	RRM_1	51,4	6,80E-14	CL0221
TRINITY_DN19405_c0_g1::TRINITY_DN19405_c0_g1_i2::g.117368::m.117368	2	13	31,419	2,2529					
TRINITY_DN19405_c2_g1::TRINITY_DN19405_c2_g1_i12::g.117378::m.117378	8	35,6	29,629	202,54	PF01370.20	Epimerase	51,6	7,80E-14	CL0063
TRINITY_DN19409_c5_g1::TRINITY_DN19409_c5_g1_i1::g.117472::m.117472	2	28,9	13,914	23,611	PF03080.14	Neprosin	72,2	3,80E-20	No_clan

TRINITY_DN19409_c5_g5::TRINITY_DN19409_c5_g5_i2::g.117475::m.117475	3	51,5	11,254	7,6393	PF03080.14	Neprosin	111,1	4,80E-32	No_clan
TRINITY_DN19413_c0_g2::TRINITY_DN19413_c0_g2_i1::g.117446::m.117446	1	7,7	23,72	2,7441	PF00085.19	Thioredoxin	31,5	1,20E-07	CL0172
TRINITY_DN19415_c0_g3::TRINITY_DN19415_c0_g3_i1::g.117477::m.117477	5	39,5	19,825	15,568	PF00182.18	Glyco_hydro_19	185,6	1,20E-54	CL0037
TRINITY_DN19418_c2_g1::TRINITY_DN19418_c2_g1_i2::g.117511::m.117511	4	4,8	151,91	7,709	PF04548.15	AIG1	73,7	1,20E-20	CL0023
TRINITY_DN19418_c3_g2::TRINITY_DN19418_c3_g2_i3::g.117519::m.117519	1	11,5	14,432	3,1523	PF00111.26	Fer2	47,9	9,20E-13	CL0486
TRINITY_DN19420_c3_g1::TRINITY_DN19420_c3_g1_i7::g.117669::m.117669	3	30,4	13,406	11,795	PF00238.18	Ribosomal_L14	123,3	5,00E-36	No_clan
TRINITY_DN19422_c2_g1::TRINITY_DN19422_c2_g1_i9::g.117599::m.117599	6	18,8	33,503	11,463	PF00153.26	Mito_carr	73,5	9,10E-21	No_clan
TRINITY_DN19425_c1_g1::TRINITY_DN19425_c1_g1_i1::g.117994::m.117994	3	12	35,458	7,0154					
TRINITY_DN19434_c0_g1::TRINITY_DN19434_c0_g1_i3::g.117932::m.117932	4	21,5	33,506	10,762	PF01429.18	MBD	39	4,30E-10	CL0081
TRINITY_DN19434_c1_g1::TRINITY_DN19434_c1_g1_i6::g.117945::m.117945	2	15,7	18,077	4,3008	PF09177.10	Syntaxin-6_N	82,9	1,70E-23	No_clan
TRINITY_DN19434_c2_g3::TRINITY_DN19434_c2_g3_i2::g.117952::m.117952	12	23,4	69,283	25,805	PF01266.23	DAO	182,2	2,00E-53	CL0063
TRINITY_DN19436_c2_g1::TRINITY_DN19436_c2_g1_i4::g.118041::m.118041	9	27,1	52,04	92,832	PF07944.11	Glyco_hydro_127	298,5	7,40E-89	CL0059
TRINITY_DN19436_c2_g2::TRINITY_DN19436_c2_g2_i1::g.118043::m.118043	4	49,5	10,635	16,09	PF07944.11	Glyco_hydro_127	69,9	1,40E-19	CL0059
TRINITY_DN19436_c2_g3::TRINITY_DN19436_c2_g3_i1::g.118048::m.118048	3	38,6	12,222	261,39					
TRINITY_DN19439_c3_g1::TRINITY_DN19439_c3_g1_i7::g.118339::m.118339	3	10,8	43,023	4,5733	PF01417.19	ENTH	155,5	5,90E-46	CL0009
TRINITY_DN19440_c4_g2::TRINITY_DN19440_c4_g2_i10::g.117992::m.117992	6	34,6	29,045	33,671	PF00085.19	Thioredoxin	115,4	9,00E-34	CL0172
TRINITY_DN19441_c2_g1::TRINITY_DN19441_c2_g1_i3::g.118113::m.118113	3	6,3	65,008	3,6173	PF13890.5	Rab3-GTPase_cat	185,7	4,40E-55	No_clan
TRINITY_DN19442_c0_g2::TRINITY_DN19442_c0_g2_i3::g.118018::m.118018	3	9,5	51,662	12,295	PF03952.15	Enolase_N	182,8	2,70E-54	CL0227
TRINITY_DN19447_c1_g1::TRINITY_DN19447_c1_g1_i4::g.118090::m.118090	6	13,5	73,765	12,327	PF06045.10	Rhamnogal_lyase	305,3	1,70E-91	CL0103
TRINITY_DN19453_c0_g2::TRINITY_DN19453_c0_g2_i2::g.118209::m.118209	2	5,8	47,479	4,3162	PF01204.17	Trehalase	518,8	1,50E-155	CL0059
TRINITY_DN19454_c4_g2::TRINITY_DN19454_c4_g2_i2::g.118225::m.118225	5	14,6	39,625	9,3708					

TRINITY_DN19462_c2_g2::TRINITY_DN19462_c2_g2_i5::g.118383::m.118383	2	1,4	209,99	2,5693	PF00613.19	PI3Ka	71,3	5,90E-20	CL0020
TRINITY_DN19464_c2_g1::TRINITY_DN19464_c2_g1_i6::g.118273::m.118273	8	38,5	28,545	18,72	PF00244.19	14.03.2003	323,7	4,60E-97	No_clan
TRINITY_DN19464_c2_g1::TRINITY_DN19464_c2_g1_i8::g.118275::m.118275	6	24,6	28,511	2,0607	PF00244.19	14.03.2003	324,8	2,10E-97	No_clan
TRINITY_DN19468_c2_g2::TRINITY_DN19468_c2_g2_i2::g.118324::m.118324	2	18,4	19,848	6,5968	PF05091.11	eIF-3_zeta	242	1,40E-71	CL0236
TRINITY_DN19468_c2_g5::TRINITY_DN19468_c2_g5_i1::g.118327::m.118327	4	46,2	12,108	99,209	PF05091.11	eIF-3_zeta	162,8	1,40E-47	CL0236
TRINITY_DN19471_c0_g1::TRINITY_DN19471_c0_g1_i4::g.118445::m.118445	1	7,8	23,267	4,8297	PF01545.20	Cation_efflux	36,3	4,30E-09	No_clan
TRINITY_DN19475_c9_g1::TRINITY_DN19475_c9_g1_i7::g.118616::m.118616	7	43,1	21,44	323,31	PF00082.21	Peptidase_S8	50,5	1,60E-13	No_clan
TRINITY_DN19475_c9_g3::TRINITY_DN19475_c9_g3_i3::g.118615::m.118615	7	24	28,682	323,31	PF00082.21	Peptidase_S8	58,4	6,30E-16	No_clan
TRINITY_DN19482_c6_g1::TRINITY_DN19482_c6_g1_i8::g.118606::m.118606	2	16,1	23,285	5,665	PF04134.11	DUF393	77,9	1,30E-21	No_clan
TRINITY_DN19485_c0_g1::TRINITY_DN19485_c0_g1_i9::g.118686::m.118686	1	3	50,87	2,0249	PF16187.4	Peptidase_M16_M	218,6	9,10E-65	CL0094
TRINITY_DN19486_c1_g2::TRINITY_DN19486_c1_g2_i2::g.118619::m.118619	3	10,2	42,142	5,4907	PF07946.13	DUF1682	263,1	3,20E-78	No_clan
TRINITY_DN19493_c1_g1::TRINITY_DN19493_c1_g1_i9::g.118079::m.118079	2	7,8	28,735	3,2106	PF03171.19	2OG-FeII_Oxy	85,2	3,30E-24	CL0029
TRINITY_DN19496_c3_g4::TRINITY_DN19496_c3_g4_i2::g.118720::m.118720	5	25,9	18,55	166,73	PF04043.14	PMEI	46,9	3,20E-12	No_clan
TRINITY_DN19497_c1_g4::TRINITY_DN19497_c1_g4_i2::g.118818::m.118818	1	5,3	45,206	2,1186	PF01554.17	MatE	108	3,40E-31	CL0222
TRINITY_DN19502_c1_g3::TRINITY_DN19502_c1_g3_i5::g.118942::m.118942	2	2,4	155,98	4,4465	PF14288.5	FKS1_dom1	139,2	5,10E-41	No_clan
TRINITY_DN19511_c1_g1::TRINITY_DN19511_c1_g1_i3::g.119032::m.119032	5	13,4	54,398	14,651	PF10602.8	RPN7	190,6	1,60E-56	CL0020
TRINITY_DN19511_c2_g3::TRINITY_DN19511_c2_g3_i2::g.119040::m.119040	2	11,6	18,635	3,9464	PF01157.17	Ribosomal_L21e	148,7	3,20E-44	CL0107
TRINITY_DN19518_c0_g1::TRINITY_DN19518_c0_g1_i2::g.119121::m.119121	6	22,9	38,173	20,704	PF00248.20	Aldo_ket_red	246	4,00E-73	No_clan
TRINITY_DN19518_c0_g1::TRINITY_DN19518_c0_g1_i8::g.119129::m.119129	5	26,1	21,961	7,5276	PF00248.20	Aldo_ket_red	150,6	4,80E-44	No_clan
TRINITY_DN19518_c0_g1::TRINITY_DN19518_c0_g1_i9::g.119130::m.119130	4	33,6	16,443	2,7634	PF00248.20	Aldo_ket_red	127,8	4,20E-37	No_clan
TRINITY_DN19519_c0_g1::TRINITY_DN19519_c0_g1_i10::g.119145::m.119145	1	5,9	25,857	2,3192	PF00514.22	Arm	27,7	1,70E-06	CL0020
TRINITY_DN19522_c5_g1::TRINITY_DN19522_c5_g1_i5::g.119202::m.119202	2	14,9	21,761	3,5121	PF00795.21	CN_hydrolase	158,5	1,80E-46	No_clan

TRINITY_DN19529_c1_g2::TRINITY_DN19529_c1_g2_i5::g.119243::m.119243	1	2	49,699	1,8812	PF03547.17	Mem_trans	313,1	1,60E-93	CL0064
TRINITY_DN19530_c3_g1::TRINITY_DN19530_c3_g1_i3::g.119253::m.119253	12	21,2	80,564	113,75	PF13510.5	Fer2_4	66,2	1,80E-18	CL0486
TRINITY_DN19531_c0_g1::TRINITY_DN19531_c0_g1_i3::g.119625::m.119625	2	2,2	246,05	2,3215	PF12234.7	Rav1p_C	252,3	8,80E-75	No_clan
TRINITY_DN19535_c2_g2::TRINITY_DN19535_c2_g2_i7::g.119393::m.119393	4	22,6	31,515	18,278	PF08241.11	Methyltransf_11	73,5	1,50E-20	CL0063
TRINITY_DN19535_c3_g1::TRINITY_DN19535_c3_g1_i2::g.119402::m.119402	1	13,5	21,497	62,765	PF00481.20	PP2C	48,3	9,40E-13	CL0238
TRINITY_DN19544_c2_g6::TRINITY_DN19544_c2_g6_i1::g.119552::m.119552	1	9,9	14,044	2,241	PF01198.18	Ribosomal_L31e	141,4	7,40E-42	No_clan
TRINITY_DN19548_c1_g1::TRINITY_DN19548_c1_g1_i5::g.119761::m.119761	3	15,2	33,805	12,553					
TRINITY_DN19548_c2_g6::TRINITY_DN19548_c2_g6_i1::g.119771::m.119771	1	7,2	21,427	2,0987	PF01554.17	MatE	83,6	1,10E-23	CL0222
TRINITY_DN19551_c4_g1::TRINITY_DN19551_c4_g1_i4::g.119678::m.119678	6	52,4	13,042	323,31					
TRINITY_DN19551_c4_g4::TRINITY_DN19551_c4_g4_i1::g.119681::m.119681	13	55,5	34,11	-2	PF00004.28	AAA	86,9	1,40E-24	CL0023
TRINITY_DN19552_c1_g2::TRINITY_DN19552_c1_g2_i2::g.119657::m.119657	2	10,9	29,454	8,2272	PF00155.20	Aminotran_1_2	100,4	1,10E-28	CL0061
TRINITY_DN19556_c1_g1::TRINITY_DN19556_c1_g1_i8::g.119918::m.119918	2	21,4	13,947	2,0658					
TRINITY_DN19559_c2_g2::TRINITY_DN19559_c2_g2_i6::g.119806::m.119806	1	8	15,013	2,4785	PF03959.12	FSH1	99,1	2,40E-28	CL0028
TRINITY_DN19562_c2_g5::TRINITY_DN19562_c2_g5_i1::g.119826::m.119826	1	2,9	41,262	2,3906	PF00012.19	HSP70	421,3	4,60E-126	CL0108
TRINITY_DN19563_c2_g1::TRINITY_DN19563_c2_g1_i4::g.120333::m.120333	28	34,7	107,74	225,74	PF00626.21	Gelsolin	60,8	7,90E-17	CL0092
TRINITY_DN19568_c0_g1::TRINITY_DN19568_c0_g1_i11::g.119880::m.119880	3	4,1	107,03	4,0655	PF00675.19	Peptidase_M16	116,7	7,80E-34	CL0094
TRINITY_DN19569_c1_g4::TRINITY_DN19569_c1_g4_i1::g.119978::m.119978	1	7,5	28,271	3,7847					
TRINITY_DN19570_c1_g1::TRINITY_DN19570_c1_g1_i2::g.119952::m.119952	5	28,9	28,493	24,143	PF13561.5	adh_short_C2	209	6,70E-62	CL0063
TRINITY_DN19576_c0_g1::TRINITY_DN19576_c0_g1_i3::g.120043::m.120043	7	24,4	43,418	19,908	PF00155.20	Aminotran_1_2	178	2,90E-52	CL0061
TRINITY_DN19577_c3_g1::TRINITY_DN19577_c3_g1_i6::g.120109::m.120109	1	8,5	17,485	2,4193	PF06708.10	DUF1195	257,8	2,20E-77	No_clan
TRINITY_DN19578_c1_g1::TRINITY_DN19578_c1_g1_i9::g.120079::m.120079	1	2,8	60,895	2,9268					

TRINITY_DN19578_c1_g1::TRINITY_DN19578_c1_g1_i2::g.120062::m.120062	10	16,2	91,66	18,245					
TRINITY_DN19579_c1_g1::TRINITY_DN19579_c1_g1_i2::g.120372::m.120372	3	9,4	52,946	16,195	PF00696.27	AA_kinase	135	2,80E-39	No_clan
TRINITY_DN19579_c1_g3::TRINITY_DN19579_c1_g3_i1::g.120400::m.120400	4	26,4	30,04	9,7578	PF00696.27	AA_kinase	126,7	9,80E-37	No_clan
TRINITY_DN19581_c3_g6::TRINITY_DN19581_c3_g6_i4::g.120132::m.120132	1	10	12,252	2,4619	PF03179.14	V-ATPase_G	116,6	6,20E-34	CL0255
TRINITY_DN19582_c3_g2::TRINITY_DN19582_c3_g2_i4::g.120114::m.120114	8	38,5	28,509	3,8442	PF01095.18	Pectinesterase	169,3	7,30E-50	CL0268
TRINITY_DN19582_c3_g2::TRINITY_DN19582_c3_g2_i6::g.120115::m.120115	8	33,5	27,743	323,31	PF01095.18	Pectinesterase	175	1,30E-51	CL0268
TRINITY_DN19587_c1_g2::TRINITY_DN19587_c1_g2_i1::g.120316::m.120316	3	14,6	36,225	32,001	PF00240.22	ubiquitin	52,3	3,30E-14	CL0072
TRINITY_DN19588_c6_g1::TRINITY_DN19588_c6_g1_i5::g.120209::m.120209	13	29,2	56,559	45,71	PF00285.20	Citrate_synt	443,2	6,30E-133	No_clan
TRINITY_DN19589_c2_g2::TRINITY_DN19589_c2_g2_i4::g.120311::m.120311	1	11,7	25,143	5,8626	PF00109.25	ketoacyl-synt	71,7	6,30E-20	CL0046
TRINITY_DN19589_c2_g2::TRINITY_DN19589_c2_g2_i4::g.120310::m.120310	3	11,7	45,551	11,737	PF00109.25	ketoacyl-synt	194,3	2,40E-57	CL0046
TRINITY_DN19595_c1_g1::TRINITY_DN19595_c1_g1_i3::g.120443::m.120443	2	28,2	11,455	4,5761	PF04137.14	ERO1	99,2	2,70E-28	No_clan
TRINITY_DN19596_c0_g2::TRINITY_DN19596_c0_g2_i1::g.120414::m.120414	9	23,1	53,422	49,875	PF00378.19	ECH_1	124,7	3,60E-36	CL0127
TRINITY_DN19596_c1_g1::TRINITY_DN19596_c1_g1_i8::g.120430::m.120430	4	20,8	31,155	67,168	PF00450.21	Peptidase_S10	252,5	9,30E-75	CL0028
TRINITY_DN19604_c2_g1::TRINITY_DN19604_c2_g1_i2::g.120755::m.120755	7	36,3	22,021	50,034	PF00759.18	Glyco_hydro_9	233,8	4,20E-69	CL0059
TRINITY_DN19604_c2_g3::TRINITY_DN19604_c2_g3_i3::g.120758::m.120758	7	57	14,112	323,31	PF00759.18	Glyco_hydro_9	89,1	3,80E-25	CL0059
TRINITY_DN19605_c0_g3::TRINITY_DN19605_c0_g3_i3::g.120913::m.120913	11	37,9	50,582	33,091	PF00183.17	HSP90	564,3	2,60E-169	No_clan
TRINITY_DN19606_c0_g1::TRINITY_DN19606_c0_g1_i15::g.121023::m.121023	6	39,8	22,413	156,58	PF00291.24	PALP	180,7	3,90E-53	No_clan
TRINITY_DN19606_c0_g1::TRINITY_DN19606_c0_g1_i4::g.121009::m.121009	5	30,3	25,137	2,4464	PF00291.24	PALP	162,5	1,30E-47	No_clan
TRINITY_DN19611_c0_g2::TRINITY_DN19611_c0_g2_i9::g.121074::m.121074	5	15,8	43,33	4,9237	PF00076.21	RRM_1	59,4	2,00E-16	CL0221
TRINITY_DN19612_c0_g2::TRINITY_DN19612_c0_g2_i5::g.120800::m.120800	2	15,4	18,983	3,0386	PF05755.11	REF	219	5,30E-65	No_clan
TRINITY_DN19612_c1_g2::TRINITY_DN19612_c1_g2_i9::g.120821::m.120821	11	42,4	38,049	20,946	PF03141.15	Methyltransf_29	413,5	1,00E-123	CL0063

TRINITY_DN19612_c1_g2::TRINITY_DN19612_c1_g2_i9::g.120822::m.120822	3	10,9	35,147	5,0534	PF03141.15	Methyltransf_29	325,5	4,80E-97	CL0063
TRINITY_DN19612_c1_g2::TRINITY_DN19612_c1_g2_i7::g.120818::m.120818	3	21,2	22,722	-2	PF03141.15	Methyltransf_29	233,1	4,90E-69	CL0063
TRINITY_DN19613_c2_g1::TRINITY_DN19613_c2_g1_i4::g.120850::m.120850	2	16,2	20,748	11,037	PF14533.5	USP7_C2	147,3	4,50E-43	CL0072
TRINITY_DN19613_c2_g2::TRINITY_DN19613_c2_g2_i3::g.120856::m.120856	2	8,9	36,918	3,6003	PF12436.7	USP7_ICP0_bdg	146,2	9,70E-43	CL0072
TRINITY_DN19614_c2_g7::TRINITY_DN19614_c2_g7_i3::g.120831::m.120831	3	13,8	33,929	7,0682	PF03195.13	LOB	137,5	1,60E-40	No_clan
TRINITY_DN19615_c0_g1::TRINITY_DN19615_c0_g1_i7::g.122149::m.122149	12	13	114,3	20,322	PF13202.5	EF-hand_5	16,6	0,0037	CL0220
TRINITY_DN19616_c4_g3::TRINITY_DN19616_c4_g3_i5::g.120935::m.120935	5	5,9	148,64	9,1928					
TRINITY_DN19617_c3_g2::TRINITY_DN19617_c3_g2_i1::g.121264::m.121264	9	51,2	27,305	323,31	PF00294.23	PfkB	158,6	2,00E-46	CL0118
TRINITY_DN19617_c3_g3::TRINITY_DN19617_c3_g3_i8::g.121273::m.121273	6	60,1	16,159	208,33	PF00294.23	PfkB	84,4	7,60E-24	CL0118
TRINITY_DN19621_c4_g1::TRINITY_DN19621_c4_g1_i2::g.120970::m.120970	1	4,2	32,657	-2	PF00487.23	FA_desaturase	40,1	3,30E-10	No_clan
TRINITY_DN19621_c6_g3::TRINITY_DN19621_c6_g3_i2::g.120980::m.120980	3	2,9	133,61	3,5115	PF11817.7	Foie-gras_1	229,8	3,70E-68	CL0020
TRINITY_DN19625_c0_g5::TRINITY_DN19625_c0_g5_i9::g.121168::m.121168	1	5	43,815	2,3366					
TRINITY_DN19627_c1_g1::TRINITY_DN19627_c1_g1_i1::g.121154::m.121154	12	20,1	87,75	25,069	PF00133.21	tRNA-synt_1	72	2,90E-20	CL0039
TRINITY_DN19627_c1_g1::TRINITY_DN19627_c1_g1_i2::g.121157::m.121157	12	20,1	87,725	5,5603	PF00133.21	tRNA-synt_1	72	2,90E-20	CL0039
TRINITY_DN19627_c1_g2::TRINITY_DN19627_c1_g2_i4::g.121159::m.121159	4	18,8	36,486	10,529	PF00133.21	tRNA-synt_1	36,3	1,80E-09	CL0039
TRINITY_DN19628_c3_g1::TRINITY_DN19628_c3_g1_i3::g.121141::m.121141	6	43,4	28,106	76,501	PF03951.18	Gln-synt_N	40,5	1,60E-10	No_clan
TRINITY_DN19634_c0_g1::TRINITY_DN19634_c0_g1_i18::g.121436::m.121436	9	4,7	316,62	21,995	PF15787.4	DUF4704	167,1	4,40E-49	No_clan
TRINITY_DN19639_c3_g1::TRINITY_DN19639_c3_g1_i7::g.121291::m.121291	2	19,4	11,265	2,0288	PF01132.19	EFP	53,2	2,10E-14	CL0021
TRINITY_DN19643_c4_g1::TRINITY_DN19643_c4_g1_i10::g.121539::m.121539	4	11,9	49,187	6,6278	PF00394.21	Cu-oxidase	154,8	1,80E-45	CL0026
TRINITY_DN19651_c4_g1::TRINITY_DN19651_c4_g1_i17::g.121374::m.121374	3	12,5	32,012	60,608	PF02878.15	PGM_PMM_I	24,3	1,90E-05	No_clan
TRINITY_DN19652_c2_g1::TRINITY_DN19652_c2_g1_i9::g.121472::m.121472	3	6,6	91,983	8,1584	PF06972.10	DUF1296	110,5	2,90E-32	CL0214
TRINITY_DN19655_c2_g1::TRINITY_DN19655_c2_g1_i11::g.121761::m.121761	7	33,3	38,712	24,075	PF00004.28	AAA	147,3	2,90E-43	CL0023

TRINITY_DN19665_c1_g1::TRINITY_DN19665_c1_g1_i7::g.121863::m.121863	7	10,1	93,295	10,333	PF00004.28	AAA	128,1	2,60E-37	CL0023
TRINITY_DN19669_c1_g1::TRINITY_DN19669_c1_g1_i10::g.121794::m.121794	4	36,2	16,351	27,691	PF01738.17	DLH	137,4	4,60E-40	CL0028
TRINITY_DN19683_c4_g2::TRINITY_DN19683_c4_g2_i1::g.121928::m.121928	1	4,6	41,629	18,209	PF01603.19	B56	456,7	6,80E-137	CL0020
TRINITY_DN19694_c1_g1::TRINITY_DN19694_c1_g1_i14::g.120695::m.120695	4	2,1	237,76	4,7166	PF00310.20	GATase_2	609,1	3,30E-183	CL0052
TRINITY_DN19696_c3_g4::TRINITY_DN19696_c3_g4_i1::g.122257::m.122257	14	36	59,628	271,96	PF00330.19	Aconitase	406	2,10E-121	No_clan
TRINITY_DN19704_c0_g1::TRINITY_DN19704_c0_g1_i3::g.122553::m.122553	1	13,9	15,013	4,8073	PF00355.25	Rieske	46,6	2,30E-12	CL0516
TRINITY_DN19704_c0_g2::TRINITY_DN19704_c0_g2_i1::g.122551::m.122551	3	23,3	18,031	28,817	PF02921.13	UCR_TM	35,3	1,10E-08	CL0300
TRINITY_DN19704_c1_g1::TRINITY_DN19704_c1_g1_i9::g.122571::m.122571	14	18	105,94	37,429	PF01602.19	Adaptin_N	296,3	3,60E-88	CL0020
TRINITY_DN19709_c5_g1::TRINITY_DN19709_c5_g1_i3::g.122591::m.122591	4	5,9	96,402	5,9888	PF00221.18	Lyase_aromatic	31,6	6,50E-08	No_clan
TRINITY_DN19710_c5_g3::TRINITY_DN19710_c5_g3_i4::g.122648::m.122648	12	33,1	55,196	247,82	PF03009.16	GDPD	87,5	1,10E-24	CL0384
TRINITY_DN19711_c3_g1::TRINITY_DN19711_c3_g1_i1::g.122630::m.122630	12	46,8	40,808	70,748	PF07992.13	Pyr_redox_2	163,6	5,50E-48	CL0063
TRINITY_DN19711_c5_g2::TRINITY_DN19711_c5_g2_i2::g.122643::m.122643	3	25,8	17,523	5,0776	PF01263.19	Aldose_epim	168,6	1,80E-49	CL0103
TRINITY_DN19714_c6_g2::TRINITY_DN19714_c6_g2_i2::g.122726::m.122726	3	7,1	45,063	4,3564	PF12819.6	Malectin_like	144,5	3,90E-42	CL0468
TRINITY_DN19715_c1_g1::TRINITY_DN19715_c1_g1_i2::g.122707::m.122707	12	16,4	110,87	36,089	PF12515.7	CaATP_NAI	71	4,30E-20	No_clan
TRINITY_DN19717_c0_g1::TRINITY_DN19717_c0_g1_i2::g.122513::m.122513	4	10,4	47,075	5,898	PF16491.4	Peptidase_M48_N	225,3	4,80E-67	No_clan
TRINITY_DN19720_c2_g1::TRINITY_DN19720_c2_g1_i1::g.122728::m.122728	1	11,3	15,188	2,8069	PF02705.15	K_trans	82,4	2,30E-23	CL0062
TRINITY_DN19723_c2_g1::TRINITY_DN19723_c2_g1_i1::g.122855::m.122855	2	16,7	15,669	5,5426	PF13649.5	Methyltransf_25	40,7	2,80E-10	CL0063
TRINITY_DN19723_c2_g3::TRINITY_DN19723_c2_g3_i3::g.122857::m.122857	10	43,3	28,197	232,84	PF13847.5	Methyltransf_31	75,7	3,00E-21	CL0063
TRINITY_DN19723_c2_g3::TRINITY_DN19723_c2_g3_i8::g.122861::m.122861	7	31,7	21,436	2,1918	PF13489.5	Methyltransf_23	70,5	1,30E-19	CL0063
TRINITY_DN19730_c4_g2::TRINITY_DN19730_c4_g2_i8::g.122947::m.122947	7	20	48,706	9,452	PF00270.28	DEAD	172	9,20E-51	CL0023
TRINITY_DN19733_c2_g1::TRINITY_DN19733_c2_g1_i4::g.123110::m.123110	3	2,6	130,54	3,7696	PF00664.22	ABC_membrane	58,6	7,00E-16	CL0241

TRINITY_DN19745_c0_g1::TRINITY_DN19745_c0_g1_i7::g.123245::m.123245	13	20,5	91,333	94,728	PF09334.10	tRNA-synt_1g	508,4	1,00E-152	CL0039
TRINITY_DN19748_c4_g1::TRINITY_DN19748_c4_g1_i8::g.123188::m.123188	5	7,5	93,789	8,5337					
TRINITY_DN19751_c2_g1::TRINITY_DN19751_c2_g1_i6::g.123209::m.123209	12	35,6	48,437	97,231	PF00108.22	Thiolase_N	268,7	4,30E-80	CL0046
TRINITY_DN19770_c1_g4::TRINITY_DN19770_c1_g4_i6::g.123393::m.123393	3	14,6	23,684	5,4089	PF00481.20	PP2C	90,2	1,50E-25	CL0238
TRINITY_DN19775_c0_g3::TRINITY_DN19775_c0_g3_i2::g.123476::m.123476	2	11,3	21,887	3,3075	PF00464.18	SHMT	209,7	4,90E-62	CL0061
TRINITY_DN19775_c1_g1::TRINITY_DN19775_c1_g1_i3::g.123478::m.123478	3	3,2	163	12,318	PF08801.10	Nucleoporin_N	278,6	7,40E-83	CL0186
TRINITY_DN19779_c3_g1::TRINITY_DN19779_c3_g1_i9::g.123559::m.123559	3	24,2	21,053	10,192	PF00152.19	tRNA-synt_2	114,4	5,10E-33	CL0040
TRINITY_DN19779_c3_g1::TRINITY_DN19779_c3_g1_i9::g.123558::m.123558	4	14,3	43,164	20,519	PF01336.24	tRNA_anti-codon	50,8	1,10E-13	CL0021
TRINITY_DN19779_c3_g3::TRINITY_DN19779_c3_g3_i2::g.123543::m.123543	3	14,8	26,758	16,725	PF00071.21	Ras	218	4,70E-65	CL0023
TRINITY_DN19781_c5_g1::TRINITY_DN19781_c5_g1_i1::g.123563::m.123563	6	22	46,355	123,02	PF01399.26	PCI	47	2,70E-12	CL0123
TRINITY_DN19783_c1_g1::TRINITY_DN19783_c1_g1_i4::g.123602::m.123602	4	21	29,441	7,112	PF00155.20	Aminotran_1_2	38,5	7,10E-10	CL0061
TRINITY_DN19790_c2_g2::TRINITY_DN19790_c2_g2_i1::g.123647::m.123647	1	11,9	10,995	3,2041	PF05368.12	NmrA	83,7	1,30E-23	CL0063
TRINITY_DN19790_c2_g2::TRINITY_DN19790_c2_g2_i6::g.123649::m.123649	4	43,8	11,598	8,2109	PF05368.12	NmrA	79,7	2,10E-22	CL0063
TRINITY_DN19791_c7_g1::TRINITY_DN19791_c7_g1_i1::g.123736::m.123736	2	6,4	42,156	70,441	PF00450.21	Peptidase_S10	364,3	1,10E-108	CL0028
TRINITY_DN19791_c7_g2::TRINITY_DN19791_c7_g2_i1::g.123738::m.123738	3	23,9	16,184	323,31	PF00450.21	Peptidase_S10	48,3	9,20E-13	CL0028
TRINITY_DN19791_c7_g4::TRINITY_DN19791_c7_g4_i2::g.123739::m.123739	1	19,2	11,648	7,8911	PF00450.21	Peptidase_S10	52	6,60E-14	CL0028
TRINITY_DN19795_c3_g1::TRINITY_DN19795_c3_g1_i8::g.123663::m.123663	2	32,7	12,347	42,584	PF03179.14	V-ATPase_G	104,2	4,60E-30	CL0255
TRINITY_DN19798_c2_g1::TRINITY_DN19798_c2_g1_i2::g.123755::m.123755	1	20,6	11,022	7,2533					
TRINITY_DN19798_c2_g2::TRINITY_DN19798_c2_g2_i18::g.123759::m.123759	6	13,9	65,23	12,828	PF02933.16	CDC48_2	25,1	1,10E-05	CL0402
TRINITY_DN19798_c2_g2::TRINITY_DN19798_c2_g2_i9::g.123749::m.123749	5	19	47,1	2,9384	PF00004.28	AAA	130,5	4,80E-38	CL0023
TRINITY_DN19806_c0_g1::TRINITY_DN19806_c0_g1_i11::g.123914::m.123914	1	7,5	38,257	2,265					

TRINITY_DN19809_c1_g1::TRINITY_DN19809_c1_g1_i1::g.124378::m.124378	2	23,6	23,241	4,3083	PF00271.30	Helicase_C	101,6	2,80E-29	CL0023
TRINITY_DN19812_c4_g1::TRINITY_DN19812_c4_g1_i1::g.124051::m.124051	18	50,3	44,439	128,73	PF00012.19	HSP70	446,8	8,70E-134	CL0108
TRINITY_DN19812_c4_g2::TRINITY_DN19812_c4_g2_i2::g.124055::m.124055	7	59,8	12,904	2,9969	PF00012.19	HSP70	158	2,60E-46	CL0108
TRINITY_DN19812_c4_g2::TRINITY_DN19812_c4_g2_i6::g.124061::m.124061	25	50,7	57,298	323,31	PF00012.19	HSP70	774	7,20E-233	CL0108
TRINITY_DN19814_c1_g2::TRINITY_DN19814_c1_g2_i2::g.124101::m.124101	5	34,3	23,59	43,451	PF01293.19	PEPCK_ATP	61,8	4,40E-17	CL0374
TRINITY_DN19814_c1_g3::TRINITY_DN19814_c1_g3_i1::g.124097::m.124097	5	54,2	15,869	1,9234	PF01293.19	PEPCK_ATP	255,9	5,80E-76	CL0374
TRINITY_DN19814_c1_g3::TRINITY_DN19814_c1_g3_i2::g.124100::m.124100	5	39,2	22,703	5,8244	PF01293.19	PEPCK_ATP	255,3	8,90E-76	CL0374
TRINITY_DN19816_c1_g2::TRINITY_DN19816_c1_g2_i10::g.124090::m.124090	1	4,6	28,356	2,7846	PF01583.19	APS_kinase	237,3	6,30E-71	CL0023
TRINITY_DN19816_c1_g2::TRINITY_DN19816_c1_g2_i9::g.124089::m.124089	2	9,5	24,363	2,6472	PF01583.19	APS_kinase	240,3	7,40E-72	CL0023
TRINITY_DN19823_c3_g2::TRINITY_DN19823_c3_g2_i6::g.124233::m.124233	1	2,5	88,488	3,2989	PF08920.9	SF3b1	143,4	3,80E-42	CL0462
TRINITY_DN19829_c1_g1::TRINITY_DN19829_c1_g1_i2::g.124633::m.124633	6	19,7	45,718	11,829	PF07712.11	SURNod19	646,6	9,00E-195	No_clan
TRINITY_DN19839_c2_g3::TRINITY_DN19839_c2_g3_i5::g.123823::m.123823	7	71,6	16,478	110,83	PF00255.18	GSHPx	132	5,40E-39	CL0172
TRINITY_DN19841_c3_g2::TRINITY_DN19841_c3_g2_i9::g.124618::m.124618	9	9,6	132,65	17,374	PF00122.19	E1-E2_ATPase	65,7	3,40E-18	No_clan
TRINITY_DN19844_c1_g2::TRINITY_DN19844_c1_g2_i1::g.124447::m.124447	3	12,4	26,077	4,3663	PF00749.20	tRNA-synt_1c	29,5	3,00E-07	CL0039
TRINITY_DN19844_c1_g3::TRINITY_DN19844_c1_g3_i3::g.124457::m.124457	2	4,6	44,134	2,6881	PF04558.14	tRNA_synt_1c_R1	183,6	2,40E-54	No_clan
TRINITY_DN19846_c6_g1::TRINITY_DN19846_c6_g1_i2::g.124495::m.124495	4	10,1	56,317	8,5949	PF00400.31	WD40	21,2	0,00037	CL0186
TRINITY_DN19846_c6_g2::TRINITY_DN19846_c6_g2_i1::g.124494::m.124494	1	4	35,233	38,515	PF14543.5	TAXi_N	169,8	6,40E-50	CL0129
TRINITY_DN19854_c2_g1::TRINITY_DN19854_c2_g1_i1::g.124554::m.124554	4	13,5	45,27	37,8	PF05470.11	eIF-3c_N	401,7	4,20E-120	No_clan
TRINITY_DN19854_c2_g2::TRINITY_DN19854_c2_g2_i3::g.124560::m.124560	10	23	59,756	44,972	PF05470.11	eIF-3c_N	374	1,10E-111	No_clan
TRINITY_DN19854_c2_g2::TRINITY_DN19854_c2_g2_i4::g.124562::m.124562	2	11,2	25,598	8,3215	PF05470.11	eIF-3c_N	320	2,30E-95	No_clan

TRINITY_DN19860_c5_g1::TRINITY_DN19860_c5_g1_i1::g.124725::m.124725	3	17,4	25,084	2,5769	PF00122.19	E1-E2_ATPase	168,5	9,80E-50	No_clan
TRINITY_DN19864_c2_g6::TRINITY_DN19864_c2_g6_i4::g.124630::m.124630	10	40,3	32,87	116,02	PF00724.19	Oxidored_FMN	236,8	3,40E-70	CL0036
TRINITY_DN19870_c1_g2::TRINITY_DN19870_c1_g2_i5::g.124748::m.124748	2	12,6	19,274	4,0442	PF00696.27	AA_kinase	26,7	3,60E-06	No_clan
TRINITY_DN19873_c6_g5::TRINITY_DN19873_c6_g5_i6::g.124897::m.124897	2	13,6	21,672	2,1374	PF03949.14	Malic_M	224	1,80E-66	CL0063
TRINITY_DN19873_c6_g8::TRINITY_DN19873_c6_g8_i6::g.124910::m.124910	4	17,1	28,436	4,8481	PF00390.18	malic	225	5,50E-67	CL0603
TRINITY_DN19874_c1_g1::TRINITY_DN19874_c1_g1_i2::g.124818::m.124818	1	6,1	21,581	2,241					
TRINITY_DN19877_c2_g3::TRINITY_DN19877_c2_g3_i1::g.125021::m.125021	6	17,3	53,45	10,404	PF01512.16	Complex1_51K	161,8	9,60E-48	CL0105
TRINITY_DN19877_c3_g4::TRINITY_DN19877_c3_g4_i1::g.125024::m.125024	1	11,2	14,344	7,9135	PF00179.25	UQ_con	59,8	2,10E-16	CL0208
TRINITY_DN19879_c1_g2::TRINITY_DN19879_c1_g2_i1::g.125068::m.125068	6	41,1	17,254	323,31	PF00295.16	Glyco_hydro_28	86,1	2,10E-24	CL0268
TRINITY_DN19879_c1_g2::TRINITY_DN19879_c1_g2_i8::g.125070::m.125070	4	53,3	11,859	10,886					
TRINITY_DN19879_c2_g1::TRINITY_DN19879_c2_g1_i5::g.125071::m.125071	3	30,3	10,647	90,204					
TRINITY_DN19880_c6_g4::TRINITY_DN19880_c6_g4_i2::g.125001::m.125001	7	56	18,87	15,915	PF14226.5	DIOX_N	88,3	5,70E-25	CL0029
TRINITY_DN19887_c8_g1::TRINITY_DN19887_c8_g1_i5::g.125402::m.125402	4	30,5	13,768	18,016					
TRINITY_DN19888_c1_g1::TRINITY_DN19888_c1_g1_i5::g.125265::m.125265	2	17,2	28,018	3,5788	PF00076.21	RRM_1	23,3	3,90E-05	CL0221
TRINITY_DN19895_c1_g1::TRINITY_DN19895_c1_g1_i3::g.125573::m.125573	1	2,2	61,181	2,1731	PF01412.17	ArfGap	91,1	4,30E-26	No_clan
TRINITY_DN19896_c1_g1::TRINITY_DN19896_c1_g1_i11::g.125427::m.125427	2	9,4	45,975	4,779	PF00288.25	GHMP_kinases_N	33,8	2,60E-08	CL0329
TRINITY_DN19896_c2_g1::TRINITY_DN19896_c2_g1_i4::g.125437::m.125437	3	8,4	50,051	7,4608	PF00462.23	Glutaredoxin	42,9	3,90E-11	CL0172
TRINITY_DN19898_c1_g2::TRINITY_DN19898_c1_g2_i1::g.123971::m.123971	4	19,5	25,014	18,578	PF01201.21	Ribosomal_S8e	196,3	2,10E-58	No_clan
TRINITY_DN19902_c1_g2::TRINITY_DN19902_c1_g2_i1::g.125828::m.125828	4	14,3	46,641	13,58	PF04811.14	Sec23_trunk	30,4	2,50E-07	CL0128
TRINITY_DN19908_c2_g1::TRINITY_DN19908_c2_g1_i5::g.125911::m.125911	2	13,5	22,643	2,2269	PF03081.14	Exo70	229,1	7,60E-68	CL0295
TRINITY_DN19909_c0_g1::TRINITY_DN19909_c0_g1_i3::g.126119::m.126119	12	28,7	61,917	323,31	PF08263.11	LRRNT_2	28,1	1,70E-06	No_clan
TRINITY_DN19911_c7_g2::TRINITY_DN19911_c7_g2_i3::g.126024::m.126024	7	56,3	14,187	181,24	PF08031.11	BBE	76,4	1,30E-21	CL0277

TRINITY_DN19911_c7_g3::TRINITY_DN19911_c7_g3_i1::g.126023::m.126023	10	61,1	18,518	114,68					
TRINITY_DN19918_c3_g1::TRINITY_DN19918_c3_g1_i11::g.126682::m.126682	6	7,7	113,94	12,371	PF04652.15	Vta1	60,7	1,30E-16	No_clan
TRINITY_DN19929_c0_g1::TRINITY_DN19929_c0_g1_i9::g.126183::m.126183	10	30,9	42,969	16,144	PF16363.4	GDP_Man_Dehyd	180,6	4,60E-53	CL0063
TRINITY_DN19935_c0_g1::TRINITY_DN19935_c0_g1_i18::g.126411::m.126411	1	7,7	17,515	1,8339					
TRINITY_DN19939_c3_g1::TRINITY_DN19939_c3_g1_i7::g.125684::m.125684	1	6	38,158	3,081	PF00069.24	Pkinase	242,6	4,00E-72	CL0016
TRINITY_DN19942_c0_g1::TRINITY_DN19942_c0_g1_i1::g.126320::m.126320	9	38,2	24,63	18,237	PF00347.22	Ribosomal_L6	44	2,50E-11	No_clan
TRINITY_DN19942_c2_g1::TRINITY_DN19942_c2_g1_i4::g.126324::m.126324	1	10,7	20,746	3,7046	PF00097.24	zf-C3HC4	25,4	8,60E-06	CL0229
TRINITY_DN19945_c1_g2::TRINITY_DN19945_c1_g2_i9::g.126623::m.126623	2	5,1	48,711	4,196	PF00083.23	Sugar_tr	322,5	4,10E-96	CL0015
TRINITY_DN19948_c1_g1::TRINITY_DN19948_c1_g1_i4::g.126476::m.126476	10	21,2	58,99	190,32	PF07991.11	IlvN	107,1	6,20E-31	CL0063
TRINITY_DN19948_c1_g1::TRINITY_DN19948_c1_g1_i7::g.126477::m.126477	4	40,4	11,605	48,05	PF07991.11	IlvN	27,7	1,60E-06	CL0063
TRINITY_DN19951_c3_g1::TRINITY_DN19951_c3_g1_i8::g.125811::m.125811	3	13,8	34,07	2,9284	PF00719.18	Pyrophosphatase	159,8	4,40E-47	No_clan
TRINITY_DN19952_c2_g2::TRINITY_DN19952_c2_g2_i3::g.126557::m.126557	4	11,8	40,023	62,982					
TRINITY_DN19957_c3_g2::TRINITY_DN19957_c3_g2_i9::g.126783::m.126783	7	63,3	16,005	56,72	PF00241.19	Cofilin_ADF	129,7	6,30E-38	CL0092
TRINITY_DN19962_c5_g2::TRINITY_DN19962_c5_g2_i2::g.126940::m.126940	1	7,4	16,792	1,8906	PF01095.18	Pectinesterase	252,5	3,30E-75	CL0268
TRINITY_DN19966_c14_g1::TRINITY_DN19966_c14_g1_i3::g.126866::m.126866	1	1,9	106,75	2,9291	PF00982.20	Glyco_transf_20	623,3	2,40E-187	CL0113
TRINITY_DN19972_c2_g1::TRINITY_DN19972_c2_g1_i3::g.126916::m.126916	5	43,3	11,54	14,217	PF00141.22	peroxidase	48,4	8,40E-13	CL0617
TRINITY_DN19979_c5_g4::TRINITY_DN19979_c5_g4_i2::g.127014::m.127014	8	50,3	19,498	41,766	PF00248.20	Aldo_ket_red	148,5	2,00E-43	No_clan
TRINITY_DN19979_c5_g4::TRINITY_DN19979_c5_g4_i5::g.127016::m.127016	8	37,3	26,15	5,7437	PF00248.20	Aldo_ket_red	202,3	8,30E-60	No_clan
TRINITY_DN19979_c5_g7::TRINITY_DN19979_c5_g7_i1::g.127021::m.127021	4	32,6	14,675	7,2217	PF00248.20	Aldo_ket_red	88	5,60E-25	No_clan
TRINITY_DN19980_c4_g3::TRINITY_DN19980_c4_g3_i6::g.127331::m.127331	10	63	13,817	323,31	PF01095.18	Pectinesterase	98	3,70E-28	CL0268
TRINITY_DN19981_c2_g1::TRINITY_DN19981_c2_g1_i2::g.127291::m.127291	5	7,4	89,455	8,4557	PF01805.19	Surp	74,7	3,90E-21	No_clan

TRINITY_DN19982_c2_g1::TRINITY_DN19982_c2_g1_i8::g.127226::m.127226	3	21,2	16,881	12,464	PF00156.26	Pribosyltran	48,2	7,70E-13	CL0533
TRINITY_DN19983_c1_g1::TRINITY_DN19983_c1_g1_i9::g.127358::m.127358	6	33	31,635	81,899	PF07933.13	DUF1681	177,6	1,10E-52	CL0266
TRINITY_DN19992_c4_g1::TRINITY_DN19992_c4_g1_i5::g.127460::m.127460	1	13,5	12,404	1,8506					
TRINITY_DN19995_c2_g1::TRINITY_DN19995_c2_g1_i2::g.127478::m.127478	3	5,9	70,874	4,5353	PF00082.21	Peptidase_S8	149	1,60E-43	No_clan
TRINITY_DN19996_c7_g1::TRINITY_DN19996_c7_g1_i2::g.127540::m.127540	13	32,5	54,181	38,355	PF07992.13	Pyr_redox_2	245,9	4,60E-73	CL0063
TRINITY_DN19997_c3_g2::TRINITY_DN19997_c3_g2_i2::g.127405::m.127405	3	23,6	14,456	4,2393	PF13414.5	TPR_11	33,4	2,40E-08	CL0020
TRINITY_DN19999_c7_g1::TRINITY_DN19999_c7_g1_i2::g.126443::m.126443	2	20	10,924	2,285	PF01230.22	HIT	67,9	9,40E-19	CL0265
TRINITY_DN20002_c1_g1::TRINITY_DN20002_c1_g1_i9::g.127794::m.127794	17	43,6	59,053	64,521	PF00118.23	Cpn60_TCP1	348,7	4,50E-104	No_clan
TRINITY_DN20003_c1_g1::TRINITY_DN20003_c1_g1_i6::g.127620::m.127620	2	13,2	14,94	2,2332	PF05347.14	Complex1_LYR	50,6	1,30E-13	CL0491
TRINITY_DN20008_c1_g1::TRINITY_DN20008_c1_g1_i8::g.127738::m.127738	6	21,3	36,597	11,237	PF00248.20	Aldo_ket_red	237,7	1,30E-70	No_clan
TRINITY_DN20011_c2_g2::TRINITY_DN20011_c2_g2_i1::g.127827::m.127827	15	35	62,757	41,162	PF00118.23	Cpn60_TCP1	512,1	1,30E-153	No_clan
TRINITY_DN20011_c3_g2::TRINITY_DN20011_c3_g2_i2::g.127835::m.127835	10	22,6	69,231	134,34	PF01301.18	Glyco_hydro_35	390,2	8,50E-117	CL0058
TRINITY_DN20016_c1_g1::TRINITY_DN20016_c1_g1_i7::g.127847::m.127847	4	20,4	31,988	8,3769	PF00248.20	Aldo_ket_red	165,8	1,10E-48	No_clan
TRINITY_DN20017_c0_g1::TRINITY_DN20017_c0_g1_i1::g.127859::m.127859	1	9,4	22,508	3,9658	PF02776.17	TPP_enzyme_N	42	6,60E-11	CL0254
TRINITY_DN20022_c2_g5::TRINITY_DN20022_c2_g5_i1::g.128013::m.128013	3	16,1	35,92	14,344	PF02338.18	OTU	113,9	6,20E-33	CL0125
TRINITY_DN20022_c3_g3::TRINITY_DN20022_c3_g3_i3::g.128023::m.128023	2	20	13,44	126,54					
TRINITY_DN20023_c4_g2::TRINITY_DN20023_c4_g2_i4::g.127985::m.127985	14	28,1	71,061	116,42	PF01602.19	Adaptin_N	438,5	2,90E-131	CL0020
TRINITY_DN20026_c6_g1::TRINITY_DN20026_c6_g1_i3::g.128057::m.128057	3	9,3	39,311	6,448	PF00067.21	p450	212,1	1,20E-62	No_clan
TRINITY_DN20029_c3_g4::TRINITY_DN20029_c3_g4_i4::g.128107::m.128107	8	76,9	15,01	269,58					
TRINITY_DN20035_c3_g1::TRINITY_DN20035_c3_g1_i3::g.128175::m.128175	6	21	43,866	13,643	PF00218.20	IGPS	271,2	6,40E-81	CL0036

TRINITY_DN20036_c5_g1::TRINITY_DN20036_c5_g1_i2::g.127587::m.127587	1	8,4	21,884	6,7506	PF02127.14	Peptidase_M18	136,2	1,20E-39	CL0035
TRINITY_DN20037_c6_g1::TRINITY_DN20037_c6_g1_i4::g.128163::m.128163	3	17,6	21,432	17,383	PF13563.5	2_5_RNA_ligase2	37,6	1,80E-09	CL0247
TRINITY_DN20043_c7_g1::TRINITY_DN20043_c7_g1_i1::g.128234::m.128234	6	56,8	19,21	27,456	PF02115.16	Rho_GDI	249,5	1,90E-74	No_clan
TRINITY_DN20043_c7_g2::TRINITY_DN20043_c7_g2_i2::g.128238::m.128238	4	55,1	12,002	8,7071	PF02115.16	Rho_GDI	53,4	2,40E-14	No_clan
TRINITY_DN20043_c8_g6::TRINITY_DN20043_c8_g6_i2::g.128243::m.128243	4	35,7	19,843	32,844	PF00400.31	WD40	21,2	0,00034	CL0186
TRINITY_DN20046_c4_g1::TRINITY_DN20046_c4_g1_i6::g.128432::m.128432	18	24,2	115,66	86,491	PF16078.4	2-oxogl_dehyd_N	59,4	1,70E-16	No_clan
TRINITY_DN20049_c7_g1::TRINITY_DN20049_c7_g1_i11::g.128495::m.128495	14	39,2	46,266	323,31	PF00370.20	FGGY_N	149,7	9,50E-44	CL0108
TRINITY_DN20189_c0_g1::TRINITY_DN20189_c0_g1_i1::g.128508::m.128508	2	11,7	22,698	6,3422	PF04398.11	DUF538	106,1	1,20E-30	No_clan
TRINITY_DN20983_c0_g1::TRINITY_DN20983_c0_g1_i1::g.128678::m.128678	3	10	44,026	5,2232	PF01535.19	PPR	14,1	0,039	CL0020
TRINITY_DN22718_c0_g1::TRINITY_DN22718_c0_g1_i1::g.128985::m.128985	1	17,4	12,447	3,9146	PF00704.27	Glyco_hydro_18	47,8	1,50E-12	CL0058
TRINITY_DN2281_c0_g1::TRINITY_DN2281_c0_g1_i1::g.635::m.635	1	5	47,454	1,8981	PF05185.15	PRMT5	243,9	7,70E-73	CL0063
TRINITY_DN27272_c0_g1::TRINITY_DN27272_c0_g1_i1::g.129628::m.129628	1	9,6	12,795	2,0604					
TRINITY_DN2866_c0_g1::TRINITY_DN2866_c0_g1_i1::g.791::m.791	2	3,1	66,703	2,5766					
TRINITY_DN3120_c0_g1::TRINITY_DN3120_c0_g1_i1::g.867::m.867	3	24,8	28,568	20,061	PF02330.15	MAM33	148,6	2,00E-43	No_clan
TRINITY_DN3188_c0_g1::TRINITY_DN3188_c0_g1_i1::g.878::m.878	4	25,5	18,311	5,6424	PF05899.11	Cupin_3	90,2	4,50E-26	CL0029
TRINITY_DN31964_c0_g1::TRINITY_DN31964_c0_g1_i1::g.130316::m.130316	2	15,1	17,406	2,3772	PF00112.22	Peptidase_C1	186	9,10E-55	CL0125
TRINITY_DN32151_c0_g1::TRINITY_DN32151_c0_g1_i1::g.130343::m.130343	1	18,4	13,002	7,4602					
TRINITY_DN32757_c0_g1::TRINITY_DN32757_c0_g1_i1::g.130429::m.130429	1	14,2	12,561	2,2704					
TRINITY_DN3292_c0_g1::TRINITY_DN3292_c0_g1_i2::g.890::m.890	1	3,8	40,868	2,0378	PF00226.30	DnaJ	91,9	1,80E-26	CL0392
TRINITY_DN34461_c0_g1::TRINITY_DN34461_c0_g1_i1::g.130651::m.130651	1	5,3	16,126	19,073	PF00295.16	Glyco_hydro_28	132,1	2,10E-38	CL0268
TRINITY_DN36067_c0_g1::TRINITY_DN36067_c0_g1_i1::g.130820::m.130820	7	70,1	19,452	323,31	PF00182.18	Glyco_hydro_19	297,9	5,70E-89	CL0037
TRINITY_DN36814_c0_g1::TRINITY_DN36814_c0_g1_i1::g.130975::m.130975	1	9	14,448	1,8789					

TRINITY_DN37045_c0_g1::TRINITY_DN37045_c0_g1_i1::g.131013::m.131013	7	25,6	45,719	12,353					
TRINITY_DN37417_c0_g1::TRINITY_DN37417_c0_g1_i1::g.131078::m.131078	1	4,8	30,577	-2	PF04564.14	U-box	57,7	9,40E-16	CL0229
TRINITY_DN44543_c0_g1::TRINITY_DN44543_c0_g1_i1::g.132115::m.132115	3	29,7	12,884	-2	PF00025.20	Arf	145,4	1,10E-42	CL0023
TRINITY_DN47853_c0_g1::TRINITY_DN47853_c0_g1_i1::g.132570::m.132570	3	22,9	14,825	2,0809	PF13499.5	EF-hand_7	56,5	2,60E-15	CL0220
TRINITY_DN48442_c0_g1::TRINITY_DN48442_c0_g1_i1::g.132681::m.132681	3	19,6	22,932	5,8424	PF00977.20	His_biosynth	91,4	5,30E-26	CL0036
TRINITY_DN49420_c0_g1::TRINITY_DN49420_c0_g1_i1::g.132815::m.132815	2	15,3	20,261	-2	PF13905.5	Thioredoxin_8	67,7	7,90E-19	CL0172
TRINITY_DN49712_c0_g1::TRINITY_DN49712_c0_g1_i1::g.132845::m.132845	1	7,1	23,772	2,205					
TRINITY_DN5236_c0_g1::TRINITY_DN5236_c0_g1_i1::g.1296::m.1296	3	15,2	29,878	6,7191					
TRINITY_DN5432_c0_g1::TRINITY_DN5432_c0_g1_i1::g.1343::m.1343	3	20,3	23,219	22,89	PF00171.21	Aldedh	250	3,20E-74	CL0099
TRINITY_DN5515_c0_g1::TRINITY_DN5515_c0_g1_i1::g.1365::m.1365	1	4,5	31,805	2,7708	PF17285.1	PRMT5_TIM	253,8	1,40E-75	CL0036
TRINITY_DN570_c0_g1::TRINITY_DN570_c0_g1_i1::g.152::m.152	3	30,7	12,836	4,7052					
TRINITY_DN6579_c0_g1::TRINITY_DN6579_c0_g1_i1::g.1685::m.1685	2	13,9	24,695	14,254	PF00394.21	Cu-oxidase	124,2	4,80E-36	CL0026
TRINITY_DN6604_c0_g1::TRINITY_DN6604_c0_g1_i1::g.1696::m.1696	1	3	36,852	1,8575	PF00394.21	Cu-oxidase	91,2	6,70E-26	CL0026
TRINITY_DN7453_c0_g1::TRINITY_DN7453_c0_g1_i1::g.2128::m.2128	4	40,6	14,984	24,668	PF00173.27	Cyt-b5	95,2	1,80E-27	No_clan
TRINITY_DN7586_c0_g2::TRINITY_DN7586_c0_g2_i1::g.2208::m.2208	2	25	13,115	41,909	PF02298.16	Cu_bind_like	85,2	2,20E-24	CL0026
TRINITY_DN7788_c0_g1::TRINITY_DN7788_c0_g1_i1::g.2350::m.2350	2	7,3	40,492	3,1878	PF00291.24	PALP	226,6	4,00E-67	No_clan
TRINITY_DN8001_c0_g1::TRINITY_DN8001_c0_g1_i1::g.2441::m.2441	2	9,4	40,571	11,136	PF16884.4	ADH_N_2	88	3,40E-25	CL0296
TRINITY_DN8833_c0_g1::TRINITY_DN8833_c0_g1_i2::g.2890::m.2890	10	49,7	20,044	323,31	PF00160.20	Pro_isomerase	164,3	2,40E-48	CL0475
TRINITY_DN8972_c0_g1::TRINITY_DN8972_c0_g1_i1::g.3000::m.3000	1	3,1	36,589	2,0405					
TRINITY_DN9198_c0_g1::TRINITY_DN9198_c0_g1_i1::g.3186::m.3186	7	20,8	51,249	21,091	PF00091.24	Tubulin	132,7	1,70E-38	CL0566
TRINITY_DN9267_c0_g1::TRINITY_DN9267_c0_g1_i1::g.3260::m.3260	2	2,1	92,534	2,257	PF00225.22	Kinesin	220,7	2,20E-65	CL0023
TRINITY_DN9332_c0_g1::TRINITY_DN9332_c0_g1_i2::g.3344::m.3344	1	4,3	26,001	5,4354	PF03099.18	BPL_LplA_LipB	102,4	1,60E-29	CL0040

TRINITY_DN9342_c0_g1::TRINITY_DN9342_c0_g1_i1::g.3304::m.3304	2	11	39,938	5,5638	PF00400.31	WD40	13,2	0,12	CL0186
TRINITY_DN9429_c0_g1::TRINITY_DN9429_c0_g1_i1::g.3466::m.3466	12	39,5	46,758	323,31	PF16499.4	Melibiase_2	264,1	1,20E-78	CL0058
TRINITY_DN9434_c0_g1::TRINITY_DN9434_c0_g1_i2::g.3474::m.3474	2	3,4	94,968	4,0387	PF00270.28	DEAD	145,7	1,10E-42	CL0023
TRINITY_DN9437_c0_g1::TRINITY_DN9437_c0_g1_i1::g.3480::m.3480	2	5,3	39,354	3,2666	PF00701.21	DHDPS	299,3	1,70E-89	CL0036
TRINITY_DN9524_c0_g1::TRINITY_DN9524_c0_g1_i2::g.3575::m.3575	1	5,2	27,594	2,8109	PF00124.18	Photo_RC	255,8	3,50E-76	No_clan
TRINITY_DN9608_c0_g1::TRINITY_DN9608_c0_g1_i2::g.3681::m.3681	1	9,2	19,921	2,6578					
TRINITY_DN9714_c0_g1::TRINITY_DN9714_c0_g1_i2::g.3800::m.3800	2	5	56,379	3,7745	PF14249.5	Tocopherol_cycl	485,9	5,30E-146	No_clan
TRINITY_DN9744_c0_g1::TRINITY_DN9744_c0_g1_i1::g.3792::m.3792	4	24,2	28,287	10,5	PF03332.12	PMM	363,5	3,70E-109	CL0137
TRINITY_DN9772_c0_g1::TRINITY_DN9772_c0_g1_i1::g.3863::m.3863	2	22,1	12,283	2,7106	PF00477.16	LEA_5	188,4	3,10E-56	CL0385
TRINITY_DN9796_c0_g1::TRINITY_DN9796_c0_g1_i1::g.3884::m.3884	2	5,2	60,627	4,0032	PF06325.12	PrmA	29,9	3,30E-07	CL0063
TRINITY_DN9822_c0_g1::TRINITY_DN9822_c0_g1_i1::g.3931::m.3931	1	6,7	24,438	3,0112	PF01425.20	Amidase	77,2	1,10E-21	No_clan
TRINITY_DN9836_c0_g1::TRINITY_DN9836_c0_g1_i2::g.3964::m.3964	1	7,6	16,286	2,4504	PF01176.18	eIF-1a	81,5	2,60E-23	CL0021
TRINITY_DN9933_c0_g1::TRINITY_DN9933_c0_g1_i1::g.4060::m.4060	5	23,4	33,594	14,661	PF13417.5	GST_N_3	71,2	6,50E-20	CL0172
TRINITY_DN9942_c0_g1::TRINITY_DN9942_c0_g1_i4::g.4015::m.4015	3	16,2	25,003	323,31	PF00314.16	Thaumatin	216,1	4,30E-64	CL0293
