# Supplementary material for: Comparative proteomics of common allergenic tree pollens of birch, alder, and hazel
Source: Allergy. 2021 Jan 15;76(6):1743–53. doi: 10.1111/all.14694 (PMC8248232; doi:10.1111/all.14694)
Supplement: Supplementary file 7 — Table S5 [file ALL-76-1743-s016.pdf]

Supplementary Table S5: Intensities of identified Betula pollen proteins in different preparations

Protein IDs	Intensity fasp1	Intensity fasp2	Intensity fasp3	Intensity fasp4	Intensity acetone1	Intensity acetone2	Intensity acetone3	Intensity acetone4	Intensity tca1	Intensity tca2	Intensity tca3	Intensity tca4	Intensity zc1	Intensity zc2	Intensity zc3
TRINITY_DN17355_c4_g1::TRINITY_DN17355_c4_g1_i11::g.82598::m.82598	189420000	29987000	146350000	120130000	99435000	42155000	119940000	110630000	402270000	226360000	432310000	542580000	36326000	39050000	39444000
TRINITY_DN15290_c1_g1::TRINITY_DN15290_c1_g1_i13::g.49834::m.49834	110660000	38697000	134280000	117170000	118120000	42819000	115410000	69355000	269050000	152650000	378760000	391940000	36789000	52218000	40488000
TRINITY_DN11319_c0_g2::TRINITY_DN11319_c0_g2_i2::g.7052::m.7052	477170000	126680000	481550000	402550000	2375600	0	692250	947470	170000000	199640000	9799200	163500000	94284	319230	108120
TRINITY_DN14555_c5_g4::TRINITY_DN14555_c5_g4_i1::g.38907::m.38907	227120000	92916000	263240000	204910000	12285000	7639600	42183000	19777000	258600000	203090000	307440000	256480000	4528200	5172800	3968200
TRINITY_DN16112_c0_g1::TRINITY_DN16112_c0_g1_i8::g.62808::m.62808	86469000	35451000	112560000	102860000	66030000	41104000	106910000	30639000	220600000	120370000	266060000	327610000	17092000	31838000	27088000
TRINITY_DN14325_c0_g1::TRINITY_DN14325_c0_g1_i1::g.35421::m.35421	86040000	24065000	73090000	67895000	63976000	26927000	68956000	42862000	243290000	192530000	274340000	344500000	4282700	12533000	7546300
TRINITY_DN19980_c4_g3::TRINITY_DN19980_c4_g3_i6::g.127331::m.127331	140890000	52861000	147390000	163800000	67496000	34355000	55697000	31444000	248070000	151840000	206930000	184970000	11306000	23104000	9771700
TRINITY_DN14455_c0_g9::TRINITY_DN14455_c0_g9_i1::g.37463::m.37463	82656000	39634000	120940000	97097000	56345000	29358000	85541000	51661000	258560000	181020000	154280000	297850000	19189000	32937000	20403000
TRINITY_DN12690_c0_g1::TRINITY_DN12690_c0_g1_i3::g.14937::m.14937	118230000	32866000	109280000	120690000	54969000	16862000	31269000	46171000	218040000	139030000	228820000	311160000	7881100	20102000	12092000
TRINITY_DN19145_c0_g2::TRINITY_DN19145_c0_g2_i2::g.112927::m.112927	120250000	40599000	136190000	93485000	48678000	34898000	6956500	7244600	155410000	109020000	291000000	291210000	7694500	12630000	10811000
TRINITY_DN18658_c1_g1::TRINITY_DN18658_c1_g1_i9::g.104122::m.104122	79435000	27169000	94335000	80620000	52902000	13952000	32911000	39006000	140140000	98845000	191560000	259930000	12388000	24118000	24589000
TRINITY_DN19475_c9_g3::TRINITY_DN19475_c9_g3_i3::g.118615::m.118615	151810000	49191000	169440000	180750000	44783000	19002000	11515000	3426600	136570000	70077000	84885000	215040000	1901100	1538000	2668200
TRINITY_DN19879_c1_g2::TRINITY_DN19879_c1_g2_i1::g.125068::m.125068	181740000	73997000	203700000	200270000	12942000	24094000	20925000	12160000	139150000	107210000	54148000	95877000	4282900	3388500	5720800
TRINITY_DN11247_c0_g1::TRINITY_DN11247_c0_g1_i5::g.6785::m.6785	172140000	67190000	207600000	171390000	547100	9706400	0	448330	99679000	28064000	58999000	123490000	3044700	5210600	4355600
TRINITY_DN15025_c0_g1::TRINITY_DN15025_c0_g1_i1::g.45918::m.45918	59973000	16072000	46323000	62081000	29832000	16697000	36551000	38312000	129680000	81993000	129210000	171700000	25515000	34132000	22978000
TRINITY_DN14511_c0_g1::TRINITY_DN14511_c0_g1_i3::g.38108::m.38108	87925000	27307000	76076000	68437000	23621000	7475500	17600000	12424000	142730000	70593000	147900000	193700000	660780	1769500	1321500
TRINITY_DN19475_c9_g1::TRINITY_DN19475_c9_g1_i7::g.118616::m.118616	37667000	10765000	58197000	48528000	48158000	14414000	67044000	31243000	70366000	55406000	169270000	189460000	1910300	8263600	11030000
TRINITY_DN16589_c2_g3::TRINITY_DN16589_c2_g3_i3::g.69229::m.69229	90217000	38334000	140200000	49626000	5496500	3968100	6801400	5766100	70202000	58683000	135790000	171030000	3221800	4238900	543640
TRINITY_DN19281_c0_g3::TRINITY_DN19281_c0_g3_i5::g.115036::m.115036	29812000	9849300	29144000	33472000	39805000	19160000	40227000	25355000	126480000	87729000	102520000	171700000	23038000	13383000	13791000

TRINITY_DN18969_c3_g1::TRINITY_DN18969_c3_g1_i1::g.109636::m.109636	39500000	11736000	48556000	54110000	29774000	14198000	26255000	25727000	94355000	44396000	138510000	152440000	23219000	19669000	41747000
TRINITY_DN17420_c0_g3::TRINITY_DN17420_c0_g3_i2::g.83576::m.83576	62921000	15018000	51564000	80463000	33847000	14881000	41940000	29650000	52646000	49601000	96883000	130440000	22674000	32052000	47883000
TRINITY_DN11273_c0_g1::TRINITY_DN11273_c0_g1_i1::g.6839::m.6839	101120000	53562000	125690000	83196000	0	32089000	0	0	85177000	77018000	95859000	95427000	0	0	0
TRINITY_DN19790_c2_g2::TRINITY_DN19790_c2_g2_i6::g.123649::m.123649	123680000	0	57332000	65473000	17882000	9585700	0	0	131570000	114650000	69773000	148080000	0	0	0
TRINITY_DN14648_c0_g1::TRINITY_DN14648_c0_g1_i1::g.39924::m.39924	37665000	11947000	31106000	35567000	32289000	13844000	36017000	26433000	104130000	76780000	153670000	167000000	2903000	4781100	2843300
TRINITY_DN13067_c1_g1::TRINITY_DN13067_c1_g1_i2::g.19355::m.19355	35578000	12475000	44436000	31749000	32039000	11564000	59066000	27609000	92964000	64057000	147990000	130170000	5529500	8034000	8038900
TRINITY_DN14120_c0_g3::TRINITY_DN14120_c0_g3_i2::g.32536::m.32536	54951000	19024000	63801000	56528000	15067000	10395000	17995000	26036000	74560000	48294000	116810000	149840000	17615000	12757000	21428000
TRINITY_DN14328_c3_g1::TRINITY_DN14328_c3_g1_i9::g.35530::m.35530	28909000	12328000	37020000	43808000	28592000	18599000	33960000	36952000	66731000	52707000	102130000	119600000	38283000	45501000	38311000
TRINITY_DN15467_c6_g1::TRINITY_DN15467_c6_g1_i1::g.52486::m.52486	33089000	6121000	33135000	34170000	17986000	11883000	29615000	26740000	77832000	32008000	119120000	121610000	20114000	12323000	14416000
TRINITY_DN14030_c0_g1::TRINITY_DN14030_c0_g1_i1::g.31442::m.31442	27868000	3534500	28231000	31661000	26210000	8204200	24201000	27027000	85169000	45140000	112570000	93588000	18352000	24300000	21678000
TRINITY_DN19909_c0_g1::TRINITY_DN19909_c0_g1_i3::g.126119::m.126119	9463300	1639300	9278500	8758500	31611000	24318000	47324000	33714000	93510000	57071000	130500000	84012000	12538000	19825000	7838000
TRINITY_DN17277_c1_g3::TRINITY_DN17277_c1_g3_i4::g.81251::m.81251	24957000	7703000	28762000	33335000	39907000	3514500	9946300	27398000	70305000	26772000	78204000	130850000	14055000	24397000	23121000
TRINITY_DN17792_c0_g2::TRINITY_DN17792_c0_g2_i8::g.89735::m.89735	21758000	5983200	22818000	14277000	18536000	5549400	28548000	23197000	82208000	43003000	125070000	121650000	5223200	10539000	9853700
TRINITY_DN14776_c1_g1::TRINITY_DN14776_c1_g1_i2::g.42049::m.42049	17053000	6343100	23584000	19513000	28893000	10352000	15909000	20772000	82960000	56211000	89274000	129820000	9621100	9057800	8470600
TRINITY_DN17630_c0_g1::TRINITY_DN17630_c0_g1_i8::g.87337::m.87337	24700000	10689000	15285000	13494000	25345000	19764000	40015000	22552000	62971000	50928000	110640000	104860000	5099000	7963800	6259800
TRINITY_DN12397_c0_g1::TRINITY_DN12397_c0_g1_i3::g.12463::m.12463	20140000	5997400	21024000	19243000	28043000	14570000	29674000	28363000	67331000	47934000	93523000	97355000	15364000	17726000	13490000
TRINITY_DN12813_c0_g1::TRINITY_DN12813_c0_g1_i4::g.16165::m.16165	18090000	4817700	18023000	25973000	13220000	5435000	28877000	24123000	62471000	33594000	82206000	109790000	33979000	25568000	19823000
TRINITY_DN16032_c0_g1::TRINITY_DN16032_c0_g1_i3::g.60899::m.60899	29537000	6596200	38272000	47760000	18175000	11082000	6381800	15557000	70383000	51033000	59314000	87954000	19850000	18659000	18818000
TRINITY_DN14204_c0_g1::TRINITY_DN14204_c0_g1_i2::g.33925::m.33925	18077000	5435200	21733000	20673000	14879000	1714500	28474000	3313900	85738000	32325000	105890000	124760000	7924500	9365300	9785700
TRINITY_DN18302_c1_g2::TRINITY_DN18302_c1_g2_i4::g.98509::m.98509	34158000	13277000	40579000	26617000	27962000	8993800	31987000	19801000	66608000	46135000	56278000	78445000	15464000	11222000	11826000

TRINITY_DN11092_c0_g1::TRINITY_DN11092_c0_g1_i5::g.6307::m.6307	32777	0	0	200020000	0	0	0	0	92673000	93803000	0	100280000	0	0	0
TRINITY_DN13033_c2_g1::TRINITY_DN13033_c2_g1_i6::g.18937::m.18937	22362000	12503000	31122000	25275000	15903000	16930000	12663000	17529000	74567000	42863000	99461000	97139000	6134000	7381000	1204500
TRINITY_DN17355_c4_g1::TRINITY_DN17355_c4_g1_i4::g.82584::m.82584	22518000	9705000	31524000	32891000	27094000	13828000	9784400	1659100	46910000	39454000	100150000	130970000	3346400	4878900	7192700
TRINITY_DN19812_c4_g2::TRINITY_DN19812_c4_g2_i6::g.124061::m.124061	10715000	5641100	10442000	14990000	18697000	10603000	28398000	25823000	56844000	38832000	70172000	54490000	41758000	45382000	42169000
TRINITY_DN11789_c0_g1::TRINITY_DN11789_c0_g1_i4::g.8830::m.8830	34820000	11344000	33898000	30667000	31481000	17451000	24844000	18204000	61233000	37858000	68537000	65128000	7756500	8284200	12105000
TRINITY_DN19911_c7_g2::TRINITY_DN19911_c7_g2_i3::g.126024::m.126024	21430000	6035500	43339000	36869000	19049000	10188000	25051000	10579000	48718000	27495000	100520000	86370000	3134200	5854700	3316100
TRINITY_DN14120_c0_g1::TRINITY_DN14120_c0_g1_i4::g.32545::m.32545	40137000	14189000	32902000	27886000	21471000	12152000	17743000	17085000	41396000	27621000	117020000	43115000	10105000	16237000	8688300
TRINITY_DN19879_c2_g1::TRINITY_DN19879_c2_g1_i5::g.125071::m.125071	74636000	40531000	58374000	67510000	29324000	15057000	2607400	2315100	73315000	60236000	11007000	10958000	0	0	0
TRINITY_DN14558_c2_g3::TRINITY_DN14558_c2_g3_i1::g.38817::m.38817	60737000	26484000	76253000	59396000	2575600	12860000	1439900	7609600	44448000	45833000	19410000	69313000	2312800	5740900	2568900
TRINITY_DN15774_c1_g1::TRINITY_DN15774_c1_g1_i3::g.56941::m.56941	9550800	6528100	27787000	6708200	18955000	10452000	7273500	9823100	71075000	46504000	84677000	110680000	3158300	0	6636000
TRINITY_DN13244_c3_g1::TRINITY_DN13244_c3_g1_i1::g.21540::m.21540	9354800	1353200	10433000	7378300	16336000	5640900	10886000	23973000	68287000	49881000	71449000	87704000	22970000	17256000	13937000
TRINITY_DN18531_c0_g1::TRINITY_DN18531_c0_g1_i4::g.102214::m.102214	15171000	3269000	16183000	17956000	13041000	3587600	11750000	14903000	39489000	30094000	54894000	88505000	23422000	35736000	37490000
TRINITY_DN16132_c1_g2::TRINITY_DN16132_c1_g2_i1::g.62633::m.62633	64400000	17625000	44223000	24541000	18235000	7283000	12178000	5094800	35657000	13939000	35752000	95687000	6997500	0	10005000
TRINITY_DN18476_c0_g1::TRINITY_DN18476_c0_g1_i4::g.100953::m.100953	29527000	14305000	30269000	28306000	10039000	0	12190000	1322200	44153000	32836000	88177000	84847000	3964600	5049300	1656800
TRINITY_DN15025_c0_g1::TRINITY_DN15025_c0_g1_i8::g.45925::m.45925	14030000	4092100	14795000	18454000	18053000	11620000	24878000	21972000	42798000	22860000	61164000	96154000	7895600	8202300	11686000
TRINITY_DN15036_c1_g1::TRINITY_DN15036_c1_g1_i9::g.46235::m.46235	68711000	15370000	68806000	43645000	0	3814900	0	0	50048000	8074800	11997000	105860000	1713400	0	0
TRINITY_DN12776_c1_g2::TRINITY_DN12776_c1_g2_i6::g.15805::m.15805	7228100	3340200	11457000	25176000	50212000	14541000	6849700	386530	94707000	48569000	44586000	56230000	2411300	2642000	3294900
TRINITY_DN17440_c0_g3::TRINITY_DN17440_c0_g3_i6::g.84091::m.84091	28843000	6792800	24420000	29651000	15756000	3857800	16947000	12377000	59281000	28181000	54381000	47617000	17282000	4000000	20632000
TRINITY_DN19281_c0_g5::TRINITY_DN19281_c0_g5_i1::g.115035::m.115035	17028000	1827800	18088000	18007000	24976000	8663000	24818000	16342000	40588000	37637000	74557000	77279000	1506300	2033000	3716100
TRINITY_DN9942_c0_g1::TRINITY_DN9942_c0_g1_i4::g.4015::m.4015	12503000	6589000	17040000	13289000	13053000	4402000	21866000	15989000	54503000	51624000	78951000	76873000	0	0	0

TRINITY_DN19839_c2_g3::TRINITY_DN19839_c2_g3_i5::g.123823::m.123823	28569000	10805000	33509000	19898000	1422500	10407000	31350000	14496000	32130000	23453000	75221000	78902000	2763200	3555700	153680
TRINITY_DN19911_c7_g3::TRINITY_DN19911_c7_g3_i1::g.126023::m.126023	40126000	13325000	35949000	30643000	6566800	3499600	5295200	2761000	46160000	20598000	52671000	79474000	1706000	1085800	1169100
TRINITY_DN15452_c6_g1::TRINITY_DN15452_c6_g1_i5::g.52129::m.52129	9635800	2968900	17457000	14593000	19376000	9009900	19751000	14123000	34755000	21959000	55936000	60788000	13268000	18220000	21612000
TRINITY_DN18205_c0_g1::TRINITY_DN18205_c0_g1_i3::g.96958::m.96958	6597900	3367500	8058100	8453100	14144000	5914200	16610000	14515000	61902000	16961000	63799000	64282000	9326200	26399000	9656200
TRINITY_DN18360_c0_g7::TRINITY_DN18360_c0_g7_i2::g.99197::m.99197	21211000	6261600	24876000	24617000	11747000	3754600	15308000	14778000	24966000	21975000	64631000	17298000	12623000	30077000	28070000
TRINITY_DN19551_c4_g1::TRINITY_DN19551_c4_g1_i4::g.119678::m.119678	15956000	5489000	19969000	19787000	14763000	6225500	11105000	6316500	35747000	19356000	67178000	80310000	6427800	10420000	2474900
TRINITY_DN18258_c0_g1::TRINITY_DN18258_c0_g1_i10::g.97660::m.97660	15851000	5826900	20969000	20628000	14596000	4884700	15992000	8990900	55746000	23154000	56925000	34641000	18140000	12578000	10624000
TRINITY_DN15932_c0_g3::TRINITY_DN15932_c0_g3_i5::g.59231::m.59231	16476000	4365200	17437000	18315000	15038000	6243200	9959100	14354000	32214000	25975000	33752000	86072000	9991500	6339700	14664000
TRINITY_DN13825_c1_g1::TRINITY_DN13825_c1_g1_i2::g.28853::m.28853	14161000	4443100	19351000	15131000	13860000	6705700	22442000	19061000	31922000	24572000	55231000	51639000	10202000	10075000	11709000
TRINITY_DN13624_c1_g6::TRINITY_DN13624_c1_g6_i6::g.26482::m.26482	20407000	6317500	15350000	16123000	6205200	7799300	15488000	8850700	30842000	18263000	44365000	29684000	14014000	34079000	30267000
TRINITY_DN18743_c1_g1::TRINITY_DN18743_c1_g1_i1::g.105300::m.105300	11350000	5516600	15150000	12689000	26255000	7689400	30616000	9951900	32710000	15496000	68356000	57526000	1246100	1553600	1231800
TRINITY_DN13291_c0_g1::TRINITY_DN13291_c0_g1_i4::g.22017::m.22017	15470000	6183800	19648000	17137000	14100000	7751900	14955000	7689800	42866000	37028000	55115000	48273000	891490	4928400	5202300
TRINITY_DN19879_c1_g2::TRINITY_DN19879_c1_g2_i8::g.125070::m.125070	48134000	6082500	0	0	0	373530	0	0	59213000	54537000	90047000	34069000	0	0	0
TRINITY_DN19354_c2_g2::TRINITY_DN19354_c2_g2_i8::g.116447::m.116447	28214000	14608000	31674000	21849000	4054000	786560	5743000	5754200	31947000	20974000	44330000	53279000	7545600	10147000	8312200
TRINITY_DN19617_c3_g2::TRINITY_DN19617_c3_g2_i1::g.121264::m.121264	13450000	4566900	17396000	16461000	11559000	2747900	11318000	3570000	34554000	23708000	63325000	69085000	2159500	5858500	3978500
TRINITY_DN36067_c0_g1::TRINITY_DN36067_c0_g1_i1::g.130820::m.130820	2197100	659000	2318400	1672100	15596000	8203500	26820000	14508000	44868000	34764000	69626000	61356000	0	0	0
TRINITY_DN18306_c2_g1::TRINITY_DN18306_c2_g1_i5::g.98735::m.98735	12931000	2751100	18068000	15507000	10194000	3545200	12562000	12372000	17449000	17117000	35294000	75854000	15007000	16055000	16768000
TRINITY_DN13776_c0_g1::TRINITY_DN13776_c0_g1_i2::g.28095::m.28095	4346900	1009600	4361000	4423800	15296000	7747900	23933000	18412000	47106000	22083000	36973000	63300000	9252100	13251000	9685200
TRINITY_DN8833_c0_g1::TRINITY_DN8833_c0_g1_i2::g.2890::m.2890	56881000	11471000	8099800	7620500	13819000	6531500	13649000	17924000	29670000	16964000	36712000	40953000	6653900	4799900	5135900

TRINITY_DN16420_c0_g2::TRINITY_DN16420_c0_g2_i1::g.67374::m.67374	18693000	6497700	19658000	18060000	12408000	6398500	18097000	21528000	21836000	8491500	26644000	39151000	14030000	14660000	24004000
TRINITY_DN13666_c1_g1::TRINITY_DN13666_c1_g1_i6::g.26985::m.26985	6481100	1345000	7258800	7162400	9283800	1048300	10739000	12707000	46952000	34598000	59502000	65243000	831160	3008900	1929000
TRINITY_DN19405_c2_g1::TRINITY_DN19405_c2_g1_i12::g.117378::m.117378	14914000	4087000	8873200	11923000	15368000	8598500	20923000	13475000	23493000	23466000	41116000	54539000	5507800	7196700	6949100
TRINITY_DN18584_c1_g5::TRINITY_DN18584_c1_g5_i6::g.103010::m.103010	6005800	2250500	12235000	7787800	13330000	5959600	16011000	14040000	36440000	29998000	55542000	54533000	515540	1482400	228810
TRINITY_DN16422_c1_g1::TRINITY_DN16422_c1_g1_i4::g.67346::m.67346	7625400	1755900	11299000	7280200	5665700	3881000	6860700	5000000	21844000	11193000	41156000	37511000	18489000	29536000	46200000
TRINITY_DN13815_c1_g2::TRINITY_DN13815_c1_g2_i8::g.28885::m.28885	50932000	35938000	88675000	71778000	0	0	0	0	0	0	0	0	0	0	0
TRINITY_DN14162_c4_g1::TRINITY_DN14162_c4_g1_i2::g.33191::m.33191	14920000	4604000	13928000	12664000	7198000	9463100	20867000	11537000	29191000	19086000	54485000	42766000	447700	2028000	1696100
TRINITY_DN15589_c0_g1::TRINITY_DN15589_c0_g1_i4::g.54099::m.54099	4487600	959080	5419400	7183400	7248400	1294700	7042800	4255200	21280000	8652400	27310000	34829000	32409000	41265000	39131000
TRINITY_DN15010_c0_g3::TRINITY_DN15010_c0_g3_i5::g.45655::m.45655	8394500	1460200	8102800	6909700	6661700	6530100	14948000	8348100	18741000	16339000	29801000	25024000	18011000	35710000	31975000
TRINITY_DN16345_c0_g1::TRINITY_DN16345_c0_g1_i2::g.66198::m.66198	9453900	2574100	12711000	12512000	2174900	3232400	9548400	6970400	20293000	10455000	34674000	44522000	21090000	23689000	21427000
TRINITY_DN17420_c0_g2::TRINITY_DN17420_c0_g2_i1::g.83570::m.83570	8478300	1909100	10027000	11649000	13314000	3456300	11754000	18853000	29877000	19617000	33945000	43182000	4674300	5830700	12404000
TRINITY_DN18333_c2_g6::TRINITY_DN18333_c2_g6_i1::g.98947::m.98947	6631400	2863400	7683300	7807200	22356000	8715200	20351000	21110000	23664000	16971000	35233000	33312000	4993000	8495600	7610700
TRINITY_DN18850_c2_g2::TRINITY_DN18850_c2_g2_i2::g.107178::m.107178	83489000	18604000	0	47044000	0	0	0	0	0	0	78451000	0	0	0	0
TRINITY_DN18145_c2_g1::TRINITY_DN18145_c2_g1_i9::g.95178::m.95178	8554000	4210600	12528000	8780100	11544000	6299700	13124000	14634000	25244000	12007000	22834000	46112000	13639000	19997000	5976600
TRINITY_DN17440_c0_g5::TRINITY_DN17440_c0_g5_i3::g.84089::m.84089	9885000	4400400	13169000	13252000	11434000	4953200	13498000	4547600	24424000	20397000	40478000	40209000	7104800	10431000	2777600
TRINITY_DN12537_c1_g2::TRINITY_DN12537_c1_g2_i4::g.13603::m.13603	19673000	7397100	21387000	19094000	10201000	4097100	9941600	7662300	14040000	17534000	41825000	39311000	2072400	2511600	1507400
TRINITY_DN15426_c0_g2::TRINITY_DN15426_c0_g2_i4::g.51819::m.51819	6603000	4028500	13423000	9189400	11871000	1276100	12893000	14694000	14429000	7803400	62424000	42190000	5402500	6449300	5194900
TRINITY_DN15464_c0_g2::TRINITY_DN15464_c0_g2_i1::g.52259::m.52259	8153700	2606400	6803200	7914200	11517000	6149200	17441000	12964000	14794000	21040000	26133000	36828000	12682000	18137000	13892000
TRINITY_DN14041_c2_g1::TRINITY_DN14041_c2_g1_i4::g.31794::m.31794	8512900	2491500	5822600	6016500	5538400	1943600	8302900	8038500	24126000	11810000	23444000	38598000	20148000	22823000	22580000
TRINITY_DN14730_c2_g2::TRINITY_DN14730_c2_g2_i1::g.41382::m.41382	30613000	12406000	13600000	4081400	4225000	140120	7009700	683820	6484500	4438900	5739900	182330	34449000	46892000	38110000

TRINITY_DN19696_c3_g4::TRINITY_DN19696_c3_g4_i1::g.122257::m.122257	7341500	1941000	7356000	6943800	6255700	1950300	10909000	9150300	12556000	4474400	26812000	36764000	26409000	24331000	23340000
TRINITY_DN16808_c0_g1::TRINITY_DN16808_c0_g1_i2::g.73724::m.73724	17724000	4676600	21690000	15783000	14976000	4532100	13036000	16255000	9335400	16591000	59030000	5097900	3922500	1171800	158190
TRINITY_DN16940_c1_g2::TRINITY_DN16940_c1_g2_i2::g.75865::m.75865	12367000	7430500	23866000	11734000	6228900	4532200	4852600	0	30153000	23034000	25083000	42680000	2885300	3397500	4832200
TRINITY_DN14508_c8_g1::TRINITY_DN14508_c8_g1_i7::g.38377::m.38377	10306000	5025800	12722000	9982200	4496400	5650300	14911000	12657000	24692000	22240000	38624000	26087000	3408100	3873900	6772200
TRINITY_DN16840_c2_g1::TRINITY_DN16840_c2_g1_i1::g.74291::m.74291	8344400	3440000	9778200	12004000	9639100	5271400	4846200	8834400	23784000	6554900	25304000	30493000	16994000	19439000	16490000
TRINITY_DN17614_c0_g2::TRINITY_DN17614_c0_g2_i5::g.87093::m.87093	7451900	1078600	4900900	6461400	9259600	3524400	9227000	5722800	15887000	10182000	18410000	34560000	23042000	22326000	25800000
TRINITY_DN18004_c1_g6::TRINITY_DN18004_c1_g6_i1::g.93462::m.93462	10930000	4401100	15506000	14783000	12377000	3803700	6405600	6079700	26836000	18371000	10216000	52472000	2410600	4731900	6031100
TRINITY_DN13376_c2_g2::TRINITY_DN13376_c2_g2_i3::g.22986::m.22986	3533800	1486500	5216200	3436200	19822000	8061000	21914000	15022000	22528000	21965000	34712000	28348000	2186400	3493200	2800500
TRINITY_DN13606_c2_g2::TRINITY_DN13606_c2_g2_i1::g.26262::m.26262	10634000	1621400	13309000	11191000	5688300	3488600	8016300	5777500	14230000	5597500	16064000	27790000	27688000	22455000	19005000
TRINITY_DN14755_c2_g2::TRINITY_DN14755_c2_g2_i1::g.41670::m.41670	7177900	3412500	8346900	3963800	6027600	3415500	2553200	8525900	26931000	14875000	36463000	44403000	5945700	10447000	7188200
TRINITY_DN13024_c1_g2::TRINITY_DN13024_c1_g2_i1::g.18973::m.18973	5548600	2367600	7463000	7737300	14381000	705330	14622000	1761000	30704000	25695000	32191000	42139000	260840	309850	316700
TRINITY_DN14909_c1_g2::TRINITY_DN14909_c1_g2_i4::g.44177::m.44177	6440700	2418500	7980700	6527900	6335100	320260	0	728280	1714000	9284700	15077000	20407000	26764000	32550000	47133000
TRINITY_DN19579_c1_g1::TRINITY_DN19579_c1_g1_i2::g.120372::m.120372	0	0	0	0	0	0	0	0	0	0	0	161070000	398520	726830	16549000
TRINITY_DN14204_c0_g1::TRINITY_DN14204_c0_g1_i1::g.33924::m.33924	9856200	4650800	12119000	10671000	15832000	4202100	8919300	2156900	38903000	0	17639000	45071000	2142400	740810	3335100
TRINITY_DN12067_c0_g1::TRINITY_DN12067_c0_g1_i1::g.10231::m.10231	6885600	1284400	5829800	4806900	11752000	5123700	12224000	13998000	18112000	5339500	41210000	44746000	2051800	1560500	980040
TRINITY_DN18581_c2_g2::TRINITY_DN18581_c2_g2_i16::g.102885::m.102885	4284700	573420	4126300	4915600	11415000	4470200	15246000	10952000	29317000	16804000	29139000	41873000	998220	969730	807750
TRINITY_DN13947_c3_g5::TRINITY_DN13947_c3_g5_i8::g.30694::m.30694	3146200	968660	13751000	13673000	9095500	4774400	10699000	9767800	23323000	19494000	31379000	32016000	1240000	0	0
TRINITY_DN18713_c3_g1::TRINITY_DN18713_c3_g1_i8::g.105061::m.105061	6965500	2941200	6999600	7975100	5629600	2416500	10137000	7248200	31677000	11856000	40471000	34019000	1469100	1211600	1931100

TRINITY_DN19957_c3_g2::TRINITY_DN19957_c3_g2_i9::g.126783::m.126783	4794100	2338400	6493500	4981900	11235000	5208800	7327000	5471100	21643000	22206000	36372000	24526000	8264600	3452700	8499200
TRINITY_DN18489_c2_g2::TRINITY_DN18489_c2_g2_i5::g.101255::m.101255	4450100	1985900	4803400	4748500	12380000	3387700	13689000	8672700	10850000	18807000	34224000	44460000	3284600	4071800	2610800
TRINITY_DN15194_c1_g2::TRINITY_DN15194_c1_g2_i1::g.48344::m.48344	32735000	14687000	35271000	35568000	7096900	3023000	1548700	4873400	7037100	6736200	5778000	1194500	6716700	7756900	287730
TRINITY_DN17177_c3_g1::TRINITY_DN17177_c3_g1_i5::g.79706::m.79706	12082000	745710	13628000	12707000	5534000	2098400	4698300	7789900	17615000	10215000	20093000	11439000	16625000	17486000	17137000
TRINITY_DN13227_c0_g1::TRINITY_DN13227_c0_g1_i8::g.21288::m.21288	4502800	1028600	3357100	5076100	4850100	2917700	8346800	9155000	23908000	12640000	33615000	40498000	5410400	5568300	7388800
TRINITY_DN18988_c0_g2::TRINITY_DN18988_c0_g2_i2::g.109900::m.109900	10574000	888270	8142700	6593100	11205000	4151500	14602000	12360000	18578000	11849000	30385000	22974000	8282800	2946600	4244800
TRINITY_DN13588_c3_g5::TRINITY_DN13588_c3_g5_i3::g.25866::m.25866	8927600	2595100	7830600	8154900	9979400	4008900	3027100	4859600	9416300	4406100	44100000	29277000	5003900	13680000	8629700
TRINITY_DN15896_c1_g3::TRINITY_DN15896_c1_g3_i4::g.58593::m.58593	3035900	1353300	4953900	3523400	5531000	2206800	5533400	10586000	21177000	17230000	38900000	25570000	2631300	8810200	7585700
TRINITY_DN15954_c0_g1::TRINITY_DN15954_c0_g1_i1::g.59385::m.59385	6597400	2465400	9006100	9429700	2035900	1918300	2877800	11252000	17417000	5245800	24309000	22708000	14563000	15163000	12571000
TRINITY_DN15441_c0_g2::TRINITY_DN15441_c0_g2_i3::g.51488::m.51488	969720	97875	1431300	1140600	302520	1599600	6566200	289720	6406800	24013000	54390000	40598000	1453600	7852600	10210000
TRINITY_DN18164_c1_g2::TRINITY_DN18164_c1_g2_i2::g.96243::m.96243	11661000	3516600	14103000	13426000	5031700	2214200	4355800	8388300	11755000	5834300	15377000	5917000	14600000	17764000	22874000
TRINITY_DN18961_c1_g1::TRINITY_DN18961_c1_g1_i1::g.109399::m.109399	10846000	4188000	14835000	11725000	2474300	2141000	7667300	8821700	8497500	10460000	30651000	26261000	5623000	6254200	5958300
TRINITY_DN12699_c0_g1::TRINITY_DN12699_c0_g1_i1::g.14170::m.14170	2738600	826400	6240900	2537600	0	1840700	7130800	1731000	17064000	23556000	70010000	15758000	2171600	2108900	2466000
TRINITY_DN16736_c3_g2::TRINITY_DN16736_c3_g2_i1::g.72521::m.72521	4809300	2294100	6321200	7308400	6686900	2518200	8367800	8258500	16980000	12119000	24195000	24744000	10140000	14639000	6175200
TRINITY_DN13916_c2_g1::TRINITY_DN13916_c2_g1_i2::g.30380::m.30380	4936600	1236100	6593100	4863600	3331700	768340	4304300	3386500	3514700	1776800	7867500	14945000	26139000	37047000	34656000
TRINITY_DN12824_c2_g2::TRINITY_DN12824_c2_g2_i6::g.16244::m.16244	12616000	2016900	11784000	7948300	7788200	787340	4202900	5227100	25942000	17581000	34774000	9325200	6250300	0	8863000
TRINITY_DN19247_c0_g1::TRINITY_DN19247_c0_g1_i9::g.114529::m.114529	9143800	2478400	10318000	9095800	6781200	4164000	7623000	4662700	14644000	10938000	21252000	19281000	11136000	12025000	11210000
TRINITY_DN12966_c3_g1::TRINITY_DN12966_c3_g1_i1::g.17946::m.17946	3542700	585400	3365700	6263700	4993700	1507500	10263000	3786900	17839000	7280400	25811000	30008000	8707200	17604000	12524000
TRINITY_DN12426_c1_g1::TRINITY_DN12426_c1_g1_i1::g.12851::m.12851	5694200	2480900	7901000	6553800	6904400	2661300	1783000	20411000	16742000	8268500	30995000	30815000	3735600	4150300	4171700
TRINITY_DN16163_c6_g1::TRINITY_DN16163_c6_g1_i3::g.63330::m.63330	7167200	2941400	8001500	8047700	8445100	4625700	11084000	10244000	15814000	13584000	23949000	20404000	1457700	2768000	14185000

TRINITY_DN19582_c3_g2::TRINITY_DN19582_c3_g2_i6::g.120115::m.120115	13032000	4528200	10054000	12530000	3544400	819700	5431000	4662200	24882000	10454000	24385000	24760000	2881700	3251700	2638800
TRINITY_DN18840_c3_g1::TRINITY_DN18840_c3_g1_i1::g.107249::m.107249	4871300	1485600	4036000	5068700	2387500	3458500	8897700	11170000	18207000	16545000	31525000	30288000	1072700	3946200	4011100
TRINITY_DN14341_c0_g1::TRINITY_DN14341_c0_g1_i1::g.35559::m.35559	3597800	930370	3835700	3396900	2761100	0	1303000	3207000	4360700	3834100	4728900	9592700	23537000	41177000	38111000
TRINITY_DN16376_c0_g2::TRINITY_DN16376_c0_g2_i2::g.66583::m.66583	7198400	1396200	7231200	8159600	4853300	1564500	1023500	4283500	9604200	3970900	21101000	36205000	12452000	11201000	13737000
TRINITY_DN14576_c1_g5::TRINITY_DN14576_c1_g5_i7::g.39012::m.39012	3503100	1759100	5356600	4992900	9495400	2911600	5574800	9195800	22259000	8444900	30508000	25259000	3952300	3892700	5032000
TRINITY_DN12837_c2_g2::TRINITY_DN12837_c2_g2_i7::g.16580::m.16580	13104000	5562800	14992000	14647000	4902000	2094700	7874100	6720000	15722000	10582000	18449000	17049000	1489500	3691400	3366400
TRINITY_DN19292_c1_g3::TRINITY_DN19292_c1_g3_i1::g.115388::m.115388	1992600	354020	2045800	1776600	4091800	2111000	4504200	5367400	6567600	7824200	19557000	23286000	19413000	20229000	20729000
TRINITY_DN19563_c2_g1::TRINITY_DN19563_c2_g1_i4::g.120333::m.120333	5234400	983130	3603100	2861000	9229700	2461000	5816300	6203900	19914000	8560400	29528000	25353000	6010400	3976100	8280100
TRINITY_DN15394_c0_g1::TRINITY_DN15394_c0_g1_i2::g.49883::m.49883	296020	49677	227410	193970	921430	308450	1157100	534710	2820900	1038200	2530700	1751400	39766000	44098000	41541000
TRINITY_DN17751_c2_g3::TRINITY_DN17751_c2_g3_i5::g.89327::m.89327	2324500	136500	807220	2360000	867240	695790	1373900	266310	831880	2131200	3515700	1646200	35771000	38025000	45932000
TRINITY_DN16246_c1_g1::TRINITY_DN16246_c1_g1_i7::g.63898::m.63898	4995700	2723400	14497000	1731300	6265500	2744200	13392000	6568100	22462000	5999900	40465000	11430000	293070	2003500	387860
TRINITY_DN17837_c2_g1::TRINITY_DN17837_c2_g1_i9::g.90611::m.90611	4428500	1333200	4409500	4918000	3129000	714930	4298800	2273300	11692000	3992900	13723000	23418000	20894000	13838000	22695000
TRINITY_DN14185_c5_g3::TRINITY_DN14185_c5_g3_i3::g.33391::m.33391	2827300	813470	4171700	4011300	3214900	140110	3804400	4577000	8988600	5089400	19298000	39040000	15393000	11631000	11835000
TRINITY_DN17690_c0_g3::TRINITY_DN17690_c0_g3_i4::g.88277::m.88277	7959800	2159600	6958800	9214900	8014200	4424900	12311000	10567000	15852000	7827800	18049000	21647000	2523500	2867300	4429800
TRINITY_DN18947_c6_g5::TRINITY_DN18947_c6_g5_i2::g.109307::m.109307	1296000	1396600	6931400	5686100	6589300	1628300	2172300	2393900	31168000	22777000	6746700	32617000	2937000	3318900	3483700
TRINITY_DN16121_c0_g1::TRINITY_DN16121_c0_g1_i8::g.62467::m.62467	305850	82777	65063000	58419000	0	107890	0	0	0	0	0	0	2132500	2633200	2310800
TRINITY_DN19791_c7_g1::TRINITY_DN19791_c7_g1_i1::g.123736::m.123736	11447000	1905200	11560000	7963200	3354900	2815900	5788300	2549700	23170000	13888000	29047000	12419000	802540	1268900	1910400
TRINITY_DN13992_c5_g1::TRINITY_DN13992_c5_g1_i1::g.31138::m.31138	1353100	0	1743800	929980	0	1284400	3692700	0	31237000	26665000	14395000	48434000	0	0	0
TRINITY_DN18360_c0_g4::TRINITY_DN18360_c0_g4_i2::g.99196::m.99196	10688000	2299400	12836000	9263200	8331100	5016100	11594000	8072200	14624000	11486000	11542000	11751000	2824500	1644800	7477300
TRINITY_DN17227_c2_g4::TRINITY_DN17227_c2_g4_i1::g.80549::m.80549	7938400	1680200	9542400	4955100	5060600	3256600	9570500	5626600	19360000	5463600	23661000	31014000	611020	759200	725020

TRINITY_DN15584_c3_g1::TRINITY_DN15584_c3_g1_i1::g.54058::m.54058	5250100	2623700	6190300	5976500	7937900	2985300	9068600	3191500	5760200	14622000	19527000	31933000	4235200	4866600	4950500
TRINITY_DN15581_c1_g3::TRINITY_DN15581_c1_g3_i1::g.54122::m.54122	2337700	573780	1838200	1817800	1419300	1415700	6348900	5943000	5289300	5396700	13812000	14169000	17713000	22910000	25417000
TRINITY_DN16112_c0_g1::TRINITY_DN16112_c0_g1_i3::g.62802::m.62802	9258800	4489900	11284000	10029000	7343000	0	7911000	6433700	10292000	6585400	19387000	19433000	4662200	4423500	4369700
TRINITY_DN14318_c1_g3::TRINITY_DN14318_c1_g3_i3::g.35377::m.35377	0	407230	3586900	0	4625600	3087700	8167200	6660800	18341000	18176000	34045000	25803000	0	1564700	0
TRINITY_DN15029_c2_g1::TRINITY_DN15029_c2_g1_i2::g.45973::m.45973	9365900	2433800	10256000	8005700	6254500	2129500	3709400	6136400	13494000	10162000	19218000	26821000	1477400	2234200	431770
TRINITY_DN13265_c1_g1::TRINITY_DN13265_c1_g1_i6::g.21679::m.21679	6660200	1379900	759260	456550	4282700	954340	1952600	2376700	11342000	6999900	22428000	11703000	18009000	13792000	18349000
TRINITY_DN17067_c1_g2::TRINITY_DN17067_c1_g2_i1::g.77892::m.77892	7708300	1523300	7199200	5579700	3702800	3245600	11510000	4566500	13176000	10332000	22705000	21343000	1891700	3264800	1566400
TRINITY_DN19292_c1_g4::TRINITY_DN19292_c1_g4_i1::g.115390::m.115390	1775100	84896	1981000	808470	2197000	725620	3926600	1902000	6871000	3485200	6550400	8982400	23590000	29629000	26491000
TRINITY_DN17861_c1_g1::TRINITY_DN17861_c1_g1_i8::g.90805::m.90805	6275900	3054100	7960000	6912900	8115200	0	10903000	2584600	16603000	6132500	20026000	17990000	2472800	3488900	2957900
TRINITY_DN18164_c1_g1::TRINITY_DN18164_c1_g1_i4::g.96244::m.96244	2599200	918700	3011500	3632300	3603500	1393000	4944400	2625100	6276300	2416900	10798000	9015600	19502000	26160000	18040000
TRINITY_DN14877_c0_g1::TRINITY_DN14877_c0_g1_i9::g.43584::m.43584	11315000	2662900	12462000	6683800	6421900	2334100	6962000	7008900	13155000	927850	21234000	17955000	1990900	2035900	1650200
TRINITY_DN12561_c1_g1::TRINITY_DN12561_c1_g1_i2::g.13897::m.13897	1503600	42190	4151800	3188600	2188800	4963000	10688000	431500	26309000	1166800	4322900	55029000	0	0	790390
TRINITY_DN20029_c3_g4::TRINITY_DN20029_c3_g4_i4::g.128107::m.128107	3685300	1940300	6232900	5336500	8132400	504690	10542000	6221700	17796000	12544000	13583000	27277000	276770	307390	135600
TRINITY_DN14766_c7_g2::TRINITY_DN14766_c7_g2_i3::g.41862::m.41862	279260	53542	133220	63906	129880	0	294210	0	382830	181310	1151300	0	27823000	45450000	37775000
TRINITY_DN16988_c0_g1::TRINITY_DN16988_c0_g1_i6::g.76602::m.76602	4727000	2270400	6210100	6524500	2154300	818860	2249700	3318500	4106900	1885900	13454000	1405100	16141000	25065000	21903000
TRINITY_DN12011_c0_g1::TRINITY_DN12011_c0_g1_i3::g.9976::m.9976	1463700	296450	1201600	1539400	8461200	2347300	5372500	8141600	12515000	10953000	23387000	18996000	4606400	6956700	5923300
TRINITY_DN15069_c0_g1::TRINITY_DN15069_c0_g1_i1::g.46535::m.46535	4542200	951690	5357000	1516400	6467000	1380100	6418200	6684400	9244700	13574000	16009000	25452000	2962200	6887900	4615800
TRINITY_DN12538_c0_g3::TRINITY_DN12538_c0_g3_i3::g.13566::m.13566	1869200	579830	2162600	2759800	2810400	2178400	2947100	5752600	6364300	6618000	14884000	11496000	17114000	18558000	14722000
TRINITY_DN18548_c0_g1::TRINITY_DN18548_c0_g1_i8::g.102421::m.102421	5202000	1721000	6420700	5792100	2630400	1828400	2962900	3462600	8323500	2676800	13723000	14728000	7460200	18084000	14413000
TRINITY_DN19710_c5_g3::TRINITY_DN19710_c5_g3_i4::g.122648::m.122648	9723300	2317900	11633000	9836500	3264100	525770	1662900	574750	8148600	4567400	15974000	20753000	8561700	5344600	5894400
TRINITY_DN13376_c2_g1::TRINITY_DN13376_c2_g1_i2::g.22983::m.22983	8122600	2608500	7958600	7379100	6106500	890400	4782200	2273400	19313000	9104000	19838000	17290000	1499100	0	856880

TRINITY_DN18686_c0_g1::TRINITY_DN18686_c0_g1_i9::g.104732::m.104732	5778900	1336700	4879600	3899000	2844100	1219500	2487100	2158600	9233700	7041400	15888000	30756000	7743100	5810000	6499100
TRINITY_DN17312_c0_g3::TRINITY_DN17312_c0_g3_i1::g.81763::m.81763	500500	88727	427060	822680	0	0	124650	278860	467210	0	0	0	36531000	38272000	29714000
TRINITY_DN19604_c2_g3::TRINITY_DN19604_c2_g3_i3::g.120758::m.120758	9931600	2356200	10863000	11433000	3018800	1153000	3158600	4763000	13406000	6212400	16475000	19637000	112560	1331200	981670
TRINITY_DN16248_c1_g2::TRINITY_DN16248_c1_g2_i8::g.64559::m.64559	6416900	1507000	9994500	6393000	2548400	124450	5248000	1959600	10455000	5650300	19787000	25396000	3279500	2046600	2880500
TRINITY_DN19596_c1_g1::TRINITY_DN19596_c1_g1_i8::g.120430::m.120430	419650	142040	429390	402670	4521200	2571400	5637700	4541900	19641000	12406000	22939000	24363000	1559000	1229000	2346400
TRINITY_DN18641_c0_g1::TRINITY_DN18641_c0_g1_i1::g.103822::m.103822	19987000	8226500	27025000	21937000	7081600	2276300	8250000	7461900	0	0	0	0	0	162830	0
TRINITY_DN14398_c0_g1::TRINITY_DN14398_c0_g1_i7::g.36405::m.36405	1143100	121550	1447600	1233100	1745500	1259200	5571000	2222700	5977400	4800000	11123000	9666200	252030	55015000	599430
TRINITY_DN12966_c1_g1::TRINITY_DN12966_c1_g1_i2::g.17932::m.17932	3271500	418970	1450800	3425900	2244200	505630	3464100	4971100	16878000	5253100	21228000	20331000	4690400	10418000	2253200
TRINITY_DN16827_c1_g1::TRINITY_DN16827_c1_g1_i8::g.73997::m.73997	2953700	572070	2304400	3357400	1199100	101690	1890400	2108400	4070400	2629800	4227600	14817000	18213000	21279000	20928000
TRINITY_DN13204_c1_g1::TRINITY_DN13204_c1_g1_i4::g.21099::m.21099	1702200	1728900	4602000	7562800	3199800	1831800	5635600	2422400	10184000	14787000	14082000	15376000	3452800	6697200	5959500
TRINITY_DN13564_c1_g1::TRINITY_DN13564_c1_g1_i6::g.26053::m.26053	1206900	575460	3405200	1189800	6308800	2157900	7228500	2234900	15676000	11253000	20259000	21634000	1573200	4233300	0
TRINITY_DN13916_c3_g1::TRINITY_DN13916_c3_g1_i2::g.30393::m.30393	216520	95164	361550	0	174680	0	648120	0	0	1144100	0	2026700	37360000	28592000	27517000
TRINITY_DN18427_c0_g1::TRINITY_DN18427_c0_g1_i8::g.100422::m.100422	1064000	911830	3169200	3830200	3722700	1437900	9630900	8019400	9762100	6535700	24580000	22851000	2582300	0	0
TRINITY_DN14460_c2_g2::TRINITY_DN14460_c2_g2_i7::g.37472::m.37472	694940	72463	817210	1021100	246400	479090	2083100	1449700	6278100	3631900	5230000	8441600	8991100	16806000	41738000
TRINITY_DN7586_c0_g2::TRINITY_DN7586_c0_g2_i1::g.2208::m.2208	4433500	1656700	6212800	2766700	143130	0	1403500	0	23412000	3919200	29033000	22978000	169590	0	477370
TRINITY_DN19791_c7_g2::TRINITY_DN19791_c7_g2_i1::g.123738::m.123738	5462300	81396	3951200	4442200	1869700	1047600	2747600	3777200	16968000	3520200	11035000	28892000	5386300	3443300	3831900
TRINITY_DN14970_c1_g1::TRINITY_DN14970_c1_g1_i3::g.45037::m.45037	48512	36878	252290	109820	0	0	163870	144080	0	0	0	510930	27430000	35770000	31921000
TRINITY_DN12692_c0_g1::TRINITY_DN12692_c0_g1_i7::g.14951::m.14951	4429100	1569300	6373200	5188700	2773300	832480	5252700	4172800	10305000	10519000	26197000	14071000	586270	1633500	1585400
TRINITY_DN14755_c2_g1::TRINITY_DN14755_c2_g1_i4::g.41671::m.41671	5053900	1641000	7953200	5278900	2347600	1373500	4425800	5334600	13731000	3430100	16107000	12902000	4544300	7941100	2954600
TRINITY_DN18875_c2_g2::TRINITY_DN18875_c2_g2_i2::g.107929::m.107929	1406100	278000	1776100	356280	868490	916920	1464600	0	1127600	1988200	2434700	3126900	20428000	28567000	30083000
TRINITY_DN16364_c0_g1::TRINITY_DN16364_c0_g1_i6::g.66368::m.66368	222050	50835	156320	136750	74000	0	98239	0	249270	483350	728860	366270	27581000	30212000	34067000

TRINITY_DN16368_c1_g2::TRINITY_DN16368_c1_g2_i3::g.66564::m.66564	8638500	0	0	0	4943400	3107800	6733100	0	13950000	11179000	21739000	22007000	0	0	1694200
TRINITY_DN16555_c1_g6::TRINITY_DN16555_c1_g6_i1::g.69342::m.69342	1067300	868310	6998300	5218600	5356800	1844200	5861300	3842000	10194000	13780000	21995000	9547600	2173000	2536500	2668500
TRINITY_DN14266_c0_g1::TRINITY_DN14266_c0_g1_i8::g.34769::m.34769	136400	53719	279940	213520	610570	320480	1183100	587790	982970	2838300	4279100	2645600	19846000	31577000	27946000
TRINITY_DN12221_c0_g1::TRINITY_DN12221_c0_g1_i1::g.11196::m.11196	2171000	2047000	3263300	2768900	6538900	3151200	4195500	6839800	7139300	6114900	24674000	22263000	521910	159320	1431200
TRINITY_DN14696_c0_g1::TRINITY_DN14696_c0_g1_i1::g.40665::m.40665	6300000	466680	8609700	7678700	5584800	942940	8051700	7061500	16233000	8637100	5529600	16046000	0	0	1187800
TRINITY_DN19169_c1_g3::TRINITY_DN19169_c1_g3_i6::g.113174::m.1131	6221200	2155600	7012300	6203600	6496700	3382000	7970900	6188200	293910	9002500	7423900	19556000	2834500	3782200	3472500
TRINITY_DN13729_c0_g1::TRINITY_DN13729_c0_g1_i8::g.27674::m.27674	1950100	933280	2593100	2406400	4548600	2990400	8414100	6671200	12266000	5847800	11498000	18284000	3650300	5383800	3720800
TRINITY_DN16649_c0_g2::TRINITY_DN16649_c0_g2_i3::g.71022::m.71022	0	0	0	0	0	0	0	0	0	0	88299000	0	104190	144590	303110
TRINITY_DN16233_c2_g1::TRINITY_DN16233_c2_g1_i1::g.64327::m.64327	0	0	0	0	0	25367000	0	47749000	0	0	0	0	1486500	2264400	11647000
TRINITY_DN16571_c1_g1::TRINITY_DN16571_c1_g1_i8::g.69835::m.69835	4554500	1120200	5044900	4762200	1641300	1843600	9156900	5279500	6180000	5208200	18821000	15636000	1526000	4852900	2058200
TRINITY_DN18328_c0_g1::TRINITY_DN18328_c0_g1_i9::g.98876::m.98876	3032000	777900	2406600	3387000	1830200	568430	2093500	3413800	3052800	2401700	7727600	14650000	11273000	16219000	14305000
TRINITY_DN16032_c0_g1::TRINITY_DN16032_c0_g1_i2::g.60897::m.60897	5150100	2392500	6936200	7418300	6311700	1575200	4868400	0	14639000	14682000	22967000	0	0	0	0
TRINITY_DN12769_c0_g3::TRINITY_DN12769_c0_g3_i1::g.15717::m.15717	2612400	1486100	4442200	4327700	4668400	4353700	9075400	6258000	4782000	7598400	10706000	0	4893600	12846000	8454100
TRINITY_DN17019_c1_g3::TRINITY_DN17019_c1_g3_i1::g.77181::m.77181	0	0	0	0	0	0	0	362260	0	0	71362000	0	4651500	5773200	3582100
TRINITY_DN17448_c2_g3::TRINITY_DN17448_c2_g3_i9::g.83903::m.83903	3355600	991620	4578300	3090500	1409100	1995500	2619100	4654100	7803800	5186500	10272000	9903000	7480200	12052000	10219000
TRINITY_DN16630_c2_g1::TRINITY_DN16630_c2_g1_i6::g.70777::m.70777	1486100	968510	2982200	685960	4081000	1442800	4940400	3263600	6639600	5851600	7316900	19826000	7067500	9342100	9108200
TRINITY_DN12516_c0_g1::TRINITY_DN12516_c0_g1_i2::g.13472::m.13472	71680000	0	0	4433700	0	0	0	0	0	0	0	0	3401400	2314200	3077500
TRINITY_DN12883_c0_g1::TRINITY_DN12883_c0_g1_i6::g.16879::m.16879	4132100	385920	661040	458900	3238300	1968000	2918500	4596400	12893000	12662000	17898000	19023000	481930	1287900	1198600
TRINITY_DN19263_c0_g3::TRINITY_DN19263_c0_g3_i4::g.114811::m.114811	1341900	503080	1688100	1851800	4636100	2437400	6079300	4392300	9401400	6678600	10150000	12919000	7043300	7928600	6016100
TRINITY_DN19354_c2_g2::TRINITY_DN19354_c2_g2_i9::g.116448::m.116448	2411300	851460	3101800	3027000	11334000	6252800	296700	0	8007900	6206800	13506000	17008000	1351700	6276400	3352400

TRINITY_DN13726_c0_g3::TRINITY_DN13726_c0_g3_i1::g.27596::m.27596	6601100	575510	6782300	4648700	3883400	1372300	5864800	967000	6002500	4274600	19224000	19386000	2045400	539400	788220
TRINITY_DN20049_c7_g1::TRINITY_DN20049_c7_g1_i11::g.128495::m.128495	4320900	1469500	7403900	5376000	3596600	918620	2012000	564520	11894000	0	11002000	24473000	4199200	2554700	2426200
TRINITY_DN11737_c0_g1::TRINITY_DN11737_c0_g1_i1::g.8601::m.8601	3633900	2322900	5994200	7354300	3612300	2022300	1691500	3872500	9006300	10902000	22333000	8134500	160070	976140	179410
TRINITY_DN15578_c0_g1::TRINITY_DN15578_c0_g1_i2::g.54153::m.54153	5100100	1216000	5333900	6755400	2506700	545250	4117300	2986900	2649700	6009900	10027000	16058000	5034400	7419200	6400300
TRINITY_DN15120_c1_g1::TRINITY_DN15120_c1_g1_i1::g.47172::m.47172	1263600	277750	4873300	1608900	3598200	378980	3416500	1797600	9931000	5617600	24137000	24597000	0	0	0
TRINITY_DN11278_c0_g2::TRINITY_DN11278_c0_g2_i1::g.6843::m.6843	4147000	2203400	4966600	4549200	4526200	2660600	6268100	4652700	7680400	8063800	15705000	14838000	749410	158640	0
TRINITY_DN19745_c0_g1::TRINITY_DN19745_c0_g1_i7::g.123245::m.123245	2759100	604620	3907600	2446800	1822400	1017900	6578500	2300400	12217000	7586200	11675000	23187000	1885800	1571500	528560
TRINITY_DN13377_c3_g2::TRINITY_DN13377_c3_g2_i9::g.23085::m.23085	4479000	597360	4282100	4557800	2332600	1253400	754100	3142700	2312900	6467900	11524000	15967000	6936000	7521200	7172300
TRINITY_DN15863_c0_g2::TRINITY_DN15863_c0_g2_i4::g.58135::m.58135	1653400	450180	1928000	1418200	5066400	1072600	5222400	6474800	15444000	6992100	12292000	17094000	756480	2078300	1013500
TRINITY_DN18548_c0_g1::TRINITY_DN18548_c0_g1_i3::g.102416::m.102416	6619300	1721500	6645400	7505000	2461700	874110	1283300	2488000	5039200	4293700	6983700	7132300	6423600	10954000	7971100
TRINITY_DN10779_c0_g1::TRINITY_DN10779_c0_g1_i3::g.5511::m.5511	6861600	1672300	6341400	7747100	3921600	2300500	3480700	5713400	12365000	3194200	8850600	8645500	2879200	4055800	303070
TRINITY_DN15451_c0_g1::TRINITY_DN15451_c0_g1_i8::g.52025::m.52025	0	0	0	0	0	0	0	0	0	76270000	0	0	517730	484910	507110
TRINITY_DN14435_c3_g2::TRINITY_DN14435_c3_g2_i6::g.36467::m.36467	958650	128590	513770	354190	736170	147820	476220	420240	304390	800680	2267100	1771700	28930000	12683000	27076000
TRINITY_DN19361_c0_g1::TRINITY_DN19361_c0_g1_i10::g.117059::m.117059	0	0	0	0	49788000	26205000	0	0	0	0	0	0	1349700	0	0
TRINITY_DN11635_c0_g1::TRINITY_DN11635_c0_g1_i7::g.8217::m.8217	239730	80161	97858	609050	1217800	0	2411900	1360100	1198600	1794200	3792400	6537400	18577000	20531000	18643000
TRINITY_DN12062_c0_g1::TRINITY_DN12062_c0_g1_i4::g.10228::m.10228	865290	211700	807620	1016200	873310	484370	1164700	1184500	2088700	694400	3063200	2813400	19720000	20827000	20876000
TRINITY_DN13692_c1_g1::TRINITY_DN13692_c1_g1_i1::g.26628::m.26628	150190	0	139270	0	0	0	0	0	0	217680	0	0	25679000	22461000	27519000
TRINITY_DN16939_c1_g2::TRINITY_DN16939_c1_g2_i8::g.75928::m.75928	4715500	1802900	7305500	5102900	3772300	549840	4048500	2896500	6178100	4949700	18890000	13512000	668250	530350	911070
TRINITY_DN13776_c0_g2::TRINITY_DN13776_c0_g2_i2::g.28097::m.28097	1704500	702180	1339300	501950	2426800	1363600	1341600	1619500	11817000	0	19406000	22568000	2681800	2406100	4654100
TRINITY_DN15671_c0_g2::TRINITY_DN15671_c0_g2_i5::g.55277::m.55277	2329500	437130	2179100	1562800	1711900	983900	325530	2849000	3059500	1485000	9133300	8161800	12931000	11878000	15007000
TRINITY_DN18192_c1_g2::TRINITY_DN18192_c1_g2_i5::g.96646::m.96646	1569600	0	1677400	1124500	4384300	2274600	8614800	4362600	7075100	5378100	9398800	12347000	5343600	5164300	5206600

TRINITY_DN18029_c5_g1::TRINITY_DN18029_c5_g1_i6::g.93985::m.93985	676430	107890	968260	1292000	193380	139380	1089400	1224100	1738100	0	3675000	21790000	13514000	13357000	13636000
TRINITY_DN14933_c0_g2::TRINITY_DN14933_c0_g2_i3::g.44460::m.44460	3779000	2237200	4340300	5875200	445660	505730	3098200	3534800	11034000	8655400	19756000	7661300	0	1353700	1018300
TRINITY_DN16437_c1_g2::TRINITY_DN16437_c1_g2_i4::g.67526::m.67526	216320	40261	85941	0	534520	308990	735800	808020	1951700	444850	3273200	0	22248000	20090000	22350000
TRINITY_DN19108_c2_g3::TRINITY_DN19108_c2_g3_i2::g.112222::m.112222	4588300	1123300	5475800	4641800	2947700	1474600	3914400	4297500	7506600	5563400	10837000	11692000	2528600	3114200	2876700
TRINITY_DN18028_c1_g1::TRINITY_DN18028_c1_g1_i5::g.93929::m.93929	4722200	1169600	5337600	4297300	1545400	1156300	2630000	3348100	8468300	2781400	5508000	9153400	7071400	8245700	6505800
TRINITY_DN12699_c0_g1::TRINITY_DN12699_c0_g1_i7::g.14174::m.14174	1775100	753470	2008300	1610300	5631100	1224900	2437700	2020400	11946000	5034000	26167000	0	3434200	3224900	4627000
TRINITY_DN19669_c1_g1::TRINITY_DN19669_c1_g1_i10::g.121794::m.121794	114550	0	180680	171750	7631100	2273500	9838700	6736800	0	7144500	19591000	9340800	4666700	2987500	1104400
TRINITY_DN14038_c1_g2::TRINITY_DN14038_c1_g2_i5::g.31646::m.31646	2546000	571500	2527200	2819600	1977900	921670	3153600	3029200	7104700	2554800	9363800	3916500	7703500	9184900	14318000
TRINITY_DN16581_c1_g3::TRINITY_DN16581_c1_g3_i2::g.69928::m.69928	997130	412160	1135900	1197100	2526800	320670	1033800	1882500	2116900	4163500	4984300	1954700	17744000	15881000	15208000
TRINITY_DN16667_c0_g2::TRINITY_DN16667_c0_g2_i1::g.71232::m.71232	780540	262260	887890	703570	0	425800	593880	472810	779270	0	4477300	3589700	14819000	24008000	19715000
TRINITY_DN16967_c3_g1::TRINITY_DN16967_c3_g1_i3::g.76257::m.76257	2693400	548980	3073300	3351700	4102000	1458300	1250400	3048600	6186500	8141900	8734200	15930000	5651900	2871000	3191300
TRINITY_DN14845_c0_g2::TRINITY_DN14845_c0_g2_i5::g.43176::m.43176	0	29713	70150	86101	0	0	0	235070	0	0	146070	562500	23430000	23449000	22159000
TRINITY_DN9429_c0_g1::TRINITY_DN9429_c0_g1_i1::g.3466::m.3466	1753300	313650	1737500	1644600	4038100	2566900	5162300	3840000	10773000	5248800	6791100	14850000	3009900	3852000	4391700
TRINITY_DN12329_c1_g1::TRINITY_DN12329_c1_g1_i1::g.11925::m.11925	4010100	946070	2774400	951870	3311700	869800	2043500	2130700	9626300	1201000	17511000	22798000	1106400	453840	0
TRINITY_DN17549_c7_g7::TRINITY_DN17549_c7_g7_i1::g.85830::m.85830	0	0	0	0	3256900	1893700	4373000	4097800	13211000	8588200	13960000	16283000	1243700	1506100	1132300
TRINITY_DN16210_c2_g2::TRINITY_DN16210_c2_g2_i2::g.64102::m.64102	2831300	713000	3303000	3120100	2895900	1513900	3618900	3814800	2356700	4340100	7616800	9568600	7668900	8178100	7904500
TRINITY_DN18132_c2_g1::TRINITY_DN18132_c2_g1_i3::g.95646::m.95646	0	0	0	0	3549700	0	3477100	3963100	2580500	8850000	22606000	17899000	2775800	3177300	0
TRINITY_DN12647_c0_g1::TRINITY_DN12647_c0_g1_i2::g.14733::m.14733	4501100	1514200	4791100	4705700	2962200	2293800	1309800	2817300	2484800	8470700	12860000	8947100	3247100	3658100	3681200
TRINITY_DN19952_c2_g2::TRINITY_DN19952_c2_g2_i3::g.126557::m.126557	4738800	1757200	4197900	3749000	0	0	2913400	2390200	4629900	6112200	16845000	7791000	2841800	4425300	4915400
TRINITY_DN17973_c0_g3::TRINITY_DN17973_c0_g3_i3::g.92497::m.92497	4397600	1100400	3807000	3265000	4237300	1045200	892930	289260	0	181930	19158000	17789000	850560	624950	9477100

TRINITY_DN16421_c0_g1::TRINITY_DN16421_c0_g1_i1::g.67240::m.67240	3856300	627490	4959400	3207700	1299000	661290	1939300	1614400	3103700	4940700	12772000	11991000	3453400	6918800	5701200
TRINITY_DN32757_c0_g1::TRINITY_DN32757_c0_g1_i1::g.130429::m.130429	0	0	0	0	0	0	0	0	0	0	0	67029000	0	0	0
TRINITY_DN14021_c1_g2::TRINITY_DN14021_c1_g2_i5::g.31431::m.31431	1657400	879610	5123600	3016600	1745100	596390	2169800	3230000	6590100	5577500	18300000	13563000	336400	1814300	2234500
TRINITY_DN20011_c3_g2::TRINITY_DN20011_c3_g2_i2::g.127835::m.127835	4251100	1073800	4183000	3771300	3530300	427000	1917500	3808700	11140000	1281400	13184000	13821000	216690	1124300	2963600
TRINITY_DN16305_c0_g1::TRINITY_DN16305_c0_g1_i3::g.65508::m.65508	7473000	2500500	6776600	3180900	4878900	2765000	0	8022700	0	9277300	7973900	9982500	3859000	0	0
TRINITY_DN12041_c0_g1::TRINITY_DN12041_c0_g1_i1::g.10132::m.10132	2978900	623360	4464400	3068400	2458200	1370500	820840	3397700	4536600	6132800	7293600	14282000	891510	6531600	7586600
TRINITY_DN15348_c2_g2::TRINITY_DN15348_c2_g2_i4::g.50676::m.50676	1899700	364950	1847400	1699200	1025700	213500	2409300	1724500	3712300	182060	10864000	8217800	3653900	17330000	10184000
TRINITY_DN14545_c0_g2::TRINITY_DN14545_c0_g2_i2::g.38716::m.38716	0	0	0	0	0	0	0	0	0	226190	0	0	19055000	22416000	23477000
TRINITY_DN13014_c6_g2::TRINITY_DN13014_c6_g2_i4::g.18867::m.18867	23910	26327	42037	0	0	100700	0	0	0	0	0	0	16678000	25060000	23165000
TRINITY_DN16814_c2_g1::TRINITY_DN16814_c2_g1_i8::g.73807::m.73807	0	0	0	0	0	0	0	0	0	0	0	0	22767000	21344000	20535000
TRINITY_DN14872_c2_g2::TRINITY_DN14872_c2_g2_i2::g.43670::m.43670	266970	0	885100	374630	216710	0	0	0	115060	2405000	2411100	2534400	14314000	22879000	18168000
TRINITY_DN13283_c0_g1::TRINITY_DN13283_c0_g1_i3::g.21813::m.21813	120090	46577	0	0	0	0	0	0	0	0	0	0	15255000	22461000	25773000
TRINITY_DN14986_c4_g1::TRINITY_DN14986_c4_g1_i9::g.45372::m.45372	1867300	602470	2262000	1962900	4089700	992090	3105900	3450200	1611500	2997100	6340400	10680000	5261900	8526000	9019100
TRINITY_DN13825_c0_g2::TRINITY_DN13825_c0_g2_i1::g.28850::m.28850	4884300	0	5161400	6246600	3198200	1913100	5115500	3335100	6287200	4387500	2594100	10635000	2586000	3179900	2884000
TRINITY_DN19496_c3_g4::TRINITY_DN19496_c3_g4_i2::g.118720::m.118720	3797500	1738700	4355500	3658900	1361300	1322700	3279100	0	15638000	1690800	23197000	1476900	541050	0	0
TRINITY_DN19996_c7_g1::TRINITY_DN19996_c7_g1_i2::g.127540::m.127540	959450	391960	915320	726480	235130	0	498240	953930	0	604260	3023000	5069400	11866000	19336000	17346000
TRINITY_DN16132_c1_g1::TRINITY_DN16132_c1_g1_i4::g.62630::m.62630	3770300	2458600	11447000	2787500	0	0	0	2670600	0	3383300	26796000	7315700	0	1228700	0
TRINITY_DN12592_c2_g1::TRINITY_DN12592_c2_g1_i5::g.14064::m.14064	715040	64332	975920	740150	3648600	2030200	3663300	3563100	7894200	5879700	10617000	11070000	2792000	4566500	3404300
TRINITY_DN15525_c0_g1::TRINITY_DN15525_c0_g1_i8::g.53299::m.53299	0	0	106630	94034	382120	0	220650	0	0	0	0	0	17897000	24054000	18685000
TRINITY_DN19723_c2_g3::TRINITY_DN19723_c2_g3_i3::g.122857::m.122857	3273200	945180	3952500	4236900	4438400	1094200	4160100	5297000	5375300	690080	11295000	7906700	2327900	2362300	3185400

TRINITY_DN18422_c0_g3::TRINITY_DN18422_c0_g3_i1::g.100190::m.100190	1772300	251220	899450	2158700	1718600	155190	971700	1085900	1332100	0	7414600	9610300	10210000	12060000	10855000
TRINITY_DN13563_c1_g1::TRINITY_DN13563_c1_g1_i8::g.25419::m.25419	78958	0	177060	12870	118810	0	0	61837	352380	0	212790	698270	17368000	20475000	20790000
TRINITY_DN18526_c1_g2::TRINITY_DN18526_c1_g2_i7::g.101942::m.101942	1525300	265410	1193800	1556900	1633100	1179100	762540	1612000	4175100	776330	5733800	9078600	6698100	15588000	8395600
TRINITY_DN13666_c1_g1::TRINITY_DN13666_c1_g1_i1::g.26956::m.26956	5093200	2262800	8114700	6701000	2596000	0	3419100	2905200	7381100	6166500	7271800	8241800	0	0	0
TRINITY_DN13901_c0_g1::TRINITY_DN13901_c0_g1_i11::g.28513::m.2851	1615500	979830	2479200	1752600	2379900	1059600	2797400	1256900	7184700	5128100	9888900	14420000	3665100	2822800	2593800
TRINITY_DN12879_c0_g1::TRINITY_DN12879_c0_g1_i1::g.16746::m.16746	1002600	517080	1527200	1560000	1314400	2299300	2631300	2358300	5710100	9518900	11147000	11615000	2327000	2794800	2882900
TRINITY_DN15261_c0_g1::TRINITY_DN15261_c0_g1_i6::g.49258::m.49258	304650	133630	1482500	1585600	554230	256100	708300	1975600	1482100	992920	2186100	2137200	10371000	15834000	16748000
TRINITY_DN11924_c0_g1::TRINITY_DN11924_c0_g1_i1::g.9513::m.9513	6992700	963270	5183900	4157200	1233200	1155000	1737000	1390100	7248000	1945900	5820400	8796200	1796500	4682200	2654500
TRINITY_DN18749_c0_g2::TRINITY_DN18749_c0_g2_i4::g.105830::m.105830	1007200	221250	1498000	815210	4260300	817280	940000	2983200	9687300	3240900	16266000	11192000	1184000	0	1592000
TRINITY_DN18258_c0_g1::TRINITY_DN18258_c0_g1_i1::g.97653::m.97653	3352600	394160	2074100	0	2476600	1436200	3409500	3414900	7354400	0	12429000	12478000	3449200	3242700	0
TRINITY_DN17853_c1_g4::TRINITY_DN17853_c1_g4_i19::g.90697::m.90697	901420	313720	1233700	3189300	3270700	123150	5620500	1462400	6435500	5785000	8484700	15711000	576190	1536000	780830
TRINITY_DN11160_c0_g1::TRINITY_DN11160_c0_g1_i1::g.6484::m.6484	1117400	92244	1474900	441800	4245900	1968700	6281100	3255500	5970400	6476000	9586100	13422000	0	522160	500130
TRINITY_DN15352_c3_g1::TRINITY_DN15352_c3_g1_i9::g.50776::m.50776	1911700	527370	3040000	2071200	3468100	1219400	2733200	3356300	5265700	5115100	12842000	8316700	1943500	1384200	2007300
TRINITY_DN19260_c1_g1::TRINITY_DN19260_c1_g1_i11::g.114784::m.114784	1669500	666690	2297500	2296600	885800	545450	1229200	1490000	6248900	3473900	6380100	8612100	5620800	5009900	7940200
TRINITY_DN18987_c4_g3::TRINITY_DN18987_c4_g3_i1::g.110065::m.110065	518710	58320	1237800	1162400	1739600	803280	2678900	2435200	7554900	3945500	9351900	11854000	2584800	5079400	3339000
TRINITY_DN12537_c1_g2::TRINITY_DN12537_c1_g2_i7::g.13611::m.13611	4305800	1326000	5489700	5055500	3638500	1123000	2064200	1289800	8724300	3200300	8766000	9239700	0	0	0
TRINITY_DN19187_c2_g2::TRINITY_DN19187_c2_g2_i1::g.113564::m.113564	641290	256680	975920	1917200	1701600	188590	3564700	1512300	7866000	3830000	6135100	11846000	4329200	3380900	5620400
TRINITY_DN12599_c0_g1::TRINITY_DN12599_c0_g1_i6::g.14058::m.14058	0	0	333110	0	169350	0	159240	91329	804460	116120	753730	724920	14629000	17090000	18524000
TRINITY_DN19040_c3_g4::TRINITY_DN19040_c3_g4_i1::g.110853::m.110853	1764800	544070	2514900	2276500	3351400	1743500	1974200	2896700	2527200	1151300	9741100	6592800	5450200	4687800	6049000
TRINITY_DN14858_c0_g1::TRINITY_DN14858_c0_g1_i1::g.43399::m.43399	447200	120960	364940	837010	559760	208020	870150	518540	1286600	0	1149900	1023300	27471000	9644800	8758200

TRINITY_DN18296_c0_g1::TRINITY_DN18296_c0_g1_i5::g.98309::m.98309	4170100	792580	4376100	3871200	602740	712410	598560	1303800	8460000	3724100	10252000	11387000	1500400	715770	739930
TRINITY_DN13876_c0_g1::TRINITY_DN13876_c0_g1_i2::g.29614::m.29614	2310700	870190	2420900	1965800	2436600	760470	3274500	3640200	3558900	4711100	10876000	15835000	0	487570	0
TRINITY_DN11399_c0_g1::TRINITY_DN11399_c0_g1_i3::g.6893::m.6893	0	0	0	0	0	0	0	0	428130	0	553130	0	14122000	15443000	22449000
TRINITY_DN16378_c0_g2::TRINITY_DN16378_c0_g2_i3::g.66649::m.66649	1440300	380700	1692300	1110000	566180	303280	1034400	816480	2438900	2521200	3696200	3707200	10263000	13460000	9441200
TRINITY_DN19790_c2_g2::TRINITY_DN19790_c2_g2_i1::g.123647::m.123647	42828	0	46816	60520	5071000	2299700	0	2697300	33914000	0	0	4220600	4482200	0	0
TRINITY_DN13975_c1_g4::TRINITY_DN13975_c1_g4_i3::g.30944::m.30944	10544000	795680	7878500	7493900	0	0	765840	0	0	0	0	24824000	0	0	0
TRINITY_DN13815_c1_g2::TRINITY_DN13815_c1_g2_i6::g.28882::m.28882	443410	146010	527250	585930	1694000	587350	793230	746080	2637200	0	952690	5659800	10088000	13751000	13633000
TRINITY_DN19396_c1_g1::TRINITY_DN19396_c1_g1_i8::g.116313::m.1163	0	0	0	0	0	0	0	0	0	0	0	0	12029000	21258000	18847000
TRINITY_DN18341_c1_g1::TRINITY_DN18341_c1_g1_i3::g.99097::m.99097	6158200	1989100	8390000	6493900	3462600	1504800	0	7082900	2616800	0	8054800	4821400	481040	1041000	0
TRINITY_DN13865_c0_g1::TRINITY_DN13865_c0_g1_i3::g.29082::m.29082	235560	39592	324780	414920	0	30223	0	0	447860	0	2155200	2587500	13080000	22125000	10398000
TRINITY_DN16815_c5_g2::TRINITY_DN16815_c5_g2_i1::g.73817::m.73817	412650	102620	564880	270140	235430	305200	622700	0	127580	0	0	0	13128000	17116000	18835000
TRINITY_DN14080_c2_g1::TRINITY_DN14080_c2_g1_i10::g.32162::m.32162	1502200	458820	1697500	2076900	2362700	1219700	2844300	2980300	8167000	3602700	8386100	6957600	2727500	3307200	3362300
TRINITY_DN15949_c1_g2::TRINITY_DN15949_c1_g2_i8::g.59514::m.59514	1802100	1088000	3576700	2818300	1141000	1240000	2871500	2208700	5396400	3538200	10012000	10640000	1013200	2516300	1455700
TRINITY_DN19409_c5_g5::TRINITY_DN19409_c5_g5_i2::g.117475::m.117475	113160	0	44842	78372	5452300	608980	0	1998000	11576000	7794400	9019900	14154000	0	0	0
TRINITY_DN19604_c2_g1::TRINITY_DN19604_c2_g1_i2::g.120755::m.120755	2428200	251960	2103000	2284700	2482800	228140	2738100	1173000	7660700	687640	12808000	15053000	336780	0	455780
TRINITY_DN17204_c0_g1::TRINITY_DN17204_c0_g1_i8::g.80281::m.80281	99248	0	25614	13332	0	0	0	0	0	0	0	0	15753000	19741000	14884000
TRINITY_DN16901_c2_g1::TRINITY_DN16901_c2_g1_i2::g.75153::m.75153	17644000	4188100	18335000	9950100	0	0	0	0	0	0	0	0	0	0	0
TRINITY_DN16836_c1_g3::TRINITY_DN16836_c1_g3_i3::g.74224::m.74224	3024900	1484800	2686000	2011900	834200	445980	1347000	0	6377700	4695800	10642000	10084000	2398500	3025600	980390
TRINITY_DN16865_c3_g3::TRINITY_DN16865_c3_g3_i2::g.74745::m.74745	1139600	336980	1417400	1367200	1983900	1254100	2985600	2451500	3886200	2009200	3900000	8357800	5321700	6701900	6821700
TRINITY_DN18894_c1_g1::TRINITY_DN18894_c1_g1_i9::g.108231::m.108231	414150	215500	817980	1739300	952720	555270	287710	1939000	928030	0	9779500	15436000	4112800	7667500	5039500

TRINITY_DN19186_c1_g1::TRINITY_DN19186_c1_g1_i2::g.113474::m.1134	15478000	1941700	8527500	17930000	0	0	308570	419370	1394900	1008900	1276700	1525100	0	0	0
TRINITY_DN20002_c1_g1::TRINITY_DN20002_c1_g1_i9::g.127794::m.127794	112610	0	18175	189080	0	0	150070	76374	215460	0	0	563040	15848000	19467000	12932000
TRINITY_DN18281_c0_g1::TRINITY_DN18281_c0_g1_i4::g.98025::m.98025	48912000	0	0	0	381880	0	0	0	0	0	0	0	0	0	0
TRINITY_DN13211_c0_g2::TRINITY_DN13211_c0_g2_i3::g.21096::m.21096	173620	22086	65650	0	0	0	157350	0	0	0	0	0	7832400	25189000	15838000
TRINITY_DN10140_c0_g1::TRINITY_DN10140_c0_g1_i2::g.4317::m.4317	1436900	213720	1757900	1200200	3903400	1047000	5406300	1208700	4181600	1248000	5868000	4498200	3718600	8069300	5493300
TRINITY_DN14435_c4_g2::TRINITY_DN14435_c4_g2_i1::g.36484::m.36484	927040	269620	1111100	733510	3266900	914650	3359700	3608600	6029900	4734800	11089000	8616400	1743300	930790	1860100
TRINITY_DN12440_c1_g2::TRINITY_DN12440_c1_g2_i1::g.13039::m.13039	0	0	0	0	116620	0	127090	0	930540	160670	470240	0	12621000	16809000	17664000
TRINITY_DN14413_c0_g1::TRINITY_DN14413_c0_g1_i13::g.36824::m.36824	173560	63242	158230	141900	0	95355	0	309350	0	0	821440	0	14512000	18578000	14023000
TRINITY_DN17675_c2_g3::TRINITY_DN17675_c2_g3_i6::g.88141::m.88141	0	0	0	0	94255	0	0	0	0	0	0	0	14320000	17229000	17176000
TRINITY_DN14025_c2_g1::TRINITY_DN14025_c2_g1_i8::g.31522::m.31522	246360	87543	490070	533370	0	0	0	89165	0	0	0	43615000	1638600	883510	1140500
TRINITY_DN11357_c0_g2::TRINITY_DN11357_c0_g2_i1::g.7096::m.7096	1205600	118850	1147500	885930	980930	2336200	9229600	4645300	4220800	4798100	5457100	10815000	486660	1104500	1199700
TRINITY_DN13606_c2_g2::TRINITY_DN13606_c2_g2_i2::g.26272::m.26272	201980	57270	274180	226330	1180000	788600	916940	1160000	1729000	1304300	1745300	2006100	12459000	8538300	15322000
TRINITY_DN15004_c1_g1::TRINITY_DN15004_c1_g1_i9::g.45691::m.45691	643160	188740	572830	542570	1458600	0	499070	331780	0	963440	210680	912770	12268000	17606000	11658000
TRINITY_DN20046_c4_g1::TRINITY_DN20046_c4_g1_i6::g.128432::m.128432	0	0	0	0	0	0	0	0	0	123640	0	0	17070000	18216000	12428000
TRINITY_DN17290_c1_g1::TRINITY_DN17290_c1_g1_i4::g.81361::m.81361	2830800	336940	2747000	2953000	2880500	27099	6156900	1385600	2705100	6916800	2670700	7810400	513820	3005500	4828700
TRINITY_DN12342_c1_g3::TRINITY_DN12342_c1_g3_i1::g.12105::m.12105	4582100	1364000	5481500	5687100	258350	1372800	4147600	2753100	0	1374400	10672000	5470100	266100	1877400	2153500
TRINITY_DN12893_c0_g1::TRINITY_DN12893_c0_g1_i1::g.17069::m.17069	2397000	425970	466790	1657000	2354000	0	2177200	816190	8952500	3645100	11842000	12702000	0	0	0
TRINITY_DN16995_c1_g3::TRINITY_DN16995_c1_g3_i3::g.76630::m.76630	3264300	800330	2135400	1857000	1076900	1243500	1241300	1336700	0	2298200	5616500	8039600	1789000	7639800	8523500
TRINITY_DN14421_c2_g1::TRINITY_DN14421_c2_g1_i4::g.36938::m.36938	579440	103130	882960	612340	0	550240	0	425670	1066100	814750	1359000	2420000	7345400	11076000	19566000
TRINITY_DN13126_c1_g4::TRINITY_DN13126_c1_g4_i1::g.20159::m.20159	3828100	1350900	4051700	4015200	1985900	485140	1480200	154390	3422600	0	4002500	7423100	2085600	7371900	4573000
TRINITY_DN16509_c1_g1::TRINITY_DN16509_c1_g1_i1::g.68768::m.68768	250000	253760	655080	1023600	343020	370590	830070	1755300	3075300	2524500	2324100	5664300	8328400	9943200	8740900
TRINITY_DN19751_c2_g1::TRINITY_DN19751_c2_g1_i6::g.123209::m.123209	574440	0	818480	823050	104500	260560	1001000	1608700	0	0	702010	350030	11150000	16027000	12283000

TRINITY_DN17891_c2_g1::TRINITY_DN17891_c2_g1_i4::g.91423::m.91423	46012	0	0	0	0	0	0	0	0	0	0	0	13680000	16692000	15175000
TRINITY_DN18954_c0_g3::TRINITY_DN18954_c0_g3_i4::g.109379::m.109379	0	0	71203	219880	645760	273420	1296000	1545900	491880	1364100	2079500	5703500	8550200	14034000	9247100
TRINITY_DN17145_c0_g3::TRINITY_DN17145_c0_g3_i2::g.79319::m.79319	125620	60027	175400	74934	167700	0	0	0	83221	86488	0	0	14800000	14609000	15248000
TRINITY_DN16061_c2_g2::TRINITY_DN16061_c2_g2_i3::g.61317::m.61317	0	0	0	0	0	0	0	0	0	0	0	0	18204000	16419000	10580000
TRINITY_DN19588_c6_g1::TRINITY_DN19588_c6_g1_i5::g.120209::m.120209	408310	92777	484450	614290	209750	168870	641080	449710	368850	638870	533920	701020	14014000	12310000	13496000
TRINITY_DN13159_c0_g1::TRINITY_DN13159_c0_g1_i1::g.20565::m.20565	3521600	1110100	0	2518200	1944300	1466600	2980100	1844900	5849300	0	6917300	6116800	3432600	3803100	3572100
TRINITY_DN13164_c0_g4::TRINITY_DN13164_c0_g4_i1::g.20741::m.20741	1282300	40082	888500	1688600	0	1378200	805420	5131500	4815400	3628600	2004200	8613500	677660	6635600	7443300
TRINITY_DN16361_c1_g1::TRINITY_DN16361_c1_g1_i5::g.66288::m.66288	0	0	0	0	0	0	0	0	0	0	0	0	18674000	17166000	9053900
TRINITY_DN15008_c0_g1::TRINITY_DN15008_c0_g1_i3::g.45737::m.45737	0	0	0	0	0	0	0	0	0	0	0	0	13717000	14136000	16982000
TRINITY_DN17275_c2_g1::TRINITY_DN17275_c2_g1_i6::g.81136::m.81136	29504	0	33394	0	0	0	0	0	0	0	0	0	15587000	15055000	13892000
TRINITY_DN18883_c1_g1::TRINITY_DN18883_c1_g1_i14::g.108273::m.108273	150630	23248	0	90986	0	0	128780	291630	547660	0	532280	0	13294000	16070000	13425000
TRINITY_DN14831_c2_g1::TRINITY_DN14831_c2_g1_i4::g.42949::m.42949	4543900	979770	4087600	3246300	2253700	0	4591700	3437600	8220000	616670	5346900	6216900	134610	0	0
TRINITY_DN17427_c0_g1::TRINITY_DN17427_c0_g1_i16::g.83642::m.83642	704650	129330	1077500	554210	1968500	1029500	3274800	916620	6020400	5665600	8363100	13348000	272060	154670	0
TRINITY_DN19711_c3_g1::TRINITY_DN19711_c3_g1_i1::g.122630::m.122630	3427100	787820	3473900	4025600	888440	0	606900	2734400	2808300	0	5230900	7614200	2230800	4055600	5503500
TRINITY_DN19077_c0_g1::TRINITY_DN19077_c0_g1_i1::g.111277::m.111277	535760	48275	730560	395910	1622600	552310	1513700	1794300	3003500	0	5800200	7897800	4106700	8015300	7358600
TRINITY_DN13164_c0_g2::TRINITY_DN13164_c0_g2_i1::g.20739::m.20739	559360	91771	2401400	892550	0	0	279030	0	677950	1195700	16360000	16107000	729460	1761800	1650900
TRINITY_DN20023_c4_g2::TRINITY_DN20023_c4_g2_i4::g.127985::m.127985	348690	47087	357510	246060	0	34709	0	53920	0	0	0	0	13279000	12003000	16213000
TRINITY_DN18086_c1_g2::TRINITY_DN18086_c1_g2_i3::g.94830::m.94830	3416500	1700200	5252500	3989900	7338800	3998700	7153100	8035400	0	0	0	0	558810	688360	259310
TRINITY_DN13268_c1_g1::TRINITY_DN13268_c1_g1_i7::g.21886::m.21886	0	0	0	0	0	0	0	0	0	0	606290	0	13129000	10503000	18061000
TRINITY_DN12877_c0_g1::TRINITY_DN12877_c0_g1_i2::g.16745::m.16745	2176000	894840	3208800	2934000	2647400	1751400	3833600	4438300	3385400	3830500	6593300	5267700	439750	461720	339700

TRINITY_DN15082_c2_g5::TRINITY_DN15082_c2_g5_i1::g.46709::m.46709	1578900	363830	1579300	1537600	2174900	1945100	2896100	3752100	5910700	3346500	6445700	8464300	668690	640100	775080
TRINITY_DN17469_c0_g1::TRINITY_DN17469_c0_g1_i8::g.84329::m.84329	0	0	0	47045	398430	0	550880	412180	0	0	1450800	2948700	14528000	6948000	14771000
TRINITY_DN16031_c0_g3::TRINITY_DN16031_c0_g3_i1::g.60905::m.60905	1483000	373090	1976900	1645700	2153100	627830	2946400	2832200	4318000	3111500	4398300	6408500	1861900	5133700	2668700
TRINITY_DN13415_c1_g3::TRINITY_DN13415_c1_g3_i4::g.23607::m.23607	140770	0	107440	44419	90405	54376	325930	161300	412680	154440	0	0	11559000	15389000	13242000
TRINITY_DN13872_c1_g1::TRINITY_DN13872_c1_g1_i2::g.29678::m.29678	1356600	267400	1882800	1355700	1082100	673300	795960	543250	2388000	3298700	4662100	9999300	3931700	2645800	6496400
TRINITY_DN14508_c8_g1::TRINITY_DN14508_c8_g1_i4::g.38373::m.38373	1946300	855660	2395400	1943700	592010	166960	3396400	2340900	1432400	5614800	9043700	8368600	788460	770190	1698800
TRINITY_DN15591_c0_g2::TRINITY_DN15591_c0_g2_i2::g.54135::m.54135	0	0	0	0	0	0	0	0	0	0	0	0	14104000	13424000	13645000
TRINITY_DN19628_c3_g1::TRINITY_DN19628_c3_g1_i3::g.121141::m.121141	101980	0	337000	0	1567100	846390	2301900	1500500	4634600	2634500	3119900	4595300	4935000	10079000	4482000
TRINITY_DN19415_c0_g3::TRINITY_DN19415_c0_g3_i1::g.117477::m.117477	1956500	0	2090100	1296500	1617400	393780	0	1580000	5703200	3524400	10138000	8201300	1086700	1345300	2093300
TRINITY_DN19617_c3_g3::TRINITY_DN19617_c3_g3_i8::g.121273::m.121273	2169600	639740	2752600	2687000	1309200	187300	2019700	926880	4740800	527910	1487600	12171000	1827000	3805300	3684700
TRINITY_DN15317_c1_g1::TRINITY_DN15317_c1_g1_i5::g.50064::m.50064	2338500	245360	775170	719770	946660	1486000	1457200	1753500	8048400	8448000	1528900	1870700	2539200	4806900	3757000
TRINITY_DN18894_c1_g1::TRINITY_DN18894_c1_g1_i5::g.108225::m.108225	857510	425760	1147800	0	3281000	0	2665700	1944800	1438900	419320	1724200	1895200	5351100	9654000	9238600
TRINITY_DN17857_c0_g1::TRINITY_DN17857_c0_g1_i1::g.90819::m.90819	106610	21061	59102	135600	367510	167180	0	701710	226630	546450	1186600	1318900	8631900	13573000	12850000
TRINITY_DN19979_c5_g4::TRINITY_DN19979_c5_g4_i2::g.127014::m.127014	1109400	376290	1201200	1157000	2755800	1414000	3428100	3420400	0	3577700	6045800	9177700	2187100	1608700	2332300
TRINITY_DN18581_c2_g1::TRINITY_DN18581_c2_g1_i1::g.102861::m.1028	274230	0	125420	448070	2107700	996580	1941000	0	3176200	2067100	15214000	12067000	157970	712090	304940
TRINITY_DN19014_c2_g2::TRINITY_DN19014_c2_g2_i4::g.110567::m.110567	0	0	0	0	0	0	0	0	224800	0	0	627280	13794000	15742000	8943900
TRINITY_DN19846_c6_g2::TRINITY_DN19846_c6_g2_i1::g.124494::m.124494	735370	139130	1058800	729400	2572000	1557400	3194200	2860100	5955300	3570300	7167700	8190400	379870	567490	422420
TRINITY_DN12374_c0_g1::TRINITY_DN12374_c0_g1_i5::g.12261::m.12261	64474	0	59344	0	0	0	0	0	0	0	0	0	10562000	15644000	12744000
TRINITY_DN14238_c0_g1::TRINITY_DN14238_c0_g1_i3::g.34351::m.34351	929540	346550	1154700	1096300	1665200	0	526010	2888200	7389400	3808400	7094900	5928300	1339800	2714600	2109400
TRINITY_DN17147_c0_g1::TRINITY_DN17147_c0_g1_i4::g.79344::m.79344	1621300	474400	1893000	1650200	3584800	1752200	4253200	4276000	2787000	3451000	7149700	6098300	0	0	0

TRINITY_DN12441_c0_g1::TRINITY_DN12441_c0_g1_i1::g.12899::m.12899	1462700	629240	3088000	1164400	2526100	410590	2947400	1041600	5423000	2823300	10998000	6320400	0	125000	0
TRINITY_DN12614_c0_g1::TRINITY_DN12614_c0_g1_i2::g.14256::m.14256	190010	44720	517990	293060	719510	150660	403150	172890	444570	1141600	1087900	1204600	9819500	12487000	10283000
TRINITY_DN11552_c0_g1::TRINITY_DN11552_c0_g1_i1::g.7817::m.7817	0	0	0	0	0	0	0	119080	265080	0	0	38054000	0	0	267460
TRINITY_DN15353_c0_g3::TRINITY_DN15353_c0_g3_i2::g.50015::m.50015	1233300	558820	1253900	1166700	1733100	994290	4122600	2012200	1476900	2233800	9449000	3527600	2623600	3029200	3148000
TRINITY_DN17847_c0_g1::TRINITY_DN17847_c0_g1_i7::g.90759::m.90759	257190	126880	131550	111910	106940	0	495710	216410	1029200	0	1592200	0	7733800	12520000	14213000
TRINITY_DN17899_c1_g1::TRINITY_DN17899_c1_g1_i20::g.90008::m.90008	1006400	227400	1235500	1076100	706760	263810	621250	233670	0	0	2425700	2912300	9493900	11124000	7114200
TRINITY_DN11696_c0_g1::TRINITY_DN11696_c0_g1_i4::g.8428::m.8428	1179400	185300	1515000	1225200	1353100	1096400	3312400	3636500	4410700	4008800	5937300	9230300	283730	724310	318150
TRINITY_DN13436_c3_g1::TRINITY_DN13436_c3_g1_i7::g.23925::m.23925	167810	69607	227260	235550	0	0	461260	784730	665800	0	0	985740	10139000	12932000	11664000
TRINITY_DN19395_c0_g1::TRINITY_DN19395_c0_g1_i9::g.117248::m.117248	2067200	276710	1710900	870130	1248000	626310	2757300	2881300	5623000	605730	5419900	5460400	2485100	3074000	2990900
TRINITY_DN17244_c0_g1::TRINITY_DN17244_c0_g1_i3::g.79946::m.79946	1374800	372580	846560	802150	2959300	233810	2188000	1015700	4438700	3967100	6707300	12011000	152690	185290	830170
TRINITY_DN11876_c0_g1::TRINITY_DN11876_c0_g1_i1::g.9282::m.9282	0	0	0	0	0	0	0	0	0	0	0	0	0	20534000	17470000
TRINITY_DN17448_c2_g2::TRINITY_DN17448_c2_g2_i8::g.83900::m.83900	0	0	89190	302060	1917200	0	2083100	1407900	6020300	803150	1372500	1600900	5876100	9188100	7174400
TRINITY_DN13409_c1_g1::TRINITY_DN13409_c1_g1_i8::g.23419::m.23419	89449	57447	221350	330630	0	86091	510290	548980	0	369900	335200	386060	12933000	7468000	14271000
TRINITY_DN13221_c2_g1::TRINITY_DN13221_c2_g1_i1::g.21206::m.21206	0	0	0	0	0	0	0	0	0	0	0	0	10524000	13435000	13252000
TRINITY_DN13244_c2_g1::TRINITY_DN13244_c2_g1_i6::g.21528::m.21528	57379	0	0	0	182650	0	0	166810	1148600	1981500	5203000	4069400	6332000	9497800	8268500
TRINITY_DN18545_c0_g2::TRINITY_DN18545_c0_g2_i6::g.102386::m.102386	96841	0	0	23717	0	47385	112680	111510	0	0	215940	303960	11954000	9854500	14134000
TRINITY_DN11123_c0_g1::TRINITY_DN11123_c0_g1_i4::g.6381::m.6381	929830	279030	1097900	1069700	1297400	595770	3298500	2025800	2606800	5005900	10911000	5462800	0	0	2072100
TRINITY_DN17109_c1_g4::TRINITY_DN17109_c1_g4_i1::g.78802::m.78802	0	0	0	0	0	0	0	0	0	0	0	0	13623000	13593000	9401400
TRINITY_DN14091_c1_g2::TRINITY_DN14091_c1_g2_i1::g.32197::m.32197	0	0	0	0	0	0	0	0	0	0	0	0	10153000	15677000	10381000
TRINITY_DN12452_c0_g1::TRINITY_DN12452_c0_g1_i1::g.12978::m.12978	1567400	380270	1937500	1395700	1033700	0	2931200	1513000	2531200	2481200	4421300	4185900	3967500	4252000	3538600
TRINITY_DN18521_c6_g6::TRINITY_DN18521_c6_g6_i1::g.101903::m.101903	1117000	312200	2901300	2351500	2236900	812910	2254600	1241700	6076400	1532200	8122900	6512600	645530	0	0

TRINITY_DN19436_c2_g1::TRINITY_DN19436_c2_g1_i4::g.118041::m.118041	2260000	718760	1909800	2224300	1362700	149900	669140	1283500	4014800	2785700	6869600	5240200	1960100	2246100	1871800
TRINITY_DN17420_c0_g1::TRINITY_DN17420_c0_g1_i7::g.83574::m.83574	543730	228070	214380	814290	657220	3022200	6189900	0	0	2966000	1659600	3550300	5803200	5921600	3595600
TRINITY_DN18973_c0_g1::TRINITY_DN18973_c0_g1_i1::g.109653::m.109653	0	0	0	0	0	0	0	0	0	0	0	0	11816000	13794000	9478700
TRINITY_DN11465_c0_g1::TRINITY_DN11465_c0_g1_i2::g.7454::m.7454	926090	357050	1650300	1874500	1532500	276300	1078200	933260	3656100	1814500	4665300	11373000	1549600	1654900	1676300
TRINITY_DN16317_c2_g1::TRINITY_DN16317_c2_g1_i4::g.65631::m.65631	1194500	513000	1212100	1387800	852930	633860	396620	1685400	3334900	1679500	4270300	3681400	3310800	6389400	4471000
TRINITY_DN16236_c1_g2::TRINITY_DN16236_c1_g2_i1::g.64353::m.64353	252270	0	264700	338380	0	0	461740	0	0	0	0	0	16274000	9416100	7936900
TRINITY_DN12721_c0_g3::TRINITY_DN12721_c0_g3_i6::g.15362::m.15362	1785300	497790	2011900	1637800	1503500	861820	2096000	1660300	3306200	2512200	9848700	3981600	560640	1426100	710010
TRINITY_DN13179_c1_g3::TRINITY_DN13179_c1_g3_i2::g.20801::m.20801	1883400	566810	2133700	1681100	0	654310	2016000	0	6745600	5438000	6022100	2615100	2126700	110570	2365400
TRINITY_DN13823_c0_g2::TRINITY_DN13823_c0_g2_i2::g.29073::m.29073	44842	24068	62670	57600	1299100	353240	1448700	0	0	0	0	0	8169500	11405000	11419000
TRINITY_DN18057_c0_g3::TRINITY_DN18057_c0_g3_i1::g.94357::m.94357	125090	52926	70155	45634	0	0	0	0	0	0	0	0	10850000	10856000	12075000
TRINITY_DN12273_c2_g3::TRINITY_DN12273_c2_g3_i1::g.11683::m.11683	254440	0	926350	529310	0	0	0	1171500	1297800	800160	5193500	6030800	2844200	9299200	5690300
TRINITY_DN14391_c2_g2::TRINITY_DN14391_c2_g2_i2::g.36282::m.36282	267970	0	1007300	178940	0	476690	0	0	4148800	0	5799500	6407700	5193400	5406300	5128300
TRINITY_DN18947_c6_g2::TRINITY_DN18947_c6_g2_i2::g.109306::m.109306	2311200	987330	3040400	2246100	1809400	1144600	0	2698100	3881900	3479900	4646100	7700800	0	0	0
TRINITY_DN18272_c2_g1::TRINITY_DN18272_c2_g1_i1::g.97904::m.97904	290410	96647	349200	266490	126990	0	0	126470	0	0	625410	0	3992800	15786000	12269000
TRINITY_DN17506_c1_g1::TRINITY_DN17506_c1_g1_i6::g.84994::m.84994	0	0	0	0	0	0	0	0	0	0	123070	0	11326000	5159600	17152000
TRINITY_DN17662_c4_g1::TRINITY_DN17662_c4_g1_i8::g.87880::m.87880	1169600	469770	1460400	2156000	572010	318340	373720	415070	4945100	1583800	5943100	8953600	610830	1558300	2571400
TRINITY_DN12028_c1_g1::TRINITY_DN12028_c1_g1_i1::g.9901::m.9901	4951200	684470	6675500	1196700	268750	158940	351360	0	6186400	4803100	6480300	0	306560	389640	388890
TRINITY_DN18870_c1_g1::TRINITY_DN18870_c1_g1_i1::g.107725::m.107725	2299100	0	2509000	1845900	3918100	781770	1604200	2670700	2270600	3514500	5550800	5559100	74615	98021	74850
TRINITY_DN17060_c1_g1::TRINITY_DN17060_c1_g1_i4::g.77740::m.77740	0	0	0	0	0	0	0	0	0	0	0	0	11836000	10663000	10264000
TRINITY_DN14188_c2_g2::TRINITY_DN14188_c2_g2_i3::g.32373::m.32373	51025	0	42841	66554	82009	0	0	127340	0	0	171320	277790	8639500	15614000	7631800

TRINITY_DN18239_c4_g4::TRINITY_DN18239_c4_g4_i1::g.97381::m.97381	0	0	0	0	0	0	0	0	0	0	0	0	10005000	13327000	9338000
TRINITY_DN19251_c1_g2::TRINITY_DN19251_c1_g2_i24::g.114984::m.114984	0	0	0	0	0	0	0	0	0	0	0	0	7579000	15460000	9463900
TRINITY_DN18181_c1_g1::TRINITY_DN18181_c1_g1_i2::g.96439::m.96439	951910	287610	1696500	1098900	1883000	317990	2989100	1279300	5504800	4706300	3836100	7134500	351150	354470	0
TRINITY_DN15085_c0_g2::TRINITY_DN15085_c0_g2_i3::g.46604::m.46604	1755200	318120	2029200	2124000	0	310020	76291	586260	0	1355900	2932700	5549300	4432800	5268500	5440200
TRINITY_DN14405_c4_g1::TRINITY_DN14405_c4_g1_i11::g.36585::m.36585	92919	0	158170	75908	156040	0	520760	154250	1300700	529460	964010	1228000	10231000	7554800	8991800
TRINITY_DN20037_c6_g1::TRINITY_DN20037_c6_g1_i4::g.128163::m.128163	143160	0	34074	34517	0	684310	2480800	1155900	6503300	4886300	9727500	6222600	0	0	0
TRINITY_DN16369_c2_g2::TRINITY_DN16369_c2_g2_i2::g.66506::m.66506	440860	88716	625830	0	0	0	528780	0	0	0	1899000	1912600	6239700	13334000	6769400
TRINITY_DN19409_c5_g1::TRINITY_DN19409_c5_g1_i1::g.117472::m.117472	2096600	630120	2461300	2520000	287870	685980	1674500	1609100	3976100	2187800	3743900	4595300	1740000	1968900	1623900
TRINITY_DN15357_c0_g1::TRINITY_DN15357_c0_g1_i2::g.50651::m.50651	0	0	0	0	0	0	0	0	0	0	0	0	10184000	11892000	9668300
TRINITY_DN17150_c2_g1::TRINITY_DN17150_c2_g1_i1::g.79329::m.79329	149300	0	368690	169860	0	211780	0	0	451130	273360	1470900	1037500	7674400	10078000	9723200
TRINITY_DN16201_c0_g1::TRINITY_DN16201_c0_g1_i1::g.62689::m.62689	0	0	0	0	0	0	0	0	0	0	0	0	8373600	8097800	15118000
TRINITY_DN14972_c1_g2::TRINITY_DN14972_c1_g2_i4::g.44982::m.44982	1615400	503080	1798300	1275000	1056400	536610	1832700	1005900	3355700	2860100	6779000	7358900	432420	266680	809830
TRINITY_DN14986_c2_g1::TRINITY_DN14986_c2_g1_i1::g.45365::m.45365	677850	568270	1118800	644890	1562800	908020	925060	1434300	4955000	4087300	6412700	6573400	489340	530120	437930
TRINITY_DN15394_c0_g2::TRINITY_DN15394_c0_g2_i6::g.49878::m.49878	0	37298	87997	217880	651120	0	376460	394490	274260	0	646720	391060	9034100	7988100	11017000
TRINITY_DN11523_c0_g1::TRINITY_DN11523_c0_g1_i1::g.7740::m.7740	0	0	0	0	0	0	0	0	0	0	0	0	10245000	13567000	6972200
TRINITY_DN14718_c0_g2::TRINITY_DN14718_c0_g2_i3::g.41293::m.41293	660470	0	707550	530600	355870	0	568310	413120	647770	0	3043000	1010300	7041100	8889100	6791600
TRINITY_DN14157_c2_g1::TRINITY_DN14157_c2_g1_i3::g.33123::m.33123	3147200	1191200	2654400	2484500	605040	434560	2882100	2059500	1446300	812430	2223000	5451600	2642000	1445200	1152500
TRINITY_DN13422_c4_g1::TRINITY_DN13422_c4_g1_i9::g.23675::m.23675	405620	138780	635220	400290	311970	0	0	0	571180	0	542960	414000	6496500	10277000	10166000
TRINITY_DN17973_c0_g2::TRINITY_DN17973_c0_g2_i4::g.92495::m.92495	0	125770	508550	294580	0	0	629150	0	0	0	0	0	6636900	12313000	9846600
TRINITY_DN14213_c1_g1::TRINITY_DN14213_c1_g1_i6::g.33993::m.33993	120960	33159	223610	222740	0	0	0	46720	0	0	202440	261020	9599500	9811700	9789600

TRINITY_DN15873_c1_g1::TRINITY_DN15873_c1_g1_i7::g.58225::m.58225	0	0	13373000	0	0	0	16727000	0	0	0	0	0	0	0	0
TRINITY_DN12030_c0_g1::TRINITY_DN12030_c0_g1_i2::g.10080::m.10080	687700	231340	550380	1076600	2420700	907680	2006500	2926400	3341700	564670	4153200	7920400	338460	1446700	1376100
TRINITY_DN16055_c0_g3::TRINITY_DN16055_c0_g3_i1::g.61219::m.61219	787300	213140	992510	856580	194700	155750	579170	316450	1237800	1489000	2180300	7067500	3807000	3954700	6030400
TRINITY_DN16644_c3_g1::TRINITY_DN16644_c3_g1_i4::g.70221::m.70221	344860	51183	35062	0	0	255740	589170	71181	0	0	0	1106800	9381400	10847000	7104600
TRINITY_DN17145_c0_g4::TRINITY_DN17145_c0_g4_i1::g.79322::m.79322	433390	125870	450910	493630	0	0	199230	141890	1178800	649990	0	0	6945100	12726000	6263400
TRINITY_DN12499_c2_g2::TRINITY_DN12499_c2_g2_i3::g.13408::m.13408	49529	0	0	0	0	0	0	0	0	0	0	0	4421500	13464000	11640000
TRINITY_DN27272_c0_g1::TRINITY_DN27272_c0_g1_i1::g.129628::m.129628	0	0	2208400	2247500	0	1784800	4016200	2682600	6624400	0	9967200	0	0	0	0
TRINITY_DN18903_c0_g1::TRINITY_DN18903_c0_g1_i9::g.108678::m.108678	0	0	0	0	0	0	0	0	0	0	0	0	0	2989600	26510000
TRINITY_DN19948_c1_g1::TRINITY_DN19948_c1_g1_i4::g.126476::m.126476	1853900	329860	1552900	2174900	323990	470990	377830	692580	2278500	827740	415430	6856500	4658700	4748800	1907900
TRINITY_DN15893_c1_g2::TRINITY_DN15893_c1_g2_i6::g.57605::m.57605	27180	0	154790	65357	0	0	143760	0	0	0	0	0	8805800	11951000	8307800
TRINITY_DN18003_c1_g3::TRINITY_DN18003_c1_g3_i3::g.93450::m.93450	0	0	72691	150390	598590	266320	586430	474610	606180	451880	426170	554690	10413000	7958600	6819700
TRINITY_DN17070_c2_g4::TRINITY_DN17070_c2_g4_i5::g.77858::m.77858	154660	101360	0	0	289410	99356	598200	0	667700	796460	1350100	308810	4958600	10631000	9328300
TRINITY_DN10166_c0_g1::TRINITY_DN10166_c0_g1_i3::g.4379::m.4379	598210	196050	633000	718340	328120	0	0	0	710160	417760	964050	4818300	6816000	4652900	8367300
TRINITY_DN19812_c4_g1::TRINITY_DN19812_c4_g1_i1::g.124051::m.1240	189060	54250	257600	95497	1150600	427920	1189700	1212700	2005600	369160	370540	3269900	3412000	10208000	4973900
TRINITY_DN11760_c0_g1::TRINITY_DN11760_c0_g1_i1::g.8722::m.8722	33997	63329	125250	160770	495560	389540	370270	823170	792390	0	1732100	859940	8061500	4403800	10804000
TRINITY_DN20022_c3_g3::TRINITY_DN20022_c3_g3_i3::g.128023::m.128023	5104200	243410	4973200	4657000	3210300	0	7596800	3241900	0	0	0	0	0	0	0
TRINITY_DN16751_c0_g2::TRINITY_DN16751_c0_g2_i11::g.72773::m.72773	785870	106180	893560	679110	155120	0	0	415360	472610	0	0	912220	5301000	8831600	10315000
TRINITY_DN15089_c0_g1::TRINITY_DN15089_c0_g1_i2::g.46688::m.46688	0	0	0	0	0	0	0	0	0	0	0	0	6429400	11363000	11022000
TRINITY_DN13431_c11_g1::TRINITY_DN13431_c11_g1_i1::g.23860::m.238	0	0	0	0	0	0	0	0	0	0	0	0	9884500	9645400	8781300
TRINITY_DN16209_c0_g5::TRINITY_DN16209_c0_g5_i1::g.64031::m.64031	0	0	0	0	0	0	0	0	0	0	0	0	15182000	4739000	8367000

TRINITY_DN18640_c0_g2::TRINITY_DN18640_c0_g2_i8::g.103688::m.103688	463210	245360	596700	332890	4147300	401810	1886500	2288900	2684900	2416500	4279400	5843500	722160	1496100	459480
TRINITY_DN11955_c0_g1::TRINITY_DN11955_c0_g1_i1::g.9671::m.9671	0	0	0	0	0	0	0	0	0	0	0	0	7964300	9561900	10553000
TRINITY_DN19422_c2_g1::TRINITY_DN19422_c2_g1_i9::g.117599::m.117599	0	0	0	0	0	0	0	0	0	0	0	0	11178000	2254800	14637000
TRINITY_DN19041_c0_g1::TRINITY_DN19041_c0_g1_i8::g.110918::m.1109	1872000	438980	1379700	1571000	313040	256520	1105200	222590	1030300	423490	0	5114500	4379200	4858100	4989400
TRINITY_DN18551_c0_g2::TRINITY_DN18551_c0_g2_i5::g.102562::m.102562	0	38074	0	0	0	0	0	0	0	0	0	0	4968200	5113800	17827000
TRINITY_DN18727_c2_g1::TRINITY_DN18727_c2_g1_i8::g.105290::m.105290	0	0	0	0	0	0	0	0	0	0	0	0	199380	14616000	12897000
TRINITY_DN14879_c3_g4::TRINITY_DN14879_c3_g4_i1::g.43729::m.43729	707660	207950	1066000	789680	0	127890	288750	187690	144130	773400	1573500	1587700	6647500	7752800	5840300
TRINITY_DN19715_c1_g1::TRINITY_DN19715_c1_g1_i2::g.122707::m.122707	0	0	0	0	0	0	0	0	0	0	0	0	9313500	8825000	9306200
TRINITY_DN11725_c0_g2::TRINITY_DN11725_c0_g2_i1::g.8540::m.8540	0	72055	197090	232350	0	124180	2001600	1795900	0	0	2072100	5107200	3116600	9752900	2877100
TRINITY_DN16136_c0_g1::TRINITY_DN16136_c0_g1_i4::g.62642::m.62642	0	0	0	0	0	0	0	0	0	0	0	0	5628100	14906000	6800200
TRINITY_DN19182_c1_g1::TRINITY_DN19182_c1_g1_i6::g.113457::m.1134	200610	95347	328900	279190	105820	125640	368560	111130	773880	439680	1610900	3235400	5882500	6965200	6798600
TRINITY_DN16339_c2_g1::TRINITY_DN16339_c2_g1_i6::g.65997::m.65997	1964000	692440	1646300	1755600	300390	0	484020	1334600	3154900	1865900	3608200	7284200	748930	1588000	861820
TRINITY_DN18576_c1_g1::TRINITY_DN18576_c1_g1_i10::g.101619::m.101619	0	0	0	0	0	0	0	0	0	0	0	0	9471400	9457200	8198800
TRINITY_DN11411_c0_g1::TRINITY_DN11411_c0_g1_i2::g.7300::m.7300	0	0	0	0	0	0	0	0	0	0	0	0	8536300	9400900	9143300
TRINITY_DN14869_c3_g5::TRINITY_DN14869_c3_g5_i3::g.43830::m.43830	77706	50458	697340	62469	1946100	721780	762380	1227400	2804300	1824700	2765000	3457800	1459500	3879400	5331300
TRINITY_DN12162_c0_g1::TRINITY_DN12162_c0_g1_i2::g.10879::m.10879	2131600	915330	2676000	2086000	2992000	472420	709680	1937400	2472700	1867800	3395500	0	1479000	2503300	1334700
TRINITY_DN15457_c4_g1::TRINITY_DN15457_c4_g1_i5::g.52200::m.52200	118190	0	97051	48934	0	0	0	0	0	0	0	0	8230700	2686900	15771000
TRINITY_DN13171_c0_g1::TRINITY_DN13171_c0_g1_i3::g.20617::m.20617	0	0	0	0	0	0	0	0	0	0	0	0	7233600	9110200	10602000
TRINITY_DN19704_c1_g1::TRINITY_DN19704_c1_g1_i9::g.122571::m.122571	0	0	0	0	0	117990	203770	121080	847540	0	0	628410	7831700	6671400	10401000
TRINITY_DN13284_c1_g1::TRINITY_DN13284_c1_g1_i7::g.21970::m.21970	958940	151950	1342700	2099000	0	495410	0	0	1214600	0	0	4027800	4909900	4994600	6549200
TRINITY_DN17067_c1_g1::TRINITY_DN17067_c1_g1_i1::g.77883::m.77883	0	0	35742	0	0	0	0	0	0	220920	0	0	7230800	10161000	9087300
TRINITY_DN12343_c1_g1::TRINITY_DN12343_c1_g1_i5::g.11822::m.11822	0	0	0	0	0	0	0	0	0	0	0	0	8845700	7710100	10144000

TRINITY_DN16029_c2_g3::TRINITY_DN16029_c2_g3_i1::g.61032::m.61032	373580	137730	226290	436340	1490700	329570	1140400	382530	2550700	1154600	1739500	3538700	3509300	5197200	4483800
TRINITY_DN12608_c0_g1::TRINITY_DN12608_c0_g1_i2::g.14177::m.14177	0	0	0	0	0	0	0	0	0	0	0	0	0	0	26510000
TRINITY_DN18182_c1_g1::TRINITY_DN18182_c1_g1_i5::g.96464::m.96464	0	0	76095	0	0	35842	122750	0	315080	0	379380	0	10568000	4598200	10267000
TRINITY_DN15675_c5_g2::TRINITY_DN15675_c5_g2_i2::g.55536::m.55536	0	0	0	0	0	0	0	0	0	0	0	0	7957000	9442800	8913000
TRINITY_DN15276_c1_g1::TRINITY_DN15276_c1_g1_i6::g.49461::m.49461	368020	336710	958120	858440	928070	292640	1258400	1362200	989670	1188500	3797700	2640500	4656700	5631800	1021200
TRINITY_DN18360_c0_g2::TRINITY_DN18360_c0_g2_i3::g.99190::m.99190	476530	34297	626180	555180	0	1387600	832090	2691900	3136100	1808300	2120900	3432200	1971200	3569200	3406600
TRINITY_DN12711_c1_g1::TRINITY_DN12711_c1_g1_i2::g.15227::m.15227	628170	392260	953680	771110	1958400	623850	1359600	713120	0	748120	1806300	2816600	4858900	4748000	3512500
TRINITY_DN15771_c0_g2::TRINITY_DN15771_c0_g2_i3::g.56846::m.56846	169060	68231	434930	215130	0	189660	0	295450	385910	0	0	245870	5993300	5653800	12089000
TRINITY_DN12201_c0_g1::TRINITY_DN12201_c0_g1_i3::g.10701::m.10701	62428	0	20656	31270	244960	133430	0	0	0	0	0	1127200	6957900	8139500	9009500
TRINITY_DN13903_c0_g3::TRINITY_DN13903_c0_g3_i6::g.30258::m.30258	346460	20506	270860	771370	0	267510	388750	80457	2589900	0	999950	1188200	5151900	7405900	6150000
TRINITY_DN16875_c1_g2::TRINITY_DN16875_c1_g2_i1::g.74175::m.74175	648560	89614	1047700	632270	899600	869760	1895700	927170	1590200	309160	4074400	5380800	2265700	3082500	1866900
TRINITY_DN12880_c1_g2::TRINITY_DN12880_c1_g2_i3::g.16816::m.16816	0	0	0	0	0	0	0	0	0	0	0	0	8623500	6656300	10294000
TRINITY_DN12853_c0_g1::TRINITY_DN12853_c0_g1_i3::g.16604::m.16604	0	0	0	0	0	0	0	0	0	0	0	0	6609300	10237000	8591300
TRINITY_DN15811_c0_g2::TRINITY_DN15811_c0_g2_i2::g.57329::m.57329	16471	0	86595	82763	320850	0	0	0	158960	0	227260	481850	7212400	8959100	7718500
TRINITY_DN12588_c4_g1::TRINITY_DN12588_c4_g1_i6::g.14071::m.14071	171210	0	194340	166340	1684900	0	1029500	1172000	1280500	0	3122000	7173800	2935500	3289800	2860700
TRINITY_DN18157_c0_g1::TRINITY_DN18157_c0_g1_i6::g.96079::m.96079	1043500	274120	1058700	938760	601520	328740	263230	675830	2140200	0	3188100	4385500	2827600	3965900	3361000
TRINITY_DN18306_c2_g1::TRINITY_DN18306_c2_g1_i6::g.98737::m.98737	1461900	648530	1461500	4699100	2262800	0	978720	2920800	0	3537300	6945600	0	125980	0	0
TRINITY_DN17921_c2_g1::TRINITY_DN17921_c2_g1_i7::g.92112::m.92112	1151400	300830	1104400	1385800	1135700	355380	811950	917460	1514400	343360	1222600	2662000	1792000	5728900	4584900
TRINITY_DN15585_c1_g1::TRINITY_DN15585_c1_g1_i4::g.54306::m.54306	1520700	69619	1679800	1509000	331060	385830	385080	948340	1790400	484900	2569400	7938700	1108800	1688500	2436100
TRINITY_DN12563_c5_g1::TRINITY_DN12563_c5_g1_i1::g.13894::m.13894	46644	0	82873	67216	0	0	0	0	0	101710	0	0	6136600	11551000	6835900
TRINITY_DN14141_c0_g1::TRINITY_DN14141_c0_g1_i5::g.32827::m.32827	0	0	0	0	0	0	0	0	0	0	0	0	7763700	7448100	9585900
TRINITY_DN19864_c2_g6::TRINITY_DN19864_c2_g6_i4::g.124630::m.124630	439750	389190	1342500	1378000	1512300	478960	1759300	1229400	2217600	1917900	5618100	4783700	703230	821460	203960

TRINITY_DN15673_c2_g2::TRINITY_DN15673_c2_g2_i2::g.55381::m.55381	0	0	0	0	0	0	0	0	0	0	248600	168560	11158000	4485000	8702400
TRINITY_DN15521_c0_g1::TRINITY_DN15521_c0_g1_i5::g.53143::m.53143	175140	44185	138830	100500	0	0	0	0	0	0	452120	0	10157000	5677800	7994500
TRINITY_DN14108_c0_g1::TRINITY_DN14108_c0_g1_i6::g.32445::m.32445	79249	0	764260	0	834910	96791	511840	218910	3115000	2498200	3975200	3043200	3142700	2572800	3495200
TRINITY_DN13599_c0_g2::TRINITY_DN13599_c0_g2_i3::g.24684::m.24684	619480	220400	595030	648730	580540	0	0	610140	0	0	0	0	5829100	9899300	5203300
TRINITY_DN12431_c0_g1::TRINITY_DN12431_c0_g1_i5::g.12785::m.12785	0	0	0	0	0	0	239100	0	0	0	456330	0	8881200	5261000	9271400
TRINITY_DN15315_c0_g3::TRINITY_DN15315_c0_g3_i4::g.50023::m.50023	191980	34397	254070	237800	297640	0	141550	61790	236770	105720	546920	933440	6126100	7801300	7099600
TRINITY_DN13825_c0_g1::TRINITY_DN13825_c0_g1_i1::g.28844::m.28844	0	0	0	0	0	0	3040100	1819200	0	3583800	6956800	8113900	0	0	417710
TRINITY_DN11213_c0_g1::TRINITY_DN11213_c0_g1_i2::g.6642::m.6642	0	0	0	0	340140	0	73750	0	0	0	355770	0	9147700	6346200	7658000
TRINITY_DN19530_c3_g1::TRINITY_DN19530_c3_g1_i3::g.119253::m.119253	0	0	0	0	0	0	0	0	0	0	0	0	8153100	7219300	8370800
TRINITY_DN16101_c1_g1::TRINITY_DN16101_c1_g1_i2::g.61978::m.61978	0	0	0	0	0	0	0	0	0	0	111990	0	7786500	7863900	7861000
TRINITY_DN16024_c0_g2::TRINITY_DN16024_c0_g2_i5::g.60694::m.60694	26407	0	0	58595	0	0	0	0	0	0	0	0	7419900	11065000	5020500
TRINITY_DN11097_c0_g1::TRINITY_DN11097_c0_g1_i1::g.6303::m.6303	200890	21513	583300	164260	453530	378250	1622800	405770	1216200	181790	4423600	5199700	3035700	4128900	1525100
TRINITY_DN18581_c2_g2::TRINITY_DN18581_c2_g2_i8::g.102871::m.102871	0	1154500	3592600	0	0	0	1475200	1046800	3167900	0	9045000	3444400	0	462290	0
TRINITY_DN15963_c2_g1::TRINITY_DN15963_c2_g1_i4::g.59609::m.59609	564170	250060	858070	668810	2224700	1166700	0	1733800	6150900	1266500	2557000	4861100	189470	445930	322000
TRINITY_DN18878_c2_g2::TRINITY_DN18878_c2_g2_i9::g.108021::m.108021	23147000	0	0	0	0	0	0	0	0	0	0	0	0	0	83674
TRINITY_DN12332_c0_g1::TRINITY_DN12332_c0_g1_i2::g.11982::m.11982	518180	378670	788870	527070	1837600	192020	2125400	1603800	2384100	2210000	4610600	4712800	0	1028700	298200
TRINITY_DN18378_c3_g4::TRINITY_DN18378_c3_g4_i1::g.99575::m.99575	0	0	0	0	0	0	0	152970	0	0	0	0	6817700	8296200	7933200
TRINITY_DN11637_c0_g1::TRINITY_DN11637_c0_g1_i3::g.8078::m.8078	651620	81412	162420	139640	0	309130	724500	0	1877600	1084400	0	1249300	3697200	6343000	6859600
TRINITY_DN16699_c3_g1::TRINITY_DN16699_c3_g1_i15::g.70256::m.70256	0	0	0	0	0	0	0	0	0	0	0	0	7110200	8183200	7872400
TRINITY_DN14704_c0_g1::TRINITY_DN14704_c0_g1_i5::g.40889::m.40889	1020500	293060	1085400	713860	679190	556790	813590	1070600	4924200	3271200	3117000	5601800	0	0	0
TRINITY_DN15232_c2_g1::TRINITY_DN15232_c2_g1_i5::g.49050::m.49050	0	0	0	0	0	0	0	0	125750	0	0	0	5934500	8891800	8127200

TRINITY_DN16447_c0_g1::TRINITY_DN16447_c0_g1_i6::g.67050::m.67050	0	0	0	0	0	0	0	0	0	0	0	0	7702800	5024800	10351000
TRINITY_DN18433_c1_g3::TRINITY_DN18433_c1_g3_i2::g.100387::m.100387	721330	0	1361900	1083600	0	60824	772560	161570	5939000	1645700	6677000	739970	1455200	1426600	949160
TRINITY_DN15610_c2_g1::TRINITY_DN15610_c2_g1_i5::g.54395::m.54395	0	0	0	0	0	0	0	0	0	0	0	0	7639700	8891800	6391700
TRINITY_DN17553_c1_g1::TRINITY_DN17553_c1_g1_i2::g.85833::m.85833	4662300	2089700	5913500	6487400	0	0	0	3513600	0	0	0	0	0	0	237900
TRINITY_DN16227_c0_g1::TRINITY_DN16227_c0_g1_i8::g.64252::m.64252	951850	229420	1302100	785760	1894300	796050	2434500	1569400	2130300	470960	771040	6173300	1371800	206380	1780000
TRINITY_DN18179_c2_g1::TRINITY_DN18179_c2_g1_i5::g.96397::m.96397	873010	0	158370	280840	583340	497260	795500	432230	1205000	927620	1629000	2716700	4026200	4809100	3878400
TRINITY_DN13194_c3_g1::TRINITY_DN13194_c3_g1_i1::g.20967::m.20967	0	0	0	0	0	0	0	0	0	0	0	0	5707200	9477800	7592900
TRINITY_DN15924_c1_g1::TRINITY_DN15924_c1_g1_i2::g.59050::m.59050	701360	224300	592290	629040	853400	662240	1015500	1683300	1039900	1854800	1490200	2667300	2280800	3515700	3496900
TRINITY_DN13535_c5_g1::TRINITY_DN13535_c5_g1_i1::g.25172::m.25172	1276800	270990	1159800	1259900	1191100	0	1326100	1122200	0	0	0	0	3947300	6386300	4552300
TRINITY_DN12971_c4_g2::TRINITY_DN12971_c4_g2_i4::g.17979::m.17979	211430	88386	157850	168050	0	0	154980	0	0	178670	437270	0	5178100	8339400	7530700
TRINITY_DN17406_c4_g4::TRINITY_DN17406_c4_g4_i2::g.83246::m.83246	170720	0	174270	113530	242520	105800	290480	306910	0	123400	0	470010	6189500	6671300	7526900
TRINITY_DN14711_c0_g2::TRINITY_DN14711_c0_g2_i2::g.40961::m.40961	0	0	0	0	0	0	0	0	0	0	0	0	6403300	6810200	9165600
TRINITY_DN12314_c0_g1::TRINITY_DN12314_c0_g1_i2::g.11797::m.11797	579730	346060	1193800	629390	1391000	0	1976100	487940	2704100	626260	4528300	2389100	2172000	2249300	1024700
TRINITY_DN19972_c2_g1::TRINITY_DN19972_c2_g1_i3::g.126916::m.126916	552380	0	159400	622930	0	0	271920	0	1494800	0	1324500	11545000	452210	2659500	3170600
TRINITY_DN12468_c0_g1::TRINITY_DN12468_c0_g1_i1::g.13082::m.13082	167260	154700	127610	216340	0	0	0	0	265030	0	173430	0	5468800	9280600	6385400
TRINITY_DN16038_c1_g1::TRINITY_DN16038_c1_g1_i5::g.61002::m.61002	0	0	0	0	0	0	0	0	0	0	0	0	6343600	8506900	7356800
TRINITY_DN14600_c0_g1::TRINITY_DN14600_c0_g1_i7::g.38565::m.38565	0	0	370050	0	0	0	0	0	0	0	117660	0	5674300	9809500	6133400
TRINITY_DN12693_c0_g1::TRINITY_DN12693_c0_g1_i3::g.14940::m.14940	1595700	805000	2417200	2158300	2122600	800220	0	1702700	0	0	6690100	0	0	1969600	1836800
TRINITY_DN12016_c0_g1::TRINITY_DN12016_c0_g1_i3::g.10082::m.10082	0	0	0	0	0	0	0	0	0	0	243830	0	6017400	6100700	9686700
TRINITY_DN12640_c0_g1::TRINITY_DN12640_c0_g1_i4::g.14587::m.14587	142730	56007	0	75961	1934100	539220	2199000	661230	3412700	1265700	3233000	2768200	1942900	1677400	1895900
TRINITY_DN18427_c1_g2::TRINITY_DN18427_c1_g2_i5::g.100438::m.100438	188320	0	128690	157880	0	0	0	0	0	0	0	0	4913000	6830600	9541400

TRINITY_DN19518_c0_g1::TRINITY_DN19518_c0_g1_i2::g.119121::m.1191	1374000	0	1343200	1118000	1351900	254950	2551900	2257900	1184400	1480600	4656700	2264000	407590	1264700	213860
TRINITY_DN17623_c2_g1::TRINITY_DN17623_c2_g1_i4::g.87423::m.87423	2008600	928140	1804600	1274100	1727700	0	2471200	1663500	3236600	0	3301800	2962100	0	0	0
TRINITY_DN14810_c0_g2::TRINITY_DN14810_c0_g2_i2::g.42832::m.42832	155950	153230	93871	752410	640540	0	1036000	796970	1960800	1774500	5004900	4977300	528540	674200	2694100
TRINITY_DN12250_c0_g1::TRINITY_DN12250_c0_g1_i2::g.11365::m.11365	0	0	0	0	0	0	0	0	0	0	0	0	6165800	7453600	7538900
TRINITY_DN16420_c0_g1::TRINITY_DN16420_c0_g1_i2::g.67378::m.67378	956410	0	1204800	1139900	577230	349680	1470300	1077900	0	0	0	4330000	3326300	5169000	1441500
TRINITY_DN16668_c3_g1::TRINITY_DN16668_c3_g1_i1::g.71264::m.71264	145920	60150	0	203580	0	0	0	0	0	373750	518890	1023400	5246900	6583300	6865700
TRINITY_DN18663_c1_g1::TRINITY_DN18663_c1_g1_i1::g.104197::m.1041	514310	350600	834710	482670	0	0	584470	0	3957000	0	6953100	3219100	1866400	563490	1687400
TRINITY_DN16719_c1_g1::TRINITY_DN16719_c1_g1_i3::g.72216::m.72216	0	0	0	0	0	0	0	0	0	0	249030	0	6156000	9363500	5210700
TRINITY_DN17040_c1_g7::TRINITY_DN17040_c1_g7_i1::g.77416::m.77416	958990	408380	1230900	1194200	0	0	0	1630900	0	0	2671300	0	3916800	4438900	4483300
TRINITY_DN14578_c1_g1::TRINITY_DN14578_c1_g1_i1::g.39115::m.39115	0	0	0	0	0	0	0	0	0	0	144990	3846200	3327500	6072600	7533300
TRINITY_DN14089_c0_g1::TRINITY_DN14089_c0_g1_i6::g.32232::m.32232	60842	0	28592	64220	0	0	0	371250	0	407290	0	1743700	4867000	7347400	5988200
TRINITY_DN14378_c0_g2::TRINITY_DN14378_c0_g2_i6::g.36119::m.36119	0	0	0	0	0	0	0	0	0	0	168700	0	5120400	5737800	9804100
TRINITY_DN19372_c1_g1::TRINITY_DN19372_c1_g1_i2::g.116684::m.116684	200170	0	0	262850	254520	404800	658180	694100	2967500	1727600	5780000	4992000	860130	1445100	547090
TRINITY_DN13787_c3_g4::TRINITY_DN13787_c3_g4_i3::g.28262::m.28262	35525	0	0	0	0	0	0	0	0	0	0	0	7533200	7729500	5461400
TRINITY_DN18760_c9_g1::TRINITY_DN18760_c9_g1_i2::g.106072::m.106072	0	0	0	0	0	0	0	0	0	0	0	0	7454600	5903100	7335100
TRINITY_DN11053_c0_g1::TRINITY_DN11053_c0_g1_i3::g.6092::m.6092	0	0	0	0	0	0	0	0	0	0	0	0	5469600	8835300	6290400
TRINITY_DN13145_c0_g1::TRINITY_DN13145_c0_g1_i9::g.20440::m.20440	0	0	0	0	0	0	0	0	0	0	0	0	5736600	5366400	9455800
TRINITY_DN16756_c2_g7::TRINITY_DN16756_c2_g7_i3::g.72811::m.72811	0	0	0	0	0	0	142020	97975	405680	476530	0	0	3358600	8709600	7326100
TRINITY_DN12713_c0_g2::TRINITY_DN12713_c0_g2_i2::g.15182::m.15182	1313200	0	0	1257700	0	0	0	1352300	4288000	0	5321600	5707900	427280	119380	513730
TRINITY_DN13140_c0_g1::TRINITY_DN13140_c0_g1_i10::g.20388::m.20388	0	0	0	0	0	0	0	0	0	0	0	0	6434900	7973600	5847400
TRINITY_DN16168_c0_g1::TRINITY_DN16168_c0_g1_i6::g.63149::m.63149	36485	0	0	0	0	0	0	0	0	0	75585	203670	6353700	5508500	7963200

TRINITY_DN18151_c0_g1::TRINITY_DN18151_c0_g1_i1::g.95922::m.95922	124290	23274	88192	0	202980	142320	464780	0	249530	0	416110	0	4649000	10309000	3470400
TRINITY_DN13594_c1_g2::TRINITY_DN13594_c1_g2_i6::g.26004::m.26004	903860	351890	1067600	721310	426520	288090	1467100	896710	1399700	1168900	5495700	4029800	0	864930	928000
TRINITY_DN16842_c1_g6::TRINITY_DN16842_c1_g6_i1::g.74458::m.74458	515720	58553	812170	383130	95971	1082800	2515400	789030	3984600	0	2466700	7247800	0	0	0
TRINITY_DN16487_c1_g1::TRINITY_DN16487_c1_g1_i9::g.68393::m.68393	0	0	0	0	0	0	0	0	0	0	0	0	6151300	5814700	7905900
TRINITY_DN17142_c0_g1::TRINITY_DN17142_c0_g1_i3::g.79276::m.79276	481210	322890	1116100	760540	2485300	664450	2514800	2575100	574930	834220	2938000	4369800	0	0	160470
TRINITY_DN18242_c1_g1::TRINITY_DN18242_c1_g1_i1::g.97405::m.97405	177520	0	209700	161060	1698800	1115300	2382200	1415200	1907300	1710600	3880700	3232400	308980	388520	1187000
TRINITY_DN18764_c1_g1::TRINITY_DN18764_c1_g1_i6::g.106059::m.106059	632070	46082	1424600	1248600	874490	0	1453400	788890	3608500	772000	2727100	6192700	0	0	0
TRINITY_DN15799_c0_g1::TRINITY_DN15799_c0_g1_i1::g.55952::m.55952	111750	61476	166580	102230	0	384300	1454800	1769900	2970300	2557400	2067000	4399400	100230	1762900	1809700
TRINITY_DN12324_c0_g2::TRINITY_DN12324_c0_g2_i2::g.11908::m.11908	0	0	0	0	0	0	0	0	0	0	0	0	6057800	5815000	7796700
TRINITY_DN14323_c0_g1::TRINITY_DN14323_c0_g1_i2::g.35566::m.35566	71428	0	59676	196490	165270	84239	0	0	0	0	0	682890	5494100	6383200	6421400
TRINITY_DN13653_c3_g1::TRINITY_DN13653_c3_g1_i1::g.26703::m.26703	0	0	0	0	0	0	0	0	0	0	0	0	7157200	6942900	5445100
TRINITY_DN20011_c2_g2::TRINITY_DN20011_c2_g2_i1::g.127827::m.127827	0	0	41031	0	0	0	0	0	309460	0	0	0	4521600	5915600	8756000
TRINITY_DN14539_c1_g2::TRINITY_DN14539_c1_g2_i5::g.38538::m.38538	0	0	0	0	0	0	0	0	0	0	0	0	4419200	7742000	7251500
TRINITY_DN15165_c0_g1::TRINITY_DN15165_c0_g1_i4::g.47616::m.47616	0	0	0	0	0	0	0	0	0	0	0	0	5818400	6927100	6636300
TRINITY_DN12436_c4_g2::TRINITY_DN12436_c4_g2_i4::g.12996::m.12996	49462	0	43585	0	0	0	0	0	0	0	0	0	7950900	4555800	6771900
TRINITY_DN14508_c8_g1::TRINITY_DN14508_c8_g1_i8::g.38378::m.38378	727600	118580	751370	314010	447720	817540	1926600	1822200	766500	1897000	4168000	4596300	703540	253290	0
TRINITY_DN14731_c0_g4::TRINITY_DN14731_c0_g4_i2::g.41282::m.41282	112110	33393	110410	92520	239980	132580	273690	206770	493520	439810	949270	732060	4507900	5216900	5750500
TRINITY_DN15108_c2_g1::TRINITY_DN15108_c2_g1_i5::g.47064::m.47064	0	0	0	0	0	0	0	0	0	0	0	0	5324400	7257800	6634700
TRINITY_DN15133_c0_g1::TRINITY_DN15133_c0_g1_i1::g.47252::m.47252	134400	65413	507680	143320	1014700	487550	2165000	1931900	3195900	882820	1702700	3026900	1618700	1599500	687250
TRINITY_DN13082_c0_g1::TRINITY_DN13082_c0_g1_i2::g.19429::m.19429	232990	95154	303660	263910	1354900	279630	1099800	721680	1427800	2899200	5021000	4831600	271550	0	341760

TRINITY_DN19812_c4_g2::TRINITY_DN19812_c4_g2_i2::g.124055::m.124055	0	0	124550	0	26232	0	0	0	0	0	3504200	3755400	3938900	4140600	3634100
TRINITY_DN13916_c3_g3::TRINITY_DN13916_c3_g3_i3::g.30403::m.30403	0	0	0	0	0	0	0	0	0	0	0	0	4509700	6205200	8288100
TRINITY_DN17369_c1_g1::TRINITY_DN17369_c1_g1_i7::g.82610::m.82610	521730	132270	417150	455800	0	63494	0	105740	271670	0	689940	299130	5978200	4952100	5105200
TRINITY_DN11073_c0_g1::TRINITY_DN11073_c0_g1_i1::g.6247::m.6247	0	0	0	0	0	0	0	0	0	0	0	0	2668200	10325000	5947300
TRINITY_DN17221_c0_g2::TRINITY_DN17221_c0_g2_i11::g.80299::m.80299	253650	26033	1092400	1075000	780800	144200	0	786060	0	0	2959000	3086000	3451700	1957900	3316800
TRINITY_DN13021_c1_g2::TRINITY_DN13021_c1_g2_i3::g.18779::m.18779	531240	177940	603140	555010	361670	0	311570	973240	3857400	1894900	4750600	4897400	0	0	0
TRINITY_DN19942_c0_g1::TRINITY_DN19942_c0_g1_i1::g.126320::m.126320	0	0	0	0	0	0	0	0	0	0	0	0	5532100	5867300	7495500
TRINITY_DN13485_c2_g3::TRINITY_DN13485_c2_g3_i2::g.24428::m.24428	154540	61426	120600	106770	462140	129740	260600	215150	862850	296790	1306800	2249100	4109900	4785800	3720000
TRINITY_DN15741_c2_g1::TRINITY_DN15741_c2_g1_i4::g.55925::m.55925	0	0	0	0	158050	0	0	2097000	4981900	3725100	4207100	3575800	0	0	0
TRINITY_DN18489_c2_g1::TRINITY_DN18489_c2_g1_i22::g.101262::m.101262	68091	52101	5736400	6078100	336690	0	408600	588320	0	0	1236400	1226300	793270	867300	1217800
TRINITY_DN13122_c2_g4::TRINITY_DN13122_c2_g4_i4::g.20179::m.20179	0	0	0	0	0	0	0	0	0	0	0	0	4447500	7559200	6595500
TRINITY_DN19434_c2_g3::TRINITY_DN19434_c2_g3_i2::g.117952::m.117952	0	0	0	0	0	0	0	0	0	0	0	0	6529100	4930600	7104800
TRINITY_DN16406_c2_g3::TRINITY_DN16406_c2_g3_i1::g.67214::m.67214	0	0	0	0	0	0	0	0	0	0	0	0	6143800	3323600	9071800
TRINITY_DN11854_c0_g1::TRINITY_DN11854_c0_g1_i1::g.9190::m.9190	465370	193600	458560	412350	1625100	776590	1261600	928440	2217700	773490	4302000	3955100	296010	348820	467690
TRINITY_DN13288_c0_g3::TRINITY_DN13288_c0_g3_i3::g.22072::m.22072	852800	322150	1084500	815850	621960	168070	1136500	411710	2044400	1025000	6627100	3054300	0	0	287670
TRINITY_DN15507_c1_g1::TRINITY_DN15507_c1_g1_i1::g.53015::m.53015	0	0	0	0	0	0	0	0	0	0	0	0	1896600	9720200	6774300
TRINITY_DN15130_c6_g3::TRINITY_DN15130_c6_g3_i3::g.47307::m.47307	737680	90878	647150	258190	749210	197030	324970	425290	1714100	2072200	3614900	3682000	1262100	1679100	931930
TRINITY_DN15473_c0_g1::TRINITY_DN15473_c0_g1_i4::g.52564::m.52564	1231600	110920	475740	1134000	2112300	1363700	0	0	845220	0	4700400	6029500	0	184520	172300
TRINITY_DN18360_c0_g5::TRINITY_DN18360_c0_g5_i1::g.99194::m.99194	93346	34741	92212	98108	0	101650	213990	166470	5134400	827470	4334100	5333000	913070	0	1006700
TRINITY_DN18610_c1_g2::TRINITY_DN18610_c1_g2_i3::g.103387::m.103387	276440	0	187360	301800	0	0	195830	369960	518530	0	1194900	893800	5002200	3465900	5927100

TRINITY_DN19948_c1_g1::TRINITY_DN19948_c1_g1_i7::g.126477::m.126477	949410	284800	1053500	1331100	790730	0	1036600	1023800	1553400	0	2122600	655020	0	3911300	3620300
TRINITY_DN15732_c1_g2::TRINITY_DN15732_c1_g2_i3::g.56333::m.56333	134320	0	78337	118060	0	0	0	0	174180	0	0	225330	3567800	6304900	7723600
TRINITY_DN19268_c1_g1::TRINITY_DN19268_c1_g1_i2::g.114841::m.1148	0	0	0	0	0	0	0	0	0	0	0	0	3892900	6699500	7669800
TRINITY_DN15694_c2_g2::TRINITY_DN15694_c2_g2_i2::g.54517::m.54517	0	0	0	0	0	0	0	0	0	0	0	0	4467100	6450400	7322600
TRINITY_DN13820_c2_g1::TRINITY_DN13820_c2_g1_i2::g.28694::m.28694	169580	64812	145060	138500	181620	193750	465180	271460	0	0	0	621640	4073700	6746100	5088200
TRINITY_DN18889_c2_g1::TRINITY_DN18889_c2_g1_i17::g.108140::m.108140	856120	47755	934160	740790	0	209320	3743400	491810	589750	395420	1884100	4305500	2072900	1019300	865000
TRINITY_DN14517_c1_g1::TRINITY_DN14517_c1_g1_i15::g.38263::m.38263	488130	184510	179060	363300	0	0	0	0	0	0	6395200	6320700	1823600	1407400	962220
TRINITY_DN15211_c1_g1::TRINITY_DN15211_c1_g1_i7::g.48526::m.48526	225570	144540	879400	434260	0	0	1432600	1213500	0	1322400	5113000	5634400	478500	574220	649840
TRINITY_DN19169_c1_g1::TRINITY_DN19169_c1_g1_i1::g.113168::m.1131	2255000	0	1703600	1343500	0	137420	0	321780	1540800	968390	1735300	1930900	1927800	2036900	2031700
TRINITY_DN18383_c2_g3::TRINITY_DN18383_c2_g3_i1::g.99654::m.99654	460130	207420	600450	742770	0	79853	0	104590	305300	248130	964320	725430	3860900	5588700	3940000
TRINITY_DN14308_c0_g1::TRINITY_DN14308_c0_g1_i1::g.35383::m.35383	0	0	0	0	263040	436120	334190	221560	1877000	0	5909300	2888400	1329400	2425500	2003600
TRINITY_DN13126_c1_g2::TRINITY_DN13126_c1_g2_i1::g.20156::m.20156	2600000	0	3065100	2616600	0	0	1091700	0	1727600	904800	2911300	0	1266500	0	1499100
TRINITY_DN18038_c0_g1::TRINITY_DN18038_c0_g1_i7::g.94082::m.94082	0	0	0	0	79914	0	0	0	150020	0	0	0	4809300	4828800	7815100
TRINITY_DN14696_c0_g1::TRINITY_DN14696_c0_g1_i2::g.40666::m.40666	376330	162050	487870	395140	1618900	1068300	2193200	1595700	2689400	2157900	3538000	0	587460	443860	358780
TRINITY_DN15303_c0_g1::TRINITY_DN15303_c0_g1_i2::g.49908::m.49908	424730	232200	621980	190740	2435500	541150	1169400	1340400	1060100	570510	2373000	3840800	541120	1340900	921920
TRINITY_DN15702_c0_g4::TRINITY_DN15702_c0_g4_i1::g.55937::m.55937	0	0	0	0	0	0	0	0	0	0	0	0	3625100	11263000	2661500
TRINITY_DN13427_c0_g2::TRINITY_DN13427_c0_g2_i5::g.23653::m.23653	0	0	53540	0	0	0	0	0	0	0	0	0	1749500	8856200	6868200
TRINITY_DN19814_c1_g2::TRINITY_DN19814_c1_g2_i2::g.124101::m.1241	281560	96749	275150	316900	351080	0	1034600	418670	0	0	1291900	2295300	4332600	5076100	1750400
TRINITY_DN12264_c2_g2::TRINITY_DN12264_c2_g2_i2::g.11522::m.11522	254610	71743	292150	188530	1702900	1316200	1758500	228810	1022100	207490	5268700	1881700	1013700	548820	1605100
TRINITY_DN13465_c1_g2::TRINITY_DN13465_c1_g2_i1::g.24163::m.24163	389600	132670	957900	1056900	935790	0	441530	658560	832930	550440	2216200	6218300	1281400	606960	1062800

TRINITY_DN19983_c1_g1::TRINITY_DN19983_c1_g1_i9::g.127358::m.127358	372730	169990	473160	381180	725560	256530	439980	1049400	1588400	1909900	3197100	3748600	819510	868030	1202300
TRINITY_DN16964_c2_g5::TRINITY_DN16964_c2_g5_i4::g.76427::m.76427	0	0	0	0	0	0	0	0	0	0	0	0	6068600	5008300	6076400
TRINITY_DN17558_c0_g1::TRINITY_DN17558_c0_g1_i9::g.85984::m.85984	0	0	0	0	0	0	0	4481600	0	0	0	0	4926200	2781400	4963200
TRINITY_DN17017_c2_g1::TRINITY_DN17017_c2_g1_i7::g.77030::m.77030	926510	467060	1174200	1176300	321670	235130	1593500	846960	1046000	611220	3085200	4835600	0	551910	270990
TRINITY_DN14717_c1_g1::TRINITY_DN14717_c1_g1_i9::g.42525::m.42525	0	0	0	0	0	0	0	0	0	0	0	0	3646700	7233000	6235500
TRINITY_DN13650_c8_g1::TRINITY_DN13650_c8_g1_i8::g.26099::m.26099	0	0	0	0	908870	606620	966480	900040	2759600	2107300	4698000	3852900	293470	0	0
TRINITY_DN18258_c0_g1::TRINITY_DN18258_c0_g1_i6::g.97657::m.97657	0	0	0	0	1074800	0	0	0	4010800	0	4562900	5903900	0	0	1514700
TRINITY_DN13419_c2_g1::TRINITY_DN13419_c2_g1_i6::g.23702::m.23702	0	0	0	0	0	0	0	0	0	0	0	0	5096700	6624100	5344900
TRINITY_DN18011_c2_g1::TRINITY_DN18011_c2_g1_i8::g.93702::m.93702	662840	217150	840470	1125800	1007200	198060	421180	1114500	0	0	1573800	4527400	603450	1909300	2833300
TRINITY_DN14942_c0_g1::TRINITY_DN14942_c0_g1_i6::g.44624::m.44624	3115200	2479800	6104300	5277700	0	0	0	0	0	0	0	0	0	0	0
TRINITY_DN17863_c0_g3::TRINITY_DN17863_c0_g3_i3::g.91035::m.91035	65325	0	0	0	247310	326250	798700	985670	1646300	1645600	2181300	968640	2083100	2812700	3167400
TRINITY_DN16933_c5_g1::TRINITY_DN16933_c5_g1_i5::g.75858::m.75858	0	0	0	0	117950	0	0	226610	260600	397520	249780	0	4437300	5347300	5886400
TRINITY_DN19887_c8_g1::TRINITY_DN19887_c8_g1_i5::g.125402::m.125402	1760800	288260	1683100	1607800	0	0	0	0	0	0	2612000	2479300	1883300	2128300	2452900
TRINITY_DN14777_c4_g4::TRINITY_DN14777_c4_g4_i3::g.42104::m.42104	0	0	0	0	1093000	1322800	0	3192900	1116100	5631100	0	0	1980100	1700300	857880
TRINITY_DN6579_c0_g1::TRINITY_DN6579_c0_g1_i1::g.1685::m.1685	510760	99654	480610	311300	0	0	0	0	1719700	0	0	2421900	3838600	5722100	1781400
TRINITY_DN16381_c2_g1::TRINITY_DN16381_c2_g1_i3::g.66693::m.66693	62439	0	157830	267640	0	0	79278	0	0	0	0	288540	4571500	5420400	5966100
TRINITY_DN14242_c1_g2::TRINITY_DN14242_c1_g2_i9::g.34423::m.34423	442150	0	342740	658670	830200	0	1702800	1879400	2965600	914330	1704500	3087500	1179600	738450	359530
TRINITY_DN18618_c2_g1::TRINITY_DN18618_c2_g1_i14::g.103865::m.103865	38699	0	0	0	0	0	0	0	223970	0	0	0	6095000	5886100	4549300
TRINITY_DN13619_c0_g1::TRINITY_DN13619_c0_g1_i2::g.26348::m.26348	273850	0	175510	323010	331790	230380	445940	557270	1036100	1546000	3021400	3271900	1379100	1850700	2336600
TRINITY_DN17578_c3_g1::TRINITY_DN17578_c3_g1_i5::g.85666::m.85666	0	0	0	0	0	0	0	0	0	142150	0	0	3608800	7325400	5678200

TRINITY_DN16993_c0_g2::TRINITY_DN16993_c0_g2_i2::g.76741::m.76741	851900	352150	1050100	849850	2053400	439340	2096500	999840	2657100	952520	2048400	2331400	0	0	0
TRINITY_DN13712_c0_g1::TRINITY_DN13712_c0_g1_i1::g.27487::m.27487	0	0	0	0	0	0	0	0	0	0	0	0	3653900	6608600	6296700
TRINITY_DN14406_c2_g1::TRINITY_DN14406_c2_g1_i7::g.36607::m.36607	156720	56274	294360	251860	242050	0	430940	225700	283920	386940	1259700	707100	4109500	3152200	4986500
TRINITY_DN18981_c5_g1::TRINITY_DN18981_c5_g1_i2::g.109832::m.109832	37127	0	961140	0	0	0	0	0	0	0	98382	0	6483500	3545800	5394900
TRINITY_DN13705_c0_g1::TRINITY_DN13705_c0_g1_i7::g.27449::m.27449	0	0	13558	0	0	0	0	0	0	0	0	0	5876100	6692700	3934800
TRINITY_DN17456_c0_g1::TRINITY_DN17456_c0_g1_i8::g.83943::m.83943	162300	78259	225070	180550	419040	0	0	516320	0	0	1286900	1422700	5463300	4577900	2161400
TRINITY_DN18817_c0_g1::TRINITY_DN18817_c0_g1_i10::g.107314::m.107314	163230	94082	528960	165450	343860	0	0	285160	0	0	0	0	6026000	6288800	2593800
TRINITY_DN19070_c1_g2::TRINITY_DN19070_c1_g2_i7::g.110348::m.110348	3519300	542240	1127900	987890	1205100	591730	993220	1361800	0	0	0	3257700	1454500	0	1442100
TRINITY_DN12832_c2_g6::TRINITY_DN12832_c2_g6_i1::g.16307::m.16307	0	0	0	0	0	0	0	0	0	0	0	0	4680200	6146400	5653300
TRINITY_DN14216_c0_g3::TRINITY_DN14216_c0_g3_i3::g.34216::m.34216	0	0	0	0	0	0	2380100	0	0	2640900	5035300	5262300	510390	0	641370
TRINITY_DN16423_c1_g3::TRINITY_DN16423_c1_g3_i3::g.67278::m.67278	178160	26662	237780	306620	989660	200850	1083600	1111000	641420	1567900	1147900	2754000	1689500	2688100	1841200
TRINITY_DN13521_c0_g4::TRINITY_DN13521_c0_g4_i1::g.24842::m.24842	45250	0	33213	44374	0	56300	0	0	0	896140	0	0	3427400	4860400	7087000
TRINITY_DN11068_c0_g1::TRINITY_DN11068_c0_g1_i1::g.6241::m.6241	0	0	0	57905	0	0	0	0	0	0	3507700	0	4845200	4475900	3541100
TRINITY_DN11631_c0_g1::TRINITY_DN11631_c0_g1_i1::g.8169::m.8169	272470	110450	347820	345470	562740	0	839950	778890	2816800	1781300	2720600	4239400	566870	430720	542000
TRINITY_DN17644_c1_g2::TRINITY_DN17644_c1_g2_i9::g.87544::m.87544	0	0	0	58364	0	0	173390	465110	0	0	774090	218850	4491300	4925400	5210800
TRINITY_DN16032_c0_g1::TRINITY_DN16032_c0_g1_i4::g.60901::m.60901	1655700	44634	433480	2197800	225990	0	0	282720	2938600	0	839530	974120	1823700	2560900	2329300
TRINITY_DN16763_c1_g3::TRINITY_DN16763_c1_g3_i3::g.72969::m.72969	0	0	0	0	0	0	0	0	0	0	0	0	5323300	6169800	4797100
TRINITY_DN13349_c1_g1::TRINITY_DN13349_c1_g1_i7::g.22584::m.22584	334040	0	341420	254020	0	145100	0	0	595880	0	0	0	5374200	6293800	2922200
TRINITY_DN14455_c0_g1::TRINITY_DN14455_c0_g1_i2::g.37451::m.37451	244210	121400	292780	350330	203010	0	0	0	563850	0	0	664810	4754800	4862700	4183600

TRINITY_DN19436_c2_g2::TRINITY_DN19436_c2_g2_i1::g.118043::m.118043	0	0	0	0	0	315230	1444200	1011000	0	2123300	1673500	5887200	151360	2241500	1379200
TRINITY_DN18302_c1_g2::TRINITY_DN18302_c1_g2_i2::g.98507::m.98507	548390	0	577510	528450	2754000	511150	0	1172800	4898700	2223300	0	2644600	323980	0	0
TRINITY_DN13203_c0_g1::TRINITY_DN13203_c0_g1_i1::g.21059::m.21059	1006000	392100	1682400	570780	656330	237250	0	641430	0	0	2297700	8318200	0	0	333790
TRINITY_DN13389_c0_g2::TRINITY_DN13389_c0_g2_i2::g.23122::m.23122	272510	104080	164160	194210	132540	105400	0	0	0	134430	636870	893030	5142700	2643500	5698900
TRINITY_DN15149_c3_g1::TRINITY_DN15149_c3_g1_i3::g.47576::m.47576	0	0	0	0	0	0	0	0	0	0	0	0	3746100	5532100	6744900
TRINITY_DN19596_c0_g2::TRINITY_DN19596_c0_g2_i1::g.120414::m.120414	419140	44339	175610	170280	0	0	226020	320790	0	338710	558710	441860	3987500	4260900	5052400
TRINITY_DN11870_c0_g1::TRINITY_DN11870_c0_g1_i2::g.9275::m.9275	0	0	0	0	0	0	0	0	0	0	0	0	6152500	3466600	6372400
TRINITY_DN18707_c2_g1::TRINITY_DN18707_c2_g1_i4::g.104948::m.104948	0	0	3567200	100390	184330	52185	0	219640	597360	346110	437630	0	2787500	5813200	1817900
TRINITY_DN18173_c2_g1::TRINITY_DN18173_c2_g1_i8::g.96624::m.96624	0	0	0	0	0	0	0	0	0	0	0	0	5116400	5779100	4945500
TRINITY_DN11898_c0_g1::TRINITY_DN11898_c0_g1_i2::g.9380::m.9380	234740	131140	526470	187860	1073200	503690	1316100	383490	1081500	1325600	2452200	2055900	1221200	966450	2325300
TRINITY_DN13113_c1_g1::TRINITY_DN13113_c1_g1_i4::g.20046::m.20046	0	0	0	0	0	0	0	0	0	0	0	0	4393100	5929400	5457900
TRINITY_DN17740_c0_g2::TRINITY_DN17740_c0_g2_i7::g.89045::m.89045	0	48752	62577	160940	0	0	434080	383830	224280	0	0	2952000	1713100	4895600	4866700
TRINITY_DN13674_c0_g1::TRINITY_DN13674_c0_g1_i2::g.26925::m.26925	0	0	0	22103	796500	0	129770	808700	0	0	128470	179120	2313800	6501400	4851900
TRINITY_DN17202_c2_g1::TRINITY_DN17202_c2_g1_i4::g.80033::m.80033	0	0	0	0	0	0	0	0	0	0	0	0	3576700	5945000	6209400
TRINITY_DN20008_c1_g1::TRINITY_DN20008_c1_g1_i8::g.127738::m.127738	71342	0	74526	60311	0	0	0	0	0	0	0	0	4315700	5947500	5257800
TRINITY_DN17814_c1_g2::TRINITY_DN17814_c1_g2_i3::g.90444::m.90444	480970	356190	1154000	753790	1224800	489560	1797500	702570	806520	246640	1437200	3986400	585170	1082600	570680
TRINITY_DN19612_c1_g2::TRINITY_DN19612_c1_g2_i9::g.120821::m.120821	0	0	0	0	0	0	0	0	0	0	0	0	4609100	5081700	5969600
TRINITY_DN13600_c1_g7::TRINITY_DN13600_c1_g7_i6::g.25108::m.25108	0	0	0	0	0	0	0	0	0	0	654330	0	1981900	8688100	4311600
TRINITY_DN18619_c0_g2::TRINITY_DN18619_c0_g2_i2::g.103437::m.103437	0	0	0	0	0	0	0	0	0	0	0	0	6034000	6497800	3073400
TRINITY_DN18061_c2_g3::TRINITY_DN18061_c2_g3_i1::g.94540::m.94540	0	0	0	0	0	0	0	0	0	0	0	0	3795400	3177100	8629100

TRINITY_DN15930_c2_g3::TRINITY_DN15930_c2_g3_i6::g.59212::m.59212	0	0	0	0	0	0	0	0	0	0	0	226940	3283800	6366400	5702200
TRINITY_DN14203_c2_g1::TRINITY_DN14203_c2_g1_i1::g.33817::m.33817	209090	111840	309320	224750	581880	176330	0	345660	0	0	341790	0	3345300	4313500	5608800
TRINITY_DN17074_c4_g2::TRINITY_DN17074_c4_g2_i4::g.77976::m.77976	0	0	0	0	0	0	0	0	0	0	0	0	5023100	6450600	4049100
TRINITY_DN17405_c1_g3::TRINITY_DN17405_c1_g3_i1::g.83287::m.83287	162650	0	716680	1116800	457320	546030	254170	356520	377290	147500	779120	3254900	1211100	3632400	2452500
TRINITY_DN16821_c3_g1::TRINITY_DN16821_c3_g1_i2::g.73875::m.73875	0	0	0	0	0	573890	0	1402800	4251600	0	6682400	0	1460000	492540	589350
TRINITY_DN13494_c0_g3::TRINITY_DN13494_c0_g3_i1::g.24527::m.24527	0	0	0	0	0	0	0	0	0	0	0	0	2248200	5715100	7458100
TRINITY_DN10893_c0_g1::TRINITY_DN10893_c0_g1_i1::g.5751::m.5751	1186900	421480	1451500	984600	1059500	0	699020	490740	3696900	1469700	1882800	2049700	0	0	0
TRINITY_DN16490_c2_g1::TRINITY_DN16490_c2_g1_i1::g.68401::m.68401	37858	0	48983	47433	0	0	238590	177320	289990	0	0	407710	5225100	4646000	4268100
TRINITY_DN16487_c1_g2::TRINITY_DN16487_c1_g2_i5::g.68395::m.68395	469520	319780	723240	645270	861580	0	449580	718080	181280	81880	0	0	2857600	4018800	4005800
TRINITY_DN17201_c0_g1::TRINITY_DN17201_c0_g1_i1::g.79917::m.79917	73574	0	47724	38680	0	0	0	0	0	280020	0	0	4872300	4299600	5623900
TRINITY_DN14870_c0_g1::TRINITY_DN14870_c0_g1_i2::g.43479::m.43479	68761	37543	111830	102540	195760	0	0	0	511260	240760	0	402490	3589700	3887400	6075000
TRINITY_DN14090_c0_g1::TRINITY_DN14090_c0_g1_i4::g.32240::m.32240	441940	159520	704290	613160	795700	0	263610	666150	1216000	1424000	918990	1668800	1235700	1989000	3074800
TRINITY_DN18902_c0_g1::TRINITY_DN18902_c0_g1_i9::g.108609::m.108609	1001100	226490	1636800	1514400	1119400	423540	2461800	1169700	475100	933840	1928500	2275200	0	0	0
TRINITY_DN19154_c1_g1::TRINITY_DN19154_c1_g1_i1::g.113024::m.1130	144310	39427	62024	0	0	0	0	0	0	0	0	0	3796900	8369200	2741500
TRINITY_DN14612_c0_g1::TRINITY_DN14612_c0_g1_i2::g.39550::m.39550	6495500	105730	1045800	663760	619210	201260	377770	382380	0	0	2517700	2492300	0	190030	0
TRINITY_DN16497_c0_g2::TRINITY_DN16497_c0_g2_i8::g.67601::m.67601	0	117380	312100	0	604800	222190	0	1001100	3583300	1175300	2477100	0	1854400	1997700	1696300
TRINITY_DN10818_c0_g1::TRINITY_DN10818_c0_g1_i2::g.5587::m.5587	380240	322960	813340	421420	3147400	149320	3391300	3096100	2121700	0	518290	602210	0	0	0
TRINITY_DN14143_c3_g2::TRINITY_DN14143_c3_g2_i2::g.32997::m.32997	192740	0	180630	314880	102250	0	0	403500	0	0	627110	1105700	3946800	3041600	5015400
TRINITY_DN16212_c0_g1::TRINITY_DN16212_c0_g1_i2::g.63955::m.63955	861360	190310	847360	850710	1059300	311930	0	830250	324790	0	2054800	2722500	705880	1952800	2182400
TRINITY_DN11356_c0_g1::TRINITY_DN11356_c0_g1_i1::g.7113::m.7113	0	0	0	0	0	0	0	0	0	0	0	0	3138000	7667500	4070400
TRINITY_DN15198_c1_g2::TRINITY_DN15198_c1_g2_i7::g.48337::m.48337	0	0	0	0	0	0	0	0	0	0	0	0	5441100	5632500	3799600

TRINITY_DN19436_c2_g3::TRINITY_DN19436_c2_g3_i1::g.118048::m.118048	404940	499840	1615500	1561500	1700400	316860	1185000	773980	1491600	0	756920	0	1908800	1276400	1372400
TRINITY_DN13757_c0_g1::TRINITY_DN13757_c0_g1_i4::g.28204::m.28204	0	0	0	0	0	0	0	0	0	269880	380840	0	3156000	4608200	6444600
TRINITY_DN14326_c0_g1::TRINITY_DN14326_c0_g1_i2::g.35500::m.35500	0	0	0	0	225490	0	0	407920	0	0	0	0	4351300	5189300	4637300
TRINITY_DN17638_c0_g5::TRINITY_DN17638_c0_g5_i1::g.87396::m.87396	114880	0	48114	108900	0	0	0	0	0	0	0	0	1733900	5589000	7212400
TRINITY_DN16307_c2_g1::TRINITY_DN16307_c2_g1_i9::g.65550::m.65550	0	0	0	0	0	0	0	0	0	0	0	0	5146700	5052100	4534500
TRINITY_DN18255_c1_g1::TRINITY_DN18255_c1_g1_i6::g.97743::m.97743	0	0	0	0	0	0	0	0	0	0	0	688900	4588000	4925300	4424800
TRINITY_DN18634_c2_g1::TRINITY_DN18634_c2_g1_i4::g.104214::m.104214	1012600	385240	1484200	1378700	136370	0	719980	0	1465300	327850	1724400	1693100	559470	1503800	2230200
TRINITY_DN12779_c0_g1::TRINITY_DN12779_c0_g1_i3::g.15740::m.15740	859740	0	133910	987850	852450	0	125430	0	0	123830	620150	0	2799400	3963200	4137300
TRINITY_DN15549_c2_g2::TRINITY_DN15549_c2_g2_i1::g.53640::m.53640	83987	25537	64674	73162	278580	82207	273190	208980	0	254040	540080	0	5757400	2120900	4834900
TRINITY_DN19606_c0_g1::TRINITY_DN19606_c0_g1_i15::g.121023::m.121023	192970	26304	212000	130670	385320	181840	583640	480700	0	0	1130000	0	2415100	5851300	2940700
TRINITY_DN16691_c0_g1::TRINITY_DN16691_c0_g1_i7::g.71695::m.71695	0	0	236180	244880	607210	0	821560	944560	1250300	1598400	1864000	2668900	0	2110100	2023600
TRINITY_DN16311_c1_g1::TRINITY_DN16311_c1_g1_i8::g.65782::m.65782	0	0	0	0	0	0	0	0	0	0	0	0	2915100	3473200	7953600
TRINITY_DN15482_c4_g1::TRINITY_DN15482_c4_g1_i5::g.52502::m.52502	59153	0	42095	0	0	0	0	0	0	0	0	0	4420400	4560300	5256000
TRINITY_DN15556_c2_g3::TRINITY_DN15556_c2_g3_i7::g.53767::m.53767	275090	34100	346230	280590	590160	407530	267740	0	0	673220	998900	2325200	3480700	2170800	2457600
TRINITY_DN12323_c0_g1::TRINITY_DN12323_c0_g1_i4::g.11964::m.11964	0	0	0	0	0	0	0	0	0	0	0	0	4256100	4654200	5382300
TRINITY_DN15236_c1_g2::TRINITY_DN15236_c1_g2_i4::g.49063::m.49063	596420	128940	0	571760	1110500	234260	621510	175000	695340	193540	0	726290	2229500	2543000	4456800
TRINITY_DN17457_c4_g1::TRINITY_DN17457_c4_g1_i4::g.83960::m.83960	955870	371610	1321600	952530	0	272360	490540	764280	804930	1109200	1181600	3254000	736800	1731900	312180
TRINITY_DN11580_c0_g1::TRINITY_DN11580_c0_g1_i1::g.7933::m.7933	445830	101280	440370	503980	1101600	0	0	148890	1030200	0	3496100	5989600	323990	0	643580
TRINITY_DN15072_c0_g1::TRINITY_DN15072_c0_g1_i2::g.46447::m.46447	452100	87934	41501	275320	0	0	463360	929950	3185100	1273300	2172100	1784000	1442700	96501	1944400
TRINITY_DN19781_c5_g1::TRINITY_DN19781_c5_g1_i1::g.123563::m.123563	0	0	0	0	0	0	0	0	0	0	207470	0	3979400	5882600	4048700

TRINITY_DN19199_c1_g1::TRINITY_DN19199_c1_g1_i2::g.113819::m.1138	0	0	0	0	0	0	0	0	0	0	0	0	0	14110000	0
TRINITY_DN11501_c0_g1::TRINITY_DN11501_c0_g1_i5::g.7580::m.7580	0	0	0	0	547250	555400	1357600	451100	4041600	793760	2120200	4127100	0	0	104070
TRINITY_DN18781_c0_g5::TRINITY_DN18781_c0_g5_i3::g.106209::m.106209	0	34426	0	0	0	0	259220	222230	0	140780	0	0	4528200	4713600	4180500
TRINITY_DN19854_c2_g2::TRINITY_DN19854_c2_g2_i3::g.124560::m.124560	0	0	0	0	0	0	0	0	0	0	0	0	4663800	4482300	4843400
TRINITY_DN14973_c1_g2::TRINITY_DN14973_c1_g2_i1::g.45015::m.45015	0	0	0	0	0	0	0	0	0	0	0	0	2809800	5322900	5843000
TRINITY_DN16462_c1_g1::TRINITY_DN16462_c1_g1_i7::g.68028::m.68028	617880	160520	0	636770	1130100	218040	1746800	1325900	988350	623270	2587600	1634200	795990	771180	696360
TRINITY_DN16061_c2_g1::TRINITY_DN16061_c2_g1_i1::g.61310::m.61310	823580	168870	962600	727550	332350	234460	1536600	1095300	467970	120950	1939600	0	1287700	2506200	1704300
TRINITY_DN15683_c2_g1::TRINITY_DN15683_c2_g1_i10::g.55639::m.55639	2948000	921330	3908900	3572000	0	0	0	2548400	0	0	0	0	0	0	0
TRINITY_DN11090_c0_g1::TRINITY_DN11090_c0_g1_i1::g.6289::m.6289	0	0	0	0	0	0	0	0	0	0	0	0	1773600	6431400	5685100
TRINITY_DN16191_c2_g1::TRINITY_DN16191_c2_g1_i7::g.63681::m.63681	587710	156210	869380	521450	536010	250550	312640	747430	0	876840	1998200	639130	1858300	1535700	2978100
TRINITY_DN19075_c0_g2::TRINITY_DN19075_c0_g2_i2::g.111296::m.111296	0	0	87205	0	0	0	0	0	0	0	0	0	4834300	5625900	3289400
TRINITY_DN11919_c0_g1::TRINITY_DN11919_c0_g1_i1::g.9495::m.9495	714430	145880	444040	499080	0	0	749480	0	2288700	0	0	0	4217600	760040	3957900
TRINITY_DN17942_c1_g1::TRINITY_DN17942_c1_g1_i6::g.91683::m.91683	0	0	0	0	0	0	0	0	0	0	0	0	4052900	4062400	5610400
TRINITY_DN15935_c0_g1::TRINITY_DN15935_c0_g1_i1::g.59258::m.59258	171770	35302	207770	346380	0	178900	427690	400010	0	0	293320	0	3730700	4838700	3084900
TRINITY_DN16842_c1_g4::TRINITY_DN16842_c1_g4_i5::g.74459::m.74459	251150	88770	288420	188530	491930	200620	1196000	414640	1357500	493540	3005900	3272200	764960	1060700	507460
TRINITY_DN14360_c0_g2::TRINITY_DN14360_c0_g2_i2::g.35887::m.35887	45908	0	0	0	63488	0	0	0	0	0	0	191640	3038100	3716100	6516700
TRINITY_DN13161_c0_g1::TRINITY_DN13161_c0_g1_i3::g.20601::m.20601	28100	0	0	63561	0	0	0	1988100	5994900	3966900	1518000	0	0	0	0
TRINITY_DN17755_c1_g2::TRINITY_DN17755_c1_g2_i5::g.89168::m.89168	50593	0	36702	0	0	0	103040	0	0	0	2934400	2875800	2381100	1038400	4084000
TRINITY_DN17545_c0_g1::TRINITY_DN17545_c0_g1_i5::g.84940::m.84940	0	0	0	0	0	0	0	0	67245	0	0	0	5203100	3444500	4776800
TRINITY_DN11691_c0_g1::TRINITY_DN11691_c0_g1_i1::g.8068::m.8068	0	0	0	0	0	0	0	0	0	0	0	0	2258700	5300000	5862800
TRINITY_DN14965_c0_g1::TRINITY_DN14965_c0_g1_i3::g.45244::m.45244	634910	35358	856640	694010	121880	193160	1001700	0	1668000	187780	3057400	0	1569900	1715500	1681800

TRINITY_DN17740_c0_g1::TRINITY_DN17740_c0_g1_i2::g.89044::m.89044	440190	163740	513380	463910	0	0	0	85191	298470	103390	0	1627100	3256400	2985100	3461700
TRINITY_DN16246_c0_g1::TRINITY_DN16246_c0_g1_i1::g.63895::m.63895	730220	111900	781460	771910	451930	502840	751850	868320	683240	0	3040600	2282100	340450	1252400	811010
TRINITY_DN18982_c0_g1::TRINITY_DN18982_c0_g1_i8::g.109877::m.109877	176290	22907	308470	276610	138290	0	530620	391240	0	245250	316650	3402200	1901700	3702100	1932600
TRINITY_DN13531_c1_g1::TRINITY_DN13531_c1_g1_i7::g.25242::m.25242	0	0	0	0	0	0	0	0	0	0	0	0	4319200	4958800	4034900
TRINITY_DN13348_c2_g1::TRINITY_DN13348_c2_g1_i3::g.22605::m.22605	0	0	0	0	0	0	0	0	0	0	0	0	3984200	5271500	4052000
TRINITY_DN19420_c3_g1::TRINITY_DN19420_c3_g1_i7::g.117669::m.117669	0	0	0	0	0	0	0	0	0	0	0	0	7143500	4895300	1247300
TRINITY_DN13350_c3_g1::TRINITY_DN13350_c3_g1_i5::g.22631::m.22631	0	0	0	0	0	0	0	0	0	0	0	0	3924000	4926300	4351300
TRINITY_DN16612_c3_g2::TRINITY_DN16612_c3_g2_i3::g.70349::m.70349	0	0	0	0	0	0	0	0	0	0	0	0	4109400	4300400	4788200
TRINITY_DN12310_c0_g1::TRINITY_DN12310_c0_g1_i3::g.11787::m.11787	1389800	534950	1607700	1389700	384020	434540	0	0	1273700	1114800	1941600	1964300	433570	507050	207370
TRINITY_DN14939_c1_g2::TRINITY_DN14939_c1_g2_i3::g.44685::m.44685	96177	0	0	489400	568410	500700	903290	0	2726200	0	0	7038200	0	358570	480910
TRINITY_DN10911_c0_g2::TRINITY_DN10911_c0_g2_i2::g.5805::m.5805	0	0	51769	47170	183400	0	0	0	445590	155170	579410	1397700	2966100	3943000	3368900
TRINITY_DN16718_c6_g1::TRINITY_DN16718_c6_g1_i4::g.72149::m.72149	84102	23206	125270	60816	661950	74851	454810	324050	2970000	1202300	2298800	4823900	0	0	0
TRINITY_DN16625_c2_g1::TRINITY_DN16625_c2_g1_i8::g.70673::m.70673	263380	124220	301440	314670	316880	164480	392790	552080	0	960500	3502400	2569600	1876800	1241800	485750
TRINITY_DN15294_c5_g1::TRINITY_DN15294_c5_g1_i4::g.49817::m.49817	38311	0	0	0	0	86749	0	157570	0	0	0	0	4505200	5076000	3136300
TRINITY_DN19873_c6_g8::TRINITY_DN19873_c6_g8_i6::g.124910::m.124910	0	0	0	0	0	0	0	0	0	0	0	0	2725600	6417200	3838200
TRINITY_DN15424_c2_g3::TRINITY_DN15424_c2_g3_i3::g.51667::m.51667	0	0	0	0	0	0	0	0	0	0	0	0	4420100	3054200	5464400
TRINITY_DN16774_c0_g1::TRINITY_DN16774_c0_g1_i9::g.73138::m.73138	657900	0	622510	442630	536610	0	0	211840	940640	675960	1955200	3428000	453890	1674500	1273900
TRINITY_DN17278_c1_g1::TRINITY_DN17278_c1_g1_i1::g.81211::m.81211	0	0	0	0	0	0	0	0	0	0	0	0	2008600	8762800	2050100
TRINITY_DN13302_c0_g2::TRINITY_DN13302_c0_g2_i1::g.22438::m.22438	82518	45681	109880	145340	0	0	0	0	0	0	0	899900	2353200	5425100	3712800
TRINITY_DN12343_c0_g1::TRINITY_DN12343_c0_g1_i5::g.11815::m.11815	0	0	0	0	0	0	0	0	0	0	0	0	4573200	3963500	4212500

TRINITY_DN16818_c0_g1::TRINITY_DN16818_c0_g1_i9::g.73983::m.73983	0	0	337950	0	365870	0	0	0	0	0	0	2204300	1663900	3549800	4600300
TRINITY_DN19014_c2_g1::TRINITY_DN19014_c2_g1_i4::g.110590::m.110590	0	0	0	0	0	0	0	0	0	112220	0	0	5653900	4482000	2461600
TRINITY_DN12914_c1_g1::TRINITY_DN12914_c1_g1_i4::g.17252::m.17252	204360	79898	174020	194430	170670	0	0	123240	0	0	855670	0	3858600	1988100	5019600
TRINITY_DN17100_c3_g1::TRINITY_DN17100_c3_g1_i1::g.78525::m.78525	0	0	0	0	0	0	0	0	0	0	0	0	2990200	3086300	6578900
TRINITY_DN14403_c4_g2::TRINITY_DN14403_c4_g2_i1::g.36526::m.36526	0	0	0	0	0	0	0	0	0	0	0	0	3475000	3956700	5211800
TRINITY_DN18412_c2_g2::TRINITY_DN18412_c2_g2_i2::g.100143::m.100143	0	0	0	0	0	0	0	0	0	0	0	0	3651700	2892100	6095700
TRINITY_DN14115_c3_g1::TRINITY_DN14115_c3_g1_i10::g.32650::m.32650	91070	0	132810	107870	0	0	0	0	477140	0	331800	0	2875400	4943300	3645400
TRINITY_DN15464_c0_g5::TRINITY_DN15464_c0_g5_i1::g.52274::m.52274	0	0	0	0	498040	0	0	0	3306800	1986600	0	3647200	0	3130800	0
TRINITY_DN15531_c1_g1::TRINITY_DN15531_c1_g1_i14::g.53405::m.53405	140730	139730	523090	117960	1096100	305560	1072500	1583500	0	835290	4316600	2167300	110580	148670	0
TRINITY_DN12587_c0_g1::TRINITY_DN12587_c0_g1_i1::g.13954::m.13954	631700	153200	708790	749860	148970	0	0	154150	862230	251780	750300	1056900	1741800	2495200	2845900
TRINITY_DN17254_c0_g1::TRINITY_DN17254_c0_g1_i5::g.81103::m.81103	105040	80586	130960	122680	272590	0	976590	590170	1633000	248180	0	1993100	1491800	3248600	1633700
TRINITY_DN17518_c0_g1::TRINITY_DN17518_c0_g1_i5::g.85741::m.85741	559530	0	439960	0	766290	0	1143600	841040	0	962490	1063500	0	2843400	3578700	311210
TRINITY_DN16163_c4_g1::TRINITY_DN16163_c4_g1_i1::g.63306::m.63306	170740	77813	301880	239840	365370	662750	1470100	1041400	1338200	0	2007600	871780	364600	2188900	1397200
TRINITY_DN16238_c3_g1::TRINITY_DN16238_c3_g1_i4::g.64390::m.64390	552670	323540	1006100	543290	266150	0	686990	548070	1402700	740080	1305200	2785600	793220	735460	794390
TRINITY_DN19244_c2_g1::TRINITY_DN19244_c2_g1_i18::g.114657::m.114657	0	0	0	0	0	0	0	0	0	0	0	0	4478200	3680600	4248100
TRINITY_DN19880_c6_g4::TRINITY_DN19880_c6_g4_i2::g.125001::m.125001	0	0	0	0	0	0	0	0	0	0	0	0	3356100	4576700	4465600
TRINITY_DN18615_c1_g3::TRINITY_DN18615_c1_g3_i2::g.103395::m.103395	165790	172370	717130	0	922180	0	0	0	2348200	1916500	3113300	3012200	0	0	0
TRINITY_DN15441_c0_g1::TRINITY_DN15441_c0_g1_i3::g.51487::m.51487	68364	0	68182	92779	199460	0	0	0	0	0	6842000	4479800	180650	204990	153370
TRINITY_DN16789_c2_g4::TRINITY_DN16789_c2_g4_i5::g.73232::m.73232	0	0	0	0	0	0	0	0	143900	0	0	0	3550700	4764600	3817500
TRINITY_DN12003_c0_g1::TRINITY_DN12003_c0_g1_i1::g.9933::m.9933	0	0	0	0	0	0	0	0	0	0	111340	0	4599200	3645700	3898000
TRINITY_DN14879_c3_g3::TRINITY_DN14879_c3_g3_i2::g.43725::m.43725	0	0	46022	42364	0	0	0	100880	0	0	230970	0	3755600	4089900	3971900

TRINITY_DN17985_c0_g4::TRINITY_DN17985_c0_g4_i1::g.93309::m.93309	0	0	0	0	0	0	0	0	0	0	0	0	4358800	5090100	2771800
TRINITY_DN13592_c0_g2::TRINITY_DN13592_c0_g2_i5::g.25790::m.25790	390320	446240	503880	417500	0	201410	0	350760	383700	1888900	2549000	3741300	92293	1228200	0
TRINITY_DN19576_c0_g1::TRINITY_DN19576_c0_g1_i3::g.120043::m.120043	435690	107530	398230	490380	572320	490160	1075600	594500	0	231700	1249500	5976000	570420	0	0
TRINITY_DN16215_c5_g2::TRINITY_DN16215_c5_g2_i7::g.63985::m.63985	0	0	43489	69405	222390	0	0	0	0	0	611550	886550	2074100	4212500	4040300
TRINITY_DN18909_c0_g1::TRINITY_DN18909_c0_g1_i4::g.108774::m.108774	0	0	0	93673	0	0	0	70100	405820	0	241900	607830	2708100	4550000	3347900
TRINITY_DN15836_c2_g1::TRINITY_DN15836_c2_g1_i8::g.57209::m.57209	25450	0	38973	22393	294700	0	302960	0	0	0	0	0	4456000	3468600	3358800
TRINITY_DN13140_c0_g3::TRINITY_DN13140_c0_g3_i1::g.20389::m.20389	0	0	0	0	0	0	0	0	0	0	0	0	4422700	5187100	2357000
TRINITY_DN16820_c1_g1::TRINITY_DN16820_c1_g1_i7::g.73896::m.73896	37482	0	47617	42419	0	0	0	0	0	0	0	412230	2586200	3388500	5354300
TRINITY_DN20043_c7_g1::TRINITY_DN20043_c7_g1_i1::g.128234::m.128234	557390	0	0	0	439260	197980	185150	1087600	785460	839730	2907300	2371900	372950	743930	1378900
TRINITY_DN13259_c0_g1::TRINITY_DN13259_c0_g1_i1::g.21585::m.21585	0	0	0	0	0	0	0	0	111710	0	0	0	2677200	4875300	4202000
TRINITY_DN18601_c0_g1::TRINITY_DN18601_c0_g1_i6::g.101598::m.101598	516090	203150	592740	677090	577760	510200	950960	463300	1625200	635900	1178900	3312600	502820	0	85130
TRINITY_DN15621_c1_g1::TRINITY_DN15621_c1_g1_i6::g.54532::m.54532	0	0	0	0	0	0	270260	498930	0	0	0	0	2935500	4642200	3451700
TRINITY_DN15203_c1_g1::TRINITY_DN15203_c1_g1_i8::g.48552::m.48552	0	0	0	0	0	0	0	0	0	0	0	0	3936400	2555800	5206300
TRINITY_DN15205_c1_g1::TRINITY_DN15205_c1_g1_i3::g.48499::m.48499	0	0	0	67884	1520200	754430	1238200	1225000	0	1437100	2953300	2481100	0	0	0
TRINITY_DN16773_c2_g1::TRINITY_DN16773_c2_g1_i3::g.73056::m.73056	0	0	0	0	0	0	0	0	0	0	0	0	3675900	2732100	5237700
TRINITY_DN16557_c0_g1::TRINITY_DN16557_c0_g1_i4::g.69370::m.69370	475650	216980	806240	638890	154720	524940	554560	673070	0	1242100	1897200	2136700	434910	729360	1121400
TRINITY_DN16644_c3_g1::TRINITY_DN16644_c3_g1_i2::g.70216::m.70216	0	0	0	0	0	0	0	0	0	0	0	0	3021100	3493000	5060100
TRINITY_DN15438_c0_g1::TRINITY_DN15438_c0_g1_i13::g.52894::m.52894	0	0	0	0	0	0	173870	171340	0	0	948570	1714900	3605000	2737500	2188200
TRINITY_DN12890_c2_g4::TRINITY_DN12890_c2_g4_i2::g.17047::m.17047	315240	302650	155660	597300	0	0	384430	0	0	0	0	289450	2685200	4074500	2708400

TRINITY_DN19235_c2_g2::TRINITY_DN19235_c2_g2_i3::g.114333::m.114333	243200	0	208180	0	654090	178890	1083700	0	2003600	323750	3041400	2768400	599060	399630	0
TRINITY_DN19013_c0_g2::TRINITY_DN19013_c0_g2_i3::g.110484::m.110484	0	0	0	0	0	58578	165440	87112	0	0	0	0	3062800	3662200	4464900
TRINITY_DN14279_c0_g2::TRINITY_DN14279_c0_g2_i1::g.34822::m.34822	50914	0	0	0	0	0	0	0	0	0	0	0	2639700	5038300	3759200
TRINITY_DN15614_c2_g1::TRINITY_DN15614_c2_g1_i7::g.54755::m.54755	252800	96011	392320	400010	0	0	108750	112930	666610	0	0	0	2779600	3321100	3335800
TRINITY_DN16133_c0_g4::TRINITY_DN16133_c0_g4_i5::g.62653::m.62653	457960	0	149370	509990	634900	222360	356570	787040	2794200	1597700	0	3718700	160710	0	0
TRINITY_DN18799_c1_g2::TRINITY_DN18799_c1_g2_i3::g.106472::m.106472	0	0	0	0	0	0	0	0	0	0	0	0	3074000	6015300	2294600
TRINITY_DN19791_c7_g4::TRINITY_DN19791_c7_g4_i2::g.123739::m.123739	0	0	0	0	177370	58359	245710	233030	4273500	0	3463500	2890800	0	0	0
TRINITY_DN14197_c0_g1::TRINITY_DN14197_c0_g1_i2::g.33493::m.33493	0	0	0	0	0	0	0	0	0	0	0	107350	2432500	5412100	3368100
TRINITY_DN16625_c3_g1::TRINITY_DN16625_c3_g1_i9::g.70678::m.70678	52825	161950	484650	0	256910	0	375880	0	1899200	750650	2008100	0	340350	4026400	958360
TRINITY_DN11558_c0_g1::TRINITY_DN11558_c0_g1_i1::g.7858::m.7858	340410	43087	362730	278080	890720	216690	204740	700010	581450	448150	1517300	5728700	0	0	0
TRINITY_DN13381_c2_g2::TRINITY_DN13381_c2_g2_i20::g.23020::m.23020	174660	102600	237730	252690	165580	0	754110	0	309780	194950	320990	449550	1859900	3926400	2532500
TRINITY_DN12837_c2_g6::TRINITY_DN12837_c2_g6_i1::g.16583::m.16583	1508100	518320	1607700	1585600	442930	0	522120	383510	950360	868390	0	1543300	589170	472320	249600
TRINITY_DN17167_c2_g1::TRINITY_DN17167_c2_g1_i1::g.79577::m.79577	0	54356	0	144520	0	0	0	0	0	0	0	0	3280800	4454000	3306500
TRINITY_DN15923_c1_g1::TRINITY_DN15923_c1_g1_i8::g.59029::m.59029	11232000	0	0	0	0	0	0	0	0	0	0	0	0	0	0
TRINITY_DN15294_c5_g3::TRINITY_DN15294_c5_g3_i1::g.49818::m.49818	0	0	0	0	0	0	0	0	0	266450	446810	2462000	5185000	2863500	
TRINITY_DN16184_c0_g3::TRINITY_DN16184_c0_g3_i5::g.63369::m.63369	0	0	0	0	0	0	0	0	0	0	0	0	2897900	4222100	4089800
TRINITY_DN17874_c2_g1::TRINITY_DN17874_c2_g1_i9::g.91168::m.91168	0	0	0	0	0	0	0	0	0	0	0	0	2664400	4287900	4199400
TRINITY_DN18048_c4_g4::TRINITY_DN18048_c4_g4_i5::g.94249::m.94249	40449	0	40845	0	0	0	0	0	1865100	0	2500500	3237600	991160	819650	1613900
TRINITY_DN19270_c0_g2::TRINITY_DN19270_c0_g2_i4::g.115031::m.115031	0	0	0	0	0	0	0	0	0	0	0	0	3480300	5137500	2485900
TRINITY_DN16102_c2_g6::TRINITY_DN16102_c2_g6_i1::g.62197::m.62197	0	136130	362010	368190	606930	326650	677390	0	1004900	714540	2566400	1660500	0	2634800	0

TRINITY_DN14814_c1_g3::TRINITY_DN14814_c1_g3_i3::g.42810::m.42810	0	0	0	0	77128	117740	0	91602	0	0	0	345370	3896500	3873900	2643100
TRINITY_DN13485_c2_g2::TRINITY_DN13485_c2_g2_i2::g.24426::m.24426	31136	0	0	0	0	0	0	0	0	0	0	0	4715300	4497600	1789100
TRINITY_DN15075_c1_g2::TRINITY_DN15075_c1_g2_i1::g.46516::m.46516	0	0	0	0	0	0	0	0	0	0	0	0	2729000	2830800	5419900
TRINITY_DN13860_c0_g1::TRINITY_DN13860_c0_g1_i1::g.29366::m.29366	293450	46675	402310	322150	162490	95982	0	465260	0	0	0	528650	1387700	4197000	3067800
TRINITY_DN19929_c0_g1::TRINITY_DN19929_c0_g1_i9::g.126183::m.126183	0	0	0	0	0	0	0	0	0	0	0	0	2915900	3801000	4247600
TRINITY_DN15983_c2_g1::TRINITY_DN15983_c2_g1_i5::g.59899::m.59899	0	0	0	0	0	0	0	0	0	0	0	0	3760500	4706500	2494400
TRINITY_DN17158_c8_g2::TRINITY_DN17158_c8_g2_i1::g.79404::m.79404	0	0	0	0	0	0	0	0	0	0	0	0	4630900	3018200	3312200
TRINITY_DN16000_c0_g1::TRINITY_DN16000_c0_g1_i10::g.60178::m.60178	0	0	0	0	0	0	0	0	0	0	0	0	3272100	2974600	4680100
TRINITY_DN15045_c2_g1::TRINITY_DN15045_c2_g1_i7::g.46215::m.46215	0	0	0	0	0	0	0	0	0	0	0	0	2823700	3992000	4056600
TRINITY_DN19898_c1_g2::TRINITY_DN19898_c1_g2_i1::g.123971::m.123971	0	0	0	0	0	0	0	0	0	0	0	0	3474800	3763800	3630100
TRINITY_DN14630_c1_g1::TRINITY_DN14630_c1_g1_i1::g.39822::m.39822	0	0	0	0	0	0	0	0	0	0	0	0	2112200	2323100	6392100
TRINITY_DN15863_c0_g1::TRINITY_DN15863_c0_g1_i12::g.58136::m.58136	510250	0	579520	0	660130	545250	0	0	2001900	1364400	2587900	2536500	0	0	0
TRINITY_DN18049_c1_g3::TRINITY_DN18049_c1_g3_i3::g.94352::m.94352	0	0	0	0	149660	89269	0	0	0	0	0	0	1890600	4251100	4404500
TRINITY_DN19093_c2_g2::TRINITY_DN19093_c2_g2_i1::g.111778::m.111778	0	0	0	0	0	0	0	0	0	0	0	0	1511000	7156000	1986800
TRINITY_DN13133_c2_g1::TRINITY_DN13133_c2_g1_i2::g.20342::m.20342	574730	0	746690	412660	223880	37601	164620	0	521130	0	168120	1880000	1526300	2700800	1692500
TRINITY_DN15315_c0_g1::TRINITY_DN15315_c0_g1_i1::g.50016::m.50016	0	0	0	0	0	0	0	0	0	0	0	0	3735100	2986800	3911700
TRINITY_DN13727_c0_g1::TRINITY_DN13727_c0_g1_i1::g.27676::m.27676	389830	195880	435010	935680	0	0	309900	163300	0	0	0	2161400	1476700	1515500	3002500
TRINITY_DN19454_c4_g2::TRINITY_DN19454_c4_g2_i2::g.118225::m.118225	0	0	0	0	0	0	0	0	0	0	0	0	3735100	4578600	2213800
TRINITY_DN15177_c0_g1::TRINITY_DN15177_c0_g1_i2::g.47833::m.47833	63224	0	63898	122780	0	0	0	0	0	0	0	484790	4059900	2240900	3447600
TRINITY_DN13215_c0_g4::TRINITY_DN13215_c0_g4_i6::g.21439::m.21439	289450	148700	527850	364250	0	0	0	0	962710	0	2042300	1988800	1323800	1442800	1358300

TRINITY_DN12615_c0_g2::TRINITY_DN12615_c0_g2_i14::g.14340::m.14340	0	0	0	0	0	0	1623000	660470	0	0	4431600	1091800	2641400	0	0
TRINITY_DN15558_c1_g3::TRINITY_DN15558_c1_g3_i8::g.53729::m.53729	32866	0	0	0	0	0	0	0	0	0	0	0	5368700	3353500	1688500
TRINITY_DN14114_c1_g1::TRINITY_DN14114_c1_g1_i9::g.32755::m.32755	0	0	0	0	0	0	0	0	0	0	0	0	3969200	3820300	2633700
TRINITY_DN18015_c3_g4::TRINITY_DN18015_c3_g4_i1::g.93638::m.93638	290840	0	569070	294130	400440	317780	247260	431260	1243300	435550	2146100	3915200	0	0	130370
TRINITY_DN19464_c2_g1::TRINITY_DN19464_c2_g1_i6::g.118273::m.118273	121930	0	57333	0	107670	0	0	0	0	0	0	0	1822900	4311600	3982900
TRINITY_DN18481_c3_g4::TRINITY_DN18481_c3_g4_i2::g.101020::m.101020	0	0	0	0	0	0	0	0	0	0	0	235140	2214000	4067200	3868500
TRINITY_DN16840_c2_g3::TRINITY_DN16840_c2_g3_i5::g.74315::m.74315	80060	0	176030	86034	0	0	77336	86782	193590	0	0	557820	2685200	3034200	3390200
TRINITY_DN16558_c0_g1::TRINITY_DN16558_c0_g1_i2::g.69315::m.69315	0	0	0	0	0	0	0	0	0	0	0	0	4395300	3085100	2882000
TRINITY_DN16629_c1_g1::TRINITY_DN16629_c1_g1_i1::g.70763::m.70763	2897900	578770	5051700	1347100	158450	0	0	0	0	0	0	0	0	0	322220
TRINITY_DN18667_c1_g2::TRINITY_DN18667_c1_g2_i6::g.104311::m.104311	0	0	0	0	0	0	0	0	0	0	0	0	3948200	3903900	2437200
TRINITY_DN19979_c5_g7::TRINITY_DN19979_c5_g7_i1::g.127021::m.127021	1062100	444240	1551400	1205700	734290	0	986960	843940	0	0	3448900	0	0	0	0
TRINITY_DN17020_c0_g1::TRINITY_DN17020_c0_g1_i5::g.77212::m.77212	0	0	0	0	0	0	0	0	0	0	0	0	2938500	2936100	4400700
TRINITY_DN17027_c2_g2::TRINITY_DN17027_c2_g2_i1::g.77239::m.77239	0	0	0	0	0	0	0	179710	0	0	0	0	2390800	3513100	4170700
TRINITY_DN19286_c0_g1::TRINITY_DN19286_c0_g1_i3::g.115330::m.115330	485460	55848	518010	634000	0	0	1393600	914710	0	556280	1235800	0	1627500	1381800	1445400
TRINITY_DN16223_c1_g2::TRINITY_DN16223_c1_g2_i2::g.64137::m.64137	0	0	0	0	915880	0	1538600	0	0	0	0	0	1406800	2212700	4161000
TRINITY_DN18521_c6_g6::TRINITY_DN18521_c6_g6_i4::g.101904::m.101904	0	0	0	0	1487800	0	0	1425800	2557800	0	0	3882800	0	842740	0
TRINITY_DN17250_c2_g1::TRINITY_DN17250_c2_g1_i27::g.80938::m.80938	481200	190970	461610	439330	554450	253770	0	517690	0	638930	958740	199440	685910	2189200	2603300
TRINITY_DN14788_c0_g4::TRINITY_DN14788_c0_g4_i2::g.42252::m.42252	0	0	0	0	0	0	0	0	0	0	0	0	1883000	5129000	3151000
TRINITY_DN16410_c3_g3::TRINITY_DN16410_c3_g3_i5::g.67165::m.67165	598890	27105	453830	513600	950520	0	316490	0	0	0	2444800	2622100	907600	954030	341020

TRINITY_DN14802_c2_g1::TRINITY_DN14802_c2_g1_i3::g.42628::m.42628	0	0	0	0	0	0	0	0	0	0	0	0	2072300	3741200	4303100
TRINITY_DN14967_c2_g1::TRINITY_DN14967_c2_g1_i8::g.44945::m.44945	0	0	0	0	0	0	0	0	0	0	0	0	2525600	3683700	3902500
TRINITY_DN16828_c0_g2::TRINITY_DN16828_c0_g2_i1::g.74105::m.74105	1914500	752230	2412700	1991300	0	391950	921730	0	0	0	0	0	467650	668100	569910
TRINITY_DN16105_c7_g3::TRINITY_DN16105_c7_g3_i3::g.62193::m.62193	0	0	0	0	310960	0	1110200	0	3251100	0	3901300	0	303520	399100	787710
TRINITY_DN15700_c1_g1::TRINITY_DN15700_c1_g1_i3::g.54890::m.54890	0	0	0	0	0	0	0	0	0	0	0	0	2332400	2490300	5195500
TRINITY_DN19704_c0_g2::TRINITY_DN19704_c0_g2_i1::g.122551::m.122551	0	0	0	0	0	0	0	0	0	0	0	0	2093200	3816400	4106600
TRINITY_DN15979_c0_g1::TRINITY_DN15979_c0_g1_i3::g.59847::m.59847	0	0	0	0	0	51811	0	0	0	0	0	0	3287300	3178800	3491300
TRINITY_DN14286_c1_g1::TRINITY_DN14286_c1_g1_i4::g.34874::m.34874	0	0	0	0	0	0	0	0	0	0	0	0	2863200	4723200	2360700
TRINITY_DN16521_c2_g4::TRINITY_DN16521_c2_g4_i1::g.68855::m.68855	0	0	0	0	0	0	0	0	0	0	0	0	3293500	2701200	3946000
TRINITY_DN18419_c1_g1::TRINITY_DN18419_c1_g1_i5::g.100150::m.1001	0	0	0	25607	0	0	0	0	0	0	117570	0	2704200	3886300	3195000
TRINITY_DN14469_c0_g1::TRINITY_DN14469_c0_g1_i2::g.37789::m.37789	0	0	0	0	0	0	0	0	0	0	0	0	2528000	2136600	5226700
TRINITY_DN19627_c1_g1::TRINITY_DN19627_c1_g1_i1::g.121154::m.1211	0	0	163780	0	0	0	0	0	0	0	0	230680	3177000	3062200	3232300
TRINITY_DN16420_c0_g2::TRINITY_DN16420_c0_g2_i4::g.67379::m.67379	656950	235190	669580	861050	270720	0	311870	0	0	0	0	1487500	1658300	1748500	1948600
TRINITY_DN12422_c0_g1::TRINITY_DN12422_c0_g1_i2::g.12726::m.12726	0	0	71023	439590	961700	38408	140330	1016300	1663700	1354800	2993500	1164600	0	0	0
TRINITY_DN16342_c0_g1::TRINITY_DN16342_c0_g1_i1::g.66087::m.66087	0	0	0	13908	0	0	0	0	0	0	82376	247840	1400900	4486400	3611300
TRINITY_DN14920_c0_g1::TRINITY_DN14920_c0_g1_i8::g.44465::m.44465	352240	172820	366290	331340	0	0	0	0	1308300	120230	0	2686600	1375500	1743300	1321700
TRINITY_DN7453_c0_g1::TRINITY_DN7453_c0_g1_i1::g.2128::m.2128	509700	215260	725050	621310	824580	266430	0	609850	783050	0	981350	1291500	0	1993800	941060
TRINITY_DN15558_c1_g3::TRINITY_DN15558_c1_g3_i7::g.53727::m.53727	0	0	0	0	0	0	0	0	0	0	0	0	3083300	3266100	3392300
TRINITY_DN17792_c0_g2::TRINITY_DN17792_c0_g2_i6::g.89728::m.89728	0	0	0	0	0	381370	0	1275600	0	0	0	6660200	0	0	1379700
TRINITY_DN19733_c2_g1::TRINITY_DN19733_c2_g1_i4::g.123110::m.123110	0	0	0	0	0	0	9303800	0	62135	0	134940	0	170130	0	0

TRINITY_DN14635_c2_g1::TRINITY_DN14635_c2_g1_i1::g.39353::m.39353	61267	0	116560	110150	0	182070	200060	57921	201170	227780	0	0	3761400	3609600	1131200
TRINITY_DN13787_c3_g4::TRINITY_DN13787_c3_g4_i2::g.28260::m.28260	25345	0	0	0	0	0	0	0	0	0	0	0	3069200	3353600	3171700
TRINITY_DN17621_c1_g3::TRINITY_DN17621_c1_g3_i2::g.87259::m.87259	0	0	0	0	0	0	0	0	169630	0	0	0	3955800	2130200	3357700
TRINITY_DN9267_c0_g1::TRINITY_DN9267_c0_g1_i1::g.3260::m.3260	0	0	136650	0	0	0	0	0	0	0	0	0	3913300	5557600	0
TRINITY_DN18406_c4_g1::TRINITY_DN18406_c4_g1_i5::g.99964::m.99964	0	0	0	0	0	0	0	0	0	0	0	0	3503500	3082900	2993000
TRINITY_DN19605_c0_g3::TRINITY_DN19605_c0_g3_i3::g.120913::m.120913	117250	24721	75285	55234	128710	0	169330	140320	0	0	0	376970	2360200	2963200	3156500
TRINITY_DN18631_c1_g1::TRINITY_DN18631_c1_g1_i3::g.103586::m.103586	0	0	0	0	0	0	0	0	0	0	0	0	3109700	2866100	3569500
TRINITY_DN18466_c3_g1::TRINITY_DN18466_c3_g1_i12::g.101047::m.101047	440390	86959	479730	315360	1811600	0	1459000	1861200	0	0	0	0	1331800	0	1707000
TRINITY_DN13201_c1_g1::TRINITY_DN13201_c1_g1_i6::g.20932::m.20932	153790	22571	206720	179100	722230	148010	0	417690	0	535610	362130	0	1947400	2475300	2319600
TRINITY_DN19814_c1_g3::TRINITY_DN19814_c1_g3_i1::g.124097::m.124097	686780	146380	545370	731790	0	0	0	0	0	849610	1532600	1649300	0	1742800	1599700
TRINITY_DN16248_c1_g2::TRINITY_DN16248_c1_g2_i1::g.64537::m.64537	525700	277120	611220	481600	1502400	460370	0	1442400	0	0	1695400	2437800	0	0	0
TRINITY_DN17313_c1_g1::TRINITY_DN17313_c1_g1_i12::g.81814::m.8181	806270	49655	678240	493870	0	277450	401760	758000	1020500	992390	1304400	2627100	0	0	0
TRINITY_DN10225_c0_g1::TRINITY_DN10225_c0_g1_i1::g.4474::m.4474	555050	0	482700	284880	0	0	0	0	994100	0	1255600	0	2550300	575610	2671800
TRINITY_DN13652_c1_g1::TRINITY_DN13652_c1_g1_i1::g.26699::m.26699	0	0	0	0	0	9363100	0	0	0	0	0	0	0	0	0
TRINITY_DN16192_c0_g2::TRINITY_DN16192_c0_g2_i1::g.63470::m.63470	0	0	0	0	0	0	0	0	0	0	0	0	3133800	2512700	3716000
TRINITY_DN18875_c2_g2::TRINITY_DN18875_c2_g2_i3::g.107932::m.107932	0	0	0	0	0	0	0	0	0	0	0	0	3203800	3158600	2941700
TRINITY_DN13691_c2_g1::TRINITY_DN13691_c2_g1_i3::g.27222::m.27222	0	0	84040	71116	0	0	0	0	0	0	0	0	1215200	5965300	1968200
TRINITY_DN15416_c2_g2::TRINITY_DN15416_c2_g2_i3::g.51536::m.51536	0	0	103620	224740	0	49481	0	0	0	261800	0	0	2304000	3755800	2602900
TRINITY_DN12883_c0_g1::TRINITY_DN12883_c0_g1_i3::g.16877::m.16877	110670	43300	152260	123070	0	258980	345690	0	0	1728200	1679300	2935000	610680	672120	593970
TRINITY_DN19511_c2_g3::TRINITY_DN19511_c2_g3_i2::g.119040::m.119040	0	0	0	0	0	0	0	0	0	0	0	0	0	4392200	4833100
TRINITY_DN11191_c0_g1::TRINITY_DN11191_c0_g1_i2::g.6575::m.6575	1274100	575260	1522600	1258700	369230	0	594330	0	0	0	0	3417300	204240	0	0

TRINITY_DN15949_c0_g1::TRINITY_DN15949_c0_g1_i6::g.59510::m.59510	0	0	180610	0	0	101120	208000	337710	0	0	516790	0	2426900	1745400	3604600
TRINITY_DN15397_c1_g1::TRINITY_DN15397_c1_g1_i5::g.51177::m.51177	0	0	0	0	0	0	0	0	0	0	0	0	3097000	3310300	2712300
TRINITY_DN11231_c0_g1::TRINITY_DN11231_c0_g1_i2::g.6700::m.6700	269140	79203	312130	467340	152270	80852	177330	255840	0	0	0	1623700	1608700	2471400	1620200
TRINITY_DN14649_c1_g6::TRINITY_DN14649_c1_g6_i3::g.39965::m.39965	0	0	0	0	0	0	0	0	0	0	0	0	2656300	2766800	3686200
TRINITY_DN13692_c0_g1::TRINITY_DN13692_c0_g1_i6::g.26626::m.26626	0	0	0	0	0	0	0	0	0	0	0	0	4401600	3862300	823920
TRINITY_DN12789_c1_g2::TRINITY_DN12789_c1_g2_i25::g.15964::m.15964	242360	144010	508860	284690	0	0	0	0	0	0	0	0	453850	3331000	4115600
TRINITY_DN17697_c2_g1::TRINITY_DN17697_c2_g1_i5::g.88410::m.88410	0	0	0	0	253460	72281	241190	189680	570800	491320	0	939850	2190500	303860	3822600
TRINITY_DN18388_c1_g1::TRINITY_DN18388_c1_g1_i7::g.99758::m.99758	0	0	0	0	0	0	0	84296	0	0	0	0	2129400	3983400	2801200
TRINITY_DN19535_c2_g2::TRINITY_DN19535_c2_g2_i7::g.119393::m.119393	0	0	0	0	0	0	0	0	0	0	0	0	3352600	3303200	2336600
TRINITY_DN11345_c0_g1::TRINITY_DN11345_c0_g1_i1::g.7064::m.7064	174110	0	441130	260350	565090	376210	1031300	186520	1277700	759960	552370	2114900	325610	662550	252610
TRINITY_DN14591_c4_g1::TRINITY_DN14591_c4_g1_i9::g.39216::m.39216	0	0	58642	0	0	0	0	0	0	0	0	0	3491800	3446200	1965300
TRINITY_DN14453_c2_g1::TRINITY_DN14453_c2_g1_i6::g.37399::m.37399	298180	136180	430260	375950	0	136570	726350	606410	1532800	1254100	1680500	1777300	0	0	0
TRINITY_DN16526_c1_g2::TRINITY_DN16526_c1_g2_i8::g.69034::m.69034	715890	206140	645390	629270	328390	363010	1212200	0	492240	1117800	330830	671020	675880	867990	696390
TRINITY_DN10373_c0_g1::TRINITY_DN10373_c0_g1_i1::g.4691::m.4691	0	93694	141520	139850	1229900	523930	1561400	381430	1138500	274680	2014100	0	999450	249940	185280
TRINITY_DN19332_c2_g1::TRINITY_DN19332_c2_g1_i2::g.116131::m.116131	760530	166590	908250	662660	0	0	0	0	0	154810	409750	0	2327400	1671600	1840000
TRINITY_DN14971_c0_g1::TRINITY_DN14971_c0_g1_i2::g.44966::m.44966	145990	0	145360	193990	321330	0	0	398100	1104300	411960	2089800	2267600	0	931850	861690
TRINITY_DN16859_c0_g3::TRINITY_DN16859_c0_g3_i1::g.74469::m.74469	499830	101030	0	237160	0	0	184630	93880	696950	1348100	2886600	2712800	0	0	105940
TRINITY_DN16399_c1_g2::TRINITY_DN16399_c1_g2_i5::g.67010::m.67010	0	0	0	0	0	0	0	190380	0	0	0	0	3146600	1942600	3583400
TRINITY_DN13466_c1_g1::TRINITY_DN13466_c1_g1_i2::g.24484::m.24484	0	0	0	0	0	0	0	0	0	0	0	0	2073700	571520	6153300
TRINITY_DN18053_c4_g1::TRINITY_DN18053_c4_g1_i4::g.94304::m.94304	0	0	0	0	0	0	0	0	0	0	0	0	3596300	2348300	2796200
TRINITY_DN18818_c1_g1::TRINITY_DN18818_c1_g1_i7::g.106901::m.1069	0	0	0	126390	216850	0	265540	283600	565890	0	964130	1859500	1319400	1568200	1569400

TRINITY_DN14578_c1_g1::TRINITY_DN14578_c1_g1_i8::g.39125::m.39125	0	0	0	0	109220	0	0	0	0	0	0	0	3498100	4498100	626260
TRINITY_DN13918_c1_g1::TRINITY_DN13918_c1_g1_i8::g.30345::m.30345	0	0	36645	0	0	0	0	0	0	0	0	0	1740500	3572000	3374200
TRINITY_DN18543_c1_g3::TRINITY_DN18543_c1_g3_i1::g.101857::m.101857	0	190490	0	0	0	0	0	0	1023000	0	0	1307300	1681800	2061900	2455500
TRINITY_DN17130_c2_g2::TRINITY_DN17130_c2_g2_i6::g.79154::m.79154	0	0	0	0	0	0	0	0	0	0	0	0	2418800	3041800	3251700
TRINITY_DN13127_c0_g1::TRINITY_DN13127_c0_g1_i1::g.20146::m.20146	0	0	0	0	0	0	0	0	0	0	0	0	2200800	3588800	2919000
TRINITY_DN11423_c0_g1::TRINITY_DN11423_c0_g1_i1::g.7308::m.7308	0	0	0	0	0	0	0	0	0	0	0	0	3160600	2577800	2966500
TRINITY_DN17687_c2_g1::TRINITY_DN17687_c2_g1_i6::g.88326::m.88326	492610	107380	65157	371980	0	39707	0	0	362050	155970	1815100	489700	1120300	1812000	1857500
TRINITY_DN19126_c0_g1::TRINITY_DN19126_c0_g1_i4::g.112486::m.112486	0	0	0	0	0	0	0	0	0	0	0	0	3108600	1856500	3708900
TRINITY_DN14181_c0_g3::TRINITY_DN14181_c0_g3_i1::g.33572::m.33572	0	0	0	0	0	0	0	0	0	0	0	0	1459100	2547200	4658700
TRINITY_DN14198_c0_g1::TRINITY_DN14198_c0_g1_i5::g.33635::m.33635	0	0	0	0	0	0	0	0	0	0	0	0	2139000	3236000	3269600
TRINITY_DN15488_c1_g5::TRINITY_DN15488_c1_g5_i3::g.52738::m.52738	93561	0	24429	128320	0	0	210940	220460	210480	833440	0	1586800	1473800	1590200	2264700
TRINITY_DN17665_c0_g2::TRINITY_DN17665_c0_g2_i3::g.87796::m.87796	2294300	864200	2703200	2221100	0	0	0	0	0	0	0	0	0	0	553020
TRINITY_DN11049_c0_g1::TRINITY_DN11049_c0_g1_i1::g.6211::m.6211	1010300	265120	1272800	670310	752330	145170	0	803750	803560	0	0	2906500	0	0	0
TRINITY_DN18004_c1_g11::TRINITY_DN18004_c1_g11_i1::g.93466::m.93466	1906300	0	1815000	1358300	0	0	0	0	0	0	0	3513400	0	0	0
TRINITY_DN17396_c1_g1::TRINITY_DN17396_c1_g1_i2::g.82990::m.82990	0	0	1254800	0	0	0	0	0	0	0	0	0	1797700	2828300	2678200
TRINITY_DN16113_c1_g1::TRINITY_DN16113_c1_g1_i2::g.62243::m.62243	0	0	0	0	0	0	0	0	0	0	0	0	2678400	2731900	3141200
TRINITY_DN12537_c5_g1::TRINITY_DN12537_c5_g1_i1::g.13612::m.13612	0	0	0	0	0	0	1142400	590180	1488700	0	5317200	0	0	0	0
TRINITY_DN13740_c0_g1::TRINITY_DN13740_c0_g1_i2::g.27757::m.27757	0	0	0	0	0	0	0	0	104170	0	0	0	1791600	4553200	2072000
TRINITY_DN13460_c3_g2::TRINITY_DN13460_c3_g2_i3::g.24327::m.24327	26102	0	50392	33972	0	0	0	0	0	0	0	0	2329100	4432900	1611000
TRINITY_DN14396_c0_g2::TRINITY_DN14396_c0_g2_i1::g.35371::m.35371	966220	222330	533290	764380	485520	0	1565300	1953600	0	1734600	0	0	117400	0	123810
TRINITY_DN18313_c3_g2::TRINITY_DN18313_c3_g2_i2::g.98848::m.98848	129480	0	90394	86623	105650	0	140010	117580	0	522850	545460	0	1766500	2996200	1964200

TRINITY_DN19187_c0_g2::TRINITY_DN19187_c0_g2_i3::g.113547::m.113547	123930	0	546100	532770	403640	0	0	0	1238300	969140	1854700	2016100	0	141920	636740
TRINITY_DN10969_c0_g1::TRINITY_DN10969_c0_g1_i1::g.5933::m.5933	228500	42942	296390	250220	335820	94268	0	212210	888450	0	1334900	3092300	577300	921530	184030
TRINITY_DN14875_c0_g2::TRINITY_DN14875_c0_g2_i7::g.43614::m.43614	0	0	0	0	0	0	0	0	0	0	0	0	2152900	4770500	1460100
TRINITY_DN16965_c3_g1::TRINITY_DN16965_c3_g1_i6::g.76283::m.76283	148010	0	3401400	3735100	0	0	133860	0	0	0	0	249900	247930	133270	238440
TRINITY_DN16752_c9_g1::TRINITY_DN16752_c9_g1_i5::g.72819::m.72819	0	0	0	0	0	0	0	0	0	0	0	2614000	3254200	2392700	
TRINITY_DN17906_c0_g1::TRINITY_DN17906_c0_g1_i4::g.91749::m.91749	434870	91295	280890	273660	0	0	0	0	0	0	3085800	2931200	0	1129600	0
TRINITY_DN10627_c0_g1::TRINITY_DN10627_c0_g1_i1::g.5151::m.5151	0	0	0	0	0	0	0	0	0	0	0	0	2838900	2657900	2721200
TRINITY_DN11182_c0_g1::TRINITY_DN11182_c0_g1_i3::g.6546::m.6546	166310	24669	45905	119760	1030200	458030	239670	925210	447440	1283100	1988000	682520	104640	513370	187990
TRINITY_DN14268_c0_g1::TRINITY_DN14268_c0_g1_i4::g.34610::m.34610	0	0	0	44317	96755	0	0	0	0	102700	627970	381380	1857500	3118200	1983200
TRINITY_DN19877_c2_g3::TRINITY_DN19877_c2_g3_i1::g.125021::m.125021	0	0	0	0	0	0	0	0	0	0	0	0	1326500	1823900	5055600
TRINITY_DN17530_c3_g12::TRINITY_DN17530_c3_g12_i1::g.85464::m.85464	0	0	0	0	0	0	0	0	122540	0	0	0	2371100	2856800	2853300
TRINITY_DN14410_c0_g6::TRINITY_DN14410_c0_g6_i1::g.36618::m.36618	0	0	0	0	0	0	0	0	0	0	0	0	2295600	3051200	2828100
TRINITY_DN12098_c3_g1::TRINITY_DN12098_c3_g1_i8::g.10503::m.10503	626610	305510	719610	692220	0	602170	0	1054500	0	1641700	0	2325600	93852	104200	0
TRINITY_DN17739_c0_g2::TRINITY_DN17739_c0_g2_i3::g.89048::m.89048	43492	0	0	110390	0	0	0	329580	159120	0	208160	0	2313500	2649400	2321400
TRINITY_DN14157_c1_g1::TRINITY_DN14157_c1_g1_i4::g.33107::m.33107	430880	142020	544300	546150	912950	378260	616760	0	645420	305710	0	3538200	0	60550	0
TRINITY_DN18293_c3_g1::TRINITY_DN18293_c3_g1_i3::g.98277::m.98277	0	0	0	0	0	0	0	0	0	0	0	0	2452500	2815400	2837600
TRINITY_DN18745_c0_g3::TRINITY_DN18745_c0_g3_i8::g.105419::m.105419	242510	27088	344520	322310	1404900	224920	928210	228340	0	1224700	0	432890	0	1309000	1412100
TRINITY_DN12590_c2_g2::TRINITY_DN12590_c2_g2_i2::g.13982::m.13982	427480	190730	447840	503580	531410	369370	0	709530	0	0	3019200	1765200	126890	0	0
TRINITY_DN14010_c5_g1::TRINITY_DN14010_c5_g1_i2::g.31420::m.31420	0	0	0	0	0	0	0	0	0	0	0	0	6892100	0	1188600
TRINITY_DN15598_c2_g1::TRINITY_DN15598_c2_g1_i7::g.54284::m.54284	0	0	0	0	0	0	0	0	0	0	0	297860	2264800	3481300	2030600

TRINITY_DN11779_c0_g1::TRINITY_DN11779_c0_g1_i1::g.8787::m.8787	288510	87038	347960	305220	0	0	0	149240	633990	0	658060	1468200	2096900	1062300	960790
TRINITY_DN13676_c3_g1::TRINITY_DN13676_c3_g1_i3::g.27135::m.27135	173360	0	226660	219360	0	0	0	0	1123700	1115700	2501500	2077700	0	317960	300150
TRINITY_DN18865_c1_g1::TRINITY_DN18865_c1_g1_i8::g.107763::m.107763	223260	95969	333070	218080	312320	213010	469890	285780	0	0	1682700	2124100	1098400	985220	0
TRINITY_DN15886_c0_g2::TRINITY_DN15886_c0_g2_i6::g.58296::m.58296	0	0	0	0	0	0	0	0	0	0	0	0	2084400	3321500	2632300
TRINITY_DN11909_c0_g1::TRINITY_DN11909_c0_g1_i2::g.9470::m.9470	122740	33730	139110	145110	0	0	0	0	0	0	312050	419820	2011600	2418400	2397200
TRINITY_DN16963_c2_g3::TRINITY_DN16963_c2_g3_i1::g.76239::m.76239	0	0	0	0	0	0	0	0	263230	0	0	0	2508200	2389100	2838600
TRINITY_DN12323_c0_g2::TRINITY_DN12323_c0_g2_i3::g.11963::m.11963	0	0	0	0	0	0	0	0	0	0	0	0	1620400	4301400	2060800
TRINITY_DN13869_c6_g1::TRINITY_DN13869_c6_g1_i2::g.29653::m.29653	0	0	0	0	0	0	0	0	0	0	0	0	2104100	3016800	2852300
TRINITY_DN18287_c3_g1::TRINITY_DN18287_c3_g1_i2::g.98102::m.98102	260900	0	104510	438200	312930	0	872390	718820	0	303870	997430	2569800	549210	93435	746430
TRINITY_DN19305_c2_g3::TRINITY_DN19305_c2_g3_i4::g.115690::m.115690	0	0	110970	86498	0	0	0	0	0	0	225260	0	3418600	0	4105600
TRINITY_DN15008_c0_g3::TRINITY_DN15008_c0_g3_i2::g.45738::m.45738	0	0	0	0	0	0	0	0	0	0	0	0	966840	3695600	3268100
TRINITY_DN18232_c0_g1::TRINITY_DN18232_c0_g1_i6::g.97390::m.97390	0	0	24260	0	160690	0	0	0	0	0	0	0	3432800	1509900	2801600
TRINITY_DN17493_c2_g1::TRINITY_DN17493_c2_g1_i7::g.84644::m.84644	0	0	0	0	0	0	0	0	0	0	0	0	1601900	3118900	3174300
TRINITY_DN11669_c0_g1::TRINITY_DN11669_c0_g1_i1::g.8337::m.8337	0	0	0	0	0	0	0	0	0	0	0	0	1039000	5742800	1101000
TRINITY_DN18124_c0_g4::TRINITY_DN18124_c0_g4_i1::g.95606::m.95606	64552	0	62007	0	0	0	0	0	44445	0	0	0	1033200	3740100	2936700
TRINITY_DN11643_c0_g1::TRINITY_DN11643_c0_g1_i1::g.8230::m.8230	0	0	0	27614	0	0	0	0	0	0	0	0	2586000	2799700	2463300
TRINITY_DN17921_c3_g2::TRINITY_DN17921_c3_g2_i1::g.92117::m.92117	0	0	0	0	0	0	0	0	0	0	115810	0	2348800	3071100	2340200
TRINITY_DN17515_c0_g1::TRINITY_DN17515_c0_g1_i3::g.85102::m.85102	0	0	58035	0	0	0	131900	0	145070	236350	0	0	2681500	2780700	1816400
TRINITY_DN17501_c0_g2::TRINITY_DN17501_c0_g2_i6::g.84659::m.84659	0	0	0	0	0	0	102760	0	198160	165970	359290	0	3715800	1144100	2130200
TRINITY_DN11905_c0_g1::TRINITY_DN11905_c0_g1_i3::g.9416::m.9416	0	0	0	0	0	0	0	0	0	0	0	0	2050200	2900500	2853600
TRINITY_DN16017_c1_g2::TRINITY_DN16017_c1_g2_i2::g.60617::m.60617	0	0	0	0	94964	46496	0	0	0	0	0	0	2988900	2196600	2445500

TRINITY_DN10830_c0_g1::TRINITY_DN10830_c0_g1_i1::g.5629::m.5629	0	0	0	33924	107610	0	0	0	0	0	0	0	1467300	3484600	2663100
TRINITY_DN13736_c4_g2::TRINITY_DN13736_c4_g2_i5::g.27391::m.27391	427220	182230	833790	213130	0	0	150330	501730	0	1784100	0	0	457580	1850800	1302700
TRINITY_DN19177_c0_g1::TRINITY_DN19177_c0_g1_i1::g.113288::m.1132	0	0	0	0	0	0	0	0	0	0	0	0	2149000	2582000	2939000
TRINITY_DN19354_c2_g2::TRINITY_DN19354_c2_g2_i2::g.116436::m.116436	383290	111010	359590	694210	0	0	0	0	3128900	0	1335100	1649200	0	0	0
TRINITY_DN17688_c2_g6::TRINITY_DN17688_c2_g6_i1::g.88272::m.88272	0	69619	143060	163660	679420	229970	675290	812160	845600	650890	1994900	1370000	0	0	0
TRINITY_DN16306_c1_g1::TRINITY_DN16306_c1_g1_i3::g.65480::m.65480	0	0	0	0	293510	0	0	414050	2642400	0	0	3336000	0	201660	730390
TRINITY_DN18894_c1_g3::TRINITY_DN18894_c1_g3_i1::g.108226::m.108226	79850	0	76743	47916	0	0	0	0	0	148580	0	0	2690400	2695500	1873200
TRINITY_DN14391_c2_g3::TRINITY_DN14391_c2_g3_i1::g.36280::m.36280	0	0	0	0	1096800	913400	0	0	0	1771500	0	2827100	1000900	0	0
TRINITY_DN12367_c0_g1::TRINITY_DN12367_c0_g1_i3::g.12189::m.12189	0	0	0	0	0	0	0	0	0	0	275450	0	2917000	1593800	2811300
TRINITY_DN14985_c3_g2::TRINITY_DN14985_c3_g2_i1::g.45232::m.45232	0	0	0	0	0	0	0	0	0	0	0	0	684830	2583600	4328700
TRINITY_DN14724_c2_g1::TRINITY_DN14724_c2_g1_i1::g.41239::m.41239	0	0	0	0	0	0	0	0	0	0	0	0	1209700	2190500	4188400
TRINITY_DN10431_c0_g1::TRINITY_DN10431_c0_g1_i2::g.4811::m.4811	64841	52117	94706	69078	0	0	0	117200	0	0	0	0	2194600	1511600	3471600
TRINITY_DN12116_c0_g1::TRINITY_DN12116_c0_g1_i3::g.10615::m.10615	0	0	0	0	0	0	0	0	0	0	0	0	3040700	2122900	2403100
TRINITY_DN12248_c0_g2::TRINITY_DN12248_c0_g2_i1::g.11152::m.11152	0	0	0	0	0	0	0	0	0	0	0	0	1978100	2214300	3367500
TRINITY_DN14430_c0_g1::TRINITY_DN14430_c0_g1_i7::g.37424::m.37424	0	0	0	0	0	0	0	0	0	0	0	0	2018700	2677700	2857000
TRINITY_DN17797_c0_g1::TRINITY_DN17797_c0_g1_i20::g.88479::m.88479	0	0	0	0	0	0	0	0	0	0	0	0	3712700	3829400	0
TRINITY_DN16328_c4_g1::TRINITY_DN16328_c4_g1_i3::g.65859::m.65859	0	0	0	0	0	0	0	0	0	0	0	0	933480	3985400	2606700
TRINITY_DN14897_c3_g5::TRINITY_DN14897_c3_g5_i1::g.43938::m.43938	0	0	0	0	0	0	0	0	0	0	0	0	3334200	0	4185700
TRINITY_DN14545_c0_g1::TRINITY_DN14545_c0_g1_i2::g.38714::m.38714	203640	95034	228310	181400	0	135280	604710	358240	323920	543150	2782400	2061100	0	0	0
TRINITY_DN18713_c3_g1::TRINITY_DN18713_c3_g1_i2::g.105054::m.105054	0	0	0	0	462640	294980	776660	594070	1302400	947220	3128400	0	0	0	0

TRINITY_DN13940_c4_g1::TRINITY_DN13940_c4_g1_i3::g.30598::m.30598	0	0	0	0	0	0	0	0	0	0	0	0	1896600	2603200	2997400
TRINITY_DN18184_c1_g1::TRINITY_DN18184_c1_g1_i15::g.96527::m.96527	0	0	0	0	441340	192980	535560	0	920760	501730	0	0	1530700	1958000	1391100
TRINITY_DN17484_c0_g3::TRINITY_DN17484_c0_g3_i1::g.84435::m.84435	163950	0	360650	249750	0	0	556920	225780	0	0	0	2417100	0	1840400	1653200
TRINITY_DN13721_c0_g1::TRINITY_DN13721_c0_g1_i7::g.27621::m.27621	0	0	0	0	0	0	0	0	0	0	0	0	1973600	3218400	2268300
TRINITY_DN17006_c0_g4::TRINITY_DN17006_c0_g4_i2::g.76859::m.76859	0	0	0	0	0	0	0	0	0	0	0	0	3325600	2437600	1691200
TRINITY_DN13463_c1_g1::TRINITY_DN13463_c1_g1_i3::g.24177::m.24177	228180	0	209470	97930	337030	131330	480420	0	1734900	0	2150900	2074700	0	0	0
TRINITY_DN17769_c1_g1::TRINITY_DN17769_c1_g1_i1::g.89345::m.89345	450860	238590	259660	163080	0	0	853280	0	272420	0	561750	0	1067600	1675500	1890000
TRINITY_DN18331_c2_g5::TRINITY_DN18331_c2_g5_i1::g.98900::m.98900	0	0	0	0	0	0	0	0	0	0	0	0	2080500	3913200	1420300
TRINITY_DN13546_c0_g1::TRINITY_DN13546_c0_g1_i3::g.25193::m.25193	0	0	0	0	0	0	0	0	0	0	0	0	3198100	3463300	739580
TRINITY_DN19711_c5_g2::TRINITY_DN19711_c5_g2_i2::g.122643::m.122643	0	0	0	0	758750	0	297370	516370	0	0	0	0	1720000	2021500	2071600
TRINITY_DN15444_c2_g1::TRINITY_DN15444_c2_g1_i7::g.52052::m.52052	0	0	0	0	0	0	0	0	0	0	0	0	1309700	3127600	2866400
TRINITY_DN18083_c4_g1::TRINITY_DN18083_c4_g1_i1::g.94854::m.94854	0	0	179680	135860	0	0	0	1336300	0	0	0	0	668200	3274000	1701200
TRINITY_DN15930_c2_g2::TRINITY_DN15930_c2_g2_i5::g.59203::m.59203	33491	0	35967	92702	0	0	0	61556	245960	0	0	0	1309300	2681200	2805100
TRINITY_DN19332_c1_g1::TRINITY_DN19332_c1_g1_i1::g.116126::m.1161	0	0	0	0	0	0	0	0	0	0	0	0	2050400	2538700	2645300
TRINITY_DN13790_c1_g1::TRINITY_DN13790_c1_g1_i1::g.28245::m.28245	0	0	0	0	0	0	0	0	0	0	0	0	2632000	3095700	1496300
TRINITY_DN9744_c0_g1::TRINITY_DN9744_c0_g1_i1::g.3792::m.3792	282410	77516	287770	171050	158710	0	167640	0	1115400	393640	2388300	963930	343290	664310	174010
TRINITY_DN13494_c0_g2::TRINITY_DN13494_c0_g2_i2::g.24524::m.24524	0	0	0	0	0	0	0	0	0	0	0	0	2080600	0	5105700
TRINITY_DN17631_c3_g6::TRINITY_DN17631_c3_g6_i1::g.87349::m.87349	199360	31230	66733	167280	705500	0	1137800	617530	227700	678670	0	0	1191600	523760	1610000
TRINITY_DN12268_c0_g1::TRINITY_DN12268_c0_g1_i1::g.11491::m.11491	0	0	0	0	0	0	0	0	0	0	0	0	1071500	2832300	3248000
TRINITY_DN19388_c2_g2::TRINITY_DN19388_c2_g2_i2::g.117003::m.117003	483650	0	580190	436760	0	0	0	713770	0	0	2049200	1835400	310910	381720	320970

TRINITY_DN13433_c0_g1::TRINITY_DN13433_c0_g1_i4::g.23725::m.23725	0	0	0	0	0	0	0	0	0	0	0	0	2550400	2296200	2263100
TRINITY_DN17014_c1_g3::TRINITY_DN17014_c1_g3_i2::g.77015::m.77015	112740	0	51527	0	0	0	0	0	0	183600	0	0	2915200	2244000	1598000
TRINITY_DN13342_c3_g2::TRINITY_DN13342_c3_g2_i4::g.22568::m.22568	0	0	0	0	0	0	0	0	0	0	0	0	1506500	3180400	2380300
TRINITY_DN15981_c2_g1::TRINITY_DN15981_c2_g1_i5::g.59872::m.59872	149410	39109	210550	236610	220450	0	0	0	0	0	0	660990	1457400	1680800	2391800
TRINITY_DN18987_c2_g1::TRINITY_DN18987_c2_g1_i1::g.110056::m.110056	269410	0	298980	372160	608810	0	0	554650	1502900	0	1510100	1865400	0	0	61121
TRINITY_DN12349_c8_g2::TRINITY_DN12349_c8_g2_i1::g.12068::m.12068	881510	432170	1132500	1280300	523920	134110	182700	0	1909800	0	0	0	560620	0	0
TRINITY_DN13325_c1_g1::TRINITY_DN13325_c1_g1_i1::g.22310::m.22310	0	0	0	0	0	0	0	0	0	0	0	0	2147700	2607200	2245100
TRINITY_DN15794_c0_g2::TRINITY_DN15794_c0_g2_i7::g.57165::m.57165	0	0	0	0	0	0	0	0	0	0	0	0	1707700	2687900	2598100
TRINITY_DN16454_c1_g1::TRINITY_DN16454_c1_g1_i2::g.67727::m.67727	398190	0	0	106140	0	0	0	0	0	657790	0	0	3039000	1383000	1391600
TRINITY_DN12161_c0_g1::TRINITY_DN12161_c0_g1_i4::g.10883::m.10883	0	0	0	0	0	0	0	0	0	0	0	0	2539200	2648800	1756800
TRINITY_DN16552_c0_g2::TRINITY_DN16552_c0_g2_i1::g.69236::m.69236	0	0	0	0	0	0	0	0	0	0	0	0	2066200	2474900	2390800
TRINITY_DN13234_c0_g1::TRINITY_DN13234_c0_g1_i7::g.21346::m.21346	336690	135070	355650	292510	327480	0	0	135400	198320	0	0	0	1855500	2243300	1040800
TRINITY_DN16040_c0_g1::TRINITY_DN16040_c0_g1_i12::g.61056::m.61056	0	0	0	0	0	0	0	0	0	0	0	0	1583700	1709200	3591500
TRINITY_DN17716_c3_g1::TRINITY_DN17716_c3_g1_i5::g.88746::m.88746	0	0	0	0	0	0	0	82097	0	139230	227790	0	977530	4347300	1105700
TRINITY_DN14659_c0_g2::TRINITY_DN14659_c0_g2_i6::g.40081::m.40081	0	0	0	0	0	0	0	0	0	0	608440	0	1209300	1723000	3296700
TRINITY_DN8972_c0_g1::TRINITY_DN8972_c0_g1_i1::g.3000::m.3000	0	0	0	0	0	0	0	0	0	0	0	0	2067600	2476700	2276800
TRINITY_DN19468_c2_g5::TRINITY_DN19468_c2_g5_i1::g.118327::m.118327	91575	51672	163630	101740	0	0	175160	217630	0	167840	0	0	1785500	1960300	2087500
TRINITY_DN16932_c1_g2::TRINITY_DN16932_c1_g2_i8::g.75832::m.75832	542210	210090	545950	449750	0	249550	0	0	0	618500	1491800	1525700	521800	597080	0
TRINITY_DN15439_c1_g1::TRINITY_DN15439_c1_g1_i4::g.51919::m.51919	344710	0	322270	0	67924	126320	68309	0	245950	0	0	362400	1658600	687350	2866700
TRINITY_DN19589_c2_g2::TRINITY_DN19589_c2_g2_i4::g.120310::m.120310	0	0	0	0	0	0	0	0	279880	0	377860	370060	782000	2704000	2161800
TRINITY_DN17548_c3_g2::TRINITY_DN17548_c3_g2_i2::g.85798::m.85798	0	0	0	0	0	0	0	0	0	0	0	0	2320600	2476500	1850700

TRINITY_DN12116_c0_g2::TRINITY_DN12116_c0_g2_i1::g.10617::m.10617	155830	46087	99852	174390	129190	0	0	0	0	0	0	0	2054600	1764500	2208600
TRINITY_DN16328_c4_g1::TRINITY_DN16328_c4_g1_i2::g.65854::m.65854	0	0	0	0	0	0	0	0	0	0	0	0	2308800	2000100	2293000
TRINITY_DN19061_c2_g1::TRINITY_DN19061_c2_g1_i2::g.111195::m.1111	0	0	0	0	0	0	0	0	0	0	0	0	1689900	2792100	2118800
TRINITY_DN17042_c1_g2::TRINITY_DN17042_c1_g2_i2::g.77496::m.77496	565280	173690	540460	612150	0	0	0	199640	0	0	0	170890	702810	1492400	2131100
TRINITY_DN16369_c2_g3::TRINITY_DN16369_c2_g3_i1::g.66518::m.66518	0	0	0	0	140710	0	0	181500	544080	368970	0	0	885490	1223500	3243300
TRINITY_DN17384_c3_g1::TRINITY_DN17384_c3_g1_i3::g.82818::m.82818	62270	0	0	76410	0	0	0	0	164450	0	363140	168240	969520	1564600	3194900
TRINITY_DN18568_c1_g1::TRINITY_DN18568_c1_g1_i19::g.102809::m.102809	56390	0	107960	86479	160410	62429	0	134510	0	187450	536670	0	1995600	1766600	1438900
TRINITY_DN19159_c0_g2::TRINITY_DN19159_c0_g2_i3::g.113047::m.113047	172930	48104	177810	120240	753240	0	836890	537570	0	896230	1471200	1061700	0	0	443080
TRINITY_DN19261_c3_g1::TRINITY_DN19261_c3_g1_i6::g.113934::m.113934	0	0	0	0	0	0	0	0	0	0	0	0	1694000	2535600	2288700
TRINITY_DN13280_c0_g2::TRINITY_DN13280_c0_g2_i2::g.21976::m.21976	456400	138390	227360	295370	211670	403780	0	0	0	0	0	734630	979460	1737300	1328700
TRINITY_DN18061_c2_g1::TRINITY_DN18061_c2_g1_i8::g.94553::m.94553	0	0	0	0	0	0	0	0	78466	0	0	0	130370	2659500	3641100
TRINITY_DN12991_c0_g2::TRINITY_DN12991_c0_g2_i1::g.18234::m.18234	0	0	0	0	0	0	0	0	0	0	0	0	1573600	3011200	1914600
TRINITY_DN19158_c0_g2::TRINITY_DN19158_c0_g2_i3::g.112794::m.112794	0	0	0	0	0	0	0	0	0	0	0	0	2089300	2314000	2062700
TRINITY_DN11523_c0_g3::TRINITY_DN11523_c0_g3_i2::g.7748::m.7748	0	0	0	0	0	0	0	0	0	0	0	0	1348500	1849600	3260400
TRINITY_DN14938_c0_g1::TRINITY_DN14938_c0_g1_i5::g.44776::m.44776	0	0	0	0	0	0	0	0	0	0	0	0	1869700	2836900	1748000
TRINITY_DN17566_c1_g1::TRINITY_DN17566_c1_g1_i8::g.86112::m.86112	0	0	0	0	0	0	0	0	0	0	0	0	1604600	2494700	2340300
TRINITY_DN19918_c3_g1::TRINITY_DN19918_c3_g1_i11::g.126682::m.126682	0	0	0	0	0	0	0	0	0	0	0	613630	1524300	2583500	1689000
TRINITY_DN12927_c2_g2::TRINITY_DN12927_c2_g2_i9::g.17583::m.17583	0	0	0	0	0	0	0	0	0	0	0	0	1286000	1753000	3354300
TRINITY_DN16523_c1_g3::TRINITY_DN16523_c1_g3_i4::g.69129::m.69129	0	0	0	0	0	0	0	0	0	0	0	0	1688900	2541900	2155900
TRINITY_DN15434_c1_g2::TRINITY_DN15434_c1_g2_i2::g.51814::m.51814	0	0	0	0	0	0	0	310360	0	0	0	0	2580300	2923000	567750

TRINITY_DN18883_c1_g1::TRINITY_DN18883_c1_g1_i17::g.108283::m.108283	0	0	0	0	0	0	0	0	0	0	0	0	2160900	2331000	1845100
TRINITY_DN15951_c5_g2::TRINITY_DN15951_c5_g2_i3::g.59457::m.59457	354700	80486	516010	401780	329830	0	296540	283020	559770	432380	1047200	1010400	0	179040	821480
TRINITY_DN17564_c2_g2::TRINITY_DN17564_c2_g2_i9::g.86086::m.86086	220870	53040	246510	82179	293070	0	417470	646480	0	568160	1128400	827240	812550	617860	390690
TRINITY_DN15444_c2_g1::TRINITY_DN15444_c2_g1_i2::g.52034::m.52034	0	0	0	0	0	0	0	0	0	0	0	0	1888300	1966000	2441400
TRINITY_DN19153_c1_g1::TRINITY_DN19153_c1_g1_i1::g.112868::m.1128	0	0	0	0	0	0	0	0	0	0	0	0	1389500	3321900	1576800
TRINITY_DN18739_c0_g2::TRINITY_DN18739_c0_g2_i10::g.105638::m.105638	0	0	0	0	0	0	0	0	0	0	0	0	3833500	1297100	1148300
TRINITY_DN17091_c0_g2::TRINITY_DN17091_c0_g2_i7::g.78224::m.78224	0	0	0	0	0	0	0	0	0	0	0	0	639580	2902800	2731300
TRINITY_DN16872_c0_g1::TRINITY_DN16872_c0_g1_i9::g.74807::m.74807	0	0	0	0	0	0	0	0	0	0	0	0	1670000	3196200	1398600
TRINITY_DN13841_c4_g1::TRINITY_DN13841_c4_g1_i2::g.28420::m.28420	0	0	0	0	0	0	0	0	0	0	0	0	2282000	2229000	1744800
TRINITY_DN11991_c0_g1::TRINITY_DN11991_c0_g1_i1::g.9823::m.9823	0	0	0	0	0	0	0	0	0	0	0	0	1919900	2674400	1657000
TRINITY_DN15464_c0_g1::TRINITY_DN15464_c0_g1_i3::g.52265::m.52265	48480	0	0	0	0	0	235130	196910	0	191070	0	0	1297200	2566900	1704100
TRINITY_DN11351_c0_g4::TRINITY_DN11351_c0_g4_i3::g.7095::m.7095	317280	107560	333230	317010	346910	180640	366080	630260	717420	0	966240	871090	504430	0	567230
TRINITY_DN19612_c1_g2::TRINITY_DN19612_c1_g2_i9::g.120822::m.120822	0	0	0	0	0	0	0	0	0	0	0	0	1808400	2176200	2240200
TRINITY_DN10989_c0_g1::TRINITY_DN10989_c0_g1_i3::g.6012::m.6012	0	0	0	0	0	0	0	0	0	0	0	0	821460	1582100	3810800
TRINITY_DN12636_c0_g1::TRINITY_DN12636_c0_g1_i3::g.14427::m.14427	258500	31614	46447	263250	345010	159530	364650	542880	1250600	462230	1087600	1019500	128350	170150	70961
TRINITY_DN15811_c0_g5::TRINITY_DN15811_c0_g5_i2::g.57331::m.57331	0	0	0	0	0	154830	0	0	0	0	0	0	2394200	1505000	2144800
TRINITY_DN19982_c2_g1::TRINITY_DN19982_c2_g1_i8::g.127226::m.127226	218460	103160	284320	239850	0	0	0	0	0	394880	888140	1880200	362960	454080	1359200
TRINITY_DN18355_c0_g1::TRINITY_DN18355_c0_g1_i6::g.99167::m.99167	0	0	0	0	0	0	1663600	0	0	0	0	0	0	165210	4320900
TRINITY_DN15264_c2_g1::TRINITY_DN15264_c2_g1_i9::g.49352::m.49352	0	0	585210	0	0	0	0	0	0	0	0	0	1538800	2051000	1944500
TRINITY_DN12375_c3_g5::TRINITY_DN12375_c3_g5_i1::g.12365::m.12365	0	0	0	0	0	0	0	0	0	0	0	0	1717600	2130500	2265900

TRINITY_DN17383_c0_g1::TRINITY_DN17383_c0_g1_i3::g.82754::m.82754	0	0	0	0	0	56124	0	0	0	0	0	3336800	1516500	828900	366190
TRINITY_DN18266_c2_g1::TRINITY_DN18266_c2_g1_i1::g.97903::m.97903	0	0	0	0	0	0	0	0	0	0	0	0	1993000	1902200	2200100
TRINITY_DN15771_c0_g1::TRINITY_DN15771_c0_g1_i5::g.56845::m.56845	0	0	132970	0	0	0	0	0	0	0	0	0	1401500	3075400	1465200
TRINITY_DN15425_c0_g1::TRINITY_DN15425_c0_g1_i1::g.51653::m.51653	327870	53572	397240	356680	202650	506790	633850	0	434170	1263100	591620	745450	0	187610	353980
TRINITY_DN19779_c3_g1::TRINITY_DN19779_c3_g1_i9::g.123558::m.123558	0	0	0	0	0	0	0	0	0	0	0	0	841940	2253900	2956300
TRINITY_DN17373_c0_g1::TRINITY_DN17373_c0_g1_i1::g.82647::m.82647	0	0	0	0	0	0	0	0	0	0	0	0	1062000	3014100	1974200
TRINITY_DN17897_c2_g2::TRINITY_DN17897_c2_g2_i2::g.91535::m.91535	0	0	0	0	0	0	0	0	0	0	0	0	1891300	2169800	1983000
TRINITY_DN17861_c1_g1::TRINITY_DN17861_c1_g1_i1::g.90803::m.90803	0	0	0	0	0	0	0	0	0	196610	0	0	2331600	3484900	0
TRINITY_DN15475_c0_g1::TRINITY_DN15475_c0_g1_i2::g.52448::m.52448	0	0	0	0	0	0	0	0	0	0	0	0	1755300	1942300	2314800
TRINITY_DN17503_c0_g1::TRINITY_DN17503_c0_g1_i2::g.84892::m.84892	0	0	0	0	0	0	0	0	0	0	0	0	1245000	2458400	2288900
TRINITY_DN14545_c0_g2::TRINITY_DN14545_c0_g2_i6::g.38726::m.38726	0	0	0	0	0	0	0	0	0	0	0	0	1566200	1846700	2547400
TRINITY_DN12477_c0_g1::TRINITY_DN12477_c0_g1_i2::g.13209::m.13209	124400	0	113070	71411	0	0	154850	0	0	0	290670	125080	678100	1862600	2496700
TRINITY_DN18895_c1_g2::TRINITY_DN18895_c1_g2_i8::g.108488::m.108488	439760	177130	555390	380470	607610	285820	0	0	919530	739860	1339700	0	423990	0	0
TRINITY_DN14210_c0_g1::TRINITY_DN14210_c0_g1_i5::g.33918::m.33918	876650	340150	1073200	934410	0	0	0	0	577390	0	784410	815000	0	441530	0
TRINITY_DN18268_c0_g1::TRINITY_DN18268_c0_g1_i8::g.98068::m.98068	0	0	0	0	0	0	86119	168360	0	0	0	0	1206300	1726500	2640000
TRINITY_DN16248_c0_g1::TRINITY_DN16248_c0_g1_i2::g.64527::m.64527	0	0	0	212110	0	0	316560	709270	0	1022600	0	2699900	295270	294230	275810
TRINITY_DN16306_c1_g2::TRINITY_DN16306_c1_g2_i2::g.65481::m.65481	0	0	0	0	0	0	0	304000	0	0	2415000	2525100	580130	0	0
TRINITY_DN19332_c2_g5::TRINITY_DN19332_c2_g5_i3::g.116136::m.116136	640840	222490	878330	673100	618930	0	780960	0	0	378540	0	1629400	0	0	0
TRINITY_DN19330_c0_g3::TRINITY_DN19330_c0_g3_i6::g.116162::m.116162	0	0	0	0	661300	0	0	0	0	1687400	3444000	0	0	0	0

TRINITY_DN19198_c2_g2::TRINITY_DN19198_c2_g2_i7::g.112135::m.112135	0	0	0	0	0	0	0	0	0	0	0	0	1932800	1485900	2369600
TRINITY_DN18055_c0_g3::TRINITY_DN18055_c0_g3_i2::g.94441::m.94441	0	0	0	0	0	0	0	0	0	0	0	0	0	0	5775500
TRINITY_DN19902_c1_g2::TRINITY_DN19902_c1_g2_i1::g.125828::m.125828	0	0	0	0	0	0	0	0	0	0	0	0	1934100	1889400	1931900
TRINITY_DN16483_c1_g1::TRINITY_DN16483_c1_g1_i8::g.68282::m.68282	0	0	0	0	0	0	0	0	0	0	0	0	1644400	2408500	1692900
TRINITY_DN13316_c0_g1::TRINITY_DN13316_c0_g1_i4::g.22236::m.22236	0	0	0	29575	0	0	0	0	0	0	0	406230	1848600	2380900	1062500
TRINITY_DN14260_c1_g1::TRINITY_DN14260_c1_g1_i4::g.34485::m.34485	437460	165750	615280	524070	669080	0	216460	724160	378870	0	982130	0	1002900	0	0
TRINITY_DN14547_c0_g1::TRINITY_DN14547_c0_g1_i3::g.38055::m.38055	0	0	0	0	0	0	0	0	0	0	0	0	1298100	2591200	1818900
TRINITY_DN17557_c0_g1::TRINITY_DN17557_c0_g1_i2::g.85865::m.85865	0	0	0	0	0	0	0	0	0	0	0	0	108260	3433300	2162600
TRINITY_DN14469_c0_g2::TRINITY_DN14469_c0_g2_i3::g.37807::m.37807	0	0	0	0	0	80722	232900	0	0	0	478040	0	1452700	1608500	1822300
TRINITY_DN13571_c3_g1::TRINITY_DN13571_c3_g1_i9::g.25607::m.25607	254780	0	0	178230	0	0	0	0	323990	0	582260	0	1707400	1424200	1193900
TRINITY_DN15222_c4_g2::TRINITY_DN15222_c4_g2_i2::g.48684::m.48684	0	0	0	0	0	0	0	0	0	0	0	0	1191300	2360700	2104100
TRINITY_DN14937_c1_g1::TRINITY_DN14937_c1_g1_i10::g.44580::m.44580	0	0	0	0	415490	0	487950	328380	1190800	0	1652600	1567000	0	0	0
TRINITY_DN11539_c0_g1::TRINITY_DN11539_c0_g1_i5::g.7794::m.7794	0	0	0	0	0	0	0	269970	0	0	0	0	1373900	2324700	1645800
TRINITY_DN15625_c0_g3::TRINITY_DN15625_c0_g3_i1::g.54608::m.54608	0	0	0	0	0	37764	87303	0	0	0	0	0	324600	2118900	3040600
TRINITY_DN17623_c4_g3::TRINITY_DN17623_c4_g3_i4::g.87434::m.87434	0	0	1066100	0	0	0	0	0	0	0	0	0	432950	2258700	1847800
TRINITY_DN19001_c1_g1::TRINITY_DN19001_c1_g1_i22::g.110256::m.110256	281210	134040	429750	350280	0	712800	0	0	569940	0	1238900	1651800	0	0	229570
TRINITY_DN14942_c2_g1::TRINITY_DN14942_c2_g1_i4::g.44640::m.44640	0	0	0	0	0	0	0	0	0	0	0	0	1766000	1855200	1956500
TRINITY_DN14097_c2_g2::TRINITY_DN14097_c2_g2_i1::g.31570::m.31570	0	0	0	0	0	0	82789	0	0	0	0	0	1468900	2287200	1735700
TRINITY_DN19080_c0_g1::TRINITY_DN19080_c0_g1_i5::g.111810::m.111810	265490	38057	288380	110130	0	125880	0	0	0	0	179930	677830	1918500	1212900	749600
TRINITY_DN18479_c0_g5::TRINITY_DN18479_c0_g5_i1::g.100968::m.100968	0	0	0	0	0	0	0	0	0	0	0	0	2089100	2342700	1127200

TRINITY_DN17219_c0_g1::TRINITY_DN17219_c0_g1_i4::g.80391::m.80391	0	0	0	0	0	0	0	0	0	0	0	0	1256100	2599100	1702600
TRINITY_DN19330_c0_g1::TRINITY_DN19330_c0_g1_i8::g.116161::m.116161	0	0	0	0	0	0	0	0	425960	0	0	0	1500800	1072600	2553500
TRINITY_DN13211_c0_g1::TRINITY_DN13211_c0_g1_i3::g.21098::m.21098	0	0	0	0	0	0	0	0	0	0	0	0	3719400	477910	1350800
TRINITY_DN13228_c1_g1::TRINITY_DN13228_c1_g1_i1::g.21382::m.21382	629550	63393	653380	330830	177800	107070	625400	184760	0	0	0	1483100	0	669670	603760
TRINITY_DN19095_c0_g1::TRINITY_DN19095_c0_g1_i1::g.111856::m.111856	204660	0	235190	163860	0	0	0	0	0	0	0	0	1182400	2154300	1563600
TRINITY_DN12846_c1_g3::TRINITY_DN12846_c1_g3_i2::g.16059::m.16059	57680	0	59420	0	376840	219730	587690	487330	153290	144130	755880	858090	903470	899600	0
TRINITY_DN16616_c1_g1::TRINITY_DN16616_c1_g1_i2::g.70640::m.70640	0	0	0	0	1347800	0	1727600	0	0	0	595470	560570	681530	320770	265180
TRINITY_DN17302_c1_g3::TRINITY_DN17302_c1_g3_i1::g.81624::m.81624	0	0	0	0	0	0	0	0	0	0	0	0	72725	0	5380600
TRINITY_DN12723_c0_g1::TRINITY_DN12723_c0_g1_i2::g.15224::m.15224	0	0	0	0	0	0	0	0	131260	0	280010	0	1018400	1358600	2660600
TRINITY_DN17461_c1_g1::TRINITY_DN17461_c1_g1_i12::g.84192::m.8419	0	0	0	0	0	0	0	0	0	0	0	0	167530	5141000	139390
TRINITY_DN17237_c2_g3::TRINITY_DN17237_c2_g3_i7::g.80360::m.80360	0	0	0	0	0	0	0	0	0	0	0	0	1343700	1601100	2488000
TRINITY_DN12529_c0_g1::TRINITY_DN12529_c0_g1_i3::g.13512::m.13512	121260	25410	0	62957	94745	0	275020	0	161010	0	1074100	447860	491780	1317600	1313400
TRINITY_DN12993_c1_g1::TRINITY_DN12993_c1_g1_i9::g.18389::m.18389	335240	104020	397360	296830	267150	0	438880	377840	0	470750	299480	1458100	0	0	921670
TRINITY_DN18316_c4_g8::TRINITY_DN18316_c4_g8_i2::g.98671::m.98671	0	66366	119290	0	0	0	0	0	0	0	0	0	1557700	2679300	917770
TRINITY_DN14932_c2_g1::TRINITY_DN14932_c2_g1_i5::g.44480::m.44480	0	0	0	0	0	0	717690	0	1909100	0	0	2701800	0	0	0
TRINITY_DN18395_c1_g2::TRINITY_DN18395_c1_g2_i5::g.99070::m.99070	219850	0	373190	269670	131880	155660	498810	372270	0	297690	0	2318200	0	0	685780
TRINITY_DN12675_c1_g1::TRINITY_DN12675_c1_g1_i2::g.14909::m.14909	0	0	0	0	65023	0	0	78676	0	0	223610	0	1638800	1556800	1748800
TRINITY_DN18566_c1_g3::TRINITY_DN18566_c1_g3_i2::g.102668::m.102668	0	0	0	0	0	0	0	0	0	0	0	0	1220300	1847300	2201000
TRINITY_DN10355_c0_g1::TRINITY_DN10355_c0_g1_i2::g.4690::m.4690	36072	0	0	0	0	0	0	0	0	0	0	0	2494900	2400100	335220
TRINITY_DN13432_c1_g1::TRINITY_DN13432_c1_g1_i19::g.23847::m.23847	67604	0	86901	49367	0	0	255860	207870	214620	436490	0	211050	1396100	1346300	988900
TRINITY_DN14200_c0_g1::TRINITY_DN14200_c0_g1_i2::g.33523::m.33523	57074	0	74932	51016	0	0	0	0	403960	0	583590	380460	1206000	791620	1705500

TRINITY_DN12445_c0_g2::TRINITY_DN12445_c0_g2_i13::g.12955::m.12955	0	0	0	0	183370	0	72654	0	0	207350	262700	0	1535800	1424600	1550300
TRINITY_DN12195_c0_g1::TRINITY_DN12195_c0_g1_i1::g.10552::m.10552	352280	136210	350770	377760	0	0	0	0	0	0	416640	0	1068300	1228500	1305600
TRINITY_DN13264_c3_g1::TRINITY_DN13264_c3_g1_i4::g.21666::m.21666	21673	79934	0	0	197180	0	196290	0	0	0	0	0	1258700	1578200	1902500
TRINITY_DN16140_c1_g1::TRINITY_DN16140_c1_g1_i18::g.63031::m.6303	0	0	0	0	0	0	0	0	273790	0	691410	787380	1242700	1050000	1187700
TRINITY_DN18120_c2_g1::TRINITY_DN18120_c2_g1_i8::g.95450::m.95450	273460	71425	273750	399270	0	74050	0	0	0	0	0	1145900	1396600	1297000	273610
TRINITY_DN19263_c0_g1::TRINITY_DN19263_c0_g1_i2::g.114802::m.114802	63955	29872	511900	0	126480	0	0	0	249420	181220	1271500	1518800	124340	0	1111000
TRINITY_DN16243_c0_g1::TRINITY_DN16243_c0_g1_i2::g.64445::m.64445	0	0	0	0	0	0	0	0	0	0	0	0	2413300	1999500	758140
TRINITY_DN13812_c2_g1::TRINITY_DN13812_c2_g1_i1::g.28623::m.28623	0	0	0	0	0	0	0	0	0	0	0	0	1600700	2033000	1527400
TRINITY_DN37045_c0_g1::TRINITY_DN37045_c0_g1_i1::g.131013::m.131013	116550	51755	148840	0	446560	87982	252400	207150	617850	0	2181200	928270	118200	0	0
TRINITY_DN17851_c1_g3::TRINITY_DN17851_c1_g3_i2::g.90156::m.90156	0	0	0	0	0	0	0	0	0	0	0	0	1580600	1596200	1956000
TRINITY_DN14011_c0_g1::TRINITY_DN14011_c0_g1_i1::g.31283::m.31283	0	0	0	0	0	0	0	0	0	0	0	0	730180	2033000	2356200
TRINITY_DN11978_c1_g4::TRINITY_DN11978_c1_g4_i1::g.9533::m.9533	0	0	0	0	0	0	0	0	0	0	0	0	2912600	0	2194300
TRINITY_DN17242_c0_g2::TRINITY_DN17242_c0_g2_i1::g.80718::m.80718	618630	195420	559940	536990	0	202300	0	796180	0	916670	443680	834830	0	0	0
TRINITY_DN16272_c1_g2::TRINITY_DN16272_c1_g2_i3::g.64808::m.64808	66777	0	33057	453650	168790	75495	142080	192660	466530	174890	355190	565990	955900	537450	882770
TRINITY_DN12654_c0_g3::TRINITY_DN12654_c0_g3_i5::g.14722::m.14722	60625	0	71095	0	347420	0	226920	85347	0	560740	1221700	0	986010	588490	918320
TRINITY_DN13334_c3_g1::TRINITY_DN13334_c3_g1_i2::g.22908::m.22908	0	0	0	0	0	0	0	0	2171600	0	2889200	0	0	0	0
TRINITY_DN16447_c0_g1::TRINITY_DN16447_c0_g1_i1::g.67047::m.67047	0	0	0	0	0	0	0	0	0	0	0	0	2225800	0	2820300
TRINITY_DN15955_c0_g1::TRINITY_DN15955_c0_g1_i1::g.59530::m.59530	0	0	0	0	0	0	0	0	0	0	0	0	452530	1987300	2580000
TRINITY_DN19579_c1_g3::TRINITY_DN19579_c1_g3_i1::g.120400::m.120400	0	0	0	0	0	0	0	0	0	0	0	0	1847900	1535700	1627000
TRINITY_DN17207_c1_g1::TRINITY_DN17207_c1_g1_i5::g.80226::m.80226	0	0	184980	0	605880	157190	703130	474110	1202200	0	0	1404500	67610	184970	0
TRINITY_DN17881_c0_g1::TRINITY_DN17881_c0_g1_i3::g.91227::m.91227	91585	66727	181340	158970	0	133950	428900	0	713290	0	2510500	683990	0	0	0

TRINITY_DN16367_c2_g1::TRINITY_DN16367_c2_g1_i2::g.66527::m.66527	86135	0	95009	110780	374550	81641	251050	546220	307730	0	0	227600	727310	1100100	1060600
TRINITY_DN12454_c0_g1::TRINITY_DN12454_c0_g1_i1::g.12984::m.12984	381700	150220	571660	403740	556190	0	620820	0	367680	0	0	931850	733730	245810	0
TRINITY_DN18113_c1_g2::TRINITY_DN18113_c1_g2_i2::g.95380::m.95380	0	0	0	0	0	0	0	0	0	0	259680	0	1307800	1850700	1538000
TRINITY_DN13825_c0_g1::TRINITY_DN13825_c0_g1_i2::g.28846::m.28846	544630	200940	749300	323850	0	149220	0	0	0	0	545620	2440600	0	0	0
TRINITY_DN15635_c1_g1::TRINITY_DN15635_c1_g1_i2::g.54767::m.54767	0	0	0	0	0	0	0	0	0	0	0	0	911550	2474800	1561500
TRINITY_DN18171_c1_g1::TRINITY_DN18171_c1_g1_i5::g.96291::m.96291	54913	29219	0	0	434670	0	586440	92683	0	875540	714450	346710	374390	439850	988110
TRINITY_DN17183_c0_g2::TRINITY_DN17183_c0_g2_i5::g.79774::m.79774	588350	76011	513980	637710	0	0	0	0	0	0	1308500	1444400	0	357340	0
TRINITY_DN16005_c0_g3::TRINITY_DN16005_c0_g3_i6::g.60403::m.60403	46421	0	42201	45561	95051	0	0	0	0	0	0	0	1763100	1298000	1625800
TRINITY_DN13194_c5_g1::TRINITY_DN13194_c5_g1_i1::g.20972::m.20972	154760	61488	203590	174280	0	0	0	0	0	0	0	0	945360	924440	2450900
TRINITY_DN19141_c0_g1::TRINITY_DN19141_c0_g1_i8::g.112746::m.1127	0	0	0	0	130530	0	149410	0	0	0	0	0	1203000	2435000	991470
TRINITY_DN19587_c1_g2::TRINITY_DN19587_c1_g2_i1::g.120316::m.120316	422500	185290	507240	467650	0	0	501140	0	0	0	1255600	0	356320	457840	750530
TRINITY_DN14259_c5_g1::TRINITY_DN14259_c5_g1_i8::g.34656::m.34656	0	0	0	0	0	0	0	0	0	0	0	0	1859500	1758300	1284100
TRINITY_DN16231_c1_g1::TRINITY_DN16231_c1_g1_i4::g.64294::m.64294	0	0	0	0	0	105700	0	0	0	0	0	206480	165390	1679300	2743300
TRINITY_DN14338_c3_g2::TRINITY_DN14338_c3_g2_i1::g.35555::m.35555	0	0	0	0	0	60453	162030	0	224070	0	53376	0	1419500	1106500	1855400
TRINITY_DN19174_c1_g3::TRINITY_DN19174_c1_g3_i1::g.113399::m.1133	0	0	0	0	0	0	0	0	0	0	0	0	1499200	1758200	1623100
TRINITY_DN19841_c3_g2::TRINITY_DN19841_c3_g2_i9::g.124618::m.124618	116210	0	89118	71887	0	0	0	0	0	0	0	0	1130700	1731700	1737000
TRINITY_DN20035_c3_g1::TRINITY_DN20035_c3_g1_i3::g.128175::m.128175	61170	29170	0	16668	226260	66433	0	173580	0	0	1062300	922350	380750	1068400	815080
TRINITY_DN18498_c3_g4::TRINITY_DN18498_c3_g4_i4::g.101572::m.101572	0	0	0	0	0	0	0	0	0	0	0	0	1003600	1872500	1921400
TRINITY_DN17440_c0_g1::TRINITY_DN17440_c0_g1_i7::g.84097::m.84097	0	0	0	0	0	0	0	0	0	0	0	0	1462400	1827000	1501800
TRINITY_DN15779_c0_g2::TRINITY_DN15779_c0_g2_i1::g.57109::m.57109	0	0	0	0	0	0	0	0	0	0	493080	0	2155400	1142600	987880
TRINITY_DN18149_c0_g1::TRINITY_DN18149_c0_g1_i3::g.95908::m.95908	0	0	0	0	82676	107690	268390	313940	0	0	177510	681800	558340	408120	2179400

TRINITY_DN18366_c0_g2::TRINITY_DN18366_c0_g2_i3::g.99417::m.99417	0	0	0	0	0	0	0	0	0	0	0	0	1938100	1770700	1051200
TRINITY_DN16745_c0_g1::TRINITY_DN16745_c0_g1_i9::g.72756::m.72756	0	0	0	0	0	0	0	0	0	0	0	0	1397000	1385000	1965500
TRINITY_DN12226_c1_g1::TRINITY_DN12226_c1_g1_i9::g.11323::m.11323	0	0	0	0	0	0	0	0	0	0	0	0	1213300	1637800	1885400
TRINITY_DN16837_c0_g2::TRINITY_DN16837_c0_g2_i4::g.74272::m.74272	0	0	0	0	0	0	0	0	0	0	0	0	1394400	2233500	1101500
TRINITY_DN14860_c0_g2::TRINITY_DN14860_c0_g2_i3::g.43462::m.43462	0	0	0	0	0	0	0	0	0	0	0	0	1373100	1571700	1759400
TRINITY_DN19634_c0_g1::TRINITY_DN19634_c0_g1_i18::g.121436::m.121436	0	0	0	0	0	0	0	0	0	0	0	0	1172800	2173300	1358000
TRINITY_DN18967_c1_g1::TRINITY_DN18967_c1_g1_i1::g.109547::m.109547	0	0	0	0	0	0	0	0	0	0	0	0	2513900	1392600	785050
TRINITY_DN10328_c0_g1::TRINITY_DN10328_c0_g1_i1::g.4642::m.4642	320740	80231	271880	235500	419200	0	311490	0	0	0	1463600	1550200	0	0	0
TRINITY_DN12931_c0_g1::TRINITY_DN12931_c0_g1_i4::g.17462::m.17462	0	0	0	0	0	0	0	0	0	0	0	0	1404900	2243200	999720
TRINITY_DN15347_c2_g1::TRINITY_DN15347_c2_g1_i4::g.50637::m.50637	248620	134630	314210	262630	1245800	312280	0	1176800	0	0	0	0	460360	468890	0
TRINITY_DN18324_c2_g1::TRINITY_DN18324_c2_g1_i6::g.99024::m.99024	0	0	0	0	0	0	0	0	0	0	0	0	656530	1827700	2139200
TRINITY_DN15702_c0_g3::TRINITY_DN15702_c0_g3_i1::g.55933::m.55933	0	0	0	0	0	0	0	0	0	0	0	0	2157700	2065800	391570
TRINITY_DN11687_c0_g1::TRINITY_DN11687_c0_g1_i2::g.8407::m.8407	30701	0	83161	143070	1220000	0	0	909510	0	0	0	0	247800	1313000	663430
TRINITY_DN12590_c1_g3::TRINITY_DN12590_c1_g3_i2::g.13976::m.13976	0	0	0	0	0	0	0	0	0	0	0	0	0	3967100	643100
TRINITY_DN15601_c2_g1::TRINITY_DN15601_c2_g1_i1::g.52933::m.52933	0	125120	119890	0	1172500	0	0	1013100	0	0	0	0	982730	1195600	0
TRINITY_DN17527_c0_g3::TRINITY_DN17527_c0_g3_i1::g.85247::m.85247	0	0	0	0	0	0	0	0	0	0	0	0	863390	0	3742000
TRINITY_DN12826_c0_g1::TRINITY_DN12826_c0_g1_i1::g.16222::m.16222	80863	43061	112660	119860	1129100	148690	1096600	660010	0	114100	0	1099500	0	0	0
TRINITY_DN19082_c3_g1::TRINITY_DN19082_c3_g1_i2::g.111610::m.111610	0	0	0	0	0	0	0	0	0	0	0	0	1383000	1622000	1597300
TRINITY_DN19829_c1_g1::TRINITY_DN19829_c1_g1_i2::g.124633::m.124633	134370	726490	93370	226700	243080	145950	0	336640	510870	428290	0	846090	0	476590	429910
TRINITY_DN16466_c0_g3::TRINITY_DN16466_c0_g3_i1::g.67992::m.67992	274650	0	326150	203880	287570	144860	0	134100	0	0	545890	0	455480	918740	1303100

TRINITY_DN12480_c0_g2::TRINITY_DN12480_c0_g2_i1::g.13216::m.13216	0	0	0	0	0	0	0	0	0	0	0	0	1701300	1932300	949360
TRINITY_DN14710_c1_g4::TRINITY_DN14710_c1_g4_i3::g.41054::m.41054	0	0	0	0	0	0	0	0	0	0	0	0	987710	2177600	1388900
TRINITY_DN17564_c2_g2::TRINITY_DN17564_c2_g2_i5::g.86079::m.86079	0	0	0	0	0	0	0	0	0	0	0	0	867000	2285800	1395900
TRINITY_DN13661_c3_g4::TRINITY_DN13661_c3_g4_i1::g.26847::m.26847	142150	56673	164810	0	241640	0	0	202040	0	0	0	656010	780390	1359000	945100
TRINITY_DN12154_c0_g1::TRINITY_DN12154_c0_g1_i6::g.10864::m.10864	0	0	0	0	0	0	0	0	0	0	0	654840	1311900	1686500	873690
TRINITY_DN19326_c2_g1::TRINITY_DN19326_c2_g1_i5::g.116051::m.116051	83618	0	123430	86463	247670	0	314060	301540	379080	0	0	1339900	433390	639440	551620
TRINITY_DN19798_c2_g2::TRINITY_DN19798_c2_g2_i18::g.123759::m.123759	0	0	0	0	0	0	0	0	0	0	0	0	2811700	907930	766000
TRINITY_DN19717_c0_g1::TRINITY_DN19717_c0_g1_i2::g.122513::m.122513	0	0	0	0	0	0	0	0	0	0	0	0	605080	2993300	886540
TRINITY_DN17321_c2_g2::TRINITY_DN17321_c2_g2_i3::g.82053::m.82053	0	0	0	0	0	0	0	0	0	0	0	0	2277200	0	2205500
TRINITY_DN16262_c1_g2::TRINITY_DN16262_c1_g2_i1::g.64873::m.64873	0	0	0	0	0	0	101780	0	227170	0	0	0	1348400	1695000	1091000
TRINITY_DN17928_c2_g1::TRINITY_DN17928_c2_g1_i2::g.92278::m.92278	387460	0	480560	318350	0	0	0	586440	231480	0	832390	1623500	0	0	0
TRINITY_DN19464_c2_g1::TRINITY_DN19464_c2_g1_i8::g.118275::m.118275	0	0	0	0	0	0	0	0	0	0	0	0	1538200	1561300	1349400
TRINITY_DN18987_c4_g5::TRINITY_DN18987_c4_g5_i1::g.110067::m.110067	0	0	0	0	651410	0	0	0	0	0	0	2338800	0	1454600	0
TRINITY_DN18350_c0_g4::TRINITY_DN18350_c0_g4_i3::g.99164::m.99164	27829	0	0	32868	222530	264700	659590	0	835610	0	1405700	411360	0	316060	259730
TRINITY_DN18760_c9_g3::TRINITY_DN18760_c9_g3_i2::g.106074::m.106074	0	0	0	0	0	0	0	0	0	0	0	0	1389300	402820	2636300
TRINITY_DN17826_c0_g3::TRINITY_DN17826_c0_g3_i6::g.90229::m.90229	42119	18444	49965	54577	0	0	197340	0	0	0	2815600	0	0	607850	638850
TRINITY_DN19627_c1_g2::TRINITY_DN19627_c1_g2_i4::g.121159::m.121159	0	0	68811	0	0	0	0	0	0	0	0	0	2331800	891320	1131600
TRINITY_DN19425_c1_g1::TRINITY_DN19425_c1_g1_i1::g.117994::m.1179	0	0	0	0	0	0	0	0	0	0	0	0	487880	2014200	1904700
TRINITY_DN13200_c1_g3::TRINITY_DN13200_c1_g3_i1::g.20941::m.20941	165640	51739	193400	154190	284560	123830	419070	376670	623280	0	0	861120	551760	320960	260550

TRINITY_DN14744_c0_g1::TRINITY_DN14744_c0_g1_i1::g.41504::m.41504	0	0	0	0	0	0	0	0	0	0	0	0	1499400	1920400	963160
TRINITY_DN16218_c1_g2::TRINITY_DN16218_c1_g2_i6::g.64375::m.64375	0	0	0	0	0	0	0	0	0	0	0	0	1778000	1530200	1073400
TRINITY_DN19518_c0_g1::TRINITY_DN19518_c0_g1_i9::g.119130::m.1191	0	0	0	0	404370	0	421750	304590	0	753700	1323100	1172800	0	0	0
TRINITY_DN19578_c1_g1::TRINITY_DN19578_c1_g1_i2::g.120062::m.120062	0	0	0	0	0	0	0	0	0	0	0	0	1219500	1055700	2099500
TRINITY_DN15774_c2_g1::TRINITY_DN15774_c2_g1_i10::g.56953::m.56953	0	0	0	0	0	0	0	0	0	0	0	0	1524400	1583700	1253100
TRINITY_DN18433_c1_g4::TRINITY_DN18433_c1_g4_i1::g.100393::m.100393	0	0	0	0	0	0	0	386430	0	0	1655700	1515500	224210	288430	290280
TRINITY_DN12393_c0_g1::TRINITY_DN12393_c0_g1_i2::g.12483::m.12483	109740	34832	122350	178500	0	0	197220	0	0	0	0	620950	1022200	973640	1075300
TRINITY_DN14525_c1_g1::TRINITY_DN14525_c1_g1_i2::g.38384::m.38384	0	0	0	0	0	0	0	0	0	0	0	0	2543900	676450	1108300
TRINITY_DN19854_c2_g1::TRINITY_DN19854_c2_g1_i1::g.124554::m.124554	0	0	0	0	0	0	0	0	0	0	675250	565200	1256000	913370	915560
TRINITY_DN16619_c0_g1::TRINITY_DN16619_c0_g1_i7::g.70514::m.70514	0	0	0	0	0	0	0	159670	0	0	0	0	1108500	1322800	1732600
TRINITY_DN11003_c0_g3::TRINITY_DN11003_c0_g3_i1::g.6096::m.6096	0	0	0	64185	0	0	0	0	0	0	223710	0	1467500	2567600	0
TRINITY_DN12512_c0_g1::TRINITY_DN12512_c0_g1_i7::g.13451::m.13451	0	0	0	0	0	0	0	0	0	0	0	0	0	2372200	1944900
TRINITY_DN12275_c0_g1::TRINITY_DN12275_c0_g1_i2::g.11548::m.11548	0	0	0	0	0	0	0	0	0	0	0	0	1658500	2092400	564180
TRINITY_DN19290_c6_g1::TRINITY_DN19290_c6_g1_i1::g.115224::m.115224	0	0	0	0	0	0	0	0	0	0	0	0	1818000	1766900	728110
TRINITY_DN13198_c0_g2::TRINITY_DN13198_c0_g2_i2::g.20890::m.20890	0	0	0	0	256000	54011	0	107320	298540	692740	802300	463130	776260	472630	384990
TRINITY_DN16577_c0_g1::TRINITY_DN16577_c0_g1_i6::g.69863::m.69863	0	0	0	0	0	0	0	371080	295790	0	409380	704110	415720	855850	1248500
TRINITY_DN18300_c1_g1::TRINITY_DN18300_c1_g1_i6::g.96808::m.96808	0	0	0	0	0	0	0	0	0	0	368900	445070	645770	1311900	1524600
TRINITY_DN14139_c1_g3::TRINITY_DN14139_c1_g3_i2::g.32774::m.32774	0	0	0	0	0	0	0	0	0	0	0	0	831950	1053100	2404300
TRINITY_DN16286_c1_g1::TRINITY_DN16286_c1_g1_i5::g.65098::m.65098	0	0	0	0	0	0	0	0	0	0	0	0	1215700	818000	2253700
TRINITY_DN15944_c0_g3::TRINITY_DN15944_c0_g3_i1::g.58792::m.58792	0	0	0	0	0	0	0	0	0	0	0	0	2379700	0	1904500
TRINITY_DN14872_c2_g1::TRINITY_DN14872_c2_g1_i7::g.43673::m.43673	319180	0	196710	375620	138760	0	173620	156410	0	0	855020	1120400	390340	0	551480
TRINITY_DN14391_c2_g1::TRINITY_DN14391_c2_g1_i3::g.36277::m.36277	0	0	0	0	0	0	0	0	0	0	0	0	0	1963700	2290800

TRINITY_DN16836_c1_g1::TRINITY_DN16836_c1_g1_i8::g.74234::m.74234	0	0	0	0	0	0	0	0	0	0	0	0	1060100	1228900	1961900
TRINITY_DN17195_c1_g1::TRINITY_DN17195_c1_g1_i2::g.79932::m.79932	0	0	0	0	0	0	0	0	0	0	0	0	1658600	917270	1674500
TRINITY_DN15687_c3_g1::TRINITY_DN15687_c3_g1_i1::g.55726::m.55726	347520	56161	324350	135630	0	0	0	0	794000	394870	934550	987680	0	0	265240
TRINITY_DN19945_c1_g2::TRINITY_DN19945_c1_g2_i9::g.126623::m.126623	0	0	0	0	0	0	0	0	0	0	0	0	1198200	1543500	1495300
TRINITY_DN16932_c1_g1::TRINITY_DN16932_c1_g1_i8::g.75833::m.75833	53963	0	43098	59741	0	266110	607180	410590	965020	0	1359200	0	0	237530	229470
TRINITY_DN18681_c1_g1::TRINITY_DN18681_c1_g1_i1::g.104540::m.104540	208150	45106	268320	287540	198290	0	362750	162520	331520	227440	0	691540	396090	525000	516910
TRINITY_DN15903_c3_g2::TRINITY_DN15903_c3_g2_i1::g.58748::m.58748	0	0	0	0	0	0	0	0	0	0	0	0	531350	2395500	1282900
TRINITY_DN17228_c0_g2::TRINITY_DN17228_c0_g2_i3::g.80435::m.80435	0	0	0	0	0	0	0	0	0	0	0	0	1587300	1335100	1278700
TRINITY_DN16216_c0_g1::TRINITY_DN16216_c0_g1_i6::g.64074::m.64074	63669	0	110880	91518	0	0	0	0	0	0	0	0	1341100	1207100	1380000
TRINITY_DN18444_c3_g2::TRINITY_DN18444_c3_g2_i4::g.100535::m.100535	0	0	0	0	0	0	0	0	0	0	0	0	1482600	796110	1911100
TRINITY_DN12460_c3_g3::TRINITY_DN12460_c3_g3_i1::g.13367::m.13367	51820	0	76786	91073	198780	48710	263140	956740	826600	0	0	1460200	215420	0	0
TRINITY_DN13616_c0_g1::TRINITY_DN13616_c0_g1_i3::g.26276::m.26276	0	65229	272650	292540	0	0	0	274790	0	706890	1023600	1356800	187270	0	0
TRINITY_DN18246_c0_g1::TRINITY_DN18246_c0_g1_i3::g.96857::m.96857	0	0	0	0	0	0	0	0	0	0	0	406940	937420	1109700	1725100
TRINITY_DN19723_c2_g1::TRINITY_DN19723_c2_g1_i1::g.122855::m.122855	81721	0	0	0	0	0	0	0	0	0	0	478080	0	2069700	1548700
TRINITY_DN13123_c3_g2::TRINITY_DN13123_c3_g2_i7::g.20202::m.20202	254550	0	226070	145520	189330	0	587390	164450	391750	0	493640	1172700	0	552360	0
TRINITY_DN18029_c5_g2::TRINITY_DN18029_c5_g2_i4::g.93978::m.93978	0	0	0	0	0	0	0	0	0	0	0	0	1101400	2529500	535410
TRINITY_DN11846_c0_g3::TRINITY_DN11846_c0_g3_i2::g.9155::m.9155	0	0	0	0	0	0	0	0	0	0	0	0	1516400	1437100	1196500
TRINITY_DN14246_c2_g1::TRINITY_DN14246_c2_g1_i1::g.34260::m.34260	243790	0	202070	268590	0	0	234770	0	0	0	1064400	1365800	0	360710	394460
TRINITY_DN18272_c3_g3::TRINITY_DN18272_c3_g3_i3::g.97910::m.97910	190310	148450	458450	219840	0	0	316150	136390	448380	631390	908650	632700	0	0	0
TRINITY_DN16015_c1_g1::TRINITY_DN16015_c1_g1_i9::g.60551::m.60551	0	0	0	0	0	0	0	0	0	0	0	0	647700	1666800	1773600

TRINITY_DN17938_c1_g3::TRINITY_DN17938_c1_g3_i1::g.92335::m.92335	112040	0	104160	376540	0	182300	0	216120	0	0	1170600	1092200	258220	282960	284080
TRINITY_DN13436_c3_g2::TRINITY_DN13436_c3_g2_i1::g.23914::m.23914	0	0	0	0	0	0	0	0	0	0	0	0	1280600	1301000	1494300
TRINITY_DN19570_c1_g1::TRINITY_DN19570_c1_g1_i2::g.119952::m.119952	146070	41986	165950	82739	0	262470	403210	376410	1018400	0	0	1248300	103360	106380	115060
TRINITY_DN19058_c2_g1::TRINITY_DN19058_c2_g1_i7::g.111114::m.1111	0	100390	0	386270	393560	0	279320	350120	768660	697710	0	1088500	0	0	0
TRINITY_DN12832_c2_g7::TRINITY_DN12832_c2_g7_i2::g.16314::m.16314	0	0	0	0	0	0	0	0	0	0	0	0	1789700	225760	2044600
TRINITY_DN17735_c1_g1::TRINITY_DN17735_c1_g1_i1::g.89033::m.89033	156580	70539	180920	172910	358910	0	486740	0	0	0	894920	1733100	0	0	0
TRINITY_DN10960_c0_g1::TRINITY_DN10960_c0_g1_i2::g.5906::m.5906	0	0	0	0	0	0	0	0	0	0	0	0	4049500	0	0
TRINITY_DN13066_c0_g1::TRINITY_DN13066_c0_g1_i8::g.19396::m.19396	53962	0	75806	61844	0	0	0	0	0	0	0	991030	0	286970	2572900
TRINITY_DN12323_c0_g2::TRINITY_DN12323_c0_g2_i9::g.11969::m.11969	0	0	0	170010	239420	85692	349370	133320	0	0	0	744860	588830	440220	1287000
TRINITY_DN18848_c2_g2::TRINITY_DN18848_c2_g2_i6::g.107389::m.107389	0	0	0	0	0	79083	0	0	364000	0	484000	0	1596000	0	1486900
TRINITY_DN19795_c3_g1::TRINITY_DN19795_c3_g1_i8::g.123663::m.123663	0	0	0	0	0	0	0	0	0	0	0	0	494300	2906000	602940
TRINITY_DN17387_c1_g1::TRINITY_DN17387_c1_g1_i3::g.82928::m.82928	0	0	0	0	13286	0	0	0	0	0	0	0	1297200	1769200	905620
TRINITY_DN13841_c4_g1::TRINITY_DN13841_c4_g1_i7::g.28424::m.28424	0	0	0	541830	176460	77908	197890	0	673430	0	213200	1000900	295550	533500	267220
TRINITY_DN12837_c2_g2::TRINITY_DN12837_c2_g2_i2::g.16573::m.16573	0	0	676020	0	0	0	899350	0	0	0	0	2357900	0	0	0
TRINITY_DN16782_c2_g2::TRINITY_DN16782_c2_g2_i4::g.73167::m.73167	0	0	0	0	0	0	0	0	1099200	0	1382400	1446200	0	0	0
TRINITY_DN18666_c0_g1::TRINITY_DN18666_c0_g1_i7::g.104181::m.104181	0	0	0	0	0	0	0	0	0	0	0	0	1509300	1942200	474950
TRINITY_DN19251_c1_g2::TRINITY_DN19251_c1_g2_i27::g.114997::m.114997	0	0	0	0	0	0	0	170570	0	0	0	0	1475700	1535900	708100
TRINITY_DN13809_c0_g1::TRINITY_DN13809_c0_g1_i2::g.28550::m.28550	120910	0	111160	151810	319320	175850	0	783280	0	0	789870	0	418510	542210	467020
TRINITY_DN19468_c2_g2::TRINITY_DN19468_c2_g2_i2::g.118324::m.118324	0	0	0	0	0	0	0	0	350790	0	431350	533780	928420	1186100	446570
TRINITY_DN16852_c0_g1::TRINITY_DN16852_c0_g1_i5::g.74328::m.74328	85005	0	82292	101480	453410	0	340840	0	519050	513900	1196200	0	0	148600	429780

TRINITY_DN16408_c1_g1::TRINITY_DN16408_c1_g1_i1::g.67131::m.67131	0	0	0	0	0	0	0	0	0	0	125020	146190	1837400	924830	829830
TRINITY_DN11560_c0_g1::TRINITY_DN11560_c0_g1_i1::g.7865::m.7865	0	0	0	0	0	0	83456	0	0	0	0	274620	552700	368600	2540100
TRINITY_DN19263_c0_g1::TRINITY_DN19263_c0_g1_i6::g.114813::m.114813	0	0	0	0	330420	0	266450	379110	0	0	1317900	1514300	0	0	0
TRINITY_DN19101_c2_g2::TRINITY_DN19101_c2_g2_i4::g.111950::m.1119	0	0	0	0	0	0	0	0	3444300	0	0	0	153030	190420	0
TRINITY_DN19615_c0_g1::TRINITY_DN19615_c0_g1_i7::g.122149::m.122149	30664	0	94758	61513	154830	0	207550	166510	0	260380	0	472020	719990	585170	1027900
TRINITY_DN18862_c1_g2::TRINITY_DN18862_c1_g2_i1::g.107502::m.107502	0	0	0	0	0	0	0	0	0	0	0	157290	1999100	859790	762420
TRINITY_DN14909_c1_g5::TRINITY_DN14909_c1_g5_i1::g.44180::m.44180	0	0	0	0	0	0	0	0	0	0	0	0	1714400	949310	1098500
TRINITY_DN17699_c0_g2::TRINITY_DN17699_c0_g2_i2::g.88416::m.88416	44207	0	0	0	0	0	0	65060	0	0	0	0	888030	860660	1903700
TRINITY_DN11338_c0_g1::TRINITY_DN11338_c0_g1_i3::g.7037::m.7037	0	0	0	0	0	0	0	0	0	0	0	0	1937100	1568400	247090
TRINITY_DN17067_c2_g2::TRINITY_DN17067_c2_g2_i1::g.77907::m.77907	0	0	0	0	0	0	0	0	0	0	0	0	780960	1303500	1651300
TRINITY_DN17136_c2_g3::TRINITY_DN17136_c2_g3_i6::g.79230::m.79230	163360	72120	180230	121410	0	0	0	0	0	269590	0	494890	857320	1142700	431820
TRINITY_DN15761_c1_g2::TRINITY_DN15761_c1_g2_i2::g.56714::m.56714	0	0	0	0	0	0	0	0	0	0	0	0	711360	2269100	750350
TRINITY_DN18490_c1_g1::TRINITY_DN18490_c1_g1_i2::g.101177::m.1011	0	0	0	0	0	0	0	0	0	0	0	0	1079400	1231100	1416700
TRINITY_DN11842_c0_g1::TRINITY_DN11842_c0_g1_i5::g.9125::m.9125	0	27219	52976	37549	351410	0	0	359310	0	0	735520	568700	345680	842030	401090
TRINITY_DN14684_c2_g1::TRINITY_DN14684_c2_g1_i9::g.40676::m.40676	0	0	0	43417	0	0	212710	0	0	0	571630	377630	1438500	725330	329250
TRINITY_DN17978_c0_g1::TRINITY_DN17978_c0_g1_i12::g.93003::m.93003	0	0	0	0	0	0	0	0	0	0	0	0	0	1477000	2210100
TRINITY_DN14251_c1_g2::TRINITY_DN14251_c1_g2_i7::g.34536::m.34536	146570	77992	198390	221240	330990	0	296810	345680	0	0	0	1484400	0	0	571710
TRINITY_DN15354_c0_g1::TRINITY_DN15354_c0_g1_i1::g.50622::m.50622	0	0	0	0	0	0	0	0	0	0	0	0	984990	1513200	1141100
TRINITY_DN14014_c3_g1::TRINITY_DN14014_c3_g1_i1::g.31362::m.31362	0	0	0	0	0	0	0	0	403590	178690	556280	529430	1583000	220180	160610
TRINITY_DN14788_c0_g2::TRINITY_DN14788_c0_g2_i4::g.42257::m.42257	0	0	28627	0	0	0	0	62416	0	0	0	0	954430	1394300	1180400
TRINITY_DN14955_c4_g2::TRINITY_DN14955_c4_g2_i6::g.44900::m.44900	0	0	0	0	0	0	0	0	0	0	0	0	379660	1725600	1508800

TRINITY_DN10990_c0_g1::TRINITY_DN10990_c0_g1_i1::g.6030::m.6030	0	0	0	0	0	0	0	0	0	0	0	0	552820	2125300	935670
TRINITY_DN10710_c0_g1::TRINITY_DN10710_c0_g1_i1::g.5355::m.5355	0	0	0	0	262890	0	0	579480	0	501960	1059500	838170	0	0	346220
TRINITY_DN17874_c2_g4::TRINITY_DN17874_c2_g4_i3::g.91179::m.91179	0	0	0	0	0	0	0	0	0	0	0	0	1119400	1566000	901300
TRINITY_DN16487_c1_g6::TRINITY_DN16487_c1_g6_i1::g.68386::m.68386	0	0	0	0	0	0	0	0	0	0	0	0	3585000	0	0
TRINITY_DN19704_c0_g1::TRINITY_DN19704_c0_g1_i3::g.122553::m.122553	0	0	0	0	0	0	0	0	0	0	0	0	1138700	994160	1451300
TRINITY_DN15899_c1_g1::TRINITY_DN15899_c1_g1_i1::g.58501::m.58501	0	0	0	0	0	0	0	0	0	0	0	0	1073800	1189500	1318500
TRINITY_DN13705_c0_g2::TRINITY_DN13705_c0_g2_i4::g.27444::m.27444	0	0	0	49597	0	0	0	138980	0	0	0	0	1232500	1723900	429940
TRINITY_DN18855_c1_g1::TRINITY_DN18855_c1_g1_i19::g.107600::m.107600	0	0	0	0	0	0	0	0	0	0	0	0	2522300	662240	378520
TRINITY_DN17745_c1_g5::TRINITY_DN17745_c1_g5_i2::g.88501::m.88501	0	0	0	0	0	0	0	0	0	0	0	0	1013400	1340000	1205300
TRINITY_DN10578_c0_g1::TRINITY_DN10578_c0_g1_i5::g.5050::m.5050	0	0	0	0	0	0	0	0	0	0	0	0	1242000	1313300	994240
TRINITY_DN13945_c0_g1::TRINITY_DN13945_c0_g1_i2::g.30107::m.30107	0	0	33753	0	326830	0	397320	462190	0	96258	0	0	812240	931510	483750
TRINITY_DN19655_c2_g1::TRINITY_DN19655_c2_g1_i11::g.121761::m.121761	0	0	0	0	0	0	0	0	0	0	0	0	1565000	1972800	0
TRINITY_DN9772_c0_g1::TRINITY_DN9772_c0_g1_i1::g.3863::m.3863	0	0	0	0	0	0	0	0	0	0	0	0	1202400	1548800	784140
TRINITY_DN14598_c1_g3::TRINITY_DN14598_c1_g3_i4::g.39295::m.39295	128190	43515	144830	102020	248130	93444	181830	322780	636930	0	1027800	494450	0	0	100760
TRINITY_DN13423_c2_g1::TRINITY_DN13423_c2_g1_i2::g.23680::m.23680	0	0	0	0	0	0	0	0	0	0	0	0	984410	1365400	1169600
TRINITY_DN13846_c2_g2::TRINITY_DN13846_c2_g2_i2::g.28548::m.28548	0	0	0	0	166640	0	0	0	364190	0	429620	0	731420	801470	1024300
TRINITY_DN13334_c3_g4::TRINITY_DN13334_c3_g4_i1::g.22910::m.22910	498330	0	433400	374430	0	0	0	0	0	0	0	0	1061300	1140200	0
TRINITY_DN17504_c0_g1::TRINITY_DN17504_c0_g1_i2::g.84827::m.84827	0	0	0	0	0	0	0	0	0	0	0	0	985850	579720	1940500
TRINITY_DN16041_c1_g2::TRINITY_DN16041_c1_g2_i6::g.61105::m.61105	0	0	0	0	0	0	0	0	0	0	0	0	1008300	1237500	1244700
TRINITY_DN13569_c0_g2::TRINITY_DN13569_c0_g2_i1::g.25677::m.25677	0	0	0	0	0	0	0	0	0	0	237710	280580	0	1599400	1364100

TRINITY_DN11464_c0_g1::TRINITY_DN11464_c0_g1_i3::g.7465::m.7465	0	0	0	0	0	0	0	0	0	0	0	0	1096300	1289200	1075600
TRINITY_DN15585_c1_g1::TRINITY_DN15585_c1_g1_i8::g.54308::m.54308	280920	90342	300770	376760	0	271100	636090	537250	957590	0	0	0	0	0	0
TRINITY_DN15428_c6_g1::TRINITY_DN15428_c6_g1_i5::g.51800::m.51800	0	0	0	0	0	0	0	0	0	0	0	0	191960	2057400	1200200
TRINITY_DN18097_c14_g3::TRINITY_DN18097_c14_g3_i5::g.95141::m.95141	0	0	0	0	0	0	0	0	0	0	0	0	840390	1332900	1269100
TRINITY_DN12611_c0_g2::TRINITY_DN12611_c0_g2_i7::g.14237::m.14237	0	0	0	0	465600	0	0	486060	0	0	0	1567600	0	608540	305950
TRINITY_DN14738_c2_g1::TRINITY_DN14738_c2_g1_i2::g.41499::m.41499	0	0	0	0	0	0	0	0	0	0	0	0	1343700	1474200	607520
TRINITY_DN14357_c5_g2::TRINITY_DN14357_c5_g2_i13::g.35817::m.35817	0	0	0	0	0	0	0	0	0	0	0	0	1053000	1236800	1122500
TRINITY_DN16879_c1_g4::TRINITY_DN16879_c1_g4_i3::g.74866::m.74866	0	0	56175	49445	0	0	0	0	0	0	0	0	1054700	1306600	922270
TRINITY_DN16280_c0_g1::TRINITY_DN16280_c0_g1_i6::g.65080::m.65080	0	0	0	0	0	0	0	0	0	0	0	0	1287200	1614800	486980
TRINITY_DN13851_c1_g1::TRINITY_DN13851_c1_g1_i8::g.29304::m.29304	157000	84420	200610	157790	0	0	0	0	0	0	0	0	885940	1168600	727890
TRINITY_DN19385_c2_g1::TRINITY_DN19385_c2_g1_i11::g.117072::m.117072	0	0	0	0	0	0	0	0	0	0	0	0	624320	1335500	1414100
TRINITY_DN14233_c0_g1::TRINITY_DN14233_c0_g1_i2::g.34181::m.34181	80119	32729	0	57331	499520	265450	92133	524290	222890	0	472790	324060	279230	171760	325550
TRINITY_DN9198_c0_g1::TRINITY_DN9198_c0_g1_i1::g.3186::m.3186	0	0	0	0	0	0	0	0	0	0	0	0	483350	1622300	1227100
TRINITY_DN15338_c2_g1::TRINITY_DN15338_c2_g1_i4::g.50559::m.50559	0	0	0	0	0	0	0	0	0	0	132490	0	1397700	511460	1289600
TRINITY_DN16719_c0_g1::TRINITY_DN16719_c0_g1_i6::g.72195::m.72195	261330	0	0	0	0	0	0	0	0	0	0	0	0	3066100	0
TRINITY_DN18047_c0_g1::TRINITY_DN18047_c0_g1_i1::g.93485::m.93485	0	0	0	0	0	0	0	0	0	0	0	0	1053800	1482300	782210
TRINITY_DN12093_c0_g1::TRINITY_DN12093_c0_g1_i8::g.10458::m.10458	133670	45380	49785	87081	396250	109760	395850	0	0	107880	423880	482460	738440	0	345140
TRINITY_DN9933_c0_g1::TRINITY_DN9933_c0_g1_i1::g.4060::m.4060	66405	0	0	58968	182570	104910	261520	209140	0	132190	1602600	0	237610	331180	126910
TRINITY_DN17539_c0_g2::TRINITY_DN17539_c0_g2_i3::g.85610::m.85610	0	0	0	0	0	0	0	0	0	0	0	0	933320	1020800	1351900
TRINITY_DN12352_c0_g2::TRINITY_DN12352_c0_g2_i4::g.12096::m.12096	0	0	44846	43140	0	0	0	0	0	0	0	0	995040	1199900	1017700
TRINITY_DN12828_c0_g1::TRINITY_DN12828_c0_g1_i2::g.16430::m.16430	0	0	0	0	0	0	0	0	0	0	0	0	1325300	0	1972500

TRINITY_DN17150_c2_g1::TRINITY_DN17150_c2_g1_i7::g.79332::m.79332	43084	0	41497	33225	0	0	0	0	0	0	0	0	1222900	1294700	657980
TRINITY_DN15988_c0_g1::TRINITY_DN15988_c0_g1_i7::g.59999::m.59999	0	0	0	0	0	0	0	0	0	0	0	0	558120	866280	1866500
TRINITY_DN19643_c4_g1::TRINITY_DN19643_c4_g1_i10::g.121539::m.121539	32967	0	40325	45014	0	0	0	0	854730	555590	0	961040	428840	196520	164380
TRINITY_DN14785_c0_g1::TRINITY_DN14785_c0_g1_i8::g.42289::m.42289	0	0	0	0	0	0	0	0	0	0	0	0	967990	1017300	1291400
TRINITY_DN16927_c0_g1::TRINITY_DN16927_c0_g1_i2::g.75647::m.75647	0	0	0	0	0	0	0	0	0	0	0	0	1010800	1322300	942060
TRINITY_DN18066_c0_g3::TRINITY_DN18066_c0_g3_i5::g.94429::m.94429	120090	63785	218630	261940	139500	0	0	164010	0	0	382780	0	484180	728940	705130
TRINITY_DN15763_c0_g1::TRINITY_DN15763_c0_g1_i1::g.56716::m.56716	0	0	0	0	0	0	0	0	0	0	0	0	779390	1304200	1182700
TRINITY_DN17933_c0_g2::TRINITY_DN17933_c0_g2_i1::g.92168::m.92168	140920	76802	199020	0	647590	0	584920	531910	0	0	0	0	179150	500130	405740
TRINITY_DN16282_c1_g3::TRINITY_DN16282_c1_g3_i1::g.64973::m.64973	0	0	0	0	0	0	0	0	0	0	0	0	692550	1594000	978350
TRINITY_DN10432_c0_g1::TRINITY_DN10432_c0_g1_i2::g.4807::m.4807	0	0	0	0	0	0	0	0	0	0	0	0	1313600	917620	1022000
TRINITY_DN18168_c0_g1::TRINITY_DN18168_c0_g1_i2::g.96274::m.96274	0	0	0	0	0	0	0	0	0	0	0	0	1166200	984940	1095800
TRINITY_DN14775_c0_g2::TRINITY_DN14775_c0_g2_i2::g.42036::m.42036	0	0	0	56203	0	263980	0	0	0	0	0	0	609500	1219600	1090900
TRINITY_DN18002_c1_g3::TRINITY_DN18002_c1_g3_i1::g.93438::m.93438	0	0	0	0	0	0	0	0	0	0	0	0	1070000	1377700	791090
TRINITY_DN16157_c0_g1::TRINITY_DN16157_c0_g1_i7::g.62500::m.62500	0	0	0	0	0	0	0	0	0	0	0	0	797030	1238100	1195600
TRINITY_DN13818_c0_g2::TRINITY_DN13818_c0_g2_i1::g.28967::m.28967	0	0	0	0	0	0	0	58396	0	0	0	173670	752880	1282200	950290
TRINITY_DN11548_c0_g1::TRINITY_DN11548_c0_g1_i1::g.7714::m.7714	0	0	0	0	0	0	0	0	0	0	0	0	292420	2007400	894250
TRINITY_DN17145_c0_g1::TRINITY_DN17145_c0_g1_i4::g.79323::m.79323	0	0	0	0	0	0	0	0	93634	0	0	0	872660	980930	1226800
TRINITY_DN17214_c1_g1::TRINITY_DN17214_c1_g1_i9::g.80183::m.80183	0	0	0	0	0	0	0	0	0	0	0	0	1202300	1344000	614300
TRINITY_DN19434_c1_g1::TRINITY_DN19434_c1_g1_i6::g.117945::m.117945	544270	158960	540310	433030	452900	119130	552320	357330	0	0	0	0	0	0	0
TRINITY_DN11638_c0_g1::TRINITY_DN11638_c0_g1_i1::g.8051::m.8051	2890400	0	0	0	0	0	0	0	0	0	0	0	0	266560	0
TRINITY_DN12478_c4_g4::TRINITY_DN12478_c4_g4_i5::g.13332::m.13332	0	0	0	0	0	0	0	0	0	0	134240	0	927850	602900	1485300

TRINITY_DN16700_c3_g3::TRINITY_DN16700_c3_g3_i4::g.71827::m.71827	0	0	0	0	0	0	0	0	0	0	0	0	1492000	990580	662590
TRINITY_DN14707_c0_g2::TRINITY_DN14707_c0_g2_i9::g.40985::m.40985	0	0	0	0	0	0	0	0	0	0	0	0	680580	1354000	1109700
TRINITY_DN19896_c2_g1::TRINITY_DN19896_c2_g1_i4::g.125437::m.125437	0	0	0	0	0	0	0	0	0	0	0	0	835000	1077900	1222400
TRINITY_DN13645_c2_g2::TRINITY_DN13645_c2_g2_i6::g.26141::m.26141	0	0	0	0	0	0	0	0	0	0	0	0	1562700	0	1570400
TRINITY_DN11142_c1_g1::TRINITY_DN11142_c1_g1_i2::g.6468::m.6468	0	0	0	0	0	0	0	0	0	0	0	0	1397000	817590	902960
TRINITY_DN15069_c1_g1::TRINITY_DN15069_c1_g1_i6::g.46552::m.46552	0	0	0	0	0	0	0	0	0	0	0	0	996950	1164100	946220
TRINITY_DN18921_c0_g1::TRINITY_DN18921_c0_g1_i2::g.108916::m.108916	0	0	0	0	0	0	0	0	0	0	0	0	856370	679230	1563500
TRINITY_DN19606_c0_g1::TRINITY_DN19606_c0_g1_i4::g.121009::m.121009	0	0	0	0	0	0	0	0	0	0	0	0	752040	899610	1441200
TRINITY_DN15436_c5_g1::TRINITY_DN15436_c5_g1_i4::g.51870::m.51870	0	0	0	0	0	0	0	0	0	0	0	0	1141900	1541000	389530
TRINITY_DN18704_c0_g1::TRINITY_DN18704_c0_g1_i1::g.104889::m.104889	0	0	0	0	0	0	0	0	0	0	0	0	529230	1451900	1069400
TRINITY_DN11290_c1_g1::TRINITY_DN11290_c1_g1_i4::g.6884::m.6884	0	0	0	0	0	0	0	0	0	0	0	0	700940	1198300	1142300
TRINITY_DN17528_c1_g2::TRINITY_DN17528_c1_g2_i6::g.85342::m.85342	0	0	0	0	0	0	0	0	0	0	0	0	871570	1151300	995700
TRINITY_DN15505_c1_g1::TRINITY_DN15505_c1_g1_i3::g.53026::m.53026	0	0	0	0	0	0	0	0	0	0	0	0	932870	959750	1120200
TRINITY_DN11648_c0_g3::TRINITY_DN11648_c0_g3_i1::g.8240::m.8240	0	0	0	0	0	0	0	0	0	0	0	0	734000	1603000	675570
TRINITY_DN14322_c1_g1::TRINITY_DN14322_c1_g1_i1::g.35379::m.35379	0	0	0	0	0	0	0	0	0	0	0	0	1350900	741840	904800
TRINITY_DN19908_c2_g1::TRINITY_DN19908_c2_g1_i5::g.125911::m.125911	0	0	0	0	0	0	0	0	0	0	0	0	1195900	926020	867780
TRINITY_DN19181_c1_g1::TRINITY_DN19181_c1_g1_i4::g.113426::m.1134	39220	0	61677	64132	0	0	0	111590	0	0	292440	0	499990	1365800	553260
TRINITY_DN16453_c2_g1::TRINITY_DN16453_c2_g1_i14::g.67807::m.67807	28217	0	0	0	144380	0	194440	0	0	0	236880	751110	750210	425060	445850
TRINITY_DN12583_c1_g3::TRINITY_DN12583_c1_g3_i1::g.14008::m.14008	0	50127	179820	148000	625520	0	841460	234350	0	320060	558160	0	0	0	0
TRINITY_DN18832_c0_g1::TRINITY_DN18832_c0_g1_i1::g.107098::m.107098	0	0	0	0	0	0	194720	199520	0	221210	0	0	677930	941690	721930

TRINITY_DN14291_c3_g7::TRINITY_DN14291_c3_g7_i2::g.35143::m.35143	495900	129600	416900	433750	0	0	351650	399660	0	0	0	0	359860	369300	0
TRINITY_DN19997_c3_g2::TRINITY_DN19997_c3_g2_i2::g.127405::m.127405	0	0	0	0	0	0	0	0	0	0	0	0	1828200	0	1126100
TRINITY_DN13437_c3_g3::TRINITY_DN13437_c3_g3_i4::g.23371::m.23371	0	0	0	0	0	0	0	0	0	0	0	0	946250	1256000	750140
TRINITY_DN16324_c2_g1::TRINITY_DN16324_c2_g1_i11::g.65876::m.65876	111560	32966	130790	115780	0	0	0	0	437740	115740	250830	0	707390	418140	618210
TRINITY_DN14723_c2_g4::TRINITY_DN14723_c2_g4_i2::g.41268::m.41268	0	0	0	0	0	0	0	0	0	0	0	0	1249300	1291300	396440
TRINITY_DN12136_c0_g1::TRINITY_DN12136_c0_g1_i2::g.10688::m.10688	0	0	0	0	0	0	0	0	0	0	0	0	738530	958760	1234800
TRINITY_DN19231_c4_g1::TRINITY_DN19231_c4_g1_i8::g.114359::m.114359	0	0	0	0	0	0	0	0	0	0	0	0	616310	1133300	1180100
TRINITY_DN15458_c1_g3::TRINITY_DN15458_c1_g3_i6::g.52102::m.52102	0	0	0	0	0	0	0	0	0	0	0	0	887440	1108600	929160
TRINITY_DN15082_c1_g1::TRINITY_DN15082_c1_g1_i1::g.46708::m.46708	0	0	0	0	0	0	133990	0	0	0	367350	1110200	711450	317450	278750
TRINITY_DN19344_c5_g1::TRINITY_DN19344_c5_g1_i3::g.115614::m.115614	0	0	0	46466	0	0	0	98982	0	0	0	0	1651700	720930	396050
TRINITY_DN14912_c1_g1::TRINITY_DN14912_c1_g1_i4::g.44268::m.44268	247530	104660	304300	381790	0	0	242320	0	0	0	1220800	0	133180	277700	0
TRINITY_DN13129_c8_g5::TRINITY_DN13129_c8_g5_i1::g.20297::m.20297	0	0	0	0	0	0	0	0	0	0	0	0	133670	1194700	1580500
TRINITY_DN12983_c5_g1::TRINITY_DN12983_c5_g1_i4::g.18189::m.18189	0	0	0	0	0	0	0	0	0	0	0	0	871910	1174700	860770
TRINITY_DN13073_c0_g1::TRINITY_DN13073_c0_g1_i2::g.19364::m.19364	0	0	0	0	0	0	0	0	0	0	0	0	816240	1128600	961000
TRINITY_DN14563_c5_g1::TRINITY_DN14563_c5_g1_i5::g.38974::m.38974	0	0	0	0	0	0	0	0	0	0	0	0	0	2899900	0
TRINITY_DN15029_c4_g1::TRINITY_DN15029_c4_g1_i3::g.45979::m.45979	0	0	0	0	0	0	0	0	0	0	0	0	848170	1009800	1041600
TRINITY_DN16123_c0_g2::TRINITY_DN16123_c0_g2_i3::g.62404::m.62404	0	0	0	0	0	0	0	0	0	0	0	0	1073900	1014800	808730
TRINITY_DN13128_c0_g1::TRINITY_DN13128_c0_g1_i1::g.20153::m.20153	0	0	0	0	0	0	0	0	0	0	0	0	596390	2082300	208630
TRINITY_DN11467_c0_g1::TRINITY_DN11467_c0_g1_i3::g.7480::m.7480	0	0	0	0	0	0	0	0	0	0	0	0	815940	992620	1066300
TRINITY_DN16134_c1_g1::TRINITY_DN16134_c1_g1_i3::g.62771::m.62771	0	0	0	0	0	0	0	0	0	0	0	196400	435310	1082100	1160600

TRINITY_DN12648_c0_g1::TRINITY_DN12648_c0_g1_i4::g.15036::m.15036	36607	30294	69845	45658	0	0	0	0	0	0	0	0	1009200	827660	844290
TRINITY_DN17776_c1_g1::TRINITY_DN17776_c1_g1_i2::g.89554::m.89554	282910	67780	213730	326150	421930	99222	275270	0	0	0	585520	386790	0	202200	0
TRINITY_DN17310_c0_g2::TRINITY_DN17310_c0_g2_i2::g.81838::m.81838	0	0	0	0	0	0	0	0	0	0	0	0	776400	929640	1152700
TRINITY_DN18211_c0_g1::TRINITY_DN18211_c0_g1_i1::g.96953::m.96953	0	0	0	0	0	0	0	0	0	0	0	0	787130	1072500	998640
TRINITY_DN16650_c2_g2::TRINITY_DN16650_c2_g2_i4::g.71040::m.71040	0	0	0	0	298670	80715	353570	87238	581510	163810	335420	608820	104440	131700	110980
TRINITY_DN17333_c5_g3::TRINITY_DN17333_c5_g3_i2::g.82453::m.82453	0	0	0	0	0	0	0	0	0	0	0	0	957920	1124100	773130
TRINITY_DN12530_c0_g1::TRINITY_DN12530_c0_g1_i1::g.13568::m.13568	0	0	0	0	0	0	0	0	0	0	0	0	729600	1467600	657840
TRINITY_DN15837_c1_g2::TRINITY_DN15837_c1_g2_i3::g.57761::m.57761	0	0	0	0	0	0	0	0	0	0	0	0	397740	918100	1531900
TRINITY_DN20022_c2_g5::TRINITY_DN20022_c2_g5_i1::g.128013::m.128013	0	0	0	0	0	0	0	0	0	0	0	0	750060	1004300	1067700
TRINITY_DN18115_c1_g1::TRINITY_DN18115_c1_g1_i4::g.95413::m.95413	0	0	0	0	0	0	0	0	0	0	0	0	1393600	891400	535700
TRINITY_DN15805_c1_g2::TRINITY_DN15805_c1_g2_i2::g.57262::m.57262	0	0	0	0	0	0	0	0	0	0	0	0	927290	706170	1184200
TRINITY_DN15457_c3_g1::TRINITY_DN15457_c3_g1_i1::g.52194::m.52194	0	0	0	0	0	0	0	0	0	0	0	0	558350	766410	1486800
TRINITY_DN14777_c1_g1::TRINITY_DN14777_c1_g1_i9::g.42089::m.42089	0	0	0	0	0	0	0	0	0	0	0	0	673450	668770	1464400
TRINITY_DN13637_c0_g1::TRINITY_DN13637_c0_g1_i7::g.26541::m.26541	0	0	0	0	0	0	0	0	0	0	0	0	945850	1018500	834090
TRINITY_DN13683_c1_g1::TRINITY_DN13683_c1_g1_i4::g.27154::m.27154	0	0	0	0	0	0	0	0	0	0	0	0	834720	1235600	716110
TRINITY_DN13930_c0_g1::TRINITY_DN13930_c0_g1_i1::g.30435::m.30435	0	0	0	0	0	0	124220	144450	0	0	0	0	907560	1601400	0
TRINITY_DN12625_c0_g2::TRINITY_DN12625_c0_g2_i2::g.14331::m.14331	0	0	0	0	0	0	0	0	0	0	0	0	880820	1485600	409510
TRINITY_DN14916_c3_g2::TRINITY_DN14916_c3_g2_i1::g.44503::m.44503	0	0	0	0	0	0	0	0	0	0	0	0	1431500	625910	715480
TRINITY_DN16821_c2_g1::TRINITY_DN16821_c2_g1_i2::g.73867::m.73867	0	0	0	0	0	0	0	0	0	0	0	0	1180500	1055600	533430
TRINITY_DN18229_c0_g2::TRINITY_DN18229_c0_g2_i1::g.97234::m.97234	0	104500	200820	314820	0	0	0	536810	1602700	0	0	0	0	0	0

TRINITY_DN19026_c0_g3::TRINITY_DN19026_c0_g3_i5::g.110636::m.110636	0	0	0	0	0	0	189650	0	0	0	0	0	816410	604850	1144400
TRINITY_DN19212_c1_g1::TRINITY_DN19212_c1_g1_i8::g.114114::m.1141	0	0	0	0	0	0	307350	215380	0	258650	0	0	380530	896180	685100
TRINITY_DN12118_c0_g1::TRINITY_DN12118_c0_g1_i1::g.10631::m.10631	0	0	0	0	0	0	0	0	0	0	0	0	1410100	625950	700810
TRINITY_DN18618_c2_g3::TRINITY_DN18618_c2_g3_i2::g.103853::m.103853	0	0	0	0	0	0	0	0	0	0	0	0	852660	1038100	843790
TRINITY_DN17695_c2_g1::TRINITY_DN17695_c2_g1_i3::g.88380::m.88380	0	0	0	0	0	0	0	0	0	0	0	0	0	2733600	0
TRINITY_DN14310_c2_g3::TRINITY_DN14310_c2_g3_i5::g.35345::m.35345	0	0	0	0	0	0	0	0	0	0	0	0	810370	970790	944290
TRINITY_DN16144_c0_g3::TRINITY_DN16144_c0_g3_i9::g.62076::m.62076	0	0	0	0	0	0	0	0	0	0	0	0	746070	637000	1331300
TRINITY_DN12285_c1_g1::TRINITY_DN12285_c1_g1_i3::g.11619::m.11619	0	0	0	0	0	0	0	0	0	0	0	0	983820	1306400	418980
TRINITY_DN19816_c1_g2::TRINITY_DN19816_c1_g2_i10::g.124090::m.124090	0	0	0	0	0	0	0	0	0	0	0	0	787430	1028100	891250
TRINITY_DN15180_c0_g2::TRINITY_DN15180_c0_g2_i2::g.47796::m.47796	0	0	0	0	0	0	0	0	0	0	0	0	579630	1189100	934080
TRINITY_DN19665_c1_g1::TRINITY_DN19665_c1_g1_i7::g.121863::m.121863	0	0	0	0	0	0	0	0	0	0	0	0	856720	947420	898440
TRINITY_DN19770_c1_g4::TRINITY_DN19770_c1_g4_i6::g.123393::m.123393	0	0	64771	72894	0	0	0	0	0	0	159490	0	984620	1066000	354360
TRINITY_DN14513_c0_g3::TRINITY_DN14513_c0_g3_i9::g.38189::m.38189	462610	175360	367590	177370	113640	69979	147980	135520	0	198010	398420	454360	0	0	0
TRINITY_DN16940_c1_g1::TRINITY_DN16940_c1_g1_i2::g.75863::m.75863	298010	151980	473430	442780	0	0	0	0	0	0	0	1326700	0	0	0
TRINITY_DN14725_c0_g1::TRINITY_DN14725_c0_g1_i4::g.41213::m.41213	667050	848730	1175500	0	0	0	0	0	0	0	0	0	0	0	0
TRINITY_DN12515_c0_g1::TRINITY_DN12515_c0_g1_i3::g.13461::m.13461	260300	0	296100	66702	285220	0	0	236240	93965	0	131570	0	731440	0	584960
TRINITY_DN16262_c1_g3::TRINITY_DN16262_c1_g3_i2::g.64875::m.64875	0	0	0	0	0	0	0	0	0	0	0	0	1058300	1159600	455010
TRINITY_DN13815_c1_g1::TRINITY_DN13815_c1_g1_i21::g.28891::m.2889	0	0	0	0	0	0	0	0	179320	0	0	0	0	2246100	240950
TRINITY_DN14602_c3_g2::TRINITY_DN14602_c3_g2_i9::g.39596::m.39596	40459	34765	143300	141870	49398	27835	0	0	0	111600	377170	0	542320	610350	579550
TRINITY_DN18669_c1_g1::TRINITY_DN18669_c1_g1_i3::g.104346::m.104346	0	0	0	0	0	0	0	0	0	0	0	0	636700	1344100	677550

TRINITY_DN16497_c0_g5::TRINITY_DN16497_c0_g5_i1::g.67600::m.67600	0	0	0	0	0	0	0	0	0	0	0	2537200	0	65960	54296
TRINITY_DN15927_c0_g1::TRINITY_DN15927_c0_g1_i2::g.59092::m.59092	129320	0	83894	42779	0	0	98739	0	0	0	0	533520	848950	0	917290
TRINITY_DN17586_c3_g1::TRINITY_DN17586_c3_g1_i5::g.86439::m.86439	0	0	0	0	0	0	0	0	0	0	0	0	605040	1093600	954940
TRINITY_DN16528_c2_g1::TRINITY_DN16528_c2_g1_i3::g.69117::m.69117	0	0	0	0	0	0	0	0	0	0	0	0	1047100	1605700	0
TRINITY_DN15498_c4_g2::TRINITY_DN15498_c4_g2_i2::g.51311::m.51311	0	0	0	0	0	0	0	0	0	0	0	0	1009200	1506500	133320
TRINITY_DN14361_c0_g1::TRINITY_DN14361_c0_g1_i5::g.35830::m.35830	0	0	0	0	0	0	0	0	0	0	0	0	1033700	661500	950100
TRINITY_DN11484_c0_g1::TRINITY_DN11484_c0_g1_i2::g.7552::m.7552	0	0	0	0	0	0	0	0	0	0	0	0	805480	745250	1076900
TRINITY_DN12658_c0_g1::TRINITY_DN12658_c0_g1_i2::g.14732::m.14732	0	0	0	0	0	0	0	0	0	0	0	0	91813	1107400	1426200
TRINITY_DN10660_c0_g1::TRINITY_DN10660_c0_g1_i1::g.5237::m.5237	543780	69922	789960	417800	0	0	0	299100	0	0	502370	0	0	0	0
TRINITY_DN3120_c0_g1::TRINITY_DN3120_c0_g1_i1::g.867::m.867	0	0	0	0	0	0	0	0	0	0	0	0	793450	1627600	195160
TRINITY_DN19581_c3_g6::TRINITY_DN19581_c3_g6_i4::g.120132::m.120132	0	0	0	0	0	0	0	0	0	0	0	0	859060	824180	928460
TRINITY_DN19709_c5_g1::TRINITY_DN19709_c5_g1_i3::g.122591::m.122591	0	0	0	0	0	0	0	0	0	0	0	0	1093400	824490	692250
TRINITY_DN16440_c0_g1::TRINITY_DN16440_c0_g1_i4::g.67503::m.67503	0	0	0	0	0	0	0	0	0	0	0	0	674660	711320	1197000
TRINITY_DN16345_c2_g1::TRINITY_DN16345_c2_g1_i1::g.66206::m.66206	0	0	95853	0	509330	180550	952820	841700	0	0	0	0	0	0	0
TRINITY_DN19239_c3_g1::TRINITY_DN19239_c3_g1_i4::g.113825::m.113825	0	0	0	0	0	0	0	0	0	0	0	0	777580	947800	853970
TRINITY_DN16612_c3_g7::TRINITY_DN16612_c3_g7_i2::g.70355::m.70355	0	0	0	0	0	0	0	0	0	0	0	0	703080	844570	1028400
TRINITY_DN19651_c4_g1::TRINITY_DN19651_c4_g1_i17::g.121374::m.121374	0	45185	110680	0	121110	163910	0	242940	0	607690	0	838380	0	280640	164110
TRINITY_DN12541_c0_g1::TRINITY_DN12541_c0_g1_i2::g.13592::m.13592	0	0	0	0	0	0	0	0	0	0	0	0	0	0	2547100
TRINITY_DN14080_c2_g2::TRINITY_DN14080_c2_g2_i1::g.32148::m.32148	0	0	0	0	0	0	0	0	0	0	0	0	1175500	1371400	0
TRINITY_DN16026_c0_g1::TRINITY_DN16026_c0_g1_i2::g.60976::m.60976	0	0	0	0	0	0	0	0	0	0	0	0	873570	695420	972240

TRINITY_DN17082_c4_g2::TRINITY_DN17082_c4_g2_i2::g.78169::m.78169	0	0	0	0	0	0	0	110660	0	0	0	0	492940	1055700	881670
TRINITY_DN19559_c2_g2::TRINITY_DN19559_c2_g2_i6::g.119806::m.119806	0	0	0	0	0	369120	0	0	0	0	0	0	998180	1171800	0
TRINITY_DN19551_c4_g4::TRINITY_DN19551_c4_g4_i1::g.119681::m.119681	0	0	0	0	0	0	0	0	0	0	0	0	0	2538600	0
TRINITY_DN12675_c1_g1::TRINITY_DN12675_c1_g1_i4::g.14912::m.14912	0	0	0	0	0	0	0	0	0	0	0	0	979750	431390	1126200
TRINITY_DN14194_c0_g2::TRINITY_DN14194_c0_g2_i9::g.33546::m.33546	0	0	0	0	0	0	0	0	0	0	0	0	677670	1121600	734720
TRINITY_DN15675_c4_g2::TRINITY_DN15675_c4_g2_i2::g.55509::m.55509	70947	0	89672	0	255430	0	0	0	0	0	809120	813820	0	492010	0
TRINITY_DN16295_c1_g2::TRINITY_DN16295_c1_g2_i1::g.65228::m.65228	0	0	0	0	0	0	0	0	0	0	0	0	924030	1096400	510520
TRINITY_DN16659_c4_g1::TRINITY_DN16659_c4_g1_i7::g.71135::m.71135	0	0	0	0	0	0	0	39660	0	0	0	0	525670	1220900	742960
TRINITY_DN15142_c0_g1::TRINITY_DN15142_c0_g1_i16::g.47469::m.47469	110240	51474	127780	117290	627650	278030	0	522200	0	0	0	0	285330	0	407950
TRINITY_DN14674_c1_g1::TRINITY_DN14674_c1_g1_i2::g.40399::m.40399	0	0	0	0	0	0	0	0	0	0	226240	0	545960	860120	892010
TRINITY_DN12462_c4_g1::TRINITY_DN12462_c4_g1_i1::g.13140::m.13140	0	0	0	0	0	0	0	0	0	0	0	0	772900	941410	809510
TRINITY_DN18768_c2_g1::TRINITY_DN18768_c2_g1_i1::g.106105::m.106105	0	0	0	0	0	0	0	0	0	0	0	0	1240300	958000	325300
TRINITY_DN19730_c4_g2::TRINITY_DN19730_c4_g2_i8::g.122947::m.122947	0	0	0	0	0	0	0	0	0	0	0	0	160610	1319700	1040700
TRINITY_DN18061_c2_g5::TRINITY_DN18061_c2_g5_i2::g.94542::m.94542	268380	0	38607	246610	233900	0	0	212050	0	0	0	0	0	1067600	453680
TRINITY_DN15458_c1_g2::TRINITY_DN15458_c1_g2_i4::g.52100::m.52100	0	0	0	0	0	0	0	0	0	0	0	0	175410	1183500	1154600
TRINITY_DN14909_c1_g1::TRINITY_DN14909_c1_g1_i3::g.44168::m.44168	0	0	0	0	0	0	0	0	0	0	0	0	1097400	649150	758590
TRINITY_DN16927_c1_g1::TRINITY_DN16927_c1_g1_i7::g.75660::m.75660	0	0	0	0	0	0	0	0	0	0	0	0	952660	804790	747080
TRINITY_DN17463_c2_g1::TRINITY_DN17463_c2_g1_i7::g.84144::m.84144	0	0	0	200020	0	0	273140	0	0	365440	871170	790360	0	0	0
TRINITY_DN19844_c1_g3::TRINITY_DN19844_c1_g3_i3::g.124457::m.124457	172780	44998	148370	134430	0	124840	0	199820	0	0	0	801300	0	474220	393290
TRINITY_DN15946_c1_g2::TRINITY_DN15946_c1_g2_i8::g.59360::m.59360	0	0	89184	0	0	0	0	118420	454150	0	0	1498100	0	171140	159420

TRINITY_DN14927_c1_g1::TRINITY_DN14927_c1_g1_i11::g.44438::m.44438	0	0	0	0	0	0	0	0	0	0	0	0	665620	1016800	796660
TRINITY_DN17612_c1_g2::TRINITY_DN17612_c1_g2_i3::g.87024::m.87024	0	0	0	0	0	0	0	0	0	0	0	0	520890	941140	1013400
TRINITY_DN18637_c1_g1::TRINITY_DN18637_c1_g1_i7::g.103721::m.103721	0	0	0	0	0	0	0	0	0	0	0	0	498930	1257600	710410
TRINITY_DN12213_c0_g1::TRINITY_DN12213_c0_g1_i3::g.11157::m.11157	0	0	0	0	0	0	0	0	0	0	0	0	435500	790840	1238100
TRINITY_DN19518_c0_g1::TRINITY_DN19518_c0_g1_i8::g.119129::m.1191	319710	78334	267040	334980	0	0	0	154310	0	0	637430	657700	0	0	0
TRINITY_DN19195_c4_g1::TRINITY_DN19195_c4_g1_i3::g.113639::m.113639	0	0	0	0	0	0	0	0	0	0	0	0	313980	949850	1185200
TRINITY_DN13292_c1_g6::TRINITY_DN13292_c1_g6_i2::g.22041::m.22041	0	0	0	0	0	0	0	0	0	0	0	0	1091000	0	1352100
TRINITY_DN14741_c0_g1::TRINITY_DN14741_c0_g1_i2::g.41477::m.41477	168940	0	467930	146120	439020	78222	0	0	0	417340	0	0	185620	538300	0
TRINITY_DN14630_c2_g8::TRINITY_DN14630_c2_g8_i1::g.39829::m.39829	0	0	0	0	0	0	0	0	0	0	0	0	753220	870330	817760
TRINITY_DN16782_c2_g1::TRINITY_DN16782_c2_g1_i3::g.73171::m.73171	152010	0	228640	232190	224000	88459	241610	0	0	0	0	1271900	0	0	0
TRINITY_DN18173_c2_g1::TRINITY_DN18173_c2_g1_i9::g.96627::m.96627	0	0	0	0	0	0	0	0	0	0	0	0	691230	781450	958640
TRINITY_DN17174_c0_g2::TRINITY_DN17174_c0_g2_i1::g.79619::m.79619	0	0	0	0	0	0	0	0	0	0	0	0	608480	820890	999770
TRINITY_DN12761_c4_g1::TRINITY_DN12761_c4_g1_i4::g.15698::m.15698	346410	79321	254830	218960	0	0	0	0	0	0	0	0	699370	816020	0
TRINITY_DN15641_c2_g1::TRINITY_DN15641_c2_g1_i3::g.54993::m.54993	135620	0	0	83453	0	0	0	0	0	400540	0	0	236300	857620	694660
TRINITY_DN16713_c1_g2::TRINITY_DN16713_c1_g2_i6::g.72096::m.72096	0	0	0	0	0	0	0	0	0	0	0	814280	269510	838000	485100
TRINITY_DN15145_c0_g2::TRINITY_DN15145_c0_g2_i2::g.46817::m.46817	0	0	0	0	0	0	0	0	0	0	0	0	886180	307230	1211400
TRINITY_DN16390_c0_g1::TRINITY_DN16390_c0_g1_i2::g.66783::m.66783	0	0	0	0	0	0	0	123110	0	0	0	350320	718450	714350	490790
TRINITY_DN16711_c0_g1::TRINITY_DN16711_c0_g1_i4::g.72030::m.72030	0	0	0	0	0	0	0	0	0	0	0	0	702840	925840	766920
TRINITY_DN15384_c0_g1::TRINITY_DN15384_c0_g1_i3::g.51070::m.51070	0	0	126090	0	0	0	0	0	0	0	0	0	1018000	797180	450490
TRINITY_DN17041_c0_g1::TRINITY_DN17041_c0_g1_i4::g.77480::m.77480	90090	0	0	78304	0	0	0	0	0	0	0	0	356660	256570	1606300
TRINITY_DN18962_c1_g1::TRINITY_DN18962_c1_g1_i3::g.109730::m.109730	0	0	0	0	0	0	0	0	0	0	0	0	335140	1075400	971540
TRINITY_DN16476_c3_g1::TRINITY_DN16476_c3_g1_i9::g.68231::m.68231	0	0	0	0	0	0	0	0	0	0	0	0	651350	783870	939170

TRINITY_DN17658_c5_g3::TRINITY_DN17658_c5_g3_i2::g.87770::m.87770	0	0	0	0	0	0	0	0	0	0	0	0	464280	768220	1139800
TRINITY_DN16523_c1_g1::TRINITY_DN16523_c1_g1_i4::g.69126::m.69126	0	0	0	0	0	0	0	0	0	0	0	0	962430	681890	726620
TRINITY_DN19779_c3_g3::TRINITY_DN19779_c3_g3_i2::g.123543::m.123543	574840	0	588840	297520	0	0	0	0	0	0	0	0	516950	390660	0
TRINITY_DN15367_c2_g1::TRINITY_DN15367_c2_g1_i7::g.50786::m.50786	0	155890	0	636170	0	260360	0	0	1312900	0	0	0	0	0	0
TRINITY_DN14788_c0_g1::TRINITY_DN14788_c0_g1_i1::g.42248::m.42248	0	0	0	0	0	0	0	0	0	0	0	0	1603900	419920	340730
TRINITY_DN12593_c2_g1::TRINITY_DN12593_c2_g1_i1::g.14128::m.14128	0	0	0	0	0	0	0	0	0	0	0	1878800	0	0	483670
TRINITY_DN18024_c1_g1::TRINITY_DN18024_c1_g1_i2::g.93774::m.93774	275170	91784	211590	175410	0	123600	210700	0	622970	0	0	650970	0	0	0
TRINITY_DN16834_c1_g1::TRINITY_DN16834_c1_g1_i1::g.74148::m.74148	0	0	0	0	91291	0	91568	0	226020	176200	0	1016600	453110	307240	0
TRINITY_DN19047_c1_g5::TRINITY_DN19047_c1_g5_i2::g.111027::m.111027	0	0	0	0	0	0	0	0	0	0	0	0	88515	1378600	891740
TRINITY_DN15127_c0_g2::TRINITY_DN15127_c0_g2_i1::g.47217::m.47217	0	0	0	0	0	0	0	0	0	0	0	0	399060	1173300	783650
TRINITY_DN13215_c0_g6::TRINITY_DN13215_c0_g6_i3::g.21426::m.21426	225020	83451	193030	152390	0	0	0	0	0	0	0	0	304560	731960	659770
TRINITY_DN16629_c2_g3::TRINITY_DN16629_c2_g3_i2::g.70768::m.70768	0	0	0	90726	480960	174370	412380	0	0	369300	818320	0	0	0	0
TRINITY_DN11853_c0_g1::TRINITY_DN11853_c0_g1_i3::g.9201::m.9201	0	0	0	0	0	0	0	0	0	0	0	0	703510	1353100	286670
TRINITY_DN12481_c0_g1::TRINITY_DN12481_c0_g1_i2::g.13223::m.13223	0	0	0	0	0	0	0	0	0	0	0	0	159080	1357900	825030
TRINITY_DN17670_c0_g2::TRINITY_DN17670_c0_g2_i7::g.87973::m.87973	0	0	0	0	0	0	0	0	0	0	0	0	171170	1665800	497640
TRINITY_DN17125_c1_g1::TRINITY_DN17125_c1_g1_i9::g.78918::m.78918	0	0	0	0	0	0	0	0	0	0	0	0	90104	1191200	1052900
TRINITY_DN16077_c0_g5::TRINITY_DN16077_c0_g5_i1::g.61752::m.61752	0	0	0	0	0	0	0	0	0	0	0	0	709560	828890	792600
TRINITY_DN17927_c1_g1::TRINITY_DN17927_c1_g1_i3::g.92125::m.92125	0	0	0	0	0	0	0	0	0	0	0	0	414540	1025200	889530
TRINITY_DN13566_c3_g2::TRINITY_DN13566_c3_g2_i6::g.25511::m.25511	0	0	0	0	0	0	0	102870	0	0	0	0	670840	801440	752740
TRINITY_DN15783_c0_g1::TRINITY_DN15783_c0_g1_i4::g.56969::m.56969	27426	0	0	166330	0	0	0	158980	0	0	0	785350	229890	549190	399150
TRINITY_DN14219_c0_g1::TRINITY_DN14219_c0_g1_i15::g.33893::m.33893	18892	0	21588	0	0	0	201910	0	0	0	0	260150	185650	801880	821030

TRINITY_DN16674_c0_g1::TRINITY_DN16674_c0_g1_i2::g.71326::m.71326	0	0	0	0	0	0	0	0	0	0	0	0	873450	839780	596900
TRINITY_DN18146_c1_g5::TRINITY_DN18146_c1_g5_i1::g.95204::m.95204	0	0	0	0	0	0	0	0	0	0	0	0	1423100	0	886770
TRINITY_DN15699_c1_g3::TRINITY_DN15699_c1_g3_i5::g.55823::m.55823	0	0	0	0	0	0	0	0	0	0	0	0	790750	376820	1139700
TRINITY_DN17055_c3_g2::TRINITY_DN17055_c3_g2_i4::g.77693::m.77693	0	0	0	0	0	53141	0	0	0	136430	441730	0	625790	336840	702280
TRINITY_DN16303_c1_g1::TRINITY_DN16303_c1_g1_i9::g.65704::m.65704	0	0	0	0	0	0	0	0	0	0	0	0	735840	0	1552500
TRINITY_DN19783_c1_g1::TRINITY_DN19783_c1_g1_i4::g.123602::m.123602	0	0	0	0	0	0	0	0	0	0	0	0	344360	772090	1171200
TRINITY_DN15141_c1_g1::TRINITY_DN15141_c1_g1_i2::g.47502::m.47502	98349	0	0	0	0	0	0	0	0	0	0	0	0	0	2186900
TRINITY_DN11784_c0_g1::TRINITY_DN11784_c0_g1_i1::g.8804::m.8804	0	0	0	0	0	0	0	0	0	0	0	0	684950	937030	660420
TRINITY_DN16035_c3_g1::TRINITY_DN16035_c3_g1_i2::g.61026::m.61026	103090	0	116550	83410	134180	0	338480	526610	309660	291510	0	364190	0	0	0
TRINITY_DN13920_c2_g1::TRINITY_DN13920_c2_g1_i2::g.30365::m.30365	0	0	0	0	0	0	0	0	0	0	111760	0	556570	819000	773680
TRINITY_DN13342_c3_g1::TRINITY_DN13342_c3_g1_i3::g.22570::m.22570	0	0	0	0	0	0	0	0	0	0	0	0	953280	488290	814960
TRINITY_DN16268_c4_g1::TRINITY_DN16268_c4_g1_i16::g.64855::m.64855	31067	0	0	0	0	0	86857	0	0	0	0	0	443260	1175400	511480
TRINITY_DN12624_c0_g1::TRINITY_DN12624_c0_g1_i1::g.14480::m.14480	0	0	0	0	0	0	0	0	0	0	0	0	504830	1424100	318540
TRINITY_DN10700_c0_g1::TRINITY_DN10700_c0_g1_i1::g.5317::m.5317	0	0	0	0	0	0	0	0	0	0	0	0	834370	393060	1019800
TRINITY_DN14461_c1_g2::TRINITY_DN14461_c1_g2_i3::g.37522::m.37522	0	0	0	0	0	0	0	0	0	0	0	0	405810	740010	1096800
TRINITY_DN11860_c0_g1::TRINITY_DN11860_c0_g1_i2::g.9213::m.9213	0	0	0	0	0	0	0	0	0	0	0	0	0	2232700	0
TRINITY_DN17343_c0_g2::TRINITY_DN17343_c0_g2_i1::g.82237::m.82237	0	0	0	0	0	0	0	0	0	0	0	0	476970	666260	1089300
TRINITY_DN17700_c1_g1::TRINITY_DN17700_c1_g1_i9::g.86845::m.86845	0	0	0	0	0	0	0	0	0	0	0	0	306760	1006000	919370
TRINITY_DN15208_c2_g1::TRINITY_DN15208_c2_g1_i1::g.48542::m.48542	0	0	0	0	0	0	0	0	0	0	0	0	120460	1640200	466200
TRINITY_DN17602_c0_g7::TRINITY_DN17602_c0_g7_i2::g.86978::m.86978	0	0	0	0	0	0	0	151740	0	0	1033100	605650	0	115890	318430
TRINITY_DN15799_c0_g1::TRINITY_DN15799_c0_g1_i13::g.55967::m.55967	82225	39138	0	0	403640	116460	0	433430	0	0	0	981920	78740	81373	0

TRINITY_DN19816_c1_g2::TRINITY_DN19816_c1_g2_i9::g.124089::m.124089	0	0	0	0	0	0	0	0	0	0	0	0	263340	1488500	463940
TRINITY_DN14106_c1_g2::TRINITY_DN14106_c1_g2_i1::g.32414::m.32414	259160	95736	397690	423200	0	0	0	124950	0	0	0	0	482780	114960	313120
TRINITY_DN19486_c1_g2::TRINITY_DN19486_c1_g2_i2::g.118619::m.118619	0	0	0	0	0	0	0	0	0	0	0	0	542370	1667800	0
TRINITY_DN16542_c2_g2::TRINITY_DN16542_c2_g2_i8::g.69479::m.69479	0	0	0	0	0	0	0	0	0	0	0	0	446330	1264500	496610
TRINITY_DN15298_c0_g1::TRINITY_DN15298_c0_g1_i1::g.49745::m.49745	0	0	0	0	0	0	0	0	0	0	0	0	955450	591330	659500
TRINITY_DN14245_c1_g1::TRINITY_DN14245_c1_g1_i8::g.34453::m.34453	0	0	0	0	0	0	0	0	0	0	0	0	752310	897130	555110
TRINITY_DN16993_c0_g1::TRINITY_DN16993_c0_g1_i5::g.76723::m.76723	91709	0	64287	60836	0	0	0	0	0	0	0	0	615300	1368600	0
TRINITY_DN18845_c2_g1::TRINITY_DN18845_c2_g1_i6::g.106645::m.106645	0	0	0	0	0	0	0	0	0	0	0	0	578860	896630	722110
TRINITY_DN17503_c1_g2::TRINITY_DN17503_c1_g2_i5::g.84903::m.84903	0	0	0	0	0	0	0	0	0	0	0	0	1126400	389630	680670
TRINITY_DN18733_c1_g1::TRINITY_DN18733_c1_g1_i16::g.105945::m.105945	0	0	0	0	0	0	0	0	0	0	0	0	706660	582330	907250
TRINITY_DN13161_c0_g2::TRINITY_DN13161_c0_g2_i6::g.20596::m.20596	0	0	0	0	0	0	0	0	0	0	0	0	1005200	0	1187900
TRINITY_DN16310_c4_g1::TRINITY_DN16310_c4_g1_i21::g.65586::m.65586	0	0	0	0	0	0	0	0	0	0	0	0	745980	873050	574090
TRINITY_DN16789_c2_g2::TRINITY_DN16789_c2_g2_i2::g.73230::m.73230	0	0	0	0	0	0	0	0	0	0	0	0	572880	119760	1496700
TRINITY_DN14198_c0_g1::TRINITY_DN14198_c0_g1_i9::g.33644::m.33644	0	0	0	0	0	0	0	0	0	0	0	0	406140	784680	996920
TRINITY_DN11382_c0_g1::TRINITY_DN11382_c0_g1_i1::g.7166::m.7166	0	0	0	0	0	0	0	0	0	0	0	0	0	0	2187100
TRINITY_DN18612_c0_g3::TRINITY_DN18612_c0_g3_i1::g.103400::m.103400	0	0	0	0	121120	0	0	113630	0	0	0	0	650270	637650	657040
TRINITY_DN13425_c1_g1::TRINITY_DN13425_c1_g1_i4::g.23719::m.23719	0	0	0	0	0	0	0	0	0	0	0	0	789230	789370	595620
TRINITY_DN18242_c3_g2::TRINITY_DN18242_c3_g2_i3::g.97426::m.97426	0	0	0	0	0	0	0	468150	0	0	0	0	0	1338600	357120
TRINITY_DN16339_c2_g2::TRINITY_DN16339_c2_g2_i2::g.65998::m.65998	0	0	0	0	24514	0	0	0	0	0	0	0	663870	487860	984930
TRINITY_DN17082_c3_g1::TRINITY_DN17082_c3_g1_i1::g.78154::m.78154	0	0	0	0	0	0	0	0	0	0	0	0	0	889840	1271300

TRINITY_DN19447_c1_g1::TRINITY_DN19447_c1_g1_i4::g.118090::m.118090	103740	40870	75844	150910	195560	0	0	91622	0	198690	0	344860	321430	393120	244450
TRINITY_DN12896_c0_g1::TRINITY_DN12896_c0_g1_i6::g.17068::m.17068	0	0	0	0	0	0	0	0	0	0	0	0	1514600	644770	0
TRINITY_DN17884_c0_g1::TRINITY_DN17884_c0_g1_i1::g.91267::m.91267	0	0	0	0	0	0	0	0	0	0	0	0	654670	672590	826630
TRINITY_DN19798_c2_g2::TRINITY_DN19798_c2_g2_i9::g.123749::m.123749	0	0	0	0	0	0	0	0	0	0	0	0	678050	420900	1054100
TRINITY_DN19714_c6_g2::TRINITY_DN19714_c6_g2_i2::g.122726::m.122726	120940	0	0	0	0	0	0	0	0	425330	686560	718780	0	0	200690
TRINITY_DN16697_c1_g2::TRINITY_DN16697_c1_g2_i3::g.70483::m.70483	0	0	0	0	0	0	0	0	0	0	0	0	0	1027500	1124700
TRINITY_DN18870_c1_g2::TRINITY_DN18870_c1_g2_i1::g.107727::m.107727	68147	0	393920	103420	598680	365780	0	193950	0	0	427830	0	0	0	0
TRINITY_DN18726_c1_g1::TRINITY_DN18726_c1_g1_i9::g.105485::m.105485	0	0	0	0	0	0	0	0	0	0	0	0	594240	986130	569620
TRINITY_DN18156_c0_g1::TRINITY_DN18156_c0_g1_i18::g.96138::m.9613	0	0	0	0	0	0	0	0	0	0	0	0	506630	887720	750490
TRINITY_DN17096_c1_g1::TRINITY_DN17096_c1_g1_i4::g.78249::m.78249	0	0	0	0	0	0	0	0	0	0	0	0	547310	518870	1078300
TRINITY_DN18240_c0_g1::TRINITY_DN18240_c0_g1_i3::g.97435::m.97435	0	0	0	0	0	0	0	0	0	0	804140	0	117770	98565	1124000
TRINITY_DN14353_c0_g2::TRINITY_DN14353_c0_g2_i2::g.35716::m.35716	0	0	0	0	0	0	0	0	0	0	0	0	0	984360	1154700
TRINITY_DN16303_c1_g2::TRINITY_DN16303_c1_g2_i1::g.65686::m.65686	0	0	0	0	0	0	0	0	0	0	0	0	700390	1163900	267500
TRINITY_DN13465_c2_g1::TRINITY_DN13465_c2_g1_i6::g.24168::m.24168	0	0	0	0	0	0	0	0	0	0	0	0	681370	407110	1031000
TRINITY_DN16506_c3_g1::TRINITY_DN16506_c3_g1_i1::g.68717::m.68717	0	0	0	0	0	0	0	0	0	0	0	0	703370	852370	560700
TRINITY_DN20189_c0_g1::TRINITY_DN20189_c0_g1_i1::g.128508::m.128508	269290	58414	229120	89218	0	0	0	0	0	0	275010	1183700	0	0	0
TRINITY_DN3188_c0_g1::TRINITY_DN3188_c0_g1_i1::g.878::m.878	0	0	245250	0	0	0	460320	0	0	0	0	1267400	0	125290	0
TRINITY_DN17908_c2_g1::TRINITY_DN17908_c2_g1_i5::g.91796::m.91796	0	0	0	0	316020	195940	209090	177610	356930	210900	226480	396440	0	0	0
TRINITY_DN13425_c1_g3::TRINITY_DN13425_c1_g3_i2::g.23712::m.23712	0	0	0	0	0	0	0	0	0	0	0	0	500520	884850	702430

TRINITY_DN14799_c0_g2::TRINITY_DN14799_c0_g2_i3::g.42387::m.42387	75398	0	102380	0	0	0	0	0	0	0	0	0	386510	1235100	284380
TRINITY_DN12952_c0_g1::TRINITY_DN12952_c0_g1_i2::g.17756::m.17756	251330	72085	170840	300780	0	0	0	138450	0	0	0	0	0	396250	753970
TRINITY_DN18755_c3_g1::TRINITY_DN18755_c3_g1_i1::g.105920::m.105920	0	0	0	0	0	0	0	0	0	0	0	0	249570	897720	935620
TRINITY_DN14404_c3_g2::TRINITY_DN14404_c3_g2_i3::g.36551::m.36551	0	0	0	0	0	0	0	0	0	0	0	0	746530	350890	982790
TRINITY_DN19358_c6_g3::TRINITY_DN19358_c6_g3_i5::g.116477::m.116477	0	0	0	0	0	0	0	0	0	0	0	0	624380	1022300	430120
TRINITY_DN12704_c0_g1::TRINITY_DN12704_c0_g1_i4::g.15091::m.15091	0	0	0	0	0	0	0	0	0	0	0	0	1303500	522760	248960
TRINITY_DN20043_c7_g2::TRINITY_DN20043_c7_g2_i2::g.128238::m.128238	0	0	0	0	0	0	0	0	0	0	0	0	104570	1395000	572070
TRINITY_DN17498_c2_g1::TRINITY_DN17498_c2_g1_i6::g.83121::m.83121	0	0	0	0	0	0	0	0	0	0	0	0	520260	1048200	500580
TRINITY_DN18857_c0_g5::TRINITY_DN18857_c0_g5_i2::g.107485::m.107485	0	0	0	0	0	0	0	0	0	0	0	0	579120	933290	546310
TRINITY_DN14751_c0_g1::TRINITY_DN14751_c0_g1_i8::g.40814::m.40814	0	0	0	0	0	0	0	0	0	0	0	0	848090	774070	433690
TRINITY_DN17602_c0_g3::TRINITY_DN17602_c0_g3_i7::g.86972::m.86972	0	0	0	0	0	0	0	0	0	0	0	0	813210	676320	563390
TRINITY_DN11500_c0_g1::TRINITY_DN11500_c0_g1_i2::g.7570::m.7570	0	0	0	2047200	0	0	0	0	0	0	0	0	0	0	0
TRINITY_DN17024_c0_g5::TRINITY_DN17024_c0_g5_i1::g.77080::m.77080	0	0	0	0	0	0	0	0	0	0	0	0	1480800	409660	150120
TRINITY_DN18652_c2_g1::TRINITY_DN18652_c2_g1_i2::g.104075::m.104075	0	0	0	0	0	0	0	0	0	164360	0	0	413330	521380	940860
TRINITY_DN19078_c0_g3::TRINITY_DN19078_c0_g3_i6::g.111344::m.111344	0	0	0	0	0	0	0	0	0	0	0	0	0	1747000	285820
TRINITY_DN13010_c2_g2::TRINITY_DN13010_c2_g2_i9::g.18708::m.18708	0	0	0	0	0	0	0	0	0	0	0	0	618080	738260	672470
TRINITY_DN18307_c0_g1::TRINITY_DN18307_c0_g1_i4::g.98617::m.98617	0	0	0	0	0	0	0	0	0	0	0	0	929590	1093400	0
TRINITY_DN14082_c1_g1::TRINITY_DN14082_c1_g1_i3::g.32100::m.32100	0	71302	0	37524	0	0	0	0	0	0	164410	0	310940	969850	459810
TRINITY_DN17986_c0_g1::TRINITY_DN17986_c0_g1_i2::g.93139::m.93139	0	0	0	0	0	0	0	0	0	0	0	0	0	598130	1412200
TRINITY_DN13091_c0_g1::TRINITY_DN13091_c0_g1_i4::g.19648::m.19648	525980	0	279350	389690	0	0	0	0	0	113130	0	0	208490	264830	228060

TRINITY_DN16278_c2_g2::TRINITY_DN16278_c2_g2_i8::g.65116::m.65116	0	0	0	0	0	0	0	0	0	0	0	0	539210	750620	717730
TRINITY_DN13098_c2_g1::TRINITY_DN13098_c2_g1_i5::g.19700::m.19700	0	0	0	0	0	0	0	0	0	0	889460	676200	0	256580	185260
TRINITY_DN11989_c0_g1::TRINITY_DN11989_c0_g1_i1::g.9805::m.9805	0	0	0	48114	0	0	0	0	0	0	0	0	100280	493450	1363800
TRINITY_DN19569_c1_g4::TRINITY_DN19569_c1_g4_i1::g.119978::m.119978	0	0	0	0	0	0	0	0	0	0	0	0	724230	636030	639700
TRINITY_DN16768_c1_g4::TRINITY_DN16768_c1_g4_i5::g.73029::m.73029	0	0	0	0	0	0	0	0	0	0	0	0	263150	753060	981240
TRINITY_DN13051_c9_g1::TRINITY_DN13051_c9_g1_i1::g.19838::m.19838	0	0	0	0	0	0	0	0	0	0	0	0	361370	880190	755320
TRINITY_DN15531_c1_g1::TRINITY_DN15531_c1_g1_i8::g.53401::m.53401	0	0	0	0	0	0	0	0	316300	0	1678600	0	0	0	0
TRINITY_DN17286_c1_g1::TRINITY_DN17286_c1_g1_i1::g.81477::m.81477	0	0	0	0	0	0	0	0	0	146530	0	0	734350	562480	545280
TRINITY_DN13833_c0_g1::TRINITY_DN13833_c0_g1_i8::g.29930::m.29930	0	0	0	0	0	0	0	0	0	0	0	0	506230	798100	682810
TRINITY_DN16501_c0_g1::TRINITY_DN16501_c0_g1_i7::g.68513::m.68513	0	0	0	0	0	0	0	0	0	0	0	0	185970	1107400	692390
TRINITY_DN14803_c0_g1::TRINITY_DN14803_c0_g1_i4::g.42638::m.42638	0	0	0	0	0	0	0	0	0	97627	0	0	0	1791500	95816
TRINITY_DN13921_c0_g1::TRINITY_DN13921_c0_g1_i2::g.30314::m.30314	0	0	0	0	0	0	0	0	0	0	0	0	445060	852960	681100
TRINITY_DN13437_c3_g1::TRINITY_DN13437_c3_g1_i7::g.23377::m.23377	0	0	0	0	0	0	0	0	0	0	0	0	634890	700350	643420
TRINITY_DN18701_c4_g10::TRINITY_DN18701_c4_g10_i2::g.103928::m.103928	0	0	0	0	386270	0	213470	348110	278710	0	0	0	116170	627700	0
TRINITY_DN19119_c0_g1::TRINITY_DN19119_c0_g1_i6::g.112459::m.1124	0	0	0	0	0	0	0	0	0	0	0	0	436090	723300	806460
TRINITY_DN17314_c1_g2::TRINITY_DN17314_c1_g2_i7::g.81924::m.81924	0	0	0	0	0	0	0	0	0	0	0	0	578220	786000	594290
TRINITY_DN13833_c0_g2::TRINITY_DN13833_c0_g2_i5::g.29925::m.29925	0	0	0	0	0	0	0	0	0	0	0	0	602820	691190	661330
TRINITY_DN13861_c0_g1::TRINITY_DN13861_c0_g1_i7::g.29399::m.29399	0	0	0	0	0	0	0	0	0	0	0	0	307130	358210	1288100
TRINITY_DN18605_c15_g2::TRINITY_DN18605_c15_g2_i1::g.103707::m.103707	0	0	0	0	0	0	0	0	0	0	0	1319000	204920	419160	0
TRINITY_DN12655_c1_g2::TRINITY_DN12655_c1_g2_i3::g.14763::m.14763	0	0	0	0	0	0	0	0	0	0	0	0	325680	1015900	589520
TRINITY_DN20026_c6_g1::TRINITY_DN20026_c6_g1_i3::g.128057::m.128057	0	0	0	0	0	0	0	0	0	0	0	0	656410	798620	475270

TRINITY_DN16058_c1_g1::TRINITY_DN16058_c1_g1_i7::g.61245::m.61245	0	0	0	0	0	0	0	0	0	0	0	0	628310	740730	557500
TRINITY_DN11865_c0_g1::TRINITY_DN11865_c0_g1_i7::g.9262::m.9262	0	0	0	0	0	0	0	0	0	0	0	0	859420	602590	457320
TRINITY_DN11961_c0_g1::TRINITY_DN11961_c0_g1_i6::g.9678::m.9678	0	0	0	0	0	0	0	0	0	0	0	0	500180	582750	827300
TRINITY_DN15627_c0_g2::TRINITY_DN15627_c0_g2_i1::g.54590::m.54590	0	0	0	0	0	0	0	0	0	0	0	0	555450	688790	665710
TRINITY_DN18478_c2_g2::TRINITY_DN18478_c2_g2_i3::g.101133::m.101133	0	0	0	0	0	0	0	0	0	0	0	0	635000	1273400	0
TRINITY_DN16690_c0_g1::TRINITY_DN16690_c0_g1_i1::g.71666::m.71666	0	0	0	0	0	0	0	0	0	0	0	0	549740	932430	421940
TRINITY_DN17879_c1_g3::TRINITY_DN17879_c1_g3_i5::g.91260::m.91260	83951	0	72679	52369	0	0	0	0	0	0	0	0	220650	693240	778300
TRINITY_DN17973_c0_g5::TRINITY_DN17973_c0_g5_i1::g.92489::m.92489	0	0	0	0	0	0	0	0	0	0	0	0	301590	365730	1222300
TRINITY_DN19252_c1_g4::TRINITY_DN19252_c1_g4_i7::g.114562::m.114562	0	0	0	0	0	0	0	0	0	0	0	0	756580	738460	390650
TRINITY_DN18739_c0_g3::TRINITY_DN18739_c0_g3_i2::g.105640::m.105640	0	0	0	0	0	0	0	0	0	0	0	0	475310	613300	796380
TRINITY_DN13888_c0_g2::TRINITY_DN13888_c0_g2_i1::g.29877::m.29877	0	0	0	0	0	0	0	0	0	0	0	0	567190	750930	565380
TRINITY_DN19344_c5_g2::TRINITY_DN19344_c5_g2_i3::g.115627::m.115627	0	0	0	0	0	0	0	0	0	0	0	0	0	743210	1134800
TRINITY_DN18418_c0_g3::TRINITY_DN18418_c0_g3_i2::g.100138::m.100138	0	0	0	0	0	0	0	0	0	0	0	0	1084800	499180	285430
TRINITY_DN18913_c2_g1::TRINITY_DN18913_c2_g1_i5::g.108928::m.108928	0	0	0	0	0	0	0	0	0	0	0	0	188870	1111500	567280
TRINITY_DN19613_c2_g1::TRINITY_DN19613_c2_g1_i4::g.120850::m.120850	58249	0	80500	0	0	53194	0	0	0	0	0	0	546760	1129000	0
TRINITY_DN17758_c1_g2::TRINITY_DN17758_c1_g2_i7::g.89243::m.89243	0	0	0	0	0	0	0	0	0	0	0	0	534580	446180	881550
TRINITY_DN14688_c0_g2::TRINITY_DN14688_c0_g2_i3::g.40630::m.40630	45967	0	65196	48398	740910	0	0	0	0	0	0	0	280030	315030	364520
TRINITY_DN16770_c0_g2::TRINITY_DN16770_c0_g2_i2::g.73031::m.73031	0	0	0	0	506790	244060	0	422260	0	0	0	0	212150	213440	258600
TRINITY_DN15617_c2_g1::TRINITY_DN15617_c2_g1_i3::g.54449::m.54449	0	0	0	0	0	0	0	0	0	0	0	0	0	1155600	698450

TRINITY_DN19193_c1_g3::TRINITY_DN19193_c1_g3_i5::g.113622::m.113622	0	0	0	0	0	0	0	0	0	0	616220	645230	220930	0	370410
TRINITY_DN11271_c0_g1::TRINITY_DN11271_c0_g1_i1::g.6832::m.6832	0	0	0	0	0	0	0	0	0	0	0	0	791460	824090	236880
TRINITY_DN17765_c0_g1::TRINITY_DN17765_c0_g1_i1::g.89341::m.89341	291520	109800	241090	238260	0	0	0	267580	193140	100940	0	404940	0	0	0
TRINITY_DN13374_c2_g1::TRINITY_DN13374_c2_g1_i4::g.22819::m.22819	0	0	0	0	0	0	0	0	0	0	0	0	897960	938380	0
TRINITY_DN15833_c5_g1::TRINITY_DN15833_c5_g1_i12::g.57896::m.57896	0	0	0	0	0	0	0	0	0	0	0	1494300	0	222660	118040
TRINITY_DN17806_c2_g2::TRINITY_DN17806_c2_g2_i4::g.90030::m.90030	0	0	0	0	0	0	0	0	0	0	0	0	101610	1286300	446730
TRINITY_DN15514_c4_g2::TRINITY_DN15514_c4_g2_i2::g.53063::m.53063	0	0	0	0	0	0	0	0	0	0	0	0	630800	463510	738720
TRINITY_DN15090_c0_g1::TRINITY_DN15090_c0_g1_i3::g.46611::m.46611	0	0	0	0	0	0	0	0	0	0	0	0	136220	1527300	168450
TRINITY_DN12620_c0_g1::TRINITY_DN12620_c0_g1_i4::g.14272::m.14272	0	0	0	0	0	0	0	0	0	0	120710	163320	0	1547600	0
TRINITY_DN10821_c0_g1::TRINITY_DN10821_c0_g1_i1::g.5589::m.5589	0	0	0	0	0	0	0	0	0	0	0	0	651600	220430	957710
TRINITY_DN12247_c0_g5::TRINITY_DN12247_c0_g5_i2::g.11476::m.11476	0	0	0	0	0	0	0	0	0	0	0	0	541670	789550	498340
TRINITY_DN17715_c1_g2::TRINITY_DN17715_c1_g2_i7::g.88682::m.88682	116000	0	115320	133030	173810	0	177630	176960	0	0	0	0	884670	0	34350
TRINITY_DN13507_c0_g2::TRINITY_DN13507_c0_g2_i4::g.24688::m.24688	0	0	0	0	0	0	0	0	0	0	0	0	0	1034000	777070
TRINITY_DN18452_c0_g2::TRINITY_DN18452_c0_g2_i5::g.100623::m.100623	216710	59296	198880	187570	0	0	0	0	0	0	0	1147000	0	0	0
TRINITY_DN18940_c3_g1::TRINITY_DN18940_c3_g1_i4::g.109190::m.109190	0	0	0	0	0	0	0	0	0	0	0	0	561520	622090	624340
TRINITY_DN19263_c0_g3::TRINITY_DN19263_c0_g3_i3::g.114810::m.114810	0	0	0	0	0	170540	0	425270	0	0	0	0	690330	521190	0
TRINITY_DN16828_c3_g2::TRINITY_DN16828_c3_g2_i1::g.74126::m.74126	0	0	0	0	0	0	0	0	0	0	0	0	143910	911030	750780
TRINITY_DN15895_c3_g2::TRINITY_DN15895_c3_g2_i1::g.57724::m.57724	26781	0	0	0	253080	0	0	0	0	0	337210	0	697750	365400	124500
TRINITY_DN18527_c2_g1::TRINITY_DN18527_c2_g1_i1::g.101980::m.101980	0	0	0	0	0	0	0	0	0	0	0	0	591990	1212200	0

TRINITY_DN18999_c4_g1::TRINITY_DN18999_c4_g1_i2::g.110202::m.110202	0	0	0	0	0	0	0	0	0	0	0	0	698810	372040	732420
TRINITY_DN16996_c2_g1::TRINITY_DN16996_c2_g1_i5::g.76760::m.76760	392480	34323	0	489270	0	0	0	0	0	0	494160	0	105810	126800	157520
TRINITY_DN17420_c0_g1::TRINITY_DN17420_c0_g1_i9::g.83584::m.83584	639120	165970	366980	627850	0	0	0	0	0	0	0	0	0	0	0
TRINITY_DN18991_c0_g1::TRINITY_DN18991_c0_g1_i4::g.110139::m.1101	0	0	0	0	0	0	0	0	0	0	0	0	233550	836930	723660
TRINITY_DN16630_c2_g2::TRINITY_DN16630_c2_g2_i3::g.70772::m.70772	0	0	0	0	0	0	0	0	0	0	0	0	481990	795330	509570
TRINITY_DN17508_c5_g4::TRINITY_DN17508_c5_g4_i2::g.85425::m.85425	0	0	0	0	0	172140	0	317510	446120	325100	0	0	190610	333720	0
TRINITY_DN16170_c0_g1::TRINITY_DN16170_c0_g1_i3::g.63133::m.63133	0	0	0	0	0	0	0	0	0	0	0	0	1165900	385990	229730
TRINITY_DN13107_c5_g1::TRINITY_DN13107_c5_g1_i1::g.19946::m.19946	0	0	0	144650	0	0	0	0	159090	119710	0	0	381630	547290	427450
TRINITY_DN17688_c2_g2::TRINITY_DN17688_c2_g2_i2::g.88270::m.88270	0	0	0	0	0	0	0	0	0	634460	0	1140700	0	0	0
TRINITY_DN12789_c1_g2::TRINITY_DN12789_c1_g2_i26::g.15965::m.15965	276170	79021	246780	249680	0	207650	0	53878	359150	0	300520	0	0	0	0
TRINITY_DN16664_c4_g1::TRINITY_DN16664_c4_g1_i4::g.71662::m.71662	0	0	0	0	0	0	0	0	0	0	0	0	557090	634690	577740
TRINITY_DN15784_c0_g1::TRINITY_DN15784_c0_g1_i2::g.57047::m.57047	0	0	0	0	0	0	0	209620	0	0	0	0	0	893660	664660
TRINITY_DN17576_c2_g1::TRINITY_DN17576_c2_g1_i10::g.86267::m.86267	0	0	0	0	0	0	0	0	0	0	0	0	264580	728610	773440
TRINITY_DN20043_c8_g6::TRINITY_DN20043_c8_g6_i2::g.128243::m.128243	201560	87962	241550	167640	0	0	0	159500	0	0	0	0	313930	0	594360
TRINITY_DN17136_c2_g4::TRINITY_DN17136_c2_g4_i1::g.79232::m.79232	0	0	0	0	0	0	0	0	0	0	0	0	441590	634030	689540
TRINITY_DN17588_c1_g2::TRINITY_DN17588_c1_g2_i1::g.86528::m.86528	0	0	0	0	0	0	0	0	0	0	0	0	480570	607740	672840
TRINITY_DN12440_c1_g1::TRINITY_DN12440_c1_g1_i2::g.13042::m.13042	0	0	0	0	0	702100	0	0	0	0	0	0	308930	692670	52637
TRINITY_DN14203_c2_g2::TRINITY_DN14203_c2_g2_i3::g.33825::m.33825	0	0	0	0	0	0	0	0	0	0	0	0	341300	782820	632140
TRINITY_DN16295_c1_g3::TRINITY_DN16295_c1_g3_i8::g.65237::m.65237	0	0	0	0	0	0	0	0	0	0	0	0	817120	937300	0

TRINITY_DN14603_c3_g1::TRINITY_DN14603_c3_g1_i1::g.39490::m.39490	0	0	0	0	0	0	0	0	0	0	0	0	336720	806280	600450
TRINITY_DN17919_c1_g4::TRINITY_DN17919_c1_g4_i5::g.91938::m.91938	0	0	0	0	0	0	0	0	0	0	0	0	765470	767440	208410
TRINITY_DN16456_c0_g2::TRINITY_DN16456_c0_g2_i7::g.67919::m.67919	0	0	0	0	0	0	0	0	0	0	0	0	548630	628620	562920
TRINITY_DN18783_c5_g1::TRINITY_DN18783_c5_g1_i3::g.106356::m.106356	0	0	88345	0	0	0	0	0	660030	0	101410	0	252620	335000	301820
TRINITY_DN14585_c2_g1::TRINITY_DN14585_c2_g1_i7::g.39202::m.39202	0	0	0	0	0	0	0	0	103410	226980	0	600390	0	0	806570
TRINITY_DN17674_c1_g2::TRINITY_DN17674_c1_g2_i8::g.88061::m.88061	0	0	0	0	0	0	0	0	0	0	0	0	301040	368970	1065700
TRINITY_DN15931_c0_g1::TRINITY_DN15931_c0_g1_i5::g.59103::m.59103	0	168270	605050	573300	0	0	0	386260	0	0	0	0	0	0	0
TRINITY_DN15984_c0_g3::TRINITY_DN15984_c0_g3_i1::g.59910::m.59910	0	0	0	0	0	0	0	0	0	0	0	0	444160	680660	607190
TRINITY_DN15696_c0_g5::TRINITY_DN15696_c0_g5_i1::g.55738::m.55738	296020	79798	218250	101800	0	0	0	0	320140	221910	0	0	0	253090	238440
TRINITY_DN13415_c1_g2::TRINITY_DN13415_c1_g2_i6::g.23608::m.23608	0	0	0	0	0	0	0	0	0	0	0	0	620490	697230	407310
TRINITY_DN10290_c0_g3::TRINITY_DN10290_c0_g3_i1::g.4573::m.4573	0	0	0	0	0	0	0	0	0	0	0	0	888520	94044	742330
TRINITY_DN17086_c3_g1::TRINITY_DN17086_c3_g1_i14::g.77558::m.77558	0	0	0	0	0	0	0	0	0	0	0	0	163980	565480	994230
TRINITY_DN15448_c0_g1::TRINITY_DN15448_c0_g1_i6::g.51979::m.51979	0	0	0	0	0	0	0	198040	0	0	0	0	399220	589930	535880
TRINITY_DN17327_c0_g4::TRINITY_DN17327_c0_g4_i4::g.82004::m.82004	0	0	0	0	0	0	0	0	0	0	0	0	376060	927420	418170
TRINITY_DN18850_c2_g1::TRINITY_DN18850_c2_g1_i14::g.107185::m.107185	266200	0	0	184070	0	0	0	0	0	0	0	0	198960	583830	487800
TRINITY_DN15339_c5_g2::TRINITY_DN15339_c5_g2_i1::g.49931::m.49931	0	0	0	0	0	0	0	0	0	0	0	0	166810	880190	668260
TRINITY_DN11883_c0_g2::TRINITY_DN11883_c0_g2_i5::g.9377::m.9377	0	0	0	0	0	0	0	0	247250	0	221080	0	481410	0	763810
TRINITY_DN15187_c1_g1::TRINITY_DN15187_c1_g1_i9::g.47932::m.47932	140870	51395	150310	151830	0	0	235880	0	0	382620	600550	0	0	0	0
TRINITY_DN18055_c0_g1::TRINITY_DN18055_c0_g1_i4::g.94445::m.94445	0	0	0	0	0	0	0	0	0	0	0	0	577390	757030	377650
TRINITY_DN17443_c0_g6::TRINITY_DN17443_c0_g6_i1::g.83849::m.83849	0	33198	81087	72163	0	0	0	120200	317260	0	553440	372840	62016	0	94921

TRINITY_DN17889_c3_g3::TRINITY_DN17889_c3_g3_i2::g.91373::m.91373	0	0	0	0	0	0	0	0	0	0	0	0	0	1220400	486070
TRINITY_DN16290_c1_g2::TRINITY_DN16290_c1_g2_i6::g.65201::m.65201	0	0	0	0	0	0	0	0	0	0	207190	0	206620	479130	810170
TRINITY_DN16914_c0_g2::TRINITY_DN16914_c0_g2_i3::g.75483::m.75483	134810	81234	214040	425750	0	245590	0	0	0	0	599930	0	0	0	0
TRINITY_DN16356_c0_g1::TRINITY_DN16356_c0_g1_i13::g.66037::m.66037	0	0	0	0	0	0	0	0	0	0	0	0	609020	0	1091900
TRINITY_DN16552_c0_g2::TRINITY_DN16552_c0_g2_i2::g.69237::m.69237	0	0	0	0	0	0	0	0	0	0	0	0	528710	626300	543760
TRINITY_DN13122_c2_g7::TRINITY_DN13122_c2_g7_i5::g.20188::m.20188	0	0	0	0	0	0	0	0	0	0	0	0	349100	964210	384860
TRINITY_DN18287_c2_g1::TRINITY_DN18287_c2_g1_i6::g.98094::m.98094	0	0	0	0	0	0	0	0	0	0	0	0	0	765200	928570
TRINITY_DN15932_c0_g5::TRINITY_DN15932_c0_g5_i5::g.59228::m.59228	0	0	0	0	0	0	0	0	0	0	0	0	454050	463750	775930
TRINITY_DN17310_c0_g1::TRINITY_DN17310_c0_g1_i2::g.81834::m.81834	25582	0	0	0	0	0	226680	0	0	241050	419790	391480	101660	139930	147100
TRINITY_DN15542_c3_g2::TRINITY_DN15542_c3_g2_i4::g.53532::m.53532	0	0	0	0	0	0	0	0	0	0	0	0	626840	704910	360830
TRINITY_DN18437_c2_g2::TRINITY_DN18437_c2_g2_i5::g.100589::m.100589	0	0	0	0	0	0	0	0	0	0	0	0	474700	752920	459940
TRINITY_DN17906_c0_g2::TRINITY_DN17906_c0_g2_i5::g.91748::m.91748	0	0	0	0	0	0	0	0	0	0	0	0	874480	289850	521330
TRINITY_DN12593_c3_g1::TRINITY_DN12593_c3_g1_i6::g.14142::m.14142	0	0	0	0	0	0	0	0	0	0	0	0	424370	533620	725380
TRINITY_DN18611_c0_g1::TRINITY_DN18611_c0_g1_i4::g.103412::m.1034	0	0	0	0	1020600	0	0	0	0	0	0	0	0	312150	349720
TRINITY_DN14624_c0_g1::TRINITY_DN14624_c0_g1_i4::g.39690::m.39690	41717	0	0	43442	0	0	0	0	0	0	0	0	732090	865000	0
TRINITY_DN19014_c2_g2::TRINITY_DN19014_c2_g2_i8::g.110573::m.110573	0	0	0	0	0	0	0	0	0	0	0	0	585240	338730	754190
TRINITY_DN16277_c1_g3::TRINITY_DN16277_c1_g3_i4::g.64929::m.64929	0	0	0	0	0	0	0	0	0	0	0	0	423810	681660	570180
TRINITY_DN17957_c0_g1::TRINITY_DN17957_c0_g1_i11::g.92631::m.92631	0	0	0	0	0	0	0	0	0	0	0	0	313640	1066400	294520
TRINITY_DN15571_c4_g3::TRINITY_DN15571_c4_g3_i4::g.54049::m.54049	0	0	0	0	0	0	0	0	0	0	0	0	253820	689250	729280

TRINITY_DN11568_c0_g1::TRINITY_DN11568_c0_g1_i1::g.7589::m.7589	0	0	0	0	0	0	0	0	0	0	0	0	205330	246510	1217200
TRINITY_DN12349_c8_g2::TRINITY_DN12349_c8_g2_i3::g.12071::m.12071	0	0	0	0	0	0	0	0	0	0	0	0	530570	190700	946230
TRINITY_DN12678_c0_g1::TRINITY_DN12678_c0_g1_i7::g.14864::m.14864	0	0	0	0	0	0	0	0	0	0	0	0	413210	852770	396630
TRINITY_DN16016_c2_g1::TRINITY_DN16016_c2_g1_i9::g.60539::m.60539	0	0	0	0	0	0	0	0	0	0	0	0	0	833690	822180
TRINITY_DN11023_c0_g2::TRINITY_DN11023_c0_g2_i1::g.6151::m.6151	504380	0	680340	468210	0	0	0	0	0	0	0	0	0	0	0
TRINITY_DN18955_c2_g1::TRINITY_DN18955_c2_g1_i2::g.109309::m.109309	0	0	0	0	0	0	0	0	0	0	0	0	626120	838930	186330
TRINITY_DN17913_c4_g1::TRINITY_DN17913_c4_g1_i1::g.91856::m.91856	92023	26569	0	0	168680	0	188520	157540	0	0	583450	427840	0	0	0
TRINITY_DN18697_c0_g2::TRINITY_DN18697_c0_g2_i2::g.103311::m.103311	0	0	0	0	0	0	0	0	0	0	0	0	486320	645020	510890
TRINITY_DN15624_c0_g1::TRINITY_DN15624_c0_g1_i1::g.54548::m.54548	0	0	0	0	0	0	0	0	0	0	0	0	701070	652920	287840
TRINITY_DN12621_c1_g1::TRINITY_DN12621_c1_g1_i2::g.14309::m.14309	221860	0	237350	180870	167120	0	0	0	0	0	0	0	272370	174320	385900
TRINITY_DN15292_c1_g3::TRINITY_DN15292_c1_g3_i1::g.49799::m.49799	0	0	0	0	0	0	0	0	0	0	0	0	284870	794880	559920
TRINITY_DN16971_c2_g1::TRINITY_DN16971_c2_g1_i8::g.76469::m.76469	0	0	0	0	0	0	0	0	0	0	466760	0	350700	416270	405480
TRINITY_DN15274_c1_g2::TRINITY_DN15274_c1_g2_i1::g.49397::m.49397	0	0	0	0	0	0	0	0	0	0	0	0	672350	191240	771260
TRINITY_DN17008_c0_g1::TRINITY_DN17008_c0_g1_i7::g.77048::m.77048	76941	0	165860	80909	0	0	0	0	0	0	0	1309600	0	0	0
TRINITY_DN18404_c1_g1::TRINITY_DN18404_c1_g1_i7::g.100040::m.100040	0	0	0	0	0	0	0	0	0	0	0	0	75515	474230	1081900
TRINITY_DN18356_c1_g1::TRINITY_DN18356_c1_g1_i9::g.99317::m.99317	0	0	0	0	0	0	0	0	0	0	0	0	315980	818860	496540
TRINITY_DN17060_c1_g1::TRINITY_DN17060_c1_g1_i6::g.77747::m.77747	0	0	0	0	0	0	0	0	0	0	0	0	0	891290	739250
TRINITY_DN14005_c1_g1::TRINITY_DN14005_c1_g1_i1::g.31333::m.31333	0	0	0	34838	0	0	0	0	0	0	0	0	444560	621970	528800
TRINITY_DN16201_c3_g1::TRINITY_DN16201_c3_g1_i2::g.62696::m.62696	0	0	0	0	0	0	0	0	0	0	0	0	308740	383200	938140
TRINITY_DN12639_c0_g1::TRINITY_DN12639_c0_g1_i2::g.14158::m.14158	0	0	0	0	0	0	0	0	0	0	0	0	599050	579870	450450
TRINITY_DN15473_c1_g1::TRINITY_DN15473_c1_g1_i2::g.52569::m.52569	24057	0	87208	45220	0	0	0	123310	0	0	0	0	386620	540070	421080
TRINITY_DN17388_c0_g2::TRINITY_DN17388_c0_g2_i7::g.82945::m.82945	0	0	34181	0	0	0	0	0	0	0	0	0	271140	711720	608260

TRINITY_DN19219_c1_g1::TRINITY_DN19219_c1_g1_i14::g.114200::m.114200	0	0	16192	0	257330	81332	331310	175850	0	0	0	0	223380	286970	244240
TRINITY_DN16821_c1_g1::TRINITY_DN16821_c1_g1_i1::g.73860::m.73860	0	0	0	0	245270	0	0	0	0	0	0	0	356860	448510	565410
TRINITY_DN18965_c3_g1::TRINITY_DN18965_c3_g1_i3::g.109455::m.109455	826240	141210	0	238790	0	0	396580	0	0	0	0	0	0	0	0
TRINITY_DN16438_c0_g4::TRINITY_DN16438_c0_g4_i1::g.67637::m.67637	0	0	0	0	0	0	0	0	0	0	0	0	582640	667340	351080
TRINITY_DN16330_c0_g1::TRINITY_DN16330_c0_g1_i8::g.66108::m.66108	0	0	0	0	0	0	0	0	0	0	0	0	556920	442890	600430
TRINITY_DN19616_c4_g3::TRINITY_DN19616_c4_g3_i5::g.120935::m.120935	0	0	0	0	0	0	0	0	0	0	0	0	101540	576210	916530
TRINITY_DN17970_c3_g1::TRINITY_DN17970_c3_g1_i6::g.92954::m.92954	41788	0	139030	64385	0	0	0	0	0	0	0	0	643120	214500	490440
TRINITY_DN16469_c0_g1::TRINITY_DN16469_c0_g1_i7::g.67034::m.67034	375460	188190	227400	245940	0	0	0	0	0	0	551850	0	0	0	0
TRINITY_DN15978_c1_g2::TRINITY_DN15978_c1_g2_i1::g.59851::m.59851	0	0	0	0	0	0	0	0	0	0	0	0	545240	705060	333770
TRINITY_DN17528_c1_g1::TRINITY_DN17528_c1_g1_i8::g.85347::m.85347	0	0	0	0	0	0	0	0	0	0	0	0	80639	741470	761960
TRINITY_DN19544_c2_g6::TRINITY_DN19544_c2_g6_i1::g.119552::m.119552	0	0	0	0	0	0	0	0	0	0	0	0	540990	494280	542990
TRINITY_DN16118_c1_g1::TRINITY_DN16118_c1_g1_i2::g.62411::m.62411	0	0	0	0	0	0	0	0	0	0	0	0	450030	549400	574950
TRINITY_DN14654_c1_g1::TRINITY_DN14654_c1_g1_i1::g.40157::m.40157	0	0	0	0	0	0	0	0	0	0	0	0	162850	1411400	0
TRINITY_DN16121_c0_g1::TRINITY_DN16121_c0_g1_i2::g.62465::m.62465	0	0	0	0	0	0	0	0	0	0	0	0	380210	734830	458620
TRINITY_DN18674_c2_g2::TRINITY_DN18674_c2_g2_i1::g.104393::m.104393	0	0	0	0	0	0	0	0	0	0	0	0	0	1569200	0
TRINITY_DN15551_c3_g3::TRINITY_DN15551_c3_g3_i8::g.53664::m.53664	0	0	0	0	0	0	0	0	0	0	0	0	655490	261810	650860
TRINITY_DN18806_c2_g1::TRINITY_DN18806_c2_g1_i13::g.106758::m.106758	0	0	0	0	0	0	0	0	0	0	0	0	443580	603930	517400
TRINITY_DN14781_c0_g1::TRINITY_DN14781_c0_g1_i21::g.42142::m.4214	0	0	0	0	124600	0	186730	129040	94635	0	295640	0	193930	349200	188930
TRINITY_DN13427_c0_g3::TRINITY_DN13427_c0_g3_i1::g.23641::m.23641	0	0	0	0	0	0	0	0	0	0	0	0	568620	650190	341760
TRINITY_DN15029_c5_g6::TRINITY_DN15029_c5_g6_i4::g.46001::m.46001	0	71873	0	0	0	0	0	0	0	0	0	0	524400	504160	455980

TRINITY_DN15434_c1_g3::TRINITY_DN15434_c1_g3_i3::g.51817::m.51817	0	0	0	0	0	0	0	0	0	0	0	0	359750	467020	724140
TRINITY_DN17008_c0_g1::TRINITY_DN17008_c0_g1_i2::g.77040::m.77040	0	0	0	103430	0	0	0	0	0	0	574690	814440	0	55868	0
TRINITY_DN17336_c2_g1::TRINITY_DN17336_c2_g1_i3::g.82080::m.82080	0	0	0	0	0	0	0	0	0	0	0	0	322000	616380	607720
TRINITY_DN15690_c0_g1::TRINITY_DN15690_c0_g1_i7::g.55685::m.55685	0	0	0	0	0	0	0	0	0	0	0	0	722500	666620	152610
TRINITY_DN13180_c0_g1::TRINITY_DN13180_c0_g1_i3::g.20757::m.20757	34755	0	29070	28951	0	0	0	0	0	0	238980	0	394320	276030	537030
TRINITY_DN11253_c0_g1::TRINITY_DN11253_c0_g1_i2::g.6760::m.6760	355910	47442	215420	164380	0	0	129420	0	0	0	465220	0	161070	0	0
TRINITY_DN16309_c0_g1::TRINITY_DN16309_c0_g1_i5::g.65915::m.65915	0	0	0	0	0	0	0	0	0	0	0	0	369550	467800	700950
TRINITY_DN11739_c0_g1::TRINITY_DN11739_c0_g1_i1::g.8613::m.8613	0	0	0	0	0	0	0	0	0	0	0	0	206240	499280	831550
TRINITY_DN13614_c0_g1::TRINITY_DN13614_c0_g1_i2::g.26309::m.26309	0	0	0	0	0	0	0	0	0	0	0	0	228070	562310	746760
TRINITY_DN18223_c0_g1::TRINITY_DN18223_c0_g1_i1::g.97248::m.97248	0	0	0	0	0	0	0	0	0	0	0	0	688140	457110	388060
TRINITY_DN19809_c1_g1::TRINITY_DN19809_c1_g1_i1::g.124378::m.124378	0	0	0	0	0	0	0	0	0	0	0	0	454520	604620	467470
TRINITY_DN15338_c3_g1::TRINITY_DN15338_c3_g1_i1::g.50560::m.50560	58647	0	60715	61168	0	0	0	75240	0	0	0	0	774410	266790	228460
TRINITY_DN18884_c1_g1::TRINITY_DN18884_c1_g1_i5::g.108202::m.108202	0	0	0	0	0	0	0	0	0	0	0	0	577040	466150	481820
TRINITY_DN12888_c1_g1::TRINITY_DN12888_c1_g1_i1::g.16988::m.16988	0	0	0	0	0	0	0	0	0	0	0	0	370880	484830	667920
TRINITY_DN18793_c0_g1::TRINITY_DN18793_c0_g1_i11::g.106461::m.106461	0	0	0	0	0	0	0	0	0	0	0	0	384630	842700	295760
TRINITY_DN14198_c0_g1::TRINITY_DN14198_c0_g1_i4::g.33633::m.33633	0	0	0	0	0	0	0	0	0	0	0	0	363610	409520	747810
TRINITY_DN13898_c1_g1::TRINITY_DN13898_c1_g1_i2::g.30051::m.30051	0	0	0	0	0	0	0	0	0	0	0	0	254160	1036000	228320
TRINITY_DN16513_c0_g3::TRINITY_DN16513_c0_g3_i2::g.68923::m.68923	0	0	0	0	0	0	0	0	0	0	0	0	506400	354050	657230
TRINITY_DN19115_c1_g2::TRINITY_DN19115_c1_g2_i10::g.112400::m.112400	0	0	0	0	0	0	0	0	0	0	0	0	167130	448220	900720
TRINITY_DN19612_c0_g2::TRINITY_DN19612_c0_g2_i5::g.120800::m.120800	0	0	0	0	0	0	0	0	0	0	0	0	467940	1046900	0
TRINITY_DN19652_c2_g1::TRINITY_DN19652_c2_g1_i9::g.121472::m.121472	0	0	0	0	0	0	0	0	0	0	0	0	647840	343780	522440

TRINITY_DN15221_c1_g3::TRINITY_DN15221_c1_g3_i1::g.48708::m.48708	86908	0	345200	0	245910	159660	237360	277510	156830	0	0	0	0	0	0
TRINITY_DN17380_c2_g1::TRINITY_DN17380_c2_g1_i4::g.82866::m.82866	0	0	0	0	0	0	0	0	0	0	0	0	488400	289530	728180
TRINITY_DN19589_c2_g2::TRINITY_DN19589_c2_g2_i4::g.120311::m.120311	0	0	0	0	0	0	0	0	0	0	0	0	0	1501500	0
TRINITY_DN12563_c3_g1::TRINITY_DN12563_c3_g1_i1::g.13893::m.13893	0	0	0	0	0	0	0	0	0	0	0	0	479270	383170	625740
TRINITY_DN14756_c11_g3::TRINITY_DN14756_c11_g3_i1::g.41922::m.41922	0	0	0	0	0	0	0	0	0	0	0	0	258850	310310	916960
TRINITY_DN16369_c2_g1::TRINITY_DN16369_c2_g1_i6::g.66520::m.66520	0	0	0	0	0	0	0	0	0	0	0	0	191580	389350	901200
TRINITY_DN14258_c0_g1::TRINITY_DN14258_c0_g1_i5::g.34520::m.34520	0	0	0	0	0	0	0	0	0	0	0	0	678210	517390	284510
TRINITY_DN16789_c2_g5::TRINITY_DN16789_c2_g5_i1::g.73225::m.73225	0	0	0	0	0	0	0	0	0	0	0	0	322180	671720	485560
TRINITY_DN17303_c1_g1::TRINITY_DN17303_c1_g1_i7::g.81652::m.81652	1381200	0	0	0	0	0	0	94469	0	0	0	0	0	0	0
TRINITY_DN19981_c2_g1::TRINITY_DN19981_c2_g1_i2::g.127291::m.127291	201110	0	224100	342570	0	0	0	0	0	0	268020	0	125380	310000	0
TRINITY_DN13121_c2_g1::TRINITY_DN13121_c2_g1_i1::g.20130::m.20130	0	0	0	0	78258	0	61386	0	200840	147430	267680	365520	265300	0	83034
TRINITY_DN17618_c2_g2::TRINITY_DN17618_c2_g2_i9::g.87148::m.87148	0	0	0	0	0	0	0	0	0	0	0	0	370760	709890	387530
TRINITY_DN18372_c2_g8::TRINITY_DN18372_c2_g8_i1::g.99519::m.99519	0	0	0	0	0	0	0	0	0	0	0	0	298670	734520	434200
TRINITY_DN18320_c3_g3::TRINITY_DN18320_c3_g3_i3::g.98813::m.98813	0	0	0	0	0	0	0	0	0	0	0	0	261350	774220	429970
TRINITY_DN15818_c1_g1::TRINITY_DN15818_c1_g1_i7::g.57419::m.57419	0	0	0	0	0	0	0	0	0	0	0	0	568970	533680	358210
TRINITY_DN17545_c0_g1::TRINITY_DN17545_c0_g1_i11::g.84946::m.84946	0	0	0	0	0	0	0	0	0	0	0	0	343950	485600	631090
TRINITY_DN18096_c1_g1::TRINITY_DN18096_c1_g1_i2::g.95119::m.95119	0	0	0	0	0	0	0	0	0	0	0	0	425120	577310	457330
TRINITY_DN13342_c2_g1::TRINITY_DN13342_c2_g1_i2::g.22558::m.22558	0	0	0	0	0	0	0	0	0	0	0	0	0	829090	630440
TRINITY_DN16074_c1_g1::TRINITY_DN16074_c1_g1_i7::g.61513::m.61513	0	0	63474	0	0	0	0	0	0	0	369200	357750	0	426510	241700
TRINITY_DN12750_c0_g2::TRINITY_DN12750_c0_g2_i1::g.15503::m.15503	0	0	0	0	200580	110350	232240	360350	0	0	554460	0	0	0	0

TRINITY_DN19511_c1_g1::TRINITY_DN19511_c1_g1_i3::g.119032::m.1190	0	0	0	0	0	0	0	0	0	0	0	0	213590	534010	707380
TRINITY_DN13260_c0_g2::TRINITY_DN13260_c0_g2_i1::g.21634::m.21634	0	0	0	0	0	0	0	0	0	0	0	0	543880	656610	251610
TRINITY_DN19385_c2_g2::TRINITY_DN19385_c2_g2_i1::g.117065::m.117065	46721	0	52728	46764	0	0	0	0	0	0	0	0	0	0	1300800
TRINITY_DN14374_c0_g1::TRINITY_DN14374_c0_g1_i4::g.36023::m.36023	93504	31556	0	312020	0	0	0	0	0	0	213900	247740	110600	0	436950
TRINITY_DN14718_c0_g3::TRINITY_DN14718_c0_g3_i4::g.41295::m.41295	0	0	0	0	0	221980	568310	0	0	538610	0	0	113960	0	0
TRINITY_DN14491_c1_g3::TRINITY_DN14491_c1_g3_i2::g.37906::m.37906	48409	0	0	0	109940	0	0	0	0	310950	0	657480	164470	0	151450
TRINITY_DN18652_c1_g2::TRINITY_DN18652_c1_g2_i5::g.104071::m.104071	0	0	0	0	0	0	129340	0	391840	0	489680	429320	0	0	0
TRINITY_DN17169_c0_g2::TRINITY_DN17169_c0_g2_i1::g.79657::m.79657	137200	0	134510	0	0	0	0	0	0	0	694920	0	158410	165510	148830
TRINITY_DN17784_c0_g1::TRINITY_DN17784_c0_g1_i3::g.89656::m.89656	0	0	0	0	0	0	0	0	0	0	0	0	301210	685050	452250
TRINITY_DN15619_c2_g2::TRINITY_DN15619_c2_g2_i4::g.54476::m.54476	0	0	0	0	0	0	0	0	0	0	0	0	0	390110	1042000
TRINITY_DN15799_c0_g1::TRINITY_DN15799_c0_g1_i8::g.55961::m.55961	0	0	340420	225900	0	0	147200	0	0	0	715280	0	0	0	0
TRINITY_DN18926_c2_g1::TRINITY_DN18926_c2_g1_i1::g.109132::m.109132	0	0	0	0	0	0	0	0	0	0	0	0	769460	438520	218190
TRINITY_DN15946_c1_g1::TRINITY_DN15946_c1_g1_i2::g.59352::m.59352	0	0	0	0	0	0	0	0	0	0	0	0	703560	460950	260280
TRINITY_DN18496_c3_g1::TRINITY_DN18496_c3_g1_i9::g.99949::m.99949	0	0	0	0	78145	0	0	0	0	0	0	0	498640	238860	607710
TRINITY_DN11371_c0_g2::TRINITY_DN11371_c0_g2_i2::g.7148::m.7148	0	33977	170620	155890	0	0	0	0	0	0	827560	0	0	111240	123710
TRINITY_DN11955_c0_g2::TRINITY_DN11955_c0_g2_i1::g.9673::m.9673	0	0	0	0	0	0	0	0	0	0	0	0	617750	803620	0
TRINITY_DN12552_c0_g1::TRINITY_DN12552_c0_g1_i2::g.13425::m.13425	165790	77251	116350	0	0	0	0	0	269110	0	231470	558630	0	0	0
TRINITY_DN19080_c0_g2::TRINITY_DN19080_c0_g2_i7::g.111803::m.111803	0	0	0	0	0	0	0	0	0	0	0	0	279550	948390	189050
TRINITY_DN19854_c2_g2::TRINITY_DN19854_c2_g2_i4::g.124562::m.124562	0	0	0	0	0	0	0	0	0	0	0	0	425190	552740	439050
TRINITY_DN19442_c0_g2::TRINITY_DN19442_c0_g2_i3::g.118018::m.118018	0	0	0	0	0	0	0	0	0	0	0	0	446980	363580	601410

TRINITY_DN18350_c0_g2::TRINITY_DN18350_c0_g2_i2::g.99155::m.99155	0	0	0	0	0	0	0	0	0	0	0	0	360830	381610	666840
TRINITY_DN12711_c1_g1::TRINITY_DN12711_c1_g1_i4::g.15231::m.15231	0	0	0	0	0	0	0	0	0	0	0	0	496020	806320	104550
TRINITY_DN14491_c1_g5::TRINITY_DN14491_c1_g5_i7::g.37912::m.37912	155880	0	164940	199740	339240	0	0	0	0	0	0	0	167270	197680	175760
TRINITY_DN18628_c2_g1::TRINITY_DN18628_c2_g1_i28::g.103627::m.103627	0	0	0	0	0	0	0	0	0	0	0	0	218660	525360	654680
TRINITY_DN14281_c0_g1::TRINITY_DN14281_c0_g1_i1::g.34852::m.34852	0	0	0	0	0	0	0	0	0	0	0	0	330830	1067400	0
TRINITY_DN16267_c0_g1::TRINITY_DN16267_c0_g1_i3::g.64801::m.64801	0	0	0	0	0	0	0	0	0	0	0	0	347330	536180	513530
TRINITY_DN14107_c0_g1::TRINITY_DN14107_c0_g1_i1::g.32583::m.32583	0	50541	154730	162230	168290	0	0	207360	0	0	0	0	87843	434830	130210
TRINITY_DN18942_c3_g3::TRINITY_DN18942_c3_g3_i8::g.109006::m.109006	0	0	0	0	0	0	0	0	0	80883	0	0	525120	668540	121210
TRINITY_DN15316_c4_g2::TRINITY_DN15316_c4_g2_i4::g.50087::m.50087	0	0	0	0	0	0	0	0	0	0	0	0	1391700	0	0
TRINITY_DN18397_c1_g1::TRINITY_DN18397_c1_g1_i2::g.99905::m.99905	0	0	0	0	0	0	0	0	0	0	0	0	0	527260	864280
TRINITY_DN14957_c1_g1::TRINITY_DN14957_c1_g1_i3::g.44723::m.44723	0	0	0	0	0	0	0	0	0	0	0	0	374710	456270	558730
TRINITY_DN5432_c0_g1::TRINITY_DN5432_c0_g1_i1::g.1343::m.1343	0	0	0	0	0	0	0	0	0	0	0	0	146710	396270	845860
TRINITY_DN10737_c0_g1::TRINITY_DN10737_c0_g1_i1::g.5423::m.5423	143810	50720	132020	77922	251530	0	0	293900	0	0	0	438330	0	0	0
TRINITY_DN18886_c3_g1::TRINITY_DN18886_c3_g1_i2::g.108068::m.108068	0	0	0	0	0	0	134920	0	0	0	201190	307870	0	357100	384200
TRINITY_DN19870_c1_g2::TRINITY_DN19870_c1_g2_i5::g.124748::m.124748	0	0	0	0	0	0	0	0	0	0	0	0	308300	566720	507660
TRINITY_DN16270_c0_g2::TRINITY_DN16270_c0_g2_i15::g.64899::m.64899	0	0	0	0	0	0	0	0	0	0	0	0	0	0	1380600
TRINITY_DN14538_c2_g2::TRINITY_DN14538_c2_g2_i3::g.38504::m.38504	0	0	0	0	0	0	0	0	0	0	0	0	275490	0	1103500
TRINITY_DN18432_c0_g1::TRINITY_DN18432_c0_g1_i9::g.99987::m.99987	0	197410	0	427760	0	0	0	0	0	0	0	0	341400	129790	276430
TRINITY_DN14697_c1_g1::TRINITY_DN14697_c1_g1_i3::g.40725::m.40725	0	0	0	0	0	0	0	0	0	0	0	0	360770	419720	591800
TRINITY_DN12130_c0_g1::TRINITY_DN12130_c0_g1_i1::g.10682::m.10682	0	0	0	0	0	0	0	0	0	0	0	0	1075600	295830	0
TRINITY_DN16549_c1_g2::TRINITY_DN16549_c1_g2_i1::g.69658::m.69658	0	0	0	0	0	0	0	0	0	0	0	0	535440	465350	366090

TRINITY_DN13928_c0_g1::TRINITY_DN13928_c0_g1_i1::g.30448::m.30448	0	0	0	0	0	0	0	0	0	0	0	0	452610	535880	378060
TRINITY_DN13623_c1_g1::TRINITY_DN13623_c1_g1_i6::g.26584::m.26584	0	0	0	0	0	0	0	0	0	0	0	0	403910	512650	448640
TRINITY_DN15579_c1_g3::TRINITY_DN15579_c1_g3_i1::g.54029::m.54029	0	0	0	0	0	0	0	0	0	0	0	0	523730	422890	416120
TRINITY_DN17845_c1_g3::TRINITY_DN17845_c1_g3_i3::g.90638::m.90638	96672	0	85003	0	125500	0	175730	111580	337630	0	156890	272960	0	0	0
TRINITY_DN18905_c1_g2::TRINITY_DN18905_c1_g2_i9::g.108635::m.108635	272210	77390	869560	142230	0	0	0	0	0	0	0	0	0	0	0
TRINITY_DN16321_c0_g3::TRINITY_DN16321_c0_g3_i5::g.65822::m.65822	0	0	0	0	0	0	0	0	0	0	0	0	395680	286910	673450
TRINITY_DN18561_c0_g1::TRINITY_DN18561_c0_g1_i3::g.102624::m.102624	0	0	0	0	0	0	0	0	0	0	0	0	646160	388440	320200
TRINITY_DN13727_c0_g2::TRINITY_DN13727_c0_g2_i11::g.27681::m.27681	8625,2	0	0	0	0	0	0	0	352440	0	374250	0	0	254330	364320
TRINITY_DN13477_c2_g3::TRINITY_DN13477_c2_g3_i1::g.24414::m.24414	43594	18944	0	64564	131850	0	0	0	0	0	0	0	0	600860	493810
TRINITY_DN11771_c0_g1::TRINITY_DN11771_c0_g1_i2::g.8609::m.8609	0	0	0	0	0	0	0	0	0	0	0	0	156830	636330	559220
TRINITY_DN11029_c0_g1::TRINITY_DN11029_c0_g1_i1::g.6154::m.6154	0	0	0	0	0	0	0	0	0	0	0	0	388220	633750	330000
TRINITY_DN19209_c0_g1::TRINITY_DN19209_c0_g1_i7::g.114040::m.114040	178230	0	0	0	0	0	0	0	0	0	0	0	131540	506470	534670
TRINITY_DN15323_c1_g4::TRINITY_DN15323_c1_g4_i7::g.50170::m.50170	0	0	0	0	0	0	0	0	0	0	0	0	0	540930	809240
TRINITY_DN14645_c3_g1::TRINITY_DN14645_c3_g1_i7::g.39449::m.39449	0	0	0	0	0	0	0	0	0	0	0	0	335080	493620	514920
TRINITY_DN14335_c0_g1::TRINITY_DN14335_c0_g1_i7::g.35543::m.35543	0	0	0	0	0	0	0	0	0	0	0	0	528780	451500	363040
TRINITY_DN17475_c1_g1::TRINITY_DN17475_c1_g1_i10::g.84374::m.84374	0	0	0	0	0	0	0	0	0	0	0	0	527860	347080	464000
TRINITY_DN18714_c0_g3::TRINITY_DN18714_c0_g3_i1::g.105033::m.105033	0	0	0	0	0	0	0	0	0	0	0	0	241110	294710	801990
TRINITY_DN18972_c2_g2::TRINITY_DN18972_c2_g2_i2::g.109605::m.109605	0	0	0	0	0	0	0	0	0	0	0	0	190790	197490	948510
TRINITY_DN12403_c0_g1::TRINITY_DN12403_c0_g1_i5::g.12601::m.12601	139600	32100	145870	85669	0	0	0	0	0	0	0	0	932880	0	0
TRINITY_DN11347_c0_g1::TRINITY_DN11347_c0_g1_i2::g.7021::m.7021	0	0	0	0	0	0	0	0	0	0	0	0	457390	453770	422770

TRINITY_DN18196_c4_g4::TRINITY_DN18196_c4_g4_i1::g.96720::m.96720	0	0	0	0	0	0	0	0	0	0	0	0	646540	0	675810
TRINITY_DN19648_c4_g4::TRINITY_DN19648_c4_g4_i4::g.120655::m.120655	0	0	0	0	0	0	0	0	0	0	0	0	100380	729320	492610
TRINITY_DN18301_c0_g2::TRINITY_DN18301_c0_g2_i7::g.97543::m.97543	0	0	0	0	0	0	0	0	0	0	0	0	400730	451080	470280
TRINITY_DN15444_c2_g2::TRINITY_DN15444_c2_g2_i4::g.52039::m.52039	0	58333	92524	128040	275480	0	0	0	0	0	0	0	377640	0	389510
TRINITY_DN19535_c3_g1::TRINITY_DN19535_c3_g1_i2::g.119402::m.119402	0	0	0	0	0	0	0	0	0	0	0	0	0	589470	727070
TRINITY_DN12020_c1_g1::TRINITY_DN12020_c1_g1_i2::g.10043::m.10043	0	0	0	0	0	0	358440	134720	0	0	0	358100	153760	156120	153450
TRINITY_DN17610_c1_g1::TRINITY_DN17610_c1_g1_i7::g.87051::m.87051	0	0	0	0	0	0	0	0	0	0	0	0	430420	515640	368490
TRINITY_DN18503_c1_g1::TRINITY_DN18503_c1_g1_i3::g.101658::m.101658	0	0	0	0	414810	0	0	310240	191090	0	0	133950	0	146000	117800
TRINITY_DN17513_c0_g1::TRINITY_DN17513_c0_g1_i8::g.85061::m.85061	0	0	0	0	0	0	0	0	0	0	0	0	380390	464630	465280
TRINITY_DN18932_c2_g2::TRINITY_DN18932_c2_g2_i2::g.109083::m.109083	77798	0	83382	76702	0	0	0	0	404510	0	0	0	0	345560	316220
TRINITY_DN18021_c3_g2::TRINITY_DN18021_c3_g2_i4::g.93827::m.93827	0	0	0	0	0	0	0	0	0	0	0	0	463670	427150	411060
TRINITY_DN13471_c1_g1::TRINITY_DN13471_c1_g1_i4::g.24277::m.24277	0	0	0	0	0	0	0	0	0	0	0	0	431470	464000	405690
TRINITY_DN14922_c2_g1::TRINITY_DN14922_c2_g1_i5::g.44553::m.44553	0	0	0	0	0	0	0	0	0	0	0	0	289040	337600	673570
TRINITY_DN15594_c4_g1::TRINITY_DN15594_c4_g1_i1::g.54180::m.54180	0	0	0	0	0	0	0	0	0	0	0	0	470100	260960	568830
TRINITY_DN11209_c0_g1::TRINITY_DN11209_c0_g1_i1::g.6616::m.6616	169530	0	174620	138080	0	0	0	0	0	0	0	0	602970	214050	0
TRINITY_DN18631_c1_g1::TRINITY_DN18631_c1_g1_i1::g.103584::m.103584	0	0	0	0	0	0	0	0	0	0	0	0	422200	468930	405410
TRINITY_DN17980_c0_g1::TRINITY_DN17980_c0_g1_i2::g.92937::m.92937	0	0	0	0	0	0	0	0	0	0	0	0	632020	658260	0
TRINITY_DN18446_c0_g1::TRINITY_DN18446_c0_g1_i3::g.100648::m.100648	0	0	0	0	0	0	0	0	0	0	0	0	247270	365090	677180
TRINITY_DN19548_c1_g1::TRINITY_DN19548_c1_g1_i5::g.119761::m.1197	0	0	0	0	0	0	0	0	0	0	0	0	678320	458760	151230
TRINITY_DN16896_c1_g1::TRINITY_DN16896_c1_g1_i19::g.75135::m.7513	16162	0	0	0	170690	0	0	0	0	0	0	0	505600	0	593240
TRINITY_DN14204_c1_g1::TRINITY_DN14204_c1_g1_i2::g.33927::m.33927	0	0	0	0	0	0	0	0	0	0	0	0	371190	493240	420140

TRINITY_DN19434_c0_g1::TRINITY_DN19434_c0_g1_i3::g.117932::m.117932	0	0	0	0	222580	72692	305140	0	101700	0	0	0	268600	166260	146060
TRINITY_DN17973_c0_g1::TRINITY_DN17973_c0_g1_i1::g.92481::m.92481	138110	70461	0	154180	0	0	0	0	0	118470	0	0	0	0	801380
TRINITY_DN18833_c4_g2::TRINITY_DN18833_c4_g2_i4::g.107207::m.107207	79051	51597	148400	89028	0	0	0	0	0	182990	342740	310080	0	0	77666
TRINITY_DN16544_c0_g2::TRINITY_DN16544_c0_g2_i7::g.68626::m.68626	0	0	0	0	0	0	0	0	0	0	0	0	168950	917260	193850
TRINITY_DN19860_c5_g1::TRINITY_DN19860_c5_g1_i1::g.124725::m.124725	0	0	0	0	0	0	0	0	0	0	0	0	0	696830	583260
TRINITY_DN48442_c0_g1::TRINITY_DN48442_c0_g1_i1::g.132681::m.132681	0	0	0	0	0	0	0	0	0	0	0	0	473960	376980	422390
TRINITY_DN19061_c2_g6::TRINITY_DN19061_c2_g6_i3::g.111201::m.111201	0	0	0	0	0	0	0	0	0	0	0	0	590040	678140	0
TRINITY_DN14323_c2_g3::TRINITY_DN14323_c2_g3_i2::g.35593::m.35593	0	0	0	0	0	0	0	0	0	0	0	0	378260	386280	495980
TRINITY_DN19440_c4_g2::TRINITY_DN19440_c4_g2_i10::g.117992::m.117992	0	0	0	0	0	0	171950	54571	0	0	0	0	274420	472830	281860
TRINITY_DN18371_c0_g1::TRINITY_DN18371_c0_g1_i2::g.99819::m.99819	0	0	0	0	0	0	0	0	0	0	0	0	477460	0	778000
TRINITY_DN14419_c1_g1::TRINITY_DN14419_c1_g1_i6::g.36874::m.36874	0	0	0	0	0	27682	0	0	201590	0	0	0	393990	472020	158850
TRINITY_DN13980_c0_g1::TRINITY_DN13980_c0_g1_i1::g.30892::m.30892	0	0	0	0	0	0	0	0	0	0	0	0	115100	421670	715120
TRINITY_DN18731_c0_g5::TRINITY_DN18731_c0_g5_i1::g.105388::m.105388	0	0	0	0	0	0	0	0	0	0	0	0	420470	828340	0
TRINITY_DN13791_c2_g2::TRINITY_DN13791_c2_g2_i3::g.28238::m.28238	0	0	0	0	0	0	0	0	0	0	0	0	592140	346120	307480
TRINITY_DN15442_c0_g2::TRINITY_DN15442_c0_g2_i1::g.51251::m.51251	0	0	0	0	0	0	0	0	0	0	0	0	0	0	1241800
TRINITY_DN1265_c0_g1::TRINITY_DN1265_c0_g1_i1::g.324::m.324	0	0	0	0	0	0	0	0	0	0	0	0	469340	456850	315150
TRINITY_DN14475_c2_g1::TRINITY_DN14475_c2_g1_i7::g.36756::m.36756	0	0	0	0	0	0	0	0	0	0	0	0	367430	629120	244140
TRINITY_DN16138_c0_g1::TRINITY_DN16138_c0_g1_i2::g.62708::m.62708	0	0	0	0	0	0	0	0	0	0	0	0	93572	908160	238590
TRINITY_DN18532_c1_g1::TRINITY_DN18532_c1_g1_i2::g.102269::m.102269	0	0	0	0	0	0	0	0	0	0	0	0	408690	152700	678360
TRINITY_DN18494_c1_g1::TRINITY_DN18494_c1_g1_i7::g.101464::m.101464	0	0	0	0	0	0	0	0	0	0	0	0	292770	0	945920

TRINITY_DN13527_c0_g1::TRINITY_DN13527_c0_g1_i14::g.25011::m.25011	0	0	0	0	0	0	0	0	0	0	0	0	326770	527580	382690
TRINITY_DN19343_c7_g5::TRINITY_DN19343_c7_g5_i1::g.116417::m.116417	182640	0	196750	165570	114800	0	0	0	0	0	575350	0	0	0	0
TRINITY_DN17435_c0_g1::TRINITY_DN17435_c0_g1_i2::g.83592::m.83592	0	0	0	0	0	0	0	0	0	0	0	0	361830	869730	0
TRINITY_DN12632_c0_g1::TRINITY_DN12632_c0_g1_i1::g.14398::m.14398	0	0	0	0	0	0	0	0	0	0	0	0	86079	604330	540690
TRINITY_DN13052_c1_g1::TRINITY_DN13052_c1_g1_i8::g.19267::m.19267	0	0	0	0	0	0	0	0	0	0	0	0	331550	0	898190
TRINITY_DN16618_c2_g1::TRINITY_DN16618_c2_g1_i4::g.70494::m.70494	0	0	0	0	0	0	0	0	0	0	0	141580	222120	259410	595700
TRINITY_DN17627_c2_g1::TRINITY_DN17627_c2_g1_i6::g.87313::m.87313	0	0	0	0	0	0	0	0	0	0	0	0	264200	474610	472230
TRINITY_DN14266_c0_g5::TRINITY_DN14266_c0_g5_i1::g.34758::m.34758	0	0	0	0	0	0	0	0	0	0	0	0	533280	509040	168100
TRINITY_DN15019_c3_g2::TRINITY_DN15019_c3_g2_i1::g.45833::m.45833	1208400	0	0	0	0	0	0	0	0	0	0	0	0	0	0
TRINITY_DN18255_c0_g1::TRINITY_DN18255_c0_g1_i22::g.97730::m.97730	0	0	0	0	0	0	0	0	0	0	0	0	363340	527810	314740
TRINITY_DN19124_c2_g3::TRINITY_DN19124_c2_g3_i2::g.112438::m.112438	0	0	0	0	0	0	0	0	0	0	0	0	95087	405950	702940
TRINITY_DN13398_c7_g1::TRINITY_DN13398_c7_g1_i2::g.23298::m.23298	23447	0	30945	0	156810	0	203060	149390	216960	0	0	305550	114160	0	0
TRINITY_DN18939_c0_g2::TRINITY_DN18939_c0_g2_i7::g.109183::m.109183	121010	45088	98479	91896	0	0	245330	0	0	0	0	598210	0	0	0
TRINITY_DN13383_c1_g4::TRINITY_DN13383_c1_g4_i2::g.23048::m.23048	0	0	0	0	0	0	0	0	0	0	0	0	300980	585350	313060
TRINITY_DN15405_c3_g1::TRINITY_DN15405_c3_g1_i6::g.51555::m.51555	0	0	0	0	0	0	0	0	0	0	0	0	546870	0	651150
TRINITY_DN12135_c0_g1::TRINITY_DN12135_c0_g1_i2::g.10684::m.10684	107950	70943	159490	109340	0	0	0	0	271020	191160	284050	0	0	0	0
TRINITY_DN17356_c4_g2::TRINITY_DN17356_c4_g2_i4::g.82383::m.82383	0	0	0	0	0	0	0	0	0	0	0	0	551540	640180	0
TRINITY_DN17524_c0_g2::TRINITY_DN17524_c0_g2_i3::g.85311::m.85311	0	0	0	0	0	0	225260	157250	489720	0	0	0	139200	0	178870
TRINITY_DN11973_c1_g1::TRINITY_DN11973_c1_g1_i7::g.9759::m.9759	104440	0	43295	182850	0	0	0	55395	0	0	0	140660	103940	431940	122610
TRINITY_DN15456_c0_g1::TRINITY_DN15456_c0_g1_i1::g.52131::m.52131	146580	63628	174900	204440	0	0	0	0	0	0	0	0	173000	169880	252010

TRINITY_DN15793_c0_g1::TRINITY_DN15793_c0_g1_i1::g.57091::m.57091	0	0	0	0	0	0	0	0	0	0	0	0	396310	434140	352640
TRINITY_DN14209_c0_g1::TRINITY_DN14209_c0_g1_i2::g.34018::m.34018	0	0	0	0	0	0	0	0	0	0	0	0	169350	224160	787270
TRINITY_DN14887_c2_g4::TRINITY_DN14887_c2_g4_i1::g.43866::m.43866	0	0	0	0	0	0	0	0	0	0	0	0	254240	252380	671620
TRINITY_DN18372_c2_g2::TRINITY_DN18372_c2_g2_i3::g.99514::m.99514	0	0	0	0	0	0	0	0	0	0	0	0	475950	354080	347580
TRINITY_DN16028_c0_g1::TRINITY_DN16028_c0_g1_i7::g.60828::m.60828	0	0	0	0	0	0	0	0	0	0	0	0	316930	367980	491430
TRINITY_DN12857_c0_g1::TRINITY_DN12857_c0_g1_i2::g.16763::m.16763	0	0	0	0	0	0	0	0	0	0	0	0	404270	771860	0
TRINITY_DN18741_c1_g1::TRINITY_DN18741_c1_g1_i9::g.105752::m.105752	104920	0	111660	172180	143990	0	178750	0	0	0	0	181520	115600	0	166600
TRINITY_DN18860_c2_g4::TRINITY_DN18860_c2_g4_i2::g.107536::m.107536	0	0	0	0	0	0	0	0	0	0	0	0	478020	261680	435330
TRINITY_DN12789_c1_g2::TRINITY_DN12789_c1_g2_i12::g.15958::m.15958	220900	0	141350	130990	243300	115400	320280	0	0	0	0	0	0	0	0
TRINITY_DN19136_c0_g1::TRINITY_DN19136_c0_g1_i3::g.112661::m.1126	0	0	0	0	0	0	0	0	0	0	0	0	370090	457700	344390
TRINITY_DN16675_c0_g2::TRINITY_DN16675_c0_g2_i3::g.71545::m.71545	0	0	0	0	0	0	0	0	0	0	0	0	373460	376000	421800
TRINITY_DN19779_c3_g1::TRINITY_DN19779_c3_g1_i9::g.123559::m.123559	0	0	0	0	0	0	0	0	0	0	0	0	468270	310300	389280
TRINITY_DN11951_c0_g1::TRINITY_DN11951_c0_g1_i4::g.9610::m.9610	36361	0	0	0	0	0	0	0	0	0	0	0	220880	690220	219420
TRINITY_DN17944_c2_g2::TRINITY_DN17944_c2_g2_i3::g.92569::m.92569	0	0	0	0	0	0	0	0	0	0	0	0	451360	222350	491300
TRINITY_DN17545_c0_g1::TRINITY_DN17545_c0_g1_i1::g.84935::m.84935	0	0	0	0	0	0	0	0	0	0	0	0	496960	174130	492500
TRINITY_DN14785_c0_g1::TRINITY_DN14785_c0_g1_i6::g.42288::m.42288	0	0	0	0	0	0	0	0	0	0	0	0	344720	381230	437540
TRINITY_DN10853_c0_g1::TRINITY_DN10853_c0_g1_i1::g.5658::m.5658	187350	0	0	0	0	0	0	0	256860	0	366270	351090	0	0	0
TRINITY_DN19439_c3_g1::TRINITY_DN19439_c3_g1_i7::g.118339::m.118339	0	0	0	0	0	0	0	0	0	0	0	0	1159100	0	0
TRINITY_DN19401_c0_g3::TRINITY_DN19401_c0_g3_i5::g.116386::m.116386	0	0	0	0	0	0	0	0	113950	0	0	0	447980	596890	0

TRINITY_DN13193_c0_g2::TRINITY_DN13193_c0_g2_i7::g.20882::m.20882	0	0	0	0	0	0	0	0	0	0	0	0	70659	514310	571950
TRINITY_DN16102_c2_g2::TRINITY_DN16102_c2_g2_i1::g.62195::m.62195	45874	0	0	0	0	0	0	0	0	0	0	0	0	1108700	0
TRINITY_DN17099_c3_g2::TRINITY_DN17099_c3_g2_i2::g.76778::m.76778	0	0	0	0	0	0	0	0	0	0	0	0	420000	564740	165470
TRINITY_DN14038_c1_g1::TRINITY_DN14038_c1_g1_i3::g.31643::m.31643	0	0	75030	74233	0	0	0	0	0	0	0	0	826170	82245	91500
TRINITY_DN16298_c0_g1::TRINITY_DN16298_c0_g1_i6::g.65227::m.65227	0	0	0	0	0	0	0	0	0	0	0	0	523750	624900	0
TRINITY_DN16403_c4_g2::TRINITY_DN16403_c4_g2_i2::g.67059::m.67059	0	0	0	0	0	0	0	0	0	0	0	0	408180	416830	323570
TRINITY_DN19748_c4_g1::TRINITY_DN19748_c4_g1_i8::g.123188::m.123188	0	0	0	0	0	0	0	0	0	0	0	0	307380	209900	629750
TRINITY_DN16008_c3_g2::TRINITY_DN16008_c3_g2_i3::g.60431::m.60431	0	0	0	0	0	0	0	0	0	0	0	0	447570	248890	443750
TRINITY_DN15365_c0_g2::TRINITY_DN15365_c0_g2_i4::g.50831::m.50831	0	0	0	0	0	0	0	0	0	0	0	0	270840	388180	481050
TRINITY_DN16706_c0_g2::TRINITY_DN16706_c0_g2_i5::g.72057::m.72057	0	0	0	0	0	0	0	0	0	0	0	0	370640	336240	431660
TRINITY_DN12693_c0_g1::TRINITY_DN12693_c0_g1_i1::g.14939::m.14939	0	0	158110	150160	0	0	0	0	0	0	0	0	313860	515620	0
TRINITY_DN13827_c1_g1::TRINITY_DN13827_c1_g1_i4::g.28842::m.28842	0	0	0	0	0	0	0	0	0	0	0	0	439890	279500	415140
TRINITY_DN11389_c0_g1::TRINITY_DN11389_c0_g1_i1::g.7193::m.7193	0	0	0	0	0	0	89965	406350	0	0	215130	211070	0	102330	109490
TRINITY_DN12755_c3_g1::TRINITY_DN12755_c3_g1_i1::g.16006::m.16006	164200	0	69002	0	195420	0	0	0	0	0	388500	315860	0	0	0
TRINITY_DN13006_c0_g1::TRINITY_DN13006_c0_g1_i3::g.18571::m.18571	0	0	0	0	0	0	0	0	0	0	0	0	553970	576290	0
TRINITY_DN31964_c0_g1::TRINITY_DN31964_c0_g1_i1::g.130316::m.130316	496160	0	323480	0	0	0	0	305880	0	0	0	0	0	0	0
TRINITY_DN10036_c0_g1::TRINITY_DN10036_c0_g1_i2::g.4202::m.4202	0	0	0	0	259110	114160	326010	226090	0	0	0	0	0	199890	0
TRINITY_DN17508_c5_g2::TRINITY_DN17508_c5_g2_i5::g.85417::m.85417	166810	53290	159210	123610	196730	0	232000	192530	0	0	0	0	0	0	0
TRINITY_DN19723_c2_g3::TRINITY_DN19723_c2_g3_i8::g.122861::m.122861	0	0	0	0	288040	0	0	174040	0	0	0	0	0	660330	0
TRINITY_DN12524_c0_g1::TRINITY_DN12524_c0_g1_i3::g.13484::m.13484	0	0	0	0	0	0	0	0	0	0	0	0	329280	362510	430240

TRINITY_DN18436_c0_g2::TRINITY_DN18436_c0_g2_i4::g.100110::m.100110	0	0	0	0	0	0	0	0	0	0	0	0	303790	154250	661290
TRINITY_DN12307_c0_g1::TRINITY_DN12307_c0_g1_i3::g.11724::m.11724	0	0	0	0	0	0	0	0	0	0	0	0	483460	0	630540
TRINITY_DN13767_c0_g1::TRINITY_DN13767_c0_g1_i2::g.28089::m.28089	0	0	0	0	0	0	0	0	0	0	0	0	106600	405510	599290
TRINITY_DN19009_c0_g1::TRINITY_DN19009_c0_g1_i8::g.110506::m.110506	0	0	0	0	0	0	0	0	0	0	0	0	422000	0	689300
TRINITY_DN17898_c0_g1::TRINITY_DN17898_c0_g1_i6::g.91458::m.91458	0	0	0	0	0	0	0	78997	0	0	0	0	81072	355320	595740
TRINITY_DN11577_c0_g1::TRINITY_DN11577_c0_g1_i3::g.7931::m.7931	0	0	0	0	0	0	0	0	0	0	0	0	0	0	1108300
TRINITY_DN17836_c0_g3::TRINITY_DN17836_c0_g3_i4::g.90474::m.90474	0	0	0	0	0	0	0	0	0	0	0	0	269830	360410	477580
TRINITY_DN19582_c3_g2::TRINITY_DN19582_c3_g2_i4::g.120114::m.120114	0	0	0	0	232070	0	632840	0	0	0	0	0	0	0	241860
TRINITY_DN15102_c1_g3::TRINITY_DN15102_c1_g3_i1::g.46949::m.46949	0	0	0	0	0	0	0	0	0	0	0	0	469570	328140	308980
TRINITY_DN18657_c1_g1::TRINITY_DN18657_c1_g1_i4::g.104087::m.104087	0	0	0	0	0	0	0	0	0	0	0	0	523010	0	582710
TRINITY_DN11984_c0_g1::TRINITY_DN11984_c0_g1_i17::g.9822::m.9822	0	0	0	0	0	0	0	0	0	0	0	0	225700	433070	446100
TRINITY_DN13083_c4_g1::TRINITY_DN13083_c4_g1_i1::g.19609::m.19609	0	0	0	0	0	145080	0	221390	298290	0	440130	0	0	0	0
TRINITY_DN11520_c0_g2::TRINITY_DN11520_c0_g2_i2::g.7719::m.7719	0	0	0	0	0	0	0	0	0	0	0	0	374730	0	726130
TRINITY_DN11695_c0_g2::TRINITY_DN11695_c0_g2_i5::g.8192::m.8192	0	0	0	0	0	0	0	0	0	0	0	0	236080	508900	354990
TRINITY_DN17518_c0_g2::TRINITY_DN17518_c0_g2_i4::g.85745::m.85745	0	0	0	0	0	0	0	0	0	0	0	0	0	0	1099000
TRINITY_DN15614_c2_g1::TRINITY_DN15614_c2_g1_i2::g.54750::m.54750	0	0	0	0	0	0	0	0	0	0	0	1098600	0	0	0
TRINITY_DN17279_c2_g2::TRINITY_DN17279_c2_g2_i30::g.80027::m.80027	0	0	0	0	0	0	0	0	0	0	0	0	252380	438020	408110
TRINITY_DN12358_c0_g1::TRINITY_DN12358_c0_g1_i4::g.12129::m.12129	0	0	66979	72349	0	0	0	0	0	0	0	0	308820	221080	428230
TRINITY_DN14089_c0_g1::TRINITY_DN14089_c0_g1_i1::g.32223::m.32223	93476	0	90897	97217	0	0	0	0	0	0	0	0	0	808130	0
TRINITY_DN13617_c1_g1::TRINITY_DN13617_c1_g1_i9::g.26343::m.26343	0	0	0	0	129240	0	385390	342580	0	0	0	0	0	0	229760
TRINITY_DN12509_c0_g1::TRINITY_DN12509_c0_g1_i1::g.13435::m.13435	0	0	0	0	0	0	0	0	0	0	0	0	602840	118960	364280

TRINITY_DN20017_c0_g1::TRINITY_DN20017_c0_g1_i1::g.127859::m.127859	0	0	0	0	0	0	0	0	0	0	0	0	0	540980	542470
TRINITY_DN15264_c3_g1::TRINITY_DN15264_c3_g1_i8::g.49356::m.49356	0	0	0	0	0	0	0	0	0	0	0	0	325840	422970	330640
TRINITY_DN19396_c1_g2::TRINITY_DN19396_c1_g2_i11::g.116356::m.116356	43758	0	0	0	0	0	0	0	0	0	0	0	201580	157940	674000
TRINITY_DN13077_c0_g1::TRINITY_DN13077_c0_g1_i9::g.19580::m.19580	0	0	0	0	0	0	0	0	0	0	0	0	223340	474230	379130
TRINITY_DN19577_c3_g1::TRINITY_DN19577_c3_g1_i6::g.120109::m.120109	0	0	0	0	0	0	0	0	0	0	0	0	348400	412150	314870
TRINITY_DN10714_c0_g1::TRINITY_DN10714_c0_g1_i4::g.5392::m.5392	0	0	0	0	0	0	0	0	0	0	0	0	282890	439020	351200
TRINITY_DN13818_c0_g2::TRINITY_DN13818_c0_g2_i15::g.28994::m.28994	0	0	0	0	0	0	0	0	0	77114	0	0	498510	0	496940
TRINITY_DN18434_c0_g1::TRINITY_DN18434_c0_g1_i17::g.100395::m.100395	61606	21071	67539	46629	0	0	120560	0	0	0	348390	0	182090	221170	0
TRINITY_DN14145_c0_g4::TRINITY_DN14145_c0_g4_i3::g.33080::m.33080	0	0	0	0	0	0	0	0	0	0	0	0	359350	357650	351400
TRINITY_DN14453_c2_g3::TRINITY_DN14453_c2_g3_i2::g.37393::m.37393	264540	0	195980	172890	0	120500	311120	0	0	0	0	0	0	0	0
TRINITY_DN12731_c3_g2::TRINITY_DN12731_c3_g2_i1::g.15534::m.15534	0	0	0	0	0	0	0	0	0	0	0	0	321190	377510	365850
TRINITY_DN19556_c1_g1::TRINITY_DN19556_c1_g1_i8::g.119918::m.1199	0	0	0	0	0	0	0	0	0	0	0	0	290440	0	770870
TRINITY_DN12315_c0_g1::TRINITY_DN12315_c0_g1_i2::g.11830::m.11830	0	0	0	0	0	0	0	0	0	0	0	0	259840	559570	241380
TRINITY_DN14308_c1_g1::TRINITY_DN14308_c1_g1_i5::g.35396::m.35396	0	0	0	0	0	0	0	0	0	0	0	0	436980	308850	313890
TRINITY_DN15701_c0_g1::TRINITY_DN15701_c0_g1_i7::g.55836::m.55836	0	0	0	0	0	0	0	0	0	0	0	0	0	0	1058700
TRINITY_DN11076_c0_g1::TRINITY_DN11076_c0_g1_i2::g.6263::m.6263	0	0	0	0	0	0	0	0	0	0	0	0	0	1055400	0
TRINITY_DN13289_c1_g1::TRINITY_DN13289_c1_g1_i2::g.22086::m.22086	0	0	0	0	0	0	0	0	0	0	0	0	290660	524660	239710
TRINITY_DN11519_c0_g1::TRINITY_DN11519_c0_g1_i2::g.7693::m.7693	0	0	0	0	0	0	0	0	0	0	0	0	198100	631960	223080
TRINITY_DN15166_c0_g1::TRINITY_DN15166_c0_g1_i7::g.47733::m.47733	0	0	0	0	0	0	0	0	0	0	0	0	169440	411200	471760
TRINITY_DN15517_c0_g1::TRINITY_DN15517_c0_g1_i10::g.53139::m.5313	0	0	0	0	0	0	0	0	0	0	0	0	740700	0	311450
TRINITY_DN15729_c0_g1::TRINITY_DN15729_c0_g1_i9::g.56378::m.56378	0	0	0	0	0	0	0	0	0	0	0	0	190100	691720	170270

TRINITY_DN15900_c2_g1::TRINITY_DN15900_c2_g1_i1::g.57240::m.57240	41462	0	0	0	0	0	0	0	0	0	0	0	0	885590	124120
TRINITY_DN13702_c0_g1::TRINITY_DN13702_c0_g1_i4::g.27394::m.27394	80372	36544	115170	114630	0	0	0	0	0	0	703840	0	0	0	0
TRINITY_DN17271_c3_g3::TRINITY_DN17271_c3_g3_i8::g.81059::m.81059	0	0	0	0	0	0	0	0	0	0	0	0	177600	461190	410520
TRINITY_DN17089_c1_g2::TRINITY_DN17089_c1_g2_i5::g.78211::m.78211	0	0	0	0	0	0	0	0	0	0	0	0	247660	311540	489470
TRINITY_DN17386_c2_g1::TRINITY_DN17386_c2_g1_i2::g.82885::m.82885	0	0	0	0	0	0	0	0	0	0	0	0	243780	261720	540790
TRINITY_DN13961_c0_g1::TRINITY_DN13961_c0_g1_i2::g.30621::m.30621	0	0	0	0	0	0	0	0	0	0	0	0	0	896820	146680
TRINITY_DN13778_c1_g3::TRINITY_DN13778_c1_g3_i1::g.28124::m.28124	0	0	0	0	0	0	0	0	0	0	0	0	391310	651330	0
TRINITY_DN13512_c0_g1::TRINITY_DN13512_c0_g1_i2::g.24707::m.24707	64510	31711	88433	56552	0	0	0	108810	236500	0	455980	0	0	0	0
TRINITY_DN19318_c3_g1::TRINITY_DN19318_c3_g1_i7::g.115859::m.115859	118440	0	0	0	0	0	0	0	293190	0	435200	0	0	195520	0
TRINITY_DN17993_c0_g1::TRINITY_DN17993_c0_g1_i2::g.93217::m.93217	0	0	0	0	0	0	0	0	0	0	0	0	178570	284260	577850
TRINITY_DN19049_c0_g1::TRINITY_DN19049_c0_g1_i9::g.110547::m.110547	0	0	0	0	0	0	0	0	0	0	0	0	189340	488300	362800
TRINITY_DN12375_c3_g1::TRINITY_DN12375_c3_g1_i9::g.12353::m.12353	0	0	0	0	0	0	0	0	0	0	0	0	456950	0	582720
TRINITY_DN18998_c2_g1::TRINITY_DN18998_c2_g1_i7::g.108559::m.108559	0	0	0	0	0	0	0	0	0	0	0	0	171830	469170	398250
TRINITY_DN18521_c6_g4::TRINITY_DN18521_c6_g4_i1::g.101901::m.101901	0	0	0	0	0	0	0	0	0	0	0	0	384680	430930	222500
TRINITY_DN17805_c0_g1::TRINITY_DN17805_c0_g1_i5::g.89980::m.89980	0	0	0	0	0	0	0	0	0	0	0	0	308960	421840	301410
TRINITY_DN19377_c2_g2::TRINITY_DN19377_c2_g2_i2::g.116852::m.116852	0	0	0	0	0	0	0	0	0	0	0	79974	363320	302970	285610
TRINITY_DN19873_c6_g5::TRINITY_DN19873_c6_g5_i6::g.124897::m.124897	0	0	0	0	0	0	0	0	0	0	0	0	0	516130	514230
TRINITY_DN16513_c0_g2::TRINITY_DN16513_c0_g2_i7::g.68926::m.68926	0	0	0	0	0	0	0	90562	0	0	0	0	411910	527850	0
TRINITY_DN12856_c0_g1::TRINITY_DN12856_c0_g1_i4::g.16613::m.16613	0	0	0	0	0	0	0	0	0	0	0	0	0	466070	562490
TRINITY_DN8001_c0_g1::TRINITY_DN8001_c0_g1_i1::g.2441::m.2441	0	0	0	0	0	0	0	0	0	0	0	0	420420	608070	0

TRINITY_DN16768_c1_g1::TRINITY_DN16768_c1_g1_i8::g.72998::m.72998	0	0	0	0	0	0	0	0	0	0	0	0	0	681600	342290
TRINITY_DN15627_c0_g1::TRINITY_DN15627_c0_g1_i7::g.54594::m.54594	0	0	0	0	0	0	0	0	0	0	0	0	344270	356500	319600
TRINITY_DN17680_c4_g1::TRINITY_DN17680_c4_g1_i22::g.88254::m.88254	0	0	0	0	0	0	0	0	0	0	0	0	385020	487610	147170
TRINITY_DN19193_c1_g1::TRINITY_DN19193_c1_g1_i1::g.113618::m.1136	102270	14786	153380	167940	120190	0	135590	323720	0	0	0	0	0	0	0
TRINITY_DN13047_c0_g2::TRINITY_DN13047_c0_g2_i4::g.19117::m.19117	0	0	0	0	0	0	0	0	0	0	0	0	597600	419740	0
TRINITY_DN16413_c0_g1::TRINITY_DN16413_c0_g1_i2::g.67167::m.67167	0	0	0	0	0	0	0	0	0	0	0	0	302640	366880	347440
TRINITY_DN16171_c0_g1::TRINITY_DN16171_c0_g1_i2::g.63190::m.63190	0	0	0	0	0	0	0	0	0	0	0	0	0	1016900	0
TRINITY_DN19552_c1_g2::TRINITY_DN19552_c1_g2_i2::g.119657::m.119657	0	0	0	0	0	0	0	0	0	0	0	0	0	503740	512480
TRINITY_DN15066_c0_g1::TRINITY_DN15066_c0_g1_i4::g.46431::m.46431	0	0	0	0	0	0	0	0	0	0	0	0	307590	0	708070
TRINITY_DN17996_c1_g1::TRINITY_DN17996_c1_g1_i2::g.93353::m.93353	0	0	0	0	0	0	0	0	0	0	0	0	352040	663540	0
TRINITY_DN19241_c0_g1::TRINITY_DN19241_c0_g1_i6::g.115140::m.1151	0	0	0	0	0	0	0	0	0	0	0	0	569440	0	443860
TRINITY_DN18797_c1_g2::TRINITY_DN18797_c1_g2_i1::g.106436::m.106436	227730	125270	233620	425790	0	0	0	0	0	0	0	0	0	0	0
TRINITY_DN10621_c0_g1::TRINITY_DN10621_c0_g1_i2::g.5142::m.5142	0	0	0	0	0	0	0	0	0	0	0	0	326710	368450	315720
TRINITY_DN18450_c3_g3::TRINITY_DN18450_c3_g3_i7::g.100614::m.100614	0	0	0	0	0	0	0	0	0	0	0	0	314180	186530	509660
TRINITY_DN12897_c1_g1::TRINITY_DN12897_c1_g1_i8::g.17082::m.17082	0	0	0	0	0	0	318140	0	244240	47853	399680	0	0	0	0
TRINITY_DN15693_c4_g1::TRINITY_DN15693_c4_g1_i3::g.55852::m.55852	0	0	0	0	0	0	0	0	0	0	0	0	266480	306460	436910
TRINITY_DN13826_c0_g2::TRINITY_DN13826_c0_g2_i4::g.28934::m.28934	0	0	0	0	0	0	0	0	0	0	0	0	372160	423660	213250
TRINITY_DN11412_c0_g1::TRINITY_DN11412_c0_g1_i1::g.7292::m.7292	0	0	0	0	0	0	0	0	0	0	0	0	0	708540	299340
TRINITY_DN11674_c0_g1::TRINITY_DN11674_c0_g1_i3::g.8359::m.8359	0	0	0	0	0	0	0	230570	0	0	769410	0	0	0	0
TRINITY_DN17979_c4_g2::TRINITY_DN17979_c4_g2_i7::g.93178::m.93178	0	0	0	0	0	20889	0	0	112930	0	0	153270	308150	0	403340
TRINITY_DN18736_c0_g1::TRINITY_DN18736_c0_g1_i7::g.105711::m.105711	0	0	0	0	0	0	0	0	0	0	0	0	0	503720	492530
TRINITY_DN13606_c2_g3::TRINITY_DN13606_c2_g3_i2::g.26267::m.26267	0	0	0	0	0	0	0	0	0	0	0	0	0	161450	834500

TRINITY_DN13348_c4_g1::TRINITY_DN13348_c4_g1_i6::g.22618::m.22618	72510	0	0	0	0	0	0	0	0	0	0	0	356110	416250	150410
TRINITY_DN17345_c4_g3::TRINITY_DN17345_c4_g3_i2::g.81576::m.81576	0	0	0	0	0	0	0	0	0	0	0	0	244510	224130	522010
TRINITY_DN11662_c0_g1::TRINITY_DN11662_c0_g1_i1::g.8281::m.8281	137590	32571	114040	75845	0	67530	288540	274150	0	0	0	0	0	0	0
TRINITY_DN15060_c1_g1::TRINITY_DN15060_c1_g1_i6::g.46413::m.46413	0	0	0	0	0	0	0	0	0	0	0	0	227220	411080	349700
TRINITY_DN15046_c3_g2::TRINITY_DN15046_c3_g2_i6::g.45963::m.45963	0	0	0	0	0	0	0	0	0	0	0	0	440880	271400	273770
TRINITY_DN17213_c1_g3::TRINITY_DN17213_c1_g3_i3::g.80204::m.80204	0	0	0	0	0	0	0	0	0	0	0	0	243840	197460	543470
TRINITY_DN18186_c3_g1::TRINITY_DN18186_c3_g1_i8::g.96539::m.96539	0	0	0	0	0	0	0	0	0	0	0	0	224420	519520	237780
TRINITY_DN19210_c1_g1::TRINITY_DN19210_c1_g1_i13::g.114068::m.114068	0	0	0	0	0	0	0	0	0	0	0	0	0	371580	608900
TRINITY_DN17483_c0_g1::TRINITY_DN17483_c0_g1_i3::g.84425::m.84425	0	0	0	0	0	0	0	0	0	0	0	0	343600	238160	396820
TRINITY_DN13893_c0_g2::TRINITY_DN13893_c0_g2_i1::g.30000::m.30000	0	0	0	0	0	0	0	0	0	0	0	0	266970	437740	273140
TRINITY_DN19502_c1_g3::TRINITY_DN19502_c1_g3_i5::g.118942::m.118942	0	0	0	0	0	0	0	0	0	0	0	0	163760	448610	364800
TRINITY_DN13503_c0_g1::TRINITY_DN13503_c0_g1_i7::g.24647::m.24647	102440	46158	104630	103110	0	0	0	0	0	0	0	618330	0	0	0
TRINITY_DN20983_c0_g1::TRINITY_DN20983_c0_g1_i1::g.128678::m.128678	0	0	0	0	0	0	0	0	0	0	0	0	182070	442580	347530
TRINITY_DN15427_c0_g1::TRINITY_DN15427_c0_g1_i2::g.51743::m.51743	0	0	0	0	0	0	0	0	0	0	0	0	137800	513210	320530
TRINITY_DN19611_c0_g2::TRINITY_DN19611_c0_g2_i9::g.121074::m.121074	0	0	0	0	0	0	0	0	0	0	325240	0	403450	0	242460
TRINITY_DN17613_c0_g1::TRINITY_DN17613_c0_g1_i11::g.87113::m.8711	0	0	0	0	0	0	0	0	0	0	0	0	155920	86127	728310
TRINITY_DN19888_c1_g1::TRINITY_DN19888_c1_g1_i5::g.125265::m.125265	0	0	0	0	0	0	0	0	0	0	0	585820	85802	296410	0
TRINITY_DN17142_c1_g1::TRINITY_DN17142_c1_g1_i7::g.79281::m.79281	0	0	0	0	0	0	0	0	0	0	0	0	0	967850	0
TRINITY_DN14016_c0_g1::TRINITY_DN14016_c0_g1_i9::g.31308::m.31308	0	0	0	0	0	0	0	0	0	0	0	0	690700	276960	0
TRINITY_DN15297_c0_g1::TRINITY_DN15297_c0_g1_i10::g.49743::m.49743	43826	0	41987	0	0	0	0	0	0	0	0	0	326070	177330	377610

TRINITY_DN18857_c0_g1::TRINITY_DN18857_c0_g1_i9::g.107487::m.107487	0	0	0	0	0	0	0	0	0	0	0	0	205780	559350	200950
TRINITY_DN14669_c0_g2::TRINITY_DN14669_c0_g2_i2::g.40246::m.40246	194790	0	410360	357920	0	0	0	0	0	0	0	0	0	0	0
TRINITY_DN11011_c0_g1::TRINITY_DN11011_c0_g1_i3::g.6100::m.6100	0	0	0	0	0	0	0	0	0	0	0	0	220080	453300	289080
TRINITY_DN13798_c2_g3::TRINITY_DN13798_c2_g3_i1::g.28387::m.28387	0	45335	136010	99859	0	0	0	0	0	0	0	680710	0	0	0
TRINITY_DN13146_c0_g1::TRINITY_DN13146_c0_g1_i3::g.20394::m.20394	0	0	0	0	0	0	0	0	0	0	0	0	255770	349570	353230
TRINITY_DN16700_c3_g5::TRINITY_DN16700_c3_g5_i2::g.71826::m.71826	0	0	0	0	0	0	0	0	0	0	0	0	404290	323640	230130
TRINITY_DN17427_c0_g1::TRINITY_DN17427_c0_g1_i3::g.83624::m.83624	171360	0	346520	440140	0	0	0	0	0	0	0	0	0	0	0
TRINITY_DN15169_c1_g1::TRINITY_DN15169_c1_g1_i14::g.47860::m.47860	0	0	0	0	0	0	0	0	0	0	0	0	265840	355550	334370
TRINITY_DN13150_c1_g1::TRINITY_DN13150_c1_g1_i6::g.20550::m.20550	83434	32967	110790	105610	141940	0	0	155260	323060	0	0	0	0	0	0
TRINITY_DN19251_c1_g2::TRINITY_DN19251_c1_g2_i17::g.114967::m.114967	0	0	0	0	0	0	0	0	0	0	0	0	271420	424310	256970
TRINITY_DN19006_c2_g1::TRINITY_DN19006_c2_g1_i11::g.110719::m.110719	0	0	0	0	0	0	0	0	0	0	0	0	506590	0	443570
TRINITY_DN18900_c0_g1::TRINITY_DN18900_c0_g1_i6::g.107340::m.107340	0	0	0	0	0	0	0	0	0	0	0	0	334340	270170	345320
TRINITY_DN15100_c0_g1::TRINITY_DN15100_c0_g1_i1::g.45506::m.45506	0	0	0	0	0	0	0	0	0	0	0	0	253680	268500	424920
TRINITY_DN11216_c0_g1::TRINITY_DN11216_c0_g1_i3::g.6674::m.6674	0	0	0	0	0	0	0	0	0	0	0	0	323670	154450	466260
TRINITY_DN19951_c3_g1::TRINITY_DN19951_c3_g1_i8::g.125811::m.1258	0	0	0	0	0	0	0	149020	0	0	290020	0	0	240510	263480
TRINITY_DN13542_c0_g1::TRINITY_DN13542_c0_g1_i7::g.24740::m.24740	0	0	0	0	0	0	0	0	0	0	0	0	171460	209100	561920
TRINITY_DN13852_c0_g1::TRINITY_DN13852_c0_g1_i7::g.28442::m.28442	0	0	0	0	0	0	0	0	0	0	0	0	246460	530410	164870
TRINITY_DN16578_c2_g1::TRINITY_DN16578_c2_g1_i1::g.69884::m.69884	0	0	0	0	0	0	0	0	0	0	0	0	356310	413570	166840
TRINITY_DN18180_c0_g1::TRINITY_DN18180_c0_g1_i6::g.96457::m.96457	0	0	0	0	0	0	0	0	0	0	0	0	0	138040	798210
TRINITY_DN16164_c3_g2::TRINITY_DN16164_c3_g2_i1::g.62975::m.62975	0	0	0	0	0	0	0	0	0	0	0	0	0	0	935440

TRINITY_DN14862_c0_g2::TRINITY_DN14862_c0_g2_i3::g.43396::m.43396	0	0	0	0	0	0	0	0	0	0	0	0	0	458480	476750
TRINITY_DN12438_c0_g1::TRINITY_DN12438_c0_g1_i11::g.12896::m.12896	0	0	0	0	0	0	0	0	0	0	0	85367	175190	346670	327470
TRINITY_DN11935_c0_g1::TRINITY_DN11935_c0_g1_i2::g.9568::m.9568	0	0	0	0	0	0	0	0	0	0	0	0	313530	275710	345280
TRINITY_DN17228_c0_g1::TRINITY_DN17228_c0_g1_i3::g.80433::m.80433	0	0	0	0	0	0	0	0	0	0	0	0	0	396530	536500
TRINITY_DN14948_c0_g1::TRINITY_DN14948_c0_g1_i2::g.43967::m.43967	0	0	0	0	0	0	0	0	0	0	0	0	382760	282020	268170
TRINITY_DN17507_c1_g2::TRINITY_DN17507_c1_g2_i9::g.85053::m.85053	0	0	0	0	0	0	0	0	0	0	0	0	361900	191520	378810
TRINITY_DN14555_c5_g1::TRINITY_DN14555_c5_g1_i2::g.38904::m.38904	0	0	0	0	137490	0	0	0	0	0	0	409300	185210	0	198410
TRINITY_DN15250_c0_g1::TRINITY_DN15250_c0_g1_i1::g.49205::m.49205	0	0	0	0	0	0	0	0	0	0	0	0	217690	152690	559950
TRINITY_DN14880_c2_g1::TRINITY_DN14880_c2_g1_i5::g.43756::m.43756	0	0	0	0	0	0	0	0	0	0	0	0	453360	237510	237020
TRINITY_DN17189_c3_g1::TRINITY_DN17189_c3_g1_i6::g.79844::m.79844	0	0	0	0	0	0	0	0	0	0	0	0	481310	204210	241460
TRINITY_DN15404_c3_g2::TRINITY_DN15404_c3_g2_i3::g.51327::m.51327	0	0	0	0	0	0	0	0	0	0	0	0	0	0	922740
TRINITY_DN18753_c1_g3::TRINITY_DN18753_c1_g3_i14::g.106335::m.106335	0	0	0	0	0	0	0	0	0	0	0	0	272990	272640	373290
TRINITY_DN19089_c3_g2::TRINITY_DN19089_c3_g2_i5::g.111660::m.111660	0	0	0	0	0	0	0	0	0	0	0	0	918750	0	0
TRINITY_DN18930_c0_g5::TRINITY_DN18930_c0_g5_i4::g.109035::m.109035	0	0	0	0	0	0	0	0	0	0	0	0	106170	544950	267260
TRINITY_DN19063_c1_g2::TRINITY_DN19063_c1_g2_i13::g.111218::m.111218	0	0	0	0	0	0	0	0	0	0	0	0	283860	467880	165270
TRINITY_DN13381_c2_g1::TRINITY_DN13381_c2_g1_i4::g.23007::m.23007	0	0	60090	70816	0	0	0	0	0	0	0	0	259940	524230	0
TRINITY_DN15027_c0_g1::TRINITY_DN15027_c0_g1_i5::g.45870::m.45870	0	0	0	0	0	0	0	0	0	0	0	0	149590	408240	354410
TRINITY_DN17756_c1_g1::TRINITY_DN17756_c1_g1_i7::g.89229::m.89229	0	0	0	0	0	0	0	0	0	0	0	0	258110	169920	483400
TRINITY_DN15011_c4_g2::TRINITY_DN15011_c4_g2_i4::g.45809::m.45809	0	0	0	0	0	0	0	0	0	0	240900	0	201840	246090	221660
TRINITY_DN12829_c0_g1::TRINITY_DN12829_c0_g1_i5::g.16229::m.16229	0	0	0	0	0	0	0	0	0	0	0	0	158120	282910	469190

TRINITY_DN19522_c5_g1::TRINITY_DN19522_c5_g1_i5::g.119202::m.119202	0	0	0	0	0	0	0	0	0	0	0	0	308140	356530	245360
TRINITY_DN19133_c0_g1::TRINITY_DN19133_c0_g1_i8::g.112559::m.112559	0	0	0	0	0	0	0	0	0	0	0	0	0	518160	391490
TRINITY_DN19814_c1_g3::TRINITY_DN19814_c1_g3_i2::g.124100::m.124100	0	0	0	0	0	0	0	172270	0	417290	0	0	0	319190	0
TRINITY_DN32151_c0_g1::TRINITY_DN32151_c0_g1_i1::g.130343::m.130343	0	0	0	0	0	0	0	0	0	0	0	0	279830	360600	266730
TRINITY_DN12363_c0_g1::TRINITY_DN12363_c0_g1_i2::g.12162::m.12162	0	0	0	0	0	0	0	0	0	0	0	0	546820	0	358980
TRINITY_DN18083_c3_g1::TRINITY_DN18083_c3_g1_i4::g.94853::m.94853	0	0	0	0	0	0	0	0	0	0	0	0	0	279600	625700
TRINITY_DN15012_c0_g1::TRINITY_DN15012_c0_g1_i2::g.45660::m.45660	0	0	0	37642	191680	0	258430	0	0	0	0	0	0	416640	0
TRINITY_DN18820_c2_g1::TRINITY_DN18820_c2_g1_i7::g.106999::m.106999	136850	0	147870	110150	0	0	0	0	0	0	0	0	0	272940	236080
TRINITY_DN15977_c4_g2::TRINITY_DN15977_c4_g2_i4::g.59843::m.59843	0	0	0	68241	0	0	183810	106040	0	0	0	0	0	295320	249990
TRINITY_DN19301_c0_g1::TRINITY_DN19301_c0_g1_i6::g.115549::m.115549	0	0	0	0	0	0	0	0	0	0	0	0	419520	405980	72849
TRINITY_DN16119_c4_g3::TRINITY_DN16119_c4_g3_i2::g.62577::m.62577	0	0	0	0	0	0	0	0	0	0	0	0	155140	288210	454180
TRINITY_DN14879_c3_g1::TRINITY_DN14879_c3_g1_i2::g.43728::m.43728	0	0	0	0	0	0	0	0	0	0	0	0	0	399200	495640
TRINITY_DN18658_c0_g1::TRINITY_DN18658_c0_g1_i8::g.104114::m.104114	0	0	58872	0	0	0	364120	0	0	0	304840	0	166540	0	0
TRINITY_DN17925_c2_g1::TRINITY_DN17925_c2_g1_i5::g.91985::m.91985	0	0	0	0	0	0	0	0	0	0	0	0	81745	472780	338840
TRINITY_DN16773_c1_g1::TRINITY_DN16773_c1_g1_i5::g.73048::m.73048	75706	47446	93914	0	0	0	0	0	0	0	0	173730	147760	168960	183240
TRINITY_DN13102_c0_g1::TRINITY_DN13102_c0_g1_i5::g.19880::m.19880	0	0	0	0	0	0	0	0	0	0	0	0	0	216380	674240
TRINITY_DN15656_c0_g5::TRINITY_DN15656_c0_g5_i2::g.55149::m.55149	0	0	0	0	0	0	0	0	0	0	0	0	134240	539130	215090
TRINITY_DN15008_c0_g2::TRINITY_DN15008_c0_g2_i1::g.45732::m.45732	0	0	0	0	0	0	0	0	0	0	0	0	287790	379800	218970
TRINITY_DN18231_c0_g2::TRINITY_DN18231_c0_g2_i7::g.97309::m.97309	0	0	0	0	0	0	0	0	0	0	88430	0	95268	459470	243270
TRINITY_DN17061_c4_g1::TRINITY_DN17061_c4_g1_i15::g.77796::m.77796	232850	76263	217250	192730	0	0	0	0	0	0	0	0	0	0	159820

TRINITY_DN17769_c1_g1::TRINITY_DN17769_c1_g1_i4::g.89348::m.89348	0	0	0	0	0	0	0	0	0	0	0	0	0	437790	437920
TRINITY_DN17821_c0_g1::TRINITY_DN17821_c0_g1_i3::g.91084::m.91084	0	0	0	0	0	0	0	0	0	0	0	0	396570	0	475520
TRINITY_DN19568_c0_g1::TRINITY_DN19568_c0_g1_i11::g.119880::m.119880	0	0	0	0	0	0	0	0	0	0	0	0	277920	407060	186460
TRINITY_DN16019_c1_g2::TRINITY_DN16019_c1_g2_i3::g.60717::m.60717	0	0	0	0	0	0	0	0	0	0	0	0	0	870620	0
TRINITY_DN13205_c0_g1::TRINITY_DN13205_c0_g1_i3::g.21053::m.21053	185670	90264	244250	200440	149030	0	0	0	0	0	0	0	0	0	0
TRINITY_DN13561_c3_g1::TRINITY_DN13561_c3_g1_i11::g.25972::m.25972	0	0	0	0	0	0	0	0	0	0	0	0	226050	288200	351760
TRINITY_DN16924_c1_g1::TRINITY_DN16924_c1_g1_i1::g.75666::m.75666	0	0	0	0	0	0	0	0	0	0	0	0	0	865770	0
TRINITY_DN10291_c0_g2::TRINITY_DN10291_c0_g2_i1::g.4575::m.4575	82669	0	119170	112040	0	0	175200	0	0	0	0	0	0	0	376510
TRINITY_DN14186_c0_g1::TRINITY_DN14186_c0_g1_i2::g.33325::m.33325	0	0	0	0	0	0	0	0	0	0	0	0	344110	248950	272520
TRINITY_DN17451_c1_g1::TRINITY_DN17451_c1_g1_i3::g.84062::m.84062	0	0	0	0	0	0	0	0	0	0	0	0	270180	351330	243030
TRINITY_DN16642_c3_g1::TRINITY_DN16642_c3_g1_i8::g.71010::m.71010	0	0	0	0	0	0	0	0	0	0	0	0	144450	277110	442920
TRINITY_DN18772_c0_g1::TRINITY_DN18772_c0_g1_i5::g.106264::m.106264	0	0	0	0	0	0	0	0	0	0	0	0	0	552760	310520
TRINITY_DN12837_c2_g1::TRINITY_DN12837_c2_g1_i5::g.16578::m.16578	388850	0	180930	289310	0	0	0	0	0	0	0	0	0	0	0
TRINITY_DN14047_c1_g1::TRINITY_DN14047_c1_g1_i4::g.31668::m.31668	0	0	0	0	0	0	0	0	0	0	0	0	188130	246440	421400
TRINITY_DN19086_c1_g1::TRINITY_DN19086_c1_g1_i6::g.111724::m.1117	0	0	0	0	0	0	0	0	0	0	0	0	198440	406080	249250
TRINITY_DN14258_c0_g5::TRINITY_DN14258_c0_g5_i3::g.34521::m.34521	0	0	0	0	0	0	0	0	0	0	0	0	136010	384710	331880
TRINITY_DN17031_c2_g2::TRINITY_DN17031_c2_g2_i1::g.77290::m.77290	0	0	0	0	0	0	0	0	0	0	0	0	107260	311960	432900
TRINITY_DN12955_c2_g1::TRINITY_DN12955_c2_g1_i5::g.17882::m.17882	0	0	0	0	0	0	0	0	0	0	0	0	149690	332380	369270
TRINITY_DN14036_c4_g1::TRINITY_DN14036_c4_g1_i2::g.31377::m.31377	0	0	0	0	0	0	0	0	0	0	0	0	313710	288210	247230
TRINITY_DN12399_c0_g1::TRINITY_DN12399_c0_g1_i1::g.12011::m.12011	0	0	46686	0	0	0	0	0	0	0	404000	398340	0	0	0
TRINITY_DN15460_c0_g1::TRINITY_DN15460_c0_g1_i9::g.52319::m.52319	0	0	0	0	0	0	0	0	0	0	0	0	105380	408200	335240
TRINITY_DN16066_c2_g3::TRINITY_DN16066_c2_g3_i1::g.61892::m.61892	0	0	0	0	0	0	0	0	0	0	0	0	312170	535660	0

TRINITY_DN15513_c2_g2::TRINITY_DN15513_c2_g2_i3::g.53087::m.53087	0	0	0	0	0	0	0	0	0	0	0	0	265170	0	581930
TRINITY_DN11403_c0_g1::TRINITY_DN11403_c0_g1_i1::g.7256::m.7256	0	0	0	0	0	0	0	0	0	0	0	0	205610	310100	330850
TRINITY_DN12993_c1_g5::TRINITY_DN12993_c1_g5_i1::g.18384::m.18384	0	0	0	0	0	0	0	0	0	0	0	0	410380	433350	0
TRINITY_DN14376_c2_g2::TRINITY_DN14376_c2_g2_i8::g.36166::m.36166	0	0	0	0	0	0	0	0	0	0	0	0	0	265720	576690
TRINITY_DN14670_c0_g1::TRINITY_DN14670_c0_g1_i1::g.40393::m.40393	140350	54292	166590	148680	0	0	0	0	0	0	0	0	0	329270	0
TRINITY_DN19844_c1_g2::TRINITY_DN19844_c1_g2_i1::g.124447::m.124447	0	0	142640	109300	0	0	0	0	0	0	0	0	189450	204310	192610
TRINITY_DN19441_c2_g1::TRINITY_DN19441_c2_g1_i3::g.118113::m.1181	0	0	0	0	0	0	0	0	0	0	0	0	0	837830	0
TRINITY_DN19418_c2_g1::TRINITY_DN19418_c2_g1_i2::g.117511::m.1175	0	0	0	0	0	0	0	0	0	0	0	0	203910	244900	388740
TRINITY_DN12196_c0_g1::TRINITY_DN12196_c0_g1_i3::g.11012::m.11012	0	0	0	0	0	0	0	0	0	0	0	0	42635	793820	0
TRINITY_DN18673_c1_g1::TRINITY_DN18673_c1_g1_i5::g.104369::m.104369	0	0	0	0	0	0	0	0	0	0	0	0	206960	354160	272900
TRINITY_DN19846_c6_g1::TRINITY_DN19846_c6_g1_i2::g.124495::m.124495	33701	0	32757	0	0	0	98002	0	0	0	113060	0	267770	159390	127000
TRINITY_DN1593_c0_g1::TRINITY_DN1593_c0_g1_i1::g.417::m.417	141150	77115	159040	43077	0	55485	0	100890	0	145760	0	0	0	105480	0
TRINITY_DN18479_c0_g3::TRINITY_DN18479_c0_g3_i1::g.100965::m.100965	0	0	0	0	0	0	0	0	0	0	0	0	371630	456230	0
TRINITY_DN12843_c0_g1::TRINITY_DN12843_c0_g1_i3::g.16447::m.16447	0	0	0	0	0	0	0	0	0	0	0	0	0	425820	401390
TRINITY_DN9796_c0_g1::TRINITY_DN9796_c0_g1_i1::g.3884::m.3884	0	0	0	0	0	0	0	0	70198	0	0	121790	195990	228680	207780
TRINITY_DN19235_c2_g1::TRINITY_DN19235_c2_g1_i2::g.114336::m.114336	0	0	0	0	0	0	0	0	0	0	314380	0	155200	193850	160780
TRINITY_DN19694_c1_g1::TRINITY_DN19694_c1_g1_i14::g.120695::m.120695	0	0	0	0	0	0	0	0	0	0	0	0	0	385050	438720
TRINITY_DN16533_c3_g1::TRINITY_DN16533_c3_g1_i1::g.69070::m.69070	0	0	0	0	0	0	0	0	0	0	0	0	164650	658740	0
TRINITY_DN11975_c0_g1::TRINITY_DN11975_c0_g1_i3::g.9738::m.9738	0	0	0	0	0	0	0	0	0	0	0	0	342110	415510	65509
TRINITY_DN12861_c0_g1::TRINITY_DN12861_c0_g1_i2::g.16621::m.16621	0	0	76076	0	0	0	0	0	0	0	219400	0	0	0	527220
TRINITY_DN14096_c0_g4::TRINITY_DN14096_c0_g4_i2::g.32317::m.32317	0	0	0	0	0	0	0	0	0	0	0	0	0	0	819530

TRINITY_DN16788_c0_g2::TRINITY_DN16788_c0_g2_i3::g.73187::m.73187	151620	26165	173820	164510	301320	0	0	0	0	0	0	0	0	0	0
TRINITY_DN17508_c4_g1::TRINITY_DN17508_c4_g1_i3::g.85406::m.85406	0	0	0	0	0	0	0	0	0	0	0	0	406000	0	409570
TRINITY_DN15658_c0_g1::TRINITY_DN15658_c0_g1_i3::g.55055::m.55055	0	0	0	0	0	0	0	0	0	0	0	0	118590	424240	269960
TRINITY_DN18925_c2_g2::TRINITY_DN18925_c2_g2_i2::g.109044::m.109044	0	0	0	0	0	0	0	0	0	0	0	0	249980	282890	279810
TRINITY_DN2281_c0_g1::TRINITY_DN2281_c0_g1_i1::g.635::m.635	0	0	0	0	0	0	0	0	0	0	0	0	419270	0	389940
TRINITY_DN14491_c1_g5::TRINITY_DN14491_c1_g5_i8::g.37914::m.37914	0	0	70386	122420	407470	0	0	0	0	0	208080	0	0	0	0
TRINITY_DN14005_c0_g1::TRINITY_DN14005_c0_g1_i3::g.31329::m.31329	0	0	0	0	0	0	0	0	0	0	0	0	227550	285250	294540
TRINITY_DN19319_c0_g3::TRINITY_DN19319_c0_g3_i9::g.115912::m.115912	196900	33940	180570	94479	0	0	0	0	301440	0	0	0	0	0	0
TRINITY_DN16713_c1_g3::TRINITY_DN16713_c1_g3_i3::g.72094::m.72094	0	0	0	0	0	0	0	0	0	0	0	0	229730	576240	0
TRINITY_DN14259_c3_g1::TRINITY_DN14259_c3_g1_i2::g.34652::m.34652	0	0	0	0	0	0	0	112470	223510	225550	244290	0	0	0	0
TRINITY_DN11562_c0_g1::TRINITY_DN11562_c0_g1_i2::g.7869::m.7869	0	54210	181550	0	0	0	0	0	0	0	0	569240	0	0	0
TRINITY_DN34461_c0_g1::TRINITY_DN34461_c0_g1_i1::g.130651::m.130651	204310	74072	274770	251660	0	0	0	0	0	0	0	0	0	0	0
TRINITY_DN11895_c0_g1::TRINITY_DN11895_c0_g1_i1::g.9352::m.9352	106410	0	123620	109300	0	0	0	0	0	0	0	0	0	0	463060
TRINITY_DN15183_c1_g3::TRINITY_DN15183_c1_g3_i4::g.48285::m.48285	124770	0	164080	91619	0	0	0	0	0	0	0	0	179950	0	241130
TRINITY_DN14482_c0_g1::TRINITY_DN14482_c0_g1_i1::g.37716::m.37716	0	0	0	0	0	0	0	0	0	0	0	0	227160	274320	295970
TRINITY_DN16600_c2_g1::TRINITY_DN16600_c2_g1_i15::g.68704::m.68704	0	0	0	0	0	0	0	0	0	0	0	0	407600	0	389810
TRINITY_DN16958_c1_g1::TRINITY_DN16958_c1_g1_i8::g.76184::m.76184	0	0	0	0	0	0	0	0	0	0	0	0	229360	326090	241020
TRINITY_DN13523_c0_g1::TRINITY_DN13523_c0_g1_i7::g.24832::m.24832	0	0	0	0	0	0	0	0	0	0	0	0	210320	298480	283130
TRINITY_DN18718_c3_g5::TRINITY_DN18718_c3_g5_i3::g.105071::m.105071	0	0	0	0	0	0	0	0	0	0	0	0	329800	150280	311410
TRINITY_DN17281_c2_g3::TRINITY_DN17281_c2_g3_i2::g.81288::m.81288	150080	0	244310	0	0	0	0	0	0	0	0	0	85758	0	311050

TRINITY_DN18452_c0_g3::TRINITY_DN18452_c0_g3_i8::g.100622::m.100622	0	56215	108950	130260	0	0	0	0	0	0	0	0	0	493980	0
TRINITY_DN14408_c0_g2::TRINITY_DN14408_c0_g2_i5::g.36629::m.36629	0	0	0	0	0	0	0	0	0	0	0	0	184870	222290	380410
TRINITY_DN19395_c1_g2::TRINITY_DN19395_c1_g2_i1::g.117249::m.117249	0	0	0	0	0	0	0	0	214230	170600	272660	0	0	0	128180
TRINITY_DN16328_c4_g2::TRINITY_DN16328_c4_g2_i8::g.65867::m.65867	0	0	0	0	0	0	0	0	0	0	0	0	454900	0	327280
TRINITY_DN16222_c1_g2::TRINITY_DN16222_c1_g2_i6::g.64132::m.64132	0	0	0	0	0	0	0	0	0	0	0	354180	137660	171880	114400
TRINITY_DN16296_c2_g1::TRINITY_DN16296_c2_g1_i8::g.65364::m.65364	0	0	0	0	0	0	0	0	0	0	0	0	381080	268720	127150
TRINITY_DN15139_c1_g2::TRINITY_DN15139_c1_g2_i1::g.47376::m.47376	0	0	41319	0	0	0	0	0	0	0	0	0	0	731420	0
TRINITY_DN17004_c0_g6::TRINITY_DN17004_c0_g6_i1::g.76895::m.76895	0	0	0	0	0	0	0	0	0	0	0	0	446850	325510	0
TRINITY_DN12855_c4_g1::TRINITY_DN12855_c4_g1_i7::g.16693::m.16693	0	0	0	0	0	0	0	0	0	0	0	0	290320	0	480650
TRINITY_DN11323_c0_g1::TRINITY_DN11323_c0_g1_i5::g.7006::m.7006	0	0	0	0	0	0	0	0	0	0	0	0	440520	161430	167750
TRINITY_DN18226_c4_g1::TRINITY_DN18226_c4_g1_i9::g.97134::m.97134	0	0	0	0	0	0	0	0	0	0	0	0	286140	190290	293050
TRINITY_DN18320_c3_g1::TRINITY_DN18320_c3_g1_i1::g.98804::m.98804	0	0	59708	0	0	0	0	0	416180	0	0	0	136720	156410	0
TRINITY_DN12400_c3_g4::TRINITY_DN12400_c3_g4_i4::g.12578::m.12578	0	0	0	0	0	0	0	0	0	0	0	0	250970	262150	255720
TRINITY_DN15264_c3_g2::TRINITY_DN15264_c3_g2_i3::g.49355::m.49355	0	245310	0	0	0	0	0	0	0	0	0	0	205410	317480	0
TRINITY_DN18252_c2_g3::TRINITY_DN18252_c2_g3_i2::g.97577::m.97577	0	0	0	0	0	0	0	0	0	0	0	0	321130	0	446190
TRINITY_DN14880_c3_g1::TRINITY_DN14880_c3_g1_i7::g.43762::m.43762	0	0	0	0	0	0	0	0	0	0	0	0	355000	211790	199550
TRINITY_DN14491_c1_g6::TRINITY_DN14491_c1_g6_i1::g.37898::m.37898	0	0	0	0	0	0	0	0	0	162080	0	357390	243600	0	0
TRINITY_DN12086_c0_g1::TRINITY_DN12086_c0_g1_i1::g.10415::m.10415	0	0	0	0	0	0	0	0	0	0	0	0	0	337440	424900
TRINITY_DN17573_c2_g1::TRINITY_DN17573_c2_g1_i14::g.86248::m.86248	0	0	0	0	0	0	0	0	0	0	0	0	544600	214550	0

TRINITY_DN15292_c0_g3::TRINITY_DN15292_c0_g3_i4::g.49797::m.49797	0	0	0	0	0	0	0	0	0	0	0	0	406370	0	352290
TRINITY_DN13144_c0_g1::TRINITY_DN13144_c0_g1_i6::g.19931::m.19931	205870	34196	151420	233450	0	0	0	0	132850	0	0	0	0	0	0
TRINITY_DN19562_c2_g5::TRINITY_DN19562_c2_g5_i1::g.119826::m.119826	0	0	0	0	0	0	0	0	0	0	0	0	0	393420	364250
TRINITY_DN13091_c0_g1::TRINITY_DN13091_c0_g1_i8::g.19651::m.19651	0	0	0	0	295670	115950	0	0	345040	0	0	0	0	0	0
TRINITY_DN14504_c3_g1::TRINITY_DN14504_c3_g1_i2::g.38234::m.38234	0	0	0	0	0	0	0	0	0	0	0	0	0	570830	185740
TRINITY_DN15895_c3_g1::TRINITY_DN15895_c3_g1_i1::g.57722::m.57722	84253	54374	115800	0	0	70574	0	0	0	0	0	0	245150	186180	0
TRINITY_DN18792_c0_g2::TRINITY_DN18792_c0_g2_i6::g.106589::m.106589	0	0	0	0	0	0	0	0	0	0	0	0	98862	308460	347310
TRINITY_DN14028_c2_g1::TRINITY_DN14028_c2_g1_i4::g.31476::m.31476	0	0	0	0	0	0	0	0	0	0	0	0	96356	304560	346180
TRINITY_DN18788_c0_g2::TRINITY_DN18788_c0_g2_i3::g.106445::m.106445	0	0	0	0	0	0	0	0	0	0	0	0	0	583590	161500
TRINITY_DN16241_c0_g1::TRINITY_DN16241_c0_g1_i2::g.63739::m.63739	0	0	0	0	0	0	0	0	0	0	0	0	246720	249580	248530
TRINITY_DN19798_c2_g1::TRINITY_DN19798_c2_g1_i2::g.123755::m.123755	0	0	0	0	0	0	0	0	0	0	0	0	210170	254040	280290
TRINITY_DN13642_c0_g1::TRINITY_DN13642_c0_g1_i1::g.26569::m.26569	0	0	0	0	0	0	0	0	0	0	0	0	551250	0	193100
TRINITY_DN15510_c3_g3::TRINITY_DN15510_c3_g3_i2::g.53102::m.53102	0	0	0	0	0	0	0	0	0	0	0	0	158660	426750	158650
TRINITY_DN14516_c0_g1::TRINITY_DN14516_c0_g1_i7::g.38023::m.38023	89321	0	65344	77030	0	0	0	0	0	0	0	511810	0	0	0
TRINITY_DN44543_c0_g1::TRINITY_DN44543_c0_g1_i1::g.132115::m.132115	0	0	0	0	0	0	0	0	0	0	0	0	0	0	742250
TRINITY_DN19344_c5_g1::TRINITY_DN19344_c5_g1_i8::g.115623::m.115623	0	0	0	0	0	0	0	0	0	0	0	0	0	378940	363180
TRINITY_DN14685_c0_g1::TRINITY_DN14685_c0_g1_i6::g.40758::m.40758	0	0	0	0	0	0	0	0	0	0	0	0	222030	318640	196150
TRINITY_DN12342_c1_g2::TRINITY_DN12342_c1_g2_i2::g.12106::m.12106	536790	0	0	199390	0	0	0	0	0	0	0	0	0	0	0
TRINITY_DN14553_c3_g1::TRINITY_DN14553_c3_g1_i5::g.38708::m.38708	233320	0	0	0	0	0	0	0	0	0	0	0	0	440400	61782
TRINITY_DN16423_c1_g1::TRINITY_DN16423_c1_g1_i12::g.67294::m.67294	0	0	0	0	99309	21392	85606	115590	0	0	0	0	103030	0	310500

TRINITY_DN5236_c0_g1::TRINITY_DN5236_c0_g1_i1::g.1296::m.1296	0	0	0	0	0	0	0	0	0	0	0	0	304550	430780	0
TRINITY_DN14069_c0_g3::TRINITY_DN14069_c0_g3_i3::g.32072::m.32072	0	0	0	0	0	0	0	0	0	0	0	0	219070	259500	255630
TRINITY_DN18518_c1_g1::TRINITY_DN18518_c1_g1_i12::g.103139::m.103139	0	0	0	0	0	0	0	0	0	0	0	0	260370	321210	149910
TRINITY_DN18275_c0_g4::TRINITY_DN18275_c0_g4_i1::g.98310::m.98310	0	0	0	0	0	0	0	0	0	0	0	0	0	0	730050
TRINITY_DN20016_c1_g1::TRINITY_DN20016_c1_g1_i7::g.127847::m.127847	83945	0	182860	155390	0	0	0	63061	0	0	243660	0	0	0	0
TRINITY_DN14420_c1_g1::TRINITY_DN14420_c1_g1_i4::g.36850::m.36850	0	0	0	0	0	0	0	0	0	0	0	0	146660	390510	191220
TRINITY_DN18968_c1_g1::TRINITY_DN18968_c1_g1_i9::g.109574::m.109574	0	0	0	0	0	0	0	0	0	0	0	0	275690	122210	330350
TRINITY_DN14219_c0_g1::TRINITY_DN14219_c0_g1_i18::g.33900::m.33900	0	0	0	0	0	0	0	0	0	0	0	0	111810	413660	201950
TRINITY_DN16257_c0_g1::TRINITY_DN16257_c0_g1_i2::g.64601::m.64601	0	0	0	0	0	0	0	0	0	0	0	0	97123	325220	303860
TRINITY_DN19366_c1_g3::TRINITY_DN19366_c1_g3_i1::g.116912::m.116912	0	0	0	0	0	0	0	0	0	0	0	725250	0	0	0
TRINITY_DN18230_c1_g3::TRINITY_DN18230_c1_g3_i4::g.97286::m.97286	0	0	0	0	0	0	0	0	0	0	0	0	0	370390	352480
TRINITY_DN16429_c0_g2::TRINITY_DN16429_c0_g2_i3::g.67310::m.67310	0	0	0	0	0	0	0	0	0	0	0	0	300740	332540	88907
TRINITY_DN18185_c0_g2::TRINITY_DN18185_c0_g2_i8::g.96676::m.96676	0	0	0	0	0	0	0	0	0	0	0	0	104690	0	616600
TRINITY_DN16928_c0_g2::TRINITY_DN16928_c0_g2_i1::g.75662::m.75662	0	0	0	0	0	0	0	0	0	0	0	0	187140	296060	235310
TRINITY_DN12765_c0_g2::TRINITY_DN12765_c0_g2_i9::g.15778::m.15778	104680	0	0	90835	0	0	0	383860	0	139110	0	0	0	0	0
TRINITY_DN16240_c0_g1::TRINITY_DN16240_c0_g1_i3::g.64405::m.64405	0	0	0	0	0	0	0	0	0	0	0	0	0	717220	0
TRINITY_DN15464_c0_g4::TRINITY_DN15464_c0_g4_i1::g.52272::m.52272	0	0	0	0	0	0	0	0	0	0	0	0	242100	253060	221670
TRINITY_DN17952_c0_g1::TRINITY_DN17952_c0_g1_i8::g.92586::m.92586	0	0	0	0	0	0	0	0	0	0	0	0	353580	0	361610
TRINITY_DN16980_c0_g3::TRINITY_DN16980_c0_g3_i2::g.76506::m.76506	0	0	0	0	0	0	0	0	0	0	0	0	197500	187700	328100

TRINITY_DN14376_c2_g1::TRINITY_DN14376_c2_g1_i4::g.36173::m.36173	0	0	0	0	0	0	0	0	0	0	0	0	163210	191950	356410
TRINITY_DN11353_c0_g1::TRINITY_DN11353_c0_g1_i1::g.7089::m.7089	0	0	0	0	0	0	0	0	0	0	0	0	643300	0	67065
TRINITY_DN17937_c1_g5::TRINITY_DN17937_c1_g5_i2::g.92219::m.92219	69954	0	104760	61983	0	0	0	0	0	0	0	0	0	472620	0
TRINITY_DN15328_c3_g2::TRINITY_DN15328_c3_g2_i4::g.50205::m.50205	0	0	0	0	0	0	0	0	0	0	0	0	0	132890	574190
TRINITY_DN19548_c2_g6::TRINITY_DN19548_c2_g6_i1::g.119771::m.119771	0	0	0	0	0	0	0	0	0	0	0	0	344820	360460	0
TRINITY_DN18997_c1_g1::TRINITY_DN18997_c1_g1_i3::g.110273::m.110273	0	0	0	0	0	0	0	0	0	0	0	0	186860	175700	342520
TRINITY_DN17307_c3_g1::TRINITY_DN17307_c3_g1_i2::g.81691::m.81691	0	0	0	0	0	0	0	152050	0	0	344170	0	208610	0	0
TRINITY_DN19257_c3_g1::TRINITY_DN19257_c3_g1_i1::g.114723::m.114723	0	0	0	0	0	0	0	0	0	0	0	0	358950	345880	0
TRINITY_DN19453_c0_g2::TRINITY_DN19453_c0_g2_i2::g.118209::m.118209	0	0	0	0	0	0	0	58119	0	0	296140	349340	0	0	0
TRINITY_DN11079_c0_g1::TRINITY_DN11079_c0_g1_i1::g.6259::m.6259	0	0	0	0	0	0	0	0	0	0	0	0	0	0	703070
TRINITY_DN18472_c4_g2::TRINITY_DN18472_c4_g2_i2::g.100983::m.100983	90127	0	124740	81903	101200	0	0	0	0	0	0	0	0	149160	155900
TRINITY_DN9342_c0_g1::TRINITY_DN9342_c0_g1_i1::g.3304::m.3304	0	0	0	0	0	0	0	0	0	0	0	0	0	351560	351330
TRINITY_DN17375_c4_g1::TRINITY_DN17375_c4_g1_i2::g.82845::m.82845	0	0	0	0	0	0	0	0	0	0	0	0	315290	182120	203730
TRINITY_DN16195_c0_g1::TRINITY_DN16195_c0_g1_i3::g.63589::m.63589	0	0	0	0	171550	0	0	0	0	0	523350	0	0	0	0
TRINITY_DN15131_c1_g3::TRINITY_DN15131_c1_g3_i1::g.47487::m.47487	0	0	0	0	0	0	0	0	0	0	0	0	351300	166840	176420
TRINITY_DN17744_c7_g1::TRINITY_DN17744_c7_g1_i4::g.89103::m.89103	0	0	0	0	0	0	0	0	0	0	0	0	0	450070	243480
TRINITY_DN18492_c0_g1::TRINITY_DN18492_c0_g1_i14::g.101403::m.101403	0	0	47498	0	141940	0	0	0	0	114070	388690	0	0	0	0
TRINITY_DN20003_c1_g1::TRINITY_DN20003_c1_g1_i6::g.127620::m.127620	0	0	0	0	0	0	0	0	0	0	0	0	0	433860	255630
TRINITY_DN14376_c2_g4::TRINITY_DN14376_c2_g4_i7::g.36164::m.36164	0	0	0	0	0	0	0	0	0	0	0	0	0	548330	139870
TRINITY_DN17923_c2_g2::TRINITY_DN17923_c2_g2_i2::g.91995::m.91995	0	0	0	0	0	0	0	0	0	0	0	0	310220	376890	0

TRINITY_DN12731_c3_g4::TRINITY_DN12731_c3_g4_i1::g.15536::m.15536	0	0	0	0	0	0	0	0	0	0	0	0	0	686970	0
TRINITY_DN11014_c0_g1::TRINITY_DN11014_c0_g1_i2::g.6114::m.6114	0	0	0	0	118570	0	0	0	402590	164640	0	0	0	0	0
TRINITY_DN17625_c1_g2::TRINITY_DN17625_c1_g2_i5::g.87298::m.87298	0	0	0	0	0	0	0	0	0	0	0	0	211940	235580	238130
TRINITY_DN15733_c1_g1::TRINITY_DN15733_c1_g1_i2::g.56414::m.56414	0	0	0	0	0	0	0	0	0	0	0	0	180540	271460	232340
TRINITY_DN18395_c1_g2::TRINITY_DN18395_c1_g2_i6::g.99075::m.99075	105400	0	65135	512330	0	0	0	0	0	0	0	0	0	0	0
TRINITY_DN15876_c0_g2::TRINITY_DN15876_c0_g2_i4::g.58231::m.58231	38652	0	32042	24887	407600	179610	0	0	0	0	0	0	0	0	0
TRINITY_DN17846_c4_g3::TRINITY_DN17846_c4_g3_i1::g.90654::m.90654	0	0	0	0	0	0	0	0	0	0	0	0	195260	116430	370420
TRINITY_DN19531_c0_g1::TRINITY_DN19531_c0_g1_i3::g.119625::m.119625	0	0	470190	0	0	0	209510	0	0	0	0	0	0	0	0
TRINITY_DN19896_c1_g1::TRINITY_DN19896_c1_g1_i11::g.125427::m.125427	0	0	0	0	0	0	0	0	0	0	0	0	299900	169450	209500
TRINITY_DN13610_c2_g1::TRINITY_DN13610_c2_g1_i1::g.26215::m.26215	0	0	0	0	0	0	0	0	0	0	0	0	216480	221790	240490
TRINITY_DN17704_c0_g1::TRINITY_DN17704_c0_g1_i3::g.88593::m.88593	0	0	0	0	0	0	0	0	0	0	0	0	326950	350870	0
TRINITY_DN13149_c0_g1::TRINITY_DN13149_c0_g1_i7::g.20402::m.20402	0	0	0	0	0	0	0	0	0	0	0	0	377770	149110	150870
TRINITY_DN16337_c1_g1::TRINITY_DN16337_c1_g1_i12::g.66068::m.66068	139960	56114	227690	170950	0	0	0	0	0	0	0	0	0	0	82501
TRINITY_DN18805_c4_g1::TRINITY_DN18805_c4_g1_i2::g.106689::m.106689	0	0	0	0	0	0	0	433360	0	0	0	0	0	0	243830
TRINITY_DN19153_c1_g3::TRINITY_DN19153_c1_g3_i2::g.112871::m.1128	0	0	0	0	0	0	0	0	0	0	0	0	312600	0	364550
TRINITY_DN12257_c1_g1::TRINITY_DN12257_c1_g1_i2::g.11446::m.11446	0	0	0	0	0	0	0	0	0	0	0	0	677080	0	0
TRINITY_DN14137_c0_g1::TRINITY_DN14137_c0_g1_i2::g.33039::m.33039	0	0	0	0	0	0	0	0	0	0	0	0	438730	116430	121450
TRINITY_DN14239_c2_g2::TRINITY_DN14239_c2_g2_i2::g.34369::m.34369	0	0	0	0	0	0	0	0	0	0	0	0	0	448280	228270
TRINITY_DN12628_c0_g1::TRINITY_DN12628_c0_g1_i15::g.14380::m.14380	0	0	52521	0	0	0	239560	147250	0	0	236630	0	0	0	0
TRINITY_DN16315_c0_g1::TRINITY_DN16315_c0_g1_i3::g.65602::m.65602	0	0	0	0	0	0	0	0	0	0	0	0	0	674200	0
TRINITY_DN11202_c0_g1::TRINITY_DN11202_c0_g1_i1::g.6604::m.6604	0	0	0	0	0	0	0	0	0	0	0	0	505230	0	168890

TRINITY_DN12134_c9_g1::TRINITY_DN12134_c9_g1_i4::g.10752::m.10752	0	0	0	0	0	0	0	0	0	0	0	0	282880	389330	0
TRINITY_DN16939_c0_g1::TRINITY_DN16939_c0_g1_i3::g.75902::m.75902	94262	47404	214770	86702	0	0	228460	0	0	0	0	0	0	0	0
TRINITY_DN17688_c2_g3::TRINITY_DN17688_c2_g3_i4::g.88274::m.88274	204510	62325	198490	0	0	0	0	205570	0	0	0	0	0	0	0
TRINITY_DN15001_c1_g2::TRINITY_DN15001_c1_g2_i1::g.45482::m.45482	211090	0	153290	137700	0	0	0	0	0	0	0	0	167200	0	0
TRINITY_DN15894_c0_g1::TRINITY_DN15894_c0_g1_i1::g.58474::m.58474	0	0	0	0	0	0	0	0	0	0	0	0	200210	237860	226960
TRINITY_DN16337_c1_g3::TRINITY_DN16337_c1_g3_i2::g.66071::m.66071	0	0	0	0	0	0	0	0	0	0	0	0	79708	272210	311840
TRINITY_DN13153_c0_g3::TRINITY_DN13153_c0_g3_i1::g.20486::m.20486	0	0	0	0	0	0	168280	132650	0	0	360040	0	0	0	0
TRINITY_DN19578_c1_g1::TRINITY_DN19578_c1_g1_i9::g.120079::m.120079	0	0	0	0	0	0	0	0	0	0	0	0	236610	241100	183140
TRINITY_DN19156_c1_g1::TRINITY_DN19156_c1_g1_i7::g.113150::m.1131	0	0	0	0	166700	0	0	134760	0	0	0	0	0	210950	148090
TRINITY_DN18639_c0_g2::TRINITY_DN18639_c0_g2_i1::g.103472::m.103472	0	0	0	0	0	0	0	0	0	0	0	0	271260	205970	183010
TRINITY_DN17531_c3_g1::TRINITY_DN17531_c3_g1_i2::g.85473::m.85473	0	0	0	0	158370	0	177470	191130	0	0	0	0	0	132650	0
TRINITY_DN9714_c0_g1::TRINITY_DN9714_c0_g1_i2::g.3800::m.3800	0	0	0	0	0	0	0	0	0	0	0	0	527130	132020	0
TRINITY_DN17192_c0_g2::TRINITY_DN17192_c0_g2_i8::g.79905::m.79905	62771	55925	155700	139520	0	0	0	0	0	0	0	0	0	0	243520
TRINITY_DN17561_c0_g1::TRINITY_DN17561_c0_g1_i2::g.86132::m.86132	0	0	0	0	0	0	0	0	0	0	0	0	324060	191350	141150
TRINITY_DN17433_c1_g2::TRINITY_DN17433_c1_g2_i1::g.83589::m.83589	0	0	0	0	0	0	164910	162040	0	0	0	0	327950	0	0
TRINITY_DN14320_c0_g6::TRINITY_DN14320_c0_g6_i4::g.35483::m.35483	0	0	0	0	0	0	175030	0	0	0	0	0	158170	179530	141070
TRINITY_DN13688_c0_g1::TRINITY_DN13688_c0_g1_i3::g.27185::m.27185	33551	0	0	0	0	37376	0	0	0	0	0	580780	0	0	0
TRINITY_DN16402_c0_g3::TRINITY_DN16402_c0_g3_i1::g.67045::m.67045	0	0	0	0	0	0	0	0	0	0	0	0	110680	259490	279080
TRINITY_DN19228_c2_g1::TRINITY_DN19228_c2_g1_i5::g.114244::m.114244	0	0	0	0	0	0	293050	0	0	0	0	0	0	180380	173200
TRINITY_DN16661_c1_g2::TRINITY_DN16661_c1_g2_i5::g.71230::m.71230	0	0	0	0	0	0	0	0	0	0	0	0	228710	0	416470
TRINITY_DN17919_c1_g1::TRINITY_DN17919_c1_g1_i2::g.91916::m.91916	0	0	0	0	0	0	0	0	0	0	0	0	95964	423890	123970

TRINITY_DN19280_c0_g1::TRINITY_DN19280_c0_g1_i5::g.115042::m.115042	0	0	0	0	0	0	0	0	0	0	0	0	146190	311350	184190
TRINITY_DN16384_c1_g4::TRINITY_DN16384_c1_g4_i4::g.66906::m.66906	0	0	0	0	0	0	0	0	0	0	0	0	178260	462930	0
TRINITY_DN11479_c0_g1::TRINITY_DN11479_c0_g1_i6::g.7503::m.7503	0	0	0	0	0	0	0	0	0	0	0	0	276700	0	362280
TRINITY_DN16861_c5_g5::TRINITY_DN16861_c5_g5_i1::g.74534::m.74534	0	0	0	0	0	0	0	0	0	0	0	0	0	638600	0
TRINITY_DN20036_c5_g1::TRINITY_DN20036_c5_g1_i2::g.127587::m.127587	0	0	0	0	0	0	0	0	0	0	0	0	204870	239600	193660
TRINITY_DN16479_c1_g2::TRINITY_DN16479_c1_g2_i4::g.68347::m.68347	216600	0	94385	158860	0	0	0	91751	0	0	0	0	0	0	75457
TRINITY_DN17999_c1_g1::TRINITY_DN17999_c1_g1_i8::g.91779::m.91779	0	0	0	0	0	0	0	0	0	0	0	0	233050	403660	0
TRINITY_DN17673_c0_g1::TRINITY_DN17673_c0_g1_i5::g.88024::m.88024	0	0	0	0	0	0	0	0	0	0	0	0	225960	190470	217180
TRINITY_DN10333_c0_g1::TRINITY_DN10333_c0_g1_i1::g.4649::m.4649	0	0	0	0	0	0	0	0	0	0	0	0	202780	312620	117710
TRINITY_DN13300_c0_g1::TRINITY_DN13300_c0_g1_i5::g.22083::m.22083	0	0	0	0	0	0	0	0	0	0	0	0	146120	158020	326300
TRINITY_DN18283_c0_g1::TRINITY_DN18283_c0_g1_i12::g.98055::m.98055	0	0	0	0	0	0	0	0	0	0	0	0	174830	190750	262460
TRINITY_DN10355_c0_g1::TRINITY_DN10355_c0_g1_i1::g.4689::m.4689	0	0	0	0	0	0	0	0	0	0	0	0	276980	350910	0
TRINITY_DN15356_c1_g1::TRINITY_DN15356_c1_g1_i4::g.50914::m.50914	0	0	0	0	0	0	0	0	0	0	0	0	0	0	627770
TRINITY_DN18321_c2_g1::TRINITY_DN18321_c2_g1_i1::g.98839::m.98839	144050	0	128300	105920	118420	0	0	129890	0	0	0	0	0	0	0
TRINITY_DN13289_c1_g2::TRINITY_DN13289_c1_g2_i4::g.22093::m.22093	0	0	0	0	0	0	0	0	0	0	0	0	193060	244970	188520
TRINITY_DN13288_c0_g1::TRINITY_DN13288_c0_g1_i6::g.22068::m.22068	0	0	59492	0	0	69687	58935	50441	266380	0	0	0	0	0	117350
TRINITY_DN17097_c0_g1::TRINITY_DN17097_c0_g1_i1::g.78356::m.78356	0	0	0	0	0	0	0	0	0	0	0	0	178990	0	442250
TRINITY_DN14079_c0_g2::TRINITY_DN14079_c0_g2_i4::g.32111::m.32111	0	0	0	0	0	0	0	0	0	0	0	0	0	621160	0
TRINITY_DN14601_c2_g1::TRINITY_DN14601_c2_g1_i8::g.39287::m.39287	0	0	0	0	0	0	0	0	0	0	0	0	0	385180	232180
TRINITY_DN17332_c3_g1::TRINITY_DN17332_c3_g1_i4::g.82072::m.82072	0	0	0	0	0	0	0	0	0	0	0	0	122210	201720	291500
TRINITY_DN18983_c0_g1::TRINITY_DN18983_c0_g1_i9::g.110074::m.110074	0	0	0	0	0	0	0	0	0	0	447390	0	0	0	166000

TRINITY_DN17234_c0_g1::TRINITY_DN17234_c0_g1_i11::g.80741::m.80741	0	0	0	0	0	0	0	0	0	0	0	0	286480	159950	165750
TRINITY_DN16222_c0_g1::TRINITY_DN16222_c0_g1_i6::g.64118::m.64118	0	0	0	0	0	0	0	0	0	0	0	0	157160	0	454170
TRINITY_DN15263_c1_g2::TRINITY_DN15263_c1_g2_i4::g.49263::m.49263	0	0	0	25469	0	0	117580	104450	0	0	0	0	181300	0	181880
TRINITY_DN13753_c0_g1::TRINITY_DN13753_c0_g1_i3::g.27858::m.27858	111210	30979	99984	0	0	0	0	166670	0	0	0	0	0	0	201760
TRINITY_DN17360_c0_g1::TRINITY_DN17360_c0_g1_i6::g.82556::m.82556	0	0	0	0	0	0	0	0	0	0	0	0	278800	245940	85324
TRINITY_DN11448_c0_g1::TRINITY_DN11448_c0_g1_i1::g.7372::m.7372	0	0	0	0	0	0	0	0	0	0	0	0	0	0	609550
TRINITY_DN13217_c3_g3::TRINITY_DN13217_c3_g3_i1::g.21250::m.21250	0	0	0	0	0	0	0	0	0	0	0	0	177190	215450	216840
TRINITY_DN13260_c0_g1::TRINITY_DN13260_c0_g1_i2::g.21630::m.21630	0	0	0	0	0	0	0	0	0	0	0	0	182850	424840	0
TRINITY_DN19222_c3_g1::TRINITY_DN19222_c3_g1_i5::g.114236::m.114236	28630	0	25788	136480	126710	0	0	95091	0	0	0	0	0	193850	0
TRINITY_DN10774_c0_g1::TRINITY_DN10774_c0_g1_i7::g.5503::m.5503	0	0	0	0	0	0	0	0	0	0	0	0	306000	0	298120
TRINITY_DN16241_c1_g4::TRINITY_DN16241_c1_g4_i5::g.63753::m.63753	0	0	0	0	0	0	0	0	0	0	0	0	357620	242810	0
TRINITY_DN19395_c1_g4::TRINITY_DN19395_c1_g4_i2::g.117259::m.117259	0	0	0	0	0	0	210380	174440	215590	0	0	0	0	0	0
TRINITY_DN22718_c0_g1::TRINITY_DN22718_c0_g1_i1::g.128985::m.128985	0	0	0	0	0	0	0	0	0	0	305940	294270	0	0	0
TRINITY_DN13284_c0_g1::TRINITY_DN13284_c0_g1_i3::g.21957::m.21957	56277	39197	61306	55399	0	0	0	0	0	0	0	154680	232350	0	0
TRINITY_DN19032_c2_g1::TRINITY_DN19032_c2_g1_i2::g.110806::m.110806	0	0	0	0	0	0	0	0	0	63085	0	0	0	535500	0
TRINITY_DN16806_c1_g1::TRINITY_DN16806_c1_g1_i6::g.73621::m.73621	0	0	0	0	0	0	0	0	0	0	0	0	0	111690	486210
TRINITY_DN14774_c0_g5::TRINITY_DN14774_c0_g5_i3::g.42116::m.42116	0	0	0	0	0	0	0	0	0	0	0	0	168850	214490	212600
TRINITY_DN11806_c0_g1::TRINITY_DN11806_c0_g1_i1::g.8989::m.8989	0	0	0	0	0	0	0	0	0	0	0	0	132760	310220	152130
TRINITY_DN15677_c1_g6::TRINITY_DN15677_c1_g6_i3::g.55549::m.55549	0	0	0	0	0	0	0	0	0	0	0	0	262110	0	331650
TRINITY_DN13179_c1_g1::TRINITY_DN13179_c1_g1_i6::g.20799::m.20799	0	16830	58834	0	316510	72817	128430	0	0	0	0	0	0	0	0

TRINITY_DN14413_c0_g2::TRINITY_DN14413_c0_g2_i2::g.36805::m.36805	0	0	0	0	0	0	0	0	0	0	0	0	114360	335260	142850
TRINITY_DN19247_c0_g1::TRINITY_DN19247_c0_g1_i8::g.114528::m.114528	0	0	0	0	0	174810	414920	0	0	0	0	0	0	0	0
TRINITY_DN17718_c6_g2::TRINITY_DN17718_c6_g2_i4::g.88774::m.88774	0	0	0	0	0	0	0	0	0	0	0	0	289100	174100	125910
TRINITY_DN18436_c0_g2::TRINITY_DN18436_c0_g2_i2::g.100108::m.100108	0	0	0	0	0	0	0	0	0	0	0	0	0	588260	0
TRINITY_DN19343_c7_g2::TRINITY_DN19343_c7_g2_i2::g.116418::m.116418	0	0	0	0	0	0	0	0	231070	0	355750	0	0	0	0
TRINITY_DN18242_c3_g1::TRINITY_DN18242_c3_g1_i7::g.97423::m.97423	0	0	0	0	0	0	0	0	0	0	0	0	263390	322690	0
TRINITY_DN14572_c4_g1::TRINITY_DN14572_c4_g1_i1::g.39016::m.39016	0	0	0	0	0	0	0	0	0	0	0	0	332980	150980	99589
TRINITY_DN13884_c2_g1::TRINITY_DN13884_c2_g1_i6::g.29871::m.29871	0	0	0	0	0	0	0	0	0	0	0	0	117990	95258	368270
TRINITY_DN17016_c4_g1::TRINITY_DN17016_c4_g1_i2::g.77096::m.77096	0	0	0	0	0	0	0	0	0	0	0	0	581220	0	0
TRINITY_DN14069_c0_g5::TRINITY_DN14069_c0_g5_i5::g.32070::m.32070	0	0	0	0	0	0	0	0	0	0	0	0	0	260930	319700
TRINITY_DN17522_c3_g1::TRINITY_DN17522_c3_g1_i3::g.85131::m.85131	0	0	0	0	0	0	0	0	0	0	0	0	262120	317680	0
TRINITY_DN18040_c0_g1::TRINITY_DN18040_c0_g1_i2::g.94127::m.94127	0	0	0	0	0	0	0	0	0	0	0	0	349170	0	229920
TRINITY_DN15831_c4_g2::TRINITY_DN15831_c4_g2_i2::g.57659::m.57659	0	0	0	0	0	0	0	0	0	0	0	0	148380	233310	195790
TRINITY_DN17167_c4_g1::TRINITY_DN17167_c4_g1_i2::g.79586::m.79586	0	0	0	0	0	0	0	0	0	0	0	0	72461	255280	249730
TRINITY_DN14538_c2_g2::TRINITY_DN14538_c2_g2_i9::g.38508::m.38508	0	0	0	0	0	0	0	0	0	0	0	0	255170	157440	159450
TRINITY_DN16535_c0_g2::TRINITY_DN16535_c0_g2_i8::g.69172::m.69172	0	0	0	0	0	0	0	0	0	0	0	0	199230	189550	183130
TRINITY_DN14304_c1_g1::TRINITY_DN14304_c1_g1_i6::g.35219::m.35219	0	0	0	0	0	0	0	0	0	0	0	0	242300	328440	0
TRINITY_DN19010_c0_g2::TRINITY_DN19010_c0_g2_i1::g.110437::m.110437	103060	45273	84574	72586	0	0	0	0	0	0	261830	0	0	0	0
TRINITY_DN16594_c0_g2::TRINITY_DN16594_c0_g2_i1::g.69960::m.69960	0	0	0	0	0	0	0	0	0	0	0	0	0	376090	190540

TRINITY_DN19775_c1_g1::TRINITY_DN19775_c1_g1_i3::g.123478::m.123478	0	0	0	0	0	0	0	0	0	0	0	0	130960	300380	135130
TRINITY_DN12386_c0_g1::TRINITY_DN12386_c0_g1_i3::g.12387::m.12387	0	28764	0	0	0	0	0	0	0	0	0	0	187930	348560	0
TRINITY_DN13587_c1_g3::TRINITY_DN13587_c1_g3_i8::g.25768::m.25768	0	0	0	0	0	0	0	0	0	0	0	0	0	338870	226010
TRINITY_DN15400_c1_g2::TRINITY_DN15400_c1_g2_i8::g.50620::m.50620	0	0	0	0	0	86058	0	197500	0	0	0	0	278690	0	0
TRINITY_DN14459_c0_g1::TRINITY_DN14459_c0_g1_i2::g.37381::m.37381	0	0	0	0	0	0	0	112730	0	0	0	0	209490	239960	0
TRINITY_DN11301_c0_g1::TRINITY_DN11301_c0_g1_i2::g.6892::m.6892	0	0	0	0	0	0	0	0	0	0	0	0	181050	138640	241810
TRINITY_DN15762_c0_g1::TRINITY_DN15762_c0_g1_i1::g.56697::m.56697	0	0	0	0	0	0	0	0	0	0	0	0	235350	0	325880
TRINITY_DN15598_c1_g1::TRINITY_DN15598_c1_g1_i2::g.54269::m.54269	0	0	0	0	0	0	0	0	0	0	0	0	264410	155130	140360
TRINITY_DN11950_c0_g1::TRINITY_DN11950_c0_g1_i3::g.9633::m.9633	0	0	0	0	0	0	0	0	0	0	0	0	132710	260450	166720
TRINITY_DN11646_c0_g1::TRINITY_DN11646_c0_g1_i1::g.8053::m.8053	0	0	0	0	0	0	0	0	0	0	0	0	137650	421240	0
TRINITY_DN15411_c1_g1::TRINITY_DN15411_c1_g1_i1::g.51593::m.51593	0	0	0	0	0	0	0	0	126300	0	0	0	0	431420	0
TRINITY_DN16682_c4_g1::TRINITY_DN16682_c4_g1_i2::g.71560::m.71560	0	0	0	0	0	0	0	0	0	0	0	0	164120	151300	242240
TRINITY_DN18037_c1_g1::TRINITY_DN18037_c1_g1_i9::g.94092::m.94092	0	0	0	0	0	0	0	0	0	0	0	0	0	358570	197460
TRINITY_DN15485_c1_g1::TRINITY_DN15485_c1_g1_i8::g.52679::m.52679	0	0	0	0	0	0	0	0	0	0	0	0	252250	300420	0
TRINITY_DN14982_c0_g1::TRINITY_DN14982_c0_g1_i9::g.45222::m.45222	0	0	0	0	0	0	0	0	0	0	0	0	88551	243430	219980
TRINITY_DN19482_c6_g1::TRINITY_DN19482_c6_g1_i8::g.118606::m.118606	0	0	0	0	0	0	0	0	0	0	0	0	240610	309380	0
TRINITY_DN14898_c0_g1::TRINITY_DN14898_c0_g1_i2::g.43888::m.43888	0	0	0	0	0	0	0	0	0	0	0	0	0	330560	219430
TRINITY_DN16344_c0_g1::TRINITY_DN16344_c0_g1_i6::g.66181::m.66181	0	0	0	0	132080	0	0	0	0	0	0	0	199870	0	217240
TRINITY_DN16793_c3_g3::TRINITY_DN16793_c3_g3_i3::g.73369::m.73369	0	0	0	0	0	0	0	0	0	0	0	0	154390	210400	184010
TRINITY_DN15444_c2_g1::TRINITY_DN15444_c2_g1_i4::g.52046::m.52046	0	0	0	0	0	0	0	0	0	0	0	0	0	0	546770
TRINITY_DN17936_c4_g1::TRINITY_DN17936_c4_g1_i9::g.92365::m.92365	0	0	0	0	0	0	0	0	0	0	0	0	257920	286780	0

TRINITY_DN18579_c2_g2::TRINITY_DN18579_c2_g2_i4::g.102900::m.102900	0	0	0	0	0	0	0	0	0	0	0	0	158800	155470	225760
TRINITY_DN14828_c0_g3::TRINITY_DN14828_c0_g3_i7::g.43009::m.43009	0	0	0	0	0	0	0	0	0	0	0	212880	161730	0	165310
TRINITY_DN16126_c1_g1::TRINITY_DN16126_c1_g1_i17::g.62053::m.62053	0	0	0	0	0	0	0	0	0	0	0	0	0	249180	287010
TRINITY_DN16055_c0_g1::TRINITY_DN16055_c0_g1_i6::g.61225::m.61225	0	0	0	0	0	0	0	0	0	0	0	0	123420	189490	222280
TRINITY_DN17454_c3_g1::TRINITY_DN17454_c3_g1_i1::g.84103::m.84103	68392	0	70501	78566	0	0	0	148780	0	0	0	0	0	0	167010
TRINITY_DN17924_c2_g1::TRINITY_DN17924_c2_g1_i1::g.91979::m.91979	0	0	0	0	186990	0	249560	0	0	0	0	0	0	0	94293
TRINITY_DN14710_c1_g2::TRINITY_DN14710_c1_g2_i5::g.41059::m.41059	0	0	0	0	0	0	0	0	0	0	0	0	0	0	530420
TRINITY_DN12060_c0_g1::TRINITY_DN12060_c0_g1_i2::g.10212::m.10212	0	0	0	0	0	0	0	0	0	0	0	0	0	187860	341990
TRINITY_DN14479_c0_g2::TRINITY_DN14479_c0_g2_i3::g.37670::m.37670	0	0	0	0	0	0	0	0	0	0	0	0	0	255400	273860
TRINITY_DN13943_c0_g2::TRINITY_DN13943_c0_g2_i1::g.30525::m.30525	0	0	0	0	0	0	0	0	0	0	0	0	0	127840	401390
TRINITY_DN17241_c0_g1::TRINITY_DN17241_c0_g1_i1::g.80827::m.80827	0	0	0	0	0	0	0	78352	0	0	0	0	161450	147650	140830
TRINITY_DN17619_c0_g2::TRINITY_DN17619_c0_g2_i5::g.87168::m.87168	0	0	0	0	0	0	0	0	0	0	0	0	0	0	527410
TRINITY_DN17406_c4_g1::TRINITY_DN17406_c4_g1_i2::g.83243::m.83243	0	0	0	0	0	0	0	0	0	0	0	0	0	526100	0
TRINITY_DN17708_c1_g1::TRINITY_DN17708_c1_g1_i3::g.88548::m.88548	0	0	0	0	0	0	0	0	0	0	0	0	105930	159570	259740
TRINITY_DN13981_c1_g3::TRINITY_DN13981_c1_g3_i1::g.30911::m.30911	0	0	0	0	0	0	0	0	0	0	0	0	0	398320	126800
TRINITY_DN15411_c1_g2::TRINITY_DN15411_c1_g2_i10::g.51619::m.5161	112760	0	122800	119700	0	0	0	0	0	0	0	0	0	83758	84834
TRINITY_DN16049_c0_g2::TRINITY_DN16049_c0_g2_i3::g.60387::m.60387	0	0	0	0	0	0	0	0	0	0	0	0	0	263440	259390
TRINITY_DN16235_c0_g1::TRINITY_DN16235_c0_g1_i5::g.64344::m.64344	0	0	0	0	0	0	0	0	0	0	0	0	0	320190	202400
TRINITY_DN19621_c6_g3::TRINITY_DN19621_c6_g3_i2::g.120980::m.120980	0	0	0	0	0	0	0	0	0	0	0	0	159520	232030	130930
TRINITY_DN11269_c0_g1::TRINITY_DN11269_c0_g1_i4::g.6758::m.6758	0	0	0	0	0	0	0	0	0	0	0	0	0	0	520710

TRINITY_DN15638_c0_g2::TRINITY_DN15638_c0_g2_i2::g.54677::m.54677	0	0	0	0	0	0	519950	0	0	0	0	0	0	0	0
TRINITY_DN10146_c0_g1::TRINITY_DN10146_c0_g1_i1::g.4308::m.4308	0	0	0	0	0	0	0	0	0	0	0	102640	0	260600	156230
TRINITY_DN13752_c3_g3::TRINITY_DN13752_c3_g3_i1::g.27967::m.27967	33909	0	0	57456	0	0	0	0	0	0	0	0	181490	246490	0
TRINITY_DN15810_c0_g1::TRINITY_DN15810_c0_g1_i2::g.57313::m.57313	0	0	0	0	0	0	0	0	0	0	0	0	151900	226370	140920
TRINITY_DN19614_c2_g7::TRINITY_DN19614_c2_g7_i3::g.120831::m.120831	0	0	0	0	0	0	0	0	0	0	0	0	54684	168640	292810
TRINITY_DN15371_c0_g2::TRINITY_DN15371_c0_g2_i1::g.50837::m.50837	0	0	0	0	0	0	0	0	0	42701	0	0	151100	166460	152880
TRINITY_DN13573_c3_g3::TRINITY_DN13573_c3_g3_i2::g.25665::m.25665	0	0	0	0	0	0	0	0	0	0	0	0	194900	173290	144470
TRINITY_DN17808_c3_g2::TRINITY_DN17808_c3_g2_i3::g.89986::m.89986	67241	25390	140560	125100	0	0	0	0	0	0	153640	0	0	0	0
TRINITY_DN17336_c6_g1::TRINITY_DN17336_c6_g1_i1::g.82089::m.82089	0	0	0	0	0	0	0	0	0	0	0	0	240550	104940	164290
TRINITY_DN17735_c1_g2::TRINITY_DN17735_c1_g2_i2::g.89035::m.89035	0	0	0	50854	0	0	0	0	0	0	0	0	133610	165110	157740
TRINITY_DN16119_c4_g1::TRINITY_DN16119_c4_g1_i1::g.62575::m.62575	0	0	0	0	0	0	0	0	0	0	0	0	446720	0	59641
TRINITY_DN16898_c0_g1::TRINITY_DN16898_c0_g1_i2::g.75056::m.75056	57446	0	47455	50957	0	0	0	0	0	0	0	0	0	348270	0
TRINITY_DN17578_c2_g1::TRINITY_DN17578_c2_g1_i2::g.85652::m.85652	0	0	0	0	0	0	0	0	0	0	0	0	139930	193280	170900
TRINITY_DN17051_c1_g1::TRINITY_DN17051_c1_g1_i6::g.77580::m.77580	0	0	0	0	0	0	0	0	0	0	0	0	193270	142120	168430
TRINITY_DN13889_c0_g1::TRINITY_DN13889_c0_g1_i2::g.29853::m.29853	0	0	0	0	0	0	0	0	0	0	0	0	363680	0	138000
TRINITY_DN15642_c0_g3::TRINITY_DN15642_c0_g3_i1::g.55086::m.55086	0	0	0	0	0	0	0	0	0	0	0	0	238820	0	261890
TRINITY_DN12917_c0_g1::TRINITY_DN12917_c0_g1_i8::g.17363::m.17363	0	0	0	0	0	0	0	0	0	0	0	0	0	115140	384800
TRINITY_DN18228_c1_g3::TRINITY_DN18228_c1_g3_i10::g.97346::m.97346	0	0	0	0	0	0	0	0	0	0	0	0	297800	0	200510
TRINITY_DN14656_c1_g3::TRINITY_DN14656_c1_g3_i2::g.40036::m.40036	0	0	0	0	0	0	0	0	0	0	0	0	0	497920	0
TRINITY_DN17427_c0_g1::TRINITY_DN17427_c0_g1_i5::g.83626::m.83626	497840	0	0	0	0	0	0	0	0	0	0	0	0	0	0

TRINITY_DN12519_c1_g2::TRINITY_DN12519_c1_g2_i2::g.13489::m.13489	0	0	35910	0	0	0	0	0	0	0	461110	0	0	0	0
TRINITY_DN16485_c1_g1::TRINITY_DN16485_c1_g1_i5::g.68172::m.68172	0	0	0	0	0	0	0	0	0	0	0	0	495680	0	0
TRINITY_DN16382_c2_g3::TRINITY_DN16382_c2_g3_i2::g.66735::m.66735	0	0	0	0	0	0	0	0	0	0	0	0	368440	122630	0
TRINITY_DN12471_c0_g1::TRINITY_DN12471_c0_g1_i6::g.13129::m.13129	0	0	0	0	0	0	0	0	0	0	0	0	251140	237490	0
TRINITY_DN13404_c2_g1::TRINITY_DN13404_c2_g1_i2::g.23444::m.23444	0	0	0	0	0	174230	0	312880	0	0	0	0	0	0	0
TRINITY_DN13849_c3_g3::TRINITY_DN13849_c3_g3_i8::g.28501::m.28501	0	0	0	0	0	0	0	0	0	0	0	0	0	0	486290
TRINITY_DN10714_c0_g1::TRINITY_DN10714_c0_g1_i7::g.5396::m.5396	0	0	0	0	0	0	0	0	0	0	0	0	304200	0	181800
TRINITY_DN17727_c1_g3::TRINITY_DN17727_c1_g3_i1::g.88887::m.88887	0	0	0	0	0	0	0	0	0	0	0	0	187600	298030	0
TRINITY_DN13894_c1_g1::TRINITY_DN13894_c1_g1_i8::g.29999::m.29999	0	0	0	0	0	0	0	0	0	0	0	0	246720	236940	0
TRINITY_DN15301_c2_g1::TRINITY_DN15301_c2_g1_i12::g.49765::m.49765	0	0	0	0	0	0	0	0	0	0	0	0	0	0	481650
TRINITY_DN17157_c0_g1::TRINITY_DN17157_c0_g1_i2::g.79386::m.79386	0	0	0	0	0	0	0	0	0	0	0	0	176700	159870	144510
TRINITY_DN19995_c2_g1::TRINITY_DN19995_c2_g1_i2::g.127478::m.127478	60059	0	53816	0	0	0	0	0	0	0	0	0	0	0	366420
TRINITY_DN13004_c2_g2::TRINITY_DN13004_c2_g2_i3::g.18474::m.18474	57276	31490	57332	57703	0	0	0	0	276200	0	0	0	0	0	0
TRINITY_DN10567_c0_g1::TRINITY_DN10567_c0_g1_i2::g.5026::m.5026	0	0	30306	0	0	0	0	0	0	0	159000	0	0	0	290320
TRINITY_DN18294_c2_g1::TRINITY_DN18294_c2_g1_i3::g.98438::m.98438	0	0	0	0	0	0	0	0	0	0	0	0	409360	69784	0
TRINITY_DN16056_c1_g1::TRINITY_DN16056_c1_g1_i8::g.61254::m.61254	0	0	0	0	0	0	0	0	0	0	0	0	185530	0	292030
TRINITY_DN13008_c2_g1::TRINITY_DN13008_c2_g1_i1::g.18585::m.18585	0	0	0	0	76183	0	0	0	0	0	0	401300	0	0	0
TRINITY_DN12353_c0_g1::TRINITY_DN12353_c0_g1_i1::g.12504::m.12504	0	0	0	0	0	0	0	0	0	0	0	0	110350	215480	149270
TRINITY_DN19066_c1_g3::TRINITY_DN19066_c1_g3_i1::g.111236::m.111236	0	0	0	0	0	0	0	0	0	0	0	0	0	114170	360450
TRINITY_DN17441_c2_g4::TRINITY_DN17441_c2_g4_i3::g.83805::m.83805	0	0	0	0	0	0	0	0	0	0	0	0	219870	0	254600
TRINITY_DN1382_c0_g1::TRINITY_DN1382_c0_g1_i1::g.348::m.348	0	0	0	0	0	74401	0	122190	276930	0	0	0	0	0	0

TRINITY_DN17113_c0_g1::TRINITY_DN17113_c0_g1_i1::g.78814::m.78814	0	0	0	0	0	0	0	0	0	0	0	0	238830	0	234500
TRINITY_DN19775_c0_g3::TRINITY_DN19775_c0_g3_i2::g.123476::m.123476	22233	0	0	0	0	0	0	0	0	0	0	0	0	0	449370
TRINITY_DN12824_c1_g1::TRINITY_DN12824_c1_g1_i1::g.16233::m.16233	0	0	0	0	0	0	0	0	0	0	0	0	237550	0	233890
TRINITY_DN11410_c0_g1::TRINITY_DN11410_c0_g1_i1::g.7283::m.7283	0	0	0	142390	0	0	0	0	0	0	0	196170	131530	0	0
TRINITY_DN14617_c1_g1::TRINITY_DN14617_c1_g1_i9::g.39676::m.39676	0	0	0	0	0	0	0	0	0	0	0	0	71627	90724	305840
TRINITY_DN11064_c0_g2::TRINITY_DN11064_c0_g2_i1::g.6238::m.6238	0	0	0	0	0	0	0	0	0	0	0	0	132910	174260	157900
TRINITY_DN14194_c1_g1::TRINITY_DN14194_c1_g1_i9::g.33557::m.33557	0	0	0	0	0	0	0	0	0	0	0	0	154650	162300	148060
TRINITY_DN9434_c0_g1::TRINITY_DN9434_c0_g1_i2::g.3474::m.3474	0	0	0	0	0	0	0	0	103420	0	0	0	104370	132440	124230
TRINITY_DN16217_c1_g2::TRINITY_DN16217_c1_g2_i5::g.64084::m.64084	0	0	0	0	0	0	0	0	0	0	0	0	0	213840	249860
TRINITY_DN16504_c2_g1::TRINITY_DN16504_c2_g1_i5::g.68713::m.68713	115050	57121	147500	143430	0	0	0	0	0	0	0	0	0	0	0
TRINITY_DN14373_c0_g1::TRINITY_DN14373_c0_g1_i5::g.36002::m.36002	0	0	0	0	175280	103450	0	0	184090	0	0	0	0	0	0
TRINITY_DN19595_c1_g1::TRINITY_DN19595_c1_g1_i3::g.120443::m.120443	0	0	0	0	0	0	0	0	0	0	0	0	0	298140	164630
TRINITY_DN16684_c1_g1::TRINITY_DN16684_c1_g1_i1::g.71521::m.71521	0	0	0	0	0	0	0	0	0	0	0	0	0	0	462620
TRINITY_DN18955_c3_g1::TRINITY_DN18955_c3_g1_i8::g.109317::m.109317	0	0	0	42187	0	0	0	0	0	0	0	0	0	205040	214120
TRINITY_DN19877_c3_g4::TRINITY_DN19877_c3_g4_i1::g.125024::m.125024	44765	0	48035	36364	0	0	0	167270	0	0	0	0	0	0	164630
TRINITY_DN11684_c0_g1::TRINITY_DN11684_c0_g1_i1::g.8363::m.8363	0	0	0	0	0	0	0	0	0	0	0	0	139740	163780	157210
TRINITY_DN16471_c14_g1::TRINITY_DN16471_c14_g1_i3::g.68132::m.68132	0	0	0	0	0	0	0	0	0	0	0	0	218860	0	241500
TRINITY_DN15705_c0_g3::TRINITY_DN15705_c0_g3_i5::g.56096::m.56096	0	0	0	0	0	0	0	0	0	0	0	0	338710	120930	0
TRINITY_DN14658_c2_g1::TRINITY_DN14658_c2_g1_i2::g.40152::m.40152	78556	0	39738	0	0	0	0	0	0	117930	0	0	110730	111720	0
TRINITY_DN16640_c0_g1::TRINITY_DN16640_c0_g1_i4::g.70902::m.70902	0	0	0	0	0	0	0	0	0	0	0	0	148050	144130	166200
TRINITY_DN12745_c0_g1::TRINITY_DN12745_c0_g1_i6::g.15122::m.15122	0	0	0	0	0	0	0	0	0	0	0	0	0	241810	215130
TRINITY_DN13091_c0_g2::TRINITY_DN13091_c0_g2_i2::g.19654::m.19654	0	0	0	0	0	0	0	0	290880	0	0	0	0	165700	0

TRINITY_DN19627_c1_g1::TRINITY_DN19627_c1_g1_i2::g.121157::m.1211	0	0	0	0	0	0	0	0	0	0	0	0	97805	204390	154130
TRINITY_DN10469_c0_g1::TRINITY_DN10469_c0_g1_i1::g.4851::m.4851	0	0	0	0	0	0	0	0	0	0	0	0	0	362670	93402
TRINITY_DN16987_c2_g1::TRINITY_DN16987_c2_g1_i9::g.76589::m.76589	93750	0	91198	111420	0	0	0	0	0	0	0	0	80547	0	78964
TRINITY_DN19240_c1_g1::TRINITY_DN19240_c1_g1_i5::g.114383::m.114383	0	0	0	0	0	0	0	0	0	0	0	0	0	0	454800
TRINITY_DN14334_c2_g6::TRINITY_DN14334_c2_g6_i1::g.35159::m.35159	0	0	0	0	0	0	0	0	0	0	0	0	306780	0	145960
TRINITY_DN18647_c1_g1::TRINITY_DN18647_c1_g1_i1::g.103820::m.103820	0	0	0	0	0	0	0	0	0	0	0	0	118140	333600	0
TRINITY_DN16308_c0_g1::TRINITY_DN16308_c0_g1_i5::g.65986::m.65986	0	0	0	0	0	0	0	0	0	0	0	0	94180	171130	181850
TRINITY_DN16705_c7_g1::TRINITY_DN16705_c7_g1_i5::g.72000::m.72000	0	0	0	0	0	0	0	0	0	0	0	0	115890	180870	150270
TRINITY_DN10617_c0_g1::TRINITY_DN10617_c0_g1_i2::g.5120::m.5120	0	0	0	0	0	0	0	0	0	445140	0	0	0	0	0
TRINITY_DN13025_c0_g1::TRINITY_DN13025_c0_g1_i1::g.18736::m.18736	0	0	0	0	0	0	0	0	0	0	0	0	0	107340	337290
TRINITY_DN16744_c0_g2::TRINITY_DN16744_c0_g2_i2::g.72603::m.72603	0	0	0	0	0	0	0	0	0	0	0	0	69525	0	373750
TRINITY_DN15178_c0_g1::TRINITY_DN15178_c0_g1_i16::g.47873::m.47873	0	0	0	0	0	0	0	0	0	0	0	0	0	343360	98824
TRINITY_DN16466_c0_g1::TRINITY_DN16466_c0_g1_i6::g.67994::m.67994	0	0	0	0	0	0	0	0	0	0	0	0	0	237750	202700
TRINITY_DN18466_c3_g1::TRINITY_DN18466_c3_g1_i19::g.101050::m.101050	0	0	0	0	440290	0	0	0	0	0	0	0	0	0	0
TRINITY_DN13825_c0_g1::TRINITY_DN13825_c0_g1_i3::g.28848::m.28848	0	0	0	140050	0	0	0	0	0	0	0	0	0	299960	0
TRINITY_DN12167_c0_g1::TRINITY_DN12167_c0_g1_i7::g.10902::m.10902	0	0	0	0	0	0	0	0	0	0	0	0	230000	0	205460
TRINITY_DN18952_c2_g1::TRINITY_DN18952_c2_g1_i9::g.109479::m.109479	0	0	0	0	0	0	0	0	0	0	0	0	203770	0	231440
TRINITY_DN13110_c0_g1::TRINITY_DN13110_c0_g1_i3::g.20012::m.20012	0	0	0	0	0	0	0	0	0	0	0	0	0	245890	187820
TRINITY_DN14898_c0_g2::TRINITY_DN14898_c0_g2_i1::g.43887::m.43887	0	0	0	0	0	0	0	0	0	0	0	0	146950	155900	129720
TRINITY_DN14096_c0_g2::TRINITY_DN14096_c0_g2_i2::g.32310::m.32310	0	0	0	0	0	0	0	0	0	0	0	0	133730	145170	153580

TRINITY_DN15203_c1_g1::TRINITY_DN15203_c1_g1_i19::g.48562::m.48562	0	0	0	0	0	0	0	0	0	0	0	0	139220	162780	129700
TRINITY_DN18497_c0_g1::TRINITY_DN18497_c0_g1_i7::g.101435::m.101435	0	0	0	0	0	0	0	0	0	0	0	0	0	277180	153850
TRINITY_DN16464_c0_g1::TRINITY_DN16464_c0_g1_i9::g.67881::m.67881	0	0	0	0	0	0	0	0	0	0	0	0	188790	241610	0
TRINITY_DN10374_c0_g1::TRINITY_DN10374_c0_g1_i1::g.4693::m.4693	0	0	0	0	0	0	0	0	0	0	0	0	121760	308600	0
TRINITY_DN16131_c0_g1::TRINITY_DN16131_c0_g1_i1::g.62590::m.62590	0	0	0	0	0	0	0	0	0	0	0	0	0	233850	195550
TRINITY_DN13858_c6_g1::TRINITY_DN13858_c6_g1_i3::g.29437::m.29437	0	0	0	0	0	0	0	0	0	0	0	0	280430	148860	0
TRINITY_DN17229_c1_g1::TRINITY_DN17229_c1_g1_i16::g.80496::m.80496	210010	0	0	0	0	0	0	0	0	0	0	0	0	0	218980
TRINITY_DN16551_c0_g1::TRINITY_DN16551_c0_g1_i1::g.69240::m.69240	0	0	0	0	0	0	0	0	0	0	0	0	0	304710	122890
TRINITY_DN14041_c3_g1::TRINITY_DN14041_c3_g1_i2::g.31810::m.31810	0	0	0	0	0	0	0	0	0	0	0	0	246150	0	181260
TRINITY_DN12986_c2_g1::TRINITY_DN12986_c2_g1_i7::g.17676::m.17676	0	0	0	0	0	0	0	0	0	0	0	0	110340	314900	0
TRINITY_DN19621_c4_g1::TRINITY_DN19621_c4_g1_i2::g.120970::m.120970	0	133940	0	291270	0	0	0	0	0	0	0	0	0	0	0
TRINITY_DN10293_c0_g1::TRINITY_DN10293_c0_g1_i1::g.4492::m.4492	0	0	0	0	0	0	0	0	0	0	0	0	199860	224850	0
TRINITY_DN14175_c1_g1::TRINITY_DN14175_c1_g1_i3::g.33680::m.33680	4300,5	0	0	0	0	0	0	0	0	0	0	0	130110	290070	0
TRINITY_DN15828_c0_g1::TRINITY_DN15828_c0_g1_i4::g.57452::m.57452	0	0	0	0	0	0	0	0	0	0	0	0	185590	0	238600
TRINITY_DN17792_c0_g5::TRINITY_DN17792_c0_g5_i3::g.89751::m.89751	0	0	0	0	0	0	240570	182860	0	0	0	0	0	0	0
TRINITY_DN12837_c2_g2::TRINITY_DN12837_c2_g2_i1::g.16572::m.16572	70472	34058	231560	84655	0	0	0	0	0	0	0	0	0	0	0
TRINITY_DN17217_c1_g1::TRINITY_DN17217_c1_g1_i9::g.80247::m.80247	0	0	0	0	0	0	0	0	0	0	0	0	225360	0	194870
TRINITY_DN14939_c1_g3::TRINITY_DN14939_c1_g3_i3::g.44686::m.44686	0	0	41661	133550	0	0	0	0	0	0	0	0	143740	0	100070
TRINITY_DN16528_c1_g1::TRINITY_DN16528_c1_g1_i4::g.69093::m.69093	111390	42882	187610	76354	0	0	0	0	0	0	0	0	0	0	0
TRINITY_DN17896_c3_g1::TRINITY_DN17896_c3_g1_i3::g.91440::m.91440	0	0	0	0	0	0	0	0	0	0	0	0	120190	156120	140280
TRINITY_DN13512_c0_g2::TRINITY_DN13512_c0_g2_i1::g.24706::m.24706	79120	0	92679	63913	180750	0	0	0	0	0	0	0	0	0	0

TRINITY_DN18396_c2_g1::TRINITY_DN18396_c2_g1_i1::g.99882::m.99882	0	0	0	0	0	0	0	0	0	0	0	0	128580	164220	123510
TRINITY_DN14316_c0_g1::TRINITY_DN14316_c0_g1_i5::g.35352::m.35352	0	0	0	0	0	0	0	0	0	0	0	0	0	277730	138170
TRINITY_DN19110_c2_g1::TRINITY_DN19110_c2_g1_i2::g.112351::m.1123	0	0	0	0	0	0	0	0	0	0	0	0	245060	0	170780
TRINITY_DN19639_c3_g1::TRINITY_DN19639_c3_g1_i7::g.121291::m.121291	0	20990	0	0	0	0	0	0	0	0	0	0	195510	0	198230
TRINITY_DN14977_c0_g1::TRINITY_DN14977_c0_g1_i14::g.45080::m.45080	0	0	0	0	0	0	0	0	0	0	0	0	295300	0	118790
TRINITY_DN19335_c0_g3::TRINITY_DN19335_c0_g3_i4::g.116290::m.116290	0	0	0	0	0	0	0	0	0	0	0	0	0	207600	206130
TRINITY_DN17394_c0_g1::TRINITY_DN17394_c0_g1_i5::g.83048::m.83048	0	0	0	0	0	0	0	0	0	0	0	0	117190	149730	146150
TRINITY_DN15312_c1_g1::TRINITY_DN15312_c1_g1_i7::g.50104::m.50104	0	0	0	0	0	0	0	0	0	0	0	0	129460	158270	124840
TRINITY_DN14520_c0_g2::TRINITY_DN14520_c0_g2_i5::g.38465::m.38465	0	0	0	0	0	0	0	0	0	0	0	0	0	320190	91926
TRINITY_DN19082_c3_g2::TRINITY_DN19082_c3_g2_i2::g.111602::m.111602	0	0	0	0	0	0	0	0	0	0	0	0	182600	0	228820
TRINITY_DN17990_c1_g1::TRINITY_DN17990_c1_g1_i2::g.93320::m.93320	0	0	0	0	0	0	0	0	0	0	0	271870	0	139280	0
TRINITY_DN10955_c0_g1::TRINITY_DN10955_c0_g1_i1::g.5913::m.5913	0	0	0	0	0	0	0	0	0	0	0	0	408980	0	0
TRINITY_DN10864_c0_g1::TRINITY_DN10864_c0_g1_i1::g.5678::m.5678	0	0	0	0	0	0	0	0	0	0	0	0	0	225210	180620
TRINITY_DN18372_c2_g6::TRINITY_DN18372_c2_g6_i1::g.99517::m.99517	0	0	0	0	0	0	0	0	0	0	0	0	0	0	405580
TRINITY_DN17198_c0_g1::TRINITY_DN17198_c0_g1_i4::g.79878::m.79878	0	0	0	0	0	0	0	0	256550	0	148950	0	0	0	0
TRINITY_DN19333_c1_g1::TRINITY_DN19333_c1_g1_i1::g.116168::m.1161	0	0	0	0	0	0	0	0	0	0	0	0	314070	0	90069
TRINITY_DN13608_c0_g1::TRINITY_DN13608_c0_g1_i3::g.26161::m.26161	0	0	0	0	0	0	0	0	0	0	0	0	159110	0	244050
TRINITY_DN17268_c0_g1::TRINITY_DN17268_c0_g1_i4::g.80463::m.80463	0	0	0	0	0	0	0	0	0	0	0	0	94198	107680	200460
TRINITY_DN11329_c0_g1::TRINITY_DN11329_c0_g1_i1::g.6996::m.6996	0	0	402040	0	0	0	0	0	0	0	0	0	0	0	0
TRINITY_DN17556_c3_g1::TRINITY_DN17556_c3_g1_i1::g.85912::m.85912	0	0	0	0	0	0	0	0	0	0	0	0	0	184370	217090
TRINITY_DN14486_c0_g4::TRINITY_DN14486_c0_g4_i1::g.37922::m.37922	0	0	0	0	0	0	0	0	0	0	0	400120	0	0	0
TRINITY_DN19016_c1_g5::TRINITY_DN19016_c1_g5_i1::g.110613::m.1106	0	0	0	0	0	0	0	0	0	0	0	0	185310	214260	0

TRINITY_DN19612_c1_g2::TRINITY_DN19612_c1_g2_i7::g.120818::m.120818	0	0	0	0	0	0	0	0	0	0	0	0	177700	0	221030
TRINITY_DN13825_c0_g2::TRINITY_DN13825_c0_g2_i4::g.28851::m.28851	0	0	0	74368	0	0	0	161140	0	0	0	0	0	162830	0
TRINITY_DN10767_c0_g1::TRINITY_DN10767_c0_g1_i1::g.5469::m.5469	0	0	0	0	0	0	0	99164	0	0	0	0	0	135050	163690
TRINITY_DN14408_c0_g1::TRINITY_DN14408_c0_g1_i2::g.36628::m.36628	0	0	0	0	0	0	0	0	0	0	0	0	185350	0	211740
TRINITY_DN16867_c0_g1::TRINITY_DN16867_c0_g1_i8::g.74659::m.74659	0	0	0	0	0	0	0	0	0	0	0	0	119100	0	277150
TRINITY_DN12184_c0_g1::TRINITY_DN12184_c0_g1_i1::g.10535::m.10535	0	0	0	0	0	0	0	0	0	0	0	0	0	96247	299760
TRINITY_DN19255_c4_g1::TRINITY_DN19255_c4_g1_i5::g.114861::m.114861	0	0	0	0	0	0	0	0	0	0	0	0	0	142060	253870
TRINITY_DN18745_c0_g4::TRINITY_DN18745_c0_g4_i1::g.105401::m.105401	0	0	0	0	0	0	0	0	0	0	0	0	175950	218160	0
TRINITY_DN15579_c2_g1::TRINITY_DN15579_c2_g1_i2::g.54035::m.54035	105310	0	157230	130000	0	0	0	0	0	0	0	0	0	0	0
TRINITY_DN19014_c2_g2::TRINITY_DN19014_c2_g2_i15::g.110588::m.110588	0	0	0	0	0	0	0	0	0	0	0	0	391660	0	0
TRINITY_DN13959_c0_g2::TRINITY_DN13959_c0_g2_i7::g.30593::m.30593	0	0	0	0	0	0	0	0	0	0	0	0	0	146560	242510
TRINITY_DN17096_c2_g1::TRINITY_DN17096_c2_g1_i4::g.78253::m.78253	0	0	0	0	0	0	0	0	0	0	0	0	124090	130140	129930
TRINITY_DN15860_c0_g1::TRINITY_DN15860_c0_g1_i3::g.57901::m.57901	0	0	0	0	0	0	0	0	0	0	0	0	0	195690	187590
TRINITY_DN12503_c2_g1::TRINITY_DN12503_c2_g1_i3::g.13440::m.13440	0	0	0	0	0	0	0	0	0	0	0	0	203690	78827	99897
TRINITY_DN14356_c4_g1::TRINITY_DN14356_c4_g1_i9::g.35853::m.35853	0	0	0	0	0	0	0	0	0	0	0	0	74330	215510	92413
TRINITY_DN14761_c1_g4::TRINITY_DN14761_c1_g4_i4::g.41839::m.41839	0	0	0	0	0	0	0	0	0	0	0	0	111490	172010	98523
TRINITY_DN16683_c2_g2::TRINITY_DN16683_c2_g2_i1::g.71583::m.71583	0	0	0	0	0	0	0	0	0	0	0	0	219590	115750	45838
TRINITY_DN13437_c2_g2::TRINITY_DN13437_c2_g2_i2::g.23355::m.23355	0	0	0	0	0	0	0	0	0	0	0	0	72228	109860	196210
TRINITY_DN13537_c0_g1::TRINITY_DN13537_c0_g1_i2::g.25155::m.25155	0	0	0	0	0	0	0	0	0	0	0	0	0	0	376440

TRINITY_DN13720_c0_g6::TRINITY_DN13720_c0_g6_i1::g.27649::m.27649	0	0	0	0	0	0	0	0	0	0	0	0	0	374920	0
TRINITY_DN15113_c1_g1::TRINITY_DN15113_c1_g1_i9::g.47525::m.47525	0	0	0	0	98205	0	0	0	0	0	0	0	0	0	274980
TRINITY_DN18151_c1_g8::TRINITY_DN18151_c1_g8_i1::g.95937::m.95937	0	0	87416	0	194720	90521	0	0	0	0	0	0	0	0	0
TRINITY_DN16542_c2_g2::TRINITY_DN16542_c2_g2_i16::g.69553::m.69553	0	0	0	0	0	0	0	0	0	0	0	0	211440	160930	0
TRINITY_DN17965_c0_g2::TRINITY_DN17965_c0_g2_i2::g.92668::m.92668	0	0	0	0	0	0	0	0	0	0	0	0	0	198990	173040
TRINITY_DN10320_c0_g1::TRINITY_DN10320_c0_g1_i1::g.4626::m.4626	0	0	0	0	0	0	0	0	0	0	0	0	0	258820	112860
TRINITY_DN19345_c3_g1::TRINITY_DN19345_c3_g1_i6::g.117087::m.117087	0	0	0	0	0	0	0	0	0	0	0	0	170190	201440	0
TRINITY_DN18504_c0_g1::TRINITY_DN18504_c0_g1_i8::g.101692::m.101692	0	0	0	0	0	0	0	0	0	0	0	0	179300	0	192100
TRINITY_DN19069_c0_g1::TRINITY_DN19069_c0_g1_i4::g.111231::m.111231	0	0	0	0	0	0	0	0	0	0	0	0	135420	233760	0
TRINITY_DN15115_c0_g4::TRINITY_DN15115_c0_g4_i3::g.47102::m.47102	0	0	0	0	0	0	0	0	0	0	0	0	0	220700	147040
TRINITY_DN18049_c1_g1::TRINITY_DN18049_c1_g1_i7::g.94346::m.94346	0	0	0	0	0	0	0	0	0	0	0	0	0	0	367640
TRINITY_DN16571_c0_g1::TRINITY_DN16571_c0_g1_i1::g.69820::m.69820	0	0	0	0	0	0	0	0	0	0	0	0	0	295860	71680
TRINITY_DN17401_c1_g1::TRINITY_DN17401_c1_g1_i4::g.83099::m.83099	0	0	0	0	0	0	0	0	0	0	0	0	0	271960	95202
TRINITY_DN11104_c0_g1::TRINITY_DN11104_c0_g1_i1::g.6331::m.6331	0	0	0	0	0	0	0	0	0	0	0	0	214780	0	151520
TRINITY_DN11331_c0_g1::TRINITY_DN11331_c0_g1_i1::g.7013::m.7013	114180	74908	0	0	113760	63371	0	0	0	0	0	0	0	0	0
TRINITY_DN19806_c0_g1::TRINITY_DN19806_c0_g1_i11::g.123914::m.123914	0	0	0	0	0	0	0	0	0	0	0	0	0	187470	177560
TRINITY_DN14324_c0_g1::TRINITY_DN14324_c0_g1_i3::g.35430::m.35430	0	0	0	0	0	0	0	0	0	0	0	0	65244	299280	0
TRINITY_DN19942_c2_g1::TRINITY_DN19942_c2_g1_i4::g.126324::m.126324	0	0	0	0	0	0	0	0	0	0	0	0	108450	133080	122750
TRINITY_DN16925_c0_g1::TRINITY_DN16925_c0_g1_i1::g.75769::m.75769	0	0	0	0	0	0	0	0	0	0	0	0	138070	69195	155670
TRINITY_DN19895_c1_g1::TRINITY_DN19895_c1_g1_i3::g.125573::m.125573	0	0	0	0	0	0	0	0	0	0	0	0	110860	123830	127260
TRINITY_DN17938_c1_g2::TRINITY_DN17938_c1_g2_i1::g.92333::m.92333	0	0	178400	0	0	0	0	0	0	183320	0	0	0	0	0

TRINITY_DN19979_c5_g4::TRINITY_DN19979_c5_g4_i5::g.127016::m.127016	0	0	0	0	0	0	0	0	0	0	0	0	0	271050	89683
TRINITY_DN17846_c4_g1::TRINITY_DN17846_c4_g1_i2::g.90658::m.90658	0	0	0	0	0	0	0	0	0	0	0	0	276480	81567	0
TRINITY_DN17717_c2_g1::TRINITY_DN17717_c2_g1_i3::g.88728::m.88728	0	0	0	0	0	0	0	0	0	0	0	0	357670	0	0
TRINITY_DN14801_c0_g1::TRINITY_DN14801_c0_g1_i4::g.42400::m.42400	0	0	0	0	0	0	0	0	0	0	0	0	0	96783	260440
TRINITY_DN14703_c2_g1::TRINITY_DN14703_c2_g1_i4::g.41069::m.41069	0	0	0	74576	0	0	0	63867	218740	0	0	0	0	0	0
TRINITY_DN11195_c0_g1::TRINITY_DN11195_c0_g1_i1::g.6571::m.6571	0	0	0	0	0	0	0	0	0	0	0	0	66434	197900	92007
TRINITY_DN10654_c0_g1::TRINITY_DN10654_c0_g1_i1::g.5213::m.5213	0	0	0	0	0	0	0	0	0	0	0	0	147140	208360	0
TRINITY_DN17314_c1_g1::TRINITY_DN17314_c1_g1_i1::g.81914::m.81914	0	0	0	0	0	0	0	0	0	0	0	0	0	209950	145380
TRINITY_DN17135_c0_g1::TRINITY_DN17135_c0_g1_i6::g.79192::m.79192	0	0	0	0	0	0	0	0	216660	0	0	0	137170	0	0
TRINITY_DN18873_c0_g1::TRINITY_DN18873_c0_g1_i7::g.107966::m.107966	0	0	0	0	0	0	0	0	0	0	0	0	137200	216070	0
TRINITY_DN15119_c0_g1::TRINITY_DN15119_c0_g1_i1::g.47105::m.47105	0	0	0	0	0	0	0	0	0	0	0	0	244010	0	106690
TRINITY_DN19022_c0_g1::TRINITY_DN19022_c0_g1_i10::g.110657::m.110657	0	0	0	0	0	0	0	0	0	0	0	0	164760	185360	0
TRINITY_DN17160_c1_g3::TRINITY_DN17160_c1_g3_i2::g.79397::m.79397	0	0	0	0	0	0	0	0	170380	0	0	0	0	179180	0
TRINITY_DN13640_c0_g3::TRINITY_DN13640_c0_g3_i9::g.26621::m.26621	0	0	0	0	0	0	0	0	0	0	0	0	0	176980	171030
TRINITY_DN19493_c1_g1::TRINITY_DN19493_c1_g1_i9::g.118079::m.118079	0	0	0	0	102930	0	0	0	0	0	0	0	0	242310	0
TRINITY_DN19823_c3_g2::TRINITY_DN19823_c3_g2_i6::g.124233::m.124233	0	0	0	0	0	0	0	0	0	0	0	0	91263	122530	130070
TRINITY_DN16244_c0_g2::TRINITY_DN16244_c0_g2_i4::g.64523::m.64523	0	0	0	0	0	0	0	0	0	0	0	0	156000	0	186040
TRINITY_DN13878_c2_g2::TRINITY_DN13878_c2_g2_i3::g.29757::m.29757	0	35970	91627	70919	0	0	0	143270	0	0	0	0	0	0	0
TRINITY_DN13194_c4_g1::TRINITY_DN13194_c4_g1_i4::g.20971::m.20971	0	0	0	0	0	0	0	0	0	0	0	0	0	90639	251090
TRINITY_DN13636_c0_g1::TRINITY_DN13636_c0_g1_i5::g.26555::m.26555	0	0	0	0	0	0	0	0	0	0	0	0	120540	0	220160
TRINITY_DN17968_c0_g2::TRINITY_DN17968_c0_g2_i1::g.92847::m.92847	0	0	0	0	0	0	0	0	0	0	0	0	0	206960	133220

TRINITY_DN10935_c0_g1::TRINITY_DN10935_c0_g1_i5::g.5847::m.5847	36225	0	37080	47738	111960	0	0	105480	0	0	0	0	0	0	0
TRINITY_DN17259_c0_g1::TRINITY_DN17259_c0_g1_i9::g.80974::m.80974	0	0	0	0	0	0	0	0	0	0	0	0	0	0	338450
TRINITY_DN14202_c2_g1::TRINITY_DN14202_c2_g1_i3::g.33869::m.33869	0	0	0	0	0	0	0	0	0	95394	0	242710	0	0	0
TRINITY_DN11729_c1_g1::TRINITY_DN11729_c1_g1_i5::g.8590::m.8590	0	0	0	0	0	0	0	0	0	0	0	0	176380	0	160870
TRINITY_DN16787_c1_g2::TRINITY_DN16787_c1_g2_i3::g.73271::m.73271	0	0	0	0	0	0	0	0	93723	0	0	0	243360	0	0
TRINITY_DN15603_c1_g3::TRINITY_DN15603_c1_g3_i1::g.54361::m.54361	0	0	0	0	0	0	0	78696	0	0	0	0	257910	0	0
TRINITY_DN13221_c3_g1::TRINITY_DN13221_c3_g1_i2::g.21212::m.21212	54636	0	31615	0	0	0	0	0	100430	0	149260	0	0	0	0
TRINITY_DN10855_c0_g2::TRINITY_DN10855_c0_g2_i3::g.5689::m.5689	0	0	0	55463	0	0	0	0	0	0	0	0	279640	0	0
TRINITY_DN47853_c0_g1::TRINITY_DN47853_c0_g1_i1::g.132570::m.132570	0	0	334720	0	0	0	0	0	0	0	0	0	0	0	0
TRINITY_DN17028_c2_g2::TRINITY_DN17028_c2_g2_i3::g.77279::m.77279	0	0	72779	81304	0	0	0	0	0	0	180340	0	0	0	0
TRINITY_DN17028_c2_g2::TRINITY_DN17028_c2_g2_i3::g.77278::m.77278	0	0	0	0	0	0	0	0	0	0	0	0	108390	0	225960
TRINITY_DN16271_c1_g1::TRINITY_DN16271_c1_g1_i4::g.64790::m.64790	0	0	0	0	0	0	0	0	0	0	0	0	0	0	333590
TRINITY_DN16125_c0_g1::TRINITY_DN16125_c0_g1_i5::g.62472::m.62472	0	0	0	0	238980	94220	0	0	0	0	0	0	0	0	0
TRINITY_DN13227_c0_g6::TRINITY_DN13227_c0_g6_i1::g.21293::m.21293	118610	0	119490	94927	0	0	0	0	0	0	0	0	0	0	0
TRINITY_DN19999_c7_g1::TRINITY_DN19999_c7_g1_i2::g.126443::m.126443	89923	0	128110	113050	0	0	0	0	0	0	0	0	0	0	0
TRINITY_DN18074_c1_g1::TRINITY_DN18074_c1_g1_i2::g.94688::m.94688	0	0	0	0	0	0	0	0	0	0	0	0	0	208520	120780
TRINITY_DN15518_c0_g1::TRINITY_DN15518_c0_g1_i2::g.53176::m.53176	0	0	0	0	0	0	0	0	0	0	0	0	186790	142110	0
TRINITY_DN14467_c3_g1::TRINITY_DN14467_c3_g1_i7::g.37643::m.37643	0	0	0	0	0	0	0	0	0	0	145280	0	0	0	182810
TRINITY_DN12438_c0_g2::TRINITY_DN12438_c0_g2_i1::g.12882::m.12882	0	0	0	0	0	0	0	0	0	0	0	0	0	106110	221420
TRINITY_DN12528_c1_g2::TRINITY_DN12528_c1_g2_i1::g.13492::m.13492	0	0	0	0	0	0	0	0	0	0	0	0	104060	121700	100410
TRINITY_DN18017_c0_g1::TRINITY_DN18017_c0_g1_i4::g.93738::m.93738	0	0	0	0	0	0	0	0	0	0	0	0	325120	0	0

TRINITY_DN13524_c0_g1::TRINITY_DN13524_c0_g1_i8::g.24870::m.24870	0	0	0	0	0	0	0	0	0	0	0	325040	0	0	0
TRINITY_DN15030_c2_g10::TRINITY_DN15030_c2_g10_i10::g.46047::m.46047	0	0	0	0	0	0	0	0	0	0	0	0	84696	85673	153780
TRINITY_DN14724_c2_g3::TRINITY_DN14724_c2_g3_i6::g.41251::m.41251	0	0	0	0	0	0	0	0	0	0	0	0	0	153200	170470
TRINITY_DN9836_c0_g1::TRINITY_DN9836_c0_g1_i2::g.3964::m.3964	0	0	0	0	0	0	0	0	0	0	0	0	83804	124400	113450
TRINITY_DN18459_c3_g1::TRINITY_DN18459_c3_g1_i2::g.100697::m.100697	0	0	0	0	0	0	0	0	0	0	0	0	0	104190	216950
TRINITY_DN14119_c2_g1::TRINITY_DN14119_c2_g1_i7::g.32525::m.32525	0	0	0	0	0	0	0	0	0	0	0	0	91077	119670	110100
TRINITY_DN17875_c1_g1::TRINITY_DN17875_c1_g1_i11::g.91219::m.9121	0	0	0	0	0	0	0	0	0	0	0	0	93981	105660	120820
TRINITY_DN10295_c0_g1::TRINITY_DN10295_c0_g1_i2::g.4577::m.4577	144290	0	45951	38088	0	91987	0	0	0	0	0	0	0	0	0
TRINITY_DN15634_c1_g2::TRINITY_DN15634_c1_g2_i1::g.54735::m.54735	0	0	0	0	0	0	0	0	0	0	0	0	0	320210	0
TRINITY_DN14193_c0_g1::TRINITY_DN14193_c0_g1_i4::g.33482::m.33482	0	0	0	0	0	0	0	0	0	0	0	0	136010	184190	0
TRINITY_DN18060_c0_g2::TRINITY_DN18060_c0_g2_i3::g.94415::m.94415	0	0	0	0	0	0	0	0	0	0	0	0	0	145200	174710
TRINITY_DN13421_c0_g4::TRINITY_DN13421_c0_g4_i1::g.23529::m.23529	0	0	0	0	0	0	0	0	0	0	0	0	97891	0	221500
TRINITY_DN15203_c1_g4::TRINITY_DN15203_c1_g4_i2::g.48560::m.48560	0	0	0	0	0	0	0	0	0	0	0	0	0	134750	184640
TRINITY_DN15462_c1_g3::TRINITY_DN15462_c1_g3_i2::g.52234::m.52234	0	0	0	0	0	0	0	81417	0	0	0	0	0	0	237060
TRINITY_DN17163_c2_g2::TRINITY_DN17163_c2_g2_i2::g.79515::m.79515	0	0	0	0	0	0	0	0	0	0	0	0	317690	0	0
TRINITY_DN14323_c3_g1::TRINITY_DN14323_c3_g1_i1::g.35598::m.35598	0	0	0	0	0	0	0	0	0	0	0	0	177660	138160	0
TRINITY_DN17612_c1_g4::TRINITY_DN17612_c1_g4_i2::g.87038::m.87038	0	0	0	0	0	0	0	0	0	0	0	0	0	170470	143770
TRINITY_DN17010_c0_g1::TRINITY_DN17010_c0_g1_i4::g.76944::m.76944	0	0	0	0	0	0	0	0	0	0	0	0	187040	126910	0
TRINITY_DN14661_c1_g1::TRINITY_DN14661_c1_g1_i2::g.40114::m.40114	0	0	0	0	0	0	0	0	0	0	0	0	125590	0	186120
TRINITY_DN19036_c0_g1::TRINITY_DN19036_c0_g1_i4::g.110800::m.110800	0	0	0	0	0	0	0	0	0	0	252500	0	0	0	59077
TRINITY_DN19720_c2_g1::TRINITY_DN19720_c2_g1_i1::g.122728::m.122728	0	0	0	0	0	0	0	0	0	0	0	0	150310	0	159970

TRINITY_DN16319_c0_g6::TRINITY_DN16319_c0_g6_i2::g.65617::m.65617	0	0	0	0	0	0	0	0	0	0	0	0	100220	111730	97736
TRINITY_DN17286_c0_g1::TRINITY_DN17286_c0_g1_i7::g.81470::m.81470	0	0	0	0	0	0	0	0	0	0	0	0	91941	0	217340
TRINITY_DN13871_c3_g1::TRINITY_DN13871_c3_g1_i4::g.29817::m.29817	0	0	0	0	0	0	0	0	0	0	0	0	173910	134870	0
TRINITY_DN13650_c9_g1::TRINITY_DN13650_c9_g1_i8::g.26115::m.26115	0	0	0	0	171270	0	0	0	0	0	0	0	0	133480	0
TRINITY_DN18482_c1_g1::TRINITY_DN18482_c1_g1_i7::g.100962::m.100962	0	0	0	0	0	0	0	0	0	0	0	0	0	90826	212590
TRINITY_DN14626_c0_g2::TRINITY_DN14626_c0_g2_i6::g.39817::m.39817	0	0	0	0	0	0	0	0	0	0	0	0	154030	0	148500
TRINITY_DN13249_c0_g1::TRINITY_DN13249_c0_g1_i1::g.21073::m.21073	0	0	0	0	0	0	0	0	0	0	0	0	150110	0	151650
TRINITY_DN15130_c6_g1::TRINITY_DN15130_c6_g1_i3::g.47299::m.47299	0	0	0	0	0	0	0	0	0	0	0	0	121860	179300	0
TRINITY_DN16370_c1_g1::TRINITY_DN16370_c1_g1_i6::g.66493::m.66493	0	0	0	0	0	0	0	0	0	0	0	0	129110	0	171210
TRINITY_DN18821_c0_g1::TRINITY_DN18821_c0_g1_i8::g.106977::m.106977	0	0	0	0	0	0	0	0	0	0	0	0	0	0	300270
TRINITY_DN2866_c0_g1::TRINITY_DN2866_c0_g1_i1::g.791::m.791	0	0	0	0	0	0	0	0	0	0	0	0	0	0	298670
TRINITY_DN13724_c5_g4::TRINITY_DN13724_c5_g4_i1::g.27589::m.27589	0	0	0	0	0	0	0	0	0	0	0	0	0	298490	0
TRINITY_DN15832_c2_g1::TRINITY_DN15832_c2_g1_i7::g.57636::m.57636	0	0	0	0	0	0	0	0	0	0	0	0	98649	108810	89331
TRINITY_DN16212_c0_g2::TRINITY_DN16212_c0_g2_i5::g.63967::m.63967	0	0	0	0	0	0	0	0	0	0	0	0	0	296300	0
TRINITY_DN18690_c2_g2::TRINITY_DN18690_c2_g2_i1::g.104786::m.104786	0	32584	0	0	0	0	0	0	0	0	0	0	0	0	263150
TRINITY_DN18367_c3_g7::TRINITY_DN18367_c3_g7_i1::g.99480::m.99480	0	0	0	0	0	0	0	0	0	0	0	0	93745	97793	102610
TRINITY_DN12696_c1_g1::TRINITY_DN12696_c1_g1_i6::g.15028::m.15028	0	0	0	0	0	0	0	0	0	0	0	0	89152	106890	97523
TRINITY_DN19683_c4_g2::TRINITY_DN19683_c4_g2_i1::g.121928::m.121928	0	0	0	0	0	0	0	0	0	0	0	0	0	153570	139310
TRINITY_DN16110_c4_g1::TRINITY_DN16110_c4_g1_i10::g.62321::m.6232	0	0	0	0	0	0	0	0	0	0	0	0	0	183610	108420
TRINITY_DN19277_c4_g1::TRINITY_DN19277_c4_g1_i8::g.115170::m.115170	0	0	0	0	0	0	0	0	0	0	0	0	0	178420	113410
TRINITY_DN16461_c2_g1::TRINITY_DN16461_c2_g1_i2::g.67850::m.67850	0	0	0	0	0	0	0	0	0	0	291760	0	0	0	0

TRINITY_DN16319_c0_g3::TRINITY_DN16319_c0_g3_i4::g.65613::m.65613	0	0	0	0	0	0	0	0	0	0	0	0	92491	112390	86759
TRINITY_DN14881_c0_g1::TRINITY_DN14881_c0_g1_i4::g.43780::m.43780	0	0	0	0	0	0	0	0	0	0	0	0	0	105130	185800
TRINITY_DN13265_c1_g2::TRINITY_DN13265_c1_g2_i3::g.21681::m.21681	0	0	0	0	0	0	0	0	0	0	0	0	0	0	290360
TRINITY_DN14049_c0_g1::TRINITY_DN14049_c0_g1_i2::g.31233::m.31233	0	0	0	0	0	0	0	0	0	0	0	0	0	0	290000
TRINITY_DN13408_c0_g1::TRINITY_DN13408_c0_g1_i4::g.23389::m.23389	0	0	0	0	0	0	0	0	0	0	0	0	0	0	289480
TRINITY_DN19519_c0_g1::TRINITY_DN19519_c0_g1_i10::g.119145::m.119145	0	0	0	0	0	0	0	0	0	0	0	0	76209	107620	105310
TRINITY_DN16032_c0_g1::TRINITY_DN16032_c0_g1_i1::g.60895::m.60895	0	0	0	0	0	0	0	0	0	0	0	0	0	0	288690
TRINITY_DN18985_c1_g6::TRINITY_DN18985_c1_g6_i3::g.109896::m.109896	0	0	0	0	0	0	0	0	0	0	0	0	0	0	288130
TRINITY_DN12405_c0_g1::TRINITY_DN12405_c0_g1_i1::g.12602::m.12602	0	0	0	0	0	0	0	0	0	0	0	0	0	287660	0
TRINITY_DN19613_c2_g2::TRINITY_DN19613_c2_g2_i3::g.120856::m.120856	24322	0	0	0	0	0	0	0	0	0	0	0	0	0	263120
TRINITY_DN17036_c2_g3::TRINITY_DN17036_c2_g3_i3::g.77409::m.77409	0	0	0	0	0	0	0	0	0	0	0	0	123550	163560	0
TRINITY_DN18208_c0_g1::TRINITY_DN18208_c0_g1_i5::g.96946::m.96946	0	0	0	0	0	0	0	0	0	0	0	0	129360	157370	0
TRINITY_DN18601_c0_g1::TRINITY_DN18601_c0_g1_i1::g.101595::m.1015	0	0	0	108390	0	0	0	0	0	0	0	0	0	0	178040
TRINITY_DN19149_c0_g1::TRINITY_DN19149_c0_g1_i16::g.113757::m.113757	0	0	0	0	0	0	0	0	0	0	0	0	114940	0	171070
TRINITY_DN18875_c2_g5::TRINITY_DN18875_c2_g5_i2::g.107944::m.107944	0	0	0	0	0	0	0	0	0	0	0	0	124110	0	159490
TRINITY_DN16082_c0_g1::TRINITY_DN16082_c0_g1_i2::g.61608::m.61608	0	0	0	0	0	0	0	0	0	0	0	0	0	130310	153230
TRINITY_DN11445_c0_g1::TRINITY_DN11445_c0_g1_i3::g.7277::m.7277	0	0	0	0	0	0	0	0	0	0	0	0	283370	0	0
TRINITY_DN13851_c1_g2::TRINITY_DN13851_c1_g2_i2::g.29292::m.29292	119660	0	0	162890	0	0	0	0	0	0	0	0	0	0	0
TRINITY_DN15110_c1_g1::TRINITY_DN15110_c1_g1_i4::g.47001::m.47001	30442	0	0	0	116200	0	135320	0	0	0	0	0	0	0	0
TRINITY_DN15121_c0_g1::TRINITY_DN15121_c0_g1_i1::g.47196::m.47196	0	0	0	0	0	0	0	0	0	0	0	0	98876	180630	0

TRINITY_DN13420_c0_g1::TRINITY_DN13420_c0_g1_i1::g.23499::m.23499	0	0	0	0	0	0	0	0	0	0	0	0	0	155280	123510
TRINITY_DN18855_c1_g1::TRINITY_DN18855_c1_g1_i51::g.107677::m.107677	0	0	0	0	0	0	0	0	0	0	0	0	0	143810	131510
TRINITY_DN16845_c1_g1::TRINITY_DN16845_c1_g1_i1::g.74565::m.74565	0	0	30059	32474	0	0	0	0	0	0	0	0	82159	0	130490
TRINITY_DN11922_c0_g1::TRINITY_DN11922_c0_g1_i1::g.9511::m.9511	0	0	0	0	0	0	0	0	0	0	0	0	88630	93819	92080
TRINITY_DN13521_c0_g1::TRINITY_DN13521_c0_g1_i1::g.24833::m.24833	0	0	0	0	0	0	0	0	0	0	0	0	0	0	273020
TRINITY_DN9608_c0_g1::TRINITY_DN9608_c0_g1_i2::g.3681::m.3681	0	0	0	0	0	0	0	0	0	0	0	0	124010	0	146960
TRINITY_DN10819_c0_g1::TRINITY_DN10819_c0_g1_i3::g.5615::m.5615	0	0	0	0	0	0	0	0	0	0	0	0	80741	93555	94420
TRINITY_DN16090_c0_g1::TRINITY_DN16090_c0_g1_i3::g.61630::m.61630	0	0	0	0	0	0	0	67964	0	0	0	0	0	0	200630
TRINITY_DN18139_c1_g1::TRINITY_DN18139_c1_g1_i7::g.95745::m.95745	18909	0	21475	38069	0	0	0	0	0	0	0	0	0	189900	0
TRINITY_DN16973_c0_g1::TRINITY_DN16973_c0_g1_i3::g.76496::m.76496	0	0	0	0	0	0	0	0	0	0	0	0	0	267770	0
TRINITY_DN19343_c7_g1::TRINITY_DN19343_c7_g1_i3::g.116415::m.116415	0	0	113630	152520	0	0	0	0	0	0	0	0	0	0	0
TRINITY_DN19191_c0_g2::TRINITY_DN19191_c0_g2_i4::g.113786::m.113786	0	0	0	0	0	0	0	0	0	0	0	265800	0	0	0
TRINITY_DN14461_c1_g4::TRINITY_DN14461_c1_g4_i3::g.37527::m.37527	0	0	0	0	0	0	0	0	0	0	0	0	0	0	265680
TRINITY_DN17541_c1_g1::TRINITY_DN17541_c1_g1_i11::g.86034::m.8603	0	0	0	0	0	0	0	0	0	0	0	0	78380	104160	82880
TRINITY_DN17922_c0_g1::TRINITY_DN17922_c0_g1_i13::g.92083::m.92083	0	173330	0	0	0	0	0	0	0	0	0	0	0	91948	0
TRINITY_DN14757_c1_g1::TRINITY_DN14757_c1_g1_i7::g.41725::m.41725	0	0	0	0	0	0	0	0	0	0	0	0	79600	92185	91804
TRINITY_DN19529_c1_g2::TRINITY_DN19529_c1_g2_i5::g.119243::m.119243	0	0	0	0	0	0	0	0	0	0	0	0	0	263460	0
TRINITY_DN19966_c14_g1::TRINITY_DN19966_c14_g1_i3::g.126866::m.126866	0	0	0	0	0	0	0	0	0	0	0	0	88753	93139	81149
TRINITY_DN15823_c4_g1::TRINITY_DN15823_c4_g1_i3::g.57508::m.57508	0	0	0	0	0	0	0	0	0	0	0	0	143310	119350	0
TRINITY_DN15546_c0_g1::TRINITY_DN15546_c0_g1_i6::g.53570::m.53570	0	0	0	0	0	0	0	0	0	0	0	0	127860	134530	0
TRINITY_DN13076_c2_g1::TRINITY_DN13076_c2_g1_i6::g.19526::m.19526	0	0	0	0	0	0	0	0	0	0	0	0	124730	0	136700

TRINITY_DN19485_c0_g1::TRINITY_DN19485_c0_g1_i9::g.118686::m.118686	0	0	0	0	0	0	0	0	0	0	0	0	0	259950	0
TRINITY_DN13021_c1_g1::TRINITY_DN13021_c1_g1_i1::g.18775::m.18775	0	0	0	0	0	0	0	0	259920	0	0	0	0	0	0
TRINITY_DN12714_c1_g2::TRINITY_DN12714_c1_g2_i1::g.15270::m.15270	0	0	0	0	0	0	0	0	0	0	0	0	259790	0	0
TRINITY_DN19212_c0_g1::TRINITY_DN19212_c0_g1_i8::g.114108::m.1141	0	0	0	0	0	0	0	0	0	0	0	0	0	137010	121550
TRINITY_DN13032_c1_g1::TRINITY_DN13032_c1_g1_i5::g.18813::m.18813	0	0	0	0	0	0	0	0	0	0	0	147300	0	111100	0
TRINITY_DN13241_c2_g1::TRINITY_DN13241_c2_g1_i4::g.21412::m.21412	0	0	0	0	0	0	0	0	0	0	0	0	0	258340	0
TRINITY_DN19075_c1_g2::TRINITY_DN19075_c1_g2_i3::g.111314::m.111314	0	0	0	0	0	0	0	0	0	0	0	0	103410	154390	0
TRINITY_DN19379_c0_g1::TRINITY_DN19379_c0_g1_i4::g.116795::m.116795	0	0	0	0	0	0	0	0	0	0	0	0	103700	154000	0
TRINITY_DN15691_c0_g1::TRINITY_DN15691_c0_g1_i9::g.55585::m.55585	0	0	0	0	134330	0	0	122990	0	0	0	0	0	0	0
TRINITY_DN10326_c0_g1::TRINITY_DN10326_c0_g1_i1::g.4634::m.4634	0	0	0	0	0	0	0	0	0	0	0	0	257160	0	0
TRINITY_DN12047_c0_g1::TRINITY_DN12047_c0_g1_i4::g.10037::m.10037	0	0	0	0	0	0	0	0	0	0	0	0	0	0	256920
TRINITY_DN18148_c1_g6::TRINITY_DN18148_c1_g6_i2::g.95979::m.95979	74754	28266	86225	67533	0	0	0	0	0	0	0	0	0	0	0
TRINITY_DN17159_c3_g1::TRINITY_DN17159_c3_g1_i2::g.79474::m.79474	0	0	0	0	0	0	0	0	0	0	0	0	81828	84743	90104
TRINITY_DN10845_c0_g1::TRINITY_DN10845_c0_g1_i1::g.5649::m.5649	0	0	0	0	0	0	0	0	0	0	0	0	124300	0	131750
TRINITY_DN15929_c0_g1::TRINITY_DN15929_c0_g1_i6::g.59159::m.59159	0	0	0	0	0	0	0	0	0	0	0	0	0	104350	151550
TRINITY_DN11125_c0_g1::TRINITY_DN11125_c0_g1_i2::g.6392::m.6392	0	0	0	0	0	0	0	0	0	0	0	0	255680	0	0
TRINITY_DN19874_c1_g1::TRINITY_DN19874_c1_g1_i2::g.124818::m.124818	0	0	0	0	0	0	0	0	0	0	0	0	0	254800	0
TRINITY_DN16609_c0_g4::TRINITY_DN16609_c0_g4_i3::g.70404::m.70404	0	0	0	0	0	0	0	150880	0	0	0	0	101070	0	0
TRINITY_DN19302_c0_g2::TRINITY_DN19302_c0_g2_i5::g.115749::m.115749	0	0	0	0	0	0	0	0	0	0	0	0	79004	92549	78992
TRINITY_DN18657_c3_g2::TRINITY_DN18657_c3_g2_i5::g.104104::m.104104	0	0	0	0	0	0	0	0	0	0	0	0	77461	90784	80537
TRINITY_DN18365_c0_g2::TRINITY_DN18365_c0_g2_i2::g.99254::m.99254	0	18199	53077	93783	0	0	0	0	0	0	0	0	0	82403	0
TRINITY_DN14704_c1_g3::TRINITY_DN14704_c1_g3_i1::g.40903::m.40903	120760	0	101980	23868	0	0	0	0	0	0	0	0	0	0	0

TRINITY_DN13379_c2_g1::TRINITY_DN13379_c2_g1_i3::g.22962::m.22962	0	0	0	0	0	0	0	0	0	0	0	0	0	118520	127480
TRINITY_DN16128_c0_g1::TRINITY_DN16128_c0_g1_i5::g.62674::m.62674	0	0	0	0	0	0	0	0	0	0	0	0	0	245380	0
TRINITY_DN15685_c2_g1::TRINITY_DN15685_c2_g1_i7::g.55504::m.55504	0	0	0	0	0	0	0	0	0	0	0	0	245210	0	0
TRINITY_DN14830_c5_g1::TRINITY_DN14830_c5_g1_i9::g.42943::m.42943	0	0	0	0	0	0	0	0	0	0	0	0	50989	70414	123750
TRINITY_DN11391_c0_g1::TRINITY_DN11391_c0_g1_i3::g.7245::m.7245	0	0	0	0	0	0	0	0	0	0	0	0	116720	126910	0
TRINITY_DN17340_c1_g1::TRINITY_DN17340_c1_g1_i17::g.82513::m.8251	0	0	0	0	0	0	0	0	0	0	0	0	118810	0	123890
TRINITY_DN15433_c2_g1::TRINITY_DN15433_c2_g1_i2::g.51849::m.51849	0	0	0	0	0	0	0	0	0	0	0	0	0	0	242450
TRINITY_DN19143_c2_g1::TRINITY_DN19143_c2_g1_i5::g.112885::m.112885	0	0	0	0	0	0	169610	0	0	0	0	0	0	0	72498
TRINITY_DN15141_c2_g2::TRINITY_DN15141_c2_g2_i7::g.47512::m.47512	0	0	0	0	0	0	0	0	0	0	0	0	0	0	241830
TRINITY_DN6604_c0_g1::TRINITY_DN6604_c0_g1_i1::g.1696::m.1696	0	0	0	0	0	0	0	0	240500	0	0	0	0	0	0
TRINITY_DN14356_c5_g1::TRINITY_DN14356_c5_g1_i2::g.35855::m.35855	0	0	66046	0	0	0	174030	0	0	0	0	0	0	0	0
TRINITY_DN17814_c1_g1::TRINITY_DN17814_c1_g1_i9::g.90451::m.90451	0	0	0	0	0	0	0	0	0	0	0	0	0	0	239870
TRINITY_DN7788_c0_g1::TRINITY_DN7788_c0_g1_i1::g.2350::m.2350	0	0	0	0	0	0	0	0	0	0	0	0	0	126890	112360
TRINITY_DN16305_c1_g1::TRINITY_DN16305_c1_g1_i3::g.65515::m.65515	0	0	0	0	0	0	0	0	0	0	0	0	0	0	238810
TRINITY_DN14505_c2_g1::TRINITY_DN14505_c2_g1_i2::g.38114::m.38114	0	0	0	0	0	0	0	0	0	0	0	0	0	119200	119440
TRINITY_DN16442_c0_g2::TRINITY_DN16442_c0_g2_i5::g.67615::m.67615	0	0	0	42894	0	0	0	0	0	0	195690	0	0	0	0
TRINITY_DN9437_c0_g1::TRINITY_DN9437_c0_g1_i1::g.3480::m.3480	0	0	0	0	143600	0	0	0	0	94249	0	0	0	0	0
TRINITY_DN10468_c0_g2::TRINITY_DN10468_c0_g2_i1::g.4854::m.4854	0	0	0	0	0	0	0	0	0	0	0	0	0	109250	128450
TRINITY_DN19289_c1_g2::TRINITY_DN19289_c1_g2_i6::g.115366::m.115366	0	0	0	0	0	0	0	0	0	0	0	0	128180	106560	0
TRINITY_DN570_c0_g1::TRINITY_DN570_c0_g1_i1::g.152::m.152	0	0	0	0	0	0	0	0	0	0	0	0	104050	0	130620
TRINITY_DN16387_c0_g7::TRINITY_DN16387_c0_g7_i3::g.66774::m.66774	0	0	0	0	0	0	0	0	0	0	0	0	0	116370	117070
TRINITY_DN19149_c0_g1::TRINITY_DN19149_c0_g1_i14::g.113756::m.113756	0	0	0	0	0	0	0	0	0	0	0	0	115930	116900	0

TRINITY_DN18020_c2_g2::TRINITY_DN18020_c2_g2_i4::g.93764::m.93764	0	0	0	0	0	0	0	0	0	0	0	0	232550	0	0
TRINITY_DN16978_c1_g3::TRINITY_DN16978_c1_g3_i2::g.76542::m.76542	139540	0	0	0	0	0	0	0	0	0	0	92972	0	0	0
TRINITY_DN17213_c1_g1::TRINITY_DN17213_c1_g1_i3::g.80196::m.80196	0	0	0	0	0	0	0	0	0	0	0	0	149410	0	81738
TRINITY_DN19992_c4_g1::TRINITY_DN19992_c4_g1_i5::g.127460::m.127460	0	0	0	0	0	0	0	0	0	0	0	0	102120	128880	0
TRINITY_DN13415_c1_g3::TRINITY_DN13415_c1_g3_i1::g.23602::m.23602	0	0	0	0	0	0	0	0	0	0	0	0	230600	0	0
TRINITY_DN12348_c0_g1::TRINITY_DN12348_c0_g1_i1::g.11710::m.11710	0	0	0	0	0	0	0	0	0	0	0	0	105110	124990	0
TRINITY_DN15405_c3_g1::TRINITY_DN15405_c3_g1_i4::g.51552::m.51552	0	0	0	0	0	0	0	0	0	0	0	0	0	226410	0
TRINITY_DN9822_c0_g1::TRINITY_DN9822_c0_g1_i1::g.3931::m.3931	39714	0	45161	44520	0	0	97018	0	0	0	0	0	0	0	0
TRINITY_DN14833_c0_g2::TRINITY_DN14833_c0_g2_i2::g.42977::m.42977	0	0	122480	0	0	0	0	0	0	0	0	0	0	0	103720
TRINITY_DN15454_c2_g1::TRINITY_DN15454_c2_g1_i4::g.52157::m.52157	0	0	0	0	0	0	225440	0	0	0	0	0	0	0	0
TRINITY_DN14480_c1_g1::TRINITY_DN14480_c1_g1_i3::g.37689::m.37689	0	0	0	0	0	0	0	0	0	0	0	0	0	0	225320
TRINITY_DN18127_c0_g1::TRINITY_DN18127_c0_g1_i1::g.95817::m.95817	0	0	0	0	0	0	0	0	0	0	0	0	45429	71349	108490
TRINITY_DN16613_c0_g1::TRINITY_DN16613_c0_g1_i5::g.70442::m.70442	0	0	0	0	0	0	0	0	0	0	0	0	131500	90390	0
TRINITY_DN17792_c0_g7::TRINITY_DN17792_c0_g7_i6::g.89759::m.89759	65324	0	69736	86082	0	0	0	0	0	0	0	0	0	0	0
TRINITY_DN19032_c3_g1::TRINITY_DN19032_c3_g1_i17::g.110826::m.110826	0	0	0	0	0	0	0	0	0	0	0	0	115380	0	104980
TRINITY_DN3292_c0_g1::TRINITY_DN3292_c0_g1_i2::g.890::m.890	0	0	0	0	0	0	0	0	0	0	0	0	0	219780	0
TRINITY_DN13487_c0_g1::TRINITY_DN13487_c0_g1_i1::g.24417::m.24417	0	0	0	0	0	0	0	0	0	0	0	0	0	134160	84644
TRINITY_DN16110_c4_g1::TRINITY_DN16110_c4_g1_i11::g.62324::m.6232	0	0	0	0	0	0	0	0	0	0	0	0	95109	122810	0
TRINITY_DN12761_c4_g2::TRINITY_DN12761_c4_g2_i1::g.15691::m.15691	0	0	0	0	0	0	0	0	0	0	0	0	0	0	217820
TRINITY_DN11277_c0_g1::TRINITY_DN11277_c0_g1_i2::g.6842::m.6842	0	0	0	0	0	0	0	0	0	0	0	0	0	124680	92794
TRINITY_DN14078_c2_g2::TRINITY_DN14078_c2_g2_i1::g.32075::m.32075	0	0	0	0	0	0	0	0	0	0	0	0	65385	88595	62180
TRINITY_DN18707_c1_g1::TRINITY_DN18707_c1_g1_i8::g.104943::m.104943	0	0	0	0	0	0	0	0	0	0	0	0	0	0	215470

TRINITY_DN14427_c0_g1::TRINITY_DN14427_c0_g1_i8::g.36957::m.36957	0	0	0	0	0	0	0	0	0	0	0	0	0	215380	0
TRINITY_DN14274_c2_g1::TRINITY_DN14274_c2_g1_i1::g.34705::m.34705	0	0	0	0	0	0	0	0	0	0	0	0	0	119920	94289
TRINITY_DN14629_c0_g3::TRINITY_DN14629_c0_g3_i2::g.40344::m.40344	0	0	0	0	0	0	0	0	0	0	0	0	101030	0	112800
TRINITY_DN17321_c2_g1::TRINITY_DN17321_c2_g1_i8::g.82045::m.82045	0	0	0	0	0	0	0	0	0	0	0	0	0	77031	136680
TRINITY_DN19385_c2_g1::TRINITY_DN19385_c2_g1_i5::g.117068::m.117068	81061	0	65734	66450	0	0	0	0	0	0	0	0	0	0	0
TRINITY_DN12251_c0_g1::TRINITY_DN12251_c0_g1_i7::g.11382::m.11382	0	0	0	0	0	0	0	0	0	0	0	0	0	212500	0
TRINITY_DN18091_c1_g1::TRINITY_DN18091_c1_g1_i6::g.94898::m.94898	0	29526	0	76711	0	0	0	0	0	0	0	0	0	0	106260
TRINITY_DN18990_c0_g1::TRINITY_DN18990_c0_g1_i1::g.110013::m.110013	0	0	0	0	0	0	0	0	0	0	0	0	0	110010	102070
TRINITY_DN19413_c0_g2::TRINITY_DN19413_c0_g2_i1::g.117446::m.117446	0	0	0	0	0	0	0	0	0	0	0	0	0	96541	114920
TRINITY_DN18349_c4_g1::TRINITY_DN18349_c4_g1_i3::g.99227::m.99227	0	0	0	0	0	0	0	0	0	0	0	0	93052	116410	0
TRINITY_DN14718_c0_g1::TRINITY_DN14718_c0_g1_i3::g.41289::m.41289	0	0	0	0	0	0	0	0	0	0	0	0	0	0	209310
TRINITY_DN18771_c1_g1::TRINITY_DN18771_c1_g1_i3::g.106137::m.1061	0	0	0	0	0	0	0	0	0	0	0	0	111670	0	97603
TRINITY_DN10875_c0_g1::TRINITY_DN10875_c0_g1_i3::g.5717::m.5717	0	0	0	0	0	0	0	0	0	0	0	0	0	104210	104460
TRINITY_DN10668_c0_g1::TRINITY_DN10668_c0_g1_i3::g.5273::m.5273	0	0	0	0	0	0	0	0	0	0	0	0	0	80446	127830
TRINITY_DN16826_c3_g1::TRINITY_DN16826_c3_g1_i6::g.74102::m.74102	0	0	0	0	0	0	0	0	0	0	0	0	0	165010	42597
TRINITY_DN19356_c0_g4::TRINITY_DN19356_c0_g4_i8::g.116588::m.116588	0	0	0	0	0	0	0	0	0	0	0	0	0	0	207440
TRINITY_DN19935_c0_g1::TRINITY_DN19935_c0_g1_i18::g.126411::m.126411	0	0	0	0	0	0	0	0	0	0	0	0	85789	121230	0
TRINITY_DN15733_c2_g3::TRINITY_DN15733_c2_g3_i4::g.56425::m.56425	0	0	0	0	0	0	0	0	0	0	0	0	78968	0	128030
TRINITY_DN11377_c0_g1::TRINITY_DN11377_c0_g1_i3::g.7158::m.7158	0	0	0	0	0	0	0	0	0	0	0	205790	0	0	0
TRINITY_DN13397_c1_g1::TRINITY_DN13397_c1_g1_i6::g.22118::m.22118	0	0	0	0	0	0	0	0	0	0	0	0	81479	123640	0
TRINITY_DN18687_c0_g1::TRINITY_DN18687_c0_g1_i17::g.103218::m.103218	0	0	0	0	0	0	0	0	0	0	0	0	102800	0	102100

TRINITY_DN15145_c0_g1::TRINITY_DN15145_c0_g1_i10::g.46819::m.4681	0	0	0	0	0	0	0	0	0	0	0	0	92698	0	110720
TRINITY_DN15345_c2_g1::TRINITY_DN15345_c2_g1_i10::g.49844::m.49844	0	0	98237	0	0	0	0	0	0	0	0	0	0	104960	0
TRINITY_DN17431_c5_g1::TRINITY_DN17431_c5_g1_i7::g.83828::m.83828	0	0	0	0	0	0	0	0	0	0	0	0	202980	0	0
TRINITY_DN16550_c0_g1::TRINITY_DN16550_c0_g1_i1::g.69273::m.69273	0	0	0	0	0	0	0	0	0	0	0	0	202890	0	0
TRINITY_DN15895_c4_g2::TRINITY_DN15895_c4_g2_i3::g.57730::m.57730	0	0	0	0	0	0	0	0	0	0	0	0	0	202520	0
TRINITY_DN12618_c1_g1::TRINITY_DN12618_c1_g1_i9::g.14449::m.14449	0	0	0	0	0	0	0	0	0	0	0	0	83741	0	116550
TRINITY_DN19462_c2_g2::TRINITY_DN19462_c2_g2_i5::g.118383::m.118383	0	0	0	0	0	0	0	0	0	0	0	0	0	0	199160
TRINITY_DN16453_c1_g1::TRINITY_DN16453_c1_g1_i1::g.67791::m.67791	0	0	0	0	0	0	0	0	0	0	0	0	61358	79943	57053
TRINITY_DN14591_c5_g1::TRINITY_DN14591_c5_g1_i3::g.39221::m.39221	0	0	0	0	0	0	0	0	0	0	0	0	196950	0	0
TRINITY_DN16615_c1_g3::TRINITY_DN16615_c1_g3_i6::g.70426::m.70426	0	0	0	0	0	0	0	0	0	0	0	0	119140	0	77225
TRINITY_DN18754_c0_g1::TRINITY_DN18754_c0_g1_i2::g.106002::m.106002	0	0	0	0	0	0	0	0	0	0	0	0	196260	0	0
TRINITY_DN17004_c0_g1::TRINITY_DN17004_c0_g1_i2::g.76878::m.76878	0	0	0	0	0	0	0	0	0	0	0	0	99291	0	96018
TRINITY_DN15390_c3_g1::TRINITY_DN15390_c3_g1_i2::g.51061::m.51061	0	0	0	0	0	0	0	0	0	0	0	0	0	0	193930
TRINITY_DN18311_c1_g1::TRINITY_DN18311_c1_g1_i2::g.98629::m.98629	0	0	0	0	0	0	0	0	0	0	0	0	0	98587	94946
TRINITY_DN17353_c2_g2::TRINITY_DN17353_c2_g2_i2::g.82341::m.82341	0	0	78672	0	0	0	0	0	0	0	0	0	114260	0	0
TRINITY_DN10747_c0_g1::TRINITY_DN10747_c0_g1_i1::g.5399::m.5399	0	0	0	0	0	0	0	0	0	0	0	192450	0	0	0
TRINITY_DN12384_c0_g1::TRINITY_DN12384_c0_g1_i2::g.12333::m.12333	0	0	0	0	0	0	0	0	0	0	0	0	0	0	189360
TRINITY_DN16358_c2_g1::TRINITY_DN16358_c2_g1_i5::g.66392::m.66392	0	0	0	0	0	0	0	0	0	0	0	0	0	0	188480
TRINITY_DN19240_c1_g2::TRINITY_DN19240_c1_g2_i4::g.114388::m.114388	0	0	0	0	0	0	0	0	0	0	0	0	99294	0	88736
TRINITY_DN17731_c6_g1::TRINITY_DN17731_c6_g1_i4::g.89011::m.89011	0	0	0	0	0	0	0	0	0	0	0	0	0	73839	114170
TRINITY_DN14931_c0_g1::TRINITY_DN14931_c0_g1_i6::g.44458::m.44458	0	0	0	0	0	0	0	0	0	0	0	0	0	187280	0
TRINITY_DN19471_c0_g1::TRINITY_DN19471_c0_g1_i4::g.118445::m.118445	0	0	0	0	0	0	0	0	0	0	0	0	0	0	187260

TRINITY_DN17953_c1_g8::TRINITY_DN17953_c1_g8_i1::g.91726::m.91726	0	0	0	0	0	0	0	0	0	0	0	0	75054	0	110930
TRINITY_DN14840_c3_g1::TRINITY_DN14840_c3_g1_i19::g.43227::m.43227	0	0	0	0	0	0	0	0	0	0	0	0	185660	0	0
TRINITY_DN16802_c5_g3::TRINITY_DN16802_c5_g3_i3::g.73661::m.73661	0	0	0	0	0	0	0	0	0	0	0	0	91959	0	93553
TRINITY_DN16580_c0_g3::TRINITY_DN16580_c0_g3_i6::g.69917::m.69917	0	0	0	0	0	0	0	0	0	0	0	0	93082	0	91161
TRINITY_DN13912_c2_g1::TRINITY_DN13912_c2_g1_i3::g.30407::m.30407	0	0	0	0	0	0	0	0	0	0	0	183980	0	0	0
TRINITY_DN19207_c4_g2::TRINITY_DN19207_c4_g2_i3::g.114212::m.114212	0	0	0	0	0	0	0	0	0	0	0	0	0	87792	96138
TRINITY_DN16740_c1_g1::TRINITY_DN16740_c1_g1_i2::g.72692::m.72692	0	0	0	0	0	0	0	0	0	0	183840	0	0	0	0
TRINITY_DN15744_c2_g4::TRINITY_DN15744_c2_g4_i2::g.56499::m.56499	0	0	0	0	0	0	0	0	0	0	0	0	0	183700	0
TRINITY_DN16211_c0_g2::TRINITY_DN16211_c0_g2_i6::g.63944::m.63944	0	0	85366	0	0	0	0	97624	0	0	0	0	0	0	0
TRINITY_DN13792_c0_g1::TRINITY_DN13792_c0_g1_i4::g.28291::m.28291	0	0	0	0	0	0	0	0	0	0	0	0	0	182840	0
TRINITY_DN14860_c0_g1::TRINITY_DN14860_c0_g1_i1::g.43455::m.43455	0	0	0	0	0	0	0	0	0	0	0	0	0	182800	0
TRINITY_DN12935_c1_g1::TRINITY_DN12935_c1_g1_i3::g.17528::m.17528	0	0	0	0	0	0	0	0	0	0	0	0	79579	102950	0
TRINITY_DN16126_c1_g1::TRINITY_DN16126_c1_g1_i14::g.62052::m.62052	0	0	0	0	0	0	0	0	0	0	0	0	0	0	181170
TRINITY_DN13480_c0_g1::TRINITY_DN13480_c0_g1_i3::g.24330::m.24330	0	0	0	0	126820	53805	0	0	0	0	0	0	0	0	0
TRINITY_DN16307_c2_g3::TRINITY_DN16307_c2_g3_i3::g.65541::m.65541	0	0	0	0	0	0	0	0	0	0	0	0	0	0	179110
TRINITY_DN17586_c3_g4::TRINITY_DN17586_c3_g4_i6::g.86457::m.86457	0	0	0	0	0	0	0	0	0	0	0	0	104950	0	73459
TRINITY_DN10182_c0_g1::TRINITY_DN10182_c0_g1_i1::g.4394::m.4394	0	0	0	0	89457	0	0	88910	0	0	0	0	0	0	0
TRINITY_DN16200_c0_g2::TRINITY_DN16200_c0_g2_i2::g.62796::m.62796	0	0	0	0	0	0	0	0	0	0	0	0	78976	0	98649
TRINITY_DN15100_c3_g1::TRINITY_DN15100_c3_g1_i6::g.45535::m.45535	0	0	0	0	0	0	0	0	0	0	0	0	177330	0	0
TRINITY_DN12087_c0_g1::TRINITY_DN12087_c0_g1_i3::g.10442::m.10442	0	0	0	0	0	0	0	0	0	0	0	0	0	0	177060

TRINITY_DN15513_c2_g1::TRINITY_DN15513_c2_g1_i6::g.53086::m.53086	0	0	0	0	0	0	0	0	0	0	0	0	54608	64191	57328
TRINITY_DN13233_c0_g4::TRINITY_DN13233_c0_g4_i3::g.21366::m.21366	0	101490	0	0	0	0	0	0	0	0	0	0	0	0	73602
TRINITY_DN17374_c2_g1::TRINITY_DN17374_c2_g1_i5::g.82712::m.82712	0	0	0	0	0	0	0	0	0	0	0	0	88941	83720	0
TRINITY_DN12068_c0_g1::TRINITY_DN12068_c0_g1_i4::g.10141::m.10141	0	0	0	0	0	0	0	0	0	0	0	171110	0	0	0
TRINITY_DN14979_c0_g1::TRINITY_DN14979_c0_g1_i9::g.45197::m.45197	0	0	0	0	0	0	0	0	0	0	0	0	170920	0	0
TRINITY_DN15527_c0_g1::TRINITY_DN15527_c0_g1_i4::g.53287::m.53287	0	0	0	0	0	0	0	0	0	0	0	0	76437	0	94133
TRINITY_DN15865_c2_g1::TRINITY_DN15865_c2_g1_i9::g.58071::m.58071	0	0	0	0	0	0	0	0	0	0	0	0	87402	0	82810
TRINITY_DN17882_c2_g1::TRINITY_DN17882_c2_g1_i14::g.91309::m.91309	0	0	0	0	0	0	0	0	0	0	0	0	0	0	169520
TRINITY_DN18694_c1_g1::TRINITY_DN18694_c1_g1_i5::g.104667::m.104667	0	0	0	0	0	0	0	0	0	0	0	0	0	86875	82359
TRINITY_DN17794_c1_g1::TRINITY_DN17794_c1_g1_i3::g.89692::m.89692	0	0	0	0	0	0	0	0	0	0	0	0	167910	0	0
TRINITY_DN15372_c1_g1::TRINITY_DN15372_c1_g1_i7::g.50862::m.50862	0	0	0	0	0	0	0	0	0	0	0	0	167350	0	0
TRINITY_DN13872_c3_g1::TRINITY_DN13872_c3_g1_i6::g.29683::m.29683	0	0	0	0	0	0	0	0	0	0	0	0	166530	0	0
TRINITY_DN14858_c0_g5::TRINITY_DN14858_c0_g5_i1::g.43408::m.43408	0	0	0	0	0	0	0	0	0	0	0	0	165770	0	0
TRINITY_DN11799_c0_g1::TRINITY_DN11799_c0_g1_i1::g.8868::m.8868	0	0	0	0	0	0	0	0	0	0	0	0	71110	0	94533
TRINITY_DN17436_c0_g1::TRINITY_DN17436_c0_g1_i7::g.83790::m.83790	0	0	0	0	0	0	0	0	0	0	0	0	57850	107340	0
TRINITY_DN15518_c0_g2::TRINITY_DN15518_c0_g2_i2::g.53174::m.53174	0	0	0	0	0	0	0	0	0	0	0	0	96545	0	67961
TRINITY_DN19390_c1_g1::TRINITY_DN19390_c1_g1_i8::g.117167::m.1171	0	0	0	0	0	0	0	0	0	0	0	0	80712	82892	0
TRINITY_DN12101_c0_g1::TRINITY_DN12101_c0_g1_i1::g.9937::m.9937	0	0	0	0	0	0	0	0	0	0	0	0	162270	0	0
TRINITY_DN11289_c0_g1::TRINITY_DN11289_c0_g1_i1::g.6862::m.6862	0	0	55069	107140	0	0	0	0	0	0	0	0	0	0	0
TRINITY_DN10610_c0_g1::TRINITY_DN10610_c0_g1_i2::g.5110::m.5110	0	0	0	0	0	0	0	0	0	0	0	0	0	160050	0
TRINITY_DN17673_c0_g1::TRINITY_DN17673_c0_g1_i8::g.88029::m.88029	0	0	0	0	0	0	0	0	0	0	0	0	0	158260	0

TRINITY_DN17467_c0_g2::TRINITY_DN17467_c0_g2_i8::g.84133::m.84133	0	0	0	0	0	0	0	0	0	0	0	0	0	157910	0
TRINITY_DN18000_c3_g4::TRINITY_DN18000_c3_g4_i1::g.93345::m.93345	0	0	0	0	0	0	0	0	0	0	0	0	76319	0	80673
TRINITY_DN13706_c2_g1::TRINITY_DN13706_c2_g1_i3::g.27413::m.27413	0	0	0	0	0	0	0	0	0	0	0	0	0	156030	0
TRINITY_DN12707_c0_g1::TRINITY_DN12707_c0_g1_i3::g.15086::m.15086	30312	0	57991	66001	0	0	0	0	0	0	0	0	0	0	0
TRINITY_DN18227_c0_g1::TRINITY_DN18227_c0_g1_i4::g.97154::m.97154	0	0	0	0	0	0	0	0	0	0	0	0	0	154000	0
TRINITY_DN17401_c2_g3::TRINITY_DN17401_c2_g3_i2::g.83107::m.83107	0	0	0	0	0	0	0	0	0	0	0	0	64490	0	89389
TRINITY_DN11447_c0_g1::TRINITY_DN11447_c0_g1_i1::g.7417::m.7417	96348	0	56786	0	0	0	0	0	0	0	0	0	0	0	0
TRINITY_DN18966_c3_g3::TRINITY_DN18966_c3_g3_i5::g.109514::m.109514	0	0	0	0	0	0	0	0	0	0	0	0	0	151700	0
TRINITY_DN18595_c0_g1::TRINITY_DN18595_c0_g1_i7::g.103121::m.103121	0	0	0	0	0	0	0	0	0	0	0	0	149250	0	0
TRINITY_DN11480_c0_g1::TRINITY_DN11480_c0_g1_i7::g.7531::m.7531	0	47952	0	0	0	0	0	0	0	98633	0	0	0	0	0
TRINITY_DN19085_c0_g1::TRINITY_DN19085_c0_g1_i22::g.111744::m.111744	0	0	0	0	0	0	0	0	0	0	0	0	0	146570	0
TRINITY_DN14786_c1_g2::TRINITY_DN14786_c1_g2_i1::g.42235::m.42235	0	0	0	0	0	0	0	0	0	0	0	0	0	0	146100
TRINITY_DN1011_c0_g1::TRINITY_DN1011_c0_g1_i1::g.255::m.255	0	0	0	0	0	0	0	0	145650	0	0	0	0	0	0
TRINITY_DN16766_c2_g2::TRINITY_DN16766_c2_g2_i3::g.73070::m.73070	0	0	0	0	0	0	0	0	0	0	0	0	0	145460	0
TRINITY_DN17471_c1_g1::TRINITY_DN17471_c1_g1_i8::g.84225::m.84225	0	0	0	0	0	0	0	0	0	0	0	0	0	145410	0
TRINITY_DN14557_c1_g1::TRINITY_DN14557_c1_g1_i7::g.38742::m.38742	0	0	0	0	0	0	0	0	0	0	0	0	0	0	144540
TRINITY_DN12733_c0_g1::TRINITY_DN12733_c0_g1_i1::g.15369::m.15369	0	0	0	0	0	0	0	0	0	0	0	0	144030	0	0
TRINITY_DN17602_c0_g2::TRINITY_DN17602_c0_g2_i1::g.86960::m.86960	0	0	0	0	0	0	0	0	82635	0	0	0	0	0	60597
TRINITY_DN13377_c3_g1::TRINITY_DN13377_c3_g1_i6::g.23084::m.23084	0	0	0	0	0	0	0	0	0	0	0	0	67684	74398	0
TRINITY_DN14518_c1_g2::TRINITY_DN14518_c1_g2_i4::g.38282::m.38282	0	0	0	0	0	0	0	0	0	0	0	0	0	139090	0

TRINITY_DN18437_c1_g1::TRINITY_DN18437_c1_g1_i4::g.100574::m.100574	0	0	0	0	0	0	0	0	0	0	0	0	71364	0	67529
TRINITY_DN15963_c1_g1::TRINITY_DN15963_c1_g1_i1::g.59605::m.59605	0	0	0	0	0	0	0	0	0	0	0	0	0	137280	0
TRINITY_DN17490_c0_g3::TRINITY_DN17490_c0_g3_i5::g.84477::m.84477	0	0	0	0	0	0	0	0	0	0	0	0	137090	0	0
TRINITY_DN19045_c3_g1::TRINITY_DN19045_c3_g1_i11::g.111100::m.111100	0	0	0	0	0	0	0	0	0	0	0	0	136860	0	0
TRINITY_DN14763_c1_g1::TRINITY_DN14763_c1_g1_i5::g.41937::m.41937	0	0	0	0	0	0	0	0	0	0	0	0	0	136110	0
TRINITY_DN18733_c1_g5::TRINITY_DN18733_c1_g5_i2::g.105940::m.105940	0	0	0	0	0	0	0	0	0	0	0	0	0	135000	0
TRINITY_DN11884_c0_g1::TRINITY_DN11884_c0_g1_i2::g.9315::m.9315	0	0	0	0	0	0	0	0	0	0	0	0	0	0	134800
TRINITY_DN14042_c0_g1::TRINITY_DN14042_c0_g1_i1::g.31618::m.31618	0	0	0	0	0	0	0	0	0	0	0	0	63398	0	70439
TRINITY_DN19087_c0_g3::TRINITY_DN19087_c0_g3_i1::g.111646::m.111646	0	0	0	0	0	0	0	0	0	0	0	0	0	73689	60065
TRINITY_DN15486_c0_g3::TRINITY_DN15486_c0_g3_i1::g.52746::m.52746	0	0	0	0	0	0	0	0	0	0	0	0	132920	0	0
TRINITY_DN16817_c0_g1::TRINITY_DN16817_c0_g1_i3::g.73774::m.73774	0	0	0	0	0	0	0	0	0	0	0	0	0	132780	0
TRINITY_DN17918_c1_g1::TRINITY_DN17918_c1_g1_i4::g.91866::m.91866	0	0	0	0	0	0	0	0	0	0	0	0	0	0	130940
TRINITY_DN19962_c5_g2::TRINITY_DN19962_c5_g2_i2::g.126940::m.126940	0	0	0	0	0	0	0	0	0	0	130940	0	0	0	0
TRINITY_DN19385_c2_g1::TRINITY_DN19385_c2_g1_i13::g.117074::m.117074	0	0	56566	72228	0	0	0	0	0	0	0	0	0	0	0
TRINITY_DN15599_c0_g1::TRINITY_DN15599_c0_g1_i1::g.54231::m.54231	71495	0	57142	0	0	0	0	0	0	0	0	0	0	0	0
TRINITY_DN13743_c6_g3::TRINITY_DN13743_c6_g3_i1::g.27816::m.27816	0	0	0	0	0	0	0	0	0	127560	0	0	0	0	0
TRINITY_DN18919_c2_g1::TRINITY_DN18919_c2_g1_i5::g.108804::m.108804	0	0	0	0	0	0	0	0	0	0	0	0	0	60561	66798
TRINITY_DN15799_c0_g1::TRINITY_DN15799_c0_g1_i6::g.55958::m.55958	65590	0	0	60146	0	0	0	0	0	0	0	0	0	0	0
TRINITY_DN15302_c0_g1::TRINITY_DN15302_c0_g1_i3::g.49970::m.49970	0	0	0	0	0	0	0	0	0	0	0	0	0	125660	0
TRINITY_DN12400_c4_g2::TRINITY_DN12400_c4_g2_i2::g.12585::m.12585	76737	0	0	48713	0	0	0	0	0	0	0	0	0	0	0

TRINITY_DN16837_c1_g1::TRINITY_DN16837_c1_g1_i3::g.74278::m.74278	0	0	0	0	0	0	0	0	0	0	0	0	0	125310	0
TRINITY_DN19176_c1_g1::TRINITY_DN19176_c1_g1_i6::g.113373::m.1133	0	0	0	0	0	0	0	0	0	0	0	0	0	123930	0
TRINITY_DN13734_c2_g1::TRINITY_DN13734_c2_g1_i1::g.27715::m.27715	0	0	0	122320	0	0	0	0	0	0	0	0	0	0	0
TRINITY_DN15824_c1_g1::TRINITY_DN15824_c1_g1_i3::g.57478::m.57478	0	0	0	0	0	0	0	0	0	0	0	0	0	121830	0
TRINITY_DN17322_c1_g1::TRINITY_DN17322_c1_g1_i1::g.82026::m.82026	0	0	0	0	0	0	0	0	0	0	0	0	120700	0	0
TRINITY_DN18188_c2_g1::TRINITY_DN18188_c2_g1_i6::g.95147::m.95147	0	0	0	0	0	0	0	0	0	0	0	0	0	0	119770
TRINITY_DN16641_c0_g1::TRINITY_DN16641_c0_g1_i7::g.70870::m.70870	0	0	0	0	0	0	0	0	0	0	0	0	118520	0	0
TRINITY_DN16217_c1_g2::TRINITY_DN16217_c1_g2_i6::g.64087::m.64087	63558	0	0	53833	0	0	0	0	0	0	0	0	0	0	0
TRINITY_DN17835_c3_g1::TRINITY_DN17835_c3_g1_i1::g.90619::m.90619	0	0	0	0	0	0	0	0	0	0	0	0	58264	0	58992
TRINITY_DN16741_c1_g1::TRINITY_DN16741_c1_g1_i4::g.72580::m.72580	0	0	0	0	0	0	0	0	0	0	0	0	0	116670	0
TRINITY_DN17827_c3_g3::TRINITY_DN17827_c3_g3_i1::g.90327::m.90327	0	0	0	0	0	0	0	0	0	0	0	0	116480	0	0
TRINITY_DN17200_c0_g1::TRINITY_DN17200_c0_g1_i7::g.79915::m.79915	0	0	0	0	0	0	0	0	0	0	0	0	54218	61992	0
TRINITY_DN11631_c1_g1::TRINITY_DN11631_c1_g1_i4::g.8173::m.8173	0	0	0	0	0	0	0	0	0	0	0	0	0	114370	0
TRINITY_DN14578_c1_g3::TRINITY_DN14578_c1_g3_i1::g.39119::m.39119	0	0	0	0	0	0	0	0	0	0	0	0	0	114360	0
TRINITY_DN13605_c0_g1::TRINITY_DN13605_c0_g1_i3::g.26153::m.26153	0	0	0	0	0	0	0	0	0	0	0	0	113560	0	0
TRINITY_DN14404_c3_g3::TRINITY_DN14404_c3_g3_i3::g.36554::m.36554	0	0	0	0	0	0	0	0	0	0	0	0	0	112910	0
TRINITY_DN15478_c1_g1::TRINITY_DN15478_c1_g1_i1::g.52474::m.52474	80946	0	31226	0	0	0	0	0	0	0	0	0	0	0	0
TRINITY_DN15217_c1_g2::TRINITY_DN15217_c1_g2_i9::g.48873::m.48873	0	0	0	0	0	0	0	0	0	0	0	0	0	111570	0
TRINITY_DN13383_c0_g1::TRINITY_DN13383_c0_g1_i9::g.23043::m.23043	0	0	0	0	0	0	0	0	0	0	0	0	111310	0	0
TRINITY_DN16086_c0_g2::TRINITY_DN16086_c0_g2_i6::g.61825::m.61825	0	0	0	0	0	0	0	0	0	0	0	0	53148	0	57315
TRINITY_DN16643_c2_g1::TRINITY_DN16643_c2_g1_i2::g.70325::m.70325	0	0	0	0	0	0	0	0	0	0	0	0	49357	0	60189
TRINITY_DN19405_c0_g1::TRINITY_DN19405_c0_g1_i2::g.117368::m.117368	0	0	0	0	74652	0	0	0	0	0	0	0	34878	0	0

TRINITY_DN13456_c0_g1::TRINITY_DN13456_c0_g1_i3::g.24091::m.24091	0	0	0	0	0	0	0	0	0	0	0	0	0	0	109500
TRINITY_DN10846_c0_g1::TRINITY_DN10846_c0_g1_i1::g.5657::m.5657	0	0	0	0	0	0	0	0	0	0	0	0	0	0	106930
TRINITY_DN17937_c1_g6::TRINITY_DN17937_c1_g6_i1::g.92221::m.92221	0	27248	0	79476	0	0	0	0	0	0	0	0	0	0	0
TRINITY_DN14048_c1_g1::TRINITY_DN14048_c1_g1_i1::g.31820::m.31820	0	0	0	0	0	0	0	0	0	0	0	0	0	0	106130
TRINITY_DN15198_c1_g3::TRINITY_DN15198_c1_g3_i1::g.48326::m.48326	0	0	0	0	0	0	0	0	0	0	0	0	0	106130	0
TRINITY_DN13739_c0_g1::TRINITY_DN13739_c0_g1_i1::g.27726::m.27726	0	0	0	0	0	0	0	0	0	0	0	0	0	0	105740
TRINITY_DN18544_c2_g2::TRINITY_DN18544_c2_g2_i1::g.102351::m.102351	0	0	0	0	0	0	0	0	0	0	0	0	105240	0	0
TRINITY_DN13630_c0_g1::TRINITY_DN13630_c0_g1_i5::g.26390::m.26390	0	0	0	0	0	0	0	0	0	0	0	0	0	104380	0
TRINITY_DN14180_c0_g1::TRINITY_DN14180_c0_g1_i2::g.33272::m.33272	0	0	0	0	0	0	0	0	0	0	0	104190	0	0	0
TRINITY_DN8216_c0_g1::TRINITY_DN8216_c0_g1_i1::g.2536::m.2536	0	0	0	0	0	0	0	0	0	0	0	104020	0	0	0
TRINITY_DN19280_c0_g1::TRINITY_DN19280_c0_g1_i3::g.115040::m.115040	0	0	0	0	0	0	0	0	0	0	0	0	0	0	103360
TRINITY_DN16993_c0_g3::TRINITY_DN16993_c0_g3_i3::g.76730::m.76730	0	0	0	0	0	0	0	0	0	0	0	0	102680	0	0
TRINITY_DN19939_c3_g1::TRINITY_DN19939_c3_g1_i7::g.125684::m.125684	0	0	0	0	0	0	0	0	0	0	0	0	0	102140	0
TRINITY_DN19123_c0_g2::TRINITY_DN19123_c0_g2_i1::g.112423::m.112423	0	0	0	0	0	0	0	0	0	0	0	0	0	0	101350
TRINITY_DN13782_c0_g3::TRINITY_DN13782_c0_g3_i2::g.28223::m.28223	0	0	0	0	0	0	0	0	0	0	0	0	0	0	100910
TRINITY_DN15101_c4_g1::TRINITY_DN15101_c4_g1_i4::g.45560::m.45560	0	0	0	0	0	0	0	0	0	0	0	0	0	0	96222
TRINITY_DN18181_c1_g1::TRINITY_DN18181_c1_g1_i3::g.96440::m.96440	0	0	96132	0	0	0	0	0	0	0	0	0	0	0	0
TRINITY_DN13858_c5_g2::TRINITY_DN13858_c5_g2_i2::g.29432::m.29432	0	0	0	0	0	0	0	0	0	0	0	0	0	95704	0
TRINITY_DN19625_c0_g5::TRINITY_DN19625_c0_g5_i9::g.121168::m.121168	0	0	0	0	0	0	0	0	0	0	0	0	0	95665	0
TRINITY_DN17351_c1_g1::TRINITY_DN17351_c1_g1_i15::g.82398::m.82398	35789	24533	32424	0	0	0	0	0	0	0	0	0	0	0	0
TRINITY_DN12181_c0_g1::TRINITY_DN12181_c0_g1_i6::g.10945::m.10945	0	0	0	0	0	0	0	0	0	0	0	0	0	92709	0

TRINITY_DN17453_c0_g6::TRINITY_DN17453_c0_g6_i1::g.83881::m.83881	0	0	0	0	0	0	0	0	0	0	0	0	0	92649	0
TRINITY_DN17009_c5_g2::TRINITY_DN17009_c5_g2_i5::g.77303::m.77303	0	0	0	0	0	0	0	0	0	0	91560	0	0	0	0
TRINITY_DN18349_c3_g1::TRINITY_DN18349_c3_g1_i4::g.99210::m.99210	0	0	0	0	0	0	0	0	0	0	0	0	0	91230	0
TRINITY_DN12974_c0_g1::TRINITY_DN12974_c0_g1_i2::g.18074::m.18074	0	0	0	0	0	0	0	0	0	0	0	0	89379	0	0
TRINITY_DN13246_c1_g2::TRINITY_DN13246_c1_g2_i1::g.21483::m.21483	0	0	0	0	0	0	0	0	0	0	0	0	87079	0	0
TRINITY_DN19497_c1_g4::TRINITY_DN19497_c1_g4_i2::g.118818::m.118818	0	0	0	0	0	0	0	0	0	0	0	0	0	0	86370
TRINITY_DN13446_c0_g3::TRINITY_DN13446_c0_g3_i1::g.23949::m.23949	0	0	0	0	0	0	0	0	0	0	0	0	0	84613	0
TRINITY_DN14491_c0_g1::TRINITY_DN14491_c0_g1_i2::g.37893::m.37893	0	0	0	0	0	0	0	0	0	0	0	0	84148	0	0
TRINITY_DN19064_c0_g1::TRINITY_DN19064_c0_g1_i3::g.111228::m.111228	0	0	0	0	0	0	0	0	0	0	0	0	0	0	82923
TRINITY_DN17932_c0_g2::TRINITY_DN17932_c0_g2_i2::g.92648::m.92648	0	0	0	0	0	0	0	0	0	0	82676	0	0	0	0
TRINITY_DN14711_c0_g4::TRINITY_DN14711_c0_g4_i1::g.40962::m.40962	0	0	0	0	0	0	0	0	0	0	0	0	0	0	82662
TRINITY_DN13915_c0_g2::TRINITY_DN13915_c0_g2_i2::g.30373::m.30373	0	0	0	0	0	0	0	0	0	80480	0	0	0	0	0
TRINITY_DN9332_c0_g1::TRINITY_DN9332_c0_g1_i2::g.3344::m.3344	0	0	39809	40082	0	0	0	0	0	0	0	0	0	0	0
TRINITY_DN10896_c0_g1::TRINITY_DN10896_c0_g1_i3::g.5759::m.5759	0	0	0	0	0	0	0	0	0	0	0	0	0	0	79117
TRINITY_DN15257_c0_g2::TRINITY_DN15257_c0_g2_i2::g.49306::m.49306	0	0	0	0	0	0	0	0	0	0	0	0	0	78757	0
TRINITY_DN14993_c0_g1::TRINITY_DN14993_c0_g1_i2::g.45400::m.45400	0	0	0	0	0	0	0	0	0	0	0	0	78433	0	0
TRINITY_DN15366_c1_g1::TRINITY_DN15366_c1_g1_i4::g.50810::m.50810	0	0	0	0	0	0	0	0	0	0	0	0	0	0	78291
TRINITY_DN11457_c0_g1::TRINITY_DN11457_c0_g1_i1::g.7427::m.7427	0	0	0	0	0	0	0	0	0	0	0	0	0	0	77714
TRINITY_DN12510_c1_g1::TRINITY_DN12510_c1_g1_i1::g.13448::m.13448	0	0	0	0	0	0	0	0	0	0	0	0	0	76797	0
TRINITY_DN17907_c0_g1::TRINITY_DN17907_c0_g1_i1::g.91817::m.91817	0	0	0	0	0	0	0	0	0	0	0	0	0	0	73405
TRINITY_DN18531_c0_g1::TRINITY_DN18531_c0_g1_i9::g.102220::m.102220	0	0	0	70362	0	0	0	0	0	0	0	0	0	0	0

TRINITY_DN13896_c0_g4::TRINITY_DN13896_c0_g4_i4::g.30042::m.30042	0	0	0	0	0	0	0	0	0	0	0	0	67399	0	0
TRINITY_DN49420_c0_g1::TRINITY_DN49420_c0_g1_i1::g.132815::m.132815	0	0	0	0	0	0	0	0	0	0	0	0	66244	0	0
TRINITY_DN1207_c0_g1::TRINITY_DN1207_c0_g1_i1::g.307::m.307	0	0	0	65810	0	0	0	0	0	0	0	0	0	0	0
TRINITY_DN16954_c2_g1::TRINITY_DN16954_c2_g1_i2::g.76223::m.76223	0	0	0	0	0	0	0	0	0	0	0	0	0	0	64970
TRINITY_DN18548_c0_g1::TRINITY_DN18548_c0_g1_i9::g.102424::m.102424	0	0	0	0	0	0	0	63915	0	0	0	0	0	0	0
TRINITY_DN14063_c0_g1::TRINITY_DN14063_c0_g1_i1::g.31854::m.31854	0	0	0	0	0	0	0	0	0	0	0	0	0	62757	0
TRINITY_DN9524_c0_g1::TRINITY_DN9524_c0_g1_i2::g.3575::m.3575	0	0	0	0	0	0	0	0	0	0	0	0	0	62293	0
TRINITY_DN14887_c2_g3::TRINITY_DN14887_c2_g3_i1::g.43865::m.43865	0	0	0	0	0	0	0	0	0	0	0	0	61541	0	0
TRINITY_DN12560_c1_g1::TRINITY_DN12560_c1_g1_i3::g.13781::m.13781	0	0	60231	0	0	0	0	0	0	0	0	0	0	0	0
TRINITY_DN12634_c0_g1::TRINITY_DN12634_c0_g1_i2::g.14412::m.14412	0	0	0	0	0	0	0	0	0	0	0	0	0	58863	0
TRINITY_DN16146_c1_g1::TRINITY_DN16146_c1_g1_i2::g.62212::m.62212	0	0	0	0	0	0	0	0	0	0	0	0	57399	0	0
TRINITY_DN16454_c2_g1::TRINITY_DN16454_c2_g1_i4::g.67739::m.67739	0	0	0	55909	0	0	0	0	0	0	0	0	0	0	0
TRINITY_DN5515_c0_g1::TRINITY_DN5515_c0_g1_i1::g.1365::m.1365	0	0	0	0	0	0	0	0	0	0	0	0	0	51909	0
TRINITY_DN37417_c0_g1::TRINITY_DN37417_c0_g1_i1::g.131078::m.131078	0	0	0	0	0	0	0	50629	0	0	0	0	0	0	0
TRINITY_DN17093_c0_g1::TRINITY_DN17093_c0_g1_i9::g.78387::m.78387	0	0	0	0	0	0	0	0	0	0	0	0	49518	0	0
TRINITY_DN16772_c0_g1::TRINITY_DN16772_c0_g1_i3::g.72983::m.72983	49167	0	0	0	0	0	0	0	0	0	0	0	0	0	0
TRINITY_DN15339_c4_g4::TRINITY_DN15339_c4_g4_i1::g.49925::m.49925	0	0	0	0	0	0	0	0	0	0	0	0	0	49033	0
TRINITY_DN15754_c0_g4::TRINITY_DN15754_c0_g4_i1::g.56661::m.56661	0	0	0	0	0	0	0	0	0	0	0	0	47967	0	0
TRINITY_DN16687_c0_g1::TRINITY_DN16687_c0_g1_i2::g.71593::m.71593	0	0	0	0	0	0	0	0	0	0	0	0	0	0	44638
TRINITY_DN49712_c0_g1::TRINITY_DN49712_c0_g1_i1::g.132845::m.132845	0	0	0	0	0	0	0	0	0	0	42848	0	0	0	0

TRINITY_DN15478_c0_g1::TRINITY_DN15478_c0_g1_i13::g.52472::m.52472	0	0	0	0	0	0	0	0	0	0	0	0	40849	0	0
TRINITY_DN13615_c2_g2::TRINITY_DN13615_c2_g2_i1::g.26300::m.26300	33665	0	0	0	0	0	0	0	0	0	0	0	0	0	0
TRINITY_DN15422_c1_g1::TRINITY_DN15422_c1_g1_i1::g.51639::m.51639	0	32564	0	0	0	0	0	0	0	0	0	0	0	0	0
TRINITY_DN17700_c2_g2::TRINITY_DN17700_c2_g2_i11::g.86871::m.86871	0	0	0	0	0	0	0	0	0	0	0	0	0	30286	0
TRINITY_DN13585_c0_g1::TRINITY_DN13585_c0_g1_i3::g.25706::m.25706	29184	0	0	0	0	0	0	0	0	0	0	0	0	0	0
TRINITY_DN16933_c4_g2::TRINITY_DN16933_c4_g2_i2::g.75851::m.75851	0	0	0	29177	0	0	0	0	0	0	0	0	0	0	0
TRINITY_DN11228_c0_g1::TRINITY_DN11228_c0_g1_i4::g.6693::m.6693	0	28633	0	0	0	0	0	0	0	0	0	0	0	0	0
TRINITY_DN17776_c0_g1::TRINITY_DN17776_c0_g1_i2::g.89550::m.89550	0	27268	0	0	0	0	0	0	0	0	0	0	0	0	0
TRINITY_DN17420_c0_g2::TRINITY_DN17420_c0_g2_i5::g.83586::m.83586	19981	0	0	0	0	0	0	0	0	0	0	0	0	0	0
TRINITY_DN11534_c0_g1::TRINITY_DN11534_c0_g1_i5::g.7773::m.7773	0	0	0	0	0	0	0	0	0	0	0	0	0	0	0
TRINITY_DN13275_c3_g1::TRINITY_DN13275_c3_g1_i7::g.21820::m.21820	0	0	0	0	0	0	0	0	0	0	0	0	0	0	0
TRINITY_DN14670_c2_g3::TRINITY_DN14670_c2_g3_i4::g.40396::m.40396	0	0	0	0	0	0	0	0	0	0	0	0	0	0	0
TRINITY_DN15660_c2_g1::TRINITY_DN15660_c2_g1_i4::g.55101::m.55101	0	0	0	0	0	0	0	0	0	0	0	0	0	0	0
TRINITY_DN15833_c5_g1::TRINITY_DN15833_c5_g1_i8::g.57893::m.57893	0	0	0	0	0	0	0	0	0	0	0	0	0	0	0
TRINITY_DN18852_c0_g2::TRINITY_DN18852_c0_g2_i1::g.107431::m.107431	0	0	0	0	0	0	0	0	0	0	0	0	0	0	0
TRINITY_DN18971_c1_g2::TRINITY_DN18971_c1_g2_i2::g.109643::m.109643	0	0	0	0	0	0	0	0	0	0	0	0	0	0	0
TRINITY_DN19418_c3_g2::TRINITY_DN19418_c3_g2_i3::g.117519::m.117519	0	0	0	0	0	0	0	0	0	0	0	0	0	0	0
TRINITY_DN36814_c0_g1::TRINITY_DN36814_c0_g1_i1::g.130975::m.130975	0	0	0	0	0	0	0	0	0	0	0	0	0	0	0
