# Supplementary material for: Comparative proteomics of common allergenic tree pollens of birch, alder, and hazel
Source: Allergy. 2021 Jan 15;76(6):1743–53. doi: 10.1111/all.14694 (PMC8248232; doi:10.1111/all.14694)
Supplement: Supplementary file 8 — Table S6 [file ALL-76-1743-s013.pdf]

Supplementary Table S6: Top BLAST hits of identified Betula pollen proteins

Protein IDs	e-value [blastx hit 1]	bitscore [blastx hit 1]	accession [blastx hit 1]	name [blastx hit 1]	organism [blastx hit 1]	accession [blastx hit 2]
TRINITY_DN17355_c4_g1::TRINITY_DN17355_c4_g1_i11::g.82598::m.82598	0	1241	ONH94299.1	hypothetical protein PRUPE_7G009200	Prunus persica	ONH94297.1
TRINITY_DN15290_c1_g1::TRINITY_DN15290_c1_g1_i13::g.49834::m.49834	0	1055	XP_018837851.1	phosphoglucomutase, cytoplasmic	Juglans regia	XP_018826631.1
TRINITY_DN11319_c0_g2::TRINITY_DN11319_c0_g2_i2::g.7052::m.7052	1,54E-99	289	AHF71027.1	glutathione S-transferase	Betula pendula	XP_018812288.1
TRINITY_DN14555_c5_g4::TRINITY_DN14555_c5_g4_i1::g.38907::m.38907	2,32E-18	79,7	XP_018837099.1	uncharacterized protein LOC109003436	Juglans regia	XP_018837039.1
TRINITY_DN16112_c0_g1::TRINITY_DN16112_c0_g1_i8::g.62808::m.62808	0	786	XP_018816885.1	UTP--glucose-1-phosphate uridylyltransferase	Juglans regia	XP_018811449.1
TRINITY_DN14325_c0_g1::TRINITY_DN14325_c0_g1_i1::g.35421::m.35421	2,58E-175	491	XP_018821921.1	peroxidase P7	Juglans regia	XP_008374580.1
TRINITY_DN19980_c4_g3::TRINITY_DN19980_c4_g3_i6::g.127331::m.127331	2,33E-55	186	XP_018859093.1	probable pectinesterase/pectinesterase inhibitor 51	Juglans regia	XP_018842710.1
TRINITY_DN14455_c0_g9::TRINITY_DN14455_c0_g9_i1::g.37463::m.37463	0	623	XP_008383158.1	phosphoglycerate kinase, cytosolic	Malus domestica	XP_004488762.1
TRINITY_DN12690_c0_g1::TRINITY_DN12690_c0_g1_i3::g.14937::m.14937	0	1054	XP_018819373.1	2,3-bisphosphoglycerate-independent phosphoglycerate mutase	Juglans regia	XP_018819374.1
TRINITY_DN19145_c0_g2::TRINITY_DN19145_c0_g2_i2::g.112927::m.112927	6,77E-31	114	XP_018837516.1	uncharacterized protein LOC109003708	Juglans regia	OAY58719.1
TRINITY_DN18658_c1_g1::TRINITY_DN18658_c1_g1_i9::g.104122::m.104122	0	680	XP_016184765.1	monodehydroascorbate reductase	Arachis ipaensis	XP_008241272.1
TRINITY_DN19475_c9_g3::TRINITY_DN19475_c9_g3_i3::g.118615::m.118615	2,51E-109	335	OAY35216.1	hypothetical protein MANES_12G081800	Manihot esculenta	OAY35218.1
TRINITY_DN19879_c1_g2::TRINITY_DN19879_c1_g2_i1::g.125068::m.125068	9,39E-98	294	XP_018847365.1	probable polygalacturonase	Juglans regia	XP_018847358.1
TRINITY_DN11247_c0_g1::TRINITY_DN11247_c0_g1_i5::g.6785::m.6785	8,51E-161	451	AAG22740.1	AF282850_1 allergenic isoflavone reductase-like protein Bet v 6.0102	Betula pendula	Q9FUW6.1
TRINITY_DN15025_c0_g1::TRINITY_DN15025_c0_g1_i1::g.45918::m.45918	0	805	XP_018839506.1	enolase 1	Juglans regia	OAY40921.1
TRINITY_DN14511_c0_g1::TRINITY_DN14511_c0_g1_i3::g.38108::m.38108	6,02E-52	164	XP_016650474.1	glycine-rich RNA-binding protein GRP1A	Prunus mume	XP_018847818.1
TRINITY_DN19475_c9_g1::TRINITY_DN19475_c9_g1_i7::g.118616::m.118616	5,78E-88	275	XP_017180254.1	subtilisin-like protease SBT5.4	Malus domestica	ONH99465.1
TRINITY_DN16589_c2_g3::TRINITY_DN16589_c2_g3_i3::g.69229::m.69229	5,44E-95	275	KDP23592.1	hypothetical protein JCGZ_23425	Jatropha curcas	XP_012089157.1
TRINITY_DN19281_c0_g3::TRINITY_DN19281_c0_g3_i5::g.115036::m.115036	1,10E-169	479	XP_018816251.1	calreticulin	Juglans regia	XP_018856844.1
TRINITY_DN18969_c3_g1::TRINITY_DN18969_c3_g1_i1::g.109636::m.109636	0	934	XP_018844063.1	adenosylhomocysteinase-like	Juglans regia	XP_018848539.1
TRINITY_DN17420_c0_g3::TRINITY_DN17420_c0_g3_i2::g.83576::m.83576	0	593	XP_018846382.1	glyceraldehyde-3-phosphate dehydrogenase, cytosolic	Juglans regia	OAY47918.1

TRINITY_DN11273_c0_g1::TRINITY_DN11273_c0_g1_i1::g.6839::m.6839	7,23E-133	377	XP_018814430.1	uncharacterized protein LOC108986306 isoform X2	Juglans regia	XP_018814429.1
TRINITY_DN19790_c2_g2::TRINITY_DN19790_c2_g2_i6::g.123649::m.123649	2,02E-32	119	AAC05116.2	isoflavone reductase homolog Bet v 6.0101, partial	Betula pendula	AAG22740.1
TRINITY_DN14648_c0_g1::TRINITY_DN14648_c0_g1_i1::g.39924::m.39924	0	1387	XP_018842163.1	beta-xylosidase/alpha-L-arabinofuranosidase 2-like	Juglans regia	XP_018836836.1
TRINITY_DN13067_c1_g1::TRINITY_DN13067_c1_g1_i2::g.19355::m.19355	6,68E-100	287	XP_018844175.1	peptidyl-prolyl cis-trans isomerase FKBP15-1	Juglans regia	OAY32871.1
TRINITY_DN14120_c0_g3::TRINITY_DN14120_c0_g3_i2::g.32536::m.32536	6,35E-157	441	XP_018823301.1	triosephosphate isomerase, cytosolic	Juglans regia	XP_008223629.1
TRINITY_DN14328_c3_g1::TRINITY_DN14328_c3_g1_i9::g.35530::m.35530	0	848	XP_018858114.1	protein disulfide-isomerase-like	Juglans regia	XP_018858121.1
TRINITY_DN15467_c6_g1::TRINITY_DN15467_c6_g1_i1::g.52486::m.52486	0	582	XP_018851336.1	2-alkenal reductase (NADP(+)-dependent)-like isoform X2	Juglans regia	XP_018839362.1
TRINITY_DN14030_c0_g1::TRINITY_DN14030_c0_g1_i1::g.31442::m.31442	0	561	XP_018834006.1	probable fructokinase-5	Juglans regia	AGH25527.1
TRINITY_DN19909_c0_g1::TRINITY_DN19909_c0_g1_i3::g.126119::m.126119	0	814	XP_018808617.1	receptor-like protein kinase HAIKU2	Juglans regia	XP_018808617.1
TRINITY_DN17277_c1_g3::TRINITY_DN17277_c1_g3_i4::g.81251::m.81251	0	627	KDP28233.1	hypothetical protein JCGZ_14004	Jatropha curcas	XP_012082863.1
TRINITY_DN17792_c0_g2::TRINITY_DN17792_c0_g2_i8::g.89735::m.89735	0	642	XP_008241286.1	fructose-bisphosphate aldolase cytoplasmic isozyme	Prunus mume	ONH96251.1
TRINITY_DN14776_c1_g1::TRINITY_DN14776_c1_g1_i2::g.42049::m.42049	0	897	XP_018825758.1	galactokinase-like isoform X1	Juglans regia	XP_018825759.1
TRINITY_DN17630_c0_g1::TRINITY_DN17630_c0_g1_i8::g.87337::m.87337	1,57E-71	214	XP_018845447.1	glutaredoxin-C4	Juglans regia	KDP46838.1
TRINITY_DN12397_c0_g1::TRINITY_DN12397_c0_g1_i3::g.12463::m.12463	0	1352	XP_018844102.1	beta-galactosidase 15-like	Juglans regia	KYP63346.1
TRINITY_DN12813_c0_g1::TRINITY_DN12813_c0_g1_i4::g.16165::m.16165	0	627	XP_018808212.1	malate dehydrogenase-like	Juglans regia	XP_018807661.1
TRINITY_DN16032_c0_g1::TRINITY_DN16032_c0_g1_i3::g.60899::m.60899	0	630	XP_018828122.1	glutelin type-B 5-like	Juglans regia	XP_018828123.1
TRINITY_DN14204_c0_g1::TRINITY_DN14204_c0_g1_i2::g.33925::m.33925	4,03E-45	147	XP_018855560.1	pectinesterase PPME1-like, partial	Juglans regia	XP_018855562.1
TRINITY_DN18302_c1_g2::TRINITY_DN18302_c1_g2_i4::g.98509::m.98509	3,75E-151	429	XP_018823047.1	2-methylene-furan-3-one reductase-like	Juglans regia	XP_018815084.1
TRINITY_DN11092_c0_g1::TRINITY_DN11092_c0_g1_i5::g.6307::m.6307	6,38E-78	251	XP_018812486.1	glycerophosphodiester phosphodiesterase GDPDL3-like	Juglans regia	XP_018812474.1
TRINITY_DN13033_c2_g1::TRINITY_DN13033_c2_g1_i6::g.18937::m.18937	5,91E-56	174	XP_018843283.1	profilin	Juglans regia	KDP40849.1
TRINITY_DN17355_c4_g1::TRINITY_DN17355_c4_g1_i4::g.82584::m.82584	0	1452	XP_018851852.1	5-methyltetrahydropteroyltriglutamate--homocysteine methyltransferase-like	Juglans regia	XP_012066300.1
TRINITY_DN19812_c4_g2::TRINITY_DN19812_c4_g2_i6::g.124061::m.124061	0	992	ALP70519.1	HSP70-type chaperone 28	Prunus persica var. persica	XP_008239207.1

TRINITY_DN11789_c0_g1::TRINITY_DN11789_c0_g1_i4::g.8830::m.8830	0	546	XP_018841935.1	putative lactoylglutathione lyase	Juglans regia	XP_015959074.1
TRINITY_DN19911_c7_g2::TRINITY_DN19911_c7_g2_i3::g.126024::m.126024	3,66E-68	207	ONI12324.1	hypothetical protein PRUPE_4G157300	Prunus persica	XP_020417826.1
TRINITY_DN14120_c0_g1::TRINITY_DN14120_c0_g1_i4::g.32545::m.32545	4,43E-128	365	XP_018814537.1	triosephosphate isomerase, cytosolic	Juglans regia	OAY46102.1
TRINITY_DN19879_c2_g1::TRINITY_DN19879_c2_g1_i5::g.125071::m.125071	1,33E-37	135	XP_018847365.1	probable polygalacturonase	Juglans regia	XP_018847358.1
TRINITY_DN14558_c2_g3::TRINITY_DN14558_c2_g3_i1::g.38817::m.38817	8,33E-93	270	XP_008241145.1	ubiquitin-NEDD8-like protein RUB2	Prunus mume	ONH95703.1
TRINITY_DN15774_c1_g1::TRINITY_DN15774_c1_g1_i3::g.56941::m.56941	4,81E-79	234	XP_018823225.1	uncharacterized protein LOC108992954	Juglans regia	XP_009353814.1
TRINITY_DN13244_c3_g1::TRINITY_DN13244_c3_g1_i1::g.21540::m.21540	0	558	XP_018849818.1	malate dehydrogenase, mitochondrial	Juglans regia	XP_018822629.1
TRINITY_DN18531_c0_g1::TRINITY_DN18531_c0_g1_i4::g.102214::m.102214	0	1554	XP_018852512.1	puromycin-sensitive aminopeptidase isoform X3	Juglans regia	XP_018852509.1
TRINITY_DN16132_c1_g2::TRINITY_DN16132_c1_g2_i1::g.62633::m.62633	1,88E-103	296	XP_018805385.1	calmodulin-like	Juglans regia	XP_018823490.1
TRINITY_DN18476_c0_g1::TRINITY_DN18476_c0_g1_i4::g.100953::m.100953	1,21E-09	57,4	ONI14780.1	hypothetical protein PRUPE_3G007900	Prunus persica	XP_007221543.2
TRINITY_DN15025_c0_g1::TRINITY_DN15025_c0_g1_i8::g.45925::m.45925	0	859	XP_008376333.1	enolase	Malus domestica	XP_009374527.1
TRINITY_DN15036_c1_g1::TRINITY_DN15036_c1_g1_i9::g.46235::m.46235	4,43E-50	161	KHN33603.1	Basic form of pathogenesis-related protein 1	Glycine soja	KRH10678.1
TRINITY_DN12776_c1_g2::TRINITY_DN12776_c1_g2_i6::g.15805::m.15805	1,32E-44	154	XP_008234902.1	probable polygalacturonase	Prunus mume	ONI17230.1
TRINITY_DN17440_c0_g3::TRINITY_DN17440_c0_g3_i6::g.84091::m.84091	8,90E-180	506	XP_018818327.1	guanosine nucleotide diphosphate dissociation inhibitor At5g09550	Juglans regia	KDP29973.1
TRINITY_DN19281_c0_g5::TRINITY_DN19281_c0_g5_i1::g.115035::m.115035	8,88E-42	146	XP_015939719.1	calreticulin isoform X2	Arachis duranensis	XP_016175239.1
TRINITY_DN9942_c0_g1::TRINITY_DN9942_c0_g1_i4::g.4015::m.4015	9,11E-126	359	XP_008238908.1	protein P21-like	Prunus mume	XP_018828872.1
TRINITY_DN19839_c2_g3::TRINITY_DN19839_c2_g3_i5::g.123823::m.123823	8,71E-97	282	XP_018852474.1	probable phospholipid hydroperoxide glutathione peroxidase	Juglans regia	OAY59821.1
TRINITY_DN19911_c7_g3::TRINITY_DN19911_c7_g3_i1::g.126023::m.126023	9,49E-87	269	XP_018808169.1	reticuline oxidase-like protein isoform X2	Juglans regia	XP_018820243.1
TRINITY_DN15452_c6_g1::TRINITY_DN15452_c6_g1_i5::g.52129::m.52129	0	764	XP_018841632.1	uncharacterized protein LOC109006717	Juglans regia	XP_018829078.1
TRINITY_DN18205_c0_g1::TRINITY_DN18205_c0_g1_i3::g.96958::m.96958	1,10E-147	417	XP_018846260.1	putative methyltransferase DDB_G0268948	Juglans regia	XP_018838664.1
TRINITY_DN18360_c0_g7::TRINITY_DN18360_c0_g7_i2::g.99197::m.99197	0	709	KRH19702.1	hypothetical protein GLYMA_13G130900	Glycine max	XP_018841713.1
TRINITY_DN19551_c4_g1::TRINITY_DN19551_c4_g1_i4::g.119678::m.119678	7,40E-71	214	XP_018823733.1	NAD(P)H dehydrogenase (quinone) FQR1-like	Juglans regia	XP_008226755.1

TRINITY_DN18258_c0_g1::TRINITY_DN18258_c0_g1_i10::g.97660::m.97660	0	546	XP_008236513.1	probable mannitol dehydrogenase	Prunus mume	ONI02574.1
TRINITY_DN15932_c0_g3::TRINITY_DN15932_c0_g3_i5::g.59231::m.59231	0	694	XP_018839886.1	alcohol dehydrogenase 1	Juglans regia	Alnus glutinosa
TRINITY_DN13825_c1_g1::TRINITY_DN13825_c1_g1_i2::g.28853::m.28853	1,26E-173	486	XP_018839113.1	aldo-keto reductase family 4 member C9-like	Juglans regia	XP_018839114.1
TRINITY_DN13624_c1_g6::TRINITY_DN13624_c1_g6_i6::g.26482::m.26482	0	1006	XP_018825745.1	heat shock cognate protein 80-like	Juglans regia	XP_018825752.1
TRINITY_DN18743_c1_g1::TRINITY_DN18743_c1_g1_i1::g.105300::m.105300	5,60E-41	139	XP_018823876.1	glycine-rich protein 2-like	Juglans regia	XP_018841456.1
TRINITY_DN13291_c0_g1::TRINITY_DN13291_c0_g1_i4::g.22017::m.22017	2,59E-82	243	XP_018837329.1	glycine cleavage system H protein 2, mitochondrial-like	Juglans regia	XP_018859042.1
TRINITY_DN19879_c1_g2::TRINITY_DN19879_c1_g2_i8::g.125070::m.125070	4,34E-55	172	XP_018853432.1	probable polygalacturonase, partial	Juglans regia	ONI25402.1
TRINITY_DN19354_c2_g2::TRINITY_DN19354_c2_g2_i8::g.116447::m.116447	6,33E-91	268	XP_018835464.1	glutathione S-transferase-like	Juglans regia	XP_018857389.1
TRINITY_DN19617_c3_g2::TRINITY_DN19617_c3_g2_i1::g.121264::m.121264	1,81E-145	414	XP_007206468.2	adenosine kinase 2	Prunus persica	XP_008243957.1
TRINITY_DN36067_c0_g1::TRINITY_DN36067_c0_g1_i1::g.130820::m.130820	4,08E-121	348	XP_018856906.1	endochitinase 2-like	Juglans regia	XP_007201290.2
TRINITY_DN18306_c2_g1::TRINITY_DN18306_c2_g1_i5::g.98735::m.98735	0	597	XP_018816808.1	isocitrate dehydrogenase [NADP]	Juglans regia	XP_018816809.1
TRINITY_DN13776_c0_g1::TRINITY_DN13776_c0_g1_i2::g.28095::m.28095	1,48E-97	288	XP_008243957.1	adenosine kinase 2	Prunus mume	KYP67010.1
TRINITY_DN8833_c0_g1::TRINITY_DN8833_c0_g1_i2::g.2890::m.2890	2,11E-104	301	XP_018824166.1	peptidyl-prolyl cis-trans isomerase CYP19-3	Juglans regia	XP_008218307.1
TRINITY_DN16420_c0_g2::TRINITY_DN16420_c0_g2_i1::g.67374::m.67374	0	716	ONI36343.1	hypothetical protein PRUPE_1G581500	Prunus persica	XP_007222927.1
TRINITY_DN13666_c1_g1::TRINITY_DN13666_c1_g1_i6::g.26985::m.26985	2,77E-163	484	XP_018823946.1	receptor protein kinase TMK1-like	Juglans regia	XP_018837902.1
TRINITY_DN19405_c2_g1::TRINITY_DN19405_c2_g1_i12::g.117378::m.117378	3,21E-167	469	XP_018839732.1	cinnamoyl-CoA reductase 1-like	Juglans regia	BAE48658.1
TRINITY_DN18584_c1_g5::TRINITY_DN18584_c1_g5_i6::g.103010::m.103010	3,63E-132	376	XP_018843858.1	cysteine-rich repeat secretory protein 38-like isoform X1	Juglans regia	XP_018843859.1
TRINITY_DN16422_c1_g1::TRINITY_DN16422_c1_g1_i4::g.67346::m.67346	0	1211	XP_018826868.1	heat shock 70 kDa protein, mitochondrial	Juglans regia	XP_018846283.1
TRINITY_DN13815_c1_g2::TRINITY_DN13815_c1_g2_i8::g.28885::m.28885	5,23E-11	53,9	XP_018810694.1	UDP-glucuronic acid decarboxylase 6-like	Juglans regia	XP_018810694.1
TRINITY_DN14162_c4_g1::TRINITY_DN14162_c4_g1_i2::g.33191::m.33191	1,48E-126	360	XP_018843523.1	haloacid dehalogenase-like hydrolase domain-containing protein Sgpp isoform X3	Juglans regia	XP_018843521.1
TRINITY_DN15589_c0_g1::TRINITY_DN15589_c0_g1_i4::g.54099::m.54099	0	992	XP_018823509.1	leucine aminopeptidase 1-like	Juglans regia	XP_018810862.1
TRINITY_DN15010_c0_g3::TRINITY_DN15010_c0_g3_i5::g.45655::m.45655	0	918	XP_018844057.1	selenium-binding protein 2-like	Juglans regia	XP_018848542.1

TRINITY_DN16345_c0_g1::TRINITY_DN16345_c0_g1_i2::g.66198::m.66198	0	1538	XP_018851707.1	phospholipase D alpha 1	Juglans regia	XP_018850650.1
TRINITY_DN17420_c0_g2::TRINITY_DN17420_c0_g2_i1::g.83570::m.83570	0	620	XP_018859983.1	glyceraldehyde-3-phosphate dehydrogenase 2, cytosolic-like	Juglans regia	OAY47918.1
TRINITY_DN18333_c2_g6::TRINITY_DN18333_c2_g6_i1::g.98947::m.98947	3,41E-32	115	XP_018837516.1	uncharacterized protein LOC109003708	Juglans regia	ONI14778.1
TRINITY_DN18850_c2_g2::TRINITY_DN18850_c2_g2_i2::g.107178::m.107178	0	554	XP_018815562.1	uncharacterized protein LOC108987149 isoform X2	Juglans regia	XP_018815561.1
TRINITY_DN18145_c2_g1::TRINITY_DN18145_c2_g1_i9::g.95178::m.95178	6,37E-158	443	XP_018851414.1	carbonic anhydrase 2-like	Juglans regia	XP_018851415.1
TRINITY_DN17440_c0_g5::TRINITY_DN17440_c0_g5_i3::g.84089::m.84089	1,49E-91	277	XP_004504841.1	guanosine nucleotide diphosphate dissociation inhibitor At5g09550	Cicer arietinum	ONH90693.1
TRINITY_DN12537_c1_g2::TRINITY_DN12537_c1_g2_i4::g.13603::m.13603	1,49E-75	239	ONI11277.1	hypothetical protein PRUPE_4G098300	Prunus persica	XP_007211965.2
TRINITY_DN15426_c0_g2::TRINITY_DN15426_c0_g2_i4::g.51819::m.51819	2,22E-68	207	XP_018831298.1	tubulin-folding cofactor A-like	Juglans regia	XP_018831299.1
TRINITY_DN15464_c0_g2::TRINITY_DN15464_c0_g2_i1::g.52259::m.52259	0	674	XP_018841713.1	heat shock cognate 70 kDa protein 2	Juglans regia	OAY38647.1
TRINITY_DN14041_c2_g1::TRINITY_DN14041_c2_g1_i4::g.31794::m.31794	0	1072	OAY48214.1	hypothetical protein MANES_06G141300	Manihot esculenta	XP_019427299.1
TRINITY_DN14730_c2_g2::TRINITY_DN14730_c2_g2_i1::g.41382::m.41382	7,10E-36	122	CAC39160.1	putative LEA III protein isoform 1	Corylus avellana	CAC39110.1
TRINITY_DN19696_c3_g4::TRINITY_DN19696_c3_g4_i1::g.122257::m.122257	0	861	XP_018841811.1	aconitate hydratase, cytoplasmic isoform X1	Juglans regia	XP_018848484.1
TRINITY_DN16808_c0_g1::TRINITY_DN16808_c0_g1_i2::g.73724::m.73724	7,22E-123	354	XP_018807198.1	uncharacterized protein LOC108980651	Juglans regia	OAY33079.1
TRINITY_DN16940_c1_g2::TRINITY_DN16940_c1_g2_i2::g.75865::m.75865	4,35E-153	429	XP_018826002.1	endo-1,3;1,4-beta-D-glucanase isoform X2	Juglans regia	XP_018826001.1
TRINITY_DN14508_c8_g1::TRINITY_DN14508_c8_g1_i7::g.38377::m.38377	8,55E-86	251	XP_018830082.1	desiccation protectant protein Lea14 homolog	Juglans regia	XP_018847006.1
TRINITY_DN16840_c2_g1::TRINITY_DN16840_c2_g1_i1::g.74291::m.74291	0	574	OAY52428.1	hypothetical protein MANES_04G082800	Manihot esculenta	KYP68282.1
TRINITY_DN17614_c0_g2::TRINITY_DN17614_c0_g2_i5::g.87093::m.87093	0	816	XP_018819528.1	protein disulfide isomerase-like 1-4	Juglans regia	KDP44055.1
TRINITY_DN18004_c1_g6::TRINITY_DN18004_c1_g6_i1::g.93462::m.93462	8,63E-84	249	XP_009365718.1	glutathione S-transferase F13 isoform X1	Pyrus x bretschneideri	XP_018505025.1
TRINITY_DN13376_c2_g2::TRINITY_DN13376_c2_g2_i3::g.22986::m.22986	0	549	XP_018806957.1	vignain-like	Juglans regia	CAA40073.1
TRINITY_DN13606_c2_g2::TRINITY_DN13606_c2_g2_i1::g.26262::m.26262	0	961	XP_018816674.1	UDP-glucose 6-dehydrogenase 1-like	Juglans regia	XP_018816673.1
TRINITY_DN14755_c2_g2::TRINITY_DN14755_c2_g2_i1::g.41670::m.41670	1,67E-120	353	XP_018824948.1	probable cinnamyl alcohol dehydrogenase 9	Juglans regia	KDP35308.1
TRINITY_DN13024_c1_g2::TRINITY_DN13024_c1_g2_i1::g.18973::m.18973	3,32E-80	249	XP_018842710.1	probable pectinesterase/pectinesterase inhibitor 51	Juglans regia	XP_018860059.1

TRINITY_DN14909_c1_g2::TRINITY_DN14909_c1_g2_i4::g.44177::m.44177	0	1237	XP_007160650.1	hypothetical protein PHAVU_001G005200g	Phaseolus vulgaris	ESW32644.1
TRINITY_DN19579_c1_g1::TRINITY_DN19579_c1_g1_i2::g.120372::m.120372	0	761	XP_018809083.1	delta-1-pyrroline-5-carboxylate synthase isoform X1	Juglans regia	OAY46148.1
TRINITY_DN14204_c0_g1::TRINITY_DN14204_c0_g1_i1::g.33924::m.33924	3,06E-51	164	XP_018855562.1	pectinesterase PPME1-like, partial	Juglans regia	XP_018855560.1
TRINITY_DN12067_c0_g1::TRINITY_DN12067_c0_g1_i1::g.10231::m.10231	4,25E-85	248	XP_008231331.1	thioredoxin-like protein Clot	Prunus mume	ONI20288.1
TRINITY_DN18581_c2_g2::TRINITY_DN18581_c2_g2_i16::g.102885::m.102885	1,06E-80	250	XP_018818966.1	serine carboxypeptidase-like	Juglans regia	OIW09125.1
TRINITY_DN13947_c3_g5::TRINITY_DN13947_c3_g5_i8::g.30694::m.30694	7,25E-176	488	XP_018833512.1	hydroxyacylglutathione hydrolase cytoplasmic	Juglans regia	XP_019430954.1
TRINITY_DN18713_c3_g1::TRINITY_DN18713_c3_g1_i8::g.105061::m.105061	0	775	XP_018849508.1	protein DJ-1 homolog B-like	Juglans regia	KDP32522.1
TRINITY_DN19957_c3_g2::TRINITY_DN19957_c3_g2_i9::g.126783::m.126783	4,55E-93	269	ONI23028.1	hypothetical protein PRUPE_2G165300	Prunus persica	XP_007218574.1
TRINITY_DN18489_c2_g2::TRINITY_DN18489_c2_g2_i5::g.101255::m.101255	3,28E-101	290	ADU56174.1	ubiquitin-conjugating family protein	Jatropha curcas	ADV04060.1
TRINITY_DN15194_c1_g2::TRINITY_DN15194_c1_g2_i1::g.48344::m.48344	6,92E-64	194	OAY50655.1	hypothetical protein MANES_05G153800	Manihot esculenta	AEC03317.1
TRINITY_DN17177_c3_g1::TRINITY_DN17177_c3_g1_i5::g.79706::m.79706	3,18E-91	271	KDP34528.1	hypothetical protein JCGZ_11078	Jatropha curcas	XP_012075988.1
TRINITY_DN13227_c0_g1::TRINITY_DN13227_c0_g1_i8::g.21288::m.21288	0	1147	XP_018839074.1	UDP-sugar pyrophosphorylase-like	Juglans regia	XP_018849179.1
TRINITY_DN18988_c0_g2::TRINITY_DN18988_c0_g2_i2::g.109900::m.109900	2,50E-66	209	KDP25066.1	hypothetical protein JCGZ_22601	Jatropha curcas	XP_012087351.1
TRINITY_DN13588_c3_g5::TRINITY_DN13588_c3_g5_i3::g.25866::m.25866	1,61E-178	499	OIW02196.1	hypothetical protein TanjilG_21849	Lupinus angustifolius	XP_019461195.1
TRINITY_DN15896_c1_g3::TRINITY_DN15896_c1_g3_i4::g.58593::m.58593	2,36E-129	372	XP_018829560.1	peroxidase 64-like	Juglans regia	OAY60924.1
TRINITY_DN15954_c0_g1::TRINITY_DN15954_c0_g1_i1::g.59385::m.59385	0	559	ONI35090.1	hypothetical protein PRUPE_1G515200	Prunus persica	XP_007227485.1
TRINITY_DN15441_c0_g2::TRINITY_DN15441_c0_g2_i3::g.51488::m.51488	3,71E-78	229	XP_018834238.1	cytochrome c	Juglans regia	OIW11132.1
TRINITY_DN18164_c1_g2::TRINITY_DN18164_c1_g2_i2::g.96243::m.96243	1,43E-170	488	KDP44975.1	hypothetical protein JCGZ_01475	Jatropha curcas	OAY46075.1
TRINITY_DN18961_c1_g1::TRINITY_DN18961_c1_g1_i1::g.109399::m.109399	2,47E-174	484	XP_018838373.1	protein CDI-like	Juglans regia	XP_016651707.1
TRINITY_DN12699_c0_g1::TRINITY_DN12699_c0_g1_i1::g.14170::m.14170	2,63E-98	283	CAA74365.1	putative Ole e 1 protein	Betula pendula	O49813.1
TRINITY_DN16736_c3_g2::TRINITY_DN16736_c3_g2_i1::g.72521::m.72521	0	737	XP_018826582.1	malate dehydrogenase, chloroplastic	Juglans regia	XP_018826583.1
TRINITY_DN13916_c2_g1::TRINITY_DN13916_c2_g1_i2::g.30380::m.30380	0	1691	XP_018832881.1	staphylococcal nuclease domain-containing protein 1-like	Juglans regia	XP_018832889.1

TRINITY_DN12824_c2_g2::TRINITY_DN12824_c2_g2_i6::g.16244::m.16244	6,11E-113	325	KDP46494.1	hypothetical protein JCGZ_08466	Jatropha curcas	XP_012067558.1
TRINITY_DN19247_c0_g1::TRINITY_DN19247_c0_g1_i9::g.114529::m.114529	0	965	XP_018859052.1	coatomer subunit delta	Juglans regia	XP_018859053.1
TRINITY_DN12966_c3_g1::TRINITY_DN12966_c3_g1_i1::g.17946::m.17946	0	1316	XP_018807321.1	aconitate hydratase 1	Juglans regia	ONH97192.1
TRINITY_DN12426_c1_g1::TRINITY_DN12426_c1_g1_i1::g.12851::m.12851	8,57E-142	407	XP_018859879.1	altered inheritance of mitochondria protein 32-like	Juglans regia	ONI19698.1
TRINITY_DN16163_c6_g1::TRINITY_DN16163_c6_g1_i3::g.63330::m.63330	4,28E-43	148	XP_007143494.1	hypothetical protein PHAVU_007G0765000g, partial	Phaseolus vulgaris	ESW15488.1
TRINITY_DN19582_c3_g2::TRINITY_DN19582_c3_g2_i6::g.120115::m.120115	1,35E-134	388	ONI24902.1	hypothetical protein PRUPE_2G268500	Prunus persica	XP_007218171.1
TRINITY_DN18840_c3_g1::TRINITY_DN18840_c3_g1_i1::g.107249::m.107249	9,76E-83	248	XP_018833669.1	chorismate mutase 2	Juglans regia	ONI32909.1
TRINITY_DN14341_c0_g1::TRINITY_DN14341_c0_g1_i1::g.35559::m.35559	0	886	XP_018820845.1	xylose isomerase	Juglans regia	XP_012065287.1
TRINITY_DN16376_c0_g2::TRINITY_DN16376_c0_g2_i2::g.66583::m.66583	0	1615	XP_018836580.1	aminopeptidase M1-like	Juglans regia	XP_018836577.1
TRINITY_DN14576_c1_g5::TRINITY_DN14576_c1_g5_i7::g.39012::m.39012	1,88E-86	252	XP_018830768.1	actin-depolymerizing factor 7	Juglans regia	XP_018828430.1
TRINITY_DN12837_c2_g2::TRINITY_DN12837_c2_g2_i7::g.16580::m.16580	1,29E-101	304	XP_018847365.1	probable polygalacturonase	Juglans regia	XP_018847358.1
TRINITY_DN19292_c1_g3::TRINITY_DN19292_c1_g3_i1::g.115388::m.115388	0	710	OAY48624.1	hypothetical protein MANES_06G172600	Manihot esculenta	OAY30259.1
TRINITY_DN19563_c2_g1::TRINITY_DN19563_c2_g1_i4::g.120333::m.120333	0	1741	XP_018839458.1	villin-4-like	Juglans regia	XP_018839459.1
TRINITY_DN15394_c0_g1::TRINITY_DN15394_c0_g1_i2::g.49883::m.49883	0	1565	XP_018837600.1	alpha-glucan phosphorylase, H isozyme-like isoform X2	Juglans regia	XP_018837598.1
TRINITY_DN17751_c2_g3::TRINITY_DN17751_c2_g3_i5::g.89327::m.89327	0	1248	XP_018848851.1	trifunctional UDP-glucose 4,6-dehydratase/UDP-4-keto-6-deoxy-D-glucose 3,5-epimerase/UDP-4-keto-L-rhamnose-reductase RHM1	Juglans regia	KHN36936.1
TRINITY_DN16246_c1_g1::TRINITY_DN16246_c1_g1_i7::g.63898::m.63898	7,06E-75	221	XP_018846792.1	peptidyl-prolyl cis-trans isomerase FKBP12	Juglans regia	AFK34981.1
TRINITY_DN17837_c2_g1::TRINITY_DN17837_c2_g1_i9::g.90611::m.90611	0	838	XP_018811769.1	aspartate aminotransferase, cytoplasmic	Juglans regia	XP_008220042.1
TRINITY_DN14185_c5_g3::TRINITY_DN14185_c5_g3_i3::g.33391::m.33391	0	624	XP_020538312.1	malate dehydrogenase, cytoplasmic isoform X1	Jatropha curcas	KDP28817.1
TRINITY_DN17690_c0_g3::TRINITY_DN17690_c0_g3_i4::g.88277::m.88277	3,47E-82	243	GAU20477.1	hypothetical protein TSUD_130350	Trifolium subterraneum	KHN13335.1
TRINITY_DN18947_c6_g5::TRINITY_DN18947_c6_g5_i2::g.109307::m.109307	1,03E-52	174	XP_008345990.1	zingipain-2-like	Malus domestica	XP_018830467.1
TRINITY_DN16121_c0_g1::TRINITY_DN16121_c0_g1_i8::g.62467::m.62467	1,31E-66	202	XP_018848559.1	coatomer subunit zeta-2-like isoform X1	Juglans regia	XP_018848561.1

TRINITY_DN19791_c7_g1::TRINITY_DN19791_c7_g1_i1::g.123736::m.123736	0	550	XP_018844468.1	serine carboxypeptidase-like 40	Juglans regia	ONI20592.1
TRINITY_DN13992_c5_g1::TRINITY_DN13992_c5_g1_i1::g.31138::m.31138	2,33E-47	153	CAA54489.1	Bet v 1 l	Betula pendula	P43185.2
TRINITY_DN18360_c0_g4::TRINITY_DN18360_c0_g4_i2::g.99196::m.99196	1,26E-173	499	XP_018814481.1	heat shock cognate 70 kDa protein 2-like	Juglans regia	ONI24313.1
TRINITY_DN17227_c2_g4::TRINITY_DN17227_c2_g4_i1::g.80549::m.80549	0	827	XP_018808164.1	flavin-dependent oxidoreductase FOX2-like	Juglans regia	XP_020230785.1
TRINITY_DN15584_c3_g1::TRINITY_DN15584_c3_g1_i1::g.54058::m.54058	3,87E-93	269	XP_018852846.1	uncharacterized protein At4g28440-like	Juglans regia	KDP26583.1
TRINITY_DN15581_c1_g3::TRINITY_DN15581_c1_g3_i1::g.54122::m.54122	0	703	XP_018855304.1	phosphoserine aminotransferase 1, chloroplastic-like	Juglans regia	XP_018852010.1
TRINITY_DN16112_c0_g1::TRINITY_DN16112_c0_g1_i3::g.62802::m.62802	1,99E-84	260	KDP35730.1	hypothetical protein JCGZ_10502	Jatropha curcas	XP_012074723.1
TRINITY_DN14318_c1_g3::TRINITY_DN14318_c1_g3_i3::g.35377::m.35377	1,35E-24	96,7	XP_008386682.1	pectinesterase inhibitor-like	Malus domestica	XP_018818001.1
TRINITY_DN15029_c2_g1::TRINITY_DN15029_c2_g1_i2::g.45973::m.45973	8,51E-60	182	XP_018847733.1	small ubiquitin-related modifier 1-like	Juglans regia	XP_018818051.1
TRINITY_DN13265_c1_g1::TRINITY_DN13265_c1_g1_i6::g.21679::m.21679	5,87E-178	493	KDP41387.1	hypothetical protein JCGZ_15794	Jatropha curcas	XP_012067879.1
TRINITY_DN17067_c1_g2::TRINITY_DN17067_c1_g2_i1::g.77892::m.77892	0	1212	XP_018851647.1	subtilisin-like protease SBT1.7 isoform X1	Juglans regia	XP_018851649.1
TRINITY_DN19292_c1_g4::TRINITY_DN19292_c1_g4_i1::g.115390::m.115390	0	1000	XP_004488810.1	elongation factor 2	Cicer arietinum	XP_004488812.1
TRINITY_DN17861_c1_g1::TRINITY_DN17861_c1_g1_i8::g.90805::m.90805	4,66E-99	289	XP_018818881.1	L-ascorbate peroxidase, cytosolic	Juglans regia	XP_018818882.1
TRINITY_DN18164_c1_g1::TRINITY_DN18164_c1_g1_i4::g.96244::m.96244	0	1229	XP_018817834.1	stromal 70 kDa heat shock-related protein, chloroplastic-like	Juglans regia	XP_018817839.1
TRINITY_DN14877_c0_g1::TRINITY_DN14877_c0_g1_i9::g.43584::m.43584	7,99E-144	409	XP_018829015.1	binding partner of ACD11 1-like	Juglans regia	KRH74377.1
TRINITY_DN12561_c1_g1::TRINITY_DN12561_c1_g1_i2::g.13897::m.13897	1,03E-35	131	XP_018842710.1	probable pectinesterase/pectinesterase inhibitor 51	Juglans regia	XP_018860059.1
TRINITY_DN20029_c3_g4::TRINITY_DN20029_c3_g4_i4::g.128107::m.128107	2,17E-75	233	XP_020218559.1	mannan endo-1,4-beta-mannosidase 7-like	Cajanus cajan	XP_018821126.1
TRINITY_DN14766_c7_g2::TRINITY_DN14766_c7_g2_i3::g.41862::m.41862	0	1092	XP_018824334.1	granule-bound starch synthase 1, chloroplastic/amyloplastic-like isoform X1	Juglans regia	XP_018824335.1
TRINITY_DN16988_c0_g1::TRINITY_DN16988_c0_g1_i6::g.76602::m.76602	0	571	XP_018850787.1	annexin D2-like	Juglans regia	XP_018834475.1
TRINITY_DN12011_c0_g1::TRINITY_DN12011_c0_g1_i3::g.9976::m.9976	3,59E-106	323	XP_018847976.1	FAM10 family protein At4g22670-like	Juglans regia	XP_018847976.1
TRINITY_DN15069_c0_g1::TRINITY_DN15069_c0_g1_i1::g.46535::m.46535	9,07E-127	360	XP_018849930.1	rho GDP-dissociation inhibitor 1-like	Juglans regia	XP_018809557.1
TRINITY_DN12538_c0_g3::TRINITY_DN12538_c0_g3_i3::g.13566::m.13566	0	657	XP_018824378.1	UDP-glucose 4-epimerase GEPI48	Juglans regia	XP_018857524.1

TRINITY_DN18548_c0_g1::TRINITY_DN18548_c0_g1_i8::g.102421::m.102421	2,33E-101	296	XP_018825058.1	elongation factor 1-delta-like	Juglans regia	XP_018825059.1
TRINITY_DN19710_c5_g3::TRINITY_DN19710_c5_g3_i4::g.122648::m.122648	0	835	XP_018850343.1	glycerophosphodiester phosphodiesterase GDPDL6-like	Juglans regia	XP_018833427.1
TRINITY_DN13376_c2_g1::TRINITY_DN13376_c2_g1_i2::g.22983::m.22983	1,59E-110	316	XP_018814979.1	peptide methionine sulfoxide reductase-like	Juglans regia	KDP30996.1
TRINITY_DN18686_c0_g1::TRINITY_DN18686_c0_g1_i9::g.104732::m.104732	0	1013	XP_018840459.1	primary amine oxidase	Juglans regia	ONI26240.1
TRINITY_DN17312_c0_g3::TRINITY_DN17312_c0_g3_i1::g.81763::m.81763	0	991	XP_018827473.1	D-3-phosphoglycerate dehydrogenase 1, chloroplastic-like	Juglans regia	XP_018851254.1
TRINITY_DN19604_c2_g3::TRINITY_DN19604_c2_g3_i3::g.120758::m.120758	1,47E-55	184	XP_008374126.1	LOW QUALITY PROTEIN: endoglucanase CX-like	Malus domestica	OAY59087.1
TRINITY_DN16248_c1_g2::TRINITY_DN16248_c1_g2_i8::g.64559::m.64559	2,37E-90	267	XP_018838677.1	inositol-phosphate phosphatase-like	Juglans regia	OAY22088.1
TRINITY_DN19596_c1_g1::TRINITY_DN19596_c1_g1_i8::g.120430::m.120430	5,17E-147	426	XP_007139719.1	hypothetical protein PHAVU_008G053600g	Phaseolus vulgaris	ESW11713.1
TRINITY_DN18641_c0_g1::TRINITY_DN18641_c0_g1_i1::g.103822::m.103822	5,35E-27	100	XP_018835725.1	EPIDERMAL PATTERNING FACTOR-like protein 6 isoform X1	Juglans regia	XP_018828520.1
TRINITY_DN14398_c0_g1::TRINITY_DN14398_c0_g1_i7::g.36405::m.36405	0	662	XP_018843013.1	NPL4-like protein 1	Juglans regia	XP_008234969.1
TRINITY_DN12966_c1_g1::TRINITY_DN12966_c1_g1_i2::g.17932::m.17932	0	859	XP_018841811.1	aconitate hydratase, cytoplasmic isoform X1	Juglans regia	XP_018841812.1
TRINITY_DN16827_c1_g1::TRINITY_DN16827_c1_g1_i8::g.73997::m.73997	0	810	XP_018827302.1	succinate--CoA ligase [ADP-forming] subunit beta, mitochondrial	Juglans regia	XP_018847166.1
TRINITY_DN13204_c1_g1::TRINITY_DN13204_c1_g1_i4::g.21099::m.21099	1,01E-55	174	XP_018821061.1	subtilisin-like protease SBT3.17	Juglans regia	XP_015953072.1
TRINITY_DN13564_c1_g1::TRINITY_DN13564_c1_g1_i6::g.26053::m.26053	1,76E-86	252	XP_018840715.1	cytochrome b5, seed isoform	Juglans regia	OAY34758.1
TRINITY_DN13916_c3_g1::TRINITY_DN13916_c3_g1_i2::g.30393::m.30393	0	705	XP_014505844.1	calnexin homolog isoform X1	Vigna radiata var. radiata	CBM36851.1
TRINITY_DN18427_c0_g1::TRINITY_DN18427_c0_g1_i8::g.100422::m.100422	1,70E-87	254	XP_018842805.1	cytochrome b5-like	Juglans regia	XP_018842806.1
TRINITY_DN14460_c2_g2::TRINITY_DN14460_c2_g2_i7::g.37472::m.37472	0	915	APO15267.1	glutathione reductase 2	Prunus avium	ONI26113.1
TRINITY_DN7586_c0_g2::TRINITY_DN7586_c0_g2_i1::g.2208::m.2208	2,69E-62	190	XP_018842089.1	basic blue protein-like	Juglans regia	OIW18081.1
TRINITY_DN19791_c7_g2::TRINITY_DN19791_c7_g2_i1::g.123738::m.123738	9,36E-33	122	GAU46802.1	hypothetical protein TSUD_268630, partial	Trifolium subterraneum	KEH17669.1
TRINITY_DN14970_c1_g1::TRINITY_DN14970_c1_g1_i3::g.45037::m.45037	0	1026	XP_018839307.1	chaperonin CPN60-2, mitochondrial	Juglans regia	XP_018850271.1
TRINITY_DN12692_c0_g1::TRINITY_DN12692_c0_g1_i7::g.14951::m.14951	0	757	XP_018838095.1	3-hydroxyisobutyryl-CoA hydrolase-like protein 3, mitochondrial isoform X1	Juglans regia	XP_018838098.1
TRINITY_DN14755_c2_g1::TRINITY_DN14755_c2_g1_i4::g.41671::m.41671	1,20E-57	186	XP_018824948.1	probable cinnamyl alcohol dehydrogenase 9	Juglans regia	ONI01833.1

TRINITY_DN18875_c2_g2::TRINITY_DN18875_c2_g2_i2::g.107929::m.107929	0	687	XP_008240928.1	actin-like	Prunus mume	XP_008240929.1
TRINITY_DN16364_c0_g1::TRINITY_DN16364_c0_g1_i6::g.66368::m.66368	0	1238	XP_018838281.1	V-type proton ATPase catalytic subunit A	Juglans regia	OAY48691.1
TRINITY_DN16368_c1_g2::TRINITY_DN16368_c1_g2_i3::g.66564::m.66564	1,61E-79	234	XP_018813964.1	ubiquitin-conjugating enzyme E2-17 kDa	Juglans regia	XP_018813970.1
TRINITY_DN16555_c1_g6::TRINITY_DN16555_c1_g6_i1::g.69342::m.69342	9,60E-71	221	ONI01209.1	hypothetical protein PRUPE_6G128200	Prunus persica	XP_007207766.1
TRINITY_DN14266_c0_g1::TRINITY_DN14266_c0_g1_i8::g.34769::m.34769	0	1471	OAY42704.1	hypothetical protein MANES_08G009500	Manihot esculenta	OAY51548.1
TRINITY_DN12221_c0_g1::TRINITY_DN12221_c0_g1_i1::g.11196::m.11196	4,06E-66	202	XP_018836953.1	early nodulin-like protein 1	Juglans regia	XP_018832258.1
TRINITY_DN14696_c0_g1::TRINITY_DN14696_c0_g1_i1::g.40665::m.40665	5,43E-61	192	XP_018810857.1	early nodulin-like protein 1	Juglans regia	KDP33508.1
TRINITY_DN19169_c1_g3::TRINITY_DN19169_c1_g3_i6::g.113174::m.113174	3,86E-72	223	XP_008237913.1	cinnamoyl-CoA reductase 2-like isoform X1	Prunus mume	KDP36740.1
TRINITY_DN13729_c0_g1::TRINITY_DN13729_c0_g1_i8::g.27674::m.27674	3,67E-169	477	XP_018816089.1	probable carboxylesterase 2	Juglans regia	XP_008220200.1
TRINITY_DN16649_c0_g2::TRINITY_DN16649_c0_g2_i3::g.71022::m.71022	0	2069	XP_018845113.1	protein TPLATE isoform X1	Juglans regia	XP_018845114.1
TRINITY_DN16233_c2_g1::TRINITY_DN16233_c2_g1_i1::g.64327::m.64327	0	509	XP_018809432.1	mitochondrial dicarboxylate/tricarboxylate transporter DTC-like	Juglans regia	OAY54741.1
TRINITY_DN16571_c1_g1::TRINITY_DN16571_c1_g1_i8::g.69835::m.69835	3,25E-132	373	XP_018807996.1	glycolipid transfer protein 1-like	Juglans regia	XP_018808001.1
TRINITY_DN18328_c0_g1::TRINITY_DN18328_c0_g1_i9::g.98876::m.98876	3,79E-145	412	XP_018823093.1	carbonic anhydrase 2-like	Juglans regia	XP_018828792.1
TRINITY_DN16032_c0_g1::TRINITY_DN16032_c0_g1_i2::g.60897::m.60897	0	627	XP_018828122.1	glutelin type-B 5-like	Juglans regia	XP_018828123.1
TRINITY_DN12769_c0_g3::TRINITY_DN12769_c0_g3_i1::g.15717::m.15717	2,81E-59	184	XP_018844068.1	glycine-rich RNA-binding protein 2, mitochondrial-like	Juglans regia	XP_018844069.1
TRINITY_DN17019_c1_g3::TRINITY_DN17019_c1_g3_i1::g.77181::m.77181	5,54E-120	343	XP_018819182.1	ran-binding protein 1 homolog a-like	Juglans regia	XP_018835624.1
TRINITY_DN17448_c2_g3::TRINITY_DN17448_c2_g3_i9::g.83903::m.83903	0	1224	XP_018857281.1	villin-3-like isoform X1	Juglans regia	XP_018857281.1
TRINITY_DN16630_c2_g1::TRINITY_DN16630_c2_g1_i6::g.70777::m.70777	5,83E-135	382	XP_018806495.1	14-3-3-like protein	Juglans regia	XP_016163651.1
TRINITY_DN12516_c0_g1::TRINITY_DN12516_c0_g1_i2::g.13472::m.13472	0	1511	XP_018824768.1	sucrose synthase 2	Juglans regia	AGM14947.1
TRINITY_DN12883_c0_g1::TRINITY_DN12883_c0_g1_i6::g.16879::m.16879	5,81E-132	375	XP_018858784.1	lactoylglutathione lyase isoform X1	Juglans regia	XP_008385523.1
TRINITY_DN19263_c0_g3::TRINITY_DN19263_c0_g3_i4::g.114811::m.114811	0	765	XP_018837044.1	probable cytosolic oligopeptidase A	Juglans regia	XP_018813437.1
TRINITY_DN19354_c2_g2::TRINITY_DN19354_c2_g2_i9::g.116448::m.116448	3,31E-104	303	XP_018835464.1	glutathione S-transferase-like	Juglans regia	XP_018857389.1

TRINITY_DN13726_c0_g3::TRINITY_DN13726_c0_g3_i1::g.27596::m.27596	0	887	XP_008380545.1	elongation factor 1-alpha	Malus domestica	XP_008365661.1
TRINITY_DN20049_c7_g1::TRINITY_DN20049_c7_g1_i11::g.128495::m.128495	0	747	XP_018806799.1	glycerol kinase	Juglans regia	XP_008372701.1
TRINITY_DN11737_c0_g1::TRINITY_DN11737_c0_g1_i1::g.8601::m.8601	1,08E-96	279	XP_018819438.1	nucleoside diphosphate kinase B	Juglans regia	OIW18826.1
TRINITY_DN15578_c0_g1::TRINITY_DN15578_c0_g1_i2::g.54153::m.54153	6,39E-111	316	GAU49703.1	hypothetical protein TSUD_182010	Trifolium subterraneum	OIW17591.1
TRINITY_DN15120_c1_g1::TRINITY_DN15120_c1_g1_i1::g.47172::m.47172	1,59E-176	496	XP_018841745.1	uncharacterized protein At5g02240-like	Juglans regia	ONI03446.1
TRINITY_DN11278_c0_g2::TRINITY_DN11278_c0_g2_i1::g.6843::m.6843	9,20E-56	178	XP_018826050.1	acyl carrier protein 1, chloroplastic-like	Juglans regia	XP_018840594.1
TRINITY_DN19745_c0_g1::TRINITY_DN19745_c0_g1_i7::g.123245::m.123245	0	1483	XP_018822921.1	methionine--tRNA ligase, cytoplasmic	Juglans regia	XP_008232482.1
TRINITY_DN13377_c3_g2::TRINITY_DN13377_c3_g2_i9::g.23085::m.23085	1,76E-135	384	XP_008221708.1	glutathione S-transferase U17-like	Prunus mume	ONI30654.1
TRINITY_DN15863_c0_g2::TRINITY_DN15863_c0_g2_i4::g.58135::m.58135	0	593	OAY46717.1	hypothetical protein MANES_06G021500	Manihot esculenta	KYP62381.1
TRINITY_DN18548_c0_g1::TRINITY_DN18548_c0_g1_i3::g.102416::m.102416	3,32E-125	358	XP_018825058.1	elongation factor 1-delta-like	Juglans regia	XP_018825059.1
TRINITY_DN10779_c0_g1::TRINITY_DN10779_c0_g1_i3::g.5511::m.5511	0	546	ABW06959.1	isopentenyl pyrophosphate isomerase	Corylus avellana	XP_018815655.1
TRINITY_DN15451_c0_g1::TRINITY_DN15451_c0_g1_i8::g.52025::m.52025	0	725	XP_018825160.1	ubiquitin-like modifier-activating enzyme 5	Juglans regia	XP_009368530.1
TRINITY_DN14435_c3_g2::TRINITY_DN14435_c3_g2_i6::g.36467::m.36467	0	934	XP_018849756.1	ATP synthase subunit beta, mitochondrial	Juglans regia	XP_008243316.1
TRINITY_DN19361_c0_g1::TRINITY_DN19361_c0_g1_i10::g.117059::m.117059	0	1853	XP_018826880.1	DNA polymerase V	Juglans regia	XP_008237045.1
TRINITY_DN11635_c0_g1::TRINITY_DN11635_c0_g1_i7::g.8217::m.8217	0	748	XP_018843179.1	fructose-bisphosphate aldolase 3, chloroplastic	Juglans regia	ONI31556.1
TRINITY_DN12062_c0_g1::TRINITY_DN12062_c0_g1_i4::g.10228::m.10228	0	931	KDP45160.1	hypothetical protein JCGZ_15025	Jatropha curcas	XP_012083963.1
TRINITY_DN13692_c1_g1::TRINITY_DN13692_c1_g1_i1::g.26628::m.26628	0	611	CAA05979.1	adenine nucleotide translocator	Lupinus albus	XP_018816668.1
TRINITY_DN16939_c1_g2::TRINITY_DN16939_c1_g2_i8::g.75928::m.75928	2,61E-127	361	XP_018813989.1	vesicle transport v-SNARE 13-like	Juglans regia	XP_018813990.1
TRINITY_DN13776_c0_g2::TRINITY_DN13776_c0_g2_i2::g.28097::m.28097	4,27E-147	417	XP_018844827.1	adenosine kinase 2-like	Juglans regia	XP_008233303.1
TRINITY_DN15671_c0_g2::TRINITY_DN15671_c0_g2_i5::g.55277::m.55277	0	1055	XP_018826687.1	glucose-6-phosphate isomerase, cytosolic	Juglans regia	KRH61180.1
TRINITY_DN18192_c1_g2::TRINITY_DN18192_c1_g2_i5::g.96646::m.96646	0	566	XP_018811421.1	polyadenylate-binding protein RBP45C-like	Juglans regia	XP_018824161.1
TRINITY_DN18029_c5_g1::TRINITY_DN18029_c5_g1_i6::g.93985::m.93985	0	849	XP_018822246.1	aspartate aminotransferase P2, mitochondrial-like	Juglans regia	XP_018822247.1

TRINITY_DN14933_c0_g2::TRINITY_DN14933_c0_g2_i3::g.44460::m.44460	7,94E-104	299	XP_018836279.1	uncharacterized protein LOC109002826	Juglans regia	XP_018836280.1
TRINITY_DN16437_c1_g2::TRINITY_DN16437_c1_g2_i4::g.67526::m.67526	0	947	XP_018844065.1	serine hydroxymethyltransferase 4	Juglans regia	XP_018848538.1
TRINITY_DN19108_c2_g3::TRINITY_DN19108_c2_g3_i2::g.112222::m.112222	1,08E-64	209	XP_018858996.1	receptor-like protein kinase FERONIA	Juglans regia	XP_018858995.1
TRINITY_DN18028_c1_g1::TRINITY_DN18028_c1_g1_i5::g.93929::m.93929	0	882	XP_018844173.1	peptidyl-prolyl cis-trans isomerase FKBP62-like isoform X1	Juglans regia	XP_018844174.1
TRINITY_DN12699_c0_g1::TRINITY_DN12699_c0_g1_i7::g.14174::m.14174	5,47E-57	178	CAA74365.1	putative Ole e 1 protein	Betula pendula	O49813.1
TRINITY_DN19669_c1_g1::TRINITY_DN19669_c1_g1_i10::g.121794::m.121794	1,19E-103	301	XP_018807198.1	uncharacterized protein LOC108980651	Juglans regia	OAY33079.1
TRINITY_DN14038_c1_g2::TRINITY_DN14038_c1_g2_i5::g.31646::m.31646	0	755	XP_018835118.1	eukaryotic initiation factor 4A-15	Juglans regia	XP_018835119.1
TRINITY_DN16581_c1_g3::TRINITY_DN16581_c1_g3_i2::g.69928::m.69928	0	1092	ONI09648.1	hypothetical protein PRUPE_4G001800	Prunus persica	XP_020418597.1
TRINITY_DN16667_c0_g2::TRINITY_DN16667_c0_g2_i1::g.71232::m.71232	0	1286	XP_018848062.1	transketolase, chloroplastic	Juglans regia	XP_018856789.1
TRINITY_DN16967_c3_g1::TRINITY_DN16967_c3_g1_i3::g.76257::m.76257	0	544	XP_018823791.1	triosephosphate isomerase, chloroplastic	Juglans regia	XP_020425754.1
TRINITY_DN14845_c0_g2::TRINITY_DN14845_c0_g2_i5::g.43176::m.43176	0	969	OAY52288.1	hypothetical protein MANES_04G071200	Manihot esculenta	XP_004492897.1
TRINITY_DN9429_c0_g1::TRINITY_DN9429_c0_g1_i1::g.3466::m.3466	0	723	XP_018857901.1	alpha-galactosidase 1-like	Juglans regia	XP_008389053.1
TRINITY_DN12329_c1_g1::TRINITY_DN12329_c1_g1_i1::g.11925::m.11925	0	656	XP_018841575.1	uncharacterized protein At2g17340-like	Juglans regia	XP_018841576.1
TRINITY_DN17549_c7_g7::TRINITY_DN17549_c7_g7_i1::g.85830::m.85830	8,26E-30	115	XP_018858816.1	probable receptor-like protein kinase At5g24010	Juglans regia	XP_018845045.1
TRINITY_DN16210_c2_g2::TRINITY_DN16210_c2_g2_i2::g.64102::m.64102	8,37E-146	409	XP_015937554.1	GTP-binding nuclear protein Ran-3	Arachis duranensis	XP_018841051.1
TRINITY_DN18132_c2_g1::TRINITY_DN18132_c2_g1_i3::g.95646::m.95646	8,41E-49	155	XP_018821975.1	thioredoxin H-type	Juglans regia	XP_018821976.1
TRINITY_DN12647_c0_g1::TRINITY_DN12647_c0_g1_i2::g.14733::m.14733	4,69E-126	380	XP_018847842.1	coatomer subunit gamma-2	Juglans regia	XP_018836922.1
TRINITY_DN19952_c2_g2::TRINITY_DN19952_c2_g2_i3::g.126557::m.126557	0	556	XP_018809774.1	protein transport protein SEC31 homolog B-like isoform X1	Juglans regia	XP_018809776.1
TRINITY_DN17973_c0_g3::TRINITY_DN17973_c0_g3_i3::g.92497::m.92497	0	1154	KYP61930.1	Heat shock protein 83	Cajanus cajan	XP_020220602.1
TRINITY_DN16421_c0_g1::TRINITY_DN16421_c0_g1_i1::g.67240::m.67240	0	736	XP_018837352.1	beta-fructofuranosidase, insoluble isoenzyme 1-like	Juglans regia	XP_009372510.1
TRINITY_DN32757_c0_g1::TRINITY_DN32757_c0_g1_i1::g.130429::m.130429	5,41E-10	60,1	XP_018831391.1	uncharacterized protein LOC108999052	Juglans regia	XP_018806439.1
TRINITY_DN14021_c1_g2::TRINITY_DN14021_c1_g2_i5::g.31431::m.31431	0	614	OAY50881.1	hypothetical protein MANES_05G169500	Manihot esculenta	XP_008220716.1

TRINITY_DN20011_c3_g2::TRINITY_DN20011_c3_g2_i2::g.127835::m.127835	0	973	XP_018845708.1	beta-galactosidase-like	Juglans regia	XP_018845720.1
TRINITY_DN16305_c0_g1::TRINITY_DN16305_c0_g1_i3::g.65508::m.65508	1,83E-69	216	XP_018813581.1	uncharacterized protein Os08g0359500	Juglans regia	OAY49527.1
TRINITY_DN12041_c0_g1::TRINITY_DN12041_c0_g1_i1::g.10132::m.10132	6,20E-174	487	XP_018826881.1	soluble inorganic pyrophosphatase 6, chloroplastic-like	Juglans regia	XP_018844455.1
TRINITY_DN15348_c2_g2::TRINITY_DN15348_c2_g2_i4::g.50676::m.50676	0	621	XP_018829808.1	probable UDP-arabinopyranose mutase 5 isoform X1	Juglans regia	XP_018829809.1
TRINITY_DN14545_c0_g2::TRINITY_DN14545_c0_g2_i2::g.38716::m.38716	0	861	XP_016204269.1	tubulin beta-1 chain	Arachis ipaensis	XP_015967408.1
TRINITY_DN13014_c6_g2::TRINITY_DN13014_c6_g2_i4::g.18867::m.18867	0	858	KDP44552.1	hypothetical protein JCGZ_16385	Jatropha curcas	OAY39347.1
TRINITY_DN16814_c2_g1::TRINITY_DN16814_c2_g1_i8::g.73807::m.73807	2,50E-102	299	XP_018807368.1	probable ATP synthase 24 kDa subunit, mitochondrial isoform X2	Juglans regia	XP_018807367.1
TRINITY_DN14872_c2_g2::TRINITY_DN14872_c2_g2_i2::g.43670::m.43670	0	2539	XP_018805215.1	alpha-glucan water dikinase, chloroplastic isoform X2	Juglans regia	XP_018805213.1
TRINITY_DN13283_c0_g1::TRINITY_DN13283_c0_g1_i3::g.21813::m.21813	0	641	XP_018822731.1	60S ribosomal protein L4	Juglans regia	XP_018850207.1
TRINITY_DN14986_c4_g1::TRINITY_DN14986_c4_g1_i9::g.45372::m.45372	0	926	XP_018822123.1	xylulose kinase	Juglans regia	XP_018822124.1
TRINITY_DN13825_c0_g2::TRINITY_DN13825_c0_g2_i1::g.28850::m.28850	5,82E-92	273	XP_020971753.1	NADPH-dependent aldo-keto reductase, chloroplastic isoform X2	Arachis ipaensis	XP_020971752.1
TRINITY_DN19496_c3_g4::TRINITY_DN19496_c3_g4_i2::g.118720::m.118720	1,28E-49	162	XP_018837516.1	uncharacterized protein LOC109003708	Juglans regia	XP_008367865.1
TRINITY_DN19996_c7_g1::TRINITY_DN19996_c7_g1_i2::g.127540::m.127540	0	941	XP_018814251.1	dihydrolipoyl dehydrogenase 1, mitochondrial	Juglans regia	XP_018814252.1
TRINITY_DN16132_c1_g1::TRINITY_DN16132_c1_g1_i4::g.62630::m.62630	2,36E-78	233		2121384A calmodulin		
TRINITY_DN12592_c2_g1::TRINITY_DN12592_c2_g1_i5::g.14064::m.14064	6,40E-47	164	XP_018847842.1	coatomer subunit gamma-2	Juglans regia	XP_018836922.1
TRINITY_DN15525_c0_g1::TRINITY_DN15525_c0_g1_i8::g.53299::m.53299	0	1168	XP_018839760.1	subtilisin-like protease SBT4.15	Juglans regia	OAY42882.1
TRINITY_DN19723_c2_g3::TRINITY_DN19723_c2_g3_i3::g.122857::m.122857	1,75E-165	469	XP_018857981.1	phosphomethylethanolamine N-methyltransferase isoform X2	Juglans regia	XP_018857980.1
TRINITY_DN18422_c0_g3::TRINITY_DN18422_c0_g3_i1::g.100190::m.100190	0	939	OAY60355.1	hypothetical protein MANES_01G105600	Manihot esculenta	OAY60356.1
TRINITY_DN13563_c1_g1::TRINITY_DN13563_c1_g1_i8::g.25419::m.25419	0	2294	XP_018815316.1	coatomer subunit alpha-1	Juglans regia	XP_018840319.1
TRINITY_DN18526_c1_g2::TRINITY_DN18526_c1_g2_i7::g.101942::m.101942	0	1061	XP_018829411.1	probable Xaa-Pro aminopeptidase P isoform X1	Juglans regia	XP_018829412.1
TRINITY_DN13666_c1_g1::TRINITY_DN13666_c1_g1_i1::g.26956::m.26956	0	1174	ONI31876.1	hypothetical protein PRUPE_1G336300	Prunus persica	XP_007225345.1
TRINITY_DN13901_c0_g1::TRINITY_DN13901_c0_g1_i11::g.28513::m.28513	1,45E-59	188	XP_018835853.1	thioredoxin O2, mitochondrial-like isoform X1	Juglans regia	XP_018835854.1

TRINITY_DN12879_c0_g1::TRINITY_DN12879_c0_g1_i1::g.16746::m.16746	1,33E-165	462	XP_018839634.1	S-formylglutathione hydrolase	Juglans regia	KDP26090.1
TRINITY_DN15261_c0_g1::TRINITY_DN15261_c0_g1_i6::g.49258::m.49258	0	744	XP_018840769.1	protein disulfide isomerase-like 2-3	Juglans regia	XP_018827031.1
TRINITY_DN11924_c0_g1::TRINITY_DN11924_c0_g1_i1::g.9513::m.9513	0	726	ONH93092.1	hypothetical protein PRUPE_8G212700	Prunus persica	XP_020425931.1
TRINITY_DN18749_c0_g2::TRINITY_DN18749_c0_g2_i4::g.105830::m.105830	0	554	XP_020221626.1	ferredoxin--NADP reductase, root isozyme, chloroplastic isoform X1	Cajanus cajan	XP_020221627.1
TRINITY_DN18258_c0_g1::TRINITY_DN18258_c0_g1_i1::g.97653::m.97653	9,01E-63	199	XP_008236492.1	probable mannitol dehydrogenase	Prunus mume	XP_008236513.1
TRINITY_DN17853_c1_g4::TRINITY_DN17853_c1_g4_i19::g.90697::m.90697	5,34E-151	422	XP_018815728.1	acylpyruvase FAHD1, mitochondrial-like	Juglans regia	XP_018806160.1
TRINITY_DN11160_c0_g1::TRINITY_DN11160_c0_g1_i1::g.6484::m.6484	7,56E-133	381	XP_018847735.1	xyloglucan endotransglucosylase/hydrolase protein 2-like	Juglans regia	OAY32796.1
TRINITY_DN15352_c3_g1::TRINITY_DN15352_c3_g1_i9::g.50776::m.50776	0	1063	XP_018819865.1	polyadenylate-binding protein 2-like	Juglans regia	XP_018822308.1
TRINITY_DN19260_c1_g1::TRINITY_DN19260_c1_g1_i11::g.114784::m.114784	0	729	XP_008394248.1	guanosine nucleotide diphosphate dissociation inhibitor 2	Malus domestica	XP_008243091.1
TRINITY_DN18987_c4_g3::TRINITY_DN18987_c4_g3_i1::g.110065::m.110065	2,42E-142	408	XP_018819069.1	uncharacterized protein LOC108989783, partial	Juglans regia	XP_018857833.1
TRINITY_DN12537_c1_g2::TRINITY_DN12537_c1_g2_i7::g.13611::m.13611	1,87E-55	185	XP_008225664.2	reticuline oxidase-like protein	Prunus mume	ONI11277.1
TRINITY_DN19187_c2_g2::TRINITY_DN19187_c2_g2_i1::g.113564::m.113564	2,05E-116	333	XP_018838134.1	nitrogen regulatory protein P-II homolog	Juglans regia	XP_018824177.1
TRINITY_DN12599_c0_g1::TRINITY_DN12599_c0_g1_i6::g.14058::m.14058	0	815	KDP44002.1	hypothetical protein JCGZ_05469	Jatropha curcas	XP_012064751.1
TRINITY_DN19040_c3_g4::TRINITY_DN19040_c3_g4_i1::g.110853::m.110853	0	592	XP_018819156.1	uncharacterized protein LOC108989851	Juglans regia	XP_018828611.1
TRINITY_DN14858_c0_g1::TRINITY_DN14858_c0_g1_i1::g.43399::m.43399	0	2059	XP_018819183.1	ubiquitin-activating enzyme E1 1-like	Juglans regia	XP_018828478.1
TRINITY_DN18296_c0_g1::TRINITY_DN18296_c0_g1_i5::g.98309::m.98309	1,36E-126	362	XP_018808145.1	uncharacterized protein LOC108981433 isoform X2	Juglans regia	XP_018808144.1
TRINITY_DN13876_c0_g1::TRINITY_DN13876_c0_g1_i2::g.29614::m.29614	1,37E-173	484	XP_018843199.1	glutathione S-transferase DHAR3, chloroplastic-like	Juglans regia	XP_018816866.1
TRINITY_DN11399_c0_g1::TRINITY_DN11399_c0_g1_i3::g.6893::m.6893	8,82E-179	496	XP_018818651.1	40S ribosomal protein S3a-like	Juglans regia	XP_018826766.1
TRINITY_DN16378_c0_g2::TRINITY_DN16378_c0_g2_i3::g.66649::m.66649	0	563	XP_018839537.1	probable protein phosphatase 2C 59	Juglans regia	XP_018839538.1
TRINITY_DN19790_c2_g2::TRINITY_DN19790_c2_g2_i1::g.123647::m.123647	3,23E-33	120	AAC05116.2	isoflavone reductase homolog Bet v 6.0101, partial	Betula pendula	AAG22740.1
TRINITY_DN13975_c1_g4::TRINITY_DN13975_c1_g4_i3::g.30944::m.30944	2,08E-64	203	XP_018813802.1	aldose 1-epimerase-like	Juglans regia	XP_008341553.1
TRINITY_DN13815_c1_g2::TRINITY_DN13815_c1_g2_i6::g.28882::m.28882	0	698	XP_018814476.1	UDP-glucuronic acid decarboxylase 6	Juglans regia	XP_018814477.1

TRINITY_DN19396_c1_g1::TRINITY_DN19396_c1_g1_i8::g.116313::m.116313	0	1807	XP_018830041.1	ABC transporter B family member 9 isoform X1	Juglans regia	XP_018830041.1
TRINITY_DN18341_c1_g1::TRINITY_DN18341_c1_g1_i3::g.99097::m.99097	3,51E-33	114	AAD10247.1	calmodulin, partial	Phaseolus vulgaris	2RO9
TRINITY_DN13865_c0_g1::TRINITY_DN13865_c0_g1_i3::g.29082::m.29082	0	975	XP_018848687.1	glucose-1-phosphate adenylyltransferase large subunit 1-like isoform X1	Juglans regia	XP_018821124.1
TRINITY_DN16815_c5_g2::TRINITY_DN16815_c5_g2_i1::g.73817::m.73817	0	764	AET62947.1	ATPase subunit 1 (mitochondrion)	Lotus japonicus	YP_005090487.1
TRINITY_DN14080_c2_g1::TRINITY_DN14080_c2_g1_i10::g.32162::m.32162	1,82E-154	439	ONI07528.1	hypothetical protein PRUPE_5G125900	Prunus persica	ONI07527.1
TRINITY_DN15949_c1_g2::TRINITY_DN15949_c1_g2_i8::g.59514::m.59514	0	559	XP_018838600.1	protein SGT1 homolog	Juglans regia	KDP22207.1
TRINITY_DN19409_c5_g5::TRINITY_DN19409_c5_g5_i2::g.117475::m.117475	8,38E-27	105	XP_004509666.1	uncharacterized protein LOC101514840	Cicer arietinum	KDP40819.1
TRINITY_DN19604_c2_g1::TRINITY_DN19604_c2_g1_i2::g.120755::m.120755	6,92E-103	309	KDP25211.1	hypothetical protein JCGZ_20367	Jatropha curcas	XP_012086616.1
TRINITY_DN17204_c0_g1::TRINITY_DN17204_c0_g1_i8::g.80281::m.80281	0	644	XP_018814614.1	probable mitochondrial-processing peptidase subunit beta, mitochondrial isoform X2	Juglans regia	XP_018814613.1
TRINITY_DN16901_c2_g1::TRINITY_DN16901_c2_g1_i2::g.75153::m.75153	0	1242	XP_018811791.1	probable LRR receptor-like serine/threonine-protein kinase At2g16250	Juglans regia	XP_018847822.1
TRINITY_DN16836_c1_g3::TRINITY_DN16836_c1_g3_i3::g.74224::m.74224	0	775	XP_018847838.1	cyclase-associated protein 1-like	Juglans regia	XP_018807064.1
TRINITY_DN16865_c3_g3::TRINITY_DN16865_c3_g3_i2::g.74745::m.74745	0	1078	XP_018850062.1	acyl-CoA-binding domain-containing protein 4	Juglans regia	OAY42302.1
TRINITY_DN18894_c1_g1::TRINITY_DN18894_c1_g1_i9::g.108231::m.108231	3,54E-69	212	XP_018816665.1	14-3-3-like protein A	Juglans regia	XP_018816666.1
TRINITY_DN19186_c1_g1::TRINITY_DN19186_c1_g1_i2::g.113474::m.113474	1,95E-82	260	XP_018837618.1	5-methyltetrahydropteroyltriglutamate--homocysteine methyltransferase-like	Juglans regia	XP_018837619.1
TRINITY_DN20002_c1_g1::TRINITY_DN20002_c1_g1_i9::g.127794::m.127794	0	1036	XP_018837398.1	ruBisCO large subunit-binding protein subunit beta, chloroplastic	Juglans regia	ONH98963.1
TRINITY_DN18281_c0_g1::TRINITY_DN18281_c0_g1_i4::g.98025::m.98025	0	2047	XP_018823942.1	DNA-directed RNA polymerase I subunit 2	Juglans regia	KRH20666.1
TRINITY_DN13211_c0_g2::TRINITY_DN13211_c0_g2_i3::g.21096::m.21096	0	734	ADR71240.1	60S ribosomal protein L3B	Hevea brasiliensis	XP_018848152.1
TRINITY_DN10140_c0_g1::TRINITY_DN10140_c0_g1_i2::g.4317::m.4317	0	553	XP_018814951.1	senescence-specific cysteine protease SAG39-like	Juglans regia	XP_018838527.1
TRINITY_DN14435_c4_g2::TRINITY_DN14435_c4_g2_i1::g.36484::m.36484	0	520	XP_018815503.1	peroxidase 65-like	Juglans regia	XP_018805662.1
TRINITY_DN12440_c1_g2::TRINITY_DN12440_c1_g2_i1::g.13039::m.13039	0	927	KDP26565.1	hypothetical protein JCGZ_17723	Jatropha curcas	XP_012085348.1
TRINITY_DN14413_c0_g1::TRINITY_DN14413_c0_g1_i13::g.36824::m.36824	0	674	XP_018806700.1	pyruvate dehydrogenase E1 component subunit beta-1, mitochondrial-like	Juglans regia	ONI29891.1
TRINITY_DN17675_c2_g3::TRINITY_DN17675_c2_g3_i6::g.88141::m.88141	7,59E-102	299	XP_018834430.1	ATP synthase subunit O, mitochondrial-like	Juglans regia	OAY40030.1

TRINITY_DN14025_c2_g1::TRINITY_DN14025_c2_g1_i8::g.31522::m.31522	0	967	XP_018848178.1	ubiquitin carboxyl-terminal hydrolase FAM188A-like isoform X1	Juglans regia	XP_018848179.1
TRINITY_DN11357_c0_g2::TRINITY_DN11357_c0_g2_i1::g.7096::m.7096	2,39E-76	226	ADB93072.1	profilin-1	Jatropha curcas	KDP33778.1
TRINITY_DN13606_c2_g2::TRINITY_DN13606_c2_g2_i2::g.26272::m.26272	0	946	XP_018818494.1	UDP-glucose 6-dehydrogenase 4	Juglans regia	XP_018831898.1
TRINITY_DN15004_c1_g1::TRINITY_DN15004_c1_g1_i9::g.45691::m.45691	0	1154	XP_018843066.1	pyrophosphate--fructose 6-phosphate 1-phosphotransferase subunit alpha	Juglans regia	XP_018832985.1
TRINITY_DN20046_c4_g1::TRINITY_DN20046_c4_g1_i6::g.128432::m.128432	0	2001	XP_018810025.1	2-oxoglutarate dehydrogenase, mitochondrial-like	Juglans regia	XP_018810026.1
TRINITY_DN17290_c1_g1::TRINITY_DN17290_c1_g1_i4::g.81361::m.81361	3,11E-97	285	XP_018818935.1	superoxide dismutase [Cu-Zn], chloroplastic	Juglans regia	XP_012064680.1
TRINITY_DN12342_c1_g3::TRINITY_DN12342_c1_g3_i1::g.12105::m.12105	1,36E-69	213	XP_018844610.1	mavicyanin-like	Juglans regia	OAY29562.1
TRINITY_DN12893_c0_g1::TRINITY_DN12893_c0_g1_i1::g.17069::m.17069	6,95E-48	151	XP_018860422.1	protein BOLA2	Juglans regia	ACU15761.1
TRINITY_DN16995_c1_g3::TRINITY_DN16995_c1_g3_i3::g.76630::m.76630	4,74E-102	294	XP_018850024.1	translationally-controlled tumor protein homolog	Juglans regia	AES61399.1
TRINITY_DN14421_c2_g1::TRINITY_DN14421_c2_g1_i4::g.36938::m.36938	1,61E-110	315	AGZ15424.1	ADP-ribosylation factor 1	Phaseolus vulgaris	XP_007151018.1
TRINITY_DN13126_c1_g4::TRINITY_DN13126_c1_g4_i1::g.20159::m.20159	2,40E-82	243	KYP67019.1	Ras-related protein RABH1B	Cajanus cajan	XP_018843528.1
TRINITY_DN16509_c1_g1::TRINITY_DN16509_c1_g1_i1::g.68768::m.68768	0	1110	XP_018839329.1	methylenetetrahydrofolate reductase 2-like	Juglans regia	XP_018858229.1
TRINITY_DN19751_c2_g1::TRINITY_DN19751_c2_g1_i6::g.123209::m.123209	0	767	XP_018817260.1	3-ketoacyl-CoA thiolase 2, peroxisomal	Juglans regia	XP_018809682.1
TRINITY_DN17891_c2_g1::TRINITY_DN17891_c2_g1_i4::g.91423::m.91423	9,90E-76	226	KDP33752.1	hypothetical protein JCGZ_07323	Jatropha curcas	XP_012076803.1
TRINITY_DN18954_c0_g3::TRINITY_DN18954_c0_g3_i4::g.109379::m.109379	0	934	XP_018807686.1	3-isopropylmalate dehydratase large subunit, chloroplastic-like	Juglans regia	KDP33327.1
TRINITY_DN17145_c0_g3::TRINITY_DN17145_c0_g3_i2::g.79319::m.79319	0	1744	XP_018846325.1	clathrin heavy chain 1	Juglans regia	XP_018835615.1
TRINITY_DN16061_c2_g2::TRINITY_DN16061_c2_g2_i3::g.61317::m.61317	0	519	OAY47507.1	hypothetical protein MANES_06G084900	Manihot esculenta	KDP31346.1
TRINITY_DN19588_c6_g1::TRINITY_DN19588_c6_g1_i5::g.120209::m.120209	0	1000	XP_018814240.1	citrate synthase, glyoxysomal	Juglans regia	XP_018828184.1
TRINITY_DN13159_c0_g1::TRINITY_DN13159_c0_g1_i1::g.20565::m.20565	8,27E-102	296	XP_018821777.1	peptidyl-prolyl cis-trans isomerase CYP20-1-like isoform X2	Juglans regia	XP_018821776.1
TRINITY_DN13164_c0_g4::TRINITY_DN13164_c0_g4_i1::g.20741::m.20741	0	811	XP_018830447.1	isocitrate dehydrogenase [NADP-like]	Juglans regia	XP_018809643.1
TRINITY_DN16361_c1_g1::TRINITY_DN16361_c1_g1_i5::g.66288::m.66288	0	885	XP_018811089.1	mitochondrial-processing peptidase subunit alpha-like isoform X1	Juglans regia	XP_018843285.1
TRINITY_DN15008_c0_g1::TRINITY_DN15008_c0_g1_i3::g.45737::m.45737	0	1804	OAY37891.1	hypothetical protein MANES_11G137000	Manihot esculenta	KDP44163.1

TRINITY_DN17275_c2_g1::TRINITY_DN17275_c2_g1_i6::g.81136::m.81136	5,57E-175	489	XP_018852330.1	60S ribosomal protein L5	Juglans regia	XP_018840429.1
TRINITY_DN18883_c1_g1::TRINITY_DN18883_c1_g1_i14::g.108273::m.108273	0	1363	OAY36779.1	hypothetical protein MANES_11G047600	Manihot esculenta	KDP37205.1
TRINITY_DN14831_c2_g1::TRINITY_DN14831_c2_g1_i4::g.42949::m.42949	1,52E-102	297	XP_008353433.1	vesicle-associated protein 1-2-like	Malus domestica	XP_018853856.1
TRINITY_DN17427_c0_g1::TRINITY_DN17427_c0_g1_i16::g.83642::m.83642	0	1152	XP_018852048.1	uncharacterized protein LOC109014152 isoform X1	Juglans regia	XP_018852049.1
TRINITY_DN19711_c3_g1::TRINITY_DN19711_c3_g1_i1::g.122630::m.122630	0	618	XP_018834985.1	thioredoxin reductase 1-like	Juglans regia	XP_004512292.1
TRINITY_DN19077_c0_g1::TRINITY_DN19077_c0_g1_i1::g.111277::m.111277	0	525	XP_018841469.1	L-ascorbate peroxidase 3, peroxisomal-like	Juglans regia	XP_018823838.1
TRINITY_DN13164_c0_g2::TRINITY_DN13164_c0_g2_i1::g.20739::m.20739	0	879	XP_018842395.1	isocitrate dehydrogenase [NADP]	Juglans regia	XP_012081175.1
TRINITY_DN20023_c4_g2::TRINITY_DN20023_c4_g2_i4::g.127985::m.127985	0	1175	XP_020425453.1	coatomer subunit gamma isoform X1	Prunus persica	OAY25507.1
TRINITY_DN18086_c1_g2::TRINITY_DN18086_c1_g2_i3::g.94830::m.94830	1,24E-38	131	XP_018825823.1	lipid transfer-like protein VAS isoform X1	Juglans regia	XP_018825824.1
TRINITY_DN13268_c1_g1::TRINITY_DN13268_c1_g1_i7::g.21886::m.21886	0	815	XP_018823304.1	argininosuccinate synthase, chloroplastic	Juglans regia	XP_018825130.1
TRINITY_DN12877_c0_g1::TRINITY_DN12877_c0_g1_i2::g.16745::m.16745	1,44E-100	296	OAY27067.1	hypothetical protein MANES_16G097000	Manihot esculenta	XP_018832824.1
TRINITY_DN15082_c2_g5::TRINITY_DN15082_c2_g5_i1::g.46709::m.46709	4,70E-119	352	XP_018805570.1	aspartyl protease family protein 2	Juglans regia	ONI11778.1
TRINITY_DN17469_c0_g1::TRINITY_DN17469_c0_g1_i8::g.84329::m.84329	4,13E-144	403	CAY07621.1	unnamed protein product	Glycine max	KRH04201.1
TRINITY_DN16031_c0_g3::TRINITY_DN16031_c0_g3_i1::g.60905::m.60905	0	1123	XP_018813002.1	actin-interacting protein 1-2-like	Juglans regia	KDP41126.1
TRINITY_DN13415_c1_g3::TRINITY_DN13415_c1_g3_i4::g.23607::m.23607	0	1951	XP_018809529.1	phosphoenolpyruvate carboxylase 4-like isoform X1	Juglans regia	XP_018853146.1
TRINITY_DN13872_c1_g1::TRINITY_DN13872_c1_g1_i2::g.29678::m.29678	1,75E-100	288	XP_018822739.1	14 kDa zinc-binding protein	Juglans regia	XP_012088435.1
TRINITY_DN14508_c8_g1::TRINITY_DN14508_c8_g1_i4::g.38373::m.38373	4,59E-92	268	XP_018830082.1	desiccation protectant protein Lea14 homolog	Juglans regia	XP_018847006.1
TRINITY_DN15591_c0_g2::TRINITY_DN15591_c0_g2_i2::g.54135::m.54135	6,42E-46	172	XP_018830686.1	low-temperature-induced 65 kDa protein-like isoform X2	Juglans regia	OAY48291.1
TRINITY_DN19628_c3_g1::TRINITY_DN19628_c3_g1_i3::g.121141::m.121141	7,44E-135	385	XP_014495844.1	glutamine synthetase nodule isozyme isoform X2	Vigna radiata var. radiata	XP_018823640.1
TRINITY_DN19415_c0_g3::TRINITY_DN19415_c0_g3_i1::g.117477::m.117477	4,05E-87	261	AFK40521.1	unknown	Lotus japonicus	KDP28348.1
TRINITY_DN19617_c3_g3::TRINITY_DN19617_c3_g3_i8::g.121273::m.121273	4,53E-99	292	XP_018844827.1	adenosine kinase 2-like	Juglans regia	XP_007206468.2
TRINITY_DN15317_c1_g1::TRINITY_DN15317_c1_g1_i5::g.50064::m.50064	7,26E-113	321	XP_016193057.1	eukaryotic translation initiation factor 5A-2	Arachis ipaensis	XP_015943065.1

TRINITY_DN18894_c1_g1::TRINITY_DN18894_c1_g1_i5::g.108225::m.108225	2,73E-112	323	KDP32938.1	hypothetical protein JCGZ_12969	Jatropha curcas	XP_012077948.1
TRINITY_DN17857_c0_g1::TRINITY_DN17857_c0_g1_i1::g.90819::m.90819	0	1026	XP_018816364.1	dolichyl-diphosphooligosaccharide--protein glycosyltransferase subunit 2-like	Juglans regia	XP_018827273.1
TRINITY_DN19979_c5_g4::TRINITY_DN19979_c5_g4_i2::g.127014::m.127014	1,22E-99	295	XP_018815924.1	probable aldo-keto reductase 1	Juglans regia	XP_018811967.1
TRINITY_DN18581_c2_g1::TRINITY_DN18581_c2_g1_i1::g.102861::m.102861	2,28E-103	311	XP_018818966.1	serine carboxypeptidase-like	Juglans regia	XP_019447818.1
TRINITY_DN19014_c2_g2::TRINITY_DN19014_c2_g2_i4::g.110567::m.110567	0	1678	XP_018849376.1	phosphoenolpyruvate carboxylase, housekeeping isozyme	Juglans regia	XP_018822521.1
TRINITY_DN19846_c6_g2::TRINITY_DN19846_c6_g2_i1::g.124494::m.124494	3,34E-108	327	KEH35638.1	aspartic proteinase nepenthesin	Medicago truncatula	XP_013461603.1
TRINITY_DN12374_c0_g1::TRINITY_DN12374_c0_g1_i5::g.12261::m.12261	3,76E-105	303	XP_008366039.1	60S ribosomal protein L11-1	Malus domestica	XP_009352738.1
TRINITY_DN14238_c0_g1::TRINITY_DN14238_c0_g1_i3::g.34351::m.34351	2,73E-129	367	XP_018813322.1	photosynthetic NDH subunit of lumenal location 5, chloroplastic-like	Juglans regia	XP_018811825.1
TRINITY_DN17147_c0_g1::TRINITY_DN17147_c0_g1_i4::g.79344::m.79344	3,07E-97	288	XP_018839732.1	cinnamoyl-CoA reductase 1-like	Juglans regia	ACK76693.1
TRINITY_DN12441_c0_g1::TRINITY_DN12441_c0_g1_i1::g.12899::m.12899	0	858	XP_018805995.1	uncharacterized protein LOC108979724	Juglans regia	XP_008372757.1
TRINITY_DN12614_c0_g1::TRINITY_DN12614_c0_g1_i2::g.14256::m.14256	0	985	XP_018836262.1	pyrophosphate--fructose 6-phosphate 1-phosphotransferase subunit beta-like	Juglans regia	XP_018829538.1
TRINITY_DN11552_c0_g1::TRINITY_DN11552_c0_g1_i1::g.7817::m.7817	0	1148	XP_018822704.1	alpha-L-arabinofuranosidase 1	Juglans regia	XP_018822706.1
TRINITY_DN15353_c0_g3::TRINITY_DN15353_c0_g3_i2::g.50015::m.50015	0	1185	XP_018823485.1	eukaryotic translation initiation factor-like isoform X2	Juglans regia	XP_018823484.1
TRINITY_DN17847_c0_g1::TRINITY_DN17847_c0_g1_i7::g.90759::m.90759	0	731	AFJ04513.1	actin7a, partial	Vernicia fordii	ADH82414.1
TRINITY_DN17899_c1_g1::TRINITY_DN17899_c1_g1_i20::g.90008::m.90008	0	932	KDP30431.1	hypothetical protein JCGZ_16670	Jatropha curcas	XP_012081034.1
TRINITY_DN11696_c0_g1::TRINITY_DN11696_c0_g1_i4::g.8428::m.8428	0	521	OAY43782.1	hypothetical protein MANES_08G097400	Manihot esculenta	OAY43781.1
TRINITY_DN13436_c3_g1::TRINITY_DN13436_c3_g1_i7::g.23925::m.23925	0	713	XP_018848751.1	acetyl-CoA acetyltransferase, cytosolic 1 isoform X2	Juglans regia	XP_018848747.1
TRINITY_DN19395_c0_g1::TRINITY_DN19395_c0_g1_i9::g.117248::m.117248	9,54E-79	233	1584322	2122374C allergen Bet v 1-Sc3	Betula pendula	CAA54696.1
TRINITY_DN17244_c0_g1::TRINITY_DN17244_c0_g1_i3::g.79946::m.79946	0	711	XP_018839811.1	glucan endo-1,3-beta-glucosidase 8	Juglans regia	XP_004502874.1
TRINITY_DN11876_c0_g1::TRINITY_DN11876_c0_g1_i1::g.9282::m.9282	2,88E-63	196	XP_018822851.1	polyadenylate-binding protein-interacting protein 6-like	Juglans regia	XP_018822852.1
TRINITY_DN17448_c2_g2::TRINITY_DN17448_c2_g2_i8::g.83900::m.83900	2,95E-91	296	XP_018857281.1	villin-3-like isoform X1	Juglans regia	XP_018857282.1
TRINITY_DN13409_c1_g1::TRINITY_DN13409_c1_g1_i8::g.23419::m.23419	0	615	XP_018840356.1	glyceraldehyde-3-phosphate dehydrogenase GAPCP2, chloroplastic-like isoform X3	Juglans regia	XP_018840355.1

TRINITY_DN13221_c2_g1::TRINITY_DN13221_c2_g1_i1::g.21206::m.21206	1,12E-171	479	XP_018845359.1	mitochondrial outer membrane protein porin 2-like	Juglans regia	XP_018849544.1
TRINITY_DN13244_c2_g1::TRINITY_DN13244_c2_g1_i6::g.21528::m.21528	2,42E-110	314	ADV04063.1	protein binding/ubiquitin-protein ligase 2	Hevea brasiliensis	AGQ57016.1
TRINITY_DN18545_c0_g2::TRINITY_DN18545_c0_g2_i6::g.102386::m.102386	0	1231	XP_018826449.1	succinate dehydrogenase [ubiquinone] flavoprotein subunit 1, mitochondrial	Juglans regia	OAY57384.1
TRINITY_DN11123_c0_g1::TRINITY_DN11123_c0_g1_i4::g.6381::m.6381	0	615	XP_018846192.1	aminoacyl tRNA synthase complex-interacting multifunctional protein 1 isoform X1	Juglans regia	XP_018846193.1
TRINITY_DN17109_c1_g4::TRINITY_DN17109_c1_g4_i1::g.78802::m.78802	1,75E-97	281	XP_008224314.1	40S ribosomal protein S18	Prunus mume	XP_008390888.1
TRINITY_DN14091_c1_g2::TRINITY_DN14091_c1_g2_i1::g.32197::m.32197	0	1207	XP_018815161.1	peroxisomal fatty acid beta-oxidation multifunctional protein AIM1	Juglans regia	ONH98171.1
TRINITY_DN12452_c0_g1::TRINITY_DN12452_c0_g1_i1::g.12978::m.12978	0	553	XP_018823000.1	formate dehydrogenase, mitochondrial	Juglans regia	OAY25819.1
TRINITY_DN18521_c6_g6::TRINITY_DN18521_c6_g6_i1::g.101903::m.101903	3,03E-91	271	XP_018831704.1	peroxidase 65-like isoform X2	Juglans regia	XP_018831703.1
TRINITY_DN19436_c2_g1::TRINITY_DN19436_c2_g1_i4::g.118041::m.118041	0	792	XP_018807473.1	uncharacterized protein LOC108980890	Juglans regia	OAY48561.1
TRINITY_DN17420_c0_g1::TRINITY_DN17420_c0_g1_i7::g.83574::m.83574	0	596	XP_018807997.1	glyceraldehyde-3-phosphate dehydrogenase, cytosolic-like	Juglans regia	ONI30151.1
TRINITY_DN18973_c0_g1::TRINITY_DN18973_c0_g1_i1::g.109653::m.109653	3,54E-121	347	XP_009371092.1	40S ribosomal protein S8-like	Pyrus x bretschneideri	XP_009373606.1
TRINITY_DN11465_c0_g1::TRINITY_DN11465_c0_g1_i2::g.7454::m.7454	0	846	XP_018829248.1	monodehydroascorbate reductase 5, mitochondrial	Juglans regia	XP_012086827.1
TRINITY_DN16317_c2_g1::TRINITY_DN16317_c2_g1_i4::g.65631::m.65631	0	760	XP_018833171.1	UDP-D-apiose/UDP-D-xylose synthase 2	Juglans regia	XP_008385469.1
TRINITY_DN16236_c1_g2::TRINITY_DN16236_c1_g2_i1::g.64353::m.64353	2,63E-76	238	XP_018815580.1	uncharacterized protein LOC108987164	Juglans regia	KOM45155.1
TRINITY_DN12721_c0_g3::TRINITY_DN12721_c0_g3_i6::g.15362::m.15362	5,64E-63	191	OAY21768.1	hypothetical protein MANES_S058900	Manihot esculenta	KDP37139.1
TRINITY_DN13179_c1_g3::TRINITY_DN13179_c1_g3_i2::g.20801::m.20801	2,84E-48	166	XP_018816425.1	L-ascorbate oxidase homolog	Juglans regia	ONH98929.1
TRINITY_DN13823_c0_g2::TRINITY_DN13823_c0_g2_i2::g.29073::m.29073	5,93E-94	291	XP_018818477.1	putative G3BP-like protein	Juglans regia	OIW10252.1
TRINITY_DN18057_c0_g3::TRINITY_DN18057_c0_g3_i1::g.94357::m.94357	0	1058	XP_018810822.1	T-complex protein 1 subunit theta	Juglans regia	XP_015946936.1
TRINITY_DN12273_c2_g3::TRINITY_DN12273_c2_g3_i1::g.11683::m.11683	6,32E-63	196	XP_008221499.1	14-3-3-like protein GF14 iota	Prunus mume	ONI30977.1
TRINITY_DN14391_c2_g2::TRINITY_DN14391_c2_g2_i2::g.36282::m.36282	1,77E-50	166	XP_018818750.1	GDSL esterase/lipase At3g48460-like	Juglans regia	OAY32374.1
TRINITY_DN18947_c6_g2::TRINITY_DN18947_c6_g2_i2::g.109306::m.109306	1,54E-50	167	XP_018830491.1	senescence-specific cysteine protease SAG39-like	Juglans regia	XP_018830467.1
TRINITY_DN18272_c2_g1::TRINITY_DN18272_c2_g1_i1::g.97904::m.97904	0	1595	XP_018836595.1	chaperone protein ClpC, chloroplastic	Juglans regia	XP_018832706.1

TRINITY_DN17506_c1_g1::TRINITY_DN17506_c1_g1_i6::g.84994::m.84994	0	1135	XP_018844383.1	probable methyltransferase PMT27	Juglans regia	XP_018844383.1
TRINITY_DN17662_c4_g1::TRINITY_DN17662_c4_g1_i8::g.87880::m.87880	0	681	XP_018809442.1	glucuronokinase 1-like	Juglans regia	XP_018846472.1
TRINITY_DN12028_c1_g1::TRINITY_DN12028_c1_g1_i1::g.9901::m.9901	1,61E-108	327	XP_018808170.1	reticuline oxidase-like protein isoform X3	Juglans regia	XP_018808169.1
TRINITY_DN18870_c1_g1::TRINITY_DN18870_c1_g1_i1::g.107725::m.107725	2,90E-172	484	XP_018818800.1	probable 6-phosphogluconolactonase 4, chloroplastic	Juglans regia	KDP37759.1
TRINITY_DN17060_c1_g1::TRINITY_DN17060_c1_g1_i4::g.77740::m.77740	0	1293	XP_018827628.1	probable methyltransferase PMT26	Juglans regia	XP_018827629.1
TRINITY_DN14188_c2_g2::TRINITY_DN14188_c2_g2_i3::g.32373::m.32373	0	610	XP_018860423.1	guanine nucleotide-binding protein subunit beta-like protein isoform X1	Juglans regia	XP_018860424.1
TRINITY_DN18239_c4_g4::TRINITY_DN18239_c4_g4_i1::g.97381::m.97381	2,17E-133	379	XP_008227948.1	60S ribosomal protein L7-2	Prunus mume	ONI14974.1
TRINITY_DN19251_c1_g2::TRINITY_DN19251_c1_g2_i24::g.114984::m.114984	0	800	ONI09316.1	hypothetical protein PRUPE_5G231400	Prunus persica	XP_007210883.1
TRINITY_DN18181_c1_g1::TRINITY_DN18181_c1_g1_i2::g.96439::m.96439	2,09E-175	494	XP_018845549.1	uncharacterized protein LOC109009507 isoform X2	Juglans regia	XP_018845548.1
TRINITY_DN15085_c0_g2::TRINITY_DN15085_c0_g2_i3::g.46604::m.46604	3,83E-42	147	OIW09879.1	hypothetical protein TanjilG_24534	Lupinus angustifolius	XP_018852509.1
TRINITY_DN14405_c4_g1::TRINITY_DN14405_c4_g1_i11::g.36585::m.36585	0	1288	XP_018825075.1	peroxisomal acyl-coenzyme A oxidase 1	Juglans regia	XP_018819219.1
TRINITY_DN20037_c6_g1::TRINITY_DN20037_c6_g1_i4::g.128163::m.128163	1,23E-99	292	XP_018830075.1	cyclic phosphodiesterase-like	Juglans regia	XP_009360792.1
TRINITY_DN16369_c2_g2::TRINITY_DN16369_c2_g2_i2::g.66506::m.66506	1,96E-60	190	AKI29078.1	iron-binding protein	Pyrus betulifolia	AFK43431.1
TRINITY_DN19409_c5_g1::TRINITY_DN19409_c5_g1_i1::g.117472::m.117472	1,65E-30	116	KDP43847.1	hypothetical protein JCGZ_20857	Jatropha curcas	XP_012065174.1
TRINITY_DN15357_c0_g1::TRINITY_DN15357_c0_g1_i2::g.50651::m.50651	1,68E-117	335	XP_018819121.1	ATP synthase subunit delta', mitochondrial-like	Juglans regia	XP_018854921.1
TRINITY_DN17150_c2_g1::TRINITY_DN17150_c2_g1_i1::g.79329::m.79329	9,46E-172	477	XP_018824212.1	proteasome subunit alpha type-4	Juglans regia	XP_008371922.1
TRINITY_DN16201_c0_g1::TRINITY_DN16201_c0_g1_i1::g.62689::m.62689	2,76E-126	358	XP_018809376.1	60S ribosomal protein L9-like	Juglans regia	XP_018809377.1
TRINITY_DN14972_c1_g2::TRINITY_DN14972_c1_g2_i4::g.44982::m.44982	1,25E-146	410	KDP39130.1	hypothetical protein JCGZ_00887	Jatropha curcas	XP_012070824.1
TRINITY_DN14986_c2_g1::TRINITY_DN14986_c2_g1_i1::g.45365::m.45365	3,67E-107	308	KDP23652.1	hypothetical protein JCGZ_23485	Jatropha curcas	XP_012089246.1
TRINITY_DN15394_c0_g2::TRINITY_DN15394_c0_g2_i6::g.49878::m.49878	0	1488	XP_018835062.1	heat shock 70 kDa protein 17	Juglans regia	ONI17954.1
TRINITY_DN11523_c0_g1::TRINITY_DN11523_c0_g1_i1::g.7740::m.7740	2,85E-170	474	AES86765.1	40S ribosomal S4-like protein	Medicago truncatula	XP_003604568.1
TRINITY_DN14718_c0_g2::TRINITY_DN14718_c0_g2_i3::g.41293::m.41293	4,06E-89	260	XP_008367681.1	NADP-dependent malic enzyme-like	Malus domestica	XP_004501098.1

TRINITY_DN14157_c2_g1::TRINITY_DN14157_c2_g1_i3::g.33123::m.33123	1,91E-32	114	XP_018811692.1	60S acidic ribosomal protein P2-like	Juglans regia	XP_018848651.1
TRINITY_DN13422_c4_g1::TRINITY_DN13422_c4_g1_i9::g.23675::m.23675	2,87E-169	471	XP_018822732.1	proteasome subunit alpha type-3-like	Juglans regia	XP_018831769.1
TRINITY_DN17973_c0_g2::TRINITY_DN17973_c0_g2_i4::g.92495::m.92495	1,35E-63	198	XP_019421657.1	40S ribosomal protein S10-1-like isoform X1	Lupinus angustifolius	XP_019421658.1
TRINITY_DN14213_c1_g1::TRINITY_DN14213_c1_g1_i6::g.33993::m.33993	0	786	XP_018812595.1	ATP sulfurylase 2-like	Juglans regia	XP_018847012.1
TRINITY_DN15873_c1_g1::TRINITY_DN15873_c1_g1_i7::g.58225::m.58225						
TRINITY_DN12030_c0_g1::TRINITY_DN12030_c0_g1_i2::g.10080::m.10080	0	788	XP_018824705.1	acetylornithine deacetylase	Juglans regia	XP_009377070.1
TRINITY_DN16055_c0_g3::TRINITY_DN16055_c0_g3_i1::g.61219::m.61219	0	918	AHJ79156.1	betaine-aldehyde dehydrogenase	Juglans regia	XP_018831659.1
TRINITY_DN16644_c3_g1::TRINITY_DN16644_c3_g1_i4::g.70221::m.70221	0	747	XP_018845321.1	vicilin-like seed storage protein At2g28490 isoform X3	Juglans regia	XP_018845319.1
TRINITY_DN17145_c0_g4::TRINITY_DN17145_c0_g4_i1::g.79322::m.79322	0	1444	XP_018846325.1	clathrin heavy chain 1	Juglans regia	KRG97711.1
TRINITY_DN12499_c2_g2::TRINITY_DN12499_c2_g2_i3::g.13408::m.13408	1,69E-60	186	XP_008232650.1	60S ribosomal protein L22-3	Prunus mume	ONI22680.1
TRINITY_DN27272_c0_g1::TRINITY_DN27272_c0_g1_i1::g.129628::m.129628	2,51E-42	152	KDP36944.1	hypothetical protein JCGZ_08235	Jatropha curcas	XP_012073826.1
TRINITY_DN18903_c0_g1::TRINITY_DN18903_c0_g1_i9::g.108678::m.108678	0	694	XP_018806250.1	eIF-2-alpha kinase GCN2 isoform X2	Juglans regia	XP_018806242.1
TRINITY_DN19948_c1_g1::TRINITY_DN19948_c1_g1_i4::g.126476::m.126476	0	868	XP_018809878.1	ketol-acid reductoisomerase, chloroplastic	Juglans regia	XP_008244478.1
TRINITY_DN15893_c1_g2::TRINITY_DN15893_c1_g2_i6::g.57605::m.57605	0	704	XP_018851483.1	isocitrate dehydrogenase [NAD] catalytic subunit 5, mitochondrial isoform X1	Juglans regia	ONI03044.1
TRINITY_DN18003_c1_g3::TRINITY_DN18003_c1_g3_i3::g.93450::m.93450	6,49E-171	481	XP_018839339.1	uncharacterized protein LOC109005031	Juglans regia	KDP29539.1
TRINITY_DN17070_c2_g4::TRINITY_DN17070_c2_g4_i5::g.77858::m.77858	5,65E-176	486	XP_018837325.1	proteasome subunit alpha type-5	Juglans regia	XP_018837326.1
TRINITY_DN10166_c0_g1::TRINITY_DN10166_c0_g1_i3::g.4379::m.4379	1,38E-162	454	XP_018844949.1	20 kDa chaperonin, chloroplastic-like	Juglans regia	XP_018844947.1
TRINITY_DN19812_c4_g1::TRINITY_DN19812_c4_g1_i1::g.124051::m.124051	0	696	KDP35832.1	hypothetical protein JCGZ_10646	Jatropha curcas	XP_012074649.1
TRINITY_DN11760_c0_g1::TRINITY_DN11760_c0_g1_i1::g.8722::m.8722	0	530	XP_018846015.1	60S acidic ribosomal protein P0-like	Juglans regia	XP_018821854.1
TRINITY_DN20022_c3_g3::TRINITY_DN20022_c3_g3_i3::g.128023::m.128023						
TRINITY_DN16751_c0_g2::TRINITY_DN16751_c0_g2_i11::g.72773::m.72773	0	1071	XP_018831656.1	probable aldehyde dehydrogenase isoform X2	Juglans regia	XP_018831655.1
TRINITY_DN15089_c0_g1::TRINITY_DN15089_c0_g1_i2::g.46688::m.46688	0	1019	XP_008230393.1	T-complex protein 1 subunit zeta 1	Prunus mume	ONI18783.1

TRINITY_DN13431_c11_g1::TRINITY_DN13431_c11_g1_i1::g.23860::m.23860	0	690	XP_018849575.1	uncharacterized protein LOC109012411 isoform X1	Juglans regia	XP_018849576.1
TRINITY_DN16209_c0_g5::TRINITY_DN16209_c0_g5_i1::g.64031::m.64031	3,53E-92	266	AFK43969.1	unknown	Medicago truncatula	KEH32217.1
TRINITY_DN18640_c0_g2::TRINITY_DN18640_c0_g2_i8::g.103688::m.103688	4,39E-102	295	XP_018835350.1	nudix hydrolase 26, chloroplastic-like	Juglans regia	XP_009351382.2
TRINITY_DN11955_c0_g1::TRINITY_DN11955_c0_g1_i1::g.9671::m.9671	0	553	AGT95889.1	beta-ketoacyl-ACP reductase	Vernicia fordii	CAV22170.1
TRINITY_DN19422_c2_g1::TRINITY_DN19422_c2_g1_i9::g.117599::m.117599	0	537	OIW03371.1	hypothetical protein TanjilG_29356	Lupinus angustifolius	XP_019458539.1
TRINITY_DN19041_c0_g1::TRINITY_DN19041_c0_g1_i8::g.110918::m.110918	7,03E-133	375	XP_018827839.1	ras-related protein RABC1 isoform X1	Juglans regia	XP_018827840.1
TRINITY_DN18551_c0_g2::TRINITY_DN18551_c0_g2_i5::g.102562::m.102562	0	1349	XP_018826765.1	pyrophosphate-energized vacuolar membrane proton pump-like	Juglans regia	ALO24186.1
TRINITY_DN18727_c2_g1::TRINITY_DN18727_c2_g1_i8::g.105290::m.105290	0	517	XP_018839964.1	protein ENHANCED DISEASE RESISTANCE 2-like isoform X3	Juglans regia	XP_018839960.1
TRINITY_DN14879_c3_g4::TRINITY_DN14879_c3_g4_i1::g.43729::m.43729	2,59E-153	427	KRH29738.1	hypothetical protein GLYMA_11G135400	Glycine max	XP_003537971.1
TRINITY_DN19715_c1_g1::TRINITY_DN19715_c1_g1_i2::g.122707::m.122707	0	1852	XP_018845356.1	calcium-transporting ATPase 2, plasma membrane-type-like isoform X1	Juglans regia	XP_018845357.1
TRINITY_DN11725_c0_g2::TRINITY_DN11725_c0_g2_i1::g.8540::m.8540	1,68E-65	200	XP_018839196.1	uncharacterized protein LOC109004950	Juglans regia	ONI26983.1
TRINITY_DN16136_c0_g1::TRINITY_DN16136_c0_g1_i4::g.62642::m.62642	0	540	XP_018848571.1	dihydrolipoyllysine-residue acetyltransferase component 2 of pyruvate dehydrogenase complex, mitochondrial-like	Juglans regia	XP_018835126.1
TRINITY_DN19182_c1_g1::TRINITY_DN19182_c1_g1_i6::g.113457::m.113457	0	846	XP_018845710.1	aspartate--tRNA ligase 2, cytoplasmic	Juglans regia	KRH05378.1
TRINITY_DN16339_c2_g1::TRINITY_DN16339_c2_g1_i6::g.65997::m.65997	0	537	XP_008236827.1	ubiquitin fusion degradation protein 1 homolog	Prunus mume	XP_016650890.1
TRINITY_DN18576_c1_g1::TRINITY_DN18576_c1_g1_i10::g.101619::m.101619	0	1068	XP_018820151.1	external alternative NAD(P)H-ubiquinone oxidoreductase B2, mitochondrial-like	Juglans regia	XP_008364132.1
TRINITY_DN11411_c0_g1::TRINITY_DN11411_c0_g1_i2::g.7300::m.7300	5,36E-130	372	XP_018498100.1	60S ribosomal protein L15-1	Pyrus x bretschneideri	XP_009334550.1
TRINITY_DN14869_c3_g5::TRINITY_DN14869_c3_g5_i3::g.43830::m.43830	5,36E-70	226	OAY56312.1	hypothetical protein MANES_02G006000	Manihot esculenta	XP_004503582.1
TRINITY_DN12162_c0_g1::TRINITY_DN12162_c0_g1_i2::g.10879::m.10879	2,65E-75	225	XP_018845602.1	AIG2-like protein	Juglans regia	OAY53495.1
TRINITY_DN15457_c4_g1::TRINITY_DN15457_c4_g1_i5::g.52200::m.52200	3,05E-111	330	BAO50884.1	ATP synthase F1 subunit 1 (mitochondrion)	Hevea brasiliensis	BAO50925.1
TRINITY_DN13171_c0_g1::TRINITY_DN13171_c0_g1_i3::g.20617::m.20617	5,86E-99	284	XP_018816977.1	40S ribosomal protein S19-3	Juglans regia	XP_018814627.1
TRINITY_DN19704_c1_g1::TRINITY_DN19704_c1_g1_i9::g.122571::m.122571	0	1804	OAY27565.1	hypothetical protein MANES_16G135200	Manihot esculenta	OAY27566.1

TRINITY_DN13284_c1_g1::TRINITY_DN13284_c1_g1_i7::g.21970::m.21970	1,51E-133	378	XP_018839423.1	stem-specific protein TSJT1-like	Juglans regia	ONI26399.1
TRINITY_DN17067_c1_g1::TRINITY_DN17067_c1_g1_i1::g.77883::m.77883	0	2208	ONI11196.1	hypothetical protein PRUPE_4G091800	Prunus persica	ONI11197.1
TRINITY_DN12343_c1_g1::TRINITY_DN12343_c1_g1_i5::g.11822::m.11822	2,94E-131	370	XP_007160383.1	hypothetical protein PHAVU_002G317200g	Phaseolus vulgaris	XP_007160384.1
TRINITY_DN16029_c2_g3::TRINITY_DN16029_c2_g3_i1::g.61032::m.61032	1,13E-127	362	XP_018840507.1	peroxiredoxin-2F, mitochondrial-like	Juglans regia	XP_018846871.1
TRINITY_DN12608_c0_g1::TRINITY_DN12608_c0_g1_i2::g.14177::m.14177	3,03E-115	334	XP_018810712.1	DNA-directed RNA polymerase V subunit 7-like	Juglans regia	XP_008353300.2
TRINITY_DN18182_c1_g1::TRINITY_DN18182_c1_g1_i5::g.96464::m.96464	1,07E-166	463	XP_018844535.1	40S ribosomal protein S3-3-like	Juglans regia	XP_018827015.1
TRINITY_DN15675_c5_g2::TRINITY_DN15675_c5_g2_i2::g.55536::m.55536	0	800	XP_018859966.1	hexokinase-1-like isoform X2	Juglans regia	KDP33801.1
TRINITY_DN15276_c1_g1::TRINITY_DN15276_c1_g1_i6::g.49461::m.49461	0	601	XP_018847873.1	serine-threonine kinase receptor-associated protein-like	Juglans regia	XP_016179643.1
TRINITY_DN18360_c0_g2::TRINITY_DN18360_c0_g2_i3::g.99190::m.99190	0	623	XP_018814451.1	heat shock cognate 70 kDa protein-like	Juglans regia	XP_018814450.1
TRINITY_DN12711_c1_g1::TRINITY_DN12711_c1_g1_i2::g.15227::m.15227	1,37E-175	498	XP_014513401.1	ubiquitin receptor RAD23d-like	Vigna radiata var. radiata	XP_020987021.1
TRINITY_DN15771_c0_g2::TRINITY_DN15771_c0_g2_i3::g.56846::m.56846	0	1549	XP_018845914.1	coatomer subunit beta'-2-like isoform X1	Juglans regia	XP_018845915.1
TRINITY_DN12201_c0_g1::TRINITY_DN12201_c0_g1_i3::g.10701::m.10701	0	674	XP_018829628.1	malate dehydrogenase, glyoxysomal	Juglans regia	XP_018819958.1
TRINITY_DN13903_c0_g3::TRINITY_DN13903_c0_g3_i6::g.30258::m.30258	1,80E-151	433	XP_018805205.1	peroxidase 5-like	Juglans regia	XP_008221883.1
TRINITY_DN16875_c1_g2::TRINITY_DN16875_c1_g2_i1::g.74175::m.74175	0	1009	XP_018813723.1	polyadenylate-binding protein 2-like	Juglans regia	BAJ53195.1
TRINITY_DN12880_c1_g2::TRINITY_DN12880_c1_g2_i3::g.16816::m.16816	1,60E-115	334	XP_018816103.1	succinate dehydrogenase subunit 5, mitochondrial-like	Juglans regia	XP_018822424.1
TRINITY_DN12853_c0_g1::TRINITY_DN12853_c0_g1_i3::g.16604::m.16604	0	639	XP_018818486.1	erlin-2-B	Juglans regia	XP_018818487.1
TRINITY_DN15811_c0_g2::TRINITY_DN15811_c0_g2_i2::g.57329::m.57329	1,58E-97	285	XP_018835046.1	proteasome subunit alpha type-6 isoform X1	Juglans regia	XP_018835047.1
TRINITY_DN12588_c4_g1::TRINITY_DN12588_c4_g1_i6::g.14071::m.14071	0	511	XP_018820882.1	glyoxylate/succinic semialdehyde reductase 1 isoform X1	Juglans regia	XP_018820891.1
TRINITY_DN18157_c0_g1::TRINITY_DN18157_c0_g1_i6::g.96079::m.96079	1,19E-125	367	XP_018837049.1	cysteine proteinase RD21A-like	Juglans regia	XP_018837050.1
TRINITY_DN18306_c2_g1::TRINITY_DN18306_c2_g1_i6::g.98737::m.98737	3,06E-64	203	KDP35739.1	hypothetical protein JCGZ_10511	Jatropha curcas	XP_012074735.1
TRINITY_DN17921_c2_g1::TRINITY_DN17921_c2_g1_i7::g.92112::m.92112	0	1697	XP_018840722.1	alanine--tRNA ligase-like	Juglans regia	XP_018840731.1
TRINITY_DN15585_c1_g1::TRINITY_DN15585_c1_g1_i4::g.54306::m.54306	7,72E-127	361	XP_018821775.1	GDSL esterase/lipase CPRD49-like	Juglans regia	XP_018810087.1

TRINITY_DN12563_c5_g1::TRINITY_DN12563_c5_g1_i1::g.13894::m.13894	0	1748	XP_018843664.1	4-alpha-glucanotransferase DPE2 isoform X2	Juglans regia	XP_018843661.1
TRINITY_DN14141_c0_g1::TRINITY_DN14141_c0_g1_i5::g.32827::m.32827	1,10E-55	174	XP_018836470.1	ATP synthase subunit d, mitochondrial-like	Juglans regia	XP_008368918.1
TRINITY_DN19864_c2_g6::TRINITY_DN19864_c2_g6_i4::g.124630::m.124630	0	546	OAY23951.1	hypothetical protein MANES_18G120400	Manihot esculenta	XP_018841148.1
TRINITY_DN15673_c2_g2::TRINITY_DN15673_c2_g2_i2::g.55381::m.55381	2,89E-75	223	XP_018820939.1	60S ribosomal protein L14-1	Juglans regia	XP_018808011.1
TRINITY_DN15521_c0_g1::TRINITY_DN15521_c0_g1_i5::g.53143::m.53143	0	1035	XP_018805935.1	pyruvate kinase 1, cytosolic isoform X2	Juglans regia	XP_018805933.1
TRINITY_DN14108_c0_g1::TRINITY_DN14108_c0_g1_i6::g.32445::m.32445	0	668	OAY60978.1	hypothetical protein MANES_01G154200	Manihot esculenta	KDP43753.1
TRINITY_DN13599_c0_g2::TRINITY_DN13599_c0_g2_i3::g.24684::m.24684	1,17E-136	385	XP_018813683.1	60S ribosomal protein L10a-like	Juglans regia	OIW20670.1
TRINITY_DN12431_c0_g1::TRINITY_DN12431_c0_g1_i5::g.12785::m.12785	6,38E-79	234	XP_014496997.1	60S ribosomal protein L23A	Vigna radiata var. radiata	XP_020224350.1
TRINITY_DN15315_c0_g3::TRINITY_DN15315_c0_g3_i4::g.50023::m.50023	0	935	XP_018850554.1	alanine aminotransferase 2-like	Juglans regia	ONI15222.1
TRINITY_DN13825_c0_g1::TRINITY_DN13825_c0_g1_i1::g.28844::m.28844	5,00E-91	268	XP_018817591.1	aldo-keto reductase family 4 member C9-like	Juglans regia	XP_020221314.1
TRINITY_DN11213_c0_g1::TRINITY_DN11213_c0_g1_i2::g.6642::m.6642	4,52E-120	345	XP_018834379.1	60S ribosomal protein L6	Juglans regia	XP_009365032.1
TRINITY_DN19530_c3_g1::TRINITY_DN19530_c3_g1_i3::g.119253::m.119253	0	1332	XP_018816636.1	NADH dehydrogenase [ubiquinone] iron-sulfur protein 1, mitochondrial	Juglans regia	ONI17532.1
TRINITY_DN16101_c1_g1::TRINITY_DN16101_c1_g1_i2::g.61978::m.61978	0	1704	XP_018836225.1	plasma membrane ATPase 4	Juglans regia	XP_018845641.1
TRINITY_DN16024_c0_g2::TRINITY_DN16024_c0_g2_i5::g.60694::m.60694	0	745	XP_018844405.1	pyruvate dehydrogenase E1 component subunit alpha-1, mitochondrial-like	Juglans regia	XP_018819117.1
TRINITY_DN11097_c0_g1::TRINITY_DN11097_c0_g1_i1::g.6303::m.6303	1,32E-155	442	XP_018847246.1	desiccation-related protein PCC13-62-like	Juglans regia	XP_018855451.1
TRINITY_DN18581_c2_g2::TRINITY_DN18581_c2_g2_i8::g.102871::m.102871	5,22E-61	198	KRH26523.1	hypothetical protein GLYMA_12G177900	Glycine max	XP_003540200.1
TRINITY_DN15963_c2_g1::TRINITY_DN15963_c2_g1_i4::g.59609::m.59609	0	665	XP_018844184.1	thiol protease aleurain-like	Juglans regia	OAY27043.1
TRINITY_DN18878_c2_g2::TRINITY_DN18878_c2_g2_i9::g.108021::m.108021	0	713	XP_018821861.1	cytochrome P450 90A1 isoform X1	Juglans regia	XP_018810085.1
TRINITY_DN12332_c0_g1::TRINITY_DN12332_c0_g1_i2::g.11982::m.11982	1,01E-119	348	XP_018809881.1	protein BOBBER 1-like	Juglans regia	XP_014503614.1
TRINITY_DN18378_c3_g4::TRINITY_DN18378_c3_g4_i1::g.99575::m.99575	4,26E-118	338	XP_018842921.1	membrane steroid-binding protein 1-like	Juglans regia	XP_018830725.1
TRINITY_DN11637_c0_g1::TRINITY_DN11637_c0_g1_i3::g.8078::m.8078	0	664	XP_018835846.1	polygalacturonase QRT3	Juglans regia	XP_018835847.1
TRINITY_DN16699_c3_g1::TRINITY_DN16699_c3_g1_i15::g.70256::m.70256	0	943	XP_018834253.1	AP-4 complex subunit epsilon-like	Juglans regia	XP_018810168.1

TRINITY_DN14704_c0_g1::TRINITY_DN14704_c0_g1_i5::g.40889::m.40889	0	624	XP_018839948.1	heparanase-like protein 3 isoform X3	Juglans regia	XP_018839937.1
TRINITY_DN15232_c2_g1::TRINITY_DN15232_c2_g1_i5::g.49050::m.49050	7,87E-98	281	XP_018848065.1	40S ribosomal protein S13	Juglans regia	XP_018815859.1
TRINITY_DN16447_c0_g1::TRINITY_DN16447_c0_g1_i6::g.67050::m.67050	1,43E-132	378	XP_018840931.1	60S ribosomal protein L7-2-like	Juglans regia	XP_018840932.1
TRINITY_DN18433_c1_g3::TRINITY_DN18433_c1_g3_i2::g.100387::m.100387	3,93E-164	462	KDP44884.1	hypothetical protein JCGZ_01384	Jatropha curcas	XP_012088585.1
TRINITY_DN15610_c2_g1::TRINITY_DN15610_c2_g1_i5::g.54395::m.54395	7,96E-163	457	OAY27542.1	hypothetical protein MANES_16G133500	Manihot esculenta	XP_008379609.1
TRINITY_DN17553_c1_g1::TRINITY_DN17553_c1_g1_i2::g.85833::m.85833	4,98E-128	362	XP_018806533.1	probable glutathione peroxidase 2	Juglans regia	XP_008380677.1
TRINITY_DN16227_c0_g1::TRINITY_DN16227_c0_g1_i8::g.64252::m.64252	4,03E-109	317	XP_018856017.1	ribosome-recycling factor, chloroplastic isoform X1	Juglans regia	OAY49710.1
TRINITY_DN18179_c2_g1::TRINITY_DN18179_c2_g1_i5::g.96397::m.96397	0	548	XP_018824867.1	ras GTPase-activating protein-binding protein 2 isoform X1	Juglans regia	XP_018824868.1
TRINITY_DN13194_c3_g1::TRINITY_DN13194_c3_g1_i1::g.20967::m.20967	1,17E-83	247	XP_018832580.1	uncharacterized protein LOC109000202 isoform X1	Juglans regia	XP_018832581.1
TRINITY_DN15924_c1_g1::TRINITY_DN15924_c1_g1_i2::g.59050::m.59050	0	760	XP_018819893.1	low-temperature-induced cysteine proteinase-like	Juglans regia	XP_018852257.1
TRINITY_DN13535_c5_g1::TRINITY_DN13535_c5_g1_i1::g.25172::m.25172	4,11E-22	95,1	XP_018838898.1	late embryogenesis abundant protein D-29 isoform X1	Juglans regia	ONI27795.1
TRINITY_DN12971_c4_g2::TRINITY_DN12971_c4_g2_i4::g.17979::m.17979	1,19E-158	444	XP_018829633.1	proteasome subunit alpha type-7	Juglans regia	XP_008337994.1
TRINITY_DN17406_c4_g4::TRINITY_DN17406_c4_g4_i2::g.83246::m.83246	0	1231	XP_018849604.1	programmed cell death protein 4-like	Juglans regia	XP_018807488.1
TRINITY_DN14711_c0_g2::TRINITY_DN14711_c0_g2_i2::g.40961::m.40961	0	717	XP_018842745.1	stomatin-like protein 2, mitochondrial	Juglans regia	XP_018828153.1
TRINITY_DN12314_c0_g1::TRINITY_DN12314_c0_g1_i2::g.11797::m.11797	0	608	XP_018855479.1	probable L-ascorbate peroxidase 6, chloroplastic isoform X2	Juglans regia	XP_018855478.1
TRINITY_DN19972_c2_g1::TRINITY_DN19972_c2_g1_i3::g.126916::m.126916	6,81E-64	197	XP_018818881.1	L-ascorbate peroxidase, cytosolic	Juglans regia	XP_018818882.1
TRINITY_DN12468_c0_g1::TRINITY_DN12468_c0_g1_i1::g.13082::m.13082	0	743	XP_018849591.1	dolichyl-diphosphooligosaccharide--protein glycosyltransferase 48 kDa subunit-like isoform X2	Juglans regia	XP_004507412.1
TRINITY_DN16038_c1_g1::TRINITY_DN16038_c1_g1_i5::g.61002::m.61002	0	942	XP_018842622.1	pyruvate kinase isozyme G, chloroplastic isoform X2	Juglans regia	XP_018842621.1
TRINITY_DN14600_c0_g1::TRINITY_DN14600_c0_g1_i7::g.38565::m.38565	0	1092	XP_018837266.1	T-complex protein 1 subunit gamma isoform X1	Juglans regia	GAU28482.1
TRINITY_DN12693_c0_g1::TRINITY_DN12693_c0_g1_i3::g.14940::m.14940	1,77E-58	187	KDP36740.1	hypothetical protein JCGZ_08031	Jatropha curcas	XP_012073565.1
TRINITY_DN12016_c0_g1::TRINITY_DN12016_c0_g1_i3::g.10082::m.10082	1,73E-129	364	XP_018850586.1	60S ribosomal protein L18a	Juglans regia	XP_018819718.1
TRINITY_DN12640_c0_g1::TRINITY_DN12640_c0_g1_i4::g.14587::m.14587	0	587	XP_018827261.1	TOM1-like protein 2	Juglans regia	XP_008235674.1

TRINITY_DN18427_c1_g2::TRINITY_DN18427_c1_g2_i5::g.100438::m.100438	1,30E-170	473	XP_018837327.1	proteasome subunit alpha type-2-B	Juglans regia	KRH13728.1
TRINITY_DN19518_c0_g1::TRINITY_DN19518_c0_g1_i2::g.119121::m.119121	0	609	XP_018824429.1	probable aldo-keto reductase 2	Juglans regia	KYP64861.1
TRINITY_DN17623_c2_g1::TRINITY_DN17623_c2_g1_i4::g.87423::m.87423	1,20E-46	152	KDP24516.1	hypothetical protein JCGZ_25080	Jatropha curcas	XP_012087959.1
TRINITY_DN14810_c0_g2::TRINITY_DN14810_c0_g2_i2::g.42832::m.42832	7,61E-112	318	XP_016171772.1	60S ribosomal protein L12	Arachis ipaensis	XP_015935785.1
TRINITY_DN12250_c0_g1::TRINITY_DN12250_c0_g1_i2::g.11365::m.11365	0	840	XP_018845963.1	elongation factor Tu, mitochondrial-like	Juglans regia	XP_018847444.1
TRINITY_DN16420_c0_g1::TRINITY_DN16420_c0_g1_i2::g.67378::m.67378	0	763	XP_018850665.1	elongation factor 1-gamma-like	Juglans regia	ONI35669.1
TRINITY_DN16668_c3_g1::TRINITY_DN16668_c3_g1_i1::g.71264::m.71264	0	1995	XP_018845797.1	glycine dehydrogenase (decarboxylating), mitochondrial	Juglans regia	XP_009361869.1
TRINITY_DN18663_c1_g1::TRINITY_DN18663_c1_g1_i1::g.104197::m.104197	2,27E-34	123	XP_018841685.1	putative invertase inhibitor	Juglans regia	XP_018806754.1
TRINITY_DN16719_c1_g1::TRINITY_DN16719_c1_g1_i3::g.72216::m.72216	0	1028	XP_018840650.1	transmembrane 9 superfamily member 11 isoform X1	Juglans regia	XP_018840651.1
TRINITY_DN17040_c1_g7::TRINITY_DN17040_c1_g7_i1::g.77416::m.77416	1,77E-87	257	XP_018849369.1	nascent polypeptide-associated complex subunit alpha-like protein 1	Juglans regia	OAY36283.1
TRINITY_DN14578_c1_g1::TRINITY_DN14578_c1_g1_i1::g.39115::m.39115	0	754	XP_008223483.1	S-adenosylmethionine synthase 5	Prunus mume	XP_012084185.1
TRINITY_DN14089_c0_g1::TRINITY_DN14089_c0_g1_i6::g.32232::m.32232	0	687	XP_018825271.1	enoyl-[acyl-carrier-protein reductase [NADH], chloroplastic-like	Juglans regia	XP_018834639.1
TRINITY_DN14378_c0_g2::TRINITY_DN14378_c0_g2_i6::g.36119::m.36119	8,15E-156	434	OAY58932.1	hypothetical protein MANES_02G217700	Manihot esculenta	AFK38655.1
TRINITY_DN19372_c1_g1::TRINITY_DN19372_c1_g1_i2::g.116684::m.116684	0	870	XP_018852464.1	oxalate--CoA ligase-like	Juglans regia	KDP25453.1
TRINITY_DN13787_c3_g4::TRINITY_DN13787_c3_g4_i3::g.28262::m.28262	0	526	XP_018812299.1	mitochondrial outer membrane protein porin of 36 kDa	Juglans regia	XP_008339931.1
TRINITY_DN18760_c9_g1::TRINITY_DN18760_c9_g1_i2::g.106072::m.106072	0	554	XP_018841471.1	beta-galactosidase 13-like	Juglans regia	XP_018823734.1
TRINITY_DN11053_c0_g1::TRINITY_DN11053_c0_g1_i3::g.6092::m.6092	1,07E-75	224	XP_018821837.1	60S ribosomal protein L35a-1	Juglans regia	XP_018821838.1
TRINITY_DN13145_c0_g1::TRINITY_DN13145_c0_g1_i9::g.20440::m.20440	0	819	OAY44035.1	hypothetical protein MANES_08G117200	Manihot esculenta	XP_018841804.1
TRINITY_DN16756_c2_g7::TRINITY_DN16756_c2_g7_i3::g.72811::m.72811	0	961	XP_018845845.1	pyruvate kinase, cytosolic isozyme	Juglans regia	ONH98946.1
TRINITY_DN12713_c0_g2::TRINITY_DN12713_c0_g2_i2::g.15182::m.15182	0	675	KYP69807.1	Alpha-1,4-glucan-protein synthase [UDP-forming 2]	Cajanus cajan	XP_020212056.1
TRINITY_DN13140_c0_g1::TRINITY_DN13140_c0_g1_i10::g.20388::m.20388	0	538	XP_018829443.1	gamma carbonic anhydrase 1, mitochondrial-like isoform X1	Juglans regia	XP_018829444.1
TRINITY_DN16168_c0_g1::TRINITY_DN16168_c0_g1_i6::g.63149::m.63149	0	1530	XP_018834279.1	26S proteasome non-ATPase regulatory subunit 2 homolog A-like	Juglans regia	XP_018843471.1

TRINITY_DN18151_c0_g1::TRINITY_DN18151_c0_g1_i1::g.95922::m.95922	2,40E-167	471	XP_018839906.1	40S ribosomal protein SA-like isoform X2	Juglans regia	XP_018839250.1
TRINITY_DN13594_c1_g2::TRINITY_DN13594_c1_g2_i6::g.26004::m.26004	8,35E-66	199	XP_018837755.1	glutaredoxin-like	Juglans regia	XP_018821252.1
TRINITY_DN16842_c1_g6::TRINITY_DN16842_c1_g6_i1::g.74458::m.74458	2,03E-92	271	XP_018842585.1	ferredoxin, root R-B2-like	Juglans regia	XP_007142856.1
TRINITY_DN16487_c1_g1::TRINITY_DN16487_c1_g1_i9::g.68393::m.68393	1,87E-112	333	XP_018856959.1	coiled-coil domain-containing protein 18-like	Juglans regia	XP_018845595.1
TRINITY_DN17142_c0_g1::TRINITY_DN17142_c0_g1_i3::g.79276::m.79276	0	593	XP_018851182.1	putative deoxyribonuclease TATDN1	Juglans regia	XP_008350215.1
TRINITY_DN18242_c1_g1::TRINITY_DN18242_c1_g1_i1::g.97405::m.97405	2,08E-154	448	XP_018847175.1	heterogeneous nuclear ribonucleoprotein 1-like	Juglans regia	XP_018847175.1
TRINITY_DN18764_c1_g1::TRINITY_DN18764_c1_g1_i6::g.106059::m.106059	0	594	XP_018819193.1	cinnamoyl-CoA reductase 1	Juglans regia	ONH99805.1
TRINITY_DN15799_c0_g1::TRINITY_DN15799_c0_g1_i1::g.55952::m.55952	4,22E-147	418	XP_018842015.1	endochitinase-like	Juglans regia	XP_018854709.1
TRINITY_DN12324_c0_g2::TRINITY_DN12324_c0_g2_i2::g.11908::m.11908	2,58E-140	396	XP_018819328.1	V-type proton ATPase subunit E2 isoform X2	Juglans regia	XP_018819326.1
TRINITY_DN14323_c0_g1::TRINITY_DN14323_c0_g1_i2::g.35566::m.35566	0	1292	XP_018818540.1	heat shock protein 90-5, chloroplastic	Juglans regia	ONI31143.1
TRINITY_DN13653_c3_g1::TRINITY_DN13653_c3_g1_i1::g.26703::m.26703	0	1288	XP_018840476.1	long chain acyl-CoA synthetase 6, peroxisomal-like	Juglans regia	XP_018846842.1
TRINITY_DN20011_c2_g2::TRINITY_DN20011_c2_g2_i1::g.127827::m.127827	0	951	KDP46998.1	hypothetical protein JCGZ_02434	Jatropha curcas	XP_012080304.1
TRINITY_DN14539_c1_g2::TRINITY_DN14539_c1_g2_i5::g.38538::m.38538	0	784	XP_008379344.1	tubulin alpha-2 chain	Malus domestica	XP_008230754.1
TRINITY_DN15165_c0_g1::TRINITY_DN15165_c0_g1_i4::g.47616::m.47616	6,28E-77	235	XP_018824934.1	late embryogenesis abundant protein D-34-like	Juglans regia	XP_018824935.1
TRINITY_DN12436_c4_g2::TRINITY_DN12436_c4_g2_i4::g.12996::m.12996	0	1014	XP_018820447.1	T-complex protein 1 subunit epsilon	Juglans regia	OAY32463.1
TRINITY_DN14508_c8_g1::TRINITY_DN14508_c8_g1_i8::g.38378::m.38378	1,49E-88	258	XP_018830082.1	desiccation protectant protein Lea14 homolog	Juglans regia	XP_018847006.1
TRINITY_DN14731_c0_g4::TRINITY_DN14731_c0_g4_i2::g.41282::m.41282	1,35E-92	273	XP_018845049.1	proteasome subunit alpha type-1-A-like	Juglans regia	XP_018821628.1
TRINITY_DN15108_c2_g1::TRINITY_DN15108_c2_g1_i5::g.47064::m.47064	0	1114	XP_018833363.1	acetyl-coenzyme A carboxylase carboxyl transferase subunit alpha, chloroplastic-like	Juglans regia	XP_018833364.1
TRINITY_DN15133_c0_g1::TRINITY_DN15133_c0_g1_i1::g.47252::m.47252	0	726	XP_018826923.1	proline iminopeptidase	Juglans regia	KRH10668.1
TRINITY_DN13082_c0_g1::TRINITY_DN13082_c0_g1_i2::g.19429::m.19429	1,79E-154	437	XP_018808845.1	GEM-like protein 1	Juglans regia	KOM41786.1
TRINITY_DN19812_c4_g2::TRINITY_DN19812_c4_g2_i2::g.124055::m.124055	7,35E-76	236	AAK21920.1	AF338252_1 BiP-isoform D, partial	Glycine max	ONI07528.1
TRINITY_DN13916_c3_g3::TRINITY_DN13916_c3_g3_i3::g.30403::m.30403	0	785	XP_018829125.1	calnexin homolog	Juglans regia	XP_018829196.1

TRINITY_DN17369_c1_g1::TRINITY_DN17369_c1_g1_i7::g.82610::m.82610	6,07E-164	456	XP_018845601.1	proteasome subunit beta type-6	Juglans regia	KDP33416.1
TRINITY_DN11073_c0_g1::TRINITY_DN11073_c0_g1_i1::g.6247::m.6247	3,34E-142	399	XP_018851128.1	40S ribosomal protein S5	Juglans regia	XP_009379661.1
TRINITY_DN17221_c0_g2::TRINITY_DN17221_c0_g2_i11::g.80299::m.80299	0	862	XP_018829691.1	eukaryotic peptide chain release factor GTP-binding subunit ERF3A isoform X2	Juglans regia	XP_018829690.1
TRINITY_DN13021_c1_g2::TRINITY_DN13021_c1_g2_i3::g.18779::m.18779	5,06E-134	386	XP_018811500.1	desiccation-related protein PCC13-62-like	Juglans regia	XP_018811498.1
TRINITY_DN19942_c0_g1::TRINITY_DN19942_c0_g1_i1::g.126320::m.126320	1,05E-129	367	XP_008234622.1	60S ribosomal protein L9-1	Prunus mume	ONI25805.1
TRINITY_DN13485_c2_g3::TRINITY_DN13485_c2_g3_i2::g.24428::m.24428	6,58E-128	364	XP_018856887.1	NADP-dependent malic enzyme-like isoform X2	Juglans regia	XP_018856888.1
TRINITY_DN15741_c2_g1::TRINITY_DN15741_c2_g1_i4::g.55925::m.55925	3,38E-36	126	XP_018811921.1	elicitor-responsive protein 3	Juglans regia	OAY56540.1
TRINITY_DN18489_c2_g1::TRINITY_DN18489_c2_g1_i22::g.101262::m.101262	0	1148	XP_018837801.1	NADPH--cytochrome P450 reductase	Juglans regia	KYP69307.1
TRINITY_DN13122_c2_g4::TRINITY_DN13122_c2_g4_i4::g.20179::m.20179	2,20E-170	474	AET62962.1	cytochrome c oxidase subunit 2 (mitochondrion)	Lotus japonicus	YP_005090502.1
TRINITY_DN19434_c2_g3::TRINITY_DN19434_c2_g3_i2::g.117952::m.117952	0	1134	XP_018851614.1	glycerol-3-phosphate dehydrogenase SDP6, mitochondrial	Juglans regia	XP_018822930.1
TRINITY_DN16406_c2_g3::TRINITY_DN16406_c2_g3_i1::g.67214::m.67214	1,99E-132	374	XP_018845301.1	60S ribosomal protein L13-1	Juglans regia	XP_018845302.1
TRINITY_DN11854_c0_g1::TRINITY_DN11854_c0_g1_i1::g.9190::m.9190	1,41E-72	219	XP_016649075.1	thioredoxin-1-like isoform X1	Prunus mume	ONI17086.1
TRINITY_DN13288_c0_g3::TRINITY_DN13288_c0_g3_i3::g.22072::m.22072	0	775	XP_018812075.1	glucan endo-1,3-beta-glucosidase 13-like	Juglans regia	XP_018812083.1
TRINITY_DN15507_c1_g1::TRINITY_DN15507_c1_g1_i1::g.53015::m.53015	7,27E-120	345	XP_012068733.1	cysteine proteinase inhibitor 6	Jatropha curcas	KDP40574.1
TRINITY_DN15130_c6_g3::TRINITY_DN15130_c6_g3_i3::g.47307::m.47307	0	572	XP_018809730.1	probable protein phosphatase 2C 76	Juglans regia	XP_018809734.1
TRINITY_DN15473_c0_g1::TRINITY_DN15473_c0_g1_i4::g.52564::m.52564	0	595	XP_018814928.1	probable lactoylglutathione lyase, chloroplastic	Juglans regia	OAY27259.1
TRINITY_DN18360_c0_g5::TRINITY_DN18360_c0_g5_i1::g.99194::m.99194	3,13E-122	361	KRG95807.1	hypothetical protein GLYMA_19G172200	Glycine max	KHN48981.1
TRINITY_DN18610_c1_g2::TRINITY_DN18610_c1_g2_i3::g.103387::m.103387	0	1684	XP_018810540.1	probable glucan 1,3-alpha-glucosidase	Juglans regia	XP_018810541.1
TRINITY_DN19948_c1_g1::TRINITY_DN19948_c1_g1_i7::g.126477::m.126477	2,57E-63	206	XP_018809878.1	ketol-acid reductoisomerase, chloroplastic	Juglans regia	XP_018840362.1
TRINITY_DN15732_c1_g2::TRINITY_DN15732_c1_g2_i3::g.56333::m.56333	0	977	XP_018812048.1	T-complex protein 1 subunit beta	Juglans regia	OAY50499.1
TRINITY_DN19268_c1_g1::TRINITY_DN19268_c1_g1_i2::g.114841::m.114841	0	790	XP_018854045.1	T-complex protein 1 subunit alpha	Juglans regia	XP_018854052.1
TRINITY_DN15694_c2_g2::TRINITY_DN15694_c2_g2_i2::g.54517::m.54517	1,82E-140	398	XP_015947071.1	cytochrome c1-2, heme protein, mitochondrial	Arachis duranensis	XP_015947072.1

TRINITY_DN13820_c2_g1::TRINITY_DN13820_c2_g1_i2::g.28694::m.28694	0	647	XP_018852657.1	dnaJ protein homolog	Juglans regia	XP_018805733.1
TRINITY_DN18889_c2_g1::TRINITY_DN18889_c2_g1_i17::g.108140::m.108140	7,89E-149	420	XP_018824888.1	phosphoserine phosphatase, chloroplastic isoform X2	Juglans regia	XP_018824889.1
TRINITY_DN14517_c1_g1::TRINITY_DN14517_c1_g1_i15::g.38263::m.38263	0	602	XP_018813794.1	ubiquitin receptor RAD23b-like	Juglans regia	ONI16551.1
TRINITY_DN15211_c1_g1::TRINITY_DN15211_c1_g1_i7::g.48526::m.48526	8,95E-156	437	XP_018841431.1	5'-methylthioadenosine/S-adenosylhomocysteine nucleosidase 2-like isoform X1	Juglans regia	XP_018815344.1
TRINITY_DN19169_c1_g1::TRINITY_DN19169_c1_g1_i1::g.113168::m.113168	7,19E-61	192	XP_018859184.1	cinnamoyl-CoA reductase 2-like isoform X3	Juglans regia	XP_018859182.1
TRINITY_DN18383_c2_g3::TRINITY_DN18383_c2_g3_i1::g.99654::m.99654	0	780	XP_018848041.1	hsp70-Hsp90 organizing protein 3-like	Juglans regia	XP_018848041.1
TRINITY_DN14308_c0_g1::TRINITY_DN14308_c0_g1_i1::g.35383::m.35383	0	587	XP_018807871.1	putative G3BP-like protein isoform X2	Juglans regia	XP_018807870.1
TRINITY_DN13126_c1_g2::TRINITY_DN13126_c1_g2_i1::g.20156::m.20156	1,64E-86	254	KYP67019.1	Ras-related protein RABH1B	Cajanus cajan	ONI22221.1
TRINITY_DN18038_c0_g1::TRINITY_DN18038_c0_g1_i7::g.94082::m.94082	8,28E-83	242	XP_018815860.1	40S ribosomal protein S20-2	Juglans regia	XP_018815861.1
TRINITY_DN14696_c0_g1::TRINITY_DN14696_c0_g1_i2::g.40666::m.40666	1,64E-61	193	XP_018810857.1	early nodulin-like protein 1	Juglans regia	KDP33508.1
TRINITY_DN15303_c0_g1::TRINITY_DN15303_c0_g1_i2::g.49908::m.49908	0	560	XP_018845848.1	DNA-damage-repair/toleration protein DRT102-like	Juglans regia	XP_018845850.1
TRINITY_DN15702_c0_g4::TRINITY_DN15702_c0_g4_i1::g.55937::m.55937	0	504	XP_018860017.1	60S ribosomal protein L8-1-like	Juglans regia	XP_018822032.1
TRINITY_DN13427_c0_g2::TRINITY_DN13427_c0_g2_i5::g.23653::m.23653	1,99E-48	155	XP_018821920.1	40S ribosomal protein S26-1-like	Juglans regia	XP_018810475.1
TRINITY_DN19814_c1_g2::TRINITY_DN19814_c1_g2_i2::g.124101::m.124101	2,98E-97	300	XP_018813183.1	phosphoenolpyruvate carboxykinase [ATP-like]	Juglans regia	XP_018821098.1
TRINITY_DN12264_c2_g2::TRINITY_DN12264_c2_g2_i2::g.11522::m.11522	0	760	XP_018811933.1	plant UBX domain-containing protein 8	Juglans regia	XP_018828814.1
TRINITY_DN13465_c1_g2::TRINITY_DN13465_c1_g2_i1::g.24163::m.24163	3,45E-163	479	XP_018807321.1	aconitate hydratase 1	Juglans regia	OAY57605.1
TRINITY_DN19983_c1_g1::TRINITY_DN19983_c1_g1_i9::g.127358::m.127358	8,84E-147	417	XP_018816369.1	uncharacterized protein At1g03900-like isoform X1	Juglans regia	XP_018816370.1
TRINITY_DN16964_c2_g5::TRINITY_DN16964_c2_g5_i4::g.76427::m.76427	3,47E-43	140	OAY45736.1	hypothetical protein MANES_07G087100	Manihot esculenta	OAY51398.1
TRINITY_DN17558_c0_g1::TRINITY_DN17558_c0_g1_i9::g.85984::m.85984	0	903	XP_018810128.1	D-3-phosphoglycerate dehydrogenase 2, chloroplastic-like	Juglans regia	KRH44645.1
TRINITY_DN17017_c2_g1::TRINITY_DN17017_c2_g1_i7::g.77030::m.77030	6,11E-169	469	XP_018821477.1	nicotinamidase 1-like	Juglans regia	XP_018826859.1
TRINITY_DN14717_c1_g1::TRINITY_DN14717_c1_g1_i9::g.42525::m.42525	1,43E-116	335	XP_018841699.1	ras-related protein RABE1c-like	Juglans regia	XP_018841700.1
TRINITY_DN13650_c8_g1::TRINITY_DN13650_c8_g1_i8::g.26099::m.26099	2,03E-77	232	XP_018844636.1	PITH domain-containing protein 1	Juglans regia	XP_020208532.1

TRINITY_DN18258_c0_g1::TRINITY_DN18258_c0_g1_i6::g.97657::m.97657	6,18E-54	176	XP_008236502.1	probable mannitol dehydrogenase	Prunus mume	KDP29800.1
TRINITY_DN13419_c2_g1::TRINITY_DN13419_c2_g1_i6::g.23702::m.23702	0	882	XP_018845472.1	ATP-dependent 6-phosphofructokinase 3-like isoform X1	Juglans regia	XP_018837558.1
TRINITY_DN18011_c2_g1::TRINITY_DN18011_c2_g1_i8::g.93702::m.93702	0	587	XP_018839987.1	fructokinase-2	Juglans regia	XP_018822554.1
TRINITY_DN14942_c0_g1::TRINITY_DN14942_c0_g1_i6::g.44624::m.44624	0	1255	XP_018815397.1	serine/threonine-protein phosphatase 7 long form homolog	Juglans regia	XP_018815398.1
TRINITY_DN17863_c0_g3::TRINITY_DN17863_c0_g3_i3::g.91035::m.91035	0	1150	XP_018843210.1	mitochondrial Rho GTPase 1-like	Juglans regia	XP_018841787.1
TRINITY_DN16933_c5_g1::TRINITY_DN16933_c5_g1_i5::g.75858::m.75858	0	818	XP_018828199.1	aspartate aminotransferase, mitochondrial-like isoform X1	Juglans regia	XP_018828201.1
TRINITY_DN19887_c8_g1::TRINITY_DN19887_c8_g1_i5::g.125402::m.125402	3,92E-41	147	XP_018825590.1	probable pectinesterase/pectinesterase inhibitor 21	Juglans regia	XP_018832135.1
TRINITY_DN14777_c4_g4::TRINITY_DN14777_c4_g4_i3::g.42104::m.42104	2,51E-127	399	XP_018819570.1	uncharacterized protein LOC108990151	Juglans regia	XP_018851886.1
TRINITY_DN6579_c0_g1::TRINITY_DN6579_c0_g1_i1::g.1685::m.1685	1,67E-142	413	OAY29021.1	hypothetical protein MANES_15G111700	Manihot esculenta	XP_008219577.1
TRINITY_DN16381_c2_g1::TRINITY_DN16381_c2_g1_i3::g.66693::m.66693	0	1840	XP_018835573.1	presequence protease 1, chloroplastic/mitochondrial-like	Juglans regia	OAY39086.1
TRINITY_DN14242_c1_g2::TRINITY_DN14242_c1_g2_i9::g.34423::m.34423	9,61E-166	466	XP_018857023.1	persulfide dioxygenase ETHE1 homolog, mitochondrial	Juglans regia	XP_018857027.1
TRINITY_DN18618_c2_g1::TRINITY_DN18618_c2_g1_i14::g.103865::m.103865	0	1961	XP_018828368.1	tripeptidyl-peptidase 2-like	Juglans regia	XP_018830702.1
TRINITY_DN13619_c0_g1::TRINITY_DN13619_c0_g1_i2::g.26348::m.26348	1,26E-97	283	XP_018825729.1	universal stress protein PHOS32-like isoform X2	Juglans regia	KDP27925.1
TRINITY_DN17578_c3_g1::TRINITY_DN17578_c3_g1_i5::g.85666::m.85666	0	1959	XP_018837083.1	probable sucrose-phosphate synthase 2	Juglans regia	ONI28761.1
TRINITY_DN16993_c0_g2::TRINITY_DN16993_c0_g2_i2::g.76741::m.76741	0	1095	XP_018808002.1	subtilisin-like protease SBT1.7	Juglans regia	OAY23008.1
TRINITY_DN13712_c0_g1::TRINITY_DN13712_c0_g1_i1::g.27487::m.27487	1,97E-168	472	XP_018838975.1	alpha-soluble NSF attachment protein 2	Juglans regia	XP_018821859.1
TRINITY_DN14406_c2_g1::TRINITY_DN14406_c2_g1_i7::g.36607::m.36607	0	1238	XP_018824245.1	acyl-coenzyme A oxidase 3, peroxisomal-like	Juglans regia	XP_018824246.1
TRINITY_DN18981_c5_g1::TRINITY_DN18981_c5_g1_i2::g.109832::m.109832	0	974	XP_018837477.1	T-complex protein 1 subunit eta isoform X1	Juglans regia	XP_016166375.1
TRINITY_DN13705_c0_g1::TRINITY_DN13705_c0_g1_i7::g.27449::m.27449	0	738	XP_018818431.1	tubulin alpha-3 chain	Juglans regia	XP_018820085.1
TRINITY_DN17456_c0_g1::TRINITY_DN17456_c0_g1_i8::g.83943::m.83943	9,66E-122	347	XP_018814564.1	soluble inorganic pyrophosphatase 1	Juglans regia	XP_018834262.1
TRINITY_DN18817_c0_g1::TRINITY_DN18817_c0_g1_i10::g.107314::m.107314	0	821	XP_018850614.1	proton pump-interactor 1-like	Juglans regia	XP_018823042.1
TRINITY_DN19070_c1_g2::TRINITY_DN19070_c1_g2_i7::g.110348::m.110348	3,68E-139	392	XP_018809757.1	calcyclin-binding protein-like	Juglans regia	XP_018819738.1

TRINITY_DN12832_c2_g6::TRINITY_DN12832_c2_g6_i1::g.16307::m.16307	0	537	XP_018849554.1	prohibitin-1, mitochondrial isoform X1	Juglans regia	XP_018849555.1
TRINITY_DN14216_c0_g3::TRINITY_DN14216_c0_g3_i3::g.34216::m.34216	1,49E-83	252	XP_018854242.1	probable aldo-keto reductase 1	Juglans regia	XP_018826312.1
TRINITY_DN16423_c1_g3::TRINITY_DN16423_c1_g3_i3::g.67278::m.67278	0	974	XP_018843166.1	proline--tRNA ligase, cytoplasmic-like isoform X1	Juglans regia	XP_018843167.1
TRINITY_DN13521_c0_g4::TRINITY_DN13521_c0_g4_i1::g.24842::m.24842	8,88E-139	408	XP_018815040.1	dihydrolipoyllysine-residue acetyltransferase component 4 of pyruvate dehydrogenase complex, chloroplastic-like	Juglans regia	XP_018825347.1
TRINITY_DN11068_c0_g1::TRINITY_DN11068_c0_g1_i1::g.6241::m.6241	5,37E-27	100	XP_018852998.1	60S acidic ribosomal protein P3-like	Juglans regia	XP_018860534.1
TRINITY_DN11631_c0_g1::TRINITY_DN11631_c0_g1_i1::g.8169::m.8169	6,62E-175	489	XP_018856419.1	uncharacterized protein LOC109018719	Juglans regia	XP_018856421.1
TRINITY_DN17644_c1_g2::TRINITY_DN17644_c1_g2_i9::g.87544::m.87544	0	849	XP_018836411.1	catalase isozyme 1	Juglans regia	CAD42908.1
TRINITY_DN16032_c0_g1::TRINITY_DN16032_c0_g1_i4::g.60901::m.60901	0	626	XP_018828122.1	glutelin type-B 5-like	Juglans regia	XP_018828123.1
TRINITY_DN16763_c1_g3::TRINITY_DN16763_c1_g3_i3::g.72969::m.72969	0	728	ONI30885.1	hypothetical protein PRUPE_1G279500	Prunus persica	XP_007222547.1
TRINITY_DN13349_c1_g1::TRINITY_DN13349_c1_g1_i7::g.22584::m.22584	1,78E-138	389	XP_018831854.1	CBS domain-containing protein CBSX3, mitochondrial-like	Juglans regia	OAY58828.1
TRINITY_DN14455_c0_g1::TRINITY_DN14455_c0_g1_i2::g.37451::m.37451	0	665	XP_018847910.1	phosphoglycerate kinase, chloroplastic	Juglans regia	XP_008371996.1
TRINITY_DN19436_c2_g2::TRINITY_DN19436_c2_g2_i1::g.118043::m.118043	2,55E-45	159	KDP22063.1	hypothetical protein JCGZ_25894	Jatropha curcas	XP_012089960.1
TRINITY_DN18302_c1_g2::TRINITY_DN18302_c1_g2_i2::g.98507::m.98507	5,50E-151	429	XP_018823047.1	2-methylene-furan-3-one reductase-like	Juglans regia	XP_018815084.1
TRINITY_DN13203_c0_g1::TRINITY_DN13203_c0_g1_i1::g.21059::m.21059	2,59E-68	204	XP_018850387.1	thioredoxin M-type, chloroplastic isoform X2	Juglans regia	XP_018822894.1
TRINITY_DN13389_c0_g2::TRINITY_DN13389_c0_g2_i2::g.23122::m.23122	0	521	OAY44160.1	hypothetical protein MANES_08G127300	Manihot esculenta	KDP23623.1
TRINITY_DN15149_c3_g1::TRINITY_DN15149_c3_g1_i3::g.47576::m.47576	0	867	KDP43990.1	hypothetical protein JCGZ_05457	Jatropha curcas	KDP43990.1
TRINITY_DN19596_c0_g2::TRINITY_DN19596_c0_g2_i1::g.120414::m.120414	0	887	XP_018815731.1	glyoxysomal fatty acid beta-oxidation multifunctional protein MFP-a-like	Juglans regia	XP_018825769.1
TRINITY_DN11870_c0_g1::TRINITY_DN11870_c0_g1_i2::g.9275::m.9275	5,33E-63	191	OIW04754.1	hypothetical protein TanjilG_08637	Lupinus angustifolius	XP_019455662.1
TRINITY_DN18707_c2_g1::TRINITY_DN18707_c2_g1_i4::g.104948::m.104948	0	2169	XP_018842317.1	nodal modulator 1	Juglans regia	XP_008227880.1
TRINITY_DN18173_c2_g1::TRINITY_DN18173_c2_g1_i8::g.96624::m.96624	0	1746	XP_018845600.1	L-arabinokinase-like	Juglans regia	XP_018845086.1
TRINITY_DN11898_c0_g1::TRINITY_DN11898_c0_g1_i2::g.9380::m.9380	0	1217	XP_018846638.1	uncharacterized protein LOC109010309	Juglans regia	ONI33699.1

TRINITY_DN13113_c1_g1::TRINITY_DN13113_c1_g1_i4::g.20046::m.20046	0	1082	XP_018839434.1	pyruvate kinase isozyme A, chloroplastic-like	Juglans regia	XP_018818630.1
TRINITY_DN17740_c0_g2::TRINITY_DN17740_c0_g2_i7::g.89045::m.89045	6,34E-164	467	XP_018847079.1	LL-diaminopimelate aminotransferase, chloroplastic-like	Juglans regia	XP_017181435.1
TRINITY_DN13674_c0_g1::TRINITY_DN13674_c0_g1_i2::g.26925::m.26925	0	1590	XP_018816788.1	alpha-1,4 glucan phosphorylase L isozyme, chloroplastic/amyloplastic	Juglans regia	XP_018503860.1
TRINITY_DN17202_c2_g1::TRINITY_DN17202_c2_g1_i4::g.80033::m.80033	0	842	XP_018816929.1	uncharacterized protein LOC108988207	Juglans regia	XP_018843198.1
TRINITY_DN20008_c1_g1::TRINITY_DN20008_c1_g1_i8::g.127738::m.127738	0	647	XP_018840255.1	probable voltage-gated potassium channel subunit beta	Juglans regia	XP_018818414.1
TRINITY_DN17814_c1_g2::TRINITY_DN17814_c1_g2_i3::g.90444::m.90444	6,78E-60	186	XP_018845324.1	protein disulfide isomerase-like 5-1	Juglans regia	XP_009338286.1
TRINITY_DN19612_c1_g2::TRINITY_DN19612_c1_g2_i9::g.120821::m.120821	0	637	XP_018809366.1	probable methyltransferase PMT18	Juglans regia	XP_018844923.1
TRINITY_DN13600_c1_g7::TRINITY_DN13600_c1_g7_i6::g.25108::m.25108	4,43E-88	265	XP_018844008.1	protein FATTY ACID EXPORT 1, chloroplastic-like	Juglans regia	XP_018847055.1
TRINITY_DN18619_c0_g2::TRINITY_DN18619_c0_g2_i2::g.103437::m.103437	1,22E-107	310	XP_018815940.1	60S ribosomal protein L18-2	Juglans regia	XP_018841792.1
TRINITY_DN18061_c2_g3::TRINITY_DN18061_c2_g3_i1::g.94540::m.94540	7,86E-87	254	XP_018806248.1	40S ribosomal protein S17-3-like	Juglans regia	XP_018806249.1
TRINITY_DN15930_c2_g3::TRINITY_DN15930_c2_g3_i6::g.59212::m.59212	0	658	ONI05269.1	hypothetical protein PRUPE_6G365100	Prunus persica	XP_007205316.1
TRINITY_DN14203_c2_g1::TRINITY_DN14203_c2_g1_i1::g.33817::m.33817	0	1793	XP_018821368.1	chaperone protein ClpB3, chloroplastic-like	Juglans regia	XP_018816799.1
TRINITY_DN17074_c4_g2::TRINITY_DN17074_c4_g2_i4::g.77976::m.77976	0	944	ONH93998.1	hypothetical protein PRUPE_8G265300	Prunus persica	XP_007200983.1
TRINITY_DN17405_c1_g3::TRINITY_DN17405_c1_g3_i1::g.83287::m.83287	0	559	XP_018841018.1	NADH-cytochrome b5 reductase-like protein	Juglans regia	XP_009368820.1
TRINITY_DN16821_c3_g1::TRINITY_DN16821_c3_g1_i2::g.73875::m.73875	0	866	XP_018828537.1	protein NETWORKED 4A-like isoform X1	Juglans regia	XP_018828539.1
TRINITY_DN13494_c0_g3::TRINITY_DN13494_c0_g3_i1::g.24527::m.24527	2,74E-132	373	XP_018817673.1	40S ribosomal protein S9-2-like	Juglans regia	XP_018845180.1
TRINITY_DN10893_c0_g1::TRINITY_DN10893_c0_g1_i1::g.5751::m.5751	0	533	XP_018825452.1	glucan endo-1,3-beta-glucosidase-like	Juglans regia	XP_018825472.1
TRINITY_DN16490_c2_g1::TRINITY_DN16490_c2_g1_i1::g.68401::m.68401	0	843	XP_018805409.1	dihydrolipoyl dehydrogenase 2, chloroplastic-like	Juglans regia	XP_016198035.1
TRINITY_DN16487_c1_g2::TRINITY_DN16487_c1_g2_i5::g.68395::m.68395	4,12E-90	263	XP_016187428.1	basic transcription factor 3	Arachis ipaensis	XP_016187427.1
TRINITY_DN17201_c0_g1::TRINITY_DN17201_c0_g1_i1::g.79917::m.79917	0	1024	XP_018819165.1	pyruvate decarboxylase 1-like	Juglans regia	XP_020977085.1
TRINITY_DN14870_c0_g1::TRINITY_DN14870_c0_g1_i2::g.43479::m.43479	3,48E-160	446	XP_018840891.1	proteasome subunit beta type-1	Juglans regia	OAY62404.1
TRINITY_DN14090_c0_g1::TRINITY_DN14090_c0_g1_i4::g.32240::m.32240	0	742	ONI11169.1	hypothetical protein PRUPE_4G091100	Prunus persica	XP_007211399.1

TRINITY_DN18902_c0_g1::TRINITY_DN18902_c0_g1_i9::g.108609::m.108609	0	628	XP_018805998.1	uncharacterized protein LOC108979726, partial	Juglans regia	XP_009375672.1
TRINITY_DN19154_c1_g1::TRINITY_DN19154_c1_g1_i1::g.113024::m.113024	2,96E-138	390	XP_018821863.1	superoxide dismutase [Mn], mitochondrial-like	Juglans regia	AEZ56249.1
TRINITY_DN14612_c0_g1::TRINITY_DN14612_c0_g1_i2::g.39550::m.39550	0	676	XP_018813652.1	probable purple acid phosphatase 20 isoform X2	Juglans regia	XP_018813650.1
TRINITY_DN16497_c0_g2::TRINITY_DN16497_c0_g2_i8::g.67601::m.67601	0	1145	OAY52363.1	hypothetical protein MANES_04G077500	Manihot esculenta	OAY52364.1
TRINITY_DN10818_c0_g1::TRINITY_DN10818_c0_g1_i2::g.5587::m.5587	3,82E-38	128	OAY30129.1	hypothetical protein MANES_14G006100	Manihot esculenta	OAY30128.1
TRINITY_DN14143_c3_g2::TRINITY_DN14143_c3_g2_i2::g.32997::m.32997	0	1114	XP_018812753.1	glucose-6-phosphate isomerase 1, chloroplastic	Juglans regia	KHN08244.1
TRINITY_DN16212_c0_g1::TRINITY_DN16212_c0_g1_i2::g.63955::m.63955	0	612	XP_018815515.1	spermidine synthase 1-like	Juglans regia	OAY34570.1
TRINITY_DN11356_c0_g1::TRINITY_DN11356_c0_g1_i1::g.7113::m.7113	3,45E-29	109	ONH95541.1	hypothetical protein PRUPE_7G076400	Prunus persica	ONH95542.1
TRINITY_DN15198_c1_g2::TRINITY_DN15198_c1_g2_i7::g.48337::m.48337	5,82E-129	379	XP_018814398.1	protein RETICULATA-RELATED 3, chloroplastic-like	Juglans regia	KDP21842.1
TRINITY_DN19436_c2_g3::TRINITY_DN19436_c2_g3_i1::g.118048::m.118048	7,58E-52	178	XP_018807473.1	uncharacterized protein LOC108980890	Juglans regia	XP_020540826.1
TRINITY_DN13757_c0_g1::TRINITY_DN13757_c0_g1_i4::g.28204::m.28204	2,85E-163	456	XP_018834440.1	40S ribosomal protein S2-4-like	Juglans regia	XP_018837209.1
TRINITY_DN14326_c0_g1::TRINITY_DN14326_c0_g1_i2::g.35500::m.35500	6,15E-22	91,7	XP_018833902.1	cytochrome c oxidase subunit 6b-1-like isoform X2	Juglans regia	XP_018833901.1
TRINITY_DN17638_c0_g5::TRINITY_DN17638_c0_g5_i1::g.87396::m.87396	1,11E-58	191	XP_004488391.1	polygalacturonase-like	Cicer arietinum	KRH14091.1
TRINITY_DN16307_c2_g1::TRINITY_DN16307_c2_g1_i9::g.65550::m.65550	0	1098	XP_018851318.1	serine/threonine-protein phosphatase 2A 65 kDa regulatory subunit A beta isoform	Juglans regia	XP_018851318.1
TRINITY_DN18255_c1_g1::TRINITY_DN18255_c1_g1_i6::g.97743::m.97743	0	2897	XP_018820347.1	myosin-11 isoform X1	Juglans regia	ONI04899.1
TRINITY_DN18634_c2_g1::TRINITY_DN18634_c2_g1_i4::g.104214::m.104214	0	1026	XP_018847003.1	subtilisin-like protease SBT1.2	Juglans regia	XP_007208895.1
TRINITY_DN12779_c0_g1::TRINITY_DN12779_c0_g1_i3::g.15740::m.15740	0	1015	XP_018813183.1	phosphoenolpyruvate carboxykinase [ATP-like]	Juglans regia	XP_018813767.1
TRINITY_DN15549_c2_g2::TRINITY_DN15549_c2_g2_i1::g.53640::m.53640	0	582	XP_018848084.1	polyadenylate-binding protein 3	Juglans regia	XP_018848084.1
TRINITY_DN19606_c0_g1::TRINITY_DN19606_c0_g1_i15::g.121023::m.121023	5,28E-136	388	XP_018842448.1	cysteine synthase	Juglans regia	XP_018842449.1
TRINITY_DN16691_c0_g1::TRINITY_DN16691_c0_g1_i7::g.71695::m.71695	0	584	XP_018849296.1	succinate--CoA ligase [ADP-forming] subunit alpha-1, mitochondrial	Juglans regia	XP_009355925.1
TRINITY_DN16311_c1_g1::TRINITY_DN16311_c1_g1_i8::g.65782::m.65782	0	851	XP_018859613.1	plastidic glucose transporter 4-like	Juglans regia	XP_018816890.1
TRINITY_DN15482_c4_g1::TRINITY_DN15482_c4_g1_i5::g.52502::m.52502	9,75E-136	385	XP_018823879.1	translocon-associated protein subunit alpha-like	Juglans regia	XP_018841445.1

TRINITY_DN15556_c2_g3::TRINITY_DN15556_c2_g3_i7::g.53767::m.53767	0	636	OAY30143.1	hypothetical protein MANES_14G007400	Manihot esculenta	XP_012087346.1
TRINITY_DN12323_c0_g1::TRINITY_DN12323_c0_g1_i4::g.11964::m.11964	0	1004	XP_018858088.1	pyruvate kinase, cytosolic isozyme-like	Juglans regia	XP_018858157.1
TRINITY_DN15236_c1_g2::TRINITY_DN15236_c1_g2_i4::g.49063::m.49063	0	804	XP_018817298.1	protein transport protein SEC31 homolog B-like isoform X2	Juglans regia	XP_018817293.1
TRINITY_DN17457_c4_g1::TRINITY_DN17457_c4_g1_i4::g.83960::m.83960	1,56E-119	341	XP_018810064.1	eukaryotic translation initiation factor	Juglans regia	XP_018807853.1
TRINITY_DN11580_c0_g1::TRINITY_DN11580_c0_g1_i1::g.7933::m.7933	0	638	XP_018824341.1	UPF0160 protein	Juglans regia	XP_018859956.1
TRINITY_DN15072_c0_g1::TRINITY_DN15072_c0_g1_i2::g.46447::m.46447	0	570	XP_018847856.1	protein transport protein SEC13 homolog B	Juglans regia	XP_018839977.1
TRINITY_DN19781_c5_g1::TRINITY_DN19781_c5_g1_i1::g.123563::m.123563	0	770	XP_018816946.1	eukaryotic translation initiation factor 3 subunit M-like	Juglans regia	ONI18991.1
TRINITY_DN19199_c1_g1::TRINITY_DN19199_c1_g1_i2::g.113819::m.113819	0	3323	XP_018845624.1	centrosomal protein of 290 kDa isoform X2	Juglans regia	XP_018845623.1
TRINITY_DN11501_c0_g1::TRINITY_DN11501_c0_g1_i5::g.7580::m.7580	4,07E-84	247	XP_018842451.1	lactoylglutathione lyase-like	Juglans regia	XP_008390907.1
TRINITY_DN18781_c0_g5::TRINITY_DN18781_c0_g5_i3::g.106209::m.106209	1,56E-142	404	XP_018828827.1	eukaryotic translation initiation factor 3 subunit I-like	Juglans regia	XP_018828827.1
TRINITY_DN19854_c2_g2::TRINITY_DN19854_c2_g2_i3::g.124560::m.124560	0	782	XP_018842400.1	eukaryotic translation initiation factor 3 subunit C isoform X1	Juglans regia	XP_018842401.1
TRINITY_DN14973_c1_g2::TRINITY_DN14973_c1_g2_i1::g.45015::m.45015	9,40E-106	303	XP_018847314.1	probable NADH dehydrogenase [ubiquinone] 1 alpha subcomplex subunit 5, mitochondrial	Juglans regia	XP_018847315.1
TRINITY_DN16462_c1_g1::TRINITY_DN16462_c1_g1_i7::g.68028::m.68028	1,32E-176	490	XP_018841038.1	acyl-protein thioesterase 2-like isoform X1	Juglans regia	XP_012078729.1
TRINITY_DN16061_c2_g1::TRINITY_DN16061_c2_g1_i1::g.61310::m.61310	0	782	XP_018817984.1	uridine 5'-monophosphate synthase-like	Juglans regia	ONI20769.1
TRINITY_DN15683_c2_g1::TRINITY_DN15683_c2_g1_i10::g.55639::m.55639	3,01E-91	290	OIW19305.1	hypothetical protein TanjilG_16839	Lupinus angustifolius	XP_019429734.1
TRINITY_DN11090_c0_g1::TRINITY_DN11090_c0_g1_i1::g.6289::m.6289	3,03E-113	326	KDP28201.1	hypothetical protein JCGZ_13972	Jatropha curcas	XP_012082823.1
TRINITY_DN16191_c2_g1::TRINITY_DN16191_c2_g1_i7::g.63681::m.63681	0	1014	XP_018838880.1	fimbrin-5-like	Juglans regia	XP_018838880.1
TRINITY_DN19075_c0_g2::TRINITY_DN19075_c0_g2_i2::g.111296::m.111296	0	1087	XP_018805414.1	delta(24)-sterol reductase	Juglans regia	XP_018805415.1
TRINITY_DN11919_c0_g1::TRINITY_DN11919_c0_g1_i1::g.9495::m.9495	1,17E-144	407	KDP25291.1	hypothetical protein JCGZ_20447	Jatropha curcas	XP_018824362.1
TRINITY_DN17942_c1_g1::TRINITY_DN17942_c1_g1_i6::g.91683::m.91683	0	754	KDP38816.1	hypothetical protein JCGZ_04973	Jatropha curcas	OAY39803.1
TRINITY_DN15935_c0_g1::TRINITY_DN15935_c0_g1_i1::g.59258::m.59258	0	805	XP_018842240.1	biotin carboxylase 1, chloroplastic isoform X1	Juglans regia	XP_018842241.1
TRINITY_DN16842_c1_g4::TRINITY_DN16842_c1_g4_i5::g.74459::m.74459	1,12E-97	282	XP_018813840.1	ferredoxin, root R-B1-like	Juglans regia	ONI01252.1

TRINITY_DN14360_c0_g2::TRINITY_DN14360_c0_g2_i2::g.35887::m.35887	0	868	XP_018833466.1	tubulin beta-1 chain isoform X1	Juglans regia	XP_018833467.1
TRINITY_DN13161_c0_g1::TRINITY_DN13161_c0_g1_i3::g.20601::m.20601	9,87E-98	288	XP_018832954.1	putative expansin-A17	Juglans regia	XP_018843121.1
TRINITY_DN17755_c1_g2::TRINITY_DN17755_c1_g2_i5::g.89168::m.89168	0	671	XP_018852266.1	dihydrolipoyllysine-residue succinyltransferase component of 2-oxoglutarate dehydrogenase complex 2, mitochondrial-like	Juglans regia	XP_018852267.1
TRINITY_DN17545_c0_g1::TRINITY_DN17545_c0_g1_i5::g.84940::m.84940	0	1329	XP_018829229.1	glycine--tRNA ligase, mitochondrial 1-like	Juglans regia	XP_018841636.1
TRINITY_DN11691_c0_g1::TRINITY_DN11691_c0_g1_i1::g.8068::m.8068	0	822	XP_018839115.1	delta-1-pyrroline-5-carboxylate synthase-like	Juglans regia	XP_018820283.1
TRINITY_DN14965_c0_g1::TRINITY_DN14965_c0_g1_i3::g.45244::m.45244	0	1043	XP_018828804.1	uncharacterized protein LOC108997121 isoform X1	Juglans regia	XP_008233319.1
TRINITY_DN17740_c0_g1::TRINITY_DN17740_c0_g1_i2::g.89044::m.89044	5,11E-120	350	XP_018847079.1	LL-diaminopimelate aminotransferase, chloroplastic-like	Juglans regia	ONH92109.1
TRINITY_DN16246_c0_g1::TRINITY_DN16246_c0_g1_i1::g.63895::m.63895	0	582	XP_018826879.1	L-galactose dehydrogenase	Juglans regia	XP_008236174.1
TRINITY_DN18982_c0_g1::TRINITY_DN18982_c0_g1_i8::g.109877::m.109877	0	659	XP_018825910.1	ERBB-3 BINDING PROTEIN 1 isoform X1	Juglans regia	KDP22867.1
TRINITY_DN13531_c1_g1::TRINITY_DN13531_c1_g1_i7::g.25242::m.25242	0	1021	XP_008338464.1	protein NETWORKED 2D-like	Malus domestica	ONI02491.1
TRINITY_DN13348_c2_g1::TRINITY_DN13348_c2_g1_i3::g.22605::m.22605	7,34E-84	246	XP_018831277.1	40S ribosomal protein S14-3	Juglans regia	XP_018814658.1
TRINITY_DN19420_c3_g1::TRINITY_DN19420_c3_g1_i7::g.117669::m.117669	1,33E-86	252	XP_008218349.1	60S ribosomal protein L23	Prunus mume	XP_008224171.1
TRINITY_DN13350_c3_g1::TRINITY_DN13350_c3_g1_i5::g.22631::m.22631	0	520	XP_018836913.1	serine carboxypeptidase-like	Juglans regia	ONI23789.1
TRINITY_DN16612_c3_g2::TRINITY_DN16612_c3_g2_i3::g.70349::m.70349	1,71E-106	332	XP_018844325.1	ATPase 9, plasma membrane-type	Juglans regia	OAY52718.1
TRINITY_DN12310_c0_g1::TRINITY_DN12310_c0_g1_i3::g.11787::m.11787	0	764	XP_018821818.1	alpha-galactosidase 3 isoform X1	Juglans regia	OAY46379.1
TRINITY_DN14939_c1_g2::TRINITY_DN14939_c1_g2_i3::g.44685::m.44685	1,28E-96	288	XP_018857924.1	GDSL esterase/lipase At1g29670-like	Juglans regia	XP_018809856.1
TRINITY_DN10911_c0_g2::TRINITY_DN10911_c0_g2_i2::g.5805::m.5805	0	526	OAY32440.1	hypothetical protein MANES_13G017700	Manihot esculenta	XP_018836697.1
TRINITY_DN16718_c6_g1::TRINITY_DN16718_c6_g1_i4::g.72149::m.72149	4,01E-124	364	XP_018834200.1	endoglucanase 9-like	Juglans regia	XP_018834199.1
TRINITY_DN16625_c2_g1::TRINITY_DN16625_c2_g1_i8::g.70673::m.70673	1,78E-102	293	XP_020233270.1	ras-related protein RABH1e-like, partial	Cajanus cajan	XP_018498817.1
TRINITY_DN15294_c5_g1::TRINITY_DN15294_c5_g1_i4::g.49817::m.49817	0	932	XP_018816807.1	glutamate decarboxylase 1	Juglans regia	OAY57229.1
TRINITY_DN19873_c6_g8::TRINITY_DN19873_c6_g8_i6::g.124910::m.124910	3,09E-177	504	XP_018845479.1	NADP-dependent malic enzyme	Juglans regia	KRH74264.1

TRINITY_DN15424_c2_g3::TRINITY_DN15424_c2_g3_i3::g.51667::m.51667	0	634	XP_018826340.1	vicilin-like seed storage protein At2g18540	Juglans regia	XP_008241881.1
TRINITY_DN16774_c0_g1::TRINITY_DN16774_c0_g1_i9::g.73138::m.73138	0	614	XP_018850953.1	dual specificity protein phosphatase 12-like	Juglans regia	XP_018850954.1
TRINITY_DN17278_c1_g1::TRINITY_DN17278_c1_g1_i1::g.81211::m.81211	2,66E-45	146	XP_018830962.1	uncharacterized protein LOC108998731 isoform X1	Juglans regia	XP_018830963.1
TRINITY_DN13302_c0_g2::TRINITY_DN13302_c0_g2_i1::g.22438::m.22438	5,47E-167	471	XP_018814733.1	ubiquinone biosynthesis protein COQ9, mitochondrial-like	Juglans regia	OAY54020.1
TRINITY_DN12343_c0_g1::TRINITY_DN12343_c0_g1_i5::g.11815::m.11815	5,96E-127	359	XP_018853133.1	40S ribosomal protein S7-like	Juglans regia	XP_018815497.1
TRINITY_DN16818_c0_g1::TRINITY_DN16818_c0_g1_i9::g.73983::m.73983	1,18E-162	464	XP_018821556.1	nucleosome assembly protein 1;4-like	Juglans regia	ONI15530.1
TRINITY_DN19014_c2_g1::TRINITY_DN19014_c2_g1_i4::g.110590::m.110590	3,20E-67	221	XP_018849376.1	phosphoenolpyruvate carboxylase, housekeeping isozyme	Juglans regia	ABM16909.1
TRINITY_DN12914_c1_g1::TRINITY_DN12914_c1_g1_i4::g.17252::m.17252	3,37E-40	132	ADD69807.1	copper transport protein ATOX1	Hevea brasiliensis	OAY45760.1
TRINITY_DN17100_c3_g1::TRINITY_DN17100_c3_g1_i1::g.78525::m.78525	1,95E-114	327	OIW18014.1	hypothetical protein TanjilG_07598	Lupinus angustifolius	XP_019460299.1
TRINITY_DN14403_c4_g2::TRINITY_DN14403_c4_g2_i1::g.36526::m.36526	0	1971	XP_018815757.1	phosphoglucan, water dikinase, chloroplastic isoform X1	Juglans regia	XP_018815758.1
TRINITY_DN18412_c2_g2::TRINITY_DN18412_c2_g2_i2::g.100143::m.100143	0	954	OAY24991.1	hypothetical protein MANES_17G059700	Manihot esculenta	OAY24992.1
TRINITY_DN14115_c3_g1::TRINITY_DN14115_c3_g1_i10::g.32650::m.32650	0	715	XP_018816053.1	mannosyl-oligosaccharide 1,2-alpha-mannosidase MNS1-like isoform X2	Juglans regia	XP_018820403.1
TRINITY_DN15464_c0_g5::TRINITY_DN15464_c0_g5_i1::g.52274::m.52274	1,44E-111	335	XP_018814481.1	heat shock cognate 70 kDa protein 2-like	Juglans regia	OAY38641.1
TRINITY_DN15531_c1_g1::TRINITY_DN15531_c1_g1_i14::g.53405::m.53405	9,01E-83	243	XP_018849412.1	nudix hydrolase 16, mitochondrial isoform X3	Juglans regia	XP_018849410.1
TRINITY_DN12587_c0_g1::TRINITY_DN12587_c0_g1_i1::g.13954::m.13954	6,89E-141	399	XP_018811145.1	glucose and ribitol dehydrogenase homolog 1-like isoform X2	Juglans regia	XP_018811144.1
TRINITY_DN17254_c0_g1::TRINITY_DN17254_c0_g1_i5::g.81103::m.81103	0	547	XP_018844641.1	far upstream element-binding protein 1 isoform X2	Juglans regia	XP_018812438.1
TRINITY_DN17518_c0_g1::TRINITY_DN17518_c0_g1_i5::g.85741::m.85741	2,50E-87	258	XP_018820076.1	SKP1-like protein 1B	Juglans regia	XP_018853688.1
TRINITY_DN16163_c4_g1::TRINITY_DN16163_c4_g1_i1::g.63306::m.63306	1,49E-90	278	XP_018850081.1	exopolygalacturonase-like	Juglans regia	XP_018856482.1
TRINITY_DN16238_c3_g1::TRINITY_DN16238_c3_g1_i4::g.64390::m.64390	0	851	KDP26669.1	hypothetical protein JCGZ_17827	Jatropha curcas	XP_020538969.1
TRINITY_DN19244_c2_g1::TRINITY_DN19244_c2_g1_i18::g.114657::m.114657	4,62E-90	271	XP_018811676.1	cold and drought-regulated protein CORA-like	Juglans regia	XP_018811677.1
TRINITY_DN19880_c6_g4::TRINITY_DN19880_c6_g4_i2::g.125001::m.125001	2,89E-81	247	XP_008233897.1	flavonol synthase/flavanone 3-hydroxylase-like	Prunus mume	XP_018820940.1
TRINITY_DN18615_c1_g3::TRINITY_DN18615_c1_g3_i2::g.103395::m.103395	3,30E-49	159	XP_018826141.1	endo-1,3;1,4-beta-D-glucanase-like isoform X2	Juglans regia	XP_015958680.1

TRINITY_DN15441_c0_g1::TRINITY_DN15441_c0_g1_i3::g.51487::m.51487	6,22E-77	226	XP_018834238.1	cytochrome c	Juglans regia	KOM36983.1
TRINITY_DN16789_c2_g4::TRINITY_DN16789_c2_g4_i5::g.73232::m.73232	1,43E-144	412	XP_018856900.1	S-adenosylmethionine synthase 2	Juglans regia	XP_018856907.1
TRINITY_DN12003_c0_g1::TRINITY_DN12003_c0_g1_i1::g.9933::m.9933	8,82E-158	444	XP_018820853.1	mitochondrial outer membrane protein porin 4	Juglans regia	XP_018831181.1
TRINITY_DN14879_c3_g3::TRINITY_DN14879_c3_g3_i2::g.43725::m.43725	0	966	XP_018847996.1	importin subunit alpha-1a-like	Juglans regia	XP_018847993.1
TRINITY_DN17985_c0_g4::TRINITY_DN17985_c0_g4_i1::g.93309::m.93309	0	663	AFK43004.1	unknown	Lotus japonicus	AFK46843.1
TRINITY_DN13592_c0_g2::TRINITY_DN13592_c0_g2_i5::g.25790::m.25790	0	706	XP_018817113.1	TOM1-like protein 2	Juglans regia	XP_018817114.1
TRINITY_DN19576_c0_g1::TRINITY_DN19576_c0_g1_i3::g.120043::m.120043	0	756	XP_018827773.1	uncharacterized protein LOC108996364	Juglans regia	XP_018827774.1
TRINITY_DN16215_c5_g2::TRINITY_DN16215_c5_g2_i7::g.63985::m.63985	0	1155	XP_018858095.1	ATP-citrate synthase beta chain protein 2	Juglans regia	XP_018858096.1
TRINITY_DN18909_c0_g1::TRINITY_DN18909_c0_g1_i4::g.108774::m.108774	0	1088	XP_018831990.1	eukaryotic translation initiation factor 3 subunit B-like	Juglans regia	OAY27531.1
TRINITY_DN15836_c2_g1::TRINITY_DN15836_c2_g1_i8::g.57209::m.57209	0	1060	XP_018834473.1	asparagine synthetase [glutamine]-hydrolyzing 3	Juglans regia	XP_018847046.1
TRINITY_DN13140_c0_g3::TRINITY_DN13140_c0_g3_i1::g.20389::m.20389	0	538	OAY42675.1	hypothetical protein MANES_08G007100	Manihot esculenta	XP_018821649.1
TRINITY_DN16820_c1_g1::TRINITY_DN16820_c1_g1_i7::g.73896::m.73896	6,83E-55	176	ONI08870.1	hypothetical protein PRUPE_5G205300	Prunus persica	XP_007209483.1
TRINITY_DN20043_c7_g1::TRINITY_DN20043_c7_g1_i1::g.128234::m.128234	4,65E-112	323	XP_018809557.1	rho GDP-dissociation inhibitor 1-like isoform X1	Juglans regia	XP_018809558.1
TRINITY_DN13259_c0_g1::TRINITY_DN13259_c0_g1_i1::g.21585::m.21585	0	674	XP_018834882.1	V-type proton ATPase subunit C	Juglans regia	ONI20047.1
TRINITY_DN18601_c0_g1::TRINITY_DN18601_c0_g1_i6::g.101598::m.101598	3,45E-149	424	XP_018844545.1	omega-amidase, chloroplastic	Juglans regia	OAY26903.1
TRINITY_DN15621_c1_g1::TRINITY_DN15621_c1_g1_i6::g.54532::m.54532	0	596	XP_018837506.1	glucosidase 2 subunit beta	Juglans regia	XP_008221020.1
TRINITY_DN15203_c1_g1::TRINITY_DN15203_c1_g1_i8::g.48552::m.48552	0	744	XP_018857609.1	UDP-glucuronic acid decarboxylase 4-like	Juglans regia	XP_018825586.1
TRINITY_DN15205_c1_g1::TRINITY_DN15205_c1_g1_i3::g.48499::m.48499	7,50E-68	214	XP_018830467.1	senescence-specific cysteine protease SAG12-like isoform X1	Juglans regia	XP_018830468.1
TRINITY_DN16773_c2_g1::TRINITY_DN16773_c2_g1_i3::g.73056::m.73056	0	1024	XP_018860642.1	dolichyl-diphosphooligosaccharide--protein glycosyltransferase subunit 1A-like isoform X1	Juglans regia	ONI05428.1
TRINITY_DN16557_c0_g1::TRINITY_DN16557_c0_g1_i4::g.69370::m.69370	0	895	XP_018819975.1	KH domain-containing protein HEN4-like isoform X2	Juglans regia	XP_018819977.1
TRINITY_DN16644_c3_g1::TRINITY_DN16644_c3_g1_i2::g.70216::m.70216	3,73E-51	172	XP_009338303.1	vicilin-like seed storage protein At2g28490	Pyrus x bretschneideri	ONI00561.1
TRINITY_DN15438_c0_g1::TRINITY_DN15438_c0_g1_i13::g.52894::m.52894	0	913	XP_018829261.1	6-phosphogluconate dehydrogenase, decarboxylating 2, chloroplastic	Juglans regia	OAY56407.1

TRINITY_DN12890_c2_g4::TRINITY_DN12890_c2_g4_i2::g.17047::m.17047						
TRINITY_DN19235_c2_g2::TRINITY_DN19235_c2_g2_i3::g.114333::m.114333	1,03E-154	433	KDP26247.1	hypothetical protein JCGZ_22493	Jatropha curcas	XP_012086001.1
TRINITY_DN19013_c0_g2::TRINITY_DN19013_c0_g2_i3::g.110484::m.110484	3,56E-84	248	XP_018819687.1	40S ribosomal protein S14-3-like	Juglans regia	XP_018831277.1
TRINITY_DN14279_c0_g2::TRINITY_DN14279_c0_g2_i1::g.34822::m.34822	0	997	XP_018823517.1	plastidic ATP/ADP-transporter-like	Juglans regia	XP_018827696.1
TRINITY_DN15614_c2_g1::TRINITY_DN15614_c2_g1_i7::g.54755::m.54755	2,24E-92	272	XP_018839125.1	(+)-neomenthol dehydrogenase-like	Juglans regia	XP_018858487.1
TRINITY_DN16133_c0_g4::TRINITY_DN16133_c0_g4_i5::g.62653::m.62653	2,27E-74	231	XP_018814589.1	biotin carboxyl carrier protein of acetyl-CoA carboxylase 2, chloroplastic-like	Juglans regia	XP_018816951.1
TRINITY_DN18799_c1_g2::TRINITY_DN18799_c1_g2_i3::g.106472::m.106472	0	1234	XP_018848760.1	long chain acyl-CoA synthetase 8	Juglans regia	XP_018848761.1
TRINITY_DN19791_c7_g4::TRINITY_DN19791_c7_g4_i2::g.123739::m.123739	1,91E-48	159	KDP43597.1	hypothetical protein JCGZ_16884	Jatropha curcas	KDP43593.1
TRINITY_DN14197_c0_g1::TRINITY_DN14197_c0_g1_i2::g.33493::m.33493	0	870	XP_018839280.1	26S protease regulatory subunit 7A	Juglans regia	XP_018859577.1
TRINITY_DN16625_c3_g1::TRINITY_DN16625_c3_g1_i9::g.70678::m.70678	2,32E-153	427	XP_018849330.1	ras-related protein Rab7	Juglans regia	CAA98171.1
TRINITY_DN11558_c0_g1::TRINITY_DN11558_c0_g1_i1::g.7858::m.7858	7,68E-106	307	ONI06685.1	hypothetical protein PRUPE_5G074400	Prunus persica	XP_020419805.1
TRINITY_DN13381_c2_g2::TRINITY_DN13381_c2_g2_i20::g.23020::m.23020	3,07E-102	294	OIV96039.1	hypothetical protein TanjilG_27143	Lupinus angustifolius	KOM53238.1
TRINITY_DN12837_c2_g6::TRINITY_DN12837_c2_g6_i1::g.16583::m.16583	6,90E-52	164	XP_018853432.1	probable polygalacturonase, partial	Juglans regia	OIW19830.1
TRINITY_DN17167_c2_g1::TRINITY_DN17167_c2_g1_i1::g.79577::m.79577	3,19E-51	170	XP_018852710.1	exopolygalacturonase-like, partial	Juglans regia	XP_018849923.1
TRINITY_DN15923_c1_g1::TRINITY_DN15923_c1_g1_i8::g.59029::m.59029	0	1015	XP_018805990.1	putative SWI/SNF-related matrix-associated actin-dependent regulator of chromatin subfamily A member 3-like 1	Juglans regia	XP_008227323.1
TRINITY_DN15294_c5_g3::TRINITY_DN15294_c5_g3_i1::g.49818::m.49818	0	859	XP_018849108.1	glutamate decarboxylase-like	Juglans regia	XP_018823508.1
TRINITY_DN16184_c0_g3::TRINITY_DN16184_c0_g3_i5::g.63369::m.63369	0	698	XP_018851843.1	protein STRICTOSIDINE SYNTHASE-LIKE 3-like	Juglans regia	XP_018844479.1
TRINITY_DN17874_c2_g1::TRINITY_DN17874_c2_g1_i9::g.91168::m.91168	0	1125	XP_018823588.1	transmembrane 9 superfamily member 8-like	Juglans regia	XP_018810860.1
TRINITY_DN18048_c4_g4::TRINITY_DN18048_c4_g4_i5::g.94249::m.94249	0	666	XP_018831343.1	probable ADP-ribosylation factor GTPase-activating protein AGD6	Juglans regia	XP_009374672.1
TRINITY_DN19270_c0_g2::TRINITY_DN19270_c0_g2_i4::g.115031::m.115031	2,34E-131	379	XP_018837139.1	metacaspase-4-like, partial	Juglans regia	ADM52185.1
TRINITY_DN16102_c2_g6::TRINITY_DN16102_c2_g6_i1::g.62197::m.62197	5,18E-126	358	XP_009338145.2	ERBB-3 BINDING PROTEIN 1, partial	Pyrus x bretschneideri	XP_009376423.1

TRINITY_DN14814_c1_g3::TRINITY_DN14814_c1_g3_i3::g.42810::m.42810	0	677	XP_018814645.1	uncharacterized protein LOC108986474	Juglans regia	XP_012072319.1
TRINITY_DN13485_c2_g2::TRINITY_DN13485_c2_g2_i2::g.24426::m.24426	1,99E-153	430	XP_018825134.1	ubiquitin carboxyl-terminal hydrolase 3-like	Juglans regia	XP_018825135.1
TRINITY_DN15075_c1_g2::TRINITY_DN15075_c1_g2_i1::g.46516::m.46516	2,54E-71	212	AAB03538.1	histone H3, partial	Glycine max	AAB03539.1
TRINITY_DN13860_c0_g1::TRINITY_DN13860_c0_g1_i1::g.29366::m.29366	0	875	XP_018837500.1	protein disulfide isomerase-like 1-6	Juglans regia	XP_008228552.1
TRINITY_DN19929_c0_g1::TRINITY_DN19929_c0_g1_i9::g.126183::m.126183	0	739	AGH25534.1	UDP-D-xylose 4-epimerase	Prunus persica	ONI11324.1
TRINITY_DN15983_c2_g1::TRINITY_DN15983_c2_g1_i5::g.59899::m.59899	0	706	XP_004503508.1	mitochondrial-processing peptidase subunit alpha-like	Cicer arietinum	KYP67499.1
TRINITY_DN17158_c8_g2::TRINITY_DN17158_c8_g2_i1::g.79404::m.79404	6,47E-144	407	XP_018817983.1	40S ribosomal protein S6-like	Juglans regia	KDP41410.1
TRINITY_DN16000_c0_g1::TRINITY_DN16000_c0_g1_i10::g.60178::m.60178	0	973	AXB70991.1	acetyl-CoA carboxylase carboxyltransferase beta subunit (chloroplast)	Betula pendula var. carelica	YP_009700057.1
TRINITY_DN15045_c2_g1::TRINITY_DN15045_c2_g1_i7::g.46215::m.46215	0	811	XP_012070239.1	V-type proton ATPase subunit H	Jatropha curcas	ONI25949.1
TRINITY_DN19898_c1_g2::TRINITY_DN19898_c1_g2_i1::g.123971::m.123971	1,94E-118	340	OAY28410.1	hypothetical protein MANES_15G064300	Manihot esculenta	OAY32872.1
TRINITY_DN14630_c1_g1::TRINITY_DN14630_c1_g1_i1::g.39822::m.39822	4,65E-111	321	XP_018805410.1	GTP-binding protein SAR1A-like	Juglans regia	XP_018805411.1
TRINITY_DN15863_c0_g1::TRINITY_DN15863_c0_g1_i12::g.58136::m.58136	9,92E-124	356	XP_018814198.1	putative glucose-6-phosphate 1-epimerase isoform X1	Juglans regia	XP_018814255.1
TRINITY_DN18049_c1_g3::TRINITY_DN18049_c1_g3_i3::g.94352::m.94352	0	829	XP_018858352.1	26S proteasome regulatory subunit 4 homolog B	Juglans regia	XP_018820323.1
TRINITY_DN19093_c2_g2::TRINITY_DN19093_c2_g2_i1::g.111778::m.111778	0	1088	XP_018843513.1	NAD-dependent malic enzyme 62 kDa isoform, mitochondrial	Juglans regia	XP_018818832.1
TRINITY_DN13133_c2_g1::TRINITY_DN13133_c2_g1_i2::g.20342::m.20342	0	702	XP_018847762.1	putative transferase At4g12130, mitochondrial	Juglans regia	XP_008238647.1
TRINITY_DN15315_c0_g1::TRINITY_DN15315_c0_g1_i1::g.50016::m.50016	0	838	XP_018840764.1	chaperonin CPN60-2, mitochondrial-like	Juglans regia	XP_014514640.1
TRINITY_DN13727_c0_g1::TRINITY_DN13727_c0_g1_i1::g.27676::m.27676	0	679	XP_018853198.1	uncharacterized protein LOC109015173 isoform X1	Juglans regia	XP_018817130.1
TRINITY_DN19454_c4_g2::TRINITY_DN19454_c4_g2_i2::g.118225::m.118225	3,07E-104	315	XP_018818310.1	uncharacterized protein LOC108989227 isoform X2	Juglans regia	OAY57847.1
TRINITY_DN15177_c0_g1::TRINITY_DN15177_c0_g1_i2::g.47833::m.47833	0	723	XP_018847636.1	ornithine aminotransferase, mitochondrial-like	Juglans regia	XP_018819712.1
TRINITY_DN13215_c0_g4::TRINITY_DN13215_c0_g4_i6::g.21439::m.21439	1,81E-91	271	XP_018843123.1	eukaryotic translation initiation factor 3 subunit J-like	Juglans regia	OAY46647.1
TRINITY_DN12615_c0_g2::TRINITY_DN12615_c0_g2_i14::g.14340::m.14340	5,06E-32	112	XP_009366604.1	60S acidic ribosomal protein P1-like	Pyrus x bretschneideri	XP_008376223.1
TRINITY_DN15558_c1_g3::TRINITY_DN15558_c1_g3_i8::g.53729::m.53729	0	608	XP_018833037.1	ADP,ATP carrier protein 3, mitochondrial-like	Juglans regia	XP_018833038.1

TRINITY_DN14114_c1_g1::TRINITY_DN14114_c1_g1_i9::g.32755::m.32755	0	821	XP_018823857.1	probable 26S proteasome non-ATPase regulatory subunit 3	Juglans regia	XP_018841498.1
TRINITY_DN18015_c3_g4::TRINITY_DN18015_c3_g4_i1::g.93638::m.93638	0	916	XP_018809797.1	eukaryotic translation initiation factor 2A	Juglans regia	XP_018809798.1
TRINITY_DN19464_c2_g1::TRINITY_DN19464_c2_g1_i6::g.118273::m.118273	1,24E-174	484	XP_018812968.1	14-3-3-like protein GF14 kappa	Juglans regia	XP_018833854.1
TRINITY_DN18481_c3_g4::TRINITY_DN18481_c3_g4_i2::g.101020::m.101020	0	953	XP_018831023.1	endo-1,3(4)-beta-glucanase 1-like	Juglans regia	KYP78466.1
TRINITY_DN16840_c2_g3::TRINITY_DN16840_c2_g3_i5::g.74315::m.74315	0	898	XP_018806299.1	enolase 1, chloroplastic	Juglans regia	XP_008244075.1
TRINITY_DN16558_c0_g1::TRINITY_DN16558_c0_g1_i2::g.69315::m.69315	0	1439	XP_018819162.1	eukaryotic translation initiation factor 4G-like isoform X1	Juglans regia	XP_018819170.1
TRINITY_DN16629_c1_g1::TRINITY_DN16629_c1_g1_i1::g.70763::m.70763	1,66E-07	50,8	AER13137.1	DHN1	Corylus avellana	BAA19768.1
TRINITY_DN18667_c1_g2::TRINITY_DN18667_c1_g2_i6::g.104311::m.104311	0	552	XP_018840009.1	ER membrane protein complex subunit 2	Juglans regia	ONI04815.1
TRINITY_DN19979_c5_g7::TRINITY_DN19979_c5_g7_i1::g.127021::m.127021	1,94E-77	236	XP_018811967.1	probable aldo-keto reductase 1	Juglans regia	XP_018854242.1
TRINITY_DN17020_c0_g1::TRINITY_DN17020_c0_g1_i5::g.77212::m.77212	0	579	ONH93127.1	hypothetical protein PRUPE_8G214700	Prunus persica	ONH93128.1
TRINITY_DN17027_c2_g2::TRINITY_DN17027_c2_g2_i1::g.77239::m.77239	4,96E-90	274	ONI14054.1	hypothetical protein PRUPE_4G259200	Prunus persica	AIX97814.1
TRINITY_DN19286_c0_g1::TRINITY_DN19286_c0_g1_i3::g.115330::m.115330	0	1451	XP_018847511.1	nudix hydrolase 3 isoform X1	Juglans regia	XP_018847513.1
TRINITY_DN16223_c1_g2::TRINITY_DN16223_c1_g2_i2::g.64137::m.64137	1,12E-94	275	XP_018848789.1	peroxiredoxin-2B	Juglans regia	XP_018857900.1
TRINITY_DN18521_c6_g6::TRINITY_DN18521_c6_g6_i4::g.101904::m.101904	2,35E-75	230	XP_018831704.1	peroxidase 65-like isoform X2	Juglans regia	XP_018831703.1
TRINITY_DN17250_c2_g1::TRINITY_DN17250_c2_g1_i27::g.80938::m.80938	2,22E-110	316	XP_018841912.1	coatomer subunit zeta-1-like	Juglans regia	XP_008225364.1
TRINITY_DN14788_c0_g4::TRINITY_DN14788_c0_g4_i2::g.42252::m.42252	0	1229	XP_018848499.1	threonine--tRNA ligase, mitochondrial 1-like	Juglans regia	XP_012092067.1
TRINITY_DN16410_c3_g3::TRINITY_DN16410_c3_g3_i5::g.67165::m.67165	0	559	ACU24192.1	unknown	Glycine max	KHN36711.1
TRINITY_DN14802_c2_g1::TRINITY_DN14802_c2_g1_i3::g.42628::m.42628	6,06E-147	416	KRH60670.1	hypothetical protein GLYMA_04G002300	Glycine max	XP_003522341.1
TRINITY_DN14967_c2_g1::TRINITY_DN14967_c2_g1_i8::g.44945::m.44945	1,26E-140	399	XP_018836119.1	reticulon-like protein B5	Juglans regia	XP_018845099.1
TRINITY_DN16828_c0_g2::TRINITY_DN16828_c0_g2_i1::g.74105::m.74105	4,29E-129	374	XP_018832807.1	velvet complex subunit B	Juglans regia	OAY44411.1
TRINITY_DN16105_c7_g3::TRINITY_DN16105_c7_g3_i3::g.62193::m.62193	5,99E-129	367	XP_018825107.1	vesicle-associated protein 2-1-like isoform X3	Juglans regia	XP_018825108.1
TRINITY_DN15700_c1_g1::TRINITY_DN15700_c1_g1_i3::g.54890::m.54890	0	1689	XP_018842577.1	ER membrane protein complex subunit 1-like	Juglans regia	XP_018845533.1

TRINITY_DN19704_c0_g2::TRINITY_DN19704_c0_g2_i1::g.122551::m.122551	1,08E-115	332	KOM26552.1	hypothetical protein LR48_Vigan293s000300	Vigna angularis	BAT84846.1
TRINITY_DN15979_c0_g1::TRINITY_DN15979_c0_g1_i3::g.59847::m.59847	0	818	XP_018822783.1	pyruvate dehydrogenase E1 component subunit alpha-3, chloroplastic	Juglans regia	OAY48935.1
TRINITY_DN14286_c1_g1::TRINITY_DN14286_c1_g1_i4::g.34874::m.34874	2,61E-120	342	XP_009350939.1	probable signal peptidase complex subunit 2 isoform X1	Pyrus x bretschneideri	XP_018501761.1
TRINITY_DN16521_c2_g4::TRINITY_DN16521_c2_g4_i1::g.68855::m.68855	1,92E-74	220	XP_018828963.1	60S ribosomal protein L30	Juglans regia	XP_018849639.1
TRINITY_DN18419_c1_g1::TRINITY_DN18419_c1_g1_i5::g.100150::m.100150	0	2144	XP_018818858.1	carbamoyl-phosphate synthase large chain, chloroplastic	Juglans regia	XP_018818859.1
TRINITY_DN14469_c0_g1::TRINITY_DN14469_c0_g1_i2::g.37789::m.37789	0	695	ONI32296.1	hypothetical protein PRUPE_1G359200	Prunus persica	XP_020423207.1
TRINITY_DN19627_c1_g1::TRINITY_DN19627_c1_g1_i1::g.121154::m.121154	0	1417	XP_018817804.1	leucine--tRNA ligase, cytoplasmic isoform X1	Juglans regia	XP_018817805.1
TRINITY_DN16420_c0_g2::TRINITY_DN16420_c0_g2_i4::g.67379::m.67379	3,40E-144	415	ADR70876.1	eukaryotic translation elongation factor 1B gamma-subunit	Hevea brasiliensis	XP_018817874.1
TRINITY_DN12422_c0_g1::TRINITY_DN12422_c0_g1_i2::g.12726::m.12726	1,89E-146	414	XP_018846809.1	haloacid dehalogenase-like hydrolase domain-containing protein At2g33255	Juglans regia	XP_012077642.1
TRINITY_DN16342_c0_g1::TRINITY_DN16342_c0_g1_i1::g.66087::m.66087	0	899	XP_018844904.1	aldehyde dehydrogenase family 7 member A1 isoform X1	Juglans regia	XP_018844905.1
TRINITY_DN14920_c0_g1::TRINITY_DN14920_c0_g1_i8::g.44465::m.44465	0	665	XP_018851637.1	fructose-1,6-bisphosphatase, cytosolic	Juglans regia	KDP42999.1
TRINITY_DN7453_c0_g1::TRINITY_DN7453_c0_g1_i1::g.2128::m.2128	1,38E-79	234	OAY55416.1	hypothetical protein MANES_03G152300	Manihot esculenta	KDP20335.1
TRINITY_DN15558_c1_g3::TRINITY_DN15558_c1_g3_i7::g.53727::m.53727	0	607	XP_018833037.1	ADP,ATP carrier protein 3, mitochondrial-like	Juglans regia	XP_018833038.1
TRINITY_DN17792_c0_g2::TRINITY_DN17792_c0_g2_i6::g.89728::m.89728	2,18E-68	214	CAA06308.1	cytosolic fructose-1,6-bisphosphate aldolase	Cicer arietinum	NP_001265896.1
TRINITY_DN19733_c2_g1::TRINITY_DN19733_c2_g1_i4::g.123110::m.123110	0	1941	XP_018828011.1	ABC transporter C family member 2-like	Juglans regia	AIU41637.1
TRINITY_DN14635_c2_g1::TRINITY_DN14635_c2_g1_i1::g.39353::m.39353	0	1011	XP_018845552.1	eukaryotic translation initiation factor 3 subunit L-like	Juglans regia	KDP34205.1
TRINITY_DN13787_c3_g4::TRINITY_DN13787_c3_g4_i2::g.28260::m.28260	0	515	XP_018835940.1	mitochondrial outer membrane protein porin of 34 kDa-like	Juglans regia	XP_018823011.1
TRINITY_DN17621_c1_g3::TRINITY_DN17621_c1_g3_i2::g.87259::m.87259	0	739	XP_018815635.1	basic leucine zipper and W2 domain-containing protein 2-like	Juglans regia	OAY34237.1
TRINITY_DN9267_c0_g1::TRINITY_DN9267_c0_g1_i1::g.3260::m.3260	0	1411	XP_018824584.1	kinesin-like protein KIN-5D	Juglans regia	XP_018824585.1
TRINITY_DN18406_c4_g1::TRINITY_DN18406_c4_g1_i5::g.99964::m.99964	0	1406	XP_018811234.1	dynamin-related protein 3A	Juglans regia	XP_018826873.1
TRINITY_DN19605_c0_g3::TRINITY_DN19605_c0_g3_i3::g.120913::m.120913	0	766	XP_014504491.1	heat shock protein 90-6, mitochondrial	Vigna radiata var. radiata	KYP48152.1
TRINITY_DN18631_c1_g1::TRINITY_DN18631_c1_g1_i3::g.103586::m.103586	0	601	XP_018822513.1	putative leucine-rich repeat receptor-like serine/threonine-protein kinase At2g24130	Juglans regia	XP_018851272.1

TRINITY_DN18466_c3_g1::TRINITY_DN18466_c3_g1_i12::g.101047::m.101047						
TRINITY_DN13201_c1_g1::TRINITY_DN13201_c1_g1_i6::g.20932::m.20932	0	575	XP_018843883.1	dnaJ protein ERDJ3B-like isoform X1	Juglans regia	XP_018843884.1
TRINITY_DN19814_c1_g3::TRINITY_DN19814_c1_g3_i1::g.124097::m.124097	7,12E-98	291	OAY23139.1	hypothetical protein MANES_18G054700	Manihot esculenta	AIW63722.1
TRINITY_DN16248_c1_g2::TRINITY_DN16248_c1_g2_i1::g.64537::m.64537	2,91E-35	126	XP_018838677.1	inositol-phosphate phosphatase-like	Juglans regia	XP_015958999.1
TRINITY_DN17313_c1_g1::TRINITY_DN17313_c1_g1_i12::g.81814::m.81814	0	530	XP_018815020.1	protein-tyrosine-phosphatase PTP1 isoform X1	Juglans regia	XP_018815022.1
TRINITY_DN10225_c0_g1::TRINITY_DN10225_c0_g1_i1::g.4474::m.4474	1,82E-166	465	XP_018813088.1	2-Cys peroxiredoxin BAS1, chloroplastic-like	Juglans regia	XP_018851224.1
TRINITY_DN13652_c1_g1::TRINITY_DN13652_c1_g1_i1::g.26699::m.26699						
TRINITY_DN16192_c0_g2::TRINITY_DN16192_c0_g2_i1::g.63470::m.63470	0	1057	XP_008240342.1	dolichyl-diphosphooligosaccharide--protein glycosyltransferase subunit STT3B	Prunus mume	ONI09533.1
TRINITY_DN18875_c2_g2::TRINITY_DN18875_c2_g2_i3::g.107932::m.107932	0	662	XP_008240928.1	actin-like	Prunus mume	XP_008240929.1
TRINITY_DN13691_c2_g1::TRINITY_DN13691_c2_g1_i3::g.27222::m.27222	2,88E-133	378	XP_018842173.1	uncharacterized protein LOC109007090	Juglans regia	ONI35859.1
TRINITY_DN15416_c2_g2::TRINITY_DN15416_c2_g2_i3::g.51536::m.51536	8,37E-146	407	XP_018839374.1	proteasome subunit beta type-2-A	Juglans regia	XP_018812168.1
TRINITY_DN12883_c0_g1::TRINITY_DN12883_c0_g1_i3::g.16877::m.16877	4,38E-134	380	XP_018858784.1	lactoylglutathione lyase isoform X1	Juglans regia	XP_008385523.1
TRINITY_DN19511_c2_g3::TRINITY_DN19511_c2_g3_i2::g.119040::m.119040	7,43E-114	323	XP_018815794.1	60S ribosomal protein L21-1-like	Juglans regia	KDP21892.1
TRINITY_DN11191_c0_g1::TRINITY_DN11191_c0_g1_i2::g.6575::m.6575	1,20E-76	228	XP_008354935.1	uncharacterized protein LOC103418606	Malus domestica	XP_018504852.1
TRINITY_DN15949_c0_g1::TRINITY_DN15949_c0_g1_i6::g.59510::m.59510	0	1131	XP_018812043.1	large proline-rich protein BAG6-like isoform X1	Juglans regia	XP_018812044.1
TRINITY_DN15397_c1_g1::TRINITY_DN15397_c1_g1_i5::g.51177::m.51177	0	1118	XP_018817514.1	transmembrane 9 superfamily member 7-like	Juglans regia	KHN39302.1
TRINITY_DN11231_c0_g1::TRINITY_DN11231_c0_g1_i2::g.6700::m.6700	0	1088	XP_018827094.1	dihydroxy-acid dehydratase, chloroplastic-like	Juglans regia	XP_008224581.1
TRINITY_DN14649_c1_g6::TRINITY_DN14649_c1_g6_i3::g.39965::m.39965	4,56E-147	413	XP_018811310.1	adenylate kinase 4	Juglans regia	OIV98165.1
TRINITY_DN13692_c0_g1::TRINITY_DN13692_c0_g1_i6::g.26626::m.26626	0	657	XP_018830936.1	ADP,ATP carrier protein, mitochondrial	Juglans regia	XP_018830937.1
TRINITY_DN12789_c1_g2::TRINITY_DN12789_c1_g2_i25::g.15964::m.15964	1,49E-51	167	XP_018850680.1	tropinone reductase homolog At5g06060-like	Juglans regia	XP_018841297.1
TRINITY_DN17697_c2_g1::TRINITY_DN17697_c2_g1_i5::g.88410::m.88410	0	1256	XP_018848022.1	sulfite reductase [ferredoxin], chloroplastic	Juglans regia	XP_018830969.1
TRINITY_DN18388_c1_g1::TRINITY_DN18388_c1_g1_i7::g.99758::m.99758						

TRINITY_DN19535_c2_g2::TRINITY_DN19535_c2_g2_i7::g.119393::m.119393	0	539	XP_018857224.1	cycloartenol-C-24-methyltransferase	Juglans regia	XP_014499710.1
TRINITY_DN11345_c0_g1::TRINITY_DN11345_c0_g1_i1::g.7064::m.7064	0	508	KDP33559.1	hypothetical protein JCGZ_07130	Jatropha curcas	XP_012076496.1
TRINITY_DN14591_c4_g1::TRINITY_DN14591_c4_g1_i9::g.39216::m.39216	0	877	XP_018807956.1	serine hydroxymethyltransferase, mitochondrial-like	Juglans regia	OAY23149.1
TRINITY_DN14453_c2_g1::TRINITY_DN14453_c2_g1_i6::g.37399::m.37399	3,46E-96	284	XP_018815776.1	pirin-like protein	Juglans regia	KDP42878.1
TRINITY_DN16526_c1_g2::TRINITY_DN16526_c1_g2_i8::g.69034::m.69034	1,27E-123	350	XP_018818615.1	1,2-dihydroxy-3-keto-5-methylthiopentene dioxygenase 2	Juglans regia	XP_009343958.1
TRINITY_DN10373_c0_g1::TRINITY_DN10373_c0_g1_i1::g.4691::m.4691	9,65E-75	226	XP_018807798.1	uncharacterized protein LOC108981167	Juglans regia	XP_018807799.1
TRINITY_DN19332_c2_g1::TRINITY_DN19332_c2_g1_i2::g.116131::m.116131	0	663	XP_018860760.1	probable pectinesterase/pectinesterase inhibitor 21	Juglans regia	KDP23449.1
TRINITY_DN14971_c0_g1::TRINITY_DN14971_c0_g1_i2::g.44966::m.44966	0	604	XP_018823086.1	probable bifunctional methylthioribulose-1-phosphate dehydratase/enolase-phosphatase E1 isoform X1	Juglans regia	XP_018823094.1
TRINITY_DN16859_c0_g3::TRINITY_DN16859_c0_g3_i1::g.74469::m.74469	1,12E-72	220	XP_018810863.1	26S proteasome non-ATPase regulatory subunit 9	Juglans regia	XP_019432553.1
TRINITY_DN16399_c1_g2::TRINITY_DN16399_c1_g2_i5::g.67010::m.67010	0	830	XP_018858191.1	citrate synthase, mitochondrial	Juglans regia	XP_018858196.1
TRINITY_DN13466_c1_g1::TRINITY_DN13466_c1_g1_i2::g.24484::m.24484	1,22E-143	402	XP_008224495.1	60S ribosomal protein L13a-4	Prunus mume	ONI26146.1
TRINITY_DN18053_c4_g1::TRINITY_DN18053_c4_g1_i4::g.94304::m.94304	0	649	XP_018807103.1	uncharacterized protein LOC108980590 isoform X1	Juglans regia	XP_018807106.1
TRINITY_DN18818_c1_g1::TRINITY_DN18818_c1_g1_i7::g.106901::m.106901	0	654	XP_018838617.1	coproporphyrinogen-III oxidase 1, chloroplastic	Juglans regia	XP_012091240.1
TRINITY_DN14578_c1_g1::TRINITY_DN14578_c1_g1_i8::g.39125::m.39125	0	769	XP_018832890.1	S-adenosylmethionine synthase 1	Juglans regia	XP_009363082.1
TRINITY_DN13918_c1_g1::TRINITY_DN13918_c1_g1_i8::g.30345::m.30345	0	1643	XP_018860275.1	26S proteasome non-ATPase regulatory subunit 1 homolog A	Juglans regia	XP_018837135.1
TRINITY_DN18543_c1_g3::TRINITY_DN18543_c1_g3_i1::g.101857::m.101857	0	1441	XP_018851882.1	ubiquitin carboxyl-terminal hydrolase 14 isoform X1	Juglans regia	OAY54976.1
TRINITY_DN17130_c2_g2::TRINITY_DN17130_c2_g2_i6::g.79154::m.79154	6,26E-76	257	XP_018835918.1	myb-like protein X	Juglans regia	XP_018835918.1
TRINITY_DN13127_c0_g1::TRINITY_DN13127_c0_g1_i1::g.20146::m.20146	0	718	XP_018805499.1	GDP-mannose 4,6 dehydratase 1-like	Juglans regia	OAY57517.1
TRINITY_DN11423_c0_g1::TRINITY_DN11423_c0_g1_i1::g.7308::m.7308	1,75E-123	352	XP_008228618.1	60S ribosomal protein L10a	Prunus mume	ONI01246.1
TRINITY_DN17687_c2_g1::TRINITY_DN17687_c2_g1_i6::g.88326::m.88326	0	576	XP_018856841.1	quinone oxidoreductase PIG3-like	Juglans regia	XP_016179633.1
TRINITY_DN19126_c0_g1::TRINITY_DN19126_c0_g1_i4::g.112486::m.112486	0	1146	XP_018852174.1	probable methyltransferase PMT21	Juglans regia	XP_018852176.1

TRINITY_DN14181_c0_g3::TRINITY_DN14181_c0_g3_i1::g.33572::m.33572	0	1027	XP_018826910.1	methylmalonate-semialdehyde dehydrogenase [acylating], mitochondrial	Juglans regia	XP_012074593.1
TRINITY_DN14198_c0_g1::TRINITY_DN14198_c0_g1_i5::g.33635::m.33635	2,18E-136	411	XP_018815969.1	granule-bound starch synthase 2, chloroplastic/amyloplastic-like	Juglans regia	XP_018813997.1
TRINITY_DN15488_c1_g5::TRINITY_DN15488_c1_g5_i3::g.52738::m.52738	0	651	XP_018829624.1	naringenin,2-oxoglutarate 3-dioxygenase	Juglans regia	ACR47976.1
TRINITY_DN17665_c0_g2::TRINITY_DN17665_c0_g2_i3::g.87796::m.87796	4,29E-65	200	XP_008218606.1	glutathione S-transferase zeta class-like isoform X1	Prunus mume	ADZ05465.1
TRINITY_DN11049_c0_g1::TRINITY_DN11049_c0_g1_i1::g.6211::m.6211	2,19E-86	253	OAY41520.1	hypothetical protein MANES_09G108600	Manihot esculenta	XP_018826192.1
TRINITY_DN18004_c1_g11::TRINITY_DN18004_c1_g11_i1::g.93466::m.93466	1,49E-27	106	XP_018835429.1	glutathione S-transferase F13	Juglans regia	XP_009365718.1
TRINITY_DN17396_c1_g1::TRINITY_DN17396_c1_g1_i2::g.82990::m.82990	0	2737	XP_018835518.1	UDP-glucose:glycoprotein glucosyltransferase	Juglans regia	XP_009348356.1
TRINITY_DN16113_c1_g1::TRINITY_DN16113_c1_g1_i2::g.62243::m.62243	0	996	XP_018821632.1	chaperonin CPN60-like 2, mitochondrial isoform X1	Juglans regia	XP_018821633.1
TRINITY_DN12537_c5_g1::TRINITY_DN12537_c5_g1_i1::g.13612::m.13612	3,29E-40	136	ONI12324.1	hypothetical protein PRUPE_4G157300	Prunus persica	XP_020417826.1
TRINITY_DN13740_c0_g1::TRINITY_DN13740_c0_g1_i2::g.27757::m.27757	0	821	XP_018834951.1	eukaryotic peptide chain release factor subunit 1-3-like	Juglans regia	XP_018834952.1
TRINITY_DN13460_c3_g2::TRINITY_DN13460_c3_g2_i3::g.24327::m.24327	4,29E-165	473	AHM02305.1	asparagine synthetase	Prunus persica	ONH99872.1
TRINITY_DN14396_c0_g2::TRINITY_DN14396_c0_g2_i1::g.35371::m.35371	1,16E-82	244	XP_018834168.1	uncharacterized protein LOC109001373	Juglans regia	XP_019441142.1
TRINITY_DN18313_c3_g2::TRINITY_DN18313_c3_g2_i2::g.98848::m.98848	0	763	XP_018829218.1	aminoacylase-1	Juglans regia	XP_009340792.1
TRINITY_DN19187_c0_g2::TRINITY_DN19187_c0_g2_i3::g.113547::m.113547	1,32E-70	212	XP_008353324.1	DNA damage-inducible protein 1-like	Malus domestica	XP_009339277.1
TRINITY_DN10969_c0_g1::TRINITY_DN10969_c0_g1_i1::g.5933::m.5933	0	844	XP_018838329.1	L-ascorbate oxidase homolog	Juglans regia	XP_018812045.1
TRINITY_DN14875_c0_g2::TRINITY_DN14875_c0_g2_i7::g.43614::m.43614	0	935	XP_018857611.1	eukaryotic translation initiation factor 2 subunit gamma-like	Juglans regia	XP_018857612.1
TRINITY_DN16965_c3_g1::TRINITY_DN16965_c3_g1_i6::g.76283::m.76283	0	1018	XP_018821933.1	indole-3-acetic acid-amido synthetase GH3.6-like	Juglans regia	KYP44609.1
TRINITY_DN16752_c9_g1::TRINITY_DN16752_c9_g1_i5::g.72819::m.72819	0	1014	XP_018825763.1	importin subunit alpha-2-like isoform X2	Juglans regia	XP_018812785.1
TRINITY_DN17906_c0_g1::TRINITY_DN17906_c0_g1_i4::g.91749::m.91749	0	630	XP_018829157.1	beta-adaptin-like protein C	Juglans regia	XP_020230079.1
TRINITY_DN10627_c0_g1::TRINITY_DN10627_c0_g1_i1::g.5151::m.5151	0	1117	XP_018825886.1	pyruvate decarboxylase 1	Juglans regia	XP_018826224.1
TRINITY_DN11182_c0_g1::TRINITY_DN11182_c0_g1_i3::g.6546::m.6546	1,80E-137	397	XP_018827577.1	SAL1 phosphatase-like isoform X2	Juglans regia	XP_018827576.1
TRINITY_DN14268_c0_g1::TRINITY_DN14268_c0_g1_i4::g.34610::m.34610	0	787	XP_018845323.1	isovaleryl-CoA dehydrogenase, mitochondrial	Juglans regia	XP_009347036.1

TRINITY_DN19877_c2_g3::TRINITY_DN19877_c2_g3_i1::g.125021::m.125021	0	914	XP_018857649.1	NADH dehydrogenase [ubiquinone] flavoprotein 1, mitochondrial-like	Juglans regia	XP_018836688.1
TRINITY_DN17530_c3_g12::TRINITY_DN17530_c3_g12_i1::g.85464::m.85464	1,32E-27	103	CBX33398.1	atp8 (mitochondrion)	Malus domestica	YP_006666136.1
TRINITY_DN14410_c0_g6::TRINITY_DN14410_c0_g6_i1::g.36618::m.36618	5,80E-79	231	CAB66329.1	defender against apoptotic cell death	Betula pendula	Q9M3T9.1
TRINITY_DN12098_c3_g1::TRINITY_DN12098_c3_g1_i8::g.10503::m.10503	1,81E-100	295	ONH90115.1	hypothetical protein PRUPE_8G035600	Prunus persica	ONH90116.1
TRINITY_DN17739_c0_g2::TRINITY_DN17739_c0_g2_i3::g.89048::m.89048	1,07E-77	233	XP_018859887.1	uncharacterized protein LOC109021657	Juglans regia	XP_008224024.1
TRINITY_DN14157_c1_g1::TRINITY_DN14157_c1_g1_i4::g.33107::m.33107	3,43E-127	374	XP_018859875.1	glycine-rich RNA-binding protein 3, mitochondrial	Juglans regia	XP_016649563.1
TRINITY_DN18293_c3_g1::TRINITY_DN18293_c3_g1_i3::g.98277::m.98277	2,21E-33	118	XP_008377491.1	ER membrane protein complex subunit 4-like	Malus domestica	XP_009359143.1
TRINITY_DN18745_c0_g3::TRINITY_DN18745_c0_g3_i8::g.105419::m.105419	0	659	XP_018812652.1	heterogeneous nuclear ribonucleoprotein 1-like	Juglans regia	XP_018812657.1
TRINITY_DN12590_c2_g2::TRINITY_DN12590_c2_g2_i2::g.13982::m.13982	1,15E-79	239	XP_018860616.1	protein canopy-1	Juglans regia	XP_008237962.1
TRINITY_DN14010_c5_g1::TRINITY_DN14010_c5_g1_i2::g.31420::m.31420	1,76E-68	204	XP_008234218.1	60S ribosomal protein L36-3-like	Prunus mume	XP_004496553.1
TRINITY_DN15598_c2_g1::TRINITY_DN15598_c2_g1_i7::g.54284::m.54284	0	563	XP_020218346.1	26S proteasome non-ATPase regulatory subunit 4 homolog	Cajanus cajan	XP_020227721.1
TRINITY_DN11779_c0_g1::TRINITY_DN11779_c0_g1_i1::g.8787::m.8787	0	783	XP_018844342.1	3-isopropylmalate dehydrogenase 2, chloroplastic-like	Juglans regia	ONI16361.1
TRINITY_DN13676_c3_g1::TRINITY_DN13676_c3_g1_i3::g.27135::m.27135	1,76E-55	174	XP_012088149.1	uncharacterized protein LOC105646819	Jatropha curcas	KDP24365.1
TRINITY_DN18865_c1_g1::TRINITY_DN18865_c1_g1_i8::g.107763::m.107763	5,33E-166	467	ONI35507.1	hypothetical protein PRUPE_1G540200	Prunus persica	XP_007222567.1
TRINITY_DN15886_c0_g2::TRINITY_DN15886_c0_g2_i6::g.58296::m.58296	0	1069	XP_018842853.1	soluble starch synthase 1, chloroplastic/amyloplastic	Juglans regia	ONI25554.1
TRINITY_DN11909_c0_g1::TRINITY_DN11909_c0_g1_i2::g.9470::m.9470	0	854	XP_018841219.1	uncharacterized protein LOC109006407	Juglans regia	ONI23255.1
TRINITY_DN16963_c2_g3::TRINITY_DN16963_c2_g3_i1::g.76239::m.76239	0	629	XP_008364974.1	26S protease regulatory subunit 10B homolog A	Malus domestica	XP_009372983.1
TRINITY_DN12323_c0_g2::TRINITY_DN12323_c0_g2_i3::g.11963::m.11963	1,18E-98	283	XP_008222957.1	40S ribosomal protein S16	Prunus mume	ONI28680.1
TRINITY_DN13869_c6_g1::TRINITY_DN13869_c6_g1_i2::g.29653::m.29653	0	552	XP_018835354.1	uricase-2 isozyme 2	Juglans regia	CAB77205.1
TRINITY_DN18287_c3_g1::TRINITY_DN18287_c3_g1_i2::g.98102::m.98102	4,08E-137	394	XP_018845913.1	cytidine deaminase 1-like	Juglans regia	ONH97802.1
TRINITY_DN19305_c2_g3::TRINITY_DN19305_c2_g3_i4::g.115690::m.115690	4,07E-81	250	XP_018857311.1	lysine histidine transporter 2-like	Juglans regia	XP_018857319.1
TRINITY_DN15008_c0_g3::TRINITY_DN15008_c0_g3_i2::g.45738::m.45738	0	1219	XP_018831592.1	ATPase 11, plasma membrane-type	Juglans regia	XP_018831860.1

TRINITY_DN18232_c0_g1::TRINITY_DN18232_c0_g1_i6::g.97390::m.97390	0	2248	XP_018806543.1	isoleucine--tRNA ligase, cytoplasmic	Juglans regia	ONI29193.1
TRINITY_DN17493_c2_g1::TRINITY_DN17493_c2_g1_i7::g.84644::m.84644	0	917	XP_008226002.1	succinate-semialdehyde dehydrogenase, mitochondrial	Prunus mume	ONI11823.1
TRINITY_DN11669_c0_g1::TRINITY_DN11669_c0_g1_i1::g.8337::m.8337	2,48E-84	249	XP_018824954.1	40S ribosomal protein S24-1-like	Juglans regia	XP_008218192.1
TRINITY_DN18124_c0_g4::TRINITY_DN18124_c0_g4_i1::g.95606::m.95606	1,08E-173	483	XP_019464492.1	26S proteasome non-ATPase regulatory subunit 8 homolog A-like	Lupinus angustifolius	XP_019464493.1
TRINITY_DN11643_c0_g1::TRINITY_DN11643_c0_g1_i1::g.8230::m.8230	4,34E-138	394	XP_018838151.1	26S proteasome non-ATPase regulatory subunit 14 homolog	Juglans regia	XP_018857189.1
TRINITY_DN17921_c3_g2::TRINITY_DN17921_c3_g2_i1::g.92117::m.92117	0	6227	XP_018859288.1	E3 ubiquitin-protein ligase UPL2-like	Juglans regia	XP_018848705.1
TRINITY_DN17515_c0_g1::TRINITY_DN17515_c0_g1_i3::g.85102::m.85102	0	1153	XP_018834612.1	formate--tetrahydrofolate ligase	Juglans regia	OAY54628.1
TRINITY_DN17501_c0_g2::TRINITY_DN17501_c0_g2_i6::g.84659::m.84659	0	1165	XP_018806016.1	vacuolar-sorting receptor 3-like	Juglans regia	XP_018846135.1
TRINITY_DN11905_c0_g1::TRINITY_DN11905_c0_g1_i3::g.9416::m.9416	0	610	XP_018815797.1	mitochondrial import receptor subunit TOM40-1-like	Juglans regia	XP_018824435.1
TRINITY_DN16017_c1_g2::TRINITY_DN16017_c1_g2_i2::g.60617::m.60617	0	810	XP_018844108.1	polyadenylate-binding protein 7 isoform X1	Juglans regia	XP_018844108.1
TRINITY_DN10830_c0_g1::TRINITY_DN10830_c0_g1_i1::g.5629::m.5629	3,05E-118	340	XP_018860721.1	glutathione S-transferase DHAR2-like	Juglans regia	XP_018860724.1
TRINITY_DN13736_c4_g2::TRINITY_DN13736_c4_g2_i5::g.27391::m.27391	5,50E-179	498	XP_018820284.1	aldo-keto reductase family 4 member C9-like	Juglans regia	XP_018820285.1
TRINITY_DN19177_c0_g1::TRINITY_DN19177_c0_g1_i1::g.113288::m.113288	0	1139	XP_018806637.1	glutamate--tRNA ligase, cytoplasmic	Juglans regia	XP_018806638.1
TRINITY_DN19354_c2_g2::TRINITY_DN19354_c2_g2_i2::g.116436::m.116436	1,02E-64	200	XP_018835464.1	glutathione S-transferase-like	Juglans regia	XP_018835471.1
TRINITY_DN17688_c2_g6::TRINITY_DN17688_c2_g6_i1::g.88272::m.88272	3,69E-50	169	XP_018834200.1	endoglucanase 9-like	Juglans regia	XP_018834199.1
TRINITY_DN16306_c1_g1::TRINITY_DN16306_c1_g1_i3::g.65480::m.65480	2,20E-61	193	XP_018822871.1	strigolactone esterase D14	Juglans regia	XP_018838099.1
TRINITY_DN18894_c1_g3::TRINITY_DN18894_c1_g3_i1::g.108226::m.108226	3,41E-170	478	XP_018810152.1	protein BTR1-like isoform X2	Juglans regia	XP_008232049.1
TRINITY_DN14391_c2_g3::TRINITY_DN14391_c2_g3_i1::g.36280::m.36280	4,39E-56	181	XP_018818750.1	GDSL esterase/lipase At3g48460-like	Juglans regia	OAY34410.1
TRINITY_DN12367_c0_g1::TRINITY_DN12367_c0_g1_i3::g.12189::m.12189	0	767	XP_018850112.1	uncharacterized protein LOC109012766	Juglans regia	KHN17427.1
TRINITY_DN14985_c3_g2::TRINITY_DN14985_c3_g2_i1::g.45232::m.45232	0	1264	ONH97993.1	hypothetical protein PRUPE_7G222300	Prunus persica	ONH97994.1
TRINITY_DN14724_c2_g1::TRINITY_DN14724_c2_g1_i1::g.41239::m.41239	0	1634	XP_018834214.1	bifunctional aspartokinase/homoserine dehydrogenase 1, chloroplastic-like	Juglans regia	XP_018831022.1
TRINITY_DN10431_c0_g1::TRINITY_DN10431_c0_g1_i2::g.4811::m.4811	8,03E-119	343	XP_018815442.1	mitochondrial import receptor subunit TOM20-like	Juglans regia	XP_018860144.1

TRINITY_DN12116_c0_g1::TRINITY_DN12116_c0_g1_i3::g.10615::m.10615	2,92E-71	216	AAT67997.1	1-cys peroxiredoxin	Medicago truncatula	Q6E2Z6.1
TRINITY_DN12248_c0_g2::TRINITY_DN12248_c0_g2_i1::g.11152::m.11152	0	527	ONH91068.1	hypothetical protein PRUPE_8G091300	Prunus persica	ONH91069.1
TRINITY_DN14430_c0_g1::TRINITY_DN14430_c0_g1_i7::g.37424::m.37424	0	974	XP_018846380.1	glucose-6-phosphate 1-dehydrogenase, cytoplasmic isoform-like isoform X2	Juglans regia	XP_018846377.1
TRINITY_DN17797_c0_g1::TRINITY_DN17797_c0_g1_i20::g.88479::m.88479	9,03E-102	308	XP_009378285.1	uncharacterized protein LOC103966791	Pyrus x bretschneideri	XP_008229423.1
TRINITY_DN16328_c4_g1::TRINITY_DN16328_c4_g1_i3::g.65859::m.65859						
TRINITY_DN14897_c3_g5::TRINITY_DN14897_c3_g5_i1::g.43938::m.43938	1,22E-56	177	OAY45814.1	hypothetical protein MANES_07G093700	Manihot esculenta	XP_016183223.1
TRINITY_DN14545_c0_g1::TRINITY_DN14545_c0_g1_i2::g.38714::m.38714	1,47E-153	435	XP_009357480.2	LOW QUALITY PROTEIN: proline synthase co-transcribed bacterial homolog protein-like	Pyrus x bretschneideri	XP_008224704.1
TRINITY_DN18713_c3_g1::TRINITY_DN18713_c3_g1_i2::g.105054::m.105054	1,60E-106	315	XP_018849508.1	protein DJ-1 homolog B-like	Juglans regia	XP_018849508.1
TRINITY_DN13940_c4_g1::TRINITY_DN13940_c4_g1_i3::g.30598::m.30598						
TRINITY_DN18184_c1_g1::TRINITY_DN18184_c1_g1_i15::g.96527::m.96527	7,76E-48	155	KRH19729.1	hypothetical protein GLYMA_13G132400	Glycine max	KRH19730.1
TRINITY_DN17484_c0_g3::TRINITY_DN17484_c0_g3_i1::g.84435::m.84435	8,47E-127	362	ALP70511.1	Fe-S cluster assembly protein 20	Prunus persica var. persica	ONI21671.1
TRINITY_DN13721_c0_g1::TRINITY_DN13721_c0_g1_i7::g.27621::m.27621	0	909	XP_018816803.1	transmembrane 9 superfamily member 3-like	Juglans regia	ONI31917.1
TRINITY_DN17006_c0_g4::TRINITY_DN17006_c0_g4_i2::g.76859::m.76859	8,20E-80	237	XP_018833404.1	40S ribosomal protein S12-like	Juglans regia	XP_018835263.1
TRINITY_DN13463_c1_g1::TRINITY_DN13463_c1_g1_i3::g.24177::m.24177	8,60E-127	363	XP_018809229.1	extradiol ring-cleavage dioxygenase-like	Juglans regia	XP_018844729.1
TRINITY_DN17769_c1_g1::TRINITY_DN17769_c1_g1_i1::g.89345::m.89345						
TRINITY_DN18331_c2_g5::TRINITY_DN18331_c2_g5_i1::g.98900::m.98900	0	865	XP_018839623.1	eukaryotic translation initiation factor 3 subunit E	Juglans regia	XP_018808879.1
TRINITY_DN13546_c0_g1::TRINITY_DN13546_c0_g1_i3::g.25193::m.25193	0	1100	XP_018842287.1	probable methyltransferase PMT3	Juglans regia	XP_018808117.1
TRINITY_DN19711_c5_g2::TRINITY_DN19711_c5_g2_i2::g.122643::m.122643	7,17E-105	307	XP_018806786.1	aldose 1-epimerase	Juglans regia	OAY32166.1
TRINITY_DN15444_c2_g1::TRINITY_DN15444_c2_g1_i7::g.52052::m.52052	5,40E-50	168	XP_018852448.1	late embryogenesis abundant protein 1-like	Juglans regia	KDP20986.1
TRINITY_DN18083_c4_g1::TRINITY_DN18083_c4_g1_i1::g.94854::m.94854	1,61E-75	238	XP_014516606.1	plasminogen activator inhibitor 1 RNA-binding protein-like	Vigna radiata var. radiata	KOM58621.1
TRINITY_DN15930_c2_g2::TRINITY_DN15930_c2_g2_i5::g.59203::m.59203	0	688	XP_018839373.1	stearoyl-[acyl-carrier-protein 9-desaturase, chloroplastic-like	Juglans regia	XP_018812159.1
TRINITY_DN19332_c1_g1::TRINITY_DN19332_c1_g1_i1::g.116126::m.116126	0	775	XP_018831291.1	inosine-5'-monophosphate dehydrogenase 2-like	Juglans regia	XP_009349337.1

TRINITY_DN13790_c1_g1::TRINITY_DN13790_c1_g1_i1::g.28245::m.28245	0	755	XP_018826982.1	GDP-mannose 3,5-epimerase 2-like	Juglans regia	XP_018826983.1
TRINITY_DN9744_c0_g1::TRINITY_DN9744_c0_g1_i1::g.3792::m.3792	1,33E-170	474	XP_018849141.1	phosphomannomutase-like	Juglans regia	XP_008232782.1
TRINITY_DN13494_c0_g2::TRINITY_DN13494_c0_g2_i2::g.24524::m.24524	1,30E-131	371	XP_018813791.1	40S ribosomal protein S9-2	Juglans regia	XP_018817673.1
TRINITY_DN17631_c3_g6::TRINITY_DN17631_c3_g6_i1::g.87349::m.87349	2,00E-78	236	XP_018832799.1	uncharacterized protein At3g03773-like	Juglans regia	KHN32820.1
TRINITY_DN12268_c0_g1::TRINITY_DN12268_c0_g1_i1::g.11491::m.11491	0	793	XP_018828790.1	dolichyl-diphosphooligosaccharide--protein glycosyltransferase subunit 1B	Juglans regia	KDP44377.1
TRINITY_DN19388_c2_g2::TRINITY_DN19388_c2_g2_i2::g.117003::m.117003	2,00E-113	330	XP_018835592.1	probable carboxylesterase 5	Juglans regia	KDP45172.1
TRINITY_DN13433_c0_g1::TRINITY_DN13433_c0_g1_i4::g.23725::m.23725	0	988	XP_018839513.1	nicalin-1 isoform X1	Juglans regia	XP_018839514.1
TRINITY_DN17014_c1_g3::TRINITY_DN17014_c1_g3_i2::g.77015::m.77015	0	608	XP_018815793.1	eukaryotic translation initiation factor 3 subunit H	Juglans regia	ONI29040.1
TRINITY_DN13342_c3_g2::TRINITY_DN13342_c3_g2_i4::g.22568::m.22568	0	1112	XP_018834193.1	dynamin-2A-like	Juglans regia	XP_018831147.1
TRINITY_DN15981_c2_g1::TRINITY_DN15981_c2_g1_i5::g.59872::m.59872	1,17E-20	83,2	XP_018857823.1	heat shock factor-binding protein 1-like	Juglans regia	XP_018811503.1
TRINITY_DN18987_c2_g1::TRINITY_DN18987_c2_g1_i1::g.110056::m.110056	0	1104	XP_018851819.1	uncharacterized protein LOC109013987	Juglans regia	XP_018851823.1
TRINITY_DN12349_c8_g2::TRINITY_DN12349_c8_g2_i1::g.12068::m.12068	3,24E-82	247	XP_018809112.1	binding partner of ACD11 1-like	Juglans regia	XP_018809602.1
TRINITY_DN13325_c1_g1::TRINITY_DN13325_c1_g1_i1::g.22310::m.22310	1,63E-43	156	XP_018823987.1	peroxisomal and mitochondrial division factor 2-like	Juglans regia	XP_018823987.1
TRINITY_DN15794_c0_g2::TRINITY_DN15794_c0_g2_i7::g.57165::m.57165	0	958	XP_018842661.1	phosphoinositide phosphatase SAC6-like	Juglans regia	XP_018842662.1
TRINITY_DN16454_c1_g1::TRINITY_DN16454_c1_g1_i2::g.67727::m.67727	0	610	XP_018849887.1	heat shock protein 90-6, mitochondrial isoform X2	Juglans regia	XP_018849886.1
TRINITY_DN12161_c0_g1::TRINITY_DN12161_c0_g1_i4::g.10883::m.10883	1,26E-34	120	XP_018845364.1	uncharacterized protein LOC109009365	Juglans regia	ONI21818.1
TRINITY_DN16552_c0_g2::TRINITY_DN16552_c0_g2_i1::g.69236::m.69236	6,19E-52	167	XP_008218348.1	peroxygenase	Prunus mume	KHN31490.1
TRINITY_DN13234_c0_g1::TRINITY_DN13234_c0_g1_i7::g.21346::m.21346	7,07E-148	424	XP_018841836.1	protein SLOW GREEN 1, chloroplastic	Juglans regia	ONI31974.1
TRINITY_DN16040_c0_g1::TRINITY_DN16040_c0_g1_i12::g.61056::m.61056	0	1771	XP_018846983.1	calcium-transporting ATPase 4, plasma membrane-type-like isoform X1	Juglans regia	XP_009368080.1
TRINITY_DN17716_c3_g1::TRINITY_DN17716_c3_g1_i5::g.88746::m.88746	0	741	XP_018811146.1	serine--tRNA ligase-like	Juglans regia	XP_018841784.1
TRINITY_DN14659_c0_g2::TRINITY_DN14659_c0_g2_i6::g.40081::m.40081	1,18E-168	469	XP_018824239.1	proteasome subunit beta type-4-like	Juglans regia	XP_018844541.1
TRINITY_DN8972_c0_g1::TRINITY_DN8972_c0_g1_i1::g.3000::m.3000	2,81E-151	431	XP_018842842.1	protein ABIL2-like isoform X3	Juglans regia	XP_018842843.1

TRINITY_DN19468_c2_g5::TRINITY_DN19468_c2_g5_i1::g.118327::m.118327	1,65E-67	211	KOM53769.1	hypothetical protein LR48_Vigan09g242800	Vigna angularis	XP_018852431.1
TRINITY_DN16932_c1_g2::TRINITY_DN16932_c1_g2_i8::g.75832::m.75832	1,78E-84	252	XP_008244645.1	uncharacterized protein LOC103342773	Prunus mume	XP_018859721.1
TRINITY_DN15439_c1_g1::TRINITY_DN15439_c1_g1_i4::g.51919::m.51919	0	881	XP_018835615.1	clathrin heavy chain 2	Juglans regia	XP_018819181.1
TRINITY_DN19589_c2_g2::TRINITY_DN19589_c2_g2_i4::g.120310::m.120310	0	717	XP_018819716.1	3-oxoacyl-[acyl-carrier-protein synthase I, chloroplastic	Juglans regia	XP_018819717.1
TRINITY_DN17548_c3_g2::TRINITY_DN17548_c3_g2_i2::g.85798::m.85798	0	582	XP_018854480.1	bifunctional dTDP-4-dehydrorhamnose 3,5-epimerase/dTDP-4-dehydrorhamnose reductase	Juglans regia	XP_018820273.1
TRINITY_DN12116_c0_g2::TRINITY_DN12116_c0_g2_i1::g.10617::m.10617	1,83E-72	220	XP_018833870.1	1-Cys peroxiredoxin	Juglans regia	XP_009372050.1
TRINITY_DN16328_c4_g1::TRINITY_DN16328_c4_g1_i2::g.65854::m.65854						
TRINITY_DN19061_c2_g1::TRINITY_DN19061_c2_g1_i2::g.111195::m.111195	1,06E-172	480	XP_018814237.1	V-type proton ATPase subunit D-like	Juglans regia	XP_018814238.1
TRINITY_DN17042_c1_g2::TRINITY_DN17042_c1_g2_i2::g.77496::m.77496	0	1953	XP_018825846.1	enhancer of mRNA-decapping protein 4-like	Juglans regia	ONH99477.1
TRINITY_DN16369_c2_g3::TRINITY_DN16369_c2_g3_i1::g.66518::m.66518	1,03E-60	193	XP_018821858.1	ferritin-3, chloroplastic	Juglans regia	OAY46317.1
TRINITY_DN17384_c3_g1::TRINITY_DN17384_c3_g1_i3::g.82818::m.82818	0	672	XP_018840433.1	cysteine synthase, chloroplastic/chromoplastic isoform X1	Juglans regia	XP_018840434.1
TRINITY_DN18568_c1_g1::TRINITY_DN18568_c1_g1_i19::g.102809::m.102809	0	1958	XP_018808786.1	valine--tRNA ligase, mitochondrial 1	Juglans regia	XP_018808787.1
TRINITY_DN19159_c0_g2::TRINITY_DN19159_c0_g2_i3::g.113047::m.113047	6,73E-94	278	XP_018858281.1	chitinase 10-like	Juglans regia	XP_015939499.1
TRINITY_DN19261_c3_g1::TRINITY_DN19261_c3_g1_i6::g.113934::m.113934	0	599	XP_018827765.1	callose synthase 5	Juglans regia	KYP67853.1
TRINITY_DN13280_c0_g2::TRINITY_DN13280_c0_g2_i2::g.21976::m.21976	0	693	XP_018820175.1	monothiol glutaredoxin-S17	Juglans regia	KDP20960.1
TRINITY_DN18061_c2_g1::TRINITY_DN18061_c2_g1_i8::g.94553::m.94553	0	695	XP_018836149.1	3-oxo-Delta(4,5)-steroid 5-beta-reductase-like	Juglans regia	XP_018833905.1
TRINITY_DN12991_c0_g2::TRINITY_DN12991_c0_g2_i1::g.18234::m.18234	0	708	XP_018847795.1	dnaJ protein P58IPK homolog	Juglans regia	XP_009377419.1
TRINITY_DN19158_c0_g2::TRINITY_DN19158_c0_g2_i3::g.112794::m.112794	0	942	XP_018857968.1	type I inositol polyphosphate 5-phosphatase 12-like isoform X2	Juglans regia	XP_018857967.1
TRINITY_DN11523_c0_g3::TRINITY_DN11523_c0_g3_i2::g.7748::m.7748	3,37E-173	482	XP_018819194.1	40S ribosomal protein S4-3	Juglans regia	AES86765.1
TRINITY_DN14938_c0_g1::TRINITY_DN14938_c0_g1_i5::g.44776::m.44776	3,68E-174	485	XP_018835690.1	eukaryotic translation initiation factor 2 subunit beta-like	Juglans regia	XP_018828541.1
TRINITY_DN17566_c1_g1::TRINITY_DN17566_c1_g1_i8::g.86112::m.86112	0	921	XP_018816061.1	2-isopropylmalate synthase 1, chloroplastic-like	Juglans regia	XP_020421790.1
TRINITY_DN19918_c3_g1::TRINITY_DN19918_c3_g1_i11::g.126682::m.126682	0	1827	XP_018827765.1	callose synthase 5	Juglans regia	XP_016648608.1

TRINITY_DN12927_c2_g2::TRINITY_DN12927_c2_g2_i9::g.17583::m.17583	0	1412	ONH96238.1	hypothetical protein PRUPE_7G115400	Prunus persica	XP_007204282.1
TRINITY_DN16523_c1_g3::TRINITY_DN16523_c1_g3_i4::g.69129::m.69129	0	764	XP_018805377.1	uncharacterized protein LOC108979207	Juglans regia	XP_018805377.1
TRINITY_DN15434_c1_g2::TRINITY_DN15434_c1_g2_i2::g.51814::m.51814	8,82E-154	429	XP_018854606.1	ras-related protein RABA2a	Juglans regia	OAY39697.1
TRINITY_DN18883_c1_g1::TRINITY_DN18883_c1_g1_i17::g.108283::m.108283	0	1147	KDP37205.1	hypothetical protein JCGZ_06261	Jatropha curcas	XP_012073334.1
TRINITY_DN15951_c5_g2::TRINITY_DN15951_c5_g2_i3::g.59457::m.59457	0	563	XP_018826547.1	aldose 1-epimerase	Juglans regia	ONI08609.1
TRINITY_DN17564_c2_g2::TRINITY_DN17564_c2_g2_i9::g.86086::m.86086	0	1044	XP_018817793.1	polyadenylate-binding protein 8-like	Juglans regia	XP_018817793.1
TRINITY_DN15444_c2_g1::TRINITY_DN15444_c2_g1_i2::g.52034::m.52034	7,78E-79	243	XP_018852448.1	late embryogenesis abundant protein 1-like	Juglans regia	OAY24830.1
TRINITY_DN19153_c1_g1::TRINITY_DN19153_c1_g1_i1::g.112868::m.112868	8,02E-113	339	XP_018858842.1	patellin-4-like	Juglans regia	XP_018857977.1
TRINITY_DN18739_c0_g2::TRINITY_DN18739_c0_g2_i10::g.105638::m.105638	0	1254	XP_018841709.1	eukaryotic translation initiation factor 3 subunit A-like	Juglans regia	XP_018809947.1
TRINITY_DN17091_c0_g2::TRINITY_DN17091_c0_g2_i7::g.78224::m.78224	0	1140	XP_018829342.1	uncharacterized protein LOC108997478 isoform X1	Juglans regia	XP_018829351.1
TRINITY_DN16872_c0_g1::TRINITY_DN16872_c0_g1_i9::g.74807::m.74807	0	692	XP_018842503.1	branched-chain-amino-acid aminotransferase 3, chloroplastic-like	Juglans regia	XP_009342188.1
TRINITY_DN13841_c4_g1::TRINITY_DN13841_c4_g1_i2::g.28420::m.28420	9,02E-145	417	XP_018821230.1	protein SLOW GREEN 1, chloroplastic-like	Juglans regia	XP_016647139.1
TRINITY_DN11991_c0_g1::TRINITY_DN11991_c0_g1_i1::g.9823::m.9823	0	841	XP_018846215.1	26S proteasome non-ATPase regulatory subunit 12 homolog A	Juglans regia	OAY33256.1
TRINITY_DN15464_c0_g1::TRINITY_DN15464_c0_g1_i3::g.52265::m.52265	0	564	XP_018823263.1	probable mediator of RNA polymerase II transcription subunit 37c	Juglans regia	GAU49026.1
TRINITY_DN11351_c0_g4::TRINITY_DN11351_c0_g4_i3::g.7095::m.7095	3,10E-122	350	XP_018815857.1	chloride conductance regulatory protein ICln isoform X1	Juglans regia	KDP22395.1
TRINITY_DN19612_c1_g2::TRINITY_DN19612_c1_g2_i9::g.120822::m.120822	0	527	XP_018809366.1	probable methyltransferase PMT18	Juglans regia	KDP25187.1
TRINITY_DN10989_c0_g1::TRINITY_DN10989_c0_g1_i3::g.6012::m.6012	2,75E-127	367	XP_018821762.1	uncharacterized protein LOC108991825	Juglans regia	XP_018838963.1
TRINITY_DN12636_c0_g1::TRINITY_DN12636_c0_g1_i3::g.14427::m.14427	1,13E-95	294	XP_018824850.1	histone deacetylase 5 isoform X2	Juglans regia	XP_018824848.1
TRINITY_DN15811_c0_g5::TRINITY_DN15811_c0_g5_i2::g.57331::m.57331	2,88E-114	328	XP_018835046.1	proteasome subunit alpha type-6 isoform X1	Juglans regia	XP_018835047.1
TRINITY_DN19982_c2_g1::TRINITY_DN19982_c2_g1_i8::g.127226::m.127226	6,55E-90	266	XP_018808748.1	adenine phosphoribosyltransferase 1-like	Juglans regia	KYP70415.1
TRINITY_DN18355_c0_g1::TRINITY_DN18355_c0_g1_i6::g.99167::m.99167	2,40E-62	195	XP_018818043.1	ankyrin repeat and SAM domain-containing protein 6-like	Juglans regia	XP_008372496.1
TRINITY_DN15264_c2_g1::TRINITY_DN15264_c2_g1_i9::g.49352::m.49352	1,39E-55	175	XP_018829795.1	uncharacterized protein At4g13230	Juglans regia	XP_020234770.1

TRINITY_DN12375_c3_g5::TRINITY_DN12375_c3_g5_i1::g.12365::m.12365	0	1150	XP_018845206.1	probable methyltransferase PMT2 isoform X1	Juglans regia	XP_018845207.1
TRINITY_DN17383_c0_g1::TRINITY_DN17383_c0_g1_i3::g.82754::m.82754	0	605	XP_018853672.1	methionine aminopeptidase 1A	Juglans regia	OAY62431.1
TRINITY_DN18266_c2_g1::TRINITY_DN18266_c2_g1_i1::g.97903::m.97903	1,81E-68	210	XP_018850051.1	REF/SRPP-like protein At3g05500	Juglans regia	XP_018850053.1
TRINITY_DN15771_c0_g1::TRINITY_DN15771_c0_g1_i5::g.56845::m.56845	0	515	XP_018845062.1	coatomer subunit beta'-2-like	Juglans regia	XP_018843771.1
TRINITY_DN15425_c0_g1::TRINITY_DN15425_c0_g1_i1::g.51653::m.51653	0	630	OAY50293.1	hypothetical protein MANES_05G124200	Manihot esculenta	KEH36332.1
TRINITY_DN19779_c3_g1::TRINITY_DN19779_c3_g1_i9::g.123558::m.123558	0	653	XP_018806897.1	lysine--tRNA ligase isoform X1	Juglans regia	XP_018806898.1
TRINITY_DN17373_c0_g1::TRINITY_DN17373_c0_g1_i1::g.82647::m.82647	0	952	XP_018837882.1	dynamin-related protein 1C	Juglans regia	XP_018814632.1
TRINITY_DN17897_c2_g2::TRINITY_DN17897_c2_g2_i2::g.91535::m.91535	0	894	XP_018834105.1	exocyst complex component EXO70B1-like	Juglans regia	XP_008231066.1
TRINITY_DN17861_c1_g1::TRINITY_DN17861_c1_g1_i1::g.90803::m.90803	1,72E-55	176	XP_018818881.1	L-ascorbate peroxidase, cytosolic	Juglans regia	XP_018818882.1
TRINITY_DN15475_c0_g1::TRINITY_DN15475_c0_g1_i2::g.52448::m.52448	0	667	XP_018808798.1	uncharacterized protein LOC108981991	Juglans regia	XP_018843302.1
TRINITY_DN17503_c0_g1::TRINITY_DN17503_c0_g1_i2::g.84892::m.84892	8,38E-30	117	XP_018842400.1	eukaryotic translation initiation factor 3 subunit C isoform X1	Juglans regia	XP_018842401.1
TRINITY_DN14545_c0_g2::TRINITY_DN14545_c0_g2_i6::g.38726::m.38726	0	854	XP_018836442.1	tubulin beta chain-like	Juglans regia	XP_018854022.1
TRINITY_DN12477_c0_g1::TRINITY_DN12477_c0_g1_i2::g.13209::m.13209	0	1000	XP_018816821.1	2-hydroxyacyl-CoA lyase	Juglans regia	XP_008229283.1
TRINITY_DN18895_c1_g2::TRINITY_DN18895_c1_g2_i8::g.108488::m.108488	3,79E-157	442	XP_018832945.1	peptidyl-prolyl cis-trans isomerase A1 isoform X1	Juglans regia	XP_018832946.1
TRINITY_DN14210_c0_g1::TRINITY_DN14210_c0_g1_i5::g.33918::m.33918	3,27E-140	395	XP_018806297.1	eukaryotic translation initiation factor 6-2	Juglans regia	XP_018806298.1
TRINITY_DN18268_c0_g1::TRINITY_DN18268_c0_g1_i8::g.98068::m.98068	0	667	XP_018836294.1	arginine--tRNA ligase, cytoplasmic-like isoform X1	Juglans regia	XP_018836295.1
TRINITY_DN16248_c0_g1::TRINITY_DN16248_c0_g1_i2::g.64527::m.64527	0	787	XP_018827585.1	fumarylacetoacetase	Juglans regia	KDP37782.1
TRINITY_DN16306_c1_g2::TRINITY_DN16306_c1_g2_i2::g.65481::m.65481	2,69E-67	208	XP_018822871.1	strigolactone esterase D14	Juglans regia	XP_018838099.1
TRINITY_DN19332_c2_g5::TRINITY_DN19332_c2_g5_i3::g.116136::m.116136	3,22E-160	447	XP_018841293.1	nicotinamidase 1-like	Juglans regia	XP_018841294.1
TRINITY_DN19330_c0_g3::TRINITY_DN19330_c0_g3_i6::g.116162::m.116162	0	733	XP_018818846.1	elongation factor 1-alpha-like	Juglans regia	XP_018818847.1
TRINITY_DN19198_c2_g2::TRINITY_DN19198_c2_g2_i7::g.112135::m.112135	3,46E-132	373	XP_008228156.1	25.3 kDa vesicle transport protein	Prunus mume	ONI15308.1
TRINITY_DN18055_c0_g3::TRINITY_DN18055_c0_g3_i2::g.94441::m.94441	9,46E-98	288	XP_008239513.1	60S ribosomal protein L7a	Prunus mume	ONI07927.1

TRINITY_DN19902_c1_g2::TRINITY_DN19902_c1_g2_i1::g.125828::m.125828	0	837	XP_018828232.1	protein transport protein SEC23-like	Juglans regia	XP_018848547.1
TRINITY_DN16483_c1_g1::TRINITY_DN16483_c1_g1_i8::g.68282::m.68282	0	3114	XP_018833570.1	brefeldin A-inhibited guanine nucleotide-exchange protein 5 isoform X2	Juglans regia	XP_018833569.1
TRINITY_DN13316_c0_g1::TRINITY_DN13316_c0_g1_i4::g.22236::m.22236	0	1325	XP_018833925.1	isoamylase 3, chloroplastic isoform X1	Juglans regia	XP_018833926.1
TRINITY_DN14260_c1_g1::TRINITY_DN14260_c1_g1_i4::g.34485::m.34485	0	561	XP_018819297.1	2-dehydro-3-deoxyphosphooctonate aldolase	Juglans regia	ONI11851.1
TRINITY_DN14547_c0_g1::TRINITY_DN14547_c0_g1_i3::g.38055::m.38055	0	518	XP_018807236.1	eukaryotic translation initiation factor 3 subunit F-like	Juglans regia	XP_018821752.1
TRINITY_DN17557_c0_g1::TRINITY_DN17557_c0_g1_i2::g.85865::m.85865	8,29E-132	384	ONH92223.1	hypothetical protein PRUPE_8G163800	Prunus persica	ONH92224.1
TRINITY_DN14469_c0_g2::TRINITY_DN14469_c0_g2_i3::g.37807::m.37807	0	542	XP_018824359.1	plant UBX domain-containing protein 10	Juglans regia	XP_018844497.1
TRINITY_DN13571_c3_g1::TRINITY_DN13571_c3_g1_i9::g.25607::m.25607	0	704	XP_018822222.1	DEAD-box ATP-dependent RNA helicase 56	Juglans regia	XP_018812870.1
TRINITY_DN15222_c4_g2::TRINITY_DN15222_c4_g2_i2::g.48684::m.48684	0	551	XP_018860327.1	probable dolichyl-diphosphooligosaccharide--protein glycosyltransferase subunit 3B	Juglans regia	ONI10057.1
TRINITY_DN14937_c1_g1::TRINITY_DN14937_c1_g1_i10::g.44580::m.44580	7,57E-117	338	XP_018842015.1	endochitinase-like	Juglans regia	ONH97234.1
TRINITY_DN11539_c0_g1::TRINITY_DN11539_c0_g1_i5::g.7794::m.7794	7,53E-62	206	XP_018825127.1	plasminogen activator inhibitor 1 RNA-binding protein	Juglans regia	OAY60106.1
TRINITY_DN15625_c0_g3::TRINITY_DN15625_c0_g3_i1::g.54608::m.54608	1,68E-134	379	KHN38389.1	Vesicle-associated membrane protein 713	Glycine soja	KRH73538.1
TRINITY_DN17623_c4_g3::TRINITY_DN17623_c4_g3_i4::g.87434::m.87434	8,64E-14	67,4	XP_018831494.1	protein PXR1	Juglans regia	XP_018831495.1
TRINITY_DN19001_c1_g1::TRINITY_DN19001_c1_g1_i22::g.110256::m.110256	1,15E-100	293	ONI16565.1	hypothetical protein PRUPE_3G106800	Prunus persica	XP_008228869.1
TRINITY_DN14942_c2_g1::TRINITY_DN14942_c2_g1_i4::g.44640::m.44640	1,96E-78	232	KRH26163.1	hypothetical protein GLYMA_12G156200	Glycine max	KDP38691.1
TRINITY_DN14097_c2_g2::TRINITY_DN14097_c2_g2_i1::g.31570::m.31570	3,39E-162	474	XP_018811830.1	exocyst complex component EXO70B1	Juglans regia	XP_009349485.1
TRINITY_DN19080_c0_g1::TRINITY_DN19080_c0_g1_i5::g.111810::m.111810	0	721	XP_018810134.1	dihydropyrimidine dehydrogenase (NADP(+)), chloroplastic-like	Juglans regia	XP_018823611.1
TRINITY_DN18479_c0_g5::TRINITY_DN18479_c0_g5_i1::g.100968::m.100968	1,37E-124	353	OIV97133.1	hypothetical protein TanjilG_00162	Lupinus angustifolius	XP_019417809.1
TRINITY_DN17219_c0_g1::TRINITY_DN17219_c0_g1_i4::g.80391::m.80391	9,74E-50	160	KDP34497.1	hypothetical protein JCGZ_11047	Jatropha curcas	XP_012075960.1
TRINITY_DN19330_c0_g1::TRINITY_DN19330_c0_g1_i8::g.116161::m.116161	4,15E-63	192	XP_018819452.1	elongation factor 1-alpha, partial	Juglans regia	XP_018860404.1
TRINITY_DN13211_c0_g1::TRINITY_DN13211_c0_g1_i3::g.21098::m.21098	0	718	XP_018806331.1	60S ribosomal protein L3-2	Juglans regia	XP_018806332.1
TRINITY_DN13228_c1_g1::TRINITY_DN13228_c1_g1_i1::g.21382::m.21382	6,93E-82	244	XP_018817862.1	thioredoxin M3, chloroplastic-like	Juglans regia	XP_008236593.1

TRINITY_DN19095_c0_g1::TRINITY_DN19095_c0_g1_i1::g.111856::m.111856	6,14E-113	321	XP_018847195.1	eukaryotic translation initiation factor 5A-2	Juglans regia	XP_018829201.1
TRINITY_DN12846_c1_g3::TRINITY_DN12846_c1_g3_i2::g.16059::m.16059	1,88E-106	305	XP_018820918.1	cyanate hydratase	Juglans regia	KDP29704.1
TRINITY_DN16616_c1_g1::TRINITY_DN16616_c1_g1_i2::g.70640::m.70640	7,45E-165	464	XP_018846161.1	uncharacterized protein LOC109009947	Juglans regia	ONH97840.1
TRINITY_DN17302_c1_g3::TRINITY_DN17302_c1_g3_i1::g.81624::m.81624	0	1804	XP_018841180.1	copper-transporting ATPase RAN1-like	Juglans regia	XP_018835852.1
TRINITY_DN12723_c0_g1::TRINITY_DN12723_c0_g1_i2::g.15224::m.15224	0	705	KDP30971.1	hypothetical protein JCGZ_11347	Jatropha curcas	XP_012079902.1
TRINITY_DN17461_c1_g1::TRINITY_DN17461_c1_g1_i12::g.84192::m.84192	1,60E-132	402	XP_018834800.1	peptidyl-prolyl cis-trans isomerase CYP95 isoform X1	Juglans regia	XP_018834801.1
TRINITY_DN17237_c2_g3::TRINITY_DN17237_c2_g3_i7::g.80360::m.80360	0	1788	KRH47017.1	hypothetical protein GLYMA_07G004100	Glycine max	XP_003529523.1
TRINITY_DN12529_c0_g1::TRINITY_DN12529_c0_g1_i3::g.13512::m.13512	0	610	XP_018812700.1	SNF1-related protein kinase regulatory subunit gamma-1-like	Juglans regia	XP_018812702.1
TRINITY_DN12993_c1_g1::TRINITY_DN12993_c1_g1_i9::g.18389::m.18389	2,73E-68	207	XP_018856764.1	cysteine proteinase inhibitor	Juglans regia	XP_018843658.1
TRINITY_DN18316_c4_g8::TRINITY_DN18316_c4_g8_i2::g.98671::m.98671	9,20E-143	402	XP_018834389.1	ras-related protein RABA4c	Juglans regia	ONI20318.1
TRINITY_DN14932_c2_g1::TRINITY_DN14932_c2_g1_i5::g.44480::m.44480	3,06E-59	182	XP_017186235.1	12-oxophytodienoate reductase 2-like	Malus domestica	OAY23951.1
TRINITY_DN18395_c1_g2::TRINITY_DN18395_c1_g2_i5::g.99070::m.99070	0	626	XP_018841544.1	ubiquitin domain-containing protein DSK2a-like isoform X3	Juglans regia	XP_018829167.1
TRINITY_DN12675_c1_g1::TRINITY_DN12675_c1_g1_i2::g.14909::m.14909	0	573	XP_018811219.1	eukaryotic translation initiation factor 2 subunit alpha homolog	Juglans regia	KDP42130.1
TRINITY_DN18566_c1_g3::TRINITY_DN18566_c1_g3_i2::g.102668::m.102668	7,64E-128	364	XP_018860411.1	coatomer subunit epsilon-1	Juglans regia	XP_018828599.1
TRINITY_DN10355_c0_g1::TRINITY_DN10355_c0_g1_i2::g.4690::m.4690						
TRINITY_DN13432_c1_g1::TRINITY_DN13432_c1_g1_i19::g.23847::m.23847	0	542	XP_018853171.1	probable protein phosphatase 2C 11	Juglans regia	XP_018857120.1
TRINITY_DN14200_c0_g1::TRINITY_DN14200_c0_g1_i2::g.33523::m.33523	0	1024	XP_008230855.1	beta-glucosidase BoGH3B-like	Prunus mume	XP_008245228.1
TRINITY_DN12445_c0_g2::TRINITY_DN12445_c0_g2_i13::g.12955::m.12955	8,62E-90	273	XP_008239439.1	probable aspartyl aminopeptidase	Prunus mume	XP_009359462.1
TRINITY_DN12195_c0_g1::TRINITY_DN12195_c0_g1_i1::g.10552::m.10552	1,52E-136	388	XP_018821879.1	tropinone reductase homolog At5g06060-like	Juglans regia	XP_008233480.1
TRINITY_DN13264_c3_g1::TRINITY_DN13264_c3_g1_i4::g.21666::m.21666	0	694	XP_018817132.1	aminoacylase-1-like	Juglans regia	XP_014505119.1
TRINITY_DN16140_c1_g1::TRINITY_DN16140_c1_g1_i18::g.63031::m.63031	1,77E-44	148	XP_004509621.1	probable calcium-binding protein CML21	Cicer arietinum	KYP50859.1
TRINITY_DN18120_c2_g1::TRINITY_DN18120_c2_g1_i8::g.95450::m.95450	6,90E-49	161	XP_018820284.1	aldo-keto reductase family 4 member C9-like	Juglans regia	XP_018820285.1

TRINITY_DN19263_c0_g1::TRINITY_DN19263_c0_g1_i2::g.114802::m.114802	6,96E-136	404	XP_018837044.1	probable cytosolic oligopeptidase A	Juglans regia	XP_018850785.1
TRINITY_DN16243_c0_g1::TRINITY_DN16243_c0_g1_i2::g.64445::m.64445	0	946	XP_018840323.1	V-type proton ATPase subunit a3-like	Juglans regia	XP_008348897.1
TRINITY_DN13812_c2_g1::TRINITY_DN13812_c2_g1_i1::g.28623::m.28623	1,96E-14	73,9	XP_018819456.1	plasminogen activator inhibitor 1 RNA-binding protein-like	Juglans regia	XP_018854638.1
TRINITY_DN37045_c0_g1::TRINITY_DN37045_c0_g1_i1::g.131013::m.131013	0	671	XP_006574889.2	PI-PLC X domain-containing protein At5g67130	Glycine max	KHN05833.1
TRINITY_DN17851_c1_g3::TRINITY_DN17851_c1_g3_i2::g.90156::m.90156	0	639	XP_008372715.1	arginase 1, mitochondrial	Malus domestica	XP_018505555.1
TRINITY_DN14011_c0_g1::TRINITY_DN14011_c0_g1_i1::g.31283::m.31283	3,39E-116	341	XP_018824670.1	uncharacterized protein LOC108994051 isoform X1	Juglans regia	ONI13454.1
TRINITY_DN11978_c1_g4::TRINITY_DN11978_c1_g4_i1::g.9533::m.9533	0	780	OAY30086.1	hypothetical protein MANES_14G002500	Manihot esculenta	XP_018830946.1
TRINITY_DN17242_c0_g2::TRINITY_DN17242_c0_g2_i1::g.80718::m.80718	0	636	XP_018838485.1	UDP-glycosyltransferase 79B30 isoform X1	Juglans regia	XP_018838486.1
TRINITY_DN16272_c1_g2::TRINITY_DN16272_c1_g2_i3::g.64808::m.64808	0	728	XP_018813395.1	ureidoglycolate hydrolase	Juglans regia	ONI26562.1
TRINITY_DN12654_c0_g3::TRINITY_DN12654_c0_g3_i5::g.14722::m.14722	0	1115	XP_018852605.1	leukotriene A-4 hydrolase homolog	Juglans regia	ONI24042.1
TRINITY_DN13334_c3_g1::TRINITY_DN13334_c3_g1_i2::g.22908::m.22908	4,30E-56	191	XP_018825817.1	subtilisin-like protease SBT5.4	Juglans regia	XP_018810465.1
TRINITY_DN16447_c0_g1::TRINITY_DN16447_c0_g1_i1::g.67047::m.67047	3,72E-106	309	KRH12348.1	hypothetical protein GLYMA_15G167400	Glycine max	XP_006596959.1
TRINITY_DN15955_c0_g1::TRINITY_DN15955_c0_g1_i1::g.59530::m.59530	2,31E-147	425	XP_018812439.1	ferrochelatase-2, chloroplastic-like	Juglans regia	ONI33279.1
TRINITY_DN19579_c1_g3::TRINITY_DN19579_c1_g3_i1::g.120400::m.120400	3,20E-147	433	XP_018839115.1	delta-1-pyrroline-5-carboxylate synthase-like	Juglans regia	XP_018820283.1
TRINITY_DN17207_c1_g1::TRINITY_DN17207_c1_g1_i5::g.80226::m.80226	0	1003	XP_018829757.1	beta-hexosaminidase 2	Juglans regia	OAY41648.1
TRINITY_DN17881_c0_g1::TRINITY_DN17881_c0_g1_i3::g.91227::m.91227	0	638	XP_018826275.1	uncharacterized protein LOC108995215	Juglans regia	ONI04868.1
TRINITY_DN16367_c2_g1::TRINITY_DN16367_c2_g1_i2::g.66527::m.66527	0	551	XP_008364783.1	LOW QUALITY PROTEIN: diaminopimelate epimerase, chloroplastic-like	Malus domestica	ONI03290.1
TRINITY_DN12454_c0_g1::TRINITY_DN12454_c0_g1_i1::g.12984::m.12984	0	877	XP_018843339.1	ubiquitin carboxyl-terminal hydrolase 6-like isoform X1	Juglans regia	XP_018843340.1
TRINITY_DN18113_c1_g2::TRINITY_DN18113_c1_g2_i2::g.95380::m.95380	0	553	XP_018851148.1	probable sucrose-phosphatase 2 isoform X1	Juglans regia	XP_009362647.1
TRINITY_DN13825_c0_g1::TRINITY_DN13825_c0_g1_i2::g.28846::m.28846	7,72E-90	265	XP_018817591.1	aldo-keto reductase family 4 member C9-like	Juglans regia	XP_020221314.1
TRINITY_DN15635_c1_g1::TRINITY_DN15635_c1_g1_i2::g.54767::m.54767	0	833	XP_018842945.1	sugar transport protein 8-like	Juglans regia	XP_018842937.1
TRINITY_DN18171_c1_g1::TRINITY_DN18171_c1_g1_i5::g.96291::m.96291	0	906	XP_018833058.1	calcium-dependent protein kinase 34-like	Juglans regia	XP_018842514.1

TRINITY_DN17183_c0_g2::TRINITY_DN17183_c0_g2_i5::g.79774::m.79774	7,87E-103	299	XP_018821677.1	maf-like protein DDB_G0281937 isoform X6	Juglans regia	XP_018821674.1
TRINITY_DN16005_c0_g3::TRINITY_DN16005_c0_g3_i6::g.60403::m.60403	0	798	XP_018831026.1	glutamate dehydrogenase A	Juglans regia	XP_018808101.1
TRINITY_DN13194_c5_g1::TRINITY_DN13194_c5_g1_i1::g.20972::m.20972	2,80E-158	454	XP_008371522.1	pollen-specific leucine-rich repeat extensin-like protein 1	Malus domestica	XP_008371522.1
TRINITY_DN19141_c0_g1::TRINITY_DN19141_c0_g1_i8::g.112746::m.112746	0	748	XP_018853594.1	dihydrolipoyllysine-residue acetyltransferase component 1 of pyruvate dehydrogenase complex, mitochondrial	Juglans regia	XP_018853594.1
TRINITY_DN19587_c1_g2::TRINITY_DN19587_c1_g2_i1::g.120316::m.120316	0	624	XP_018825827.1	DNA damage-inducible protein 1 isoform X1	Juglans regia	XP_018825835.1
TRINITY_DN14259_c5_g1::TRINITY_DN14259_c5_g1_i8::g.34656::m.34656	9,89E-75	224	XP_018860648.1	uncharacterized protein LOC109022250	Juglans regia	XP_018860649.1
TRINITY_DN16231_c1_g1::TRINITY_DN16231_c1_g1_i4::g.64294::m.64294	0	731	XP_018816814.1	mannose-6-phosphate isomerase 1	Juglans regia	KDP32959.1
TRINITY_DN14338_c3_g2::TRINITY_DN14338_c3_g2_i1::g.35555::m.35555	0	709	XP_018832863.1	beta-glucosidase 42 isoform X1	Juglans regia	ONI05292.1
TRINITY_DN19174_c1_g3::TRINITY_DN19174_c1_g3_i1::g.113399::m.113399	0	537	XP_018818703.1	cycloartenol synthase 2	Juglans regia	XP_009370034.1
TRINITY_DN19841_c3_g2::TRINITY_DN19841_c3_g2_i9::g.124618::m.124618	0	2182	XP_018833940.1	probable manganese-transporting ATPase PDR2	Juglans regia	XP_018833946.1
TRINITY_DN20035_c3_g1::TRINITY_DN20035_c3_g1_i3::g.128175::m.128175	0	652	XP_018848755.1	indole-3-glycerol phosphate synthase, chloroplastic-like	Juglans regia	XP_012081510.1
TRINITY_DN18498_c3_g4::TRINITY_DN18498_c3_g4_i4::g.101572::m.101572	1,05E-97	281	XP_018850281.1	NADH dehydrogenase [ubiquinone] 1 alpha subcomplex subunit 13-B-like	Juglans regia	XP_008361715.1
TRINITY_DN17440_c0_g1::TRINITY_DN17440_c0_g1_i7::g.84097::m.84097	0	1154	XP_018848330.1	dnaJ protein ERDJ2A	Juglans regia	XP_018848331.1
TRINITY_DN15779_c0_g2::TRINITY_DN15779_c0_g2_i1::g.57109::m.57109	2,19E-84	256	XP_018840900.1	probable prolyl 4-hydroxylase 10	Juglans regia	XP_018807322.1
TRINITY_DN18149_c0_g1::TRINITY_DN18149_c0_g1_i3::g.95908::m.95908	5,67E-178	499	XP_018840325.1	cysteine protease RD19A-like	Juglans regia	KDP41743.1
TRINITY_DN18366_c0_g2::TRINITY_DN18366_c0_g2_i3::g.99417::m.99417	2,79E-101	291	XP_018808968.1	40S ribosomal protein S11	Juglans regia	XP_018822277.1
TRINITY_DN16745_c0_g1::TRINITY_DN16745_c0_g1_i9::g.72756::m.72756	0	1115	XP_018830674.1	uncharacterized protein LOC108998567 isoform X2	Juglans regia	XP_018830673.1
TRINITY_DN12226_c1_g1::TRINITY_DN12226_c1_g1_i9::g.11323::m.11323	1,45E-51	162	XP_018836815.1	uncharacterized protein LOC109003223	Juglans regia	XP_018836816.1
TRINITY_DN16837_c0_g2::TRINITY_DN16837_c0_g2_i4::g.74272::m.74272	0	952	XP_018809133.1	cleft lip and palate transmembrane protein 1 homolog	Juglans regia	XP_009379523.1
TRINITY_DN14860_c0_g2::TRINITY_DN14860_c0_g2_i3::g.43462::m.43462	0	2170	XP_018825838.1	uncharacterized protein LOC108994897 isoform X1	Juglans regia	XP_018825838.1
TRINITY_DN19634_c0_g1::TRINITY_DN19634_c0_g1_i18::g.121436::m.121436	0	4563	XP_018846305.1	BEACH domain-containing protein C2 isoform X2	Juglans regia	XP_018846304.1

TRINITY_DN18967_c1_g1::TRINITY_DN18967_c1_g1_i1::g.109547::m.109547	0	870	XP_018833063.1	dihydropyrimidinase-like	Juglans regia	XP_018842508.1
TRINITY_DN10328_c0_g1::TRINITY_DN10328_c0_g1_i1::g.4642::m.4642	8,93E-67	204	ONI01573.1	hypothetical protein PRUPE_6G147100	Prunus persica	XP_020422670.1
TRINITY_DN12931_c0_g1::TRINITY_DN12931_c0_g1_i4::g.17462::m.17462	2,39E-110	318	XP_018813434.1	ER membrane protein complex subunit 8/9 homolog	Juglans regia	XP_018825614.1
TRINITY_DN15347_c2_g1::TRINITY_DN15347_c2_g1_i4::g.50637::m.50637	3,37E-52	174	XP_018852304.1	putative HVA22-like protein g	Juglans regia	KRH51714.1
TRINITY_DN18324_c2_g1::TRINITY_DN18324_c2_g1_i6::g.99024::m.99024	0	850	XP_018841744.1	oxysterol-binding protein-related protein 3A	Juglans regia	XP_018807417.1
TRINITY_DN15702_c0_g3::TRINITY_DN15702_c0_g3_i1::g.55933::m.55933	5,39E-177	491	KDP41064.1	hypothetical protein JCGZ_03170	Jatropha curcas	XP_012068495.1
TRINITY_DN11687_c0_g1::TRINITY_DN11687_c0_g1_i2::g.8407::m.8407	0	647	XP_018837102.1	chromatin assembly factor 1 subunit A isoform X1	Juglans regia	XP_018837103.1
TRINITY_DN12590_c1_g3::TRINITY_DN12590_c1_g3_i2::g.13976::m.13976	6,75E-161	451	XP_018825081.1	gamma carbonic anhydrase-like 2, mitochondrial	Juglans regia	KDP22602.1
TRINITY_DN15601_c2_g1::TRINITY_DN15601_c2_g1_i1::g.52933::m.52933						
TRINITY_DN17527_c0_g3::TRINITY_DN17527_c0_g3_i1::g.85247::m.85247	0	831	XP_018818792.1	molybdate-anion transporter-like	Juglans regia	XP_018842813.1
TRINITY_DN12826_c0_g1::TRINITY_DN12826_c0_g1_i1::g.16222::m.16222	0	526	XP_018807335.1	endonuclease 4-like	Juglans regia	XP_008371307.1
TRINITY_DN19082_c3_g1::TRINITY_DN19082_c3_g1_i2::g.111610::m.111610	3,79E-109	313	XP_016176151.1	transmembrane protein 205	Arachis ipaensis	XP_015938890.1
TRINITY_DN19829_c1_g1::TRINITY_DN19829_c1_g1_i2::g.124633::m.124633	0	697	XP_018826456.1	uncharacterized protein LOC108995353	Juglans regia	OAY48056.1
TRINITY_DN16466_c0_g3::TRINITY_DN16466_c0_g3_i1::g.67992::m.67992	0	567	XP_018823768.1	protein EARLY-RESPONSIVE TO DEHYDRATION 7, chloroplastic-like	Juglans regia	XP_018823769.1
TRINITY_DN12480_c0_g2::TRINITY_DN12480_c0_g2_i1::g.13216::m.13216	7,75E-73	216	XP_018809915.1	uncharacterized protein LOC108982896	Juglans regia	XP_018809916.1
TRINITY_DN14710_c1_g4::TRINITY_DN14710_c1_g4_i3::g.41054::m.41054	0	1422	XP_018843379.1	protein ROOT HAIR DEFECTIVE 3-like	Juglans regia	XP_008228296.1
TRINITY_DN17564_c2_g2::TRINITY_DN17564_c2_g2_i5::g.86079::m.86079	0	740	XP_018851337.1	acetolactate synthase small subunit 2, chloroplastic-like isoform X1	Juglans regia	XP_018851338.1
TRINITY_DN13661_c3_g4::TRINITY_DN13661_c3_g4_i1::g.26847::m.26847	3,44E-112	319	XP_008394324.1	eukaryotic translation initiation factor 5A-2-like	Malus domestica	XP_008343897.1
TRINITY_DN12154_c0_g1::TRINITY_DN12154_c0_g1_i6::g.10864::m.10864	1,71E-103	311	XP_018849569.1	multiple organellar RNA editing factor 8, chloroplastic/mitochondrial	Juglans regia	KDP36270.1
TRINITY_DN19326_c2_g1::TRINITY_DN19326_c2_g1_i5::g.116051::m.116051	0	1711	ONI05682.1	hypothetical protein PRUPE_5G018900	Prunus persica	ONI05683.1
TRINITY_DN19798_c2_g2::TRINITY_DN19798_c2_g2_i18::g.123759::m.123759	0	1006	OAY26193.1	hypothetical protein MANES_16G027900	Manihot esculenta	KDP20974.1
TRINITY_DN19717_c0_g1::TRINITY_DN19717_c0_g1_i2::g.122513::m.122513	0	797	XP_018837241.1	CAAX prenyl protease 1 homolog	Juglans regia	ONI23213.1

TRINITY_DN17321_c2_g2::TRINITY_DN17321_c2_g2_i3::g.82053::m.82053	1,06E-132	381	XP_018825826.1	ferritin-3, chloroplastic-like	Juglans regia	AJK93885.1
TRINITY_DN16262_c1_g2::TRINITY_DN16262_c1_g2_i1::g.64873::m.64873	5,74E-58	187	KHN32164.1	Endoglucanase 16	Glycine soja	KRH52260.1
TRINITY_DN17928_c2_g1::TRINITY_DN17928_c2_g1_i2::g.92278::m.92278	2,08E-160	449	XP_018827791.1	probable carboxylesterase SOBER1-like	Juglans regia	OAY25864.1
TRINITY_DN19464_c2_g1::TRINITY_DN19464_c2_g1_i8::g.118275::m.118275	7,00E-169	470	KDP41459.1	hypothetical protein JCGZ_15866	Jatropha curcas	XP_012067968.1
TRINITY_DN18987_c4_g5::TRINITY_DN18987_c4_g5_i1::g.110067::m.110067	2,91E-52	176	XP_018857833.1	uncharacterized protein LOC109019909, partial	Juglans regia	XP_018852053.1
TRINITY_DN18350_c0_g4::TRINITY_DN18350_c0_g4_i3::g.99164::m.99164	0	531	XP_018813115.1	fasciclin-like arabinogalactan protein 1	Juglans regia	OAY28350.1
TRINITY_DN18760_c9_g3::TRINITY_DN18760_c9_g3_i2::g.106074::m.106074	2,13E-98	305	XP_018841471.1	beta-galactosidase 13-like	Juglans regia	XP_008347518.1
TRINITY_DN17826_c0_g3::TRINITY_DN17826_c0_g3_i6::g.90229::m.90229	3,04E-93	275	XP_018841452.1	dirigent protein 23-like	Juglans regia	KDP27569.1
TRINITY_DN19627_c1_g2::TRINITY_DN19627_c1_g2_i4::g.121159::m.121159	0	582	XP_018817804.1	leucine--tRNA ligase, cytoplasmic isoform X1	Juglans regia	XP_018817805.1
TRINITY_DN19425_c1_g1::TRINITY_DN19425_c1_g1_i1::g.117994::m.117994	0	561	XP_018842494.1	uncharacterized protein LOC109007319	Juglans regia	ONH94022.1
TRINITY_DN13200_c1_g3::TRINITY_DN13200_c1_g3_i1::g.20941::m.20941	2,77E-108	310	XP_018835316.1	peptidyl-prolyl cis-trans isomerase CYP18-2	Juglans regia	ONH96008.1
TRINITY_DN14744_c0_g1::TRINITY_DN14744_c0_g1_i1::g.41504::m.41504	0	688	XP_018848695.1	protein disulfide-isomerase 5-2-like	Juglans regia	XP_018821144.1
TRINITY_DN16218_c1_g2::TRINITY_DN16218_c1_g2_i6::g.64375::m.64375	6,02E-140	395	XP_018824217.1	CBS domain-containing protein CBSX1, chloroplastic	Juglans regia	XP_018824481.1
TRINITY_DN19518_c0_g1::TRINITY_DN19518_c0_g1_i9::g.119130::m.119130	3,00E-91	268	XP_018858776.1	IN2-2 protein-like, partial	Juglans regia	KYP64861.1
TRINITY_DN19578_c1_g1::TRINITY_DN19578_c1_g1_i2::g.120062::m.120062	0	724	XP_018843078.1	calponin homology domain-containing protein DDB_G0272472	Juglans regia	XP_018843078.1
TRINITY_DN15774_c2_g1::TRINITY_DN15774_c2_g1_i10::g.56953::m.56953	0	1817	XP_012082774.1	inositol hexakisphosphate and diphosphoinositol-pentakisphosphate kinase 1 isoform X5	Jatropha curcas	XP_012082766.1
TRINITY_DN18433_c1_g4::TRINITY_DN18433_c1_g4_i1::g.100393::m.100393	2,17E-169	474	XP_018830580.1	eukaryotic translation initiation factor 3 subunit G-A-like	Juglans regia	XP_018835374.1
TRINITY_DN12393_c0_g1::TRINITY_DN12393_c0_g1_i2::g.12483::m.12483	0	651	XP_018835130.1	uncharacterized oxidoreductase At4g09670-like	Juglans regia	XP_018835132.1
TRINITY_DN14525_c1_g1::TRINITY_DN14525_c1_g1_i2::g.38384::m.38384	0	1318	XP_018845402.1	acyl-coenzyme A oxidase 2, peroxisomal	Juglans regia	XP_009345476.1
TRINITY_DN19854_c2_g1::TRINITY_DN19854_c2_g1_i1::g.124554::m.124554	6,17E-175	516	XP_018842400.1	eukaryotic translation initiation factor 3 subunit C isoform X1	Juglans regia	XP_018842401.1
TRINITY_DN16619_c0_g1::TRINITY_DN16619_c0_g1_i7::g.70514::m.70514	0	917	XP_018835103.1	threonine synthase, chloroplastic-like	Juglans regia	XP_020964169.1
TRINITY_DN11003_c0_g3::TRINITY_DN11003_c0_g3_i1::g.6096::m.6096	3,84E-59	182	XP_018812411.1	10 kDa chaperonin-like	Juglans regia	XP_018812412.1

TRINITY_DN12512_c0_g1::TRINITY_DN12512_c0_g1_i7::g.13451::m.13451	2,09E-80	236	XP_018812440.1	uncharacterized protein LOC108984828	Juglans regia	XP_018812441.1
TRINITY_DN12275_c0_g1::TRINITY_DN12275_c0_g1_i2::g.11548::m.11548	1,51E-153	432	XP_018818651.1	40S ribosomal protein S3a-like	Juglans regia	XP_018826766.1
TRINITY_DN19290_c6_g1::TRINITY_DN19290_c6_g1_i1::g.115224::m.115224	0	822	XP_018835920.1	cysteine desulfurase, mitochondrial	Juglans regia	KRH28308.1
TRINITY_DN13198_c0_g2::TRINITY_DN13198_c0_g2_i2::g.20890::m.20890	5,58E-52	170	KDP41286.1	hypothetical protein JCGZ_15693	Jatropha curcas	AGL39707.1
TRINITY_DN16577_c0_g1::TRINITY_DN16577_c0_g1_i6::g.69863::m.69863	2,79E-148	419	ONH97761.1	hypothetical protein PRUPE_7G208200	Prunus persica	XP_007202368.1
TRINITY_DN18300_c1_g1::TRINITY_DN18300_c1_g1_i6::g.96808::m.96808	1,44E-82	244	XP_018858686.1	mitochondrial fission 1 protein A-like	Juglans regia	XP_018858687.1
TRINITY_DN14139_c1_g3::TRINITY_DN14139_c1_g3_i2::g.32774::m.32774	0	850	XP_018846328.1	asparagine--tRNA ligase, cytoplasmic 1 isoform X1	Juglans regia	XP_019436209.1
TRINITY_DN16286_c1_g1::TRINITY_DN16286_c1_g1_i5::g.65098::m.65098	0	640	XP_018817568.1	probable ADP-ribosylation factor GTPase-activating protein AGD9 isoform X1	Juglans regia	XP_018817570.1
TRINITY_DN15944_c0_g3::TRINITY_DN15944_c0_g3_i1::g.58792::m.58792	1,54E-54	171	XP_018825951.1	40S ribosomal protein S21-2-like	Juglans regia	XP_018825952.1
TRINITY_DN14872_c2_g1::TRINITY_DN14872_c2_g1_i7::g.43673::m.43673	0	950	XP_018826109.1	bifunctional 3-dehydroquinate dehydratase/shikimate dehydrogenase, chloroplastic-like isoform X1	Juglans regia	XP_018840587.1
TRINITY_DN14391_c2_g1::TRINITY_DN14391_c2_g1_i3::g.36277::m.36277	3,05E-10	59,3	XP_018832312.1	GDSL esterase/lipase At3g48460-like, partial	Juglans regia	XP_018855251.1
TRINITY_DN16836_c1_g1::TRINITY_DN16836_c1_g1_i8::g.74234::m.74234	0	2199	XP_018817814.1	cullin-associated NEDD8-dissociated protein 1	Juglans regia	OAY45096.1
TRINITY_DN17195_c1_g1::TRINITY_DN17195_c1_g1_i2::g.79932::m.79932	0	1309	XP_018848394.1	phosphatidylinositol 4-phosphate 5-kinase 4-like	Juglans regia	XP_018860125.1
TRINITY_DN15687_c3_g1::TRINITY_DN15687_c3_g1_i1::g.55726::m.55726	5,17E-87	256	XP_018816906.1	uncharacterized protein LOC108988199 isoform X2	Juglans regia	XP_018816905.1
TRINITY_DN19945_c1_g2::TRINITY_DN19945_c1_g2_i9::g.126623::m.126623	0	798	XP_018815121.1	probable plastidic glucose transporter 2	Juglans regia	KDP33696.1
TRINITY_DN16932_c1_g1::TRINITY_DN16932_c1_g1_i8::g.75833::m.75833	3,03E-80	243	XP_020218942.1	uncharacterized protein LOC109802131	Cajanus cajan	XP_020996584.1
TRINITY_DN18681_c1_g1::TRINITY_DN18681_c1_g1_i1::g.104540::m.104540	2,26E-134	383	XP_018826389.1	probable NAD(P)H dehydrogenase (quinone) FQR1-like 2	Juglans regia	OAY33932.1
TRINITY_DN15903_c3_g2::TRINITY_DN15903_c3_g2_i1::g.58748::m.58748	2,68E-49	159	XP_018818244.1	uncharacterized protein LOC108989175	Juglans regia	KDP45236.1
TRINITY_DN17228_c0_g2::TRINITY_DN17228_c0_g2_i3::g.80435::m.80435	3,84E-78	230	XP_019444292.1	60S ribosomal protein L24-like isoform X2	Lupinus angustifolius	KRH20034.1
TRINITY_DN16216_c0_g1::TRINITY_DN16216_c0_g1_i6::g.64074::m.64074	0	1790	XP_018813272.1	AP-2 complex subunit alpha-1-like	Juglans regia	XP_018833174.1
TRINITY_DN18444_c3_g2::TRINITY_DN18444_c3_g2_i4::g.100535::m.100535	0	1096	XP_018844348.1	N-alpha-acetyltransferase 16, NatA auxiliary subunit-like	Juglans regia	XP_018845079.1

TRINITY_DN12460_c3_g3::TRINITY_DN12460_c3_g3_i1::g.13367::m.13367	1,62E-57	190	XP_018831476.1	uncharacterized protein At4g06744-like	Juglans regia	XP_018859113.1
TRINITY_DN13616_c0_g1::TRINITY_DN13616_c0_g1_i3::g.26276::m.26276	3,74E-98	284	XP_018830449.1	UPF0678 fatty acid-binding protein-like protein At1g79260 isoform X1	Juglans regia	XP_018830450.1
TRINITY_DN18246_c0_g1::TRINITY_DN18246_c0_g1_i3::g.96857::m.96857	0	854	XP_018842344.1	adenylosuccinate synthetase 2, chloroplastic	Juglans regia	KDP26008.1
TRINITY_DN19723_c2_g1::TRINITY_DN19723_c2_g1_i1::g.122855::m.122855	4,85E-81	250	XP_018814264.1	phosphomethylethanolamine N-methyltransferase-like	Juglans regia	ONI09335.1
TRINITY_DN13123_c3_g2::TRINITY_DN13123_c3_g2_i7::g.20202::m.20202	3,87E-165	459	XP_018820822.1	(DL)-glycerol-3-phosphatase 2	Juglans regia	XP_008223698.1
TRINITY_DN18029_c5_g2::TRINITY_DN18029_c5_g2_i4::g.93978::m.93978	0	1050	XP_018839707.1	phospholipid:diacylglycerol acyltransferase 1 isoform X1	Juglans regia	OAY36448.1
TRINITY_DN11846_c0_g3::TRINITY_DN11846_c0_g3_i2::g.9155::m.9155	6,71E-17	79,7	XP_018806898.1	lysine--tRNA ligase, cytoplasmic isoform X2	Juglans regia	XP_018806897.1
TRINITY_DN14246_c2_g1::TRINITY_DN14246_c2_g1_i1::g.34260::m.34260	0	541	XP_018846970.1	ADP-glucose phosphorylase-like isoform X1	Juglans regia	OAY41942.1
TRINITY_DN18272_c3_g3::TRINITY_DN18272_c3_g3_i3::g.97910::m.97910	0	765	XP_018845584.1	cytokinin dehydrogenase 2-like	Juglans regia	XP_008242393.1
TRINITY_DN16015_c1_g1::TRINITY_DN16015_c1_g1_i9::g.60551::m.60551	3,54E-144	408	XP_018809603.1	syntaxin-22-like	Juglans regia	XP_018809113.1
TRINITY_DN17938_c1_g3::TRINITY_DN17938_c1_g3_i1::g.92335::m.92335	1,49E-140	406	XP_018829254.1	glutamate--cysteine ligase, chloroplastic	Juglans regia	XP_018829255.1
TRINITY_DN13436_c3_g2::TRINITY_DN13436_c3_g2_i1::g.23914::m.23914	0	696	XP_018837387.1	acetyl-CoA acetyltransferase, cytosolic 1	Juglans regia	OAY34999.1
TRINITY_DN19570_c1_g1::TRINITY_DN19570_c1_g1_i2::g.119952::m.119952	4,47E-136	388	XP_018831496.1	(-)-isopiperitenol/(-)-carveol dehydrogenase, mitochondrial-like	Juglans regia	XP_018816008.1
TRINITY_DN19058_c2_g1::TRINITY_DN19058_c2_g1_i7::g.111114::m.111114	3,56E-163	469	ONI04889.1	hypothetical protein PRUPE_6G346300	Prunus persica	XP_007208154.2
TRINITY_DN12832_c2_g7::TRINITY_DN12832_c2_g7_i2::g.16314::m.16314	5,76E-168	471	KDP24066.1	hypothetical protein JCGZ_25723	Jatropha curcas	XP_012088199.1
TRINITY_DN17735_c1_g1::TRINITY_DN17735_c1_g1_i1::g.89033::m.89033	3,91E-101	296	OAY34830.1	hypothetical protein MANES_12G050000	Manihot esculenta	KDP45282.1
TRINITY_DN10960_c0_g1::TRINITY_DN10960_c0_g1_i2::g.5906::m.5906	1,05E-138	405	XP_018823263.1	probable mediator of RNA polymerase II transcription subunit 37c	Juglans regia	XP_018823263.1
TRINITY_DN13066_c0_g1::TRINITY_DN13066_c0_g1_i8::g.19396::m.19396	9,47E-144	402	XP_008237447.1	ras-related protein Rab7	Prunus mume	ONH90275.1
TRINITY_DN12323_c0_g2::TRINITY_DN12323_c0_g2_i9::g.11969::m.11969	0	873	XP_018846853.1	RAN GTPase-activating protein 2	Juglans regia	XP_018846854.1
TRINITY_DN18848_c2_g2::TRINITY_DN18848_c2_g2_i6::g.107389::m.107389	1,27E-43	152	XP_019452405.1	ubiquitin receptor RAD23c-like isoform X5	Lupinus angustifolius	XP_019441388.1
TRINITY_DN19795_c3_g1::TRINITY_DN19795_c3_g1_i8::g.123663::m.123663	3,07E-56	174	XP_018852827.1	V-type proton ATPase subunit G-like	Juglans regia	XP_018852828.1
TRINITY_DN17387_c1_g1::TRINITY_DN17387_c1_g1_i3::g.82928::m.82928	0	2407	XP_018834376.1	clustered mitochondria protein	Juglans regia	XP_008231340.1

TRINITY_DN13841_c4_g1::TRINITY_DN13841_c4_g1_i7::g.28424::m.28424	0	711	XP_018816748.1	protein DJ-1 homolog D-like	Juglans regia	XP_018816749.1
TRINITY_DN12837_c2_g2::TRINITY_DN12837_c2_g2_i2::g.16573::m.16573	1,38E-113	336	XP_018847365.1	probable polygalacturonase	Juglans regia	XP_018847358.1
TRINITY_DN16782_c2_g2::TRINITY_DN16782_c2_g2_i4::g.73167::m.73167	5,83E-62	200	KDP43847.1	hypothetical protein JCGZ_20857	Jatropha curcas	XP_012065174.1
TRINITY_DN18666_c0_g1::TRINITY_DN18666_c0_g1_i7::g.104181::m.104181	4,75E-113	332	XP_018815210.1	UDP-glucuronic acid decarboxylase 2-like	Juglans regia	XP_008386777.1
TRINITY_DN19251_c1_g2::TRINITY_DN19251_c1_g2_i27::g.114997::m.114997	2,28E-119	352	XP_018851524.1	mitogen-activated protein kinase 9-like isoform X2	Juglans regia	XP_018851523.1
TRINITY_DN13809_c0_g1::TRINITY_DN13809_c0_g1_i2::g.28550::m.28550	0	717	XP_018829285.1	tyrosine--tRNA ligase 1, cytoplasmic	Juglans regia	XP_007156912.1
TRINITY_DN19468_c2_g2::TRINITY_DN19468_c2_g2_i2::g.118324::m.118324	2,81E-113	331	KOM53769.1	hypothetical protein LR48_Vigan09g242800	Vigna angularis	XP_018852431.1
TRINITY_DN16852_c0_g1::TRINITY_DN16852_c0_g1_i5::g.74328::m.74328	1,07E-86	256	XP_018849247.1	ribulose-phosphate 3-epimerase, cytoplasmic isoform	Juglans regia	XP_008341322.1
TRINITY_DN16408_c1_g1::TRINITY_DN16408_c1_g1_i1::g.67131::m.67131	0	853	XP_018844988.1	nicotinate phosphoribosyltransferase 1	Juglans regia	XP_008241476.1
TRINITY_DN11560_c0_g1::TRINITY_DN11560_c0_g1_i1::g.7865::m.7865	0	678	XP_018830668.1	sorbitol dehydrogenase	Juglans regia	OAY48230.1
TRINITY_DN19263_c0_g1::TRINITY_DN19263_c0_g1_i6::g.114813::m.114813	0	535	XP_018837044.1	probable cytosolic oligopeptidase A	Juglans regia	XP_018850785.1
TRINITY_DN19101_c2_g2::TRINITY_DN19101_c2_g2_i4::g.111950::m.111950	0	880	XP_018808839.1	pyridoxine/pyridoxamine 5'-phosphate oxidase 1, chloroplastic isoform X1	Juglans regia	XP_018808840.1
TRINITY_DN19615_c0_g1::TRINITY_DN19615_c0_g1_i7::g.122149::m.122149	0	1467	XP_018841554.1	actin cytoskeleton-regulatory complex protein PAN1-like isoform X1	Juglans regia	XP_018841555.1
TRINITY_DN18862_c1_g2::TRINITY_DN18862_c1_g2_i1::g.107502::m.107502	0	704	XP_018837796.1	argininosuccinate lyase, chloroplastic	Juglans regia	XP_009350549.1
TRINITY_DN14909_c1_g5::TRINITY_DN14909_c1_g5_i1::g.44180::m.44180	0	1631	XP_018828503.1	protein TIC110, chloroplastic isoform X1	Juglans regia	XP_018828504.1
TRINITY_DN17699_c0_g2::TRINITY_DN17699_c0_g2_i2::g.88416::m.88416	0	3119	XP_018826298.1	ferredoxin-dependent glutamate synthase, chloroplastic-like isoform X1	Juglans regia	XP_018848396.1
TRINITY_DN11338_c0_g1::TRINITY_DN11338_c0_g1_i3::g.7037::m.7037	0	793	XP_018827777.1	carbamoyl-phosphate synthase small chain, chloroplastic	Juglans regia	XP_009364736.1
TRINITY_DN17067_c2_g2::TRINITY_DN17067_c2_g2_i1::g.77907::m.77907	0	568	XP_008381007.1	dihydrolipoyllysine-residue acetyltransferase component 5 of pyruvate dehydrogenase complex, chloroplastic	Malus domestica	OAY58590.1
TRINITY_DN17136_c2_g3::TRINITY_DN17136_c2_g3_i6::g.79230::m.79230	8,16E-85	249	ONH97139.1	hypothetical protein PRUPE_7G171700	Prunus persica	ACM68949.1
TRINITY_DN15761_c1_g2::TRINITY_DN15761_c1_g2_i2::g.56714::m.56714	0	863	OAY48299.1	hypothetical protein MANES_06G147900	Manihot esculenta	XP_008234243.1
TRINITY_DN18490_c1_g1::TRINITY_DN18490_c1_g1_i2::g.101177::m.101177	0	736	ADZ96378.1	hexokinase 1	Eriobotrya japonica	ONI15732.1

TRINITY_DN11842_c0_g1::TRINITY_DN11842_c0_g1_i5::g.9125::m.9125	0	941	XP_018808643.1	serine/threonine-protein phosphatase 5 isoform X2	Juglans regia	XP_012084998.1
TRINITY_DN14684_c2_g1::TRINITY_DN14684_c2_g1_i9::g.40676::m.40676	5,19E-149	416	XP_018810806.1	adenylyl-sulfate kinase 3	Juglans regia	XP_018810807.1
TRINITY_DN17978_c0_g1::TRINITY_DN17978_c0_g1_i12::g.93003::m.93003	0	681	XP_018847824.1	phenylalanine--tRNA ligase alpha subunit, cytoplasmic	Juglans regia	XP_014494097.1
TRINITY_DN14251_c1_g2::TRINITY_DN14251_c1_g2_i7::g.34536::m.34536	8,83E-156	438	XP_018837663.1	HD domain-containing protein C4G3.17	Juglans regia	ONI31245.1
TRINITY_DN15354_c0_g1::TRINITY_DN15354_c0_g1_i1::g.50622::m.50622	0	1526	XP_018857605.1	urease	Juglans regia	XP_018857607.1
TRINITY_DN14014_c3_g1::TRINITY_DN14014_c3_g1_i1::g.31362::m.31362	7,58E-24	98,6	XP_012069043.1	uncharacterized protein LOC105631511	Jatropha curcas	KDP43847.1
TRINITY_DN14788_c0_g2::TRINITY_DN14788_c0_g2_i4::g.42257::m.42257	6,41E-103	301	XP_018825937.1	GDSL esterase/lipase CPRD49-like	Juglans regia	AFK45704.1
TRINITY_DN14955_c4_g2::TRINITY_DN14955_c4_g2_i6::g.44900::m.44900	1,08E-121	352	XP_018839624.1	probable calcium-binding protein CML49	Juglans regia	XP_018808876.1
TRINITY_DN10990_c0_g1::TRINITY_DN10990_c0_g1_i1::g.6030::m.6030	0	526	XP_018821285.1	triose phosphate/phosphate translocator, non-green plastid, chloroplastic-like	Juglans regia	XP_008239775.1
TRINITY_DN10710_c0_g1::TRINITY_DN10710_c0_g1_i1::g.5355::m.5355	1,93E-121	348	XP_018820917.1	multiple organellar RNA editing factor 2, chloroplastic-like	Juglans regia	OAY35808.1
TRINITY_DN17874_c2_g4::TRINITY_DN17874_c2_g4_i3::g.91179::m.91179	0	1060	XP_018839574.1	transmembrane 9 superfamily member 8-like	Juglans regia	ONI32921.1
TRINITY_DN16487_c1_g6::TRINITY_DN16487_c1_g6_i1::g.68386::m.68386	1,61E-53	180	ONI33944.1	hypothetical protein PRUPE_1G455000	Prunus persica	XP_020410106.1
TRINITY_DN19704_c0_g1::TRINITY_DN19704_c0_g1_i3::g.122553::m.122553	1,03E-89	265	OAY52558.1	hypothetical protein MANES_04G093300	Manihot esculenta	KOM26552.1
TRINITY_DN15899_c1_g1::TRINITY_DN15899_c1_g1_i1::g.58501::m.58501	2,93E-88	285	XP_018809774.1	protein transport protein SEC31 homolog B-like isoform X1	Juglans regia	XP_018809776.1
TRINITY_DN13705_c0_g2::TRINITY_DN13705_c0_g2_i4::g.27444::m.27444	0	531	ONH98930.1	hypothetical protein PRUPE_6G000600	Prunus persica	ONH98930.1
TRINITY_DN18855_c1_g1::TRINITY_DN18855_c1_g1_i19::g.107600::m.107600	0	1277	XP_018836237.1	probable sucrose-phosphate synthase 1	Juglans regia	ABV32550.1
TRINITY_DN17745_c1_g5::TRINITY_DN17745_c1_g5_i2::g.88501::m.88501	5,55E-33	115	XP_018859166.1	60S acidic ribosomal protein P1-like	Juglans regia	XP_018842776.1
TRINITY_DN10578_c0_g1::TRINITY_DN10578_c0_g1_i5::g.5050::m.5050	0	867	XP_018835696.1	DEAD-box ATP-dependent RNA helicase 52A-like isoform X1	Juglans regia	OAY24033.1
TRINITY_DN13945_c0_g1::TRINITY_DN13945_c0_g1_i2::g.30107::m.30107	0	516	XP_018840568.1	probable prolyl 4-hydroxylase 4	Juglans regia	KDP40054.1
TRINITY_DN19655_c2_g1::TRINITY_DN19655_c2_g1_i11::g.121761::m.121761	0	619	XP_018841063.1	26S protease regulatory subunit 8 homolog A	Juglans regia	OAY47083.1
TRINITY_DN9772_c0_g1::TRINITY_DN9772_c0_g1_i1::g.3863::m.3863	1,01E-60	185	XP_018826159.1	protein SLE1	Juglans regia	OAY41498.1
TRINITY_DN14598_c1_g3::TRINITY_DN14598_c1_g3_i4::g.39295::m.39295	0	698	XP_018830095.1	glycerol-3-phosphate acyltransferase, chloroplastic isoform X2	Juglans regia	XP_018830093.1

TRINITY_DN13423_c2_g1::TRINITY_DN13423_c2_g1_i2::g.23680::m.23680	4,85E-88	271	XP_018808540.1	TBCC domain-containing protein 1-like	Juglans regia	XP_018843632.1
TRINITY_DN13846_c2_g2::TRINITY_DN13846_c2_g2_i2::g.28548::m.28548	5,50E-148	413	XP_018844705.1	proteasome subunit beta type-3-A	Juglans regia	XP_016203278.1
TRINITY_DN13334_c3_g4::TRINITY_DN13334_c3_g4_i1::g.22910::m.22910	4,06E-97	298	XP_017180254.1	subtilisin-like protease SBT5.4	Malus domestica	XP_008353281.1
TRINITY_DN17504_c0_g1::TRINITY_DN17504_c0_g1_i2::g.84827::m.84827	8,29E-86	250	XP_018834779.1	probable histone H2A variant 3	Juglans regia	OAY31632.1
TRINITY_DN16041_c1_g2::TRINITY_DN16041_c1_g2_i6::g.61105::m.61105	0	520	XP_018824063.1	tryptophan synthase beta chain 1	Juglans regia	XP_018824072.1
TRINITY_DN13569_c0_g2::TRINITY_DN13569_c0_g2_i1::g.25677::m.25677	1,78E-179	501	XP_018827184.1	ABC transporter I family member 19-like	Juglans regia	AIU41666.1
TRINITY_DN11464_c0_g1::TRINITY_DN11464_c0_g1_i3::g.7465::m.7465	0	1373	XP_018843439.1	ALG-2 interacting protein X-like	Juglans regia	XP_018849759.1
TRINITY_DN15585_c1_g1::TRINITY_DN15585_c1_g1_i8::g.54308::m.54308	1,91E-69	213	XP_018810087.1	GDSL esterase/lipase CPRD49-like	Juglans regia	XP_018810088.1
TRINITY_DN15428_c6_g1::TRINITY_DN15428_c6_g1_i5::g.51800::m.51800	1,69E-78	233	XP_018835348.1	uncharacterized protein LOC109002166	Juglans regia	XP_018816039.1
TRINITY_DN18097_c14_g3::TRINITY_DN18097_c14_g3_i5::g.95141::m.95141	0	740	XP_018844419.1	3-oxoacyl-[acyl-carrier-protein synthase 3 A, chloroplastic-like	Juglans regia	XP_018848180.1
TRINITY_DN12611_c0_g2::TRINITY_DN12611_c0_g2_i7::g.14237::m.14237	3,40E-157	442	XP_018833326.1	protein-L-isoaspartate O-methyltransferase 1-like	Juglans regia	XP_018814971.1
TRINITY_DN14738_c2_g1::TRINITY_DN14738_c2_g1_i2::g.41499::m.41499	0	1063	XP_018827500.1	acetolactate synthase 2, chloroplastic	Juglans regia	XP_008376595.1
TRINITY_DN14357_c5_g2::TRINITY_DN14357_c5_g2_i13::g.35817::m.35817	1,67E-66	205	XP_007137680.1	hypothetical protein PHAVU_009G146600g	Phaseolus vulgaris	ESW09674.1
TRINITY_DN16879_c1_g4::TRINITY_DN16879_c1_g4_i3::g.74866::m.74866	0	1537	XP_018809958.1	chaperone protein ClpB1	Juglans regia	XP_015943335.1
TRINITY_DN16280_c0_g1::TRINITY_DN16280_c0_g1_i6::g.65080::m.65080	2,97E-161	454	AES76041.2	glutathione S-transferase	Medicago truncatula	XP_003619823.2
TRINITY_DN13851_c1_g1::TRINITY_DN13851_c1_g1_i8::g.29304::m.29304	0	900	XP_018811868.1	aspartic proteinase-like isoform X2	Juglans regia	XP_018811875.1
TRINITY_DN19385_c2_g1::TRINITY_DN19385_c2_g1_i11::g.117072::m.117072	8,62E-132	393	XP_018812895.1	uncharacterized protein LOC108985160 isoform X4	Juglans regia	XP_018812894.1
TRINITY_DN14233_c0_g1::TRINITY_DN14233_c0_g1_i2::g.34181::m.34181	0	865	XP_018807730.1	3-phosphoshikimate 1-carboxyvinyltransferase 2	Juglans regia	ONI22671.1
TRINITY_DN9198_c0_g1::TRINITY_DN9198_c0_g1_i1::g.3186::m.3186	0	800	XP_018813681.1	cell division protein FtsZ homolog 2-2, chloroplastic	Juglans regia	XP_018813682.1
TRINITY_DN15338_c2_g1::TRINITY_DN15338_c2_g1_i4::g.50559::m.50559	2,51E-106	303	OIV92449.1	hypothetical protein TanjilG_02212	Lupinus angustifolius	XP_019425255.1
TRINITY_DN16719_c0_g1::TRINITY_DN16719_c0_g1_i6::g.72195::m.72195	2,69E-94	292	XP_018822459.1	uncharacterized protein LOC108992363 isoform X5	Juglans regia	XP_018822460.1
TRINITY_DN18047_c0_g1::TRINITY_DN18047_c0_g1_i1::g.93485::m.93485	0	756	XP_018850838.1	MICOS complex subunit MIC60 isoform X1	Juglans regia	XP_018850839.1

TRINITY_DN12093_c0_g1::TRINITY_DN12093_c0_g1_i8::g.10458::m.10458	0	957	XP_018827269.1	glutathione reductase, chloroplastic-like	Juglans regia	XP_018827270.1
TRINITY_DN9933_c0_g1::TRINITY_DN9933_c0_g1_i1::g.4060::m.4060	8,73E-154	436	ONI24059.1	hypothetical protein PRUPE_2G221700	Prunus persica	XP_007218731.1
TRINITY_DN17539_c0_g2::TRINITY_DN17539_c0_g2_i3::g.85610::m.85610	0	1326	XP_018855488.1	protein TOC75-3, chloroplastic-like	Juglans regia	XP_008356781.1
TRINITY_DN12352_c0_g2::TRINITY_DN12352_c0_g2_i4::g.12096::m.12096	1,14E-149	432	XP_018844632.1	vacuolar-processing enzyme-like	Juglans regia	XP_018844633.1
TRINITY_DN12828_c0_g1::TRINITY_DN12828_c0_g1_i2::g.16430::m.16430	6,84E-80	238	XP_017185326.1	ras-related protein RABF1 isoform X2	Malus domestica	XP_008362670.1
TRINITY_DN17150_c2_g1::TRINITY_DN17150_c2_g1_i7::g.79332::m.79332	8,66E-172	477	XP_018824212.1	proteasome subunit alpha type-4	Juglans regia	XP_008371922.1
TRINITY_DN15988_c0_g1::TRINITY_DN15988_c0_g1_i7::g.59999::m.59999	0	693	XP_018832925.1	protein ABC transporter 1, mitochondrial	Juglans regia	OAY48812.1
TRINITY_DN19643_c4_g1::TRINITY_DN19643_c4_g1_i10::g.121539::m.121539	0	728	XP_018838778.1	L-ascorbate oxidase-like	Juglans regia	XP_018850832.1
TRINITY_DN14785_c0_g1::TRINITY_DN14785_c0_g1_i8::g.42289::m.42289	0	606	XP_018810986.1	protein disulfide-isomerase 5-3-like	Juglans regia	XP_009378812.1
TRINITY_DN16927_c0_g1::TRINITY_DN16927_c0_g1_i2::g.75647::m.75647	2,40E-142	402	XP_018805885.1	derlin-2.2-like	Juglans regia	XP_018807331.1
TRINITY_DN18066_c0_g3::TRINITY_DN18066_c0_g3_i5::g.94429::m.94429	3,60E-117	333	XP_018815941.1	malignant T-cell-amplified sequence 1 homolog	Juglans regia	OAY44020.1
TRINITY_DN15763_c0_g1::TRINITY_DN15763_c0_g1_i1::g.56716::m.56716	0	1172	OAY34927.1	hypothetical protein MANES_12G058500	Manihot esculenta	XP_012084017.1
TRINITY_DN17933_c0_g2::TRINITY_DN17933_c0_g2_i1::g.92168::m.92168	8,98E-75	222	XP_018852394.1	cytochrome b5	Juglans regia	OAY59358.1
TRINITY_DN16282_c1_g3::TRINITY_DN16282_c1_g3_i1::g.64973::m.64973	0	915	XP_008386115.1	phospho-2-dehydro-3-deoxyheptonate aldolase 1, chloroplastic-like	Malus domestica	ALE18236.1
TRINITY_DN10432_c0_g1::TRINITY_DN10432_c0_g1_i2::g.4807::m.4807	1,20E-83	245	XP_008235643.1	60S ribosomal protein L14-1	Prunus mume	ONH93236.1
TRINITY_DN18168_c0_g1::TRINITY_DN18168_c0_g1_i2::g.96274::m.96274	2,00E-09	58,2	XP_018827742.1	protein starmaker isoform X1	Juglans regia	XP_018827747.1
TRINITY_DN14775_c0_g2::TRINITY_DN14775_c0_g2_i2::g.42036::m.42036	0	804	XP_018811216.1	multiple inositol polyphosphate phosphatase 1	Juglans regia	XP_008233804.1
TRINITY_DN18002_c1_g3::TRINITY_DN18002_c1_g3_i1::g.93438::m.93438	0	795	XP_018852306.1	endoglucanase 10	Juglans regia	OAY50597.1
TRINITY_DN16157_c0_g1::TRINITY_DN16157_c0_g1_i7::g.62500::m.62500	0	926	XP_018829077.1	sterol 14-demethylase	Juglans regia	XP_018841630.1
TRINITY_DN13818_c0_g2::TRINITY_DN13818_c0_g2_i1::g.28967::m.28967	0	765	XP_018857848.1	ATP-citrate synthase alpha chain protein 2	Juglans regia	XP_018805227.1
TRINITY_DN11548_c0_g1::TRINITY_DN11548_c0_g1_i1::g.7714::m.7714	0	727	XP_018818640.1	uncharacterized protein LOC108989478	Juglans regia	XP_016171978.1
TRINITY_DN17145_c0_g1::TRINITY_DN17145_c0_g1_i4::g.79323::m.79323	0	1529	XP_018835615.1	clathrin heavy chain 2	Juglans regia	XP_018819181.1

TRINITY_DN17214_c1_g1::TRINITY_DN17214_c1_g1_i9::g.80183::m.80183	0	521	XP_018836625.1	tryptophan--tRNA ligase, cytoplasmic	Juglans regia	XP_019462109.1
TRINITY_DN19434_c1_g1::TRINITY_DN19434_c1_g1_i6::g.117945::m.117945	2,17E-99	290	XP_018840400.1	syntaxin-61-like	Juglans regia	XP_018840401.1
TRINITY_DN11638_c0_g1::TRINITY_DN11638_c0_g1_i1::g.8051::m.8051	0	657	XP_008233415.1	uncharacterized aarF domain-containing protein kinase At1g79600, chloroplastic	Prunus mume	ONI05293.1
TRINITY_DN12478_c4_g4::TRINITY_DN12478_c4_g4_i5::g.13332::m.13332	1,81E-65	213	XP_018836693.1	uncharacterized protein LOC109003142	Juglans regia	XP_018836693.1
TRINITY_DN16700_c3_g3::TRINITY_DN16700_c3_g3_i4::g.71827::m.71827	5,89E-71	216	XP_016176378.1	40S ribosomal protein S8	Arachis ipaensis	XP_014496519.1
TRINITY_DN14707_c0_g2::TRINITY_DN14707_c0_g2_i9::g.40985::m.40985	0	823	XP_018837001.1	RAN GTPase-activating protein 1	Juglans regia	XP_018837002.1
TRINITY_DN19896_c2_g1::TRINITY_DN19896_c2_g1_i4::g.125437::m.125437	8,53E-169	495	XP_018842420.1	uncharacterized protein LOC109007267	Juglans regia	XP_018842421.1
TRINITY_DN13645_c2_g2::TRINITY_DN13645_c2_g2_i6::g.26141::m.26141	1,18E-84	254	XP_018851775.1	ribonuclease P protein subunit p25-like protein	Juglans regia	KYP44772.1
TRINITY_DN11142_c1_g1::TRINITY_DN11142_c1_g1_i2::g.6468::m.6468	4,79E-134	379	XP_018811802.1	eukaryotic translation initiation factor 3 subunit K-like	Juglans regia	KDP33976.1
TRINITY_DN15069_c1_g1::TRINITY_DN15069_c1_g1_i6::g.46552::m.46552	1,93E-52	169	XP_018809557.1	rho GDP-dissociation inhibitor 1-like isoform X1	Juglans regia	XP_018809558.1
TRINITY_DN18921_c0_g1::TRINITY_DN18921_c0_g1_i2::g.108916::m.108916	0	1135	XP_018809732.1	SEC1 family transport protein SLY1-like	Juglans regia	OAY57009.1
TRINITY_DN19606_c0_g1::TRINITY_DN19606_c0_g1_i4::g.121009::m.121009	8,82E-134	384	XP_018842448.1	cysteine synthase	Juglans regia	XP_018842449.1
TRINITY_DN15436_c5_g1::TRINITY_DN15436_c5_g1_i4::g.51870::m.51870	0	606	XP_018839647.1	eukaryotic translation initiation factor 3 subunit A-like	Juglans regia	XP_018839648.1
TRINITY_DN18704_c0_g1::TRINITY_DN18704_c0_g1_i1::g.104889::m.104889	0	844	XP_018843891.1	DEAD-box ATP-dependent RNA helicase 53-like	Juglans regia	XP_008229751.1
TRINITY_DN11290_c1_g1::TRINITY_DN11290_c1_g1_i4::g.6884::m.6884	4,84E-75	223	XP_018841009.1	uncharacterized protein At2g34160-like	Juglans regia	XP_018858938.1
TRINITY_DN17528_c1_g2::TRINITY_DN17528_c1_g2_i6::g.85342::m.85342	0	1179	XP_018824324.1	dynamin-related protein 5A	Juglans regia	XP_018857541.1
TRINITY_DN15505_c1_g1::TRINITY_DN15505_c1_g1_i3::g.53026::m.53026	1,13E-37	134	XP_018828132.1	maltose excess protein 1-like, chloroplastic isoform X2	Juglans regia	XP_018828131.1
TRINITY_DN11648_c0_g3::TRINITY_DN11648_c0_g3_i1::g.8240::m.8240	1,36E-59	183	XP_018823473.1	prefoldin subunit 1	Juglans regia	OAY58676.1
TRINITY_DN14322_c1_g1::TRINITY_DN14322_c1_g1_i1::g.35379::m.35379	0	818	XP_018809613.1	alanine--glyoxylate aminotransferase 2 homolog 1, mitochondrial	Juglans regia	OAY33360.1
TRINITY_DN19908_c2_g1::TRINITY_DN19908_c2_g1_i5::g.125911::m.125911	1,72E-119	356	XP_018811830.1	exocyst complex component EXO70B1	Juglans regia	ONI29902.1
TRINITY_DN19181_c1_g1::TRINITY_DN19181_c1_g1_i4::g.113426::m.113426	0	542	XP_018857004.1	4-hydroxyphenylpyruvate dioxygenase	Juglans regia	BAH10638.1
TRINITY_DN16453_c2_g1::TRINITY_DN16453_c2_g1_i14::g.67807::m.67807	0	892	XP_018819043.1	xaa-Pro dipeptidase	Juglans regia	XP_008242428.1

TRINITY_DN12583_c1_g3::TRINITY_DN12583_c1_g3_i1::g.14008::m.14008	0	554	OAY29057.1	hypothetical protein MANES_15G114600	Manihot esculenta	AMK38013.1
TRINITY_DN18832_c0_g1::TRINITY_DN18832_c0_g1_i1::g.107098::m.107098	9,14E-117	333	XP_018850349.1	temperature-induced lipocalin-1	Juglans regia	XP_018850350.1
TRINITY_DN14291_c3_g7::TRINITY_DN14291_c3_g7_i2::g.35143::m.35143	1,92E-118	341	XP_018828853.1	probable acetyltransferase NATA1-like	Juglans regia	OIW01482.1
TRINITY_DN19997_c3_g2::TRINITY_DN19997_c3_g2_i2::g.127405::m.127405	1,27E-72	221	GAU13824.1	hypothetical protein TSUD_261510	Trifolium subterraneum	KYP36617.1
TRINITY_DN13437_c3_g3::TRINITY_DN13437_c3_g3_i4::g.23371::m.23371	0	970	XP_019463977.1	transmembrane 9 superfamily member 3-like	Lupinus angustifolius	KHN28892.1
TRINITY_DN16324_c2_g1::TRINITY_DN16324_c2_g1_i11::g.65876::m.65876	0	1330	XP_018835648.1	6-phosphofructo-2-kinase/fructose-2,6-bisphosphatase-like isoform X1	Juglans regia	XP_018819189.1
TRINITY_DN14723_c2_g4::TRINITY_DN14723_c2_g4_i2::g.41268::m.41268	0	638	XP_018838198.1	uncharacterized protein LOC109004190	Juglans regia	XP_018829345.1
TRINITY_DN12136_c0_g1::TRINITY_DN12136_c0_g1_i2::g.10688::m.10688	0	774	XP_018833108.1	cysteine desulfurase 1, chloroplastic	Juglans regia	XP_012076008.1
TRINITY_DN19231_c4_g1::TRINITY_DN19231_c4_g1_i8::g.114359::m.114359	0	958	AIR95612.1	4-coumarate:CoA ligase-like protein	Betula pendula	XP_018858801.1
TRINITY_DN15458_c1_g3::TRINITY_DN15458_c1_g3_i6::g.52102::m.52102	1,19E-101	298	XP_018842307.1	ATP-dependent Clp protease proteolytic subunit 5, chloroplastic	Juglans regia	OIW12999.1
TRINITY_DN15082_c1_g1::TRINITY_DN15082_c1_g1_i1::g.46708::m.46708	0	1021	XP_018823898.1	L-ascorbate oxidase homolog	Juglans regia	OAY52251.1
TRINITY_DN19344_c5_g1::TRINITY_DN19344_c5_g1_i3::g.115614::m.115614	9,98E-33	128	XP_018845634.1	suppressor protein SRP40-like	Juglans regia	XP_008219941.1
TRINITY_DN14912_c1_g1::TRINITY_DN14912_c1_g1_i4::g.44268::m.44268	2,88E-164	464	XP_018839982.1	phosphoglycolate phosphatase 1B, chloroplastic-like	Juglans regia	KRH00963.1
TRINITY_DN13129_c8_g5::TRINITY_DN13129_c8_g5_i1::g.20297::m.20297	0	874	XP_018812297.1	sorting and assembly machinery component 50 homolog B-like	Juglans regia	XP_018821909.1
TRINITY_DN12983_c5_g1::TRINITY_DN12983_c5_g1_i4::g.18189::m.18189	0	669	XP_018835689.1	26S protease regulatory subunit 6B homolog	Juglans regia	XP_018828576.1
TRINITY_DN13073_c0_g1::TRINITY_DN13073_c0_g1_i2::g.19364::m.19364	0	1022	XP_018850821.1	inositol-3-phosphate synthase	Juglans regia	XP_018850822.1
TRINITY_DN14563_c5_g1::TRINITY_DN14563_c5_g1_i5::g.38974::m.38974	7,98E-116	334	XP_018854482.1	glucose-6-phosphate/phosphate translocator 1, chloroplastic-like	Juglans regia	XP_018854483.1
TRINITY_DN15029_c4_g1::TRINITY_DN15029_c4_g1_i3::g.45979::m.45979	5,53E-82	242	XP_018849696.1	probable NADH dehydrogenase [ubiquinone] 1 alpha subcomplex subunit 12	Juglans regia	XP_008234575.1
TRINITY_DN16123_c0_g2::TRINITY_DN16123_c0_g2_i3::g.62404::m.62404	0	826	XP_018812654.1	eukaryotic initiation factor 4A-3	Juglans regia	XP_018826866.1
TRINITY_DN13128_c0_g1::TRINITY_DN13128_c0_g1_i1::g.20153::m.20153	0	826	XP_018844115.1	DEAD-box ATP-dependent RNA helicase 38	Juglans regia	KDP29205.1
TRINITY_DN11467_c0_g1::TRINITY_DN11467_c0_g1_i3::g.7480::m.7480	0	1010	XP_018833292.1	uncharacterized protein LOC109000757	Juglans regia	OAY35008.1
TRINITY_DN16134_c1_g1::TRINITY_DN16134_c1_g1_i3::g.62771::m.62771	0	540	XP_018816315.1	ABC transporter I family member 20-like isoform X1	Juglans regia	AIU41667.1

TRINITY_DN12648_c0_g1::TRINITY_DN12648_c0_g1_i4::g.15036::m.15036	2,58E-149	416	XP_018847846.1	ras-related protein Rab7 isoform X1	Juglans regia	XP_018847847.1
TRINITY_DN17776_c1_g1::TRINITY_DN17776_c1_g1_i2::g.89554::m.89554	8,76E-70	216	XP_018835592.1	probable carboxylesterase 5	Juglans regia	KDP45172.1
TRINITY_DN17310_c0_g2::TRINITY_DN17310_c0_g2_i2::g.81838::m.81838	9,68E-134	390	XP_018845068.1	nuclear pore complex protein NUP50A-like	Juglans regia	XP_018845069.1
TRINITY_DN18211_c0_g1::TRINITY_DN18211_c0_g1_i1::g.96953::m.96953	1,84E-103	312	XP_018846385.1	probable nucleoredoxin 1	Juglans regia	XP_018846385.1
TRINITY_DN16650_c2_g2::TRINITY_DN16650_c2_g2_i4::g.71040::m.71040	9,15E-123	352	XP_018813211.1	uncharacterized protein LOC108985386 isoform X1	Juglans regia	AFK37701.1
TRINITY_DN17333_c5_g3::TRINITY_DN17333_c5_g3_i2::g.82453::m.82453	5,80E-148	419	XP_018826593.1	NAP1-related protein 2	Juglans regia	XP_018833970.1
TRINITY_DN12530_c0_g1::TRINITY_DN12530_c0_g1_i1::g.13568::m.13568	1,51E-68	213	XP_018833432.1	nascent polypeptide-associated complex subunit alpha-like protein 2	Juglans regia	XP_018842755.1
TRINITY_DN15837_c1_g2::TRINITY_DN15837_c1_g2_i3::g.57761::m.57761	0	791	XP_018830113.1	glutamate-1-semialdehyde 2,1-aminomutase 2, chloroplastic	Juglans regia	OAY47995.1
TRINITY_DN20022_c2_g5::TRINITY_DN20022_c2_g5_i1::g.128013::m.128013	2,56E-166	469	XP_018843798.1	OTU domain-containing protein 6B	Juglans regia	XP_018843799.1
TRINITY_DN18115_c1_g1::TRINITY_DN18115_c1_g1_i4::g.95413::m.95413	2,50E-101	296	KRH40130.1	hypothetical protein GLYMA_09G240300	Glycine max	KHN11589.1
TRINITY_DN15805_c1_g2::TRINITY_DN15805_c1_g2_i2::g.57262::m.57262	0	2385	XP_018813899.1	proteasome activator subunit 4-like	Juglans regia	KDP36327.1
TRINITY_DN15457_c3_g1::TRINITY_DN15457_c3_g1_i1::g.52194::m.52194	2,78E-14	67,4	XP_018856978.1	uncharacterized protein LOC109019191 isoform X2	Juglans regia	XP_018856977.1
TRINITY_DN14777_c1_g1::TRINITY_DN14777_c1_g1_i9::g.42089::m.42089	0	946	XP_018819005.1	glutamate--glyoxylate aminotransferase 2	Juglans regia	XP_018813477.1
TRINITY_DN13637_c0_g1::TRINITY_DN13637_c0_g1_i7::g.26541::m.26541	7,90E-14	69,7	ONI32249.1	hypothetical protein PRUPE_1G356400	Prunus persica	XP_007225997.1
TRINITY_DN13683_c1_g1::TRINITY_DN13683_c1_g1_i4::g.27154::m.27154	0	1169	XP_018840095.1	quinolinate synthase, chloroplastic	Juglans regia	ALP70495.1
TRINITY_DN13930_c0_g1::TRINITY_DN13930_c0_g1_i1::g.30435::m.30435	0	593	XP_008220830.1	ornithine carbamoyltransferase, chloroplastic	Prunus mume	ONI32617.1
TRINITY_DN12625_c0_g2::TRINITY_DN12625_c0_g2_i2::g.14331::m.14331	0	910	XP_018828658.1	BTB/POZ domain-containing protein At5g03250-like	Juglans regia	XP_018844832.1
TRINITY_DN14916_c3_g2::TRINITY_DN14916_c3_g2_i1::g.44503::m.44503	0	578	XP_018821750.1	26S proteasome non-ATPase regulatory subunit 7 homolog A	Juglans regia	KHN34740.1
TRINITY_DN16821_c2_g1::TRINITY_DN16821_c2_g1_i2::g.73867::m.73867	0	1735	XP_018808675.1	protein argonaute 1-like	Juglans regia	XP_018808676.1
TRINITY_DN18229_c0_g2::TRINITY_DN18229_c0_g2_i1::g.97234::m.97234	4,39E-82	245	XP_009345067.1	caffeoyl-CoA O-methyltransferase	Pyrus x bretschneideri	XP_009345068.1
TRINITY_DN19026_c0_g3::TRINITY_DN19026_c0_g3_i5::g.110636::m.110636	0	1281	XP_018825566.1	cellulose synthase-like protein D4	Juglans regia	XP_018826134.1
TRINITY_DN19212_c1_g1::TRINITY_DN19212_c1_g1_i8::g.114114::m.114114	1,58E-146	432	XP_018811352.1	heat shock 70 kDa protein 16-like	Juglans regia	XP_018811359.1

TRINITY_DN12118_c0_g1::TRINITY_DN12118_c0_g1_i1::g.10631::m.10631	0	872	ONH91092.1	hypothetical protein PRUPE_8G092300	Prunus persica	XP_007199807.1
TRINITY_DN18618_c2_g3::TRINITY_DN18618_c2_g3_i2::g.103853::m.103853	1,24E-105	335	XP_018830702.1	LOW QUALITY PROTEIN: tripeptidyl-peptidase 2-like	Juglans regia	XP_018828368.1
TRINITY_DN17695_c2_g1::TRINITY_DN17695_c2_g1_i3::g.88380::m.88380	0	3508	XP_018844516.1	DNA polymerase epsilon catalytic subunit A-like	Juglans regia	XP_012075886.1
TRINITY_DN14310_c2_g3::TRINITY_DN14310_c2_g3_i5::g.35345::m.35345	3,28E-89	272	XP_018846976.1	LOW QUALITY PROTEIN: apyrase 1-like	Juglans regia	GAU47627.1
TRINITY_DN16144_c0_g3::TRINITY_DN16144_c0_g3_i9::g.62076::m.62076	0	802	XP_018807977.1	uncharacterized protein LOC108981316	Juglans regia	XP_008236697.1
TRINITY_DN12285_c1_g1::TRINITY_DN12285_c1_g1_i3::g.11619::m.11619	0	622	XP_018848409.1	protein STRICTOSIDINE SYNTHASE-LIKE 10-like	Juglans regia	KDP37825.1
TRINITY_DN19816_c1_g2::TRINITY_DN19816_c1_g2_i10::g.124090::m.124090	2,25E-155	438	XP_018806025.1	adenylyl-sulfate kinase 3-like isoform X2	Juglans regia	XP_018806026.1
TRINITY_DN15180_c0_g2::TRINITY_DN15180_c0_g2_i2::g.47796::m.47796	0	550	XP_018835257.1	uncharacterized protein LOC109002109	Juglans regia	XP_008243353.1
TRINITY_DN19665_c1_g1::TRINITY_DN19665_c1_g1_i7::g.121863::m.121863	0	1376	XP_018859316.1	putative cell division cycle ATPase	Juglans regia	XP_018829264.1
TRINITY_DN19770_c1_g4::TRINITY_DN19770_c1_g4_i6::g.123393::m.123393	3,04E-106	311	XP_018850794.1	probable protein phosphatase 2C 39	Juglans regia	XP_018823175.1
TRINITY_DN14513_c0_g3::TRINITY_DN14513_c0_g3_i9::g.38189::m.38189	2,84E-83	246	XP_018838152.1	major allergen Pru ar 1-like	Juglans regia	ONI28174.1
TRINITY_DN16940_c1_g1::TRINITY_DN16940_c1_g1_i2::g.75863::m.75863	2,48E-60	189	XP_018826000.1	endo-1,3;1,4-beta-D-glucanase-like	Juglans regia	XP_018826002.1
TRINITY_DN14725_c0_g1::TRINITY_DN14725_c0_g1_i4::g.41213::m.41213	5,80E-50	159	XP_018834322.1	uncharacterized protein LOC109001480	Juglans regia	XP_018834323.1
TRINITY_DN12515_c0_g1::TRINITY_DN12515_c0_g1_i3::g.13461::m.13461	0	789	XP_018849865.1	aminomethyltransferase, mitochondrial	Juglans regia	XP_018849809.1
TRINITY_DN16262_c1_g3::TRINITY_DN16262_c1_g3_i2::g.64875::m.64875	1,62E-62	202	XP_018845260.1	endoglucanase 16	Juglans regia	XP_012077550.1
TRINITY_DN13815_c1_g1::TRINITY_DN13815_c1_g1_i21::g.28891::m.28891	0	687	XP_008223236.1	UDP-glucuronic acid decarboxylase 6 isoform X1	Prunus mume	ONI00298.1
TRINITY_DN14602_c3_g2::TRINITY_DN14602_c3_g2_i9::g.39596::m.39596	0	1033	XP_018827690.1	acetyl-coenzyme A synthetase, chloroplastic/glyoxysomal isoform X1	Juglans regia	XP_018827691.1
TRINITY_DN18669_c1_g1::TRINITY_DN18669_c1_g1_i3::g.104346::m.104346	2,37E-160	451	XP_018805220.1	signal recognition particle receptor subunit beta-like	Juglans regia	XP_018805221.1
TRINITY_DN16497_c0_g5::TRINITY_DN16497_c0_g5_i1::g.67600::m.67600	0	701	XP_018845018.1	tRNA-dihydrouridine(16/17) synthase [NAD(P)(+)-like]	Juglans regia	XP_018845019.1
TRINITY_DN15927_c0_g1::TRINITY_DN15927_c0_g1_i2::g.59092::m.59092	0	857	XP_018833437.1	hexokinase-2, chloroplastic	Juglans regia	AHY84730.1
TRINITY_DN17586_c3_g1::TRINITY_DN17586_c3_g1_i5::g.86439::m.86439	0	1323	XP_018828049.1	serine/threonine-protein phosphatase 6 regulatory subunit 3-like	Juglans regia	XP_018847866.1
TRINITY_DN16528_c2_g1::TRINITY_DN16528_c2_g1_i3::g.69117::m.69117	9,38E-125	372	XP_018823734.1	beta-galactosidase 13-like	Juglans regia	XP_018841471.1

TRINITY_DN15498_c4_g2::TRINITY_DN15498_c4_g2_i2::g.51311::m.51311	2,21E-122	354	XP_018820852.1	protein YIPF6 homolog	Juglans regia	OAY46745.1
TRINITY_DN14361_c0_g1::TRINITY_DN14361_c0_g1_i5::g.35830::m.35830	0	1411	XP_018836327.1	uncharacterized protein LOC109002866	Juglans regia	XP_008361927.1
TRINITY_DN11484_c0_g1::TRINITY_DN11484_c0_g1_i2::g.7552::m.7552	8,62E-34	117	XP_008364885.1	60S ribosomal protein L22-2	Malus domestica	XP_018858309.1
TRINITY_DN12658_c0_g1::TRINITY_DN12658_c0_g1_i2::g.14732::m.14732	8,70E-73	216	OAY52437.1	hypothetical protein MANES_04G083400	Manihot esculenta	KYP64937.1
TRINITY_DN10660_c0_g1::TRINITY_DN10660_c0_g1_i1::g.5237::m.5237	5,48E-128	369	XP_018809935.1	RWD domain-containing protein 1	Juglans regia	XP_012069022.1
TRINITY_DN3120_c0_g1::TRINITY_DN3120_c0_g1_i1::g.867::m.867	1,24E-100	297	XP_018821736.1	uncharacterized protein At2g39795, mitochondrial-like	Juglans regia	XP_008372078.1
TRINITY_DN19581_c3_g6::TRINITY_DN19581_c3_g6_i4::g.120132::m.120132	2,36E-45	146	XP_018850188.1	V-type proton ATPase subunit G-like	Juglans regia	XP_018850189.1
TRINITY_DN19709_c5_g1::TRINITY_DN19709_c5_g1_i3::g.122591::m.122591	0	1299	XP_018834202.1	histidine--tRNA ligase, cytoplasmic	Juglans regia	XP_008237753.1
TRINITY_DN16440_c0_g1::TRINITY_DN16440_c0_g1_i4::g.67503::m.67503	0	1600	XP_018812523.1	probable alpha,alpha-trehalose-phosphate synthase [UDP-forming 7]	Juglans regia	XP_018841187.1
TRINITY_DN16345_c2_g1::TRINITY_DN16345_c2_g1_i1::g.66206::m.66206	1,05E-33	115	XP_018835558.1	cx9C motif-containing protein 4	Juglans regia	KYP52067.1
TRINITY_DN19239_c3_g1::TRINITY_DN19239_c3_g1_i4::g.113825::m.113825	0	656	ONI03239.1	hypothetical protein PRUPE_6G246500	Prunus persica	XP_007205216.1
TRINITY_DN16612_c3_g7::TRINITY_DN16612_c3_g7_i2::g.70355::m.70355	0	579	XP_018844325.1	ATPase 9, plasma membrane-type	Juglans regia	ONI16393.1
TRINITY_DN19651_c4_g1::TRINITY_DN19651_c4_g1_i17::g.121374::m.121374	2,58E-169	485	XP_018849269.1	phosphoacetylglucosamine mutase-like	Juglans regia	XP_018838256.1
TRINITY_DN12541_c0_g1::TRINITY_DN12541_c0_g1_i2::g.13592::m.13592	2,79E-86	256	XP_018834081.1	translocon-associated protein subunit beta-like	Juglans regia	XP_018834886.1
TRINITY_DN14080_c2_g2::TRINITY_DN14080_c2_g2_i1::g.32148::m.32148	4,51E-115	332	OIW09901.1	hypothetical protein TanjilG_32050	Lupinus angustifolius	XP_019447243.1
TRINITY_DN16026_c0_g1::TRINITY_DN16026_c0_g1_i2::g.60976::m.60976	0	4237	XP_012089387.1	protein ILITYHIA	Jatropha curcas	ONI05051.1
TRINITY_DN17082_c4_g2::TRINITY_DN17082_c4_g2_i2::g.78169::m.78169	0	1003	XP_018847198.1	protein CASP	Juglans regia	OAY50476.1
TRINITY_DN19559_c2_g2::TRINITY_DN19559_c2_g2_i6::g.119806::m.119806	1,65E-77	233	XP_018808841.1	esterase CG5412-like	Juglans regia	OAY58075.1
TRINITY_DN19551_c4_g4::TRINITY_DN19551_c4_g4_i1::g.119681::m.119681	0	603	XP_004493989.1	cell division control protein 48 homolog D	Cicer arietinum	XP_004493989.1
TRINITY_DN12675_c1_g1::TRINITY_DN12675_c1_g1_i4::g.14912::m.14912	1,74E-165	467	KDP42130.1	hypothetical protein JCGZ_01918	Jatropha curcas	XP_012067122.1
TRINITY_DN14194_c0_g2::TRINITY_DN14194_c0_g2_i9::g.33546::m.33546	0	771	ONI11967.1	hypothetical protein PRUPE_4G137500	Prunus persica	XP_007213801.1
TRINITY_DN15675_c4_g2::TRINITY_DN15675_c4_g2_i2::g.55509::m.55509	7,92E-111	321	XP_018848086.1	uncharacterized protein LOC109011377 isoform X1	Juglans regia	KDP36026.1

TRINITY_DN16295_c1_g2::TRINITY_DN16295_c1_g2_i1::g.65228::m.65228	2,03E-178	499	XP_018811162.1	serine/threonine-protein kinase SAPK7-like	Juglans regia	XP_014509517.1
TRINITY_DN16659_c4_g1::TRINITY_DN16659_c4_g1_i7::g.71135::m.71135	0	1109	XP_018843251.1	VHS domain-containing protein At3g16270	Juglans regia	XP_018843252.1
TRINITY_DN15142_c0_g1::TRINITY_DN15142_c0_g1_i16::g.47469::m.47469	1,07E-117	345	XP_018812490.1	uncharacterized protein LOC108984861 isoform X5	Juglans regia	XP_018842938.1
TRINITY_DN14674_c1_g1::TRINITY_DN14674_c1_g1_i2::g.40399::m.40399	2,73E-125	363	XP_018822743.1	uncharacterized protein LOC108992601 isoform X2	Juglans regia	ONH94692.1
TRINITY_DN12462_c4_g1::TRINITY_DN12462_c4_g1_i1::g.13140::m.13140	1,40E-75	239	XP_018814632.1	dynamin-related protein 1C-like	Juglans regia	OAY25314.1
TRINITY_DN18768_c2_g1::TRINITY_DN18768_c2_g1_i1::g.106105::m.106105	3,50E-162	471	XP_018821713.1	eukaryotic translation initiation factor 4B2-like	Juglans regia	XP_018821321.1
TRINITY_DN19730_c4_g2::TRINITY_DN19730_c4_g2_i8::g.122947::m.122947	0	726	XP_018834571.1	DEAD-box ATP-dependent RNA helicase 37-like	Juglans regia	OAY28940.1
TRINITY_DN18061_c2_g5::TRINITY_DN18061_c2_g5_i2::g.94542::m.94542	0	775	XP_018846848.1	leucine-rich repeat receptor-like serine/threonine-protein kinase At2g14510	Juglans regia	XP_018840482.1
TRINITY_DN15458_c1_g2::TRINITY_DN15458_c1_g2_i4::g.52100::m.52100	1,76E-74	221	XP_018823645.1	uncharacterized protein LOC108993249	Juglans regia	XP_018857024.1
TRINITY_DN14909_c1_g1::TRINITY_DN14909_c1_g1_i3::g.44168::m.44168	0	1806	XP_018838035.1	conserved oligomeric Golgi complex subunit 1	Juglans regia	KDP32491.1
TRINITY_DN16927_c1_g1::TRINITY_DN16927_c1_g1_i7::g.75660::m.75660	2,06E-160	456	XP_018824340.1	uncharacterized protein LOC108993774	Juglans regia	OAY37742.1
TRINITY_DN17463_c2_g1::TRINITY_DN17463_c2_g1_i7::g.84144::m.84144	0	636	XP_018824020.1	alpha-amylase-like isoform X1	Juglans regia	XP_018824021.1
TRINITY_DN19844_c1_g3::TRINITY_DN19844_c1_g3_i3::g.124457::m.124457	0	683	XP_018849698.1	glutamine--tRNA ligase-like	Juglans regia	XP_018849699.1
TRINITY_DN15946_c1_g2::TRINITY_DN15946_c1_g2_i8::g.59360::m.59360	0	855	XP_018848567.1	glycylpeptide N-tetradecanoyltransferase 1-like	Juglans regia	XP_018848568.1
TRINITY_DN14927_c1_g1::TRINITY_DN14927_c1_g1_i11::g.44438::m.44438	1,21E-96	283	XP_018842815.1	RNA-binding protein 1-like	Juglans regia	XP_018842816.1
TRINITY_DN17612_c1_g2::TRINITY_DN17612_c1_g2_i3::g.87024::m.87024	0	1634	OAY59550.1	hypothetical protein MANES_01G040200	Manihot esculenta	XP_012066298.1
TRINITY_DN18637_c1_g1::TRINITY_DN18637_c1_g1_i7::g.103721::m.103721	0	842	XP_018852639.1	serine/threonine protein phosphatase 2A regulatory subunit B''beta-like	Juglans regia	XP_018855937.1
TRINITY_DN12213_c0_g1::TRINITY_DN12213_c0_g1_i3::g.11157::m.11157	3,11E-104	309	XP_018846823.1	prostaglandin E synthase 2-like	Juglans regia	XP_008221232.1
TRINITY_DN19518_c0_g1::TRINITY_DN19518_c0_g1_i8::g.119129::m.119129	1,51E-134	380	XP_018810318.1	IN2-2 protein-like isoform X4	Juglans regia	XP_018810314.1
TRINITY_DN19195_c4_g1::TRINITY_DN19195_c4_g1_i3::g.113639::m.113639	0	554	KYP78448.1	Beta-galactosidase	Cajanus cajan	XP_020208721.1
TRINITY_DN13292_c1_g6::TRINITY_DN13292_c1_g6_i2::g.22041::m.22041	1,18E-63	194	XP_018824894.1	uncharacterized protein LOC108994217	Juglans regia	OAY47489.1
TRINITY_DN14741_c0_g1::TRINITY_DN14741_c0_g1_i2::g.41477::m.41477	0	884	XP_018849265.1	peptide-N4-(N-acetyl-beta-glucosaminyl)asparagine amidase A	Juglans regia	XP_018849272.1

TRINITY_DN14630_c2_g8::TRINITY_DN14630_c2_g8_i1::g.39829::m.39829	1,62E-114	327	XP_018846146.1	GTP-binding protein SAR1A isoform X1	Juglans regia	KDP28245.1
TRINITY_DN16782_c2_g1::TRINITY_DN16782_c2_g1_i3::g.73171::m.73171	3,53E-43	150	ONI15658.1	hypothetical protein PRUPE_3G054100	Prunus persica	KDP43847.1
TRINITY_DN18173_c2_g1::TRINITY_DN18173_c2_g1_i9::g.96627::m.96627	0	1547	XP_018845600.1	L-arabinokinase-like	Juglans regia	XP_018845086.1
TRINITY_DN17174_c0_g2::TRINITY_DN17174_c0_g2_i1::g.79619::m.79619	1,05E-42	139	XP_004485978.1	60S ribosomal protein L38-like	Cicer arietinum	XP_004507353.1
TRINITY_DN12761_c4_g1::TRINITY_DN12761_c4_g1_i4::g.15698::m.15698	2,04E-92	275	ACU16831.1	unknown	Glycine max	XP_018813170.1
TRINITY_DN15641_c2_g1::TRINITY_DN15641_c2_g1_i3::g.54993::m.54993	0	545	XP_018817143.1	uncharacterized protein LOC108988355	Juglans regia	XP_018828950.1
TRINITY_DN16713_c1_g2::TRINITY_DN16713_c1_g2_i6::g.72096::m.72096	0	590	XP_018837262.1	protein FREE1	Juglans regia	KDP36892.1
TRINITY_DN15145_c0_g2::TRINITY_DN15145_c0_g2_i2::g.46817::m.46817	0	729	XP_018859548.1	protein AUXIN RESPONSE 4	Juglans regia	XP_018859549.1
TRINITY_DN16390_c0_g1::TRINITY_DN16390_c0_g1_i2::g.66783::m.66783	0	639	XP_018836609.1	RNA-binding KH domain-containing protein PEPPER	Juglans regia	XP_008223873.1
TRINITY_DN16711_c0_g1::TRINITY_DN16711_c0_g1_i4::g.72030::m.72030	0	1150	XP_018826782.1	TBC1 domain family member 15-like isoform X2	Juglans regia	XP_018826781.1
TRINITY_DN15384_c0_g1::TRINITY_DN15384_c0_g1_i3::g.51070::m.51070	0	664	XP_018850136.1	developmentally-regulated G-protein 3	Juglans regia	XP_016162010.1
TRINITY_DN17041_c0_g1::TRINITY_DN17041_c0_g1_i4::g.77480::m.77480	0	2041	XP_018849426.1	uncharacterized protein LOC109012316	Juglans regia	ONI20827.1
TRINITY_DN18962_c1_g1::TRINITY_DN18962_c1_g1_i3::g.109730::m.109730	0	1260	XP_018833756.1	protein argonaute 5	Juglans regia	XP_018833165.1
TRINITY_DN16476_c3_g1::TRINITY_DN16476_c3_g1_i9::g.68231::m.68231	0	881	XP_018842650.1	probable arabinosyltransferase ARAD1	Juglans regia	XP_018842651.1
TRINITY_DN17658_c5_g3::TRINITY_DN17658_c5_g3_i2::g.87770::m.87770	5,12E-101	297	XP_018821628.1	proteasome subunit alpha type-1-B-like	Juglans regia	XP_018845049.1
TRINITY_DN16523_c1_g1::TRINITY_DN16523_c1_g1_i4::g.69126::m.69126	7,51E-28	118	XP_018805377.1	uncharacterized protein LOC108979207	Juglans regia	XP_018805377.1
TRINITY_DN19779_c3_g3::TRINITY_DN19779_c3_g3_i2::g.123543::m.123543	1,30E-149	420	OAY39696.1	hypothetical protein MANES_10G115800	Manihot esculenta	KDP42463.1
TRINITY_DN15367_c2_g1::TRINITY_DN15367_c2_g1_i7::g.50786::m.50786	3,66E-57	178	XP_018851174.1	putative phosphatidylglycerol/phosphatidylinositol transfer protein DDB_G0282179	Juglans regia	XP_018836592.1
TRINITY_DN14788_c0_g1::TRINITY_DN14788_c0_g1_i1::g.42248::m.42248	4,39E-25	95,5	XP_018852386.1	protein SPIRAL1-like 1	Juglans regia	XP_018852387.1
TRINITY_DN12593_c2_g1::TRINITY_DN12593_c2_g1_i1::g.14128::m.14128	7,00E-59	193	XP_018817520.1	glycosyltransferase-like KOBITO 1 isoform X2	Juglans regia	KRH62919.1
TRINITY_DN18024_c1_g1::TRINITY_DN18024_c1_g1_i2::g.93774::m.93774	5,47E-137	389	XP_018841297.1	tropinone reductase homolog At5g06060-like	Juglans regia	XP_018850680.1

TRINITY_DN16834_c1_g1::TRINITY_DN16834_c1_g1_i1::g.74148::m.74148	0	935	XP_018821057.1	calcium-dependent protein kinase 11-like	Juglans regia	XP_018848690.1
TRINITY_DN19047_c1_g5::TRINITY_DN19047_c1_g5_i2::g.111027::m.111027	0	610	XP_018845017.1	probable 2-oxoglutarate-dependent dioxygenase At3g49630 isoform X2	Juglans regia	XP_018845016.1
TRINITY_DN15127_c0_g2::TRINITY_DN15127_c0_g2_i1::g.47217::m.47217	0	1672	XP_018823060.1	alpha-mannosidase 2	Juglans regia	ONI19395.1
TRINITY_DN13215_c0_g6::TRINITY_DN13215_c0_g6_i3::g.21426::m.21426	3,41E-46	153	XP_018843123.1	eukaryotic translation initiation factor 3 subunit J-like	Juglans regia	OAY46647.1
TRINITY_DN16629_c2_g3::TRINITY_DN16629_c2_g3_i2::g.70768::m.70768	1,09E-79	240	XP_018828615.1	gamma-interferon-inducible lysosomal thiol reductase-like	Juglans regia	XP_008234981.2
TRINITY_DN11853_c0_g1::TRINITY_DN11853_c0_g1_i3::g.9201::m.9201	0	630	XP_018829679.1	2-methyl-6-phytyl-1,4-hydroquinone methyltransferase, chloroplastic-like isoform X1	Juglans regia	XP_018829680.1
TRINITY_DN12481_c0_g1::TRINITY_DN12481_c0_g1_i2::g.13223::m.13223	1,92E-82	243	XP_012074113.1	microsomal glutathione S-transferase 3	Jatropha curcas	OAY57255.1
TRINITY_DN17670_c0_g2::TRINITY_DN17670_c0_g2_i7::g.87973::m.87973	0	883	XP_018837095.1	uncharacterized protein At1g04910	Juglans regia	ONI05883.1
TRINITY_DN17125_c1_g1::TRINITY_DN17125_c1_g1_i9::g.78918::m.78918	0	533	XP_018834513.1	clathrin interactor EPSIN 1	Juglans regia	XP_018834519.1
TRINITY_DN16077_c0_g5::TRINITY_DN16077_c0_g5_i1::g.61752::m.61752	2,55E-130	370	GAU32398.1	hypothetical protein TSUD_44410, partial	Trifolium subterraneum	XP_008234243.1
TRINITY_DN17927_c1_g1::TRINITY_DN17927_c1_g1_i3::g.92125::m.92125	0	563	XP_018810997.1	electron transfer flavoprotein subunit alpha, mitochondrial	Juglans regia	OAY28546.1
TRINITY_DN13566_c3_g2::TRINITY_DN13566_c3_g2_i6::g.25511::m.25511	4,81E-112	334	XP_018833217.1	uncharacterized protein LOC109000707	Juglans regia	XP_018833218.1
TRINITY_DN15783_c0_g1::TRINITY_DN15783_c0_g1_i4::g.56969::m.56969	6,13E-110	317	XP_018853288.1	triphosphate tunel metalloenzyme 3-like	Juglans regia	XP_018816133.1
TRINITY_DN14219_c0_g1::TRINITY_DN14219_c0_g1_i15::g.33893::m.33893	0	549	XP_018848890.1	activator of 90 kDa heat shock protein ATPase homolog	Juglans regia	XP_018809717.1
TRINITY_DN16674_c0_g1::TRINITY_DN16674_c0_g1_i2::g.71326::m.71326	0	1047	XP_018836506.1	uncharacterized protein LOC109003012	Juglans regia	XP_008233533.1
TRINITY_DN18146_c1_g5::TRINITY_DN18146_c1_g5_i1::g.95204::m.95204	2,37E-78	233	XP_018808548.1	outer envelope pore protein 16-3, chloroplastic/mitochondrial	Juglans regia	XP_008367365.1
TRINITY_DN15699_c1_g3::TRINITY_DN15699_c1_g3_i5::g.55823::m.55823	0	1258	XP_018816462.1	protein transport protein Sec24-like At3g07100	Juglans regia	XP_018816462.1
TRINITY_DN17055_c3_g2::TRINITY_DN17055_c3_g2_i4::g.77693::m.77693	0	2892	XP_018850497.1	uncharacterized protein LOC109013033	Juglans regia	XP_018850498.1
TRINITY_DN16303_c1_g1::TRINITY_DN16303_c1_g1_i9::g.65704::m.65704	1,83E-169	490	XP_018817229.1	uncharacterized protein LOC108988420	Juglans regia	XP_008387749.1
TRINITY_DN19783_c1_g1::TRINITY_DN19783_c1_g1_i4::g.123602::m.123602	3,37E-130	399	XP_018817838.1	methionine S-methyltransferase	Juglans regia	XP_008379013.1
TRINITY_DN15141_c1_g1::TRINITY_DN15141_c1_g1_i2::g.47502::m.47502	0	540	XP_018846483.1	proteasome subunit beta type-7-B	Juglans regia	KDP46386.1
TRINITY_DN11784_c0_g1::TRINITY_DN11784_c0_g1_i1::g.8804::m.8804	8,77E-83	247	XP_018825585.1	uncharacterized protein LOC108994714	Juglans regia	OAY27757.1

TRINITY_DN16035_c3_g1::TRINITY_DN16035_c3_g1_i2::g.61026::m.61026	0	573	XP_018825295.1	BSD domain-containing protein 1	Juglans regia	OAY49651.1
TRINITY_DN13920_c2_g1::TRINITY_DN13920_c2_g1_i2::g.30365::m.30365	1,41E-112	337	XP_018823489.1	putative 3,4-dihydroxy-2-butanone kinase	Juglans regia	XP_018859162.1
TRINITY_DN13342_c3_g1::TRINITY_DN13342_c3_g1_i3::g.22570::m.22570	1,83E-64	215	XP_018831147.1	dynamin-2A-like	Juglans regia	XP_018834193.1
TRINITY_DN16268_c4_g1::TRINITY_DN16268_c4_g1_i16::g.64855::m.64855	0	1142	XP_018821646.1	uncharacterized protein LOC108991727 isoform X2	Juglans regia	XP_018821645.1
TRINITY_DN12624_c0_g1::TRINITY_DN12624_c0_g1_i1::g.14480::m.14480	0	839	XP_018844325.1	ATPase 9, plasma membrane-type	Juglans regia	XP_009334638.1
TRINITY_DN10700_c0_g1::TRINITY_DN10700_c0_g1_i1::g.5317::m.5317	6,93E-169	475	XP_018835415.1	elongation factor Ts, mitochondrial isoform X1	Juglans regia	OAY32958.1
TRINITY_DN14461_c1_g2::TRINITY_DN14461_c1_g2_i3::g.37522::m.37522	1,15E-106	319	KDP33945.1	hypothetical protein JCGZ_07516	Jatropha curcas	XP_020208720.1
TRINITY_DN11860_c0_g1::TRINITY_DN11860_c0_g1_i2::g.9213::m.9213	1,60E-67	204	AFP43694.1	actin 2, partial	Eriobotrya japonica	AFP43696.1
TRINITY_DN17343_c0_g2::TRINITY_DN17343_c0_g2_i1::g.82237::m.82237	0	1542	XP_018825849.1	AMP deaminase	Juglans regia	XP_018825850.1
TRINITY_DN17700_c1_g1::TRINITY_DN17700_c1_g1_i9::g.86845::m.86845	6,51E-176	489	XP_018859347.1	probable enoyl-CoA hydratase 1, peroxisomal	Juglans regia	ONI27997.1
TRINITY_DN15208_c2_g1::TRINITY_DN15208_c2_g1_i1::g.48542::m.48542	0	1009	XP_018847349.1	plastidial pyruvate kinase 2	Juglans regia	OAY48391.1
TRINITY_DN17602_c0_g7::TRINITY_DN17602_c0_g7_i2::g.86978::m.86978	0	2972	XP_018816643.1	auxin transport protein BIG	Juglans regia	ONI18935.1
TRINITY_DN15799_c0_g1::TRINITY_DN15799_c0_g1_i13::g.55967::m.55967	1,61E-116	338	XP_018842015.1	endochitinase-like	Juglans regia	XP_018854709.1
TRINITY_DN19816_c1_g2::TRINITY_DN19816_c1_g2_i9::g.124089::m.124089	8,65E-138	392	XP_018806025.1	adenylyl-sulfate kinase 3-like isoform X2	Juglans regia	XP_018806026.1
TRINITY_DN14106_c1_g2::TRINITY_DN14106_c1_g2_i1::g.32414::m.32414	0	728	XP_018829584.1	3-dehydroquinate synthase, chloroplastic-like	Juglans regia	XP_018832026.1
TRINITY_DN19486_c1_g2::TRINITY_DN19486_c1_g2_i2::g.118619::m.118619	0	587	XP_018809158.1	uncharacterized protein At5g49945-like	Juglans regia	XP_018817873.1
TRINITY_DN16542_c2_g2::TRINITY_DN16542_c2_g2_i8::g.69479::m.69479	0	1722	XP_018807838.1	uncharacterized protein LOC108981175	Juglans regia	XP_009358942.1
TRINITY_DN15298_c0_g1::TRINITY_DN15298_c0_g1_i1::g.49745::m.49745	1,10E-70	216	XP_018848462.1	B-cell receptor-associated protein 31	Juglans regia	AFK41440.1
TRINITY_DN14245_c1_g1::TRINITY_DN14245_c1_g1_i8::g.34453::m.34453	7,10E-90	264	KYP70294.1	Stromal cell-derived factor 2-like protein	Cajanus cajan	XP_020213613.1
TRINITY_DN16993_c0_g1::TRINITY_DN16993_c0_g1_i5::g.76723::m.76723	7,96E-68	211	XP_018828640.1	bifunctional UDP-glucose 4-epimerase and UDP-xylose 4-epimerase 1	Juglans regia	XP_018811946.1
TRINITY_DN18845_c2_g1::TRINITY_DN18845_c2_g1_i6::g.106645::m.106645	0	1388	XP_018823809.1	transportin-1-like	Juglans regia	XP_018823810.1
TRINITY_DN17503_c1_g2::TRINITY_DN17503_c1_g2_i5::g.84903::m.84903	4,83E-149	446	XP_009374844.1	WEB family protein At1g12150-like	Pyrus x bretschneideri	XP_018837389.1

TRINITY_DN18733_c1_g1::TRINITY_DN18733_c1_g1_i16::g.105945::m.105945	9,97E-88	276	XP_018821030.1	transmembrane and coiled-coil domain-containing protein 4-like isoform X1	Juglans regia	XP_017186447.1
TRINITY_DN13161_c0_g2::TRINITY_DN13161_c0_g2_i6::g.20596::m.20596	4,86E-100	289	XP_016205498.1	signal peptidase complex subunit 3B	Arachis ipaensis	XP_015968566.1
TRINITY_DN16310_c4_g1::TRINITY_DN16310_c4_g1_i21::g.65586::m.65586	5,18E-138	387	AEX31757.1	clp protease proteolytic subunit (chloroplast)	Pentactina rupicola	YP_005296125.1
TRINITY_DN16789_c2_g2::TRINITY_DN16789_c2_g2_i2::g.73230::m.73230	0	559	XP_018812975.1	mitochondrial import inner membrane translocase subunit TIM50	Juglans regia	XP_018825124.1
TRINITY_DN14198_c0_g1::TRINITY_DN14198_c0_g1_i9::g.33644::m.33644	1,84E-106	325	XP_018815969.1	granule-bound starch synthase 2, chloroplastic/amyloplastic-like	Juglans regia	OIW21160.1
TRINITY_DN11382_c0_g1::TRINITY_DN11382_c0_g1_i1::g.7166::m.7166	0	730	XP_018810396.1	pyruvate dehydrogenase E1 component subunit beta-3, chloroplastic	Juglans regia	XP_018810397.1
TRINITY_DN18612_c0_g3::TRINITY_DN18612_c0_g3_i1::g.103400::m.103400	1,73E-131	373	XP_018831192.1	polyadenylate-binding protein 1 isoform X1	Juglans regia	XP_018808102.1
TRINITY_DN13425_c1_g1::TRINITY_DN13425_c1_g1_i4::g.23719::m.23719	2,03E-178	496	XP_018842653.1	NADH--cytochrome b5 reductase 1	Juglans regia	OAY41871.1
TRINITY_DN18242_c3_g2::TRINITY_DN18242_c3_g2_i3::g.97426::m.97426	2,32E-124	362	XP_018807889.1	heterogeneous nuclear ribonucleoprotein 1-like	Juglans regia	XP_018810068.1
TRINITY_DN16339_c2_g2::TRINITY_DN16339_c2_g2_i2::g.65998::m.65998	0	1748	XP_018807888.1	lon protease homolog 1, mitochondrial-like	Juglans regia	OAY43962.1
TRINITY_DN17082_c3_g1::TRINITY_DN17082_c3_g1_i1::g.78154::m.78154	0	902	XP_018848774.1	cystathionine gamma-synthase 1, chloroplastic-like isoform X1	Juglans regia	XP_018848775.1
TRINITY_DN19447_c1_g1::TRINITY_DN19447_c1_g1_i4::g.118090::m.118090	0	1191	XP_018839054.1	probable rhamnogalacturonate lyase B	Juglans regia	XP_018842495.1
TRINITY_DN12896_c0_g1::TRINITY_DN12896_c0_g1_i6::g.17068::m.17068	1,58E-172	484	ONI22818.1	hypothetical protein PRUPE_2G153000	Prunus persica	XP_007218197.1
TRINITY_DN17884_c0_g1::TRINITY_DN17884_c0_g1_i1::g.91267::m.91267	3,04E-25	97,8	OAY59405.1	hypothetical protein MANES_01G030100	Manihot esculenta	XP_018836344.1
TRINITY_DN19798_c2_g2::TRINITY_DN19798_c2_g2_i9::g.123749::m.123749	0	801	OAY26193.1	hypothetical protein MANES_16G027900	Manihot esculenta	XP_018860049.1
TRINITY_DN19714_c6_g2::TRINITY_DN19714_c6_g2_i2::g.122726::m.122726	0	664	XP_018815318.1	probable receptor-like protein kinase At4g39110	Juglans regia	XP_018850919.1
TRINITY_DN16697_c1_g2::TRINITY_DN16697_c1_g2_i3::g.70483::m.70483	3,70E-112	340	XP_018836272.1	cell division cycle protein 48 homolog	Juglans regia	XP_018836273.1
TRINITY_DN18870_c1_g2::TRINITY_DN18870_c1_g2_i1::g.107727::m.107727	1,37E-161	454	XP_018818800.1	probable 6-phosphogluconolactonase 4, chloroplastic	Juglans regia	XP_018842795.1
TRINITY_DN18726_c1_g1::TRINITY_DN18726_c1_g1_i9::g.105485::m.105485	0	1835	XP_018837729.1	protein MODIFIER OF SNC1 1-like isoform X1	Juglans regia	XP_018837730.1
TRINITY_DN18156_c0_g1::TRINITY_DN18156_c0_g1_i18::g.96138::m.96138	0	853	XP_018824574.1	E3 UFM1-protein ligase 1 homolog isoform X1	Juglans regia	XP_018824575.1
TRINITY_DN17096_c1_g1::TRINITY_DN17096_c1_g1_i4::g.78249::m.78249	7,59E-57	175	XP_018805734.1	huntingtin-interacting protein K-like	Juglans regia	KDP27909.1
TRINITY_DN18240_c0_g1::TRINITY_DN18240_c0_g1_i3::g.97435::m.97435	0	582	XP_018808481.1	AP-1 complex subunit gamma-2-like isoform X2	Juglans regia	XP_018808480.1

TRINITY_DN14353_c0_g2::TRINITY_DN14353_c0_g2_i2::g.35716::m.35716	1,44E-41	146	OAY26155.1	hypothetical protein MANES_16G025400	Manihot esculenta	KDP20942.1
TRINITY_DN16303_c1_g2::TRINITY_DN16303_c1_g2_i1::g.65686::m.65686	2,09E-71	232	XP_018817229.1	uncharacterized protein LOC108988420	Juglans regia	XP_012569741.1
TRINITY_DN13465_c2_g1::TRINITY_DN13465_c2_g1_i6::g.24168::m.24168	4,48E-105	313	XP_018854479.1	signal recognition particle 54 kDa protein 2	Juglans regia	XP_018851743.1
TRINITY_DN16506_c3_g1::TRINITY_DN16506_c3_g1_i1::g.68717::m.68717	3,07E-66	200	XP_018846320.1	60S ribosomal protein L31	Juglans regia	XP_008242444.1
TRINITY_DN20189_c0_g1::TRINITY_DN20189_c0_g1_i1::g.128508::m.128508	1,69E-87	260	XP_018834573.1	uncharacterized protein LOC109001661	Juglans regia	ONH99077.1
TRINITY_DN3188_c0_g1::TRINITY_DN3188_c0_g1_i1::g.878::m.878	1,67E-69	211	XP_018834960.1	uncharacterized protein LOC109001911	Juglans regia	XP_008238399.1
TRINITY_DN17908_c2_g1::TRINITY_DN17908_c2_g1_i5::g.91796::m.91796	0	660	XP_018830811.1	pectin acetylesterase 9	Juglans regia	XP_008234234.1
TRINITY_DN13425_c1_g3::TRINITY_DN13425_c1_g3_i2::g.23712::m.23712	0	513	KDP42913.1	hypothetical protein JCGZ_23855	Jatropha curcas	XP_012066294.1
TRINITY_DN14799_c0_g2::TRINITY_DN14799_c0_g2_i3::g.42387::m.42387	1,26E-132	383	XP_018828255.1	LOW QUALITY PROTEIN: protein TIC 22-like, chloroplastic	Juglans regia	ONI28937.1
TRINITY_DN12952_c0_g1::TRINITY_DN12952_c0_g1_i2::g.17756::m.17756	0	541	XP_018847067.1	branched-chain-amino-acid aminotransferase 6-like isoform X2	Juglans regia	XP_018847068.1
TRINITY_DN18755_c3_g1::TRINITY_DN18755_c3_g1_i1::g.105920::m.105920	0	568	XP_018809691.1	probable protein kinase At2g41970 isoform X1	Juglans regia	XP_018848920.1
TRINITY_DN14404_c3_g2::TRINITY_DN14404_c3_g2_i3::g.36551::m.36551	0	1050	XP_018854140.1	elongation factor G-2, mitochondrial	Juglans regia	ONI22518.1
TRINITY_DN19358_c6_g3::TRINITY_DN19358_c6_g3_i5::g.116477::m.116477	1,13E-106	310	XP_018824738.1	phytanoyl-CoA dioxygenase	Juglans regia	XP_020411582.1
TRINITY_DN12704_c0_g1::TRINITY_DN12704_c0_g1_i4::g.15091::m.15091	0	552	XP_018811469.1	mitochondrial carnitine/acylcarnitine carrier-like protein	Juglans regia	XP_018811470.1
TRINITY_DN20043_c7_g2::TRINITY_DN20043_c7_g2_i2::g.128238::m.128238	1,49E-54	174	XP_018849930.1	rho GDP-dissociation inhibitor 1-like	Juglans regia	XP_018809557.1
TRINITY_DN17498_c2_g1::TRINITY_DN17498_c2_g1_i6::g.83121::m.83121	0	1230	XP_018825631.1	translation initiation factor eIF-2B subunit epsilon isoform X1	Juglans regia	XP_018825633.1
TRINITY_DN18857_c0_g5::TRINITY_DN18857_c0_g5_i2::g.107485::m.107485	8,21E-34	126	XP_018835872.1	SEC12-like protein 2	Juglans regia	XP_018835880.1
TRINITY_DN14751_c0_g1::TRINITY_DN14751_c0_g1_i8::g.40814::m.40814	0	1451	XP_018828780.1	endoplasmic reticulum metallopeptidase 1 isoform X1	Juglans regia	ONI23872.1
TRINITY_DN17602_c0_g3::TRINITY_DN17602_c0_g3_i7::g.86972::m.86972	0	744	XP_018834060.1	glutamine--fructose-6-phosphate aminotransferase [isomerizing 2]	Juglans regia	OAY34226.1
TRINITY_DN11500_c0_g1::TRINITY_DN11500_c0_g1_i2::g.7570::m.7570	0	560	XP_018846417.1	beta-amylase-like	Juglans regia	ONI18541.1
TRINITY_DN17024_c0_g5::TRINITY_DN17024_c0_g5_i1::g.77080::m.77080	7,06E-161	450	XP_018816519.1	uncharacterized protein At3g49720-like	Juglans regia	XP_018816520.1
TRINITY_DN18652_c2_g1::TRINITY_DN18652_c2_g1_i2::g.104075::m.104075	0	1033	XP_018817490.1	mannosyl-oligosaccharide 1,2-alpha-mannosidase MNS3	Juglans regia	ONI10982.1

TRINITY_DN19078_c0_g3::TRINITY_DN19078_c0_g3_i6::g.111344::m.111344	1,90E-52	177	XP_018836309.1	aspartic proteinase-like	Juglans regia	XP_018836310.1
TRINITY_DN13010_c2_g2::TRINITY_DN13010_c2_g2_i9::g.18708::m.18708	7,65E-77	230	XP_018818664.1	transmembrane emp24 domain-containing protein p24beta3-like	Juglans regia	XP_018839419.1
TRINITY_DN18307_c0_g1::TRINITY_DN18307_c0_g1_i4::g.98617::m.98617	0	741	XP_018828232.1	protein transport protein SEC23-like	Juglans regia	XP_018848547.1
TRINITY_DN14082_c1_g1::TRINITY_DN14082_c1_g1_i3::g.32100::m.32100	2,08E-136	395	XP_018859992.1	protein TIC 40, chloroplastic-like	Juglans regia	XP_004500418.1
TRINITY_DN17986_c0_g1::TRINITY_DN17986_c0_g1_i2::g.93139::m.93139	5,35E-148	446	XP_018809776.1	protein transport protein SEC31 homolog B-like isoform X2	Juglans regia	XP_018809774.1
TRINITY_DN13091_c0_g1::TRINITY_DN13091_c0_g1_i4::g.19648::m.19648	1,94E-116	339	OAY23951.1	hypothetical protein MANES_18G120400	Manihot esculenta	OAY23950.1
TRINITY_DN16278_c2_g2::TRINITY_DN16278_c2_g2_i8::g.65116::m.65116	6,06E-132	380	XP_018825844.1	dnaJ homolog subfamily B member 1-like	Juglans regia	ACJ84895.1
TRINITY_DN13098_c2_g1::TRINITY_DN13098_c2_g1_i5::g.19700::m.19700	0	817	XP_018816542.1	uncharacterized protein LOC108987901 isoform X1	Juglans regia	KDP21813.1
TRINITY_DN11989_c0_g1::TRINITY_DN11989_c0_g1_i1::g.9805::m.9805	0	784	XP_018850824.1	bifunctional aspartate aminotransferase and glutamate/aspartate-prephenate aminotransferase-like	Juglans regia	XP_018816075.1
TRINITY_DN19569_c1_g4::TRINITY_DN19569_c1_g4_i1::g.119978::m.119978	1,41E-124	359	ONI06354.1	hypothetical protein PRUPE_5G055300	Prunus persica	ONI06351.1
TRINITY_DN16768_c1_g4::TRINITY_DN16768_c1_g4_i5::g.73029::m.73029	0	1145	XP_018829197.1	monosaccharide-sensing protein 2-like	Juglans regia	XP_018829198.1
TRINITY_DN13051_c9_g1::TRINITY_DN13051_c9_g1_i1::g.19838::m.19838						
TRINITY_DN15531_c1_g1::TRINITY_DN15531_c1_g1_i8::g.53401::m.53401	1,21E-53	170	ONI20907.1	hypothetical protein PRUPE_2G040100	Prunus persica	XP_007218492.1
TRINITY_DN17286_c1_g1::TRINITY_DN17286_c1_g1_i1::g.81477::m.81477	0	1874	XP_018834056.1	probable alpha-mannosidase At5g13980 isoform X4	Juglans regia	XP_018834055.1
TRINITY_DN13833_c0_g1::TRINITY_DN13833_c0_g1_i8::g.29930::m.29930	0	613	XP_018856733.1	serine/threonine-protein phosphatase PP2A-3 catalytic subunit	Juglans regia	XP_008387207.1
TRINITY_DN16501_c0_g1::TRINITY_DN16501_c0_g1_i7::g.68513::m.68513	0	748	XP_018848063.1	UDP-glucuronate 4-epimerase 3	Juglans regia	XP_008364369.1
TRINITY_DN14803_c0_g1::TRINITY_DN14803_c0_g1_i4::g.42638::m.42638	0	689	XP_018832781.1	thiosulfate/3-mercaptopyruvate sulfurtransferase 1, mitochondrial-like isoform X1	Juglans regia	KDP25080.1
TRINITY_DN13921_c0_g1::TRINITY_DN13921_c0_g1_i2::g.30314::m.30314	1,33E-136	391	XP_018860709.1	secretory carrier-associated membrane protein 1 isoform X1	Juglans regia	OAY34103.1
TRINITY_DN13437_c3_g1::TRINITY_DN13437_c3_g1_i7::g.23377::m.23377	0	905	XP_018849128.1	transmembrane 9 superfamily member 3	Juglans regia	XP_018823528.1
TRINITY_DN18701_c4_g10::TRINITY_DN18701_c4_g10_i2::g.103928::m.103928	8,80E-161	461	XP_018849298.1	E3 ubiquitin-protein ligase RING1-like	Juglans regia	ONI09528.1
TRINITY_DN19119_c0_g1::TRINITY_DN19119_c0_g1_i6::g.112459::m.112459	1,16E-140	415	KRH76261.1	hypothetical protein GLYMA_01G142600	Glycine max	XP_014630670.1

TRINITY_DN17314_c1_g2::TRINITY_DN17314_c1_g2_i7::g.81924::m.81924	2,05E-104	313	OIV96749.1	hypothetical protein TanjilG_11745	Lupinus angustifolius	XP_019417566.1
TRINITY_DN13833_c0_g2::TRINITY_DN13833_c0_g2_i5::g.29925::m.29925	5,84E-83	248	AET04180.2	serine/threonine protein phosphatase 2A	Medicago truncatula	XP_003629704.2
TRINITY_DN13861_c0_g1::TRINITY_DN13861_c0_g1_i7::g.29399::m.29399	2,69E-153	429	XP_018830330.1	peroxisomal membrane protein 11C	Juglans regia	OAY62356.1
TRINITY_DN18605_c15_g2::TRINITY_DN18605_c15_g2_i1::g.103707::m.103707	2,61E-90	276	XP_018816425.1	L-ascorbate oxidase homolog	Juglans regia	XP_018819385.1
TRINITY_DN12655_c1_g2::TRINITY_DN12655_c1_g2_i3::g.14763::m.14763	0	737	XP_008228324.1	serine/threonine protein phosphatase 2A 55 kDa regulatory subunit B beta isoform isoform X3	Prunus mume	XP_020415190.1
TRINITY_DN20026_c6_g1::TRINITY_DN20026_c6_g1_i3::g.128057::m.128057	0	526	XP_018837633.1	cytochrome P450 89A2-like	Juglans regia	XP_018837634.1
TRINITY_DN16058_c1_g1::TRINITY_DN16058_c1_g1_i7::g.61245::m.61245	0	671	KDP45754.1	hypothetical protein JCGZ_17361	Jatropha curcas	XP_012078672.1
TRINITY_DN11865_c0_g1::TRINITY_DN11865_c0_g1_i7::g.9262::m.9262	0	1818	XP_018824098.1	alpha-aminoadipic semialdehyde synthase isoform X1	Juglans regia	OAY26961.1
TRINITY_DN11961_c0_g1::TRINITY_DN11961_c0_g1_i6::g.9678::m.9678	1,10E-130	371	OAY41624.1	hypothetical protein MANES_09G116800	Manihot esculenta	XP_018812032.1
TRINITY_DN15627_c0_g2::TRINITY_DN15627_c0_g2_i1::g.54590::m.54590	0	757	XP_018821636.1	arabinosyltransferase RRA3-like	Juglans regia	ONI32725.1
TRINITY_DN18478_c2_g2::TRINITY_DN18478_c2_g2_i3::g.101133::m.101133	0	1084	XP_018815548.1	long chain acyl-CoA synthetase 4-like	Juglans regia	XP_018824382.1
TRINITY_DN16690_c0_g1::TRINITY_DN16690_c0_g1_i1::g.71666::m.71666	0	974	XP_018806310.1	signal recognition particle subunit SRP72-like	Juglans regia	KDP26748.1
TRINITY_DN17879_c1_g3::TRINITY_DN17879_c1_g3_i5::g.91260::m.91260	0	968	XP_018835197.1	ABC transporter F family member 3	Juglans regia	XP_018835197.1
TRINITY_DN17973_c0_g5::TRINITY_DN17973_c0_g5_i1::g.92489::m.92489	1,00E-129	383	OAY48362.1	hypothetical protein MANES_06G152800	Manihot esculenta	XP_009377558.1
TRINITY_DN19252_c1_g4::TRINITY_DN19252_c1_g4_i7::g.114562::m.114562	9,47E-154	436	XP_018842516.1	rhodanese-like/PpiC domain-containing protein 12, chloroplastic	Juglans regia	XP_018842517.1
TRINITY_DN18739_c0_g3::TRINITY_DN18739_c0_g3_i2::g.105640::m.105640	0	896	KDP23427.1	hypothetical protein JCGZ_23260	Jatropha curcas	XP_012088953.1
TRINITY_DN13888_c0_g2::TRINITY_DN13888_c0_g2_i1::g.29877::m.29877	4,83E-37	124	XP_018850773.1	uncharacterized protein LOC109013215 isoform X2	Juglans regia	XP_018850772.1
TRINITY_DN19344_c5_g2::TRINITY_DN19344_c5_g2_i3::g.115627::m.115627	2,49E-28	114	XP_018845634.1	suppressor protein SRP40-like	Juglans regia	KHN16458.1
TRINITY_DN18418_c0_g3::TRINITY_DN18418_c0_g3_i2::g.100138::m.100138	0	566	XP_018812300.1	uncharacterized protein LOC108984712	Juglans regia	XP_008339938.1
TRINITY_DN18913_c2_g1::TRINITY_DN18913_c2_g1_i5::g.108928::m.108928	0	1357	XP_018829609.1	vacuolar protein sorting-associated protein 35A-like	Juglans regia	KDP23991.1
TRINITY_DN19613_c2_g1::TRINITY_DN19613_c2_g1_i4::g.120850::m.120850	6,26E-119	344	XP_017184434.1	ubiquitin carboxyl-terminal hydrolase 13-like, partial	Malus domestica	OAY40681.1
TRINITY_DN17758_c1_g2::TRINITY_DN17758_c1_g2_i7::g.89243::m.89243	0	635	XP_018830975.1	uncharacterized protein LOC108998739	Juglans regia	OAY48516.1

TRINITY_DN14688_c0_g2::TRINITY_DN14688_c0_g2_i3::g.40630::m.40630	1,95E-178	505	XP_004485538.1	serine decarboxylase 1	Cicer arietinum	XP_004485539.1
TRINITY_DN16770_c0_g2::TRINITY_DN16770_c0_g2_i2::g.73031::m.73031	8,47E-78	235	XP_018849016.1	glutathione S-transferase U8-like	Juglans regia	XP_018849018.1
TRINITY_DN15617_c2_g1::TRINITY_DN15617_c2_g1_i3::g.54449::m.54449	1,40E-67	203	XP_018805495.1	peptidyl-prolyl cis-trans isomerase Pin1 isoform X2	Juglans regia	XP_018822042.1
TRINITY_DN19193_c1_g3::TRINITY_DN19193_c1_g3_i5::g.113622::m.113622	1,86E-120	350	XP_018818624.1	3-hydroxyisobutyryl-CoA hydrolase 1-like	Juglans regia	ONI22132.1
TRINITY_DN11271_c0_g1::TRINITY_DN11271_c0_g1_i1::g.6832::m.6832	1,54E-109	316	XP_018820087.1	tankyrase	Juglans regia	OIV89168.1
TRINITY_DN17765_c0_g1::TRINITY_DN17765_c0_g1_i1::g.89341::m.89341	0	580	XP_008368822.1	homocysteine S-methyltransferase 2-like	Malus domestica	XP_009370794.1
TRINITY_DN13374_c2_g1::TRINITY_DN13374_c2_g1_i4::g.22819::m.22819	0	890	XP_018806906.1	3-oxoacyl-[acyl-carrier-protein synthase II, chloroplastic-like	Juglans regia	XP_018820205.1
TRINITY_DN15833_c5_g1::TRINITY_DN15833_c5_g1_i12::g.57896::m.57896	3,05E-102	298	XP_018503189.1	probable ADP-ribosylation factor GTPase-activating protein AGD15	Pyrus x bretschneideri	XP_008346894.1
TRINITY_DN17806_c2_g2::TRINITY_DN17806_c2_g2_i4::g.90030::m.90030	0	1158	XP_018858563.1	protein transport protein SEC16B homolog	Juglans regia	XP_018859927.1
TRINITY_DN15514_c4_g2::TRINITY_DN15514_c4_g2_i2::g.53063::m.53063	0	992	KDP45840.1	hypothetical protein JCGZ_17447	Jatropha curcas	XP_012079840.1
TRINITY_DN15090_c0_g1::TRINITY_DN15090_c0_g1_i3::g.46611::m.46611	0	2083	XP_018816763.1	protease Do-like 7	Juglans regia	ONI17440.1
TRINITY_DN12620_c0_g1::TRINITY_DN12620_c0_g1_i4::g.14272::m.14272	0	875	XP_018820021.1	E3 ubiquitin-protein ligase BRE1-like 1 isoform X2	Juglans regia	XP_018820012.1
TRINITY_DN10821_c0_g1::TRINITY_DN10821_c0_g1_i1::g.5589::m.5589	6,42E-116	332	XP_018836742.1	mitochondrial import receptor subunit TOM20-like	Juglans regia	XP_018852624.1
TRINITY_DN12247_c0_g5::TRINITY_DN12247_c0_g5_i2::g.11476::m.11476	0	756	XP_018844607.1	cation/H(+) antiporter 15-like	Juglans regia	ONI14265.1
TRINITY_DN17715_c1_g2::TRINITY_DN17715_c1_g2_i7::g.88682::m.88682	0	534	XP_018848132.1	uncharacterized protein LOC109011398	Juglans regia	XP_009343901.1
TRINITY_DN13507_c0_g2::TRINITY_DN13507_c0_g2_i4::g.24688::m.24688	2,46E-72	215	XP_018833220.1	60S ribosomal protein L32-1-like	Juglans regia	XP_018833221.1
TRINITY_DN18452_c0_g2::TRINITY_DN18452_c0_g2_i5::g.100623::m.100623	3,84E-73	232	XP_018859162.1	putative 3,4-dihydroxy-2-butanone kinase, partial	Juglans regia	XP_008240398.1
TRINITY_DN18940_c3_g1::TRINITY_DN18940_c3_g1_i4::g.109190::m.109190	3,91E-68	221	XP_018835985.1	ATP-dependent zinc metalloprotease FTSH 10, mitochondrial-like	Juglans regia	XP_018850454.1
TRINITY_DN19263_c0_g3::TRINITY_DN19263_c0_g3_i3::g.114810::m.114810	0	684	XP_018813437.1	probable cytosolic oligopeptidase A	Juglans regia	XP_018837044.1
TRINITY_DN16828_c3_g2::TRINITY_DN16828_c3_g2_i1::g.74126::m.74126	3,04E-92	274	XP_018830672.1	choline-phosphate cytidylyltransferase 2-like	Juglans regia	ONI17886.1
TRINITY_DN15895_c3_g2::TRINITY_DN15895_c3_g2_i1::g.57724::m.57724	2,95E-132	385	XP_018846484.1	28 kDa ribonucleoprotein, chloroplastic-like	Juglans regia	ONI19760.1
TRINITY_DN18527_c2_g1::TRINITY_DN18527_c2_g1_i1::g.101980::m.101980	6,83E-55	181	XP_018828437.1	UDP-glycosyltransferase 87A1-like	Juglans regia	XP_018828459.1

TRINITY_DN18999_c4_g1::TRINITY_DN18999_c4_g1_i2::g.110202::m.110202	1,93E-151	430	XP_018820983.1	GLABRA2 expression modulator-like	Juglans regia	XP_009376597.1
TRINITY_DN16996_c2_g1::TRINITY_DN16996_c2_g1_i5::g.76760::m.76760	0	1170	XP_018856630.1	prolyl endopeptidase-like	Juglans regia	XP_018812062.1
TRINITY_DN17420_c0_g1::TRINITY_DN17420_c0_g1_i9::g.83584::m.83584	0	617	XP_018807997.1	glyceraldehyde-3-phosphate dehydrogenase, cytosolic-like	Juglans regia	AES72079.1
TRINITY_DN18991_c0_g1::TRINITY_DN18991_c0_g1_i4::g.110139::m.110139	3,76E-154	456	XP_018834221.1	far upstream element-binding protein 2-like isoform X2	Juglans regia	XP_018834220.1
TRINITY_DN16630_c2_g2::TRINITY_DN16630_c2_g2_i3::g.70772::m.70772	0	714	XP_018823451.1	bifunctional D-cysteine desulfhydrase/1-aminocyclopropane-1-carboxylate deaminase, mitochondrial	Juglans regia	ONI09471.1
TRINITY_DN17508_c5_g4::TRINITY_DN17508_c5_g4_i2::g.85425::m.85425	2,82E-20	88,2	XP_018817991.1	exopolygalacturonase-like	Juglans regia	XP_018817992.1
TRINITY_DN16170_c0_g1::TRINITY_DN16170_c0_g1_i3::g.63133::m.63133	0	1321	XP_018817103.1	long chain acyl-CoA synthetase 9, chloroplastic	Juglans regia	XP_018812536.1
TRINITY_DN13107_c5_g1::TRINITY_DN13107_c5_g1_i1::g.19946::m.19946	1,19E-50	167	XP_018837699.1	oleosin 18.2 kDa-like	Juglans regia	OAY47824.1
TRINITY_DN17688_c2_g2::TRINITY_DN17688_c2_g2_i2::g.88270::m.88270	1,49E-42	145	GAU16385.1	hypothetical protein TSUD_117340	Trifolium subterraneum	OIW17390.1
TRINITY_DN12789_c1_g2::TRINITY_DN12789_c1_g2_i26::g.15965::m.15965	2,19E-86	257	XP_018806698.1	somatic embryogenesis receptor kinase 2-like	Juglans regia	XP_008389932.1
TRINITY_DN16664_c4_g1::TRINITY_DN16664_c4_g1_i4::g.71662::m.71662	1,67E-45	160	XP_018851780.1	la-related protein 1C-like	Juglans regia	XP_018856743.1
TRINITY_DN15784_c0_g1::TRINITY_DN15784_c0_g1_i2::g.57047::m.57047	0	1315	XP_018842610.1	neutral ceramidase-like	Juglans regia	XP_018837606.1
TRINITY_DN17576_c2_g1::TRINITY_DN17576_c2_g1_i10::g.86267::m.86267	8,28E-49	161	XP_018821662.1	desumoylating isopeptidase 1-like	Juglans regia	XP_018821664.1
TRINITY_DN20043_c8_g6::TRINITY_DN20043_c8_g6_i2::g.128243::m.128243	3,30E-118	355	XP_018858135.1	LOW QUALITY PROTEIN: phospholipase A-2-activating protein	Juglans regia	OAY25488.1
TRINITY_DN17136_c2_g4::TRINITY_DN17136_c2_g4_i1::g.79232::m.79232	3,78E-147	421	XP_018826990.1	uncharacterized protein LOC108995816	Juglans regia	OAY42107.1
TRINITY_DN17588_c1_g2::TRINITY_DN17588_c1_g2_i1::g.86528::m.86528	4,99E-126	358	XP_018855277.1	peptide methionine sulfoxide reductase B5-like	Juglans regia	XP_018844456.1
TRINITY_DN12440_c1_g1::TRINITY_DN12440_c1_g1_i2::g.13042::m.13042	0	968	XP_018831593.1	aldehyde dehydrogenase family 2 member B4, mitochondrial isoform X1	Juglans regia	XP_018831594.1
TRINITY_DN14203_c2_g2::TRINITY_DN14203_c2_g2_i3::g.33825::m.33825	0	1623	XP_018839604.1	chaperone protein ClpB4, mitochondrial	Juglans regia	OIW14979.1
TRINITY_DN16295_c1_g3::TRINITY_DN16295_c1_g3_i8::g.65237::m.65237	0	625	XP_018821643.1	uncharacterized protein LOC108991725	Juglans regia	XP_018848582.1
TRINITY_DN14603_c3_g1::TRINITY_DN14603_c3_g1_i1::g.39490::m.39490	0	1598	XP_018830142.1	peroxisomal ATPase pex6-like	Juglans regia	XP_018821222.1
TRINITY_DN17919_c1_g4::TRINITY_DN17919_c1_g4_i5::g.91938::m.91938	0	1759	ONH94219.1	hypothetical protein PRUPE_7G004800	Prunus persica	XP_020423622.1

TRINITY_DN16456_c0_g2::TRINITY_DN16456_c0_g2_i7::g.67919::m.67919	3,94E-160	462	XP_018859944.1	uncharacterized protein LOC109021706	Juglans regia	XP_018824342.1
TRINITY_DN18783_c5_g1::TRINITY_DN18783_c5_g1_i3::g.106356::m.106356	0	887	XP_018818227.1	serine carboxypeptidase-like 20	Juglans regia	AFK43057.1
TRINITY_DN14585_c2_g1::TRINITY_DN14585_c2_g1_i7::g.39202::m.39202	8,66E-118	337	XP_018821284.1	GDSL esterase/lipase At5g62930 isoform X2	Juglans regia	XP_018821283.1
TRINITY_DN17674_c1_g2::TRINITY_DN17674_c1_g2_i8::g.88061::m.88061	0	841	XP_018841870.1	uncharacterized protein LOC109006898	Juglans regia	XP_018826237.1
TRINITY_DN15931_c0_g1::TRINITY_DN15931_c0_g1_i5::g.59103::m.59103	9,19E-165	472	XP_008237677.1	serine carboxypeptidase-like 42	Prunus mume	ONH89835.1
TRINITY_DN15984_c0_g3::TRINITY_DN15984_c0_g3_i1::g.59910::m.59910	2,29E-109	319	XP_018859330.1	protein STRICTOSIDINE SYNTHASE-LIKE 5-like, partial	Juglans regia	XP_018859332.1
TRINITY_DN15696_c0_g5::TRINITY_DN15696_c0_g5_i1::g.55738::m.55738	1,22E-161	470	XP_018858135.1	LOW QUALITY PROTEIN: phospholipase A-2-activating protein	Juglans regia	XP_018858135.1
TRINITY_DN13415_c1_g2::TRINITY_DN13415_c1_g2_i6::g.23608::m.23608	0	619	XP_018831119.1	putative lipase ROG1 isoform X2	Juglans regia	XP_018831117.1
TRINITY_DN10290_c0_g3::TRINITY_DN10290_c0_g3_i1::g.4573::m.4573	0	531	XP_018844550.1	tryptophan synthase alpha chain-like	Juglans regia	XP_018827345.1
TRINITY_DN17086_c3_g1::TRINITY_DN17086_c3_g1_i14::g.77558::m.77558	0	709	XP_008372070.1	PTI1-like tyrosine-protein kinase 1 isoform X1	Malus domestica	XP_009345628.1
TRINITY_DN15448_c0_g1::TRINITY_DN15448_c0_g1_i6::g.51979::m.51979	3,78E-172	489	XP_018807641.1	arginine biosynthesis bifunctional protein ArgJ, chloroplastic	Juglans regia	XP_020990889.1
TRINITY_DN17327_c0_g4::TRINITY_DN17327_c0_g4_i4::g.82004::m.82004	1,42E-102	302	XP_018848101.1	dehydrogenase/reductase SDR family member 12 isoform X1	Juglans regia	XP_018848102.1
TRINITY_DN18850_c2_g1::TRINITY_DN18850_c2_g1_i14::g.107185::m.107185	0	810	XP_018809451.1	acetate/butyrate--CoA ligase AAE7, peroxisomal	Juglans regia	XP_008340623.1
TRINITY_DN15339_c5_g2::TRINITY_DN15339_c5_g2_i1::g.49931::m.49931	0	1270	XP_018827878.1	uncharacterized protein LOC108996435 isoform X1	Juglans regia	XP_018827896.1
TRINITY_DN11883_c0_g2::TRINITY_DN11883_c0_g2_i5::g.9377::m.9377	0	1063	XP_004515814.1	probable inactive purple acid phosphatase 27	Cicer arietinum	XP_016190655.1
TRINITY_DN15187_c1_g1::TRINITY_DN15187_c1_g1_i9::g.47932::m.47932	1,75E-69	213	XP_018822762.1	RNA-binding protein 38-like isoform X1	Juglans regia	XP_018822763.1
TRINITY_DN18055_c0_g1::TRINITY_DN18055_c0_g1_i4::g.94445::m.94445	0	3810	XP_018843075.1	DExH-box ATP-dependent RNA helicase DExH12	Juglans regia	OAY22523.1
TRINITY_DN17443_c0_g6::TRINITY_DN17443_c0_g6_i1::g.83849::m.83849	0	923	XP_018842578.1	L-gulonolactone oxidase 3-like	Juglans regia	XP_008220057.1
TRINITY_DN17889_c3_g3::TRINITY_DN17889_c3_g3_i2::g.91373::m.91373	4,91E-80	243	XP_018836516.1	caffeoylshikimate esterase-like	Juglans regia	ONI24143.1
TRINITY_DN16290_c1_g2::TRINITY_DN16290_c1_g2_i6::g.65201::m.65201	0	1093	XP_018831786.1	NAD-dependent malic enzyme 59 kDa isoform, mitochondrial	Juglans regia	KDP28665.1
TRINITY_DN16914_c0_g2::TRINITY_DN16914_c0_g2_i3::g.75483::m.75483	4,28E-68	206	XP_018814519.1	peptidyl-prolyl cis-trans isomerase FKBP20-1	Juglans regia	KDP44932.1
TRINITY_DN16356_c0_g1::TRINITY_DN16356_c0_g1_i13::g.66037::m.66037	4,72E-140	406	XP_018825908.1	LIMR family protein At5g01460	Juglans regia	KHN07715.1

TRINITY_DN16552_c0_g2::TRINITY_DN16552_c0_g2_i2::g.69237::m.69237	7,13E-79	237	KHN31490.1	Peroxygenase 2	Glycine soja	KRH68777.1
TRINITY_DN13122_c2_g7::TRINITY_DN13122_c2_g7_i5::g.20188::m.20188	5,89E-58	179	XP_008229999.1	uncharacterized protein LOC103329321	Prunus mume	ONI01557.1
TRINITY_DN18287_c2_g1::TRINITY_DN18287_c2_g1_i6::g.98094::m.98094	0	701	XP_018826105.1	glutamine synthetase leaf isozyme, chloroplastic	Juglans regia	XP_018826106.1
TRINITY_DN15932_c0_g5::TRINITY_DN15932_c0_g5_i5::g.59228::m.59228	0	558	XP_018856751.1	eukaryotic translation initiation factor 5B-like, partial	Juglans regia	XP_018848181.1
TRINITY_DN17310_c0_g1::TRINITY_DN17310_c0_g1_i2::g.81834::m.81834	0	655	XP_018829410.1	splicing factor U2af large subunit B-like isoform X6	Juglans regia	XP_018826445.1
TRINITY_DN15542_c3_g2::TRINITY_DN15542_c3_g2_i4::g.53532::m.53532	3,52E-81	246	XP_018835712.1	uncharacterized membrane protein At1g06890-like	Juglans regia	XP_018835713.1
TRINITY_DN18437_c2_g2::TRINITY_DN18437_c2_g2_i5::g.100589::m.100589	0	2755	XP_018829159.1	brefeldin A-inhibited guanine nucleotide-exchange protein 1 like isoform X1	Juglans regia	XP_018841580.1
TRINITY_DN17906_c0_g2::TRINITY_DN17906_c0_g2_i5::g.91748::m.91748	0	904	KDP36969.1	hypothetical protein JCGZ_08561	Jatropha curcas	XP_012073486.1
TRINITY_DN12593_c3_g1::TRINITY_DN12593_c3_g1_i6::g.14142::m.14142	0	827	XP_018820957.1	ATP-dependent 6-phosphofructokinase 5, chloroplastic-like isoform X1	Juglans regia	XP_018830337.1
TRINITY_DN18611_c0_g1::TRINITY_DN18611_c0_g1_i4::g.103412::m.103412	0	858	XP_018832851.1	ABC transporter F family member 4	Juglans regia	XP_018832851.1
TRINITY_DN14624_c0_g1::TRINITY_DN14624_c0_g1_i4::g.39690::m.39690	1,62E-128	366	XP_018845530.1	6,7-dimethyl-8-ribityllumazine synthase, chloroplastic	Juglans regia	XP_018845612.1
TRINITY_DN19014_c2_g2::TRINITY_DN19014_c2_g2_i8::g.110573::m.110573	0	1840	XP_018808616.1	phosphoenolpyruvate carboxylase 2	Juglans regia	OAY57364.1
TRINITY_DN16277_c1_g3::TRINITY_DN16277_c1_g3_i4::g.64929::m.64929	3,70E-65	209	XP_018835532.1	multiple organellar RNA editing factor 1, mitochondrial	Juglans regia	XP_008339499.1
TRINITY_DN17957_c0_g1::TRINITY_DN17957_c0_g1_i11::g.92631::m.92631	0	5894	XP_018813247.1	E3 ubiquitin-protein ligase UPL1-like	Juglans regia	XP_018813253.1
TRINITY_DN15571_c4_g3::TRINITY_DN15571_c4_g3_i4::g.54049::m.54049	0	753	XP_018831543.1	mannose-1-phosphate guanyltransferase alpha-like isoform X2	Juglans regia	XP_018831541.1
TRINITY_DN11568_c0_g1::TRINITY_DN11568_c0_g1_i1::g.7589::m.7589	1,90E-93	275	XP_018811558.1	outer envelope pore protein 16-2, chloroplastic isoform X1	Juglans regia	ONI28085.1
TRINITY_DN12349_c8_g2::TRINITY_DN12349_c8_g2_i3::g.12071::m.12071	1,09E-89	267	XP_018809112.1	binding partner of ACD11 1-like	Juglans regia	ONI05935.1
TRINITY_DN12678_c0_g1::TRINITY_DN12678_c0_g1_i7::g.14864::m.14864	0	786	XP_018824522.1	signal recognition particle subunit SRP68	Juglans regia	XP_018824523.1
TRINITY_DN16016_c2_g1::TRINITY_DN16016_c2_g1_i9::g.60539::m.60539	2,65E-71	213	XP_018812852.1	uncharacterized protein At2g34160-like	Juglans regia	OAY27368.1
TRINITY_DN11023_c0_g2::TRINITY_DN11023_c0_g2_i1::g.6151::m.6151	0	574	XP_020422962.1	putative E3 ubiquitin-protein ligase LIN-1	Prunus persica	OAY54637.1
TRINITY_DN18955_c2_g1::TRINITY_DN18955_c2_g1_i2::g.109309::m.109309	2,08E-78	231	OAY42085.1	hypothetical protein MANES_09G151800	Manihot esculenta	OAY42086.1
TRINITY_DN17913_c4_g1::TRINITY_DN17913_c4_g1_i1::g.91856::m.91856	0	701	XP_018824898.1	glycerophosphodiester phosphodiesterase GDPD6	Juglans regia	ONI08875.1

TRINITY_DN18697_c0_g2::TRINITY_DN18697_c0_g2_i2::g.103311::m.103311	0	852	XP_018845274.1	probable nucleolar protein 5-2	Juglans regia	XP_018845272.1
TRINITY_DN15624_c0_g1::TRINITY_DN15624_c0_g1_i1::g.54548::m.54548	0	1511	XP_018815732.1	probable acyl-CoA dehydrogenase IBR3	Juglans regia	OAY56055.1
TRINITY_DN12621_c1_g1::TRINITY_DN12621_c1_g1_i2::g.14309::m.14309	1,17E-143	407	XP_018846418.1	acid phosphatase 1-like	Juglans regia	XP_008242463.1
TRINITY_DN15292_c1_g3::TRINITY_DN15292_c1_g3_i1::g.49799::m.49799	2,61E-84	259	XP_018832707.1	uncharacterized protein LOC109000333	Juglans regia	KDP23730.1
TRINITY_DN16971_c2_g1::TRINITY_DN16971_c2_g1_i8::g.76469::m.76469	1,01E-78	240	OAY27875.1	hypothetical protein MANES_15G022900	Manihot esculenta	XP_018846892.1
TRINITY_DN15274_c1_g2::TRINITY_DN15274_c1_g2_i1::g.49397::m.49397	8,30E-55	184	XP_018831636.1	phosphatidylinositol 3,4,5-trisphosphate 3-phosphatase and protein-tyrosine-phosphatase PTEN2A-like	Juglans regia	XP_018831637.1
TRINITY_DN17008_c0_g1::TRINITY_DN17008_c0_g1_i7::g.77048::m.77048	1,66E-126	365	XP_018860153.1	sulfite oxidase	Juglans regia	XP_018860154.1
TRINITY_DN18404_c1_g1::TRINITY_DN18404_c1_g1_i7::g.100040::m.100040	0	887	KDP29923.1	hypothetical protein JCGZ_18492	Jatropha curcas	XP_012081460.1
TRINITY_DN18356_c1_g1::TRINITY_DN18356_c1_g1_i9::g.99317::m.99317	0	876	XP_018856983.1	conserved oligomeric Golgi complex subunit 5	Juglans regia	XP_008229436.1
TRINITY_DN17060_c1_g1::TRINITY_DN17060_c1_g1_i6::g.77747::m.77747	0	566	XP_018835418.1	protein-lysine N-methyltransferase Mettl10-like	Juglans regia	XP_018817005.1
TRINITY_DN14005_c1_g1::TRINITY_DN14005_c1_g1_i1::g.31333::m.31333	0	769	XP_018816505.1	probable L-cysteine desulfhydrase, chloroplastic	Juglans regia	XP_018816506.1
TRINITY_DN16201_c3_g1::TRINITY_DN16201_c3_g1_i2::g.62696::m.62696	2,57E-61	186	KYP67103.1	60S ribosomal protein L37a	Cajanus cajan	XP_012075387.1
TRINITY_DN12639_c0_g1::TRINITY_DN12639_c0_g1_i2::g.14158::m.14158	9,33E-111	323	CAX05443.1	unnamed protein product	Glycine max	CAX05471.1
TRINITY_DN15473_c1_g1::TRINITY_DN15473_c1_g1_i2::g.52569::m.52569	0	762	XP_018811842.1	uncharacterized protein LOC108984369	Juglans regia	XP_018811842.1
TRINITY_DN17388_c0_g2::TRINITY_DN17388_c0_g2_i7::g.82945::m.82945	7,91E-177	493	XP_018850886.1	ribulose-phosphate 3-epimerase, chloroplastic	Juglans regia	KYP55554.1
TRINITY_DN19219_c1_g1::TRINITY_DN19219_c1_g1_i14::g.114200::m.114200	0	919	XP_018838318.1	protein STRUBBELIG-RECEPTOR FAMILY 5-like	Juglans regia	KDP25030.1
TRINITY_DN16821_c1_g1::TRINITY_DN16821_c1_g1_i1::g.73860::m.73860	2,50E-45	151	XP_018836814.1	uncharacterized protein LOC109003222	Juglans regia	XP_018805269.1
TRINITY_DN18965_c3_g1::TRINITY_DN18965_c3_g1_i3::g.109455::m.109455						
TRINITY_DN16438_c0_g4::TRINITY_DN16438_c0_g4_i1::g.67637::m.67637	0	1035	XP_018826754.1	probable galactinol--sucrose galactosyltransferase 6 isoform X1	Juglans regia	XP_018826755.1
TRINITY_DN16330_c0_g1::TRINITY_DN16330_c0_g1_i8::g.66108::m.66108	0	526	XP_018832022.1	exocyst complex component EXO70A1-like, partial	Juglans regia	XP_012074334.1
TRINITY_DN19616_c4_g3::TRINITY_DN19616_c4_g3_i5::g.120935::m.120935	0	1283	XP_018851380.1	uncharacterized protein LOC109013676	Juglans regia	XP_018856765.1

TRINITY_DN17970_c3_g1::TRINITY_DN17970_c3_g1_i6::g.92954::m.92954	0	810	OAY33567.1	hypothetical protein MANES_13G107500	Manihot esculenta	KDP32128.1
TRINITY_DN16469_c0_g1::TRINITY_DN16469_c0_g1_i7::g.67034::m.67034	0	1204	ONH98575.1	hypothetical protein PRUPE_7G254900	Prunus persica	ONH98576.1
TRINITY_DN15978_c1_g2::TRINITY_DN15978_c1_g2_i1::g.59851::m.59851	0	1037	XP_018850452.1	ATP-dependent zinc metalloprotease FTSH 10, mitochondrial-like isoform X1	Juglans regia	XP_018850453.1
TRINITY_DN17528_c1_g1::TRINITY_DN17528_c1_g1_i8::g.85347::m.85347	1,79E-100	300	OAY49013.1	hypothetical protein MANES_05G022800	Manihot esculenta	OAY49012.1
TRINITY_DN19544_c2_g6::TRINITY_DN19544_c2_g6_i1::g.119552::m.119552	8,90E-61	186	XP_018830984.1	60S ribosomal protein L31-like	Juglans regia	XP_008219353.1
TRINITY_DN16118_c1_g1::TRINITY_DN16118_c1_g1_i2::g.62411::m.62411	2,72E-149	422	KDP24500.1	hypothetical protein JCGZ_25064	Jatropha curcas	XP_012087936.1
TRINITY_DN14654_c1_g1::TRINITY_DN14654_c1_g1_i1::g.40157::m.40157	0	1136	XP_018859772.1	uncharacterized protein LOC109021570	Juglans regia	XP_018834514.1
TRINITY_DN16121_c0_g1::TRINITY_DN16121_c0_g1_i2::g.62465::m.62465	7,17E-64	196	XP_009372405.1	coatomer subunit zeta-2-like	Pyrus x bretschneideri	XP_009340672.1
TRINITY_DN18674_c2_g2::TRINITY_DN18674_c2_g2_i1::g.104393::m.104393	8,68E-139	399	XP_018828995.1	ER membrane protein complex subunit 10	Juglans regia	OAY32255.1
TRINITY_DN15551_c3_g3::TRINITY_DN15551_c3_g3_i8::g.53664::m.53664	1,49E-126	369	XP_018846833.1	vacuole membrane protein KMS1-like	Juglans regia	XP_018840464.1
TRINITY_DN18806_c2_g1::TRINITY_DN18806_c2_g1_i13::g.106758::m.106758	8,03E-167	466	XP_008239808.1	microtubule-associated protein RP/EB family member 1A	Prunus mume	ONI08630.1
TRINITY_DN14781_c0_g1::TRINITY_DN14781_c0_g1_i21::g.42142::m.42142	0	702	XP_018810936.1	kinesin-4-like isoform X1	Juglans regia	XP_018810937.1
TRINITY_DN13427_c0_g3::TRINITY_DN13427_c0_g3_i1::g.23641::m.23641	9,43E-38	129	XP_008384671.1	40S ribosomal protein S26-3-like	Malus domestica	XP_009354778.1
TRINITY_DN15029_c5_g6::TRINITY_DN15029_c5_g6_i4::g.46001::m.46001	1,48E-64	204	XP_018824102.1	protein FATTY ACID EXPORT 2, chloroplastic-like	Juglans regia	XP_018806598.1
TRINITY_DN15434_c1_g3::TRINITY_DN15434_c1_g3_i3::g.51817::m.51817	1,04E-147	414	XP_015949837.1	ras-related protein RABA4d	Arachis duranensis	ONH95452.1
TRINITY_DN17008_c0_g1::TRINITY_DN17008_c0_g1_i2::g.77040::m.77040	2,47E-128	367	OAY29635.1	hypothetical protein MANES_15G160200	Manihot esculenta	ONI19931.1
TRINITY_DN17336_c2_g1::TRINITY_DN17336_c2_g1_i3::g.82080::m.82080	0	1008	XP_018833558.1	uncharacterized protein LOC109000940	Juglans regia	OAY57262.1
TRINITY_DN15690_c0_g1::TRINITY_DN15690_c0_g1_i7::g.55685::m.55685	3,45E-163	459	XP_018845290.1	probable uridine nucleosidase 2	Juglans regia	XP_008243078.1
TRINITY_DN13180_c0_g1::TRINITY_DN13180_c0_g1_i3::g.20757::m.20757	0	774	XP_009373714.1	uncharacterized protein LOC103962691	Pyrus x bretschneideri	XP_009373715.1
TRINITY_DN11253_c0_g1::TRINITY_DN11253_c0_g1_i2::g.6760::m.6760	1,66E-94	276	XP_018821966.1	frataxin, mitochondrial-like isoform X2	Juglans regia	XP_018821965.1
TRINITY_DN16309_c0_g1::TRINITY_DN16309_c0_g1_i5::g.65915::m.65915	7,15E-61	190	XP_018830835.1	protein phosphatase inhibitor 2 isoform X2	Juglans regia	XP_018830836.1
TRINITY_DN11739_c0_g1::TRINITY_DN11739_c0_g1_i1::g.8613::m.8613	2,12E-171	483	XP_018849967.1	probable mitochondrial import inner membrane translocase subunit TIM21 isoform X1	Juglans regia	XP_018849968.1

TRINITY_DN13614_c0_g1::TRINITY_DN13614_c0_g1_i2::g.26309::m.26309	8,54E-171	478	XP_018843402.1	ubiquinol-cytochrome-c reductase complex assembly factor 1	Juglans regia	XP_018843404.1
TRINITY_DN18223_c0_g1::TRINITY_DN18223_c0_g1_i1::g.97248::m.97248	0	1688	XP_018815773.1	importin beta-like SAD2	Juglans regia	OAY52150.1
TRINITY_DN19809_c1_g1::TRINITY_DN19809_c1_g1_i1::g.124378::m.124378	2,60E-98	296	XP_016182572.1	DEAD-box ATP-dependent RNA helicase 52C	Arachis ipaensis	XP_018834571.1
TRINITY_DN15338_c3_g1::TRINITY_DN15338_c3_g1_i1::g.50560::m.50560	9,20E-122	349	XP_018814792.1	nucleoside diphosphate kinase 2, chloroplastic	Juglans regia	ONI08060.1
TRINITY_DN18884_c1_g1::TRINITY_DN18884_c1_g1_i5::g.108202::m.108202	0	999	XP_018819118.1	exocyst complex component EXO84A	Juglans regia	KDP21256.1
TRINITY_DN12888_c1_g1::TRINITY_DN12888_c1_g1_i1::g.16988::m.16988	7,48E-109	314	XP_018838204.1	heme-binding protein 1	Juglans regia	XP_020215778.1
TRINITY_DN18793_c0_g1::TRINITY_DN18793_c0_g1_i11::g.106461::m.106461	0	522	OAY52792.1	hypothetical protein MANES_04G111700	Manihot esculenta	CAY09187.1
TRINITY_DN14198_c0_g1::TRINITY_DN14198_c0_g1_i4::g.33633::m.33633	3,27E-134	398	XP_014513658.1	granule-bound starch synthase 2, chloroplastic/amyloplastic-like	Vigna radiata var. radiata	KOM56651.1
TRINITY_DN13898_c1_g1::TRINITY_DN13898_c1_g1_i2::g.30051::m.30051	1,76E-157	445	ONI16083.1	hypothetical protein PRUPE_3G078000	Prunus persica	ONI16084.1
TRINITY_DN16513_c0_g3::TRINITY_DN16513_c0_g3_i2::g.68923::m.68923	0	520	XP_018815697.1	syntaxin-124	Juglans regia	OIV91639.1
TRINITY_DN19115_c1_g2::TRINITY_DN19115_c1_g2_i10::g.112400::m.112400	0	778	XP_018824165.1	uridine kinase-like protein 1, chloroplastic isoform X1	Juglans regia	KDP46297.1
TRINITY_DN19612_c0_g2::TRINITY_DN19612_c0_g2_i5::g.120800::m.120800	1,37E-83	250	XP_008235050.1	stress-related protein	Prunus mume	ONH93881.1
TRINITY_DN19652_c2_g1::TRINITY_DN19652_c2_g1_i9::g.121472::m.121472	0	1211	XP_018859492.1	putative uncharacterized protein DDB_G0277255 isoform X5	Juglans regia	XP_018859489.1
TRINITY_DN15221_c1_g3::TRINITY_DN15221_c1_g3_i1::g.48708::m.48708	0	955	XP_018821744.1	probable alpha-galactosidase B	Juglans regia	XP_018821744.1
TRINITY_DN17380_c2_g1::TRINITY_DN17380_c2_g1_i4::g.82866::m.82866	0	1395	KDP22848.1	hypothetical protein JCGZ_00435	Jatropha curcas	XP_012089796.1
TRINITY_DN19589_c2_g2::TRINITY_DN19589_c2_g2_i4::g.120311::m.120311	1,21E-169	473	KHN28969.1	3-oxoacyl-[acyl-carrier-protein synthase I, chloroplastic	Glycine soja	ABJ90468.1
TRINITY_DN12563_c3_g1::TRINITY_DN12563_c3_g1_i1::g.13893::m.13893	2,15E-76	228	XP_018834275.1	uncharacterized protein At4g28440	Juglans regia	XP_018805198.1
TRINITY_DN14756_c11_g3::TRINITY_DN14756_c11_g3_i1::g.41922::m.41922	0	621	XP_018808557.1	pectinesterase-like	Juglans regia	XP_008223522.1
TRINITY_DN16369_c2_g1::TRINITY_DN16369_c2_g1_i6::g.66520::m.66520	2,14E-80	278	XP_018823799.1	translocase of chloroplast 120, chloroplastic-like	Juglans regia	XP_018823799.1
TRINITY_DN14258_c0_g1::TRINITY_DN14258_c0_g1_i5::g.34520::m.34520	0	993	XP_018851343.1	LETM1 and EF-hand domain-containing protein 1, mitochondrial	Juglans regia	XP_018851344.1
TRINITY_DN16789_c2_g5::TRINITY_DN16789_c2_g5_i1::g.73225::m.73225	0	798	XP_018852491.1	uncharacterized protein LOC109014469	Juglans regia	OAY42749.1
TRINITY_DN17303_c1_g1::TRINITY_DN17303_c1_g1_i7::g.81652::m.81652	0	536	ONI18361.1	hypothetical protein PRUPE_3G211200	Prunus persica	XP_020416553.1

TRINITY_DN19981_c2_g1::TRINITY_DN19981_c2_g1_i2::g.127291::m.127291	0	1149	XP_018842158.1	probable splicing factor 3A subunit 1 isoform X1	Juglans regia	XP_018842159.1
TRINITY_DN13121_c2_g1::TRINITY_DN13121_c2_g1_i1::g.20130::m.20130	0	684	XP_018807788.1	pre-mRNA-processing factor 19-like	Juglans regia	XP_018857360.1
TRINITY_DN17618_c2_g2::TRINITY_DN17618_c2_g2_i9::g.87148::m.87148	2,18E-64	207	XP_018837633.1	cytochrome P450 89A2-like	Juglans regia	XP_018837634.1
TRINITY_DN18372_c2_g8::TRINITY_DN18372_c2_g8_i1::g.99519::m.99519	2,92E-49	171	XP_018845025.1	phosphatidylinositol 3,4,5-trisphosphate 3-phosphatase and protein-tyrosine-phosphatase PTEN2A-like	Juglans regia	KDP31989.1
TRINITY_DN18320_c3_g3::TRINITY_DN18320_c3_g3_i3::g.98813::m.98813	1,07E-157	447	XP_018808205.1	polyadenylate-binding protein RBP45-like isoform X1	Juglans regia	XP_018808205.1
TRINITY_DN15818_c1_g1::TRINITY_DN15818_c1_g1_i7::g.57419::m.57419	4,14E-95	280	XP_018835166.1	uncharacterized protein LOC109002047	Juglans regia	ONI23151.1
TRINITY_DN17545_c0_g1::TRINITY_DN17545_c0_g1_i11::g.84946::m.84946	0	1341	XP_018829229.1	glycine--tRNA ligase, mitochondrial 1-like	Juglans regia	XP_018841636.1
TRINITY_DN18096_c1_g1::TRINITY_DN18096_c1_g1_i2::g.95119::m.95119	0	1057	XP_018806781.1	ERAD-associated E3 ubiquitin-protein ligase component HRD3A	Juglans regia	ONI09356.1
TRINITY_DN13342_c2_g1::TRINITY_DN13342_c2_g1_i2::g.22558::m.22558	9,54E-146	417	CAA69726.1	mitochondrial phosphate translocator	Betula pendula	XP_018832657.1
TRINITY_DN16074_c1_g1::TRINITY_DN16074_c1_g1_i7::g.61513::m.61513	0	1577	XP_018817529.1	serine/threonine-protein phosphatase BSL3-like	Juglans regia	XP_018829734.1
TRINITY_DN12750_c0_g2::TRINITY_DN12750_c0_g2_i1::g.15503::m.15503	0	590	XP_018849186.1	pectin acetylesterase 8-like	Juglans regia	XP_018832508.1
TRINITY_DN19511_c1_g1::TRINITY_DN19511_c1_g1_i3::g.119032::m.119032	0	795	XP_018810471.1	COP9 signalosome complex subunit 1-like	Juglans regia	XP_018810472.1
TRINITY_DN13260_c0_g2::TRINITY_DN13260_c0_g2_i1::g.21634::m.21634	4,88E-59	197	XP_018821273.1	peroxisomal membrane protein PEX14	Juglans regia	XP_018821274.1
TRINITY_DN19385_c2_g2::TRINITY_DN19385_c2_g2_i1::g.117065::m.117065	1,73E-36	135	KDP39665.1	hypothetical protein JCGZ_02685	Jatropha curcas	XP_012070406.1
TRINITY_DN14374_c0_g1::TRINITY_DN14374_c0_g1_i4::g.36023::m.36023	1,33E-119	342	XP_018856668.1	protein LURP-one-related 10-like isoform X1	Juglans regia	KRH00381.1
TRINITY_DN14718_c0_g3::TRINITY_DN14718_c0_g3_i4::g.41295::m.41295	5,98E-152	436	ONH97960.1	hypothetical protein PRUPE_7G221100	Prunus persica	KDP33810.1
TRINITY_DN14491_c1_g3::TRINITY_DN14491_c1_g3_i2::g.37906::m.37906	1,72E-133	399	XP_018858250.1	uncharacterized protein LOC109020252	Juglans regia	XP_020421355.1
TRINITY_DN18652_c1_g2::TRINITY_DN18652_c1_g2_i5::g.104071::m.104071	9,86E-74	231	KYP71032.1	UBA and UBX domain-containing protein At4g15410 family	Cajanus cajan	XP_020212365.1
TRINITY_DN17169_c0_g2::TRINITY_DN17169_c0_g2_i1::g.79657::m.79657	1,28E-67	209	XP_018836881.1	anamorsin homolog	Juglans regia	XP_018836882.1
TRINITY_DN17784_c0_g1::TRINITY_DN17784_c0_g1_i3::g.89656::m.89656	0	509	XP_018845293.1	outer envelope pore protein 37, chloroplastic-like	Juglans regia	XP_018839352.1
TRINITY_DN15619_c2_g2::TRINITY_DN15619_c2_g2_i4::g.54476::m.54476	4,42E-63	202	XP_018844917.1	transmembrane protein 120 homolog	Juglans regia	XP_012086582.1

TRINITY_DN15799_c0_g1::TRINITY_DN15799_c0_g1_i8::g.55961::m.55961	1,32E-115	335	XP_018842015.1	endochitinase-like	Juglans regia	XP_008241979.1
TRINITY_DN18926_c2_g1::TRINITY_DN18926_c2_g1_i1::g.109132::m.109132	0	1313	XP_018822784.1	conserved oligomeric Golgi complex subunit 4	Juglans regia	OAY62076.1
TRINITY_DN15946_c1_g1::TRINITY_DN15946_c1_g1_i2::g.59352::m.59352	0	804	XP_009361638.1	glycylpeptide N-tetradecanoyltransferase 1-like	Pyrus x bretschneideri	XP_009361640.1
TRINITY_DN18496_c3_g1::TRINITY_DN18496_c3_g1_i9::g.99949::m.99949	0	1005	XP_018805796.1	protein WEAK CHLOROPLAST MOVEMENT UNDER BLUE LIGHT 1-like	Juglans regia	XP_018805797.1
TRINITY_DN11371_c0_g2::TRINITY_DN11371_c0_g2_i2::g.7148::m.7148	0	627	XP_018813685.1	protein CIA1-like	Juglans regia	XP_018835731.1
TRINITY_DN11955_c0_g2::TRINITY_DN11955_c0_g2_i1::g.9673::m.9673	6,36E-162	461	XP_018814780.1	3-oxoacyl-[acyl-carrier-protein reductase 4 isoform X2	Juglans regia	XP_018814779.1
TRINITY_DN12552_c0_g1::TRINITY_DN12552_c0_g1_i2::g.13425::m.13425	2,29E-82	245	XP_018806727.1	magnesium-dependent phosphatase 1-like	Juglans regia	KDP29765.1
TRINITY_DN19080_c0_g2::TRINITY_DN19080_c0_g2_i7::g.111803::m.111803	0	1586	XP_018842470.1	probable glutamyl endopeptidase, chloroplastic isoform X2	Juglans regia	XP_018842472.1
TRINITY_DN19854_c2_g2::TRINITY_DN19854_c2_g2_i4::g.124562::m.124562	4,69E-142	424	XP_018842400.1	eukaryotic translation initiation factor 3 subunit C isoform X1	Juglans regia	XP_018842401.1
TRINITY_DN19442_c0_g2::TRINITY_DN19442_c0_g2_i3::g.118018::m.118018	0	912	XP_018842611.1	cytosolic enolase 3	Juglans regia	KDP46567.1
TRINITY_DN18350_c0_g2::TRINITY_DN18350_c0_g2_i2::g.99155::m.99155	2,34E-84	250	XP_018829252.1	uncharacterized protein LOC108997431	Juglans regia	XP_008390244.1
TRINITY_DN12711_c1_g1::TRINITY_DN12711_c1_g1_i4::g.15231::m.15231	1,20E-140	405	ACU22776.1	unknown	Glycine max	KHN47572.1
TRINITY_DN14491_c1_g5::TRINITY_DN14491_c1_g5_i7::g.37912::m.37912	0	892	XP_018858250.1	uncharacterized protein LOC109020252	Juglans regia	XP_020421355.1
TRINITY_DN18628_c2_g1::TRINITY_DN18628_c2_g1_i28::g.103627::m.103627	0	842	AET62908.1	NADH dehydrogenase subunit 5 (mitochondrion)	Millettia pinnata	YP_005090448.1
TRINITY_DN14281_c0_g1::TRINITY_DN14281_c0_g1_i1::g.34852::m.34852	0	752	XP_018827095.1	ubiquinone biosynthesis monooxygenase COQ6, mitochondrial isoform X1	Juglans regia	XP_018827096.1
TRINITY_DN16267_c0_g1::TRINITY_DN16267_c0_g1_i3::g.64801::m.64801	0	699	XP_018821343.1	IST1 homolog	Juglans regia	XP_018809491.1
TRINITY_DN14107_c0_g1::TRINITY_DN14107_c0_g1_i1::g.32583::m.32583	0	654	XP_018859242.1	flowering locus K homology domain-like	Juglans regia	OAY44268.1
TRINITY_DN18942_c3_g3::TRINITY_DN18942_c3_g3_i8::g.109006::m.109006	0	839	XP_018854773.1	heterogeneous nuclear ribonucleoprotein R-like isoform X2	Juglans regia	XP_018854773.1
TRINITY_DN15316_c4_g2::TRINITY_DN15316_c4_g2_i4::g.50087::m.50087	2,82E-108	310	KHN26395.1	Signal peptidase complex catalytic subunit SEC11C	Glycine soja	KRH56207.1
TRINITY_DN18397_c1_g1::TRINITY_DN18397_c1_g1_i2::g.99905::m.99905	0	1845	XP_018846667.1	brefeldin A-inhibited guanine nucleotide-exchange protein 2	Juglans regia	KDP28742.1
TRINITY_DN14957_c1_g1::TRINITY_DN14957_c1_g1_i3::g.44723::m.44723	2,64E-74	237	XP_018811765.1	la protein 1	Juglans regia	XP_008220038.1
TRINITY_DN5432_c0_g1::TRINITY_DN5432_c0_g1_i1::g.1343::m.1343	7,67E-128	364	XP_008360830.1	aldehyde dehydrogenase family 2 member B4, mitochondrial-like, partial	Malus domestica	XP_004508854.1

TRINITY_DN10737_c0_g1::TRINITY_DN10737_c0_g1_i1::g.5423::m.5423	3,79E-111	317	XP_018834778.1	probable N-acetyltransferase san	Juglans regia	OAY27343.1
TRINITY_DN18886_c3_g1::TRINITY_DN18886_c3_g1_i2::g.108068::m.108068	4,46E-147	420	XP_018850608.1	caffeic acid 3-O-methyltransferase	Juglans regia	KDP29212.1
TRINITY_DN19870_c1_g2::TRINITY_DN19870_c1_g2_i5::g.124748::m.124748	1,30E-52	180	XP_018853563.1	aspartokinase 1, chloroplastic-like	Juglans regia	XP_018840015.1
TRINITY_DN16270_c0_g2::TRINITY_DN16270_c0_g2_i15::g.64899::m.64899	0	518	XP_018837992.1	uncharacterized protein LOC109004054	Juglans regia	XP_018837993.1
TRINITY_DN14538_c2_g2::TRINITY_DN14538_c2_g2_i3::g.38504::m.38504	0	583	XP_018820232.1	very-long-chain 3-oxoacyl-CoA reductase 1	Juglans regia	OAY32455.1
TRINITY_DN18432_c0_g1::TRINITY_DN18432_c0_g1_i9::g.99987::m.99987	0	7453	XP_018814243.1	uncharacterized protein LOC108986177 isoform X1	Juglans regia	XP_018814244.1
TRINITY_DN14697_c1_g1::TRINITY_DN14697_c1_g1_i3::g.40725::m.40725	0	604	XP_018819392.1	ethanolamine-phosphate cytidylyltransferase	Juglans regia	XP_018819392.1
TRINITY_DN12130_c0_g1::TRINITY_DN12130_c0_g1_i1::g.10682::m.10682	9,29E-85	248	XP_018849401.1	NADH dehydrogenase [ubiquinone] 1 alpha subcomplex subunit 6	Juglans regia	OAY36255.1
TRINITY_DN16549_c1_g2::TRINITY_DN16549_c1_g2_i1::g.69658::m.69658	2,68E-136	384	XP_018827308.1	uncharacterized protein LOC108996034 isoform X1	Juglans regia	XP_018827309.1
TRINITY_DN13928_c0_g1::TRINITY_DN13928_c0_g1_i1::g.30448::m.30448	0	1334	XP_018841693.1	myosin heavy chain, non-muscle isoform X1	Juglans regia	XP_018841693.1
TRINITY_DN13623_c1_g1::TRINITY_DN13623_c1_g1_i6::g.26584::m.26584	0	629	AES82113.1	potassium transporter-like protein	Medicago truncatula	XP_003625895.1
TRINITY_DN15579_c1_g3::TRINITY_DN15579_c1_g3_i1::g.54029::m.54029	0	877	XP_018845393.1	DEAD-box ATP-dependent RNA helicase 8-like	Juglans regia	XP_008232788.1
TRINITY_DN17845_c1_g3::TRINITY_DN17845_c1_g3_i3::g.90638::m.90638	0	583	XP_018823299.1	glucan endo-1,3-beta-glucosidase-like	Juglans regia	OAY61233.1
TRINITY_DN18905_c1_g2::TRINITY_DN18905_c1_g2_i9::g.108635::m.108635	5,50E-67	205	XP_018835503.1	uncharacterized protein LOC109002285	Juglans regia	XP_015943687.1
TRINITY_DN16321_c0_g3::TRINITY_DN16321_c0_g3_i5::g.65822::m.65822	0	696	XP_018810395.1	peroxisomal (S)-2-hydroxy-acid oxidase GLO1	Juglans regia	XP_018819691.1
TRINITY_DN18561_c0_g1::TRINITY_DN18561_c0_g1_i3::g.102624::m.102624	0	1995	XP_018826677.1	uncharacterized protein LOC108995553 isoform X1	Juglans regia	XP_018826678.1
TRINITY_DN13727_c0_g2::TRINITY_DN13727_c0_g2_i11::g.27681::m.27681	6,27E-50	169	XP_018817131.1	uncharacterized protein LOC108988346 isoform X2	Juglans regia	XP_018817130.1
TRINITY_DN13477_c2_g3::TRINITY_DN13477_c2_g3_i1::g.24414::m.24414	0	507	XP_018842612.1	proliferating cell nuclear antigen	Juglans regia	XP_004500211.1
TRINITY_DN11771_c0_g1::TRINITY_DN11771_c0_g1_i2::g.8609::m.8609	0	517	XP_018837754.1	anthranilate phosphoribosyltransferase, chloroplastic-like	Juglans regia	OAY47794.1
TRINITY_DN11029_c0_g1::TRINITY_DN11029_c0_g1_i1::g.6154::m.6154	0	982	XP_018843241.1	malonate--CoA ligase-like isoform X1	Juglans regia	XP_018843242.1
TRINITY_DN19209_c0_g1::TRINITY_DN19209_c0_g1_i7::g.114040::m.114040	6,65E-179	503	XP_018848680.1	eukaryotic translation initiation factor 5B-like	Juglans regia	XP_020533049.1
TRINITY_DN15323_c1_g4::TRINITY_DN15323_c1_g4_i7::g.50170::m.50170	1,22E-82	246	XP_008234339.1	uncharacterized protein LOC103333300	Prunus mume	XP_020411795.1

TRINITY_DN14645_c3_g1::TRINITY_DN14645_c3_g1_i7::g.39449::m.39449	0	1182	XP_018836467.1	mitochondrial substrate carrier family protein C isoform X1	Juglans regia	XP_018836468.1
TRINITY_DN14335_c0_g1::TRINITY_DN14335_c0_g1_i7::g.35543::m.35543	0	597	XP_018842811.1	vacuolar protein sorting-associated protein 26B-like	Juglans regia	XP_018842812.1
TRINITY_DN17475_c1_g1::TRINITY_DN17475_c1_g1_i10::g.84374::m.84374	0	1205	XP_018815848.1	probable ubiquitin conjugation factor E4	Juglans regia	ONI19115.1
TRINITY_DN18714_c0_g3::TRINITY_DN18714_c0_g3_i1::g.105033::m.105033	8,39E-71	214	XP_018850228.1	uncharacterized protein At4g28440-like	Juglans regia	XP_009340594.1
TRINITY_DN18972_c2_g2::TRINITY_DN18972_c2_g2_i2::g.109605::m.109605	0	624	XP_018829145.1	ankyrin repeat domain-containing protein 2B-like	Juglans regia	XP_018841596.1
TRINITY_DN12403_c0_g1::TRINITY_DN12403_c0_g1_i5::g.12601::m.12601	4,89E-43	144	XP_018813172.1	small heat shock protein, chloroplastic-like	Juglans regia	AKP06201.1
TRINITY_DN11347_c0_g1::TRINITY_DN11347_c0_g1_i2::g.7021::m.7021	3,53E-71	216	XP_018831072.1	uncharacterized protein LOC108998821	Juglans regia	XP_009370500.1
TRINITY_DN18196_c4_g4::TRINITY_DN18196_c4_g4_i1::g.96720::m.96720	1,42E-59	185	XP_008374043.1	LIM domain-containing protein WLIM2b-like	Malus domestica	XP_008374043.1
TRINITY_DN19648_c4_g4::TRINITY_DN19648_c4_g4_i4::g.120655::m.120655	1,80E-106	304	XP_008388680.1	40S ribosomal protein S15-4	Malus domestica	XP_008345721.1
TRINITY_DN18301_c0_g2::TRINITY_DN18301_c0_g2_i7::g.97543::m.97543	0	512	XP_018841131.1	protein AIG1-like	Juglans regia	XP_012073509.1
TRINITY_DN15444_c2_g2::TRINITY_DN15444_c2_g2_i4::g.52039::m.52039	7,85E-122	358	XP_018825622.1	heterogeneous nuclear ribonucleoprotein 1	Juglans regia	KDP39313.1
TRINITY_DN19535_c3_g1::TRINITY_DN19535_c3_g1_i2::g.119402::m.119402	1,30E-83	256	XP_018811706.1	probable protein phosphatase 2C 47	Juglans regia	XP_018811707.1
TRINITY_DN12020_c1_g1::TRINITY_DN12020_c1_g1_i2::g.10043::m.10043	1,27E-172	484	OAY25879.1	hypothetical protein MANES_16G002900	Manihot esculenta	KYP62724.1
TRINITY_DN17610_c1_g1::TRINITY_DN17610_c1_g1_i7::g.87051::m.87051	3,09E-78	241	XP_018848057.1	phosphoglucan phosphatase DSP4, amyloplastic-like isoform X1	Juglans regia	XP_018848058.1
TRINITY_DN18503_c1_g1::TRINITY_DN18503_c1_g1_i3::g.101658::m.101658	1,94E-173	484	XP_018832031.1	probable ribose-5-phosphate isomerase 3, chloroplastic	Juglans regia	XP_015968505.1
TRINITY_DN17513_c0_g1::TRINITY_DN17513_c0_g1_i8::g.85061::m.85061	0	2076	XP_018820451.1	tubulin-folding cofactor D	Juglans regia	XP_008232610.1
TRINITY_DN18932_c2_g2::TRINITY_DN18932_c2_g2_i2::g.109083::m.109083	5,94E-176	495	XP_018849565.1	inositol-tetrakisphosphate 1-kinase 1-like	Juglans regia	XP_018849566.1
TRINITY_DN18021_c3_g2::TRINITY_DN18021_c3_g2_i4::g.93827::m.93827	3,88E-32	121	KDP23970.1	hypothetical protein JCGZ_25358	Jatropha curcas	XP_012088477.1
TRINITY_DN13471_c1_g1::TRINITY_DN13471_c1_g1_i4::g.24277::m.24277	2,93E-80	235	XP_018827356.1	bet1-like SNARE 1-1	Juglans regia	XP_018825210.1
TRINITY_DN14922_c2_g1::TRINITY_DN14922_c2_g1_i5::g.44553::m.44553	0	4474	XP_018859294.1	protein SABRE isoform X1	Juglans regia	XP_018859295.1
TRINITY_DN15594_c4_g1::TRINITY_DN15594_c4_g1_i1::g.54180::m.54180	0	694	XP_018839609.1	UDP-N-acetylglucosamine--dolichyl-phosphate N-acetylglucosaminephosphotransferase-like	Juglans regia	XP_008392344.1
TRINITY_DN11209_c0_g1::TRINITY_DN11209_c0_g1_i1::g.6616::m.6616	5,02E-165	463	ALB76795.1	enoyl-CoA hydratase, partial	Jatropha curcas	XP_018815731.1

TRINITY_DN18631_c1_g1::TRINITY_DN18631_c1_g1_i1::g.103584::m.103584	1,29E-166	465	XP_018839985.1	protein YIF1B-like	Juglans regia	XP_018839986.1
TRINITY_DN17980_c0_g1::TRINITY_DN17980_c0_g1_i2::g.92937::m.92937	3,23E-104	324	XP_018845571.1	beta-adaptin-like protein A	Juglans regia	ONI33920.1
TRINITY_DN18446_c0_g1::TRINITY_DN18446_c0_g1_i3::g.100648::m.100648	1,78E-60	197	BAT86921.1	hypothetical protein VIGAN_05025400	Vigna angularis var. angularis	BAA36972.1
TRINITY_DN19548_c1_g1::TRINITY_DN19548_c1_g1_i5::g.119761::m.119761	1,94E-140	403	XP_018837590.1	uncharacterized protein LOC109003755 isoform X2	Juglans regia	XP_018837582.1
TRINITY_DN16896_c1_g1::TRINITY_DN16896_c1_g1_i19::g.75135::m.75135	5,41E-94	273	XP_018826083.1	peptide methionine sulfoxide reductase B5-like isoform X1	Juglans regia	XP_018826084.1
TRINITY_DN14204_c1_g1::TRINITY_DN14204_c1_g1_i2::g.33927::m.33927	2,96E-73	237	XP_018847401.1	TOM1-like protein 2	Juglans regia	XP_018847402.1
TRINITY_DN19434_c0_g1::TRINITY_DN19434_c0_g1_i3::g.117932::m.117932	2,19E-115	340	XP_018830436.1	methyl-CpG-binding domain-containing protein 11-like isoform X1	Juglans regia	XP_018851719.1
TRINITY_DN17973_c0_g1::TRINITY_DN17973_c0_g1_i1::g.92481::m.92481	0	738	OAY30335.1	hypothetical protein MANES_14G022300	Manihot esculenta	OAY48361.1
TRINITY_DN18833_c4_g2::TRINITY_DN18833_c4_g2_i4::g.107207::m.107207	3,48E-145	410	XP_018827478.1	probable carbohydrate esterase At4g34215	Juglans regia	XP_008236370.1
TRINITY_DN16544_c0_g2::TRINITY_DN16544_c0_g2_i7::g.68626::m.68626	3,41E-85	257	ONI25827.1	hypothetical protein PRUPE_2G322200	Prunus persica	XP_007218831.1
TRINITY_DN19860_c5_g1::TRINITY_DN19860_c5_g1_i1::g.124725::m.124725	1,54E-148	441	XP_018833676.1	ATPase 8, plasma membrane-type	Juglans regia	XP_009347012.1
TRINITY_DN48442_c0_g1::TRINITY_DN48442_c0_g1_i1::g.132681::m.132681	3,71E-146	420	XP_018811850.1	imidazole glycerol phosphate synthase hisHF, chloroplastic isoform X3	Juglans regia	XP_018811844.1
TRINITY_DN19061_c2_g6::TRINITY_DN19061_c2_g6_i3::g.111201::m.111201	5,24E-170	474	XP_018821200.1	V-type proton ATPase subunit D-like	Juglans regia	XP_008229383.1
TRINITY_DN14323_c2_g3::TRINITY_DN14323_c2_g3_i2::g.35593::m.35593	0	700	XP_018818781.1	ATPase family AAA domain-containing protein 1-B-like	Juglans regia	XP_018842807.1
TRINITY_DN19440_c4_g2::TRINITY_DN19440_c4_g2_i10::g.117992::m.117992	4,93E-168	473	XP_018816304.1	probable protein disulfide-isomerase A6	Juglans regia	XP_018816304.1
TRINITY_DN18371_c0_g1::TRINITY_DN18371_c0_g1_i2::g.99819::m.99819	7,25E-33	122	OAY37422.1	hypothetical protein MANES_11G100600	Manihot esculenta	OAY37423.1
TRINITY_DN14419_c1_g1::TRINITY_DN14419_c1_g1_i6::g.36874::m.36874	0	1082	XP_018812504.1	probable inactive purple acid phosphatase 2	Juglans regia	AGL44406.1
TRINITY_DN13980_c0_g1::TRINITY_DN13980_c0_g1_i1::g.30892::m.30892	4,85E-180	505	XP_018812444.1	ubiquinone biosynthesis O-methyltransferase, mitochondrial	Juglans regia	XP_012077529.1
TRINITY_DN18731_c0_g5::TRINITY_DN18731_c0_g5_i1::g.105388::m.105388	3,23E-108	315	XP_004485968.1	eukaryotic translation initiation factor 4E-1-like	Cicer arietinum	KHN06937.1
TRINITY_DN13791_c2_g2::TRINITY_DN13791_c2_g2_i3::g.28238::m.28238	0	570	XP_018845774.1	pentatricopeptide repeat-containing protein At4g35850, mitochondrial-like isoform X1	Juglans regia	XP_018845776.1
TRINITY_DN15442_c0_g2::TRINITY_DN15442_c0_g2_i1::g.51251::m.51251	3,99E-77	228	ACU15022.1	unknown	Glycine max	KHN12783.1
TRINITY_DN1265_c0_g1::TRINITY_DN1265_c0_g1_i1::g.324::m.324	2,85E-152	436	XP_018851722.1	SNF1-related protein kinase regulatory subunit gamma-like PV42a	Juglans regia	KDP37073.1

TRINITY_DN14475_c2_g1::TRINITY_DN14475_c2_g1_i7::g.36756::m.36756	9,10E-124	359	XP_018815354.1	squalene synthase-like	Juglans regia	KRH29366.1
TRINITY_DN16138_c0_g1::TRINITY_DN16138_c0_g1_i2::g.62708::m.62708	0	726	XP_018844889.1	ruvB-like protein 1 isoform X1	Juglans regia	XP_018844890.1
TRINITY_DN18532_c1_g1::TRINITY_DN18532_c1_g1_i2::g.102269::m.102269	0	1207	XP_018837526.1	golgin candidate 5 isoform X1	Juglans regia	XP_018837528.1
TRINITY_DN18494_c1_g1::TRINITY_DN18494_c1_g1_i7::g.101464::m.101464	3,35E-140	399	XP_018844403.1	short-chain type dehydrogenase/reductase	Juglans regia	OAY44433.1
TRINITY_DN13527_c0_g1::TRINITY_DN13527_c0_g1_i14::g.25011::m.25011	0	1081	KDP37982.1	hypothetical protein JCGZ_04625	Jatropha curcas	XP_012072113.1
TRINITY_DN19343_c7_g5::TRINITY_DN19343_c7_g5_i1::g.116417::m.116417	4,16E-81	253	KRH09479.1	hypothetical protein GLYMA_16G217900	Glycine max	XP_003548316.1
TRINITY_DN17435_c0_g1::TRINITY_DN17435_c0_g1_i2::g.83592::m.83592	3,43E-142	400	XP_018857020.1	peptidyl-prolyl cis-trans isomerase CYP23	Juglans regia	KDP41170.1
TRINITY_DN12632_c0_g1::TRINITY_DN12632_c0_g1_i1::g.14398::m.14398	3,11E-133	384	XP_018827780.1	protein TIC 21, chloroplastic	Juglans regia	XP_018827781.1
TRINITY_DN13052_c1_g1::TRINITY_DN13052_c1_g1_i8::g.19267::m.19267	7,01E-96	285	XP_018823251.1	THO complex subunit 4A-like isoform X1	Juglans regia	XP_018836017.1
TRINITY_DN16618_c2_g1::TRINITY_DN16618_c2_g1_i4::g.70494::m.70494	0	813	XP_018820267.1	ubiquitin carboxyl-terminal hydrolase MINDY-2-like	Juglans regia	XP_018820490.1
TRINITY_DN17627_c2_g1::TRINITY_DN17627_c2_g1_i6::g.87313::m.87313	2,47E-153	431	XP_018858478.1	uracil phosphoribosyltransferase isoform X1	Juglans regia	XP_018858479.1
TRINITY_DN14266_c0_g5::TRINITY_DN14266_c0_g5_i1::g.34758::m.34758	7,37E-162	469	OAY41017.1	hypothetical protein MANES_09G067300	Manihot esculenta	OAY41017.1
TRINITY_DN15019_c3_g2::TRINITY_DN15019_c3_g2_i1::g.45833::m.45833	0	589	XP_018849372.1	uncharacterized protein LOC109012285	Juglans regia	XP_018818523.1
TRINITY_DN18255_c0_g1::TRINITY_DN18255_c0_g1_i22::g.97730::m.97730	0	547	XP_007150083.1	hypothetical protein PHAVU_005G125100g	Phaseolus vulgaris	ESW22077.1
TRINITY_DN19124_c2_g3::TRINITY_DN19124_c2_g3_i2::g.112438::m.112438	0	539	XP_018816447.1	DUF21 domain-containing protein At2g14520-like isoform X1	Juglans regia	XP_012079460.1
TRINITY_DN13398_c7_g1::TRINITY_DN13398_c7_g1_i2::g.23298::m.23298	4,16E-104	320	XP_018826435.1	acidic leucine-rich nuclear phosphoprotein 32-related protein-like	Juglans regia	XP_018829399.1
TRINITY_DN18939_c0_g2::TRINITY_DN18939_c0_g2_i7::g.109183::m.109183	3,08E-119	353	XP_018828549.1	alpha-glucosidase	Juglans regia	XP_018828549.1
TRINITY_DN13383_c1_g4::TRINITY_DN13383_c1_g4_i2::g.23048::m.23048	0	811	KYP42121.1	hypothetical protein KK1_036495	Cajanus cajan	XP_020239762.1
TRINITY_DN15405_c3_g1::TRINITY_DN15405_c3_g1_i6::g.51555::m.51555	2,58E-90	278	XP_018818346.1	dynamin-related protein 5A	Juglans regia	OAY49012.1
TRINITY_DN12135_c0_g1::TRINITY_DN12135_c0_g1_i2::g.10684::m.10684	7,23E-142	402	XP_018807787.1	GDSL esterase/lipase 7-like	Juglans regia	XP_018806668.1
TRINITY_DN17356_c4_g2::TRINITY_DN17356_c4_g2_i4::g.82383::m.82383	0	835	XP_018811934.1	lysophospholipid acyltransferase 1-like	Juglans regia	XP_018828813.1
TRINITY_DN17524_c0_g2::TRINITY_DN17524_c0_g2_i3::g.85311::m.85311	3,29E-168	474	XP_018816770.1	pyridoxal kinase-like isoform X1	Juglans regia	OAY45362.1

TRINITY_DN11973_c1_g1::TRINITY_DN11973_c1_g1_i7::g.9759::m.9759	0	918	XP_018826789.1	bifunctional purple acid phosphatase 26-like	Juglans regia	XP_008242837.1
TRINITY_DN15456_c0_g1::TRINITY_DN15456_c0_g1_i1::g.52131::m.52131	0	571	XP_018826206.1	acyl-coenzyme A oxidase 4, peroxisomal isoform X1	Juglans regia	XP_018826207.1
TRINITY_DN15793_c0_g1::TRINITY_DN15793_c0_g1_i1::g.57091::m.57091	7,09E-64	194	AFK36986.1	unknown	Lotus japonicus	KHN13429.1
TRINITY_DN14209_c0_g1::TRINITY_DN14209_c0_g1_i2::g.34018::m.34018	3,41E-96	294	XP_018827296.1	uncharacterized protein LOC108996024	Juglans regia	XP_018807874.1
TRINITY_DN14887_c2_g4::TRINITY_DN14887_c2_g4_i1::g.43866::m.43866	0	525	XP_018808420.1	uridine-cytidine kinase C-like isoform X1	Juglans regia	XP_018808421.1
TRINITY_DN18372_c2_g2::TRINITY_DN18372_c2_g2_i3::g.99514::m.99514	0	694	XP_018845025.1	phosphatidylinositol 3,4,5-trisphosphate 3-phosphatase and protein-tyrosine-phosphatase PTEN2A-like	Juglans regia	KOM28908.1
TRINITY_DN16028_c0_g1::TRINITY_DN16028_c0_g1_i7::g.60828::m.60828	0	770	XP_018820165.1	trafficking protein particle complex subunit 13 isoform X1	Juglans regia	XP_018820166.1
TRINITY_DN12857_c0_g1::TRINITY_DN12857_c0_g1_i2::g.16763::m.16763	1,30E-105	318	XP_018852253.1	plastoglobulin-1, chloroplastic-like	Juglans regia	KDP30383.1
TRINITY_DN18741_c1_g1::TRINITY_DN18741_c1_g1_i9::g.105752::m.105752	5,97E-127	366	XP_018845112.1	phosducin-like protein 3	Juglans regia	KDP46320.1
TRINITY_DN18860_c2_g4::TRINITY_DN18860_c2_g4_i2::g.107536::m.107536	2,16E-105	325	XP_018856430.1	uncharacterized protein LOC109018725	Juglans regia	OAY23752.1
TRINITY_DN12789_c1_g2::TRINITY_DN12789_c1_g2_i12::g.15958::m.15958	5,26E-52	169	XP_018850680.1	tropinone reductase homolog At5g06060-like	Juglans regia	XP_018841297.1
TRINITY_DN19136_c0_g1::TRINITY_DN19136_c0_g1_i3::g.112661::m.112661	4,06E-76	228	XP_018831755.1	ubiquitin-conjugating enzyme E2 27	Juglans regia	KDP22561.1
TRINITY_DN16675_c0_g2::TRINITY_DN16675_c0_g2_i3::g.71545::m.71545	0	809	XP_018844867.1	anthranilate synthase alpha subunit 2, chloroplastic-like	Juglans regia	XP_004497238.1
TRINITY_DN19779_c3_g1::TRINITY_DN19779_c3_g1_i9::g.123559::m.123559	2,11E-121	360	XP_018806898.1	lysine--tRNA ligase, cytoplasmic isoform X2	Juglans regia	XP_018806897.1
TRINITY_DN11951_c0_g1::TRINITY_DN11951_c0_g1_i4::g.9610::m.9610	0	830	XP_018848980.1	folylpolyglutamate synthase	Juglans regia	OAY38548.1
TRINITY_DN17944_c2_g2::TRINITY_DN17944_c2_g2_i3::g.92569::m.92569	0	875	XP_018824286.1	SUN domain-containing protein 2	Juglans regia	XP_009358629.1
TRINITY_DN17545_c0_g1::TRINITY_DN17545_c0_g1_i1::g.84935::m.84935	0	1335	XP_018829229.1	glycine--tRNA ligase, mitochondrial 1-like	Juglans regia	XP_018841636.1
TRINITY_DN14785_c0_g1::TRINITY_DN14785_c0_g1_i6::g.42288::m.42288	2,20E-66	211	XP_018810986.1	protein disulfide-isomerase 5-3-like	Juglans regia	KDP39071.1
TRINITY_DN10853_c0_g1::TRINITY_DN10853_c0_g1_i1::g.5658::m.5658	7,18E-76	228	XP_016205608.1	uncharacterized protein LOC107645960	Arachis ipaensis	XP_014495882.1
TRINITY_DN19439_c3_g1::TRINITY_DN19439_c3_g1_i7::g.118339::m.118339	0	546	XP_018858974.1	clathrin interactor EPSIN 2 isoform X2	Juglans regia	XP_018858973.1
TRINITY_DN19401_c0_g3::TRINITY_DN19401_c0_g3_i5::g.116386::m.116386	1,54E-152	435	XP_018845609.1	oligouridylate-binding protein 1B-like	Juglans regia	XP_018845609.1

TRINITY_DN13193_c0_g2::TRINITY_DN13193_c0_g2_i7::g.20882::m.20882	1,62E-157	448	AAD51625.1	AF169022_1 seed maturation protein PM37	Glycine max	KHN21201.1
TRINITY_DN16102_c2_g2::TRINITY_DN16102_c2_g2_i1::g.62195::m.62195	4,96E-69	217	XP_008243362.1	ERBB-3 BINDING PROTEIN 1	Prunus mume	ONI03759.1
TRINITY_DN17099_c3_g2::TRINITY_DN17099_c3_g2_i2::g.76778::m.76778	0	605	XP_018850843.1	apoptotic chromatin condensation inducer in the nucleus-like isoform X3	Juglans regia	XP_018850842.1
TRINITY_DN14038_c1_g1::TRINITY_DN14038_c1_g1_i3::g.31643::m.31643	0	680	XP_008337727.1	eukaryotic initiation factor 4A-11	Malus domestica	XP_018845033.1
TRINITY_DN16298_c0_g1::TRINITY_DN16298_c0_g1_i6::g.65227::m.65227	6,67E-174	484	ONH95000.1	hypothetical protein PRUPE_7G045500	Prunus persica	XP_007202429.1
TRINITY_DN16403_c4_g2::TRINITY_DN16403_c4_g2_i2::g.67059::m.67059	2,50E-68	205	AET62921.1	NADH dehydrogenase subunit 3 (mitochondrion)	Millettia pinnata	YP_005090461.1
TRINITY_DN19748_c4_g1::TRINITY_DN19748_c4_g1_i8::g.123188::m.123188	0	1194	XP_018819079.1	uncharacterized protein LOC108989795 isoform X1	Juglans regia	XP_018819080.1
TRINITY_DN16008_c3_g2::TRINITY_DN16008_c3_g2_i3::g.60431::m.60431	9,33E-49	155	OAY45897.1	hypothetical protein MANES_07G101100	Manihot esculenta	KYP35949.1
TRINITY_DN15365_c0_g2::TRINITY_DN15365_c0_g2_i4::g.50831::m.50831	7,71E-146	414	OAY38567.1	hypothetical protein MANES_10G025000	Manihot esculenta	XP_018857484.1
TRINITY_DN16706_c0_g2::TRINITY_DN16706_c0_g2_i5::g.72057::m.72057	3,57E-145	423	XP_018815234.1	UBP1-associated protein 2B-like	Juglans regia	XP_018815235.1
TRINITY_DN12693_c0_g1::TRINITY_DN12693_c0_g1_i1::g.14939::m.14939	3,52E-49	162	XP_008237914.1	cinnamoyl-CoA reductase 2-like isoform X2	Prunus mume	XP_018859183.1
TRINITY_DN13827_c1_g1::TRINITY_DN13827_c1_g1_i4::g.28842::m.28842	1,21E-84	268	XP_018817970.1	stress protein DDR48	Juglans regia	OAY27720.1
TRINITY_DN11389_c0_g1::TRINITY_DN11389_c0_g1_i1::g.7193::m.7193	0	654	XP_018857199.1	protein RAE1 isoform X1	Juglans regia	XP_018857200.1
TRINITY_DN12755_c3_g1::TRINITY_DN12755_c3_g1_i1::g.16006::m.16006	1,02E-71	236	XP_018807970.1	pollen-specific leucine-rich repeat extensin-like protein 3	Juglans regia	XP_018807970.1
TRINITY_DN13006_c0_g1::TRINITY_DN13006_c0_g1_i3::g.18571::m.18571	1,55E-79	235	XP_018847546.1	40S ribosomal protein S12-like	Juglans regia	XP_018853126.1
TRINITY_DN31964_c0_g1::TRINITY_DN31964_c0_g1_i1::g.130316::m.130316	1,61E-85	258	XP_018824464.1	ervatamin-B-like	Juglans regia	XP_018824181.1
TRINITY_DN10036_c0_g1::TRINITY_DN10036_c0_g1_i2::g.4202::m.4202	1,16E-98	291	XP_018828138.1	uncharacterized protein LOC108996608	Juglans regia	OAY44531.1
TRINITY_DN17508_c5_g2::TRINITY_DN17508_c5_g2_i5::g.85417::m.85417	4,35E-49	166	XP_018824758.1	exopolygalacturonase clone GBGE184-like	Juglans regia	XP_018817991.1
TRINITY_DN19723_c2_g3::TRINITY_DN19723_c2_g3_i8::g.122861::m.122861	8,40E-126	366	XP_018857981.1	phosphomethylethanolamine N-methyltransferase isoform X2	Juglans regia	XP_018857980.1
TRINITY_DN12524_c0_g1::TRINITY_DN12524_c0_g1_i3::g.13484::m.13484	1,02E-175	493	XP_018822923.1	plastid-lipid-associated protein, chloroplastic-like	Juglans regia	ONI09855.1
TRINITY_DN18436_c0_g2::TRINITY_DN18436_c0_g2_i4::g.100110::m.100110	0	880	XP_018815864.1	conserved oligomeric Golgi complex subunit 7	Juglans regia	AIU51136.1
TRINITY_DN12307_c0_g1::TRINITY_DN12307_c0_g1_i3::g.11724::m.11724	0	956	XP_018850957.1	pentatricopeptide repeat-containing protein At1g80270, mitochondrial-like	Juglans regia	OAY40209.1

TRINITY_DN13767_c0_g1::TRINITY_DN13767_c0_g1_i2::g.28089::m.28089	2,66E-50	166	XP_008345965.2	vesicle-fusing ATPase-like	Malus domestica	KDP20974.1
TRINITY_DN19009_c0_g1::TRINITY_DN19009_c0_g1_i8::g.110506::m.110506	1,97E-110	317	XP_008393051.1	ubiquitin-conjugating enzyme E2-23 kDa-like	Malus domestica	XP_008393052.1
TRINITY_DN17898_c0_g1::TRINITY_DN17898_c0_g1_i6::g.91458::m.91458	0	2876	XP_018805563.1	nuclear-pore anchor-like isoform X3	Juglans regia	XP_018805561.1
TRINITY_DN11577_c0_g1::TRINITY_DN11577_c0_g1_i3::g.7931::m.7931	1,81E-76	226	KHN35208.1	40S ribosomal protein S20-2	Glycine soja	KRH27587.1
TRINITY_DN17836_c0_g3::TRINITY_DN17836_c0_g3_i4::g.90474::m.90474	3,42E-51	164	XP_018813688.1	iron-sulfur assembly protein IscA-like 2, mitochondrial isoform X1	Juglans regia	KDP42748.1
TRINITY_DN19582_c3_g2::TRINITY_DN19582_c3_g2_i4::g.120114::m.120114	1,28E-130	378	ONI24902.1	hypothetical protein PRUPE_2G268500	Prunus persica	XP_007218171.1
TRINITY_DN15102_c1_g3::TRINITY_DN15102_c1_g3_i1::g.46949::m.46949	0	659	XP_018860326.1	thiamine thiazole synthase 2, chloroplastic-like	Juglans regia	XP_018820652.1
TRINITY_DN18657_c1_g1::TRINITY_DN18657_c1_g1_i4::g.104087::m.104087	1,94E-143	410	XP_018821666.1	COP9 signalosome complex subunit 4 isoform X2	Juglans regia	XP_018826700.1
TRINITY_DN11984_c0_g1::TRINITY_DN11984_c0_g1_i17::g.9822::m.9822	0	663	XP_018840513.1	ATP-dependent (S)-NAD(P)H-hydrate dehydratase isoform X2	Juglans regia	XP_018840512.1
TRINITY_DN13083_c4_g1::TRINITY_DN13083_c4_g1_i1::g.19609::m.19609	1,29E-140	397	XP_018848377.1	uncharacterized protein LOC109011572 isoform X1	Juglans regia	XP_008238559.1
TRINITY_DN11520_c0_g2::TRINITY_DN11520_c0_g2_i2::g.7719::m.7719	1,66E-135	390	XP_018828701.1	ATPase ASNA1 homolog 2-like	Juglans regia	XP_018828702.1
TRINITY_DN11695_c0_g2::TRINITY_DN11695_c0_g2_i5::g.8192::m.8192	1,15E-152	430	OAY46091.1	hypothetical protein MANES_07G115800	Manihot esculenta	XP_018814522.1
TRINITY_DN17518_c0_g2::TRINITY_DN17518_c0_g2_i4::g.85745::m.85745	5,16E-83	245	XP_020996771.1	SKP1-like protein 1B	Arachis duranensis	XP_018847894.1
TRINITY_DN15614_c2_g1::TRINITY_DN15614_c2_g1_i2::g.54750::m.54750	3,96E-94	280	XP_018858487.1	(+)-neomenthol dehydrogenase-like isoform X1	Juglans regia	XP_018839129.1
TRINITY_DN17279_c2_g2::TRINITY_DN17279_c2_g2_i30::g.80027::m.80027	0	1639	XP_018821562.1	exocyst complex component SEC3A	Juglans regia	ONI32787.1
TRINITY_DN12358_c0_g1::TRINITY_DN12358_c0_g1_i4::g.12129::m.12129	0	795	XP_018841273.1	plant UBX domain-containing protein 7	Juglans regia	OAY25414.1
TRINITY_DN14089_c0_g1::TRINITY_DN14089_c0_g1_i1::g.32223::m.32223	0	693	XP_018825271.1	enoyl-[acyl-carrier-protein reductase [NADH], chloroplastic-like	Juglans regia	XP_018834639.1
TRINITY_DN13617_c1_g1::TRINITY_DN13617_c1_g1_i9::g.26343::m.26343	0	616	XP_018836164.1	protein decapping 5 isoform X2	Juglans regia	XP_018836156.1
TRINITY_DN12509_c0_g1::TRINITY_DN12509_c0_g1_i1::g.13435::m.13435	0	524	XP_018859854.1	phosphoglycerate mutase-like protein 1 isoform X6	Juglans regia	XP_018859855.1
TRINITY_DN20017_c0_g1::TRINITY_DN20017_c0_g1_i1::g.127859::m.127859	3,58E-137	400	XP_018830495.1	pyruvate decarboxylase 1	Juglans regia	OAY26295.1
TRINITY_DN15264_c3_g1::TRINITY_DN15264_c3_g1_i8::g.49356::m.49356	3,31E-83	256	XP_018814264.1	phosphomethylethanolamine N-methyltransferase-like	Juglans regia	XP_018857981.1
TRINITY_DN19396_c1_g2::TRINITY_DN19396_c1_g2_i11::g.116356::m.116356	0	1694	XP_018830041.1	ABC transporter B family member 9 isoform X1	Juglans regia	XP_018830041.1

TRINITY_DN13077_c0_g1::TRINITY_DN13077_c0_g1_i9::g.19580::m.19580	2,57E-127	365	XP_018846540.1	uncharacterized protein LOC109010229 isoform X1	Juglans regia	XP_018846541.1
TRINITY_DN19577_c3_g1::TRINITY_DN19577_c3_g1_i6::g.120109::m.120109	7,51E-91	265	XP_018826455.1	uncharacterized protein LOC108995352	Juglans regia	XP_018829401.1
TRINITY_DN10714_c0_g1::TRINITY_DN10714_c0_g1_i4::g.5392::m.5392	3,35E-38	128	XP_018853464.1	uncharacterized protein LOC109015450	Juglans regia	ONI35449.1
TRINITY_DN13818_c0_g2::TRINITY_DN13818_c0_g2_i15::g.28994::m.28994	0	683	XP_018857848.1	ATP-citrate synthase alpha chain protein 2	Juglans regia	XP_018805227.1
TRINITY_DN18434_c0_g1::TRINITY_DN18434_c0_g1_i17::g.100395::m.100395	5,46E-81	258	XP_008236777.1	phospholipase A-2-activating protein	Prunus mume	XP_007131862.1
TRINITY_DN14145_c0_g4::TRINITY_DN14145_c0_g4_i3::g.33080::m.33080	2,97E-100	298	XP_018858935.1	chaperone protein dnaJ 49-like	Juglans regia	XP_018858936.1
TRINITY_DN14453_c2_g3::TRINITY_DN14453_c2_g3_i2::g.37393::m.37393	7,72E-66	206	KRH01821.1	hypothetical protein GLYMA_18G300500	Glycine max	XP_003552752.1
TRINITY_DN12731_c3_g2::TRINITY_DN12731_c3_g2_i1::g.15534::m.15534	2,55E-34	126	XP_018809534.1	exopolygalacturonase-like	Juglans regia	XP_018825627.1
TRINITY_DN19556_c1_g1::TRINITY_DN19556_c1_g1_i8::g.119918::m.119918	2,05E-80	236	AFK34423.1	unknown	Lotus japonicus	AFK37880.1
TRINITY_DN12315_c0_g1::TRINITY_DN12315_c0_g1_i2::g.11830::m.11830	0	859	XP_018838722.1	splicing factor SF3a60 homolog	Juglans regia	XP_018850767.1
TRINITY_DN14308_c1_g1::TRINITY_DN14308_c1_g1_i5::g.35396::m.35396	1,57E-169	483	XP_018822425.1	polyadenylate-binding protein RBP47-like	Juglans regia	XP_018822425.1
TRINITY_DN15701_c0_g1::TRINITY_DN15701_c0_g1_i7::g.55836::m.55836	0	1057	XP_018831403.1	MAP3K epsilon protein kinase 1-like	Juglans regia	XP_018831405.1
TRINITY_DN11076_c0_g1::TRINITY_DN11076_c0_g1_i2::g.6263::m.6263	0	912	XP_018842528.1	pentatricopeptide repeat-containing protein At4g28010	Juglans regia	XP_018842528.1
TRINITY_DN13289_c1_g1::TRINITY_DN13289_c1_g1_i2::g.22086::m.22086	0	647	XP_018850158.1	protein SAD1/UNC-84 domain protein 1-like	Juglans regia	XP_018842411.1
TRINITY_DN11519_c0_g1::TRINITY_DN11519_c0_g1_i2::g.7693::m.7693	0	1062	XP_018815603.1	ATPase family AAA domain-containing protein 3-like	Juglans regia	OAY57408.1
TRINITY_DN15166_c0_g1::TRINITY_DN15166_c0_g1_i7::g.47733::m.47733	1,13E-137	392	XP_018826641.1	peroxisomal membrane protein 11A	Juglans regia	XP_018826642.1
TRINITY_DN15517_c0_g1::TRINITY_DN15517_c0_g1_i10::g.53139::m.53139	0	1569	XP_018809437.1	golgin candidate 6 isoform X2	Juglans regia	XP_018809435.1
TRINITY_DN15729_c0_g1::TRINITY_DN15729_c0_g1_i9::g.56378::m.56378	4,14E-139	400	XP_018825366.1	SNF1-related protein kinase regulatory subunit gamma-1	Juglans regia	XP_008239461.1
TRINITY_DN15900_c2_g1::TRINITY_DN15900_c2_g1_i1::g.57240::m.57240	0	1379	XP_018818626.1	acylamino-acid-releasing enzyme isoform X2	Juglans regia	XP_018818625.1
TRINITY_DN13702_c0_g1::TRINITY_DN13702_c0_g1_i4::g.27394::m.27394	1,44E-47	152	XP_008373046.1	histone deacetylase 6	Malus domestica	XP_008356371.1
TRINITY_DN17271_c3_g3::TRINITY_DN17271_c3_g3_i8::g.81059::m.81059	3,94E-72	219	XP_018843992.1	vacuolar protein sorting-associated protein 32 homolog 2	Juglans regia	XP_018846598.1
TRINITY_DN17089_c1_g2::TRINITY_DN17089_c1_g2_i5::g.78211::m.78211	0	880	XP_018851837.1	outer envelope protein 61	Juglans regia	ONI01600.1

TRINITY_DN17386_c2_g1::TRINITY_DN17386_c2_g1_i2::g.82885::m.82885	0	1059	XP_018816335.1	LOW QUALITY PROTEIN: cation/H(+) antiporter 14-like	Juglans regia	ONH99745.1
TRINITY_DN13961_c0_g1::TRINITY_DN13961_c0_g1_i2::g.30621::m.30621	0	683	XP_018835559.1	ATP phosphoribosyltransferase 2, chloroplastic isoform X1	Juglans regia	OAY44990.1
TRINITY_DN13778_c1_g3::TRINITY_DN13778_c1_g3_i1::g.28124::m.28124	0	855	XP_018817409.1	ruvB-like 2	Juglans regia	KRH46465.1
TRINITY_DN13512_c0_g1::TRINITY_DN13512_c0_g1_i2::g.24707::m.24707	2,62E-54	172	XP_018827511.1	uncharacterized protein LOC108996194	Juglans regia	XP_018827510.1
TRINITY_DN19318_c3_g1::TRINITY_DN19318_c3_g1_i7::g.115859::m.115859	6,32E-93	274	XP_018819521.1	yrdC domain-containing protein, mitochondrial isoform X4	Juglans regia	XP_018819522.1
TRINITY_DN17993_c0_g1::TRINITY_DN17993_c0_g1_i2::g.93217::m.93217	0	568	XP_018841527.1	heterodimeric geranylgeranyl pyrophosphate synthase small subunit, chloroplastic-like	Juglans regia	XP_018853159.1
TRINITY_DN19049_c0_g1::TRINITY_DN19049_c0_g1_i9::g.110547::m.110547	0	592	XP_018815364.1	SH3 domain-containing protein 2-like	Juglans regia	XP_018841364.1
TRINITY_DN12375_c3_g1::TRINITY_DN12375_c3_g1_i9::g.12353::m.12353	0	973	XP_018826576.1	probable methyltransferase PMT2	Juglans regia	OAY31024.1
TRINITY_DN18998_c2_g1::TRINITY_DN18998_c2_g1_i7::g.108559::m.108559	0	1970	XP_018818695.1	uncharacterized protein LOC108989521 isoform X1	Juglans regia	XP_018818696.1
TRINITY_DN18521_c6_g4::TRINITY_DN18521_c6_g4_i1::g.101901::m.101901	6,35E-62	191	XP_018843076.1	calvin cycle protein CP12-3, chloroplastic	Juglans regia	OAY50363.1
TRINITY_DN17805_c0_g1::TRINITY_DN17805_c0_g1_i5::g.89980::m.89980	3,11E-91	266	OIW00325.1	hypothetical protein TanjilG_27576	Lupinus angustifolius	XP_019462763.1
TRINITY_DN19377_c2_g2::TRINITY_DN19377_c2_g2_i2::g.116852::m.116852	9,90E-100	299	XP_018823596.1	TPR repeat-containing thioredoxin TDX	Juglans regia	ONI09381.1
TRINITY_DN19873_c6_g5::TRINITY_DN19873_c6_g5_i6::g.124897::m.124897	2,05E-107	324	KRH23403.1	hypothetical protein GLYMA_13G354900	Glycine max	XP_018839142.1
TRINITY_DN16513_c0_g2::TRINITY_DN16513_c0_g2_i7::g.68926::m.68926	0	662	XP_018818176.1	bifunctional riboflavin kinase/FMN phosphatase isoform X1	Juglans regia	XP_018818177.1
TRINITY_DN12856_c0_g1::TRINITY_DN12856_c0_g1_i4::g.16613::m.16613	5,23E-109	336	XP_018827682.1	uncharacterized protein LOC108996311	Juglans regia	XP_016175320.1
TRINITY_DN8001_c0_g1::TRINITY_DN8001_c0_g1_i1::g.2441::m.2441	0	624	XP_018856560.1	2-alkenal reductase (NADP(+)-dependent)-like	Juglans regia	XP_018829693.1
TRINITY_DN16768_c1_g1::TRINITY_DN16768_c1_g1_i8::g.72998::m.72998	0	1284	XP_018850254.1	pyrophosphate-energized membrane proton pump 3 isoform X1	Juglans regia	XP_018850256.1
TRINITY_DN15627_c0_g1::TRINITY_DN15627_c0_g1_i7::g.54594::m.54594	6,85E-171	479	XP_018805589.1	cinnamoyl-CoA reductase 2	Juglans regia	XP_018805590.1
TRINITY_DN17680_c4_g1::TRINITY_DN17680_c4_g1_i22::g.88254::m.88254	0	847	XP_018836626.1	putative clathrin assembly protein At5g57200 isoform X1	Juglans regia	XP_018836627.1
TRINITY_DN19193_c1_g1::TRINITY_DN19193_c1_g1_i1::g.113618::m.113618	1,70E-47	160	XP_018818624.1	3-hydroxyisobutyryl-CoA hydrolase 1-like	Juglans regia	OAY38989.1
TRINITY_DN13047_c0_g2::TRINITY_DN13047_c0_g2_i4::g.19117::m.19117	0	644	XP_007212263.1	aspartate carbamoyltransferase, chloroplastic	Prunus persica	XP_020417020.1
TRINITY_DN16413_c0_g1::TRINITY_DN16413_c0_g1_i2::g.67167::m.67167	2,41E-120	350	XP_018844572.1	uncharacterized protein LOC109008795	Juglans regia	KYP50055.1

TRINITY_DN16171_c0_g1::TRINITY_DN16171_c0_g1_i2::g.63190::m.63190	0	791	XP_018859392.1	acyl-lipid (9-3)-desaturase-like	Juglans regia	ABP01349.1
TRINITY_DN19552_c1_g2::TRINITY_DN19552_c1_g2_i2::g.119657::m.119657	2,19E-171	486	XP_018838516.1	long chain base biosynthesis protein 1	Juglans regia	XP_018838517.1
TRINITY_DN15066_c0_g1::TRINITY_DN15066_c0_g1_i4::g.46431::m.46431	0	845	XP_018822081.1	uridine kinase-like protein 1, chloroplastic	Juglans regia	XP_008231136.1
TRINITY_DN17996_c1_g1::TRINITY_DN17996_c1_g1_i2::g.93353::m.93353	3,73E-95	284	XP_018815856.1	hypersensitive-induced response protein 4	Juglans regia	XP_018815856.1
TRINITY_DN19241_c0_g1::TRINITY_DN19241_c0_g1_i6::g.115140::m.115140	1,03E-17	78,2	OAY36830.1	hypothetical protein MANES_11G052200	Manihot esculenta	XP_014620654.1
TRINITY_DN18797_c1_g2::TRINITY_DN18797_c1_g2_i1::g.106436::m.106436	1,94E-60	190	ONH91725.1	hypothetical protein PRUPE_8G133400	Prunus persica	XP_007201309.1
TRINITY_DN10621_c0_g1::TRINITY_DN10621_c0_g1_i2::g.5142::m.5142	8,80E-120	342	XP_018826580.1	uncharacterized endoplasmic reticulum membrane protein YGL010W-like	Juglans regia	ONI08705.1
TRINITY_DN18450_c3_g3::TRINITY_DN18450_c3_g3_i7::g.100614::m.100614	0	565	XP_018810529.1	auxilin-related protein 2-like	Juglans regia	XP_018810529.1
TRINITY_DN12897_c1_g1::TRINITY_DN12897_c1_g1_i8::g.17082::m.17082	1,91E-172	482	XP_018827762.1	TIP41-like protein	Juglans regia	XP_018809405.1
TRINITY_DN15693_c4_g1::TRINITY_DN15693_c4_g1_i3::g.55852::m.55852	0	521	XP_018852058.1	ABC transporter I family member 6, chloroplastic-like	Juglans regia	XP_018852059.1
TRINITY_DN13826_c0_g2::TRINITY_DN13826_c0_g2_i4::g.28934::m.28934	2,11E-63	206	XP_018826436.1	U1 small nuclear ribonucleoprotein 70 kDa-like	Juglans regia	XP_018829413.1
TRINITY_DN11412_c0_g1::TRINITY_DN11412_c0_g1_i1::g.7292::m.7292	0	660	XP_018821557.1	glyoxylate/succinic semialdehyde reductase 2, chloroplastic	Juglans regia	XP_008228276.1
TRINITY_DN11674_c0_g1::TRINITY_DN11674_c0_g1_i3::g.8359::m.8359	5,62E-134	378	XP_018810148.1	ras-related protein RABC2a-like isoform X1	Juglans regia	XP_018848039.1
TRINITY_DN17979_c4_g2::TRINITY_DN17979_c4_g2_i7::g.93178::m.93178	0	909	XP_018831141.1	NADPH--cytochrome P450 reductase-like	Juglans regia	XP_018820842.1
TRINITY_DN18736_c0_g1::TRINITY_DN18736_c0_g1_i7::g.105711::m.105711	0	1938	XP_018822938.1	exocyst complex component SEC8-like	Juglans regia	XP_018822939.1
TRINITY_DN13606_c2_g3::TRINITY_DN13606_c2_g3_i2::g.26267::m.26267	0	1394	XP_018857657.1	exocyst complex component SEC15A-like	Juglans regia	XP_018857659.1
TRINITY_DN13348_c4_g1::TRINITY_DN13348_c4_g1_i6::g.22618::m.22618	2,95E-146	414	OAY42657.1	hypothetical protein MANES_08G005400	Manihot esculenta	XP_016207932.1
TRINITY_DN17345_c4_g3::TRINITY_DN17345_c4_g3_i2::g.81576::m.81576	1,56E-73	222	XP_018853705.1	Golgi SNAP receptor complex member 1-2	Juglans regia	OAY62436.1
TRINITY_DN11662_c0_g1::TRINITY_DN11662_c0_g1_i1::g.8281::m.8281	5,82E-121	343	XP_018818270.1	peptidyl-prolyl cis-trans isomerase CYP22	Juglans regia	OAY53361.1
TRINITY_DN15060_c1_g1::TRINITY_DN15060_c1_g1_i6::g.46413::m.46413	0	990	XP_018851735.1	E3 ubiquitin-protein ligase KEG-like	Juglans regia	XP_018851736.1
TRINITY_DN15046_c3_g2::TRINITY_DN15046_c3_g2_i6::g.45963::m.45963	2,39E-67	209	XP_018854585.1	tRNA wybutosine-synthesizing protein 4	Juglans regia	KDP28857.1
TRINITY_DN17213_c1_g3::TRINITY_DN17213_c1_g3_i3::g.80204::m.80204	0	892	XP_018849388.1	dol-P-Man:Man(6)GlcNAc(2)-PP-Dol alpha-1,2-mannosyltransferase	Juglans regia	KDP32537.1

TRINITY_DN18186_c3_g1::TRINITY_DN18186_c3_g1_i8::g.96539::m.96539	0	1712	XP_018816812.1	transportin MOS14 isoform X1	Juglans regia	XP_018816813.1
TRINITY_DN19210_c1_g1::TRINITY_DN19210_c1_g1_i13::g.114068::m.114068	0	1599	XP_018821055.1	exocyst complex component SEC5A-like	Juglans regia	XP_009361420.1
TRINITY_DN17483_c0_g1::TRINITY_DN17483_c0_g1_i3::g.84425::m.84425	0	2270	XP_018833087.1	proteasome-associated protein ECM29 homolog	Juglans regia	ONI14235.1
TRINITY_DN13893_c0_g2::TRINITY_DN13893_c0_g2_i1::g.30000::m.30000	9,70E-177	496	XP_018849256.1	sorting nexin 1-like isoform X1	Juglans regia	XP_018849257.1
TRINITY_DN19502_c1_g3::TRINITY_DN19502_c1_g3_i5::g.118942::m.118942	0	2339	XP_018834616.1	callose synthase 9	Juglans regia	XP_018834617.1
TRINITY_DN13503_c0_g1::TRINITY_DN13503_c0_g1_i7::g.24647::m.24647	3,11E-130	375	XP_018827313.1	renalase isoform X2	Juglans regia	XP_018827334.1
TRINITY_DN20983_c0_g1::TRINITY_DN20983_c0_g1_i1::g.128678::m.128678	0	530	XP_018824706.1	pentatricopeptide repeat-containing protein At3g49240	Juglans regia	XP_018824706.1
TRINITY_DN15427_c0_g1::TRINITY_DN15427_c0_g1_i2::g.51743::m.51743	0	727	XP_018815673.1	golgin candidate 1-like isoform X2	Juglans regia	XP_018815674.1
TRINITY_DN19611_c0_g2::TRINITY_DN19611_c0_g2_i9::g.121074::m.121074	0	613	XP_018849912.1	heterogeneous nuclear ribonucleoprotein 1 isoform X3	Juglans regia	XP_018849904.1
TRINITY_DN17613_c0_g1::TRINITY_DN17613_c0_g1_i11::g.87113::m.87113	0	1254	XP_018805408.1	ATP-dependent zinc metalloprotease FTSH 9, chloroplastic-like	Juglans regia	XP_018842944.1
TRINITY_DN19888_c1_g1::TRINITY_DN19888_c1_g1_i5::g.125265::m.125265	6,57E-149	427	XP_018811673.1	polypyrimidine tract-binding protein homolog 3	Juglans regia	XP_018811674.1
TRINITY_DN17142_c1_g1::TRINITY_DN17142_c1_g1_i7::g.79281::m.79281	7,23E-102	301	XP_018819539.1	uncharacterized protein LOC108990124 isoform X2	Juglans regia	XP_018819538.1
TRINITY_DN14016_c0_g1::TRINITY_DN14016_c0_g1_i9::g.31308::m.31308	2,17E-63	201	XP_018814804.1	protein phosphatase 1 regulatory subunit pprA	Juglans regia	XP_018814804.1
TRINITY_DN15297_c0_g1::TRINITY_DN15297_c0_g1_i10::g.49743::m.49743	0	604	XP_018842133.1	fructose-1,6-bisphosphatase, cytosolic	Juglans regia	XP_018842134.1
TRINITY_DN18857_c0_g1::TRINITY_DN18857_c0_g1_i9::g.107487::m.107487	2,56E-142	408	XP_018835872.1	SEC12-like protein 2	Juglans regia	XP_018835880.1
TRINITY_DN14669_c0_g2::TRINITY_DN14669_c0_g2_i2::g.40246::m.40246	0	1158	XP_018822356.1	uncharacterized protein LOC108992291 isoform X1	Juglans regia	XP_018822358.1
TRINITY_DN11011_c0_g1::TRINITY_DN11011_c0_g1_i3::g.6100::m.6100	0	1773	XP_018818368.1	exportin-2 isoform X1	Juglans regia	XP_018818369.1
TRINITY_DN13798_c2_g3::TRINITY_DN13798_c2_g3_i1::g.28387::m.28387	1,70E-90	266	XP_018829803.1	uncharacterized protein LOC108997870	Juglans regia	XP_008218429.1
TRINITY_DN13146_c0_g1::TRINITY_DN13146_c0_g1_i3::g.20394::m.20394	4,53E-59	188	XP_018835416.1	uncharacterized protein LOC109002223	Juglans regia	XP_018835424.1
TRINITY_DN16700_c3_g5::TRINITY_DN16700_c3_g5_i2::g.71826::m.71826	6,34E-87	263	XP_018833107.1	feruloyl CoA ortho-hydroxylase 1-like	Juglans regia	XP_018833023.1
TRINITY_DN17427_c0_g1::TRINITY_DN17427_c0_g1_i3::g.83624::m.83624	0	671	XP_018852048.1	uncharacterized protein LOC109014152 isoform X1	Juglans regia	XP_018852049.1
TRINITY_DN15169_c1_g1::TRINITY_DN15169_c1_g1_i14::g.47860::m.47860	6,47E-136	397	OAY48824.1	hypothetical protein MANES_05G008200	Manihot esculenta	OIV91038.1

TRINITY_DN13150_c1_g1::TRINITY_DN13150_c1_g1_i6::g.20550::m.20550	1,55E-61	195	XP_018851019.1	glycine-rich RNA-binding protein RZ1A-like	Juglans regia	KDP37561.1
TRINITY_DN19251_c1_g2::TRINITY_DN19251_c1_g2_i17::g.114967::m.114967	2,40E-71	224	XP_018851523.1	mitogen-activated protein kinase 9-like isoform X1	Juglans regia	XP_018851524.1
TRINITY_DN19006_c2_g1::TRINITY_DN19006_c2_g1_i11::g.110719::m.110719	1,58E-138	407	XP_018847843.1	proline-rich receptor-like protein kinase PERK7	Juglans regia	XP_018847844.1
TRINITY_DN18900_c0_g1::TRINITY_DN18900_c0_g1_i6::g.107340::m.107340	0	558	XP_018836395.1	uncharacterized protein LOC109002925 isoform X1	Juglans regia	XP_007222499.2
TRINITY_DN15100_c0_g1::TRINITY_DN15100_c0_g1_i1::g.45506::m.45506	0	533	XP_018833509.1	uncharacterized protein LOC109000915 isoform X1	Juglans regia	ONI24868.1
TRINITY_DN11216_c0_g1::TRINITY_DN11216_c0_g1_i3::g.6674::m.6674	0	1003	XP_018831623.1	beta-glucosidase-like SFR2, chloroplastic isoform X2	Juglans regia	XP_018831622.1
TRINITY_DN19951_c3_g1::TRINITY_DN19951_c3_g1_i8::g.125811::m.125811	9,43E-160	451	XP_018826881.1	soluble inorganic pyrophosphatase 6, chloroplastic-like	Juglans regia	XP_004485644.1
TRINITY_DN13542_c0_g1::TRINITY_DN13542_c0_g1_i7::g.24740::m.24740	0	570	XP_018817784.1	ubiquitin carboxyl-terminal hydrolase 2-like	Juglans regia	XP_018838029.1
TRINITY_DN13852_c0_g1::TRINITY_DN13852_c0_g1_i7::g.28442::m.28442	0	577	XP_009377023.1	FAD synthase-like	Pyrus x bretschneideri	XP_009377024.1
TRINITY_DN16578_c2_g1::TRINITY_DN16578_c2_g1_i1::g.69884::m.69884	0	1560	XP_018825470.1	nuclear cap-binding protein subunit 1	Juglans regia	XP_008227217.1
TRINITY_DN18180_c0_g1::TRINITY_DN18180_c0_g1_i6::g.96457::m.96457	0	1917	XP_018828508.1	E3 ubiquitin-protein ligase listerin	Juglans regia	XP_018828509.1
TRINITY_DN16164_c3_g2::TRINITY_DN16164_c3_g2_i1::g.62975::m.62975	2,57E-104	305	XP_018845967.1	REF/SRPP-like protein At1g67360 isoform X2	Juglans regia	XP_018806821.1
TRINITY_DN14862_c0_g2::TRINITY_DN14862_c0_g2_i3::g.43396::m.43396	2,20E-82	255	ONI25929.1	hypothetical protein PRUPE_2G327500	Prunus persica	XP_020413087.1
TRINITY_DN12438_c0_g1::TRINITY_DN12438_c0_g1_i11::g.12896::m.12896	3,50E-161	462	XP_018821455.1	poly(U)-specific endoribonuclease-B-like isoform X1	Juglans regia	XP_018821456.1
TRINITY_DN11935_c0_g1::TRINITY_DN11935_c0_g1_i2::g.9568::m.9568	0	825	XP_018817346.1	dihydroorotate dehydrogenase (quinone), mitochondrial isoform X1	Juglans regia	XP_018817347.1
TRINITY_DN17228_c0_g1::TRINITY_DN17228_c0_g1_i3::g.80433::m.80433	3,22E-75	226	XP_018853627.1	60S ribosomal protein L24-like	Juglans regia	XP_018811954.1
TRINITY_DN14948_c0_g1::TRINITY_DN14948_c0_g1_i2::g.43967::m.43967	0	827	XP_018836286.1	protein trichome birefringence-like 16	Juglans regia	XP_018836287.1
TRINITY_DN17507_c1_g2::TRINITY_DN17507_c1_g2_i9::g.85053::m.85053	0	998	XP_018812888.1	putative ATPase N2B isoform X1	Juglans regia	XP_018812889.1
TRINITY_DN14555_c5_g1::TRINITY_DN14555_c5_g1_i2::g.38904::m.38904	2,01E-75	229	KYP53704.1	Tetratricopeptide repeat protein 1	Cajanus cajan	XP_020229034.1
TRINITY_DN15250_c0_g1::TRINITY_DN15250_c0_g1_i1::g.49205::m.49205	0	528	XP_018826883.1	beta-ureidopropionase-like	Juglans regia	ONH90705.1
TRINITY_DN14880_c2_g1::TRINITY_DN14880_c2_g1_i5::g.43756::m.43756	1,20E-89	275	XP_018848045.1	acyl-CoA-binding domain-containing protein 4	Juglans regia	XP_018848046.1
TRINITY_DN17189_c3_g1::TRINITY_DN17189_c3_g1_i6::g.79844::m.79844	2,50E-99	296	OAY39563.1	hypothetical protein MANES_10G104500	Manihot esculenta	KDP40925.1

TRINITY_DN15404_c3_g2::TRINITY_DN15404_c3_g2_i3::g.51327::m.51327	7,96E-175	496	XP_018841021.1	nudix hydrolase 19, chloroplastic isoform X2	Juglans regia	XP_018841020.1
TRINITY_DN18753_c1_g3::TRINITY_DN18753_c1_g3_i14::g.106335::m.106335	0	1038	XP_018856581.1	carotenoid 9,10(9',10')-cleavage dioxygenase 1	Juglans regia	ONI20422.1
TRINITY_DN19089_c3_g2::TRINITY_DN19089_c3_g2_i5::g.111660::m.111660	0	703	XP_018852211.1	26S proteasome non-ATPase regulatory subunit 13 homolog A	Juglans regia	XP_018852212.1
TRINITY_DN18930_c0_g5::TRINITY_DN18930_c0_g5_i4::g.109035::m.109035	2,27E-125	371	XP_018810643.1	cytochrome P450 71D9-like	Juglans regia	XP_018810634.1
TRINITY_DN19063_c1_g2::TRINITY_DN19063_c1_g2_i13::g.111218::m.111218	0	595	XP_018858396.1	uncharacterized protein LOC109020402 isoform X1	Juglans regia	XP_018806239.1
TRINITY_DN13381_c2_g1::TRINITY_DN13381_c2_g1_i4::g.23007::m.23007	4,18E-155	432	OAY25631.1	hypothetical protein MANES_17G110100	Manihot esculenta	XP_018845779.1
TRINITY_DN15027_c0_g1::TRINITY_DN15027_c0_g1_i5::g.45870::m.45870	0	768	XP_018843544.1	geranylgeranyl transferase type-2 subunit alpha 1	Juglans regia	XP_008219243.1
TRINITY_DN17756_c1_g1::TRINITY_DN17756_c1_g1_i7::g.89229::m.89229	0	1026	XP_018857193.1	cell division cycle and apoptosis regulator protein 1 isoform X2	Juglans regia	XP_018857193.1
TRINITY_DN15011_c4_g2::TRINITY_DN15011_c4_g2_i4::g.45809::m.45809	9,78E-123	357	XP_018818639.1	serine/threonine-protein kinase STY17-like	Juglans regia	XP_008351239.1
TRINITY_DN12829_c0_g1::TRINITY_DN12829_c0_g1_i5::g.16229::m.16229	1,64E-84	256	XP_018850153.1	reticulon-4-interacting protein 1, mitochondrial-like isoform X1	Juglans regia	XP_018822796.1
TRINITY_DN19522_c5_g1::TRINITY_DN19522_c5_g1_i5::g.119202::m.119202	3,05E-133	381	XP_009344569.2	bifunctional nitrilase/nitrile hydratase NIT4A-like	Pyrus x bretschneideri	XP_008361132.1
TRINITY_DN19133_c0_g1::TRINITY_DN19133_c0_g1_i8::g.112559::m.112559	3,59E-101	298	XP_018845249.1	agamous-like MADS-box protein AGL30 isoform X5	Juglans regia	XP_018845245.1
TRINITY_DN19814_c1_g3::TRINITY_DN19814_c1_g3_i2::g.124100::m.124100	1,51E-95	289	XP_008352742.2	LOW QUALITY PROTEIN: phosphoenolpyruvate carboxykinase [ATP-like]	Malus domestica	XP_008352742.2
TRINITY_DN32151_c0_g1::TRINITY_DN32151_c0_g1_i1::g.130343::m.130343						
TRINITY_DN12363_c0_g1::TRINITY_DN12363_c0_g1_i2::g.12162::m.12162	0	1007	XP_018850129.1	4-alpha-glucanotransferase, chloroplastic/amyloplastic	Juglans regia	XP_008232738.1
TRINITY_DN18083_c3_g1::TRINITY_DN18083_c3_g1_i4::g.94853::m.94853	0	521	XP_018814713.1	ATP-dependent Clp protease proteolytic subunit-related protein 4, chloroplastic-like	Juglans regia	XP_018821438.1
TRINITY_DN15012_c0_g1::TRINITY_DN15012_c0_g1_i2::g.45660::m.45660	9,76E-171	480	XP_018835247.1	uncharacterized protein YMR315W	Juglans regia	KDP20201.1
TRINITY_DN18820_c2_g1::TRINITY_DN18820_c2_g1_i7::g.106999::m.106999	0	594	XP_018848045.1	acyl-CoA-binding domain-containing protein 4	Juglans regia	XP_018848046.1
TRINITY_DN15977_c4_g2::TRINITY_DN15977_c4_g2_i4::g.59843::m.59843	5,54E-105	302	XP_018818738.1	NADH dehydrogenase [ubiquinone] 1 alpha subcomplex assembly factor 3-like	Juglans regia	XP_009363132.1
TRINITY_DN19301_c0_g1::TRINITY_DN19301_c0_g1_i6::g.115549::m.115549	1,99E-69	218	XP_018847165.1	CRIB domain-containing protein RIC7	Juglans regia	XP_012569758.1
TRINITY_DN16119_c4_g3::TRINITY_DN16119_c4_g3_i2::g.62577::m.62577	1,06E-124	356	XP_008360906.1	uncharacterized protein LOC103424589	Malus domestica	XP_018858950.1
TRINITY_DN14879_c3_g1::TRINITY_DN14879_c3_g1_i2::g.43728::m.43728	0	959	XP_018824811.1	importin subunit alpha-like isoform X1	Juglans regia	XP_018824812.1

TRINITY_DN18658_c0_g1::TRINITY_DN18658_c0_g1_i8::g.104114::m.104114	0	610	XP_018814981.1	manganese-dependent ADP-ribose/CDP-alcohol diphosphatase	Juglans regia	XP_018814982.1
TRINITY_DN17925_c2_g1::TRINITY_DN17925_c2_g1_i5::g.91985::m.91985	1,54E-82	245	XP_018821657.1	outer envelope pore protein 24, chloroplastic	Juglans regia	OAY22817.1
TRINITY_DN16773_c1_g1::TRINITY_DN16773_c1_g1_i5::g.73048::m.73048	0	548	XP_008225596.1	ADP-ribosylation factor GTPase-activating protein AGD12-like	Prunus mume	XP_016648708.1
TRINITY_DN13102_c0_g1::TRINITY_DN13102_c0_g1_i5::g.19880::m.19880	0	754	XP_018813877.1	plant intracellular Ras-group-related LRR protein 9-like	Juglans regia	XP_018808183.1
TRINITY_DN15656_c0_g5::TRINITY_DN15656_c0_g5_i2::g.55149::m.55149	0	673	XP_018812277.1	cell division protein FtsZ homolog 1, chloroplastic-like isoform X1	Juglans regia	XP_018824764.1
TRINITY_DN15008_c0_g2::TRINITY_DN15008_c0_g2_i1::g.45732::m.45732	0	1860	XP_018817247.1	calcium-transporting ATPase 1, endoplasmic reticulum-type-like	Juglans regia	XP_018818320.1
TRINITY_DN18231_c0_g2::TRINITY_DN18231_c0_g2_i7::g.97309::m.97309	0	3332	XP_018853999.1	CCR4-NOT transcription complex subunit 1-like isoform X1	Juglans regia	XP_018854006.1
TRINITY_DN17061_c4_g1::TRINITY_DN17061_c4_g1_i15::g.77796::m.77796	1,80E-104	303	XP_018833575.1	uncharacterized protein LOC109000956 isoform X1	Juglans regia	XP_008380623.1
TRINITY_DN17769_c1_g1::TRINITY_DN17769_c1_g1_i4::g.89348::m.89348						
TRINITY_DN17821_c0_g1::TRINITY_DN17821_c0_g1_i3::g.91084::m.91084	1,62E-63	201	XP_019417486.1	CLP protease regulatory subunit CLPX1, mitochondrial isoform X2	Lupinus angustifolius	XP_004502652.1
TRINITY_DN19568_c0_g1::TRINITY_DN19568_c0_g1_i11::g.119880::m.119880	0	1519	XP_018828527.1	nardilysin-like	Juglans regia	XP_008241920.1
TRINITY_DN16019_c1_g2::TRINITY_DN16019_c1_g2_i3::g.60717::m.60717	2,53E-77	235	XP_018852770.1	CRAL-TRIO domain-containing protein C3H8.02-like isoform X1	Juglans regia	XP_018852771.1
TRINITY_DN13205_c0_g1::TRINITY_DN13205_c0_g1_i3::g.21053::m.21053	1,12E-54	177	XP_018835591.1	2-hydroxyisoflavanone dehydratase-like	Juglans regia	XP_018835592.1
TRINITY_DN13561_c3_g1::TRINITY_DN13561_c3_g1_i11::g.25972::m.25972	0	886	XP_018858585.1	tyrosine decarboxylase 1-like isoform X2	Juglans regia	XP_018858584.1
TRINITY_DN16924_c1_g1::TRINITY_DN16924_c1_g1_i1::g.75666::m.75666	3,71E-180	501	XP_012089070.1	protein YIPF1 homolog	Jatropha curcas	OAY42270.1
TRINITY_DN10291_c0_g2::TRINITY_DN10291_c0_g2_i1::g.4575::m.4575	1,59E-73	226	XP_018807754.1	superoxide dismutase [Fe], chloroplastic-like isoform X1	Juglans regia	XP_018807755.1
TRINITY_DN14186_c0_g1::TRINITY_DN14186_c0_g1_i2::g.33325::m.33325	1,28E-132	381	XP_018842628.1	ribose-phosphate pyrophosphokinase 1, chloroplastic-like isoform X3	Juglans regia	XP_018842627.1
TRINITY_DN17451_c1_g1::TRINITY_DN17451_c1_g1_i3::g.84062::m.84062	3,05E-87	275	XP_018818209.1	uncharacterized protein LOC108989149	Juglans regia	XP_018840454.1
TRINITY_DN16642_c3_g1::TRINITY_DN16642_c3_g1_i8::g.71010::m.71010	2,29E-60	197	OAY48824.1	hypothetical protein MANES_05G008200	Manihot esculenta	OIV91038.1
TRINITY_DN18772_c0_g1::TRINITY_DN18772_c0_g1_i5::g.106264::m.106264	0	1103	XP_018814927.1	phenylalanine--tRNA ligase beta subunit, cytoplasmic-like	Juglans regia	XP_018806301.1
TRINITY_DN12837_c2_g1::TRINITY_DN12837_c2_g1_i5::g.16578::m.16578	6,68E-56	184	XP_018847358.1	probable polygalacturonase	Juglans regia	XP_018809647.1
TRINITY_DN14047_c1_g1::TRINITY_DN14047_c1_g1_i4::g.31668::m.31668	9,28E-147	417	XP_018839416.1	perakine reductase-like	Juglans regia	XP_018818666.1

TRINITY_DN19086_c1_g1::TRINITY_DN19086_c1_g1_i6::g.111724::m.111724	7,45E-75	227	XP_008363998.1	EH domain-containing protein 1-like	Malus domestica	XP_009334157.1
TRINITY_DN14258_c0_g5::TRINITY_DN14258_c0_g5_i3::g.34521::m.34521	0	1019	XP_018856587.1	LETM1 and EF-hand domain-containing protein 1, mitochondrial-like isoform X1	Juglans regia	XP_018856593.1
TRINITY_DN17031_c2_g2::TRINITY_DN17031_c2_g2_i1::g.77290::m.77290	1,02E-140	395	XP_018833476.1	VAMP-like protein YKT61	Juglans regia	XP_018850344.1
TRINITY_DN12955_c2_g1::TRINITY_DN12955_c2_g1_i5::g.17882::m.17882	0	1433	XP_018832818.1	general negative regulator of transcription subunit 3 isoform X2	Juglans regia	XP_018832816.1
TRINITY_DN14036_c4_g1::TRINITY_DN14036_c4_g1_i2::g.31377::m.31377	2,38E-82	245	XP_018842727.1	protein BOLA4, chloroplastic/mitochondrial-like	Juglans regia	XP_018828128.1
TRINITY_DN12399_c0_g1::TRINITY_DN12399_c0_g1_i1::g.12011::m.12011	0	664	XP_018833157.1	serine carboxypeptidase-like 51	Juglans regia	XP_018833716.1
TRINITY_DN15460_c0_g1::TRINITY_DN15460_c0_g1_i9::g.52319::m.52319	1,93E-144	412	XP_018807734.1	probable proteasome inhibitor isoform X1	Juglans regia	XP_008240153.1
TRINITY_DN16066_c2_g3::TRINITY_DN16066_c2_g3_i1::g.61892::m.61892	1,85E-30	111	GAU19610.1	hypothetical protein TSUD_383050	Trifolium subterraneum	XP_018831010.1
TRINITY_DN15513_c2_g2::TRINITY_DN15513_c2_g2_i3::g.53087::m.53087	0	625	ONH96192.1	hypothetical protein PRUPE_7G112400	Prunus persica	XP_007204364.1
TRINITY_DN11403_c0_g1::TRINITY_DN11403_c0_g1_i1::g.7256::m.7256	1,83E-164	469	OAY24624.1	hypothetical protein MANES_17G030600	Manihot esculenta	XP_008224890.1
TRINITY_DN12993_c1_g5::TRINITY_DN12993_c1_g5_i1::g.18384::m.18384	0	865	XP_018818643.1	ATP sulfurylase 1, chloroplastic-like	Juglans regia	XP_018839429.1
TRINITY_DN14376_c2_g2::TRINITY_DN14376_c2_g2_i8::g.36166::m.36166	0	516	XP_018835931.1	hypersensitive-induced response protein 2-like	Juglans regia	XP_018835932.1
TRINITY_DN14670_c0_g1::TRINITY_DN14670_c0_g1_i1::g.40393::m.40393	1,02E-65	203	XP_018806103.1	12-oxophytodienoate reductase 1-like, partial	Juglans regia	ONI35666.1
TRINITY_DN19844_c1_g2::TRINITY_DN19844_c1_g2_i1::g.124447::m.124447	1,38E-150	442	XP_018849698.1	glutamine--tRNA ligase-like	Juglans regia	XP_018849699.1
TRINITY_DN19441_c2_g1::TRINITY_DN19441_c2_g1_i3::g.118113::m.118113	0	951	XP_018824493.1	uncharacterized protein LOC108993891	Juglans regia	ONI13349.1
TRINITY_DN19418_c2_g1::TRINITY_DN19418_c2_g1_i2::g.117511::m.117511	0	1656	XP_018857825.1	translocase of chloroplast 159, chloroplastic-like	Juglans regia	KDP28139.1
TRINITY_DN12196_c0_g1::TRINITY_DN12196_c0_g1_i3::g.11012::m.11012	1,11E-142	404	XP_018849277.1	probable aquaporin TIP3-2	Juglans regia	XP_018851532.1
TRINITY_DN18673_c1_g1::TRINITY_DN18673_c1_g1_i5::g.104369::m.104369	3,18E-64	199	AFK48658.1	unknown	Lotus japonicus	XP_012068990.1
TRINITY_DN19846_c6_g1::TRINITY_DN19846_c6_g1_i2::g.124495::m.124495	0	737	XP_016199270.1	LOW QUALITY PROTEIN: topless-related protein 4-like	Arachis ipaensis	XP_015935744.1
TRINITY_DN1593_c0_g1::TRINITY_DN1593_c0_g1_i1::g.417::m.417	2,68E-75	225	XP_018838135.1	uncharacterized protein LOC109004143 isoform X1	Juglans regia	XP_018838136.1
TRINITY_DN18479_c0_g3::TRINITY_DN18479_c0_g3_i1::g.100965::m.100965	4,35E-139	391	OIV93234.1	hypothetical protein TanjilG_27413	Lupinus angustifolius	XP_019423713.1
TRINITY_DN12843_c0_g1::TRINITY_DN12843_c0_g1_i3::g.16447::m.16447	0	1650	XP_018841898.1	probable starch synthase 4, chloroplastic/amyloplastic	Juglans regia	KDP37946.1

TRINITY_DN9796_c0_g1::TRINITY_DN9796_c0_g1_i1::g.3884::m.3884	0	943	XP_018826128.1	probable histone-arginine methyltransferase 1.3	Juglans regia	XP_018825751.1
TRINITY_DN19235_c2_g1::TRINITY_DN19235_c2_g1_i2::g.114336::m.114336	7,83E-103	319	XP_018826267.1	receptor-like protein kinase ANXUR2	Juglans regia	XP_018859750.1
TRINITY_DN19694_c1_g1::TRINITY_DN19694_c1_g1_i14::g.120695::m.120695	0	3939	XP_018850769.1	glutamate synthase [NADH], amyloplastic isoform X1	Juglans regia	XP_018838716.1
TRINITY_DN16533_c3_g1::TRINITY_DN16533_c3_g1_i1::g.69070::m.69070	0	508	XP_018835895.1	uncharacterized protein LOC109002555	Juglans regia	XP_018850123.1
TRINITY_DN11975_c0_g1::TRINITY_DN11975_c0_g1_i3::g.9738::m.9738	0	711	XP_018835079.1	developmentally-regulated G-protein 2	Juglans regia	KDP40713.1
TRINITY_DN12861_c0_g1::TRINITY_DN12861_c0_g1_i2::g.16621::m.16621	8,44E-97	282	XP_018831702.1	protein NUCLEAR FUSION DEFECTIVE 2	Juglans regia	ONI08410.1
TRINITY_DN14096_c0_g4::TRINITY_DN14096_c0_g4_i2::g.32317::m.32317	1,29E-50	161	XP_018834937.1	nascent polypeptide-associated complex subunit beta-like	Juglans regia	XP_018834149.1
TRINITY_DN16788_c0_g2::TRINITY_DN16788_c0_g2_i3::g.73187::m.73187	0	889	OAY36500.1	hypothetical protein MANES_11G025700	Manihot esculenta	XP_012068968.1
TRINITY_DN17508_c4_g1::TRINITY_DN17508_c4_g1_i3::g.85406::m.85406	8,74E-102	300	XP_008232561.1	uncharacterized protein LOC103331692	Prunus mume	ONI22544.1
TRINITY_DN15658_c0_g1::TRINITY_DN15658_c0_g1_i3::g.55055::m.55055	0	1873	XP_018856627.1	nuclear export mediator factor Nemf isoform X1	Juglans regia	XP_012084140.1
TRINITY_DN18925_c2_g2::TRINITY_DN18925_c2_g2_i2::g.109044::m.109044	4,60E-64	204	XP_018823005.1	probable pectate lyase P59	Juglans regia	XP_018812687.1
TRINITY_DN2281_c0_g1::TRINITY_DN2281_c0_g1_i1::g.635::m.635	0	698	XP_018813360.1	protein arginine N-methyltransferase 1.5-like	Juglans regia	ONI34114.1
TRINITY_DN14491_c1_g5::TRINITY_DN14491_c1_g5_i8::g.37914::m.37914	3,12E-62	192	XP_012068558.1	mitochondrial intermembrane space import and assembly protein 40 homolog	Jatropha curcas	KDP40464.1
TRINITY_DN14005_c0_g1::TRINITY_DN14005_c0_g1_i3::g.31329::m.31329	2,14E-54	176	XP_018825692.1	ubiquitin-conjugating enzyme E2 32-like	Juglans regia	OAY27662.1
TRINITY_DN19319_c0_g3::TRINITY_DN19319_c0_g3_i9::g.115912::m.115912	2,54E-56	180	XP_018857758.1	serine/arginine-rich splicing factor RSZ21	Juglans regia	XP_016190407.1
TRINITY_DN16713_c1_g3::TRINITY_DN16713_c1_g3_i3::g.72094::m.72094	1,17E-97	294	XP_018843093.1	uncharacterized protein LOC109007740 isoform X1	Juglans regia	KDP36714.1
TRINITY_DN14259_c3_g1::TRINITY_DN14259_c3_g1_i2::g.34652::m.34652	0	665	ONI09011.1	hypothetical protein PRUPE_5G211900	Prunus persica	ONI09012.1
TRINITY_DN11562_c0_g1::TRINITY_DN11562_c0_g1_i2::g.7869::m.7869	6,01E-83	246	XP_018848154.1	thioredoxin Y1, chloroplastic-like	Juglans regia	ONH90075.1
TRINITY_DN34461_c0_g1::TRINITY_DN34461_c0_g1_i1::g.130651::m.130651	6,32E-61	197	XP_018817991.1	exopolygalacturonase-like	Juglans regia	XP_018817992.1
TRINITY_DN11895_c0_g1::TRINITY_DN11895_c0_g1_i1::g.9352::m.9352	0	735	XP_018844051.1	probable glucan endo-1,3-beta-glucosidase A6	Juglans regia	ONI28912.1
TRINITY_DN15183_c1_g3::TRINITY_DN15183_c1_g3_i4::g.48285::m.48285	8,17E-132	379	XP_018822954.1	uncharacterized protein LOC108992770	Juglans regia	XP_017179652.1
TRINITY_DN14482_c0_g1::TRINITY_DN14482_c0_g1_i1::g.37716::m.37716	0	699	XP_018858563.1	protein transport protein SEC16B homolog	Juglans regia	XP_018859925.1

TRINITY_DN16600_c2_g1::TRINITY_DN16600_c2_g1_i15::g.68704::m.68704	5,56E-44	149	XP_018814379.1	protein FATTY ACID EXPORT 3, chloroplastic	Juglans regia	XP_016198771.1
TRINITY_DN16958_c1_g1::TRINITY_DN16958_c1_g1_i8::g.76184::m.76184	2,96E-70	213	XP_018855746.1	HMG1/2-like protein	Juglans regia	XP_018809064.1
TRINITY_DN13523_c0_g1::TRINITY_DN13523_c0_g1_i7::g.24832::m.24832	6,62E-105	303	XP_018849422.1	NADPH:quinone oxidoreductase-like	Juglans regia	XP_018818174.1
TRINITY_DN18718_c3_g5::TRINITY_DN18718_c3_g5_i3::g.105071::m.105071	2,61E-86	252	KYP55926.1	Multiprotein-bridging factor 1a	Cajanus cajan	KYP67761.1
TRINITY_DN17281_c2_g3::TRINITY_DN17281_c2_g3_i2::g.81288::m.81288	1,90E-78	234	XP_018845596.1	translation machinery-associated protein 22	Juglans regia	OAY53925.1
TRINITY_DN18452_c0_g3::TRINITY_DN18452_c0_g3_i8::g.100622::m.100622	1,82E-128	379	XP_018859162.1	putative 3,4-dihydroxy-2-butanone kinase, partial	Juglans regia	XP_018823489.1
TRINITY_DN14408_c0_g2::TRINITY_DN14408_c0_g2_i5::g.36629::m.36629	0	937	XP_018847688.1	SNARE-interacting protein KEULE isoform X2	Juglans regia	XP_018847679.1
TRINITY_DN19395_c1_g2::TRINITY_DN19395_c1_g2_i1::g.117249::m.117249	1,47E-76	226	XP_018840332.1	purple acid phosphatase 10-like	Juglans regia	ANG56537.1
TRINITY_DN16328_c4_g2::TRINITY_DN16328_c4_g2_i8::g.65867::m.65867	1,31E-157	442	XP_018815495.1	uncharacterized protein LOC108987089	Juglans regia	XP_009344930.1
TRINITY_DN16222_c1_g2::TRINITY_DN16222_c1_g2_i6::g.64132::m.64132	0	1014	XP_018851146.1	calcium-dependent protein kinase 20-like	Juglans regia	BAT96505.1
TRINITY_DN16296_c2_g1::TRINITY_DN16296_c2_g1_i8::g.65364::m.65364	0	527	XP_018852504.1	short-chain dehydrogenase TIC 32, chloroplastic-like	Juglans regia	XP_009374991.1
TRINITY_DN15139_c1_g2::TRINITY_DN15139_c1_g2_i1::g.47376::m.47376	0	926	XP_018841587.1	probable L-type lectin-domain containing receptor kinase S.5	Juglans regia	XP_018829183.1
TRINITY_DN17004_c0_g6::TRINITY_DN17004_c0_g6_i1::g.76895::m.76895	0	1931	XP_018833828.1	110 kDa U5 small nuclear ribonucleoprotein component CLO	Juglans regia	XP_008367763.1
TRINITY_DN12855_c4_g1::TRINITY_DN12855_c4_g1_i7::g.16693::m.16693	0	882	XP_018825775.1	uridine kinase-like protein 3	Juglans regia	XP_018813670.1
TRINITY_DN11323_c0_g1::TRINITY_DN11323_c0_g1_i5::g.7006::m.7006	2,36E-107	312	XP_018856589.1	vacuolar protein sorting-associated protein 20 homolog 2-like	Juglans regia	XP_018827788.1
TRINITY_DN18226_c4_g1::TRINITY_DN18226_c4_g1_i9::g.97134::m.97134	0	576	XP_018834921.1	protein FLX-like 2	Juglans regia	OAY60372.1
TRINITY_DN18320_c3_g1::TRINITY_DN18320_c3_g1_i1::g.98804::m.98804	0	595	ONI14643.1	hypothetical protein PRUPE_3G000200	Prunus persica	XP_007221486.1
TRINITY_DN12400_c3_g4::TRINITY_DN12400_c3_g4_i4::g.12578::m.12578	0	1130	XP_018833256.1	GBF-interacting protein 1-like isoform X2	Juglans regia	XP_018833257.1
TRINITY_DN15264_c3_g2::TRINITY_DN15264_c3_g2_i3::g.49355::m.49355	7,97E-87	255	OAY23775.1	hypothetical protein MANES_18G106200	Manihot esculenta	KDP26978.1
TRINITY_DN18252_c2_g3::TRINITY_DN18252_c2_g3_i2::g.97577::m.97577	0	898	XP_018858503.1	UDP-sulfoquinovose synthase, chloroplastic	Juglans regia	XP_018858504.1
TRINITY_DN14880_c3_g1::TRINITY_DN14880_c3_g1_i7::g.43762::m.43762	0	579	XP_018807006.1	ribosome maturation protein SBDS	Juglans regia	KDP43068.1
TRINITY_DN14491_c1_g6::TRINITY_DN14491_c1_g6_i1::g.37898::m.37898	4,69E-118	339	XP_018819044.1	inorganic pyrophosphatase 3-like	Juglans regia	XP_020536633.1

TRINITY_DN12086_c0_g1::TRINITY_DN12086_c0_g1_i1::g.10415::m.10415	6,64E-77	227	XP_018815656.1	acyl carrier protein 2, mitochondrial-like	Juglans regia	XP_018828996.1
TRINITY_DN17573_c2_g1::TRINITY_DN17573_c2_g1_i14::g.86248::m.86248	1,17E-89	268	XP_008229761.1	uncharacterized protein LOC103329111	Prunus mume	ONI18025.1
TRINITY_DN15292_c0_g3::TRINITY_DN15292_c0_g3_i4::g.49797::m.49797	5,68E-113	333	XP_018852566.1	uncharacterized protein LOC109014492 isoform X1	Juglans regia	XP_018852634.1
TRINITY_DN13144_c0_g1::TRINITY_DN13144_c0_g1_i6::g.19931::m.19931	2,64E-96	280	XP_018838514.1	uncharacterized protein LOC109004430	Juglans regia	OIV93225.1
TRINITY_DN19562_c2_g5::TRINITY_DN19562_c2_g5_i1::g.119826::m.119826	0	701	XP_018811352.1	heat shock 70 kDa protein 16-like	Juglans regia	XP_018811359.1
TRINITY_DN13091_c0_g1::TRINITY_DN13091_c0_g1_i8::g.19651::m.19651	2,85E-115	336	OAY23951.1	hypothetical protein MANES_18G120400	Manihot esculenta	OAY23950.1
TRINITY_DN14504_c3_g1::TRINITY_DN14504_c3_g1_i2::g.38234::m.38234	0	603	XP_018811788.1	dihydrolipoyllysine-residue succinyltransferase component of 2-oxoglutarate dehydrogenase complex 2, mitochondrial-like	Juglans regia	XP_008226865.1
TRINITY_DN15895_c3_g1::TRINITY_DN15895_c3_g1_i1::g.57722::m.57722	1,10E-149	428	XP_018833327.1	28 kDa ribonucleoprotein, chloroplastic-like	Juglans regia	KDP31006.1
TRINITY_DN18792_c0_g2::TRINITY_DN18792_c0_g2_i6::g.106589::m.106589	0	873	XP_008235650.1	serine/threonine-protein kinase tricorner-like	Prunus mume	XP_016650733.1
TRINITY_DN14028_c2_g1::TRINITY_DN14028_c2_g1_i4::g.31476::m.31476	0	1435	XP_018820782.1	respiratory burst oxidase homolog protein E isoform X1	Juglans regia	XP_018820783.1
TRINITY_DN18788_c0_g2::TRINITY_DN18788_c0_g2_i3::g.106445::m.106445	0	1233	XP_018822020.1	uncharacterized protein LOC108992033	Juglans regia	KDP32668.1
TRINITY_DN16241_c0_g1::TRINITY_DN16241_c0_g1_i2::g.63739::m.63739	7,70E-131	377	XP_018846318.1	cinnamoyl-CoA reductase 1-like	Juglans regia	KDP41979.1
TRINITY_DN19798_c2_g1::TRINITY_DN19798_c2_g1_i2::g.123755::m.123755	9,72E-38	138	XP_018860049.1	vesicle-fusing ATPase	Juglans regia	XP_018860050.1
TRINITY_DN13642_c0_g1::TRINITY_DN13642_c0_g1_i1::g.26569::m.26569	0	1066	XP_018814175.1	mitochondrial Rho GTPase 2-like isoform X1	Juglans regia	ONH93599.1
TRINITY_DN15510_c3_g3::TRINITY_DN15510_c3_g3_i2::g.53102::m.53102	0	855	XP_018818867.1	V-type proton ATPase subunit a1-like	Juglans regia	XP_018817442.1
TRINITY_DN14516_c0_g1::TRINITY_DN14516_c0_g1_i7::g.38023::m.38023	9,08E-90	267	XP_018805307.1	ATP-dependent Clp protease ATP-binding subunit CLPT1, chloroplastic	Juglans regia	XP_008231397.1
TRINITY_DN44543_c0_g1::TRINITY_DN44543_c0_g1_i1::g.132115::m.132115	3,86E-69	209	ACU13131.1	unknown	Glycine max	XP_014513266.1
TRINITY_DN19344_c5_g1::TRINITY_DN19344_c5_g1_i8::g.115623::m.115623						
TRINITY_DN14685_c0_g1::TRINITY_DN14685_c0_g1_i6::g.40758::m.40758	0	5051	XP_018835909.1	uncharacterized protein LOC109002567	Juglans regia	ONI06280.1
TRINITY_DN12342_c1_g2::TRINITY_DN12342_c1_g2_i2::g.12106::m.12106	3,61E-77	233	XP_018839161.1	early nodulin-like protein 1	Juglans regia	OAY53057.1
TRINITY_DN14553_c3_g1::TRINITY_DN14553_c3_g1_i5::g.38708::m.38708	0	867	XP_018850893.1	probable receptor-like protein kinase At5g61350	Juglans regia	XP_016651358.1

TRINITY_DN16423_c1_g1::TRINITY_DN16423_c1_g1_i12::g.67294::m.67294	0	1651	KDP46148.1	hypothetical protein JCGZ_06659	Jatropha curcas	XP_020535342.1
TRINITY_DN5236_c0_g1::TRINITY_DN5236_c0_g1_i1::g.1296::m.1296	4,90E-125	358	OAY49094.1	hypothetical protein MANES_05G029000	Manihot esculenta	XP_018841223.1
TRINITY_DN14069_c0_g3::TRINITY_DN14069_c0_g3_i3::g.32072::m.32072	0	710	XP_018858469.1	uncharacterized protein LOC109020435	Juglans regia	KDP40469.1
TRINITY_DN18518_c1_g1::TRINITY_DN18518_c1_g1_i12::g.103139::m.103139	0	1098	XP_018836708.1	mannosyl-oligosaccharide glucosidase GCS1-like isoform X6	Juglans regia	XP_018836709.1
TRINITY_DN18275_c0_g4::TRINITY_DN18275_c0_g4_i1::g.98310::m.98310	0	2842	XP_018852332.1	probable phosphoinositide phosphatase SAC9	Juglans regia	XP_008243038.1
TRINITY_DN20016_c1_g1::TRINITY_DN20016_c1_g1_i7::g.127847::m.127847	1,94E-169	476	XP_018844177.1	non-functional NADPH-dependent codeinone reductase 2-like isoform X1	Juglans regia	ONI30338.1
TRINITY_DN14420_c1_g1::TRINITY_DN14420_c1_g1_i4::g.36850::m.36850	4,13E-77	229	XP_018849484.1	uncharacterized protein LOC109012353	Juglans regia	XP_008219081.1
TRINITY_DN18968_c1_g1::TRINITY_DN18968_c1_g1_i9::g.109574::m.109574	0	828	XP_018845038.1	probable cadmium/zinc-transporting ATPase HMA1, chloroplastic	Juglans regia	XP_008238762.1
TRINITY_DN14219_c0_g1::TRINITY_DN14219_c0_g1_i18::g.33900::m.33900	1,67E-169	470	XP_018836403.1	dolichol-phosphate mannosyltransferase subunit 1	Juglans regia	KDP36797.1
TRINITY_DN16257_c0_g1::TRINITY_DN16257_c0_g1_i2::g.64601::m.64601	0	822	XP_018815156.1	uncharacterized protein LOC108986841 isoform X3	Juglans regia	XP_018815155.1
TRINITY_DN19366_c1_g3::TRINITY_DN19366_c1_g3_i1::g.116912::m.116912	4,62E-76	225	XP_018847221.1	NHP2-like protein 1	Juglans regia	XP_020204814.1
TRINITY_DN18230_c1_g3::TRINITY_DN18230_c1_g3_i4::g.97286::m.97286	8,97E-82	248	XP_018838439.1	uncharacterized protein LOC109004361	Juglans regia	KDP24159.1
TRINITY_DN16429_c0_g2::TRINITY_DN16429_c0_g2_i3::g.67310::m.67310	4,53E-103	306	XP_018824717.1	acyl-CoA-binding domain-containing protein 1-like isoform X1	Juglans regia	XP_018824718.1
TRINITY_DN18185_c0_g2::TRINITY_DN18185_c0_g2_i8::g.96676::m.96676	0	1256	XP_018833351.1	protein DEFECTIVE IN EXINE FORMATION 1	Juglans regia	XP_009370211.1
TRINITY_DN16928_c0_g2::TRINITY_DN16928_c0_g2_i1::g.75662::m.75662	9,53E-101	298	XP_018851859.1	2-oxoglutarate-dependent dioxygenase DAO-like	Juglans regia	XP_018851860.1
TRINITY_DN12765_c0_g2::TRINITY_DN12765_c0_g2_i9::g.15778::m.15778	1,43E-111	323	XP_018822168.1	peptidyl-prolyl cis-trans isomerase CYP21-4-like	Juglans regia	XP_018842322.1
TRINITY_DN16240_c0_g1::TRINITY_DN16240_c0_g1_i3::g.64405::m.64405	0	1309	XP_018836405.1	1,4-alpha-glucan-branching enzyme 1, chloroplastic/amyloplastic-like	Juglans regia	OAY50415.1
TRINITY_DN15464_c0_g4::TRINITY_DN15464_c0_g4_i1::g.52272::m.52272	8,06E-148	431	XP_018823263.1	probable mediator of RNA polymerase II transcription subunit 37c	Juglans regia	XP_018831319.1
TRINITY_DN17952_c0_g1::TRINITY_DN17952_c0_g1_i8::g.92586::m.92586	0	1387	XP_018835441.1	cullin-1-like	Juglans regia	XP_018835442.1
TRINITY_DN16980_c0_g3::TRINITY_DN16980_c0_g3_i2::g.76506::m.76506	0	1122	XP_018821637.1	putative pentatricopeptide repeat-containing protein At2g02150	Juglans regia	XP_018821639.1
TRINITY_DN14376_c2_g1::TRINITY_DN14376_c2_g1_i4::g.36173::m.36173	2,41E-75	228	OAY61455.1	hypothetical protein MANES_01G190000	Manihot esculenta	OAY61456.1
TRINITY_DN11353_c0_g1::TRINITY_DN11353_c0_g1_i1::g.7089::m.7089	2,47E-138	395	XP_018829313.1	U2 small nuclear ribonucleoprotein A'	Juglans regia	XP_008222301.1

TRINITY_DN17937_c1_g5::TRINITY_DN17937_c1_g5_i2::g.92219::m.92219	3,40E-46	155	XP_018858704.1	uncharacterized protein LOC109020641 isoform X2	Juglans regia	XP_018858710.1
TRINITY_DN15328_c3_g2::TRINITY_DN15328_c3_g2_i4::g.50205::m.50205	2,61E-148	431	XP_018852000.1	uncharacterized protein LOC109014122 isoform X1	Juglans regia	XP_018852001.1
TRINITY_DN19548_c2_g6::TRINITY_DN19548_c2_g6_i1::g.119771::m.119771	7,31E-105	316	XP_012083840.2	protein DETOXIFICATION 34	Jatropha curcas	XP_008233023.1
TRINITY_DN18997_c1_g1::TRINITY_DN18997_c1_g1_i3::g.110273::m.110273	0	1599	XP_018835198.1	ABC transporter G family member 31	Juglans regia	XP_018835198.1
TRINITY_DN17307_c3_g1::TRINITY_DN17307_c3_g1_i2::g.81691::m.81691	0	631	ONI16935.1	hypothetical protein PRUPE_3G131200	Prunus persica	XP_007198834.2
TRINITY_DN19257_c3_g1::TRINITY_DN19257_c3_g1_i1::g.114723::m.114723	0	532	XP_018849919.1	cytochrome b561 and DOMON domain-containing protein At5g47530-like	Juglans regia	OAY41119.1
TRINITY_DN19453_c0_g2::TRINITY_DN19453_c0_g2_i2::g.118209::m.118209	0	746	XP_018835196.1	probable trehalase	Juglans regia	XP_018824320.1
TRINITY_DN11079_c0_g1::TRINITY_DN11079_c0_g1_i1::g.6259::m.6259	0	566	XP_018847493.1	nitrile-specifier protein 5 isoform X1	Juglans regia	XP_018847494.1
TRINITY_DN18472_c4_g2::TRINITY_DN18472_c4_g2_i2::g.100983::m.100983	2,57E-166	476	XP_018822502.1	calcium-dependent protein kinase 24-like	Juglans regia	OIV99428.1
TRINITY_DN9342_c0_g1::TRINITY_DN9342_c0_g1_i1::g.3304::m.3304	0	598	XP_018844750.1	WD repeat-containing protein VIP3	Juglans regia	KEH32469.1
TRINITY_DN17375_c4_g1::TRINITY_DN17375_c4_g1_i2::g.82845::m.82845	0	1077	XP_018851853.1	molybdopterin biosynthesis protein CNX1	Juglans regia	XP_018851854.1
TRINITY_DN16195_c0_g1::TRINITY_DN16195_c0_g1_i3::g.63589::m.63589	5,83E-28	108	KDP43847.1	hypothetical protein JCGZ_20857	Jatropha curcas	XP_012065174.1
TRINITY_DN15131_c1_g3::TRINITY_DN15131_c1_g3_i1::g.47487::m.47487	2,43E-53	185	XP_018817838.1	methionine S-methyltransferase	Juglans regia	XP_009355389.1
TRINITY_DN17744_c7_g1::TRINITY_DN17744_c7_g1_i4::g.89103::m.89103	1,80E-140	397	XP_018852761.1	gamma-soluble NSF attachment protein-like, partial	Juglans regia	XP_018806434.1
TRINITY_DN18492_c0_g1::TRINITY_DN18492_c0_g1_i14::g.101403::m.101403	2,11E-88	275	XP_018822688.1	uncharacterized protein LOC108992543	Juglans regia	XP_018822693.1
TRINITY_DN20003_c1_g1::TRINITY_DN20003_c1_g1_i6::g.127620::m.127620	1,22E-52	165	XP_018846616.1	succinate dehydrogenase assembly factor 1, mitochondrial-like	Juglans regia	XP_018834218.1
TRINITY_DN14376_c2_g4::TRINITY_DN14376_c2_g4_i7::g.36164::m.36164	6,54E-104	302	XP_018831584.1	hypersensitive-induced response protein 1	Juglans regia	XP_018831584.1
TRINITY_DN17923_c2_g2::TRINITY_DN17923_c2_g2_i2::g.91995::m.91995	0	1453	ONI18007.1	hypothetical protein PRUPE_3G191700	Prunus persica	XP_007217043.1
TRINITY_DN12731_c3_g4::TRINITY_DN12731_c3_g4_i1::g.15536::m.15536	8,59E-67	214	XP_018825627.1	exopolygalacturonase-like	Juglans regia	XP_018809534.1
TRINITY_DN11014_c0_g1::TRINITY_DN11014_c0_g1_i2::g.6114::m.6114	1,25E-107	312	KDP34029.1	hypothetical protein JCGZ_07600	Jatropha curcas	XP_012077195.1
TRINITY_DN17625_c1_g2::TRINITY_DN17625_c1_g2_i5::g.87298::m.87298	0	676	XP_018810439.1	uncharacterized protein LOC108983304	Juglans regia	XP_018810614.1
TRINITY_DN15733_c1_g1::TRINITY_DN15733_c1_g1_i2::g.56414::m.56414	2,31E-97	281	XP_018828972.1	probable prefoldin subunit 5	Juglans regia	KDP37534.1

TRINITY_DN18395_c1_g2::TRINITY_DN18395_c1_g2_i6::g.99075::m.99075	1,53E-94	291	XP_018841544.1	ubiquitin domain-containing protein DSK2a-like isoform X3	Juglans regia	XP_018841544.1
TRINITY_DN15876_c0_g2::TRINITY_DN15876_c0_g2_i4::g.58231::m.58231	7,48E-57	186	XP_018808206.1	polyadenylate-binding protein RBP45-like isoform X2	Juglans regia	XP_018808206.1
TRINITY_DN17846_c4_g3::TRINITY_DN17846_c4_g3_i1::g.90654::m.90654	0	620	XP_018844673.1	protein bem46	Juglans regia	XP_020228885.1
TRINITY_DN19531_c0_g1::TRINITY_DN19531_c0_g1_i3::g.119625::m.119625	0	3323	XP_018818361.1	uncharacterized protein LOC108989269 isoform X1	Juglans regia	XP_018818363.1
TRINITY_DN19896_c1_g1::TRINITY_DN19896_c1_g1_i11::g.125427::m.125427	0	781	XP_018813340.1	diphosphomevalonate decarboxylase MVD2-like isoform X2	Juglans regia	XP_018813339.1
TRINITY_DN13610_c2_g1::TRINITY_DN13610_c2_g1_i1::g.26215::m.26215	0	641	XP_018844048.1	xylulose 5-phosphate/phosphate translocator, chloroplastic-like	Juglans regia	XP_008232176.1
TRINITY_DN17704_c0_g1::TRINITY_DN17704_c0_g1_i3::g.88593::m.88593	0	1362	XP_018823799.1	translocase of chloroplast 120, chloroplastic-like	Juglans regia	XP_018841453.1
TRINITY_DN13149_c0_g1::TRINITY_DN13149_c0_g1_i7::g.20402::m.20402	0	675	XP_018813478.1	ribose-phosphate pyrophosphokinase 4-like isoform X1	Juglans regia	ONI30368.1
TRINITY_DN16337_c1_g1::TRINITY_DN16337_c1_g1_i12::g.66068::m.66068	8,19E-139	407	XP_018816062.1	plant UBX domain-containing protein 11	Juglans regia	XP_018816063.1
TRINITY_DN18805_c4_g1::TRINITY_DN18805_c4_g1_i2::g.106689::m.106689	8,95E-105	303	XP_018815840.1	UMP-CMP kinase isoform X5	Juglans regia	XP_018815838.1
TRINITY_DN19153_c1_g3::TRINITY_DN19153_c1_g3_i2::g.112871::m.112871	2,12E-53	180	XP_018858842.1	patellin-4-like	Juglans regia	KRH36854.1
TRINITY_DN12257_c1_g1::TRINITY_DN12257_c1_g1_i2::g.11446::m.11446	0	802	XP_018806921.1	AP-2 complex subunit mu	Juglans regia	XP_018824667.1
TRINITY_DN14137_c0_g1::TRINITY_DN14137_c0_g1_i2::g.33039::m.33039	0	675	XP_018844104.1	threonine dehydratase biosynthetic, chloroplastic	Juglans regia	XP_009340966.1
TRINITY_DN14239_c2_g2::TRINITY_DN14239_c2_g2_i2::g.34369::m.34369	0	675	XP_018830097.1	exocyst complex component SEC10-like	Juglans regia	ONI23250.1
TRINITY_DN12628_c0_g1::TRINITY_DN12628_c0_g1_i15::g.14380::m.14380	2,10E-94	275	XP_008365972.1	AB hydrolase superfamily protein YfhM-like	Malus domestica	XP_009336925.1
TRINITY_DN16315_c0_g1::TRINITY_DN16315_c0_g1_i3::g.65602::m.65602	0	885	XP_018819948.1	3beta-hydroxysteroid-dehydrogenase/decarboxylase	Juglans regia	XP_018819949.1
TRINITY_DN11202_c0_g1::TRINITY_DN11202_c0_g1_i1::g.6604::m.6604	0	551	XP_018837250.1	annexin D5	Juglans regia	XP_004503249.1
TRINITY_DN12134_c9_g1::TRINITY_DN12134_c9_g1_i4::g.10752::m.10752	0	1008	XP_018816904.1	probable galacturonosyltransferase 9	Juglans regia	XP_008230332.1
TRINITY_DN16939_c0_g1::TRINITY_DN16939_c0_g1_i3::g.75902::m.75902	3,53E-90	266	XP_018849964.1	monothiol glutaredoxin-S10-like	Juglans regia	XP_008382672.1
TRINITY_DN17688_c2_g3::TRINITY_DN17688_c2_g3_i4::g.88274::m.88274	2,21E-46	159	XP_008237744.1	endoglucanase 9	Prunus mume	ONH89638.1
TRINITY_DN15001_c1_g2::TRINITY_DN15001_c1_g2_i1::g.45482::m.45482	1,18E-77	245	XP_018846208.1	polypyrimidine tract-binding protein homolog 3-like	Juglans regia	XP_017187311.1
TRINITY_DN15894_c0_g1::TRINITY_DN15894_c0_g1_i1::g.58474::m.58474	5,15E-102	299	XP_018856631.1	enoyl-CoA hydratase 2, peroxisomal	Juglans regia	ONI32267.1

TRINITY_DN16337_c1_g3::TRINITY_DN16337_c1_g3_i2::g.66071::m.66071	0	562	XP_018859271.1	28S ribosomal protein S9, mitochondrial	Juglans regia	XP_018859272.1
TRINITY_DN13153_c0_g3::TRINITY_DN13153_c0_g3_i1::g.20486::m.20486	1,05E-83	251	XP_018812118.1	beta-amylase 1, chloroplastic-like, partial	Juglans regia	XP_018858814.1
TRINITY_DN19578_c1_g1::TRINITY_DN19578_c1_g1_i9::g.120079::m.120079	4,11E-152	474	XP_018843078.1	calponin homology domain-containing protein DDB_G0272472	Juglans regia	XP_018843079.1
TRINITY_DN19156_c1_g1::TRINITY_DN19156_c1_g1_i7::g.113150::m.113150	8,47E-116	334	XP_018849297.1	ribonuclease 2	Juglans regia	OAY34914.1
TRINITY_DN18639_c0_g2::TRINITY_DN18639_c0_g2_i1::g.103472::m.103472	6,99E-62	207	XP_008374168.1	ADP-ribosylation factor GTPase-activating protein AGD3	Malus domestica	OAY48031.1
TRINITY_DN17531_c3_g1::TRINITY_DN17531_c3_g1_i2::g.85473::m.85473	3,83E-45	160	XP_018850837.1	germinal center kinase 1-like isoform X2	Juglans regia	XP_018850820.1
TRINITY_DN9714_c0_g1::TRINITY_DN9714_c0_g1_i2::g.3800::m.3800	0	865	XP_018842576.1	tocopherol cyclase, chloroplastic-like	Juglans regia	XP_018845525.1
TRINITY_DN17192_c0_g2::TRINITY_DN17192_c0_g2_i8::g.79905::m.79905	2,09E-84	256	ONI17464.1	hypothetical protein PRUPE_3G160800	Prunus persica	XP_020415028.1
TRINITY_DN17561_c0_g1::TRINITY_DN17561_c0_g1_i2::g.86132::m.86132	0	4642	XP_018851728.1	pre-mRNA-processing-splicing factor 8A-like	Juglans regia	XP_018851726.1
TRINITY_DN17433_c1_g2::TRINITY_DN17433_c1_g2_i1::g.83589::m.83589	6,59E-52	166	XP_018811251.1	hemiasterlin resistant protein 1	Juglans regia	XP_018826924.1
TRINITY_DN14320_c0_g6::TRINITY_DN14320_c0_g6_i4::g.35483::m.35483	1,98E-98	302	XP_018811770.1	target of Myb protein 1-like	Juglans regia	XP_018845550.1
TRINITY_DN13688_c0_g1::TRINITY_DN13688_c0_g1_i3::g.27185::m.27185	1,03E-178	501	XP_018816582.1	uncharacterized protein LOC108987951	Juglans regia	KDP43433.1
TRINITY_DN16402_c0_g3::TRINITY_DN16402_c0_g3_i1::g.67045::m.67045	4,78E-95	286	XP_018823907.1	uncharacterized protein LOC108993437	Juglans regia	XP_009355644.1
TRINITY_DN19228_c2_g1::TRINITY_DN19228_c2_g1_i5::g.114244::m.114244	2,49E-81	243	XP_018857124.1	membrane-associated progesterone-binding protein 4 isoform X1	Juglans regia	XP_018857125.1
TRINITY_DN16661_c1_g2::TRINITY_DN16661_c1_g2_i5::g.71230::m.71230	0	1176	XP_018816466.1	alkaline/neutral invertase A, mitochondrial isoform X1	Juglans regia	XP_018816467.1
TRINITY_DN17919_c1_g1::TRINITY_DN17919_c1_g1_i2::g.91916::m.91916	0	1969	XP_018834545.1	importin-5-like	Juglans regia	XP_018809428.1
TRINITY_DN19280_c0_g1::TRINITY_DN19280_c0_g1_i5::g.115042::m.115042	0	527	XP_018838909.1	uncharacterized protein LOC109004724 isoform X2	Juglans regia	XP_018838910.1
TRINITY_DN16384_c1_g4::TRINITY_DN16384_c1_g4_i4::g.66906::m.66906	0	822	XP_018808629.1	synaptotagmin-5-like	Juglans regia	OAY49277.1
TRINITY_DN11479_c0_g1::TRINITY_DN11479_c0_g1_i6::g.7503::m.7503	5,28E-93	272	XP_018834185.1	uncharacterized protein LOC109001389 isoform X1	Juglans regia	AFK43060.1
TRINITY_DN16861_c5_g5::TRINITY_DN16861_c5_g5_i1::g.74534::m.74534	3,61E-132	377	XP_018859928.1	peroxisome biogenesis protein 19-2-like	Juglans regia	ONI06608.1
TRINITY_DN20036_c5_g1::TRINITY_DN20036_c5_g1_i2::g.127587::m.127587	2,75E-97	295	XP_018811308.1	probable aspartyl aminopeptidase	Juglans regia	XP_018814786.1
TRINITY_DN16479_c1_g2::TRINITY_DN16479_c1_g2_i4::g.68347::m.68347	0	1342	XP_018821143.1	uncharacterized TPR repeat-containing protein At1g05150-like	Juglans regia	OAY50637.1

TRINITY_DN17999_c1_g1::TRINITY_DN17999_c1_g1_i8::g.91779::m.91779	7,52E-92	285	XP_018857443.1	aspartic proteinase-like protein 2 isoform X1	Juglans regia	XP_018857444.1
TRINITY_DN17673_c0_g1::TRINITY_DN17673_c0_g1_i5::g.88024::m.88024	0	991	XP_018816325.1	cellulose synthase-like protein D1	Juglans regia	OAY28231.1
TRINITY_DN10333_c0_g1::TRINITY_DN10333_c0_g1_i1::g.4649::m.4649	0	733	XP_016187654.1	elongation factor Tu, chloroplastic	Arachis ipaensis	CAA61444.1
TRINITY_DN13300_c0_g1::TRINITY_DN13300_c0_g1_i5::g.22083::m.22083	0	618	XP_018838659.1	glutathione synthetase, chloroplastic	Juglans regia	XP_008246528.1
TRINITY_DN18283_c0_g1::TRINITY_DN18283_c0_g1_i12::g.98055::m.98055	1,18E-108	318	XP_018858693.1	traB domain-containing protein-like	Juglans regia	ONI18511.1
TRINITY_DN10355_c0_g1::TRINITY_DN10355_c0_g1_i1::g.4689::m.4689						
TRINITY_DN15356_c1_g1::TRINITY_DN15356_c1_g1_i4::g.50914::m.50914	2,48E-91	282	XP_018849693.1	ERAD-associated E3 ubiquitin-protein ligase HRD1B-like	Juglans regia	XP_018849695.1
TRINITY_DN18321_c2_g1::TRINITY_DN18321_c2_g1_i1::g.98839::m.98839	9,75E-88	260	XP_018853745.1	vesicle-associated protein 1-3-like	Juglans regia	XP_018820386.1
TRINITY_DN13289_c1_g2::TRINITY_DN13289_c1_g2_i4::g.22093::m.22093	1,64E-36	125	XP_018856681.1	uncharacterized protein LOC109018939 isoform X2	Juglans regia	XP_018850003.1
TRINITY_DN13288_c0_g1::TRINITY_DN13288_c0_g1_i6::g.22068::m.22068	0	620	XP_018840790.1	glucan endo-1,3-beta-D-glucosidase	Juglans regia	ONI18348.1
TRINITY_DN17097_c0_g1::TRINITY_DN17097_c0_g1_i1::g.78356::m.78356	0	644	XP_008219547.1	uncharacterized protein LOC103319739	Prunus mume	XP_018822242.1
TRINITY_DN14079_c0_g2::TRINITY_DN14079_c0_g2_i4::g.32111::m.32111	2,95E-72	218	XP_008360788.1	probable aspartyl aminopeptidase, partial	Malus domestica	XP_018811308.1
TRINITY_DN14601_c2_g1::TRINITY_DN14601_c2_g1_i8::g.39287::m.39287	0	567	XP_018827750.1	zinc finger protein ZPR1-like	Juglans regia	XP_018827750.1
TRINITY_DN17332_c3_g1::TRINITY_DN17332_c3_g1_i4::g.82072::m.82072	0	601	XP_018846580.1	inositol oxygenase 4-like	Juglans regia	XP_008361306.1
TRINITY_DN18983_c0_g1::TRINITY_DN18983_c0_g1_i9::g.110074::m.110074	0	1150	XP_018808201.1	uncharacterized protein LOC108981462 isoform X2	Juglans regia	XP_018808192.1
TRINITY_DN17234_c0_g1::TRINITY_DN17234_c0_g1_i11::g.80741::m.80741	2,70E-47	168	XP_012080994.1	pullulanase 1, chloroplastic	Jatropha curcas	XP_018808474.1
TRINITY_DN16222_c0_g1::TRINITY_DN16222_c0_g1_i6::g.64118::m.64118	2,06E-63	201	XP_018845736.1	syntaxin-32-like	Juglans regia	XP_018845737.1
TRINITY_DN15263_c1_g2::TRINITY_DN15263_c1_g2_i4::g.49263::m.49263	0	690	XP_018851345.1	UDP-glycosyltransferase 74E2-like isoform X1	Juglans regia	XP_018851346.1
TRINITY_DN13753_c0_g1::TRINITY_DN13753_c0_g1_i3::g.27858::m.27858	0	566	XP_018827509.1	methylthioribose kinase-like	Juglans regia	KHN42353.1
TRINITY_DN17360_c0_g1::TRINITY_DN17360_c0_g1_i6::g.82556::m.82556	5,19E-77	246	XP_018820105.1	uncharacterized protein LOC108990561	Juglans regia	XP_018820105.1
TRINITY_DN11448_c0_g1::TRINITY_DN11448_c0_g1_i1::g.7372::m.7372	2,65E-56	178	XP_018819823.1	MFP1 attachment factor 1-like	Juglans regia	XP_018824955.1
TRINITY_DN13217_c3_g3::TRINITY_DN13217_c3_g3_i1::g.21250::m.21250	1,01E-135	396	XP_018840528.1	DUF21 domain-containing protein At4g14240-like	Juglans regia	XP_018826137.1

TRINITY_DN13260_c0_g1::TRINITY_DN13260_c0_g1_i2::g.21630::m.21630	1,75E-37	137	XP_018821273.1	peroxisomal membrane protein PEX14	Juglans regia	XP_018821274.1
TRINITY_DN19222_c3_g1::TRINITY_DN19222_c3_g1_i5::g.114236::m.114236	0	608	XP_018820828.1	anthocyanidin 3-O-glucosyltransferase 7-like isoform X2	Juglans regia	XP_018820827.1
TRINITY_DN10774_c0_g1::TRINITY_DN10774_c0_g1_i7::g.5503::m.5503	1,11E-121	350	XP_018814173.1	COP9 signalosome complex subunit 6a	Juglans regia	KDP29518.1
TRINITY_DN16241_c1_g4::TRINITY_DN16241_c1_g4_i5::g.63753::m.63753	0	1030	XP_018809511.1	pantothenate kinase 2 isoform X1	Juglans regia	XP_018809513.1
TRINITY_DN19395_c1_g4::TRINITY_DN19395_c1_g4_i2::g.117259::m.117259	0	823	XP_018821163.1	purple acid phosphatase 2-like	Juglans regia	XP_018821164.1
TRINITY_DN22718_c0_g1::TRINITY_DN22718_c0_g1_i1::g.128985::m.128985	2,27E-76	227	CAB66334.1	acidic endochitinase, partial	Betula pendula	XP_018838170.1
TRINITY_DN13284_c0_g1::TRINITY_DN13284_c0_g1_i3::g.21957::m.21957	0	601	XP_018835583.1	polyadenylate-binding protein RBP47-like isoform X1	Juglans regia	XP_018835584.1
TRINITY_DN19032_c2_g1::TRINITY_DN19032_c2_g1_i2::g.110806::m.110806	0	598	XP_018805195.1	probable serine/threonine-protein kinase DDB_G0291350	Juglans regia	ONI09009.1
TRINITY_DN16806_c1_g1::TRINITY_DN16806_c1_g1_i6::g.73621::m.73621	0	726	XP_018815003.1	histidinol-phosphate aminotransferase, chloroplastic-like	Juglans regia	XP_018815004.1
TRINITY_DN14774_c0_g5::TRINITY_DN14774_c0_g5_i3::g.42116::m.42116	0	2417	XP_018836518.1	splicing factor 3B subunit 3-like	Juglans regia	XP_018836519.1
TRINITY_DN11806_c0_g1::TRINITY_DN11806_c0_g1_i1::g.8989::m.8989	0	829	XP_018841303.1	acyl-coenzyme A thioesterase 9, mitochondrial-like isoform X3	Juglans regia	XP_018841302.1
TRINITY_DN15677_c1_g6::TRINITY_DN15677_c1_g6_i3::g.55549::m.55549	4,57E-69	221	XP_018825904.1	uncharacterized protein LOC108994927 isoform X2	Juglans regia	XP_018825905.1
TRINITY_DN13179_c1_g1::TRINITY_DN13179_c1_g1_i6::g.20799::m.20799	1,13E-100	291	OAY33789.1	hypothetical protein MANES_13G124900	Manihot esculenta	XP_009375705.1
TRINITY_DN14413_c0_g2::TRINITY_DN14413_c0_g2_i2::g.36805::m.36805	0	793	XP_018824373.1	methionine gamma-lyase	Juglans regia	OAY21242.1
TRINITY_DN19247_c0_g1::TRINITY_DN19247_c0_g1_i8::g.114528::m.114528	1,56E-161	461	XP_018859052.1	coatomer subunit delta	Juglans regia	XP_018859053.1
TRINITY_DN17718_c6_g2::TRINITY_DN17718_c6_g2_i4::g.88774::m.88774	0	1478	XP_018852141.1	serine/threonine-protein kinase EDR1-like isoform X4	Juglans regia	XP_018852140.1
TRINITY_DN18436_c0_g2::TRINITY_DN18436_c0_g2_i2::g.100108::m.100108	0	592	XP_018815864.1	conserved oligomeric Golgi complex subunit 7	Juglans regia	XP_008376080.1
TRINITY_DN19343_c7_g2::TRINITY_DN19343_c7_g2_i2::g.116418::m.116418	5,57E-63	204	XP_007152599.1	hypothetical protein PHAVU_004G143600g	Phaseolus vulgaris	ESW24593.1
TRINITY_DN18242_c3_g1::TRINITY_DN18242_c3_g1_i7::g.97423::m.97423	4,20E-16	82,8	XP_018807889.1	heterogeneous nuclear ribonucleoprotein 1-like	Juglans regia	XP_018807889.1
TRINITY_DN14572_c4_g1::TRINITY_DN14572_c4_g1_i1::g.39016::m.39016	2,02E-59	191	XP_018851581.1	oleoyl-acyl carrier protein thioesterase 1, chloroplastic-like	Juglans regia	XP_018830356.1
TRINITY_DN13884_c2_g1::TRINITY_DN13884_c2_g1_i6::g.29871::m.29871	0	585	XP_018842779.1	polyadenylate-binding protein-interacting protein 12-like	Juglans regia	XP_018842779.1
TRINITY_DN17016_c4_g1::TRINITY_DN17016_c4_g1_i2::g.77096::m.77096	4,10E-58	191	KYP53201.1	Sugar transporter ERD6-like 6	Cajanus cajan	XP_020229565.1

TRINITY_DN14069_c0_g5::TRINITY_DN14069_c0_g5_i5::g.32070::m.32070	7,94E-72	224	XP_018834116.1	probable isoprenylcysteine alpha-carbonyl methylesterase ICMEL2	Juglans regia	XP_008230375.1
TRINITY_DN17522_c3_g1::TRINITY_DN17522_c3_g1_i3::g.85131::m.85131	2,24E-67	204	XP_018840440.1	50S ribosomal protein HLP, mitochondrial-like isoform X2	Juglans regia	XP_018840439.1
TRINITY_DN18040_c0_g1::TRINITY_DN18040_c0_g1_i2::g.94127::m.94127	5,90E-167	470	KDP40635.1	hypothetical protein JCGZ_24634	Jatropha curcas	XP_012068802.1
TRINITY_DN15831_c4_g2::TRINITY_DN15831_c4_g2_i2::g.57659::m.57659	0	920	XP_018825496.1	non-specific phospholipase C2	Juglans regia	XP_009337051.1
TRINITY_DN17167_c4_g1::TRINITY_DN17167_c4_g1_i2::g.79586::m.79586	4,62E-97	286	XP_018812413.1	uncharacterized protein LOC108984806	Juglans regia	XP_020540194.1
TRINITY_DN14538_c2_g2::TRINITY_DN14538_c2_g2_i9::g.38508::m.38508	2,70E-136	385	XP_018834170.1	membrin-11-like	Juglans regia	XP_018839683.1
TRINITY_DN16535_c0_g2::TRINITY_DN16535_c0_g2_i8::g.69172::m.69172	0	775	XP_018846814.1	ammonium transporter 1 member 1-like	Juglans regia	XP_008224012.1
TRINITY_DN14304_c1_g1::TRINITY_DN14304_c1_g1_i6::g.35219::m.35219	1,18E-18	84,7	XP_018845734.1	probable serine/threonine-protein kinase kinX	Juglans regia	XP_008219845.1
TRINITY_DN19010_c0_g2::TRINITY_DN19010_c0_g2_i1::g.110437::m.110437	2,83E-65	198	XP_018835303.1	glycine-rich RNA-binding protein 4, mitochondrial-like	Juglans regia	XP_018835298.1
TRINITY_DN16594_c0_g2::TRINITY_DN16594_c0_g2_i1::g.69960::m.69960	3,15E-176	494	XP_018847248.1	copper chaperone for superoxide dismutase, chloroplastic/cytosolic isoform X1	Juglans regia	XP_018847253.1
TRINITY_DN19775_c1_g1::TRINITY_DN19775_c1_g1_i3::g.123478::m.123478	0	2670	XP_018829523.1	nuclear pore complex protein NUP155	Juglans regia	ONI31831.1
TRINITY_DN12386_c0_g1::TRINITY_DN12386_c0_g1_i3::g.12387::m.12387	0	1063	XP_018839879.1	vacuolar protein sorting-associated protein 45 homolog isoform X1	Juglans regia	XP_009348500.1
TRINITY_DN13587_c1_g3::TRINITY_DN13587_c1_g3_i8::g.25768::m.25768	0	543	XP_018858731.1	(+)-neomenthol dehydrogenase-like	Juglans regia	KDP42906.1
TRINITY_DN15400_c1_g2::TRINITY_DN15400_c1_g2_i8::g.50620::m.50620	1,01E-116	331	XP_018826216.1	uncharacterized protein At5g01610-like	Juglans regia	XP_018809179.1
TRINITY_DN14459_c0_g1::TRINITY_DN14459_c0_g1_i2::g.37381::m.37381	0	617	XP_018811142.1	mevalonate kinase	Juglans regia	KDP23529.1
TRINITY_DN11301_c0_g1::TRINITY_DN11301_c0_g1_i2::g.6892::m.6892	1,03E-126	370	XP_018841848.1	notchless protein homolog	Juglans regia	XP_018841848.1
TRINITY_DN15762_c0_g1::TRINITY_DN15762_c0_g1_i1::g.56697::m.56697	0	2657	XP_018814445.1	probable phosphoribosylformylglycinamidine synthase, chloroplastic/mitochondrial	Juglans regia	XP_018814446.1
TRINITY_DN15598_c1_g1::TRINITY_DN15598_c1_g1_i2::g.54269::m.54269	0	877	XP_018816759.1	uncharacterized protein LOC108988098 isoform X1	Juglans regia	XP_018816760.1
TRINITY_DN11950_c0_g1::TRINITY_DN11950_c0_g1_i3::g.9633::m.9633	1,23E-166	468	XP_018847037.1	ubiquitin carboxyl-terminal hydrolase 4	Juglans regia	OAY22015.1
TRINITY_DN11646_c0_g1::TRINITY_DN11646_c0_g1_i1::g.8053::m.8053	1,16E-83	270	XP_018828527.1	nardilysin-like	Juglans regia	XP_016646759.1
TRINITY_DN15411_c1_g1::TRINITY_DN15411_c1_g1_i1::g.51593::m.51593	0	619	XP_018814289.1	topless-related protein 4-like	Juglans regia	XP_018814290.1
TRINITY_DN16682_c4_g1::TRINITY_DN16682_c4_g1_i2::g.71560::m.71560	2,62E-50	174	XP_018846385.1	probable nucleoredoxin 1	Juglans regia	XP_018846385.1

TRINITY_DN18037_c1_g1::TRINITY_DN18037_c1_g1_i9::g.94092::m.94092	0	924	XP_018830065.1	uncharacterized protein LOC108998071	Juglans regia	AAO14626.1
TRINITY_DN15485_c1_g1::TRINITY_DN15485_c1_g1_i8::g.52679::m.52679	9,13E-62	201	XP_018823837.1	eukaryotic translation initiation factor 4B3	Juglans regia	XP_018841447.1
TRINITY_DN14982_c0_g1::TRINITY_DN14982_c0_g1_i9::g.45222::m.45222	3,77E-173	488	XP_018836146.1	uncharacterized protein LOC109002731	Juglans regia	XP_018833900.1
TRINITY_DN19482_c6_g1::TRINITY_DN19482_c6_g1_i8::g.118606::m.118606	2,20E-123	352	XP_018856489.1	uncharacterized protein At5g50100, mitochondrial	Juglans regia	XP_008239718.1
TRINITY_DN14898_c0_g1::TRINITY_DN14898_c0_g1_i2::g.43888::m.43888	0	600	XP_018808112.1	zinc-binding alcohol dehydrogenase domain-containing protein 2 isoform X1	Juglans regia	OAY39079.1
TRINITY_DN16344_c0_g1::TRINITY_DN16344_c0_g1_i6::g.66181::m.66181	0	541	XP_018857864.1	7-methylguanosine phosphate-specific 5'-nucleotidase A-like	Juglans regia	XP_018857865.1
TRINITY_DN16793_c3_g3::TRINITY_DN16793_c3_g3_i3::g.73369::m.73369	0	515	XP_018850746.1	uncharacterized protein LOC109013191 isoform X2	Juglans regia	ONI12657.1
TRINITY_DN15444_c2_g1::TRINITY_DN15444_c2_g1_i4::g.52046::m.52046	7,92E-40	139	XP_018852448.1	late embryogenesis abundant protein 1-like	Juglans regia	XP_008371385.1
TRINITY_DN17936_c4_g1::TRINITY_DN17936_c4_g1_i9::g.92365::m.92365	1,01E-76	236	XP_018816070.1	CMP-sialic acid transporter 3	Juglans regia	XP_018816071.1
TRINITY_DN18579_c2_g2::TRINITY_DN18579_c2_g2_i4::g.102900::m.102900	0	514	XP_018831176.1	uncharacterized Rho GTPase-activating protein At5g61530-like isoform X2	Juglans regia	XP_018831175.1
TRINITY_DN14828_c0_g3::TRINITY_DN14828_c0_g3_i7::g.43009::m.43009	2,68E-151	441	XP_018820769.1	probable inactive purple acid phosphatase 27 isoform X3	Juglans regia	XP_018820768.1
TRINITY_DN16126_c1_g1::TRINITY_DN16126_c1_g1_i17::g.62053::m.62053	1,89E-132	385	XP_018813062.1	alanine--glyoxylate aminotransferase 2 homolog 3, mitochondrial-like	Juglans regia	XP_018819405.1
TRINITY_DN16055_c0_g1::TRINITY_DN16055_c0_g1_i6::g.61225::m.61225	0	933	XP_018806130.1	guanylate-binding protein 1-like	Juglans regia	XP_018811300.1
TRINITY_DN17454_c3_g1::TRINITY_DN17454_c3_g1_i1::g.84103::m.84103	5,72E-174	489	XP_018808627.1	probable carboxylesterase 8	Juglans regia	XP_008232822.1
TRINITY_DN17924_c2_g1::TRINITY_DN17924_c2_g1_i1::g.91979::m.91979	2,68E-155	438	XP_018850530.1	replication protein A 32 kDa subunit A-like isoform X1	Juglans regia	XP_018850532.1
TRINITY_DN14710_c1_g2::TRINITY_DN14710_c1_g2_i5::g.41059::m.41059	6,24E-95	282	XP_018828823.1	rhomboid-like protein 19	Juglans regia	ONH91897.1
TRINITY_DN12060_c0_g1::TRINITY_DN12060_c0_g1_i2::g.10212::m.10212	1,72E-163	475	XP_018824402.1	protein NLRC3	Juglans regia	XP_018824402.1
TRINITY_DN14479_c0_g2::TRINITY_DN14479_c0_g2_i3::g.37670::m.37670	6,58E-40	135	XP_018817075.1	uncharacterized protein LOC108988301, partial	Juglans regia	XP_018851360.1
TRINITY_DN13943_c0_g2::TRINITY_DN13943_c0_g2_i1::g.30525::m.30525	2,69E-66	199	XP_008371027.1	mitochondrial pyruvate carrier 4	Malus domestica	XP_008355920.1
TRINITY_DN17241_c0_g1::TRINITY_DN17241_c0_g1_i1::g.80827::m.80827	0	1265	XP_018835052.1	subtilisin-like protease SBT1.4	Juglans regia	OAY53224.1
TRINITY_DN17619_c0_g2::TRINITY_DN17619_c0_g2_i5::g.87168::m.87168	0	1042	XP_018809157.1	protein CYPRO4-like	Juglans regia	XP_018817815.1
TRINITY_DN17406_c4_g1::TRINITY_DN17406_c4_g1_i2::g.83243::m.83243	3,67E-156	445	XP_018847666.1	solanesyl diphosphate synthase 3, chloroplastic/mitochondrial isoform X2	Juglans regia	XP_018847665.1

TRINITY_DN17708_c1_g1::TRINITY_DN17708_c1_g1_i3::g.88548::m.88548	0	845	XP_018840171.1	actin-related protein 4-like	Juglans regia	XP_018825389.1
TRINITY_DN13981_c1_g3::TRINITY_DN13981_c1_g3_i1::g.30911::m.30911	0	559	XP_018859227.1	uncharacterized protein At1g04910	Juglans regia	XP_008353648.1
TRINITY_DN15411_c1_g2::TRINITY_DN15411_c1_g2_i10::g.51619::m.51619	1,18E-87	270	XP_018846220.1	RNA-binding protein 39-like	Juglans regia	XP_018824937.1
TRINITY_DN16049_c0_g2::TRINITY_DN16049_c0_g2_i3::g.60387::m.60387	1,94E-162	453	XP_018817906.1	NADPH-dependent pterin aldehyde reductase	Juglans regia	OAY45073.1
TRINITY_DN16235_c0_g1::TRINITY_DN16235_c0_g1_i5::g.64344::m.64344	0	1255	XP_018845886.1	long chain acyl-CoA synthetase 7, peroxisomal-like isoform X1	Juglans regia	XP_018845887.1
TRINITY_DN19621_c6_g3::TRINITY_DN19621_c6_g3_i2::g.120980::m.120980	0	1964	XP_018818762.1	trafficking protein particle complex subunit 11 isoform X2	Juglans regia	XP_018818761.1
TRINITY_DN11269_c0_g1::TRINITY_DN11269_c0_g1_i4::g.6758::m.6758	1,19E-50	159	XP_018823952.1	NADH dehydrogenase	ubiquinone 1 beta subcomplex subunit 7-like	XP_004497173.1
TRINITY_DN15638_c0_g2::TRINITY_DN15638_c0_g2_i2::g.54677::m.54677	4,67E-142	401	XP_018815216.1	U1 small nuclear ribonucleoprotein A-like	Juglans regia	XP_018843609.1
TRINITY_DN10146_c0_g1::TRINITY_DN10146_c0_g1_i1::g.4308::m.4308	7,04E-40	132	XP_018830814.1	mitochondrial import inner membrane translocase subunit TIM8	Juglans regia	XP_008239525.1
TRINITY_DN13752_c3_g3::TRINITY_DN13752_c3_g3_i1::g.27967::m.27967	0	819	XP_018831645.1	protein NRT1/ PTR FAMILY 2.8	Juglans regia	XP_019462778.1
TRINITY_DN15810_c0_g1::TRINITY_DN15810_c0_g1_i2::g.57313::m.57313	8,48E-78	236	XP_018850528.1	novel plant SNARE 13-like	Juglans regia	XP_018833856.1
TRINITY_DN19614_c2_g7::TRINITY_DN19614_c2_g7_i3::g.120831::m.120831	4,56E-111	329	XP_018856691.1	LOB domain-containing protein 36-like	Juglans regia	OAY35348.1
TRINITY_DN15371_c0_g2::TRINITY_DN15371_c0_g2_i1::g.50837::m.50837	0	630	XP_018806029.1	zinc finger protein-like 1 homolog isoform X2	Juglans regia	XP_018806028.1
TRINITY_DN13573_c3_g3::TRINITY_DN13573_c3_g3_i2::g.25665::m.25665	2,68E-107	320	XP_018845677.1	uncharacterized protein At2g24330	Juglans regia	XP_018813351.1
TRINITY_DN17808_c3_g2::TRINITY_DN17808_c3_g2_i3::g.89986::m.89986	4,62E-38	127	XP_015962951.1	uncharacterized protein LOC107486893	Arachis duranensis	KHN17310.1
TRINITY_DN17336_c6_g1::TRINITY_DN17336_c6_g1_i1::g.82089::m.82089	0	949	XP_018817860.1	methylcrotonoyl-CoA carboxylase beta chain, mitochondrial isoform X2	Juglans regia	XP_018817859.1
TRINITY_DN17735_c1_g2::TRINITY_DN17735_c1_g2_i2::g.89035::m.89035	1,01E-89	269	XP_018844199.1	putative quinone-oxidoreductase homolog, chloroplastic	Juglans regia	XP_018850172.1
TRINITY_DN16119_c4_g1::TRINITY_DN16119_c4_g1_i1::g.62575::m.62575	0	1020	XP_018858950.1	protein transport protein SEC24	Juglans regia	XP_018858951.1
TRINITY_DN16898_c0_g1::TRINITY_DN16898_c0_g1_i2::g.75056::m.75056	1,27E-96	285	XP_018837111.1	uncharacterized protein LOC109003443 isoform X1	Juglans regia	XP_018837120.1
TRINITY_DN17578_c2_g1::TRINITY_DN17578_c2_g1_i2::g.85652::m.85652	0	650	XP_018845644.1	COP9 signalosome complex subunit 5b-like	Juglans regia	KDP33444.1
TRINITY_DN17051_c1_g1::TRINITY_DN17051_c1_g1_i6::g.77580::m.77580	0	889	XP_018807346.1	uncharacterized protein LOC108980794	Juglans regia	XP_015953619.1

TRINITY_DN13889_c0_g1::TRINITY_DN13889_c0_g1_i2::g.29853::m.29853	0	1376	XP_018837769.1	pentatricopeptide repeat-containing protein At2g37230	Juglans regia	XP_018837770.1
TRINITY_DN15642_c0_g3::TRINITY_DN15642_c0_g3_i1::g.55086::m.55086	3,37E-91	265	AFK46227.1	unknown	Lotus japonicus	XP_018809666.1
TRINITY_DN12917_c0_g1::TRINITY_DN12917_c0_g1_i8::g.17363::m.17363	1,77E-71	223	XP_018849705.1	chaperone protein dnaJ 15-like	Juglans regia	KHN06676.1
TRINITY_DN18228_c1_g3::TRINITY_DN18228_c1_g3_i10::g.97346::m.97346	0	620	XP_018833878.1	DEAD-box ATP-dependent RNA helicase 40 isoform X1	Juglans regia	XP_018833878.1
TRINITY_DN14656_c1_g3::TRINITY_DN14656_c1_g3_i2::g.40036::m.40036	0	1633	XP_018849148.1	K(+) efflux antiporter 2, chloroplastic-like	Juglans regia	XP_018845392.1
TRINITY_DN17427_c0_g1::TRINITY_DN17427_c0_g1_i5::g.83626::m.83626	1,93E-173	501	XP_018852051.1	uncharacterized protein LOC109014152 isoform X4	Juglans regia	XP_018852051.1
TRINITY_DN12519_c1_g2::TRINITY_DN12519_c1_g2_i2::g.13489::m.13489	2,39E-70	226	XP_018820767.1	probable inactive purple acid phosphatase 27 isoform X1	Juglans regia	XP_008231569.2
TRINITY_DN16485_c1_g1::TRINITY_DN16485_c1_g1_i5::g.68172::m.68172	0	677	XP_018831170.1	ATP-dependent 6-phosphofructokinase 4, chloroplastic	Juglans regia	ONI24965.1
TRINITY_DN16382_c2_g3::TRINITY_DN16382_c2_g3_i2::g.66735::m.66735	1,24E-139	404	XP_018845036.1	putative oxidoreductase TDA3	Juglans regia	ONI02917.1
TRINITY_DN12471_c0_g1::TRINITY_DN12471_c0_g1_i6::g.13129::m.13129	0	679	XP_018852537.1	mitochondrial import inner membrane translocase subunit TIM44-2-like	Juglans regia	OAY34596.1
TRINITY_DN13404_c2_g1::TRINITY_DN13404_c2_g1_i2::g.23444::m.23444	1,80E-61	198	XP_018857362.1	tetratricopeptide repeat protein 38	Juglans regia	XP_018857363.1
TRINITY_DN13849_c3_g3::TRINITY_DN13849_c3_g3_i8::g.28501::m.28501	6,55E-153	436	XP_018828830.1	zinc finger CCCH domain-containing protein 11-like	Juglans regia	ONI24039.1
TRINITY_DN10714_c0_g1::TRINITY_DN10714_c0_g1_i7::g.5396::m.5396	0	1032	XP_018815300.1	amidophosphoribosyltransferase, chloroplastic	Juglans regia	XP_018841407.1
TRINITY_DN17727_c1_g3::TRINITY_DN17727_c1_g3_i1::g.88887::m.88887	0	550	XP_018818699.1	serine carboxypeptidase II-2	Juglans regia	XP_008243035.1
TRINITY_DN13894_c1_g1::TRINITY_DN13894_c1_g1_i8::g.29999::m.29999	2,00E-112	336	XP_008223354.1	aldehyde dehydrogenase 22A1	Prunus mume	XP_016648043.1
TRINITY_DN15301_c2_g1::TRINITY_DN15301_c2_g1_i12::g.49765::m.49765	3,05E-117	334	XP_008234264.1	ADP-ribosylation factor-related protein 1	Prunus mume	XP_018830856.1
TRINITY_DN17157_c0_g1::TRINITY_DN17157_c0_g1_i2::g.79386::m.79386	6,13E-69	211	ONI18405.1	hypothetical protein PRUPE_3G213900	Prunus persica	XP_007217271.1
TRINITY_DN19995_c2_g1::TRINITY_DN19995_c2_g1_i2::g.127478::m.127478	0	926	XP_018847585.1	subtilisin-like protease SBT4.14	Juglans regia	XP_008232921.1
TRINITY_DN13004_c2_g2::TRINITY_DN13004_c2_g2_i3::g.18474::m.18474	1,35E-134	386	XP_018856033.1	fatty-acid-binding protein 1	Juglans regia	KDP21455.1
TRINITY_DN10567_c0_g1::TRINITY_DN10567_c0_g1_i2::g.5026::m.5026	2,30E-26	99	KRH07998.1	hypothetical protein GLYMA_16G123000	Glycine max	KDP23968.1
TRINITY_DN18294_c2_g1::TRINITY_DN18294_c2_g1_i3::g.98438::m.98438	0	1554	XP_018860622.1	epidermal growth factor receptor substrate 15-like 1 isoform X2	Juglans regia	XP_018860621.1
TRINITY_DN16056_c1_g1::TRINITY_DN16056_c1_g1_i8::g.61254::m.61254	1,96E-153	433	XP_018843911.1	secretory carrier-associated membrane protein 4	Juglans regia	XP_018852208.1

TRINITY_DN13008_c2_g1::TRINITY_DN13008_c2_g1_i1::g.18585::m.18585	2,37E-161	468	XP_018827529.1	beta-galactosidase 6-like isoform X3	Juglans regia	XP_018827527.1
TRINITY_DN12353_c0_g1::TRINITY_DN12353_c0_g1_i1::g.12504::m.12504	1,75E-111	341	XP_018807539.1	scopoletin glucosyltransferase-like	Juglans regia	XP_018807539.1
TRINITY_DN19066_c1_g3::TRINITY_DN19066_c1_g3_i1::g.111236::m.111236	7,62E-158	444	XP_018850582.1	uncharacterized protein At2g34460, chloroplastic	Juglans regia	OAY25119.1
TRINITY_DN17441_c2_g4::TRINITY_DN17441_c2_g4_i3::g.83805::m.83805	0	688	XP_018833274.1	endoplasmic reticulum-Golgi intermediate compartment protein 3 isoform X1	Juglans regia	XP_018833275.1
TRINITY_DN1382_c0_g1::TRINITY_DN1382_c0_g1_i1::g.348::m.348	9,24E-134	384	XP_016194705.1	actin-97-like	Arachis ipaensis	XP_015962997.1
TRINITY_DN17113_c0_g1::TRINITY_DN17113_c0_g1_i1::g.78814::m.78814	0	2048	XP_018849357.1	exportin-7 isoform X1	Juglans regia	XP_018849358.1
TRINITY_DN19775_c0_g3::TRINITY_DN19775_c0_g3_i2::g.123476::m.123476	3,79E-138	400	XP_018839164.1	serine hydroxymethyltransferase 3, chloroplastic-like	Juglans regia	XP_018822579.1
TRINITY_DN12824_c1_g1::TRINITY_DN12824_c1_g1_i1::g.16233::m.16233	3,87E-116	346	XP_018841524.1	serine/threonine-protein kinase STY46-like isoform X2	Juglans regia	XP_018841522.1
TRINITY_DN11410_c0_g1::TRINITY_DN11410_c0_g1_i1::g.7283::m.7283	0	518	XP_018806031.1	mitotic checkpoint protein BUB3.2	Juglans regia	XP_018852123.1
TRINITY_DN14617_c1_g1::TRINITY_DN14617_c1_g1_i9::g.39676::m.39676	0	891	XP_008218919.1	probable alkaline/neutral invertase D	Prunus mume	XP_016647230.1
TRINITY_DN11064_c0_g2::TRINITY_DN11064_c0_g2_i1::g.6238::m.6238	2,47E-164	462	KDP37227.1	hypothetical protein JCGZ_06283	Jatropha curcas	XP_012073347.1
TRINITY_DN14194_c1_g1::TRINITY_DN14194_c1_g1_i9::g.33557::m.33557	1,78E-94	286	XP_018812532.1	small glutamine-rich tetratricopeptide repeat-containing protein isoform X3	Juglans regia	XP_018812531.1
TRINITY_DN9434_c0_g1::TRINITY_DN9434_c0_g1_i2::g.3474::m.3474	0	1154	XP_018827923.1	DEAD-box ATP-dependent RNA helicase 31-like isoform X1	Juglans regia	XP_018827924.1
TRINITY_DN16217_c1_g2::TRINITY_DN16217_c1_g2_i5::g.64084::m.64084	0	709	XP_018830819.1	uncharacterized protein LOC108998648	Juglans regia	ONI25150.1
TRINITY_DN16504_c2_g1::TRINITY_DN16504_c2_g1_i5::g.68713::m.68713	4,42E-111	315	XP_008389513.1	ubiquitin-conjugating enzyme E2 2-like	Malus domestica	XP_018819103.1
TRINITY_DN14373_c0_g1::TRINITY_DN14373_c0_g1_i5::g.36002::m.36002	8,35E-65	198	XP_018855445.1	dCTP pyrophosphatase 1-like, partial	Juglans regia	XP_018848325.1
TRINITY_DN19595_c1_g1::TRINITY_DN19595_c1_g1_i3::g.120443::m.120443	3,43E-56	173	XP_018805857.1	endoplasmic reticulum oxidoreductin-2-like	Juglans regia	OAY29178.1
TRINITY_DN16684_c1_g1::TRINITY_DN16684_c1_g1_i1::g.71521::m.71521	2,87E-57	181	KHN24180.1	Ras-related protein RHN1	Glycine soja	CAX86753.1
TRINITY_DN18955_c3_g1::TRINITY_DN18955_c3_g1_i8::g.109317::m.109317	0	1008	XP_018824253.1	uncharacterized protein LOC108993713	Juglans regia	XP_012079025.1
TRINITY_DN19877_c3_g4::TRINITY_DN19877_c3_g4_i1::g.125024::m.125024	1,91E-53	167	ONI32563.1	hypothetical protein PRUPE_1G373500	Prunus persica	XP_018822047.1
TRINITY_DN11684_c0_g1::TRINITY_DN11684_c0_g1_i1::g.8363::m.8363	6,24E-117	341	OAY27542.1	hypothetical protein MANES_16G133500	Manihot esculenta	OAY57022.1
TRINITY_DN16471_c14_g1::TRINITY_DN16471_c14_g1_i3::g.68132::m.68132	0	712	XP_018831158.1	conserved oligomeric Golgi complex subunit 3	Juglans regia	XP_016651945.1

TRINITY_DN15705_c0_g3::TRINITY_DN15705_c0_g3_i5::g.56096::m.56096	1,39E-52	171	XP_018840945.1	Golgi to ER traffic protein 4 homolog	Juglans regia	XP_008233370.1
TRINITY_DN14658_c2_g1::TRINITY_DN14658_c2_g1_i2::g.40152::m.40152	0	885	XP_018822003.1	probable serine protease EDA2	Juglans regia	OAY60835.1
TRINITY_DN16640_c0_g1::TRINITY_DN16640_c0_g1_i4::g.70902::m.70902	6,63E-138	397	XP_018840777.1	zinc finger CCCH domain-containing protein 12-like isoform X3	Juglans regia	XP_018840777.1
TRINITY_DN12745_c0_g1::TRINITY_DN12745_c0_g1_i6::g.15122::m.15122	7,85E-73	220	OAY50757.1	hypothetical protein MANES_05G160700	Manihot esculenta	OAY50756.1
TRINITY_DN13091_c0_g2::TRINITY_DN13091_c0_g2_i2::g.19654::m.19654	1,59E-93	279	XP_018841148.1	12-oxophytodienoate reductase 2-like	Juglans regia	ONI35666.1
TRINITY_DN19627_c1_g1::TRINITY_DN19627_c1_g1_i2::g.121157::m.121157	0	1407	XP_018817804.1	leucine--tRNA ligase, cytoplasmic isoform X1	Juglans regia	XP_018817805.1
TRINITY_DN10469_c0_g1::TRINITY_DN10469_c0_g1_i1::g.4851::m.4851	3,63E-98	283	XP_018832728.1	protein FAM136A-like	Juglans regia	XP_018840436.1
TRINITY_DN16987_c2_g1::TRINITY_DN16987_c2_g1_i9::g.76589::m.76589	0	620	XP_018824727.1	uncharacterized protein LOC108994089	Juglans regia	XP_018842839.1
TRINITY_DN19240_c1_g1::TRINITY_DN19240_c1_g1_i5::g.114383::m.114383	8,91E-84	258	XP_018827562.1	UDP-N-acetylglucosamine diphosphorylase 2 isoform X1	Juglans regia	XP_018827564.1
TRINITY_DN14334_c2_g6::TRINITY_DN14334_c2_g6_i1::g.35159::m.35159	2,77E-29	108	XP_009357663.1	uncharacterized protein LOC103948371	Pyrus x bretschneideri	ONI24174.1
TRINITY_DN18647_c1_g1::TRINITY_DN18647_c1_g1_i1::g.103820::m.103820	1,37E-169	475	XP_018849036.1	ATP-dependent Clp protease proteolytic subunit 6, chloroplastic	Juglans regia	XP_008237184.1
TRINITY_DN16308_c0_g1::TRINITY_DN16308_c0_g1_i5::g.65986::m.65986	6,74E-131	376	XP_018839810.1	S-adenosylmethionine carrier 1, chloroplastic/mitochondrial isoform X2	Juglans regia	XP_018839807.1
TRINITY_DN16705_c7_g1::TRINITY_DN16705_c7_g1_i5::g.72000::m.72000	0	1532	XP_018826088.1	isoleucine--tRNA ligase, chloroplastic/mitochondrial	Juglans regia	XP_008223184.1
TRINITY_DN10617_c0_g1::TRINITY_DN10617_c0_g1_i2::g.5120::m.5120	2,22E-124	352	XP_018838369.1	DNA replication complex GINS protein PSF3-like	Juglans regia	GAU37013.1
TRINITY_DN13025_c0_g1::TRINITY_DN13025_c0_g1_i1::g.18736::m.18736	0	759	XP_018824599.1	adenylyltransferase and sulfurtransferase MOCS3	Juglans regia	OAY55162.1
TRINITY_DN16744_c0_g2::TRINITY_DN16744_c0_g2_i2::g.72603::m.72603	5,52E-174	488	XP_018806535.1	syntaxin-81	Juglans regia	XP_018806536.1
TRINITY_DN15178_c0_g1::TRINITY_DN15178_c0_g1_i16::g.47873::m.47873	0	559	XP_018818948.1	protein argonaute 4-like isoform X2	Juglans regia	XP_018818945.1
TRINITY_DN16466_c0_g1::TRINITY_DN16466_c0_g1_i6::g.67994::m.67994	0	653	XP_018809719.1	protein EARLY-RESPONSIVE TO DEHYDRATION 7, chloroplastic-like	Juglans regia	XP_018829645.1
TRINITY_DN18466_c3_g1::TRINITY_DN18466_c3_g1_i19::g.101050::m.101050						
TRINITY_DN13825_c0_g1::TRINITY_DN13825_c0_g1_i3::g.28848::m.28848	1,45E-91	269	XP_018817591.1	aldo-keto reductase family 4 member C9-like	Juglans regia	OAY43567.1
TRINITY_DN12167_c0_g1::TRINITY_DN12167_c0_g1_i7::g.10902::m.10902	2,29E-169	471	XP_018842205.1	ER membrane protein complex subunit 3-like	Juglans regia	XP_018809559.1
TRINITY_DN18952_c2_g1::TRINITY_DN18952_c2_g1_i9::g.109479::m.109479	6,65E-180	516	XP_018846613.1	cytochrome P450 CYP82D47-like	Juglans regia	ONI32807.1

TRINITY_DN13110_c0_g1::TRINITY_DN13110_c0_g1_i3::g.20012::m.20012	5,44E-103	300	XP_018831224.1	replication protein A 32 kDa subunit B isoform X1	Juglans regia	XP_018831225.1
TRINITY_DN14898_c0_g2::TRINITY_DN14898_c0_g2_i1::g.43887::m.43887	5,42E-124	359	OAY39080.1	hypothetical protein MANES_10G065700	Manihot esculenta	ONH91559.1
TRINITY_DN14096_c0_g2::TRINITY_DN14096_c0_g2_i2::g.32310::m.32310	0	728	XP_018820061.1	3-hydroxyisobutyryl-CoA hydrolase-like protein 1, mitochondrial	Juglans regia	ONI22521.1
TRINITY_DN15203_c1_g1::TRINITY_DN15203_c1_g1_i19::g.48562::m.48562	1,22E-72	227	XP_018840818.1	uncharacterized protein LOC109006107	Juglans regia	ONI15943.1
TRINITY_DN18497_c0_g1::TRINITY_DN18497_c0_g1_i7::g.101435::m.101435	1,37E-74	228	XP_009335066.1	probable pyridoxal 5'-phosphate synthase subunit PDX2 isoform X1	Pyrus x bretschneideri	XP_008225516.1
TRINITY_DN16464_c0_g1::TRINITY_DN16464_c0_g1_i9::g.67881::m.67881	8,82E-144	426	XP_018856777.1	auxilin-related protein 2-like isoform X1	Juglans regia	XP_018856778.1
TRINITY_DN10374_c0_g1::TRINITY_DN10374_c0_g1_i1::g.4693::m.4693	0	803	XP_018850329.1	uncharacterized protein LOC109012887 isoform X3	Juglans regia	XP_018850309.1
TRINITY_DN16131_c0_g1::TRINITY_DN16131_c0_g1_i1::g.62590::m.62590	0	974	XP_018831162.1	uncharacterized protein LOC108998880	Juglans regia	XP_008231341.1
TRINITY_DN13858_c6_g1::TRINITY_DN13858_c6_g1_i3::g.29437::m.29437	7,54E-115	337	XP_018807257.1	calcium-transporting ATPase 8, plasma membrane-type-like	Juglans regia	XP_018807258.1
TRINITY_DN17229_c1_g1::TRINITY_DN17229_c1_g1_i16::g.80496::m.80496	1,90E-131	372	XP_018807308.1	probable glutathione S-transferase	Juglans regia	XP_008382602.1
TRINITY_DN16551_c0_g1::TRINITY_DN16551_c0_g1_i1::g.69240::m.69240	1,04E-154	438	XP_018849757.1	fruit protein pKIWI502-like	Juglans regia	OAY31413.1
TRINITY_DN14041_c3_g1::TRINITY_DN14041_c3_g1_i2::g.31810::m.31810	5,59E-175	496	XP_018835707.1	transcription factor RF2b-like	Juglans regia	XP_018828447.1
TRINITY_DN12986_c2_g1::TRINITY_DN12986_c2_g1_i7::g.17676::m.17676	0	1441	XP_018834443.1	probable galactinol--sucrose galactosyltransferase 2	Juglans regia	XP_018834445.1
TRINITY_DN19621_c4_g1::TRINITY_DN19621_c4_g1_i2::g.120970::m.120970	2,65E-126	369	XP_018830401.1	palmitoyl-monogalactosyldiacylglycerol delta-7 desaturase, chloroplastic-like	Juglans regia	ONI16238.1
TRINITY_DN10293_c0_g1::TRINITY_DN10293_c0_g1_i1::g.4492::m.4492	6,27E-153	432	XP_018845106.1	uncharacterized protein LOC109009176	Juglans regia	KHN37697.1
TRINITY_DN14175_c1_g1::TRINITY_DN14175_c1_g1_i3::g.33680::m.33680	0	622	XP_018845916.1	uncharacterized protein LOC109009760	Juglans regia	XP_018845070.1
TRINITY_DN15828_c0_g1::TRINITY_DN15828_c0_g1_i4::g.57452::m.57452	2,74E-94	273	XP_018816641.1	centromere protein V	Juglans regia	XP_018816642.1
TRINITY_DN17792_c0_g5::TRINITY_DN17792_c0_g5_i3::g.89751::m.89751	4,12E-99	295	XP_018844982.1	PI-PLC X domain-containing protein At5g67130	Juglans regia	KDP31946.1
TRINITY_DN12837_c2_g2::TRINITY_DN12837_c2_g2_i1::g.16572::m.16572	1,67E-101	304	XP_018847365.1	probable polygalacturonase	Juglans regia	XP_018847358.1
TRINITY_DN17217_c1_g1::TRINITY_DN17217_c1_g1_i9::g.80247::m.80247	1,78E-98	291	XP_018835586.1	uncharacterized protein LOC109002344 isoform X2	Juglans regia	XP_018835588.1
TRINITY_DN14939_c1_g3::TRINITY_DN14939_c1_g3_i3::g.44686::m.44686	0	557	XP_018851554.1	probable pinoresinol-lariciresinol reductase 3 isoform X1	Juglans regia	XP_018851555.1
TRINITY_DN16528_c1_g1::TRINITY_DN16528_c1_g1_i4::g.69093::m.69093	4,40E-131	394	XP_018819501.1	beta-galactosidase 8	Juglans regia	ONI00641.1

TRINITY_DN17896_c3_g1::TRINITY_DN17896_c3_g1_i3::g.91440::m.91440	3,75E-111	319	KDP38526.1	hypothetical protein JCGZ_04451	Jatropha curcas	XP_012071890.1
TRINITY_DN13512_c0_g2::TRINITY_DN13512_c0_g2_i1::g.24706::m.24706	7,41E-73	220	KDP45917.1	hypothetical protein JCGZ_15477	Jatropha curcas	XP_012076020.1
TRINITY_DN18396_c2_g1::TRINITY_DN18396_c2_g1_i1::g.99882::m.99882	8,09E-56	185	XP_018849050.1	polyol transporter 5-like	Juglans regia	XP_018836941.1
TRINITY_DN14316_c0_g1::TRINITY_DN14316_c0_g1_i5::g.35352::m.35352	4,12E-44	147	XP_018842596.1	tobamovirus multiplication protein 2A-like, partial	Juglans regia	XP_018838258.1
TRINITY_DN19110_c2_g1::TRINITY_DN19110_c2_g1_i2::g.112351::m.112351	3,50E-12	70,5	XP_018848181.1	eukaryotic translation initiation factor 5B	Juglans regia	XP_018848182.1
TRINITY_DN19639_c3_g1::TRINITY_DN19639_c3_g1_i7::g.121291::m.121291	4,29E-64	198	XP_018814411.1	uncharacterized protein LOC108986289	Juglans regia	KHN40629.1
TRINITY_DN14977_c0_g1::TRINITY_DN14977_c0_g1_i14::g.45080::m.45080	1,54E-109	317	XP_017192425.1	phytochrome-associated serine/threonine-protein phosphatase-like isoform X2	Malus domestica	AES60048.1
TRINITY_DN19335_c0_g3::TRINITY_DN19335_c0_g3_i4::g.116290::m.116290	4,39E-122	351	XP_018841149.1	pectinesterase 31 isoform X1	Juglans regia	XP_008341600.1
TRINITY_DN17394_c0_g1::TRINITY_DN17394_c0_g1_i5::g.83048::m.83048	0	531	XP_018848619.1	protein FLX-like 1	Juglans regia	XP_018813162.1
TRINITY_DN15312_c1_g1::TRINITY_DN15312_c1_g1_i7::g.50104::m.50104	7,17E-58	200	XP_018851910.1	phospholipase A I-like isoform X2	Juglans regia	XP_018851909.1
TRINITY_DN14520_c0_g2::TRINITY_DN14520_c0_g2_i5::g.38465::m.38465	0	1427	KDP41183.1	hypothetical protein JCGZ_15590	Jatropha curcas	XP_012067623.1
TRINITY_DN19082_c3_g2::TRINITY_DN19082_c3_g2_i2::g.111602::m.111602	8,17E-151	441	XP_018825221.1	protein NRT1/ PTR FAMILY 7.1-like isoform X1	Juglans regia	XP_018825222.1
TRINITY_DN17990_c1_g1::TRINITY_DN17990_c1_g1_i2::g.93320::m.93320	7,13E-114	332	XP_018826263.1	BAG family molecular chaperone regulator 4	Juglans regia	XP_018825895.1
TRINITY_DN10955_c0_g1::TRINITY_DN10955_c0_g1_i1::g.5913::m.5913	1,59E-69	207	XP_018807121.1	signal recognition particle 9 kDa protein	Juglans regia	XP_016187179.1
TRINITY_DN10864_c0_g1::TRINITY_DN10864_c0_g1_i1::g.5678::m.5678	0	612	XP_018846091.1	pentatricopeptide repeat-containing protein At1g61870, mitochondrial-like	Juglans regia	XP_018810112.1
TRINITY_DN18372_c2_g6::TRINITY_DN18372_c2_g6_i1::g.99517::m.99517	8,87E-135	395	XP_018827694.1	phosphatidylinositol 3,4,5-trisphosphate 3-phosphatase and protein-tyrosine-phosphatase PTEN2A-like	Juglans regia	XP_018827695.1
TRINITY_DN17198_c0_g1::TRINITY_DN17198_c0_g1_i4::g.79878::m.79878	0	708	XP_018847416.1	basic 7S globulin-like	Juglans regia	XP_014512687.1
TRINITY_DN19333_c1_g1::TRINITY_DN19333_c1_g1_i1::g.116168::m.116168	0	1215	XP_018806296.1	probable acyl-activating enzyme 17, peroxisomal isoform X2	Juglans regia	XP_018806295.1
TRINITY_DN13608_c0_g1::TRINITY_DN13608_c0_g1_i3::g.26161::m.26161	0	584	XP_018849418.1	uncharacterized protein LOC109012311	Juglans regia	XP_018849419.1
TRINITY_DN17268_c0_g1::TRINITY_DN17268_c0_g1_i4::g.80463::m.80463	0	712	XP_008222441.1	TBC1 domain family member 22B-like	Prunus mume	XP_018806881.1
TRINITY_DN11329_c0_g1::TRINITY_DN11329_c0_g1_i1::g.6996::m.6996	0	508	XP_018823732.1	uncharacterized protein At2g29880-like	Juglans regia	OAY37362.1

TRINITY_DN17556_c3_g1::TRINITY_DN17556_c3_g1_i1::g.85912::m.85912	3,96E-66	202	KRH69486.1	hypothetical protein GLYMA_02G030900	Glycine max	KRH74669.1
TRINITY_DN14486_c0_g4::TRINITY_DN14486_c0_g4_i1::g.37922::m.37922						
TRINITY_DN19016_c1_g5::TRINITY_DN19016_c1_g5_i1::g.110613::m.110613	8,53E-61	193	XP_018813512.1	farnesyl pyrophosphate synthase 1-like	Juglans regia	XP_018813518.1
TRINITY_DN19612_c1_g2::TRINITY_DN19612_c1_g2_i7::g.120818::m.120818	2,81E-103	314	XP_018809366.1	probable methyltransferase PMT18	Juglans regia	XP_018844923.1
TRINITY_DN13825_c0_g2::TRINITY_DN13825_c0_g2_i4::g.28851::m.28851	7,11E-90	268	AFK48248.1	unknown	Lotus japonicus	XP_019420805.1
TRINITY_DN10767_c0_g1::TRINITY_DN10767_c0_g1_i1::g.5469::m.5469	0	652	XP_018838226.1	probable low-specificity L-threonine aldolase 1	Juglans regia	XP_018838227.1
TRINITY_DN14408_c0_g1::TRINITY_DN14408_c0_g1_i2::g.36628::m.36628	4,90E-82	255	XP_018840883.1	protein transport Sec1a-like	Juglans regia	XP_016186669.1
TRINITY_DN16867_c0_g1::TRINITY_DN16867_c0_g1_i8::g.74659::m.74659	0	591	OAY50624.1	hypothetical protein MANES_05G151200	Manihot esculenta	OAY50625.1
TRINITY_DN12184_c0_g1::TRINITY_DN12184_c0_g1_i1::g.10535::m.10535	0	608	XP_018819584.1	AUGMIN subunit 7	Juglans regia	XP_018819585.1
TRINITY_DN19255_c4_g1::TRINITY_DN19255_c4_g1_i5::g.114861::m.114861	4,50E-78	254	XP_018819113.1	alpha-amylase 3, chloroplastic-like	Juglans regia	XP_018812497.1
TRINITY_DN18745_c0_g4::TRINITY_DN18745_c0_g4_i1::g.105401::m.105401	0	726	XP_018843925.1	LOW QUALITY PROTEIN: phosphomevalonate kinase	Juglans regia	KDP21278.1
TRINITY_DN15579_c2_g1::TRINITY_DN15579_c2_g1_i2::g.54035::m.54035	1,78E-68	215	XP_016648983.1	probable polygalacturonase	Prunus mume	XP_018847365.1
TRINITY_DN19014_c2_g2::TRINITY_DN19014_c2_g2_i15::g.110588::m.110588	0	1648	XP_018822521.1	phosphoenolpyruvate carboxylase, housekeeping isozyme-like	Juglans regia	XP_018849376.1
TRINITY_DN13959_c0_g2::TRINITY_DN13959_c0_g2_i7::g.30593::m.30593	0	769	XP_018838404.1	importin-9 isoform X1	Juglans regia	XP_018838405.1
TRINITY_DN17096_c2_g1::TRINITY_DN17096_c2_g1_i4::g.78253::m.78253	0	775	XP_018845117.1	COP9 signalosome complex subunit 3 isoform X1	Juglans regia	XP_018845118.1
TRINITY_DN15860_c0_g1::TRINITY_DN15860_c0_g1_i3::g.57901::m.57901	9,76E-178	510	XP_018849435.1	protein NRT1/ PTR FAMILY 5.4-like	Juglans regia	ONI20821.1
TRINITY_DN12503_c2_g1::TRINITY_DN12503_c2_g1_i3::g.13440::m.13440	3,57E-77	229	XP_009347705.1	probable glutathione peroxidase 5 isoform X2	Pyrus x bretschneideri	XP_018501031.1
TRINITY_DN14356_c4_g1::TRINITY_DN14356_c4_g1_i9::g.35853::m.35853	2,15E-166	466	XP_018811873.1	peroxisome biogenesis protein 22-like isoform X1	Juglans regia	XP_018836339.1
TRINITY_DN14761_c1_g4::TRINITY_DN14761_c1_g4_i4::g.41839::m.41839	0	2033	XP_018849114.1	uncharacterized protein LOC109012094	Juglans regia	XP_008240391.1
TRINITY_DN16683_c2_g2::TRINITY_DN16683_c2_g2_i1::g.71583::m.71583	5,02E-51	170	XP_018848829.1	rhomboid-like protein 15	Juglans regia	XP_018851134.1
TRINITY_DN13437_c2_g2::TRINITY_DN13437_c2_g2_i2::g.23355::m.23355	5,63E-160	489	XP_018846195.1	uncharacterized protein LOC109009964	Juglans regia	XP_018819031.1
TRINITY_DN13537_c0_g1::TRINITY_DN13537_c0_g1_i2::g.25155::m.25155	1,55E-102	302	XP_018822955.1	uncharacterized protein LOC108992771	Juglans regia	ONI19571.1

TRINITY_DN13720_c0_g6::TRINITY_DN13720_c0_g6_i1::g.27649::m.27649	1,04E-126	366	XP_018836373.1	uncharacterized protein LOC109002903 isoform X1	Juglans regia	ONI32161.1
TRINITY_DN15113_c1_g1::TRINITY_DN15113_c1_g1_i9::g.47525::m.47525	1,63E-100	288	XP_018823137.1	ferredoxin-thioredoxin reductase catalytic chain, chloroplastic	Juglans regia	XP_008230669.1
TRINITY_DN18151_c1_g8::TRINITY_DN18151_c1_g8_i1::g.95937::m.95937	8,81E-61	193	XP_018806470.1	protein BOBBER 2-like	Juglans regia	XP_015934358.1
TRINITY_DN16542_c2_g2::TRINITY_DN16542_c2_g2_i16::g.69553::m.69553	0	1903	XP_018807838.1	uncharacterized protein LOC108981175	Juglans regia	XP_008235875.1
TRINITY_DN17965_c0_g2::TRINITY_DN17965_c0_g2_i2::g.92668::m.92668	6,06E-85	258	XP_018846002.1	histidinol dehydrogenase, chloroplastic isoform X2	Juglans regia	XP_018846003.1
TRINITY_DN10320_c0_g1::TRINITY_DN10320_c0_g1_i1::g.4626::m.4626	5,91E-59	181	XP_018858854.1	auxin-repressed 12.5 kDa protein-like	Juglans regia	XP_018811601.1
TRINITY_DN19345_c3_g1::TRINITY_DN19345_c3_g1_i6::g.117087::m.117087	1,97E-25	102	XP_018829287.1	heparan-alpha-glucosaminide N-acetyltransferase isoform X1	Juglans regia	XP_018829289.1
TRINITY_DN18504_c0_g1::TRINITY_DN18504_c0_g1_i8::g.101692::m.101692	0	977	XP_018826310.1	peptide-N(4)-(N-acetyl-beta-glucosaminyl)asparagine amidase isoform X1	Juglans regia	XP_018826311.1
TRINITY_DN19069_c0_g1::TRINITY_DN19069_c0_g1_i4::g.111231::m.111231	4,56E-158	446	XP_018821010.1	protein FLX-like 3	Juglans regia	XP_018821011.1
TRINITY_DN15115_c0_g4::TRINITY_DN15115_c0_g4_i3::g.47102::m.47102	0	548	XP_018830763.1	bifunctional epoxide hydrolase 2-like	Juglans regia	XP_018812192.1
TRINITY_DN18049_c1_g1::TRINITY_DN18049_c1_g1_i7::g.94346::m.94346	0	813	XP_018831178.1	26S proteasome regulatory subunit 4 homolog B-like	Juglans regia	XP_012065309.1
TRINITY_DN16571_c0_g1::TRINITY_DN16571_c0_g1_i1::g.69820::m.69820	4,96E-138	394	XP_018839156.1	nuclear pore complex protein NUP35	Juglans regia	XP_018843261.1
TRINITY_DN17401_c1_g1::TRINITY_DN17401_c1_g1_i4::g.83099::m.83099	0	600	XP_008234506.1	methionine aminopeptidase 2B-like	Prunus mume	XP_008234507.1
TRINITY_DN11104_c0_g1::TRINITY_DN11104_c0_g1_i1::g.6331::m.6331	0	1097	XP_018815365.1	probable alkaline/neutral invertase B	Juglans regia	XP_018815366.1
TRINITY_DN11331_c0_g1::TRINITY_DN11331_c0_g1_i1::g.7013::m.7013	2,70E-151	430	OAY37035.1	hypothetical protein MANES_11G069800	Manihot esculenta	XP_008228852.1
TRINITY_DN19806_c0_g1::TRINITY_DN19806_c0_g1_i11::g.123914::m.123914	0	550	XP_018835905.1	protein MON2 homolog isoform X1	Juglans regia	XP_018835907.1
TRINITY_DN14324_c0_g1::TRINITY_DN14324_c0_g1_i3::g.35430::m.35430	0	692	XP_018823621.1	lys-63-specific deubiquitinase BRCC36-like isoform X1	Juglans regia	XP_018827792.1
TRINITY_DN19942_c2_g1::TRINITY_DN19942_c2_g1_i4::g.126324::m.126324	1,46E-97	298	XP_018815844.1	E3 ubiquitin protein ligase RIN2-like	Juglans regia	XP_018851934.1
TRINITY_DN16925_c0_g1::TRINITY_DN16925_c0_g1_i1::g.75769::m.75769	0	760	XP_018824318.1	ubiquitin carboxyl-terminal hydrolase 9-like	Juglans regia	XP_018817088.1
TRINITY_DN19895_c1_g1::TRINITY_DN19895_c1_g1_i3::g.125573::m.125573	0	581	XP_018845718.1	probable ADP-ribosylation factor GTPase-activating protein AGD14 isoform X2	Juglans regia	XP_018845717.1
TRINITY_DN17938_c1_g2::TRINITY_DN17938_c1_g2_i1::g.92333::m.92333	0	594	XP_018829254.1	glutamate--cysteine ligase, chloroplastic	Juglans regia	XP_018829255.1
TRINITY_DN19979_c5_g4::TRINITY_DN19979_c5_g4_i5::g.127016::m.127016	2,25E-125	362	XP_018854242.1	probable aldo-keto reductase 1	Juglans regia	XP_018826312.1

TRINITY_DN17846_c4_g1::TRINITY_DN17846_c4_g1_i2::g.90658::m.90658	0	514	XP_018845405.1	casein kinase 1-like protein 1	Juglans regia	XP_018817582.1
TRINITY_DN17717_c2_g1::TRINITY_DN17717_c2_g1_i3::g.88728::m.88728	1,24E-34	123	XP_018822663.1	UDP-galactose transporter 2-like isoform X3	Juglans regia	XP_018822662.1
TRINITY_DN14801_c0_g1::TRINITY_DN14801_c0_g1_i4::g.42400::m.42400	0	557	XP_018846155.1	vacuolar protein sorting-associated protein 51 homolog	Juglans regia	ONI05445.1
TRINITY_DN14703_c2_g1::TRINITY_DN14703_c2_g1_i4::g.41069::m.41069	1,22E-139	398	XP_018839330.1	aminoacyl tRNA synthase complex-interacting multifunctional protein 1	Juglans regia	XP_004513672.1
TRINITY_DN11195_c0_g1::TRINITY_DN11195_c0_g1_i1::g.6571::m.6571	0	1720	XP_018849399.1	uncharacterized protein LOC109012301	Juglans regia	XP_018849400.1
TRINITY_DN10654_c0_g1::TRINITY_DN10654_c0_g1_i1::g.5213::m.5213	2,46E-127	363	XP_018807078.1	uncharacterized protein At2g39795, mitochondrial	Juglans regia	ONI21593.1
TRINITY_DN17314_c1_g1::TRINITY_DN17314_c1_g1_i1::g.81914::m.81914	3,04E-79	246	XP_018851869.1	diacylglycerol kinase 5-like	Juglans regia	KRH55430.1
TRINITY_DN17135_c0_g1::TRINITY_DN17135_c0_g1_i6::g.79192::m.79192	0	634	XP_018807500.1	probable Ufm1-specific protease isoform X2	Juglans regia	XP_018807499.1
TRINITY_DN18873_c0_g1::TRINITY_DN18873_c0_g1_i7::g.107966::m.107966	0	964	XP_018822797.1	phosphoribosylaminoimidazole carboxylase, chloroplastic isoform X1	Juglans regia	OAY28979.1
TRINITY_DN15119_c0_g1::TRINITY_DN15119_c0_g1_i1::g.47105::m.47105	0	764	XP_018844840.1	chorismate synthase, chloroplastic-like	Juglans regia	XP_018828862.1
TRINITY_DN19022_c0_g1::TRINITY_DN19022_c0_g1_i10::g.110657::m.110657	0	518	XP_018809736.1	isocitrate dehydrogenase [NAD] regulatory subunit 1, mitochondrial	Juglans regia	OAY57636.1
TRINITY_DN17160_c1_g3::TRINITY_DN17160_c1_g3_i2::g.79397::m.79397	0	896	XP_018834194.1	uncharacterized protein slp1	Juglans regia	XP_018834195.1
TRINITY_DN13640_c0_g3::TRINITY_DN13640_c0_g3_i9::g.26621::m.26621	0	723	XP_018818703.1	cycloartenol synthase 2	Juglans regia	OIW03083.1
TRINITY_DN19493_c1_g1::TRINITY_DN19493_c1_g1_i9::g.118079::m.118079	1,78E-150	428	XP_018829509.1	1-aminocyclopropane-1-carboxylate oxidase homolog 4-like	Juglans regia	XP_018829510.1
TRINITY_DN19823_c3_g2::TRINITY_DN19823_c3_g2_i6::g.124233::m.124233	0	1438	XP_018832246.1	splicing factor 3B subunit 1, partial	Juglans regia	ONH90625.1
TRINITY_DN16244_c0_g2::TRINITY_DN16244_c0_g2_i4::g.64523::m.64523	1,93E-90	270	KRH63709.1	hypothetical protein GLYMA_04G1923001, partial	Glycine max	XP_008337074.1
TRINITY_DN13878_c2_g2::TRINITY_DN13878_c2_g2_i3::g.29757::m.29757	2,48E-44	148	XP_018837336.1	14 kDa zinc-binding protein	Juglans regia	ONI11561.1
TRINITY_DN13194_c4_g1::TRINITY_DN13194_c4_g1_i4::g.20971::m.20971	5,70E-70	211	XP_018833650.1	30S ribosomal protein S16-2, chloroplastic/mitochondrial	Juglans regia	XP_016651601.1
TRINITY_DN13636_c0_g1::TRINITY_DN13636_c0_g1_i5::g.26555::m.26555	2,29E-107	311	XP_018827848.1	tropinone reductase-like 3	Juglans regia	XP_009357508.1
TRINITY_DN17968_c0_g2::TRINITY_DN17968_c0_g2_i1::g.92847::m.92847	0	2322	XP_018822813.1	ARF guanine-nucleotide exchange factor GNL2	Juglans regia	XP_008242641.1
TRINITY_DN10935_c0_g1::TRINITY_DN10935_c0_g1_i5::g.5847::m.5847	7,17E-46	147	XP_018813206.1	uncharacterized protein LOC108985380	Juglans regia	XP_008394294.1
TRINITY_DN17259_c0_g1::TRINITY_DN17259_c0_g1_i9::g.80974::m.80974	4,74E-141	398	XP_018849009.1	syntaxin-52-like isoform X2	Juglans regia	XP_018849010.1

TRINITY_DN14202_c2_g1::TRINITY_DN14202_c2_g1_i3::g.33869::m.33869	6,01E-69	212	XP_018836821.1	vesicle transport v-SNARE 12	Juglans regia	XP_018836822.1
TRINITY_DN11729_c1_g1::TRINITY_DN11729_c1_g1_i5::g.8590::m.8590	3,63E-82	241	XP_018831693.1	39S ribosomal protein L47, mitochondrial	Juglans regia	XP_014495313.1
TRINITY_DN16787_c1_g2::TRINITY_DN16787_c1_g2_i3::g.73271::m.73271	0	578	XP_008229214.1	probable fructokinase-6, chloroplastic	Prunus mume	XP_018860003.1
TRINITY_DN15603_c1_g3::TRINITY_DN15603_c1_g3_i1::g.54361::m.54361	1,31E-79	234	XP_018819607.1	prefoldin subunit 6-like	Juglans regia	OAY36902.1
TRINITY_DN13221_c3_g1::TRINITY_DN13221_c3_g1_i2::g.21212::m.21212	3,24E-134	383	XP_018817492.1	probable aldo-keto reductase 1, partial	Juglans regia	XP_018837027.1
TRINITY_DN10855_c0_g2::TRINITY_DN10855_c0_g2_i3::g.5689::m.5689	9,68E-48	155	XP_018830706.1	CDGSH iron-sulfur domain-containing protein NEET	Juglans regia	XP_008234341.1
TRINITY_DN47853_c0_g1::TRINITY_DN47853_c0_g1_i1::g.132570::m.132570	4,08E-79	234	KOM56098.1	hypothetical protein LR48_Vigan10g199000	Vigna angularis	KOM56098.1
TRINITY_DN17028_c2_g2::TRINITY_DN17028_c2_g2_i3::g.77279::m.77279	1,82E-118	340	XP_018829348.1	uncharacterized protein At4g14100-like isoform X1	Juglans regia	XP_018841627.1
TRINITY_DN17028_c2_g2::TRINITY_DN17028_c2_g2_i3::g.77278::m.77278	0	1595	XP_018841626.1	uncharacterized protein LOC109006713	Juglans regia	OAY43812.1
TRINITY_DN16271_c1_g1::TRINITY_DN16271_c1_g1_i4::g.64790::m.64790	2,74E-60	200	XP_018834169.1	CLP protease regulatory subunit CLPX1, mitochondrial-like	Juglans regia	XP_018834959.1
TRINITY_DN16125_c0_g1::TRINITY_DN16125_c0_g1_i5::g.62472::m.62472	2,71E-95	279	XP_018833227.1	HD domain-containing protein 2 isoform X1	Juglans regia	KDP38749.1
TRINITY_DN13227_c0_g6::TRINITY_DN13227_c0_g6_i1::g.21293::m.21293	3,30E-67	202	XP_018836608.1	macrophage migration inhibitory factor homolog	Juglans regia	OAY46685.1
TRINITY_DN19999_c7_g1::TRINITY_DN19999_c7_g1_i2::g.126443::m.126443	1,45E-60	187	XP_018837336.1	14 kDa zinc-binding protein	Juglans regia	XP_019434854.1
TRINITY_DN18074_c1_g1::TRINITY_DN18074_c1_g1_i2::g.94688::m.94688	0	941	XP_018812875.1	probable clathrin assembly protein At4g32285	Juglans regia	XP_018822255.1
TRINITY_DN15518_c0_g1::TRINITY_DN15518_c0_g1_i2::g.53176::m.53176	0	2582	XP_018841155.1	ARF guanine-nucleotide exchange factor GNOM-like	Juglans regia	XP_018813978.1
TRINITY_DN14467_c3_g1::TRINITY_DN14467_c3_g1_i7::g.37643::m.37643	0	750	XP_018820274.1	hydroxymethylglutaryl-CoA synthase-like	Juglans regia	XP_018820512.1
TRINITY_DN12438_c0_g2::TRINITY_DN12438_c0_g2_i1::g.12882::m.12882	7,38E-71	217	XP_018837090.1	uncharacterized protein LOC109003431 isoform X1	Juglans regia	ONI28753.1
TRINITY_DN12528_c1_g2::TRINITY_DN12528_c1_g2_i1::g.13492::m.13492	2,19E-153	436	XP_018812229.1	uncharacterized protein LOC108984656	Juglans regia	OAY58206.1
TRINITY_DN18017_c0_g1::TRINITY_DN18017_c0_g1_i4::g.93738::m.93738	6,21E-148	438	XP_018835088.1	polyadenylate-binding protein-interacting protein 3-like isoform X2	Juglans regia	XP_018835089.1
TRINITY_DN13524_c0_g1::TRINITY_DN13524_c0_g1_i8::g.24870::m.24870	1,97E-79	257	XP_018857854.1	ubiquitin carboxyl-terminal hydrolase 13-like	Juglans regia	XP_018852065.1
TRINITY_DN15030_c2_g10::TRINITY_DN15030_c2_g10_i10::g.46047::m.46047	0	1557	XP_018825490.1	protein VACUOLELESS1	Juglans regia	KRG98955.1
TRINITY_DN14724_c2_g3::TRINITY_DN14724_c2_g3_i6::g.41251::m.41251	0	609	XP_018824151.1	CBS domain-containing protein CBSX6	Juglans regia	XP_015962149.1

TRINITY_DN9836_c0_g1::TRINITY_DN9836_c0_g1_i2::g.3964::m.3964	9,19E-82	241	XP_018827889.1	eukaryotic translation initiation factor 1A	Juglans regia	XP_007135187.1
TRINITY_DN18459_c3_g1::TRINITY_DN18459_c3_g1_i2::g.100697::m.100697	0	1620	XP_018844089.1	DNA gyrase subunit A, chloroplastic/mitochondrial isoform X1	Juglans regia	XP_018844090.1
TRINITY_DN14119_c2_g1::TRINITY_DN14119_c2_g1_i7::g.32525::m.32525	6,49E-89	265	KRH52793.1	hypothetical protein GLYMA_06G088000	Glycine max	KHN10178.1
TRINITY_DN17875_c1_g1::TRINITY_DN17875_c1_g1_i11::g.91219::m.91219	2,89E-47	164	ONH93446.1	hypothetical protein PRUPE_8G233000	Prunus persica	XP_020426420.1
TRINITY_DN10295_c0_g1::TRINITY_DN10295_c0_g1_i2::g.4577::m.4577	6,91E-124	351	XP_018822169.1	gamma-glutamylcyclotransferase 2-3-like	Juglans regia	ONI34811.1
TRINITY_DN15634_c1_g2::TRINITY_DN15634_c1_g2_i1::g.54735::m.54735	0	976	XP_018841694.1	myosin heavy chain, non-muscle isoform X2	Juglans regia	XP_018841693.1
TRINITY_DN14193_c0_g1::TRINITY_DN14193_c0_g1_i4::g.33482::m.33482	1,11E-69	217	XP_018810280.1	interferon-related developmental regulator 1 isoform X3	Juglans regia	XP_018810278.1
TRINITY_DN18060_c0_g2::TRINITY_DN18060_c0_g2_i3::g.94415::m.94415	2,28E-106	310	KDP29395.1	hypothetical protein JCGZ_18316	Jatropha curcas	XP_012082078.1
TRINITY_DN13421_c0_g4::TRINITY_DN13421_c0_g4_i1::g.23529::m.23529	0	1767	XP_018805749.1	uncharacterized protein LOC108979512	Juglans regia	XP_018845508.1
TRINITY_DN15203_c1_g4::TRINITY_DN15203_c1_g4_i2::g.48560::m.48560	0	750	XP_018841198.1	UDP-glucuronic acid decarboxylase 1	Juglans regia	XP_009377117.1
TRINITY_DN15462_c1_g3::TRINITY_DN15462_c1_g3_i2::g.52234::m.52234	1,24E-45	165	XP_018805377.1	uncharacterized protein LOC108979207	Juglans regia	
TRINITY_DN17163_c2_g2::TRINITY_DN17163_c2_g2_i2::g.79515::m.79515	5,34E-77	232	KYP35787.1	Decaprenyl-diphosphate synthase subunit 1	Cajanus cajan	XP_006600931.1
TRINITY_DN14323_c3_g1::TRINITY_DN14323_c3_g1_i1::g.35598::m.35598	7,21E-88	267	XP_018844889.1	ruvB-like protein 1 isoform X1	Juglans regia	XP_018844890.1
TRINITY_DN17612_c1_g4::TRINITY_DN17612_c1_g4_i2::g.87038::m.87038	0	1021	XP_018824311.1	importin subunit beta-1-like	Juglans regia	XP_018835204.1
TRINITY_DN17010_c0_g1::TRINITY_DN17010_c0_g1_i4::g.76944::m.76944	0	2299	AES59623.2	DNA-directed RNA polymerase subunit beta	Medicago truncatula	XP_003589372.2
TRINITY_DN14661_c1_g1::TRINITY_DN14661_c1_g1_i2::g.40114::m.40114	9,92E-64	198	XP_018811471.1	probable phosphopantothenoylcysteine decarboxylase	Juglans regia	XP_018811472.1
TRINITY_DN19036_c0_g1::TRINITY_DN19036_c0_g1_i4::g.110800::m.110800	1,26E-95	286	XP_018827794.1	leucine-rich repeat extensin-like protein 5	Juglans regia	OAY39225.1
TRINITY_DN19720_c2_g1::TRINITY_DN19720_c2_g1_i1::g.122728::m.122728	1,04E-43	156	XP_018835461.1	potassium transporter 5-like	Juglans regia	AJA36505.1
TRINITY_DN16319_c0_g6::TRINITY_DN16319_c0_g6_i2::g.65617::m.65617	0	598	XP_020211310.1	activating signal cointegrator 1 complex subunit 2 isoform X2	Cajanus cajan	KRH61401.1
TRINITY_DN17286_c0_g1::TRINITY_DN17286_c0_g1_i7::g.81470::m.81470	0	1898	XP_018834052.1	alpha-mannosidase At3g26720-like	Juglans regia	KDP41856.1
TRINITY_DN13871_c3_g1::TRINITY_DN13871_c3_g1_i4::g.29817::m.29817	0	1918	XP_018843784.1	trafficking protein particle complex II-specific subunit 120 homolog	Juglans regia	XP_009355810.1
TRINITY_DN13650_c9_g1::TRINITY_DN13650_c9_g1_i8::g.26115::m.26115	0	1131	XP_018832961.1	stromal processing peptidase, chloroplastic-like	Juglans regia	XP_008385802.1

TRINITY_DN18482_c1_g1::TRINITY_DN18482_c1_g1_i7::g.100962::m.100962	3,69E-71	218	XP_018821291.1	uncharacterized protein LOC108991486	Juglans regia	XP_008364609.1
TRINITY_DN14626_c0_g2::TRINITY_DN14626_c0_g2_i6::g.39817::m.39817	4,70E-93	278	XP_018852162.1	ankyrin-3	Juglans regia	XP_018808658.1
TRINITY_DN13249_c0_g1::TRINITY_DN13249_c0_g1_i1::g.21073::m.21073	4,51E-125	361	KDP36167.1	hypothetical protein JCGZ_08811	Jatropha curcas	XP_012074377.1
TRINITY_DN15130_c6_g1::TRINITY_DN15130_c6_g1_i3::g.47299::m.47299	8,51E-94	290	XP_018826728.1	uncharacterized protein LOC108995594	Juglans regia	XP_020237202.1
TRINITY_DN16370_c1_g1::TRINITY_DN16370_c1_g1_i6::g.66493::m.66493	0	801	XP_018841559.1	alpha-1,3-mannosyl-glycoprotein 2-beta-N-acetylglucosaminyltransferase-like isoform X1	Juglans regia	XP_020237698.1
TRINITY_DN18821_c0_g1::TRINITY_DN18821_c0_g1_i8::g.106977::m.106977	0	823	XP_018856249.1	methylcrotonoyl-CoA carboxylase subunit alpha, mitochondrial isoform X2	Juglans regia	XP_018856242.1
TRINITY_DN2866_c0_g1::TRINITY_DN2866_c0_g1_i1::g.791::m.791	0	966	ONI24548.1	hypothetical protein PRUPE_2G246000	Prunus persica	XP_007220435.1
TRINITY_DN13724_c5_g4::TRINITY_DN13724_c5_g4_i1::g.27589::m.27589	0	688	ONI01623.1	hypothetical protein PRUPE_6G149500	Prunus persica	ONI01622.1
TRINITY_DN15832_c2_g1::TRINITY_DN15832_c2_g1_i7::g.57636::m.57636	0	641	XP_008240351.1	retinol dehydrogenase 11-like	Prunus mume	ONI09521.1
TRINITY_DN16212_c0_g2::TRINITY_DN16212_c0_g2_i5::g.63967::m.63967	1,64E-130	372	XP_018840728.1	desumoylating isopeptidase 1-like isoform X1	Juglans regia	XP_018821662.1
TRINITY_DN18690_c2_g2::TRINITY_DN18690_c2_g2_i1::g.104786::m.104786	0	771	XP_018826114.1	xyloglucan 6-xylosyltransferase 2-like	Juglans regia	XP_018825733.1
TRINITY_DN18367_c3_g7::TRINITY_DN18367_c3_g7_i1::g.99480::m.99480	9,39E-83	265	XP_018840248.1	cation/H(+) antiporter 19	Juglans regia	KRH19427.1
TRINITY_DN12696_c1_g1::TRINITY_DN12696_c1_g1_i6::g.15028::m.15028	1,17E-127	363	XP_018821066.1	uncharacterized protein LOC108991339	Juglans regia	XP_018821068.1
TRINITY_DN19683_c4_g2::TRINITY_DN19683_c4_g2_i1::g.121928::m.121928	0	557	XP_018848959.1	serine/threonine protein phosphatase 2A 57 kDa regulatory subunit B' beta isoform-like	Juglans regia	XP_018848960.1
TRINITY_DN16110_c4_g1::TRINITY_DN16110_c4_g1_i10::g.62321::m.62321	1,29E-157	456	XP_018814959.1	aldehyde oxidase GLOX1-like	Juglans regia	XP_018812716.1
TRINITY_DN19277_c4_g1::TRINITY_DN19277_c4_g1_i8::g.115170::m.115170	0	698	KDP20774.1	hypothetical protein JCGZ_21245	Jatropha curcas	XP_012091380.1
TRINITY_DN16461_c2_g1::TRINITY_DN16461_c2_g1_i2::g.67850::m.67850	1,70E-108	325	XP_018843899.1	4-coumarate--CoA ligase-like 6 isoform X1	Juglans regia	XP_018843900.1
TRINITY_DN16319_c0_g3::TRINITY_DN16319_c0_g3_i4::g.65613::m.65613	1,00E-29	118	XP_008220318.2	LOW QUALITY PROTEIN: uncharacterized protein LOC103320417	Prunus mume	XP_020416470.1
TRINITY_DN14881_c0_g1::TRINITY_DN14881_c0_g1_i4::g.43780::m.43780	0	856	XP_018829140.1	histone deacetylase 19-like	Juglans regia	XP_018829142.1
TRINITY_DN13265_c1_g2::TRINITY_DN13265_c1_g2_i3::g.21681::m.21681	6,16E-159	446	XP_018808599.1	14-3-3-like protein D	Juglans regia	BAT92355.1
TRINITY_DN14049_c0_g1::TRINITY_DN14049_c0_g1_i2::g.31233::m.31233	3,62E-124	361	XP_018849220.1	uncharacterized protein C57A10.07-like	Juglans regia	XP_018839870.1
TRINITY_DN13408_c0_g1::TRINITY_DN13408_c0_g1_i4::g.23389::m.23389	8,97E-174	482	XP_018812734.1	haloacid dehalogenase-like hydrolase domain-containing protein 3	Juglans regia	XP_018812735.1

TRINITY_DN19519_c0_g1::TRINITY_DN19519_c0_g1_i10::g.119145::m.119145	1,60E-123	364	XP_018840957.1	importin subunit alpha-9	Juglans regia	XP_008387618.1
TRINITY_DN16032_c0_g1::TRINITY_DN16032_c0_g1_i1::g.60895::m.60895	0	549	XP_018828122.1	glutelin type-B 5-like	Juglans regia	XP_018828123.1
TRINITY_DN18985_c1_g6::TRINITY_DN18985_c1_g6_i3::g.109896::m.109896	0	978	XP_018823882.1	synaptotagmin-1-like isoform X1	Juglans regia	XP_018841468.1
TRINITY_DN12405_c0_g1::TRINITY_DN12405_c0_g1_i1::g.12602::m.12602	0	809	XP_018806866.1	uncharacterized protein LOC108980411	Juglans regia	XP_008234511.1
TRINITY_DN19613_c2_g2::TRINITY_DN19613_c2_g2_i3::g.120856::m.120856	4,13E-116	368	OIW16247.1	hypothetical protein TanjilG_18962	Lupinus angustifolius	OIW16247.1
TRINITY_DN17036_c2_g3::TRINITY_DN17036_c2_g3_i3::g.77409::m.77409	7,40E-62	192	XP_018807851.1	dephospho-CoA kinase-like	Juglans regia	XP_008345240.1
TRINITY_DN18208_c0_g1::TRINITY_DN18208_c0_g1_i5::g.96946::m.96946	0	537	XP_018837640.1	probable tocopherol O-methyltransferase, chloroplastic isoform X4	Juglans regia	XP_018837639.1
TRINITY_DN18601_c0_g1::TRINITY_DN18601_c0_g1_i1::g.101595::m.101595	6,78E-60	195	XP_018844545.1	omega-amidase, chloroplastic	Juglans regia	XP_008377810.1
TRINITY_DN19149_c0_g1::TRINITY_DN19149_c0_g1_i16::g.113757::m.113757	1,04E-174	519	XP_018842267.1	uncharacterized protein LOC109007158 isoform X3	Juglans regia	XP_018842265.1
TRINITY_DN18875_c2_g5::TRINITY_DN18875_c2_g5_i2::g.107944::m.107944	8,79E-118	334	XP_018854702.1	actin	Juglans regia	XP_018858821.1
TRINITY_DN16082_c0_g1::TRINITY_DN16082_c0_g1_i2::g.61608::m.61608	0	920	XP_018807705.1	glucose-1-phosphate adenylyltransferase large subunit 1-like	Juglans regia	XP_018858808.1
TRINITY_DN11445_c0_g1::TRINITY_DN11445_c0_g1_i3::g.7277::m.7277	9,96E-120	348	XP_018826264.1	probable envelope ADP,ATP carrier protein, chloroplastic	Juglans regia	XP_018826264.1
TRINITY_DN13851_c1_g2::TRINITY_DN13851_c1_g2_i2::g.29292::m.29292	3,34E-151	437	XP_018836309.1	aspartic proteinase-like	Juglans regia	XP_018836310.1
TRINITY_DN15110_c1_g1::TRINITY_DN15110_c1_g1_i4::g.47001::m.47001	0	592	XP_015956091.1	probable cinnamyl alcohol dehydrogenase 1	Arachis duranensis	XP_016189854.1
TRINITY_DN15121_c0_g1::TRINITY_DN15121_c0_g1_i1::g.47196::m.47196	0	1231	OAY25308.1	hypothetical protein MANES_17G083800	Manihot esculenta	ONI31830.1
TRINITY_DN13420_c0_g1::TRINITY_DN13420_c0_g1_i1::g.23499::m.23499	1,42E-127	362	XP_018829987.1	ESCRT-related protein CHMP1B	Juglans regia	XP_007150545.1
TRINITY_DN18855_c1_g1::TRINITY_DN18855_c1_g1_i51::g.107677::m.107677	0	1539	XP_018826787.1	probable sucrose-phosphate synthase 1	Juglans regia	XP_018814107.1
TRINITY_DN16845_c1_g1::TRINITY_DN16845_c1_g1_i1::g.74565::m.74565	3,17E-132	374	XP_016650847.1	ras-related protein RABC1-like	Prunus mume	KDP43080.1
TRINITY_DN11922_c0_g1::TRINITY_DN11922_c0_g1_i1::g.9511::m.9511	2,17E-153	436	XP_018808628.1	probable carboxylesterase 9	Juglans regia	XP_018846314.1
TRINITY_DN13521_c0_g1::TRINITY_DN13521_c0_g1_i1::g.24833::m.24833	6,07E-66	209	XP_018815040.1	dihydrolipoyllysine-residue acetyltransferase component 4 of pyruvate dehydrogenase complex, chloroplastic-like	Juglans regia	XP_018825347.1
TRINITY_DN9608_c0_g1::TRINITY_DN9608_c0_g1_i2::g.3681::m.3681	7,39E-85	255	XP_018838663.1	uncharacterized protein LOC109004542 isoform X2	Juglans regia	XP_018838662.1

TRINITY_DN10819_c0_g1::TRINITY_DN10819_c0_g1_i3::g.5615::m.5615	5,35E-51	160	XP_018853827.1	uncharacterized protein LOC109015827	Juglans regia	XP_018853937.1
TRINITY_DN16090_c0_g1::TRINITY_DN16090_c0_g1_i3::g.61630::m.61630	0	622	XP_018806282.1	uncharacterized protein LOC108979939 isoform X1	Juglans regia	XP_018806285.1
TRINITY_DN18139_c1_g1::TRINITY_DN18139_c1_g1_i7::g.95745::m.95745	5,77E-73	230	XP_018847038.1	tubulin-folding cofactor C	Juglans regia	XP_017188064.1
TRINITY_DN16973_c0_g1::TRINITY_DN16973_c0_g1_i3::g.76496::m.76496	0	980	XP_018853040.1	probable galacturonosyltransferase 10	Juglans regia	XP_018852295.1
TRINITY_DN19343_c7_g1::TRINITY_DN19343_c7_g1_i3::g.116415::m.116415	4,25E-135	385	XP_018812118.1	beta-amylase 1, chloroplastic-like, partial	Juglans regia	XP_018858814.1
TRINITY_DN19191_c0_g2::TRINITY_DN19191_c0_g2_i4::g.113786::m.113786	5,33E-60	195	KDP28382.1	hypothetical protein JCGZ_14153	Jatropha curcas	XP_012083068.1
TRINITY_DN14461_c1_g4::TRINITY_DN14461_c1_g4_i3::g.37527::m.37527	1,10E-55	190	GAU22242.1	hypothetical protein TSUD_227780	Trifolium subterraneum	KYP78448.1
TRINITY_DN17541_c1_g1::TRINITY_DN17541_c1_g1_i11::g.86034::m.86034	0	1488	XP_018822432.1	uncharacterized protein LOC108992351 isoform X2	Juglans regia	XP_018822433.1
TRINITY_DN17922_c0_g1::TRINITY_DN17922_c0_g1_i13::g.92083::m.92083	0	1782	XP_018852299.1	THO complex subunit 2	Juglans regia	XP_012072357.1
TRINITY_DN14757_c1_g1::TRINITY_DN14757_c1_g1_i7::g.41725::m.41725	0	563	XP_018839043.1	cell division cycle 5-like protein	Juglans regia	XP_008218318.1
TRINITY_DN19529_c1_g2::TRINITY_DN19529_c1_g2_i5::g.119243::m.119243	0	739	XP_018855962.1	protein PIN-LIKES 2-like	Juglans regia	XP_018853142.1
TRINITY_DN19966_c14_g1::TRINITY_DN19966_c14_g1_i3::g.126866::m.126866	0	1580	XP_018830052.1	alpha,alpha-trehalose-phosphate synthase [UDP-forming] 1-like isoform X1	Juglans regia	XP_018830053.1
TRINITY_DN15823_c4_g1::TRINITY_DN15823_c4_g1_i3::g.57508::m.57508	0	988	XP_018833579.1	uncharacterized protein LOC109000958 isoform X1	Juglans regia	XP_018833580.1
TRINITY_DN15546_c0_g1::TRINITY_DN15546_c0_g1_i6::g.53570::m.53570	0	721	XP_018818948.1	protein argonaute 4-like isoform X2	Juglans regia	XP_018818945.1
TRINITY_DN13076_c2_g1::TRINITY_DN13076_c2_g1_i6::g.19526::m.19526	6,28E-138	397	XP_018848411.1	cystathionine beta-lyase, chloroplastic-like	Juglans regia	XP_018848412.1
TRINITY_DN19485_c0_g1::TRINITY_DN19485_c0_g1_i9::g.118686::m.118686	0	796	XP_018838814.1	insulin-degrading enzyme-like 1, peroxisomal	Juglans regia	KDP26057.1
TRINITY_DN13021_c1_g1::TRINITY_DN13021_c1_g1_i1::g.18775::m.18775	4,12E-130	376	XP_018811500.1	desiccation-related protein PCC13-62-like	Juglans regia	XP_018811498.1
TRINITY_DN12714_c1_g2::TRINITY_DN12714_c1_g2_i1::g.15270::m.15270	0	717	XP_018819378.1	protein GPR107-like	Juglans regia	XP_020205462.1
TRINITY_DN19212_c0_g1::TRINITY_DN19212_c0_g1_i8::g.114108::m.114108	0	715	XP_018841522.1	serine/threonine-protein kinase STY46-like isoform X1	Juglans regia	XP_018841523.1
TRINITY_DN13032_c1_g1::TRINITY_DN13032_c1_g1_i5::g.18813::m.18813	2,87E-105	312	XP_018842766.1	uncharacterized protein LOC109007529	Juglans regia	ONI25762.1
TRINITY_DN13241_c2_g1::TRINITY_DN13241_c2_g1_i4::g.21412::m.21412	7,88E-98	281	XP_018807480.1	DNA-directed RNA polymerases II and V subunit 8A-like isoform X2	Juglans regia	XP_018807487.1
TRINITY_DN19075_c1_g2::TRINITY_DN19075_c1_g2_i3::g.111314::m.111314	2,19E-62	196	XP_018812502.1	putative SNAP25 homologous protein SNAP30	Juglans regia	KHN03632.1

TRINITY_DN19379_c0_g1::TRINITY_DN19379_c0_g1_i4::g.116795::m.116795	0	1106	XP_008388543.1	luminal-binding protein 5-like	Malus domestica	ALP70520.1
TRINITY_DN15691_c0_g1::TRINITY_DN15691_c0_g1_i9::g.55585::m.55585	0	903	XP_016646826.1	diphthine--ammonia ligase isoform X3	Prunus mume	XP_008218334.1
TRINITY_DN10326_c0_g1::TRINITY_DN10326_c0_g1_i1::g.4634::m.4634	0	634	XP_018852365.1	pyruvate kinase, cytosolic isozyme-like	Juglans regia	XP_020210350.1
TRINITY_DN12047_c0_g1::TRINITY_DN12047_c0_g1_i4::g.10037::m.10037	0	569	XP_018815870.1	BAG family molecular chaperone regulator 7-like	Juglans regia	XP_018851941.1
TRINITY_DN18148_c1_g6::TRINITY_DN18148_c1_g6_i2::g.95979::m.95979	2,75E-109	322	XP_018832080.1	purple acid phosphatase 15-like, partial	Juglans regia	XP_014619149.1
TRINITY_DN17159_c3_g1::TRINITY_DN17159_c3_g1_i2::g.79474::m.79474	5,46E-72	218	XP_018847089.1	cytochrome c oxidase assembly protein COX11, mitochondrial-like isoform X2	Juglans regia	XP_018847090.1
TRINITY_DN10845_c0_g1::TRINITY_DN10845_c0_g1_i1::g.5649::m.5649	2,32E-91	270	XP_018831140.1	uncharacterized protein LOC108998861	Juglans regia	ONH89548.1
TRINITY_DN15929_c0_g1::TRINITY_DN15929_c0_g1_i6::g.59159::m.59159	0	1036	XP_018809403.1	homeobox protein HAT3.1	Juglans regia	ONH91822.1
TRINITY_DN11125_c0_g1::TRINITY_DN11125_c0_g1_i2::g.6392::m.6392	0	792	OAY49554.1	hypothetical protein MANES_05G065400	Manihot esculenta	OAY49556.1
TRINITY_DN19874_c1_g1::TRINITY_DN19874_c1_g1_i2::g.124818::m.124818	4,53E-84	260	XP_018844870.1	UDP-glycosyltransferase 71K1-like	Juglans regia	OAY61088.1
TRINITY_DN16609_c0_g4::TRINITY_DN16609_c0_g4_i3::g.70404::m.70404	0	2727	XP_018818212.1	E3 ubiquitin-protein ligase KEG isoform X1	Juglans regia	XP_018818212.1
TRINITY_DN19302_c0_g2::TRINITY_DN19302_c0_g2_i5::g.115749::m.115749	3,02E-115	356	XP_014512034.1	myosin-11	Vigna radiata var. radiata	KHN44988.1
TRINITY_DN18657_c3_g2::TRINITY_DN18657_c3_g2_i5::g.104104::m.104104	2,26E-64	197	XP_018837566.1	glutamyl-tRNA(Gln) amidotransferase subunit C, chloroplastic/mitochondrial	Juglans regia	ONI33729.1
TRINITY_DN18365_c0_g2::TRINITY_DN18365_c0_g2_i2::g.99254::m.99254	0	701	XP_018834399.1	uncharacterized protein LOC109001525	Juglans regia	XP_018816504.1
TRINITY_DN14704_c1_g3::TRINITY_DN14704_c1_g3_i1::g.40903::m.40903	0	866	XP_018844220.1	heparanase-like protein 1, partial	Juglans regia	XP_018835860.1
TRINITY_DN13379_c2_g1::TRINITY_DN13379_c2_g1_i3::g.22962::m.22962	8,35E-47	156	XP_018841785.1	peroxisomal adenine nucleotide carrier 1-like	Juglans regia	XP_018841786.1
TRINITY_DN16128_c0_g1::TRINITY_DN16128_c0_g1_i5::g.62674::m.62674	0	939	XP_018821773.1	phosphoinositide phospholipase C 6-like isoform X1	Juglans regia	XP_018821774.1
TRINITY_DN15685_c2_g1::TRINITY_DN15685_c2_g1_i7::g.55504::m.55504	0	881	XP_018851702.1	polygalacturonate 4-alpha-galacturonosyltransferase-like isoform X1	Juglans regia	XP_018851703.1
TRINITY_DN14830_c5_g1::TRINITY_DN14830_c5_g1_i9::g.42943::m.42943	0	798	XP_018829528.1	histone-lysine N-methyltransferase setd3 isoform X1	Juglans regia	OAY29294.1
TRINITY_DN11391_c0_g1::TRINITY_DN11391_c0_g1_i3::g.7245::m.7245	0	680	XP_018857638.1	cytosolic Fe-S cluster assembly factor NBP35	Juglans regia	XP_008219249.1
TRINITY_DN17340_c1_g1::TRINITY_DN17340_c1_g1_i17::g.82513::m.82513	1,17E-71	226	XP_018822229.1	protein WVD2-like 6 isoform X2	Juglans regia	XP_018822227.1
TRINITY_DN15433_c2_g1::TRINITY_DN15433_c2_g1_i2::g.51849::m.51849	4,22E-133	388	XP_018822723.1	aladin isoform X1	Juglans regia	XP_018822724.1

TRINITY_DN19143_c2_g1::TRINITY_DN19143_c2_g1_i5::g.112885::m.112885	0	1681	XP_018810738.1	uncharacterized protein LOC108983527 isoform X1	Juglans regia	XP_018810747.1
TRINITY_DN15141_c2_g2::TRINITY_DN15141_c2_g2_i7::g.47512::m.47512	2,88E-56	176	OAY52767.1	hypothetical protein MANES_04G109500	Manihot esculenta	AFC88294.1
TRINITY_DN6604_c0_g1::TRINITY_DN6604_c0_g1_i1::g.1696::m.1696	0	599	XP_018856707.1	L-ascorbate oxidase homolog	Juglans regia	XP_018856708.1
TRINITY_DN14356_c5_g1::TRINITY_DN14356_c5_g1_i2::g.35855::m.35855	1,79E-143	417	XP_018842638.1	double-stranded RNA-binding protein 1	Juglans regia	XP_018842639.1
TRINITY_DN17814_c1_g1::TRINITY_DN17814_c1_g1_i9::g.90451::m.90451	5,67E-122	358	XP_018851655.1	probable ADP-ribosylation factor GTPase-activating protein AGD5 isoform X4	Juglans regia	XP_018851632.1
TRINITY_DN7788_c0_g1::TRINITY_DN7788_c0_g1_i1::g.2350::m.2350	0	690	CBF70796.1	unnamed protein product	Betula pendula	CBF86074.1
TRINITY_DN16305_c1_g1::TRINITY_DN16305_c1_g1_i3::g.65515::m.65515	0	781	XP_018860345.1	chaperone protein dnaJ A6, chloroplastic-like	Juglans regia	XP_018834540.1
TRINITY_DN14505_c2_g1::TRINITY_DN14505_c2_g1_i2::g.38114::m.38114	1,03E-64	199	XP_008219047.1	charged multivesicular body protein 5-like	Prunus mume	ONI35685.1
TRINITY_DN16442_c0_g2::TRINITY_DN16442_c0_g2_i5::g.67615::m.67615	2,76E-162	458	XP_018830828.1	probable fructokinase-7	Juglans regia	XP_008234319.1
TRINITY_DN9437_c0_g1::TRINITY_DN9437_c0_g1_i1::g.3480::m.3480	0	631	XP_018846669.1	4-hydroxy-tetrahydrodipicolinate synthase, chloroplastic-like	Juglans regia	XP_018806961.1
TRINITY_DN10468_c0_g2::TRINITY_DN10468_c0_g2_i1::g.4854::m.4854	0	862	XP_018847630.1	bifunctional 3-dehydroquinate dehydratase/shikimate dehydrogenase, chloroplastic-like	Juglans regia	XP_012089153.1
TRINITY_DN19289_c1_g2::TRINITY_DN19289_c1_g2_i6::g.115366::m.115366	0	556	XP_018847765.1	putative WEB family protein At1g65010, chloroplastic	Juglans regia	XP_018847766.1
TRINITY_DN570_c0_g1::TRINITY_DN570_c0_g1_i1::g.152::m.152	2,09E-34	119	XP_018810557.1	uncharacterized protein LOC108983399	Juglans regia	XP_008385848.1
TRINITY_DN16387_c0_g7::TRINITY_DN16387_c0_g7_i3::g.66774::m.66774	1,80E-71	219	XP_018821449.1	novel plant SNARE 11-like	Juglans regia	XP_018821450.1
TRINITY_DN19149_c0_g1::TRINITY_DN19149_c0_g1_i14::g.113756::m.113756	9,35E-52	179	XP_018842267.1	uncharacterized protein LOC109007158 isoform X3	Juglans regia	XP_018842265.1
TRINITY_DN18020_c2_g2::TRINITY_DN18020_c2_g2_i4::g.93764::m.93764	3,09E-153	436	XP_018850691.1	beta-1,3-galactosyltransferase 7-like	Juglans regia	ONI34929.1
TRINITY_DN16978_c1_g3::TRINITY_DN16978_c1_g3_i2::g.76542::m.76542	0	815	XP_018832674.1	anaphase-promoting complex subunit 8	Juglans regia	XP_004516815.1
TRINITY_DN17213_c1_g1::TRINITY_DN17213_c1_g1_i3::g.80196::m.80196	6,06E-123	352	XP_018830852.1	cysteine and histidine-rich domain-containing protein RAR1	Juglans regia	XP_009346938.1
TRINITY_DN19992_c4_g1::TRINITY_DN19992_c4_g1_i5::g.127460::m.127460	1,93E-58	187	XP_018805839.1	dolichyl-phosphate beta-glucosyltransferase-like	Juglans regia	XP_018814490.1
TRINITY_DN13415_c1_g3::TRINITY_DN13415_c1_g3_i1::g.23602::m.23602	0	1023	XP_018809529.1	phosphoenolpyruvate carboxylase 4-like isoform X1	Juglans regia	XP_018853146.1
TRINITY_DN12348_c0_g1::TRINITY_DN12348_c0_g1_i1::g.11710::m.11710	1,60E-54	172	XP_018826323.1	uncharacterized protein LOC108995253	Juglans regia	XP_018832869.1

TRINITY_DN15405_c3_g1::TRINITY_DN15405_c3_g1_i4::g.51552::m.51552	7,42E-43	152	XP_018818346.1	dynamin-related protein 5A	Juglans regia	XP_007135262.1
TRINITY_DN9822_c0_g1::TRINITY_DN9822_c0_g1_i1::g.3931::m.3931	1,39E-127	371	XP_018811141.1	amidase 1 isoform X2	Juglans regia	XP_018811139.1
TRINITY_DN14833_c0_g2::TRINITY_DN14833_c0_g2_i2::g.42977::m.42977	0	760	XP_018842090.1	uncharacterized protein LOC109007046	Juglans regia	XP_018851050.1
TRINITY_DN15454_c2_g1::TRINITY_DN15454_c2_g1_i4::g.52157::m.52157	0	800	XP_018854589.1	PAP-specific phosphatase HAL2-like	Juglans regia	XP_018859020.1
TRINITY_DN14480_c1_g1::TRINITY_DN14480_c1_g1_i3::g.37689::m.37689	2,33E-61	190	XP_018813027.1	vacuolar protein-sorting-associated protein 37 homolog 1-like	Juglans regia	XP_018836180.1
TRINITY_DN18127_c0_g1::TRINITY_DN18127_c0_g1_i1::g.95817::m.95817	0	1445	XP_018811836.1	uncharacterized protein LOC108984363	Juglans regia	XP_018813316.1
TRINITY_DN16613_c0_g1::TRINITY_DN16613_c0_g1_i5::g.70442::m.70442	0	773	XP_018822837.1	FRIGIDA-like protein 4a	Juglans regia	ONH98105.1
TRINITY_DN17792_c0_g7::TRINITY_DN17792_c0_g7_i6::g.89759::m.89759	5,49E-55	179	XP_018844982.1	PI-PLC X domain-containing protein At5g67130	Juglans regia	KHN05833.1
TRINITY_DN19032_c3_g1::TRINITY_DN19032_c3_g1_i17::g.110826::m.110826	0	769	XP_018850270.1	U-box domain-containing protein 35 isoform X3	Juglans regia	XP_018850268.1
TRINITY_DN3292_c0_g1::TRINITY_DN3292_c0_g1_i2::g.890::m.890	0	517	XP_018836995.1	dnaJ homolog subfamily B member 4-like isoform X1	Juglans regia	XP_018836996.1
TRINITY_DN13487_c0_g1::TRINITY_DN13487_c0_g1_i1::g.24417::m.24417	0	883	XP_018845396.1	diacylglycerol O-acyltransferase 1-like	Juglans regia	XP_018846200.1
TRINITY_DN16110_c4_g1::TRINITY_DN16110_c4_g1_i11::g.62324::m.62324	3,73E-43	154	XP_018814959.1	aldehyde oxidase GLOX1-like	Juglans regia	XP_018853951.1
TRINITY_DN12761_c4_g2::TRINITY_DN12761_c4_g2_i1::g.15691::m.15691	2,43E-64	206	XP_018851305.1	switch-associated protein 70	Juglans regia	XP_012088449.1
TRINITY_DN11277_c0_g1::TRINITY_DN11277_c0_g1_i2::g.6842::m.6842	1,97E-70	213	XP_018825580.1	uncharacterized protein LOC108994710 isoform X3	Juglans regia	XP_018826010.1
TRINITY_DN14078_c2_g2::TRINITY_DN14078_c2_g2_i1::g.32075::m.32075	6,12E-54	170	XP_018835557.1	ubiquitin-like domain-containing CTD phosphatase	Juglans regia	XP_018842013.1
TRINITY_DN18707_c1_g1::TRINITY_DN18707_c1_g1_i8::g.104943::m.104943	8,49E-80	242	XP_009371858.1	B-cell receptor-associated protein 31	Pyrus x bretschneideri	XP_008235454.1
TRINITY_DN14427_c0_g1::TRINITY_DN14427_c0_g1_i8::g.36957::m.36957	0	633	XP_018821599.1	lysophospholipid acyltransferase LPEAT1-like isoform X1	Juglans regia	XP_018821600.1
TRINITY_DN14274_c2_g1::TRINITY_DN14274_c2_g1_i1::g.34705::m.34705	4,33E-148	427	XP_018836672.1	sphingosine-1-phosphate lyase	Juglans regia	ONI29929.1
TRINITY_DN14629_c0_g3::TRINITY_DN14629_c0_g3_i2::g.40344::m.40344	2,36E-43	149	XP_018849379.1	dr1-associated corepressor-like isoform X2	Juglans regia	XP_018849378.1
TRINITY_DN17321_c2_g1::TRINITY_DN17321_c2_g1_i8::g.82045::m.82045	1,59E-73	232	XP_018816046.1	uncharacterized protein LOC108987537	Juglans regia	OAY41680.1
TRINITY_DN19385_c2_g1::TRINITY_DN19385_c2_g1_i5::g.117068::m.117068	1,68E-107	313	XP_008386967.1	uncharacterized protein LOC103449426	Malus domestica	XP_008359455.1
TRINITY_DN12251_c0_g1::TRINITY_DN12251_c0_g1_i7::g.11382::m.11382	1,97E-60	184	XP_008234567.1	NADH dehydrogenase [ubiquinone] iron-sulfur protein 6, mitochondrial	Prunus mume	ONI25909.1

TRINITY_DN18091_c1_g1::TRINITY_DN18091_c1_g1_i6::g.94898::m.94898	1,27E-146	415	XP_018850569.1	nitrilase-like protein 2	Juglans regia	XP_016176841.1
TRINITY_DN18990_c0_g1::TRINITY_DN18990_c0_g1_i1::g.110013::m.110013	2,77E-105	327	XP_018832888.1	uncharacterized protein LOC109000473	Juglans regia	XP_018840661.1
TRINITY_DN19413_c0_g2::TRINITY_DN19413_c0_g2_i1::g.117446::m.117446	1,12E-129	367	OIW12873.1	hypothetical protein TanjilG_24806	Lupinus angustifolius	XP_019441486.1
TRINITY_DN18349_c4_g1::TRINITY_DN18349_c4_g1_i3::g.99227::m.99227	7,42E-164	480	ONI08508.1	hypothetical protein PRUPE_5G182400	Prunus persica	XP_008239683.1
TRINITY_DN14718_c0_g1::TRINITY_DN14718_c0_g1_i3::g.41289::m.41289	4,71E-179	511	XP_018839142.1	NADP-dependent malic enzyme-like	Juglans regia	XP_018843270.1
TRINITY_DN18771_c1_g1::TRINITY_DN18771_c1_g1_i3::g.106137::m.106137	0	710	XP_018853620.1	3-phosphoinositide-dependent protein kinase 1	Juglans regia	XP_018820470.1
TRINITY_DN10875_c0_g1::TRINITY_DN10875_c0_g1_i3::g.5717::m.5717	0	822	XP_018832960.1	histone deacetylase 19-like	Juglans regia	XP_018843098.1
TRINITY_DN10668_c0_g1::TRINITY_DN10668_c0_g1_i3::g.5273::m.5273	0	1159	XP_018848649.1	peptidyl-prolyl cis-trans isomerase CYP71	Juglans regia	XP_014498242.1
TRINITY_DN16826_c3_g1::TRINITY_DN16826_c3_g1_i6::g.74102::m.74102	0	1278	XP_018811212.1	microtubule-associated protein TORTIFOLIA1-like isoform X1	Juglans regia	XP_018811221.1
TRINITY_DN19356_c0_g4::TRINITY_DN19356_c0_g4_i8::g.116588::m.116588	7,96E-154	437	XP_018830880.1	1-acyl-sn-glycerol-3-phosphate acyltransferase 3-like	Juglans regia	XP_009375057.2
TRINITY_DN19935_c0_g1::TRINITY_DN19935_c0_g1_i18::g.126411::m.126411	9,83E-56	176	XP_017188793.1	protein MOR1-like	Malus domestica	XP_018834726.1
TRINITY_DN15733_c2_g3::TRINITY_DN15733_c2_g3_i4::g.56425::m.56425	0	1648	XP_018860544.1	phospholipase D beta 2-like	Juglans regia	XP_018847586.1
TRINITY_DN11377_c0_g1::TRINITY_DN11377_c0_g1_i3::g.7158::m.7158	1,51E-107	311	OAY36849.1	hypothetical protein MANES_11G053800	Manihot esculenta	XP_018812067.1
TRINITY_DN13397_c1_g1::TRINITY_DN13397_c1_g1_i6::g.22118::m.22118	3,29E-27	108	XP_018821852.1	uroporphyrinogen decarboxylase-like	Juglans regia	XP_018838987.1
TRINITY_DN18687_c0_g1::TRINITY_DN18687_c0_g1_i17::g.103218::m.103218	0	929	XP_018852272.1	nuclear pore complex protein NUP93A-like	Juglans regia	OAY36979.1
TRINITY_DN15145_c0_g1::TRINITY_DN15145_c0_g1_i10::g.46819::m.46819	5,10E-35	131	XP_018850946.1	DNA gyrase subunit B, chloroplastic/mitochondrial-like	Juglans regia	XP_008387565.1
TRINITY_DN15345_c2_g1::TRINITY_DN15345_c2_g1_i10::g.49844::m.49844	0	984	XP_018813804.1	probable galactinol--sucrose galactosyltransferase 1	Juglans regia	XP_008219010.1
TRINITY_DN17431_c5_g1::TRINITY_DN17431_c5_g1_i7::g.83828::m.83828	0	1278	XP_018815904.1	squamous cell carcinoma antigen recognized by T-cells 3 isoform X2	Juglans regia	XP_018815903.1
TRINITY_DN16550_c0_g1::TRINITY_DN16550_c0_g1_i1::g.69273::m.69273	0	988	XP_018837556.1	uncharacterized protein LOC109003736	Juglans regia	OAY26770.1
TRINITY_DN15895_c4_g2::TRINITY_DN15895_c4_g2_i3::g.57730::m.57730	1,87E-94	303	XP_018816385.1	uncharacterized protein LOC108987817 isoform X3	Juglans regia	XP_018816382.1
TRINITY_DN12618_c1_g1::TRINITY_DN12618_c1_g1_i9::g.14449::m.14449	3,36E-149	427	XP_018820745.1	serine/arginine-rich splicing factor RS40-like isoform X6	Juglans regia	XP_018820747.1
TRINITY_DN19462_c2_g2::TRINITY_DN19462_c2_g2_i5::g.118383::m.118383	0	3246	XP_018836938.1	phosphatidylinositol 4-kinase alpha 1 isoform X4	Juglans regia	XP_018836937.1

TRINITY_DN16453_c1_g1::TRINITY_DN16453_c1_g1_i1::g.67791::m.67791	1,85E-104	300	XP_018852393.1	peptidyl-prolyl cis-trans isomerase E	Juglans regia	KDP41325.1
TRINITY_DN14591_c5_g1::TRINITY_DN14591_c5_g1_i3::g.39221::m.39221	3,80E-149	425	OIV92338.1	hypothetical protein TanjilG_10548	Lupinus angustifolius	XP_019426028.1
TRINITY_DN16615_c1_g3::TRINITY_DN16615_c1_g3_i6::g.70426::m.70426	0	732	XP_018833896.1	isoflavone 2'-hydroxylase-like	Juglans regia	XP_018854609.1
TRINITY_DN18754_c0_g1::TRINITY_DN18754_c0_g1_i2::g.106002::m.106002	0	720	XP_018832099.1	actin cytoskeleton-regulatory complex protein PAN1-like	Juglans regia	XP_018838276.1
TRINITY_DN17004_c0_g1::TRINITY_DN17004_c0_g1_i2::g.76878::m.76878	8,84E-78	237	XP_018852079.1	uncharacterized protein LOC109014173	Juglans regia	OAY30302.1
TRINITY_DN15390_c3_g1::TRINITY_DN15390_c3_g1_i2::g.51061::m.51061	6,37E-129	366	XP_018822751.1	probable aquaporin SIP2-1 isoform X1	Juglans regia	XP_018839934.1
TRINITY_DN18311_c1_g1::TRINITY_DN18311_c1_g1_i2::g.98629::m.98629	3,67E-109	315	XP_018857636.1	adenylate kinase 4-like	Juglans regia	OAY32425.1
TRINITY_DN17353_c2_g2::TRINITY_DN17353_c2_g2_i2::g.82341::m.82341	3,14E-142	429	XP_018849205.1	putative transcription elongation factor SPT5 homolog 1	Juglans regia	XP_008237688.1
TRINITY_DN10747_c0_g1::TRINITY_DN10747_c0_g1_i1::g.5399::m.5399	9,95E-144	410	XP_018844066.1	Werner Syndrome-like exonuclease	Juglans regia	ONI28848.1
TRINITY_DN12384_c0_g1::TRINITY_DN12384_c0_g1_i2::g.12333::m.12333	4,18E-118	345	XP_018807146.1	nicotinate-nucleotide pyrophosphorylase [carboxylating], chloroplastic isoform X2	Juglans regia	XP_018807147.1
TRINITY_DN16358_c2_g1::TRINITY_DN16358_c2_g1_i5::g.66392::m.66392	5,94E-152	433	XP_018858103.1	delta-aminolevulinic acid dehydratase, chloroplastic-like	Juglans regia	XP_008378481.1
TRINITY_DN19240_c1_g2::TRINITY_DN19240_c1_g2_i4::g.114388::m.114388	0	633	XP_018833674.1	muscle M-line assembly protein unc-89-like isoform X2	Juglans regia	XP_018833673.1
TRINITY_DN17731_c6_g1::TRINITY_DN17731_c6_g1_i4::g.89011::m.89011	1,26E-86	259	XP_018815778.1	prolyl 4-hydroxylase 1-like isoform X2	Juglans regia	XP_018815777.1
TRINITY_DN14931_c0_g1::TRINITY_DN14931_c0_g1_i6::g.44458::m.44458	0	928	ONI23221.1	hypothetical protein PRUPE_2G176000	Prunus persica	XP_020412098.1
TRINITY_DN19471_c0_g1::TRINITY_DN19471_c0_g1_i4::g.118445::m.118445	7,31E-69	219	XP_018818273.1	metal tolerance protein 4-like	Juglans regia	XP_018834606.1
TRINITY_DN17953_c1_g8::TRINITY_DN17953_c1_g8_i1::g.91726::m.91726	1,72E-171	492	XP_008391350.1	phosphatidylserine decarboxylase proenzyme 3-like	Malus domestica	XP_018842147.1
TRINITY_DN14840_c3_g1::TRINITY_DN14840_c3_g1_i19::g.43227::m.43227	2,57E-64	203	XP_018820465.1	N-acetyl-D-glucosamine kinase-like isoform X1	Juglans regia	OAY57353.1
TRINITY_DN16802_c5_g3::TRINITY_DN16802_c5_g3_i3::g.73661::m.73661	1,55E-151	442	XP_018833887.1	uncharacterized protein LOC109001170 isoform X2	Juglans regia	XP_018833884.1
TRINITY_DN16580_c0_g3::TRINITY_DN16580_c0_g3_i6::g.69917::m.69917	0	880	XP_018847635.1	KH domain-containing protein At4g18375	Juglans regia	XP_008225505.1
TRINITY_DN13912_c2_g1::TRINITY_DN13912_c2_g1_i3::g.30407::m.30407	0	816	XP_018832822.1	glucan endo-1,3-beta-glucosidase 8-like	Juglans regia	XP_018832823.1
TRINITY_DN19207_c4_g2::TRINITY_DN19207_c4_g2_i3::g.114212::m.114212	0	852	XP_018820332.1	uncharacterized protein LOC108990725 isoform X2	Juglans regia	XP_018820331.1
TRINITY_DN16740_c1_g1::TRINITY_DN16740_c1_g1_i2::g.72692::m.72692	0	685	XP_018814289.1	topless-related protein 4-like	Juglans regia	XP_018814290.1

TRINITY_DN15744_c2_g4::TRINITY_DN15744_c2_g4_i2::g.56499::m.56499	1,11E-101	300	XP_018807462.1	membrane-associated 30 kDa protein, chloroplastic-like	Juglans regia	XP_018806924.1
TRINITY_DN16211_c0_g2::TRINITY_DN16211_c0_g2_i6::g.63944::m.63944	0	682	XP_018817248.1	phosphatidate phosphatase PAH1	Juglans regia	XP_009340900.1
TRINITY_DN13792_c0_g1::TRINITY_DN13792_c0_g1_i4::g.28291::m.28291	5,09E-43	154	XP_018825898.1	importin-11 isoform X1	Juglans regia	XP_018825900.1
TRINITY_DN14860_c0_g1::TRINITY_DN14860_c0_g1_i1::g.43455::m.43455	0	1256	XP_018810118.1	cation-chloride cotransporter 1 isoform X1	Juglans regia	XP_018810124.1
TRINITY_DN12935_c1_g1::TRINITY_DN12935_c1_g1_i3::g.17528::m.17528	2,40E-120	348	XP_018807963.1	2-alkenal reductase (NADP(+)-dependent)-like	Juglans regia	XP_018855810.1
TRINITY_DN16126_c1_g1::TRINITY_DN16126_c1_g1_i14::g.62052::m.62052	0	877	XP_018828572.1	alanine--glyoxylate aminotransferase 2 homolog 2, mitochondrial-like	Juglans regia	OAY41602.1
TRINITY_DN13480_c0_g1::TRINITY_DN13480_c0_g1_i3::g.24330::m.24330	0	1069	XP_018830309.1	uncharacterized protein LOC108998264 isoform X1	Juglans regia	XP_018830310.1
TRINITY_DN16307_c2_g3::TRINITY_DN16307_c2_g3_i3::g.65541::m.65541	0	1040	OAY31284.1	hypothetical protein MANES_14G099600	Manihot esculenta	KDP29385.1
TRINITY_DN17586_c3_g4::TRINITY_DN17586_c3_g4_i6::g.86457::m.86457	0	826	XP_018819504.1	serine/threonine-protein phosphatase 6 regulatory subunit 3-like isoform X1	Juglans regia	XP_018819504.1
TRINITY_DN10182_c0_g1::TRINITY_DN10182_c0_g1_i1::g.4394::m.4394	3,09E-83	247	XP_020215321.1	protein C2-DOMAIN ABA-RELATED 9-like	Cajanus cajan	ACU19693.1
TRINITY_DN16200_c0_g2::TRINITY_DN16200_c0_g2_i2::g.62796::m.62796	2,78E-145	414	XP_018827350.1	deoxyhypusine hydroxylase	Juglans regia	XP_008235715.1
TRINITY_DN15100_c3_g1::TRINITY_DN15100_c3_g1_i6::g.45535::m.45535	4,01E-93	280	XP_018838088.1	palmitoyl-acyl carrier protein thioesterase, chloroplastic-like isoform X2	Juglans regia	XP_018838086.1
TRINITY_DN12087_c0_g1::TRINITY_DN12087_c0_g1_i3::g.10442::m.10442	9,79E-90	266	XP_018822178.1	transmembrane emp24 domain-containing protein p24delta3-like	Juglans regia	XP_012078211.1
TRINITY_DN15513_c2_g1::TRINITY_DN15513_c2_g1_i6::g.53086::m.53086	2,55E-29	115	XP_018822483.1	uncharacterized protein DDB_G0283697-like	Juglans regia	XP_018826899.1
TRINITY_DN13233_c0_g4::TRINITY_DN13233_c0_g4_i3::g.21366::m.21366	0	621	XP_018850089.1	patellin-3-like	Juglans regia	XP_018850089.1
TRINITY_DN17374_c2_g1::TRINITY_DN17374_c2_g1_i5::g.82712::m.82712	0	664	XP_008225034.1	probable polyol transporter 4	Prunus mume	ONI10351.1
TRINITY_DN12068_c0_g1::TRINITY_DN12068_c0_g1_i4::g.10141::m.10141	0	754	XP_018857491.1	uncharacterized protein LOC109019613	Juglans regia	ONI03867.1
TRINITY_DN14979_c0_g1::TRINITY_DN14979_c0_g1_i9::g.45197::m.45197	0	1204	KDP31060.1	hypothetical protein JCGZ_11436	Jatropha curcas	XP_012080012.1
TRINITY_DN15527_c0_g1::TRINITY_DN15527_c0_g1_i4::g.53287::m.53287	0	615	XP_018828485.1	LRR repeats and ubiquitin-like domain-containing protein At2g30105	Juglans regia	ONH99825.1
TRINITY_DN15865_c2_g1::TRINITY_DN15865_c2_g1_i9::g.58071::m.58071	2,48E-92	267	XP_018812078.1	cyclin-B1-2-like	Juglans regia	XP_018815474.1
TRINITY_DN17882_c2_g1::TRINITY_DN17882_c2_g1_i14::g.91309::m.91309	2,33E-142	421	XP_018844053.1	probable acyl-activating enzyme 16, chloroplastic isoform X2	Juglans regia	XP_018844052.1
TRINITY_DN18694_c1_g1::TRINITY_DN18694_c1_g1_i5::g.104667::m.104667	1,23E-108	318	XP_018837715.1	short-chain dehydrogenase TIC 32, chloroplastic	Juglans regia	ONI08287.1

TRINITY_DN17794_c1_g1::TRINITY_DN17794_c1_g1_i3::g.89692::m.89692	5,06E-113	326	KDP44031.1	hypothetical protein JCGZ_05498	Jatropha curcas	XP_012064793.1
TRINITY_DN15372_c1_g1::TRINITY_DN15372_c1_g1_i7::g.50862::m.50862	3,83E-64	200	XP_018846517.1	uncharacterized protein LOC109010214 isoform X2	Juglans regia	XP_018846516.1
TRINITY_DN13872_c3_g1::TRINITY_DN13872_c3_g1_i6::g.29683::m.29683	2,83E-70	212	XP_018823244.1	outer envelope pore protein 16, chloroplastic-like	Juglans regia	KDP34497.1
TRINITY_DN14858_c0_g5::TRINITY_DN14858_c0_g5_i1::g.43408::m.43408	0	1933	XP_018825828.1	ubiquitin-activating enzyme E1 1-like isoform X1	Juglans regia	XP_018825829.1
TRINITY_DN11799_c0_g1::TRINITY_DN11799_c0_g1_i1::g.8868::m.8868	0	795	XP_018835856.1	phenylalanine--tRNA ligase, chloroplastic/mitochondrial	Juglans regia	ONI11601.1
TRINITY_DN17436_c0_g1::TRINITY_DN17436_c0_g1_i7::g.83790::m.83790	0	1876	XP_018826777.1	WD repeat-containing protein 11	Juglans regia	XP_008242799.1
TRINITY_DN15518_c0_g2::TRINITY_DN15518_c0_g2_i2::g.53174::m.53174	0	2721	XP_018821790.1	ARF guanine-nucleotide exchange factor GNOM	Juglans regia	XP_018821791.1
TRINITY_DN19390_c1_g1::TRINITY_DN19390_c1_g1_i8::g.117167::m.117167	0	2262	XP_018852083.1	ABC transporter C family member 10-like	Juglans regia	XP_018852084.1
TRINITY_DN12101_c0_g1::TRINITY_DN12101_c0_g1_i1::g.9937::m.9937	5,23E-133	382	XP_018842452.1	late embryogenesis abundant protein D-34-like	Juglans regia	XP_018840448.1
TRINITY_DN11289_c0_g1::TRINITY_DN11289_c0_g1_i1::g.6862::m.6862	0	592	XP_018831263.1	N-carbamoylputrescine amidase-like	Juglans regia	XP_018831264.1
TRINITY_DN10610_c0_g1::TRINITY_DN10610_c0_g1_i2::g.5110::m.5110	6,15E-164	460	XP_020228837.1	uncharacterized protein LOC109809844 isoform X2	Cajanus cajan	KYP55377.1
TRINITY_DN17673_c0_g1::TRINITY_DN17673_c0_g1_i8::g.88029::m.88029	5,88E-61	204	XP_018816325.1	cellulose synthase-like protein D1	Juglans regia	OAY28231.1
TRINITY_DN17467_c0_g2::TRINITY_DN17467_c0_g2_i8::g.84133::m.84133	2,60E-110	328	XP_018816311.1	RNA polymerase II-associated protein 3 isoform X3	Juglans regia	XP_018816310.1
TRINITY_DN18000_c3_g4::TRINITY_DN18000_c3_g4_i1::g.93345::m.93345	1,41E-13	69,3	XP_018836207.1	uncharacterized protein LOC109002773	Juglans regia	XP_012068578.1
TRINITY_DN13706_c2_g1::TRINITY_DN13706_c2_g1_i3::g.27413::m.27413	0	639	XP_018808593.1	serine/threonine-protein phosphatase 2A activator-like	Juglans regia	XP_018807165.1
TRINITY_DN12707_c0_g1::TRINITY_DN12707_c0_g1_i3::g.15086::m.15086	2,60E-168	475	XP_018807745.1	uridine nucleosidase 1	Juglans regia	XP_008241263.1
TRINITY_DN18227_c0_g1::TRINITY_DN18227_c0_g1_i4::g.97154::m.97154	2,09E-75	246	XP_018846461.1	apoptotic chromatin condensation inducer in the nucleus-like isoform X2	Juglans regia	XP_018846458.1
TRINITY_DN17401_c2_g3::TRINITY_DN17401_c2_g3_i2::g.83107::m.83107	0	628	XP_018816020.1	probable sodium-coupled neutral amino acid transporter 6	Juglans regia	XP_018846083.1
TRINITY_DN11447_c0_g1::TRINITY_DN11447_c0_g1_i1::g.7417::m.7417	0	600	XP_018841012.1	probable ethanolamine kinase	Juglans regia	ONH98290.1
TRINITY_DN18966_c3_g3::TRINITY_DN18966_c3_g3_i5::g.109514::m.109514	5,10E-180	510	XP_018855526.1	transcription initiation factor IIA large subunit isoform X2	Juglans regia	XP_018855521.1
TRINITY_DN18595_c0_g1::TRINITY_DN18595_c0_g1_i7::g.103121::m.103121	0	1931	XP_018814465.1	AP3-complex subunit beta-A	Juglans regia	XP_018814466.1
TRINITY_DN11480_c0_g1::TRINITY_DN11480_c0_g1_i7::g.7531::m.7531	8,88E-132	377	XP_018824591.1	nudix hydrolase 9 isoform X2	Juglans regia	XP_018824590.1

TRINITY_DN19085_c0_g1::TRINITY_DN19085_c0_g1_i22::g.111744::m.111744	1,77E-162	483	XP_018810627.1	AMP deaminase-like	Juglans regia	XP_016647605.1
TRINITY_DN14786_c1_g2::TRINITY_DN14786_c1_g2_i1::g.42235::m.42235	0	4422	XP_018822821.1	dnaJ homolog subfamily C GRV2 isoform X1	Juglans regia	ONH98186.1
TRINITY_DN1011_c0_g1::TRINITY_DN1011_c0_g1_i1::g.255::m.255	3,56E-158	469	XP_018829558.1	alpha-mannosidase	Juglans regia	XP_018858492.1
TRINITY_DN16766_c2_g2::TRINITY_DN16766_c2_g2_i3::g.73070::m.73070	9,90E-75	229	ONI09830.1	hypothetical protein PRUPE_4G012100	Prunus persica	XP_007213842.1
TRINITY_DN17471_c1_g1::TRINITY_DN17471_c1_g1_i8::g.84225::m.84225	0	1121	XP_018838401.1	putative phospholipid-transporting ATPase 9	Juglans regia	XP_018837664.1
TRINITY_DN14557_c1_g1::TRINITY_DN14557_c1_g1_i7::g.38742::m.38742	0	628	XP_018828900.1	uncharacterized protein LOC108997197	Juglans regia	XP_018828901.1
TRINITY_DN12733_c0_g1::TRINITY_DN12733_c0_g1_i1::g.15369::m.15369	3,79E-124	352	XP_018849174.1	ras-related protein RABA1b-like	Juglans regia	XP_018832029.1
TRINITY_DN17602_c0_g2::TRINITY_DN17602_c0_g2_i1::g.86960::m.86960	0	3516	XP_018816643.1	auxin transport protein BIG	Juglans regia	XP_012088111.1
TRINITY_DN13377_c3_g1::TRINITY_DN13377_c3_g1_i6::g.23084::m.23084	1,72E-93	279	XP_018833470.1	BRCA1-A complex subunit BRE isoform X1	Juglans regia	OIV91900.1
TRINITY_DN14518_c1_g2::TRINITY_DN14518_c1_g2_i4::g.38282::m.38282	0	902	XP_018846982.1	probable glycerol-3-phosphate dehydrogenase [NAD(+)] 1, cytosolic	Juglans regia	OAY36176.1
TRINITY_DN18437_c1_g1::TRINITY_DN18437_c1_g1_i4::g.100574::m.100574	0	834	XP_018839607.1	U4/U6 small nuclear ribonucleoprotein PRP4-like protein	Juglans regia	XP_004516365.1
TRINITY_DN15963_c1_g1::TRINITY_DN15963_c1_g1_i1::g.59605::m.59605	0	776	XP_018836810.1	glucose-6-phosphate 1-dehydrogenase, chloroplastic-like isoform X2	Juglans regia	XP_018836809.1
TRINITY_DN17490_c0_g3::TRINITY_DN17490_c0_g3_i5::g.84477::m.84477	5,85E-110	333	XP_018842238.1	pollen receptor-like kinase 1	Juglans regia	KYP67551.1
TRINITY_DN19045_c3_g1::TRINITY_DN19045_c3_g1_i11::g.111100::m.111100	0	597	XP_018837119.1	uncharacterized protein LOC109003448 isoform X2	Juglans regia	XP_018837117.1
TRINITY_DN14763_c1_g1::TRINITY_DN14763_c1_g1_i5::g.41937::m.41937	0	655	XP_018844406.1	paired amphipathic helix protein Sin3-like 4	Juglans regia	XP_018844407.1
TRINITY_DN18733_c1_g5::TRINITY_DN18733_c1_g5_i2::g.105940::m.105940	0	816	XP_018821996.1	transmembrane and coiled-coil domain-containing protein 4	Juglans regia	OAY60830.1
TRINITY_DN11884_c0_g1::TRINITY_DN11884_c0_g1_i2::g.9315::m.9315	1,14E-155	442	XP_018837945.1	ATP-dependent Clp protease proteolytic subunit 4, chloroplastic	Juglans regia	XP_015963188.1
TRINITY_DN14042_c0_g1::TRINITY_DN14042_c0_g1_i1::g.31618::m.31618	0	1109	XP_018818763.1	ATPase family AAA domain-containing protein 3-like	Juglans regia	XP_018817154.1
TRINITY_DN19087_c0_g3::TRINITY_DN19087_c0_g3_i1::g.111646::m.111646	2,07E-61	188	XP_018850000.1	uncharacterized protein LOC109012689	Juglans regia	OAY59575.1
TRINITY_DN15486_c0_g3::TRINITY_DN15486_c0_g3_i1::g.52746::m.52746	4,89E-98	299	ONH92992.1	hypothetical protein PRUPE_8G206600	Prunus persica	XP_007201728.1
TRINITY_DN16817_c0_g1::TRINITY_DN16817_c0_g1_i3::g.73774::m.73774	0	1031	XP_018844424.1	acid beta-fructofuranosidase-like	Juglans regia	XP_018848119.1
TRINITY_DN17918_c1_g1::TRINITY_DN17918_c1_g1_i4::g.91866::m.91866	2,62E-43	149	XP_018840390.1	carbonyl reductase [NADPH 1-like]	Juglans regia	XP_018859664.1

TRINITY_DN19962_c5_g2::TRINITY_DN19962_c5_g2_i2::g.126940::m.126940	3,38E-86	267	XP_018824082.1	pectinesterase-like	Juglans regia	KDP33981.1
TRINITY_DN19385_c2_g1::TRINITY_DN19385_c2_g1_i13::g.117074::m.117074	6,19E-97	286	XP_008386967.1	uncharacterized protein LOC103449426	Malus domestica	XP_008359455.1
TRINITY_DN15599_c0_g1::TRINITY_DN15599_c0_g1_i1::g.54231::m.54231	1,58E-40	135	ONI03937.1	hypothetical protein PRUPE_6G292800	Prunus persica	XP_020420889.1
TRINITY_DN13743_c6_g3::TRINITY_DN13743_c6_g3_i1::g.27816::m.27816	1,34E-180	525	XP_018808771.1	phosphatidylinositol 4-phosphate 5-kinase 2-like	Juglans regia	XP_018842196.1
TRINITY_DN18919_c2_g1::TRINITY_DN18919_c2_g1_i5::g.108804::m.108804	0	1169	XP_018825045.1	uncharacterized protein LOC108994347 isoform X2	Juglans regia	XP_018825044.1
TRINITY_DN15799_c0_g1::TRINITY_DN15799_c0_g1_i6::g.55958::m.55958	1,22E-140	401	XP_018842015.1	endochitinase-like	Juglans regia	XP_018854709.1
TRINITY_DN15302_c0_g1::TRINITY_DN15302_c0_g1_i3::g.49970::m.49970	1,26E-163	470	XP_018817752.1	branched-chain-amino-acid aminotransferase-like protein 1	Juglans regia	XP_020212375.1
TRINITY_DN12400_c4_g2::TRINITY_DN12400_c4_g2_i2::g.12585::m.12585	5,27E-45	146	XP_018815679.1	cytochrome B5-like protein	Juglans regia	XP_018815680.1
TRINITY_DN16837_c1_g1::TRINITY_DN16837_c1_g1_i3::g.74278::m.74278	0	732	XP_018815872.1	probable mitochondrial saccharopine dehydrogenase-like oxidoreductase At5g39410	Juglans regia	ONI19128.1
TRINITY_DN19176_c1_g1::TRINITY_DN19176_c1_g1_i6::g.113373::m.113373						
TRINITY_DN13734_c2_g1::TRINITY_DN13734_c2_g1_i1::g.27715::m.27715	2,57E-167	466	XP_018820201.1	stem-specific protein TSJT1-like	Juglans regia	OAY24788.1
TRINITY_DN15824_c1_g1::TRINITY_DN15824_c1_g1_i3::g.57478::m.57478	0	535	XP_018808979.1	uncharacterized protein LOC108982137 isoform X1	Juglans regia	XP_018808980.1
TRINITY_DN17322_c1_g1::TRINITY_DN17322_c1_g1_i1::g.82026::m.82026	0	867	XP_018851184.1	trafficking protein particle complex subunit 8 isoform X1	Juglans regia	XP_018851185.1
TRINITY_DN18188_c2_g1::TRINITY_DN18188_c2_g1_i6::g.95147::m.95147	0	905	XP_018841822.1	golgin candidate 2-like	Juglans regia	XP_009356049.1
TRINITY_DN16641_c0_g1::TRINITY_DN16641_c0_g1_i7::g.70870::m.70870	0	527	XP_018828226.1	probable anion transporter 5	Juglans regia	XP_018828227.1
TRINITY_DN16217_c1_g2::TRINITY_DN16217_c1_g2_i6::g.64087::m.64087	0	629	XP_018835470.1	autophagy-related protein 18a-like isoform X2	Juglans regia	XP_018835469.1
TRINITY_DN17835_c3_g1::TRINITY_DN17835_c3_g1_i1::g.90619::m.90619	0	768	XP_018823556.1	myosin-2-like	Juglans regia	XP_018851729.1
TRINITY_DN16741_c1_g1::TRINITY_DN16741_c1_g1_i4::g.72580::m.72580	0	1082	XP_018807479.1	pre-mRNA-splicing factor CWC22 homolog	Juglans regia	XP_018830941.1
TRINITY_DN17827_c3_g3::TRINITY_DN17827_c3_g3_i1::g.90327::m.90327	0	541	XP_018839217.1	pollen receptor-like kinase 3	Juglans regia	XP_020414890.1
TRINITY_DN17200_c0_g1::TRINITY_DN17200_c0_g1_i7::g.79915::m.79915	1,18E-56	185	XP_018811115.1	uncharacterized protein LOC108983804 isoform X1	Juglans regia	XP_018811116.1
TRINITY_DN11631_c1_g1::TRINITY_DN11631_c1_g1_i4::g.8173::m.8173	2,95E-163	455	XP_018809788.1	glucose-induced degradation protein 8 homolog	Juglans regia	XP_018809789.1
TRINITY_DN14578_c1_g3::TRINITY_DN14578_c1_g3_i1::g.39119::m.39119	0	765	XP_018816480.1	S-adenosylmethionine synthase 5-like	Juglans regia	KDP23344.1

TRINITY_DN13605_c0_g1::TRINITY_DN13605_c0_g1_i3::g.26153::m.26153	1,84E-164	464	XP_018833855.1	probable sugar phosphate/phosphate translocator At3g17430	Juglans regia	OIV97317.1
TRINITY_DN14404_c3_g3::TRINITY_DN14404_c3_g3_i3::g.36554::m.36554	2,77E-133	395	XP_018854140.1	elongation factor G-2, mitochondrial	Juglans regia	GAU12992.1
TRINITY_DN15478_c1_g1::TRINITY_DN15478_c1_g1_i1::g.52474::m.52474	0	676	XP_018837780.1	3-hydroxyisobutyryl-CoA hydrolase-like protein 5 isoform X1	Juglans regia	XP_018837782.1
TRINITY_DN15217_c1_g2::TRINITY_DN15217_c1_g2_i9::g.48873::m.48873	0	542	XP_018818499.1	probable methyltransferase PMT5 isoform X3	Juglans regia	XP_018818500.1
TRINITY_DN13383_c0_g1::TRINITY_DN13383_c0_g1_i9::g.23043::m.23043	2,12E-115	336	XP_018834855.1	syntaxin-22-like	Juglans regia	KHN46828.1
TRINITY_DN16086_c0_g2::TRINITY_DN16086_c0_g2_i6::g.61825::m.61825	0	717	XP_018836235.1	uncharacterized protein LOC109002795 isoform X1	Juglans regia	XP_018836235.1
TRINITY_DN16643_c2_g1::TRINITY_DN16643_c2_g1_i2::g.70325::m.70325	1,22E-67	216	XP_018840718.1	E3 ubiquitin-protein ligase PRT1	Juglans regia	XP_008348988.1
TRINITY_DN19405_c0_g1::TRINITY_DN19405_c0_g1_i2::g.117368::m.117368	2,14E-119	345	XP_018825743.1	multiple organellar RNA editing factor 3, mitochondrial-like	Juglans regia	XP_018825744.1
TRINITY_DN13456_c0_g1::TRINITY_DN13456_c0_g1_i3::g.24091::m.24091	0	619	XP_018814087.1	WPP domain-interacting protein 2	Juglans regia	XP_018814096.1
TRINITY_DN10846_c0_g1::TRINITY_DN10846_c0_g1_i1::g.5657::m.5657	1,21E-95	279	XP_018847950.1	uncharacterized protein LOC109011278	Juglans regia	ONI14076.1
TRINITY_DN17937_c1_g6::TRINITY_DN17937_c1_g6_i1::g.92221::m.92221	5,56E-70	216	XP_018858704.1	uncharacterized protein LOC109020641 isoform X2	Juglans regia	XP_018858710.1
TRINITY_DN14048_c1_g1::TRINITY_DN14048_c1_g1_i1::g.31820::m.31820	5,99E-98	287	XP_018836255.1	ER membrane protein complex subunit 7 homolog	Juglans regia	XP_012068149.1
TRINITY_DN15198_c1_g3::TRINITY_DN15198_c1_g3_i1::g.48326::m.48326	0	932	XP_018843375.1	WD-40 repeat-containing protein MSI4-like	Juglans regia	XP_008228297.1
TRINITY_DN13739_c0_g1::TRINITY_DN13739_c0_g1_i1::g.27726::m.27726	5,02E-77	244	XP_018820691.1	protein S-acyltransferase 24	Juglans regia	XP_004508416.1
TRINITY_DN18544_c2_g2::TRINITY_DN18544_c2_g2_i1::g.102351::m.102351	0	1192	KDP36832.1	hypothetical protein JCGZ_08123	Jatropha curcas	XP_012073680.1
TRINITY_DN13630_c0_g1::TRINITY_DN13630_c0_g1_i5::g.26390::m.26390	0	1311	XP_018830564.1	uncharacterized protein LOC108998470	Juglans regia	XP_018830564.1
TRINITY_DN14180_c0_g1::TRINITY_DN14180_c0_g1_i2::g.33272::m.33272	0	556	XP_008236505.1	alpha-amylase-like isoform X2	Prunus mume	XP_016650792.1
TRINITY_DN8216_c0_g1::TRINITY_DN8216_c0_g1_i1::g.2536::m.2536	1,58E-83	274	XP_018853114.1	uncharacterized protein LOC109015083, partial	Juglans regia	XP_018816058.1
TRINITY_DN19280_c0_g1::TRINITY_DN19280_c0_g1_i3::g.115040::m.115040	4,57E-44	155	XP_018838909.1	uncharacterized protein LOC109004724 isoform X2	Juglans regia	XP_018838910.1
TRINITY_DN16993_c0_g3::TRINITY_DN16993_c0_g3_i3::g.76730::m.76730	0	768	XP_018812834.1	probable galacturonosyltransferase 4 isoform X1	Juglans regia	XP_018812835.1
TRINITY_DN19939_c3_g1::TRINITY_DN19939_c3_g1_i7::g.125684::m.125684	0	601	KDP30449.1	hypothetical protein JCGZ_17137	Jatropha curcas	XP_012081018.1
TRINITY_DN19123_c0_g2::TRINITY_DN19123_c0_g2_i1::g.112423::m.112423	0	952	XP_018810077.1	ubiquitin fusion degradation protein 1	Juglans regia	XP_008229992.1

TRINITY_DN13782_c0_g3::TRINITY_DN13782_c0_g3_i2::g.28223::m.28223	4,16E-64	197	XP_018822750.1	28 kDa heat- and acid-stable phosphoprotein-like isoform X1	Juglans regia	XP_018850063.1
TRINITY_DN15101_c4_g1::TRINITY_DN15101_c4_g1_i4::g.45560::m.45560	0	752	XP_014501058.1	probable 26S proteasome non-ATPase regulatory subunit 3	Vigna radiata var. radiata	XP_016167375.1
TRINITY_DN18181_c1_g1::TRINITY_DN18181_c1_g1_i3::g.96440::m.96440	2,19E-178	502	XP_018845549.1	uncharacterized protein LOC109009507 isoform X2	Juglans regia	XP_018845548.1
TRINITY_DN13858_c5_g2::TRINITY_DN13858_c5_g2_i2::g.29432::m.29432	6,45E-140	394	XP_018850336.1	trafficking protein particle complex subunit 5	Juglans regia	KDP38658.1
TRINITY_DN19625_c0_g5::TRINITY_DN19625_c0_g5_i9::g.121168::m.121168	4,53E-60	212	XP_018824531.1	altered inheritance of mitochondria protein 44	Juglans regia	XP_018824531.1
TRINITY_DN17351_c1_g1::TRINITY_DN17351_c1_g1_i15::g.82398::m.82398	6,03E-109	318	KDP35530.1	hypothetical protein JCGZ_08968	Jatropha curcas	XP_012074814.1
TRINITY_DN12181_c0_g1::TRINITY_DN12181_c0_g1_i6::g.10945::m.10945	7,92E-99	292	XP_018834236.1	protein phosphatase methylesterase 1	Juglans regia	XP_018834237.1
TRINITY_DN17453_c0_g6::TRINITY_DN17453_c0_g6_i1::g.83881::m.83881	2,39E-47	159	XP_018829738.1	apoptosis-inducing factor homolog B-like	Juglans regia	KYP68674.1
TRINITY_DN17009_c5_g2::TRINITY_DN17009_c5_g2_i5::g.77303::m.77303	4,86E-159	462	XP_018833426.1	cationic amino acid transporter 2, vacuolar-like isoform X2	Juglans regia	XP_018833425.1
TRINITY_DN18349_c3_g1::TRINITY_DN18349_c3_g1_i4::g.99210::m.99210	0	578	XP_018823061.1	DEAD-box ATP-dependent RNA helicase 14 isoform X1	Juglans regia	XP_018823062.1
TRINITY_DN12974_c0_g1::TRINITY_DN12974_c0_g1_i2::g.18074::m.18074	0	830	XP_018812515.1	MAR-binding filament-like protein 1-1 isoform X1	Juglans regia	XP_018812517.1
TRINITY_DN13246_c1_g2::TRINITY_DN13246_c1_g2_i1::g.21483::m.21483	0	575	OAY47012.1	hypothetical protein MANES_06G045500	Manihot esculenta	ONH98475.1
TRINITY_DN19497_c1_g4::TRINITY_DN19497_c1_g4_i2::g.118818::m.118818	0	695	XP_018807250.1	protein DETOXIFICATION 27-like	Juglans regia	XP_018807251.1
TRINITY_DN13446_c0_g3::TRINITY_DN13446_c0_g3_i1::g.23949::m.23949	0	624	XP_018848451.1	2-alkenal reductase (NADP(+)-dependent)-like	Juglans regia	XP_018848449.1
TRINITY_DN14491_c0_g1::TRINITY_DN14491_c0_g1_i2::g.37893::m.37893	1,59E-101	304	ONH90030.1	hypothetical protein PRUPE_8G031200	Prunus persica	XP_020426098.1
TRINITY_DN19064_c0_g1::TRINITY_DN19064_c0_g1_i3::g.111228::m.111228	0	651	XP_018851432.1	ABC transporter G family member 9	Juglans regia	XP_018851432.1
TRINITY_DN17932_c0_g2::TRINITY_DN17932_c0_g2_i2::g.92648::m.92648	2,32E-152	449	XP_018837790.1	kinesin-like protein KIN-4C, partial	Juglans regia	OAY23187.1
TRINITY_DN14711_c0_g4::TRINITY_DN14711_c0_g4_i1::g.40962::m.40962	1,06E-160	462	XP_018842745.1	stomatin-like protein 2, mitochondrial	Juglans regia	XP_018828153.1
TRINITY_DN13915_c0_g2::TRINITY_DN13915_c0_g2_i2::g.30373::m.30373	1,85E-57	189	XP_018849269.1	phosphoacetylglucosamine mutase-like	Juglans regia	XP_018838256.1
TRINITY_DN9332_c0_g1::TRINITY_DN9332_c0_g1_i2::g.3344::m.3344	2,29E-146	417	XP_018851371.1	biotin--protein ligase 2-like	Juglans regia	ONI34885.1
TRINITY_DN10896_c0_g1::TRINITY_DN10896_c0_g1_i3::g.5759::m.5759	1,24E-61	195	XP_018858553.1	beta carbonic anhydrase 5, chloroplastic-like	Juglans regia	KHN26133.1
TRINITY_DN15257_c0_g2::TRINITY_DN15257_c0_g2_i2::g.49306::m.49306	4,12E-111	323	XP_018844469.1	N-alpha-acetyltransferase daf-31	Juglans regia	XP_016171058.1

TRINITY_DN14993_c0_g1::TRINITY_DN14993_c0_g1_i2::g.45400::m.45400	0	552	XP_018844413.1	uncharacterized protein LOC109008682	Juglans regia	ONH97583.1
TRINITY_DN15366_c1_g1::TRINITY_DN15366_c1_g1_i4::g.50810::m.50810	0	864	XP_018851051.1	transmembrane protein 87B	Juglans regia	XP_018851052.1
TRINITY_DN11457_c0_g1::TRINITY_DN11457_c0_g1_i1::g.7427::m.7427	1,55E-145	425	XP_018823191.1	nucleolar protein 56-like	Juglans regia	XP_018811309.1
TRINITY_DN12510_c1_g1::TRINITY_DN12510_c1_g1_i1::g.13448::m.13448	0	1038	XP_018836549.1	uncharacterized protein LOC109003048	Juglans regia	OAY23471.1
TRINITY_DN17907_c0_g1::TRINITY_DN17907_c0_g1_i1::g.91817::m.91817	0	974	XP_018819524.1	protein SAND	Juglans regia	ONI00709.1
TRINITY_DN18531_c0_g1::TRINITY_DN18531_c0_g1_i9::g.102220::m.102220	9,95E-76	225	XP_007160081.1	hypothetical protein PHAVU_002G2910001g, partial	Phaseolus vulgaris	ESW32075.1
TRINITY_DN13896_c0_g4::TRINITY_DN13896_c0_g4_i4::g.30042::m.30042	8,80E-155	446	XP_018826785.1	putative clathrin assembly protein At5g35200	Juglans regia	XP_018826786.1
TRINITY_DN49420_c0_g1::TRINITY_DN49420_c0_g1_i1::g.132815::m.132815	1,86E-97	295	XP_018851398.1	probable nucleoredoxin 1 isoform X2	Juglans regia	XP_018851398.1
TRINITY_DN1207_c0_g1::TRINITY_DN1207_c0_g1_i1::g.307::m.307	0	1017	XP_018838954.1	cytosolic endo-beta-N-acetylglucosaminidase 1 isoform X1	Juglans regia	XP_009345357.1
TRINITY_DN16954_c2_g1::TRINITY_DN16954_c2_g1_i2::g.76223::m.76223	1,08E-113	349	XP_018840969.1	uncharacterized protein LOC109006211	Juglans regia	ONI31989.1
TRINITY_DN18548_c0_g1::TRINITY_DN18548_c0_g1_i9::g.102424::m.102424	3,98E-87	261	XP_018825058.1	elongation factor 1-delta-like	Juglans regia	XP_018825059.1
TRINITY_DN14063_c0_g1::TRINITY_DN14063_c0_g1_i1::g.31854::m.31854	1,26E-163	463	XP_018825639.1	GPN-loop GTPase 1-like	Juglans regia	XP_018825640.1
TRINITY_DN9524_c0_g1::TRINITY_DN9524_c0_g1_i2::g.3575::m.3575	2,27E-168	473	AFK35142.1	unknown	Medicago truncatula	AJE71963.1
TRINITY_DN14887_c2_g3::TRINITY_DN14887_c2_g3_i1::g.43865::m.43865	0	767	XP_018808420.1	uridine-cytidine kinase C-like isoform X1	Juglans regia	XP_018808421.1
TRINITY_DN12560_c1_g1::TRINITY_DN12560_c1_g1_i3::g.13781::m.13781	1,36E-127	367	XP_018808547.1	UBP1-associated protein 2A-like	Juglans regia	XP_018808547.1
TRINITY_DN12634_c0_g1::TRINITY_DN12634_c0_g1_i2::g.14412::m.14412	0	874	XP_018840672.1	uncharacterized protein LOC109005996 isoform X2	Juglans regia	XP_018840671.1
TRINITY_DN16146_c1_g1::TRINITY_DN16146_c1_g1_i2::g.62212::m.62212	0	796	XP_018816881.1	WPP domain-interacting tail-anchored protein 1-like	Juglans regia	XP_018816882.1
TRINITY_DN16454_c2_g1::TRINITY_DN16454_c2_g1_i4::g.67739::m.67739	1,22E-46	182	XP_018822557.1	nuclear pore complex protein NUP98A	Juglans regia	XP_018822557.1
TRINITY_DN5515_c0_g1::TRINITY_DN5515_c0_g1_i1::g.1365::m.1365	0	523	XP_018813360.1	protein arginine N-methyltransferase 1.5-like	Juglans regia	XP_018845692.1
TRINITY_DN37417_c0_g1::TRINITY_DN37417_c0_g1_i1::g.131078::m.131078	6,76E-117	369	XP_018814633.1	putative E3 ubiquitin-protein ligase LIN-1	Juglans regia	XP_018502155.1
TRINITY_DN17093_c0_g1::TRINITY_DN17093_c0_g1_i9::g.78387::m.78387	1,18E-109	337	XP_018845097.1	golgin candidate 3-like isoform X2	Juglans regia	XP_018845095.1
TRINITY_DN16772_c0_g1::TRINITY_DN16772_c0_g1_i3::g.72983::m.72983	0	1217	XP_018814980.1	probable galactinol--sucrose galactosyltransferase 2	Juglans regia	XP_012079949.1

TRINITY_DN15339_c4_g4::TRINITY_DN15339_c4_g4_i1::g.49925::m.49925	8,79E-54	177	XP_018825639.1	GPN-loop GTPase 1-like	Juglans regia	XP_018825640.1
TRINITY_DN15754_c0_g4::TRINITY_DN15754_c0_g4_i1::g.56661::m.56661	2,31E-59	186	XP_018859034.1	protein YLS9-like	Juglans regia	XP_018851215.1
TRINITY_DN16687_c0_g1::TRINITY_DN16687_c0_g1_i2::g.71593::m.71593	0	963	XP_018815434.1	HEAT repeat-containing protein 5B isoform X2	Juglans regia	XP_018815433.1
TRINITY_DN49712_c0_g1::TRINITY_DN49712_c0_g1_i1::g.132845::m.132845	5,74E-70	231	XP_018807961.1	long-chain-alcohol oxidase FAO1	Juglans regia	XP_008224323.1
TRINITY_DN15478_c0_g1::TRINITY_DN15478_c0_g1_i13::g.52472::m.52472	7,72E-69	221	XP_018808171.1	putative E3 ubiquitin-protein ligase XBAT35	Juglans regia	XP_018842288.1
TRINITY_DN13615_c2_g2::TRINITY_DN13615_c2_g2_i1::g.26300::m.26300	6,19E-79	233	OAY55698.1	hypothetical protein MANES_03G173600	Manihot esculenta	OAY55700.1
TRINITY_DN15422_c1_g1::TRINITY_DN15422_c1_g1_i1::g.51639::m.51639	0	554	XP_018850906.1	lanC-like protein GCL2	Juglans regia	ONI17390.1
TRINITY_DN17700_c2_g2::TRINITY_DN17700_c2_g2_i11::g.86871::m.86871	3,15E-58	187	XP_018825560.1	DNA-(apurinic or apyrimidinic site) lyase	Juglans regia	ONI08144.1
TRINITY_DN13585_c0_g1::TRINITY_DN13585_c0_g1_i3::g.25706::m.25706	3,56E-129	369	XP_018830849.1	uncharacterized protein LOC108998659	Juglans regia	OAY30439.1
TRINITY_DN16933_c4_g2::TRINITY_DN16933_c4_g2_i2::g.75851::m.75851	4,77E-94	281	XP_009373531.1	phosphoribosylaminoimidazole-succinocarboxamide synthase, chloroplastic	Pyrus x bretschneideri	XP_018844624.1
TRINITY_DN11228_c0_g1::TRINITY_DN11228_c0_g1_i4::g.6693::m.6693	2,32E-38	129	KOM25909.1	hypothetical protein LR48_Vigan205s004600	Vigna angularis	BAT74108.1
TRINITY_DN17776_c0_g1::TRINITY_DN17776_c0_g1_i2::g.89550::m.89550	2,18E-150	427	XP_018835582.1	probable carboxylesterase 13	Juglans regia	XP_009358071.1
TRINITY_DN17420_c0_g2::TRINITY_DN17420_c0_g2_i5::g.83586::m.83586	6,01E-142	403	CAI83772.1	glyceraldehyde-3-phosphate-dehydrogenase	Lupinus albus	KEH35270.1
TRINITY_DN11534_c0_g1::TRINITY_DN11534_c0_g1_i5::g.7773::m.7773	1,91E-165	463	XP_018850845.1	NADPH-dependent 1-acyldihydroxyacetone phosphate reductase-like	Juglans regia	XP_018846462.1
TRINITY_DN13275_c3_g1::TRINITY_DN13275_c3_g1_i7::g.21820::m.21820	1,45E-147	416	XP_018814423.1	thaumatin-like protein	Juglans regia	ONI24401.1
TRINITY_DN14670_c2_g3::TRINITY_DN14670_c2_g3_i4::g.40396::m.40396	5,87E-72	216	XP_018816108.1	uncharacterized protein At3g03773 isoform X2	Juglans regia	XP_018816104.1
TRINITY_DN15660_c2_g1::TRINITY_DN15660_c2_g1_i4::g.55101::m.55101	9,97E-163	458	XP_018814132.1	rhomboid-like protein 20	Juglans regia	XP_018850955.1
TRINITY_DN15833_c5_g1::TRINITY_DN15833_c5_g1_i8::g.57893::m.57893	1,33E-57	184	XP_008346894.1	probable ADP-ribosylation factor GTPase-activating protein AGD15	Malus domestica	XP_019434874.1
TRINITY_DN18852_c0_g2::TRINITY_DN18852_c0_g2_i1::g.107431::m.107431	0	532	XP_018807382.1	putative G3BP-like protein	Juglans regia	XP_018850163.1
TRINITY_DN18971_c1_g2::TRINITY_DN18971_c1_g2_i2::g.109643::m.109643	3,99E-56	183	XP_018818482.1	ureide permease 1-like	Juglans regia	XP_018818483.1
TRINITY_DN19418_c3_g2::TRINITY_DN19418_c3_g2_i3::g.117519::m.117519	1,43E-80	237	KRH19367.1	hypothetical protein GLYMA_13G113400	Glycine max	XP_006593963.1
TRINITY_DN36814_c0_g1::TRINITY_DN36814_c0_g1_i1::g.130975::m.130975	7,37E-51	176	XP_018819496.1	serine/threonine-protein kinase D6PK-like	Juglans regia	XP_018819503.1
