# Supplementary material for: Comparative proteomics of common allergenic tree pollens of birch, alder, and hazel
Source: Allergy. 2021 Jan 15;76(6):1743–53. doi: 10.1111/all.14694 (PMC8248232; doi:10.1111/all.14694)
Supplement: Supplementary file 9 — Table S7 [file ALL-76-1743-s007.pdf]

Supplementary Table S7: Pfam annotation of identified Alnus pollen proteins

Protein IDs	Peptide counts (all)	Sequence coverage [%]	Mol. weight [kDa]	Score	Pfam accession	Pfam family name	bitscore	e-value [Pfam]	clan
ARUBRA_DN3160_c0_g1_i1_1	7	34,1	24,216	323,31	PF00407.18	Bet_v_1	91,9	3,20E-26	CL0209
ARHOMBI_DN6124_c0_g1_i1_4	21	50,8	53,381	323,31	PF00162.18	PGK	558,1	8,00E-168	No_clan
ARUBRA_DN4553_c0_g1_i1_3	10	45,8	29,745	323,31	PF00160.20	Pro_isomerase	161,8	1,40E-47	CL0475
ARUBRA_DN4458_c0_g1_i1_5	29	58,1	54,26	323,31	PF07992.13	Pyr_redox_2	192,7	7,40E-57	CL0063
ARHOMBI_DN14767_c0_g1_i1_6	1	11,6	9,3606	6,3174					
ARUBRA_DN4969_c0_g1_i11_2	9	34,4	29,337	323,31	PF00076.21	RRM_1	78,6	2,20E-22	CL0221
ARUBRA_DN4696_c0_g1_i2_3	17	53,3	36,513	323,31	PF13417.5	GST_N_3	78,9	2,70E-22	CL0172
ARUBRA_DN4914_c0_g1_i1_2	14	40,3	45,648	323,31	PF01370.20	Epimerase	56,4	2,70E-15	CL0063
ARUBRA_DN3195_c0_g1_i1_6	15	44,9	42,543	323,31	PF05368.12	NmrA	288,1	4,50E-86	CL0063
ARUBRA_DN2052_c0_g1_i1_5	15	43,9	37,581	323,31	PF00141.22	peroxidase	150	7,70E-44	CL0617
ARUBRA_DN17603_c0_g1_i1_4	8	34,6	22,986	323,31	PF00240.22	ubiquitin	115,3	7,10E-34	CL0072
ARUBRA_DN23118_c0_g1_i1_1	8	31,7	26,501	323,31	PF00235.18	Profilin	146,8	3,50E-43	CL0431
ARHOMBI_DN3404_c0_g1_i1_5	11	37,8	42,66	323,31	PF00903.24	Glyoxalase	80,4	1,10E-22	CL0104
ARHOMBI_DN8875_c0_g1_i1_3	17	63,3	39,824	323,31	PF01263.19	Aldose_epim	296,8	1,60E-88	CL0103
ARUBRA_DN10871_c0_g1_i1_1	9	55,8	18,214	323,31	PF00462.23	Glutaredoxin	75,4	2,70E-21	CL0172
ARHOMBI_DN3889_c0_g1_i1_5	16	51,7	45,246	323,31	PF00248.20	Aldo_ket_red	166,9	5,20E-49	No_clan
ARHOMBI_DN5373_c0_g1_i1_4	27	44,5	74,905	323,31	PF01676.17	Metalloenzyme	341,7	1,90E-102	CL0088
ARUBRA_DN4854_c0_g1_i1_3	8	35,1	35,259	227,12	PF00255.18	GSHPx	140	1,80E-41	CL0172
ARUBRA_DN1249_c0_g2_i1_2	6	34,9	20,83	323,31	PF00254.27	FKBP_C	114,7	1,70E-33	CL0487
ARHOMBI_DN5306_c0_g1_i1_6	11	38,5	39,025	323,31	PF00121.17	TIM	291,6	3,80E-87	CL0036
ARUBRA_DN5302_c0_g1_i1_5	20	59,3	43,443	323,31	PF01704.17	UDPGP	443,5	5,40E-133	CL0110
ARHOMBI_DN26299_c0_g1_i1_4	5	55,2	9,6625	190,49					
ARHOMBI_DN5459_c0_g1_i1_2	20	42,7	57,574	323,31	PF00923.18	TAL_FSA	257,3	1,60E-76	CL0036
ARHOMBI_DN5273_c0_g1_i1_3	6	28	26,759	97,01	PF00080.19	Sod_Cu	161,2	1,50E-47	No_clan
ARHOMBI_DN1417_c0_g1_i1_5	3	62,6	9,3579	323,31	PF00313.21	CSD	93,9	3,60E-27	CL0021
ARHOMBI_DN5581_c0_g1_i1_3	10	21,5	33,285	323,31	PF13499.5	EF-hand_7	54,4	1,20E-14	CL0220
ARUBRA_DN2313_c0_g1_i1_3	3	22,8	19,653	323,31					
ARUBRA_DN9810_c0_g1_i1_4	7	34,6	29,588	163,28	PF01738.17	DLH	190,8	2,10E-56	CL0028

ARHOMBI_DN6211_c0_g2_i1_6	7	22,3	32,503	262,42	PF00838.16	TCTP	193,1	3,80E-57	CL0080
ARUBRA_DN22898_c0_g1_i1_5	15	35,6	46,645	323,31	PF00056.22	Ldh_1_N	112,9	1,10E-32	CL0063
ARUBRA_DN2253_c0_g1_i1_1	17	50,4	43,625	323,31	PF02880.15	PGM_PMM_III	102	1,90E-29	No_clan
ARHOMBI_DN4873_c0_g1_i1_4	19	47,6	47,456	323,31	PF16884.4	ADH_N_2	84,7	3,40E-24	CL0296
ARUBRA_DN18206_c0_g1_i1_4	2	23,4	12,962	323,31	PF04043.14	PMEI	41,9	1,10E-10	No_clan
ARUBRA_DN6222_c0_g1_i1_5	13	46,3	29,984	323,31	PF13405.5	EF-hand_6	27,9	1,20E-06	CL0220
ARHOMBI_DN5949_c0_g2_i1_6	23	48,3	58,331	323,31	PF03952.15	Enolase_N	182,4	3,60E-54	CL0227
ARHOMBI_DN4456_c0_g1_i1_5	12	32,3	52,071	323,31	PF00903.24	Glyoxalase	78,8	3,60E-22	CL0104
ARUBRA_DN390_c0_g1_i1_5	8	55,9	19,043	323,31	PF01704.17	UDPGP	158,5	1,80E-46	CL0110
ARUBRA_DN4992_c0_g1_i1_3	12	31,9	41,564	237,26	PF08241.11	Methyltransf_11	40,6	2,80E-10	CL0063
ARHOMBI_DN5841_c0_g1_i1_5	6	21,3	38,228	196,77	PF00071.21	Ras	167,6	1,50E-49	CL0023
ARHOMBI_DN6196_c0_g1_i2_5	13	41,8	43,772	323,31	PF08240.11	ADH_N	81,1	4,40E-23	CL0296
ARHOMBI_DN3043_c0_g1_i1_4	4	22,4	21,321	266,82	PF00254.27	FKBP_C	86,3	1,20E-24	CL0487
ARUBRA_DN4475_c0_g1_i1_2	12	51,3	29,532	323,31	PF08718.10	GLTP	151,9	1,40E-44	No_clan
ARUBRA_DN1141_c0_g1_i1_1	4	24	16,446	323,31	PF11976.7	Rad60-SLD	65,7	2,30E-18	CL0072
ARHOMBI_DN3285_c0_g1_i1_6	11	58,9	28,375	233,05	PF00248.20	Aldo_ket_red	148,2	2,60E-43	No_clan
ARUBRA_DN10174_c0_g1_i1_6	9	43,9	20,368	323,31	PF06110.10	DUF953	129	5,90E-38	CL0172
ARHOMBI_DN7524_c0_g1_i1_6	7	26,3	28,025	127,34	PF00160.20	Pro_isomerase	157,9	2,20E-46	CL0475
ARHOMBI_DN4459_c0_g1_i1_3	3	20,4	23,091	304,7	PF01187.17	MIF	78,4	4,70E-22	CL0082
ARHOMBI_DN6146_c1_g1_i1_4	25	35,6	103,77	323,31	PF08267.11	Meth_synt_1	394,1	4,90E-118	CL0160
ARUBRA_DN4074_c0_g1_i1_4	2	6,2	27,262	106,21	PF00550.24	PP-binding	44,4	1,40E-11	CL0314
ARHOMBI_DN3575_c0_g1_i1_3	11	48,9	29,074	323,31	PF13419.5	HAD_2	101,7	4,40E-29	CL0137
ARHOMBI_DN4328_c0_g1_i1_6	12	44,7	36,339	323,31	PF00106.24	adh_short	90,5	8,80E-26	CL0063
ARUBRA_DN411_c0_g1_i1_5	3	14,1	30,356	147,32					
ARHOMBI_DN3323_c0_g1_i1_4	5	20,5	28,15	109,76	PF00034.20	Cytochrom_C	47,2	3,40E-12	CL0318
ARHOMBI_DN5928_c0_g3_i2_6	2	24,2	13,458	27,654	PF00179.25	UQ_con	139,8	4,20E-41	CL0208
ARUBRA_DN4841_c0_g1_i1_6	13	31,8	49,329	246,1	PF13460.5	NAD_binding_10	116	1,60E-33	CL0063
ARHOMBI_DN6080_c1_g1_i1_1	8	37,6	19,325	323,31	PF16845.4	SQAPI	81	5,60E-23	CL0121
ARUBRA_DN16244_c0_g1_i1_2	13	41	47,717	323,31	PF00294.23	PfkB	262,3	5,10E-78	CL0118
ARUBRA_DN18719_c0_g1_i1_2	6	51	16,126	142,18	PF00121.17	TIM	177,7	2,20E-52	CL0036
ARHOMBI_DN4484_c0_g1_i1_2	3	23,8	26,54	118,88	PF00235.18	Profilin	146,5	4,20E-43	CL0431
ARHOMBI_DN5698_c2_g1_i1_5	4	35,5	12,189	323,31	PF02970.15	TBCA	96,9	6,30E-28	No_clan

ARHOMBI_DN15379_c0_g1_i1_6	8	23,8	44,252	217,5					
ARUBRA_DN10745_c0_g1_i1_4	1	5,9	18,314	80,065	PF00168.29	C2	55,8	4,10E-15	CL0154
ARUBRA_DN17745_c0_g1_i1_5	5	24,5	32,7	323,31	PF06521.10	PAR1	244,9	2,20E-73	No_clan
ARUBRA_DN16936_c0_g1_i1_5	6	22,5	31,256	241,25	PF00179.25	UQ_con	48,4	6,50E-13	CL0208
ARUBRA_DN4620_c0_g1_i1_1	25	34,2	89,51	323,31	PF02518.25	HATPase_c	59,2	4,80E-16	CL0025
ARUBRA_DN4102_c0_g1_i1_3	18	34,9	59,731	309,56	PF00262.17	Calreticulin	202,1	1,10E-59	CL0004
ARHOMBI_DN9782_c0_g1_i1_2	3	59,3	8,5917	93,118					
ARUBRA_DN4847_c0_g2_i2_1	12	34,3	43,301	323,31	PF00248.20	Aldo_ket_red	247,8	1,10E-73	No_clan
ARUBRA_DN5827_c0_g1_i1_2	4	63,6	8,2416	54,372	PF01263.19	Aldose_epim	73,7	1,40E-20	CL0103
ARHOMBI_DN3150_c0_g1_i1_6	16	71,5	24,822	323,31	PF08544.12	GHMP_kinases_C	49,9	3,00E-13	No_clan
ARHOMBI_DN3869_c0_g1_i1_1	7	30,7	28,284	132,27	PF00160.20	Pro_isomerase	169,2	7,50E-50	CL0475
ARHOMBI_DN4569_c0_g1_i1_2	5	25,7	21,434	60,77	PF00887.18	ACBP	97,1	4,70E-28	CL0632
ARHOMBI_DN8000_c0_g1_i1_4	6	53,1	15,239	323,31					
ARHOMBI_DN10026_c0_g1_i1_5	5	31,9	22,804	323,31	PF00462.23	Glutaredoxin	66,7	1,50E-18	CL0172
ARHOMBI_DN3561_c0_g2_i1_2	5	23,8	21,619	323,31	PF00173.27	Cyt-b5	57,5	1,00E-15	No_clan
ARHOMBI_DN18818_c0_g1_i1_2	7	26,6	29,367	126,72	PF01597.18	GCV_H	161,6	6,50E-48	CL0105
ARUBRA_DN19744_c0_g1_i1_2	12	38,4	34,272	323,31					
ARHOMBI_DN5210_c0_g1_i1_5	8	28,5	49,455	122,42	PF00400.31	WD40	14,6	0,044	CL0186
ARHOMBI_DN5071_c0_g1_i1_6	9	24,3	42,408	187,27	PF00141.22	peroxidase	171,5	2,00E-50	CL0617
ARUBRA_DN24356_c0_g1_i1_4	4	37,6	15,641	323,31	PF02136.19	NTF2	124,8	2,40E-36	CL0051
ARHOMBI_DN25271_c0_g1_i1_5	1	11,2	10,999	6,4204					
ARUBRA_DN4454_c0_g1_i1_2	6	24,5	33,738	169,57	PF01095.18	Pectinesterase	373,5	4,40E-112	CL0268
ARHOMBI_DN6208_c0_g1_i2_5	15	27,8	67,483	161,74	PF05221.16	AdoHcyase	476,7	2,20E-143	CL0325
ARHOMBI_DN1745_c0_g1_i1_2	12	27,2	58,334	323,31	PF02847.16	MA3	89,5	1,20E-25	CL0020
ARUBRA_DN21566_c0_g1_i1_2	9	25,2	37,928	323,31	PF00481.20	PP2C	226,4	4,20E-67	CL0238
ARUBRA_DN4108_c0_g1_i1_5	7	21,5	42,883	323,31	PF00719.18	Pyrophosphatase	158,3	1,20E-46	No_clan
ARHOMBI_DN5571_c0_g1_i1_5	10	39,3	36,829	323,31	PF00736.18	EF1_GNE	112	1,10E-32	No_clan
ARUBRA_DN4060_c0_g2_i1_2	7	28,9	28,423	323,31	PF01042.20	Ribonuc_L-PSP	137,3	2,30E-40	CL0534
ARHOMBI_DN6185_c1_g1_i1_5	3	8,9	29,918	49,476	PF13410.5	GST_C_2	38,1	1,10E-09	CL0497
ARHOMBI_DN5260_c0_g1_i1_6	8	27,8	34,435	323,31	PF00071.21	Ras	221,2	4,80E-66	CL0023

ARHOMBI_DN6086_c1_g1_i1_6	13	43,2	48,26	116,6	PF00022.18	Actin	490,5	2,80E-147	CL0108
ARUBRA_DN4124_c0_g1_i1_3	17	41,6	62,39	274,65	PF02874.22	ATP-synt_ab_N	72,4	3,10E-20	CL0275
ARHOMBI_DN6236_c1_g1_i3_6	9	25,1	48,97	323,31	PF00248.20	Aldo_ket_red	264	1,30E-78	No_clan
ARHOMBI_DN5869_c0_g1_i1_6	12	35,4	49,496	323,31	PF00085.19	Thioredoxin	106,1	7,50E-31	CL0172
ARUBRA_DN4641_c0_g1_i1_2	23	36,8	84,837	253,95	PF00012.19	HSP70	868,6	1,70E-261	CL0108
ARUBRA_DN23856_c0_g1_i1_3	4	42,6	16,9	323,31	PF00076.21	RRM_1	83,4	6,60E-24	CL0221
ARHOMBI_DN5685_c0_g2_i1_3	15	41,4	51,652	323,31	PF00274.18	Glycolytic	572,1	2,80E-172	CL0035
ARUBRA_DN3590_c0_g2_i1_3	15	43	41,919	323,31	PF00121.17	TIM	301,2	4,30E-90	CL0036
ARHOMBI_DN3136_c0_g1_i1_1	7	26	33,914	308,89	PF00071.21	Ras	192,2	4,00E-57	CL0023
ARUBRA_DN4556_c0_g1_i2_4	15	32,4	59,027	323,31	PF02798.19	GST_N	59,1	3,90E-16	CL0172
ARHOMBI_DN24422_c0_g1_i1_5	5	35,9	16,794	142,1	PF00121.17	TIM	141,8	2,20E-41	CL0036
ARUBRA_DN990_c0_g1_i1_1	5	28,2	24,546	323,31	PF00173.27	Cyt-b5	91,6	2,30E-26	No_clan
ARUBRA_DN1071_c0_g1_i1_2	8	55,7	21,957	323,31	PF01965.23	DJ-1_PfpI	54,9	8,40E-15	CL0014
ARUBRA_DN2969_c0_g1_i1_3	7	74,7	10,875	323,31	PF00248.20	Aldo_ket_red	52,4	4,00E-14	No_clan
ARHOMBI_DN4165_c0_g1_i1_1	1	4,2	26,385	8,7766	PF02136.19	NTF2	127,2	4,40E-37	CL0051
ARHOMBI_DN5934_c0_g1_i1_3	17	32,3	71,276	323,31	PF00085.19	Thioredoxin	102,7	8,10E-30	CL0172
ARHOMBI_DN5622_c0_g1_i1_3	17	48,1	38,072	323,31					
ARHOMBI_DN4656_c0_g1_i1_6	7	33,6	30,257	185,23	PF08534.9	Redoxin	110,8	4,10E-32	CL0172
ARHOMBI_DN6034_c0_g1_i2_3	25	33,3	93,341	323,31	PF00012.19	HSP70	872,1	1,50E-262	CL0108
ARHOMBI_DN6068_c0_g1_i2_3	21	24,5	106,57	219,42	PF02518.25	HATPase_c	47,4	2,30E-12	CL0025
ARHOMBI_DN1849_c0_g1_i1_3	13	34,8	45,107	323,31	PF00996.17	GDI	682,6	1,60E-205	CL0063
ARUBRA_DN8471_c0_g1_i1_3	1	4,2	17,571	17,945					
ARUBRA_DN2937_c0_g1_i2_1	12	56,7	33,525	148,61	PF00044.23	Gp_dh_N	36,4	4,90E-09	CL0063
ARUBRA_DN6062_c0_g1_i1_1	7	32,2	25,189	71,801	PF01230.22	HIT	100,5	6,10E-29	CL0265
ARHOMBI_DN5785_c0_g1_i1_4	6	19,1	48,462	323,31	PF12819.6	Malectin_like	129,1	1,90E-37	CL0468
ARHOMBI_DN3876_c0_g1_i1_6	12	34,7	50,683	323,31	PF14226.5	DIOX_N	75,6	4,70E-21	CL0029
ARHOMBI_DN6149_c0_g1_i1_6	15	20,5	93,054	323,31	PF00076.21	RRM_1	60,7	8,40E-17	CL0221
ARUBRA_DN6670_c0_g1_i1_3	1	9,8	8,5503	6,1904	PF00295.16	Glyco_hydro_28	52,7	3,00E-14	CL0268

ARHOMBI_DN6225_c0_g2_i1_5	10	18,5	60,986	136,48	PF00153.26	Mito_carr	80,9	4,60E-23	No_clan
ARUBRA_DN4747_c0_g1_i1_6	13	35,7	43,782	323,31	PF08240.11	ADH_N	37	2,30E-09	CL0296
ARHOMBI_DN5137_c0_g1_i1_5	6	25,7	31,083	292,09	PF01625.20	PMSR	198,9	4,70E-59	No_clan
ARUBRA_DN18006_c0_g1_i1_4	11	39,8	43,657	241,93	PF00082.21	Peptidase_S8	86,1	2,40E-24	No_clan
ARHOMBI_DN2797_c0_g1_i1_2	7	32,1	35,33	323,31	PF00903.24	Glyoxalase	75,2	4,70E-21	CL0104
ARHOMBI_DN3646_c0_g1_i2_4	9	36	29,356	171,59	PF00406.21	ADK	163,3	3,80E-48	CL0023
ARHOMBI_DN6264_c0_g1_i4_2	15	38,3	50,984	323,31	PF00724.19	Oxidored_FMN	299,1	4,10E-89	CL0036
ARUBRA_DN1274_c0_g1_i1_4	11	35,7	50,469	323,31	PF00928.20	Adap_comp_sub	112,4	2,20E-32	CL0448
ARHOMBI_DN7316_c0_g1_i1_3	4	27,1	19,629	36,896	PF00141.22	peroxidase	51,2	1,20E-13	CL0617
ARUBRA_DN13722_c0_g1_i1_3	1	14,1	9,0017	6,4528					
ARUBRA_DN4746_c1_g2_i1_3	5	22,8	29,539	323,31	PF07876.11	Dabb	72,1	4,10E-20	CL0032
ARHOMBI_DN11262_c0_g1_i1_4	1	12,8	8,6779	30,902	PF00160.20	Pro_isomerase	37,6	2,40E-09	CL0475
ARHOMBI_DN4307_c0_g1_i1_5	20	30,3	89,321	147,89	PF16363.4	GDP_Man_Dehyd	234,7	1,60E-69	CL0063
ARUBRA_DN1657_c0_g1_i1_5	5	26,3	24,684	212,95	PF00085.19	Thioredoxin	107,2	3,40E-31	CL0172
ARHOMBI_DN16963_c0_g2_i1_3	5	41,7	15,453	123,16	PF01738.17	DLH	58,9	4,70E-16	CL0028
ARHOMBI_DN5659_c0_g1_i1_3	8	41,3	31,359	323,31					
ARHOMBI_DN9830_c0_g1_i1_4	2	16,8	15,697	16,517	PF00160.20	Pro_isomerase	50,8	2,10E-13	CL0475
ARHOMBI_DN7974_c0_g1_i1_2	4	26,1	22,226	37,642	PF10509.8	GalKase_gal_bdg	80	6,10E-23	CL0329
ARHOMBI_DN5417_c0_g1_i1_4	14	34,8	55,136	131,4	PF00108.22	Thiolase_N	263,1	2,30E-78	CL0046
ARHOMBI_DN753_c0_g1_i1_1	11	32,9	52,08	197,63	PF00190.21	Cupin_1	103,3	8,40E-30	CL0029
ARUBRA_DN4963_c0_g1_i2_5	6	15,8	35,303	323,31	PF02798.19	GST_N	61,4	7,10E-17	CL0172
ARHOMBI_DN24139_c0_g1_i1_4	2	28,3	10,315	74,316	PF00141.22	peroxidase	88,2	6,10E-25	CL0617
ARUBRA_DN18820_c0_g1_i1_4	6	32,4	27,309	186,23	PF00179.25	UQ_con	162,7	3,70E-48	CL0208
ARUBRA_DN325_c0_g2_i1_1	8	33,6	33,291	217,59	PF01287.19	eIF-5a	99,5	7,50E-29	CL0021
ARHOMBI_DN13323_c0_g1_i1_2	2	31,9	7,3443	13,022	PF02985.21	HEAT	23,1	5,00E-05	CL0020
ARUBRA_DN1786_c0_g1_i1_1	7	29,3	33,118	169,08	PF12481.7	DUF3700	369,2	6,20E-111	CL0052
ARUBRA_DN2535_c0_g1_i1_5	18	37,6	70,536	185,02	PF08323.10	Glyco_transf_5	244,2	1,50E-72	CL0113
ARHOMBI_DN6004_c0_g1_i1_1	8	26,2	41,877	273,13	PF00071.21	Ras	219,3	1,90E-65	CL0023
ARHOMBI_DN6257_c0_g1_i1_3	26	40,1	90,784	206,15	PF00009.26	GTP_EFTU	98,2	4,00E-28	CL0023
ARHOMBI_DN4827_c0_g1_i1_1	3	15,9	29,581	18,849					
ARHOMBI_DN5844_c0_g1_i1_3	1	6	12,914	16,144	PF01190.16	Pollen_Ole_e_I	34,2	2,40E-08	No_clan
ARHOMBI_DN5686_c0_g1_i1_6	13	33,9	65,702	281,28	PF03721.13	UDPG_MGDP_dh_N	223	2,10E-66	CL0063

ARUBRA_DN3412_c0_g1_i1_6	8	43,9	34,538	164,46	PF08534.9	Redoxin	118,7	1,50E-34	CL0172
ARHOMBI_DN9161_c0_g1_i1_5	2	22,1	11,238	11,144	PF09787.8	Golgin_A5	37,9	1,30E-09	No_clan
ARUBRA_DN21065_c0_g1_i1_6	2	42,6	10,555	302,53	PF00182.18	Glyco_hydro_19	122,1	2,80E-35	CL0037
ARUBRA_DN11174_c0_g1_i1_2	4	25,6	17,879	52,085	PF00076.21	RRM_1	27,3	2,10E-06	CL0221
ARUBRA_DN8598_c0_g1_i1_5	4	26,1	14,959	163,74	PF02878.15	PGM_PMM_I	56	3,10E-15	No_clan
ARHOMBI_DN3049_c0_g1_i1_5	8	31,9	38,862	323,31	PF02338.18	OTU	27,9	2,50E-06	CL0125
ARUBRA_DN16568_c0_g1_i1_3	10	47,7	29,027	291,71	PF01344.24	Kelch_1	39,7	2,40E-10	CL0186
ARUBRA_DN5078_c0_g1_i1_6	15	25	72,584	115,26	PF00118.23	Cpn60_TCP1	324,6	9,20E-97	No_clan
ARHOMBI_DN6227_c0_g1_i3_4	3	11,3	34,055	7,8956	PF02798.19	GST_N	60	2,00E-16	CL0172
ARHOMBI_DN4048_c0_g1_i1_3	15	25,9	72,536	152,95	PF00262.17	Calreticulin	488,1	1,30E-146	CL0004
ARHOMBI_DN294_c0_g2_i1_2	1	7,7	9,9256	11,325					
ARUBRA_DN335_c0_g1_i1_4	6	21,6	34,894	323,31	PF03079.13	ARD	211,7	5,70E-63	CL0029
ARUBRA_DN4552_c0_g2_i1_4	6	41,1	21,745	323,31	PF00025.20	Arf	229,5	1,60E-68	CL0023
ARHOMBI_DN19000_c0_g1_i1_1	2	16,4	15,295	21,334					
ARUBRA_DN4655_c0_g2_i1_6	8	29,7	33,191	151,62	PF00071.21	Ras	208,2	4,90E-62	CL0023
ARHOMBI_DN5421_c0_g1_i1_1	7	25,4	37,208	271,82	PF00736.18	EF1_GNE	111,9	1,10E-32	No_clan
ARUBRA_DN2639_c0_g2_i1_5	10	51,3	33,033	249,42	PF02209.18	VHP	58,7	4,00E-16	No_clan
ARHOMBI_DN6203_c0_g1_i1_4	6	30,4	34,055	297,09	PF00694.18	Aconitase_C	64,1	1,40E-17	CL0364
ARHOMBI_DN5924_c0_g1_i1_3	13	39,7	45,151	56,083	PF01370.20	Epimerase	85,8	2,70E-24	CL0063
ARUBRA_DN4385_c0_g1_i1_6	1	12	20,314	225,37	PF00188.25	CAP	88,4	6,00E-25	CL0659
ARHOMBI_DN16907_c0_g1_i1_2	5	21,9	26,188	241,55	PF01965.23	DJ-1_PfpI	111,8	2,50E-32	CL0014
ARUBRA_DN23412_c0_g1_i1_1	4	33,7	10,435	80,334	PF01965.23	DJ-1_PfpI	81,3	6,40E-23	CL0014
ARHOMBI_DN4949_c0_g1_i1_4	3	23,9	22,578	31,467	PF00173.27	Cyt-b5	54,6	8,40E-15	No_clan
ARHOMBI_DN9199_c0_g1_i1_3	3	34,6	13,761	146,87	PF02879.15	PGM_PMM_II	40,2	3,80E-10	No_clan
ARUBRA_DN20664_c0_g1_i1_6	4	41,9	18,551	103,86	PF00080.19	Sod_Cu	108,1	3,80E-31	No_clan
ARHOMBI_DN9737_c0_g1_i1_1	1	16,7	10,681	-2					
ARHOMBI_DN5509_c0_g1_i1_1	17	39,6	53,99	177,48	PF03214.12	RGP	592,8	1,80E-178	CL0110
ARUBRA_DN4741_c0_g1_i1_4	6	18,6	48,049	231,57	PF02900.17	LigB	190,7	2,40E-56	CL0283
ARUBRA_DN1142_c0_g1_i1_1	7	26	41,879	107,51	PF02265.15	S1-P1_nuclease	266,7	2,20E-79	CL0368
ARUBRA_DN2596_c0_g1_i1_5	7	26,8	27,526	223,64	PF02036.16	SCP2	68,2	6,50E-19	CL0311
ARUBRA_DN3451_c0_g2_i1_2	2	13,4	23,044	11,947	PF04420.13	CHD5	34,7	1,30E-08	No_clan

ARHOMBI_DN5949_c0_g1_i1_6	20	49,4	59,343	323,31	PF03952.15	Enolase_N	179,2	3,40E-53	CL0227
ARUBRA_DN25800_c0_g1_i1_2	6	53,3	13,716	44,569					
ARUBRA_DN2944_c0_g2_i1_1	11	21,7	60,328	323,31	PF00270.28	DEAD	135,5	1,50E-39	CL0023
ARUBRA_DN3626_c0_g1_i1_4	7	32,1	34,056	184,04	PF09280.10	XPC-binding	78,9	1,60E-22	No_clan
ARUBRA_DN1658_c0_g1_i1_3	12	26,8	53,178	187,82	PF16363.4	GDP_Man_Dehyd	179,4	1,10E-52	CL0063
ARUBRA_DN5995_c0_g2_i1_4	7	34,8	20,908	132,05	PF00160.20	Pro_isomerase	164,8	1,70E-48	CL0475
ARUBRA_DN17764_c0_g1_i1_4	6	31,2	21,707	51,148	PF01182.19	Glucosamine_iso	83,8	1,50E-23	CL0246
ARUBRA_DN1399_c0_g1_i1_3	10	33,6	39,107	323,31	PF01263.19	Aldose_epim	236,1	4,90E-70	CL0103
ARHOMBI_DN5222_c0_g2_i1_6	8	19,4	43,422	273,99	PF00638.17	Ran_BP1	139,9	4,30E-41	CL0266
ARUBRA_DN4539_c0_g1_i1_1	4	34,6	23,944	242,69	PF01849.17	NAC	81,1	4,00E-23	No_clan
ARHOMBI_DN6283_c0_g15_i1_6	9	34,1	35,619	74,369	PF00009.26	GTP_EFTU	181,6	1,10E-53	CL0023
ARUBRA_DN14209_c0_g1_i1_5	1	16,7	10,996	-2	PF00083.23	Sugar_tr	27,7	1,10E-06	CL0015
ARHOMBI_DN3140_c0_g1_i1_4	4	35,3	16,423	35,711	PF03671.13	Ufm1	140,4	1,40E-41	CL0072
ARUBRA_DN3913_c0_g2_i2_6	10	24,1	60,615	75,666	PF00464.18	SHMT	625,6	2,40E-188	CL0061
ARHOMBI_DN5648_c0_g1_i1_6	13	29	58,057	210,26	PF06999.11	Suc_Fer-like	147,2	4,90E-43	No_clan
ARHOMBI_DN9622_c0_g1_i1_2	8	42,6	27,392	141,37	PF00171.21	Aldedh	324	1,10E-96	CL0099
ARUBRA_DN4716_c0_g2_i1_1	9	24,7	48,85	74,724	PF08240.11	ADH_N	96,5	7,60E-28	CL0296
ARHOMBI_DN5959_c0_g1_i1_1	15	35,5	61,478	323,31	PF00180.19	Iso_dh	304,4	9,70E-91	CL0270
ARUBRA_DN7093_c0_g1_i1_1	7	48,5	18,599	49,574	PF04969.15	CS	40,3	4,70E-10	CL0190
ARHOMBI_DN5317_c0_g1_i1_1	11	32,8	57,594	323,31	PF00056.22	Ldh_1_N	162,1	7,60E-48	CL0063
ARHOMBI_DN17873_c0_g1_i1_6	3	22,3	19,749	323,31					
ARHOMBI_DN15725_c0_g1_i1_6	11	35,8	47,2	323,31	PF16499.4	Melibiase_2	205,9	6,50E-61	CL0058
ARUBRA_DN3134_c0_g2_i1_6	5	33,9	26,564	160,83	PF02431.14	Chalcone	99,1	2,00E-28	CL0560
ARUBRA_DN8237_c0_g1_i1_4	6	22	35,516	163,24	PF05008.14	V-SNARE	88,5	2,50E-25	CL0147
ARHOMBI_DN5100_c0_g1_i1_3	13	21,7	88,616	323,31	PF00076.21	RRM_1	68,5	3,00E-19	CL0221
ARUBRA_DN4014_c0_g3_i1_3	7	56,2	16,825	127,38	PF00248.20	Aldo_ket_red	77,9	6,60E-22	No_clan
ARUBRA_DN2976_c1_g1_i1_5	12	33,6	45,103	323,31	PF00244.19	14.03.2003	345,6	9,40E-104	No_clan
ARHOMBI_DN5768_c0_g1_i1_6	7	39,6	22,006	323,31					
ARUBRA_DN6355_c0_g1_i1_3	9	33,2	32,271	91,577	PF00085.19	Thioredoxin	74,2	6,10E-21	CL0172
ARHOMBI_DN2325_c0_g2_i1_5	5	15,7	45,173	43,946	PF03152.13	UFD1	269,2	9,90E-81	CL0402
ARHOMBI_DN9876_c0_g1_i1_3	6	33,2	24,938	53,067	PF00076.21	RRM_1	57,1	1,10E-15	CL0221

ARUBRA_DN1667_c0_g1_i1_4	5	24	20,714	93,215	PF00085.19	Thioredoxin	113	5,10E-33	CL0172
ARHOMBI_DN2829_c0_g1_i1_5	4	20,4	22,813	206,38	PF06108.11	DUF952	82,5	1,50E-23	CL0084
ARUBRA_DN2825_c0_g1_i1_4	1	6,1	18,087	7,4482					
ARUBRA_DN1403_c0_g1_i1_4	16	33,7	69,954	111,74	PF02874.22	ATP-synt_ab_N	51,1	1,30E-13	CL0275
ARHOMBI_DN5577_c0_g1_i1_6	10	23,1	64,748	71,631	PF09334.10	tRNA-synt_1g	212,8	6,20E-63	CL0039
ARHOMBI_DN20240_c0_g1_i1_6	4	44,3	12,491	37,57	PF13415.5	Kelch_3	42,7	4,20E-11	CL0186
ARUBRA_DN5882_c0_g1_i1_4	5	15,7	50,666	323,31	PF00112.22	Peptidase_C1	259,8	2,50E-77	CL0125
ARUBRA_DN4352_c0_g3_i1_5	3	22,3	20,41	323,31	PF06825.11	HSBP1	78	3,40E-22	No_clan
ARHOMBI_DN5282_c0_g1_i1_5	11	26,5	54,06	100,38	PF00012.19	HSP70	469,6	1,10E-140	CL0108
ARHOMBI_DN5162_c0_g1_i1_6	3	16,9	34,585	215,33	PF00834.18	Ribul_P_3_epim	234,9	4,70E-70	CL0036
ARHOMBI_DN1661_c0_g1_i1_5	13	35,9	48,211	323,31	PF00244.19	14.03.2003	350,5	3,00E-105	No_clan
ARUBRA_DN4331_c0_g1_i1_1	7	32	29,531	196,28	PF13499.5	EF-hand_7	47,7	1,50E-12	CL0220
ARHOMBI_DN5855_c0_g1_i1_3	15	35,9	61,828	114,51	PF00343.19	Phosphorylase	561,3	2,90E-168	CL0113
ARUBRA_DN19380_c0_g2_i1_4	5	23,7	30,928	104,89	PF03358.14	FMN_red	46,8	2,30E-12	CL0042
ARUBRA_DN337_c0_g2_i1_5	8	23,8	41,853	323,31	PF01765.18	RRF	209,2	3,10E-62	No_clan
ARUBRA_DN4321_c0_g2_i1_1	22	35,5	90,017	142,93	PF02933.16	CDC48_2	44,1	1,20E-11	CL0402
ARUBRA_DN1238_c0_g1_i1_5	16	40,9	54,037	112,45	PF16886.4	ATP-synt_ab_Xtn	48,2	8,10E-13	No_clan
ARHOMBI_DN4982_c0_g1_i1_1	9	18,7	62,635	44,6	PF00996.17	GDI	772,1	1,10E-232	CL0063
ARHOMBI_DN4944_c0_g1_i1_3	7	22,2	42,828	54,603	PF00160.20	Pro_isomerase	163,1	5,40E-48	CL0475
ARUBRA_DN1865_c0_g1_i1_1	11	25,7	58,56	323,31	PF00155.20	Aminotran_1_2	323,4	1,80E-96	CL0061
ARUBRA_DN7363_c0_g1_i1_1	8	27,3	36,422	56,788	PF00459.24	Inositol_P	248	1,10E-73	CL0171
ARHOMBI_DN6048_c0_g1_i2_2	9	31,3	40,196	294,77	PF00484.18	Pro_CA	139,5	9,80E-41	No_clan
ARHOMBI_DN5714_c0_g2_i1_1	10	26	51,265	145,4	PF00266.18	Aminotran_5	176,9	5,50E-52	CL0061
ARUBRA_DN11957_c0_g1_i1_4	3	35,5	11,464	49,646	PF04043.14	PMEI	45,6	8,10E-12	No_clan
ARUBRA_DN26276_c0_g1_i1_1	1	13,3	9,549	6,5559	PF13839.5	PC-Esterase	59,7	3,40E-16	CL0264
ARUBRA_DN5081_c0_g1_i1_6	7	41,4	21,926	70,115	PF00996.17	GDI	324,7	6,30E-97	CL0063
ARUBRA_DN11233_c0_g1_i1_2	2	20,8	12,218	11,209					
ARHOMBI_DN5854_c0_g1_i1_3	6	11,5	67,358	323,31	PF00150.17	Cellulase	81,7	5,40E-23	CL0058
ARHOMBI_DN6063_c0_g1_i1_4	3	11	29,128	291,18	PF00085.19	Thioredoxin	108,1	1,70E-31	CL0172

ARHOMBI_DN2876_c0_g2_i1_3	10	39,8	39,138	147,86	PF00141.22	peroxidase	130,3	8,10E-38	CL0617
ARUBRA_DN22470_c0_g1_i1_3	1	18,3	8,4963	6,51					
ARHOMBI_DN3221_c0_g1_i1_3	11	39,5	40,599	323,31	PF00293.27	NUDIX	79,7	1,80E-22	CL0261
ARUBRA_DN4811_c0_g2_i1_3	2	7,9	36,926	11,685					
ARUBRA_DN1006_c1_g1_i1_5	4	16,7	22,186	323,31	PF13499.5	EF-hand_7	34,5	2,00E-08	CL0220
ARHOMBI_DN15853_c0_g1_i1_3	10	39,7	36,649	217,38	PF16113.4	ECH_2	213,5	5,00E-63	CL0127
ARHOMBI_DN15373_c0_g1_i1_5	14	27	65,562	97,266	PF00883.20	Peptidase_M17	384,1	4,40E-115	CL0035
ARUBRA_DN10605_c0_g1_i1_2	6	39,5	22,273	115,4	PF11543.7	UN_NPL4	26,6	5,80E-06	CL0072
ARUBRA_DN10035_c0_g1_i1_2	1	10,1	18,225	6,4405					
ARUBRA_DN9263_c0_g1_i1_6	1	12	8,9073	40,985	PF02298.16	Cu_bind_like	47,2	1,60E-12	CL0026
ARUBRA_DN10200_c0_g1_i1_2	7	18,1	45,475	323,31	PF00561.19	Abhydrolase_1	74,6	8,80E-21	CL0028
ARUBRA_DN7621_c0_g1_i1_2	5	23,4	31,922	250,02	PF13181.5	TPR_8	16	0,0098	CL0020
ARUBRA_DN1568_c0_g2_i1_4	2	13,8	23,447	248,27	PF00111.26	Fer2	65,6	2,70E-18	CL0486
ARUBRA_DN3840_c0_g1_i1_2	11	17	72,455	180,39	PF00254.27	FKBP_C	118,2	1,40E-34	CL0487
ARUBRA_DN4409_c0_g1_i1_6	12	42,3	46,837	75,813	PF00091.24	Tubulin	234,4	1,20E-69	CL0566
ARUBRA_DN4436_c0_g1_i1_5	1	11,5	14,076	90,134	PF00141.22	peroxidase	116,4	1,40E-33	CL0617
ARHOMBI_DN5595_c0_g1_i1_6	8	37,9	31,323	142,27	PF00248.20	Aldo_ket_red	190,2	3,90E-56	No_clan
ARUBRA_DN4227_c1_g2_i1_2	4	19,4	21,124	323,31	PF07876.11	Dabb	102,2	1,70E-29	CL0032
ARHOMBI_DN4937_c0_g3_i1_5	5	23,6	22,529	43,487	PF05922.15	Inhibitor_I9	45,1	1,10E-11	CL0570
ARUBRA_DN143_c0_g2_i1_4	14	41,8	52,305	95,101	PF00343.19	Phosphorylase	434,2	7,80E-130	CL0113
ARHOMBI_DN21168_c0_g1_i1_5	4	22,2	21,038	184,01	PF02209.18	VHP	59,9	1,70E-16	No_clan
ARHOMBI_DN4920_c0_g1_i1_6	13	25,8	77,867	87,383	PF02219.16	MTHFR	407,1	3,60E-122	CL0086
ARUBRA_DN26231_c0_g1_i1_1	1	19,4	7,3913	6,4454	PF00198.22	2-oxoacid_dh	75,4	4,10E-21	CL0149
ARUBRA_DN304_c0_g1_i1_5	1	2,9	41,836	6,8371					
ARUBRA_DN3080_c0_g1_i1_1	11	13	114,27	77,666	PF00456.20	Transketolase_N	499,9	2,60E-150	CL0254
ARUBRA_DN10173_c0_g1_i1_6	10	27,2	50,74	59,815	PF00056.22	Ldh_1_N	166,2	4,10E-49	CL0063
ARHOMBI_DN1063_c0_g2_i1_1	7	15,8	75,898	118,53	PF00614.21	PLDc	41,1	1,10E-10	CL0479
ARHOMBI_DN4718_c0_g1_i1_2	9	25,5	53,981	275,91	PF09229.10	Aha1_N	101,3	4,60E-29	CL0648
ARUBRA_DN4883_c0_g1_i1_3	7	24,9	38,877	47,182	PF01015.17	Ribosomal_S3Ae	324	3,10E-97	No_clan

ARUBRA_DN4330_c0_g2_i1_4	2	15,7	17,128	12,755	PF14705.5	Costars	66,5	1,60E-18	No_clan
ARHOMBI_DN5519_c0_g1_i2_6	7	61,7	16,704	33,018	PF00240.22	ubiquitin	93,6	4,00E-27	CL0072
ARHOMBI_DN5140_c0_g1_i2_1	4	28,9	19,623	58,584	PF00334.18	NDK	71,6	5,70E-20	No_clan
ARHOMBI_DN6061_c0_g1_i1_5	4	18	28,422	176,83	PF00334.18	NDK	182,3	3,70E-54	No_clan
ARHOMBI_DN1760_c0_g1_i1_4	8	29,9	34,798	85,484					
ARUBRA_DN16482_c0_g1_i1_1	3	33,3	15,377	218,32	PF02878.15	PGM_PMM_I	53,4	2,00E-14	No_clan
ARUBRA_DN4585_c0_g2_i2_2	6	23,4	51,862	218,08	PF00076.21	RRM_1	46,7	1,90E-12	CL0221
ARUBRA_DN4349_c0_g1_i1_5	9	20,4	69,585	103,39	PF00365.19	PFK	124,9	3,40E-36	CL0240
ARHOMBI_DN5054_c0_g1_i1_1	6	19,5	51,288	49,988	PF08752.9	COP-gamma_platf	182,7	3,90E-54	CL0159
ARUBRA_DN18216_c0_g1_i1_2	5	47,2	17,268	92,439	PF00459.24	Inositol_P	63,6	1,90E-17	CL0171
ARHOMBI_DN3394_c0_g1_i1_1	9	31,3	47,982	289,03	PF00753.26	Lactamase_B	40,3	3,10E-10	CL0381
ARHOMBI_DN20977_c0_g1_i1_5	2	37,5	10,674	13,377	PF02115.16	Rho_GDI	149,4	9,30E-44	No_clan
ARHOMBI_DN6641_c0_g2_i1_5	6	19,3	41,047	323,31	PF00248.20	Aldo_ket_red	161,4	2,40E-47	No_clan
ARHOMBI_DN5627_c0_g2_i1_2	4	45,5	11,889	323,31	PF06825.11	HSBP1	83	9,50E-24	No_clan
ARHOMBI_DN2280_c0_g1_i1_6	9	51,2	30,328	81,751	PF00290.19	Trp_syntA	316,4	8,10E-95	CL0036
ARHOMBI_DN5380_c0_g1_i1_2	7	25,4	44,352	72,412	PF02737.17	3HCDH_N	118,6	2,60E-34	CL0063
ARUBRA_DN6789_c0_g1_i1_6	1	8,2	21,051	6,6407					
ARUBRA_DN17519_c0_g1_i1_3	5	43,3	13,826	45,489					
ARHOMBI_DN940_c0_g1_i1_3	6	69,8	10,72	58,642	PF13848.5	Thioredoxin_6	33,5	3,50E-08	CL0172
ARHOMBI_DN2154_c0_g1_i1_4	5	33,8	24,001	71,435	PF00173.27	Cyt-b5	92,2	1,50E-26	No_clan
ARHOMBI_DN16490_c0_g1_i1_2	7	23,3	35,056	46,644	PF01842.24	ACT	34,5	1,20E-08	CL0070
ARHOMBI_DN6177_c0_g2_i1_3	2	9,7	19,045	6,4235					
ARHOMBI_DN24802_c0_g1_i1_2	4	50	9,1395	323,31	PF16113.4	ECH_2	72,4	4,30E-20	CL0127
ARUBRA_DN18898_c0_g1_i1_2	8	29	22,525	84,691	PF00240.22	ubiquitin	100,8	2,40E-29	CL0072
ARUBRA_DN10159_c0_g1_i1_5	9	22,3	49,863	60,401	PF00675.19	Peptidase_M16	186,8	2,00E-55	CL0094
ARUBRA_DN147_c0_g1_i1_6	5	48,2	15,384	69,435	PF07944.11	Glyco_hydro_127	106,2	1,40E-30	CL0059
ARHOMBI_DN5600_c0_g1_i1_6	14	25,5	74,607	323,31	PF07719.16	TPR_2	24,8	1,30E-05	CL0020
ARUBRA_DN18800_c0_g2_i1_4	6	23,6	37,705	177,89	PF07859.12	Abhydrolase_3	166,1	8,60E-49	CL0028
ARHOMBI_DN4017_c0_g1_i1_1	8	38,4	28,962	323,31					
ARUBRA_DN4582_c0_g1_i1_5	5	21,1	32,985	33,27	PF00561.19	Abhydrolase_1	70	2,30E-19	CL0028
ARUBRA_DN1643_c0_g2_i1_1	4	27,6	18,01	42,385	PF00481.20	PP2C	21,5	0,00014	CL0238
ARUBRA_DN5911_c0_g1_i1_4	4	19,6	28,269	128,07	PF00293.27	NUDIX	94,5	4,60E-27	CL0261
ARUBRA_DN3180_c0_g1_i1_4	6	22,6	40,695	125,44	PF01625.20	PMSR	204,5	8,90E-61	No_clan

ARHOMBI_DN12129_c0_g1_i1_2	6	27,5	26,608	68,428	PF00462.23	Glutaredoxin	57,3	1,30E-15	CL0172
ARHOMBI_DN4888_c0_g1_i1_6	5	26,3	24,539	110,29	PF16363.4	GDP_Man_Dehyd	30	3,10E-07	CL0063
ARUBRA_DN6309_c0_g1_i1_5	8	27,2	42,983	93,181	PF00318.19	Ribosomal_S2	49,2	3,40E-13	CL0067
ARUBRA_DN3885_c0_g1_i1_5	1	4	19,214	20,679					
ARHOMBI_DN667_c0_g2_i1_1	2	16,7	10,023	12,631	PF13561.5	adh_short_C2	110,5	8,30E-32	CL0063
ARUBRA_DN18848_c0_g1_i1_3	2	6,8	25,651	6,3297					
ARUBRA_DN4554_c0_g1_i1_4	5	17	38,143	33,901	PF13417.5	GST_N_3	45,3	7,80E-12	CL0172
ARUBRA_DN9538_c0_g1_i1_1	6	56,2	16,13	137,59	PF12146.7	Hydrolase_4	37,5	1,40E-09	CL0028
ARHOMBI_DN4282_c0_g1_i1_5	7	30,7	37,231	70,683	PF03214.12	RGP	483,1	4,30E-145	CL0110
ARHOMBI_DN22144_c0_g1_i1_6	3	26,6	15,26	323,31	PF00079.19	Serpin	121,1	6,10E-35	No_clan
ARUBRA_DN3174_c0_g1_i1_1	4	11,4	39,664	26,401	PF00213.17	OSCP	145	2,20E-42	CL0255
ARHOMBI_DN19334_c0_g1_i1_6	1	14,8	8,3641	24,327					
ARHOMBI_DN8571_c0_g1_i1_5	7	41,3	26,296	153,33	PF05694.10	SBP56	314	1,50E-93	CL0186
ARUBRA_DN4684_c0_g2_i1_5	12	35,3	53,532	54,291	PF00044.23	Gp_dh_N	111,2	2,30E-32	CL0063
ARHOMBI_DN1355_c0_g2_i1_2	6	21,3	35,266	74,946	PF00071.21	Ras	189,6	2,60E-56	CL0023
ARUBRA_DN15624_c0_g1_i1_4	2	22	12,846	173,96	PF06404.11	PSK	54,1	2,30E-14	No_clan
ARUBRA_DN2409_c0_g2_i1_4	6	22,3	39,086	51,57	PF00210.23	Ferritin	100,8	5,40E-29	CL0044
ARHOMBI_DN5337_c0_g1_i1_4	5	22,2	26,7	59,563	PF00281.18	Ribosomal_L5	70	1,50E-19	CL0652
ARHOMBI_DN6227_c0_g1_i4_5	6	18,8	38,849	47,761	PF02798.19	GST_N	59,7	2,50E-16	CL0172
ARHOMBI_DN4176_c0_g1_i1_6	5	12,2	51,954	132,94	PF13181.5	TPR_8	11,8	0,2	CL0020
ARUBRA_DN22728_c0_g1_i1_2	11	31,2	45,585	123,36	PF00294.23	PfkB	254,5	1,30E-75	CL0118
ARUBRA_DN18837_c0_g1_i1_3	3	29,4	18,087	197,16	PF00076.21	RRM_1	56,8	1,40E-15	CL0221
ARHOMBI_DN6122_c0_g1_i1_5	7	23,5	51,775	46,042	PF00573.21	Ribosomal_L4	133,6	5,90E-39	No_clan
ARHOMBI_DN6266_c0_g2_i3_6	6	17,8	41,661	323,31	PF00248.20	Aldo_ket_red	175,1	1,60E-51	No_clan
ARUBRA_DN4148_c0_g1_i1_5	3	11,7	33,757	46,35	PF13774.5	Longin	79,1	1,80E-22	No_clan
ARHOMBI_DN15512_c0_g1_i1_6	3	15,3	28,571	28,859	PF00332.17	Glyco_hydro_17	93	2,10E-26	CL0058
ARHOMBI_DN6104_c0_g1_i1_6	1	4,5	30,182	6,6843	PF00190.21	Cupin_1	137,9	1,90E-40	CL0029
ARUBRA_DN4076_c0_g1_i1_5	2	6,4	53,214	14,208	PF04526.12	DUF568	102,2	2,00E-29	No_clan
ARHOMBI_DN6065_c0_g3_i2_6	5	34,8	19,384	98,737	PF08241.11	Methyltransf_11	34,5	2,20E-08	CL0063
ARUBRA_DN4576_c0_g1_i1_2	13	42,9	31,225	137,93					
ARHOMBI_DN3224_c0_g1_i1_2	5	31,2	31,322	36,423	PF11566.7	PI31_Prot_N	43	3,70E-11	No_clan
ARHOMBI_DN6597_c0_g1_i1_6	7	27,5	32,998	122,53	PF00179.25	UQ_con	145,7	6,20E-43	CL0208

ARUBRA_DN16167_c0_g1_i1_3	3	15,5	20,86	166,12	PF00481.20	PP2C	183,3	5,80E-54	CL0238
ARHOMBI_DN4850_c0_g1_i1_5	5	22,2	27,758	66,533	PF00411.18	Ribosomal_S11	157,9	9,20E-47	CL0267
ARHOMBI_DN641_c0_g1_i1_6	9	51,9	17,589	56,207	PF00012.19	HSP70	251,8	9,80E-75	CL0108
ARUBRA_DN9455_c0_g1_i1_2	7	32	34,13	118,72	PF01048.19	PNP_UDP_1	79,7	1,70E-22	CL0408
ARUBRA_DN4936_c0_g1_i1_3	7	32,3	30,07	323,31	PF03931.14	Skp1_POZ	106,4	5,60E-31	CL0033
ARUBRA_DN11512_c0_g1_i1_6	4	36,4	16,811	75,816	PF00702.25	Hydrolase	54,4	1,90E-14	CL0137
ARHOMBI_DN13390_c0_g1_i1_2	2	28,2	12,006	13,639	PF00076.21	RRM_1	65,8	2,10E-18	CL0221
ARUBRA_DN13261_c0_g1_i1_6	3	38,8	11,133	142,82	PF00085.19	Thioredoxin	64,1	8,50E-18	CL0172
ARUBRA_DN7781_c0_g1_i1_6	2	20,3	12,996	12,003	PF02817.16	E3_binding	60,3	1,50E-16	No_clan
ARHOMBI_DN15181_c0_g1_i1_3	4	29,2	18,267	22,107					
ARUBRA_DN4108_c0_g1_i1_4	1	3,6	43,563	6,7805					
ARHOMBI_DN2905_c0_g1_i1_2	7	33,8	32,66	72,144	PF13417.5	GST_N_3	65,7	3,40E-18	CL0172
ARUBRA_DN4026_c0_g1_i1_5	9	16,6	81,321	100,11	PF01433.19	Peptidase_M1	311,7	2,50E-93	CL0126
ARHOMBI_DN6235_c0_g1_i4_4	8	19,2	60,587	51,123	PF00199.18	Catalase	577,7	1,00E-173	No_clan
ARUBRA_DN25569_c0_g1_i1_3	4	58,1	11,286	200,77	PF01557.17	FAA_hydrolase	111,8	3,40E-32	CL0377
ARUBRA_DN4711_c0_g1_i2_5	7	29,9	32,427	50,986	PF00022.18	Actin	265	8,10E-79	CL0108
ARUBRA_DN2993_c0_g1_i1_2	6	22,7	31,111	85,969	PF04450.11	BSP	243,9	1,20E-72	CL0126
ARUBRA_DN3089_c0_g2_i1_6	6	19,9	42,009	43,486	PF01459.21	Porin_3	253,7	2,00E-75	CL0193
ARUBRA_DN8636_c0_g1_i1_1	2	22,7	13,141	18,188					
ARHOMBI_DN4932_c0_g1_i1_1	7	22,6	47,135	51,272	PF00231.18	ATP-synt	246,4	4,10E-73	No_clan
ARUBRA_DN5237_c0_g1_i1_6	6	17,1	36,187	37,186	PF07650.16	KH_2	47,9	8,10E-13	CL0007
ARUBRA_DN4540_c0_g1_i2_6	7	46	23,584	50,864	PF00118.23	Cpn60_TCP1	176,1	9,90E-52	No_clan
ARUBRA_DN830_c0_g1_i1_6	6	43,5	16,521	83,269	PF00288.25	GHMP_kinases_N	56,2	2,80E-15	CL0329
ARHOMBI_DN5602_c0_g2_i1_1	11	16,1	112,09	75,624	PF00690.25	Cation_ATPase_N	41,3	8,50E-11	No_clan
ARHOMBI_DN6202_c0_g1_i2_4	5	9,8	66,705	221,08	PF00450.21	Peptidase_S10	444,9	3,60E-133	CL0028
ARUBRA_DN2984_c0_g1_i2_6	1	6,4	23,59	6,1984					
ARUBRA_DN6258_c0_g1_i1_5	11	29,6	54,714	164,62	PF00565.16	SNase	54,4	1,40E-14	CL0049
ARUBRA_DN1424_c0_g2_i1_3	4	9	69,888	31,748	PF03030.15	H_PPase	717,2	2,00E-215	No_clan
ARHOMBI_DN6163_c0_g1_i2_6	6	19,3	37,649	37,642	PF08079.11	Ribosomal_L30_N	87,5	4,70E-25	No_clan
ARHOMBI_DN2855_c0_g1_i1_2	7	23,9	36,764	46,575	PF01201.21	Ribosomal_S8e	196,9	1,30E-58	No_clan

ARHOMBI_DN4907_c0_g3_i3_4	8	33,9	32,396	11,504	PF01287.19	eIF-5a	99,6	7,40E-29	CL0021
ARUBRA_DN20327_c0_g1_i1_5	8	18,6	52,962	206,54	PF02879.15	PGM_PMM_II	44,5	1,70E-11	No_clan
ARHOMBI_DN3004_c0_g1_i1_6	8	37,4	38,878	58,043	PF00171.21	Aldedh	286,2	3,40E-85	CL0099
ARUBRA_DN20570_c0_g1_i1_4	6	23,5	37,902	127,11	PF07933.13	DUF1681	177,4	1,30E-52	CL0266
ARHOMBI_DN4754_c0_g1_i1_5	10	25,9	68,541	70,265	PF00483.22	NTP_transferase	225,9	5,20E-67	CL0110
ARUBRA_DN2276_c0_g1_i1_5	3	20	16,67	41,065	PF00708.17	Acylphosphatase	76,1	2,00E-21	CL0622
ARHOMBI_DN6055_c0_g1_i1_6	3	20,7	20,974	23,251	PF03501.14	S10_plectin	146,2	2,20E-43	No_clan
ARHOMBI_DN1472_c0_g1_i1_6	10	38	51,248	323,31	PF01412.17	ArfGap	119,5	6,90E-35	No_clan
ARUBRA_DN2452_c0_g2_i1_2	4	13,4	36,861	28,006	PF01092.18	Ribosomal_S6e	191,5	4,00E-57	No_clan
ARUBRA_DN4724_c0_g1_i1_5	8	42	26,165	156,82	PF01287.19	eIF-5a	99,7	6,90E-29	CL0021
ARHOMBI_DN7816_c0_g1_i1_3	4	13,4	29,403	323,31	PF01370.20	Epimerase	47,2	1,70E-12	CL0063
ARHOMBI_DN4989_c0_g1_i1_1	8	25,7	47,294	49,806	PF00153.26	Mito_carr	56	2,50E-15	No_clan
ARUBRA_DN8722_c0_g1_i1_2	2	18	11,658	38,286					
ARUBRA_DN19739_c0_g2_i1_3	6	23,8	39,11	59,136	PF07859.12	Abhydrolase_3	178,4	1,50E-52	CL0028
ARUBRA_DN5052_c0_g1_i2_2	6	37,8	19,07	48,268	PF01370.20	Epimerase	62,9	2,70E-17	CL0063
ARHOMBI_DN19084_c0_g1_i1_5	5	27,2	29,094	292,85	PF00240.22	ubiquitin	68,4	3,00E-19	CL0072
ARHOMBI_DN4789_c0_g1_i1_3	7	15,9	63,126	53,628	PF07992.13	Pyr_redox_2	246,4	3,20E-73	CL0063
ARUBRA_DN14251_c0_g1_i1_2	3	45,6	12,461	57,162	PF01182.19	Glucosamine_iso	126,8	1,00E-36	CL0246
ARUBRA_DN1618_c0_g1_i1_5	1	5,6	24,039	279,64					
ARHOMBI_DN277_c0_g1_i1_2	4	32	16,384	29,331	PF05907.12	DUF866	84,5	6,60E-24	No_clan
ARUBRA_DN4780_c0_g3_i1_6	8	27,2	57,527	66,603	PF00091.24	Tubulin	225,1	8,40E-67	CL0566
ARHOMBI_DN4043_c0_g1_i1_2	6	40,3	13,665	115,59	PF00012.19	HSP70	140,6	4,60E-41	CL0108
ARUBRA_DN4785_c0_g1_i3_6	10	32,9	53,604	106,36	PF12796.6	Ank_2	58,5	6,90E-16	CL0465
ARHOMBI_DN5765_c0_g1_i1_5	5	27,2	28,717	50,787	PF06172.10	Cupin_5	142,1	9,90E-42	CL0029
ARHOMBI_DN4921_c0_g1_i1_3	8	18,7	58,557	171,27	PF00012.19	HSP70	575,2	1,10E-172	CL0108
ARUBRA_DN4799_c0_g1_i1_4	2	12,8	20,682	36,403	PF16845.4	SQAPI	129	5,90E-38	CL0121
ARUBRA_DN2524_c0_g1_i1_6	4	69,8	11,971	45,337	PF00330.19	Aconitase	115,3	3,30E-33	No_clan
ARUBRA_DN830_c1_g1_i1_2	13	63,1	24,585	323,31	PF08544.12	GHMP_kinases_C	48,2	9,40E-13	No_clan
ARUBRA_DN5581_c0_g1_i1_2	3	15,8	29,809	323,31	PF03647.12	Tmemb_14	62,1	6,10E-17	No_clan
ARUBRA_DN18248_c0_g1_i1_4	3	34	10,591	323,31	PF00635.25	Motile_Sperm	104,4	2,50E-30	CL0556
ARHOMBI_DN15811_c0_g1_i1_3	3	33	11,797	20,659	PF02826.18	2-Hacid_dh_C	138,8	1,10E-40	CL0063
ARHOMBI_DN5696_c0_g1_i1_3	5	18,8	35,125	30,83	PF00181.22	Ribosomal_L2	59,8	1,70E-16	CL0021

ARHOMBI_DN6039_c0_g1_i3_2	4	24,2	25,564	34,004	PF03946.13	Ribosomal_L11_N	79	1,50E-22	No_clan
ARHOMBI_DN787_c0_g2_i1_5	7	26	41,256	85,607	PF00175.20	NAD_binding_1	96,8	1,00E-27	CL0091
ARUBRA_DN19178_c0_g1_i1_4	4	18,4	28,113	26,607	PF01090.18	Ribosomal_S19e	191	6,50E-57	CL0123
ARUBRA_DN202_c0_g1_i1_3	5	13	56,257	57,484	PF00274.18	Glycolytic	521,7	6,00E-157	CL0035
ARHOMBI_DN5950_c0_g1_i1_2	1	2,3	68,807	6,6261					
ARHOMBI_DN2248_c0_g1_i1_5	6	35,7	24,143	40,416	PF00224.20	PK	103,2	1,20E-29	CL0151
ARUBRA_DN11411_c0_g1_i1_4	3	20,5	20,493	22,012	PF17135.3	Ribosomal_L18	309,9	4,10E-93	CL0588
ARUBRA_DN17927_c0_g1_i1_6	6	18,7	43,562	40,532	PF15704.4	Mt_ATP_synt	260,8	6,80E-78	No_clan
ARHOMBI_DN5374_c1_g1_i1_6	7	28,4	44,426	48,244	PF00004.28	AAA	145,2	1,30E-42	CL0023
ARUBRA_DN763_c0_g1_i1_4	4	15,3	28,776	150,55	PF01778.16	Ribosomal_L28e	114,9	2,90E-33	No_clan
ARUBRA_DN2819_c0_g1_i1_3	3	39,2	10,988	20,068					
ARUBRA_DN4688_c0_g1_i1_1	7	17,1	55,479	55,372	PF00330.19	Aconitase	95,1	4,20E-27	No_clan
ARHOMBI_DN4778_c0_g1_i1_3	4	11,3	58,585	57,882	PF00400.31	WD40	21,1	0,00037	CL0186
ARHOMBI_DN777_c0_g1_i1_1	5	33,3	21,812	207,57					
ARHOMBI_DN18025_c0_g1_i1_2	6	37,7	23,252	161,14	PF01661.20	Macro	126,9	3,70E-37	CL0223
ARHOMBI_DN5069_c0_g2_i1_3	10	24,6	57,4	65,862	PF08442.9	ATP-grasp_2	199	5,80E-59	CL0179
ARUBRA_DN4080_c0_g1_i1_6	2	28,1	14,437	88,962	PF00235.18	Profilin	73,5	1,60E-20	CL0431
ARUBRA_DN19946_c0_g1_i1_3	3	14,5	24,521	25,021	PF00252.17	Ribosomal_L16	116,8	5,80E-34	No_clan
ARUBRA_DN4991_c0_g1_i1_6	7	18	54,513	78,592	PF08241.11	Methyltransf_11	27,3	3,90E-06	CL0063
ARHOMBI_DN17727_c0_g1_i1_3	7	19,3	44,353	44,044	PF04053.13	Coatomer_WDAD	451,2	4,20E-135	CL0186
ARHOMBI_DN4645_c0_g2_i1_4	9	21,2	65,011	77,85	PF01433.19	Peptidase_M1	49,1	4,40E-13	CL0126
ARHOMBI_DN2411_c0_g1_i1_5	10	33,8	49,209	68,833	PF00118.23	Cpn60_TCP1	347,8	8,30E-104	No_clan
ARUBRA_DN4641_c0_g2_i1_2	23	39,3	82,022	55,052	PF00012.19	HSP70	870,8	3,60E-262	CL0108
ARHOMBI_DN1922_c0_g1_i1_3	7	33,1	30,616	323,31	PF00300.21	His_Phos_1	77,3	1,10E-21	CL0071
ARUBRA_DN17998_c0_g1_i1_1	5	30,7	23,976	31,006	PF02737.17	3HCDH_N	53,9	1,90E-14	CL0063
ARHOMBI_DN5775_c0_g1_i1_2	8	24	53,295	323,31	PF00112.22	Peptidase_C1	272	4,70E-81	CL0125
ARUBRA_DN5604_c0_g1_i1_1	6	18,6	29,58	39,027	PF05873.11	Mt_ATP-synt_D	66,3	2,50E-18	No_clan
ARUBRA_DN4856_c0_g1_i2_3	3	15,7	25,088	47,15					
ARHOMBI_DN478_c0_g2_i1_6	5	21,6	35,291	246,56	PF00756.19	Esterase	202	1,10E-59	CL0028

ARHOMBI_DN5072_c0_g1_i1_5	4	14,1	36,656	32,216	PF00081.21	Sod_Fe_N	116,8	4,10E-34	No_clan
ARUBRA_DN162_c0_g1_i1_1	8	27,3	38,773	322,46	PF12697.6	Abhydrolase_6	31	3,50E-07	CL0028
ARHOMBI_DN5339_c0_g1_i1_5	7	19	60,99	65,774	PF03405.13	FA_desaturase_2	532,9	2,00E-160	CL0044
ARHOMBI_DN5797_c1_g1_i2_6	3	30,6	15,196	127,02	PF00248.20	Aldo_ket_red	46,1	3,30E-12	No_clan
ARHOMBI_DN5453_c0_g1_i1_1	9	20	71,222	54,202	PF00262.17	Calreticulin	485,8	6,30E-146	CL0004
ARHOMBI_DN4158_c0_g1_i1_4	4	12,6	49,83	73,516	PF08240.11	ADH_N	85,9	1,50E-24	CL0296
ARHOMBI_DN3362_c0_g1_i1_5	11	30,4	61,534	37,094	PF00091.24	Tubulin	235,4	5,70E-70	CL0566
ARUBRA_DN20595_c0_g1_i1_3	8	22,6	51,732	52,579	PF00012.19	HSP70	50,6	8,40E-14	CL0108
ARUBRA_DN427_c0_g2_i1_5	5	29,9	31,234	51,355	PF00438.19	S-AdoMet_synt_N	144,8	9,70E-43	No_clan
ARHOMBI_DN19156_c0_g1_i1_5	2	25,5	10,462	7,552	PF00459.24	Inositol_P	78,5	5,50E-22	CL0171
ARHOMBI_DN3290_c0_g1_i1_1	6	29,8	33,783	112,94	PF00156.26	Pribosyltran	65,6	3,40E-18	CL0533
ARUBRA_DN2245_c0_g2_i1_4	4	17,5	34,082	30,461	PF13848.5	Thioredoxin_6	35,8	7,00E-09	CL0172
ARHOMBI_DN19115_c0_g1_i1_6	6	36,9	21,403	37,619	PF00118.23	Cpn60_TCP1	118,4	3,00E-34	No_clan
ARHOMBI_DN5316_c0_g1_i1_2	8	18,4	45,003	74,282	PF01680.16	SOR_SNZ	372,1	6,90E-112	CL0036
ARUBRA_DN8606_c0_g1_i1_1	7	24,3	27,177	25,814	PF00647.18	EF1G	142,9	3,10E-42	No_clan
ARHOMBI_DN5381_c0_g1_i1_4	5	38,1	15,596	43,501	PF00238.18	Ribosomal_L14	120,2	4,70E-35	No_clan
ARUBRA_DN43_c0_g2_i1_6	3	13,8	21,186	29,571	PF02704.13	GASA	81,7	2,80E-23	No_clan
ARHOMBI_DN3956_c0_g1_i1_1	4	10	44,014	36,249	PF00141.22	peroxidase	273,7	1,10E-81	CL0617
ARUBRA_DN609_c0_g1_i1_6	4	14,9	55,095	32,919	PF00091.24	Tubulin	227,7	1,30E-67	CL0566
ARUBRA_DN2_c0_g1_i1_4	6	16,3	44,937	37,436	PF08241.11	Methyltransf_11	78	5,90E-22	CL0063
ARUBRA_DN1844_c0_g1_i1_1	6	22,7	41,194	39,176	PF01459.21	Porin_3	216,9	3,20E-64	CL0193
ARHOMBI_DN3609_c0_g2_i1_1	6	38,4	19,489	323,31	PF00248.20	Aldo_ket_red	51,8	6,10E-14	No_clan
ARHOMBI_DN3185_c0_g1_i1_4	6	19,9	49,274	39,456	PF05193.20	Peptidase_M16_C	116,9	9,20E-34	CL0094
ARUBRA_DN24520_c0_g1_i1_2	5	33,3	16,257	29,824	PF04832.11	SOUL	66,1	3,70E-18	CL0319
ARUBRA_DN4994_c0_g1_i2_3	2	7,2	37,575	113,76	PF01912.17	eIF-6	271,7	2,50E-81	CL0197
ARUBRA_DN19637_c0_g1_i1_2	9	28,2	49,492	103,37	PF00009.26	GTP_EFTU	145,6	1,20E-42	CL0023
ARUBRA_DN18881_c0_g1_i1_2	7	29,7	37,276	57,707	PF10584.8	Proteasome_A_N	49,6	2,10E-13	CL0052
ARHOMBI_DN5176_c0_g1_i1_6	4	20,8	26,81	323,31	PF01641.17	SelR	158,6	5,70E-47	CL0080
ARUBRA_DN13710_c0_g1_i1_3	1	11,8	15,126	8,3666	PF00182.18	Glyco_hydro_19	101	8,20E-29	CL0037
ARUBRA_DN18453_c0_g1_i1_3	4	37,3	17,808	302,26	PF00240.22	ubiquitin	70,8	5,30E-20	CL0072

ARHOMBI_DN5422_c0_g1_i1_5	10	42,8	43,186	142,06	PF07992.13	Pyr_redox_2	165,2	1,80E-48	CL0063
ARHOMBI_DN15946_c0_g1_i1_6	2	9,6	22,098	19,001					
ARHOMBI_DN4424_c0_g1_i1_1	5	14	52,641	31,574	PF00297.21	Ribosomal_L3	650,9	4,70E-196	CL0575
ARUBRA_DN3570_c0_g1_i1_3	6	38,2	18,429	42,251	PF00347.22	Ribosomal_L6	43,9	2,60E-11	No_clan
ARUBRA_DN2080_c0_g1_i1_2	4	36,8	16,871	85,141	PF00887.18	ACBP	58,5	5,30E-16	CL0632
ARUBRA_DN2407_c0_g1_i1_1	6	18,7	47,29	37,679	PF01370.20	Epimerase	200,1	3,40E-59	CL0063
ARUBRA_DN6096_c0_g1_i1_5	4	13,7	33,25	23,013	PF00687.20	Ribosomal_L1	145,1	2,00E-42	No_clan
ARUBRA_DN5878_c0_g1_i1_4	8	22,4	60,569	67,581	PF00224.20	PK	519,2	3,80E-156	CL0151
ARUBRA_DN462_c0_g2_i1_3	2	22,4	16,897	144,9					
ARHOMBI_DN8302_c0_g1_i1_6	4	27,9	24,207	28,552	PF00890.23	FAD_binding_2	217,1	4,10E-64	CL0063
ARHOMBI_DN6204_c0_g1_i2_1	8	27,2	45,65	49,563	PF00118.23	Cpn60_TCP1	200,5	3,80E-59	No_clan
ARUBRA_DN935_c0_g1_i1_4	2	8,6	32,411	12,578	PF02823.15	ATP-synt_DE_N	55,4	4,00E-15	No_clan
ARHOMBI_DN6019_c0_g1_i1_2	3	8	50,503	19,684	PF03168.12	LEA_2	51,8	8,50E-14	CL0159
ARHOMBI_DN4558_c0_g2_i1_1	2	14	30,967	24,155	PF00108.22	Thiolase_N	163,7	4,80E-48	CL0046
ARUBRA_DN6207_c0_g1_i1_4	3	13,3	26,65	19,255	PF00900.19	Ribosomal_S4e	123,9	1,70E-36	No_clan
ARUBRA_DN17796_c0_g1_i1_2	8	23	60,008	49,115	PF00676.19	E1_dh	395,5	1,10E-118	CL0254
ARUBRA_DN4392_c0_g3_i1_4	7	20,4	44,199	323,31	PF00481.20	PP2C	183,1	6,90E-54	CL0238
ARUBRA_DN2862_c0_g2_i1_5	3	48,8	8,4652	23,867	PF03492.14	Methyltransf_7	30,8	1,60E-07	CL0063
ARUBRA_DN18480_c0_g1_i1_4	3	15,1	37,121	26,899	PF00108.22	Thiolase_N	305,8	2,10E-91	CL0046
ARHOMBI_DN6139_c0_g1_i1_4	5	23,7	32,564	35,476	PF10584.8	Proteasome_A_N	50,2	1,40E-13	CL0052
ARHOMBI_DN5986_c0_g1_i2_6	3	20,4	19,903	204,97	PF00403.25	HMA	63	2,40E-17	No_clan
ARHOMBI_DN20063_c0_g1_i1_4	3	21,5	17,959	48,411	PF00240.22	ubiquitin	62,5	2,20E-17	CL0072
ARHOMBI_DN5759_c0_g1_i1_6	7	23,2	45,034	49,693	PF17144.3	Ribosomal_L5e	274,6	2,40E-82	CL0267
ARHOMBI_DN3534_c0_g1_i1_3	4	16,4	31,226	25,23	PF00210.23	Ferritin	112,9	1,00E-32	CL0044
ARUBRA_DN11326_c0_g1_i1_6	4	19,8	22,616	139,57	PF08694.10	UFC1	276,4	4,50E-83	CL0208
ARUBRA_DN2423_c0_g1_i1_1	4	13,9	31,518	23,715	PF00827.16	Ribosomal_L15e	314,1	2,80E-94	CL0652
ARUBRA_DN3020_c0_g1_i1_1	4	18,9	29,357	29,709	PF16845.4	SQAPI	73,1	1,60E-20	CL0121
ARUBRA_DN12109_c0_g1_i1_6	1	19,4	11,074	11,689	PF00627.30	UBA	58,7	3,40E-16	CL0214
ARUBRA_DN16721_c0_g1_i1_2	7	45,3	29,673	76,668	PF01588.19	tRNA_bind	86,6	8,20E-25	CL0021
ARUBRA_DN4896_c0_g1_i1_4	5	16,5	35,872	48,838	PF01937.18	DUF89	185,1	1,70E-54	No_clan

ARHOMBI_DN347_c0_g1_i1_1	3	7,4	51,312	101,51	PF12708.6	Pectate_lyase_3	27,7	2,20E-06	CL0268
ARUBRA_DN3353_c0_g1_i1_5	5	17,5	38,1	323,31	PF04774.14	HABP4_PAI-RBP1	103,9	8,40E-30	No_clan
ARHOMBI_DN1690_c0_g1_i1_4	7	21,3	32,837	95,551					
ARUBRA_DN4036_c0_g1_i1_5	3	21,8	20,041	51,319	PF03179.14	V-ATPase_G	110,2	5,90E-32	CL0255
ARHOMBI_DN3961_c0_g1_i1_1	2	5,7	46,254	39,981	PF08597.9	eIF3_subunit	170,3	5,90E-50	No_clan
ARUBRA_DN6223_c0_g1_i1_2	6	31,1	29,551	43,23	PF03446.14	NAD_binding_2	87,4	1,00E-24	CL0063
ARUBRA_DN17757_c0_g1_i1_6	7	26,9	37,184	168,59	PF00071.21	Ras	203,1	1,90E-60	CL0023
ARUBRA_DN1661_c0_g1_i1_3	5	24,4	24,171	99,853	PF06094.11	GGACT	83,7	1,30E-23	CL0278
ARUBRA_DN2570_c0_g2_i1_5	3	42,2	9,515	239,4	PF00085.19	Thioredoxin	76,9	8,90E-22	CL0172
ARUBRA_DN2915_c0_g1_i1_5	12	29,6	58,034	72,307	PF00155.20	Aminotran_1_2	315	6,30E-94	CL0061
ARUBRA_DN4959_c0_g1_i4_6	5	19	26,362	32,621	PF00338.21	Ribosomal_S10	94,7	2,80E-27	No_clan
ARUBRA_DN4738_c0_g1_i1_6	4	23,2	25,794	115,26	PF01248.25	Ribosomal_L7Ae	89,8	6,70E-26	CL0101
ARHOMBI_DN5099_c0_g2_i1_3	5	11	56,252	30,594	PF00153.26	Mito_carr	73	1,30E-20	No_clan
ARHOMBI_DN6124_c0_g2_i1_6	16	32	64,078	121,2	PF00162.18	PGK	541,5	9,10E-163	No_clan
ARUBRA_DN22130_c0_g1_i1_6	5	19,9	32,133	323,31	PF01472.19	PUA	63	1,70E-17	CL0178
ARHOMBI_DN17024_c0_g1_i1_5	5	28,1	22,094	41,641	PF13410.5	GST_C_2	26,2	5,50E-06	CL0497
ARHOMBI_DN3923_c0_g2_i1_3	3	40	10,99	123,48					
ARUBRA_DN16253_c0_g1_i1_2	7	32,5	31,391	49,669	PF00224.20	PK	157,5	3,80E-46	CL0151
ARUBRA_DN18651_c0_g1_i1_6	9	22,2	41,473	94,552					
ARHOMBI_DN16154_c0_g1_i1_2	4	16,5	31,129	216,53	PF06201.12	PITH	135,7	1,30E-39	CL0202
ARUBRA_DN5051_c0_g1_i1_2	10	29,3	42,057	98,688	PF00244.19	14.03.2003	345,8	8,30E-104	No_clan
ARHOMBI_DN2561_c0_g1_i1_3	4	23	22,139	24,375	PF01776.16	Ribosomal_L22e	155,6	4,70E-46	No_clan
ARUBRA_DN1380_c0_g2_i1_2	9	16,7	76,246	59,965	PF07991.11	IlvN	107,4	5,20E-31	CL0063
ARUBRA_DN2595_c0_g3_i1_4	2	11,2	26,673	12,886	PF00828.18	Ribosomal_L27A	71,9	6,60E-20	CL0588
ARUBRA_DN3028_c0_g1_i1_1	5	17,1	32,766	124,25	PF02297.16	COX6B	58	7,90E-16	CL0351
ARUBRA_DN3878_c0_g1_i1_3	5	34,9	21,128	33,932	PF02785.18	Biotin_carb_C	124,3	2,00E-36	CL0105
ARUBRA_DN2248_c0_g1_i1_6	6	19,1	50,708	42,914	PF03088.15	Str_synth	108,6	1,20E-31	CL0186
ARUBRA_DN3983_c0_g1_i1_4	8	19,5	70,613	56,116	PF00330.19	Aconitase	254,7	1,70E-75	No_clan
ARUBRA_DN7521_c0_g1_i1_3	3	15	20,257	18,985	PF07944.11	Glyco_hydro_127	81,8	3,60E-23	CL0059
ARUBRA_DN15944_c0_g1_i1_3	3	34,1	8,6768	20,625	PF05694.10	SBP56	118	4,50E-34	CL0186
ARUBRA_DN4965_c10_g1_i1_3	11	29,5	60,264	69,862	PF02847.16	MA3	99,9	7,40E-29	CL0020

ARHOMBI_DN2725_c0_g1_i1_2	1	4,2	25,872	6,3587	PF00079.19	Serpin	122,2	2,90E-35	No_clan
ARHOMBI_DN4252_c0_g1_i1_5	6	20,9	31,694	19,232	PF00347.22	Ribosomal_L6	45	1,20E-11	No_clan
ARHOMBI_DN5741_c0_g1_i1_3	3	10,9	35,566	18,373	PF03868.14	Ribosomal_L6e_N	60,7	1,00E-16	No_clan
ARHOMBI_DN4907_c0_g1_i1_4	8	32,6	33,16	129,01	PF01287.19	eIF-5a	99,8	6,10E-29	CL0021
ARHOMBI_DN3938_c0_g1_i1_4	2	8,3	26,85	23,729	PF02230.15	Abhydrolase_2	68,5	6,30E-19	CL0028
ARUBRA_DN17582_c0_g1_i1_4	2	18,3	11,566	284,45	PF13419.5	HAD_2	24,5	2,20E-05	CL0137
ARUBRA_DN9146_c0_g1_i1_2	2	10,6	25,802	174,16	PF01984.19	dsDNA_bind	106,5	7,80E-31	No_clan
ARHOMBI_DN15390_c0_g1_i1_4	3	11,6	34,688	30,865	PF13181.5	TPR_8	18,2	0,0018	CL0020
ARHOMBI_DN4487_c0_g1_i1_2	10	26,6	54,658	263,05	PF00790.18	VHS	62,5	3,50E-17	CL0009
ARUBRA_DN1889_c0_g1_i1_3	4	17,9	34,422	31,856	PF01294.17	Ribosomal_L13e	260,1	9,00E-78	No_clan
ARUBRA_DN4712_c0_g2_i1_6	2	8,5	27,272	12,996	PF16906.4	Ribosomal_L26	127,4	2,00E-37	No_clan
ARUBRA_DN4939_c0_g1_i3_5	3	32	17,488	26,585	PF00428.18	Ribosomal_60s	85,7	2,20E-24	No_clan
ARUBRA_DN4833_c0_g1_i2_5	1	9,5	10,583	50,398	PF04568.11	IATP	21,7	0,0002	No_clan
ARUBRA_DN2992_c0_g1_i1_3	3	11,4	32,037	205,25	PF01652.17	IF4E	175,5	5,90E-52	CL0625
ARUBRA_DN7857_c0_g1_i1_1	3	37	14,059	307,39	PF02115.16	Rho_GDI	72,3	3,90E-20	No_clan
ARUBRA_DN22505_c0_g1_i1_3	4	34,4	13,351	24,222	PF15511.5	CENP-T_C	33,8	2,60E-08	CL0012
ARUBRA_DN21075_c0_g1_i1_5	4	31	17,924	77,158	PF01088.20	Peptidase_C12	78,8	4,40E-22	CL0125
ARHOMBI_DN5548_c0_g1_i1_5	5	14,1	68,555	35,791	PF02776.17	TPP_enzyme_N	117,4	4,70E-34	CL0254
ARUBRA_DN2771_c0_g1_i1_5	10	24,8	57,338	60,875					
ARUBRA_DN4858_c0_g1_i1_4	3	19	13,507	21,042	PF01200.17	Ribosomal_S28e	109,8	4,10E-32	CL0021
ARUBRA_DN25882_c0_g1_i1_2	4	45,9	11,102	25,424	PF00330.19	Aconitase	57,4	1,20E-15	No_clan
ARHOMBI_DN6034_c0_g1_i3_3	14	30,6	50,254	12,895	PF00012.19	HSP70	589,8	4,30E-177	CL0108
ARHOMBI_DN5395_c0_g1_i1_3	7	24,2	52,701	46,979	PF07992.13	Pyr_redox_2	179,4	8,50E-53	CL0063
ARUBRA_DN2691_c0_g1_i1_5	3	11,3	28,564	36,752	PF00254.27	FKBP_C	94,9	2,60E-27	CL0487
ARUBRA_DN16143_c0_g1_i1_5	2	9	24,729	13,068	PF01929.16	Ribosomal_L14e	93	1,20E-26	CL0107
ARUBRA_DN7703_c0_g1_i1_6	7	20,1	47,201	43,973	PF00071.21	Ras	183,4	2,10E-54	CL0023
ARHOMBI_DN5124_c0_g1_i1_4	2	11,5	23,675	12,377	PF01283.18	Ribosomal_S26e	180,2	1,10E-53	No_clan
ARHOMBI_DN3648_c0_g1_i1_4	4	24,8	18,323	23,525	PF08069.11	Ribosomal_S13_N	109,6	5,00E-32	No_clan
ARHOMBI_DN1734_c0_g1_i1_3	4	25,3	26,032	31,606	PF01145.24	Band_7	86,4	2,10E-24	CL0433
ARUBRA_DN9894_c0_g1_i1_4	6	22,8	46,923	51,349	PF00112.22	Peptidase_C1	247,7	1,30E-73	CL0125
ARUBRA_DN2456_c0_g1_i1_2	4	16,9	37,115	27,781	PF03492.14	Methyltransf_7	280,4	1,90E-83	CL0063
ARUBRA_DN6971_c0_g1_i1_6	4	14,4	33,704	27,587	PF10584.8	Proteasome_A_N	49,4	2,30E-13	CL0052

ARHOMBI_DN516_c0_g1_i1_6	3	10,8	27,326	18,121	PF00190.21	Cupin_1	166,9	2,10E-49	CL0029
ARUBRA_DN3974_c0_g1_i1_1	3	26,7	20,476	37,37	PF01722.17	BolA	69,2	2,50E-19	No_clan
ARUBRA_DN19869_c0_g1_i1_3	3	31,3	18,067	15,8	PF01370.20	Epimerase	90,2	1,30E-25	CL0063
ARUBRA_DN5948_c0_g1_i1_2	6	30,9	22,521	37,908	PF00342.18	PGI	240,5	2,70E-71	CL0067
ARUBRA_DN20440_c0_g1_i1_3	1	26,1	7,6086	18,27					
ARUBRA_DN20805_c0_g1_i1_5	1	5,6	15,732	-2					
ARUBRA_DN1508_c0_g1_i1_1	5	42,6	17,104	25,585	PF00080.19	Sod_Cu	167,5	1,70E-49	No_clan
ARUBRA_DN8181_c0_g1_i1_4	6	22,7	37,542	37,092	PF10584.8	Proteasome_A_N	56,4	1,50E-15	CL0052
ARHOMBI_DN5015_c0_g1_i1_3	4	18	36,022	126,87	PF01168.19	Ala_racemase_N	81,6	5,90E-23	CL0036
ARUBRA_DN11787_c0_g1_i1_3	1	7,8	21,039	7,438	PF06201.12	PITH	100,7	8,30E-29	CL0202
ARHOMBI_DN5946_c1_g1_i1_6	2	5,6	45,041	12,964	PF03951.18	Gln-synt_N	36,3	3,20E-09	No_clan
ARUBRA_DN23186_c0_g1_i1_4	3	31	14,453	77,123	PF00076.21	RRM_1	78,1	3,00E-22	CL0221
ARUBRA_DN3089_c0_g1_i1_6	5	17,7	39,876	29,39	PF01459.21	Porin_3	252,1	6,00E-75	CL0193
ARUBRA_DN1937_c0_g1_i1_4	1	1,9	65,374	6,2402					
ARHOMBI_DN9002_c0_g1_i1_5	2	28,6	16,218	164,34	PF00076.21	RRM_1	83,2	7,70E-24	CL0221
ARHOMBI_DN10315_c0_g1_i1_4	4	28,5	20,106	28,574	PF02786.16	CPSase_L_D2	231,8	5,60E-69	CL0179
ARUBRA_DN20947_c0_g1_i1_6	4	32,9	23,201	98,524	PF01722.17	BolA	49,4	3,80E-13	No_clan
ARUBRA_DN18979_c0_g1_i1_5	5	23	30,312	100,72	PF02878.15	PGM_PMM_I	127	3,80E-37	No_clan
ARUBRA_DN6370_c0_g1_i1_6	4	10,5	43,063	26,737	PF00248.20	Aldo_ket_red	237,8	1,30E-70	No_clan
ARHOMBI_DN16206_c0_g1_i1_4	3	11,8	32,306	119,24	PF00543.21	P-II	114,6	2,50E-33	CL0089
ARHOMBI_DN6026_c0_g2_i1_4	6	24,7	47,652	46,369	PF00438.19	S-AdoMet_synt_N	143,2	3,00E-42	No_clan
ARHOMBI_DN11466_c0_g1_i1_1	2	26	11,079	27,055	PF00171.21	Aldedh	76,5	1,40E-21	CL0099
ARUBRA_DN6756_c0_g1_i1_3	5	38,6	24,931	47,312	PF03727.15	Hexokinase_2	204,3	1,90E-60	CL0108
ARHOMBI_DN7040_c0_g1_i1_2	1	18,4	9,5738	-2					
ARHOMBI_DN11661_c0_g1_i1_3	2	17,5	17,435	24,384	PF00171.21	Aldedh	142,7	1,10E-41	CL0099
ARHOMBI_DN23618_c0_g1_i1_3	2	18,8	13,116	11,931	PF00076.21	RRM_1	61,9	3,50E-17	CL0221
ARUBRA_DN6507_c0_g1_i1_3	3	41,8	11,817	42,041					
ARHOMBI_DN21306_c0_g1_i1_1	3	21,7	18,775	20,888					
ARHOMBI_DN16200_c0_g1_i1_5	4	33,7	17,934	116,32	PF08244.11	Glyco_hydro_32C	66,6	2,30E-18	CL0004
ARHOMBI_DN18668_c0_g1_i1_4	2	17,6	14,786	15,471	PF00109.25	ketoacyl-synt	26,8	3,20E-06	CL0046
ARHOMBI_DN7785_c0_g1_i1_5	4	30	11,457	140,03	PF08991.9	MTCP1	48,3	8,10E-13	CL0351
ARHOMBI_DN2296_c0_g2_i1_6	12	27,1	58,327	323,31	PF00152.19	tRNA-synt_2	202,6	7,40E-60	CL0040
ARUBRA_DN4291_c0_g1_i1_3	2	11,1	22,884	14,075	PF00276.19	Ribosomal_L23	51,9	7,00E-14	No_clan

ARHOMBI_DN4083_c0_g1_i1_6	4	23,8	29,009	22,423	PF05193.20	Peptidase_M16_C	59	5,40E-16	CL0094
ARHOMBI_DN5446_c0_g1_i1_5	5	9,6	61,735	30,393	PF02020.17	W2	69	2,70E-19	CL0020
ARUBRA_DN8529_c0_g1_i1_1	5	38,4	21,774	35,157	PF06957.10	COPI_C	217,6	2,30E-64	CL0020
ARUBRA_DN4933_c0_g1_i2_4	3	9,9	43,297	19,289	PF00333.19	Ribosomal_S5	107,6	2,10E-31	CL0196
ARUBRA_DN3170_c0_g1_i1_4	7	19,2	48,573	161,78	PF00782.19	DSPc	41,6	8,90E-11	CL0031
ARUBRA_DN4127_c0_g2_i1_4	5	15,3	54,996	33,545	PF00180.19	Iso_dh	349,8	1,50E-104	CL0270
ARUBRA_DN6378_c0_g1_i1_2	6	21,5	43,707	13,206	PF00614.21	PLDc	42,1	5,40E-11	CL0479
ARUBRA_DN2301_c0_g1_i1_1	3	22,5	19,679	20,041					
ARUBRA_DN16958_c0_g1_i1_6	3	21,5	31,854	26,614	PF00224.20	PK	88,3	4,00E-25	CL0151
ARUBRA_DN256_c0_g2_i1_1	8	20	63,607	261,7	PF00789.19	UBX	63,2	1,80E-17	CL0072
ARHOMBI_DN2281_c0_g1_i1_1	4	19,4	29,855	26,74	PF00719.18	Pyrophosphatase	181,1	1,20E-53	No_clan
ARHOMBI_DN6231_c0_g2_i2_2	4	11,4	35,859	24,312	PF13561.5	adh_short_C2	199	7,60E-59	CL0063
ARHOMBI_DN835_c0_g1_i1_4	2	14,7	20,804	18,442	PF00177.20	Ribosomal_S7	110,5	5,20E-32	No_clan
ARUBRA_DN4252_c0_g2_i2_4	9	12,3	125,44	55,439	PF02347.15	GDC-P	610,6	1,30E-183	CL0061
ARHOMBI_DN11662_c0_g1_i1_4	1	47,1	7,3263	6,4307					
ARUBRA_DN21714_c0_g1_i1_3	3	13,8	21,729	18,25	PF01199.17	Ribosomal_L34e	129,2	5,00E-38	No_clan
ARHOMBI_DN4867_c0_g1_i1_5	5	18,5	36,761	33,158	PF00227.25	Proteasome	158,6	1,10E-46	CL0052
ARHOMBI_DN5538_c0_g1_i1_5	5	17,1	39,593	39,671	PF14290.5	DUF4370	390,8	2,50E-117	No_clan
ARUBRA_DN9182_c0_g1_i1_2	1	9,8	13,492	12,093	PF00012.19	HSP70	130,5	5,20E-38	CL0108
ARUBRA_DN4826_c0_g1_i1_4	3	21,7	29,1	97,683	PF06747.12	CHCH	35	1,00E-08	CL0351
ARHOMBI_DN21064_c0_g1_i1_2	3	43,3	10,887	17,926	PF00349.20	Hexokinase_1	94,6	7,00E-27	CL0108
ARHOMBI_DN50_c0_g3_i1_6	4	13,3	40,404	25,74	PF03345.13	DDOST_48kD	317,3	1,30E-94	No_clan
ARUBRA_DN4961_c0_g1_i2_4	2	26	10,676	6,9694	PF00450.21	Peptidase_S10	68,8	5,50E-19	CL0028
ARHOMBI_DN5474_c0_g1_i1_5	4	25,3	25,725	47,288	PF00257.18	Dehydrin	52,8	6,00E-14	No_clan
ARUBRA_DN3097_c0_g2_i1_2	6	44,2	24,21	43,508	PF00012.19	HSP70	245,2	9,90E-73	CL0108
ARUBRA_DN7003_c0_g1_i1_4	1	10,8	25,514	6,8674					
ARHOMBI_DN20442_c0_g1_i1_1	1	7,2	11,432	6,515					
ARUBRA_DN3105_c0_g1_i1_1	3	19,8	21,694	20,201					
ARUBRA_DN18186_c0_g1_i1_5	1	12,2	7,7414	15,67					

ARUBRA_DN3014_c0_g1_i1_2	12	17,1	99,257	83,13	PF02446.16	Glyco_hydro_77	504,9	1,90E-151	CL0058
ARUBRA_DN12532_c0_g1_i1_5	2	32,9	7,5016	31,206	PF13668.5	Ferritin_2	30,4	3,40E-07	CL0044
ARHOMBI_DN5429_c0_g1_i1_2	4	13,6	35,298	33,011	PF01738.17	DLH	101,3	5,00E-29	CL0028
ARHOMBI_DN16845_c0_g1_i1_5	5	26,8	20,611	30,724	PF00637.19	Clathrin	77,9	6,30E-22	CL0020
ARUBRA_DN720_c0_g1_i1_6	4	9,3	37,759	23,267	PF00163.18	Ribosomal_S4	29	1,40E-06	CL0492
ARHOMBI_DN17235_c0_g1_i1_3	5	19,7	42,656	99,462	PF00890.23	FAD_binding_2	112,6	2,20E-32	CL0063
ARUBRA_DN2328_c0_g2_i1_4	2	11,9	20,093	12,208	PF01667.16	Ribosomal_S27e	89,9	5,60E-26	CL0167
ARUBRA_DN4073_c0_g1_i1_2	4	14,8	46,244	27,491	PF10584.8	Proteasome_A_N	51,2	6,20E-14	CL0052
ARHOMBI_DN1161_c0_g1_i1_2	4	11,7	49,293	27,908	PF02990.15	EMP70	445,5	2,30E-133	No_clan
ARHOMBI_DN22444_c0_g1_i1_1	5	18,8	33,968	315,36	PF00248.20	Aldo_ket_red	163,3	6,50E-48	No_clan
ARHOMBI_DN5200_c0_g2_i1_5	3	14,6	33,099	20,107	PF01280.19	Ribosomal_L19e	220,8	6,10E-66	No_clan
ARHOMBI_DN19654_c0_g1_i1_2	3	16,2	19,656	18,932					
ARHOMBI_DN2987_c0_g1_i1_5	7	15,8	63,886	45,288	PF00285.20	Citrate_synt	344,2	7,90E-103	No_clan
ARHOMBI_DN6144_c0_g1_i1_4	2	5,1	60,854	14,946	PF00112.22	Peptidase_C1	283,2	1,70E-84	CL0125
ARUBRA_DN4862_c0_g1_i1_4	19	45,9	53,371	-2	PF00162.18	PGK	558,8	4,90E-168	No_clan
ARHOMBI_DN1127_c0_g2_i1_4	1	6,6	30,719	8,243	PF03151.15	TPT	237,3	2,00E-70	CL0184
ARUBRA_DN17228_c1_g1_i1_1	5	22,8	38,06	47,628	PF10584.8	Proteasome_A_N	33,9	1,70E-08	CL0052
ARHOMBI_DN4125_c0_g1_i1_5	4	25,7	23,261	17,067	PF01248.25	Ribosomal_L7Ae	88,6	1,50E-25	CL0101
ARHOMBI_DN4270_c0_g1_i1_2	2	11,6	24,15	11,047	PF00393.18	6PGD	151,5	2,90E-44	CL0106
ARUBRA_DN4844_c0_g1_i1_2	3	18,8	21,664	20,843	PF03297.14	Ribosomal_S25	133,9	1,90E-39	CL0123
ARUBRA_DN13846_c0_g1_i1_5	2	33,8	7,9931	75,424	PF00026.22	Asp	74,3	1,00E-20	CL0129
ARUBRA_DN5052_c0_g2_i1_1	3	17,5	18,941	55,134					
ARUBRA_DN4025_c0_g2_i1_5	2	7,1	26,94	11,052	PF00410.18	Ribosomal_S8	75,4	3,40E-21	No_clan
ARUBRA_DN4599_c2_g1_i1_4	3	14,2	37,277	264,84	PF01217.19	Clat_adaptor_s	78,2	5,40E-22	CL0212
ARUBRA_DN21659_c0_g1_i1_3	3	31,2	15,796	18,009	PF04969.15	CS	35,5	1,50E-08	CL0190
ARHOMBI_DN17850_c0_g1_i1_6	4	13,1	37,482	73,312	PF00857.19	Isochorismatase	116,4	1,40E-33	No_clan
ARUBRA_DN2529_c0_g2_i1_6	5	15,4	54,738	323,31	PF13519.5	VWA_2	96,1	1,70E-27	CL0128
ARHOMBI_DN6264_c0_g1_i5_2	13	40,9	43,731	23,453	PF00724.19	Oxidored_FMN	300,7	1,30E-89	CL0036
ARHOMBI_DN5393_c0_g1_i1_1	7	18,9	46,12	8,9466	PF00561.19	Abhydrolase_1	78,2	7,10E-22	CL0028

ARUBRA_DN3140_c0_g1_i1_5	3	8,9	39,003	18,32	PF00166.20	Cpn10	97,7	2,80E-28	CL0296
ARHOMBI_DN5550_c0_g1_i1_2	5	18,8	36,189	32,364	PF12353.7	eIF3g	117,3	4,80E-34	CL0511
ARUBRA_DN3107_c0_g1_i1_6	5	16,6	38,069	109,01	PF08059.12	SEP	94,6	3,60E-27	No_clan
ARUBRA_DN4381_c0_g1_i1_5	5	13,5	62,602	323,31	PF00282.18	Pyridoxal_deC	349,2	2,00E-104	CL0061
ARUBRA_DN3552_c0_g1_i1_4	5	25,2	29,698	29,959	PF00656.21	Peptidase_C14	78,3	8,00E-22	CL0093
ARUBRA_DN3608_c0_g1_i1_6	3	34	16,317	17,88	PF02221.14	E1_DerP2_DerF2	57,6	1,70E-15	CL0532
ARHOMBI_DN17898_c0_g1_i1_3	5	20,9	35,515	30,948	PF01432.19	Peptidase_M3	284,1	2,50E-84	CL0126
ARHOMBI_DN17178_c0_g1_i1_4	2	13,5	18,352	192,46	PF02879.15	PGM_PMM_II	51,3	1,30E-13	No_clan
ARUBRA_DN4547_c0_g1_i1_6	4	13,4	36,103	323,31					
ARHOMBI_DN486_c0_g1_i1_1	3	44,7	8,3352	182,69					
ARHOMBI_DN5664_c0_g1_i1_5	3	13,3	21,63	29,407	PF00234.21	Tryp_alpha_amyl	23,8	4,30E-05	CL0482
ARHOMBI_DN7133_c0_g1_i1_1	4	28,8	17,483	25,079	PF00122.19	E1-E2_ATPase	134,8	2,10E-39	No_clan
ARHOMBI_DN5590_c0_g1_i1_4	3	12,9	36,912	25,138	PF13085.5	Fer2_3	111,6	1,60E-32	CL0486
ARHOMBI_DN3067_c0_g1_i1_1	6	19,1	40,357	27,017	PF00244.19	14.03.2003	324,2	3,40E-97	No_clan
ARUBRA_DN3632_c0_g1_i1_6	4	21,2	25,831	190,94	PF00370.20	FGGY_N	192,1	1,10E-56	CL0108
ARUBRA_DN5040_c0_g4_i8_2	2	8,4	29,402	8,8548	PF13561.5	adh_short_C2	196,9	3,30E-58	CL0063
ARHOMBI_DN9099_c0_g1_i1_1	1	9,6	14,493	6,3659					
ARHOMBI_DN1783_c0_g2_i1_3	2	18,9	21,263	78,401	PF00481.20	PP2C	136,8	9,10E-40	CL0238
ARUBRA_DN5025_c0_g1_i3_3	8	10,6	116,05	52,143	PF00690.25	Cation_ATPase_N	50,3	1,30E-13	No_clan
ARUBRA_DN1938_c0_g1_i1_1	4	14,1	38,634	25,322	PF10584.8	Proteasome_A_N	50,4	1,20E-13	CL0052
ARHOMBI_DN16492_c0_g1_i1_4	6	14,8	67,27	125,53	PF07732.14	Cu-oxidase_3	120,1	4,70E-35	CL0026
ARUBRA_DN5418_c0_g1_i1_4	5	12,8	50,686	38,244	PF00285.20	Citrate_synt	445,8	1,00E-133	No_clan
ARHOMBI_DN13783_c0_g1_i1_2	2	22,2	10,28	158,79					
ARHOMBI_DN16013_c0_g1_i1_5	5	35,8	14,171	11,652	PF08267.11	Meth_synt_1	67,4	1,40E-18	CL0160
ARUBRA_DN19786_c0_g1_i1_6	1	7,9	16,911	7,4601	PF16113.4	ECH_2	76,2	3,00E-21	CL0127
ARUBRA_DN5795_c0_g1_i1_1	3	12,9	36,853	22,69	PF07992.13	Pyr_redox_2	44,1	1,40E-11	CL0063
ARHOMBI_DN6222_c0_g1_i1_3	4	16,7	29,394	34,257	PF00462.23	Glutaredoxin	61,5	6,00E-17	CL0172
ARHOMBI_DN3998_c0_g1_i1_1	2	8,3	39,426	16,329	PF02921.13	UCR_TM	45,9	5,20E-12	CL0300
ARUBRA_DN25017_c0_g1_i1_1	1	19,8	10,941	7,6609	PF01263.19	Aldose_epim	74,4	8,90E-21	CL0103
ARUBRA_DN2627_c0_g1_i1_2	5	12,2	60,456	34,91	PF00450.21	Peptidase_S10	427,1	9,10E-128	CL0028

ARHOMBI_DN2324_c0_g1_i1_5	4	41,6	14,952	144,57					
ARUBRA_DN6463_c0_g1_i1_2	2	10,5	26,181	12,973					
ARUBRA_DN18383_c0_g1_i1_3	1	6,3	22,675	7,7285					
ARUBRA_DN4889_c0_g1_i1_6	10	35	47,493	32,403	PF00004.28	AAA	159,8	4,10E-47	CL0023
ARHOMBI_DN6134_c0_g1_i1_6	8	18,8	55,473	142,61	PF01370.20	Epimerase	78,9	3,60E-22	CL0063
ARHOMBI_DN3483_c0_g3_i1_5	3	9,2	38,101	29,997	PF11891.7	RETICULATA-like	220,9	9,50E-66	No_clan
ARUBRA_DN11125_c0_g1_i1_4	3	45,7	8,1552	19,036	PF01991.17	vATP-synt_E	61,5	6,50E-17	CL0255
ARHOMBI_DN13455_c0_g1_i1_1	2	16,8	11,885	12,992	PF00462.23	Glutaredoxin	77,3	7,30E-22	CL0172
ARHOMBI_DN5719_c0_g1_i1_2	3	16,6	22,909	19,045	PF00380.18	Ribosomal_S9	94,4	6,50E-27	CL0329
ARHOMBI_DN25944_c0_g1_i1_6	1	13,9	7,7497	7,8502	PF02866.17	Ldh_1_C	41,8	9,70E-11	CL0341
ARHOMBI_DN1498_c0_g1_i1_1	1	6,6	12,785	6,5246	PF03168.12	LEA_2	41,2	1,70E-10	CL0159
ARUBRA_DN3905_c0_g1_i1_4	3	11,7	37,441	19,148	PF13561.5	adh_short_C2	226,7	2,50E-67	CL0063
ARUBRA_DN8419_c0_g1_i1_3	3	15,9	40,508	25,135	PF00342.18	PGI	438,7	2,20E-131	CL0067
ARUBRA_DN11134_c0_g1_i1_6	6	26,6	28,094	35,744	PF01145.24	Band_7	30,2	3,80E-07	CL0433
ARHOMBI_DN2322_c0_g1_i1_2	4	14,9	42,322	40,736	PF01263.19	Aldose_epim	136,8	8,50E-40	CL0103
ARHOMBI_DN20297_c0_g1_i1_1	6	30,9	30,991	36,52	PF00044.23	Gp_dh_N	31,2	1,90E-07	CL0063
ARUBRA_DN4829_c0_g2_i2_4	5	21,9	25,396	32,662	PF01775.16	Ribosomal_L18A	180,7	8,60E-54	No_clan
ARUBRA_DN18092_c0_g1_i1_5	6	47,2	19,353	17,089	PF08240.11	ADH_N	81,4	3,70E-23	CL0296
ARHOMBI_DN10619_c0_g1_i1_4	4	25,3	18,219	25,183	PF01602.19	Adaptin_N	121,8	2,80E-35	CL0020
ARHOMBI_DN3191_c0_g1_i1_2	3	14,1	33,923	22,986	PF00025.20	Arf	217,2	9,40E-65	CL0023
ARUBRA_DN19085_c0_g1_i1_1	8	19,9	49,225	53,288	PF02990.15	EMP70	343,9	1,40E-102	No_clan
ARHOMBI_DN5770_c0_g2_i2_6	1	3	46,542	6,5095	PF00466.19	Ribosomal_L10	66	2,40E-18	No_clan
ARHOMBI_DN3157_c0_g1_i1_6	5	23,7	24,638	30,759	PF13561.5	adh_short_C2	135,9	1,50E-39	CL0063
ARUBRA_DN21700_c0_g1_i1_4	5	16,4	33,124	31,842	PF00571.27	CBS	30,4	3,60E-07	No_clan
ARHOMBI_DN16801_c0_g1_i1_1	3	25,8	23,307	13,912					
ARUBRA_DN192_c0_g1_i1_1	4	17	25,801	6,4273	PF00153.26	Mito_carr	82,6	1,30E-23	No_clan
ARHOMBI_DN24575_c0_g1_i1_1	1	18,3	10,314	11,6	PF01432.19	Peptidase_M3	50,8	1,50E-13	CL0126
ARHOMBI_DN5209_c2_g1_i1_4	8	36,1	30,283	53,638	PF13246.5	Cation_ATPase	38,3	9,80E-10	CL0137
ARUBRA_DN1890_c0_g2_i1_3	2	18,5	21,767	13,459	PF00085.19	Thioredoxin	103,9	3,60E-30	CL0172
ARUBRA_DN3867_c0_g1_i1_1	3	12,9	42,284	30,764	PF03896.15	TRAP_alpha	87,6	7,50E-25	No_clan
ARHOMBI_DN23168_c0_g1_i1_4	2	17,9	17,57	42,428	PF12710.6	HAD	50,8	2,40E-13	CL0137

ARHOMBI_DN3469_c0_g1_i1_3	4	18,2	39,019	29,478	PF00501.27	AMP-binding	143,5	6,50E-42	CL0378
ARUBRA_DN4948_c0_g2_i1_5	3	17,6	23,925	19,446	PF01655.17	Ribosomal_L32e	167,7	7,70E-50	No_clan
ARUBRA_DN3297_c0_g1_i1_1	3	23,3	20,216	310,5	PF01204.17	Trehalase	156,2	1,30E-45	CL0059
ARUBRA_DN2696_c0_g1_i1_3	4	40,6	11,92	6,2207	PF01370.20	Epimerase	69	3,80E-19	CL0063
ARHOMBI_DN15716_c0_g1_i1_4	4	40,1	21,665	29,022	PF02136.19	NTF2	41	2,30E-10	CL0051
ARHOMBI_DN4884_c0_g1_i1_2	4	12,7	43,801	25,102	PF16363.4	GDP_Man_Dehyd	183,9	4,80E-54	CL0063
ARUBRA_DN20045_c0_g1_i1_4	3	14,8	28,253	62,734	PF03912.13	Psb28	144,6	1,00E-42	No_clan
ARHOMBI_DN1594_c0_g1_i1_2	7	45,9	24,958	47,049	PF00012.19	HSP70	195,2	1,40E-57	CL0108
ARUBRA_DN4593_c0_g2_i1_4	5	22,4	27,636	13,123	PF00411.18	Ribosomal_S11	155,8	4,00E-46	CL0267
ARUBRA_DN4903_c0_g1_i1_3	3	14,6	22,968	19,017	PF01777.17	Ribosomal_L27e	105,4	1,40E-30	No_clan
ARHOMBI_DN18322_c0_g1_i1_3	3	69,9	8,8828	7,618	PF02878.15	PGM_PMM_I	56,9	1,70E-15	No_clan
ARUBRA_DN11129_c0_g1_i1_5	3	26,1	13,111	19,759					
ARUBRA_DN24080_c0_g1_i1_2	8	31,5	33,928	45,917	PF00224.20	PK	382,8	1,20E-114	CL0151
ARUBRA_DN10280_c0_g1_i1_1	5	46,3	15,144	323,31	PF09032.10	Siah-Interact_N	29,8	5,00E-07	No_clan
ARUBRA_DN19519_c0_g1_i1_5	3	37,8	12,471	45,578	PF14560.5	Ubiquitin_2	99,6	9,20E-29	CL0072
ARUBRA_DN5259_c0_g1_i1_6	6	22,2	50,999	235,43	PF02469.21	Fasciclin	24,7	2,00E-05	No_clan
ARUBRA_DN4393_c0_g2_i1_4	6	14,4	64,697	39,357					
ARHOMBI_DN5839_c1_g1_i1_5	4	21,3	21,658	39,983	PF00416.21	Ribosomal_S13	163,7	2,40E-48	CL0303
ARUBRA_DN10773_c0_g1_i1_4	3	27,2	19,907	74,029	PF01182.19	Glucosamine_iso	195,4	1,10E-57	CL0246
ARUBRA_DN21519_c0_g1_i1_5	4	19,5	26,789	12,909	PF08079.11	Ribosomal_L30_N	94,2	3,80E-27	No_clan
ARUBRA_DN6882_c0_g1_i1_3	3	23,9	16,386	30,664	PF00023.29	Ank	26,5	5,40E-06	CL0465
ARHOMBI_DN22543_c0_g1_i1_3	1	16,5	13,466	12,885	PF02298.16	Cu_bind_like	83,6	6,70E-24	CL0026
ARUBRA_DN9648_c0_g1_i1_1	3	14,3	31,248	22,447	PF04969.15	CS	36	1,00E-08	CL0190
ARUBRA_DN5039_c0_g1_i1_2	7	26,1	44,142	39,238	PF16884.4	ADH_N_2	89,4	1,20E-25	CL0296
ARHOMBI_DN18646_c0_g1_i1_5	2	15,1	18,271	12,607	PF06592.12	DUF1138	146,7	1,40E-43	No_clan
ARHOMBI_DN4447_c0_g1_i1_4	5	17,3	42,441	33,663	PF02629.18	CoA_binding	110,4	4,70E-32	CL0063
ARUBRA_DN2978_c0_g1_i1_6	4	17,8	25,242	27,369	PF04716.13	ETC_C1_NDUFA5	91,4	2,10E-26	No_clan
ARUBRA_DN7999_c0_g1_i1_2	6	24,7	40,174	37,7	PF00118.23	Cpn60_TCP1	283,6	2,50E-84	No_clan
ARHOMBI_DN5214_c0_g1_i1_4	3	18,7	28,192	179,77	PF00314.16	Thaumatin	215,6	6,40E-64	CL0293
ARUBRA_DN4195_c0_g1_i1_4	2	7,5	29,231	11,636	PF16205.4	Ribosomal_S17_N	109,5	6,90E-32	No_clan
ARHOMBI_DN6230_c0_g2_i3_1	3	10,2	24,599	17,199	PF00833.17	Ribosomal_S17e	163,9	1,30E-48	No_clan
ARUBRA_DN8665_c0_g1_i1_5	4	34,3	19,052	30,033	PF00400.31	WD40	25,6	1,40E-05	CL0186

ARHOMBI_DN19852_c0_g1_i1_3	4	59,8	9,8781	27,227	PF03141.15	Methyltransf_29	70,4	1,00E-19	CL0063
ARUBRA_DN7713_c0_g2_i1_1	4	17,8	32,342	25,178	PF01145.24	Band_7	72,2	4,80E-20	CL0433
ARHOMBI_DN15828_c0_g1_i1_6	5	21,8	31,261	33,137	PF09668.9	Asp_protease	184,1	6,40E-55	CL0129
ARHOMBI_DN965_c0_g1_i1_1	2	14,8	27,164	32,717	PF01217.19	Clat_adaptor_s	76,9	1,30E-21	CL0212
ARHOMBI_DN4105_c0_g1_i1_1	6	19,5	43,071	26,399	PF00244.19	14.03.2003	350,9	2,30E-105	No_clan
ARUBRA_DN6657_c0_g1_i1_6	3	51,3	12,425	25,926	PF05817.13	Ribophorin_II	56,9	1,40E-15	No_clan
ARUBRA_DN13405_c0_g1_i1_1	3	57,9	8,5656	17,981	PF00501.27	AMP-binding	27,4	1,10E-06	CL0378
ARUBRA_DN1195_c0_g1_i1_2	4	14,6	32,658	25,294	PF03737.14	RraA-like	147,7	2,50E-43	CL0364
ARUBRA_DN2242_c0_g1_i1_5	3	15,2	31,712	20,109	PF00311.16	PEPcase	247,9	1,80E-73	CL0151
ARHOMBI_DN3366_c0_g1_i1_4	3	12,7	39,707	19,095	PF01459.21	Porin_3	211	2,00E-62	CL0193
ARUBRA_DN6838_c0_g1_i1_3	5	21,9	28,2	30,863					
ARUBRA_DN10378_c0_g1_i1_1	4	47,9	10,759	23,903	PF00224.20	PK	25,8	4,20E-06	CL0151
ARHOMBI_DN5914_c0_g1_i1_4	4	9,2	62,314	26,291	PF01370.20	Epimerase	156,5	7,20E-46	CL0063
ARHOMBI_DN5134_c0_g1_i1_6	7	12,2	78,495	43,253	PF00005.26	ABC_tran	81,5	7,50E-23	CL0023
ARUBRA_DN16002_c0_g1_i1_2	1	25,4	7,2218	133,63					
ARUBRA_DN6617_c0_g1_i1_2	6	25,4	36,503	42,719	PF01182.19	Glucosamine_iso	239,6	3,40E-71	CL0246
ARHOMBI_DN7393_c0_g1_i1_3	6	28,8	30,312	137,53	PF00071.21	Ras	217,5	7,00E-65	CL0023
ARHOMBI_DN1339_c0_g1_i1_3	5	35,4	17,735	69,586	PF00481.20	PP2C	21,3	0,00016	CL0238
ARHOMBI_DN16218_c0_g1_i1_1	2	19	12,981	116,39	PF01179.19	Cu_amine_oxid	82,2	3,20E-23	No_clan
ARUBRA_DN3982_c0_g2_i1_1	7	22,3	43,955	40,496	PF05470.11	eIF-3c_N	94,2	5,80E-27	No_clan
ARHOMBI_DN2364_c0_g1_i1_5	7	32,7	30,42	45,486	PF14938.5	SNAP	253,2	2,90E-75	CL0020
ARHOMBI_DN5683_c0_g1_i2_5	7	18,3	58,574	45,292	PF01399.26	PCI	77	1,30E-21	CL0123
ARUBRA_DN14982_c0_g1_i1_3	2	12,9	17,651	173,26	PF13193.5	AMP-binding_C	64,4	1,30E-17	CL0531
ARHOMBI_DN21103_c0_g1_i1_1	2	17,7	21,383	51,846	PF00332.17	Glyco_hydro_17	173,6	6,10E-51	CL0058
ARUBRA_DN18762_c0_g1_i1_4	5	14,8	58,769	323,31	PF01363.20	FYVE	72,8	1,80E-20	CL0390
ARUBRA_DN4063_c0_g1_i2_5	4	13,5	45,814	27,697	PF00575.22	S1	49,3	4,40E-13	CL0021
ARUBRA_DN11481_c0_g1_i1_6	6	14,2	52,171	35,569	PF13460.5	NAD_binding_10	43,9	2,20E-11	CL0063
ARUBRA_DN934_c0_g1_i1_3	6	25,6	35,861	37,027	PF00227.25	Proteasome	113,1	1,00E-32	CL0052
ARUBRA_DN3268_c0_g1_i1_2	5	23,1	33,3	30,834	PF00224.20	PK	312,2	3,40E-93	CL0151
ARHOMBI_DN2479_c0_g1_i1_1	2	33	11,867	14,009					
ARUBRA_DN1490_c0_g1_i1_3	3	10,3	49,882	20,997	PF02779.23	Transket_pyr	157,6	2,30E-46	CL0254
ARUBRA_DN20284_c0_g1_i1_5	4	50,4	15,289	28,594	PF00565.16	SNase	35	1,50E-08	CL0049

ARUBRA_DN12380_c0_g1_i1_5	1	4,4	19,896	6,2972					
ARHOMBI_DN16116_c0_g1_i1_3	4	16,4	34,465	23,558					
ARHOMBI_DN6092_c0_g1_i1_1	8	24,3	47,805	242,95	PF00291.24	PALP	223,2	4,20E-66	No_clan
ARHOMBI_DN15878_c0_g1_i1_3	2	11,2	16,442	22,632	PF00076.21	RRM_1	45,8	3,80E-12	CL0221
ARHOMBI_DN2833_c0_g1_i1_1	5	18,8	36,889	42,483	PF01412.17	ArfGap	115,2	1,50E-33	No_clan
ARUBRA_DN16146_c0_g1_i1_4	6	28,2	31,374	43,344	PF06552.11	TOM20_plant	320,5	3,00E-96	CL0020
ARHOMBI_DN10766_c0_g1_i1_6	2	16,1	20,747	301,25	PF01928.20	CYTH	40,8	2,10E-10	CL0273
ARHOMBI_DN5588_c0_g1_i1_3	5	15,9	40,901	7,3621	PF00210.23	Ferritin	108	3,40E-31	CL0044
ARUBRA_DN22820_c0_g1_i1_3	2	22,2	10,068	127,39	PF06094.11	GGACT	28	2,50E-06	CL0278
ARHOMBI_DN5983_c0_g1_i1_5	4	10,1	50,69	27,057	PF00389.29	2-Hacid_dh	56,1	2,80E-15	CL0325
ARUBRA_DN20709_c0_g1_i1_5	5	47,3	18,835	18,225	PF01432.19	Peptidase_M3	133,7	1,00E-38	CL0126
ARUBRA_DN1983_c0_g1_i1_4	2	10,9	27,181	14,038	PF06417.11	DUF1077	129,5	5,70E-38	No_clan
ARHOMBI_DN3005_c0_g2_i1_6	3	14,8	26,537	21,43	PF01282.18	Ribosomal_S24e	128,6	6,20E-38	No_clan
ARHOMBI_DN5420_c0_g1_i1_2	7	17,8	57,974	50,383	PF00004.28	AAA	134,2	3,30E-39	CL0023
ARUBRA_DN3944_c0_g1_i1_3	5	25	30,442	34,027	PF00364.21	Biotin_lipoyl	52,6	2,80E-14	CL0105
ARHOMBI_DN18815_c0_g1_i1_6	2	18	16,872	37,865	PF16035.4	Chalcone_2	27,2	2,80E-06	CL0560
ARHOMBI_DN5059_c0_g1_i1_5	3	9,9	52,884	23,322	PF00012.19	HSP70	28	5,70E-07	CL0108
ARHOMBI_DN5953_c0_g1_i1_4	10	17,9	84,803	40,109	PF00076.21	RRM_1	70,1	9,60E-20	CL0221
ARUBRA_DN193_c0_g1_i1_1	3	13,8	25,423	17,937	PF01246.19	Ribosomal_L24e	100,7	3,60E-29	CL0175
ARUBRA_DN7445_c0_g1_i1_4	4	23,1	21,995	48,254					
ARHOMBI_DN18779_c0_g1_i1_3	2	11,2	29,815	51,239	PF00076.21	RRM_1	58,3	4,50E-16	CL0221
ARUBRA_DN19198_c0_g1_i1_2	6	45,2	21,454	34,835	PF00183.17	HSP90	157,1	6,60E-46	No_clan
ARHOMBI_DN5769_c0_g1_i1_5	3	12,3	32,01	17,65	PF00572.17	Ribosomal_L13	33,6	3,60E-08	No_clan
ARHOMBI_DN18804_c0_g1_i1_4	2	16,5	12,198	12,197	PF01704.17	UDPGP	21,7	6,00E-05	CL0110
ARHOMBI_DN7_c0_g2_i1_2	3	23,6	19,249	22,83	PF00113.21	Enolase_C	183,5	4,70E-54	CL0256
ARUBRA_DN3923_c0_g1_i1_5	2	11,7	19,948	323,31					
ARHOMBI_DN6177_c0_g2_i2_3	3	10,3	17,915	98,829					
ARHOMBI_DN24695_c0_g1_i1_4	2	26,3	12,228	67,773					
ARHOMBI_DN2476_c0_g1_i1_4	8	53	26,826	23,473	PF02874.22	ATP-synt_ab_N	53,3	2,70E-14	CL0275
ARHOMBI_DN22511_c0_g1_i1_1	3	22,8	22,025	120,62					
ARHOMBI_DN3114_c0_g1_i1_1	5	12,6	49,444	29,855	PF10559.8	Plug_translocon	65,8	2,10E-18	No_clan
ARUBRA_DN16322_c0_g1_i1_4	6	20,7	50,417	36,351	PF00491.20	Arginase	223,5	3,50E-66	CL0302
ARUBRA_DN3916_c0_g1_i1_5	6	43,3	30,628	22,901	PF00009.26	GTP_EFTU	218,8	4,20E-65	CL0023

ARUBRA_DN4811_c0_g2_i1_4	1	2,9	38,002	40,375	PF05184.14	SapB_1	36	4,80E-09	No_clan
ARUBRA_DN18543_c0_g1_i1_6	6	25,8	31,875	38,576					
ARHOMBI_DN3540_c0_g1_i1_2	6	28,9	34,333	31,812	PF00071.21	Ras	208,5	4,10E-62	CL0023
ARUBRA_DN478_c0_g1_i1_2	2	11,5	27,917	13,829	PF01641.17	SelR	156,4	2,90E-46	CL0080
ARUBRA_DN20269_c0_g1_i1_6	3	50,5	10,606	21,207	PF00400.31	WD40	26,5	7,40E-06	CL0186
ARUBRA_DN20878_c0_g1_i1_1	5	17,3	47,866	23,692	PF00694.18	Aconitase_C	167,4	1,70E-49	CL0364
ARHOMBI_DN912_c0_g1_i1_6	9	29,1	52,016	58,724	PF07718.11	Coatamer_beta_C	203,7	9,60E-61	CL0159
ARUBRA_DN11488_c0_g1_i1_6	3	37,5	12,487	22,053	PF03952.15	Enolase_N	163,1	3,20E-48	CL0227
ARUBRA_DN19442_c0_g1_i1_2	4	25,7	21,862	272,59					
ARUBRA_DN4301_c0_g1_i1_3	5	26,9	26,827	34,582	PF05564.11	Auxin_repressed	187,5	9,00E-56	No_clan
ARUBRA_DN16241_c0_g1_i1_5	2	9,7	32,015	11,671	PF05753.13	TRAP_beta	184,6	9,90E-55	CL0159
ARUBRA_DN6197_c0_g1_i1_1	1	12,9	9,5467	7,5631	PF01095.18	Pectinesterase	125,3	1,80E-36	CL0268
ARUBRA_DN5359_c0_g1_i1_3	9	21,2	72,652	54,842	PF01268.18	FTHFS	833,8	4,40E-251	CL0023
ARUBRA_DN20048_c0_g1_i1_1	1	21,5	8,3254	21,097	PF16363.4	GDP_Man_Dehyd	51,6	8,50E-14	CL0063
ARHOMBI_DN6095_c0_g2_i1_5	6	12,8	56,418	40,821	PF00226.30	DnaJ	79,1	1,80E-22	CL0392
ARUBRA_DN4711_c0_g2_i1_6	7	54,8	20,893	10,474	PF00022.18	Actin	201,9	1,10E-59	CL0108
ARUBRA_DN2566_c0_g2_i1_5	7	13,2	85,713	49,139	PF01326.18	PPDK_N	47,5	1,40E-12	CL0179
ARHOMBI_DN6176_c1_g1_i1_5	2	5,1	43,05	23,197	PF00141.22	peroxidase	270,7	9,80E-81	CL0617
ARUBRA_DN413_c0_g1_i1_3	1	8,2	9,7039	35,484					
ARUBRA_DN16443_c0_g1_i1_3	3	31,2	12,059	18,226	PF13838.5	Clathrin_H_link	73	1,30E-20	CL0020
ARHOMBI_DN15325_c0_g1_i1_4	2	11,2	26,47	12,959	PF06747.12	CHCH	27,6	2,10E-06	CL0351
ARHOMBI_DN5371_c0_g1_i1_2	2	16,5	24,572	16,64	PF02271.15	UCR_14kD	95,8	1,10E-27	No_clan
ARUBRA_DN4439_c0_g1_i1_3	3	14,7	23,629	13,774	PF01776.16	Ribosomal_L22e	156	3,50E-46	No_clan
ARHOMBI_DN24520_c0_g1_i1_6	1	15	8,8213	6,4672	PF00171.21	Aldedh	34,3	8,90E-09	CL0099
ARHOMBI_DN900_c0_g1_i1_2	5	24,6	48,212	32,779	PF00106.24	adh_short	92,5	2,10E-26	CL0063
ARUBRA_DN2910_c0_g1_i1_5	2	6,8	36,93	11,76	PF01248.25	Ribosomal_L7Ae	87,5	3,40E-25	CL0101
ARHOMBI_DN5383_c0_g1_i1_2	1	7	18,353	6,2213					
ARUBRA_DN1777_c0_g1_i1_3	1	10,7	13,556	6,6706	PF02672.14	CP12	29,5	8,40E-07	No_clan
ARHOMBI_DN4470_c0_g1_i1_6	6	26,4	32,328	39,531	PF00224.20	PK	173,9	3,90E-51	CL0151
ARUBRA_DN22163_c0_g1_i1_3	5	30,9	24,325	94,899	PF07992.13	Pyr_redox_2	30,7	1,70E-07	CL0063
ARHOMBI_DN13192_c0_g1_i1_2	3	41,3	11,413	16,151	PF00282.18	Pyridoxal_deC	79,4	1,90E-22	CL0061
ARHOMBI_DN54_c0_g1_i1_2	4	19,4	21,645	26,294	PF00012.19	HSP70	313,5	2,00E-93	CL0108

ARHOMBI_DN4900_c0_g1_i1_5	5	24,2	26,365	255,82	PF04398.11	DUF538	115,4	1,60E-33	No_clan
ARHOMBI_DN13072_c0_g1_i1_6	2	37,5	9,0194	33,411	PF00215.23	OMPdecase	85,6	3,40E-24	CL0036
ARUBRA_DN14237_c0_g1_i1_6	2	42,2	8,2377	11,384					
ARHOMBI_DN18431_c0_g1_i1_1	3	21,1	27,792	19,062	PF02779.23	Transket_pyr	204,2	1,10E-60	CL0254
ARUBRA_DN8433_c0_g1_i1_6	2	26,4	12,105	14,449	PF02545.13	Maf	51,7	8,00E-14	CL0269
ARHOMBI_DN19091_c0_g1_i1_5	4	19,8	28,447	127,4	PF01928.20	CYTH	77,1	1,40E-21	CL0273
ARUBRA_DN18317_c0_g1_i1_5	5	26,8	29,471	52,914	PF01323.19	DSBA	119,3	1,50E-34	CL0172
ARHOMBI_DN22440_c0_g1_i1_6	2	28,6	13,458	14,047	PF00069.24	Pkinase	57,2	1,40E-15	CL0016
ARHOMBI_DN8601_c0_g1_i1_4	3	23,5	18,29	36,818	PF05817.13	Ribophorin_II	154,1	5,00E-45	No_clan
ARHOMBI_DN25934_c0_g1_i1_3	6	38,9	19,991	29,815	PF00071.21	Ras	211,6	4,30E-63	CL0023
ARHOMBI_DN13008_c0_g1_i1_6	2	23,3	10,156	132,42	PF00179.25	UQ_con	60	1,70E-16	CL0208
ARUBRA_DN1845_c0_g1_i1_5	4	11,9	50,73	26,257	PF02629.18	CoA_binding	23,8	5,20E-05	CL0063
ARUBRA_DN4305_c0_g2_i1_6	6	13,9	56,864	39,173	PF00004.28	AAA	137,2	4,10E-40	CL0023
ARUBRA_DN13152_c0_g1_i1_3	2	35,2	9,6997	14,15					
ARUBRA_DN3999_c0_g1_i1_6	2	12	27,331	-2	PF02776.17	TPP_enzyme_N	41,6	9,00E-11	CL0254
ARUBRA_DN5726_c0_g1_i1_1	5	15,1	45,973	30,927	PF01756.18	ACOX	183	3,60E-54	CL0087
ARHOMBI_DN3987_c0_g1_i1_4	4	12,5	43,638	31,956	PF00627.30	UBA	34,5	1,30E-08	CL0214
ARHOMBI_DN9641_c0_g1_i1_3	5	22,1	29,777	29,716	PF00270.28	DEAD	171	1,80E-50	CL0023
ARUBRA_DN16264_c0_g1_i1_5	5	11,5	62,316	30,731	PF00676.19	E1_dh	271,7	5,30E-81	CL0254
ARHOMBI_DN15803_c0_g1_i1_2	2	10	29,856	7,089	PF04752.11	ChaC	140,9	4,00E-41	CL0278
ARUBRA_DN1574_c0_g2_i1_2	2	33,9	12,379	14,961					
ARHOMBI_DN1369_c0_g1_i1_5	1	4,9	21,215	65,035	PF00428.18	Ribosomal_60s	82,8	1,90E-23	No_clan
ARUBRA_DN8743_c0_g1_i1_3	2	29,5	9,8755	12,738					
ARHOMBI_DN1764_c0_g1_i1_5	4	11	52,226	24,016	PF00733.20	Asn_synthase	140,6	9,00E-41	CL0039
ARHOMBI_DN5253_c0_g2_i1_2	5	17,8	46,707	30,677	PF00316.19	FBPase	263,8	6,20E-79	CL0171
ARHOMBI_DN5557_c0_g1_i1_6	5	10,3	60,562	29,243	PF16363.4	GDP_Man_Dehyd	177	5,90E-52	CL0063
ARHOMBI_DN27310_c0_g1_i1_4	3	56,9	7,7525	43,696					
ARUBRA_DN99_c0_g1_i1_2	6	27,7	32,754	37,202	PF16191.4	E1_4HB	82,3	1,90E-23	No_clan
ARUBRA_DN3903_c0_g1_i1_1	8	20,9	69,201	55,538	PF00587.24	tRNA-synt_2b	64,9	8,10E-18	CL0040
ARUBRA_DN620_c0_g1_i1_5	1	11,2	14,549	7,5385	PF09298.10	FAA_hydrolase_N	121,5	1,50E-35	No_clan
ARUBRA_DN7726_c0_g1_i1_6	3	33,8	17,244	20,573	PF00483.22	NTP_transferase	76,5	2,20E-21	CL0110
ARHOMBI_DN12388_c0_g1_i1_3	3	36,4	15,395	28,011	PF00400.31	WD40	22,1	0,00019	CL0186
ARHOMBI_DN5442_c0_g1_i1_6	11	23,7	59,167	38,085	PF00270.28	DEAD	138,2	2,20E-40	CL0023

ARHOMBI_DN97_c0_g1_i1_1	4	26,3	24,18	106,36					
ARHOMBI_DN6380_c0_g1_i1_4	1	16,4	7,8938	6,4686					
ARUBRA_DN7925_c0_g1_i1_2	3	33,6	13,904	20,238					
ARHOMBI_DN4703_c0_g1_i1_3	5	21,4	42,678	34,293	PF00132.23	Hexapep	22,2	7,60E-05	CL0536
ARUBRA_DN2228_c0_g2_i1_2	5	25,3	38,882	35,626	PF00984.18	UDPG_MGDP_dh	110,5	3,10E-32	CL0106
ARUBRA_DN23264_c0_g1_i1_3	2	58,3	7,3703	18,538					
ARUBRA_DN22823_c0_g1_i1_2	6	22	33,482	323,31	PF00218.20	IGPS	253,2	1,90E-75	CL0036
ARHOMBI_DN3499_c0_g1_i1_2	5	9,2	80,152	34,603	PF01749.19	IBB	86,8	8,60E-25	CL0020
ARHOMBI_DN6537_c0_g1_i1_4	3	38,9	16,461	21,501	PF00637.19	Clathrin	77,3	9,70E-22	CL0020
ARUBRA_DN16598_c0_g1_i1_4	2	27,7	10,328	12,37	PF00626.21	Gelsolin	30,1	3,10E-07	CL0092
ARUBRA_DN3848_c0_g2_i1_1	3	11,3	36,94	19,151	PF00179.25	UQ_con	127,4	2,70E-37	CL0208
ARUBRA_DN16655_c0_g1_i1_1	4	16	43,455	27,57	PF13510.5	Fer2_4	67	1,10E-18	CL0486
ARHOMBI_DN2235_c0_g1_i1_5	6	21,3	37,176	181,48	PF00013.28	KH_1	51,8	5,00E-14	CL0007
ARUBRA_DN19778_c0_g1_i1_3	4	11,7	40,646	23,015	PF10151.8	TMEM214	54,5	6,60E-15	No_clan
ARUBRA_DN5666_c0_g1_i1_2	5	18,4	43,464	33,499	PF09440.9	eIF3_N	46,6	4,10E-12	No_clan
ARUBRA_DN5508_c0_g1_i1_2	3	16,1	29,053	45,034	PF00173.27	Cyt-b5	87,1	6,00E-25	No_clan
ARUBRA_DN3962_c0_g1_i1_6	4	10,1	43,425	26,292	PF00400.31	WD40	12,3	0,23	CL0186
ARUBRA_DN20257_c0_g1_i1_3	3	37,7	11,988	15,196	PF01263.19	Aldose_epim	70,4	1,40E-19	CL0103
ARUBRA_DN11024_c0_g1_i1_6	3	52,3	11,851	106,34	PF05822.11	UMPH-1	89,5	2,20E-25	CL0137
ARUBRA_DN21569_c0_g1_i1_4	3	9,4	48,848	19,713	PF01398.20	JAB	110,4	4,40E-32	CL0366
ARUBRA_DN965_c0_g1_i1_3	2	23,4	12,265	7,8947	PF00349.20	Hexokinase_1	115,2	3,50E-33	CL0108
ARHOMBI_DN27321_c0_g1_i1_2	4	55,1	11,267	32,034					
ARUBRA_DN1379_c0_g2_i1_4	3	9,5	56,571	20,429	PF00282.18	Pyridoxal_deC	72,6	2,20E-20	CL0061
ARUBRA_DN21229_c0_g1_i1_6	2	17	18,325	11,9	PF04597.13	Ribophorin_I	114,2	7,90E-33	No_clan
ARUBRA_DN16619_c0_g1_i1_3	4	13,8	35,744	25,599	PF00294.23	PfkB	253,4	2,80E-75	CL0118
ARHOMBI_DN17281_c0_g1_i1_1	3	46,7	9,9854	20,871	PF13848.5	Thioredoxin_6	28,8	9,80E-07	CL0172
ARHOMBI_DN4623_c0_g1_i1_3	5	26	21,686	32,753					
ARUBRA_DN6168_c0_g1_i1_1	2	21,2	11,573	12,075	PF07944.11	Glyco_hydro_127	77,4	7,70E-22	CL0059
ARHOMBI_DN24755_c0_g1_i1_2	1	9,7	9,6641	6,5751	PF00330.19	Aconitase	119,8	1,40E-34	No_clan
ARUBRA_DN4123_c0_g1_i1_5	6	21,4	52,362	37,91	PF02403.21	Seryl_tRNA_N	67,2	1,20E-18	CL0298
ARUBRA_DN9_c0_g1_i1_4	2	6,9	41,65	12,603	PF03141.15	Methyltransf_29	382,9	2,00E-114	CL0063
ARHOMBI_DN6166_c0_g1_i1_6	7	11,4	80,836	41,72	PF00587.24	tRNA-synt_2b	39,1	6,70E-10	CL0040

ARUBRA_DN1535_c0_g1_i1_5	1	4,6	26,147	6,5532	PF00343.19	Phosphorylase	235	1,50E-69	CL0113
ARUBRA_DN17285_c0_g1_i1_2	3	34,1	18,249	24,232	PF16189.4	Creatinase_N_2	128,6	2,10E-37	CL0356
ARHOMBI_DN20415_c0_g1_i1_5	2	24,7	10,169	17,24					
ARHOMBI_DN6137_c0_g1_i1_4	8	11	116,17	51,111	PF02861.19	Clp_N	62,4	3,00E-17	No_clan
ARUBRA_DN20044_c0_g1_i1_6	2	33	10,908	15,463	PF00118.23	Cpn60_TCP1	80,8	7,50E-23	No_clan
ARUBRA_DN20625_c0_g1_i1_2	5	47,2	13,637	278,57	PF00977.20	His_biosynth	54,7	8,80E-15	CL0036
ARUBRA_DN21250_c0_g1_i1_6	3	33,3	9,2003	12,099	PF02518.25	HATPase_c	34,3	2,60E-08	CL0025
ARUBRA_DN4028_c0_g1_i2_4	3	16,1	27,868	12,578	PF01201.21	Ribosomal_S8e	192,7	2,60E-57	No_clan
ARUBRA_DN12697_c0_g1_i1_5	2	36,1	7,8577	12,813	PF00675.19	Peptidase_M16	74,2	9,80E-21	CL0094
ARUBRA_DN17667_c0_g1_i1_4	2	9,8	26,161	12,433					
ARHOMBI_DN16026_c0_g1_i1_1	6	15,8	51,25	34,403					
ARUBRA_DN16494_c0_g1_i1_3	4	22,6	27,546	24,604	PF00118.23	Cpn60_TCP1	211,5	1,80E-62	No_clan
ARUBRA_DN6330_c0_g1_i1_4	5	11,8	62,363	29,6	PF01979.19	Amidohydro_1	100,6	9,80E-29	CL0034
ARUBRA_DN13172_c0_g1_i1_1	1	5,4	16,774	17,8	PF00924.17	MS_channel	24,8	1,20E-05	No_clan
ARHOMBI_DN6108_c0_g1_i2_2	2	29,2	11,46	8,6131					
ARUBRA_DN20543_c0_g1_i1_1	4	34,2	17,514	36,914	PF00722.20	Glyco_hydro_16	112	2,00E-32	CL0004
ARUBRA_DN3626_c0_g2_i1_5	6	23,9	38,802	206,96	PF00627.30	UBA	55,2	4,10E-15	CL0214
ARHOMBI_DN3946_c0_g1_i1_1	4	12,8	41,386	25,871	PF05755.11	REF	317,9	2,80E-95	No_clan
ARUBRA_DN17212_c0_g1_i1_1	3	25,1	19,346	19,606	PF02770.18	Acyl-CoA_dh_M	47,3	1,70E-12	No_clan
ARHOMBI_DN1738_c0_g2_i1_2	4	38,3	13,735	93,511					
ARUBRA_DN4695_c0_g1_i1_1	1	4,8	30,724	6,3665					
ARUBRA_DN6843_c0_g1_i1_2	5	11,2	65,388	29,657	PF00171.21	Aldedh	575,6	5,70E-173	CL0099
ARHOMBI_DN565_c0_g1_i1_1	1	8,4	11,099	6,5463	PF02826.18	2-Hacid_dh_C	27,2	2,00E-06	CL0063
ARUBRA_DN12655_c0_g1_i1_2	3	21,4	15,64	18,352					
ARHOMBI_DN7486_c0_g1_i1_4	1	6,8	16,408	6,1653					
ARHOMBI_DN3745_c0_g1_i1_6	2	20,2	13,843	33,613					
ARHOMBI_DN161_c0_g2_i1_2	6	26,3	39,079	39,22	PF10075.8	CSN8_PSD8_EIF3K	89,1	2,40E-25	CL0123
ARHOMBI_DN4381_c0_g1_i1_4	3	12,2	26,839	21,148	PF09066.9	B2-adapt-app_C	108,1	2,30E-31	CL0545
ARUBRA_DN4254_c0_g2_i1_1	5	22,7	43,236	141,1	PF00270.28	DEAD	132,6	1,20E-38	CL0023
ARUBRA_DN4783_c0_g1_i1_3	3	10,1	44,797	18,828	PF00155.20	Aminotran_1_2	128,1	4,20E-37	CL0061
ARHOMBI_DN4848_c0_g1_i1_6	6	19,8	40,991	40,275	PF00227.25	Proteasome	163	5,00E-48	CL0052
ARHOMBI_DN16223_c0_g1_i1_5	3	19,6	24,372	21,791	PF00675.19	Peptidase_M16	118	3,10E-34	CL0094

ARHOMBI_DN19081_c0_g1_i1_3	3	42,5	12,592	18,237	PF00343.19	Phosphorylase	106,7	9,50E-31	CL0113
ARUBRA_DN24202_c0_g1_i1_3	2	14	15,138	31,535	PF01928.20	CYTH	33,4	3,80E-08	CL0273
ARUBRA_DN3211_c0_g1_i1_4	2	7	25,618	101,66	PF00505.18	HMG_box	79,7	1,50E-22	CL0114
ARUBRA_DN20320_c0_g1_i1_4	4	19,3	30,042	25,507	PF13561.5	adh_short_C2	134,7	3,30E-39	CL0063
ARUBRA_DN4936_c0_g2_i1_2	4	23,3	30,64	7,2864	PF03931.14	Skp1_POZ	106,6	4,80E-31	CL0033
ARUBRA_DN9672_c0_g1_i1_4	2	14,2	22,372	8,167	PF03030.15	H_PPase	82,7	2,20E-23	No_clan
ARUBRA_DN19765_c0_g1_i1_2	2	9,1	27,73	14,71	PF04979.13	IPP-2	95,4	3,90E-27	No_clan
ARUBRA_DN3204_c0_g2_i1_1	4	19,4	23,652	24,149	PF13774.5	Longin	54,7	7,20E-15	No_clan
ARUBRA_DN3663_c0_g1_i1_5	5	16,5	37,839	29,233	PF00118.23	Cpn60_TCP1	293,4	2,60E-87	No_clan
ARHOMBI_DN23207_c0_g1_i1_1	1	6,1	16,02	15,938					
ARHOMBI_DN10568_c0_g1_i1_2	3	12,4	42,349	20,757	PF00118.23	Cpn60_TCP1	275,9	5,40E-82	No_clan
ARUBRA_DN26415_c0_g1_i1_1	2	26,1	10,609	12,511	PF01918.20	Alba	46,6	2,10E-12	CL0441
ARHOMBI_DN4088_c0_g1_i1_3	6	14,9	54,48	41,777	PF01571.20	GCV_T	293,3	1,40E-87	CL0289
ARHOMBI_DN5585_c0_g1_i1_3	8	25	59,652	109,32	PF00790.18	VHS	90,3	9,40E-26	CL0009
ARHOMBI_DN14389_c0_g1_i1_3	3	35,2	10,247	17,94	PF00311.16	PEPcase	132,1	1,80E-38	CL0151
ARUBRA_DN11036_c0_g1_i1_1	3	24	18,207	86,269	PF07992.13	Pyr_redox_2	68,4	5,70E-19	CL0063
ARHOMBI_DN6168_c0_g1_i1_4	5	6,5	87,454	28,302	PF03219.13	TLC	655,6	4,20E-197	CL0015
ARHOMBI_DN1051_c0_g1_i1_2	2	20,7	17,658	18,1	PF00080.19	Sod_Cu	100,7	7,30E-29	No_clan
ARHOMBI_DN4618_c0_g1_i1_5	1	4,4	30,634	17,938	PF00255.18	GSHPx	140	1,70E-41	CL0172
ARUBRA_DN5779_c0_g1_i1_6	5	23,6	38,481	13,38	PF14290.5	DUF4370	390,9	2,30E-117	No_clan
ARHOMBI_DN11483_c0_g1_i1_6	1	8,7	21,087	9,5529	PF08498.9	Sterol_MT_C	115	1,30E-33	No_clan
ARHOMBI_DN15111_c0_g1_i1_2	4	24,7	29,131	26,473	PF00956.17	NAP	196,3	4,80E-58	No_clan
ARUBRA_DN343_c0_g2_i1_5	1	7,5	22,088	6,4253	PF00428.18	Ribosomal_60s	90,5	7,40E-26	No_clan
ARHOMBI_DN12998_c0_g1_i1_2	3	43,8	8,4186	18,055	PF00400.31	WD40	13,3	0,11	CL0186
ARHOMBI_DN1655_c0_g1_i1_1	8	18	63,654	145,52	PF00275.19	EPSP_synthase	483,4	4,60E-145	CL0290
ARUBRA_DN8461_c0_g1_i1_4	2	12,7	22,871	13,63	PF04398.11	DUF538	111,7	2,20E-32	No_clan
ARHOMBI_DN17992_c0_g1_i1_1	4	15,2	43,231	33,839	PF03141.15	Methyltransf_29	355,6	3,70E-106	CL0063
ARUBRA_DN796_c0_g1_i1_5	5	22,4	31,831	30,027					
ARHOMBI_DN11775_c0_g1_i1_1	2	22,6	11,121	12,693					

ARUBRA_DN3727_c0_g1_i1_4	2	17,1	12,926	107,48	PF13774.5	Longin	76,6	1,00E-21	No_clan
ARUBRA_DN7635_c0_g1_i1_4	7	18,1	55,815	44,323	PF00342.18	PGI	157,3	4,60E-46	CL0067
ARHOMBI_DN17384_c0_g1_i1_1	4	17,6	36,601	27,513	PF13774.5	Longin	84	5,30E-24	No_clan
ARHOMBI_DN8440_c0_g1_i1_5	5	30	33,177	36,441	PF02127.14	Peptidase_M18	316,1	3,20E-94	CL0035
ARHOMBI_DN4904_c0_g1_i1_1	3	11	35,448	13,034	PF00071.21	Ras	203,6	1,30E-60	CL0023
ARUBRA_DN24171_c0_g1_i1_6	1	37,3	8,3212	13,44	PF10585.8	UBA_e1_thiolCys	50,9	1,80E-13	No_clan
ARHOMBI_DN15505_c0_g1_i1_1	1	15,2	11,368	22,093	PF00083.23	Sugar_tr	80,3	1,20E-22	CL0015
ARUBRA_DN19884_c0_g1_i1_4	2	10,5	29,93	43,567	PF12796.6	Ank_2	35	1,50E-08	CL0465
ARHOMBI_DN2480_c0_g1_i1_6	5	26,3	27,748	51,935	PF14622.5	Ribonucleas_3_3	42	9,00E-11	CL0539
ARHOMBI_DN5911_c0_g2_i1_1	5	9,8	70,017	33,862	PF01474.15	DAHP_synth_2	651,4	4,90E-196	CL0036
ARHOMBI_DN11895_c0_g1_i1_5	1	14,4	9,5969	193,62					
ARHOMBI_DN10040_c0_g1_i1_3	2	12,4	23,537	12,847					
ARHOMBI_DN20237_c0_g1_i1_3	3	13,3	51,954	29,411	PF05602.11	CLPTM1	448,6	2,30E-134	No_clan
ARHOMBI_DN13255_c0_g1_i1_6	2	26	8,3045	11,281					
ARUBRA_DN1076_c0_g1_i1_4	4	11,6	47,362	323,31	PF00581.19	Rhodanese	52,9	4,10E-14	CL0031
ARHOMBI_DN7071_c0_g1_i1_2	2	9,1	30,863	63,238	PF00085.19	Thioredoxin	61,3	6,60E-17	CL0172
ARUBRA_DN13122_c0_g1_i1_4	1	15,7	12,184	6,2918					
ARHOMBI_DN1703_c0_g1_i1_6	3	15	39,453	24,566	PF01398.20	JAB	73,8	1,00E-20	CL0366
ARUBRA_DN8641_c0_g1_i1_4	3	20,6	14,395	17,428	PF04718.14	ATP-synt_G	79,8	1,90E-22	No_clan
ARUBRA_DN2071_c0_g3_i1_4	4	24,9	20,824	16,855	PF01778.16	Ribosomal_L28e	116,6	8,70E-34	No_clan
ARUBRA_DN24590_c0_g1_i1_2	3	67,9	7,5189	176,7					
ARUBRA_DN9637_c0_g1_i1_6	1	15,9	7,2531	6,9515					
ARUBRA_DN12612_c0_g1_i1_4	1	13	12,065	12,684	PF00160.20	Pro_isomerase	128,8	2,00E-37	CL0475
ARHOMBI_DN3999_c0_g1_i1_1	7	16,3	79,747	43,27	PF00350.22	Dynamin_N	187,9	1,30E-55	CL0023
ARHOMBI_DN5284_c0_g1_i1_4	2	24,3	19,284	18,146	PF02782.15	FGGY_C	100,4	9,80E-29	CL0108
ARUBRA_DN7275_c0_g1_i1_1	3	28	15,99	18,783	PF00171.21	Aldedh	145,6	1,50E-42	CL0099
ARHOMBI_DN3047_c0_g1_i1_6	3	7,5	68,871	19,311	PF00202.20	Aminotran_3	224,4	1,70E-66	CL0061
ARUBRA_DN12194_c0_g1_i1_6	4	26,9	26,79	17,004	PF01602.19	Adaptin_N	88,6	3,20E-25	CL0020
ARHOMBI_DN20434_c0_g1_i1_2	2	13,5	28,828	13,429	PF01429.18	MBD	48,3	5,70E-13	CL0081
ARHOMBI_DN3438_c0_g1_i1_6	9	26,8	51,562	57,142	PF00291.24	PALP	222,3	8,20E-66	No_clan
ARHOMBI_DN4924_c0_g1_i1_5	5	32,6	19,863	14,007	PF00071.21	Ras	102,1	2,20E-29	CL0023

ARUBRA_DN11906_c0_g1_i1_6	2	23,1	11,987	12,298	PF01756.18	ACOX	25,4	8,30E-06	CL0087
ARUBRA_DN21459_c0_g1_i1_4	2	21,5	13,312	19,962	PF01370.20	Epimerase	43,9	1,70E-11	CL0063
ARUBRA_DN23079_c0_g1_i1_1	2	19,5	13,468	6,9225	PF00022.18	Actin	134,7	3,00E-39	CL0108
ARUBRA_DN15741_c0_g1_i1_6	2	35,8	10,057	323,31	PF01182.19	Glucosamine_iso	108,7	3,50E-31	CL0246
ARHOMBI_DN3883_c0_g1_i1_5	6	26,4	41,795	36,99	PF03446.14	NAD_binding_2	157,1	3,80E-46	CL0063
ARUBRA_DN4014_c0_g2_i1_3	3	36,2	8,7079	13,538	PF00248.20	Aldo_ket_red	48,7	5,40E-13	No_clan
ARUBRA_DN4532_c0_g1_i1_4	7	12,7	89,112	46,145	PF13589.5	HATPase_c_3	39,8	3,40E-10	CL0025
ARUBRA_DN7957_c0_g1_i1_1	4	18,8	40,697	28,605	PF06026.13	Rib_5-P_isom_A	207,6	8,80E-62	CL0246
ARHOMBI_DN22787_c0_g1_i1_4	3	7,9	33,686	17,896	PF02806.17	Alpha-amylase_C	95,6	1,70E-27	CL0369
ARUBRA_DN18867_c0_g1_i1_5	5	22,3	40,623	44,331	PF00400.31	WD40	16,3	0,012	CL0186
ARHOMBI_DN17883_c0_g1_i1_2	3	23,2	22,465	20,183	PF16114.4	Citrate_bind	271,7	1,90E-81	CL0506
ARHOMBI_DN14524_c0_g1_i1_1	3	50,6	9,8551	18,437	PF00076.21	RRM_1	64,1	7,40E-18	CL0221
ARUBRA_DN2441_c0_g1_i1_2	1	4,1	31,983	6,7146	PF13664.5	DUF4149	100	7,10E-29	CL0430
ARUBRA_DN25648_c0_g1_i1_1	1	13,3	7,8548	7,2342	PF00389.29	2-Hacid_dh	81,4	4,10E-23	CL0325
ARUBRA_DN4730_c0_g1_i1_2	3	17	23,641	48,006	PF01370.20	Epimerase	28,8	7,40E-07	CL0063
ARUBRA_DN4181_c0_g1_i1_5	2	8,9	31,249	11,192	PF00237.18	Ribosomal_L22	134,2	1,60E-39	No_clan
ARHOMBI_DN26738_c0_g1_i1_4	3	27,8	10,073	17,98	PF02990.15	EMP70	29,7	2,50E-07	No_clan
ARUBRA_DN5147_c0_g1_i1_4	2	45,7	8,6504	14,634					
ARHOMBI_DN5499_c0_g1_i1_3	3	6,6	69,354	18,225	PF00781.23	DAGK_cat	83,7	7,00E-24	CL0240
ARUBRA_DN8113_c0_g1_i1_3	4	22,2	20,438	24,143	PF00626.21	Gelsolin	64,1	7,50E-18	CL0092
ARHOMBI_DN1623_c0_g1_i1_6	5	18,4	50,364	31,362	PF00118.23	Cpn60_TCP1	366,3	2,00E-109	No_clan
ARUBRA_DN4329_c0_g1_i1_6	2	7,9	54,885	13,595	PF02167.14	Cytochrom_C1	324,2	3,80E-97	CL0318
ARUBRA_DN4117_c0_g1_i1_6	2	9,1	28,428	6,6575	PF03939.12	Ribosomal_L23eN	67,1	1,10E-18	No_clan
ARUBRA_DN18085_c0_g1_i1_1	2	18,6	10,835	11,085					
ARUBRA_DN5456_c0_g1_i1_2	4	10,5	63,501	42,477	PF13905.5	Thioredoxin_8	92,9	1,10E-26	CL0172
ARHOMBI_DN23974_c0_g1_i1_1	1	12	10,409	6,8858					
ARHOMBI_DN3770_c0_g1_i1_1	5	12,5	55,488	13,121	PF00330.19	Aconitase	47,7	1,00E-12	No_clan
ARUBRA_DN4031_c0_g1_i1_2	1	12,4	11,169	33,818	PF01204.17	Trehalase	106,5	1,40E-30	CL0059
ARUBRA_DN4198_c0_g2_i2_4	2	11,7	27,026	13,57	PF01789.15	PsbP	135,7	1,30E-39	CL0619
ARHOMBI_DN16331_c0_g1_i1_2	4	24,6	21,769	23,79					
ARHOMBI_DN8849_c0_g1_i1_2	3	37,9	14,184	18,729	PF00742.18	Homoserine_dh	71,1	9,40E-20	No_clan
ARUBRA_DN6372_c0_g1_i1_2	2	12,6	17,716	6,6693					

ARUBRA_DN16718_c0_g1_i1_3	1	16,5	8,4125	6,6754	PF00005.26	ABC_tran	32,1	1,30E-07	CL0023
ARHOMBI_DN10686_c0_g1_i1_3	2	19	16,091	323,31	PF00013.28	KH_1	45,2	5,70E-12	CL0007
ARUBRA_DN8773_c0_g1_i1_4	2	21,2	18,108	12,353					
ARUBRA_DN1981_c0_g1_i1_6	2	8	24,313	10,888	PF00725.21	3HCDH	25	2,10E-05	CL0106
ARHOMBI_DN6947_c0_g1_i1_1	2	38,7	12,807	13,767	PF02801.21	Ketoacyl-synt_C	58,9	4,20E-16	CL0046
ARHOMBI_DN4632_c0_g1_i1_3	3	13,7	40,744	21,19	PF01293.19	PEPCK_ATP	34,1	1,10E-08	CL0374
ARHOMBI_DN14482_c0_g1_i1_6	1	21,1	7,5838	6,5475					
ARHOMBI_DN3429_c0_g1_i1_6	5	13,1	52,948	32,668	PF05879.11	RHD3	324,9	8,70E-97	CL0023
ARHOMBI_DN3622_c0_g1_i1_4	4	9,9	59,821	23,985	PF02776.17	TPP_enzyme_N	73,4	1,50E-20	CL0254
ARUBRA_DN4615_c0_g2_i1_3	1	9,2	16,315	6,2303					
ARHOMBI_DN17829_c0_g1_i1_1	4	17,5	30,894	24,702	PF03224.13	V-ATPase_H_N	183	7,70E-54	CL0020
ARUBRA_DN6205_c0_g1_i1_1	2	27,4	15,355	13,094	PF03223.14	V-ATPase_C	122,4	2,70E-35	No_clan
ARUBRA_DN24096_c0_g1_i1_3	2	13,6	12,258	11,361					
ARUBRA_DN1065_c0_g2_i1_2	4	24,8	27,539	25,31	PF03949.14	Malic_M	267,6	8,40E-80	CL0063
ARHOMBI_DN12564_c0_g1_i1_6	4	36,1	14,729	25,541	PF00579.24	tRNA-synt_1b	41,8	7,20E-11	CL0039
ARHOMBI_DN3278_c0_g1_i1_3	6	24,3	31,517	37,561	PF01253.21	SUI1	71,7	5,00E-20	No_clan
ARUBRA_DN16058_c0_g1_i1_4	4	61,1	10,117	25,629	PF00565.16	SNase	28,3	1,70E-06	CL0049
ARUBRA_DN9760_c0_g1_i1_3	3	20	16,834	17,415	PF01399.26	PCI	34,6	2,00E-08	CL0123
ARHOMBI_DN6246_c0_g1_i3_3	3	20,9	25,098	20,945	PF00182.18	Glyco_hydro_19	365,6	1,20E-109	CL0037
ARHOMBI_DN3643_c0_g1_i1_2	2	18,8	12,246	15,31					
ARUBRA_DN18439_c0_g1_i1_3	4	11,9	56,817	27,208	PF00169.28	PH	43,9	2,60E-11	CL0266
ARUBRA_DN4784_c0_g2_i3_2	4	22,1	33,141	26,829	PF00227.25	Proteasome	108,4	2,90E-31	CL0052
ARHOMBI_DN16305_c0_g1_i1_3	1	31	7,9648	12,761					
ARUBRA_DN1937_c0_g2_i1_6	3	6,6	65,508	19,189	PF01399.26	PCI	72,8	2,50E-20	CL0123
ARHOMBI_DN3129_c0_g1_i1_2	1	8,8	11,18	8,7348	PF01990.16	ATP-synt_F	61,1	8,90E-17	No_clan
ARUBRA_DN1180_c0_g1_i1_3	5	10,7	70,352	31,229	PF10255.8	Paf67	500,8	2,70E-150	CL0020
ARHOMBI_DN24079_c0_g1_i1_2	4	35,7	13,828	59,371	PF00218.20	IGPS	70,1	1,40E-19	CL0036
ARHOMBI_DN17905_c0_g1_i1_4	4	29,9	17,303	23,133	PF00071.21	Ras	58,1	7,20E-16	CL0023
ARHOMBI_DN13275_c0_g1_i1_1	3	29,6	18,382	15,072	PF06957.10	COPI_C	143	1,00E-41	CL0020
ARHOMBI_DN17238_c0_g1_i1_3	3	41,3	13,839	29,125	PF07944.11	Glyco_hydro_127	81	6,20E-23	CL0059
ARHOMBI_DN6259_c0_g2_i7_3	2	13,6	16,71	12,769	PF02798.19	GST_N	69	3,00E-19	CL0172

ARHOMBI_DN4135_c0_g2_i1_5	3	16,5	24,938	53,231	PF02943.14	FeThRed_B	137	1,90E-40	No_clan
ARUBRA_DN4552_c1_g2_i1_5	6	42,5	21,008	12,683	PF00025.20	Arf	244,7	3,40E-73	CL0023
ARHOMBI_DN6298_c0_g1_i1_1	1	12,4	10,604	6,4577	PF00343.19	Phosphorylase	132,4	1,70E-38	CL0113
ARHOMBI_DN2163_c0_g1_i1_5	5	19,9	40,956	34,45	PF03141.15	Methyltransf_29	427,6	5,50E-128	CL0063
ARHOMBI_DN6274_c0_g2_i10_6	2	7,3	34,412	62,376	PF00314.16	Thaumatin	261,3	6,70E-78	CL0293
ARUBRA_DN6417_c0_g1_i1_1	1	27,7	9,5659	7,4626	PF10585.8	UBA_e1_thiolCys	110,7	1,00E-31	No_clan
ARUBRA_DN16375_c0_g1_i1_3	2	8,4	34,831	13,06	PF07986.11	TBCC	62,3	3,20E-17	CL0391
ARUBRA_DN20085_c0_g1_i1_4	3	43,8	12,424	20,633					
ARHOMBI_DN7053_c0_g1_i1_6	4	47	11,442	30,457					
ARHOMBI_DN4215_c0_g1_i1_6	3	8,8	47,803	17,733	PF13432.5	TPR_16	27,6	3,10E-06	CL0020
ARUBRA_DN6101_c0_g1_i1_3	3	8,3	53,861	20,162	PF02990.15	EMP70	422,3	2,40E-126	No_clan
ARHOMBI_DN5908_c0_g1_i1_6	6	13,7	56,907	38,842	PF01761.19	DHQ_synthase	355,5	1,30E-106	CL0224
ARHOMBI_DN12316_c0_g1_i1_3	2	27,9	9,4137	11,912	PF00012.19	HSP70	27,4	8,50E-07	CL0108
ARHOMBI_DN1573_c0_g2_i1_5	2	14,2	18,794	12	PF04756.12	OST3_OST6	120,2	1,00E-34	CL0172
ARUBRA_DN3281_c0_g1_i1_4	3	20,7	27,258	20,86	PF00241.19	Cofilin_ADF	125,3	1,40E-36	CL0092
ARHOMBI_DN5746_c0_g1_i1_4	2	7,8	25,916	11,381	PF01157.17	Ribosomal_L21e	147	1,10E-43	CL0107
ARUBRA_DN17629_c0_g1_i1_1	2	42,4	9,2651	13,704					
ARHOMBI_DN1692_c0_g1_i1_5	5	14,8	49,836	24,94	PF00004.28	AAA	143,4	4,80E-42	CL0023
ARUBRA_DN15157_c0_g1_i1_2	2	30,4	8,5838	40,901					
ARHOMBI_DN17586_c0_g1_i1_5	3	9,8	56,262	20,032	PF00291.24	PALP	162,7	1,10E-47	No_clan
ARUBRA_DN16576_c0_g1_i1_6	1	29,9	7,5443	18,105					
ARHOMBI_DN12404_c0_g1_i1_3	3	26,6	15,663	19,735	PF04053.13	Coatomer_WDAD	152,4	1,90E-44	CL0186
ARHOMBI_DN5690_c0_g1_i1_6	1	6	31,522	12,588	PF00179.25	UQ_con	175,1	5,30E-52	CL0208
ARHOMBI_DN14107_c0_g1_i1_6	3	67,4	9,0923	7,4681	PF02800.19	Gp_dh_C	100,4	6,70E-29	CL0139
ARUBRA_DN21696_c0_g1_i1_3	1	7,6	18,739	6,6917	PF07859.12	Abhydrolase_3	91,4	6,40E-26	CL0028
ARUBRA_DN12039_c0_g1_i1_4	3	31,8	16,746	18,428	PF13460.5	NAD_binding_10	42,5	6,20E-11	CL0063
ARHOMBI_DN17617_c0_g1_i1_1	3	24,7	18,09	19,472	PF00004.28	AAA	126,9	6,10E-37	CL0023
ARUBRA_DN25457_c0_g1_i1_2	1	21,4	7,5285	6,4573					
ARUBRA_DN9520_c0_g1_i1_3	3	19,2	30,044	19,423	PF00587.24	tRNA-synt_2b	38,5	1,10E-09	CL0040
ARUBRA_DN12747_c0_g1_i1_4	1	15,6	8,7158	12,533	PF08267.11	Meth_synt_1	112	3,60E-32	CL0160

ARHOMBI_DN27277_c0_g1_i1_1	2	39,1	7,5622	15,071					
ARUBRA_DN21434_c0_g1_i1_3	3	24,5	15,623	6,5301	PF00012.19	HSP70	122	1,90E-35	CL0108
ARUBRA_DN4990_c0_g1_i1_1	4	21,2	30,203	26,978	PF00314.16	Thaumatin	213,7	2,40E-63	CL0293
ARHOMBI_DN18290_c0_g1_i1_4	4	22,4	20,579	86,968					
ARUBRA_DN8515_c0_g1_i1_2	2	15,5	17,972	12,836	PF00106.24	adh_short	115,9	1,40E-33	CL0063
ARUBRA_DN7009_c0_g1_i1_3	2	6,4	55,665	13,288	PF02779.23	Transket_pyr	158	1,70E-46	CL0254
ARUBRA_DN16706_c0_g1_i1_4	1	15,6	10,087	6,9079	PF08544.12	GHMP_kinases_C	23,1	6,50E-05	No_clan
ARUBRA_DN8627_c0_g1_i1_5	2	10,8	28,612	12,825	PF04573.11	SPC22	179,7	2,90E-53	No_clan
ARUBRA_DN19094_c0_g1_i1_2	2	9,9	24,337	12,302	PF01918.20	Alba	46,3	2,60E-12	CL0441
ARUBRA_DN4464_c0_g2_i1_2	6	26,9	28,382	38,292	PF00749.20	tRNA-synt_1c	36,6	2,20E-09	CL0039
ARUBRA_DN19978_c0_g1_i1_6	2	14,2	25,017	13,306	PF02516.13	STT3	82,9	2,20E-23	CL0111
ARHOMBI_DN26182_c0_g1_i1_3	1	19	9,5445	7,1576					
ARUBRA_DN5518_c0_g1_i1_3	3	27,3	14,662	19,704					
ARHOMBI_DN6030_c1_g5_i1_6	13	32,7	45,14	6,3822	PF08240.11	ADH_N	37,5	1,60E-09	CL0296
ARHOMBI_DN15479_c0_g1_i1_2	2	4,8	44,321	11,615					
ARHOMBI_DN21367_c0_g1_i1_4	2	30,2	15,46	17,445	PF00180.19	Iso_dh	109,7	1,50E-31	CL0270
ARUBRA_DN9892_c0_g1_i1_6	1	6,7	30,931	10,675	PF00005.26	ABC_tran	56,6	3,50E-15	CL0023
ARHOMBI_DN8750_c0_g1_i1_3	1	24	8,3094	7,3608	PF01321.17	Creatinase_N	35,2	1,60E-08	CL0356
ARHOMBI_DN13150_c0_g1_i1_1	2	24	10,406	32,582	PF00248.20	Aldo_ket_red	30,4	1,90E-07	No_clan
ARHOMBI_DN3473_c0_g1_i1_4	1	5	25,249	6,2247					
ARUBRA_DN22076_c0_g1_i1_2	4	17,4	32,587	26,308					
ARUBRA_DN11215_c0_g1_i1_3	2	33,6	13,458	20,14	PF16078.4	2-oxogl_dehyd_N	49,3	2,40E-13	No_clan
ARHOMBI_DN1047_c0_g1_i1_6	2	11,2	27,889	12,444	PF00085.19	Thioredoxin	49,9	2,20E-13	CL0172
ARUBRA_DN1728_c0_g1_i1_2	2	37,2	11,981	21,866					
ARHOMBI_DN5812_c1_g3_i1_1	3	17,7	26,353	244,67	PF02020.17	W2	81,4	3,50E-23	CL0020
ARUBRA_DN17307_c0_g1_i1_2	2	23,5	13,259	12,832					
ARHOMBI_DN4645_c1_g1_i1_2	3	15,6	20,475	11,52	PF01433.19	Peptidase_M1	124,5	4,10E-36	CL0126
ARUBRA_DN8669_c0_g1_i1_4	4	23,7	23,811	23,22					
ARUBRA_DN5513_c0_g1_i1_1	3	28,5	16,413	20,758					
ARUBRA_DN4813_c0_g1_i1_1	6	13,8	69,749	39,306	PF00109.25	ketoacyl-synt	203,1	5,00E-60	CL0046
ARUBRA_DN10983_c0_g1_i1_4	2	20,9	14,135	14,461	PF03144.24	GTP_EFTU_D2	66,8	1,50E-18	CL0575
ARUBRA_DN4671_c1_g1_i1_5	11	22,3	60,896	31,171	PF00012.19	HSP70	581,7	1,20E-174	CL0108

ARUBRA_DN5698_c0_g1_i1_2	2	8,5	39,894	12,07	PF00970.23	FAD_binding_6	86,2	1,50E-24	CL0076
ARUBRA_DN8516_c0_g1_i1_2	3	18,1	28,715	21,15	PF00206.19	Lyase_1	215,7	9,40E-64	No_clan
ARHOMBI_DN3145_c0_g1_i1_3	2	3,5	62,899	11,937	PF01565.22	FAD_binding_4	55,5	4,40E-15	CL0077
ARUBRA_DN26369_c0_g1_i1_1	3	57,8	11,612	8,7748	PF02874.22	ATP-synt_ab_N	55,9	4,30E-15	CL0275
ARUBRA_DN16851_c0_g1_i1_2	2	9,5	22,175	13,38	PF01477.22	PLAT	47,9	1,60E-12	CL0321
ARHOMBI_DN22852_c0_g1_i1_6	1	13,8	12,28	7,5985	PF00311.16	PEPcase	156,2	9,10E-46	CL0151
ARHOMBI_DN25003_c0_g1_i1_6	2	25,4	12,753	12,512	PF01433.19	Peptidase_M1	28,9	6,20E-07	CL0126
ARUBRA_DN17081_c0_g1_i1_5	1	6,2	27,951	7,8819					
ARHOMBI_DN4475_c0_g1_i1_1	6	21	39,842	70,433					
ARUBRA_DN6562_c0_g1_i1_2	1	28,4	7,012	7,7009					
ARUBRA_DN9315_c0_g1_i1_1	2	30,7	8,4084	13,647	PF00012.19	HSP70	42,7	2,00E-11	CL0108
ARUBRA_DN5821_c0_g1_i1_4	4	58,9	9,7211	26,817	PF01063.18	Aminotran_4	36,7	3,70E-09	No_clan
ARHOMBI_DN15559_c0_g1_i1_1	3	18,6	17,605	17,713	PF00656.21	Peptidase_C14	176,4	9,10E-52	CL0093
ARHOMBI_DN3769_c0_g1_i1_4	6	36	23,933	37,885	PF01451.20	LMWPc	135,9	1,20E-39	CL0031
ARUBRA_DN6679_c0_g1_i1_2	3	10,2	31,735	16,849					
ARUBRA_DN3045_c0_g1_i1_5	3	8,4	38,606	18,091	PF01813.16	ATP-synt_D	236,5	2,10E-70	No_clan
ARUBRA_DN21269_c0_g1_i1_2	3	19,9	32,753	18,097	PF00862.18	Sucrose_synth	292,9	3,40E-87	CL0113
ARHOMBI_DN3422_c0_g1_i1_5	2	10,9	30,005	16,024	PF01849.17	NAC	68,5	3,40E-19	No_clan
ARUBRA_DN11946_c0_g1_i1_3	3	33,5	20,24	23,341	PF00091.24	Tubulin	42,3	8,80E-11	CL0566
ARUBRA_DN428_c0_g1_i1_5	2	21,7	14,942	15,387					
ARHOMBI_DN1985_c0_g2_i1_1	3	22,5	17,669	18,468	PF06957.10	COPI_C	214,8	1,60E-63	CL0020
ARUBRA_DN11238_c0_g1_i1_6	3	15,3	21,096	68,026	PF10185.8	Mesd	27,5	2,40E-06	No_clan
ARHOMBI_DN11429_c0_g1_i1_1	3	24,1	11,811	17,785	PF05051.12	COX17	47,3	1,90E-12	CL0351
ARUBRA_DN2583_c0_g2_i1_4	1	8,3	12,165	7,0631	PF01253.21	SUI1	89,5	1,40E-25	No_clan
ARHOMBI_DN5242_c0_g1_i1_4	3	9,6	55,894	19,147	PF08241.11	Methyltransf_11	63,4	2,20E-17	CL0063
ARUBRA_DN3984_c0_g1_i1_3	3	8,6	57,477	19,532	PF00026.22	Asp	466,6	4,00E-140	CL0129
ARHOMBI_DN13132_c0_g1_i1_4	3	42	8,4598	124,71	PF05907.12	DUF866	37,9	1,50E-09	No_clan
ARHOMBI_DN17057_c0_g1_i1_3	1	5,3	25,711	6,465	PF00479.21	G6PD_N	195,1	1,40E-57	CL0063
ARHOMBI_DN328_c0_g1_i1_6	3	25	25,132	20,014	PF00198.22	2-oxoacid_dh	156,7	5,80E-46	CL0149
ARUBRA_DN4992_c0_g1_i2_1	2	8,5	19,492	16,521					
ARHOMBI_DN10598_c0_g1_i1_3	3	19,6	21,697	20,542					
ARHOMBI_DN4392_c0_g1_i1_3	2	5,5	47,629	12,626	PF01399.26	PCI	39,8	4,90E-10	CL0123

ARUBRA_DN6255_c0_g1_i1_2	2	23	18,083	82,812	PF00076.21	RRM_1	39,5	3,50E-10	CL0221
ARUBRA_DN8387_c0_g1_i1_5	2	11,3	29,769	13,359					
ARUBRA_DN2057_c0_g1_i1_6	3	16,4	33,242	20,654	PF00118.23	Cpn60_TCP1	221,3	1,90E-65	No_clan
ARHOMBI_DN11616_c0_g1_i1_3	2	29,7	12,317	7,074	PF04969.15	CS	66,1	4,00E-18	CL0190
ARHOMBI_DN15140_c0_g1_i1_3	1	13,4	8,6341	6,1503					
ARUBRA_DN186_c0_g1_i1_4	5	16,6	49,913	32,965	PF00076.21	RRM_1	71,7	3,00E-20	CL0221
ARUBRA_DN18420_c0_g1_i1_3	1	9,3	11,58	9,2792	PF03662.13	Glyco_hydro_79n	126,1	1,30E-36	CL0058
ARHOMBI_DN736_c0_g2_i1_6	4	11,5	60,535	27,43	PF01370.20	Epimerase	76	2,70E-21	CL0063
ARHOMBI_DN8058_c0_g1_i1_5	3	18,1	28,103	19,262	PF03141.15	Methyltransf_29	348	7,50E-104	CL0063
ARHOMBI_DN13310_c0_g1_i1_4	1	18	10,42	6,5963	PF00311.16	PEPcase	76,5	1,20E-21	CL0151
ARUBRA_DN19795_c0_g1_i1_2	1	13,9	10,847	6,9498	PF00118.23	Cpn60_TCP1	123,1	1,10E-35	No_clan
ARHOMBI_DN15499_c0_g1_i1_1	3	14,6	38,394	66,919	PF00635.25	Motile_Sperm	104,4	2,50E-30	CL0556
ARHOMBI_DN5591_c0_g1_i1_2	5	11,2	56,858	34,026	PF01238.20	PMI_typeI	363	1,70E-108	CL0029
ARHOMBI_DN2375_c0_g1_i1_6	3	28,2	13,706	17,712					
ARHOMBI_DN15343_c0_g1_i1_2	4	6,4	72,604	80,555	PF04734.12	Ceramidase_alk	696,1	2,30E-209	No_clan
ARUBRA_DN1522_c0_g1_i1_5	3	17,4	32,268	21,139	PF00561.19	Abhydrolase_1	28,5	9,90E-07	CL0028
ARHOMBI_DN6216_c0_g1_i1_4	3	7,2	67,272	20,981	PF00464.18	SHMT	696,6	6,70E-210	CL0061
ARHOMBI_DN5056_c0_g1_i1_4	2	16	22,658	6,6541	PF00298.18	Ribosomal_L11	58,3	7,70E-16	No_clan
ARUBRA_DN4756_c0_g1_i1_6	2	5,6	34,463	11,036	PF01251.17	Ribosomal_S7e	265,8	1,70E-79	CL0652
ARHOMBI_DN3633_c0_g1_i1_2	4	10,9	64,345	25,689	PF00270.28	DEAD	121,3	3,50E-35	CL0023
ARHOMBI_DN16542_c0_g1_i1_1	1	2,8	36,285	9,0516	PF06244.11	Ccdc124	122,5	1,50E-35	CL0114
ARHOMBI_DN2386_c0_g1_i1_2	1	12,3	21,377	7,585	PF08541.9	ACP_syn_III_C	127,4	1,60E-37	CL0046
ARHOMBI_DN2790_c0_g2_i1_3	1	5,4	23,214	6,3584	PF05470.11	eIF-3c_N	309,3	4,30E-92	No_clan
ARHOMBI_DN5912_c0_g2_i4_5	1	5,7	15,395	90,398	PF01439.17	Metallothio_2	101,7	3,00E-29	No_clan
ARUBRA_DN9466_c0_g1_i1_6	1	5,4	20,511	7,5411	PF00118.23	Cpn60_TCP1	149,1	1,50E-43	No_clan
ARHOMBI_DN5625_c0_g1_i1_5	4	13,4	33,436	25,473	PF00085.19	Thioredoxin	79,7	1,20E-22	CL0172
ARUBRA_DN26355_c0_g1_i1_1	2	19,7	18,766	12,317	PF00920.20	ILVD_EDD	247,5	2,30E-73	No_clan
ARUBRA_DN17795_c0_g1_i1_3	2	13	31,063	15,522	PF16363.4	GDP_Man_Dehyd	352,3	2,80E-105	CL0063

ARUBRA_DN4209_c0_g1_i1_6	1	12,5	14,541	8,7809					
ARHOMBI_DN13841_c0_g1_i1_1	2	31	9,3941	6,4361					
ARUBRA_DN7189_c0_g1_i1_4	3	7,5	54,48	18,726	PF02889.15	Sec63	81,3	5,70E-23	No_clan
ARUBRA_DN17439_c0_g1_i1_4	3	32,3	11,177	7,9704	PF16113.4	ECH_2	44,9	9,80E-12	CL0127
ARUBRA_DN23385_c0_g1_i1_4	5	17,8	28,539	29,929	PF01417.19	ENTH	102,2	1,80E-29	CL0009
ARUBRA_DN3392_c0_g2_i1_2	6	21	35,991	135,66	PF00175.20	NAD_binding_1	57,7	1,40E-15	CL0091
ARUBRA_DN16420_c0_g1_i1_5	3	24,3	24,468	27,514					
ARUBRA_DN5780_c0_g1_i1_6	2	12,7	23,359	12,238	PF01248.25	Ribosomal_L7Ae	82,8	1,00E-23	CL0101
ARHOMBI_DN9968_c0_g1_i1_3	1	14,5	8,8089	7,883					
ARUBRA_DN22029_c0_g1_i1_1	3	21,5	18,732	17,526	PF00012.19	HSP70	129,7	8,90E-38	CL0108
ARHOMBI_DN6264_c0_g1_i2_1	11	47,4	29,279	20,189	PF00724.19	Oxidored_FMN	238,4	1,10E-70	CL0036
ARUBRA_DN9942_c0_g1_i1_2	3	14	24,378	166,72	PF01230.22	HIT	94,1	6,10E-27	CL0265
ARHOMBI_DN10129_c0_g1_i1_4	1	7,2	22,494	6,891	PF10602.8	RPN7	182,3	5,70E-54	CL0020
ARUBRA_DN4816_c0_g4_i1_1	1	14,5	8,2291	6,2036	PF00164.24	Ribosom_S12_S23	22,1	8,40E-05	CL0021
ARHOMBI_DN9150_c0_g1_i1_2	4	14	39,692	25,201	PF02847.16	MA3	87	7,60E-25	CL0020
ARHOMBI_DN18526_c0_g1_i1_5	3	22	19,741	13,431	PF00244.19	14.03.2003	155,1	1,70E-45	No_clan
ARHOMBI_DN5512_c0_g1_i1_3	4	20,2	36,658	29,003	PF04733.13	Coatomer_E	461,2	1,30E-138	CL0020
ARUBRA_DN18242_c0_g1_i1_1	3	22,7	23,956	21,015					
ARUBRA_DN6103_c0_g1_i1_5	3	13,2	40,802	100,49	PF01652.17	IF4E	181,6	7,90E-54	CL0625
ARHOMBI_DN4464_c0_g1_i1_6	4	27,8	28,444	24,321	PF00582.25	Usp	101,4	5,60E-29	CL0039
ARUBRA_DN21777_c0_g1_i1_4	6	15,3	57,966	45,932	PF01411.18	tRNA-synt_2c	233,5	4,00E-69	CL0040
ARHOMBI_DN6373_c0_g1_i1_1	1	12,7	14,763	6,3316					
ARUBRA_DN4890_c0_g2_i1_5	5	14,6	38,059	34,421	PF08212.11	Lipocalin_2	181,5	7,90E-54	CL0116
ARHOMBI_DN3410_c0_g1_i1_4	2	9,3	30,017	21,433	PF01204.17	Trehalase	221,2	2,50E-65	CL0059
ARUBRA_DN1360_c0_g2_i1_4	1	3,5	38,87	7,6886	PF00076.21	RRM_1	59,1	2,60E-16	CL0221
ARUBRA_DN20255_c0_g1_i1_4	2	14,7	20,596	13,579					
ARUBRA_DN12071_c0_g1_i1_2	2	21,5	23,88	13,474	PF00108.22	Thiolase_N	52,2	5,00E-14	CL0046
ARUBRA_DN25817_c0_g1_i1_2	1	17,5	10,627	7,9341	PF02453.16	Reticulon	85,8	2,80E-24	No_clan
ARUBRA_DN24145_c0_g1_i1_1	3	49,1	11,126	18,151	PF00180.19	Iso_dh	92,3	2,90E-26	CL0270
ARHOMBI_DN6283_c0_g6_i1_6	1	15,5	14,249	7,7389	PF03143.16	GTP_EFTU_D3	118,3	1,80E-34	No_clan
ARUBRA_DN3001_c0_g1_i1_6	2	10,9	28,228	12,812					
ARUBRA_DN18461_c0_g1_i1_6	1	17,3	8,7276	7,2809					

ARUBRA_DN10353_c1_g1_i1_5	4	20,1	31,811	69,125					
ARHOMBI_DN25343_c0_g1_i1_2	2	12	18,895	16,256	PF00450.21	Peptidase_S10	102,4	3,30E-29	CL0028
ARHOMBI_DN7398_c0_g1_i1_3	2	17,1	20,404	13,123	PF00762.18	Ferrochelatase	173,6	4,90E-51	CL0043
ARHOMBI_DN6728_c0_g1_i1_4	3	15,6	30,413	19,44					
ARUBRA_DN3802_c0_g1_i1_4	2	13,1	30,942	19,009	PF02985.21	HEAT	22,3	9,00E-05	CL0020
ARHOMBI_DN13057_c0_g1_i1_1	2	14,8	22,484	18,466	PF02338.18	OTU	89,9	1,70E-25	CL0125
ARUBRA_DN9924_c0_g1_i1_4	3	10,6	47,164	18,566	PF01926.22	MMR_HSR1	57,9	8,70E-16	CL0023
ARHOMBI_DN430_c0_g1_i1_3	2	9,9	44,898	12,716	PF00574.22	CLP_protease	268,3	3,00E-80	CL0127
ARUBRA_DN3936_c0_g2_i1_4	3	38,4	12,587	19,224	PF03405.13	FA_desaturase_2	184,8	1,70E-54	CL0044
ARUBRA_DN4814_c0_g1_i3_4	2	11,3	26,74	18,577	PF00314.16	Thaumatin	186,4	5,50E-55	CL0293
ARUBRA_DN2381_c0_g1_i1_1	5	14,2	56,843	71,837	PF00150.17	Cellulase	55	7,60E-15	CL0058
ARHOMBI_DN6212_c0_g1_i2_6	2	5,9	27,41	50,022	PF02966.15	DIM1	222,4	1,20E-66	CL0172
ARUBRA_DN20443_c0_g1_i1_3	5	30,5	24,872	32,488	PF00155.20	Aminotran_1_2	30,7	1,70E-07	CL0061
ARHOMBI_DN11311_c0_g1_i1_5	4	44,1	20,107	21,605	PF00702.25	Hydrolase	55,5	8,40E-15	CL0137
ARHOMBI_DN17429_c0_g1_i1_6	4	34,8	17,975	26,088					
ARUBRA_DN3578_c0_g1_i1_4	2	7,8	34,934	12,538	PF00071.21	Ras	193,6	1,50E-57	CL0023
ARUBRA_DN11131_c0_g1_i1_3	2	29,5	10,646	12,876					
ARHOMBI_DN4521_c1_g1_i1_1	4	41,5	16,696	20,238	PF02359.17	CDC48_N	40,1	2,80E-10	CL0332
ARHOMBI_DN17261_c0_g1_i1_1	4	17,8	27,943	30,131	PF04321.16	RmlD_sub_bind	120,7	5,90E-35	CL0063
ARUBRA_DN1069_c0_g2_i1_1	1	10	15,247	6,7657	PF03151.15	TPT	130,6	6,70E-38	CL0184
ARHOMBI_DN5132_c0_g2_i1_6	1	6,1	21,584	6,4715					
ARUBRA_DN3311_c0_g1_i1_6	2	29,3	10,826	13,625					
ARUBRA_DN8175_c0_g1_i1_5	2	7,3	60,872	15,53	PF01326.18	PPDK_N	115,1	3,60E-33	CL0179
ARUBRA_DN16564_c0_g1_i1_6	1	16,1	12,362	-2					
ARUBRA_DN19149_c0_g1_i1_6	6	16,2	54,373	35,799	PF01591.17	6PF2K	283,5	8,10E-85	CL0023
ARUBRA_DN5180_c0_g1_i1_3	1	17,8	16,588	6,5217	PF07970.11	COPIIcoated_ERV	136	1,60E-39	No_clan
ARUBRA_DN20917_c0_g1_i1_3	2	21,1	12,343	15,785	PF00160.20	Pro_isomerase	135,1	2,30E-39	CL0475
ARHOMBI_DN20733_c0_g1_i1_3	2	22,1	12,141	18,454	PF00155.20	Aminotran_1_2	37,6	1,30E-09	CL0061
ARUBRA_DN10088_c0_g1_i1_1	4	29,7	21,701	6,7465	PF00171.21	Aldedh	175,8	1,10E-51	CL0099
ARHOMBI_DN19569_c0_g1_i1_5	5	20,6	24,872	34,379	PF00160.20	Pro_isomerase	151,7	1,80E-44	CL0475
ARUBRA_DN2998_c0_g1_i1_1	1	3,9	41,652	7,9373	PF01086.16	Clathrin_lg_ch	51,2	1,60E-13	No_clan
ARUBRA_DN1494_c0_g1_i1_3	5	8,5	91,55	34,044	PF01645.16	Glu_synthase	530	3,00E-159	CL0036

ARHOMBI_DN4699_c0_g1_i1_3	5	18,5	46,568	30,335	PF01975.16	SurE	179	8,30E-53	No_clan
ARHOMBI_DN5814_c0_g1_i1_4	4	10,6	49,099	17,535	PF00056.22	Ldh_1_N	155,9	6,00E-46	CL0063
ARHOMBI_DN21914_c0_g1_i1_1	5	62,9	11,508	18,391	PF02800.19	Gp_dh_C	120,9	3,20E-35	CL0139
ARHOMBI_DN5567_c0_g1_i1_3	5	11,3	67,977	29,634	PF01237.17	Oxysterol_BP	333,7	1,10E-99	No_clan
ARUBRA_DN1463_c0_g2_i1_5	4	13,7	48,585	22,676	PF02990.15	EMP70	350,9	1,00E-104	No_clan
ARHOMBI_DN17982_c0_g1_i1_5	3	20,3	19,915	165,03	PF01263.19	Aldose_epim	161,6	2,40E-47	CL0103
ARUBRA_DN2123_c0_g2_i1_3	2	16,6	25,649	13,959	PF10585.8	UBA_e1_thiolCys	122,1	3,40E-35	No_clan
ARHOMBI_DN2126_c0_g2_i1_6	4	14,4	48,859	31,834	PF01429.18	MBD	23,4	3,30E-05	CL0081
ARUBRA_DN8853_c0_g1_i1_6	1	24,4	9,695	9,2321	PF00390.18	malic	70,7	1,20E-19	CL0603
ARUBRA_DN1745_c0_g2_i1_1	2	26,1	11,882	13,994					
ARHOMBI_DN3877_c0_g1_i1_1	3	22,6	20,053	19,785	PF02230.15	Abhydrolase_2	105,3	3,60E-30	CL0028
ARUBRA_DN3955_c0_g2_i1_2	1	7,4	16,756	6,1647					
ARUBRA_DN8757_c0_g1_i1_5	2	7,8	30,871	311,75					
ARHOMBI_DN1665_c0_g1_i1_2	5	24,5	34,182	32,624	PF01459.21	Porin_3	228,2	1,20E-67	CL0193
ARHOMBI_DN11418_c0_g1_i1_2	1	34,6	8,3135	9,5086					
ARHOMBI_DN8306_c0_g1_i1_4	2	22,4	16,339	12,722	PF01088.20	Peptidase_C12	110,8	6,90E-32	CL0125
ARUBRA_DN20020_c0_g1_i1_2	1	6,7	13,44	6,3961					
ARUBRA_DN148_c0_g1_i1_1	3	15,5	25,479	24,685	PF09360.9	zf-CDGSH	37	2,60E-09	No_clan
ARHOMBI_DN25089_c0_g1_i1_1	2	13,7	17,453	11,842	PF00152.19	tRNA-synt_2	57,2	1,30E-15	CL0040
ARUBRA_DN11014_c0_g1_i1_6	2	17	10,333	16,917	PF01633.19	Choline_kinase	95,3	3,60E-27	CL0016
ARHOMBI_DN18155_c0_g1_i1_2	2	13,9	25,302	13,501	PF00085.19	Thioredoxin	30,4	2,70E-07	CL0172
ARUBRA_DN5032_c2_g1_i2_2	3	16,8	21,223	24,264	PF13602.5	ADH_zinc_N_2	73,4	3,20E-20	CL0063
ARHOMBI_DN5326_c0_g1_i1_3	1	15,1	18,547	10,086	PF08240.11	ADH_N	36,1	4,40E-09	CL0296
ARHOMBI_DN15674_c0_g1_i1_5	4	51,9	11,597	26,217	PF01467.25	CTP_transf_like	37,1	3,00E-09	CL0039
ARHOMBI_DN5386_c1_g1_i2_5	3	10,3	48,015	17,737	PF00076.21	RRM_1	50,7	1,10E-13	CL0221
ARUBRA_DN4407_c0_g4_i1_6	1	7,7	28,82	6,3448	PF06351.10	Allene_ox_cyc	310	3,30E-93	CL0650
ARHOMBI_DN17588_c0_g1_i1_4	2	35,2	10,293	12,959					
ARHOMBI_DN7219_c0_g1_i1_3	1	14,8	8,3152	23,909					
ARHOMBI_DN7958_c0_g1_i1_4	2	25,6	13,921	6,8398	PF01459.21	Porin_3	36,3	3,80E-09	CL0193
ARUBRA_DN4721_c0_g1_i2_1	2	4,3	51,797	12,951	PF09598.9	Stm1_N	75,8	3,30E-21	No_clan
ARHOMBI_DN5441_c0_g1_i1_5	2	5,2	38,853	11,376	PF00582.25	Usp	96,5	1,80E-27	CL0039
ARUBRA_DN7416_c0_g1_i1_6	5	16,7	45,175	28,989	PF00202.20	Aminotran_3	326,2	2,30E-97	CL0061

ARUBRA_DN6341_c0_g1_i1_1	2	10,6	27,843	12,444	PF00384.21	Molybdopterin	89,1	2,70E-25	No_clan
ARUBRA_DN25250_c0_g1_i1_2	1	20,4	11,55	7,4991	PF01399.26	PCI	53,2	3,10E-14	CL0123
ARUBRA_DN12031_c0_g1_i1_3	2	28,7	11,061	13,655					
ARUBRA_DN9279_c0_g1_i1_5	1	18,9	10,152	9,1826	PF02990.15	EMP70	95,3	3,10E-27	No_clan
ARHOMBI_DN3515_c0_g1_i1_3	2	12,2	24,229	9,4823	PF13561.5	adh_short_C2	109,8	1,30E-31	CL0063
ARHOMBI_DN4682_c0_g1_i1_3	1	8,7	21,342	11,418	PF00428.18	Ribosomal_60s	45,6	7,40E-12	No_clan
ARHOMBI_DN23760_c0_g1_i1_6	1	29,4	6,9045	197,11					
ARHOMBI_DN15221_c0_g1_i1_1	3	21,9	24,242	14,433	PF01417.19	ENTH	70,6	1,10E-19	CL0009
ARHOMBI_DN9078_c0_g1_i1_6	4	29,7	15,697	26,462	PF05450.14	Nicastrin	25	1,00E-05	CL0035
ARUBRA_DN11010_c0_g1_i1_3	4	50,8	14,573	25,145	PF05091.11	eIF-3_zeta	169,8	1,00E-49	CL0236
ARUBRA_DN17427_c0_g1_i1_4	4	43,5	14,824	24,118	PF00160.20	Pro_isomerase	67,4	1,60E-18	CL0475
ARUBRA_DN20283_c0_g1_i1_1	4	21,4	27,776	36,508	PF16499.4	Melibiase_2	46,6	2,30E-12	CL0058
ARUBRA_DN18073_c0_g1_i1_6	3	13,9	32,856	19,347	PF13774.5	Longin	42,8	3,70E-11	No_clan
ARUBRA_DN4594_c0_g2_i1_4	3	7,5	56,172	19,191	PF00450.21	Peptidase_S10	484,5	3,60E-145	CL0028
ARUBRA_DN4871_c0_g2_i1_3	2	4,1	46,579	11,311	PF13855.5	LRR_8	37,8	1,10E-09	CL0022
ARHOMBI_DN5577_c1_g1_i1_5	2	15,3	21,88	12,405	PF09334.10	tRNA-synt_1g	208,3	1,50E-61	CL0039
ARHOMBI_DN21271_c0_g1_i1_1	1	8,3	16,084	6,8443	PF14226.5	DIOX_N	105,2	3,20E-30	CL0029
ARUBRA_DN8542_c0_g1_i1_4	3	16,3	32,441	23,684	PF00076.21	RRM_1	50,2	1,50E-13	CL0221
ARHOMBI_DN10118_c0_g1_i1_1	2	24,8	13,424	13,007	PF00478.24	IMPDH	59,7	2,20E-16	CL0036
ARUBRA_DN18680_c0_g1_i1_3	2	13,2	24,486	12,122					
ARUBRA_DN2284_c0_g2_i1_3	3	12,7	34,771	24,409	PF13616.5	Rotamase_3	52,7	5,00E-14	CL0487
ARUBRA_DN7415_c0_g1_i1_6	4	19,1	29,96	7,6139	PF03721.13	UDPG_MGDP_dh_N	227,1	1,10E-67	CL0063
ARUBRA_DN113_c0_g1_i2_3	2	7,3	33,309	12,307	PF00227.25	Proteasome	134,7	2,50E-39	CL0052
ARHOMBI_DN26770_c0_g1_i1_3	3	39,4	11,655	14,701	PF00330.19	Aconitase	69	3,70E-19	No_clan
ARHOMBI_DN16112_c0_g1_i1_2	1	7,4	18,73	6,4644	PF03223.14	V-ATPase_C	221	2,90E-65	No_clan
ARUBRA_DN17277_c0_g1_i1_1	2	18,1	16,066	63,061	PF05564.11	Auxin_repressed	35,2	1,50E-08	No_clan
ARHOMBI_DN1288_c0_g1_i1_2	2	37,7	8,0983	12,254					
ARUBRA_DN70_c0_g1_i1_1	2	8	29,312	12,177	PF02668.15	TauD	71,2	1,10E-19	CL0029
ARUBRA_DN18307_c0_g1_i1_1	4	12,9	41,37	24,285					
ARUBRA_DN17020_c0_g1_i1_1	3	9,2	55,23	19,233	PF02847.16	MA3	53,8	1,60E-14	CL0020
ARUBRA_DN4917_c0_g1_i2_1	2	7,1	45,87	13,128	PF00834.18	Ribul_P_3_epim	292,3	1,20E-87	CL0036
ARHOMBI_DN5372_c0_g1_i1_3	2	8,7	29,5	12,427	PF06703.10	SPC25	137	4,30E-40	No_clan

ARHOMBI_DN24609_c0_g1_i1_6	1	8,3	15,256	6,3395	PF05368.12	NmrA	132,2	2,00E-38	CL0063
ARHOMBI_DN2660_c0_g1_i1_5	2	8,7	40,115	25,355	PF00076.21	RRM_1	47,9	8,10E-13	CL0221
ARUBRA_DN16475_c0_g1_i1_2	2	25,5	11,368	160,58					
ARUBRA_DN23765_c0_g1_i1_1	3	38,6	9,3818	26,592					
ARHOMBI_DN22879_c0_g1_i1_2	4	26,9	21,241	28,105					
ARUBRA_DN8139_c0_g1_i1_1	2	14,7	24,074	14,901	PF03358.14	FMN_red	39	5,70E-10	CL0042
ARUBRA_DN1221_c0_g2_i1_5	1	12,8	15,933	9,5032	PF01247.17	Ribosomal_L35Ae	151,3	6,00E-45	CL0575
ARHOMBI_DN14041_c0_g1_i1_6	1	14,7	8,1881	6,3956	PF13528.5	Glyco_trans_1_3	28,2	9,90E-07	CL0113
ARUBRA_DN4848_c0_g1_i1_3	1	7,9	16,312	7,0753					
ARUBRA_DN25967_c0_g1_i1_1	5	53,9	14,38	13,985	PF00179.25	UQ_con	127,6	2,40E-37	CL0208
ARUBRA_DN6775_c0_g1_i1_1	4	21,7	27,843	23,92	PF11938.7	DUF3456	120,1	1,10E-34	No_clan
ARHOMBI_DN4127_c0_g1_i1_6	3	9,6	45,416	17,078	PF00479.21	G6PD_N	34,3	3,10E-08	CL0063
ARUBRA_DN6005_c0_g1_i1_2	1	20,2	9,0331	7,1899	PF10509.8	GalKase_gal_bdg	21,7	9,90E-05	CL0329
ARUBRA_DN9533_c0_g1_i1_6	1	12,9	14,34	7,6961	PF02776.17	TPP_enzyme_N	41,7	8,40E-11	CL0254
ARUBRA_DN4945_c1_g1_i1_4	1	3,5	40,86	7,0728	PF00365.19	PFK	151	4,00E-44	CL0240
ARUBRA_DN2632_c0_g1_i1_5	6	22,6	39,791	39,123	PF08240.11	ADH_N	34	1,90E-08	CL0296
ARUBRA_DN253_c0_g2_i1_2	1	10,3	11,351	6,4257	PF01249.17	Ribosomal_S21e	67,6	6,80E-19	No_clan
ARUBRA_DN4035_c0_g2_i1_1	1	3,5	56,36	7,2777	PF00450.21	Peptidase_S10	476	1,30E-142	CL0028
ARHOMBI_DN5174_c0_g1_i1_1	2	10	22,859	13,25	PF05368.12	NmrA	105,9	2,10E-30	CL0063
ARUBRA_DN3267_c0_g1_i1_6	2	4,8	44,604	11,544	PF02453.16	Reticulon	196,2	2,90E-58	No_clan
ARUBRA_DN12853_c0_g1_i1_1	5	51,4	11,934	120,46	PF01717.17	Meth_synt_2	171,3	2,80E-50	CL0160
ARHOMBI_DN20424_c0_g1_i1_6	2	13,8	16,868	91,812	PF01172.17	SBDS	100	5,40E-29	No_clan
ARUBRA_DN4281_c0_g1_i1_1	7	20,4	60,466	38,242	PF00004.28	AAA	146,1	7,10E-43	CL0023
ARHOMBI_DN3485_c0_g1_i1_4	4	19,1	29,543	24,888	PF02575.15	YbaB_DNA_bd	61,2	8,00E-17	No_clan
ARUBRA_DN18869_c0_g1_i1_5	3	10,7	39,232	17,937	PF00750.18	tRNA-synt_1d	257,9	1,10E-76	CL0039
ARHOMBI_DN804_c0_g1_i1_6	2	17,8	15,979	12,25	PF02747.14	PCNA_C	189,7	1,80E-56	CL0060
ARUBRA_DN1537_c0_g1_i1_2	2	13	23,395	156,05	PF00076.21	RRM_1	66,4	1,40E-18	CL0221
ARUBRA_DN1957_c0_g2_i1_6	2	26,7	17,92	14,705	PF03485.15	Arg_tRNA_synt_N	62,7	3,50E-17	No_clan
ARUBRA_DN6807_c0_g1_i1_3	3	15,1	29,373	19,815	PF02784.15	Orn_Arg_deC_N	116,4	1,20E-33	CL0036
ARUBRA_DN4637_c0_g2_i1_1	4	17,1	43,982	6,1697	PF00438.19	S-AdoMet_synt_N	143,1	3,20E-42	No_clan
ARHOMBI_DN6131_c0_g1_i4_6	2	5,7	47,861	55,407	PF08240.11	ADH_N	31,6	1,10E-07	CL0296
ARUBRA_DN5357_c0_g1_i1_1	2	16,1	23,186	15,699	PF00152.19	tRNA-synt_2	112	2,70E-32	CL0040

ARUBRA_DN2637_c0_g1_i1_4	2	10	29,997	20,893	PF12796.6	Ank_2	46,8	3,10E-12	CL0465
ARHOMBI_DN16197_c0_g1_i1_4	2	19,3	22,223	13,878	PF00790.18	VHS	35,8	5,90E-09	CL0009
ARUBRA_DN10661_c0_g1_i1_4	3	13,7	34,616	17,716	PF03141.15	Methyltransf_29	346,8	1,70E-103	CL0063
ARHOMBI_DN4011_c0_g2_i1_5	2	14,6	24,719	7,1231					
ARUBRA_DN4545_c0_g1_i1_5	2	12,1	21,943	11,611	PF01738.17	DLH	84,1	9,40E-24	CL0028
ARUBRA_DN22266_c0_g1_i1_1	2	13,3	23,186	11,535	PF00450.21	Peptidase_S10	100,2	1,60E-28	CL0028
ARUBRA_DN9362_c0_g1_i1_5	2	25,9	14,321	18,371	PF02594.15	DUF167	79,7	1,20E-22	No_clan
ARUBRA_DN22269_c0_g1_i1_4	1	16,9	9,2842	6,9197	PF13855.5	LRR_8	27,8	1,50E-06	CL0022
ARHOMBI_DN3395_c0_g2_i1_6	2	8,7	38,216	15,169	PF01875.16	Memo	281,9	3,90E-84	CL0283
ARUBRA_DN18997_c0_g1_i1_6	4	9,3	69,918	26,477	PF03055.14	RPE65	397,7	6,30E-119	No_clan
ARHOMBI_DN6095_c0_g1_i1_4	6	12	62,395	20,332	PF00226.30	DnaJ	80,2	8,00E-23	CL0392
ARHOMBI_DN1066_c0_g2_i1_3	1	7,6	17,816	6,4667	PF06814.12	Lung_7-TM_R	86,6	1,60E-24	CL0192
ARHOMBI_DN14495_c0_g1_i1_4	2	50	7,953	15,665	PF01496.18	V_ATPase_I	104,2	4,30E-30	No_clan
ARUBRA_DN10359_c0_g1_i1_4	2	7,4	39,654	13,919	PF02990.15	EMP70	416,2	1,80E-124	No_clan
ARUBRA_DN20926_c0_g1_i1_1	2	18,7	13,391	39,505					
ARHOMBI_DN3507_c0_g1_i1_4	3	4,3	85,747	20,339	PF08240.11	ADH_N	33,6	2,70E-08	CL0296
ARHOMBI_DN25279_c0_g1_i1_2	1	21,1	7,6393	6,8664	PF00091.24	Tubulin	61,6	1,10E-16	CL0566
ARHOMBI_DN4819_c0_g1_i1_1	3	11	43,915	19,532	PF00118.23	Cpn60_TCP1	328,7	5,30E-98	No_clan
ARHOMBI_DN6082_c0_g1_i3_5	9	36,9	31,243	7,7695	PF00076.21	RRM_1	78,4	2,40E-22	CL0221
ARUBRA_DN24896_c0_g1_i1_3	2	43,8	9,3148	12,541	PF00343.19	Phosphorylase	109	1,90E-31	CL0113
ARUBRA_DN6755_c0_g1_i1_2	3	15,9	33,094	13,664	PF00514.22	Arm	32	7,90E-08	CL0020
ARHOMBI_DN5218_c0_g1_i2_1	3	9	45,94	18,664					
ARUBRA_DN25837_c0_g1_i1_3	3	20,2	19,63	12,631	PF00393.18	6PGD	170,9	3,60E-50	CL0106
ARUBRA_DN24388_c0_g1_i1_4	3	61,8	8,1689	18,223					
ARHOMBI_DN11066_c0_g1_i1_4	1	12,4	11,704	6,6599	PF00067.21	p450	39,7	2,40E-10	No_clan
ARUBRA_DN3745_c0_g2_i1_2	2	25	14,089	15,527	PF04398.11	DUF538	30,9	2,80E-07	No_clan
ARUBRA_DN14017_c0_g1_i1_3	2	31,1	8,2946	12,075					
ARUBRA_DN145_c0_g1_i1_4	2	22,4	19,187	16,152					
ARUBRA_DN11007_c0_g1_i1_2	3	16,3	23,02	18,394	PF05529.11	Bap31	40,4	2,10E-10	No_clan
ARUBRA_DN9096_c0_g1_i1_3	1	17,4	6,9715	6,4692					

ARHOMBI_DN4782_c0_g1_i1_2	4	7,6	71,132	24,205	PF00888.21	Cullin	456,8	1,20E-136	No_clan
ARHOMBI_DN2849_c0_g1_i1_3	4	10,9	49,682	25,865	PF00226.30	DnaJ	98	2,30E-28	CL0392
ARHOMBI_DN7054_c0_g1_i1_3	3	16,1	28,253	20,048	PF00632.24	HECT	256,6	3,20E-76	CL0552
ARHOMBI_DN2798_c0_g1_i1_2	3	14,3	44,219	20,584	PF01103.22	Bac_surface_Ag	63,8	1,70E-17	CL0193
ARUBRA_DN18224_c0_g1_i1_4	2	20,6	15,268	11,942	PF00626.21	Gelsolin	42,3	4,80E-11	CL0092
ARHOMBI_DN25584_c0_g1_i1_5	3	44,1	10,237	18,304	PF03911.15	Sec61_beta	42,7	3,80E-11	No_clan
ARHOMBI_DN3781_c0_g2_i1_5	2	7,5	30,967	16,075	PF02861.19	Clp_N	35,6	7,10E-09	No_clan
ARHOMBI_DN7337_c0_g1_i1_1	1	9,1	19,938	6,6419	PF02178.18	AT_hook	11,5	0,21	No_clan
ARHOMBI_DN5812_c2_g1_i1_3	2	12,7	19,167	37,547	PF01873.16	eIF-5_eIF-2B	128	1,50E-37	No_clan
ARUBRA_DN4150_c2_g1_i1_4	6	45,5	17,631	55,624	PF00071.21	Ras	186,9	1,70E-55	CL0023
ARHOMBI_DN1574_c0_g1_i1_4	2	16,1	17,817	13,643	PF00118.23	Cpn60_TCP1	154,9	2,60E-45	No_clan
ARUBRA_DN8202_c0_g1_i1_4	1	4,4	27,357	6,3797					
ARUBRA_DN1551_c0_g1_i1_5	2	52,7	9,6886	16,051					
ARUBRA_DN12626_c0_g1_i1_3	1	3,9	36,744	25,179	PF03517.12	Voldacs	89,5	2,00E-25	CL0266
ARHOMBI_DN4054_c0_g1_i1_3	2	10,1	24,998	24,162	PF13616.5	Rotamase_3	70	2,20E-19	CL0487
ARUBRA_DN9286_c0_g1_i1_1	3	17,1	24,466	18,77	PF12481.7	DUF3700	301	4,40E-90	CL0052
ARHOMBI_DN3767_c0_g1_i1_1	1	13,8	18,12	12,779	PF01920.19	Prefoldin_2	78,1	4,00E-22	CL0200
ARUBRA_DN20292_c0_g1_i1_6	1	5,9	32,143	7,9822	PF00903.24	Glyoxalase	31	2,20E-07	CL0104
ARHOMBI_DN2005_c0_g1_i1_5	2	17,2	14,026	14,681	PF01132.19	EFP	46	3,70E-12	CL0021
ARUBRA_DN4908_c0_g2_i1_1	2	6,2	47,224	50,189	PF00300.21	His_Phos_1	23,4	3,80E-05	CL0071
ARUBRA_DN1276_c0_g1_i1_4	3	13,5	39,769	21,1	PF00149.27	Metallophos	77,3	2,10E-21	CL0163
ARUBRA_DN4583_c0_g1_i2_5	3	14,6	28,541	18,91	PF04667.16	Endosulfine	73,5	1,00E-20	No_clan
ARHOMBI_DN4896_c0_g1_i3_2	3	18,6	23,197	17,998	PF10604.8	Polyketide_cyc2	78,9	4,20E-22	CL0209
ARUBRA_DN12695_c0_g1_i1_2	2	17,5	8,7048	12,771	PF05633.10	BPS1	81,4	6,00E-23	CL0133
ARHOMBI_DN15171_c0_g1_i1_3	3	22,4	20,63	18,939	PF00198.22	2-oxoacid_dh	153,7	5,00E-45	CL0149
ARHOMBI_DN7103_c0_g1_i1_1	2	32,5	8,84	13,682					
ARHOMBI_DN5209_c1_g1_i1_4	5	31,4	23,882	13,992	PF00702.25	Hydrolase	67,6	1,70E-18	CL0137
ARHOMBI_DN22071_c0_g1_i1_3	3	28,3	13,644	182,67	PF02879.15	PGM_PMM_II	59,9	2,80E-16	No_clan
ARHOMBI_DN1841_c0_g2_i1_2	7	47,1	24,547	6,9735	PF00004.28	AAA	140,4	4,10E-41	CL0023
ARUBRA_DN563_c0_g1_i1_2	1	7,6	22,668	6,178	PF00106.24	adh_short	34,6	1,20E-08	CL0063
ARUBRA_DN24632_c0_g1_i1_4	1	19,4	10,068	7,5131	PF00162.18	PGK	142,7	1,30E-41	No_clan
ARUBRA_DN3539_c0_g1_i1_2	5	19,1	36,747	37,778					

ARUBRA_DN1361_c0_g2_i1_4	3	14,4	25,178	16,954	PF06212.11	GRIM-19	140,6	2,50E-41	No_clan
ARUBRA_DN11150_c0_g1_i1_6	3	54,8	10,773	19,321	PF10609.8	ParA	39,9	2,80E-10	CL0023
ARHOMBI_DN1996_c0_g2_i1_5	1	4,6	28,494	12,363	PF00627.30	UBA	40,5	1,60E-10	CL0214
ARHOMBI_DN2999_c0_g1_i1_4	2	10,1	45,646	13,554	PF01398.20	JAB	52	5,60E-14	CL0366
ARHOMBI_DN2060_c0_g1_i1_5	4	24,8	22,352	25,569	PF06957.10	COPI_C	330	1,70E-98	CL0020
ARUBRA_DN9709_c0_g1_i1_6	1	7,7	16,605	43,557	PF02136.19	NTF2	51,8	9,70E-14	CL0051
ARHOMBI_DN23173_c0_g1_i1_6	1	8,7	20,215	6,8898	PF00781.23	DAGK_cat	48,9	4,30E-13	CL0240
ARHOMBI_DN10290_c0_g1_i1_3	1	9,9	10,917	6,4007	PF00658.17	PABP	47,6	1,30E-12	No_clan
ARUBRA_DN2965_c0_g1_i2_5	4	15,7	38,094	25,641	PF01135.18	PCMT	284,9	3,50E-85	CL0063
ARUBRA_DN3863_c1_g1_i1_5	3	12,7	37,796	19,183	PF07714.16	Pkinase_Tyr	128,5	2,40E-37	CL0016
ARHOMBI_DN10379_c0_g1_i1_2	4	23,1	22,858	-2	PF01717.17	Meth_synt_2	181,5	2,20E-53	CL0160
ARHOMBI_DN2726_c0_g1_i1_1	1	11,4	16,551	13,623	PF00364.21	Biotin_lipoyl	32,8	4,20E-08	CL0105
ARUBRA_DN7712_c0_g1_i1_6	1	13,6	11,186	6,9883	PF13646.5	HEAT_2	38,1	1,50E-09	CL0020
ARUBRA_DN4320_c0_g1_i1_6	4	13,3	37,764	24,657	PF01873.16	eIF-5_eIF-2B	134,1	1,90E-39	No_clan
ARUBRA_DN11275_c0_g1_i1_5	2	21,7	17,702	12,144					
ARHOMBI_DN8255_c0_g1_i1_2	3	17,2	26,212	18,543	PF07992.13	Pyr_redox_2	132,1	2,20E-38	CL0063
ARUBRA_DN13834_c0_g1_i1_4	2	14,4	15,085	12,893	PF03141.15	Methyltransf_29	98,4	3,30E-28	CL0063
ARUBRA_DN19618_c0_g1_i1_4	3	13	33,67	36,785	PF04734.12	Ceramidase_alk	195,9	1,00E-57	No_clan
ARUBRA_DN1773_c0_g2_i1_6	2	19,7	13,217	12,933					
ARUBRA_DN1305_c0_g1_i1_6	3	8,3	38,309	6,375	PF00297.21	Ribosomal_L3	446,5	6,30E-134	CL0575
ARHOMBI_DN3128_c0_g1_i1_1	4	14,3	46,784	39,06	PF01678.18	DAP_epimerase	97,1	7,20E-28	CL0288
ARHOMBI_DN20118_c0_g1_i1_1	1	9,4	18,271	37,752	PF06101.10	Vps62	215,2	1,20E-63	No_clan
ARHOMBI_DN2022_c0_g1_i1_1	2	10,8	20,822	11,208	PF01918.20	Alba	47,1	1,50E-12	CL0441
ARHOMBI_DN3079_c0_g1_i1_3	5	12	48,584	34,438	PF01379.19	Porphobil_deam	243,9	1,00E-72	CL0177
ARHOMBI_DN8476_c0_g1_i1_3	2	8,3	24,441	11,029					
ARHOMBI_DN4305_c0_g1_i2_6	1	3,8	33,608	6,7651	PF01106.16	NifU	79,8	1,10E-22	CL0232
ARUBRA_DN10474_c0_g1_i1_2	3	20,2	23,216	19,739					
ARHOMBI_DN6299_c0_g1_i1_1	2	22	13,66	12,169					
ARHOMBI_DN3992_c0_g1_i1_6	3	8,3	58,288	18,843					
ARUBRA_DN8631_c0_g1_i1_6	1	11,3	16,462	-2					
ARUBRA_DN158_c0_g1_i1_2	3	12,5	30,9	19,551	PF00013.28	KH_1	63,8	9,00E-18	CL0007
ARUBRA_DN6110_c0_g1_i1_1	3	21,1	20,345	18,002					

ARUBRA_DN4152_c0_g1_i1_5	2	14,8	22,251	39,341	PF13637.5	Ank_4	60,9	1,00E-16	CL0465
ARHOMBI_DN20386_c0_g1_i1_1	2	14,2	18,74	12,607	PF00378.19	ECH_1	48,9	4,80E-13	CL0127
ARUBRA_DN3314_c0_g1_i1_4	1	6,5	24,857	323,31	PF00550.24	PP-binding	43,9	2,20E-11	CL0314
ARHOMBI_DN19207_c0_g1_i1_4	2	26,4	9,4598	11,943					
ARUBRA_DN13376_c0_g1_i1_2	1	8,1	21,143	6,4787	PF00349.20	Hexokinase_1	172,8	7,80E-51	CL0108
ARHOMBI_DN16745_c0_g1_i1_1	4	43,5	13,505	25,223					
ARUBRA_DN4722_c0_g1_i1_3	3	8,8	65,59	17,948	PF01512.16	Complex1_51K	161,7	1,00E-47	CL0105
ARUBRA_DN881_c0_g1_i1_1	3	18	32,262	19,585	PF00227.25	Proteasome	144,8	2,00E-42	CL0052
ARHOMBI_DN16363_c0_g1_i1_2	1	20	7,7477	36,765	PF04770.11	ZF-HD_dimer	83,2	1,10E-23	No_clan
ARHOMBI_DN18734_c0_g1_i1_1	3	18,1	30,142	18,652	PF03807.16	F420_oxidored	49,1	6,20E-13	CL0063
ARUBRA_DN3334_c0_g1_i1_1	2	13,1	19,185	12,332	PF03949.14	Malic_M	98,1	4,40E-28	CL0063
ARHOMBI_DN421_c0_g1_i1_5	2	11,4	35,001	14,213	PF00291.24	PALP	74,2	1,10E-20	No_clan
ARUBRA_DN416_c0_g1_i1_2	2	14,6	20,049	12,987	PF00085.19	Thioredoxin	97,8	2,70E-28	CL0172
ARHOMBI_DN25721_c0_g1_i1_4	2	18,8	12,78	11,25					
ARUBRA_DN11356_c0_g1_i1_6	2	7,5	30,25	11,415	PF01105.23	EMP24_GP25L	168,7	1,10E-49	CL0521
ARUBRA_DN18553_c0_g1_i1_5	1	10	14,895	173,12	PF00459.24	Inositol_P	76,7	1,90E-21	CL0171
ARHOMBI_DN3435_c0_g2_i1_3	2	9,6	29,797	13,926	PF00005.26	ABC_tran	40	4,80E-10	CL0023
ARHOMBI_DN13700_c0_g1_i1_6	2	32,2	9,7638	15,621	PF06552.11	TOM20_plant	160,8	2,80E-47	CL0020
ARHOMBI_DN10592_c0_g1_i1_1	1	12,5	11,6	6,6575					
ARUBRA_DN9072_c0_g1_i1_5	2	27,7	10,489	7,6584	PF00464.18	SHMT	144,8	2,60E-42	CL0061
ARUBRA_DN3824_c0_g1_i1_5	1	18,3	8,977	7,9284	PF00076.21	RRM_1	28,8	7,60E-07	CL0221
ARUBRA_DN4832_c0_g1_i1_2	2	18,2	16,52	9,918	PF00450.21	Peptidase_S10	20,7	0,00022	CL0028
ARUBRA_DN9069_c0_g1_i1_4	2	11,1	25,065	14,431	PF00076.21	RRM_1	53,3	1,70E-14	CL0221
ARHOMBI_DN8777_c0_g1_i1_4	1	10,8	11,739	6,4281	PF00400.31	WD40	29,5	8,50E-07	CL0186
ARHOMBI_DN23553_c0_g1_i1_1	1	8,8	13,791	6,1647	PF03345.13	DDOST_48kD	66,5	1,80E-18	No_clan
ARHOMBI_DN3610_c0_g1_i1_3	4	31,3	22,09	201,71	PF03332.12	PMM	231,9	6,60E-69	CL0137
ARHOMBI_DN5807_c0_g2_i1_3	1	9,9	16,409	7,1361	PF00428.18	Ribosomal_60s	84,1	7,30E-24	No_clan
ARUBRA_DN4275_c0_g1_i1_5	5	10,1	68,647	32,442	PF06273.10	eIF-4B	724	1,10E-217	No_clan
ARUBRA_DN24862_c0_g1_i1_4	2	20	13,545	11,215	PF00005.26	ABC_tran	32,4	1,10E-07	CL0023
ARUBRA_DN17478_c0_g1_i1_6	4	17	29,03	6,7739	PF00244.19	14.03.2003	332,4	1,00E-99	No_clan
ARUBRA_DN2215_c0_g1_i1_4	1	7,8	16,643	85,324					
ARUBRA_DN7908_c0_g1_i1_1	2	6,6	50,559	12,739	PF01634.17	HisG	126,5	8,30E-37	CL0177

ARHOMBI_DN4809_c0_g2_i1_2	4	13,4	30,15	12,861	PF00107.25	ADH_zinc_N	91,1	4,80E-26	CL0063
ARUBRA_DN7769_c0_g1_i1_1	4	20,9	38,05	26,91	PF03446.14	NAD_binding_2	140,9	3,50E-41	CL0063
ARUBRA_DN4322_c0_g1_i1_1	2	9,9	32,432	12,196	PF04774.14	HABP4_PAI-RBP1	27,3	4,70E-06	No_clan
ARUBRA_DN25612_c0_g1_i1_4	2	19	22,06	14,638	PF13669.5	Glyoxalase_4	34,3	2,20E-08	CL0104
ARHOMBI_DN11791_c0_g1_i1_2	1	12,6	20,961	7,7996	PF00224.20	PK	183,7	3,90E-54	CL0151
ARHOMBI_DN5868_c0_g1_i1_6	3	10,3	39,115	18,437	PF01946.16	Thi4	367,4	2,10E-110	CL0063
ARUBRA_DN2981_c0_g1_i1_3	3	6,4	56,435	16,743	PF10250.8	O-FucT	101,3	8,10E-29	CL0113
ARHOMBI_DN24401_c0_g1_i1_3	1	27	9,6158	19,969					
ARUBRA_DN23505_c0_g1_i1_1	1	8,3	15,567	6,4588					
ARUBRA_DN17618_c0_g1_i1_6	1	11,9	12,048	6,21					
ARUBRA_DN1478_c0_g1_i1_1	4	13,5	40,15	23,245	PF01180.20	DHO_dh	91,9	3,70E-26	CL0036
ARHOMBI_DN2506_c0_g1_i1_6	3	24	22,284	18,241	PF04845.12	PurA	74,5	6,70E-21	CL0609
ARHOMBI_DN8898_c0_g1_i1_5	2	23,3	16,267	16,613	PF16113.4	ECH_2	192,8	1,00E-56	CL0127
ARUBRA_DN3963_c0_g2_i1_4	1	10,1	26,207	7,2339					
ARUBRA_DN210_c0_g1_i1_5	2	20,6	12,127	153,61	PF08207.11	EFP_N	25,2	1,20E-05	CL0107
ARUBRA_DN12757_c0_g1_i1_6	2	26,5	11,539	11,942	PF04053.13	Coatomer_WDAD	111,7	4,20E-32	CL0186
ARHOMBI_DN1108_c0_g2_i1_1	1	12,9	22,962	6,2775	PF00403.25	HMA	40,3	3,00E-10	No_clan
ARHOMBI_DN11591_c0_g1_i1_2	2	26,1	13,254	13,219	PF08241.11	Methyltransf_11	73,7	1,30E-20	CL0063
ARHOMBI_DN10607_c0_g1_i1_1	2	21,3	19,282	14,029	PF12580.7	TPPII	46,4	2,80E-12	No_clan
ARUBRA_DN6875_c0_g1_i1_6	2	24,9	19,451	14,464	PF14306.5	PUA_2	96,5	1,20E-27	CL0178
ARUBRA_DN25440_c0_g1_i1_2	2	25,8	15,11	12,586	PF14308.5	DnaJ-X	96,8	1,10E-27	No_clan
ARUBRA_DN7688_c0_g1_i1_2	2	11,9	25,075	14,227					
ARHOMBI_DN19744_c0_g1_i1_2	3	16,4	29,337	18,423					
ARUBRA_DN24337_c0_g1_i1_6	5	30,8	18,28	15,571	PF00071.21	Ras	127,8	2,60E-37	CL0023
ARUBRA_DN20434_c0_g1_i1_2	2	8,2	32,635	11,987	PF10602.8	RPN7	143	6,90E-42	CL0020
ARHOMBI_DN5476_c0_g1_i1_6	3	9,9	54,811	18,029	PF01851.21	PC_rep	21,3	0,00024	CL0020
ARHOMBI_DN9888_c0_g1_i1_2	2	19,3	16,545	13,172	PF00390.18	malic	192,7	4,30E-57	CL0603
ARHOMBI_DN2706_c0_g1_i1_5	2	14,5	28,262	13,361	PF00557.23	Peptidase_M24	105,4	2,90E-30	No_clan
ARUBRA_DN5778_c0_g1_i1_4	3	8,9	46,55	17,812	PF03009.16	GDPD	224,2	2,10E-66	CL0384
ARUBRA_DN3967_c0_g1_i1_4	3	17	22,308	19,886	PF00076.21	RRM_1	74,6	3,80E-21	CL0221
ARUBRA_DN26097_c0_g1_i1_3	2	39,7	8,9162	12,882	PF00332.17	Glyco_hydro_17	63,7	1,80E-17	CL0058
ARHOMBI_DN2700_c0_g2_i1_5	3	10,6	30,958	25,713	PF02861.19	Clp_N	32,6	6,00E-08	No_clan

ARHOMBI_DN8688_c0_g1_i1_2	2	21,6	12,532	11,049	PF05470.11	eIF-3c_N	88,1	3,90E-25	No_clan
ARHOMBI_DN15526_c0_g1_i1_1	3	8,2	41,364	27,679	PF02893.19	GRAM	103,6	5,00E-30	CL0266
ARHOMBI_DN12805_c0_g1_i1_1	1	8,6	13,557	12,575					
ARHOMBI_DN10876_c0_g1_i1_3	2	20,7	9,839	12,901					
ARUBRA_DN5971_c0_g1_i1_2	3	11	30,588	30,026	PF00085.19	Thioredoxin	103,9	3,40E-30	CL0172
ARHOMBI_DN18415_c0_g1_i1_4	1	27,5	7,4083	7,594					
ARUBRA_DN4814_c0_g1_i2_6	2	12,9	34,291	7,1391	PF00314.16	Thaumatin	206,8	3,10E-61	CL0293
ARUBRA_DN21824_c0_g1_i1_6	1	9,7	23,534	7,0223					
ARUBRA_DN10839_c0_g1_i1_5	4	34,9	18,493	7,2314					
ARUBRA_DN4729_c0_g1_i1_2	2	32,8	13,798	14,672	PF00107.25	ADH_zinc_N	97,2	6,00E-28	CL0063
ARUBRA_DN20854_c0_g1_i1_1	2	9,2	23,866	12,643					
ARUBRA_DN8756_c0_g1_i1_5	2	38	7,5675	11,673	PF00920.20	ILVD_EDD	80,5	9,40E-23	No_clan
ARUBRA_DN18126_c0_g1_i1_2	1	5,7	18,722	40,588					
ARUBRA_DN7462_c0_g1_i1_6	1	13,6	13,516	6,3541					
ARUBRA_DN18563_c0_g1_i1_3	3	29,1	16,217	18,284	PF00501.27	AMP-binding	98,1	4,00E-28	CL0378
ARUBRA_DN10939_c0_g1_i1_3	2	22,6	14,892	13,248	PF00862.18	Sucrose_synth	234,7	1,50E-69	CL0113
ARHOMBI_DN21563_c0_g1_i1_5	2	11,6	24,366	12,03	PF01602.19	Adaptin_N	217,6	2,50E-64	CL0020
ARHOMBI_DN6102_c0_g2_i1_1	4	16,1	29,178	25,236	PF02431.14	Chalcone	356,7	2,50E-107	CL0560
ARUBRA_DN9246_c0_g1_i1_4	1	6,4	19,021	17,135	PF01583.19	APS_kinase	231,7	3,40E-69	CL0023
ARHOMBI_DN26256_c0_g1_i1_3	1	14,3	8,5566	6,5089					
ARUBRA_DN22761_c0_g1_i1_6	1	9,1	17,825	6,9079	PF00107.25	ADH_zinc_N	57,2	1,50E-15	CL0063
ARHOMBI_DN20260_c0_g1_i1_3	2	19,3	21,289	12,121	PF12999.6	PRKCSH-like	125,6	1,70E-36	No_clan
ARHOMBI_DN19503_c0_g1_i1_3	1	7,5	21,896	6,4534	PF01150.16	GDA1_CD39	57,3	1,00E-15	CL0108
ARHOMBI_DN10131_c0_g1_i1_2	2	13	18,228	13,378	PF03255.13	ACCA	73,3	1,70E-20	CL0127
ARHOMBI_DN19928_c0_g1_i1_4	3	28,4	12,938	17,924	PF01344.24	Kelch_1	39,4	3,00E-10	CL0186
ARUBRA_DN1385_c0_g1_i1_2	4	11,4	65,244	24,396	PF00155.20	Aminotran_1_2	125,7	2,20E-36	CL0061
ARHOMBI_DN2055_c1_g1_i1_1	3	68,2	9,9682	6,6055	PF00022.18	Actin	85,4	2,70E-24	CL0108
ARUBRA_DN22675_c0_g1_i1_5	1	8,6	19,038	6,8702					
ARHOMBI_DN1643_c0_g1_i1_5	3	17,1	32,081	13,26	PF00004.28	AAA	151,8	1,30E-44	CL0023
ARHOMBI_DN1170_c0_g1_i1_5	2	10,2	30,374	7,0929	PF05529.11	Bap31	54,9	7,70E-15	No_clan
ARHOMBI_DN22076_c0_g1_i1_3	1	12,2	12,327	34,262					
ARUBRA_DN16830_c0_g1_i1_1	2	9,7	40,401	13,366	PF01956.15	DUF106	174,4	1,50E-51	CL0376

ARUBRA_DN17659_c0_g1_i1_1	3	16,1	27,18	16,553	PF09409.9	PUB	80,2	7,10E-23	No_clan
ARHOMBI_DN21263_c0_g1_i1_6	2	18,5	20,633	15,851	PF01593.23	Amino_oxidase	102,6	2,80E-29	CL0063
ARHOMBI_DN22368_c0_g1_i1_3	2	21,1	16,23	13,052					
ARHOMBI_DN20690_c0_g1_i1_2	2	18,7	24,344	15,241	PF03141.15	Methyltransf_29	228,4	1,30E-67	CL0063
ARUBRA_DN2528_c0_g1_i1_6	1	9,6	19,713	8,2355					
ARHOMBI_DN1508_c0_g1_i1_5	2	8,2	30,235	10,96	PF01399.26	PCI	58,1	9,90E-16	CL0123
ARHOMBI_DN4881_c0_g1_i1_3	2	5,8	50,285	184,9	PF00076.21	RRM_1	30,2	2,60E-07	CL0221
ARHOMBI_DN24692_c0_g1_i1_6	2	21,9	16,428	11,351	PF00118.23	Cpn60_TCP1	149,4	1,20E-43	No_clan
ARUBRA_DN51_c0_g1_i1_2	3	17,8	23,431	12,523	PF00244.19	14.03.2003	215,8	4,60E-64	No_clan
ARUBRA_DN24099_c0_g1_i1_5	2	11,5	20,684	16,292	PF00156.26	Pribosyltran	81,6	3,90E-23	CL0533
ARUBRA_DN7587_c0_g1_i1_6	1	10	23,632	7,6114					
ARUBRA_DN5361_c0_g1_i1_2	1	5,3	20,398	116,44	PF06747.12	CHCH	38,3	9,00E-10	CL0351
ARHOMBI_DN2590_c0_g1_i1_1	4	21,6	24,466	78,181	PF00071.21	Ras	124,3	3,10E-36	CL0023
ARHOMBI_DN12163_c0_g1_i1_3	5	45,5	13,972	6,6304	PF00004.28	AAA	105,6	2,40E-30	CL0023
ARHOMBI_DN15490_c0_g1_i1_6	2	9,9	23,086	12,596	PF00534.19	Glycos_transf_1	39,5	3,90E-10	CL0113
ARUBRA_DN24778_c0_g1_i1_1	1	24	11,844	6,437	PF00232.17	Glyco_hydro_1	128,3	2,50E-37	CL0058
ARUBRA_DN20929_c0_g1_i1_2	1	8	19,715	7,2617	PF01106.16	NifU	102,3	1,10E-29	CL0232
ARUBRA_DN16965_c0_g1_i1_3	2	20,8	12,299	12,96	PF04752.11	ChaC	112	3,00E-32	CL0278
ARUBRA_DN20307_c0_g1_i1_3	2	31,8	9,0798	13,905					
ARHOMBI_DN24428_c0_g1_i1_4	2	16	14,836	30,33					
ARUBRA_DN2923_c0_g2_i1_5	2	11,5	38,633	17,47	PF00227.25	Proteasome	158,2	1,50E-46	CL0052
ARHOMBI_DN581_c0_g1_i1_2	3	12,3	34,301	21,134	PF00635.25	Motile_Sperm	94,1	3,90E-27	CL0556
ARHOMBI_DN6817_c0_g1_i1_1	2	6,7	39,587	12,584	PF02678.15	Pirin	112,6	7,50E-33	CL0029
ARUBRA_DN10993_c0_g1_i1_2	2	27,9	13,544	13,579	PF00501.27	AMP-binding	96,9	8,90E-28	CL0378
ARUBRA_DN12715_c0_g1_i1_2	1	11,5	14,156	6,712	PF13620.5	CarboxypepD_reg	39,6	4,40E-10	CL0287
ARUBRA_DN9682_c0_g1_i1_4	2	24,8	15,803	13,783	PF08543.11	Phos_pyr_kin	53,9	1,50E-14	CL0118
ARHOMBI_DN18725_c0_g1_i1_3	3	31,8	14,154	19,338	PF01394.19	Clathrin_propel	44,2	1,20E-11	CL0020
ARHOMBI_DN20294_c0_g1_i1_3	1	15,2	10,761	6,8744					
ARHOMBI_DN4086_c0_g1_i1_4	3	10,5	36,398	17,638	PF03665.12	UPF0172	190,5	2,90E-56	CL0366
ARUBRA_DN19093_c0_g1_i1_3	2	10,5	22,764	12,471	PF08712.10	Nfu_N	87,9	3,20E-25	No_clan
ARHOMBI_DN13465_c0_g2_i1_6	3	24,3	16,196	17,475	PF00107.25	ADH_zinc_N	70,6	1,10E-19	CL0063
ARUBRA_DN20855_c0_g1_i1_2	1	10,9	19,485	6,8294					
ARUBRA_DN2934_c0_g2_i2_4	3	11,7	41,91	18,868	PF05670.12	DUF814	80,8	5,50E-23	No_clan

ARUBRA_DN17957_c0_g1_i1_3	2	34,6	9,1102	14,635	PF05822.11	UMPH-1	78,1	6,70E-22	CL0137
ARUBRA_DN189_c0_g2_i1_5	3	8,6	51,905	18,078	PF01063.18	Aminotran_4	143,6	7,50E-42	No_clan
ARUBRA_DN12373_c0_g1_i1_4	5	26,8	30,158	29,991	PF00091.24	Tubulin	134,4	5,00E-39	CL0566
ARHOMBI_DN26479_c0_g1_i1_3	1	11,5	11,875	103,3	PF00300.21	His_Phos_1	30,2	3,10E-07	CL0071
ARUBRA_DN1156_c0_g1_i1_5	2	12,4	28,794	13,005	PF03643.14	Vps26	225,5	6,00E-67	CL0135
ARHOMBI_DN715_c0_g1_i1_6	5	22,4	38,931	30,384	PF00255.18	GSHPx	144,5	6,90E-43	CL0172
ARUBRA_DN18388_c0_g1_i1_5	3	44,5	12,402	152,35	PF00251.19	Glyco_hydro_32N	70,9	1,30E-19	CL0143
ARUBRA_DN24939_c0_g1_i1_6	1	20,5	9,8581	7,0823	PF13855.5	LRR_8	38,1	8,80E-10	CL0022
ARUBRA_DN1579_c0_g1_i1_3	3	19,6	26,019	14,03	PF00719.18	Pyrophosphatase	177,1	2,00E-52	No_clan
ARUBRA_DN3088_c1_g1_i1_2	2	9	29,631	6,7411	PF00118.23	Cpn60_TCP1	251,9	1,00E-74	No_clan
ARUBRA_DN18995_c0_g1_i1_4	2	20,2	12,597	11,531					
ARUBRA_DN6428_c0_g1_i1_3	1	12,7	20,411	7,9797	PF05116.12	S6PP	56,8	2,20E-15	CL0137
ARUBRA_DN24802_c0_g1_i1_6	2	34,2	7,8127	11,614					
ARHOMBI_DN6811_c0_g1_i1_3	2	22,4	16,227	12,778	PF00311.16	PEPcase	211,6	1,70E-62	CL0151
ARHOMBI_DN23413_c0_g1_i1_2	1	9,2	13,539	54,976	PF08324.10	PUL	72,5	3,00E-20	No_clan
ARUBRA_DN26636_c0_g1_i1_3	1	13,6	10,418	6,46	PF14533.5	USP7_C2	70,5	1,40E-19	CL0072
ARUBRA_DN24535_c0_g1_i1_1	3	48,2	12,581	13,267	PF01459.21	Porin_3	79,8	2,00E-22	CL0193
ARUBRA_DN5752_c0_g1_i1_3	2	20,7	16,055	13,599					
ARUBRA_DN12277_c0_g1_i1_4	1	12,4	11,227	17,623	PF13943.5	WPP	45,2	8,60E-12	No_clan
ARUBRA_DN12763_c0_g1_i1_6	2	57,9	7,9399	37,799	PF01213.18	CAP_N	34,9	1,00E-08	No_clan
ARUBRA_DN23296_c0_g1_i1_1	2	21,8	16,121	13,438	PF00995.22	Sec1	63,8	1,60E-17	No_clan
ARHOMBI_DN6174_c0_g1_i1_5	1	2,3	67,099	7,1721	PF00483.22	NTP_transferase	245,1	7,10E-73	CL0110
ARHOMBI_DN3360_c0_g1_i1_6	2	13	22,226	12,453	PF05047.15	L51_S25_CI-B8	52,4	3,40E-14	CL0172
ARUBRA_DN4576_c0_g3_i1_3	4	22,4	23,249	14,368					
ARUBRA_DN27180_c0_g1_i1_6	2	31,8	9,4948	30,794					
ARUBRA_DN3154_c0_g1_i1_6	6	17,3	31,568	49,128	PF02298.16	Cu_bind_like	76,4	1,20E-21	CL0026
ARUBRA_DN6360_c0_g1_i1_2	4	15,6	51,344	29,009	PF00076.21	RRM_1	48	7,60E-13	CL0221
ARUBRA_DN24250_c0_g1_i1_6	1	15,3	8,1819	6,2028					
ARUBRA_DN9113_c0_g1_i1_5	2	25,2	13,715	12,366					
ARUBRA_DN18999_c0_g1_i1_5	7	30,2	31,369	6,4819	PF00736.18	EF1_GNE	110,8	2,60E-32	No_clan
ARUBRA_DN23874_c0_g1_i1_1	2	12,9	17,229	13,179	PF01012.20	ETF	93,7	1,10E-26	CL0039
ARHOMBI_DN8339_c0_g1_i1_2	1	9,4	17,11	8,8387	PF00005.26	ABC_tran	62,7	4,70E-17	CL0023
ARHOMBI_DN1833_c0_g1_i1_4	3	25,2	14,182	10,829	PF00347.22	Ribosomal_L6	51,7	9,70E-14	No_clan

ARHOMBI_DN187_c0_g2_i1_1	2	8,6	37,733	14,388	PF00005.26	ABC_tran	126,9	6,90E-37	CL0023
ARHOMBI_DN18091_c0_g1_i1_1	1	8,5	13,121	98,262	PF04558.14	tRNA_synt_1c_R1	99,4	1,90E-28	No_clan
ARHOMBI_DN11853_c0_g1_i1_1	3	25,1	20,997	6,4571	PF06957.10	COPI_C	112,4	2,00E-32	CL0020
ARUBRA_DN898_c0_g1_i1_1	1	5	27,267	6,3427	PF01636.22	APH	59,1	5,50E-16	CL0016
ARHOMBI_DN5055_c0_g1_i1_3	2	29,5	15,101	11,508	PF00012.19	HSP70	50,3	1,00E-13	CL0108
ARUBRA_DN21162_c0_g1_i1_4	3	15,1	37,497	23,393	PF00443.28	UCH	85,7	3,30E-24	CL0125
ARUBRA_DN19451_c0_g1_i1_2	1	13,9	9,3824	6,4278					
ARHOMBI_DN17114_c0_g1_i1_5	2	6,7	42,041	11,485	PF04815.14	Sec23_helical	38,2	9,00E-10	No_clan
ARHOMBI_DN4946_c0_g1_i2_4	2	5,7	35,191	12,613	PF03227.15	GILT	105	2,10E-30	CL0172
ARUBRA_DN9563_c0_g1_i1_2	2	12,7	13,135	78,691	PF00226.30	DnaJ	93,6	5,60E-27	CL0392
ARUBRA_DN3083_c0_g1_i1_4	2	9,5	38,808	12,425	PF02453.16	Reticulon	197,6	1,00E-58	No_clan
ARUBRA_DN10864_c0_g1_i1_2	1	25,6	9,6275	7,1376	PF02338.18	OTU	63,9	1,80E-17	CL0125
ARHOMBI_DN8832_c0_g1_i1_6	5	24,4	30,018	29,84	PF01433.19	Peptidase_M1	44,4	1,20E-11	CL0126
ARHOMBI_DN16126_c0_g1_i1_1	2	26,2	16,705	6,252	PF02359.17	CDC48_N	83,5	8,20E-24	CL0332
ARUBRA_DN11418_c0_g1_i1_5	3	13,7	37,256	19,64	PF00118.23	Cpn60_TCP1	207,5	3,00E-61	No_clan
ARUBRA_DN711_c0_g1_i1_2	2	11,3	30,34	12,77	PF01992.15	vATP-synt_AC39	214,9	1,90E-63	No_clan
ARUBRA_DN23248_c0_g1_i1_5	1	7,3	12,2	-2	PF00069.24	Pkinase	29,9	3,30E-07	CL0016
ARHOMBI_DN11424_c0_g1_i1_1	3	22,9	15,82	17,263					
ARUBRA_DN13078_c0_g1_i1_4	1	12,1	14,34	7,3432					
ARUBRA_DN22748_c0_g1_i1_3	3	40,5	8,9801	18,242	PF02127.14	Peptidase_M18	39,9	2,10E-10	CL0035
ARUBRA_DN19446_c0_g1_i1_1	2	17,1	25,438	13,31	PF00202.20	Aminotran_3	161,7	1,90E-47	CL0061
ARHOMBI_DN25338_c0_g1_i1_6	1	18,5	9,2703	8,1949					
ARUBRA_DN1375_c0_g1_i1_4	3	4,2	111,09	18,285	PF02037.26	SAP	37	1,80E-09	CL0306
ARHOMBI_DN5366_c0_g1_i2_2	2	6,4	37,895	12,332	PF05648.13	PEX11	165,4	1,20E-48	No_clan
ARUBRA_DN26151_c0_g1_i1_1	1	11,4	8,5572	11,758					
ARHOMBI_DN16439_c0_g1_i1_3	3	10,9	42,493	17,327	PF02383.17	Syja_N	78,8	4,20E-22	CL0031
ARHOMBI_DN17772_c0_g1_i1_2	2	32,1	9,7959	13,88	PF01496.18	V_ATPase_I	34,2	6,40E-09	No_clan
ARHOMBI_DN3967_c0_g1_i1_1	4	15,7	30,965	51,814	PF13419.5	HAD_2	84,6	8,20E-24	CL0137
ARUBRA_DN18178_c0_g1_i1_2	2	26,5	13,538	14,915	PF08375.10	Rpn3_C	98,4	2,10E-28	No_clan
ARUBRA_DN4925_c0_g1_i1_4	10	28,9	47,542	6,6624	PF00883.20	Peptidase_M17	385,4	1,70E-115	CL0035
ARUBRA_DN14063_c0_g1_i1_3	1	25,2	12,817	9,7476	PF04053.13	Coatomer_WDAD	56,5	2,30E-15	CL0186
ARHOMBI_DN2499_c0_g1_i1_5	2	7	30,619	10,7					

ARUBRA_DN2486_c0_g1_i1_3	2	31	14,19	6,2265	PF16363.4	GDP_Man_Dehyd	27,9	1,30E-06	CL0063
ARUBRA_DN9169_c0_g1_i1_1	1	13	10,981	6,4603					
ARHOMBI_DN2036_c0_g2_i1_1	2	6,7	32,345	24,526	PF04969.15	CS	37,5	3,30E-09	CL0190
ARUBRA_DN20749_c0_g1_i1_4	1	23,5	7,6194	6,2992					
ARHOMBI_DN13970_c0_g1_i1_4	2	36,6	8,0775	11,83	PF05470.11	eIF-3c_N	64,6	5,30E-18	No_clan
ARHOMBI_DN16110_c0_g1_i1_4	1	15,7	9,3367	6,6686	PF00069.24	Pkinase	96	2,10E-27	CL0016
ARUBRA_DN16649_c0_g1_i1_5	1	5,4	30,331	6,6958	PF06964.11	Alpha-L-AF_C	130,8	4,90E-38	CL0369
ARHOMBI_DN26305_c0_g1_i1_3	2	26,4	10,346	6,5427					
ARUBRA_DN7710_c0_g1_i1_3	8	30,3	42,72	8,0926	PF08442.9	ATP-grasp_2	80,8	9,00E-23	CL0179
ARUBRA_DN9775_c0_g1_i1_2	2	21,8	12,387	11,224					
ARUBRA_DN9988_c0_g1_i1_3	1	25	9,6059	7,8691					
ARHOMBI_DN18064_c0_g1_i1_4	1	27,9	7,0031	7,7104	PF13840.5	ACT_7	37,5	1,40E-09	CL0070
ARHOMBI_DN4801_c0_g1_i1_1	5	12,4	51,365	13,939	PF06273.10	eIF-4B	580,3	4,00E-174	No_clan
ARUBRA_DN5397_c0_g1_i1_4	1	27	8,1635	7,2611					
ARHOMBI_DN3803_c0_g2_i1_6	1	15,1	7,6849	6,3171					
ARUBRA_DN4646_c0_g1_i1_4	2	16,7	20,258	17,623	PF05899.11	Cupin_3	101,1	1,90E-29	CL0029
ARUBRA_DN16654_c0_g1_i1_4	1	14	21,199	9,3521	PF01532.19	Glyco_hydro_47	164,6	3,40E-48	CL0059
ARUBRA_DN20972_c0_g1_i1_2	2	11,3	32,266	12,255	PF03141.15	Methyltransf_29	254,4	1,70E-75	CL0063
ARHOMBI_DN23602_c0_g1_i1_6	1	9,6	15,415	6,3786	PF14686.5	fn3_3	100,2	3,70E-29	CL0287
ARUBRA_DN20900_c0_g1_i1_2	3	18,2	20,626	11,77	PF03224.13	V-ATPase_H_N	124,2	5,90E-36	CL0020
ARUBRA_DN17183_c0_g1_i1_5	2	13,9	32,312	14,047	PF02771.15	Acyl-CoA_dh_N	121,1	3,20E-35	CL0544
ARHOMBI_DN3229_c0_g1_i1_4	3	16	19,061	17,606					
ARUBRA_DN12858_c0_g1_i1_4	1	17,5	12,717	11,957	PF01145.24	Band_7	30,4	3,20E-07	CL0433
ARUBRA_DN20_c0_g1_i1_4	3	23,7	16,615	61,145	PF12799.6	LRR_4	36,4	4,30E-09	CL0022
ARUBRA_DN2891_c0_g1_i1_5	1	7,7	16,616	6,4659	PF00118.23	Cpn60_TCP1	50,3	1,40E-13	No_clan
ARHOMBI_DN4641_c0_g1_i1_6	2	8,5	40,986	15,502	PF01105.23	EMP24_GP25L	108,9	2,60E-31	CL0521
ARUBRA_DN5439_c0_g1_i1_5	3	19,3	26,279	18,153	PF13640.5	2OG-FeII_Oxy_3	77,6	9,50E-22	CL0029
ARUBRA_DN2788_c0_g1_i1_6	2	19,1	20,511	19,221					
ARHOMBI_DN24746_c0_g1_i1_1	1	6,3	20,077	6,5949					
ARUBRA_DN12422_c0_g1_i1_2	1	12,5	10,536	6,7975	PF07859.12	Abhydrolase_3	51	1,50E-13	CL0028
ARUBRA_DN19709_c0_g1_i1_5	3	10	33,286	17,133	PF01213.18	CAP_N	126,7	1,20E-36	No_clan

ARUBRA_DN4950_c1_g1_i1_4	2	8,9	41,684	51,398	PF00332.17	Glyco_hydro_17	415,8	1,10E-124	CL0058
ARHOMBI_DN8627_c0_g1_i1_4	2	27,8	10,36	12,166	PF00920.20	ILVD_EDD	116,5	1,20E-33	No_clan
ARHOMBI_DN8139_c0_g1_i1_2	2	27,7	18,196	12,098	PF05193.20	Peptidase_M16_C	48,2	1,10E-12	CL0094
ARUBRA_DN9097_c0_g1_i1_3	1	10,5	27,745	8,1002					
ARUBRA_DN23250_c0_g1_i1_1	2	27,6	12,523	12,348					
ARHOMBI_DN13828_c0_g1_i1_2	1	16,2	11,251	8,6984	PF00113.21	Enolase_C	89,2	2,50E-25	CL0256
ARHOMBI_DN16373_c0_g1_i1_2	1	7,2	29,114	7,2454	PF00160.20	Pro_isomerase	41,6	1,30E-10	CL0475
ARUBRA_DN12629_c0_g1_i1_4	1	20	11,951	8,1281					
ARHOMBI_DN6215_c0_g1_i2_2	2	8,3	36,36	12,13	PF03937.15	Sdh5	79,3	1,50E-22	No_clan
ARUBRA_DN4706_c0_g1_i1_4	1	3,2	38,183	6,288					
ARUBRA_DN6348_c0_g1_i1_2	3	13,8	29,441	19,616	PF00076.21	RRM_1	53,4	1,60E-14	CL0221
ARHOMBI_DN4431_c0_g3_i1_1	5	29,1	27,549	29,223	PF00071.21	Ras	223,6	9,00E-67	CL0023
ARHOMBI_DN2426_c0_g1_i1_5	1	6,7	18,869	102,99	PF01187.17	MIF	88,3	3,80E-25	CL0082
ARUBRA_DN2608_c0_g1_i1_4	1	4,8	36,804	6,4768	PF01842.24	ACT	44,9	6,30E-12	CL0070
ARHOMBI_DN5630_c0_g2_i1_5	2	19,8	21,357	11,377	PF01435.17	Peptidase_M48	57	2,00E-15	CL0126
ARHOMBI_DN2188_c0_g1_i1_4	2	9,2	39,371	12,513	PF01055.25	Glyco_hydro_31	100,3	1,30E-28	CL0058
ARUBRA_DN16537_c0_g1_i1_2	2	7,7	46,138	13,505	PF04321.16	RmlD_sub_bind	48,8	4,70E-13	CL0063
ARHOMBI_DN6250_c0_g2_i3_5	1	4,8	29,631	6,5467	PF01657.16	Stress-antifung	56,8	2,50E-15	No_clan
ARUBRA_DN12906_c0_g1_i1_4	1	14,7	11,177	7,0944					
ARHOMBI_DN15675_c0_g1_i1_3	3	40,7	15,851	6,2152	PF00118.23	Cpn60_TCP1	120,1	9,40E-35	No_clan
ARHOMBI_DN20830_c0_g1_i1_1	2	22,5	16,579	14,828	PF00271.30	Helicase_C	72,4	3,30E-20	CL0023
ARHOMBI_DN6132_c0_g5_i1_3	2	17,5	13,762	23,565	PF16845.4	SQAPI	76,5	1,40E-21	CL0121
ARHOMBI_DN13938_c0_g1_i1_3	2	27,5	10,846	12,349					
ARHOMBI_DN343_c0_g2_i1_6	3	11,3	34,484	18,217	PF01412.17	ArfGap	138,2	1,10E-40	No_clan
ARHOMBI_DN20204_c0_g1_i1_6	1	11,8	16,48	6,437					
ARHOMBI_DN5653_c0_g1_i1_4	3	12,4	39,058	17,918	PF00187.18	Chitin_bind_1	53,3	2,60E-14	No_clan
ARUBRA_DN9043_c0_g1_i1_6	1	14,3	9,6912	6,6105	PF00350.22	Dynamin_N	60,6	1,70E-16	CL0023
ARUBRA_DN8452_c0_g1_i1_1	2	15,2	25,387	12,931	PF04909.13	Amidohydro_2	77,7	1,20E-21	CL0034
ARHOMBI_DN5207_c0_g1_i1_5	2	6,8	35,876	6,2682	PF04734.12	Ceramidase_alk	74,3	7,10E-21	No_clan
ARHOMBI_DN5473_c0_g1_i1_2	2	13,9	24,858	12,651					
ARHOMBI_DN15547_c1_g1_i1_2	2	7,7	44,959	46,358	PF03127.13	GAT	46,1	4,20E-12	No_clan
ARUBRA_DN21709_c0_g1_i1_1	1	26,4	8,2863	6,7109					

ARUBRA_DN7051_c0_g1_i1_2	2	11,9	20,629	11,664	PF00378.19	ECH_1	108,2	3,70E-31	CL0127
ARUBRA_DN25464_c0_g1_i1_1	2	51,9	8,6279	13,413	PF00004.28	AAA	31,6	1,70E-07	CL0023
ARHOMBI_DN4589_c0_g1_i1_6	1	6,1	23,49	35,196	PF04398.11	DUF538	117,2	4,30E-34	No_clan
ARUBRA_DN931_c0_g1_i1_3	1	3,7	30,303	160,37	PF00462.23	Glutaredoxin	69,9	1,50E-19	CL0172
ARHOMBI_DN10963_c0_g1_i1_2	2	24,5	9,9705	18,859					
ARUBRA_DN14101_c0_g1_i1_6	2	28,3	10,89	13,173					
ARHOMBI_DN18821_c0_g1_i1_3	2	26,4	15,236	14,942					
ARHOMBI_DN14763_c0_g1_i1_1	2	47,3	12,153	11,173					
ARUBRA_DN7233_c0_g1_i1_1	2	20,7	12,858	11,553					
ARUBRA_DN3582_c0_g2_i1_3	2	19,5	25,038	13,542					
ARUBRA_DN3740_c0_g1_i1_2	4	26	16,475	6,4057	PF00637.19	Clathrin	75,7	2,90E-21	CL0020
ARUBRA_DN2116_c0_g1_i1_1	1	4,9	37,6	7,644	PF13266.5	DUF4057	84,8	7,70E-24	No_clan
ARHOMBI_DN6367_c0_g1_i1_3	1	21,9	15,169	11,36	PF00118.23	Cpn60_TCP1	78,4	3,90E-22	No_clan
ARUBRA_DN9714_c0_g1_i1_3	2	37,5	8,749	12,663	PF08033.11	Sec23_BS	32,7	8,80E-08	No_clan
ARHOMBI_DN5629_c0_g1_i1_3	3	3,6	132,93	18,857	PF01061.23	ABC2_membrane	147	4,50E-43	CL0181
ARUBRA_DN17225_c0_g1_i1_6	2	13,3	25,42	12,949					
ARUBRA_DN5760_c0_g1_i1_1	9	17,6	78,545	49,207	PF00076.21	RRM_1	72	2,50E-20	CL0221
ARHOMBI_DN13303_c0_g1_i1_5	2	19,6	21,209	14,889					
ARHOMBI_DN44_c0_g1_i1_5	3	8,8	50,15	17,951	PF00155.20	Aminotran_1_2	173	9,20E-51	CL0061
ARUBRA_DN2012_c0_g1_i1_3	2	4,7	56,159	11,922	PF00483.22	NTP_transferase	96	2,50E-27	CL0110
ARHOMBI_DN18373_c0_g1_i1_1	3	18,8	26,712	22,446	PF01588.19	tRNA_bind	99,1	1,10E-28	CL0021
ARUBRA_DN22899_c0_g1_i1_4	2	14,2	24,436	16,68	PF00005.26	ABC_tran	62,6	5,10E-17	CL0023
ARUBRA_DN19779_c0_g1_i1_2	4	8,5	56,441	17,062	PF00270.28	DEAD	144,1	3,30E-42	CL0023
ARHOMBI_DN2168_c0_g1_i1_5	3	20	26,034	-2	PF00108.22	Thiolase_N	237,8	1,10E-70	CL0046
ARHOMBI_DN18268_c0_g1_i1_5	2	39,8	10,408	13,167	PF00183.17	HSP90	108,9	2,60E-31	No_clan
ARUBRA_DN20895_c0_g1_i1_4	1	18,2	8,6619	6,3259	PF02383.17	Syja_N	42,7	4,00E-11	CL0031
ARHOMBI_DN9816_c0_g1_i1_3	1	23,9	7,7208	6,9972					
ARUBRA_DN6711_c0_g1_i1_1	2	8,8	23,275	13,404	PF00828.18	Ribosomal_L27A	75,8	4,10E-21	CL0588
ARUBRA_DN1256_c0_g2_i1_1	2	8	33,51	11,251	PF01145.24	Band_7	88,3	5,40E-25	CL0433
ARHOMBI_DN4768_c0_g1_i1_5	1	12,5	19,911	7,9084	PF01487.14	DHquinase_I	129,2	2,60E-37	CL0036
ARUBRA_DN1822_c0_g1_i1_4	3	13,8	45,408	18,383	PF05116.12	S6PP	46,7	2,60E-12	CL0137
ARUBRA_DN2382_c0_g1_i1_2	1	11,7	24,602	6,2416	PF01095.18	Pectinesterase	365,1	1,60E-109	CL0268

ARUBRA_DN6119_c0_g1_i1_1	1	9,6	16,025	6,937	PF02953.14	zf-Tim10_DDP	88,1	2,00E-25	No_clan
ARUBRA_DN19079_c0_g1_i1_2	3	14,3	34,725	18,555	PF02775.20	TPP_enzyme_C	58,1	7,90E-16	CL0254
ARUBRA_DN14154_c0_g1_i1_1	1	22,9	13,013	7,9347	PF00005.26	ABC_tran	24,9	2,20E-05	CL0023
ARUBRA_DN6181_c0_g1_i1_5	1	7,2	29,651	7,16	PF01592.15	NifU_N	182,4	3,30E-54	CL0233
ARHOMBI_DN5536_c0_g1_i1_1	2	4,8	60,555	14,042	PF04043.14	PMEI	69	5,20E-19	No_clan
ARHOMBI_DN25582_c0_g1_i1_3	1	12,7	11,321	25,929	PF04414.11	tRNA_deacylase	23,3	3,70E-05	CL0408
ARUBRA_DN1176_c0_g1_i1_5	1	5,8	25,733	6,8226					
ARUBRA_DN766_c0_g1_i1_6	2	23,9	15,104	12,235					
ARHOMBI_DN20645_c0_g1_i1_1	2	23,3	13,03	12,937	PF00005.26	ABC_tran	32,2	1,20E-07	CL0023
ARHOMBI_DN985_c0_g1_i1_6	2	17,2	31,56	14,233	PF03949.14	Malic_M	196,3	4,90E-58	CL0063
ARUBRA_DN14493_c0_g1_i1_2	1	7,1	19,197	6,3618	PF09334.10	tRNA-synt_1g	39	3,90E-10	CL0039
ARHOMBI_DN18281_c0_g1_i1_5	1	20,6	8,1479	6,9911	PF03909.16	BSD	56,9	1,50E-15	No_clan
ARHOMBI_DN18082_c0_g1_i1_2	2	25,8	17,233	11,274					
ARUBRA_DN3448_c0_g1_i1_6	2	9,1	25,046	11,569	PF01176.18	eIF-1a	74,9	3,00E-21	CL0021
ARHOMBI_DN19564_c0_g1_i1_4	2	12,1	26,898	11,81	PF04815.14	Sec23_helical	86,2	1,10E-24	No_clan
ARHOMBI_DN18202_c0_g1_i1_6	1	9,4	14,473	32,041					
ARUBRA_DN4562_c0_g1_i1_3	2	7,3	36,3	13,324	PF00805.21	Pentapeptide	46,3	2,10E-12	CL0505
ARUBRA_DN756_c0_g1_i1_1	2	4,7	62,521	12,78	PF00009.26	GTP_EFTU	190,5	2,00E-56	CL0023
ARHOMBI_DN5795_c0_g1_i1_6	1	5,1	24,658	6,3797	PF13774.5	Longin	87,2	5,40E-25	No_clan
ARUBRA_DN18766_c0_g1_i1_6	1	11,8	13,747	6,4918	PF13714.5	PEP_mutase	65,6	4,00E-18	CL0151
ARHOMBI_DN11368_c0_g1_i1_5	3	21,6	23,403	19,841	PF01218.17	Coprogen_oxidas	193,2	3,80E-57	No_clan
ARHOMBI_DN19194_c0_g1_i1_2	3	19	24,971	6,245	PF00224.20	PK	119,9	1,00E-34	CL0151
ARUBRA_DN21311_c0_g1_i1_6	1	29	7,9398	7,4946					
ARUBRA_DN19687_c0_g1_i1_4	1	5,1	26,614	6,3025	PF05676.12	NDUF_B7	96,8	4,20E-28	CL0351
ARHOMBI_DN1072_c0_g1_i1_2	1	7,3	19,616	7,6426	PF08207.11	EFP_N	69,6	1,50E-19	CL0107
ARHOMBI_DN4606_c0_g2_i1_3	2	24,6	15,648	7,5575	PF00928.20	Adap_comp_sub	126,6	1,00E-36	CL0448
ARHOMBI_DN3865_c0_g1_i1_4	2	13	26,592	10,947	PF01918.20	Alba	67,1	8,30E-19	CL0441
ARHOMBI_DN3210_c0_g1_i1_3	4	30,1	22,798	25,544	PF02996.16	Prefoldin	102,3	1,40E-29	CL0200
ARHOMBI_DN127_c0_g1_i1_4	2	13,5	39,021	14,676					
ARUBRA_DN6664_c0_g1_i1_5	3	12,3	37,742	17,057	PF02466.18	Tim17	46,4	3,80E-12	No_clan
ARUBRA_DN17807_c0_g1_i1_6	2	27,7	9,3035	18,948					
ARHOMBI_DN26518_c0_g1_i1_6	1	19	9,035	6,3891					
ARHOMBI_DN26272_c0_g1_i1_3	3	26,1	14,195	18,247					

ARUBRA_DN24367_c0_g1_i1_3	2	21,3	9,7019	12,348	PF12796.6	Ank_2	45,7	6,70E-12	CL0465
ARHOMBI_DN10852_c0_g1_i1_2	2	21,1	12,914	12,104	PF05633.10	BPS1	141,2	4,00E-41	CL0133
ARUBRA_DN17980_c0_g1_i1_5	2	10,1	44,463	14,455	PF01429.18	MBD	37,2	1,60E-09	CL0081
ARHOMBI_DN178_c0_g2_i1_4	2	6,2	49,476	12,632	PF00887.18	ACBP	94,4	3,40E-27	CL0632
ARUBRA_DN3098_c0_g1_i1_4	3	15,1	45,35	20,013	PF00970.23	FAD_binding_6	109,8	6,10E-32	CL0076
ARHOMBI_DN8417_c0_g1_i1_6	1	11,4	17,712	6,6004					
ARUBRA_DN16531_c0_g1_i1_2	1	13	12,797	6,3939	PF00166.20	Cpn10	77,5	5,60E-22	CL0296
ARUBRA_DN201_c0_g2_i1_4	6	27,3	31,401	8,2436	PF00071.21	Ras	188,2	7,10E-56	CL0023
ARUBRA_DN4976_c0_g1_i1_4	13	38,6	43,075	-2	PF16884.4	ADH_N_2	85,4	2,10E-24	CL0296
ARUBRA_DN22472_c0_g1_i1_1	1	17,2	13,438	7,3004					
ARHOMBI_DN22711_c0_g1_i1_4	1	26,2	9,2329	6,4879					
ARHOMBI_DN5461_c0_g1_i2_3	2	9	38,711	10,952	PF03357.20	Snf7	153,6	3,50E-45	CL0235
ARUBRA_DN1871_c0_g1_i1_5	3	15,6	23,914	20,545					
ARHOMBI_DN11140_c0_g1_i1_4	1	18,1	8,8601	6,4501	PF00365.19	PFK	61	1,00E-16	CL0240
ARUBRA_DN23048_c0_g1_i1_5	1	13	22,469	24,125	PF00111.26	Fer2	66,8	1,10E-18	CL0486
ARHOMBI_DN2451_c0_g1_i1_1	1	8,4	19,287	6,3129					
ARUBRA_DN2562_c0_g2_i2_3	2	11,8	23,329	49,706	PF00581.19	Rhodanese	38,9	9,00E-10	CL0031
ARHOMBI_DN20377_c0_g1_i1_1	1	4,7	28,365	6,1986	PF16901.4	DAO_C	109,5	8,90E-32	No_clan
ARUBRA_DN15849_c0_g1_i1_3	2	30,3	8,3733	12,136	PF02136.19	NTF2	45,2	1,10E-11	CL0051
ARUBRA_DN24185_c0_g1_i1_2	2	31,6	10,604	16,395	PF00933.20	Glyco_hydro_3	71,9	5,30E-20	CL0058
ARUBRA_DN3137_c0_g1_i1_1	1	4,1	53,21	6,2905	PF02812.17	ELFV_dehydrog_N	167,1	1,50E-49	CL0603
ARUBRA_DN18794_c0_g1_i1_1	2	18,8	23,556	15,813	PF02880.15	PGM_PMM_III	31,7	1,30E-07	No_clan
ARHOMBI_DN16770_c0_g1_i1_3	1	10,3	16,398	6,4994	PF00171.21	Aldedh	153	8,90E-45	CL0099
ARUBRA_DN8214_c1_g1_i1_2	2	15,1	27,258	17,334					
ARHOMBI_DN5351_c0_g3_i2_2	2	14,8	18,837	11,046					
ARUBRA_DN26670_c0_g1_i1_1	1	23,7	7,6304	8,6107	PF01915.21	Glyco_hydro_3_C	40,9	1,90E-10	No_clan
ARHOMBI_DN14110_c0_g1_i1_4	1	12,7	12,518	6,6816	PF14580.5	LRR_9	51,9	5,70E-14	CL0022
ARHOMBI_DN3010_c0_g2_i1_1	1	11,5	11,724	6,5909					
ARUBRA_DN14051_c0_g1_i1_3	1	18,8	13,136	7,8622	PF02734.16	Dak2	80,9	9,20E-23	No_clan
ARUBRA_DN20086_c0_g1_i1_3	1	6,7	21,538	6,5095					
ARUBRA_DN11076_c0_g1_i1_6	1	8,3	21,112	6,7422	PF04080.12	Per1	100,7	9,80E-29	CL0192
ARHOMBI_DN25990_c0_g1_i1_4	3	18,8	16,832	11,915	PF09280.10	XPC-binding	85,7	1,20E-24	No_clan
ARHOMBI_DN2521_c0_g1_i1_6	2	13,4	27,182	12,462	PF00156.26	Pribosyltran	34,1	1,60E-08	CL0533

ARUBRA_DN3144_c0_g1_i1_5	1	54,2	7,4191	6,1762	PF00627.30	UBA	30,2	2,80E-07	CL0214
ARUBRA_DN524_c0_g2_i1_3	2	3,5	99,804	12,896	PF00564.23	PB1	35,6	6,20E-09	CL0072
ARUBRA_DN4339_c0_g1_i2_1	1	2,8	51,322	6,4827	PF01373.16	Glyco_hydro_14	315,6	4,30E-94	CL0058
ARHOMBI_DN2257_c0_g1_i1_1	2	8,1	43,214	12,037	PF07946.13	DUF1682	246,4	3,90E-73	No_clan
ARUBRA_DN17897_c0_g1_i1_5	1	4,8	33,04	46,344	PF00082.21	Peptidase_S8	51,1	1,00E-13	No_clan
ARHOMBI_DN2123_c0_g2_i1_4	2	15,1	28,193	12,655					
ARUBRA_DN11421_c0_g1_i1_5	1	16,3	10,683	6,3604	PF02817.16	E3_binding	31,8	1,20E-07	No_clan
ARHOMBI_DN13369_c0_g1_i1_6	1	16	10,753	6,4372	PF00205.21	TPP_enzyme_M	86,8	1,00E-24	CL0085
ARUBRA_DN10411_c0_g1_i1_2	1	11,7	13,909	6,5075	PF03690.12	UPF0160	144,8	4,30E-42	No_clan
ARHOMBI_DN5006_c0_g1_i1_1	1	6,2	19,538	6,2096	PF00202.20	Aminotran_3	37,1	1,30E-09	CL0061
ARHOMBI_DN17209_c0_g1_i1_1	2	46,7	8,4437	11,434					
ARUBRA_DN6201_c0_g1_i1_4	2	11,3	22,844	11,421	PF05832.11	DUF846	132	1,40E-38	No_clan
ARHOMBI_DN4317_c0_g1_i1_2	2	7,5	50,122	14,52	PF00155.20	Aminotran_1_2	155	2,80E-45	CL0061
ARHOMBI_DN5264_c0_g1_i2_1	1	3,7	43,465	6,8721	PF00483.22	NTP_transferase	111,8	3,70E-32	CL0110
ARUBRA_DN3956_c0_g2_i2_6	2	8,7	39,904	11,651	PF00248.20	Aldo_ket_red	213,3	3,80E-63	No_clan
ARUBRA_DN7892_c0_g1_i1_4	4	15,4	43,286	25,193	PF00076.21	RRM_1	51,8	4,90E-14	CL0221
ARUBRA_DN5621_c0_g1_i1_1	3	8,3	72,66	17,167	PF00501.27	AMP-binding	262	7,20E-78	CL0378
ARUBRA_DN6290_c0_g1_i1_6	1	3,2	37,556	6,2249	PF01025.18	GrpE	158,4	1,10E-46	No_clan
ARUBRA_DN8437_c0_g1_i1_2	2	16,8	19,542	14,186	PF03357.20	Snf7	62,5	3,30E-17	CL0235
ARHOMBI_DN2197_c0_g1_i1_2	2	33,8	9,2092	12,249					
ARUBRA_DN20773_c0_g1_i1_2	2	26,4	11,732	9,3316	PF02773.15	S-AdoMet_synt_C	67,3	1,30E-18	No_clan
ARUBRA_DN19652_c0_g1_i1_5	1	13,6	11,932	6,4723					
ARUBRA_DN23301_c0_g1_i1_2	2	25	12,785	12,146					
ARHOMBI_DN6000_c0_g1_i1_4	2	5,1	65,215	12,076	PF00026.22	Asp	449,9	4,80E-135	CL0129
ARHOMBI_DN27227_c0_g1_i1_2	1	21,5	8,4065	8,9583					
ARHOMBI_DN3035_c0_g1_i1_2	1	8,2	18,818	6,8482	PF02953.14	zf-Tim10_DDP	71	4,40E-20	No_clan
ARUBRA_DN7465_c0_g1_i1_1	2	31	7,6837	11,993					
ARHOMBI_DN25461_c0_g1_i1_3	2	13,6	21,158	19,412					
ARUBRA_DN23449_c0_g1_i1_4	1	18,1	9,5651	9,3541	PF00108.22	Thiolase_N	27	2,50E-06	CL0046
ARUBRA_DN4751_c0_g1_i2_6	1	6,4	21,419	8,8368	PF00187.18	Chitin_bind_1	54,1	1,40E-14	No_clan
ARHOMBI_DN23037_c0_g1_i1_6	1	12,3	18,181	6,1985	PF01434.17	Peptidase_M41	111,5	3,90E-32	CL0126
ARHOMBI_DN17541_c0_g1_i1_1	2	20,9	12,098	7,3735	PF01717.17	Meth_synt_2	37	2,20E-09	CL0160

ARUBRA_DN10528_c0_g1_i1_4	1	12,4	14,604	7,1868					
ARUBRA_DN13908_c0_g1_i1_4	1	11,6	10,849	6,2096	PF04832.11	SOUL	45	1,10E-11	CL0319
ARHOMBI_DN13282_c0_g1_i1_1	1	18,4	9,845	7,1413	PF00202.20	Aminotran_3	40,9	9,00E-11	CL0061
ARHOMBI_DN3944_c0_g1_i2_2	3	11,9	48,772	19,041	PF00069.24	Pkinase	178,4	1,60E-52	CL0016
ARHOMBI_DN19974_c0_g1_i1_4	2	21,5	14,524	7,243	PF07992.13	Pyr_redox_2	83,4	1,50E-23	CL0063
ARUBRA_DN7017_c0_g1_i1_3	2	7,7	29,709	10,954	PF00701.21	DHDPS	216,6	2,60E-64	CL0036
ARUBRA_DN1910_c0_g1_i1_5	1	4,7	32,508	6,5155	PF02941.14	FeThRed_A	96,8	5,60E-28	CL0610
ARUBRA_DN4277_c0_g1_i1_2	2	6,3	40,618	12,468	PF00571.27	CBS	46,7	3,00E-12	No_clan
ARUBRA_DN22918_c0_g1_i1_6	2	9,8	21,966	11,044	PF01501.19	Glyco_transf_8	216,1	5,30E-64	CL0110
ARUBRA_DN9150_c0_g1_i1_5	2	17,1	18,471	13,69					
ARHOMBI_DN6685_c1_g1_i1_6	1	14,5	14,027	6,2835					
ARHOMBI_DN26281_c0_g1_i1_5	1	14,7	10,691	6,8638	PF00122.19	E1-E2_ATPase	111,4	3,10E-32	No_clan
ARUBRA_DN3358_c0_g1_i2_1	2	18,5	19,889	13,612	PF00579.24	tRNA-synt_1b	52,5	4,20E-14	CL0039
ARHOMBI_DN4457_c0_g1_i1_1	2	11,7	38,234	14,396					
ARHOMBI_DN10001_c0_g1_i1_3	2	23,8	18,279	13,157	PF00800.17	PDT	57,5	1,40E-15	CL0177
ARHOMBI_DN13618_c0_g2_i1_3	1	9,3	15,006	7,0626					
ARHOMBI_DN3625_c0_g1_i1_3	1	6,5	25,25	6,4285	PF00557.23	Peptidase_M24	61,1	1,10E-16	No_clan
ARHOMBI_DN23530_c0_g1_i1_1	2	17,1	13,135	13,529	PF00076.21	RRM_1	32,1	7,00E-08	CL0221
ARUBRA_DN21885_c0_g1_i1_1	1	9	14,714	6,4819	PF07992.13	Pyr_redox_2	48,7	5,40E-13	CL0063
ARHOMBI_DN4759_c0_g2_i1_2	1	4,3	28,596	6,3148	PF03465.14	eRF1_3	127,2	3,60E-37	CL0101
ARHOMBI_DN995_c0_g2_i1_5	3	15,9	28,266	7,2538					
ARHOMBI_DN19127_c0_g1_i1_2	1	26,5	9,1815	7,6577	PF01583.19	APS_kinase	99,1	2,00E-28	CL0023
ARUBRA_DN19640_c0_g1_i1_4	2	14	19,8	11,283	PF01145.24	Band_7	46,9	2,90E-12	CL0433
ARUBRA_DN16258_c0_g1_i1_4	1	20,4	10,985	7,2326	PF02922.17	CBM_48	69,6	2,00E-19	CL0369
ARUBRA_DN19843_c0_g1_i1_1	1	5,2	27,239	6,675					
ARHOMBI_DN4707_c0_g1_i1_6	4	14,6	46,752	23,961	PF04012.11	PspA_IM30	178,1	1,60E-52	CL0235
ARUBRA_DN6269_c0_g1_i1_1	2	36,4	13,076	12,882	PF00793.19	DAHP_synth_1	78,1	4,50E-22	CL0036
ARUBRA_DN14413_c0_g1_i1_1	1	40,8	7,098	7,7662					
ARUBRA_DN23541_c0_g1_i1_3	2	26,2	11,296	14,573	PF05903.13	Peptidase_C97	92,2	2,50E-26	CL0125
ARHOMBI_DN21586_c0_g1_i1_2	2	14,7	19,884	12,923	PF00012.19	HSP70	24,9	4,90E-06	CL0108
ARUBRA_DN20454_c0_g1_i1_1	3	23,1	17,238	6,3033	PF13246.5	Cation_ATPase	47,3	1,50E-12	CL0137
ARUBRA_DN25494_c0_g1_i1_2	1	16,5	9,5818	7,7141					
ARUBRA_DN1726_c0_g1_i1_1	1	5,7	24,967	12,266	PF00583.24	Acetyltransf_1	55,2	7,00E-15	CL0257

ARUBRA_DN4369_c0_g1_i1_1	2	11,8	21,812	11,95	PF06364.11	DUF1068	243,2	1,10E-72	No_clan
ARHOMBI_DN7832_c0_g1_i1_1	2	14,9	18,066	12,054					
ARHOMBI_DN3921_c0_g1_i1_3	1	4,4	39,394	8,6818	PF00702.25	Hydrolase	34,6	2,10E-08	CL0137
ARUBRA_DN729_c0_g1_i1_6	1	4,8	31,563	6,5128	PF00085.19	Thioredoxin	86,4	9,80E-25	CL0172
ARUBRA_DN17182_c0_g1_i1_3	3	16,1	30,206	59,403					
ARUBRA_DN5225_c0_g1_i1_3	2	19,4	15,622	30,992	PF00248.20	Aldo_ket_red	101,1	5,70E-29	No_clan
ARHOMBI_DN26642_c0_g1_i1_3	3	38,8	9,6676	17,865					
ARHOMBI_DN6091_c0_g1_i3_6	1	3,9	38,056	6,1784	PF00067.21	p450	210,5	3,50E-62	No_clan
ARHOMBI_DN2397_c0_g1_i1_3	2	10,3	23,805	11,457	PF13561.5	adh_short_C2	110,5	8,70E-32	CL0063
ARUBRA_DN19230_c0_g1_i1_6	2	11,6	24,37	12,043	PF00583.24	Acetyltransf_1	57,5	1,30E-15	CL0257
ARUBRA_DN10511_c0_g1_i1_4	1	24,6	13,935	6,1854	PF07802.10	GCK	45,3	8,10E-12	No_clan
ARHOMBI_DN18648_c0_g1_i1_2	1	14,1	7,7327	7,6921	PF00156.26	Pribosyltran	27,2	2,20E-06	CL0533
ARHOMBI_DN20035_c0_g1_i1_4	3	23,5	22,689	11,763	PF13015.5	PRKCSH_1	100,3	7,60E-29	CL0226
ARHOMBI_DN22203_c0_g1_i1_5	2	15,7	27,896	12,007					
ARUBRA_DN8245_c0_g1_i1_1	2	38,1	11,389	12,523					
ARUBRA_DN18674_c0_g1_i1_6	1	13,1	12,068	6,5075	PF16969.4	SRP68	73,5	1,70E-20	No_clan
ARUBRA_DN1052_c0_g1_i1_4	4	22,1	22,311	6,6414	PF00071.21	Ras	148	1,60E-43	CL0023
ARUBRA_DN4497_c0_g1_i1_6	1	5,7	26,726	6,756	PF00348.16	polyprenyl_synt	84,3	6,40E-24	CL0613
ARHOMBI_DN23074_c0_g1_i1_6	1	14,4	16,778	7,3889					
ARUBRA_DN17867_c0_g1_i1_4	2	13,8	26,647	15,177					
ARUBRA_DN26207_c0_g1_i1_1	1	34,8	7,507	6,9643					
ARUBRA_DN993_c0_g1_i1_2	3	13,1	28,408	14,15	PF01092.18	Ribosomal_S6e	189,1	2,30E-56	No_clan
ARUBRA_DN5001_c0_g1_i1_4	1	2,2	55,74	-2					
ARUBRA_DN16126_c0_g1_i1_1	1	4,5	44,878	-2					
ARUBRA_DN18434_c0_g1_i1_5	1	7,1	23,628	6,3997	PF13883.5	Pyrid_oxidase_2	146,7	5,70E-43	CL0336
ARHOMBI_DN8608_c0_g1_i1_5	1	4,6	25,568	6,1496	PF00013.28	KH_1	45,8	3,70E-12	CL0007
ARUBRA_DN21219_c0_g1_i1_3	1	28,6	8,5218	6,3255					
ARUBRA_DN10906_c0_g1_i1_1	2	26,1	13,5	13,008					
ARUBRA_DN24391_c0_g1_i1_1	1	14,8	15,745	6,2282					
ARHOMBI_DN6909_c0_g1_i1_1	2	17,7	19,591	6,4241	PF00076.21	RRM_1	58,9	3,00E-16	CL0221
ARUBRA_DN24666_c0_g1_i1_6	1	25,6	8,5159	7,0361	PF01575.18	MaoC_dehydratas	60,2	1,20E-16	CL0050
ARUBRA_DN6630_c0_g1_i1_2	3	17,9	20,615	21,771	PF13472.5	Lipase_GDSL_2	72,2	6,30E-20	CL0264
ARUBRA_DN9370_c0_g1_i1_1	1	22,4	7,7645	14,179	PF06747.12	CHCH	29,4	5,90E-07	CL0351

ARUBRA_DN10905_c0_g1_i1_2	2	29,7	14,042	13,543	PF00009.26	GTP_EFTU	73,2	1,80E-20	CL0023
ARHOMBI_DN13448_c0_g1_i1_5	2	16,1	21,11	11,326	PF01725.15	Ham1p_like	131,6	2,50E-38	CL0269
ARUBRA_DN20978_c0_g1_i1_4	1	15,1	11,731	6,4678	PF02540.16	NAD_synthase	48	7,90E-13	CL0039
ARUBRA_DN6384_c0_g1_i1_5	2	20,4	17,439	12,162	PF08367.10	M16C_assoc	131,5	2,60E-38	CL0094
ARHOMBI_DN981_c0_g1_i1_4	2	9	34,525	12,923	PF00291.24	PALP	88,7	4,20E-25	No_clan
ARUBRA_DN22585_c0_g1_i1_2	1	14,9	14,94	7,5448	PF02774.17	Semialdhyde_dhC	91,9	4,80E-26	CL0139
ARHOMBI_DN4343_c0_g1_i1_6	2	10,8	46,592	15,645	PF05093.12	CIAPIN1	57	2,10E-15	No_clan
ARHOMBI_DN796_c0_g1_i1_1	1	24,2	10,29	20,229	PF01704.17	UDPGP	32,8	2,60E-08	CL0110
ARUBRA_DN3285_c0_g1_i1_2	3	19,5	23,051	11,483	PF04667.16	Endosulfine	85,6	1,70E-24	No_clan
ARUBRA_DN5446_c0_g1_i1_1	2	19,8	17,712	10,944	PF08502.9	LeuA_dimer	105,9	1,30E-30	No_clan
ARUBRA_DN10803_c0_g1_i1_2	2	14,7	19,664	12,129	PF00224.20	PK	136,9	6,90E-40	CL0151
ARUBRA_DN16402_c0_g1_i1_6	1	27,5	8,7038	7,4246	PF16363.4	GDP_Man_Dehyd	59,6	3,00E-16	CL0063
ARUBRA_DN19030_c0_g1_i1_1	1	19,4	12,15	6,7793					
ARHOMBI_DN6155_c0_g1_i1_1	3	13,7	45,992	17,279	PF08241.11	Methyltransf_11	71	9,20E-20	CL0063
ARUBRA_DN13110_c0_g1_i1_1	1	14,6	9,1684	6,1933	PF13419.5	HAD_2	29,4	7,10E-07	CL0137
ARUBRA_DN25449_c0_g1_i1_2	1	15	8,5346	24,001					
ARHOMBI_DN4728_c0_g3_i1_1	2	5,7	55,378	13,105	PF04499.14	SAPS	81,7	4,40E-23	No_clan
ARHOMBI_DN6142_c0_g1_i3_4	2	5,9	39,24	11,342	PF01070.17	FMN_dh	389,2	1,50E-116	CL0036
ARHOMBI_DN5011_c0_g1_i1_4	3	12,4	44,746	19,011					
ARHOMBI_DN7836_c0_g1_i1_2	2	6	40,782	6,1933	PF08240.11	ADH_N	36,3	3,90E-09	CL0296
ARHOMBI_DN4465_c0_g1_i1_1	2	13,9	39,204	6,5629	PF03141.15	Methyltransf_29	300,6	1,70E-89	CL0063
ARHOMBI_DN2932_c0_g2_i1_5	2	6,7	47,388	12,401	PF00462.23	Glutaredoxin	57,5	1,10E-15	CL0172
ARHOMBI_DN6286_c0_g1_i1_6	1	5,5	29,588	6,2214					
ARUBRA_DN17025_c0_g1_i1_2	1	18,1	13,389	6,7199	PF00132.23	Hexapep	22	8,60E-05	CL0536
ARHOMBI_DN7489_c1_g1_i1_4	1	14,1	9,5648	7,1577	PF00933.20	Glyco_hydro_3	55,2	6,60E-15	CL0058
ARUBRA_DN13369_c0_g1_i1_3	2	28	10,219	12,944	PF03283.12	PAE	154,5	3,60E-45	CL0028
ARUBRA_DN18574_c0_g1_i1_2	1	14	14,358	9,705	PF02990.15	EMP70	106,9	9,60E-31	No_clan
ARUBRA_DN21886_c0_g1_i1_1	2	13,4	17,839	18,203	PF13023.5	HD_3	92,6	2,20E-26	CL0237
ARHOMBI_DN17203_c0_g1_i1_2	1	8	34,191	8,1191	PF01031.19	Dynamin_M	55,2	5,90E-15	No_clan
ARHOMBI_DN13122_c0_g1_i1_4	1	21,8	9,0942	6,707					
ARHOMBI_DN25341_c0_g1_i1_1	5	50,5	12,551	72,257	PF00071.21	Ras	126,7	5,70E-37	CL0023
ARHOMBI_DN3340_c0_g1_i1_2	5	15,5	38,355	16,576	PF00270.28	DEAD	50,6	1,70E-13	CL0023

ARUBRA_DN3419_c0_g1_i1_6	3	17,3	22,02	56,497	PF04107.12	GCS2	52,9	3,20E-14	CL0286
ARHOMBI_DN8889_c0_g1_i1_1	1	22,2	9,2029	6,9407					
ARHOMBI_DN4948_c0_g1_i1_2	1	8,5	26,422	7,0684					
ARUBRA_DN23749_c0_g1_i1_2	1	13,4	18,052	6,6646					
ARUBRA_DN23540_c0_g1_i1_4	1	15,6	9,9182	6,7706	PF00185.23	OTCace	94,6	5,90E-27	CL0399
ARHOMBI_DN658_c0_g1_i1_6	1	10,9	25,172	7,9678	PF10609.8	ParA	133,5	7,80E-39	CL0023
ARUBRA_DN1712_c0_g1_i1_3	3	12,9	45,907	18,62	PF00722.20	Glyco_hydro_16	174	1,80E-51	CL0004
ARUBRA_DN9464_c0_g1_i1_3	1	19,4	10,343	6,3799					
ARUBRA_DN8289_c0_g1_i1_1	3	12,5	32,311	24,86					
ARHOMBI_DN11041_c0_g1_i1_3	1	25,4	7,7108	6,5038	PF00288.25	GHMP_kinases_N	36,3	4,50E-09	CL0329
ARHOMBI_DN8091_c0_g1_i1_4	1	4,7	25,197	6,237	PF00378.19	ECH_1	72	4,20E-20	CL0127
ARHOMBI_DN4244_c0_g1_i2_2	2	8,9	30,265	12,057	PF00574.22	CLP_protease	136,1	1,10E-39	CL0127
ARUBRA_DN313_c0_g2_i1_6	2	16,5	18,125	11,492	PF02320.15	UCR_hinge	81,4	3,70E-23	No_clan
ARUBRA_DN707_c0_g1_i1_4	1	10,8	23,53	6,8732	PF01198.18	Ribosomal_L31e	141,1	9,30E-42	No_clan
ARHOMBI_DN3837_c0_g1_i1_6	1	2,5	48,387	6,2783	PF03106.14	WRKY	85,2	2,70E-24	CL0274
ARUBRA_DN4588_c0_g1_i2_1	2	6,3	37,706	11,368	PF05193.20	Peptidase_M16_C	48,7	8,10E-13	CL0094
ARHOMBI_DN6158_c0_g1_i1_6	6	22,3	31,612	11,875	PF02798.19	GST_N	32,3	9,00E-08	CL0172
ARHOMBI_DN6709_c0_g1_i1_2	1	7,8	33,559	10,951	PF08662.10	eIF2A	105,7	2,60E-30	CL0186
ARHOMBI_DN12465_c0_g1_i1_1	1	19,7	8,1736	19,167					
ARHOMBI_DN26923_c0_g1_i1_1	1	13,5	10,564	6,6575	PF00571.27	CBS	40,6	2,50E-10	No_clan
ARUBRA_DN1422_c0_g2_i1_2	3	7	64,285	19,596	PF04212.17	MIT	75,3	2,70E-21	No_clan
ARHOMBI_DN23444_c0_g1_i1_1	2	26,3	10,94	11,874	PF07766.12	LETM1	93	1,80E-26	No_clan
ARUBRA_DN2399_c0_g1_i1_4	2	16,2	16,168	12,384	PF09585.9	Lin0512_fam	75,7	2,60E-21	No_clan
ARUBRA_DN2263_c0_g2_i1_1	1	4,1	31,838	6,3843	PF00956.17	NAP	62,3	3,60E-17	No_clan
ARHOMBI_DN15914_c0_g1_i1_1	2	10	29,085	12,612	PF02492.18	cobW	24,8	1,30E-05	CL0023
ARUBRA_DN1557_c0_g1_i1_4	1	10,3	18,734	7,4376					
ARUBRA_DN4786_c0_g1_i1_2	2	8,3	37,581	7,064					
ARHOMBI_DN18534_c0_g1_i1_3	1	7,8	22,894	6,9727	PF01502.17	PRA-CH	92,3	1,10E-26	No_clan
ARHOMBI_DN10516_c0_g1_i1_6	2	25,3	8,8909	14,948					
ARUBRA_DN178_c0_g1_i1_1	1	6,1	31,485	8,3951	PF01105.23	EMP24_GP25L	170,9	2,50E-50	CL0521
ARUBRA_DN24618_c0_g1_i1_5	1	15	15,449	7,3505					
ARUBRA_DN23959_c0_g1_i1_1	1	25	8,841	6,8437	PF00202.20	Aminotran_3	61,1	6,70E-17	CL0061
ARUBRA_DN19174_c0_g1_i1_2	2	15,8	24,39	6,7472					

ARHOMBI_DN4479_c0_g1_i1_4	1	2,5	39,53	6,3505	PF13640.5	2OG-FeII_Oxy_3	77,6	9,20E-22	CL0029
ARHOMBI_DN3958_c0_g1_i1_6	4	12	47,61	18,709	PF00076.21	RRM_1	70,8	5,90E-20	CL0221
ARHOMBI_DN2738_c0_g1_i1_2	6	24,7	39,917	6,4311	PF07973.13	tRNA_SAD	63,4	1,40E-17	CL0094
ARUBRA_DN1466_c0_g2_i1_4	2	7,3	27,716	13,999	PF02374.14	ArsA_ATPase	82,9	2,10E-23	CL0023
ARHOMBI_DN3523_c0_g1_i1_4	2	14,7	15,216	10,76	PF01233.18	NMT	44,1	1,70E-11	CL0257
ARHOMBI_DN1869_c0_g2_i1_5	2	9	24,024	14,841	PF00805.21	Pentapeptide	37,2	1,50E-09	CL0505
ARUBRA_DN22416_c0_g1_i1_5	1	17	10,579	7,7452					
ARUBRA_DN446_c0_g1_i1_2	1	13,6	18,866	7,1012					
ARUBRA_DN1177_c0_g2_i1_1	2	5,3	42,27	10,947	PF02781.15	G6PD_C	299,3	2,40E-89	No_clan
ARUBRA_DN22100_c0_g1_i1_5	2	22,8	12,269	11,863	PF00441.23	Acyl-CoA_dh_1	70,7	1,40E-19	CL0087
ARHOMBI_DN9092_c0_g1_i1_2	1	15,9	13,628	7,1432	PF06552.11	TOM20_plant	137,3	4,60E-40	CL0020
ARHOMBI_DN5351_c0_g2_i1_2	1	11,8	13,56	6,798	PF01215.18	COX5B	59,5	2,40E-16	CL0045
ARUBRA_DN3055_c0_g1_i2_3	2	14,7	23,507	10,978					
ARHOMBI_DN21638_c0_g1_i1_6	1	9,7	17,701	6,8237					
ARUBRA_DN4234_c0_g2_i1_6	2	7,3	46,985	11,232	PF02544.15	Steroid_dh	99,1	2,10E-28	CL0115
ARUBRA_DN2493_c0_g1_i1_1	1	7,2	27,846	7,6323	PF13710.5	ACT_5	39,7	3,00E-10	CL0070
ARUBRA_DN7662_c0_g1_i1_2	2	13,2	23,602	13,201	PF16752.4	TBCC_N	103,8	6,90E-30	No_clan
ARUBRA_DN12348_c0_g1_i1_6	3	47,3	12,006	18,91	PF00173.27	Cyt-b5	92	1,70E-26	No_clan
ARHOMBI_DN3285_c0_g2_i1_4	3	22,2	13,177	8,615	PF00248.20	Aldo_ket_red	28,5	7,40E-07	No_clan
ARHOMBI_DN8957_c0_g1_i1_1	2	13,1	24,85	12,295	PF00291.24	PALP	136,8	8,80E-40	No_clan
ARHOMBI_DN21971_c0_g1_i1_1	2	16,6	21,167	11,135	PF05542.10	DUF760	26,4	6,10E-06	No_clan
ARUBRA_DN13989_c0_g1_i1_2	1	12,8	12,088	6,446					
ARUBRA_DN26201_c0_g1_i1_1	1	20,2	9,9904	8,6423	PF17004.4	SRP_TPR_like	57,2	1,40E-15	CL0020
ARHOMBI_DN16098_c0_g1_i1_2	2	27,6	11,054	13,365	PF13409.5	GST_N_2	44,3	1,50E-11	CL0172
ARHOMBI_DN2295_c0_g1_i1_1	2	9,6	31,262	30,291					
ARHOMBI_DN8630_c0_g1_i1_2	1	10,3	19,192	9,1835	PF01756.18	ACOX	39,9	2,90E-10	CL0087
ARUBRA_DN15491_c0_g1_i1_1	1	20,9	7,3062	6,8992					
ARUBRA_DN7176_c0_g1_i1_6	1	19,1	10,109	8,323					
ARUBRA_DN22998_c0_g1_i1_5	2	9,7	29,275	13,513	PF14938.5	SNAP	43,5	2,40E-11	CL0020
ARHOMBI_DN1819_c0_g1_i1_2	1	9,6	18,94	7,3028	PF00107.25	ADH_zinc_N	70,3	1,30E-19	CL0063
ARUBRA_DN1769_c0_g2_i1_6	2	27,3	12,298	11,013	PF01014.17	Uricase	79,2	3,20E-22	CL0334
ARHOMBI_DN7578_c0_g1_i1_4	4	29,6	23,486	7,6208	PF00676.19	E1_dh	109,8	1,20E-31	CL0254
ARHOMBI_DN4440_c0_g1_i1_4	4	20,9	30,104	6,8337	PF01557.17	FAA_hydrolase	199	7,10E-59	CL0377

ARUBRA_DN16297_c0_g1_i1_4	2	10,5	22,167	12,3					
ARUBRA_DN7697_c0_g1_i1_2	1	23,6	11,776	8,2064	PF12068.7	DUF3548	76,1	2,70E-21	No_clan
ARUBRA_DN19516_c0_g1_i1_2	1	6,2	36,817	6,6111	PF01915.21	Glyco_hydro_3_C	121,7	3,70E-35	No_clan
ARUBRA_DN9248_c0_g1_i1_4	2	8,8	32,829	64,616	PF03662.13	Glyco_hydro_79n	131,9	2,20E-38	CL0058
ARHOMBI_DN26238_c0_g1_i1_4	1	16,2	7,9361	8,9362	PF00190.21	Cupin_1	62	4,40E-17	CL0029
ARHOMBI_DN1217_c0_g2_i1_1	1	18,4	10,796	8,5566	PF00198.22	2-oxoacid_dh	85,1	4,40E-24	CL0149
ARUBRA_DN22060_c0_g1_i1_4	2	11,3	28,402	12,129					
ARHOMBI_DN3796_c0_g1_i1_3	2	6,3	41,408	11,944	PF00082.21	Peptidase_S8	81,8	4,80E-23	No_clan
ARUBRA_DN3884_c0_g1_i1_3	1	6,9	25,872	6,2226	PF01124.17	MAPEG	80,5	9,20E-23	No_clan
ARHOMBI_DN11238_c0_g1_i1_1	1	20,6	10,519	7,6285	PF00118.23	Cpn60_TCP1	120,4	7,40E-35	No_clan
ARUBRA_DN20512_c0_g1_i1_5	2	15,5	18,742	12,805					
ARUBRA_DN2494_c0_g1_i1_5	3	18,8	25,999	18,735	PF00300.21	His_Phos_1	204,4	1,30E-60	CL0071
ARHOMBI_DN25808_c0_g1_i1_4	1	14,9	10,222	6,6593	PF08264.12	Anticodon_1	44,1	1,80E-11	CL0258
ARHOMBI_DN5409_c0_g1_i1_1	1	3,4	58,681	8,9167					
ARUBRA_DN4725_c0_g1_i1_2	3	9,7	50,798	24,625	PF00112.22	Peptidase_C1	216,3	5,20E-64	CL0125
ARUBRA_DN19472_c0_g1_i1_2	1	6,7	30,933	6,1814					
ARUBRA_DN9681_c0_g1_i1_1	1	7,7	24,63	6,5465	PF05193.20	Peptidase_M16_C	32	1,10E-07	CL0094
ARUBRA_DN21358_c0_g1_i1_1	1	13,9	15,254	6,9574	PF00642.23	zf-CCCH	38,9	5,00E-10	CL0537
ARUBRA_DN2192_c0_g1_i1_5	1	9,5	16,886	6,9411	PF04398.11	DUF538	59,2	4,60E-16	No_clan
ARUBRA_DN14031_c0_g1_i1_6	2	21,8	16,879	12,517	PF00013.28	KH_1	31,4	1,20E-07	CL0007
ARUBRA_DN22638_c0_g1_i1_6	1	31,8	8,9872	9,2824	PF00288.25	GHMP_kinases_N	24,3	2,40E-05	CL0329
ARUBRA_DN3929_c0_g1_i2_5	3	22,2	20,053	6,4529	PF00248.20	Aldo_ket_red	54,7	7,50E-15	No_clan
ARUBRA_DN3241_c0_g1_i1_4	2	6	51,805	11,573	PF00153.26	Mito_carr	80,6	5,50E-23	No_clan
ARUBRA_DN2358_c0_g1_i1_4	1	8,4	21,869	6,5968					
ARUBRA_DN24043_c0_g1_i1_4	2	23,5	13,599	10,943					
ARHOMBI_DN24632_c0_g1_i1_3	1	20,3	8,2083	6,6182	PF00501.27	AMP-binding	44,5	7,20E-12	CL0378
ARHOMBI_DN26919_c0_g1_i1_4	2	38,8	8,7346	12,102	PF00933.20	Glyco_hydro_3	42,7	4,20E-11	CL0058
ARUBRA_DN23841_c0_g1_i1_4	1	21,5	8,5869	6,6839					
ARUBRA_DN19491_c0_g1_i1_2	1	15,3	10,098	6,9552					
ARUBRA_DN22396_c0_g1_i1_1	2	23,8	12,083	12,966					
ARHOMBI_DN19578_c0_g1_i1_4	2	9,5	25,229	11,649	PF00013.28	KH_1	42,6	3,60E-11	CL0007
ARHOMBI_DN3163_c0_g1_i1_3	2	7,7	32,48	10,889	PF00005.26	ABC_tran	63,5	2,50E-17	CL0023
ARUBRA_DN19450_c0_g1_i1_4	1	14,9	22,519	6,6805					

ARUBRA_DN130_c0_g1_i1_4	2	11,5	19,645	11,959	PF00731.19	AIRC	70,7	8,80E-20	No_clan
ARHOMBI_DN4296_c0_g1_i1_1	2	7,7	40,689	13,492	PF00326.20	Peptidase_S9	68,6	4,90E-19	CL0028
ARUBRA_DN6440_c0_g1_i1_4	2	7,9	24,237	11,748	PF00403.25	HMA	44,5	1,50E-11	No_clan
ARHOMBI_DN6362_c0_g1_i1_1	1	18,2	8,6977	6,446	PF00183.17	HSP90	84,4	7,00E-24	No_clan
ARHOMBI_DN23114_c0_g1_i1_1	2	33	11,29	13,485					
ARHOMBI_DN4923_c0_g1_i1_6	1	6,6	22,743	6,6414	PF07876.11	Dabb	90,3	8,60E-26	CL0032
ARUBRA_DN6647_c0_g1_i1_3	1	18,6	10,958	6,4637					
ARHOMBI_DN5696_c0_g1_i1_6	1	5,5	34,462	6,2343					
ARUBRA_DN8888_c0_g1_i1_4	1	9,1	19,702	8,1362					
ARHOMBI_DN5174_c0_g2_i1_1	1	15,8	8,017	7,0407	PF05368.12	NmrA	73	2,40E-20	CL0063
ARUBRA_DN16605_c0_g1_i1_4	2	12,2	28,215	12,363	PF00464.18	SHMT	318,5	4,40E-95	CL0061
ARHOMBI_DN18922_c0_g1_i1_3	1	13,6	14,527	7,3279					
ARHOMBI_DN5270_c0_g1_i1_5	2	11	36,244	11,781	PF03767.13	Acid_phosphat_B	239,5	3,10E-71	CL0137
ARHOMBI_DN9484_c0_g2_i1_3	2	17	24,136	14,36	PF16550.4	RPN13_C	82,9	1,60E-23	No_clan
ARHOMBI_DN498_c0_g1_i1_1	2	8,8	38,703	11,994	PF00076.21	RRM_1	41,6	7,60E-11	CL0221
ARHOMBI_DN1736_c0_g1_i1_6	1	11,5	23,27	8,6961	PF02775.20	TPP_enzyme_C	70,6	1,20E-19	CL0254
ARUBRA_DN171_c0_g2_i1_3	1	15,6	15,49	7,0487	PF02990.15	EMP70	161,5	2,70E-47	No_clan
ARUBRA_DN23798_c0_g1_i1_1	1	9,3	17,28	6,8614					
ARHOMBI_DN9171_c0_g1_i1_1	1	8,5	14,124	6,4228	PF12580.7	TPPII	61,3	7,70E-17	No_clan
ARUBRA_DN3301_c0_g2_i1_5	1	3,5	51,149	-2	PF00400.31	WD40	12,9	0,15	CL0186
ARUBRA_DN2204_c0_g1_i1_6	2	21,6	22,946	16,207	PF13639.5	zf-RING_2	44	1,80E-11	CL0229
ARHOMBI_DN1709_c0_g2_i1_6	1	5,3	24,73	24,024	PF00407.18	Bet_v_1	120,8	4,00E-35	CL0209
ARUBRA_DN4715_c0_g1_i1_5	1	6,6	28,954	6,3023	PF00326.20	Peptidase_S9	124,4	4,00E-36	CL0028
ARHOMBI_DN17044_c0_g1_i1_2	1	5,2	31,66	6,7911	PF02770.18	Acyl-CoA_dh_M	54,7	8,10E-15	No_clan
ARHOMBI_DN6819_c0_g1_i1_2	1	5,4	40,541	6,6085	PF00155.20	Aminotran_1_2	207,1	3,90E-61	CL0061
ARHOMBI_DN5985_c0_g1_i1_4	2	10,8	39,561	9,1714	PF03951.18	Gln-synt_N	39,4	3,30E-10	No_clan
ARUBRA_DN24121_c0_g1_i1_6	1	22,2	10,083	6,2905					
ARUBRA_DN4657_c0_g1_i1_6	1	5,4	22,034	7,6778	PF00407.18	Bet_v_1	166	4,90E-49	CL0209
ARUBRA_DN290_c0_g1_i1_5	3	8,3	55,427	17,6	PF00574.22	CLP_protease	145,2	1,80E-42	CL0127
ARHOMBI_DN5862_c0_g1_i1_2	1	3,8	35,175	6,3122	PF04526.12	DUF568	88,7	3,00E-25	No_clan
ARHOMBI_DN6231_c0_g3_i5_2	3	9,5	36,732	11,594	PF13561.5	adh_short_C2	196,3	4,90E-58	CL0063
ARUBRA_DN5145_c0_g1_i1_2	1	13,2	21,795	6,236	PF01501.19	Glyco_transf_8	27,8	1,60E-06	CL0110
ARUBRA_DN20610_c0_g1_i1_6	1	26,1	7,0769	6,5278					

ARHOMBI_DN19734_c0_g1_i1_3	1	6,5	20,692	6,4664	PF01399.26	PCI	42,2	8,50E-11	CL0123
ARHOMBI_DN13808_c0_g1_i1_5	2	20,9	18,634	11,564	PF00400.31	WD40	33,5	4,50E-08	CL0186
ARUBRA_DN19459_c0_g1_i1_5	2	13,5	26,27	12,781	PF00255.18	GSHPx	137,4	1,10E-40	CL0172
ARHOMBI_DN4450_c0_g1_i1_5	2	6,6	30,995	11,071	PF13419.5	HAD_2	66,4	3,00E-18	CL0137
ARUBRA_DN2002_c0_g2_i1_5	1	7,6	27,101	9,9246	PF14159.5	CAAD	89	1,20E-25	No_clan
ARHOMBI_DN22399_c0_g1_i1_2	1	21,8	11,832	6,5664					
ARHOMBI_DN16397_c0_g1_i1_6	1	8	18,606	6,5601	PF01920.19	Prefoldin_2	67,8	6,20E-19	CL0200
ARHOMBI_DN3020_c0_g2_i1_1	4	30	16,985	7,6027	PF01599.18	Ribosomal_S27	95,4	1,50E-27	CL0167
ARHOMBI_DN6884_c0_g1_i1_5	2	18	16,421	12,388	PF01399.26	PCI	71	9,50E-20	CL0123
ARUBRA_DN21439_c0_g1_i1_3	1	13,3	10,765	6,7576	PF00314.16	Thaumatin	122,2	2,30E-35	CL0293
ARHOMBI_DN5298_c0_g1_i1_5	1	6,9	37,915	6,6631	PF04051.15	TRAPP	143,7	3,00E-42	CL0210
ARHOMBI_DN6134_c0_g1_i2_6	3	12,5	35,544	6,9924					
ARHOMBI_DN21703_c0_g1_i1_5	2	15,2	18,591	12,699	PF12265.7	CAF1C_H4-bd	57,6	1,10E-15	No_clan
ARHOMBI_DN1691_c0_g1_i1_5	1	10,2	21,316	6,4353					
ARHOMBI_DN20074_c0_g1_i1_1	3	28,9	16,121	6,1647	PF01433.19	Peptidase_M1	206,7	3,10E-61	CL0126
ARUBRA_DN16643_c0_g1_i1_2	2	34	10,028	13,225	PF08240.11	ADH_N	35,3	7,70E-09	CL0296
ARHOMBI_DN16129_c0_g1_i1_4	1	10,9	13,506	6,358					
ARUBRA_DN4512_c0_g1_i1_2	2	14,5	29,226	6,4173					
ARHOMBI_DN8916_c0_g1_i1_1	2	10,2	25,164	13,253	PF00106.24	adh_short	167,6	2,00E-49	CL0063
ARHOMBI_DN12802_c0_g1_i1_2	1	15,5	10,741	6,387	PF02446.16	Glyco_hydro_77	85,3	3,80E-24	CL0058
ARUBRA_DN14943_c0_g1_i1_6	1	17,4	9,6437	6,9221	PF02786.16	CPSase_L_D2	107,7	5,30E-31	CL0179
ARHOMBI_DN21499_c0_g1_i1_5	1	10,8	17,931	7,4363	PF00289.21	Biotin_carb_N	77,3	1,10E-21	CL0483
ARHOMBI_DN16804_c0_g1_i1_2	3	16,8	26,858	6,2036	PF13415.5	Kelch_3	30,7	2,60E-07	CL0186
ARHOMBI_DN3081_c0_g1_i2_3	2	5,4	46,038	11,868	PF04548.15	AIG1	275	3,00E-82	CL0023
ARHOMBI_DN26634_c0_g1_i1_6	1	26,9	9,1554	7,3345	PF00240.22	ubiquitin	45,6	4,10E-12	CL0072
ARUBRA_DN16199_c0_g1_i1_3	2	9,5	30,428	90,411	PF03959.12	FSH1	165,5	1,20E-48	CL0028
ARHOMBI_DN10954_c0_g1_i1_5	2	46,8	10,308	13,013					
ARHOMBI_DN8652_c0_g1_i1_2	1	9,9	15,135	6,2184	PF05562.10	WCOR413	49	5,90E-13	No_clan
ARUBRA_DN26687_c0_g1_i1_2	2	41,2	8,6436	11,855	PF00266.18	Aminotran_5	46,9	1,70E-12	CL0061
ARHOMBI_DN3151_c0_g2_i1_5	1	13,2	11,662	6,822	PF00226.30	DnaJ	71,4	4,60E-20	CL0392
ARHOMBI_DN12758_c0_g1_i1_4	1	20,9	7,3782	6,7553					
ARUBRA_DN6423_c0_g1_i1_6	1	5,8	18,26	6,2546	PF00515.27	TPR_1	35,4	5,60E-09	CL0020
ARUBRA_DN11367_c0_g1_i1_2	2	45,2	8,3044	12,84					

ARHOMBI_DN22357_c0_g1_i1_4	2	9	34,779	11,413	PF00585.17	Thr_dehydrat_C	84,3	3,50E-24	CL0070
ARUBRA_DN20780_c0_g1_i1_5	2	12,3	30,256	12,239					
ARHOMBI_DN17128_c0_g1_i1_2	1	7,7	27,466	14,114	PF06232.10	ATS3	182,4	2,50E-54	CL0321
ARHOMBI_DN21148_c0_g1_i1_2	1	9,5	15,145	6,3595	PF04548.15	AIG1	83,6	1,10E-23	CL0023
ARHOMBI_DN4969_c0_g1_i1_6	1	4	38,245	6,5747					
ARHOMBI_DN2477_c0_g2_i1_4	2	8,8	48,225	11,968	PF00930.20	DPPIV_N	254,2	1,40E-75	CL0186
ARUBRA_DN2574_c0_g1_i1_1	1	11,8	16,862	6,1711	PF00237.18	Ribosomal_L22	104,2	3,60E-30	No_clan
ARHOMBI_DN1345_c0_g1_i1_3	1	5,9	32,549	6,1641	PF16656.4	Pur_ac_phosph_N	70,5	1,30E-19	CL0159
ARUBRA_DN7816_c0_g1_i1_3	3	21,4	15,11	17,754	PF01053.19	Cys_Met_Meta_PP	155,8	1,10E-45	CL0061
ARHOMBI_DN4238_c0_g1_i1_6	2	7,5	39,326	11,486	PF02492.18	cobW	109,3	1,40E-31	CL0023
ARUBRA_DN17859_c0_g1_i1_6	1	20	8,5224	6,4726					
ARHOMBI_DN6130_c1_g1_i2_6	2	19,7	17,777	10,938	PF12220.7	U1snRNP70_N	74,4	8,00E-21	CL0221
ARUBRA_DN15205_c0_g1_i1_1	1	11,6	12,487	6,733					
ARUBRA_DN17787_c0_g1_i1_5	1	10,8	15,768	6,3661					
ARHOMBI_DN21125_c0_g1_i1_2	1	17,3	8,2669	123,52	PF09070.10	PFU	68,8	4,10E-19	No_clan
ARUBRA_DN22796_c0_g1_i1_1	2	54,1	7,8158	11,054					
ARHOMBI_DN2978_c0_g1_i1_5	3	20,7	27,923	18,471	PF01593.23	Amino_oxidase	83,1	2,30E-23	CL0063
ARHOMBI_DN8760_c0_g1_i1_1	1	14,3	10,356	6,2449	PF01602.19	Adaptin_N	62,3	3,10E-17	CL0020
ARUBRA_DN11549_c0_g1_i1_5	4	27	17,467	-2	PF00044.23	Gp_dh_N	116,3	6,00E-34	CL0063
ARHOMBI_DN22363_c0_g1_i1_3	2	20,7	14,357	12,463					
ARUBRA_DN7615_c0_g1_i1_5	2	16,9	16,708	13,497					
ARHOMBI_DN10838_c0_g1_i1_3	1	5,8	22,043	12,324	PF01221.17	Dynein_light	120,2	3,20E-35	No_clan
ARHOMBI_DN1649_c0_g1_i1_5	1	12,8	16,638	8,8188	PF00705.17	PCNA_N	200,5	5,60E-60	CL0060
ARHOMBI_DN20781_c0_g1_i1_6	3	20,7	19,737	16,836	PF01152.20	Bac_globin	146	5,10E-43	CL0090
ARHOMBI_DN6024_c0_g1_i2_5	3	9,9	51,498	12,021	PF01873.16	eIF-5_eIF-2B	133,3	3,30E-39	No_clan
ARHOMBI_DN6092_c0_g3_i3_3	3	11,1	51,385	6,7924	PF00127.19	Copper-bind	116,9	4,10E-34	CL0026
ARUBRA_DN23408_c0_g1_i1_6	1	13,9	11,962	6,9742	PF07393.10	Sec10	44,2	8,80E-12	CL0294
ARHOMBI_DN17360_c0_g1_i1_3	1	15,9	9,2932	6,1969	PF00013.28	KH_1	45,4	5,00E-12	CL0007
ARUBRA_DN6106_c0_g1_i1_6	1	16,2	8,0193	6,5761					
ARUBRA_DN8839_c0_g1_i1_5	1	7,3	17,329	6,6801					
ARUBRA_DN5290_c0_g1_i1_5	2	11,6	28,202	12,655	PF00383.22	dCMP_cyt_deam_1	85,6	1,70E-24	CL0109
ARHOMBI_DN16684_c0_g1_i1_4	2	10,2	30,686	11,964	PF09177.10	Syntaxin-6_N	81,7	4,20E-23	No_clan
ARUBRA_DN16761_c0_g1_i1_6	2	12	18,081	6,2027	PF00450.21	Peptidase_S10	115,6	3,40E-33	CL0028

ARUBRA_DN18234_c0_g1_i1_6	1	11,7	12,588	6,6633					
ARUBRA_DN483_c0_g1_i1_1	1	8,3	21,982	8,0191					
ARHOMBI_DN20642_c0_g1_i1_2	1	30,7	8,3154	7,9418	PF02737.17	3HCDH_N	100,6	8,30E-29	CL0063
ARHOMBI_DN1985_c0_g1_i1_1	3	22,5	17,653	6,4688	PF06957.10	COPI_C	215,2	1,20E-63	CL0020
ARUBRA_DN4720_c0_g1_i2_2	2	9,3	38,272	12,201	PF13640.5	2OG-FeII_Oxy_3	77,5	9,90E-22	CL0029
ARHOMBI_DN8466_c0_g1_i1_2	1	15,8	9,6251	6,3659					
ARUBRA_DN26386_c0_g1_i1_2	2	40,5	7,8694	12,535					
ARHOMBI_DN16500_c0_g1_i1_6	1	20,3	8,794	6,307	PF06068.12	TIP49	130,2	8,80E-38	CL0023
ARUBRA_DN7170_c0_g1_i1_6	1	12,5	20,109	7,6213	PF00657.21	Lipase_GDSL	60,4	2,10E-16	CL0264
ARUBRA_DN828_c0_g1_i1_6	2	4,6	55,994	11,676	PF00310.20	GATase_2	415,4	2,00E-124	CL0052
ARHOMBI_DN8693_c1_g1_i1_5	1	11,4	13,134	6,3588					
ARHOMBI_DN19277_c0_g1_i1_4	2	11,3	24,764	12,545	PF00638.17	Ran_BP1	68,3	6,40E-19	CL0266
ARUBRA_DN11258_c0_g1_i1_3	1	23,3	9,0959	7,3004	PF00350.22	Dynamin_N	45	1,10E-11	CL0023
ARHOMBI_DN9241_c0_g1_i1_6	1	12,3	17,031	6,8424	PF04871.12	Uso1_p115_C	72,5	3,60E-20	No_clan
ARHOMBI_DN2715_c0_g1_i1_5	2	12,4	26,91	12,518					
ARUBRA_DN18267_c0_g1_i1_4	1	7,7	23,344	7,3312	PF14555.5	UBA_4	45,6	4,10E-12	CL0214
ARUBRA_DN17506_c0_g1_i1_4	1	7,7	34,033	7,4464	PF04371.14	PAD_porph	298,5	5,60E-89	CL0197
ARUBRA_DN5389_c0_g1_i1_3	4	17,5	32,15	24,776	PF00141.22	peroxidase	204,5	1,60E-60	CL0617
ARHOMBI_DN3097_c0_g1_i2_1	2	13,9	18,454	6,1988	PF01095.18	Pectinesterase	155,1	1,50E-45	CL0268
ARUBRA_DN5867_c0_g1_i1_1	1	18,9	8,0631	6,5362	PF13507.5	GATase_5	76,7	1,40E-21	CL0014
ARUBRA_DN18169_c0_g1_i1_2	1	16,3	15,828	17,158	PF00627.30	UBA	34,2	1,60E-08	CL0214
ARUBRA_DN4178_c0_g2_i1_3	1	2,6	65,345	6,2857	PF00226.30	DnaJ	91,1	3,40E-26	CL0392
ARUBRA_DN6773_c0_g1_i1_3	1	5,9	30,718	6,8483	PF03248.12	Rer1	240,5	7,50E-72	No_clan
ARHOMBI_DN20737_c0_g1_i1_3	1	13,5	14,089	7,4946	PF01411.18	tRNA-synt_2c	176,2	9,20E-52	CL0040
ARUBRA_DN1212_c0_g2_i1_5	1	8,7	18,501	10,318					
ARUBRA_DN22616_c0_g1_i1_2	1	18,4	8,5847	6,7576	PF00854.20	PTR2	28,5	6,00E-07	CL0015
ARHOMBI_DN4651_c0_g1_i1_3	4	16,7	26,842	6,5167	PF00071.21	Ras	183,2	2,50E-54	CL0023
ARUBRA_DN4817_c0_g1_i2_6	2	5,3	74,899	12,256	PF00258.24	Flavodoxin_1	110,7	6,20E-32	CL0042
ARUBRA_DN5232_c0_g1_i1_6	1	16,7	12,407	7,0278					
ARUBRA_DN8160_c0_g1_i1_1	1	16,9	9,3335	6,8914	PF00642.23	zf-CCCH	36,7	2,50E-09	CL0537
ARHOMBI_DN25761_c0_g1_i1_2	1	12,9	13,463	6,3588	PF02824.20	TGS	89,1	1,30E-25	CL0072
ARUBRA_DN18459_c0_g1_i1_4	1	8,2	20,529	6,158					

ARUBRA_DN23641_c0_g1_i1_3	2	15,2	18,586	10,997	PF01425.20	Amidase	23	3,20E-05	No_clan
ARHOMBI_DN15083_c0_g1_i1_2	1	38	8,6406	8,6349					
ARHOMBI_DN5292_c0_g1_i1_5	1	8,4	27,615	6,3585	PF02560.13	Cyanate_lyase	101,6	1,40E-29	No_clan
ARUBRA_DN13177_c0_g1_i1_4	1	16,5	9,0923	6,6614	PF01008.16	IF-2B	22,6	4,80E-05	CL0246
ARUBRA_DN25572_c0_g1_i1_1	1	19,5	9,667	6,1978	PF03367.12	zf-ZPR1	64	1,30E-17	CL0167
ARHOMBI_DN10905_c0_g1_i1_3	2	19,6	15,279	11,288	PF00266.18	Aminotran_5	141,6	2,80E-41	CL0061
ARHOMBI_DN4521_c0_g1_i1_6	2	21,7	17,838	9,711	PF02933.16	CDC48_2	48,9	3,80E-13	CL0402
ARUBRA_DN16776_c0_g1_i1_3	1	5,4	25,143	6,7786	PF01633.19	Choline_kinase	139	1,50E-40	CL0016
ARUBRA_DN3345_c0_g2_i1_6	2	5,1	50,049	12,097					
ARHOMBI_DN3792_c0_g1_i2_2	2	11,1	34,612	14,561	PF07720.11	TPR_3	12,5	0,1	CL0020
ARHOMBI_DN2492_c0_g1_i1_2	1	14,9	11,308	6,8678					
ARHOMBI_DN17916_c0_g1_i1_3	1	8,2	20,463	6,4253	PF07724.13	AAA_2	120,9	5,60E-35	CL0023
ARUBRA_DN23363_c0_g1_i1_1	1	28,4	7,9559	6,315	PF06101.10	Vps62	93,4	9,60E-27	No_clan
ARUBRA_DN6829_c0_g1_i1_2	1	14,1	14,172	7,4625					
ARUBRA_DN14638_c0_g1_i1_5	1	11,4	13,453	6,3931					
ARUBRA_DN20092_c0_g1_i1_5	1	29,4	7,1471	7,803					
ARHOMBI_DN4417_c0_g1_i1_4	3	10,5	45,752	18,764	PF00076.21	RRM_1	33,2	3,10E-08	CL0221
ARHOMBI_DN22894_c0_g1_i1_5	1	13,8	9,5616	43,754	PF00332.17	Glyco_hydro_17	48,5	7,50E-13	CL0058
ARUBRA_DN7549_c0_g1_i1_1	1	6,8	18,206	6,5665	PF00076.21	RRM_1	48,6	4,80E-13	CL0221
ARHOMBI_DN3368_c0_g1_i1_3	1	8,2	24,509	7,3759	PF02201.17	SWIB	93,5	4,90E-27	No_clan
ARUBRA_DN1922_c0_g2_i1_6	2	20,2	13,106	11,357					
ARHOMBI_DN7605_c0_g1_i1_1	1	16,1	9,3108	6,7048					
ARUBRA_DN7746_c0_g1_i1_5	3	24,8	16,671	33,07	PF00248.20	Aldo_ket_red	94,4	6,30E-27	No_clan
ARUBRA_DN23775_c0_g1_i1_5	1	19,4	7,4052	6,1496					
ARUBRA_DN22204_c0_g1_i1_5	1	8,6	20,305	6,2137					
ARHOMBI_DN16579_c0_g1_i1_6	1	14,5	8,6148	6,237	PF05770.10	Ins134_P3_kin	90,6	1,00E-25	CL0179
ARUBRA_DN23978_c0_g1_i1_3	1	25,4	6,999	6,7763	PF08241.11	Methyltransf_11	33,5	4,70E-08	CL0063
ARUBRA_DN293_c0_g1_i1_1	2	12,8	33,521	13,782	PF00022.18	Actin	308,7	4,40E-92	CL0108
ARHOMBI_DN14395_c0_g1_i1_5	1	20,3	7,9662	6,8664					
ARHOMBI_DN18669_c0_g1_i1_3	1	27	7,476	6,4823					
ARUBRA_DN18022_c0_g1_i1_6	1	7,2	18,117	7,0859	PF01603.19	B56	227,4	2,90E-67	CL0020
ARUBRA_DN784_c0_g2_i1_6	2	8,4	26,324	12,022	PF12697.6	Abhydrolase_6	41,4	2,30E-10	CL0028
ARUBRA_DN7117_c0_g1_i1_3	1	21,1	8,1472	6,8134	PF00982.20	Glyco_transf_20	82	3,20E-23	CL0113

ARUBRA_DN13794_c0_g1_i1_1	1	20	8,4608	6,8237					
ARHOMBI_DN2834_c0_g2_i1_4	1	7,3	21,053	6,2597					
ARUBRA_DN7201_c0_g1_i1_6	1	9,3	24,553	6,724					
ARUBRA_DN20463_c0_g1_i1_4	1	10,3	16,892	6,8699	PF00152.19	tRNA-synt_2	125,6	1,90E-36	CL0040
ARUBRA_DN12786_c0_g1_i1_6	2	9,7	32,484	11,08					
ARUBRA_DN4014_c0_g1_i1_1	4	53,5	11,135	6,6691	PF00248.20	Aldo_ket_red	35	7,90E-09	No_clan
ARUBRA_DN21_c0_g1_i1_6	1	6,4	22,193	6,1533					
ARUBRA_DN14287_c0_g1_i1_2	2	21	18,398	12,179	PF16488.4	ArgoL2	54	1,50E-14	No_clan
ARUBRA_DN3405_c0_g2_i1_2	1	4,6	28,573	6,169					
ARHOMBI_DN17003_c0_g1_i1_2	1	16,7	10,302	7,5534	PF06972.10	DUF1296	121,4	1,20E-35	CL0214
ARUBRA_DN1302_c0_g1_i1_2	2	21,4	13,79	12,14					
ARHOMBI_DN20638_c0_g1_i1_2	1	23,6	7,8589	6,2011					
ARHOMBI_DN3957_c0_g2_i1_3	2	8	43,056	6,5403	PF00069.24	Pkinase	253,4	2,10E-75	CL0016
ARUBRA_DN19773_c0_g1_i1_2	1	4	26,838	10,88					
ARHOMBI_DN15739_c0_g1_i1_6	1	10,4	20,569	6,3265	PF00069.24	Pkinase	178,1	2,00E-52	CL0016
ARUBRA_DN1164_c0_g1_i1_2	1	14,2	12,277	6,3663					
ARUBRA_DN12959_c0_g1_i1_2	2	34,4	13,363	16,835	PF00076.21	RRM_1	21,6	0,00013	CL0221
ARHOMBI_DN11084_c0_g1_i1_6	1	19,1	7,5293	6,2669	PF02574.15	S-methyl_trans	28,2	1,40E-06	No_clan
ARUBRA_DN24628_c0_g1_i1_6	2	29,3	11,022	11,16	PF00118.23	Cpn60_TCP1	72,4	2,70E-20	No_clan
ARHOMBI_DN5257_c0_g1_i1_3	1	4,2	36,161	6,9239	PF01058.21	Oxidored_q6	77,7	6,10E-22	No_clan
ARUBRA_DN15806_c0_g1_i1_3	2	18	14,669	11,051					
ARHOMBI_DN21683_c0_g1_i1_3	1	12,8	16,565	6,6976	PF02887.15	PK_C	46,2	4,20E-12	No_clan
ARHOMBI_DN7504_c0_g1_i1_3	1	36,4	8,3284	8,459	PF00330.19	Aconitase	115,9	2,20E-33	No_clan
ARUBRA_DN13027_c0_g1_i1_5	1	10,9	13,456	6,7656	PF12796.6	Ank_2	53,3	2,90E-14	CL0465
ARUBRA_DN22863_c0_g1_i1_4	1	15,2	8,6093	6,4741					
ARUBRA_DN19918_c0_g1_i1_5	1	7,5	22,849	6,5598	PF01070.17	FMN_dh	251,3	1,20E-74	CL0036
ARHOMBI_DN4736_c0_g1_i1_4	2	4,9	60,17	12,613	PF00232.17	Glyco_hydro_1	573,1	2,80E-172	CL0058
ARUBRA_DN24058_c0_g1_i1_4	2	20,1	17,669	11,252	PF00076.21	RRM_1	72,3	1,90E-20	CL0221
ARUBRA_DN4090_c0_g1_i2_2	2	13,7	20,984	11,462	PF04190.12	DUF410	52	7,60E-14	No_clan
ARUBRA_DN13119_c0_g1_i1_6	1	10,3	17,304	6,5818	PF13812.5	PPR_3	24,3	2,20E-05	CL0020
ARHOMBI_DN13499_c0_g1_i1_4	2	16,2	18,946	12,513					
ARUBRA_DN19563_c0_g1_i1_5	1	6,7	23,298	6,446	PF05577.11	Peptidase_S28	69,7	1,90E-19	CL0028

ARUBRA_DN14032_c0_g1_i1_2	2	31,4	11,586	6,2904	PF09668.9	Asp_protease	125	1,30E-36	CL0129
ARHOMBI_DN5062_c0_g1_i1_6	2	4,8	71,263	12,15	PF07994.11	NAD_binding_5	473,8	2,80E-142	CL0063
ARHOMBI_DN24773_c0_g1_i1_3	2	15,4	19,078	11,712					
ARHOMBI_DN4958_c0_g1_i1_3	1	10,4	8,3698	6,2423					
ARHOMBI_DN4594_c0_g1_i1_1	2	5,3	48,56	11,158					
ARHOMBI_DN27276_c0_g1_i1_4	1	17,2	9,5307	6,3893					
ARHOMBI_DN3256_c0_g1_i1_1	1	7,8	19,498	6,822	PF00153.26	Mito_carr	35,6	6,10E-09	No_clan
ARHOMBI_DN20557_c0_g1_i1_4	1	14,7	14,89	6,1693	PF00226.30	DnaJ	86,2	1,10E-24	CL0392
ARHOMBI_DN186_c0_g2_i1_4	2	11,9	23,81	13,407	PF03662.13	Glyco_hydro_79n	335,2	2,90E-100	CL0058
ARUBRA_DN19452_c0_g1_i1_6	1	16,3	15,376	6,3339					
ARUBRA_DN26125_c0_g1_i1_2	1	18,4	8,4016	6,5212	PF02545.13	Maf	42,5	5,30E-11	CL0269
ARHOMBI_DN20462_c0_g1_i1_3	1	12,6	16,517	6,5961					
ARUBRA_DN3192_c0_g1_i1_2	1	3,6	35,589	6,7929	PF13023.5	HD_3	162,2	8,80E-48	CL0237
ARUBRA_DN5035_c0_g1_i1_2	1	3,9	43,83	6,6204	PF00332.17	Glyco_hydro_17	408,5	1,90E-122	CL0058
ARHOMBI_DN1419_c0_g1_i1_6	1	8,1	26,473	6,2471					
ARHOMBI_DN19246_c0_g1_i1_1	1	10,3	21,563	6,4711	PF04597.13	Ribophorin_I	125,1	4,00E-36	No_clan
ARHOMBI_DN17996_c0_g1_i1_4	1	18,1	7,9208	6,4865					
ARUBRA_DN18443_c0_g1_i1_6	1	30,9	8,6278	6,8841	PF00270.28	DEAD	42,7	4,60E-11	CL0023
ARHOMBI_DN20793_c0_g1_i1_6	1	10,7	13,653	6,3632	PF03179.14	V-ATPase_G	59,2	4,50E-16	CL0255
ARUBRA_DN6539_c0_g2_i1_2	1	3	47,84	6,21	PF16940.4	Tic110	33,4	1,70E-08	No_clan
ARUBRA_DN2046_c0_g2_i1_2	2	19,3	17,683	13,59	PF08240.11	ADH_N	75,3	2,90E-21	CL0296
ARUBRA_DN3646_c0_g1_i1_3	1	5,1	30,782	6,6268	PF02466.18	Tim17	74,6	6,30E-21	No_clan
ARUBRA_DN6310_c0_g1_i1_4	5	54	12,438	-2	PF00012.19	HSP70	157,4	3,70E-46	CL0108
ARUBRA_DN1384_c0_g1_i1_3	1	5	26,167	6,2667	PF08523.9	MBF1	85,2	2,80E-24	No_clan
ARHOMBI_DN14955_c0_g1_i1_2	1	24,3	8,2814	6,202					
ARHOMBI_DN49_c0_g2_i1_6	1	6	39,873	6,742	PF00574.22	CLP_protease	234,1	9,40E-70	CL0127
ARUBRA_DN8685_c0_g1_i1_3	1	9,6	19,437	7,6424	PF04371.14	PAD_porph	114,6	5,30E-33	CL0197
ARHOMBI_DN17696_c0_g1_i1_3	1	6,5	27,949	6,8387	PF02225.21	PA	44,5	1,10E-11	CL0364
ARUBRA_DN25420_c0_g1_i1_2	1	16	14,077	6,4161					
ARHOMBI_DN24508_c0_g1_i1_4	1	20,2	9,5998	7,3109	PF03952.15	Enolase_N	99,4	1,60E-28	CL0227

ARUBRA_DN13680_c0_g1_i1_6	1	12,6	12,762	6,1823					
ARUBRA_DN4241_c0_g1_i1_3	1	4,9	29,596	6,2449	PF03764.17	EFG_IV	137,3	2,00E-40	CL0329
ARHOMBI_DN1471_c0_g1_i1_6	3	12	34,74	-2	PF00635.25	Motile_Sperm	104,3	2,70E-30	CL0556
ARHOMBI_DN7801_c0_g1_i1_5	1	34,2	7,8541	6,9947					
ARHOMBI_DN4606_c0_g1_i1_1	1	11,2	22,089	6,6327	PF00928.20	Adap_comp_sub	210,3	3,00E-62	CL0448
ARUBRA_DN17255_c0_g1_i1_6	1	10,1	14,789	6,4207	PF08294.10	TIM21	61,1	9,60E-17	CL0455
ARUBRA_DN17467_c0_g1_i1_6	3	65,8	8,1818	8,5794					
ARUBRA_DN17174_c0_g1_i1_5	1	18,4	9,9715	7,3734	PF00406.21	ADK	39,7	4,60E-10	CL0023
ARHOMBI_DN2723_c0_g1_i1_1	3	21	19,538	6,8522	PF03949.14	Malic_M	131,4	3,20E-38	CL0063
ARUBRA_DN13395_c0_g1_i1_4	3	27,6	18,736	12,862	PF00189.19	Ribosomal_S3_C	88,7	2,60E-25	No_clan
ARUBRA_DN5089_c0_g1_i1_5	4	25,1	20,681	6,1823					
ARHOMBI_DN3292_c0_g1_i1_4	2	16,1	20,504	6,7303	PF00160.20	Pro_isomerase	74,3	1,20E-20	CL0475
ARHOMBI_DN8290_c0_g2_i1_4	3	17,6	37,05	6,3289	PF00270.28	DEAD	130,5	5,10E-38	CL0023
ARHOMBI_DN22455_c0_g1_i1_1	1	11,3	17,262	7,2025	PF00076.21	RRM_1	52,2	3,60E-14	CL0221
ARUBRA_DN3317_c0_g1_i1_3	1	4,5	31,578	24,759	PF07002.15	Copine	167,8	2,20E-49	CL0128
ARHOMBI_DN8153_c0_g1_i1_4	1	11	18,42	6,4581	PF02540.16	NAD_synthase	22,6	4,50E-05	CL0039
ARUBRA_DN19644_c0_g1_i1_4	1	23	11,385	7,0155					
ARUBRA_DN17529_c0_g1_i1_1	1	1,4	70,505	6,2776					
ARHOMBI_DN390_c0_g1_i1_5	3	10,9	40,935	11,564	PF00514.22	Arm	23,4	3,90E-05	CL0020
ARHOMBI_DN10092_c0_g1_i1_1	1	11,9	14,392	6,4283	PF00626.21	Gelsolin	24,1	2,20E-05	CL0092
ARHOMBI_DN15307_c0_g1_i1_4	1	20	12,351	6,3877					
ARHOMBI_DN9011_c0_g1_i1_2	1	14,3	9,7511	6,6545	PF06454.10	DUF1084	59,8	2,50E-16	No_clan
ARHOMBI_DN18489_c0_g2_i1_2	5	55,6	10,006	12,988	PF00071.21	Ras	105,3	2,20E-30	CL0023
ARHOMBI_DN1634_c0_g1_i1_6	2	19,1	21,237	7,4475	PF00155.20	Aminotran_1_2	44,1	1,40E-11	CL0061
ARUBRA_DN8885_c0_g1_i1_2	1	11	24,095	7,3531	PF01301.18	Glyco_hydro_35	157,6	5,40E-46	CL0058
ARHOMBI_DN16188_c0_g1_i1_3	1	20,4	11,374	8,1772	PF16507.4	BLM10_mid	52,8	2,40E-14	No_clan
ARUBRA_DN21545_c0_g1_i1_4	1	11,9	12,82	6,7613					
ARHOMBI_DN16333_c1_g1_i1_1	1	7,5	22,558	6,4841	PF00627.30	UBA	33,4	2,70E-08	CL0214
ARUBRA_DN4684_c0_g2_i2_5	12	36,1	53,758	-2	PF00009.26	GTP_EFTU	39,9	3,00E-10	CL0023
ARHOMBI_DN25309_c0_g1_i1_1	1	19,1	9,9887	7,2781					
ARHOMBI_DN7295_c0_g1_i1_3	1	6,3	24,798	6,3635	PF03446.14	NAD_binding_2	119,7	1,20E-34	CL0063
ARHOMBI_DN3332_c0_g1_i1_5	1	7,4	21,356	6,8744					
ARUBRA_DN12402_c0_g1_i1_2	2	31,4	16,625	13,173	PF00459.24	Inositol_P	45,2	7,70E-12	CL0171

ARHOMBI_DN9886_c0_g1_i1_3	1	11,6	14,258	6,865					
ARHOMBI_DN16830_c0_g1_i1_4	1	7,4	22,563	6,3149	PF03208.18	PRA1	145,9	5,70E-43	No_clan
ARHOMBI_DN19962_c0_g1_i1_1	2	11,8	26,617	11,936					
ARHOMBI_DN7050_c0_g1_i1_3	1	8,6	21,152	6,5836	PF00789.19	UBX	32,4	7,40E-08	CL0072
ARUBRA_DN8811_c0_g1_i1_6	3	19,2	26,126	6,6142	PF03030.15	H_PPase	362,2	5,70E-108	No_clan
ARUBRA_DN9001_c0_g1_i1_4	1	5	34,233	6,9077	PF13460.5	NAD_binding_10	67	1,80E-18	CL0063
ARHOMBI_DN17895_c0_g1_i1_5	1	17,8	11,181	6,402					
ARHOMBI_DN1601_c0_g1_i1_6	1	2,8	53,101	6,1496	PF13949.5	ALIX_LYPXL_bnd	219,7	4,60E-65	No_clan
ARHOMBI_DN22439_c0_g1_i1_5	1	17,1	15,406	6,538					
ARHOMBI_DN3123_c0_g1_i2_2	1	6,4	21,637	6,8971	PF14709.6	DND1_DSRM	58,2	7,00E-16	CL0196
ARUBRA_DN26477_c0_g1_i1_2	1	15	8,8939	6,4681					
ARHOMBI_DN15728_c0_g1_i1_3	1	10	22,607	7,1165	PF13839.5	PC-Esterase	117,7	7,10E-34	CL0264
ARUBRA_DN14653_c0_g1_i1_5	2	42,7	8,7008	11,16	PF14543.5	TAXi_N	58,1	1,20E-15	CL0129
ARUBRA_DN239_c0_g1_i1_1	2	35,2	7,4676	24,762					
ARUBRA_DN981_c0_g1_i1_2	1	7,2	24,327	6,5818					
ARHOMBI_DN6224_c0_g1_i3_2	2	7,1	46,864	17,519	PF00128.23	Alpha-amylase	57,5	1,60E-15	CL0058
ARHOMBI_DN43_c0_g1_i1_1	1	5,8	26,627	6,7552					
ARHOMBI_DN10061_c0_g1_i1_1	1	16,4	7,8677	6,9878					
ARUBRA_DN860_c0_g1_i1_4	1	11	17,424	6,1959					
ARUBRA_DN4926_c0_g2_i1_2	1	11,6	19,101	9,479	PF02466.18	Tim17	41,7	1,10E-10	No_clan
ARHOMBI_DN442_c0_g2_i1_6	1	9,2	22,454	7,8188	PF13489.5	Methyltransf_23	41,8	8,60E-11	CL0063
ARUBRA_DN7677_c0_g1_i1_5	2	6,8	57,302	11,22	PF00067.21	p450	188,5	1,80E-55	No_clan
ARHOMBI_DN23943_c0_g1_i1_5	2	21,3	20,566	11,913	PF12134.7	PRP8_domainIV	91,5	5,70E-26	No_clan
ARHOMBI_DN5334_c0_g2_i1_6	1	8,2	15,818	-2					
ARHOMBI_DN2144_c0_g1_i1_2	1	6,1	26,42	7,5768	PF02878.15	PGM_PMM_I	27,4	2,10E-06	No_clan
ARUBRA_DN17900_c0_g1_i1_4	1	9	17,183	7,4569	PF00240.22	ubiquitin	47,1	1,30E-12	CL0072
ARUBRA_DN7215_c0_g1_i1_2	1	9,9	20,176	6,8789	PF10236.8	DAP3	86,4	1,80E-24	CL0023
ARHOMBI_DN10966_c0_g1_i1_1	2	30,1	9,2931	15,175	PF00085.19	Thioredoxin	23,4	4,00E-05	CL0172
ARUBRA_DN4852_c0_g1_i2_5	5	9,3	70,485	6,2873	PF01474.15	DAHP_synth_2	652,1	2,90E-196	CL0036
ARUBRA_DN7820_c0_g1_i1_3	1	8,1	19,263	6,9581					
ARUBRA_DN3218_c0_g2_i1_1	1	3,5	39,702	6,3907	PF13561.5	adh_short_C2	200,1	3,60E-59	CL0063

ARUBRA_DN14060_c0_g1_i1_6	1	15,7	7,6455	6,3892					
ARHOMBI_DN3766_c0_g1_i1_2	1	9,2	20,274	6,5963	PF13561.5	adh_short_C2	53,9	1,70E-14	CL0063
ARHOMBI_DN19865_c0_g1_i1_4	1	21,5	9,9581	6,9255	PF08911.10	NUP50	36,8	4,20E-09	No_clan
ARHOMBI_DN27008_c0_g1_i1_5	1	15,5	9,4964	6,3907					
ARHOMBI_DN14983_c0_g1_i1_5	1	14	10,758	6,7056					
ARUBRA_DN23880_c0_g1_i1_2	1	6,4	23,594	6,4725	PF01039.21	Carboxyl_trans	181,5	2,30E-53	CL0127
ARHOMBI_DN17419_c0_g1_i1_1	1	14,2	12,325	6,4053					
ARUBRA_DN16616_c0_g1_i1_4	1	12,2	12,95	6,446	PF04815.14	Sec23_helical	41,8	6,90E-11	No_clan
ARUBRA_DN17646_c0_g1_i1_5	1	6,3	24,135	6,7583					
ARUBRA_DN22151_c0_g1_i1_2	3	62,2	9,2845	26,008	PF16113.4	ECH_2	46	4,70E-12	CL0127
ARHOMBI_DN14825_c0_g1_i1_2	2	44,3	7,5654	67,163					
ARHOMBI_DN16314_c0_g1_i1_1	1	17,2	14,138	6,3882	PF00226.30	DnaJ	86,8	7,20E-25	CL0392
ARHOMBI_DN3532_c0_g1_i1_4	1	8,9	22,194	6,6199					
ARHOMBI_DN4371_c0_g1_i1_5	1	7,3	22,767	6,4422	PF04564.14	U-box	104,4	2,50E-30	CL0229
ARUBRA_DN13912_c0_g1_i1_3	1	28,2	8,1233	6,3255					
ARHOMBI_DN17734_c0_g1_i1_4	1	13,2	10,417	6,3302	PF03129.19	HGTP_anticodon	26,4	5,40E-06	CL0458
ARHOMBI_DN21284_c0_g1_i1_3	1	9,7	16,547	6,712	PF13246.5	Cation_ATPase	57,8	8,30E-16	CL0137
ARUBRA_DN8408_c0_g1_i1_4	2	42	8,9988	13,897					
ARUBRA_DN8779_c0_g1_i1_1	1	8,8	18,007	6,861	PF14683.5	CBM-like	137,8	2,80E-40	CL0202
ARUBRA_DN85_c0_g1_i1_3	1	5	33,38	6,8716	PF00156.26	Pribosyltran	35,9	4,50E-09	CL0533
ARHOMBI_DN16378_c0_g1_i1_1	2	20,3	16,336	11,963					
ARHOMBI_DN22637_c0_g1_i1_5	1	20,5	9,0635	6,6632					
ARHOMBI_DN16961_c0_g1_i1_3	1	29,2	7,8485	6,2638					
ARUBRA_DN6264_c0_g1_i1_1	1	10,7	17,653	7,9226	PF00709.20	Adenylsucc_synt	69,2	2,80E-19	CL0023
ARHOMBI_DN6677_c0_g1_i1_3	1	16,7	13,701	6,4307	PF00293.27	NUDIX	26,4	5,20E-06	CL0261
ARHOMBI_DN15571_c0_g1_i1_5	1	5,3	30,892	6,6958					
ARUBRA_DN2280_c0_g1_i1_3	1	8,9	21,439	6,5476	PF00445.17	Ribonuclease_T2	131,2	4,50E-38	No_clan
ARHOMBI_DN5894_c0_g1_i1_5	1	6,7	22,51	6,2838	PF00888.21	Cullin	77,5	8,00E-22	No_clan
ARHOMBI_DN18104_c0_g1_i1_1	1	17,5	11,564	6,4867					
ARUBRA_DN9413_c0_g1_i1_3	1	4,8	36,98	6,7122	PF00175.20	NAD_binding_1	58,5	8,00E-16	CL0091
ARUBRA_DN8576_c0_g1_i1_3	1	23,3	7,0348	7,3319					
ARHOMBI_DN4497_c0_g1_i1_6	1	6,8	26,019	28,193	PF03931.14	Skp1_POZ	57,6	1,00E-15	CL0033
ARUBRA_DN7437_c0_g1_i1_1	2	21,3	17,202	11,989	PF06480.14	FtsH_ext	33,5	3,80E-08	No_clan

ARUBRA_DN6344_c0_g1_i1_3	1	7,3	20,866	6,2916	PF00696.27	AA_kinase	31,9	9,00E-08	No_clan
ARHOMBI_DN142_c0_g1_i2_6	1	4,8	23,367	7,1713	PF08534.9	Redoxin	61,1	8,70E-17	CL0172
ARHOMBI_DN2804_c0_g1_i1_5	1	10,9	14,343	-2					
ARUBRA_DN14140_c0_g1_i1_5	1	9,1	18,054	6,5197					
ARUBRA_DN20165_c0_g1_i1_6	1	12,8	13,721	6,6591					
ARUBRA_DN11522_c0_g1_i1_2	1	6,2	23,721	6,573					
ARUBRA_DN7178_c0_g1_i1_1	1	19,4	7,6033	6,2046	PF00928.20	Adap_comp_sub	57,9	9,10E-16	CL0448
ARUBRA_DN495_c0_g1_i1_6	1	15,7	10,573	-2	PF01138.20	RNase_PH	53,9	2,40E-14	CL0329
ARUBRA_DN15567_c0_g1_i1_3	1	24,7	8,121	6,4867	PF06419.10	COG6	72,8	1,60E-20	CL0295
ARHOMBI_DN20913_c0_g1_i1_5	1	8,7	20,006	6,5659	PF00270.28	DEAD	37,1	2,40E-09	CL0023
ARUBRA_DN19959_c0_g1_i1_6	1	9,3	20,642	6,3356					
ARUBRA_DN8310_c0_g1_i1_2	1	7,2	26,575	6,9499	PF01077.21	NIR_SIR	33	3,50E-08	No_clan
ARHOMBI_DN11012_c0_g1_i1_4	1	7,2	17,751	6,1995	PF02212.17	GED	89	1,50E-25	No_clan
ARUBRA_DN6936_c0_g1_i1_4	1	17,4	8,3042	6,7975					
ARHOMBI_DN21368_c0_g1_i1_6	2	31,4	8,2533	11,667					
ARUBRA_DN5364_c0_g1_i1_3	2	8	32,556	11,068	PF00400.31	WD40	25,6	1,50E-05	CL0186
ARUBRA_DN568_c0_g1_i1_3	1	8,3	22,292	6,5655	PF05193.20	Peptidase_M16_C	44,8	1,30E-11	CL0094
ARHOMBI_DN24822_c0_g1_i1_5	1	13,3	10,405	6,4663	PF00314.16	Thaumatin	81,5	6,70E-23	CL0293
ARHOMBI_DN8308_c0_g1_i1_3	1	6,8	21,184	6,2449	PF01602.19	Adaptin_N	53,6	1,30E-14	CL0020
ARHOMBI_DN249_c0_g1_i1_6	2	17,5	19,092	12,117	PF01423.21	LSM	39,8	2,50E-10	CL0527
ARHOMBI_DN12088_c0_g1_i1_5	2	43,6	10,684	13,395					
ARHOMBI_DN16872_c0_g1_i1_5	1	8,4	20,654	6,2507	PF01423.21	LSM	61,4	4,50E-17	CL0527
ARHOMBI_DN12473_c0_g1_i1_6	1	16,3	9,7599	6,6676					
ARUBRA_DN9724_c0_g1_i1_1	1	15,5	9,1646	6,6569					
ARHOMBI_DN11959_c0_g1_i1_2	1	4,6	21,225	6,1497	PF12327.7	FtsZ_C	102,5	1,10E-29	CL0442
ARUBRA_DN3899_c0_g2_i1_5	1	4,8	28,236	6,4706	PF00637.19	Clathrin	108,9	1,70E-31	CL0020
ARUBRA_DN9135_c0_g1_i1_4	1	6,5	21,71	6,6748	PF03997.11	VPS28	140,9	3,50E-41	CL0596
ARUBRA_DN3321_c0_g1_i1_1	1	9,5	16,885	6,2798					
ARUBRA_DN3221_c0_g1_i1_6	2	5,4	50,232	10,754	PF00490.20	ALAD	447,1	3,00E-134	CL0036
ARUBRA_DN7240_c0_g1_i1_1	1	7,2	26,543	6,4132	PF03178.14	CPSF_A	233,6	3,20E-69	CL0186
ARUBRA_DN25966_c0_g1_i1_5	1	16,2	12,456	6,3217	PF05701.10	WEMBL	140,8	5,30E-41	No_clan
ARHOMBI_DN2599_c0_g1_i1_4	1	11,9	14,603	6,1525	PF16656.4	Pur_ac_phosph_N	41,5	1,40E-10	CL0159

ARUBRA_DN13308_c0_g1_i1_1	1	23,7	7,966	32,423					
ARUBRA_DN10910_c0_g1_i1_6	1	12,1	14,706	6,4285	PF00282.18	Pyridoxal_deC	140,3	6,10E-41	CL0061
ARUBRA_DN24761_c0_g1_i1_3	1	12,3	12,023	7,4966					
ARHOMBI_DN11254_c0_g1_i1_1	1	12,5	9,5835	12,764					
ARUBRA_DN24634_c0_g1_i1_5	1	17,3	8,3904	6,764					
ARHOMBI_DN4130_c0_g1_i1_2	1	4,3	41,108	6,4323	PF04597.13	Ribophorin_I	311,8	8,80E-93	No_clan
ARHOMBI_DN2201_c0_g1_i1_2	1	17,4	13,95	8,2245	PF00542.18	Ribosomal_L12	80,6	7,20E-23	No_clan
ARUBRA_DN19418_c0_g1_i1_3	1	25,6	10,153	6,1954					
ARUBRA_DN1850_c0_g1_i1_3	2	8,1	34,434	11,265					
ARHOMBI_DN9657_c0_g1_i1_1	2	16,8	17,398	11,829					
ARUBRA_DN22015_c0_g1_i1_3	1	10,4	13,372	6,4662					
ARUBRA_DN21497_c0_g1_i1_5	1	8,6	19,698	6,6055					
ARHOMBI_DN17553_c0_g1_i1_3	4	64,1	9,9823	6,1707	PF00004.28	AAA	50,4	2,70E-13	CL0023
ARUBRA_DN23958_c0_g1_i1_3	1	7,9	20,589	6,4468					
ARHOMBI_DN6893_c0_g1_i1_1	1	6,1	27,657	6,8678					
ARUBRA_DN9664_c0_g1_i1_5	1	10,6	16,082	6,2247					
ARUBRA_DN3808_c0_g2_i1_1	1	4,3	21,007	6,3007	PF00255.18	GSHPx	121	1,40E-35	CL0172
ARUBRA_DN14331_c0_g1_i1_6	2	16,8	22,887	11,989	PF00013.28	KH_1	20,8	0,00023	CL0007
ARUBRA_DN209_c0_g1_i1_5	1	8	18,188	6,7994					
ARHOMBI_DN16878_c0_g1_i1_1	2	24,1	9,5058	11,342					
ARUBRA_DN3885_c0_g1_i1_1	1	8,1	18,963	6,3879	PF08695.9	Coa1	29,3	5,20E-07	CL0455
ARHOMBI_DN9575_c0_g1_i1_5	2	22,8	15,103	11,88					
ARHOMBI_DN4563_c0_g2_i1_3	1	8,3	20,174	6,5397	PF02806.17	Alpha-amylase_C	65	6,10E-18	CL0369
ARHOMBI_DN5164_c0_g1_i1_4	1	3,7	45,441	6,9002	PF00076.21	RRM_1	48,5	5,20E-13	CL0221
ARUBRA_DN2931_c0_g1_i1_4	1	2,8	50,96	6,4684	PF00128.23	Alpha-amylase	55,7	5,40E-15	CL0058
ARUBRA_DN8810_c0_g1_i1_3	2	9	44,499	12,328					
ARUBRA_DN27193_c0_g1_i1_3	1	11,9	13,929	6,8696					
ARHOMBI_DN2247_c0_g1_i1_2	1	5,3	33,402	6,4841	PF06775.13	Seipin	119,4	1,90E-34	No_clan
ARUBRA_DN25129_c0_g1_i1_1	1	11,7	11,983	6,6603	PF03061.21	4HBT	29,5	6,70E-07	CL0050
ARUBRA_DN7581_c0_g1_i1_1	1	14,6	11,164	-2					
ARUBRA_DN26635_c0_g1_i1_3	2	29,4	9,4868	11,889	PF00928.20	Adap_comp_sub	63,7	1,60E-17	CL0448
ARUBRA_DN25922_c0_g1_i1_4	1	16,2	8,7749	6,2866	PF08597.9	eIF3_subunit	48,7	8,40E-13	No_clan
ARHOMBI_DN16586_c0_g1_i1_6	1	11,6	17,46	7,3039	PF08356.11	EF_assoc_2	91,9	1,80E-26	No_clan

ARUBRA_DN23667_c0_g1_i1_3	1	15,7	9,4883	6,6917	PF00433.23	Pkinase_C	30,9	3,00E-07	No_clan
ARHOMBI_DN1477_c0_g1_i1_3	2	18,5	16,795	11,54	PF00749.20	tRNA-synt_1c	74,5	6,20E-21	CL0039
ARUBRA_DN23915_c0_g1_i1_1	1	9,8	13,538	6,6051	PF00153.26	Mito_carr	25,2	1,10E-05	No_clan
ARUBRA_DN16207_c0_g1_i1_4	1	7,5	31,015	7,1358	PF00149.27	Metallophos	38,2	2,00E-09	CL0163
ARUBRA_DN12100_c0_g1_i1_6	1	15,2	12,538	6,4841					
ARHOMBI_DN5013_c0_g1_i1_5	1	5,3	30,693	6,5098	PF03357.20	Snf7	122,7	1,00E-35	CL0235
ARHOMBI_DN17278_c0_g1_i1_1	2	19,8	19,199	11,345					
ARUBRA_DN8077_c0_g1_i1_3	1	9,2	14,428	6,6208	PF01521.19	Fe-S_biosyn	28,9	9,50E-07	No_clan
ARHOMBI_DN12156_c0_g1_i1_6	3	34,6	11,504	6,4207	PF00071.21	Ras	44,3	1,30E-11	CL0023
ARHOMBI_DN19746_c0_g1_i1_3	1	11,1	21,706	6,7482	PF02824.20	TGS	86,4	8,80E-25	CL0072
ARHOMBI_DN5681_c0_g1_i1_3	1	4,8	40,235	6,4383	PF12838.6	Fer4_7	48,4	9,40E-13	CL0344
ARHOMBI_DN9772_c0_g1_i1_3	1	6,3	20,752	6,7064	PF03091.14	CutA1	129,4	3,30E-38	CL0089
ARUBRA_DN2587_c0_g1_i1_2	1	25,3	9,027	7,6962					
ARHOMBI_DN14925_c0_g1_i1_1	1	29,9	7,5888	6,3313	PF00682.18	HMGL-like	43,9	1,90E-11	CL0036
ARHOMBI_DN13424_c0_g1_i1_2	1	26	8,5053	6,8061	PF05770.10	Ins134_P3_kin	57,5	1,20E-15	CL0179
ARHOMBI_DN17556_c0_g1_i1_2	1	20,8	14,731	6,4271	PF04526.12	DUF568	33,2	5,90E-08	No_clan
ARHOMBI_DN23552_c0_g1_i1_5	1	17,3	8,1703	6,2531					
ARHOMBI_DN2495_c0_g2_i1_3	1	11,5	16,742	6,9922	PF00076.21	RRM_1	24,7	1,40E-05	CL0221
ARUBRA_DN16642_c0_g1_i1_1	1	25,5	11,387	7,233					
ARUBRA_DN8504_c0_g1_i1_5	1	17,5	10,817	6,245	PF00076.21	RRM_1	36,2	3,60E-09	CL0221
ARHOMBI_DN3218_c0_g1_i1_5	2	8,4	37,493	12,088					
ARHOMBI_DN6191_c0_g1_i1_5	2	15,4	22,5	6,5758	PF01777.17	Ribosomal_L27e	104,5	2,70E-30	No_clan
ARUBRA_DN13074_c0_g1_i1_4	1	12,6	15,277	6,211	PF04442.13	CtaG_Cox11	168,6	8,50E-50	No_clan
ARHOMBI_DN2701_c0_g1_i1_1	1	12,7	12,95	6,8683	PF06414.11	Zeta_toxin	32,1	5,90E-08	CL0023
ARUBRA_DN13385_c0_g1_i1_1	1	22,1	8,134	6,2272	PF13839.5	PC-Esterase	80,3	1,80E-22	CL0264
ARHOMBI_DN4455_c0_g2_i1_5	1	9,9	20,603	6,9595	PF00962.21	A_deaminase	100,3	1,20E-28	CL0034
ARUBRA_DN1705_c0_g1_i1_4	1	7,6	20,946	6,4207	PF06046.12	Sec6	71,8	3,60E-20	CL0295
ARHOMBI_DN14374_c0_g1_i1_3	1	16,2	8,1622	6,2193					
ARUBRA_DN3623_c0_g1_i1_6	1	5,3	36,627	10,078	PF02020.17	W2	67,4	8,10E-19	CL0020
ARUBRA_DN6227_c0_g1_i1_3	1	8,2	17,531	6,4329					
ARUBRA_DN14347_c0_g1_i1_6	1	7,6	18,595	6,1784					
ARUBRA_DN4655_c0_g1_i1_4	7	25	35,913	6,8231	PF00071.21	Ras	209,9	1,50E-62	CL0023
ARUBRA_DN152_c0_g1_i1_6	1	6,5	27,194	6,6047	PF01039.21	Carboxyl_trans	270	3,30E-80	CL0127

ARUBRA_DN21454_c0_g1_i1_1	1	16,9	9,5277	6,1969	PF06427.10	UDP-g_GGTase	68,2	4,10E-19	No_clan
ARHOMBI_DN14749_c0_g1_i1_1	1	12,8	13,523	6,9694	PF04086.12	SRP-alpha_N	37,5	2,30E-09	No_clan
ARUBRA_DN1738_c0_g1_i1_5	1	16	9,3457	6,498					
ARHOMBI_DN6745_c0_g1_i1_6	3	13	32,115	6,4056	PF00378.19	ECH_1	60,4	1,50E-16	CL0127
ARHOMBI_DN9761_c0_g1_i1_5	1	7,5	21,222	6,2321	PF03016.14	Exostosin	107,8	5,50E-31	No_clan
ARHOMBI_DN16459_c0_g1_i1_4	1	5,5	32,489	6,6584	PF08540.9	HMG_CoA_synt_C	275	7,30E-82	CL0046
ARHOMBI_DN630_c0_g2_i1_1	1	6,5	27,41	7,1479	PF01798.17	Nop	46,7	2,50E-12	No_clan
ARUBRA_DN10120_c0_g1_i1_3	1	11,3	14,015	6,4501					
ARHOMBI_DN26737_c0_g1_i1_6	1	15,1	9,8642	6,254					
ARHOMBI_DN20055_c0_g1_i1_2	1	9	19,225	6,1838					
ARUBRA_DN68_c0_g2_i1_3	1	17,7	8,3284	6,71					
ARUBRA_DN18173_c0_g1_i1_5	1	11,3	15,645	6,9124	PF01625.20	PMSR	121,6	2,90E-35	No_clan
ARHOMBI_DN11266_c0_g1_i1_5	1	11,1	14,552	6,3406	PF16123.4	HAGH_C	90,4	7,60E-26	CL0381
ARUBRA_DN15293_c0_g1_i1_1	1	17,6	10,131	6,5968					
ARUBRA_DN16348_c0_g1_i1_1	1	11,8	12,08	33,456					
ARUBRA_DN5557_c0_g1_i1_3	1	9,8	13,697	56,422	PF00076.21	RRM_1	23,1	4,60E-05	CL0221
ARUBRA_DN807_c0_g1_i1_6	2	26,3	10,609	7,288	PF06172.10	Cupin_5	71,8	5,10E-20	CL0029
ARHOMBI_DN22251_c0_g1_i1_1	1	8	27,755	6,6448	PF03909.16	BSD	42,5	4,30E-11	No_clan
ARHOMBI_DN6086_c0_g2_i1_5	6	23,8	32,963	6,4425	PF00022.18	Actin	231,4	1,30E-68	CL0108
ARUBRA_DN15584_c0_g1_i1_1	1	21,3	8,2592	6,6421					
ARUBRA_DN23559_c0_g1_i1_2	1	18,5	10,896	6,7932					
ARHOMBI_DN3575_c0_g2_i1_3	11	48,9	29,076	7,4335	PF13419.5	HAD_2	102,8	2,10E-29	CL0137
ARUBRA_DN18865_c0_g1_i1_4	1	7,2	23,411	6,8237					
ARUBRA_DN81_c0_g1_i1_5	1	5,3	29,915	6,4207	PF13869.5	NUDIX_2	276,3	9,40E-83	CL0261
ARUBRA_DN17068_c0_g1_i1_4	1	8,9	17,743	6,8721	PF00180.19	Iso_dh	104,6	5,40E-30	CL0270
ARHOMBI_DN11773_c0_g1_i1_2	1	32,8	7,6231	7,3473					
ARHOMBI_DN15266_c0_g1_i1_2	1	8,2	18,191	6,2817	PF00226.30	DnaJ	86	1,30E-24	CL0392
ARHOMBI_DN19693_c0_g1_i1_6	1	7,2	21,691	6,2814	PF07859.12	Abhydrolase_3	40,6	2,30E-10	CL0028
ARHOMBI_DN21801_c0_g1_i1_4	1	21,1	7,4332	6,8725					
ARHOMBI_DN676_c0_g2_i1_2	1	6,4	25,13	6,4258					
ARHOMBI_DN6521_c0_g1_i1_4	1	21,2	9,2753	6,2244					
ARHOMBI_DN4350_c0_g1_i1_4	1	3,7	34,578	-2					
ARHOMBI_DN6943_c0_g1_i1_2	1	12,2	15,069	7,4946					

ARHOMBI_DN16004_c0_g1_i1_3	2	16,8	20,311	6,5234	PF00071.21	Ras	130,8	3,10E-38	CL0023
ARUBRA_DN26911_c0_g1_i1_3	2	25,2	12,892	12,088	PF14829.5	GPAT_N	126	3,90E-37	No_clan
ARHOMBI_DN7765_c0_g1_i1_6	1	6,4	21,016	6,4962	PF00149.27	Metallophos	89,7	3,40E-25	CL0163
ARUBRA_DN9287_c0_g1_i1_4	1	7,1	19,114	6,4731	PF00190.21	Cupin_1	129,9	5,40E-38	CL0029
ARUBRA_DN10301_c0_g1_i1_3	1	16,5	8,7287	6,1625					
ARHOMBI_DN25736_c0_g1_i1_6	1	25,3	9,1081	7,4809					
ARHOMBI_DN25307_c0_g1_i1_1	1	18,3	9,9898	7,9261					
ARHOMBI_DN827_c0_g1_i1_1	1	8,5	17,625	6,5368					
ARUBRA_DN5154_c0_g1_i1_1	1	6,7	18,756	6,1823					
ARHOMBI_DN10484_c0_g1_i1_1	1	13,7	10,751	6,6661	PF09753.8	Use1	114,7	5,20E-33	No_clan
ARUBRA_DN4965_c9_g2_i1_4	1	3,6	38,129	6,7056	PF01596.16	Methyltransf_3	342,2	7,00E-103	CL0063
ARHOMBI_DN650_c0_g2_i1_3	1	14,4	15,157	7,1951					
ARUBRA_DN19110_c0_g1_i1_2	1	8	25,226	10,201	PF01230.22	HIT	88,5	3,40E-25	CL0265
ARHOMBI_DN5597_c0_g2_i1_5	1	10,8	19,589	6,2488	PF00203.20	Ribosomal_S19	118,9	5,90E-35	No_clan
ARUBRA_DN21139_c0_g1_i1_2	1	17,8	12,067	7,0844	PF04050.13	Upf2	109,6	1,80E-31	CL0020
ARUBRA_DN3651_c0_g1_i1_3	1	9	15,6	6,572					
ARUBRA_DN460_c0_g1_i1_6	1	5,8	23,971	-2					
ARUBRA_DN26660_c0_g1_i1_1	1	12,5	15,151	-2					
ARHOMBI_DN2961_c0_g1_i1_6	1	7,7	21,766	6,1779	PF07011.10	DUF1313	134,5	8,00E-40	No_clan
ARUBRA_DN15712_c0_g1_i1_3	1	13,2	7,7689	6,3127					
ARHOMBI_DN5284_c0_g3_i1_5	2	28,8	16,054	6,8256	PF02782.15	FGGY_C	62,6	3,80E-17	CL0108
ARHOMBI_DN12176_c0_g1_i1_1	1	24,3	7,9746	6,8286					
ARUBRA_DN4419_c0_g1_i1_1	2	9,9	28,431	6,3895					
ARUBRA_DN3086_c0_g1_i1_4	1	6	24,843	6,4082	PF01126.19	Heme_oxygenase	46,6	3,10E-12	CL0230
ARUBRA_DN5853_c0_g1_i1_3	1	18,4	8,5119	-2					
ARHOMBI_DN12506_c0_g1_i1_1	1	15,5	8,52	6,4374					
ARUBRA_DN10624_c0_g1_i1_5	3	17,6	16,384	24,582	PF00179.25	UQ_con	82,5	1,90E-23	CL0208
ARHOMBI_DN13880_c0_g1_i1_4	1	8,6	11,755	8,7837	PF00079.19	Serpin	77,8	8,90E-22	No_clan
ARHOMBI_DN23964_c0_g1_i1_2	1	17,5	8,2383	9,5011	PF01915.21	Glyco_hydro_3_C	44,8	1,30E-11	No_clan
ARUBRA_DN7424_c0_g1_i1_6	1	4	33,979	-2					
ARHOMBI_DN5795_c0_g1_i1_5	1	6	23,31	6,4617					
ARHOMBI_DN6070_c0_g2_i1_6	1	6,6	26,148	-2					

ARUBRA_DN5014_c0_g1_i1_2	1	4	28,8	9,0182	PF00487.23	FA_desaturase	35,6	8,10E-09	No_clan
ARHOMBI_DN8315_c0_g1_i1_3	1	11,9	13,213	6,1592					
ARUBRA_DN13376_c0_g1_i1_4	1	7,6	20,426	-2					
ARUBRA_DN21106_c0_g1_i1_3	1	11,3	13,019	6,8302					
ARUBRA_DN22162_c0_g1_i1_5	1	5	21,493	-2					
