# Supplementary material for: Comparative proteomics of common allergenic tree pollens of birch, alder, and hazel
Source: Allergy. 2021 Jan 15;76(6):1743–53. doi: 10.1111/all.14694 (PMC8248232; doi:10.1111/all.14694)
Supplement: Supplementary file 10 — Table S8 [file ALL-76-1743-s014.pdf]

Supplementary Table S8: Intensities of identified Alnus pollen proteins in different preparations

Protein IDs	Intensity fasp1	Intensity fasp2	Intensity fasp3	Intensity fasp4	Intensity acetone1	Intensity acetone2	Intensity acetone3	Intensity acetone 4	Intensity tca1	Intensity tca2	Intensity tca3	Intensity tca4	Intensity zc1	Intensity zc2	Intensity zc3
ARUBRA_DN3160_c0_g1_i1_1	1061100000	2641200000	1206400000	2006300000	0	11585000	36396000	894610	180950000	0	0	0	8461500	5028700	3068500
ARHOMBI_DN6124_c0_g1_i1_4	371020000	455130000	288120000	263420000	152230000	80628000	132610000	98223000	471800000	568810000	294330000	445140000	39707000	33589000	26616000
ARUBRA_DN4553_c0_g1_i1_3	432810000	419920000	463850000	345050000	75385000	86913000	248290000	82937000	560750000	192840000	216980000	265850000	12263000	29634000	7550500
ARUBRA_DN4458_c0_g1_i1_5	392540000	393380000	279150000	271110000	54934000	48500000	153020000	49986000	631750000	344100000	377220000	389190000	14191000	19954000	20328000
ARHOMBI_DN14767_c0_g1_i1_6	1039600000	654050000	838610000	806830000	0	0	0	0	0	0	0	0	0	0	0
ARUBRA_DN4969_c0_g1_i11_2	395680000	436250000	512780000	450530000	19613000	13258000	134010000	6035800	473150000	254230000	244210000	257480000	10247000	3716400	3988900
ARUBRA_DN4696_c0_g1_i2_3	321470000	331240000	300860000	480090000	80763000	77592000	203550000	14894000	665250000	139740000	82910000	140190000	9979000	25765000	6637300
ARUBRA_DN4914_c0_g1_i1_2	452360000	371130000	414840000	318400000	78445000	30760000	157160000	28947000	442640000	63171000	60370000	25151000	40006000	35232000	32582000
ARUBRA_DN3195_c0_g1_i1_6	361310000	236020000	326970000	190050000	9890100	40214000	81867000	19500000	300140000	247870000	386930000	246300000	15240000	2575600	0
ARUBRA_DN2052_c0_g1_i1_5	340510000	362180000	362860000	273850000	66137000	65653000	122740000	49046000	493450000	44273000	34762000	33307000	24538000	23466000	18860000
ARUBRA_DN17603_c0_g1_i1_4	385160000	357290000	387510000	342020000	20919000	14143000	19354000	8420700	138760000	103360000	88890000	95578000	2553200	1217700	3400100
ARUBRA_DN23118_c0_g1_i1_1	61447000	63331000	94898000	78802000	56170000	40050000	145550000	27335000	274720000	285850000	334170000	259480000	3216800	4893400	4636000
ARHOMBI_DN3404_c0_g1_i1_5	178080000	169780000	149540000	83203000	62908000	30610000	71573000	53214000	219580000	187970000	225080000	179710000	8049000	8846400	9912300
ARHOMBI_DN8875_c0_g1_i1_3	235280000	201350000	176840000	124030000	85340000	53410000	115820000	75799000	265050000	41671000	37655000	31854000	24419000	42316000	13452000
ARUBRA_DN10871_c0_g1_i1_1	154790000	154340000	124570000	96929000	77477000	46010000	110650000	57486000	322810000	47595000	56542000	8884400	11791000	18524000	15627000
ARHOMBI_DN3889_c0_g1_i1_5	103030000	134330000	95783000	78817000	30083000	29954000	62253000	34364000	240490000	155540000	86481000	143960000	21513000	24219000	20695000
ARHOMBI_DN5373_c0_g1_i1_4	108700000	125670000	137370000	88604000	50410000	24241000	23358000	32703000	146500000	113820000	156500000	152570000	11414000	19850000	17841000
ARUBRA_DN4854_c0_g1_i1_3	113400000	186530000	262460000	104380000	68381000	19316000	25489000	17503000	265080000	28836000	31602000	23823000	13448000	8605600	2569500
ARUBRA_DN1249_c0_g2_i1_2	81555000	82668000	81034000	68471000	5472700	24770000	60942000	30107000	86199000	198690000	207690000	179650000	11368000	5353200	9505700
ARHOMBI_DN5306_c0_g1_i1_6	112020000	114550000	142080000	89300000	49132000	40923000	35760000	18088000	214120000	107290000	57851000	64540000	19326000	15515000	22112000
ARUBRA_DN5302_c0_g1_i1_5	78767000	94417000	93671000	48957000	45421000	19905000	45125000	14395000	108260000	159010000	130990000	120020000	32651000	19668000	6106400
ARHOMBI_DN26299_c0_g1_i1_4	156570000	180110000	176540000	196630000	3681900	12200000	25668000	42360000	165300000	0	0	0	4321600	5126200	0
ARHOMBI_DN5459_c0_g1_i1_2	115170000	132660000	130700000	67730000	54173000	30167000	63178000	30286000	85989000	71333000	60497000	52248000	15065000	20501000	17543000
ARHOMBI_DN5273_c0_g1_i1_3	67265000	202870000	26145000	72658000	2675300	35784000	48867000	1779800	163450000	5990600	89952000	8658700	276510	642330	408640
ARHOMBI_DN1417_c0_g1_i1_5	30830000	26115000	49724000	25564000	1486400	19638000	18436000	2554100	63359000	159150000	153780000	120950000	9069500	10021000	5329600
ARHOMBI_DN5581_c0_g1_i1_3	112120000	102060000	122980000	79202000	24373000	9183000	17160000	8849300	76745000	31966000	50896000	53575000	1076100	2452700	662240

ARUBRA_DN2313_c0_g1_i1_3	0	187880000	162520000	13176000	976170	164360	91125	0	100630000	0	0	0	59539000	27919000	27250000
ARUBRA_DN9810_c0_g1_i1_4	44356000	18005000	43688000	10535000	20609000	23938000	24407000	45322000	83884000	65962000	59561000	102850000	5585900	7064300	3328500
ARHOMBI_DN6211_c0_g2_i1_6	68323000	72259000	66397000	38722000	24517000	13176000	33010000	15120000	52636000	42920000	53477000	51809000	4524700	5057500	5242000
ARUBRA_DN22898_c0_g1_i1_5	46532000	45715000	45783000	30955000	8583400	4099400	12619000	4536100	35601000	29022000	62853000	60653000	44286000	50862000	37653000
ARUBRA_DN2253_c0_g1_i1_1	31400000	37161000	45465000	17998000	14713000	5196800	13377000	19864000	71171000	90443000	66373000	51194000	25067000	17084000	12335000
ARHOMBI_DN4873_c0_g1_i1_4	34251000	53163000	52503000	52523000	24885000	13950000	23766000	25258000	81219000	8223300	6874700	5468800	36518000	35418000	20077000
ARUBRA_DN18206_c0_g1_i1_4	49764000	49073000	57719000	51238000	21681000	11724000	65496000	6611000	120890000	0	0	0	7557100	15483000	9468200
ARUBRA_DN6222_c0_g1_i1_5	40235000	43601000	43221000	38619000	22470000	9371200	31322000	29511000	64675000	42271000	46071000	27557000	5688400	3880400	5617300
ARHOMBI_DN5949_c0_g2_i1_6	17089000	15019000	16329000	14292000	10580000	3841300	8862900	10514000	49406000	77089000	62272000	45398000	44606000	40617000	37029000
ARHOMBI_DN4456_c0_g1_i1_5	40564000	41802000	40220000	27606000	19045000	11842000	28903000	19354000	62297000	48283000	51866000	34385000	6489900	6214700	6193400
ARUBRA_DN390_c0_g1_i1_5	16271000	23876000	34268000	13369000	23118000	11518000	24490000	15539000	50193000	88859000	39882000	70311000	8995800	13455000	5833800
ARUBRA_DN4992_c0_g1_i1_3	23845000	26470000	27480000	34217000	22661000	7968900	23288000	13591000	98104000	44663000	49111000	44429000	2613900	6738700	3738300
ARHOMBI_DN5841_c0_g1_i1_5	51193000	50882000	39024000	28296000	12654000	6898900	14915000	6539600	32422000	47530000	44138000	42503000	15586000	16163000	11837000
ARHOMBI_DN6196_c0_g1_i2_5	51772000	60847000	62352000	69479000	2815000	5171600	7099500	12066000	53793000	17980000	21375000	17825000	2181200	1285100	15833000
ARHOMBI_DN3043_c0_g1_i1_4	43013000	38438000	26615000	50506000	13811000	5959400	11948000	25237000	58947000	7563000	55701000	49773000	626390	0	0
ARUBRA_DN4475_c0_g1_i1_2	52294000	51715000	55577000	41210000	3910900	3952600	33320000	14951000	75241000	4840200	12782000	13111000	3283000	7622800	9896800
ARUBRA_DN1141_c0_g1_i1_1	28515000	26734000	25609000	18311000	10657000	7363900	14922000	12946000	36131000	57015000	58997000	63515000	0	2116600	0
ARHOMBI_DN3285_c0_g1_i1_6	27553000	35247000	32364000	29150000	4922200	8071100	13696000	6169000	56560000	40917000	43694000	40254000	5196000	5584500	2469500
ARUBRA_DN10174_c0_g1_i1_6	29945000	33669000	33440000	38753000	14580000	8961800	20730000	38190000	123590000	0	0	0	3508600	2473100	2469100
ARHOMBI_DN7524_c0_g1_i1_6	24002000	24423000	22655000	22384000	14551000	5277900	6252400	7983900	70035000	31335000	31983000	65423000	2133200	3314600	11417000
ARHOMBI_DN4459_c0_g1_i1_3	23985000	26044000	23403000	23336000	32955000	10656000	14361000	21859000	122820000	10833000	0	9073300	4015000	4351400	4525700
ARHOMBI_DN6146_c1_g1_i1_4	11196000	13825000	10603000	8820400	9285300	3362800	2478900	7955000	40390000	43718000	21563000	46917000	29935000	33930000	46186000
ARUBRA_DN4074_c0_g1_i1_4	36920000	33098000	62677000	41122000	4361600	2221800	29640000	372300	93714000	0	0	0	6839100	1542600	2885100
ARHOMBI_DN3575_c0_g1_i1_3	9024600	9261700	9646400	19496000	6771500	6101800	10916000	25087000	60145000	54421000	33970000	59021000	1576500	0	1916800
ARHOMBI_DN4328_c0_g1_i1_6	36525000	38359000	20172000	41033000	13187000	8516000	23340000	28382000	62555000	0	0	0	9894100	10518000	8336100
ARUBRA_DN411_c0_g1_i1_5	22323000	17260000	25327000	25022000	10720000	5439700	15894000	1244300	24479000	62547000	45682000	38736000	1249400	2007800	2174000
ARHOMBI_DN3323_c0_g1_i1_4	9370700	7698600	9910200	11714000	0	0	3310200	4830900	67762000	52235000	66415000	62730000	459890	0	1441400

ARHOMBI_DN5928_c0_g3_i2_6	26272000	26395000	33886000	26205000	8624000	0	8665300	8285900	34953000	39548000	43358000	34602000	0	0	0
ARUBRA_DN4841_c0_g1_i1_6	25329000	20634000	15335000	13292000	17409000	10873000	21728000	9639600	93083000	33209000	4281800	12177000	1934800	1037800	2454000
ARHOMBI_DN6080_c1_g1_i1_1	35087000	29397000	94885000	26409000	16167000	7318200	12233000	10813000	35506000	2618800	0	0	3336100	1428300	4746100
ARUBRA_DN16244_c0_g1_i1_2	12669000	13945000	15490000	7609700	6576100	3896900	5139000	5860100	24190000	55357000	33903000	39537000	13454000	20068000	19580000
ARUBRA_DN18719_c0_g1_i1_2	17955000	40686000	36592000	22555000	5065900	6378400	4306000	0	30831000	32670000	60971000	0	5850500	6079700	6910900
ARHOMBI_DN4484_c0_g1_i1_2	21353000	21394000	22109000	23191000	15776000	6467300	11514000	13302000	55486000	22455000	32746000	23030000	1549200	2938200	2969200
ARHOMBI_DN5698_c2_g1_i1_5	11061000	13110000	13186000	12764000	2388800	3994500	8266900	6212200	10669000	55078000	64897000	57089000	0	301390	0
ARHOMBI_DN15379_c0_g1_i1_6	23129000	24540000	25164000	15742000	6063700	3977300	8877700	9978100	31876000	36323000	29045000	36584000	2195800	310300	1588000
ARUBRA_DN10745_c0_g1_i1_4	43220000	42459000	51260000	48273000	10345000	5504600	0	0	41599000	0	0	0	2549600	0	0
ARUBRA_DN17745_c0_g1_i1_5	26581000	28479000	30373000	41494000	15871000	7298000	18346000	20489000	50673000	232440	0	0	0	0	0
ARUBRA_DN16936_c0_g1_i1_5	13519000	11231000	14106000	6898300	19295000	4679300	11247000	19202000	32737000	33892000	31245000	38055000	1530500	1445300	134410
ARUBRA_DN4620_c0_g1_i1_1	8687100	9064900	10392000	8573500	2507000	683740	3458000	6090000	15845000	25611000	39455000	31768000	24564000	27657000	24154000
ARUBRA_DN4102_c0_g1_i1_3	3749300	6992000	4495100	4696600	5967000	1564600	2009800	5160400	16229000	22792000	34741000	64071000	25618000	22175000	15751000
ARHOMBI_DN9782_c0_g1_i1_2	38656000	26059000	39750000	22076000	4538700	2293600	0	8134200	57954000	0	0	0	11525000	11381000	2217100
ARUBRA_DN4847_c0_g2_i2_1	19444000	20798000	21633000	18344000	10612000	7574100	16043000	15833000	35146000	8676500	12419000	11206000	7140700	6765300	6295200
ARUBRA_DN5827_c0_g1_i1_2	0	0	0	0	24467000	19401000	35166000	2262600	75554000	1480300	16003000	15129000	3250300	5301600	6773500
ARHOMBI_DN3150_c0_g1_i1_6	22682000	18572000	17421000	11006000	12122000	1576600	4175600	5409000	25395000	24899000	24992000	17082000	4114900	6449500	6451600
ARHOMBI_DN3869_c0_g1_i1_1	16734000	15040000	14646000	14210000	7914700	4063900	15206000	11424000	44967000	19508000	12096000	18440000	0	1335300	1189300
ARHOMBI_DN4569_c0_g1_i1_2	15006000	15418000	16752000	19229000	6795200	5303600	11518000	9013100	20761000	19879000	25412000	23748000	4684400	2976300	0
ARHOMBI_DN8000_c0_g1_i1_4	10119000	10738000	8437600	3420700	5573100	1098100	7007500	2858400	16895000	39684000	27512000	26790000	9784600	9837000	11614000
ARHOMBI_DN10026_c0_g1_i1_5	12948000	6289700	8026100	4238700	17441000	8390600	6788300	6167300	34351000	23745000	35896000	21571000	351540	2988400	555210
ARHOMBI_DN3561_c0_g2_i1_2	14873000	13785000	15237000	12225000	16833000	4464300	23936000	25190000	37751000	9125900	0	10031000	1417600	1967100	1006700
ARHOMBI_DN18818_c0_g1_i1_2	9897100	9985300	6506200	4068300	0	4001900	10592000	6131500	13468000	35542000	39517000	32655000	4195100	3969900	6018400
ARUBRA_DN19744_c0_g1_i1_2	22940000	23568000	29379000	20677000	6448200	2172000	16301000	8631400	35073000	7607000	3155700	5852900	217150	1950700	1539400
ARHOMBI_DN5210_c0_g1_i1_5	5816300	8525300	0	32689	0	0	0	0	0	63302000	0	83343000	7033800	7757200	9063500
ARHOMBI_DN5071_c0_g1_i1_6	1962700	2989000	1805600	1668100	1381900	140680	724080	655050	2191300	42679000	46407000	42900000	9113500	12781000	8399000
ARUBRA_DN24356_c0_g1_i1_4	25767000	29058000	26337000	13351000	4333400	5296000	10102000	2585100	19749000	12193000	14250000	6558100	3141200	1896900	1096700

ARHOMBI_DN25271_c0_g1_i1_5	0	0	0	0	0	0	0	0	0	61827000	59941000	51342000	0	0	0
ARUBRA_DN4454_c0_g1_i1_2	16297000	15365000	16295000	37390000	3914700	1112200	4010700	7703100	38443000	10973000	0	0	3927800	8268000	6548000
ARHOMBI_DN6208_c0_g1_i2_5	2433600	3676500	2920400	4124700	197980	331550	0	142140	5553300	1573500	2934600	1923400	41400000	62119000	39178000
ARHOMBI_DN1745_c0_g1_i1_2	12666000	23898000	25776000	21061000	11833000	8693500	17659000	16482000	20686000	0	4940900	0	1137000	1343600	409580
ARUBRA_DN21566_c0_g1_i1_2	18312000	18549000	19662000	16240000	3673900	1010000	9674500	5799300	26495000	12295000	9269900	12716000	3519200	2716300	3001300
ARUBRA_DN4108_c0_g1_i1_5	18883000	20919000	23548000	10699000	11276000	5371800	13688000	9442500	20305000	6967400	7196500	6599100	1304100	1207600	3275000
ARHOMBI_DN5571_c0_g1_i1_5	4553800	4323800	5658700	3491800	8366800	616870	9525600	10342000	15072000	9656400	8195700	23388000	16075000	11149000	28902000
ARUBRA_DN4060_c0_g2_i1_2	15653000	12846000	19260000	13310000	9919700	4726600	12824000	11646000	30301000	10294000	9035500	8375600	0	0	691880
ARHOMBI_DN6185_c1_g1_i1_5	24830000	0	24099000	80878000	0	0	0	5053300	16912000	0	0	516620	0	0	0
ARHOMBI_DN5260_c0_g1_i1_6	19465000	24039000	28099000	13754000	2036500	2183400	6291500	2183900	14955000	6983500	8196600	3291700	3221200	8881000	8582000
ARHOMBI_DN6086_c1_g1_i1_6	12347000	14152000	7447700	17543000	387140	0	0	4429400	2592000	1096300	0	0	23369000	40119000	27956000
ARUBRA_DN4124_c0_g1_i1_3	563360	468010	4530700	2199400	5918800	0	12439000	1956200	253010	0	0	0	49349000	50676000	22732000
ARHOMBI_DN6236_c1_g1_i3_6	11775000	15454000	16201000	12980000	10937000	6252000	9927600	7960500	25811000	8108200	5126700	6381500	3668200	3127200	6290900
ARHOMBI_DN5869_c0_g1_i1_6	11907000	9499400	7792900	13445000	713710	0	4760000	8227700	22647000	4045700	6575200	3765400	17119000	19685000	13545000
ARUBRA_DN4641_c0_g1_i1_2	3486100	2573800	3506400	5327700	0	662980	692060	1210400	12626000	2763000	2524000	3381000	29936000	48844000	25544000
ARUBRA_DN23856_c0_g1_i1_3	17471000	16178000	10086000	4763600	8535900	7997700	16950000	6890600	15223000	6186000	12990000	10825000	1303000	2571200	3685500
ARHOMBI_DN5685_c0_g2_i1_3	2705800	3101900	3127500	4926400	1398500	176090	289540	1930700	11281000	1301500	1819300	0	27258000	26510000	55536000
ARUBRA_DN3590_c0_g2_i1_3	14992000	14280000	18615000	7238400	3634400	3073800	7817400	8534200	22179000	0	996550	33166000	1617800	1548700	3456900
ARHOMBI_DN3136_c0_g1_i1_1	19751000	21551000	26833000	14574000	3057100	1321100	3178900	791370	12062000	8954000	1717100	8148500	5707700	6281800	7075800
ARUBRA_DN4556_c0_g1_i2_4	17444000	23618000	22519000	12758000	3290700	1646200	3038000	347880	12218000	0	6280600	2289200	8336000	13830000	13380000
ARHOMBI_DN24422_c0_g1_i1_5	10857000	9504700	15841000	4934400	0	3369600	6121700	3155700	13056000	35132000	0	21920000	5485500	5739600	4099000
ARUBRA_DN990_c0_g1_i1_1	3154500	3772900	6205200	6086100	6879400	1767200	7612100	14997000	28491000	0	19541000	24953000	7483500	2331300	5369400
ARUBRA_DN1071_c0_g1_i1_2	16086000	9443400	14825000	13264000	3645900	1448500	5756000	6738800	28516000	14840000	8102100	12529000	79696	1703900	1029000
ARUBRA_DN2969_c0_g1_i1_3	19920000	13928000	20471000	12811000	5882900	8832300	3943100	15080000	27703000	0	0	0	4507700	2832200	1333800
ARHOMBI_DN4165_c0_g1_i1_1	12432000	15798000	9022500	0	0	0	3971300	0	16034000	24778000	27775000	22168000	0	0	1484100
ARHOMBI_DN5934_c0_g1_i1_3	2484000	2416600	2999200	4641300	0	873390	193030	1520800	5613900	8807500	8487100	4995100	35958000	28228000	25371000
ARHOMBI_DN5622_c0_g1_i1_3	2022200	2005000	2506900	4028800	5508700	1041500	13010000	14175000	23346000	0	0	0	25687000	28048000	11004000

ARHOMBI_DN4656_c0_g1_i1_6	8853600	12477000	11233000	3064900	4274300	0	1003100	2035700	16581000	24524000	24208000	16440000	426950	3311400	3269100
ARHOMBI_DN6034_c0_g1_i2_3	1008700	1077800	305850	2534200	1535900	128240	292220	1620500	0	731250	1431800	610300	50105000	44007000	24779000
ARHOMBI_DN6068_c0_g1_i2_3	450280	155170	0	133990	0	521610	1180200	164050	0	0	0	0	34703000	48763000	43800000
ARHOMBI_DN1849_c0_g1_i1_3	10204000	10147000	8950300	9088900	762630	0	0	716760	5031100	4499800	30778000	18266000	7115200	14361000	8593200
ARUBRA_DN8471_c0_g1_i1_3	22585000	12497000	18926000	30589000	0	0	0	0	0	0	43053000	0	0	0	0
ARUBRA_DN2937_c0_g1_i2_1	3610100	3072300	2868600	6840900	607730	599540	672570	2315300	4614300	1472800	0	0	39593000	37665000	23567000
ARUBRA_DN6062_c0_g1_i1_1	3547500	5089300	3054600	3586700	18379000	6305100	12182000	13205000	17950000	13918000	21082000	1348500	2788900	983880	3127000
ARHOMBI_DN5785_c0_g1_i1_4	9735500	10468000	10682000	21373000	1717100	2373700	1305700	10980000	49130000	0	0	0	3447900	614810	3710000
ARHOMBI_DN3876_c0_g1_i1_6	12642000	12690000	9158700	10811000	9729200	7733400	6984500	6048600	23145000	7358500	4621200	5583800	2702600	1500900	2075600
ARHOMBI_DN6149_c0_g1_i1_6	9490100	7846700	3747000	12387000	850140	211660	3898800	3712100	30501000	19669000	14932000	10647000	936680	2810700	814990
ARUBRA_DN6670_c0_g1_i1_3	0	0	0	0	0	0	0	0	0	61617000	60664000	0	0	0	0
ARHOMBI_DN6225_c0_g2_i1_5	854780	728410	206830	839310	0	0	0	0	905960	0	448820	0	36354000	46573000	32535000
ARUBRA_DN4747_c0_g1_i1_6	6345300	6503800	6589500	7374200	2167500	1331100	1185900	5629000	13505000	47342000	9539300	8976500	514780	534450	91377
ARHOMBI_DN5137_c0_g1_i1_5	16330000	16926000	11894000	11803000	0	3591000	3748400	1134000	19545000	7788900	15632000	6741100	0	221780	226920
ARUBRA_DN18006_c0_g1_i1_4	15640000	18289000	19387000	10651000	8249800	3370700	4252000	2578500	29116000	0	0	0	1399300	1652300	875050
ARHOMBI_DN2797_c0_g1_i1_2	7820000	5800800	7666200	4049400	7759000	2952400	2907100	1705500	20021000	23618000	13235000	12732000	1504900	1434200	1783500
ARHOMBI_DN3646_c0_g1_i2_4	12370000	8138200	9070100	9551300	8060800	3903200	3142800	2366400	12563000	9696300	18192000	5833700	5842500	0	5216700
ARHOMBI_DN6264_c0_g1_i4_2	9836000	10316000	10235000	23206000	1370100	0	1484100	6206500	46842000	1446300	0	1292900	350890	554480	508340
ARUBRA_DN1274_c0_g1_i1_4	15303000	16160000	18874000	12472000	1369800	0	5493400	1570400	7784000	4679500	9564000	8215000	2394800	3374400	6224700
ARHOMBI_DN7316_c0_g1_i1_3	0	55098	0	0	0	0	0	0	0	43203000	38689000	31356000	0	0	0
ARUBRA_DN13722_c0_g1_i1_3	0	0	0	0	41630000	0	0	49299000	0	0	0	0	10140000	0	10019000
ARUBRA_DN4746_c1_g2_i1_3	12107000	15627000	19773000	19102000	4107000	4429400	5896200	5884200	22099000	0	0	0	717970	617230	732280
ARHOMBI_DN11262_c0_g1_i1_4	13939000	14943000	24445000	13665000	0	0	0	0	6784400	24503000	0	0	4115600	3978600	4467600
ARHOMBI_DN4307_c0_g1_i1_5	70550	221250	0	949800	0	0	0	213120	0	0	0	0	26439000	46209000	36590000
ARUBRA_DN1657_c0_g1_i1_5	18204000	19366000	15822000	785200	0	0	1473700	18543000	27867000	304220	0	5981300	1598300	0	0
ARHOMBI_DN16963_c0_g2_i1_3	18688000	17994000	21585000	9590900	9789300	2918700	0	8268600	17627000	0	0	0	0	0	1185900
ARHOMBI_DN5659_c0_g1_i1_3	17480000	15623000	16603000	7743200	6776500	4204600	10389000	6605300	19064000	0	0	0	410500	1052700	1414100

ARHOMBI_DN9830_c0_g1_i1_4	0	0	0	0	0	0	0	0	0	36800000	36412000	31623000	0	0	0
ARHOMBI_DN7974_c0_g1_i1_2	12964000	11556000	11244000	0	1972300	0	3836500	382100	4560200	30936000	11409000	8089700	0	4156600	0
ARHOMBI_DN5417_c0_g1_i1_4	54502	2330800	34663	130140	408920	0	0	230600	1295000	0	0	0	28022000	36045000	32452000
ARHOMBI_DN753_c0_g1_i1_1	1818700	3698700	2922200	4284500	1052500	0	1383200	2325400	14356000	0	1213200	0	19227000	19423000	29129000
ARUBRA_DN4963_c0_g1_i2_5	24234000	20185000	17966000	11065000	637270	1474100	3555100	0	3759900	0	0	0	14959000	2985200	0
ARHOMBI_DN24139_c0_g1_i1_4	4526300	4611400	9242900	18037000	3806800	3250800	11450000	16783000	24730000	0	0	0	1914800	1270400	0
ARUBRA_DN18820_c0_g1_i1_4	1513000	3385100	2694300	1515900	6404900	863140	2131400	5725100	20713000	11438000	15666000	10860000	5874600	5181700	5242500
ARUBRA_DN325_c0_g2_i1_1	7183100	8047800	8672300	6214100	4700100	4173100	0	3195600	21983000	22750000	0	65314	4394800	4652800	2693000
ARHOMBI_DN13323_c0_g1_i1_2	0	0	0	0	0	0	0	0	0	48797000	0	48423000	0	245900	0
ARUBRA_DN1786_c0_g1_i1_1	14259000	13558000	14949000	5847200	5299500	2679400	4634700	5314800	12107000	0	0	0	6006200	5969300	6420400
ARUBRA_DN2535_c0_g1_i1_5	165870	71957	0	352370	0	0	0	127170	0	0	0	0	34700000	40475000	21051000
ARHOMBI_DN6004_c0_g1_i1_1	8882000	8529000	9913600	3298100	8575000	3121800	7761900	6067200	14145000	1045400	0	9592600	6595200	5975000	2312700
ARHOMBI_DN6257_c0_g1_i1_3	277110	503580	646150	0	0	0	677230	791020	580170	461380	0	0	27046000	37088000	27568000
ARHOMBI_DN4827_c0_g1_i1_1	0	0	0	0	0	0	0	0	93910000	0	0	0	121550	460050	370100
ARHOMBI_DN5844_c0_g1_i1_3	7066600	4999600	11265000	9443300	0	2105400	0	0	59695000	0	0	0	0	0	0
ARHOMBI_DN5686_c0_g1_i1_6	607850	691940	749730	723620	668740	0	482850	0	0	0	0	0	33724000	35963000	20489000
ARUBRA_DN3412_c0_g1_i1_6	2985000	3181500	7402000	466860	8733400	4111100	11098000	11536000	19707000	6532700	5780000	7241700	2290700	462750	1414000
ARHOMBI_DN9161_c0_g1_i1_5	0	88838000	0	0	0	0	0	0	0	0	0	0	0	0	1116100
ARUBRA_DN21065_c0_g1_i1_6	6049600	5537300	7718700	6158500	0	0	10920000	15795000	30874000	0	0	0	4851400	1368500	44595
ARUBRA_DN11174_c0_g1_i1_2	628740	88742	710100	119510	1594200	491940	968130	1316400	8952200	33956000	35561000	0	1993000	566240	2128800
ARUBRA_DN8598_c0_g1_i1_5	5863300	6414900	5938000	4983600	4877600	1053900	5659200	3930700	14972000	0	12552000	13498000	3211400	0	3779400
ARHOMBI_DN3049_c0_g1_i1_5	5244300	6742500	7316200	4136200	2604700	1920500	5014300	3630200	12247000	2520400	15990000	17243000	912660	605380	0
ARUBRA_DN16568_c0_g1_i1_3	15293000	12816000	20748000	5505400	3140700	3586500	1581200	4806800	17163000	0	0	0	0	1027500	0
ARUBRA_DN5078_c0_g1_i1_6	0	0	0	290080	0	0	0	0	0	0	0	0	28481000	31268000	25205000
ARHOMBI_DN6227_c0_g1_i3_4	0	0	0	0	0	0	0	0	0	36280000	25589000	23284000	0	0	0
ARHOMBI_DN4048_c0_g1_i1_3	341850	132100	0	160200	0	0	0	0	0	0	0	0	21053000	32809000	29972000
ARHOMBI_DN294_c0_g2_i1_2	16998000	14316000	13194000	38143000	0	0	0	0	0	0	0	0	0	0	0

ARUBRA_DN335_c0_g1_i1_4	7607900	7765600	8081100	7570500	4329200	1722700	2714500	8100000	23211000	8212700	0	0	1113100	840100	996040
ARUBRA_DN4552_c0_g2_i1_4	4068100	5389600	4917200	5792900	0	658860	1475100	3887200	12203000	6467100	4659900	1937000	9753600	9760800	11105000
ARHOMBI_DN19000_c0_g1_i1_1	587880	0	0	893870	0	0	0	4171600	12836000	22837000	39790000	0	413820	338500	0
ARUBRA_DN4655_c0_g2_i1_6	10982000	6166700	12340000	7493500	0	504810	1051100	1393300	7674500	3686700	3363000	729420	5176800	9935000	10502000
ARHOMBI_DN5421_c0_g1_i1_1	771720	968930	6697100	532810	5069400	2728900	6927700	4650400	6987400	5260000	5224800	4568800	12291000	11149000	7070400
ARUBRA_DN2639_c0_g2_i1_5	10217000	8542500	9169600	12851000	3559100	239700	1365700	3544700	17696000	1725300	2081700	1922700	1346600	2247600	2481500
ARHOMBI_DN6203_c0_g1_i1_4	3937600	3083400	5514300	2493600	7054500	5572000	5008200	5719200	13792000	7554700	8189200	1437000	1148800	3097600	5313800
ARHOMBI_DN5924_c0_g1_i1_3	2292600	2552000	4176600	3314500	1826600	3195500	7839900	10280000	32504000	8612400	0	0	0	547930	938870
ARUBRA_DN4385_c0_g1_i1_6	3121800	2204900	4742000	3255000	18382000	4918000	19246000	9165000	7362700	0	0	0	1639600	1557500	1676500
ARHOMBI_DN16907_c0_g1_i1_2	15329000	14832000	12253000	3914200	2631100	2878600	6551500	4629700	13252000	0	0	0	0	883270	0
ARUBRA_DN23412_c0_g1_i1_1	11173000	13584000	10975000	12535000	3783200	504440	1179200	4284400	4303700	7796700	6352700	0	0	0	372570
ARHOMBI_DN4949_c0_g1_i1_4	135820	111560	204770	0	3656600	0	5114000	3257500	9895200	16084000	14459000	3396600	5929400	6392300	6862800
ARHOMBI_DN9199_c0_g1_i1_3	931870	1524200	705400	760280	3759200	2704900	4314000	4686000	3658100	17486000	15350000	12251000	1188300	3830200	2159400
ARUBRA_DN20664_c0_g1_i1_6	4158200	4666400	3175100	5649300	0	301540	2324400	3512800	10518000	21491000	19018000	0	0	0	0
ARHOMBI_DN9737_c0_g1_i1_1	0	0	0	0	0	0	0	59163000	0	0	0	0	0	0	14800000
ARHOMBI_DN5509_c0_g1_i1_1	1558700	1370400	821860	2245800	610860	468010	799200	450800	0	0	0	0	15793000	31177000	18147000
ARUBRA_DN4741_c0_g1_i1_4	5457500	4968000	6803400	3378800	4258900	584080	2563800	5014300	40348000	0	0	0	0	0	0
ARUBRA_DN1142_c0_g1_i1_1	6265400	8985400	5853100	2172700	16806000	4553100	12572000	2946300	10350000	0	0	516280	779450	267890	562090
ARUBRA_DN2596_c0_g1_i1_5	3730700	7245400	6477200	1328600	10619000	2628700	11184000	5890800	6369100	8662200	0	6068800	0	447790	1141300
ARUBRA_DN3451_c0_g2_i1_2	0	0	0	0	0	0	70916000	0	0	0	0	0	0	181080	233630
ARHOMBI_DN5949_c0_g1_i1_6	2748400	2376100	2635200	4363100	3263700	250230	396460	1986400	11017000	7591200	7959800	6339000	9267800	7381400	3595500
ARUBRA_DN25800_c0_g1_i1_2	0	272280	381790	648790	0	0	0	0	0	0	0	0	22209000	31298000	16099000
ARUBRA_DN2944_c0_g2_i1_1	2623500	2746100	4085700	2196000	2013100	845530	1924300	2681600	6798400	0	0	0	11270000	15286000	18219000
ARUBRA_DN3626_c0_g1_i1_4	11857000	5130400	8958800	3641300	3366400	3673600	4133000	3678200	13138000	0	3509200	2984100	2535600	1124600	2300800
ARUBRA_DN1658_c0_g1_i1_3	799550	696700	692640	547220	470310	0	0	161750	1011200	0	0	0	26898000	18535000	19672000
ARUBRA_DN5995_c0_g2_i1_4	4686400	1598000	4666100	1407000	744840	996500	4887600	638310	8005200	10123000	10045000	19019000	448510	1603900	535850
ARUBRA_DN17764_c0_g1_i1_4	11804000	1205500	14157000	7288800	5467500	825220	6071200	3419800	5328900	6009200	0	7271600	0	217560	197490

ARUBRA_DN1399_c0_g1_i1_3	7619600	5157500	6452100	2897200	6450900	1430500	5007700	2479800	15156000	7002200	4963300	2189200	759640	780950	904440
ARHOMBI_DN5222_c0_g2_i1_6	2393600	3338400	4373400	4612500	1692400	688330	4191300	1627400	17027000	10270000	3515000	9138000	2278600	1225100	2467100
ARUBRA_DN4539_c0_g1_i1_1	5554000	5857000	7652300	4165100	3212400	1539000	3830400	4298400	5780100	2060700	2321100	0	4479200	8386100	9423400
ARHOMBI_DN6283_c0_g15_i1_6	12618000	9908100	10126000	2444300	2500200	1028700	1556600	3141700	4121700	0	3899800	2782700	4714500	4524700	5096100
ARUBRA_DN14209_c0_g1_i1_5	0	0	54268000	0	0	0	0	13595000	0	0	0	0	0	0	0
ARHOMBI_DN3140_c0_g1_i1_4	278470	3357700	288570	3400300	5317000	0	2641300	2503400	20159000	3854500	10346000	8277000	2538500	2249600	2438400
ARUBRA_DN3913_c0_g2_i2_6	0	0	0	0	282940	0	172310	597910	0	0	0	0	26970000	27240000	12334000
ARHOMBI_DN5648_c0_g1_i1_6	7183900	7621100	8504900	6727900	1203600	0	2003400	949290	11957000	0	0	8107900	4614200	4714600	3408300
ARHOMBI_DN9622_c0_g1_i1_2	0	0	0	319090	236130	0	188590	0	1397000	0	0	0	20225000	27075000	17164000
ARUBRA_DN4716_c0_g2_i1_1	2844100	1313900	2720600	2578000	898460	612060	881830	1294000	4012600	7734700	7570000	7324800	9094300	12127000	5390000
ARHOMBI_DN5959_c0_g1_i1_1	796020	1172200	625560	1625800	631330	0	193930	659910	0	499310	0	0	17280000	24689000	18078000
ARUBRA_DN7093_c0_g1_i1_1	11609000	7927800	13151000	7759300	1288800	337990	118530	0	22718000	0	0	0	384560	299130	239390
ARHOMBI_DN5317_c0_g1_i1_1	4388200	4971300	3534300	1942400	0	0	687720	646710	5539600	6119200	4683700	4166200	7962600	12689000	8226700
ARHOMBI_DN17873_c0_g1_i1_6	4199700	4747300	4616200	12018000	3493900	1497700	4654800	5460600	12974000	0	0	0	3306500	4324600	2682200
ARHOMBI_DN15725_c0_g1_i1_6	4050500	4050400	4428200	1616800	4509600	437290	3246100	1223400	6503300	0	1961100	0	7642600	12617000	11338000
ARUBRA_DN3134_c0_g2_i1_6	0	0	0	0	0	0	0	0	0	23144000	19287000	21124000	0	0	0
ARUBRA_DN8237_c0_g1_i1_4	7306600	5215700	6444900	5597900	2666800	352380	2991400	3486600	2455100	8085000	9303300	8520000	464350	286780	358410
ARHOMBI_DN5100_c0_g1_i1_3	4316800	3836000	5186400	1955700	4484600	0	1359400	8248600	7181100	7660600	11174000	4087300	888580	1440600	1520100
ARUBRA_DN4014_c0_g3_i1_3	6455400	0	5415800	11513000	3238900	2838000	0	5431200	21314000	0	0	0	3299400	0	3484400
ARUBRA_DN2976_c1_g1_i1_5	1080700	391010	832340	185780	1559300	0	531080	2640000	6496700	0	2591800	1152900	17545000	18165000	9342400
ARHOMBI_DN5768_c0_g1_i1_6	5118500	6687300	5204200	8708200	3186900	366760	4627700	4159200	8213700	0	0	0	8896600	5634300	1627900
ARUBRA_DN6355_c0_g1_i1_3	0	210170	98294	366630	0	149500	0	334010	905950	0	0	0	20291000	24042000	15993000
ARUBRA_DN2325_c0_g2_i1_5	4960400	6074700	7026700	3809700	0	201630	590210	621070	6849600	11754000	12578000	5816200	575660	604500	729000
ARHOMBI_DN9876_c0_g1_i1_3	483450	1101700	473630	328960	2597800	1715700	1459200	4790400	8009700	11372000	15771000	13558000	0	167080	0
ARUBRA_DN1667_c0_g1_i1_4	9860800	6633400	6926900	10256000	0	0	0	0	10527000	5403000	6873700	4870600	0	0	0
ARHOMBI_DN2829_c0_g1_i1_5	7947400	9456900	8265800	6885400	2029000	1041900	5502100	2755200	13835000	1861200	0	1376400	225020	0	0
ARUBRA_DN2825_c0_g1_i1_4	0	0	0	0	0	0	0	0	0	16512000	24692000	19787000	0	0	0

ARUBRA_DN1403_c0_g1_i1_4	0	0	0	142660	0	0	0	0	0	0	0	0	22390000	14046000	23917000
ARHOMBI_DN5577_c0_g1_i1_6	3454000	8460100	5756300	5951900	0	931380	1816200	2756600	11274000	7192400	2440800	6944200	544250	350360	1947800
ARHOMBI_DN20240_c0_g1_i1_6	12089000	15905000	13594000	5000200	1910100	1056700	2070800	0	7977100	0	0	0	0	0	0
ARUBRA_DN5882_c0_g1_i1_4	2157900	1936500	3045200	3049500	3848400	888670	3391700	11043000	28455000	0	0	0	418800	371090	892840
ARUBRA_DN4352_c0_g3_i1_5	5673900	5939400	7605600	1799800	2098400	803190	4530800	1901600	3146400	6738000	8454500	0	3359400	2859200	3734800
ARHOMBI_DN5282_c0_g1_i1_5	79689	118830	107850	159070	178890	0	0	0	463040	0	0	0	17966000	18899000	20583000
ARHOMBI_DN5162_c0_g1_i1_6	4085000	4637200	3016500	1697300	4292100	4133100	2039700	6671100	10564000	3242700	3428000	6789200	1648400	314510	1902600
ARHOMBI_DN1661_c0_g1_i1_5	761340	801460	269060	838700	239100	0	222240	0	0	2335100	3106000	2570300	16650000	16334000	12883000
ARUBRA_DN4331_c0_g1_i1_1	505880	518420	771450	2806800	518220	240370	1274200	2957700	10261000	14308000	10334000	7184200	1063400	1536200	2324900
ARHOMBI_DN5855_c0_g1_i1_3	0	0	0	0	177360	0	0	0	0	0	0	0	16709000	21582000	18079000
ARUBRA_DN19380_c0_g2_i1_4	1150600	1369400	1105800	1514000	0	0	0	1035000	3066800	4780200	6836300	5740800	9970300	8177100	11565000
ARUBRA_DN337_c0_g2_i1_5	6798600	7925400	7531400	4230400	4676200	2104200	7639100	3244900	8275200	1102700	0	1082400	381730	0	892900
ARUBRA_DN4321_c0_g2_i1_1	0	0	0	0	0	0	0	0	0	0	0	0	20083000	15580000	19947000
ARUBRA_DN1238_c0_g1_i1_5	0	0	0	0	0	0	0	124880	202800	0	0	0	14904000	18110000	22149000
ARHOMBI_DN4982_c0_g1_i1_1	440140	627170	197920	168210	541930	0	0	0	2804200	11735000	11804000	9093600	4963400	5054700	7399800
ARHOMBI_DN4944_c0_g1_i1_3	5052500	5615700	7461600	8701700	3793400	2711200	5209500	1022500	14469000	0	0	0	0	677110	0
ARUBRA_DN1865_c0_g1_i1_1	167380	183490	0	727960	0	0	0	0	0	0	0	0	17751000	23011000	12365000
ARUBRA_DN7363_c0_g1_i1_1	4849500	2717300	3997600	2851500	2145100	825380	0	779940	11222000	10772000	7527000	4583000	0	0	1443900
ARHOMBI_DN6048_c0_g1_i2_2	1249200	1295000	857060	2929900	0	0	1116800	779360	2761300	4233400	0	4102500	10800000	11497000	11291000
ARHOMBI_DN5714_c0_g2_i1_1	2641300	2058700	1890100	196480	867210	507700	0	673720	0	0	0	0	12213000	15678000	15249000
ARUBRA_DN11957_c0_g1_i1_4	2888100	2888000	3487200	4765600	1479700	1550600	2291200	3145700	17525000	0	0	0	2657300	3633400	4294400
ARUBRA_DN26276_c0_g1_i1_1	0	0	0	0	0	0	0	0	0	14930000	18703000	16611000	0	0	0
ARUBRA_DN5081_c0_g1_i1_6	2568200	3479800	2579400	2763500	0	0	2236100	1831100	6365600	14321000	0	0	7198700	6895300	0
ARUBRA_DN11233_c0_g1_i1_2	0	170490	0	0	0	0	0	0	0	0	49182000	0	0	0	0
ARHOMBI_DN5854_c0_g1_i1_3	2013200	1954300	5164100	4272000	978420	365800	4757100	2415500	12012000	6312200	7785700	267710	404890	217350	260620
ARHOMBI_DN6063_c0_g1_i1_4	4010400	1985100	3674000	2343700	2424400	0	2051400	1990300	10900000	10345000	4854900	4140600	0	0	449320
ARHOMBI_DN2876_c0_g2_i1_3	3177000	6393500	7076300	10191000	2844200	328930	3350800	2643000	8115100	0	0	0	750410	409150	3423600

ARUBRA_DN22470_c0_g1_i1_3	0	0	0	0	0	0	0	0	0	0	0	0	23421000	0	24909000
ARHOMBI_DN3221_c0_g1_i1_3	4295400	4228900	7293600	10473000	1321700	2139900	2601100	3423200	8835400	0	1726900	406660	430950	0	523960
ARUBRA_DN4811_c0_g2_i1_3	0	0	0	0	0	0	0	0	0	0	0	39347000	0	8339700	0
ARUBRA_DN1006_c1_g1_i1_5	7594300	7212300	9451700	3155900	2179600	1110800	2569500	2649200	2863500	4552300	0	4293700	0	0	0
ARHOMBI_DN15853_c0_g1_i1_3	4006000	3590700	2921300	2153500	0	0	1717500	0	1972300	12937000	4595800	13657000	0	0	0
ARHOMBI_DN15373_c0_g1_i1_5	91175	0	0	0	0	0	0	171440	0	0	0	0	15406000	10751000	20579000
ARUBRA_DN10605_c0_g1_i1_2	5362200	8972200	7694600	8241800	2061200	0	1933700	0	5627800	0	0	4713700	96535	770390	1164300
ARUBRA_DN10035_c0_g1_i1_2	0	0	0	0	0	0	0	0	46553000	0	0	0	0	0	0
ARUBRA_DN9263_c0_g1_i1_6	4240200	6362300	4750500	11279000	0	646070	0	0	18883000	0	0	0	0	0	0
ARUBRA_DN10200_c0_g1_i1_2	3235100	2686500	3024300	8566900	0	0	0	2594400	19158000	2245000	2201200	1101700	164990	192390	212130
ARUBRA_DN7621_c0_g1_i1_2	3401300	4292200	8417200	3699900	1383200	1282100	1736600	2580400	7477600	4860900	3320300	0	0	1384100	1438100
ARUBRA_DN1568_c0_g2_i1_4	1596000	995700	2307000	1250300	1960600	0	3645800	3604900	16006000	5380900	5847300	1403600	378050	0	759400
ARUBRA_DN3840_c0_g1_i1_2	297710	422890	301410	1705000	1525000	189130	1089500	2007000	1845600	0	0	0	8193000	13960000	13575000
ARUBRA_DN4409_c0_g1_i1_6	0	0	0	0	0	0	0	0	0	0	0	0	15266000	14867000	14942000
ARUBRA_DN4436_c0_g1_i1_5	6762200	5171500	6959600	3253600	4012500	2749700	6457200	5445700	4195600	0	0	0	0	0	0
ARHOMBI_DN5595_c0_g1_i1_6	8029200	9197900	9417000	3752400	2123800	561610	2419400	1369300	4323500	1070600	0	0	1824000	0	828850
ARUBRA_DN4227_c1_g2_i1_2	6054400	6516400	7715300	4483800	0	1877900	3361100	4069000	7386800	0	0	0	1172100	1105300	1158400
ARHOMBI_DN4937_c0_g3_i1_5	2846500	3289200	3272900	3506900	2769200	951840	3123400	0	8974500	0	0	0	4366700	4376000	6615600
ARUBRA_DN143_c0_g2_i1_4	0	0	0	0	0	0	0	0	0	0	0	0	12509000	13400000	18055000
ARHOMBI_DN21168_c0_g1_i1_5	2454300	2199700	4040300	3933200	4513700	1981400	4820000	6573800	11068000	0	0	0	0	1020600	1214500
ARHOMBI_DN4920_c0_g1_i1_6	0	0	0	18115	0	0	0	228660	0	5291100	3402500	1185700	12043000	9714200	11705000
ARUBRA_DN26231_c0_g1_i1_1	0	0	0	0	0	4881100	8130500	10629000	19915000	0	0	0	0	0	0
ARUBRA_DN304_c0_g1_i1_5	0	0	0	42957000	0	0	0	0	0	0	0	0	0	0	0
ARUBRA_DN3080_c0_g1_i1_1	85318	0	0	0	0	0	0	0	0	0	0	0	12073000	13898000	16645000
ARUBRA_DN10173_c0_g1_i1_6	1383700	1455400	1438900	290930	1353100	920810	1561800	0	3414000	1829100	2249300	0	9572700	13899000	3195200
ARHOMBI_DN1063_c0_g2_i1_1	45559	165700	134810	281770	0	0	0	525480	329080	367400	0	0	10964000	15043000	14110000
ARHOMBI_DN4718_c0_g1_i1_2	4006000	4430700	5727300	4435500	0	1136300	3074700	1947800	10586000	1675400	3346900	866790	0	648620	0

ARUBRA_DN4883_c0_g1_i1_3	0	0	277190	0	0	0	0	0	0	0	0	0	14681000	12763000	13403000
ARUBRA_DN4330_c0_g2_i1_4	6764500	6732200	5478700	5040100	0	0	4022900	4463100	7607100	0	0	0	0	0	829740
ARHOMBI_DN5519_c0_g1_i2_6	368560	299850	0	1747700	0	0	0	2739100	4474300	14718000	16564000	0	0	0	0
ARHOMBI_DN5140_c0_g1_i2_1	0	0	0	0	0	0	0	0	0	0	0	0	15905000	4985100	19927000
ARHOMBI_DN6061_c0_g1_i1_5	1739800	2232400	1646500	2346600	0	683560	0	922050	2756000	9370300	9561200	8726500	316410	122470	147740
ARHOMBI_DN1760_c0_g1_i1_4	656220	983680	546490	587380	251020	0	0	0	0	0	0	0	12999000	11562000	12817000
ARHOMBI_DN16482_c0_g1_i1_1	605810	522720	1171600	679750	431590	0	2580300	1726500	4617800	5121100	7274000	5371600	3026300	3639200	3625600
ARUBRA_DN4585_c0_g2_i2_2	2752900	2750800	2966800	758710	2280300	943270	1511000	5449900	6858400	1936400	2249500	2245500	2488400	2766500	2212200
ARUBRA_DN4349_c0_g1_i1_5	0	83821	84536	0	0	0	0	0	0	0	0	0	14798000	11033000	14104000
ARHOMBI_DN5054_c0_g1_i1_1	891790	336250	693940	826340	0	0	0	1564600	4002800	5226800	3058400	2604100	7197500	6235500	7460200
ARUBRA_DN18216_c0_g1_i1_2	2208900	2761700	2316100	1867200	1312400	1275200	651700	2325700	14931000	2594700	359380	2450100	1644600	1039100	2353500
ARHOMBI_DN3394_c0_g1_i1_1	2780900	2605000	3073600	5643200	4204800	591630	3313100	2171300	14459000	0	875860	0	342930	0	0
ARHOMBI_DN20977_c0_g1_i1_5	2384300	0	0	0	0	368180	0	0	0	11815000	13231000	11149000	0	476200	414760
ARHOMBI_DN6641_c0_g2_i1_5	3774400	2076600	759800	4538600	973620	0	1000700	0	1920400	2879800	0	2963700	5534500	6905100	6264400
ARHOMBI_DN5627_c0_g2_i1_2	6790000	6839300	7753700	1939700	2364900	1158500	2773200	1060100	1642500	619890	0	0	1381000	1658900	3222700
ARHOMBI_DN2280_c0_g1_i1_6	7533900	8052900	7136000	2696500	2072300	1115900	2819000	0	4215000	0	0	0	1869800	0	1611000
ARHOMBI_DN5380_c0_g1_i1_2	0	40586	0	170230	0	0	0	0	0	0	0	0	14086000	12056000	12595000
ARUBRA_DN6789_c0_g1_i1_6	0	0	0	414680	0	0	0	0	38477000	0	0	0	0	0	0
ARUBRA_DN17519_c0_g1_i1_3	9368700	7981500	5973100	255310	0	768070	4926800	0	0	0	0	0	2349400	2405400	4149800
ARHOMBI_DN940_c0_g1_i1_3	309930	230690	255910	0	0	0	0	202600	379820	0	0	0	12854000	15544000	8397300
ARHOMBI_DN2154_c0_g1_i1_4	902580	262040	1727300	757510	2655300	1823200	2198700	7697200	12914000	0	0	0	1523300	3084400	1842000
ARHOMBI_DN16490_c0_g1_i1_2	193230	213000	146490	242800	0	0	0	0	0	0	0	0	11370000	14071000	11092000
ARHOMBI_DN6177_c0_g2_i1_3	9690400	0	21859000	5633900	0	0	0	0	0	0	0	0	0	0	0
ARHOMBI_DN24802_c0_g1_i1_2	2625900	2628200	2676000	1534300	0	0	0	0	5411500	6303900	6443100	9128700	0	0	231830
ARUBRA_DN18898_c0_g1_i1_2	0	0	0	0	360530	140490	580180	3555500	5380300	8459100	1602100	12509000	0	1933900	2184500
ARUBRA_DN10159_c0_g1_i1_5	0	59634	10030	39886	0	0	0	0	0	0	0	0	15319000	9868600	11315000
ARUBRA_DN147_c0_g1_i1_6	8413800	7623400	9241900	3943800	2296300	182990	2331600	0	192040	0	0	0	1042000	154020	1075800

ARHOMBI_DN5600_c0_g1_i1_6	4239200	3578500	4754900	4574700	302150	0	1224800	877640	4010000	1129700	0	5235700	1365700	2354500	2728600
ARUBRA_DN18800_c0_g2_i1_4	3865500	4492700	5280500	4414100	1191000	758060	3407900	2984900	1104200	0	0	0	1825900	3205900	3380500
ARHOMBI_DN4017_c0_g1_i1_1	2036600	3711700	5230200	1115100	1097300	2085300	3625700	2805600	5980700	0	0	0	2544100	3219300	2452400
ARUBRA_DN4582_c0_g1_i1_5	2145100	0	178260	687090	0	0	2145200	2785100	2821800	9825900	0	14433000	0	734850	0
ARUBRA_DN1643_c0_g2_i1_1	3706900	2717300	2978700	2023000	671210	218120	598080	1359000	8411200	4138300	4450900	4025500	0	171940	0
ARUBRA_DN5911_c0_g1_i1_4	1553800	1569700	1989800	1561800	4135900	815080	3888400	2628500	5788800	3185200	4322100	3163200	334470	0	449920
ARUBRA_DN3180_c0_g1_i1_4	3469100	2590900	4057700	3287600	0	0	2255500	1344400	10521000	4167500	0	2308700	525950	313420	464490
ARHOMBI_DN12129_c0_g1_i1_2	4687900	3671300	4792600	3343200	0	0	1512700	1450500	2176000	3363300	3067500	7227200	0	0	0
ARHOMBI_DN4888_c0_g1_i1_6	474460	529400	994780	740740	0	0	0	97809	2916400	0	0	0	10308000	10465000	8553500
ARUBRA_DN6309_c0_g1_i1_5	110790	126870	77979	0	184700	0	234630	0	0	0	0	0	6097100	15792000	12362000
ARUBRA_DN3885_c0_g1_i1_5	9237400	6279400	11483000	7915800	0	0	0	0	0	0	0	0	0	0	0
ARHOMBI_DN667_c0_g2_i1_1	0	0	0	0	0	0	0	0	0	0	0	0	12141000	10460000	12251000
ARUBRA_DN18848_c0_g1_i1_3	0	0	0	0	0	0	0	0	0	6455300	14565000	13762000	0	0	0
ARUBRA_DN4554_c0_g1_i1_4	5494600	1190900	12099000	1064900	1416500	1241300	0	0	4944100	0	0	0	2652600	2348900	2230100
ARUBRA_DN9538_c0_g1_i1_1	3621000	4383000	5438000	3359800	1560300	0	1715700	1725000	5646900	0	2612700	2132500	725930	862450	823450
ARHOMBI_DN4282_c0_g1_i1_5	0	0	0	0	0	0	100780	0	0	0	0	0	13405000	13155000	7663200
ARHOMBI_DN22144_c0_g1_i1_6	3170800	3620000	4235700	3659800	1970900	503200	1369500	1195600	7568300	4180800	0	0	0	1685800	1076600
ARUBRA_DN3174_c0_g1_i1_1	0	0	0	0	0	0	0	0	0	0	0	0	10274000	9287500	14377000
ARHOMBI_DN19334_c0_g1_i1_6	3845400	3804000	5314300	5072500	2136300	1276600	2502700	2532600	3039600	0	0	0	1357300	1164000	1482900
ARHOMBI_DN8571_c0_g1_i1_5	6389600	4955900	6290600	2328400	3367200	1492600	664090	1246000	0	0	0	0	753760	3174300	2825200
ARUBRA_DN4684_c0_g2_i1_5	102650	0	65716	363350	984280	0	0	1378300	448210	0	0	0	9642300	8204400	12096000
ARHOMBI_DN1355_c0_g2_i1_2	1079000	8432300	892250	1116500	277400	131010	298400	0	8011000	12245000	0	0	223180	219770	267700
ARUBRA_DN15624_c0_g1_i1_4	3948100	5116200	1422000	672360	0	1429100	2570900	7713600	2619300	0	0	0	2765200	2254400	2592700
ARUBRA_DN2409_c0_g2_i1_4	71936	110150	79070	166130	0	0	0	0	272320	0	0	0	14179000	10293000	7692900
ARHOMBI_DN5337_c0_g1_i1_4	0	111170	0	0	0	0	0	0	0	0	0	0	11186000	7776100	13553000
ARHOMBI_DN6227_c0_g1_i4_5	7497000	6090400	3744000	4510600	0	0	1079700	0	7298500	0	0	0	0	1596600	712470
ARHOMBI_DN4176_c0_g1_i1_6	2611000	2934800	3591500	1876000	2604900	1068400	3139200	2092700	8401900	2966400	0	0	386310	345860	468920

ARUBRA_DN22728_c0_g1_i1_2	2332800	2118200	1192100	1604100	1781000	169000	957750	1042000	3151700	1030500	0	0	5649500	5478200	5944900
ARUBRA_DN18837_c0_g1_i1_3	1974100	2512600	1982500	743450	623660	1388600	3987000	1002700	5928000	4891500	5067100	2042900	0	0	0
ARHOMBI_DN6122_c0_g1_i1_5	0	81881	0	0	0	0	0	0	0	0	0	0	15332000	8323800	8292200
ARHOMBI_DN6266_c0_g2_i3_6	9432200	9055100	7571400	1734200	183170	468220	650080	190620	2736100	0	0	0	0	0	0
ARUBRA_DN4148_c0_g1_i1_5	1533500	4144200	5928500	5305100	560360	1122700	973160	1114700	1706900	0	4340700	4523400	0	0	502020
ARHOMBI_DN15512_c0_g1_i1_6	883510	1109200	1195700	1907100	0	329190	463210	0	1261200	15001000	4357300	3895600	509690	214080	259290
ARHOMBI_DN6104_c0_g1_i1_6	3911500	3766100	2444400	1767500	3079500	1100100	3091300	0	5587500	0	0	0	5267100	414810	489750
ARUBRA_DN4076_c0_g1_i1_5	0	0	0	0	0	0	0	0	0	0	0	0	266910	16024000	14617000
ARHOMBI_DN6065_c0_g3_i2_6	1292500	0	0	1483700	0	0	0	269100	0	6963000	11376000	9059500	0	234060	148320
ARUBRA_DN4576_c0_g1_i1_2	1108800	670840	960930	2466100	1891000	0	2592700	4808700	7145800	0	3444000	3006000	1371200	1262600	0
ARHOMBI_DN3224_c0_g1_i1_2	6540300	6864500	8354500	3314900	0	0	0	0	4930300	0	0	0	258820	0	387300
ARHOMBI_DN6597_c0_g1_i1_6	5125200	4727200	5559300	1665600	0	82476	0	875390	6537800	1044700	3331600	529630	0	0	1111000
ARUBRA_DN16167_c0_g1_i1_3	2883800	3950100	2948500	3461900	0	0	0	0	4737900	3892100	4255600	2947100	588990	0	729560
ARHOMBI_DN4850_c0_g1_i1_5	512800	289360	392820	396520	0	0	378120	0	0	0	0	0	8201900	7995400	12177000
ARHOMBI_DN641_c0_g1_i1_6	1030200	843150	824060	1583700	0	182080	212000	688170	3072200	0	0	0	3356800	7144000	11321000
ARUBRA_DN9455_c0_g1_i1_2	1046600	817510	921810	1405700	2183000	494870	1152100	1016500	2469300	6209800	6763900	3679900	781890	0	989300
ARUBRA_DN4936_c0_g1_i1_3	5631400	4995500	1194700	3124500	1831900	317960	1105300	2192200	605720	0	1333600	2853100	2177500	1327300	1235000
ARUBRA_DN11512_c0_g1_i1_6	2392200	1998700	2687200	2208400	3335500	2535500	4715300	2202000	6448100	0	1219900	0	0	0	0
ARHOMBI_DN13390_c0_g1_i1_2	210470	194600	103320	87757	2625600	1307100	2942100	3132700	3959700	4870600	5358300	4835400	0	0	0
ARUBRA_DN13261_c0_g1_i1_6	3911500	3851200	4593100	2166900	2681600	1036000	2091800	2519300	4489100	0	0	0	731490	633510	723370
ARUBRA_DN7781_c0_g1_i1_6	0	0	29171000	0	0	0	0	0	0	0	0	0	0	217930	0
ARHOMBI_DN15181_c0_g1_i1_3	272510	248160	270400	38113	0	0	0	0	0	0	0	0	10908000	12714000	4746700
ARUBRA_DN4108_c0_g1_i1_4	0	0	0	0	0	0	0	0	0	0	19204000	9982300	0	0	0
ARHOMBI_DN2905_c0_g1_i1_2	4551700	4785000	5207000	1888800	1528700	0	1935200	217480	4675400	0	0	0	1020300	2121800	1233700
ARUBRA_DN4026_c0_g1_i1_5	469260	376210	423250	509120	0	0	0	0	0	490930	926270	0	6662400	7001100	12128000
ARHOMBI_DN6235_c0_g1_i4_4	0	0	0	0	0	0	0	0	0	0	0	0	11225000	6647900	10935000
ARUBRA_DN25569_c0_g1_i1_3	4016100	5154300	3694500	1757200	3169800	1513400	0	2560500	3808400	0	0	0	1312800	0	1651300

ARUBRA_DN4711_c0_g1_i2_5	1369300	1157500	832500	1457600	342720	0	0	0	2889800	0	0	0	4962200	10252000	5295400
ARUBRA_DN2993_c0_g1_i1_2	0	0	0	5947700	0	0	0	6877100	15515000	0	0	0	0	0	0
ARUBRA_DN3089_c0_g2_i1_6	0	0	0	0	0	0	0	0	0	0	0	0	8314100	10236000	9779800
ARUBRA_DN8636_c0_g1_i1_1	0	0	0	0	1147700	0	276810	0	2000500	4286800	5546700	4957100	2216400	7465900	383800
ARHOMBI_DN4932_c0_g1_i1_1	0	0	0	0	0	0	0	0	0	0	0	0	11257000	11190000	5615600
ARUBRA_DN5237_c0_g1_i1_6	0	0	0	0	0	0	0	0	0	0	0	0	8557000	9649300	9394500
ARUBRA_DN4540_c0_g1_i2_6	0	0	0	0	0	0	0	0	0	0	0	0	8470400	11935000	7086900
ARUBRA_DN830_c0_g1_i1_6	4794300	4124300	2288500	2887700	0	0	3284600	1969700	2976800	0	0	0	1021300	1910800	2184700
ARHOMBI_DN5602_c0_g2_i1_1	0	0	0	0	0	0	0	0	0	0	0	0	9749000	8126900	9489000
ARHOMBI_DN6202_c0_g1_i2_4	4543200	4059000	2531500	958650	1820500	586900	1960300	0	211650	0	0	0	5448200	1097700	4020200
ARUBRA_DN2984_c0_g1_i2_6	701820	0	0	0	5083300	0	3044100	14488000	3896300	0	0	0	0	0	0
ARUBRA_DN6258_c0_g1_i1_5	1453000	1156600	1410400	1836200	435610	0	0	809880	2545400	0	0	918980	4572900	4307600	7454500
ARUBRA_DN1424_c0_g2_i1_3	0	0	0	0	0	0	0	0	0	0	0	0	12374000	6730400	7773200
ARHOMBI_DN6163_c0_g1_i2_6	0	0	0	62610	0	0	0	0	0	0	0	0	10433000	8738000	7517000
ARHOMBI_DN2855_c0_g1_i1_2	0	0	0	0	0	0	0	0	0	0	0	0	7957900	3655500	14881000
ARHOMBI_DN4907_c0_g3_i3_4	2302400	0	0	0	0	1704800	0	0	9755200	0	0	12677000	0	0	0
ARUBRA_DN20327_c0_g1_i1_5	3952200	607050	2461300	1737600	773600	0	0	0	5130800	257900	0	0	3734900	2415800	4800900
ARHOMBI_DN3004_c0_g1_i1_6	0	0	0	0	0	0	0	0	0	0	0	0	4092200	10962000	10665000
ARUBRA_DN20570_c0_g1_i1_4	2236500	2115600	2784500	1728000	2132100	0	1585100	1288100	6694000	1723200	1478400	0	856720	477050	530740
ARHOMBI_DN4754_c0_g1_i1_5	0	0	0	0	0	0	0	0	0	0	0	0	7510600	9652900	8425500
ARUBRA_DN2276_c0_g1_i1_5	0	0	0	0	0	63764	0	0	968250	7035900	9899200	7592400	0	0	0
ARHOMBI_DN6055_c0_g1_i1_6	106890	114690	0	0	0	0	0	0	0	0	0	0	7045500	8881200	9396900
ARHOMBI_DN1472_c0_g1_i1_6	3093500	5120000	4068200	1056500	1150900	0	1400800	784650	0	1659700	0	1730500	2003300	1777000	1698300
ARUBRA_DN2452_c0_g2_i1_2	384810	0	232120	208560	0	0	172780	272800	0	0	0	0	8194700	7297500	8741600
ARUBRA_DN4724_c0_g1_i1_5	3484000	3561300	3496100	2091200	1139900	1701100	0	812800	4604700	1356900	1680300	0	635670	862050	0
ARHOMBI_DN7816_c0_g1_i1_3	2396900	6170100	5202500	752810	1881400	268140	2119400	0	3336800	0	0	0	562380	547650	2100500
ARHOMBI_DN4989_c0_g1_i1_1	128920	158800	0	97659	0	0	0	0	0	0	0	0	9742000	9406500	5031200

ARUBRA_DN8722_c0_g1_i1_2	2126200	2045800	2117000	6263800	685180	0	0	0	11314000	0	0	0	0	0	0
ARUBRA_DN19739_c0_g2_i1_3	1923100	1897800	2671800	2648700	2024400	0	1517400	2027200	4300000	4572800	0	0	499840	455900	0
ARUBRA_DN5052_c0_g1_i2_2	1701600	1627200	2410300	495280	2276800	781210	2303100	1036300	1960900	3016200	3092800	2737400	360970	312370	321530
ARHOMBI_DN19084_c0_g1_i1_5	763790	1047500	1145400	897040	0	494530	0	968720	0	6830600	5862300	5721000	256390	351010	0
ARHOMBI_DN4789_c0_g1_i1_3	176500	138640	0	197380	0	0	0	0	0	0	0	0	7569500	5333600	10873000
ARUBRA_DN14251_c0_g1_i1_2	1808600	2018200	869310	0	1677400	798830	1351100	0	1858800	4149700	4595800	4004000	406260	566220	163380
ARUBRA_DN1618_c0_g1_i1_5	3752300	4252800	3836300	4130600	0	1630800	0	0	2622900	0	0	0	1341300	944870	1726100
ARHOMBI_DN277_c0_g1_i1_2	1401200	1162200	1638300	767680	1573400	857030	1809600	1793900	2831900	5015000	5271800	0	0	0	0
ARUBRA_DN4780_c0_g3_i1_6	43664	0	0	0	0	202270	384270	0	782780	0	0	0	5244700	8666500	8723800
ARHOMBI_DN4043_c0_g1_i1_2	381560	423890	296000	814580	0	213390	0	0	0	0	1081300	0	9781800	8812800	2210900
ARUBRA_DN4785_c0_g1_i3_6	2716700	1126900	3575600	4930200	798560	0	1187300	0	6700200	0	0	0	1374500	112990	1475200
ARHOMBI_DN5765_c0_g1_i1_5	4674200	4982700	5517100	1143700	1058300	0	4467100	868640	1203800	0	0	0	0	0	0
ARHOMBI_DN4921_c0_g1_i1_3	682530	766860	708440	1022200	0	0	0	0	0	0	0	0	7483600	6642600	6564100
ARUBRA_DN4799_c0_g1_i1_4	1198600	1457700	2105300	635840	197140	0	0	0	0	4727500	4547100	8956800	0	0	0
ARUBRA_DN2524_c0_g1_i1_6	0	0	0	0	0	0	0	0	0	0	0	0	8133000	7994700	7592300
ARUBRA_DN830_c1_g1_i1_2	5155500	1600800	1626200	1020600	0	823040	0	0	0	5639100	6274100	0	598570	0	899780
ARUBRA_DN5581_c0_g1_i1_2	1502000	1251400	2698800	733860	1679400	555460	2126700	1849800	238430	0	0	0	2506200	3455000	5024500
ARUBRA_DN18248_c0_g1_i1_4	4589900	4938500	4693300	2592200	0	453130	0	0	4301700	0	1610400	0	208360	214390	0
ARHOMBI_DN15811_c0_g1_i1_3	0	0	0	0	0	0	0	0	0	0	0	0	9926100	3941300	9717700
ARHOMBI_DN5696_c0_g1_i1_3	0	0	0	0	0	0	0	0	0	0	0	0	5611400	8412100	9451000
ARHOMBI_DN6039_c0_g1_i3_2	207400	166480	218070	205130	0	0	0	0	0	0	0	351210	6290600	9711800	5975900
ARHOMBI_DN787_c0_g2_i1_5	5257800	4318100	4216000	1538600	2433300	513400	0	921910	3885800	0	0	0	0	0	0
ARUBRA_DN19178_c0_g1_i1_4	0	0	0	0	0	0	0	0	0	0	0	0	10016000	6329800	6725700
ARUBRA_DN202_c0_g1_i1_3	0	0	0	0	308760	0	0	0	0	0	0	0	7413600	8687500	6604900
ARHOMBI_DN5950_c0_g1_i1_2	0	0	0	0	18493000	0	0	0	0	0	0	0	0	0	4345600
ARHOMBI_DN2248_c0_g1_i1_5	0	0	0	0	0	0	0	0	0	0	0	0	8944800	8189800	5666900
ARUBRA_DN11411_c0_g1_i1_4	0	0	0	69689	0	0	0	0	0	0	0	0	6252300	7762600	8585100

ARUBRA_DN17927_c0_g1_i1_6	0	0	0	0	0	0	0	0	0	0	0	0	9574100	7765300	4944600
ARHOMBI_DN5374_c1_g1_i1_6	0	0	0	0	0	0	0	0	0	0	0	0	7387700	6677100	8217000
ARUBRA_DN763_c0_g1_i1_4	0	0	0	0	0	0	0	0	0	0	0	0	12067000	6787100	3276700
ARUBRA_DN2819_c0_g1_i1_3	1714700	2160400	2083600	147890	897010	0	1031700	0	372960	9769500	0	3838100	0	0	0
ARUBRA_DN4688_c0_g1_i1_1	0	119340	0	56078	0	0	0	0	0	0	0	0	5300700	5990400	10547000
ARHOMBI_DN4778_c0_g1_i1_3	405750	569900	636120	607000	406230	139240	538690	928510	2148800	3365000	1669200	3037600	1755400	2973800	2562800
ARHOMBI_DN777_c0_g1_i1_1	181120	148500	6474600	7012300	1791800	447990	1978300	407170	2017700	0	0	0	0	658050	622710
ARHOMBI_DN18025_c0_g1_i1_2	896220	732060	629230	979190	1042300	383410	1020400	1686200	3413500	1886500	5562200	3239300	0	106580	153470
ARHOMBI_DN5069_c0_g2_i1_3	0	0	0	0	0	0	0	0	0	0	0	0	6019100	8457800	7222300
ARUBRA_DN4080_c0_g1_i1_6	0	0	0	0	0	0	0	435210	0	6848100	7474300	6785200	0	0	0
ARUBRA_DN19946_c0_g1_i1_3	125120	162840	0	0	0	114100	259020	0	0	0	0	0	7179200	5618000	8074300
ARUBRA_DN4991_c0_g1_i1_6	1973300	1893400	2670400	1151200	1127000	0	1085000	751210	1006100	1290300	1261100	1222100	490050	3783500	1822200
ARHOMBI_DN17727_c0_g1_i1_3	0	0	0	0	0	0	0	0	0	0	0	0	7429000	5844200	8227800
ARHOMBI_DN4645_c0_g2_i1_4	169400	0	121400	254390	0	0	0	192130	0	0	0	0	7144500	4284100	9189000
ARHOMBI_DN2411_c0_g1_i1_5	0	0	0	0	0	0	0	0	0	0	0	0	5075200	5323500	10868000
ARUBRA_DN4641_c0_g2_i1_2	255310	154640	193330	193890	0	0	0	0	1236300	0	0	0	3315400	10101000	5631100
ARHOMBI_DN1922_c0_g1_i1_3	2027000	3733100	2360500	534250	842590	1336500	3031000	2216900	4403400	0	0	0	285240	0	276300
ARUBRA_DN17998_c0_g1_i1_1	0	0	0	0	0	0	0	0	0	0	0	0	3320200	5171200	12553000
ARHOMBI_DN5775_c0_g1_i1_2	50722	193980	513240	536980	231270	0	347610	0	1437200	0	0	11266000	2093900	1981100	2357200
ARUBRA_DN5604_c0_g1_i1_1	0	0	0	0	135160	0	0	150240	0	0	0	0	7115500	6348500	7238100
ARUBRA_DN4856_c0_g1_i2_3	8799700	3842700	4265300	3787200	0	0	0	0	0	0	0	0	0	0	278890
ARHOMBI_DN478_c0_g2_i1_6	3325800	3390000	2451600	933590	303870	660380	1351400	196200	0	419700	2367800	0	2605900	1046000	1881600
ARHOMBI_DN5072_c0_g1_i1_5	0	0	0	0	0	0	0	0	0	0	0	0	7010000	5644200	8203000
ARUBRA_DN162_c0_g1_i1_1	2385300	2380900	1959800	2008200	2058900	1750200	1487900	1941600	3392400	0	0	0	318800	691010	421280
ARHOMBI_DN5339_c0_g1_i1_5	0	0	0	0	0	0	0	0	0	0	0	0	7755700	6731500	6278400
ARHOMBI_DN5797_c1_g1_i2_6	1466500	1517700	1773100	973040	529990	0	826910	0	1540900	6069300	3389400	2156200	0	218400	249130
ARHOMBI_DN5453_c0_g1_i1_1	0	0	0	0	0	0	0	0	144650	0	0	0	5752600	7179000	7515300

ARHOMBI_DN4158_c0_g1_i1_4	1238400	1639000	1694200	844990	0	0	0	0	1273700	2417100	3220800	733250	3444700	2340600	1633400
ARHOMBI_DN3362_c0_g1_i1_5	0	0	0	0	0	0	0	0	0	0	0	0	8526800	7977300	3902500
ARUBRA_DN20595_c0_g1_i1_3	0	0	0	140950	0	0	158430	0	0	0	0	0	5658700	5916300	8524100
ARUBRA_DN427_c0_g2_i1_5	382760	326750	200840	340240	1500200	552280	1869900	817830	610440	1156200	0	0	1523400	4772300	6269100
ARHOMBI_DN19156_c0_g1_i1_5	596900	666310	313310	274940	0	1569900	2506700	0	6079500	2514700	2623100	2463300	0	0	628570
ARHOMBI_DN3290_c0_g1_i1_1	245290	0	4286500	0	2300700	0	2657000	549720	0	4852300	0	4634000	249840	0	328650
ARUBRA_DN2245_c0_g2_i1_4	0	0	0	0	0	0	0	0	0	0	340070	0	5441000	8632200	5680400
ARHOMBI_DN19115_c0_g1_i1_6	0	0	0	0	0	0	0	0	0	0	0	0	4493200	7848100	7645000
ARHOMBI_DN5316_c0_g1_i1_2	0	85572	90071	0	0	0	0	0	0	0	0	0	7671800	6947500	5159100
ARUBRA_DN8606_c0_g1_i1_1	2751900	2743800	2719600	852710	0	0	316040	406840	0	1141800	4853500	3078100	1068700	0	0
ARHOMBI_DN5381_c0_g1_i1_4	2012700	2417600	1160600	2795500	0	0	0	0	0	0	0	0	3639000	5759300	2058100
ARUBRA_DN43_c0_g2_i1_6	1487900	1316700	2024000	1389700	0	3460700	4091300	3534400	2538100	0	0	0	0	0	0
ARHOMBI_DN3956_c0_g1_i1_1	4868000	4090100	4922000	5535700	0	0	0	183710	0	0	0	0	0	0	0
ARUBRA_DN609_c0_g1_i1_6	0	0	0	0	244130	0	0	0	0	0	0	0	6598900	6226300	6526100
ARUBRA_DN2_c0_g1_i1_4	0	0	0	0	0	0	0	0	0	0	0	0	5417900	7698100	6474900
ARUBRA_DN1844_c0_g1_i1_1	0	0	0	0	0	0	0	0	0	0	0	0	6053300	8238300	5290200
ARHOMBI_DN3609_c0_g2_i1_1	1240100	2934900	4078600	995330	2116100	0	1292900	1511400	2134300	0	0	0	673040	1056300	1492800
ARHOMBI_DN3185_c0_g1_i1_4	0	0	0	0	0	0	0	0	0	0	0	0	5390500	7545600	6389000
ARUBRA_DN24520_c0_g1_i1_2	0	0	0	0	0	0	0	0	0	0	0	0	6955600	6327100	5998900
ARUBRA_DN4994_c0_g1_i2_3	3205400	4105900	4604700	2297000	0	696170	0	0	0	2089500	0	1735800	497920	0	0
ARUBRA_DN19637_c0_g1_i1_2	316190	323800	803660	272460	1233700	214380	2633500	523950	2447000	393650	590840	924920	3695400	3199100	1623300
ARUBRA_DN18881_c0_g1_i1_2	0	0	0	0	0	0	0	0	0	0	0	135430	5457700	7716900	5843200
ARHOMBI_DN5176_c0_g1_i1_6	500310	620550	405530	4404100	1885400	608170	2211700	1898800	6418200	0	0	0	0	0	0
ARUBRA_DN13710_c0_g1_i1_3	0	0	0	0	1509300	0	1801700	871690	7586100	0	2322600	2327700	0	1239900	1238000
ARUBRA_DN18453_c0_g1_i1_3	2104100	2486700	2883700	2535600	872780	1133500	276130	1313800	4829200	0	0	0	0	0	353060
ARHOMBI_DN5422_c0_g1_i1_5	504510	926640	455170	557660	0	141870	0	0	0	0	0	0	6487700	2935100	6748900
ARHOMBI_DN15946_c0_g1_i1_6	0	0	0	0	0	0	0	0	0	0	8174000	10575000	0	0	0

ARHOMBI_DN4424_c0_g1_i1_1	60894	45273	0	175470	0	0	0	228150	0	0	0	0	4431500	6112500	7586100
ARUBRA_DN3570_c0_g1_i1_3	281930	221990	323800	182290	625870	121510	0	0	0	0	0	0	4384400	8242000	4250000
ARUBRA_DN2080_c0_g1_i1_2	202260	266960	498720	813480	0	0	1211800	0	2943200	5771000	0	3185800	1003200	1609600	1085000
ARUBRA_DN2407_c0_g1_i1_1	0	0	0	0	0	0	0	0	0	0	0	0	5840700	4212800	8432800
ARUBRA_DN6096_c0_g1_i1_5	0	0	242870	135910	214960	0	198900	0	0	0	0	0	3340200	3376100	10941000
ARUBRA_DN5878_c0_g1_i1_4	0	0	66232	0	0	0	0	254240	0	0	0	0	5302700	7531600	5187900
ARUBRA_DN462_c0_g2_i1_3	504670	589840	495170	241820	1134300	248660	0	0	0	4275500	7022200	3766400	0	0	0
ARHOMBI_DN8302_c0_g1_i1_6	0	0	0	0	0	0	0	0	0	0	0	0	8660200	4932400	4640100
ARHOMBI_DN6204_c0_g1_i2_1	0	0	0	0	0	0	0	0	0	0	0	0	4639100	6073500	7505500
ARUBRA_DN935_c0_g1_i1_4	0	0	0	0	0	0	0	0	0	0	0	0	4655500	9123700	4403300
ARHOMBI_DN6019_c0_g1_i1_2	43088	50269	0	0	0	0	0	0	402540	0	0	0	3705400	7640300	6327100
ARHOMBI_DN4558_c0_g2_i1_1	0	0	0	0	0	0	0	0	0	0	0	0	6132500	5469800	6561900
ARUBRA_DN6207_c0_g1_i1_4	0	90489	0	0	0	0	0	0	0	0	0	0	4364300	7625300	6023300
ARUBRA_DN17796_c0_g1_i1_2	0	0	0	0	0	0	0	0	0	0	0	0	6262200	6034900	5680200
ARUBRA_DN4392_c0_g3_i1_4	2311800	2780300	2555000	410470	0	0	1126600	475610	1406300	0	0	0	1162400	3096000	2570000
ARUBRA_DN2862_c0_g2_i1_5	158660	108480	93844	0	0	0	0	0	0	0	0	0	7155500	6708000	3627300
ARUBRA_DN18480_c0_g1_i1_4	0	0	0	0	0	0	0	0	0	0	0	0	6421700	5506500	5880500
ARHOMBI_DN6139_c0_g1_i1_4	60586	62101	0	35572	0	0	0	0	146020	0	0	0	5397000	5120600	6916600
ARHOMBI_DN5986_c0_g1_i2_6	1467100	1593900	1797500	3108500	0	161920	512830	1279200	4534000	0	0	0	236140	1292600	1690500
ARHOMBI_DN20063_c0_g1_i1_4	388820	328120	115450	146090	0	0	0	0	0	5250000	6136000	5234700	0	0	0
ARHOMBI_DN5759_c0_g1_i1_6	43481	36206	0	16029	278960	0	0	0	0	0	0	0	3112900	10904000	3203400
ARHOMBI_DN3534_c0_g1_i1_3	0	0	0	0	0	0	0	0	159130	0	0	0	8600400	5526000	3082100
ARUBRA_DN11326_c0_g1_i1_6	607870	1510300	706570	1548100	0	496910	0	0	0	3857900	4921200	3504300	0	191470	0
ARUBRA_DN2423_c0_g1_i1_1	55748	0	0	0	0	0	0	0	0	0	0	0	7509600	2716500	7006600
ARUBRA_DN3020_c0_g1_i1_1	770250	596500	1166500	289410	970950	0	0	0	0	0	0	0	4826100	2637200	5864400
ARUBRA_DN12109_c0_g1_i1_6	127950	192090	163590	80239	4038000	1252700	4048800	971870	2094200	0	0	1252300	735600	939670	1120300
ARUBRA_DN16721_c0_g1_i1_2	1019900	838700	730620	1104800	801990	187890	447700	1015500	644640	4387100	1556100	3787500	0	197360	176230

ARUBRA_DN4896_c0_g1_i1_4	500200	1129900	362680	354980	454320	420470	1002400	647130	3331700	4175900	0	3636100	0	788380	0
ARHOMBI_DN347_c0_g1_i1_1	293110	443590	605510	1284000	0	0	392470	0	2749400	0	5849200	0	1648300	1678300	1734400
ARUBRA_DN3353_c0_g1_i1_5	727910	669780	1300200	505340	2982200	763590	4303000	1126800	1314400	0	0	0	962090	1039000	971680
ARHOMBI_DN1690_c0_g1_i1_4	1587300	3740600	1085900	4329200	0	105950	0	1643600	4103500	0	0	0	0	0	0
ARUBRA_DN4036_c0_g1_i1_5	0	0	0	0	0	0	0	0	0	0	0	0	6788000	2041100	7737500
ARHOMBI_DN3961_c0_g1_i1_1	1653100	1939100	2259000	1879900	301070	181850	0	1589100	3552000	0	0	0	866870	1365600	977250
ARUBRA_DN6223_c0_g1_i1_2	0	0	0	0	0	0	0	0	0	0	0	0	3873200	5726000	6918600
ARUBRA_DN17757_c0_g1_i1_6	2978700	2762900	1897000	1451400	1435300	0	0	291270	1702100	0	0	714270	1270100	240070	1652400
ARUBRA_DN1661_c0_g1_i1_3	553020	576820	971960	838620	2112100	0	2684100	1602300	4048100	1276200	891640	0	0	0	825100
ARUBRA_DN2570_c0_g2_i1_5	1223100	1397000	1876000	942260	1147500	939950	2829300	737710	3006400	708590	0	0	451820	466200	571470
ARUBRA_DN2915_c0_g1_i1_5	52504	854740	0	47338	0	0	0	0	0	0	0	0	6466400	7058400	1795300
ARUBRA_DN4959_c0_g1_i4_6	82336	105370	0	0	0	0	0	0	0	0	0	0	7423500	1463500	7159500
ARUBRA_DN4738_c0_g1_i1_6	2360100	2224300	1865800	0	2149900	418090	2366500	307690	0	0	0	0	1877000	0	2571400
ARHOMBI_DN5099_c0_g2_i1_3	0	117020	0	0	0	0	0	0	0	0	0	0	5233300	7875000	2903000
ARHOMBI_DN6124_c0_g2_i1_6	1350800	1388800	634190	1731800	270180	120960	326430	1451600	1605500	1752100	0	0	2003500	2037700	1374700
ARUBRA_DN22130_c0_g1_i1_6	2196100	2412200	4921500	2554700	100460	701890	929360	0	2195600	0	0	0	0	0	0
ARHOMBI_DN17024_c0_g1_i1_5	1451600	1641400	2208100	1720900	1597200	699800	847460	1444900	1934600	0	0	2365200	0	0	0
ARHOMBI_DN3923_c0_g2_i1_3	1243700	1639300	784010	760620	0	0	0	0	11367000	0	0	0	0	0	0
ARUBRA_DN16253_c0_g1_i1_2	0	0	0	0	0	0	0	0	0	0	0	0	5213100	5075300	5491900
ARUBRA_DN18651_c0_g1_i1_6	187750	145830	159050	249400	0	0	0	0	327340	0	0	0	1861100	8666200	4127100
ARHOMBI_DN16154_c0_g1_i1_2	1849000	1638600	2590100	1075700	1416500	615020	1048900	856860	2429800	1409600	0	0	112010	292160	380140
ARUBRA_DN5051_c0_g1_i1_2	0	0	0	0	0	0	148290	0	0	0	0	0	6193200	8237900	1117800
ARHOMBI_DN2561_c0_g1_i1_3	0	0	0	0	0	0	0	0	0	0	0	0	5460800	4788900	5440700
ARUBRA_DN1380_c0_g2_i1_2	0	0	0	0	0	0	0	0	0	0	0	0	2865700	4621700	8176000
ARUBRA_DN2595_c0_g3_i1_4	38210	55833	0	0	0	0	0	0	0	0	0	0	5337900	4718000	5361300
ARUBRA_DN3028_c0_g1_i1_1	614830	781290	1890500	1817200	937840	509840	1067900	1585100	0	0	0	0	1639200	3314900	1246900
ARUBRA_DN3878_c0_g1_i1_3	63758	0	0	0	0	0	0	0	0	0	0	0	4764600	4873300	5649100

ARUBRA_DN2248_c0_g1_i1_6	0	0	0	0	0	0	0	0	0	0	0	0	4763600	5160200	5421100
ARUBRA_DN3983_c0_g1_i1_4	37509	103930	0	0	0	0	223490	497460	0	0	0	0	3120500	5865100	5476900
ARUBRA_DN7521_c0_g1_i1_3	0	0	1429800	0	1017100	529620	0	2293400	8881400	0	0	0	586350	0	574080
ARUBRA_DN15944_c0_g1_i1_3	2819800	3048100	2245500	0	1235400	0	0	841170	0	0	0	0	1640400	1946600	1513300
ARUBRA_DN4965_c10_g1_i1_3	0	81721	0	0	0	0	0	296730	0	0	0	0	3291900	6279100	5296500
ARHOMBI_DN2725_c0_g1_i1_2	2188700	2851800	2550300	0	826960	352210	1014200	0	4187300	0	0	0	0	507000	528600
ARHOMBI_DN4252_c0_g1_i1_5	0	73680	0	0	0	0	0	0	0	0	0	0	3976700	4229200	6580000
ARHOMBI_DN5741_c0_g1_i1_3	0	0	0	0	0	0	0	0	0	0	0	0	4617600	3870100	6369700
ARHOMBI_DN4907_c0_g1_i1_4	1605200	2337100	2273100	1265400	699950	1049500	1066400	0	1472800	0	0	758480	567810	814120	846200
ARHOMBI_DN3938_c0_g1_i1_4	811120	0	0	13488000	446400	0	0	0	0	0	0	0	0	0	0
ARUBRA_DN17582_c0_g1_i1_4	4246600	4144600	4089300	713920	0	0	532960	0	0	998290	0	0	0	0	0
ARUBRA_DN9146_c0_g1_i1_2	3759400	4099000	3675900	137920	0	1631400	443290	0	0	504870	0	0	250800	0	181130
ARHOMBI_DN15390_c0_g1_i1_4	713020	524120	588070	282270	2555700	0	2684300	1912100	3796200	0	0	0	787780	0	805860
ARHOMBI_DN4487_c0_g1_i1_2	962750	1313100	1769100	756040	1546800	471010	71346	1318000	4069800	388280	532190	0	498530	136800	773700
ARUBRA_DN1889_c0_g1_i1_3	0	0	0	0	0	0	0	0	0	0	0	0	1822300	5873500	6885300
ARUBRA_DN4712_c0_g2_i1_6	0	0	0	0	0	0	0	0	0	0	0	0	7304700	3054000	4216900
ARUBRA_DN4939_c0_g1_i3_5	0	0	0	0	0	0	0	0	0	0	0	0	2647500	3983200	7910900
ARUBRA_DN4833_c0_g1_i2_5	3036200	4814900	2871500	1503300	0	1041700	0	0	0	0	0	0	479590	0	684710
ARUBRA_DN2992_c0_g1_i1_3	1150400	0	1716500	1418700	357420	369340	549870	796270	3432800	1255800	0	1457400	593700	589850	665340
ARUBRA_DN7857_c0_g1_i1_1	548000	534540	882710	999920	0	0	430510	1420400	0	0	0	0	1246700	4068500	4203000
ARUBRA_DN22505_c0_g1_i1_3	0	0	0	0	0	0	0	0	0	0	0	0	4405400	5033600	4873800
ARUBRA_DN21075_c0_g1_i1_5	797410	894120	972960	149830	322460	0	0	0	254570	2908300	3146000	1277900	1090500	1216200	1254000
ARHOMBI_DN5548_c0_g1_i1_5	0	0	0	0	0	0	0	0	401820	0	0	0	3558800	6897300	3384600
ARUBRA_DN2771_c0_g1_i1_5	668910	158660	0	0	0	0	793870	145440	0	0	0	0	4027100	3183100	5128000
ARUBRA_DN4858_c0_g1_i1_4	294880	243070	158010	31333	0	0	1784500	0	896740	0	0	0	1648800	6148500	2829500
ARUBRA_DN25882_c0_g1_i1_2	0	172580	182440	243400	0	0	0	0	0	0	0	0	3798600	3524900	6083900
ARHOMBI_DN6034_c0_g1_i3_3	0	0	0	0	0	0	0	0	0	0	0	0	6662900	0	7291100

ARHOMBI_DN5395_c0_g1_i1_3	0	0	0	0	0	0	0	0	0	0	0	0	5558400	4341700	4042100
ARUBRA_DN2691_c0_g1_i1_5	1051300	1382500	580910	4218700	0	494220	0	0	3806400	0	0	2393800	0	0	0
ARUBRA_DN16143_c0_g1_i1_5	0	187690	0	236710	0	0	0	0	0	0	0	0	5298600	6337200	1709500
ARUBRA_DN7703_c0_g1_i1_6	0	489880	1924800	187050	0	0	0	0	0	1092200	0	0	3551900	2617300	3895900
ARHOMBI_DN5124_c0_g1_i1_4	105860	105060	0	75966	0	0	0	0	0	0	0	0	0	5762800	7682700
ARHOMBI_DN3648_c0_g1_i1_4	0	0	0	0	0	0	0	0	0	0	0	0	2984700	7212800	3501100
ARHOMBI_DN1734_c0_g1_i1_3	0	0	0	0	0	0	0	0	0	0	0	0	3838500	4807700	5042600
ARUBRA_DN9894_c0_g1_i1_4	0	0	609290	255690	836420	0	969460	1128500	3379300	0	1584800	0	1176400	1199500	2546300
ARUBRA_DN2456_c0_g1_i1_2	0	0	0	0	0	0	0	0	0	0	0	0	5546500	4111000	4016100
ARUBRA_DN6971_c0_g1_i1_6	0	0	0	0	0	0	0	0	0	0	0	0	4991200	4659000	4020900
ARHOMBI_DN516_c0_g1_i1_6	1314200	1572100	313870	807480	0	448910	866640	717830	2633200	0	0	0	1867600	1522300	1604700
ARUBRA_DN3974_c0_g1_i1_1	702810	978270	1179600	1169500	1137400	688220	0	0	327500	3891900	381060	3167100	0	0	0
ARUBRA_DN19869_c0_g1_i1_3	0	0	0	0	0	0	0	0	0	0	0	0	5160400	2982100	5332900
ARUBRA_DN5948_c0_g1_i1_2	0	0	0	88967	0	0	0	0	0	0	0	0	3493800	5114100	4749800
ARUBRA_DN20440_c0_g1_i1_3	238520	200120	95199	95429	1742600	0	1226500	0	0	2591100	2916700	2918500	459070	437950	474000
ARUBRA_DN20805_c0_g1_i1_5	0	0	13380000	0	0	0	0	0	0	0	0	0	0	0	0
ARUBRA_DN1508_c0_g1_i1_1	0	0	0	244450	543600	164610	0	1084000	3728800	2409600	2568000	2240200	235110	0	144400
ARUBRA_DN8181_c0_g1_i1_4	0	0	0	0	0	0	0	0	0	0	0	0	5297200	283110	7763000
ARHOMBI_DN5015_c0_g1_i1_3	1079600	1157800	1396600	573980	708910	0	305600	709230	1467200	1686000	2497800	1432400	161810	121320	0
ARUBRA_DN11787_c0_g1_i1_3	0	0	0	0	0	0	0	0	0	4568600	4557600	4112400	0	0	0
ARHOMBI_DN5946_c1_g1_i1_6	0	0	0	0	0	0	0	0	0	0	0	0	5037800	8185900	0
ARUBRA_DN23186_c0_g1_i1_4	1985100	2054500	2153900	1215800	0	0	0	1769700	2733500	1257900	0	0	0	0	0
ARUBRA_DN3089_c0_g1_i1_6	0	0	0	0	0	0	0	0	0	0	0	0	5230500	4400100	3509700
ARUBRA_DN1937_c0_g1_i1_4	0	0	0	0	0	0	0	0	0	12988000	0	0	0	0	0
ARHOMBI_DN9002_c0_g1_i1_5	843940	1192700	619790	246040	3489500	0	0	870610	1664900	0	0	0	1010300	1510100	1464300
ARHOMBI_DN10315_c0_g1_i1_4	0	0	0	0	0	0	0	0	0	0	0	0	4244100	3883800	4781000
ARUBRA_DN20947_c0_g1_i1_6	940750	1338600	949750	1379400	2019200	366600	1049900	1096300	3625500	0	0	0	0	117070	0

ARUBRA_DN18979_c0_g1_i1_5	1145500	525410	2272700	472990	1911500	0	0	0	2851400	0	0	0	1723300	0	1906400
ARUBRA_DN6370_c0_g1_i1_6	0	0	0	0	0	0	0	0	0	0	0	0	7206300	3128900	2404500
ARHOMBI_DN16206_c0_g1_i1_4	393230	513810	527370	240350	1017700	0	0	0	1542800	0	0	0	2892500	2172100	3235700
ARHOMBI_DN6026_c0_g2_i1_4	289990	92058	146270	67763	762380	0	678020	671720	203290	0	0	1483300	1652800	3898900	2483900
ARHOMBI_DN11466_c0_g1_i1_1	0	0	0	0	0	124790	0	0	0	0	0	0	7925100	2069400	2292500
ARUBRA_DN6756_c0_g1_i1_3	0	0	0	0	0	0	0	0	0	0	0	0	4477200	3406300	4499400
ARHOMBI_DN7040_c0_g1_i1_2	0	0	0	0	0	0	0	0	0	0	0	0	0	0	12382000
ARHOMBI_DN11661_c0_g1_i1_3	0	0	0	0	179360	48706	0	201810	0	0	324470	0	3656600	3744900	4204200
ARHOMBI_DN23618_c0_g1_i1_3	311610	66789	0	1254200	0	0	0	377080	0	0	10342000	0	0	0	0
ARUBRA_DN6507_c0_g1_i1_3	1273900	1284700	1151500	579790	1440500	0	837010	0	500640	0	0	1499000	1094900	1346100	1342300
ARHOMBI_DN21306_c0_g1_i1_1	0	0	0	0	0	0	0	0	0	0	0	0	4407200	2615500	5321000
ARHOMBI_DN16200_c0_g1_i1_5	1916500	1883600	3415300	1298400	423220	518630	1415900	363670	1103000	0	0	0	0	0	0
ARHOMBI_DN18668_c0_g1_i1_4	175840	262470	0	47621	189640	0	0	0	0	0	0	0	3989500	2696900	4965200
ARHOMBI_DN7785_c0_g1_i1_5	1285300	976610	715840	2325500	2067000	1328000	2007000	492580	788270	0	0	307880	0	0	0
ARHOMBI_DN2296_c0_g2_i1_6	1256800	1317300	1012300	744760	0	0	0	0	1100000	0	0	0	2848800	2376400	1607200
ARUBRA_DN4291_c0_g1_i1_3	0	0	0	0	0	0	0	0	0	0	0	0	6147600	4445900	1662000
ARHOMBI_DN4083_c0_g1_i1_6	0	0	0	0	0	0	0	0	0	0	0	0	4936000	3760000	3549700
ARHOMBI_DN5446_c0_g1_i1_5	269600	643970	440200	0	0	0	238210	0	0	1825100	2396600	1518500	3583300	0	1305600
ARUBRA_DN8529_c0_g1_i1_1	0	0	0	0	0	0	0	0	0	0	0	0	4071800	2890200	5188600
ARUBRA_DN4933_c0_g1_i2_4	0	0	0	0	0	0	0	233520	0	0	0	0	1964800	7522500	2402200
ARUBRA_DN3170_c0_g1_i1_4	776620	871550	1257300	228410	0	0	0	0	0	0	0	0	3702900	2302300	2969900
ARUBRA_DN4127_c0_g2_i1_4	0	0	0	0	0	0	0	0	0	0	0	0	2940700	2637100	6498500
ARUBRA_DN6378_c0_g1_i1_2	0	0	0	194870	0	0	0	0	0	0	0	0	2765900	2965300	6039700
ARUBRA_DN2301_c0_g1_i1_1	0	0	0	0	0	0	0	0	0	0	0	0	2614500	3900600	5449200
ARUBRA_DN16958_c0_g1_i1_6	0	0	0	0	0	0	0	0	0	0	0	0	4197400	2932000	4741500
ARUBRA_DN256_c0_g2_i1_1	417810	411660	584540	145860	2337000	1419200	1126100	1676100	508870	0	467400	1660200	263020	259000	572670
ARHOMBI_DN2281_c0_g1_i1_1	337100	321630	440930	0	575560	0	0	0	0	0	0	0	2166200	5687600	2267700

ARHOMBI_DN6231_c0_g2_i2_2	0	0	0	0	0	0	0	0	0	0	0	0	4969100	2610100	4212500
ARHOMBI_DN835_c0_g1_i1_4	0	92712	0	0	0	0	0	0	0	0	0	0	4784900	4813500	2044400
ARUBRA_DN4252_c0_g2_i2_4	0	0	0	0	0	0	0	0	0	0	0	0	5257900	3102900	3335200
ARHOMBI_DN11662_c0_g1_i1_4	0	0	0	0	0	0	0	0	0	0	11617000	0	0	0	0
ARUBRA_DN21714_c0_g1_i1_3	0	0	0	0	0	0	0	0	0	0	0	0	6372500	2368500	2866700
ARHOMBI_DN4867_c0_g1_i1_5	0	0	0	0	0	0	0	0	0	0	0	0	4195700	2488900	4917600
ARHOMBI_DN5538_c0_g1_i1_5	357990	303270	217650	0	0	0	0	0	0	0	0	0	1646400	2417400	6629600
ARUBRA_DN9182_c0_g1_i1_2	70943	66244	0	60616	258100	0	323830	0	0	0	0	0	3132200	3732700	3896600
ARUBRA_DN4826_c0_g1_i1_4	440160	321550	743710	0	1529100	717740	483950	0	0	2160000	2388900	1980700	293400	0	480310
ARHOMBI_DN21064_c0_g1_i1_2	0	0	0	0	0	0	0	0	0	0	0	0	3692300	2991700	4837300
ARHOMBI_DN50_c0_g3_i1_6	0	0	0	0	0	0	0	0	0	0	0	0	4606800	3460900	3399100
ARUBRA_DN4961_c0_g1_i2_4	2349200	1729400	2116400	0	0	395170	1463800	0	1251900	0	0	0	0	2146800	0
ARHOMBI_DN5474_c0_g1_i1_5	2297400	477500	3281000	850230	229620	602220	549800	359980	2146000	0	0	0	166530	0	415040
ARUBRA_DN3097_c0_g2_i1_2	0	0	0	114420	0	0	0	0	0	0	0	0	4190200	1869800	5194200
ARUBRA_DN7003_c0_g1_i1_4	0	0	0	0	0	0	0	0	0	0	11359000	0	0	0	0
ARHOMBI_DN20442_c0_g1_i1_1	0	0	0	0	0	0	0	0	0	11349000	0	0	0	0	0
ARUBRA_DN3105_c0_g1_i1_1	0	0	0	0	0	0	0	0	0	0	0	0	4286300	2979600	4008600
ARUBRA_DN18186_c0_g1_i1_5	2339100	2824500	1752800	0	0	0	0	0	3493600	0	0	0	822420	0	0
ARUBRA_DN3014_c0_g1_i1_2	0	0	0	0	0	0	0	0	0	0	0	0	3827400	1688900	5713400
ARUBRA_DN12532_c0_g1_i1_5	0	0	383280	0	0	693290	4513500	910780	1277200	0	0	0	1708300	0	1739300
ARHOMBI_DN5429_c0_g1_i1_2	572240	669770	866250	1503200	0	0	0	453740	7144400	0	0	0	0	0	0
ARHOMBI_DN16845_c0_g1_i1_5	0	0	0	0	0	0	0	0	0	0	0	0	996210	6017000	4191800
ARUBRA_DN720_c0_g1_i1_6	0	0	0	120190	0	0	0	0	0	0	0	0	2561600	5011900	3450100
ARHOMBI_DN17235_c0_g1_i1_3	90497	59057	0	0	0	0	0	0	0	0	0	0	3737700	4370300	2839200
ARUBRA_DN2328_c0_g2_i1_4	0	0	0	0	0	0	0	0	0	0	0	0	525060	4512300	6033900
ARUBRA_DN4073_c0_g1_i1_2	0	0	0	0	0	0	0	0	143740	0	0	0	3739000	3310400	3798000
ARHOMBI_DN1161_c0_g1_i1_2	0	0	0	0	0	0	0	0	0	0	0	0	2677200	3190000	5027000

ARHOMBI_DN22444_c0_g1_i1_1	1080200	769070	585580	279900	524480	0	409530	371580	1686100	0	1802300	3383800	0	0	0
ARHOMBI_DN5200_c0_g2_i1_5	0	0	0	0	0	0	0	0	0	0	0	0	2109700	5781900	3001300
ARHOMBI_DN19654_c0_g1_i1_2	0	0	0	52363	352400	0	0	0	0	0	1704100	0	4007000	1379900	3373200
ARHOMBI_DN2987_c0_g1_i1_5	0	0	0	0	0	0	0	0	0	0	0	0	3384900	4148700	3334700
ARHOMBI_DN6144_c0_g1_i1_4	180060	161680	185060	600710	0	0	0	154180	457250	0	0	0	3083800	2737200	3281300
ARUBRA_DN4862_c0_g1_i1_4	2856500	0	3697600	4275800	0	0	0	0	0	0	0	0	0	0	0
ARHOMBI_DN1127_c0_g2_i1_4	0	0	0	0	0	0	0	0	0	0	0	0	3429000	3305800	4043300
ARUBRA_DN17228_c1_g1_i1_1	0	0	0	0	0	0	0	0	0	0	0	0	3117500	3443700	4204800
ARHOMBI_DN4125_c0_g1_i1_5	694370	602350	863540	197380	648100	225270	0	285390	0	0	0	0	3704800	1624100	1852500
ARHOMBI_DN4270_c0_g1_i1_2	0	0	0	0	0	0	0	0	0	0	0	0	3149700	3125700	4407800
ARUBRA_DN4844_c0_g1_i1_2	0	0	0	0	0	0	0	0	0	0	0	0	5414700	1659400	3607500
ARUBRA_DN13846_c0_g1_i1_5	230080	248220	111620	210550	0	0	0	0	679840	0	0	0	3102200	2877400	3141100
ARUBRA_DN5052_c0_g2_i1_1	2805000	1106700	6448800	154140	0	0	0	0	0	0	0	0	0	0	0
ARUBRA_DN4025_c0_g2_i1_5	0	0	0	0	0	0	0	0	0	0	0	0	2210900	2685400	5613400
ARUBRA_DN4599_c2_g1_i1_4	1089800	1148400	1462500	392560	0	172170	0	0	1299300	0	0	0	1317600	2145900	1454900
ARUBRA_DN21659_c0_g1_i1_3	0	2972300	2765000	0	1740400	0	322120	0	2661300	0	0	0	0	0	0
ARHOMBI_DN17850_c0_g1_i1_6	1982600	2111800	1039200	760770	1080500	427740	0	690480	2322300	0	0	0	0	0	0
ARUBRA_DN2529_c0_g2_i1_6	444720	474470	738010	640520	0	0	1020100	1102400	0	0	0	0	1380300	1452100	3160300
ARHOMBI_DN6264_c0_g1_i5_2	0	0	41942	0	0	0	0	0	10245000	125270	0	0	0	0	0
ARHOMBI_DN5393_c0_g1_i1_1	44081	0	0	102130	0	641190	819280	3242900	5456400	0	0	0	0	0	0
ARUBRA_DN3140_c0_g1_i1_5	108280	0	0	0	0	0	0	0	0	0	0	0	1386800	2543100	6256900
ARHOMBI_DN5550_c0_g1_i1_2	518060	1616400	388420	1392400	0	0	0	0	2825500	0	0	2380000	0	0	1166700
ARUBRA_DN3107_c0_g1_i1_6	446060	552850	495940	387890	1427800	487100	2016300	1811900	1426100	385800	0	353460	0	0	475310
ARUBRA_DN4381_c0_g1_i1_5	239890	354990	178560	110900	372370	0	0	0	881340	0	0	0	1382900	3392900	3348900
ARUBRA_DN3552_c0_g1_i1_4	0	0	0	0	0	0	0	0	0	0	0	0	3026700	4714300	2483400
ARUBRA_DN3608_c0_g1_i1_6	487270	1205900	1208000	439870	2231400	1733700	0	1179500	1706500	0	0	0	0	0	0
ARHOMBI_DN17898_c0_g1_i1_3	25415	28578	0	0	0	0	0	0	0	0	0	0	3771800	3631500	2718300

ARHOMBI_DN17178_c0_g1_i1_4	1426600	1585700	983710	379940	0	501550	825180	632640	1351100	0	0	0	807750	789490	837450
ARUBRA_DN4547_c0_g1_i1_6	1813600	2469500	2968200	2514600	0	0	0	0	0	0	0	0	0	344770	0
ARHOMBI_DN486_c0_g1_i1_1	1259000	1745800	1914600	37095	702710	0	0	0	1373100	0	0	0	1541400	1480500	0
ARHOMBI_DN5664_c0_g1_i1_5	154690	103970	138510	84512	8300100	436070	0	826550	0	0	0	0	0	0	0
ARHOMBI_DN7133_c0_g1_i1_1	0	0	0	0	0	0	0	0	0	0	0	0	3182000	3398400	3463500
ARHOMBI_DN5590_c0_g1_i1_4	0	0	0	0	0	0	0	0	0	0	0	0	2781500	2584200	4671300
ARHOMBI_DN3067_c0_g1_i1_1	0	0	0	0	0	0	0	0	0	0	0	0	3269700	3353400	3412500
ARUBRA_DN3632_c0_g1_i1_6	298420	327100	313440	0	0	174030	0	0	865750	0	0	0	1426700	3416000	3193400
ARUBRA_DN5040_c0_g4_i8_2	66665	67610	0	0	462240	0	405730	422250	836820	0	0	0	3957000	3792200	0
ARHOMBI_DN9099_c0_g1_i1_1	0	0	0	0	0	0	0	0	0	5383800	0	4610900	0	0	0
ARHOMBI_DN1783_c0_g2_i1_3	314190	342690	718630	360480	1428500	959410	1530000	1136400	2077300	0	0	0	0	486800	636040
ARUBRA_DN5025_c0_g1_i3_3	0	0	0	0	0	0	0	0	0	0	0	0	3397800	2293400	4293500
ARUBRA_DN1938_c0_g1_i1_1	0	0	0	0	0	0	0	0	0	0	0	0	3576900	2515200	3878500
ARHOMBI_DN16492_c0_g1_i1_4	1945800	1790000	2164900	1273300	680850	0	733640	856750	525000	0	0	0	0	0	0
ARUBRA_DN5418_c0_g1_i1_4	0	0	0	0	0	0	0	0	0	0	0	0	3428900	2948700	3583400
ARHOMBI_DN13783_c0_g1_i1_2	1996800	2101000	2296000	566680	0	0	0	0	0	0	0	0	1460700	1517000	0
ARHOMBI_DN16013_c0_g1_i1_5	319160	851460	947140	0	0	0	0	0	0	0	0	0	2490300	2555500	2762600
ARUBRA_DN19786_c0_g1_i1_6	0	0	0	0	0	0	0	0	0	4888400	5007800	0	0	0	0
ARUBRA_DN5795_c0_g1_i1_1	0	0	0	0	0	0	0	0	0	0	0	0	3094500	3028900	3746600
ARHOMBI_DN6222_c0_g1_i1_3	1060400	1915500	1768000	0	712450	0	694470	0	632490	0	0	0	2345000	0	730870
ARHOMBI_DN3998_c0_g1_i1_1	117330	148190	200500	0	0	0	413790	0	0	0	0	0	3638800	1127700	4197500
ARUBRA_DN25017_c0_g1_i1_1	0	0	0	0	0	0	0	1028900	4244900	0	0	0	1428200	1607300	1497200
ARUBRA_DN2627_c0_g1_i1_2	0	0	0	26489	0	0	0	0	0	0	0	0	2865800	2595500	4309800
ARHOMBI_DN2324_c0_g1_i1_5	143430	94411	95627	40262	0	0	0	0	0	0	0	0	1013700	4093300	4284200
ARUBRA_DN6463_c0_g1_i1_2	0	0	0	0	0	0	0	0	0	0	0	0	2571100	2251800	4929800
ARUBRA_DN18383_c0_g1_i1_3	253160	255540	352290	452570	0	0	0	810770	1483200	2641800	3478900	0	0	0	0
ARUBRA_DN4889_c0_g1_i1_6	0	0	0	0	0	0	0	0	0	0	0	0	3262300	2593400	3845900

ARHOMBI_DN6134_c0_g1_i1_6	1104300	2162600	1181300	5079700	0	0	0	0	170890	0	0	0	0	0	0
ARHOMBI_DN3483_c0_g3_i1_5	269290	308980	295240	459170	0	0	0	0	0	0	0	0	0	3361700	4946600
ARUBRA_DN11125_c0_g1_i1_4	0	0	0	0	0	0	0	0	0	0	0	0	2425200	2553500	4646100
ARHOMBI_DN13455_c0_g1_i1_1	842500	662930	923980	1486500	0	0	0	0	0	0	5700900	0	0	0	0
ARHOMBI_DN5719_c0_g1_i1_2	69556	59516	0	34520	0	0	0	0	0	0	0	0	3410700	1601300	4435200
ARHOMBI_DN25944_c0_g1_i1_6	1168300	712610	793380	220560	1735400	0	0	0	0	0	0	4980100	0	0	0
ARHOMBI_DN1498_c0_g1_i1_1	0	0	0	0	0	0	9603900	0	0	0	0	0	0	0	0
ARUBRA_DN3905_c0_g1_i1_4	0	0	0	0	0	0	0	0	0	0	0	0	2493200	3144700	3932900
ARUBRA_DN8419_c0_g1_i1_3	0	0	30363	0	0	0	288650	0	0	0	0	0	4014700	2524300	2659200
ARUBRA_DN11134_c0_g1_i1_6	0	0	0	0	0	0	0	0	0	0	0	0	5591800	1737200	2129900
ARHOMBI_DN2322_c0_g1_i1_2	1365900	1599500	1726600	1265300	974310	0	0	852220	1648600	0	0	0	0	0	0
ARHOMBI_DN20297_c0_g1_i1_1	0	0	0	0	0	0	0	0	0	0	0	0	4067700	1764600	3596700
ARUBRA_DN4829_c0_g2_i2_4	0	0	0	0	0	0	0	0	0	0	0	0	628710	2054200	6720000
ARUBRA_DN18092_c0_g1_i1_5	0	0	156330	0	2171100	0	222660	2505900	1679000	2393400	0	0	0	0	264090
ARHOMBI_DN10619_c0_g1_i1_4	0	0	0	0	0	0	0	0	207750	0	0	0	2643300	2229500	4303400
ARHOMBI_DN3191_c0_g1_i1_2	78749	73277	0	43614	0	0	0	0	0	0	0	0	3024800	4142600	1974200
ARUBRA_DN19085_c0_g1_i1_1	0	0	0	0	0	0	0	0	0	0	0	0	482940	4039000	4814500
ARHOMBI_DN5770_c0_g2_i2_6	104280	113650	0	0	0	0	0	0	0	0	0	0	2850900	2837200	3430300
ARHOMBI_DN3157_c0_g1_i1_6	0	0	0	0	0	0	0	0	0	0	0	0	4437300	0	4894400
ARUBRA_DN21700_c0_g1_i1_4	2064000	2403100	1107200	282940	627510	0	0	0	0	0	0	0	896310	473010	1474800
ARHOMBI_DN16801_c0_g1_i1_1	0	0	0	0	0	0	0	0	0	0	0	0	3755100	3469800	2103000
ARUBRA_DN192_c0_g1_i1_1	153660	134310	55441	55376	0	0	0	0	0	0	0	0	2854900	3099300	2950200
ARHOMBI_DN24575_c0_g1_i1_1	80089	66225	36618	28791	295900	0	0	316650	0	0	1171900	1042400	2730500	2381200	1150000
ARHOMBI_DN5209_c2_g1_i1_4	0	0	0	0	0	0	0	0	0	0	0	0	3283100	3467700	2483500
ARUBRA_DN1890_c0_g2_i1_3	0	0	0	0	0	0	0	0	0	0	0	0	3192300	3221100	2801800
ARUBRA_DN3867_c0_g1_i1_1	0	0	0	0	0	0	0	0	0	0	0	0	4303700	2462400	2448400
ARHOMBI_DN23168_c0_g1_i1_4	832550	813880	1173900	755260	1042200	354230	379900	0	3214700	0	0	0	169220	237770	230280

ARHOMBI_DN3469_c0_g1_i1_3	0	0	0	0	0	0	0	0	0	0	0	0	2984100	2843300	3367800
ARUBRA_DN4948_c0_g2_i1_5	0	0	0	0	0	0	0	0	0	0	0	0	2629600	3507600	3013600
ARUBRA_DN3297_c0_g1_i1_1	1227800	1029000	1355300	1298900	0	0	0	537780	2592900	0	0	0	249930	0	853440
ARUBRA_DN2696_c0_g1_i1_3	0	0	0	0	0	0	0	0	0	0	0	0	3575500	3814200	1725000
ARHOMBI_DN15716_c0_g1_i1_4	0	0	0	0	0	0	0	0	0	0	0	0	3256100	1201500	4544800
ARHOMBI_DN4884_c0_g1_i1_2	0	0	0	0	0	0	0	0	0	0	0	0	2633100	2731500	3599100
ARUBRA_DN20045_c0_g1_i1_4	1398700	845040	1019900	1100800	567960	0	632950	505050	0	1406500	1445400	0	0	0	0
ARHOMBI_DN1594_c0_g1_i1_2	0	0	0	0	0	0	0	0	0	0	0	0	1991900	2801400	4123200
ARUBRA_DN4593_c0_g2_i1_4	0	138410	0	0	0	119760	0	0	0	0	0	0	2484200	2858700	3289900
ARUBRA_DN4903_c0_g1_i1_3	0	0	0	0	0	0	0	0	0	0	0	0	820720	4223300	3831400
ARHOMBI_DN18322_c0_g1_i1_3	0	0	0	0	398660	0	347700	624210	1814600	1911600	2150100	1614000	0	0	0
ARUBRA_DN11129_c0_g1_i1_5	2243000	2173500	1804600	325110	852220	490900	949700	0	0	0	0	0	0	0	0
ARUBRA_DN24080_c0_g1_i1_2	0	0	0	0	0	0	0	0	0	0	0	0	2463700	4161500	2164000
ARUBRA_DN10280_c0_g1_i1_1	1782000	1458600	1710100	888910	0	0	1412400	1242200	0	0	0	0	0	0	256470
ARUBRA_DN19519_c0_g1_i1_5	1291000	1010000	1332000	794140	690620	0	0	1514700	1318100	0	0	0	302890	267950	221370
ARUBRA_DN5259_c0_g1_i1_6	360350	273120	506330	383980	0	151380	1374000	269180	5243100	0	0	0	0	180340	0
ARUBRA_DN4393_c0_g2_i1_4	0	0	0	0	0	0	0	0	0	0	0	0	2289900	3955900	2479400
ARHOMBI_DN5839_c1_g1_i1_5	0	0	0	0	0	0	0	0	0	0	0	0	1962800	5834000	878520
ARUBRA_DN10773_c0_g1_i1_4	0	1081400	216140	0	314210	960310	1139100	869160	4088900	0	0	0	0	0	0
ARUBRA_DN21519_c0_g1_i1_5	0	0	0	0	0	0	0	0	0	0	0	0	3697400	2250000	2698100
ARUBRA_DN6882_c0_g1_i1_3	1711700	1721300	1636200	855050	986780	748670	985870	0	0	0	0	0	0	0	0
ARHOMBI_DN22543_c0_g1_i1_3	0	0	0	0	0	655040	858330	1081300	5710400	0	0	0	0	145060	149080
ARUBRA_DN9648_c0_g1_i1_1	0	73088	464860	134400	0	0	182130	0	0	3554300	3820700	0	255600	0	108240
ARUBRA_DN5039_c0_g1_i1_2	0	0	0	0	0	0	0	0	0	0	0	0	485310	3851600	4214800
ARHOMBI_DN18646_c0_g1_i1_5	0	0	0	0	0	0	0	0	0	0	0	0	3026000	4310600	1196700
ARHOMBI_DN4447_c0_g1_i1_4	0	0	0	0	0	0	0	0	329300	0	0	0	3785500	1914300	2503800
ARUBRA_DN2978_c0_g1_i1_6	0	0	0	0	0	0	0	0	0	0	0	0	871850	3346200	4297700

ARUBRA_DN7999_c0_g1_i1_2	0	0	0	0	0	0	0	0	0	0	0	0	3870200	1470600	3169900
ARHOMBI_DN5214_c0_g1_i1_4	139600	253950	190530	0	0	0	89565	0	561480	2379700	2238700	2302900	164730	0	166000
ARUBRA_DN4195_c0_g1_i1_4	0	0	0	0	0	0	0	0	150080	0	0	0	1161100	3367500	3736700
ARHOMBI_DN6230_c0_g2_i3_1	372910	0	639560	40668	0	0	0	0	0	0	0	0	2101100	2543900	2695000
ARUBRA_DN8665_c0_g1_i1_5	0	0	0	0	0	0	0	0	0	0	0	0	3299700	2392200	2691800
ARHOMBI_DN19852_c0_g1_i1_3	0	0	0	0	0	0	0	0	0	0	0	0	3336200	1424600	3614900
ARUBRA_DN7713_c0_g2_i1_1	0	0	0	0	0	0	0	0	0	0	0	0	3473700	1401400	3484300
ARHOMBI_DN15828_c0_g1_i1_6	910980	1074400	1138300	1110800	0	0	0	0	0	0	1949300	1578700	324580	267410	0
ARHOMBI_DN965_c0_g1_i1_1	352550	349690	374620	304890	0	0	0	0	0	0	0	0	2399300	1875500	2646000
ARHOMBI_DN4105_c0_g1_i1_1	0	0	0	0	0	0	0	0	0	0	0	0	2401300	2032700	3856500
ARUBRA_DN6657_c0_g1_i1_6	0	0	0	0	0	0	0	0	0	0	0	0	269430	5756400	2249200
ARUBRA_DN13405_c0_g1_i1_1	291260	144560	253630	0	0	0	0	567940	932430	0	0	217840	2099600	1559700	2182900
ARUBRA_DN1195_c0_g1_i1_2	0	227360	315130	0	0	0	0	0	0	0	0	0	2735100	1771200	3199600
ARUBRA_DN2242_c0_g1_i1_5	0	0	0	0	0	0	0	0	0	0	0	0	3317500	3415000	1507000
ARHOMBI_DN3366_c0_g1_i1_4	0	0	0	0	0	0	0	0	0	0	0	0	2185400	3138000	2900700
ARUBRA_DN6838_c0_g1_i1_3	171000	167930	168590	0	0	0	0	0	0	0	0	0	2666500	1494600	3459000
ARUBRA_DN10378_c0_g1_i1_1	0	0	0	0	0	0	0	0	0	0	0	0	2654800	3038000	2423900
ARHOMBI_DN5914_c0_g1_i1_4	0	0	0	0	0	0	0	0	0	0	0	0	3087100	1949500	3054600
ARHOMBI_DN5134_c0_g1_i1_6	0	0	0	0	0	0	0	0	0	0	0	0	2738200	2228500	3123400
ARUBRA_DN16002_c0_g1_i1_2	634910	801240	854090	558450	451140	273500	1160000	782840	1921500	0	0	0	319470	323540	0
ARUBRA_DN6617_c0_g1_i1_2	1491300	1675700	1563400	694440	1519800	0	191570	210030	383920	0	0	0	328970	0	0
ARHOMBI_DN7393_c0_g1_i1_3	939170	1236100	2820400	1718300	0	0	0	0	0	0	0	482140	289790	543860	0
ARHOMBI_DN1339_c0_g1_i1_3	616810	1037100	2396900	430140	1740800	155920	0	1632200	0	0	0	0	0	0	0
ARHOMBI_DN16218_c0_g1_i1_1	2395600	2127300	2493700	977160	0	0	0	0	0	0	0	0	0	0	0
ARUBRA_DN3982_c0_g2_i1_1	0	0	0	0	0	0	0	0	0	0	0	0	1740200	2990900	3253900
ARHOMBI_DN2364_c0_g1_i1_5	0	0	0	0	0	0	0	0	0	0	0	0	2092900	2996000	2891500
ARHOMBI_DN5683_c0_g1_i2_5	0	0	0	0	0	0	0	0	0	0	0	0	1262400	2206900	4497300

ARUBRA_DN14982_c0_g1_i1_3	991720	1230200	1634400	1358900	917030	0	1067800	0	0	0	0	0	0	613180	152880
ARHOMBI_DN21103_c0_g1_i1_1	462140	0	446820	877630	0	0	0	469800	2234800	0	0	0	1275100	995420	1185700
ARUBRA_DN18762_c0_g1_i1_4	707980	645170	1016500	555420	0	215860	0	0	663990	1939900	0	1786500	187030	209110	0
ARUBRA_DN4063_c0_g1_i2_5	0	0	0	0	0	0	104710	0	268480	0	0	90001	1923200	2594800	2932500
ARUBRA_DN11481_c0_g1_i1_6	0	0	0	0	0	0	0	0	0	0	0	0	247360	4805200	2850000
ARUBRA_DN934_c0_g1_i1_3	0	0	0	0	0	0	0	0	0	0	0	0	1110800	1803200	4971800
ARUBRA_DN3268_c0_g1_i1_2	97261	137100	0	0	0	0	0	0	0	0	0	0	3052900	2406300	2181700
ARHOMBI_DN2479_c0_g1_i1_1	385820	195430	482600	255850	899790	0	0	1398600	3202300	0	0	0	521520	0	525680
ARUBRA_DN1490_c0_g1_i1_3	0	0	0	0	0	0	0	0	0	0	0	0	1287700	1429000	5145900
ARUBRA_DN20284_c0_g1_i1_5	0	0	0	0	0	0	0	0	0	0	0	0	2984300	2399700	2469900
ARUBRA_DN12380_c0_g1_i1_5	0	0	0	0	0	0	0	0	0	0	0	0	0	0	7808300
ARHOMBI_DN16116_c0_g1_i1_3	0	0	0	0	0	0	0	0	0	0	0	0	2619600	2738700	2448700
ARHOMBI_DN6092_c0_g1_i1_1	374350	255160	289960	208330	0	0	0	0	448420	0	0	0	1996300	2384600	1812300
ARHOMBI_DN15878_c0_g1_i1_3	92447	163350	51539	113080	740810	0	0	1228300	0	1591600	1944600	1728700	0	0	0
ARHOMBI_DN2833_c0_g1_i1_1	0	214010	0	1405100	0	0	0	0	5418800	0	0	0	0	0	605070
ARUBRA_DN16146_c0_g1_i1_4	92515	124560	267090	74800	0	0	0	0	0	0	0	0	2784100	1863900	2409900
ARHOMBI_DN10766_c0_g1_i1_6	433700	328560	430360	227820	0	331600	2207800	1343300	2132800	0	0	0	0	79609	89868
ARHOMBI_DN5588_c0_g1_i1_3	0	0	0	0	0	0	0	0	0	0	0	0	2144100	2628900	2831100
ARUBRA_DN22820_c0_g1_i1_3	1140500	2177000	1761200	1465500	785360	0	0	0	0	0	0	0	0	0	269700
ARHOMBI_DN5983_c0_g1_i1_5	581810	527070	186610	110690	407360	0	0	0	221520	0	0	0	1390900	1813000	2354200
ARUBRA_DN20709_c0_g1_i1_5	0	0	0	0	0	0	0	0	0	0	2095000	0	1054000	3249500	1154000
ARUBRA_DN1983_c0_g1_i1_4	0	0	0	0	0	0	0	0	0	0	0	0	2308800	2251300	2953400
ARHOMBI_DN3005_c0_g2_i1_6	122910	122060	0	0	0	0	0	0	0	0	0	0	3312300	3318000	606000
ARHOMBI_DN5420_c0_g1_i1_2	0	0	0	0	0	0	0	0	0	0	0	0	2774600	2459500	2228900
ARUBRA_DN3944_c0_g1_i1_3	0	0	0	0	0	0	0	0	0	0	0	0	2532800	1936700	2984400
ARHOMBI_DN18815_c0_g1_i1_6	345910	599690	471090	379290	0	0	0	0	0	2823600	0	2470700	0	0	343840
ARHOMBI_DN5059_c0_g1_i1_5	0	0	0	0	0	0	0	0	0	0	0	0	3612800	1867200	1919100

ARHOMBI_DN5953_c0_g1_i1_4	355690	301750	191460	1402200	227960	0	190670	419690	0	1037800	1262500	1265300	235640	490550	0
ARUBRA_DN193_c0_g1_i1_1	0	0	0	0	0	0	0	0	0	0	0	0	3087200	3923600	356620
ARUBRA_DN7445_c0_g1_i1_4	1954400	1783600	848670	1257900	0	0	796440	700540	0	0	0	0	0	0	0
ARHOMBI_DN18779_c0_g1_i1_3	515840	580010	478540	212270	502450	0	0	676300	0	2771600	1578100	0	0	0	0
ARUBRA_DN19198_c0_g1_i1_2	0	0	0	0	0	0	0	0	0	0	0	0	2794300	1926200	2521900
ARHOMBI_DN5769_c0_g1_i1_5	0	73428	44849	0	0	0	0	0	0	0	0	0	0	435670	6648500
ARHOMBI_DN18804_c0_g1_i1_4	512040	428000	283710	764500	0	0	0	0	1664100	0	0	0	1237600	1054500	1219200
ARHOMBI_DN7_c0_g2_i1_2	301910	141130	315270	201150	0	0	0	0	382410	0	0	0	1778000	1935400	2080400
ARUBRA_DN3923_c0_g1_i1_5	283920	0	552220	0	1816800	799560	1359400	1734400	423510	0	0	0	145690	0	0
ARHOMBI_DN6177_c0_g2_i2_3	0	2859100	0	1583600	0	0	0	0	0	0	0	0	0	406260	2262600
ARHOMBI_DN24695_c0_g1_i1_4	432820	383130	643460	414620	1861300	0	0	0	0	0	0	0	1160400	942640	1236500
ARHOMBI_DN2476_c0_g1_i1_4	0	0	0	0	0	0	0	0	0	0	0	0	4681000	966980	1423100
ARHOMBI_DN22511_c0_g1_i1_1	428160	343200	614690	270810	397400	288340	0	0	0	0	0	0	1587700	1184900	1948600
ARHOMBI_DN3114_c0_g1_i1_1	0	0	0	0	0	0	0	0	0	0	0	0	2528300	1842200	2670400
ARUBRA_DN16322_c0_g1_i1_4	0	0	0	0	0	0	0	0	0	0	0	0	2238400	2155500	2644700
ARUBRA_DN3916_c0_g1_i1_5	0	145300	225550	165390	0	0	0	0	0	0	0	0	2008600	2154100	2335600
ARUBRA_DN4811_c0_g2_i1_4	690560	701260	758840	598870	786420	0	0	944040	2545000	0	0	0	0	0	0
ARUBRA_DN18543_c0_g1_i1_6	0	94933	0	0	0	0	0	492400	0	0	0	0	2388000	851960	3194800
ARHOMBI_DN3540_c0_g1_i1_2	0	182840	274530	0	0	0	414490	0	1405900	417720	0	0	1169300	1303600	1845200
ARUBRA_DN478_c0_g1_i1_2	0	130010	0	149920	485490	0	705500	785800	4278900	0	0	0	0	0	435600
ARUBRA_DN20269_c0_g1_i1_6	0	0	0	0	0	0	0	0	0	0	0	0	1278900	2490500	3197600
ARUBRA_DN20878_c0_g1_i1_1	0	0	0	0	0	0	0	0	0	622640	0	0	3289800	1796300	1220900
ARHOMBI_DN912_c0_g1_i1_6	0	0	0	0	0	0	0	0	0	0	0	0	1375000	2369800	3161500
ARUBRA_DN11488_c0_g1_i1_6	157660	99610	0	0	0	0	0	0	0	0	0	0	1894400	2220100	2524900
ARUBRA_DN19442_c0_g1_i1_2	471870	495420	355290	176750	1584600	0	1587100	962770	1259000	0	0	0	0	0	0
ARUBRA_DN4301_c0_g1_i1_3	0	0	0	0	0	0	0	0	0	0	0	0	2105200	1886300	2887500
ARUBRA_DN16241_c0_g1_i1_5	0	0	0	0	0	0	0	0	0	0	0	0	0	5081700	1785900

ARUBRA_DN6197_c0_g1_i1_1	1873600	2014300	2446000	514310	0	0	0	0	0	0	0	0	0	0	0
ARUBRA_DN5359_c0_g1_i1_3	0	0	0	0	0	0	0	0	0	0	0	0	3577500	803660	2451200
ARUBRA_DN20048_c0_g1_i1_1	93837	0	135570	0	0	0	0	0	0	0	0	0	1705700	2259500	2637800
ARHOMBI_DN6095_c0_g2_i1_5	262880	197940	252080	289920	0	0	411820	908590	240330	0	835320	0	835510	867810	1681900
ARUBRA_DN4711_c0_g2_i1_6	0	0	0	0	0	0	0	0	0	0	0	0	1362700	2723000	2651700
ARUBRA_DN2566_c0_g2_i1_5	75010	0	0	141750	0	0	0	0	0	0	0	105370	2516300	1093100	2801000
ARHOMBI_DN6176_c1_g1_i1_5	326810	231400	174480	0	2026200	1615400	2308400	0	0	32250	0	0	0	0	0
ARUBRA_DN413_c0_g1_i1_3	0	0	0	0	0	547910	0	0	0	2958600	3201600	0	0	0	0
ARUBRA_DN16443_c0_g1_i1_3	0	0	0	0	0	0	0	0	0	0	0	0	1472500	2582500	2632400
ARHOMBI_DN15325_c0_g1_i1_4	473320	0	0	718170	0	0	0	1812700	2155800	0	831260	682590	0	0	0
ARHOMBI_DN5371_c0_g1_i1_2	0	0	0	0	0	0	0	0	0	0	0	0	2180600	2048800	2415700
ARUBRA_DN4439_c0_g1_i1_3	214220	231720	185950	205310	0	0	0	0	0	0	0	0	0	3847400	1951900
ARHOMBI_DN24520_c0_g1_i1_6	0	0	0	0	0	0	0	0	0	0	0	0	2324200	2114500	2189500
ARHOMBI_DN900_c0_g1_i1_2	0	0	0	0	0	0	0	0	0	0	0	0	2616400	867790	3142200
ARUBRA_DN2910_c0_g1_i1_5	0	0	0	0	0	0	0	0	0	0	0	0	0	1085300	5535100
ARHOMBI_DN5383_c0_g1_i1_2	0	0	0	0	6606800	0	0	0	0	0	0	0	0	0	0
ARUBRA_DN1777_c0_g1_i1_3	0	0	0	0	0	0	0	0	849320	1821400	2042500	1748100	0	0	92194
ARHOMBI_DN4470_c0_g1_i1_6	115480	109480	0	104510	0	0	0	0	0	0	0	0	0	3199400	3018300
ARUBRA_DN22163_c0_g1_i1_3	390050	1265100	1472400	181970	330030	0	646370	0	0	0	0	0	864180	593890	802930
ARHOMBI_DN13192_c0_g1_i1_2	0	0	0	0	0	0	0	0	0	0	0	0	2176600	2492300	1805100
ARHOMBI_DN54_c0_g1_i1_2	0	0	0	43861	0	0	0	0	0	0	0	0	2040000	946940	3430100
ARHOMBI_DN4900_c0_g1_i1_5	1934800	1397500	1523300	279090	0	0	985550	0	0	339680	0	0	0	0	0
ARHOMBI_DN13072_c0_g1_i1_6	117150	126450	0	112720	0	0	0	286320	482050	337980	0	0	1805800	1124200	2059700
ARUBRA_DN14237_c0_g1_i1_6	0	0	0	0	680030	0	0	0	5771700	0	0	0	0	0	0
ARHOMBI_DN18431_c0_g1_i1_1	0	0	0	0	0	0	0	0	0	0	0	0	2840600	1895600	1679200
ARUBRA_DN8433_c0_g1_i1_6	1305500	1355100	1529200	1043300	0	0	0	0	1170900	0	0	0	0	0	0
ARHOMBI_DN19091_c0_g1_i1_5	1098800	1272500	1605300	600040	919700	351750	0	0	0	0	0	0	194750	163230	186780

ARUBRA_DN18317_c0_g1_i1_5	674990	750470	799730	1095400	310980	0	0	0	2705900	0	0	0	0	0	0
ARHOMBI_DN22440_c0_g1_i1_6	0	0	0	0	0	0	0	0	0	0	0	0	1497800	3166700	1650000
ARHOMBI_DN8601_c0_g1_i1_4	0	0	0	0	0	0	0	0	0	0	0	0	6311300	0	0
ARHOMBI_DN25934_c0_g1_i1_3	1090600	538560	595110	506040	0	0	0	0	1648600	0	0	0	974510	955170	0
ARHOMBI_DN13008_c0_g1_i1_6	418680	463700	1320500	308690	0	0	0	582540	2387200	0	0	805970	0	0	0
ARUBRA_DN1845_c0_g1_i1_5	0	0	0	0	0	0	0	0	0	0	0	0	1940500	1415600	2921600
ARUBRA_DN4305_c0_g2_i1_6	0	0	0	0	0	0	0	0	0	0	0	0	2620700	2398400	1250800
ARUBRA_DN13152_c0_g1_i1_3	0	0	0	0	0	174490	505440	0	108280	0	0	0	2726400	644540	2107100
ARUBRA_DN3999_c0_g1_i1_6	0	0	0	0	0	0	0	0	0	0	0	0	1850600	2005600	2392000
ARUBRA_DN5726_c0_g1_i1_1	0	0	0	0	0	0	0	0	0	0	0	0	1750900	2224500	2270500
ARHOMBI_DN3987_c0_g1_i1_4	376980	319750	361890	0	0	0	0	0	0	0	0	0	1753500	1553000	1872200
ARHOMBI_DN9641_c0_g1_i1_3	0	0	0	0	0	0	0	0	0	0	0	0	2916900	1416200	1902000
ARUBRA_DN16264_c0_g1_i1_5	0	0	0	0	0	0	0	0	0	0	0	0	3196200	1373300	1657200
ARHOMBI_DN15803_c0_g1_i1_2	0	0	0	0	0	141250	0	0	0	3064800	0	3009600	0	0	0
ARUBRA_DN1574_c0_g2_i1_2	0	0	0	0	0	0	0	0	0	0	0	0	3203200	3011600	0
ARHOMBI_DN1369_c0_g1_i1_5	193560	132770	125570	184440	0	0	0	0	0	0	0	0	1072100	2041800	2447600
ARUBRA_DN8743_c0_g1_i1_3	0	0	0	0	0	0	0	0	0	0	0	0	1760400	2426700	1970300
ARHOMBI_DN1764_c0_g1_i1_5	0	0	159490	0	0	0	0	0	0	0	0	0	1383700	2317800	2291900
ARHOMBI_DN5253_c0_g2_i1_2	0	0	0	0	0	0	0	0	0	0	0	0	2389300	1833900	1916700
ARHOMBI_DN5557_c0_g1_i1_6	0	0	0	0	0	0	0	0	0	0	0	0	1161800	2727000	2226700
ARHOMBI_DN27310_c0_g1_i1_4	225800	88525	269000	96005	0	0	465170	0	725460	0	2270700	1967400	0	0	0
ARUBRA_DN99_c0_g1_i1_2	0	0	0	0	0	0	0	0	0	0	0	0	1993200	1793700	2302200
ARUBRA_DN3903_c0_g1_i1_1	0	0	0	0	0	0	0	0	0	0	0	0	2339800	2677700	1068700
ARUBRA_DN620_c0_g1_i1_5	0	0	0	0	0	0	0	0	573070	0	0	0	1596000	1580800	2333500
ARUBRA_DN7726_c0_g1_i1_6	0	0	0	0	0	0	0	0	0	0	0	0	2725000	422760	2934300
ARHOMBI_DN12388_c0_g1_i1_3	0	0	0	0	0	0	0	0	0	0	0	0	1340700	1881100	2853000
ARHOMBI_DN5442_c0_g1_i1_6	582430	732620	501780	1263300	0	0	0	0	0	0	0	0	580830	600930	1806600

ARHOMBI_DN97_c0_g1_i1_1	268750	719950	921590	576390	420480	138280	1762100	920850	0	0	0	0	145610	0	179590
ARHOMBI_DN6380_c0_g1_i1_4	0	0	0	0	0	142950	0	0	4226300	0	0	0	0	1675400	0
ARUBRA_DN7925_c0_g1_i1_2	0	0	0	0	0	0	0	0	0	0	0	0	1432800	2050700	2556200
ARHOMBI_DN4703_c0_g1_i1_3	0	0	0	0	0	0	0	0	0	0	0	0	1180700	1905000	2933500
ARUBRA_DN2228_c0_g2_i1_2	0	0	0	0	0	0	0	0	0	0	0	0	1849900	2177600	1989600
ARUBRA_DN23264_c0_g1_i1_3	0	0	0	0	0	0	0	0	0	0	0	0	1875900	1737900	2391600
ARUBRA_DN22823_c0_g1_i1_2	1310100	1373100	1010400	1585600	422970	0	0	0	0	0	0	0	0	129990	171040
ARHOMBI_DN3499_c0_g1_i1_2	0	0	0	0	0	0	0	0	0	0	0	0	1647600	1656900	2684300
ARHOMBI_DN6537_c0_g1_i1_4	0	0	0	0	0	0	0	0	0	0	0	0	1780200	2315600	1865800
ARUBRA_DN16598_c0_g1_i1_4	0	0	0	0	0	0	0	0	0	2722500	0	3075400	143790	0	0
ARUBRA_DN3848_c0_g2_i1_1	0	2424600	2972600	219310	0	0	0	0	0	0	0	237360	0	0	0
ARUBRA_DN16655_c0_g1_i1_1	0	0	0	0	0	0	0	0	0	0	0	0	2393600	1434600	2018100
ARHOMBI_DN2235_c0_g1_i1_5	663660	835690	1595300	270350	0	140470	323280	0	0	0	0	0	408260	519890	1041900
ARUBRA_DN19778_c0_g1_i1_3	0	0	0	0	0	0	0	0	0	0	0	0	1365200	2185000	2231400
ARUBRA_DN5666_c0_g1_i1_2	0	0	0	0	0	0	0	0	0	0	0	0	1336500	2178400	2262800
ARUBRA_DN5508_c0_g1_i1_2	322370	0	720160	536160	0	0	0	2700400	1466000	0	0	0	0	0	0
ARUBRA_DN3962_c0_g1_i1_6	0	0	0	0	0	0	0	0	0	0	0	0	3072400	0	2645600
ARUBRA_DN20257_c0_g1_i1_3	1632200	858020	113400	60808	879160	522120	0	909650	229650	0	0	0	0	489500	0
ARUBRA_DN11024_c0_g1_i1_6	256190	411030	439400	115560	338980	0	0	0	0	1262200	1508800	1356400	0	0	0
ARUBRA_DN21569_c0_g1_i1_4	0	0	0	0	0	0	0	0	0	0	0	0	2602900	1269400	1812900
ARUBRA_DN965_c0_g1_i1_3	0	0	0	0	0	0	0	0	0	0	0	0	1851000	1764700	2068300
ARHOMBI_DN27321_c0_g1_i1_2	0	0	0	0	0	0	0	0	0	0	0	0	1982500	2105400	1580200
ARUBRA_DN1379_c0_g2_i1_4	0	0	0	0	2212600	0	2819300	0	0	0	0	0	163260	251480	217140
ARUBRA_DN21229_c0_g1_i1_6	0	0	0	0	0	0	0	0	0	0	0	0	1822300	1944200	1885500
ARUBRA_DN16619_c0_g1_i1_3	747480	881400	761900	431230	0	0	408620	0	858000	0	0	0	327630	576000	642730
ARHOMBI_DN17281_c0_g1_i1_1	0	0	0	0	0	0	0	0	0	0	0	0	1981800	1790000	1831800
ARHOMBI_DN4623_c0_g1_i1_3	114080	90009	90690	0	0	0	0	0	0	0	0	0	3074300	1081100	1145600

ARUBRA_DN6168_c0_g1_i1_1	2325000	1470000	1220900	578280	0	0	0	0	0	0	0	0	0	0	0
ARHOMBI_DN24755_c0_g1_i1_2	333720	332350	0	0	0	0	0	0	0	0	0	0	2389200	0	2528300
ARUBRA_DN4123_c0_g1_i1_5	0	0	0	0	0	0	0	0	0	0	0	0	2384300	1639800	1558900
ARUBRA_DN9_c0_g1_i1_4	0	0	0	0	0	0	0	0	0	0	0	0	1348000	2041600	2176500
ARHOMBI_DN6166_c0_g1_i1_6	164380	0	0	172830	0	0	0	0	0	0	0	0	377550	3329000	1503000
ARUBRA_DN1535_c0_g1_i1_5	0	0	0	0	0	0	0	0	0	0	0	0	1734300	1773700	2033200
ARUBRA_DN17285_c0_g1_i1_2	0	0	0	0	0	0	0	0	0	0	0	0	1564200	1739100	2227000
ARHOMBI_DN20415_c0_g1_i1_5	0	0	0	0	0	0	0	0	0	0	0	0	2533900	2529100	457410
ARHOMBI_DN6137_c0_g1_i1_4	354780	254900	472730	313350	0	0	0	198940	0	0	0	0	1099400	1245200	1571300
ARUBRA_DN20044_c0_g1_i1_6	0	0	0	0	0	0	0	0	0	0	0	0	2127600	1315400	2061500
ARUBRA_DN20625_c0_g1_i1_2	553980	1447600	801840	764030	503230	59200	0	349620	891070	0	0	0	0	0	121810
ARUBRA_DN21250_c0_g1_i1_6	0	0	0	0	0	0	0	0	0	0	0	0	2231500	3260300	0
ARUBRA_DN4028_c0_g1_i2_4	0	0	0	0	0	0	0	0	0	0	0	0	827910	1713600	2945900
ARUBRA_DN12697_c0_g1_i1_5	0	0	0	0	0	0	0	0	0	0	0	0	3118600	1105700	1262300
ARUBRA_DN17667_c0_g1_i1_4	0	0	0	0	0	0	0	0	0	0	0	0	1044800	2013800	2423100
ARHOMBI_DN16026_c0_g1_i1_1	0	0	0	0	0	0	0	0	0	0	0	0	2045500	1749300	1679900
ARUBRA_DN16494_c0_g1_i1_3	0	0	0	0	0	0	0	0	0	0	0	0	1307300	2305000	1838100
ARUBRA_DN6330_c0_g1_i1_4	0	0	0	0	0	0	0	0	0	0	0	0	4902100	543300	0
ARUBRA_DN13172_c0_g1_i1_1	1109700	1948900	1271500	1109900	0	0	0	0	0	0	0	0	0	0	0
ARHOMBI_DN6108_c0_g1_i2_2	100490	121260	102410	27297	2027900	0	3051200	0	0	0	0	0	0	0	0
ARUBRA_DN20543_c0_g1_i1_1	732330	471830	1116100	295150	796260	0	839350	202570	865640	0	0	0	0	83624	0
ARUBRA_DN3626_c0_g2_i1_5	1462800	897990	1051000	427620	287490	0	767160	0	0	0	0	0	0	226450	272700
ARHOMBI_DN3946_c0_g1_i1_1	111590	145180	110760	0	0	0	0	0	0	0	0	0	798970	1085400	3139000
ARUBRA_DN17212_c0_g1_i1_1	0	0	0	0	0	0	0	0	0	0	0	0	1345300	1501700	2532100
ARHOMBI_DN1738_c0_g2_i1_2	892980	1588500	2354500	537820	0	0	0	0	0	0	0	0	0	0	0
ARUBRA_DN4695_c0_g1_i1_1	1214000	2032100	2107500	0	0	0	0	0	0	0	0	0	0	0	0
ARUBRA_DN6843_c0_g1_i1_2	0	0	0	0	0	0	0	0	0	0	0	0	1480500	1446300	2410400

ARHOMBI_DN565_c0_g1_i1_1	0	0	0	0	0	0	0	0	0	5333100	0	0	0	0	0
ARUBRA_DN12655_c0_g1_i1_2	0	0	0	0	0	0	0	0	0	0	0	0	1031200	1953500	2340700
ARHOMBI_DN7486_c0_g1_i1_4	1271700	1629700	1810800	587310	0	0	0	0	0	0	0	0	0	0	0
ARHOMBI_DN3745_c0_g1_i1_6	1452500	827220	1331800	506890	545330	304810	0	0	0	0	0	244310	85758	0	0
ARHOMBI_DN161_c0_g2_i1_2	0	0	0	0	0	0	0	0	0	0	0	0	613780	1944100	2737200
ARHOMBI_DN4381_c0_g1_i1_4	103700	118720	0	97745	0	0	0	0	0	0	2736100	2236100	0	0	0
ARUBRA_DN4254_c0_g2_i1_1	1207600	1139100	1474500	757090	0	0	0	0	0	0	0	0	102380	487100	116570
ARUBRA_DN4783_c0_g1_i1_3	0	0	0	0	0	0	0	0	0	0	0	0	1395900	1913600	1967700
ARHOMBI_DN4848_c0_g1_i1_6	0	0	0	0	0	0	0	0	0	0	0	0	1499500	1831500	1941900
ARHOMBI_DN16223_c0_g1_i1_5	0	0	0	0	0	0	0	0	0	0	0	0	2658500	1614400	992490
ARHOMBI_DN19081_c0_g1_i1_3	0	0	0	0	0	0	0	0	0	0	0	0	2301300	1039500	1905200
ARUBRA_DN24202_c0_g1_i1_3	892100	1876600	1291400	1183900	0	0	0	0	0	0	0	0	0	0	0
ARUBRA_DN3211_c0_g1_i1_4	152740	126860	281590	302370	0	0	0	2441100	1451200	0	0	0	92610	114420	251030
ARUBRA_DN20320_c0_g1_i1_4	0	0	0	0	0	0	0	0	0	0	0	0	924740	900040	3380600
ARUBRA_DN4936_c0_g2_i1_2	0	0	27486	0	808630	0	610060	0	0	589050	690070	607180	0	793010	1060800
ARUBRA_DN9672_c0_g1_i1_4	0	0	0	0	0	0	0	0	0	0	0	0	1123000	1133500	2929400
ARUBRA_DN19765_c0_g1_i1_2	461970	597490	403530	962730	0	0	0	0	1003300	0	0	0	734880	0	995860
ARUBRA_DN3204_c0_g2_i1_1	174190	0	213200	66850	0	0	0	0	0	0	0	0	1849700	1899300	949420
ARUBRA_DN3663_c0_g1_i1_5	0	0	0	0	0	0	0	0	0	0	0	0	1276900	2663400	1189800
ARHOMBI_DN23207_c0_g1_i1_1	690300	617030	993130	0	758940	0	0	0	816520	0	0	0	0	585680	628150
ARHOMBI_DN10568_c0_g1_i1_2	0	0	0	0	0	0	0	0	0	0	0	0	1512100	1774900	1785100
ARUBRA_DN26415_c0_g1_i1_1	0	317370	0	0	0	0	0	0	0	0	0	0	637570	1874000	2238000
ARHOMBI_DN4088_c0_g1_i1_3	568130	236990	587250	0	163460	0	0	0	0	0	0	0	1355800	1285500	866880
ARHOMBI_DN5585_c0_g1_i1_3	308490	847000	874900	363750	116680	0	224220	238210	0	1090300	0	278830	0	395580	313180
ARHOMBI_DN14389_c0_g1_i1_3	0	0	0	0	0	0	0	0	0	0	0	0	1359700	0	3665400
ARUBRA_DN11036_c0_g1_i1_1	203270	443880	147330	30133	489640	0	0	0	0	0	0	0	1594600	381390	1730700
ARHOMBI_DN6168_c0_g1_i1_4	0	0	0	0	0	0	0	0	0	0	0	0	1118800	2438800	1462600

ARHOMBI_DN1051_c0_g1_i1_2	408410	291280	681120	417780	0	438040	815500	0	1505400	0	0	0	146830	128250	146400
ARHOMBI_DN4618_c0_g1_i1_5	0	0	0	649880	0	0	0	0	0	0	0	4323700	0	0	0
ARUBRA_DN5779_c0_g1_i1_6	0	0	0	0	0	0	0	0	0	0	0	0	2166800	590630	2203200
ARHOMBI_DN11483_c0_g1_i1_6	0	0	0	0	0	0	0	0	0	0	0	0	1573300	1573400	1807200
ARHOMBI_DN15111_c0_g1_i1_2	0	0	0	0	0	0	0	0	0	0	0	0	2325200	1160100	1460000
ARUBRA_DN343_c0_g2_i1_5	0	0	0	0	0	0	0	0	0	0	0	0	2475500	2444200	0
ARHOMBI_DN12998_c0_g1_i1_2	0	0	0	0	0	0	0	0	0	0	0	0	1206200	2069800	1641800
ARHOMBI_DN1655_c0_g1_i1_1	467690	753500	1352400	240650	220150	532960	155460	0	284300	0	0	0	358140	110030	382170
ARUBRA_DN8461_c0_g1_i1_4	110960	424920	400980	301550	470210	520590	0	789750	920590	0	0	0	260060	339320	304900
ARHOMBI_DN17992_c0_g1_i1_1	0	0	0	0	0	0	0	0	0	0	0	0	1056600	2697000	1079500
ARUBRA_DN796_c0_g1_i1_5	0	0	0	0	0	0	0	0	0	0	0	0	2391700	781340	1651500
ARHOMBI_DN11775_c0_g1_i1_1	0	0	0	0	0	0	0	0	743380	0	0	0	1301600	1243800	1529700
ARUBRA_DN3727_c0_g1_i1_4	0	647930	595500	1755600	0	0	0	0	1815600	0	0	0	0	0	0
ARUBRA_DN7635_c0_g1_i1_4	0	0	0	0	0	0	0	0	0	0	0	0	1914700	1192400	1707400
ARHOMBI_DN17384_c0_g1_i1_1	301160	368790	276530	0	629760	0	706600	0	835590	0	0	0	791560	614970	286760
ARHOMBI_DN8440_c0_g1_i1_5	0	0	0	0	0	0	0	0	0	0	0	0	953970	1895900	1957000
ARHOMBI_DN4904_c0_g1_i1_1	1250700	1165300	1577800	717780	0	0	93508	0	0	0	0	0	0	0	0
ARUBRA_DN24171_c0_g1_i1_6	0	0	0	0	0	0	0	0	0	0	0	0	1612500	1485300	1698900
ARHOMBI_DN15505_c0_g1_i1_1	0	0	0	0	0	0	0	0	0	0	0	0	1518400	1410200	1846700
ARUBRA_DN19884_c0_g1_i1_4	761700	572330	786480	281450	519230	0	526860	0	0	0	1327100	0	0	0	0
ARHOMBI_DN2480_c0_g1_i1_6	1118500	1150100	1247300	458360	287860	0	338770	0	0	0	0	0	0	162850	0
ARHOMBI_DN5911_c0_g2_i1_1	0	0	0	0	0	0	0	0	0	0	0	0	1927500	1473100	1349200
ARHOMBI_DN11895_c0_g1_i1_5	804710	838870	1252100	500290	0	629140	0	703550	0	0	0	0	0	0	0
ARHOMBI_DN10040_c0_g1_i1_3	0	0	0	0	0	0	0	0	0	0	0	0	2285200	2430500	0
ARHOMBI_DN20237_c0_g1_i1_3	0	0	0	0	0	0	0	0	0	0	0	0	1318200	1996700	1388100
ARHOMBI_DN13255_c0_g1_i1_6	0	0	0	0	0	0	0	0	0	0	0	0	3209400	672650	814550
ARUBRA_DN1076_c0_g1_i1_4	652870	573710	790130	0	0	0	169290	0	0	0	0	0	659380	473870	1334600

ARHOMBI_DN7071_c0_g1_i1_2	323700	376190	230290	38776	692400	0	765980	268120	428610	0	0	0	1063700	463750	0
ARUBRA_DN13122_c0_g1_i1_4	0	0	0	0	0	0	4640900	0	0	0	0	0	0	0	0
ARHOMBI_DN1703_c0_g1_i1_6	0	0	0	0	0	0	0	0	0	0	0	0	1273900	1775300	1588000
ARUBRA_DN8641_c0_g1_i1_4	0	0	0	0	0	0	0	0	0	0	0	0	1130800	2430000	1071300
ARUBRA_DN2071_c0_g3_i1_4	0	0	0	0	0	0	0	0	0	0	0	0	1698100	864620	2067500
ARUBRA_DN24590_c0_g1_i1_2	752700	854170	1020000	757100	0	0	726510	373680	0	0	0	0	133690	0	0
ARUBRA_DN9637_c0_g1_i1_6	0	0	0	0	0	0	0	0	0	0	0	0	1523100	1459200	1627700
ARUBRA_DN12612_c0_g1_i1_4	404210	400930	465900	381600	0	0	453330	0	1194200	0	0	1111900	182910	0	0
ARHOMBI_DN3999_c0_g1_i1_1	0	0	0	0	0	0	0	0	0	0	0	0	1569200	1119400	1888800
ARHOMBI_DN5284_c0_g1_i1_4	99351	101580	64183	0	0	0	0	0	0	0	0	0	731920	747690	2819900
ARUBRA_DN7275_c0_g1_i1_1	0	0	0	0	0	0	0	0	0	0	0	0	455330	1889000	2214200
ARHOMBI_DN3047_c0_g1_i1_6	0	0	0	0	0	0	0	0	0	0	0	0	1776400	1311500	1464200
ARUBRA_DN12194_c0_g1_i1_6	0	0	0	0	0	0	0	0	0	0	0	0	0	1440100	3107600
ARHOMBI_DN20434_c0_g1_i1_2	108620	0	210970	0	825920	309550	903690	656950	1258200	0	0	0	0	253250	0
ARHOMBI_DN3438_c0_g1_i1_6	423800	315700	355500	0	0	106040	0	0	0	0	0	0	504170	645420	2139700
ARHOMBI_DN4924_c0_g1_i1_5	115050	0	0	0	0	0	0	0	0	0	0	0	1385100	1403700	1580100
ARUBRA_DN11906_c0_g1_i1_6	0	0	0	0	0	0	0	0	0	0	0	0	1858300	2622000	0
ARUBRA_DN21459_c0_g1_i1_4	368280	2792200	410710	906860	0	0	0	0	0	0	0	0	0	0	0
ARUBRA_DN23079_c0_g1_i1_1	0	0	0	0	0	0	0	0	0	0	0	0	1868500	2582300	0
ARUBRA_DN15741_c0_g1_i1_6	747610	1056400	777850	694780	0	0	0	0	1151700	0	0	0	0	0	0
ARHOMBI_DN3883_c0_g1_i1_5	0	0	0	0	0	0	0	0	0	0	0	0	1023700	979800	2415700
ARUBRA_DN4014_c0_g2_i1_3	1824800	1979000	610480	0	0	0	0	0	0	0	0	0	0	0	0
ARUBRA_DN4532_c0_g1_i1_4	0	0	0	0	0	0	0	0	0	0	0	0	98849	3478800	833200
ARUBRA_DN7957_c0_g1_i1_1	79005	60090	63035	25318	0	186540	1259600	1477300	341680	0	0	0	426260	489900	0
ARHOMBI_DN22787_c0_g1_i1_4	0	0	0	0	0	0	0	0	0	0	0	0	860630	1137300	2390800
ARUBRA_DN18867_c0_g1_i1_5	0	78258	337000	119720	641700	0	0	0	0	0	0	0	2051600	0	1157300
ARHOMBI_DN17883_c0_g1_i1_2	0	0	0	0	0	0	0	0	0	0	0	0	1167700	1361300	1853000

ARHOMBI_DN14524_c0_g1_i1_1	181490	166460	42888	49932	0	0	0	302200	0	1642400	1982700	0	0	0	0
ARUBRA_DN2441_c0_g1_i1_2	0	0	0	0	0	0	0	0	0	0	0	0	1349900	1389200	1599000
ARUBRA_DN25648_c0_g1_i1_1	0	0	0	0	0	0	0	0	0	0	0	0	1376000	1390400	1567600
ARUBRA_DN4730_c0_g1_i1_2	259070	177740	331240	0	1385400	0	816070	307680	533230	0	0	0	0	361410	155240
ARUBRA_DN4181_c0_g1_i1_5	0	0	0	0	0	0	0	0	0	0	0	0	0	1051600	3273900
ARHOMBI_DN26738_c0_g1_i1_4	0	0	0	0	0	0	0	0	0	0	0	0	825810	2469800	1029300
ARUBRA_DN5147_c0_g1_i1_4	0	0	0	0	0	0	0	0	0	0	0	0	1300900	1305500	1709000
ARHOMBI_DN5499_c0_g1_i1_3	0	0	0	0	0	0	0	0	0	0	0	0	1748600	1893500	664760
ARUBRA_DN8113_c0_g1_i1_3	0	0	0	0	0	0	0	0	0	0	0	0	1201000	816480	2282400
ARHOMBI_DN1623_c0_g1_i1_6	0	0	0	0	0	0	0	0	0	0	0	0	726810	1806000	1761600
ARUBRA_DN4329_c0_g1_i1_6	0	0	0	0	0	0	0	0	0	0	0	0	1710200	713560	1870300
ARUBRA_DN4117_c0_g1_i1_6	0	0	0	0	0	0	0	0	0	0	0	0	2106400	0	2168600
ARUBRA_DN18085_c0_g1_i1_1	0	0	0	0	0	0	0	0	0	0	0	0	2504300	1768100	0
ARUBRA_DN5456_c0_g1_i1_2	265730	242380	459680	310070	0	0	395020	0	0	0	0	0	745050	1029500	824830
ARHOMBI_DN23974_c0_g1_i1_1	0	0	0	0	0	0	0	0	0	0	0	0	1406100	1294200	1569200
ARHOMBI_DN3770_c0_g1_i1_1	0	0	0	0	0	0	0	0	0	0	0	0	1979200	100340	2176000
ARUBRA_DN4031_c0_g1_i1_2	516110	409560	763780	770440	0	0	0	449200	1338800	0	0	0	0	0	0
ARUBRA_DN4198_c0_g2_i2_4	258350	245750	330100	187810	835660	238670	205070	0	1945400	0	0	0	0	0	0
ARHOMBI_DN16331_c0_g1_i1_2	0	66377	0	0	0	0	0	0	0	0	0	0	989360	2714200	467260
ARHOMBI_DN8849_c0_g1_i1_2	0	0	0	0	0	0	0	0	0	0	0	0	1555300	980300	1695600
ARUBRA_DN6372_c0_g1_i1_2	0	111360	0	0	0	0	0	0	0	0	0	0	1226600	1478000	1408500
ARUBRA_DN16718_c0_g1_i1_3	0	0	0	0	0	0	0	0	366340	0	0	0	1396000	1162800	1291100
ARHOMBI_DN10686_c0_g1_i1_3	531150	619880	696960	374510	0	0	669500	0	0	0	0	0	368850	0	938610
ARUBRA_DN8773_c0_g1_i1_4	0	0	0	0	0	0	0	0	0	0	0	0	4074600	103760	0
ARUBRA_DN1981_c0_g1_i1_6	0	0	0	0	0	0	0	0	0	0	0	0	0	1623700	2552700
ARHOMBI_DN6947_c0_g1_i1_1	0	0	0	0	0	0	0	0	0	0	0	0	1737500	2278400	153130
ARHOMBI_DN4632_c0_g1_i1_3	0	0	0	0	0	0	0	0	0	0	0	0	926680	1239100	2000000

ARHOMBI_DN14482_c0_g1_i1_6	70952	0	71480	41380	0	0	629640	737870	0	0	0	0	1153800	0	1456700
ARHOMBI_DN3429_c0_g1_i1_6	0	0	0	0	0	0	0	0	0	0	0	0	923680	1272500	1952200
ARHOMBI_DN3622_c0_g1_i1_4	0	0	0	0	0	0	0	0	0	0	0	0	1498000	1238900	1410700
ARUBRA_DN4615_c0_g2_i1_3	0	0	0	0	0	0	0	0	0	0	0	0	0	0	4132300
ARHOMBI_DN17829_c0_g1_i1_1	0	0	0	0	0	0	0	0	0	0	0	0	2139600	469730	1491300
ARUBRA_DN6205_c0_g1_i1_1	0	0	0	0	0	0	0	0	0	0	0	0	1785200	1188400	1103900
ARUBRA_DN24096_c0_g1_i1_3	792800	0	0	0	0	0	0	0	0	0	3279700	0	0	0	0
ARUBRA_DN1065_c0_g2_i1_2	0	0	0	0	0	0	0	0	0	0	0	0	988090	1481200	1600000
ARHOMBI_DN12564_c0_g1_i1_6	0	0	0	0	0	0	0	0	0	0	0	0	1034400	992000	2034400
ARHOMBI_DN3278_c0_g1_i1_3	1181200	820440	365240	350630	0	0	269650	184070	0	0	0	0	0	563120	317540
ARUBRA_DN16058_c0_g1_i1_4	278290	327450	175010	336310	0	45268	0	168230	0	0	0	0	0	1488200	1232800
ARUBRA_DN9760_c0_g1_i1_3	0	0	0	0	0	0	0	0	0	0	0	0	1330100	1646800	1069200
ARHOMBI_DN6246_c0_g1_i3_3	0	0	0	837140	0	0	0	1276400	1923200	0	0	0	0	0	0
ARHOMBI_DN3643_c0_g1_i1_2	0	0	0	0	0	0	0	0	0	0	0	0	1081200	0	2946000
ARUBRA_DN18439_c0_g1_i1_3	0	0	0	0	0	0	0	0	0	0	0	0	1305600	1264200	1452300
ARUBRA_DN4784_c0_g2_i3_2	0	0	0	0	0	0	0	0	0	0	0	0	1507900	1333000	1175000
ARHOMBI_DN16305_c0_g1_i1_3	0	0	0	0	126810	0	0	0	0	0	0	0	1706100	2179400	0
ARUBRA_DN1937_c0_g2_i1_6	0	0	0	0	0	0	0	0	0	0	0	0	1141600	1440300	1430200
ARHOMBI_DN3129_c0_g1_i1_2	955320	869730	779840	312240	0	0	0	0	0	0	0	0	0	0	1094500
ARUBRA_DN1180_c0_g1_i1_3	0	0	0	0	0	0	0	0	0	0	0	0	2211400	745200	1026700
ARHOMBI_DN24079_c0_g1_i1_2	293110	341600	157170	249330	529400	136120	0	339960	1734900	0	0	0	194700	0	0
ARHOMBI_DN17905_c0_g1_i1_4	0	196550	198150	466770	0	0	0	0	0	0	1633100	1468700	0	0	0
ARHOMBI_DN13275_c0_g1_i1_1	0	0	0	0	0	0	0	0	0	0	0	0	2509800	0	1438400
ARHOMBI_DN17238_c0_g1_i1_3	319720	322890	320170	112790	0	108850	310590	414460	1789800	0	0	0	0	0	221380
ARHOMBI_DN6259_c0_g2_i7_3	0	67615	0	0	0	0	0	0	0	1985800	1863700	0	0	0	0
ARHOMBI_DN4135_c0_g2_i1_5	410610	0	572490	431510	989190	338450	503190	666930	0	0	0	0	0	0	0
ARUBRA_DN4552_c1_g2_i1_5	1289300	1416400	478570	719190	0	0	0	0	0	0	0	0	0	0	0

ARHOMBI_DN6298_c0_g1_i1_1	0	0	0	0	0	0	0	0	0	0	0	0	0	1878300	2024600
ARHOMBI_DN2163_c0_g1_i1_5	0	0	0	0	0	0	0	0	0	0	0	0	1501900	1362700	1032900
ARHOMBI_DN6274_c0_g2_i10_6	797130	492330	109330	578920	325580	803480	785020	0	0	0	0	0	0	0	0
ARUBRA_DN6417_c0_g1_i1_1	0	0	0	0	0	0	0	0	0	0	0	0	1803000	2083800	0
ARUBRA_DN16375_c0_g1_i1_3	0	0	0	0	0	0	0	0	0	0	0	0	1299500	1149200	1436700
ARUBRA_DN20085_c0_g1_i1_4	0	0	0	0	0	0	0	0	0	0	0	0	2363700	841520	679260
ARHOMBI_DN7053_c0_g1_i1_6	227550	479010	300260	458270	998960	0	0	245500	0	0	0	264090	907570	0	0
ARHOMBI_DN4215_c0_g1_i1_6	0	0	0	0	0	0	0	0	0	0	0	0	2005800	1093900	779470
ARUBRA_DN6101_c0_g1_i1_3	0	0	0	0	0	0	0	0	0	0	0	0	2135600	892210	845570
ARHOMBI_DN5908_c0_g1_i1_6	89155	0	0	99559	0	50143	0	0	0	0	0	0	771600	1023400	1831300
ARHOMBI_DN12316_c0_g1_i1_3	0	0	0	0	0	0	0	0	0	0	0	0	0	1563900	2293400
ARHOMBI_DN1573_c0_g2_i1_5	0	0	0	0	0	0	0	0	0	0	0	0	1175800	1640100	1028900
ARUBRA_DN3281_c0_g1_i1_4	75227	0	127210	0	854270	364940	956280	870580	406630	189670	0	0	0	0	0
ARHOMBI_DN5746_c0_g1_i1_4	0	0	0	0	0	0	0	0	0	0	0	0	0	1753600	2086200
ARUBRA_DN17629_c0_g1_i1_1	0	0	0	0	0	0	0	0	0	0	0	0	1582100	477780	1775000
ARHOMBI_DN1692_c0_g1_i1_5	0	65144	0	101840	0	0	0	0	0	0	0	0	2031700	273470	1333000
ARUBRA_DN15157_c0_g1_i1_2	0	193730	0	512720	0	0	0	0	1312600	0	943460	834860	0	0	0
ARHOMBI_DN17586_c0_g1_i1_5	0	0	0	0	0	0	0	0	0	0	0	0	1785800	1745300	258500
ARUBRA_DN16576_c0_g1_i1_6	0	0	0	0	0	0	0	0	0	0	0	0	1110900	1228200	1441900
ARHOMBI_DN12404_c0_g1_i1_3	0	0	0	0	0	0	0	0	0	0	0	0	588820	632250	2554500
ARHOMBI_DN5690_c0_g1_i1_6	0	0	0	0	0	0	0	677850	0	999300	991370	935680	0	161220	0
ARHOMBI_DN14107_c0_g1_i1_6	0	0	0	0	0	0	0	0	0	0	0	0	1363000	1155300	1247000
ARUBRA_DN21696_c0_g1_i1_3	0	0	0	0	0	0	0	0	0	0	2106000	1655500	0	0	0
ARUBRA_DN12039_c0_g1_i1_4	0	0	0	0	0	0	0	0	0	0	0	0	1067200	729410	1961400
ARHOMBI_DN17617_c0_g1_i1_1	0	0	0	0	0	0	0	0	0	0	0	0	1399700	610070	1740100
ARUBRA_DN25457_c0_g1_i1_2	0	0	0	0	0	0	0	0	0	0	0	0	1317200	1086600	1337400
ARUBRA_DN9520_c0_g1_i1_3	0	0	0	0	0	0	0	0	0	0	0	0	1372100	1135400	1229900

ARUBRA_DN12747_c0_g1_i1_4	177270	0	0	0	0	0	0	0	436800	0	0	0	1555500	0	1561700
ARHOMBI_DN27277_c0_g1_i1_1	0	0	0	0	0	0	0	0	0	0	0	0	1737800	383880	1604100
ARUBRA_DN21434_c0_g1_i1_3	0	0	0	0	0	0	0	0	0	0	0	0	1219100	1205500	1282100
ARUBRA_DN4990_c0_g1_i1_1	0	0	0	218840	0	0	0	0	3467800	0	0	0	0	0	0
ARHOMBI_DN18290_c0_g1_i1_4	773910	587560	937930	627860	0	53023	0	0	0	0	0	0	183000	514980	0
ARUBRA_DN8515_c0_g1_i1_2	0	0	0	0	0	0	0	0	0	0	0	0	701320	705780	2259800
ARUBRA_DN7009_c0_g1_i1_3	0	0	0	0	0	0	0	0	0	0	0	0	899440	1889400	877480
ARUBRA_DN16706_c0_g1_i1_4	0	0	0	0	0	0	0	0	0	0	0	0	1222300	1102600	1341300
ARUBRA_DN8627_c0_g1_i1_5	0	0	0	0	0	0	0	0	0	0	0	0	0	2510400	1151200
ARUBRA_DN19094_c0_g1_i1_2	291040	0	24611	0	0	0	183640	0	0	0	0	206850	548680	1570900	802800
ARUBRA_DN4464_c0_g2_i1_2	0	0	0	0	0	0	0	0	0	0	0	0	1317100	945760	1361100
ARUBRA_DN19978_c0_g1_i1_6	0	0	0	0	0	0	0	0	0	0	0	0	346990	562150	2711000
ARHOMBI_DN26182_c0_g1_i1_3	0	0	0	0	0	0	0	0	0	0	0	0	1134200	1124800	1360900
ARUBRA_DN5518_c0_g1_i1_3	82065	50023	222650	270770	0	0	0	0	0	0	0	0	976870	881050	1125700
ARHOMBI_DN6030_c1_g5_i1_6	777260	1175400	0	1653300	0	0	0	0	0	0	0	0	0	0	0
ARHOMBI_DN15479_c0_g1_i1_2	0	0	0	0	0	0	0	0	0	0	0	0	266190	0	3315900
ARHOMBI_DN21367_c0_g1_i1_4	0	0	0	0	0	0	0	0	0	0	0	0	834730	1201100	1545800
ARUBRA_DN9892_c0_g1_i1_6	0	0	0	0	0	0	0	0	0	0	0	0	779120	1392500	1409700
ARHOMBI_DN8750_c0_g1_i1_3	0	0	0	0	0	0	0	0	0	0	0	0	1425300	1007200	1139200
ARHOMBI_DN13150_c0_g1_i1_1	0	0	0	80748	0	0	0	0	0	0	0	3462000	0	0	0
ARHOMBI_DN3473_c0_g1_i1_4	1590000	0	1447300	504360	0	0	0	0	0	0	0	0	0	0	0
ARUBRA_DN22076_c0_g1_i1_2	340520	0	1381700	0	403540	248780	0	0	429110	0	0	0	0	427410	297820
ARUBRA_DN11215_c0_g1_i1_3	0	0	0	0	0	0	0	0	0	0	0	0	912090	1192000	1422600
ARHOMBI_DN1047_c0_g1_i1_6	0	0	0	0	0	0	0	0	0	0	0	0	572230	2485000	468870
ARUBRA_DN1728_c0_g1_i1_2	0	0	0	152680	0	0	0	0	0	0	0	0	1841700	1531300	0
ARHOMBI_DN5812_c1_g3_i1_1	365630	304120	171000	240070	0	276710	435910	0	1551700	0	0	0	0	0	173450
ARUBRA_DN17307_c0_g1_i1_2	0	0	0	0	0	0	741100	1196600	1579000	0	0	0	0	0	0

ARHOMBI_DN4645_c1_g1_i1_2	0	0	0	123840	0	0	0	0	0	0	0	0	791230	1155400	1446100
ARUBRA_DN8669_c0_g1_i1_4	0	0	0	0	0	0	0	0	0	0	0	0	0	1489200	2020700
ARUBRA_DN5513_c0_g1_i1_1	0	0	0	0	0	0	0	0	0	0	0	0	821990	408130	2278500
ARUBRA_DN4813_c0_g1_i1_1	0	0	0	0	0	0	0	0	0	0	0	0	1553600	1252500	697370
ARUBRA_DN10983_c0_g1_i1_4	0	0	0	0	0	0	0	0	0	0	0	0	408720	1227000	1866900
ARUBRA_DN4671_c1_g1_i1_5	0	0	0	0	0	0	0	0	0	0	0	0	204690	2008900	1287300
ARUBRA_DN5698_c0_g1_i1_2	0	0	0	0	0	0	0	0	0	0	0	0	615710	2311000	572390
ARUBRA_DN8516_c0_g1_i1_2	0	0	0	0	0	0	0	0	0	0	0	0	946810	913080	1633000
ARHOMBI_DN3145_c0_g1_i1_3	0	0	0	1455300	0	0	0	0	0	0	0	0	944530	0	1059800
ARUBRA_DN26369_c0_g1_i1_1	0	0	0	0	0	0	0	0	0	0	0	0	1557900	0	1893400
ARUBRA_DN16851_c0_g1_i1_2	305850	135210	0	0	0	0	0	0	0	0	0	0	0	0	3001800
ARHOMBI_DN22852_c0_g1_i1_6	0	0	0	0	0	0	0	0	0	0	0	0	1110500	1232900	1095300
ARHOMBI_DN25003_c0_g1_i1_6	0	0	0	0	0	0	0	0	181090	0	0	0	1315200	1166200	771220
ARUBRA_DN17081_c0_g1_i1_5	0	0	0	0	0	0	0	0	0	0	0	0	932980	1138000	1357700
ARHOMBI_DN4475_c0_g1_i1_1	248580	245900	0	0	253740	135780	732700	0	257020	0	176620	0	512050	631120	229060
ARUBRA_DN6562_c0_g1_i1_2	0	0	0	0	0	0	0	0	0	0	0	0	642990	2773900	0
ARUBRA_DN9315_c0_g1_i1_1	0	0	0	0	0	0	0	0	0	0	0	0	1474000	1619900	305870
ARUBRA_DN5821_c0_g1_i1_4	958470	897440	595090	132730	0	0	0	0	0	0	0	0	0	342690	469410
ARHOMBI_DN15559_c0_g1_i1_1	1035800	1283200	385510	192060	0	249980	0	0	0	0	0	0	0	246940	0
ARHOMBI_DN3769_c0_g1_i1_4	994860	577550	610900	0	866600	0	0	333600	0	0	0	0	0	0	0
ARUBRA_DN6679_c0_g1_i1_2	0	0	0	0	0	0	0	0	0	0	0	0	2986400	0	396000
ARUBRA_DN3045_c0_g1_i1_5	0	0	0	0	0	0	0	0	0	0	0	0	988660	1509000	874800
ARUBRA_DN21269_c0_g1_i1_2	0	0	0	0	0	0	0	0	0	0	0	0	587420	1352000	1432300
ARHOMBI_DN3422_c0_g1_i1_5	0	1404300	0	444920	0	0	0	0	0	0	0	168770	211620	211820	923450
ARUBRA_DN11946_c0_g1_i1_3	56505	0	0	0	0	0	0	0	0	0	0	0	1376900	269580	1638700
ARUBRA_DN428_c0_g1_i1_5	0	0	0	0	0	0	0	0	0	0	0	0	398600	1485900	1456200
ARHOMBI_DN1985_c0_g2_i1_1	0	0	0	0	0	0	0	0	0	0	0	0	1593500	433440	1285900

ARUBRA_DN11238_c0_g1_i1_6	713230	536820	492570	891280	0	0	0	0	0	0	0	0	0	0	677130
ARHOMBI_DN11429_c0_g1_i1_1	0	64572	0	0	0	436330	0	1223600	1433400	0	0	0	0	0	142420
ARUBRA_DN2583_c0_g2_i1_4	1546600	1748900	0	0	0	0	0	0	0	0	0	0	0	0	0
ARHOMBI_DN5242_c0_g1_i1_4	0	0	0	0	0	0	0	0	0	0	0	0	562090	605530	2105600
ARUBRA_DN3984_c0_g1_i1_3	313520	392970	366590	388780	0	0	0	0	0	0	0	0	0	851250	959040
ARHOMBI_DN13132_c0_g1_i1_4	661920	372410	507780	545460	0	0	0	0	0	0	1072400	0	0	0	106490
ARHOMBI_DN17057_c0_g1_i1_3	0	0	0	0	0	0	0	0	0	0	0	0	1089900	992120	1166000
ARHOMBI_DN328_c0_g1_i1_6	0	0	0	0	0	0	0	0	0	0	0	0	248150	729760	2267800
ARUBRA_DN4992_c0_g1_i2_1	218270	183010	138230	44819	699920	219260	802980	0	700260	0	0	0	228390	0	0
ARHOMBI_DN10598_c0_g1_i1_3	435250	435990	492490	78918	173750	237250	326980	346270	0	0	0	0	245180	0	461530
ARHOMBI_DN4392_c0_g1_i1_3	0	0	0	0	0	0	0	0	0	0	0	0	911340	1287100	1034900
ARUBRA_DN6255_c0_g1_i1_2	158150	137310	192350	263250	0	0	0	0	761880	0	0	0	424080	307550	984350
ARUBRA_DN8387_c0_g1_i1_5	0	0	0	0	0	0	0	0	0	0	0	0	223710	1359900	1632800
ARUBRA_DN2057_c0_g1_i1_6	0	0	0	0	0	0	0	0	0	0	0	0	2015100	1200700	0
ARHOMBI_DN11616_c0_g1_i1_3	0	0	0	0	297910	0	0	0	2907800	0	0	0	0	0	0
ARHOMBI_DN15140_c0_g1_i1_3	0	0	0	0	0	0	0	0	0	0	0	0	0	3185500	0
ARUBRA_DN186_c0_g1_i1_4	293870	183390	176900	0	469900	169000	336760	256960	221610	0	0	593130	141110	178910	163610
ARUBRA_DN18420_c0_g1_i1_3	527920	410840	416170	354860	370010	0	0	0	1103300	0	0	0	0	0	0
ARHOMBI_DN736_c0_g2_i1_6	0	0	0	0	0	0	0	0	0	0	0	0	235760	1522200	1423100
ARHOMBI_DN8058_c0_g1_i1_5	0	0	0	0	0	0	0	0	0	0	0	0	1407800	627790	1143000
ARHOMBI_DN13310_c0_g1_i1_4	0	0	0	0	0	0	0	0	0	0	0	0	916480	993280	1256900
ARUBRA_DN19795_c0_g1_i1_2	0	0	0	0	0	0	0	0	0	0	0	0	1048200	908800	1205000
ARHOMBI_DN15499_c0_g1_i1_1	496990	573150	554940	477110	325760	0	0	389200	328590	0	0	0	0	0	0
ARHOMBI_DN5591_c0_g1_i1_2	0	0	0	395900	0	150250	269150	515050	847140	0	0	0	155380	0	809930
ARHOMBI_DN2375_c0_g1_i1_6	0	0	0	81688	0	0	0	0	0	0	0	0	108790	1047200	1901600
ARHOMBI_DN15343_c0_g1_i1_2	663900	258840	548140	524270	593220	0	0	187330	0	0	0	0	0	0	343520
ARUBRA_DN1522_c0_g1_i1_5	0	50797	66573	0	0	0	0	0	306830	679800	756200	0	389510	402410	438950

ARHOMBI_DN6216_c0_g1_i1_4	0	0	0	0	0	0	0	0	0	0	0	0	1154500	641550	1283200
ARHOMBI_DN5056_c0_g1_i1_4	0	0	0	0	0	0	0	0	0	0	0	0	974130	934210	1161500
ARUBRA_DN4756_c0_g1_i1_6	0	0	0	0	0	0	0	0	0	0	0	0	0	2052200	1017300
ARHOMBI_DN3633_c0_g1_i1_2	0	0	0	0	0	0	462640	0	0	0	0	0	529860	1315600	757720
ARHOMBI_DN16542_c0_g1_i1_1	564010	505190	564270	371990	0	0	1058900	0	0	0	0	0	0	0	0
ARHOMBI_DN2386_c0_g1_i1_2	0	0	0	0	0	0	0	0	0	0	0	0	1185600	847730	1028100
ARHOMBI_DN2790_c0_g2_i1_3	0	0	0	0	0	0	0	0	0	0	0	0	979010	984440	1094600
ARHOMBI_DN5912_c0_g2_i4_5	594220	429030	1455700	578670	0	0	0	0	0	0	0	0	0	0	0
ARUBRA_DN9466_c0_g1_i1_6	0	0	0	0	0	0	0	0	0	0	0	0	1496100	0	1560000
ARHOMBI_DN5625_c0_g1_i1_5	432460	296360	347870	185470	692580	768400	331320	0	0	0	0	0	0	0	0
ARUBRA_DN26355_c0_g1_i1_1	0	0	0	0	0	0	0	0	0	0	0	0	0	1454200	1593600
ARUBRA_DN17795_c0_g1_i1_3	0	0	0	0	0	0	0	0	0	0	0	0	1203600	1171200	662240
ARUBRA_DN4209_c0_g1_i1_6	257560	269180	308240	337570	0	0	0	0	1749400	0	0	0	0	0	112160
ARHOMBI_DN13841_c0_g1_i1_1	0	0	0	0	0	0	0	0	0	0	0	0	3027200	0	0
ARUBRA_DN7189_c0_g1_i1_4	0	0	0	0	0	0	0	0	0	0	0	0	455730	1528300	1043000
ARUBRA_DN17439_c0_g1_i1_4	737640	920060	768910	155000	0	444740	0	0	0	0	0	0	0	0	0
ARUBRA_DN23385_c0_g1_i1_4	108030	206320	746340	0	0	0	0	0	0	600870	0	0	723620	640720	0
ARUBRA_DN3392_c0_g2_i1_2	925930	666420	641940	605740	0	172170	0	0	0	0	0	0	0	0	0
ARUBRA_DN16420_c0_g1_i1_5	0	0	0	0	0	0	0	0	0	0	0	0	532150	1297200	1181900
ARUBRA_DN5780_c0_g1_i1_6	0	0	0	0	0	0	0	0	0	0	0	0	2577700	429600	0
ARHOMBI_DN9968_c0_g1_i1_3	353620	573950	364240	815090	0	0	898140	0	0	0	0	0	0	0	0
ARUBRA_DN22029_c0_g1_i1_1	0	0	0	0	0	0	0	0	0	0	0	0	438570	350160	2210300
ARHOMBI_DN6264_c0_g1_i2_1	0	124690	0	217230	0	141590	0	0	2515000	0	0	0	0	0	0
ARUBRA_DN9942_c0_g1_i1_2	0	810850	998880	311930	557410	0	0	0	0	0	0	0	0	303850	0
ARHOMBI_DN10129_c0_g1_i1_4	0	0	0	0	0	0	0	0	0	0	0	0	885870	981630	1114600
ARUBRA_DN4816_c0_g4_i1_1	0	0	0	0	0	0	0	0	0	0	0	0	957110	2018200	0
ARHOMBI_DN9150_c0_g1_i1_2	733040	626240	685010	101610	0	0	488320	0	0	0	0	0	338840	0	0

ARHOMBI_DN18526_c0_g1_i1_5	0	0	0	0	0	0	0	0	0	0	0	0	466140	1154100	1351500
ARHOMBI_DN5512_c0_g1_i1_3	0	52009	0	0	0	0	0	0	0	0	0	0	618850	1361600	929930
ARUBRA_DN18242_c0_g1_i1_1	0	208020	79820	29076	0	0	0	0	0	0	0	0	1486600	0	1158200
ARUBRA_DN6103_c0_g1_i1_5	480620	314030	578230	0	0	0	0	0	0	0	0	0	232650	505870	830170
ARHOMBI_DN4464_c0_g1_i1_6	0	0	0	0	0	0	0	0	0	0	0	0	1424500	394030	1121300
ARUBRA_DN21777_c0_g1_i1_4	0	74304	0	0	0	0	0	0	0	0	0	274560	829920	1106900	647700
ARHOMBI_DN6373_c0_g1_i1_1	0	0	0	0	0	0	0	0	0	0	0	0	843450	1033900	1054900
ARUBRA_DN4890_c0_g2_i1_5	53510	111680	0	0	376800	0	0	140880	341030	0	0	0	748040	582560	572700
ARHOMBI_DN3410_c0_g1_i1_4	793750	645020	782930	695000	0	0	0	0	0	0	0	0	0	0	0
ARUBRA_DN1360_c0_g2_i1_4	0	0	0	0	0	152680	471530	407980	1074200	0	0	0	251920	272390	285200
ARUBRA_DN20255_c0_g1_i1_4	0	0	0	0	0	0	925030	0	0	0	0	0	266680	529770	1192200
ARUBRA_DN12071_c0_g1_i1_2	0	0	0	0	0	0	0	0	0	0	0	0	717480	793980	1401300
ARUBRA_DN25817_c0_g1_i1_2	0	0	0	0	0	0	0	0	0	0	0	0	1503400	981020	415710
ARUBRA_DN24145_c0_g1_i1_1	0	0	0	0	0	0	0	0	0	0	0	0	1319100	0	1568600
ARHOMBI_DN6283_c0_g6_i1_6	0	0	0	0	0	0	0	0	0	0	0	0	0	1473900	1413100
ARUBRA_DN3001_c0_g1_i1_6	0	0	0	0	0	54538	0	290440	0	0	0	0	826210	833990	870730
ARUBRA_DN18461_c0_g1_i1_6	0	0	0	0	199330	0	215870	0	418070	729950	759810	545920	0	0	0
ARUBRA_DN10353_c1_g1_i1_5	630150	155490	376480	82436	506150	162050	950960	0	0	0	0	0	0	0	0
ARHOMBI_DN25343_c0_g1_i1_2	398500	464170	46720	267330	106240	0	0	0	0	0	0	0	0	0	1565300
ARHOMBI_DN7398_c0_g1_i1_3	0	0	0	0	0	0	0	0	0	0	0	0	1240400	690500	917030
ARHOMBI_DN6728_c0_g1_i1_4	0	0	0	0	0	0	0	0	0	0	0	0	692380	653150	1499900
ARUBRA_DN3802_c0_g1_i1_4	0	0	0	0	0	0	0	0	0	0	0	0	779420	1090400	973600
ARHOMBI_DN13057_c0_g1_i1_1	173140	211640	378770	92483	0	0	467390	0	575030	0	0	201090	242920	216900	276630
ARUBRA_DN9924_c0_g1_i1_4	0	0	0	0	0	0	0	0	0	0	0	0	1922600	234710	676860
ARHOMBI_DN430_c0_g1_i1_3	0	0	257660	0	0	0	0	0	0	0	0	0	871020	810290	890540
ARUBRA_DN3936_c0_g2_i1_4	0	0	0	0	0	0	0	0	0	0	0	0	918900	644010	1254200
ARUBRA_DN4814_c0_g1_i3_4	307920	280170	274710	181000	0	0	0	0	0	0	1773100	0	0	0	0

ARUBRA_DN2381_c0_g1_i1_1	0	0	0	1124400	0	0	0	469220	1212700	0	0	0	0	0	0
ARHOMBI_DN6212_c0_g1_i2_6	128020	145800	137270	76207	637880	372060	657030	647920	0	0	0	0	0	0	0
ARUBRA_DN20443_c0_g1_i1_3	0	876880	0	0	0	0	0	0	0	0	0	0	493790	579660	850070
ARHOMBI_DN11311_c0_g1_i1_5	0	0	0	0	0	0	0	0	0	0	0	0	877780	1010700	911750
ARHOMBI_DN17429_c0_g1_i1_6	0	0	0	0	0	0	0	297930	504340	0	0	1187500	251730	297670	255330
ARUBRA_DN3578_c0_g1_i1_4	449710	452650	531930	0	0	0	0	0	0	0	0	0	1360000	0	0
ARUBRA_DN11131_c0_g1_i1_3	0	0	147790	105080	0	193980	0	0	0	0	0	0	993130	725030	622990
ARHOMBI_DN4521_c1_g1_i1_1	0	0	0	0	0	0	0	0	0	0	0	0	799900	1069600	904360
ARHOMBI_DN17261_c0_g1_i1_1	334320	272760	359540	343210	0	0	0	0	867980	595180	0	0	0	0	0
ARUBRA_DN1069_c0_g2_i1_1	0	0	0	0	0	0	0	0	0	0	0	0	831460	862660	1078500
ARHOMBI_DN5132_c0_g2_i1_6	0	0	0	0	0	0	0	0	0	0	0	0	798100	956890	1011600
ARUBRA_DN3311_c0_g1_i1_6	0	0	0	0	0	0	0	0	0	607240	0	564080	523540	469350	598510
ARUBRA_DN8175_c0_g1_i1_5	0	0	0	0	0	0	0	0	0	0	0	0	694300	1000900	1060400
ARUBRA_DN16564_c0_g1_i1_6	0	0	0	0	0	0	0	0	0	0	0	2753100	0	0	0
ARUBRA_DN19149_c0_g1_i1_6	0	0	0	0	0	0	0	0	0	221160	827790	484940	423350	114150	681090
ARUBRA_DN5180_c0_g1_i1_3	2029700	713820	0	0	0	0	0	0	0	0	0	0	0	0	0
ARUBRA_DN20917_c0_g1_i1_3	1013100	625580	690550	409050	0	0	0	0	0	0	0	0	0	0	0
ARHOMBI_DN20733_c0_g1_i1_3	0	0	0	0	0	0	0	0	0	0	0	0	242930	1168300	1317400
ARUBRA_DN10088_c0_g1_i1_1	0	0	0	0	0	0	0	0	0	0	0	0	777280	978340	972550
ARHOMBI_DN19569_c0_g1_i1_5	545820	280690	175000	348120	0	0	0	0	0	0	0	1374900	0	0	0
ARUBRA_DN2998_c0_g1_i1_1	0	0	0	0	0	0	0	0	0	0	0	0	1318900	1400400	0
ARUBRA_DN1494_c0_g1_i1_3	0	0	0	0	0	0	0	0	0	0	0	0	782510	1285800	641320
ARHOMBI_DN4699_c0_g1_i1_3	0	0	0	0	131410	0	0	0	0	0	0	0	1059100	542060	974950
ARHOMBI_DN5814_c0_g1_i1_4	0	0	0	0	0	0	0	0	0	0	0	0	219630	1041800	1437800
ARHOMBI_DN21914_c0_g1_i1_1	0	0	0	0	0	0	0	0	0	0	0	0	530070	1539400	628960
ARHOMBI_DN5567_c0_g1_i1_3	0	0	0	0	0	0	0	0	0	0	0	0	615320	1314700	760240
ARUBRA_DN1463_c0_g2_i1_5	0	0	0	0	0	0	0	0	0	0	0	0	1068000	1053500	562970

ARHOMBI_DN17982_c0_g1_i1_5	784320	555510	690220	380380	0	0	0	0	0	0	0	0	0	272170	0
ARUBRA_DN2123_c0_g2_i1_3	0	0	0	0	0	0	0	0	0	0	0	0	0	1481400	1195100
ARHOMBI_DN2126_c0_g2_i1_6	0	68829	83892	0	230910	0	375240	613610	1303100	0	0	0	0	0	0
ARUBRA_DN8853_c0_g1_i1_6	0	0	0	0	0	0	0	0	0	0	0	0	1203800	306330	1159800
ARUBRA_DN1745_c0_g2_i1_1	0	0	0	126490	0	0	0	0	0	0	0	0	188870	963860	1380800
ARHOMBI_DN3877_c0_g1_i1_1	77387	113100	124940	662460	0	174920	0	0	1009100	495570	0	0	0	0	0
ARUBRA_DN3955_c0_g2_i1_2	0	0	0	0	0	0	0	0	0	0	0	0	1253300	0	1386100
ARUBRA_DN8757_c0_g1_i1_5	720810	686040	1083400	0	145520	0	0	0	0	0	0	0	0	0	0
ARHOMBI_DN1665_c0_g1_i1_2	0	0	0	0	0	0	0	0	0	0	0	0	614190	605470	1404600
ARHOMBI_DN11418_c0_g1_i1_2	0	0	0	0	0	0	0	0	0	0	347880	0	749490	774290	749290
ARHOMBI_DN8306_c0_g1_i1_4	558890	670120	688630	174500	0	0	0	0	0	0	0	0	0	524230	0
ARUBRA_DN20020_c0_g1_i1_2	437830	577470	698400	899060	0	0	0	0	0	0	0	0	0	0	0
ARUBRA_DN148_c0_g1_i1_1	0	154800	910130	1045800	0	291590	0	0	0	0	0	0	0	205220	0
ARHOMBI_DN25089_c0_g1_i1_1	0	0	0	0	0	0	0	0	0	0	0	0	2604100	0	0
ARUBRA_DN11014_c0_g1_i1_6	538280	836570	559940	658920	0	0	0	0	0	0	0	0	0	0	0
ARHOMBI_DN18155_c0_g1_i1_2	696160	673330	745760	233900	0	0	232100	0	0	0	0	0	0	0	0
ARUBRA_DN5032_c2_g1_i2_2	278420	200680	413070	71101	0	0	0	0	0	0	0	0	0	874770	738870
ARHOMBI_DN5326_c0_g1_i1_3	0	0	0	0	0	0	0	0	0	0	0	0	809750	737590	1021400
ARHOMBI_DN15674_c0_g1_i1_5	518310	374410	284040	0	1241500	148370	0	0	0	0	0	0	0	0	0
ARHOMBI_DN5386_c1_g1_i2_5	0	0	0	0	0	0	0	0	0	1139600	0	561710	158600	242080	455500
ARUBRA_DN4407_c0_g4_i1_6	0	0	0	0	463270	195060	544720	0	872640	0	0	0	481630	0	0
ARHOMBI_DN17588_c0_g1_i1_4	0	0	0	0	0	0	0	0	0	0	0	0	110110	1091500	1355300
ARHOMBI_DN7219_c0_g1_i1_3	696640	741670	716670	395820	0	0	0	0	0	0	0	0	0	0	0
ARHOMBI_DN7958_c0_g1_i1_4	0	0	0	0	0	0	0	0	0	0	0	0	796590	882050	864950
ARUBRA_DN4721_c0_g1_i2_1	0	865220	376180	1300300	0	0	0	0	0	0	0	0	0	0	0
ARHOMBI_DN5441_c0_g1_i1_5	0	0	0	0	272900	0	0	0	0	0	0	0	2266600	0	0
ARUBRA_DN7416_c0_g1_i1_6	0	0	0	0	0	0	0	0	0	0	0	0	1626000	498250	402750

ARUBRA_DN6341_c0_g1_i1_1	0	0	0	0	0	0	0	0	0	0	0	0	1118300	299790	1108400
ARUBRA_DN25250_c0_g1_i1_2	0	0	0	0	0	0	0	0	0	0	0	0	850770	770390	901900
ARUBRA_DN12031_c0_g1_i1_3	0	0	44950	0	294600	0	0	293140	934430	0	0	0	419430	528390	0
ARUBRA_DN9279_c0_g1_i1_5	0	0	0	0	0	0	0	0	0	0	0	0	833340	881990	793290
ARHOMBI_DN3515_c0_g1_i1_3	0	0	0	0	0	0	0	0	0	0	0	0	1244500	0	1260900
ARHOMBI_DN4682_c0_g1_i1_3	0	0	0	0	0	0	0	0	0	0	0	0	1085000	523990	895010
ARHOMBI_DN23760_c0_g1_i1_6	173330	144660	263940	123800	0	0	589940	537350	179260	0	0	0	0	231240	255120
ARHOMBI_DN15221_c0_g1_i1_1	194510	288450	313680	87325	0	0	0	0	0	0	0	0	238950	0	1363600
ARHOMBI_DN9078_c0_g1_i1_6	0	0	0	0	0	0	0	0	0	0	0	0	623920	1306500	555670
ARUBRA_DN11010_c0_g1_i1_3	0	0	0	0	0	0	0	0	0	0	0	0	720460	833560	928570
ARUBRA_DN17427_c0_g1_i1_4	0	0	0	0	0	0	0	0	0	0	0	0	284720	725810	1456800
ARUBRA_DN20283_c0_g1_i1_1	806110	761590	441030	370300	0	0	0	0	0	0	0	0	80913	0	0
ARUBRA_DN18073_c0_g1_i1_6	0	0	548670	0	0	0	0	151370	1732800	0	0	0	0	0	0
ARUBRA_DN4594_c0_g2_i1_4	437370	405750	407970	0	0	0	0	269030	0	0	0	0	0	439590	466440
ARUBRA_DN4871_c0_g2_i1_3	0	0	0	0	0	0	0	0	0	0	0	0	0	0	2424700
ARHOMBI_DN5577_c1_g1_i1_5	0	0	0	0	0	0	0	0	275840	0	0	0	715530	615410	816710
ARHOMBI_DN21271_c0_g1_i1_1	0	222670	353680	304150	0	0	0	0	333610	0	0	0	360540	382340	465440
ARUBRA_DN8542_c0_g1_i1_4	517620	604910	196140	369800	175530	0	0	280690	272000	0	0	0	0	0	0
ARHOMBI_DN10118_c0_g1_i1_1	0	0	0	0	0	0	0	0	0	0	0	0	888500	265660	1258200
ARUBRA_DN18680_c0_g1_i1_3	0	0	0	0	0	0	0	0	0	0	0	0	785320	709060	917920
ARUBRA_DN2284_c0_g2_i1_3	158030	127610	226070	0	0	0	0	0	0	0	0	0	637380	596880	665740
ARUBRA_DN7415_c0_g1_i1_6	0	0	0	0	0	0	0	0	0	0	0	0	954210	201430	1254400
ARUBRA_DN113_c0_g1_i2_3	0	0	0	0	0	0	0	0	0	0	0	0	237650	0	2161900
ARHOMBI_DN26770_c0_g1_i1_3	173860	128070	0	0	0	0	0	0	0	0	0	0	683270	730150	672170
ARHOMBI_DN16112_c0_g1_i1_2	0	0	0	0	0	0	0	0	0	0	0	0	780460	703990	900570
ARUBRA_DN17277_c0_g1_i1_1	756400	762660	863680	0	0	0	0	0	0	0	0	0	0	0	0
ARHOMBI_DN1288_c0_g1_i1_2	0	0	0	0	0	0	0	0	0	0	0	0	1257500	1123500	0

ARUBRA_DN70_c0_g1_i1_1	0	94657	0	117420	0	0	0	0	618400	712330	837570	0	0	0	0
ARUBRA_DN18307_c0_g1_i1_1	0	0	0	0	0	0	0	0	0	0	0	0	1631500	139260	602220
ARUBRA_DN17020_c0_g1_i1_1	0	0	0	0	0	0	0	0	0	0	0	0	794960	695480	881710
ARUBRA_DN4917_c0_g1_i2_1	66787	209420	58817	0	0	0	0	0	0	0	0	0	387860	693150	949360
ARHOMBI_DN5372_c0_g1_i1_3	0	0	0	0	0	0	0	0	0	0	0	0	1761600	596480	0
ARHOMBI_DN24609_c0_g1_i1_6	311940	320050	451600	0	449160	0	500310	322150	0	0	0	0	0	0	0
ARHOMBI_DN2660_c0_g1_i1_5	267210	242690	149510	131620	345530	0	0	0	661390	0	0	0	554470	0	0
ARUBRA_DN16475_c0_g1_i1_2	130120	923950	818220	68096	0	0	0	0	410500	0	0	0	0	0	0
ARUBRA_DN23765_c0_g1_i1_1	65626	92240	0	80742	0	0	0	0	0	0	0	0	2109000	0	0
ARHOMBI_DN22879_c0_g1_i1_2	0	0	0	0	0	0	0	0	0	0	0	0	979050	294200	1072100
ARUBRA_DN8139_c0_g1_i1_1	0	126970	0	0	0	0	0	0	0	0	0	396420	283180	734210	798700
ARUBRA_DN1221_c0_g2_i1_5	0	0	0	0	0	0	0	0	0	0	0	0	706370	778310	850680
ARHOMBI_DN14041_c0_g1_i1_6	0	0	0	0	0	0	0	0	0	0	0	0	574160	781890	975840
ARUBRA_DN4848_c0_g1_i1_3	0	0	0	0	0	0	0	0	0	0	0	0	0	1077800	1250700
ARUBRA_DN25967_c0_g1_i1_1	0	0	0	0	0	0	0	336820	519050	0	0	0	931250	541080	0
ARUBRA_DN6775_c0_g1_i1_1	92015	516940	54102	0	1227900	340820	0	0	0	0	0	0	0	95002	0
ARHOMBI_DN4127_c0_g1_i1_6	0	0	0	0	0	0	0	0	0	0	0	0	0	2325300	0
ARUBRA_DN6005_c0_g1_i1_2	0	0	0	0	0	0	0	0	0	0	0	0	1233900	0	1074000
ARUBRA_DN9533_c0_g1_i1_6	0	0	0	0	0	0	0	0	0	0	0	0	742880	717520	843130
ARUBRA_DN4945_c1_g1_i1_4	0	0	0	0	0	0	0	0	0	0	0	0	832950	658410	799340
ARUBRA_DN2632_c0_g1_i1_5	363530	680660	368920	47385	0	0	0	0	0	0	0	0	0	0	822320
ARUBRA_DN253_c0_g2_i1_2	0	0	0	0	0	0	0	0	0	0	0	0	0	2279600	0
ARUBRA_DN4035_c0_g2_i1_1	0	0	0	0	0	0	0	0	0	0	0	0	726860	694180	843930
ARHOMBI_DN5174_c0_g1_i1_1	672360	626650	728170	232630	0	0	0	0	0	0	0	0	0	0	0
ARUBRA_DN3267_c0_g1_i1_6	1231400	0	0	0	0	0	0	0	0	0	0	0	0	351970	671310
ARUBRA_DN12853_c0_g1_i1_1	314080	328240	140040	99063	0	0	0	0	0	352890	0	0	0	469720	542540
ARHOMBI_DN20424_c0_g1_i1_6	276170	421360	550930	0	0	0	532680	0	0	0	0	0	196760	0	266760

ARUBRA_DN4281_c0_g1_i1_1	0	0	0	0	0	0	0	0	0	0	0	0	464210	1071300	705890
ARHOMBI_DN3485_c0_g1_i1_4	275330	0	107040	0	0	0	328420	0	0	0	0	0	281740	0	1248800
ARUBRA_DN18869_c0_g1_i1_5	0	0	0	0	0	0	0	0	0	0	0	0	456020	1315400	466930
ARHOMBI_DN804_c0_g1_i1_6	0	0	0	0	0	0	0	0	0	0	0	0	1141700	286300	806820
ARUBRA_DN1537_c0_g1_i1_2	188260	578860	169700	82550	0	329420	434760	437310	0	0	0	0	0	0	0
ARUBRA_DN1957_c0_g2_i1_6	0	0	0	0	0	0	0	0	0	0	0	0	691920	719410	808730
ARUBRA_DN6807_c0_g1_i1_3	0	0	0	0	0	0	0	0	0	0	0	0	576850	527120	1109200
ARUBRA_DN4637_c0_g2_i1_1	0	0	0	0	0	0	0	0	0	0	0	0	0	2210600	0
ARHOMBI_DN6131_c0_g1_i4_6	238250	285410	0	0	0	138760	0	0	0	0	0	0	0	0	1548100
ARUBRA_DN5357_c0_g1_i1_1	0	0	0	0	0	0	0	0	0	0	0	0	647930	573000	989110
ARUBRA_DN2637_c0_g1_i1_4	358200	0	250340	0	0	0	272850	224940	0	0	0	0	293670	807530	0
ARHOMBI_DN16197_c0_g1_i1_4	0	0	0	0	124940	271240	0	654880	526190	244940	315860	0	0	69235	0
ARUBRA_DN10661_c0_g1_i1_4	0	0	0	0	0	0	0	0	0	0	0	0	0	599570	1604400
ARHOMBI_DN4011_c0_g2_i1_5	0	0	205820	0	0	0	0	0	0	0	0	0	1000900	996480	0
ARUBRA_DN4545_c0_g1_i1_5	0	0	125560	269560	0	0	0	0	1802700	0	0	0	0	0	0
ARUBRA_DN22266_c0_g1_i1_1	0	0	0	0	0	0	0	0	0	0	909370	1279000	0	0	0
ARUBRA_DN9362_c0_g1_i1_5	219030	221620	478520	0	0	367640	650160	232950	0	0	0	0	0	0	0
ARUBRA_DN22269_c0_g1_i1_4	0	0	0	0	0	0	263820	280410	496320	0	0	0	364000	344890	417010
ARHOMBI_DN3395_c0_g2_i1_6	354310	0	360660	223520	0	0	0	255280	0	0	0	0	244280	239270	486520
ARUBRA_DN18997_c0_g1_i1_6	0	0	0	0	0	0	0	0	0	0	0	0	514760	668040	980930
ARHOMBI_DN6095_c0_g1_i1_4	0	0	0	0	0	0	0	0	0	0	0	0	859340	907400	395390
ARHOMBI_DN1066_c0_g2_i1_3	0	0	0	0	0	0	0	0	0	0	0	0	591880	670080	893890
ARHOMBI_DN14495_c0_g1_i1_4	0	0	0	0	0	0	0	0	0	0	0	0	0	1281100	871150
ARUBRA_DN10359_c0_g1_i1_4	0	0	0	0	0	0	0	0	0	0	0	0	535100	575160	1032300
ARUBRA_DN20926_c0_g1_i1_1	136820	121630	125040	234760	0	0	165220	0	0	0	0	0	1357600	0	0
ARHOMBI_DN3507_c0_g1_i1_4	0	263200	262640	0	0	0	0	0	0	0	0	0	0	776280	836390
ARHOMBI_DN25279_c0_g1_i1_2	0	0	0	0	0	0	0	0	0	0	0	0	0	2134200	0

ARHOMBI_DN4819_c0_g1_i1_1	0	0	0	0	0	0	0	0	0	0	0	0	0	315880	1814400
ARHOMBI_DN6082_c0_g1_i3_5	0	0	0	0	0	0	0	913170	0	0	0	0	456490	276750	467890
ARUBRA_DN24896_c0_g1_i1_3	0	0	0	0	0	0	0	0	0	0	0	0	252380	1231400	625340
ARUBRA_DN6755_c0_g1_i1_2	0	0	0	0	0	0	0	0	0	0	0	0	0	891490	1213400
ARHOMBI_DN5218_c0_g1_i2_1	0	0	0	0	0	0	0	0	0	0	0	0	242050	0	1862300
ARUBRA_DN25837_c0_g1_i1_3	0	0	0	0	0	0	0	0	0	0	0	0	626440	441290	1028300
ARUBRA_DN24388_c0_g1_i1_4	236390	0	0	277410	469220	212840	733210	0	0	0	0	0	0	0	165450
ARHOMBI_DN11066_c0_g1_i1_4	0	0	0	0	0	0	0	0	0	0	0	0	714520	645600	733240
ARUBRA_DN3745_c0_g2_i1_2	0	0	0	0	0	0	0	0	0	1528400	0	0	126680	267320	170310
ARUBRA_DN14017_c0_g1_i1_3	0	0	0	0	0	0	0	0	0	0	0	0	648010	505590	934070
ARUBRA_DN145_c0_g1_i1_4	0	0	0	0	0	0	0	0	0	0	0	0	526680	927680	626330
ARUBRA_DN11007_c0_g1_i1_2	0	0	0	0	0	0	0	0	0	0	0	0	722240	0	1357600
ARUBRA_DN9096_c0_g1_i1_3	0	0	0	0	0	140190	0	552930	0	0	0	0	407620	439030	539410
ARHOMBI_DN4782_c0_g1_i1_2	0	0	0	0	0	0	0	0	0	0	0	0	1070900	515120	491110
ARHOMBI_DN2849_c0_g1_i1_3	0	0	0	0	0	0	0	0	0	0	0	0	793810	110100	1170400
ARHOMBI_DN7054_c0_g1_i1_3	0	0	0	0	0	0	0	0	0	0	0	0	398090	377500	1285900
ARHOMBI_DN2798_c0_g1_i1_2	0	0	0	0	0	0	0	0	0	0	0	0	356700	796960	889690
ARUBRA_DN18224_c0_g1_i1_4	0	0	0	0	0	0	0	0	0	0	0	166610	956500	916190	0
ARHOMBI_DN25584_c0_g1_i1_5	0	0	0	0	0	0	0	0	0	0	0	0	703750	0	1334800
ARHOMBI_DN3781_c0_g2_i1_5	685470	621370	729210	0	0	0	0	0	0	0	0	0	0	0	0
ARHOMBI_DN7337_c0_g1_i1_1	0	0	0	0	0	0	0	0	0	0	0	0	662210	612310	754280
ARHOMBI_DN5812_c2_g1_i1_3	481990	556060	423310	563180	0	0	0	0	0	0	0	0	0	0	0
ARUBRA_DN4150_c2_g1_i1_4	565040	534630	376080	467410	0	0	0	0	0	0	0	0	0	0	73739
ARHOMBI_DN1574_c0_g1_i1_4	0	0	0	0	0	0	0	0	0	0	0	0	1379900	290890	345430
ARUBRA_DN8202_c0_g1_i1_4	0	2015100	0	0	0	0	0	0	0	0	0	0	0	0	0
ARUBRA_DN1551_c0_g1_i1_5	0	0	0	0	346180	0	0	0	0	0	0	0	309590	847020	510310
ARUBRA_DN12626_c0_g1_i1_3	386480	374550	402530	347890	252090	0	0	0	0	0	0	0	244050	0	0

ARHOMBI_DN4054_c0_g1_i1_3	473130	391230	376940	0	0	0	246650	235620	0	0	0	0	280440	0	0
ARUBRA_DN9286_c0_g1_i1_1	72960	65801	19929	0	0	0	0	0	0	0	0	0	916970	490320	436170
ARHOMBI_DN3767_c0_g1_i1_1	0	0	0	0	0	0	0	0	0	0	0	0	704930	631160	661690
ARUBRA_DN20292_c0_g1_i1_6	0	0	0	0	0	0	0	0	0	0	0	0	547980	798940	646820
ARHOMBI_DN2005_c0_g1_i1_5	588810	312930	613440	306120	170040	0	0	0	0	0	0	0	0	0	0
ARUBRA_DN4908_c0_g2_i1_1	449940	283610	259990	273260	0	110850	0	610050	0	0	0	0	0	0	0
ARUBRA_DN1276_c0_g1_i1_4	0	0	0	0	0	0	0	0	0	0	0	0	444620	549420	987100
ARUBRA_DN4583_c0_g1_i2_5	0	0	153070	144850	0	197850	0	220470	0	0	0	0	387970	867380	0
ARHOMBI_DN4896_c0_g1_i3_2	281250	49698	306670	274980	0	0	0	0	1049600	0	0	0	0	0	0
ARUBRA_DN12695_c0_g1_i1_2	0	0	0	0	0	0	0	0	0	0	0	0	1142300	803820	0
ARHOMBI_DN15171_c0_g1_i1_3	0	0	0	0	0	0	0	0	0	0	0	0	70013	329110	1546900
ARHOMBI_DN7103_c0_g1_i1_1	205180	91124	0	50007	0	0	463700	0	546700	0	0	0	299110	290170	0
ARHOMBI_DN5209_c1_g1_i1_4	0	0	0	0	0	0	0	0	0	0	0	0	136430	763310	1045900
ARHOMBI_DN22071_c0_g1_i1_3	449060	538790	56863	565400	0	0	0	0	0	0	0	0	326880	0	0
ARHOMBI_DN1841_c0_g2_i1_2	0	0	0	0	0	0	0	0	0	0	0	0	424850	1015800	489660
ARUBRA_DN563_c0_g1_i1_2	0	0	0	0	0	0	0	0	0	0	0	0	560860	647260	721550
ARUBRA_DN24632_c0_g1_i1_4	0	0	0	0	0	0	0	0	0	0	0	0	572270	721830	634550
ARUBRA_DN3539_c0_g1_i1_2	1446900	237850	69274	54180	119090	0	0	0	0	0	0	0	0	0	0
ARUBRA_DN1361_c0_g2_i1_4	0	0	0	0	0	0	0	0	0	0	0	0	0	0	1924500
ARUBRA_DN11150_c0_g1_i1_6	0	0	0	0	0	0	0	0	0	0	0	0	658290	457150	806390
ARHOMBI_DN1996_c0_g2_i1_5	551190	215710	1008900	144000	0	0	0	0	0	0	0	0	0	0	0
ARHOMBI_DN2999_c0_g1_i1_4	0	0	0	0	0	0	0	0	0	0	0	0	355610	704580	859160
ARHOMBI_DN2060_c0_g1_i1_5	0	0	0	0	0	0	0	0	0	0	0	0	491460	753650	669980
ARUBRA_DN9709_c0_g1_i1_6	153520	167920	129290	179540	0	0	0	0	0	0	0	0	0	573710	709840
ARHOMBI_DN23173_c0_g1_i1_6	0	0	0	0	0	0	0	0	0	0	0	0	835810	1075600	0
ARHOMBI_DN10290_c0_g1_i1_3	421310	457530	614920	415060	0	0	0	0	0	0	0	0	0	0	0
ARUBRA_DN2965_c0_g1_i2_5	211090	406470	0	447260	0	344250	0	0	496920	0	0	0	0	0	0

ARUBRA_DN3863_c1_g1_i1_5	375640	462220	396020	0	0	0	375190	0	0	0	0	0	0	145450	150840
ARHOMBI_DN10379_c0_g1_i1_2	0	0	0	0	0	0	0	0	0	0	0	0	1900100	0	0
ARHOMBI_DN2726_c0_g1_i1_1	35777	41732	17372	39430	0	0	0	0	693710	0	0	0	414080	658020	0
ARUBRA_DN7712_c0_g1_i1_6	0	0	0	0	0	0	0	0	0	0	0	0	0	948290	950520
ARUBRA_DN4320_c0_g1_i1_6	0	126230	0	0	0	0	0	0	0	0	0	0	494390	143970	1121800
ARUBRA_DN11275_c0_g1_i1_5	0	485550	0	514570	0	0	0	0	675200	0	0	0	210070	0	0
ARHOMBI_DN8255_c0_g1_i1_2	0	0	0	0	0	0	0	0	0	0	0	0	172300	1014500	688630
ARUBRA_DN13834_c0_g1_i1_4	0	0	0	0	0	0	0	0	0	0	0	0	0	836650	1038700
ARUBRA_DN19618_c0_g1_i1_4	458060	498380	592200	51873	0	0	96168	171140	0	0	0	0	0	0	0
ARUBRA_DN1773_c0_g2_i1_6	0	0	0	0	0	0	0	0	0	0	0	0	498630	0	1364900
ARUBRA_DN1305_c0_g1_i1_6	0	0	0	0	0	0	0	0	0	0	0	0	0	1863500	0
ARHOMBI_DN3128_c0_g1_i1_1	315290	0	201900	0	0	0	0	0	0	0	0	0	858460	265280	221080
ARHOMBI_DN20118_c0_g1_i1_1	220670	258990	318570	0	571820	0	0	0	482480	0	0	0	0	0	0
ARHOMBI_DN2022_c0_g1_i1_1	0	0	0	0	0	0	0	0	0	0	0	0	1241400	0	606850
ARHOMBI_DN3079_c0_g1_i1_3	703610	677080	309880	0	150680	0	0	0	0	0	0	0	0	0	0
ARHOMBI_DN8476_c0_g1_i1_3	0	0	0	0	0	0	0	0	0	0	0	0	0	796540	1039700
ARHOMBI_DN4305_c0_g1_i2_6	557900	562860	704410	0	0	0	0	0	0	0	0	0	0	0	0
ARUBRA_DN10474_c0_g1_i1_2	0	0	0	0	0	0	0	0	0	0	0	0	724620	618970	474030
ARHOMBI_DN6299_c0_g1_i1_1	0	0	0	0	0	0	0	0	0	0	0	0	260360	470030	1086500
ARHOMBI_DN3992_c0_g1_i1_6	0	0	0	0	0	0	0	0	0	0	0	0	421960	1055000	337730
ARUBRA_DN8631_c0_g1_i1_6	0	0	0	1809300	0	0	0	0	0	0	0	0	0	0	0
ARUBRA_DN158_c0_g1_i1_2	109320	114310	0	109890	0	0	0	0	0	0	0	0	888450	0	585370
ARUBRA_DN6110_c0_g1_i1_1	0	46824	96195	0	0	0	0	0	0	0	1534400	0	129840	0	0
ARUBRA_DN4152_c0_g1_i1_5	0	560600	809700	433880	0	0	0	0	0	0	0	0	0	0	0
ARHOMBI_DN20386_c0_g1_i1_1	0	0	0	0	0	0	0	0	0	0	0	0	1226300	572290	0
ARUBRA_DN3314_c0_g1_i1_4	0	0	447410	278930	0	0	1065400	0	0	0	0	0	0	0	0
ARHOMBI_DN19207_c0_g1_i1_4	0	0	0	0	0	0	0	0	0	0	0	0	0	323720	1464500

ARUBRA_DN13376_c0_g1_i1_2	0	0	0	0	0	0	0	0	0	0	0	0	545390	577560	665180
ARHOMBI_DN16745_c0_g1_i1_1	0	0	0	0	0	0	0	127820	0	0	0	0	839700	344700	475330
ARUBRA_DN4722_c0_g1_i1_3	0	0	0	0	0	0	0	0	0	0	0	0	0	1782200	0
ARUBRA_DN881_c0_g1_i1_1	0	0	0	0	0	0	0	0	0	0	0	0	285460	1055200	440650
ARHOMBI_DN16363_c0_g1_i1_2	0	0	0	0	370180	183060	0	0	1227000	0	0	0	0	0	0
ARHOMBI_DN18734_c0_g1_i1_1	0	0	0	0	0	0	0	0	0	0	0	0	591490	167820	1015300
ARUBRA_DN3334_c0_g1_i1_1	0	0	0	0	0	0	0	0	0	0	0	0	365460	639200	760580
ARHOMBI_DN421_c0_g1_i1_5	0	0	0	0	0	0	0	0	0	0	0	0	562090	159700	1041600
ARUBRA_DN416_c0_g1_i1_2	530400	0	0	0	0	0	0	0	1232300	0	0	0	0	0	0
ARHOMBI_DN25721_c0_g1_i1_4	0	0	0	0	0	0	0	0	0	0	0	0	449130	399600	912580
ARUBRA_DN11356_c0_g1_i1_6	0	0	0	0	0	0	0	0	0	0	0	0	1275000	478760	0
ARUBRA_DN18553_c0_g1_i1_5	404480	389040	329540	401420	0	226280	0	0	0	0	0	0	0	0	0
ARHOMBI_DN3435_c0_g2_i1_3	0	0	108030	0	381220	152160	0	0	0	0	0	0	446820	426520	232860
ARHOMBI_DN13700_c0_g1_i1_6	405690	298970	486800	259270	0	0	285950	0	0	0	0	0	0	0	0
ARHOMBI_DN10592_c0_g1_i1_1	0	0	0	0	0	0	0	0	0	0	0	0	797400	0	936580
ARUBRA_DN9072_c0_g1_i1_5	0	0	0	0	0	0	0	0	0	0	0	0	906410	0	826260
ARUBRA_DN3824_c0_g1_i1_5	118450	100320	113660	0	0	0	485750	0	0	0	910690	0	0	0	0
ARUBRA_DN4832_c0_g1_i1_2	552300	490500	439670	0	0	0	245200	0	0	0	0	0	0	0	0
ARUBRA_DN9069_c0_g1_i1_4	178310	148010	220900	169310	0	112020	0	0	365320	0	531490	0	0	0	0
ARHOMBI_DN8777_c0_g1_i1_4	0	0	0	0	0	0	0	0	0	0	0	0	866100	0	855610
ARHOMBI_DN23553_c0_g1_i1_1	24600	0	0	0	0	0	0	0	0	0	0	0	0	0	1695700
ARHOMBI_DN3610_c0_g1_i1_3	802660	0	0	142640	0	0	314620	317800	0	0	0	0	0	0	142610
ARHOMBI_DN5807_c0_g2_i1_3	0	0	0	0	0	0	0	0	0	0	0	0	0	0	1714600
ARUBRA_DN4275_c0_g1_i1_5	281040	153940	396700	216530	0	0	0	0	0	0	0	0	116370	95266	444730
ARUBRA_DN24862_c0_g1_i1_4	0	0	0	0	0	0	0	0	0	0	0	0	733750	0	970360
ARUBRA_DN17478_c0_g1_i1_6	0	0	0	0	0	0	0	0	0	0	0	0	551190	535370	616510
ARUBRA_DN2215_c0_g1_i1_4	305220	454920	348190	323240	0	0	0	0	271310	0	0	0	0	0	0

ARUBRA_DN7908_c0_g1_i1_1	0	0	0	0	0	0	0	0	0	0	0	0	340180	1005900	354570
ARHOMBI_DN4809_c0_g2_i1_2	91095	55858	0	0	0	48718	0	0	0	0	0	0	0	727570	777030
ARUBRA_DN7769_c0_g1_i1_1	0	0	0	0	0	0	0	0	0	0	0	0	178630	659620	857870
ARUBRA_DN4322_c0_g1_i1_1	0	0	0	0	0	0	0	0	0	0	0	0	1586100	0	109680
ARUBRA_DN25612_c0_g1_i1_4	0	42064	57106	48860	0	491620	0	1046000	0	0	0	0	0	0	0
ARHOMBI_DN11791_c0_g1_i1_2	0	0	0	0	0	0	0	0	0	0	0	0	846860	0	837840
ARHOMBI_DN5868_c0_g1_i1_6	0	0	0	0	0	0	0	0	0	0	0	0	819810	863930	0
ARUBRA_DN2981_c0_g1_i1_3	0	0	0	0	0	0	0	0	0	0	0	0	188610	928640	553830
ARHOMBI_DN24401_c0_g1_i1_3	0	0	0	0	0	0	0	0	0	0	0	0	443600	777500	447080
ARUBRA_DN23505_c0_g1_i1_1	0	0	0	0	0	0	0	0	0	0	0	0	563680	475910	626080
ARUBRA_DN17618_c0_g1_i1_6	0	0	0	0	0	0	0	0	0	0	0	0	578920	511410	567240
ARUBRA_DN1478_c0_g1_i1_1	61064	0	0	0	0	0	0	0	0	0	0	0	0	387570	1208200
ARHOMBI_DN2506_c0_g1_i1_6	0	0	0	143500	0	0	0	0	0	0	0	0	0	831150	681980
ARHOMBI_DN8898_c0_g1_i1_5	0	0	0	0	0	0	0	0	0	0	0	0	780870	871920	0
ARUBRA_DN3963_c0_g2_i1_4	0	0	0	0	0	0	0	0	0	0	0	0	882550	0	764510
ARUBRA_DN210_c0_g1_i1_5	285950	693400	310080	355430	0	0	0	0	0	0	0	0	0	0	0
ARUBRA_DN12757_c0_g1_i1_6	0	0	0	0	0	0	0	0	0	0	0	0	208730	687060	744850
ARHOMBI_DN1108_c0_g2_i1_1	0	0	0	0	0	0	0	483090	742930	409780	0	0	0	0	0
ARHOMBI_DN11591_c0_g1_i1_2	0	0	0	0	0	0	0	0	0	0	0	0	457570	554850	621620
ARHOMBI_DN10607_c0_g1_i1_1	0	0	0	0	0	0	0	0	0	0	0	0	600970	637870	380330
ARUBRA_DN6875_c0_g1_i1_6	0	0	0	0	0	0	0	0	0	0	0	0	239640	383930	995590
ARUBRA_DN25440_c0_g1_i1_2	0	0	0	0	0	0	0	0	0	0	0	0	613330	1003900	0
ARUBRA_DN7688_c0_g1_i1_2	188440	167780	260830	405210	0	0	0	288020	0	0	0	0	0	147180	157950
ARHOMBI_DN19744_c0_g1_i1_2	0	0	0	0	0	0	0	0	0	0	0	0	440080	789980	384860
ARUBRA_DN24337_c0_g1_i1_6	0	100190	147210	0	0	0	0	0	0	0	0	0	426010	472810	458600
ARUBRA_DN20434_c0_g1_i1_2	0	0	0	0	0	0	0	0	0	0	0	0	639640	248970	714960
ARHOMBI_DN5476_c0_g1_i1_6	0	0	0	0	0	0	0	0	0	0	0	0	0	1601900	0

ARHOMBI_DN9888_c0_g1_i1_2	0	0	0	0	0	0	0	0	0	0	0	0	698620	580570	318460
ARHOMBI_DN2706_c0_g1_i1_5	0	0	0	0	0	0	0	0	0	0	0	0	84422	1513000	0
ARUBRA_DN5778_c0_g1_i1_4	0	124140	99324	89795	175260	0	0	0	0	0	0	0	250190	565660	292610
ARUBRA_DN3967_c0_g1_i1_4	244210	193790	138110	398430	0	0	0	381030	237550	0	0	0	0	0	0
ARUBRA_DN26097_c0_g1_i1_3	65607	96090	0	131650	0	0	0	0	0	0	0	0	883710	0	414530
ARHOMBI_DN2700_c0_g2_i1_5	478620	565080	543130	0	0	0	0	0	0	0	0	0	0	0	0
ARHOMBI_DN8688_c0_g1_i1_2	0	0	0	0	0	0	0	0	0	0	0	0	462040	500060	622750
ARHOMBI_DN15526_c0_g1_i1_1	0	111020	147040	293390	0	0	0	0	0	0	0	0	1028500	0	0
ARHOMBI_DN12805_c0_g1_i1_1	114980	87330	0	1374900	0	0	0	0	0	0	0	0	0	0	0
ARHOMBI_DN10876_c0_g1_i1_3	0	0	0	0	0	0	0	0	0	0	0	0	0	642440	930180
ARUBRA_DN5971_c0_g1_i1_2	192880	850940	272120	256410	0	0	0	0	0	0	0	0	0	0	0
ARHOMBI_DN18415_c0_g1_i1_4	0	0	0	0	0	0	0	0	0	0	0	0	791490	0	778020
ARUBRA_DN4814_c0_g1_i2_6	0	0	0	0	900610	0	656730	0	0	0	0	0	0	0	0
ARUBRA_DN21824_c0_g1_i1_6	0	0	0	0	0	0	0	0	0	0	0	0	439720	474170	642950
ARUBRA_DN10839_c0_g1_i1_5	0	0	0	0	0	0	0	0	0	0	484680	0	0	0	1066900
ARUBRA_DN4729_c0_g1_i1_2	0	0	0	0	0	0	0	0	0	0	0	0	221500	715310	614800
ARUBRA_DN20854_c0_g1_i1_1	0	139000	97970	76328	0	0	0	0	0	0	0	0	0	589340	646600
ARUBRA_DN8756_c0_g1_i1_5	0	0	0	0	0	0	0	0	0	0	0	0	643810	903610	0
ARUBRA_DN18126_c0_g1_i1_2	303590	385780	289960	541790	0	0	0	0	0	0	0	0	0	0	0
ARUBRA_DN7462_c0_g1_i1_6	0	0	1520500	0	0	0	0	0	0	0	0	0	0	0	0
ARUBRA_DN18563_c0_g1_i1_3	0	0	0	0	0	0	0	0	0	0	0	0	121380	1085800	313060
ARUBRA_DN10939_c0_g1_i1_3	0	0	0	0	0	0	0	0	0	0	0	0	0	172550	1345000
ARHOMBI_DN21563_c0_g1_i1_5	0	0	0	0	0	0	0	0	0	0	0	0	584090	222360	704630
ARHOMBI_DN6102_c0_g2_i1_1	80139	88722	194410	543710	0	0	111480	489730	0	0	0	0	0	0	0
ARUBRA_DN9246_c0_g1_i1_4	119580	218280	0	114780	132070	0	127880	0	0	0	0	0	339450	0	454200
ARHOMBI_DN26256_c0_g1_i1_3	0	370600	522470	263510	0	0	0	0	348430	0	0	0	0	0	0
ARUBRA_DN22761_c0_g1_i1_6	0	0	0	0	0	0	0	0	0	0	0	0	498520	438950	555750

ARHOMBI_DN20260_c0_g1_i1_3	0	0	0	0	0	0	0	0	0	0	0	0	322940	0	1170000
ARHOMBI_DN19503_c0_g1_i1_3	0	0	0	0	0	0	0	0	0	0	0	0	472270	479780	537890
ARHOMBI_DN10131_c0_g1_i1_2	0	0	0	0	0	0	0	0	0	0	0	0	1254100	0	234640
ARHOMBI_DN19928_c0_g1_i1_4	0	0	0	0	0	0	0	0	0	0	0	178550	344130	467600	497220
ARUBRA_DN1385_c0_g1_i1_2	0	0	0	0	0	0	0	0	0	0	0	0	658910	400840	422720
ARHOMBI_DN2055_c1_g1_i1_1	0	0	0	0	0	0	0	0	0	0	0	0	408290	571820	500640
ARUBRA_DN22675_c0_g1_i1_5	0	0	0	0	0	0	0	0	0	0	0	0	480310	461020	527570
ARHOMBI_DN1643_c0_g1_i1_5	0	0	0	0	0	0	0	0	0	0	0	0	0	334830	1129400
ARHOMBI_DN1170_c0_g1_i1_5	0	0	0	0	0	0	0	0	0	0	0	0	362750	1098500	0
ARHOMBI_DN22076_c0_g1_i1_3	0	0	138690	113290	0	0	316800	322410	0	0	0	0	150040	177670	239430
ARUBRA_DN16830_c0_g1_i1_1	0	0	0	0	0	0	0	0	0	0	0	0	726780	0	730790
ARUBRA_DN17659_c0_g1_i1_1	94261	0	0	0	0	0	0	0	0	0	0	0	407360	391840	558520
ARHOMBI_DN21263_c0_g1_i1_6	0	0	0	0	0	0	0	0	0	0	0	0	354710	298430	789880
ARHOMBI_DN22368_c0_g1_i1_3	0	0	0	0	0	0	0	0	0	0	0	0	414290	490850	537480
ARHOMBI_DN20690_c0_g1_i1_2	0	0	0	0	0	0	0	0	0	0	0	0	509690	437610	492770
ARUBRA_DN2528_c0_g1_i1_6	0	0	0	0	0	0	0	0	0	0	0	0	277580	481810	675820
ARHOMBI_DN1508_c0_g1_i1_5	0	0	0	0	0	0	0	0	0	0	0	0	998160	434360	0
ARHOMBI_DN4881_c0_g1_i1_3	140640	139330	249520	35921	0	0	280920	0	0	0	0	584420	0	0	0
ARHOMBI_DN24692_c0_g1_i1_6	0	0	0	0	0	0	0	0	0	0	0	0	520200	904310	0
ARUBRA_DN51_c0_g1_i1_2	0	0	0	0	0	0	0	0	0	0	0	0	0	365070	1059000
ARUBRA_DN24099_c0_g1_i1_5	353090	331070	558040	0	0	0	0	0	0	0	0	0	0	0	180600
ARUBRA_DN7587_c0_g1_i1_6	0	0	0	0	0	0	0	0	0	0	0	0	616050	0	804430
ARUBRA_DN5361_c0_g1_i1_2	247470	200710	188950	423370	0	0	0	0	0	0	0	0	0	359400	0
ARHOMBI_DN2590_c0_g1_i1_1	227910	239090	148630	110140	0	0	0	0	0	91236	0	0	408800	190840	0
ARHOMBI_DN12163_c0_g1_i1_3	0	0	0	0	0	0	0	0	0	0	0	0	1414600	0	0
ARHOMBI_DN15490_c0_g1_i1_6	0	0	0	0	0	0	0	0	0	0	0	0	1040900	0	372800
ARUBRA_DN24778_c0_g1_i1_1	0	0	0	0	0	0	0	0	0	0	1413300	0	0	0	0

ARUBRA_DN20929_c0_g1_i1_2	0	0	0	0	0	0	0	0	0	0	599120	0	0	385150	419850
ARUBRA_DN16965_c0_g1_i1_3	268080	266540	97510	238180	0	0	0	0	0	533750	0	0	0	0	0
ARUBRA_DN20307_c0_g1_i1_3	0	0	0	0	0	0	0	231100	0	0	0	0	881580	131910	159180
ARHOMBI_DN24428_c0_g1_i1_4	0	0	0	0	0	0	0	0	0	0	0	0	110700	1114600	178150
ARUBRA_DN2923_c0_g2_i1_5	0	0	0	0	0	0	0	0	0	0	0	0	1191600	0	204680
ARHOMBI_DN581_c0_g1_i1_2	111320	0	0	421550	0	0	0	0	0	0	0	0	398740	463370	0
ARHOMBI_DN6817_c0_g1_i1_1	404710	362220	0	386100	0	0	0	0	0	0	0	0	85702	154850	0
ARUBRA_DN10993_c0_g1_i1_2	0	0	0	0	0	0	0	0	0	0	0	0	462510	451930	477130
ARUBRA_DN12715_c0_g1_i1_2	0	0	0	0	0	0	0	0	0	0	0	0	483780	434400	470760
ARUBRA_DN9682_c0_g1_i1_4	0	0	0	0	328640	0	0	0	0	0	0	0	0	331300	728000
ARHOMBI_DN18725_c0_g1_i1_3	0	0	0	0	0	0	0	0	0	0	0	0	308840	466620	611120
ARHOMBI_DN20294_c0_g1_i1_3	0	0	0	0	0	0	0	0	0	0	0	0	544080	364030	476160
ARHOMBI_DN4086_c0_g1_i1_4	0	0	0	0	0	0	0	0	0	0	0	0	0	314100	1061700
ARUBRA_DN19093_c0_g1_i1_3	321380	320730	162140	107400	0	0	0	0	0	0	0	0	462900	0	0
ARHOMBI_DN13465_c0_g2_i1_6	0	0	0	0	0	0	0	0	0	0	0	0	659440	526710	185680
ARUBRA_DN20855_c0_g1_i1_2	0	0	0	0	0	0	0	0	0	0	0	0	451550	431030	488950
ARUBRA_DN2934_c0_g2_i2_4	154590	170400	351660	94339	201160	0	0	0	397040	0	0	0	0	0	0
ARUBRA_DN17957_c0_g1_i1_3	298130	204490	394040	0	0	0	284240	173270	0	0	0	0	0	0	0
ARUBRA_DN189_c0_g2_i1_5	0	0	0	0	0	0	0	0	0	0	0	0	891230	461990	0
ARUBRA_DN12373_c0_g1_i1_4	0	0	0	0	0	0	0	0	0	0	0	0	0	444660	904740
ARHOMBI_DN26479_c0_g1_i1_3	319240	411840	311240	306600	0	0	0	0	0	0	0	0	0	0	0
ARUBRA_DN1156_c0_g1_i1_5	0	0	0	0	0	0	0	0	0	0	0	0	368790	616850	361810
ARHOMBI_DN715_c0_g1_i1_6	270150	511690	199100	0	182360	0	0	0	0	0	0	0	0	0	181610
ARUBRA_DN18388_c0_g1_i1_5	333390	271760	273240	270600	0	0	0	0	195450	0	0	0	0	0	0
ARUBRA_DN24939_c0_g1_i1_6	0	0	0	0	0	0	0	0	0	0	0	0	0	649700	694540
ARUBRA_DN1579_c0_g1_i1_3	0	0	0	0	0	0	0	0	0	0	0	0	374440	810450	158240
ARUBRA_DN3088_c1_g1_i1_2	0	0	0	0	0	0	0	0	0	0	0	0	0	0	1342500

ARUBRA_DN18995_c0_g1_i1_4	0	0	0	0	0	0	0	0	0	0	0	0	598750	311670	431360
ARUBRA_DN6428_c0_g1_i1_3	0	0	0	0	0	0	0	0	0	0	0	0	457980	423770	456440
ARUBRA_DN24802_c0_g1_i1_6	0	0	0	0	0	0	0	0	0	0	0	0	1335400	0	0
ARHOMBI_DN6811_c0_g1_i1_3	0	0	0	0	0	0	0	0	0	0	0	0	0	1097900	234610
ARHOMBI_DN23413_c0_g1_i1_2	231380	216580	189000	213990	479510	0	0	0	0	0	0	0	0	0	0
ARUBRA_DN26636_c0_g1_i1_3	0	0	0	0	0	0	0	0	0	0	0	0	0	624130	703430
ARUBRA_DN24535_c0_g1_i1_1	0	0	0	0	0	0	0	0	0	0	0	0	0	1110600	215410
ARUBRA_DN5752_c0_g1_i1_3	0	0	0	0	0	0	0	0	0	0	0	0	582830	512400	227960
ARUBRA_DN12277_c0_g1_i1_4	311380	0	0	170120	291200	0	0	0	0	0	0	0	0	541370	0
ARUBRA_DN12763_c0_g1_i1_6	216160	211740	261340	208240	0	0	0	0	0	0	0	0	161840	252410	0
ARUBRA_DN23296_c0_g1_i1_1	0	0	0	0	0	0	0	0	0	0	0	0	296580	610130	403780
ARHOMBI_DN6174_c0_g1_i1_5	0	0	0	0	0	0	0	0	0	0	0	0	622680	682000	0
ARHOMBI_DN3360_c0_g1_i1_6	0	0	0	0	0	0	0	0	0	0	0	0	1015100	0	288250
ARUBRA_DN4576_c0_g3_i1_3	0	0	0	0	0	0	405590	0	0	0	0	0	278910	276470	339370
ARUBRA_DN27180_c0_g1_i1_6	117750	120120	128350	91320	0	0	0	0	0	0	573640	267850	0	0	0
ARUBRA_DN3154_c0_g1_i1_6	118260	0	274480	688280	0	0	0	0	213660	0	0	0	0	0	0
ARUBRA_DN6360_c0_g1_i1_2	281520	191510	285700	160960	188370	0	0	0	0	0	0	0	0	182370	0
ARUBRA_DN24250_c0_g1_i1_6	0	0	0	0	0	0	0	0	0	0	0	0	447730	373740	468620
ARUBRA_DN9113_c0_g1_i1_5	0	0	0	0	0	0	0	0	0	0	0	0	506880	328160	448420
ARUBRA_DN18999_c0_g1_i1_5	0	0	0	0	0	0	0	376310	905870	0	0	0	0	0	0
ARUBRA_DN23874_c0_g1_i1_1	0	0	0	0	0	0	0	0	0	0	0	0	506220	448650	324700
ARHOMBI_DN8339_c0_g1_i1_2	153940	206230	262210	72957	0	0	0	0	0	0	0	0	0	247670	336510
ARHOMBI_DN1833_c0_g1_i1_4	0	0	0	0	0	0	0	0	0	0	0	0	0	0	1278500
ARHOMBI_DN187_c0_g2_i1_1	0	0	0	0	0	0	0	0	0	0	0	0	303670	973030	0
ARHOMBI_DN18091_c0_g1_i1_1	287520	281140	335850	371710	0	0	0	0	0	0	0	0	0	0	0
ARHOMBI_DN11853_c0_g1_i1_1	0	0	0	0	0	0	0	0	0	0	0	0	0	0	1272000
ARUBRA_DN898_c0_g1_i1_1	0	0	0	0	0	0	0	0	0	0	0	0	622570	649070	0

ARHOMBI_DN5055_c0_g1_i1_3	0	0	0	0	0	0	0	0	0	0	0	0	531600	345170	388360
ARUBRA_DN21162_c0_g1_i1_4	0	0	0	0	0	0	0	0	0	0	0	0	737810	252040	272540
ARUBRA_DN19451_c0_g1_i1_2	0	0	0	0	0	0	0	0	0	0	0	0	0	594730	666510
ARHOMBI_DN17114_c0_g1_i1_5	0	0	0	0	0	0	0	0	0	0	0	0	537780	345790	377530
ARHOMBI_DN4946_c0_g1_i2_4	141320	219610	213130	0	256290	0	0	0	0	0	0	0	0	235960	193040
ARUBRA_DN9563_c0_g1_i1_2	0	0	315470	138920	0	0	0	242570	0	0	0	0	0	194800	367430
ARUBRA_DN3083_c0_g1_i1_4	0	0	0	0	0	0	0	0	0	0	0	0	0	1254000	0
ARUBRA_DN10864_c0_g1_i1_2	0	39541	35170	0	0	0	1175900	0	0	0	0	0	0	0	0
ARHOMBI_DN8832_c0_g1_i1_6	0	0	236790	0	0	0	78842	0	0	0	0	0	0	278540	655950
ARHOMBI_DN16126_c0_g1_i1_1	0	0	0	0	0	0	0	0	0	0	0	0	127260	535730	586670
ARUBRA_DN11418_c0_g1_i1_5	0	0	0	0	0	0	0	0	0	0	0	0	536660	712650	0
ARUBRA_DN711_c0_g1_i1_2	0	0	0	0	0	0	0	0	0	0	0	0	308640	580180	356630
ARUBRA_DN23248_c0_g1_i1_5	0	0	469470	775950	0	0	0	0	0	0	0	0	0	0	0
ARHOMBI_DN11424_c0_g1_i1_1	189180	217960	121800	176760	0	0	0	536380	0	0	0	0	0	0	0
ARUBRA_DN13078_c0_g1_i1_4	0	0	0	0	0	0	0	0	0	0	0	0	568660	670690	0
ARUBRA_DN22748_c0_g1_i1_3	0	0	0	0	0	0	0	0	0	0	0	0	888510	132810	216830
ARUBRA_DN19446_c0_g1_i1_1	0	0	0	0	0	0	0	0	0	0	0	0	442910	428170	366450
ARHOMBI_DN25338_c0_g1_i1_6	0	0	0	0	0	0	0	0	0	0	0	0	0	0	1236400
ARUBRA_DN1375_c0_g1_i1_4	0	0	0	0	0	0	0	0	0	0	0	0	744530	0	491090
ARHOMBI_DN5366_c0_g1_i2_2	0	0	0	0	0	0	0	0	0	0	0	0	383710	0	851830
ARUBRA_DN26151_c0_g1_i1_1	0	336230	312940	584610	0	0	0	0	0	0	0	0	0	0	0
ARHOMBI_DN16439_c0_g1_i1_3	0	0	0	0	0	0	0	0	0	0	0	0	0	629040	604070
ARHOMBI_DN17772_c0_g1_i1_2	0	0	0	0	0	0	0	0	0	0	0	0	474740	341280	415900
ARHOMBI_DN3967_c0_g1_i1_1	259080	63307	81566	755910	0	71704	0	0	0	0	0	0	0	0	0
ARUBRA_DN18178_c0_g1_i1_2	0	0	0	0	0	0	0	0	0	0	0	0	315340	540220	375950
ARUBRA_DN4925_c0_g1_i1_4	0	0	0	0	0	0	0	0	0	0	0	0	479360	746330	0
ARUBRA_DN14063_c0_g1_i1_3	0	0	0	0	0	0	0	0	0	0	0	0	985340	238840	0

ARHOMBI_DN2499_c0_g1_i1_5	0	0	0	0	0	0	0	0	0	0	0	0	0	0	1223900
ARUBRA_DN2486_c0_g1_i1_3	0	0	0	0	0	0	0	0	0	0	0	0	239800	983900	0
ARUBRA_DN9169_c0_g1_i1_1	0	0	0	0	0	0	115820	0	0	0	0	0	383640	334730	386130
ARHOMBI_DN2036_c0_g2_i1_1	380260	342230	299500	198100	0	0	0	0	0	0	0	0	0	0	0
ARUBRA_DN20749_c0_g1_i1_4	0	0	0	0	0	0	0	0	0	0	0	0	330060	438230	447190
ARHOMBI_DN13970_c0_g1_i1_4	0	0	0	0	0	0	0	0	0	0	0	0	614310	599610	0
ARHOMBI_DN16110_c0_g1_i1_4	0	0	0	0	0	0	0	0	0	0	0	0	588720	0	623760
ARUBRA_DN16649_c0_g1_i1_5	67263	0	0	0	0	0	0	0	0	0	0	0	361250	323180	458160
ARHOMBI_DN26305_c0_g1_i1_3	0	0	0	0	0	0	0	0	0	0	0	0	525730	0	672720
ARUBRA_DN7710_c0_g1_i1_3	0	0	0	0	0	0	0	0	0	0	0	0	429420	362100	403330
ARUBRA_DN9775_c0_g1_i1_2	0	0	0	78591	0	0	0	0	0	0	0	0	0	0	1115500
ARUBRA_DN9988_c0_g1_i1_3	0	0	0	0	0	0	0	0	0	0	0	0	235810	229800	722870
ARHOMBI_DN18064_c0_g1_i1_4	0	0	0	0	0	0	0	0	0	0	0	0	424000	351210	412820
ARHOMBI_DN4801_c0_g1_i1_1	0	0	0	0	192710	0	124940	0	0	0	0	0	268910	305400	295780
ARUBRA_DN5397_c0_g1_i1_4	0	0	0	0	0	0	0	0	0	0	0	0	414630	384060	388120
ARHOMBI_DN3803_c0_g2_i1_6	0	0	0	0	0	0	0	0	0	0	0	0	0	514230	669750
ARUBRA_DN4646_c0_g1_i1_4	166570	58513	621910	143280	189840	0	0	0	0	0	0	0	0	0	0
ARUBRA_DN16654_c0_g1_i1_4	0	0	0	0	0	0	0	0	0	0	0	0	472290	354720	352690
ARUBRA_DN20972_c0_g1_i1_2	0	0	0	0	0	0	0	0	0	0	0	0	947470	231020	0
ARHOMBI_DN23602_c0_g1_i1_6	0	0	0	0	0	0	0	0	0	0	1178400	0	0	0	0
ARUBRA_DN20900_c0_g1_i1_2	0	0	0	0	0	0	0	0	0	0	0	0	343460	828880	0
ARUBRA_DN17183_c0_g1_i1_5	0	0	0	0	0	0	0	0	0	0	0	0	275990	577270	316800
ARHOMBI_DN3229_c0_g1_i1_4	111230	131080	518180	0	0	0	0	0	0	0	0	407830	0	0	0
ARUBRA_DN12858_c0_g1_i1_4	0	0	0	0	0	0	0	0	0	0	0	0	347890	473030	342940
ARUBRA_DN20_c0_g1_i1_4	131750	204110	45246	179820	243910	0	261020	0	0	0	0	0	0	96384	0
ARUBRA_DN2891_c0_g1_i1_5	0	0	0	0	0	0	0	0	0	0	0	0	0	596300	563160
ARHOMBI_DN4641_c0_g1_i1_6	0	0	0	0	0	0	0	0	0	0	0	0	0	1155400	0

ARUBRA_DN5439_c0_g1_i1_5	0	0	0	0	0	0	0	0	0	0	0	0	453270	0	699690
ARUBRA_DN2788_c0_g1_i1_6	0	0	0	0	0	0	0	0	0	0	0	0	470590	336340	344100
ARHOMBI_DN24746_c0_g1_i1_1	243980	376490	0	0	0	0	0	529120	0	0	0	0	0	0	0
ARUBRA_DN12422_c0_g1_i1_2	206940	0	387920	0	0	553830	0	0	0	0	0	0	0	0	0
ARUBRA_DN19709_c0_g1_i1_5	0	0	0	0	0	0	0	0	0	0	0	0	761600	0	383110
ARUBRA_DN4950_c1_g1_i1_4	182430	175900	0	215690	251260	0	0	317860	0	0	0	0	0	0	0
ARHOMBI_DN8627_c0_g1_i1_4	0	0	0	0	0	0	0	0	0	0	0	0	749880	0	387220
ARHOMBI_DN8139_c0_g1_i1_2	0	0	0	0	0	0	0	0	0	0	0	0	381060	341300	413580
ARUBRA_DN9097_c0_g1_i1_3	0	0	0	0	0	0	0	0	0	0	0	0	359680	369550	406090
ARUBRA_DN23250_c0_g1_i1_1	0	0	0	0	0	0	0	0	0	0	0	0	466430	194810	467510
ARHOMBI_DN13828_c0_g1_i1_2	0	0	0	0	0	0	0	0	0	0	0	0	344580	328470	454960
ARHOMBI_DN16373_c0_g1_i1_2	0	0	0	0	0	0	0	0	0	0	0	0	386110	390950	350580
ARUBRA_DN12629_c0_g1_i1_4	0	0	0	0	0	0	0	0	0	0	0	0	380170	330180	413580
ARHOMBI_DN6215_c0_g1_i2_2	0	0	0	0	0	0	0	0	0	0	0	0	354570	245450	519680
ARUBRA_DN4706_c0_g1_i1_4	0	0	0	0	0	0	0	0	0	0	1119400	0	0	0	0
ARUBRA_DN6348_c0_g1_i1_2	217010	592680	0	61818	0	247180	0	0	0	0	0	0	0	0	0
ARHOMBI_DN4431_c0_g3_i1_1	332750	528550	208080	45388	0	0	0	0	0	0	0	0	0	0	0
ARHOMBI_DN2426_c0_g1_i1_5	298350	301900	295520	209120	0	0	0	0	0	0	0	0	0	0	0
ARUBRA_DN2608_c0_g1_i1_4	0	0	0	0	0	0	0	0	0	0	0	0	507150	0	597770
ARHOMBI_DN5630_c0_g2_i1_5	0	0	0	0	0	0	0	0	0	0	0	0	0	184480	919190
ARHOMBI_DN2188_c0_g1_i1_4	0	0	0	0	0	0	0	0	0	0	0	0	347020	156990	599230
ARUBRA_DN16537_c0_g1_i1_2	0	0	0	0	0	0	0	0	0	0	0	0	451850	113220	536160
ARHOMBI_DN6250_c0_g2_i3_5	0	0	0	325510	0	0	0	239290	532740	0	0	0	0	0	0
ARUBRA_DN12906_c0_g1_i1_4	0	0	0	0	0	0	0	0	0	0	0	0	351370	341940	403310
ARHOMBI_DN15675_c0_g1_i1_3	0	0	0	0	0	0	0	0	0	0	0	0	1095200	0	0
ARHOMBI_DN20830_c0_g1_i1_1	0	0	0	0	0	0	0	0	0	0	0	0	0	250160	841790
ARHOMBI_DN6132_c0_g5_i1_3	239430	291250	309030	251450	0	0	0	0	0	0	0	0	0	0	0

ARHOMBI_DN13938_c0_g1_i1_3	0	0	0	0	0	0	0	0	0	0	0	0	273850	365840	450150
ARHOMBI_DN343_c0_g2_i1_6	282590	100900	174360	109700	0	0	0	0	0	0	0	0	192120	0	228120
ARHOMBI_DN20204_c0_g1_i1_6	0	0	0	0	0	0	0	0	0	0	0	0	352170	341390	394010
ARHOMBI_DN5653_c0_g1_i1_4	0	0	0	217430	0	0	0	396530	473530	0	0	0	0	0	0
ARUBRA_DN9043_c0_g1_i1_6	0	0	0	0	0	0	0	0	0	0	0	0	0	519020	567910
ARUBRA_DN8452_c0_g1_i1_1	0	0	0	0	179910	0	151470	0	339980	0	412110	0	0	0	0
ARHOMBI_DN5207_c0_g1_i1_5	397020	0	0	337440	0	0	0	0	0	0	0	0	0	162200	186550
ARHOMBI_DN5473_c0_g1_i1_2	0	0	0	0	0	0	0	0	0	0	0	0	132270	140340	810460
ARHOMBI_DN15547_c1_g1_i1_2	391560	235020	380780	0	0	72034	0	0	0	0	0	0	0	0	0
ARUBRA_DN21709_c0_g1_i1_1	0	0	0	0	0	0	0	0	0	0	0	0	203100	616410	256500
ARUBRA_DN7051_c0_g1_i1_2	0	0	0	0	0	0	0	0	0	0	0	0	506350	286980	280620
ARUBRA_DN25464_c0_g1_i1_1	0	0	0	0	0	0	0	0	0	0	0	0	379840	353340	334710
ARHOMBI_DN4589_c0_g1_i1_6	112710	150850	0	158860	0	0	0	168430	473540	0	0	0	0	0	0
ARUBRA_DN931_c0_g1_i1_3	234250	331630	257140	241260	0	0	0	0	0	0	0	0	0	0	0
ARHOMBI_DN10963_c0_g1_i1_2	213110	184000	308320	355740	0	0	0	0	0	0	0	0	0	0	0
ARUBRA_DN14101_c0_g1_i1_6	96499	0	0	0	0	88352	0	0	0	0	0	0	230670	360950	284140
ARHOMBI_DN18821_c0_g1_i1_3	0	94388	0	0	0	0	153270	0	0	0	0	0	109760	138150	561310
ARHOMBI_DN14763_c0_g1_i1_1	0	0	0	0	0	0	0	0	0	0	0	0	851780	201400	0
ARUBRA_DN7233_c0_g1_i1_1	0	0	0	0	0	0	0	0	0	0	0	0	112410	0	937680
ARUBRA_DN3582_c0_g2_i1_3	0	105780	0	176230	0	0	0	0	0	0	0	0	388810	377980	0
ARUBRA_DN3740_c0_g1_i1_2	0	0	0	0	0	0	0	0	0	0	0	0	1047700	0	0
ARUBRA_DN2116_c0_g1_i1_1	0	0	0	0	0	0	0	0	0	0	0	0	342230	336730	368210
ARHOMBI_DN6367_c0_g1_i1_3	0	0	0	0	0	0	0	0	0	0	0	0	1046900	0	0
ARUBRA_DN9714_c0_g1_i1_3	0	0	0	0	0	0	0	0	0	0	0	0	705530	341230	0
ARHOMBI_DN5629_c0_g1_i1_3	0	0	0	0	0	0	0	0	0	0	0	0	267560	514780	264090
ARUBRA_DN17225_c0_g1_i1_6	0	0	0	0	0	0	0	0	0	0	0	0	76544	0	965980
ARUBRA_DN5760_c0_g1_i1_1	0	0	0	70795	0	0	0	0	675630	0	0	0	134730	0	160750

ARHOMBI_DN13303_c0_g1_i1_5	0	0	0	0	0	0	0	0	0	0	0	0	556450	246170	237700
ARHOMBI_DN44_c0_g1_i1_5	0	0	0	0	0	0	0	0	183290	0	0	0	437890	415200	0
ARUBRA_DN2012_c0_g1_i1_3	0	0	0	0	0	0	0	0	0	0	0	0	392650	171380	467900
ARHOMBI_DN18373_c0_g1_i1_1	307460	218860	116050	0	180990	0	205110	0	0	0	0	0	0	0	0
ARUBRA_DN22899_c0_g1_i1_4	144480	119960	150940	63277	0	0	0	0	0	0	0	0	239590	0	309430
ARUBRA_DN19779_c0_g1_i1_2	0	0	0	0	0	0	0	0	0	0	0	0	0	851410	175810
ARHOMBI_DN2168_c0_g1_i1_5	0	0	0	0	0	0	0	0	0	0	0	0	0	1027100	0
ARHOMBI_DN18268_c0_g1_i1_5	0	0	0	0	0	0	0	0	0	0	0	0	0	620400	405490
ARUBRA_DN20895_c0_g1_i1_4	0	0	0	0	0	0	0	0	0	0	0	0	438120	400020	183800
ARHOMBI_DN9816_c0_g1_i1_3	0	0	0	0	0	0	0	0	0	0	0	0	0	546470	475270
ARUBRA_DN6711_c0_g1_i1_1	0	0	0	0	0	0	0	0	0	0	0	0	888490	0	132970
ARUBRA_DN1256_c0_g2_i1_1	0	0	0	0	0	0	0	0	0	0	0	0	343550	356180	319840
ARHOMBI_DN4768_c0_g1_i1_5	0	0	0	0	0	0	0	0	0	0	0	0	322140	331170	364460
ARUBRA_DN1822_c0_g1_i1_4	0	0	0	0	0	0	0	0	0	0	0	0	142810	234030	639190
ARUBRA_DN2382_c0_g1_i1_2	0	0	0	0	0	0	138090	0	475680	0	0	0	188180	0	212300
ARUBRA_DN6119_c0_g1_i1_1	0	0	0	0	0	0	0	0	0	0	0	0	372840	287700	350890
ARUBRA_DN19079_c0_g1_i1_2	0	0	0	0	0	0	0	0	0	0	0	0	0	279920	731010
ARUBRA_DN14154_c0_g1_i1_1	0	0	0	0	0	0	0	0	0	0	0	0	280770	424820	298020
ARUBRA_DN6181_c0_g1_i1_5	0	0	0	0	0	0	0	0	0	0	0	0	0	460470	542890
ARHOMBI_DN5536_c0_g1_i1_1	0	486340	262010	0	0	0	0	0	251830	0	0	0	0	0	0
ARHOMBI_DN25582_c0_g1_i1_3	249510	286440	335080	0	0	0	0	0	0	0	0	0	0	128260	0
ARUBRA_DN1176_c0_g1_i1_5	0	0	0	0	0	0	0	0	0	0	0	0	546120	0	453010
ARUBRA_DN766_c0_g1_i1_6	0	0	0	0	0	0	0	0	0	0	0	0	0	320520	678600
ARHOMBI_DN20645_c0_g1_i1_1	0	0	0	0	0	0	0	0	0	0	0	0	468080	530820	0
ARHOMBI_DN985_c0_g1_i1_6	0	0	0	0	0	0	0	0	0	0	0	0	359470	271010	366650
ARUBRA_DN14493_c0_g1_i1_2	0	0	0	0	0	0	0	0	0	0	0	0	519340	0	476500
ARHOMBI_DN18281_c0_g1_i1_5	0	0	0	0	0	192780	0	0	388820	414230	0	0	0	0	0

ARHOMBI_DN18082_c0_g1_i1_2	0	0	0	0	0	0	0	0	0	0	0	0	477750	119370	396100
ARUBRA_DN3448_c0_g1_i1_6	0	0	0	0	0	0	0	0	0	0	0	0	0	852040	140450
ARHOMBI_DN19564_c0_g1_i1_4	0	0	0	0	0	0	0	0	0	0	0	0	352240	637850	0
ARHOMBI_DN18202_c0_g1_i1_6	321270	388940	278130	0	0	0	0	0	0	0	0	0	0	0	0
ARUBRA_DN4562_c0_g1_i1_3	93293	170350	97123	323540	0	0	0	0	302860	0	0	0	0	0	0
ARUBRA_DN756_c0_g1_i1_1	0	0	0	0	0	0	0	0	0	0	0	0	172940	376530	436670
ARHOMBI_DN5795_c0_g1_i1_6	232820	172510	188890	390860	0	0	0	0	0	0	0	0	0	0	0
ARUBRA_DN18766_c0_g1_i1_6	0	0	0	0	0	0	0	0	0	0	0	0	387660	596060	0
ARHOMBI_DN11368_c0_g1_i1_5	0	0	0	0	0	0	0	0	0	0	0	0	789400	0	192360
ARHOMBI_DN19194_c0_g1_i1_2	0	0	0	0	0	0	0	0	0	0	0	0	396400	582450	0
ARUBRA_DN21311_c0_g1_i1_6	0	0	0	0	0	0	0	0	0	0	0	0	296260	344040	337900
ARUBRA_DN19687_c0_g1_i1_4	0	0	0	0	0	0	0	0	0	0	0	0	546870	0	430090
ARHOMBI_DN1072_c0_g1_i1_2	295660	399600	0	280240	0	0	0	0	0	0	0	0	0	0	0
ARHOMBI_DN4606_c0_g2_i1_3	0	0	0	0	0	0	0	0	0	0	0	0	327970	304080	341270
ARHOMBI_DN3865_c0_g1_i1_4	0	0	0	0	0	0	0	0	0	0	0	0	730690	240080	0
ARHOMBI_DN3210_c0_g1_i1_3	0	0	0	0	0	0	0	0	0	0	0	0	202300	590700	177080
ARHOMBI_DN127_c0_g1_i1_4	0	0	0	0	0	0	0	0	0	0	0	0	242690	511250	212190
ARUBRA_DN6664_c0_g1_i1_5	0	0	0	0	0	0	0	0	0	0	0	0	848380	116860	0
ARUBRA_DN17807_c0_g1_i1_6	114970	132930	713190	0	0	0	0	0	0	0	0	0	0	0	0
ARHOMBI_DN26518_c0_g1_i1_6	0	0	0	0	0	0	0	0	0	0	0	0	196150	360830	403020
ARHOMBI_DN26272_c0_g1_i1_3	0	0	0	0	0	0	0	0	0	0	0	0	0	90345	869000
ARUBRA_DN24367_c0_g1_i1_3	292230	296950	0	0	0	0	0	0	0	0	0	0	365870	0	0
ARHOMBI_DN10852_c0_g1_i1_2	0	0	0	0	0	0	0	0	0	0	0	0	0	765290	187390
ARUBRA_DN17980_c0_g1_i1_5	57364	0	0	0	182710	0	492270	0	217160	0	0	0	0	0	0
ARHOMBI_DN178_c0_g2_i1_4	0	0	0	0	0	0	0	0	0	0	0	0	251050	223140	474380
ARUBRA_DN3098_c0_g1_i1_4	0	0	0	0	0	0	0	0	0	0	0	0	0	523540	424330
ARHOMBI_DN8417_c0_g1_i1_6	0	0	0	0	0	0	0	0	0	0	0	0	307030	298470	341620

ARUBRA_DN16531_c0_g1_i1_2	0	0	0	0	0	0	0	0	0	0	0	0	222530	321190	400060
ARUBRA_DN201_c0_g2_i1_4	0	0	0	0	0	0	0	0	0	0	0	0	253720	322170	367300
ARUBRA_DN4976_c0_g1_i1_4	0	0	0	0	0	0	0	0	0	0	0	0	0	0	942100
ARUBRA_DN22472_c0_g1_i1_1	0	0	0	0	0	0	0	0	0	0	0	0	320930	335550	284490
ARHOMBI_DN22711_c0_g1_i1_4	0	0	0	0	0	0	0	0	0	0	0	0	0	0	939830
ARHOMBI_DN5461_c0_g1_i2_3	0	0	0	0	0	0	0	0	0	0	0	0	763180	0	175510
ARUBRA_DN1871_c0_g1_i1_5	135400	116980	100340	0	0	0	0	0	0	0	0	0	585860	0	0
ARHOMBI_DN11140_c0_g1_i1_4	0	0	0	0	0	0	0	0	0	0	0	0	245750	297540	394690
ARUBRA_DN23048_c0_g1_i1_5	134050	107100	493770	0	0	202810	0	0	0	0	0	0	0	0	0
ARHOMBI_DN2451_c0_g1_i1_1	0	0	0	0	0	0	143450	0	0	0	0	0	785840	0	0
ARUBRA_DN2562_c0_g2_i2_3	353560	404430	0	0	0	0	0	0	0	0	0	0	168860	0	0
ARHOMBI_DN20377_c0_g1_i1_1	0	0	0	0	0	0	0	0	0	0	0	0	926790	0	0
ARUBRA_DN15849_c0_g1_i1_3	0	0	0	78871	0	0	0	0	0	0	0	0	844760	0	0
ARUBRA_DN24185_c0_g1_i1_2	0	315900	189030	0	0	0	416430	0	0	0	0	0	0	0	0
ARUBRA_DN3137_c0_g1_i1_1	0	0	0	0	0	0	0	0	0	0	0	0	0	432780	487870
ARUBRA_DN18794_c0_g1_i1_1	0	24927	0	0	0	0	102510	170440	542380	0	0	0	79268	0	0
ARHOMBI_DN16770_c0_g1_i1_3	0	0	0	0	0	0	0	0	0	0	0	0	384820	0	529920
ARUBRA_DN8214_c1_g1_i1_2	130380	142980	234200	106610	0	0	0	0	206000	0	0	0	0	89761	0
ARHOMBI_DN5351_c0_g3_i2_2	0	0	0	0	0	0	0	234650	0	0	0	0	312340	361920	0
ARUBRA_DN26670_c0_g1_i1_1	76581	99847	0	49478	0	0	0	0	682070	0	0	0	0	0	0
ARHOMBI_DN14110_c0_g1_i1_4	0	0	0	0	0	0	0	0	0	0	0	0	0	408720	495600
ARHOMBI_DN3010_c0_g2_i1_1	0	0	264470	0	294880	0	343370	0	0	0	0	0	0	0	0
ARUBRA_DN14051_c0_g1_i1_3	0	0	0	0	0	0	0	0	0	0	0	0	0	527600	373540
ARUBRA_DN20086_c0_g1_i1_3	0	0	0	0	0	0	0	0	0	0	0	0	0	376140	524650
ARUBRA_DN11076_c0_g1_i1_6	0	0	0	0	0	0	0	0	0	0	0	0	280750	363540	255790
ARHOMBI_DN25990_c0_g1_i1_4	0	687220	0	0	0	0	209570	0	0	0	0	0	0	0	0
ARHOMBI_DN2521_c0_g1_i1_6	0	0	0	0	0	0	0	0	0	0	0	0	643240	0	253000

ARUBRA_DN3144_c0_g1_i1_5	0	0	0	0	0	0	0	0	0	0	0	0	271080	260420	364060
ARUBRA_DN524_c0_g2_i1_3	0	0	122520	0	177400	0	0	0	0	0	0	0	281250	0	313320
ARUBRA_DN4339_c0_g1_i2_1	296130	0	330380	0	0	0	265370	0	0	0	0	0	0	0	0
ARHOMBI_DN2257_c0_g1_i1_1	0	0	0	0	0	0	0	0	0	0	0	0	0	575940	315860
ARUBRA_DN17897_c0_g1_i1_5	191710	152110	0	0	0	105670	0	0	0	0	0	0	336440	0	104810
ARHOMBI_DN2123_c0_g2_i1_4	0	0	0	0	0	0	0	0	0	0	0	0	501900	387270	0
ARUBRA_DN11421_c0_g1_i1_5	0	0	0	0	0	0	0	0	0	0	0	0	306670	256410	325250
ARHOMBI_DN13369_c0_g1_i1_6	0	0	0	0	0	0	0	0	0	0	0	0	888170	0	0
ARUBRA_DN10411_c0_g1_i1_2	75200	0	0	0	0	0	0	0	0	0	0	0	0	357480	452530
ARHOMBI_DN5006_c0_g1_i1_1	0	0	0	0	0	0	0	0	0	0	0	0	0	0	883460
ARHOMBI_DN17209_c0_g1_i1_1	0	0	0	0	0	0	0	0	0	0	0	0	0	402850	480230
ARUBRA_DN6201_c0_g1_i1_4	0	0	0	0	0	0	0	0	0	0	0	0	496200	0	386600
ARHOMBI_DN4317_c0_g1_i1_2	0	0	0	0	0	0	0	0	0	0	0	0	218740	373100	290950
ARHOMBI_DN5264_c0_g1_i2_1	0	0	0	0	0	0	0	0	0	0	0	0	198640	374010	307880
ARUBRA_DN3956_c0_g2_i2_6	0	0	0	0	0	0	0	0	0	0	0	0	214620	0	662640
ARUBRA_DN7892_c0_g1_i1_4	0	65129	65759	0	0	0	0	0	0	0	0	0	502640	0	243650
ARUBRA_DN5621_c0_g1_i1_1	0	0	0	0	0	0	0	0	0	0	0	0	574030	0	298020
ARUBRA_DN6290_c0_g1_i1_6	0	0	0	0	0	0	0	0	0	0	0	0	0	0	870920
ARUBRA_DN8437_c0_g1_i1_2	0	0	0	0	0	0	0	0	0	0	0	0	0	564960	303960
ARHOMBI_DN2197_c0_g1_i1_2	0	0	0	0	0	0	0	0	0	0	0	0	868280	0	0
ARUBRA_DN20773_c0_g1_i1_2	0	145770	63100	0	351240	0	0	307890	0	0	0	0	0	0	0
ARUBRA_DN19652_c0_g1_i1_5	0	0	0	0	0	0	0	0	0	0	0	0	0	0	866150
ARUBRA_DN23301_c0_g1_i1_2	0	0	0	0	0	0	0	0	0	0	0	0	350520	332220	181640
ARHOMBI_DN6000_c0_g1_i1_4	0	0	0	0	0	0	0	0	0	0	0	0	0	134880	727880
ARHOMBI_DN27227_c0_g1_i1_2	0	0	0	0	0	0	0	0	0	0	0	0	240660	299330	321980
ARHOMBI_DN3035_c0_g1_i1_2	0	0	0	0	0	0	0	0	0	0	0	0	304010	272680	282500
ARUBRA_DN7465_c0_g1_i1_1	0	0	0	0	0	0	0	0	0	0	0	0	230580	325710	302590

ARHOMBI_DN25461_c0_g1_i1_3	0	250420	168240	252050	0	0	0	183950	0	0	0	0	0	0	0
ARUBRA_DN23449_c0_g1_i1_4	0	0	0	0	0	0	0	0	0	0	0	0	295450	296480	261100
ARUBRA_DN4751_c0_g1_i2_6	0	157780	0	691110	0	0	0	0	0	0	0	0	0	0	0
ARHOMBI_DN23037_c0_g1_i1_6	0	0	0	0	0	0	0	0	0	0	0	0	0	848530	0
ARHOMBI_DN17541_c0_g1_i1_1	79750	59592	0	0	0	0	0	0	0	0	0	0	332970	0	375990
ARUBRA_DN10528_c0_g1_i1_4	66949	74420	118680	0	368070	0	0	218860	0	0	0	0	0	0	0
ARUBRA_DN13908_c0_g1_i1_4	0	0	0	0	0	0	0	0	0	0	0	0	410950	0	435980
ARHOMBI_DN13282_c0_g1_i1_1	0	0	0	0	0	0	0	0	0	0	0	0	310820	327150	207970
ARHOMBI_DN3944_c0_g1_i2_2	0	0	0	0	0	0	0	0	0	0	0	0	0	339320	505110
ARHOMBI_DN19974_c0_g1_i1_4	241900	385490	216050	0	0	0	0	0	0	0	0	0	0	0	0
ARUBRA_DN7017_c0_g1_i1_3	0	0	0	0	0	0	0	0	0	0	0	0	842140	0	0
ARUBRA_DN1910_c0_g1_i1_5	0	0	0	0	310530	0	341590	0	0	0	0	0	91661	98114	0
ARUBRA_DN4277_c0_g1_i1_2	0	0	0	0	0	0	0	0	0	0	0	0	290890	248490	300690
ARUBRA_DN22918_c0_g1_i1_6	0	0	0	0	0	0	0	0	0	0	0	0	0	0	840040
ARUBRA_DN9150_c0_g1_i1_5	120940	119410	0	157080	0	0	166680	0	0	0	0	164840	0	0	106080
ARHOMBI_DN6685_c1_g1_i1_6	0	0	0	0	0	0	0	0	0	0	0	0	0	394820	440190
ARHOMBI_DN26281_c0_g1_i1_5	0	0	0	0	0	0	0	0	0	0	0	0	393590	0	440710
ARUBRA_DN3358_c0_g1_i2_1	0	0	0	0	0	0	0	0	0	0	0	0	0	501640	330620
ARHOMBI_DN4457_c0_g1_i1_1	0	30200	0	0	0	0	0	0	0	0	0	0	202970	311060	286200
ARHOMBI_DN10001_c0_g1_i1_3	0	0	0	0	0	0	0	0	0	0	0	0	363340	231730	227370
ARHOMBI_DN13618_c0_g2_i1_3	0	0	0	0	0	0	0	0	0	0	0	0	0	414300	408120
ARHOMBI_DN3625_c0_g1_i1_3	0	0	0	0	0	0	0	0	0	0	0	0	269870	257090	294390
ARHOMBI_DN23530_c0_g1_i1_1	118340	188000	101390	175260	0	75964	0	161330	0	0	0	0	0	0	0
ARUBRA_DN21885_c0_g1_i1_1	0	0	0	0	0	0	0	0	0	0	0	0	819570	0	0
ARHOMBI_DN4759_c0_g2_i1_2	0	0	141770	136530	0	0	0	0	540830	0	0	0	0	0	0
ARHOMBI_DN995_c0_g2_i1_5	0	0	0	0	0	0	0	0	0	0	0	0	577710	237030	0
ARHOMBI_DN19127_c0_g1_i1_2	0	0	0	0	0	0	0	0	0	0	0	0	0	371660	440370

ARUBRA_DN19640_c0_g1_i1_4	0	0	0	0	0	0	0	0	0	0	0	0	0	297410	514040
ARUBRA_DN16258_c0_g1_i1_4	0	0	0	0	0	0	0	0	0	0	0	0	0	0	811010
ARUBRA_DN19843_c0_g1_i1_1	0	0	0	0	0	0	0	0	0	0	0	0	224840	249640	335830
ARHOMBI_DN4707_c0_g1_i1_6	0	0	0	0	170500	0	0	0	0	0	0	0	144740	340720	153540
ARUBRA_DN6269_c0_g1_i1_1	0	0	0	0	0	0	0	0	0	0	0	0	334700	473670	0
ARUBRA_DN14413_c0_g1_i1_1	0	0	0	0	0	0	0	0	0	0	0	0	611300	0	196420
ARUBRA_DN23541_c0_g1_i1_3	71521	243910	146790	95682	0	0	0	0	248440	0	0	0	0	0	0
ARHOMBI_DN21586_c0_g1_i1_2	0	0	0	0	0	0	0	0	0	0	0	0	256870	0	548920
ARUBRA_DN20454_c0_g1_i1_1	0	0	0	0	0	0	0	0	0	0	0	0	282040	0	520450
ARUBRA_DN25494_c0_g1_i1_2	0	0	0	0	0	0	0	0	0	0	0	0	240160	267950	291580
ARUBRA_DN1726_c0_g1_i1_1	170960	139540	169650	104840	211810	0	0	0	0	0	0	0	0	0	0
ARUBRA_DN4369_c0_g1_i1_1	0	0	0	0	0	0	0	0	0	0	0	0	498750	148540	148910
ARHOMBI_DN7832_c0_g1_i1_1	0	0	0	0	0	0	0	0	0	0	0	0	292960	86170	413880
ARHOMBI_DN3921_c0_g1_i1_3	0	0	0	0	0	0	0	0	0	0	0	0	0	357570	434200
ARUBRA_DN729_c0_g1_i1_6	0	0	0	0	0	0	0	0	0	0	0	0	366610	0	423170
ARUBRA_DN17182_c0_g1_i1_3	86052	143280	187970	0	0	370720	0	0	0	0	0	0	0	0	0
ARUBRA_DN5225_c0_g1_i1_3	253730	293330	240190	0	0	0	0	0	0	0	0	0	0	0	0
ARHOMBI_DN26642_c0_g1_i1_3	79517	107670	0	0	0	0	0	0	0	0	0	0	121430	0	477620
ARHOMBI_DN6091_c0_g1_i3_6	0	0	0	0	0	0	0	0	0	0	0	0	261180	236300	288470
ARHOMBI_DN2397_c0_g1_i1_3	0	0	0	0	0	0	0	0	0	0	0	0	0	492080	289380
ARUBRA_DN19230_c0_g1_i1_6	0	0	0	0	0	0	0	0	0	0	0	0	346960	347170	87218
ARUBRA_DN10511_c0_g1_i1_4	0	0	0	0	0	0	0	0	0	0	0	0	238430	271680	270360
ARHOMBI_DN18648_c0_g1_i1_2	94116	105450	0	0	0	0	0	0	0	0	0	0	0	580800	0
ARHOMBI_DN20035_c0_g1_i1_4	0	0	0	0	0	0	0	0	0	0	0	0	392530	385540	0
ARHOMBI_DN22203_c0_g1_i1_5	0	0	0	0	0	0	0	0	0	0	0	0	239950	298310	239300
ARUBRA_DN8245_c0_g1_i1_1	0	0	0	0	0	0	0	0	0	0	0	0	612690	0	164880
ARUBRA_DN18674_c0_g1_i1_6	0	0	0	0	0	0	0	0	0	0	0	0	249470	218910	308930

ARUBRA_DN1052_c0_g1_i1_4	0	0	0	0	0	0	0	0	0	0	0	0	0	0	776700
ARUBRA_DN4497_c0_g1_i1_6	0	0	0	0	0	0	0	0	0	0	0	0	217120	270490	287250
ARHOMBI_DN23074_c0_g1_i1_6	0	0	0	0	0	0	0	0	0	0	0	0	361530	0	412980
ARUBRA_DN17867_c0_g1_i1_4	170010	114060	214650	150960	0	0	0	0	0	0	0	0	0	124130	0
ARUBRA_DN26207_c0_g1_i1_1	0	0	0	0	0	0	0	0	0	0	0	0	277010	495050	0
ARUBRA_DN993_c0_g1_i1_2	0	0	0	0	0	0	0	0	0	0	0	0	212830	185360	373030
ARUBRA_DN5001_c0_g1_i1_4	332500	0	438220	0	0	0	0	0	0	0	0	0	0	0	0
ARUBRA_DN16126_c0_g1_i1_1	0	0	0	0	0	0	0	0	0	0	0	0	0	0	770450
ARUBRA_DN18434_c0_g1_i1_5	139250	190290	0	0	234660	0	0	206000	0	0	0	0	0	0	0
ARHOMBI_DN8608_c0_g1_i1_5	0	0	0	0	0	0	0	0	0	0	0	0	0	766770	0
ARUBRA_DN21219_c0_g1_i1_3	0	0	0	0	0	0	0	0	0	0	0	0	389680	376880	0
ARUBRA_DN10906_c0_g1_i1_1	0	0	0	0	0	0	0	0	535340	0	0	0	0	98649	131450
ARUBRA_DN24391_c0_g1_i1_1	0	0	0	0	0	0	0	0	0	0	0	0	378200	0	386360
ARHOMBI_DN6909_c0_g1_i1_1	55798	49322	20862	0	635430	0	0	0	0	0	0	0	0	0	0
ARUBRA_DN24666_c0_g1_i1_6	0	0	0	0	0	0	0	0	0	0	0	0	0	336330	421480
ARUBRA_DN6630_c0_g1_i1_2	155480	127520	49261	26198	0	0	0	0	0	0	0	0	0	165450	232870
ARUBRA_DN9370_c0_g1_i1_1	79116	0	0	219720	0	0	0	456740	0	0	0	0	0	0	0
ARUBRA_DN10905_c0_g1_i1_2	0	0	0	0	0	0	0	0	0	0	0	0	265660	0	489620
ARHOMBI_DN13448_c0_g1_i1_5	62654	0	76204	0	0	0	0	0	613740	0	0	0	0	0	0
ARUBRA_DN20978_c0_g1_i1_4	0	0	0	0	0	0	0	0	0	0	0	0	0	339890	412100
ARUBRA_DN6384_c0_g1_i1_5	0	0	0	0	0	0	0	0	0	0	0	0	507060	244870	0
ARHOMBI_DN981_c0_g1_i1_4	0	0	0	0	0	0	0	0	0	0	0	0	0	234260	515840
ARUBRA_DN22585_c0_g1_i1_2	0	0	0	0	0	0	0	0	0	0	0	0	223460	241100	285300
ARHOMBI_DN4343_c0_g1_i1_6	0	0	0	0	0	0	218670	275500	0	0	0	0	0	0	255370
ARHOMBI_DN796_c0_g1_i1_1	0	0	0	0	0	0	0	0	0	0	0	0	295410	0	453670
ARUBRA_DN3285_c0_g1_i1_2	0	0	0	0	0	0	0	246720	0	0	0	0	0	0	502150
ARUBRA_DN5446_c0_g1_i1_1	0	0	0	0	0	0	0	0	0	0	0	0	0	236550	511050

ARUBRA_DN10803_c0_g1_i1_2	0	0	0	0	0	0	0	0	0	0	0	0	0	747080	0
ARUBRA_DN16402_c0_g1_i1_6	0	0	0	0	0	0	0	0	0	0	0	0	193440	248280	302820
ARUBRA_DN19030_c0_g1_i1_1	0	0	0	0	0	0	0	0	0	0	0	0	205180	248160	289830
ARHOMBI_DN6155_c0_g1_i1_1	0	0	0	0	0	0	0	0	0	0	0	0	742790	0	0
ARUBRA_DN13110_c0_g1_i1_1	0	0	0	0	368310	0	374430	0	0	0	0	0	0	0	0
ARUBRA_DN25449_c0_g1_i1_2	240970	244530	255220	0	0	0	0	0	0	0	0	0	0	0	0
ARHOMBI_DN4728_c0_g3_i1_1	85222	0	121190	113600	0	0	0	0	240580	0	0	0	0	0	179100
ARHOMBI_DN6142_c0_g1_i3_4	0	0	0	0	0	0	0	0	0	0	0	0	144690	591150	0
ARHOMBI_DN5011_c0_g1_i1_4	0	0	0	0	0	0	0	0	0	0	0	0	282550	453220	0
ARHOMBI_DN7836_c0_g1_i1_2	0	0	0	0	0	0	0	0	0	0	0	0	0	0	723850
ARHOMBI_DN4465_c0_g1_i1_1	0	0	0	0	0	0	0	0	0	0	0	0	0	342700	376680
ARHOMBI_DN2932_c0_g2_i1_5	0	0	0	0	0	0	0	0	0	0	0	0	0	449810	268570
ARHOMBI_DN6286_c0_g1_i1_6	0	0	0	0	0	0	0	0	0	0	0	0	238580	193680	286110
ARUBRA_DN17025_c0_g1_i1_2	0	0	0	0	0	0	0	0	0	0	0	0	0	374470	337540
ARHOMBI_DN7489_c1_g1_i1_4	164840	163620	70645	41735	269070	0	0	0	0	0	0	0	0	0	0
ARUBRA_DN13369_c0_g1_i1_3	0	0	0	0	0	0	0	0	0	0	0	0	214330	186450	304630
ARUBRA_DN18574_c0_g1_i1_2	0	0	0	0	0	0	0	0	0	0	0	0	168280	0	535740
ARUBRA_DN21886_c0_g1_i1_1	69578	117680	0	58994	0	0	0	220650	0	0	0	0	236060	0	0
ARHOMBI_DN17203_c0_g1_i1_2	0	0	0	0	0	0	0	0	0	0	0	0	235670	214040	253210
ARHOMBI_DN13122_c0_g1_i1_4	0	0	0	0	0	0	0	0	0	0	0	0	345690	0	357090
ARHOMBI_DN25341_c0_g1_i1_1	216320	216790	122850	143840	0	0	0	0	0	0	0	0	0	0	0
ARHOMBI_DN3340_c0_g1_i1_2	0	0	0	0	0	0	0	0	0	0	0	0	0	536410	163230
ARUBRA_DN3419_c0_g1_i1_6	238830	236090	147490	75571	0	0	0	0	0	0	0	0	0	0	0
ARHOMBI_DN8889_c0_g1_i1_1	0	0	0	0	0	0	0	0	0	0	0	0	0	324000	372190
ARHOMBI_DN4948_c0_g1_i1_2	0	0	0	0	0	0	0	0	0	0	0	0	361060	333620	0
ARUBRA_DN23749_c0_g1_i1_2	0	0	0	0	0	0	0	0	0	0	0	0	410520	0	283930
ARUBRA_DN23540_c0_g1_i1_4	0	0	0	0	0	0	0	0	0	0	0	0	0	335680	358530

ARHOMBI_DN658_c0_g1_i1_6	0	0	0	0	0	0	0	0	0	0	0	0	326140	0	368070
ARUBRA_DN1712_c0_g1_i1_3	0	0	0	0	0	0	0	0	0	0	0	0	287570	0	403750
ARUBRA_DN9464_c0_g1_i1_3	0	0	0	0	0	0	0	0	0	0	0	0	0	690180	0
ARUBRA_DN8289_c0_g1_i1_1	258880	187280	114390	129360	0	0	0	0	0	0	0	0	0	0	0
ARHOMBI_DN11041_c0_g1_i1_3	0	0	0	0	0	0	0	0	0	0	0	0	0	369780	319670
ARHOMBI_DN8091_c0_g1_i1_4	0	0	0	0	0	0	0	0	0	0	0	0	221630	218970	245250
ARHOMBI_DN4244_c0_g1_i2_2	0	0	0	0	0	0	0	0	0	0	0	0	0	436640	248010
ARUBRA_DN313_c0_g2_i1_6	0	0	0	0	0	0	0	0	0	0	0	0	0	165300	519300
ARUBRA_DN707_c0_g1_i1_4	0	0	0	0	0	0	0	0	0	0	0	0	309110	0	375210
ARHOMBI_DN3837_c0_g1_i1_6	0	0	680580	0	0	0	0	0	0	0	0	0	0	0	0
ARUBRA_DN4588_c0_g1_i2_1	0	0	0	0	0	0	0	0	0	0	0	0	0	163560	514320
ARHOMBI_DN6158_c0_g1_i1_6	0	0	113890	0	0	562330	0	0	0	0	0	0	0	0	0
ARHOMBI_DN6709_c0_g1_i1_2	0	0	0	0	0	0	0	0	0	0	0	0	331010	0	344820
ARHOMBI_DN12465_c0_g1_i1_1	139140	77529	80717	89921	0	0	286060	0	0	0	0	0	0	0	0
ARHOMBI_DN26923_c0_g1_i1_1	0	0	0	0	0	0	0	0	0	0	0	0	307450	0	365410
ARUBRA_DN1422_c0_g2_i1_2	0	0	0	0	0	0	0	0	0	0	0	0	0	312670	359330
ARHOMBI_DN23444_c0_g1_i1_1	0	0	0	0	0	0	0	0	0	0	0	0	0	0	671170
ARUBRA_DN2399_c0_g1_i1_4	0	0	0	0	0	0	0	0	0	301740	0	0	0	0	367640
ARUBRA_DN2263_c0_g2_i1_1	0	0	0	0	0	0	0	0	0	0	0	0	0	0	668920
ARHOMBI_DN15914_c0_g1_i1_1	0	0	0	0	0	0	0	0	0	0	0	0	324510	174540	169700
ARUBRA_DN1557_c0_g1_i1_4	0	0	0	0	0	0	0	0	0	0	0	0	243240	200140	217040
ARUBRA_DN4786_c0_g1_i1_2	0	0	0	0	209810	0	0	449410	0	0	0	0	0	0	0
ARHOMBI_DN18534_c0_g1_i1_3	0	0	0	0	0	0	0	0	0	0	0	0	183230	253700	221810
ARHOMBI_DN10516_c0_g1_i1_6	186760	166780	0	0	0	0	0	0	0	0	0	0	304810	0	0
ARUBRA_DN178_c0_g1_i1_1	0	0	0	0	0	0	0	0	0	0	0	0	0	289250	367400
ARUBRA_DN24618_c0_g1_i1_5	0	0	0	0	0	0	0	0	0	0	0	0	0	365600	288560
ARUBRA_DN23959_c0_g1_i1_1	0	0	0	0	0	0	0	0	0	0	0	0	445210	0	207590

ARUBRA_DN19174_c0_g1_i1_2	0	0	0	0	123750	0	0	0	0	0	0	0	0	528250	0
ARHOMBI_DN4479_c0_g1_i1_4	0	0	0	0	0	0	0	0	0	0	0	0	640760	0	0
ARHOMBI_DN3958_c0_g1_i1_6	97297	126370	79866	37532	0	0	0	0	0	0	0	0	146110	152130	0
ARHOMBI_DN2738_c0_g1_i1_2	0	0	0	0	0	0	0	0	0	0	0	0	0	638520	0
ARUBRA_DN1466_c0_g2_i1_4	0	0	0	0	0	0	0	0	0	0	0	0	112190	384530	141690
ARHOMBI_DN3523_c0_g1_i1_4	0	0	0	0	0	0	0	0	0	0	0	0	0	638350	0
ARHOMBI_DN1869_c0_g2_i1_5	137960	217950	205900	73253	0	0	0	0	0	0	0	0	0	0	0
ARUBRA_DN22416_c0_g1_i1_5	0	0	0	0	0	0	0	0	0	0	0	0	0	341900	292670
ARUBRA_DN446_c0_g1_i1_2	0	0	0	0	0	0	0	0	0	0	0	0	111260	244240	278200
ARUBRA_DN1177_c0_g2_i1_1	0	0	0	0	0	0	0	0	0	0	0	0	191670	156650	284140
ARUBRA_DN22100_c0_g1_i1_5	0	0	0	0	0	0	0	0	0	0	0	0	629850	0	0
ARHOMBI_DN9092_c0_g1_i1_2	0	0	0	0	0	0	0	0	0	0	0	0	0	304320	325200
ARHOMBI_DN5351_c0_g2_i1_2	0	0	0	0	0	0	0	0	0	0	0	0	310640	0	317960
ARUBRA_DN3055_c0_g1_i2_3	0	0	0	0	0	0	0	0	0	0	0	0	124910	0	503480
ARHOMBI_DN21638_c0_g1_i1_6	0	0	0	0	0	0	0	0	0	0	0	0	166800	223140	236120
ARUBRA_DN4234_c0_g2_i1_6	0	0	0	0	0	0	0	0	0	0	0	0	114940	339370	171600
ARUBRA_DN2493_c0_g1_i1_1	0	0	0	0	0	0	0	0	0	0	0	0	185690	241010	197000
ARUBRA_DN7662_c0_g1_i1_2	0	181240	70141	0	0	0	0	0	0	0	0	0	123340	125750	122760
ARUBRA_DN12348_c0_g1_i1_6	0	0	0	0	0	42130	0	211660	0	0	0	0	0	0	367750
ARHOMBI_DN3285_c0_g2_i1_4	0	0	621160	0	0	0	0	0	0	0	0	0	0	0	0
ARHOMBI_DN8957_c0_g1_i1_1	0	0	0	0	0	0	0	0	0	0	0	0	182150	163630	275330
ARHOMBI_DN21971_c0_g1_i1_1	0	0	0	0	0	0	619300	0	0	0	0	0	0	0	0
ARUBRA_DN13989_c0_g1_i1_2	0	0	0	0	0	0	0	0	0	0	0	0	291550	0	327270
ARUBRA_DN26201_c0_g1_i1_1	0	0	0	0	0	0	0	0	0	0	0	0	288250	330140	0
ARHOMBI_DN16098_c0_g1_i1_2	0	0	0	0	0	0	0	0	0	0	0	0	229630	284510	102580
ARHOMBI_DN2295_c0_g1_i1_1	171780	172410	177930	0	0	0	0	0	0	0	0	0	0	0	92339
ARHOMBI_DN8630_c0_g1_i1_2	0	0	0	0	0	0	0	0	0	0	0	0	336460	0	274930

ARUBRA_DN15491_c0_g1_i1_1	0	0	0	0	0	0	0	0	212540	0	0	0	136570	126840	131850
ARUBRA_DN7176_c0_g1_i1_6	0	0	0	0	0	0	0	0	0	0	0	0	173570	231990	198420
ARUBRA_DN22998_c0_g1_i1_5	0	0	0	0	0	0	0	0	0	0	0	0	0	0	603940
ARHOMBI_DN1819_c0_g1_i1_2	0	0	0	0	0	0	0	0	0	0	0	0	294150	0	309560
ARUBRA_DN1769_c0_g2_i1_6	0	0	0	0	0	0	0	0	0	0	0	0	239090	0	364310
ARHOMBI_DN7578_c0_g1_i1_4	0	0	0	0	0	0	0	0	0	0	0	0	173640	222510	203170
ARHOMBI_DN4440_c0_g1_i1_4	0	0	0	0	0	0	0	0	0	0	0	0	0	310810	287040
ARUBRA_DN16297_c0_g1_i1_4	88269	0	97487	123500	0	0	0	0	286600	0	0	0	0	0	0
ARUBRA_DN7697_c0_g1_i1_2	0	0	0	0	0	0	0	0	0	0	0	0	189140	181520	225190
ARUBRA_DN19516_c0_g1_i1_2	0	0	0	0	0	0	340330	254610	0	0	0	0	0	0	0
ARUBRA_DN9248_c0_g1_i1_4	202410	190030	198820	0	0	0	0	0	0	0	0	0	0	0	0
ARHOMBI_DN26238_c0_g1_i1_4	237140	164360	93415	94931	0	0	0	0	0	0	0	0	0	0	0
ARHOMBI_DN1217_c0_g2_i1_1	0	0	0	0	0	0	0	0	0	0	0	0	589600	0	0
ARUBRA_DN22060_c0_g1_i1_4	0	0	0	0	0	0	0	0	0	0	0	0	125070	210250	253340
ARHOMBI_DN3796_c0_g1_i1_3	0	0	0	0	0	0	0	0	0	0	0	0	218770	0	367370
ARUBRA_DN3884_c0_g1_i1_3	0	0	0	0	0	0	0	0	0	0	0	0	585650	0	0
ARHOMBI_DN11238_c0_g1_i1_1	0	0	0	0	0	0	0	0	0	0	0	0	0	297000	287970
ARUBRA_DN20512_c0_g1_i1_5	0	0	0	0	0	0	0	0	0	0	0	0	0	273840	309720
ARUBRA_DN2494_c0_g1_i1_5	0	0	0	0	142340	0	187880	252480	0	0	0	0	0	0	0
ARHOMBI_DN25808_c0_g1_i1_4	0	0	0	0	0	0	0	0	0	0	0	0	133920	184780	263480
ARHOMBI_DN5409_c0_g1_i1_1	0	0	0	0	0	0	0	0	0	0	0	0	177490	186370	218230
ARUBRA_DN4725_c0_g1_i1_2	65219	214580	68366	59001	0	0	172850	0	0	0	0	0	0	0	0
ARUBRA_DN19472_c0_g1_i1_2	0	0	0	0	0	0	0	0	0	0	0	0	305000	0	273560
ARUBRA_DN9681_c0_g1_i1_1	0	0	0	0	0	0	0	0	0	0	0	0	0	0	578160
ARUBRA_DN21358_c0_g1_i1_1	0	0	0	0	0	0	0	0	0	0	0	0	238960	185950	152130
ARUBRA_DN2192_c0_g1_i1_5	161450	124890	141320	149010	0	0	0	0	0	0	0	0	0	0	0
ARUBRA_DN14031_c0_g1_i1_6	0	0	0	0	0	0	0	0	0	0	0	0	0	399410	176390

ARUBRA_DN22638_c0_g1_i1_6	0	0	0	0	0	0	0	0	0	0	0	0	238360	337190	0
ARUBRA_DN3929_c0_g1_i2_5	0	0	0	0	0	0	0	0	0	0	0	0	262600	0	312030
ARUBRA_DN3241_c0_g1_i1_4	0	0	0	0	0	0	0	0	0	0	0	0	397570	175260	0
ARUBRA_DN2358_c0_g1_i1_4	0	0	0	0	0	0	0	0	0	0	0	0	571400	0	0
ARUBRA_DN24043_c0_g1_i1_4	0	0	0	0	0	0	0	0	0	0	0	0	0	0	571100
ARHOMBI_DN24632_c0_g1_i1_3	0	0	0	0	0	0	0	0	0	0	0	0	0	264570	305130
ARHOMBI_DN26919_c0_g1_i1_4	0	0	0	0	0	0	0	0	0	567410	0	0	0	0	0
ARUBRA_DN23841_c0_g1_i1_4	0	0	0	0	0	0	0	0	0	0	0	0	170770	0	395130
ARUBRA_DN19491_c0_g1_i1_2	0	0	0	0	0	0	0	0	0	0	0	0	0	267560	295360
ARUBRA_DN22396_c0_g1_i1_1	0	0	0	0	0	0	0	0	0	0	0	0	184040	377590	0
ARHOMBI_DN19578_c0_g1_i1_4	0	0	0	0	0	0	0	0	0	0	0	0	0	362710	193430
ARHOMBI_DN3163_c0_g1_i1_3	0	0	0	0	0	0	0	0	0	0	0	0	373890	180370	0
ARUBRA_DN19450_c0_g1_i1_4	0	0	0	0	0	0	0	0	0	0	0	0	0	0	553660
ARUBRA_DN130_c0_g1_i1_4	0	0	0	0	0	0	0	0	0	0	0	0	370040	182410	0
ARHOMBI_DN4296_c0_g1_i1_1	0	0	0	0	0	0	0	0	0	0	0	0	446970	104990	0
ARUBRA_DN6440_c0_g1_i1_4	0	0	361690	188210	0	0	0	0	0	0	0	0	0	0	0
ARHOMBI_DN6362_c0_g1_i1_1	0	0	0	0	0	0	0	0	0	0	0	0	0	281910	267820
ARHOMBI_DN23114_c0_g1_i1_1	0	0	0	0	0	0	549480	0	0	0	0	0	0	0	0
ARHOMBI_DN4923_c0_g1_i1_6	0	0	0	48086	0	0	0	0	500470	0	0	0	0	0	0
ARUBRA_DN6647_c0_g1_i1_3	0	0	0	0	0	0	133650	0	178280	0	0	0	126410	107980	0
ARHOMBI_DN5696_c0_g1_i1_6	0	0	0	545040	0	0	0	0	0	0	0	0	0	0	0
ARUBRA_DN8888_c0_g1_i1_4	0	0	0	0	0	0	0	0	0	0	0	0	0	271680	271820
ARHOMBI_DN5174_c0_g2_i1_1	187290	183370	172610	0	0	0	0	0	0	0	0	0	0	0	0
ARUBRA_DN16605_c0_g1_i1_4	0	0	0	0	0	0	0	0	0	0	0	0	179420	169340	194030
ARHOMBI_DN18922_c0_g1_i1_3	0	0	0	0	0	0	0	0	0	0	0	0	186810	189340	164530
ARHOMBI_DN5270_c0_g1_i1_5	0	0	0	0	0	98157	221780	0	0	0	0	0	0	220530	0
ARHOMBI_DN9484_c0_g2_i1_3	0	0	0	0	0	0	0	0	0	0	0	0	0	107970	431290

ARHOMBI_DN498_c0_g1_i1_1	0	0	0	0	0	0	0	0	0	0	0	0	158040	381100	0
ARHOMBI_DN1736_c0_g1_i1_6	0	0	0	0	0	0	0	0	0	0	0	0	0	151540	382990
ARUBRA_DN171_c0_g2_i1_3	0	0	0	0	0	0	0	0	0	0	0	0	163150	151850	217550
ARUBRA_DN23798_c0_g1_i1_1	0	0	0	0	0	0	0	0	0	0	0	0	284730	247520	0
ARHOMBI_DN9171_c0_g1_i1_1	0	0	0	0	0	0	0	0	0	0	0	0	0	0	531740
ARUBRA_DN3301_c0_g2_i1_5	0	531250	0	0	0	0	0	0	0	0	0	0	0	0	0
ARUBRA_DN2204_c0_g1_i1_6	0	0	0	0	0	0	0	0	0	0	0	0	190860	93540	244400
ARHOMBI_DN1709_c0_g2_i1_6	110710	135720	103870	177470	0	0	0	0	0	0	0	0	0	0	0
ARUBRA_DN4715_c0_g1_i1_5	0	0	0	0	0	0	0	0	0	0	0	0	0	525840	0
ARHOMBI_DN17044_c0_g1_i1_2	0	0	0	0	0	0	0	0	0	0	0	0	210340	0	314400
ARHOMBI_DN6819_c0_g1_i1_2	0	0	0	0	0	0	0	0	0	0	0	0	0	0	522450
ARHOMBI_DN5985_c0_g1_i1_4	0	0	0	0	0	0	0	0	0	0	0	0	0	0	521510
ARUBRA_DN24121_c0_g1_i1_6	0	0	0	0	0	0	0	0	0	0	0	0	282310	238600	0
ARUBRA_DN4657_c0_g1_i1_6	185110	161800	0	169980	0	0	0	0	0	0	0	0	0	0	0
ARUBRA_DN290_c0_g1_i1_5	0	0	0	0	0	0	0	0	0	0	0	0	0	305960	209940
ARHOMBI_DN5862_c0_g1_i1_2	0	0	0	513990	0	0	0	0	0	0	0	0	0	0	0
ARHOMBI_DN6231_c0_g3_i5_2	0	0	0	0	0	0	0	0	209450	0	0	0	0	0	303990
ARUBRA_DN5145_c0_g1_i1_2	0	0	0	0	0	0	0	0	0	0	0	0	0	214970	296570
ARUBRA_DN20610_c0_g1_i1_6	0	0	0	0	0	0	0	0	0	0	0	0	0	233490	275490
ARHOMBI_DN19734_c0_g1_i1_3	0	0	0	0	0	0	0	0	0	0	0	0	151200	178360	178970
ARHOMBI_DN13808_c0_g1_i1_5	0	0	0	0	0	0	0	0	0	0	0	0	0	329420	175340
ARUBRA_DN19459_c0_g1_i1_5	0	0	0	0	0	0	0	0	0	0	0	0	0	374810	129800
ARHOMBI_DN4450_c0_g1_i1_5	140550	0	165030	197350	0	0	0	0	0	0	0	0	0	0	0
ARUBRA_DN2002_c0_g2_i1_5	0	0	0	0	0	0	0	0	0	0	0	0	158600	160170	183360
ARHOMBI_DN22399_c0_g1_i1_2	0	0	0	0	246910	0	251600	0	0	0	0	0	0	0	0
ARHOMBI_DN16397_c0_g1_i1_6	0	0	0	0	0	0	0	0	0	0	0	0	0	252760	243530
ARHOMBI_DN3020_c0_g2_i1_1	0	0	0	0	0	0	0	0	0	0	0	0	317430	178440	0

ARHOMBI_DN6884_c0_g1_i1_5	0	0	0	0	0	0	0	0	0	0	0	0	230900	115260	149550
ARUBRA_DN21439_c0_g1_i1_3	0	0	0	0	0	0	0	0	0	283360	0	212170	0	0	0
ARHOMBI_DN5298_c0_g1_i1_5	0	0	0	0	0	0	0	0	0	0	0	0	172750	156160	166510
ARHOMBI_DN6134_c0_g1_i2_6	0	0	0	0	0	0	0	0	0	0	491340	0	0	0	0
ARHOMBI_DN21703_c0_g1_i1_5	0	0	0	0	0	0	0	0	0	0	0	0	179050	178540	133130
ARHOMBI_DN1691_c0_g1_i1_5	0	0	0	0	0	0	0	0	0	0	0	0	207140	0	281600
ARHOMBI_DN20074_c0_g1_i1_1	44180	32597	0	40541	0	0	0	0	0	0	0	0	0	0	370030
ARUBRA_DN16643_c0_g1_i1_2	0	0	0	0	0	0	0	0	0	0	0	0	0	486660	0
ARHOMBI_DN16129_c0_g1_i1_4	0	0	0	0	0	114280	0	272340	0	0	0	0	0	0	99699
ARUBRA_DN4512_c0_g1_i1_2	0	0	0	0	0	0	0	0	0	0	0	0	0	486240	0
ARHOMBI_DN8916_c0_g1_i1_1	0	0	0	0	0	0	0	0	0	0	0	0	229750	256070	0
ARHOMBI_DN12802_c0_g1_i1_2	0	0	0	0	0	0	0	0	0	0	0	0	483040	0	0
ARUBRA_DN14943_c0_g1_i1_6	0	0	0	0	0	0	0	0	0	0	0	0	0	235650	246510
ARHOMBI_DN21499_c0_g1_i1_5	0	0	0	0	0	0	0	0	0	0	0	0	0	247490	234650
ARHOMBI_DN16804_c0_g1_i1_2	0	0	0	0	0	0	0	0	0	0	0	0	0	481900	0
ARHOMBI_DN3081_c0_g1_i2_3	0	0	0	0	0	0	0	0	0	0	0	0	80076	184420	212100
ARHOMBI_DN26634_c0_g1_i1_6	0	89717	0	0	178850	0	0	206240	0	0	0	0	0	0	0
ARUBRA_DN16199_c0_g1_i1_3	165380	112960	51915	0	0	0	0	0	142990	0	0	0	0	0	0
ARHOMBI_DN10954_c0_g1_i1_5	0	0	0	0	0	0	0	0	0	0	0	0	0	472300	0
ARHOMBI_DN8652_c0_g1_i1_2	0	0	0	0	0	0	0	0	0	0	0	0	0	181360	290920
ARUBRA_DN26687_c0_g1_i1_2	0	0	0	0	0	0	0	0	0	0	0	0	221920	249210	0
ARHOMBI_DN3151_c0_g2_i1_5	0	0	0	0	0	0	0	0	0	0	0	0	203580	264750	0
ARHOMBI_DN12758_c0_g1_i1_4	0	0	0	0	0	0	0	0	0	0	0	0	132870	148380	184000
ARUBRA_DN6423_c0_g1_i1_6	0	169780	291160	0	0	0	0	0	0	0	0	0	0	0	0
ARUBRA_DN11367_c0_g1_i1_2	0	0	0	0	0	0	0	0	0	0	0	0	180500	0	278310
ARHOMBI_DN22357_c0_g1_i1_4	0	0	0	0	0	0	0	0	0	0	0	0	0	0	458160
ARUBRA_DN20780_c0_g1_i1_5	0	0	98259	84269	0	0	0	174800	0	0	0	0	99975	0	0

ARHOMBI_DN17128_c0_g1_i1_2	0	72645	40353	26871	0	0	0	0	315880	0	0	0	0	0	0
ARHOMBI_DN21148_c0_g1_i1_2	0	0	0	0	0	0	0	0	0	0	0	0	0	0	454230
ARHOMBI_DN4969_c0_g1_i1_6	37649	37093	14588	0	245680	0	118570	0	0	0	0	0	0	0	0
ARHOMBI_DN2477_c0_g2_i1_4	0	0	0	0	0	0	0	0	0	0	0	0	179360	273300	0
ARUBRA_DN2574_c0_g1_i1_1	0	0	0	0	0	0	0	0	0	0	0	0	450670	0	0
ARHOMBI_DN1345_c0_g1_i1_3	0	0	0	0	0	0	0	0	0	0	0	0	228400	221870	0
ARUBRA_DN7816_c0_g1_i1_3	0	0	0	0	0	0	0	0	0	0	0	0	0	328200	121750
ARHOMBI_DN4238_c0_g1_i1_6	0	0	0	0	0	0	0	0	0	0	0	0	449880	0	0
ARUBRA_DN17859_c0_g1_i1_6	0	0	0	0	0	0	0	0	0	0	0	0	0	222650	223830
ARHOMBI_DN6130_c1_g1_i2_6	0	0	0	0	0	0	0	0	0	0	0	0	117930	0	328110
ARUBRA_DN15205_c0_g1_i1_1	138450	166260	140830	0	0	0	0	0	0	0	0	0	0	0	0
ARUBRA_DN17787_c0_g1_i1_5	0	0	0	0	0	0	0	0	0	0	0	0	152550	0	292820
ARHOMBI_DN21125_c0_g1_i1_2	180590	0	203320	61294	0	0	0	0	0	0	0	0	0	0	0
ARUBRA_DN22796_c0_g1_i1_1	355720	0	0	0	0	0	88801	0	0	0	0	0	0	0	0
ARHOMBI_DN2978_c0_g1_i1_5	0	0	0	0	0	0	0	0	0	0	0	0	200320	0	243750
ARHOMBI_DN8760_c0_g1_i1_1	0	0	0	0	0	0	0	0	0	0	0	0	0	443660	0
ARUBRA_DN11549_c0_g1_i1_5	0	0	0	0	0	0	0	0	0	0	0	0	443560	0	0
ARHOMBI_DN22363_c0_g1_i1_3	0	0	0	0	0	0	0	0	0	0	0	0	291950	0	148500
ARUBRA_DN7615_c0_g1_i1_5	71921	86145	67212	50177	161450	0	0	0	0	0	0	0	0	0	0
ARHOMBI_DN10838_c0_g1_i1_3	111050	122700	129400	72777	0	0	0	0	0	0	0	0	0	0	0
ARHOMBI_DN1649_c0_g1_i1_5	0	0	0	0	0	0	0	0	0	0	0	0	0	433040	0
ARHOMBI_DN20781_c0_g1_i1_6	163540	0	97311	0	0	0	0	0	171860	0	0	0	0	0	0
ARHOMBI_DN6024_c0_g1_i2_5	0	0	0	0	0	0	101510	0	0	0	0	0	0	0	330450
ARHOMBI_DN6092_c0_g3_i3_3	0	0	0	0	0	0	0	0	429270	0	0	0	0	0	0
ARUBRA_DN23408_c0_g1_i1_6	0	0	0	0	0	0	0	0	0	0	0	0	108290	153260	167670
ARHOMBI_DN17360_c0_g1_i1_3	0	0	0	0	0	0	0	0	0	0	0	0	205780	0	222620
ARUBRA_DN6106_c0_g1_i1_6	142620	0	85362	198960	0	0	0	0	0	0	0	0	0	0	0

ARUBRA_DN8839_c0_g1_i1_5	0	0	0	0	0	0	0	0	0	0	0	0	426000	0	0
ARUBRA_DN5290_c0_g1_i1_5	0	0	195960	106510	0	0	0	0	0	0	0	0	123210	0	0
ARHOMBI_DN16684_c0_g1_i1_4	0	0	0	327230	0	0	0	0	0	0	0	0	0	98383	0
ARUBRA_DN16761_c0_g1_i1_6	0	225430	199810	0	0	0	0	0	0	0	0	0	0	0	0
ARUBRA_DN18234_c0_g1_i1_6	0	0	0	0	0	0	0	0	0	0	0	0	117530	137420	170010
ARUBRA_DN483_c0_g1_i1_1	0	0	0	0	0	0	0	0	0	0	0	0	151170	103830	169170
ARHOMBI_DN20642_c0_g1_i1_2	0	0	0	0	0	0	0	0	0	0	0	0	117850	178990	126440
ARHOMBI_DN1985_c0_g1_i1_1	0	0	0	0	0	0	0	0	0	0	0	0	0	215210	205930
ARUBRA_DN4720_c0_g1_i2_2	0	0	0	0	0	0	0	0	0	0	0	0	0	166560	253110
ARHOMBI_DN8466_c0_g1_i1_2	0	0	0	0	0	0	0	0	0	0	0	0	0	419140	0
ARUBRA_DN26386_c0_g1_i1_2	0	0	0	0	0	0	0	0	0	0	0	0	0	144070	273970
ARHOMBI_DN16500_c0_g1_i1_6	0	0	0	0	0	0	0	0	0	0	0	0	0	197470	220110
ARUBRA_DN7170_c0_g1_i1_6	0	0	0	0	0	0	0	0	0	0	0	0	99790	190760	126890
ARUBRA_DN828_c0_g1_i1_6	0	0	0	0	0	0	0	0	0	0	0	0	0	0	417190
ARHOMBI_DN8693_c1_g1_i1_5	0	0	0	0	0	0	0	0	0	0	0	0	98401	150320	167960
ARHOMBI_DN19277_c0_g1_i1_4	82882	57157	91728	0	0	0	0	0	0	0	0	0	184590	0	0
ARUBRA_DN11258_c0_g1_i1_3	0	0	0	0	0	0	0	0	0	0	0	0	136070	122420	157470
ARHOMBI_DN9241_c0_g1_i1_6	0	0	0	0	0	0	0	0	0	0	0	0	158890	0	255700
ARHOMBI_DN2715_c0_g1_i1_5	86336	0	89653	0	0	0	0	0	0	0	0	0	0	0	230900
ARUBRA_DN18267_c0_g1_i1_4	0	0	0	0	0	0	0	0	0	0	0	0	0	203370	203350
ARUBRA_DN17506_c0_g1_i1_4	0	0	0	0	0	0	0	0	0	0	0	0	0	200900	205140
ARUBRA_DN5389_c0_g1_i1_3	0	51854	21097	57778	0	0	0	272940	0	0	0	0	0	0	0
ARHOMBI_DN3097_c0_g1_i2_1	0	0	0	403520	0	0	0	0	0	0	0	0	0	0	0
ARUBRA_DN5867_c0_g1_i1_1	0	0	0	0	0	0	0	0	0	0	0	0	0	207300	195400
ARUBRA_DN18169_c0_g1_i1_2	0	0	300580	100520	0	0	0	0	0	0	0	0	0	0	0
ARUBRA_DN4178_c0_g2_i1_3	0	0	0	0	0	0	0	0	0	0	0	0	0	208770	192240
ARUBRA_DN6773_c0_g1_i1_3	0	0	0	0	0	0	0	0	0	0	0	0	169970	0	228180

ARHOMBI_DN20737_c0_g1_i1_3	0	0	0	0	0	0	0	0	0	0	0	0	0	171080	225280
ARUBRA_DN1212_c0_g2_i1_5	115730	108160	113260	59120	0	0	0	0	0	0	0	0	0	0	0
ARUBRA_DN22616_c0_g1_i1_2	0	0	0	0	0	0	0	0	0	0	0	0	188060	0	208160
ARHOMBI_DN4651_c0_g1_i1_3	136810	0	174410	0	0	0	0	0	0	0	0	0	0	84275	0
ARUBRA_DN4817_c0_g1_i2_6	0	0	0	0	0	0	0	0	0	0	0	0	0	0	394110
ARUBRA_DN5232_c0_g1_i1_6	0	0	0	0	0	0	0	0	0	0	0	0	128940	120970	143720
ARUBRA_DN8160_c0_g1_i1_1	0	0	0	0	0	0	0	0	0	0	0	0	143380	100620	149620
ARHOMBI_DN25761_c0_g1_i1_2	0	0	0	0	0	0	0	0	0	0	0	0	0	188430	204210
ARUBRA_DN18459_c0_g1_i1_4	0	0	0	0	0	0	0	0	0	0	0	0	392280	0	0
ARUBRA_DN23641_c0_g1_i1_3	0	0	0	0	0	0	0	0	0	0	0	0	0	282360	109130
ARHOMBI_DN15083_c0_g1_i1_2	0	0	0	0	0	0	0	0	0	0	0	0	179880	0	210740
ARHOMBI_DN5292_c0_g1_i1_5	0	0	0	0	0	0	0	0	0	0	0	0	390440	0	0
ARUBRA_DN13177_c0_g1_i1_4	0	0	0	0	0	0	0	0	0	0	0	0	188770	200360	0
ARUBRA_DN25572_c0_g1_i1_1	0	0	0	0	0	0	0	0	0	0	0	0	118550	122770	147480
ARHOMBI_DN10905_c0_g1_i1_3	0	0	0	0	0	0	0	0	0	0	0	0	0	0	388030
ARHOMBI_DN4521_c0_g1_i1_6	0	0	0	0	0	0	0	0	0	0	0	0	0	386950	0
ARUBRA_DN16776_c0_g1_i1_3	177430	0	0	208430	0	0	0	0	0	0	0	0	0	0	0
ARUBRA_DN3345_c0_g2_i1_6	0	0	0	0	0	0	0	0	0	0	0	0	0	0	385540
ARHOMBI_DN3792_c0_g1_i2_2	0	0	0	0	0	0	0	0	0	0	0	0	108190	274380	0
ARHOMBI_DN2492_c0_g1_i1_2	0	0	0	0	0	0	0	0	0	0	0	0	185260	0	195640
ARHOMBI_DN17916_c0_g1_i1_3	0	0	0	0	0	0	0	0	0	0	0	0	198280	0	181980
ARUBRA_DN23363_c0_g1_i1_1	0	0	0	0	0	0	214060	0	164870	0	0	0	0	0	0
ARUBRA_DN6829_c0_g1_i1_2	0	0	0	0	0	0	0	0	0	0	0	0	188820	0	189230
ARUBRA_DN14638_c0_g1_i1_5	0	0	0	0	0	0	0	0	0	0	0	0	109310	120480	148100
ARUBRA_DN20092_c0_g1_i1_5	0	0	0	0	0	0	0	0	0	0	0	0	104070	171590	101620
ARHOMBI_DN4417_c0_g1_i1_4	0	0	0	0	0	0	0	0	0	0	0	0	189730	186240	0
ARHOMBI_DN22894_c0_g1_i1_5	164470	210920	0	0	0	0	0	0	0	0	0	0	0	0	0

ARUBRA_DN7549_c0_g1_i1_1	0	71311	103020	77693	122860	0	0	0	0	0	0	0	0	0	0
ARHOMBI_DN3368_c0_g1_i1_3	0	0	0	0	0	0	0	0	0	0	0	0	0	185770	188910
ARUBRA_DN1922_c0_g2_i1_6	0	0	0	0	0	0	0	0	0	0	0	0	0	374270	0
ARHOMBI_DN7605_c0_g1_i1_1	0	0	0	0	0	0	0	0	0	0	0	0	187710	185770	0
ARUBRA_DN7746_c0_g1_i1_5	131390	122200	86392	32886	0	0	0	0	0	0	0	0	0	0	0
ARUBRA_DN23775_c0_g1_i1_5	0	0	0	0	0	0	0	0	0	0	0	0	129720	114410	127870
ARUBRA_DN22204_c0_g1_i1_5	0	0	18710	0	0	0	0	0	0	0	0	0	353130	0	0
ARHOMBI_DN16579_c0_g1_i1_6	0	0	0	0	0	0	0	0	0	0	0	0	0	193190	175320
ARUBRA_DN23978_c0_g1_i1_3	0	0	0	0	0	0	0	0	0	0	0	0	117240	109960	140380
ARUBRA_DN293_c0_g1_i1_1	0	0	0	0	0	0	0	0	0	0	0	0	121080	154750	90138
ARHOMBI_DN14395_c0_g1_i1_5	0	0	0	0	0	0	0	0	0	0	0	0	141330	0	224220
ARHOMBI_DN18669_c0_g1_i1_3	0	0	0	0	0	0	0	0	0	0	0	0	0	182800	182740
ARUBRA_DN18022_c0_g1_i1_6	0	0	0	0	0	0	0	0	0	0	0	0	191090	173400	0
ARUBRA_DN784_c0_g2_i1_6	0	44455	0	0	0	0	136010	0	0	0	0	0	180320	0	0
ARUBRA_DN7117_c0_g1_i1_3	0	0	0	0	0	0	0	0	0	0	0	0	0	169590	190700
ARUBRA_DN13794_c0_g1_i1_1	0	0	0	0	0	0	0	0	0	0	0	0	163390	0	196630
ARHOMBI_DN2834_c0_g2_i1_4	0	0	0	0	0	0	0	0	0	0	0	0	0	150520	208980
ARUBRA_DN7201_c0_g1_i1_6	0	0	0	0	0	0	0	0	0	0	0	0	0	0	358090
ARUBRA_DN20463_c0_g1_i1_4	0	0	0	0	0	0	0	0	0	0	0	0	103420	113740	140810
ARUBRA_DN12786_c0_g1_i1_6	0	0	0	0	0	0	0	0	0	0	0	0	0	357470	0
ARUBRA_DN4014_c0_g1_i1_1	97507	137320	0	121510	0	0	0	0	0	0	0	0	0	0	0
ARUBRA_DN21_c0_g1_i1_6	0	92853	34526	0	0	0	0	0	0	0	0	0	0	0	228410
ARUBRA_DN14287_c0_g1_i1_2	0	0	0	0	0	0	0	0	0	0	0	0	0	249980	105360
ARUBRA_DN3405_c0_g2_i1_2	0	0	0	354300	0	0	0	0	0	0	0	0	0	0	0
ARHOMBI_DN17003_c0_g1_i1_2	0	0	0	0	177520	0	176680	0	0	0	0	0	0	0	0
ARUBRA_DN1302_c0_g1_i1_2	0	0	0	53955	0	0	0	0	299840	0	0	0	0	0	0
ARHOMBI_DN20638_c0_g1_i1_2	0	0	0	0	0	0	0	0	0	0	0	0	0	178580	174760

ARHOMBI_DN3957_c0_g2_i1_3	0	0	0	0	0	0	0	0	0	0	0	0	0	351340	0
ARUBRA_DN19773_c0_g1_i1_2	132120	0	86104	132480	0	0	0	0	0	0	0	0	0	0	0
ARHOMBI_DN15739_c0_g1_i1_6	0	0	0	0	0	0	0	0	0	0	0	0	0	148360	200790
ARUBRA_DN1164_c0_g1_i1_2	0	0	0	0	0	0	0	0	0	0	0	0	0	345360	0
ARUBRA_DN12959_c0_g1_i1_2	0	0	0	101060	0	0	0	243000	0	0	0	0	0	0	0
ARHOMBI_DN11084_c0_g1_i1_6	0	0	0	0	0	0	0	0	141230	0	0	200470	0	0	0
ARUBRA_DN24628_c0_g1_i1_6	0	0	0	0	0	0	0	0	0	0	0	0	0	143970	196680
ARHOMBI_DN5257_c0_g1_i1_3	0	0	0	0	0	0	0	0	0	0	0	0	0	0	340200
ARUBRA_DN15806_c0_g1_i1_3	0	0	0	0	0	0	0	0	0	0	0	0	0	136240	202660
ARHOMBI_DN21683_c0_g1_i1_3	0	0	0	0	0	0	0	0	0	0	0	0	338510	0	0
ARHOMBI_DN7504_c0_g1_i1_3	0	0	0	0	0	0	0	0	0	0	0	0	0	338460	0
ARUBRA_DN13027_c0_g1_i1_5	0	0	0	0	0	0	0	0	0	0	0	0	104020	111240	122770
ARUBRA_DN22863_c0_g1_i1_4	0	0	0	0	0	0	0	0	0	0	0	0	173760	163240	0
ARUBRA_DN19918_c0_g1_i1_5	0	0	0	0	0	0	0	0	0	0	0	0	162410	174440	0
ARHOMBI_DN4736_c0_g1_i1_4	0	0	0	0	0	92113	0	0	0	0	0	0	244410	0	0
ARUBRA_DN24058_c0_g1_i1_4	0	0	0	0	0	0	0	0	0	0	0	0	180840	0	155140
ARUBRA_DN4090_c0_g1_i2_2	0	0	0	0	0	0	0	0	0	0	0	0	334370	0	0
ARUBRA_DN13119_c0_g1_i1_6	0	0	0	0	0	0	0	0	0	0	0	0	111100	101510	121400
ARHOMBI_DN13499_c0_g1_i1_4	0	0	76627	0	0	128150	0	0	0	0	0	0	0	124900	0
ARUBRA_DN19563_c0_g1_i1_5	0	0	0	0	0	0	0	0	0	0	0	0	99476	103370	125700
ARUBRA_DN14032_c0_g1_i1_2	0	0	0	0	0	0	0	0	0	0	0	0	0	0	328280
ARHOMBI_DN5062_c0_g1_i1_6	0	0	0	0	0	0	0	0	0	0	0	0	213130	0	113770
ARHOMBI_DN24773_c0_g1_i1_3	0	0	0	0	0	0	0	0	0	0	0	0	0	135860	190880
ARHOMBI_DN4958_c0_g1_i1_3	133390	93069	100210	0	0	0	0	0	0	0	0	0	0	0	0
ARHOMBI_DN4594_c0_g1_i1_1	0	0	0	0	0	0	0	0	0	0	0	0	0	326580	0
ARHOMBI_DN27276_c0_g1_i1_4	0	0	0	0	0	0	0	0	0	0	0	0	107960	80910	135490
ARHOMBI_DN3256_c0_g1_i1_1	0	0	0	0	0	0	0	0	0	0	0	0	0	0	324060

ARHOMBI_DN20557_c0_g1_i1_4	0	0	0	0	0	0	0	0	0	0	0	0	0	150330	171600
ARHOMBI_DN186_c0_g2_i1_4	157080	75372	89309	0	0	0	0	0	0	0	0	0	0	0	0
ARUBRA_DN19452_c0_g1_i1_6	0	0	0	0	0	0	0	0	0	0	0	0	0	0	319830
ARUBRA_DN26125_c0_g1_i1_2	103370	107930	85763	21380	0	0	0	0	0	0	0	0	0	0	0
ARHOMBI_DN20462_c0_g1_i1_3	0	0	0	0	0	0	0	0	0	0	0	0	144120	173210	0
ARUBRA_DN3192_c0_g1_i1_2	53242	0	40489	0	220890	0	0	0	0	0	0	0	0	0	0
ARUBRA_DN5035_c0_g1_i1_2	127230	186500	0	0	0	0	0	0	0	0	0	0	0	0	0
ARHOMBI_DN1419_c0_g1_i1_6	0	0	0	0	0	0	0	0	0	0	0	0	0	313340	0
ARHOMBI_DN19246_c0_g1_i1_1	0	0	0	0	0	0	0	0	0	0	0	0	160290	151220	0
ARHOMBI_DN17996_c0_g1_i1_4	0	0	0	0	0	0	0	0	0	0	0	0	0	141880	169070
ARUBRA_DN18443_c0_g1_i1_6	0	0	0	0	0	0	0	0	0	0	0	0	180270	130660	0
ARHOMBI_DN20793_c0_g1_i1_6	0	0	0	0	0	0	0	0	0	0	0	0	141820	0	168260
ARUBRA_DN6539_c0_g2_i1_2	0	0	0	0	0	0	0	0	0	0	0	0	0	138450	171260
ARUBRA_DN2046_c0_g2_i1_2	0	0	0	0	0	0	0	0	176090	0	0	0	0	0	132880
ARUBRA_DN3646_c0_g1_i1_3	0	0	0	0	0	0	0	0	0	0	0	0	92645	110280	105440
ARUBRA_DN6310_c0_g1_i1_4	0	0	0	0	0	0	0	0	0	0	0	0	308290	0	0
ARUBRA_DN1384_c0_g1_i1_3	0	0	0	0	0	307480	0	0	0	0	0	0	0	0	0
ARHOMBI_DN14955_c0_g1_i1_2	0	0	0	0	0	0	0	0	0	0	0	0	307270	0	0
ARHOMBI_DN49_c0_g2_i1_6	0	0	0	0	0	0	0	0	0	0	0	0	0	187350	119670
ARUBRA_DN8685_c0_g1_i1_3	0	0	0	0	0	0	0	0	0	0	0	0	0	0	303930
ARHOMBI_DN17696_c0_g1_i1_3	0	0	0	0	0	0	0	0	0	0	0	0	0	153200	148340
ARUBRA_DN25420_c0_g1_i1_2	0	0	0	0	0	0	0	0	0	0	0	0	0	127560	173060
ARHOMBI_DN24508_c0_g1_i1_4	0	0	0	0	0	0	0	0	0	0	0	0	0	133190	166300
ARUBRA_DN13680_c0_g1_i1_6	0	0	0	0	0	0	0	0	0	0	0	0	98578	0	199440
ARUBRA_DN4241_c0_g1_i1_3	0	0	0	0	0	0	0	0	0	0	0	0	152680	144870	0
ARHOMBI_DN1471_c0_g1_i1_6	0	0	0	0	0	0	0	296570	0	0	0	0	0	0	0
ARHOMBI_DN7801_c0_g1_i1_5	0	0	0	0	0	0	0	0	0	0	0	0	0	296210	0

ARHOMBI_DN4606_c0_g1_i1_1	0	0	0	0	0	0	0	0	0	0	0	0	148640	0	147180
ARUBRA_DN17255_c0_g1_i1_6	0	0	0	0	0	0	0	0	0	0	0	0	92946	0	202480
ARUBRA_DN17467_c0_g1_i1_6	0	0	77297	24761	0	0	0	0	0	0	0	0	0	0	192140
ARUBRA_DN17174_c0_g1_i1_5	0	0	0	0	0	0	0	0	0	0	0	0	150680	141340	0
ARHOMBI_DN2723_c0_g1_i1_1	0	0	0	0	0	0	0	0	0	0	0	0	97702	86711	106610
ARUBRA_DN13395_c0_g1_i1_4	0	0	0	0	0	0	0	0	0	0	0	0	0	200340	89769
ARUBRA_DN5089_c0_g1_i1_5	0	0	0	0	0	0	0	0	0	0	0	0	288580	0	0
ARHOMBI_DN3292_c0_g1_i1_4	0	0	0	0	0	0	0	0	0	0	0	0	222060	63938	0
ARHOMBI_DN8290_c0_g2_i1_4	0	0	0	0	0	0	0	0	0	0	0	0	122860	0	162870
ARHOMBI_DN22455_c0_g1_i1_1	0	0	0	0	0	0	0	0	0	0	0	0	0	0	284040
ARUBRA_DN3317_c0_g1_i1_3	0	59839	94172	129960	0	0	0	0	0	0	0	0	0	0	0
ARHOMBI_DN8153_c0_g1_i1_4	0	0	0	0	0	0	0	0	0	0	0	0	0	283720	0
ARUBRA_DN19644_c0_g1_i1_4	0	0	0	0	0	0	0	0	0	0	0	0	0	154490	125960
ARUBRA_DN17529_c0_g1_i1_1	279250	0	0	0	0	0	0	0	0	0	0	0	0	0	0
ARHOMBI_DN390_c0_g1_i1_5	0	0	0	0	0	0	0	0	0	0	0	0	114460	163970	0
ARHOMBI_DN10092_c0_g1_i1_1	95387	111240	0	70113	0	0	0	0	0	0	0	0	0	0	0
ARHOMBI_DN15307_c0_g1_i1_4	0	0	0	0	0	0	0	0	0	0	0	0	276450	0	0
ARHOMBI_DN9011_c0_g1_i1_2	0	0	0	0	0	0	0	0	0	0	0	0	120140	153560	0
ARHOMBI_DN18489_c0_g2_i1_2	0	0	0	113580	0	0	0	0	0	0	0	0	159080	0	0
ARHOMBI_DN1634_c0_g1_i1_6	0	0	0	0	0	0	0	0	0	0	0	0	0	0	270710
ARUBRA_DN8885_c0_g1_i1_2	0	0	0	0	270040	0	0	0	0	0	0	0	0	0	0
ARHOMBI_DN16188_c0_g1_i1_3	0	0	0	0	0	0	0	0	0	0	0	0	0	106560	163210
ARUBRA_DN21545_c0_g1_i1_4	0	0	0	0	0	0	0	0	0	0	0	0	0	126700	142570
ARHOMBI_DN16333_c1_g1_i1_1	0	0	0	0	0	0	0	0	0	0	0	0	169040	0	99709
ARUBRA_DN4684_c0_g2_i2_5	0	0	0	0	0	0	0	0	0	0	0	0	267710	0	0
ARHOMBI_DN25309_c0_g1_i1_1	0	0	0	0	0	0	0	0	0	0	0	0	0	131390	136150
ARHOMBI_DN7295_c0_g1_i1_3	0	0	0	0	0	0	0	0	0	0	0	0	0	0	267370

ARHOMBI_DN3332_c0_g1_i1_5	0	0	0	0	0	0	0	0	0	0	0	0	0	121760	144650
ARUBRA_DN12402_c0_g1_i1_2	0	0	0	0	0	0	0	0	0	0	0	0	0	113450	152500
ARHOMBI_DN9886_c0_g1_i1_3	0	0	0	0	0	0	0	0	0	0	0	0	0	120580	144620
ARHOMBI_DN16830_c0_g1_i1_4	0	0	0	0	0	0	0	0	0	0	0	0	0	0	263960
ARHOMBI_DN19962_c0_g1_i1_1	0	0	0	0	0	0	0	0	0	0	0	0	263400	0	0
ARHOMBI_DN7050_c0_g1_i1_3	0	0	0	0	0	0	0	262890	0	0	0	0	0	0	0
ARUBRA_DN8811_c0_g1_i1_6	0	0	0	0	0	0	0	0	0	0	0	0	0	0	262840
ARUBRA_DN9001_c0_g1_i1_4	0	0	0	0	0	0	0	0	0	0	0	0	125440	0	137240
ARHOMBI_DN17895_c0_g1_i1_5	0	0	0	0	0	0	0	0	0	0	0	0	0	0	262290
ARHOMBI_DN1601_c0_g1_i1_6	0	0	0	0	0	0	0	0	0	0	0	0	0	262250	0
ARHOMBI_DN22439_c0_g1_i1_5	0	0	0	0	261270	0	0	0	0	0	0	0	0	0	0
ARHOMBI_DN3123_c0_g1_i2_2	0	0	0	0	0	0	0	0	0	0	0	0	0	0	261180
ARUBRA_DN26477_c0_g1_i1_2	0	0	0	0	0	121400	0	0	0	0	0	0	139650	0	0
ARHOMBI_DN15728_c0_g1_i1_3	0	0	0	0	0	0	0	0	0	0	0	0	138390	121980	0
ARUBRA_DN14653_c0_g1_i1_5	0	0	0	23029	0	0	0	0	236230	0	0	0	0	0	0
ARUBRA_DN239_c0_g1_i1_1	0	0	0	121000	0	0	0	0	138120	0	0	0	0	0	0
ARUBRA_DN981_c0_g1_i1_2	0	0	0	0	0	0	0	0	0	0	0	0	0	258380	0
ARHOMBI_DN6224_c0_g1_i3_2	0	0	15743	39095	202860	0	0	0	0	0	0	0	0	0	0
ARHOMBI_DN43_c0_g1_i1_1	39536	44189	0	0	0	0	0	0	0	0	0	0	0	0	171670
ARHOMBI_DN10061_c0_g1_i1_1	114000	139600	0	0	0	0	0	0	0	0	0	0	0	0	0
ARUBRA_DN860_c0_g1_i1_4	0	0	0	0	0	0	0	0	0	0	0	0	0	0	247780
ARUBRA_DN4926_c0_g2_i1_2	0	0	0	0	0	0	0	0	0	0	0	0	0	247350	0
ARHOMBI_DN442_c0_g2_i1_6	0	0	0	0	0	0	0	0	0	0	0	0	0	247290	0
ARUBRA_DN7677_c0_g1_i1_5	0	0	0	0	0	0	0	0	0	0	0	0	0	245290	0
ARHOMBI_DN23943_c0_g1_i1_5	0	0	0	0	0	0	0	0	0	0	0	0	86235	108320	48468
ARHOMBI_DN5334_c0_g2_i1_6	242910	0	0	0	0	0	0	0	0	0	0	0	0	0	0
ARHOMBI_DN2144_c0_g1_i1_2	0	0	0	0	0	0	0	0	0	0	0	0	0	241990	0

ARUBRA_DN17900_c0_g1_i1_4	90168	89266	61556	0	0	0	0	0	0	0	0	0	0	0	0
ARUBRA_DN7215_c0_g1_i1_2	0	0	0	0	0	0	0	0	0	0	0	0	104700	0	135010
ARHOMBI_DN10966_c0_g1_i1_1	0	0	0	0	0	0	0	0	0	0	0	0	140670	98047	0
ARUBRA_DN4852_c0_g1_i2_5	0	0	0	0	0	0	0	0	0	0	0	0	237990	0	0
ARUBRA_DN7820_c0_g1_i1_3	0	0	0	0	0	0	0	0	0	0	0	0	0	123570	113750
ARUBRA_DN3218_c0_g2_i1_1	0	0	0	0	0	0	0	0	0	0	0	0	0	0	237060
ARUBRA_DN14060_c0_g1_i1_6	236500	0	0	0	0	0	0	0	0	0	0	0	0	0	0
ARHOMBI_DN3766_c0_g1_i1_2	0	0	0	0	0	0	0	0	0	0	0	0	0	102220	134090
ARHOMBI_DN19865_c0_g1_i1_4	0	0	0	0	0	0	0	236020	0	0	0	0	0	0	0
ARHOMBI_DN27008_c0_g1_i1_5	0	0	0	0	0	0	0	0	0	0	0	0	0	0	234090
ARHOMBI_DN14983_c0_g1_i1_5	0	0	0	0	0	0	0	0	0	0	0	0	0	0	233400
ARUBRA_DN23880_c0_g1_i1_2	0	0	0	0	0	0	0	0	0	0	0	0	0	126760	106610
ARHOMBI_DN17419_c0_g1_i1_1	0	0	0	0	0	0	0	0	0	0	0	0	233010	0	0
ARUBRA_DN16616_c0_g1_i1_4	0	0	0	0	0	0	0	0	0	0	0	0	0	0	232430
ARUBRA_DN17646_c0_g1_i1_5	0	0	0	0	0	0	0	0	0	0	0	0	111750	0	119790
ARUBRA_DN22151_c0_g1_i1_2	0	0	0	0	0	0	0	0	0	0	0	0	0	231480	0
ARHOMBI_DN14825_c0_g1_i1_2	45563	158020	26918	0	0	0	0	0	0	0	0	0	0	0	0
ARHOMBI_DN16314_c0_g1_i1_1	0	0	0	0	0	0	0	0	0	0	0	0	0	230260	0
ARHOMBI_DN3532_c0_g1_i1_4	0	0	0	0	0	0	0	0	0	0	0	0	0	229610	0
ARHOMBI_DN4371_c0_g1_i1_5	0	0	0	0	0	0	0	0	0	0	0	0	0	228840	0
ARUBRA_DN13912_c0_g1_i1_3	0	0	0	0	0	0	0	0	0	0	0	0	0	108990	119700
ARHOMBI_DN17734_c0_g1_i1_4	0	0	0	0	0	0	0	0	0	0	0	0	92264	133610	0
ARHOMBI_DN21284_c0_g1_i1_3	0	0	0	0	0	0	0	0	0	0	0	0	225710	0	0
ARUBRA_DN8408_c0_g1_i1_4	0	0	0	0	0	0	0	0	0	0	0	0	0	0	224030
ARUBRA_DN8779_c0_g1_i1_1	0	0	0	0	0	0	0	0	0	0	0	0	0	223770	0
ARUBRA_DN85_c0_g1_i1_3	0	0	0	0	0	0	0	0	0	0	0	0	223610	0	0
ARHOMBI_DN16378_c0_g1_i1_1	0	0	0	0	0	0	0	0	0	0	0	0	0	54624	168480

ARHOMBI_DN22637_c0_g1_i1_5	0	0	0	0	0	0	0	0	0	0	0	0	103780	0	117480
ARHOMBI_DN16961_c0_g1_i1_3	0	0	0	0	0	0	0	0	0	0	0	0	106500	114130	0
ARUBRA_DN6264_c0_g1_i1_1	0	0	0	0	0	0	0	0	0	0	0	0	0	220220	0
ARHOMBI_DN6677_c0_g1_i1_3	0	0	0	0	0	0	219890	0	0	0	0	0	0	0	0
ARHOMBI_DN15571_c0_g1_i1_5	0	0	0	0	0	0	0	0	0	0	0	0	0	85044	134650
ARUBRA_DN2280_c0_g1_i1_3	0	0	0	0	0	0	0	0	0	0	0	0	0	100110	119430
ARHOMBI_DN5894_c0_g1_i1_5	0	0	0	0	0	0	0	0	0	0	0	0	0	0	219470
ARHOMBI_DN18104_c0_g1_i1_1	0	0	0	0	0	0	0	0	0	0	0	0	120160	99117	0
ARUBRA_DN9413_c0_g1_i1_3	0	0	0	0	0	0	0	0	0	0	0	0	219240	0	0
ARUBRA_DN8576_c0_g1_i1_3	0	0	0	0	0	0	0	0	0	0	0	0	94391	0	124410
ARHOMBI_DN4497_c0_g1_i1_6	78701	0	139960	0	0	0	0	0	0	0	0	0	0	0	0
ARUBRA_DN7437_c0_g1_i1_1	0	0	0	0	0	0	0	0	0	0	0	0	0	171260	45949
ARUBRA_DN6344_c0_g1_i1_3	0	0	0	0	0	0	0	0	0	0	0	0	216750	0	0
ARHOMBI_DN142_c0_g1_i2_6	71229	97461	45075	0	0	0	0	0	0	0	0	0	0	0	0
ARHOMBI_DN2804_c0_g1_i1_5	0	0	0	0	0	0	0	0	0	0	0	0	0	0	213730
ARUBRA_DN14140_c0_g1_i1_5	0	0	0	0	0	0	0	213190	0	0	0	0	0	0	0
ARUBRA_DN20165_c0_g1_i1_6	0	0	50314	0	0	0	0	0	0	0	0	0	0	162730	0
ARUBRA_DN11522_c0_g1_i1_2	0	0	0	0	0	0	0	0	0	0	0	0	0	0	211460
ARUBRA_DN7178_c0_g1_i1_1	0	0	0	0	0	0	0	0	0	0	0	0	0	211360	0
ARUBRA_DN495_c0_g1_i1_6	208950	0	0	0	0	0	0	0	0	0	0	0	0	0	0
ARUBRA_DN15567_c0_g1_i1_3	0	0	0	0	0	0	0	0	0	0	0	0	115580	93072	0
ARHOMBI_DN20913_c0_g1_i1_5	0	0	0	0	0	0	0	0	0	0	0	0	0	91887	115820
ARUBRA_DN19959_c0_g1_i1_6	0	0	0	0	0	0	0	0	0	0	0	0	207520	0	0
ARUBRA_DN8310_c0_g1_i1_2	0	0	0	0	0	0	0	0	0	0	0	0	207300	0	0
ARHOMBI_DN11012_c0_g1_i1_4	0	0	0	0	0	0	0	0	0	0	0	0	0	205620	0
ARUBRA_DN6936_c0_g1_i1_4	81418	91104	32759	0	0	0	0	0	0	0	0	0	0	0	0
ARHOMBI_DN21368_c0_g1_i1_6	47601	157440	0	0	0	0	0	0	0	0	0	0	0	0	0

ARUBRA_DN5364_c0_g1_i1_3	0	0	0	0	0	0	0	0	0	0	0	0	79509	0	125240
ARUBRA_DN568_c0_g1_i1_3	0	0	0	0	0	0	0	0	0	0	0	0	0	0	203970
ARHOMBI_DN24822_c0_g1_i1_5	0	203870	0	0	0	0	0	0	0	0	0	0	0	0	0
ARHOMBI_DN8308_c0_g1_i1_3	0	0	0	0	0	0	0	0	0	0	0	0	202970	0	0
ARHOMBI_DN249_c0_g1_i1_6	0	0	0	0	0	0	0	0	0	0	0	0	0	121010	81453
ARHOMBI_DN12088_c0_g1_i1_5	0	0	0	0	0	0	0	0	0	0	0	0	0	0	200410
ARHOMBI_DN16872_c0_g1_i1_5	0	0	0	0	0	0	0	0	0	0	0	0	200080	0	0
ARHOMBI_DN12473_c0_g1_i1_6	0	199290	0	0	0	0	0	0	0	0	0	0	0	0	0
ARUBRA_DN9724_c0_g1_i1_1	0	0	0	0	0	0	0	0	0	0	0	0	0	199200	0
ARHOMBI_DN11959_c0_g1_i1_2	0	199030	0	0	0	0	0	0	0	0	0	0	0	0	0
ARUBRA_DN3899_c0_g2_i1_5	0	0	0	0	0	0	0	0	0	0	0	0	0	197750	0
ARUBRA_DN9135_c0_g1_i1_4	0	0	0	0	0	0	0	0	0	0	0	197120	0	0	0
ARUBRA_DN3321_c0_g1_i1_1	71663	65066	60372	0	0	0	0	0	0	0	0	0	0	0	0
ARUBRA_DN3221_c0_g1_i1_6	0	0	0	0	0	0	0	0	0	0	0	0	0	0	196990
ARUBRA_DN7240_c0_g1_i1_1	0	0	0	0	0	0	0	0	0	0	0	0	0	0	196730
ARUBRA_DN25966_c0_g1_i1_5	0	0	0	0	0	0	0	0	0	0	0	0	0	0	196690
ARHOMBI_DN2599_c0_g1_i1_4	0	0	0	0	0	0	0	0	0	0	0	0	0	195150	0
ARUBRA_DN13308_c0_g1_i1_1	0	0	191770	0	0	0	0	0	0	0	0	0	0	0	0
ARUBRA_DN10910_c0_g1_i1_6	0	0	0	0	0	0	0	0	0	0	0	0	0	190840	0
ARUBRA_DN24761_c0_g1_i1_3	0	0	0	106620	0	0	0	0	0	0	0	0	0	0	83808
ARHOMBI_DN11254_c0_g1_i1_1	0	0	0	189440	0	0	0	0	0	0	0	0	0	0	0
ARUBRA_DN24634_c0_g1_i1_5	0	0	0	0	0	0	0	0	0	0	0	0	0	0	189430
ARHOMBI_DN4130_c0_g1_i1_2	0	0	0	0	0	0	0	0	0	0	0	0	0	189240	0
ARHOMBI_DN2201_c0_g1_i1_2	0	0	0	0	0	0	0	0	0	0	0	0	188980	0	0
ARUBRA_DN19418_c0_g1_i1_3	0	0	0	0	0	0	0	0	0	0	0	0	0	188370	0
ARUBRA_DN1850_c0_g1_i1_3	0	0	0	0	0	0	0	0	0	0	0	0	0	185070	0
ARHOMBI_DN9657_c0_g1_i1_1	0	0	0	0	0	0	0	0	0	0	0	0	0	0	184280

ARUBRA_DN22015_c0_g1_i1_3	0	0	0	0	0	0	0	0	0	0	0	0	0	0	184120
ARUBRA_DN21497_c0_g1_i1_5	0	0	0	0	0	0	0	0	0	0	0	0	184050	0	0
ARHOMBI_DN17553_c0_g1_i1_3	0	0	0	0	0	0	0	0	0	0	0	0	0	182640	0
ARUBRA_DN23958_c0_g1_i1_3	0	0	0	0	0	0	0	0	0	0	0	0	0	0	182250
ARHOMBI_DN6893_c0_g1_i1_1	0	0	0	0	0	0	0	0	0	0	0	0	0	87350	93811
ARUBRA_DN9664_c0_g1_i1_5	0	0	0	0	0	0	0	0	0	0	0	0	0	0	180900
ARUBRA_DN3808_c0_g2_i1_1	0	0	0	180640	0	0	0	0	0	0	0	0	0	0	0
ARUBRA_DN14331_c0_g1_i1_6	0	0	0	0	0	0	0	0	0	0	0	0	0	100410	79622
ARUBRA_DN209_c0_g1_i1_5	0	0	0	0	0	0	0	0	0	0	0	0	179310	0	0
ARHOMBI_DN16878_c0_g1_i1_1	0	0	44362	0	0	0	0	0	0	0	0	0	0	0	133910
ARUBRA_DN3885_c0_g1_i1_1	0	0	0	0	0	0	0	0	0	0	0	0	77910	0	99438
ARHOMBI_DN9575_c0_g1_i1_5	0	0	0	0	0	0	0	0	0	0	0	0	0	0	177170
ARHOMBI_DN4563_c0_g2_i1_3	0	0	0	0	0	0	0	0	0	0	0	0	176860	0	0
ARHOMBI_DN5164_c0_g1_i1_4	0	0	0	0	0	0	0	0	0	0	0	0	74245	0	102070
ARUBRA_DN2931_c0_g1_i1_4	70959	104890	0	0	0	0	0	0	0	0	0	0	0	0	0
ARUBRA_DN8810_c0_g1_i1_3	0	0	171560	0	0	0	0	0	0	0	0	0	0	0	0
ARUBRA_DN27193_c0_g1_i1_3	0	0	0	0	0	0	0	0	0	0	0	0	0	95387	75726
ARHOMBI_DN2247_c0_g1_i1_2	0	0	0	0	0	0	0	0	0	0	0	171060	0	0	0
ARUBRA_DN25129_c0_g1_i1_1	0	0	0	0	0	0	0	0	0	0	0	0	170590	0	0
ARUBRA_DN7581_c0_g1_i1_1	0	0	0	0	0	0	0	0	0	169930	0	0	0	0	0
ARUBRA_DN26635_c0_g1_i1_3	0	0	0	0	0	0	0	0	0	0	0	0	0	0	165680
ARUBRA_DN25922_c0_g1_i1_4	0	0	0	162810	0	0	0	0	0	0	0	0	0	0	0
ARHOMBI_DN16586_c0_g1_i1_6	0	0	0	0	0	0	0	0	0	0	0	0	0	162590	0
ARUBRA_DN23667_c0_g1_i1_3	0	0	0	0	0	0	0	0	0	0	0	0	77269	0	85156
ARHOMBI_DN1477_c0_g1_i1_3	0	0	0	0	0	0	0	0	0	0	0	0	0	160660	0
ARUBRA_DN23915_c0_g1_i1_1	0	0	0	0	0	0	0	0	0	0	0	0	0	160530	0
ARUBRA_DN16207_c0_g1_i1_4	0	0	0	0	0	0	0	0	0	0	0	0	78905	81261	0

ARUBRA_DN12100_c0_g1_i1_6	0	0	0	0	0	0	0	0	0	0	0	0	158500	0	0
ARHOMBI_DN5013_c0_g1_i1_5	0	0	0	0	0	0	0	0	0	0	0	0	151370	0	0
ARHOMBI_DN17278_c0_g1_i1_1	0	0	0	0	150240	0	0	0	0	0	0	0	0	0	0
ARUBRA_DN8077_c0_g1_i1_3	84301	0	64244	0	0	0	0	0	0	0	0	0	0	0	0
ARHOMBI_DN12156_c0_g1_i1_6	0	73132	37676	36612	0	0	0	0	0	0	0	0	0	0	0
ARHOMBI_DN19746_c0_g1_i1_3	0	0	0	0	0	0	0	0	0	0	0	0	0	0	146270
ARHOMBI_DN5681_c0_g1_i1_3	0	0	0	0	0	0	0	0	0	0	0	0	0	145070	0
ARHOMBI_DN9772_c0_g1_i1_3	0	100030	44052	0	0	0	0	0	0	0	0	0	0	0	0
ARUBRA_DN2587_c0_g1_i1_2	0	0	0	0	0	0	0	0	0	0	0	0	0	0	139610
ARHOMBI_DN14925_c0_g1_i1_1	0	0	0	0	0	0	0	0	0	0	0	0	0	138890	0
ARHOMBI_DN13424_c0_g1_i1_2	0	0	0	0	0	0	0	0	0	0	0	0	135640	0	0
ARHOMBI_DN17556_c0_g1_i1_2	0	0	0	0	0	0	0	0	0	0	0	0	0	135160	0
ARHOMBI_DN23552_c0_g1_i1_5	0	0	0	0	0	0	0	0	0	0	0	0	82453	0	52122
ARHOMBI_DN2495_c0_g2_i1_3	0	0	0	0	0	0	0	134050	0	0	0	0	0	0	0
ARUBRA_DN16642_c0_g1_i1_1	0	0	0	0	0	0	0	0	0	0	0	0	0	0	131220
ARUBRA_DN8504_c0_g1_i1_5	0	0	0	0	0	0	0	0	0	0	0	0	0	0	130270
ARHOMBI_DN3218_c0_g1_i1_5	58661	70626	0	0	0	0	0	0	0	0	0	0	0	0	0
ARHOMBI_DN6191_c0_g1_i1_5	0	0	0	0	0	0	0	0	0	0	0	0	0	128270	0
ARUBRA_DN13074_c0_g1_i1_4	0	0	0	0	0	0	0	0	0	0	0	0	0	123960	0
ARHOMBI_DN2701_c0_g1_i1_1	0	0	0	0	0	0	0	0	0	0	0	0	0	0	123900
ARUBRA_DN13385_c0_g1_i1_1	0	0	0	0	0	0	0	0	0	0	0	0	0	123850	0
ARHOMBI_DN4455_c0_g2_i1_5	0	0	0	0	0	0	0	0	0	0	0	0	0	0	123390
ARUBRA_DN1705_c0_g1_i1_4	0	0	0	0	0	0	0	0	0	0	0	0	0	123130	0
ARHOMBI_DN14374_c0_g1_i1_3	0	0	0	0	0	0	0	0	0	0	0	0	0	122770	0
ARUBRA_DN3623_c0_g1_i1_6	52788	0	33190	33712	0	0	0	0	0	0	0	0	0	0	0
ARUBRA_DN6227_c0_g1_i1_3	0	0	0	0	0	0	0	0	0	0	0	0	0	116150	0
ARUBRA_DN14347_c0_g1_i1_6	0	0	0	0	0	0	0	0	0	0	0	0	114770	0	0

ARUBRA_DN4655_c0_g1_i1_4	0	58978	55464	0	0	0	0	0	0	0	0	0	0	0	0
ARUBRA_DN152_c0_g1_i1_6	0	0	0	0	0	0	0	0	0	0	0	0	0	0	113980
ARUBRA_DN21454_c0_g1_i1_1	0	0	0	0	0	0	0	0	0	0	0	0	112230	0	0
ARHOMBI_DN14749_c0_g1_i1_1	0	0	0	0	0	0	0	0	0	0	0	0	0	0	111960
ARUBRA_DN1738_c0_g1_i1_5	0	0	0	0	0	0	0	0	0	0	0	0	0	111160	0
ARHOMBI_DN6745_c0_g1_i1_6	0	0	0	0	0	0	0	0	0	0	0	0	0	67169	42512
ARHOMBI_DN9761_c0_g1_i1_5	0	0	0	0	0	0	0	0	0	0	0	0	0	0	109060
ARHOMBI_DN16459_c0_g1_i1_4	0	0	0	0	0	0	0	0	0	0	0	0	0	108340	0
ARHOMBI_DN630_c0_g2_i1_1	0	0	0	0	0	0	0	0	0	0	0	0	0	0	108170
ARUBRA_DN10120_c0_g1_i1_3	0	0	0	0	0	0	0	0	0	0	0	0	0	0	107370
ARHOMBI_DN26737_c0_g1_i1_6	0	0	0	0	0	0	0	0	0	0	0	0	0	0	106280
ARHOMBI_DN20055_c0_g1_i1_2	0	0	0	0	0	0	0	0	0	0	0	0	0	103020	0
ARUBRA_DN68_c0_g2_i1_3	0	0	0	0	0	0	0	0	0	0	0	0	102730	0	0
ARUBRA_DN18173_c0_g1_i1_5	0	0	0	0	0	0	0	0	0	0	0	0	0	102430	0
ARHOMBI_DN11266_c0_g1_i1_5	0	0	102420	0	0	0	0	0	0	0	0	0	0	0	0
ARUBRA_DN15293_c0_g1_i1_1	0	0	0	0	0	0	0	0	0	0	0	0	0	102190	0
ARUBRA_DN16348_c0_g1_i1_1	36567	44933	20158	0	0	0	0	0	0	0	0	0	0	0	0
ARUBRA_DN5557_c0_g1_i1_3	0	0	0	100790	0	0	0	0	0	0	0	0	0	0	0
ARUBRA_DN807_c0_g1_i1_6	60395	40009	0	0	0	0	0	0	0	0	0	0	0	0	0
ARHOMBI_DN22251_c0_g1_i1_1	0	0	0	0	0	0	0	0	0	0	0	0	0	0	95226
ARHOMBI_DN6086_c0_g2_i1_5	0	0	0	0	0	0	0	0	0	0	0	0	0	0	92008
ARUBRA_DN15584_c0_g1_i1_1	0	0	0	0	0	0	0	0	0	0	0	0	0	0	90612
ARUBRA_DN23559_c0_g1_i1_2	0	0	0	0	0	0	0	0	0	0	0	0	88737	0	0
ARHOMBI_DN3575_c0_g2_i1_3	0	0	0	88700	0	0	0	0	0	0	0	0	0	0	0
ARUBRA_DN18865_c0_g1_i1_4	0	0	0	0	0	0	0	0	0	0	0	0	0	0	87991
ARUBRA_DN81_c0_g1_i1_5	0	0	0	0	0	0	0	0	0	0	0	0	0	0	87238
ARUBRA_DN17068_c0_g1_i1_4	0	0	0	0	0	0	0	0	0	0	0	0	0	86393	0

ARHOMBI_DN11773_c0_g1_i1_2	0	0	0	0	0	0	0	0	0	0	0	0	0	0	85784
ARHOMBI_DN15266_c0_g1_i1_2	0	0	0	0	0	0	0	0	0	0	0	0	84031	0	0
ARHOMBI_DN19693_c0_g1_i1_6	0	63798	19842	0	0	0	0	0	0	0	0	0	0	0	0
ARHOMBI_DN21801_c0_g1_i1_4	0	0	0	0	0	0	0	0	0	0	0	0	0	0	80917
ARHOMBI_DN676_c0_g2_i1_2	0	0	0	80200	0	0	0	0	0	0	0	0	0	0	0
ARHOMBI_DN6521_c0_g1_i1_4	0	0	0	0	0	0	0	0	0	0	0	0	0	0	77047
ARHOMBI_DN4350_c0_g1_i1_4	0	0	0	76957	0	0	0	0	0	0	0	0	0	0	0
ARHOMBI_DN6943_c0_g1_i1_2	0	0	0	0	0	0	0	0	0	0	0	0	0	0	75806
ARHOMBI_DN16004_c0_g1_i1_3	0	0	0	0	0	0	0	0	0	0	0	0	0	0	74367
ARUBRA_DN26911_c0_g1_i1_3	40216	32823	0	0	0	0	0	0	0	0	0	0	0	0	0
ARHOMBI_DN7765_c0_g1_i1_6	0	0	73037	0	0	0	0	0	0	0	0	0	0	0	0
ARUBRA_DN9287_c0_g1_i1_4	0	0	0	72878	0	0	0	0	0	0	0	0	0	0	0
ARUBRA_DN10301_c0_g1_i1_3	0	0	0	0	0	0	0	0	0	0	0	0	0	0	72069
ARHOMBI_DN25736_c0_g1_i1_6	0	0	0	0	0	0	0	0	0	0	0	0	0	0	70246
ARHOMBI_DN25307_c0_g1_i1_1	0	0	0	0	0	0	0	0	0	0	0	0	0	0	67918
ARHOMBI_DN827_c0_g1_i1_1	0	0	0	0	0	0	0	0	0	0	0	0	65802	0	0
ARUBRA_DN5154_c0_g1_i1_1	0	64391	0	0	0	0	0	0	0	0	0	0	0	0	0
ARHOMBI_DN10484_c0_g1_i1_1	0	0	0	0	0	0	0	0	0	0	0	0	63357	0	0
ARUBRA_DN4965_c9_g2_i1_4	0	60470	0	0	0	0	0	0	0	0	0	0	0	0	0
ARHOMBI_DN650_c0_g2_i1_3	0	0	0	0	0	0	0	0	0	0	0	0	0	0	59287
ARUBRA_DN19110_c0_g1_i1_2	0	0	58833	0	0	0	0	0	0	0	0	0	0	0	0
ARHOMBI_DN5597_c0_g2_i1_5	0	0	0	0	0	0	0	0	0	0	0	0	0	0	54195
ARUBRA_DN21139_c0_g1_i1_2	0	0	0	0	0	0	0	0	0	0	0	0	0	0	51723
ARUBRA_DN3651_c0_g1_i1_3	0	0	0	0	0	0	0	0	0	0	0	0	0	0	50612
ARUBRA_DN460_c0_g1_i1_6	0	0	0	0	14176	0	0	31364	0	0	0	0	0	0	0
ARUBRA_DN26660_c0_g1_i1_1	0	0	0	0	0	0	0	44771	0	0	0	0	0	0	0
ARHOMBI_DN2961_c0_g1_i1_6	0	0	0	0	0	0	0	0	0	0	0	0	0	0	44529

ARUBRA_DN15712_c0_g1_i1_3	0	0	0	0	0	0	0	0	0	0	0	43050	0	0	0
ARHOMBI_DN5284_c0_g3_i1_5	0	0	0	41516	0	0	0	0	0	0	0	0	0	0	0
ARHOMBI_DN12176_c0_g1_i1_1	0	0	0	0	0	0	0	0	0	0	0	0	0	0	40333
ARUBRA_DN4419_c0_g1_i1_1	38263	0	0	0	0	0	0	0	0	0	0	0	0	0	0
ARUBRA_DN3086_c0_g1_i1_4	0	26960	0	0	0	0	0	0	0	0	0	0	0	0	0
ARUBRA_DN5853_c0_g1_i1_3	0	0	0	0	0	0	0	0	0	0	0	0	0	0	22364
ARHOMBI_DN12506_c0_g1_i1_1	0	0	0	0	0	0	0	0	0	0	0	0	0	0	0
ARUBRA_DN10624_c0_g1_i1_5	0	0	0	0	0	0	0	0	0	0	0	0	0	0	0
ARHOMBI_DN13880_c0_g1_i1_4	0	0	0	0	0	0	0	0	0	0	0	0	0	0	0
ARHOMBI_DN23964_c0_g1_i1_2	0	0	0	0	0	0	0	0	0	0	0	0	0	0	0
ARUBRA_DN7424_c0_g1_i1_6	0	0	0	0	0	0	0	0	0	0	0	0	0	0	0
ARHOMBI_DN5795_c0_g1_i1_5	0	0	0	0	0	0	0	0	0	0	0	0	0	0	0
ARHOMBI_DN6070_c0_g2_i1_6	0	0	0	0	0	0	0	0	0	0	0	0	0	0	0
ARUBRA_DN5014_c0_g1_i1_2	0	0	0	0	0	0	0	0	0	0	0	0	0	0	0
ARHOMBI_DN8315_c0_g1_i1_3	0	0	0	0	0	0	0	0	0	0	0	0	0	0	0
ARUBRA_DN13376_c0_g1_i1_4	0	0	0	0	0	0	0	0	0	0	0	0	0	0	0
ARUBRA_DN21106_c0_g1_i1_3	0	0	0	0	0	0	0	0	0	0	0	0	0	0	0
ARUBRA_DN22162_c0_g1_i1_5	0	0	0	0	0	0	0	0	0	0	0	0	0	0	0
