# Supplementary material for: Comparative proteomics of common allergenic tree pollens of birch, alder, and hazel
Source: Allergy. 2021 Jan 15;76(6):1743–53. doi: 10.1111/all.14694 (PMC8248232; doi:10.1111/all.14694)
Supplement: Supplementary file 11 — Table S9 [file ALL-76-1743-s019.pdf]

Supplementary Table S9: Top BLAST hits of identified Alnus pollen proteins

Protein IDs	e-value [blastx hit 1]	bitscore [blastx hit 1]	accession [blastx hit 1]	name [blastx hit 1]	organism [blastx hit 1]	accession [blastx hit 2]
ARUBRA_DN3160_c0_g1_i1_1	5,11E-102	296	AAB24432.1	Aln g I	Alnus glutinosa	P38948.2
ARHOMBI_DN6124_c0_g1_i1_4	0	729	XP_008226131.1	phosphoglycerate kinase, cytosolic	Prunus mume	ONI11910.1
ARUBRA_DN4553_c0_g1_i1_3	1,95E-112	325	CAC84116.1	peptidylprolyl isomerase (cyclophilin)	Betula pendula	CAW66894.1
ARUBRA_DN4458_c0_g1_i1_5	0	753	XP_008241272.1	monodehydroascorbate reductase	Prunus mume	ONH96231.1
ARHOMBI_DN14767_c0_g1_i1_6						
ARUBRA_DN4969_c0_g1_i11_2	5,46E-50	165	XP_016650474.1	glycine-rich RNA-binding protein GRP1A	Prunus mume	ONH91156.1
ARUBRA_DN4696_c0_g1_i2_3	9,58E-161	452	AHF71027.1	glutathione S-transferase	Betula pendula	XP_018812288.1
ARUBRA_DN4914_c0_g1_i1_2	2,11E-172	489	XP_018839732.1	cinnamoyl-CoA reductase 1-like	Juglans regia	XP_018839732.1
ARUBRA_DN3195_c0_g1_i1_6	0	583	AGU09563.1	isoflavone reductase	Corylus avellana	AAG22740.1
ARUBRA_DN2052_c0_g1_i1_5	5,02E-160	451	XP_018818881.1	L-ascorbate peroxidase, cytosolic	Juglans regia	XP_018818882.1
ARUBRA_DN17603_c0_g1_i1_4	3,63E-92	270	XP_008241145.1	ubiquitin-NEDD8-like protein RUB2	Prunus mume	ONH95703.1
ARUBRA_DN23118_c0_g1_i1_1	1,34E-79	239	OAY27195.1	hypothetical protein MANES_16G107500	Manihot esculenta	ABG81302.1
ARHOMBI_DN3404_c0_g1_i1_5	0	553	XP_018841935.1	putative lactoylglutathione lyase	Juglans regia	KHN33024.1
ARHOMBI_DN8875_c0_g1_i1_3	0	538	XP_018806786.1	aldose 1-epimerase	Juglans regia	OAY32166.1
ARUBRA_DN10871_c0_g1_i1_1	3,86E-63	194	XP_018842397.1	glutaredoxin-like	Juglans regia	XP_009334103.1
ARHOMBI_DN3889_c0_g1_i1_5	0	558	XP_018839113.1	aldo-keto reductase family 4 member C9-like	Juglans regia	XP_018839114.1
ARHOMBI_DN5373_c0_g1_i1_4	0	1008	XP_018819373.1	2,3-bisphosphoglycerate-independent phosphoglycerate mutase	Juglans regia	XP_018819374.1
ARUBRA_DN4854_c0_g1_i1_3	1,49E-124	360	XP_018852474.1	probable phospholipid hydroperoxide glutathione peroxidase	Juglans regia	KDP25447.1
ARUBRA_DN1249_c0_g2_i1_2	2,94E-98	285	XP_018844175.1	peptidyl-prolyl cis-trans isomerase FKBP15-1	Juglans regia	OAY32871.1
ARHOMBI_DN5306_c0_g1_i1_6	3,49E-156	442	XP_018814537.1	triosephosphate isomerase, cytosolic	Juglans regia	OAY46102.1
ARUBRA_DN5302_c0_g1_i1_5	0	615	XP_018811449.1	UTP--glucose-1-phosphate uridylyltransferase	Juglans regia	XP_018816885.1
ARHOMBI_DN26299_c0_g1_i1_4	6,82E-15	70,1	XP_018837516.1	uncharacterized protein LOC109003708	Juglans regia	
ARHOMBI_DN5459_c0_g1_i1_2	0	761	XP_018841632.1	uncharacterized protein LOC109006717	Juglans regia	XP_018829078.1
ARHOMBI_DN5273_c0_g1_i1_3	5,59E-84	251	KDP23592.1	hypothetical protein JCGZ_23425	Jatropha curcas	XP_012089157.1
ARHOMBI_DN1417_c0_g1_i1_5	1,76E-41	139	XP_008235991.1	glycine-rich protein 2-like	Prunus mume	XP_018823876.1
ARHOMBI_DN5581_c0_g1_i1_3	5,93E-103	303	ONI14208.1	hypothetical protein PRUPE_4G269100	Prunus persica	KDP45998.1
ARUBRA_DN2313_c0_g1_i1_3	1,61E-31	114	CAC39160.1	putative LEA III protein isoform 1	Corylus avellana	CAC39110.1
ARUBRA_DN9810_c0_g1_i1_4	4,12E-152	429	XP_018807198.1	uncharacterized protein LOC108980651	Juglans regia	XP_020973954.1
ARHOMBI_DN6211_c0_g2_i1_6	1,64E-98	290	XP_018850024.1	translationally-controlled tumor protein homolog	Juglans regia	XP_015939773.1
ARUBRA_DN22898_c0_g1_i1_5	0	581	XP_018807661.1	malate dehydrogenase	Juglans regia	XP_018808212.1
ARUBRA_DN2253_c0_g1_i1_1	0	645	XP_018837851.1	phosphoglucomutase, cytoplasmic	Juglans regia	XP_018826631.1
ARHOMBI_DN4873_c0_g1_i1_4	0	593	XP_018851336.1	2-alkenal reductase (NADP(+)-dependent)-like isoform X2	Juglans regia	XP_018851335.1

ARUBRA_DN18206_c0_g1_i1_4	6,41E-20	84,7	XP_018837516.1	uncharacterized protein LOC109003708	Juglans regia	XP_008367865.1
ARUBRA_DN6222_c0_g1_i1_5	5,46E-99	290	XP_018847774.1	probable calcium-binding protein CML13	Juglans regia	XP_018831699.1
ARHOMBI_DN5949_c0_g2_i1_6	0	857	CAA63121.1	enolase	Alnus glutinosa	CBN61481.1
ARHOMBI_DN4456_c0_g1_i1_5	0	616	XP_018814928.1	probable lactoylglutathione lyase, chloroplastic	Juglans regia	XP_009352517.1
ARUBRA_DN390_c0_g1_i1_5	1,33E-74	235	XP_018816885.1	UTP--glucose-1-phosphate uridylyltransferase	Juglans regia	XP_018811449.1
ARUBRA_DN4992_c0_g1_i1_3	4,12E-145	415	XP_018846260.1	putative methyltransferase DDB_G0268948	Juglans regia	XP_018838664.1
ARHOMBI_DN5841_c0_g1_i1_5	1,57E-153	434	XP_018851982.1	GTP-binding nuclear protein Ran-3-like	Juglans regia	KOM41958.1
ARHOMBI_DN6196_c0_g1_i2_5	0	662	CAJ21172.1	alcohol dehydrogenase	Alnus glutinosa	XP_018839886.1
ARHOMBI_DN3043_c0_g1_i1_4	9,15E-73	219	XP_018846792.1	peptidyl-prolyl cis-trans isomerase FKBP12	Juglans regia	XP_016182032.1
ARUBRA_DN4475_c0_g1_i1_2	6,35E-131	373	XP_018807996.1	glycolipid transfer protein 1-like	Juglans regia	XP_018808001.1
ARUBRA_DN1141_c0_g1_i1_1	7,07E-52	164	XP_008385845.1	small ubiquitin-related modifier 1	Malus domestica	XP_008359255.1
ARHOMBI_DN3285_c0_g1_i1_6	2,53E-148	421	XP_018839113.1	aldo-keto reductase family 4 member C9-like	Juglans regia	XP_018839114.1
ARUBRA_DN10174_c0_g1_i1_6	5,86E-85	250	XP_008231331.1	thioredoxin-like protein Clot	Prunus mume	ONI20288.1
ARHOMBI_DN7524_c0_g1_i1_6	1,32E-122	351	XP_018810050.1	peptidyl-prolyl cis-trans isomerase CYP20-1	Juglans regia	ONH99912.1
ARHOMBI_DN4459_c0_g1_i1_3	5,08E-72	218	XP_012089770.1	macrophage migration inhibitory factor homolog isoform X2	Jatropha curcas	OAY41554.1
ARHOMBI_DN6146_c1_g1_i1_4	0	1481	ONH94297.1	hypothetical protein PRUPE_7G009200	Prunus persica	ONH94298.1
ARUBRA_DN4074_c0_g1_i1_4	9,63E-55	177	XP_018826050.1	acyl carrier protein 1, chloroplastic-like	Juglans regia	XP_018840595.1
ARHOMBI_DN3575_c0_g1_i1_3	4,20E-152	427	XP_018860753.1	haloacid dehalogenase-like hydrolase domain-containing protein Sgpp	Juglans regia	XP_008229524.1
ARHOMBI_DN4328_c0_g1_i1_6	3,76E-162	458	XP_018858487.1	(+)-neomenthol dehydrogenase-like isoform X1	Juglans regia	XP_020218534.1
ARUBRA_DN411_c0_g1_i1_5	1,32E-83	250	XP_018852846.1	uncharacterized protein At4g28440-like	Juglans regia	KDP26583.1
ARHOMBI_DN3323_c0_g1_i1_4	3,39E-75	228	XP_018834238.1	cytochrome c	Juglans regia	ONI35591.1
ARHOMBI_DN5928_c0_g3_i2_6	2,69E-64	195	XP_018830469.1	ubiquitin-conjugating enzyme E2-17 kDa-like, partial	Juglans regia	XP_008233222.1
ARUBRA_DN4841_c0_g1_i1_6	1,85E-178	506	XP_018841745.1	uncharacterized protein At5g02240-like	Juglans regia	ONI03446.1
ARHOMBI_DN6080_c1_g1_i1_1	1,23E-56	177	XP_018833110.1	cysteine proteinase inhibitor A-like	Juglans regia	XP_018833111.1
ARUBRA_DN16244_c0_g1_i1_2	0	650	ONI03043.1	hypothetical protein PRUPE_6G235100	Prunus persica	XP_008243957.1
ARUBRA_DN18719_c0_g1_i1_2	1,83E-94	277	XP_009353661.1	triosephosphate isomerase, cytosolic	Pyrus x bretschneideri	XP_018823301.1
ARHOMBI_DN4484_c0_g1_i1_2	7,81E-87	257	XP_018833372.1	profilin-3	Juglans regia	XP_018808763.1
ARHOMBI_DN5698_c2_g1_i1_5	4,37E-57	176	XP_018831298.1	tubulin-folding cofactor A-like	Juglans regia	XP_018831299.1
ARHOMBI_DN15379_c0_g1_i1_6	1,24E-166	470	XP_018838373.1	protein CDI-like	Juglans regia	XP_016202283.1
ARUBRA_DN10745_c0_g1_i1_4	5,21E-79	235	XP_018811921.1	elicitor-responsive protein 3	Juglans regia	OAY56540.1
ARUBRA_DN17745_c0_g1_i1_5	1,39E-81	248	XP_016183838.1	uncharacterized protein LOC107625656	Arachis ipaensis	KHN12948.1
ARUBRA_DN16936_c0_g1_i1_5	2,73E-100	294	ADU56174.1	ubiquitin-conjugating family protein	Jatropha curcas	ADV04060.1
ARUBRA_DN4620_c0_g1_i1_1	0	1153	KDP32613.1	hypothetical protein JCGZ_13163	Jatropha curcas	XP_012078471.1
ARUBRA_DN4102_c0_g1_i1_3	0	627	XP_018856844.1	calreticulin-like	Juglans regia	XP_018816251.1

ARHOMBI_DN9782_c0_g1_i1_2	1,65E-39	131	XP_018830082.1	desiccation protectant protein Lea14 homolog	Juglans regia	XP_018847006.1
ARUBRA_DN4847_c0_g2_i2_1	0	631	XP_018824429.1	probable aldo-keto reductase 2	Juglans regia	KHN15572.1
ARUBRA_DN5827_c0_g1_i1_2	4,73E-45	151	XP_018806786.1	aldose 1-epimerase	Juglans regia	XP_009360073.1
ARHOMBI_DN3150_c0_g1_i1_6	8,73E-111	331	XP_018825758.1	galactokinase-like isoform X1	Juglans regia	XP_018825759.1
ARHOMBI_DN3869_c0_g1_i1_1	6,09E-125	360	XP_018811825.1	photosynthetic NDH subunit of lumenal location 5, chloroplastic-like	Juglans regia	XP_018813322.1
ARHOMBI_DN4569_c0_g1_i1_2	3,83E-54	171	OAY29922.1	hypothetical protein MANES_15G182400	Manihot esculenta	OAY24334.1
ARHOMBI_DN8000_c0_g1_i1_4	6,45E-90	274	XP_018825758.1	galactokinase-like isoform X1	Juglans regia	XP_018825759.1
ARHOMBI_DN10026_c0_g1_i1_5	4,06E-70	214	XP_018845447.1	glutaredoxin-C4	Juglans regia	KDP46838.1
ARHOMBI_DN3561_c0_g2_i1_2	1,90E-61	190	XP_018839522.1	probable steroid-binding protein 3	Juglans regia	XP_018839569.1
ARHOMBI_DN18818_c0_g1_i1_2	1,12E-99	292	XP_018837329.1	glycine cleavage system H protein 2, mitochondrial-like	Juglans regia	XP_018859042.1
ARUBRA_DN19744_c0_g1_i1_2	2,83E-126	371	XP_018828611.1	uncharacterized protein LOC108996994	Juglans regia	XP_018819156.1
ARHOMBI_DN5210_c0_g1_i1_5	0	615	XP_018829694.1	guanine nucleotide-binding protein subunit beta-like protein	Juglans regia	XP_018860423.1
ARHOMBI_DN5071_c0_g1_i1_6	0	525	XP_018841469.1	L-ascorbate peroxidase 3, peroxisomal-like	Juglans regia	XP_018823838.1
ARUBRA_DN24356_c0_g1_i1_4	2,96E-81	239	XP_018840197.1	nuclear transport factor 2-like	Juglans regia	KDP26818.1
ARHOMBI_DN25271_c0_g1_i1_5	1,99E-43	152	XP_018819144.1	hydroxyproline O-galactosyltransferase GALT6-like isoform X2	Juglans regia	XP_018819143.1
ARUBRA_DN4454_c0_g1_i1_2	1,60E-175	502	ONI21983.1	hypothetical protein PRUPE_2G099900	Prunus persica	XP_007218975.2
ARHOMBI_DN6208_c0_g1_i2_5	0	934	XP_018844063.1	adenosylhomocysteinase-like	Juglans regia	OAY55304.1
ARHOMBI_DN1745_c0_g1_i1_2	0	588	XP_018823484.1	eukaryotic translation initiation factor-like isoform X1	Juglans regia	XP_018823485.1
ARUBRA_DN21566_c0_g1_i1_2	0	565	XP_018839537.1	probable protein phosphatase 2C 59	Juglans regia	XP_018839538.1
ARUBRA_DN4108_c0_g1_i1_5	0	519	XP_018826881.1	soluble inorganic pyrophosphatase 6, chloroplastic-like	Juglans regia	XP_018844455.1
ARHOMBI_DN5571_c0_g1_i1_5	4,39E-108	319	XP_018825058.1	elongation factor 1-delta-like	Juglans regia	XP_018825059.1
ARUBRA_DN4060_c0_g2_i1_2	1,58E-106	310	XP_018824314.1	reactive Intermediate Deaminase A, chloroplastic-like	Juglans regia	XP_018811490.1
ARHOMBI_DN6185_c1_g1_i1_5	1,98E-90	274	XP_017188304.1	protein IN2-1 homolog B-like	Malus domestica	ONI24059.1
ARHOMBI_DN5260_c0_g1_i1_6	2,46E-125	361	XP_018841699.1	ras-related protein RABE1c-like	Juglans regia	XP_018841700.1
ARHOMBI_DN6086_c1_g1_i1_6	0	784	ADT91622.1	actin7a	Hevea brasiliensis	AEF33711.1
ARUBRA_DN4124_c0_g1_i1_3	0	910	XP_018849756.1	ATP synthase subunit beta, mitochondrial	Juglans regia	XP_008243316.1
ARHOMBI_DN6236_c1_g1_i3_6	0	616	XP_018811967.1	probable aldo-keto reductase 1	Juglans regia	XP_018826312.1
ARHOMBI_DN5869_c0_g1_i1_6	0	621	ACU23175.1	unknown	Glycine max	NP_001236300.2
ARUBRA_DN4641_c0_g1_i1_2	0	1201	XP_018814481.1	heat shock cognate 70 kDa protein 2-like	Juglans regia	OAY38641.1
ARUBRA_DN23856_c0_g1_i1_3	1,44E-67	206	XP_018844068.1	glycine-rich RNA-binding protein 2, mitochondrial-like	Juglans regia	ACI15745.1
ARHOMBI_DN5685_c0_g2_i1_3	0	650	ONH96252.1	hypothetical protein PRUPE_7G116600	Prunus persica	XP_007202259.1

ARUBRA_DN3590_c0_g2_i1_3	0	525	XP_018823791.1	triosephosphate isomerase, chloroplastic	Juglans regia	XP_020425754.1
ARHOMBI_DN3136_c0_g1_i1_1	1,89E-145	411	XP_012083760.1	ras-related protein RABH1b	Jatropha curcas	ONI22221.1
ARUBRA_DN4556_c0_g1_i2_4	0	728	ADR70876.1	eukaryotic translation elongation factor 1B gamma-subunit	Hevea brasiliensis	XP_018817874.1
ARHOMBI_DN24422_c0_g1_i1_5	3,47E-65	203	XP_018823301.1	triosephosphate isomerase, cytosolic	Juglans regia	ONI00223.1
ARUBRA_DN990_c0_g1_i1_1	2,57E-84	250	XP_018842805.1	cytochrome b5-like	Juglans regia	XP_018842806.1
ARUBRA_DN1071_c0_g1_i1_2	1,60E-132	382	XP_018849508.1	protein DJ-1 homolog B-like	Juglans regia	XP_018849508.1
ARUBRA_DN2969_c0_g1_i1_3	4,89E-57	182	XP_014516627.1	aldo-keto reductase family 4 member C9-like Vigna radiata var. radiata	Vigna radiata var. radiata	BAT97190.1
ARHOMBI_DN4165_c0_g1_i1_1	2,93E-81	243	XP_018836787.1	nuclear transport factor 2-like	Juglans regia	XP_018807037.1
ARHOMBI_DN5934_c0_g1_i1_3	0	832	XP_018858114.1	protein disulfide-isomerase-like	Juglans regia	XP_018858121.1
ARHOMBI_DN5622_c0_g1_i1_3	1,50E-108	327	XP_018848770.1	uncharacterized protein At5g39570-like	Juglans regia	XP_018848770.1
ARHOMBI_DN4656_c0_g1_i1_6	3,73E-92	273	XP_018848789.1	peroxiredoxin-2B	Juglans regia	XP_018857900.1
ARHOMBI_DN6034_c0_g1_i2_3	0	1249	CAC14168.1	putative luminal binding protein	Corylus avellana	CAW63975.1
ARHOMBI_DN6068_c0_g1_i2_3	0	1238	KYP38780.1	Endoplasmin isogeny	Cajanus cajan	XP_020203653.1
ARHOMBI_DN1849_c0_g1_i1_3	0	768	XP_018818327.1	guanosine nucleotide diphosphate dissociation inhibitor At5g09550	Juglans regia	ONH90692.1
ARUBRA_DN8471_c0_g1_i1_3	3,89E-64	197	XP_018847877.1	EG45-like domain containing protein	Juglans regia	XP_018858754.1
ARUBRA_DN2937_c0_g1_i2_1	0	511	XP_014494377.1	glyceraldehyde-3-phosphate dehydrogenase GAPC1, cytosolic	Vigna radiata var. radiata	AES72079.1
ARUBRA_DN6062_c0_g1_i1_1	6,35E-98	285	XP_018822739.1	14 kDa zinc-binding protein	Juglans regia	XP_020204134.1
ARHOMBI_DN5785_c0_g1_i1_4	1,81E-162	468	XP_018845045.1	probable receptor-like protein kinase At5g24010	Juglans regia	XP_018851677.1
ARHOMBI_DN3876_c0_g1_i1_6	0	689	XP_018829624.1	naringenin,2-oxoglutarate 3-dioxygenase	Juglans regia	ARV78456.1
ARHOMBI_DN6149_c0_g1_i1_6	0	1086	XP_018819865.1	polyadenylate-binding protein 2-like	Juglans regia	XP_018822308.1
ARUBRA_DN6670_c0_g1_i1_3	4,24E-46	155	AIU50414.1	pectin lyase-like superfamily protein, partial	Glycine max	CBD34387.1
ARHOMBI_DN6225_c0_g2_i1_5	0	691	XP_018816668.1	ADP,ATP carrier protein 1, mitochondrial	Juglans regia	CAA05979.1
ARUBRA_DN4747_c0_g1_i1_6	6,82E-172	487	XP_018823047.1	2-methylene-furan-3-one reductase-like	Juglans regia	XP_018823045.1
ARHOMBI_DN5137_c0_g1_i1_5	1,29E-122	352	KDP30996.1	hypothetical protein JCGZ_11372	Jatropha curcas	XP_012079937.1
ARUBRA_DN18006_c0_g1_i1_4	0	624	XP_018851647.1	subtilisin-like protease SBT1.7 isoform X1	Juglans regia	XP_018851649.1
ARHOMBI_DN2797_c0_g1_i1_2	5,56E-133	381	ONI00873.1	hypothetical protein PRUPE_6G109100	Prunus persica	ONI00874.1
ARHOMBI_DN3646_c0_g1_i2_4	9,03E-127	362	XP_018843515.1	UMP-CMP kinase 3-like	Juglans regia	XP_018818963.1
ARHOMBI_DN6264_c0_g1_i4_2	0	712	OAY23951.1	hypothetical protein MANES_18G120400	Manihot esculenta	XP_018841148.1
ARUBRA_DN1274_c0_g1_i1_4	0	822	XP_018859052.1	coatomer subunit delta	Juglans regia	XP_018859053.1
ARHOMBI_DN7316_c0_g1_i1_3	9,90E-63	193	XP_017191848.1	L-ascorbate peroxidase 1, cytosolic-like	Malus domestica	AGG14282.1
ARUBRA_DN13722_c0_g1_i1_3	4,00E-06	47,4	XP_008365922.1	DDB1- and CUL4-associated factor 8-like isoform X1	Malus domestica	XP_008365923.1
ARUBRA_DN4746_c1_g2_i1_3	2,63E-109	321	XP_018844473.1	stress-response A/B barrel domain-containing protein UP3-like	Juglans regia	XP_008242209.2

ARHOMBI_DN11262_c0_g1_i1_4	1,93E-23	89,4	XP_014490343.1	peptidyl-prolyl cis-trans isomerase CYP20-1-like Vigna radiata var. radiata		XP_004500488.1
ARHOMBI_DN4307_c0_g1_i1_5	0	1310	XP_018848851.1	trifunctional UDP-glucose 4,6-dehydratase/UDP-4-keto-6-deoxy-D-glucose 3,5-epimerase/UDP-4-keto-L-rhamnose-reductase RHM1	Juglans regia	XP_020226307.1
ARUBRA_DN1657_c0_g1_i1_5	4,81E-62	193	AEC03317.1	thioredoxin H-type 3	Hevea brasiliensis	OAY50655.1
ARHOMBI_DN16963_c0_g2_i1_3	5,92E-78	236	XP_018807198.1	uncharacterized protein LOC108980651	Juglans regia	ONI18422.1
ARHOMBI_DN5659_c0_g1_i1_3	3,64E-65	206	XP_018858259.1	uncharacterized protein At5g48480-like	Juglans regia	XP_018837465.1
ARHOMBI_DN9830_c0_g1_i1_4	2,37E-48	113	XP_018821777.1	peptidyl-prolyl cis-trans isomerase CYP20-1-like isoform X2	Juglans regia	XP_018821777.1
ARHOMBI_DN7974_c0_g1_i1_2	5,35E-107	320	XP_018825758.1	galactokinase-like isoform X1	Juglans regia	XP_018825759.1
ARHOMBI_DN5417_c0_g1_i1_4	0	792	XP_018817260.1	3-ketoacyl-CoA thiolase 2, peroxisomal	Juglans regia	XP_018809682.1
ARHOMBI_DN753_c0_g1_i1_1	0	610	XP_018828122.1	glutelin type-B 5-like	Juglans regia	XP_018828123.1
ARUBRA_DN4963_c0_g1_i2_5	1,39E-116	340	XP_008221708.1	glutathione S-transferase U17-like	Prunus mume	ONI30654.1
ARHOMBI_DN24139_c0_g1_i1_4	1,08E-51	168	XP_018821921.1	peroxidase P7	Juglans regia	XP_008374580.1
ARUBRA_DN18820_c0_g1_i1_4	6,43E-108	311	XP_004489035.1	ubiquitin-conjugating enzyme E2 35	Cicer arietinum	OIW06071.1
ARUBRA_DN325_c0_g2_i1_1	8,51E-109	317	XP_014507002.1	eukaryotic translation initiation factor 5A-2	Vigna radiata var. radiata	AAQ08191.1
ARHOMBI_DN13323_c0_g1_i1_2	1,64E-35	130	KDP33292.1	hypothetical protein JCGZ_13079	Jatropha curcas	XP_012077722.1
ARUBRA_DN1786_c0_g1_i1_1	4,49E-165	462	XP_018839423.1	stem-specific protein TSJT1-like	Juglans regia	XP_008224229.1
ARUBRA_DN2535_c0_g1_i1_5	0	1016	XP_018824334.1	granule-bound starch synthase 1, chloroplastic/amyloplastic-like isoform X1	Juglans regia	XP_018824335.1
ARHOMBI_DN6004_c0_g1_i1_1	5,43E-147	418	XP_019424523.1	ras-related protein RABD2b	Lupinus angustifolius	XP_008234451.1
ARHOMBI_DN6257_c0_g1_i1_3	0	1466	OAY30259.1	hypothetical protein MANES_14G016900	Manihot esculenta	OAY48624.1
ARHOMBI_DN4827_c0_g1_i1_1	9,41E-92	296	XP_018835573.1	presequence protease 1, chloroplastic/mitochondrial-like	Juglans regia	OAY39086.1
ARHOMBI_DN5844_c0_g1_i1_3	2,49E-30	110	XP_018849651.1	protein DOWNSTREAM OF FLC-like	Juglans regia	XP_018828917.1
ARHOMBI_DN5686_c0_g1_i1_6	0	944	XP_018816674.1	UDP-glucose 6-dehydrogenase 1-like	Juglans regia	XP_018816673.1
ARUBRA_DN3412_c0_g1_i1_6	7,57E-115	335	XP_018812389.1	peroxiredoxin-2E-2, chloroplastic-like	Juglans regia	XP_018839507.1
ARHOMBI_DN9161_c0_g1_i1_5	1,09E-54	184	KDP33733.1	hypothetical protein JCGZ_07304	Jatropha curcas	XP_012076781.1
ARUBRA_DN21065_c0_g1_i1_6	6,68E-55	176	ADF87393.1	class II chitinase	Arachis hypogaea	XP_018858281.1
ARUBRA_DN11174_c0_g1_i1_2	1,63E-64	203	XP_018829015.1	binding partner of ACD11 1-like	Juglans regia	AFK43849.1
ARUBRA_DN8598_c0_g1_i1_5	9,14E-54	182	XP_018837851.1	phosphoglucomutase, cytoplasmic	Juglans regia	XP_018826631.1
ARHOMBI_DN3049_c0_g1_i1_5	2,78E-142	405	KDP39130.1	hypothetical protein JCGZ_00887	Jatropha curcas	XP_012070824.1
ARUBRA_DN16568_c0_g1_i1_3	3,72E-159	449	XP_018847493.1	nitrile-specifier protein 5 isoform X1	Juglans regia	XP_018847493.1
ARUBRA_DN5078_c0_g1_i1_6	0	1054	XP_018815415.1	ruBisCO large subunit-binding protein subunit alpha	Juglans regia	XP_009376066.1

ARHOMBI_DN6227_c0_g1_i3_4	2,23E-112	328	XP_020995365.1	glutathione S-transferase U17-like	Arachis duranensis	XP_020995366.1
ARHOMBI_DN4048_c0_g1_i1_3	0	758	OAY48061.1	hypothetical protein MANES_06G128200	Manihot esculenta	OAY30593.1
ARHOMBI_DN294_c0_g2_i1_2	2,95E-56	189	XP_018858995.1	receptor-like protein kinase FERONIA	Juglans regia	ACM89471.1
ARUBRA_DN335_c0_g1_i1_4	1,28E-131	377	ONI26383.1	hypothetical protein PRUPE_1G021100	Prunus persica	XP_018818615.1
ARUBRA_DN4552_c0_g2_i1_4	6,87E-101	293	XP_014513266.1	ADP-ribosylation factor	Vigna radiata var. radiata	AGZ15424.1
ARHOMBI_DN19000_c0_g1_i1_1	7,47E-43	141	KDP42468.1	hypothetical protein JCGZ_00265	Jatropha curcas	XP_012066709.1
ARUBRA_DN4655_c0_g2_i1_6	2,20E-153	431	OIV93234.1	hypothetical protein TanjilG_27413	Lupinus angustifolius	XP_019423713.1
ARHOMBI_DN5421_c0_g1_i1_1	3,95E-101	301	XP_018833734.1	elongation factor 1-beta 2	Juglans regia	KDP34528.1
ARUBRA_DN2639_c0_g2_i1_5	8,00E-53	190	XP_018857281.1	villin-3-like isoform X1	Juglans regia	XP_018857281.1
ARHOMBI_DN6203_c0_g1_i1_4	1,22E-116	341	XP_018829689.1	3-isopropylmalate dehydratase small subunit 3-like	Juglans regia	KDP39892.1
ARHOMBI_DN5924_c0_g1_i1_3	0	592	XP_018839732.1	cinnamoyl-CoA reductase 1-like	Juglans regia	XP_008349860.1
ARUBRA_DN4385_c0_g1_i1_6	7,32E-62	192	XP_007148307.1	hypothetical protein PHAVU_006G197500g	Phaseolus vulgaris	ESW20301.1
ARHOMBI_DN16907_c0_g1_i1_2	1,23E-86	268	XP_018849508.1	protein DJ-1 homolog B-like	Juglans regia	XP_018849508.1
ARUBRA_DN23412_c0_g1_i1_1	6,36E-56	182	XP_018849508.1	protein DJ-1 homolog B-like	Juglans regia	XP_018849508.1
ARHOMBI_DN4949_c0_g1_i1_4	1,46E-105	306	KDP22298.1	hypothetical protein JCGZ_26129	Jatropha curcas	XP_012090276.1
ARHOMBI_DN9199_c0_g1_i1_3	1,16E-77	244	OAY25273.1	hypothetical protein MANES_17G081000	Manihot esculenta	XP_018826631.1
ARUBRA_DN20664_c0_g1_i1_6	3,59E-64	200	KRH30558.1	hypothetical protein GLYMA_11G192700	Glycine max	XP_003538169.1
ARHOMBI_DN9737_c0_g1_i1_1	1,71E-19	83,2	ACU16029.1	unknown	Glycine max	NP_001236978.1
ARHOMBI_DN5509_c0_g1_i1_1	0	707	XP_018817015.1	alpha-1,4-glucan-protein synthase UDP-forming 2-like	Juglans regia	XP_018818659.1
ARUBRA_DN4741_c0_g1_i1_4	3,20E-170	481	XP_018809229.1	extradiol ring-cleavage dioxygenase-like	Juglans regia	XP_018844729.1
ARUBRA_DN1142_c0_g1_i1_1	0	519	XP_018807335.1	endonuclease 4-like	Juglans regia	XP_008371307.1
ARUBRA_DN2596_c0_g1_i1_5	5,17E-75	228	ACU15022.1	unknown	Glycine max	KHN12783.1
ARUBRA_DN3451_c0_g2_i1_2	3,88E-103	299	XP_018853969.1	uncharacterized protein LOC109015966	Juglans regia	XP_018853993.1
ARHOMBI_DN5949_c0_g1_i1_6	0	867	XP_008376333.1	enolase	Malus domestica	KDP22386.1
ARUBRA_DN25800_c0_g1_i1_2	3,51E-74	231	XP_018820845.1	xylose isomerase	Juglans regia	OAY46789.1
ARUBRA_DN2944_c0_g2_i1_1	0	845	XP_018845033.1	eukaryotic initiation factor 4A-15-like	Juglans regia	XP_018504074.1
ARUBRA_DN3626_c0_g1_i1_4	1,16E-112	332	OAY31304.1	hypothetical protein MANES_14G101300	Manihot esculenta	KRH45284.1
ARUBRA_DN1658_c0_g1_i1_3	0	692	XP_018814476.1	UDP-glucuronic acid decarboxylase 6	Juglans regia	XP_018814477.1
ARUBRA_DN5995_c0_g2_i1_4	5,16E-103	298	CAW66836.1	unnamed protein product	Glycine max	CAW52404.1
ARUBRA_DN17764_c0_g1_i1_4	2,73E-77	238	XP_018818800.1	probable 6-phosphogluconolactonase 4, chloroplastic	Juglans regia	OAY59533.1
ARUBRA_DN1399_c0_g1_i1_3	0	538	XP_018805947.1	putative glucose-6-phosphate 1-epimerase isoform X1	Juglans regia	XP_018805948.1
ARHOMBI_DN5222_c0_g2_i1_6	2,63E-118	347	XP_018819182.1	ran-binding protein 1 homolog a-like	Juglans regia	XP_018835624.1
ARUBRA_DN4539_c0_g1_i1_1	1,21E-88	263	XP_018849369.1	nascent polypeptide-associated complex subunit alpha-like protein 1	Juglans regia	OAY36283.1

ARHOMBI_DN6283_c0_g15_i1_6	0	595	XP_008385284.1	elongation factor 1-alpha-like	Malus domestica	XP_018498857.1
ARUBRA_DN14209_c0_g1_i1_5	2,78E-43	151	XP_018842945.1	sugar transport protein 8-like	Juglans regia	XP_018842937.1
ARHOMBI_DN3140_c0_g1_i1_4	3,85E-54	170	XP_018851363.1	ubiquitin-fold modifier 1	Juglans regia	XP_004505718.1
ARUBRA_DN3913_c0_g2_i2_6	0	938	XP_018844065.1	serine hydroxymethyltransferase 4	Juglans regia	XP_018848538.1
ARHOMBI_DN5648_c0_g1_i1_6	0	545	XP_018859879.1	altered inheritance of mitochondria protein 32-like	Juglans regia	ONI19697.1
ARHOMBI_DN9622_c0_g1_i1_2	4,39E-164	469	KHN12100.1	Aldehyde dehydrogenase family 2 member B7, mitochondrial	Glycine soja	KRH74615.1
ARUBRA_DN4716_c0_g2_i1_1	0	597	ONI02574.1	hypothetical protein PRUPE_6G207500	Prunus persica	XP_007205414.1
ARHOMBI_DN5959_c0_g1_i1_1	0	810	KDP35739.1	hypothetical protein JCGZ_10511	Jatropha curcas	XP_012074735.1
ARUBRA_DN7093_c0_g1_i1_1	3,00E-68	211	XP_018813581.1	uncharacterized protein Os08g0359500	Juglans regia	XP_020959531.1
ARHOMBI_DN5317_c0_g1_i1_1	0	748	XP_018826582.1	malate dehydrogenase, chloroplastic	Juglans regia	XP_018826583.1
ARHOMBI_DN17873_c0_g1_i1_6	7,18E-12	67,4	XP_018838903.1	late embryogenesis abundant protein D-29-like isoform X1	Juglans regia	XP_018838904.1
ARHOMBI_DN15725_c0_g1_i1_6	0	596	XP_018857901.1	alpha-galactosidase 1-like	Juglans regia	XP_008389053.1
ARUBRA_DN3134_c0_g2_i1_6	3,00E-105	307	KDP21650.1	hypothetical protein JCGZ_03321	Jatropha curcas	XP_012091217.1
ARUBRA_DN8237_c0_g1_i1_4	3,75E-135	386	XP_018813989.1	vesicle transport v-SNARE 13-like	Juglans regia	XP_018813990.1
ARHOMBI_DN5100_c0_g1_i1_3	0	971	XP_018813723.1	polyadenylate-binding protein 2-like	Juglans regia	BAJ53195.1
ARUBRA_DN4014_c0_g3_i1_3	8,66E-90	268	XP_009358774.1	aldo-keto reductase family 4 member C9-like	Pyrus x bretschneideri	ONI03512.1
ARUBRA_DN2976_c1_g1_i1_5	4,09E-169	478	XP_018806495.1	14-3-3-like protein	Juglans regia	XP_018808590.1
ARHOMBI_DN5768_c0_g1_i1_6	8,44E-15	71,2	KDP41684.1	hypothetical protein JCGZ_16091	Jatropha curcas	OAY22978.1
ARUBRA_DN6355_c0_g1_i1_3	7,09E-121	362	KDP44055.1	hypothetical protein JCGZ_05522	Jatropha curcas	KDP44055.1
ARHOMBI_DN2325_c0_g2_i1_5	0	511	XP_008236827.1	ubiquitin fusion degradation protein 1 homolog	Prunus mume	XP_016650890.1
ARHOMBI_DN9876_c0_g1_i1_3	2,26E-113	336	XP_018807889.1	heterogeneous nuclear ribonucleoprotein 1-like	Juglans regia	XP_018807889.1
ARUBRA_DN1667_c0_g1_i1_4	2,04E-74	223	XP_018821975.1	thioredoxin H-type	Juglans regia	XP_018821976.1
ARHOMBI_DN2829_c0_g1_i1_5	4,97E-64	198	XP_018838136.1	uncharacterized protein LOC109004143 isoform X2	Juglans regia	XP_018838135.1
ARUBRA_DN2825_c0_g1_i1_4	7,51E-76	246	XP_018836580.1	aminopeptidase M1-like	Juglans regia	XP_018836578.1
ARUBRA_DN1403_c0_g1_i1_4	0	972	XP_004492897.1	V-type proton ATPase subunit B 2-like	Cicer arietinum	AES80476.1
ARHOMBI_DN5577_c0_g1_i1_6	0	1016	XP_018822921.1	methionine--tRNA ligase, cytoplasmic	Juglans regia	XP_008232482.1
ARHOMBI_DN20240_c0_g1_i1_6	1,85E-65	199	XP_018856302.1	nitrile-specifier protein 5-like, partial	Juglans regia	XP_018847493.1
ARUBRA_DN5882_c0_g1_i1_4	0	652	XP_018844184.1	thiol protease aleurain-like	Juglans regia	KDP33917.1
ARUBRA_DN4352_c0_g3_i1_5	7,34E-45	147	XP_008368986.1	heat shock factor-binding protein 1-like	Malus domestica	XP_012082726.1
ARHOMBI_DN5282_c0_g1_i1_5	0	674	KYP54525.1	hypothetical protein KK1_000715	Cajanus cajan	XP_020227385.1
ARHOMBI_DN5162_c0_g1_i1_6	1,05E-155	439	XP_018849247.1	ribulose-phosphate 3-epimerase, cytoplasmic isoform	Juglans regia	XP_008379127.1

ARHOMBI_DN1661_c0_g1_i1_5	0	517	XP_018818507.1	14-3-3-like protein GF14 iota isoform X1	Juglans regia	XP_018852339.1
ARUBRA_DN4331_c0_g1_i1_1	3,92E-89	266	XP_018855792.1	probable calcium-binding protein CML27	Juglans regia	XP_018810240.1
ARHOMBI_DN5855_c0_g1_i1_3	0	819	XP_018849803.1	alpha-glucan phosphorylase, H isozyme	Juglans regia	OAY57768.1
ARUBRA_DN19380_c0_g2_i1_4	2,75E-127	364	OAY54996.1	hypothetical protein MANES_03G119600	Manihot esculenta	XP_018823733.1
ARUBRA_DN337_c0_g2_i1_5	4,20E-138	399	XP_018856023.1	ribosome-recycling factor, chloroplastic isoform X2	Juglans regia	XP_018856017.1
ARUBRA_DN4321_c0_g2_i1_1	0	1333	KDP29157.1	hypothetical protein JCGZ_16546	Jatropha curcas	AHA83580.1
ARUBRA_DN1238_c0_g1_i1_5	0	927	XP_018838281.1	V-type proton ATPase catalytic subunit A	Juglans regia	KDP25051.1
ARHOMBI_DN4982_c0_g1_i1_1	0	886	XP_008394248.1	guanosine nucleotide diphosphate dissociation inhibitor 2	Malus domestica	OAY58939.1
ARHOMBI_DN4944_c0_g1_i1_3	1,09E-160	456	XP_018832945.1	peptidyl-prolyl cis-trans isomerase A1 isoform X1	Juglans regia	XP_018832946.1
ARUBRA_DN1865_c0_g1_i1_1	0	861	XP_018811769.1	aspartate aminotransferase, cytoplasmic	Juglans regia	XP_008220042.1
ARUBRA_DN7363_c0_g1_i1_1	0	513	XP_018838677.1	inositol-phosphate phosphatase-like	Juglans regia	KDP32911.1
ARHOMBI_DN6048_c0_g1_i2_2	0	529	XP_018823093.1	carbonic anhydrase 2-like	Juglans regia	KDP45339.1
ARHOMBI_DN5714_c0_g2_i1_1	0	677	XP_018855304.1	phosphoserine aminotransferase 1, chloroplastic-like	Juglans regia	AGF95094.1
ARUBRA_DN11957_c0_g1_i1_4	2,96E-46	159	XP_018842710.1	probable pectinesterase/pectinesterase inhibitor 51	Juglans regia	XP_018860059.1
ARUBRA_DN26276_c0_g1_i1_1	2,85E-46	155	XP_008237059.1	LOW QUALITY PROTEIN: protein trichome birefringence-like 12	Prunus mume	ONH90835.1
ARUBRA_DN5081_c0_g1_i1_6	3,25E-133	384	XP_018818327.1	guanosine nucleotide diphosphate dissociation inhibitor At5g09550	Juglans regia	OAY45180.1
ARUBRA_DN11233_c0_g1_i1_2	2,25E-27	108	KDP25957.1	hypothetical protein JCGZ_22947	Jatropha curcas	XP_012086291.1
ARHOMBI_DN5854_c0_g1_i1_3	0	819	XP_018832184.1	uncharacterized protein LOC108999736	Juglans regia	XP_018821522.1
ARHOMBI_DN6063_c0_g1_i1_4	4,96E-86	258	XP_018822894.1	uncharacterized protein LOC108992718	Juglans regia	XP_018850381.1
ARHOMBI_DN2876_c0_g2_i1_3	0	534	XP_018855478.1	probable L-ascorbate peroxidase 6, chloroplastic isoform X1	Juglans regia	XP_008219619.1
ARUBRA_DN22470_c0_g1_i1_3	4,52E-29	112	XP_018811384.1	protein kinase PVPK-1-like	Juglans regia	XP_018811385.1
ARHOMBI_DN3221_c0_g1_i1_3	4,50E-180	505	ABW06959.1	isopentenyl pyrophosphate isomerase	Corylus avellana	XP_018815655.1
ARUBRA_DN4811_c0_g2_i1_3	4,70E-135	388	XP_018819317.1	proactivator polypeptide-like 1	Juglans regia	XP_018819318.1
ARUBRA_DN1006_c1_g1_i1_5	6,64E-76	229	XP_008354935.1	uncharacterized protein LOC103418606	Malus domestica	XP_018805179.1
ARHOMBI_DN15853_c0_g1_i1_3	1,03E-166	472	XP_018838097.1	3-hydroxyisobutyryl-CoA hydrolase-like protein 3, mitochondrial isoform X3	Juglans regia	XP_018838098.1
ARHOMBI_DN15373_c0_g1_i1_5	0	941	XP_018823509.1	leucine aminopeptidase 1-like	Juglans regia	XP_018810862.1
ARUBRA_DN10605_c0_g1_i1_2	2,08E-118	346	XP_018843013.1	NPL4-like protein 1	Juglans regia	XP_012092346.1
ARUBRA_DN10035_c0_g1_i1_2	5,86E-96	303	XP_018805582.1	subtilisin-like protease SBT6.1 isoform X4	Juglans regia	XP_018805579.1
ARUBRA_DN9263_c0_g1_i1_6	1,84E-30	107	XP_018842089.1	basic blue protein-like	Juglans regia	XP_008218855.1
ARUBRA_DN10200_c0_g1_i1_2	0	543	XP_018850910.1	bifunctional epoxide hydrolase 2-like	Juglans regia	ONI17394.1
ARUBRA_DN7621_c0_g1_i1_2	3,93E-115	339	XP_018841836.1	protein SLOW GREEN 1, chloroplastic	Juglans regia	OAY39056.1

ARUBRA_DN1568_c0_g2_i1_4	3,07E-95	278	XP_018813840.1	ferredoxin, root R-B1-like	Juglans regia	ONI01252.1
ARUBRA_DN3840_c0_g1_i1_2	0	933	XP_018844173.1	peptidyl-prolyl cis-trans isomerase FKBP62-like isoform X1	Juglans regia	XP_018844174.1
ARUBRA_DN4409_c0_g1_i1_6	0	830	XP_008234243.1	tubulin beta-1 chain	Prunus mume	XP_009371335.1
ARUBRA_DN4436_c0_g1_i1_5	2,48E-67	210	KDP45728.1	hypothetical protein JCGZ_17335	Jatropha curcas	XP_020537678.1
ARHOMBI_DN5595_c0_g1_i1_6	7,11E-174	488	XP_018839416.1	perakine reductase-like	Juglans regia	KHN02754.1
ARUBRA_DN4227_c1_g2_i1_2	5,35E-60	186	ONI27924.1	hypothetical protein PRUPE_1G111200	Prunus persica	ONI27925.1
ARHOMBI_DN4937_c0_g3_i1_5	7,72E-53	170	XP_018821061.1	subtilisin-like protease SBT3.17	Juglans regia	XP_015953072.1
ARUBRA_DN143_c0_g2_i1_4	0	792	XP_018837598.1	alpha-glucan phosphorylase, H isozyme-like isoform X1	Juglans regia	XP_018837601.1
ARHOMBI_DN21168_c0_g1_i1_5	1,15E-43	150	XP_018832214.1	villin-4-like	Juglans regia	XP_018832215.1
ARHOMBI_DN4920_c0_g1_i1_6	0	1110	XP_018839329.1	methylenetetrahydrofolate reductase 2-like	Juglans regia	XP_018858229.1
ARUBRA_DN26231_c0_g1_i1_1	7,15E-42	145	XP_008237316.1	LOW QUALITY PROTEIN: dihydrolipoyllysine-residue acetyltransferase component 5 of pyruvate dehydrogenase complex, chloroplastic	Prunus mume	XP_009345952.1
ARUBRA_DN304_c0_g1_i1_5	1,08E-176	498	XP_018806446.1	uncharacterized protein LOC108980071 isoform X2	Juglans regia	XP_018806435.1
ARUBRA_DN3080_c0_g1_i1_1	0	1266	XP_018856789.1	transketolase, chloroplastic-like	Juglans regia	XP_018848062.1
ARUBRA_DN10173_c0_g1_i1_6	0	591	XP_018849818.1	malate dehydrogenase, mitochondrial	Juglans regia	KDP32500.1
ARHOMBI_DN1063_c0_g2_i1_1	0	1140	XP_018851707.1	phospholipase D alpha 1	Juglans regia	XP_018850650.1
ARHOMBI_DN4718_c0_g1_i1_2	0	541	XP_018848890.1	activator of 90 kDa heat shock protein ATPase homolog	Juglans regia	XP_018809717.1
ARUBRA_DN4883_c0_g1_i1_3	1,46E-176	494	XP_018818651.1	40S ribosomal protein S3a-like	Juglans regia	XP_018826766.1
ARUBRA_DN4330_c0_g2_i1_4	1,34E-29	107	OAY45213.1	hypothetical protein MANES_07G041500	Manihot esculenta	KDP29995.1
ARHOMBI_DN5519_c0_g1_i2_6	2,83E-103	297	AAZ32851.1	pentameric polyubiquitin, partial	Medicago sativa	AAZ32851.1
ARHOMBI_DN5140_c0_g1_i2_1	4,25E-46	152	ACU16188.1	unknown	Glycine max	NP_001235503.1
ARHOMBI_DN6061_c0_g1_i1_5	2,74E-98	288	XP_018819438.1	nucleoside diphosphate kinase B	Juglans regia	ADB85102.1
ARHOMBI_DN1760_c0_g1_i1_4	9,37E-136	397	XP_018820845.1	xylose isomerase	Juglans regia	KDP43806.1
ARUBRA_DN16482_c0_g1_i1_1	2,79E-88	272	XP_018826631.1	phosphoglucomutase, cytoplasmic-like	Juglans regia	XP_018826632.1
ARUBRA_DN4585_c0_g2_i2_2	0	563	XP_018811421.1	polyadenylate-binding protein RBP45C-like	Juglans regia	XP_018824161.1
ARUBRA_DN4349_c0_g1_i1_5	0	975	XP_018829538.1	pyrophosphate--fructose 6-phosphate 1-phosphotransferase subunit beta	Juglans regia	XP_018836262.1
ARHOMBI_DN5054_c0_g1_i1_1	0	647	XP_018836922.1	coatomer subunit gamma-2-like	Juglans regia	XP_018847842.1
ARUBRA_DN18216_c0_g1_i1_2	2,78E-79	244	XP_018827577.1	SAL1 phosphatase-like isoform X2	Juglans regia	OAY36111.1
ARHOMBI_DN3394_c0_g1_i1_1	3,26E-153	438	XP_018833512.1	hydroxyacylglutathione hydrolase cytoplasmic	Juglans regia	XP_019430954.1
ARHOMBI_DN20977_c0_g1_i1_5	1,66E-59	185	KYP48129.1	Rho GDP-dissociation inhibitor 1	Cajanus cajan	XP_020234344.1

ARHOMBI_DN6641_c0_g2_i1_5	0	513	XP_018815367.1	NADP-dependent D-sorbitol-6-phosphate dehydrogenase-like	Juglans regia	OAY52428.1
ARHOMBI_DN5627_c0_g2_i1_2	1,22E-33	116	XP_008223181.1	heat shock factor-binding protein 1	Prunus mume	ONI28303.1
ARHOMBI_DN2280_c0_g1_i1_6	4,79E-163	459	AFK33699.1	unknown	Lotus japonicus	KDP21376.1
ARHOMBI_DN5380_c0_g1_i1_2	0	543	XP_018815161.1	peroxisomal fatty acid beta-oxidation multifunctional protein AIM1	Juglans regia	OAY53774.1
ARUBRA_DN6789_c0_g1_i1_6	8,47E-12	63,5	XP_018827113.1	mitochondrial import receptor subunit TOM9-2-like	Juglans regia	XP_008235602.1
ARUBRA_DN17519_c0_g1_i1_3	6,27E-57	183	XP_018847246.1	desiccation-related protein PCC13-62-like	Juglans regia	XP_018855451.1
ARHOMBI_DN940_c0_g1_i1_3	3,90E-35	124	ABR13309.1	putative ATPDIL1-4 electron transporter, partial	Prunus dulcis	XP_018819528.1
ARHOMBI_DN2154_c0_g1_i1_4	2,78E-80	240	OAY55416.1	hypothetical protein MANES_03G152300	Manihot esculenta	AAT84459.1
ARHOMBI_DN16490_c0_g1_i1_2	0	519	XP_018827473.1	D-3-phosphoglycerate dehydrogenase 1, chloroplastic-like	Juglans regia	XP_018851254.1
ARHOMBI_DN6177_c0_g2_i1_3	1,09E-27	104	CAC39160.1	putative LEA III protein isoform 1	Corylus avellana	CAC39110.1
ARHOMBI_DN24802_c0_g1_i1_2	2,88E-47	159	XP_018838095.1	3-hydroxyisobutyryl-CoA hydrolase-like protein 3, mitochondrial isoform X1	Juglans regia	KHN16590.1
ARUBRA_DN18898_c0_g1_i1_2	4,01E-84	249	XP_018854928.1	ubiquitin-40S ribosomal protein S27a-like	Juglans regia	XP_018805239.1
ARUBRA_DN10159_c0_g1_i1_5	0	743	XP_018814614.1	probable mitochondrial-processing peptidase subunit beta, mitochondrial isoform X2	Juglans regia	XP_018814613.1
ARUBRA_DN147_c0_g1_i1_6	1,19E-81	257	ONH99280.1	hypothetical protein PRUPE_6G022600	Prunus persica	XP_018807473.1
ARHOMBI_DN5600_c0_g1_i1_6	0	958	XP_018812243.1	hsp70-Hsp90 organizing protein 3-like isoform X2	Juglans regia	XP_018812243.1
ARUBRA_DN18800_c0_g2_i1_4	3,02E-178	499	XP_018835592.1	probable carboxylesterase 5	Juglans regia	KDP45172.1
ARHOMBI_DN4017_c0_g1_i1_1	1,17E-122	374	XP_018807473.1	uncharacterized protein LOC108980890	Juglans regia	KDP22063.1
ARUBRA_DN4582_c0_g1_i1_5	5,36E-146	415	XP_018822871.1	strigolactone esterase D14	Juglans regia	XP_018838099.1
ARUBRA_DN1643_c0_g2_i1_1	1,27E-94	281	XP_018809730.1	probable protein phosphatase 2C 76	Juglans regia	XP_018809734.1
ARUBRA_DN5911_c0_g1_i1_4	2,31E-104	306	XP_009351382.2	nudix hydrolase 26, chloroplastic-like	Pyrus x bretschneideri	XP_009351383.2
ARUBRA_DN3180_c0_g1_i1_4	3,87E-155	441	XP_018810444.1	peptide methionine sulfoxide reductase A1-like	Juglans regia	KHN38281.1
ARHOMBI_DN12129_c0_g1_i1_2	8,52E-89	264	XP_018849964.1	monothiol glutaredoxin-S10-like	Juglans regia	ONH93069.1
ARHOMBI_DN4888_c0_g1_i1_6	7,16E-69	219	XP_018824378.1	UDP-glucose 4-epimerase GEPI48	Juglans regia	XP_018857524.1
ARUBRA_DN6309_c0_g1_i1_5	0	514	XP_018839910.1	40S ribosomal protein SA-like	Juglans regia	XP_018839250.1
ARUBRA_DN3885_c0_g1_i1_5	6,99E-75	227	XP_018827114.1	uncharacterized protein LOC108995901	Juglans regia	XP_018807876.1
ARHOMBI_DN667_c0_g2_i1_1	5,17E-50	158	XP_018830562.1	tropinone reductase homolog At5g06060-like	Juglans regia	XP_018841297.1
ARUBRA_DN18848_c0_g1_i1_3	2,73E-71	218	XP_018811596.1	uncharacterized protein At4g28440-like	Juglans regia	KDP21003.1
ARUBRA_DN4554_c0_g1_i1_4	2,62E-114	335	OAY46654.1	hypothetical protein MANES_06G016600	Manihot esculenta	XP_008218606.1
ARUBRA_DN9538_c0_g1_i1_1	3,92E-79	240	OAY27120.1	hypothetical protein MANES_16G101100	Manihot esculenta	ONH97761.1

ARHOMBI_DN4282_c0_g1_i1_5	2,73E-160	456	XP_018829808.1	probable UDP-arabinopyranose mutase 5 isoform X1	Juglans regia	XP_018829809.1
ARHOMBI_DN22144_c0_g1_i1_6	1,10E-72	226	XP_008220716.1	serpin-ZX	Prunus mume	XP_016647842.1
ARUBRA_DN3174_c0_g1_i1_1	7,24E-125	363	XP_018834430.1	ATP synthase subunit O, mitochondrial-like	Juglans regia	XP_008231281.1
ARHOMBI_DN19334_c0_g1_i1_6	3,62E-34	126	XP_018823042.1	proton pump-interactor 1-like	Juglans regia	XP_018850614.1
ARHOMBI_DN8571_c0_g1_i1_5	4,39E-138	400	XP_018848542.1	selenium-binding protein 2-like	Juglans regia	XP_018844057.1
ARUBRA_DN4684_c0_g2_i1_5	0	615	XP_018859983.1	glyceraldehyde-3-phosphate dehydrogenase 2, cytosolic-like	Juglans regia	XP_019434027.1
ARHOMBI_DN1355_c0_g2_i1_2	1,27E-138	395	XP_019426063.1	ras-related protein RABC1	Lupinus angustifolius	XP_016650847.1
ARUBRA_DN15624_c0_g1_i1_4	2,13E-21	85,5	XP_008237502.1	phytosulfokines 3-like	Prunus mume	XP_018857368.1
ARUBRA_DN2409_c0_g2_i1_4	1,08E-131	381	XP_018821858.1	ferritin-3, chloroplastic	Juglans regia	OAY46317.1
ARHOMBI_DN5337_c0_g1_i1_4	5,92E-112	323	XP_018857107.1	60S ribosomal protein L11	Juglans regia	OAY26364.1
ARHOMBI_DN6227_c0_g1_i4_5	2,25E-99	297	XP_020995365.1	glutathione S-transferase U17-like	Arachis duranensis	XP_020995366.1
ARHOMBI_DN4176_c0_g1_i1_6	0	528	XP_018838600.1	protein SGT1 homolog	Juglans regia	XP_020540786.1
ARUBRA_DN22728_c0_g1_i1_2	0	605	XP_018822554.1	probable fructokinase-4	Juglans regia	XP_018839987.1
ARUBRA_DN18837_c0_g1_i1_3	4,36E-89	270	XP_018847175.1	heterogeneous nuclear ribonucleoprotein 1-like	Juglans regia	XP_018847175.1
ARHOMBI_DN6122_c0_g1_i1_5	0	652	XP_018850207.1	60S ribosomal protein L4-like	Juglans regia	XP_018822731.1
ARHOMBI_DN6266_c0_g2_i3_6	0	540	XP_018844177.1	non-functional NADPH-dependent codeinone reductase 2-like isoform X1	Juglans regia	ONI30338.1
ARUBRA_DN4148_c0_g1_i1_5	2,32E-141	401	OAY32771.1	hypothetical protein MANES_13G044600	Manihot esculenta	XP_018840734.1
ARHOMBI_DN15512_c0_g1_i1_6	8,03E-142	410	XP_018812075.1	glucan endo-1,3-beta-glucosidase 13-like	Juglans regia	XP_018812083.1
ARHOMBI_DN6104_c0_g1_i1_6	5,16E-102	301	XP_008239677.1	germin-like protein subfamily T member 2	Prunus mume	ONI08515.1
ARUBRA_DN4076_c0_g1_i1_5	0	593	XP_018844149.1	cytochrome b561 and DOMON domain-containing protein At3g25290-like	Juglans regia	XP_018827547.1
ARHOMBI_DN6065_c0_g3_i2_6	2,68E-66	207	XP_018846260.1	putative methyltransferase DDB_G0268948	Juglans regia	XP_018838664.1
ARUBRA_DN4576_c0_g1_i1_2	4,10E-20	94	XP_018848770.1	uncharacterized protein At5g39570-like	Juglans regia	XP_018848770.1
ARHOMBI_DN3224_c0_g1_i1_2	1,15E-79	247	XP_018807734.1	probable proteasome inhibitor isoform X1	Juglans regia	XP_008240153.1
ARHOMBI_DN6597_c0_g1_i1_6	5,86E-135	384	KDP22561.1	hypothetical protein JCGZ_26392	Jatropha curcas	XP_012090621.1
ARUBRA_DN16167_c0_g1_i1_3	5,95E-88	266	XP_018809730.1	probable protein phosphatase 2C 76	Juglans regia	XP_018809734.1
ARHOMBI_DN4850_c0_g1_i1_5	8,15E-84	251	XP_018814658.1	40S ribosomal protein S14-3-like	Juglans regia	XP_018814659.1
ARHOMBI_DN641_c0_g1_i1_6	6,00E-105	317	OAY46075.1	hypothetical protein MANES_07G114800	Manihot esculenta	OAY38641.1
ARUBRA_DN9455_c0_g1_i1_2	4,87E-157	444	XP_018841431.1	5'-methylthioadenosine/S-adenosylhomocysteine nucleosidase 2-like isoform X1	Juglans regia	XP_018815344.1
ARUBRA_DN4936_c0_g1_i1_3	5,14E-101	295	XP_020996771.1	SKP1-like protein 1B	Arachis duranensis	XP_018820076.1
ARUBRA_DN11512_c0_g1_i1_6	7,87E-101	293	XP_018820822.1	(DL)-glycerol-3-phosphatase 2	Juglans regia	GAU35204.1

ARHOMBI_DN13390_c0_g1_i1_2	5,28E-54	178	XP_018847175.1	heterogeneous nuclear ribonucleoprotein 1-like	Juglans regia	XP_018847175.1
ARUBRA_DN13261_c0_g1_i1_6	1,85E-51	163	XP_018817862.1	thioredoxin M3, chloroplastic-like	Juglans regia	XP_004505233.1
ARUBRA_DN7781_c0_g1_i1_6	2,80E-52	172	XP_018806990.1	lipoamide acyltransferase component of branched-chain alpha-keto acid dehydrogenase complex, mitochondrial-like, partial	Juglans regia	XP_018815726.1
ARHOMBI_DN15181_c0_g1_i1_3	9,48E-100	299	XP_018820845.1	xylose isomerase	Juglans regia	XP_008223675.1
ARUBRA_DN4108_c0_g1_i1_4	0	519	XP_018826881.1	soluble inorganic pyrophosphatase 6, chloroplastic-like	Juglans regia	XP_018844455.1
ARHOMBI_DN2905_c0_g1_i1_2	4,79E-117	339	XP_018860721.1	glutathione S-transferase DHAR2-like	Juglans regia	XP_018860724.1
ARUBRA_DN4026_c0_g1_i1_5	0	1216	XP_018836580.1	aminopeptidase M1-like	Juglans regia	XP_018836577.1
ARHOMBI_DN6235_c0_g1_i4_4	0	990	XP_018836411.1	catalase isozyme 1	Juglans regia	XP_008237987.1
ARUBRA_DN25569_c0_g1_i1_3	2,24E-65	200	XP_018815728.1	acylpyruvase FAHD1, mitochondrial-like	Juglans regia	XP_008223221.1
ARUBRA_DN4711_c0_g1_i2_5	3,98E-156	442	KOM38842.1	hypothetical protein LR48_Vigan03g222400	Vigna angularis	KYP70609.1
ARUBRA_DN2993_c0_g1_i1_2	1,26E-137	391	XP_018827511.1	uncharacterized protein LOC108996194	Juglans regia	KDP45917.1
ARUBRA_DN3089_c0_g2_i1_6	0	521	XP_018823011.1	mitochondrial outer membrane protein porin of 34 kDa	Juglans regia	XP_018835940.1
ARUBRA_DN8636_c0_g1_i1_1	1,13E-74	226	OAY23502.1	hypothetical protein MANES_18G083200	Manihot esculenta	XP_018839074.1
ARHOMBI_DN4932_c0_g1_i1_1	0	551	OAY47507.1	hypothetical protein MANES_06G084900	Manihot esculenta	XP_018824861.1
ARUBRA_DN5237_c0_g1_i1_6	1,22E-164	461	XP_018844535.1	40S ribosomal protein S3-3-like	Juglans regia	XP_018827015.1
ARUBRA_DN4540_c0_g1_i2_6	8,86E-150	431	KRH43862.1	hypothetical protein GLYMA_08G175900	Glycine max	XP_018837398.1
ARUBRA_DN830_c0_g1_i1_6	2,35E-102	306	OAY56156.1	hypothetical protein MANES_03G206800	Manihot esculenta	OAY27625.1
ARHOMBI_DN5602_c0_g2_i1_1	0	1678	XP_018845356.1	calcium-transporting ATPase 2, plasma membrane-type-like isoform X1	Juglans regia	XP_018845357.1
ARHOMBI_DN6202_c0_g1_i2_4	0	662	XP_018844468.1	serine carboxypeptidase-like 40	Juglans regia	ONI20592.1
ARUBRA_DN2984_c0_g1_i2_6	3,44E-44	149	XP_004513370.1	protein EARLY RESPONSIVE TO DEHYDRATION 15-like isoform X1	Cicer arietinum	XP_004513371.1
ARUBRA_DN6258_c0_g1_i1_5	0	781	XP_018832881.1	staphylococcal nuclease domain-containing protein 1-like	Juglans regia	XP_018832881.1
ARUBRA_DN1424_c0_g2_i1_3	0	960	KDP23377.1	hypothetical protein JCGZ_23210	Jatropha curcas	XP_012088873.1
ARHOMBI_DN6163_c0_g1_i2_6	1,69E-133	384	XP_018840931.1	60S ribosomal protein L7-2-like	Juglans regia	XP_018840932.1
ARHOMBI_DN2855_c0_g1_i1_2	1,41E-113	332	KDP39216.1	hypothetical protein JCGZ_00973	Jatropha curcas	XP_012070941.1
ARHOMBI_DN4907_c0_g3_i3_4	1,77E-110	321	XP_016193057.1	eukaryotic translation initiation factor 5A-2	Arachis ipaensis	XP_015943065.1
ARUBRA_DN20327_c0_g1_i1_5	0	719	XP_018830726.1	phosphoglucomutase, chloroplastic	Juglans regia	OIV91473.1
ARHOMBI_DN3004_c0_g1_i1_6	0	572	XP_018844904.1	aldehyde dehydrogenase family 7 member A1 isoform X1	Juglans regia	XP_018844905.1
ARUBRA_DN20570_c0_g1_i1_4	7,54E-140	402	XP_018816369.1	uncharacterized protein At1g03900-like isoform X1	Juglans regia	XP_018816370.1

ARHOMBI_DN4754_c0_g1_i1_5	0	935	XP_018833268.1	glucose-1-phosphate adenylyltransferase small subunit, chloroplastic/amyloplastic	Juglans regia	AES71731.1
ARUBRA_DN2276_c0_g1_i1_5	1,76E-52	168	XP_018814019.1	uncharacterized protein LOC108985979	Juglans regia	XP_018820585.1
ARHOMBI_DN6055_c0_g1_i1_6	6,34E-66	204	XP_008387404.1	40S ribosomal protein S10-1-like	Malus domestica	XP_008392399.1
ARHOMBI_DN1472_c0_g1_i1_6	0	650	XP_018817568.1	probable ADP-ribosylation factor GTPase-activating protein AGD9 isoform X1	Juglans regia	XP_018817570.1
ARUBRA_DN2452_c0_g2_i1_2	3,95E-138	395	XP_009372980.1	40S ribosomal protein S6	Pyrus x bretschneideri	XP_008386113.1
ARUBRA_DN4724_c0_g1_i1_5	5,38E-98	286	AGV54677.1	eukaryotic translation initiation factor 5A	Phaseolus vulgaris	XP_008394324.1
ARHOMBI_DN7816_c0_g1_i1_3	3,85E-136	391	KDP28756.1	hypothetical protein JCGZ_14527	Jatropha curcas	XP_012083577.1
ARHOMBI_DN4989_c0_g1_i1_1	0	575	XP_018809432.1	mitochondrial dicarboxylate/tricarboxylate transporter DTC-like	Juglans regia	OAY54741.1
ARUBRA_DN8722_c0_g1_i1_2	2,79E-41	146	XP_008225664.2	reticuline oxidase-like protein	Prunus mume	ONI11277.1
ARUBRA_DN19739_c0_g2_i1_3	0	510	XP_018835577.1	probable carboxylesterase 12	Juglans regia	XP_008236606.1
ARUBRA_DN5052_c0_g1_i2_2	1,79E-101	299	XP_018819193.1	cinnamoyl-CoA reductase 1	Juglans regia	ONH99807.1
ARHOMBI_DN19084_c0_g1_i1_5	5,15E-93	284	OAY57066.1	hypothetical protein MANES_02G067800	Manihot esculenta	XP_008230399.1
ARHOMBI_DN4789_c0_g1_i1_3	0	942	XP_018814251.1	dihydrolipoyl dehydrogenase 1, mitochondrial	Juglans regia	XP_018814252.1
ARUBRA_DN14251_c0_g1_i1_2	1,06E-76	233	XP_018818800.1	probable 6-phosphogluconolactonase 4, chloroplastic	Juglans regia	XP_018842795.1
ARUBRA_DN1618_c0_g1_i1_5	4,36E-95	305	XP_018817298.1	protein transport protein SEC31 homolog B-like isoform X2	Juglans regia	XP_018817293.1
ARHOMBI_DN277_c0_g1_i1_2	7,12E-54	171	XP_018852574.1	UPF0587 protein C1orf123 homolog	Juglans regia	XP_007160977.1
ARUBRA_DN4780_c0_g3_i1_6	0	861	CAB66336.1	alpha-tubulin	Betula pendula	KRH62139.1
ARHOMBI_DN4043_c0_g1_i1_2	1,80E-65	198	AFU10994.1	HSP70, partial	Malus zumi	XP_016187299.1
ARUBRA_DN4785_c0_g1_i3_6	0	646	XP_018829145.1	ankyrin repeat domain-containing protein 2B-like	Juglans regia	XP_018841596.1
ARHOMBI_DN5765_c0_g1_i1_5	1,33E-112	325	XP_018836279.1	uncharacterized protein LOC109002826	Juglans regia	XP_018836280.1
ARHOMBI_DN4921_c0_g1_i1_3	0	791	XP_018817834.1	stromal 70 kDa heat shock-related protein, chloroplastic-like	Juglans regia	XP_018817839.1
ARUBRA_DN4799_c0_g1_i1_4	4,45E-49	159	XP_018838679.1	cysteine proteinase inhibitor 5	Juglans regia	XP_014508270.1
ARUBRA_DN2524_c0_g1_i1_6	8,08E-67	217	XP_018831924.1	aconitate hydratase, cytoplasmic-like, partial	Juglans regia	XP_018848484.1
ARUBRA_DN830_c1_g1_i1_2	2,25E-133	388	XP_018825758.1	galactokinase-like isoform X1	Juglans regia	XP_018825759.1
ARUBRA_DN5581_c0_g1_i1_2	5,84E-93	278	XP_018844008.1	protein FATTY ACID EXPORT 1, chloroplastic-like	Juglans regia	XP_018847055.1
ARUBRA_DN18248_c0_g1_i1_4	1,23E-61	191	XP_018813170.1	vesicle-associated protein 1-2-like	Juglans regia	XP_008377496.1
ARHOMBI_DN15811_c0_g1_i1_3	1,89E-70	224	KDP47143.1	hypothetical protein JCGZ_22139	Jatropha curcas	XP_012067521.1
ARHOMBI_DN5696_c0_g1_i1_3	0	507	XP_018860017.1	60S ribosomal protein L8-1-like	Juglans regia	XP_018855871.1
ARHOMBI_DN6039_c0_g1_i3_2	1,88E-106	308	KDP44051.1	hypothetical protein JCGZ_05518	Jatropha curcas	XP_012064820.1

ARHOMBI_DN787_c0_g2_i1_5	0	577	XP_020221626.1	ferredoxin--NADP reductase, root isozyme, chloroplastic isoform X1	Cajanus cajan	XP_020221627.1
ARUBRA_DN19178_c0_g1_i1_4	1,45E-95	281	XP_018816977.1	40S ribosomal protein S19-3	Juglans regia	KHN12325.1
ARUBRA_DN202_c0_g1_i1_3	0	755	XP_018843179.1	fructose-bisphosphate aldolase 3, chloroplastic	Juglans regia	OAY31394.1
ARHOMBI_DN5950_c0_g1_i1_2	0	865	XP_018839429.1	ATP sulfurylase 1, chloroplastic	Juglans regia	XP_018818643.1
ARHOMBI_DN2248_c0_g1_i1_5	2,94E-158	445	KHN18718.1	Pyruvate kinase, cytosolic isozyme	Glycine soja	XP_018805935.1
ARUBRA_DN11411_c0_g1_i1_4	2,81E-113	324	XP_018815940.1	60S ribosomal protein L18-2	Juglans regia	XP_018841792.1
ARUBRA_DN17927_c0_g1_i1_6	3,57E-144	412	XP_018807368.1	probable ATP synthase 24 kDa subunit, mitochondrial isoform X2	Juglans regia	XP_018807367.1
ARHOMBI_DN5374_c1_g1_i1_6	0	674	OAY42321.1	hypothetical protein MANES_09G170700	Manihot esculenta	OAY44035.1
ARUBRA_DN763_c0_g1_i1_4	5,75E-89	264	KDP33752.1	hypothetical protein JCGZ_07323	Jatropha curcas	XP_012076803.1
ARUBRA_DN2819_c0_g1_i1_3	1,26E-39	137	XP_018830611.1	carboxylesterase 1-like	Juglans regia	ONI25181.1
ARUBRA_DN4688_c0_g1_i1_1	0	810	XP_018841811.1	aconitate hydratase, cytoplasmic isoform X1	Juglans regia	XP_018841812.1
ARHOMBI_DN4778_c0_g1_i1_3	0	689	XP_018847873.1	serine-threonine kinase receptor-associated protein-like	Juglans regia	XP_016179643.1
ARHOMBI_DN777_c0_g1_i1_1	2,18E-07	53,9	AGC51777.1	dehydrin protein	Manihot esculenta	OAY50493.1
ARHOMBI_DN18025_c0_g1_i1_2	9,83E-102	299	XP_018813211.1	uncharacterized protein LOC108985386 isoform X1	Juglans regia	AFK37701.1
ARHOMBI_DN5069_c0_g2_i1_3	0	776	XP_018827302.1	succinate--CoA ligase ADP-forming subunit beta, mitochondrial	Juglans regia	XP_018847166.1
ARUBRA_DN4080_c0_g1_i1_6	1,83E-26	100	XP_018846356.1	profilin-1	Juglans regia	AAK01235.1
ARUBRA_DN19946_c0_g1_i1_3	5,25E-115	331	XP_015961744.1	60S ribosomal protein L10	Arachis duranensis	AFK38655.1
ARUBRA_DN4991_c0_g1_i1_6	0	776	XP_018814264.1	phosphomethylethanolamine N-methyltransferase-like	Juglans regia	XP_018857980.1
ARHOMBI_DN17727_c0_g1_i1_3	0	796	XP_018845060.1	coatomer subunit beta'-2-like	Juglans regia	XP_018845914.1
ARHOMBI_DN4645_c0_g2_i1_4	0	944	XP_018852509.1	puromycin-sensitive aminopeptidase isoform X2	Juglans regia	XP_018852512.1
ARHOMBI_DN2411_c0_g1_i1_5	0	714	XP_018810822.1	T-complex protein 1 subunit theta	Juglans regia	GAU30876.1
ARUBRA_DN4641_c0_g2_i1_2	0	1205	OAY58361.1	hypothetical protein MANES_02G171300	Manihot esculenta	BAT95374.1
ARHOMBI_DN1922_c0_g1_i1_3	1,59E-139	395	XP_018841531.1	metal-independent phosphoserine phosphatase-like	Juglans regia	XP_018841533.1
ARUBRA_DN17998_c0_g1_i1_1	4,39E-133	391	ONH98173.1	hypothetical protein PRUPE_7G233700	Prunus persica	ONH98172.1
ARHOMBI_DN5775_c0_g1_i1_2	0	612	XP_018813719.1	cysteine proteinase COT44-like	Juglans regia	XP_018845023.1
ARUBRA_DN5604_c0_g1_i1_1	1,38E-95	281	XP_018836470.1	ATP synthase subunit d, mitochondrial-like	Juglans regia	XP_018858751.1
ARUBRA_DN4856_c0_g1_i2_3	1,13E-22	93,2	AER13137.1	DHN1	Corylus avellana	ACU15585.1
ARHOMBI_DN478_c0_g2_i1_6	0	551	XP_018839634.1	S-formylglutathione hydrolase	Juglans regia	KDP26090.1
ARHOMBI_DN5072_c0_g1_i1_5	1,46E-141	404	AFH08811.1	chloroplast Mn-superoxide dismutase 1A-c	Prunus persica	AFH08818.1
ARUBRA_DN162_c0_g1_i1_1	0	583	XP_018845549.1	uncharacterized protein LOC109009507 isoform X2	Juglans regia	XP_018845548.1

ARHOMBI_DN5339_c0_g1_i1_5	0	764	XP_018839373.1	stearoyl-[acyl-carrier-protein 9-desaturase, chloroplastic-like	Juglans regia	XP_018812159.1
ARHOMBI_DN5797_c1_g1_i2_6	7,80E-53	166	XP_018855864.1	probable aldo-keto reductase 2, partial	Juglans regia	XP_008337505.1
ARHOMBI_DN5453_c0_g1_i1_1	0	778	XP_018829125.1	calnexin homolog	Juglans regia	XP_018829196.1
ARHOMBI_DN4158_c0_g1_i1_4	0	724	OAY32695.1	hypothetical protein MANES_13G038500	Manihot esculenta	ONH93092.1
ARHOMBI_DN3362_c0_g1_i1_5	0	869	XP_008221256.1	tubulin beta chain-like	Prunus mume	ONI32387.1
ARUBRA_DN20595_c0_g1_i1_3	0	555	XP_018812010.1	heat shock 70 kDa protein 15-like	Juglans regia	XP_018812011.1
ARUBRA_DN427_c0_g2_i1_5	0	511	XP_008223483.1	S-adenosylmethionine synthase 5	Prunus mume	XP_018833868.1
ARHOMBI_DN19156_c0_g1_i1_5	5,48E-59	189	XP_018827577.1	SAL1 phosphatase-like isoform X2	Juglans regia	XP_018827576.1
ARHOMBI_DN3290_c0_g1_i1_1	4,22E-117	342	XP_018808748.1	adenine phosphoribosyltransferase 1-like	Juglans regia	XP_020212312.1
ARUBRA_DN2245_c0_g2_i1_4	8,22E-122	360	XP_018840769.1	protein disulfide isomerase-like 2-3	Juglans regia	KDP30825.1
ARHOMBI_DN19115_c0_g1_i1_6	1,52E-96	296	XP_018850271.1	chaperonin CPN60-2, mitochondrial-like	Juglans regia	XP_018839307.1
ARHOMBI_DN5316_c0_g1_i1_2	0	561	XP_018810049.1	probable pyridoxal 5'-phosphate synthase subunit PDX1	Juglans regia	XP_018835387.1
ARUBRA_DN8606_c0_g1_i1_1	6,76E-109	324	XP_018850665.1	elongation factor 1-gamma-like	Juglans regia	XP_020221597.1
ARHOMBI_DN5381_c0_g1_i1_4	1,84E-96	278	XP_008218349.1	60S ribosomal protein L23	Prunus mume	XP_008224171.1
ARUBRA_DN43_c0_g2_i1_6	5,30E-37	127	XP_009354123.1	peamaclein-like isoform X1	Pyrus x bretschneideri	XP_008377985.1
ARHOMBI_DN3956_c0_g1_i1_1	2,10E-164	469	XP_018820730.1	cationic peroxidase 1-like	Juglans regia	XP_009373126.1
ARUBRA_DN609_c0_g1_i1_6	0	820	OAY54743.1	hypothetical protein MANES_03G098100	Manihot esculenta	XP_018834544.1
ARUBRA_DN2_c0_g1_i1_4	0	705	XP_008221201.1	24-methylenesterol C-methyltransferase 2	Prunus mume	ONI32296.1
ARUBRA_DN1844_c0_g1_i1_1	6,10E-172	484	XP_018845359.1	mitochondrial outer membrane protein porin 2-like	Juglans regia	XP_018849544.1
ARHOMBI_DN3609_c0_g2_i1_1	1,40E-69	218	XP_018818666.1	perakine reductase-like	Juglans regia	XP_018839416.1
ARHOMBI_DN3185_c0_g1_i1_4	0	607	XP_018843285.1	mitochondrial-processing peptidase subunit alpha-like	Juglans regia	XP_018811090.1
ARUBRA_DN24520_c0_g1_i1_2	5,10E-64	200	XP_020215778.1	heme-binding-like protein At3g10130, chloroplastic	Cajanus cajan	XP_018838204.1
ARUBRA_DN4994_c0_g1_i2_3	3,09E-173	485	OIV90699.1	hypothetical protein TanjilG_15085	Lupinus angustifolius	OIW07225.1
ARUBRA_DN19637_c0_g1_i1_2	0	660	XP_018829691.1	eukaryotic peptide chain release factor GTP-binding subunit ERF3A isoform X2	Juglans regia	XP_018829690.1
ARUBRA_DN18881_c0_g1_i1_2	1,06E-174	490	XP_018841289.1	proteasome subunit alpha type-5-like isoform X1	Juglans regia	XP_018837325.1
ARHOMBI_DN5176_c0_g1_i1_6	7,42E-93	275	XP_018826083.1	peptide methionine sulfoxide reductase B5-like isoform X1	Juglans regia	XP_018826084.1
ARUBRA_DN13710_c0_g1_i1_3	6,14E-91	268	XP_018807334.1	endochitinase EP3-like	Juglans regia	XP_009368176.1
ARUBRA_DN18453_c0_g1_i1_3	3,05E-50	171	XP_018841545.1	ubiquitin domain-containing protein DSK2b-like isoform X4	Juglans regia	XP_018841543.1
ARHOMBI_DN5422_c0_g1_i1_5	0	604	XP_018834985.1	thioredoxin reductase 1-like	Juglans regia	XP_004512292.1

ARHOMBI_DN15946_c0_g1_i1_6	1,57E-110	322	XP_018850456.1	uncharacterized protein LOC109013009	Juglans regia	OAY61203.1
ARHOMBI_DN4424_c0_g1_i1_1	0	738	ADR71240.1	60S ribosomal protein L3B	Hevea brasiliensis	XP_018848152.1
ARUBRA_DN3570_c0_g1_i1_3	9,13E-83	246	XP_018809376.1	60S ribosomal protein L9-like	Juglans regia	XP_018809377.1
ARUBRA_DN2080_c0_g1_i1_2	5,57E-83	261	XP_018850062.1	acyl-CoA-binding domain-containing protein 4	Juglans regia	KEH24514.1
ARUBRA_DN2407_c0_g1_i1_1	0	750	XP_018833171.1	UDP-D-apiose/UDP-D-xylose synthase 2	Juglans regia	XP_008385469.1
ARUBRA_DN6096_c0_g1_i1_5	2,59E-136	388	XP_018813683.1	60S ribosomal protein L10a-like	Juglans regia	CAX02806.1
ARUBRA_DN5878_c0_g1_i1_4	0	961	XP_018845845.1	pyruvate kinase, cytosolic isozyme	Juglans regia	XP_004505347.1
ARUBRA_DN462_c0_g2_i1_3	4,14E-94	279	XP_018815980.1	uncharacterized protein LOC108987512	Juglans regia	XP_008241278.1
ARHOMBI_DN8302_c0_g1_i1_6	6,88E-162	465	OAY60729.1	hypothetical protein MANES_01G134800	Manihot esculenta	GAU40004.1
ARHOMBI_DN6204_c0_g1_i2_1	0	685	XP_018837398.1	ruBisCO large subunit-binding protein subunit beta, chloroplastic	Juglans regia	OAY29001.1
ARUBRA_DN935_c0_g1_i1_4	1,78E-117	340	XP_018819121.1	ATP synthase subunit delta', mitochondrial-like	Juglans regia	XP_018854921.1
ARHOMBI_DN6019_c0_g1_i1_2	1,08E-172	491	XP_018839339.1	uncharacterized protein LOC109005031	Juglans regia	KDP29539.1
ARHOMBI_DN4558_c0_g2_i1_1	0	518	XP_018848751.1	acetyl-CoA acetyltransferase, cytosolic 1 isoform X2	Juglans regia	XP_018848747.1
ARUBRA_DN6207_c0_g1_i1_4	5,29E-104	303	KHN10552.1	40S ribosomal protein S4	Glycine soja	XP_006576177.1
ARUBRA_DN17796_c0_g1_i1_2	0	744	XP_018844405.1	pyruvate dehydrogenase E1 component subunit alpha-1, mitochondrial-like	Juglans regia	KDP21276.1
ARUBRA_DN4392_c0_g3_i1_4	9,12E-180	505	XP_018850794.1	probable protein phosphatase 2C 39	Juglans regia	ONI12649.1
ARUBRA_DN2862_c0_g2_i1_5	2,86E-26	102	XP_018829662.1	probable S-adenosylmethionine-dependent methyltransferase At5g38100	Juglans regia	XP_018829663.1
ARUBRA_DN18480_c0_g1_i1_4	0	601	XP_018848751.1	acetyl-CoA acetyltransferase, cytosolic 1 isoform X2	Juglans regia	XP_018848747.1
ARHOMBI_DN6139_c0_g1_i1_4	1,15E-169	474	XP_018824212.1	proteasome subunit alpha type-4	Juglans regia	XP_008371922.1
ARHOMBI_DN5986_c0_g1_i2_6	8,67E-39	132	ADD69807.1	copper transport protein ATOX1	Hevea brasiliensis	OAY45760.1
ARHOMBI_DN20063_c0_g1_i1_4	1,44E-61	199	XP_018813794.1	ubiquitin receptor RAD23b-like	Juglans regia	XP_009365249.1
ARHOMBI_DN5759_c0_g1_i1_6	4,35E-149	428	XP_018852330.1	60S ribosomal protein L5	Juglans regia	XP_018840429.1
ARHOMBI_DN3534_c0_g1_i1_3	3,49E-107	316	XP_018825826.1	ferritin-3, chloroplastic-like	Juglans regia	XP_008370177.1
ARUBRA_DN11326_c0_g1_i1_6	1,25E-127	360	XP_018808681.1	ubiquitin-fold modifier-conjugating enzyme 1	Juglans regia	KDP41535.1
ARUBRA_DN2423_c0_g1_i1_1	1,22E-124	360	XP_018498100.1	60S ribosomal protein L15-1	Pyrus x bretschneideri	XP_009334550.1
ARUBRA_DN3020_c0_g1_i1_1	9,16E-117	337	XP_018818478.1	cysteine proteinase inhibitor 6-like	Juglans regia	XP_012068733.1
ARUBRA_DN12109_c0_g1_i1_6	1,27E-47	160	XP_018813794.1	ubiquitin receptor RAD23b-like	Juglans regia	XP_016197096.1
ARUBRA_DN16721_c0_g1_i1_2	4,30E-137	395	XP_018846193.1	aminoacyl tRNA synthase complex-interacting multifunctional protein 1 isoform X2	Juglans regia	XP_018846192.1
ARUBRA_DN4896_c0_g1_i1_4	0	533	XP_018841575.1	uncharacterized protein At2g17340-like	Juglans regia	XP_018841576.1
ARHOMBI_DN347_c0_g1_i1_1	0	684	XP_008376628.1	probable polygalacturonase	Malus domestica	ONI18043.1
ARUBRA_DN3353_c0_g1_i1_5	2,81E-81	256	KRH01060.1	hypothetical protein GLYMA_18G251400	Glycine max	XP_003552493.1

ARHOMBI_DN1690_c0_g1_i1_4	5,09E-126	365	XP_018832807.1	velvet complex subunit B	Juglans regia	KDP20282.1
ARUBRA_DN4036_c0_g1_i1_5	6,55E-43	143	XP_018850188.1	V-type proton ATPase subunit G-like	Juglans regia	XP_018850189.1
ARHOMBI_DN3961_c0_g1_i1_1	4,34E-97	293	XP_018843123.1	eukaryotic translation initiation factor 3 subunit J-like	Juglans regia	OAY46647.1
ARUBRA_DN6223_c0_g1_i1_2	0	534	KDP24308.1	hypothetical protein JCGZ_25604	Jatropha curcas	XP_012088080.1
ARUBRA_DN17757_c0_g1_i1_6	2,34E-153	432	OAY25631.1	hypothetical protein MANES_17G110100	Manihot esculenta	XP_018845779.1
ARUBRA_DN1661_c0_g1_i1_3	3,87E-89	264	XP_018845602.1	AIG2-like protein	Juglans regia	OAY53495.1
ARUBRA_DN2570_c0_g2_i1_5	1,12E-46	150	KRH26085.1	hypothetical protein GLYMA_12G151400	Glycine max	KRH26086.1
ARUBRA_DN2915_c0_g1_i1_5	0	875	XP_018822246.1	aspartate aminotransferase P2, mitochondrial-like	Juglans regia	XP_018822247.1
ARUBRA_DN4959_c0_g1_i4_6	6,38E-80	239	XP_018851971.1	40S ribosomal protein S20-2-like	Juglans regia	XP_018851972.1
ARUBRA_DN4738_c0_g1_i1_6	3,53E-84	252	XP_018833404.1	40S ribosomal protein S12-like	Juglans regia	XP_018835263.1
ARHOMBI_DN5099_c0_g2_i1_3	0	585	XP_018834071.1	mitochondrial phosphate carrier protein 3, mitochondrial-like	Juglans regia	XP_008341244.1
ARHOMBI_DN6124_c0_g2_i1_6	0	815	XP_018847910.1	phosphoglycerate kinase, chloroplastic	Juglans regia	OAY30163.1
ARUBRA_DN22130_c0_g1_i1_6	1,25E-107	314	XP_018815941.1	malignant T-cell-amplified sequence 1 homolog	Juglans regia	OAY44020.1
ARHOMBI_DN17024_c0_g1_i1_5	1,88E-96	285	XP_018843199.1	glutathione S-transferase DHAR3, chloroplastic-like	Juglans regia	XP_018816866.1
ARHOMBI_DN3923_c0_g2_i1_3	8,31E-30	105	XP_018807625.1	aldo-keto reductase family 4 member C9-like	Juglans regia	XP_018846254.1
ARUBRA_DN16253_c0_g1_i1_2	0	549	XP_018839434.1	pyruvate kinase isozyme A, chloroplastic-like	Juglans regia	XP_018818630.1
ARUBRA_DN18651_c0_g1_i1_6	2,26E-164	489	XP_018852509.1	puromycin-sensitive aminopeptidase isoform X2	Juglans regia	ONI07154.1
ARHOMBI_DN16154_c0_g1_i1_2	5,65E-107	311	KDP23652.1	hypothetical protein JCGZ_23485	Jatropha curcas	XP_012089246.1
ARUBRA_DN5051_c0_g1_i1_2	0	509	XP_018816665.1	14-3-3-like protein A	Juglans regia	XP_018816666.1
ARHOMBI_DN2561_c0_g1_i1_3	3,62E-52	168	XP_009348814.2	60S ribosomal protein L22-2-like isoform X1	Pyrus x bretschneideri	XP_018815191.1
ARUBRA_DN1380_c0_g2_i1_2	0	1042	XP_018840362.1	ketol-acid reductoisomerase, chloroplastic-like	Juglans regia	XP_018809878.1
ARUBRA_DN2595_c0_g3_i1_4	2,05E-89	264	XP_018823169.1	60S ribosomal protein L27a-3-like	Juglans regia	KDP31039.1
ARUBRA_DN3028_c0_g1_i1_1	1,65E-72	226	XP_018833902.1	cytochrome c oxidase subunit 6b-1-like isoform X2	Juglans regia	XP_018833901.1
ARUBRA_DN3878_c0_g1_i1_3	9,57E-80	251	XP_018842240.1	biotin carboxylase 1, chloroplastic isoform X1	Juglans regia	ONI14054.1
ARUBRA_DN2248_c0_g1_i1_6	0	706	XP_018851843.1	protein STRICTOSIDINE SYNTHASE-LIKE 3-like	Juglans regia	XP_018844479.1
ARUBRA_DN3983_c0_g1_i1_4	0	1009	XP_018807686.1	3-isopropylmalate dehydratase large subunit, chloroplastic-like	Juglans regia	XP_018820100.1
ARUBRA_DN7521_c0_g1_i1_3	1,60E-91	288	KDP22063.1	hypothetical protein JCGZ_25894	Jatropha curcas	XP_012089960.1
ARUBRA_DN15944_c0_g1_i1_3	1,50E-50	169	XP_018844057.1	selenium-binding protein 2-like	Juglans regia	XP_018848542.1
ARUBRA_DN4965_c10_g1_i1_3	0	882	XP_018807488.1	uncharacterized protein LOC108980899	Juglans regia	XP_018807488.1
ARHOMBI_DN2725_c0_g1_i1_2	8,56E-80	248	OAY50881.1	hypothetical protein MANES_05G169500	Manihot esculenta	XP_009364054.1
ARHOMBI_DN4252_c0_g1_i1_5	1,59E-128	367	XP_008234622.1	60S ribosomal protein L9-1	Prunus mume	ONI25805.1

ARHOMBI_DN5741_c0_g1_i1_3	7,83E-121	350	XP_018834379.1	60S ribosomal protein L6	Juglans regia	XP_018816601.1
ARHOMBI_DN4907_c0_g1_i1_4	3,43E-108	315	AAQ08191.1	eukaryotic translation initiation factor 5A isoform I	Hevea brasiliensis	AAQ08192.1
ARHOMBI_DN3938_c0_g1_i1_4	4,89E-110	321	XP_018841038.1	acyl-protein thioesterase 2-like isoform X1	Juglans regia	KYP34215.1
ARUBRA_DN17582_c0_g1_i1_4	4,47E-46	152	XP_018820822.1	(DL)-glycerol-3-phosphatase 2	Juglans regia	XP_008223698.1
ARUBRA_DN9146_c0_g1_i1_2	2,74E-50	164	XP_018841906.1	DNA-binding protein DDB_G0278111-like isoform X2	Juglans regia	XP_018841907.1
ARHOMBI_DN15390_c0_g1_i1_4	1,70E-127	373	XP_018831678.1	FAM10 family protein At4g22670-like	Juglans regia	XP_018847976.1
ARHOMBI_DN4487_c0_g1_i1_2	0	588	XP_018827261.1	TOM1-like protein 2	Juglans regia	XP_008235674.1
ARUBRA_DN1889_c0_g1_i1_3	2,79E-130	373	XP_018845301.1	60S ribosomal protein L13-1	Juglans regia	XP_018845302.1
ARUBRA_DN4712_c0_g2_i1_6	2,05E-72	221	XP_008392865.1	60S ribosomal protein L26-1	Malus domestica	XP_008393851.1
ARUBRA_DN4939_c0_g1_i3_5	8,72E-33	117	ABH03379.1	60S acidic ribosomal protein	Prunus dulcis	AAL91663.1
ARUBRA_DN4833_c0_g1_i2_5	2,34E-15	68,9	OAY45733.1	hypothetical protein MANES_07G086800	Manihot esculenta	KDP43375.1
ARUBRA_DN2992_c0_g1_i1_3	2,50E-116	336	XP_018810064.1	eukaryotic translation initiation factor	Juglans regia	XP_018807853.1
ARUBRA_DN7857_c0_g1_i1_1	3,29E-58	184	XP_018809557.1	rho GDP-dissociation inhibitor 1-like isoform X1	Juglans regia	XP_018809558.1
ARUBRA_DN22505_c0_g1_i1_3	2,48E-52	164	XP_007133241.1	hypothetical protein PHAVU_011G163600g, partial	Phaseolus vulgaris	ESW05235.1
ARUBRA_DN21075_c0_g1_i1_5	8,68E-60	189	XP_018825134.1	ubiquitin carboxyl-terminal hydrolase 3-like	Juglans regia	XP_018825135.1
ARHOMBI_DN5548_c0_g1_i1_5	0	1054	XP_020977085.1	pyruvate decarboxylase 2	Arachis ipaensis	XP_020977086.1
ARUBRA_DN2771_c0_g1_i1_5	0	698	XP_018850614.1	proton pump-interactor 1-like	Juglans regia	XP_018823042.1
ARUBRA_DN4858_c0_g1_i1_4	1,32E-29	105	XP_018848846.1	40S ribosomal protein S28	Juglans regia	XP_018848847.1
ARUBRA_DN25882_c0_g1_i1_2	1,74E-61	205	XP_016188426.1	aconitate hydratase, cytoplasmic	Arachis ipaensis	XP_015953278.1
ARHOMBI_DN6034_c0_g1_i3_3	0	805	XP_018835203.1	luminal-binding protein	Juglans regia	ALP70519.1
ARHOMBI_DN5395_c0_g1_i1_3	0	662	APO15267.1	glutathione reductase 2	Prunus avium	XP_008224600.1
ARUBRA_DN2691_c0_g1_i1_5	3,13E-92	274	XP_018814519.1	peptidyl-prolyl cis-trans isomerase FKBP20-1	Juglans regia	KDP44932.1
ARUBRA_DN16143_c0_g1_i1_5	5,19E-86	254	XP_018820939.1	60S ribosomal protein L14-1	Juglans regia	XP_018808011.1
ARUBRA_DN7703_c0_g1_i1_6	3,36E-149	425	CAA98171.1	RAB7D	Lotus japonicus	ACU13699.1
ARHOMBI_DN5124_c0_g1_i1_4	1,31E-36	129	XP_008384671.1	40S ribosomal protein S26-3-like	Malus domestica	XP_009354778.1
ARHOMBI_DN3648_c0_g1_i1_4	1,82E-96	279	XP_018848065.1	40S ribosomal protein S13	Juglans regia	XP_008227708.1
ARHOMBI_DN1734_c0_g1_i1_3	2,48E-122	352	XP_018846383.1	prohibitin-3, mitochondrial-like	Juglans regia	XP_018857639.1
ARUBRA_DN9894_c0_g1_i1_4	0	653	XP_018840325.1	cysteine protease RD19A-like	Juglans regia	OAY52466.1
ARUBRA_DN2456_c0_g1_i1_2	1,29E-121	358	XP_018829662.1	probable S-adenosylmethionine-dependent methyltransferase At5g38100	Juglans regia	XP_018829663.1
ARUBRA_DN6971_c0_g1_i1_6	5,33E-168	470	XP_018822732.1	proteasome subunit alpha type-3-like	Juglans regia	XP_018831769.1
ARHOMBI_DN516_c0_g1_i1_6	2,85E-125	358	XP_018837970.1	germin-like protein subfamily 2 member 1	Juglans regia	XP_018806639.1
ARUBRA_DN3974_c0_g1_i1_1	1,70E-45	149	XP_018860422.1	protein BOLA2	Juglans regia	ACU15761.1
ARUBRA_DN19869_c0_g1_i1_3	9,38E-113	328	XP_018824378.1	UDP-glucose 4-epimerase GEPI48	Juglans regia	XP_018857524.1
ARUBRA_DN5948_c0_g1_i1_2	5,16E-132	386	XP_018826687.1	glucose-6-phosphate isomerase, cytosolic	Juglans regia	KRH61181.1
ARUBRA_DN20440_c0_g1_i1_3						

ARUBRA_DN20805_c0_g1_i1_5	4,31E-90	271	XP_018827585.1	fumarylacetoacetase	Juglans regia	KDP37782.1
ARUBRA_DN1508_c0_g1_i1_1	1,36E-92	270	XP_018820088.1	superoxide dismutase Cu-Zn 2	Juglans regia	ONH94232.1
ARUBRA_DN8181_c0_g1_i1_4	5,26E-180	501	XP_018835046.1	proteasome subunit alpha type-6 isoform X1	Juglans regia	XP_018835047.1
ARHOMBI_DN5015_c0_g1_i1_3	1,79E-151	432	XP_009357480.2	LOW QUALITY PROTEIN: proline synthase co-transcribed bacterial homolog protein-like	Pyrus x bretschneideri	XP_018857393.1
ARUBRA_DN11787_c0_g1_i1_3	5,04E-101	295	XP_018844636.1	PITH domain-containing protein 1	Juglans regia	XP_020208532.1
ARHOMBI_DN5946_c1_g1_i1_6	0	622	XP_018817332.1	glutamine synthetase nodule isozyme-like	Juglans regia	AAB61597.1
ARUBRA_DN23186_c0_g1_i1_4	1,02E-46	154	XP_018842145.1	glycine-rich RNA-binding protein RZ1A-like	Juglans regia	XP_018842146.1
ARUBRA_DN3089_c0_g1_i1_6	0	525	XP_018812299.1	mitochondrial outer membrane protein porin of 36 kDa	Juglans regia	XP_008339931.1
ARUBRA_DN1937_c0_g1_i1_4	0	843	XP_018846215.1	26S proteasome non-ATPase regulatory subunit 12 homolog A	Juglans regia	OIW17846.1
ARHOMBI_DN9002_c0_g1_i1_5	3,87E-67	210	XP_018824896.1	glycine-rich RNA-binding protein 3, mitochondrial-like	Juglans regia	ONI08870.1
ARHOMBI_DN10315_c0_g1_i1_4	2,92E-124	364	OIV93977.1	hypothetical protein TanjilG_05680	Lupinus angustifolius	XP_019422038.1
ARUBRA_DN20947_c0_g1_i1_6	1,67E-89	265	XP_018842727.1	protein BOLA4, chloroplastic/mitochondrial-like	Juglans regia	XP_018828128.1
ARUBRA_DN18979_c0_g1_i1_5	1,13E-120	363	OAY48214.1	hypothetical protein MANES_06G141300	Manihot esculenta	KDP22352.1
ARUBRA_DN6370_c0_g1_i1_6	0	650	XP_018818414.1	probable voltage-gated potassium channel subunit beta	Juglans regia	XP_018840255.1
ARHOMBI_DN16206_c0_g1_i1_4	1,34E-115	335	XP_018838134.1	nitrogen regulatory protein P-II homolog	Juglans regia	XP_018824177.1
ARHOMBI_DN6026_c0_g2_i1_4	0	766	XP_018832890.1	S-adenosylmethionine synthase 1	Juglans regia	XP_009363082.1
ARHOMBI_DN11466_c0_g1_i1_1	4,29E-55	182	XP_009363534.1	aldehyde dehydrogenase family 2 member B7, mitochondrial-like isoform X2 Pyrus x bretschneideri		XP_018813280.1
ARUBRA_DN6756_c0_g1_i1_3	7,59E-152	435	XP_018859966.1	hexokinase-1-like isoform X2	Juglans regia	XP_018859964.1
ARHOMBI_DN7040_c0_g1_i1_2	4,47E-20	87,4	XP_018809958.1	chaperone protein ClpB1	Juglans regia	GAU24636.1
ARHOMBI_DN11661_c0_g1_i1_3	6,30E-105	310	XP_007138652.1	hypothetical protein PHAVU_009G226700g	Phaseolus vulgaris	ESW10646.1
ARHOMBI_DN23618_c0_g1_i1_3	5,60E-45	148	XP_018857758.1	serine/arginine-rich splicing factor RSZ21	Juglans regia	XP_004509838.1
ARUBRA_DN6507_c0_g1_i1_3	1,39E-57	191	XP_018839074.1	UDP-sugar pyrophosphorylase-like	Juglans regia	XP_018849179.1
ARHOMBI_DN21306_c0_g1_i1_1	2,91E-72	233	XP_018843066.1	pyrophosphate--fructose 6-phosphate 1-phosphotransferase subunit alpha	Juglans regia	XP_018832985.1
ARHOMBI_DN16200_c0_g1_i1_5	2,45E-57	194	XP_018815884.1	acid beta-fructofuranosidase-like	Juglans regia	XP_008376436.1
ARHOMBI_DN18668_c0_g1_i1_4	2,88E-43	152	OAY29130.1	hypothetical protein MANES_15G120000	Manihot esculenta	XP_018850581.1
ARHOMBI_DN7785_c0_g1_i1_5	6,40E-30	105	XP_018835558.1	cx9C motif-containing protein 4	Juglans regia	OAY39770.1

ARHOMBI_DN2296_c0_g2_i1_6	0	841	XP_018845710.1	aspartate--tRNA ligase 2, cytoplasmic	Juglans regia	KRH15638.1
ARUBRA_DN4291_c0_g1_i1_3	2,95E-54	174	XP_016171684.1	60S ribosomal protein L23a	Arachis ipaensis	XP_015935618.1
ARHOMBI_DN4083_c0_g1_i1_6	6,46E-123	352	AFK45884.1	unknown	Lotus japonicus	XP_007134165.1
ARHOMBI_DN5446_c0_g1_i1_5	0	746	XP_018815635.1	basic leucine zipper and W2 domain-containing protein 2-like	Juglans regia	OAY34237.1
ARUBRA_DN8529_c0_g1_i1_1	1,08E-92	298	XP_018815316.1	coatomer subunit alpha-1	Juglans regia	XP_018840319.1
ARUBRA_DN4933_c0_g1_i2_4	9,90E-159	451	XP_018834440.1	40S ribosomal protein S2-4-like	Juglans regia	XP_018837209.1
ARUBRA_DN3170_c0_g1_i1_4	0	597	XP_018848058.1	phosphoglucan phosphatase DSP4, amyloplastic-like isoform X2	Juglans regia	XP_018848057.1
ARUBRA_DN4127_c0_g2_i1_4	0	712	XP_018851483.1	isocitrate dehydrogenase NAD catalytic subunit 5, mitochondrial isoform X1	Juglans regia	ONI03044.1
ARUBRA_DN6378_c0_g1_i1_2	0	750	XP_018851707.1	phospholipase D alpha 1	Juglans regia	XP_018850650.1
ARUBRA_DN2301_c0_g1_i1_1	1,33E-50	172	ONI33944.1	hypothetical protein PRUPE_1G455000	Prunus persica	XP_020410106.1
ARUBRA_DN16958_c0_g1_i1_6	4,74E-137	401	OIV94179.1	hypothetical protein TanjilG_13796	Lupinus angustifolius	XP_019421126.1
ARUBRA_DN256_c0_g2_i1_1	0	654	XP_018811933.1	plant UBX domain-containing protein 8	Juglans regia	XP_018828814.1
ARHOMBI_DN2281_c0_g1_i1_1	5,06E-152	427	XP_018814564.1	soluble inorganic pyrophosphatase 1	Juglans regia	XP_018834262.1
ARHOMBI_DN6231_c0_g2_i2_2	3,62E-145	414	XP_018850680.1	tropinone reductase homolog At5g06060-like	Juglans regia	ONI00132.1
ARHOMBI_DN835_c0_g1_i1_4	4,29E-85	251	ACU20582.1	unknown	Glycine max	XP_018851128.1
ARUBRA_DN4252_c0_g2_i2_4	0	2005	XP_018845797.1	glycine dehydrogenase (decarboxylating), mitochondrial	Juglans regia	XP_008393646.1
ARHOMBI_DN11662_c0_g1_i1_4	2,35E-34	127	XP_018835052.1	subtilisin-like protease SBT1.4	Juglans regia	XP_015939299.1
ARUBRA_DN21714_c0_g1_i1_3	9,15E-62	191	XP_014507151.1	60S ribosomal protein L34	Vigna radiata var. radiata	KOM31413.1
ARHOMBI_DN4867_c0_g1_i1_5	5,79E-163	458	XP_018845601.1	proteasome subunit beta type-6	Juglans regia	KDP33416.1
ARHOMBI_DN5538_c0_g1_i1_5	3,53E-117	343	XP_018816103.1	succinate dehydrogenase subunit 5, mitochondrial-like	Juglans regia	XP_018822424.1
ARUBRA_DN9182_c0_g1_i1_2	8,08E-53	179	XP_018814451.1	heat shock cognate 70 kDa protein-like	Juglans regia	XP_018814450.1
ARUBRA_DN4826_c0_g1_i1_4	1,33E-48	162	XP_018811251.1	hemiasterlin resistant protein 1	Juglans regia	XP_018826924.1
ARHOMBI_DN21064_c0_g1_i1_2	2,16E-60	196	XP_018815658.1	hexokinase-1-like	Juglans regia	KDP33801.1
ARHOMBI_DN50_c0_g3_i1_6	0	544	XP_018849591.1	dolichyl-diphosphooligosaccharide--protein glycosyltransferase 48 kDa subunit-like isoform X2	Juglans regia	ONH91270.1
ARUBRA_DN4961_c0_g1_i2_4	1,98E-38	130	XP_017185883.1	serine carboxypeptidase-like 40, partial	Malus domestica	XP_009368057.1
ARHOMBI_DN5474_c0_g1_i1_5	1,23E-05	49,7	XP_018859442.1	phosphoprotein ECPP44-like	Juglans regia	AAL32035.1
ARUBRA_DN3097_c0_g2_i1_2	2,04E-121	363	OAY39428.1	hypothetical protein MANES_10G094100	Manihot esculenta	XP_018826868.1
ARUBRA_DN7003_c0_g1_i1_4	1,54E-130	378	XP_018835563.1	protein PMR5-like	Juglans regia	XP_008385046.2
ARHOMBI_DN20442_c0_g1_i1_1	2,36E-32	122	XP_018828540.1	protein NETWORKED 4A-like isoform X4	Juglans regia	XP_018828538.1

ARUBRA_DN3105_c0_g1_i1_1	3,15E-82	249	XP_018821649.1	gamma carbonic anhydrase 1, mitochondrial-like	Juglans regia	XP_018854607.1
ARUBRA_DN18186_c0_g1_i1_5	2,02E-36	126	XP_018836212.1	26S proteasome non-ATPase regulatory subunit 10	Juglans regia	XP_007157422.1
ARUBRA_DN3014_c0_g1_i1_2	0	1369	XP_018843664.1	4-alpha-glucanotransferase DPE2 isoform X2	Juglans regia	XP_018843661.1
ARUBRA_DN12532_c0_g1_i1_5	2,33E-34	122	XP_018847246.1	desiccation-related protein PCC13-62-like	Juglans regia	XP_018855451.1
ARHOMBI_DN5429_c0_g1_i1_2	1,54E-150	426	XP_018826002.1	endo-1,3;1,4-beta-D-glucanase isoform X2	Juglans regia	XP_018826001.1
ARHOMBI_DN16845_c0_g1_i1_5	1,35E-117	353	XP_018846323.1	clathrin heavy chain 1-like isoform X1	Juglans regia	XP_018846324.1
ARUBRA_DN720_c0_g1_i1_6	4,94E-126	363	XP_018845180.1	40S ribosomal protein S9-2-like	Juglans regia	XP_018826572.1
ARHOMBI_DN17235_c0_g1_i1_3	0	645	OAY57384.1	hypothetical protein MANES_02G093200	Manihot esculenta	XP_015964026.1
ARUBRA_DN2328_c0_g2_i1_4	6,54E-46	149	XP_007161552.1	hypothetical protein PHAVU_001G079100g	Phaseolus vulgaris	ESW33546.1
ARUBRA_DN4073_c0_g1_i1_2	8,01E-172	486	XP_018821628.1	proteasome subunit alpha type-1-B-like	Juglans regia	XP_018845049.1
ARHOMBI_DN1161_c0_g1_i1_2	0	793	XP_018823588.1	transmembrane 9 superfamily member 8-like	Juglans regia	XP_018810860.1
ARHOMBI_DN22444_c0_g1_i1_1	0	513	XP_018826879.1	L-galactose dehydrogenase	Juglans regia	KDP35919.1
ARHOMBI_DN5200_c0_g2_i1_5	6,96E-114	331	KDP28201.1	hypothetical protein JCGZ_13972	Jatropha curcas	XP_012082823.1
ARHOMBI_DN19654_c0_g1_i1_2	1,18E-94	293	ONI01918.1	hypothetical protein PRUPE_6G167100	Prunus persica	XP_007204964.1
ARHOMBI_DN2987_c0_g1_i1_5	0	826	XP_018858191.1	citrate synthase, mitochondrial	Juglans regia	XP_018858196.1
ARHOMBI_DN6144_c0_g1_i1_4	0	771	XP_018819893.1	low-temperature-induced cysteine proteinase-like	Juglans regia	XP_018852257.1
ARUBRA_DN4862_c0_g1_i1_4	0	739	XP_008226131.1	phosphoglycerate kinase, cytosolic	Prunus mume	ONI11910.1
ARHOMBI_DN1127_c0_g2_i1_4	3,04E-125	367	XP_018821285.1	triose phosphate/phosphate translocator, non-green plastid, chloroplastic-like	Juglans regia	ONI08336.1
ARUBRA_DN17228_c1_g1_i1_1	1,34E-168	473	KHM98889.1	Proteasome subunit alpha type-2-A	Glycine soja	KRH75738.1
ARHOMBI_DN4125_c0_g1_i1_5	7,47E-81	241	XP_018847546.1	40S ribosomal protein S12-like	Juglans regia	XP_018853126.1
ARHOMBI_DN4270_c0_g1_i1_2	3,95E-75	230	XP_018816612.1	6-phosphogluconate dehydrogenase, decarboxylating 3-like	Juglans regia	AFK47475.1
ARUBRA_DN4844_c0_g1_i1_2	1,97E-42	142	OAY45736.1	hypothetical protein MANES_07G087100	Manihot esculenta	XP_018842587.1
ARUBRA_DN13846_c0_g1_i1_5	3,94E-45	145	AAK55849.1	AF266465_1 aspartic protease, partial	Manihot esculenta	XP_018836309.1
ARUBRA_DN5052_c0_g2_i1_1	2,21E-61	189	ONH99801.1	hypothetical protein PRUPE_6G051200	Prunus persica	XP_018819193.1
ARUBRA_DN4025_c0_g2_i1_5	1,79E-90	266	XP_018836004.1	40S ribosomal protein S15a-1	Juglans regia	XP_018857112.1
ARUBRA_DN4599_c2_g1_i1_4	2,36E-103	305	XP_009372405.1	coatomer subunit zeta-2-like	Pyrus x bretschneideri	XP_009340672.1
ARUBRA_DN21659_c0_g1_i1_3	1,31E-83	251	XP_018850753.1	protein BOBBER 1	Juglans regia	KYP69897.1
ARHOMBI_DN17850_c0_g1_i1_6	8,70E-161	453	XP_018841293.1	nicotinamidase 1-like	Juglans regia	XP_018841294.1
ARUBRA_DN2529_c0_g2_i1_6	0	580	XP_020218346.1	26S proteasome non-ATPase regulatory subunit 4 homolog	Cajanus cajan	XP_020227721.1
ARHOMBI_DN6264_c0_g1_i5_2	0	717	XP_018841148.1	12-oxophytodienoate reductase 2-like	Juglans regia	OAY23951.1

ARHOMBI_DN5393_c0_g1_i1_1	0	543	XP_018850910.1	bifunctional epoxide hydrolase 2-like	Juglans regia	ONI17394.1
ARUBRA_DN3140_c0_g1_i1_5	1,84E-158	449	XP_018844947.1	20 kDa chaperonin, chloroplastic-like	Juglans regia	XP_018844949.1
ARHOMBI_DN5550_c0_g1_i1_2	1,73E-168	474	KDP44884.1	hypothetical protein JCGZ_01384	Jatropha curcas	XP_012088585.1
ARUBRA_DN3107_c0_g1_i1_6	1,09E-172	486	OAY25879.1	hypothetical protein MANES_16G002900	Manihot esculenta	KYP62724.1
ARUBRA_DN4381_c0_g1_i1_5	0	830	XP_018849108.1	glutamate decarboxylase-like	Juglans regia	XP_018823508.1
ARUBRA_DN3552_c0_g1_i1_4	1,02E-101	303	XP_018837139.1	metacaspase-4-like, partial	Juglans regia	KDP27423.1
ARUBRA_DN3608_c0_g1_i1_6	3,06E-48	157	XP_008230471.1	putative phosphatidylglycerol/phosphatidylinositol transfer protein DDB_G0282179	Prunus mume	XP_018858667.1
ARHOMBI_DN17898_c0_g1_i1_3	0	550	XP_018837044.1	probable cytosolic oligopeptidase A	Juglans regia	XP_018850785.1
ARHOMBI_DN17178_c0_g1_i1_4	8,45E-104	315	OAY30441.1	hypothetical protein MANES_14G031100	Manihot esculenta	KDP22352.1
ARUBRA_DN4547_c0_g1_i1_6	1,97E-127	367	XP_018808145.1	uncharacterized protein LOC108981433 isoform X2	Juglans regia	XP_018808144.1
ARHOMBI_DN486_c0_g1_i1_1	6,53E-08	52,4	KYP42200.1	Putative DNA repair protein RAD23-3	Cajanus cajan	XP_020239676.1
ARHOMBI_DN5664_c0_g1_i1_5	3,43E-39	135	AGW21340.1	lipid transfer protein	Prunus kansuensis	AGH27866.1
ARHOMBI_DN7133_c0_g1_i1_1	2,51E-94	298	OAY37891.1	hypothetical protein MANES_11G137000	Manihot esculenta	KDP44163.1
ARHOMBI_DN5590_c0_g1_i1_4	9,19E-177	494	XP_018814321.1	succinate dehydrogenase ubiquinone iron-sulfur subunit 2, mitochondrial-like	Juglans regia	XP_018844747.1
ARHOMBI_DN3067_c0_g1_i1_1	9,80E-174	487	XP_018812968.1	14-3-3-like protein GF14 kappa	Juglans regia	OAY23218.1
ARUBRA_DN3632_c0_g1_i1_6	2,28E-143	415	XP_018806799.1	glycerol kinase	Juglans regia	ONI16317.1
ARUBRA_DN5040_c0_g4_i8_2	2,70E-139	396	XP_018841297.1	tropinone reductase homolog At5g06060-like	Juglans regia	XP_008221978.1
ARHOMBI_DN9099_c0_g1_i1_1	5,30E-66	204	XP_018807016.1	putative methyltransferase DDB_G0268948	Juglans regia	XP_018817184.1
ARHOMBI_DN1783_c0_g2_i1_3	3,37E-84	254	XP_018851375.1	probable protein phosphatase 2C 11	Juglans regia	XP_018857896.1
ARUBRA_DN5025_c0_g1_i3_3	0	1696	XP_018845641.1	plasma membrane ATPase 4-like	Juglans regia	XP_018854375.1
ARUBRA_DN1938_c0_g1_i1_1	3,29E-159	449	XP_018829633.1	proteasome subunit alpha type-7	Juglans regia	XP_018809418.1
ARHOMBI_DN16492_c0_g1_i1_4	0	1021	XP_018812045.1	L-ascorbate oxidase homolog	Juglans regia	XP_018838329.1
ARUBRA_DN5418_c0_g1_i1_4	0	818	XP_018814240.1	citrate synthase, glyoxysomal	Juglans regia	OAY57307.1
ARHOMBI_DN13783_c0_g1_i1_2	1,35E-20	83,2	KHN37210.1	Xylulose kinase	Glycine soja	XP_014631757.1
ARHOMBI_DN16013_c0_g1_i1_5	3,60E-79	253	XP_018851852.1	5-methyltetrahydropteroyltriglutamate--homocysteine methyltransferase-like	Juglans regia	ONH94299.1
ARUBRA_DN19786_c0_g1_i1_6	9,61E-63	201	XP_018837782.1	3-hydroxyisobutyryl-CoA hydrolase-like protein 5 isoform X2	Juglans regia	XP_018837783.1
ARUBRA_DN5795_c0_g1_i1_1	4,10E-164	474	XP_018805409.1	dihydrolipoyl dehydrogenase 2, chloroplastic-like	Juglans regia	ONI28088.1
ARHOMBI_DN6222_c0_g1_i1_3	2,58E-104	304	XP_018827597.1	monothiol glutaredoxin-S15, mitochondrial	Juglans regia	XP_018827598.1
ARHOMBI_DN3998_c0_g1_i1_1	2,00E-168	474	XP_018834872.1	cytochrome b-c1 complex subunit Rieske-4, mitochondrial-like isoform X2	Juglans regia	XP_018834050.1

ARUBRA_DN25017_c0_g1_i1_1	1,23E-52	172	XP_018813802.1	aldose 1-epimerase-like	Juglans regia	XP_009347058.1
ARUBRA_DN2627_c0_g1_i1_2	0	910	XP_018836913.1	serine carboxypeptidase-like	Juglans regia	ONI23789.1
ARHOMBI_DN2324_c0_g1_i1_5	3,67E-90	276	XP_018809339.1	serine/threonine-protein phosphatase 2A 65 kDa regulatory subunit A beta isoform-like	Juglans regia	XP_018829949.1
ARUBRA_DN6463_c0_g1_i1_2	8,46E-68	210	XP_018832580.1	uncharacterized protein LOC109000202 isoform X1	Juglans regia	XP_018832581.1
ARUBRA_DN18383_c0_g1_i1_3	1,83E-63	204	XP_018834911.1	transmembrane protein 33 homolog	Juglans regia	XP_018834117.1
ARUBRA_DN4889_c0_g1_i1_6	0	695	XP_020226595.1	cell division cycle protein 48 homolog	Cajanus cajan	XP_020226595.1
ARHOMBI_DN6134_c0_g1_i1_6	0	560	XP_018819193.1	cinnamoyl-CoA reductase 1	Juglans regia	XP_009369570.1
ARHOMBI_DN3483_c0_g3_i1_5	4,24E-129	379	XP_018814398.1	protein RETICULATA-RELATED 3, chloroplastic-like	Juglans regia	XP_008245279.1
ARUBRA_DN11125_c0_g1_i1_4	1,36E-34	121	XP_018819326.1	V-type proton ATPase subunit E2 isoform X1	Juglans regia	XP_018819328.1
ARHOMBI_DN13455_c0_g1_i1_1	1,91E-53	167	XP_018821252.1	glutaredoxin-like	Juglans regia	XP_018837755.1
ARHOMBI_DN5719_c0_g1_i1_2	3,77E-85	252	XP_008236476.1	40S ribosomal protein S16-like	Prunus mume	OAY45072.1
ARHOMBI_DN25944_c0_g1_i1_6	6,60E-41	140	XP_018854134.1	malate dehydrogenase-like isoform X2	Juglans regia	KDP28817.1
ARHOMBI_DN1498_c0_g1_i1_1	5,14E-39	132	XP_018847006.1	desiccation protectant protein Lea14 homolog isoform X2	Juglans regia	XP_018830082.1
ARUBRA_DN3905_c0_g1_i1_4	1,03E-159	453	XP_018814780.1	3-oxoacyl-[acyl-carrier-protein reductase 4 isoform X2	Juglans regia	XP_018814779.1
ARUBRA_DN8419_c0_g1_i1_3	0	598	XP_004489356.1	glucose-6-phosphate isomerase, cytosolic	Cicer arietinum	XP_004489357.1
ARUBRA_DN11134_c0_g1_i1_6	1,20E-132	382	XP_018818486.1	erlin-2-B	Juglans regia	XP_018818487.1
ARHOMBI_DN2322_c0_g1_i1_2	0	581	XP_018829441.1	putative glucose-6-phosphate 1-epimerase	Juglans regia	ONH96870.1
ARHOMBI_DN20297_c0_g1_i1_1	9,85E-166	470	XP_018840356.1	glyceraldehyde-3-phosphate dehydrogenase GAPCP2, chloroplastic-like isoform X3	Juglans regia	XP_018840355.1
ARUBRA_DN4829_c0_g2_i2_4	5,08E-129	365	XP_018850586.1	60S ribosomal protein L18a	Juglans regia	XP_018819718.1
ARUBRA_DN18092_c0_g1_i1_5	2,06E-84	253	XP_018809253.1	alcohol dehydrogenase-like	Juglans regia	XP_018839888.1
ARHOMBI_DN10619_c0_g1_i1_4	1,94E-107	330	OAY25507.1	hypothetical protein MANES_17G100400	Manihot esculenta	OAY29681.1
ARHOMBI_DN3191_c0_g1_i1_2	1,24E-119	345	XP_018805410.1	GTP-binding protein SAR1A-like	Juglans regia	XP_018805411.1
ARUBRA_DN19085_c0_g1_i1_1	0	817	XP_018840650.1	transmembrane 9 superfamily member 11 isoform X1	Juglans regia	XP_018840651.1
ARHOMBI_DN5770_c0_g2_i2_6	0	511	OAY46226.1	hypothetical protein MANES_07G127000	Manihot esculenta	XP_018806389.1
ARHOMBI_DN3157_c0_g1_i1_6	6,66E-145	414	XP_018825271.1	enoyl-[acyl-carrier-protein reductase NADH , chloroplastic-like	Juglans regia	AIS93131.1
ARUBRA_DN21700_c0_g1_i1_4	9,08E-138	392	XP_018831854.1	CBS domain-containing protein CBSX3, mitochondrial-like	Juglans regia	XP_018815110.1
ARHOMBI_DN16801_c0_g1_i1_1	7,66E-116	356	XP_018845862.1	staphylococcal nuclease domain-containing protein 1-like	Juglans regia	XP_018832881.1
ARUBRA_DN192_c0_g1_i1_1	3,45E-120	351	XP_018833037.1	ADP,ATP carrier protein 3, mitochondrial-like	Juglans regia	XP_018833038.1

ARHOMBI_DN24575_c0_g1_i1_1	6,41E-54	182	KRH39503.1	hypothetical protein GLYMA_09G202100	Glycine max	XP_003534255.2
ARHOMBI_DN5209_c2_g1_i1_4	7,04E-158	470	OAY37891.1	hypothetical protein MANES_11G137000	Manihot esculenta	KDP44163.1
ARUBRA_DN1890_c0_g2_i1_3	2,46E-137	395	XP_018827031.1	protein disulfide isomerase-like 2-3 isoform X1	Juglans regia	XP_018827031.1
ARUBRA_DN3867_c0_g1_i1_1	2,28E-152	434	XP_018823879.1	translocon-associated protein subunit alpha-like	Juglans regia	XP_018841445.1
ARHOMBI_DN23168_c0_g1_i1_4	4,67E-105	306	XP_018824887.1	phosphoserine phosphatase, chloroplastic isoform X1	Juglans regia	XP_018824888.1
ARHOMBI_DN3469_c0_g1_i1_3	0	602	XP_018848760.1	long chain acyl-CoA synthetase 8	Juglans regia	XP_018848761.1
ARUBRA_DN4948_c0_g2_i1_5	1,98E-81	243	XP_018833220.1	60S ribosomal protein L32-1-like	Juglans regia	XP_018833221.1
ARUBRA_DN3297_c0_g1_i1_1	3,04E-93	287	XP_018835196.1	probable trehalase	Juglans regia	XP_008239192.1
ARUBRA_DN2696_c0_g1_i1_3	6,83E-72	217	ONI00303.1	hypothetical protein PRUPE_6G081100	Prunus persica	XP_008223244.1
ARHOMBI_DN15716_c0_g1_i1_4	8,21E-57	189	XP_018818477.1	putative G3BP-like protein	Juglans regia	XP_019421899.1
ARHOMBI_DN4884_c0_g1_i1_2	0	619	XP_018815210.1	UDP-glucuronic acid decarboxylase 2-like	Juglans regia	XP_008369016.1
ARUBRA_DN20045_c0_g1_i1_4	1,21E-102	300	XP_018827351.1	photosystem II reaction center PSB28 protein, chloroplastic	Juglans regia	XP_008235587.1
ARHOMBI_DN1594_c0_g1_i1_2	2,23E-141	420	OAY36779.1	hypothetical protein MANES_11G047600	Manihot esculenta	KDP37205.1
ARUBRA_DN4593_c0_g2_i1_4	7,86E-81	243	XP_018819687.1	40S ribosomal protein S14-3-like	Juglans regia	XP_018831277.1
ARUBRA_DN4903_c0_g1_i1_3	6,09E-79	236	XP_018813383.1	60S ribosomal protein L27	Juglans regia	XP_018827822.1
ARHOMBI_DN18322_c0_g1_i1_3	4,24E-49	158	AFK46078.1	unknown	Lotus japonicus	XP_018837851.1
ARUBRA_DN11129_c0_g1_i1_5	4,49E-61	189	XP_008365972.1	AB hydrolase superfamily protein YfhM-like	Malus domestica	XP_009336925.1
ARUBRA_DN24080_c0_g1_i1_2	0	569	XP_008340410.1	pyruvate kinase, cytosolic isozyme	Malus domestica	XP_008340411.1
ARUBRA_DN10280_c0_g1_i1_1	1,85E-56	179	XP_018809757.1	calcyclin-binding protein-like	Juglans regia	XP_018819738.1
ARUBRA_DN19519_c0_g1_i1_5	5,54E-69	207	XP_018857103.1	tubulin-folding cofactor B-like, partial	Juglans regia	XP_018835787.1
ARUBRA_DN5259_c0_g1_i1_6	3,28E-179	512	XP_018813115.1	fasciclin-like arabinogalactan protein 1	Juglans regia	KDP39270.1
ARUBRA_DN4393_c0_g2_i1_4	0	972	XP_018844988.1	nicotinate phosphoribosyltransferase 1	Juglans regia	XP_008241476.1
ARHOMBI_DN5839_c1_g1_i1_5	5,26E-72	218	XP_008224314.1	40S ribosomal protein S18	Prunus mume	XP_008390888.1
ARUBRA_DN10773_c0_g1_i1_4	1,34E-113	329	XP_018818519.1	probable 6-phosphogluconolactonase 1 isoform X1	Juglans regia	XP_018818520.1
ARUBRA_DN21519_c0_g1_i1_5	1,36E-109	318	XP_008227948.1	60S ribosomal protein L7-2	Prunus mume	KYP44357.1
ARUBRA_DN6882_c0_g1_i1_3	8,80E-79	237	XP_018836212.1	26S proteasome non-ATPase regulatory subunit 10	Juglans regia	XP_015965524.1
ARHOMBI_DN22543_c0_g1_i1_3	1,02E-71	214	XP_018842089.1	basic blue protein-like	Juglans regia	XP_008218855.1
ARUBRA_DN9648_c0_g1_i1_1	9,71E-74	228	XP_018832799.1	uncharacterized protein At3g03773-like	Juglans regia	XP_008237430.1
ARUBRA_DN5039_c0_g1_i1_2	0	639	XP_018856560.1	2-alkenal reductase (NADP(+)-dependent)-like	Juglans regia	XP_018829693.1

ARHOMBI_DN18646_c0_g1_i1_5	1,12E-43	143	XP_018830962.1	uncharacterized protein LOC108998731 isoform X1	Juglans regia	XP_018830963.1
ARHOMBI_DN4447_c0_g1_i1_4	0	576	XP_018849296.1	succinate--CoA ligase ADP-forming subunit alpha-1, mitochondrial	Juglans regia	XP_009355925.1
ARUBRA_DN2978_c0_g1_i1_6	2,03E-103	300	XP_018847314.1	probable NADH dehydrogenase ubiquinone 1 alpha subcomplex subunit 5, mitochondrial	Juglans regia	XP_018847315.1
ARUBRA_DN7999_c0_g1_i1_2	0	556	XP_018837266.1	T-complex protein 1 subunit gamma isoform X1	Juglans regia	XP_018837267.1
ARHOMBI_DN5214_c0_g1_i1_4	1,05E-135	385	XP_008238908.1	protein P21-like	Prunus mume	XP_018828872.1
ARUBRA_DN4195_c0_g1_i1_4	1,97E-99	291	XP_018808968.1	40S ribosomal protein S11	Juglans regia	XP_018822277.1
ARHOMBI_DN6230_c0_g2_i3_1	4,10E-76	230	XP_018835344.1	40S ribosomal protein S17-like	Juglans regia	XP_018855694.1
ARUBRA_DN8665_c0_g1_i1_5	1,22E-123	374	GAU11024.1	hypothetical protein TSUD_113170	Trifolium subterraneum	GAU11024.1
ARHOMBI_DN19852_c0_g1_i1_3	6,88E-49	167	XP_018809366.1	probable methyltransferase PMT18	Juglans regia	XP_018844923.1
ARUBRA_DN7713_c0_g2_i1_1	1,34E-151	429	KYP53345.1	Prohibitin-2	Cajanus cajan	XP_020229475.1
ARHOMBI_DN15828_c0_g1_i1_6	3,08E-142	409	XP_017180251.1	DNA damage-inducible protein 1-like	Malus domestica	XP_008384617.1
ARHOMBI_DN965_c0_g1_i1_1	4,84E-111	321	XP_018841912.1	coatomer subunit zeta-1-like	Juglans regia	XP_012072050.1
ARHOMBI_DN4105_c0_g1_i1_1	3,94E-175	491	KDP41387.1	hypothetical protein JCGZ_15794	Jatropha curcas	XP_012067879.1
ARUBRA_DN6657_c0_g1_i1_6	2,81E-46	162	XP_018816364.1	dolichyl-diphosphooligosaccharide--protein glycosyltransferase subunit 2-like	Juglans regia	XP_018827273.1
ARUBRA_DN13405_c0_g1_i1_1	1,19E-41	145	XP_008365420.1	oxalate--CoA ligase-like	Malus domestica	KDP25453.1
ARUBRA_DN1195_c0_g1_i1_2	8,28E-111	322	XP_004492839.1	putative 4-hydroxy-4-methyl-2-oxoglutarate aldolase 2	Cicer arietinum	GAU49703.1
ARUBRA_DN2242_c0_g1_i1_5	3,09E-148	443	XP_018849376.1	phosphoenolpyruvate carboxylase, housekeeping isozyme	Juglans regia	XP_018822521.1
ARHOMBI_DN3366_c0_g1_i1_4	7,26E-165	466	XP_018820853.1	mitochondrial outer membrane protein porin 4	Juglans regia	XP_018831181.1
ARUBRA_DN6838_c0_g1_i1_3	2,73E-55	194	XP_018838202.1	acetyl-coenzyme A carboxylase carboxyl transferase subunit alpha, chloroplastic-like	Juglans regia	XP_018833363.1
ARUBRA_DN10378_c0_g1_i1_1	5,72E-60	196	XP_018818630.1	pyruvate kinase isozyme A, chloroplastic-like	Juglans regia	OIW13099.1
ARHOMBI_DN5914_c0_g1_i1_4	0	771	XP_018830218.1	GDP-mannose 3,5-epimerase 2	Juglans regia	XP_018826982.1
ARHOMBI_DN5134_c0_g1_i1_6	0	1020	KOM44158.1	hypothetical protein LR48_Vigan05g176300	Vigna angularis	BAT92001.1
ARUBRA_DN16002_c0_g1_i1_2	2,09E-29	110	XP_018857924.1	GDSL esterase/lipase At1g29670-like	Juglans regia	KEH36671.1
ARUBRA_DN6617_c0_g1_i1_2	3,19E-159	452	XP_018818800.1	probable 6-phosphogluconolactonase 4, chloroplastic	Juglans regia	XP_018842795.1
ARHOMBI_DN7393_c0_g1_i1_3	6,06E-147	413	KDP42463.1	hypothetical protein JCGZ_00260	Jatropha curcas	XP_012066701.1
ARHOMBI_DN1339_c0_g1_i1_3	8,81E-68	213	XP_018853171.1	probable protein phosphatase 2C 11	Juglans regia	XP_018835371.1
ARHOMBI_DN16218_c0_g1_i1_1	1,00E-32	124	ONI26240.1	hypothetical protein PRUPE_1G012100	Prunus persica	XP_007225123.1

ARUBRA_DN3982_c0_g2_i1_1	1,06E-172	510	XP_018842400.1	eukaryotic translation initiation factor 3 subunit C isoform X1	Juglans regia	XP_018842401.1
ARHOMBI_DN2364_c0_g1_i1_5	1,86E-106	314	XP_018821859.1	alpha-soluble NSF attachment protein 2-like	Juglans regia	KYP44939.1
ARHOMBI_DN5683_c0_g1_i2_5	0	733	ONI30885.1	hypothetical protein PRUPE_1G279500	Prunus persica	XP_007222547.1
ARUBRA_DN14982_c0_g1_i1_3	2,00E-76	240	OAY59825.1	hypothetical protein MANES_01G062800	Manihot esculenta	OAY30371.1
ARHOMBI_DN21103_c0_g1_i1_1	3,33E-110	325	XP_018824621.1	glucan endo-1,3-beta-glucosidase 14-like	Juglans regia	XP_018824593.1
ARUBRA_DN18762_c0_g1_i1_4	0	716	XP_018837262.1	protein FREE1	Juglans regia	KDP36892.1
ARUBRA_DN4063_c0_g1_i2_5	0	573	XP_018811219.1	eukaryotic translation initiation factor 2 subunit alpha homolog	Juglans regia	KDP42130.1
ARUBRA_DN11481_c0_g1_i1_6	0	719	XP_018827303.1	NADH dehydrogenase ubiquinone 1 alpha subcomplex subunit 9, mitochondrial	Juglans regia	ONH93127.1
ARUBRA_DN934_c0_g1_i1_3	2,37E-159	448	XP_018840891.1	proteasome subunit beta type-1	Juglans regia	OAY62404.1
ARUBRA_DN3268_c0_g1_i1_2	0	613	OAY48391.1	hypothetical protein MANES_06G155200	Manihot esculenta	KDP22191.1
ARHOMBI_DN2479_c0_g1_i1_1	1,13E-50	165	ONH97761.1	hypothetical protein PRUPE_7G208200	Prunus persica	XP_007202368.1
ARUBRA_DN1490_c0_g1_i1_3	0	677	ONI29891.1	hypothetical protein PRUPE_1G220200	Prunus persica	XP_007223276.1
ARUBRA_DN20284_c0_g1_i1_5	5,96E-89	281	XP_018845862.1	staphylococcal nuclease domain-containing protein 1-like	Juglans regia	XP_004503032.1
ARUBRA_DN12380_c0_g1_i1_5	3,89E-101	304	AAW65140.1	3-dehydroquinate dehydratase/shikimate 5-dehydrogenase, partial	Juglans regia	XP_018840587.1
ARHOMBI_DN16116_c0_g1_i1_3	0	556	XP_018842494.1	uncharacterized protein LOC109007319	Juglans regia	XP_008235146.1
ARHOMBI_DN6092_c0_g1_i1_1	0	555	KDP23623.1	hypothetical protein JCGZ_23456	Jatropha curcas	OAY44160.1
ARHOMBI_DN15878_c0_g1_i1_3	2,17E-69	220	XP_018814628.1	putative G3BP-like protein	Juglans regia	XP_018816596.1
ARHOMBI_DN2833_c0_g1_i1_1	4,90E-162	464	XP_018831343.1	probable ADP-ribosylation factor GTPase-activating protein AGD6	Juglans regia	KRH32301.1
ARUBRA_DN16146_c0_g1_i1_4	1,08E-112	327	XP_018836742.1	mitochondrial import receptor subunit TOM20-like	Juglans regia	XP_018852624.1
ARHOMBI_DN10766_c0_g1_i1_6	4,84E-52	169	XP_018853288.1	triphosphate tunel metalloenzyme 3-like	Juglans regia	XP_018816133.1
ARHOMBI_DN5588_c0_g1_i1_3	3,54E-119	350	XP_018821858.1	ferritin-3, chloroplastic	Juglans regia	OAY46317.1
ARUBRA_DN22820_c0_g1_i1_3	2,31E-40	135	XP_016651326.1	putative gamma-glutamylcyclotransferase At3g02910	Prunus mume	XP_009346482.1
ARHOMBI_DN5983_c0_g1_i1_5	0	701	XP_018823000.1	formate dehydrogenase, mitochondrial	Juglans regia	XP_018828647.1
ARUBRA_DN20709_c0_g1_i1_5	2,00E-104	320	XP_018813437.1	probable cytosolic oligopeptidase A	Juglans regia	XP_018837044.1
ARUBRA_DN1983_c0_g1_i1_4	8,97E-121	345	XP_018833647.1	ER membrane protein complex subunit 4-like	Juglans regia	XP_008220646.1
ARHOMBI_DN3005_c0_g2_i1_6	1,80E-82	246	KYP71828.1	40S ribosomal protein S24-2	Cajanus cajan	XP_020210999.1
ARHOMBI_DN5420_c0_g1_i1_2	0	870	XP_018839280.1	26S protease regulatory subunit 7A	Juglans regia	XP_018859577.1

ARUBRA_DN3944_c0_g1_i1_3	5,69E-128	377	XP_018815040.1	dihydrolipoyllysine-residue acetyltransferase component 4 of pyruvate dehydrogenase complex, chloroplastic-like	Juglans regia	XP_018825347.1
ARHOMBI_DN18815_c0_g1_i1_6	3,82E-73	224	KDP21455.1	hypothetical protein JCGZ_21926	Jatropha curcas	XP_012092235.1
ARHOMBI_DN5059_c0_g1_i1_5	8,58E-153	461	XP_018835062.1	heat shock 70 kDa protein 17	Juglans regia	ONI17954.1
ARHOMBI_DN5953_c0_g1_i1_4	0	1040	XP_018817793.1	polyadenylate-binding protein 8-like	Juglans regia	XP_016179797.1
ARUBRA_DN193_c0_g1_i1_1	7,29E-78	235	KDP29235.1	hypothetical protein JCGZ_16624	Jatropha curcas	XP_012082538.1
ARUBRA_DN7445_c0_g1_i1_4	6,56E-93	273	XP_018822137.1	uncharacterized protein LOC108992124	Juglans regia	XP_007199293.2
ARHOMBI_DN18779_c0_g1_i1_3	2,83E-175	496	XP_018812652.1	heterogeneous nuclear ribonucleoprotein 1-like	Juglans regia	XP_018812652.1
ARUBRA_DN19198_c0_g1_i1_2	1,02E-92	291	XP_018849887.1	heat shock protein 90-6, mitochondrial isoform X2	Juglans regia	XP_018849886.1
ARHOMBI_DN5769_c0_g1_i1_5	4,28E-143	404	XP_018860302.1	60S ribosomal protein L13a-4	Juglans regia	XP_018860308.1
ARHOMBI_DN18804_c0_g1_i1_4	6,21E-65	210	XP_008394163.1	UDP-sugar pyrophosphorylase	Malus domestica	XP_009355829.1
ARHOMBI_DN7_c0_g2_i1_2	6,27E-71	226	OAY59318.1	hypothetical protein MANES_01G023200	Manihot esculenta	KDP20053.1
ARUBRA_DN3923_c0_g1_i1_5	5,07E-39	132	XP_018821552.1	uncharacterized protein LOC108991675 isoform X1	Juglans regia	XP_018821553.1
ARHOMBI_DN6177_c0_g2_i2_3	2,36E-20	85,5	CAC39160.1	putative LEA III protein isoform 1	Corylus avellana	XP_008391284.1
ARHOMBI_DN24695_c0_g1_i1_4						
ARHOMBI_DN2476_c0_g1_i1_4	4,40E-165	474	XP_018838281.1	V-type proton ATPase catalytic subunit A	Juglans regia	OAY48691.1
ARHOMBI_DN22511_c0_g1_i1_1	4,79E-105	312	XP_018851523.1	mitogen-activated protein kinase 9-like isoform X1	Juglans regia	XP_018851524.1
ARHOMBI_DN3114_c0_g1_i1_1	0	842	XP_018840407.1	protein transport protein Sec61 subunit alpha-like	Juglans regia	XP_018840408.1
ARUBRA_DN16322_c0_g1_i1_4	0	640	XP_008372715.1	arginase 1, mitochondrial	Malus domestica	XP_018505555.1
ARUBRA_DN3916_c0_g1_i1_5	0	538	XP_018815587.1	elongation factor 2	Juglans regia	XP_018815588.1
ARUBRA_DN4811_c0_g2_i1_4	4,70E-135	388	XP_018819317.1	proactivator polypeptide-like 1	Juglans regia	XP_018819318.1
ARUBRA_DN18543_c0_g1_i1_6	1,92E-80	260	XP_018850062.1	acyl-CoA-binding domain-containing protein 4	Juglans regia	ONI04458.1
ARHOMBI_DN3540_c0_g1_i1_2	4,63E-144	408	KRH29738.1	hypothetical protein GLYMA_11G135400	Glycine max	XP_003537971.1
ARUBRA_DN478_c0_g1_i1_2	4,12E-130	371	XP_018844456.1	peptide methionine sulfoxide reductase B5-like	Juglans regia	XP_018855277.1
ARUBRA_DN20269_c0_g1_i1_6	5,51E-57	192	OAY52464.1	hypothetical protein MANES_04G085400	Manihot esculenta	XP_018815316.1
ARUBRA_DN20878_c0_g1_i1_1	0	647	XP_018807321.1	aconitate hydratase 1	Juglans regia	KDP23953.1
ARHOMBI_DN912_c0_g1_i1_6	0	736	XP_018828219.1	coatomer subunit beta-1	Juglans regia	XP_018843747.1
ARUBRA_DN11488_c0_g1_i1_6	1,11E-72	228	XP_004488376.1	enolase 1, chloroplastic	Cicer arietinum	KEH38028.1
ARUBRA_DN19442_c0_g1_i1_2	2,38E-56	190	XP_018832184.1	uncharacterized protein LOC108999736	Juglans regia	KHN06535.1
ARUBRA_DN4301_c0_g1_i1_3	1,26E-55	178	XP_018858854.1	auxin-repressed 12.5 kDa protein-like	Juglans regia	XP_018811601.1

ARUBRA_DN16241_c0_g1_i1_5	1,37E-89	269	XP_018834886.1	translocon-associated protein subunit beta-like isoform X1	Juglans regia	XP_018834081.1
ARUBRA_DN6197_c0_g1_i1_1	2,66E-52	167	XP_008386773.1	pectinesterase-like, partial	Malus domestica	XP_018824905.1
ARUBRA_DN5359_c0_g1_i1_3	0	1179	XP_018834612.1	formate--tetrahydrofolate ligase	Juglans regia	XP_008227769.1
ARUBRA_DN20048_c0_g1_i1_1	5,98E-38	133	XP_018857524.1	UDP-glucose 4-epimerase GEPI48-like	Juglans regia	XP_004511515.1
ARHOMBI_DN6095_c0_g2_i1_5	0	669	XP_018852657.1	dnaJ protein homolog	Juglans regia	XP_018805733.1
ARUBRA_DN4711_c0_g2_i1_6	1,38E-131	377	XP_008345452.1	actin	Malus domestica	KYP68187.1
ARUBRA_DN2566_c0_g2_i1_5	0	1183	XP_018805213.1	alpha-glucan water dikinase, chloroplastic isoform X1	Juglans regia	XP_018805214.1
ARHOMBI_DN6176_c1_g1_i1_5	0	546	XP_018828606.1	peroxidase 4-like	Juglans regia	XP_020537678.1
ARUBRA_DN413_c0_g1_i1_3	2,91E-41	142	XP_018841575.1	uncharacterized protein At2g17340-like	Juglans regia	XP_018841576.1
ARUBRA_DN16443_c0_g1_i1_3	3,93E-71	214	XP_020968128.1	clathrin heavy chain 2-like	Arachis ipaensis	XP_020968129.1
ARHOMBI_DN15325_c0_g1_i1_4	2,48E-56	181	XP_018836402.1	mitochondrial intermembrane space import and assembly protein 40	Juglans regia	KDP40464.1
ARHOMBI_DN5371_c0_g1_i1_2	5,86E-63	195	OAY53712.1	hypothetical protein MANES_03G017600	Manihot esculenta	XP_018834799.1
ARUBRA_DN4439_c0_g1_i1_3	2,86E-54	174	XP_008232650.1	60S ribosomal protein L22-3	Prunus mume	ONI22680.1
ARHOMBI_DN24520_c0_g1_i1_6	2,24E-27	107	KDP26565.1	hypothetical protein JCGZ_17723	Jatropha curcas	XP_012085348.1
ARHOMBI_DN900_c0_g1_i1_2	0	544	XP_018838640.1	short-chain dehydrogenase TIC 32, chloroplastic-like	Juglans regia	ONI25356.1
ARUBRA_DN2910_c0_g1_i1_5	1,27E-137	394	XP_008231080.1	60S ribosomal protein L7a isoform X2	Prunus mume	ONI19998.1
ARHOMBI_DN5383_c0_g1_i1_2	8,48E-82	253	XP_018847079.1	LL-diaminopimelate aminotransferase, chloroplastic-like	Juglans regia	XP_008382390.1
ARUBRA_DN1777_c0_g1_i1_3	8,08E-34	118	XP_018843076.1	calvin cycle protein CP12-3, chloroplastic	Juglans regia	KDP36727.1
ARHOMBI_DN4470_c0_g1_i1_6	0	516	KDP29239.1	hypothetical protein JCGZ_16628	Jatropha curcas	XP_012082542.1
ARUBRA_DN22163_c0_g1_i1_3	1,15E-112	335	CBM39040.1	unnamed protein product	Glycine max	CBM39131.1
ARHOMBI_DN13192_c0_g1_i1_2	2,88E-67	203	ACU23663.1	unknown, partial	Glycine max	OAY60567.1
ARHOMBI_DN54_c0_g1_i1_2	4,69E-141	398	KRH26302.1	hypothetical protein GLYMA_12G1662002, partial	Glycine max	XP_016194848.2
ARHOMBI_DN4900_c0_g1_i1_5	1,28E-68	214	XP_008246159.1	uncharacterized protein LOC103344327	Prunus mume	OAY43942.1
ARHOMBI_DN13072_c0_g1_i1_6	1,91E-48	160	XP_018820370.1	uridine 5'-monophosphate synthase-like	Juglans regia	XP_018817984.1
ARUBRA_DN14237_c0_g1_i1_6						
ARHOMBI_DN18431_c0_g1_i1_1	2,64E-159	472	OAY46048.1	hypothetical protein MANES_07G112300	Manihot esculenta	OAY46049.1
ARUBRA_DN8433_c0_g1_i1_6	5,56E-39	133	XP_018821673.1	maf-like protein DDB_G0281937 isoform X3	Juglans regia	XP_018821675.1
ARHOMBI_DN19091_c0_g1_i1_5	3,15E-126	360	XP_018819054.1	triphosphate tunel metalloenzyme 3-like	Juglans regia	XP_014496452.1
ARUBRA_DN18317_c0_g1_i1_5	5,07E-160	448	XP_018816905.1	uncharacterized protein LOC108988199 isoform X1	Juglans regia	XP_018816906.1

ARHOMBI_DN22440_c0_g1_i1_6	5,10E-73	231	OIV93350.1	hypothetical protein TanjilG_08763	Lupinus angustifolius	XP_019424386.1
ARHOMBI_DN8601_c0_g1_i1_4	1,62E-94	292	XP_018827273.1	dolichyl-diphosphooligosaccharide--protein glycosyltransferase subunit 2-like	Juglans regia	XP_018816364.1
ARHOMBI_DN25934_c0_g1_i1_3	1,48E-125	355	XP_008351114.1	ras-related protein RABB1c-like	Malus domestica	OAY41077.1
ARHOMBI_DN13008_c0_g1_i1_6	5,97E-54	167	ONI32563.1	hypothetical protein PRUPE_1G373500	Prunus persica	XP_018822047.1
ARUBRA_DN1845_c0_g1_i1_5	0	859	XP_018858095.1	ATP-citrate synthase beta chain protein 2	Juglans regia	XP_018858096.1
ARUBRA_DN4305_c0_g2_i1_6	0	830	XP_018858352.1	26S proteasome regulatory subunit 4 homolog B	Juglans regia	XP_018820323.1
ARUBRA_DN13152_c0_g1_i1_3	8,46E-32	120	XP_018853146.1	phosphoenolpyruvate carboxylase 4-like	Juglans regia	OAY31242.1
ARUBRA_DN3999_c0_g1_i1_6	2,76E-170	487	KYP46666.1	Pyruvate decarboxylase isozyme 2	Cajanus cajan	XP_020235713.1
ARUBRA_DN5726_c0_g1_i1_1	0	694	XP_018825075.1	peroxisomal acyl-coenzyme A oxidase 1	Juglans regia	XP_018819219.1
ARHOMBI_DN3987_c0_g1_i1_4	2,11E-152	446	KDP43753.1	hypothetical protein JCGZ_22380	Jatropha curcas	OAY60978.1
ARHOMBI_DN9641_c0_g1_i1_3	3,39E-180	513	XP_018848856.1	DEAD-box ATP-dependent RNA helicase 37-like	Juglans regia	XP_018848857.1
ARUBRA_DN16264_c0_g1_i1_5	0	775	XP_018822783.1	pyruvate dehydrogenase E1 component subunit alpha-3, chloroplastic	Juglans regia	OAY48935.1
ARHOMBI_DN15803_c0_g1_i1_2	5,91E-110	319	XP_018822169.1	gamma-glutamylcyclotransferase 2-3-like	Juglans regia	ONI34811.1
ARUBRA_DN1574_c0_g2_i1_2	1,72E-46	161	XP_009372651.1	pyrophosphate--fructose 6-phosphate 1-phosphotransferase subunit alpha	Pyrus x bretschneideri	XP_018832985.1
ARHOMBI_DN1369_c0_g1_i1_5	9,34E-31	113	OIW05856.1	hypothetical protein TanjilG_23642	Lupinus angustifolius	XP_019451066.1
ARUBRA_DN8743_c0_g1_i1_3	2,48E-51	160	GAU16914.1	hypothetical protein TSUD_36720, partial	Trifolium subterraneum	KDP33373.1
ARHOMBI_DN1764_c0_g1_i1_5	0	540	XP_018847046.1	asparagine synthetase glutamine-hydrolyzing 3-like	Juglans regia	XP_018847046.1
ARHOMBI_DN5253_c0_g2_i1_2	0	666	XP_018851637.1	fructose-1,6-bisphosphatase, cytosolic	Juglans regia	KDP42999.1
ARHOMBI_DN5557_c0_g1_i1_6	0	768	XP_008225692.1	UDP-arabinose 4-epimerase 1	Prunus mume	AGH25534.1
ARHOMBI_DN27310_c0_g1_i1_4	2,53E-36	129	XP_018837102.1	chromatin assembly factor 1 subunit A isoform X1	Juglans regia	XP_018837103.1
ARUBRA_DN99_c0_g1_i1_2	0	563	XP_018819183.1	ubiquitin-activating enzyme E1 1-like	Juglans regia	XP_018819183.1
ARUBRA_DN3903_c0_g1_i1_1	0	950	XP_018843166.1	proline--tRNA ligase, cytoplasmic-like isoform X1	Juglans regia	XP_018843167.1
ARUBRA_DN620_c0_g1_i1_5	3,06E-80	246	XP_018827585.1	fumarylacetoacetase	Juglans regia	XP_015959013.1
ARUBRA_DN7726_c0_g1_i1_6	1,35E-99	300	XP_008220832.1	glucose-1-phosphate adenylyltransferase large subunit, chloroplastic/amyloplastic isoform X1	Prunus mume	ONI32613.1
ARHOMBI_DN12388_c0_g1_i1_3	7,42E-80	244	XP_017188574.1	protein transport protein SEC31 homolog B-like	Malus domestica	XP_018817298.1

ARHOMBI_DN5442_c0_g1_i1_6	0	840	XP_018835118.1	eukaryotic initiation factor 4A-15	Juglans regia	XP_018835119.1
ARHOMBI_DN97_c0_g1_i1_1	1,38E-43	153	XP_018833217.1	uncharacterized protein LOC109000707	Juglans regia	XP_018833218.1
ARHOMBI_DN6380_c0_g1_i1_4	3,71E-32	120	XP_018859093.1	probable pectinesterase/pectinesterase inhibitor 51	Juglans regia	XP_018859084.1
ARUBRA_DN7925_c0_g1_i1_2	1,73E-72	236	XP_018849376.1	phosphoenolpyruvate carboxylase, housekeeping isozyme	Juglans regia	XP_018822521.1
ARHOMBI_DN4703_c0_g1_i1_3	0	535	XP_018829443.1	gamma carbonic anhydrase 1, mitochondrial-like isoform X1	Juglans regia	XP_018829444.1
ARUBRA_DN2228_c0_g2_i1_2	0	522	XP_018818494.1	UDP-glucose 6-dehydrogenase 4	Juglans regia	KDP41285.1
ARUBRA_DN23264_c0_g1_i1_3	1,44E-36	133	XP_019432775.1	calcium-transporting ATPase 2, plasma membrane-type-like	Lupinus angustifolius	XP_007155834.1
ARUBRA_DN22823_c0_g1_i1_2	2,88E-162	461	XP_018848755.1	indole-3-glycerol phosphate synthase, chloroplastic-like	Juglans regia	KDP29953.1
ARHOMBI_DN3499_c0_g1_i1_2	0	962	XP_018812785.1	importin subunit alpha-2-like	Juglans regia	XP_018825763.1
ARHOMBI_DN6537_c0_g1_i1_4	5,59E-87	281	BAT86449.1	hypothetical protein VIGAN_04410200	Vigna angularis var. angularis	XP_017418308.1
ARUBRA_DN16598_c0_g1_i1_4	4,75E-53	180	XP_018816352.1	villin-3-like isoform X3	Juglans regia	XP_018816349.1
ARUBRA_DN3848_c0_g2_i1_1	1,61E-127	366	XP_018836944.1	NEDD8-conjugating enzyme Ubc12-like	Juglans regia	XP_018836945.1
ARUBRA_DN16655_c0_g1_i1_1	0	742	XP_018816636.1	NADH dehydrogenase ubiquinone iron-sulfur protein 1, mitochondrial	Juglans regia	ONI17532.1
ARHOMBI_DN2235_c0_g1_i1_5	1,61E-116	343	XP_018810152.1	protein BTR1-like isoform X2	Juglans regia	XP_018810152.1
ARUBRA_DN19778_c0_g1_i1_3	1,92E-144	426	XP_018816929.1	uncharacterized protein LOC108988207	Juglans regia	XP_018843198.1
ARUBRA_DN5666_c0_g1_i1_2	0	711	XP_018839623.1	eukaryotic translation initiation factor 3 subunit E	Juglans regia	XP_018808879.1
ARUBRA_DN5508_c0_g1_i1_2	2,16E-83	249	XP_018840715.1	cytochrome b5, seed isoform	Juglans regia	XP_018821473.1
ARUBRA_DN3962_c0_g1_i1_6	0	639	XP_018828827.1	eukaryotic translation initiation factor 3 subunit I-like	Juglans regia	XP_018818014.1
ARUBRA_DN20257_c0_g1_i1_3	8,90E-67	207	XP_018814198.1	putative glucose-6-phosphate 1-epimerase isoform X1	Juglans regia	XP_018814255.1
ARUBRA_DN11024_c0_g1_i1_6	2,58E-49	163	XP_008379431.1	7-methylguanosine phosphate-specific 5'-nucleotidase	Malus domestica	XP_008356844.1
ARUBRA_DN21569_c0_g1_i1_4	0	596	XP_018838151.1	26S proteasome non-ATPase regulatory subunit 14 homolog	Juglans regia	XP_018857189.1
ARUBRA_DN965_c0_g1_i1_3	6,73E-65	207	XP_018815658.1	hexokinase-1-like	Juglans regia	KDP33801.1
ARHOMBI_DN27321_c0_g1_i1_2	7,18E-60	201	KDP33418.1	hypothetical protein JCGZ_06989	Jatropha curcas	XP_012076297.1
ARUBRA_DN1379_c0_g2_i1_4	0	821	XP_004485538.1	serine decarboxylase 1	Cicer arietinum	XP_004485539.1
ARUBRA_DN21229_c0_g1_i1_6	8,36E-93	284	XP_018860643.1	dolichyl-diphosphooligosaccharide--protein glycosyltransferase subunit 1A-like isoform X2	Juglans regia	XP_018860642.1
ARUBRA_DN16619_c0_g1_i1_3	8,52E-179	502	XP_018830828.1	probable fructokinase-7	Juglans regia	XP_008234319.1

ARHOMBI_DN17281_c0_g1_i1_1	1,03E-32	121	XP_008344156.1	protein disulfide-isomerase 5-2-like	Malus domestica	XP_009379564.1
ARHOMBI_DN4623_c0_g1_i1_3	7,91E-93	276	XP_018829015.1	binding partner of ACD11 1-like	Juglans regia	XP_020203072.1
ARUBRA_DN6168_c0_g1_i1_1	1,93E-57	193	XP_018807473.1	uncharacterized protein LOC108980890	Juglans regia	OAY48561.1
ARHOMBI_DN24755_c0_g1_i1_2	2,77E-56	189	XP_008393748.1	aconitate hydratase 1-like	Malus domestica	XP_008363078.1
ARUBRA_DN4123_c0_g1_i1_5	0	823	XP_018841784.1	serine--tRNA ligase-like isoform X2	Juglans regia	XP_018811146.1
ARUBRA_DN9_c0_g1_i1_4	0	613	XP_018827628.1	probable methyltransferase PMT26	Juglans regia	XP_018827629.1
ARHOMBI_DN6166_c0_g1_i1_6	0	1151	XP_018829229.1	glycine--tRNA ligase, mitochondrial 1-like	Juglans regia	XP_018841636.1
ARUBRA_DN1535_c0_g1_i1_5	4,71E-123	354	ACU21482.1	unknown	Glycine max	OAY21182.1
ARUBRA_DN17285_c0_g1_i1_2	1,44E-75	242	XP_018829411.1	probable Xaa-Pro aminopeptidase P isoform X1	Juglans regia	XP_018829412.1
ARHOMBI_DN20415_c0_g1_i1_5	3,21E-57	183	KHN01580.1	Pyrophosphate--fructose 6-phosphate 1-phosphotransferase subunit alpha	Glycine soja	AIE47261.1
ARHOMBI_DN6137_c0_g1_i1_4	0	1572	XP_018836595.1	chaperone protein ClpC, chloroplastic	Juglans regia	XP_018832706.1
ARUBRA_DN20044_c0_g1_i1_6	1,96E-46	160	XP_018854045.1	T-complex protein 1 subunit alpha	Juglans regia	XP_018854052.1
ARUBRA_DN20625_c0_g1_i1_2	7,75E-73	220	XP_018806116.1	1-(5-phosphoribosyl)-5-[(5-phosphoribosylamino)methylideneamino imidazole-4-carboxamide isomerase, chloroplastic-like	Juglans regia	XP_018813677.1
ARUBRA_DN21250_c0_g1_i1_6	8,43E-43	151	KOM46326.1	hypothetical protein LR48_Vigan07g003000	Vigna angularis	BAT80521.1
ARUBRA_DN4028_c0_g1_i2_4	2,61E-105	308	KHM99496.1	40S ribosomal protein S8	Glycine soja	KRH46288.1
ARUBRA_DN12697_c0_g1_i1_5	4,19E-42	146	XP_018843285.1	mitochondrial-processing peptidase subunit alpha-like	Juglans regia	KOM27410.1
ARUBRA_DN17667_c0_g1_i1_4	2,92E-70	215	XP_018823645.1	uncharacterized protein LOC108993249	Juglans regia	XP_018857024.1
ARHOMBI_DN16026_c0_g1_i1_1	0	718	XP_018843471.1	26S proteasome non-ATPase regulatory subunit 2 homolog A	Juglans regia	XP_018834279.1
ARUBRA_DN16494_c0_g1_i1_3	4,00E-147	426	KYP61474.1	T-complex protein 1 subunit delta	Cajanus cajan	XP_020220387.1
ARUBRA_DN6330_c0_g1_i1_4	0	937	XP_018833063.1	dihydropyrimidinase-like	Juglans regia	XP_018842508.1
ARUBRA_DN13172_c0_g1_i1_1	1,45E-57	195	XP_018844544.1	mechanosensitive ion channel protein 10-like	Juglans regia	XP_018827036.1
ARHOMBI_DN6108_c0_g1_i2_2	4,28E-18	80,1	XP_018828533.1	glutathione S-transferase zeta class-like isoform X1	Juglans regia	OAY46654.1
ARUBRA_DN20543_c0_g1_i1_1	1,21E-89	264	XP_018807447.1	probable xyloglucan endotransglucosylase/hydrolase protein 27	Juglans regia	XP_018807448.1
ARUBRA_DN3626_c0_g2_i1_5	2,22E-150	433	XP_018815486.1	ubiquitin receptor RAD23c-like	Juglans regia	XP_018815467.1
ARHOMBI_DN3946_c0_g1_i1_1	3,41E-139	400	XP_018850051.1	REF/SRPP-like protein At3g05500	Juglans regia	XP_018850053.1
ARUBRA_DN17212_c0_g1_i1_1	9,79E-119	354	XP_018825075.1	peroxisomal acyl-coenzyme A oxidase 1	Juglans regia	XP_018819219.1
ARHOMBI_DN1738_c0_g2_i1_2	7,15E-62	199	XP_018857981.1	phosphomethylethanolamine N-methyltransferase isoform X2	Juglans regia	XP_018857980.1
ARUBRA_DN4695_c0_g1_i1_1	6,68E-89	265	XP_018858810.1	uncharacterized protein LOC109020739	Juglans regia	XP_018851562.1

ARUBRA_DN6843_c0_g1_i1_2	0	944	AHJ79156.1	betaine-aldehyde dehydrogenase	Juglans regia	XP_018831659.1
ARHOMBI_DN565_c0_g1_i1_1	1,34E-48	162	XP_018824948.1	probable cinnamyl alcohol dehydrogenase 9	Juglans regia	ONI01829.1
ARUBRA_DN12655_c0_g1_i1_2	4,90E-86	270	XP_018815731.1	glyoxysomal fatty acid beta-oxidation multifunctional protein MFP-a-like	Juglans regia	OIV95201.1
ARHOMBI_DN7486_c0_g1_i1_4	3,00E-37	130	XP_018820703.1	GTP-binding protein SAR1A-like	Juglans regia	XP_018846147.1
ARHOMBI_DN3745_c0_g1_i1_6	1,45E-49	160	XP_018820994.1	phosphopantetheine adenylyltransferase	Juglans regia	ONH96872.1
ARHOMBI_DN161_c0_g2_i1_2	4,70E-172	482	XP_019464492.1	26S proteasome non-ATPase regulatory subunit 8 homolog A-like	Lupinus angustifolius	XP_019464493.1
ARHOMBI_DN4381_c0_g1_i1_4	2,55E-114	352	XP_018829157.1	beta-adaptin-like protein C	Juglans regia	KYP52643.1
ARUBRA_DN4254_c0_g2_i1_1	0	744	XP_018822222.1	DEAD-box ATP-dependent RNA helicase 56	Juglans regia	XP_018812870.1
ARUBRA_DN4783_c0_g1_i1_3	0	737	XP_018847079.1	LL-diaminopimelate aminotransferase, chloroplastic-like	Juglans regia	XP_009378578.1
ARHOMBI_DN4848_c0_g1_i1_6	0	529	XP_018836697.1	proteasome subunit beta type-5	Juglans regia	XP_018836698.1
ARHOMBI_DN16223_c0_g1_i1_5	1,82E-130	381	KHM99824.1	Mitochondrial-processing peptidase subunit alpha	Glycine soja	KRH60182.1
ARHOMBI_DN19081_c0_g1_i1_3	1,11E-60	198	OAY56891.1	hypothetical protein MANES_02G053100	Manihot esculenta	XP_018816788.1
ARUBRA_DN24202_c0_g1_i1_3	1,71E-39	133	XP_018856129.1	triphosphate tunel metalloenzyme 3-like, partial	Juglans regia	XP_018853288.1
ARUBRA_DN3211_c0_g1_i1_4	4,60E-71	218	XP_018855746.1	HMG1/2-like protein	Juglans regia	XP_018809064.1
ARUBRA_DN20320_c0_g1_i1_4	8,87E-153	431	XP_018815495.1	uncharacterized protein LOC108987089	Juglans regia	XP_009344930.1
ARUBRA_DN4936_c0_g2_i1_2	5,24E-95	280	XP_018847894.1	SKP1-like protein 1B	Juglans regia	OAY50541.1
ARUBRA_DN9672_c0_g1_i1_4	5,58E-105	323	XP_018826765.1	pyrophosphate-energized vacuolar membrane proton pump-like	Juglans regia	XP_009358552.1
ARUBRA_DN19765_c0_g1_i1_2	1,26E-85	257	XP_018830835.1	protein phosphatase inhibitor 2 isoform X2	Juglans regia	XP_018830836.1
ARUBRA_DN3204_c0_g2_i1_1	1,41E-105	306	XP_018835006.1	25.3 kDa vesicle transport protein-like	Juglans regia	XP_018835007.1
ARUBRA_DN3663_c0_g1_i1_5	0	590	XP_018854045.1	T-complex protein 1 subunit alpha	Juglans regia	XP_018854052.1
ARHOMBI_DN23207_c0_g1_i1_1	4,90E-11	63,9	ONI29014.1	hypothetical protein PRUPE_1G175600	Prunus persica	XP_007222406.1
ARHOMBI_DN10568_c0_g1_i1_2	0	558	XP_018837477.1	T-complex protein 1 subunit eta isoform X1	Juglans regia	OIW02779.1
ARUBRA_DN26415_c0_g1_i1_1	1,21E-35	125	XP_007137680.1	hypothetical protein PHAVU_009G146600g	Phaseolus vulgaris	ESW09674.1
ARHOMBI_DN4088_c0_g1_i1_3	0	783	XP_018849865.1	aminomethyltransferase, mitochondrial	Juglans regia	XP_018849809.1
ARHOMBI_DN5585_c0_g1_i1_3	0	701	XP_018817113.1	TOM1-like protein 2	Juglans regia	XP_018817114.1
ARHOMBI_DN14389_c0_g1_i1_3	3,00E-52	179	XP_018849376.1	phosphoenolpyruvate carboxylase, housekeeping isozyme	Juglans regia	XP_018822521.1
ARUBRA_DN11036_c0_g1_i1_1	4,24E-107	318	XP_018829248.1	monodehydroascorbate reductase 5, mitochondrial	Juglans regia	XP_016651062.1
ARHOMBI_DN6168_c0_g1_i1_4	0	1054	XP_018827696.1	plastidic ATP/ADP-transporter-like	Juglans regia	XP_018823517.1
ARHOMBI_DN1051_c0_g1_i1_2	6,00E-63	197	XP_018818935.1	superoxide dismutase Cu-Zn , chloroplastic	Juglans regia	XP_018841942.1
ARHOMBI_DN4618_c0_g1_i1_5	9,50E-126	360	XP_018806533.1	probable glutathione peroxidase 2	Juglans regia	XP_008380677.1

ARUBRA_DN5779_c0_g1_i1_6	1,82E-116	341	XP_018816103.1	succinate dehydrogenase subunit 5, mitochondrial-like	Juglans regia	XP_018822424.1
ARHOMBI_DN11483_c0_g1_i1_6	8,54E-74	230	XP_018857224.1	cycloartenol-C-24-methyltransferase	Juglans regia	XP_018857224.1
ARHOMBI_DN15111_c0_g1_i1_2	1,57E-131	380	XP_018845516.1	nucleosome assembly protein 1;2-like	Juglans regia	XP_018845505.1
ARUBRA_DN343_c0_g2_i1_5	2,58E-30	112	XP_008229222.1	60S acidic ribosomal protein P1-like	Prunus mume	XP_008233051.1
ARHOMBI_DN12998_c0_g1_i1_2	1,77E-47	153	KRH23486.1	hypothetical protein GLYMA_13G3602002, partial	Glycine max	AAZ32864.1
ARHOMBI_DN1655_c0_g1_i1_1	0	834	XP_018807730.1	3-phosphoshikimate 1-carboxyvinyltransferase 2	Juglans regia	ONI22671.1
ARUBRA_DN8461_c0_g1_i1_4	1,25E-93	275	XP_018817485.1	uncharacterized protein LOC108988625	Juglans regia	OAY29144.1
ARHOMBI_DN17992_c0_g1_i1_1	0	537	XP_018839045.1	probable methyltransferase PMT26	Juglans regia	XP_018839046.1
ARUBRA_DN796_c0_g1_i1_5	1,92E-141	427	XP_018837135.1	26S proteasome non-ATPase regulatory subunit 1 homolog A-like	Juglans regia	OAY28247.1
ARHOMBI_DN11775_c0_g1_i1_1	2,52E-59	196	XP_018822530.1	subtilisin-like protease SBT3.5, partial	Juglans regia	XP_008392653.1
ARUBRA_DN3727_c0_g1_i1_4	7,34E-79	235	XP_018827104.1	vesicle-associated membrane protein 722	Juglans regia	XP_018816327.1
ARUBRA_DN7635_c0_g1_i1_4	0	899	OAY23212.1	hypothetical protein MANES_18G060600	Manihot esculenta	OAY23211.1
ARHOMBI_DN17384_c0_g1_i1_1	2,31E-141	402	XP_008219517.1	vesicle-associated membrane protein 711	Prunus mume	ONI34446.1
ARHOMBI_DN8440_c0_g1_i1_5	2,67E-171	488	XP_018811308.1	probable aspartyl aminopeptidase	Juglans regia	XP_018814786.1
ARHOMBI_DN4904_c0_g1_i1_1	1,70E-151	428	XP_018852621.1	ras-related protein Rab11A	Juglans regia	GAU36334.1
ARUBRA_DN24171_c0_g1_i1_6	2,73E-44	155	XP_004490631.1	ubiquitin-activating enzyme E1 1	Cicer arietinum	XP_004490632.1
ARHOMBI_DN15505_c0_g1_i1_1	1,20E-64	207	XP_018823615.1	sugar transport protein 14-like	Juglans regia	XP_018823616.1
ARUBRA_DN19884_c0_g1_i1_4	1,18E-113	332	XP_018824108.1	hepatocyte growth factor-regulated tyrosine kinase substrate isoform X3	Juglans regia	XP_018824107.1
ARHOMBI_DN2480_c0_g1_i1_6	1,46E-80	244	XP_018831702.1	protein NUCLEAR FUSION DEFECTIVE 2	Juglans regia	ONI08410.1
ARHOMBI_DN5911_c0_g2_i1_1	0	932	ALE18236.1	DAHPS	Pyrus x bretschneideri	NP_001306742.1
ARHOMBI_DN11895_c0_g1_i1_5	2,86E-26	102	ANG56496.1	cinnamoyl-CoA reductase	Hevea brasiliensis subsp. brasiliensis	XP_009355409.1
ARHOMBI_DN10040_c0_g1_i1_3	4,45E-105	318	XP_018851318.1	serine/threonine-protein phosphatase 2A 65 kDa regulatory subunit A beta isoform	Juglans regia	XP_018851318.1
ARHOMBI_DN20237_c0_g1_i1_3	0	744	XP_018809133.1	cleft lip and palate transmembrane protein 1 homolog	Juglans regia	XP_009379523.1
ARHOMBI_DN13255_c0_g1_i1_6	1,21E-29	114	XP_007135745.1	hypothetical protein PHAVU_010G155000g	Phaseolus vulgaris	ESW07739.1

ARUBRA_DN1076_c0_g1_i1_4	0	637	XP_018832781.1	thiosulfate/3-mercaptopyruvate sulfurtransferase 1, mitochondrial-like isoform X1	Juglans regia	KDP25080.1
ARHOMBI_DN7071_c0_g1_i1_2	2,25E-89	268	XP_018835854.1	thioredoxin O2, mitochondrial-like isoform X2	Juglans regia	XP_018835853.1
ARUBRA_DN13122_c0_g1_i1_4	3,90E-39	142	XP_018850823.1	sulfate transporter 2.1	Juglans regia	XP_012064904.1
ARHOMBI_DN1703_c0_g1_i1_6	0	514	XP_018807236.1	eukaryotic translation initiation factor 3 subunit F-like	Juglans regia	XP_018821752.1
ARUBRA_DN8641_c0_g1_i1_4	2,10E-74	221	XP_018809915.1	uncharacterized protein LOC108982896	Juglans regia	XP_018809916.1
ARUBRA_DN2071_c0_g3_i1_4	1,49E-96	280	XP_018843327.1	60S ribosomal protein L28-2-like	Juglans regia	XP_018843328.1
ARUBRA_DN24590_c0_g1_i1_2	2,89E-12	64,7	XP_018812044.1	large proline-rich protein BAG6-like isoform X2	Juglans regia	XP_018812043.1
ARUBRA_DN9637_c0_g1_i1_6	1,64E-43	152	XP_018824245.1	acyl-coenzyme A oxidase 3, peroxisomal-like	Juglans regia	XP_018824246.1
ARUBRA_DN12612_c0_g1_i1_4	2,69E-64	196	XP_018835316.1	peptidyl-prolyl cis-trans isomerase CYP18-2	Juglans regia	ONH96008.1
ARHOMBI_DN3999_c0_g1_i1_1	0	1180	XP_018824324.1	dynamin-related protein 5A	Juglans regia	XP_018857541.1
ARHOMBI_DN5284_c0_g1_i1_4	2,65E-103	310	XP_018806799.1	glycerol kinase	Juglans regia	XP_008372701.1
ARUBRA_DN7275_c0_g1_i1_1	1,11E-93	285	KDP35967.1	hypothetical protein JCGZ_09939	Jatropha curcas	XP_012074593.1
ARHOMBI_DN3047_c0_g1_i1_6	0	814	XP_018830113.1	glutamate-1-semialdehyde 2,1-aminomutase 2, chloroplastic	Juglans regia	OAY47995.1
ARUBRA_DN12194_c0_g1_i1_6	1,19E-149	443	XP_018836922.1	coatomer subunit gamma-2-like	Juglans regia	XP_018847842.1
ARHOMBI_DN20434_c0_g1_i1_2	5,55E-40	143	XP_008337280.1	methyl-CpG-binding domain-containing protein 11-like	Malus domestica	XP_008337277.1
ARHOMBI_DN3438_c0_g1_i1_6	0	691	XP_018840433.1	cysteine synthase, chloroplastic/chromoplastic isoform X1	Juglans regia	XP_018840434.1
ARHOMBI_DN4924_c0_g1_i1_5	2,20E-76	228	OAY43528.1	hypothetical protein MANES_08G077000	Manihot esculenta	XP_018823305.1
ARUBRA_DN11906_c0_g1_i1_6	5,41E-57	181	XP_008353771.1	acyl-coenzyme A oxidase 3, peroxisomal-like	Malus domestica	XP_018824245.1
ARUBRA_DN21459_c0_g1_i1_4	2,94E-72	222	XP_018805589.1	cinnamoyl-CoA reductase 2	Juglans regia	XP_018805590.1
ARUBRA_DN23079_c0_g1_i1_1	5,01E-59	181	CAA10126.1	actin, partial	Cicer arietinum	AFP43694.1
ARUBRA_DN15741_c0_g1_i1_6	4,91E-48	159	XP_018818800.1	probable 6-phosphogluconolactonase 4, chloroplastic	Juglans regia	XP_018842795.1
ARHOMBI_DN3883_c0_g1_i1_5	0	682	XP_018829261.1	6-phosphogluconate dehydrogenase, decarboxylating 2, chloroplastic	Juglans regia	ONI07307.1
ARUBRA_DN4014_c0_g2_i1_3	4,30E-43	142	XP_018817591.1	aldo-keto reductase family 4 member C9-like	Juglans regia	KRH75709.1
ARUBRA_DN4532_c0_g1_i1_4	0	1174	OAY47426.1	hypothetical protein MANES_06G078500	Manihot esculenta	ONI31143.1
ARUBRA_DN7957_c0_g1_i1_1	4,02E-174	490	XP_018832031.1	probable ribose-5-phosphate isomerase 3, chloroplastic	Juglans regia	XP_015968505.1
ARHOMBI_DN22787_c0_g1_i1_4	1,43E-111	345	GAU30183.1	hypothetical protein TSUD_311340	Trifolium subterraneum	GAU30182.1
ARUBRA_DN18867_c0_g1_i1_5	0	586	XP_018847856.1	protein transport protein SEC13 homolog B	Juglans regia	XP_018839977.1
ARHOMBI_DN17883_c0_g1_i1_2	1,08E-112	332	XP_018816525.1	ATP-citrate synthase alpha chain protein 3	Juglans regia	XP_018859551.1

ARHOMBI_DN14524_c0_g1_i1_1	3,07E-46	158	XP_018849912.1	heterogeneous nuclear ribonucleoprotein 1 isoform X3	Juglans regia	XP_018849912.1
ARUBRA_DN2441_c0_g1_i1_2	1,51E-143	406	XP_018821207.1	transmembrane protein 205	Juglans regia	XP_008235189.1
ARUBRA_DN25648_c0_g1_i1_1	2,88E-43	151	KDP41683.1	hypothetical protein JCGZ_16090	Jatropha curcas	XP_012068288.1
ARUBRA_DN4730_c0_g1_i1_2	3,62E-91	274	OAY62412.1	hypothetical protein MANES_01G266200	Manihot esculenta	OAY49309.1
ARUBRA_DN4181_c0_g1_i1_5	4,05E-112	325	XP_008222138.1	60S ribosomal protein L17-2	Prunus mume	XP_018809465.1
ARHOMBI_DN26738_c0_g1_i1_4	4,50E-40	143	GAU48115.1	hypothetical protein TSUD_351210	Trifolium subterraneum	KEH18347.1
ARUBRA_DN5147_c0_g1_i1_4	1,17E-31	120	XP_018832881.1	staphylococcal nuclease domain-containing protein 1-like	Juglans regia	XP_018832889.1
ARHOMBI_DN5499_c0_g1_i1_3	0	815	XP_018839119.1	diacylglycerol kinase 5 isoform X1	Juglans regia	XP_018839120.1
ARUBRA_DN8113_c0_g1_i1_3	2,27E-104	320	XP_018857284.1	villin-2-like isoform X2	Juglans regia	XP_018857284.1
ARHOMBI_DN1623_c0_g1_i1_6	0	637	XP_016194788.1	T-complex protein 1 subunit zeta 1	Arachis ipaensis	XP_015962928.1
ARUBRA_DN4329_c0_g1_i1_6	0	552	XP_008231164.1	cytochrome c1-2, heme protein, mitochondrial	Prunus mume	ONI19859.1
ARUBRA_DN4117_c0_g1_i1_6	7,42E-75	228	XP_014496997.1	60S ribosomal protein L23A Vigna radiata var. radiata	Vigna radiata var. radiata	XP_020224350.1
ARUBRA_DN18085_c0_g1_i1_1	9,21E-60	188	XP_018827184.1	ABC transporter I family member 19-like	Juglans regia	XP_008235651.1
ARUBRA_DN5456_c0_g1_i1_2	0	715	XP_018846385.1	probable nucleoredoxin 1	Juglans regia	XP_018846385.1
ARHOMBI_DN23974_c0_g1_i1_1	1,53E-52	175	XP_008352317.1	coatomer subunit beta-1-like, partial	Malus domestica	XP_009371101.1
ARHOMBI_DN3770_c0_g1_i1_1	0	760	XP_018807321.1	aconitate hydratase 1	Juglans regia	KDP23953.1
ARUBRA_DN4031_c0_g1_i1_2	3,07E-40	144	XP_018835196.1	probable trehalase	Juglans regia	XP_018824320.1
ARUBRA_DN4198_c0_g2_i2_4	5,40E-85	257	KDP37390.1	hypothetical protein JCGZ_08401	Jatropha curcas	XP_012072933.1
ARHOMBI_DN16331_c0_g1_i1_2	1,96E-107	319	XP_018850555.1	alanine aminotransferase 1, mitochondrial-like	Juglans regia	XP_018850554.1
ARHOMBI_DN8849_c0_g1_i1_2	4,16E-68	214	OAY23899.1	hypothetical protein MANES_18G116300	Manihot esculenta	XP_008358263.1
ARUBRA_DN6372_c0_g1_i1_2	1,05E-33	118	GAU46388.1	hypothetical protein TSUD_375680	Trifolium subterraneum	XP_018844689.1
ARUBRA_DN16718_c0_g1_i1_3	2,82E-37	130	XP_018827184.1	ABC transporter I family member 19-like	Juglans regia	AIU41666.1
ARHOMBI_DN10686_c0_g1_i1_3	7,69E-39	137	ONI21854.1	hypothetical protein PRUPE_2G093900	Prunus persica	ONI21854.1
ARUBRA_DN8773_c0_g1_i1_4	3,50E-68	210	AFK36987.1	unknown	Lotus japonicus	BAT72624.1
ARUBRA_DN1981_c0_g1_i1_6	1,01E-111	325	ALB76795.1	enoyl-CoA hydratase, partial	Jatropha curcas	XP_018815731.1
ARHOMBI_DN6947_c0_g1_i1_1	4,19E-66	206	KHN28969.1	3-oxoacyl-[acyl-carrier-protein synthase I, chloroplastic	Glycine soja	XP_018819716.1
ARHOMBI_DN4632_c0_g1_i1_3	1,08E-160	461	XP_018849576.1	uncharacterized protein LOC109012411 isoform X2	Juglans regia	XP_018849575.1
ARHOMBI_DN14482_c0_g1_i1_6	2,65E-20	87	XP_018815032.1	dihydrolipoyllysine-residue acetyltransferase component 5 of pyruvate dehydrogenase complex, chloroplastic	Juglans regia	XP_008381007.1
ARHOMBI_DN3429_c0_g1_i1_6	0	607	XP_018843379.1	protein ROOT HAIR DEFECTIVE 3-like	Juglans regia	XP_017189277.1

ARHOMBI_DN3622_c0_g1_i1_4	0	942	XP_018827500.1	acetolactate synthase 2, chloroplastic	Juglans regia	ONH89544.1
ARUBRA_DN4615_c0_g2_i1_3						
ARHOMBI_DN17829_c0_g1_i1_1	0	510	XP_012070239.1	V-type proton ATPase subunit H	Jatropha curcas	XP_017405700.1
ARUBRA_DN6205_c0_g1_i1_1	4,17E-73	226	XP_018834882.1	V-type proton ATPase subunit C	Juglans regia	ONI20048.1
ARUBRA_DN24096_c0_g1_i1_3	1,21E-46	162	XP_018832228.1	actin cytoskeleton-regulatory complex protein PAN1-like	Juglans regia	XP_018841554.1
ARUBRA_DN1065_c0_g2_i1_2	5,42E-142	416	XP_018839142.1	NADP-dependent malic enzyme-like	Juglans regia	ONI15755.1
ARHOMBI_DN12564_c0_g1_i1_6	1,69E-65	208	XP_018836625.1	tryptophan--tRNA ligase, cytoplasmic	Juglans regia	KDP42894.1
ARHOMBI_DN3278_c0_g1_i1_3	1,67E-112	327	XP_018845596.1	translation machinery-associated protein 22	Juglans regia	OAY53925.1
ARUBRA_DN16058_c0_g1_i1_4	6,15E-51	175	XP_018845862.1	staphylococcal nuclease domain-containing protein 1-like	Juglans regia	BAC06183.1
ARUBRA_DN9760_c0_g1_i1_3	2,58E-99	297	XP_018823857.1	probable 26S proteasome non-ATPase regulatory subunit 3	Juglans regia	KRH51667.1
ARHOMBI_DN6246_c0_g1_i3_3	2,23E-132	379	XP_018842015.1	endochitinase-like	Juglans regia	XP_018854709.1
ARHOMBI_DN3643_c0_g1_i1_2	3,85E-65	209	XP_018814240.1	citrate synthase, glyoxysomal	Juglans regia	XP_018828184.1
ARUBRA_DN18439_c0_g1_i1_3	0	632	XP_018831147.1	dynamin-2A-like	Juglans regia	XP_018834193.1
ARUBRA_DN4784_c0_g2_i3_2	1,38E-168	471	XP_018824239.1	proteasome subunit beta type-4-like	Juglans regia	XP_018844541.1
ARHOMBI_DN16305_c0_g1_i1_3	1,26E-41	136	ACF74298.1	glutamine synthetase GS56, partial	Arachis hypogaea	XP_014495844.1
ARUBRA_DN1937_c0_g2_i1_6	0	843	XP_018846215.1	26S proteasome non-ATPase regulatory subunit 12 homolog A	Juglans regia	OIW17846.1
ARHOMBI_DN3129_c0_g1_i1_2	1,02E-52	165	XP_018830250.1	V-type proton ATPase subunit F isoform X1	Juglans regia	XP_008237928.1
ARUBRA_DN1180_c0_g1_i1_3	0	1021	XP_018845552.1	eukaryotic translation initiation factor 3 subunit L-like	Juglans regia	KDP34205.1
ARHOMBI_DN24079_c0_g1_i1_2	3,11E-55	181	XP_018848755.1	indole-3-glycerol phosphate synthase, chloroplastic-like	Juglans regia	OAY35002.1
ARHOMBI_DN17905_c0_g1_i1_4	2,63E-60	189	XP_004502943.1	ras-related protein RABC1-like	Cicer arietinum	ACU20062.1
ARHOMBI_DN13275_c0_g1_i1_1	1,76E-49	175	XP_008346150.1	coatomer subunit alpha-1-like	Malus domestica	XP_008349149.1
ARHOMBI_DN17238_c0_g1_i1_3	9,94E-69	225	XP_018807473.1	uncharacterized protein LOC108980890	Juglans regia	ONH99280.1
ARHOMBI_DN6259_c0_g2_i7_3	6,26E-72	219	XP_018807308.1	probable glutathione S-transferase	Juglans regia	OIW11645.1
ARHOMBI_DN4135_c0_g2_i1_5	7,92E-99	288	XP_018823137.1	ferredoxin-thioredoxin reductase catalytic chain, chloroplastic	Juglans regia	XP_008230669.1
ARUBRA_DN4552_c1_g2_i1_5	8,59E-120	342	KRH69176.1	hypothetical protein GLYMA_02G009600	Glycine max	AAB91395.1
ARHOMBI_DN6298_c0_g1_i1_1	3,04E-52	179	OAY56882.1	hypothetical protein MANES_02G052300	Manihot esculenta	XP_016180001.1
ARHOMBI_DN2163_c0_g1_i1_5	0	673	XP_018826576.1	probable methyltransferase PMT2	Juglans regia	OAY31024.1

ARHOMBI_DN6274_c0_g2_i10_6	5,06E-148	420	XP_018850618.1	thaumatin-like protein 1	Juglans regia	XP_018851248.1
ARUBRA_DN6417_c0_g1_i1_1	3,30E-50	173	XP_018828478.1	ubiquitin-activating enzyme E1 1	Juglans regia	XP_018828479.1
ARUBRA_DN16375_c0_g1_i1_3	3,20E-129	382	XP_018843632.1	TBCC domain-containing protein 1-like isoform X2	Juglans regia	XP_018808540.1
ARUBRA_DN20085_c0_g1_i1_4	5,11E-70	219	OAY24401.1	hypothetical protein MANES_17G013000	Manihot esculenta	XP_018824705.1
ARHOMBI_DN7053_c0_g1_i1_6	5,29E-45	158	XP_018828383.1	eukaryotic translation initiation factor-like	Juglans regia	XP_018823485.1
ARHOMBI_DN4215_c0_g1_i1_6	0	557	XP_018840009.1	ER membrane protein complex subunit 2	Juglans regia	ONI04815.1
ARUBRA_DN6101_c0_g1_i1_3	0	719	ONI36176.1	hypothetical protein PRUPE_1G573400	Prunus persica	ONI36177.1
ARHOMBI_DN5908_c0_g1_i1_6	0	718	XP_018829584.1	3-dehydroquinate synthase, chloroplastic-like	Juglans regia	XP_018834582.1
ARHOMBI_DN12316_c0_g1_i1_3	1,20E-45	160	XP_018835062.1	heat shock 70 kDa protein 17	Juglans regia	OAY41090.1
ARHOMBI_DN1573_c0_g2_i1_5	1,74E-98	291	XP_018860327.1	probable dolichyl-diphosphooligosaccharide--protein glycosyltransferase subunit 3B	Juglans regia	OAY25973.1
ARUBRA_DN3281_c0_g1_i1_4	1,26E-81	244	XP_018831984.1	actin-depolymerizing factor 2-like	Juglans regia	XP_018814539.1
ARHOMBI_DN5746_c0_g1_i1_4	8,92E-113	324	XP_018815794.1	60S ribosomal protein L21-1-like	Juglans regia	KDP21892.1
ARUBRA_DN17629_c0_g1_i1_1	8,50E-20	86,3	XP_018825904.1	uncharacterized protein LOC108994927 isoform X2	Juglans regia	XP_018825903.1
ARHOMBI_DN1692_c0_g1_i1_5	0	533	KDP38557.1	hypothetical protein JCGZ_04482	Jatropha curcas	OAY50668.1
ARUBRA_DN15157_c0_g1_i1_2	1,53E-23	94,7	XP_012083109.1	RNA-binding protein 38 isoform X1	Jatropha curcas	KDP28419.1
ARHOMBI_DN17586_c0_g1_i1_5	0	795	XP_018811056.1	tryptophan synthase beta chain 1-like	Juglans regia	XP_018824063.1
ARUBRA_DN16576_c0_g1_i1_6	3,27E-38	137	XP_018845086.1	L-arabinokinase-like	Juglans regia	XP_018845600.1
ARHOMBI_DN12404_c0_g1_i1_3	8,10E-88	281	XP_018840319.1	coatomer subunit alpha-1-like	Juglans regia	XP_018840320.1
ARHOMBI_DN5690_c0_g1_i1_6	5,90E-110	319	KEH19791.1	ubiquitin-conjugating enzyme	Medicago truncatula	XP_013445765.1
ARHOMBI_DN14107_c0_g1_i1_6	7,88E-56	174	ACU27389.1	glyceraldehyde-3-phosphate dehydrogenase, partial	Codiaeum variegatum	ABP88103.1
ARUBRA_DN21696_c0_g1_i1_3	7,88E-94	278	XP_018835591.1	2-hydroxyisoflavanone dehydratase-like	Juglans regia	XP_018835592.1
ARUBRA_DN12039_c0_g1_i1_4	1,87E-72	223	OAY27542.1	hypothetical protein MANES_16G133500	Manihot esculenta	XP_008220617.1
ARHOMBI_DN17617_c0_g1_i1_1	1,09E-108	324	OAY24816.1	hypothetical protein MANES_17G045800	Manihot esculenta	XP_008383736.1
ARUBRA_DN25457_c0_g1_i1_2	2,44E-24	98,6	XP_018816929.1	uncharacterized protein LOC108988207	Juglans regia	XP_018843198.1
ARUBRA_DN9520_c0_g1_i1_3	2,36E-140	415	XP_018848499.1	threonine--tRNA ligase, mitochondrial 1-like	Juglans regia	KDP21323.1
ARUBRA_DN12747_c0_g1_i1_4	3,71E-48	166	OAY51887.1	hypothetical protein MANES_04G040700	Manihot esculenta	XP_008237251.1
ARHOMBI_DN27277_c0_g1_i1_1	1,38E-24	99,4	XP_018809774.1	protein transport protein SEC31 homolog B-like isoform X1	Juglans regia	XP_018809776.1
ARUBRA_DN21434_c0_g1_i1_3	7,56E-65	208	ONI07528.1	hypothetical protein PRUPE_5G125900	Prunus persica	OIW09901.1
ARUBRA_DN4990_c0_g1_i1_1	1,68E-144	409	XP_018811920.1	thaumatin-like protein 1	Juglans regia	AHA83528.1

ARHOMBI_DN18290_c0_g1_i1_4	1,50E-90	293	XP_018825846.1	enhancer of mRNA-decapping protein 4-like	Juglans regia	XP_017182550.1
ARUBRA_DN8515_c0_g1_i1_2	9,31E-94	277	XP_018821879.1	tropinone reductase homolog At5g06060-like	Juglans regia	OAY38586.1
ARUBRA_DN7009_c0_g1_i1_3	0	719	XP_018810396.1	pyruvate dehydrogenase E1 component subunit beta-3, chloroplastic	Juglans regia	XP_018810397.1
ARUBRA_DN16706_c0_g1_i1_4	2,80E-40	145	XP_018845086.1	L-arabinokinase-like	Juglans regia	XP_020536405.1
ARUBRA_DN8627_c0_g1_i1_5	3,49E-118	338	KRG96854.1	hypothetical protein GLYMA_19G236500	Glycine max	KYP71247.1
ARUBRA_DN19094_c0_g1_i1_2	1,16E-75	228	XP_018841009.1	uncharacterized protein At2g34160-like	Juglans regia	XP_018858938.1
ARUBRA_DN4464_c0_g2_i1_2	7,47E-163	472	XP_018806637.1	glutamate--tRNA ligase, cytoplasmic	Juglans regia	XP_018806638.1
ARUBRA_DN19978_c0_g1_i1_6	2,78E-122	369	ONH97993.1	hypothetical protein PRUPE_7G222300	Prunus persica	ONH97994.1
ARHOMBI_DN26182_c0_g1_i1_3	7,93E-08	52,4	XP_014495779.1	calcium-transporting ATPase 4, endoplasmic reticulum-type-like	Vigna radiata var. radiata	KOM38945.1
ARUBRA_DN5518_c0_g1_i1_3	7,24E-41	145	XP_018844497.1	plant UBX domain-containing protein 10-like	Juglans regia	OIW07211.1
ARHOMBI_DN6030_c1_g5_i1_6	4,56E-170	483	XP_018823047.1	2-methylene-furan-3-one reductase-like	Juglans regia	XP_018823045.1
ARHOMBI_DN15479_c0_g1_i1_2	0	573	XP_008236172.1	uncharacterized protein LOC103334966	Prunus mume	XP_008372892.1
ARHOMBI_DN21367_c0_g1_i1_4	1,10E-95	283	KHN09360.1	Isocitrate dehydrogenase NAD regulatory subunit 1, mitochondrial	Glycine soja	OAY57637.1
ARUBRA_DN9892_c0_g1_i1_6	3,11E-137	391	XP_018816316.1	ABC transporter I family member 20-like isoform X2	Juglans regia	XP_018816315.1
ARHOMBI_DN8750_c0_g1_i1_3	2,33E-44	154	XP_018826381.1	probable Xaa-Pro aminopeptidase P	Juglans regia	XP_018829411.1
ARHOMBI_DN13150_c0_g1_i1_1	1,38E-55	175	XP_018817591.1	aldo-keto reductase family 4 member C9-like	Juglans regia	XP_018846256.1
ARHOMBI_DN3473_c0_g1_i1_4	8,46E-76	229	XP_020210745.1	iron-sulfur assembly protein IscA-like 1, mitochondrial	Cajanus cajan	KYP71118.1
ARUBRA_DN22076_c0_g1_i1_2	3,90E-101	303	XP_018821230.1	protein SLOW GREEN 1, chloroplastic-like	Juglans regia	XP_016647139.1
ARUBRA_DN11215_c0_g1_i1_3	3,07E-74	239	XP_020977896.1	2-oxoglutarate dehydrogenase, mitochondrial isoform X2	Arachis ipaensis	OAY38674.1
ARHOMBI_DN1047_c0_g1_i1_6	2,53E-138	403	XP_018837500.1	protein disulfide isomerase-like 1-6	Juglans regia	KDP37240.1
ARUBRA_DN1728_c0_g1_i1_2	1,74E-25	96,3	XP_008391131.1	uncharacterized protein At2g27730, mitochondrial-like isoform X1	Malus domestica	XP_008391132.1
ARHOMBI_DN5812_c1_g3_i1_1	6,96E-70	225	XP_018835420.1	eukaryotic translation initiation factor 5-like isoform X1	Juglans regia	XP_018835421.1
ARUBRA_DN17307_c0_g1_i1_2	2,62E-21	84,7	XP_009352165.1	cytochrome c oxidase assembly factor 4 homolog, mitochondrial	Pyrus x bretschneideri	XP_008223056.1
ARHOMBI_DN4645_c1_g1_i1_2	3,84E-125	365	ONI07154.1	hypothetical protein PRUPE_5G103100	Prunus persica	XP_018852508.1
ARUBRA_DN8669_c0_g1_i1_4	1,19E-80	254	XP_018831655.1	probable aldehyde dehydrogenase isoform X1	Juglans regia	XP_018831656.1
ARUBRA_DN5513_c0_g1_i1_1	1,71E-54	176	XP_018839531.1	uncharacterized protein At1g32220, chloroplastic	Juglans regia	OAY27542.1

ARUBRA_DN4813_c0_g1_i1_1	0	814	XP_018806906.1	3-oxoacyl-[acyl-carrier-protein synthase II, chloroplastic-like	Juglans regia	XP_018848626.1
ARUBRA_DN10983_c0_g1_i1_4	5,82E-82	251	XP_018825255.1	elongation factor Tu, mitochondrial-like	Juglans regia	XP_018847441.1
ARUBRA_DN4671_c1_g1_i1_5	0	828	KDP35832.1	hypothetical protein JCGZ_10646	Jatropha curcas	XP_012074649.1
ARUBRA_DN5698_c0_g1_i1_2	0	553	XP_018841018.1	NADH-cytochrome b5 reductase-like protein	Juglans regia	XP_009368820.1
ARUBRA_DN8516_c0_g1_i1_2	1,70E-171	486	XP_018824803.1	fumarate hydratase 1, mitochondrial	Juglans regia	ONH93997.1
ARHOMBI_DN3145_c0_g1_i1_3	0	1053	XP_018805414.1	delta(24)-sterol reductase	Juglans regia	XP_018805415.1
ARUBRA_DN26369_c0_g1_i1_1	2,31E-58	191	AHA83579.1	V-type proton ATPase subunit A	Hevea brasiliensis	OAY48691.1
ARUBRA_DN16851_c0_g1_i1_2	2,73E-88	261	XP_018856574.1	PLAT domain-containing protein 2-like	Juglans regia	AES72232.1
ARHOMBI_DN22852_c0_g1_i1_6	6,71E-62	194	XP_017179549.1	phosphoenolpyruvate carboxylase, housekeeping isozyme-like	Malus domestica	AAL26863.1
ARHOMBI_DN25003_c0_g1_i1_6	7,79E-62	202	XP_018852605.1	leukotriene A-4 hydrolase homolog	Juglans regia	OAY24194.1
ARUBRA_DN17081_c0_g1_i1_5	1,94E-80	243	XP_018860648.1	uncharacterized protein LOC109022250	Juglans regia	XP_018860649.1
ARHOMBI_DN4475_c0_g1_i1_1	4,64E-164	464	XP_018846161.1	uncharacterized protein LOC109009947	Juglans regia	KHN32908.1
ARUBRA_DN6562_c0_g1_i1_2	1,20E-24	92	XP_008344138.1	aldehyde dehydrogenase family 7 member A1-like, partial	Malus domestica	XP_018844904.1
ARUBRA_DN9315_c0_g1_i1_1	1,72E-41	147	XP_018835062.1	heat shock 70 kDa protein 17	Juglans regia	ONI17954.1
ARUBRA_DN5821_c0_g1_i1_4	3,31E-37	132	XP_008236152.1	branched-chain-amino-acid aminotransferase 6-like	Prunus mume	XP_016650586.1
ARHOMBI_DN15559_c0_g1_i1_1	4,89E-88	260	XP_018805571.1	metacaspase-4-like, partial	Juglans regia	XP_018809771.1
ARHOMBI_DN3769_c0_g1_i1_4	5,04E-127	362	XP_018844530.1	uncharacterized protein LOC109008761	Juglans regia	ONI01120.1
ARUBRA_DN6679_c0_g1_i1_2	3,02E-54	182	XP_018823987.1	peroxisomal and mitochondrial division factor 2-like	Juglans regia	XP_018823987.1
ARUBRA_DN3045_c0_g1_i1_5	6,36E-171	479	XP_018814237.1	V-type proton ATPase subunit D-like	Juglans regia	XP_018814238.1
ARUBRA_DN21269_c0_g1_i1_2	0	564	XP_018824768.1	sucrose synthase 2	Juglans regia	OAY61856.1
ARHOMBI_DN3422_c0_g1_i1_5	1,67E-83	251	XP_020419740.1	basic transcription factor 3 isoform X1	Prunus persica	ONI09552.1
ARUBRA_DN11946_c0_g1_i1_3	8,40E-118	342	KRH42843.1	hypothetical protein GLYMA_08G115100	Glycine max	KRH42842.1
ARUBRA_DN428_c0_g1_i1_5	2,79E-38	140	XP_018842622.1	pyruvate kinase isozyme G, chloroplastic isoform X2	Juglans regia	XP_018842621.1
ARHOMBI_DN1985_c0_g2_i1_1	4,04E-89	286	KDP41738.1	hypothetical protein JCGZ_26756	Jatropha curcas	XP_012067196.1
ARUBRA_DN11238_c0_g1_i1_6	4,72E-84	251	XP_018835166.1	uncharacterized protein LOC109002047	Juglans regia	ONI23151.1
ARHOMBI_DN11429_c0_g1_i1_1	4,56E-35	119	XP_018822933.1	cytochrome c oxidase copper chaperone 1-like	Juglans regia	XP_018850576.1
ARUBRA_DN2583_c0_g2_i1_4	3,20E-70	209	XP_018821680.1	protein translation factor SUI1 homolog 1	Juglans regia	XP_018853744.1
ARHOMBI_DN5242_c0_g1_i1_4	0	617	XP_018829679.1	2-methyl-6-phytyl-1,4-hydroquinone methyltransferase, chloroplastic-like isoform X1	Juglans regia	XP_018829680.1
ARUBRA_DN3984_c0_g1_i1_3	0	903	XP_018811862.1	aspartic proteinase-like isoform X1	Juglans regia	XP_018811868.1

ARHOMBI_DN13132_c0_g1_i1_4	2,13E-26	99	XP_018852574.1	UPF0587 protein C1orf123 homolog	Juglans regia	ACU15162.1
ARHOMBI_DN17057_c0_g1_i1_3	4,30E-130	380	XP_018846380.1	glucose-6-phosphate 1-dehydrogenase, cytoplasmic isoform-like isoform X2	Juglans regia	XP_018846377.1
ARHOMBI_DN328_c0_g1_i1_6	2,34E-81	257	XP_018848571.1	dihydrolipoyllysine-residue acetyltransferase component 2 of pyruvate dehydrogenase complex, mitochondrial-like	Juglans regia	ONI15482.1
ARUBRA_DN4992_c0_g1_i2_1	1,17E-42	147	XP_018807016.1	putative methyltransferase DDB_G0268948	Juglans regia	XP_018817184.1
ARHOMBI_DN10598_c0_g1_i1_3	7,20E-82	253	XP_018826282.1	protein WVD2-like 1 isoform X2	Juglans regia	XP_018826281.1
ARHOMBI_DN4392_c0_g1_i1_3	0	699	XP_018852211.1	26S proteasome non-ATPase regulatory subunit 13 homolog A	Juglans regia	XP_018852212.1
ARUBRA_DN6255_c0_g1_i1_2	2,99E-77	241	XP_018856080.1	UBP1-associated protein 2C-like	Juglans regia	XP_018856080.1
ARUBRA_DN8387_c0_g1_i1_5	4,79E-160	449	XP_018825081.1	gamma carbonic anhydrase-like 2, mitochondrial	Juglans regia	OAY47968.1
ARUBRA_DN2057_c0_g1_i1_6	5,22E-154	439	XP_018805636.1	T-complex protein 1 subunit beta-like, partial	Juglans regia	XP_018812048.1
ARHOMBI_DN11616_c0_g1_i1_3	1,39E-50	166	XP_018850753.1	protein BOBBER 1	Juglans regia	XP_018838704.1
ARHOMBI_DN15140_c0_g1_i1_3	5,26E-30	115	XP_018834434.1	protein DETOXIFICATION 43-like	Juglans regia	XP_018834435.1
ARUBRA_DN186_c0_g1_i1_4	2,46E-155	447	XP_018833327.1	28 kDa ribonucleoprotein, chloroplastic-like	Juglans regia	KDP31006.1
ARUBRA_DN18420_c0_g1_i1_3	1,12E-49	169	XP_018839948.1	heparanase-like protein 3 isoform X3	Juglans regia	XP_018839937.1
ARHOMBI_DN736_c0_g2_i1_6	0	880	XP_018858503.1	UDP-sulfoquinovose synthase, chloroplastic	Juglans regia	XP_018858504.1
ARHOMBI_DN8058_c0_g1_i1_5	3,05E-174	496	XP_018852174.1	probable methyltransferase PMT21	Juglans regia	XP_018852176.1
ARHOMBI_DN13310_c0_g1_i1_4	2,47E-56	188	ONI16737.1	hypothetical protein PRUPE_3G118300	Prunus persica	XP_016168070.2
ARUBRA_DN19795_c0_g1_i1_2	1,43E-63	205	XP_008230393.1	T-complex protein 1 subunit zeta 1	Prunus mume	ONI18783.1
ARHOMBI_DN15499_c0_g1_i1_1	6,38E-116	339	XP_018820386.1	vesicle-associated protein 1-3-like	Juglans regia	XP_018853745.1
ARHOMBI_DN5591_c0_g1_i1_2	0	717	XP_018816814.1	mannose-6-phosphate isomerase 1	Juglans regia	OAY57080.1
ARHOMBI_DN2375_c0_g1_i1_6	1,79E-76	246	XP_018836580.1	aminopeptidase M1-like	Juglans regia	XP_018836578.1
ARHOMBI_DN15343_c0_g1_i1_2	0	974	XP_018842610.1	neutral ceramidase-like	Juglans regia	XP_018837606.1
ARUBRA_DN1522_c0_g1_i1_5	9,93E-141	404	KOM53906.1	hypothetical protein LR48_Vigan09g256500	Vigna angularis	XP_017434190.1
ARHOMBI_DN6216_c0_g1_i1_4	0	957	OAY33522.1	hypothetical protein MANES_13G103800	Manihot esculenta	OAY35689.1
ARHOMBI_DN5056_c0_g1_i1_4	1,67E-48	160	KDP44051.1	hypothetical protein JCGZ_05518	Jatropha curcas	XP_012064820.1
ARUBRA_DN4756_c0_g1_i1_6	1,26E-123	355	XP_018816816.1	40S ribosomal protein S7-like	Juglans regia	XP_018853133.1
ARHOMBI_DN3633_c0_g1_i1_2	0	792	XP_018844115.1	DEAD-box ATP-dependent RNA helicase 38	Juglans regia	KYP40304.1
ARHOMBI_DN16542_c0_g1_i1_1	3,75E-59	193	XP_018823842.1	coiled-coil domain-containing protein 124	Juglans regia	XP_007137314.1

ARHOMBI_DN2386_c0_g1_i1_2	1,29E-96	290	XP_018844419.1	3-oxoacyl-[acyl-carrier-protein synthase 3 A, chloroplastic-like	Juglans regia	OAY56674.1
ARHOMBI_DN2790_c0_g2_i1_3	1,13E-134	404	XP_018842400.1	eukaryotic translation initiation factor 3 subunit C isoform X1	Juglans regia	XP_018842401.1
ARHOMBI_DN5912_c0_g2_i4_5	1,67E-23	92	KYP74286.1	Metallothionein-like protein type 2	Cajanus cajan	XP_020237961.1
ARUBRA_DN9466_c0_g1_i1_6	5,24E-90	277	XP_018820447.1	T-complex protein 1 subunit epsilon	Juglans regia	OAY34423.1
ARHOMBI_DN5625_c0_g1_i1_5	1,52E-75	233	XP_009340388.1	thioredoxin F-type, chloroplastic-like isoform X2	Pyrus x bretschneideri	XP_009340351.1
ARUBRA_DN26355_c0_g1_i1_1	3,97E-105	318	XP_018827094.1	dihydroxy-acid dehydratase, chloroplastic-like	Juglans regia	XP_008224581.1
ARUBRA_DN17795_c0_g1_i1_3	2,71E-180	504	ONH97049.1	hypothetical protein PRUPE_7G166700	Prunus persica	XP_007204374.1
ARUBRA_DN4209_c0_g1_i1_6	2,72E-52	178	XP_018824320.1	probable trehalase	Juglans regia	XP_018835196.1
ARHOMBI_DN13841_c0_g1_i1_1	9,84E-52	172	ACN41352.2	protein phosphatase 2A regulatory subunit A, partial	Betula pendula	239586196
ARUBRA_DN7189_c0_g1_i1_4	0	704	XP_018848330.1	dnaJ protein ERDJ2A	Juglans regia	XP_018848331.1
ARUBRA_DN17439_c0_g1_i1_4	3,15E-51	170	XP_018838095.1	3-hydroxyisobutyryl-CoA hydrolase-like protein 3, mitochondrial isoform X1	Juglans regia	XP_018838096.1
ARUBRA_DN23385_c0_g1_i1_4	7,30E-128	377	XP_018834513.1	clathrin interactor EPSIN 1	Juglans regia	XP_018834519.1
ARUBRA_DN3392_c0_g2_i1_2	7,75E-158	447	XP_018849757.1	fruit protein pKIWI502-like	Juglans regia	KDP34990.1
ARUBRA_DN16420_c0_g1_i1_5	5,18E-83	263	XP_018837506.1	glucosidase 2 subunit beta	Juglans regia	XP_004505348.1
ARUBRA_DN5780_c0_g1_i1_6	3,56E-75	226	OAY37574.1	hypothetical protein MANES_11G112000	Manihot esculenta	OAY52073.1
ARHOMBI_DN9968_c0_g1_i1_3	9,76E-09	54,7	XP_006606594.1	clathrin interactor EPSIN 2-like isoform X4	Glycine max	XP_018499212.1
ARUBRA_DN22029_c0_g1_i1_1	8,01E-80	257	XP_018840813.1	heat shock 70 kDa protein 15-like	Juglans regia	XP_018812010.1
ARHOMBI_DN6264_c0_g1_i2_1	1,07E-165	467	OAY23950.1	hypothetical protein MANES_18G120300	Manihot esculenta	OAY23951.1
ARUBRA_DN9942_c0_g1_i1_2	1,74E-91	270	XP_018837336.1	14 kDa zinc-binding protein	Juglans regia	ONI11561.1
ARHOMBI_DN10129_c0_g1_i1_4	2,20E-124	360	KDP30971.1	hypothetical protein JCGZ_11347	Jatropha curcas	XP_012079902.1
ARUBRA_DN4816_c0_g4_i1_1	3,98E-09	52,8	XP_004493946.1	40S ribosomal protein S23-2-like	Cicer arietinum	AAL50317.1
ARHOMBI_DN9150_c0_g1_i1_2	4,16E-179	518	XP_018849604.1	programmed cell death protein 4-like	Juglans regia	XP_018849604.1
ARHOMBI_DN18526_c0_g1_i1_5	1,14E-72	223	XP_018812655.1	14-3-3 protein 2-like	Juglans regia	XP_009375314.1
ARHOMBI_DN5512_c0_g1_i1_3	0	568	XP_018860411.1	coatomer subunit epsilon-1	Juglans regia	XP_018828599.1
ARUBRA_DN18242_c0_g1_i1_1	4,41E-117	350	XP_018839074.1	UDP-sugar pyrophosphorylase-like	Juglans regia	XP_018849179.1
ARUBRA_DN6103_c0_g1_i1_5	1,97E-111	328	XP_018815959.1	eukaryotic translation initiation factor 4E-1-like	Juglans regia	XP_004485968.1
ARHOMBI_DN4464_c0_g1_i1_6	1,10E-90	269	XP_018825729.1	universal stress protein PHOS32-like isoform X2	Juglans regia	KDP27925.1
ARUBRA_DN21777_c0_g1_i1_4	0	906	XP_018840722.1	alanine--tRNA ligase-like	Juglans regia	XP_018840731.1
ARHOMBI_DN6373_c0_g1_i1_1	2,32E-37	135	XP_018836625.1	tryptophan--tRNA ligase, cytoplasmic	Juglans regia	XP_019462109.1
ARUBRA_DN4890_c0_g2_i1_5	1,57E-118	343	XP_018850349.1	temperature-induced lipocalin-1	Juglans regia	XP_018850350.1
ARHOMBI_DN3410_c0_g1_i1_4	4,33E-127	379	XP_018835196.1	probable trehalase	Juglans regia	XP_018824320.1

ARUBRA_DN1360_c0_g2_i1_4	6,64E-133	381	XP_018808102.1	polyadenylate-binding protein 2-like isoform X1	Juglans regia	XP_018831192.1
ARUBRA_DN20255_c0_g1_i1_4	5,14E-51	176	XP_018807977.1	uncharacterized protein LOC108981316	Juglans regia	OAY29434.1
ARUBRA_DN12071_c0_g1_i1_2	3,03E-107	319	XP_018837387.1	acetyl-CoA acetyltransferase, cytosolic 1	Juglans regia	XP_008384188.1
ARUBRA_DN25817_c0_g1_i1_2	2,15E-28	106	XP_015942168.2	LOW QUALITY PROTEIN: reticulon-like protein B11	Arachis duranensis	XP_018827369.1
ARUBRA_DN24145_c0_g1_i1_1	1,15E-64	204	XP_018844342.1	3-isopropylmalate dehydrogenase 2, chloroplastic-like	Juglans regia	KDP37016.1
ARHOMBI_DN6283_c0_g6_i1_6	3,10E-89	261	ABS10822.1	elongation factor 1-alpha, partial	Phaseolus vulgaris	AAT72900.1
ARUBRA_DN3001_c0_g1_i1_6	2,17E-80	242	XP_018850228.1	uncharacterized protein At4g28440-like	Juglans regia	XP_009340594.1
ARUBRA_DN18461_c0_g1_i1_6	2,08E-31	111	XP_018852112.1	histone deacetylase 6	Juglans regia	XP_008373046.1
ARUBRA_DN10353_c1_g1_i1_5	1,16E-98	296	OIV95683.1	hypothetical protein TanjilG_01477	Lupinus angustifolius	XP_004500418.1
ARHOMBI_DN25343_c0_g1_i1_2	7,75E-106	311	KRH64548.1	hypothetical protein GLYMA_04G240600	Glycine max	XP_006578942.1
ARHOMBI_DN7398_c0_g1_i1_3	3,34E-112	332	XP_018812439.1	ferrochelatase-2, chloroplastic-like	Juglans regia	KDP34118.1
ARHOMBI_DN6728_c0_g1_i1_4	4,76E-119	357	XP_018820151.1	external alternative NAD(P)H-ubiquinone oxidoreductase B2, mitochondrial-like	Juglans regia	OAY24726.1
ARUBRA_DN3802_c0_g1_i1_4	0	553	ACN41352.2	protein phosphatase 2A regulatory subunit A, partial	Betula pendula	ACN41352.2
ARHOMBI_DN13057_c0_g1_i1_1	3,36E-133	380	XP_018843798.1	OTU domain-containing protein 6B	Juglans regia	XP_018843799.1
ARUBRA_DN9924_c0_g1_i1_4	0	709	XP_018850292.1	obg-like ATPase 1	Juglans regia	OAY60642.1
ARHOMBI_DN430_c0_g1_i1_3	0	539	XP_008235142.1	ATP-dependent Clp protease proteolytic subunit 5, chloroplastic	Prunus mume	ONH94009.1
ARUBRA_DN3936_c0_g2_i1_4	1,54E-74	229	XP_008218541.1	stearoyl-[acyl-carrier-protein 9-desaturase, chloroplastic-like	Prunus mume	ONI05270.1
ARUBRA_DN4814_c0_g1_i3_4	3,85E-108	315	XP_009345456.1	thaumatin-like protein	Pyrus x bretschneideri	XP_018828872.1
ARUBRA_DN2381_c0_g1_i1_1	0	696	XP_018821126.1	mannan endo-1,4-beta-mannosidase 7-like	Juglans regia	ABV32547.1
ARHOMBI_DN6212_c0_g1_i2_6	1,03E-102	298	KDP42594.1	hypothetical protein JCGZ_24368	Jatropha curcas	XP_012066576.1
ARUBRA_DN20443_c0_g1_i1_3	2,51E-109	325	XP_018850555.1	alanine aminotransferase 1, mitochondrial-like	Juglans regia	XP_018850554.1
ARHOMBI_DN11311_c0_g1_i1_5	6,88E-116	354	XP_018846984.1	calcium-transporting ATPase 4, plasma membrane-type-like isoform X2	Juglans regia	XP_018846983.1
ARHOMBI_DN17429_c0_g1_i1_6	3,84E-96	296	XP_018846638.1	uncharacterized protein LOC109010309	Juglans regia	XP_008393841.1
ARUBRA_DN3578_c0_g1_i1_4	2,20E-132	379	XP_008362670.1	ras-related protein RABF1 isoform X1	Malus domestica	XP_009350234.1
ARUBRA_DN11131_c0_g1_i1_3	3,39E-27	107	XP_018847349.1	plastidial pyruvate kinase 2	Juglans regia	OAY48391.1
ARHOMBI_DN4521_c1_g1_i1_1	1,93E-98	305	XP_020974231.1	cell division control protein 48 homolog E isoform X5	Arachis ipaensis	XP_020974232.1
ARHOMBI_DN17261_c0_g1_i1_1	1,90E-154	436	XP_018859762.1	LOW QUALITY PROTEIN: methionine adenosyltransferase 2 subunit beta	Juglans regia	OAY62334.1

ARUBRA_DN1069_c0_g2_i1_1	8,75E-54	178	XP_018838239.1	triose phosphate/phosphate translocator, non-green plastid, chloroplastic-like	Juglans regia	XP_018821285.1
ARHOMBI_DN5132_c0_g2_i1_6	8,22E-28	103	XP_018824900.1	mitochondrial import receptor subunit TOM5 homolog	Juglans regia	OAY47495.1
ARUBRA_DN3311_c0_g1_i1_6	1,33E-31	118	XP_018827585.1	fumarylacetoacetase	Juglans regia	KDP37782.1
ARUBRA_DN8175_c0_g1_i1_5	0	831	XP_018815758.1	phosphoglucan, water dikinase, chloroplastic isoform X2	Juglans regia	XP_018815757.1
ARUBRA_DN16564_c0_g1_i1_6	3,67E-73	226	XP_018839578.1	3-hydroxyisobutyryl-CoA hydrolase-like protein 2, mitochondrial	Juglans regia	ONI32914.1
ARUBRA_DN19149_c0_g1_i1_6	0	911	XP_018835655.1	6-phosphofructo-2-kinase/fructose-2,6-bisphosphatase-like isoform X2	Juglans regia	XP_018835648.1
ARUBRA_DN5180_c0_g1_i1_3	1,45E-75	233	XP_008237176.1	endoplasmic reticulum-Golgi intermediate compartment protein 3-like	Prunus mume	XP_018836846.1
ARUBRA_DN20917_c0_g1_i1_3	5,13E-69	208	XP_018846675.1	peptidyl-prolyl cis-trans isomerase CYP18-1-like	Juglans regia	XP_018806960.1
ARHOMBI_DN20733_c0_g1_i1_3	6,90E-70	219	OAY60676.1	hypothetical protein MANES_01G130700	Manihot esculenta	XP_018828199.1
ARUBRA_DN10088_c0_g1_i1_1	1,28E-136	395	XP_018844904.1	aldehyde dehydrogenase family 7 member A1 isoform X1	Juglans regia	XP_018844905.1
ARHOMBI_DN19569_c0_g1_i1_5	5,52E-116	333	OAY53361.1	hypothetical protein MANES_04G157000	Manihot esculenta	XP_018818270.1
ARUBRA_DN2998_c0_g1_i1_1	5,41E-97	296	XP_018858960.1	clathrin light chain 2-like	Juglans regia	AFK42367.1
ARUBRA_DN1494_c0_g1_i1_3	0	1521	XP_018848396.1	ferredoxin-dependent glutamate synthase, chloroplastic	Juglans regia	XP_018826303.1
ARHOMBI_DN4699_c0_g1_i1_3	0	515	XP_018856419.1	uncharacterized protein LOC109018719	Juglans regia	XP_018856421.1
ARHOMBI_DN5814_c0_g1_i1_4	0	683	XP_018819958.1	malate dehydrogenase, glyoxysomal-like	Juglans regia	XP_018829628.1
ARHOMBI_DN21914_c0_g1_i1_1	2,60E-53	170	AFH88395.1	glyceraldehyde-3-phosphate dehydrogenase, partial	Eriobotrya japonica	XP_007137942.1
ARHOMBI_DN5567_c0_g1_i1_3	0	844	XP_018841744.1	oxysterol-binding protein-related protein 3A	Juglans regia	XP_018807417.1
ARUBRA_DN1463_c0_g2_i1_5	0	805	XP_012084017.1	transmembrane 9 superfamily member 12	Jatropha curcas	KDP45164.1
ARHOMBI_DN17982_c0_g1_i1_5	5,98E-89	268	XP_018826547.1	aldose 1-epimerase	Juglans regia	XP_008239819.1
ARUBRA_DN2123_c0_g2_i1_3	7,72E-134	407	XP_018828478.1	ubiquitin-activating enzyme E1 1	Juglans regia	XP_018828479.1
ARHOMBI_DN2126_c0_g2_i1_6	1,10E-77	248	XP_018814287.1	methyl-CpG-binding domain-containing protein 11-like isoform X1	Juglans regia	XP_018814287.1
ARUBRA_DN8853_c0_g1_i1_6	2,77E-52	165	XP_008347076.1	NAD-dependent malic enzyme 1, mitochondrial-like	Malus domestica	XP_019462740.1
ARUBRA_DN1745_c0_g2_i1_1	7,41E-67	211	XP_018824705.1	acetylornithine deacetylase	Juglans regia	XP_008238268.1
ARHOMBI_DN3877_c0_g1_i1_1	1,51E-96	284	XP_018827791.1	probable carboxylesterase SOBER1-like	Juglans regia	OAY25864.1
ARUBRA_DN3955_c0_g2_i1_2	2,29E-31	110	XP_018836020.1	uncharacterized protein LOC109002634	Juglans regia	XP_018823193.1

ARUBRA_DN8757_c0_g1_i1_5	4,77E-127	363	XP_018829348.1	uncharacterized protein At4g14100-like isoform X1	Juglans regia	XP_018841627.1
ARHOMBI_DN1665_c0_g1_i1_2	5,03E-169	476	XP_018824435.1	mitochondrial import receptor subunit TOM40-1-like	Juglans regia	XP_018815797.1
ARHOMBI_DN11418_c0_g1_i1_2	1,65E-44	155	XP_018806016.1	vacuolar-sorting receptor 3-like	Juglans regia	XP_018846135.1
ARHOMBI_DN8306_c0_g1_i1_4	9,10E-86	254	XP_018825134.1	ubiquitin carboxyl-terminal hydrolase 3-like	Juglans regia	XP_018825135.1
ARUBRA_DN20020_c0_g1_i1_2	2,06E-41	149	XP_018817298.1	protein transport protein SEC31 homolog B-like isoform X2	Juglans regia	XP_018817293.1
ARUBRA_DN148_c0_g1_i1_1	1,52E-58	185	XP_018830706.1	CDGSH iron-sulfur domain-containing protein NEET	Juglans regia	XP_020221377.1
ARHOMBI_DN25089_c0_g1_i1_1	1,60E-89	276	XP_018806898.1	lysine--tRNA ligase, cytoplasmic isoform X2	Juglans regia	XP_018806897.1
ARUBRA_DN11014_c0_g1_i1_6	8,02E-50	164	XP_018818410.1	probable choline kinase 2	Juglans regia	OAY36267.1
ARHOMBI_DN18155_c0_g1_i1_2	1,00E-99	293	XP_018827856.1	thioredoxin domain-containing protein 9 homolog	Juglans regia	OAY45497.1
ARUBRA_DN5032_c2_g1_i2_2	9,35E-74	226	KDP45286.1	hypothetical protein JCGZ_15151	Jatropha curcas	OAY34830.1
ARHOMBI_DN5326_c0_g1_i1_3	1,98E-83	249	XP_018813019.1	uncharacterized protein LOC108985261	Juglans regia	XP_018844572.1
ARHOMBI_DN15674_c0_g1_i1_5	3,28E-48	155	XP_009371619.1	phosphopantetheine adenylyltransferase-like isoform X1	Pyrus x bretschneideri	XP_018820994.1
ARHOMBI_DN5386_c1_g1_i2_5	4,38E-97	295	XP_018823251.1	THO complex subunit 4A-like isoform X1	Juglans regia	XP_018836017.1
ARUBRA_DN4407_c0_g4_i1_6	4,07E-115	332	ONI18797.1	hypothetical protein PRUPE_3G239900	Prunus persica	XP_007215041.1
ARHOMBI_DN17588_c0_g1_i1_4	1,24E-53	176	XP_018816946.1	eukaryotic translation initiation factor 3 subunit M-like	Juglans regia	XP_008231016.1
ARHOMBI_DN7219_c0_g1_i1_3	9,54E-43	150	XP_018807664.1	subtilisin-like protease SBT1.7, partial	Juglans regia	OAY33654.1
ARHOMBI_DN7958_c0_g1_i1_4	1,40E-39	138	XP_018815797.1	mitochondrial import receptor subunit TOM40-1-like	Juglans regia	XP_018824435.1
ARUBRA_DN4721_c0_g1_i2_1	7,12E-76	246	XP_018825127.1	plasminogen activator inhibitor 1 RNA-binding protein	Juglans regia	OAY60106.1
ARHOMBI_DN5441_c0_g1_i1_5	1,62E-89	273	XP_018851989.1	universal stress protein PHOS32-like isoform X2	Juglans regia	XP_018851981.1
ARUBRA_DN7416_c0_g1_i1_6	0	680	XP_018847636.1	ornithine aminotransferase, mitochondrial-like	Juglans regia	XP_018819712.1
ARUBRA_DN6341_c0_g1_i1_1	5,71E-105	324	XP_018816636.1	NADH dehydrogenase ubiquinone iron-sulfur protein 1, mitochondrial	Juglans regia	XP_008381363.1
ARUBRA_DN25250_c0_g1_i1_2	4,21E-55	187	XP_018809947.1	eukaryotic translation initiation factor 3 subunit A-like	Juglans regia	XP_018841709.1
ARUBRA_DN12031_c0_g1_i1_3	2,08E-37	132	XP_018816770.1	pyridoxal kinase-like isoform X1	Juglans regia	ONI17422.1
ARUBRA_DN9279_c0_g1_i1_5	3,18E-42	149	KDP38191.1	hypothetical protein JCGZ_04834	Jatropha curcas	XP_012072397.1
ARHOMBI_DN3515_c0_g1_i1_3	2,96E-71	217	XP_018857344.1	3-oxoacyl-[acyl-carrier-protein reductase, chloroplastic	Juglans regia	OAY34372.1

ARHOMBI_DN4682_c0_g1_i1_3	6,04E-26	101	XP_018852998.1	60S acidic ribosomal protein P3-like	Juglans regia	XP_018860534.1
ARHOMBI_DN23760_c0_g1_i1_6						
ARHOMBI_DN15221_c0_g1_i1_1	1,45E-55	182	OIW15548.1	hypothetical protein TanjilG_01071	Lupinus angustifolius	OIW15548.1
ARHOMBI_DN9078_c0_g1_i1_6	6,22E-94	286	XP_018839513.1	nicalin-1 isoform X1	Juglans regia	XP_018839514.1
ARUBRA_DN11010_c0_g1_i1_3	1,32E-82	248	KHN24774.1	Eukaryotic translation initiation factor 3 subunit D	Glycine soja	KOM53769.1
ARUBRA_DN17427_c0_g1_i1_4	6,10E-71	216	XP_018822168.1	peptidyl-prolyl cis-trans isomerase CYP21-4-like	Juglans regia	XP_018842322.1
ARUBRA_DN20283_c0_g1_i1_1	1,84E-154	448	XP_018821744.1	probable alpha-galactosidase B	Juglans regia	OAY32489.1
ARUBRA_DN18073_c0_g1_i1_6	7,15E-137	389	XP_018850344.1	VAMP-like protein YKT61	Juglans regia	XP_018833476.1
ARUBRA_DN4594_c0_g2_i1_4	0	839	XP_018818227.1	serine carboxypeptidase-like 20	Juglans regia	XP_004510209.1
ARUBRA_DN4871_c0_g2_i1_3	0	616	XP_018808617.1	receptor-like protein kinase HAIKU2	Juglans regia	XP_018808617.1
ARHOMBI_DN5577_c1_g1_i1_5	9,11E-115	348	XP_018822921.1	methionine--tRNA ligase, cytoplasmic	Juglans regia	KDP39347.1
ARHOMBI_DN21271_c0_g1_i1_1	2,29E-68	213	XP_018845017.1	probable 2-oxoglutarate-dependent dioxygenase At3g49630 isoform X2	Juglans regia	XP_018845016.1
ARUBRA_DN8542_c0_g1_i1_4	1,28E-118	348	XP_018825622.1	heterogeneous nuclear ribonucleoprotein 1	Juglans regia	KDP39313.1
ARHOMBI_DN10118_c0_g1_i1_1	6,38E-53	166	AAC19381.1	inosine-5'-monophosphate dehydrogenase, partial	Prunus persica	XP_020963350.1
ARUBRA_DN18680_c0_g1_i1_3	5,83E-137	393	XP_018825911.1	ERBB-3 BINDING PROTEIN 1 isoform X2	Juglans regia	XP_018825910.1
ARUBRA_DN2284_c0_g2_i1_3	4,42E-150	427	XP_018842516.1	rhodanese-like/PpiC domain-containing protein 12, chloroplastic	Juglans regia	XP_018842517.1
ARUBRA_DN7415_c0_g1_i1_6	6,48E-170	483	XP_018831898.1	UDP-glucose 6-dehydrogenase 3	Juglans regia	XP_018818494.1
ARUBRA_DN113_c0_g1_i2_3	8,46E-143	404	XP_018839374.1	proteasome subunit beta type-2-A	Juglans regia	XP_018812168.1
ARHOMBI_DN26770_c0_g1_i1_3	6,65E-63	209	XP_018807321.1	aconitate hydratase 1	Juglans regia	KDP23953.1
ARHOMBI_DN16112_c0_g1_i1_2	5,91E-107	314	KDP41839.1	hypothetical protein JCGZ_26857	Jatropha curcas	XP_012067332.1
ARUBRA_DN17277_c0_g1_i1_1	4,93E-51	162	XP_018839925.1	dormancy-associated protein homolog 4 isoform X1	Juglans regia	XP_008228141.1
ARHOMBI_DN1288_c0_g1_i1_2	1,35E-22	92,4	AFK35200.1	unknown	Medicago truncatula	KEH24737.1
ARUBRA_DN70_c0_g1_i1_1	1,62E-135	390	XP_018843274.1	clavaminate synthase-like protein At3g21360	Juglans regia	XP_009353541.1
ARUBRA_DN18307_c0_g1_i1_1	0	526	XP_018833905.1	3-oxo-Delta(4,5)-steroid 5-beta-reductase-like	Juglans regia	XP_018836149.1
ARUBRA_DN17020_c0_g1_i1_1	0	669	XP_018819162.1	eukaryotic translation initiation factor 4G-like isoform X1	Juglans regia	XP_018819170.1
ARUBRA_DN4917_c0_g1_i2_1	1,77E-170	483	XP_018850886.1	ribulose-phosphate 3-epimerase, chloroplastic	Juglans regia	KYP55554.1
ARHOMBI_DN5372_c0_g1_i1_3	8,30E-121	347	XP_018836138.1	probable signal peptidase complex subunit 2	Juglans regia	XP_009350939.1

ARHOMBI_DN24609_c0_g1_i1_6	5,10E-55	179	OAY25201.1	hypothetical protein MANES_17G074700	Manihot esculenta	XP_018820972.1
ARHOMBI_DN2660_c0_g1_i1_5	1,00E-141	412	XP_018845609.1	oligouridylate-binding protein 1B-like	Juglans regia	XP_018845609.1
ARUBRA_DN16475_c0_g1_i1_2	6,91E-41	147	XP_018807473.1	uncharacterized protein LOC108980890	Juglans regia	ONH99277.1
ARUBRA_DN23765_c0_g1_i1_1	1,60E-46	157	XP_018822676.1	GDSL esterase/lipase At3g26430-like	Juglans regia	XP_012085878.1
ARHOMBI_DN22879_c0_g1_i1_2	5,36E-128	381	XP_018860049.1	vesicle-fusing ATPase	Juglans regia	XP_018860050.1
ARUBRA_DN8139_c0_g1_i1_1	4,44E-97	287	XP_018826389.1	probable NAD(P)H dehydrogenase (quinone) FQR1-like 2	Juglans regia	XP_019422117.1
ARUBRA_DN1221_c0_g2_i1_5	2,52E-75	223	KDP44839.1	hypothetical protein JCGZ_01339	Jatropha curcas	XP_012088066.1
ARHOMBI_DN14041_c0_g1_i1_6	1,43E-33	125	OAY27593.1	hypothetical protein MANES_16G137600	Manihot esculenta	XP_018845600.1
ARUBRA_DN4848_c0_g1_i1_3	3,60E-18	82	XP_016181529.1	stromal cell-derived factor 2-like protein	Arachis ipaensis	XP_016181529.1
ARUBRA_DN25967_c0_g1_i1_1	9,64E-73	218	OIW11578.1	hypothetical protein TanjilG_26944	Lupinus angustifolius	XP_019443882.1
ARUBRA_DN6775_c0_g1_i1_1	6,04E-98	288	XP_018860616.1	protein canopy-1	Juglans regia	XP_008237962.1
ARHOMBI_DN4127_c0_g1_i1_6	0	640	AIE47268.1	glucose-6-phosphate dehydrogenase	Hevea brasiliensis	XP_018846380.1
ARUBRA_DN6005_c0_g1_i1_2	5,11E-36	132	XP_018845600.1	L-arabinokinase-like	Juglans regia	OAY27593.1
ARUBRA_DN9533_c0_g1_i1_6	7,02E-59	194	XP_018816821.1	2-hydroxyacyl-CoA lyase	Juglans regia	OAY60450.1
ARUBRA_DN4945_c1_g1_i1_4	3,62E-160	465	KDP33769.1	hypothetical protein JCGZ_07340	Jatropha curcas	XP_012076828.1
ARUBRA_DN2632_c0_g1_i1_5	0	568	XP_018856841.1	quinone oxidoreductase PIG3-like	Juglans regia	XP_016179633.1
ARUBRA_DN253_c0_g2_i1_2	7,88E-30	106	XP_004487852.2	40S ribosomal protein S21-2	Cicer arietinum	XP_004504058.1
ARUBRA_DN4035_c0_g2_i1_1	0	852	XP_018818699.1	serine carboxypeptidase II-2	Juglans regia	XP_008243035.1
ARHOMBI_DN5174_c0_g1_i1_1	6,43E-96	285	XP_018820980.1	isoflavone reductase-like protein	Juglans regia	XP_018820986.1
ARUBRA_DN3267_c0_g1_i1_6	9,60E-124	362	XP_018829231.1	reticulon-like protein B2	Juglans regia	XP_014510434.1
ARUBRA_DN12853_c0_g1_i1_1	5,14E-68	209	BAT89111.1	hypothetical protein VIGAN_05280500	Vigna angularis var. angularis	XP_007146040.1
ARHOMBI_DN20424_c0_g1_i1_6	5,08E-65	206	KDP43068.1	hypothetical protein JCGZ_25254	Jatropha curcas	XP_012066099.1
ARUBRA_DN4281_c0_g1_i1_1	0	751	OAY47083.1	hypothetical protein MANES_06G050800	Manihot esculenta	XP_008242710.1
ARHOMBI_DN3485_c0_g1_i1_4	1,51E-103	303	XP_018828370.1	nucleoid-associated protein At4g30620, chloroplastic-like	Juglans regia	XP_015933002.1
ARUBRA_DN18869_c0_g1_i1_5	0	612	XP_018836294.1	arginine--tRNA ligase, cytoplasmic-like isoform X1	Juglans regia	XP_018836295.1
ARHOMBI_DN804_c0_g1_i1_6	3,08E-85	254	XP_018837605.1	proliferating cell nuclear antigen-like	Juglans regia	XP_018842612.1
ARUBRA_DN1537_c0_g1_i1_2	1,36E-66	205	XP_018852254.1	glycine-rich RNA-binding protein 4, mitochondrial	Juglans regia	XP_008226798.1
ARUBRA_DN1957_c0_g2_i1_6	1,14E-95	293	XP_018836294.1	arginine--tRNA ligase, cytoplasmic-like isoform X1	Juglans regia	XP_008242476.1
ARUBRA_DN6807_c0_g1_i1_3	8,80E-165	466	XP_018857179.1	diaminopimelate decarboxylase 2, chloroplastic-like, partial	Juglans regia	XP_008236927.1
ARUBRA_DN4637_c0_g2_i1_1	0	747	XP_018816480.1	S-adenosylmethionine synthase 5-like	Juglans regia	XP_020223541.1

ARHOMBI_DN6131_c0_g1_i4_6	0	523	XP_018844199.1	putative quinone-oxidoreductase homolog, chloroplastic	Juglans regia	XP_018850172.1
ARUBRA_DN5357_c0_g1_i1_1	6,48E-95	293	XP_008223754.1	lysine--tRNA ligase, cytoplasmic	Prunus mume	XP_018806898.1
ARUBRA_DN2637_c0_g1_i1_4	1,14E-119	345	XP_018820087.1	tankyrase	Juglans regia	OIV89168.1
ARHOMBI_DN16197_c0_g1_i1_4	1,63E-114	344	XP_018811770.1	target of Myb protein 1-like	Juglans regia	XP_018845550.1
ARUBRA_DN10661_c0_g1_i1_4	2,48E-163	472	XP_018842287.1	probable methyltransferase PMT3	Juglans regia	XP_018808117.1
ARHOMBI_DN4011_c0_g2_i1_5						
ARUBRA_DN4545_c0_g1_i1_5	4,32E-85	255	XP_018826140.1	endo-1,3;1,4-beta-D-glucanase-like isoform X1	Juglans regia	XP_018826141.1
ARUBRA_DN22266_c0_g1_i1_1	8,87E-108	321	XP_018833157.1	serine carboxypeptidase-like 51	Juglans regia	XP_018833716.1
ARUBRA_DN9362_c0_g1_i1_5	2,08E-61	189	XP_018813900.1	UPF0235 protein C15orf40 homolog	Juglans regia	ACU16361.1
ARUBRA_DN22269_c0_g1_i1_4	8,53E-40	137	XP_018814804.1	protein phosphatase 1 regulatory subunit pprA	Juglans regia	XP_008242516.1
ARHOMBI_DN3395_c0_g2_i1_6	2,54E-172	484	XP_012076496.1	protein MEMO1	Jatropha curcas	OAY27428.1
ARUBRA_DN18997_c0_g1_i1_6	0	1020	XP_018856581.1	carotenoid 9,10(9',10')-cleavage dioxygenase 1	Juglans regia	ONI20422.1
ARHOMBI_DN6095_c0_g1_i1_4	0	668	XP_018852657.1	dnaJ protein homolog	Juglans regia	XP_018805733.1
ARHOMBI_DN1066_c0_g2_i1_3	1,88E-72	228	XP_008350414.1	protein GPR107-like	Malus domestica	XP_008366712.1
ARHOMBI_DN14495_c0_g1_i1_4	2,21E-41	143	XP_018815296.1	V-type proton ATPase subunit a3-like	Juglans regia	XP_018840323.1
ARUBRA_DN10359_c0_g1_i1_4	0	612	OAY29373.1	hypothetical protein MANES_15G139700	Manihot esculenta	XP_019463977.1
ARUBRA_DN20926_c0_g1_i1_1	1,48E-38	141	XP_018832881.1	staphylococcal nuclease domain-containing protein 1-like	Juglans regia	XP_018832881.1
ARHOMBI_DN3507_c0_g1_i1_4	0	644	XP_018848804.1	E3 ubiquitin-protein ligase RHF2A-like	Juglans regia	XP_018851155.1
ARHOMBI_DN25279_c0_g1_i1_2	1,51E-45	154	XP_018808619.1	tubulin alpha chain-like isoform X2	Juglans regia	XP_018822099.1
ARHOMBI_DN4819_c0_g1_i1_1	0	689	XP_018820447.1	T-complex protein 1 subunit epsilon	Juglans regia	KDP26716.1
ARHOMBI_DN6082_c0_g1_i3_5	2,42E-49	164	XP_016650474.1	glycine-rich RNA-binding protein GRP1A	Prunus mume	ONH91156.1
ARUBRA_DN24896_c0_g1_i1_3	3,60E-47	156	OAY56694.1	hypothetical protein MANES_02G037700	Manihot esculenta	OAY56891.1
ARUBRA_DN6755_c0_g1_i1_2	7,34E-93	289	XP_018847996.1	importin subunit alpha-1a-like	Juglans regia	XP_018847993.1
ARHOMBI_DN5218_c0_g1_i2_1	0	519	XP_018837992.1	uncharacterized protein LOC109004054	Juglans regia	XP_018837993.1
ARUBRA_DN25837_c0_g1_i1_3	4,34E-88	261	KHN16764.1	6-phosphogluconate dehydrogenase, decarboxylating	Glycine soja	OAY56407.1
ARUBRA_DN24388_c0_g1_i1_4	8,41E-20	85,9	XP_018851380.1	uncharacterized protein LOC109013676	Juglans regia	KHN34605.1
ARHOMBI_DN11066_c0_g1_i1_4	3,77E-64	205	OIW06914.1	hypothetical protein TanjilG_19563	Lupinus angustifolius	XP_019452505.1
ARUBRA_DN3745_c0_g2_i1_2	7,70E-54	170	XP_018826216.1	uncharacterized protein At5g01610-like	Juglans regia	XP_018809179.1
ARUBRA_DN14017_c0_g1_i1_3	2,50E-15	73,2	OAY48578.1	hypothetical protein MANES_06G168500	Manihot esculenta	XP_018851510.1
ARUBRA_DN145_c0_g1_i1_4	1,03E-57	187	XP_018828823.1	rhomboid-like protein 19	Juglans regia	XP_008236446.1
ARUBRA_DN11007_c0_g1_i1_2	9,09E-52	170	XP_018836308.1	uncharacterized protein LOC109002851	Juglans regia	XP_008224696.1

ARUBRA_DN9096_c0_g1_i1_3	1,70E-18	82	ONH97426.1	hypothetical protein PRUPE_7G189500	Prunus persica	ONH97424.1
ARHOMBI_DN4782_c0_g1_i1_2	0	1031	XP_018840640.1	cullin-1-like	Juglans regia	XP_018826086.1
ARHOMBI_DN2849_c0_g1_i1_3	0	580	XP_018821210.1	dnaJ protein ERDJ3B	Juglans regia	OIW17431.1
ARHOMBI_DN7054_c0_g1_i1_3	3,31E-168	493	XP_007141284.1	hypothetical protein PHAVU_008G183200g	Phaseolus vulgaris	ESW13278.1
ARHOMBI_DN2798_c0_g1_i1_2	0	718	XP_018855488.1	protein TOC75-3, chloroplastic-like	Juglans regia	ONI00157.1
ARUBRA_DN18224_c0_g1_i1_4	1,33E-86	271	XP_018857284.1	villin-2-like isoform X2	Juglans regia	XP_018857284.1
ARHOMBI_DN25584_c0_g1_i1_5	4,87E-21	84,3	OIW09151.1	hypothetical protein TanjilG_11289	Lupinus angustifolius	XP_019447899.1
ARHOMBI_DN3781_c0_g2_i1_5	2,12E-102	303	XP_018805307.1	ATP-dependent Clp protease ATP-binding subunit CLPT1, chloroplastic	Juglans regia	XP_008231397.1
ARHOMBI_DN7337_c0_g1_i1_1	2,12E-26	109	XP_018821135.1	protein RCC2 homolog	Juglans regia	KDP36956.1
ARHOMBI_DN5812_c2_g1_i1_3	9,59E-96	288	XP_018835421.1	eukaryotic translation initiation factor 5-like isoform X2	Juglans regia	XP_018835420.1
ARUBRA_DN4150_c2_g1_i1_4	7,03E-110	315	KYP67019.1	Ras-related protein RABH1B	Cajanus cajan	XP_018843528.1
ARHOMBI_DN1574_c0_g1_i1_4	2,17E-101	294	XP_008366394.1	T-complex protein 1 subunit eta-like	Malus domestica	XP_008363557.2
ARUBRA_DN8202_c0_g1_i1_4	2,73E-95	285	XP_018860318.1	protein PHR1-LIKE 2-like	Juglans regia	XP_018837130.1
ARUBRA_DN1551_c0_g1_i1_5	1,74E-27	107	XP_018824867.1	ras GTPase-activating protein-binding protein 2 isoform X1	Juglans regia	XP_018824868.1
ARUBRA_DN12626_c0_g1_i1_3	7,28E-118	343	XP_018815857.1	chloride conductance regulatory protein ICln isoform X1	Juglans regia	KDP22395.1
ARHOMBI_DN4054_c0_g1_i1_3	4,16E-76	230	ACJ86035.1	unknown	Medicago truncatula	AFK46052.1
ARUBRA_DN9286_c0_g1_i1_1	1,47E-134	382	XP_018820201.1	stem-specific protein TSJT1-like	Juglans regia	ONI10376.1
ARHOMBI_DN3767_c0_g1_i1_1	5,33E-72	216	ONI04863.1	hypothetical protein PRUPE_6G344500	Prunus persica	XP_007206144.1
ARUBRA_DN20292_c0_g1_i1_6	3,59E-168	479	XP_018857004.1	4-hydroxyphenylpyruvate dioxygenase	Juglans regia	OAY57333.1
ARHOMBI_DN2005_c0_g1_i1_5	3,61E-63	196	XP_018814411.1	uncharacterized protein LOC108986289	Juglans regia	KDP21869.1
ARUBRA_DN4908_c0_g2_i1_1	2,22E-132	386	XP_018854291.1	uncharacterized protein LOC109016313	Juglans regia	OAY62495.1
ARUBRA_DN1276_c0_g1_i1_4	1,21E-156	446	XP_008351677.1	serine/threonine-protein phosphatase PP2A catalytic subunit	Malus domestica	XP_008384402.1
ARUBRA_DN4583_c0_g1_i2_5	2,03E-46	155	XP_018836815.1	uncharacterized protein LOC109003223	Juglans regia	XP_018836816.1
ARHOMBI_DN4896_c0_g1_i3_2	1,41E-65	203	ONI33798.1	hypothetical protein PRUPE_1G446400	Prunus persica	XP_007226749.2
ARUBRA_DN12695_c0_g1_i1_2	1,75E-41	143	XP_008238951.1	uncharacterized protein LOC103337565	Prunus mume	ONI07201.1
ARHOMBI_DN15171_c0_g1_i1_3	8,58E-102	305	XP_008237316.1	LOW QUALITY PROTEIN: dihydrolipoyllysine-residue acetyltransferase component 5 of pyruvate dehydrogenase complex, chloroplastic	Prunus mume	KDP47113.1
ARHOMBI_DN7103_c0_g1_i1_1	5,99E-45	150	XP_018845017.1	probable 2-oxoglutarate-dependent dioxygenase At3g49630 isoform X2	Juglans regia	KRH04548.1
ARHOMBI_DN5209_c1_g1_i1_4	8,03E-139	419	XP_018817247.1	calcium-transporting ATPase 1, endoplasmic reticulum-type-like	Juglans regia	XP_018818320.1
ARHOMBI_DN22071_c0_g1_i1_3	2,92E-81	252	XP_009344764.1	uncharacterized protein LOC103936642 isoform X3	Pyrus x bretschneideri	XP_008368102.1

ARHOMBI_DN1841_c0_g2_i1_2	2,86E-147	434	XP_018836272.1	cell division cycle protein 48 homolog	Juglans regia	XP_018836272.1
ARUBRA_DN563_c0_g1_i1_2	2,54E-123	356	XP_018848102.1	dehydrogenase/reductase SDR family member 12 isoform X2	Juglans regia	XP_018848101.1
ARUBRA_DN24632_c0_g1_i1_4	9,96E-58	188	OAY48675.1	hypothetical protein MANES_06G176400	Manihot esculenta	ACU18322.1
ARUBRA_DN3539_c0_g1_i1_2	4,93E-175	489	XP_018859721.1	uncharacterized protein LOC109021532	Juglans regia	XP_008244645.1
ARUBRA_DN1361_c0_g2_i1_4	1,33E-97	285	XP_018850281.1	NADH dehydrogenase ubiquinone 1 alpha subcomplex subunit 13-B-like	Juglans regia	XP_018839306.1
ARUBRA_DN11150_c0_g1_i1_6	3,56E-55	179	KDP46339.1	hypothetical protein JCGZ_10179	Jatropha curcas	XP_012071167.1
ARHOMBI_DN1996_c0_g2_i1_5	3,47E-90	281	XP_018841544.1	ubiquitin domain-containing protein DSK2a-like isoform X3	Juglans regia	XP_018841544.1
ARHOMBI_DN2999_c0_g1_i1_4	0	622	XP_018815793.1	eukaryotic translation initiation factor 3 subunit H	Juglans regia	OAY60536.1
ARHOMBI_DN2060_c0_g1_i1_5	1,59E-127	391	KDP41738.1	hypothetical protein JCGZ_26756	Jatropha curcas	XP_012067196.1
ARUBRA_DN9709_c0_g1_i1_6	2,25E-61	200	XP_018824867.1	ras GTPase-activating protein-binding protein 2 isoform X1	Juglans regia	XP_018824868.1
ARHOMBI_DN23173_c0_g1_i1_6	4,60E-70	224	XP_018851869.1	diacylglycerol kinase 5-like	Juglans regia	KRH26026.1
ARHOMBI_DN10290_c0_g1_i1_3	2,82E-23	97,1	XP_018848084.1	polyadenylate-binding protein 3	Juglans regia	XP_018807796.1
ARUBRA_DN2965_c0_g1_i2_5	2,60E-165	467	XP_018833326.1	protein-L-isoaspartate O-methyltransferase 1-like	Juglans regia	XP_008239363.1
ARUBRA_DN3863_c1_g1_i1_5	0	541	XP_009345628.1	PTI1-like tyrosine-protein kinase 1 isoform X1	Pyrus x bretschneideri	XP_009345630.1
ARHOMBI_DN10379_c0_g1_i1_2	6,88E-86	273	XP_008386852.1	5-methyltetrahydropteroyltriglutamate--homocysteine methyltransferase-like	Malus domestica	XP_008386853.1
ARHOMBI_DN2726_c0_g1_i1_1	5,05E-66	207	XP_018821762.1	uncharacterized protein LOC108991825	Juglans regia	XP_018838963.1
ARUBRA_DN7712_c0_g1_i1_6	6,70E-64	212	OAY28247.1	hypothetical protein MANES_15G052800	Manihot esculenta	OAY55369.1
ARUBRA_DN4320_c0_g1_i1_6	2,69E-175	491	XP_018835690.1	eukaryotic translation initiation factor 2 subunit beta-like	Juglans regia	XP_018828541.1
ARUBRA_DN11275_c0_g1_i1_5	7,60E-55	181	XP_018850953.1	dual specificity protein phosphatase 12-like	Juglans regia	XP_018850954.1
ARHOMBI_DN8255_c0_g1_i1_2	3,04E-160	460	XP_018820151.1	external alternative NAD(P)H-ubiquinone oxidoreductase B2, mitochondrial-like	Juglans regia	ONI10254.1
ARUBRA_DN13834_c0_g1_i1_4	1,37E-55	174	XP_008392677.1	probable methyltransferase PMT18	Malus domestica	OAY59057.1
ARUBRA_DN19618_c0_g1_i1_4	0	533	XP_018842610.1	neutral ceramidase-like	Juglans regia	ONI00029.1
ARUBRA_DN1773_c0_g2_i1_6	4,52E-66	211	XP_018816505.1	probable L-cysteine desulfhydrase, chloroplastic	Juglans regia	XP_018816506.1
ARUBRA_DN1305_c0_g1_i1_6	0	526	XP_018806331.1	60S ribosomal protein L3-2	Juglans regia	XP_018806332.1
ARHOMBI_DN3128_c0_g1_i1_1	1,26E-163	469	ONI03290.1	hypothetical protein PRUPE_6G249100	Prunus persica	XP_007205407.1
ARHOMBI_DN20118_c0_g1_i1_1	2,44E-75	239	XP_008235570.1	uncharacterized protein LOC103334389	Prunus mume	ONI20178.1
ARHOMBI_DN2022_c0_g1_i1_1	3,25E-69	210	XP_018812852.1	uncharacterized protein At2g34160-like	Juglans regia	OAY27368.1

ARHOMBI_DN3079_c0_g1_i1_3	0	656	XP_018808362.1	porphobilinogen deaminase, chloroplastic isoform X1	Juglans regia	KDP37560.1
ARHOMBI_DN8476_c0_g1_i1_3	3,21E-91	268	XP_018851840.1	succinate dehydrogenase subunit 6, mitochondrial	Juglans regia	OAY37464.1
ARHOMBI_DN4305_c0_g1_i2_6	3,35E-98	292	XP_018808868.1	nifU-like protein 1, chloroplastic	Juglans regia	ALP70497.1
ARUBRA_DN10474_c0_g1_i1_2	6,58E-96	284	AES60048.1	serine/threonine protein phosphatase 2A	Medicago truncatula	XP_003589797.1
ARHOMBI_DN6299_c0_g1_i1_1	2,38E-74	238	OAY50416.1	hypothetical protein MANES_05G133800	Manihot esculenta	OAY50415.1
ARHOMBI_DN3992_c0_g1_i1_6	0	700	XP_018846135.1	vacuolar-sorting receptor 3-like	Juglans regia	XP_018846136.1
ARUBRA_DN8631_c0_g1_i1_6	1,95E-74	233	XP_018841097.1	probable WRKY transcription factor 3 isoform X2	Juglans regia	XP_018841096.1
ARUBRA_DN158_c0_g1_i1_2	2,83E-156	456	XP_018844637.1	far upstream element-binding protein 2 isoform X1	Juglans regia	XP_018844638.1
ARUBRA_DN6110_c0_g1_i1_1	3,31E-64	199	XP_018807798.1	uncharacterized protein LOC108981167	Juglans regia	XP_018807799.1
ARUBRA_DN4152_c0_g1_i1_5	2,52E-72	220	XP_018825467.1	ankyrin repeat-containing protein P16F5.05c isoform X2	Juglans regia	XP_018825466.1
ARHOMBI_DN20386_c0_g1_i1_1	1,02E-78	239	ONI27997.1	hypothetical protein PRUPE_1G116000	Prunus persica	XP_007223205.1
ARUBRA_DN3314_c0_g1_i1_4	3,45E-62	195	XP_018833952.1	acyl carrier protein 1, chloroplastic-like	Juglans regia	XP_018840027.1
ARHOMBI_DN19207_c0_g1_i1_4	1,29E-36	133	XP_018816929.1	uncharacterized protein LOC108988207	Juglans regia	XP_018843198.1
ARUBRA_DN13376_c0_g1_i1_2	4,62E-120	343	AAL16968.1	AF367452_1 hexokinase, partial	Prunus persica	XP_009338735.1
ARHOMBI_DN16745_c0_g1_i1_1	6,77E-43	154	XP_018819170.1	eukaryotic translation initiation factor 4G-like isoform X2	Juglans regia	XP_018819162.1
ARUBRA_DN4722_c0_g1_i1_3	0	899	XP_018857649.1	NADH dehydrogenase ubiquinone flavoprotein 1, mitochondrial-like	Juglans regia	XP_018836688.1
ARUBRA_DN881_c0_g1_i1_1	2,00E-143	405	XP_018844705.1	proteasome subunit beta type-3-A	Juglans regia	GAU21784.1
ARHOMBI_DN16363_c0_g1_i1_2	1,30E-29	104	XP_012080548.1	mini zinc finger protein 3	Jatropha curcas	KDP31250.1
ARHOMBI_DN18734_c0_g1_i1_1	3,98E-155	437	ONH95000.1	hypothetical protein PRUPE_7G045500	Prunus persica	XP_007202429.1
ARUBRA_DN3334_c0_g1_i1_1	9,52E-74	230	OAY62541.1	hypothetical protein MANES_01G275200, partial	Manihot esculenta	OAY62541.1
ARHOMBI_DN421_c0_g1_i1_5	6,48E-147	427	OAY36457.1	hypothetical protein MANES_11G022700	Manihot esculenta	XP_020964169.1
ARUBRA_DN416_c0_g1_i1_2	6,32E-74	223	XP_018823025.1	thioredoxin H2-like	Juglans regia	XP_009335817.1
ARHOMBI_DN25721_c0_g1_i1_4	1,68E-42	141	XP_018838371.1	ADP-glucose phosphorylase-like	Juglans regia	XP_018846971.1
ARUBRA_DN11356_c0_g1_i1_6	1,46E-114	332	XP_018836820.1	transmembrane emp24 domain-containing protein p24delta9-like	Juglans regia	XP_018823192.1
ARUBRA_DN18553_c0_g1_i1_5	9,78E-71	220	KDP39401.1	hypothetical protein JCGZ_03683	Jatropha curcas	XP_012070587.1
ARHOMBI_DN3435_c0_g2_i1_3	1,06E-165	480	KDP44499.1	hypothetical protein JCGZ_16332	Jatropha curcas	KDP44499.1

ARHOMBI_DN13700_c0_g1_i1_6	1,09E-49	159	XP_018815442.1	mitochondrial import receptor subunit TOM20-like	Juglans regia	XP_018860144.1
ARHOMBI_DN10592_c0_g1_i1_1	1,49E-44	149	XP_018821649.1	gamma carbonic anhydrase 1, mitochondrial-like	Juglans regia	OAY50705.1
ARUBRA_DN9072_c0_g1_i1_5	3,50E-61	190	XP_012567690.1	serine hydroxymethyltransferase, mitochondrial-like	Cicer arietinum	ONI35495.1
ARUBRA_DN3824_c0_g1_i1_5	1,70E-36	131	XP_008342177.1	heterogeneous nuclear ribonucleoprotein 1-like	Malus domestica	XP_008342177.1
ARUBRA_DN4832_c0_g1_i1_2	1,13E-31	122	XP_018844468.1	serine carboxypeptidase-like 40	Juglans regia	XP_018817239.1
ARUBRA_DN9069_c0_g1_i1_4	1,95E-28	115	ONI13583.1	hypothetical protein PRUPE_4G231900	Prunus persica	ONI13583.1
ARHOMBI_DN8777_c0_g1_i1_4	4,28E-65	216	XP_018815316.1	coatomer subunit alpha-1	Juglans regia	XP_018815316.1
ARHOMBI_DN23553_c0_g1_i1_1	4,03E-38	136	XP_018849589.1	dolichyl-diphosphooligosaccharide--protein glycosyltransferase 48 kDa subunit-like isoform X1	Juglans regia	XP_018849591.1
ARHOMBI_DN3610_c0_g1_i1_3	6,94E-117	336	XP_018849141.1	phosphomannomutase-like	Juglans regia	AGH25530.1
ARHOMBI_DN5807_c0_g2_i1_3	4,73E-31	112	XP_018859166.1	60S acidic ribosomal protein P1-like	Juglans regia	XP_018842776.1
ARUBRA_DN4275_c0_g1_i1_5	0	548	XP_018821713.1	eukaryotic translation initiation factor 4B2-like	Juglans regia	XP_018821321.1
ARUBRA_DN24862_c0_g1_i1_4	3,89E-83	250	XP_008377063.1	ABC transporter E family member 2-like	Malus domestica	XP_008377064.1
ARUBRA_DN17478_c0_g1_i1_6	1,12E-168	469	XP_018820977.1	14-3-3-like protein GF14 kappa	Juglans regia	KDP41459.1
ARUBRA_DN2215_c0_g1_i1_4	1,83E-35	122	XP_015962951.1	uncharacterized protein LOC107486893	Arachis duranensis	KDP28251.1
ARUBRA_DN7908_c0_g1_i1_1	0	678	XP_018835559.1	ATP phosphoribosyltransferase 2, chloroplastic isoform X1	Juglans regia	OAY44990.1
ARHOMBI_DN4809_c0_g2_i1_2	3,95E-137	394	XP_018807963.1	2-alkenal reductase (NADP(+)-dependent)-like	Juglans regia	XP_018855810.1
ARUBRA_DN7769_c0_g1_i1_1	1,47E-169	478	XP_018820882.1	glyoxylate/succinic semialdehyde reductase 1 isoform X1	Juglans regia	XP_018820891.1
ARUBRA_DN4322_c0_g1_i1_1	1,00E-58	199	XP_018827682.1	uncharacterized protein LOC108996311	Juglans regia	XP_016175320.1
ARUBRA_DN25612_c0_g1_i1_4	8,33E-84	248	XP_018842451.1	lactoylglutathione lyase-like	Juglans regia	XP_008224306.1
ARHOMBI_DN11791_c0_g1_i1_2	1,71E-123	364	OAY29759.1	hypothetical protein MANES_15G170100	Manihot esculenta	XP_018842623.1
ARHOMBI_DN5868_c0_g1_i1_6	0	567	CAA66064.1	thaizole biosynthetic enzmye	Alnus glutinosa	Q38709.1
ARUBRA_DN2981_c0_g1_i1_3	0	744	XP_018859227.1	uncharacterized protein At1g04910	Juglans regia	XP_008353648.1
ARHOMBI_DN24401_c0_g1_i1_3	1,22E-42	145	XP_018828339.1	gamma aminobutyrate transaminase 3, chloroplastic-like, partial	Juglans regia	KDP30431.1
ARUBRA_DN23505_c0_g1_i1_1	6,03E-49	161	XP_018854405.1	ribosome maturation protein SBDS-like	Juglans regia	XP_018807006.1
ARUBRA_DN17618_c0_g1_i1_6	1,14E-09	58,5	XP_018806025.1	adenylyl-sulfate kinase 3-like isoform X2	Juglans regia	XP_018860443.1
ARUBRA_DN1478_c0_g1_i1_1	0	581	XP_018810134.1	dihydropyrimidine dehydrogenase (NADP(+)), chloroplastic-like	Juglans regia	XP_018823611.1
ARHOMBI_DN2506_c0_g1_i1_6	8,84E-136	386	ONH98930.1	hypothetical protein PRUPE_6G000600	Prunus persica	ONH98930.1
ARHOMBI_DN8898_c0_g1_i1_5	2,39E-82	251	XP_018818624.1	3-hydroxyisobutyryl-CoA hydrolase 1-like	Juglans regia	OAY38989.1

ARUBRA_DN3963_c0_g2_i1_4	1,34E-150	440	XP_008218420.1	eukaryotic translation initiation factor 3 subunit B-like	Prunus mume	ONI05147.1
ARUBRA_DN210_c0_g1_i1_5	5,30E-69	210	XP_018814411.1	uncharacterized protein LOC108986289	Juglans regia	KDP21869.1
ARUBRA_DN12757_c0_g1_i1_6	3,09E-58	197	XP_009362204.1	coatomer subunit alpha-1-like	Pyrus x bretschneideri	XP_018504237.1
ARHOMBI_DN1108_c0_g2_i1_1	1,00E-51	171	XP_008348820.1	copper-transporting ATPase PAA1, chloroplastic-like isoform X2	Malus domestica	XP_017181418.1
ARHOMBI_DN11591_c0_g1_i1_2	3,36E-80	243	XP_016180263.1	cycloartenol-C-24-methyltransferase	Arachis ipaensis	KYP60330.1
ARHOMBI_DN10607_c0_g1_i1_1	7,88E-85	276	XP_018828368.1	tripeptidyl-peptidase 2-like	Juglans regia	XP_018830702.1
ARUBRA_DN6875_c0_g1_i1_6	4,07E-101	303	XP_018812595.1	ATP sulfurylase 2-like	Juglans regia	XP_018847012.1
ARUBRA_DN25440_c0_g1_i1_2	1,15E-75	231	XP_018844548.1	chaperone protein dnaJ 10-like, partial	Juglans regia	ONH91071.1
ARUBRA_DN7688_c0_g1_i1_2	5,61E-77	233	XP_018834275.1	uncharacterized protein At4g28440	Juglans regia	XP_018805198.1
ARHOMBI_DN19744_c0_g1_i1_2	1,23E-154	463	XP_018815758.1	phosphoglucan, water dikinase, chloroplastic isoform X2	Juglans regia	XP_018815757.1
ARUBRA_DN24337_c0_g1_i1_6	3,09E-88	259	OIV96039.1	hypothetical protein TanjilG_27143	Lupinus angustifolius	KOM53238.1
ARUBRA_DN20434_c0_g1_i1_2	4,88E-140	406	XP_018810471.1	COP9 signalosome complex subunit 1-like	Juglans regia	XP_018810472.1
ARHOMBI_DN5476_c0_g1_i1_6	0	757	XP_018834279.1	26S proteasome non-ATPase regulatory subunit 2 homolog A-like	Juglans regia	XP_018843471.1
ARHOMBI_DN9888_c0_g1_i1_2	9,65E-101	306	XP_018843270.1	NADP-dependent malic enzyme	Juglans regia	KRH74264.1
ARHOMBI_DN2706_c0_g1_i1_5	1,13E-137	406	XP_018826381.1	probable Xaa-Pro aminopeptidase P	Juglans regia	OAY22143.1
ARUBRA_DN5778_c0_g1_i1_4	0	702	XP_018824898.1	glycerophosphodiester phosphodiesterase GDPD6	Juglans regia	ONI08875.1
ARUBRA_DN3967_c0_g1_i1_4	6,31E-49	164	ONI23764.1	hypothetical protein PRUPE_2G206800	Prunus persica	XP_007218796.1
ARUBRA_DN26097_c0_g1_i1_3	5,07E-44	150	XP_018814434.1	glucan endo-1,3-beta-glucosidase-like	Juglans regia	XP_018806507.1
ARHOMBI_DN2700_c0_g2_i1_5	2,37E-111	325	XP_018847499.1	ATP-dependent Clp protease ATP-binding subunit CLPT2, chloroplastic	Juglans regia	XP_008238671.1
ARHOMBI_DN8688_c0_g1_i1_2	1,65E-36	135	XP_018842400.1	eukaryotic translation initiation factor 3 subunit C isoform X1	Juglans regia	XP_018842401.1
ARHOMBI_DN15526_c0_g1_i1_1	1,94E-150	431	XP_018808845.1	GEM-like protein 1	Juglans regia	KOM41786.1
ARHOMBI_DN12805_c0_g1_i1_1	1,69E-35	129	XP_018858232.1	probable L-ascorbate peroxidase 6, chloroplastic	Juglans regia	XP_018855479.1
ARHOMBI_DN10876_c0_g1_i1_3	1,14E-45	158	XP_018829351.1	uncharacterized protein LOC108997478 isoform X2	Juglans regia	XP_018829342.1
ARUBRA_DN5971_c0_g1_i1_2	3,17E-77	236	XP_018848154.1	thioredoxin Y1, chloroplastic-like	Juglans regia	ONH90075.1
ARHOMBI_DN18415_c0_g1_i1_4	5,59E-38	132	XP_018828827.1	eukaryotic translation initiation factor 3 subunit I-like	Juglans regia	XP_018818014.1
ARUBRA_DN4814_c0_g1_i2_6	8,27E-114	333	XP_018828872.1	protein P21-like	Juglans regia	OIV93525.1
ARUBRA_DN21824_c0_g1_i1_6	2,64E-83	247	XP_018845962.1	uncharacterized protein LOC109009802	Juglans regia	XP_018845960.1

ARUBRA_DN10839_c0_g1_i1_5	1,73E-88	277	XP_018846638.1	uncharacterized protein LOC109010309	Juglans regia	XP_008393841.1
ARUBRA_DN4729_c0_g1_i1_2	2,34E-69	218	XP_018844572.1	uncharacterized protein LOC109008795	Juglans regia	AFK34731.1
ARUBRA_DN20854_c0_g1_i1_1	3,51E-118	371	XP_018805213.1	alpha-glucan water dikinase, chloroplastic isoform X1	Juglans regia	XP_018805214.1
ARUBRA_DN8756_c0_g1_i1_5	7,34E-40	141	XP_018827094.1	dihydroxy-acid dehydratase, chloroplastic-like	Juglans regia	XP_009376350.1
ARUBRA_DN18126_c0_g1_i1_2	5,38E-32	125	KEH41799.1	calmodulin-domain kinase CDPK protein	Medicago truncatula	XP_013467762.1
ARUBRA_DN7462_c0_g1_i1_6	7,54E-28	108	XP_018810692.1	geranylgeranyl transferase type-1 subunit beta	Juglans regia	ONI24309.1
ARUBRA_DN18563_c0_g1_i1_3	4,42E-90	271	XP_016202338.1	long chain acyl-CoA synthetase 6, peroxisomal	Arachis ipaensis	XP_018840476.1
ARUBRA_DN10939_c0_g1_i1_3	2,60E-78	236	BAD91191.1	sucrose synthase, partial	Pyrus communis	XP_008246136.2
ARHOMBI_DN21563_c0_g1_i1_5	7,22E-133	397	ONI07170.1	hypothetical protein PRUPE_5G103900	Prunus persica	XP_008392596.1
ARHOMBI_DN6102_c0_g2_i1_1	2,38E-129	371	XP_018828033.1	chalcone--flavonone isomerase-like isoform X2	Juglans regia	XP_018828032.1
ARUBRA_DN9246_c0_g1_i1_4	1,05E-122	348	XP_018810806.1	adenylyl-sulfate kinase 3	Juglans regia	XP_018810807.1
ARHOMBI_DN26256_c0_g1_i1_3	1,58E-23	95,1	XP_018847248.1	copper chaperone for superoxide dismutase, chloroplastic/cytosolic isoform X1	Juglans regia	XP_018847253.1
ARUBRA_DN22761_c0_g1_i1_6	1,54E-76	239	XP_018850112.1	uncharacterized protein LOC109012766	Juglans regia	KDP25741.1
ARHOMBI_DN20260_c0_g1_i1_3	7,28E-101	308	XP_018837506.1	glucosidase 2 subunit beta	Juglans regia	XP_009339210.1
ARHOMBI_DN19503_c0_g1_i1_3	7,77E-64	208	XP_018846976.1	LOW QUALITY PROTEIN: apyrase 1-like	Juglans regia	KHN00658.1
ARHOMBI_DN10131_c0_g1_i1_2	2,62E-90	266	XP_018808584.1	acetyl-coenzyme A carboxylase carboxyl transferase subunit alpha, chloroplastic-like	Juglans regia	XP_018833363.1
ARHOMBI_DN19928_c0_g1_i1_4	7,03E-71	228	XP_018850062.1	acyl-CoA-binding domain-containing protein 4	Juglans regia	XP_018850062.1
ARUBRA_DN1385_c0_g1_i1_2	0	944	XP_018819005.1	glutamate--glyoxylate aminotransferase 2	Juglans regia	XP_018813477.1
ARHOMBI_DN2055_c1_g1_i1_1	1,31E-58	181	AFP43694.1	actin 2, partial	Eriobotrya japonica	XP_020541461.1
ARUBRA_DN22675_c0_g1_i1_5	2,40E-25	101	ONI03924.1	hypothetical protein PRUPE_6G291700	Prunus persica	XP_007205848.1
ARHOMBI_DN1643_c0_g1_i1_5	3,00E-161	458	XP_019419657.1	26S protease regulatory subunit 6B homolog	Lupinus angustifolius	KHN18648.1
ARHOMBI_DN1170_c0_g1_i1_5	3,00E-102	301	XP_018848462.1	B-cell receptor-associated protein 31	Juglans regia	AFK41440.1
ARHOMBI_DN22076_c0_g1_i1_3	3,39E-35	132	XP_018854869.1	eukaryotic translation initiation factor 4G-like isoform X1	Juglans regia	XP_018854875.1
ARUBRA_DN16830_c0_g1_i1_1	1,68E-171	481	XP_018809559.1	ER membrane protein complex subunit 3-like	Juglans regia	XP_018842205.1
ARUBRA_DN17659_c0_g1_i1_1	1,55E-88	271	KDP39354.1	hypothetical protein JCGZ_01111	Jatropha curcas	XP_012071121.1
ARHOMBI_DN21263_c0_g1_i1_6	8,08E-106	313	OIW07052.1	hypothetical protein TanjilG_02686	Lupinus angustifolius	OAY48825.1
ARHOMBI_DN22368_c0_g1_i1_3	1,90E-46	165	XP_018830674.1	uncharacterized protein LOC108998567 isoform X2	Juglans regia	XP_018830673.1

ARHOMBI_DN20690_c0_g1_i1_2	2,69E-120	358	XP_018817683.1	probable methyltransferase PMT2	Juglans regia	XP_018845208.1
ARUBRA_DN2528_c0_g1_i1_6	1,72E-104	315	XP_018836136.1	coronatine-insensitive protein 1-like	Juglans regia	XP_018821796.1
ARHOMBI_DN1508_c0_g1_i1_5	6,72E-117	342	XP_018814791.1	26S proteasome non-ATPase regulatory subunit 6	Juglans regia	OIW21859.1
ARHOMBI_DN4881_c0_g1_i1_3	0	544	XP_018835583.1	polyadenylate-binding protein RBP47-like isoform X1	Juglans regia	XP_018835583.1
ARHOMBI_DN24692_c0_g1_i1_6	3,74E-98	296	XP_018810822.1	T-complex protein 1 subunit theta	Juglans regia	GAU32577.1
ARUBRA_DN51_c0_g1_i1_2	2,74E-96	285	KDP40925.1	hypothetical protein JCGZ_24924	Jatropha curcas	XP_012069158.1
ARUBRA_DN24099_c0_g1_i1_5	2,64E-106	306	XP_018848086.1	uncharacterized protein LOC109011377 isoform X1	Juglans regia	ABL85241.1
ARUBRA_DN7587_c0_g1_i1_6	8,31E-135	382	XP_018834676.1	eukaryotic translation initiation factor 3 subunit M-like	Juglans regia	XP_018816946.1
ARUBRA_DN5361_c0_g1_i1_2	4,60E-69	209	XP_018826018.1	NADH dehydrogenase ubiquinone 1 alpha subcomplex subunit 8-B-like	Juglans regia	XP_018840578.1
ARHOMBI_DN2590_c0_g1_i1_1	1,22E-107	312	XP_008237447.1	ras-related protein Rab7	Prunus mume	ONH90275.1
ARHOMBI_DN12163_c0_g1_i1_3	2,37E-76	244	OAY41017.1	hypothetical protein MANES_09G067300	Manihot esculenta	OAY41017.1
ARHOMBI_DN15490_c0_g1_i1_6	8,05E-136	410	XP_018826787.1	probable sucrose-phosphate synthase 1	Juglans regia	KDP30238.1
ARUBRA_DN24778_c0_g1_i1_1	2,53E-68	217	XP_016647327.1	beta-glucosidase 40-like	Prunus mume	ONI30961.1
ARUBRA_DN20929_c0_g1_i1_2	9,09E-60	191	XP_015959760.1	nifU-like protein 4, mitochondrial	Arachis duranensis	XP_016176058.1
ARUBRA_DN16965_c0_g1_i1_3	6,56E-54	170	XP_018822169.1	gamma-glutamylcyclotransferase 2-3-like	Juglans regia	KDP32786.1
ARUBRA_DN20307_c0_g1_i1_3	2,85E-36	133	XP_018827628.1	probable methyltransferase PMT26	Juglans regia	XP_018827629.1
ARHOMBI_DN24428_c0_g1_i1_4	5,73E-49	170	XP_018829342.1	uncharacterized protein LOC108997478 isoform X1	Juglans regia	XP_009339143.1
ARUBRA_DN2923_c0_g2_i1_5	0	539	XP_018846483.1	proteasome subunit beta type-7-B	Juglans regia	KDP46386.1
ARHOMBI_DN581_c0_g1_i1_2	8,20E-118	342	XP_018825107.1	vesicle-associated protein 2-1-like isoform X3	Juglans regia	XP_018825108.1
ARHOMBI_DN6817_c0_g1_i1_1	0	528	KDP42878.1	hypothetical protein JCGZ_23820	Jatropha curcas	XP_012066253.1
ARUBRA_DN10993_c0_g1_i1_2	6,44E-77	244	XP_018817103.1	long chain acyl-CoA synthetase 9, chloroplastic	Juglans regia	XP_018812541.1
ARUBRA_DN12715_c0_g1_i1_2	5,22E-67	223	OAY48901.1	hypothetical protein MANES_05G014200	Manihot esculenta	ONI14812.1
ARUBRA_DN9682_c0_g1_i1_4	7,39E-93	274	XP_018816771.1	pyridoxal kinase-like isoform X2	Juglans regia	XP_018816770.1
ARHOMBI_DN18725_c0_g1_i1_3	1,03E-78	256	KHN38581.1	Clathrin heavy chain 2	Glycine soja	KRH73205.1
ARHOMBI_DN20294_c0_g1_i1_3	6,99E-57	186	XP_018859548.1	protein AUXIN RESPONSE 4	Juglans regia	XP_018859549.1
ARHOMBI_DN4086_c0_g1_i1_4	2,07E-117	341	XP_018813434.1	ER membrane protein complex subunit 8/9 homolog	Juglans regia	XP_018825614.1

ARUBRA_DN19093_c0_g1_i1_3	1,57E-52	174	XP_018851786.1	nifU-like protein 4, mitochondrial	Juglans regia	KDP30075.1
ARHOMBI_DN13465_c0_g2_i1_6	3,71E-79	242	XP_015956091.1	probable cinnamyl alcohol dehydrogenase 1	Arachis duranensis	XP_016189854.1
ARUBRA_DN20855_c0_g1_i1_2	4,19E-111	336	OAY48760.1	hypothetical protein MANES_05G003600	Manihot esculenta	XP_018842470.1
ARUBRA_DN2934_c0_g2_i2_4	2,69E-139	399	XP_008220037.1	coiled-coil domain-containing protein 25 isoform X2	Prunus mume	ONI33902.1
ARUBRA_DN17957_c0_g1_i1_3	3,16E-35	121	XP_018847027.1	7-methylguanosine phosphate-specific 5'-nucleotidase A like, partial	Juglans regia	XP_018857864.1
ARUBRA_DN189_c0_g2_i1_5	0	696	XP_018842503.1	branched-chain-amino-acid aminotransferase 3, chloroplastic-like	Juglans regia	XP_009342188.1
ARUBRA_DN12373_c0_g1_i1_4	9,48E-157	450	KDP42743.1	hypothetical protein JCGZ_23683	Jatropha curcas	XP_012066493.1
ARHOMBI_DN26479_c0_g1_i1_3	1,54E-43	144	XP_018828106.1	uncharacterized protein LOC108996580 isoform X2	Juglans regia	XP_018828105.1
ARUBRA_DN1156_c0_g1_i1_5	5,02E-100	297	XP_018842811.1	vacuolar protein sorting-associated protein 26B-like	Juglans regia	XP_018842812.1
ARHOMBI_DN715_c0_g1_i1_6	4,87E-142	406	XP_018833655.1	phospholipid hydroperoxide glutathione peroxidase 1, chloroplastic-like	Juglans regia	XP_018839583.1
ARUBRA_DN18388_c0_g1_i1_5	1,32E-60	200	XP_018815884.1	acid beta-fructofuranosidase-like	Juglans regia	OAY48150.1
ARUBRA_DN24939_c0_g1_i1_6	5,71E-51	171	XP_018813877.1	plant intracellular Ras-group-related LRR protein 9-like	Juglans regia	XP_018808183.1
ARUBRA_DN1579_c0_g1_i1_3	1,44E-110	320	XP_018829416.1	soluble inorganic pyrophosphatase-like isoform X1	Juglans regia	XP_018829417.1
ARUBRA_DN3088_c1_g1_i1_2	0	538	XP_018820447.1	T-complex protein 1 subunit epsilon	Juglans regia	KDP26716.1
ARUBRA_DN18995_c0_g1_i1_4	2,35E-67	223	XP_018833940.1	probable manganese-transporting ATPase PDR2	Juglans regia	XP_018833946.1
ARUBRA_DN6428_c0_g1_i1_3	1,86E-113	350	XP_018837083.1	probable sucrose-phosphate synthase 2	Juglans regia	XP_008222839.1
ARUBRA_DN24802_c0_g1_i1_6	3,64E-36	132	XP_007162188.1	hypothetical protein PHAVU_001G131700g	Phaseolus vulgaris	XP_007162189.1
ARHOMBI_DN6811_c0_g1_i1_3	3,98E-87	275	XP_018809531.1	phosphoenolpyruvate carboxylase 4-like isoform X2	Juglans regia	XP_008389332.1
ARHOMBI_DN23413_c0_g1_i1_2	1,06E-67	205	XP_018860744.1	phospholipase A-2-activating protein-like isoform X2	Juglans regia	KHN25352.1
ARUBRA_DN26636_c0_g1_i1_3	6,43E-53	172	XP_017184434.1	ubiquitin carboxyl-terminal hydrolase 13-like, partial	Malus domestica	XP_016165934.1
ARUBRA_DN24535_c0_g1_i1_1	1,32E-73	225	XP_018824435.1	mitochondrial import receptor subunit TOM40-1-like	Juglans regia	XP_018815797.1
ARUBRA_DN5752_c0_g1_i1_3	3,29E-61	191	XP_008388576.1	mitochondrial Rho GTPase 1-like	Malus domestica	XP_008246476.1
ARUBRA_DN12277_c0_g1_i1_4	7,48E-21	85,9	XP_018819823.1	MFP1 attachment factor 1-like	Juglans regia	XP_018824955.1
ARUBRA_DN12763_c0_g1_i1_6	1,08E-24	99,4	XP_018807064.1	cyclase-associated protein 1-like	Juglans regia	XP_018847838.1
ARUBRA_DN23296_c0_g1_i1_1	1,92E-90	278	XP_018809732.1	SEC1 family transport protein SLY1-like	Juglans regia	OAY57009.1
ARHOMBI_DN6174_c0_g1_i1_5	0	867	XP_020226848.1	glucose-1-phosphate adenylyltransferase large subunit 3, chloroplastic/amyloplastic	Cajanus cajan	ACU20108.1

ARHOMBI_DN3360_c0_g1_i1_6	3,60E-62	192	XP_018829244.1	NADH dehydrogenase ubiquinone 1 alpha subcomplex subunit 2-like	Juglans regia	XP_018841602.1
ARUBRA_DN4576_c0_g3_i1_3						
ARUBRA_DN27180_c0_g1_i1_6	6,43E-35	123	XP_017182136.1	uncharacterized protein LOC103414782	Malus domestica	XP_018843340.1
ARUBRA_DN3154_c0_g1_i1_6	2,88E-59	194	XP_018850710.1	early nodulin-like protein 2	Juglans regia	XP_018807542.1
ARUBRA_DN6360_c0_g1_i1_2	0	580	XP_018857622.1	polyadenylate-binding protein RBP45-like	Juglans regia	XP_018808205.1
ARUBRA_DN24250_c0_g1_i1_6	6,02E-35	128	XP_018847198.1	protein CASP	Juglans regia	KDP20098.1
ARUBRA_DN9113_c0_g1_i1_5	7,75E-51	171	XP_018845393.1	DEAD-box ATP-dependent RNA helicase 8-like	Juglans regia	XP_008347150.1
ARUBRA_DN18999_c0_g1_i1_5	5,21E-99	293	XP_018833734.1	elongation factor 1-beta 2	Juglans regia	XP_016187218.1
ARUBRA_DN23874_c0_g1_i1_1	6,04E-101	298	XP_018810997.1	electron transfer flavoprotein subunit alpha, mitochondrial	Juglans regia	KDP39073.1
ARHOMBI_DN8339_c0_g1_i1_2	1,33E-109	315	XP_018852886.1	ABC transporter F family member 3-like, partial	Juglans regia	XP_018835197.1
ARHOMBI_DN1833_c0_g1_i1_4	7,17E-72	217	KDP36139.1	hypothetical protein JCGZ_08783	Jatropha curcas	XP_012074351.1
ARHOMBI_DN187_c0_g2_i1_1	1,26E-168	508	XP_018810975.1	ABC transporter B family member 11-like	Juglans regia	XP_018810975.1
ARHOMBI_DN18091_c0_g1_i1_1	3,67E-33	125	XP_018849698.1	glutamine--tRNA ligase-like	Juglans regia	XP_018849699.1
ARHOMBI_DN11853_c0_g1_i1_1	3,37E-85	277	XP_018815316.1	coatomer subunit alpha-1	Juglans regia	XP_018840319.1
ARUBRA_DN898_c0_g1_i1_1	3,22E-163	461	XP_008236384.1	methylthioribose kinase	Prunus mume	AGF95119.1
ARHOMBI_DN5055_c0_g1_i1_3	9,84E-57	191	XP_018823263.1	probable mediator of RNA polymerase II transcription subunit 37c	Juglans regia	KOM26183.1
ARUBRA_DN21162_c0_g1_i1_4	5,22E-161	462	XP_018843339.1	ubiquitin carboxyl-terminal hydrolase 6-like isoform X1	Juglans regia	XP_018843340.1
ARUBRA_DN19451_c0_g1_i1_2	1,57E-40	145	XP_018830674.1	uncharacterized protein LOC108998567 isoform X2	Juglans regia	XP_018830673.1
ARHOMBI_DN17114_c0_g1_i1_5	2,70E-126	392	XP_018857395.1	protein transport protein Sec24-like At3g07100	Juglans regia	XP_018857396.1
ARHOMBI_DN4946_c0_g1_i2_4	7,28E-134	384	XP_018828615.1	gamma-interferon-inducible lysosomal thiol reductase-like	Juglans regia	XP_008234981.2
ARUBRA_DN9563_c0_g1_i1_2	3,34E-58	186	XP_018836996.1	dnaJ homolog subfamily B member 1-like isoform X2	Juglans regia	XP_018836995.1
ARUBRA_DN3083_c0_g1_i1_4	4,01E-145	414	XP_018836119.1	reticulon-like protein B5	Juglans regia	XP_018845099.1
ARUBRA_DN10864_c0_g1_i1_2	2,20E-55	176	XP_018854469.1	OTU domain-containing protein 6B-like, partial	Juglans regia	XP_018843798.1
ARHOMBI_DN8832_c0_g1_i1_6	1,05E-174	499	XP_018852605.1	leukotriene A-4 hydrolase homolog	Juglans regia	KDP46145.1
ARHOMBI_DN16126_c0_g1_i1_1	4,59E-61	189	KEH17133.1	cell division control-like protein	Medicago truncatula	XP_013443108.1
ARUBRA_DN11418_c0_g1_i1_5	4,61E-154	441	XP_018838327.1	T-complex protein 1 subunit beta-like	Juglans regia	XP_018838327.1
ARUBRA_DN711_c0_g1_i1_2	4,11E-146	414	KRH40130.1	hypothetical protein GLYMA_09G240300	Glycine max	KHN11589.1
ARUBRA_DN23248_c0_g1_i1_5	1,28E-59	194	XP_018808130.1	calcium-dependent protein kinase 29	Juglans regia	XP_018808131.1

ARHOMBI_DN11424_c0_g1_i1_1	4,43E-35	122	XP_018839525.1	chorismate mutase 2-like	Juglans regia	XP_018833669.1
ARUBRA_DN13078_c0_g1_i1_4	1,12E-56	187	XP_018857004.1	4-hydroxyphenylpyruvate dioxygenase	Juglans regia	KDP45435.1
ARUBRA_DN22748_c0_g1_i1_3	3,22E-37	134	XP_018811308.1	probable aspartyl aminopeptidase	Juglans regia	XP_018814786.1
ARUBRA_DN19446_c0_g1_i1_1	2,65E-165	469	XP_018809613.1	alanine--glyoxylate aminotransferase 2 homolog 1, mitochondrial	Juglans regia	XP_008218566.1
ARHOMBI_DN25338_c0_g1_i1_6	6,08E-46	160	OAY38388.1	hypothetical protein MANES_10G010500	Manihot esculenta	KDP44739.1
ARUBRA_DN1375_c0_g1_i1_4	0	776	XP_018846461.1	apoptotic chromatin condensation inducer in the nucleus-like isoform X2	Juglans regia	XP_018850842.1
ARHOMBI_DN5366_c0_g1_i2_2	5,22E-152	431	XP_018830330.1	peroxisomal membrane protein 11C	Juglans regia	XP_020219477.1
ARUBRA_DN26151_c0_g1_i1_1	9,38E-25	100	XP_008387982.1	uncharacterized TPR repeat-containing protein At1g05150-like	Malus domestica	XP_008387984.1
ARHOMBI_DN16439_c0_g1_i1_3	0	570	XP_018842661.1	phosphoinositide phosphatase SAC6-like	Juglans regia	XP_018842662.1
ARHOMBI_DN17772_c0_g1_i1_2	3,39E-41	147	XP_018840323.1	V-type proton ATPase subunit a3-like	Juglans regia	ONH91014.1
ARHOMBI_DN3967_c0_g1_i1_1	5,42E-174	486	XP_018842902.1	haloacid dehalogenase-like hydrolase domain-containing protein At4g39970 isoform X1	Juglans regia	XP_018860336.1
ARUBRA_DN18178_c0_g1_i1_2	1,86E-29	108	AFK34954.1	unknown	Lotus japonicus	KRH51667.1
ARUBRA_DN4925_c0_g1_i1_4	0	687	XP_018823509.1	leucine aminopeptidase 1-like	Juglans regia	XP_018810862.1
ARUBRA_DN14063_c0_g1_i1_3	9,40E-61	204	XP_018815316.1	coatomer subunit alpha-1	Juglans regia	XP_018840319.1
ARHOMBI_DN2499_c0_g1_i1_5	1,65E-131	376	XP_018809664.1	uncharacterized protein LOC108982679	Juglans regia	ONI25352.1
ARUBRA_DN2486_c0_g1_i1_3	1,22E-62	194	ONI00303.1	hypothetical protein PRUPE_6G081100	Prunus persica	XP_018823316.1
ARUBRA_DN9169_c0_g1_i1_1	2,72E-51	171	XP_018808643.1	serine/threonine-protein phosphatase 5 isoform X2	Juglans regia	XP_018808625.1
ARHOMBI_DN2036_c0_g2_i1_1	1,85E-71	222	XP_018816108.1	uncharacterized protein At3g03773 isoform X2	Juglans regia	KDP39664.1
ARUBRA_DN20749_c0_g1_i1_4						
ARHOMBI_DN13970_c0_g1_i1_4	1,26E-24	99,4	OAY43070.1	hypothetical protein MANES_08G039600	Manihot esculenta	KDP35933.1
ARHOMBI_DN16110_c0_g1_i1_4	1,36E-57	184	XP_007148531.1	hypothetical protein PHAVU_006G216400g	Phaseolus vulgaris	ESW20525.1
ARUBRA_DN16649_c0_g1_i1_5	1,49E-112	343	XP_018822704.1	alpha-L-arabinofuranosidase 1	Juglans regia	XP_018822706.1
ARHOMBI_DN26305_c0_g1_i1_3	1,63E-26	90,5	ONI06071.1	hypothetical protein PRUPE_5G038300	Prunus persica	ONI06071.1
ARUBRA_DN7710_c0_g1_i1_3	0	536	XP_018827302.1	succinate--CoA ligase ADP-forming subunit beta, mitochondrial	Juglans regia	XP_018847166.1
ARUBRA_DN9775_c0_g1_i1_2	7,06E-50	164	XP_018816315.1	ABC transporter I family member 20-like isoform X1	Juglans regia	KHN02819.1

ARUBRA_DN9988_c0_g1_i1_3	1,87E-43	150	XP_018846051.1	diphosphomevalonate decarboxylase MVD2-like	Juglans regia	XP_018813340.1
ARHOMBI_DN18064_c0_g1_i1_4	7,78E-38	137	XP_019433597.1	bifunctional aspartokinase/homoserine dehydrogenase, chloroplastic-like isoform X1	Lupinus angustifolius	OIV89670.1
ARHOMBI_DN4801_c0_g1_i1_1	6,32E-172	497	XP_018821713.1	eukaryotic translation initiation factor 4B2-like	Juglans regia	XP_018821321.1
ARUBRA_DN5397_c0_g1_i1_4	2,92E-28	109	XP_018842090.1	uncharacterized protein LOC109007046	Juglans regia	XP_018851050.1
ARHOMBI_DN3803_c0_g2_i1_6	4,61E-16	70,5	XP_008372389.1	mitochondrial import receptor subunit TOM9-2	Malus domestica	XP_009370408.1
ARUBRA_DN4646_c0_g1_i1_4	1,44E-85	252	XP_018834168.1	uncharacterized protein LOC109001373	Juglans regia	XP_019441142.1
ARUBRA_DN16654_c0_g1_i1_4	6,04E-127	372	XP_019456385.1	mannosyl-oligosaccharide 1,2-alpha-mannosidase MNS1-like isoform X2	Lupinus angustifolius	OIW04981.1
ARUBRA_DN20972_c0_g1_i1_2	3,14E-119	358	XP_018826576.1	probable methyltransferase PMT2	Juglans regia	ONI08658.1
ARHOMBI_DN23602_c0_g1_i1_6	2,39E-81	255	XP_018839054.1	probable rhamnogalacturonate lyase B	Juglans regia	XP_018842495.1
ARUBRA_DN20900_c0_g1_i1_2	4,53E-115	338	OAY41777.1	hypothetical protein MANES_09G128600	Manihot esculenta	KDP39537.1
ARUBRA_DN17183_c0_g1_i1_5	1,02E-144	417	XP_018845323.1	isovaleryl-CoA dehydrogenase, mitochondrial	Juglans regia	XP_008225328.1
ARHOMBI_DN3229_c0_g1_i1_4	9,13E-58	184	XP_018824069.1	thioredoxin domain-containing protein PLP3B-like	Juglans regia	XP_018824070.1
ARUBRA_DN12858_c0_g1_i1_4	5,15E-70	218	XP_018818486.1	erlin-2-B	Juglans regia	XP_018818487.1
ARUBRA_DN20_c0_g1_i1_4	2,50E-77	231	OIV94287.1	hypothetical protein TanjilG_00036	Lupinus angustifolius	XP_004503473.1
ARUBRA_DN2891_c0_g1_i1_5	6,05E-46	161	KRH60476.1	hypothetical protein GLYMA_05G242700	Glycine max	XP_003525207.1
ARHOMBI_DN4641_c0_g1_i1_6	2,37E-131	378	XP_018815526.1	transmembrane emp24 domain-containing protein p24beta2-like isoform X1	Juglans regia	XP_018815527.1
ARUBRA_DN5439_c0_g1_i1_5	1,67E-151	427	XP_018840568.1	probable prolyl 4-hydroxylase 4	Juglans regia	OAY27838.1
ARUBRA_DN2788_c0_g1_i1_6	3,90E-34	132	XP_018835518.1	UDP-glucose:glycoprotein glucosyltransferase	Juglans regia	XP_016179809.2
ARHOMBI_DN24746_c0_g1_i1_1	5,61E-64	201	XP_018828105.1	uncharacterized protein LOC108996580 isoform X1	Juglans regia	ONI09614.1
ARUBRA_DN12422_c0_g1_i1_2	2,14E-47	154	GAU28872.1	hypothetical protein TSUD_293240	Trifolium subterraneum	XP_016649976.1
ARUBRA_DN19709_c0_g1_i1_5	1,83E-176	500	XP_018847838.1	cyclase-associated protein 1-like	Juglans regia	XP_018807064.1
ARUBRA_DN4950_c1_g1_i1_4	3,20E-166	473	XP_018839618.1	glucan endo-1,3-beta-glucosidase, basic isoform-like	Juglans regia	XP_008240769.1
ARHOMBI_DN8627_c0_g1_i1_4	4,02E-56	186	XP_018827094.1	dihydroxy-acid dehydratase, chloroplastic-like	Juglans regia	KYP72842.1
ARHOMBI_DN8139_c0_g1_i1_2	8,21E-105	327	XP_018835573.1	presequence protease 1, chloroplastic/mitochondrial-like	Juglans regia	KDP42318.1
ARUBRA_DN9097_c0_g1_i1_3	6,48E-107	313	XP_018841597.1	uncharacterized protein LOC109006689	Juglans regia	XP_018829186.1
ARUBRA_DN23250_c0_g1_i1_1	3,77E-37	137	XP_018839600.1	uncharacterized protein DDB_G0286299-like isoform X2	Juglans regia	XP_018839599.1

ARHOMBI_DN13828_c0_g1_i1_2	9,62E-31	117	XP_018842611.1	cytosolic enolase 3	Juglans regia	KDP46567.1
ARHOMBI_DN16373_c0_g1_i1_2	2,26E-156	442	XP_018810174.1	uncharacterized protein LOC108983094	Juglans regia	XP_009346159.1
ARUBRA_DN12629_c0_g1_i1_4	4,05E-46	157	XP_008235829.1	aminoacylase-1 isoform X2	Prunus mume	ONH92575.1
ARHOMBI_DN6215_c0_g1_i2_2	3,03E-98	291	XP_018845871.1	succinate dehydrogenase assembly factor 2, mitochondrial isoform X1	Juglans regia	KDP22524.1
ARUBRA_DN4706_c0_g1_i1_4	2,84E-134	388	XP_018857385.1	probable ribosomal protein S11, mitochondrial	Juglans regia	XP_018857386.1
ARUBRA_DN6348_c0_g1_i1_2	2,64E-127	370	XP_018859875.1	glycine-rich RNA-binding protein 3, mitochondrial	Juglans regia	XP_016649563.1
ARHOMBI_DN4431_c0_g3_i1_1	5,38E-138	390	AGL52581.1	Rab6	Hevea brasiliensis	OAY62531.1
ARHOMBI_DN2426_c0_g1_i1_5	6,59E-74	221	XP_018836608.1	macrophage migration inhibitory factor homolog	Juglans regia	XP_020227962.1
ARUBRA_DN2608_c0_g1_i1_4	1,29E-116	350	XP_018851337.1	acetolactate synthase small subunit 2, chloroplastic-like isoform X1	Juglans regia	XP_018851337.1
ARHOMBI_DN5630_c0_g2_i1_5	1,97E-42	151	KYP64251.1	CAAX prenyl protease 1 isogeny	Cajanus cajan	XP_020219597.1
ARHOMBI_DN2188_c0_g1_i1_4	0	604	XP_018837016.1	uncharacterized protein LOC109003359, partial	Juglans regia	ONI05682.1
ARUBRA_DN16537_c0_g1_i1_2	0	586	XP_018854480.1	bifunctional dTDP-4-dehydrorhamnose 3,5-epimerase/dTDP-4-dehydrorhamnose reductase	Juglans regia	XP_018820521.1
ARHOMBI_DN6250_c0_g2_i3_5	2,72E-66	211	KHN02664.1	Cysteine-rich receptor-like protein kinase 29	Glycine soja	KRH35616.1
ARUBRA_DN12906_c0_g1_i1_4	1,30E-24	97,8	XP_018839985.1	protein YIF1B-like	Juglans regia	XP_018839986.1
ARHOMBI_DN15675_c0_g1_i1_3	8,61E-94	284	XP_018837267.1	T-complex protein 1 subunit gamma isoform X2	Juglans regia	XP_018837266.1
ARHOMBI_DN20830_c0_g1_i1_1	3,07E-58	183	XP_008344672.1	DEAD-box ATP-dependent RNA helicase 52C-like, partial	Malus domestica	KDP30456.1
ARHOMBI_DN6132_c0_g5_i1_3	1,44E-55	174	XP_018856764.1	cysteine proteinase inhibitor	Juglans regia	XP_018843658.1
ARHOMBI_DN13938_c0_g1_i1_3	1,03E-55	189	KDP29853.1	hypothetical protein JCGZ_18428	Jatropha curcas	XP_012081551.1
ARHOMBI_DN343_c0_g2_i1_6	4,54E-159	456	XP_018851632.1	probable ADP-ribosylation factor GTPase-activating protein AGD5 isoform X1	Juglans regia	XP_018823524.1
ARHOMBI_DN20204_c0_g1_i1_6	1,02E-91	279	XP_018807977.1	uncharacterized protein LOC108981316	Juglans regia	XP_008236697.1
ARHOMBI_DN5653_c0_g1_i1_4	0	546	XP_018856906.1	endochitinase 2-like	Juglans regia	XP_007201290.2
ARUBRA_DN9043_c0_g1_i1_6	1,46E-54	184	XP_018811234.1	dynamin-related protein 3A	Juglans regia	XP_018826873.1
ARUBRA_DN8452_c0_g1_i1_1	8,37E-158	444	XP_018811826.1	uncharacterized protein LOC108984353	Juglans regia	ONI29911.1
ARHOMBI_DN5207_c0_g1_i1_5	2,76E-129	390	XP_015972778.1	neutral ceramidase	Arachis duranensis	KDP46568.1
ARHOMBI_DN5473_c0_g1_i1_2	6,44E-82	244	XP_018849401.1	NADH dehydrogenase ubiquinone 1 alpha subcomplex subunit 6	Juglans regia	OAY36255.1

ARHOMBI_DN15547_c1_g1_i1_2	2,21E-152	441	XP_018850096.1	TOM1-like protein 2	Juglans regia	XP_018850097.1
ARUBRA_DN21709_c0_g1_i1_1	4,59E-38	134	AGV54246.1	COP9 signalosome complex subunit 2-like protein	Phaseolus vulgaris	KOM38655.1
ARUBRA_DN7051_c0_g1_i1_2	2,17E-97	287	XP_018838997.1	delta(3,5)-Delta(2,4)-dienoyl-CoA isomerase, peroxisomal	Juglans regia	OAY34252.1
ARUBRA_DN25464_c0_g1_i1_1	9,76E-51	162	ACJ84811.1	unknown, partial	Medicago truncatula	XP_018855205.1
ARHOMBI_DN4589_c0_g1_i1_6	3,27E-83	248	XP_018806135.1	uncharacterized protein LOC108979821	Juglans regia	XP_018824745.1
ARUBRA_DN931_c0_g1_i1_3	1,59E-97	287	XP_018829803.1	uncharacterized protein LOC108997870	Juglans regia	XP_008218429.1
ARHOMBI_DN10963_c0_g1_i1_2	2,76E-33	120	XP_018845186.1	haloacid dehalogenase-like hydrolase domain-containing protein 3 isoform X2	Juglans regia	XP_018845190.1
ARUBRA_DN14101_c0_g1_i1_6	1,89E-33	121	XP_018845848.1	DNA-damage-repair/toleration protein DRT102-like	Juglans regia	XP_018845850.1
ARHOMBI_DN18821_c0_g1_i1_3	2,36E-40	140	XP_018811389.1	1,4-alpha-glucan-branching enzyme 1, chloroplastic/amyloplastic-like	Juglans regia	XP_018845815.1
ARHOMBI_DN14763_c0_g1_i1_1	3,70E-54	185	XP_018860275.1	26S proteasome non-ATPase regulatory subunit 1 homolog A	Juglans regia	XP_018837135.1
ARUBRA_DN7233_c0_g1_i1_1	3,42E-52	180	XP_018805213.1	alpha-glucan water dikinase, chloroplastic isoform X1	Juglans regia	XP_018805213.1
ARUBRA_DN3582_c0_g2_i1_3	1,17E-85	260	XP_018820983.1	GLABRA2 expression modulator-like	Juglans regia	XP_018830258.1
ARUBRA_DN3740_c0_g1_i1_2	1,26E-90	282	XP_018846323.1	clathrin heavy chain 1-like isoform X1	Juglans regia	XP_018846324.1
ARUBRA_DN2116_c0_g1_i1_1	1,26E-122	345	XP_018834471.1	uncharacterized protein LOC109001574 isoform X1	Juglans regia	XP_018834471.1
ARHOMBI_DN6367_c0_g1_i1_3	4,54E-61	200	XP_018820447.1	T-complex protein 1 subunit epsilon	Juglans regia	OAY34423.1
ARUBRA_DN9714_c0_g1_i1_3	1,35E-44	156	XP_018816462.1	protein transport protein Sec24-like At3g07100	Juglans regia	XP_018816463.1
ARHOMBI_DN5629_c0_g1_i1_3	0	1868	XP_018827627.1	pleiotropic drug resistance protein 1-like	Juglans regia	XP_018827627.1
ARUBRA_DN17225_c0_g1_i1_6	4,43E-105	311	AFK44312.1	unknown	Lotus japonicus	XP_020211438.1
ARUBRA_DN5760_c0_g1_i1_1	0	596	XP_018813723.1	polyadenylate-binding protein 2-like	Juglans regia	XP_018813723.1
ARHOMBI_DN13303_c0_g1_i1_5	4,68E-109	342	XP_018842317.1	nodal modulator 1	Juglans regia	XP_008227880.1
ARHOMBI_DN44_c0_g1_i1_5	0	756	XP_018827773.1	uncharacterized protein LOC108996364	Juglans regia	XP_018827774.1
ARUBRA_DN2012_c0_g1_i1_3	0	791	XP_008239702.1	mannose-1-phosphate guanyltransferase alpha	Prunus mume	ONI08471.1
ARHOMBI_DN18373_c0_g1_i1_1	4,79E-124	358	XP_018839330.1	aminoacyl tRNA synthase complex-interacting multifunctional protein 1	Juglans regia	XP_008228858.1
ARUBRA_DN22899_c0_g1_i1_4	9,00E-119	358	XP_016180595.1	ABC transporter F family member 4	Arachis ipaensis	XP_015944239.1
ARUBRA_DN19779_c0_g1_i1_2	0	815	XP_018812654.1	eukaryotic initiation factor 4A-3	Juglans regia	XP_018826866.1
ARHOMBI_DN2168_c0_g1_i1_5	1,35E-136	394	XP_018837387.1	acetyl-CoA acetyltransferase, cytosolic 1	Juglans regia	OAY34999.1
ARHOMBI_DN18268_c0_g1_i1_5	2,45E-40	144	OAY34976.1	hypothetical protein MANES_12G061400	Manihot esculenta	XP_018849887.1

ARUBRA_DN20895_c0_g1_i1_4	5,41E-47	154	XP_018855286.1	phosphoinositide phosphatase SAC7-like	Juglans regia	XP_018855293.1
ARHOMBI_DN9816_c0_g1_i1_3	3,28E-05	43,9	XP_018835918.1	myb-like protein X	Juglans regia	XP_018835919.1
ARUBRA_DN6711_c0_g1_i1_1	1,90E-97	283	KDP26654.1	hypothetical protein JCGZ_17812	Jatropha curcas	XP_012085480.1
ARUBRA_DN1256_c0_g2_i1_1	0	542	OAY48170.1	hypothetical protein MANES_06G137400	Manihot esculenta	KDP22391.1
ARHOMBI_DN4768_c0_g1_i1_5	3,95E-112	333	XP_018826110.1	bifunctional 3-dehydroquinate dehydratase/shikimate dehydrogenase, chloroplastic-like isoform X2	Juglans regia	AAW65140.1
ARUBRA_DN1822_c0_g1_i1_4	0	697	XP_018826787.1	probable sucrose-phosphate synthase 1	Juglans regia	ABV32551.1
ARUBRA_DN2382_c0_g1_i1_2	5,61E-154	430	XP_018849834.1	pectinesterase 2.2-like	Juglans regia	XP_018860325.1
ARUBRA_DN6119_c0_g1_i1_1	1,92E-39	132	XP_018830814.1	mitochondrial import inner membrane translocase subunit TIM8	Juglans regia	XP_008239525.1
ARUBRA_DN19079_c0_g1_i1_2	3,58E-165	476	XP_018830495.1	pyruvate decarboxylase 1	Juglans regia	XP_018838507.1
ARUBRA_DN14154_c0_g1_i1_1	7,14E-64	200	OAY34614.1	hypothetical protein MANES_12G033800	Manihot esculenta	OAY34615.1
ARUBRA_DN6181_c0_g1_i1_5	8,28E-94	278	ADM67612.1	iron-sulfur cluster scaffold protein	Hevea brasiliensis	KDP31091.1
ARHOMBI_DN5536_c0_g1_i1_1	0	884	XP_018839273.1	pectinesterase-like	Juglans regia	XP_018850544.1
ARHOMBI_DN25582_c0_g1_i1_3	6,68E-26	95,9	XP_008389377.1	D-aminoacyl-tRNA deacylase-like	Malus domestica	ONI31116.1
ARUBRA_DN1176_c0_g1_i1_5	7,08E-34	133	XP_018844348.1	N-alpha-acetyltransferase 16, NatA auxiliary subunit-like	Juglans regia	XP_012073296.1
ARUBRA_DN766_c0_g1_i1_6	5,06E-46	161	XP_018849693.1	ERAD-associated E3 ubiquitin-protein ligase HRD1B-like	Juglans regia	XP_018849695.1
ARHOMBI_DN20645_c0_g1_i1_1	2,12E-72	223	XP_008377063.1	ABC transporter E family member 2-like	Malus domestica	XP_008377064.1
ARHOMBI_DN985_c0_g1_i1_6	2,52E-124	372	XP_018831786.1	NAD-dependent malic enzyme 59 kDa isoform, mitochondrial	Juglans regia	CAB95832.1
ARUBRA_DN14493_c0_g1_i1_2	2,46E-106	331	XP_018817805.1	leucine--tRNA ligase, cytoplasmic isoform X2	Juglans regia	XP_018817804.1
ARHOMBI_DN18281_c0_g1_i1_5	1,13E-23	96,3	XP_018825295.1	BSD domain-containing protein 1	Juglans regia	XP_008386574.1
ARHOMBI_DN18082_c0_g1_i1_2	2,91E-74	239	XP_018820267.1	ubiquitin carboxyl-terminal hydrolase MINDY-2-like	Juglans regia	XP_018820491.1
ARUBRA_DN3448_c0_g1_i1_6	8,16E-71	217	XP_018849946.1	eukaryotic translation initiation factor 1A-like	Juglans regia	XP_018849947.1
ARHOMBI_DN19564_c0_g1_i1_4	1,89E-147	433	XP_018849881.1	protein transport protein SEC23	Juglans regia	XP_015938826.1
ARHOMBI_DN18202_c0_g1_i1_6	1,04E-26	105	XP_018850353.1	RNA-binding protein 25	Juglans regia	KYP40983.1
ARUBRA_DN4562_c0_g1_i1_3	6,17E-70	222	XP_009351427.1	thylakoid lumenal 15 kDa protein 1, chloroplastic	Pyrus x bretschneideri	XP_009351427.1
ARUBRA_DN756_c0_g1_i1_1	0	729	XP_016187654.1	elongation factor Tu, chloroplastic	Arachis ipaensis	CAA61444.1
ARHOMBI_DN5795_c0_g1_i1_6	6,07E-104	303	XP_014518805.1	vesicle-associated membrane protein 722-like	Vigna radiata var. radiata	XP_007147528.1

ARUBRA_DN18766_c0_g1_i1_6	3,01E-79	243	XP_018858979.1	uncharacterized protein LOC109020906 isoform X4	Juglans regia	XP_018858977.1
ARHOMBI_DN11368_c0_g1_i1_5	8,47E-116	340	XP_018838617.1	coproporphyrinogen-III oxidase 1, chloroplastic	Juglans regia	XP_012091240.1
ARHOMBI_DN19194_c0_g1_i1_2	1,56E-153	442	XP_018847349.1	plastidial pyruvate kinase 2	Juglans regia	OAY48391.1
ARUBRA_DN21311_c0_g1_i1_6	7,39E-18	79,7	XP_018835689.1	26S protease regulatory subunit 6B homolog	Juglans regia	XP_018814878.1
ARUBRA_DN19687_c0_g1_i1_4	3,26E-48	158	XP_018823952.1	NADH dehydrogenase ubiquinone 1 beta subcomplex subunit 7-like	Juglans regia	ONI31936.1
ARHOMBI_DN1072_c0_g1_i1_2	1,20E-30	115	XP_018814411.1	uncharacterized protein LOC108986289	Juglans regia	AFK47180.1
ARHOMBI_DN4606_c0_g2_i1_3	3,52E-91	267	KHN13429.1	AP-1 complex subunit mu-2	Glycine soja	XP_018813476.1
ARHOMBI_DN3865_c0_g1_i1_4	1,10E-92	276	XP_018851775.1	ribonuclease P protein subunit p25-like protein	Juglans regia	XP_014504552.1
ARHOMBI_DN3210_c0_g1_i1_3	7,69E-93	272	XP_018828972.1	probable prefoldin subunit 5	Juglans regia	KDP37534.1
ARHOMBI_DN127_c0_g1_i1_4	1,55E-138	399	XP_018828995.1	ER membrane protein complex subunit 10	Juglans regia	ONI05218.1
ARUBRA_DN6664_c0_g1_i1_5	7,86E-121	353	XP_009351884.1	mitochondrial import inner membrane translocase subunit TIM22-like	Pyrus x bretschneideri	XP_008381679.1
ARUBRA_DN17807_c0_g1_i1_6	5,20E-18	80,5	XP_018815021.1	protein-tyrosine-phosphatase PTP1 isoform X2	Juglans regia	XP_018815024.1
ARHOMBI_DN26518_c0_g1_i1_6	1,33E-46	154	XP_018815403.1	ribose-phosphate pyrophosphokinase 4-like isoform X1	Juglans regia	XP_018815404.1
ARHOMBI_DN26272_c0_g1_i1_3	3,66E-63	211	XP_018831147.1	dynamin-2A-like	Juglans regia	XP_018834193.1
ARUBRA_DN24367_c0_g1_i1_3	3,73E-50	157	XP_008345769.1	ankyrin repeat domain-containing protein 39-like	Malus domestica	OAY42079.1
ARHOMBI_DN10852_c0_g1_i1_2	2,63E-45	154	XP_018829345.1	uncharacterized protein LOC108997482	Juglans regia	XP_018829346.1
ARUBRA_DN17980_c0_g1_i1_5	5,37E-130	381	XP_018830436.1	methyl-CpG-binding domain-containing protein 11-like isoform X1	Juglans regia	XP_018851719.1
ARHOMBI_DN178_c0_g2_i1_4	0	546	XP_018842828.1	acyl-CoA-binding domain-containing protein 1-like isoform X1	Juglans regia	XP_018824717.1
ARUBRA_DN3098_c0_g1_i1_4	1,04E-177	500	XP_018842653.1	NADH--cytochrome b5 reductase 1	Juglans regia	OAY41871.1
ARHOMBI_DN8417_c0_g1_i1_6	1,21E-35	134	XP_018806897.1	lysine--tRNA ligase isoform X1	Juglans regia	XP_018806898.1
ARUBRA_DN16531_c0_g1_i1_2	6,44E-52	163	XP_018842878.1	10 kDa chaperonin-like	Juglans regia	XP_008339549.1
ARUBRA_DN201_c0_g2_i1_4	3,45E-152	427	XP_018847846.1	ras-related protein Rab7 isoform X1	Juglans regia	XP_018847847.1
ARUBRA_DN4976_c0_g1_i1_4	0	595	XP_018851336.1	2-alkenal reductase (NADP(+)-dependent)-like isoform X2	Juglans regia	XP_018839362.1
ARUBRA_DN22472_c0_g1_i1_1	2,63E-35	130	XP_018847012.1	ATP sulfurylase 2-like	Juglans regia	XP_018812595.1
ARHOMBI_DN22711_c0_g1_i1_4	1,00E-23	96,3	XP_018833291.1	U-box domain-containing protein 2 isoform X2	Juglans regia	XP_018833290.1

ARHOMBI_DN5461_c0_g1_i2_3	4,68E-98	291	XP_018843992.1	vacuolar protein sorting-associated protein 32 homolog 2	Juglans regia	XP_018846598.1
ARUBRA_DN1871_c0_g1_i1_5	5,51E-116	334	XP_018849009.1	syntaxin-52-like isoform X2	Juglans regia	XP_018849010.1
ARHOMBI_DN11140_c0_g1_i1_4	1,36E-36	132	XP_008235410.1	ATP-dependent 6-phosphofructokinase 5, chloroplastic	Prunus mume	ONI02403.1
ARUBRA_DN23048_c0_g1_i1_5	7,18E-78	234	XP_018842585.1	ferredoxin, root R-B2-like	Juglans regia	XP_007142856.1
ARHOMBI_DN2451_c0_g1_i1_1	1,65E-78	242	XP_018835130.1	uncharacterized oxidoreductase At4g09670-like	Juglans regia	KHN00711.1
ARUBRA_DN2562_c0_g2_i2_3	4,14E-73	222	XP_018857050.1	thiosulfate sulfurtransferase 18-like isoform X1	Juglans regia	XP_018857051.1
ARHOMBI_DN20377_c0_g1_i1_1	1,32E-73	240	XP_018851614.1	glycerol-3-phosphate dehydrogenase SDP6, mitochondrial	Juglans regia	XP_014514280.1
ARUBRA_DN15849_c0_g1_i1_3	4,52E-28	108	XP_018818477.1	putative G3BP-like protein	Juglans regia	GAU19860.1
ARUBRA_DN24185_c0_g1_i1_2	1,83E-59	194	XP_017185477.1	beta-glucosidase BoGH3B-like isoform X3	Malus domestica	XP_008375543.1
ARUBRA_DN3137_c0_g1_i1_1	0	825	XP_018810823.1	glutamate dehydrogenase 1 isoform X1	Juglans regia	XP_018810824.1
ARUBRA_DN18794_c0_g1_i1_1	1,81E-116	346	XP_018828806.1	uncharacterized protein LOC108997121 isoform X3	Juglans regia	XP_018828804.1
ARHOMBI_DN16770_c0_g1_i1_3	6,66E-86	266	XP_018825750.1	aldehyde dehydrogenase 22A1	Juglans regia	ARJ55426.1
ARUBRA_DN8214_c1_g1_i1_2	1,71E-157	445	XP_018806029.1	zinc finger protein-like 1 homolog isoform X2	Juglans regia	XP_018806028.1
ARHOMBI_DN5351_c0_g3_i2_2	6,93E-21	75,9	XP_018827819.1	cytochrome c oxidase subunit 5b-1, mitochondrial-like	Juglans regia	XP_018827819.1
ARUBRA_DN26670_c0_g1_i1_1	1,74E-27	107	ONI35887.1	hypothetical protein PRUPE_1G559400	Prunus persica	XP_007225245.1
ARHOMBI_DN14110_c0_g1_i1_4	1,12E-56	186	OAY57375.1	hypothetical protein MANES_02G092300	Manihot esculenta	KYP34374.1
ARHOMBI_DN3010_c0_g2_i1_1						
ARUBRA_DN14051_c0_g1_i1_3	2,08E-45	159	XP_018859162.1	putative 3,4-dihydroxy-2-butanone kinase, partial	Juglans regia	XP_018823489.1
ARUBRA_DN20086_c0_g1_i1_3	1,25E-68	209	XP_018827356.1	bet1-like SNARE 1-1	Juglans regia	XP_018825210.1
ARUBRA_DN11076_c0_g1_i1_6	3,35E-114	332	XP_018830707.1	post-GPI attachment to proteins factor 3-like	Juglans regia	XP_018810418.1
ARHOMBI_DN25990_c0_g1_i1_4	4,97E-89	268	XP_018813794.1	ubiquitin receptor RAD23b-like	Juglans regia	XP_020226843.1
ARHOMBI_DN2521_c0_g1_i1_6	1,85E-139	407	XP_018815300.1	amidophosphoribosyltransferase, chloroplastic	Juglans regia	AFK35899.1
ARUBRA_DN3144_c0_g1_i1_5	3,18E-32	120	XP_018851892.1	ubiquitin carboxyl-terminal hydrolase 14 isoform X3	Juglans regia	XP_018851888.1
ARUBRA_DN524_c0_g2_i1_3	0	879	XP_018840074.1	protein NBR1 homolog isoform X1	Juglans regia	XP_018840075.1
ARUBRA_DN4339_c0_g1_i2_1	0	684	KDP33360.1	hypothetical protein JCGZ_12909	Jatropha curcas	KDP33360.1
ARHOMBI_DN2257_c0_g1_i1_1	3,48E-173	496	XP_018809158.1	uncharacterized protein At5g49945-like	Juglans regia	XP_018817873.1
ARUBRA_DN17897_c0_g1_i1_5	3,02E-123	375	XP_018835052.1	subtilisin-like protease SBT1.4	Juglans regia	AET01799.1
ARHOMBI_DN2123_c0_g2_i1_4	2,06E-80	249	XP_018839141.1	uncharacterized protein LOC109004890	Juglans regia	XP_018843271.1

ARUBRA_DN11421_c0_g1_i1_5	8,65E-30	115	XP_018853594.1	dihydrolipoyllysine-residue acetyltransferase component 1 of pyruvate dehydrogenase complex, mitochondrial	Juglans regia	XP_020966051.1
ARHOMBI_DN13369_c0_g1_i1_6	4,11E-59	194	XP_018816821.1	2-hydroxyacyl-CoA lyase	Juglans regia	XP_008353818.1
ARUBRA_DN10411_c0_g1_i1_2	1,42E-73	226	XP_018824341.1	UPF0160 protein	Juglans regia	ONI07578.1
ARHOMBI_DN5006_c0_g1_i1_1	4,60E-75	233	XP_018846608.1	LOW QUALITY PROTEIN: gamma aminobutyrate transaminase 3, chloroplastic	Juglans regia	XP_015952737.1
ARHOMBI_DN17209_c0_g1_i1_1	1,03E-41	147	XP_018845402.1	acyl-coenzyme A oxidase 2, peroxisomal	Juglans regia	ALB76816.1
ARUBRA_DN6201_c0_g1_i1_4	4,70E-94	276	XP_018832001.1	Golgi apparatus membrane protein-like protein ECHIDNA isoform X1	Juglans regia	XP_018816227.1
ARHOMBI_DN4317_c0_g1_i1_2	0	705	XP_018838516.1	long chain base biosynthesis protein 1	Juglans regia	XP_018838517.1
ARHOMBI_DN5264_c0_g1_i2_1	0	594	XP_018857484.1	mannose-1-phosphate guanylyltransferase 1	Juglans regia	XP_018857485.1
ARUBRA_DN3956_c0_g2_i2_6	0	657	XP_018844514.1	uncharacterized protein LOC109008754	Juglans regia	XP_018844515.1
ARUBRA_DN7892_c0_g1_i1_4	3,84E-101	306	XP_018816820.1	THO complex subunit 4D	Juglans regia	XP_018811304.1
ARUBRA_DN5621_c0_g1_i1_1	0	921	XP_018815548.1	long chain acyl-CoA synthetase 4-like	Juglans regia	XP_018824382.1
ARUBRA_DN6290_c0_g1_i1_6	8,44E-129	375	XP_018824678.1	uncharacterized protein LOC108994051 isoform X2	Juglans regia	XP_018824670.1
ARUBRA_DN8437_c0_g1_i1_2	4,54E-59	187	XP_018856589.1	vacuolar protein sorting-associated protein 20 homolog 2-like	Juglans regia	XP_018827788.1
ARHOMBI_DN2197_c0_g1_i1_2	6,92E-30	114	XP_018832979.1	calponin homology domain-containing protein DDB_G0272472-like	Juglans regia	XP_018843078.1
ARUBRA_DN20773_c0_g1_i1_2	3,57E-53	173	KYP70903.1	S-adenosylmethionine synthetase 2	Cajanus cajan	OAY42731.1
ARUBRA_DN19652_c0_g1_i1_5	8,57E-55	183	ONI31767.1	hypothetical protein PRUPE_1G329900	Prunus persica	XP_020425549.1
ARUBRA_DN23301_c0_g1_i1_2	3,44E-51	177	XP_018817805.1	leucine--tRNA ligase, cytoplasmic isoform X2	Juglans regia	XP_018817804.1
ARHOMBI_DN6000_c0_g1_i1_4	0	852	XP_018848460.1	aspartic proteinase A1-like	Juglans regia	ONI18425.1
ARHOMBI_DN27227_c0_g1_i1_2	1,19E-36	131	XP_018825366.1	SNF1-related protein kinase regulatory subunit gamma-1	Juglans regia	XP_014630956.1
ARHOMBI_DN3035_c0_g1_i1_2	1,42E-54	171	XP_018830593.1	mitochondrial import inner membrane translocase subunit TIM10-like	Juglans regia	XP_018830594.1
ARUBRA_DN7465_c0_g1_i1_1	9,27E-40	142	XP_009378530.1	acyl-coenzyme A oxidase, peroxisomal-like	Pyrus x bretschneideri	XP_008370191.1
ARHOMBI_DN25461_c0_g1_i1_3	2,50E-63	200	XP_018852310.1	protein MODIFIER OF SNC1 11-like	Juglans regia	XP_018858651.1
ARUBRA_DN23449_c0_g1_i1_4	3,90E-15	73,6	XP_018837387.1	acetyl-CoA acetyltransferase, cytosolic 1	Juglans regia	KHN43976.1
ARUBRA_DN4751_c0_g1_i2_6	5,19E-98	287	XP_018826274.1	barwin-like	Juglans regia	XP_018826287.1
ARHOMBI_DN23037_c0_g1_i1_6	1,39E-108	329	XP_018835985.1	ATP-dependent zinc metalloprotease FTSH 10, mitochondrial-like	Juglans regia	XP_020992956.1
ARHOMBI_DN17541_c0_g1_i1_1	2,67E-48	167	ONH94299.1	hypothetical protein PRUPE_7G009200	Prunus persica	XP_009366484.1
ARUBRA_DN10528_c0_g1_i1_4	2,39E-55	176	OAY60795.1	hypothetical protein MANES_01G139600	Manihot esculenta	XP_018824069.1

ARUBRA_DN13908_c0_g1_i1_4	4,22E-25	97,1	ONI03605.1	hypothetical protein PRUPE_6G268400	Prunus persica	XP_007205928.1
ARHOMBI_DN13282_c0_g1_i1_1	3,28E-57	177	XP_018828328.1	gamma aminobutyrate transaminase 3, chloroplastic-like	Juglans regia	XP_015952737.1
ARHOMBI_DN3944_c0_g1_i2_2	0	691	XP_018811976.1	CBL-interacting serine/threonine-protein kinase 9	Juglans regia	XP_008376207.1
ARHOMBI_DN19974_c0_g1_i1_4	1,13E-79	245	XP_016651062.1	monodehydroascorbate reductase 5, mitochondrial	Prunus mume	XP_018829248.1
ARUBRA_DN7017_c0_g1_i1_3	8,82E-155	439	XP_018846669.1	4-hydroxy-tetrahydrodipicolinate synthase, chloroplastic like	Juglans regia	XP_018806961.1
ARUBRA_DN1910_c0_g1_i1_5	1,18E-52	174	XP_018836471.1	ferredoxin-thioredoxin reductase, variable chain, chloroplastic	Juglans regia	XP_018836479.1
ARUBRA_DN4277_c0_g1_i1_2	1,28E-111	330	XP_018817798.1	CBS domain-containing protein CBSX1, chloroplastic-like	Juglans regia	XP_018817799.1
ARUBRA_DN22918_c0_g1_i1_6	2,08E-133	388	KDP26352.1	hypothetical protein JCGZ_17510	Jatropha curcas	XP_012085051.1
ARUBRA_DN9150_c0_g1_i1_5	1,01E-106	323	KOM29550.1	hypothetical protein LR48_Vigan727s000900	Vigna angularis	XP_018850497.1
ARHOMBI_DN6685_c1_g1_i1_6	3,63E-57	176	XP_004512667.1	protein CYPRO4-like	Cicer arietinum	KYP76357.1
ARHOMBI_DN26281_c0_g1_i1_5	6,17E-61	204	XP_018810126.1	ATPase 10, plasma membrane-type-like	Juglans regia	XP_007139050.1
ARUBRA_DN3358_c0_g1_i2_1	4,17E-95	285	XP_018829285.1	tyrosine--tRNA ligase 1, cytoplasmic	Juglans regia	XP_008380680.1
ARHOMBI_DN4457_c0_g1_i1_1	6,73E-152	440	XP_018848980.1	folylpolyglutamate synthase	Juglans regia	OAY38549.1
ARHOMBI_DN10001_c0_g1_i1_3	6,17E-78	239	XP_018818889.1	arogenate dehydratase/prephenate dehydratase 2, chloroplastic isoform X3	Juglans regia	XP_018818888.1
ARHOMBI_DN13618_c0_g2_i1_3	3,26E-68	150	XP_018841787.1	mitochondrial Rho GTPase 1-like	Juglans regia	XP_018841787.1
ARHOMBI_DN3625_c0_g1_i1_3	2,81E-104	315	XP_018819043.1	xaa-Pro dipeptidase	Juglans regia	KDP33743.1
ARHOMBI_DN23530_c0_g1_i1_1	7,23E-36	128	XP_018859899.1	dnaJ homolog subfamily C member 17-like	Juglans regia	XP_018859900.1
ARUBRA_DN21885_c0_g1_i1_1	3,96E-64	209	XP_008223314.1	dihydrolipoyl dehydrogenase 1, chloroplastic-like	Prunus mume	ONI28088.1
ARHOMBI_DN4759_c0_g2_i1_2	2,89E-115	341	XP_018843312.1	eukaryotic peptide chain release factor subunit 1-3	Juglans regia	OIW06484.1
ARHOMBI_DN995_c0_g2_i1_5	4,12E-110	328	XP_018810134.1	dihydropyrimidine dehydrogenase (NADP(+)), chloroplastic-like	Juglans regia	XP_018810134.1
ARHOMBI_DN19127_c0_g1_i1_2	1,20E-53	169	XP_018810806.1	adenylyl-sulfate kinase 3	Juglans regia	XP_018810807.1
ARUBRA_DN19640_c0_g1_i1_4	2,29E-112	330	XP_018842745.1	stomatin-like protein 2, mitochondrial	Juglans regia	XP_018828153.1
ARUBRA_DN16258_c0_g1_i1_4	3,90E-62	195	KYP71087.1	hypothetical protein KK1_010330	Cajanus cajan	XP_018502255.1
ARUBRA_DN19843_c0_g1_i1_1	1,92E-26	108	ONI36174.1	hypothetical protein PRUPE_1G573300	Prunus persica	XP_009355252.1
ARHOMBI_DN4707_c0_g1_i1_6	0	601	XP_018807462.1	membrane-associated 30 kDa protein, chloroplastic-like	Juglans regia	XP_018806924.1
ARUBRA_DN6269_c0_g1_i1_1	2,91E-68	210	OAY22084.1	hypothetical protein MANES_S031600	Manihot esculenta	XP_018819297.1

ARUBRA_DN14413_c0_g1_i1_1	1,99E-32	120	OAY26173.1	hypothetical protein MANES_16G026500	Manihot esculenta	KDP20960.1
ARUBRA_DN23541_c0_g1_i1_3	3,72E-65	201	XP_018821662.1	desumoylating isopeptidase 1-like	Juglans regia	XP_018821664.1
ARHOMBI_DN21586_c0_g1_i1_2	5,75E-91	285	XP_018811352.1	heat shock 70 kDa protein 16-like	Juglans regia	XP_018811359.1
ARUBRA_DN20454_c0_g1_i1_1	1,87E-82	266	KDP44163.1	hypothetical protein JCGZ_05630	Jatropha curcas	XP_012064944.1
ARUBRA_DN25494_c0_g1_i1_2	1,71E-45	159	XP_012066299.1	importin subunit beta-1	Jatropha curcas	KDP42918.1
ARUBRA_DN1726_c0_g1_i1_1	1,77E-107	310	OAY27343.1	hypothetical protein MANES_16G118300	Manihot esculenta	XP_018834778.1
ARUBRA_DN4369_c0_g1_i1_1	1,18E-80	241	ONH98640.1	hypothetical protein PRUPE_7G259500	Prunus persica	XP_007202663.1
ARHOMBI_DN7832_c0_g1_i1_1	2,55E-70	232	XP_018839647.1	eukaryotic translation initiation factor 3 subunit A-like	Juglans regia	XP_018839648.1
ARHOMBI_DN3921_c0_g1_i1_3	0	547	XP_018823086.1	probable bifunctional methylthioribulose-1-phosphate dehydratase/enolase-phosphatase E1 isoform X1	Juglans regia	XP_018823094.1
ARUBRA_DN729_c0_g1_i1_6	2,96E-103	310	XP_008240130.1	TPR repeat-containing thioredoxin TDX	Prunus mume	XP_008374854.1
ARUBRA_DN17182_c0_g1_i1_3	6,90E-106	317	XP_018843013.1	NPL4-like protein 1	Juglans regia	XP_008234969.1
ARUBRA_DN5225_c0_g1_i1_3	9,69E-85	255	XP_018837027.1	probable aldo-keto reductase 1	Juglans regia	XP_018817492.1
ARHOMBI_DN26642_c0_g1_i1_3	2,56E-30	115	XP_018825904.1	uncharacterized protein LOC108994927 isoform X2	Juglans regia	XP_018825905.1
ARHOMBI_DN6091_c0_g1_i3_6	1,93E-172	485	XP_018837625.1	cytochrome P450 89A2-like, partial	Juglans regia	XP_018837633.1
ARHOMBI_DN2397_c0_g1_i1_3	2,15E-87	264	XP_018812413.1	uncharacterized protein LOC108984806	Juglans regia	XP_009371362.1
ARUBRA_DN19230_c0_g1_i1_6	2,54E-109	316	XP_018844469.1	N-alpha-acetyltransferase daf-31	Juglans regia	XP_016171058.1
ARUBRA_DN10511_c0_g1_i1_4	2,44E-20	85,1	XP_018836814.1	uncharacterized protein LOC109003222	Juglans regia	XP_018805269.1
ARHOMBI_DN18648_c0_g1_i1_2	1,37E-35	125	XP_018842895.1	uridine 5'-monophosphate synthase-like	Juglans regia	XP_008348657.1
ARHOMBI_DN20035_c0_g1_i1_4	9,31E-115	343	XP_018837506.1	glucosidase 2 subunit beta	Juglans regia	KOM31758.1
ARHOMBI_DN22203_c0_g1_i1_5	9,48E-94	284	XP_018850953.1	dual specificity protein phosphatase 12-like	Juglans regia	XP_018850954.1
ARUBRA_DN8245_c0_g1_i1_1	2,35E-17	80,5	XP_018835918.1	myb-like protein X	Juglans regia	XP_018835919.1
ARUBRA_DN18674_c0_g1_i1_6	1,07E-47	164	XP_018824522.1	signal recognition particle subunit SRP68	Juglans regia	XP_018824523.1
ARUBRA_DN1052_c0_g1_i1_4	1,85E-112	324	OIV93234.1	hypothetical protein TanjilG_27413	Lupinus angustifolius	XP_019423713.1
ARUBRA_DN4497_c0_g1_i1_6	7,72E-65	203	XP_008364292.1	farnesyl pyrophosphate synthase 1-like, partial	Malus domestica	XP_018813512.1
ARHOMBI_DN23074_c0_g1_i1_6	3,58E-86	265	XP_018859944.1	uncharacterized protein LOC109021706	Juglans regia	XP_018824342.1
ARUBRA_DN17867_c0_g1_i1_4	4,44E-131	387	XP_018824851.1	histone deacetylase 5 isoform X3	Juglans regia	XP_018824848.1
ARUBRA_DN26207_c0_g1_i1_1	2,02E-18	81,6	XP_018843078.1	calponin homology domain-containing protein DDB_G0272472	Juglans regia	XP_018843079.1
ARUBRA_DN993_c0_g1_i1_2	1,10E-125	360	XP_018847771.1	40S ribosomal protein S6-like	Juglans regia	XP_008235060.1
ARUBRA_DN5001_c0_g1_i1_4	0	821	XP_012066228.2	LOW QUALITY PROTEIN: polyubiquitin-A	Jatropha curcas	XP_012066228.2

ARUBRA_DN16126_c0_g1_i1_1	0	540	XP_018850824.1	bifunctional aspartate aminotransferase and glutamate/aspartate-prephenate aminotransferase-like	Juglans regia	XP_018816075.1
ARUBRA_DN18434_c0_g1_i1_5	1,26E-76	234	GAU13040.1	hypothetical protein TSUD_173410	Trifolium subterraneum	XP_018828854.1
ARHOMBI_DN8608_c0_g1_i1_5	2,76E-143	412	XP_018827207.1	flowering locus K homology domain-like	Juglans regia	XP_018827208.1
ARUBRA_DN21219_c0_g1_i1_3	1,16E-20	87	XP_018835354.1	uricase-2 isozyme 2	Juglans regia	XP_008231809.1
ARUBRA_DN10906_c0_g1_i1_1	2,93E-53	144	XP_018846638.1	uncharacterized protein LOC109010309	Juglans regia	XP_018846638.1
ARUBRA_DN24391_c0_g1_i1_1	8,46E-42	145	OAY56557.1	hypothetical protein MANES_02G026500	Manihot esculenta	KDP25494.1
ARHOMBI_DN6909_c0_g1_i1_1	6,14E-102	305	XP_018823852.1	heterogeneous nuclear ribonucleoprotein 1-like isoform X1	Juglans regia	XP_018823852.1
ARUBRA_DN24666_c0_g1_i1_6	1,38E-48	159	XP_018856631.1	enoyl-CoA hydratase 2, peroxisomal	Juglans regia	KRH51640.1
ARUBRA_DN6630_c0_g1_i1_2	5,71E-123	351	XP_018821775.1	GDSL esterase/lipase CPRD49-like	Juglans regia	XP_018810087.1
ARUBRA_DN9370_c0_g1_i1_1	4,44E-30	105	XP_018819229.1	cytochrome c oxidase-assembly factor COX23, mitochondrial	Juglans regia	XP_007140753.1
ARUBRA_DN10905_c0_g1_i1_2	5,20E-87	265	OIW21593.1	hypothetical protein TanjilG_06439	Lupinus angustifolius	XP_019433374.1
ARHOMBI_DN13448_c0_g1_i1_5	1,11E-103	301	XP_018840252.1	inosine triphosphate pyrophosphatase isoform X2	Juglans regia	XP_018840253.1
ARUBRA_DN20978_c0_g1_i1_4	7,89E-67	215	KRH60088.1	hypothetical protein GLYMA_05G219500	Glycine max	KRH60089.1
ARUBRA_DN6384_c0_g1_i1_5	3,21E-90	283	XP_009348649.2	presequence protease 1, chloroplastic/mitochondrial-like	Pyrus x bretschneideri	XP_018835573.1
ARHOMBI_DN981_c0_g1_i1_4	0	531	ONI09471.1	hypothetical protein PRUPE_5G240500	Prunus persica	ONI09472.1
ARUBRA_DN22585_c0_g1_i1_2	3,30E-83	252	OAY29318.1	hypothetical protein MANES_15G135500	Manihot esculenta	XP_012072319.1
ARHOMBI_DN4343_c0_g1_i1_6	2,17E-138	401	XP_018821130.1	anamorsin homolog isoform X1	Juglans regia	XP_018821131.1
ARHOMBI_DN796_c0_g1_i1_1	6,79E-57	186	XP_018827562.1	UDP-N-acetylglucosamine diphosphorylase 2 isoform X1	Juglans regia	XP_018827564.1
ARUBRA_DN3285_c0_g1_i1_2	7,20E-66	202	XP_018805629.1	uncharacterized protein LOC108979405, partial	Juglans regia	XP_008339881.1
ARUBRA_DN5446_c0_g1_i1_1	4,68E-94	289	XP_008232677.1	2-isopropylmalate synthase 1, chloroplastic-like isoform X2	Prunus mume	XP_016652519.1
ARUBRA_DN10803_c0_g1_i1_2	1,57E-115	339	XP_007145516.1	hypothetical protein PHAVU_007G2449000g, partial	Phaseolus vulgaris	ESW17510.1
ARUBRA_DN16402_c0_g1_i1_6	7,62E-49	163	XP_018848063.1	UDP-glucuronate 4-epimerase 3	Juglans regia	XP_018807731.1
ARUBRA_DN19030_c0_g1_i1_1	2,88E-72	221	XP_018853893.1	EH domain-containing protein 1	Juglans regia	XP_018812205.1
ARHOMBI_DN6155_c0_g1_i1_1	0	534	XP_018837640.1	probable tocopherol O-methyltransferase, chloroplastic isoform X4	Juglans regia	XP_018837638.1
ARUBRA_DN13110_c0_g1_i1_1	1,22E-33	119	XP_018821056.1	uncharacterized protein LOC108991326	Juglans regia	XP_008388789.1
ARUBRA_DN25449_c0_g1_i1_2	3,84E-34	121	XP_018817492.1	probable aldo-keto reductase 1, partial	Juglans regia	XP_018837027.1
ARHOMBI_DN4728_c0_g3_i1_1	0	651	XP_018828049.1	serine/threonine-protein phosphatase 6 regulatory subunit 3-like	Juglans regia	XP_018847866.1

ARHOMBI_DN6142_c0_g1_i3_4	0	587	XP_009354832.1	peroxisomal (S)-2-hydroxy-acid oxidase-like isoform X1	Pyrus x bretschneideri	XP_009344324.1
ARHOMBI_DN5011_c0_g1_i1_4	1,18E-134	409	XP_018819304.1	heterogeneous nuclear ribonucleoprotein R isoform X1	Juglans regia	XP_018819308.1
ARHOMBI_DN7836_c0_g1_i1_2	0	572	XP_018849878.1	putative quinone-oxidoreductase homolog, chloroplastic	Juglans regia	XP_018844198.1
ARHOMBI_DN4465_c0_g1_i1_1	2,45E-158	461	XP_018826576.1	probable methyltransferase PMT2	Juglans regia	ONI08658.1
ARHOMBI_DN2932_c0_g2_i1_5	0	634	XP_018820175.1	monothiol glutaredoxin-S17	Juglans regia	KDP20960.1
ARHOMBI_DN6286_c0_g1_i1_6	5,62E-144	414	XP_018835872.1	SEC12-like protein 2	Juglans regia	XP_018835880.1
ARUBRA_DN17025_c0_g1_i1_2	3,74E-32	112	XP_014504331.1	glucose-1-phosphate adenylyltransferase large subunit 1 like	Vigna radiata var. radiata	XP_014504330.1
ARHOMBI_DN7489_c1_g1_i1_4	2,75E-48	166	KDP34274.1	hypothetical protein JCGZ_12843	Jatropha curcas	XP_012076264.1
ARUBRA_DN13369_c0_g1_i1_3	3,39E-56	179	KRG96658.1	hypothetical protein GLYMA_19G224500	Glycine max	KRG96659.1
ARUBRA_DN18574_c0_g1_i1_2	1,31E-63	207	OIW18923.1	hypothetical protein TanjilG_25366	Lupinus angustifolius	XP_019448161.1
ARUBRA_DN21886_c0_g1_i1_1	1,71E-76	233	XP_018837663.1	HD domain-containing protein C4G3.17	Juglans regia	XP_008243131.1
ARHOMBI_DN17203_c0_g1_i1_2	4,54E-170	496	XP_009368315.1	dynamin-related protein 3A-like	Pyrus x bretschneideri	XP_008382203.1
ARHOMBI_DN13122_c0_g1_i1_4	1,73E-15	74,3	XP_018835920.1	cysteine desulfurase, mitochondrial	Juglans regia	XP_016201576.1
ARHOMBI_DN25341_c0_g1_i1_1	1,33E-71	216	KOM30716.1	hypothetical protein LR48_Vigan01g027000	Vigna angularis	BAT73392.1
ARHOMBI_DN3340_c0_g1_i1_2	4,08E-172	496	XP_018848856.1	DEAD-box ATP-dependent RNA helicase 37-like	Juglans regia	XP_018848857.1
ARUBRA_DN3419_c0_g1_i1_6	1,04E-102	310	XP_020539645.1	glutamate--cysteine ligase, chloroplastic	Jatropha curcas	KDP25439.1
ARHOMBI_DN8889_c0_g1_i1_1	2,31E-36	133	XP_018831158.1	conserved oligomeric Golgi complex subunit 3	Juglans regia	XP_020225745.1
ARHOMBI_DN4948_c0_g1_i1_2	1,34E-55	182	XP_008229761.1	uncharacterized protein LOC103329111	Prunus mume	ONI18026.1
ARUBRA_DN23749_c0_g1_i1_2	2,46E-51	163	XP_018853827.1	uncharacterized protein LOC109015827	Juglans regia	XP_018853937.1
ARUBRA_DN23540_c0_g1_i1_4	3,71E-53	173	XP_008220830.1	ornithine carbamoyltransferase, chloroplastic	Prunus mume	ONI32617.1
ARHOMBI_DN658_c0_g1_i1_6	4,58E-138	395	XP_018857638.1	cytosolic Fe-S cluster assembly factor NBP35	Juglans regia	XP_008219249.1
ARUBRA_DN1712_c0_g1_i1_3	0	562	XP_018852252.1	probable xyloglucan endotransglucosylase/hydrolase protein 30	Juglans regia	XP_008373536.1
ARUBRA_DN9464_c0_g1_i1_3	6,94E-20	86,7	XP_008242736.1	nudix hydrolase 19, chloroplastic	Prunus mume	OAY47072.1
ARUBRA_DN8289_c0_g1_i1_1	0	531	XP_018844991.1	PI-PLC X domain-containing protein At5g67130	Juglans regia	KRH57924.1
ARHOMBI_DN11041_c0_g1_i1_3	6,72E-40	137	XP_018809442.1	glucuronokinase 1-like	Juglans regia	XP_018846473.1
ARHOMBI_DN8091_c0_g1_i1_4	1,25E-109	318	XP_018825523.1	enoyl-CoA delta isomerase 1, peroxisomal-like	Juglans regia	XP_018825524.1
ARHOMBI_DN4244_c0_g1_i2_2	1,12E-93	282	XP_008238370.1	ATP-dependent Clp protease proteolytic subunit-related protein 4, chloroplastic	Prunus mume	XP_008238371.1

ARUBRA_DN313_c0_g2_i1_6	1,14E-41	138	XP_012066031.1	cytochrome b-c1 complex subunit 6	Jatropha curcas	XP_008218833.1
ARUBRA_DN707_c0_g1_i1_4	1,42E-62	194	XP_018846320.1	60S ribosomal protein L31	Juglans regia	XP_008242444.1
ARHOMBI_DN3837_c0_g1_i1_6	9,10E-148	427	XP_018805247.1	probable WRKY transcription factor 40	Juglans regia	XP_018847539.1
ARUBRA_DN4588_c0_g1_i2_1	0	535	XP_018838814.1	insulin-degrading enzyme-like 1, peroxisomal	Juglans regia	XP_018837211.1
ARHOMBI_DN6158_c0_g1_i1_6	3,94E-161	458	XP_018850665.1	elongation factor 1-gamma-like	Juglans regia	ONI35669.1
ARHOMBI_DN6709_c0_g1_i1_2	0	574	XP_018831990.1	eukaryotic translation initiation factor 3 subunit B-like	Juglans regia	XP_015945461.1
ARHOMBI_DN12465_c0_g1_i1_1	3,18E-17	74,3	KDP22143.1	hypothetical protein JCGZ_25974	Jatropha curcas	XP_020540656.1
ARHOMBI_DN26923_c0_g1_i1_1	7,16E-59	189	XP_018825366.1	SNF1-related protein kinase regulatory subunit gamma-1	Juglans regia	XP_008239461.1
ARUBRA_DN1422_c0_g2_i1_2	0	781	XP_018813832.1	protein SUPPRESSOR OF K(+) TRANSPORT GROWTH DEFECT 1 isoform X1	Juglans regia	XP_018813833.1
ARHOMBI_DN23444_c0_g1_i1_1	1,68E-57	190	KRH00969.1	hypothetical protein GLYMA_18G245200	Glycine max	XP_015962138.1
ARUBRA_DN2399_c0_g1_i1_4	1,26E-73	222	XP_018838514.1	uncharacterized protein LOC109004430	Juglans regia	XP_015956379.1
ARUBRA_DN2263_c0_g2_i1_1	8,37E-69	219	XP_018826593.1	NAP1-related protein 2	Juglans regia	XP_018833970.1
ARHOMBI_DN15914_c0_g1_i1_1	2,58E-106	319	XP_008228667.1	COBW domain-containing protein 1	Prunus mume	ONI16329.1
ARUBRA_DN1557_c0_g1_i1_4	1,41E-62	204	XP_018851305.1	switch-associated protein 70	Juglans regia	XP_008229573.1
ARUBRA_DN4786_c0_g1_i1_2	1,32E-124	380	XP_018828049.1	serine/threonine-protein phosphatase 6 regulatory subunit 3-like	Juglans regia	XP_018847866.1
ARHOMBI_DN18534_c0_g1_i1_3	2,93E-116	336	XP_018822017.1	histidine biosynthesis bifunctional protein hisIE, chloroplastic isoform X1	Juglans regia	XP_018822018.1
ARHOMBI_DN10516_c0_g1_i1_6	9,77E-37	134	XP_018843251.1	VHS domain-containing protein At3g16270	Juglans regia	XP_018843252.1
ARUBRA_DN178_c0_g1_i1_1	8,76E-123	353	ONI19203.1	hypothetical protein PRUPE_3G264000	Prunus persica	XP_007216546.1
ARUBRA_DN24618_c0_g1_i1_5	7,76E-24	97,4	KDP26618.1	hypothetical protein JCGZ_17776	Jatropha curcas	XP_012085428.1
ARUBRA_DN23959_c0_g1_i1_1	1,13E-46	158	XP_008372028.1	acetylornithine aminotransferase, mitochondrial-like	Malus domestica	XP_008383084.1
ARUBRA_DN19174_c0_g1_i1_2	5,55E-98	300	KDP43753.1	hypothetical protein JCGZ_22380	Jatropha curcas	XP_020532739.1
ARHOMBI_DN4479_c0_g1_i1_4	5,68E-165	466	XP_018840900.1	probable prolyl 4-hydroxylase 10	Juglans regia	XP_018807322.1
ARHOMBI_DN3958_c0_g1_i1_6	8,77E-136	397	XP_018846484.1	28 kDa ribonucleoprotein, chloroplastic-like	Juglans regia	ONI19760.1
ARHOMBI_DN2738_c0_g1_i1_2	6,77E-160	479	KDP43289.1	hypothetical protein JCGZ_24210	Jatropha curcas	XP_012065800.1
ARUBRA_DN1466_c0_g2_i1_4	4,96E-84	258	XP_018844826.1	ATPase ASNA1 homolog isoform X2	Juglans regia	XP_016176650.1
ARHOMBI_DN3523_c0_g1_i1_4	1,34E-62	201	XP_018835121.1	glycylpeptide N-tetradecanoyltransferase 1-like	Juglans regia	OAY53172.1
ARHOMBI_DN1869_c0_g2_i1_5	5,21E-137	387	XP_018842827.1	thylakoid lumenal 17.4 kDa protein, chloroplastic	Juglans regia	ONI25676.1
ARUBRA_DN22416_c0_g1_i1_5	1,82E-36	129	XP_018807587.1	mitogen-activated protein kinase 8-like	Juglans regia	ONI09316.1

ARUBRA_DN446_c0_g1_i1_2	1,14E-12	64,7	XP_008387535.1	uncharacterized protein LOC103449996	Malus domestica	ACU17160.1
ARUBRA_DN1177_c0_g2_i1_1	3,32E-173	494	OAY34264.1	hypothetical protein MANES_12G007900	Manihot esculenta	OAY34266.1
ARUBRA_DN22100_c0_g1_i1_5	6,50E-67	204	XP_008246109.1	probable acyl-CoA dehydrogenase IBR3	Prunus mume	XP_018815732.1
ARHOMBI_DN9092_c0_g1_i1_2	4,99E-45	149	XP_018815442.1	mitochondrial import receptor subunit TOM20-like	Juglans regia	XP_018860144.1
ARHOMBI_DN5351_c0_g2_i1_2	1,89E-56	177	XP_020228142.1	cytochrome c oxidase subunit 5b-2, mitochondrial-like	Cajanus cajan	ACU18970.1
ARUBRA_DN3055_c0_g1_i2_3	2,45E-15	74,7	OAY59405.1	hypothetical protein MANES_01G030100	Manihot esculenta	XP_018836344.1
ARHOMBI_DN21638_c0_g1_i1_6	7,77E-72	229	XP_018841304.1	uncharacterized protein LOC109006469 isoform X4	Juglans regia	XP_018841303.1
ARUBRA_DN4234_c0_g2_i1_6	0	584	XP_008382818.1	very-long-chain enoyl-CoA reductase	Malus domestica	AGJ00072.1
ARUBRA_DN2493_c0_g1_i1_1	6,05E-104	313	XP_014509695.1	acetolactate synthase small subunit 2, chloroplastic-like	Vigna radiata var. radiata	XP_014509695.1
ARUBRA_DN7662_c0_g1_i1_2	1,21E-65	210	XP_018847038.1	tubulin-folding cofactor C	Juglans regia	XP_017188064.1
ARUBRA_DN12348_c0_g1_i1_6	5,17E-63	191	XP_018852394.1	cytochrome b5	Juglans regia	OAY59358.1
ARHOMBI_DN3285_c0_g2_i1_4	1,15E-56	180	XP_018846254.1	aldo-keto reductase family 4 member C11-like	Juglans regia	XP_018817591.1
ARHOMBI_DN8957_c0_g1_i1_1	7,21E-94	247	GAU30588.1	hypothetical protein TSUD_392790	Trifolium subterraneum	GAU30588.1
ARHOMBI_DN21971_c0_g1_i1_1	7,90E-117	336	XP_018836396.1	uncharacterized protein LOC109002925 isoform X2	Juglans regia	XP_018836396.1
ARUBRA_DN13989_c0_g1_i1_2	6,06E-33	122	XP_018825160.1	ubiquitin-like modifier-activating enzyme 5	Juglans regia	XP_009368530.1
ARUBRA_DN26201_c0_g1_i1_1	1,28E-46	161	XP_018806310.1	signal recognition particle subunit SRP72-like	Juglans regia	OAY32389.1
ARHOMBI_DN16098_c0_g1_i1_2	1,84E-58	183	XP_018811551.1	uncharacterized protein LOC108984148 isoform X2	Juglans regia	XP_018811550.1
ARHOMBI_DN2295_c0_g1_i1_1	3,50E-129	372	XP_018848880.1	uncharacterized protein LOC109011934	Juglans regia	XP_018848881.1
ARHOMBI_DN8630_c0_g1_i1_2	9,35E-58	196	XP_018845402.1	acyl-coenzyme A oxidase 2, peroxisomal	Juglans regia	OAY35867.1
ARUBRA_DN15491_c0_g1_i1_1	1,24E-34	126	XP_018809797.1	eukaryotic translation initiation factor 2A	Juglans regia	XP_018809798.1
ARUBRA_DN7176_c0_g1_i1_6	9,21E-12	63,5	XP_018813351.1	uncharacterized protein At2g24330-like	Juglans regia	XP_018813352.1
ARUBRA_DN22998_c0_g1_i1_5	9,28E-136	389	XP_018806434.1	gamma-soluble NSF attachment protein-like	Juglans regia	XP_018828462.1
ARHOMBI_DN1819_c0_g1_i1_2	6,00E-105	309	XP_018830668.1	sorbitol dehydrogenase	Juglans regia	AFK33603.1
ARUBRA_DN1769_c0_g2_i1_6	1,84E-63	199	XP_018835354.1	uricase-2 isozyme 2	Juglans regia	BAB18540.1
ARHOMBI_DN7578_c0_g1_i1_4	1,75E-93	284	XP_018822783.1	pyruvate dehydrogenase E1 component subunit alpha-3, chloroplastic	Juglans regia	OAY62077.1
ARHOMBI_DN4440_c0_g1_i1_4	9,66E-139	394	XP_018815728.1	acylpyruvase FAHD1, mitochondrial-like	Juglans regia	XP_018806160.1
ARUBRA_DN16297_c0_g1_i1_4	1,05E-106	334	XP_008237688.1	putative transcription elongation factor SPT5 homolog 1	Prunus mume	ONH89815.1
ARUBRA_DN7697_c0_g1_i1_2	1,99E-62	202	XP_018826783.1	TBC1 domain family member 17-like isoform X3	Juglans regia	XP_018826782.1
ARUBRA_DN19516_c0_g1_i1_2	0	545	XP_018852048.1	uncharacterized protein LOC109014152 isoform X1	Juglans regia	XP_018852049.1

ARUBRA_DN9248_c0_g1_i1_4	5,08E-153	438	OAY31102.1	hypothetical protein MANES_14G083500	Manihot esculenta	OAY47555.1
ARHOMBI_DN26238_c0_g1_i1_4	1,48E-34	118	XP_018819561.1	germin-like protein subfamily T member 2, partial	Juglans regia	XP_018832652.1
ARHOMBI_DN1217_c0_g2_i1_1	2,28E-61	197	ONI23731.1	hypothetical protein PRUPE_2G204600	Prunus persica	ONI23729.1
ARUBRA_DN22060_c0_g1_i1_4	2,22E-135	384	XP_018829252.1	uncharacterized protein LOC108997431	Juglans regia	ONI29700.1
ARHOMBI_DN3796_c0_g1_i1_3	0	551	XP_018823763.1	subtilisin-like protease SBT1.6	Juglans regia	XP_015952465.2
ARUBRA_DN3884_c0_g1_i1_3	4,35E-82	246	XP_012074113.1	microsomal glutathione S-transferase 3	Jatropha curcas	OAY57255.1
ARHOMBI_DN11238_c0_g1_i1_1	1,45E-58	192	XP_018854693.1	T-complex protein 1 subunit delta	Juglans regia	KDP46998.1
ARUBRA_DN20512_c0_g1_i1_5	2,14E-44	160	XP_018826937.1	uncharacterized protein LOC108995769	Juglans regia	XP_008380047.1
ARUBRA_DN2494_c0_g1_i1_5	3,58E-145	411	XP_018857760.1	phosphoglycerate mutase-like protein 4	Juglans regia	XP_018856718.1
ARHOMBI_DN25808_c0_g1_i1_4	2,83E-48	167	XP_018808786.1	valine--tRNA ligase, mitochondrial 1	Juglans regia	XP_018808787.1
ARHOMBI_DN5409_c0_g1_i1_1	0	625	XP_018841570.1	calcium-dependent protein kinase 26	Juglans regia	XP_018841571.1
ARUBRA_DN4725_c0_g1_i1_2	0	595	XP_018843117.1	cathepsin B-like	Juglans regia	ONI14643.1
ARUBRA_DN19472_c0_g1_i1_2	1,39E-110	346	XP_018810168.1	AP-4 complex subunit epsilon-like	Juglans regia	XP_018834253.1
ARUBRA_DN9681_c0_g1_i1_1	1,51E-75	229	KRH41466.1	hypothetical protein GLYMA_08G0318001, partial	Glycine max	XP_015954179.1
ARUBRA_DN21358_c0_g1_i1_1	8,33E-87	264	XP_018827029.1	zinc finger CCCH domain-containing protein 3-like	Juglans regia	XP_018827029.1
ARUBRA_DN2192_c0_g1_i1_5	8,13E-39	134	XP_018834573.1	uncharacterized protein LOC109001661	Juglans regia	XP_008244735.1
ARUBRA_DN14031_c0_g1_i1_6	1,29E-95	290	XP_009376284.1	KH domain-containing protein HEN4 isoform X2	Pyrus x bretschneideri	XP_009376284.1
ARUBRA_DN22638_c0_g1_i1_6	1,97E-31	119	OAY27593.1	hypothetical protein MANES_16G137600	Manihot esculenta	KHN08417.1
ARUBRA_DN3929_c0_g1_i2_5	1,84E-74	231	XP_018815924.1	probable aldo-keto reductase 1	Juglans regia	XP_018815922.1
ARUBRA_DN3241_c0_g1_i1_4	0	649	XP_018815559.1	mitochondrial adenine nucleotide transporter ADNT1-like isoform X1	Juglans regia	KDP28516.1
ARUBRA_DN2358_c0_g1_i1_4	2,08E-98	308	KRH45625.1	hypothetical protein GLYMA_08G283700	Glycine max	XP_016652593.1
ARUBRA_DN24043_c0_g1_i1_4	2,91E-70	218	XP_009372763.1	uncharacterized protein LOC103961851	Pyrus x bretschneideri	XP_009372764.1
ARHOMBI_DN24632_c0_g1_i1_3	8,74E-46	159	XP_017186079.1	LOW QUALITY PROTEIN: acetyl-coenzyme A synthetase, chloroplastic/glyoxysomal-like	Malus domestica	XP_016194125.1
ARHOMBI_DN26919_c0_g1_i1_4	3,46E-49	169	XP_018842163.1	beta-xylosidase/alpha-L-arabinofuranosidase 2-like	Juglans regia	KDP30704.1
ARUBRA_DN23841_c0_g1_i1_4	1,13E-44	146	XP_020206789.1	dynamin-2B-like	Cajanus cajan	XP_009348247.1
ARUBRA_DN19491_c0_g1_i1_2	6,52E-37	132	XP_018845677.1	uncharacterized protein At2g24330	Juglans regia	XP_018813351.1
ARUBRA_DN22396_c0_g1_i1_1	2,85E-54	180	XP_018819428.1	phenylalanine--tRNA ligase beta subunit, cytoplasmic-like	Juglans regia	XP_018806301.1
ARHOMBI_DN19578_c0_g1_i1_4	3,61E-102	309	XP_018836609.1	RNA-binding KH domain-containing protein PEPPER	Juglans regia	XP_008223873.1
ARHOMBI_DN3163_c0_g1_i1_3	2,92E-140	402	XP_018852058.1	ABC transporter I family member 6, chloroplastic-like	Juglans regia	XP_018852059.1

ARUBRA_DN19450_c0_g1_i1_4	6,52E-126	361	ACU22732.1	unknown, partial	Glycine max	XP_016167639.1
ARUBRA_DN130_c0_g1_i1_4	1,16E-32	126	XP_018822798.1	phosphoribosylaminoimidazole carboxylase, chloroplastic isoform X2	Juglans regia	XP_008218293.1
ARHOMBI_DN4296_c0_g1_i1_1	2,21E-155	466	KDP28258.1	hypothetical protein JCGZ_14029	Jatropha curcas	XP_012082896.1
ARUBRA_DN6440_c0_g1_i1_4	6,72E-141	401	XP_018847248.1	copper chaperone for superoxide dismutase, chloroplastic/cytosolic isoform X1	Juglans regia	XP_018847253.1
ARHOMBI_DN6362_c0_g1_i1_1	1,94E-36	125	XP_016192392.1	heat shock protein 90-6, mitochondrial-like	Arachis ipaensis	XP_018849887.1
ARHOMBI_DN23114_c0_g1_i1_1	8,39E-50	161	XP_018810863.1	26S proteasome non-ATPase regulatory subunit 9	Juglans regia	BAT79725.1
ARHOMBI_DN4923_c0_g1_i1_6	8,80E-56	176	OAY21768.1	hypothetical protein MANES_S058900	Manihot esculenta	KDP37139.1
ARUBRA_DN6647_c0_g1_i1_3	7,07E-35	123	XP_018853705.1	Golgi SNAP receptor complex member 1-2	Juglans regia	OAY62436.1
ARHOMBI_DN5696_c0_g1_i1_6	0	507	XP_018860017.1	60S ribosomal protein L8-1-like	Juglans regia	XP_018855871.1
ARUBRA_DN8888_c0_g1_i1_4	6,15E-124	350	XP_018851087.1	uncharacterized protein At3g49720-like	Juglans regia	XP_018816519.1
ARHOMBI_DN5174_c0_g2_i1_1	3,49E-34	122	XP_018820980.1	isoflavone reductase-like protein	Juglans regia	AES74192.1
ARUBRA_DN16605_c0_g1_i1_4	9,33E-166	473	XP_018822579.1	serine hydroxymethyltransferase 3, chloroplastic-like	Juglans regia	XP_018822580.1
ARHOMBI_DN18922_c0_g1_i1_3	2,50E-69	217	XP_018843094.1	uncharacterized protein LOC109007740 isoform X2	Juglans regia	XP_018843093.1
ARHOMBI_DN5270_c0_g1_i1_5	3,71E-135	388	XP_018846418.1	acid phosphatase 1-like	Juglans regia	OAY27189.1
ARHOMBI_DN9484_c0_g2_i1_3	1,23E-101	301	XP_018818704.1	26S proteasome regulatory subunit RPN13 isoform X1	Juglans regia	XP_018818705.1
ARHOMBI_DN498_c0_g1_i1_1	8,66E-126	364	XP_018842815.1	RNA-binding protein 1-like	Juglans regia	XP_018842816.1
ARHOMBI_DN1736_c0_g1_i1_6	1,09E-134	394	XP_018816821.1	2-hydroxyacyl-CoA lyase	Juglans regia	KDP33027.1
ARUBRA_DN171_c0_g2_i1_3	1,25E-81	251	XP_018843376.1	transmembrane 9 superfamily member 7	Juglans regia	XP_004510372.1
ARUBRA_DN23798_c0_g1_i1_1	8,89E-40	144	XP_018826137.1	DUF21 domain-containing protein At4g14240-like	Juglans regia	XP_018840528.1
ARHOMBI_DN9171_c0_g1_i1_1	8,34E-70	231	XP_018830702.1	LOW QUALITY PROTEIN: tripeptidyl-peptidase 2-like	Juglans regia	XP_018828368.1
ARUBRA_DN3301_c0_g2_i1_5	0	666	XP_018835470.1	autophagy-related protein 18a-like isoform X2	Juglans regia	XP_018835463.1
ARUBRA_DN2204_c0_g1_i1_6	6,79E-77	239	XP_018849298.1	E3 ubiquitin-protein ligase RING1-like	Juglans regia	KOM54584.1
ARHOMBI_DN1709_c0_g2_i1_6	1,76E-78	236	XP_018815501.1	MLP-like protein 423	Juglans regia	XP_018813307.1
ARUBRA_DN4715_c0_g1_i1_5	1,62E-126	381	XP_018818627.1	acylamino-acid-releasing enzyme isoform X3	Juglans regia	XP_018818626.1
ARHOMBI_DN17044_c0_g1_i1_2	8,59E-173	486	XP_018826207.1	acyl-coenzyme A oxidase 4, peroxisomal isoform X2	Juglans regia	XP_018826206.1
ARHOMBI_DN6819_c0_g1_i1_2	0	644	XP_018817130.1	uncharacterized protein LOC108988346 isoform X1	Juglans regia	XP_018817131.1
ARHOMBI_DN5985_c0_g1_i1_4	0	539	CAA69937.1	glutamate synthetase	Alnus glutinosa	CAY39013.1
ARUBRA_DN24121_c0_g1_i1_6	1,03E-51	166	XP_020239064.1	probable inactive purple acid phosphatase 1, partial	Cajanus cajan	XP_008243847.1
ARUBRA_DN4657_c0_g1_i1_6	3,53E-65	201	XP_018824643.1	MLP-like protein 328	Juglans regia	XP_018824604.1

ARUBRA_DN290_c0_g1_i1_5	0	564	XP_018841808.1	ATP-dependent Clp protease proteolytic subunit-related protein 3, chloroplastic	Juglans regia	XP_008246420.1
ARHOMBI_DN5862_c0_g1_i1_2	6,13E-79	246	XP_018844150.1	auxin-induced in root cultures protein 12-like	Juglans regia	XP_018827548.1
ARHOMBI_DN6231_c0_g3_i5_2	5,20E-124	361	XP_018841297.1	tropinone reductase homolog At5g06060-like	Juglans regia	XP_018850680.1
ARUBRA_DN5145_c0_g1_i1_2	2,22E-122	360	XP_018832761.1	inositol phosphorylceramide glucuronosyltransferase 1	Juglans regia	OAY44337.1
ARUBRA_DN20610_c0_g1_i1_6	1,70E-24	99	XP_018859613.1	plastidic glucose transporter 4-like	Juglans regia	XP_018816890.1
ARHOMBI_DN19734_c0_g1_i1_3	5,70E-70	222	XP_018816946.1	eukaryotic translation initiation factor 3 subunit M-like	Juglans regia	XP_020538900.1
ARHOMBI_DN13808_c0_g1_i1_5	3,08E-87	283	XP_008232710.1	BEACH domain-containing protein C2	Prunus mume	XP_016650092.1
ARUBRA_DN19459_c0_g1_i1_5	2,32E-104	303	XP_009347705.1	probable glutathione peroxidase 5 isoform X2	Pyrus x bretschneideri	XP_018501031.1
ARHOMBI_DN4450_c0_g1_i1_5	3,91E-167	470	XP_018812633.1	haloacid dehalogenase-like hydrolase domain-containing protein At3g48420	Juglans regia	XP_018812634.1
ARUBRA_DN2002_c0_g2_i1_5	5,50E-83	251	XP_018830307.1	protein CURVATURE THYLAKOID 1D, chloroplastic	Juglans regia	ONI02409.1
ARHOMBI_DN22399_c0_g1_i1_2	7,90E-57	182	XP_018814981.1	manganese-dependent ADP-ribose/CDP-alcohol diphosphatase	Juglans regia	XP_018814982.1
ARHOMBI_DN16397_c0_g1_i1_6	2,32E-70	212	XP_018823473.1	prefoldin subunit 1	Juglans regia	XP_008351369.1
ARHOMBI_DN3020_c0_g2_i1_1	1,29E-51	165	XP_008227802.1	ubiquitin-40S ribosomal protein S27a	Prunus mume	XP_008389677.1
ARHOMBI_DN6884_c0_g1_i1_5	4,70E-58	198	XP_018839647.1	eukaryotic translation initiation factor 3 subunit A-like	Juglans regia	XP_018839647.1
ARUBRA_DN21439_c0_g1_i1_3	4,24E-58	184	XP_018851362.1	pathogenesis-related protein 5	Juglans regia	XP_007141088.1
ARHOMBI_DN5298_c0_g1_i1_5	5,10E-122	352	XP_018859435.1	trafficking protein particle complex subunit 6B	Juglans regia	XP_020208519.1
ARHOMBI_DN6134_c0_g1_i2_6	1,22E-97	295	XP_018819193.1	cinnamoyl-CoA reductase 1	Juglans regia	XP_016166897.1
ARHOMBI_DN21703_c0_g1_i1_5	9,11E-69	220	ONH97794.1	hypothetical protein PRUPE_7G211000	Prunus persica	XP_007202010.1
ARHOMBI_DN1691_c0_g1_i1_5	7,23E-39	142	XP_018846051.1	diphosphomevalonate decarboxylase MVD2-like	Juglans regia	XP_018835329.1
ARHOMBI_DN20074_c0_g1_i1_1	1,01E-94	295	XP_018836580.1	aminopeptidase M1-like	Juglans regia	XP_018844701.1
ARUBRA_DN16643_c0_g1_i1_2	1,40E-53	174	XP_018850161.1	reticulon-4-interacting protein 1, mitochondrial-like isoform X2	Juglans regia	KDP34858.1
ARHOMBI_DN16129_c0_g1_i1_4	1,11E-16	79,7	XP_018809515.1	pantothenate kinase 2 isoform X2	Juglans regia	XP_018809511.1
ARUBRA_DN4512_c0_g1_i1_2	6,86E-129	375	XP_018826234.1	ERBB-3 BINDING PROTEIN 1-like	Juglans regia	XP_008243362.1
ARHOMBI_DN8916_c0_g1_i1_1	2,01E-134	385	AGT95889.1	beta-ketoacyl-ACP reductase	Vernicia fordii	XP_018852608.1

ARHOMBI_DN12802_c0_g1_i1_2	3,80E-54	181	XP_018850129.1	4-alpha-glucanotransferase, chloroplastic/amyloplastic	Juglans regia	OAY35931.1
ARUBRA_DN14943_c0_g1_i1_6	9,01E-53	167	BAC00778.1	3-methylcrotonyl CoA carboxylase, partial	Pisum sativum	XP_009367597.1
ARHOMBI_DN21499_c0_g1_i1_5	4,28E-83	258	XP_018842244.1	biotin carboxylase 1, chloroplastic isoform X3	Juglans regia	XP_018842245.1
ARHOMBI_DN16804_c0_g1_i1_2	2,51E-167	482	XP_018850062.1	acyl-CoA-binding domain-containing protein 4	Juglans regia	XP_018850062.1
ARHOMBI_DN3081_c0_g1_i2_3	0	576	XP_018841131.1	protein AIG1-like	Juglans regia	XP_018850667.1
ARHOMBI_DN26634_c0_g1_i1_6	4,00E-28	104	AFK48240.1	unknown	Lotus japonicus	XP_018825827.1
ARUBRA_DN16199_c0_g1_i1_3	1,22E-140	398	XP_018808841.1	esterase CG5412-like	Juglans regia	OAY58075.1
ARHOMBI_DN10954_c0_g1_i1_5	4,03E-50	169	XP_018841219.1	uncharacterized protein LOC109006407	Juglans regia	XP_017179882.1
ARHOMBI_DN8652_c0_g1_i1_2	4,28E-38	133	XP_018834465.1	cold-regulated 413 inner membrane protein 1, chloroplastic-like isoform X1	Juglans regia	XP_008383519.1
ARUBRA_DN26687_c0_g1_i1_2	4,20E-44	152	XP_018833108.1	cysteine desulfurase 1, chloroplastic	Juglans regia	XP_012076009.1
ARHOMBI_DN3151_c0_g2_i1_5	2,29E-24	100	XP_018847795.1	dnaJ protein P58IPK homolog	Juglans regia	XP_016207930.1
ARHOMBI_DN12758_c0_g1_i1_4	1,38E-25	102	XP_018853594.1	dihydrolipoyllysine-residue acetyltransferase component 1 of pyruvate dehydrogenase complex, mitochondrial	Juglans regia	GAU40873.1
ARUBRA_DN6423_c0_g1_i1_6	9,01E-57	183	XP_018819402.1	tetratricopeptide repeat protein 1 isoform X1	Juglans regia	XP_018819403.1
ARUBRA_DN11367_c0_g1_i1_2	3,80E-41	137	XP_018825585.1	uncharacterized protein LOC108994714	Juglans regia	XP_018826055.1
ARHOMBI_DN22357_c0_g1_i1_4	9,77E-118	355	XP_009340966.1	threonine dehydratase biosynthetic, chloroplastic	Pyrus x bretschneideri	XP_018844104.1
ARUBRA_DN20780_c0_g1_i1_5	1,19E-106	312	XP_018852079.1	uncharacterized protein LOC109014173	Juglans regia	OAY30302.1
ARHOMBI_DN17128_c0_g1_i1_2	1,89E-86	258	XP_018830864.1	uncharacterized protein LOC108998669	Juglans regia	ONI25146.1
ARHOMBI_DN21148_c0_g1_i1_2	1,00E-77	236	ONI16083.1	hypothetical protein PRUPE_3G078000	Prunus persica	ONI16084.1
ARHOMBI_DN4969_c0_g1_i1_6	1,86E-167	472	XP_018855654.1	survival of motor neuron-related-splicing factor 30 isoform X1	Juglans regia	XP_008218638.1
ARHOMBI_DN2477_c0_g2_i1_4	0	815	XP_018824671.1	uncharacterized protein LOC108994054 isoform X1	Juglans regia	XP_018824672.1
ARUBRA_DN2574_c0_g1_i1_1	7,20E-68	207	XP_018825039.1	60S ribosomal protein L17-2-like	Juglans regia	GAU16630.1
ARHOMBI_DN1345_c0_g1_i1_3	0	531	XP_018812504.1	probable inactive purple acid phosphatase 2	Juglans regia	AGL44406.1
ARUBRA_DN7816_c0_g1_i1_3	8,90E-91	270	XP_018855616.1	cystathionine beta-lyase, chloroplastic-like	Juglans regia	XP_019442365.1
ARHOMBI_DN4238_c0_g1_i1_6	4,69E-175	491	XP_018850584.1	urease accessory protein G	Juglans regia	XP_008225465.1
ARUBRA_DN17859_c0_g1_i1_6	4,74E-30	114	XP_018842287.1	probable methyltransferase PMT3	Juglans regia	GAU25817.1
ARHOMBI_DN6130_c1_g1_i2_6	8,32E-67	215	XP_018829413.1	U1 small nuclear ribonucleoprotein 70 kDa	Juglans regia	XP_018829414.1
ARUBRA_DN15205_c0_g1_i1_1	1,85E-69	224	XP_018822704.1	alpha-L-arabinofuranosidase 1	Juglans regia	XP_018822706.1

ARUBRA_DN17787_c0_g1_i1_5	6,81E-73	223	XP_018856886.1	NADP-dependent malic enzyme-like isoform X1	Juglans regia	XP_018856887.1
ARHOMBI_DN21125_c0_g1_i1_2	1,46E-45	158	XP_018858135.1	LOW QUALITY PROTEIN: phospholipase A-2-activating protein	Juglans regia	OAY25488.1
ARUBRA_DN22796_c0_g1_i1_1	1,24E-31	115	XP_018827350.1	deoxyhypusine hydroxylase	Juglans regia	XP_020213989.1
ARHOMBI_DN2978_c0_g1_i1_5	2,03E-104	317	XP_018843153.1	protoporphyrinogen oxidase 1, chloroplastic	Juglans regia	GAU40565.1
ARHOMBI_DN8760_c0_g1_i1_1	4,28E-53	181	XP_009374177.1	coatomer subunit gamma	Pyrus x bretschneideri	XP_020216389.1
ARUBRA_DN11549_c0_g1_i1_5	5,69E-98	290	XP_018846382.1	glyceraldehyde-3-phosphate dehydrogenase, cytosolic	Juglans regia	ONI30153.1
ARHOMBI_DN22363_c0_g1_i1_3	8,05E-71	229	XP_018824493.1	uncharacterized protein LOC108993891	Juglans regia	KRH14371.1
ARUBRA_DN7615_c0_g1_i1_5	1,03E-83	254	XP_018827313.1	renalase isoform X2	Juglans regia	XP_018827304.1
ARHOMBI_DN10838_c0_g1_i1_3	2,60E-64	198	XP_018815734.1	dynein light chain 2, cytoplasmic	Juglans regia	XP_018825770.1
ARHOMBI_DN1649_c0_g1_i1_5	7,75E-84	251	XP_008220194.1	proliferating cell nuclear antigen	Prunus mume	ONI00025.1
ARHOMBI_DN20781_c0_g1_i1_6	9,39E-103	296	XP_018805864.1	two-on-two hemoglobin-3-like	Juglans regia	XP_008393905.1
ARHOMBI_DN6024_c0_g1_i2_5	2,28E-167	476	XP_018835690.1	eukaryotic translation initiation factor 2 subunit beta-like	Juglans regia	XP_018828541.1
ARHOMBI_DN6092_c0_g3_i3_3	3,11E-85	269	KHN15751.1	Cysteine synthase	Glycine soja	KRG91324.1
ARUBRA_DN23408_c0_g1_i1_6	5,96E-64	211	XP_018830097.1	exocyst complex component SEC10-like	Juglans regia	XP_018819250.1
ARHOMBI_DN17360_c0_g1_i1_3	1,46E-39	140	XP_018819975.1	KH domain-containing protein HEN4-like isoform X2	Juglans regia	XP_018819977.1
ARUBRA_DN6106_c0_g1_i1_6	2,67E-34	126	XP_018828962.1	uncharacterized protein LOC108997234	Juglans regia	KDP32699.1
ARUBRA_DN8839_c0_g1_i1_5	1,37E-94	281	ABR88113.1	mitochondrial L-galactono-1,4-lactone dehydrogenase, partial	Malus domestica	XP_018807818.1
ARUBRA_DN5290_c0_g1_i1_5	1,15E-108	315	AFK37233.1	unknown	Lotus japonicus	XP_004508073.1
ARHOMBI_DN16684_c0_g1_i1_4	6,13E-134	382	XP_018840400.1	syntaxin-61-like	Juglans regia	XP_018840401.1
ARUBRA_DN16761_c0_g1_i1_6	9,22E-103	299	XP_018839849.1	serine carboxypeptidase-like 42	Juglans regia	KRH53392.1
ARUBRA_DN18234_c0_g1_i1_6	2,35E-51	163	KHN41212.1	DUF246 domain-containing protein	Glycine soja	XP_018837095.1
ARUBRA_DN483_c0_g1_i1_1	4,08E-60	206	XP_018838332.1	large proline-rich protein BAG6-like isoform X2	Juglans regia	XP_018838333.1
ARHOMBI_DN20642_c0_g1_i1_2	1,35E-43	153	XP_016197543.1	peroxisomal fatty acid beta-oxidation multifunctional protein MFP2	Arachis ipaensis	XP_015938351.1
ARHOMBI_DN1985_c0_g1_i1_1	1,50E-89	287	KDP41738.1	hypothetical protein JCGZ_26756	Jatropha curcas	XP_012067196.1
ARUBRA_DN4720_c0_g1_i2_2	3,55E-179	503	XP_018823126.1	probable prolyl 4-hydroxylase 6	Juglans regia	XP_018844780.1
ARHOMBI_DN8466_c0_g1_i1_2	3,34E-34	122	XP_018819297.1	2-dehydro-3-deoxyphosphooctonate aldolase	Juglans regia	KYP39349.1
ARUBRA_DN26386_c0_g1_i1_2	1,36E-33	125	XP_016650129.1	LOW QUALITY PROTEIN: enhancer of mRNA-decapping protein 4	Prunus mume	ONH99477.1

ARHOMBI_DN16500_c0_g1_i1_6	4,94E-50	160	XP_017183078.1	ruvB-like 2	Malus domestica	KHN01798.1
ARUBRA_DN7170_c0_g1_i1_6	4,43E-101	295	XP_018821284.1	GDSL esterase/lipase At5g62930 isoform X2	Juglans regia	XP_018821283.1
ARUBRA_DN828_c0_g1_i1_6	0	968	XP_018826303.1	ferredoxin-dependent glutamate synthase, chloroplastic-like isoform X2	Juglans regia	XP_018826298.1
ARHOMBI_DN8693_c1_g1_i1_5	7,35E-11	63,2	ONI18008.1	hypothetical protein PRUPE_3G191800	Prunus persica	ONI18009.1
ARHOMBI_DN19277_c0_g1_i1_4	5,81E-95	288	XP_018845068.1	nuclear pore complex protein NUP50A-like	Juglans regia	XP_018845069.1
ARUBRA_DN11258_c0_g1_i1_3	1,02E-39	131	OIW15117.1	hypothetical protein TanjilG_08604	Lupinus angustifolius	XP_018834193.1
ARHOMBI_DN9241_c0_g1_i1_6	3,04E-40	147	XP_018809439.1	golgin candidate 6 isoform X4	Juglans regia	XP_018809438.1
ARHOMBI_DN2715_c0_g1_i1_5	2,79E-58	188	XP_018827700.1	uncharacterized protein LOC108996318	Juglans regia	ONI09830.1
ARUBRA_DN18267_c0_g1_i1_4	4,79E-76	241	XP_018824359.1	plant UBX domain-containing protein 10	Juglans regia	ONI07554.1
ARUBRA_DN17506_c0_g1_i1_4	4,78E-157	447	XP_018805194.1	agmatine deiminase	Juglans regia	ONI09011.1
ARUBRA_DN5389_c0_g1_i1_3	3,12E-160	450	XP_018809237.1	peroxidase 51-like, partial	Juglans regia	XP_018838180.1
ARHOMBI_DN3097_c0_g1_i2_1	9,60E-78	245	XP_012087640.1	probable pectinesterase/pectinesterase inhibitor 51 isoform X2	Jatropha curcas	KDP24790.1
ARUBRA_DN5867_c0_g1_i1_1	7,54E-31	117	XP_009371085.1	probable phosphoribosylformylglycinamidine synthase, chloroplastic/mitochondrial	Pyrus x bretschneideri	XP_009371087.1
ARUBRA_DN18169_c0_g1_i1_2	5,30E-39	140	XP_018848829.1	rhomboid-like protein 15	Juglans regia	XP_018851134.1
ARUBRA_DN4178_c0_g2_i1_3	0	777	XP_018860345.1	chaperone protein dnaJ A6, chloroplastic-like	Juglans regia	XP_018834540.1
ARUBRA_DN6773_c0_g1_i1_3	2,47E-124	358	XP_018829415.1	protein RER1B-like	Juglans regia	OAY60767.1
ARHOMBI_DN20737_c0_g1_i1_3	1,11E-78	254	XP_018840722.1	alanine--tRNA ligase-like	Juglans regia	XP_018840731.1
ARUBRA_DN1212_c0_g2_i1_5	2,22E-34	129	XP_018826443.1	splicing factor U2af large subunit B isoform X4	Juglans regia	XP_018826446.1
ARUBRA_DN22616_c0_g1_i1_2	3,65E-43	150	XP_018811480.1	protein NRT1/ PTR FAMILY 8.1-like	Juglans regia	XP_018811482.1
ARHOMBI_DN4651_c0_g1_i1_3	1,83E-135	384	XP_018854606.1	ras-related protein RABA2a	Juglans regia	OAY39697.1
ARUBRA_DN4817_c0_g1_i2_6	0	998	XP_018820842.1	NADPH--cytochrome P450 reductase-like	Juglans regia	XP_018831141.1
ARUBRA_DN5232_c0_g1_i1_6						
ARUBRA_DN8160_c0_g1_i1_1	1,31E-30	113	XP_018834630.1	zinc finger CCCH domain-containing protein 14-like	Juglans regia	XP_018834630.1
ARHOMBI_DN25761_c0_g1_i1_2	1,40E-69	216	XP_020212655.1	developmentally-regulated G-protein 3	Cajanus cajan	KYP69358.1
ARUBRA_DN18459_c0_g1_i1_4	2,29E-65	200	XP_018824894.1	uncharacterized protein LOC108994217	Juglans regia	OAY47489.1
ARUBRA_DN23641_c0_g1_i1_3	1,03E-98	298	KEH21426.1	translocon at the outer membrane ofs-like protein	Medicago truncatula	XP_013447399.1
ARHOMBI_DN15083_c0_g1_i1_2	1,38E-44	149	KHN42877.1	Exocyst complex component SEC3A-like protein	Glycine soja	ONI32790.1
ARHOMBI_DN5292_c0_g1_i1_5	1,54E-101	296	XP_018820918.1	cyanate hydratase	Juglans regia	KDP29704.1
ARUBRA_DN13177_c0_g1_i1_4	9,40E-49	160	OIW04544.1	hypothetical protein TanjilG_13926	Lupinus angustifolius	XP_019457082.1

ARUBRA_DN25572_c0_g1_i1_1	3,95E-49	162	KOM31785.1	hypothetical protein LR48_Vigan01g134000	Vigna angularis	XP_020202613.1
ARHOMBI_DN10905_c0_g1_i1_3	5,82E-79	245	XP_018833108.1	cysteine desulfurase 1, chloroplastic	Juglans regia	KHN14359.1
ARHOMBI_DN4521_c0_g1_i1_6	4,16E-105	325	XP_015953920.2	dolichol kinase EVAN isoform X2	Arachis duranensis	XP_020993688.1
ARUBRA_DN16776_c0_g1_i1_3	1,73E-94	285	XP_018841012.1	probable ethanolamine kinase	Juglans regia	ONH98289.1
ARUBRA_DN3345_c0_g2_i1_6	0	673	XP_018819391.1	vacuolar-sorting receptor 1-like	Juglans regia	XP_018814376.1
ARHOMBI_DN3792_c0_g1_i2_2	3,54E-162	461	XP_018820105.1	uncharacterized protein LOC108990561	Juglans regia	XP_018820105.1
ARHOMBI_DN2492_c0_g1_i1_2	6,35E-05	44,7	XP_018818640.1	uncharacterized protein LOC108989478	Juglans regia	ONI26425.1
ARHOMBI_DN17916_c0_g1_i1_3	1,65E-109	332	KRH44980.1	hypothetical protein GLYMA_08G242100	Glycine max	CBM37992.1
ARUBRA_DN23363_c0_g1_i1_1	9,28E-38	130	XP_018847498.1	uncharacterized protein LOC109010971, partial	Juglans regia	KDP32271.1
ARUBRA_DN6829_c0_g1_i1_2	4,17E-30	117	XP_018859048.1	probable arabinosyltransferase ARAD1	Juglans regia	XP_009353973.1
ARUBRA_DN14638_c0_g1_i1_5	6,83E-33	123	XP_018842112.1	pinin	Juglans regia	OAY39908.1
ARUBRA_DN20092_c0_g1_i1_5	8,36E-14	68,6	OAY39576.1	hypothetical protein MANES_10G105800	Manihot esculenta	KDP40898.1
ARHOMBI_DN4417_c0_g1_i1_4	3,47E-146	428	XP_018811765.1	la protein 1	Juglans regia	XP_008220038.1
ARHOMBI_DN22894_c0_g1_i1_5	2,40E-49	166	XP_018832822.1	glucan endo-1,3-beta-glucosidase 8-like	Juglans regia	XP_018832823.1
ARUBRA_DN7549_c0_g1_i1_1	1,76E-68	217	XP_020981273.1	polyadenylate-binding protein RBP47C isoform X2	Arachis duranensis	XP_020981273.1
ARHOMBI_DN3368_c0_g1_i1_3	9,18E-43	145	OAY41842.1	hypothetical protein MANES_09G133600	Manihot esculenta	ONI22099.1
ARUBRA_DN1922_c0_g2_i1_6	2,56E-08	55,5	KDP46216.1	hypothetical protein JCGZ_10056	Jatropha curcas	XP_012069525.1
ARHOMBI_DN7605_c0_g1_i1_1	1,33E-20	89	XP_018817970.1	stress protein DDR48	Juglans regia	KDP27843.1
ARUBRA_DN7746_c0_g1_i1_5	1,19E-72	224	XP_018826879.1	L-galactose dehydrogenase	Juglans regia	OAY45294.1
ARUBRA_DN23775_c0_g1_i1_5	1,35E-25	102	XP_017182641.1	uncharacterized protein LOC103416653	Malus domestica	XP_009360399.1
ARUBRA_DN22204_c0_g1_i1_5	1,54E-56	177	XP_018805734.1	huntingtin-interacting protein K-like	Juglans regia	KDP27909.1
ARHOMBI_DN16579_c0_g1_i1_6	1,29E-43	142	XP_018853518.1	inositol-tetrakisphosphate 1-kinase 1-like, partial	Juglans regia	XP_018808816.1
ARUBRA_DN23978_c0_g1_i1_3	2,08E-39	135	XP_018812444.1	ubiquinone biosynthesis O-methyltransferase, mitochondrial	Juglans regia	XP_012077529.1
ARUBRA_DN293_c0_g1_i1_1	0	587	XP_018840171.1	actin-related protein 4-like	Juglans regia	XP_018825395.1
ARHOMBI_DN14395_c0_g1_i1_5	3,02E-42	149	XP_009378530.1	acyl-coenzyme A oxidase, peroxisomal-like	Pyrus x bretschneideri	XP_009345476.1
ARHOMBI_DN18669_c0_g1_i1_3	4,79E-10	58,2	XP_018812044.1	large proline-rich protein BAG6-like isoform X2	Juglans regia	XP_018812043.1
ARUBRA_DN18022_c0_g1_i1_6	5,84E-101	302	KDP29180.1	hypothetical protein JCGZ_16569	Jatropha curcas	XP_020538168.1
ARUBRA_DN784_c0_g2_i1_6	3,53E-131	377	XP_018834236.1	protein phosphatase methylesterase 1	Juglans regia	XP_018834237.1
ARUBRA_DN7117_c0_g1_i1_3	1,37E-35	130	ALN13344.1	trehalose-6-phosphate synthase-11	Hevea brasiliensis	XP_008371505.1
ARUBRA_DN13794_c0_g1_i1_1	3,95E-32	120	XP_018817805.1	leucine--tRNA ligase, cytoplasmic isoform X2	Juglans regia	XP_018817804.1
ARHOMBI_DN2834_c0_g2_i1_4	1,39E-80	248	OAY42106.1	hypothetical protein MANES_09G153400	Manihot esculenta	OAY44238.1
ARUBRA_DN7201_c0_g1_i1_6	5,56E-89	270	ONI24868.1	hypothetical protein PRUPE_2G266500	Prunus persica	XP_007218249.1

ARUBRA_DN20463_c0_g1_i1_4	2,84E-77	243	XP_018847042.1	asparagine--tRNA ligase, chloroplastic/mitochondrial isoform X2	Juglans regia	XP_020995975.1
ARUBRA_DN12786_c0_g1_i1_6	2,66E-76	235	XP_018832787.1	uncharacterized protein LOC109000390	Juglans regia	KDP27508.1
ARUBRA_DN4014_c0_g1_i1_1	7,04E-54	174	OAY41365.1	hypothetical protein MANES_09G096000	Manihot esculenta	XP_009358774.1
ARUBRA_DN21_c0_g1_i1_6	3,80E-54	183	XP_018857362.1	tetratricopeptide repeat protein 38	Juglans regia	XP_018857363.1
ARUBRA_DN14287_c0_g1_i1_2	1,90E-90	286	XP_018818948.1	protein argonaute 4-like isoform X2	Juglans regia	XP_018818945.1
ARUBRA_DN3405_c0_g2_i1_2	4,04E-15	79	KDP26787.1	hypothetical protein JCGZ_17945	Jatropha curcas	XP_012085659.1
ARHOMBI_DN17003_c0_g1_i1_2	1,18E-36	135	XP_018859492.1	putative uncharacterized protein DDB_G0277255 isoform X5	Juglans regia	XP_018834450.1
ARUBRA_DN1302_c0_g1_i1_2	3,88E-49	166	XP_018845036.1	putative oxidoreductase TDA3	Juglans regia	ONI02917.1
ARHOMBI_DN20638_c0_g1_i1_2	1,17E-37	136	XP_020421355.1	uncharacterized protein LOC18789583 isoform X1	Prunus persica	XP_008221096.1
ARHOMBI_DN3957_c0_g2_i1_3	0	726	XP_018847659.1	CBL-interacting serine/threonine-protein kinase 23 isoform X1	Juglans regia	XP_018847660.1
ARUBRA_DN19773_c0_g1_i1_2	7,31E-82	253	XP_018830985.1	DNA-damage-repair/toleration protein DRT100-like	Juglans regia	XP_018830985.1
ARHOMBI_DN15739_c0_g1_i1_6	9,93E-125	369	XP_018851146.1	calcium-dependent protein kinase 20-like	Juglans regia	XP_007145755.1
ARUBRA_DN1164_c0_g1_i1_2	4,66E-36	129	XP_008356645.1	serine/threonine-protein kinase 16-like	Malus domestica	XP_009337751.1
ARUBRA_DN12959_c0_g1_i1_2	1,51E-24	97,8	XP_018843610.1	U1 small nuclear ribonucleoprotein A-like isoform X2	Juglans regia	XP_018843609.1
ARHOMBI_DN11084_c0_g1_i1_6	7,00E-35	121	AFK39355.1	unknown	Medicago truncatula	KEH23795.1
ARUBRA_DN24628_c0_g1_i1_6	1,86E-61	199	XP_018854693.1	T-complex protein 1 subunit delta	Juglans regia	KEH35974.1
ARHOMBI_DN5257_c0_g1_i1_3	3,77E-102	303	XP_018844945.1	NADH dehydrogenase ubiquinone iron-sulfur protein 7, mitochondrial	Juglans regia	OIV89995.1
ARUBRA_DN15806_c0_g1_i1_3	1,92E-62	201	XP_018824151.1	CBS domain-containing protein CBSX6	Juglans regia	ONI05173.1
ARHOMBI_DN21683_c0_g1_i1_3	5,48E-56	189	XP_018842622.1	pyruvate kinase isozyme G, chloroplastic isoform X2	Juglans regia	XP_018842623.1
ARHOMBI_DN7504_c0_g1_i1_3	1,30E-44	145	XP_006592692.1	putative aconitate hydratase, cytoplasmic isoform X2	Glycine max	KRH26402.1
ARUBRA_DN13027_c0_g1_i1_5	2,89E-68	220	XP_018820691.1	protein S-acyltransferase 24	Juglans regia	XP_008242880.1
ARUBRA_DN22863_c0_g1_i1_4	4,93E-13	67	XP_018816062.1	plant UBX domain-containing protein 11	Juglans regia	XP_018816063.1
ARUBRA_DN19918_c0_g1_i1_5	1,00E-139	399	XP_018810395.1	peroxisomal (S)-2-hydroxy-acid oxidase GLO1	Juglans regia	AEP95753.1
ARHOMBI_DN4736_c0_g1_i1_4	0	810	XP_018832863.1	beta-glucosidase 42 isoform X1	Juglans regia	XP_009336228.1
ARUBRA_DN24058_c0_g1_i1_4	2,84E-82	261	OAY49444.1	hypothetical protein MANES_05G056900	Manihot esculenta	OAY49446.1
ARUBRA_DN4090_c0_g1_i2_2	2,69E-74	230	XP_018840945.1	Golgi to ER traffic protein 4 homolog	Juglans regia	XP_009333738.1
ARUBRA_DN13119_c0_g1_i1_6	7,07E-99	301	XP_018824706.1	pentatricopeptide repeat-containing protein At3g49240	Juglans regia	KDP32431.1
ARHOMBI_DN13499_c0_g1_i1_4	2,61E-85	258	XP_018826607.1	uncharacterized protein LOC108995484 isoform X2	Juglans regia	XP_018826606.1

ARUBRA_DN19563_c0_g1_i1_5	8,68E-135	389	XP_018848019.1	lysosomal Pro-X carboxypeptidase-like	Juglans regia	KDP32128.1
ARUBRA_DN14032_c0_g1_i1_2	4,26E-69	214	XP_017180251.1	DNA damage-inducible protein 1-like	Malus domestica	XP_020990866.1
ARHOMBI_DN5062_c0_g1_i1_6	0	1024	XP_018850821.1	inositol-3-phosphate synthase	Juglans regia	XP_018850822.1
ARHOMBI_DN24773_c0_g1_i1_3	5,95E-67	218	OAY51315.1	hypothetical protein MANES_05G204700	Manihot esculenta	XP_018826037.1
ARHOMBI_DN4958_c0_g1_i1_3	9,34E-10	55,1	XP_018815679.1	cytochrome B5-like protein	Juglans regia	XP_018815680.1
ARHOMBI_DN4594_c0_g1_i1_1	0	525	XP_018836506.1	uncharacterized protein LOC109003012	Juglans regia	ONI24131.1
ARHOMBI_DN27276_c0_g1_i1_4	2,49E-50	171	KDP41126.1	hypothetical protein JCGZ_03617	Jatropha curcas	XP_012068431.1
ARHOMBI_DN3256_c0_g1_i1_1	6,10E-111	323	XP_018839810.1	S-adenosylmethionine carrier 1, chloroplastic/mitochondrial isoform X2	Juglans regia	XP_018839807.1
ARHOMBI_DN20557_c0_g1_i1_4	3,73E-69	214	XP_008371126.1	chaperone protein dnaJ 50-like	Malus domestica	XP_020966851.1
ARHOMBI_DN186_c0_g2_i1_4	4,56E-134	389	XP_018844220.1	heparanase-like protein 1, partial	Juglans regia	XP_018835860.1
ARUBRA_DN19452_c0_g1_i1_6	2,65E-74	233	XP_018820107.1	putative G3BP-like protein	Juglans regia	XP_018807711.1
ARUBRA_DN26125_c0_g1_i1_2	8,35E-34	117	XP_018826490.1	maf-like protein DDB_G0281937 isoform X6	Juglans regia	XP_018826486.1
ARHOMBI_DN20462_c0_g1_i1_3	1,80E-60	187	XP_018850262.1	uncharacterized protein LOC109012860	Juglans regia	ONI15074.1
ARUBRA_DN3192_c0_g1_i1_2	2,27E-125	363	XP_018833227.1	HD domain-containing protein 2 isoform X1	Juglans regia	KDP38749.1
ARUBRA_DN5035_c0_g1_i1_2	3,34E-162	464	XP_018825513.1	glucan endo-1,3-beta-glucosidase-like	Juglans regia	XP_018859110.1
ARHOMBI_DN1419_c0_g1_i1_6	2,15E-101	310	XP_018834513.1	clathrin interactor EPSIN 1	Juglans regia	XP_018834519.1
ARHOMBI_DN19246_c0_g1_i1_1	1,60E-94	286	XP_018828790.1	dolichyl-diphosphooligosaccharide--protein glycosyltransferase subunit 1B	Juglans regia	XP_009344769.1
ARHOMBI_DN17996_c0_g1_i1_4	1,53E-33	123	XP_008339723.1	probable isoprenylcysteine alpha-carbonyl methylesterase ICMEL1	Malus domestica	XP_008348265.1
ARUBRA_DN18443_c0_g1_i1_6	8,39E-44	151	XP_018835697.1	ATP-dependent RNA helicase DBP2-like isoform X2	Juglans regia	XP_018835698.1
ARHOMBI_DN20793_c0_g1_i1_6	3,96E-36	123	OAY45897.1	hypothetical protein MANES_07G101100	Manihot esculenta	KYP35949.1
ARUBRA_DN6539_c0_g2_i1_2	0	625	XP_018828503.1	protein TIC110, chloroplastic isoform X1	Juglans regia	XP_018828504.1
ARUBRA_DN2046_c0_g2_i1_2	3,61E-102	301	XP_016189854.1	probable cinnamyl alcohol dehydrogenase 1	Arachis ipaensis	XP_015956091.1
ARUBRA_DN3646_c0_g1_i1_3	1,15E-76	236	XP_018848573.1	mitochondrial import inner membrane translocase subunit TIM23-1-like	Juglans regia	XP_018835127.1
ARUBRA_DN6310_c0_g1_i1_4	5,36E-72	230	XP_016187299.1	heat shock cognate 70 kDa protein 2	Arachis ipaensis	XP_015952265.1
ARUBRA_DN1384_c0_g1_i1_3	4,51E-78	235	KYP55926.1	Multiprotein-bridging factor 1a	Cajanus cajan	KYP67761.1
ARHOMBI_DN14955_c0_g1_i1_2	3,96E-20	86,7	XP_018832979.1	calponin homology domain-containing protein DDB_G0272472-like	Juglans regia	XP_018843078.1
ARHOMBI_DN49_c0_g2_i1_6	4,21E-164	465	XP_018837945.1	ATP-dependent Clp protease proteolytic subunit 4, chloroplastic	Juglans regia	KYP66493.1
ARUBRA_DN8685_c0_g1_i1_3	6,75E-75	231	XP_008367592.2	agmatine deiminase-like	Malus domestica	XP_008240052.1

ARHOMBI_DN17696_c0_g1_i1_3	5,78E-105	319	XP_018815772.1	signal peptide peptidase-like 3	Juglans regia	XP_018824430.1
ARUBRA_DN25420_c0_g1_i1_2	1,80E-70	229	GAU45051.1	hypothetical protein TSUD_198390	Trifolium subterraneum	ONI27993.1
ARHOMBI_DN24508_c0_g1_i1_4	1,70E-51	171	KDP46567.1	hypothetical protein JCGZ_08539	Jatropha curcas	XP_012068432.1
ARUBRA_DN13680_c0_g1_i1_6	5,30E-56	185	ONI35298.1	hypothetical protein PRUPE_1G528400	Prunus persica	ONI35297.1
ARUBRA_DN4241_c0_g1_i1_3	1,76E-133	384	ACU20301.1	unknown	Glycine max	XP_018848015.1
ARHOMBI_DN1471_c0_g1_i1_6	6,17E-69	181	KHN38822.1	Vesicle-associated protein 1-2	Glycine soja	KHN38822.1
ARHOMBI_DN7801_c0_g1_i1_5	7,26E-32	120	XP_018846729.1	exportin-2-like	Juglans regia	XP_018846730.1
ARHOMBI_DN4606_c0_g1_i1_1	1,43E-133	385	KDP31052.1	hypothetical protein JCGZ_11428	Jatropha curcas	XP_012080000.1
ARUBRA_DN17255_c0_g1_i1_6	4,58E-80	242	XP_018849968.1	probable mitochondrial import inner membrane translocase subunit TIM21 isoform X2	Juglans regia	XP_018849967.1
ARUBRA_DN17467_c0_g1_i1_6	1,30E-29	114	XP_018819162.1	eukaryotic translation initiation factor 4G-like isoform X1	Juglans regia	XP_018819170.1
ARUBRA_DN17174_c0_g1_i1_5	2,88E-49	159	XP_018815840.1	UMP-CMP kinase isoform X5	Juglans regia	XP_018815835.1
ARHOMBI_DN2723_c0_g1_i1_1	1,18E-77	231	KHN13597.1	NADP-dependent malic enzyme	Glycine soja	KRH01213.1
ARUBRA_DN13395_c0_g1_i1_4	4,61E-90	266	XP_018808381.1	40S ribosomal protein S3-2	Juglans regia	XP_018827015.1
ARUBRA_DN5089_c0_g1_i1_5	8,73E-99	300	XP_018839513.1	nicalin-1 isoform X1	Juglans regia	XP_018839514.1
ARHOMBI_DN3292_c0_g1_i1_4	7,78E-52	170	XP_018822168.1	peptidyl-prolyl cis-trans isomerase CYP21-4-like	Juglans regia	XP_018842322.1
ARHOMBI_DN8290_c0_g2_i1_4	0	542	XP_018850655.1	DEAD-box ATP-dependent RNA helicase 56-like	Juglans regia	XP_018851705.1
ARHOMBI_DN22455_c0_g1_i1_1	9,61E-74	230	XP_018848228.1	serine/arginine-rich splicing factor SR45 isoform X2	Juglans regia	XP_018848227.1
ARUBRA_DN3317_c0_g1_i1_3	4,40E-124	363	XP_009340215.1	E3 ubiquitin-protein ligase RGLG1 isoform X2 Pyrus x bretschneideri	Pyrus x bretschneideri	XP_009340213.1
ARHOMBI_DN8153_c0_g1_i1_4	8,26E-107	319	XP_018852491.1	uncharacterized protein LOC109014469	Juglans regia	ONI07118.1
ARUBRA_DN19644_c0_g1_i1_4	1,03E-61	191	KYP50859.1	putative calcium-binding protein CML21	Cajanus cajan	XP_020231881.1
ARUBRA_DN17529_c0_g1_i1_1	0	1061	XP_018834884.1	protein AUXIN SIGNALING F-BOX 2-like	Juglans regia	XP_018834885.1
ARHOMBI_DN390_c0_g1_i1_5	0	567	XP_018816499.1	importin subunit alpha-4	Juglans regia	XP_018816499.1
ARHOMBI_DN10092_c0_g1_i1_1	7,96E-63	208	XP_018816352.1	villin-3-like isoform X3	Juglans regia	XP_018857284.1
ARHOMBI_DN15307_c0_g1_i1_4	1,12E-65	207	XP_008347805.1	probable rhamnogalacturonate lyase B, partial	Malus domestica	KOM41808.1
ARHOMBI_DN9011_c0_g1_i1_2	5,70E-10	58,5	XP_018841106.1	tobamovirus multiplication protein 3-like	Juglans regia	KDP29476.1
ARHOMBI_DN18489_c0_g2_i1_2	7,68E-57	178	XP_007153889.1	hypothetical protein PHAVU_003G073500g	Phaseolus vulgaris	ESW25883.1
ARHOMBI_DN1634_c0_g1_i1_6	1,83E-125	365	XP_018847079.1	LL-diaminopimelate aminotransferase, chloroplastic-like	Juglans regia	XP_017181435.1

ARUBRA_DN8885_c0_g1_i1_2	9,16E-140	409	KRH22912.1	hypothetical protein GLYMA_13G327000	Glycine max	XP_018819501.1
ARHOMBI_DN16188_c0_g1_i1_3	2,62E-55	188	XP_018813899.1	proteasome activator subunit 4-like	Juglans regia	XP_016651528.1
ARUBRA_DN21545_c0_g1_i1_4	2,34E-12	66,6	XP_018827617.1	auxilin-related protein 2-like	Juglans regia	XP_018827618.1
ARHOMBI_DN16333_c1_g1_i1_1	1,35E-51	176	XP_018848829.1	rhomboid-like protein 15	Juglans regia	XP_018851134.1
ARUBRA_DN4684_c0_g2_i2_5	6,71E-178	506	XP_018859983.1	glyceraldehyde-3-phosphate dehydrogenase 2, cytosolic-like	Juglans regia	OAY47918.1
ARHOMBI_DN25309_c0_g1_i1_1	5,92E-50	172	XP_018846895.1	calcium-transporting ATPase 4, plasma membrane-type-like	Juglans regia	XP_004498043.1
ARHOMBI_DN7295_c0_g1_i1_3	1,76E-129	374	XP_018821557.1	glyoxylate/succinic semialdehyde reductase 2, chloroplastic	Juglans regia	XP_008228276.1
ARHOMBI_DN3332_c0_g1_i1_5	4,94E-82	250	XP_018825187.1	SUMO-activating enzyme subunit 1B-1-like	Juglans regia	OAY47973.1
ARUBRA_DN12402_c0_g1_i1_2	4,86E-88	264	XP_018850920.1	bifunctional phosphatase IMPL2, chloroplastic-like	Juglans regia	XP_018815280.1
ARHOMBI_DN9886_c0_g1_i1_3	3,00E-44	143	XP_018806695.1	uncharacterized protein LOC108980286	Juglans regia	OAY37163.1
ARHOMBI_DN16830_c0_g1_i1_4	1,38E-53	174	XP_018821765.1	PRA1 family protein B1-like	Juglans regia	XP_008374366.1
ARHOMBI_DN19962_c0_g1_i1_1	3,05E-60	206	XP_018843251.1	VHS domain-containing protein At3g16270	Juglans regia	XP_018843252.1
ARHOMBI_DN7050_c0_g1_i1_3	1,29E-103	309	XP_018841273.1	plant UBX domain-containing protein 7	Juglans regia	XP_008220984.1
ARUBRA_DN8811_c0_g1_i1_6	5,44E-166	481	XP_017187956.1	pyrophosphate-energized vacuolar membrane proton pump-like	Malus domestica	BAC41250.1
ARUBRA_DN9001_c0_g1_i1_4	3,89E-120	351	OAY57022.1	hypothetical protein MANES_02G064300	Manihot esculenta	OAY27542.1
ARHOMBI_DN17895_c0_g1_i1_5	2,58E-50	174	OAY43754.1	hypothetical protein MANES_08G095300	Manihot esculenta	KDP22848.1
ARHOMBI_DN1601_c0_g1_i1_6	0	638	XP_018843439.1	ALG-2 interacting protein X-like	Juglans regia	XP_018849759.1
ARHOMBI_DN22439_c0_g1_i1_5	1,13E-13	71,6	XP_018838880.1	fimbrin-5-like	Juglans regia	
ARHOMBI_DN3123_c0_g1_i2_2	9,73E-36	137	KDP28399.1	hypothetical protein JCGZ_14170	Jatropha curcas	XP_012083084.1
ARUBRA_DN26477_c0_g1_i1_2	5,13E-46	151	XP_018812734.1	haloacid dehalogenase-like hydrolase domain-containing protein 3	Juglans regia	XP_018812735.1
ARHOMBI_DN15728_c0_g1_i1_3	3,45E-81	256	KEH42818.1	trichome birefringence-like protein	Medicago truncatula	XP_013468781.1
ARUBRA_DN14653_c0_g1_i1_5	2,89E-43	148	XP_018810480.1	aspartic proteinase-like protein 2 isoform X2	Juglans regia	XP_009356738.1
ARUBRA_DN239_c0_g1_i1_1						
ARUBRA_DN981_c0_g1_i1_2	1,82E-96	286	XP_018831584.1	hypersensitive-induced response protein 1	Juglans regia	XP_018831585.1
ARHOMBI_DN6224_c0_g1_i3_2	0	713	XP_018824020.1	alpha-amylase-like isoform X1	Juglans regia	XP_018824021.1
ARHOMBI_DN43_c0_g1_i1_1	2,41E-86	259	XP_015951555.1	uncharacterized protein LOC107476272	Arachis duranensis	KYP39795.1
ARHOMBI_DN10061_c0_g1_i1_1	1,59E-23	91,7	XP_018848607.1	chorismate mutase 2-like	Juglans regia	XP_018860755.1

ARUBRA_DN860_c0_g1_i1_4	1,30E-35	124	XP_018845364.1	uncharacterized protein LOC109009365	Juglans regia	XP_008232032.1
ARUBRA_DN4926_c0_g2_i1_2	4,10E-54	172	XP_009364401.1	outer envelope pore protein 16, chloroplastic-like	Pyrus x bretschneideri	XP_009364415.1
ARHOMBI_DN442_c0_g2_i1_6	1,60E-96	286	XP_017428249.1	ubiquinone biosynthesis O-methyltransferase, mitochondrial	Vigna angularis	KOM46488.1
ARUBRA_DN7677_c0_g1_i1_5	0	838	XP_018821861.1	cytochrome P450 90A1 isoform X1	Juglans regia	XP_018810085.1
ARHOMBI_DN23943_c0_g1_i1_5	1,66E-111	353	KDP37070.1	hypothetical protein JCGZ_06126	Jatropha curcas	XP_012073164.1
ARHOMBI_DN5334_c0_g2_i1_6	8,88E-29	111	XP_018839364.1	chitinase-like protein 1	Juglans regia	ONI29079.1
ARHOMBI_DN2144_c0_g1_i1_2	4,35E-121	360	XP_018849269.1	phosphoacetylglucosamine mutase-like	Juglans regia	XP_018838256.1
ARUBRA_DN17900_c0_g1_i1_4	1,38E-57	179	XP_018813206.1	uncharacterized protein LOC108985380	Juglans regia	XP_008394294.1
ARUBRA_DN7215_c0_g1_i1_2	8,35E-115	337	XP_018811258.1	28S ribosomal protein S29, mitochondrial-like isoform X1	Juglans regia	XP_018828432.1
ARHOMBI_DN10966_c0_g1_i1_1	4,32E-43	149	XP_018810986.1	protein disulfide-isomerase 5-3-like	Juglans regia	ONI13146.1
ARUBRA_DN4852_c0_g1_i2_5	0	901	OAY23274.1	hypothetical protein MANES_18G065500	Manihot esculenta	KDP41408.1
ARUBRA_DN7820_c0_g1_i1_3	1,53E-92	288	XP_018836549.1	uncharacterized protein LOC109003048	Juglans regia	KDP32822.1
ARUBRA_DN3218_c0_g2_i1_1	1,12E-139	401	XP_018827848.1	tropinone reductase-like 3	Juglans regia	KDP20683.1
ARUBRA_DN14060_c0_g1_i1_6	6,16E-34	121	XP_018827350.1	deoxyhypusine hydroxylase	Juglans regia	ONH93348.1
ARHOMBI_DN3766_c0_g1_i1_2	1,50E-69	217	XP_018847917.1	peroxisomal 2,4-dienoyl-CoA reductase	Juglans regia	XP_008383145.1
ARHOMBI_DN19865_c0_g1_i1_4	4,96E-21	90,1	OAY36786.1	hypothetical protein MANES_11G048200	Manihot esculenta	XP_018845068.1
ARHOMBI_DN27008_c0_g1_i1_5	1,97E-20	88,2	XP_018816929.1	uncharacterized protein LOC108988207	Juglans regia	XP_018843198.1
ARHOMBI_DN14983_c0_g1_i1_5	4,25E-28	110	CAC82184.1	pyrroline-5-carboxylate synthetase 1	Medicago truncatula	CAC82184.1
ARUBRA_DN23880_c0_g1_i1_2	1,06E-139	436	XP_018849647.1	acetyl-CoA carboxylase 1-like	Juglans regia	XP_018849648.1
ARHOMBI_DN17419_c0_g1_i1_1	4,91E-13	67,8	ONH91506.1	hypothetical protein PRUPE_8G119400	Prunus persica	XP_007201229.1
ARUBRA_DN16616_c0_g1_i1_4	1,77E-70	215	XP_008360906.1	uncharacterized protein LOC103424589	Malus domestica	XP_018858950.1
ARUBRA_DN17646_c0_g1_i1_5	5,35E-19	89,7	OIW01530.1	hypothetical protein TanjilG_19456	Lupinus angustifolius	XP_019461657.1
ARUBRA_DN22151_c0_g1_i1_2	2,36E-34	128	XP_018815731.1	glyoxysomal fatty acid beta-oxidation multifunctional protein MFP-a-like	Juglans regia	XP_018825769.1
ARHOMBI_DN14825_c0_g1_i1_2	8,82E-36	125	XP_018819460.1	beta-galactosidase 6-like	Juglans regia	KHN22059.1
ARHOMBI_DN16314_c0_g1_i1_1	2,32E-40	136	KRH77986.1	hypothetical protein GLYMA_01G245700	Glycine max	KRH77984.1
ARHOMBI_DN3532_c0_g1_i1_4	4,79E-04	45,4	XP_018847175.1	heterogeneous nuclear ribonucleoprotein 1-like	Juglans regia	
ARHOMBI_DN4371_c0_g1_i1_5	3,07E-84	273	ONI19115.1	hypothetical protein PRUPE_3G259400	Prunus persica	XP_007214914.1
ARUBRA_DN13912_c0_g1_i1_3	1,07E-43	152	XP_018834192.1	eukaryotic translation initiation factor 2D	Juglans regia	XP_020537372.1

ARHOMBI_DN17734_c0_g1_i1_4	2,29E-15	74,3	XP_018834202.1	histidine--tRNA ligase, cytoplasmic	Juglans regia	ONH89649.1
ARHOMBI_DN21284_c0_g1_i1_3	2,01E-80	261	XP_018847260.1	calcium-transporting ATPase 10, plasma membrane-type-like	Juglans regia	XP_018847261.1
ARUBRA_DN8408_c0_g1_i1_4	2,61E-17	79,7	XP_018854869.1	eukaryotic translation initiation factor 4G-like isoform X1	Juglans regia	XP_018854875.1
ARUBRA_DN8779_c0_g1_i1_1	1,49E-88	260	XP_018853118.1	uncharacterized protein LOC109015091	Juglans regia	XP_018859289.1
ARUBRA_DN85_c0_g1_i1_3	6,61E-177	495	XP_018815404.1	ribose-phosphate pyrophosphokinase 4-like isoform X2	Juglans regia	XP_018815403.1
ARHOMBI_DN16378_c0_g1_i1_1	2,12E-50	176	XP_018849270.1	uncharacterized protein LOC109012201 isoform X1	Juglans regia	XP_018849271.1
ARHOMBI_DN22637_c0_g1_i1_5	1,42E-48	161	XP_009345032.1	1-acyl-sn-glycerol-3-phosphate acyltransferase 2-like	Pyrus x bretschneideri	XP_008392182.1
ARHOMBI_DN16961_c0_g1_i1_3	3,53E-14	69,7	XP_018817970.1	stress protein DDR48	Juglans regia	KDP27843.1
ARUBRA_DN6264_c0_g1_i1_1	4,80E-38	140	XP_018842344.1	adenylosuccinate synthetase 2, chloroplastic	Juglans regia	XP_017437496.1
ARHOMBI_DN6677_c0_g1_i1_3	1,12E-67	205	XP_018835881.1	nudix hydrolase 25	Juglans regia	XP_004498300.1
ARHOMBI_DN15571_c0_g1_i1_5	2,55E-109	332	XP_018851837.1	outer envelope protein 61	Juglans regia	ONI01600.1
ARUBRA_DN2280_c0_g1_i1_3	1,53E-97	288	XP_018849297.1	ribonuclease 2	Juglans regia	OAY34914.1
ARHOMBI_DN5894_c0_g1_i1_5	4,38E-114	345	XP_018835441.1	cullin-1-like	Juglans regia	XP_018835442.1
ARHOMBI_DN18104_c0_g1_i1_1	1,56E-54	184	XP_018836549.1	uncharacterized protein LOC109003048	Juglans regia	XP_008338671.1
ARUBRA_DN9413_c0_g1_i1_3	1,19E-140	418	XP_018837801.1	NADPH--cytochrome P450 reductase	Juglans regia	KYP69307.1
ARUBRA_DN8576_c0_g1_i1_3	3,71E-25	100	XP_018848959.1	serine/threonine protein phosphatase 2A 57 kDa regulatory subunit B' beta isoform-like	Juglans regia	XP_018848960.1
ARHOMBI_DN4497_c0_g1_i1_6	1,95E-58	188	XP_018847929.1	E3 ubiquitin ligase complex SCF subunit sconC-like	Juglans regia	XP_018806824.1
ARUBRA_DN7437_c0_g1_i1_1	4,08E-64	214	XP_018842944.1	ATP-dependent zinc metalloprotease FTSH 9, chloroplastic-like	Juglans regia	XP_018805408.1
ARUBRA_DN6344_c0_g1_i1_3	2,03E-62	206	XP_018853563.1	aspartokinase 1, chloroplastic-like	Juglans regia	XP_018840015.1
ARHOMBI_DN142_c0_g1_i2_6	2,86E-78	237	XP_018840507.1	peroxiredoxin-2F, mitochondrial-like	Juglans regia	XP_018846871.1
ARHOMBI_DN2804_c0_g1_i1_5	3,07E-65	204	XP_018814379.1	protein FATTY ACID EXPORT 3, chloroplastic	Juglans regia	XP_008244826.1
ARUBRA_DN14140_c0_g1_i1_5	6,20E-76	242	XP_018831636.1	phosphatidylinositol 3,4,5-trisphosphate 3-phosphatase and protein-tyrosine-phosphatase PTEN2A-like	Juglans regia	XP_018831637.1
ARUBRA_DN20165_c0_g1_i1_6	3,02E-20	89,7	XP_018824848.1	histone deacetylase 5 isoform X1	Juglans regia	XP_018824850.1
ARUBRA_DN11522_c0_g1_i1_2	1,88E-86	272	XP_008240143.2	LOW QUALITY PROTEIN: ERAD-associated E3 ubiquitin-protein ligase component HRD3A	Prunus mume	ONI09356.1
ARUBRA_DN7178_c0_g1_i1_1	9,57E-26	102	XP_018851429.1	AP-4 complex subunit mu	Juglans regia	ACJ85581.1

ARUBRA_DN495_c0_g1_i1_6	2,21E-49	162	XP_018811366.1	exosome complex component RRP42 isoform X3	Juglans regia	XP_018811367.1
ARUBRA_DN15567_c0_g1_i1_3	1,28E-38	139	OAY30010.1	hypothetical protein MANES_15G189800	Manihot esculenta	XP_018814583.1
ARHOMBI_DN20913_c0_g1_i1_5	3,65E-79	248	XP_018843358.1	DEAD-box ATP-dependent RNA helicase 20	Juglans regia	OAY36514.1
ARUBRA_DN19959_c0_g1_i1_6	7,76E-79	253	XP_018840095.1	quinolinate synthase, chloroplastic	Juglans regia	ALP70495.1
ARUBRA_DN8310_c0_g1_i1_2	1,30E-114	331	XP_018501879.1	sulfite reductase ferredoxin , chloroplastic-like, partial	Pyrus x bretschneideri	XP_018830969.1
ARHOMBI_DN11012_c0_g1_i1_4	1,52E-83	261	XP_018837882.1	dynamin-related protein 1C	Juglans regia	XP_018814632.1
ARUBRA_DN6936_c0_g1_i1_4	3,30E-41	142	XP_018844624.1	phosphoribosylaminoimidazole-succinocarboxamide synthase, chloroplastic isoform X2	Juglans regia	XP_018844623.1
ARHOMBI_DN21368_c0_g1_i1_6	3,36E-35	122	XP_008246189.1	alpha-L-fucosidase 1-like, partial	Prunus mume	XP_016649953.1
ARUBRA_DN5364_c0_g1_i1_3	1,54E-155	447	XP_018858534.1	glutamate-rich WD repeat-containing protein 1-like	Juglans regia	ONH97796.1
ARUBRA_DN568_c0_g1_i1_3	4,45E-101	319	XP_018828527.1	nardilysin-like	Juglans regia	ONH99660.1
ARHOMBI_DN24822_c0_g1_i1_5	3,87E-53	169	KDP46638.1	hypothetical protein JCGZ_04572	Jatropha curcas	XP_012066839.1
ARHOMBI_DN8308_c0_g1_i1_3	6,30E-91	195	XP_018808481.1	AP-1 complex subunit gamma-2-like isoform X2	Juglans regia	XP_018808481.1
ARHOMBI_DN249_c0_g1_i1_6	7,93E-49	163	XP_018833182.1	small nuclear ribonucleoprotein-associated proteins B and B'	Juglans regia	KDP34864.1
ARHOMBI_DN12088_c0_g1_i1_5	1,54E-13	69,3	XP_018818640.1	uncharacterized protein LOC108989478	Juglans regia	KDP40251.1
ARHOMBI_DN16872_c0_g1_i1_5	2,93E-51	164	ACU13705.1	unknown	Glycine max	KHN26805.1
ARHOMBI_DN12473_c0_g1_i1_6	7,31E-20	85,1	XP_018835591.1	2-hydroxyisoflavanone dehydratase-like	Juglans regia	XP_012084123.2
ARUBRA_DN9724_c0_g1_i1_1	2,72E-50	173	XP_018818858.1	carbamoyl-phosphate synthase large chain, chloroplastic	Juglans regia	XP_018818858.1
ARHOMBI_DN11959_c0_g1_i1_2	1,61E-96	290	KDP39208.1	hypothetical protein JCGZ_00965	Jatropha curcas	XP_012070932.1
ARUBRA_DN3899_c0_g2_i1_5	3,72E-131	391	XP_018846323.1	clathrin heavy chain 1-like isoform X1	Juglans regia	XP_018846324.1
ARUBRA_DN9135_c0_g1_i1_4	1,62E-70	217	XP_018815961.1	vacuolar protein sorting-associated protein 28 homolog 2	Juglans regia	XP_018815962.1
ARUBRA_DN3321_c0_g1_i1_1	2,73E-48	159	KDP26247.1	hypothetical protein JCGZ_22493	Jatropha curcas	XP_012086001.1
ARUBRA_DN3221_c0_g1_i1_6	0	715	XP_018858103.1	delta-aminolevulinic acid dehydratase, chloroplastic-like	Juglans regia	KDP32813.1
ARUBRA_DN7240_c0_g1_i1_1	2,82E-163	486	XP_018836518.1	splicing factor 3B subunit 3-like	Juglans regia	XP_018836519.1

ARUBRA_DN25966_c0_g1_i1_5	1,85E-41	149	XP_018805796.1	protein WEAK CHLOROPLAST MOVEMENT UNDER BLUE LIGHT 1-like	Juglans regia	XP_018805797.1
ARHOMBI_DN2599_c0_g1_i1_4	9,84E-67	213	XP_018826789.1	bifunctional purple acid phosphatase 26-like	Juglans regia	XP_008242837.1
ARUBRA_DN13308_c0_g1_i1_1	8,43E-18	80,1	ONI22285.1	hypothetical protein PRUPE_2G118700	Prunus persica	XP_020413518.1
ARUBRA_DN10910_c0_g1_i1_6	4,04E-80	247	XP_008235581.1	tyrosine decarboxylase 1	Prunus mume	XP_020425168.1
ARUBRA_DN24761_c0_g1_i1_3	1,57E-58	182	XP_018849082.1	Golgi SNAP receptor complex member 1-1-like isoform X1	Juglans regia	XP_018849085.1
ARHOMBI_DN11254_c0_g1_i1_1	3,03E-27	98,6	ACH58419.1	chloroplast aldolase, partial	Prunus dulcis	ONI31556.1
ARUBRA_DN24634_c0_g1_i1_5	2,22E-22	93,6	XP_018851021.1	plant intracellular Ras-group-related LRR protein 6 isoform X2	Juglans regia	XP_018851013.1
ARHOMBI_DN4130_c0_g1_i1_2	5,24E-172	491	XP_018828790.1	dolichyl-diphosphooligosaccharide--protein glycosyltransferase subunit 1B	Juglans regia	KDP44377.1
ARHOMBI_DN2201_c0_g1_i1_2	3,09E-40	137	XP_018821291.1	uncharacterized protein LOC108991486	Juglans regia	OIV95972.1
ARUBRA_DN19418_c0_g1_i1_3	3,75E-31	119	XP_018830673.1	uncharacterized protein LOC108998567 isoform X1	Juglans regia	XP_018830674.1
ARUBRA_DN1850_c0_g1_i1_3	1,67E-158	458	XP_018833292.1	uncharacterized protein LOC109000757	Juglans regia	XP_008229779.1
ARHOMBI_DN9657_c0_g1_i1_1	5,94E-46	152	XP_008228306.1	uncharacterized protein LOC103327716	Prunus mume	XP_012068990.1
ARUBRA_DN22015_c0_g1_i1_3	2,04E-68	225	XP_018815773.1	importin beta-like SAD2	Juglans regia	ONI29088.1
ARUBRA_DN21497_c0_g1_i1_5	1,66E-98	313	XP_018833940.1	probable manganese-transporting ATPase PDR2	Juglans regia	XP_018833946.1
ARHOMBI_DN17553_c0_g1_i1_3	3,03E-57	192	XP_020974231.1	cell division control protein 48 homolog E isoform X5	Arachis ipaensis	XP_020974232.1
ARUBRA_DN23958_c0_g1_i1_3	1,98E-83	273	XP_018813247.1	E3 ubiquitin-protein ligase UPL1-like	Juglans regia	XP_018813253.1
ARHOMBI_DN6893_c0_g1_i1_1	2,40E-154	459	KDP34897.1	hypothetical protein JCGZ_09185	Jatropha curcas	XP_018834728.1
ARUBRA_DN9664_c0_g1_i1_5	1,23E-80	255	KRH44980.1	hypothetical protein GLYMA_08G242100	Glycine max	XP_004491879.1
ARUBRA_DN3808_c0_g2_i1_1	2,16E-91	269	XP_018806533.1	probable glutathione peroxidase 2	Juglans regia	BAT72709.1
ARUBRA_DN14331_c0_g1_i1_6	9,88E-84	269	XP_020230001.1	LOW QUALITY PROTEIN: far upstream element-binding protein 2	Cajanus cajan	XP_020230001.1
ARUBRA_DN209_c0_g1_i1_5	3,39E-06	50,8	XP_018819971.1	KH domain-containing protein HEN4-like isoform X1	Juglans regia	XP_018819972.1
ARHOMBI_DN16878_c0_g1_i1_1	1,70E-41	145	XP_018847824.1	phenylalanine--tRNA ligase alpha subunit, cytoplasmic	Juglans regia	GAU11740.1
ARUBRA_DN3885_c0_g1_i1_1	6,99E-75	227	XP_018827114.1	uncharacterized protein LOC108995901	Juglans regia	XP_018807876.1
ARHOMBI_DN9575_c0_g1_i1_5	1,23E-84	260	XP_018844642.1	serine/threonine protein phosphatase 2A 55 kDa regulatory subunit B beta isoform-like isoform X1	Juglans regia	XP_018844643.1
ARHOMBI_DN4563_c0_g2_i1_3	2,78E-47	155	XP_018839487.1	1,4-alpha-glucan-branching enzyme 1, chloroplastic/amyloplastic-like, partial	Juglans regia	GAU16049.1
ARHOMBI_DN5164_c0_g1_i1_4	5,48E-106	320	XP_018820745.1	serine/arginine-rich splicing factor RS40-like isoform X6	Juglans regia	XP_018820747.1

ARUBRA_DN2931_c0_g1_i1_4	0	580	XP_008236505.1	alpha-amylase-like isoform X2	Prunus mume	XP_016650792.1
ARUBRA_DN8810_c0_g1_i1_3	0	607	XP_018846016.1	non-specific phospholipase C1-like	Juglans regia	XP_018844883.1
ARUBRA_DN27193_c0_g1_i1_3	2,83E-52	181	XP_018846305.1	BEACH domain-containing protein C2 isoform X2	Juglans regia	XP_018846304.1
ARHOMBI_DN2247_c0_g1_i1_2	7,58E-139	405	XP_018849981.1	seipin-2-like	Juglans regia	XP_008371515.1
ARUBRA_DN25129_c0_g1_i1_1	1,16E-43	145	XP_018810319.1	uncharacterized protein LOC108983212	Juglans regia	XP_009376900.1
ARUBRA_DN7581_c0_g1_i1_1	1,33E-54	179	XP_018854057.1	protein NRT1/ PTR FAMILY 5.2-like, partial	Juglans regia	XP_018820905.1
ARUBRA_DN26635_c0_g1_i1_3	2,82E-46	149	AFK36986.1	unknown	Lotus japonicus	XP_018805240.1
ARUBRA_DN25922_c0_g1_i1_4	7,49E-11	59,7	KDP32458.1	hypothetical protein JCGZ_13383	Jatropha curcas	XP_012078853.1
ARHOMBI_DN16586_c0_g1_i1_6	6,19E-83	260	XP_018841787.1	mitochondrial Rho GTPase 1-like	Juglans regia	XP_018843210.1
ARUBRA_DN23667_c0_g1_i1_3	2,14E-43	151	XP_020206492.1	serine/threonine-protein kinase 38-like	Cajanus cajan	KEH42501.1
ARHOMBI_DN1477_c0_g1_i1_3	1,67E-88	278	XP_018849698.1	glutamine--tRNA ligase-like	Juglans regia	XP_018849699.1
ARUBRA_DN23915_c0_g1_i1_1	2,93E-59	189	ONI18977.1	hypothetical protein PRUPE_3G250600	Prunus persica	XP_020416394.1
ARUBRA_DN16207_c0_g1_i1_4	1,00E-125	370	XP_018826789.1	bifunctional purple acid phosphatase 26-like	Juglans regia	XP_008242837.1
ARUBRA_DN12100_c0_g1_i1_6	3,12E-64	211	XP_018818703.1	cycloartenol synthase 2	Juglans regia	BAE53431.1
ARHOMBI_DN5013_c0_g1_i1_5	8,52E-119	343	ONH93231.1	hypothetical protein PRUPE_8G220500	Prunus persica	XP_020425518.1
ARHOMBI_DN17278_c0_g1_i1_1	7,09E-14	73,2	XP_018818025.1	E3 ubiquitin-protein ligase RNF8-like isoform X2	Juglans regia	XP_018818024.1
ARUBRA_DN8077_c0_g1_i1_3	1,88E-43	144	AFK37566.1	unknown	Lotus japonicus	ACU16958.1
ARHOMBI_DN12156_c0_g1_i1_6	7,47E-59	182	XP_008353923.2	ras-related protein RABH1e	Malus domestica	XP_018498817.1
ARHOMBI_DN19746_c0_g1_i1_3	4,97E-86	263	XP_018835079.1	developmentally-regulated G-protein 2	Juglans regia	OAY53197.1
ARHOMBI_DN5681_c0_g1_i1_3	2,47E-143	408	OAY29673.1	hypothetical protein MANES_15G163400	Manihot esculenta	XP_018847825.1
ARHOMBI_DN9772_c0_g1_i1_3	2,31E-107	309	XP_018812971.1	protein CutA, chloroplastic isoform X2	Juglans regia	XP_018812969.1
ARUBRA_DN2587_c0_g1_i1_2	2,15E-17	74,7	XP_018825755.1	ER membrane protein complex subunit 6-like	Juglans regia	XP_018825756.1
ARHOMBI_DN14925_c0_g1_i1_1	2,70E-37	134	ARE67147.1	isopropylmalate synthase	Aeschynomene evenia	ARE67148.1
ARHOMBI_DN13424_c0_g1_i1_2	3,06E-27	104	XP_018849565.1	inositol-tetrakisphosphate 1-kinase 1-like	Juglans regia	XP_018849566.1
ARHOMBI_DN17556_c0_g1_i1_2	1,50E-12	67,4	XP_018844150.1	auxin-induced in root cultures protein 12-like	Juglans regia	OAY34851.1
ARHOMBI_DN23552_c0_g1_i1_5						
ARHOMBI_DN2495_c0_g2_i1_3	2,85E-56	179	KYP43658.1	hypothetical protein KK1_034886	Cajanus cajan	XP_007157820.1
ARUBRA_DN16642_c0_g1_i1_1	1,54E-52	166	XP_007147177.1	hypothetical protein PHAVU_006G1024001g, partial	Phaseolus vulgaris	ESW19171.1
ARUBRA_DN8504_c0_g1_i1_5	8,04E-52	172	XP_018811765.1	la protein 1	Juglans regia	ONI33901.1
ARHOMBI_DN3218_c0_g1_i1_5	2,93E-162	459	XP_008228852.1	uncharacterized protein LOC103328234 isoform X1	Prunus mume	ONI16530.1

ARHOMBI_DN6191_c0_g1_i1_5	3,76E-78	233	XP_018813383.1	60S ribosomal protein L27	Juglans regia	XP_018827822.1
ARUBRA_DN13074_c0_g1_i1_4	5,70E-93	273	XP_018847089.1	cytochrome c oxidase assembly protein COX11, mitochondrial-like isoform X2	Juglans regia	XP_018847090.1
ARHOMBI_DN2701_c0_g1_i1_1	3,12E-37	136	XP_009347800.2	uncharacterized protein LOC103939438	Pyrus x bretschneideri	XP_014627166.1
ARUBRA_DN13385_c0_g1_i1_1	4,33E-33	122	KDP33932.1	hypothetical protein JCGZ_07503	Jatropha curcas	XP_020536455.1
ARHOMBI_DN4455_c0_g2_i1_5	6,37E-80	243	GAU40699.1	hypothetical protein TSUD_263490	Trifolium subterraneum	KDP24426.1
ARUBRA_DN1705_c0_g1_i1_4	1,65E-85	253	AFK46629.1	unknown	Lotus japonicus	XP_018821350.1
ARHOMBI_DN14374_c0_g1_i1_3	3,43E-34	127	XP_018835573.1	presequence protease 1, chloroplastic/mitochondrial-like	Juglans regia	XP_008373169.1
ARUBRA_DN3623_c0_g1_i1_6	8,74E-91	282	XP_018860284.1	probable eukaryotic translation initiation factor 5-1	Juglans regia	XP_008338806.1
ARUBRA_DN6227_c0_g1_i1_3	1,21E-39	144	XP_018829140.1	histone deacetylase 19-like	Juglans regia	XP_018829142.1
ARUBRA_DN14347_c0_g1_i1_6	9,28E-62	201	XP_018829161.1	uncharacterized protein LOC108997384 isoform X1	Juglans regia	XP_018829162.1
ARUBRA_DN4655_c0_g1_i1_4	1,83E-153	432	XP_018838488.1	ras-related protein RABA1f	Juglans regia	XP_018860734.1
ARUBRA_DN152_c0_g1_i1_6	7,16E-167	471	AAB63199.2	acetyl-CoA carboxylase, partial	Phaseolus vulgaris	XP_018828918.1
ARUBRA_DN21454_c0_g1_i1_1	5,61E-25	101	XP_018835518.1	UDP-glucose:glycoprotein glucosyltransferase	Juglans regia	XP_020420190.1
ARHOMBI_DN14749_c0_g1_i1_1	5,08E-37	136	XP_008243006.1	signal recognition particle receptor subunit alpha homolog	Prunus mume	OAY31837.1
ARUBRA_DN1738_c0_g1_i1_5	1,94E-04	42,7	XP_018833269.1	vacuolar cation/proton exchanger 5-like isoform X1	Juglans regia	
ARHOMBI_DN6745_c0_g1_i1_6	1,09E-56	188	XP_018829791.1	peroxisomal fatty acid beta-oxidation multifunctional protein AIM1-like isoform X1	Juglans regia	XP_018829791.1
ARHOMBI_DN9761_c0_g1_i1_5	1,48E-103	310	XP_008371893.1	probable arabinosyltransferase ARAD1	Malus domestica	XP_018859048.1
ARHOMBI_DN16459_c0_g1_i1_4	8,87E-144	416	XP_018820274.1	hydroxymethylglutaryl-CoA synthase-like	Juglans regia	XP_018820512.1
ARHOMBI_DN630_c0_g2_i1_1	2,74E-50	176	XP_018811309.1	nucleolar protein 56-like	Juglans regia	XP_018823191.1
ARUBRA_DN10120_c0_g1_i1_3	9,17E-75	231	XP_018811142.1	mevalonate kinase	Juglans regia	XP_012089081.1
ARHOMBI_DN26737_c0_g1_i1_6	2,81E-31	110	XP_018851348.1	uncharacterized protein LOC109013650	Juglans regia	XP_018851349.1
ARHOMBI_DN20055_c0_g1_i1_2	9,11E-81	265	XP_018852332.1	probable phosphoinositide phosphatase SAC9	Juglans regia	KDP35146.1
ARUBRA_DN68_c0_g2_i1_3	1,81E-11	62,4	XP_018849371.1	protein ELC-like	Juglans regia	XP_018818514.1
ARUBRA_DN18173_c0_g1_i1_5	4,84E-85	253	XP_018822055.1	peptide methionine sulfoxide reductase A5	Juglans regia	XP_004509351.1
ARHOMBI_DN11266_c0_g1_i1_5	1,60E-62	198	XP_018812777.1	hydroxyacylglutathione hydrolase 2, mitochondrial-like	Juglans regia	XP_018812778.1
ARUBRA_DN15293_c0_g1_i1_1	1,22E-54	186	XP_016183417.1	translocase of chloroplast 120, chloroplastic	Arachis ipaensis	XP_016183418.1
ARUBRA_DN16348_c0_g1_i1_1	4,15E-54	172	XP_018809955.1	eukaryotic initiation factor 4A	Juglans regia	OAY26452.1
ARUBRA_DN5557_c0_g1_i1_3	5,53E-46	157	OAY52293.1	hypothetical protein MANES_04G071600	Manihot esculenta	OAY52294.1
ARUBRA_DN807_c0_g1_i1_6	9,20E-42	139	XP_018836279.1	uncharacterized protein LOC109002826	Juglans regia	XP_018836280.1

ARHOMBI_DN22251_c0_g1_i1_1	7,87E-105	309	XP_018860390.1	uncharacterized protein LOC109022048 isoform X2	Juglans regia	XP_018860389.1
ARHOMBI_DN6086_c0_g2_i1_5	1,63E-122	352	AFP43695.1	actin 3, partial	Eriobotrya japonica	AFP43693.1
ARUBRA_DN15584_c0_g1_i1_1	1,34E-34	122	XP_018859934.1	embryogenesis-associated protein EMB8-like	Juglans regia	XP_018806108.1
ARUBRA_DN23559_c0_g1_i1_2	3,74E-09	57,4	XP_018857825.1	translocase of chloroplast 159, chloroplastic-like	Juglans regia	
ARHOMBI_DN3575_c0_g2_i1_3	3,16E-152	428	XP_018860753.1	haloacid dehalogenase-like hydrolase domain-containing protein Sgpp	Juglans regia	XP_018843522.1
ARUBRA_DN18865_c0_g1_i1_4	3,63E-65	202	XP_018825580.1	uncharacterized protein LOC108994710 isoform X3	Juglans regia	XP_018826010.1
ARUBRA_DN81_c0_g1_i1_5	2,07E-145	409	XP_018828415.1	pre-mRNA cleavage factor Im 25 kDa subunit 2-like	Juglans regia	XP_018830816.1
ARUBRA_DN17068_c0_g1_i1_4	7,90E-63	201	OAY60965.1	hypothetical protein MANES_01G153600	Manihot esculenta	KDP43747.1
ARHOMBI_DN11773_c0_g1_i1_2	2,11E-35	129	ONI09460.1	hypothetical protein PRUPE_5G239700	Prunus persica	ONI09461.1
ARHOMBI_DN15266_c0_g1_i1_2	7,17E-83	251	XP_018810253.1	chaperone protein dnaJ 49	Juglans regia	XP_018810254.1
ARHOMBI_DN19693_c0_g1_i1_6	5,30E-55	181	XP_008220200.1	probable carboxylesterase 2	Prunus mume	XP_018816089.1
ARHOMBI_DN21801_c0_g1_i1_4	1,03E-21	89,7	XP_018848003.1	syntaxin-41-like	Juglans regia	XP_018856670.1
ARHOMBI_DN676_c0_g2_i1_2	9,74E-116	336	XP_018828952.1	uncharacterized protein LOC108997227 isoform X3	Juglans regia	XP_018828950.1
ARHOMBI_DN6521_c0_g1_i1_4	7,69E-07	48,9	XP_018834883.1	nucleolin-like	Juglans regia	XP_018854830.1
ARHOMBI_DN4350_c0_g1_i1_4	4,55E-125	370	XP_018807257.1	calcium-transporting ATPase 8, plasma membrane-type-like	Juglans regia	XP_018807258.1
ARHOMBI_DN6943_c0_g1_i1_2						
ARHOMBI_DN16004_c0_g1_i1_3	2,78E-92	272	XP_018834389.1	ras-related protein RABA4c	Juglans regia	OAY44766.1
ARUBRA_DN26911_c0_g1_i1_3	1,48E-49	167	XP_018830093.1	glycerol-3-phosphate acyltransferase, chloroplastic isoform X1	Juglans regia	XP_018830095.1
ARHOMBI_DN7765_c0_g1_i1_6	7,87E-128	372	XP_018808643.1	serine/threonine-protein phosphatase 5 isoform X2	Juglans regia	OIW02858.1
ARUBRA_DN9287_c0_g1_i1_4	8,79E-72	220	XP_008239677.1	germin-like protein subfamily T member 2	Prunus mume	ONI08515.1
ARUBRA_DN10301_c0_g1_i1_3	1,31E-19	85,5	ONI10141.1	hypothetical protein PRUPE_4G030000	Prunus persica	ONI10139.1
ARHOMBI_DN25736_c0_g1_i1_6	3,21E-30	116	XP_018838333.1	large proline-rich protein BAG6-like isoform X3	Juglans regia	XP_018838332.1
ARHOMBI_DN25307_c0_g1_i1_1	3,18E-21	90,9	XP_018809157.1	protein CYPRO4-like	Juglans regia	OIW14875.1
ARHOMBI_DN827_c0_g1_i1_1	1,27E-16	80,9	XP_018839647.1	eukaryotic translation initiation factor 3 subunit A-like	Juglans regia	XP_018839648.1

ARUBRA_DN5154_c0_g1_i1_1	8,34E-96	288	OAY49286.1	hypothetical protein MANES_05G043800	Manihot esculenta	XP_008232692.1
ARHOMBI_DN10484_c0_g1_i1_1	1,45E-53	171	XP_018845444.1	uncharacterized protein LOC109009446	Juglans regia	XP_020534697.1
ARUBRA_DN4965_c9_g2_i1_4	1,01E-172	483	AKC02191.1	caffeoyl-CoA O-methyltransferase	Prunus mume	NP_001313438.1
ARHOMBI_DN650_c0_g2_i1_3	6,85E-60	198	XP_018840543.1	uncharacterized protein LOC109005908 isoform X1	Juglans regia	XP_018840544.1
ARUBRA_DN19110_c0_g1_i1_2	1,27E-82	248	XP_018818253.1	bifunctional bis(5'-adenosyl)-triphosphatase/adenylylsulfatase FHIT	Juglans regia	XP_019439570.1
ARHOMBI_DN5597_c0_g2_i1_5	3,62E-105	301	XP_018810746.1	40S ribosomal protein S15	Juglans regia	XP_018813415.1
ARUBRA_DN21139_c0_g1_i1_2	5,75E-54	185	XP_008376320.1	regulator of nonsense transcripts UPF2-like	Malus domestica	XP_008233383.1
ARUBRA_DN3651_c0_g1_i1_3	4,99E-19	81,6	XP_018818788.1	GDP-mannose transporter GONST3-like isoform X2	Juglans regia	XP_018818787.1
ARUBRA_DN460_c0_g1_i1_6	1,95E-115	340	XP_018847561.1	D-glycerate 3-kinase, chloroplastic	Juglans regia	XP_020966505.1
ARUBRA_DN26660_c0_g1_i1_1	4,53E-57	193	XP_018820213.1	putative U-box domain-containing protein 50	Juglans regia	XP_018839024.1
ARHOMBI_DN2961_c0_g1_i1_6	4,80E-75	225	KDP33899.1	hypothetical protein JCGZ_07470	Jatropha curcas	XP_020536326.1
ARUBRA_DN15712_c0_g1_i1_3						
ARHOMBI_DN5284_c0_g3_i1_5	8,19E-78	244	XP_018806799.1	glycerol kinase	Juglans regia	XP_009336317.1
ARHOMBI_DN12176_c0_g1_i1_1	7,16E-17	77,4	XP_018815618.1	uncharacterized protein LOC108987198 isoform X1	Juglans regia	XP_018815619.1
ARUBRA_DN4419_c0_g1_i1_1	4,31E-123	360	XP_018852158.1	26S proteasome non-ATPase regulatory subunit 11 homolog	Juglans regia	ONI30885.1
ARUBRA_DN3086_c0_g1_i1_4	5,48E-122	351	XP_018841000.1	heme oxygenase 1, chloroplastic	Juglans regia	ONH98326.1
ARUBRA_DN5853_c0_g1_i1_3	6,86E-33	119	XP_018836110.1	acidic endochitinase-like	Juglans regia	XP_018817162.1
ARHOMBI_DN12506_c0_g1_i1_1						
ARUBRA_DN10624_c0_g1_i1_5	1,41E-50	162	ADV04063.1	protein binding/ubiquitin-protein ligase 2	Hevea brasiliensis	AGQ57016.1
ARHOMBI_DN13880_c0_g1_i1_4	8,43E-29	108	XP_018846626.1	serpin-ZX-like isoform X2	Juglans regia	XP_018846625.1
ARHOMBI_DN23964_c0_g1_i1_2	4,72E-45	157	KDP30704.1	hypothetical protein JCGZ_16402	Jatropha curcas	XP_012080785.1
ARUBRA_DN7424_c0_g1_i1_6	1,32E-106	323	XP_018825737.1	protein pleiotropic regulatory locus 1	Juglans regia	XP_018825738.1
ARHOMBI_DN5795_c0_g1_i1_5	6,07E-104	303	XP_014518805.1	vesicle-associated membrane protein 722-like	Vigna radiata var. radiata	XP_007147528.1
ARHOMBI_DN6070_c0_g2_i1_6	2,79E-59	189	XP_018824389.1	uncharacterized protein LOC108993809 isoform X1	Juglans regia	XP_018824390.1
ARUBRA_DN5014_c0_g1_i1_2	1,51E-57	183	XP_017186549.1	omega-3 fatty acid desaturase, chloroplastic-like	Malus domestica	AGC51776.1
ARHOMBI_DN8315_c0_g1_i1_3	2,01E-66	211	XP_018831134.1	CBL-interacting serine/threonine-protein kinase 21-like	Juglans regia	XP_018820844.1
ARUBRA_DN13376_c0_g1_i1_4	4,62E-120	343	AAL16968.1	AF367452_1 hexokinase, partial	Prunus persica	XP_009338735.1
ARUBRA_DN21106_c0_g1_i1_3	4,84E-66	219	XP_018821055.1	exocyst complex component SEC5A-like	Juglans regia	XP_008375648.1

ARUBRA_DN22162_c0_g1_i1_5	4,08E-15	77,4	XP_018813392.1	death-associated protein kinase 1-like	Juglans regia	XP_018825087.1
