# Supplementary material for: Comparative proteomics of common allergenic tree pollens of birch, alder, and hazel
Source: Allergy. 2021 Jan 15;76(6):1743–53. doi: 10.1111/all.14694 (PMC8248232; doi:10.1111/all.14694)
Supplement: Supplementary file 12 — Table S10 [file ALL-76-1743-s009.pdf]

Protein IDs	found in soluble proteome	found in total proteome	name [blastx hit 1]	allergome code	Description	Code	Score	e-value	%Identity
TRINITY_DN8608_c2_g1_i2_5	x	x	major allergen	235	Cor a 1.0103	uniprot:Q08407	321	2.0e-89	98
TRINITY_DN11207_c0_g1_i2_3	x	x	major allergen Cor a 1	238	Cor a 1.0301	uniprot:Q39454	290	8,00E-80	92
TRINITY_DN8608_c2_g1_i1_5	x	x	major allergen	234	Cor a 1.0102	uniprot:Q08407	229	1,00E-61	99
TRINITY_DN11207_c0_g1_i1_3	x	x	major allergen Cor a 1	238	Cor a 1.0301	uniprot:Q39454	298	3,00E-82	96
TRINITY_DN8608_c1_g1_i1_4	x		AF323974_1 major allergen variant Cor a 1.0403	241	Cor a 1.0403	uniprot:Q9FPK3	327	5,00E-91	99
TRINITY_DN9775_c0_g1_i1_1	x		hypothetical protein MANES_09G127700	10102	Vig r 6.0101	uniprot:Q9ZWP8	61	9,00E-11	28
TRINITY_DN3503_c0_g1_i2_1	x	x	Bet v 4	3056	Aln g 4.0101	uniprot:O81701	130	4,00E-32	78
TRINITY_DN17339_c0_g1_i1_3	x		Bet v 4	356	Fra e 3	uniprot:W8PPL7	77	3,00E-16	87
TRINITY_DN13307_c0_g1_i1_1	x	x	pollen profilin variant 7	244	Cor a 2	uniprot:A4KA45	275	1,00E-75	100
TRINITY_DN2899_c0_g1_i1_1	x	x	pollen profilin variant 3	248	Cor a 2	uniprot:A4KA41	248	2,00E-67	97
TRINITY_DN6901_c0_g1_i1_3	x	x	AF327622_1 minor allergen hazelnut profilin	3218	Cor a 2.0102	uniprot:Q9AXH4	267	4,00E-73	98
TRINITY_DN10821_c0_g1_i5_2	x	x	isoflavone reductase	10995	Cor a 6.0101	uniprot:A0A0U1VZC8	623	1,00E-180	99
TRINITY_DN11225_c0_g1_i1_3	x	x	isoflavone reductase-like protein	133	Bet v 6.0102	uniprot:Q9FUW6	444	1,00E-126	69
TRINITY_DN2745_c0_g1_i1_1		x	bifunctional pinoresinol-lariciresinol reductase-like	9234	Fra e 12	uniprot:E6Y2L7	167	5,00E-43	46
TRINITY_DN18817_c0_g1_i1_6		x	eugenol synthase 1-like	3460	Pyr c 5.0101	uniprot:O81355	73,2	4,00E-15	47
TRINITY_DN6201_c0_g1_i1_1	x	x	hypothetical protein PRUPE_4G269100	3105	Art v 5.0101	uniprot:Q2KM81	140	1,00E-34	48
TRINITY_DN10616_c0_g1_i2_2	x	x	probable calcium-binding protein CML27	3389	Ole e 8.0101	uniprot:Q9M7R0	173	1,00E-44	57
TRINITY_DN3503_c0_g1_i1_2	x	x	Bet v 4	3056	Aln g 4.0101	uniprot:O81701	120	3,00E-29	75
TRINITY_DN8849_c0_g2_i3_2	x		uncharacterized protein LOC103418606	2861	Bla g 8	uniprot:G9JWG6	30,8	0,09	33
TRINITY_DN21254_c0_g1_i1_2		x	calmodulin-like protein 3	3066	Amb a 10.0101	uniprot:Q2KN25	110	6,00E-26	41
