# Supplementary material for: Comparative proteomics of common allergenic tree pollens of birch, alder, and hazel
Source: Allergy. 2021 Jan 15;76(6):1743–53. doi: 10.1111/all.14694 (PMC8248232; doi:10.1111/all.14694)
Supplement: Supplementary file 13 — Table S11 [file ALL-76-1743-s015.pdf]

Protein IDs	found in soluble proteome	found in total proteome	name [blastx hit 1]	allergome code	Description	Code	Score	e-value	%Identity
TRINITY_DN13992_c5_g1::TRINITY_DN13992_c5_g1_i1::g.31138::m.31138	x		Bet v 1 l	1255	Bet p 1	uniprot:Q9AYS4	154	2,00E-39	77
TRINITY_DN19395_c0_g1::TRINITY_DN19395_c0_g1_i9::g.117248::m.117248	x	x	2122374C allergen Bet v 1-Sc3	103	Bet v 1.0301	uniprot:Q39415	261	1,00E-71	100
TRINITY_DN14513_c0_g3::TRINITY_DN14513_c0_g3_i9::g.38189::m.38189	x		major allergen Pru ar 1-like	10702	Vit v 8	uniprot:A5C113	206	1,00E-54	61
TRINITY_DN11247_c0_g1::TRINITY_DN11247_c0_g1_i5::g.6785::m.6785	x	x	AF282850_1 allergenic isoflavone reductase-like protein Bet v 6.0102	133	Bet v 6.0102	uniprot:Q9FUW6	451	1,00E-128	90
TRINITY_DN19790_c2_g2::TRINITY_DN19790_c2_g2_i6::g.123649::m.123649	x		isoflavone reductase homolog Bet v 6.0101, partial	133	Bet v 6.0102	uniprot:Q9FUW6	155	8,00E-40	97
TRINITY_DN19790_c2_g2::TRINITY_DN19790_c2_g2_i1::g.123647::m.123647	x	x	isoflavone reductase homolog Bet v 6.0101, partial	133	Bet v 6.0102	uniprot:Q9FUW6	150	2,00E-38	92
TRINITY_DN14939_c1_g3::TRINITY_DN14939_c1_g3_i3::g.44686::m.44686	x	x	probable pinoresinol-lariciresinol reductase 3 isoform X1	133	Bet v 6.0102	uniprot:Q9FUW6	340	1,00E-94	54
TRINITY_DN16132_c1_g2::TRINITY_DN16132_c1_g2_i1::g.62633::m.62633	x	x	calmodulin-like	3105	Art v 5.0101	uniprot:Q2KM81	147	4,00E-37	48
TRINITY_DN11191_c0_g1::TRINITY_DN11191_c0_g1_i2::g.6575::m.6575	x	x	uncharacterized protein LOC103418606	2861	Bla g 8	uniprot:G9JWG6	31,2	0,037	34
TRINITY_DN18637_c1_g1::TRINITY_DN18637_c1_g1_i7::g.103721::m.103721		x	serine/threonine protein phosphatase 2A regulatory subunit B''beta-like	797	Ani s Troponin	uniprot:Q9U3U5	32,7	0,061	26
TRINITY_DN18492_c0_g1::TRINITY_DN18492_c0_g1_i14::g.101403::m.101403	x		uncharacterized protein LOC108992543	3066	Amb a 10.0101	uniprot:Q2KN25	30,8	0,078	44
TRINITY_DN13179_c1_g1::TRINITY_DN13179_c1_g1_i6::g.20799::m.20799	x		hypothetical protein MANES_13G124900	3066	Amb a 10.0101	uniprot:Q2KN25	99,4	1,00E-22	34
TRINITY_DN47853_c0_g1::TRINITY_DN47853_c0_g1_i1::g.132570::m.132570	x		hypothetical protein LR48_Vigan10g199000	12218	Der f 39.0101	uniprot:A0A411P9C3	124	2,00E-30	45
TRINITY_DN13033_c2_g1::TRINITY_DN13033_c2_g1_i6::g.18937::m.18937	x	x	profilin	1607	Jug r 7	uniprot:A0A2I4GHA9	174	2,00E-45	90
TRINITY_DN11357_c0_g2::TRINITY_DN11357_c0_g2_i1::g.7096::m.7096	x	x	profilin-1	1607	Jug r 7	uniprot:A0A2I4GHA9	253	3,00E-69	93
