# Supplementary material for: Comparative proteomics of common allergenic tree pollens of birch, alder, and hazel
Source: Allergy. 2021 Jan 15;76(6):1743–53. doi: 10.1111/all.14694 (PMC8248232; doi:10.1111/all.14694)
Supplement: Supplementary file 14 — Table S12 [file ALL-76-1743-s023.pdf]

| Protein IDs | found in soluble proteome | found in total proteome | name [blastx hit 1] | allergome code | Description | Code | Score | e-value | %Identity |
|---|---|---|---|---|---|---|---|---|---|
| ARUBRA_DN3160_c0_g1_i1_1 | x | x | Aln g I | 3055 | Aln g 1.0101 | uniprot:P38948 | 298 | 3,00E-82 | 90 |
| ARHOMBI_DN1709_c0_g2_i1_6 | x | | MLP-like protein 423 | 10131 | Pan g 1 | uniprot:E9M220 | 84,3 | 7,00E-18 | 36 |
| ARUBRA_DN4657_c0_g1_i1_6 | x | | MLP-like protein 328 | 6135 | Act d 11.0101 | uniprot:P85524 | 99,4 | 2,00E-22 | 37 |
| ARUBRA_DN3195_c0_g1_i1_6 | x | x | isoflavone reductase | 10995 | Cor a 6.0101 | uniprot:A0A0U1VZC8 | 585 | 1,00E-168 | 92 |
| ARHOMBI_DN24609_c0_g1_i1_6 | x | | hypothetical protein MANES_17G074700 | 132 | Bet v 6.0101 | uniprot:O65002 | 193 | 5,00E-51 | 74 |
| ARHOMBI_DN5174_c0_g1_i1_1 | x | | isoflavone reductase-like protein | 3460 | Pyr c 5.0101 | uniprot:O81355 | 259 | 9,00E-71 | 69 |
| ARHOMBI_DN5174_c0_g2_i1_1 | x | | isoflavone reductase-like protein | 3460 | Pyr c 5.0101 | uniprot:O81355 | 110 | 2,00E-26 | 71 |
| ARUBRA_DN23118_c0_g1_i1_1 | x | x | hypothetical protein MANES_16G107500 | 244 | Cor a 2 | uniprot:A4KA45 | 269 | 1,00E-73 | 96 |
| ARHOMBI_DN4484_c0_g1_i1_2 | x | x | profilin-3 | 12076 | Jug r 7.0101 | uniprot:A0A2I4DNN6 | 258 | 2,00E-70 | 93 |
| ARUBRA_DN4080_c0_g1_i1_6 | x | | profilin-1 | 1607 | Jug r 7 | uniprot:A0A2I4GR27 | 132 | 1,00E-32 | 98 |
