# Supplementary material for: Comparative proteomics of common allergenic tree pollens of birch, alder, and hazel
Source: Allergy. 2021 Jan 15;76(6):1743–53. doi: 10.1111/all.14694 (PMC8248232; doi:10.1111/all.14694)
Supplement: Supplementary file 15 — Table S13 [file ALL-76-1743-s006.pdf]

Supplementary Table S10: Identified peptidases in Betula pollen

Protein IDs	Pfam accession	Pfam family name	Merops accession	Merops family	Merops peptidase ID	Merops subfamily
TRINITY_DN19058_c2_g1::TRINITY_DN19058_c2_g1_i7::g.111114::m.111114	PF14543.5	TAXi_N	MER0372282	CDR1 peptidase	A01.069	A01B
TRINITY_DN13851_c1_g1::TRINITY_DN13851_c1_g1_i8::g.29304::m.29304	PF00026.22	Asp	MER0680933	At1g62290	A01.A02	A01A
TRINITY_DN19078_c0_g3::TRINITY_DN19078_c0_g3_i6::g.111344::m.111344	PF05184.14	SapB_1	MER0412334	At1g62290	A01.A02	A01A
TRINITY_DN15082_c2_g5::TRINITY_DN15082_c2_g5_i1::g.46709::m.46709	PF14543.5	TAXi_N	MER0106023	At3g12700	A01.A30	A01B
TRINITY_DN17999_c1_g1::TRINITY_DN17999_c1_g1_i8::g.91779::m.91779	PF14541.5	TAXi_C	MER0650368	At5g43100	A01.A59	A01B
TRINITY_DN17198_c0_g1::TRINITY_DN17198_c0_g1_i4::g.79878::m.79878	PF14543.5	TAXi_N	MER0155847	subfamily A1B non-peptidase homologues	A01.UNB	A01B
TRINITY_DN13851_c1_g2::TRINITY_DN13851_c1_g2_i2::g.29292::m.29292	PF00026.22	Asp	MER1131201	subfamily A1A unassigned peptidases	A01.UPA	A01A
TRINITY_DN15036_c1_g1::TRINITY_DN15036_c1_g1_i9::g.46235::m.46235	PF00188.25	CAP	MER0228949	subfamily A1A unassigned peptidases	A01.UPA	A01A
TRINITY_DN19846_c6_g2::TRINITY_DN19846_c6_g2_i1::g.124494::m.124494	PF14543.5	TAXi_N	MER0250417	subfamily A1A unassigned peptidases	A01.UPA	A01A
TRINITY_DN19335_c0_g3::TRINITY_DN19335_c0_g3_i4::g.116290::m.116290	PF01095.18	Pectinesterase	MER0572141	family A2 unassigned peptidases	A02.UPW	A02X
TRINITY_DN19962_c5_g2::TRINITY_DN19962_c5_g2_i2::g.126940::m.126940	PF01095.18	Pectinesterase	MER0570148	family A2 unassigned peptidases	A02.UPW	A02X
TRINITY_DN13852_c0_g1::TRINITY_DN13852_c0_g1_i7::g.28442::m.28442	PF01507.18	PAPS_reduct	MER0639549	subfamily A11A unassigned peptidases	A11.UPA	A11A
TRINITY_DN19187_c0_g2::TRINITY_DN19187_c0_g2_i3::g.113547::m.113547	PF00627.30	UBA	MER0628276	subfamily A28A unassigned peptidases	A28.UPA	A28A
TRINITY_DN19587_c1_g2::TRINITY_DN19587_c1_g2_i1::g.120316::m.120316	PF00240.22	ubiquitin	MER0242394	subfamily A28A unassigned peptidases	A28.UPA	A28A
TRINITY_DN13666_c1_g1::TRINITY_DN13666_c1_g1_i1::g.26956::m.26956	PF08263.11	LRRNT_2	MER1360781	family A31 non-peptidase homologues	A31.UNW	A31
TRINITY_DN14553_c3_g1::TRINITY_DN14553_c3_g1_i5::g.38708::m.38708	PF12819.6	Malectin_like	MER1360781	family A31 non-peptidase homologues	A31.UNW	A31
TRINITY_DN14761_c1_g4::TRINITY_DN14761_c1_g4_i4::g.41839::m.41839	PF00069.24	Pkinase	MER1360781	family A31 non-peptidase homologues	A31.UNW	A31
TRINITY_DN15139_c1_g2::TRINITY_DN15139_c1_g2_i1::g.47376::m.47376	PF00139.18	Lectin_legB	MER1360781	family A31 non-peptidase homologues	A31.UNW	A31
TRINITY_DN16295_c1_g2::TRINITY_DN16295_c1_g2_i1::g.65228::m.65228	PF00069.24	Pkinase	MER1360781	family A31 non-peptidase homologues	A31.UNW	A31

TRINITY_DN16834_c1_g1::TRINITY_DN16834_c1_g1_i1::g.74148::m.74148	PF00069.24	Pkinase	MER1360781	family A31 non-peptidase homologues	A31.UNW	A31
TRINITY_DN17086_c3_g1::TRINITY_DN17086_c3_g1_i14::g.77558::m.77558	PF07714.16	Pkinase_Tyr	MER1360781	family A31 non-peptidase homologues	A31.UNW	A31
TRINITY_DN17490_c0_g3::TRINITY_DN17490_c0_g3_i5::g.84477::m.84477	PF00069.24	Pkinase	MER1360781	family A31 non-peptidase homologues	A31.UNW	A31
TRINITY_DN17718_c6_g2::TRINITY_DN17718_c6_g2_i4::g.88774::m.88774	PF14381.5	EDR1	MER1360781	family A31 non-peptidase homologues	A31.UNW	A31
TRINITY_DN18755_c3_g1::TRINITY_DN18755_c3_g1_i1::g.105920::m.105920	PF07714.16	Pkinase_Tyr	MER1360781	family A31 non-peptidase homologues	A31.UNW	A31
TRINITY_DN18771_c1_g1::TRINITY_DN18771_c1_g1_i3::g.106137::m.106137	PF00069.24	Pkinase	MER1360781	family A31 non-peptidase homologues	A31.UNW	A31
TRINITY_DN18792_c0_g2::TRINITY_DN18792_c0_g2_i6::g.106589::m.106589	PF00069.24	Pkinase	MER1360781	family A31 non-peptidase homologues	A31.UNW	A31
TRINITY_DN19006_c2_g1::TRINITY_DN19006_c2_g1_i11::g.110719::m.110719	PF00069.24	Pkinase	MER1360781	family A31 non-peptidase homologues	A31.UNW	A31
TRINITY_DN19032_c3_g1::TRINITY_DN19032_c3_g1_i17::g.110826::m.110826	PF00069.24	Pkinase	MER1360781	family A31 non-peptidase homologues	A31.UNW	A31
TRINITY_DN19212_c0_g1::TRINITY_DN19212_c0_g1_i8::g.114108::m.114108	PF07714.16	Pkinase_Tyr	MER1360781	family A31 non-peptidase homologues	A31.UNW	A31
TRINITY_DN19251_c1_g2::TRINITY_DN19251_c1_g2_i24::g.114984::m.114984	PF00069.24	Pkinase	MER1360781	family A31 non-peptidase homologues	A31.UNW	A31
TRINITY_DN19939_c3_g1::TRINITY_DN19939_c3_g1_i7::g.125684::m.125684	PF00069.24	Pkinase	MER1360781	family A31 non-peptidase homologues	A31.UNW	A31
TRINITY_DN18320_c3_g1::TRINITY_DN18320_c3_g1_i1::g.98804::m.98804	PF00112.22	Peptidase_C1	MER0640046	cathepsin B, plant form	C01.049	C01A
TRINITY_DN10140_c0_g1::TRINITY_DN10140_c0_g1_i2::g.4317::m.4317	PF00112.22	Peptidase_C1	MER1161383	subfamily C1A unassigned peptidases	C01.UPA	C01A
TRINITY_DN13376_c2_g2::TRINITY_DN13376_c2_g2_i3::g.22986::m.22986	PF00112.22	Peptidase_C1	MER1161143	subfamily C1A unassigned peptidases	C01.UPA	C01A
TRINITY_DN15205_c1_g1::TRINITY_DN15205_c1_g1_i3::g.48499::m.48499	PF00112.22	Peptidase_C1	MER1160913	subfamily C1A unassigned peptidases	C01.UPA	C01A
TRINITY_DN15924_c1_g1::TRINITY_DN15924_c1_g1_i2::g.59050::m.59050	PF00112.22	Peptidase_C1	MER1161707	subfamily C1A unassigned peptidases	C01.UPA	C01A
TRINITY_DN15963_c2_g1::TRINITY_DN15963_c2_g1_i4::g.59609::m.59609	PF00112.22	Peptidase_C1	MER1164780	subfamily C1A unassigned peptidases	C01.UPA	C01A
TRINITY_DN18149_c0_g1::TRINITY_DN18149_c0_g1_i3::g.95908::m.95908	PF00112.22	Peptidase_C1	MER1164799	subfamily C1A unassigned peptidases	C01.UPA	C01A
TRINITY_DN18157_c0_g1::TRINITY_DN18157_c0_g1_i6::g.96079::m.96079	PF00112.22	Peptidase_C1	MER1161753	subfamily C1A unassigned peptidases	C01.UPA	C01A

TRINITY_DN18947_c6_g2::TRINITY_DN18947_c6_g2_i2::g.109306::m.109306	PF00112.22	Peptidase_C1	MER1161493	subfamily C1A unassigned peptidases	C01.UPA	C01A
TRINITY_DN18947_c6_g5::TRINITY_DN18947_c6_g5_i2::g.109307::m.109307	PF00112.22	Peptidase_C1	MER1162079	subfamily C1A unassigned peptidases	C01.UPA	C01A
TRINITY_DN31964_c0_g1::TRINITY_DN31964_c0_g1_i1::g.130316::m.130316	PF00112.22	Peptidase_C1	MER1161346	subfamily C1A unassigned peptidases	C01.UPA	C01A
TRINITY_DN19396_c1_g1::TRINITY_DN19396_c1_g1_i8::g.116313::m.116313	PF00664.22	ABC_membrane	MER1363071	subfamily C2A non-peptidase homologues	C02.UNA	C02A
TRINITY_DN19396_c1_g2::TRINITY_DN19396_c1_g2_i11::g.116356::m.116356	PF00664.22	ABC_membrane	MER1363071	subfamily C2A non-peptidase homologues	C02.UNA	C02A
TRINITY_DN16618_c2_g1::TRINITY_DN16618_c2_g1_i4::g.70494::m.70494	PF04424.12	MINDY_DUB	MER0934028	FAM63B g.p.	C115.002	C115
TRINITY_DN13542_c0_g1::TRINITY_DN13542_c0_g1_i7::g.24740::m.24740	PF01088.20	Peptidase_C12	MER0630401	ubiquitinyl hydrolase-L5	C12.005	C12
TRINITY_DN13485_c2_g2::TRINITY_DN13485_c2_g2_i2::g.24426::m.24426	PF01088.20	Peptidase_C12	MER1170789	family C12 unassigned peptidases	C12.UPW	C12
TRINITY_DN14025_c2_g1::TRINITY_DN14025_c2_g1_i8::g.31522::m.31522	PF13898.5	DUF4205	MER1253126		C121.UNW	C121
TRINITY_DN12352_c0_g2::TRINITY_DN12352_c0_g2_i4::g.12096::m.12096	PF01650.17	Peptidase_C13	MER1173188	family C13 unassigned peptidases	C13.UPW	C13
TRINITY_DN19270_c0_g2::TRINITY_DN19270_c0_g2_i4::g.115031::m.115031	PF00656.21	Peptidase_C14	MER0611140	subfamily C14B unassigned peptidases	C14.UPB	C14B
TRINITY_DN11950_c0_g1::TRINITY_DN11950_c0_g1_i3::g.9633::m.9633	PF00443.28	UCH	MER0546412	Ubp3 ubiquitin peptidase	C19.008	C19
TRINITY_DN12454_c0_g1::TRINITY_DN12454_c0_g1_i1::g.12984::m.12984	PF00240.22	ubiquitin	MER0109705	UBP6 peptidase	C19.094	C19
TRINITY_DN16925_c0_g1::TRINITY_DN16925_c0_g1_i1::g.75769::m.75769	PF00443.28	UCH	MER0430051	At4g10590	C19.A03	C19
TRINITY_DN11687_c0_g1::TRINITY_DN11687_c0_g1_i2::g.8407::m.8407	PF00627.30	UBA	MER0552051	family C19 non-peptidase homologues	C19.UNW	C19
TRINITY_DN13724_c5_g4::TRINITY_DN13724_c5_g4_i1::g.27589::m.27589	PF00917.25	MATH	MER0550185	family C19 non-peptidase homologues	C19.UNW	C19
TRINITY_DN17921_c3_g2::TRINITY_DN17921_c3_g2_i1::g.92117::m.92117	PF06012.11	DUF908	MER0588271	family C19 non-peptidase homologues	C19.UNW	C19
TRINITY_DN17957_c0_g1::TRINITY_DN17957_c0_g1_i11::g.92631::m.92631	PF06012.11	DUF908	MER0588271	family C19 non-peptidase homologues	C19.UNW	C19
TRINITY_DN13524_c0_g1::TRINITY_DN13524_c0_g1_i8::g.24870::m.24870	PF00917.25	MATH	MER0712608	family C19 unassigned peptidases	C19.UPW	C19
TRINITY_DN18543_c1_g3::TRINITY_DN18543_c1_g3_i1::g.101857::m.101857	PF02148.18	zf-UBP	MER0546657	family C19 unassigned peptidases	C19.UPW	C19

TRINITY_DN19613_c2_g2::TRINITY_DN19613_c2_g2_i3::g.120856::m.120856	PF12436.7	USP7_ICP0_bdg	MER0715954	family C19 unassigned peptidases	C19.UPW	C19
TRINITY_DN10578_c0_g1::TRINITY_DN10578_c0_g1_i5::g.5050::m.5050	PF00270.28	DEAD	MER0295850	family C26 non-peptidase homologues	C26.UNW	C26
TRINITY_DN15579_c1_g3::TRINITY_DN15579_c1_g3_i1::g.54029::m.54029	PF00270.28	DEAD	MER0295850	family C26 non-peptidase homologues	C26.UNW	C26
TRINITY_DN18349_c3_g1::TRINITY_DN18349_c3_g1_i4::g.99210::m.99210	PF00270.28	DEAD	MER0295850	family C26 non-peptidase homologues	C26.UNW	C26
TRINITY_DN18704_c0_g1::TRINITY_DN18704_c0_g1_i1::g.104889::m.104889	PF00270.28	DEAD	MER0295850	family C26 non-peptidase homologues	C26.UNW	C26
TRINITY_DN19730_c4_g2::TRINITY_DN19730_c4_g2_i8::g.122947::m.122947	PF00270.28	DEAD	MER0295850	family C26 non-peptidase homologues	C26.UNW	C26
TRINITY_DN11338_c0_g1::TRINITY_DN11338_c0_g1_i3::g.7037::m.7037	PF00988.21	CPSase_sm_chain	MER0584525	family C26 unassigned peptidases	C26.UPW	C26
TRINITY_DN15762_c0_g1::TRINITY_DN15762_c0_g1_i1::g.56697::m.56697	PF02769.21	AIRS_C	MER0087959	family C26 unassigned peptidases	C26.UPW	C26
TRINITY_DN16789_c2_g5::TRINITY_DN16789_c2_g5_i1::g.73225::m.73225	PF00117.27	GATase	MER0571210	family C26 unassigned peptidases	C26.UPW	C26
TRINITY_DN18497_c0_g1::TRINITY_DN18497_c0_g1_i7::g.101435::m.101435	PF01174.18	SNO	MER0562247	family C26 unassigned peptidases	C26.UPW	C26
TRINITY_DN17699_c0_g2::TRINITY_DN17699_c0_g2_i2::g.88416::m.88416	PF00310.20	GATase_2	MER0511740	glutamate synthase	C44.003	C44
TRINITY_DN13460_c3_g2::TRINITY_DN13460_c3_g2_i3::g.24327::m.24327	PF13537.5	GATase_7	MER0457154	family C44 non-peptidase homologues	C44.UNW	C44
TRINITY_DN17602_c0_g3::TRINITY_DN17602_c0_g3_i7::g.86972::m.86972	PF01380.21	SIS	MER0411888	family C44 non-peptidase homologues	C44.UNW	C44
TRINITY_DN10714_c0_g1::TRINITY_DN10714_c0_g1_i7::g.5396::m.5396	PF13537.5	GATase_7	MER0650964	family C44 unassigned peptidases	C44.UPW	C44
TRINITY_DN13284_c1_g1::TRINITY_DN13284_c1_g1_i7::g.21970::m.21970	PF12481.7	DUF3700	MER0614899	family C44 unassigned peptidases	C44.UPW	C44
TRINITY_DN13734_c2_g1::TRINITY_DN13734_c2_g1_i1::g.27715::m.27715	PF12481.7	DUF3700	MER0571885	family C44 unassigned peptidases	C44.UPW	C44
TRINITY_DN15836_c2_g1::TRINITY_DN15836_c2_g1_i8::g.57209::m.57209	PF13537.5	GATase_7	MER0037116	family C44 unassigned peptidases	C44.UPW	C44
TRINITY_DN19694_c1_g1::TRINITY_DN19694_c1_g1_i14::g.120695::m.120695	PF00310.20	GATase_2	MER0502151	family C44 unassigned peptidases	C44.UPW	C44
TRINITY_DN18713_c3_g1::TRINITY_DN18713_c3_g1_i2::g.105054::m.105054	PF01965.23	DJ-1_PfpI	MER0571867	family C56 non-peptidase homologues	C56.UNW	C56
TRINITY_DN18713_c3_g1::TRINITY_DN18713_c3_g1_i8::g.105061::m.105061	PF01965.23	DJ-1_PfpI	MER0589129	family C56 non-peptidase homologues	C56.UNW	C56

TRINITY_DN13841_c4_g1::TRINITY_DN13841_c4_g1_i7::g.28424::m.28424	PF01965.23	DJ-1_PfpI	MER0891420	family C56 unassigned peptidases	C56.UPW	C56
TRINITY_DN17135_c0_g1::TRINITY_DN17135_c0_g1_i6::g.79192::m.79192	PF07910.12	Peptidase_C78	MER0737213	At3g48380 protein	C78.A02	C78A
TRINITY_DN20022_c2_g5::TRINITY_DN20022_c2_g5_i1::g.128013::m.128013	PF02338.18	OTU	MER1128346	subfamily C85A unassigned peptidases	C85.UPA	C85A
TRINITY_DN14972_c1_g2::TRINITY_DN14972_c1_g2_i4::g.44982::m.44982	PF02338.18	OTU	MER0744567	subfamily C85B unassigned peptidases	C85.UPB	C85B
TRINITY_DN16212_c0_g2::TRINITY_DN16212_c0_g2_i5::g.63967::m.63967	PF05903.13	Peptidase_C97	MER0660526	family C97 unassigned peptidases	C97.UPW	C97
TRINITY_DN18196_c4_g4::TRINITY_DN18196_c4_g4_i1::g.96720::m.96720	PF00412.21	LIM	MER0689923	family I2 unassigned peptidase inhibitors	I02.UPW	I02
TRINITY_DN11558_c0_g1::TRINITY_DN11558_c0_g1_i1::g.7858::m.7858	PF00197.17	Kunitz_legume	MER0453920	inhibitor MtTi2	I03.030	I03A
TRINITY_DN14021_c1_g2::TRINITY_DN14021_c1_g2_i5::g.31431::m.31431	PF00079.19	Serpin	MER0510912	AtSerpin1	I04.087	I04
TRINITY_DN17174_c0_g2::TRINITY_DN17174_c0_g2_i1::g.79619::m.79619	PF01781.17	Ribosomal_L38e	MER0621953	family I4 unassigned peptidase inhibitors	I04.UPW	I04
TRINITY_DN17835_c3_g1::TRINITY_DN17835_c3_g1_i1::g.90619::m.90619	PF00063.20	Myosin_head	MER1118174	family I4 unassigned peptidase inhibitors	I04.UPW	I04
TRINITY_DN18255_c1_g1::TRINITY_DN18255_c1_g1_i6::g.97743::m.97743	PF02736.18	Myosin_N	MER1118174	family I4 unassigned peptidase inhibitors	I04.UPW	I04
TRINITY_DN19302_c0_g2::TRINITY_DN19302_c0_g2_i5::g.115749::m.115749	PF00063.20	Myosin_head	MER1118174	family I4 unassigned peptidase inhibitors	I04.UPW	I04
TRINITY_DN13204_c1_g1::TRINITY_DN13204_c1_g1_i4::g.21099::m.21099	PF05922.15	Inhibitor_I9	MER0628798	family I9 unassigned peptidase inhibitors	I09.UPW	I09
TRINITY_DN12914_c1_g1::TRINITY_DN12914_c1_g1_i4::g.17252::m.17252	PF00403.25	HMA	MER0592272	family I13 unassigned peptidase inhibitors	I13.UPW	I13
TRINITY_DN17501_c0_g2::TRINITY_DN17501_c0_g2_i6::g.84659::m.84659	PF02225.21	PA	MER0642455	family I15 unassigned peptidase inhibitors	I15.UPW	I15
TRINITY_DN12993_c1_g1::TRINITY_DN12993_c1_g1_i9::g.18389::m.18389	PF16845.4	SQAPI	MER0628337	phytocystatin	I25.014	I25B
TRINITY_DN13289_c1_g2::TRINITY_DN13289_c1_g2_i4::g.22093::m.22093			MER0046563	phytocystatin	I25.014	I25B
TRINITY_DN13585_c0_g1::TRINITY_DN13585_c0_g1_i3::g.25706::m.25706			MER0680968	subfamily I25A unassigned peptidase inhibitors	I25.UPA	I25A
TRINITY_DN15507_c1_g1::TRINITY_DN15507_c1_g1_i1::g.53015::m.53015	PF16845.4	SQAPI	MER0622052	subfamily I25B unassigned peptidase inhibitors	I25.UPB	I25B
TRINITY_DN17227_c2_g4::TRINITY_DN17227_c2_g4_i1::g.80549::m.80549	PF01565.22	FAD_binding_4	MER0659912	family I29 unassigned peptidase inhibitors	I29.UPW	I29

TRINITY_DN19911_c7_g2::TRINITY_DN19911_c7_g2_i3::g.126024::m.126024	PF08031.11	BBE	MER0659912	family I29 unassigned peptidase inhibitors	I29.UPW	I29
TRINITY_DN11277_c0_g1::TRINITY_DN11277_c0_g1_i2::g.6842::m.6842			MER0583275	family I34 unassigned peptidase inhibitors	I34.UPW	I34
TRINITY_DN11854_c0_g1::TRINITY_DN11854_c0_g1_i1::g.9190::m.9190	PF00085.19	Thioredoxin	MER0546787	family I39 unassigned peptidase inhibitors	I39.UPW	I39
TRINITY_DN11049_c0_g1::TRINITY_DN11049_c0_g1_i1::g.6211::m.6211	PF01161.19	PBP	MER0785675	family I51 unassigned peptidase inhibitors	I51.UPW	I51
TRINITY_DN17130_c2_g2::TRINITY_DN17130_c2_g2_i6::g.79154::m.79154			MER0571023	family I71 unassigned peptidase inhibitors	I71.UPW	I71
TRINITY_DN17913_c4_g1::TRINITY_DN17913_c4_g1_i1::g.91856::m.91856	PF03009.16	GDPD	MER0604943	family I71 unassigned peptidase inhibitors	I71.UPW	I71
TRINITY_DN12832_c2_g6::TRINITY_DN12832_c2_g6_i1::g.16307::m.16307	PF01145.24	Band_7	MER0797839	family I87 unassigned peptidase inhibitors	I87.UPW	I87
TRINITY_DN12832_c2_g7::TRINITY_DN12832_c2_g7_i2::g.16314::m.16314	PF01145.24	Band_7	MER0797839	family I87 unassigned peptidase inhibitors	I87.UPW	I87
TRINITY_DN12853_c0_g1::TRINITY_DN12853_c0_g1_i3::g.16604::m.16604	PF01145.24	Band_7	MER0801850	family I87 unassigned peptidase inhibitors	I87.UPW	I87
TRINITY_DN14376_c2_g1::TRINITY_DN14376_c2_g1_i4::g.36173::m.36173			MER0571692	family I87 unassigned peptidase inhibitors	I87.UPW	I87
TRINITY_DN14376_c2_g2::TRINITY_DN14376_c2_g2_i8::g.36166::m.36166	PF01145.24	Band_7	MER0577440	family I87 unassigned peptidase inhibitors	I87.UPW	I87
TRINITY_DN14376_c2_g4::TRINITY_DN14376_c2_g4_i7::g.36164::m.36164	PF01145.24	Band_7	MER0637058	family I87 unassigned peptidase inhibitors	I87.UPW	I87
TRINITY_DN14711_c0_g2::TRINITY_DN14711_c0_g2_i2::g.40961::m.40961	PF01145.24	Band_7	MER0517269	family I87 unassigned peptidase inhibitors	I87.UPW	I87
TRINITY_DN14711_c0_g4::TRINITY_DN14711_c0_g4_i1::g.40962::m.40962	PF01145.24	Band_7	MER0517269	family I87 unassigned peptidase inhibitors	I87.UPW	I87
TRINITY_DN17996_c1_g1::TRINITY_DN17996_c1_g1_i2::g.93353::m.93353			MER0680924	family I87 unassigned peptidase inhibitors	I87.UPW	I87
TRINITY_DN10864_c0_g1::TRINITY_DN10864_c0_g1_i1::g.5678::m.5678	PF17177.3	PPR_long	MER0680915	family I93 unassigned peptidase inhibitors	I93.UPW	I93
TRINITY_DN11076_c0_g1::TRINITY_DN11076_c0_g1_i2::g.6263::m.6263	PF13812.5	PPR_3	MER0680915	family I93 unassigned peptidase inhibitors	I93.UPW	I93
TRINITY_DN13889_c0_g1::TRINITY_DN13889_c0_g1_i2::g.29853::m.29853	PF13812.5	PPR_3	MER0680915	family I93 unassigned peptidase inhibitors	I93.UPW	I93
TRINITY_DN15085_c0_g2::TRINITY_DN15085_c0_g2_i3::g.46604::m.46604			MER0134791	alanyl aminopeptidase	M01.005	M01
TRINITY_DN12654_c0_g3::TRINITY_DN12654_c0_g3_i5::g.14722::m.14722	PF01433.19	Peptidase_M1	MER0336326	family M1 unassigned peptidases	M01.UPW	M01

TRINITY_DN16376_c0_g2::TRINITY_DN16376_c0_g2_i2::g.66583::m.66583	PF01433.19	Peptidase_M1	MER0412570	family M1 unassigned peptidases	M01.UPW	M01
TRINITY_DN18531_c0_g1::TRINITY_DN18531_c0_g1_i4::g.102214::m.102214	PF01433.19	Peptidase_M1	MER0171524	family M1 unassigned peptidases	M01.UPW	M01
TRINITY_DN19263_c0_g1::TRINITY_DN19263_c0_g1_i6::g.114813::m.114813	PF01432.19	Peptidase_M3	MER0546417	oligopeptidase A	M03.004	M03A
TRINITY_DN19263_c0_g3::TRINITY_DN19263_c0_g3_i4::g.114811::m.114811	PF01432.19	Peptidase_M3	MER0511610	oligopeptidase A	M03.004	M03A
TRINITY_DN19263_c0_g1::TRINITY_DN19263_c0_g1_i2::g.114802::m.114802	PF01432.19	Peptidase_M3	MER0818462	subfamily M3A unassigned peptidases	M03.UPA	M03A
TRINITY_DN19263_c0_g3::TRINITY_DN19263_c0_g3_i3::g.114810::m.114810	PF01432.19	Peptidase_M3	MER0820311	subfamily M3A unassigned peptidases	M03.UPA	M03A
TRINITY_DN18434_c0_g1::TRINITY_DN18434_c0_g1_i17::g.100395::m.100395	PF08324.10	PUL	MER0273962	griselysin	M04.017	M04
TRINITY_DN13918_c1_g1::TRINITY_DN13918_c1_g1_i8::g.30345::m.30345	PF01851.21	PC_rep	MER1206231	subfamily M10A unassigned peptidases	M10.UPA	M10A
TRINITY_DN13739_c0_g1::TRINITY_DN13739_c0_g1_i1::g.27726::m.27726	PF12796.6	Ank_2	MER0053345	Kell blood-group peptidase	M13.090	M13
TRINITY_DN16222_c1_g2::TRINITY_DN16222_c1_g2_i6::g.64132::m.64132	PF00069.24	Pkinase	MER1154309	subfamily M14B non-peptidase homologues	M14.UNB	M14B
TRINITY_DN18171_c1_g1::TRINITY_DN18171_c1_g1_i5::g.96291::m.96291	PF00069.24	Pkinase	MER1154309	subfamily M14B non-peptidase homologues	M14.UNB	M14B
TRINITY_DN18472_c4_g2::TRINITY_DN18472_c4_g2_i2::g.100983::m.100983	PF00069.24	Pkinase	MER1154309	subfamily M14B non-peptidase homologues	M14.UNB	M14B
TRINITY_DN18707_c2_g1::TRINITY_DN18707_c2_g1_i4::g.104948::m.104948	PF13620.5	CarboxypepD_reg	MER0469406	subfamily M14B non-peptidase homologues	M14.UNB	M14B
TRINITY_DN11646_c0_g1::TRINITY_DN11646_c0_g1_i1::g.8053::m.8053			MER0586073	subfamily M16A non-peptidase homologues	M16.UNA	M16A
TRINITY_DN19568_c0_g1::TRINITY_DN19568_c0_g1_i11::g.119880::m.119880	PF00675.19	Peptidase_M16	MER0511546	subfamily M16A non-peptidase homologues	M16.UNA	M16A
TRINITY_DN13650_c9_g1::TRINITY_DN13650_c9_g1_i8::g.26115::m.26115	PF05193.20	Peptidase_M16_C	MER0160376	subfamily M16B non-peptidase homologues	M16.UNB	M16B
TRINITY_DN15983_c2_g1::TRINITY_DN15983_c2_g1_i5::g.59899::m.59899	PF00675.19	Peptidase_M16	MER0923642	subfamily M16B non-peptidase homologues	M16.UNB	M16B
TRINITY_DN16361_c1_g1::TRINITY_DN16361_c1_g1_i5::g.66288::m.66288	PF00675.19	Peptidase_M16	MER0922066	subfamily M16B non-peptidase homologues	M16.UNB	M16B
TRINITY_DN17204_c0_g1::TRINITY_DN17204_c0_g1_i8::g.80281::m.80281	PF00675.19	Peptidase_M16	MER0923033	subfamily M16B unassigned peptidases	M16.UPB	M16B
TRINITY_DN16381_c2_g1::TRINITY_DN16381_c2_g1_i3::g.66693::m.66693	PF05193.20	Peptidase_M16_C	MER0504856	subfamily M16C unassigned peptidases	M16.UPC	M16C

TRINITY_DN19485_c0_g1::TRINITY_DN19485_c0_g1_i9::g.118686::m.118686	PF16187.4	Peptidase_M16_M	MER0628558	subfamily M16C unassigned peptidases	M16.UPC	M16C
TRINITY_DN15589_c0_g1::TRINITY_DN15589_c0_g1_i4::g.54099::m.54099	PF00883.20	Peptidase_M17	MER0659646	leucyl aminopeptidase	M17.002	M17
TRINITY_DN17805_c0_g1::TRINITY_DN17805_c0_g1_i5::g.89980::m.89980	PF01592.15	NifU_N	MER0474067	PepB aminopeptidase	M17.004	M17
TRINITY_DN14079_c0_g2::TRINITY_DN14079_c0_g2_i4::g.32111::m.32111	PF02127.14	Peptidase_M18	MER0546943	family M18 non-peptidase homologues	M18.UNW	M18
TRINITY_DN12445_c0_g2::TRINITY_DN12445_c0_g2_i13::g.12955::m.12955	PF02127.14	Peptidase_M18	MER0900398	family M18 unassigned peptidases	M18.UPW	M18
TRINITY_DN20036_c5_g1::TRINITY_DN20036_c5_g1_i2::g.127587::m.127587	PF02127.14	Peptidase_M18	MER0901680	family M18 unassigned peptidases	M18.UPW	M18
TRINITY_DN12030_c0_g1::TRINITY_DN12030_c0_g1_i2::g.10080::m.10080	PF01546.27	Peptidase_M20	MER0015308	subfamily M20A unassigned peptidases	M20.UPA	M20A
TRINITY_DN13264_c3_g1::TRINITY_DN13264_c3_g1_i4::g.21666::m.21666	PF01546.27	Peptidase_M20	MER0622135	subfamily M20A unassigned peptidases	M20.UPA	M20A
TRINITY_DN18313_c3_g2::TRINITY_DN18313_c3_g2_i2::g.98848::m.98848	PF01546.27	Peptidase_M20	MER0550674	subfamily M20A unassigned peptidases	M20.UPA	M20A
TRINITY_DN16272_c1_g2::TRINITY_DN16272_c1_g2_i3::g.64808::m.64808	PF01546.27	Peptidase_M20	MER0588973	family M20 unassigned peptidases	M20.UPW	M20X
TRINITY_DN16513_c0_g3::TRINITY_DN16513_c0_g3_i2::g.68923::m.68923	PF00804.24	Syntaxin	MER1151076	subfamily M23B unassigned peptidases	M23.UPB	M23B
TRINITY_DN17082_c4_g2::TRINITY_DN17082_c4_g2_i2::g.78169::m.78169	PF08172.11	CASP_C	MER1150565	subfamily M23B unassigned peptidases	M23.UPB	M23B
TRINITY_DN19972_c2_g1::TRINITY_DN19972_c2_g1_i3::g.126916::m.126916	PF00141.22	peroxidase	MER0233833	subfamily M23B unassigned peptidases	M23.UPB	M23B
TRINITY_DN17383_c0_g1::TRINITY_DN17383_c0_g1_i3::g.82754::m.82754	PF00557.23	Peptidase_M24	MER0536976	methionyl aminopeptidase 1	M24.001	M24A
TRINITY_DN16453_c2_g1::TRINITY_DN16453_c2_g1_i14::g.67807::m.67807	PF00557.23	Peptidase_M24	MER0189826	Xaa-Pro dipeptidase	M24.007	M24B
TRINITY_DN18526_c1_g2::TRINITY_DN18526_c1_g2_i7::g.101942::m.101942	PF01321.17	Creatinase_N	MER0650756	At4g36760 g.p.	M24.037	M24B
TRINITY_DN16102_c2_g2::TRINITY_DN16102_c2_g2_i1::g.62195::m.62195			MER0168264	proliferation-association protein 1	M24.973	M24X
TRINITY_DN18982_c0_g1::TRINITY_DN18982_c0_g1_i8::g.109877::m.109877	PF00557.23	Peptidase_M24	MER0176444	proliferation-association protein 1	M24.973	M24X
TRINITY_DN17401_c1_g1::TRINITY_DN17401_c1_g1_i4::g.83099::m.83099	PF00557.23	Peptidase_M24	MER0551690	At2g44180	M24.A02	M24A
TRINITY_DN16102_c2_g6::TRINITY_DN16102_c2_g6_i1::g.62197::m.62197	PF00557.23	Peptidase_M24	MER0586503	family M24 non-peptidase homologues	M24.UNW	M24X

TRINITY_DN11213_c0_g1::TRINITY_DN11213_c0_g1_i2::g.6642::m.6642	PF03868.14	Ribosomal_L6e_N	MER0512373	subfamily M24B unassigned peptidases	M24.UPB	M24B
TRINITY_DN13433_c0_g1::TRINITY_DN13433_c0_g1_i4::g.23725::m.23725	PF05450.14	Nicastrin	MER0622161	family M28 non-peptidase homologues	M28.UNW	M28X
TRINITY_DN14751_c0_g1::TRINITY_DN14751_c0_g1_i8::g.40814::m.40814	PF04389.16	Peptidase_M28	MER0645168	family M28 unassigned peptidases	M28.UPW	M28X
TRINITY_DN15354_c0_g1::TRINITY_DN15354_c0_g1_i1::g.50622::m.50622	PF00547.17	Urease_gamma	MER0413426	urease	M38.982	M38
TRINITY_DN15473_c1_g1::TRINITY_DN15473_c1_g1_i2::g.52569::m.52569	PF01979.19	Amidohydro_1	MER0411921	family M38 non-peptidase homologues	M38.UNW	M38
TRINITY_DN18967_c1_g1::TRINITY_DN18967_c1_g1_i1::g.109547::m.109547	PF01979.19	Amidohydro_1	MER0585097	family M38 non-peptidase homologues	M38.UNW	M38
TRINITY_DN17307_c3_g1::TRINITY_DN17307_c3_g1_i2::g.81691::m.81691	PF01979.19	Amidohydro_1	MER0622001	family M38 unassigned peptidases	M38.UPW	M38
TRINITY_DN18544_c2_g2::TRINITY_DN18544_c2_g2_i1::g.102351::m.102351	PF01434.17	Peptidase_M41	MER0588329	AtFtsH2 peptidase	M41.005	M41
TRINITY_DN12983_c5_g1::TRINITY_DN12983_c5_g1_i4::g.18189::m.18189	PF00004.28	AAA	MER0278187	family M41 non-peptidase homologues	M41.UNW	M41
TRINITY_DN13145_c0_g1::TRINITY_DN13145_c0_g1_i9::g.20440::m.20440	PF00004.28	AAA	MER0278187	family M41 non-peptidase homologues	M41.UNW	M41
TRINITY_DN14197_c0_g1::TRINITY_DN14197_c0_g1_i2::g.33493::m.33493	PF00004.28	AAA	MER0278187	family M41 non-peptidase homologues	M41.UNW	M41
TRINITY_DN14266_c0_g1::TRINITY_DN14266_c0_g1_i8::g.34769::m.34769	PF02359.17	CDC48_N	MER0413614	family M41 non-peptidase homologues	M41.UNW	M41
TRINITY_DN14266_c0_g5::TRINITY_DN14266_c0_g5_i1::g.34758::m.34758	PF02933.16	CDC48_2	MER0413614	family M41 non-peptidase homologues	M41.UNW	M41
TRINITY_DN14603_c3_g1::TRINITY_DN14603_c3_g1_i1::g.39490::m.39490	PF00004.28	AAA	MER0224153	family M41 non-peptidase homologues	M41.UNW	M41
TRINITY_DN14979_c0_g1::TRINITY_DN14979_c0_g1_i9::g.45197::m.45197	PF00004.28	AAA	MER0413614	family M41 non-peptidase homologues	M41.UNW	M41
TRINITY_DN16963_c2_g3::TRINITY_DN16963_c2_g3_i1::g.76239::m.76239	PF00004.28	AAA	MER0258487	family M41 non-peptidase homologues	M41.UNW	M41
TRINITY_DN18049_c1_g1::TRINITY_DN18049_c1_g1_i7::g.94346::m.94346	PF00004.28	AAA	MER0413614	family M41 non-peptidase homologues	M41.UNW	M41
TRINITY_DN18049_c1_g3::TRINITY_DN18049_c1_g3_i3::g.94352::m.94352	PF00004.28	AAA	MER0413614	family M41 non-peptidase homologues	M41.UNW	M41
TRINITY_DN18255_c0_g1::TRINITY_DN18255_c0_g1_i22::g.97730::m.97730	PF04212.17	MIT	MER0258487	family M41 non-peptidase homologues	M41.UNW	M41
TRINITY_DN18940_c3_g1::TRINITY_DN18940_c3_g1_i4::g.109190::m.109190	PF06480.14	FtsH_ext	MER0037053	family M41 non-peptidase homologues	M41.UNW	M41

TRINITY_DN19551_c4_g4::TRINITY_DN19551_c4_g4_i1::g.119681::m.119681	PF00004.28	AAA	MER0264995	family M41 non-peptidase homologues	M41.UNW	M41
TRINITY_DN19655_c2_g1::TRINITY_DN19655_c2_g1_i11::g.121761::m.121761	PF00004.28	AAA	MER0278187	family M41 non-peptidase homologues	M41.UNW	M41
TRINITY_DN19665_c1_g1::TRINITY_DN19665_c1_g1_i7::g.121863::m.121863	PF00004.28	AAA	MER0412267	family M41 non-peptidase homologues	M41.UNW	M41
TRINITY_DN19798_c2_g2::TRINITY_DN19798_c2_g2_i18::g.123759::m.123759	PF02933.16	CDC48_2	MER0273571	family M41 non-peptidase homologues	M41.UNW	M41
TRINITY_DN19798_c2_g2::TRINITY_DN19798_c2_g2_i9::g.123749::m.123749	PF00004.28	AAA	MER0273571	family M41 non-peptidase homologues	M41.UNW	M41
TRINITY_DN15978_c1_g2::TRINITY_DN15978_c1_g2_i1::g.59851::m.59851	PF01434.17	Peptidase_M41	MER0511566	family M41 unassigned peptidases	M41.UPW	M41
TRINITY_DN17613_c0_g1::TRINITY_DN17613_c0_g1_i11::g.87113::m.87113	PF01434.17	Peptidase_M41	MER0190576	family M41 unassigned peptidases	M41.UPW	M41
TRINITY_DN17821_c0_g1::TRINITY_DN17821_c0_g1_i3::g.91084::m.91084	PF07724.13	AAA_2	MER0285623	family M41 unassigned peptidases	M41.UPW	M41
TRINITY_DN19717_c0_g1::TRINITY_DN19717_c0_g1_i2::g.122513::m.122513	PF16491.4	Peptidase_M48_N	MER0171229	farnesylated-protein converting enzyme 1	M48.003	M48A
TRINITY_DN16217_c1_g2::TRINITY_DN16217_c1_g2_i5::g.64084::m.64084	PF01435.17	Peptidase_M48	MER0570784	At5g51740	M48.A01	M48C
TRINITY_DN19286_c0_g1::TRINITY_DN19286_c0_g1_i3::g.115330::m.115330	PF00293.27	NUDIX	MER0627730	family M49 unassigned peptidases	M49.UPW	M49
TRINITY_DN13349_c1_g1::TRINITY_DN13349_c1_g1_i7::g.22584::m.22584	PF00571.27	CBS	MER0236128	subfamily M50B non-peptidase homologues	M50.UNB	M50B
TRINITY_DN16859_c0_g3::TRINITY_DN16859_c0_g3_i1::g.74469::m.74469	PF13180.5	PDZ_2	MER0270354	subfamily M50B non-peptidase homologues	M50.UNB	M50B
TRINITY_DN16218_c1_g2::TRINITY_DN16218_c1_g2_i6::g.64375::m.64375	PF00571.27	CBS	MER0231653	family M50 non-peptidase homologues	M50.UNW	M50
TRINITY_DN17578_c2_g1::TRINITY_DN17578_c2_g1_i2::g.85652::m.85652	PF01398.20	JAB	MER0637000	Csn5 peptidase	M67.002	M67A
TRINITY_DN17014_c1_g3::TRINITY_DN17014_c1_g3_i2::g.77015::m.77015	PF01398.20	JAB	MER0570062	Mername-AA168 protein	M67.971	M67X
TRINITY_DN14916_c3_g2::TRINITY_DN14916_c3_g2_i1::g.44503::m.44503	PF01398.20	JAB	MER0592313	26S proteasome non-ATPase regulatory subunit 7	M67.973	M67A
TRINITY_DN14547_c0_g1::TRINITY_DN14547_c0_g1_i3::g.38055::m.38055	PF01398.20	JAB	MER0571370	eukaryotic translation initiation factor 3 subunit F	M67.974	M67X
TRINITY_DN14324_c0_g1::TRINITY_DN14324_c0_g1_i3::g.35430::m.35430	PF01398.20	JAB	MER0191219	At1g80210	M67.A03	M67A
TRINITY_DN14398_c0_g1::TRINITY_DN14398_c0_g1_i7::g.36405::m.36405	PF11543.7	UN_NPL4	MER0393371	subfamily M67A non-peptidase homologues	M67.UNA	M67A

TRINITY_DN10774_c0_g1::TRINITY_DN10774_c0_g1_i7::g.5503::m.5503	PF13012.5	MitMem_reg	MER0535737	family M67 non-peptidase homologues	M67.UNW	M67X
TRINITY_DN11643_c0_g1::TRINITY_DN11643_c0_g1_i1::g.8230::m.8230	PF01398.20	JAB	MER0905581	subfamily M67A unassigned peptidases	M67.UPA	M67A
TRINITY_DN19333_c1_g1::TRINITY_DN19333_c1_g1_i1::g.116168::m.116168	PF00501.27	AMP-binding	MER0681296	subfamily M67C unassigned peptidases	M67.UPC	M67C
TRINITY_DN17010_c0_g1::TRINITY_DN17010_c0_g1_i4::g.76944::m.76944	PF04563.14	RNA_pol_Rpb2_1	MER0195833	family N11 unassigned peptide lyases	N11.UPW	N11
TRINITY_DN18281_c0_g1::TRINITY_DN18281_c0_g1_i4::g.98025::m.98025	PF04563.14	RNA_pol_Rpb2_1	MER0195833	family N11 unassigned peptide lyases	N11.UPW	N11
TRINITY_DN15090_c0_g1::TRINITY_DN15090_c0_g1_i3::g.46611::m.46611	PF13365.5	Trypsin_2	MER0217205	Nma111 peptidase	S01.434	S01D
TRINITY_DN11519_c0_g1::TRINITY_DN11519_c0_g1_i2::g.7693::m.7693	PF12037.7	DUF3523	MER0413366	subfamily S1A non-peptidase homologues	S01.UNA	S01A
TRINITY_DN14042_c0_g1::TRINITY_DN14042_c0_g1_i1::g.31618::m.31618	PF12037.7	DUF3523	MER0411906	subfamily S1A non-peptidase homologues	S01.UNA	S01A
TRINITY_DN14323_c2_g3::TRINITY_DN14323_c2_g3_i2::g.35593::m.35593	PF00004.28	AAA	MER0413366	subfamily S1A non-peptidase homologues	S01.UNA	S01A
TRINITY_DN11079_c0_g1::TRINITY_DN11079_c0_g1_i1::g.6259::m.6259	PF01344.24	Kelch_1	MER0366613	subfamily S1A unassigned peptidases	S01.UPA	S01A
TRINITY_DN13916_c2_g1::TRINITY_DN13916_c2_g1_i2::g.30380::m.30380	PF00565.16	SNase	MER0573000	subfamily S1A unassigned peptidases	S01.UPA	S01A
TRINITY_DN16993_c0_g2::TRINITY_DN16993_c0_g2_i2::g.76741::m.76741	PF00082.21	Peptidase_S8	MER0621382	ARA12 peptidase	S08.112	S08A
TRINITY_DN17067_c1_g2::TRINITY_DN17067_c1_g2_i1::g.77892::m.77892	PF00082.21	Peptidase_S8	MER0570101	ARA12 peptidase	S08.112	S08A
TRINITY_DN13334_c3_g4::TRINITY_DN13334_c3_g4_i1::g.22910::m.22910	PF00082.21	Peptidase_S8	MER0551416	AIR3 peptidase	S08.119	S08A
TRINITY_DN19475_c9_g3::TRINITY_DN19475_c9_g3_i3::g.118615::m.118615	PF00082.21	Peptidase_S8	MER0511553	AIR3 peptidase	S08.119	S08A
TRINITY_DN19995_c2_g1::TRINITY_DN19995_c2_g1_i2::g.127478::m.127478	PF00082.21	Peptidase_S8	MER0500511	At4g00230	S08.A14	S08A
TRINITY_DN16581_c1_g3::TRINITY_DN16581_c1_g3_i2::g.69928::m.69928	PF00082.21	Peptidase_S8	MER0511501	At5g59810	S08.A26	S08A
TRINITY_DN13334_c3_g1::TRINITY_DN13334_c3_g1_i2::g.22908::m.22908	PF00082.21	Peptidase_S8	MER0039101	At1g32980	S08.A31	S08A
TRINITY_DN19475_c9_g1::TRINITY_DN19475_c9_g1_i7::g.118616::m.118616	PF00082.21	Peptidase_S8	MER0039101	At1g32980	S08.A31	S08A
TRINITY_DN16867_c0_g1::TRINITY_DN16867_c0_g1_i8::g.74659::m.74659	PF00415.17	RCC1	MER0987837	subfamily S8A non-peptidase homologues	S08.UNA	S08A

TRINITY_DN18983_c0_g1::TRINITY_DN18983_c0_g1_i9::g.110074::m.110074	PF00415.17	RCC1	MER0987837	subfamily S8A non-peptidase homologues	S08.UNA	S08A
TRINITY_DN10710_c0_g1::TRINITY_DN10710_c0_g1_i1::g.5355::m.5355			MER0622172	subfamily S8A unassigned peptidases	S08.UPA	S08A
TRINITY_DN12154_c0_g1::TRINITY_DN12154_c0_g1_i6::g.10864::m.10864			MER0592913	subfamily S8A unassigned peptidases	S08.UPA	S08A
TRINITY_DN15525_c0_g1::TRINITY_DN15525_c0_g1_i8::g.53299::m.53299	PF00082.21	Peptidase_S8	MER0544355	subfamily S8A unassigned peptidases	S08.UPA	S08A
TRINITY_DN17241_c0_g1::TRINITY_DN17241_c0_g1_i1::g.80827::m.80827	PF00082.21	Peptidase_S8	MER0621966	subfamily S8A unassigned peptidases	S08.UPA	S08A
TRINITY_DN18618_c2_g1::TRINITY_DN18618_c2_g1_i14::g.103865::m.103865	PF00082.21	Peptidase_S8	MER0979912	subfamily S8A unassigned peptidases	S08.UPA	S08A
TRINITY_DN18634_c2_g1::TRINITY_DN18634_c2_g1_i4::g.104214::m.104214	PF00082.21	Peptidase_S8	MER0495288	subfamily S8A unassigned peptidases	S08.UPA	S08A
TRINITY_DN19405_c0_g1::TRINITY_DN19405_c0_g1_i2::g.117368::m.117368			MER0527005	subfamily S8A unassigned peptidases	S08.UPA	S08A
TRINITY_DN16996_c2_g1::TRINITY_DN16996_c2_g1_i5::g.76760::m.76760	PF02897.14	Peptidase_S9_N	MER0180801	prolyl oligopeptidase	S09.001	S09A
TRINITY_DN19080_c0_g2::TRINITY_DN19080_c0_g2_i7::g.111803::m.111803	PF00326.20	Peptidase_S9	MER0547062	glutamyl endopeptidase C	S09.021	S09D
TRINITY_DN11898_c0_g1::TRINITY_DN11898_c0_g1_i2::g.9380::m.9380	PF00326.20	Peptidase_S9	MER0627651	tyrosyl aminopeptidase	S09.074	S09C
TRINITY_DN12879_c0_g1::TRINITY_DN12879_c0_g1_i1::g.16746::m.16746	PF00756.19	Esterase	MER0622192	S-formylglutathione hydrolase FrmB	S09.940	S09B
TRINITY_DN17454_c3_g1::TRINITY_DN17454_c3_g1_i1::g.84103::m.84103	PF07859.12	Abhydrolase_3	MER0500509	At2g45600	S09.A07	S09X
TRINITY_DN11922_c0_g1::TRINITY_DN11922_c0_g1_i1::g.9511::m.9511	PF07859.12	Abhydrolase_3	MER0500510	At2g45610	S09.A11	S09X
TRINITY_DN14069_c0_g5::TRINITY_DN14069_c0_g5_i5::g.32070::m.32070	PF00326.20	Peptidase_S9	MER0510411	At1g26120	S09.A21	S09X
TRINITY_DN17846_c4_g3::TRINITY_DN17846_c4_g3_i1::g.90654::m.90654	PF00326.20	Peptidase_S9	MER0571840	At5g20520	S09.A24	S09C
TRINITY_DN16462_c1_g1::TRINITY_DN16462_c1_g1_i7::g.68028::m.68028	PF02230.15	Abhydrolase_2	MER0209135	AT5G20060 protein	S09.A56	S09X
TRINITY_DN11301_c0_g1::TRINITY_DN11301_c0_g1_i2::g.6892::m.6892	PF08154.11	NLE	MER0149148	family S9 non-peptidase homologues	S09.UNW	S09X
TRINITY_DN11371_c0_g2::TRINITY_DN11371_c0_g2_i2::g.7148::m.7148	PF00400.31	WD40	MER0183518	family S9 non-peptidase homologues	S09.UNW	S09X
TRINITY_DN11389_c0_g1::TRINITY_DN11389_c0_g1_i1::g.7193::m.7193	PF00400.31	WD40	MER0148915	family S9 non-peptidase homologues	S09.UNW	S09X

TRINITY_DN11410_c0_g1::TRINITY_DN11410_c0_g1_i1::g.7283::m.7283	PF00400.31	WD40	MER0156739	family S9 non-peptidase homologues	S09.UNW	S09X
TRINITY_DN13121_c2_g1::TRINITY_DN13121_c2_g1_i1::g.20130::m.20130	PF08606.10	Prp19	MER0137389	family S9 non-peptidase homologues	S09.UNW	S09X
TRINITY_DN13563_c1_g1::TRINITY_DN13563_c1_g1_i8::g.25419::m.25419	PF00400.31	WD40	MER0156766	family S9 non-peptidase homologues	S09.UNW	S09X
TRINITY_DN14188_c2_g2::TRINITY_DN14188_c2_g2_i3::g.32373::m.32373	PF00400.31	WD40	MER0057464	family S9 non-peptidase homologues	S09.UNW	S09X
TRINITY_DN15198_c1_g3::TRINITY_DN15198_c1_g3_i1::g.48326::m.48326	PF12265.7	CAF1C_H4-bd	MER0157466	family S9 non-peptidase homologues	S09.UNW	S09X
TRINITY_DN15236_c1_g2::TRINITY_DN15236_c1_g2_i4::g.49063::m.49063	PF00400.31	WD40	MER0146853	family S9 non-peptidase homologues	S09.UNW	S09X
TRINITY_DN15276_c1_g1::TRINITY_DN15276_c1_g1_i6::g.49461::m.49461	PF00400.31	WD40	MER0158050	family S9 non-peptidase homologues	S09.UNW	S09X
TRINITY_DN15696_c0_g5::TRINITY_DN15696_c0_g5_i1::g.55738::m.55738	PF00400.31	WD40	MER0136116	family S9 non-peptidase homologues	S09.UNW	S09X
TRINITY_DN15771_c0_g1::TRINITY_DN15771_c0_g1_i5::g.56845::m.56845	PF00400.31	WD40	MER0156555	family S9 non-peptidase homologues	S09.UNW	S09X
TRINITY_DN15771_c0_g2::TRINITY_DN15771_c0_g2_i3::g.56846::m.56846	PF00400.31	WD40	MER0156555	family S9 non-peptidase homologues	S09.UNW	S09X
TRINITY_DN16031_c0_g3::TRINITY_DN16031_c0_g3_i1::g.60905::m.60905	PF00400.31	WD40	MER0158050	family S9 non-peptidase homologues	S09.UNW	S09X
TRINITY_DN16808_c0_g1::TRINITY_DN16808_c0_g1_i2::g.73724::m.73724	PF01738.17	DLH	MER0092863	family S9 non-peptidase homologues	S09.UNW	S09X
TRINITY_DN16940_c1_g1::TRINITY_DN16940_c1_g1_i2::g.75863::m.75863	PF01738.17	DLH	MER0209990	family S9 non-peptidase homologues	S09.UNW	S09X
TRINITY_DN16940_c1_g2::TRINITY_DN16940_c1_g2_i2::g.75865::m.75865	PF01738.17	DLH	MER0213725	family S9 non-peptidase homologues	S09.UNW	S09X
TRINITY_DN17067_c1_g1::TRINITY_DN17067_c1_g1_i1::g.77883::m.77883	PF00400.31	WD40	MER0156766	family S9 non-peptidase homologues	S09.UNW	S09X
TRINITY_DN18437_c1_g1::TRINITY_DN18437_c1_g1_i4::g.100574::m.100574	PF08799.10	PRP4	MER0137389	family S9 non-peptidase homologues	S09.UNW	S09X
TRINITY_DN18615_c1_g3::TRINITY_DN18615_c1_g3_i2::g.103395::m.103395	PF01738.17	DLH	MER0210444	family S9 non-peptidase homologues	S09.UNW	S09X
TRINITY_DN18781_c0_g5::TRINITY_DN18781_c0_g5_i3::g.106209::m.106209	PF00400.31	WD40	MER0156586	family S9 non-peptidase homologues	S09.UNW	S09X
TRINITY_DN18857_c0_g1::TRINITY_DN18857_c0_g1_i9::g.107487::m.107487	PF00400.31	WD40	MER0159213	family S9 non-peptidase homologues	S09.UNW	S09X
TRINITY_DN19669_c1_g1::TRINITY_DN19669_c1_g1_i10::g.121794::m.121794	PF01738.17	DLH	MER0135138	family S9 non-peptidase homologues	S09.UNW	S09X

TRINITY_DN20043_c8_g6::TRINITY_DN20043_c8_g6_i2::g.128243::m.128243	PF00400.31	WD40	MER0136116	family S9 non-peptidase homologues	S09.UNW	S09X
TRINITY_DN9342_c0_g1::TRINITY_DN9342_c0_g1_i1::g.3304::m.3304	PF00400.31	WD40	MER0158050	family S9 non-peptidase homologues	S09.UNW	S09X
TRINITY_DN14491_c1_g5::TRINITY_DN14491_c1_g5_i7::g.37912::m.37912	PF00326.20	Peptidase_S9	MER0413191	subfamily S9A unassigned peptidases	S09.UPA	S09A
TRINITY_DN12510_c1_g1::TRINITY_DN12510_c1_g1_i1::g.13448::m.13448	PF07676.11	PD40	MER0404572	subfamily S9B unassigned peptidases	S09.UPB	S09B
TRINITY_DN13205_c0_g1::TRINITY_DN13205_c0_g1_i3::g.21053::m.21053	PF07859.12	Abhydrolase_3	MER0588552	subfamily S9C unassigned peptidases	S09.UPC	S09C
TRINITY_DN13729_c0_g1::TRINITY_DN13729_c0_g1_i8::g.27674::m.27674	PF07859.12	Abhydrolase_3	MER0511717	subfamily S9C unassigned peptidases	S09.UPC	S09C
TRINITY_DN15900_c2_g1::TRINITY_DN15900_c2_g1_i1::g.57240::m.57240	PF00326.20	Peptidase_S9	MER1300318	subfamily S9C unassigned peptidases	S09.UPC	S09C
TRINITY_DN17776_c0_g1::TRINITY_DN17776_c0_g1_i2::g.89550::m.89550	PF07859.12	Abhydrolase_3	MER0621913	subfamily S9C unassigned peptidases	S09.UPC	S09C
TRINITY_DN17776_c1_g1::TRINITY_DN17776_c1_g1_i2::g.89554::m.89554	PF07859.12	Abhydrolase_3	MER0621926	subfamily S9C unassigned peptidases	S09.UPC	S09C
TRINITY_DN19388_c2_g2::TRINITY_DN19388_c2_g2_i2::g.117003::m.117003	PF07859.12	Abhydrolase_3	MER0621926	subfamily S9C unassigned peptidases	S09.UPC	S09C
TRINITY_DN15072_c0_g1::TRINITY_DN15072_c0_g1_i2::g.46447::m.46447	PF00400.31	WD40	MER0156515	family S9 unassigned peptidases	S09.UPW	S09X
TRINITY_DN16577_c0_g1::TRINITY_DN16577_c0_g1_i6::g.69863::m.69863	PF12146.7	Hydrolase_4	MER0583385	family S9 unassigned peptidases	S09.UPW	S09X
TRINITY_DN17928_c2_g1::TRINITY_DN17928_c2_g1_i2::g.92278::m.92278	PF02230.15	Abhydrolase_2	MER0588084	family S9 unassigned peptidases	S09.UPW	S09X
TRINITY_DN18181_c1_g1::TRINITY_DN18181_c1_g1_i2::g.96439::m.96439	PF12697.6	Abhydrolase_6	MER0639174	family S9 unassigned peptidases	S09.UPW	S09X
TRINITY_DN18181_c1_g1::TRINITY_DN18181_c1_g1_i3::g.96440::m.96440	PF12697.6	Abhydrolase_6	MER0639174	family S9 unassigned peptidases	S09.UPW	S09X
TRINITY_DN18783_c5_g1::TRINITY_DN18783_c5_g1_i3::g.106356::m.106356	PF00450.21	Peptidase_S10	MER0629092	serine carboxypeptidase C	S10.004	S10
TRINITY_DN15931_c0_g1::TRINITY_DN15931_c0_g1_i5::g.59103::m.59103	PF00450.21	Peptidase_S10	MER0425718	serine carboxypeptidase D	S10.005	S10
TRINITY_DN13350_c3_g1::TRINITY_DN13350_c3_g1_i5::g.22631::m.22631	PF00450.21	Peptidase_S10	MER0525671	serine carboxypeptidase III	S10.009	S10
TRINITY_DN18581_c2_g1::TRINITY_DN18581_c2_g1_i1::g.102861::m.102861	PF00450.21	Peptidase_S10	MER0571188	serine carboxypeptidase III	S10.009	S10
TRINITY_DN12399_c0_g1::TRINITY_DN12399_c0_g1_i1::g.12011::m.12011	PF00450.21	Peptidase_S10	MER0637468	OsBISCPL1-type putative carboxypeptidase	S10.017	S10

TRINITY_DN17727_c1_g3::TRINITY_DN17727_c1_g3_i1::g.88887::m.88887	PF00450.21	Peptidase_S10	MER0511739	At4g30810	S10.A32	S10
TRINITY_DN19791_c7_g1::TRINITY_DN19791_c7_g1_i1::g.123736::m.123736	PF00450.21	Peptidase_S10	MER0546785	At3g63470	S10.A41	S10
TRINITY_DN19791_c7_g4::TRINITY_DN19791_c7_g4_i2::g.123739::m.123739	PF00450.21	Peptidase_S10	MER0660317	At3g63470	S10.A41	S10
TRINITY_DN19791_c7_g2::TRINITY_DN19791_c7_g2_i1::g.123738::m.123738	PF00450.21	Peptidase_S10	MER0628063	family S10 non-peptidase homologues	S10.UNW	S10
TRINITY_DN13374_c2_g1::TRINITY_DN13374_c2_g1_i4::g.22819::m.22819	PF00109.25	ketoacyl-synt	MER0947272	family S10 unassigned peptidases	S10.UPW	S10
TRINITY_DN18581_c2_g2::TRINITY_DN18581_c2_g2_i8::g.102871::m.102871	PF00450.21	Peptidase_S10	MER0943604	family S10 unassigned peptidases	S10.UPW	S10
TRINITY_DN18581_c2_g2::TRINITY_DN18581_c2_g2_i16::g.102885::m.102885	PF00450.21	Peptidase_S10	MER0943604	family S10 unassigned peptidases	S10.UPW	S10
TRINITY_DN19589_c2_g2::TRINITY_DN19589_c2_g2_i4::g.120310::m.120310	PF00109.25	ketoacyl-synt	MER0947272	family S10 unassigned peptidases	S10.UPW	S10
TRINITY_DN19596_c1_g1::TRINITY_DN19596_c1_g1_i8::g.120430::m.120430	PF00450.21	Peptidase_S10	MER0943128	family S10 unassigned peptidases	S10.UPW	S10
TRINITY_DN19909_c0_g1::TRINITY_DN19909_c0_g1_i3::g.126119::m.126119	PF08263.11	LRRNT_2	MER0350329	family S10 unassigned peptidases	S10.UPW	S10
TRINITY_DN15458_c1_g3::TRINITY_DN15458_c1_g3_i6::g.52102::m.52102	PF00574.22	CLP_protease	MER0639468	peptidase Clp	S14.001	S14
TRINITY_DN18083_c3_g1::TRINITY_DN18083_c3_g1_i4::g.94853::m.94853	PF00574.22	CLP_protease	MER0547056	peptidase Clp	S14.001	S14
TRINITY_DN16310_c4_g1::TRINITY_DN16310_c4_g1_i21::g.65586::m.65586	PF00574.22	CLP_protease	MER0423286	peptidase Clp	S14.002	S14
TRINITY_DN18647_c1_g1::TRINITY_DN18647_c1_g1_i1::g.103820::m.103820	PF00574.22	CLP_protease	MER0411733	peptidase Clp	S14.006	S14
TRINITY_DN11884_c0_g1::TRINITY_DN11884_c0_g1_i2::g.9315::m.9315	PF00574.22	CLP_protease	MER0663832	ClpP4 peptidase	S14.010	S14
TRINITY_DN16339_c2_g2::TRINITY_DN16339_c2_g2_i2::g.65998::m.65998	PF02190.15	LON_substr_bdg	MER1036228	family S16 unassigned peptidases	S16.UPW	S16
TRINITY_DN15316_c4_g2::TRINITY_DN15316_c4_g2_i4::g.50087::m.50087	PF00717.22	Peptidase_S24	MER0659784	signalase	S26.010	S26B
TRINITY_DN16549_c1_g2::TRINITY_DN16549_c1_g2_i1::g.69658::m.69658			MER0411784	subfamily S26A non-peptidase homologues	S26.UNA	S26A
TRINITY_DN17970_c3_g1::TRINITY_DN17970_c3_g1_i6::g.92954::m.92954	PF05577.11	Peptidase_S28	MER0143936	AT5g65760	S28.A02	S28
TRINITY_DN14658_c2_g1::TRINITY_DN14658_c2_g1_i2::g.40152::m.40152	PF05577.11	Peptidase_S28	MER1054347	family S28 unassigned peptidases	S28.UPW	S28

TRINITY_DN17889_c3_g3::TRINITY_DN17889_c3_g3_i2::g.91373::m.91373	PF12146.7	Hydrolase_4	MER0036047	At2g39420	S33.A20	S33
TRINITY_DN12628_c0_g1::TRINITY_DN12628_c0_g1_i15::g.14380::m.14380			MER0579732	family S33 non-peptidase homologues	S33.UNW	S33
TRINITY_DN12692_c0_g1::TRINITY_DN12692_c0_g1_i7::g.14951::m.14951	PF16113.4	ECH_2	MER0663876	family S33 non-peptidase homologues	S33.UNW	S33
TRINITY_DN15115_c0_g4::TRINITY_DN15115_c0_g4_i3::g.47102::m.47102	PF00561.19	Abhydrolase_1	MER0588542	family S33 non-peptidase homologues	S33.UNW	S33
TRINITY_DN15145_c0_g2::TRINITY_DN15145_c0_g2_i2::g.46817::m.46817	PF00561.19	Abhydrolase_1	MER1140018	family S33 non-peptidase homologues	S33.UNW	S33
TRINITY_DN11909_c0_g1::TRINITY_DN11909_c0_g1_i2::g.9470::m.9470	PF00561.19	Abhydrolase_1	MER0622092	family S33 unassigned peptidases	S33.UPW	S33
TRINITY_DN12181_c0_g1::TRINITY_DN12181_c0_g1_i6::g.10945::m.10945	PF12146.7	Hydrolase_4	MER0570602	family S33 unassigned peptidases	S33.UPW	S33
TRINITY_DN12384_c0_g1::TRINITY_DN12384_c0_g1_i2::g.12333::m.12333	PF01729.18	QRPTase_C	MER0411618	family S33 unassigned peptidases	S33.UPW	S33
TRINITY_DN15133_c0_g1::TRINITY_DN15133_c0_g1_i1::g.47252::m.47252	PF00561.19	Abhydrolase_1	MER1334561	family S33 unassigned peptidases	S33.UPW	S33
TRINITY_DN15513_c2_g2::TRINITY_DN15513_c2_g2_i3::g.53087::m.53087	PF12697.6	Abhydrolase_6	MER0622197	family S33 unassigned peptidases	S33.UPW	S33
TRINITY_DN16306_c1_g1::TRINITY_DN16306_c1_g1_i3::g.65480::m.65480			MER0551075	family S33 unassigned peptidases	S33.UPW	S33
TRINITY_DN16306_c1_g2::TRINITY_DN16306_c1_g2_i2::g.65481::m.65481	PF00561.19	Abhydrolase_1	MER0639178	family S33 unassigned peptidases	S33.UPW	S33
TRINITY_DN16971_c2_g1::TRINITY_DN16971_c2_g1_i8::g.76469::m.76469	PF08538.9	DUF1749	MER0624846	family S33 unassigned peptidases	S33.UPW	S33
TRINITY_DN16987_c2_g1::TRINITY_DN16987_c2_g1_i9::g.76589::m.76589	PF00561.19	Abhydrolase_1	MER0633901	family S33 unassigned peptidases	S33.UPW	S33
TRINITY_DN14091_c1_g2::TRINITY_DN14091_c1_g2_i1::g.32197::m.32197	PF00378.19	ECH_1	MER1073240	subfamily S49C non-peptidase homologues	S49.UNC	S49C
TRINITY_DN15478_c1_g1::TRINITY_DN15478_c1_g1_i1::g.52474::m.52474	PF16113.4	ECH_2	MER1072352	subfamily S49C non-peptidase homologues	S49.UNC	S49C
TRINITY_DN17700_c1_g1::TRINITY_DN17700_c1_g1_i9::g.86845::m.86845	PF00378.19	ECH_1	MER1073240	subfamily S49C non-peptidase homologues	S49.UNC	S49C
TRINITY_DN19596_c0_g2::TRINITY_DN19596_c0_g2_i1::g.120414::m.120414	PF00378.19	ECH_1	MER1073240	subfamily S49C non-peptidase homologues	S49.UNC	S49C
TRINITY_DN12704_c0_g1::TRINITY_DN12704_c0_g1_i4::g.15091::m.15091	PF00153.26	Mito_carr	MER1363300	family S54 non-peptidase homologues	S54.UNW	S54
TRINITY_DN13692_c0_g1::TRINITY_DN13692_c0_g1_i6::g.26626::m.26626	PF00153.26	Mito_carr	MER1363300	family S54 non-peptidase homologues	S54.UNW	S54

TRINITY_DN13692_c1_g1::TRINITY_DN13692_c1_g1_i1::g.26628::m.26628	PF00153.26	Mito_carr	MER1363300	family S54 non-peptidase homologues	S54.UNW	S54
TRINITY_DN14710_c1_g2::TRINITY_DN14710_c1_g2_i5::g.41059::m.41059			MER1080788	family S54 non-peptidase homologues	S54.UNW	S54
TRINITY_DN15558_c1_g3::TRINITY_DN15558_c1_g3_i8::g.53729::m.53729	PF00153.26	Mito_carr	MER1363300	family S54 non-peptidase homologues	S54.UNW	S54
TRINITY_DN15558_c1_g3::TRINITY_DN15558_c1_g3_i7::g.53727::m.53727	PF00153.26	Mito_carr	MER1363300	family S54 non-peptidase homologues	S54.UNW	S54
TRINITY_DN15660_c2_g1::TRINITY_DN15660_c2_g1_i4::g.55101::m.55101	PF01694.21	Rhomboid	MER1082091	family S54 non-peptidase homologues	S54.UNW	S54
TRINITY_DN16118_c1_g1::TRINITY_DN16118_c1_g1_i2::g.62411::m.62411	PF00153.26	Mito_carr	MER1363300	family S54 non-peptidase homologues	S54.UNW	S54
TRINITY_DN16308_c0_g1::TRINITY_DN16308_c0_g1_i5::g.65986::m.65986	PF00153.26	Mito_carr	MER1363300	family S54 non-peptidase homologues	S54.UNW	S54
TRINITY_DN16927_c0_g1::TRINITY_DN16927_c0_g1_i2::g.75647::m.75647	PF04511.14	DER1	MER0125609	family S54 non-peptidase homologues	S54.UNW	S54
TRINITY_DN17369_c1_g1::TRINITY_DN17369_c1_g1_i7::g.82610::m.82610	PF00227.25	Proteasome	MER0172841	proteasome subunit beta1c	T01.010	T01A
TRINITY_DN15811_c0_g5::TRINITY_DN15811_c0_g5_i2::g.57331::m.57331	PF00227.25	Proteasome	MER0411684	proteasome subunit alpha 6	T01.971	T01A
TRINITY_DN18427_c1_g2::TRINITY_DN18427_c1_g2_i5::g.100438::m.100438	PF10584.8	Proteasome_A_N	MER0570712	proteasome subunit alpha 2	T01.972	T01A
TRINITY_DN17658_c5_g3::TRINITY_DN17658_c5_g3_i2::g.87770::m.87770	PF00227.25	Proteasome	MER0571498	proteasome subunit alpha 1	T01.976	T01A
TRINITY_DN13422_c4_g1::TRINITY_DN13422_c4_g1_i9::g.23675::m.23675	PF10584.8	Proteasome_A_N	MER0020043	proteasome subunit alpha 3	T01.977	T01A
TRINITY_DN17070_c2_g4::TRINITY_DN17070_c2_g4_i5::g.77858::m.77858	PF10584.8	Proteasome_A_N	MER0505546	Mername-AA242 peptidase homologue	T01.995	T01A
TRINITY_DN15141_c1_g1::TRINITY_DN15141_c1_g1_i2::g.47502::m.47502	PF00227.25	Proteasome	MER0173164	proteasome subunit beta2	T01.A02	T01A
TRINITY_DN10911_c0_g2::TRINITY_DN10911_c0_g2_i2::g.5805::m.5805	PF00227.25	Proteasome	MER0126199	PBE2 g.p.	T01.A10	T01A
TRINITY_DN14659_c0_g2::TRINITY_DN14659_c0_g2_i6::g.40081::m.40081	PF00227.25	Proteasome	MER0588910	psmB4-2 g.p.	T01.A13	T01X
TRINITY_DN13846_c2_g2::TRINITY_DN13846_c2_g2_i2::g.28548::m.28548	PF00227.25	Proteasome	MER1093865	subfamily T1A non-peptidase homologues	T01.UNA	T01A
TRINITY_DN14731_c0_g4::TRINITY_DN14731_c0_g4_i2::g.41282::m.41282	PF10584.8	Proteasome_A_N	MER0650520	subfamily T1A non-peptidase homologues	T01.UNA	T01A
TRINITY_DN14870_c0_g1::TRINITY_DN14870_c0_g1_i2::g.43479::m.43479	PF00227.25	Proteasome	MER1088634	subfamily T1A non-peptidase homologues	T01.UNA	T01A

TRINITY_DN15416_c2_g2::TRINITY_DN15416_c2_g2_i3::g.51536::m.51536	PF00227.25	Proteasome	MER1090404	subfamily T1A non-peptidase homologues	T01.UNA	T01A
TRINITY_DN12971_c4_g2::TRINITY_DN12971_c4_g2_i4::g.17979::m.17979	PF10584.8	Proteasome_A_N	MER0584369	subfamily T1A unassigned peptidases	T01.UPA	T01A
TRINITY_DN15811_c0_g2::TRINITY_DN15811_c0_g2_i2::g.57329::m.57329	PF10584.8	Proteasome_A_N	MER0637297	subfamily T1A unassigned peptidases	T01.UPA	T01A
TRINITY_DN17150_c2_g1::TRINITY_DN17150_c2_g1_i1::g.79329::m.79329	PF10584.8	Proteasome_A_N	MER0576836	subfamily T1A unassigned peptidases	T01.UPA	T01A
TRINITY_DN17150_c2_g1::TRINITY_DN17150_c2_g1_i7::g.79332::m.79332	PF10584.8	Proteasome_A_N	MER0576836	subfamily T1A unassigned peptidases	T01.UPA	T01A
TRINITY_DN16964_c2_g5::TRINITY_DN16964_c2_g5_i4::g.76427::m.76427	PF03297.14	Ribosomal_S25	MER1365022	family T3 non-peptidase homologues	T03.UNW	T03
TRINITY_DN15448_c0_g1::TRINITY_DN15448_c0_g1_i6::g.51979::m.51979	PF01960.17	ArgJ	MER0122168	ArgJ protein	T05.002	T05
TRINITY_DN15488_c1_g5::TRINITY_DN15488_c1_g5_i3::g.52738::m.52738	PF14226.5	DIOX_N	MER0576457	family T7 unassigned peptidases	T07.UPW	T07
TRINITY_DN19047_c1_g5::TRINITY_DN19047_c1_g5_i2::g.111027::m.111027	PF14226.5	DIOX_N	MER0576457	family T7 unassigned peptidases	T07.UPW	T07
TRINITY_DN19493_c1_g1::TRINITY_DN19493_c1_g1_i9::g.118079::m.118079	PF03171.19	2OG-FeII_Oxy	MER0576457	family T7 unassigned peptidases	T07.UPW	T07
TRINITY_DN19552_c1_g2::TRINITY_DN19552_c1_g2_i2::g.119657::m.119657	PF00155.20	Aminotran_1_2	MER0501945	family U32 unassigned peptidases	U32.UPW	U32
TRINITY_DN14014_c3_g1::TRINITY_DN14014_c3_g1_i1::g.31362::m.31362	PF03080.14	Neprosin	MER0973935	family U74 unassigned peptidases	U74.UPW	U74
TRINITY_DN16195_c0_g1::TRINITY_DN16195_c0_g1_i3::g.63589::m.63589	PF03080.14	Neprosin	MER0973935	family U74 unassigned peptidases	U74.UPW	U74
TRINITY_DN16782_c2_g1::TRINITY_DN16782_c2_g1_i3::g.73171::m.73171	PF03080.14	Neprosin	MER1001725	family U74 unassigned peptidases	U74.UPW	U74
TRINITY_DN16782_c2_g2::TRINITY_DN16782_c2_g2_i4::g.73167::m.73167	PF03080.14	Neprosin	MER0973935	family U74 unassigned peptidases	U74.UPW	U74
TRINITY_DN19409_c5_g1::TRINITY_DN19409_c5_g1_i1::g.117472::m.117472	PF03080.14	Neprosin	MER0973935	family U74 unassigned peptidases	U74.UPW	U74
TRINITY_DN19409_c5_g5::TRINITY_DN19409_c5_g5_i2::g.117475::m.117475	PF03080.14	Neprosin	MER1001725	family U74 unassigned peptidases	U74.UPW	U74
