# Supplementary material for: Comparative proteomics of common allergenic tree pollens of birch, alder, and hazel
Source: Allergy. 2021 Jan 15;76(6):1743–53. doi: 10.1111/all.14694 (PMC8248232; doi:10.1111/all.14694)
Supplement: Supplementary file 16 — Table S14 [file ALL-76-1743-s010.pdf]

Supplementary Table S11:  Identified peptidases in Alnus pollen

| Protein IDs | Pfam accession | Pfam family name | Merops accession | Merops family | source organism | Merops peptidase ID | Merops subfamily |
|---|---|---|---|---|---|---|---|
| ARHOMBI_DN6000_c0_g1_i1_4 | PF00026.22 | Asp | MER0225868 | At1g62290 | Arabidopsis thaliana-Coffea canephora | A01.A02 | A01A |
| ARUBRA_DN3984_c0_g1_i1_3 | PF00026.22 | Asp | MER0680933 | At1g62290 | Arabidopsis thaliana-Gossypium arboreum | A01.A02 | A01A |
| ARUBRA_DN14653_c0_g1_i1_5 | PF14543.5 | TAXi_N | MER0584129 | At1g05840 | Arabidopsis thaliana-type peptidase | A01.A34 | A01B |
| ARUBRA_DN13846_c0_g1_i1_5 | PF00026.22 | Asp | MER1133951 | subfamily A1A unassigned peptidases | Juglans regia | A01.UPA | A01A |
| ARUBRA_DN4385_c0_g1_i1_6 | PF00188.25 | CAP | MER0228949 | subfamily A1A unassigned peptidases | Debaryomyces hansenii | A01.UPA | A01A |
| ARUBRA_DN2382_c0_g1_i1_2 | PF01095.18 | Pectinesterase | MER0570148 | family A2 unassigned peptidases | Cucumis sativus | A02.UPW | A02X |
| ARHOMBI_DN3097_c0_g1_i2_1 | PF01095.18 | Pectinesterase | MER0572141 | family A2 unassigned peptidases | Cucumis melo | A02.UPW | A02X |
| ARHOMBI_DN5536_c0_g1_i1_1 | PF04043.14 | PMEI | MER0570148 | family A2 unassigned peptidases | Cucumis sativus | A02.UPW | A02X |
| ARUBRA_DN4454_c0_g1_i1_2 | PF01095.18 | Pectinesterase | MER0572141 | family A2 unassigned peptidases | Cucumis melo | A02.UPW | A02X |
| ARUBRA_DN6197_c0_g1_i1_1 | PF01095.18 | Pectinesterase | MER0570148 | family A2 unassigned peptidases | Cucumis sativus | A02.UPW | A02X |
| ARHOMBI_DN17696_c0_g1_i1_3 | PF02225.21 | PA | MER0143143 | subfamily A22B unassigned peptidases | Vitis vinifera | A22.UPB | A22B |
| ARHOMBI_DN15828_c0_g1_i1_6 | PF09668.9 | Asp_protease | MER0628276 | subfamily A28A unassigned peptidases | Populus euphratica | A28.UPA | A28A |
| ARUBRA_DN14032_c0_g1_i1_2 | PF09668.9 | Asp_protease | MER0242426 | subfamily A28A unassigned peptidases | | A28.UPA | A28A |
| ARHOMBI_DN16110_c0_g1_i1_4 | PF00069.24 | Pkinase | MER1360781 | family A31 non-peptidase homologues | Thecamonas trahens | A31.UNW | A31 |
| ARHOMBI_DN15739_c0_g1_i1_6 | PF00069.24 | Pkinase | MER1360781 | family A31 non-peptidase homologues | Thecamonas trahens | A31.UNW | A31 |
| ARUBRA_DN3863_c1_g1_i1_5 | PF07714.16 | Pkinase_Tyr | MER1360781 | family A31 non-peptidase homologues | Thecamonas trahens | A31.UNW | A31 |
| ARHOMBI_DN3944_c0_g1_i2_2 | PF00069.24 | Pkinase | MER1360781 | family A31 non-peptidase homologues | Thecamonas trahens | A31.UNW | A31 |
| ARHOMBI_DN3957_c0_g2_i1_3 | PF00069.24 | Pkinase | MER1360781 | family A31 non-peptidase homologues | Thecamonas trahens | A31.UNW | A31 |
| ARHOMBI_DN5409_c0_g1_i1_1 | | | MER1360781 | family A31 non-peptidase homologues | Thecamonas trahens | A31.UNW | A31 |
| ARUBRA_DN9894_c0_g1_i1_4 | PF00112.22 | Peptidase_C1 | MER0660253 | glycinain | Eucalyptus grandis | C01.022 | C01A |
| ARUBRA_DN4725_c0_g1_i1_2 | PF00112.22 | Peptidase_C1 | MER0640046 | cathepsin B, plant form | Nelumbo nucifera | C01.049 | C01A |
| ARHOMBI_DN5653_c0_g1_i1_4 | PF00187.18 | Chitin_bind_1 | MER0428589 | Cwp84 peptidase | Limonium bicolor | C01.125 | C01A |

| | | | | | | | |
|---|---|---|---|---|---|---|---|
| ARUBRA_DN5882_c0_g1_i1_4 | PF00112.22 | Peptidase_C1 | MER1164780 | subfamily C1A unassigned peptidases | Juglans regia | C01.UPA | C01A |
| ARHOMBI_DN5775_c0_g1_i1_2 | PF00112.22 | Peptidase_C1 | MER1160989 | subfamily C1A unassigned peptidases | Juglans regia | C01.UPA | C01A |
| ARHOMBI_DN6144_c0_g1_i1_4 | PF00112.22 | Peptidase_C1 | MER1161707 | subfamily C1A unassigned peptidases | Quercus suber | C01.UPA | C01A |
| ARHOMBI_DN18082_c0_g1_i1_2 | | | MER0956974 | family C115 homologues, unassigned | Genlisea aurea | C115.UPW | C115 |
| ARHOMBI_DN8306_c0_g1_i1_4 | PF01088.20 | Peptidase_C12 | MER1170789 | family C12 unassigned peptidases | Juglans regia | C12.UPW | C12 |
| ARUBRA_DN21075_c0_g1_i1_5 | PF01088.20 | Peptidase_C12 | MER1170789 | family C12 unassigned peptidases | Juglans regia | C12.UPW | C12 |
| ARHOMBI_DN15559_c0_g1_i1_1 | PF00656.21 | Peptidase_C14 | MER0627629 | subfamily C14B unassigned peptidases | Populus euphratica | C14.UPB | C14B |
| ARUBRA_DN3552_c0_g1_i1_4 | PF00656.21 | Peptidase_C14 | MER0611140 | subfamily C14B unassigned peptidases | Populus euphratica | C14.UPB | C14B |
| ARUBRA_DN21162_c0_g1_i1_4 | PF00443.28 | UCH | MER0109705 | UBP6 peptidase | Vitis vinifera | C19.094 | C19 |
| ARHOMBI_DN27310_c0_g1_i1_4 | | | MER0592791 | family C19 non-peptidase homologues | Cicer arietinum | C19.UNW | C19 |
| ARUBRA_DN3144_c0_g1_i1_5 | PF00627.30 | UBA | MER0650471 | family C19 unassigned peptidases | Jatropha curcas | C19.UPW | C19 |
| ARHOMBI_DN20913_c0_g1_i1_5 | PF00270.28 | DEAD | MER0295850 | family C26 non-peptidase homologues | Nematostella vectensis | C26.UNW | C26 |
| ARHOMBI_DN9641_c0_g1_i1_3 | PF00270.28 | DEAD | MER0295850 | family C26 non-peptidase homologues | Nematostella vectensis | C26.UNW | C26 |
| ARUBRA_DN18443_c0_g1_i1_6 | PF00270.28 | DEAD | MER0295850 | family C26 non-peptidase homologues | Nematostella vectensis | C26.UNW | C26 |
| ARHOMBI_DN8153_c0_g1_i1_4 | PF02540.16 | NAD_synthase | MER0588421 | family C26 unassigned peptidases | Citrus sinensis | C26.UPW | C26 |
| ARUBRA_DN9286_c0_g1_i1_1 | PF12481.7 | DUF3700 | MER0038019 | At4g27450 | Arabidopsis thaliana-Arabidopsis thaliana | C44.A04 | C44 |
| ARUBRA_DN1786_c0_g1_i1_1 | PF12481.7 | DUF3700 | MER0570230 | family C44 unassigned peptidases | Cucumis sativus | C44.UPW | C44 |
| ARHOMBI_DN1764_c0_g1_i1_5 | PF00733.20 | Asn_synthase | MER0037116 | family C44 unassigned peptidases | Solanum lycopersicum | C44.UPW | C44 |
| ARHOMBI_DN2521_c0_g1_i1_6 | PF00156.26 | Pribosyltran | MER0223378 | family C44 unassigned peptidases | Ignisphaera aggregans | C44.UPW | C44 |
| ARUBRA_DN828_c0_g1_i1_6 | PF00310.20 | GATase_2 | MER0628622 | family C44 unassigned peptidases | Populus euphratica | C44.UPW | C44 |
| ARHOMBI_DN16907_c0_g1_i1_2 | PF01965.23 | DJ-1_PfpI | MER0733396 | family C56 non-peptidase homologues | Prunus persica | C56.UNW | C56 |
| ARUBRA_DN5867_c0_g1_i1_1 | PF13507.5 | GATase_5 | MER0511682 | family C56 non-peptidase homologues | Prunus mume | C56.UNW | C56 |
| ARUBRA_DN1071_c0_g1_i1_2 | PF01965.23 | DJ-1_PfpI | MER0733395 | family C56 non-peptidase homologues | Prunus persica | C56.UNW | C56 |

| | | | | | | | |
|---|---|---|---|---|---|---|---|
| ARUBRA_DN23412_c0_g1_i1_1 | PF01965.23 | DJ-1_PfpI | MER0570317 | family C56 non-peptidase homologues | Cucumis sativus | C56.UNW | C56 |
| ARHOMBI_DN13057_c0_g1_i1_1 | PF02338.18 | OTU | MER0622165 | OTU2 peptidase | | C85.008 | C85A |
| ARUBRA_DN10864_c0_g1_i1_2 | PF02338.18 | OTU | MER0546486 | OTU2 peptidase | Saccharomyces cerevisiae}-type) | C85.008 | C85A |
| ARHOMBI_DN3049_c0_g1_i1_5 | PF02338.18 | OTU | MER0744567 | subfamily C85B unassigned peptidases | Prunus persica | C85.UPB | C85B |
| ARUBRA_DN23541_c0_g1_i1_3 | PF05903.13 | Peptidase_C97 | MER0636972 | family C97 unassigned peptidases | Nicotiana sylvestris | C97.UPW | C97 |
| ARHOMBI_DN13880_c0_g1_i1_4 | PF00079.19 | Serpin | MER0756064 | AtSerpin1 | Arabidopsis thaliana-Vitis vinifera | I04.087 | I04 |
| ARHOMBI_DN22144_c0_g1_i1_6 | PF00079.19 | Serpin | MER0758854 | AtSerpin1 | Arabidopsis thaliana-Morus notabilis | I04.087 | I04 |
| ARHOMBI_DN2725_c0_g1_i1_2 | PF00079.19 | Serpin | MER0180116 | AtSerpin1 | Arabidopsis thaliana-Ricinus communis | I04.087 | I04 |
| ARUBRA_DN43_c0_g2_i1_6 | PF02704.13 | GASA | MER0526833 | family I8 unassigned peptidase inhibitors | Oryza brachyantha | I08.UPW | I08 |
| ARUBRA_DN4751_c0_g1_i2_6 | PF00187.18 | Chitin_bind_1 | MER0449312 | family I8 unassigned peptidase inhibitors | Tarenaya hassleriana | I08.UPW | I08 |
| ARHOMBI_DN4937_c0_g3_i1_5 | PF05922.15 | Inhibitor_I9 | MER0628798 | family I9 unassigned peptidase inhibitors | Populus euphratica | I09.UPW | I09 |
| ARHOMBI_DN5986_c0_g1_i2_6 | PF00403.25 | HMA | MER0592272 | family I13 unassigned peptidase inhibitors | Cicer arietinum | I13.UPW | I13 |
| ARHOMBI_DN3992_c0_g1_i1_6 | | | MER0642455 | family I15 unassigned peptidase inhibitors | Musa acuminata | I15.UPW | I15 |
| ARUBRA_DN3345_c0_g2_i1_6 | | | MER0458007 | family I15 unassigned peptidase inhibitors | Setaria italica | I15.UPW | I15 |
| ARHOMBI_DN6080_c1_g1_i1_1 | PF16845.4 | SQAPI | MER0178254 | phytocystatin | Ricinus communis | I25.014 | I25B |
| ARHOMBI_DN6132_c0_g5_i1_3 | PF16845.4 | SQAPI | MER0628337 | phytocystatin | Populus euphratica | I25.014 | I25B |
| ARUBRA_DN3020_c0_g1_i1_1 | PF16845.4 | SQAPI | MER0622052 | subfamily I25B unassigned peptidase inhibitors | Vitis vinifera | I25.UPB | I25B |
| ARUBRA_DN4799_c0_g1_i1_4 | PF16845.4 | SQAPI | MER0172552 | subfamily I25B unassigned peptidase inhibitors | Vitis vinifera | I25.UPB | I25B |
| ARUBRA_DN18865_c0_g1_i1_4 | | | MER0583275 | family I34 unassigned peptidase inhibitors | | I34.UPW | I34 |
| ARUBRA_DN5778_c0_g1_i1_4 | PF03009.16 | GDPD | MER0604943 | family I71 unassigned peptidase inhibitors | Cryptosporidium parvum | I71.UPW | I71 |
| ARUBRA_DN981_c0_g1_i1_2 | | | MER0571692 | family I87 unassigned peptidase inhibitors | Cucumis melo | I87.UPW | I87 |

| | | | | | | | |
|---|---|---|---|---|---|---|---|
| ARHOMBI_DN1734_c0_g1_i1_3 | PF01145.24 | Band_7 | MER0803110 | family I87 unassigned peptidase inhibitors | Erythranthe guttata | I87.UPW | I87 |
| ARUBRA_DN1256_c0_g2_i1_1 | PF01145.24 | Band_7 | MER0680924 | family I87 unassigned peptidase inhibitors | Beta vulgaris | I87.UPW | I87 |
| ARUBRA_DN11134_c0_g1_i1_6 | PF01145.24 | Band_7 | MER0808409 | family I87 unassigned peptidase inhibitors | Medicago truncatula | I87.UPW | I87 |
| ARUBRA_DN12858_c0_g1_i1_4 | PF01145.24 | Band_7 | MER0809431 | family I87 unassigned peptidase inhibitors | Brassica oleracea | I87.UPW | I87 |
| ARUBRA_DN19640_c0_g1_i1_4 | PF01145.24 | Band_7 | MER0517269 | family I87 unassigned peptidase inhibitors | Glycine max | I87.UPW | I87 |
| ARUBRA_DN7713_c0_g2_i1_1 | PF01145.24 | Band_7 | MER0797839 | family I87 unassigned peptidase inhibitors | Amborella trichopoda | I87.UPW | I87 |
| ARHOMBI_DN20074_c0_g1_i1_1 | PF01433.19 | Peptidase_M1 | MER1182093 | family M1 unassigned peptidases | Corchorus capsularis | M01.UPW | M01 |
| ARHOMBI_DN2375_c0_g1_i1_6 | | | MER0412570 | family M1 unassigned peptidases | Solanum tuberosum | M01.UPW | M01 |
| ARHOMBI_DN25003_c0_g1_i1_6 | PF01433.19 | Peptidase_M1 | MER0484433 | family M1 unassigned peptidases | Tarenaya hassleriana | M01.UPW | M01 |
| ARHOMBI_DN4645_c0_g2_i1_4 | PF01433.19 | Peptidase_M1 | MER1116733 | family M1 unassigned peptidases | Brachypodium distachyon | M01.UPW | M01 |
| ARHOMBI_DN4645_c1_g1_i1_2 | PF01433.19 | Peptidase_M1 | MER0812872 | family M1 unassigned peptidases | Gossypium raimondii | M01.UPW | M01 |
| ARHOMBI_DN8832_c0_g1_i1_6 | PF01433.19 | Peptidase_M1 | MER0336326 | family M1 unassigned peptidases | Sorghum bicolor | M01.UPW | M01 |
| ARUBRA_DN18651_c0_g1_i1_6 | | | MER0135621 | family M1 unassigned peptidases | Populus trichocarpa | M01.UPW | M01 |
| ARUBRA_DN2825_c0_g1_i1_4 | | | MER0412570 | family M1 unassigned peptidases | Solanum tuberosum | M01.UPW | M01 |
| ARUBRA_DN4026_c0_g1_i1_5 | PF01433.19 | Peptidase_M1 | MER0413560 | family M1 unassigned peptidases | Solanum lycopersicum | M01.UPW | M01 |
| ARHOMBI_DN19654_c0_g1_i1_2 | | | MER0628637 | subfamily M3A non-peptidase homologues | Populus euphratica | M03.UNA | M03A |
| ARHOMBI_DN24575_c0_g1_i1_1 | PF01432.19 | Peptidase_M3 | MER0660426 | subfamily M3A non-peptidase homologues | Eucalyptus grandis | M03.UNA | M03A |
| ARHOMBI_DN17898_c0_g1_i1_3 | PF01432.19 | Peptidase_M3 | MER0817958 | subfamily M3A unassigned peptidases | Prunus persica | M03.UPA | M03A |
| ARUBRA_DN20709_c0_g1_i1_5 | PF01432.19 | Peptidase_M3 | MER0817154 | subfamily M3A unassigned peptidases | Morus notabilis | M03.UPA | M03A |
| ARUBRA_DN7712_c0_g1_i1_6 | PF13646.5 | HEAT_2 | MER1206231 | subfamily M10A unassigned peptidases | Parasteatoda tepidariorum | M10.UPA | M10A |
| ARUBRA_DN6882_c0_g1_i1_3 | PF00023.29 | Ank | MER0263582 | family M13 non-peptidase homologues | Pteropus vampyrus | M13.UNW | M13 |
| ARUBRA_DN5666_c0_g1_i1_2 | PF09440.9 | eIF3_N | MER1224224 | family M13 unassigned peptidases | Piromyces sp. E2 | M13.UPW | M13 |

| | | | | | | | |
|---|---|---|---|---|---|---|---|
| ARUBRA_DN12715_c0_g1_i1_2 | PF13620.5 | CarboxypepD_reg | MER0469406 | subfamily M14B non-peptidase homologues | Brevundimonas sp. BAL3 | M14.UNB | M14B |
| ARUBRA_DN23248_c0_g1_i1_5 | PF00069.24 | Pkinase | MER1154309 | subfamily M14B non-peptidase homologues | Strongylocentrotus purpuratus | M14.UNB | M14B |
| ARHOMBI_DN4083_c0_g1_i1_6 | PF05193.20 | Peptidase_M16_C | MER0411679 | ubiquinol-cytochrome c reductase core protein domain 2 | Nicotiana benthamiana | M16.981 | M16B |
| ARUBRA_DN4588_c0_g1_i2_1 | PF05193.20 | Peptidase_M16_C | MER0628559 | subfamily M16A non-peptidase homologues | Populus euphratica | M16.UNA | M16A |
| ARUBRA_DN568_c0_g1_i1_3 | PF05193.20 | Peptidase_M16_C | MER0579826 | subfamily M16A non-peptidase homologues | | M16.UNA | M16A |
| ARHOMBI_DN16223_c0_g1_i1_5 | PF00675.19 | Peptidase_M16 | MER0123729 | subfamily M16B non-peptidase homologues | Vitis vinifera | M16.UNB | M16B |
| ARUBRA_DN9681_c0_g1_i1_1 | PF05193.20 | Peptidase_M16_C | MER0659899 | subfamily M16B non-peptidase homologues | Eucalyptus grandis | M16.UNB | M16B |
| ARHOMBI_DN3185_c0_g1_i1_4 | PF05193.20 | Peptidase_M16_C | MER0922066 | subfamily M16B non-peptidase homologues | Glycine soja | M16.UNB | M16B |
| ARUBRA_DN12697_c0_g1_i1_5 | PF00675.19 | Peptidase_M16 | MER0592993 | subfamily M16B non-peptidase homologues | Cicer arietinum | M16.UNB | M16B |
| ARUBRA_DN10159_c0_g1_i1_5 | PF00675.19 | Peptidase_M16 | MER0923033 | subfamily M16B unassigned peptidases | Gossypium arboreum | M16.UPB | M16B |
| ARHOMBI_DN8139_c0_g1_i1_2 | PF05193.20 | Peptidase_M16_C | MER0920360 | subfamily M16C unassigned peptidases | Theobroma cacao | M16.UPC | M16C |
| ARUBRA_DN6384_c0_g1_i1_5 | PF08367.10 | M16C_assoc | MER0588858 | subfamily M16C unassigned peptidases | Citrus sinensis | M16.UPC | M16C |
| ARHOMBI_DN15373_c0_g1_i1_5 | PF00883.20 | Peptidase_M17 | MER0659646 | leucyl aminopeptidase | Eucalyptus grandis | M17.002 | M17 |
| ARUBRA_DN4925_c0_g1_i1_4 | PF00883.20 | Peptidase_M17 | MER0659646 | leucyl aminopeptidase | Eucalyptus grandis | M17.002 | M17 |
| ARUBRA_DN6181_c0_g1_i1_5 | PF01592.15 | NifU_N | MER0474067 | PepB aminopeptidase | Ceratitis capitata | M17.004 | M17 |
| ARHOMBI_DN8440_c0_g1_i1_5 | PF02127.14 | Peptidase_M18 | MER0588432 | At5g60160 | Arabidopsis thaliana})-like peptidase | M18.A01 | M18 |
| ARUBRA_DN22748_c0_g1_i1_3 | PF02127.14 | Peptidase_M18 | MER0901680 | family M18 unassigned peptidases | Juglans regia | M18.UPW | M18 |
| ARUBRA_DN12629_c0_g1_i1_4 | | | MER0550674 | subfamily M20A unassigned peptidases | Malus domestica | M20.UPA | M20A |
| ARUBRA_DN1745_c0_g2_i1_1 | | | MER0621905 | subfamily M20A unassigned peptidases | Vitis vinifera | M20.UPA | M20A |
| ARUBRA_DN20085_c0_g1_i1_4 | | | MER0803824 | subfamily M20A unassigned peptidases | Populus trichocarpa | M20.UPA | M20A |
| ARHOMBI_DN26305_c0_g1_i1_3 | | | MER0547052 | subfamily M20F unassigned peptidases | Fragaria vesca | M20.UPF | M20F |
| ARHOMBI_DN3625_c0_g1_i1_3 | PF00557.23 | Peptidase_M24 | MER0628925 | Xaa-Pro dipeptidase | eukaryote-type | M24.007 | M24B |

| | | | | | | | |
|---|---|---|---|---|---|---|---|
| ARUBRA_DN4512_c0_g1_i1_2 | | | MER0170633 | proliferation-association protein 1 | Medicago truncatula | M24.973 | M24X |
| ARUBRA_DN18680_c0_g1_i1_3 | | | MER0176444 | proliferation-association protein 1 | Ricinus communis | M24.973 | M24X |
| ARHOMBI_DN8750_c0_g1_i1_3 | PF01321.17 | Creatinase_N | MER0411863 | subfamily M24B non-peptidase homologues | Nicotiana benthamiana | M24.UNB | M24B |
| ARHOMBI_DN2706_c0_g1_i1_5 | PF00557.23 | Peptidase_M24 | MER0481621 | subfamily M24B unassigned peptidases | Populus trichocarpa | M24.UPB | M24B |
| ARHOMBI_DN5741_c0_g1_i1_3 | PF03868.14 | Ribosomal_L6e_N | MER0512373 | subfamily M24B unassigned peptidases | Saimiri boliviensis | M24.UPB | M24B |
| ARUBRA_DN17285_c0_g1_i1_2 | PF16189.4 | Creatinase_N_2 | MER0032950 | subfamily M24B unassigned peptidases | Magnaporthe grisea | M24.UPB | M24B |
| ARHOMBI_DN9078_c0_g1_i1_6 | PF05450.14 | Nicastrin | MER0637502 | family M28 non-peptidase homologues | Nicotiana sylvestris | M28.UNW | M28X |
| ARUBRA_DN5089_c0_g1_i1_5 | | | MER0572089 | family M28 non-peptidase homologues | Cucumis melo | M28.UNW | M28X |
| ARUBRA_DN6330_c0_g1_i1_4 | PF01979.19 | Amidohydro_1 | MER0585097 | family M38 non-peptidase homologues | Glycine max | M38.UNW | M38 |
| ARHOMBI_DN1643_c0_g1_i1_5 | PF00004.28 | AAA | MER0412697 | family M41 non-peptidase homologues | Solanum tuberosum | M41.UNW | M41 |
| ARHOMBI_DN1692_c0_g1_i1_5 | PF00004.28 | AAA | MER0286041 | family M41 non-peptidase homologues | Echinococcus multilocularis | M41.UNW | M41 |
| ARHOMBI_DN17553_c0_g1_i1_3 | PF00004.28 | AAA | MER0413614 | family M41 non-peptidase homologues | Solanum lycopersicum | M41.UNW | M41 |
| ARHOMBI_DN17617_c0_g1_i1_1 | PF00004.28 | AAA | MER0273571 | family M41 non-peptidase homologues | Paenibacillus mucilaginosus | M41.UNW | M41 |
| ARHOMBI_DN1841_c0_g2_i1_2 | PF00004.28 | AAA | MER0413614 | family M41 non-peptidase homologues | Solanum lycopersicum | M41.UNW | M41 |
| ARUBRA_DN1422_c0_g2_i1_2 | PF04212.17 | MIT | MER0258487 | family M41 non-peptidase homologues | Loxodonta africana | M41.UNW | M41 |
| ARHOMBI_DN5374_c1_g1_i1_6 | PF00004.28 | AAA | MER0278187 | family M41 non-peptidase homologues | Glossina morsitans | M41.UNW | M41 |
| ARHOMBI_DN5420_c0_g1_i1_2 | PF00004.28 | AAA | MER0278187 | family M41 non-peptidase homologues | Glossina morsitans | M41.UNW | M41 |
| ARUBRA_DN4305_c0_g2_i1_6 | PF00004.28 | AAA | MER0413614 | family M41 non-peptidase homologues | Solanum lycopersicum | M41.UNW | M41 |
| ARUBRA_DN4281_c0_g1_i1_1 | PF00004.28 | AAA | MER0278187 | family M41 non-peptidase homologues | Glossina morsitans | M41.UNW | M41 |
| ARUBRA_DN4321_c0_g2_i1_1 | PF02933.16 | CDC48_2 | MER0413614 | family M41 non-peptidase homologues | Solanum lycopersicum | M41.UNW | M41 |
| ARUBRA_DN4889_c0_g1_i1_6 | PF00004.28 | AAA | MER0278187 | family M41 non-peptidase homologues | Glossina morsitans | M41.UNW | M41 |

| | | | | | | | |
|---|---|---|---|---|---|---|---|
| ARHOMBI_DN23037_c0_g1_i1_6 | PF01434.17 | Peptidase_M41 | MER0172803 | family M41 unassigned peptidases | Vitis vinifera | M41.UPW | M41 |
| ARHOMBI_DN5630_c0_g2_i1_5 | PF01435.17 | Peptidase_M48 | MER0142399 | farnesylated-protein converting enzyme 1 | Vitis vinifera | M48.003 | M48A |
| ARUBRA_DN21700_c0_g1_i1_4 | PF00571.27 | CBS | MER0236128 | subfamily M50B non-peptidase homologues | Thermus scotoductus | M50.UNB | M50B |
| ARUBRA_DN4277_c0_g1_i1_2 | PF00571.27 | CBS | MER0231653 | family M50 non-peptidase homologues | Desulfovibrio aespoeensis | M50.UNW | M50 |
| ARHOMBI_DN2999_c0_g1_i1_4 | PF01398.20 | JAB | MER0551083 | Mername-AA168 protein | Malus domestica | M67.971 | M67X |
| ARHOMBI_DN1703_c0_g1_i1_6 | PF01398.20 | JAB | MER0571370 | eukaryotic translation initiation factor 3 subunit F | Cucumis melo | M67.974 | M67X |
| ARUBRA_DN10605_c0_g1_i1_2 | PF11543.7 | UN_NPL4 | MER0393371 | subfamily M67A non-peptidase homologues | Dictyostelium fasciculatum | M67.UNA | M67A |
| ARUBRA_DN21569_c0_g1_i1_4 | PF01398.20 | JAB | MER0905094 | subfamily M67A unassigned peptidases | Theobroma cacao | M67.UPA | M67A |
| ARUBRA_DN19450_c0_g1_i1_4 | | | MER0411906 | subfamily S1A non-peptidase homologues | Nicotiana benthamiana | S01.UNA | S01A |
| ARUBRA_DN25464_c0_g1_i1_1 | PF00004.28 | AAA | MER0411906 | subfamily S1A non-peptidase homologues | Nicotiana benthamiana | S01.UNA | S01A |
| ARUBRA_DN16568_c0_g1_i1_3 | PF01344.24 | Kelch_1 | MER0366613 | subfamily S1A unassigned peptidases | Bos taurus | S01.UPA | S01A |
| ARHOMBI_DN7219_c0_g1_i1_3 | | | MER0975537 | ARA12 peptidase | Ricinus communis | S08.112 | S08A |
| ARUBRA_DN18006_c0_g1_i1_4 | PF00082.21 | Peptidase_S8 | MER0975537 | ARA12 peptidase | Ricinus communis | S08.112 | S08A |
| ARHOMBI_DN11662_c0_g1_i1_4 | | | MER0546828 | At3g14067 | Arabidopsis thaliana-Fragaria vesca | S08.A28 | S08A |
| ARHOMBI_DN11775_c0_g1_i1_1 | | | MER0024767 | At1g32980 | Arabidopsis thaliana-Arabidopsis thaliana | S08.A31 | S08A |
| ARUBRA_DN17897_c0_g1_i1_5 | PF00082.21 | Peptidase_S8 | MER0039101 | At1g32980 | Arabidopsis thaliana-Arachis hypogaea | S08.A31 | S08A |
| ARHOMBI_DN3796_c0_g1_i1_3 | PF00082.21 | Peptidase_S8 | MER0177896 | At4g34980 | Arabidopsis thaliana-Vitis vinifera | S08.A39 | S08A |
| ARHOMBI_DN20638_c0_g1_i1_2 | | | MER0551610 | oligopeptidase B | Malus domestica | S09.010 | S09A |
| ARHOMBI_DN4296_c0_g1_i1_1 | PF00326.20 | Peptidase_S9 | MER0639469 | glutamyl endopeptidase C | Nelumbo nucifera | S09.021 | S09D |
| ARUBRA_DN2819_c0_g1_i1_3 | | | MER0536974 | At5g23530 | Arabidopsis thaliana-Glycine max | S09.A10 | S09C |
| ARUBRA_DN12422_c0_g1_i1_2 | PF07859.12 | Abhydrolase_3 | MER0500863 | At5g62180 | Arabidopsis thaliana-Prunus mume | S09.A14 | S09X |

| | | | | | | | |
|---|---|---|---|---|---|---|---|
| ARHOMBI_DN17996_c0_g1_i1_4 | | | MER0551592 | At1g26120 | Malus domestica | S09.A21 | S09X |
| ARHOMBI_DN2479_c0_g1_i1_1 | | | MER0499110 | At3g47560 | Arabidopsis thaliana-Prunus mume | S09.A31 | S09X |
| ARHOMBI_DN3938_c0_g1_i1_4 | PF02230.15 | Abhydrolase_2 | MER0209135 | AT5G20060 protein | Arabidopsis thaliana-Ricinus communis | S09.A56 | S09X |
| ARUBRA_DN9538_c0_g1_i1_1 | PF12146.7 | Hydrolase_4 | MER0621993 | F14F18_80 protein | Arabidopsis thaliana-Vitis vinifera | S09.A58 | S09B |
| ARHOMBI_DN12388_c0_g1_i1_3 | PF00400.31 | WD40 | MER0149649 | family S9 non-peptidase homologues | Ciona intestinalis | S09.UNW | S09X |
| ARHOMBI_DN12998_c0_g1_i1_2 | PF00400.31 | WD40 | MER0156555 | family S9 non-peptidase homologues | Dipodomys ordii | S09.UNW | S09X |
| ARHOMBI_DN13808_c0_g1_i1_5 | PF00400.31 | WD40 | MER0129460 | family S9 non-peptidase homologues | Pedobacter heparinus | S09.UNW | S09X |
| ARHOMBI_DN16963_c0_g2_i1_3 | PF01738.17 | DLH | MER0061658 | family S9 non-peptidase homologues | Burkholderia  xenovorans | S09.UNW | S09X |
| ARHOMBI_DN27276_c0_g1_i1_4 | | | MER0183170 | family S9 non-peptidase homologues | Ochotona princeps | S09.UNW | S09X |
| ARUBRA_DN7424_c0_g1_i1_6 | | | MER0183210 | family S9 non-peptidase homologues | Ochotona princeps | S09.UNW | S09X |
| ARHOMBI_DN4778_c0_g1_i1_3 | PF00400.31 | WD40 | MER0158050 | family S9 non-peptidase homologues | Dipodomys ordii | S09.UNW | S09X |
| ARHOMBI_DN5210_c0_g1_i1_5 | PF00400.31 | WD40 | MER0057464 | family S9 non-peptidase homologues | Thermobispora bispora | S09.UNW | S09X |
| ARHOMBI_DN5429_c0_g1_i1_2 | PF01738.17 | DLH | MER0209990 | family S9 non-peptidase homologues | Vitis vinifera | S09.UNW | S09X |
| ARUBRA_DN4545_c0_g1_i1_5 | PF01738.17 | DLH | MER0210444 | family S9 non-peptidase homologues | Vitis vinifera | S09.UNW | S09X |
| ARHOMBI_DN6286_c0_g1_i1_6 | | | MER0159213 | family S9 non-peptidase homologues | Nostoc sp. PCC 7120 | S09.UNW | S09X |
| ARHOMBI_DN8777_c0_g1_i1_4 | PF00400.31 | WD40 | MER0156766 | family S9 non-peptidase homologues | Dipodomys ordii | S09.UNW | S09X |
| ARUBRA_DN20269_c0_g1_i1_6 | PF00400.31 | WD40 | MER0156766 | family S9 non-peptidase homologues | Dipodomys ordii | S09.UNW | S09X |
| ARUBRA_DN3962_c0_g1_i1_6 | PF00400.31 | WD40 | MER0156586 | family S9 non-peptidase homologues | Dipodomys ordii | S09.UNW | S09X |
| ARUBRA_DN5364_c0_g1_i1_3 | PF00400.31 | WD40 | MER0146162 | family S9 non-peptidase homologues | Ciona savignyi | S09.UNW | S09X |
| ARUBRA_DN7820_c0_g1_i1_3 | | | MER0161056 | family S9 non-peptidase homologues | Gemmata obscuriglobus | S09.UNW | S09X |

| | | | | | | | |
|---|---|---|---|---|---|---|---|
| ARUBRA_DN8665_c0_g1_i1_5 | PF00400.31 | WD40 | MER0156555 | family S9 non-peptidase homologues | Dipodomys ordii | S09.UNW | S09X |
| ARUBRA_DN9810_c0_g1_i1_4 | PF01738.17 | DLH | MER0092863 | family S9 non-peptidase homologues | Polynucleobacter necessarius | S09.UNW | S09X |
| ARHOMBI_DN2477_c0_g2_i1_4 | PF00930.20 | DPPIV_N | MER0192406 | subfamily S9B unassigned peptidases | Sorghum bicolor | S09.UPB | S09B |
| ARHOMBI_DN19693_c0_g1_i1_6 | PF07859.12 | Abhydrolase_3 | MER0511717 | subfamily S9C unassigned peptidases | Prunus mume | S09.UPC | S09C |
| ARUBRA_DN4715_c0_g1_i1_5 | PF00326.20 | Peptidase_S9 | MER1300318 | subfamily S9C unassigned peptidases | Quercus suber | S09.UPC | S09C |
| ARUBRA_DN18800_c0_g2_i1_4 | PF07859.12 | Abhydrolase_3 | MER0621926 | subfamily S9C unassigned peptidases | Vitis vinifera | S09.UPC | S09C |
| ARUBRA_DN19739_c0_g2_i1_3 | PF07859.12 | Abhydrolase_3 | MER0592815 | subfamily S9C unassigned peptidases | Cicer arietinum | S09.UPC | S09C |
| ARUBRA_DN21696_c0_g1_i1_3 | PF07859.12 | Abhydrolase_3 | MER0588552 | subfamily S9C unassigned peptidases | Citrus sinensis | S09.UPC | S09C |
| ARHOMBI_DN3877_c0_g1_i1_1 | PF02230.15 | Abhydrolase_2 | MER0588084 | family S9 unassigned peptidases | Citrus sinensis | S09.UPW | S09X |
| ARHOMBI_DN478_c0_g2_i1_6 | PF00756.19 | Esterase | MER0651049 | family S9 unassigned peptidases | Jatropha curcas | S09.UPW | S09X |
| ARUBRA_DN162_c0_g1_i1_1 | PF12697.6 | Abhydrolase_6 | MER0650695 | family S9 unassigned peptidases | Jatropha curcas | S09.UPW | S09X |
| ARUBRA_DN18867_c0_g1_i1_5 | PF00400.31 | WD40 | MER0156515 | family S9 unassigned peptidases | Dipodomys ordii | S09.UPW | S09X |
| ARUBRA_DN4594_c0_g2_i1_4 | PF00450.21 | Peptidase_S10 | MER0592987 | serine carboxypeptidase C | Cicer arietinum | S10.004 | S10 |
| ARUBRA_DN16761_c0_g1_i1_6 | PF00450.21 | Peptidase_S10 | MER0539743 | serine carboxypeptidase D | Sesamum indicum | S10.005 | S10 |
| ARUBRA_DN2627_c0_g1_i1_2 | PF00450.21 | Peptidase_S10 | MER0660303 | serine carboxypeptidase III | Eucalyptus grandis | S10.009 | S10 |
| ARUBRA_DN22266_c0_g1_i1_1 | PF00450.21 | Peptidase_S10 | MER0177780 | OsBISCPL1-type putative carboxypeptidase | Ricinus communis | S10.017 | S10 |
| ARUBRA_DN4035_c0_g2_i1_1 | PF00450.21 | Peptidase_S10 | MER0581564 | At4g30810 | Arabidopsis thaliana- | S10.A32 | S10 |
| ARHOMBI_DN6202_c0_g1_i2_4 | PF00450.21 | Peptidase_S10 | MER0551973 | At3g63470 | Arabidopsis thaliana-Malus domestica | S10.A41 | S10 |
| ARUBRA_DN4832_c0_g1_i1_2 | PF00450.21 | Peptidase_S10 | MER0637561 | At3g63470 | Arabidopsis thaliana-Nicotiana sylvestris | S10.A41 | S10 |
| ARUBRA_DN4961_c0_g1_i2_4 | PF00450.21 | Peptidase_S10 | MER0551973 | At3g63470 | Arabidopsis thaliana-Malus domestica | S10.A41 | S10 |
| ARHOMBI_DN25343_c0_g1_i1_2 | PF00450.21 | Peptidase_S10 | MER0942946 | family S10 unassigned peptidases | Erythranthe guttata | S10.UPW | S10 |
| ARUBRA_DN4813_c0_g1_i1_1 | PF00109.25 | ketoacyl-synt | MER0947272 | family S10 unassigned peptidases | Brassica napus | S10.UPW | S10 |
| ARHOMBI_DN430_c0_g1_i1_3 | PF00574.22 | CLP_protease | MER0639468 | peptidase Clp | Nelumbo nucifera | S14.001 | S14 |
| ARHOMBI_DN49_c0_g2_i1_6 | PF00574.22 | CLP_protease | MER0663832 | ClpP4 peptidase | Elaeis guineensis | S14.010 | S14 |
| ARUBRA_DN290_c0_g1_i1_5 | PF00574.22 | CLP_protease | MER0171106 | At5g23140 | Arabidopsis thaliana})-type peptidase | S14.A02 | S14 |

| | | | | | | | |
|---|---|---|---|---|---|---|---|
| ARHOMBI_DN4244_c0_g1_i2_2 | PF00574.22 | CLP_protease | MER1012784 | family S14 non-peptidase homologues | Prunus persica | S14.UNW | S14 |
| ARUBRA_DN19563_c0_g1_i1_5 | PF05577.11 | Peptidase_S28 | MER0650784 | AT5g65760 | Arabidopsis thaliana-Jatropha curcas | S28.A02 | S28 |
| ARUBRA_DN13110_c0_g1_i1_1 | PF13419.5 | HAD_2 | MER0201475 | cytosolic epoxide hydrolase | | S33.973 | S33 |
| ARHOMBI_DN24802_c0_g1_i1_2 | PF16113.4 | ECH_2 | MER0663876 | family S33 non-peptidase homologues | Elaeis guineensis | S33.UNW | S33 |
| ARHOMBI_DN5393_c0_g1_i1_1 | PF00561.19 | Abhydrolase_1 | MER0584079 | family S33 non-peptidase homologues | Cicer arietinum | S33.UNW | S33 |
| ARUBRA_DN784_c0_g2_i1_6 | PF12697.6 | Abhydrolase_6 | MER0570161 | family S33 non-peptidase homologues | Cucumis sativus | S33.UNW | S33 |
| ARUBRA_DN10200_c0_g1_i1_2 | PF00561.19 | Abhydrolase_1 | MER0584079 | family S33 non-peptidase homologues | Cicer arietinum | S33.UNW | S33 |
| ARUBRA_DN11129_c0_g1_i1_5 | | | MER0579732 | family S33 non-peptidase homologues | | S33.UNW | S33 |
| ARUBRA_DN17439_c0_g1_i1_4 | PF16113.4 | ECH_2 | MER0663876 | family S33 non-peptidase homologues | Elaeis guineensis | S33.UNW | S33 |
| ARHOMBI_DN10954_c0_g1_i1_5 | | | MER0622092 | family S33 unassigned peptidases | Vitis vinifera | S33.UPW | S33 |
| ARHOMBI_DN3967_c0_g1_i1_1 | PF13419.5 | HAD_2 | MER0230625 | family S33 unassigned peptidases | Micromonospora sp. L5 | S33.UPW | S33 |
| ARUBRA_DN4582_c0_g1_i1_5 | PF00561.19 | Abhydrolase_1 | MER0588391 | family S33 unassigned peptidases | Citrus sinensis | S33.UPW | S33 |
| ARUBRA_DN1522_c0_g1_i1_5 | PF00561.19 | Abhydrolase_1 | MER1336005 | family S33 unassigned peptidases | Vigna radiata | S33.UPW | S33 |
| ARUBRA_DN15584_c0_g1_i1_1 | | | MER0627781 | family S33 unassigned peptidases | Populus euphratica | S33.UPW | S33 |
| ARUBRA_DN2934_c0_g2_i2_4 | PF05670.12 | DUF814 | MER0599089 | family S33 unassigned peptidases | Echinops telfairi | S33.UPW | S33 |
| ARUBRA_DN7781_c0_g1_i1_6 | PF02817.16 | E3_binding | MER0094490 | family S33 unassigned peptidases | Sinorhizobium medicae | S33.UPW | S33 |
| ARHOMBI_DN20386_c0_g1_i1_1 | PF00378.19 | ECH_1 | MER1072342 | subfamily S49C non-peptidase homologues | Geomicrobium sp. JCM 19055 | S49.UNC | S49C |
| ARHOMBI_DN6745_c0_g1_i1_6 | PF00378.19 | ECH_1 | MER1073240 | subfamily S49C non-peptidase homologues | Acinetobacter bohemicus | S49.UNC | S49C |
| ARUBRA_DN7051_c0_g1_i1_2 | PF00378.19 | ECH_1 | MER1073240 | subfamily S49C non-peptidase homologues | Acinetobacter bohemicus | S49.UNC | S49C |
| ARUBRA_DN24391_c0_g1_i1_1 | | | MER0681183 | RBL2 peptidase | Beta vulgaris | S54.015 | S54 |
| ARUBRA_DN145_c0_g1_i1_4 | | | MER1082728 | family S54 non-peptidase homologues | Vitis vinifera | S54.UNW | S54 |
| ARHOMBI_DN3256_c0_g1_i1_1 | PF00153.26 | Mito_carr | MER1363300 | family S54 non-peptidase homologues | Thecamonas trahens | S54.UNW | S54 |
| ARHOMBI_DN6225_c0_g2_i1_5 | PF00153.26 | Mito_carr | MER1363300 | family S54 non-peptidase homologues | Thecamonas trahens | S54.UNW | S54 |
| ARUBRA_DN3241_c0_g1_i1_4 | PF00153.26 | Mito_carr | MER1363300 | family S54 non-peptidase homologues | Thecamonas trahens | S54.UNW | S54 |

| | | | | | | | |
|---|---|---|---|---|---|---|---|
| ARHOMBI_DN4867_c0_g1_i1_5 | PF00227.25 | Proteasome | MER0172841 | proteasome subunit beta1c | Ricinus communis | T01.010 | T01A |
| ARUBRA_DN8181_c0_g1_i1_4 | PF10584.8 | Proteasome_A_N | MER0411684 | proteasome subunit alpha 6 | Nicotiana benthamiana | T01.971 | T01A |
| ARUBRA_DN17228_c1_g1_i1_1 | PF10584.8 | Proteasome_A_N | MER0570712 | proteasome subunit alpha 2 | Cucumis sativus | T01.972 | T01A |
| ARUBRA_DN1938_c0_g1_i1_1 | PF10584.8 | Proteasome_A_N | MER0079890 | proteasome subunit alpha 7 | Nicotiana benthamiana | T01.974 | T01A |
| ARUBRA_DN4073_c0_g1_i1_2 | PF10584.8 | Proteasome_A_N | MER0461296 | proteasome subunit alpha 1 | Glycine max | T01.976 | T01A |
| ARUBRA_DN6971_c0_g1_i1_6 | PF10584.8 | Proteasome_A_N | MER0020043 | proteasome subunit alpha 3 | Nicotiana tabacum | T01.977 | T01A |
| ARUBRA_DN18881_c0_g1_i1_2 | PF10584.8 | Proteasome_A_N | MER0505546 | Mername-AA242 peptidase homologue | Mus musculus-Prunus mume | T01.995 | T01A |
| ARUBRA_DN2923_c0_g2_i1_5 | PF00227.25 | Proteasome | MER0173164 | proteasome subunit beta2 | Drosophila melanogaster-Ricinus communis | T01.A02 | T01A |
| ARHOMBI_DN4848_c0_g1_i1_6 | PF00227.25 | Proteasome | MER0126199 | PBE2 g.p. | Arabidopsis thaliana-Vitis vinifera | T01.A10 | T01A |
| ARUBRA_DN4784_c0_g2_i3_2 | PF00227.25 | Proteasome | MER0588910 | psmB4-2 g.p. | Dictyostelium discoideum-Citrus sinensis | T01.A13 | T01X |
| ARUBRA_DN881_c0_g1_i1_1 | PF00227.25 | Proteasome | MER0639619 | subfamily T1A non-peptidase homologues | Nelumbo nucifera | T01.UNA | T01A |
| ARUBRA_DN113_c0_g1_i2_3 | PF00227.25 | Proteasome | MER1090404 | subfamily T1A non-peptidase homologues | Hevea brasiliensis | T01.UNA | T01A |
| ARUBRA_DN934_c0_g1_i1_3 | PF00227.25 | Proteasome | MER1088634 | subfamily T1A non-peptidase homologues | Gossypium raimondii | T01.UNA | T01A |
| ARHOMBI_DN6139_c0_g1_i1_4 | PF10584.8 | Proteasome_A_N | MER0576836 | subfamily T1A unassigned peptidases | | T01.UPA | T01A |
| ARUBRA_DN4844_c0_g1_i1_2 | PF03297.14 | Ribosomal_S25 | MER1365022 | family T3 non-peptidase homologues | Thecamonas trahens | T03.UNW | T03 |
| ARHOMBI_DN3876_c0_g1_i1_6 | PF14226.5 | DIOX_N | MER0576457 | family T7 unassigned peptidases | | T07.UPW | T07 |
| ARHOMBI_DN4317_c0_g1_i1_2 | PF00155.20 | Aminotran_1_2 | MER0501945 | family U32 unassigned peptidases | Chthonomonas calidirosea | U32.UPW | U32 |
| | | | | | | | |
| | | | | | | | |
| | | | | | | | |
| | | | | | | | |
| | | | | | | | |
