# Supplementary material for: Comparative proteomics of common allergenic tree pollens of birch, alder, and hazel
Source: Allergy. 2021 Jan 15;76(6):1743–53. doi: 10.1111/all.14694 (PMC8248232; doi:10.1111/all.14694)
Supplement: Supplementary file 17 — Table S15 [file ALL-76-1743-s021.pdf]

Supplementary Table S12: Identified peptidases in Corylus pollen

Protein IDs	Pfam accession	Pfam family name	Merops accession	Merops family	source organism	Merops peptidase ID	Merops subfamily
TRINITY_DN10467_c0_g1_i1_1	PF14543.5	TAXi_N	MER0184554	nepenthesin	Vitis vinifera	A01.040	A01B
TRINITY_DN10590_c0_g2_i1_2	PF14543.5	TAXi_N	MER0372282	CDR1 peptidase	Prunus persica	A01.069	A01B
TRINITY_DN1993_c0_g1_i1_1			MER0511659	CDR1 peptidase	Prunus mume	A01.069	A01B
TRINITY_DN10194_c0_g1_i1_3	PF00026.22	Asp	MER0398222	At1g62290	Gossypium raimondii	A01.A02	A01A
TRINITY_DN10241_c0_g2_i1_1	PF00026.22	Asp	MER0693223	At1g62290	Glycine soja	A01.A02	A01A
TRINITY_DN11009_c0_g1_i1_1	PF14541.5	TAXi_C	MER0535750	At3g12700	Sesamum indicum	A01.A30	A01B
TRINITY_DN1283_c0_g1_i1_3	PF00188.25	CAP	MER0228949	subfamily A1A unassigned peptidases	Debaryomyces hansenii	A01.UPA	A01A
TRINITY_DN6029_c0_g1_i1_1	PF00026.22	Asp	MER1131201	subfamily A1A unassigned peptidases	Populus trichocarpa	A01.UPA	A01A
TRINITY_DN10179_c0_g1_i1_1	PF04043.14	PMEI	MER0570148	family A2 unassigned peptidases	Cucumis sativus	A02.UPW	A02X
TRINITY_DN6919_c0_g2_i1_3	PF01095.18	Pectinesterase	MER0570148	family A2 unassigned peptidases	Cucumis sativus	A02.UPW	A02X
TRINITY_DN9802_c0_g1_i3_2	PF01095.18	Pectinesterase	MER0572141	family A2 unassigned peptidases	Cucumis melo	A02.UPW	A02X
TRINITY_DN9853_c0_g2_i1_2	PF01095.18	Pectinesterase	MER0572141	family A2 unassigned peptidases	Cucumis melo	A02.UPW	A02X
TRINITY_DN10448_c0_g1_i2_3	PF04258.12	Peptidase_A22B	MER0143143	subfamily A22B unassigned peptidases	Vitis vinifera	A22.UPB	A22B
TRINITY_DN5112_c0_g2_i3_3	PF00240.22	ubiquitin	MER0628276	subfamily A28A unassigned peptidases	Populus euphratica	A28.UPA	A28A
TRINITY_DN10085_c0_g1_i1_3	PF12796.6	Ank_2	MER1360781	family A31 non-peptidase homologues	Thecamonas trahens	A31.UNW	A31
TRINITY_DN10206_c0_g8_i1_3	PF00069.24	Pkinase	MER1360781	family A31 non-peptidase homologues	Thecamonas trahens	A31.UNW	A31
TRINITY_DN10474_c1_g1_i1_2	PF00069.24	Pkinase	MER1360781	family A31 non-peptidase homologues	Thecamonas trahens	A31.UNW	A31
TRINITY_DN11081_c0_g1_i2_3			MER1360781	family A31 non-peptidase homologues	Thecamonas trahens	A31.UNW	A31
TRINITY_DN11146_c0_g1_i2_3	PF07714.16	Pkinase_Tyr	MER1360781	family A31 non-peptidase homologues	Thecamonas trahens	A31.UNW	A31
TRINITY_DN17813_c0_g1_i1_1	PF00069.24	Pkinase	MER1360781	family A31 non-peptidase homologues	Thecamonas trahens	A31.UNW	A31
TRINITY_DN17826_c0_g2_i1_1	PF00069.24	Pkinase	MER1360781	family A31 non-peptidase homologues	Thecamonas trahens	A31.UNW	A31
TRINITY_DN18596_c0_g1_i1_5	PF00069.24	Pkinase	MER1360781	family A31 non-peptidase homologues	Thecamonas trahens	A31.UNW	A31
TRINITY_DN18912_c0_g1_i1_2	PF00069.24	Pkinase	MER1360781	family A31 non-peptidase homologues	Thecamonas trahens	A31.UNW	A31

TRINITY_DN20973_c0_g1_i1_1	PF00069.24	Pkinase	MER1360781	family A31 non-peptidase homologues	Thecamonas trahens	A31.UNW	A31
TRINITY_DN2355_c0_g1_i1_2	PF07714.16	Pkinase_Tyr	MER1360781	family A31 non-peptidase homologues	Thecamonas trahens	A31.UNW	A31
TRINITY_DN30_c0_g1_i1_2	PF00069.24	Pkinase	MER1360781	family A31 non-peptidase homologues	Thecamonas trahens	A31.UNW	A31
TRINITY_DN4286_c0_g1_i1_4	PF00069.24	Pkinase	MER1360781	family A31 non-peptidase homologues	Thecamonas trahens	A31.UNW	A31
TRINITY_DN4765_c0_g3_i1_1	PF00069.24	Pkinase	MER1360781	family A31 non-peptidase homologues	Thecamonas trahens	A31.UNW	A31
TRINITY_DN5356_c0_g1_i1_3	PF00069.24	Pkinase	MER1360781	family A31 non-peptidase homologues	Thecamonas trahens	A31.UNW	A31
TRINITY_DN5635_c0_g1_i1_5	PF00069.24	Pkinase	MER1360781	family A31 non-peptidase homologues	Thecamonas trahens	A31.UNW	A31
TRINITY_DN6123_c0_g1_i1_4	PF00069.24	Pkinase	MER1360781	family A31 non-peptidase homologues	Thecamonas trahens	A31.UNW	A31
TRINITY_DN7127_c0_g1_i1_1	PF00069.24	Pkinase	MER1360781	family A31 non-peptidase homologues	Thecamonas trahens	A31.UNW	A31
TRINITY_DN7164_c0_g1_i2_1	PF00069.24	Pkinase	MER1360781	family A31 non-peptidase homologues	Thecamonas trahens	A31.UNW	A31
TRINITY_DN7964_c0_g4_i1_6	PF07714.16	Pkinase_Tyr	MER1360781	family A31 non-peptidase homologues	Thecamonas trahens	A31.UNW	A31
TRINITY_DN9376_c1_g3_i1_4			MER1360781	family A31 non-peptidase homologues	Thecamonas trahens	A31.UNW	A31
TRINITY_DN9503_c0_g1_i1_2	PF07714.16	Pkinase_Tyr	MER1360781	family A31 non-peptidase homologues	Thecamonas trahens	A31.UNW	A31
TRINITY_DN11279_c0_g2_i3_5	PF00112.22	Peptidase_C1	MER0660253	glycinain	Eucalyptus grandis	C01.022	C01A
TRINITY_DN9814_c0_g1_i2_2	PF00112.22	Peptidase_C1	MER0640046	cathepsin B, plant form	Nelumbo nucifera	C01.049	C01A
TRINITY_DN10303_c0_g1_i1_3	PF00112.22	Peptidase_C1	MER1161753	subfamily C1A unassigned peptidases	Juglans regia	C01.UPA	C01A
TRINITY_DN10919_c0_g1_i5_1	PF00112.22	Peptidase_C1	MER1161891	subfamily C1A unassigned peptidases	Cynara cardunculus	C01.UPA	C01A
TRINITY_DN11174_c0_g1_i5_1	PF00112.22	Peptidase_C1	MER1161383	subfamily C1A unassigned peptidases	Ziziphus jujuba	C01.UPA	C01A
TRINITY_DN11576_c1_g1_i1_2	PF00112.22	Peptidase_C1	MER0166880	subfamily C1A unassigned peptidases	Actinidia deliciosa	C01.UPA	C01A
TRINITY_DN7727_c0_g2_i1_3	PF00112.22	Peptidase_C1	MER1161346	subfamily C1A unassigned peptidases	Quercus suber	C01.UPA	C01A
TRINITY_DN8308_c0_g2_i1_2	PF00112.22	Peptidase_C1	MER1161707	subfamily C1A unassigned peptidases	Quercus suber	C01.UPA	C01A
TRINITY_DN9489_c0_g2_i1_2	PF00112.22	Peptidase_C1	MER1160989	subfamily C1A unassigned peptidases	Juglans regia	C01.UPA	C01A
TRINITY_DN10600_c0_g1_i1_5	PF04424.12	MINDY_DUB	MER0934028	FAM63B g.p.	Capsicum annuum	C115.002	C115

TRINITY_DN12921_c0_g1_i1_3			MER1170789	family C12 unassigned peptidases	Juglans regia	C12.UPW	C12
TRINITY_DN12921_c0_g1_i1_6	PF01088.20	Peptidase_C12	MER1170789	family C12 unassigned peptidases	Juglans regia	C12.UPW	C12
TRINITY_DN6323_c0_g1_i1_4	PF01650.17	Peptidase_C13	MER0691713	family C13 unassigned peptidases	Morus notabilis	C13.UPW	C13
TRINITY_DN8225_c0_g1_i1_3	PF01650.17	Peptidase_C13	MER1173236	family C13 unassigned peptidases	Vigna radiata	C13.UPW	C13
TRINITY_DN7265_c0_g2_i5_2	PF00656.21	Peptidase_C14	MER0659855	subfamily C14B unassigned peptidases	Eucalyptus grandis	C14.UPB	C14B
TRINITY_DN9010_c0_g1_i2_3	PF01470.16	Peptidase_C15	MER0584791	At1g56700		C15.A02	C15
TRINITY_DN5492_c0_g2_i1_3	PF02148.18	zf-UBP	MER0430041	deubiquitinating enzyme 14	Vitis vinifera	C19.084	C19
TRINITY_DN9214_c0_g1_i3_1	PF00240.22	ubiquitin	MER0109705	UBP6 peptidase	Vitis vinifera	C19.094	C19
TRINITY_DN294_c0_g1_i1_2	PF00443.28	UCH	MER0430051	At4g10590	Vitis vinifera	C19.A03	C19
TRINITY_DN2894_c0_g1_i1_6	PF00443.28	UCH	MER0640023	At4g30890	Nelumbo nucifera	C19.A14	C19
TRINITY_DN3113_c0_g1_i1_2	PF00443.28	UCH	MER0650391	At4g30890	Jatropha curcas	C19.A14	C19
TRINITY_DN20326_c0_g1_i1_2	PF00443.28	UCH	MER0550185	family C19 non-peptidase homologues	Malus domestica	C19.UNW	C19
TRINITY_DN2462_c0_g2_i1_2	PF00627.30	UBA	MER0552051	family C19 non-peptidase homologues	Malus domestica	C19.UNW	C19
TRINITY_DN8882_c0_g1_i3_2	PF00917.25	MATH	MER0433352	family C19 unassigned peptidases	Ricinus communis	C19.UPW	C19
TRINITY_DN12458_c0_g3_i1_3	PF00270.28	DEAD	MER0295850	family C26 non-peptidase homologues	Nematostella vectensis	C26.UNW	C26
TRINITY_DN2528_c0_g1_i2_1	PF00270.28	DEAD	MER0295850	family C26 non-peptidase homologues	Nematostella vectensis	C26.UNW	C26
TRINITY_DN6675_c0_g1_i4_2	PF00270.28	DEAD	MER0295850	family C26 non-peptidase homologues	Nematostella vectensis	C26.UNW	C26
TRINITY_DN11898_c0_g1_i1_4	PF00117.27	GATase	MER0502488	family C26 unassigned peptidases	Prunus mume	C26.UPW	C26
TRINITY_DN3450_c0_g1_i1_1	PF13507.5	GATase_5	MER0087959	family C26 unassigned peptidases	Cellvibrio japonicus	C26.UPW	C26
TRINITY_DN7736_c0_g1_i2_1	PF00988.21	CPSase_sm_chain	MER0584525	family C26 unassigned peptidases		C26.UPW	C26
TRINITY_DN14633_c0_g1_i1_2	PF01380.21	SIS	MER0176590	family C44 non-peptidase homologues	Populus trichocarpa	C44.UNW	C44
TRINITY_DN10738_c0_g1_i2_5	PF13537.5	GATase_7	MER0037116	family C44 unassigned peptidases	Solanum lycopersicum	C44.UPW	C44
TRINITY_DN11267_c0_g1_i1_2	PF12481.7	DUF3700	MER0570230	family C44 unassigned peptidases	Cucumis sativus	C44.UPW	C44
TRINITY_DN15062_c0_g1_i1_1	PF00733.20	Asn_synthase	MER0037116	family C44 unassigned peptidases	Solanum lycopersicum	C44.UPW	C44
TRINITY_DN16264_c0_g1_i1_2	PF00733.20	Asn_synthase	MER0037116	family C44 unassigned peptidases	Solanum lycopersicum	C44.UPW	C44

TRINITY_DN6495_c0_g1_i1_1	PF00156.26	Pribosyltran	MER0223378	family C44 unassigned peptidases	Ignisphaera aggregans	C44.UPW	C44
TRINITY_DN11339_c0_g1_i3_1	PF01965.23	DJ-1_PfpI	MER0511793	PfpI peptidase	Prunus mume	C56.001	C56
TRINITY_DN10618_c0_g1_i1_3	PF01965.23	DJ-1_PfpI	MER0589129	family C56 non-peptidase homologues	Citrus sinensis	C56.UNW	C56
TRINITY_DN8002_c0_g1_i1_2	PF02338.18	OTU	MER1128346	subfamily C85A unassigned peptidases	Glycine max	C85.UPA	C85A
TRINITY_DN6792_c0_g1_i2_5	PF02338.18	OTU	MER0744567	subfamily C85B unassigned peptidases	Prunus persica	C85.UPB	C85B
TRINITY_DN4761_c0_g1_i1_3	PF05903.13	Peptidase_C97	MER0660526	family C97 unassigned peptidases	Eucalyptus grandis	C97.UPW	C97
TRINITY_DN11097_c0_g1_i2_6	PF00079.19	Serpin	MER0758854	AtSerpin1	Morus notabilis	I04.087	I04
TRINITY_DN6915_c0_g2_i2_2	PF02704.13	GASA	MER0526833	family I8 unassigned peptidase inhibitors	Oryza brachyantha	I08.UPW	I08
TRINITY_DN9357_c0_g1_i1_4			MER0609806	family I8 unassigned peptidase inhibitors	Xenopus tropicalis	I08.UPW	I08
TRINITY_DN11161_c0_g2_i1_1	PF05922.15	Inhibitor_I9	MER0646935	family I9 unassigned peptidase inhibitors	Jatropha curcas	I09.UPW	I09
TRINITY_DN5978_c0_g1_i1_4	PF00403.25	HMA	MER0592272	family I13 unassigned peptidase inhibitors	Cicer arietinum	I13.UPW	I13
TRINITY_DN6677_c0_g2_i1_6	PF00280.17	potato_inhibit	MER0511491	family I13 unassigned peptidase inhibitors	Prunus mume	I13.UPW	I13
TRINITY_DN11259_c0_g1_i2_5	PF02225.21	PA	MER0642455	family I15 unassigned peptidase inhibitors	Musa acuminata	I15.UPW	I15
TRINITY_DN9261_c0_g1_i2_1	PF02225.21	PA	MER0458007	family I15 unassigned peptidase inhibitors	Setaria italica	I15.UPW	I15
TRINITY_DN5250_c0_g1_i1_1	PF16845.4	SQAPI	MER0195773	phytocystatin	Hevea brasiliensis	I25.014	I25B
TRINITY_DN9738_c1_g1_i1_3	PF00031.20	Cystatin	MER0135396	phytocystatin	Populus trichocarpa	I25.014	I25B
TRINITY_DN10831_c0_g1_i1_3	PF16845.4	SQAPI	MER0511446	cystatin Hv-CPI5	Prunus mume	I25.054	I25B
TRINITY_DN8644_c0_g2_i1_1	PF16845.4	SQAPI	MER0593269	subfamily I25B unassigned peptidase inhibitors	Cicer arietinum	I25.UPB	I25B
TRINITY_DN9738_c2_g1_i2_3	PF16845.4	SQAPI	MER0622052	subfamily I25B unassigned peptidase inhibitors	Vitis vinifera	I25.UPB	I25B
TRINITY_DN11574_c0_g1_i5_3	PF01565.22	FAD_binding_4	MER0659912	family I29 unassigned peptidase inhibitors	Eucalyptus grandis	I29.UPW	I29
TRINITY_DN9962_c0_g2_i1_2	PF01565.22	FAD_binding_4	MER0659912	family I29 unassigned peptidase inhibitors	Eucalyptus grandis	I29.UPW	I29
TRINITY_DN17430_c0_g1_i1_5	PF01161.19	PBP	MER0785675	family I51 unassigned peptidase inhibitors	Citrus clementina	I51.UPW	I51
TRINITY_DN20395_c0_g2_i1_2			MER0571023	family I71 unassigned peptidase inhibitors	Cucumis sativus	I71.UPW	I71
TRINITY_DN10858_c0_g1_i1_5	PF01145.24	Band_7	MER0801850	family I87 unassigned peptidase inhibitors	Brassica napus	I87.UPW	I87
TRINITY_DN17896_c0_g1_i1_5	PF01145.24	Band_7	MER0517269	family I87 unassigned peptidase inhibitors	Glycine max	I87.UPW	I87
TRINITY_DN5908_c0_g1_i1_3	PF01145.24	Band_7	MER0680924	family I87 unassigned peptidase inhibitors	Beta vulgaris	I87.UPW	I87
TRINITY_DN9819_c1_g2_i1_2	PF01145.24	Band_7	MER0797839	family I87 unassigned peptidase inhibitors	Amborella trichopoda	I87.UPW	I87
TRINITY_DN9822_c0_g1_i2_3	PF01145.24	Band_7	MER0577440	family I87 unassigned peptidase inhibitors	Glycine max	I87.UPW	I87

TRINITY_DN3307_c0_g1_i1_1	PF01535.19	PPR	MER0680915	family I93 unassigned peptidase inhibitors	Beta vulgaris	I93.UPW	I93
TRINITY_DN11601_c0_g2_i2_5	PF01433.19	Peptidase_M1	MER0412087	alanyl aminopeptidase	Nicotiana benthamiana	M01.005	M01
TRINITY_DN11240_c0_g2_i1_2	PF01433.19	Peptidase_M1	MER0412570	family M1 unassigned peptidases	Solanum tuberosum	M01.UPW	M01
TRINITY_DN3173_c0_g3_i1_1			MER0628627	oligopeptidase A	Populus euphratica	M03.004	M03A
TRINITY_DN3111_c0_g2_i1_2	PF01432.19	Peptidase_M3	MER0817154	subfamily M3A unassigned peptidases	Morus notabilis	M03.UPA	M03A
TRINITY_DN10370_c0_g1_i1_3	PF13646.5	HEAT_2	MER1206231	subfamily M10A unassigned peptidases	Parasteatoda tepidariorum	M10.UPA	M10A
TRINITY_DN8417_c0_g1_i2_2	PF12796.6	Ank_2	MER0263582	family M13 non-peptidase homologues	Pteropus vampyrus	M13.UNW	M13
TRINITY_DN18724_c0_g1_i1_4	PF00069.24	Pkinase	MER1154309	subfamily M14B non-peptidase homologues	Strongylocentrotus purpuratus	M14.UNB	M14B
TRINITY_DN5937_c0_g1_i1_6	PF13620.5	CarboxypepD_reg	MER0469406	subfamily M14B non-peptidase homologues	Brevundimonas sp. BAL3	M14.UNB	M14B
TRINITY_DN18164_c0_g1_i1_4	PF05193.20	Peptidase_M16_C	MER0511546	subfamily M16A non-peptidase homologues	Prunus mume	M16.UNA	M16A
TRINITY_DN10095_c0_g1_i1_3	PF00675.19	Peptidase_M16	MER0922066	subfamily M16B non-peptidase homologues	Glycine soja	M16.UNB	M16B
TRINITY_DN17262_c0_g1_i1_3	PF00675.19	Peptidase_M16	MER0923642	subfamily M16B non-peptidase homologues	Citrus clementina	M16.UNB	M16B
TRINITY_DN17228_c0_g1_i1_3			MER0685626	subfamily M16A unassigned peptidases	Rosa chinensis	M16.UPA	M16A
TRINITY_DN19686_c0_g1_i1_5	PF00675.19	Peptidase_M16	MER0674993	subfamily M16A unassigned peptidases	Juglans regia	M16.UPA	M16A
TRINITY_DN11427_c0_g1_i3_2	PF05193.20	Peptidase_M16_C	MER0922059	subfamily M16B unassigned peptidases	Medicago truncatula	M16.UPB	M16B
TRINITY_DN15269_c0_g1_i1_3	PF00675.19	Peptidase_M16	MER0921599	subfamily M16B unassigned peptidases	Phaseolus vulgaris	M16.UPB	M16B
TRINITY_DN11446_c0_g1_i1_2	PF05193.20	Peptidase_M16_C	MER0504856	subfamily M16C unassigned peptidases	Prunus mume	M16.UPC	M16C
TRINITY_DN16500_c0_g1_i1_3			MER0179067	subfamily M16C unassigned peptidases	Ricinus communis	M16.UPC	M16C
TRINITY_DN11191_c0_g1_i2_6	PF00883.20	Peptidase_M17	MER0659646	leucyl aminopeptidase	Eucalyptus grandis	M17.002	M17
TRINITY_DN11579_c0_g1_i2_1	PF01592.15	NifU_N	MER0474067	PepB aminopeptidase	Ceratitis capitata	M17.004	M17
TRINITY_DN2292_c0_g3_i1_5	PF00428.18	Ribosomal_60s	MER0890057	family M18 unassigned peptidases	Lachancea quebecensis	M18.UPW	M18
TRINITY_DN9714_c0_g1_i1_1	PF02127.14	Peptidase_M18	MER0901680	family M18 unassigned peptidases	Juglans regia	M18.UPW	M18
TRINITY_DN1790_c0_g1_i1_2			MER0627913	subfamily M20A non-peptidase homologues	Populus euphratica	M20.UNA	M20A

TRINITY_DN8163_c0_g1_i1_1	PF01546.27	Peptidase_M20	MER0628810	subfamily M20A unassigned peptidases	Populus euphratica	M20.UPA	M20A
TRINITY_DN9076_c0_g2_i1_2	PF01546.27	Peptidase_M20	MER0015308	subfamily M20A unassigned peptidases	Arabidopsis thaliana	M20.UPA	M20A
TRINITY_DN8497_c0_g1_i1_2	PF01546.27	Peptidase_M20	MER0511719	family M20D unassigned peptidases	Prunus mume	M20.UPD	M20D
TRINITY_DN6447_c0_g1_i1_4	PF01546.27	Peptidase_M20	MER0621877	family M20 unassigned peptidases	Vitis vinifera	M20.UPW	M20X
TRINITY_DN9756_c0_g1_i3_1	PF01546.27	Peptidase_M20	MER0622142	family M20 unassigned peptidases	Vitis vinifera	M20.UPW	M20X
TRINITY_DN3078_c0_g1_i1_3	PF00557.23	Peptidase_M24	MER0180027	methionyl aminopeptidase 1	Ricinus communis	M24.001	M24A
TRINITY_DN10562_c0_g1_i5_3	PF00557.23	Peptidase_M24	MER0189826	Xaa-Pro dipeptidase	Glycine max	M24.007	M24B
TRINITY_DN4354_c0_g1_i1_4	PF15801.4	zf-C6H2	MER0161105	methionyl aminopeptidase 1	Drosophila simulans	M24.017	M24A
TRINITY_DN4347_c0_g1_i1_6	PF01321.17	Creatinase_N	MER0498075	At4g36760 g.p.	Prunus mume	M24.037	M24B
TRINITY_DN10807_c0_g1_i5_1	PF00557.23	Peptidase_M24	MER0093675	proliferation-association protein 1	Ammopiptanthus mongolicus	M24.973	M24X
TRINITY_DN3498_c0_g1_i1_3	PF00557.23	Peptidase_M24	MER0176444	proliferation-association protein 1	Ricinus communis	M24.973	M24X
TRINITY_DN10361_c1_g2_i1_1	PF03868.14	Ribosomal_L6e_N	MER0512373	subfamily M24B unassigned peptidases	Saimiri boliviensis	M24.UPB	M24B
TRINITY_DN10218_c0_g1_i2_2	PF05450.14	Nicastrin	MER0622161	family M28 non-peptidase homologues	Vitis vinifera	M28.UNW	M28X
TRINITY_DN13262_c0_g1_i1_3	PF01979.19	Amidohydro_1	MER0042999	urease	Arabidopsis thaliana	M38.982	M38
TRINITY_DN20109_c0_g1_i1_4	PF00699.19	Urease_beta	MER0004826	urease	Klebsiella aerogenes	M38.982	M38
TRINITY_DN11049_c0_g1_i2_6	PF01979.19	Amidohydro_1	MER0585097	family M38 non-peptidase homologues	Glycine max	M38.UNW	M38
TRINITY_DN19298_c0_g1_i1_1	PF01979.19	Amidohydro_1	MER0859048	family M38 unassigned peptidases	Citrus clementina	M38.UPW	M38
TRINITY_DN1050_c0_g1_i1_3	PF00004.28	AAA	MER0412267	family M41 non-peptidase homologues	Nicotiana benthamiana	M41.UNW	M41
TRINITY_DN10875_c0_g1_i2_2	PF00004.28	AAA	MER0258487	family M41 non-peptidase homologues	Loxodonta africana	M41.UNW	M41
TRINITY_DN11412_c0_g1_i3_3	PF00004.28	AAA	MER0413614	family M41 non-peptidase homologues	Solanum lycopersicum	M41.UNW	M41
TRINITY_DN11515_c0_g1_i3_1	PF00004.28	AAA	MER0278187	family M41 non-peptidase homologues	Glossina morsitans	M41.UNW	M41
TRINITY_DN11618_c0_g1_i1_2	PF02359.17	CDC48_N	MER0413614	family M41 non-peptidase homologues	Solanum lycopersicum	M41.UNW	M41
TRINITY_DN19006_c0_g1_i1_5	PF02933.16	CDC48_2	MER0273571	family M41 non-peptidase homologues	Paenibacillus mucilaginosus	M41.UNW	M41
TRINITY_DN4382_c0_g1_i1_1	PF00004.28	AAA	MER0278187	family M41 non-peptidase homologues	Glossina morsitans	M41.UNW	M41

TRINITY_DN7324_c0_g1_i1_3	PF00004.28	AAA	MER0412267	family M41 non-peptidase homologues	Nicotiana benthamiana	M41.UNW	M41
TRINITY_DN7885_c0_g1_i8_1	PF00004.28	AAA	MER0278187	family M41 non-peptidase homologues	Glossina morsitans	M41.UNW	M41
TRINITY_DN8933_c1_g1_i1_2	PF00004.28	AAA	MER0412130	family M41 non-peptidase homologues	Nicotiana benthamiana	M41.UNW	M41
TRINITY_DN9788_c0_g1_i1_3	PF00004.28	AAA	MER0278187	family M41 non-peptidase homologues	Glossina morsitans	M41.UNW	M41
TRINITY_DN9826_c0_g1_i1_3	PF00004.28	AAA	MER0278187	family M41 non-peptidase homologues	Glossina morsitans	M41.UNW	M41
TRINITY_DN7466_c0_g3_i1_3	PF01434.17	Peptidase_M41	MER0659563	family M41 unassigned peptidases	Eucalyptus grandis	M41.UPW	M41
TRINITY_DN9825_c0_g1_i1_3	PF01434.17	Peptidase_M41	MER0369857	family M41 unassigned peptidases	Cucumis sativus	M41.UPW	M41
TRINITY_DN9943_c0_g1_i2_6	PF06480.14	FtsH_ext	MER0511566	family M41 unassigned peptidases	Prunus mume	M41.UPW	M41
TRINITY_DN10995_c0_g2_i4_3	PF16491.4	Peptidase_M48_N	MER0171229	farnesylated-protein converting enzyme 1	Ricinus communis	M48.003	M48A
TRINITY_DN4227_c0_g1_i1_3	PF01435.17	Peptidase_M48	MER0570784	At5g51740	Cucumis sativus	M48.A01	M48C
TRINITY_DN1809_c0_g1_i1_3	PF03571.14	Peptidase_M49	MER0627730	family M49 unassigned peptidases	Populus euphratica	M49.UPW	M49
TRINITY_DN10628_c0_g1_i1_3	PF00571.27	CBS	MER0236128	subfamily M50B non-peptidase homologues	Thermus scotoductus	M50.UNB	M50B
TRINITY_DN17153_c0_g1_i1_1			MER0270354	subfamily M50B non-peptidase homologues	Ailuropoda melanoleuca	M50.UNB	M50B
TRINITY_DN8157_c0_g1_i1_3	PF00571.27	CBS	MER0231653	family M50 non-peptidase homologues	Desulfovibrio aespoeensis	M50.UNW	M50
TRINITY_DN8158_c0_g1_i1_1	PF01398.20	JAB	MER0637000	Csn5 peptidase	Nicotiana sylvestris	M67.002	M67A
TRINITY_DN10774_c0_g1_i1_1	PF01398.20	JAB	MER0570062	Mername-AA168 protein	Cucumis sativus	M67.971	M67X
TRINITY_DN11397_c0_g1_i5_2	PF13012.5	MitMem_reg	MER0571370	eukaryotic translation initiation factor 3 subunit F	Cucumis melo	M67.974	M67X
TRINITY_DN4385_c0_g1_i1_2	PF11543.7	UN_NPL4	MER0393371	subfamily M67A non-peptidase homologues	Dictyostelium fasciculatum	M67.UNA	M67A
TRINITY_DN6126_c0_g1_i2_6	PF01398.20	JAB	MER0621841	family M67 non-peptidase homologues	Vitis vinifera	M67.UNW	M67X
TRINITY_DN11353_c0_g1_i2_3	PF01398.20	JAB	MER0905581	subfamily M67A unassigned peptidases	Gossypium raimondii	M67.UPA	M67A
TRINITY_DN8579_c0_g2_i1_1	PF00562.27	RNA_pol_Rpb2_6	MER0195833	family N11 unassigned peptide lyases		N11.UPW	N11
TRINITY_DN19832_c0_g1_i1_3			MER0217205	Nma111 peptidase	Vitis vinifera	S01.434	S01D
TRINITY_DN4033_c0_g2_i1_3	PF12812.6	PDZ_1	MER0217205	Nma111 peptidase	Vitis vinifera	S01.434	S01D

TRINITY_DN10701_c0_g1_i2_1	PF00333.19	Ribosomal_S5	MER0486762	subfamily S1A unassigned peptidases	Nomascus leucogenys	S01.UPA	S01A
TRINITY_DN12510_c0_g1_i1_1	PF13964.5	Kelch_6	MER0366613	subfamily S1A unassigned peptidases	Bos taurus	S01.UPA	S01A
TRINITY_DN9813_c0_g1_i1_2	PF00565.16	SNase	MER0573000	subfamily S1A unassigned peptidases	Corvus brachyrhynchos	S01.UPA	S01A
TRINITY_DN4739_c0_g2_i1_6	PF13365.5	Trypsin_2	MER0412287	subfamily S1C unassigned peptidases	Nicotiana benthamiana	S01.UPC	S01C
TRINITY_DN3347_c0_g1_i1_2	PF00082.21	Peptidase_S8	MER0570101	ARA12 peptidase	Citrus sinensis	S08.112	S08A
TRINITY_DN10388_c0_g2_i6_4	PF00082.21	Peptidase_S8	MER0511553	AIR3 peptidase	Prunus mume	S08.119	S08A
TRINITY_DN10388_c0_g2_i5_4	PF00082.21	Peptidase_S8	MER0511553	AIR3 peptidase	Prunus mume	S08.119	S08A
TRINITY_DN53_c0_g1_i1_5	PF00082.21	Peptidase_S8	MER0551416	AIR3 peptidase	Malus domestica	S08.119	S08A
TRINITY_DN10388_c0_g2_i1_5	PF00082.21	Peptidase_S8	MER0550217	At5g59810	Malus domestica	S08.A26	S08A
TRINITY_DN10903_c0_g1_i6_2	PF00082.21	Peptidase_S8	MER0039101	At1g32980	Arachis hypogaea	S08.A31	S08A
TRINITY_DN10961_c0_g1_i3_3			MER0039101	At1g32980	Arachis hypogaea	S08.A31	S08A
TRINITY_DN9173_c0_g1_i1_4	PF00082.21	Peptidase_S8	MER0039101	At1g32980	Arachis hypogaea	S08.A31	S08A
TRINITY_DN12081_c0_g1_i1_6	PF13540.5	RCC1_2	MER0987837	subfamily S8A non-peptidase homologues	Actinokineospora spheciospongiae	S08.UNA	S08A
TRINITY_DN23044_c0_g1_i1_1			MER0978777	subfamily S8A non-peptidase homologues	Gossypium arboreum	S08.UNA	S08A
TRINITY_DN10039_c0_g1_i2_2			MER0600777	subfamily S8A unassigned peptidases	Zea mays	S08.UPA	S08A
TRINITY_DN12695_c0_g2_i1_3			MER0622014	subfamily S8A unassigned peptidases	Vitis vinifera	S08.UPA	S08A
TRINITY_DN18123_c0_g2_i1_2			MER0535213	subfamily S8A unassigned peptidases	Sesamum indicum	S08.UPA	S08A
TRINITY_DN8107_c0_g1_i1_3	PF00082.21	Peptidase_S8	MER0989878	subfamily S8A unassigned peptidases	Citrus clementina	S08.UPA	S08A
TRINITY_DN9297_c0_g3_i2_1			MER0571725	subfamily S8A unassigned peptidases	Cucumis melo	S08.UPA	S08A
TRINITY_DN9993_c0_g1_i2_4	PF00082.21	Peptidase_S8	MER0544355	subfamily S8A unassigned peptidases	Glycine max	S08.UPA	S08A
TRINITY_DN9993_c0_g1_i4_4			MER0550407	subfamily S8A unassigned peptidases	Malus domestica	S08.UPA	S08A
TRINITY_DN10694_c0_g1_i1_2	PF00756.19	Esterase	MER0622192	S-formylglutathione hydrolase FrmB	Vitis vinifera	S09.940	S09B
TRINITY_DN10196_c0_g1_i1_1	PF07859.12	Abhydrolase_3	MER0500863	At5g62180	Prunus mume	S09.A14	S09X
TRINITY_DN8778_c0_g1_i2_2	PF12146.7	Hydrolase_4	MER0499110	At3g47560	Prunus mume	S09.A31	S09X
TRINITY_DN8570_c0_g1_i2_1	PF02230.15	Abhydrolase_2	MER0209135	AT5G20060 protein	Ricinus communis	S09.A56	S09X

TRINITY_DN7057_c1_g1_i1_2	PF07859.12	Abhydrolase_3	MER0036044	F16b3.4 protein	Arabidopsis thaliana	S09.A62	S09X
TRINITY_DN10509_c0_g1_i1_3	PF01738.17	DLH	MER0210195	family S9 non-peptidase homologues	Vitis vinifera	S09.UNW	S09X
TRINITY_DN10693_c1_g1_i1_3	PF00400.31	WD40	MER0137389	family S9 non-peptidase homologues	Ciona savignyi	S09.UNW	S09X
TRINITY_DN10708_c0_g1_i1_2	PF00400.31	WD40	MER0156586	family S9 non-peptidase homologues	Dipodomys ordii	S09.UNW	S09X
TRINITY_DN10708_c1_g1_i1_1	PF00400.31	WD40	MER0146540	family S9 non-peptidase homologues	Ciona savignyi	S09.UNW	S09X
TRINITY_DN11227_c0_g1_i5_5	PF01738.17	DLH	MER0092863	family S9 non-peptidase homologues	Polynucleobacter necessarius	S09.UNW	S09X
TRINITY_DN11355_c0_g1_i2_1	PF00400.31	WD40	MER0057464	family S9 non-peptidase homologues	Thermobispora bispora	S09.UNW	S09X
TRINITY_DN11433_c0_g1_i2_1	PF00400.31	WD40	MER0156555	family S9 non-peptidase homologues	Dipodomys ordii	S09.UNW	S09X
TRINITY_DN11500_c0_g1_i1_3	PF00400.31	WD40	MER0156586	family S9 non-peptidase homologues	Dipodomys ordii	S09.UNW	S09X
TRINITY_DN16390_c0_g1_i1_1	PF00400.31	WD40	MER0156766	family S9 non-peptidase homologues	Dipodomys ordii	S09.UNW	S09X
TRINITY_DN2267_c0_g1_i1_5	PF00400.31	WD40	MER0157926	family S9 non-peptidase homologues	Dipodomys ordii	S09.UNW	S09X
TRINITY_DN2864_c0_g1_i1_4	PF00400.31	WD40	MER0156766	family S9 non-peptidase homologues	Dipodomys ordii	S09.UNW	S09X
TRINITY_DN3258_c0_g1_i1_2	PF00400.31	WD40	MER0136116	family S9 non-peptidase homologues	Batrachochytrium dendrobatidis	S09.UNW	S09X
TRINITY_DN4661_c0_g1_i3_3	PF00400.31	WD40	MER0145804	family S9 non-peptidase homologues	Ciona savignyi	S09.UNW	S09X
TRINITY_DN5067_c0_g1_i1_3	PF00400.31	WD40	MER0183518	family S9 non-peptidase homologues	Ochotona princeps	S09.UNW	S09X
TRINITY_DN6356_c0_g1_i1_3	PF00400.31	WD40	MER0183170	family S9 non-peptidase homologues	Ochotona princeps	S09.UNW	S09X
TRINITY_DN6371_c0_g1_i2_5			MER0159213	family S9 non-peptidase homologues	Nostoc sp. PCC 7120	S09.UNW	S09X
TRINITY_DN709_c0_g2_i1_2	PF00400.31	WD40	MER0135310	family S9 non-peptidase homologues	Batrachochytrium dendrobatidis	S09.UNW	S09X
TRINITY_DN8751_c1_g1_i1_3	PF00400.31	WD40	MER0157482	family S9 non-peptidase homologues	Dipodomys ordii	S09.UNW	S09X
TRINITY_DN9170_c0_g1_i1_1	PF01738.17	DLH	MER0209990	family S9 non-peptidase homologues	Vitis vinifera	S09.UNW	S09X
TRINITY_DN9218_c0_g1_i7_3	PF08606.10	Prp19	MER0137389	family S9 non-peptidase homologues	Ciona savignyi	S09.UNW	S09X
TRINITY_DN2451_c0_g1_i1_2	PF00326.20	Peptidase_S9	MER0413191	subfamily S9A unassigned peptidases	Solanum lycopersicum	S09.UPA	S09A

TRINITY_DN10971_c0_g1_i2_2	PF07676.11	PD40	MER0404572	subfamily S9B unassigned peptidases	Nectria haematococca	S09.UPB	S09B
TRINITY_DN3963_c0_g1_i1_1	PF00326.20	Peptidase_S9	MER0650926	subfamily S9B unassigned peptidases	Jatropha curcas	S09.UPB	S09B
TRINITY_DN11503_c0_g1_i1_1	PF07859.12	Abhydrolase_3	MER0588552	subfamily S9C unassigned peptidases	Citrus sinensis	S09.UPC	S09C
TRINITY_DN11503_c0_g1_i5_1	PF07859.12	Abhydrolase_3	MER0588552	subfamily S9C unassigned peptidases	Citrus sinensis	S09.UPC	S09C
TRINITY_DN8311_c0_g1_i1_6	PF07859.12	Abhydrolase_3	MER0659141	subfamily S9C unassigned peptidases	Eucalyptus grandis	S09.UPC	S09C
TRINITY_DN11588_c0_g1_i3_2	PF00400.31	WD40	MER0156515	family S9 unassigned peptidases	Dipodomys ordii	S09.UPW	S09X
TRINITY_DN6770_c0_g1_i2_3	PF12697.6	Abhydrolase_6	MER0650695	family S9 unassigned peptidases	Jatropha curcas	S09.UPW	S09X
TRINITY_DN8280_c0_g2_i1_2	PF02230.15	Abhydrolase_2	MER0588084	family S9 unassigned peptidases	Citrus sinensis	S09.UPW	S09X
TRINITY_DN17892_c1_g1_i1_5	PF00450.21	Peptidase_S10	MER0660349	serine carboxypeptidase C	Eucalyptus grandis	S10.004	S10
TRINITY_DN21541_c0_g1_i1_4	PF00450.21	Peptidase_S10	MER0581883	serine carboxypeptidase C		S10.004	S10
TRINITY_DN10461_c0_g1_i3_3	PF00450.21	Peptidase_S10	MER0525671	serine carboxypeptidase III	Malus domestica	S10.009	S10
TRINITY_DN2711_c0_g1_i1_3	PF00450.21	Peptidase_S10	MER0637468	OsBISCPL1-type putative carboxypeptidase	Nicotiana sylvestris	S10.017	S10
TRINITY_DN9708_c0_g1_i1_2	PF00450.21	Peptidase_S10	MER0581564	At4g30810		S10.A32	S10
TRINITY_DN22082_c0_g1_i1_3	PF00450.21	Peptidase_S10	MER0629038	At3g63470	Populus euphratica	S10.A41	S10
TRINITY_DN2708_c0_g2_i1_1	PF00450.21	Peptidase_S10	MER0627870	At3g63470	Populus euphratica	S10.A41	S10
TRINITY_DN14006_c0_g1_i1_3	PF00109.25	ketoacyl-synt	MER0947272	family S10 unassigned peptidases	Brassica napus	S10.UPW	S10
TRINITY_DN17601_c0_g1_i1_6	PF00109.25	ketoacyl-synt	MER0947272	family S10 unassigned peptidases	Brassica napus	S10.UPW	S10
TRINITY_DN10262_c0_g2_i1_1	PF00574.22	CLP_protease	MER0639468	peptidase Clp	Nelumbo nucifera	S14.001	S14
TRINITY_DN17662_c0_g2_i1_1	PF00574.22	CLP_protease	MER0663832	ClpP4 peptidase	Elaeis guineensis	S14.010	S14
TRINITY_DN10960_c0_g1_i1_3	PF00574.22	CLP_protease	MER0171106	At5g23140	Ricinus communis	S14.A02	S14
TRINITY_DN9518_c0_g2_i2_1	PF00574.22	CLP_protease	MER0588742	At5g23140	Citrus sinensis	S14.A02	S14
TRINITY_DN8532_c0_g1_i1_2	PF00574.22	CLP_protease	MER1014788	family S14 non-peptidase homologues	Morus notabilis	S14.UNW	S14
TRINITY_DN128_c0_g1_i1_1	PF00240.22	ubiquitin	MER0053503	family S16 unassigned peptidases	Pan troglodytes	S16.UPW	S16
TRINITY_DN10594_c0_g1_i4_3	PF00717.22	Peptidase_S24	MER0232593	signalase	Glycine max	S26.010	S26B
TRINITY_DN7152_c0_g1_i2_3			MER0411784	subfamily S26A non-peptidase homologues	Nicotiana benthamiana	S26.UNA	S26A
TRINITY_DN9538_c0_g1_i1_2	PF05577.11	Peptidase_S28	MER0143936	AT5g65760	Vitis vinifera	S28.A02	S28
TRINITY_DN10611_c0_g2_i1_3	PF05577.11	Peptidase_S28	MER1054347	family S28 unassigned peptidases	Theobroma cacao	S28.UPW	S28

TRINITY_DN11102_c0_g1_i4_6	PF00561.19	Abhydrolase_1	MER0511511	family S33 non-peptidase homologues	Prunus mume	S33.UNW	S33
TRINITY_DN11102_c1_g1_i1_1			MER0511511	family S33 non-peptidase homologues	Prunus mume	S33.UNW	S33
TRINITY_DN7295_c0_g1_i1_1	PF00155.20	Aminotran_1_2	MER1363070	family S33 non-peptidase homologues	Thecamonas trahens	S33.UNW	S33
TRINITY_DN8211_c0_g1_i2_3	PF00561.19	Abhydrolase_1	MER0584079	family S33 non-peptidase homologues	Cicer arietinum	S33.UNW	S33
TRINITY_DN9475_c0_g1_i1_3	PF16113.4	ECH_2	MER0663876	family S33 non-peptidase homologues	Elaeis guineensis	S33.UNW	S33
TRINITY_DN17041_c0_g1_i1_1	PF12146.7	Hydrolase_4	MER0660114	family S33 unassigned peptidases	Eucalyptus grandis	S33.UPW	S33
TRINITY_DN20857_c0_g1_i1_2	PF13419.5	HAD_2	MER0230625	family S33 unassigned peptidases	Micromonospora sp. L5	S33.UPW	S33
TRINITY_DN2750_c0_g2_i1_2	PF13419.5	HAD_2	MER0230625	family S33 unassigned peptidases	Micromonospora sp. L5	S33.UPW	S33
TRINITY_DN5337_c0_g1_i1_4	PF00561.19	Abhydrolase_1	MER0577056	family S33 unassigned peptidases		S33.UPW	S33
TRINITY_DN5779_c0_g1_i1_2	PF00561.19	Abhydrolase_1	MER0588391	family S33 unassigned peptidases	Citrus sinensis	S33.UPW	S33
TRINITY_DN8864_c0_g1_i1_1	PF00561.19	Abhydrolase_1	MER0152083	family S33 unassigned peptidases	Desulfatibacillum alkenivorans	S33.UPW	S33
TRINITY_DN9585_c0_g1_i1_2	PF00561.19	Abhydrolase_1	MER1334561	family S33 unassigned peptidases	Juglans regia	S33.UPW	S33
TRINITY_DN10216_c0_g1_i1_3	PF00378.19	ECH_1	MER1072352	subfamily S49C non-peptidase homologues	Thermus thermophilus	S49.UNC	S49C
TRINITY_DN11585_c0_g1_i2_3	PF00378.19	ECH_1	MER1073240	subfamily S49C non-peptidase homologues	Acinetobacter bohemicus	S49.UNC	S49C
TRINITY_DN7857_c0_g1_i4_2	PF00378.19	ECH_1	MER1073240	subfamily S49C non-peptidase homologues	Acinetobacter bohemicus	S49.UNC	S49C
TRINITY_DN9598_c0_g1_i1_1	PF00378.19	ECH_1	MER1073240	subfamily S49C non-peptidase homologues	Acinetobacter bohemicus	S49.UNC	S49C
TRINITY_DN7535_c0_g2_i1_1	PF01694.21	Rhomboid	MER0578051	RBL2 peptidase		S54.015	S54
TRINITY_DN11173_c0_g2_i3_4	PF00153.26	Mito_carr	MER1363300	family S54 non-peptidase homologues	Thecamonas trahens	S54.UNW	S54
TRINITY_DN11173_c0_g3_i1_4	PF00153.26	Mito_carr	MER1363300	family S54 non-peptidase homologues	Thecamonas trahens	S54.UNW	S54
TRINITY_DN11525_c0_g1_i1_2	PF00153.26	Mito_carr	MER1363300	family S54 non-peptidase homologues	Thecamonas trahens	S54.UNW	S54
TRINITY_DN17861_c0_g1_i1_6	PF00153.26	Mito_carr	MER1363300	family S54 non-peptidase homologues	Thecamonas trahens	S54.UNW	S54
TRINITY_DN9097_c0_g1_i1_2	PF00153.26	Mito_carr	MER1363300	family S54 non-peptidase homologues	Thecamonas trahens	S54.UNW	S54
TRINITY_DN6573_c0_g1_i1_4	PF01694.21	Rhomboid	MER1081552	family S54 unassigned peptidases	Brassica oleracea	S54.UPW	S54

TRINITY_DN10750_c0_g1_i1_2	PF00227.25	Proteasome	MER0172841	proteasome subunit beta1c	Ricinus communis	T01.010	T01A
TRINITY_DN11620_c0_g1_i1_2	PF10584.8	Proteasome_A_N	MER0411684	proteasome subunit alpha 6	Nicotiana benthamiana	T01.971	T01A
TRINITY_DN6332_c0_g2_i1_3	PF10584.8	Proteasome_A_N	MER0570712	proteasome subunit alpha 2	Cucumis sativus	T01.972	T01A
TRINITY_DN3942_c0_g1_i3_3	PF10584.8	Proteasome_A_N	MER0180218	proteasome subunit alpha 1	Ricinus communis	T01.976	T01A
TRINITY_DN11086_c0_g1_i1_1	PF10584.8	Proteasome_A_N	MER0505546	Mername-AA242 peptidase homologue	Prunus mume	T01.995	T01A
TRINITY_DN10558_c0_g1_i2_4	PF00227.25	Proteasome	MER0173164	proteasome subunit beta2	Ricinus communis	T01.A02	T01A
TRINITY_DN9582_c0_g2_i1_5	PF00227.25	Proteasome	MER0126199	PBE2 g.p.	Vitis vinifera	T01.A10	T01A
TRINITY_DN11422_c0_g1_i2_2	PF00227.25	Proteasome	MER1088634	subfamily T1A non-peptidase homologues	Gossypium raimondii	T01.UNA	T01A
TRINITY_DN3481_c0_g1_i1_6	PF00227.25	Proteasome	MER0570686	subfamily T1A non-peptidase homologues	Cucumis sativus	T01.UNA	T01A
TRINITY_DN5812_c0_g1_i1_1	PF00227.25	Proteasome	MER1088605	subfamily T1A non-peptidase homologues	Theobroma cacao	T01.UNA	T01A
TRINITY_DN11283_c0_g1_i3_1	PF10584.8	Proteasome_A_N	MER0576836	subfamily T1A unassigned peptidases		T01.UPA	T01A
TRINITY_DN3139_c0_g1_i1_2	PF00227.25	Proteasome	MER0584369	subfamily T1A unassigned peptidases		T01.UPA	T01A
TRINITY_DN9816_c0_g1_i2_1	PF00227.25	Proteasome	MER0538291	subfamily T1A unassigned peptidases	Sesamum indicum	T01.UPA	T01A
TRINITY_DN11439_c0_g1_i3_4	PF00227.25	Proteasome	MER0645901	family T1 unassigned peptidases	Jatropha curcas	T01.UPW	T01X
TRINITY_DN11439_c0_g1_i2_4	PF00227.25	Proteasome	MER0645901	family T1 unassigned peptidases	Jatropha curcas	T01.UPW	T01X
TRINITY_DN9206_c0_g2_i1_1	PF01112.17	Asparaginase_2	MER0179633	family T2 unassigned peptidases	Ricinus communis	T02.UPW	T02
TRINITY_DN10152_c0_g1_i1_1	PF03297.14	Ribosomal_S25	MER1365022	family T3 non-peptidase homologues	Thecamonas trahens	T03.UNW	T03
TRINITY_DN10152_c0_g1_i1_3			MER1365022	family T3 non-peptidase homologues	Thecamonas trahens	T03.UNW	T03
TRINITY_DN3322_c0_g2_i1_2	PF01960.17	ArgJ	MER0122168	ArgJ protein	Vitis vinifera	T05.002	T05
TRINITY_DN10088_c0_g2_i1_3	PF14226.5	DIOX_N	MER0576457	family T7 unassigned peptidases		T07.UPW	T07
TRINITY_DN11452_c0_g2_i2_2	PF14226.5	DIOX_N	MER0576457	family T7 unassigned peptidases		T07.UPW	T07
TRINITY_DN4171_c0_g2_i2_3	PF14226.5	DIOX_N	MER0576457	family T7 unassigned peptidases		T07.UPW	T07
TRINITY_DN8889_c0_g1_i1_1	PF14226.5	DIOX_N	MER0576457	family T7 unassigned peptidases		T07.UPW	T07
TRINITY_DN6342_c0_g1_i2_3	PF00155.20	Aminotran_1_2	MER0501945	family U32 unassigned peptidases	Chthonomonas calidirosea	U32.UPW	U32
TRINITY_DN2392_c0_g1_i1_2			MER0901042	family U74 unassigned peptidases	Gossypium arboreum	U74.UPW	U74
