# Supplementary material for: Comparative proteomics of common allergenic tree pollens of birch, alder, and hazel
Source: Allergy. 2021 Jan 15;76(6):1743–53. doi: 10.1111/all.14694 (PMC8248232; doi:10.1111/all.14694)
Supplement: Supplementary file 18 — Table S16 [file ALL-76-1743-s004.pdf]

Supplementary Table S13: Water soluble peptidases of Betula pollen

Protein IDs	Pfam accession	Pfam family name	Merops accession	Merops family	Merops peptidase ID	Merops subfamily
TRINITY_DN19058_c2_g1::TRINITY_DN19058_c2_g1_i7::g.111114::m.111114	PF14543.5	TAXi_N	MER0372282	CDR1 peptidase	A01.069	A01B
TRINITY_DN13851_c1_g1::TRINITY_DN13851_c1_g1_i8::g.29304::m.29304	PF00026.22	Asp	MER0680933	At1g62290	A01.A02	A01A
TRINITY_DN15082_c2_g5::TRINITY_DN15082_c2_g5_i1::g.46709::m.46709	PF14543.5	TAXi_N	MER0106023	At3g12700	A01.A30	A01B
TRINITY_DN13851_c1_g2::TRINITY_DN13851_c1_g2_i2::g.29292::m.29292	PF00026.22	Asp	MER1131201	subfamily A1A unassigned peptidases	A01.UPA	A01A
TRINITY_DN15036_c1_g1::TRINITY_DN15036_c1_g1_i9::g.46235::m.46235	PF00188.25	CAP	MER0228949	subfamily A1A unassigned peptidases	A01.UPA	A01A
TRINITY_DN19846_c6_g2::TRINITY_DN19846_c6_g2_i1::g.124494::m.124494	PF14543.5	TAXi_N	MER0250417	subfamily A1A unassigned peptidases	A01.UPA	A01A
TRINITY_DN19962_c5_g2::TRINITY_DN19962_c5_g2_i2::g.126940::m.126940	PF01095.18	Pectinesterase	MER0570148	family A2 unassigned peptidases	A02.UPW	A02X
TRINITY_DN19187_c0_g2::TRINITY_DN19187_c0_g2_i3::g.113547::m.113547	PF00627.30	UBA	MER0628276	subfamily A28A unassigned peptidases	A28.UPA	A28A
TRINITY_DN19587_c1_g2::TRINITY_DN19587_c1_g2_i1::g.120316::m.120316	PF00240.22	ubiquitin	MER0242394	subfamily A28A unassigned peptidases	A28.UPA	A28A
TRINITY_DN18320_c3_g1::TRINITY_DN18320_c3_g1_i1::g.98804::m.98804	PF00112.22	Peptidase_C1	MER0640046	cathepsin B, plant form	C01.049	C01A
TRINITY_DN10140_c0_g1::TRINITY_DN10140_c0_g1_i2::g.4317::m.4317	PF00112.22	Peptidase_C1	MER1161383	subfamily C1A unassigned peptidases	C01.UPA	C01A
TRINITY_DN13376_c2_g2::TRINITY_DN13376_c2_g2_i3::g.22986::m.22986	PF00112.22	Peptidase_C1	MER1161143	subfamily C1A unassigned peptidases	C01.UPA	C01A
TRINITY_DN15205_c1_g1::TRINITY_DN15205_c1_g1_i3::g.48499::m.48499	PF00112.22	Peptidase_C1	MER1160913	subfamily C1A unassigned peptidases	C01.UPA	C01A
TRINITY_DN15924_c1_g1::TRINITY_DN15924_c1_g1_i2::g.59050::m.59050	PF00112.22	Peptidase_C1	MER1161707	subfamily C1A unassigned peptidases	C01.UPA	C01A
TRINITY_DN15963_c2_g1::TRINITY_DN15963_c2_g1_i4::g.59609::m.59609	PF00112.22	Peptidase_C1	MER1164780	subfamily C1A unassigned peptidases	C01.UPA	C01A
TRINITY_DN18149_c0_g1::TRINITY_DN18149_c0_g1_i3::g.95908::m.95908	PF00112.22	Peptidase_C1	MER1164799	subfamily C1A unassigned peptidases	C01.UPA	C01A
TRINITY_DN18157_c0_g1::TRINITY_DN18157_c0_g1_i6::g.96079::m.96079	PF00112.22	Peptidase_C1	MER1161753	subfamily C1A unassigned peptidases	C01.UPA	C01A
TRINITY_DN18947_c6_g2::TRINITY_DN18947_c6_g2_i2::g.109306::m.109306	PF00112.22	Peptidase_C1	MER1161493	subfamily C1A unassigned peptidases	C01.UPA	C01A
TRINITY_DN18947_c6_g5::TRINITY_DN18947_c6_g5_i2::g.109307::m.109307	PF00112.22	Peptidase_C1	MER1162079	subfamily C1A unassigned peptidases	C01.UPA	C01A

TRINITY_DN31964_c0_g1::TRINITY_DN31964_c0_g1_i1::g.130316::m.130316	PF00112.22	Peptidase_C1	MER1161346	subfamily C1A unassigned peptidases	C01.UPA	C01A
TRINITY_DN16618_c2_g1::TRINITY_DN16618_c2_g1_i4::g.70494::m.70494	PF04424.12	MINDY_DUB	MER0934028	FAM63B g.p.	C115.002	C115
TRINITY_DN13485_c2_g2::TRINITY_DN13485_c2_g2_i2::g.24426::m.24426	PF01088.20	Peptidase_C12	MER1170789	family C12 unassigned peptidases	C12.UPW	C12
TRINITY_DN14025_c2_g1::TRINITY_DN14025_c2_g1_i8::g.31522::m.31522	PF13898.5	DUF4205	MER1253126		C121.UNW	C121
TRINITY_DN12352_c0_g2::TRINITY_DN12352_c0_g2_i4::g.12096::m.12096	PF01650.17	Peptidase_C13	MER1173188	family C13 unassigned peptidases	C13.UPW	C13
TRINITY_DN12454_c0_g1::TRINITY_DN12454_c0_g1_i1::g.12984::m.12984	PF00240.22	ubiquitin	MER0109705	UBP6 peptidase	C19.094	C19
TRINITY_DN13524_c0_g1::TRINITY_DN13524_c0_g1_i8::g.24870::m.24870	PF00917.25	MATH	MER0712608	family C19 unassigned peptidases	C19.UPW	C19
TRINITY_DN18543_c1_g3::TRINITY_DN18543_c1_g3_i1::g.101857::m.101857	PF02148.18	zf-UBP	MER0546657	family C19 unassigned peptidases	C19.UPW	C19
TRINITY_DN19613_c2_g2::TRINITY_DN19613_c2_g2_i3::g.120856::m.120856	PF12436.7	USP7_ICP0_bdg	MER0715954	family C19 unassigned peptidases	C19.UPW	C19
TRINITY_DN17699_c0_g2::TRINITY_DN17699_c0_g2_i2::g.88416::m.88416	PF00310.20	GATase_2	MER0511740	glutamate synthase	C44.003	C44
TRINITY_DN13284_c1_g1::TRINITY_DN13284_c1_g1_i7::g.21970::m.21970	PF12481.7	DUF3700	MER0614899	family C44 unassigned peptidases	C44.UPW	C44
TRINITY_DN13734_c2_g1::TRINITY_DN13734_c2_g1_i1::g.27715::m.27715	PF12481.7	DUF3700	MER0571885	family C44 unassigned peptidases	C44.UPW	C44
TRINITY_DN15836_c2_g1::TRINITY_DN15836_c2_g1_i8::g.57209::m.57209	PF13537.5	GATase_7	MER0037116	family C44 unassigned peptidases	C44.UPW	C44
TRINITY_DN13841_c4_g1::TRINITY_DN13841_c4_g1_i7::g.28424::m.28424	PF01965.23	DJ-1_PfpI	MER0891420	family C56 unassigned peptidases	C56.UPW	C56
TRINITY_DN17135_c0_g1::TRINITY_DN17135_c0_g1_i6::g.79192::m.79192	PF07910.12	Peptidase_C78	MER0737213	At3g48380 protein	C78.A02	C78A
TRINITY_DN14972_c1_g2::TRINITY_DN14972_c1_g2_i4::g.44982::m.44982	PF02338.18	OTU	MER0744567	subfamily C85B unassigned peptidases	C85.UPB	C85B
TRINITY_DN11558_c0_g1::TRINITY_DN11558_c0_g1_i1::g.7858::m.7858	PF00197.17	Kunitz_legume	MER0453920	inhibitor MtTi2	I03.030	I03A
TRINITY_DN14021_c1_g2::TRINITY_DN14021_c1_g2_i5::g.31431::m.31431	PF00079.19	Serpin	MER0510912	AtSerpin1	I04.087	I04
TRINITY_DN18255_c1_g1::TRINITY_DN18255_c1_g1_i6::g.97743::m.97743	PF02736.18	Myosin_N	MER1118174	family I4 unassigned peptidase inhibitors	I04.UPW	I04
TRINITY_DN13204_c1_g1::TRINITY_DN13204_c1_g1_i4::g.21099::m.21099	PF05922.15	Inhibitor_I9	MER0628798	family I9 unassigned peptidase inhibitors	I09.UPW	I09

TRINITY_DN12914_c1_g1::TRINITY_DN12914_c1_g1_i4::g.17252::m.17252	PF00403.25	HMA	MER0592272	family I13 unassigned peptidase inhibitors	I13.UPW	I13
TRINITY_DN17501_c0_g2::TRINITY_DN17501_c0_g2_i6::g.84659::m.84659	PF02225.21	PA	MER0642455	family I15 unassigned peptidase inhibitors	I15.UPW	I15
TRINITY_DN12993_c1_g1::TRINITY_DN12993_c1_g1_i9::g.18389::m.18389	PF16845.4	SQAPI	MER0628337	phytocystatin	I25.014	I25B
TRINITY_DN13585_c0_g1::TRINITY_DN13585_c0_g1_i3::g.25706::m.25706			MER0680968	subfamily I25A unassigned peptidase inhibitors	I25.UPA	I25A
TRINITY_DN17227_c2_g4::TRINITY_DN17227_c2_g4_i1::g.80549::m.80549	PF01565.22	FAD_binding_4	MER0659912	family I29 unassigned peptidase inhibitors	I29.UPW	I29
TRINITY_DN19911_c7_g2::TRINITY_DN19911_c7_g2_i3::g.126024::m.126024	PF08031.11	BBE	MER0659912	family I29 unassigned peptidase inhibitors	I29.UPW	I29
TRINITY_DN11854_c0_g1::TRINITY_DN11854_c0_g1_i1::g.9190::m.9190	PF00085.19	Thioredoxin	MER0546787	family I39 unassigned peptidase inhibitors	I39.UPW	I39
TRINITY_DN11049_c0_g1::TRINITY_DN11049_c0_g1_i1::g.6211::m.6211	PF01161.19	PBP	MER0785675	family I51 unassigned peptidase inhibitors	I51.UPW	I51
TRINITY_DN17913_c4_g1::TRINITY_DN17913_c4_g1_i1::g.91856::m.91856	PF03009.16	GDPD	MER0604943	family I71 unassigned peptidase inhibitors	I71.UPW	I71
TRINITY_DN15085_c0_g2::TRINITY_DN15085_c0_g2_i3::g.46604::m.46604			MER0134791	alanyl aminopeptidase	M01.005	M01
TRINITY_DN12654_c0_g3::TRINITY_DN12654_c0_g3_i5::g.14722::m.14722	PF01433.19	Peptidase_M1	MER0336326	family M1 unassigned peptidases	M01.UPW	M01
TRINITY_DN16376_c0_g2::TRINITY_DN16376_c0_g2_i2::g.66583::m.66583	PF01433.19	Peptidase_M1	MER0412570	family M1 unassigned peptidases	M01.UPW	M01
TRINITY_DN18531_c0_g1::TRINITY_DN18531_c0_g1_i4::g.102214::m.102214	PF01433.19	Peptidase_M1	MER0171524	family M1 unassigned peptidases	M01.UPW	M01
TRINITY_DN19263_c0_g1::TRINITY_DN19263_c0_g1_i6::g.114813::m.114813	PF01432.19	Peptidase_M3	MER0546417	oligopeptidase A	M03.004	M03A
TRINITY_DN19263_c0_g3::TRINITY_DN19263_c0_g3_i4::g.114811::m.114811	PF01432.19	Peptidase_M3	MER0511610	oligopeptidase A	M03.004	M03A
TRINITY_DN19263_c0_g1::TRINITY_DN19263_c0_g1_i2::g.114802::m.114802	PF01432.19	Peptidase_M3	MER0818462	subfamily M3A unassigned peptidases	M03.UPA	M03A
TRINITY_DN19263_c0_g3::TRINITY_DN19263_c0_g3_i3::g.114810::m.114810	PF01432.19	Peptidase_M3	MER0820311	subfamily M3A unassigned peptidases	M03.UPA	M03A
TRINITY_DN18434_c0_g1::TRINITY_DN18434_c0_g1_i17::g.100395::m.100395	PF08324.10	PUL	MER0273962	griselysin	M04.017	M04
TRINITY_DN13918_c1_g1::TRINITY_DN13918_c1_g1_i8::g.30345::m.30345	PF01851.21	PC_rep	MER1206231	subfamily M10A unassigned peptidases	M10.UPA	M10A
TRINITY_DN17204_c0_g1::TRINITY_DN17204_c0_g1_i8::g.80281::m.80281	PF00675.19	Peptidase_M16	MER0923033	subfamily M16B unassigned peptidases	M16.UPB	M16B

TRINITY_DN16381_c2_g1::TRINITY_DN16381_c2_g1_i3::g.66693::m.66693	PF05193.20	Peptidase_M16_C	MER0504856	subfamily M16C unassigned peptidases	M16.UPC	M16C
TRINITY_DN15589_c0_g1::TRINITY_DN15589_c0_g1_i4::g.54099::m.54099	PF00883.20	Peptidase_M17	MER0659646	leucyl aminopeptidase	M17.002	M17
TRINITY_DN12445_c0_g2::TRINITY_DN12445_c0_g2_i13::g.12955::m.12955	PF02127.14	Peptidase_M18	MER0900398	family M18 unassigned peptidases	M18.UPW	M18
TRINITY_DN12030_c0_g1::TRINITY_DN12030_c0_g1_i2::g.10080::m.10080	PF01546.27	Peptidase_M20	MER0015308	subfamily M20A unassigned peptidases	M20.UPA	M20A
TRINITY_DN13264_c3_g1::TRINITY_DN13264_c3_g1_i4::g.21666::m.21666	PF01546.27	Peptidase_M20	MER0622135	subfamily M20A unassigned peptidases	M20.UPA	M20A
TRINITY_DN18313_c3_g2::TRINITY_DN18313_c3_g2_i2::g.98848::m.98848	PF01546.27	Peptidase_M20	MER0550674	subfamily M20A unassigned peptidases	M20.UPA	M20A
TRINITY_DN16272_c1_g2::TRINITY_DN16272_c1_g2_i3::g.64808::m.64808	PF01546.27	Peptidase_M20	MER0588973	family M20 unassigned peptidases	M20.UPW	M20X
TRINITY_DN17082_c4_g2::TRINITY_DN17082_c4_g2_i2::g.78169::m.78169	PF08172.11	CASP_C	MER1150565	subfamily M23B unassigned peptidases	M23.UPB	M23B
TRINITY_DN19972_c2_g1::TRINITY_DN19972_c2_g1_i3::g.126916::m.126916	PF00141.22	peroxidase	MER0233833	subfamily M23B unassigned peptidases	M23.UPB	M23B
TRINITY_DN17383_c0_g1::TRINITY_DN17383_c0_g1_i3::g.82754::m.82754	PF00557.23	Peptidase_M24	MER0536976	methionyl aminopeptidase 1	M24.001	M24A
TRINITY_DN16453_c2_g1::TRINITY_DN16453_c2_g1_i14::g.67807::m.67807	PF00557.23	Peptidase_M24	MER0189826	Xaa-Pro dipeptidase	M24.007	M24B
TRINITY_DN18526_c1_g2::TRINITY_DN18526_c1_g2_i7::g.101942::m.101942	PF01321.17	Creatinase_N	MER0650756	At4g36760 g.p.	M24.037	M24B
TRINITY_DN16102_c2_g2::TRINITY_DN16102_c2_g2_i1::g.62195::m.62195			MER0168264	proliferation-association protein 1	M24.973	M24X
TRINITY_DN18982_c0_g1::TRINITY_DN18982_c0_g1_i8::g.109877::m.109877	PF00557.23	Peptidase_M24	MER0176444	proliferation-association protein 1	M24.973	M24X
TRINITY_DN11213_c0_g1::TRINITY_DN11213_c0_g1_i2::g.6642::m.6642	PF03868.14	Ribosomal_L6e_N	MER0512373	subfamily M24B unassigned peptidases	M24.UPB	M24B
TRINITY_DN17307_c3_g1::TRINITY_DN17307_c3_g1_i2::g.81691::m.81691	PF01979.19	Amidohydro_1	MER0622001	family M38 unassigned peptidases	M38.UPW	M38
TRINITY_DN19286_c0_g1::TRINITY_DN19286_c0_g1_i3::g.115330::m.115330	PF00293.27	NUDIX	MER0627730	family M49 unassigned peptidases	M49.UPW	M49
TRINITY_DN17014_c1_g3::TRINITY_DN17014_c1_g3_i2::g.77015::m.77015	PF01398.20	JAB	MER0570062	Mername-AA168 protein	M67.971	M67X
TRINITY_DN11643_c0_g1::TRINITY_DN11643_c0_g1_i1::g.8230::m.8230	PF01398.20	JAB	MER0905581	subfamily M67A unassigned peptidases	M67.UPA	M67A
TRINITY_DN18281_c0_g1::TRINITY_DN18281_c0_g1_i4::g.98025::m.98025	PF04563.14	RNA_pol_Rpb2_1	MER0195833	family N11 unassigned peptide lyases	N11.UPW	N11

TRINITY_DN13916_c2_g1::TRINITY_DN13916_c2_g1_i2::g.30380::m.30380	PF00565.16	SNase	MER0573000	subfamily S1A unassigned peptidases	S01.UPA	S01A
TRINITY_DN16993_c0_g2::TRINITY_DN16993_c0_g2_i2::g.76741::m.76741	PF00082.21	Peptidase_S8	MER0621382	ARA12 peptidase	S08.112	S08A
TRINITY_DN17067_c1_g2::TRINITY_DN17067_c1_g2_i1::g.77892::m.77892	PF00082.21	Peptidase_S8	MER0570101	ARA12 peptidase	S08.112	S08A
TRINITY_DN13334_c3_g4::TRINITY_DN13334_c3_g4_i1::g.22910::m.22910	PF00082.21	Peptidase_S8	MER0551416	AIR3 peptidase	S08.119	S08A
TRINITY_DN19475_c9_g3::TRINITY_DN19475_c9_g3_i3::g.118615::m.118615	PF00082.21	Peptidase_S8	MER0511553	AIR3 peptidase	S08.119	S08A
TRINITY_DN19995_c2_g1::TRINITY_DN19995_c2_g1_i2::g.127478::m.127478	PF00082.21	Peptidase_S8	MER0500511	At4g00230	S08.A14	S08A
TRINITY_DN16581_c1_g3::TRINITY_DN16581_c1_g3_i2::g.69928::m.69928	PF00082.21	Peptidase_S8	MER0511501	At5g59810	S08.A26	S08A
TRINITY_DN13334_c3_g1::TRINITY_DN13334_c3_g1_i2::g.22908::m.22908	PF00082.21	Peptidase_S8	MER0039101	At1g32980	S08.A31	S08A
TRINITY_DN19475_c9_g1::TRINITY_DN19475_c9_g1_i7::g.118616::m.118616	PF00082.21	Peptidase_S8	MER0039101	At1g32980	S08.A31	S08A
TRINITY_DN10710_c0_g1::TRINITY_DN10710_c0_g1_i1::g.5355::m.5355			MER0622172	subfamily S8A unassigned peptidases	S08.UPA	S08A
TRINITY_DN12154_c0_g1::TRINITY_DN12154_c0_g1_i6::g.10864::m.10864			MER0592913	subfamily S8A unassigned peptidases	S08.UPA	S08A
TRINITY_DN15525_c0_g1::TRINITY_DN15525_c0_g1_i8::g.53299::m.53299	PF00082.21	Peptidase_S8	MER0544355	subfamily S8A unassigned peptidases	S08.UPA	S08A
TRINITY_DN17241_c0_g1::TRINITY_DN17241_c0_g1_i1::g.80827::m.80827	PF00082.21	Peptidase_S8	MER0621966	subfamily S8A unassigned peptidases	S08.UPA	S08A
TRINITY_DN18618_c2_g1::TRINITY_DN18618_c2_g1_i14::g.103865::m.103865	PF00082.21	Peptidase_S8	MER0979912	subfamily S8A unassigned peptidases	S08.UPA	S08A
TRINITY_DN18634_c2_g1::TRINITY_DN18634_c2_g1_i4::g.104214::m.104214	PF00082.21	Peptidase_S8	MER0495288	subfamily S8A unassigned peptidases	S08.UPA	S08A
TRINITY_DN19405_c0_g1::TRINITY_DN19405_c0_g1_i2::g.117368::m.117368			MER0527005	subfamily S8A unassigned peptidases	S08.UPA	S08A
TRINITY_DN16996_c2_g1::TRINITY_DN16996_c2_g1_i5::g.76760::m.76760	PF02897.14	Peptidase_S9_N	MER0180801	prolyl oligopeptidase	S09.001	S09A
TRINITY_DN11898_c0_g1::TRINITY_DN11898_c0_g1_i2::g.9380::m.9380	PF00326.20	Peptidase_S9	MER0627651	tyrosyl aminopeptidase	S09.074	S09C
TRINITY_DN12879_c0_g1::TRINITY_DN12879_c0_g1_i1::g.16746::m.16746	PF00756.19	Esterase	MER0622192	S-formylglutathione hydrolase FrmB	S09.940	S09B
TRINITY_DN17454_c3_g1::TRINITY_DN17454_c3_g1_i1::g.84103::m.84103	PF07859.12	Abhydrolase_3	MER0500509	At2g45600	S09.A07	S09X

TRINITY_DN16462_c1_g1::TRINITY_DN16462_c1_g1_i7::g.68028::m.68028	PF02230.15	Abhydrolase_2	MER0209135	AT5G20060 protein	S09.A56	S09X
TRINITY_DN14491_c1_g5::TRINITY_DN14491_c1_g5_i7::g.37912::m.37912	PF00326.20	Peptidase_S9	MER0413191	subfamily S9A unassigned peptidases	S09.UPA	S09A
TRINITY_DN13205_c0_g1::TRINITY_DN13205_c0_g1_i3::g.21053::m.21053	PF07859.12	Abhydrolase_3	MER0588552	subfamily S9C unassigned peptidases	S09.UPC	S09C
TRINITY_DN13729_c0_g1::TRINITY_DN13729_c0_g1_i8::g.27674::m.27674	PF07859.12	Abhydrolase_3	MER0511717	subfamily S9C unassigned peptidases	S09.UPC	S09C
TRINITY_DN15900_c2_g1::TRINITY_DN15900_c2_g1_i1::g.57240::m.57240	PF00326.20	Peptidase_S9	MER1300318	subfamily S9C unassigned peptidases	S09.UPC	S09C
TRINITY_DN17776_c0_g1::TRINITY_DN17776_c0_g1_i2::g.89550::m.89550	PF07859.12	Abhydrolase_3	MER0621913	subfamily S9C unassigned peptidases	S09.UPC	S09C
TRINITY_DN17776_c1_g1::TRINITY_DN17776_c1_g1_i2::g.89554::m.89554	PF07859.12	Abhydrolase_3	MER0621926	subfamily S9C unassigned peptidases	S09.UPC	S09C
TRINITY_DN19388_c2_g2::TRINITY_DN19388_c2_g2_i2::g.117003::m.117003	PF07859.12	Abhydrolase_3	MER0621926	subfamily S9C unassigned peptidases	S09.UPC	S09C
TRINITY_DN15072_c0_g1::TRINITY_DN15072_c0_g1_i2::g.46447::m.46447	PF00400.31	WD40	MER0156515	family S9 unassigned peptidases	S09.UPW	S09X
TRINITY_DN16577_c0_g1::TRINITY_DN16577_c0_g1_i6::g.69863::m.69863	PF12146.7	Hydrolase_4	MER0583385	family S9 unassigned peptidases	S09.UPW	S09X
TRINITY_DN17928_c2_g1::TRINITY_DN17928_c2_g1_i2::g.92278::m.92278	PF02230.15	Abhydrolase_2	MER0588084	family S9 unassigned peptidases	S09.UPW	S09X
TRINITY_DN18181_c1_g1::TRINITY_DN18181_c1_g1_i2::g.96439::m.96439	PF12697.6	Abhydrolase_6	MER0639174	family S9 unassigned peptidases	S09.UPW	S09X
TRINITY_DN18181_c1_g1::TRINITY_DN18181_c1_g1_i3::g.96440::m.96440	PF12697.6	Abhydrolase_6	MER0639174	family S9 unassigned peptidases	S09.UPW	S09X
TRINITY_DN18783_c5_g1::TRINITY_DN18783_c5_g1_i3::g.106356::m.106356	PF00450.21	Peptidase_S10	MER0629092	serine carboxypeptidase C	S10.004	S10
TRINITY_DN15931_c0_g1::TRINITY_DN15931_c0_g1_i5::g.59103::m.59103	PF00450.21	Peptidase_S10	MER0425718	serine carboxypeptidase D	S10.005	S10
TRINITY_DN18581_c2_g1::TRINITY_DN18581_c2_g1_i1::g.102861::m.102861	PF00450.21	Peptidase_S10	MER0571188	serine carboxypeptidase III	S10.009	S10
TRINITY_DN12399_c0_g1::TRINITY_DN12399_c0_g1_i1::g.12011::m.12011	PF00450.21	Peptidase_S10	MER0637468	OsBISCPL1-type putative carboxypeptidase	S10.017	S10
TRINITY_DN19791_c7_g1::TRINITY_DN19791_c7_g1_i1::g.123736::m.123736	PF00450.21	Peptidase_S10	MER0546785	At3g63470	S10.A41	S10
TRINITY_DN19791_c7_g4::TRINITY_DN19791_c7_g4_i2::g.123739::m.123739	PF00450.21	Peptidase_S10	MER0660317	At3g63470	S10.A41	S10
TRINITY_DN18581_c2_g2::TRINITY_DN18581_c2_g2_i8::g.102871::m.102871	PF00450.21	Peptidase_S10	MER0943604	family S10 unassigned peptidases	S10.UPW	S10

TRINITY_DN18581_c2_g2::TRINITY_DN18581_c2_g2_i16::g.102885::m.102885	PF00450.21	Peptidase_S10	MER0943604	family S10 unassigned peptidases	S10.UPW	S10
TRINITY_DN19589_c2_g2::TRINITY_DN19589_c2_g2_i4::g.120310::m.120310	PF00109.25	ketoacyl-synt	MER0947272	family S10 unassigned peptidases	S10.UPW	S10
TRINITY_DN19596_c1_g1::TRINITY_DN19596_c1_g1_i8::g.120430::m.120430	PF00450.21	Peptidase_S10	MER0943128	family S10 unassigned peptidases	S10.UPW	S10
TRINITY_DN19909_c0_g1::TRINITY_DN19909_c0_g1_i3::g.126119::m.126119	PF08263.11	LRRNT_2	MER0350329	family S10 unassigned peptidases	S10.UPW	S10
TRINITY_DN16339_c2_g2::TRINITY_DN16339_c2_g2_i2::g.65998::m.65998	PF02190.15	LON_substr_bdg	MER1036228	family S16 unassigned peptidases	S16.UPW	S16
TRINITY_DN17970_c3_g1::TRINITY_DN17970_c3_g1_i6::g.92954::m.92954	PF05577.11	Peptidase_S28	MER0143936	AT5g65760	S28.A02	S28
TRINITY_DN14658_c2_g1::TRINITY_DN14658_c2_g1_i2::g.40152::m.40152	PF05577.11	Peptidase_S28	MER1054347	family S28 unassigned peptidases	S28.UPW	S28
TRINITY_DN11909_c0_g1::TRINITY_DN11909_c0_g1_i2::g.9470::m.9470	PF00561.19	Abhydrolase_1	MER0622092	family S33 unassigned peptidases	S33.UPW	S33
TRINITY_DN15133_c0_g1::TRINITY_DN15133_c0_g1_i1::g.47252::m.47252	PF00561.19	Abhydrolase_1	MER1334561	family S33 unassigned peptidases	S33.UPW	S33
TRINITY_DN16306_c1_g1::TRINITY_DN16306_c1_g1_i3::g.65480::m.65480			MER0551075	family S33 unassigned peptidases	S33.UPW	S33
TRINITY_DN16306_c1_g2::TRINITY_DN16306_c1_g2_i2::g.65481::m.65481	PF00561.19	Abhydrolase_1	MER0639178	family S33 unassigned peptidases	S33.UPW	S33
TRINITY_DN16971_c2_g1::TRINITY_DN16971_c2_g1_i8::g.76469::m.76469	PF08538.9	DUF1749	MER0624846	family S33 unassigned peptidases	S33.UPW	S33
TRINITY_DN16987_c2_g1::TRINITY_DN16987_c2_g1_i9::g.76589::m.76589	PF00561.19	Abhydrolase_1	MER0633901	family S33 unassigned peptidases	S33.UPW	S33
TRINITY_DN17369_c1_g1::TRINITY_DN17369_c1_g1_i7::g.82610::m.82610	PF00227.25	Proteasome	MER0172841	proteasome subunit beta1c	T01.010	T01A
TRINITY_DN15811_c0_g5::TRINITY_DN15811_c0_g5_i2::g.57331::m.57331	PF00227.25	Proteasome	MER0411684	proteasome subunit alpha 6	T01.971	T01A
TRINITY_DN18427_c1_g2::TRINITY_DN18427_c1_g2_i5::g.100438::m.100438	PF10584.8	Proteasome_A_N	MER0570712	proteasome subunit alpha 2	T01.972	T01A
TRINITY_DN13422_c4_g1::TRINITY_DN13422_c4_g1_i9::g.23675::m.23675	PF10584.8	Proteasome_A_N	MER0020043	proteasome subunit alpha 3	T01.977	T01A
TRINITY_DN17070_c2_g4::TRINITY_DN17070_c2_g4_i5::g.77858::m.77858	PF10584.8	Proteasome_A_N	MER0505546	Mername-AA242 peptidase homologue	T01.995	T01A
TRINITY_DN15141_c1_g1::TRINITY_DN15141_c1_g1_i2::g.47502::m.47502	PF00227.25	Proteasome	MER0173164	proteasome subunit beta2	T01.A02	T01A
TRINITY_DN10911_c0_g2::TRINITY_DN10911_c0_g2_i2::g.5805::m.5805	PF00227.25	Proteasome	MER0126199	PBE2 g.p.	T01.A10	T01A

TRINITY_DN14659_c0_g2::TRINITY_DN14659_c0_g2_i6::g.40081::m.40081	PF00227.25	Proteasome	MER0588910	psmB4-2 g.p.	T01.A13	T01X
TRINITY_DN12971_c4_g2::TRINITY_DN12971_c4_g2_i4::g.17979::m.17979	PF10584.8	Proteasome_A_N	MER0584369	subfamily T1A unassigned peptidases	T01.UPA	T01A
TRINITY_DN15811_c0_g2::TRINITY_DN15811_c0_g2_i2::g.57329::m.57329	PF10584.8	Proteasome_A_N	MER0637297	subfamily T1A unassigned peptidases	T01.UPA	T01A
TRINITY_DN17150_c2_g1::TRINITY_DN17150_c2_g1_i1::g.79329::m.79329	PF10584.8	Proteasome_A_N	MER0576836	subfamily T1A unassigned peptidases	T01.UPA	T01A
TRINITY_DN17150_c2_g1::TRINITY_DN17150_c2_g1_i7::g.79332::m.79332	PF10584.8	Proteasome_A_N	MER0576836	subfamily T1A unassigned peptidases	T01.UPA	T01A
TRINITY_DN15448_c0_g1::TRINITY_DN15448_c0_g1_i6::g.51979::m.51979	PF01960.17	ArgJ	MER0122168	ArgJ protein	T05.002	T05
TRINITY_DN15488_c1_g5::TRINITY_DN15488_c1_g5_i3::g.52738::m.52738	PF14226.5	DIOX_N	MER0576457	family T7 unassigned peptidases	T07.UPW	T07
TRINITY_DN19493_c1_g1::TRINITY_DN19493_c1_g1_i9::g.118079::m.118079	PF03171.19	2OG-FeII_Oxy	MER0576457	family T7 unassigned peptidases	T07.UPW	T07
TRINITY_DN14014_c3_g1::TRINITY_DN14014_c3_g1_i1::g.31362::m.31362	PF03080.14	Neprosin	MER0973935	family U74 unassigned peptidases	U74.UPW	U74
TRINITY_DN16195_c0_g1::TRINITY_DN16195_c0_g1_i3::g.63589::m.63589	PF03080.14	Neprosin	MER0973935	family U74 unassigned peptidases	U74.UPW	U74
TRINITY_DN16782_c2_g1::TRINITY_DN16782_c2_g1_i3::g.73171::m.73171	PF03080.14	Neprosin	MER1001725	family U74 unassigned peptidases	U74.UPW	U74
TRINITY_DN16782_c2_g2::TRINITY_DN16782_c2_g2_i4::g.73167::m.73167	PF03080.14	Neprosin	MER0973935	family U74 unassigned peptidases	U74.UPW	U74
TRINITY_DN19409_c5_g1::TRINITY_DN19409_c5_g1_i1::g.117472::m.117472	PF03080.14	Neprosin	MER0973935	family U74 unassigned peptidases	U74.UPW	U74
TRINITY_DN19409_c5_g5::TRINITY_DN19409_c5_g5_i2::g.117475::m.117475	PF03080.14	Neprosin	MER1001725	family U74 unassigned peptidases	U74.UPW	U74
