# Supplementary material for: Comparative proteomics of common allergenic tree pollens of birch, alder, and hazel
Source: Allergy. 2021 Jan 15;76(6):1743–53. doi: 10.1111/all.14694 (PMC8248232; doi:10.1111/all.14694)
Supplement: Supplementary file 19 — Table S17 [file ALL-76-1743-s011.pdf]

Supplementary Table S17: Water soluble peptidases identified in Corylus pollen

Protein IDs	Pfam accession	Pfam family name	Merops accession	Merops family	source organism	Merops peptidase ID	Merops subfamily
TRINITY_DN10467_c0_g1_i1_1	PF14543.5	TAXi_N	MER0184554	nepenthesin	Vitis vinifera	A01.040	A01B
TRINITY_DN10590_c0_g2_i1_2	PF14543.5	TAXi_N	MER0372282	CDR1 peptidase	Prunus persica	A01.069	A01B
TRINITY_DN1993_c0_g1_i1_1			MER0511659	CDR1 peptidase	Prunus mume	A01.069	A01B
TRINITY_DN10194_c0_g1_i1_3	PF00026.22	Asp	MER0398222	At1g62290	Gossypium raimondii	A01.A02	A01A
TRINITY_DN1283_c0_g1_i1_3	PF00188.25	CAP	MER0228949	subfamily A1A unassigned peptidases	Debaryomyces hansenii	A01.UPA	A01A
TRINITY_DN10179_c0_g1_i1_1	PF04043.14	PMEI	MER0570148	family A2 unassigned peptidases	Cucumis sativus	A02.UPW	A02X
TRINITY_DN9853_c0_g2_i1_2	PF01095.18	Pectinesterase	MER0572141	family A2 unassigned peptidases	Cucumis melo	A02.UPW	A02X
TRINITY_DN5112_c0_g2_i3_3	PF00240.22	ubiquitin	MER0628276	subfamily A28A unassigned peptidases	Populus euphratica	A28.UPA	A28A
TRINITY_DN11279_c0_g2_i3_5	PF00112.22	Peptidase_C1	MER0660253	glycinain	Eucalyptus grandis	C01.022	C01A
TRINITY_DN9814_c0_g1_i2_2	PF00112.22	Peptidase_C1	MER0640046	cathepsin B, plant form	Nelumbo nucifera	C01.049	C01A
TRINITY_DN10303_c0_g1_i1_3	PF00112.22	Peptidase_C1	MER1161753	subfamily C1A unassigned peptidases	Juglans regia	C01.UPA	C01A
TRINITY_DN10919_c0_g1_i5_1	PF00112.22	Peptidase_C1	MER1161891	subfamily C1A unassigned peptidases	Cynara cardunculus	C01.UPA	C01A
TRINITY_DN11174_c0_g1_i5_1	PF00112.22	Peptidase_C1	MER1161383	subfamily C1A unassigned peptidases	Ziziphus jujuba	C01.UPA	C01A
TRINITY_DN11576_c1_g1_i1_2	PF00112.22	Peptidase_C1	MER0166880	subfamily C1A unassigned peptidases	Actinidia deliciosa	C01.UPA	C01A
TRINITY_DN7727_c0_g2_i1_3	PF00112.22	Peptidase_C1	MER1161346	subfamily C1A unassigned peptidases	Quercus suber	C01.UPA	C01A
TRINITY_DN8308_c0_g2_i1_2	PF00112.22	Peptidase_C1	MER1161707	subfamily C1A unassigned peptidases	Quercus suber	C01.UPA	C01A
TRINITY_DN9489_c0_g2_i1_2	PF00112.22	Peptidase_C1	MER1160989	subfamily C1A unassigned peptidases	Juglans regia	C01.UPA	C01A
TRINITY_DN10600_c0_g1_i1_5	PF04424.12	MINDY_DUB	MER0934028	FAM63B g.p.	Capsicum annuum	C115.002	C115
TRINITY_DN12921_c0_g1_i1_3			MER1170789	family C12 unassigned peptidases	Juglans regia	C12.UPW	C12
TRINITY_DN12921_c0_g1_i1_6	PF01088.20	Peptidase_C12	MER1170789	family C12 unassigned peptidases	Juglans regia	C12.UPW	C12
TRINITY_DN6323_c0_g1_i1_4	PF01650.17	Peptidase_C13	MER0691713	family C13 unassigned peptidases	Morus notabilis	C13.UPW	C13
TRINITY_DN7265_c0_g2_i5_2	PF00656.21	Peptidase_C14	MER0659855	subfamily C14B unassigned peptidases	Eucalyptus grandis	C14.UPB	C14B

TRINITY_DN9214_c0_g1_i3_1	PF00240.22	ubiquitin	MER0109705	UBP6 peptidase	Vitis vinifera	C19.094	C19
TRINITY_DN11267_c0_g1_i1_2	PF12481.7	DUF3700	MER0570230	family C44 unassigned peptidases	Cucumis sativus	C44.UPW	C44
TRINITY_DN8002_c0_g1_i1_2	PF02338.18	OTU	MER1128346	subfamily C85A unassigned peptidases	Glycine max	C85.UPA	C85A
TRINITY_DN6792_c0_g1_i2_5	PF02338.18	OTU	MER0744567	subfamily C85B unassigned peptidases	Prunus persica	C85.UPB	C85B
TRINITY_DN4761_c0_g1_i1_3	PF05903.13	Peptidase_C97	MER0660526	family C97 unassigned peptidases	Eucalyptus grandis	C97.UPW	C97
TRINITY_DN11097_c0_g1_i2_6	PF00079.19	Serpin	MER0758854	AtSerpin1	Morus notabilis	I04.087	I04
TRINITY_DN6915_c0_g2_i2_2	PF02704.13	GASA	MER0526833	family I8 unassigned peptidase inhibitors	Oryza brachyantha	I08.UPW	I08
TRINITY_DN9357_c0_g1_i1_4			MER0609806	family I8 unassigned peptidase inhibitors	Xenopus tropicalis	I08.UPW	I08
TRINITY_DN11161_c0_g2_i1_1	PF05922.15	Inhibitor_I9	MER0646935	family I9 unassigned peptidase inhibitors	Jatropha curcas	I09.UPW	I09
TRINITY_DN5978_c0_g1_i1_4	PF00403.25	HMA	MER0592272	family I13 unassigned peptidase inhibitors	Cicer arietinum	I13.UPW	I13
TRINITY_DN6677_c0_g2_i1_6	PF00280.17	potato_inhibit	MER0511491	family I13 unassigned peptidase inhibitors	Prunus mume	I13.UPW	I13
TRINITY_DN11259_c0_g1_i2_5	PF02225.21	PA	MER0642455	family I15 unassigned peptidase inhibitors	Musa acuminata	I15.UPW	I15
TRINITY_DN5250_c0_g1_i1_1	PF16845.4	SQAPI	MER0195773	phytocystatin	Hevea brasiliensis	I25.014	I25B
TRINITY_DN9738_c1_g1_i1_3	PF00031.20	Cystatin	MER0135396	phytocystatin	Populus trichocarpa	I25.014	I25B
TRINITY_DN10831_c0_g1_i1_3	PF16845.4	SQAPI	MER0511446	cystatin Hv-CPI5	Prunus mume	I25.054	I25B
TRINITY_DN8644_c0_g2_i1_1	PF16845.4	SQAPI	MER0593269	subfamily I25B unassigned peptidase inhibitors	Cicer arietinum	I25.UPB	I25B
TRINITY_DN9738_c2_g1_i2_3	PF16845.4	SQAPI	MER0622052	subfamily I25B unassigned peptidase inhibitors	Vitis vinifera	I25.UPB	I25B
TRINITY_DN11574_c0_g1_i5_3	PF01565.22	FAD_binding_4	MER0659912	family I29 unassigned peptidase inhibitors	Eucalyptus grandis	I29.UPW	I29
TRINITY_DN9962_c0_g2_i1_2	PF01565.22	FAD_binding_4	MER0659912	family I29 unassigned peptidase inhibitors	Eucalyptus grandis	I29.UPW	I29
TRINITY_DN17430_c0_g1_i1_5	PF01161.19	PBP	MER0785675	family I51 unassigned peptidase inhibitors	Citrus clementina	I51.UPW	I51
TRINITY_DN20395_c0_g2_i1_2			MER0571023	family I71 unassigned peptidase inhibitors	Cucumis sativus	I71.UPW	I71
TRINITY_DN5908_c0_g1_i1_3	PF01145.24	Band_7	MER0680924	family I87 unassigned peptidase inhibitors	Beta vulgaris	I87.UPW	I87
TRINITY_DN11601_c0_g2_i2_5	PF01433.19	Peptidase_M1	MER0412087	alanyl aminopeptidase	Nicotiana benthamiana	M01.005	M01

TRINITY_DN11240_c0_g2_i1_2	PF01433.19	Peptidase_M1	MER0412570	family M1 unassigned peptidases	Solanum tuberosum	M01.UPW	M01
TRINITY_DN3173_c0_g3_i1_1			MER0628627	oligopeptidase A	Populus euphratica	M03.004	M03A
TRINITY_DN3111_c0_g2_i1_2	PF01432.19	Peptidase_M3	MER0817154	subfamily M3A unassigned peptidases	Morus notabilis	M03.UPA	M03A
TRINITY_DN11191_c0_g1_i2_6	PF00883.20	Peptidase_M17	MER0659646	leucyl aminopeptidase	Eucalyptus grandis	M17.002	M17
TRINITY_DN11579_c0_g1_i2_1	PF01592.15	NifU_N	MER0474067	PepB aminopeptidase	Ceratitis capitata	M17.004	M17
TRINITY_DN2292_c0_g3_i1_5	PF00428.18	Ribosomal_60s	MER0890057	family M18 unassigned peptidases	Lachancea quebecensis	M18.UPW	M18
TRINITY_DN9076_c0_g2_i1_2	PF01546.27	Peptidase_M20	MER0015308	subfamily M20A unassigned peptidases	Arabidopsis thaliana	M20.UPA	M20A
TRINITY_DN4354_c0_g1_i1_4	PF15801.4	zf-C6H2	MER0161105	methionyl aminopeptidase 1	Drosophila simulans	M24.017	M24A
TRINITY_DN10807_c0_g1_i5_1	PF00557.23	Peptidase_M24	MER0093675	proliferation-association protein 1	Ammopiptanthus mongolicus	M24.973	M24X
TRINITY_DN12510_c0_g1_i1_1	PF13964.5	Kelch_6	MER0366613	subfamily S1A unassigned peptidases	Bos taurus	S01.UPA	S01A
TRINITY_DN9813_c0_g1_i1_2	PF00565.16	SNase	MER0573000	subfamily S1A unassigned peptidases	Corvus brachyrhynchos	S01.UPA	S01A
TRINITY_DN3347_c0_g1_i1_2	PF00082.21	Peptidase_S8	MER0570101	ARA12 peptidase	Citrus sinensis	S08.112	S08A
TRINITY_DN10388_c0_g2_i6_4	PF00082.21	Peptidase_S8	MER0511553	AIR3 peptidase	Prunus mume	S08.119	S08A
TRINITY_DN10388_c0_g2_i5_4	PF00082.21	Peptidase_S8	MER0511553	AIR3 peptidase	Prunus mume	S08.119	S08A
TRINITY_DN53_c0_g1_i1_5	PF00082.21	Peptidase_S8	MER0551416	AIR3 peptidase	Malus domestica	S08.119	S08A
TRINITY_DN10903_c0_g1_i6_2	PF00082.21	Peptidase_S8	MER0039101	At1g32980	Arachis hypogaea	S08.A31	S08A
TRINITY_DN9173_c0_g1_i1_4	PF00082.21	Peptidase_S8	MER0039101	At1g32980	Arachis hypogaea	S08.A31	S08A
TRINITY_DN10039_c0_g1_i2_2			MER0600777	subfamily S8A unassigned peptidases	Zea mays	S08.UPA	S08A
TRINITY_DN9297_c0_g3_i2_1			MER0571725	subfamily S8A unassigned peptidases	Cucumis melo	S08.UPA	S08A
TRINITY_DN9993_c0_g1_i2_4	PF00082.21	Peptidase_S8	MER0544355	subfamily S8A unassigned peptidases	Glycine max	S08.UPA	S08A
TRINITY_DN10694_c0_g1_i1_2	PF00756.19	Esterase	MER0622192	S-formylglutathione hydrolase FrmB	Vitis vinifera	S09.940	S09B

TRINITY_DN10196_c0_g1_i1_1	PF07859.12	Abhydrolase_3	MER0500863	At5g62180	Prunus mume	S09.A14	S09X
TRINITY_DN8778_c0_g1_i2_2	PF12146.7	Hydrolase_4	MER0499110	At3g47560	Prunus mume	S09.A31	S09X
TRINITY_DN8570_c0_g1_i2_1	PF02230.15	Abhydrolase_2	MER0209135	AT5G20060 protein	Ricinus communis	S09.A56	S09X
TRINITY_DN2451_c0_g1_i1_2	PF00326.20	Peptidase_S9	MER0413191	subfamily S9A unassigned peptidases	Solanum lycopersicum	S09.UPA	S09A
TRINITY_DN10971_c0_g1_i2_2	PF07676.11	PD40	MER0404572	subfamily S9B unassigned peptidases	Nectria haematococca	S09.UPB	S09B
TRINITY_DN11503_c0_g1_i1_1	PF07859.12	Abhydrolase_3	MER0588552	subfamily S9C unassigned peptidases	Citrus sinensis	S09.UPC	S09C
TRINITY_DN11503_c0_g1_i5_1	PF07859.12	Abhydrolase_3	MER0588552	subfamily S9C unassigned peptidases	Citrus sinensis	S09.UPC	S09C
TRINITY_DN8311_c0_g1_i1_6	PF07859.12	Abhydrolase_3	MER0659141	subfamily S9C unassigned peptidases	Eucalyptus grandis	S09.UPC	S09C
TRINITY_DN11588_c0_g1_i3_2	PF00400.31	WD40	MER0156515	family S9 unassigned peptidases	Dipodomys ordii	S09.UPW	S09X
TRINITY_DN6770_c0_g1_i2_3	PF12697.6	Abhydrolase_6	MER0650695	family S9 unassigned peptidases	Jatropha curcas	S09.UPW	S09X
TRINITY_DN8280_c0_g2_i1_2	PF02230.15	Abhydrolase_2	MER0588084	family S9 unassigned peptidases	Citrus sinensis	S09.UPW	S09X
TRINITY_DN2711_c0_g1_i1_3	PF00450.21	Peptidase_S10	MER0637468	OsBISCPL1-type putative carboxypeptidase	Nicotiana sylvestris	S10.017	S10
TRINITY_DN22082_c0_g1_i1_3	PF00450.21	Peptidase_S10	MER0629038	At3g63470	Populus euphratica	S10.A41	S10
TRINITY_DN2708_c0_g2_i1_1	PF00450.21	Peptidase_S10	MER0627870	At3g63470	Populus euphratica	S10.A41	S10
TRINITY_DN128_c0_g1_i1_1	PF00240.22	ubiquitin	MER0053503	family S16 unassigned peptidases	Pan troglodytes	S16.UPW	S16
TRINITY_DN20857_c0_g1_i1_2	PF13419.5	HAD_2	MER0230625	family S33 unassigned peptidases	Micromonospora sp. L5	S33.UPW	S33
TRINITY_DN2750_c0_g2_i1_2	PF13419.5	HAD_2	MER0230625	family S33 unassigned peptidases	Micromonospora sp. L5	S33.UPW	S33
TRINITY_DN5779_c0_g1_i1_2	PF00561.19	Abhydrolase_1	MER0588391	family S33 unassigned peptidases	Citrus sinensis	S33.UPW	S33
TRINITY_DN9585_c0_g1_i1_2	PF00561.19	Abhydrolase_1	MER1334561	family S33 unassigned peptidases	Juglans regia	S33.UPW	S33
TRINITY_DN6573_c0_g1_i1_4	PF01694.21	Rhomboid	MER1081552	family S54 unassigned peptidases	Brassica oleracea	S54.UPW	S54
TRINITY_DN9206_c0_g2_i1_1	PF01112.17	Asparaginase_2	MER0179633	family T2 unassigned peptidases	Ricinus communis	T02.UPW	T02
TRINITY_DN10088_c0_g2_i1_3	PF14226.5	DIOX_N	MER0576457	family T7 unassigned peptidases		T07.UPW	T07

TRINITY_DN11452_c0_g2_i2_2	PF14226.5	DIOX_N	MER0576457	family T7 unassigned peptidases		T07.UPW	T07
TRINITY_DN4171_c0_g2_i2_3	PF14226.5	DIOX_N	MER0576457	family T7 unassigned peptidases		T07.UPW	T07
TRINITY_DN2392_c0_g1_i1_2			MER0901042	family U74 unassigned peptidases	Gossypium arboreum	U74.UPW	U74
