# Supplementary material for: Comparative proteomics of common allergenic tree pollens of birch, alder, and hazel
Source: Allergy. 2021 Jan 15;76(6):1743–53. doi: 10.1111/all.14694 (PMC8248232; doi:10.1111/all.14694)
Supplement: Supplementary file 20 — Table S18 [file ALL-76-1743-s012.pdf]

Supplementary Table S15: Water soluble peptidases identified in Alnus pollen

Protein IDs	Pfam accession	Pfam family name	Merops accession	Merops family	source organism	Merops peptidase ID	Merops subfamily
ARUBRA_DN3984_c0_g1_i1_3	PF00026.22	Asp	MER0680933	At1g62290	Arabidopsis thaliana-Gossypium arboreum	A01.A02	A01A
ARUBRA_DN14653_c0_g1_i1_5	PF14543.5	TAXi_N	MER0584129	At1g05840	Arabidopsis thaliana-type peptidase	A01.A34	A01B
ARUBRA_DN13846_c0_g1_i1_5	PF00026.22	Asp	MER1133951	subfamily A1A unassigned peptidases	Juglans regia	A01.UPA	A01A
ARUBRA_DN4385_c0_g1_i1_6	PF00188.25	CAP	MER0228949	subfamily A1A unassigned peptidases	Debaryomyces hansenii	A01.UPA	A01A
ARUBRA_DN2382_c0_g1_i1_2	PF01095.18	Pectinesterase	MER0570148	family A2 unassigned peptidases	Cucumis sativus	A02.UPW	A02X
ARHOMBI_DN3097_c0_g1_i2_1	PF01095.18	Pectinesterase	MER0572141	family A2 unassigned peptidases	Cucumis melo	A02.UPW	A02X
ARHOMBI_DN5536_c0_g1_i1_1	PF04043.14	PMEI	MER0570148	family A2 unassigned peptidases	Cucumis sativus	A02.UPW	A02X
ARUBRA_DN4454_c0_g1_i1_2	PF01095.18	Pectinesterase	MER0572141	family A2 unassigned peptidases	Cucumis melo	A02.UPW	A02X
ARUBRA_DN6197_c0_g1_i1_1	PF01095.18	Pectinesterase	MER0570148	family A2 unassigned peptidases	Cucumis sativus	A02.UPW	A02X
ARHOMBI_DN15828_c0_g1_i1_6	PF09668.9	Asp_protease	MER0628276	subfamily A28A unassigned peptidases	Populus euphratica	A28.UPA	A28A
ARUBRA_DN9894_c0_g1_i1_4	PF00112.22	Peptidase_C1	MER0660253	glycinain	Eucalyptus grandis	C01.022	C01A
ARUBRA_DN4725_c0_g1_i1_2	PF00112.22	Peptidase_C1	MER0640046	cathepsin B, plant form	Nelumbo nucifera	C01.049	C01A
ARHOMBI_DN5653_c0_g1_i1_4	PF00187.18	Chitin_bind_1	MER0428589	Cwp84 peptidase	Limonium bicolor	C01.125	C01A
ARUBRA_DN5882_c0_g1_i1_4	PF00112.22	Peptidase_C1	MER1164780	subfamily C1A unassigned peptidases	Juglans regia	C01.UPA	C01A
ARHOMBI_DN5775_c0_g1_i1_2	PF00112.22	Peptidase_C1	MER1160989	subfamily C1A unassigned peptidases	Juglans regia	C01.UPA	C01A
ARHOMBI_DN6144_c0_g1_i1_4	PF00112.22	Peptidase_C1	MER1161707	subfamily C1A unassigned peptidases	Quercus suber	C01.UPA	C01A
ARHOMBI_DN8306_c0_g1_i1_4	PF01088.20	Peptidase_C12	MER1170789	family C12 unassigned peptidases	Juglans regia	C12.UPW	C12
ARUBRA_DN21075_c0_g1_i1_5	PF01088.20	Peptidase_C12	MER1170789	family C12 unassigned peptidases	Juglans regia	C12.UPW	C12
ARHOMBI_DN15559_c0_g1_i1_1	PF00656.21	Peptidase_C14	MER0627629	subfamily C14B unassigned peptidases	Populus euphratica	C14.UPB	C14B

ARUBRA_DN9286_c0_g1_i1_1	PF12481.7	DUF3700	MER0038019	At4g27450	Arabidopsis thaliana-Arabidopsis thaliana	C44.A04	C44
ARUBRA_DN1786_c0_g1_i1_1	PF12481.7	DUF3700	MER0570230	family C44 unassigned peptidases	Cucumis sativus	C44.UPW	C44
ARHOMBI_DN1764_c0_g1_i1_5	PF00733.20	Asn_synthase	MER0037116	family C44 unassigned peptidases	Solanum lycopersicum	C44.UPW	C44
ARHOMBI_DN13057_c0_g1_i1_1	PF02338.18	OTU	MER0622165	OTU2 peptidase		C85.008	C85A
ARUBRA_DN10864_c0_g1_i1_2	PF02338.18	OTU	MER0546486	OTU2 peptidase	Saccharomyces cerevisiae}-type)	C85.008	C85A
ARHOMBI_DN3049_c0_g1_i1_5	PF02338.18	OTU	MER0744567	subfamily C85B unassigned peptidases	Prunus persica	C85.UPB	C85B
ARUBRA_DN23541_c0_g1_i1_3	PF05903.13	Peptidase_C97	MER0636972	family C97 unassigned peptidases	Nicotiana sylvestris	C97.UPW	C97
ARHOMBI_DN22144_c0_g1_i1_6	PF00079.19	Serpin	MER0758854	AtSerpin1	Arabidopsis thaliana-Morus notabilis	I04.087	I04
ARHOMBI_DN2725_c0_g1_i1_2	PF00079.19	Serpin	MER0180116	AtSerpin1	Arabidopsis thaliana-Ricinus communis	I04.087	I04
ARUBRA_DN43_c0_g2_i1_6	PF02704.13	GASA	MER0526833	family I8 unassigned peptidase inhibitors	Oryza brachyantha	I08.UPW	I08
ARUBRA_DN4751_c0_g1_i2_6	PF00187.18	Chitin_bind_1	MER0449312	family I8 unassigned peptidase inhibitors	Tarenaya hassleriana	I08.UPW	I08
ARHOMBI_DN4937_c0_g3_i1_5	PF05922.15	Inhibitor_I9	MER0628798	family I9 unassigned peptidase inhibitors	Populus euphratica	I09.UPW	I09
ARHOMBI_DN5986_c0_g1_i2_6	PF00403.25	HMA	MER0592272	family I13 unassigned peptidase inhibitors	Cicer arietinum	I13.UPW	I13
ARHOMBI_DN6080_c1_g1_i1_1	PF16845.4	SQAPI	MER0178254	phytocystatin	Ricinus communis	I25.014	I25B
ARHOMBI_DN6132_c0_g5_i1_3	PF16845.4	SQAPI	MER0628337	phytocystatin	Populus euphratica	I25.014	I25B
ARUBRA_DN3020_c0_g1_i1_1	PF16845.4	SQAPI	MER0622052	subfamily I25B unassigned peptidase inhibitors	Vitis vinifera	I25.UPB	I25B
ARUBRA_DN4799_c0_g1_i1_4	PF16845.4	SQAPI	MER0172552	subfamily I25B unassigned peptidase inhibitors	Vitis vinifera	I25.UPB	I25B
ARUBRA_DN5778_c0_g1_i1_4	PF03009.16	GDPD	MER0604943	family I71 unassigned peptidase inhibitors	Cryptosporidium parvum	I71.UPW	I71
ARHOMBI_DN20074_c0_g1_i1_1	PF01433.19	Peptidase_M1	MER1182093	family M1 unassigned peptidases	Corchorus capsularis	M01.UPW	M01

ARHOMBI_DN2375_c0_g1_i1_6			MER0412570	family M1 unassigned peptidases	Solanum tuberosum	M01.UPW	M01
ARHOMBI_DN25003_c0_g1_i1_6	PF01433.19	Peptidase_M1	MER0484433	family M1 unassigned peptidases	Tarenaya hassleriana	M01.UPW	M01
ARHOMBI_DN4645_c0_g2_i1_4	PF01433.19	Peptidase_M1	MER1116733	family M1 unassigned peptidases	Brachypodium distachyon	M01.UPW	M01
ARHOMBI_DN4645_c1_g1_i1_2	PF01433.19	Peptidase_M1	MER0812872	family M1 unassigned peptidases	Gossypium raimondii	M01.UPW	M01
ARHOMBI_DN8832_c0_g1_i1_6	PF01433.19	Peptidase_M1	MER0336326	family M1 unassigned peptidases	Sorghum bicolor	M01.UPW	M01
ARUBRA_DN18651_c0_g1_i1_6			MER0135621	family M1 unassigned peptidases	Populus trichocarpa	M01.UPW	M01
ARUBRA_DN2825_c0_g1_i1_4			MER0412570	family M1 unassigned peptidases	Solanum tuberosum	M01.UPW	M01
ARUBRA_DN4026_c0_g1_i1_5	PF01433.19	Peptidase_M1	MER0413560	family M1 unassigned peptidases	Solanum lycopersicum	M01.UPW	M01
ARHOMBI_DN17898_c0_g1_i1_3	PF01432.19	Peptidase_M3	MER0817958	subfamily M3A unassigned peptidases	Prunus persica	M03.UPA	M03A
ARUBRA_DN20709_c0_g1_i1_5	PF01432.19	Peptidase_M3	MER0817154	subfamily M3A unassigned peptidases	Morus notabilis	M03.UPA	M03A
ARUBRA_DN10159_c0_g1_i1_5	PF00675.19	Peptidase_M16	MER0923033	subfamily M16B unassigned peptidases	Gossypium arboreum	M16.UPB	M16B
ARHOMBI_DN15373_c0_g1_i1_5	PF00883.20	Peptidase_M17	MER0659646	leucyl aminopeptidase	Eucalyptus grandis	M17.002	M17
ARUBRA_DN1745_c0_g2_i1_1			MER0621905	subfamily M20A unassigned peptidases	Vitis vinifera	M20.UPA	M20A
ARUBRA_DN16568_c0_g1_i1_3	PF01344.24	Kelch_1	MER0366613	subfamily S1A unassigned peptidases	Bos taurus	S01.UPA	S01A
ARHOMBI_DN7219_c0_g1_i1_3			MER0975537	ARA12 peptidase	Ricinus communis	S08.112	S08A
ARUBRA_DN18006_c0_g1_i1_4	PF00082.21	Peptidase_S8	MER0975537	ARA12 peptidase	Ricinus communis	S08.112	S08A
ARHOMBI_DN11662_c0_g1_i1_4			MER0546828	At3g14067	Arabidopsis thaliana-Fragaria vesca	S08.A28	S08A
ARHOMBI_DN11775_c0_g1_i1_1			MER0024767	At1g32980	Arabidopsis thaliana-Arabidopsis thaliana	S08.A31	S08A

ARUBRA_DN17897_c0_g1_i1_5	PF00082.21	Peptidase_S8	MER0039101	At1g32980	Arabidopsis thaliana-Arachis hypogaea	S08.A31	S08A
ARUBRA_DN2819_c0_g1_i1_3			MER0536974	At5g23530	Arabidopsis thaliana-Glycine max	S09.A10	S09C
ARUBRA_DN12422_c0_g1_i1_2	PF07859.12	Abhydrolase_3	MER0500863	At5g62180	Arabidopsis thaliana-Prunus mume	S09.A14	S09X
ARHOMBI_DN2479_c0_g1_i1_1			MER0499110	At3g47560	Arabidopsis thaliana-Prunus mume	S09.A31	S09X
ARHOMBI_DN3938_c0_g1_i1_4	PF02230.15	Abhydrolase_2	MER0209135	AT5G20060 protein	Arabidopsis thaliana-Ricinus communis	S09.A56	S09X
ARUBRA_DN9538_c0_g1_i1_1	PF12146.7	Hydrolase_4	MER0621993	F14F18_80 protein	Arabidopsis thaliana-Vitis vinifera	S09.A58	S09B
ARHOMBI_DN19693_c0_g1_i1_6	PF07859.12	Abhydrolase_3	MER0511717	subfamily S9C unassigned peptidases	Prunus mume	S09.UPC	S09C
ARUBRA_DN18800_c0_g2_i1_4	PF07859.12	Abhydrolase_3	MER0621926	subfamily S9C unassigned peptidases	Vitis vinifera	S09.UPC	S09C
ARUBRA_DN19739_c0_g2_i1_3	PF07859.12	Abhydrolase_3	MER0592815	subfamily S9C unassigned peptidases	Cicer arietinum	S09.UPC	S09C
ARUBRA_DN21696_c0_g1_i1_3	PF07859.12	Abhydrolase_3	MER0588552	subfamily S9C unassigned peptidases	Citrus sinensis	S09.UPC	S09C
ARHOMBI_DN3877_c0_g1_i1_1	PF02230.15	Abhydrolase_2	MER0588084	family S9 unassigned peptidases	Citrus sinensis	S09.UPW	S09X
ARHOMBI_DN478_c0_g2_i1_6	PF00756.19	Esterase	MER0651049	family S9 unassigned peptidases	Jatropha curcas	S09.UPW	S09X
ARUBRA_DN162_c0_g1_i1_1	PF12697.6	Abhydrolase_6	MER0650695	family S9 unassigned peptidases	Jatropha curcas	S09.UPW	S09X
ARUBRA_DN18867_c0_g1_i1_5	PF00400.31	WD40	MER0156515	family S9 unassigned peptidases	Dipodomys ordii	S09.UPW	S09X
ARUBRA_DN4594_c0_g2_i1_4	PF00450.21	Peptidase_S10	MER0592987	serine carboxypeptidase C	Cicer arietinum	S10.004	S10
ARUBRA_DN16761_c0_g1_i1_6	PF00450.21	Peptidase_S10	MER0539743	serine carboxypeptidase D	Sesamum indicum	S10.005	S10
ARUBRA_DN2627_c0_g1_i1_2	PF00450.21	Peptidase_S10	MER0660303	serine carboxypeptidase III	Eucalyptus grandis	S10.009	S10
ARUBRA_DN22266_c0_g1_i1_1	PF00450.21	Peptidase_S10	MER0177780	OsBISCPL1-type putative carboxypeptidase	Ricinus communis	S10.017	S10
ARHOMBI_DN6202_c0_g1_i2_4	PF00450.21	Peptidase_S10	MER0551973	At3g63470	Arabidopsis thaliana-Malus domestica	S10.A41	S10

ARUBRA_DN4832_c0_g1_i1_2	PF00450.21	Peptidase_S10	MER0637561	At3g63470	Arabidopsis thaliana-Nicotiana sylvestris	S10.A41	S10
ARUBRA_DN4961_c0_g1_i2_4	PF00450.21	Peptidase_S10	MER0551973	At3g63470	Arabidopsis thaliana-Malus domestica	S10.A41	S10
ARHOMBI_DN25343_c0_g1_i1_2	PF00450.21	Peptidase_S10	MER0942946	family S10 unassigned peptidases	Erythranthe guttata	S10.UPW	S10
ARHOMBI_DN430_c0_g1_i1_3	PF00574.22	CLP_protease	MER0639468	peptidase Clp	Nelumbo nucifera	S14.001	S14
ARUBRA_DN13110_c0_g1_i1_1	PF13419.5	HAD_2	MER0201475	cytosolic epoxide hydrolase		S33.973	S33
ARHOMBI_DN3967_c0_g1_i1_1	PF13419.5	HAD_2	MER0230625	family S33 unassigned peptidases	Micromonospora sp. L5	S33.UPW	S33
ARUBRA_DN4582_c0_g1_i1_5	PF00561.19	Abhydrolase_1	MER0588391	family S33 unassigned peptidases	Citrus sinensis	S33.UPW	S33
ARUBRA_DN1522_c0_g1_i1_5	PF00561.19	Abhydrolase_1	MER1336005	family S33 unassigned peptidases	Vigna radiata	S33.UPW	S33
ARUBRA_DN2934_c0_g2_i2_4	PF05670.12	DUF814	MER0599089	family S33 unassigned peptidases	Echinops telfairi	S33.UPW	S33
ARUBRA_DN7781_c0_g1_i1_6	PF02817.16	E3_binding	MER0094490	family S33 unassigned peptidases	Sinorhizobium medicae	S33.UPW	S33
ARUBRA_DN4073_c0_g1_i1_2	PF10584.8	Proteasome_A_N	MER0461296	proteasome subunit alpha 1	Glycine max	T01.976	T01A
ARUBRA_DN18881_c0_g1_i1_2	PF10584.8	Proteasome_A_N	MER0505546	Mername-AA242 peptidase homologue	Mus musculus-Prunus mume	T01.995	T01A
ARHOMBI_DN6139_c0_g1_i1_4	PF10584.8	Proteasome_A_N	MER0576836	subfamily T1A unassigned peptidases		T01.UPA	T01A
ARHOMBI_DN3876_c0_g1_i1_6	PF14226.5	DIOX_N	MER0576457	family T7 unassigned peptidases		T07.UPW	T07
