# Supplementary material for: Comparative proteomics of common allergenic tree pollens of birch, alder, and hazel
Source: Allergy. 2021 Jan 15;76(6):1743–53. doi: 10.1111/all.14694 (PMC8248232; doi:10.1111/all.14694)
Supplement: Supplementary file 21 — Table S19 [file ALL-76-1743-s020.pdf]

Supplementary Table S16: Peptidases exclusively identified in water extracted Alnus pollen

Protein IDs	Pfam accession	Pfam family name	Merops accession	Merops family	source organism	Merops peptidase ID	Merops subfamily
ARUBRA_DN14653_c0_g1_i1_5	PF14543.5	TAXi_N	MER0584129	At1g05840	Arabidopsis thaliana-type peptidase	A01.A34	A01B
ARHOMBI_DN3097_c0_g1_i2_1	PF01095.18	Pectinesterase	MER0572141	family A2 unassigned peptidases	Cucumis melo	A02.UPW	A02X
ARHOMBI_DN5536_c0_g1_i1_1	PF04043.14	PMEI	MER0570148	family A2 unassigned peptidases	Cucumis sativus	A02.UPW	A02X
ARUBRA_DN6197_c0_g1_i1_1	PF01095.18	Pectinesterase	MER0570148	family A2 unassigned peptidases	Cucumis sativus	A02.UPW	A02X
ARUBRA_DN4725_c0_g1_i1_2	PF00112.22	Peptidase_C1	MER0640046	cathepsin B, plant form	Nelumbo nucifera	C01.049	C01A
ARHOMBI_DN5653_c0_g1_i1_4	PF00187.18	Chitin_bind_1	MER0428589	Cwp84 peptidase	Limonium bicolor	C01.125	C01A
ARUBRA_DN10864_c0_g1_i1_2	PF02338.18	OTU	MER0546486	OTU2 peptidase	Saccharomyces cerevisiae}-type)	C85.008	C85A
ARUBRA_DN23541_c0_g1_i1_3	PF05903.13	Peptidase_C97	MER0636972	family C97 unassigned peptidases	Nicotiana sylvestris	C97.UPW	C97
ARHOMBI_DN13880_c0_g1_i1_4	PF00079.19	Serpin	MER0756064	AtSerpin1	Arabidopsis thaliana-Vitis vinifera	I04.087	I04
ARUBRA_DN43_c0_g2_i1_6	PF02704.13	GASA	MER0526833	family I8 unassigned peptidase inhibitors	Oryza brachyantha	I08.UPW	I08
ARUBRA_DN4751_c0_g1_i2_6	PF00187.18	Chitin_bind_1	MER0449312	family I8 unassigned peptidase inhibitors	Tarenaya hassleriana	I08.UPW	I08
ARHOMBI_DN6132_c0_g5_i1_3	PF16845.4	SQAPI	MER0628337	phytocystatin	Populus euphratica	I25.014	I25B
ARUBRA_DN4799_c0_g1_i1_4	PF16845.4	SQAPI	MER0172552	subfamily I25B unassigned peptidase inhibitors	Vitis vinifera	I25.UPB	I25B
ARUBRA_DN2825_c0_g1_i1_4			MER0412570	family M1 unassigned peptidases	Solanum tuberosum	M01.UPW	M01
ARHOMBI_DN7219_c0_g1_i1_3			MER0975537	ARA12 peptidase	Ricinus communis	S08.112	S08A
ARHOMBI_DN11662_c0_g1_i1_4			MER0546828	At3g14067	Arabidopsis thaliana-Fragaria vesca	S08.A28	S08A
ARUBRA_DN2819_c0_g1_i1_3			MER0536974	At5g23530	Arabidopsis thaliana-Glycine max	S09.A10	S09C
ARUBRA_DN12422_c0_g1_i1_2	PF07859.12	Abhydrolase_3	MER0500863	At5g62180	Arabidopsis thaliana-Prunus mume	S09.A14	S09X

ARHOMBI_DN3938_c0_g1_i1_4	PF02230.15	Abhydrolase_2	MER0209135	AT5G20060 protein	Arabidopsis thaliana-Ricinus communis	S09.A56	S09X
ARHOMBI_DN19693_c0_g1_i1_6	PF07859.12	Abhydrolase_3	MER0511717	subfamily S9C unassigned peptidases	Prunus mume	S09.UPC	S09C
ARUBRA_DN21696_c0_g1_i1_3	PF07859.12	Abhydrolase_3	MER0588552	subfamily S9C unassigned peptidases	Citrus sinensis	S09.UPC	S09C
ARHOMBI_DN3877_c0_g1_i1_1	PF02230.15	Abhydrolase_2	MER0588084	family S9 unassigned peptidases	Citrus sinensis	S09.UPW	S09X
ARUBRA_DN16761_c0_g1_i1_6	PF00450.21	Peptidase_S10	MER0539743	serine carboxypeptidase D	Sesamum indicum	S10.005	S10
ARUBRA_DN22266_c0_g1_i1_1	PF00450.21	Peptidase_S10	MER0177780	OsBISCPL1-type putative carboxypeptidase	Ricinus communis	S10.017	S10
ARUBRA_DN4832_c0_g1_i1_2	PF00450.21	Peptidase_S10	MER0637561	At3g63470	Arabidopsis thaliana-Nicotiana sylvestris	S10.A41	S10
ARUBRA_DN13110_c0_g1_i1_1	PF13419.5	HAD_2	MER0201475	cytosolic epoxide hydrolase		S33.973	S33
ARHOMBI_DN3967_c0_g1_i1_1	PF13419.5	HAD_2	MER0230625	family S33 unassigned peptidases	Micromonospora sp. L5	S33.UPW	S33
ARUBRA_DN2934_c0_g2_i2_4	PF05670.12	DUF814	MER0599089	family S33 unassigned peptidases	Echinops telfairi	S33.UPW	S33
