# Supplementary material for: Comparative proteomics of common allergenic tree pollens of birch, alder, and hazel
Source: Allergy. 2021 Jan 15;76(6):1743–53. doi: 10.1111/all.14694 (PMC8248232; doi:10.1111/all.14694)
Supplement: Supplementary file 22 — Table S20 [file ALL-76-1743-s002.pdf]

Supplementary Table S14: Peptidases exclusively identified in the water extracted Betula pollen

Protein IDs	Pfam accession	Pfam family name	Merops accession	Merops family	Merops peptidase ID	Merops subfamily
TRINITY_DN19058_c2_g1::TRINITY_DN19058_c2_g1_i7::g.111114::m.111114	PF14543.5	TAXi_N	MER0372282	CDR1 peptidase	A01.069	A01B
TRINITY_DN13851_c1_g2::TRINITY_DN13851_c1_g2_i2::g.29292::m.29292	PF00026.22	Asp	MER1131201	subfamily A1A unassigned peptidases	A01.UPA	A01A
TRINITY_DN19962_c5_g2::TRINITY_DN19962_c5_g2_i2::g.126940::m.126940	PF01095.18	Pectinesterase	MER0570148	family A2 unassigned peptidases	A02.UPW	A02X
TRINITY_DN15205_c1_g1::TRINITY_DN15205_c1_g1_i3::g.48499::m.48499	PF00112.22	Peptidase_C1	MER1160913	subfamily C1A unassigned peptidases	C01.UPA	C01A
TRINITY_DN18947_c6_g2::TRINITY_DN18947_c6_g2_i2::g.109306::m.109306	PF00112.22	Peptidase_C1	MER1161493	subfamily C1A unassigned peptidases	C01.UPA	C01A
TRINITY_DN31964_c0_g1::TRINITY_DN31964_c0_g1_i1::g.130316::m.130316	PF00112.22	Peptidase_C1	MER1161346	subfamily C1A unassigned peptidases	C01.UPA	C01A
TRINITY_DN13524_c0_g1::TRINITY_DN13524_c0_g1_i8::g.24870::m.24870	PF00917.25	MATH	MER0712608	family C19 unassigned peptidases	C19.UPW	C19
TRINITY_DN13734_c2_g1::TRINITY_DN13734_c2_g1_i1::g.27715::m.27715	PF12481.7	DUF3700	MER0571885	family C44 unassigned peptidases	C44.UPW	C44
TRINITY_DN11558_c0_g1::TRINITY_DN11558_c0_g1_i1::g.7858::m.7858	PF00197.17	Kunitz_legume	MER0453920	inhibitor MtTi2	I03.030	I03A
TRINITY_DN13585_c0_g1::TRINITY_DN13585_c0_g1_i3::g.25706::m.25706			MER0680968	subfamily I25A unassigned peptidase inhibitors	I25.UPA	I25A
TRINITY_DN11049_c0_g1::TRINITY_DN11049_c0_g1_i1::g.6211::m.6211	PF01161.19	PBP	MER0785675	family I51 unassigned peptidase inhibitors	I51.UPW	I51
TRINITY_DN17913_c4_g1::TRINITY_DN17913_c4_g1_i1::g.91856::m.91856	PF03009.16	GDPD	MER0604943	family I71 unassigned peptidase inhibitors	I71.UPW	I71
TRINITY_DN19263_c0_g1::TRINITY_DN19263_c0_g1_i6::g.114813::m.114813	PF01432.19	Peptidase_M3	MER0546417	oligopeptidase A	M03.004	M03A
TRINITY_DN18281_c0_g1::TRINITY_DN18281_c0_g1_i4::g.98025::m.98025	PF04563.14	RNA_pol_Rpb2_1	MER0195833	family N11 unassigned peptide lyases	N11.UPW	N11
TRINITY_DN16993_c0_g2::TRINITY_DN16993_c0_g2_i2::g.76741::m.76741	PF00082.21	Peptidase_S8	MER0621382	ARA12 peptidase	S08.112	S08A
TRINITY_DN13334_c3_g1::TRINITY_DN13334_c3_g1_i2::g.22908::m.22908	PF00082.21	Peptidase_S8	MER0039101	At1g32980	S08.A31	S08A
TRINITY_DN13205_c0_g1::TRINITY_DN13205_c0_g1_i3::g.21053::m.21053	PF07859.12	Abhydrolase_3	MER0588552	subfamily S9C unassigned peptidases	S09.UPC	S09C
TRINITY_DN17776_c0_g1::TRINITY_DN17776_c0_g1_i2::g.89550::m.89550	PF07859.12	Abhydrolase_3	MER0621913	subfamily S9C unassigned peptidases	S09.UPC	S09C
TRINITY_DN17928_c2_g1::TRINITY_DN17928_c2_g1_i2::g.92278::m.92278	PF02230.15	Abhydrolase_2	MER0588084	family S9 unassigned peptidases	S09.UPW	S09X

TRINITY_DN18181_c1_g1::TRINITY_DN18181_c1_g1_i3::g.96440::m.96440	PF12697.6	Abhydrolase_6	MER0639174	family S9 unassigned peptidases	S09.UPW	S09X
TRINITY_DN15931_c0_g1::TRINITY_DN15931_c0_g1_i5::g.59103::m.59103	PF00450.21	Peptidase_S10	MER0425718	serine carboxypeptidase D	S10.005	S10
TRINITY_DN12399_c0_g1::TRINITY_DN12399_c0_g1_i1::g.12011::m.12011	PF00450.21	Peptidase_S10	MER0637468	OsBISCPL1-type putative carboxypeptidase	S10.017	S10
TRINITY_DN19791_c7_g4::TRINITY_DN19791_c7_g4_i2::g.123739::m.123739	PF00450.21	Peptidase_S10	MER0660317	At3g63470	S10.A41	S10
TRINITY_DN16195_c0_g1::TRINITY_DN16195_c0_g1_i3::g.63589::m.63589	PF03080.14	Neprosin	MER0973935	family U74 unassigned peptidases	U74.UPW	U74
TRINITY_DN16782_c2_g1::TRINITY_DN16782_c2_g1_i3::g.73171::m.73171	PF03080.14	Neprosin	MER1001725	family U74 unassigned peptidases	U74.UPW	U74
TRINITY_DN16782_c2_g2::TRINITY_DN16782_c2_g2_i4::g.73167::m.73167	PF03080.14	Neprosin	MER0973935	family U74 unassigned peptidases	U74.UPW	U74
TRINITY_DN19409_c5_g5::TRINITY_DN19409_c5_g5_i2::g.117475::m.117475	PF03080.14	Neprosin	MER1001725	family U74 unassigned peptidases	U74.UPW	U74
