# Supplementary material for: Comparative proteomics of common allergenic tree pollens of birch, alder, and hazel
Source: Allergy. 2021 Jan 15;76(6):1743–53. doi: 10.1111/all.14694 (PMC8248232; doi:10.1111/all.14694)
Supplement: Supplementary file 23 — Table S21 [file ALL-76-1743-s008.pdf]

Supplementary Table S18: Peptidases exclusively identified in water extracted Corylus pollen

Protein IDs	Pfam accession	Pfam family name	Merops accession	Merops family	source organism	Merops peptidase ID	Merops subfamily
TRINITY_DN1283_c0_g1_i1_3	PF00188.25	CAP	MER0228949	subfamily A1A unassigned peptidases	Debaryomyces hansenii	A01.UPA	A01A
TRINITY_DN10919_c0_g1_i5_1	PF00112.22	Peptidase_C1	MER1161891	subfamily C1A unassigned peptidases	Cynara cardunculus	C01.UPA	C01A
TRINITY_DN8308_c0_g2_i1_2	PF00112.22	Peptidase_C1	MER1161707	subfamily C1A unassigned peptidases	Quercus suber	C01.UPA	C01A
TRINITY_DN9489_c0_g2_i1_2	PF00112.22	Peptidase_C1	MER1160989	subfamily C1A unassigned peptidases	Juglans regia	C01.UPA	C01A
TRINITY_DN12921_c0_g1_i1_3			MER1170789	family C12 unassigned peptidases	Juglans regia	C12.UPW	C12
TRINITY_DN6323_c0_g1_i1_4	PF01650.17	Peptidase_C13	MER0691713	family C13 unassigned peptidases	Morus notabilis	C13.UPW	C13
TRINITY_DN9010_c0_g1_i2_3	PF01470.16	Peptidase_C15	MER0584791	At1g56700		C15.A02	C15
TRINITY_DN4761_c0_g1_i1_3	PF05903.13	Peptidase_C97	MER0660526	family C97 unassigned peptidases	Eucalyptus grandis	C97.UPW	C97
TRINITY_DN6915_c0_g2_i2_2	PF02704.13	GASA	MER0526833	family I8 unassigned peptidase inhibitors	Oryza brachyantha	I08.UPW	I08
TRINITY_DN9357_c0_g1_i1_4			MER0609806	family I8 unassigned peptidase inhibitors	Xenopus tropicalis	I08.UPW	I08
TRINITY_DN6677_c0_g2_i1_6	PF00280.17	potato_inhibit	MER0511491	family I13 unassigned peptidase inhibitors	Prunus mume	I13.UPW	I13
TRINITY_DN2292_c0_g3_i1_5	PF00428.18	Ribosomal_60s	MER0890057	family M18 unassigned peptidases	Lachancea quebecensis	M18.UPW	M18
TRINITY_DN4354_c0_g1_i1_4	PF15801.4	zf-C6H2	MER0161105	methionyl aminopeptidase 1	Drosophila simulans	M24.017	M24A
TRINITY_DN3347_c0_g1_i1_2	PF00082.21	Peptidase_S8	MER0570101	ARA12 peptidase	Citrus sinensis	S08.112	S08A
TRINITY_DN53_c0_g1_i1_5	PF00082.21	Peptidase_S8	MER0551416	AIR3 peptidase	Malus domestica	S08.119	S08A
TRINITY_DN10903_c0_g1_i6_2	PF00082.21	Peptidase_S8	MER0039101	At1g32980	Arachis hypogaea	S08.A31	S08A
TRINITY_DN18123_c0_g2_i1_2			MER0535213	subfamily S8A unassigned peptidases	Sesamum indicum	S08.UPA	S08A
TRINITY_DN8778_c0_g1_i2_2	PF12146.7	Hydrolase_4	MER0499110	At3g47560	Prunus mume	S09.A31	S09X
TRINITY_DN11503_c0_g1_i5_1	PF07859.12	Abhydrolase_3	MER0588552	subfamily S9C unassigned peptidases	Citrus sinensis	S09.UPC	S09C
TRINITY_DN8280_c0_g2_i1_2	PF02230.15	Abhydrolase_2	MER0588084	family S9 unassigned peptidases	Citrus sinensis	S09.UPW	S09X
TRINITY_DN128_c0_g1_i1_1	PF00240.22	ubiquitin	MER0053503	family S16 unassigned peptidases	Pan troglodytes	S16.UPW	S16

TRINITY_DN20857_c0_g1_i1_2	PF13419.5	HAD_2	MER0230625	family S33 unassigned peptidases	Micromonospora sp. L5	S33.UPW	S33
TRINITY_DN2750_c0_g2_i1_2	PF13419.5	HAD_2	MER0230625	family S33 unassigned peptidases	Micromonospora sp. L5	S33.UPW	S33
TRINITY_DN2392_c0_g1_i1_2			MER0901042	family U74 unassigned peptidases	Gossypium arboreum	U74.UPW	U74
